# Integrated Physiological, Transcriptomic, and Proteomic Analyses Reveal Alkali Stress Response and Tolerance Mechanisms of *Rhododendron obtusum* ‘Fenzhuanglou’ Seedlings

**DOI:** 10.3390/plants15193053

**Published:** 2026-10-06

**Authors:** Riwen Fei, Xiao Yang, Xinyue Liu, Xinyu Li, Siyang Duan, Jiansong You, Xiaoyu Li

**Affiliations:** 1 School of Agriculture, Liaodong University, Dandong 118003, China; 15714252112@163.com (X.Y.); 18441502710@163.com (X.L.); 18642787589@163.com (X.L.); duansiyang1994@126.com (S.D.); 2 Postdoctoral Workstation, Aim Honesty Biopharmaceutical Co., Ltd., Dalian 116620, China; ruser@126.com; 3 School of Bioengineering, Dalian University of Technology, Dalian 116024, China; xiaoyuli@dlut.edu.cn

**Keywords:** *Rhododendron obtusum* ‘Fenzhuanglou’, alkali stress, transcriptome, DIA proteomics, integrated multi-omics, antioxidant defense, flavonoid biosynthesis

## Abstract

Soil alkalization limits the cultivation of *Rhododendron*, an acidophilic ornamental genus, yet the alkali tolerance threshold of *R. obtusum* ‘Fenzhuanglou’ remains undefined. Seedlings were exposed for 40 d to mixed alkali (NaHCO_3_:Na_2_CO_3_ = 9:1; 0, 50, 60, and 70 mmol·L^−1^), combining physiological assays with RNA-seq and DIA proteomics of control and 60 mmol·L^−1^ leaves. The 50 mmol·L^−1^ treatment caused no visible injury, 60 mmol·L^−1^ defined the visible-injury threshold, and 70 mmol·L^−1^ caused severe, progressive damage. Chlorophyll b fell to about 20% of the control by 40 d under 60–70 mmol·L^−1^; antioxidant enzymes peaked at 20 d and then collapsed, while malondialdehyde accumulated dose-dependently, indicating late-stage failure of reactive oxygen species scavenging. Transcriptomics identified 7214 differentially expressed genes enriched in MAPK signaling, hormone signal transduction, and circadian rhythm; proteomics quantified 7452 proteins, including 1093 differentially expressed proteins enriched in flavonoid biosynthesis and α-linolenic acid metabolism. The low mRNA–protein correlation (ρ = 0.14–0.20) is consistent with transcript–protein decoupling potentially involving post-transcriptional regulation, protein stability, and turnover. These results define 60 mmol·L^−1^ as the tolerance limit of this cultivar and, to our knowledge, provide the first integrated multi-omics framework for alkali adaptation in *Rhododendron*.

## 1. Introduction

Soil salinization and alkalization, which are frequently coupled, already affect more than 800 million hectares of land globally and continue to expand through aridification, irrigation with alkaline water, and inappropriate land management [1,2]. Alkali stress, imposed by alkaline salts such as NaHCO_3_ and Na_2_CO_3_, is mechanistically distinct from neutral salt stress (e.g., NaCl) [1,3]. Beyond Na^+^ toxicity and osmotic imbalance, it imposes the additional burden of a high-pH environment. Elevated rhizosphere pH precipitates phosphate and essential metal micronutrients (Fe, Mn, Zn, Cu), damages root cell structure directly, and disrupts intracellular pH and ion homeostasis [3,4]. Plants must therefore cope simultaneously with ion toxicity, osmotic stress, high-pH injury, and secondary nutrient deficiency, so that alkali stress is generally more damaging than neutral salt stress at equivalent Na^+^ concentrations [1,3]. Understanding how this compound stress injures plants is a prerequisite for improving the tolerance of susceptible species.

At the whole-plant level, alkali injury follows a recognizable physiological chain that begins in the roots. Alkaline stress provokes rapid accumulation of reactive oxygen species (ROS) in roots, and this oxidative burst is directly associated with root damage in rice (*Oryza sativa* L.) [5]. Excess ROS attack membrane lipids, causing peroxidation that is conventionally tracked through malondialdehyde (MDA) accumulation and that compromises membrane integrity and compartmentation. These disturbances are then propagated to the shoot, where chloroplast structure deteriorates, pigment pools are degraded, and photosynthesis is inhibited. In cucumber seedlings, photosynthetic capacity, ion homeostasis, and reactive oxygen metabolism were jointly implicated in alkali-stress injury and its alleviation [6]. Plants counteract these injuries through three interlocking defense layers. First, the photosynthetic apparatus is protected by maintaining chlorophyll content and engaging photoprotective pigments, which are central to the alkaline-stress tolerance of willow species [7]. Second, osmotic adjustment is achieved by accumulating compatible solutes: soluble sugars, soluble proteins, and proline accumulated coordinately with antioxidant responses in sugar beet (*Beta vulgaris* L.) under alkaline stress [8]. Osmolyte pools were likewise metabolically reprogrammed in the roots of oat cultivars under complex saline-alkali stress [9]. Third, the antioxidant system—superoxide dismutase (SOD), peroxidase (POD), catalase (CAT), and associated redox metabolites—scavenges excess ROS. Priming with silicon in maize [10], abscisic acid in rice [11], or melatonin in rice and *Fraxinus mandshurica* seedlings [12,13] each enhanced alkaline-stress tolerance specifically by upregulating antioxidant defense. Together with hormone signaling and transcriptional regulation, these systems constitute the core of plant saline-alkali tolerance and are increasingly targeted in breeding programs [14,15]. Whether they operate similarly in acidophilic ornamentals, however, remains unclear.

The genus *Rhododendron* (Ericaceae) comprises more than one thousand species of evergreen and deciduous shrubs and ranks among the most economically important groups of ornamental woody plants worldwide. Rhododendrons are valued for their abundant and diverse floral display in gardens, urban landscaping, and potted production. They are also classic calcifuges: field surveys of natural populations in Yunnan, China, showed that *Rhododendron* species are consistently associated with acidic soils, typically within a pH range of 4.5–6.0 [16]. This strict acidophily reflects physiological specializations, including root systems adapted to ammonium-based nutrition and iron acquisition under acidic conditions. Hydroponic experiments have confirmed that even moderately elevated pH alters root morphology and disrupts the nutritional status of rhododendron plants [17]. Consequently, in calcareous and alkaline soils, rhododendrons characteristically develop lime-induced chlorosis, growth retardation, and premature decline, which restricts the geographic deployment of this otherwise highly marketable genus. Encouragingly, breeding-oriented screening of evergreen azaleas (*Rhododendron* spp.) has revealed substantial genetic variation in adaptation to iron deficiency and high pH, indicating that tolerance resources exist within the genus but remain largely unexploited [18]. Geochemical analyses of herbarium specimens have likewise been used to predict lime tolerance across *Rhododendron* taxa, confirming that alkalinity tolerance is a quantifiable, species-specific trait [19]. ‘Fenzhuanglou’, an introduced *Rhododendron obtusum* cultivar officially registered in Zhejiang Province, China, is valued for its profuse deep-pink flowers and is extensively used in landscape greening and pot cultivation, with commercial production extending to greenhouse nurseries in northern China [20]. It is commercially propagated by cuttings, which ensures the genetic uniformity of experimental materials [20,21]. Because low-temperature and other abiotic stresses are major constraints on the northward expansion of azalea cultivars [22], elucidating the stress-tolerance mechanisms of this cultivar is of direct practical relevance; however, its molecular responses to stress remain largely uncharacterized.

Resolving the molecular basis of alkali tolerance in this cultivar requires evidence at both the transcript and protein levels. Transcriptome profiling captures genome-wide transcriptional responses, but mRNA abundance is an imperfect proxy for protein output, because post-transcriptional regulation, differences in translation efficiency, and protein turnover decouple the two layers. Quantitative proteomics, together with protein-to-transcript ratio (PTR) metrics of relative protein output per transcript, can expose these otherwise invisible regulatory layers, and integrated transcriptome–proteome analysis has become a powerful strategy for dissecting complex stress responses. Mechanistic studies of alkali stress have nevertheless focused predominantly on crops and vegetables—rice, maize, sugar beet, oat, tomato, and cucumber [5,6,8,9,10,23]—whereas ornamental woody plants, and *Rhododendron* in particular, have received far less attention. Wang et al. [24] developed a greenhouse screening protocol for pH stress across *Rhododendron* genotypes, providing physiological but not molecular evidence. Zhou et al. [25] combined biochemical and proteomic analyses of antioxidant enzymes in *R. chrysanthum* Pall., but without transcriptomic coverage and without concentration-resolved tolerance thresholds. Wang et al. [26] detected alkalinity stress in *Rhododendron* species through germination kinetics and chlorophyll fluorescence imaging, again at the physiological level only. Zhang et al. [27] performed a comparative root transcriptome analysis of alkali-tolerant and -sensitive rhododendron genotypes and identified candidate tolerance genes, yet without proteomic validation and without linkage to a defined physiological injury gradient. Thus, for ‘Fenzhuanglou’, the tolerance threshold to mixed alkaline salts has not been defined, no proteome-wide dataset exists, and no study has integrated physiology, transcriptomics, and proteomics within a single experimental design.

In this study, *R. obtusum* ‘Fenzhuanglou’ seedlings were exposed to a 40-day gradient of a mixed alkali solution (NaHCO_3_:Na_2_CO_3_ = 9:1 molar ratio, pH 8.94 ± 0.12; 0, 50, 60, and 70 mmol·L^−1^). The physiological, transcriptomic, and proteomic responses of the seedlings were investigated to define the alkali tolerance threshold of this cultivar and to elucidate the physiological and molecular mechanisms underlying its alkali response. The defined threshold is expected to provide a practical benchmark for substrate management and cultivation-site selection, and the resulting multi-omics resource may offer candidate targets for breeding alkali-tolerant rhododendrons.

## 2. Results

### 2.1. Phenotypic Responses and Alkali Tolerance Threshold

**Figure 1:**
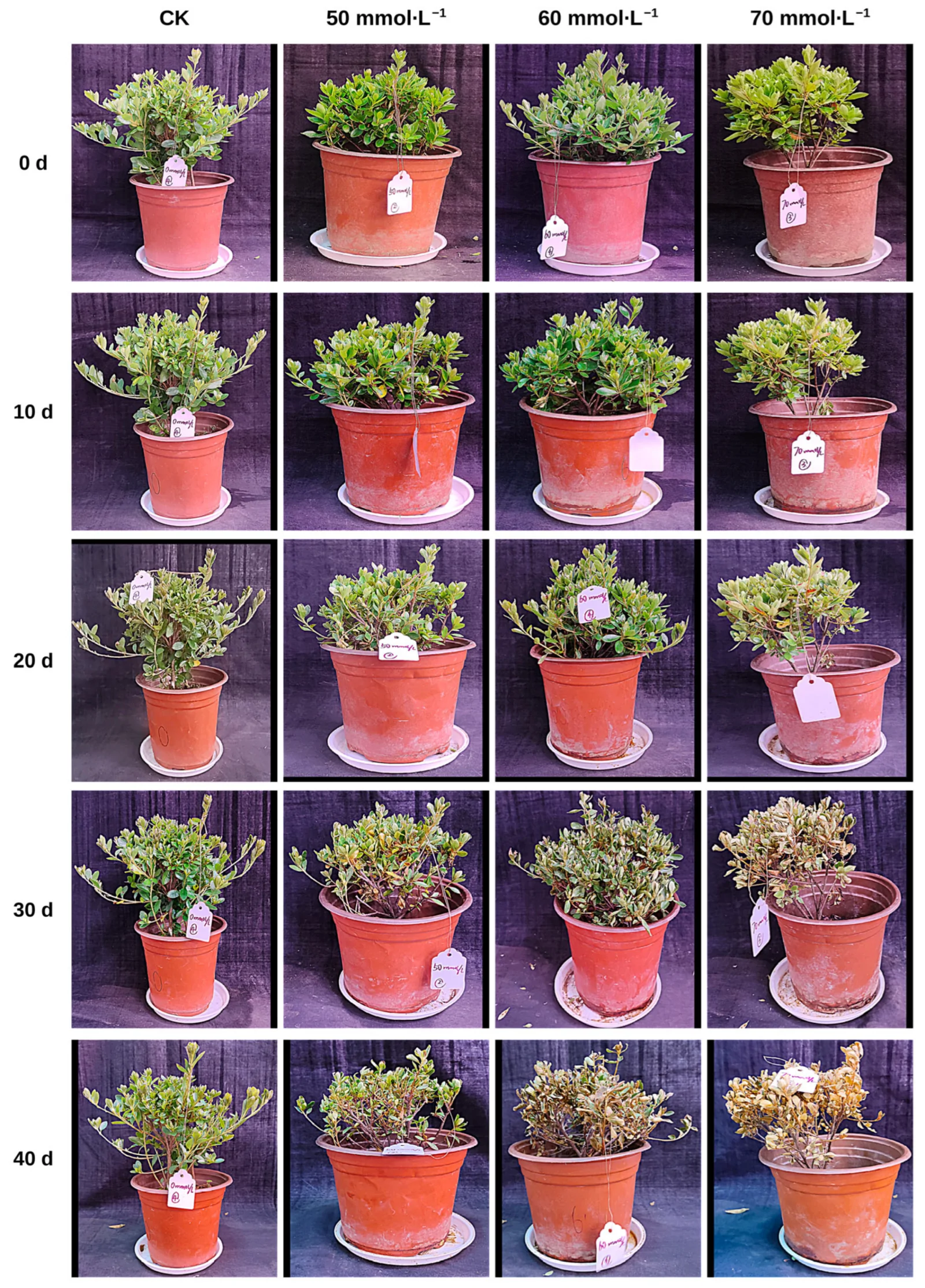
Phenotypic responses of *R. obtusum* ‘Fenzhuanglou’ seedlings to alkali stress. Within each panel, plants were photographed at 0, 10, 20, 30, and 40 days (d) of treatment. Consistent with the physiological measurements, no visible injury was discernible in any treatment at 0–10 d; chlorosis and marginal necrosis emerged from approximately 20 d onward at 60–70 mmol·L^−1^ and progressed to severe leaf withering at 70 mmol·L^−1^ by 40 d, whereas 50 mmol·L^−1^ remained largely symptomless throughout.

Throughout the 40-day experimental period, seedlings irrigated with distilled water (CK) maintained fully expanded, dark-green leaves with no visible injury. In contrast, the alkali-treated plants displayed damage symptoms whose onset and severity were graded by concentration (Figure 1).

At 50 mmol·L^−1^, the leaf phenotype remained indistinguishable from that of the CK throughout the 40-day period: no leaf-tip curling, chlorosis, or wilting was observed. This visual integrity indicates that 50 mmol·L^−1^ alkali stress remained within the compensatory capacity of ‘Fenzhuanglou’ seedlings. On this basis, 50 mmol·L^−1^ was defined as a concentration tolerated for at least 40 d without visible injury.

At 60 mmol·L^−1^, the first visible symptom appeared at 30 d, when the tips of individual leaves began to curl slightly. By 40 d, some leaves showed chlorosis, and many exhibited mild water loss with apical shrinkage and wrinkling. The damage was partial rather than lethal, and its onset was later than at 70 mmol·L^−1^. On the basis of these symptoms, 60 mmol·L^−1^ was designated the visible-injury threshold of alkali stress for this cultivar under the present experimental conditions, defined here operationally as the lowest concentration at which visible foliar symptoms (leaf-tip curling and chlorosis) appeared during the 40 d exposure while the plants nonetheless survived. Because physiological indices already changed markedly at 50 mmol·L^−1^ and mortality, biomass loss, and recovery were not measured, this term refers strictly to the onset of visible injury; it is used in place of “semi-lethal” and does not imply an LC_50_-type estimate.

At 70 mmol·L^−1^, symptoms appeared earliest and progressed fastest. Slight curling and yellowing of individual leaf tips were visible by 20 d. By 30 d, most leaves exhibited shrinkage, chlorosis, and pronounced water loss with curled margins; by 40 d, leaves showed severe chlorosis and curling, with entire leaves desiccated and necrotic. This rapid progression from first symptoms to whole-leaf desiccation within 20 d suggests that the injury at 70 mmol·L^−1^ was severe and progressive under prolonged exposure; recovery after desiccation was not assessed. Taken together, the phenotypic gradient—50 mmol·L^−1^ tolerant, 60 mmol·L^−1^ at the visible-injury threshold, and 70 mmol·L^−1^ severe-injury—provided the framework against which the subsequent physiological and molecular measurements were interpreted.

### 2.2. Photosynthetic Pigments

Two-way ANOVA (treatment × time) of the full 4 × 5 data set revealed significant main effects of treatment for all indicators except lutein (only weak for Chl a, *p* < 0.05) and significant main effects of sampling time for all indicators except Chl a, together with significant treatment × time interactions for Chl b, soluble sugar, soluble protein, proline, SOD, POD, CAT, and MDA (all *p* < 0.01; Supplementary Tables S1 and S2), indicating that the treatment effect intensified with exposure duration in a concentration-dependent manner.

**Figure 2:**
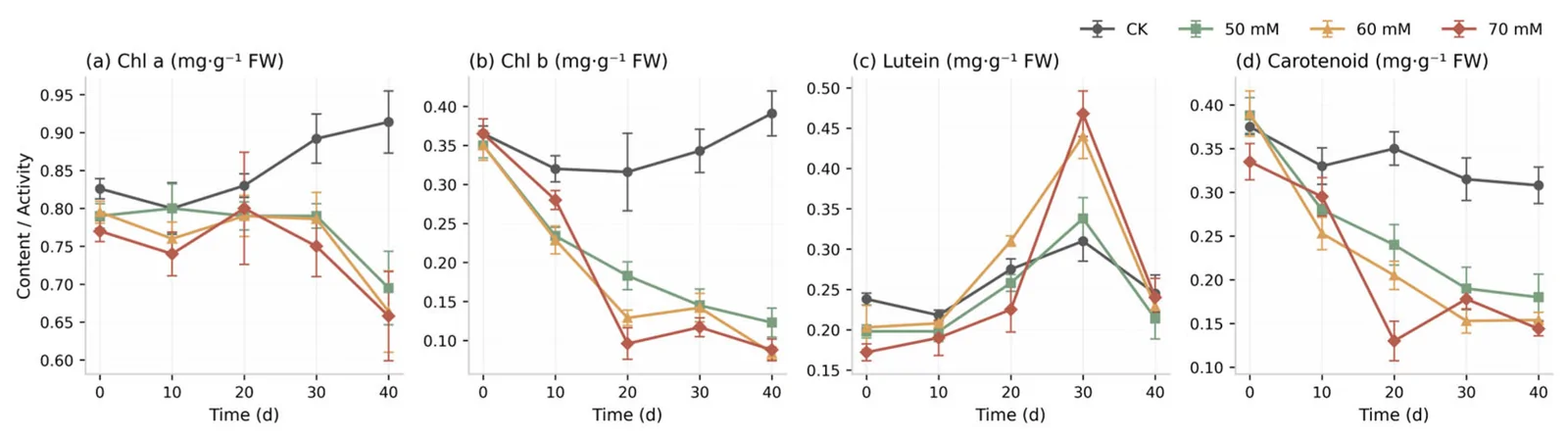
Effects of alkali stress on photosynthetic pigment contents in leaves of *R.obtusum* ‘Fenzhuanglou’. (**a**) Chl a; (**b**) Chl b; (**c**) lutein; (**d**) carotenoid. Values are means ± SE of three biological replicates (*n* = 3); statistical groupings (Duncan’s multiple range test, *p* < 0.05) are provided in Supplementary Tables S1 and S2.

The four measured pigments responded to alkali stress with markedly different sensitivities and temporal patterns (Figure 2).

Chl a was the most stable of the four pigments (Figure 2a). In the CK, Chl a ranged from 0.800 to 0.914 mg·g^−1^ FW over the 40 d. During the first 20 d, no treatment differed significantly from the CK, all values remaining within 0.740–0.830 mg·g^−1^ FW. Divergence emerged at 30 d: the 60 mmol·L^−1^ group declined to 0.786 mg·g^−1^ FW, significantly below the CK (0.892 mg·g^−1^ FW), while the 50 and 70 mmol·L^−1^ groups (0.790 and 0.750 mg·g^−1^ FW) remained intermediate. At 40 d, the decline was concentration-dependent. Chl a fell to 0.664 mg·g^−1^ FW at 60 mmol·L^−1^, significantly below the CK (0.914 mg·g^−1^ FW); the 50 and 70 mmol·L^−1^ groups remained at 0.695 and 0.658 mg·g^−1^ FW, respectively (76.0% and 72.0% of the CK). These results indicate that Chl a degradation became measurable only after 20–30 d of exposure and that the decline at 60–70 mmol·L^−1^ coincided with the onset of leaf-tip curling and chlorosis.

Chl b was the earliest and most severely affected pigment (Figure 2b). In the CK, Chl b remained between 0.316 and 0.391 mg·g^−1^ FW. At 0–10 d, the treated groups did not yet differ significantly from the CK, although mean values had already fallen (e.g., 0.234 mg·g^−1^ FW at 50 mmol·L^−1^, 10 d). From 20 d onward, the reduction became significant and concentration-dependent: at 20 d, Chl b dropped to 0.183 (50 mmol·L^−1^), 0.129 (60 mmol·L^−1^), and 0.096 mg·g^−1^ FW (70 mmol·L^−1^) versus 0.316 in the CK. At 30 d, all treated groups (0.145, 0.142, and 0.117 mg·g^−1^ FW) were significantly below the CK (0.343). At 40 d, Chl b was only 0.123, 0.084, and 0.088 mg·g^−1^ FW at 50, 60, and 70 mmol·L^−1^—31.5%, 21.5%, and 22.5% of the CK (0.391 mg·g^−1^ FW), respectively. These results identify Chl b as the most sensitive pigment marker of alkali injury in this cultivar, with depletion beginning well before any visible symptom.

In contrast to the chlorophylls, lutein increased under alkali stress (Figure 2c). In the CK, lutein ranged from 0.218 to 0.310 mg·g^−1^ FW. At 20 d, lutein in the 60 mmol·L^−1^ group (0.310 mg·g^−1^ FW) already exceeded the CK (0.275). At 30 d, the treated groups reached their maxima—0.338, 0.440, and 0.468 mg·g^−1^ FW at 50, 60, and 70 mmol·L^−1^, respectively, versus 0.310 in the CK. These maxima corresponded to increases of 41.9% and 51.0% over the CK at 60 and 70 mmol·L^−1^, respectively. With the exception of the 70 mmol·L^−1^ group at 10 d, these differences were not statistically significant. By 40 d, the treated groups had declined from their peaks to 0.214, 0.229, and 0.240 mg·g^−1^ FW, close to the CK (0.245). The mid-stress accumulation, strongest at the two higher concentrations, is consistent with the engagement of xanthophyll-cycle-associated photoprotection under constrained carbon assimilation [28,29]. The return toward control levels by 40 d coincided with visible injury and the late decline of antioxidant enzyme activities.

Carotenoid content declined progressively and dose-dependently throughout the stress period (Figure 2d). In the CK, carotenoid remained between 0.308 and 0.375 mg·g^−1^ FW. A significant reduction was already detectable at 10 d at 50 mmol·L^−1^ (0.280 mg·g^−1^ FW versus 0.330 in the CK). At 20 d, the concentration gradient was fully resolved: 0.240 (50 mmol·L^−1^), 0.205 (60 mmol·L^−1^), and 0.130 mg·g^−1^ FW (70 mmol·L^−1^) versus 0.350 in the CK. The treated groups remained significantly below the CK thereafter. At 40 d, carotenoid had fallen to 0.180, 0.154, and 0.144 mg·g^−1^ FW at 50, 60, and 70 mmol·L^−1^—58.4%, 50.0%, and 46.8% of the CK (0.308 mg·g^−1^ FW), respectively. These results suggest a progressive, dose-dependent loss of photoprotective capacity, with nearly 60% of the control level retained at 50 mmol·L^−1^ versus less than half at 70 mmol·L^−1^.

### 2.3. Osmotic Adjustment Substances

**Figure 3:**
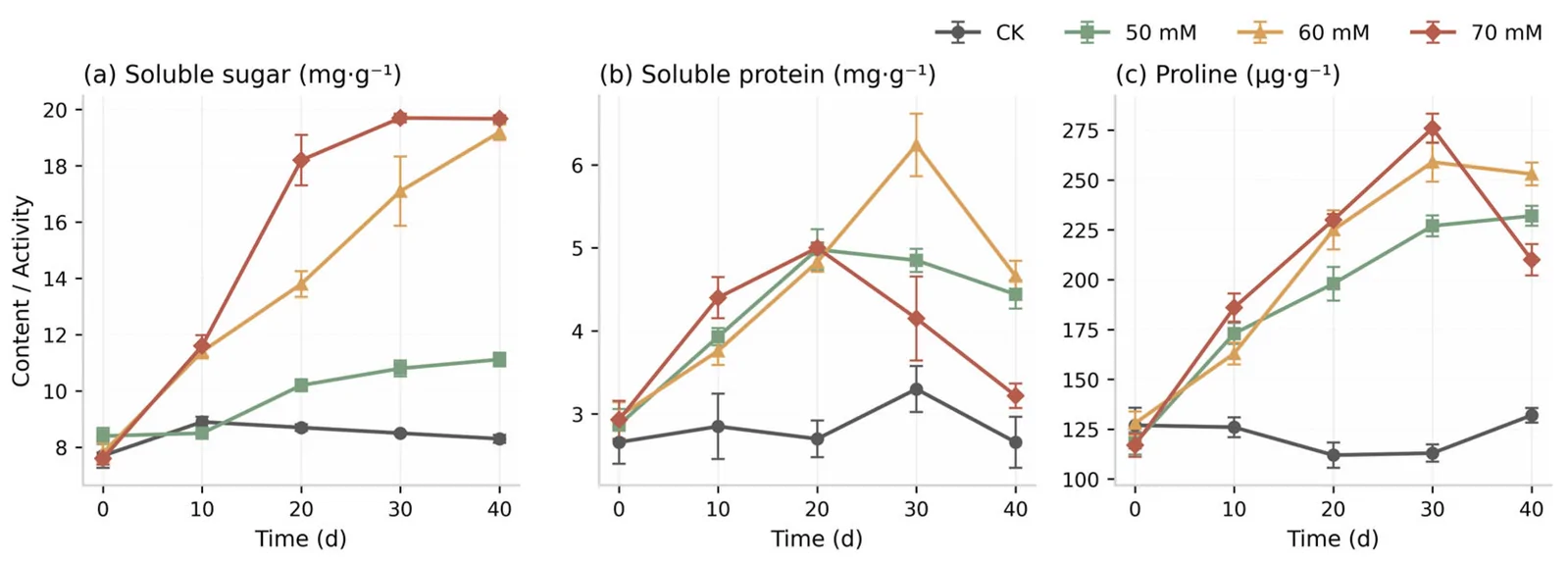
Effects of alkali stress on osmotic adjustment substance contents in leaves of *R. obtusum* ‘Fenzhuanglou’. (**a**) Soluble sugar; (**b**) soluble protein; (**c**) proline. Values are means ± SE of three biological replicates (*n* = 3); statistical groupings (Duncan’s multiple range test, *p* < 0.05) are provided in Supplementary Tables S1 and S2.

The three measured osmolytes all increased under alkali stress in a concentration-dependent manner (Figure 3). Their temporal patterns differed: continuous accumulation (soluble sugar), rise-then-fall (soluble protein), and rapid accumulation followed by partial decline at the highest concentration (proline).

In the CK, soluble sugar remained within 7.70–8.90 mg·g^−1^ with no consistent temporal trend (Figure 3a). All treated groups accumulated soluble sugar continuously and at concentration-graded rates. A significant divergence was already evident at 10 d, when the 60 and 70 mmol·L^−1^ groups (11.40 and 11.60 mg·g^−1^) exceeded the CK (8.90). The gradient widened with time: at 20 d, soluble sugar rose to 10.20, 13.80, and 18.20 mg·g^−1^ at 50, 60, and 70 mmol·L^−1^ versus 8.70 in the CK, every pairwise contrast being significant. At 30 d, the corresponding values were 10.80, 17.10, and 19.70 versus 8.50. At 40 d, the 60 and 70 mmol·L^−1^ groups reached 19.19 and 19.67 mg·g^−1^, approximately 2.4-fold the CK (8.30 mg·g^−1^), while the 50 mmol·L^−1^ group accumulated more moderately, to 11.12 mg·g^−1^. At the same stress time point, soluble sugar content was significantly positively correlated with alkali concentration (r = 0.57, *p* < 0.01; *n* = 20 across the full concentration × time series). These results identify soluble sugar as the principal osmotic effector in *R. obtusum* ‘Fenzhuanglou’ under alkali stress, although the near-plateau of the 70 mmol·L^−1^ group after 30 d suggests a metabolic ceiling to osmotic adjustment under severe stress.

Soluble protein, determined by the Coomassie Brilliant Blue G-250 method [30], followed a rise-then-fall trajectory against a narrow CK range of 2.66–3.30 mg·g^−1^ (Figure 3b). All treatments increased during the first 30 d at concentration-dependent rates. At 10 d, the treated groups (3.76–4.40 mg·g^−1^) already significantly exceeded the CK (2.85); at 20 d, they reached 4.83–5.00 mg·g^−1^ versus 2.70 in the CK. The 60 mmol·L^−1^ group accumulated fastest and peaked at 6.24 mg·g^−1^ at 30 d, significantly higher than the 50 mmol·L^−1^ group (4.85) and the CK (3.30). In contrast, the 70 mmol·L^−1^ group had already begun to decline, to 4.15 mg·g^−1^. From 30 to 40 d, the high-concentration groups declined further, to 4.66 mg·g^−1^ at 60 mmol·L^−1^ and 3.22 at 70 mmol·L^−1^, whereas the 50 mmol·L^−1^ group remained comparatively stable at 4.44 mg·g^−1^. All three treatments nonetheless remained significantly above the CK (2.66) at 40 d. These results indicate that soluble protein accumulation was maximal at the injury-threshold concentration (60 mmol·L^−1^), whereas the late decline at 60–70 mmol·L^−1^ coincided with pigment loss and visible desiccation.

In the CK, proline remained low and stable at 112–132 µg·g^−1^ (Figure 3c). After a significant initial rise at 10 d (163–186 µg·g^−1^ in the treated groups versus 126 in the CK), proline accumulated rapidly and dose-dependently between 10 and 30 d. At 30 d, it reached 227, 259, and 276 µg·g^−1^ in the 50, 60, and 70 mmol·L^−1^ groups, respectively (CK: 113 µg·g^−1^); the concentration gradient was significant at both 20 and 30 d. Between 30 and 40 d, the 70 mmol·L^−1^ group partially declined, to 210 µg·g^−1^, and the 60 mmol·L^−1^ group remained essentially stable at 253 µg·g^−1^, the highest value at 40 d (1.92-fold the CK). In contrast, the 50 mmol·L^−1^ group continued to rise slightly to 232 µg·g^−1^. At 40 d, all treatments remained significantly above the CK (132 µg·g^−1^). These results identify proline as a rapidly induced osmoprotectant whose accumulation at 70 mmol·L^−1^ could not be sustained beyond 30 d, whereas at 50 mmol·L^−1^ the adjustment capacity was not exhausted within 40 d.

### 2.4. Antioxidant Enzymes and Malondialdehyde

**Figure 4:**
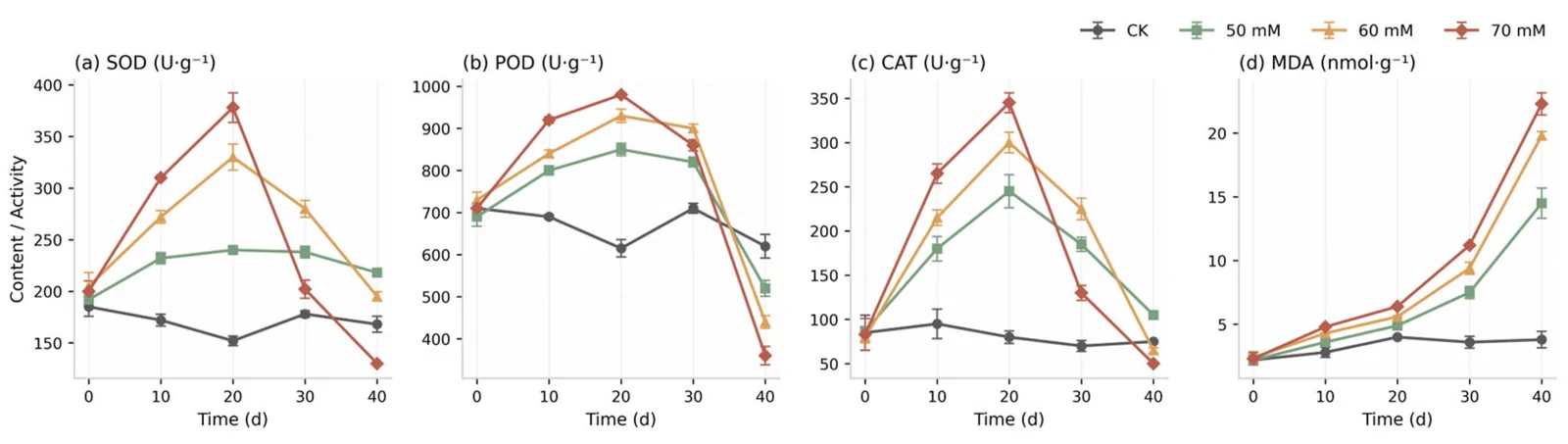
Effects of alkali stress on antioxidant enzyme activities and MDA contents in leaves of *R. obtusum* ‘Fenzhuanglou’. (**a**) SOD; (**b**) POD; (**c**) CAT; (**d**) MDA. Values are means ± SE of three biological replicates (*n* = 3); statistical groupings (Duncan’s multiple range test, *p* < 0.05) are provided in Supplementary Tables S1 and S2.

The activities of superoxide dismutase (SOD), peroxidase (POD), and catalase (CAT) all followed a rise-then-fall pattern under alkali stress, peaking at 20 d (Figure 4). In contrast, malondialdehyde (MDA) accumulated continuously in a dose- and time-dependent manner.

At 0 d, the four groups were statistically indistinguishable for all three enzymes (Figure 4a–c). During the first 10 d, all three enzyme activities rose significantly above the CK in every treated group. The increase was concentration-graded (e.g., SOD: 232, 272, and 310 U·g^−1^ at 50, 60, and 70 mmol·L^−1^ versus 172 in the CK). At 20 d, SOD, POD, and CAT activities all reached their maxima at 70 mmol·L^−1^ (378, 980, and 345 U·g^−1^, respectively). The 50 and 60 mmol·L^−1^ groups were significantly lower but remained far above the CK (152, 615, and 80 U·g^−1^).

From 30 d onward, activities declined in all groups, with concentration-dependent severity. At 30 d, the 70 mmol·L^−1^ group already showed the steepest drop, its SOD activity (202 U·g^−1^) falling significantly below those of the 50 and 60 mmol·L^−1^ groups (238 and 280 U·g^−1^). By 40 d, the divergence was fully resolved and differed among enzymes. SOD activity remained highest at 50 mmol·L^−1^ (218 U·g^−1^, a), was intermediate at 60 mmol·L^−1^ (195, b), and fell significantly below the CK (168, c) only at 70 mmol·L^−1^ (130, d). POD activity declined below the CK (620 U·g^−1^, a) in all treated groups, in a strict concentration gradient (50 mmol·L^−1^: 520, b; 60 mmol·L^−1^: 440, c; 70 mmol·L^−1^: 360, d). CAT activity remained significantly above the CK at 50 mmol·L^−1^ (105 versus 75 U·g^−1^) but fell significantly below the CK at 60 and 70 mmol·L^−1^ (65 and 50 U·g^−1^, respectively). These results indicate that the antioxidant system remained functional over the full 40 d at 50 mmol·L^−1^, whereas at 60–70 mmol·L^−1^ it was transiently over-activated and then exhausted. This biphasic pattern has also been reported in *R*. *chrysanthum* under stress [25].

MDA content increased continuously throughout the stress period in all treated groups (Figure 4d). At 0 d, all groups were statistically indistinguishable (2.2–2.4 nmol·g^−1^). From 10 d onward, the treated groups were significantly higher than the CK, and the divergence widened progressively. At 10 and 20 d, the concentration gradient was already fully resolved (20 d: CK 4.0 < 50 mmol·L^−1^ 4.9 < 60 mmol·L^−1^ 5.6 < 70 mmol·L^−1^ 6.4 nmol·g^−1^, every pairwise contrast significant). After 20 d, accumulation accelerated: by 40 d, MDA had reached 14.5, 19.8, and 22.3 nmol·g^−1^ at 50, 60, and 70 mmol·L^−1^, all significantly above the CK (3.8 nmol·g^−1^), with the 70 mmol·L^−1^ group at 5.9-fold the control. These results suggest that membrane lipid peroxidation accelerated once antioxidant enzyme activities declined after 20 d. The MDA gradient at 40 d (CK d < 50 c < 60 b < 70 a) paralleled the phenotypic severity gradient.

### 2.5. Transcriptome Sequencing Quality and Differentially Expressed Genes

#### 2.5.1. Sequencing Quality, Read Mapping, and Sample-Level Relationships

Twelve leaf RNA-seq libraries (four sample groups × three biological replicates) were constructed and sequenced on the Illumina platform. After quality filtering, a total of 90.41 Gb of clean data was obtained, with 6.39–8.69 Gb per sample (Table 1). The Q30 base percentage ranged from 93.51% to 93.78%, and the GC content ranged from 46.68% to 47.88%. Clean reads were aligned to the *R. simsii* reference genome (GCA_014282245.1): the total mapping rate ranged from 76.87% to 80.05%, of which 72.74–76.40% were uniquely mapped reads (Table 1). Gene-level read counts were generated after transcript assembly, and expression was normalized as FPKM. These metrics indicate high and consistent sequencing quality across all libraries. Although the study species and the reference belong to different species of the same subgenus (*Tsutsusi*), the observed mapping rates are within the range commonly reported for alignment against a congeneric reference genome, and all downstream quantification and differential expression analyses were restricted to uniquely mapped reads (72.74–76.40%) to exclude ambiguously aligned sequences. Moreover, the qRT-PCR primers designed from the *R. simsii* reference coding sequences amplified their targets specifically and efficiently in *R. obtusum* cDNA, and the resulting expression values correlated strongly with the RNA-seq data, providing independent experimental evidence that coding regions are sufficiently conserved between the two species to support reliable quantification.

**Figure 5:**
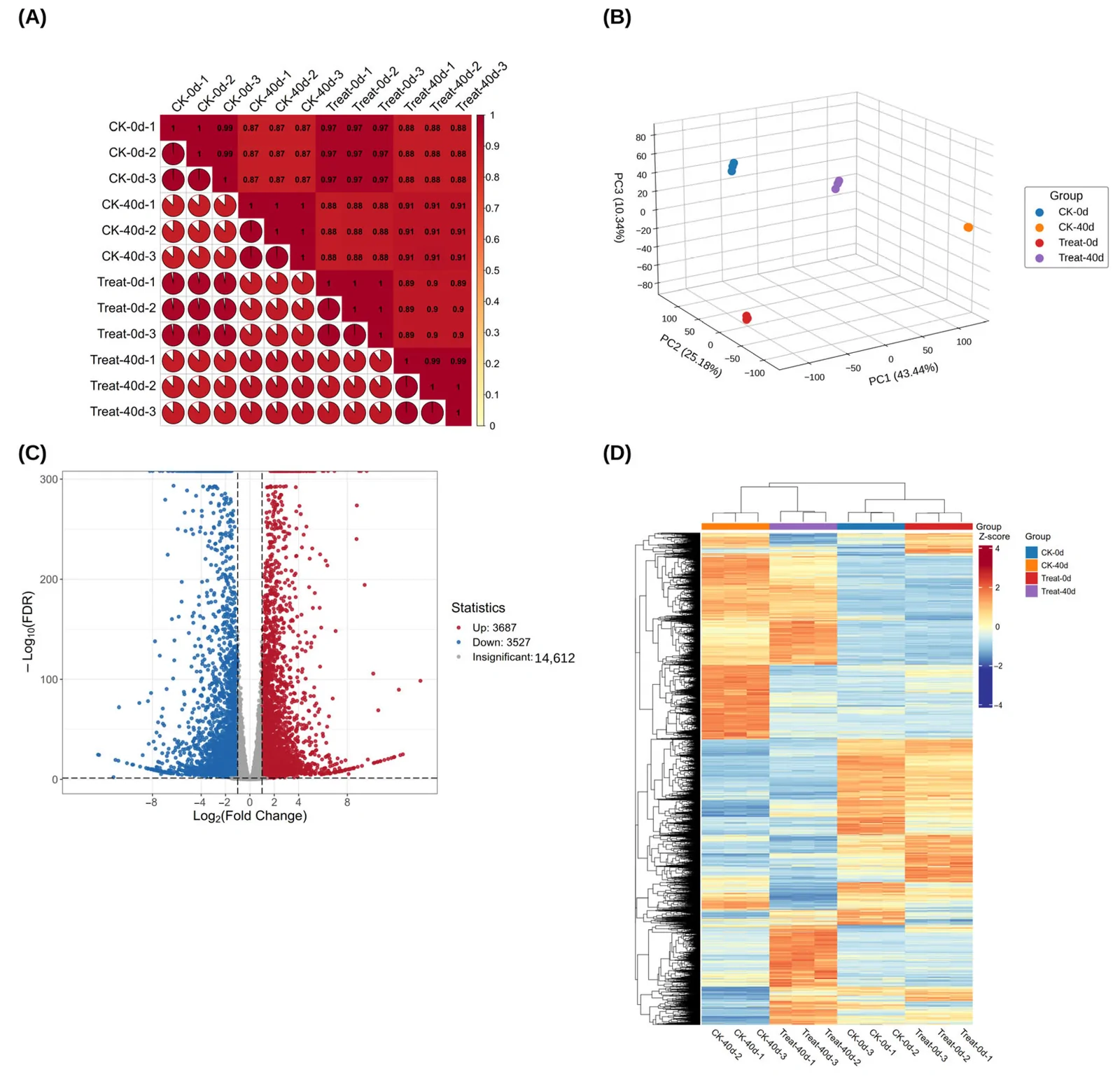
Overview of the leaf transcriptome of *R. obtusum* ‘Fenzhuanglou’ under alkali stress. (**A**) Pearson correlation heatmap of the 12 samples; correlation coefficients among biological replicates within each group were ≥0.87. (**B**) Three-dimensional PCA score plot based on FPKM values (PC1, PC2, and PC3 explain 43.44%, 25.18%, and 10.34% of the variance, respectively). (**C**) Volcano plot of DEGs in Treat-40d vs. CK-40d. (**D**) Expression heatmap of all DEGs across the 12 samples.

Sample-level relationships were examined by Pearson correlation and PCA on FPKM values (Figure 5A,B). Correlation coefficients among the three biological replicates within each group were all ≥ 0.87 (Figure 5A). In the PCA, PC1 and PC2 explained 43.44% and 25.18% of the variance, respectively, and the twelve samples separated clearly by treatment and sampling time (Figure 5B). These results indicate that treatment duration (0 d vs. 40 d) was the dominant source of expression divergence (PC1), that PC2 primarily captured the alkali effect, and that the dataset was sufficiently reproducible for the subsequent differential expression comparisons.

#### 2.5.2. Differentially Expressed Genes in the Four Pairwise Comparisons

Differential expression analysis of the four pairwise comparisons was performed with DESeq2 [31] to identify DEGs.

In the baseline comparison Treat-0d vs. CK-0d, 2441 DEGs (1372 up-regulated and 1069 down-regulated) were detected (Table 2), reflecting baseline variation between the plant batches and/or an immediate early response at sampling rather than prolonged exposure. A total of 7214 DEGs (|log_2_FC| ≥ 1, FDR < 0.05), including 3687 up-regulated and 3527 down-regulated, were identified in the stress-specific comparison Treat-40d vs. CK-40d, which controls for sampling time (Table 2; Figure 5C). The two time-course comparisons yielded the largest DEG sets: 9041 DEGs in CK-40d vs. CK-0d (3737 up-regulated, 5304 down-regulated) and 9211 DEGs in Treat-40d vs. Treat-0d (4405 up-regulated, 4806 down-regulated), both dominated by down-regulation (58.7% and 52.2%, respectively). The DEG heatmap showed distinct group-specific expression blocks, consistent with the PCA (Figure 5D). These results indicate that 40 d of 60 mmol·L^−1^ alkali treatment triggered transcriptome-wide reprogramming well beyond the developmental background captured by the control time course. Because the CK-40d vs. CK-0d comparison (9041 DEGs) captures the natural developmental drift over the 40 d experimental period, all stress-related inferences in this study are drawn from Treat-40d vs. CK-40d, in which the two groups are of identical age and developmental stage, so that developmental aging is controlled by experimental design rather than inferred post hoc. The two time-course comparisons were used only to describe temporal trajectories and were always interpreted as a pair: expression changes shared by the control and treated time courses were attributed to development, and only the excess change in the treated series was attributed to alkali stress. This excess-change reasoning is here corroborated by a formal treatment × time interaction test.

#### 2.5.3. Formal Treatment × Time Interaction Analysis

To test formally whether alkali stress alters the temporal expression trajectory, an NB-GLM with design~time + treatment + time:treatment was fitted to the count matrix and the interaction term was evaluated by a likelihood-ratio test. Of the 28,643 genes tested, 16,307 showed a significant treatment × time interaction (BH-adjusted *p* < 0.05), and 9549 of these also exhibited a large interaction effect (|log_2_ interaction fold change| ≥ 1): 5187 with an amplified temporal trajectory under alkali stress (positive interaction) and 4362 with a buffered trajectory (negative interaction). The interaction genes strongly overlapped the stress-specific DEG set: genes with a significant interaction were 14.4-fold enriched among the DEGs of Treat-40d vs. CK-40d (6354 overlapping genes; Fisher’s exact test, *p* < 1 × 10^−300^), confirming that the stress-specific DEG set is dominated by genes whose temporal behavior is genuinely altered by alkali stress rather than by baseline differences between plant batches. Because the model absorbs any 0 d baseline difference in the treatment main effect, the interaction signals cannot be attributed to baseline imbalance between the plant batches. The full interaction test results are provided in Supplementary Table S3.

#### 2.5.4. Representative DEGs and Transcription Factor Analysis in Treat-40d vs. CK-40d

The most strongly differentially expressed genes in Treat-40d vs. CK-40d are listed in Table 3. The most highly induced gene encoded an early light-induced protein 1 (ELIP, +8.97), a chloroplastic stress protein of the chlorophyll a/b-binding family, followed by an uncharacterized YtxH-like protein (+5.97). Other annotated up-regulated genes included transcription factor bHLH92 (+5.14), a late embryogenesis abundant protein (LEA D-34, +5.09), glucan endo-1,3-β-glucosidase (+4.94; starch and sucrose metabolism), and an ethylene-responsive transcription factor ERF017 (+3.63), lactoylglutathione lyase *RoGLO1* (+2.65; glyoxalase system), galactinol–sucrose galactosyltransferase (+2.60; raffinose-family oligosaccharide synthesis), a mitogen-activated protein kinase *RoMAPK3* (+2.29), and a Tify protein *RoTIFY2* (+2.22). The down-regulated set was headed by an extracellular ribonuclease LE of the RNase T2 family (−8.21) and ethylene receptor 2 (ETR2, −6.40), followed by the homeobox–leucine zipper transcription factor ATHB-6 (−5.48) and NAC domain-containing protein 83 (−4.24). The remaining down-regulated genes were chloroplastic glutamate–cysteine ligase *RoGCL* (−4.16; rate-limiting in glutathione biosynthesis), a CONSTANS-like CCT-domain protein *RoCCT* (−3.72; circadian rhythm), cinnamyl alcohol dehydrogenase 1 (CAD1, −3.56; phenylpropanoid biosynthesis), a WRKY protein *RoWRKY1* (−3.30), and a DELLA protein *RoDELLA* (−3.26; gibberellin signaling).

Transcription factor (TF) annotation of the most significant DEGs showed that hormone- and stress-related families were represented in all four comparisons. bHLH (2), WRKY (2), and AP2/ERF-ERF (1) members were detected in Treat-40d vs. CK-40d; C2C2-CO-like (2), GNAT (1), GRAS (1), and AP2/ERF-ERF (1) members in Treat-40d vs. Treat-0d; C2C2-CO-like (1), HB-HD-ZIP (1), and AP2/ERF-ERF (1) members in CK-40d vs. CK-0d; and HB-HD-ZIP (1) and Others (1) in Treat-0d vs. CK-0d. In Treat-40d vs. CK-40d, the differentially expressed TFs spanned the major stress- and hormone-associated families in both directions: bHLH RHSIM_Rhsim02G0032900 (+5.14); WRKY *RoWRKY2* (RHSIM_Rhsim02G0213700, +2.48) and *RoWRKY1* (RHSIM_Rhsim03G0192300, −3.30); AP2/ERF RHSIM_Rhsim05G0004400 (+3.63), *RoERF1* (RHSIM_Rhsim04G0184600, +2.49), and *RoERF2* (RHSIM_Rhsim01G0287500, −1.93); NAC *RoNAC* (RHSIM_Rhsim13G0113000, +2.45) and RHSIM_Rhsim12G0021700 (−4.24); and DELLA/GRAS *RoDELLA* (RHSIM_Rhsim08G0018400, −3.26). Repressed TFs further included C2C2-CO-like/CCT *RoCCT* (RHSIM_Rhsim03G0208000, −3.72), HD-ZIP/homeobox RHSIM_Rhsim10G0167600 (−5.48), BZIP RHSIM_Rhsim05G0071600 (−4.11), and LHW/bHLH156 RHSIM_Rhsim01G0201100 (−3.11).

The predominance of WRKY, AP2/ERF, NAC, bHLH, BZIP, GRAS, and C2C2-CO-like transcription factors among the alkali-responsive DEGs is consistent with the established roles of these families as central regulators of abiotic stress responses [3,28]. The opposite directions within the WRKY, AP2/ERF, and NAC families suggest a rebalancing of regulatory modules rather than uniform activation.

### 2.6. KEGG Enrichment Analysis of DEGs

#### 2.6.1. Overview of Enriched Pathways in the Four Comparisons

**Figure 6:**
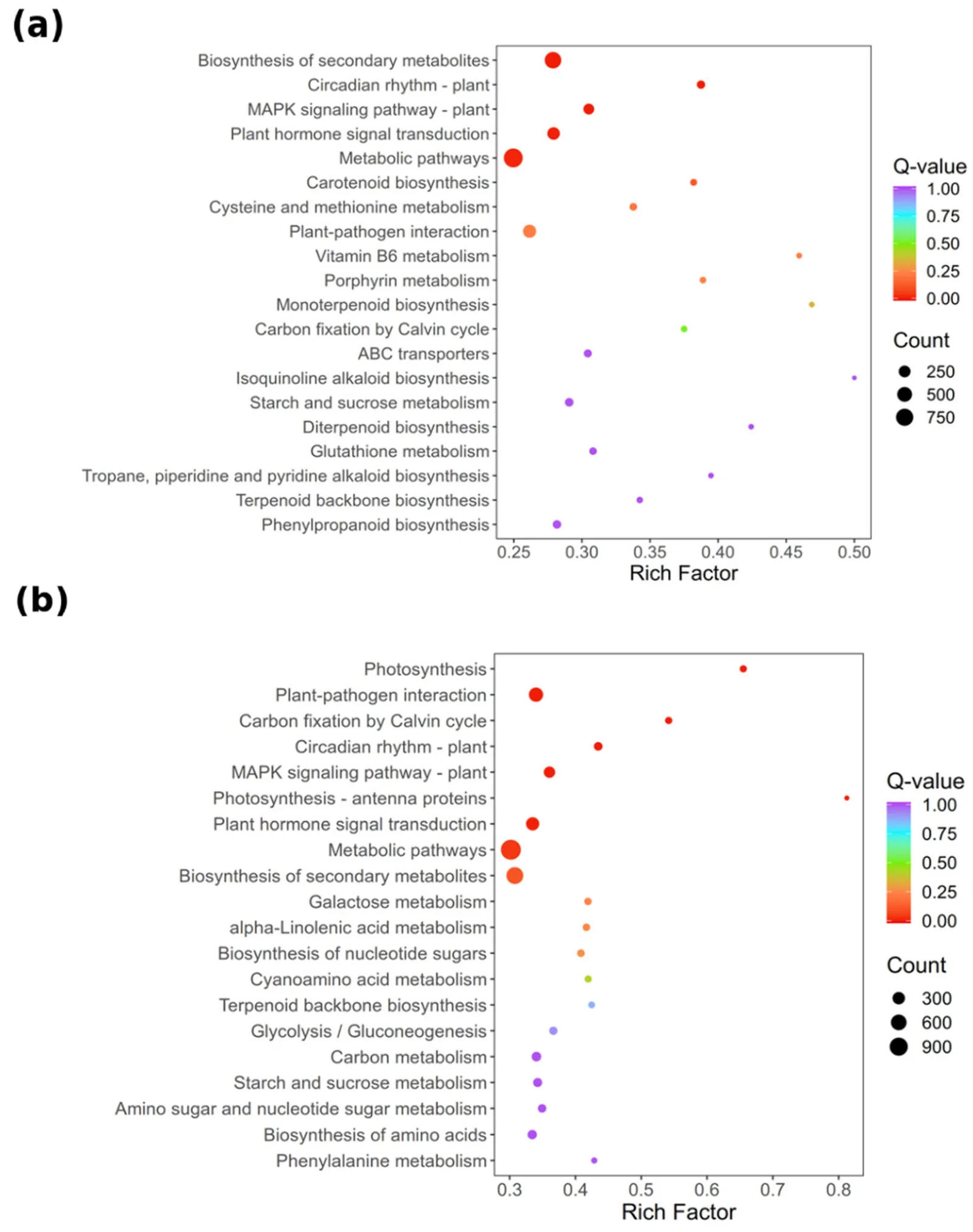
KEGG enrichment analysis of DEGs. (**a**) Enrichment dot plot for Treat-40d vs. CK-40d; biosynthesis of secondary metabolites, circadian rhythm–plant, MAPK signaling pathway–plant, plant hormone signal transduction, and metabolic pathways were significantly enriched (adjusted *p* < 0.05); carotenoid biosynthesis, glutathione metabolism, starch and sucrose metabolism, and ABC transporters were represented but did not reach significance. (**b**) Enrichment dot plot for Treat-40d vs. Treat-0d; photosynthesis, plant–pathogen interaction, carbon fixation by the Calvin cycle, circadian rhythm–plant, MAPK signaling, photosynthesis–antenna proteins, and plant hormone signal transduction were the top enriched pathways.

KEGG enrichment analysis of the DEGs was conducted with clusterProfiler [32] against the KEGG database [33], and pathways with an adjusted *p* < 0.05 were considered significantly enriched. In total, 7, 13, 5, and 8 pathways were significantly enriched in Treat-0d vs. CK-0d, CK-40d vs. CK-0d, Treat-40d vs. CK-40d, and Treat-40d vs. Treat-0d, respectively (Table 4). The enrichment dot plots for the two treatment-focused comparisons are shown in Figure 6.

In Treat-0d vs. CK-0d, the enriched pathways were dominated by up-regulated secondary-metabolite biosynthesis. These were biosynthesis of secondary metabolites (305 DEGs, 231 up; adjusted *p* = 1.23 × 10^−15^), phenylpropanoid biosynthesis (48 DEGs, 38 up), flavonoid biosynthesis (28 DEGs, 27 up), flavone and flavonol biosynthesis (10 DEGs, all up), and indole alkaloid biosynthesis (7 DEGs, all up). In the control time course CK-40d vs. CK-0d, the enriched pathways were instead dominated by down-regulation of the photosynthetic apparatus. This pattern comprised photosynthesis (43 DEGs, 41 down), photosynthesis–antenna proteins (13 DEGs, all down), porphyrin metabolism (48 DEGs, 38 down), and carbon fixation by the Calvin cycle (39 DEGs, 32 down). These results indicate that phenylpropanoid/flavonoid defenses differed between the two day-0 groups—reflecting batch variation, an immediate early response to the first alkali application, or both—whereas the photosynthetic decline over the 40-day period was partly developmental.

#### 2.6.2. KEGG Enrichment in the Late Stress-Specific Comparison (Treat-40d vs. CK-40d)

In the stress-specific comparison Treat-40d vs. CK-40d (Figure 6a), five pathways were significantly enriched (Table 4). The most significant was biosynthesis of secondary metabolites (671 DEGs, 308 up/363 down; adjusted *p* = 5.97 × 10^−9^), followed by circadian rhythm–plant (74 DEGs, 35 up/39 down; adjusted *p* = 7.54 × 10^−5^) and MAPK signaling pathway–plant (187 DEGs, 93 up/94 down; adjusted *p* = 6.77 × 10^−4^). Plant hormone signal transduction (301 DEGs, 135 up/166 down; adjusted *p* = 4.18 × 10^−3^) and metabolic pathways (965 DEGs, 499 up/466 down; adjusted *p* = 1.05 × 10^−2^) were also significantly enriched. Carotenoid biosynthesis (34 DEGs), glutathione metabolism (49 DEGs), starch and sucrose metabolism (84 DEGs), and ABC transporters (63 DEGs) were represented but did not reach significance (adjusted *p* > 0.05). Plant–pathogen interaction was likewise represented by 350 DEGs. At the Gene Ontology level, this comparison was further characterized by significant enrichment of photoprotection (25 DEGs, 24 up-regulated; *p* = 5.4 × 10^−11^), rhythmic process (101 DEGs), and response to blue light (53 DEGs, 37 up-regulated), indicating that light-acclimation and circadian programs were specifically mobilized under alkali stress.

In contrast to the time-course comparisons, which were dominated by repression of photosynthesis and carbon assimilation, the stress-specific comparison was dominated by secondary metabolism (671 DEGs). The enriched signaling pathways were underpinned by named genes (Table 3). A mitogen-activated protein kinase (*RoMAPK3*) was induced in MAPK signaling (+2.29). In plant hormone signal transduction, a Tify/JAZ protein (*RoTIFY2*) was induced (+2.22), whereas a second Tify/JAZ member (*RoTIFY1*, RHSIM_Rhsim09G0091800, −2.08), the DELLA protein *RoDELLA* (−3.26), and the ethylene receptor (−6.40) were repressed. In circadian rhythm–plant, the CCT-domain protein *RoCCT* was repressed (−3.72). The repression of chloroplastic glutamate–cysteine ligase *RoGCL* (−4.16) additionally linked these signaling changes to the glutathione/antioxidant module. These results suggest that signal transduction, rather than any single metabolic endpoint, constitutes the core of the transcriptomic alkali response.

The enrichment of the plant–pathogen interaction pathway in both comparisons (455 and 350 DEGs) warrants clarification, as no biotic stress was applied in this study. In the KEGG framework, this pathway is a composite of generic immune- and stress-signaling modules—including Ca^2+^ signaling, MAPK cascades, respiratory burst oxidase homolog (RBOH)-mediated ROS production, WRKY transcription factors, and defense-related hormone signaling—that are extensively shared with abiotic stress responses. Its enrichment here therefore most plausibly reflects the recruitment of these shared ROS/MAPK/Ca^2+^ modules by alkali stress rather than any pathogen-related process, an interpretation consistent with the strong enrichment of MAPK signaling and with the oxidative injury documented at the physiological level. Similar enrichment of plant–pathogen interaction has been commonly reported in abiotic-stress transcriptome studies.

#### 2.6.3. KEGG Enrichment in the Alkali Time Course (Treat-40d vs. Treat-0d)

In Treat-40d vs. Treat-0d (Figure 6b), the most significantly enriched pathway was photosynthesis (38 DEGs, of which 35 were down-regulated; adjusted *p* = 5.03 × 10^−7^). Photosynthesis–antenna proteins was also significant (13 DEGs, all down-regulated; adjusted *p* = 2.26 × 10^−3^). The remaining enriched pathways were carbon fixation by the Calvin cycle (39 DEGs, 31 down), plant–pathogen interaction (455 DEGs, 338 down), and circadian rhythm–plant (83 DEGs, 55 down). MAPK signaling pathway–plant (221 DEGs, 149 down), plant hormone signal transduction (361 DEGs, 253 down), and metabolic pathways (1167 DEGs, 694 down) were likewise significantly enriched (Table 4).

Photosynthesis (35 of 38 DEGs), carbon fixation by the Calvin cycle (31 of 39), and photosynthesis–antenna proteins (13 of 13) were all strongly skewed toward down-regulation. This skew indicates systemic repression of light capture and carbon assimilation after 40 d of alkali exposure, consistent with the leaf chlorosis and the decline of chlorophyll a and b contents. Because the Treat-40d vs. Treat-0d comparison spans 40 d of growth, these changes integrate alkali-stress progression with normal development; accordingly, this time course is interpreted throughout this study against the developmental baseline defined by the CK-40d vs. CK-0d comparison, and the alkali-specific component of these changes is isolated in the time-matched Treat-40d vs. CK-40d comparison. The plant–pathogen interaction, MAPK signaling, and plant hormone signal transduction pathways, which share calcium- and kinase-based signaling modules between biotic and abiotic stress responses [3], were also extensively remodeled.

### 2.7. Proteome Identification and Differentially Expressed Proteins

#### 2.7.1. Proteome Identification Depth and Data Quality

Data-independent acquisition (DIA) proteomics was performed on the same twelve leaf samples (four groups × three biological replicates) used for transcriptome sequencing, and the spectra were processed with a library-free search in DIA-NN v1.8.1 [34]. In total, 62,181 peptides were detected, corresponding to 7452 proteins quantified across all twelve samples at a FDR below 1%; within-group replicate correlations of at least 0.94 confirmed reproducible quantification.

**Figure 7:**
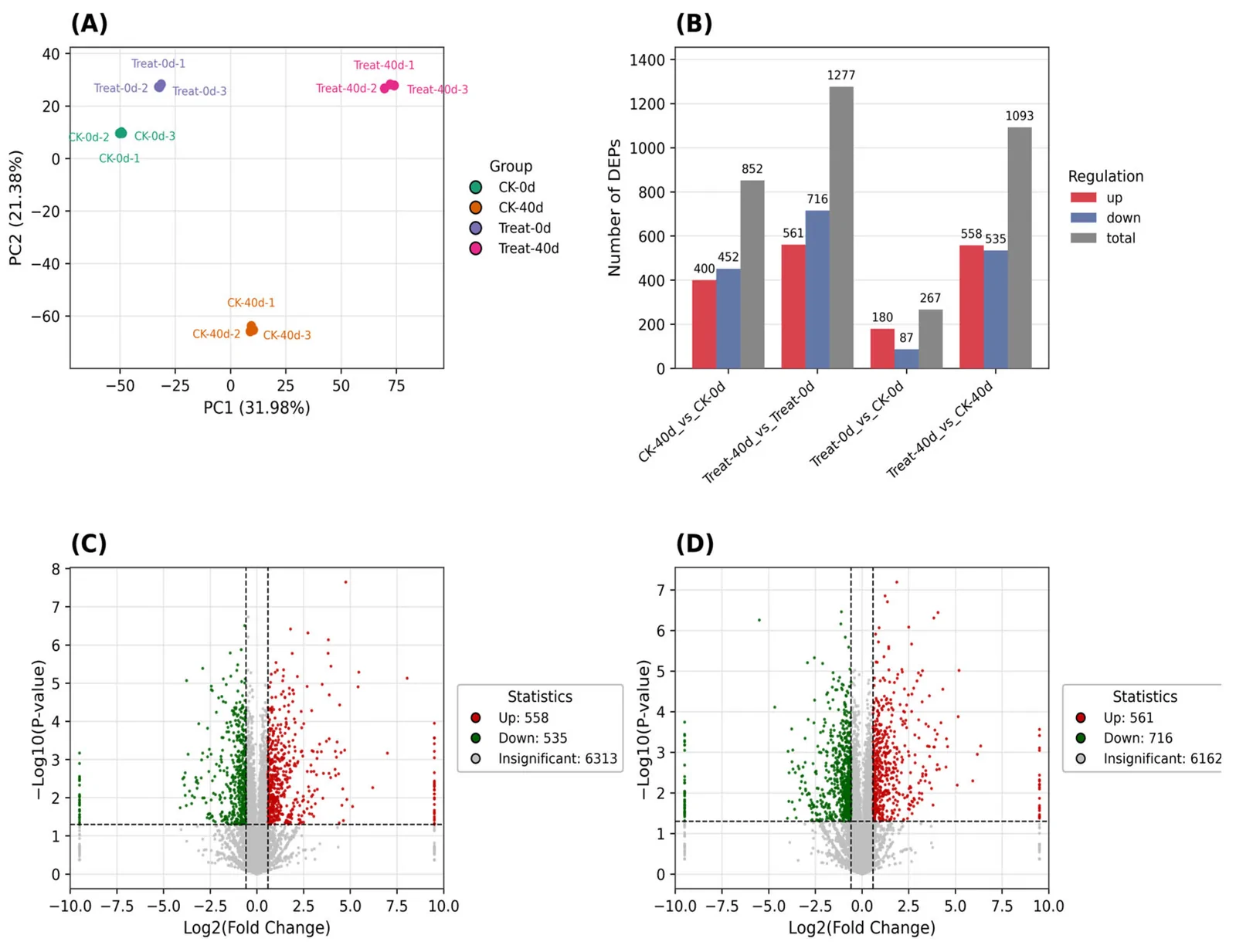
Overview of the DIA proteome of *R. obtusum* ‘Fenzhuanglou’ leaves under alkali stress. (**A**) Two-dimensional PCA score plot of the quantified proteome (PC1, 31.98%; PC2, 21.38%), showing separation of the four sample groups. (**B**) Numbers of up- and down-regulated DEPs (FC ≥ 1.5 or ≤0.6667, *p* ≤ 0.05) in the four pairwise comparisons. (**C**) Volcano plot of DEPs in Treat-40d vs. CK-40d. (**D**) Volcano plot of DEPs in Treat-40d vs. Treat-0d.

PCA of the quantified proteome resolved the four groups into distinct clusters (Figure 7A), with PC1 and PC2 explaining 31.98% and 21.38% of the total variance. The Treat-40d samples were clearly separated from the other three groups, whereas CK-0d, CK-40d, and Treat-0d occupied relatively closer positions, indicating that 40 d of 60 mmol·L^−1^ alkali exposure, rather than sampling time or initial treatment status, was the dominant driver of proteome remodeling.

#### 2.7.2. Statistics of Differentially Expressed Proteins

DEPs were defined by a fold change (FC) ≥ 1.5 or ≤0.6667 with *p* ≤ 0.05. A total of 1093 DEPs were identified in Treat-40d vs. CK-40d, of which 558 were up-regulated and 535 down-regulated (Table 5). The remaining comparisons yielded 267 DEPs in Treat-0d vs. CK-0d (180 up-regulated and 87 down-regulated), 852 DEPs in CK-40d vs. CK-0d (400 up-regulated and 452 down-regulated), and 1277 DEPs in Treat-40d vs. Treat-0d (561 up-regulated and 716 down-regulated) (Table 5; Figure 7B–D). A Benjamini–Hochberg FDR sensitivity analysis showed that the DEP sets were robust to multiple-testing correction: with the same FC thresholds, 674 of the 1093 DEPs in Treat-40d vs. CK-40d (61.7%; 349 up- and 325 down-regulated) remained significant at q ≤ 0.05, and 927 (84.8%) at q ≤ 0.10 (Supplementary Table S4). The baseline comparison Treat-0d vs. CK-0d yielded the smallest and least robust set (48 DEPs at q ≤ 0.05), consistent with its interpretation as reflecting plant-to-plant batch variation and/or an immediate early response rather than the sustained alkali response. Unless stated otherwise, the DEP numbers reported below and the downstream enrichment analyses based on them refer to the nominal-*p* set and are therefore exploratory; the q ≤ 0.05 subset provides the confirmatory core (Supplementary Table S4).

The DEP pattern was strongly time-dependent. The smallest change occurred between the two day-0 groups, and the 267 DEPs detected there were far fewer than the 2441 DEGs identified in the same comparison at the transcript level, consistent with the narrower dynamic range of protein abundance. The largest change occurred in the treatment time course (Treat-40d vs. Treat-0d), exceeding that of the water-control time course (CK-40d vs. CK-0d). Because developmental drift alone accounted for 852 DEPs, the DEPs in Treat-40d vs. CK-40d—nearly balanced between up- and down-regulation and spanning a wide range of fold changes (Figure 7C,D)—most directly represent the alkali-specific proteome response after 40 d.

Representative DEPs of Treat-40d vs. CK-40d are listed in Table 6. The most strongly up-regulated protein was an Fe2OG dioxygenase domain-containing protein (FC = 19.14, q = 1.41 × 10^−2^). Other up-regulated proteins supported at q ≤ 0.05 included a cytochrome P450 (FC = 7.42, q = 1.78 × 10^−2^), a CBS domain-containing protein (FC = 3.90, q = 1.42 × 10^−2^), a UspA domain-containing universal stress protein (FC = 2.83, q = 2.68 × 10^−3^), and chalcone synthase (FC = 2.67, q = 1.29 × 10^−2^), the committed enzyme of flavonoid biosynthesis. Two further up-regulated proteins—a second cytochrome P450 (FC = 3.45, q = 0.13) and a major facilitator superfamily (MFS) transporter (FC = 6.67, q = 6.02 × 10^−2^)—are supported only at nominal *p* ≤ 0.05 and are therefore regarded as tentative.

### 2.8. KEGG Enrichment Analysis of DEPs

#### 2.8.1. Enrichment Profile of Treat-40d vs. CK-40d

**Figure 8:**
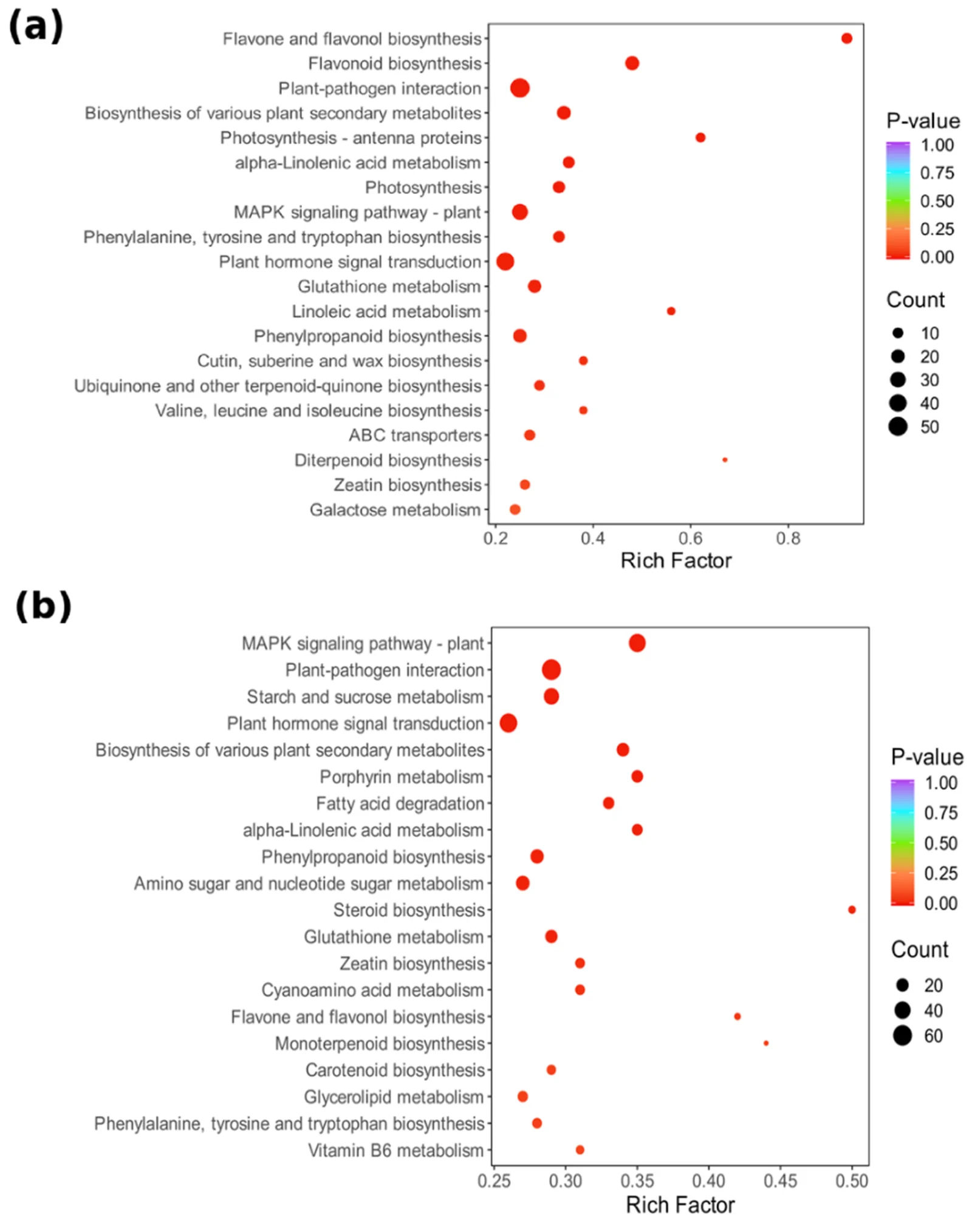
KEGG enrichment bubble plots of DEPs. (**a**) Treat-40d vs. CK-40d; (**b**) Treat-40d vs. Treat-0d. Bubble size indicates the number of enriched DEPs and color indicates the enrichment significance.

Pathway enrichment in this section was computed on the nominal-*p* DEP set and is therefore regarded as exploratory. The alkali-specific comparison was dominated by secondary-metabolite pathways and by photosynthetic suppression (Table 7; Figure 8a). Flavone and flavonol biosynthesis (11/511 DEPs, FDR = 1.0 × 10^−6^) and flavonoid biosynthesis (22/511 DEPs, FDR = 9.0 × 10^−6^) were the most significantly enriched pathways, consistent with the up-regulation of chalcone synthase (Table 6); biosynthesis of various plant secondary metabolites contributed a further 21 DEPs (FDR = 4.09 × 10^−3^). Phenylalanine, tyrosine and tryptophan biosynthesis (13/511 DEPs, FDR = 4.80 × 10^−2^) supplies the shikimate-derived precursors of phenylpropanoid and flavonoid metabolism. In contrast, the enrichment of photosynthesis (15/511 DEPs, FDR = 2.81 × 10^−2^) provides a proteomic explanation for the chlorophyll b decline and leaf chlorosis observed at day 40. MAPK signaling pathway–plant (32/511 DEPs, FDR = 2.81 × 10^−2^) and α-linolenic acid metabolism (14/511 DEPs, FDR = 2.81 × 10^−2^) were also enriched, implicating kinase signaling and jasmonate-precursor metabolism in the chronic alkali response.

#### 2.8.2. Enrichment Profiles of the Remaining Comparisons

The day-0 comparison yielded only two significantly enriched pathways—protein processing in endoplasmic reticulum (16/136) and flavonoid biosynthesis (8/136), both at FDR = 1.98 × 10^−2^—mirroring the limited proteomic divergence at the start of treatment (Table 7). Developmental drift in the water control (CK-40d vs. CK-0d) was dominated by starch and sucrose metabolism (33/397, FDR = 6.3 × 10^−5^) and phenylpropanoid biosynthesis (27/397, FDR = 6.3 × 10^−5^). The treatment time course (Treat-40d vs. Treat-0d; Figure 8b) highlighted signal-transduction and carbon-metabolism adjustments, led by MAPK signaling pathway–plant (45/575 DEPs, FDR = 3.5 × 10^−5^), plant hormone signal transduction (50/575, FDR = 1.72 × 10^−2^), and starch and sucrose metabolism (35/575, FDR = 1.72 × 10^−2^). The starch and sucrose enrichment agrees with the soluble-sugar accumulation measured at day 40, whereas porphyrin-metabolism enrichment (17/575, FDR = 3.31 × 10^−2^) parallels the suppression of photosynthesis, because chlorophyll synthesis depends on the porphyrin pathway. At the GO level, the treatment time course was further marked by broad suppression of signaling and light-capture functions: protein phosphorylation (60 of 64 DEPs down-regulated in Treat-40d vs. CK-40d, *p* = 2.1 × 10^−6^; 62 of 67 down-regulated in Treat-40d vs. Treat-0d, *p* = 1.2 × 10^−4^), photosynthesis light harvesting (8 DEPs, all down-regulated; *p* = 9.0 × 10^−5^), and defense response to fungus (14 DEPs, 13 down-regulated), whereas glutathione metabolic process was enriched with 11 of 16 DEPs up-regulated (*p* = 7.9 × 10^−5^).

### 2.9. Integrated Transcriptome–Proteome Analysis

**Figure 9:**
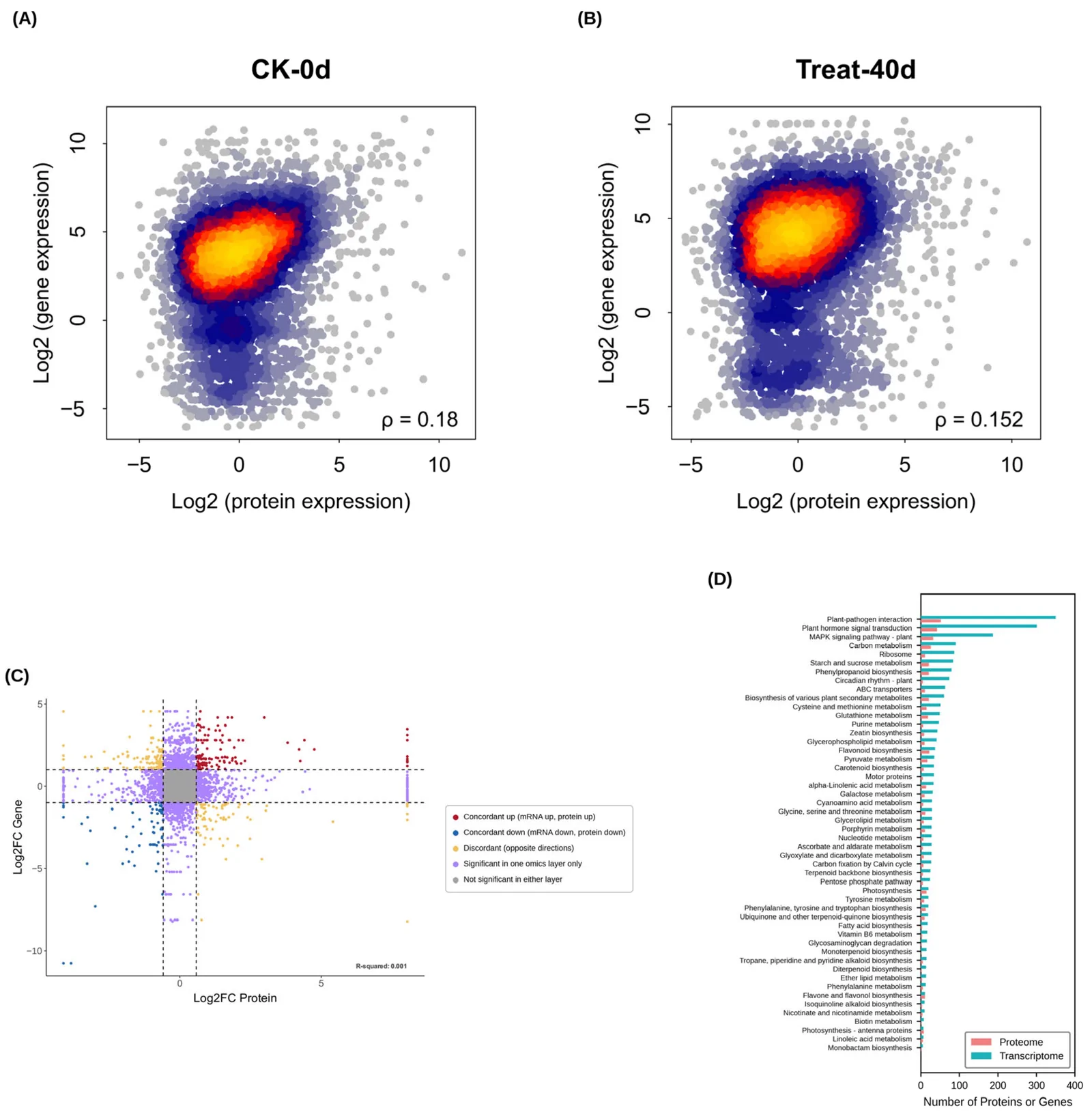
Integrated transcriptome–proteome analysis. (**A**) Heat-scatter plot of mRNA–protein expression correlation in the CK-0d group (Pearson ρ = 0.18); point colors indicate local point density, increasing from gray (lowest) through blue and purple to red and yellow (highest). (**B**) Corresponding plot for the Treat-40d group (ρ = 0.152), with the same density color scheme. (**C**) Nine-quadrant plot of differential changes at the transcript and protein levels; dot colors indicate concordance between transcript and protein changes: red, concordant up-regulation at both levels; dark blue, concordant down-regulation at both levels; gold, changes in opposite directions; purple, significant change in only one omics layer; gray, no significant change in either layer. (**D**) Bar chart of KEGG pathways commonly enriched in both omics layers.

Pearson correlations between transcript and protein abundance were low in all four groups: ρ = 0.18 (CK-0d), 0.137 (CK-40d), 0.198 (Treat-0d), and 0.152 (Treat-40d) (Figure 9A,B). Nine-quadrant analysis of differential changes yielded similarly low concordance (R^2^ = 0.001–0.009; Figure 9C). Such low correlations are common in plant stress studies and may reflect extensive post-transcriptional, translational, and protein-turnover regulation, together with methodological differences between RNA-seq (FPKM) and DIA-MaxLFQ quantification. Alkali stress did not further weaken the correlation, suggesting that the modest agreement is intrinsic to this system rather than a stress-induced decoupling. Nevertheless, the two layers converged at the pathway level (Figure 9D; Table 8).

MAPK signaling pathway–plant was significantly enriched in both omics layers in both key stress comparisons (Table 8), identifying the kinase cascade as the most robust convergent regulatory axis of the alkali response. Plant hormone signal transduction was jointly enriched in the time-course comparison, in line with the ACC-oxidase and ethylene-receptor suppression described above. Beyond these jointly enriched pathways, each layer independently pointed to the same physiological phenotypes: at the protein level, starch and sucrose metabolism (35 DEPs) and photosynthesis (15 DEPs in Treat-40d vs. CK-40d) were enriched, whereas at the transcript level the corresponding pathways were represented (84 DEGs in starch and sucrose metabolism; 38 DEGs in photosynthesis in the time-course comparison) without reaching pathway-level significance. This one-layer enrichment, supported at the gene/protein level, mirrors the soluble-sugar accumulation and pigment-degradation phenotypes. α-Linolenic acid metabolism, significant at the protein level (FDR = 2.81 × 10^−2^), was further supported at the transcript level by differentially expressed chloroplast envelope quinone oxidoreductase (ceQORH) and jasmonate-related CYP94C1 genes. Because pathway-level agreement was observed even where gene-level correlation was weak, these commonly enriched pathways are the most robust candidates for the mechanistic model developed in the Section 3.

To link the molecular changes directly to the physiological injury gradient, we examined the correspondence between the fifteen qRT-PCR candidate genes, their protein products where quantified, and four physiological markers (SOD, POD, CAT, and MDA) across the four sample groups. The directions of change were concordant with the physiological interpretation. In the glutathione/glyoxalase redox module, the chloroplastic glutamate–cysteine ligase *RoGCL* was repressed at the transcript level (log_2_FC = −4.16) and glutathione S-transferases were down-regulated at the protein level, paralleling the collapse of POD and CAT activities and the 5.2-fold accumulation of MDA in Treat-40d vs. CK-40d; the glyoxalase *RoGLO1* was induced (log_2_FC = +2.65), consistent with enhanced detoxification of methylglyoxal under severe oxidative injury. For the five candidate genes with detectable expression at all four sampling points (*RoMAPK3*, the ethylene receptor, *RoWRKY2*, *RoCCT*, and *RoERF1*), the directions of change across the four groups were qualitatively concordant with the physiological trajectories—for example, the induction pattern of RoERF1 paralleled MDA accumulation and ran opposite to the POD/SOD decline, whereas *RoMAPK3* changed oppositely to MDA and in parallel with CAT. Because only four group-level observations are available, no inferential correlation test is appropriate (*n* = 4 cannot support statistical inference), and these concordances are therefore presented strictly as qualitative associations rather than statistical evidence; a replicate-paired correlation analysis (*n* = 12) and a denser time-course design would be required to establish quantitative gene–phenotype relationships, which we identify as future work.

### 2.10. PTR Analysis

**Figure 10:**
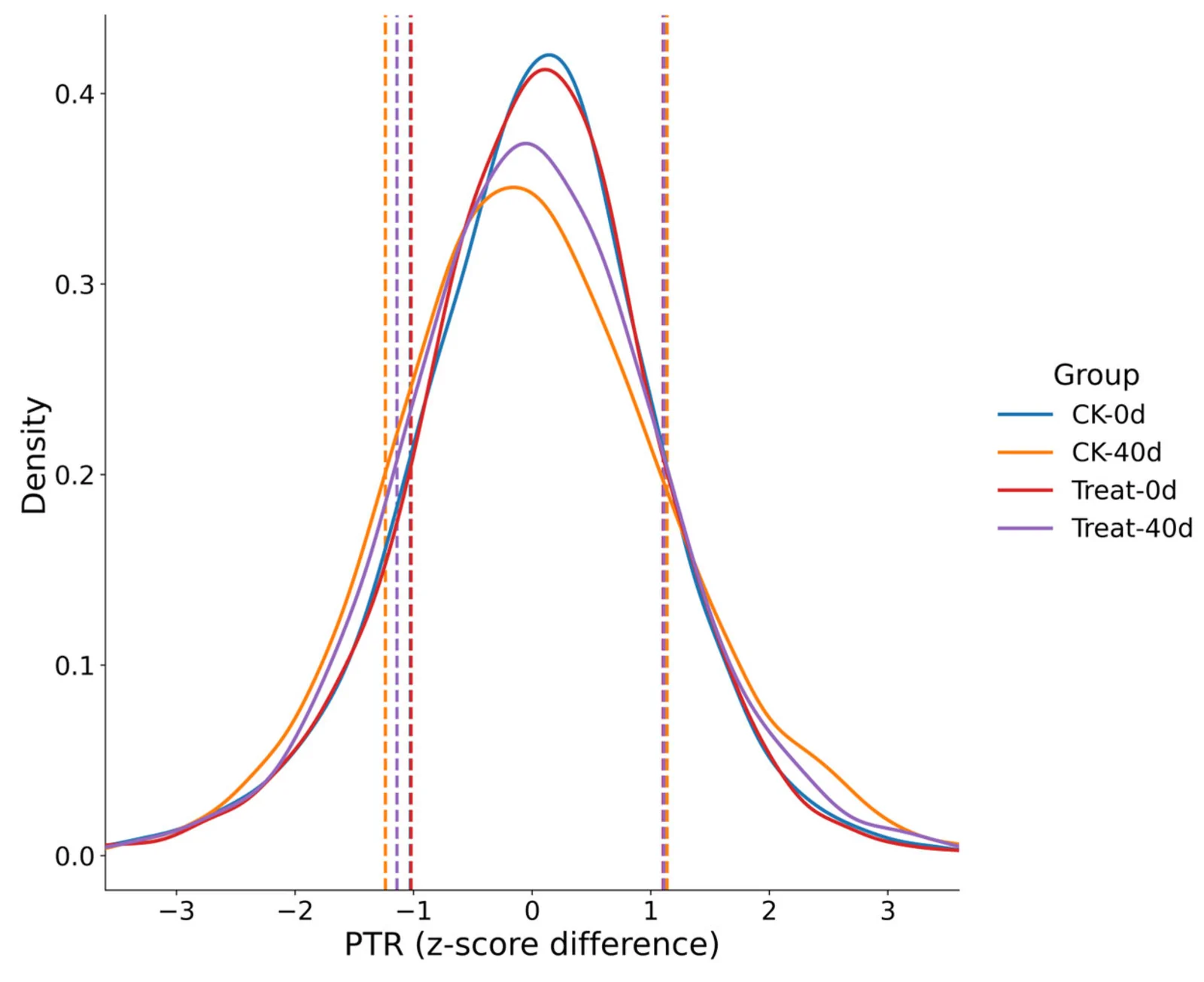
Distribution of the PTR, computed as the difference in within-group z-scores of log_2_(LFQ) and log_2_(FPKM), across the four sample groups. Dashed lines mark the median ± 1 SD thresholds defining extreme-PTR genes.

To characterize post-transcriptional regulation under alkali stress, the PTR was computed for each gene and group as the difference in within-group z-scores, PTR = z(log_2_ LFQ) − z(log_2_ FPKM), replacing the previously used ratio-of-logs score, which is mathematically distorted by the arbitrary zero of the log scale. Between 6510 and 6726 genes per group were quantified at both layers and retained for analysis. PTR is used here as a descriptive, group-internal ranking metric of relative protein output per transcript, jointly reflecting translation efficiency, protein stability, and turnover rather than translation efficiency alone. The PTR distributions of the four groups were broadly similar: each was unimodal and centered near zero, and the median ± 1 SD thresholds defining extreme-PTR genes are marked by dashed lines (Figure 10). The extreme-PTR sets comprised 1832 genes in CK-0d (847 high, 985 low), 1957 in CK-40d (1063 high, 894 low), 1856 in Treat-0d (886 high, 970 low), and 1877 in Treat-40d (958 high, 919 low)—approximately 28% of the quantified genes per group, as expected for a ±1 SD two-sided criterion. Fisher’s exact tests showed that genes differentially expressed at either the transcript or the protein layer were consistently over-represented among extreme-PTR genes in all four comparisons and both groups of each comparison (odds ratios 1.49–2.65; all *p* < 5 × 10^−11^; e.g., Treat-40d vs. CK-40d: OR = 1.65 in Treat-40d, *p* = 1.2 × 10^−18^; OR = 2.19 in CK-40d, *p* = 1.8 × 10^−44^), indicating that transcript–protein divergence is concentrated among stress-responsive loci (full PTR scores, extreme-PTR gene lists, and Fisher test results in Supplementary Table S5). GO enrichment of the extreme-PTR sets was deliberately conservative: few terms reached significance (q < 0.05), with photosynthesis (GO:0015979) recurring in three of the four groups—consistent with post-transcriptional control of photosynthetic components under alkali stress—and protein phosphorylation (GO:0006468) enriched in CK-0d. The transcript–protein divergence captured by PTR is consistent with transcript–protein decoupling potentially involving post-transcriptional regulation, protein stability, and turnover as contributors to the alkali-stress response.

### 2.11. qRT-PCR Validation of the Transcriptome Data

To verify the reliability of the RNA-seq data, fifteen DEGs (eight up-regulated and seven down-regulated in Treat-40d vs. CK-40d) were selected for qRT-PCR validation across the four sample groups (CK-0d, CK-40d, Treat-0d, and Treat-40d; Table 9). The candidate genes span the principal regulatory and metabolic themes identified by the transcriptome analysis: MAPK signaling (*RoMAPK3*, log_2_FC +2.29), hormone signal transduction (*RoTIFY1* and *RoTIFY2*, −2.08 and +2.22; *RoDELLA*, −3.26), stress-responsive transcription factors (*RoWRKY1* and *RoWRKY2*, −3.30 and +2.48; *RoNAC*, +2.45; *RoERF1* and *RoERF2*, +2.49 and −1.93; and the ethylene-responsive transcription factor *RoERF110*, −2.14), circadian regulation (*RoCCT*, −3.72), and protective metabolism (*RoRS*, +2.23; *RoGLO1*, +2.65; *RoGCL*, −4.16; *RoQORH*, +2.44). The ethylene receptor gene (RHSIM_Rhsim04G0188600, log_2_FC = −6.40), although the most strongly repressed annotated DEG in hormone signal transduction, was excluded from the validation set because its expression under alkali stress was too low for reliable quantification (Treat-40d FPKM ≈ 0.70); in its place, *RoERF110* (RHSIM_Rhsim11G0086800), a downstream ethylene-signaling transcription factor with comparable repression (log_2_FC = −2.14) and robust expression (CK-40d FPKM ≈ 672; Treat-40d FPKM ≈ 147), was selected to represent the ethylene pathway.

**Figure 11:**
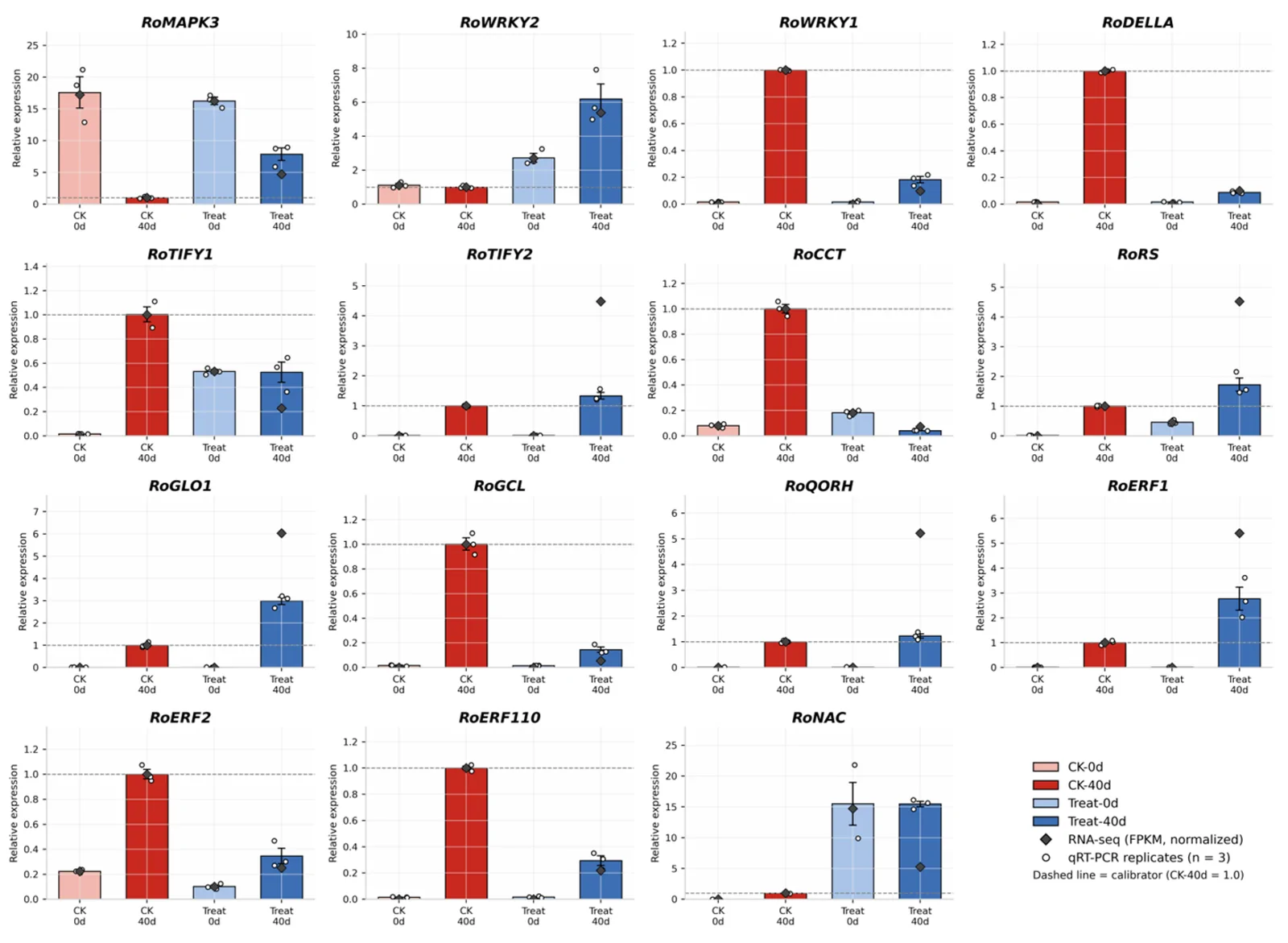
qRT-PCR validation of the RNA-seq data for 15 selected DEGs across the four sample groups (CK-0d, CK-40d, Treat-0d, Treat-40d). Bars show qRT-PCR relative expression (2^−ΔΔCt^, calibrator CK-40d = 1.0, dashed line; mean ± SE, *n* = 3 biological replicates; open circles = individual replicates); black diamonds show the corresponding RNA-seq FPKM values normalized identically. qPCR and RNA-seq fold changes (Treat-40d vs. CK-40d) were highly concordant (Pearson r = 0.908, R^2^ = 0.825).

The relative expression levels measured by qRT-PCR (2^−ΔΔCT^ method, normalized to *RoEF1*-α; three biological replicates per group) agreed with the RNA-seq expression trends for all 15 candidate genes across the four groups (Figure 11). Every gene retained its RNA-seq-defined direction of change in Treat-40d vs. CK-40d (eight up-regulated and seven down-regulated), and the fold-change magnitudes were of the same order: for example, *RoMAPK3* (+2.95 by qRT-PCR vs. +2.29 by RNA-seq, log_2_scale), *RoDELLA* (−3.53 vs. −3.26), *RoWRKY1* (−2.48 vs. −3.30), *RoCCT* (−4.60 vs. −3.72), *RoGLO1* (+1.58 vs. +2.65), and *RoERF110* (−1.79 vs. −2.14), which confirms the reliability of the transcriptome data and their suitability for downstream biological interpretation.

## 3. Discussion

Soil alkalization constrains the cultivation of acidophilic ornamentals, and rhododendrons rank among the most pH-sensitive woody genera in horticulture [17,18]. In this study, a graded physiological assay was combined with parallel transcriptome and DIA proteome analyses of the same leaf samples to characterize how *R. obtusum* ‘Fenzhuanglou’ perceives, buffers, and ultimately succumbs to mixed NaHCO_3_/Na_2_CO_3_ stress. The findings are interpreted below with emphasis on the tolerance threshold, the photosynthetic apparatus, osmotic and redox adjustment, signaling networks, and the discordance between the transcript and protein layers. Because the evidence is correlative in nature—physiological time courses coupled with transcriptomic and proteomic profiles—the mechanisms discussed below are presented as proposed models and associations consistent with the data, not as demonstrated causal relationships; functional validation of the implicated genes and pathways is required to establish causality.

### 3.1. Alkali Tolerance Threshold of R. obtusum ‘Fenzhuanglou’ in Context

The phenotypic gradient—tolerance of 50 mmol·L^−1^ through 40 d, the visible-injury threshold at 60 mmol·L^−1^, and severe damage at 70 mmol·L^−1^—defines a narrow alkali-tolerance window for *R. obtusum* ‘Fenzhuanglou’. Consistent with cultivar-dependent thresholds reported for other *Rhododendron* cultivars [24,26], inter-cultivar divergence in alkali tolerance appears to reside in the amplitude and timing of shared defense modules rather than in qualitatively different mechanisms [27]. Acidophilic *Rhododendron* species have a limited capacity for rhizosphere acidification and iron acquisition at high pH [17,18]. The 60 mmol·L^−1^ threshold therefore likely marks the point at which osmotic, ionic, and high-pH burdens jointly exceed compensatory capacity [1,4]. Within the conditions of this greenhouse pot experiment, substrate-solution alkali concentrations at or below 50 mmol·L^−1^ sustained normal growth for at least 40 d; this value may therefore serve as a preliminary reference for the cultivation of this cultivar. As it derives from a single pot trial using pine-needle soil and one mixed-alkali composition, its applicability as a practical cultivation guideline requires validation under field conditions and across different substrates.

### 3.2. The Photosynthetic Apparatus as the Primary Target of Alkali Stress

#### 3.2.1. Chl b Sensitivity and Repression of Porphyrin Metabolism at the Gene and Protein Levels

Chl b was the earliest and most severely affected pigment. It fell by 12.5–28.4% at 10 d (a stage at which the differences were not yet statistically significant), well before any visible symptom, and reached only about 20% of the control at 40 d under 60–70 mmol·L^−1^. Because Chl b is confined to the peripheral light-harvesting antenna complexes, its selective depletion suggests that the antenna system is a primary target of alkali injury. Both omics layers support this interpretation: all 13 DEGs of photosynthesis–antenna proteins were down-regulated, and the eight photosynthesis light-harvesting DEPs, which included chlorophyll a/b-binding proteins, were uniformly down-regulated at day 40. Preferential loss of antenna pigments relative to reaction-center Chl a has likewise been reported in willow and other ornamental species under alkaline or salt stress [7,35,36,37]. Porphyrin metabolism, which supplies the chlorophyll precursor pool, was enriched in both omics layers—at the transcript level in CK-40d vs. CK-0d (48 DEGs, 38 down) and at the protein level in Treat-40d vs. Treat-0d, with 12 of 17 DEPs declining over the stress period. Correspondingly, protection of chlorophyll biosynthesis was central to salicylic-acid-mediated alkali tolerance in cucumber [6]. Together, these physiological and omics results suggest that alkali stress represses chlorophyll biosynthetic supply while destabilizing the Chl-b-dependent antenna, so that light capture declines before bulk photochemistry. This early lesion may set the pace of the whole stress syndrome in this cultivar.

#### 3.2.2. Lutein and Xanthophyll Buffering with Induction of Carotenoid Biosynthesis

In contrast to the monotonic decline of chlorophylls and total carotenoids, lutein accumulated transiently, peaking at 30 d under 60 mmol·L^−1^ before receding. This pattern is consistent with engagement of xanthophyll-cycle-associated photoprotection, which dissipates excess excitation energy as heat when carbon assimilation is constrained [28,29]. The transcriptome points to an active, regulated basis for this buffering: carotenoid biosynthesis was represented by 34 DEGs (23 up-regulated), although the pathway did not reach pathway-level enrichment significance (adjusted *p* > 0.05), and the Gene Ontology term photoprotection was significantly enriched with 24 of 25 DEGs up-regulated (*p* = 5.4 × 10^−11^). Enhanced xanthophyll-cycle activity has been linked to stress resistance in spinach and to photosynthetic acclimation in sugar beet under alkaline stress [8,28]; our results extend this pattern to an acidophilic woody ornamental. The post-30 d decline of the lutein pool, most pronounced at the injury-threshold concentration, coincided with the collapse of antioxidant enzyme activities. This coincidence suggests that pigment-based photoprotection can be sustained only while enzymatic defense remains functional; thereafter, the carotenoid pool was presumably consumed faster than the induced machinery could replenish it. Xanthophyll buffering thus appears to provide only transient protection rather than lasting acclimation at supra-threshold alkalinity.

#### 3.2.3. Coordination Between Pigment Loss and Down-Regulation of Photosynthesis Genes

The pigment measurements and the expression data were tightly coordinated. Photosynthesis was repressed in parallel with the loss of Chl a, Chl b, and carotenoids (35 of 38 DEGs down-regulated in the stress time course; 15 DEPs enriched in Treat-40d vs. CK-40d). Calvin-cycle carbon fixation was likewise repressed (31 of 39 DEGs down-regulated). This coordination suggests that pigment decline was not solely a result of oxidative destruction but was reinforced by programmed down-scaling of the photosynthetic apparatus. Such a dual mechanism is regarded as a hallmark of chronic alkaline stress, in which plants trade carbon gain for reduced excitation pressure on a damaged electron transport chain [8,28,36]. Because part of the photosynthetic-gene decline also occurred in the water controls over 40 d, a developmental component exists, and the alkali-specific repression is correctly isolated only in the time-matched Treat-40d vs. CK-40d comparison. The same developmental component is embedded in the stress time course (Treat-40d vs. Treat-0d), which is therefore interpreted here as stress progression superimposed on development rather than as a purely alkali-driven change. Taken together, these results place repression of photosynthetic capacity at the core of the alkali-tolerance limit of *R. obtusum* ‘Fenzhuanglou’.

### 3.3. Osmotic Adjustment from Solute Accumulation to Sugar Metabolism Reprogramming

#### 3.3.1. Soluble Sugar and Proline Dynamics Versus Starch–Sucrose Remodeling

Soluble sugar accumulated continuously and dose-dependently (r = 0.57 with concentration), suggesting that it acts as the principal osmotic effector, whereas proline accumulated rapidly until 30 d and then partially declined. This division of labor—proline as an early emergency osmoprotectant and sugar as the chronic-phase effector—mirrors observations in oat roots [9]; in *Elaeagnus angustifolia*, maintenance of photosynthetic function under salt stress likewise supports sustained osmotic adjustment [38]. The proteome provides a plausible mechanistic substrate. Starch and sucrose metabolism was enriched at the protein level (35 DEPs, 20 up-regulated); at the transcript level the pathway was represented by 84 DEGs but did not reach enrichment significance. In addition, trehalose-phosphate synthase was down-regulated among the representative DEPs (FC = 0.335; nominal *p* = 0.046, q = 0.133, supported only at the nominal level and therefore tentative). Remodeling of starch–sucrose interconversion under alkalization has been linked to osmotic adjustment and energy supply in several crop systems [15,39]. The plateau of sugar accumulation after 30 d at 70 mmol·L^−1^, coincident with severe desiccation, suggests that the carbon supply for osmolyte synthesis became limiting once photosynthesis collapsed. The osmotic ceiling of this cultivar is thus coupled directly to its residual assimilatory capacity.

#### 3.3.2. Soluble Protein Rise–Fall Is Consistent with Induced Defense-Protein Synthesis Followed by Net Protein Loss

Soluble protein peaked at 30 d at the injury-threshold 60 mmol·L^−1^ concentration (2.11-fold the initial value) and declined at 60–70 mmol·L^−1^ by 40 d. The early accumulation is consistent with induced synthesis of stress-related proteins and agrees with the proteomic evidence of induced flavonoid, glutathione, and defense enzymes. The maximal induction at the intermediate concentration could indicate that 60 mmol·L^−1^ approaches the upper limit at which defense-protein mobilization remains effective; whether higher concentrations impair the translational machinery itself cannot be resolved from bulk protein measurements. Declines in soluble protein under severe or prolonged stress have likewise been reported in grapevine, tree peony, guava, and canola under salt and saline–alkali conditions [40,41,42,43]. The shift from protein synthesis to proteolysis has been proposed as a general marker of stress exceeding the compensatory range [37]. The late decline at 70 mmol·L^−1^, coincident with MDA acceleration, is therefore consistent with a shift in the synthesis–degradation balance toward net protein loss accompanying the threshold-level injury phenotype. Because total soluble protein is a bulk measure that cannot separate the synthesis and degradation of individual proteins, these mechanistic interpretations remain inferential; targeted measurements of protein turnover or of specific defense proteins would be required to confirm them.

### 3.4. Redox Homeostasis Involving Antioxidant Enzymes, Glutathione, and the α-Linolenic Acid–Jasmonate Axis

#### 3.4.1. SOD/POD/CAT Peak at 20 d Then Collapse Under High Alkali, with MDA Accumulation

All three antioxidant enzymes peaked at 20 d and then declined, but the late-stage pattern was strongly concentration-dependent. At 40 d, CAT activity remained above the control only at 50 mmol·L^−1^ and fell significantly below it at 60 and 70 mmol·L^−1^. POD activity dropped significantly below the control in all alkali treatments, and SOD stayed above the control only at 50 mmol·L^−1^ while falling significantly below it at 70 mmol·L^−1^. MDA, by contrast, accumulated continuously and dose-dependently; at 40 d all alkali treatments significantly exceeded the control, reaching ~22 nmol·g^−1^ at 70 mmol·L^−1^. This biphasic mobilization–exhaustion pattern has been documented in *Rhododendron* leaves under alkali stress [25] and accords with evidence that alkaline-stress injury is driven by ROS accumulation once scavenging capacity is exceeded [1,44]. Treatments that sustain antioxidant defense—silicon or abscisic acid priming and melatonin application—consistently delay the collapse phase in maize, rice, and *Fraxinus* [10,11,12,13]. In tree peony, alkali stress likewise drives ROS imbalance that overwhelms antioxidant defense [41]. Late-stage injury coupled with rising MDA has been widely documented under salt stress [37], and salt-responsive transcriptional reprogramming of stress-defense genes has been reported in *Caragana korshinskii* seedlings [45]. The inverse temporal coupling between enzyme activities and MDA in our data indicates that membrane lipid peroxidation was governed by the production–scavenging balance rather than by ROS production alone. We therefore interpret the early (≤20 d) induction of SOD, POD, and CAT as a functional mobilization of the antioxidant system. Conversely, the fall of CAT and POD—and, at 70 mmol·L^−1^, of SOD—below the control at day 40 marks the collapse of H_2_O_2_-scavenging capacity at higher alkali concentrations. The resulting unchecked peroxidation, reflected in the dose-dependent MDA accumulation, is consistent with the progressive membrane damage underlying the leaf senescence and death phenotype observed at 60 and 70 mmol·L^−1^.

A potential alternative contributor to the late-stage enzyme decline is natural leaf senescence over the 40 d experimental period, but several observations argue that this is at most a minor component. First, the age-matched control showed only modest drift over the same period, whereas the treated groups diverged from the control in a dose-dependent manner (at 40 d, POD fell to 520, 440, and 360 U·g^−1^ at 50, 60, and 70 mmol·L^−1^, respectively, and MDA rose 3.8-, 5.2-, and 5.9-fold over the control). Second, the decline was concentration-graded rather than uniform across groups, which is incompatible with a purely developmental explanation. Third, the molecular evidence underpinning the exhaustion interpretation—the repression of *RoGCL* and glutathione S-transferases—was obtained against the age-matched CK-40d control, which cancels the developmental component by design. We therefore attribute the day-40 enzyme decline predominantly to stress-induced exhaustion of the antioxidant system, superimposed on a minor, quantifiable developmental drift captured by the control time course.

#### 3.4.2. Glutathione-Linked Machinery Shows Transcript-Level Engagement but Protein-Level Repression of GSTs

Glutathione-linked redox metabolism was engaged at both omics layers, but with divergent directionality. At the transcript level, glutathione metabolism was among the notable pathways in Treat-40d vs. CK-40d (49 DEGs). Its representative members included an up-regulated lactoylglutathione lyase (*RoGLO1*, log_2_FC = 2.65) but a strongly repressed chloroplastic glutamate–cysteine ligase (*RoGCL*, log_2_FC = −4.16), the rate-limiting enzyme of glutathione biosynthesis. At the protein level, two glutathione S-transferases (GSTs) were significantly down-regulated (FC = 0.209 and 0.275). Proteomic work on *R. chrysanthum* identified antioxidant enzymes, including GSTs, as central to stress tolerance [25]. Integrated omics of *Malus halliana* under saline–alkali stress likewise highlighted glutathione-linked redox buffering [46], and abscisic-acid alleviation of saline–alkaline injury in tomato was similarly associated with glutathione-dependent scavenging [23]. The late repression of glutamate–cysteine ligase and of GSTs suggests that glutathione biosynthetic and conjugative capacity was progressively exhausted during chronic alkali stress rather than expanded. This pattern is consistent with the collapse of enzymatic antioxidant defense described above. Restoration of glutathione metabolism therefore emerges as a candidate target for improving alkali tolerance in *Rhododendron*.

#### 3.4.3. α-Linolenic Acid Metabolism as Jasmonate-Precursor Lipid Signaling

Alpha-linolenic acid metabolism was significantly enriched at the protein level in Treat-40d vs. CK-40d (14 DEPs; FDR = 2.81 × 10^−2^) and recurred among the pathways jointly implicated by both omics layers. α-Linolenic acid released from membrane galactolipids is the precursor of 12-oxo-phytodienoic acid and jasmonates, which orchestrate defense gene expression under abiotic stress [3,47]. In addition, differentially expressed Tify-family transcription factors (the family that includes JAZ repressors; *RoTIFY1* and *RoTIFY2* among the validated DEGs) provide a downstream signature of jasmonate-pathway engagement. These results suggest that membrane lipid peroxidation in alkali-stressed leaves is not purely destructive. The lipid remodeling that compromises membrane integrity may simultaneously feed an oxylipin signaling stream that participates in defense activation, a duality proposed for saline–alkali responses generally [1,3]. This interpretation is anchored by broader evidence in woody plants and by the established biochemistry of the pathway. α-Linolenic acid released from membrane lipids by regulated lipase activity is the established precursor of phyto-oxylipins, including jasmonates [48,49], and membrane-lipid remodeling with fatty-acid mobilization is a documented acclimation response of woody and herbaceous species to drought, salinity, and temperature stress [48]. In poplar (*Populus simonii* × *P. nigra*) leaves under herbivore stress, the α-linolenic acid pathway was simultaneously enriched at the transcriptome, proteome, and metabolome levels, with up-regulation of LOX, AOS, AOC, and OPR and accumulation of JA, MeJA, and 12-OPDA [50]—a multi-layered pattern paralleling the protein-level enrichment and Tify/JAZ engagement observed here. These precedents support reading the α-linolenic acid enrichment in alkali-stressed ‘Fenzhuanglou’ leaves as engagement of a conserved oxylipin defense stream rather than a pathway-level artifact.

#### 3.4.4. Flavonoid Biosynthesis at the Protein Level as a Candidate Non-Enzymatic Protective Response

Whereas enzymatic antioxidant defense collapsed late in stress at higher concentrations, the proteome revealed concurrent induction of the flavonoid biosynthetic pathway, a candidate non-enzymatic protective layer. In Treat-40d vs. CK-40d, flavone and flavonol biosynthesis (11 DEPs; FDR = 1.0 × 10^−6^) and flavonoid biosynthesis (22 DEPs; FDR = 9.0 × 10^−6^) were the two most significantly enriched protein-level pathways. Representative up-regulated DEPs included chalcone synthase (FC = 2.67), the committed enzyme of flavonoid biosynthesis, and two cytochrome P450 proteins (FC = 7.42 and 3.45). An Fe2OG dioxygenase domain-containing protein (FC = 19.14) and a universal stress protein (UspA; FC = 2.83) were also up-regulated. The induction of the phenylpropanoid–flavonoid branch while SOD/POD/CAT activities fell below the control is consistent with a partial shift in late-stage defense from enzymatic ROS scavenging toward flavonoid-based chemical protection in *R. obtusum* ‘Fenzhuanglou’. This interpretation is consistent with the prominent antioxidant role attributed to secondary-metabolite accumulation in proteomic analyses of stress-tolerant *Rhododendron* [25]. Because flavonoid biosynthesis draws on phenylalanine and photosynthetic carbon, such a protective layer, if established, would itself likely become vulnerable once photosynthesis collapses, which may help explain why it could not prevent severe injury at 70 mmol·L^−1^. It should be noted, however, that protein-level pathway enrichment demonstrates induction of the biosynthetic machinery rather than flavonoid accumulation or antioxidant activity per se; targeted flavonoid metabolite profiling and in vitro antioxidant assays would be required to confirm that this candidate protective layer was functionally realized.

### 3.5. Signaling Networks Involving MAPK, Hormones, Circadian Rhythm, and Defense Cross-Talk

#### 3.5.1. MAPK and Hormone Signal Transduction Co-Enriched in Both Omics Across Comparisons

MAPK signaling pathway–plant and plant hormone signal transduction were enriched in both key stress comparisons (Treat-40d vs. CK-40d and Treat-40d vs. Treat-0d) and in both omics layers. Together, they accounted for 221 and 361 DEGs in the stress time course and 45 and 50 DEPs. These two pathways thus constitute the most recurrent signal-transduction themes of the dataset, consistent with the established role of kinase cascades and hormone pathways as integrators of saline–alkali perception [3,4]. Notably, the direction of change at the protein level was predominantly negative. Among these changes, 30 of 45 MAPK DEPs and 36 of 50 hormone-transduction DEPs were down-regulated, and protein phosphorylation was enriched with 60 of 64 DEPs down-regulated in Treat-40d vs. CK-40d and 62 of 67 down-regulated in Treat-40d vs. Treat-0d. This pattern indicates broad attenuation of phosphorylation-dependent signaling after 40 d; whether it represents adaptive de-escalation or signaling damage cannot be resolved from the present data. Representative signaling genes sharpen this picture. In Treat-40d vs. CK-40d, the mitogen-activated protein kinase gene *RoMAPK3* was induced at the transcript level (log_2_FC = 2.29). By contrast, a WRKY transcription factor annotated to MAPK signaling (*RoWRKY1*, log_2_FC = −3.30) and a DELLA protein of the gibberellin pathway (*RoDELLA*, log_2_FC = −3.26) were down-regulated. An ethylene receptor (log_2_FC = −6.40) was the most strongly repressed annotated DEG. Notably, the down-regulation of the ethylene receptor at the transcript level coincided with the down-regulation of ACC oxidase (FC = 0.313, q = 3.16 × 10^−3^), the terminal enzyme of ethylene biosynthesis, at the protein level. This parallel suppression across both omics layers suggests coordinated repression of ethylene production and perception, consistent with reprogrammed hormone cross-talk under chronic saline–alkali stress [3,47].

#### 3.5.2. Plant–Pathogen Interaction Enrichment Reflects Shared Stress and Defense Machinery

Plant–pathogen interaction ranked among the most significantly enriched transcriptomic pathways (455 DEGs in the stress time course) but did not reach significance among the differentially expressed proteins, despite the absence of any pathogen challenge. This apparent paradox is largely resolved by the pathway’s composition: Ca^2+^-signaling components, respiratory burst oxidase homologs, MAPK cascades, and defense transcription factors are modules shared between biotic and abiotic stress responses [3,47]. The enrichment therefore more plausibly reflects recruitment of a generic Ca^2+^/ROS/MAPK defense scaffold than any pathogen-like process. Consistent with this reading, defense response was enriched at the protein level, whereas defense response to fungus was largely down-regulated (13 of 14 DEPs), arguing against an actual biotic component. Cross-usage of immune machinery by abiotic stress has been recognized in saline–alkali contexts [3], and the present dataset provides a further example in an ornamental woody species.

#### 3.5.3. Circadian Rhythm as an Alkali-Response Modulator and the Transcription Factor Landscape

Circadian rhythm–plant was enriched in both key transcriptomic comparisons (83 and 74 DEGs), with rhythmic process (101 DEGs) and response to blue light enriched at the GO level. The circadian clock gates stress responsiveness by modulating stomatal behavior, ROS homeostasis, and hormone sensitivity according to the time of day, and its perturbation under saline–alkali stress has been reported previously [3,47]. Because all samples were collected at a fixed time of day (9:00–10:00 a.m.), the enrichment is unlikely to be a circadian-sampling artifact; it more plausibly reflects stress-induced reprogramming of clock components, potentially linking the repression of light-harvesting genes to altered diel regulation. We note, however, that with only two sampling timepoints this study cannot distinguish a phase shift in the circadian clock from sustained changes in clock-gene expression; resolving this distinction would require sampling across a full diel cycle and is identified as future work. The sampling window itself (9:00–10:00 a.m.) places all measurements in the mid-morning phase, when photosynthetic gene expression is typically near its daily maximum and clock components occupy a defined phase of their diel cycle. The expression values reported here therefore represent this specific circadian phase: the magnitude—and possibly the direction—of stress-induced changes in clock and light-harvesting genes may differ at other times of day, and the down-regulation of photosynthesis genes in morning-collected Treat-40d samples is best interpreted as suppression of the daytime expression peak rather than a uniform reduction across the diel cycle. At the regulatory core, AP2/ERF, WRKY, NAC, MYB, and bHLH transcription factors dominated the differentially expressed TF set. These families have repeatedly been identified as central regulators of saline–alkali tolerance [3,47], with GRAS and Tify members connecting gibberellin and jasmonate signaling to the response. Among the named regulators in Treat-40d vs. CK-40d, a bHLH factor was strongly induced (log_2_FC = 5.14) and two WRKY members changed in opposite directions (+2.48 and −3.30). NAC (+2.45 and −4.24) and AP2/ERF (+3.63, +2.49, and −1.93) factors showed family-internal divergence. In contrast, the GRAS-family DELLA protein (log_2_FC = −3.26) and a CCT-domain clock component (log_2_FC = −3.72) were repressed. These TF families constitute rational entry points for candidate-gene prioritization, and fifteen representative DEGs spanning the MAPK, hormone-signaling, circadian, and transcription-factor categories were selected for qRT-PCR validation.

### 3.6. Uncoupling of Transcription and Translation with Post-Transcriptional Regulation

#### 3.6.1. Causes and Implications of the Low Correlation Between mRNA and Protein Abundance

Transcript–protein abundance correlation was modest (ρ = 0.137–0.198), and nine-quadrant concordance was minimal (R^2^ ≤ 0.009). Weak mRNA–protein coupling is commonly observed in plant stress multi-omics studies [46]. In line with previous interpretations, the low correlation observed here may be attributable to the following factors. (i) Post-transcriptional and post-translational modification can decouple protein abundance from transcript levels. (ii) The different rates of transcription and translation, and the different half-lives of mRNA and proteins, shift the timing of the two layers. (iii) The thresholds used to identify DEGs and DEPs differ between the two platforms. (iv) Cross-species mapping artifacts may also have contributed: reads from diverged *R. obtusum* loci fail to align, and the DIA search database, derived from the same reference, cannot represent cultivar-specific proteins, so the two layers are restricted to conserved—and only partially overlapping—measurable subsets, which injects uncorrelated noise between them. Two considerations nevertheless indicate that mapping artifacts are not the dominant driver of the divergence: within-layer replicate correlations were high in both omics (≥0.87 for transcripts and ≥0.94 for proteins), and confining both layers to conserved loci would, if anything, bias the cross-layer correlation upward. Cross-species effects therefore likely deflate the observed correlation modestly without accounting for its magnitude. These factors are further compounded by the narrower dynamic range of protein abundance measurements and by the annotation gaps introduced by the congeneric *R. simsii* reference. The same layer asymmetry is evident at the differential level: the day-0 comparison yielded 2441 DEGs but only 267 DEPs. This difference is attributable to the narrower dynamic range of protein abundance and to the predominantly immediate-early character of the day-0 transcript response, which is expected to precede measurable proteomic change. Stress did not worsen the correlation (the highest ρ occurred at Treat-0d), indicating intrinsic uncoupling rather than stress-induced failure. Transcript fold changes therefore cannot reliably proxy protein outcomes, and mechanistic conclusions in this study therefore rest primarily on the pathways jointly enriched in both layers (MAPK signaling and plant hormone signal transduction), complemented by pathways enriched in one layer with concordant gene- and protein-level changes (glutathione, porphyrin, starch and sucrose, α-linolenic acid, and photosynthesis).

#### 3.6.2. PTR Scores Are Consistent with Translational Control at Specific Loci

To resolve where this transcript–protein uncoupling arises, we computed a PTR score for each gene in every group as the difference in within-group z-scores of log_2_-transformed protein and transcript abundances, a scale-free formulation that avoids the zero-point artifact of ratio-of-logs scores. Extreme-PTR genes were defined as those whose values fell beyond one standard deviation from the median of each group’s PTR distribution, corresponding to loci with unusually high or low protein output per transcript. The PTR density distributions were broadly similar in shape across the four groups, but the identity of extreme-PTR genes differed between control and alkali-treated samples, and genes differentially expressed at either layer were consistently over-represented among extreme-PTR genes in all comparisons (odds ratios 1.49–2.65, all *p* < 5 × 10^−11^), indicating that transcript–protein divergence is concentrated among stress-responsive loci in a functionally selective, rather than random, manner under alkali stress. Together with the low global mRNA–protein correlation, the PTR analysis is consistent with transcript–protein decoupling potentially involving post-transcriptional regulation, differential protein stability, and turnover, rather than pointing to any single mechanism. It also nominates specific loci at which translational control may contribute to alkali tolerance in this cultivar. We emphasize, however, that PTR conflates translation efficiency with protein stability and turnover and that the median ± 1 SD thresholds are descriptive rather than calibrated, so these inferences remain hypothesis-generating and await direct experimental validation (e.g., polysome profiling or targeted protein quantification).

### 3.7. Integrated Model, Limitations, and Perspectives

#### 3.7.1. A Working Model of the Alkali Response and Candidate Pathways for Breeding

The combined evidence suggests an integrated working model of the alkali response of *R. obtusum* ‘Fenzhuanglou’, which we present as a testable hypothesis rather than a confirmed causal mechanism. Mixed alkali imposes osmotic, ionic, and high-pH burdens that appear to be perceived through Ca^2+^/MAPK signaling and transduced by AP2/ERF, WRKY, NAC, MYB, and bHLH factors. Within this model, the light-harvesting antenna and the porphyrin-based chlorophyll supply fail first. Compensation then proceeds through three modules. The first is sugar-dominated osmotic adjustment underpinned by starch–sucrose remodeling, with proline acting early. The second is redox defense, which combines transient SOD/POD/CAT mobilization with glutathione-linked buffering and induced flavonoid biosynthesis, both progressively exhausted at supra-threshold concentrations. The third is photoprotection and chemical defense via carotenoid/xanthophyll and phenylpropanoid–flavonoid pathways, potentially fed by jasmonate signaling. At 60 mmol·L^−1^, compensation merely delays enzyme collapse, pigment-buffer exhaustion, and MDA acceleration, which together define the injury-threshold state. The recurrently co-enriched pathways and dominant TF families identified here are therefore prime candidates for breeding alkali-tolerant *Rhododendron* [14,18,19]; however, the causal links proposed by this working model require experimental validation—for example, through functional characterization of the candidate genes and pathway components—before they can be regarded as established.

#### 3.7.2. Limitations

Several limitations qualify these conclusions. First, a single cultivar was examined; given the documented inter-cultivar variation in *Rhododendron* alkali tolerance [24,26], the 60 mmol·L^−1^ threshold and the pathway priorities proposed here require testing across a broader germplasm panel. Second, only leaf tissue was profiled, although roots are the primary site of alkali perception and injury [5,27]; root-targeted omics are needed to complete the organ-level picture. Third, although fifteen representative DEGs were subjected to qRT-PCR validation, a single reference gene (*RoEF1-α*) was used for normalization, and protein-level confirmation by targeted methods such as parallel reaction monitoring (PRM) is still lacking. Representative DEPs—including GSTs, ACC oxidase, chalcone synthase, and trehalose-phosphate synthase—merit such validation. Fourth, the pot experiment was conducted under greenhouse conditions with a defined NaHCO_3_:Na_2_CO_3_ mixture and cannot capture the heterogeneous ionic composition and fluctuating water regimes of field soils. The threshold must therefore be verified in field trials. Fifth, the exposure gradient was defined by the input dosing regimen, and substrate pH and EC dynamics were not tracked; actual rhizosphere concentrations may therefore have differed from the nominal input concentrations, although the dose-ordered physiological and molecular responses indicate that four distinct and stable exposure levels were maintained throughout the experiment. Sixth, the physiological time course relied on three pooled biological replicates per treatment–time combination, so each measurement integrates over the plants of one replicate rather than resolving individual plants, and with *n* = 3 replicates per cell the statistical power to detect small effects is limited. The five time points are statistically independent—a different set of plants was sampled within each replicate at every event—so the pooling design does not compromise the factorial ANOVA. Seventh, the classification of 60 mmol·L^−1^ as the visible-injury threshold rests on visible injury symptoms and physiological measurements only; mortality, biomass loss, and recovery were not quantified, so the threshold should not be interpreted as a quantitatively determined, LC50-type lethal benchmark. Finally, the use of a congeneric reference genome may have limited annotation completeness for *R. obtusum*-specific loci. These limitations notwithstanding, the convergence of physiology, transcriptomics, and DIA proteomics on a coherent set of pathways provides a defensible mechanistic framework for the alkali response of this acidophilic ornamental.

## 4. Materials and Methods

### 4.1. Plant Materials and Alkali Stress Treatment

#### 4.1.1. Plant Materials and Growth Conditions

Two-year-old cuttings of *R. obtusum* ‘Fenzhuanglou’, selected for uniform growth vigor and the absence of visible pest or disease symptoms, were obtained from the Rhododendron germplasm nursery of the Agricultural College, Liaodong University, Dandong, China (40°11′7.758″ N, 124°21′58.765″ E). The cuttings were cultivated individually in plastic pots (top diameter 14 cm, bottom diameter 9.5 cm, height 12.5 cm) filled with pine-needle soil. The experiment was conducted in a greenhouse under natural lighting without supplemental illumination. Over the March–June experimental period, the natural photoperiod at this latitude (40° N) increased from approximately 12 h to 15 h, and the midday photosynthetic photon flux density at canopy level on clear days ranged from 800 to 1200 µmol·m^−2^·s^−1^. Daytime and nighttime temperatures were maintained at 25 ± 3 °C and 15 ± 3 °C, respectively, and the relative humidity ranged from 60% to 80%. The pine-needle soil substrate had an initial pH of 6.93 and an initial electrical conductivity of 1.150 mS·cm^−1^ (1:5 soil-to-water extract). No fertilizer was applied during the experimental period.

#### 4.1.2. Alkali Stress Treatment

To characterize the concentration-dependent response of *R. obtusum* ‘Fenzhuanglou’ to alkali stress, four alkali concentration treatments were applied from March to June 2025: 0 (distilled-water control, CK), 50, 60, and 70 mmol·L^−1^. The alkaline solution consisted of NaHCO_3_ and Na_2_CO_3_ mixed at a molar ratio of 9:1, and the mixed solutions had a pH of 8.94 ± 0.12. At each application, 500 mL of the corresponding solution (or distilled water for CK) was poured onto the substrate of each plant; the leachate draining from the pot base was collected in a tray and re-applied to the substrate to ensure complete uptake of the salts, so that the full intended dose was delivered to each pot and the cumulative input dose was reproducible across replicates. Treatment concentrations throughout this study refer to the concentrations of the applied input solutions, and the repeated-application design mimics irrigation with alkaline water under field conditions, in which salts progressively accumulate in the rhizosphere. Treatments were applied once every 3 d and served as the sole source of irrigation during the treatment period: apart from these applications no additional watering was given, and the CK plants received 500 mL of distilled water on the same 3 d schedule. Each treatment comprised 3 replicates, with 15 plants per replicate; the 180 plants, selected for uniform growth vigor, were randomly assigned to the four treatment groups, and the replicate (15 plants) served as the experimental unit, with leaves from multiple plants within a replicate pooled for each measurement.

#### 4.1.3. Sample Collection

For the physiological measurements, leaves were sampled from plants of all four concentration treatments at 0, 10, 20, 30, and 40 d of treatment, for a total of five sampling events. All sampling events were performed at a fixed time of day, between 9:00 and 10:00 a.m., to control for diurnal variation in photosynthesis- and circadian-related traits and gene expression; alkali solutions were likewise applied in the morning. At each event, leaves were collected from a different set of plants within each replicate, so that no individual plant contributed leaf tissue at more than one time point; the five time points therefore constitute independent, non-repeated measurements. At each event, approximately 0.3 g of fully expanded leaves from the middle portion of each sampled plant was randomly collected, and the leaves of multiple plants within a replicate were pooled to constitute one biological replicate sample, immediately frozen, and stored at −80 °C until analysis.

For the transcriptomic and proteomic analyses, leaf samples were collected concurrently from the same batch of plants, but only from the CK and 60 mmol·L^−1^ treatments and only at 0 and 40 d. The 60 mmol·L^−1^ concentration was selected because it was identified as the visible-injury threshold in the physiological experiment. This threshold concentration was prioritized because it is the point at which tolerance mechanisms are maximally engaged while the leaf tissue remains alive and acclimating; by contrast, 50 mM caused no visible injury and was expected to yield mainly homeostatic molecular changes, whereas at 70 mM the molecular profile at day 40 would be dominated by non-specific senescence and cell-death signals that would confound the identification of specific tolerance mechanisms. The two omics timepoints were chosen to bracket the chronic phase of the stress response: day 0 provides the pre-stress baseline, and day 40 is the endpoint at which the physiological injury phenotypes had fully differentiated among treatments and at which plants had entered a stabilized acclimation state. Our objective was to characterize the sustained molecular reprogramming underlying chronic alkali acclimation—most directly relevant to long-term cultivation on alkaline soils—rather than the transient early signaling events, which have been widely documented in other species and decay within days of stress onset. The 10-day physiological monitoring series was used to delineate the injury trajectory and to verify that day 40 captured the stabilized endpoint. We acknowledge that intermediate dynamics, such as the transient antioxidant enzyme peak at day 20, are not resolved at the omics level; a fine-scale time-course analysis is identified as a direction for future work. Four sample groups were thereby defined and are referred to throughout this study as follows: CK-0d (water control, day 0), CK-40d (water control, day 40), Treat-0d (60 mmol·L^−1^ alkali, day 0), and Treat-40d (60 mmol·L^−1^ alkali, day 40). The Treat-0d samples were collected immediately after the first alkali application; consequently, differences in Treat-0d vs. CK-0d may reflect batch variation between plants, an immediate early response, or both, and this comparison was interpreted accordingly. Each group comprised three biological replicates (12 samples in total). Leaf tissues were frozen and stored at −80 °C.

### 4.2. Physiological Measurements

#### 4.2.1. Measurement of Photosynthetic Pigment Contents

Photosynthetic pigment contents were determined by ethanol extraction followed by spectrophotometry. Fresh leaf tissue (0.1 g) with the midrib removed was cut into pieces and immersed in 10 mL of 95% ethanol for 24 h in the dark until the tissue was completely bleached. Absorbance of the extract was recorded with a UV-1800 spectrophotometer (Shimadzu, Kyoto, Japan) at 470, 485, 649, and 665 nm. Chlorophyll a (Chl a), chlorophyll b (Chl b), lutein, and total carotenoid contents (mg·g^−1^ FW) were calculated as follows:

Chl a = (9.784 × A_665_ − 0.99 × A_649_) × V/FW (1)

Chl b = (12.6 × A_649_ − 4.65 × A_665_) × V/FW (2)

Lutein = (10.2 × A_470_ − 11.5 × A_485_ − 0.0036 × a − 0.652 × b) × V/FW (3)

Carotene = (12.6 × A_485_ − 6.00 × A_470_ − 0.0298 × a + 0.336 × b) × V/FW (4)

where A_470_, A_485_, A_649_, and A_665_ are the absorbance values at the respective wavelengths; V is the total volume of the extraction solution (L); FW is the fresh weight of the sample (g); and a and b denote the Chl a and Chl b contents, respectively. All measurements were performed with three replicates.

#### 4.2.2. Osmolytes

Soluble sugar and proline contents were determined with commercial assay kits (BC0030 and BC0290, respectively; Solarbio Science & Technology Co., Ltd., Beijing, China) according to the manufacturer’s instructions. Soluble protein content was determined by the Coomassie Brilliant Blue G-250 dye-binding method [30], with bovine serum albumin as the standard; absorbance was recorded at 595 nm.

#### 4.2.3. Assays of Antioxidant Enzyme Activities and Malondialdehyde Content

The activities of superoxide dismutase (SOD), peroxidase (POD), and catalase (CAT) and the content of malondialdehyde (MDA) were determined with commercial assay kits (Solarbio, Beijing, China): BC0170 for SOD, BC0090 for POD, BC0200 for CAT, and BC0020 for MDA. All assays were performed according to the manufacturer’s protocols, and each measurement was performed with three replicates.

### 4.3. RNA-Seq and Transcriptomic Analysis

#### 4.3.1. RNA Extraction, Library Construction, and Sequencing

To profile the transcriptional response of *R. obtusum* ‘Fenzhuanglou’ to alkali stress, total RNA was extracted from the 12 leaf samples (four groups × three biological replicates). Messenger RNA was enriched with Oligo (dT) magnetic beads and used for the construction of paired-end sequencing libraries. Sequencing was performed on an Illumina platform with 150 bp paired-end reads (PE150) by Wuhan Metware Biotechnology Co., Ltd. (Wuhan, China; https://www.metware.cn (accessed on 21 August 2025)).

#### 4.3.2. Read Quality Control, Alignment, and Quantification

Raw reads were quality-filtered with fastp [51] to remove adapter-containing reads, reads with excessive ambiguous bases (N), and low-quality reads, yielding clean data for downstream analysis. The Q30 base percentage and GC content of the clean data were calculated for each sample. Because no chromosome-level reference genome is currently available for *R. obtusum*, clean reads were aligned to the *R. simsii* reference genome (GenBank accession GCA_014282245.1) using HISAT2 [52]; *R. simsii* and *R. obtusum* are closely related evergreen azaleas of *R*. subgen. *Tsutsusi*, and a congeneric reference is the standard practice in azalea transcriptomics when a species-specific genome is lacking. Transcript assembly and quantification were performed with StringTie [53], and gene-level read counts were generated with featureCounts [54]; gene expression levels were normalized as fragments per kilobase of transcript per million mapped reads (FPKM). As quality-control checks, Pearson correlation coefficients among biological replicates were computed from the FPKM values, and principal component analysis (PCA) was performed across all 12 samples.

#### 4.3.3. Differential Expression and Functional Enrichment Analysis

Differentially expressed genes (DEGs) were identified with DESeq2 [31], using the raw gene-level read counts generated by featureCounts (Section 2.3.2) as input, with thresholds of |log_2_fold change (FC)| ≥ 1 and false discovery rate (FDR) < 0.05. FPKM values were used only for sample-level quality control and visualization, not as input for differential expression analysis. The four sample groups (CK/Treat × 0 d/40 d) constitute a 2 × 2 treatment-by-time factorial. Four pairwise comparisons were analyzed: CK-40d vs. CK-0d, Treat-0d vs. CK-0d, Treat-40d vs. CK-40d, and Treat-40d vs. Treat-0d. Within this factorial, Treat-40d vs. CK-40d estimates the simple (alkali-specific) effect at 40 d against age-matched controls, and CK-40d vs. CK-0d estimates the pure time/developmental effect. To test the treatment × time interaction formally rather than by approximation of pairwise contrasts, a negative binomial generalized linear model (NB-GLM) equivalent to the DESeq2 design ~ time + treatment + time:treatment was additionally fitted to the prefiltered count matrix (genes with ≥10 total reads retained; 28,643 genes tested), using DESeq2 median-of-ratios size factors and gene-wise dispersions estimated under the full design; the interaction term was evaluated with a likelihood-ratio test (LRT) against the reduced model~time + treatment (1 df), and the resulting *p* values were Benjamini–Hochberg adjusted. Genes with adjusted *p* < 0.05 and |log2 interaction fold change| ≥ 1 were considered to show a significant and biologically meaningful interaction. Size factors and dispersions were estimated with pydeseq2 v0.5.4; the LRT was computed with a custom NB-GLM implementation validated against independent statsmodels fits. In this model, any baseline imbalance between treatment batches at 0 d is absorbed by the treatment main effect, so interaction signals cannot arise from baseline differences. Gene Ontology (GO) [55] and Kyoto Encyclopedia of Genes and Genomes (KEGG) [33] enrichment analyses of the DEGs were performed with the hypergeometric test implemented in clusterProfiler [32]. Transcription factors (TFs) were predicted and classified with iTAK.

### 4.4. DIA Proteomic Analysis

#### 4.4.1. Protein Extraction and Digestion

To obtain peptides for mass spectrometry, proteins were extracted from the same 12 samples used for the transcriptomic analysis. Leaf tissue was lysed with SDT lysis buffer (4% SDS, 100 mmol·L^−1^ Tris-HCl, 1 mmol·L^−1^ dithiothreitol, pH 7.6), and proteins were recovered by acetone precipitation. The protein solutions were digested with trypsin, and the resulting peptides were desalted on C18 cartridges, dried under vacuum, and stored at −20 °C until liquid chromatography–tandem mass spectrometry (LC-MS/MS) analysis.

#### 4.4.2. LC-MS/MS Data Acquisition

Peptides were separated on a Vanquish Neo ultra-high-performance liquid chromatography (UHPLC) system (Thermo Fisher Scientific, Waltham, MA, USA) coupled online to an Orbitrap Astral mass spectrometer (Thermo Fisher Scientific, Waltham, MA, USA) operated in data-independent acquisition (DIA) mode. The DIA covered a precursor mass range of 380–980 m/z using 299 variable isolation windows with an isolation width of 2 Th.

#### 4.4.3. Database Search, Quantification, and Enrichment Analysis

DIA raw files were processed with DIA-NN v1.8.1 [34] in library-free mode against the UniProt reference proteome (proteome ID UP000626092; 32,714 protein sequences). Match-between-runs (MBR) was enabled, and both precursor-level and protein-level FDR were controlled below 1%. Protein intensities were quantified with the MaxLFQ algorithm. Differentially expressed proteins (DEPs) were defined as proteins with FC ≥ 1.5 or ≤0.6667 and *p* ≤ 0.05 for the same four pairwise comparisons as in the transcriptomic analysis. Defining DEPs by combining a fold-change threshold with an uncorrected Student’s *t*-test *p* value, rather than an FDR-adjusted criterion, follows established practice in quantitative plant proteomics, in which protein-level abundance changes are typically modest [56,57]; in the present pipeline, stringency was ensured at the identification stage (FDR < 1% at both the precursor and protein levels) and by the relatively strict FC threshold (≥1.5). In addition, as a sensitivity analysis, Benjamini–Hochberg (BH) FDR correction was applied to the *p* values of all quantified proteins within each comparison, and DEP counts were re-derived at q ≤ 0.05 and q ≤ 0.10 with the same FC thresholds (Supplementary Table S4). The nominal-*p* DEP set is retained as the primary criterion for comparability with standard quantitative plant-proteomics practice, but it is explicitly treated as exploratory and hypothesis-generating throughout this study, and the BH-adjusted subset (q ≤ 0.05) constitutes the confirmatory core of the proteomic results. Within-group replicate correlations (Pearson) were computed as a quality-control step. Functional annotation and enrichment analyses of the DEPs were performed with clusterProfiler (v4.6.0) [32] against the GO [55], KEGG [33], euKaryotic Orthologous Groups (KOG), and InterPro (IPR) databases.

### 4.5. Transcriptome–Proteome Integration

#### 4.5.1. Correlation, Nine-Quadrant, and Common-KEGG Analysis

To integrate the two omics datasets, protein identifiers were mapped to their corresponding gene identifiers. For each sample group, the correlation between transcript and protein abundance was assessed by Pearson correlation analysis of log-transformed expression values. For each pairwise comparison, the log_2_FC values of transcripts and proteins were jointly evaluated in a nine-quadrant analysis to classify genes according to the concordance of their mRNA and protein changes; the coefficient of determination (R^2^) of the linear fit between the two fold-change vectors was used to quantify the overall concordance in each comparison. KEGG pathways significantly enriched in both the transcriptome and the proteome were identified to define common pathways.

#### 4.5.2. PTR Calculation

To assess post-transcriptional regulation, a PTR score was calculated for each gene and sample group as the difference in within-group z-scores, PTR = z(log_2_ LFQ) − z(log_2_ FPKM), where log_2_-transformed mean LFQ intensity and mean FPKM of the three biological replicates were each standardized (z-scored) across all genes quantified in that group. This z-score-difference PTR is a dimensionless, scale-free metric that avoids the mathematical artifact of the previously used ratio of log-transformed abundances (log_2_LFQ/log_2_FPKM), whose value is distorted by the arbitrary zero of the log scale; the z-score formulation expresses, for each gene, how far its protein abundance rank deviates from its transcript abundance rank within the same group. Genes were retained when the protein was detected (LFQ > 0) in at least two of the three replicates and the transcript had mean FPKM ≥ 1 in that group (6510–6726 genes per group). Genes whose PTR fell beyond the median ± 1 standard deviation (SD) of the corresponding group distribution were defined as extreme-PTR genes; this distribution-based heuristic nominates the upper and lower tails (approximately the outer 16% per tail under approximate normality) as candidates with unusually high or low protein output per transcript, and applying the criterion independently within each group prevents group-level shifts from confounding the definition. We emphasize that PTR is used only as a relative, group-internal ranking metric of protein output per transcript—jointly reflecting translation efficiency, protein stability, and turnover—and is not a direct measure of translation efficiency. A stricter robust cutoff (median ± 2 × MAD) yielded extreme-PTR gene sets fully nested within the SD-based sets (100% nested; 22–28% of their size), indicating that the tail-ranking structure is insensitive to the choice of dispersion measure. Extreme-PTR genes were subjected to GO and KEGG enrichment analysis; overlaps between differentially expressed genes/proteins and extreme-PTR genes were assessed with Fisher’s exact test (2 × 2 tables; genes differentially expressed at either layer versus extreme-PTR status in the respective group).

### 4.6. Statistical Analysis

Physiological data were organized in Microsoft Excel. Because the experiment combined four alkali concentrations with five sampling times, the full data set of each physiological indicator was first analyzed by two-way analysis of variance (ANOVA) with treatment concentration, sampling time, and their interaction as fixed factors. Because each sampling event collected leaves from a different set of plants within each replicate, observations at the five time points are statistically independent rather than repeated measurements of the same individuals; a two-way factorial ANOVA is therefore the appropriate model, and no repeated-measures correction or subject-level random effect is required. Treatment means within each sampling time were then compared by one-way ANOVA followed by Duncan’s multiple range test, and differences were considered significant at *p* < 0.05. Analyses were performed with IBM SPSS Statistics 26. The experimental unit was the replicate: each treatment comprised 3 replicates of 15 plants each, and each measurement was performed on a pooled leaf sample collected from the plants of one replicate (*n* = 3 replicates). Figures were generated with GraphPad Prism 8.

### 4.7. qRT-PCR Validation of RNA-Seq Data

To validate the RNA-seq results, fifteen DEGs were selected for quantitative real-time PCR (qRT-PCR) analysis (Table 9). Total RNA was re-extracted from the same leaf samples used for RNA-seq (three biological replicates per group) with TRIzol reagent. First-strand cDNA was synthesized with the PrimeScript^TM^ RT reagent Kit with gDNA Eraser (TaKaRa, Dalian, China), and residual genomic DNA was removed during reverse transcription. qRT-PCR was performed with SYBR^®^ Premix Ex Taq^TM^ II (TaKaRa) on a QuantStudio^TM^ 3 Real-Time PCR System (Applied Biosystems, Foster City, CA, USA). Gene-specific primers (Table 9) were designed from the coding sequences of the *R. simsii* reference genome (GCA_014282245.1) with Primer3 (v2.3.0), targeting amplicons of 80–200 bp, a melting temperature (Tm) of 58–62 °C with ≤1 °C difference within pairs, and a GC content of 40–60%. *RoEF1-*α (elongation factor 1-α) served as the internal reference gene. *RoEF1-α* was chosen because *EF1-α* has been validated as one of the most stably expressed reference genes under abiotic stress in multiple plant species, including salt-stressed potato [58,59]. Consistent with this, *EF1-α* transcripts were constitutively expressed in all twelve RNA-seq samples of this study, and the two most abundant *EF1-α* homologs varied by less than two-fold among the four treatment groups, supporting the suitability of *RoEF1-α* as a reference under the present experimental conditions. Each sample was analyzed with three biological and three technical replicates, and amplification specificity was verified by melt-curve analysis. Relative expression levels were calculated using the 2^−ΔΔCT^ method [60].

## 5. Conclusions

To our knowledge, this is the first integrated physiological, transcriptomic, and proteomic characterization of the alkali stress response of *R. obtusum* ‘Fenzhuanglou’. A graded mixed NaHCO_3_/Na_2_CO_3_ pot experiment was combined with parallel transcriptome and DIA proteome profiling of the same leaf samples, covering a 40-day stress time course. The tolerance threshold of this cultivar was defined for the first time. Plants tolerated 50 mmol·L^−1^ without visible injury through 40 d, reached the visible-injury threshold at 60 mmol·L^−1^—the concentration adopted for omics sampling—and suffered severe, progressive damage at 70 mmol·L^−1^. The physiological time course of pigments, osmolytes, antioxidant enzymes, and malondialdehyde revealed a reproducible temporal defense pattern. Early chlorophyll b depletion and transient mobilization of antioxidant enzymes (SOD/POD/CAT, peaking at 20 d), lutein, and proline (both peaking at 30 d) gave way to soluble-sugar-dominated osmotic adjustment. By day 40, however, the antioxidant system had collapsed at higher concentrations: POD fell below the control in all alkali treatments and CAT fell significantly below the control at 60–70 mmol·L^−1^. Malondialdehyde accumulated dose-dependently, reaching ~22 nmol·g^−1^ at 70 mmol·L^−1^. This work also generated the transcriptome (90.41 Gb clean data) and DIA proteome (7452 quantified proteins) of the cultivar. In Treat-40d vs. CK-40d, 7214 differentially expressed genes (3687 up, 3527 down) and 1093 differentially expressed proteins (558 up, 535 down) were identified. The two omics layers jointly implicated MAPK and plant hormone signal transduction, and each layer independently supported the suppression of photosynthesis and porphyrin metabolism. Key signaling components included an induced MAPK gene (*RoMAPK3*, log_2_FC = 2.29) and a repressed ethylene receptor (log_2_FC = −6.40), DELLA protein (*RoDELLA*, log_2_FC = −3.26), and ACC oxidase (FC = 0.313). At the protein level, flavonoid and flavone/flavonol biosynthesis and α-linolenic acid metabolism were enriched, with chalcone synthase up-regulated (FC = 2.67). The modest transcript–protein correlation (ρ = 0.14–0.20), together with PTR analysis, is consistent with transcript–protein decoupling potentially involving post-transcriptional regulation, protein stability, and turnover, and fifteen representative DEGs were subjected to qRT-PCR validation. These findings support an integrated working model in which the 60 mmol·L^−1^ visible-injury threshold marks the compensation limit of a multi-layered defense network spanning osmotic, redox, photoprotective, and signaling modules. Beyond this point, the network can no longer offset the combined osmotic, ionic, and high-pH burdens of alkali stress. This model is proposed as a testable hypothesis, and the future work outlined below is designed to validate or refute its components. Future work should extend validation of key candidates to the protein level by parallel reaction monitoring and profile root tissue as the primary site of alkali perception. The threshold should also be tested across a multi-cultivar germplasm panel, and candidate genes such as glutathione S-transferases and chalcone synthase should be functionally characterized to support the breeding of alkali-tolerant *Rhododendron* cultivars.

## Figures and Tables

**Table 1:** Summary of transcriptome sequencing quality and read mapping for the 12 libraries.

Metric	Value
Total clean data	90.41 Gb
Clean data per sample	6.39–8.69 Gb
Q30 (%)	93.51–93.78
GC content (%)	46.68–47.88
Total mapping rate (%)	76.87–80.05
Uniquely mapped reads (%)	72.74–76.40

**Table 2:** Statistics of DEGs in the four pairwise comparisons.

Comparison	Total DEGs	Up-Regulated	Down-Regulated
Treat-0d vs. CK-0d	2441	1372	1069
CK-40d vs. CK-0d	9041	3737	5304
Treat-40d vs. CK-40d	7214	3687	3527
Treat-40d vs. Treat-0d	9211	4405	4806

**Table 3:** Representative differentially expressed genes in Treat-40d vs. CK-40d.

Gene ID	Annotation	log_2_FC	Regulation	KEGG Pathway
RHSIM_Rhsim02G0029100	Early light-induced protein 1, chloroplastic (*ELIP*)	8.97	up	–
RHSIM_Rhsim02G0132400	Uncharacterized protein (YtxH-like domain)	5.97	up	–
RHSIM_Rhsim02G0032900	Transcription factor bHLH92	5.14	up	–
RHSIM_Rhsim01G0273700	Late embryogenesis abundant protein D-34 (*LEA*)	5.09	up	–
RHSIM_Rhsim01G0192800	Glucan endo-1,3-β-glucosidase	4.94	up	Starch and sucrose metabolism
RHSIM_Rhsim05G0004400	Ethylene-responsive transcription factor ERF017 (*AP2/ERF*)	3.63	up	–
RHSIM_Rhsim02G0106400	Lactoylglutathione lyase (*RoGLO1*)	2.65	up	Pyruvate metabolism
RHSIM_Rhsim02G0088400	Galactinol–sucrose galactosyltransferase 2 (*RFS2*)	2.60	up	Galactose metabolism
RHSIM_Rhsim01G0221500	Mitogen-activated protein kinase (*RoMAPK3*)	2.29	up	MAPK signaling pathway–plant
RHSIM_Rhsim12G0019500	Tify domain-containing protein (*RoTIFY2*)	2.22	up	Plant hormone signal transduction
RHSIM_Rhsim08G0018400	DELLA protein (*RoDELLA*)	−3.26	down	Plant hormone signal transduction
RHSIM_Rhsim03G0192300	WRKY transcription factor 22 (*RoWRKY1*)	−3.30	down	MAPK signaling pathway–plant; Plant–pathogen interaction
RHSIM_Rhsim01G0246400	Cinnamyl alcohol dehydrogenase 1 (*CAD1*)	−3.56	down	Phenylpropanoid biosynthesis
RHSIM_Rhsim03G0208000	CONSTANS-like zinc finger protein 3 (*RoCCT*)	−3.72	down	Circadian rhythm–plant
RHSIM_Rhsim02G0230000	Glutamate–cysteine ligase, chloroplastic (*RoGCL*)	−4.16	down	Cysteine and methionine metabolism; Glutathione metabolism
RHSIM_Rhsim12G0021700	NAC domain-containing protein 83	−4.24	down	–
RHSIM_Rhsim10G0167600	Homeobox–leucine zipper protein ATHB-6 (*HD-ZIP*)	−5.48	down	–
RHSIM_Rhsim04G0188600	Ethylene receptor 2 (*ETR2*)	−6.40	down	MAPK signaling pathway–plant; Plant hormone signal transduction
RHSIM_Rhsim01G0145400	Extracellular ribonuclease LE (RNase T2 family)	−8.21	down	–

**Table 4:** Significantly enriched KEGG pathways of DEGs.

Comparison	KEGG Pathway	DEG Count (Up/Down)	Adjusted *p*
Treat-0d vs. CK-0d	Biosynthesis of secondary metabolites	305 (231/74)	1.23 × 10^−15^
Treat-0d vs. CK-0d	Metabolic pathways	407 (268/139)	2.03 × 10^−8^
Treat-0d vs. CK-0d	Circadian rhythm–plant	40 (27/13)	3.95 × 10^−6^
Treat-0d vs. CK-0d	Phenylpropanoid biosynthesis	48 (38/10)	1.69 × 10^−4^
Treat-0d vs. CK-0d	Flavonoid biosynthesis	28 (27/1)	1.17 × 10^−3^
Treat-0d vs. CK-0d	Indole alkaloid biosynthesis	7 (7/0)	1.17 × 10^−2^
Treat-0d vs. CK-0d	Flavone and flavonol biosynthesis	10 (10/0)	1.18 × 10^−2^
CK-40d vs. CK-0d	Photosynthesis	43 (2/41)	4.89 × 10^−10^
CK-40d vs. CK-0d	Metabolic pathways	1299 (528/771)	2.92 × 10^−9^
CK-40d vs. CK-0d	Biosynthesis of secondary metabolites	846 (378/468)	4.57 × 10^−9^
CK-40d vs. CK-0d	Plant–pathogen interaction	502 (85/417)	5.05 × 10^−9^
CK-40d vs. CK-0d	Porphyrin metabolism	48 (10/38)	1.35 × 10^−8^
CK-40d vs. CK-0d	Carbon metabolism	150 (62/88)	1.15 × 10^−4^
CK-40d vs. CK-0d	Carbon fixation by Calvin cycle	39 (7/32)	1.78 × 10^−3^
CK-40d vs. CK-0d	Photosynthesis–antenna proteins	13 (0/13)	4.29 × 10^−3^
Treat-40d vs. CK-40d	Biosynthesis of secondary metabolites	671 (308/363)	5.97 × 10^−9^
Treat-40d vs. CK-40d	Circadian rhythm–plant	74 (35/39)	7.54 × 10^−5^
Treat-40d vs. CK-40d	MAPK signaling pathway–plant	187 (93/94)	6.77 × 10^−4^
Treat-40d vs. CK-40d	Plant hormone signal transduction	301 (135/166)	4.18 × 10^−3^
Treat-40d vs. CK-40d	Metabolic pathways	965 (499/466)	1.05 × 10^−2^
Treat-40d vs. Treat-0d	Photosynthesis	38 (3/35)	5.03 × 10^−7^
Treat-40d vs. Treat-0d	Plant–pathogen interaction	455 (117/338)	5.26 × 10^−5^
Treat-40d vs. Treat-0d	Carbon fixation by Calvin cycle	39 (8/31)	4.31 × 10^−4^
Treat-40d vs. Treat-0d	Circadian rhythm–plant	83 (28/55)	5.53 × 10^−4^
Treat-40d vs. Treat-0d	MAPK signaling pathway–plant	221 (72/149)	1.14 × 10^−3^
Treat-40d vs. Treat-0d	Photosynthesis–antenna proteins	13 (0/13)	2.26 × 10^−3^
Treat-40d vs. Treat-0d	Plant hormone signal transduction	361 (108/253)	4.84 × 10^−3^
Treat-40d vs. Treat-0d	Metabolic pathways	1167 (473/694)	4.09 × 10^−2^

**Table 5:** Statistics of DEPs in the four pairwise comparisons.

Comparison	Up-Regulated	Down-Regulated	Total DEPs
Treat-0d vs. CK-0d	180	87	267
CK-40d vs. CK-0d	400	452	852
Treat-40d vs. CK-40d	558	535	1093
Treat-40d vs. Treat-0d	561	716	1277

**Table 6:** Representative differentially expressed proteins in Treat-40d vs. CK-40d. FDR denotes the Benjamini–Hochberg-adjusted q value; entries not meeting q ≤ 0.05 are supported only at nominal *p* ≤ 0.05 and should be regarded as tentative.

Gene	Description	FC (Treat/CK)	*p*-Value	FDR	Regulation
RHSIM_RhsimUnG0066200	Fe2OG dioxygenase domain-containing protein	19.14	8.68 × 10^−4^	1.41 × 10^−2^	up
RHSIM_RhsimUnG0070500	Cytochrome P450	7.42	1.40 × 10^−3^	1.78 × 10^−2^	up
RHSIM_RhsimUnG0141100	Major facilitator superfamily (MFS) protein	6.67	1.28 × 10^−2^	6.02 × 10^−2^	up
RHSIM_Rhsim13G0033100	Non-haem dioxygenase N-terminal domain protein	5.26	4.36 × 10^−2^	1.30 × 10^−1^	up
RHSIM_RhsimUnG0231700	CBS domain-containing protein	3.90	9.02 × 10^−4^	1.42 × 10^−2^	up
RHSIM_Rhsim13G0059400	Cytochrome P450	3.45	4.37 × 10^−2^	1.30 × 10^−1^	up
RHSIM_RhsimUnG0197600	UspA domain-containing protein	2.83	2.30 × 10^−5^	2.68 × 10^−3^	up
RHSIM_RhsimUnG0180800	Chalcone synthase	2.67	7.39 × 10^−4^	1.29 × 10^−2^	up
RHSIM_RhsimUnG0211100	Glutathione S-transferase	0.209	2.78 × 10^−3^	2.56 × 10^−2^	down
RHSIM_RhsimUnG0210300	Glutathione S-transferase	0.275	2.40 × 10^−2^	8.92 × 10^−2^	down
RHSIM_RhsimUnG0169900	Protein kinase domain-containing protein	0.275	2.35 × 10^−2^	8.81 × 10^−2^	down
RHSIM_Rhsim13G0149200	1-aminocyclopropane-1-carboxylate oxidase (ACC oxidase)	0.313	3.20 × 10^−5^	3.16 × 10^−3^	down
RHSIM_RhsimUnG0158600	Protein kinase domain-containing protein	0.332	2.56 × 10^−3^	2.45 × 10^−2^	down
RHSIM_RhsimUnG0206600	alpha,alpha-trehalose-phosphate synthase (UDP-forming)	0.335	4.58 × 10^−2^	1.33 × 10^−1^	down
RHSIM_RhsimUnG0047600	Protein kinase domain-containing protein	0.339	5.07 × 10^−3^	3.56 × 10^−2^	down
RHSIM_RhsimUnG0075900	Bifunctional inhibitor/plant lipid transfer protein	0.359	5.74 × 10^−3^	3.79 × 10^−2^	down

**Table 7:** Significantly enriched KEGG pathways of DEPs.

Comparison	KEGG Pathway	DEPs/Total	FDR
Treat-0d vs. CK-0d	Protein processing in endoplasmic reticulum	16/136	1.98 × 10^−2^
Treat-0d vs. CK-0d	Flavonoid biosynthesis	8/136	1.98 × 10^−2^
CK-40d vs. CK-0d	Starch and sucrose metabolism	33/397	6.3 × 10^−5^
CK-40d vs. CK-0d	Phenylpropanoid biosynthesis	27/397	6.3 × 10^−5^
CK-40d vs. CK-0d	Biosynthesis of various plant secondary metabolites	21/397	9.0 × 10^−5^
CK-40d vs. CK-0d	Cyanoamino acid metabolism	15/397	9.0 × 10^−5^
CK-40d vs. CK-0d	Flavonoid biosynthesis	16/397	8.1 × 10^−4^
CK-40d vs. CK-0d	MAPK signaling pathway–plant	28/397	1.13 × 10^−2^
CK-40d vs. CK-0d	Flavone and flavonol biosynthesis	6/397	2.07 × 10^−2^
CK-40d vs. CK-0d	Anthocyanin biosynthesis	6/397	3.03 × 10^−2^
Treat-40d vs. CK-40d	Flavone and flavonol biosynthesis	11/511	1.0 × 10^−6^
Treat-40d vs. CK-40d	Flavonoid biosynthesis	22/511	9.0 × 10^−6^
Treat-40d vs. CK-40d	Biosynthesis of various plant secondary metabolites	21/511	4.09 × 10^−3^
Treat-40d vs. CK-40d	MAPK signaling pathway–plant	32/511	2.81 × 10^−2^
Treat-40d vs. CK-40d	Photosynthesis	15/511	2.81 × 10^−2^
Treat-40d vs. CK-40d	α-Linolenic acid metabolism	14/511	2.81 × 10^−2^
Treat-40d vs. CK-40d	Phenylalanine, tyrosine and tryptophan biosynthesis	13/511	4.80 × 10^−2^
Treat-40d vs. Treat-0d	MAPK signaling pathway–plant	45/575	3.5 × 10^−5^
Treat-40d vs. Treat-0d	Plant hormone signal transduction	50/575	1.72 × 10^−2^
Treat-40d vs. Treat-0d	Starch and sucrose metabolism	35/575	1.72 × 10^−2^
Treat-40d vs. Treat-0d	Biosynthesis of various plant secondary metabolites	21/575	1.79 × 10^−2^
Treat-40d vs. Treat-0d	Porphyrin metabolism	17/575	3.31 × 10^−2^

**Table 8:** KEGG pathways commonly enriched in both transcriptome and proteome.

Comparison	KEGG Pathway	Transcriptome Adjusted *p*	Proteome FDR
Treat-40d vs. CK-40d	MAPK signaling pathway–plant	6.77 × 10^−4^	2.81 × 10^−2^
Treat-40d vs. Treat-0d	MAPK signaling pathway–plant	1.14 × 10^−3^	3.5 × 10^−5^
Treat-40d vs. Treat-0d	Plant hormone signal transduction	4.84 × 10^−3^	1.72 × 10^−2^

**Table 9:** Candidate genes selected for qRT-PCR validation of the RNA-seq data and their primer sequences.

Gene	Forward Primer (5′→3′)	Reverse Primer (5′→3′)
*RoMAPK3*	CGGAGGGCAGTTCATTCAGT	ACCGCAACCATCTCATTCGT
*RoERF110*	GGGCAGAAGAGAGAGAGGGA	CCGGACGCCTCTGTATCTTC
*RoWRKY1*	ACAAACGATGGTGCAAGTGC	ATCTGACGCACCAACTTCGT
*RoWRKY2*	GCATAACCACCTCCAACCCA	GCTCGAGACCGAACAAGGAA
*RoDELLA*	CTACTTCGCCGAAGCCCTAG	TACGGGCAGGTCTCGTAGAA
*RoTIFY1*	CTTCTCTTGGACCTGCTGCA	ACTTTCCTTGCGCTTCTCGA
*RoTIFY2*	AGAGGTTTCTGGAGAAGCGC	GGACCGATAACTTGGTGCCA
*RoCCT*	CCAAAGAGTGTCAGCTCGGA	TCATCACCAAGGCCCATTCC
*RoRS*	GAGCAATACGGGTCGTCGAT	GCTATCGCATCGGTCTTCCA
*RoGLO1*	GGCAGAGAGACATTCCGGAG	GCCCAAAGCCAGTTCCAATG
*RoGCL*	GGCTGCAGCTTTGTTTCCAA	TCTGCATTGTCCCTGTCACC
*RoQORH*	AGGGAAGCTCAAGACGGTTG	TAGCGTGACCATCGATGCTC
*RoERF1*	AATGCTTCTCTTCGGGCTCC	AATCCCTTATCTCGGCAGCG
*RoERF2*	CTGTGTCTCCACCTTCGACC	TACAGGTTCTTGCGTTGCCT
*RoNAC*	ACTGGAAGGCTACCGGATCT	CAGCTTTGGGACAGCACAAC
*RoEF1-*α	AAGGGAGGCAGCGAATTTCA	TTTCGGCAAACTTGACAGCG

## Data Availability

The transcriptome sequencing data reported in this paper have been deposited in the NCBI Sequence Read Archive (SRA) under BioProject accession number PRJNA1500028. The mass spectrometry proteomics data have been deposited to the ProteomeXchange Consortium via the iProX partner repository with the dataset identifier PXD082840. All other data supporting the findings of this study are included within this article.

## Supplementary Materials

The following supporting information can be downloaded at: https://www.mdpi.com/article/10.3390/plants15193053/s1, Table S1: Time courses of physiological indicators in leaves of *Rhododendron obtusum* ‘Fenzhuanglou’ seedlings under alkali stress (NaHCO_3_:Na_2_CO_3_ = 9:1 molar ratio, pH 8.94 ± 0.12; CK, 50, 60 and 70 mmol·L^−1^, applied once every 3 d for 40 d); Table S2: Two-way ANOVA (treatment concentration × sampling time) of the eleven physiological indicators (F values; degrees of freedom in parentheses; error df = 40); Table S3: Full results of the treatment × time interaction likelihood-ratio test (28,643 genes); Table S4: Benjamini–Hochberg FDR sensitivity analysis of DEP sets in the four pairwise comparisons; Table S5: Recomputed z-score-based PTR values, extreme-PTR gene lists, and Fisher’s exact tests.
