# Supplementary material for: Integrated Physiological, Transcriptomic, and Proteomic Analyses Reveal Alkali Stress Response and Tolerance Mechanisms of Rhododendron obtusum ‘Fenzhuanglou’ Seedlings
Source: Plants (Basel). 2026 Oct 6;15(19):3053. doi: 10.3390/plants15193053 (PMC13666605; doi:10.3390/plants15193053)
Supplement: Supplementary file 1 [file plants-15-03053-s001.zip › Supplementary Table S5.pdf]

Supplementary Table S5. Recomputed z-score-based PTR values, extreme-PTR gene lists, and Fisher's exact tests.

Group	Gene	PTR (z-score difference)	z(log2 LFQ)	z(log2 FPKM)	extreme-PTR
CK-0d	RHSIM_RhsimUnG0249100	-3.103	0.149	3.252	low
CK-0d	RHSIM_RhsimUnG0254800	-2.531	-0.831	1.700	low
CK-0d	RHSIM_RhsimUnG0258600	2.807	2.909	0.102	high
CK-0d	RHSIM_RhsimUnG0249600	-1.969	-0.432	1.537	low
CK-0d	RHSIM_RhsimUnG0251500	0.637	0.133	-0.504	no
CK-0d	RHSIM_RhsimUnG0252300	0.608	-0.122	-0.730	no
CK-0d	RHSIM_RhsimUnG0228100	0.173	-1.567	-1.740	no
CK-0d	RHSIM_RhsimUnG0251200	-0.811	0.468	1.279	no
CK-0d	RHSIM_RhsimUnG0226800	0.505	-0.587	-1.092	no
CK-0d	RHSIM_RhsimUnG0245900	1.582	3.519	1.937	high
CK-0d	RHSIM_RhsimUnG0241900	1.733	0.768	-0.965	high
CK-0d	RHSIM_RhsimUnG0250300	-3.878	-1.079	2.799	low
CK-0d	RHSIM_RhsimUnG0250600	-0.067	-0.084	-0.017	no
CK-0d	RHSIM_RhsimUnG0216900	0.489	1.511	1.022	no
CK-0d	RHSIM_RhsimUnG0239100	1.699	0.351	-1.349	high
CK-0d	RHSIM_RhsimUnG0233600	-0.566	-2.530	-1.963	no
CK-0d	RHSIM_RhsimUnG0212900	2.325	0.227	-2.098	high
CK-0d	RHSIM_RhsimUnG0231900	1.001	-0.241	-1.242	no
CK-0d	RHSIM_RhsimUnG0209400	1.488	0.937	-0.551	high
CK-0d	RHSIM_RhsimUnG0228800	2.090	0.250	-1.840	high
CK-0d	RHSIM_RhsimUnG0219900	0.490	-0.909	-1.399	no
CK-0d	RHSIM_RhsimUnG0217500	1.469	0.541	-0.929	high
CK-0d	RHSIM_RhsimUnG0226200	0.032	0.148	0.117	no
CK-0d	RHSIM_RhsimUnG0210000	0.809	0.243	-0.566	no
CK-0d	RHSIM_RhsimUnG0190800	1.360	0.325	-1.035	high
CK-0d	RHSIM_RhsimUnG0217400	0.360	-0.229	-0.589	no
CK-0d	RHSIM_RhsimUnG0220800	0.566	-0.412	-0.978	no
CK-0d	RHSIM_RhsimUnG0253500	2.257	1.706	-0.550	high
CK-0d	RHSIM_RhsimUnG0248900	0.525	0.969	0.444	no
CK-0d	RHSIM_RhsimUnG0211400	-0.138	1.221	1.359	no
CK-0d	RHSIM_RhsimUnG0209900	2.422	0.644	-1.778	high

Group	Gene	PTR (z-score difference)	z(log2 LFQ)	z(log2 FPKM)	extreme-PTR
CK-0d	RHSIM_RhsimUnG0205800	-0.307	1.726	2.033	no
CK-0d	RHSIM_Rhsim09G0148400	-2.199	-0.632	1.568	low
CK-0d	RHSIM_RhsimUnG0209800	0.371	-0.338	-0.709	no
CK-0d	RHSIM_RhsimUnG0225600	-0.818	-0.327	0.491	no
CK-0d	RHSIM_RhsimUnG0255000	-0.854	0.595	1.450	no
CK-0d	RHSIM_RhsimUnG0156400	0.505	-1.774	-2.279	no
CK-0d	RHSIM_RhsimUnG0205900	-1.215	0.945	2.160	low
CK-0d	RHSIM_RhsimUnG0197600	2.554	0.704	-1.850	high
CK-0d	RHSIM_RhsimUnG0206500	0.233	-0.806	-1.039	no
CK-0d	RHSIM_Rhsim09G0069900	1.621	1.841	0.220	high
CK-0d	RHSIM_RhsimUnG0169900	-0.259	-0.823	-0.563	no
CK-0d	RHSIM_RhsimUnG0208500	-0.712	-1.652	-0.940	no
CK-0d	RHSIM_RhsimUnG0196500	-1.037	-1.299	-0.262	low
CK-0d	RHSIM_RhsimUnG0185400	0.113	-0.264	-0.377	no
CK-0d	RHSIM_RhsimUnG0158200	1.607	1.712	0.105	high
CK-0d	RHSIM_RhsimUnG0197500	1.427	-0.279	-1.706	high
CK-0d	RHSIM_Rhsim09G0021500	1.442	1.607	0.165	high
CK-0d	RHSIM_RhsimUnG0136600	0.190	0.333	0.143	no
CK-0d	RHSIM_RhsimUnG0226700	0.687	-0.334	-1.021	no
CK-0d	RHSIM_RhsimUnG0126200	1.404	1.048	-0.355	high
CK-0d	RHSIM_RhsimUnG0217000	0.521	-0.284	-0.805	no
CK-0d	RHSIM_RhsimUnG0109300	0.958	1.517	0.559	no
CK-0d	RHSIM_RhsimUnG0122900	1.222	-0.152	-1.374	high
CK-0d	RHSIM_RhsimUnG0158600	1.520	0.215	-1.305	high
CK-0d	RHSIM_RhsimUnG0137100	0.849	0.494	-0.355	no
CK-0d	RHSIM_RhsimUnG0190300	1.019	-0.227	-1.245	no
CK-0d	RHSIM_RhsimUnG0256800	0.175	0.089	-0.085	no
CK-0d	RHSIM_RhsimUnG0185700	0.004	-1.804	-1.808	no
CK-0d	RHSIM_RhsimUnG0180800	-0.537	0.015	0.552	no
CK-0d	RHSIM_RhsimUnG0124400	1.716	-0.321	-2.037	high
CK-0d	RHSIM_RhsimUnG0254600	-1.568	-0.977	0.591	low
CK-0d	RHSIM_RhsimUnG0242200	-0.753	-0.811	-0.058	no

Group	Gene	PTR (z-score difference)	z(log2 LFQ)	z(log2 FPKM)	extreme-PTR
CK-0d	RHSIM_RhsimUnG0253800	-0.508	-1.113	-0.605	no
CK-0d	RHSIM_RhsimUnG0093800	0.057	-0.066	-0.124	no
CK-0d	RHSIM_RhsimUnG0257500	1.889	0.740	-1.150	high
CK-0d	RHSIM_RhsimUnG0118900	-0.248	1.340	1.587	no
CK-0d	RHSIM_RhsimUnG0120200	-0.379	-0.813	-0.434	no
CK-0d	RHSIM_RhsimUnG0111200	-0.131	-1.108	-0.977	no
CK-0d	RHSIM_RhsimUnG0092300	0.127	0.888	0.761	no
CK-0d	RHSIM_RhsimUnG0085600	2.429	0.218	-2.212	high
CK-0d	RHSIM_RhsimUnG0124200	0.080	-0.709	-0.790	no
CK-0d	RHSIM_RhsimUnG0094500	0.444	0.221	-0.223	no
CK-0d	RHSIM_RhsimUnG0059700	0.449	-0.205	-0.654	no
CK-0d	RHSIM_RhsimUnG0145200	0.473	-1.207	-1.680	no
CK-0d	RHSIM_RhsimUnG0118400	-1.547	-2.169	-0.622	low
CK-0d	RHSIM_RhsimUnG0138800	2.435	1.114	-1.321	high
CK-0d	RHSIM_RhsimUnG0054600	-1.152	-0.596	0.556	low
CK-0d	RHSIM_RhsimUnG0216100	-2.795	-0.219	2.576	low
CK-0d	RHSIM_RhsimUnG0141400	-0.634	-2.076	-1.443	no
CK-0d	RHSIM_RhsimUnG0213100	-0.349	-1.444	-1.094	no
CK-0d	RHSIM_RhsimUnG0112300	0.201	-1.420	-1.621	no
CK-0d	RHSIM_RhsimUnG0112800	1.204	-0.789	-1.992	high
CK-0d	RHSIM_RhsimUnG0111000	-0.397	-0.521	-0.124	no
CK-0d	RHSIM_RhsimUnG0205000	0.208	1.571	1.363	no
CK-0d	RHSIM_RhsimUnG0221600	0.374	0.938	0.564	no
CK-0d	RHSIM_RhsimUnG0060900	-0.344	-0.238	0.106	no
CK-0d	RHSIM_RhsimUnG0195900	0.342	-0.927	-1.269	no
CK-0d	RHSIM_RhsimUnG0215100	1.407	-0.170	-1.577	high
CK-0d	RHSIM_RhsimUnG0124800	0.210	-0.961	-1.172	no
CK-0d	RHSIM_RhsimUnG0098600	0.978	-0.189	-1.167	no
CK-0d	RHSIM_RhsimUnG0055200	-0.118	-0.082	0.036	no
CK-0d	RHSIM_RhsimUnG0096900	0.768	0.178	-0.590	no
CK-0d	RHSIM_RhsimUnG0119200	-0.313	-0.289	0.024	no
CK-0d	RHSIM_RhsimUnG0032200	-0.726	-0.943	-0.217	no

Group	Gene	PTR (z-score difference)	z(log2 LFQ)	z(log2 FPKM)	extreme-PTR
CK-0d	RHSIM_RhsimUnG0119600	-1.012	-0.809	0.203	no
CK-0d	RHSIM_RhsimUnG0087900	2.094	1.403	-0.690	high
CK-0d	RHSIM_RhsimUnG0206600	0.334	0.022	-0.312	no
CK-0d	RHSIM_RhsimUnG0086700	0.698	-0.977	-1.675	no
CK-0d	RHSIM_RhsimUnG0065700	0.650	0.087	-0.563	no
CK-0d	RHSIM_RhsimUnG0113700	0.312	-0.491	-0.803	no
CK-0d	RHSIM_RhsimUnG0109400	1.337	1.270	-0.067	high
CK-0d	RHSIM_RhsimUnG0080300	0.683	0.684	0.001	no
CK-0d	RHSIM_RhsimUnG0020600	0.173	-0.406	-0.580	no
CK-0d	RHSIM_RhsimUnG0019600	-0.246	0.575	0.821	no
CK-0d	RHSIM_RhsimUnG0039700	-0.814	-1.656	-0.841	no
CK-0d	RHSIM_RhsimUnG0117900	0.766	0.727	-0.039	no
CK-0d	RHSIM_RhsimUnG0021000	0.866	-0.514	-1.379	no
CK-0d	RHSIM_RhsimUnG0055900	-0.832	-0.288	0.544	no
CK-0d	RHSIM_RhsimUnG0098000	0.991	-0.044	-1.035	no
CK-0d	RHSIM_RhsimUnG0194300	-0.427	-0.001	0.426	no
CK-0d	RHSIM_RhsimUnG0119800	-0.103	-1.104	-1.001	no
CK-0d	RHSIM_RhsimUnG0094700	0.633	0.718	0.085	no
CK-0d	RHSIM_RhsimUnG0109200	0.700	0.332	-0.368	no
CK-0d	RHSIM_RhsimUnG0107500	0.512	0.341	-0.171	no
CK-0d	RHSIM_RhsimUnG0105000	1.389	1.246	-0.143	high
CK-0d	RHSIM_RhsimUnG0188600	-0.400	-0.802	-0.402	no
CK-0d	RHSIM_RhsimUnG0006400	1.035	-0.251	-1.286	no
CK-0d	RHSIM_RhsimPtG0002900	2.231	0.710	-1.521	high
CK-0d	RHSIM_RhsimUnG0096000	0.550	-0.960	-1.510	no
CK-0d	RHSIM_RhsimUnG0079300	-0.288	-1.494	-1.206	no
CK-0d	RHSIM_RhsimUnG0021300	-1.169	-1.040	0.129	low
CK-0d	RHSIM_Rhsim13G0142900	-1.393	-1.533	-0.141	low
CK-0d	RHSIM_RhsimUnG0026400	1.663	0.271	-1.392	high
CK-0d	RHSIM_RhsimUnG0045900	2.458	1.558	-0.900	high
CK-0d	RHSIM_RhsimUnG0139700	0.183	-0.616	-0.799	no
CK-0d	RHSIM_RhsimUnG0085800	-0.619	-0.544	0.075	no

Group	Gene	PTR (z-score difference)	z(log2 LFQ)	z(log2 FPKM)	extreme-PTR
CK-0d	RHSIM_RhsimUnG0044200	-1.078	-1.252	-0.175	low
CK-0d	RHSIM_Rhsim13G0005000	0.491	0.738	0.247	no
CK-0d	RHSIM_RhsimUnG0021800	1.670	2.042	0.372	high
CK-0d	RHSIM_Rhsim13G0010100	-1.255	-0.758	0.498	low
CK-0d	RHSIM_RhsimUnG0022700	0.958	-1.044	-2.002	no
CK-0d	RHSIM_RhsimUnG0073900	0.552	0.151	-0.402	no
CK-0d	RHSIM_RhsimUnG0070500	1.016	-1.095	-2.111	no
CK-0d	RHSIM_Rhsim13G0064900	0.652	0.482	-0.169	no
CK-0d	RHSIM_RhsimUnG0027200	0.502	0.017	-0.485	no
CK-0d	RHSIM_Rhsim13G0234100	-0.179	-0.580	-0.402	no
CK-0d	RHSIM_RhsimUnG0060500	-0.496	-0.340	0.155	no
CK-0d	RHSIM_Rhsim13G0198200	1.060	0.831	-0.229	no
CK-0d	RHSIM_RhsimUnG0014100	-0.814	-0.521	0.293	no
CK-0d	RHSIM_RhsimMtG0006700	1.359	-0.266	-1.626	high
CK-0d	RHSIM_Rhsim13G0018300	0.621	-0.115	-0.736	no
CK-0d	RHSIM_RhsimUnG0022900	1.088	0.147	-0.941	no
CK-0d	RHSIM_Rhsim05G0087400	-0.164	0.430	0.594	no
CK-0d	RHSIM_RhsimUnG0051400	1.084	0.925	-0.160	no
CK-0d	RHSIM_RhsimUnG0059400	0.593	-0.201	-0.794	no
CK-0d	RHSIM_RhsimUnG0005600	-0.621	-0.659	-0.038	no
CK-0d	RHSIM_Rhsim13G0009100	-0.467	1.277	1.745	no
CK-0d	RHSIM_RhsimPtG0000900	3.518	2.241	-1.277	high
CK-0d	RHSIM_RhsimUnG0015500	0.653	0.036	-0.617	no
CK-0d	RHSIM_RhsimUnG0055000	1.073	-0.262	-1.335	no
CK-0d	RHSIM_RhsimUnG0120400	-0.473	-1.726	-1.253	no
CK-0d	RHSIM_Rhsim13G0140400	-0.645	-0.755	-0.110	no
CK-0d	RHSIM_Rhsim13G0104300	0.772	0.783	0.011	no
CK-0d	RHSIM_Rhsim13G0149200	-0.036	2.065	2.101	no
CK-0d	RHSIM_RhsimUnG0052000	2.774	0.472	-2.302	high
CK-0d	RHSIM_Rhsim13G0215900	-0.783	1.761	2.544	no
CK-0d	RHSIM_Rhsim13G0000400	-0.233	0.578	0.811	no
CK-0d	RHSIM_Rhsim13G0111800	-1.575	0.108	1.683	low

Group	Gene	PTR (z-score difference)	z(log2 LFQ)	z(log2 FPKM)	extreme-PTR
CK-0d	RHSIM_RhsimUnG0008400	0.394	1.691	1.297	no
CK-0d	RHSIM_Rhsim13G0157800	1.507	1.489	-0.018	high
CK-0d	RHSIM_RhsimUnG0043200	0.173	2.802	2.629	no
CK-0d	RHSIM_RhsimUnG0002100	-0.072	0.367	0.439	no
CK-0d	RHSIM_Rhsim13G0110800	0.587	0.693	0.106	no
CK-0d	RHSIM_Rhsim13G0009200	0.399	-0.338	-0.737	no
CK-0d	RHSIM_Rhsim13G0150700	0.041	0.364	0.323	no
CK-0d	RHSIM_Rhsim13G0112500	1.461	1.499	0.038	high
CK-0d	RHSIM_Rhsim13G0088500	0.443	-0.311	-0.754	no
CK-0d	RHSIM_Rhsim13G0186600	0.724	0.184	-0.540	no
CK-0d	RHSIM_Rhsim13G0177500	-2.212	-1.399	0.812	low
CK-0d	RHSIM_Rhsim13G0205900	0.381	0.445	0.064	no
CK-0d	RHSIM_Rhsim13G0156100	0.324	-0.477	-0.801	no
CK-0d	RHSIM_RhsimUnG0018800	2.197	1.390	-0.806	high
CK-0d	RHSIM_Rhsim13G0099400	0.128	-1.166	-1.294	no
CK-0d	RHSIM_RhsimUnG0023000	-1.125	-0.891	0.234	low
CK-0d	RHSIM_RhsimUnG0094300	0.951	0.704	-0.247	no
CK-0d	RHSIM_RhsimUnG0025700	0.699	-0.269	-0.968	no
CK-0d	RHSIM_RhsimUnG0254000	0.584	1.504	0.920	no
CK-0d	RHSIM_Rhsim13G0077700	-0.293	-0.433	-0.140	no
CK-0d	RHSIM_Rhsim13G0088800	-1.010	-0.571	0.439	no
CK-0d	RHSIM_Rhsim13G0030500	-0.168	-1.267	-1.099	no
CK-0d	RHSIM_RhsimUnG0252800	0.743	0.072	-0.671	no
CK-0d	RHSIM_Rhsim13G0201300	0.114	-0.041	-0.156	no
CK-0d	RHSIM_Rhsim13G0179600	-1.127	-0.201	0.925	low
CK-0d	RHSIM_Rhsim13G0140500	0.182	-0.229	-0.411	no
CK-0d	RHSIM_Rhsim13G0175200	0.158	0.570	0.411	no
CK-0d	RHSIM_Rhsim13G0069400	1.505	1.935	0.430	high
CK-0d	RHSIM_Rhsim13G0169400	0.462	0.639	0.177	no
CK-0d	RHSIM_Rhsim13G0211800	0.948	0.744	-0.205	no
CK-0d	RHSIM_RhsimUnG0139600	0.537	0.312	-0.225	no
CK-0d	RHSIM_RhsimUnG0004000	1.347	0.272	-1.075	high

Group	Gene	PTR (z-score difference)	z(log2 LFQ)	z(log2 FPKM)	extreme-PTR
CK-0d	RHSIM_RhsimUnG0248600	0.655	-0.740	-1.396	no
CK-0d	RHSIM_Rhsim13G0014400	-1.119	-1.074	0.044	low
CK-0d	RHSIM_RhsimUnG0006200	1.338	0.733	-0.605	high
CK-0d	RHSIM_Rhsim13G0147900	0.857	0.105	-0.752	no
CK-0d	RHSIM_Rhsim13G0091700	-0.789	0.898	1.687	no
CK-0d	RHSIM_RhsimUnG0135600	0.941	-0.750	-1.691	no
CK-0d	RHSIM_Rhsim13G0140200	-0.367	-0.057	0.310	no
CK-0d	RHSIM_Rhsim13G0039100	-0.318	-0.744	-0.427	no
CK-0d	RHSIM_RhsimUnG0133400	-0.538	-2.212	-1.675	no
CK-0d	RHSIM_Rhsim13G0012100	0.794	0.929	0.135	no
CK-0d	RHSIM_Rhsim13G0162700	2.057	2.341	0.284	high
CK-0d	RHSIM_Rhsim13G0201600	0.580	-0.628	-1.207	no
CK-0d	RHSIM_Rhsim13G0170400	-0.812	-0.001	0.810	no
CK-0d	RHSIM_Rhsim13G0218000	-1.052	-1.549	-0.497	low
CK-0d	RHSIM_Rhsim13G0024600	0.823	0.325	-0.499	no
CK-0d	RHSIM_Rhsim13G0024200	-3.305	-2.340	0.965	low
CK-0d	RHSIM_RhsimUnG0077000	1.950	0.418	-1.532	high
CK-0d	RHSIM_Rhsim13G0122800	1.786	2.088	0.302	high
CK-0d	RHSIM_Rhsim13G0115500	0.042	-0.363	-0.405	no
CK-0d	RHSIM_RhsimUnG0237100	0.500	-1.212	-1.713	no
CK-0d	RHSIM_RhsimUnG0120500	-1.815	-1.411	0.403	low
CK-0d	RHSIM_Rhsim13G0030900	2.929	3.398	0.469	high
CK-0d	RHSIM_Rhsim13G0202300	-2.642	-2.418	0.224	low
CK-0d	RHSIM_Rhsim13G0203400	0.205	1.565	1.360	no
CK-0d	RHSIM_Rhsim13G0233700	-0.731	0.263	0.994	no
CK-0d	RHSIM_Rhsim13G0057000	-0.364	-0.695	-0.331	no
CK-0d	RHSIM_Rhsim13G0044900	0.262	-0.881	-1.143	no
CK-0d	RHSIM_Rhsim13G0155800	-1.101	-0.159	0.942	low
CK-0d	RHSIM_Rhsim13G0060100	-0.231	-0.493	-0.262	no
CK-0d	RHSIM_Rhsim13G0062700	0.685	-0.370	-1.054	no
CK-0d	RHSIM_Rhsim13G0140900	0.280	-0.123	-0.403	no
CK-0d	RHSIM_Rhsim13G0111500	-0.076	1.341	1.417	no

Group	Gene	PTR (z-score difference)	z(log2 LFQ)	z(log2 FPKM)	extreme-PTR
CK-0d	RHSIM_Rhsim13G0111400	-0.002	1.034	1.037	no
CK-0d	RHSIM_RhsimUnG0102900	-0.258	-0.493	-0.235	no
CK-0d	RHSIM_Rhsim13G0208400	-0.530	-0.435	0.096	no
CK-0d	RHSIM_Rhsim13G0217600	-0.399	-0.345	0.055	no
CK-0d	RHSIM_Rhsim13G0195100	-1.382	-1.170	0.213	low
CK-0d	RHSIM_Rhsim13G0216400	0.121	-0.326	-0.447	no
CK-0d	RHSIM_Rhsim13G0024400	0.357	-0.017	-0.374	no
CK-0d	RHSIM_Rhsim13G0040400	-1.325	-1.457	-0.132	low
CK-0d	RHSIM_Rhsim13G0059900	0.318	0.110	-0.208	no
CK-0d	RHSIM_Rhsim13G0125900	0.664	0.443	-0.221	no
CK-0d	RHSIM_Rhsim13G0121000	-0.695	-0.663	0.032	no
CK-0d	RHSIM_Rhsim13G0189200	-3.443	-1.881	1.562	low
CK-0d	RHSIM_Rhsim13G0013600	0.447	1.820	1.372	no
CK-0d	RHSIM_Rhsim13G0071000	0.062	-0.063	-0.125	no
CK-0d	RHSIM_RhsimUnG0086000	-0.021	0.831	0.852	no
CK-0d	RHSIM_Rhsim13G0047200	-2.034	-0.482	1.552	low
CK-0d	RHSIM_Rhsim13G0067900	-4.956	-2.228	2.727	low
CK-0d	RHSIM_Rhsim13G0120300	-1.433	-1.778	-0.346	low
CK-0d	RHSIM_Rhsim13G0191600	-0.013	0.409	0.422	no
CK-0d	RHSIM_Rhsim13G0119400	0.748	3.112	2.364	no
CK-0d	RHSIM_Rhsim13G0174200	0.612	0.221	-0.391	no
CK-0d	RHSIM_Rhsim13G0216600	-1.210	-2.302	-1.093	low
CK-0d	RHSIM_Rhsim13G0205000	1.174	-0.090	-1.264	high
CK-0d	RHSIM_Rhsim13G0191100	-1.163	-0.478	0.685	low
CK-0d	RHSIM_Rhsim13G0146300	-0.932	-0.935	-0.003	no
CK-0d	RHSIM_Rhsim13G0163000	0.531	-0.492	-1.023	no
CK-0d	RHSIM_RhsimUnG0079400	-1.106	-0.652	0.454	low
CK-0d	RHSIM_Rhsim13G0194200	1.329	2.182	0.854	high
CK-0d	RHSIM_Rhsim13G0052700	-0.573	-0.361	0.212	no
CK-0d	RHSIM_Rhsim13G0072700	0.453	0.657	0.204	no
CK-0d	RHSIM_RhsimUnG0206200	-1.094	-1.257	-0.163	low
CK-0d	RHSIM_Rhsim13G0094700	-0.329	0.291	0.620	no

Group	Gene	PTR (z-score difference)	z(log2 LFQ)	z(log2 FPKM)	extreme-PTR
CK-0d	RHSIM_Rhsim13G0201700	-0.120	0.530	0.650	no
CK-0d	RHSIM_Rhsim13G0056300	-0.536	-1.440	-0.904	no
CK-0d	RHSIM_RhsimUnG0046000	0.814	-0.284	-1.098	no
CK-0d	RHSIM_Rhsim13G0169800	1.017	1.042	0.025	no
CK-0d	RHSIM_Rhsim13G0152000	0.222	0.493	0.271	no
CK-0d	RHSIM_Rhsim13G0011700	-0.510	1.163	1.673	no
CK-0d	RHSIM_Rhsim13G0056400	0.972	0.933	-0.039	no
CK-0d	RHSIM_RhsimUnG0066200	-1.170	-1.048	0.121	low
CK-0d	RHSIM_Rhsim13G0098900	-2.367	-0.169	2.198	low
CK-0d	RHSIM_RhsimUnG0065400	1.577	2.473	0.896	high
CK-0d	RHSIM_Rhsim13G0226700	-0.580	-0.079	0.500	no
CK-0d	RHSIM_Rhsim13G0061400	-1.482	-1.981	-0.499	low
CK-0d	RHSIM_Rhsim13G0129400	0.002	-0.506	-0.507	no
CK-0d	RHSIM_Rhsim13G0050600	0.202	-0.156	-0.358	no
CK-0d	RHSIM_Rhsim13G0020400	-1.907	-1.364	0.543	low
CK-0d	RHSIM_Rhsim13G0009900	0.093	-1.395	-1.488	no
CK-0d	RHSIM_Rhsim13G0098700	-0.385	0.452	0.837	no
CK-0d	RHSIM_Rhsim13G0198700	-1.150	-0.354	0.796	low
CK-0d	RHSIM_Rhsim13G0057400	-0.420	-0.412	0.008	no
CK-0d	RHSIM_Rhsim13G0179900	-2.144	-1.321	0.824	low
CK-0d	RHSIM_Rhsim13G0094300	1.206	0.075	-1.132	high
CK-0d	RHSIM_Rhsim13G0035600	-0.558	-0.080	0.478	no
CK-0d	RHSIM_Rhsim13G0234800	0.689	0.729	0.040	no
CK-0d	RHSIM_Rhsim13G0001000	1.202	0.390	-0.812	high
CK-0d	RHSIM_Rhsim13G0221000	-1.333	-1.021	0.312	low
CK-0d	RHSIM_RhsimUnG0053100	-0.917	-1.187	-0.270	no
CK-0d	RHSIM_Rhsim13G0107100	-2.443	-0.082	2.361	low
CK-0d	RHSIM_Rhsim13G0024900	0.950	0.407	-0.542	no
CK-0d	RHSIM_Rhsim13G0045300	0.673	1.557	0.884	no
CK-0d	RHSIM_Rhsim13G0180000	-2.938	-2.338	0.599	low
CK-0d	RHSIM_Rhsim13G0037500	0.004	0.049	0.045	no
CK-0d	RHSIM_RhsimUnG0048500	-0.536	-1.420	-0.884	no

Group	Gene	PTR (z-score difference)	z(log2 LFQ)	z(log2 FPKM)	extreme-PTR
CK-0d	RHSIM_Rhsim13G0211100	-0.222	0.153	0.374	no
CK-0d	RHSIM_Rhsim13G0072000	-0.704	-0.243	0.462	no
CK-0d	RHSIM_Rhsim13G0027700	0.089	-0.094	-0.183	no
CK-0d	RHSIM_Rhsim13G0018600	-0.083	0.267	0.349	no
CK-0d	RHSIM_Rhsim13G0127200	1.316	3.868	2.552	high
CK-0d	RHSIM_RhsimUnG0023900	1.814	0.032	-1.781	high
CK-0d	RHSIM_Rhsim13G0231000	0.154	-0.052	-0.206	no
CK-0d	RHSIM_Rhsim13G0036500	-1.802	-0.388	1.414	low
CK-0d	RHSIM_Rhsim13G0031600	0.872	0.579	-0.293	no
CK-0d	RHSIM_Rhsim13G0065500	0.500	0.996	0.496	no
CK-0d	RHSIM_RhsimUnG0021400	-0.973	-0.224	0.748	no
CK-0d	RHSIM_Rhsim13G0015500	0.532	0.132	-0.401	no
CK-0d	RHSIM_Rhsim13G0056900	-0.567	0.779	1.346	no
CK-0d	RHSIM_Rhsim13G0115200	0.504	0.679	0.175	no
CK-0d	RHSIM_RhsimUnG0172300	0.440	-0.209	-0.648	no
CK-0d	RHSIM_Rhsim13G0209000	-0.976	-1.550	-0.575	no
CK-0d	RHSIM_Rhsim13G0121400	0.012	-0.065	-0.076	no
CK-0d	RHSIM_Rhsim13G0208500	-3.703	-2.343	1.360	low
CK-0d	RHSIM_Rhsim13G0058000	0.015	0.863	0.849	no
CK-0d	RHSIM_Rhsim13G0224400	2.157	-0.022	-2.179	high
CK-0d	RHSIM_Rhsim13G0004800	0.554	-0.214	-0.768	no
CK-0d	RHSIM_Rhsim13G0221900	-0.024	-0.317	-0.293	no
CK-0d	RHSIM_Rhsim13G0104500	0.009	-1.086	-1.095	no
CK-0d	RHSIM_RhsimUnG0033300	-0.084	-0.307	-0.222	no
CK-0d	RHSIM_Rhsim13G0129600	-0.623	-0.459	0.164	no
CK-0d	RHSIM_Rhsim13G0098500	-0.489	-0.455	0.035	no
CK-0d	RHSIM_Rhsim13G0228700	1.951	1.477	-0.474	high
CK-0d	RHSIM_Rhsim13G0152700	2.258	2.756	0.498	high
CK-0d	RHSIM_Rhsim13G0228600	0.211	0.844	0.633	no
CK-0d	RHSIM_Rhsim13G0196900	-1.181	-1.300	-0.118	low
CK-0d	RHSIM_Rhsim13G0116700	-1.590	-1.980	-0.390	low
CK-0d	RHSIM_RhsimUnG0009300	-0.215	0.852	1.067	no

Group	Gene	PTR (z-score difference)	z(log2 LFQ)	z(log2 FPKM)	extreme-PTR
CK-0d	RHSIM_Rhsim13G0017200	-0.365	-0.529	-0.164	no
CK-0d	RHSIM_Rhsim13G0000200	-0.534	0.548	1.082	no
CK-0d	RHSIM_Rhsim13G0073600	0.074	-0.606	-0.681	no
CK-0d	RHSIM_Rhsim13G0209100	-0.813	-2.002	-1.189	no
CK-0d	RHSIM_Rhsim13G0056100	-1.380	-1.426	-0.046	low
CK-0d	RHSIM_Rhsim13G0056500	0.396	0.454	0.058	no
CK-0d	RHSIM_Rhsim13G0109800	-1.393	-1.934	-0.540	low
CK-0d	RHSIM_RhsimUnG0023200	-0.497	-0.945	-0.448	no
CK-0d	RHSIM_Rhsim13G0200400	0.441	-0.825	-1.266	no
CK-0d	RHSIM_Rhsim13G0127700	0.763	0.157	-0.606	no
CK-0d	RHSIM_Rhsim13G0230700	0.713	-0.301	-1.014	no
CK-0d	RHSIM_Rhsim13G0046300	-0.796	-0.394	0.402	no
CK-0d	RHSIM_Rhsim13G0112600	-0.065	-0.520	-0.456	no
CK-0d	RHSIM_Rhsim13G0193900	-3.078	-1.587	1.491	low
CK-0d	RHSIM_RhsimUnG0015300	1.560	1.082	-0.478	high
CK-0d	RHSIM_Rhsim13G0053000	-1.596	-1.513	0.083	low
CK-0d	RHSIM_Rhsim13G0082500	0.589	2.837	2.248	no
CK-0d	RHSIM_Rhsim13G0200800	-0.454	-0.447	0.007	no
CK-0d	RHSIM_Rhsim13G0094400	1.335	2.511	1.175	high
CK-0d	RHSIM_Rhsim13G0084100	-0.118	2.478	2.596	no
CK-0d	RHSIM_Rhsim13G0011000	-0.800	0.654	1.455	no
CK-0d	RHSIM_Rhsim13G0137100	0.568	0.298	-0.270	no
CK-0d	RHSIM_Rhsim13G0104400	0.182	-0.049	-0.231	no
CK-0d	RHSIM_RhsimUnG0009100	0.591	0.828	0.237	no
CK-0d	RHSIM_Rhsim13G0065400	0.031	-0.757	-0.788	no
CK-0d	RHSIM_Rhsim13G0077000	-1.964	-1.628	0.336	low
CK-0d	RHSIM_RhsimUnG0153300	-0.738	-0.483	0.255	no
CK-0d	RHSIM_Rhsim13G0088300	0.337	-0.503	-0.840	no
CK-0d	RHSIM_Rhsim13G0035500	-0.474	-0.107	0.367	no
CK-0d	RHSIM_Rhsim13G0225600	1.197	3.238	2.040	high
CK-0d	RHSIM_Rhsim13G0225300	1.352	0.649	-0.703	high
CK-0d	RHSIM_Rhsim13G0210700	-1.727	-0.520	1.207	low

Group	Gene	PTR (z-score difference)	z(log2 LFQ)	z(log2 FPKM)	extreme-PTR
CK-0d	RHSIM_Rhsim13G0075700	0.611	1.238	0.628	no
CK-0d	RHSIM_Rhsim13G0141400	-1.158	-0.629	0.529	low
CK-0d	RHSIM_Rhsim13G0021200	0.028	0.628	0.600	no
CK-0d	RHSIM_Rhsim13G0163700	-0.141	0.576	0.716	no
CK-0d	RHSIM_RhsimUnG0150500	3.622	1.437	-2.185	high
CK-0d	RHSIM_Rhsim13G0095400	-1.556	-1.678	-0.123	low
CK-0d	RHSIM_Rhsim13G0209300	-0.978	-0.957	0.021	no
CK-0d	RHSIM_Rhsim13G0104000	0.618	1.761	1.142	no
CK-0d	RHSIM_Rhsim13G0034500	0.397	-0.406	-0.803	no
CK-0d	RHSIM_Rhsim13G0117100	-1.164	-0.744	0.419	low
CK-0d	RHSIM_Rhsim13G0089200	2.290	2.182	-0.108	high
CK-0d	RHSIM_Rhsim13G0232600	-0.338	-1.234	-0.897	no
CK-0d	RHSIM_Rhsim13G0105300	0.443	0.378	-0.065	no
CK-0d	RHSIM_Rhsim13G0207700	1.406	1.762	0.356	high
CK-0d	RHSIM_Rhsim13G0110000	-1.024	0.235	1.259	no
CK-0d	RHSIM_Rhsim13G0128200	-0.702	-0.667	0.035	no
CK-0d	RHSIM_Rhsim12G0014500	0.023	-0.403	-0.426	no
CK-0d	RHSIM_Rhsim13G0201400	-2.627	-1.467	1.160	low
CK-0d	RHSIM_Rhsim13G0203300	-0.098	-0.326	-0.228	no
CK-0d	RHSIM_Rhsim12G0044300	-0.458	0.071	0.529	no
CK-0d	RHSIM_Rhsim13G0051800	0.469	0.157	-0.312	no
CK-0d	RHSIM_Rhsim13G0196400	-1.681	-0.591	1.090	low
CK-0d	RHSIM_Rhsim13G0181500	0.282	-0.763	-1.045	no
CK-0d	RHSIM_Rhsim13G0066200	0.057	-0.796	-0.854	no
CK-0d	RHSIM_Rhsim13G0093000	0.726	-0.515	-1.241	no
CK-0d	RHSIM_Rhsim12G0190000	0.719	0.213	-0.506	no
CK-0d	RHSIM_Rhsim13G0123900	-0.013	2.034	2.048	no
CK-0d	RHSIM_RhsimUnG0144200	0.357	1.819	1.463	no
CK-0d	RHSIM_Rhsim12G0144400	-1.029	-1.033	-0.004	low
CK-0d	RHSIM_Rhsim13G0044300	0.433	-0.596	-1.029	no
CK-0d	RHSIM_Rhsim13G0018200	0.290	0.359	0.069	no
CK-0d	RHSIM_Rhsim12G0188700	-1.298	-0.251	1.048	low

Group	Gene	PTR (z-score difference)	z(log2 LFQ)	z(log2 FPKM)	extreme-PTR
CK-0d	RHSIM_Rhsim13G0099900	-0.548	-0.719	-0.171	no
CK-0d	RHSIM_Rhsim12G0145400	0.818	0.238	-0.580	no
CK-0d	RHSIM_Rhsim12G0089400	-0.752	-0.844	-0.092	no
CK-0d	RHSIM_Rhsim13G0064700	1.136	0.580	-0.556	high
CK-0d	RHSIM_Rhsim13G0002600	-0.707	-0.514	0.192	no
CK-0d	RHSIM_Rhsim12G0136900	-0.052	0.195	0.247	no
CK-0d	RHSIM_Rhsim13G0102500	1.635	0.968	-0.667	high
CK-0d	RHSIM_Rhsim13G0225400	0.836	0.635	-0.201	no
CK-0d	RHSIM_Rhsim13G0018900	-0.901	0.337	1.238	no
CK-0d	RHSIM_Rhsim13G0191300	1.199	-0.479	-1.678	high
CK-0d	RHSIM_Rhsim13G0178100	0.368	-0.286	-0.654	no
CK-0d	RHSIM_Rhsim13G0077300	0.886	1.194	0.308	no
CK-0d	RHSIM_Rhsim13G0072100	-0.958	-0.480	0.478	no
CK-0d	RHSIM_Rhsim13G0227500	1.247	1.185	-0.062	high
CK-0d	RHSIM_Rhsim13G0162300	-0.993	-0.296	0.698	no
CK-0d	RHSIM_Rhsim13G0134900	0.717	0.503	-0.214	no
CK-0d	RHSIM_Rhsim13G0067700	0.040	1.292	1.252	no
CK-0d	RHSIM_Rhsim12G0057500	0.828	1.833	1.005	no
CK-0d	RHSIM_Rhsim13G0229700	-0.453	0.376	0.829	no
CK-0d	RHSIM_Rhsim13G0170300	0.764	0.181	-0.583	no
CK-0d	RHSIM_Rhsim13G0002500	0.292	0.451	0.160	no
CK-0d	RHSIM_Rhsim13G0077400	-0.437	3.590	4.027	no
CK-0d	RHSIM_Rhsim13G0013500	-0.054	0.293	0.347	no
CK-0d	RHSIM_Rhsim13G0211600	0.126	-0.629	-0.755	no
CK-0d	RHSIM_Rhsim13G0102300	0.304	-0.404	-0.708	no
CK-0d	RHSIM_Rhsim13G0015800	-0.377	-1.015	-0.638	no
CK-0d	RHSIM_Rhsim13G0023600	0.311	0.369	0.058	no
CK-0d	RHSIM_Rhsim12G0205600	0.962	-0.572	-1.534	no
CK-0d	RHSIM_Rhsim13G0198600	0.252	-0.535	-0.788	no
CK-0d	RHSIM_Rhsim13G0126100	-2.320	0.471	2.792	low
CK-0d	RHSIM_Rhsim13G0029500	1.176	-0.884	-2.060	high
CK-0d	RHSIM_Rhsim13G0216500	0.079	0.615	0.536	no

Group	Gene	PTR (z-score difference)	z(log2 LFQ)	z(log2 FPKM)	extreme-PTR
CK-0d	RHSIM_Rhsim13G0115600	-0.202	-0.029	0.173	no
CK-0d	RHSIM_Rhsim13G0044100	-0.246	-0.721	-0.475	no
CK-0d	RHSIM_Rhsim13G0073300	-0.127	-0.780	-0.654	no
CK-0d	RHSIM_RhsimUnG0124700	-0.901	-0.756	0.145	no
CK-0d	RHSIM_Rhsim13G0234200	-1.587	-0.523	1.064	low
CK-0d	RHSIM_Rhsim13G0049400	-0.186	0.203	0.389	no
CK-0d	RHSIM_Rhsim13G0155900	-0.558	-1.230	-0.672	no
CK-0d	RHSIM_Rhsim13G0065900	0.382	-0.828	-1.210	no
CK-0d	RHSIM_Rhsim12G0054400	1.508	0.436	-1.072	high
CK-0d	RHSIM_Rhsim13G0125300	1.399	-0.284	-1.683	high
CK-0d	RHSIM_Rhsim13G0013000	0.209	-0.151	-0.359	no
CK-0d	RHSIM_RhsimUnG0118000	0.366	1.270	0.904	no
CK-0d	RHSIM_Rhsim13G0150000	0.068	-0.524	-0.591	no
CK-0d	RHSIM_Rhsim13G0100500	0.793	1.318	0.524	no
CK-0d	RHSIM_Rhsim12G0041400	-0.359	0.225	0.585	no
CK-0d	RHSIM_Rhsim13G0198800	-0.818	-0.398	0.420	no
CK-0d	RHSIM_Rhsim13G0071100	-0.690	0.200	0.891	no
CK-0d	RHSIM_Rhsim13G0151100	-0.036	0.162	0.198	no
CK-0d	RHSIM_Rhsim13G0000500	-2.134	-1.972	0.162	low
CK-0d	RHSIM_Rhsim13G0143800	1.186	0.236	-0.950	high
CK-0d	RHSIM_RhsimUnG0113900	-0.946	-1.854	-0.907	no
CK-0d	RHSIM_Rhsim13G0126200	-3.151	-1.042	2.109	low
CK-0d	RHSIM_Rhsim12G0094700	0.095	-0.451	-0.546	no
CK-0d	RHSIM_Rhsim12G0069500	0.040	-0.554	-0.594	no
CK-0d	RHSIM_Rhsim13G0228400	-0.210	0.667	0.876	no
CK-0d	RHSIM_Rhsim13G0218100	-0.168	-0.613	-0.445	no
CK-0d	RHSIM_Rhsim13G0127300	-0.716	-1.533	-0.816	no
CK-0d	RHSIM_Rhsim13G0026200	0.096	1.306	1.210	no
CK-0d	RHSIM_Rhsim13G0088000	-0.039	-1.035	-0.996	no
CK-0d	RHSIM_Rhsim12G0000600	2.189	4.388	2.200	high
CK-0d	RHSIM_Rhsim12G0123000	1.055	-0.381	-1.436	no
CK-0d	RHSIM_Rhsim13G0191900	0.436	-0.651	-1.087	no

Group	Gene	PTR (z-score difference)	z(log2 LFQ)	z(log2 FPKM)	extreme-PTR
CK-0d	RHSIM_Rhsim13G0065600	1.350	1.326	-0.024	high
CK-0d	RHSIM_Rhsim13G0180600	0.452	-0.068	-0.520	no
CK-0d	RHSIM_Rhsim12G0124700	0.481	-0.080	-0.561	no
CK-0d	RHSIM_Rhsim12G0173700	0.171	1.622	1.451	no
CK-0d	RHSIM_Rhsim12G0061300	-0.033	-0.808	-0.775	no
CK-0d	RHSIM_Rhsim13G0224500	-0.857	-1.133	-0.276	no
CK-0d	RHSIM_Rhsim13G0199800	1.023	0.026	-0.998	no
CK-0d	RHSIM_Rhsim13G0076400	-0.198	-0.312	-0.114	no
CK-0d	RHSIM_Rhsim13G0063200	-3.877	-0.996	2.881	low
CK-0d	RHSIM_Rhsim13G0202500	0.944	0.292	-0.652	no
CK-0d	RHSIM_Rhsim12G0204500	-1.728	-0.295	1.433	low
CK-0d	RHSIM_Rhsim13G0235300	0.039	0.551	0.512	no
CK-0d	RHSIM_Rhsim13G0085200	1.832	2.966	1.134	high
CK-0d	RHSIM_Rhsim12G0195700	0.395	0.807	0.412	no
CK-0d	RHSIM_Rhsim12G0197300	0.663	0.478	-0.185	no
CK-0d	RHSIM_Rhsim12G0060800	-2.762	-1.556	1.206	low
CK-0d	RHSIM_Rhsim12G0130500	-0.625	0.090	0.715	no
CK-0d	RHSIM_Rhsim13G0058700	-0.903	-1.385	-0.481	no
CK-0d	RHSIM_Rhsim12G0210700	2.270	1.204	-1.066	high
CK-0d	RHSIM_Rhsim13G0121900	0.066	-1.011	-1.078	no
CK-0d	RHSIM_Rhsim12G0178300	1.794	0.273	-1.520	high
CK-0d	RHSIM_Rhsim13G0000600	-1.936	-1.014	0.922	low
CK-0d	RHSIM_Rhsim13G0128900	-0.664	-1.171	-0.507	no
CK-0d	RHSIM_RhsimUnG0093000	-0.479	-0.268	0.210	no
CK-0d	RHSIM_Rhsim12G0182600	-0.499	-0.170	0.329	no
CK-0d	RHSIM_Rhsim13G0152200	1.959	0.214	-1.744	high
CK-0d	RHSIM_Rhsim12G0127000	1.243	0.238	-1.005	high
CK-0d	RHSIM_Rhsim13G0138500	-0.091	0.766	0.857	no
CK-0d	RHSIM_Rhsim13G0072300	1.725	-0.070	-1.795	high
CK-0d	RHSIM_Rhsim13G0097900	0.670	-0.795	-1.465	no
CK-0d	RHSIM_Rhsim12G0143900	0.495	-0.136	-0.630	no
CK-0d	RHSIM_Rhsim13G0154700	0.674	-0.021	-0.694	no

Group	Gene	PTR (z-score difference)	z(log2 LFQ)	z(log2 FPKM)	extreme-PTR
CK-0d	RHSIM_RhsimUnG0089100	-1.643	0.132	1.775	low
CK-0d	RHSIM_Rhsim12G0035800	0.085	0.060	-0.025	no
CK-0d	RHSIM_Rhsim13G0017300	-0.348	-1.477	-1.128	no
CK-0d	RHSIM_Rhsim12G0047600	-0.225	0.661	0.886	no
CK-0d	RHSIM_RhsimUnG0088100	0.479	-1.589	-2.068	no
CK-0d	RHSIM_Rhsim13G0200500	0.496	-0.157	-0.653	no
CK-0d	RHSIM_Rhsim13G0016000	0.146	-0.289	-0.435	no
CK-0d	RHSIM_Rhsim13G0199600	0.242	0.242	-0.001	no
CK-0d	RHSIM_Rhsim12G0112500	0.046	-0.605	-0.650	no
CK-0d	RHSIM_Rhsim13G0008000	1.897	0.900	-0.997	high
CK-0d	RHSIM_Rhsim13G0197500	-0.897	-0.185	0.712	no
CK-0d	RHSIM_Rhsim13G0074100	-1.667	0.458	2.125	low
CK-0d	RHSIM_Rhsim12G0077600	0.146	-0.037	-0.184	no
CK-0d	RHSIM_Rhsim12G0116500	-0.898	-0.693	0.205	no
CK-0d	RHSIM_Rhsim13G0040100	-4.850	-1.551	3.299	low
CK-0d	RHSIM_Rhsim13G0075400	0.728	0.244	-0.484	no
CK-0d	RHSIM_Rhsim12G0207400	0.580	-0.045	-0.626	no
CK-0d	RHSIM_Rhsim12G0135500	-0.735	-0.929	-0.194	no
CK-0d	RHSIM_Rhsim12G0035400	1.898	0.037	-1.861	high
CK-0d	RHSIM_Rhsim13G0037600	-1.024	-0.149	0.875	no
CK-0d	RHSIM_Rhsim13G0173900	-0.523	-0.656	-0.133	no
CK-0d	RHSIM_Rhsim13G0102400	0.350	-0.227	-0.577	no
CK-0d	RHSIM_Rhsim12G0130100	0.610	1.070	0.459	no
CK-0d	RHSIM_Rhsim12G0113000	0.958	1.150	0.191	no
CK-0d	RHSIM_Rhsim12G0013800	0.225	0.424	0.199	no
CK-0d	RHSIM_Rhsim13G0223300	1.562	0.945	-0.617	high
CK-0d	RHSIM_Rhsim13G0223000	0.493	2.692	2.199	no
CK-0d	RHSIM_Rhsim13G0154600	-1.166	-0.632	0.534	low
CK-0d	RHSIM_RhsimUnG0067300	1.185	-1.082	-2.267	high
CK-0d	RHSIM_Rhsim12G0212100	0.708	-0.186	-0.894	no
CK-0d	RHSIM_Rhsim13G0012200	0.785	0.701	-0.084	no
CK-0d	RHSIM_Rhsim12G0143400	-0.371	0.748	1.119	no

Group	Gene	PTR (z-score difference)	z(log2 LFQ)	z(log2 FPKM)	extreme-PTR
CK-0d	RHSIM_Rhsim13G0076300	-0.886	-0.388	0.498	no
CK-0d	RHSIM_Rhsim13G0029400	0.217	-0.161	-0.377	no
CK-0d	RHSIM_Rhsim13G0149600	0.885	2.568	1.683	no
CK-0d	RHSIM_Rhsim12G0021800	0.005	0.051	0.046	no
CK-0d	RHSIM_Rhsim12G0133000	-0.334	-0.600	-0.266	no
CK-0d	RHSIM_Rhsim13G0081700	1.767	-0.037	-1.804	high
CK-0d	RHSIM_Rhsim13G0224600	-0.276	-0.428	-0.152	no
CK-0d	RHSIM_Rhsim12G0149400	1.076	1.724	0.648	no
CK-0d	RHSIM_Rhsim13G0070200	0.121	-1.217	-1.338	no
CK-0d	RHSIM_Rhsim12G0076200	-1.288	-0.636	0.652	low
CK-0d	RHSIM_Rhsim12G0157800	-0.317	0.498	0.816	no
CK-0d	RHSIM_Rhsim13G0023900	0.260	1.126	0.866	no
CK-0d	RHSIM_Rhsim12G0116400	-2.712	0.676	3.389	low
CK-0d	RHSIM_Rhsim13G0116000	0.826	1.583	0.757	no
CK-0d	RHSIM_Rhsim13G0119800	-0.698	-1.298	-0.599	no
CK-0d	RHSIM_Rhsim12G0163200	-0.439	-0.308	0.131	no
CK-0d	RHSIM_Rhsim12G0193800	0.470	-0.408	-0.878	no
CK-0d	RHSIM_Rhsim12G0214100	-0.584	-0.441	0.143	no
CK-0d	RHSIM_Rhsim12G0015600	0.515	3.808	3.293	no
CK-0d	RHSIM_Rhsim12G0186700	-0.587	-1.353	-0.766	no
CK-0d	RHSIM_Rhsim12G0134700	-1.025	-0.677	0.348	no
CK-0d	RHSIM_Rhsim13G0198500	0.974	1.554	0.580	no
CK-0d	RHSIM_Rhsim12G0026000	-0.252	-0.038	0.214	no
CK-0d	RHSIM_Rhsim12G0078900	0.033	-0.526	-0.558	no
CK-0d	RHSIM_Rhsim13G0037700	0.980	0.242	-0.738	no
CK-0d	RHSIM_Rhsim12G0140200	-0.047	0.630	0.677	no
CK-0d	RHSIM_Rhsim12G0137600	0.174	0.072	-0.102	no
CK-0d	RHSIM_Rhsim13G0039600	0.614	-0.077	-0.691	no
CK-0d	RHSIM_Rhsim12G0108100	-1.053	-1.002	0.051	low
CK-0d	RHSIM_Rhsim12G0009900	0.936	-0.009	-0.945	no
CK-0d	RHSIM_Rhsim13G0095800	-0.318	-0.680	-0.362	no
CK-0d	RHSIM_Rhsim12G0144200	-1.260	-1.665	-0.405	low

Group	Gene	PTR (z-score difference)	z(log2 LFQ)	z(log2 FPKM)	extreme-PTR
CK-0d	RHSIM_Rhsim13G0119500	0.379	0.310	-0.070	no
CK-0d	RHSIM_Rhsim13G0031400	2.096	0.303	-1.794	high
CK-0d	RHSIM_Rhsim13G0017500	0.455	-0.939	-1.393	no
CK-0d	RHSIM_Rhsim12G0195800	-0.168	0.404	0.572	no
CK-0d	RHSIM_Rhsim12G0152200	-0.034	-0.503	-0.469	no
CK-0d	RHSIM_Rhsim12G0170600	-1.364	0.413	1.777	low
CK-0d	RHSIM_Rhsim13G0210400	1.334	0.887	-0.447	high
CK-0d	RHSIM_Rhsim13G0044700	-0.744	-1.622	-0.878	no
CK-0d	RHSIM_Rhsim12G0165900	-0.050	0.481	0.531	no
CK-0d	RHSIM_Rhsim12G0208900	-0.049	-0.738	-0.690	no
CK-0d	RHSIM_Rhsim13G0222300	0.199	-0.745	-0.944	no
CK-0d	RHSIM_Rhsim13G0077600	0.603	1.165	0.562	no
CK-0d	RHSIM_Rhsim12G0085200	-1.029	-1.911	-0.882	low
CK-0d	RHSIM_Rhsim12G0150200	-0.894	-1.948	-1.054	no
CK-0d	RHSIM_Rhsim12G0152500	0.197	-0.329	-0.526	no
CK-0d	RHSIM_Rhsim13G0233300	0.647	1.600	0.952	no
CK-0d	RHSIM_Rhsim12G0182800	0.149	1.897	1.747	no
CK-0d	RHSIM_Rhsim12G0195100	0.402	-0.495	-0.897	no
CK-0d	RHSIM_Rhsim12G0169500	1.017	1.430	0.413	no
CK-0d	RHSIM_Rhsim12G0171400	-1.367	-1.420	-0.053	low
CK-0d	RHSIM_Rhsim12G0109000	3.477	1.240	-2.238	high
CK-0d	RHSIM_Rhsim13G0089100	0.525	0.925	0.400	no
CK-0d	RHSIM_Rhsim12G0041100	-0.369	-0.701	-0.332	no
CK-0d	RHSIM_Rhsim12G0180700	0.514	0.741	0.227	no
CK-0d	RHSIM_Rhsim12G0155700	1.516	3.349	1.833	high
CK-0d	RHSIM_Rhsim12G0188800	-0.651	-1.475	-0.824	no
CK-0d	RHSIM_RhsimUnG0039500	2.838	0.664	-2.174	high
CK-0d	RHSIM_Rhsim12G0140600	0.727	0.639	-0.088	no
CK-0d	RHSIM_Rhsim12G0195900	-1.411	-1.160	0.251	low
CK-0d	RHSIM_Rhsim12G0015500	-0.859	0.770	1.629	no
CK-0d	RHSIM_Rhsim12G0090000	-0.693	0.257	0.950	no
CK-0d	RHSIM_Rhsim12G0182500	-0.361	-0.982	-0.621	no

Group	Gene	PTR (z-score difference)	z(log2 LFQ)	z(log2 FPKM)	extreme-PTR
CK-0d	RHSIM_Rhsim12G0169800	-0.180	0.340	0.520	no
CK-0d	RHSIM_Rhsim12G0074100	-0.165	-0.414	-0.249	no
CK-0d	RHSIM_Rhsim12G0190900	-0.305	-0.466	-0.161	no
CK-0d	RHSIM_Rhsim12G0124000	-1.301	-1.596	-0.295	low
CK-0d	RHSIM_Rhsim13G0230600	0.128	0.163	0.035	no
CK-0d	RHSIM_Rhsim12G0168400	-1.117	-0.071	1.046	low
CK-0d	RHSIM_Rhsim12G0208200	-1.668	-0.338	1.331	low
CK-0d	RHSIM_Rhsim12G0167500	-0.739	0.430	1.169	no
CK-0d	RHSIM_Rhsim12G0209100	-0.426	-0.471	-0.045	no
CK-0d	RHSIM_Rhsim12G0086400	-1.213	-1.458	-0.246	low
CK-0d	RHSIM_Rhsim12G0132700	0.597	0.378	-0.219	no
CK-0d	RHSIM_Rhsim12G0175800	0.550	1.282	0.732	no
CK-0d	RHSIM_Rhsim12G0163900	1.269	0.395	-0.873	high
CK-0d	RHSIM_Rhsim12G0149700	0.541	-1.135	-1.677	no
CK-0d	RHSIM_Rhsim12G0212300	0.636	-0.588	-1.224	no
CK-0d	RHSIM_Rhsim12G0197400	-1.800	0.669	2.469	low
CK-0d	RHSIM_Rhsim12G0076500	-0.459	0.308	0.767	no
CK-0d	RHSIM_Rhsim12G0127200	-0.985	-1.150	-0.165	no
CK-0d	RHSIM_RhsimUnG0027400	1.071	-0.420	-1.491	no
CK-0d	RHSIM_Rhsim13G0064000	-0.207	-0.539	-0.332	no
CK-0d	RHSIM_Rhsim13G0071700	0.234	1.795	1.561	no
CK-0d	RHSIM_Rhsim12G0137100	-2.271	-1.842	0.430	low
CK-0d	RHSIM_Rhsim12G0003100	-1.103	-0.279	0.825	low
CK-0d	RHSIM_Rhsim12G0003800	-0.591	-0.541	0.050	no
CK-0d	RHSIM_Rhsim12G0116900	0.713	0.504	-0.209	no
CK-0d	RHSIM_Rhsim12G0013200	0.657	-1.172	-1.830	no
CK-0d	RHSIM_Rhsim12G0099000	0.436	-0.081	-0.517	no
CK-0d	RHSIM_Rhsim12G0013400	0.736	1.351	0.616	no
CK-0d	RHSIM_Rhsim12G0000200	0.823	-0.667	-1.490	no
CK-0d	RHSIM_Rhsim12G0017500	-0.779	-1.170	-0.390	no
CK-0d	RHSIM_RhsimUnG0019800	-0.399	-0.425	-0.026	no
CK-0d	RHSIM_Rhsim12G0208000	1.523	1.965	0.442	high

Group	Gene	PTR (z-score difference)	z(log2 LFQ)	z(log2 FPKM)	extreme-PTR
CK-0d	RHSIM_Rhsim13G0117600	0.309	-0.635	-0.944	no
CK-0d	RHSIM_Rhsim12G0190700	-0.370	0.082	0.452	no
CK-0d	RHSIM_Rhsim12G0003400	0.260	0.523	0.263	no
CK-0d	RHSIM_Rhsim12G0053900	-3.864	-1.365	2.499	low
CK-0d	RHSIM_RhsimUnG0020300	0.307	0.300	-0.007	no
CK-0d	RHSIM_Rhsim13G0102900	0.036	-0.693	-0.729	no
CK-0d	RHSIM_Rhsim12G0192900	-3.209	-1.640	1.569	low
CK-0d	RHSIM_Rhsim12G0151800	-0.256	0.495	0.751	no
CK-0d	RHSIM_Rhsim13G0204000	0.173	-0.287	-0.461	no
CK-0d	RHSIM_Rhsim12G0129700	0.466	-0.823	-1.289	no
CK-0d	RHSIM_Rhsim12G0144700	-1.178	0.607	1.784	low
CK-0d	RHSIM_Rhsim12G0164400	-0.798	-0.849	-0.052	no
CK-0d	RHSIM_Rhsim12G0106200	1.596	3.281	1.685	high
CK-0d	RHSIM_Rhsim13G0136300	1.634	1.683	0.050	high
CK-0d	RHSIM_Rhsim12G0194200	-0.381	0.920	1.302	no
CK-0d	RHSIM_Rhsim12G0004500	-0.668	-1.297	-0.628	no
CK-0d	RHSIM_Rhsim12G0137400	0.515	-0.367	-0.882	no
CK-0d	RHSIM_Rhsim12G0074200	0.352	-1.259	-1.611	no
CK-0d	RHSIM_Rhsim12G0107100	-0.297	1.300	1.597	no
CK-0d	RHSIM_Rhsim12G0087800	0.258	0.579	0.320	no
CK-0d	RHSIM_Rhsim12G0043300	0.558	0.452	-0.107	no
CK-0d	RHSIM_Rhsim12G0138800	1.049	0.358	-0.691	no
CK-0d	RHSIM_Rhsim12G0191200	-0.117	1.407	1.524	no
CK-0d	RHSIM_Rhsim12G0099400	-0.374	-0.784	-0.410	no
CK-0d	RHSIM_Rhsim12G0146800	-0.248	0.486	0.733	no
CK-0d	RHSIM_Rhsim12G0025000	0.169	-1.452	-1.622	no
CK-0d	RHSIM_Rhsim13G0057500	-1.210	0.598	1.808	low
CK-0d	RHSIM_Rhsim12G0071500	-0.281	-1.017	-0.736	no
CK-0d	RHSIM_Rhsim12G0013500	1.064	4.283	3.220	no
CK-0d	RHSIM_Rhsim12G0116700	1.971	-0.055	-2.026	high
CK-0d	RHSIM_Rhsim13G0228300	0.998	0.408	-0.590	no
CK-0d	RHSIM_Rhsim12G0063600	0.736	0.402	-0.334	no

Group	Gene	PTR (z-score difference)	z(log2 LFQ)	z(log2 FPKM)	extreme-PTR
CK-0d	RHSIM_Rhsim12G0167900	-2.660	-0.999	1.661	low
CK-0d	RHSIM_Rhsim12G0071800	-0.542	-0.721	-0.179	no
CK-0d	RHSIM_Rhsim12G0047500	-0.150	0.624	0.775	no
CK-0d	RHSIM_Rhsim12G0124800	-2.909	-0.006	2.903	low
CK-0d	RHSIM_Rhsim12G0046600	-0.674	-0.182	0.492	no
CK-0d	RHSIM_Rhsim12G0189200	-0.282	-0.002	0.279	no
CK-0d	RHSIM_Rhsim12G0182200	0.225	-0.532	-0.757	no
CK-0d	RHSIM_Rhsim12G0190600	0.935	0.393	-0.542	no
CK-0d	RHSIM_Rhsim13G0131000	-0.846	0.295	1.141	no
CK-0d	RHSIM_Rhsim11G0139400	0.549	-0.051	-0.600	no
CK-0d	RHSIM_Rhsim12G0139100	-1.746	0.387	2.133	low
CK-0d	RHSIM_Rhsim12G0127600	-0.547	0.875	1.422	no
CK-0d	RHSIM_Rhsim12G0193000	0.570	-0.157	-0.727	no
CK-0d	RHSIM_Rhsim12G0058100	-1.831	-0.129	1.702	low
CK-0d	RHSIM_Rhsim13G0116200	2.385	0.106	-2.280	high
CK-0d	RHSIM_Rhsim12G0004800	0.626	1.301	0.676	no
CK-0d	RHSIM_Rhsim12G0179700	0.247	-0.709	-0.956	no
CK-0d	RHSIM_Rhsim13G0166600	-0.581	-0.415	0.167	no
CK-0d	RHSIM_Rhsim12G0197200	-0.003	0.148	0.151	no
CK-0d	RHSIM_Rhsim12G0137300	-1.912	-0.881	1.031	low
CK-0d	RHSIM_Rhsim12G0126700	0.846	0.187	-0.659	no
CK-0d	RHSIM_Rhsim12G0025400	0.810	0.278	-0.532	no
CK-0d	RHSIM_Rhsim12G0028200	-0.706	-0.703	0.004	no
CK-0d	RHSIM_Rhsim12G0204700	0.249	0.476	0.228	no
CK-0d	RHSIM_Rhsim13G0047800	-0.409	-0.909	-0.500	no
CK-0d	RHSIM_Rhsim12G0199300	0.163	0.956	0.793	no
CK-0d	RHSIM_Rhsim12G0214300	-0.510	0.001	0.511	no
CK-0d	RHSIM_Rhsim13G0039000	-2.026	-1.346	0.680	low
CK-0d	RHSIM_Rhsim12G0139200	-0.958	-1.725	-0.767	no
CK-0d	RHSIM_Rhsim12G0162500	-1.190	-1.554	-0.364	low
CK-0d	RHSIM_Rhsim11G0006900	-0.952	-1.398	-0.446	no
CK-0d	RHSIM_Rhsim12G0053200	1.991	0.217	-1.774	high

Group	Gene	PTR (z-score difference)	z(log2 LFQ)	z(log2 FPKM)	extreme-PTR
CK-0d	RHSIM_Rhsim12G0191400	-0.795	0.398	1.192	no
CK-0d	RHSIM_Rhsim13G0098600	0.405	1.730	1.325	no
CK-0d	RHSIM_Rhsim12G0193700	1.396	0.890	-0.505	high
CK-0d	RHSIM_Rhsim12G0109300	-1.749	-1.105	0.644	low
CK-0d	RHSIM_Rhsim12G0121200	0.294	-0.176	-0.470	no
CK-0d	RHSIM_Rhsim13G0042200	-0.368	0.881	1.249	no
CK-0d	RHSIM_Rhsim12G0213600	2.500	2.691	0.190	high
CK-0d	RHSIM_Rhsim13G0138000	1.444	0.994	-0.450	high
CK-0d	RHSIM_Rhsim13G0234000	-1.155	-0.969	0.186	low
CK-0d	RHSIM_Rhsim13G0074400	1.091	0.257	-0.834	no
CK-0d	RHSIM_Rhsim13G0100300	-0.451	-1.003	-0.552	no
CK-0d	RHSIM_Rhsim12G0144100	0.859	0.566	-0.293	no
CK-0d	RHSIM_Rhsim12G0070500	1.741	1.156	-0.584	high
CK-0d	RHSIM_Rhsim12G0185700	0.470	-0.998	-1.468	no
CK-0d	RHSIM_Rhsim12G0104600	0.256	0.429	0.173	no
CK-0d	RHSIM_Rhsim12G0075100	0.943	-1.113	-2.056	no
CK-0d	RHSIM_Rhsim13G0081400	-0.391	-1.165	-0.775	no
CK-0d	RHSIM_Rhsim13G0045200	0.544	0.283	-0.261	no
CK-0d	RHSIM_Rhsim12G0085800	-1.954	-1.882	0.072	low
CK-0d	RHSIM_Rhsim12G0206900	-1.317	0.098	1.415	low
CK-0d	RHSIM_Rhsim13G0009500	0.124	-0.959	-1.083	no
CK-0d	RHSIM_Rhsim12G0070100	-0.558	-1.428	-0.870	no
CK-0d	RHSIM_Rhsim12G0144000	-2.121	-0.605	1.516	low
CK-0d	RHSIM_Rhsim12G0004100	0.555	0.802	0.247	no
CK-0d	RHSIM_Rhsim13G0199500	0.810	-0.769	-1.580	no
CK-0d	RHSIM_Rhsim11G0191500	-2.440	0.647	3.086	low
CK-0d	RHSIM_Rhsim12G0055000	-2.480	-0.893	1.587	low
CK-0d	RHSIM_Rhsim12G0005700	-1.027	-1.239	-0.212	low
CK-0d	RHSIM_Rhsim13G0050700	-0.622	1.453	2.075	no
CK-0d	RHSIM_Rhsim13G0194300	0.105	1.560	1.455	no
CK-0d	RHSIM_Rhsim12G0027600	-0.510	0.449	0.959	no
CK-0d	RHSIM_Rhsim12G0087900	0.509	0.838	0.329	no

Group	Gene	PTR (z-score difference)	z(log2 LFQ)	z(log2 FPKM)	extreme-PTR
CK-0d	RHSIM_Rhsim11G0052100	1.332	0.940	-0.392	high
CK-0d	RHSIM_Rhsim13G0026100	0.301	-0.154	-0.456	no
CK-0d	RHSIM_Rhsim12G0048100	0.529	0.709	0.180	no
CK-0d	RHSIM_Rhsim12G0002800	0.930	-0.006	-0.936	no
CK-0d	RHSIM_Rhsim12G0038100	-0.395	-0.655	-0.260	no
CK-0d	RHSIM_Rhsim12G0168200	1.127	-0.665	-1.792	high
CK-0d	RHSIM_Rhsim12G0148300	-0.783	0.704	1.487	no
CK-0d	RHSIM_Rhsim12G0177900	-0.593	1.140	1.733	no
CK-0d	RHSIM_Rhsim13G0174400	1.401	0.090	-1.312	high
CK-0d	RHSIM_Rhsim12G0138000	-0.074	0.485	0.559	no
CK-0d	RHSIM_Rhsim13G0119700	1.960	0.201	-1.758	high
CK-0d	RHSIM_Rhsim12G0025300	-1.164	-0.629	0.535	low
CK-0d	RHSIM_Rhsim12G0113100	-1.247	-0.814	0.433	low
CK-0d	RHSIM_Rhsim13G0148600	-1.538	0.085	1.624	low
CK-0d	RHSIM_Rhsim12G0203300	0.830	-0.989	-1.819	no
CK-0d	RHSIM_Rhsim13G0163200	0.595	-0.462	-1.057	no
CK-0d	RHSIM_Rhsim12G0126600	0.309	-0.767	-1.076	no
CK-0d	RHSIM_Rhsim12G0205400	-0.179	-0.418	-0.239	no
CK-0d	RHSIM_Rhsim12G0006200	1.289	1.682	0.394	high
CK-0d	RHSIM_Rhsim12G0070300	2.250	0.600	-1.650	high
CK-0d	RHSIM_Rhsim12G0165000	-0.284	-0.506	-0.222	no
CK-0d	RHSIM_Rhsim12G0006800	-0.133	-0.739	-0.606	no
CK-0d	RHSIM_Rhsim12G0172100	-0.253	-0.681	-0.428	no
CK-0d	RHSIM_Rhsim12G0110200	1.008	0.772	-0.236	no
CK-0d	RHSIM_Rhsim12G0048500	-1.000	-0.715	0.285	no
CK-0d	RHSIM_Rhsim12G0137200	1.095	0.595	-0.500	no
CK-0d	RHSIM_Rhsim12G0161200	0.228	0.318	0.090	no
CK-0d	RHSIM_Rhsim12G0003000	-0.935	0.319	1.254	no
CK-0d	RHSIM_Rhsim11G0167100	1.160	1.312	0.152	high
CK-0d	RHSIM_Rhsim12G0052000	0.797	1.387	0.590	no
CK-0d	RHSIM_Rhsim12G0198100	-0.121	-1.057	-0.935	no
CK-0d	RHSIM_Rhsim12G0147400	-0.218	-0.725	-0.507	no

Group	Gene	PTR (z-score difference)	z(log2 LFQ)	z(log2 FPKM)	extreme-PTR
CK-0d	RHSIM_Rhsim11G0124300	-0.670	0.029	0.699	no
CK-0d	RHSIM_Rhsim12G0115900	0.708	1.953	1.245	no
CK-0d	RHSIM_Rhsim12G0192800	0.586	-0.701	-1.287	no
CK-0d	RHSIM_Rhsim13G0126800	-0.307	-0.493	-0.186	no
CK-0d	RHSIM_Rhsim12G0171000	1.151	-0.217	-1.368	high
CK-0d	RHSIM_Rhsim11G0045800	-2.031	-0.129	1.903	low
CK-0d	RHSIM_Rhsim12G0192300	0.881	1.080	0.199	no
CK-0d	RHSIM_Rhsim11G0195600	-3.245	-1.383	1.862	low
CK-0d	RHSIM_Rhsim12G0102400	0.288	1.145	0.857	no
CK-0d	RHSIM_Rhsim11G0129600	-0.410	0.227	0.636	no
CK-0d	RHSIM_Rhsim12G0133400	0.039	2.112	2.074	no
CK-0d	RHSIM_Rhsim12G0043200	-0.784	0.291	1.075	no
CK-0d	RHSIM_Rhsim12G0153900	0.065	0.359	0.295	no
CK-0d	RHSIM_Rhsim12G0015200	-0.305	0.458	0.763	no
CK-0d	RHSIM_Rhsim11G0167300	1.905	1.559	-0.346	high
CK-0d	RHSIM_Rhsim11G0127100	-0.912	-0.488	0.424	no
CK-0d	RHSIM_Rhsim12G0138500	-0.141	1.543	1.684	no
CK-0d	RHSIM_Rhsim11G0100300	-1.608	1.300	2.909	low
CK-0d	RHSIM_Rhsim11G0188300	-1.633	-0.754	0.878	low
CK-0d	RHSIM_Rhsim12G0099800	-0.281	2.480	2.761	no
CK-0d	RHSIM_Rhsim12G0135900	-0.224	-0.375	-0.151	no
CK-0d	RHSIM_Rhsim11G0132400	-2.145	-1.891	0.254	low
CK-0d	RHSIM_Rhsim12G0047700	-0.004	0.353	0.357	no
CK-0d	RHSIM_Rhsim11G0129000	1.585	-0.257	-1.842	high
CK-0d	RHSIM_Rhsim12G0214500	-0.669	-1.192	-0.524	no
CK-0d	RHSIM_Rhsim11G0034700	0.479	1.075	0.595	no
CK-0d	RHSIM_Rhsim11G0172800	0.837	1.707	0.870	no
CK-0d	RHSIM_Rhsim12G0017000	1.090	0.863	-0.228	no
CK-0d	RHSIM_Rhsim12G0152700	-0.176	-1.414	-1.238	no
CK-0d	RHSIM_Rhsim12G0054500	-0.059	1.556	1.615	no
CK-0d	RHSIM_Rhsim12G0183500	1.106	1.938	0.832	no
CK-0d	RHSIM_Rhsim11G0011600	0.510	0.762	0.252	no

Group	Gene	PTR (z-score difference)	z(log2 LFQ)	z(log2 FPKM)	extreme-PTR
CK-0d	RHSIM_Rhsim13G0178500	-0.930	-1.260	-0.330	no
CK-0d	RHSIM_Rhsim12G0168600	-0.070	0.156	0.225	no
CK-0d	RHSIM_Rhsim12G0065100	-0.983	-0.744	0.239	no
CK-0d	RHSIM_Rhsim11G0129800	-1.123	-0.571	0.553	low
CK-0d	RHSIM_Rhsim11G0092300	-0.738	-1.204	-0.467	no
CK-0d	RHSIM_Rhsim12G0132900	0.071	-0.101	-0.172	no
CK-0d	RHSIM_Rhsim13G0080100	-0.501	-0.014	0.487	no
CK-0d	RHSIM_Rhsim12G0063100	0.237	0.050	-0.187	no
CK-0d	RHSIM_Rhsim11G0076700	-0.056	-1.478	-1.422	no
CK-0d	RHSIM_Rhsim12G0166300	0.471	0.246	-0.226	no
CK-0d	RHSIM_Rhsim11G0025100	-0.000	-0.894	-0.893	no
CK-0d	RHSIM_Rhsim12G0124500	-0.691	-1.312	-0.621	no
CK-0d	RHSIM_Rhsim12G0207300	0.603	0.648	0.044	no
CK-0d	RHSIM_Rhsim11G0101800	0.034	-0.814	-0.848	no
CK-0d	RHSIM_Rhsim12G0134100	-0.234	-0.788	-0.555	no
CK-0d	RHSIM_Rhsim12G0197600	0.880	0.644	-0.236	no
CK-0d	RHSIM_Rhsim11G0114600	1.249	-0.007	-1.256	high
CK-0d	RHSIM_Rhsim12G0164700	-0.203	0.114	0.317	no
CK-0d	RHSIM_Rhsim12G0089200	-0.467	-0.852	-0.386	no
CK-0d	RHSIM_Rhsim12G0064700	-0.493	-0.559	-0.066	no
CK-0d	RHSIM_Rhsim11G0093300	-1.233	0.176	1.409	low
CK-0d	RHSIM_Rhsim12G0042000	0.376	0.392	0.016	no
CK-0d	RHSIM_Rhsim12G0171100	0.410	1.600	1.190	no
CK-0d	RHSIM_Rhsim11G0034400	0.657	0.021	-0.635	no
CK-0d	RHSIM_Rhsim12G0182900	0.250	0.116	-0.134	no
CK-0d	RHSIM_Rhsim13G0038100	-0.946	-0.399	0.548	no
CK-0d	RHSIM_Rhsim11G0188100	-0.274	1.359	1.633	no
CK-0d	RHSIM_Rhsim12G0096200	-0.759	-0.367	0.392	no
CK-0d	RHSIM_Rhsim13G0074500	-0.706	-0.568	0.138	no
CK-0d	RHSIM_Rhsim11G0135800	1.589	2.070	0.481	high
CK-0d	RHSIM_Rhsim12G0046400	0.032	1.876	1.843	no
CK-0d	RHSIM_Rhsim11G0001200	0.005	-0.084	-0.089	no

Group	Gene	PTR (z-score difference)	z(log2 LFQ)	z(log2 FPKM)	extreme-PTR
CK-0d	RHSIM_Rhsim11G0136900	-0.342	1.280	1.622	no
CK-0d	RHSIM_Rhsim12G0065000	2.020	1.569	-0.451	high
CK-0d	RHSIM_Rhsim11G0182800	1.483	-0.458	-1.941	high
CK-0d	RHSIM_Rhsim11G0039200	-2.807	-0.367	2.439	low
CK-0d	RHSIM_Rhsim12G0161700	-0.103	-1.483	-1.380	no
CK-0d	RHSIM_Rhsim11G0125200	0.001	-0.076	-0.077	no
CK-0d	RHSIM_Rhsim11G0098400	1.429	1.659	0.230	high
CK-0d	RHSIM_Rhsim12G0047800	-0.892	-1.813	-0.921	no
CK-0d	RHSIM_Rhsim11G0186300	-0.371	-0.031	0.341	no
CK-0d	RHSIM_Rhsim11G0072000	2.112	0.495	-1.616	high
CK-0d	RHSIM_Rhsim12G0073200	1.050	0.584	-0.466	no
CK-0d	RHSIM_Rhsim12G0129600	-1.118	-1.603	-0.485	low
CK-0d	RHSIM_Rhsim11G0176000	0.954	1.041	0.087	no
CK-0d	RHSIM_Rhsim11G0133600	-0.827	-0.092	0.735	no
CK-0d	RHSIM_Rhsim12G0130600	0.225	0.564	0.339	no
CK-0d	RHSIM_Rhsim11G0197000	-0.463	0.294	0.757	no
CK-0d	RHSIM_Rhsim11G0113300	-0.236	0.016	0.252	no
CK-0d	RHSIM_Rhsim11G0192400	-0.506	0.994	1.500	no
CK-0d	RHSIM_Rhsim12G0016200	-0.346	-0.894	-0.548	no
CK-0d	RHSIM_Rhsim12G0079100	-0.478	-0.075	0.403	no
CK-0d	RHSIM_Rhsim11G0095300	0.253	-0.630	-0.883	no
CK-0d	RHSIM_Rhsim13G0229400	1.314	-0.257	-1.571	high
CK-0d	RHSIM_Rhsim11G0016500	-0.481	0.668	1.149	no
CK-0d	RHSIM_Rhsim12G0206500	1.107	1.533	0.426	no
CK-0d	RHSIM_Rhsim11G0121500	0.826	1.234	0.408	no
CK-0d	RHSIM_Rhsim13G0197700	0.444	1.275	0.830	no
CK-0d	RHSIM_Rhsim13G0015200	-0.694	-0.706	-0.012	no
CK-0d	RHSIM_Rhsim11G0160100	-0.109	-0.675	-0.566	no
CK-0d	RHSIM_Rhsim12G0201500	0.273	0.361	0.088	no
CK-0d	RHSIM_Rhsim11G0177100	-0.036	0.592	0.628	no
CK-0d	RHSIM_Rhsim13G0133100	3.398	1.186	-2.212	high
CK-0d	RHSIM_Rhsim11G0161200	-0.497	-0.001	0.496	no

Group	Gene	PTR (z-score difference)	z(log2 LFQ)	z(log2 FPKM)	extreme-PTR
CK-0d	RHSIM_Rhsim12G0128500	1.226	-0.066	-1.292	high
CK-0d	RHSIM_Rhsim11G0121900	-0.553	-0.949	-0.396	no
CK-0d	RHSIM_Rhsim11G0196200	0.227	-0.718	-0.945	no
CK-0d	RHSIM_Rhsim13G0234500	0.584	0.076	-0.508	no
CK-0d	RHSIM_Rhsim13G0201500	0.577	2.562	1.986	no
CK-0d	RHSIM_Rhsim11G0148100	-0.476	-0.300	0.176	no
CK-0d	RHSIM_Rhsim13G0052600	-0.943	-1.284	-0.341	no
CK-0d	RHSIM_Rhsim11G0122800	-0.197	-0.181	0.016	no
CK-0d	RHSIM_Rhsim11G0017900	0.497	-0.770	-1.268	no
CK-0d	RHSIM_Rhsim11G0130100	0.426	1.796	1.371	no
CK-0d	RHSIM_Rhsim13G0010400	-0.190	-0.489	-0.299	no
CK-0d	RHSIM_Rhsim12G0087000	-0.294	-0.214	0.080	no
CK-0d	RHSIM_Rhsim11G0167000	0.817	1.584	0.767	no
CK-0d	RHSIM_Rhsim11G0153500	0.497	0.344	-0.153	no
CK-0d	RHSIM_Rhsim12G0080800	1.459	0.229	-1.230	high
CK-0d	RHSIM_Rhsim11G0106600	0.171	-1.008	-1.179	no
CK-0d	RHSIM_Rhsim11G0165700	-0.842	0.101	0.943	no
CK-0d	RHSIM_Rhsim13G0153900	0.378	0.196	-0.182	no
CK-0d	RHSIM_Rhsim11G0097900	0.066	-0.660	-0.725	no
CK-0d	RHSIM_Rhsim12G0010700	1.909	1.792	-0.116	high
CK-0d	RHSIM_Rhsim11G0099800	0.787	0.054	-0.733	no
CK-0d	RHSIM_Rhsim13G0049600	-2.365	-0.671	1.694	low
CK-0d	RHSIM_Rhsim12G0076700	-0.282	-0.342	-0.060	no
CK-0d	RHSIM_Rhsim12G0209000	0.371	0.654	0.283	no
CK-0d	RHSIM_Rhsim12G0090500	-0.328	-0.405	-0.076	no
CK-0d	RHSIM_Rhsim11G0132600	-0.488	-0.575	-0.088	no
CK-0d	RHSIM_Rhsim11G0099600	-2.020	-0.589	1.431	low
CK-0d	RHSIM_Rhsim11G0121300	-0.080	1.216	1.296	no
CK-0d	RHSIM_Rhsim11G0123700	0.255	-0.002	-0.257	no
CK-0d	RHSIM_Rhsim11G0161300	0.971	-0.687	-1.658	no
CK-0d	RHSIM_Rhsim11G0097400	-0.700	-0.914	-0.214	no
CK-0d	RHSIM_Rhsim11G0145600	-0.817	1.236	2.054	no

Group	Gene	PTR (z-score difference)	z(log2 LFQ)	z(log2 FPKM)	extreme-PTR
CK-0d	RHSIM_Rhsim11G0045300	1.770	2.176	0.406	high
CK-0d	RHSIM_Rhsim12G0206700	0.040	-0.291	-0.331	no
CK-0d	RHSIM_Rhsim11G0058800	-0.887	1.175	2.061	no
CK-0d	RHSIM_Rhsim12G0077400	0.704	-1.225	-1.929	no
CK-0d	RHSIM_Rhsim11G0191300	-1.383	-1.301	0.082	low
CK-0d	RHSIM_Rhsim12G0215400	-0.189	0.229	0.418	no
CK-0d	RHSIM_Rhsim11G0142100	1.584	1.375	-0.208	high
CK-0d	RHSIM_Rhsim11G0199200	-0.139	-0.329	-0.190	no
CK-0d	RHSIM_Rhsim13G0037000	0.407	0.440	0.033	no
CK-0d	RHSIM_Rhsim11G0044200	2.014	1.027	-0.988	high
CK-0d	RHSIM_Rhsim12G0025900	0.288	0.168	-0.120	no
CK-0d	RHSIM_Rhsim12G0200000	0.931	-0.577	-1.508	no
CK-0d	RHSIM_Rhsim11G0142400	1.980	0.968	-1.012	high
CK-0d	RHSIM_Rhsim11G0036800	-0.850	-0.886	-0.036	no
CK-0d	RHSIM_Rhsim11G0105300	-0.817	-1.001	-0.184	no
CK-0d	RHSIM_Rhsim11G0149600	0.711	0.047	-0.665	no
CK-0d	RHSIM_Rhsim12G0149800	-0.066	1.612	1.678	no
CK-0d	RHSIM_Rhsim11G0053800	1.372	-0.802	-2.174	high
CK-0d	RHSIM_Rhsim11G0197800	-1.415	-0.948	0.467	low
CK-0d	RHSIM_Rhsim11G0006500	-0.179	-0.688	-0.510	no
CK-0d	RHSIM_Rhsim12G0003500	1.494	1.745	0.250	high
CK-0d	RHSIM_Rhsim11G0179400	0.217	0.244	0.027	no
CK-0d	RHSIM_Rhsim11G0008100	2.560	1.355	-1.205	high
CK-0d	RHSIM_Rhsim11G0084200	-1.400	2.063	3.463	low
CK-0d	RHSIM_Rhsim04G0146800	-0.455	0.567	1.023	no
CK-0d	RHSIM_Rhsim11G0166500	-0.708	-0.526	0.182	no
CK-0d	RHSIM_Rhsim11G0036600	0.498	-0.754	-1.252	no
CK-0d	RHSIM_Rhsim11G0087200	-0.380	0.707	1.087	no
CK-0d	RHSIM_Rhsim12G0157100	-0.601	-0.932	-0.331	no
CK-0d	RHSIM_Rhsim12G0151900	-0.585	1.877	2.462	no
CK-0d	RHSIM_Rhsim11G0088900	0.066	0.364	0.298	no
CK-0d	RHSIM_Rhsim11G0108000	0.713	-1.517	-2.230	no

Group	Gene	PTR (z-score difference)	z(log2 LFQ)	z(log2 FPKM)	extreme-PTR
CK-0d	RHSIM_Rhsim13G0225000	0.260	-1.439	-1.699	no
CK-0d	RHSIM_Rhsim11G0168200	0.151	-1.238	-1.389	no
CK-0d	RHSIM_Rhsim11G0003700	0.218	0.887	0.669	no
CK-0d	RHSIM_Rhsim11G0136600	0.763	0.185	-0.578	no
CK-0d	RHSIM_Rhsim11G0168600	-1.039	-1.429	-0.390	low
CK-0d	RHSIM_Rhsim12G0179900	0.043	-0.891	-0.934	no
CK-0d	RHSIM_Rhsim11G0193300	0.152	-0.976	-1.128	no
CK-0d	RHSIM_Rhsim11G0105100	0.173	1.100	0.926	no
CK-0d	RHSIM_Rhsim11G0088000	-0.316	-0.146	0.171	no
CK-0d	RHSIM_Rhsim12G0090900	-0.856	-0.985	-0.129	no
CK-0d	RHSIM_Rhsim12G0193400	-1.244	1.166	2.410	low
CK-0d	RHSIM_Rhsim11G0183800	0.225	3.777	3.552	no
CK-0d	RHSIM_Rhsim13G0213200	-0.858	1.390	2.249	no
CK-0d	RHSIM_Rhsim11G0066500	1.178	-0.586	-1.765	high
CK-0d	RHSIM_Rhsim11G0008300	1.167	-0.047	-1.214	high
CK-0d	RHSIM_Rhsim12G0145800	0.358	0.162	-0.196	no
CK-0d	RHSIM_Rhsim13G0229300	0.192	1.590	1.397	no
CK-0d	RHSIM_Rhsim11G0098600	-0.140	0.028	0.168	no
CK-0d	RHSIM_Rhsim11G0180700	-0.079	0.295	0.374	no
CK-0d	RHSIM_Rhsim12G0201700	0.179	1.072	0.893	no
CK-0d	RHSIM_Rhsim13G0029900	0.787	1.292	0.506	no
CK-0d	RHSIM_Rhsim12G0180500	0.313	-0.270	-0.583	no
CK-0d	RHSIM_Rhsim11G0065900	-1.366	-0.493	0.872	low
CK-0d	RHSIM_Rhsim11G0159300	1.191	0.611	-0.580	high
CK-0d	RHSIM_Rhsim12G0167000	-0.926	-1.175	-0.249	no
CK-0d	RHSIM_Rhsim11G0055100	0.070	-0.012	-0.082	no
CK-0d	RHSIM_Rhsim11G0016400	-0.097	-0.842	-0.746	no
CK-0d	RHSIM_Rhsim11G0022000	-0.561	-0.371	0.189	no
CK-0d	RHSIM_Rhsim11G0184900	-0.397	0.084	0.481	no
CK-0d	RHSIM_Rhsim11G0063600	1.671	0.831	-0.840	high
CK-0d	RHSIM_Rhsim13G0166900	0.652	-0.633	-1.285	no
CK-0d	RHSIM_Rhsim11G0010400	-0.412	-0.989	-0.577	no

Group	Gene	PTR (z-score difference)	z(log2 LFQ)	z(log2 FPKM)	extreme-PTR
CK-0d	RHSIM_Rhsim11G0063800	-1.586	-1.848	-0.261	low
CK-0d	RHSIM_Rhsim11G0174300	0.905	0.009	-0.896	no
CK-0d	RHSIM_Rhsim11G0085900	-0.129	1.703	1.832	no
CK-0d	RHSIM_Rhsim11G0041300	1.879	1.845	-0.035	high
CK-0d	RHSIM_Rhsim12G0209200	0.098	-0.026	-0.124	no
CK-0d	RHSIM_Rhsim12G0134600	1.107	0.526	-0.582	no
CK-0d	RHSIM_Rhsim12G0024700	-1.583	-0.722	0.860	low
CK-0d	RHSIM_Rhsim11G0198200	-0.085	0.285	0.370	no
CK-0d	RHSIM_Rhsim12G0025500	-1.352	-0.673	0.680	low
CK-0d	RHSIM_Rhsim12G0109900	0.145	-1.270	-1.415	no
CK-0d	RHSIM_Rhsim11G0112000	-0.342	-0.732	-0.390	no
CK-0d	RHSIM_Rhsim12G0133300	0.429	-0.176	-0.605	no
CK-0d	RHSIM_Rhsim12G0173900	-1.310	-1.397	-0.087	low
CK-0d	RHSIM_Rhsim11G0193600	0.796	0.283	-0.513	no
CK-0d	RHSIM_Rhsim12G0016800	0.652	0.495	-0.156	no
CK-0d	RHSIM_Rhsim12G0112100	-0.684	1.511	2.195	no
CK-0d	RHSIM_Rhsim11G0042300	0.498	-0.246	-0.744	no
CK-0d	RHSIM_Rhsim12G0212900	-0.218	-0.248	-0.029	no
CK-0d	RHSIM_Rhsim12G0144900	-0.125	-0.336	-0.211	no
CK-0d	RHSIM_Rhsim11G0090900	1.032	-0.226	-1.258	no
CK-0d	RHSIM_Rhsim11G0196900	-0.304	-0.676	-0.372	no
CK-0d	RHSIM_Rhsim11G0157200	-0.350	0.067	0.417	no
CK-0d	RHSIM_Rhsim12G0162200	0.918	-0.237	-1.156	no
CK-0d	RHSIM_Rhsim11G0176400	0.331	0.002	-0.330	no
CK-0d	RHSIM_Rhsim11G0036900	1.194	-0.186	-1.379	high
CK-0d	RHSIM_Rhsim11G0177200	-0.046	0.427	0.472	no
CK-0d	RHSIM_Rhsim11G0051900	1.629	-0.298	-1.927	high
CK-0d	RHSIM_Rhsim12G0137000	0.873	0.033	-0.839	no
CK-0d	RHSIM_Rhsim11G0045000	0.413	-0.297	-0.710	no
CK-0d	RHSIM_Rhsim12G0203700	1.885	-0.130	-2.015	high
CK-0d	RHSIM_Rhsim12G0130000	0.086	0.592	0.506	no
CK-0d	RHSIM_Rhsim11G0122200	1.109	2.574	1.466	no

Group	Gene	PTR (z-score difference)	z(log2 LFQ)	z(log2 FPKM)	extreme-PTR
CK-0d	RHSIM_Rhsim12G0005000	0.880	0.087	-0.793	no
CK-0d	RHSIM_Rhsim11G0114400	0.488	-0.641	-1.129	no
CK-0d	RHSIM_Rhsim11G0194700	0.463	0.954	0.491	no
CK-0d	RHSIM_Rhsim12G0003200	-0.005	-0.613	-0.608	no
CK-0d	RHSIM_Rhsim11G0182000	0.728	0.745	0.017	no
CK-0d	RHSIM_Rhsim11G0134200	1.253	0.509	-0.744	high
CK-0d	RHSIM_Rhsim11G0177800	-1.750	1.139	2.889	low
CK-0d	RHSIM_Rhsim11G0046400	0.525	-0.122	-0.647	no
CK-0d	RHSIM_Rhsim12G0041700	-2.404	-0.975	1.429	low
CK-0d	RHSIM_Rhsim11G0195000	0.091	-1.234	-1.325	no
CK-0d	RHSIM_Rhsim11G0054800	-1.072	-1.552	-0.481	low
CK-0d	RHSIM_Rhsim12G0002100	0.117	-0.983	-1.100	no
CK-0d	RHSIM_Rhsim11G0065700	-1.058	1.006	2.064	low
CK-0d	RHSIM_Rhsim11G0167500	1.393	0.850	-0.542	high
CK-0d	RHSIM_Rhsim11G0045700	0.388	-0.651	-1.039	no
CK-0d	RHSIM_Rhsim11G0118400	-0.117	0.441	0.558	no
CK-0d	RHSIM_Rhsim12G0204600	-0.189	1.795	1.984	no
CK-0d	RHSIM_Rhsim12G0051400	-0.960	-0.413	0.547	no
CK-0d	RHSIM_Rhsim11G0147400	-0.405	-0.417	-0.013	no
CK-0d	RHSIM_Rhsim12G0075500	0.740	1.799	1.060	no
CK-0d	RHSIM_Rhsim11G0184600	-1.116	-1.392	-0.275	low
CK-0d	RHSIM_Rhsim11G0199100	0.020	-0.716	-0.736	no
CK-0d	RHSIM_Rhsim11G0053000	-2.164	-1.402	0.762	low
CK-0d	RHSIM_Rhsim11G0020200	1.003	2.192	1.190	no
CK-0d	RHSIM_Rhsim11G0197500	0.220	0.197	-0.024	no
CK-0d	RHSIM_Rhsim12G0203600	3.236	1.141	-2.095	high
CK-0d	RHSIM_Rhsim11G0121700	0.566	1.051	0.486	no
CK-0d	RHSIM_Rhsim12G0017200	-0.460	0.528	0.987	no
CK-0d	RHSIM_Rhsim10G0183500	0.583	0.395	-0.189	no
CK-0d	RHSIM_Rhsim12G0037000	-0.593	-1.511	-0.918	no
CK-0d	RHSIM_Rhsim11G0167700	2.247	0.738	-1.509	high
CK-0d	RHSIM_Rhsim11G0088700	0.391	-1.298	-1.689	no

Group	Gene	PTR (z-score difference)	z(log2 LFQ)	z(log2 FPKM)	extreme-PTR
CK-0d	RHSIM_Rhsim10G0007500	-0.325	-0.154	0.171	no
CK-0d	RHSIM_Rhsim12G0000100	1.228	0.808	-0.420	high
CK-0d	RHSIM_Rhsim11G0054200	-0.380	-0.705	-0.324	no
CK-0d	RHSIM_Rhsim11G0163600	0.663	-0.086	-0.749	no
CK-0d	RHSIM_Rhsim10G0040200	0.666	0.879	0.213	no
CK-0d	RHSIM_Rhsim11G0195400	0.316	0.169	-0.147	no
CK-0d	RHSIM_Rhsim11G0156300	2.289	0.754	-1.535	high
CK-0d	RHSIM_Rhsim10G0102500	0.258	0.216	-0.042	no
CK-0d	RHSIM_Rhsim11G0122000	1.521	0.169	-1.352	high
CK-0d	RHSIM_Rhsim12G0102200	-0.347	-0.756	-0.409	no
CK-0d	RHSIM_Rhsim11G0161100	-3.428	-1.886	1.542	low
CK-0d	RHSIM_Rhsim11G0028700	-0.432	-1.786	-1.354	no
CK-0d	RHSIM_Rhsim11G0052000	0.474	-0.066	-0.540	no
CK-0d	RHSIM_Rhsim10G0183700	-0.053	1.650	1.703	no
CK-0d	RHSIM_Rhsim11G0175900	-0.669	-1.245	-0.576	no
CK-0d	RHSIM_Rhsim10G0204000	0.769	0.423	-0.345	no
CK-0d	RHSIM_Rhsim11G0125500	-0.191	-1.014	-0.822	no
CK-0d	RHSIM_Rhsim11G0173600	-2.437	-1.853	0.584	low
CK-0d	RHSIM_Rhsim10G0079900	0.899	1.203	0.304	no
CK-0d	RHSIM_Rhsim12G0090700	0.305	0.879	0.574	no
CK-0d	RHSIM_Rhsim11G0010500	0.155	0.383	0.228	no
CK-0d	RHSIM_Rhsim11G0095500	0.602	0.106	-0.496	no
CK-0d	RHSIM_Rhsim10G0133700	1.952	3.298	1.346	high
CK-0d	RHSIM_Rhsim10G0079400	0.696	0.114	-0.581	no
CK-0d	RHSIM_Rhsim10G0135700	2.150	1.383	-0.766	high
CK-0d	RHSIM_Rhsim12G0205300	-0.183	-0.098	0.084	no
CK-0d	RHSIM_Rhsim12G0036900	0.548	-0.591	-1.139	no
CK-0d	RHSIM_Rhsim10G0196000	-1.321	1.021	2.342	low
CK-0d	RHSIM_Rhsim11G0190300	0.235	0.399	0.164	no
CK-0d	RHSIM_Rhsim12G0105500	2.018	1.310	-0.708	high
CK-0d	RHSIM_Rhsim10G0045000	-1.073	-0.210	0.863	low
CK-0d	RHSIM_Rhsim12G0185400	-1.883	-1.168	0.715	low

Group	Gene	PTR (z-score difference)	z(log2 LFQ)	z(log2 FPKM)	extreme-PTR
CK-0d	RHSIM_Rhsim10G0210800	1.569	-0.421	-1.991	high
CK-0d	RHSIM_Rhsim12G0110600	-1.380	-0.656	0.724	low
CK-0d	RHSIM_Rhsim12G0080300	-0.709	-1.682	-0.973	no
CK-0d	RHSIM_Rhsim10G0121300	-0.271	-0.643	-0.372	no
CK-0d	RHSIM_Rhsim11G0008500	-0.034	0.066	0.099	no
CK-0d	RHSIM_Rhsim10G0069400	-1.726	-1.479	0.247	low
CK-0d	RHSIM_Rhsim12G0168500	0.202	-0.199	-0.400	no
CK-0d	RHSIM_Rhsim11G0196800	0.360	-0.098	-0.458	no
CK-0d	RHSIM_Rhsim10G0029000	0.186	-0.076	-0.262	no
CK-0d	RHSIM_Rhsim10G0155900	0.868	-1.104	-1.972	no
CK-0d	RHSIM_Rhsim10G0009900	0.061	-1.383	-1.444	no
CK-0d	RHSIM_Rhsim10G0164700	-0.377	-0.754	-0.377	no
CK-0d	RHSIM_Rhsim12G0166000	-0.680	-0.626	0.054	no
CK-0d	RHSIM_Rhsim10G0197300	-0.868	0.919	1.788	no
CK-0d	RHSIM_Rhsim11G0097800	-0.106	0.736	0.841	no
CK-0d	RHSIM_Rhsim12G0007900	-0.419	0.513	0.932	no
CK-0d	RHSIM_Rhsim12G0139700	1.284	0.432	-0.852	high
CK-0d	RHSIM_Rhsim10G0171300	-3.373	-1.969	1.405	low
CK-0d	RHSIM_Rhsim12G0159900	-0.869	-0.884	-0.015	no
CK-0d	RHSIM_Rhsim11G0167600	1.001	1.298	0.298	no
CK-0d	RHSIM_Rhsim10G0067100	-0.571	-0.588	-0.016	no
CK-0d	RHSIM_Rhsim11G0126200	-0.182	-0.485	-0.303	no
CK-0d	RHSIM_Rhsim11G0023700	0.288	0.737	0.449	no
CK-0d	RHSIM_Rhsim12G0068000	-0.086	0.608	0.695	no
CK-0d	RHSIM_Rhsim11G0198400	0.817	0.983	0.166	no
CK-0d	RHSIM_Rhsim11G0052800	-0.416	-1.364	-0.947	no
CK-0d	RHSIM_Rhsim10G0051200	-1.007	-0.637	0.370	no
CK-0d	RHSIM_Rhsim10G0191900	0.154	-0.540	-0.693	no
CK-0d	RHSIM_Rhsim10G0026400	0.454	0.810	0.355	no
CK-0d	RHSIM_Rhsim10G0030500	-0.392	-1.998	-1.606	no
CK-0d	RHSIM_Rhsim10G0005500	-0.159	0.101	0.260	no
CK-0d	RHSIM_Rhsim10G0105200	0.300	-0.074	-0.374	no

Group	Gene	PTR (z-score difference)	z(log2 LFQ)	z(log2 FPKM)	extreme-PTR
CK-0d	RHSIM_Rhsim12G0114000	-0.843	-0.759	0.084	no
CK-0d	RHSIM_Rhsim10G0005700	-0.014	-0.417	-0.403	no
CK-0d	RHSIM_Rhsim10G0117200	0.137	1.209	1.072	no
CK-0d	RHSIM_Rhsim10G0191600	0.089	-0.851	-0.940	no
CK-0d	RHSIM_Rhsim10G0194400	0.502	0.475	-0.028	no
CK-0d	RHSIM_Rhsim11G0053100	1.451	1.613	0.162	high
CK-0d	RHSIM_Rhsim11G0198000	0.113	-0.401	-0.514	no
CK-0d	RHSIM_Rhsim11G0113700	-1.136	0.317	1.454	low
CK-0d	RHSIM_Rhsim10G0180700	1.061	-0.273	-1.333	no
CK-0d	RHSIM_Rhsim10G0199000	1.208	-0.203	-1.412	high
CK-0d	RHSIM_Rhsim12G0008000	0.933	0.017	-0.916	no
CK-0d	RHSIM_Rhsim10G0035600	-0.073	-0.421	-0.348	no
CK-0d	RHSIM_Rhsim10G0116000	-1.156	-1.060	0.096	low
CK-0d	RHSIM_Rhsim11G0048900	-0.789	-1.167	-0.378	no
CK-0d	RHSIM_Rhsim11G0167200	2.673	0.829	-1.844	high
CK-0d	RHSIM_Rhsim12G0070400	1.658	0.714	-0.943	high
CK-0d	RHSIM_Rhsim10G0148300	0.548	0.560	0.012	no
CK-0d	RHSIM_Rhsim10G0099800	-0.490	1.415	1.905	no
CK-0d	RHSIM_Rhsim10G0136800	-0.526	-0.306	0.219	no
CK-0d	RHSIM_Rhsim12G0115000	-2.173	-1.537	0.636	low
CK-0d	RHSIM_Rhsim10G0186500	0.146	0.433	0.287	no
CK-0d	RHSIM_Rhsim12G0166200	-0.203	-1.162	-0.958	no
CK-0d	RHSIM_Rhsim11G0135900	1.620	3.119	1.500	high
CK-0d	RHSIM_Rhsim12G0189800	-0.500	-0.324	0.176	no
CK-0d	RHSIM_Rhsim10G0108600	0.021	-0.100	-0.121	no
CK-0d	RHSIM_Rhsim10G0162300	0.903	-0.568	-1.471	no
CK-0d	RHSIM_Rhsim12G0014000	0.804	-0.004	-0.808	no
CK-0d	RHSIM_Rhsim10G0161200	-0.476	-0.950	-0.474	no
CK-0d	RHSIM_Rhsim11G0125700	-1.614	-1.586	0.028	low
CK-0d	RHSIM_Rhsim10G0192800	-0.456	-0.688	-0.232	no
CK-0d	RHSIM_Rhsim10G0197900	0.195	0.754	0.560	no
CK-0d	RHSIM_Rhsim12G0136800	-0.360	-1.045	-0.685	no

Group	Gene	PTR (z-score difference)	z(log2 LFQ)	z(log2 FPKM)	extreme-PTR
CK-0d	RHSIM_Rhsim10G0014000	-0.240	-0.719	-0.479	no
CK-0d	RHSIM_Rhsim10G0094500	0.071	-0.400	-0.471	no
CK-0d	RHSIM_Rhsim11G0173400	-2.425	-0.850	1.575	low
CK-0d	RHSIM_Rhsim12G0150700	0.254	0.689	0.435	no
CK-0d	RHSIM_Rhsim10G0069000	0.819	1.071	0.252	no
CK-0d	RHSIM_Rhsim11G0135200	-0.268	-0.246	0.022	no
CK-0d	RHSIM_Rhsim10G0019600	-0.763	-0.050	0.713	no
CK-0d	RHSIM_Rhsim12G0001500	1.137	0.356	-0.780	high
CK-0d	RHSIM_Rhsim11G0106300	1.604	1.373	-0.231	high
CK-0d	RHSIM_Rhsim10G0126200	1.196	1.725	0.528	high
CK-0d	RHSIM_Rhsim11G0074300	-1.125	0.669	1.794	low
CK-0d	RHSIM_Rhsim11G0155300	2.496	1.068	-1.428	high
CK-0d	RHSIM_Rhsim12G0128400	0.641	0.284	-0.357	no
CK-0d	RHSIM_Rhsim12G0213700	0.732	1.261	0.529	no
CK-0d	RHSIM_Rhsim10G0141300	1.557	3.237	1.680	high
CK-0d	RHSIM_Rhsim10G0115500	-0.087	-0.883	-0.796	no
CK-0d	RHSIM_Rhsim11G0130000	-0.234	-0.665	-0.430	no
CK-0d	RHSIM_Rhsim11G0064900	0.248	-0.856	-1.105	no
CK-0d	RHSIM_Rhsim10G0202500	-0.518	1.295	1.813	no
CK-0d	RHSIM_Rhsim10G0183800	-0.299	-1.512	-1.213	no
CK-0d	RHSIM_Rhsim10G0183200	-0.669	-0.651	0.018	no
CK-0d	RHSIM_Rhsim11G0192900	0.294	0.841	0.547	no
CK-0d	RHSIM_Rhsim11G0015100	-0.992	-1.657	-0.665	no
CK-0d	RHSIM_Rhsim10G0078300	-0.826	-0.739	0.086	no
CK-0d	RHSIM_Rhsim11G0164000	1.506	-0.811	-2.317	high
CK-0d	RHSIM_Rhsim10G0014400	-0.985	-1.077	-0.092	no
CK-0d	RHSIM_Rhsim10G0101200	0.232	1.548	1.316	no
CK-0d	RHSIM_Rhsim11G0159000	-0.429	1.288	1.717	no
CK-0d	RHSIM_Rhsim10G0099300	0.741	0.217	-0.524	no
CK-0d	RHSIM_Rhsim11G0099900	-0.501	-0.861	-0.360	no
CK-0d	RHSIM_Rhsim11G0075200	-0.806	-0.345	0.460	no
CK-0d	RHSIM_Rhsim11G0094000	0.470	1.754	1.283	no

Group	Gene	PTR (z-score difference)	z(log2 LFQ)	z(log2 FPKM)	extreme-PTR
CK-0d	RHSIM_Rhsim10G0156300	1.122	0.545	-0.577	high
CK-0d	RHSIM_Rhsim11G0003600	-0.643	0.068	0.711	no
CK-0d	RHSIM_Rhsim11G0077900	0.081	0.244	0.162	no
CK-0d	RHSIM_Rhsim10G0167100	2.598	1.735	-0.863	high
CK-0d	RHSIM_Rhsim11G0075500	-4.318	-1.232	3.086	low
CK-0d	RHSIM_Rhsim10G0071100	1.276	1.494	0.218	high
CK-0d	RHSIM_Rhsim10G0006900	-0.944	-2.076	-1.132	no
CK-0d	RHSIM_Rhsim11G0133300	2.869	1.483	-1.386	high
CK-0d	RHSIM_Rhsim11G0078100	1.482	1.004	-0.478	high
CK-0d	RHSIM_Rhsim11G0121800	0.283	1.673	1.390	no
CK-0d	RHSIM_Rhsim11G0094400	-0.148	0.901	1.049	no
CK-0d	RHSIM_Rhsim11G0093100	-0.154	-0.495	-0.340	no
CK-0d	RHSIM_Rhsim11G0159200	-1.295	-1.181	0.114	low
CK-0d	RHSIM_Rhsim10G0198000	-1.039	-1.692	-0.654	low
CK-0d	RHSIM_Rhsim10G0211400	-0.145	2.262	2.407	no
CK-0d	RHSIM_Rhsim10G0188500	-1.126	-0.039	1.087	low
CK-0d	RHSIM_Rhsim10G0172500	0.295	-0.288	-0.583	no
CK-0d	RHSIM_Rhsim11G0117700	-0.268	0.387	0.655	no
CK-0d	RHSIM_Rhsim11G0196300	0.388	1.453	1.065	no
CK-0d	RHSIM_Rhsim11G0019600	-0.138	0.093	0.231	no
CK-0d	RHSIM_Rhsim10G0006300	1.014	0.019	-0.995	no
CK-0d	RHSIM_Rhsim10G0112600	0.487	-0.526	-1.013	no
CK-0d	RHSIM_Rhsim11G0136500	-0.370	-0.481	-0.111	no
CK-0d	RHSIM_Rhsim10G0107900	0.665	1.015	0.350	no
CK-0d	RHSIM_Rhsim11G0090400	0.241	0.087	-0.154	no
CK-0d	RHSIM_Rhsim11G0190500	0.512	0.156	-0.356	no
CK-0d	RHSIM_Rhsim10G0008800	-1.068	-1.262	-0.194	low
CK-0d	RHSIM_Rhsim11G0136000	0.648	0.591	-0.057	no
CK-0d	RHSIM_Rhsim10G0014200	-0.260	-0.721	-0.461	no
CK-0d	RHSIM_Rhsim11G0141900	-0.299	0.036	0.335	no
CK-0d	RHSIM_Rhsim11G0131600	0.119	-0.946	-1.064	no
CK-0d	RHSIM_Rhsim10G0141900	0.050	-0.196	-0.246	no

Group	Gene	PTR (z-score difference)	z(log2 LFQ)	z(log2 FPKM)	extreme-PTR
CK-0d	RHSIM_Rhsim11G0072400	-0.785	-0.597	0.188	no
CK-0d	RHSIM_Rhsim10G0022700	-0.231	0.403	0.634	no
CK-0d	RHSIM_Rhsim11G0008000	-0.147	-0.723	-0.576	no
CK-0d	RHSIM_Rhsim11G0077600	-0.406	-0.793	-0.387	no
CK-0d	RHSIM_Rhsim11G0128300	0.282	0.348	0.066	no
CK-0d	RHSIM_Rhsim10G0068700	-1.749	-1.890	-0.142	low
CK-0d	RHSIM_Rhsim11G0172600	0.099	-0.609	-0.707	no
CK-0d	RHSIM_Rhsim11G0178400	-0.191	-0.445	-0.255	no
CK-0d	RHSIM_Rhsim10G0171700	0.911	2.583	1.672	no
CK-0d	RHSIM_Rhsim11G0165400	0.292	-0.039	-0.331	no
CK-0d	RHSIM_Rhsim10G0006400	0.186	0.780	0.594	no
CK-0d	RHSIM_Rhsim11G0180400	1.112	-0.194	-1.306	high
CK-0d	RHSIM_Rhsim11G0132900	-1.689	-1.164	0.525	low
CK-0d	RHSIM_Rhsim10G0072100	-1.115	0.043	1.158	low
CK-0d	RHSIM_Rhsim10G0134600	1.650	-0.323	-1.972	high
CK-0d	RHSIM_Rhsim10G0061400	-0.328	-0.028	0.300	no
CK-0d	RHSIM_Rhsim10G0039800	0.047	1.246	1.198	no
CK-0d	RHSIM_Rhsim10G0151800	-0.098	-0.660	-0.562	no
CK-0d	RHSIM_Rhsim10G0127000	-2.668	-1.624	1.044	low
CK-0d	RHSIM_Rhsim11G0184700	-1.123	-0.176	0.947	low
CK-0d	RHSIM_Rhsim11G0111500	-0.513	-0.396	0.117	no
CK-0d	RHSIM_Rhsim10G0021200	-0.205	3.419	3.624	no
CK-0d	RHSIM_Rhsim10G0013900	-0.176	0.096	0.272	no
CK-0d	RHSIM_Rhsim10G0015700	1.060	-1.015	-2.075	no
CK-0d	RHSIM_Rhsim10G0149000	0.892	0.461	-0.431	no
CK-0d	RHSIM_Rhsim10G0179500	-0.789	0.444	1.232	no
CK-0d	RHSIM_Rhsim10G0178400	-0.307	3.068	3.376	no
CK-0d	RHSIM_Rhsim10G0177300	0.397	1.433	1.036	no
CK-0d	RHSIM_Rhsim10G0127200	0.423	0.177	-0.245	no
CK-0d	RHSIM_Rhsim10G0019900	0.062	0.948	0.886	no
CK-0d	RHSIM_Rhsim10G0060000	-1.069	-1.402	-0.334	low
CK-0d	RHSIM_Rhsim10G0079500	1.425	0.867	-0.558	high

Group	Gene	PTR (z-score difference)	z(log2 LFQ)	z(log2 FPKM)	extreme-PTR
CK-0d	RHSIM_Rhsim10G0046600	0.588	0.102	-0.486	no
CK-0d	RHSIM_Rhsim10G0104000	-0.873	-1.650	-0.777	no
CK-0d	RHSIM_Rhsim10G0078600	0.073	0.047	-0.026	no
CK-0d	RHSIM_Rhsim11G0028300	1.231	1.126	-0.105	high
CK-0d	RHSIM_Rhsim10G0174600	-1.545	-1.240	0.306	low
CK-0d	RHSIM_Rhsim11G0045600	-1.145	0.153	1.298	low
CK-0d	RHSIM_Rhsim11G0197700	0.139	-0.855	-0.994	no
CK-0d	RHSIM_Rhsim10G0082000	-0.451	-0.519	-0.068	no
CK-0d	RHSIM_Rhsim10G0048900	0.513	1.119	0.606	no
CK-0d	RHSIM_Rhsim10G0022600	1.588	1.178	-0.409	high
CK-0d	RHSIM_Rhsim11G0169400	1.178	-0.220	-1.398	high
CK-0d	RHSIM_Rhsim10G0199600	1.622	0.938	-0.684	high
CK-0d	RHSIM_Rhsim10G0175100	0.972	1.057	0.085	no
CK-0d	RHSIM_Rhsim11G0173300	0.895	0.383	-0.513	no
CK-0d	RHSIM_Rhsim11G0014400	0.093	0.610	0.516	no
CK-0d	RHSIM_Rhsim11G0071200	-0.199	-0.682	-0.484	no
CK-0d	RHSIM_Rhsim11G0069400	0.778	1.905	1.128	no
CK-0d	RHSIM_Rhsim10G0163500	0.181	1.718	1.537	no
CK-0d	RHSIM_Rhsim11G0031700	-0.170	-0.690	-0.520	no
CK-0d	RHSIM_Rhsim11G0133700	-0.814	-1.715	-0.901	no
CK-0d	RHSIM_Rhsim10G0152000	1.255	1.278	0.023	high
CK-0d	RHSIM_Rhsim10G0185900	-1.598	-1.312	0.286	low
CK-0d	RHSIM_Rhsim10G0062200	2.824	1.442	-1.381	high
CK-0d	RHSIM_Rhsim11G0184000	-0.083	0.326	0.408	no
CK-0d	RHSIM_Rhsim10G0100700	-0.359	-1.103	-0.743	no
CK-0d	RHSIM_Rhsim11G0158600	-0.321	-1.164	-0.843	no
CK-0d	RHSIM_Rhsim11G0077800	1.445	0.463	-0.982	high
CK-0d	RHSIM_Rhsim10G0154300	2.443	0.682	-1.761	high
CK-0d	RHSIM_Rhsim10G0012800	0.650	-0.217	-0.867	no
CK-0d	RHSIM_Rhsim10G0207900	0.512	0.839	0.327	no
CK-0d	RHSIM_Rhsim09G0201000	0.052	0.230	0.178	no
CK-0d	RHSIM_Rhsim10G0035200	0.301	0.888	0.588	no

Group	Gene	PTR (z-score difference)	z(log2 LFQ)	z(log2 FPKM)	extreme-PTR
CK-0d	RHSIM_Rhsim10G0043800	1.486	2.053	0.567	high
CK-0d	RHSIM_Rhsim10G0128200	0.004	-0.700	-0.704	no
CK-0d	RHSIM_Rhsim10G0181800	1.992	0.142	-1.850	high
CK-0d	RHSIM_Rhsim11G0021700	0.817	1.149	0.332	no
CK-0d	RHSIM_Rhsim10G0209100	0.101	0.098	-0.003	no
CK-0d	RHSIM_Rhsim10G0006600	1.025	1.122	0.098	no
CK-0d	RHSIM_Rhsim11G0113500	1.296	1.223	-0.072	high
CK-0d	RHSIM_Rhsim10G0064900	0.522	-0.596	-1.118	no
CK-0d	RHSIM_Rhsim10G0202400	-0.588	0.350	0.938	no
CK-0d	RHSIM_Rhsim10G0213600	-1.495	-1.175	0.319	low
CK-0d	RHSIM_Rhsim10G0072600	-0.073	-1.155	-1.082	no
CK-0d	RHSIM_Rhsim09G0032200	0.209	0.062	-0.147	no
CK-0d	RHSIM_Rhsim10G0008600	1.299	0.383	-0.916	high
CK-0d	RHSIM_Rhsim10G0131300	-0.963	-1.264	-0.300	no
CK-0d	RHSIM_Rhsim10G0102800	-0.092	-1.117	-1.025	no
CK-0d	RHSIM_Rhsim11G0098900	0.144	-0.180	-0.323	no
CK-0d	RHSIM_Rhsim11G0100500	-1.485	-0.067	1.418	low
CK-0d	RHSIM_Rhsim10G0138000	1.713	1.647	-0.066	high
CK-0d	RHSIM_Rhsim09G0097600	0.024	0.034	0.010	no
CK-0d	RHSIM_Rhsim10G0102700	-0.097	-0.485	-0.388	no
CK-0d	RHSIM_Rhsim11G0088600	-0.250	-0.018	0.232	no
CK-0d	RHSIM_Rhsim10G0034800	1.846	0.112	-1.734	high
CK-0d	RHSIM_Rhsim11G0178900	-0.357	-0.378	-0.020	no
CK-0d	RHSIM_Rhsim10G0030200	-0.948	-0.068	0.880	no
CK-0d	RHSIM_Rhsim09G0168000	0.918	-0.738	-1.656	no
CK-0d	RHSIM_Rhsim10G0036600	0.069	-0.091	-0.160	no
CK-0d	RHSIM_Rhsim11G0030300	-0.275	-0.332	-0.057	no
CK-0d	RHSIM_Rhsim10G0109900	-1.965	-0.345	1.620	low
CK-0d	RHSIM_Rhsim11G0134800	-1.238	0.131	1.369	low
CK-0d	RHSIM_Rhsim11G0104600	1.421	-0.449	-1.870	high
CK-0d	RHSIM_Rhsim10G0077000	-1.111	-1.331	-0.220	low
CK-0d	RHSIM_Rhsim10G0019400	-1.348	-1.442	-0.094	low

Group	Gene	PTR (z-score difference)	z(log2 LFQ)	z(log2 FPKM)	extreme-PTR
CK-0d	RHSIM_Rhsim09G0144400	2.028	0.581	-1.447	high
CK-0d	RHSIM_Rhsim10G0084700	-0.388	-0.707	-0.320	no
CK-0d	RHSIM_Rhsim09G0121900	-0.159	-0.296	-0.137	no
CK-0d	RHSIM_Rhsim09G0169200	-1.370	-0.466	0.904	low
CK-0d	RHSIM_Rhsim11G0184800	0.797	1.477	0.679	no
CK-0d	RHSIM_Rhsim10G0202900	2.577	0.900	-1.676	high
CK-0d	RHSIM_Rhsim10G0138300	-1.218	-1.262	-0.044	low
CK-0d	RHSIM_Rhsim11G0058900	-0.131	0.173	0.304	no
CK-0d	RHSIM_Rhsim09G0150400	2.630	0.771	-1.859	high
CK-0d	RHSIM_Rhsim10G0028000	0.096	1.753	1.657	no
CK-0d	RHSIM_Rhsim09G0123000	-0.255	-0.051	0.204	no
CK-0d	RHSIM_Rhsim10G0210700	-0.645	-0.727	-0.082	no
CK-0d	RHSIM_Rhsim10G0023700	-0.369	-1.067	-0.698	no
CK-0d	RHSIM_Rhsim10G0156100	-0.777	-0.263	0.514	no
CK-0d	RHSIM_Rhsim09G0140200	-0.252	-0.388	-0.136	no
CK-0d	RHSIM_Rhsim11G0089800	1.421	0.089	-1.332	high
CK-0d	RHSIM_Rhsim09G0147900	0.799	-0.180	-0.979	no
CK-0d	RHSIM_Rhsim09G0181100	-0.123	-0.884	-0.761	no
CK-0d	RHSIM_Rhsim09G0069500	0.086	0.638	0.552	no
CK-0d	RHSIM_Rhsim10G0209800	0.636	1.393	0.758	no
CK-0d	RHSIM_Rhsim10G0009000	0.000	0.144	0.144	no
CK-0d	RHSIM_Rhsim09G0208200	0.473	0.628	0.156	no
CK-0d	RHSIM_Rhsim10G0029200	-0.362	0.273	0.635	no
CK-0d	RHSIM_Rhsim09G0208400	0.512	1.271	0.759	no
CK-0d	RHSIM_Rhsim10G0180300	0.241	-0.460	-0.701	no
CK-0d	RHSIM_Rhsim10G0212700	0.770	0.690	-0.080	no
CK-0d	RHSIM_Rhsim10G0185200	0.038	-1.270	-1.308	no
CK-0d	RHSIM_Rhsim10G0056100	0.460	0.308	-0.152	no
CK-0d	RHSIM_Rhsim10G0176600	1.348	2.048	0.700	high
CK-0d	RHSIM_Rhsim09G0128400	1.219	-0.986	-2.205	high
CK-0d	RHSIM_Rhsim09G0110200	-0.118	-1.047	-0.930	no
CK-0d	RHSIM_Rhsim09G0157700	0.159	1.347	1.188	no

Group	Gene	PTR (z-score difference)	z(log2 LFQ)	z(log2 FPKM)	extreme-PTR
CK-0d	RHSIM_Rhsim09G0003700	1.684	1.589	-0.095	high
CK-0d	RHSIM_Rhsim10G0189100	-0.669	-0.834	-0.165	no
CK-0d	RHSIM_Rhsim10G0011300	-0.409	-0.772	-0.363	no
CK-0d	RHSIM_Rhsim09G0208000	0.056	-0.607	-0.663	no
CK-0d	RHSIM_Rhsim09G0060100	-2.636	-1.233	1.403	low
CK-0d	RHSIM_Rhsim09G0141900	-0.254	0.807	1.061	no
CK-0d	RHSIM_Rhsim11G0099400	-0.288	0.342	0.630	no
CK-0d	RHSIM_Rhsim10G0034700	-0.625	-0.168	0.458	no
CK-0d	RHSIM_Rhsim10G0029400	-1.084	-0.934	0.150	low
CK-0d	RHSIM_Rhsim10G0005000	-2.156	-1.064	1.092	low
CK-0d	RHSIM_Rhsim09G0111300	-0.079	-0.453	-0.374	no
CK-0d	RHSIM_Rhsim09G0207500	-0.498	0.125	0.623	no
CK-0d	RHSIM_Rhsim10G0030100	0.764	-1.117	-1.881	no
CK-0d	RHSIM_Rhsim10G0064100	-2.383	-1.199	1.184	low
CK-0d	RHSIM_Rhsim10G0026800	0.995	-0.874	-1.869	no
CK-0d	RHSIM_Rhsim10G0113200	-0.962	0.520	1.481	no
CK-0d	RHSIM_Rhsim09G0018700	0.038	-1.247	-1.285	no
CK-0d	RHSIM_Rhsim10G0067800	-0.051	0.291	0.342	no
CK-0d	RHSIM_Rhsim09G0141100	-0.305	0.012	0.317	no
CK-0d	RHSIM_Rhsim10G0018300	-0.826	0.569	1.395	no
CK-0d	RHSIM_Rhsim09G0004300	-0.660	-1.652	-0.992	no
CK-0d	RHSIM_Rhsim10G0015600	1.413	0.647	-0.766	high
CK-0d	RHSIM_Rhsim11G0141800	-1.357	-0.942	0.415	low
CK-0d	RHSIM_Rhsim09G0167500	-0.919	-1.417	-0.498	no
CK-0d	RHSIM_Rhsim09G0187300	0.588	0.463	-0.124	no
CK-0d	RHSIM_Rhsim10G0126900	-2.034	-1.417	0.617	low
CK-0d	RHSIM_Rhsim09G0208500	-0.154	-0.445	-0.290	no
CK-0d	RHSIM_Rhsim11G0137800	0.517	0.045	-0.472	no
CK-0d	RHSIM_Rhsim09G0005100	0.635	0.655	0.021	no
CK-0d	RHSIM_Rhsim10G0011500	-0.570	-1.429	-0.859	no
CK-0d	RHSIM_Rhsim11G0040600	0.566	0.148	-0.418	no
CK-0d	RHSIM_Rhsim10G0077900	-0.492	-0.898	-0.406	no

Group	Gene	PTR (z-score difference)	z(log2 LFQ)	z(log2 FPKM)	extreme-PTR
CK-0d	RHSIM_Rhsim10G0102900	-0.951	-0.606	0.345	no
CK-0d	RHSIM_Rhsim09G0168600	-0.588	-0.855	-0.267	no
CK-0d	RHSIM_Rhsim11G0145300	1.407	1.074	-0.333	high
CK-0d	RHSIM_Rhsim10G0065800	-0.308	-0.855	-0.547	no
CK-0d	RHSIM_Rhsim09G0155300	-0.322	-0.963	-0.641	no
CK-0d	RHSIM_Rhsim10G0212500	0.553	-1.002	-1.555	no
CK-0d	RHSIM_Rhsim09G0151400	0.826	0.626	-0.200	no
CK-0d	RHSIM_Rhsim09G0003600	0.352	1.857	1.505	no
CK-0d	RHSIM_Rhsim10G0130500	0.298	-0.821	-1.119	no
CK-0d	RHSIM_Rhsim10G0162800	1.068	-0.250	-1.318	no
CK-0d	RHSIM_Rhsim09G0071300	0.540	-1.462	-2.002	no
CK-0d	RHSIM_Rhsim10G0187700	1.085	-0.979	-2.065	no
CK-0d	RHSIM_Rhsim10G0134900	0.471	1.499	1.029	no
CK-0d	RHSIM_Rhsim09G0043400	-0.505	0.519	1.024	no
CK-0d	RHSIM_Rhsim10G0057900	0.408	2.000	1.591	no
CK-0d	RHSIM_Rhsim10G0162400	0.309	-0.665	-0.974	no
CK-0d	RHSIM_Rhsim09G0165400	0.471	-0.549	-1.019	no
CK-0d	RHSIM_Rhsim09G0152300	-1.155	-1.248	-0.093	low
CK-0d	RHSIM_Rhsim10G0144800	0.099	-0.880	-0.978	no
CK-0d	RHSIM_Rhsim10G0081500	0.319	-0.038	-0.357	no
CK-0d	RHSIM_Rhsim09G0199500	-1.211	1.254	2.465	low
CK-0d	RHSIM_Rhsim10G0043600	0.115	0.602	0.487	no
CK-0d	RHSIM_Rhsim09G0146200	0.014	1.617	1.603	no
CK-0d	RHSIM_Rhsim10G0162700	-0.138	-0.552	-0.414	no
CK-0d	RHSIM_Rhsim09G0146500	-1.387	-0.812	0.575	low
CK-0d	RHSIM_Rhsim09G0137600	0.444	0.757	0.313	no
CK-0d	RHSIM_Rhsim10G0165500	0.347	-0.487	-0.834	no
CK-0d	RHSIM_Rhsim10G0133000	-0.006	0.939	0.945	no
CK-0d	RHSIM_Rhsim10G0034600	-0.968	-0.735	0.233	no
CK-0d	RHSIM_Rhsim09G0128000	2.247	0.508	-1.740	high
CK-0d	RHSIM_Rhsim09G0025300	0.379	1.327	0.948	no
CK-0d	RHSIM_Rhsim09G0136800	0.669	0.161	-0.507	no

Group	Gene	PTR (z-score difference)	z(log2 LFQ)	z(log2 FPKM)	extreme-PTR
CK-0d	RHSIM_Rhsim09G0010200	-0.068	0.476	0.544	no
CK-0d	RHSIM_Rhsim11G0153300	0.649	0.439	-0.209	no
CK-0d	RHSIM_Rhsim09G0037000	-0.957	-1.200	-0.243	no
CK-0d	RHSIM_Rhsim10G0019000	-0.421	0.915	1.336	no
CK-0d	RHSIM_Rhsim09G0113200	-0.943	0.470	1.413	no
CK-0d	RHSIM_Rhsim10G0186700	0.231	-0.451	-0.681	no
CK-0d	RHSIM_Rhsim10G0167000	0.173	-1.129	-1.302	no
CK-0d	RHSIM_Rhsim10G0141700	-0.752	-1.117	-0.365	no
CK-0d	RHSIM_Rhsim09G0165600	0.854	0.477	-0.377	no
CK-0d	RHSIM_Rhsim10G0177900	0.324	0.185	-0.139	no
CK-0d	RHSIM_Rhsim11G0183100	-0.801	-1.525	-0.725	no
CK-0d	RHSIM_Rhsim09G0209400	-0.524	0.788	1.312	no
CK-0d	RHSIM_Rhsim10G0000500	2.238	0.504	-1.734	high
CK-0d	RHSIM_Rhsim09G0067900	0.636	1.050	0.413	no
CK-0d	RHSIM_Rhsim09G0001400	0.475	1.560	1.086	no
CK-0d	RHSIM_Rhsim09G0140900	-2.859	-1.204	1.655	low
CK-0d	RHSIM_Rhsim09G0197000	-2.870	-0.853	2.016	low
CK-0d	RHSIM_Rhsim09G0129700	0.078	0.401	0.323	no
CK-0d	RHSIM_Rhsim10G0003500	-0.849	-0.732	0.117	no
CK-0d	RHSIM_Rhsim10G0040000	1.618	1.482	-0.136	high
CK-0d	RHSIM_Rhsim11G0056000	0.820	-0.262	-1.081	no
CK-0d	RHSIM_Rhsim09G0213100	-0.022	-0.508	-0.486	no
CK-0d	RHSIM_Rhsim10G0145200	0.292	-0.379	-0.671	no
CK-0d	RHSIM_Rhsim10G0001300	0.487	-0.085	-0.572	no
CK-0d	RHSIM_Rhsim09G0066100	0.128	-0.874	-1.002	no
CK-0d	RHSIM_Rhsim10G0049300	-0.190	-0.260	-0.070	no
CK-0d	RHSIM_Rhsim11G0134900	-0.122	-0.409	-0.286	no
CK-0d	RHSIM_Rhsim09G0165800	1.003	0.105	-0.898	no
CK-0d	RHSIM_Rhsim09G0027800	0.278	0.267	-0.012	no
CK-0d	RHSIM_Rhsim11G0197300	-0.309	1.145	1.454	no
CK-0d	RHSIM_Rhsim10G0113000	-0.270	-0.409	-0.139	no
CK-0d	RHSIM_Rhsim09G0072200	0.377	-1.108	-1.485	no

Group	Gene	PTR (z-score difference)	z(log2 LFQ)	z(log2 FPKM)	extreme-PTR
CK-0d	RHSIM_Rhsim10G0053700	-0.186	-1.057	-0.871	no
CK-0d	RHSIM_Rhsim09G0107700	0.444	2.002	1.557	no
CK-0d	RHSIM_Rhsim09G0112700	-0.355	-0.334	0.021	no
CK-0d	RHSIM_Rhsim09G0103400	-1.580	-0.320	1.260	low
CK-0d	RHSIM_Rhsim11G0111700	0.259	-0.357	-0.616	no
CK-0d	RHSIM_Rhsim09G0058600	-0.543	-1.222	-0.679	no
CK-0d	RHSIM_Rhsim09G0199000	0.614	0.503	-0.110	no
CK-0d	RHSIM_Rhsim09G0157900	2.159	1.662	-0.497	high
CK-0d	RHSIM_Rhsim11G0060100	0.810	0.008	-0.802	no
CK-0d	RHSIM_Rhsim10G0105000	-1.265	-1.789	-0.524	low
CK-0d	RHSIM_Rhsim11G0049400	-1.309	0.332	1.640	low
CK-0d	RHSIM_Rhsim09G0061400	-0.035	0.896	0.931	no
CK-0d	RHSIM_Rhsim09G0012100	-2.460	-0.468	1.992	low
CK-0d	RHSIM_Rhsim09G0207600	0.164	0.204	0.040	no
CK-0d	RHSIM_Rhsim09G0037600	-0.478	0.265	0.743	no
CK-0d	RHSIM_Rhsim09G0195600	-0.249	-0.173	0.076	no
CK-0d	RHSIM_Rhsim10G0020400	0.808	0.065	-0.742	no
CK-0d	RHSIM_Rhsim10G0111500	-1.147	0.296	1.444	low
CK-0d	RHSIM_Rhsim10G0121000	0.864	1.010	0.145	no
CK-0d	RHSIM_Rhsim09G0166500	-0.110	-1.102	-0.992	no
CK-0d	RHSIM_Rhsim10G0006500	-0.805	-0.991	-0.186	no
CK-0d	RHSIM_Rhsim09G0199700	1.127	-0.188	-1.315	high
CK-0d	RHSIM_Rhsim09G0214500	-1.153	-0.320	0.833	low
CK-0d	RHSIM_Rhsim10G0044100	-0.982	1.122	2.104	no
CK-0d	RHSIM_Rhsim09G0044400	1.212	0.306	-0.906	high
CK-0d	RHSIM_Rhsim09G0179000	-1.254	-0.986	0.268	low
CK-0d	RHSIM_Rhsim09G0213800	-0.553	0.896	1.449	no
CK-0d	RHSIM_Rhsim09G0071800	0.027	0.095	0.068	no
CK-0d	RHSIM_Rhsim09G0158600	-0.823	-0.855	-0.032	no
CK-0d	RHSIM_Rhsim09G0051700	0.014	0.444	0.430	no
CK-0d	RHSIM_Rhsim09G0126300	-0.514	-0.672	-0.158	no
CK-0d	RHSIM_Rhsim09G0140700	-1.676	1.250	2.926	low

Group	Gene	PTR (z-score difference)	z(log2 LFQ)	z(log2 FPKM)	extreme-PTR
CK-0d	RHSIM_Rhsim09G0064600	0.169	0.242	0.073	no
CK-0d	RHSIM_Rhsim10G0183900	0.659	1.488	0.828	no
CK-0d	RHSIM_Rhsim10G0072700	-0.847	-0.681	0.166	no
CK-0d	RHSIM_Rhsim10G0020900	0.523	-0.380	-0.904	no
CK-0d	RHSIM_Rhsim09G0132000	0.076	1.447	1.371	no
CK-0d	RHSIM_Rhsim10G0176900	-0.308	0.356	0.664	no
CK-0d	RHSIM_Rhsim09G0208100	0.141	-0.074	-0.214	no
CK-0d	RHSIM_Rhsim09G0073200	-0.143	-1.023	-0.880	no
CK-0d	RHSIM_Rhsim09G0140100	0.110	-1.593	-1.703	no
CK-0d	RHSIM_Rhsim10G0180400	-0.792	-1.125	-0.333	no
CK-0d	RHSIM_Rhsim10G0080000	2.288	0.975	-1.313	high
CK-0d	RHSIM_Rhsim09G0020400	0.051	-0.133	-0.184	no
CK-0d	RHSIM_Rhsim09G0105400	-0.278	-0.198	0.080	no
CK-0d	RHSIM_Rhsim09G0005500	1.331	0.707	-0.624	high
CK-0d	RHSIM_Rhsim10G0128500	0.039	0.289	0.251	no
CK-0d	RHSIM_Rhsim10G0192700	-1.340	0.168	1.508	low
CK-0d	RHSIM_Rhsim10G0002200	-2.781	-0.607	2.174	low
CK-0d	RHSIM_Rhsim09G0188800	0.028	1.013	0.985	no
CK-0d	RHSIM_Rhsim10G0002800	-0.906	-0.595	0.311	no
CK-0d	RHSIM_Rhsim09G0092400	-2.419	-1.477	0.942	low
CK-0d	RHSIM_Rhsim09G0154300	-0.080	-0.656	-0.576	no
CK-0d	RHSIM_Rhsim09G0184000	0.179	0.797	0.618	no
CK-0d	RHSIM_Rhsim09G0195100	0.516	0.188	-0.328	no
CK-0d	RHSIM_Rhsim09G0087000	0.265	-0.318	-0.584	no
CK-0d	RHSIM_Rhsim09G0172200	-0.950	-0.447	0.503	no
CK-0d	RHSIM_Rhsim09G0206900	1.136	2.085	0.949	high
CK-0d	RHSIM_Rhsim10G0041000	-1.457	-0.728	0.729	low
CK-0d	RHSIM_Rhsim09G0037900	-1.236	-1.402	-0.166	low
CK-0d	RHSIM_Rhsim09G0107200	0.736	-0.671	-1.407	no
CK-0d	RHSIM_Rhsim09G0040700	3.378	1.691	-1.687	high
CK-0d	RHSIM_Rhsim09G0179700	0.517	-1.215	-1.732	no
CK-0d	RHSIM_Rhsim10G0083200	-0.549	0.562	1.111	no

Group	Gene	PTR (z-score difference)	z(log2 LFQ)	z(log2 FPKM)	extreme-PTR
CK-0d	RHSIM_Rhsim09G0212900	-0.823	-1.015	-0.192	no
CK-0d	RHSIM_Rhsim09G0096600	-0.580	-0.377	0.203	no
CK-0d	RHSIM_Rhsim09G0109400	0.682	0.551	-0.131	no
CK-0d	RHSIM_Rhsim09G0058800	0.615	0.161	-0.454	no
CK-0d	RHSIM_Rhsim10G0185700	0.083	0.307	0.224	no
CK-0d	RHSIM_Rhsim09G0190900	0.257	-0.643	-0.901	no
CK-0d	RHSIM_Rhsim09G0199400	-0.852	-1.426	-0.574	no
CK-0d	RHSIM_Rhsim09G0214300	-0.010	0.266	0.276	no
CK-0d	RHSIM_Rhsim09G0110100	-0.263	1.001	1.264	no
CK-0d	RHSIM_Rhsim09G0043700	1.020	0.235	-0.785	no
CK-0d	RHSIM_Rhsim09G0176800	-0.420	-1.142	-0.722	no
CK-0d	RHSIM_Rhsim07G0015900	-0.194	1.019	1.214	no
CK-0d	RHSIM_Rhsim09G0028200	-1.034	-0.718	0.315	low
CK-0d	RHSIM_Rhsim10G0076800	0.956	1.419	0.463	no
CK-0d	RHSIM_Rhsim10G0021400	1.128	0.899	-0.229	high
CK-0d	RHSIM_Rhsim09G0143800	-0.020	-0.139	-0.120	no
CK-0d	RHSIM_Rhsim09G0066000	0.485	0.903	0.417	no
CK-0d	RHSIM_Rhsim09G0087100	0.288	0.603	0.315	no
CK-0d	RHSIM_Rhsim09G0058700	-0.933	-1.343	-0.410	no
CK-0d	RHSIM_Rhsim10G0127700	-1.503	-0.468	1.035	low
CK-0d	RHSIM_Rhsim09G0204400	-1.251	-0.747	0.503	low
CK-0d	RHSIM_Rhsim09G0066200	-1.399	-0.597	0.802	low
CK-0d	RHSIM_Rhsim10G0034300	0.619	0.434	-0.185	no
CK-0d	RHSIM_Rhsim09G0211500	-0.573	-0.272	0.301	no
CK-0d	RHSIM_Rhsim09G0084400	0.243	-0.468	-0.710	no
CK-0d	RHSIM_Rhsim09G0154600	0.366	0.007	-0.359	no
CK-0d	RHSIM_Rhsim10G0214200	-0.316	-0.243	0.073	no
CK-0d	RHSIM_Rhsim09G0130100	-0.187	1.551	1.738	no
CK-0d	RHSIM_Rhsim09G0011700	-3.039	0.174	3.213	low
CK-0d	RHSIM_Rhsim09G0103000	-1.052	-0.535	0.517	low
CK-0d	RHSIM_Rhsim10G0186800	-3.140	-1.047	2.093	low
CK-0d	RHSIM_Rhsim09G0146700	-1.100	-1.278	-0.178	low

Group	Gene	PTR (z-score difference)	z(log2 LFQ)	z(log2 FPKM)	extreme-PTR
CK-0d	RHSIM_Rhsim09G0063000	1.095	-0.399	-1.494	no
CK-0d	RHSIM_Rhsim09G0214600	-2.195	-1.072	1.124	low
CK-0d	RHSIM_Rhsim10G0084800	-0.917	0.170	1.087	no
CK-0d	RHSIM_Rhsim09G0122300	0.292	-0.456	-0.748	no
CK-0d	RHSIM_Rhsim10G0167600	-2.328	-1.042	1.286	low
CK-0d	RHSIM_Rhsim09G0151300	-1.924	0.297	2.220	low
CK-0d	RHSIM_Rhsim09G0012200	0.352	1.104	0.752	no
CK-0d	RHSIM_Rhsim09G0061700	0.903	-0.594	-1.497	no
CK-0d	RHSIM_Rhsim09G0166000	-2.617	-0.165	2.452	low
CK-0d	RHSIM_Rhsim09G0043800	0.532	0.304	-0.228	no
CK-0d	RHSIM_Rhsim10G0185500	1.157	0.638	-0.519	high
CK-0d	RHSIM_Rhsim09G0150500	0.706	0.123	-0.583	no
CK-0d	RHSIM_Rhsim09G0152400	-0.087	0.546	0.633	no
CK-0d	RHSIM_Rhsim10G0163100	-0.272	0.114	0.386	no
CK-0d	RHSIM_Rhsim09G0035000	-0.004	-0.720	-0.716	no
CK-0d	RHSIM_Rhsim09G0117700	0.956	1.517	0.561	no
CK-0d	RHSIM_Rhsim09G0196800	-0.537	-0.414	0.123	no
CK-0d	RHSIM_Rhsim09G0033400	1.220	0.827	-0.392	high
CK-0d	RHSIM_Rhsim09G0087600	-1.174	-1.927	-0.753	low
CK-0d	RHSIM_Rhsim09G0084100	-1.782	0.590	2.372	low
CK-0d	RHSIM_Rhsim09G0090200	0.526	-0.547	-1.073	no
CK-0d	RHSIM_Rhsim09G0094700	-0.161	0.163	0.323	no
CK-0d	RHSIM_Rhsim09G0143200	-1.165	2.226	3.391	low
CK-0d	RHSIM_Rhsim10G0125000	-0.414	-0.604	-0.189	no
CK-0d	RHSIM_Rhsim09G0004100	0.424	1.347	0.923	no
CK-0d	RHSIM_Rhsim09G0140500	0.757	-0.451	-1.208	no
CK-0d	RHSIM_Rhsim10G0004100	-0.565	-0.546	0.019	no
CK-0d	RHSIM_Rhsim09G0196200	-1.613	-0.824	0.789	low
CK-0d	RHSIM_Rhsim09G0127700	-1.215	-0.820	0.395	low
CK-0d	RHSIM_Rhsim09G0065100	-0.933	-1.110	-0.177	no
CK-0d	RHSIM_Rhsim09G0202400	0.314	1.120	0.806	no
CK-0d	RHSIM_Rhsim09G0148900	0.578	0.198	-0.381	no

Group	Gene	PTR (z-score difference)	z(log2 LFQ)	z(log2 FPKM)	extreme-PTR
CK-0d	RHSIM_Rhsim09G0210400	1.358	0.901	-0.458	high
CK-0d	RHSIM_Rhsim09G0011500	-1.367	-1.137	0.230	low
CK-0d	RHSIM_Rhsim09G0184800	0.788	-0.420	-1.208	no
CK-0d	RHSIM_Rhsim10G0174500	-2.137	-0.264	1.873	low
CK-0d	RHSIM_Rhsim09G0105600	0.089	-0.014	-0.102	no
CK-0d	RHSIM_Rhsim10G0004300	-0.372	0.453	0.826	no
CK-0d	RHSIM_Rhsim09G0132100	-0.083	-0.499	-0.416	no
CK-0d	RHSIM_Rhsim09G0078200	-0.062	-0.899	-0.838	no
CK-0d	RHSIM_Rhsim09G0206100	-0.468	-0.467	0.001	no
CK-0d	RHSIM_Rhsim10G0166700	-1.151	-0.555	0.596	low
CK-0d	RHSIM_Rhsim09G0049700	1.333	-0.186	-1.519	high
CK-0d	RHSIM_Rhsim08G0034300	-0.496	-0.425	0.072	no
CK-0d	RHSIM_Rhsim09G0030100	-0.268	-0.603	-0.335	no
CK-0d	RHSIM_Rhsim10G0020100	0.341	2.535	2.195	no
CK-0d	RHSIM_Rhsim09G0044600	0.467	0.702	0.235	no
CK-0d	RHSIM_Rhsim10G0092800	0.075	-0.156	-0.231	no
CK-0d	RHSIM_Rhsim10G0064800	1.234	0.415	-0.818	high
CK-0d	RHSIM_Rhsim09G0034100	-1.121	-0.111	1.010	low
CK-0d	RHSIM_Rhsim08G0095100	0.050	-1.190	-1.240	no
CK-0d	RHSIM_Rhsim09G0173200	-1.148	0.373	1.522	low
CK-0d	RHSIM_Rhsim10G0019700	-2.449	-0.517	1.933	low
CK-0d	RHSIM_Rhsim09G0004500	-0.419	-1.453	-1.034	no
CK-0d	RHSIM_Rhsim08G0223900	2.766	2.356	-0.410	high
CK-0d	RHSIM_Rhsim08G0096200	-0.575	0.556	1.131	no
CK-0d	RHSIM_Rhsim09G0140000	0.382	0.400	0.018	no
CK-0d	RHSIM_Rhsim10G0138100	-1.982	-1.930	0.052	low
CK-0d	RHSIM_Rhsim08G0116000	-0.035	-0.313	-0.278	no
CK-0d	RHSIM_Rhsim09G0208800	1.205	1.498	0.293	high
CK-0d	RHSIM_Rhsim08G0081800	-0.283	0.052	0.335	no
CK-0d	RHSIM_Rhsim08G0039900	0.882	2.179	1.296	no
CK-0d	RHSIM_Rhsim09G0091600	0.773	-0.907	-1.680	no
CK-0d	RHSIM_Rhsim08G0000800	-1.074	-0.456	0.618	low

Group	Gene	PTR (z-score difference)	z(log2 LFQ)	z(log2 FPKM)	extreme-PTR
CK-0d	RHSIM_Rhsim09G0034800	-1.371	-0.250	1.120	low
CK-0d	RHSIM_Rhsim10G0008400	-0.687	-0.987	-0.299	no
CK-0d	RHSIM_Rhsim09G0154900	-0.271	-0.487	-0.216	no
CK-0d	RHSIM_Rhsim09G0206000	-0.667	-0.404	0.264	no
CK-0d	RHSIM_Rhsim09G0013300	1.030	0.301	-0.729	no
CK-0d	RHSIM_Rhsim10G0104900	0.503	-0.098	-0.601	no
CK-0d	RHSIM_Rhsim08G0244500	1.764	1.417	-0.348	high
CK-0d	RHSIM_Rhsim08G0231100	0.092	0.383	0.291	no
CK-0d	RHSIM_Rhsim10G0119200	-1.358	-0.224	1.134	low
CK-0d	RHSIM_Rhsim08G0237400	0.392	-0.484	-0.876	no
CK-0d	RHSIM_Rhsim08G0102500	-0.059	-0.598	-0.540	no
CK-0d	RHSIM_Rhsim09G0097800	-0.492	-0.725	-0.232	no
CK-0d	RHSIM_Rhsim08G0128100	-0.147	-0.820	-0.673	no
CK-0d	RHSIM_Rhsim10G0016800	1.452	-0.023	-1.475	high
CK-0d	RHSIM_Rhsim09G0128800	0.665	-0.355	-1.020	no
CK-0d	RHSIM_Rhsim09G0005700	0.923	0.106	-0.817	no
CK-0d	RHSIM_Rhsim09G0081900	-0.747	0.574	1.321	no
CK-0d	RHSIM_Rhsim08G0128800	0.623	0.084	-0.539	no
CK-0d	RHSIM_Rhsim09G0137900	-1.466	-0.716	0.751	low
CK-0d	RHSIM_Rhsim09G0187600	0.050	-0.562	-0.612	no
CK-0d	RHSIM_Rhsim09G0214000	0.306	0.861	0.554	no
CK-0d	RHSIM_Rhsim08G0039400	-1.787	1.880	3.667	low
CK-0d	RHSIM_Rhsim09G0005000	0.631	0.125	-0.507	no
CK-0d	RHSIM_Rhsim09G0145300	0.574	0.276	-0.298	no
CK-0d	RHSIM_Rhsim09G0193000	-0.030	0.203	0.233	no
CK-0d	RHSIM_Rhsim09G0203700	0.195	-0.972	-1.167	no
CK-0d	RHSIM_Rhsim08G0027400	-2.006	-0.573	1.434	low
CK-0d	RHSIM_Rhsim09G0204300	-0.984	-0.853	0.130	no
CK-0d	RHSIM_Rhsim10G0151200	-0.027	-0.235	-0.207	no
CK-0d	RHSIM_Rhsim10G0049800	-1.287	-0.881	0.405	low
CK-0d	RHSIM_Rhsim09G0129900	-1.556	0.234	1.790	low
CK-0d	RHSIM_Rhsim08G0146000	1.229	0.324	-0.904	high

Group	Gene	PTR (z-score difference)	z(log2 LFQ)	z(log2 FPKM)	extreme-PTR
CK-0d	RHSIM_Rhsim09G0122200	0.129	-0.644	-0.773	no
CK-0d	RHSIM_Rhsim09G0142800	-1.862	-0.600	1.262	low
CK-0d	RHSIM_Rhsim09G0141500	0.013	0.745	0.732	no
CK-0d	RHSIM_Rhsim08G0115800	-1.174	-0.171	1.003	low
CK-0d	RHSIM_Rhsim09G0177600	-3.875	-0.443	3.432	low
CK-0d	RHSIM_Rhsim09G0153300	-0.841	-0.364	0.477	no
CK-0d	RHSIM_Rhsim08G0250700	-0.071	-0.321	-0.250	no
CK-0d	RHSIM_Rhsim09G0201300	-0.591	-0.643	-0.052	no
CK-0d	RHSIM_Rhsim09G0142500	-1.075	0.157	1.231	low
CK-0d	RHSIM_Rhsim09G0074600	0.076	0.444	0.367	no
CK-0d	RHSIM_Rhsim09G0089600	-0.119	-0.633	-0.514	no
CK-0d	RHSIM_Rhsim09G0030700	0.642	-0.151	-0.794	no
CK-0d	RHSIM_Rhsim08G0043800	0.140	0.233	0.093	no
CK-0d	RHSIM_Rhsim09G0169300	0.461	-0.449	-0.910	no
CK-0d	RHSIM_Rhsim09G0127200	0.870	0.063	-0.807	no
CK-0d	RHSIM_Rhsim08G0125200	-0.260	-0.733	-0.473	no
CK-0d	RHSIM_Rhsim08G0191700	0.017	0.034	0.016	no
CK-0d	RHSIM_Rhsim09G0069100	1.448	2.773	1.324	high
CK-0d	RHSIM_Rhsim08G0067800	1.245	0.512	-0.733	high
CK-0d	RHSIM_Rhsim09G0201100	-1.380	-0.618	0.761	low
CK-0d	RHSIM_Rhsim09G0029200	-1.480	-0.815	0.665	low
CK-0d	RHSIM_Rhsim09G0061500	0.565	0.247	-0.318	no
CK-0d	RHSIM_Rhsim08G0227500	-1.759	-1.422	0.337	low
CK-0d	RHSIM_Rhsim08G0041900	-2.031	-1.739	0.292	low
CK-0d	RHSIM_Rhsim09G0171700	1.522	0.331	-1.190	high
CK-0d	RHSIM_Rhsim08G0183700	0.715	0.015	-0.700	no
CK-0d	RHSIM_Rhsim09G0028800	0.010	-0.457	-0.468	no
CK-0d	RHSIM_Rhsim09G0179300	-0.562	-0.975	-0.413	no
CK-0d	RHSIM_Rhsim08G0006400	2.301	0.555	-1.746	high
CK-0d	RHSIM_Rhsim09G0039400	0.713	-0.546	-1.259	no
CK-0d	RHSIM_Rhsim09G0145500	-0.585	-0.357	0.229	no
CK-0d	RHSIM_Rhsim10G0145000	0.390	1.044	0.653	no

Group	Gene	PTR (z-score difference)	z(log2 LFQ)	z(log2 FPKM)	extreme-PTR
CK-0d	RHSIM_Rhsim09G0178000	-0.460	-1.606	-1.146	no
CK-0d	RHSIM_Rhsim09G0113100	1.773	-0.015	-1.788	high
CK-0d	RHSIM_Rhsim08G0121400	0.575	-0.049	-0.624	no
CK-0d	RHSIM_Rhsim08G0202500	0.534	0.705	0.171	no
CK-0d	RHSIM_Rhsim08G0084400	0.000	-0.374	-0.374	no
CK-0d	RHSIM_Rhsim08G0167000	-0.231	-0.239	-0.008	no
CK-0d	RHSIM_Rhsim09G0023000	0.971	-0.485	-1.456	no
CK-0d	RHSIM_Rhsim08G0224700	0.348	2.371	2.023	no
CK-0d	RHSIM_Rhsim09G0151500	0.806	-0.089	-0.894	no
CK-0d	RHSIM_Rhsim09G0126900	-2.355	-2.044	0.311	low
CK-0d	RHSIM_Rhsim09G0125900	-0.228	-0.479	-0.251	no
CK-0d	RHSIM_Rhsim09G0197100	-0.915	0.723	1.638	no
CK-0d	RHSIM_Rhsim09G0117300	0.653	0.027	-0.625	no
CK-0d	RHSIM_Rhsim08G0028900	-0.870	-1.526	-0.656	no
CK-0d	RHSIM_Rhsim10G0212000	-1.603	0.943	2.546	low
CK-0d	RHSIM_Rhsim09G0181400	-0.083	0.134	0.217	no
CK-0d	RHSIM_Rhsim09G0088400	-0.077	-0.440	-0.363	no
CK-0d	RHSIM_Rhsim08G0214200	0.573	0.085	-0.488	no
CK-0d	RHSIM_Rhsim08G0063900	-0.358	0.171	0.530	no
CK-0d	RHSIM_Rhsim09G0082300	-0.075	-0.873	-0.798	no
CK-0d	RHSIM_Rhsim09G0172400	-0.090	0.646	0.735	no
CK-0d	RHSIM_Rhsim08G0055000	0.806	1.441	0.635	no
CK-0d	RHSIM_Rhsim09G0040800	-0.162	-0.415	-0.253	no
CK-0d	RHSIM_Rhsim08G0044300	0.480	-0.414	-0.894	no
CK-0d	RHSIM_Rhsim08G0006500	0.500	0.793	0.292	no
CK-0d	RHSIM_Rhsim09G0171600	-0.886	-0.417	0.470	no
CK-0d	RHSIM_Rhsim09G0168800	1.160	0.232	-0.928	high
CK-0d	RHSIM_Rhsim08G0043500	-1.348	-1.087	0.261	low
CK-0d	RHSIM_Rhsim09G0004000	-1.936	-1.625	0.310	low
CK-0d	RHSIM_Rhsim09G0183900	-0.480	-0.332	0.147	no
CK-0d	RHSIM_Rhsim08G0005200	-2.112	-2.564	-0.452	low
CK-0d	RHSIM_Rhsim09G0152700	0.886	3.274	2.388	no

Group	Gene	PTR (z-score difference)	z(log2 LFQ)	z(log2 FPKM)	extreme-PTR
CK-0d	RHSIM_Rhsim09G0046600	0.439	0.015	-0.424	no
CK-0d	RHSIM_Rhsim08G0014900	0.374	0.585	0.211	no
CK-0d	RHSIM_Rhsim08G0081400	-0.961	-0.829	0.132	no
CK-0d	RHSIM_Rhsim08G0159000	0.955	0.431	-0.524	no
CK-0d	RHSIM_Rhsim08G0171800	-1.251	-0.398	0.853	low
CK-0d	RHSIM_Rhsim08G0033300	-0.485	-0.504	-0.018	no
CK-0d	RHSIM_Rhsim10G0045100	0.816	0.654	-0.162	no
CK-0d	RHSIM_Rhsim09G0077000	-0.793	-0.748	0.045	no
CK-0d	RHSIM_Rhsim09G0198800	1.720	0.301	-1.419	high
CK-0d	RHSIM_Rhsim08G0141300	-0.874	-0.993	-0.119	no
CK-0d	RHSIM_Rhsim08G0196800	0.109	1.534	1.426	no
CK-0d	RHSIM_Rhsim08G0037100	-1.259	-0.941	0.319	low
CK-0d	RHSIM_Rhsim08G0042900	0.293	-0.084	-0.378	no
CK-0d	RHSIM_Rhsim08G0181700	0.537	1.610	1.074	no
CK-0d	RHSIM_Rhsim10G0122600	0.103	-0.756	-0.858	no
CK-0d	RHSIM_Rhsim08G0243400	1.453	-0.776	-2.229	high
CK-0d	RHSIM_Rhsim08G0114500	-0.816	-0.460	0.355	no
CK-0d	RHSIM_Rhsim08G0240000	-0.954	-0.086	0.868	no
CK-0d	RHSIM_Rhsim09G0133500	1.518	0.475	-1.043	high
CK-0d	RHSIM_Rhsim08G0100200	0.160	0.328	0.168	no
CK-0d	RHSIM_Rhsim08G0248700	-0.344	-0.647	-0.303	no
CK-0d	RHSIM_Rhsim08G0026000	0.427	-0.173	-0.600	no
CK-0d	RHSIM_Rhsim09G0141000	-0.878	-0.860	0.018	no
CK-0d	RHSIM_Rhsim08G0088900	-0.701	-1.364	-0.663	no
CK-0d	RHSIM_Rhsim08G0229600	-1.095	-0.007	1.087	low
CK-0d	RHSIM_Rhsim08G0241300	0.297	-0.157	-0.453	no
CK-0d	RHSIM_Rhsim09G0164300	-0.658	-2.196	-1.538	no
CK-0d	RHSIM_Rhsim09G0145400	-0.453	-0.160	0.293	no
CK-0d	RHSIM_Rhsim08G0209300	1.101	1.116	0.015	no
CK-0d	RHSIM_Rhsim08G0122300	-1.243	-0.472	0.771	low
CK-0d	RHSIM_Rhsim09G0189300	-1.306	0.308	1.614	low
CK-0d	RHSIM_Rhsim08G0054900	2.271	1.590	-0.681	high

Group	Gene	PTR (z-score difference)	z(log2 LFQ)	z(log2 FPKM)	extreme-PTR
CK-0d	RHSIM_Rhsim09G0117800	1.303	0.504	-0.798	high
CK-0d	RHSIM_Rhsim08G0102400	0.456	-1.443	-1.899	no
CK-0d	RHSIM_Rhsim08G0002000	1.509	-0.666	-2.175	high
CK-0d	RHSIM_Rhsim08G0230600	-3.033	0.039	3.072	low
CK-0d	RHSIM_Rhsim09G0000900	0.637	0.122	-0.515	no
CK-0d	RHSIM_Rhsim08G0150200	0.785	-0.232	-1.017	no
CK-0d	RHSIM_Rhsim08G0067900	0.680	0.780	0.100	no
CK-0d	RHSIM_Rhsim10G0129000	0.531	-0.040	-0.570	no
CK-0d	RHSIM_Rhsim08G0121500	-1.542	-0.856	0.686	low
CK-0d	RHSIM_Rhsim08G0040400	0.364	0.749	0.385	no
CK-0d	RHSIM_Rhsim09G0010600	-0.240	0.214	0.454	no
CK-0d	RHSIM_Rhsim10G0125300	0.135	-0.426	-0.561	no
CK-0d	RHSIM_Rhsim08G0223400	-0.938	1.544	2.482	no
CK-0d	RHSIM_Rhsim08G0103000	0.602	0.846	0.245	no
CK-0d	RHSIM_Rhsim10G0139000	-0.245	-0.335	-0.090	no
CK-0d	RHSIM_Rhsim09G0008600	-0.752	0.705	1.457	no
CK-0d	RHSIM_Rhsim08G0183000	-0.188	0.534	0.722	no
CK-0d	RHSIM_Rhsim08G0215400	1.647	0.272	-1.374	high
CK-0d	RHSIM_Rhsim09G0210600	1.127	1.547	0.420	high
CK-0d	RHSIM_Rhsim08G0020800	-0.015	-0.544	-0.529	no
CK-0d	RHSIM_Rhsim09G0111700	0.186	0.572	0.387	no
CK-0d	RHSIM_Rhsim08G0188900	-0.448	-0.226	0.223	no
CK-0d	RHSIM_Rhsim10G0078400	0.633	-0.473	-1.106	no
CK-0d	RHSIM_Rhsim08G0053600	0.754	0.086	-0.667	no
CK-0d	RHSIM_Rhsim09G0099400	-0.568	-0.564	0.004	no
CK-0d	RHSIM_Rhsim09G0106300	0.430	0.533	0.102	no
CK-0d	RHSIM_Rhsim08G0008500	-0.478	-0.535	-0.057	no
CK-0d	RHSIM_Rhsim10G0211900	1.435	0.591	-0.844	high
CK-0d	RHSIM_Rhsim08G0033600	0.260	-0.748	-1.008	no
CK-0d	RHSIM_Rhsim09G0142300	0.413	-0.887	-1.300	no
CK-0d	RHSIM_Rhsim09G0008200	-0.349	-0.593	-0.245	no
CK-0d	RHSIM_Rhsim08G0083400	-2.248	-0.658	1.590	low

Group	Gene	PTR (z-score difference)	z(log2 LFQ)	z(log2 FPKM)	extreme-PTR
CK-0d	RHSIM_Rhsim09G0138400	0.631	0.946	0.315	no
CK-0d	RHSIM_Rhsim08G0156200	-0.823	-0.546	0.277	no
CK-0d	RHSIM_Rhsim08G0156700	2.130	0.956	-1.173	high
CK-0d	RHSIM_Rhsim08G0153100	-0.824	1.087	1.911	no
CK-0d	RHSIM_Rhsim09G0203300	-1.164	-0.680	0.483	low
CK-0d	RHSIM_Rhsim09G0207900	1.023	1.381	0.358	no
CK-0d	RHSIM_Rhsim08G0208100	-0.015	-1.151	-1.136	no
CK-0d	RHSIM_Rhsim08G0034400	-0.539	-0.870	-0.331	no
CK-0d	RHSIM_Rhsim08G0205700	0.669	1.177	0.508	no
CK-0d	RHSIM_Rhsim09G0155500	0.064	-0.905	-0.969	no
CK-0d	RHSIM_Rhsim08G0145900	-1.029	-0.096	0.933	low
CK-0d	RHSIM_Rhsim08G0041500	-0.555	1.028	1.582	no
CK-0d	RHSIM_Rhsim08G0082100	-0.172	-0.270	-0.098	no
CK-0d	RHSIM_Rhsim10G0049400	-0.119	0.563	0.682	no
CK-0d	RHSIM_Rhsim08G0174000	0.455	0.852	0.397	no
CK-0d	RHSIM_Rhsim10G0104100	-1.594	-1.432	0.162	low
CK-0d	RHSIM_Rhsim09G0056700	1.002	-0.127	-1.129	no
CK-0d	RHSIM_Rhsim09G0069000	0.369	1.645	1.276	no
CK-0d	RHSIM_Rhsim09G0200500	0.478	-0.144	-0.622	no
CK-0d	RHSIM_Rhsim09G0115400	0.930	0.547	-0.383	no
CK-0d	RHSIM_Rhsim09G0085500	-1.443	-1.278	0.165	low
CK-0d	RHSIM_Rhsim08G0069100	0.543	-0.081	-0.624	no
CK-0d	RHSIM_Rhsim08G0202300	-3.014	-1.801	1.213	low
CK-0d	RHSIM_Rhsim09G0024000	-0.386	-0.727	-0.340	no
CK-0d	RHSIM_Rhsim09G0022900	-1.294	-0.632	0.662	low
CK-0d	RHSIM_Rhsim08G0038100	0.036	0.461	0.425	no
CK-0d	RHSIM_Rhsim08G0045400	1.615	1.490	-0.125	high
CK-0d	RHSIM_Rhsim08G0180500	0.666	2.060	1.395	no
CK-0d	RHSIM_Rhsim09G0128100	0.087	0.827	0.740	no
CK-0d	RHSIM_Rhsim08G0109700	0.216	3.928	3.712	no
CK-0d	RHSIM_Rhsim08G0047900	3.070	1.089	-1.981	high
CK-0d	RHSIM_Rhsim08G0013000	1.177	-0.682	-1.859	high

Group	Gene	PTR (z-score difference)	z(log2 LFQ)	z(log2 FPKM)	extreme-PTR
CK-0d	RHSIM_Rhsim08G0088400	0.407	1.096	0.689	no
CK-0d	RHSIM_Rhsim09G0002200	1.562	1.340	-0.222	high
CK-0d	RHSIM_Rhsim08G0030700	0.898	0.593	-0.305	no
CK-0d	RHSIM_Rhsim08G0020500	0.963	-1.229	-2.192	no
CK-0d	RHSIM_Rhsim08G0045800	-1.760	0.649	2.409	low
CK-0d	RHSIM_Rhsim08G0245700	0.344	1.125	0.781	no
CK-0d	RHSIM_Rhsim09G0114500	-3.031	0.453	3.483	low
CK-0d	RHSIM_Rhsim09G0109300	-1.511	-1.003	0.508	low
CK-0d	RHSIM_Rhsim08G0043600	-0.373	1.059	1.433	no
CK-0d	RHSIM_Rhsim09G0090700	0.476	-0.769	-1.244	no
CK-0d	RHSIM_Rhsim09G0069700	-0.937	-1.438	-0.501	no
CK-0d	RHSIM_Rhsim09G0201200	0.072	0.801	0.728	no
CK-0d	RHSIM_Rhsim08G0195500	-0.912	-0.180	0.732	no
CK-0d	RHSIM_Rhsim08G0008300	-0.124	-1.475	-1.351	no
CK-0d	RHSIM_Rhsim09G0149700	1.160	0.821	-0.339	high
CK-0d	RHSIM_Rhsim09G0067700	0.403	0.448	0.046	no
CK-0d	RHSIM_Rhsim09G0144000	0.869	-0.830	-1.700	no
CK-0d	RHSIM_Rhsim08G0044100	-4.403	-1.315	3.088	low
CK-0d	RHSIM_Rhsim09G0146300	0.778	1.332	0.554	no
CK-0d	RHSIM_Rhsim08G0188400	1.289	1.766	0.477	high
CK-0d	RHSIM_Rhsim08G0096400	-0.377	-0.890	-0.513	no
CK-0d	RHSIM_Rhsim09G0197500	1.501	1.103	-0.398	high
CK-0d	RHSIM_Rhsim09G0168700	0.357	0.949	0.591	no
CK-0d	RHSIM_Rhsim09G0025000	0.393	-0.874	-1.267	no
CK-0d	RHSIM_Rhsim08G0037000	-0.101	0.485	0.585	no
CK-0d	RHSIM_Rhsim08G0072300	1.157	0.553	-0.604	high
CK-0d	RHSIM_Rhsim09G0000500	-0.001	0.035	0.037	no
CK-0d	RHSIM_Rhsim08G0176100	-0.047	-1.069	-1.023	no
CK-0d	RHSIM_Rhsim08G0250400	-1.591	-1.356	0.235	low
CK-0d	RHSIM_Rhsim09G0052100	0.622	0.412	-0.210	no
CK-0d	RHSIM_Rhsim08G0185000	-0.924	-1.101	-0.177	no
CK-0d	RHSIM_Rhsim09G0074800	0.036	-0.160	-0.196	no

Group	Gene	PTR (z-score difference)	z(log2 LFQ)	z(log2 FPKM)	extreme-PTR
CK-0d	RHSIM_Rhsim09G0032100	0.814	0.115	-0.699	no
CK-0d	RHSIM_Rhsim09G0143900	1.346	0.270	-1.077	high
CK-0d	RHSIM_Rhsim08G0103200	1.282	0.283	-1.000	high
CK-0d	RHSIM_Rhsim08G0114200	-0.156	-0.414	-0.258	no
CK-0d	RHSIM_Rhsim08G0012200	-0.685	-0.244	0.441	no
CK-0d	RHSIM_Rhsim08G0086400	-0.997	-1.036	-0.039	no
CK-0d	RHSIM_Rhsim09G0100400	-0.166	-0.428	-0.262	no
CK-0d	RHSIM_Rhsim09G0114100	1.550	0.485	-1.066	high
CK-0d	RHSIM_Rhsim09G0109100	-0.077	0.695	0.772	no
CK-0d	RHSIM_Rhsim09G0000200	0.421	0.494	0.073	no
CK-0d	RHSIM_Rhsim09G0206600	0.459	-0.568	-1.027	no
CK-0d	RHSIM_Rhsim08G0141600	1.963	1.805	-0.158	high
CK-0d	RHSIM_Rhsim08G0221100	0.045	-0.397	-0.442	no
CK-0d	RHSIM_Rhsim08G0185200	0.616	0.052	-0.564	no
CK-0d	RHSIM_Rhsim08G0231900	-0.457	-0.888	-0.432	no
CK-0d	RHSIM_Rhsim08G0002400	0.889	0.609	-0.280	no
CK-0d	RHSIM_Rhsim08G0239200	-0.277	1.304	1.581	no
CK-0d	RHSIM_Rhsim08G0069300	-0.148	0.898	1.045	no
CK-0d	RHSIM_Rhsim08G0131700	0.414	-0.905	-1.318	no
CK-0d	RHSIM_Rhsim09G0008700	0.328	-0.394	-0.721	no
CK-0d	RHSIM_Rhsim08G0241200	1.064	0.898	-0.167	no
CK-0d	RHSIM_Rhsim08G0167100	-0.056	-0.184	-0.128	no
CK-0d	RHSIM_Rhsim08G0186100	-0.853	-0.859	-0.007	no
CK-0d	RHSIM_Rhsim09G0000700	-0.702	-0.840	-0.138	no
CK-0d	RHSIM_Rhsim08G0214400	0.076	0.875	0.799	no
CK-0d	RHSIM_Rhsim08G0230200	-0.348	-0.797	-0.449	no
CK-0d	RHSIM_Rhsim08G0046300	-0.336	-0.781	-0.445	no
CK-0d	RHSIM_Rhsim08G0241900	-1.559	-0.511	1.048	low
CK-0d	RHSIM_Rhsim08G0242600	1.072	0.208	-0.864	no
CK-0d	RHSIM_Rhsim08G0244800	2.461	0.618	-1.843	high
CK-0d	RHSIM_Rhsim08G0046700	1.665	1.283	-0.382	high
CK-0d	RHSIM_Rhsim08G0026100	-0.224	-0.285	-0.061	no

Group	Gene	PTR (z-score difference)	z(log2 LFQ)	z(log2 FPKM)	extreme-PTR
CK-0d	RHSIM_Rhsim09G0212200	-0.743	-0.363	0.380	no
CK-0d	RHSIM_Rhsim09G0099700	0.544	-1.399	-1.943	no
CK-0d	RHSIM_Rhsim09G0171300	-0.501	-1.409	-0.908	no
CK-0d	RHSIM_Rhsim08G0084800	1.176	-0.023	-1.198	high
CK-0d	RHSIM_Rhsim09G0119600	-0.476	-1.022	-0.546	no
CK-0d	RHSIM_Rhsim09G0213700	0.636	0.585	-0.051	no
CK-0d	RHSIM_Rhsim08G0057900	1.735	0.934	-0.801	high
CK-0d	RHSIM_Rhsim09G0188300	0.091	-1.099	-1.190	no
CK-0d	RHSIM_Rhsim08G0127800	1.037	1.219	0.183	no
CK-0d	RHSIM_Rhsim08G0030100	0.251	0.467	0.217	no
CK-0d	RHSIM_Rhsim08G0102200	0.329	-0.010	-0.340	no
CK-0d	RHSIM_Rhsim08G0214000	0.814	1.618	0.804	no
CK-0d	RHSIM_Rhsim08G0030200	-0.534	-0.189	0.345	no
CK-0d	RHSIM_Rhsim09G0185000	1.452	-0.009	-1.461	high
CK-0d	RHSIM_Rhsim09G0171800	0.966	0.703	-0.264	no
CK-0d	RHSIM_Rhsim08G0211400	0.323	0.879	0.556	no
CK-0d	RHSIM_Rhsim09G0064200	-1.406	0.258	1.664	low
CK-0d	RHSIM_Rhsim08G0094100	-1.554	0.496	2.050	low
CK-0d	RHSIM_Rhsim09G0105100	0.935	-0.169	-1.103	no
CK-0d	RHSIM_Rhsim08G0029600	2.927	3.253	0.326	high
CK-0d	RHSIM_Rhsim09G0111100	0.178	0.303	0.124	no
CK-0d	RHSIM_Rhsim09G0179800	-0.056	-1.204	-1.148	no
CK-0d	RHSIM_Rhsim08G0019700	0.527	1.042	0.514	no
CK-0d	RHSIM_Rhsim09G0093800	0.014	-0.548	-0.562	no
CK-0d	RHSIM_Rhsim09G0209300	-1.331	-0.309	1.022	low
CK-0d	RHSIM_Rhsim08G0094000	1.115	1.842	0.727	high
CK-0d	RHSIM_Rhsim09G0110000	1.736	-0.182	-1.919	high
CK-0d	RHSIM_Rhsim08G0018700	0.839	-1.263	-2.102	no
CK-0d	RHSIM_Rhsim08G0173300	0.540	-0.533	-1.073	no
CK-0d	RHSIM_Rhsim09G0164800	-0.100	-0.188	-0.088	no
CK-0d	RHSIM_Rhsim08G0195100	-1.163	0.540	1.703	low
CK-0d	RHSIM_Rhsim08G0199200	-3.285	-1.840	1.445	low

Group	Gene	PTR (z-score difference)	z(log2 LFQ)	z(log2 FPKM)	extreme-PTR
CK-0d	RHSIM_Rhsim08G0034000	-2.076	-0.928	1.148	low
CK-0d	RHSIM_Rhsim09G0164900	0.184	-0.359	-0.543	no
CK-0d	RHSIM_Rhsim09G0000100	-1.751	-0.883	0.869	low
CK-0d	RHSIM_Rhsim08G0045600	0.270	-0.819	-1.089	no
CK-0d	RHSIM_Rhsim08G0025900	0.740	-0.033	-0.772	no
CK-0d	RHSIM_Rhsim08G0174600	-0.258	-0.502	-0.244	no
CK-0d	RHSIM_Rhsim09G0142700	0.044	-0.143	-0.187	no
CK-0d	RHSIM_Rhsim08G0237100	-0.719	-1.250	-0.531	no
CK-0d	RHSIM_Rhsim08G0032100	0.285	0.582	0.297	no
CK-0d	RHSIM_Rhsim08G0148900	0.628	0.269	-0.358	no
CK-0d	RHSIM_Rhsim08G0246900	-0.830	-0.577	0.253	no
CK-0d	RHSIM_Rhsim09G0103800	-0.007	-1.703	-1.696	no
CK-0d	RHSIM_Rhsim07G0073300	-0.373	0.477	0.850	no
CK-0d	RHSIM_Rhsim08G0045900	1.019	1.363	0.344	no
CK-0d	RHSIM_Rhsim08G0075800	-0.561	0.481	1.042	no
CK-0d	RHSIM_Rhsim08G0109800	0.029	-1.151	-1.179	no
CK-0d	RHSIM_Rhsim07G0082300	1.524	0.900	-0.623	high
CK-0d	RHSIM_Rhsim08G0074500	0.087	-0.276	-0.363	no
CK-0d	RHSIM_Rhsim09G0152100	0.175	0.715	0.540	no
CK-0d	RHSIM_Rhsim08G0203900	0.944	1.214	0.270	no
CK-0d	RHSIM_Rhsim08G0028600	0.973	0.083	-0.889	no
CK-0d	RHSIM_Rhsim07G0065200	2.462	2.135	-0.326	high
CK-0d	RHSIM_Rhsim08G0232500	0.701	1.003	0.301	no
CK-0d	RHSIM_Rhsim08G0233200	-0.070	-0.124	-0.054	no
CK-0d	RHSIM_Rhsim07G0063000	0.177	-0.006	-0.184	no
CK-0d	RHSIM_Rhsim08G0062700	0.428	1.304	0.876	no
CK-0d	RHSIM_Rhsim08G0195300	0.811	0.790	-0.021	no
CK-0d	RHSIM_Rhsim08G0144000	-0.433	-1.053	-0.619	no
CK-0d	RHSIM_Rhsim08G0190900	-0.002	0.568	0.569	no
CK-0d	RHSIM_Rhsim07G0109500	1.148	0.197	-0.951	high
CK-0d	RHSIM_Rhsim09G0111900	0.954	0.904	-0.049	no
CK-0d	RHSIM_Rhsim07G0077100	-0.364	-0.053	0.312	no

Group	Gene	PTR (z-score difference)	z(log2 LFQ)	z(log2 FPKM)	extreme-PTR
CK-0d	RHSIM_Rhsim09G0003200	0.563	0.291	-0.272	no
CK-0d	RHSIM_Rhsim08G0051500	-0.795	-0.790	0.005	no
CK-0d	RHSIM_Rhsim08G0206900	0.128	-0.634	-0.763	no
CK-0d	RHSIM_Rhsim09G0013500	-0.394	-1.354	-0.960	no
CK-0d	RHSIM_Rhsim09G0004800	0.977	1.283	0.305	no
CK-0d	RHSIM_Rhsim08G0152700	-0.922	0.221	1.143	no
CK-0d	RHSIM_Rhsim08G0150000	0.203	0.713	0.510	no
CK-0d	RHSIM_Rhsim09G0192600	1.041	0.824	-0.217	no
CK-0d	RHSIM_Rhsim09G0073000	0.145	0.100	-0.045	no
CK-0d	RHSIM_Rhsim07G0227100	0.900	-1.267	-2.167	no
CK-0d	RHSIM_Rhsim08G0232800	0.356	0.061	-0.295	no
CK-0d	RHSIM_Rhsim09G0069200	1.233	0.822	-0.411	high
CK-0d	RHSIM_Rhsim08G0250100	0.649	1.520	0.871	no
CK-0d	RHSIM_Rhsim08G0072500	-0.229	-0.041	0.188	no
CK-0d	RHSIM_Rhsim07G0142300	0.354	2.530	2.176	no
CK-0d	RHSIM_Rhsim08G0125600	0.009	0.455	0.446	no
CK-0d	RHSIM_Rhsim07G0079800	-0.312	-0.214	0.097	no
CK-0d	RHSIM_Rhsim08G0106000	1.652	-0.510	-2.162	high
CK-0d	RHSIM_Rhsim08G0225600	0.047	-0.956	-1.003	no
CK-0d	RHSIM_Rhsim07G0095900	-0.820	0.415	1.235	no
CK-0d	RHSIM_Rhsim08G0008000	0.853	-0.585	-1.439	no
CK-0d	RHSIM_Rhsim08G0005000	1.694	0.260	-1.435	high
CK-0d	RHSIM_Rhsim09G0102400	0.129	-0.487	-0.616	no
CK-0d	RHSIM_Rhsim08G0109400	0.047	-0.235	-0.282	no
CK-0d	RHSIM_Rhsim09G0106700	1.161	0.405	-0.756	high
CK-0d	RHSIM_Rhsim08G0161300	-0.921	-1.662	-0.741	no
CK-0d	RHSIM_Rhsim08G0003400	-0.051	-0.518	-0.466	no
CK-0d	RHSIM_Rhsim09G0138500	-0.709	-0.022	0.687	no
CK-0d	RHSIM_Rhsim09G0066700	1.175	0.794	-0.382	high
CK-0d	RHSIM_Rhsim08G0054100	1.456	1.427	-0.029	high
CK-0d	RHSIM_Rhsim07G0212400	0.531	-0.343	-0.873	no
CK-0d	RHSIM_Rhsim07G0257800	0.226	0.011	-0.215	no

Group	Gene	PTR (z-score difference)	z(log2 LFQ)	z(log2 FPKM)	extreme-PTR
CK-0d	RHSIM_Rhsim07G0038700	1.324	1.208	-0.116	high
CK-0d	RHSIM_Rhsim07G0074200	0.374	-0.766	-1.139	no
CK-0d	RHSIM_Rhsim08G0079800	0.434	-0.767	-1.201	no
CK-0d	RHSIM_Rhsim08G0205400	-0.149	-0.450	-0.301	no
CK-0d	RHSIM_Rhsim08G0185100	1.044	1.273	0.229	no
CK-0d	RHSIM_Rhsim08G0160300	0.422	0.240	-0.182	no
CK-0d	RHSIM_Rhsim08G0240600	-0.042	-0.899	-0.856	no
CK-0d	RHSIM_Rhsim07G0106500	1.397	-0.148	-1.545	high
CK-0d	RHSIM_Rhsim09G0203200	-0.426	-1.529	-1.104	no
CK-0d	RHSIM_Rhsim08G0202600	0.150	-0.427	-0.577	no
CK-0d	RHSIM_Rhsim07G0011800	0.207	0.986	0.779	no
CK-0d	RHSIM_Rhsim08G0242400	0.245	-1.282	-1.527	no
CK-0d	RHSIM_Rhsim08G0003500	0.174	0.895	0.721	no
CK-0d	RHSIM_Rhsim08G0136400	0.025	0.306	0.282	no
CK-0d	RHSIM_Rhsim09G0074000	-0.054	-0.916	-0.862	no
CK-0d	RHSIM_Rhsim08G0044500	-0.055	-0.732	-0.677	no
CK-0d	RHSIM_Rhsim09G0190400	-0.435	-0.474	-0.038	no
CK-0d	RHSIM_Rhsim08G0207400	0.202	1.954	1.753	no
CK-0d	RHSIM_Rhsim07G0010300	0.696	-0.108	-0.804	no
CK-0d	RHSIM_Rhsim07G0204900	1.282	0.069	-1.212	high
CK-0d	RHSIM_Rhsim08G0190700	0.089	0.029	-0.060	no
CK-0d	RHSIM_Rhsim07G0096600	-0.496	-0.497	-0.001	no
CK-0d	RHSIM_Rhsim08G0118900	-0.906	-0.272	0.634	no
CK-0d	RHSIM_Rhsim08G0241100	-1.895	-0.826	1.070	low
CK-0d	RHSIM_Rhsim07G0225100	-0.307	0.212	0.519	no
CK-0d	RHSIM_Rhsim08G0086000	-1.622	-0.340	1.281	low
CK-0d	RHSIM_Rhsim07G0087000	-0.960	-0.562	0.397	no
CK-0d	RHSIM_Rhsim08G0076000	-1.157	-0.571	0.586	low
CK-0d	RHSIM_Rhsim07G0203200	-0.487	-0.054	0.433	no
CK-0d	RHSIM_Rhsim08G0222800	2.117	0.525	-1.592	high
CK-0d	RHSIM_Rhsim09G0132300	0.196	-0.613	-0.809	no
CK-0d	RHSIM_Rhsim08G0071500	-0.530	-0.354	0.176	no

Group	Gene	PTR (z-score difference)	z(log2 LFQ)	z(log2 FPKM)	extreme-PTR
CK-0d	RHSIM_Rhsim07G0062900	-0.789	-0.797	-0.008	no
CK-0d	RHSIM_Rhsim08G0088200	-1.238	1.252	2.490	low
CK-0d	RHSIM_Rhsim09G0031700	-0.643	0.040	0.682	no
CK-0d	RHSIM_Rhsim09G0090500	-0.380	-0.630	-0.250	no
CK-0d	RHSIM_Rhsim08G0208000	-2.173	-0.657	1.516	low
CK-0d	RHSIM_Rhsim08G0075200	0.026	0.477	0.450	no
CK-0d	RHSIM_Rhsim08G0197500	0.046	-0.544	-0.590	no
CK-0d	RHSIM_Rhsim07G0077300	0.080	0.546	0.466	no
CK-0d	RHSIM_Rhsim08G0184700	-1.721	-2.142	-0.420	low
CK-0d	RHSIM_Rhsim07G0006700	0.552	-0.687	-1.240	no
CK-0d	RHSIM_Rhsim08G0209600	-0.411	-0.940	-0.529	no
CK-0d	RHSIM_Rhsim07G0119700	1.525	1.448	-0.077	high
CK-0d	RHSIM_Rhsim08G0189500	-1.164	-1.785	-0.621	low
CK-0d	RHSIM_Rhsim07G0100700	-0.534	-1.135	-0.601	no
CK-0d	RHSIM_Rhsim07G0215500	-0.368	-0.389	-0.021	no
CK-0d	RHSIM_Rhsim08G0212100	-0.076	-1.181	-1.105	no
CK-0d	RHSIM_Rhsim08G0216500	1.094	0.131	-0.963	no
CK-0d	RHSIM_Rhsim07G0017000	-1.051	0.730	1.781	low
CK-0d	RHSIM_Rhsim09G0003500	-0.138	-0.448	-0.310	no
CK-0d	RHSIM_Rhsim08G0189100	-0.638	0.185	0.823	no
CK-0d	RHSIM_Rhsim08G0052700	-1.631	-1.763	-0.133	low
CK-0d	RHSIM_Rhsim09G0181600	0.540	-0.404	-0.944	no
CK-0d	RHSIM_Rhsim08G0132500	-0.429	0.521	0.950	no
CK-0d	RHSIM_Rhsim09G0182800	1.306	-0.236	-1.542	high
CK-0d	RHSIM_Rhsim07G0123700	-0.456	-0.466	-0.009	no
CK-0d	RHSIM_Rhsim08G0199800	0.234	0.673	0.439	no
CK-0d	RHSIM_Rhsim09G0146100	0.017	-0.561	-0.579	no
CK-0d	RHSIM_Rhsim08G0228400	0.524	0.286	-0.238	no
CK-0d	RHSIM_Rhsim08G0146100	1.374	2.068	0.694	high
CK-0d	RHSIM_Rhsim07G0165600	0.842	1.573	0.732	no
CK-0d	RHSIM_Rhsim09G0211400	0.191	-0.983	-1.174	no
CK-0d	RHSIM_Rhsim08G0086100	1.533	1.091	-0.442	high

Group	Gene	PTR (z-score difference)	z(log2 LFQ)	z(log2 FPKM)	extreme-PTR
CK-0d	RHSIM_Rhsim09G0084200	-1.475	0.176	1.652	low
CK-0d	RHSIM_Rhsim07G0010500	-0.130	-1.267	-1.138	no
CK-0d	RHSIM_Rhsim07G0118600	-0.092	-0.698	-0.606	no
CK-0d	RHSIM_Rhsim08G0067700	-1.239	-1.056	0.182	low
CK-0d	RHSIM_Rhsim09G0185500	1.903	0.525	-1.377	high
CK-0d	RHSIM_Rhsim08G0080100	-0.831	-0.476	0.355	no
CK-0d	RHSIM_Rhsim08G0002700	-0.143	-0.092	0.051	no
CK-0d	RHSIM_Rhsim08G0029700	2.339	1.188	-1.151	high
CK-0d	RHSIM_Rhsim09G0126400	-2.106	-1.199	0.907	low
CK-0d	RHSIM_Rhsim09G0199100	-1.557	-1.405	0.152	low
CK-0d	RHSIM_Rhsim09G0210100	1.323	0.569	-0.754	high
CK-0d	RHSIM_Rhsim08G0157100	-0.160	-0.343	-0.183	no
CK-0d	RHSIM_Rhsim08G0199500	-0.376	-0.222	0.154	no
CK-0d	RHSIM_Rhsim09G0079200	-0.307	0.437	0.744	no
CK-0d	RHSIM_Rhsim08G0225700	-1.037	-0.979	0.058	low
CK-0d	RHSIM_Rhsim08G0191000	1.911	0.720	-1.191	high
CK-0d	RHSIM_Rhsim07G0037800	0.398	0.110	-0.288	no
CK-0d	RHSIM_Rhsim08G0113200	-1.506	-1.141	0.365	low
CK-0d	RHSIM_Rhsim08G0187700	0.170	0.596	0.426	no
CK-0d	RHSIM_Rhsim08G0033400	0.416	2.858	2.442	no
CK-0d	RHSIM_Rhsim08G0135900	1.827	0.711	-1.115	high
CK-0d	RHSIM_Rhsim08G0081500	0.902	2.223	1.322	no
CK-0d	RHSIM_Rhsim08G0233000	-0.281	-1.658	-1.377	no
CK-0d	RHSIM_Rhsim08G0013100	-2.596	-1.594	1.002	low
CK-0d	RHSIM_Rhsim08G0176300	-0.385	-0.973	-0.588	no
CK-0d	RHSIM_Rhsim08G0221200	1.071	1.685	0.614	no
CK-0d	RHSIM_Rhsim07G0115500	0.035	0.257	0.222	no
CK-0d	RHSIM_Rhsim08G0009100	-0.217	0.026	0.242	no
CK-0d	RHSIM_Rhsim08G0233300	-0.069	0.231	0.300	no
CK-0d	RHSIM_Rhsim07G0204400	0.401	1.411	1.010	no
CK-0d	RHSIM_Rhsim08G0015000	-0.416	-0.750	-0.333	no
CK-0d	RHSIM_Rhsim07G0074900	-1.306	-0.690	0.616	low

Group	Gene	PTR (z-score difference)	z(log2 LFQ)	z(log2 FPKM)	extreme-PTR
CK-0d	RHSIM_Rhsim08G0100700	1.383	0.965	-0.418	high
CK-0d	RHSIM_Rhsim08G0012800	0.202	0.467	0.265	no
CK-0d	RHSIM_Rhsim08G0000900	-0.548	0.164	0.712	no
CK-0d	RHSIM_Rhsim08G0009800	1.027	-0.235	-1.262	no
CK-0d	RHSIM_Rhsim08G0202900	1.363	-0.518	-1.881	high
CK-0d	RHSIM_Rhsim08G0118800	-0.545	-0.380	0.166	no
CK-0d	RHSIM_Rhsim08G0243800	-2.181	-1.668	0.513	low
CK-0d	RHSIM_Rhsim08G0020200	0.931	-0.364	-1.295	no
CK-0d	RHSIM_Rhsim07G0118400	-0.092	-1.327	-1.235	no
CK-0d	RHSIM_Rhsim07G0173200	0.410	0.461	0.052	no
CK-0d	RHSIM_Rhsim08G0210300	1.200	0.055	-1.145	high
CK-0d	RHSIM_Rhsim07G0124000	2.741	0.737	-2.004	high
CK-0d	RHSIM_Rhsim08G0151900	-0.094	-0.339	-0.245	no
CK-0d	RHSIM_Rhsim08G0070300	0.359	0.651	0.292	no
CK-0d	RHSIM_Rhsim08G0096300	-0.172	-0.300	-0.128	no
CK-0d	RHSIM_Rhsim07G0016900	-1.104	-0.591	0.513	low
CK-0d	RHSIM_Rhsim08G0249800	-1.549	-2.078	-0.529	low
CK-0d	RHSIM_Rhsim07G0131600	0.247	-0.579	-0.825	no
CK-0d	RHSIM_Rhsim08G0056900	-0.469	-0.967	-0.498	no
CK-0d	RHSIM_Rhsim08G0170400	0.500	1.899	1.399	no
CK-0d	RHSIM_Rhsim07G0175400	0.186	0.199	0.013	no
CK-0d	RHSIM_Rhsim07G0170000	0.122	1.119	0.997	no
CK-0d	RHSIM_Rhsim07G0057300	0.460	0.250	-0.210	no
CK-0d	RHSIM_Rhsim08G0071700	0.447	0.788	0.342	no
CK-0d	RHSIM_Rhsim07G0035700	-0.207	-0.676	-0.468	no
CK-0d	RHSIM_Rhsim08G0077000	-0.688	0.255	0.943	no
CK-0d	RHSIM_Rhsim08G0161000	0.772	0.180	-0.593	no
CK-0d	RHSIM_Rhsim07G0257200	1.430	1.216	-0.214	high
CK-0d	RHSIM_Rhsim07G0024200	0.950	-0.379	-1.329	no
CK-0d	RHSIM_Rhsim08G0088300	-0.474	1.962	2.436	no
CK-0d	RHSIM_Rhsim08G0188700	-0.534	0.356	0.890	no
CK-0d	RHSIM_Rhsim08G0200500	-2.187	-0.416	1.770	low

Group	Gene	PTR (z-score difference)	z(log2 LFQ)	z(log2 FPKM)	extreme-PTR
CK-0d	RHSIM_Rhsim08G0157300	0.266	-0.572	-0.838	no
CK-0d	RHSIM_Rhsim07G0017400	-1.001	0.657	1.658	no
CK-0d	RHSIM_Rhsim07G0085300	0.489	1.397	0.908	no
CK-0d	RHSIM_Rhsim08G0216800	-0.721	0.494	1.215	no
CK-0d	RHSIM_Rhsim08G0126200	0.207	0.616	0.408	no
CK-0d	RHSIM_Rhsim07G0011500	-0.467	1.291	1.758	no
CK-0d	RHSIM_Rhsim08G0222300	-1.119	-0.973	0.146	low
CK-0d	RHSIM_Rhsim07G0216000	-1.242	-0.432	0.810	low
CK-0d	RHSIM_Rhsim08G0245600	1.468	-0.223	-1.691	high
CK-0d	RHSIM_Rhsim08G0153200	-0.114	1.065	1.179	no
CK-0d	RHSIM_Rhsim07G0101700	0.157	-0.461	-0.618	no
CK-0d	RHSIM_Rhsim08G0248400	2.981	3.350	0.370	high
CK-0d	RHSIM_Rhsim08G0153400	-0.384	-0.532	-0.148	no
CK-0d	RHSIM_Rhsim08G0215500	0.898	-1.146	-2.044	no
CK-0d	RHSIM_Rhsim07G0071000	1.684	-0.532	-2.216	high
CK-0d	RHSIM_Rhsim08G0240500	0.125	0.034	-0.091	no
CK-0d	RHSIM_Rhsim07G0004600	-1.647	-1.456	0.191	low
CK-0d	RHSIM_Rhsim08G0157700	-1.149	-0.808	0.341	low
CK-0d	RHSIM_Rhsim07G0053800	0.100	0.210	0.110	no
CK-0d	RHSIM_Rhsim08G0230900	-0.019	0.286	0.305	no
CK-0d	RHSIM_Rhsim07G0248600	-0.263	-0.723	-0.460	no
CK-0d	RHSIM_Rhsim07G0144500	-0.672	-0.304	0.368	no
CK-0d	RHSIM_Rhsim07G0201100	0.134	-1.185	-1.319	no
CK-0d	RHSIM_Rhsim08G0012300	0.513	1.613	1.100	no
CK-0d	RHSIM_Rhsim07G0023600	-0.039	-0.750	-0.712	no
CK-0d	RHSIM_Rhsim08G0241600	-0.021	-0.563	-0.542	no
CK-0d	RHSIM_Rhsim07G0025600	2.655	0.560	-2.095	high
CK-0d	RHSIM_Rhsim08G0123400	-1.137	-0.031	1.106	low
CK-0d	RHSIM_Rhsim08G0040600	0.988	-0.517	-1.505	no
CK-0d	RHSIM_Rhsim08G0119200	0.241	0.731	0.490	no
CK-0d	RHSIM_Rhsim08G0058800	-0.569	-0.325	0.245	no
CK-0d	RHSIM_Rhsim08G0089400	-0.477	-0.418	0.058	no

Group	Gene	PTR (z-score difference)	z(log2 LFQ)	z(log2 FPKM)	extreme-PTR
CK-0d	RHSIM_Rhsim07G0019000	1.159	0.785	-0.374	high
CK-0d	RHSIM_Rhsim07G0251100	-0.918	1.550	2.468	no
CK-0d	RHSIM_Rhsim08G0169300	-0.749	-1.076	-0.327	no
CK-0d	RHSIM_Rhsim08G0093900	-0.898	0.755	1.653	no
CK-0d	RHSIM_Rhsim08G0098500	-0.002	-0.591	-0.589	no
CK-0d	RHSIM_Rhsim08G0187400	1.114	1.684	0.570	high
CK-0d	RHSIM_Rhsim08G0212600	-0.694	-0.373	0.321	no
CK-0d	RHSIM_Rhsim07G0258200	-0.491	-1.211	-0.720	no
CK-0d	RHSIM_Rhsim07G0146900	-0.248	-0.975	-0.727	no
CK-0d	RHSIM_Rhsim08G0035600	1.787	-0.273	-2.060	high
CK-0d	RHSIM_Rhsim08G0078700	0.399	-0.141	-0.541	no
CK-0d	RHSIM_Rhsim08G0043300	0.632	0.410	-0.221	no
CK-0d	RHSIM_Rhsim08G0184400	0.657	0.162	-0.495	no
CK-0d	RHSIM_Rhsim08G0119000	1.070	1.094	0.024	no
CK-0d	RHSIM_Rhsim07G0209200	-3.740	-1.516	2.225	low
CK-0d	RHSIM_Rhsim08G0068000	-0.813	0.059	0.872	no
CK-0d	RHSIM_Rhsim07G0250500	-0.477	0.849	1.326	no
CK-0d	RHSIM_Rhsim08G0199900	0.354	0.381	0.027	no
CK-0d	RHSIM_Rhsim07G0188400	-2.773	-2.290	0.484	low
CK-0d	RHSIM_Rhsim07G0184400	1.001	-0.460	-1.461	no
CK-0d	RHSIM_Rhsim08G0207200	-0.519	0.543	1.063	no
CK-0d	RHSIM_Rhsim08G0216200	-0.146	-0.127	0.019	no
CK-0d	RHSIM_Rhsim08G0227300	-2.971	-1.250	1.722	low
CK-0d	RHSIM_Rhsim08G0124200	0.887	0.341	-0.546	no
CK-0d	RHSIM_Rhsim07G0186000	-0.066	-0.164	-0.098	no
CK-0d	RHSIM_Rhsim08G0027500	-0.310	-0.121	0.189	no
CK-0d	RHSIM_Rhsim08G0000300	-0.291	-0.464	-0.173	no
CK-0d	RHSIM_Rhsim08G0155400	-0.754	-0.162	0.591	no
CK-0d	RHSIM_Rhsim08G0125000	-0.253	-0.033	0.220	no
CK-0d	RHSIM_Rhsim07G0256400	0.083	0.353	0.269	no
CK-0d	RHSIM_Rhsim08G0146900	0.767	0.119	-0.648	no
CK-0d	RHSIM_Rhsim07G0125300	0.544	1.089	0.545	no

Group	Gene	PTR (z-score difference)	z(log2 LFQ)	z(log2 FPKM)	extreme-PTR
CK-0d	RHSIM_Rhsim08G0041400	-0.022	-1.505	-1.483	no
CK-0d	RHSIM_Rhsim08G0198600	0.429	1.344	0.915	no
CK-0d	RHSIM_Rhsim08G0124400	-0.962	-0.939	0.023	no
CK-0d	RHSIM_Rhsim08G0106700	1.555	-0.309	-1.864	high
CK-0d	RHSIM_Rhsim08G0095400	-0.840	-1.036	-0.197	no
CK-0d	RHSIM_Rhsim07G0102300	-0.204	-1.134	-0.930	no
CK-0d	RHSIM_Rhsim08G0189200	-1.528	-1.662	-0.134	low
CK-0d	RHSIM_Rhsim08G0198900	-0.975	-0.850	0.125	no
CK-0d	RHSIM_Rhsim07G0187600	-0.893	1.020	1.913	no
CK-0d	RHSIM_Rhsim08G0038400	-0.837	-1.537	-0.701	no
CK-0d	RHSIM_Rhsim08G0066200	-0.105	2.684	2.788	no
CK-0d	RHSIM_Rhsim08G0129000	0.608	1.879	1.272	no
CK-0d	RHSIM_Rhsim08G0076500	0.466	0.715	0.249	no
CK-0d	RHSIM_Rhsim08G0171400	0.156	-0.299	-0.454	no
CK-0d	RHSIM_Rhsim08G0047100	1.891	0.333	-1.558	high
CK-0d	RHSIM_Rhsim08G0206000	0.097	-0.730	-0.827	no
CK-0d	RHSIM_Rhsim07G0219200	-0.099	-1.152	-1.053	no
CK-0d	RHSIM_Rhsim08G0227800	1.091	0.147	-0.944	no
CK-0d	RHSIM_Rhsim08G0249600	0.292	-0.807	-1.099	no
CK-0d	RHSIM_Rhsim07G0251700	1.100	1.853	0.753	no
CK-0d	RHSIM_Rhsim08G0038900	-0.030	0.896	0.926	no
CK-0d	RHSIM_Rhsim08G0096700	-0.172	-0.436	-0.264	no
CK-0d	RHSIM_Rhsim07G0067800	-0.370	-0.259	0.111	no
CK-0d	RHSIM_Rhsim08G0006300	0.688	-0.001	-0.689	no
CK-0d	RHSIM_Rhsim07G0215000	-0.274	-0.973	-0.699	no
CK-0d	RHSIM_Rhsim07G0007400	-0.551	-1.216	-0.665	no
CK-0d	RHSIM_Rhsim08G0115600	-0.473	0.103	0.576	no
CK-0d	RHSIM_Rhsim07G0241500	0.886	0.688	-0.198	no
CK-0d	RHSIM_Rhsim08G0005400	-2.402	-1.145	1.257	low
CK-0d	RHSIM_Rhsim07G0208900	-0.433	-0.486	-0.052	no
CK-0d	RHSIM_Rhsim08G0044400	0.005	-0.760	-0.765	no
CK-0d	RHSIM_Rhsim08G0018500	1.332	0.608	-0.724	high

Group	Gene	PTR (z-score difference)	z(log2 LFQ)	z(log2 FPKM)	extreme-PTR
CK-0d	RHSIM_Rhsim08G0090300	0.259	0.997	0.738	no
CK-0d	RHSIM_Rhsim07G0052800	0.807	-0.922	-1.729	no
CK-0d	RHSIM_Rhsim07G0108500	-0.658	-0.905	-0.246	no
CK-0d	RHSIM_Rhsim07G0063200	-2.214	-1.151	1.063	low
CK-0d	RHSIM_Rhsim07G0080500	1.860	0.557	-1.303	high
CK-0d	RHSIM_Rhsim08G0074900	-0.423	-0.263	0.161	no
CK-0d	RHSIM_Rhsim08G0010200	-0.055	-0.703	-0.648	no
CK-0d	RHSIM_Rhsim07G0032500	0.784	0.919	0.135	no
CK-0d	RHSIM_Rhsim08G0171600	-0.317	3.022	3.339	no
CK-0d	RHSIM_Rhsim07G0096500	0.233	2.157	1.924	no
CK-0d	RHSIM_Rhsim08G0252300	-0.753	-0.634	0.119	no
CK-0d	RHSIM_Rhsim07G0181400	0.495	0.219	-0.276	no
CK-0d	RHSIM_Rhsim08G0175700	-0.917	-1.273	-0.357	no
CK-0d	RHSIM_Rhsim08G0173200	-1.821	0.193	2.014	low
CK-0d	RHSIM_Rhsim07G0091300	-0.771	-0.040	0.731	no
CK-0d	RHSIM_Rhsim07G0054000	-2.185	-1.783	0.402	low
CK-0d	RHSIM_Rhsim07G0068700	0.547	-0.259	-0.806	no
CK-0d	RHSIM_Rhsim08G0054600	2.235	0.066	-2.169	high
CK-0d	RHSIM_Rhsim07G0106900	0.464	-1.729	-2.193	no
CK-0d	RHSIM_Rhsim08G0016700	-0.431	1.036	1.468	no
CK-0d	RHSIM_Rhsim07G0080100	0.228	0.185	-0.043	no
CK-0d	RHSIM_Rhsim07G0245700	0.346	-0.326	-0.672	no
CK-0d	RHSIM_Rhsim08G0080000	0.327	-0.776	-1.104	no
CK-0d	RHSIM_Rhsim08G0140900	-0.573	-0.420	0.152	no
CK-0d	RHSIM_Rhsim08G0046200	0.139	0.481	0.342	no
CK-0d	RHSIM_Rhsim08G0010700	0.643	-0.753	-1.395	no
CK-0d	RHSIM_Rhsim08G0116700	1.591	0.743	-0.848	high
CK-0d	RHSIM_Rhsim07G0145700	-0.462	0.405	0.867	no
CK-0d	RHSIM_Rhsim07G0006200	-0.820	0.336	1.156	no
CK-0d	RHSIM_Rhsim07G0013500	-1.178	-1.338	-0.160	low
CK-0d	RHSIM_Rhsim07G0053100	-0.538	0.652	1.189	no
CK-0d	RHSIM_Rhsim08G0249200	-1.444	-0.680	0.764	low

Group	Gene	PTR (z-score difference)	z(log2 LFQ)	z(log2 FPKM)	extreme-PTR
CK-0d	RHSIM_Rhsim07G0096000	0.331	0.777	0.445	no
CK-0d	RHSIM_Rhsim08G0245800	-1.115	-0.345	0.770	low
CK-0d	RHSIM_Rhsim08G0019400	0.614	-0.952	-1.566	no
CK-0d	RHSIM_Rhsim08G0102300	0.514	-0.804	-1.318	no
CK-0d	RHSIM_Rhsim07G0005700	-0.341	-1.160	-0.819	no
CK-0d	RHSIM_Rhsim07G0056700	0.554	-0.220	-0.773	no
CK-0d	RHSIM_Rhsim08G0210500	0.131	0.772	0.641	no
CK-0d	RHSIM_Rhsim07G0045400	1.017	0.873	-0.144	no
CK-0d	RHSIM_Rhsim07G0188300	-0.509	-1.031	-0.522	no
CK-0d	RHSIM_Rhsim08G0037900	0.473	0.181	-0.291	no
CK-0d	RHSIM_Rhsim07G0023500	0.127	-0.281	-0.408	no
CK-0d	RHSIM_Rhsim07G0068500	-0.247	0.267	0.515	no
CK-0d	RHSIM_Rhsim07G0081300	2.564	0.491	-2.073	high
CK-0d	RHSIM_Rhsim07G0117800	-0.608	1.066	1.674	no
CK-0d	RHSIM_Rhsim08G0119100	-0.016	0.445	0.461	no
CK-0d	RHSIM_Rhsim07G0186700	0.104	-0.591	-0.696	no
CK-0d	RHSIM_Rhsim08G0034600	-0.992	0.502	1.494	no
CK-0d	RHSIM_Rhsim07G0083300	-0.284	-0.941	-0.657	no
CK-0d	RHSIM_Rhsim08G0139800	-0.241	-0.419	-0.178	no
CK-0d	RHSIM_Rhsim07G0240400	-1.039	-0.624	0.414	low
CK-0d	RHSIM_Rhsim07G0126200	-0.502	0.097	0.598	no
CK-0d	RHSIM_Rhsim07G0251400	-0.422	2.286	2.708	no
CK-0d	RHSIM_Rhsim07G0127400	-0.868	-0.597	0.271	no
CK-0d	RHSIM_Rhsim07G0022300	-0.689	-0.046	0.643	no
CK-0d	RHSIM_Rhsim07G0094300	0.202	-0.997	-1.199	no
CK-0d	RHSIM_Rhsim07G0170800	-1.677	-1.115	0.562	low
CK-0d	RHSIM_Rhsim08G0013500	1.630	-0.673	-2.303	high
CK-0d	RHSIM_Rhsim08G0031200	1.743	1.803	0.061	high
CK-0d	RHSIM_Rhsim07G0071200	1.289	0.132	-1.157	high
CK-0d	RHSIM_Rhsim08G0248600	-0.253	-0.250	0.003	no
CK-0d	RHSIM_Rhsim08G0067400	1.136	0.553	-0.583	high
CK-0d	RHSIM_Rhsim08G0175500	-0.265	-0.782	-0.517	no

Group	Gene	PTR (z-score difference)	z(log2 LFQ)	z(log2 FPKM)	extreme-PTR
CK-0d	RHSIM_Rhsim07G0172400	-0.272	-1.399	-1.127	no
CK-0d	RHSIM_Rhsim08G0249300	-1.002	-0.010	0.992	no
CK-0d	RHSIM_Rhsim07G0155300	-0.873	-0.817	0.056	no
CK-0d	RHSIM_Rhsim07G0181600	-1.113	-1.280	-0.167	low
CK-0d	RHSIM_Rhsim07G0202000	1.015	1.031	0.016	no
CK-0d	RHSIM_Rhsim08G0221900	0.051	-0.408	-0.459	no
CK-0d	RHSIM_Rhsim07G0040000	0.540	1.661	1.121	no
CK-0d	RHSIM_Rhsim08G0000400	-0.641	0.182	0.823	no
CK-0d	RHSIM_Rhsim08G0050800	1.133	0.612	-0.520	high
CK-0d	RHSIM_Rhsim07G0236600	-0.193	-0.839	-0.646	no
CK-0d	RHSIM_Rhsim07G0219500	-1.275	0.087	1.362	low
CK-0d	RHSIM_Rhsim07G0059300	-0.795	-1.047	-0.252	no
CK-0d	RHSIM_Rhsim07G0209000	1.285	0.286	-1.000	high
CK-0d	RHSIM_Rhsim08G0126600	-0.166	-0.283	-0.118	no
CK-0d	RHSIM_Rhsim07G0168300	-1.263	-1.923	-0.660	low
CK-0d	RHSIM_Rhsim07G0244100	-0.679	-1.246	-0.568	no
CK-0d	RHSIM_Rhsim07G0163600	0.757	-0.814	-1.571	no
CK-0d	RHSIM_Rhsim07G0173800	-0.151	-0.504	-0.353	no
CK-0d	RHSIM_Rhsim07G0239800	-0.637	-0.391	0.246	no
CK-0d	RHSIM_Rhsim07G0031300	-0.283	-0.537	-0.254	no
CK-0d	RHSIM_Rhsim07G0016500	0.425	0.194	-0.230	no
CK-0d	RHSIM_Rhsim07G0250100	0.849	2.336	1.487	no
CK-0d	RHSIM_Rhsim07G0107500	-2.607	-1.141	1.466	low
CK-0d	RHSIM_Rhsim07G0068000	-0.028	-0.900	-0.873	no
CK-0d	RHSIM_Rhsim07G0024600	-0.835	-1.420	-0.585	no
CK-0d	RHSIM_Rhsim07G0038000	0.494	0.360	-0.134	no
CK-0d	RHSIM_Rhsim07G0086400	0.015	-0.879	-0.895	no
CK-0d	RHSIM_Rhsim07G0052400	0.308	-0.228	-0.535	no
CK-0d	RHSIM_Rhsim07G0029300	0.513	-1.084	-1.597	no
CK-0d	RHSIM_Rhsim08G0068500	-0.944	0.569	1.513	no
CK-0d	RHSIM_Rhsim07G0069700	1.659	-0.656	-2.315	high
CK-0d	RHSIM_Rhsim07G0033300	0.187	-1.322	-1.508	no

Group	Gene	PTR (z-score difference)	z(log2 LFQ)	z(log2 FPKM)	extreme-PTR
CK-0d	RHSIM_Rhsim07G0105400	0.345	-0.099	-0.444	no
CK-0d	RHSIM_Rhsim08G0085100	0.878	1.046	0.168	no
CK-0d	RHSIM_Rhsim07G0173600	-0.865	0.213	1.078	no
CK-0d	RHSIM_Rhsim08G0016400	-0.201	-0.901	-0.700	no
CK-0d	RHSIM_Rhsim08G0152500	0.502	2.759	2.258	no
CK-0d	RHSIM_Rhsim07G0027900	-1.506	-1.046	0.460	low
CK-0d	RHSIM_Rhsim07G0026600	-0.884	-1.374	-0.490	no
CK-0d	RHSIM_Rhsim07G0055300	0.748	1.548	0.801	no
CK-0d	RHSIM_Rhsim08G0176400	-1.131	1.310	2.441	low
CK-0d	RHSIM_Rhsim07G0179300	-0.223	-1.794	-1.571	no
CK-0d	RHSIM_Rhsim08G0232200	0.864	-0.776	-1.640	no
CK-0d	RHSIM_Rhsim07G0061300	-1.047	-0.574	0.474	low
CK-0d	RHSIM_Rhsim07G0125000	0.800	-0.234	-1.035	no
CK-0d	RHSIM_Rhsim07G0228300	0.522	-0.668	-1.190	no
CK-0d	RHSIM_Rhsim07G0020700	-0.753	0.276	1.029	no
CK-0d	RHSIM_Rhsim07G0132100	-0.870	-0.643	0.227	no
CK-0d	RHSIM_Rhsim07G0074100	-0.210	-0.102	0.108	no
CK-0d	RHSIM_Rhsim07G0198900	0.569	-0.426	-0.995	no
CK-0d	RHSIM_Rhsim07G0007900	0.689	3.134	2.445	no
CK-0d	RHSIM_Rhsim07G0243800	-0.245	1.686	1.931	no
CK-0d	RHSIM_Rhsim07G0239400	-1.000	-1.247	-0.248	no
CK-0d	RHSIM_Rhsim07G0025200	-0.059	-0.736	-0.677	no
CK-0d	RHSIM_Rhsim07G0143700	-0.668	-0.140	0.528	no
CK-0d	RHSIM_Rhsim08G0094700	-0.893	-0.241	0.652	no
CK-0d	RHSIM_Rhsim07G0214200	0.839	-0.670	-1.509	no
CK-0d	RHSIM_Rhsim07G0109900	-0.895	-1.507	-0.612	no
CK-0d	RHSIM_Rhsim08G0173100	-0.797	-0.139	0.658	no
CK-0d	RHSIM_Rhsim07G0049200	-0.274	0.587	0.861	no
CK-0d	RHSIM_Rhsim08G0224200	-0.355	-1.003	-0.648	no
CK-0d	RHSIM_Rhsim08G0112300	-0.317	-0.236	0.081	no
CK-0d	RHSIM_Rhsim07G0172200	-0.581	-0.181	0.401	no
CK-0d	RHSIM_Rhsim08G0226500	-0.271	-0.577	-0.306	no

Group	Gene	PTR (z-score difference)	z(log2 LFQ)	z(log2 FPKM)	extreme-PTR
CK-0d	RHSIM_Rhsim07G0215100	-1.353	-0.490	0.863	low
CK-0d	RHSIM_Rhsim07G0082400	-0.293	-0.343	-0.050	no
CK-0d	RHSIM_Rhsim07G0106300	-0.035	0.877	0.912	no
CK-0d	RHSIM_Rhsim07G0200600	-1.198	-1.022	0.176	low
CK-0d	RHSIM_Rhsim07G0029400	0.437	-0.065	-0.502	no
CK-0d	RHSIM_Rhsim07G0171600	0.655	0.515	-0.140	no
CK-0d	RHSIM_Rhsim08G0080600	0.523	0.044	-0.478	no
CK-0d	RHSIM_Rhsim06G0201500	0.568	-0.316	-0.884	no
CK-0d	RHSIM_Rhsim08G0006200	1.482	1.606	0.124	high
CK-0d	RHSIM_Rhsim07G0076300	-1.941	-0.781	1.160	low
CK-0d	RHSIM_Rhsim06G0223900	-0.113	-0.959	-0.846	no
CK-0d	RHSIM_Rhsim08G0157000	0.610	-0.805	-1.415	no
CK-0d	RHSIM_Rhsim08G0248900	-1.479	-1.661	-0.182	low
CK-0d	RHSIM_Rhsim06G0029100	-1.745	-0.641	1.104	low
CK-0d	RHSIM_Rhsim07G0102800	0.351	0.065	-0.286	no
CK-0d	RHSIM_Rhsim08G0031800	-1.918	-1.147	0.771	low
CK-0d	RHSIM_Rhsim07G0054400	-0.833	-0.181	0.652	no
CK-0d	RHSIM_Rhsim07G0252800	-1.557	-1.038	0.519	low
CK-0d	RHSIM_Rhsim07G0222500	-0.140	-0.088	0.052	no
CK-0d	RHSIM_Rhsim07G0255000	-0.457	0.134	0.591	no
CK-0d	RHSIM_Rhsim06G0046600	0.907	0.214	-0.692	no
CK-0d	RHSIM_Rhsim08G0240200	0.727	0.344	-0.383	no
CK-0d	RHSIM_Rhsim07G0237100	0.398	-0.228	-0.626	no
CK-0d	RHSIM_Rhsim07G0136400	0.794	0.099	-0.695	no
CK-0d	RHSIM_Rhsim07G0048200	-0.679	-1.351	-0.672	no
CK-0d	RHSIM_Rhsim08G0064800	-0.071	1.745	1.815	no
CK-0d	RHSIM_Rhsim07G0045800	1.792	2.691	0.899	high
CK-0d	RHSIM_Rhsim07G0137100	-1.412	0.163	1.574	low
CK-0d	RHSIM_Rhsim06G0084500	-0.101	-0.472	-0.371	no
CK-0d	RHSIM_Rhsim07G0178600	-0.470	-0.000	0.469	no
CK-0d	RHSIM_Rhsim07G0143500	0.898	0.642	-0.255	no
CK-0d	RHSIM_Rhsim06G0122400	-1.069	-0.090	0.979	low

Group	Gene	PTR (z-score difference)	z(log2 LFQ)	z(log2 FPKM)	extreme-PTR
CK-0d	RHSIM_Rhsim07G0022700	-0.685	-0.994	-0.309	no
CK-0d	RHSIM_Rhsim06G0170400	-0.968	0.228	1.196	no
CK-0d	RHSIM_Rhsim07G0065100	1.531	-0.521	-2.052	high
CK-0d	RHSIM_Rhsim07G0124300	1.176	0.593	-0.584	high
CK-0d	RHSIM_Rhsim07G0006000	-0.630	0.596	1.226	no
CK-0d	RHSIM_Rhsim07G0130900	0.177	-0.116	-0.293	no
CK-0d	RHSIM_Rhsim08G0163100	1.117	-0.189	-1.306	high
CK-0d	RHSIM_Rhsim06G0208000	-0.143	-0.513	-0.370	no
CK-0d	RHSIM_Rhsim07G0201800	-0.321	-0.764	-0.442	no
CK-0d	RHSIM_Rhsim06G0046900	1.970	1.460	-0.510	high
CK-0d	RHSIM_Rhsim06G0184300	0.612	0.304	-0.308	no
CK-0d	RHSIM_Rhsim07G0205400	0.742	0.741	-0.000	no
CK-0d	RHSIM_Rhsim07G0137000	0.873	1.017	0.143	no
CK-0d	RHSIM_Rhsim06G0007800	-0.414	0.346	0.759	no
CK-0d	RHSIM_Rhsim07G0014000	-1.190	-0.622	0.568	low
CK-0d	RHSIM_Rhsim06G0140600	0.627	-0.214	-0.841	no
CK-0d	RHSIM_Rhsim07G0120800	-1.787	-1.092	0.694	low
CK-0d	RHSIM_Rhsim08G0230400	-1.884	0.346	2.231	low
CK-0d	RHSIM_Rhsim07G0134100	-0.551	-0.436	0.115	no
CK-0d	RHSIM_Rhsim06G0008600	-1.938	-0.894	1.044	low
CK-0d	RHSIM_Rhsim07G0244700	-0.610	-0.655	-0.045	no
CK-0d	RHSIM_Rhsim07G0068100	-0.589	0.368	0.957	no
CK-0d	RHSIM_Rhsim06G0227300	-0.213	1.719	1.932	no
CK-0d	RHSIM_Rhsim06G0231100	-0.015	0.600	0.615	no
CK-0d	RHSIM_Rhsim08G0152000	-0.064	-1.028	-0.964	no
CK-0d	RHSIM_Rhsim06G0121500	0.845	-0.200	-1.045	no
CK-0d	RHSIM_Rhsim06G0180800	0.340	-0.426	-0.766	no
CK-0d	RHSIM_Rhsim07G0037100	-0.187	-0.506	-0.319	no
CK-0d	RHSIM_Rhsim07G0075100	-0.026	-0.087	-0.061	no
CK-0d	RHSIM_Rhsim07G0243000	0.350	-0.257	-0.607	no
CK-0d	RHSIM_Rhsim06G0009200	0.841	0.220	-0.621	no
CK-0d	RHSIM_Rhsim07G0193800	-0.327	-0.386	-0.058	no

Group	Gene	PTR (z-score difference)	z(log2 LFQ)	z(log2 FPKM)	extreme-PTR
CK-0d	RHSIM_Rhsim07G0175800	0.399	0.811	0.412	no
CK-0d	RHSIM_Rhsim07G0214800	-1.827	-0.715	1.112	low
CK-0d	RHSIM_Rhsim07G0244600	0.716	0.531	-0.185	no
CK-0d	RHSIM_Rhsim07G0011100	-0.555	-1.232	-0.676	no
CK-0d	RHSIM_Rhsim07G0069200	-1.028	-1.065	-0.037	low
CK-0d	RHSIM_Rhsim08G0163500	0.504	-0.055	-0.559	no
CK-0d	RHSIM_Rhsim06G0229900	-0.416	-0.261	0.154	no
CK-0d	RHSIM_Rhsim07G0104500	1.629	0.992	-0.637	high
CK-0d	RHSIM_Rhsim07G0136600	0.051	-0.939	-0.990	no
CK-0d	RHSIM_Rhsim06G0035500	-0.186	-0.312	-0.125	no
CK-0d	RHSIM_Rhsim07G0018800	0.174	0.961	0.788	no
CK-0d	RHSIM_Rhsim06G0089000	0.145	0.741	0.596	no
CK-0d	RHSIM_Rhsim07G0221800	-3.369	-1.343	2.026	low
CK-0d	RHSIM_Rhsim07G0200400	-0.177	0.080	0.257	no
CK-0d	RHSIM_Rhsim08G0038600	1.219	0.267	-0.952	high
CK-0d	RHSIM_Rhsim07G0183300	0.420	0.595	0.175	no
CK-0d	RHSIM_Rhsim07G0039600	0.326	1.125	0.799	no
CK-0d	RHSIM_Rhsim07G0229800	2.378	0.911	-1.467	high
CK-0d	RHSIM_Rhsim06G0079300	-0.908	-0.666	0.242	no
CK-0d	RHSIM_Rhsim08G0146700	0.038	-0.837	-0.875	no
CK-0d	RHSIM_Rhsim07G0172500	0.302	0.160	-0.142	no
CK-0d	RHSIM_Rhsim06G0198700	-6.093	-2.678	3.415	low
CK-0d	RHSIM_Rhsim07G0134000	0.233	0.071	-0.162	no
CK-0d	RHSIM_Rhsim07G0078200	0.168	-0.320	-0.488	no
CK-0d	RHSIM_Rhsim07G0179200	0.816	-0.092	-0.908	no
CK-0d	RHSIM_Rhsim06G0203800	-2.284	-0.086	2.198	low
CK-0d	RHSIM_Rhsim06G0204600	-0.542	0.649	1.191	no
CK-0d	RHSIM_Rhsim06G0229700	-2.025	-0.739	1.286	low
CK-0d	RHSIM_Rhsim06G0003900	-0.936	0.115	1.051	no
CK-0d	RHSIM_Rhsim07G0176900	1.123	-1.004	-2.127	high
CK-0d	RHSIM_Rhsim06G0211400	-0.570	1.138	1.708	no
CK-0d	RHSIM_Rhsim07G0093600	-1.650	-1.031	0.619	low

Group	Gene	PTR (z-score difference)	z(log2 LFQ)	z(log2 FPKM)	extreme-PTR
CK-0d	RHSIM_Rhsim07G0086200	-0.093	0.567	0.660	no
CK-0d	RHSIM_Rhsim07G0017700	-0.917	-0.978	-0.060	no
CK-0d	RHSIM_Rhsim07G0102000	0.264	0.493	0.229	no
CK-0d	RHSIM_Rhsim06G0069100	-0.675	0.622	1.297	no
CK-0d	RHSIM_Rhsim07G0122900	0.716	0.513	-0.203	no
CK-0d	RHSIM_Rhsim07G0141400	-0.191	0.183	0.375	no
CK-0d	RHSIM_Rhsim06G0026300	1.985	1.673	-0.312	high
CK-0d	RHSIM_Rhsim08G0011300	0.074	0.109	0.035	no
CK-0d	RHSIM_Rhsim06G0050800	-0.289	-0.813	-0.523	no
CK-0d	RHSIM_Rhsim06G0013500	-1.551	-0.090	1.460	low
CK-0d	RHSIM_Rhsim06G0131700	-0.550	-0.026	0.524	no
CK-0d	RHSIM_Rhsim07G0243900	-0.284	-0.378	-0.094	no
CK-0d	RHSIM_Rhsim07G0213800	-1.122	-0.911	0.211	low
CK-0d	RHSIM_Rhsim07G0075700	0.243	0.402	0.159	no
CK-0d	RHSIM_Rhsim06G0175500	-0.260	-0.337	-0.077	no
CK-0d	RHSIM_Rhsim06G0068700	0.325	1.344	1.019	no
CK-0d	RHSIM_Rhsim07G0257700	-0.112	-0.169	-0.057	no
CK-0d	RHSIM_Rhsim06G0032100	0.312	-0.702	-1.014	no
CK-0d	RHSIM_Rhsim06G0227500	1.148	0.480	-0.668	high
CK-0d	RHSIM_Rhsim06G0077700	-1.259	-2.057	-0.799	low
CK-0d	RHSIM_Rhsim06G0082900	-0.263	-0.054	0.209	no
CK-0d	RHSIM_Rhsim07G0144100	-0.227	0.332	0.559	no
CK-0d	RHSIM_Rhsim07G0039900	0.015	-0.963	-0.978	no
CK-0d	RHSIM_Rhsim07G0239500	1.859	0.847	-1.013	high
CK-0d	RHSIM_Rhsim06G0027200	-1.075	-1.308	-0.233	low
CK-0d	RHSIM_Rhsim07G0252900	-0.879	-1.590	-0.711	no
CK-0d	RHSIM_Rhsim06G0210400	0.794	0.369	-0.425	no
CK-0d	RHSIM_Rhsim06G0079600	-2.880	-0.581	2.299	low
CK-0d	RHSIM_Rhsim06G0050100	0.004	-1.106	-1.110	no
CK-0d	RHSIM_Rhsim07G0211300	-0.292	-0.266	0.025	no
CK-0d	RHSIM_Rhsim06G0231000	0.084	-0.485	-0.570	no
CK-0d	RHSIM_Rhsim07G0039700	-0.468	0.525	0.992	no

Group	Gene	PTR (z-score difference)	z(log2 LFQ)	z(log2 FPKM)	extreme-PTR
CK-0d	RHSIM_Rhsim07G0139700	-2.575	0.260	2.835	low
CK-0d	RHSIM_Rhsim07G0016400	-0.048	-0.892	-0.843	no
CK-0d	RHSIM_Rhsim06G0154000	0.824	-0.354	-1.178	no
CK-0d	RHSIM_Rhsim07G0045700	-0.815	-0.730	0.085	no
CK-0d	RHSIM_Rhsim06G0162500	1.073	2.176	1.103	no
CK-0d	RHSIM_Rhsim06G0242100	0.235	-1.117	-1.352	no
CK-0d	RHSIM_Rhsim06G0213100	1.487	-0.481	-1.968	high
CK-0d	RHSIM_Rhsim07G0213700	-1.160	-1.300	-0.140	low
CK-0d	RHSIM_Rhsim07G0030600	1.299	0.337	-0.961	high
CK-0d	RHSIM_Rhsim07G0177400	-0.621	-0.478	0.144	no
CK-0d	RHSIM_Rhsim07G0047700	-0.734	0.557	1.291	no
CK-0d	RHSIM_Rhsim07G0201200	0.470	-1.526	-1.996	no
CK-0d	RHSIM_Rhsim06G0219700	0.613	0.370	-0.243	no
CK-0d	RHSIM_Rhsim08G0244100	0.273	-0.126	-0.399	no
CK-0d	RHSIM_Rhsim07G0253800	-1.220	0.429	1.649	low
CK-0d	RHSIM_Rhsim07G0137800	-1.157	-0.525	0.632	low
CK-0d	RHSIM_Rhsim07G0209300	0.241	-0.293	-0.533	no
CK-0d	RHSIM_Rhsim06G0207200	0.787	2.167	1.380	no
CK-0d	RHSIM_Rhsim07G0175500	-0.269	-0.835	-0.566	no
CK-0d	RHSIM_Rhsim06G0142500	0.883	0.733	-0.150	no
CK-0d	RHSIM_Rhsim07G0141900	-0.114	0.587	0.701	no
CK-0d	RHSIM_Rhsim07G0054800	1.871	1.858	-0.014	high
CK-0d	RHSIM_Rhsim07G0236700	-0.585	-1.319	-0.734	no
CK-0d	RHSIM_Rhsim07G0179600	0.978	2.754	1.776	no
CK-0d	RHSIM_Rhsim06G0198600	-3.668	0.733	4.401	low
CK-0d	RHSIM_Rhsim06G0232600	0.493	2.336	1.843	no
CK-0d	RHSIM_Rhsim07G0043400	-0.774	-0.842	-0.068	no
CK-0d	RHSIM_Rhsim07G0018900	0.878	-0.914	-1.792	no
CK-0d	RHSIM_Rhsim07G0028400	-0.714	-0.294	0.421	no
CK-0d	RHSIM_Rhsim07G0072700	0.658	0.375	-0.282	no
CK-0d	RHSIM_Rhsim07G0075600	0.220	1.084	0.863	no
CK-0d	RHSIM_Rhsim07G0155800	-1.384	-0.877	0.507	low

Group	Gene	PTR (z-score difference)	z(log2 LFQ)	z(log2 FPKM)	extreme-PTR
CK-0d	RHSIM_Rhsim07G0039800	0.650	-0.044	-0.695	no
CK-0d	RHSIM_Rhsim08G0001800	0.714	0.759	0.045	no
CK-0d	RHSIM_Rhsim07G0024100	0.492	-0.320	-0.812	no
CK-0d	RHSIM_Rhsim07G0012700	0.761	-0.717	-1.479	no
CK-0d	RHSIM_Rhsim07G0048100	-0.418	1.000	1.418	no
CK-0d	RHSIM_Rhsim07G0227800	1.747	0.245	-1.502	high
CK-0d	RHSIM_Rhsim08G0022300	-1.047	-0.034	1.013	low
CK-0d	RHSIM_Rhsim06G0063500	-1.461	-1.359	0.102	low
CK-0d	RHSIM_Rhsim07G0034100	-0.272	0.383	0.655	no
CK-0d	RHSIM_Rhsim06G0003400	-0.539	-1.375	-0.836	no
CK-0d	RHSIM_Rhsim06G0057600	-1.330	-1.595	-0.265	low
CK-0d	RHSIM_Rhsim06G0027900	0.486	-0.082	-0.568	no
CK-0d	RHSIM_Rhsim06G0018200	0.552	-0.814	-1.367	no
CK-0d	RHSIM_Rhsim07G0161800	-1.423	-1.134	0.289	low
CK-0d	RHSIM_Rhsim08G0049700	0.470	1.163	0.693	no
CK-0d	RHSIM_Rhsim06G0078300	1.095	1.280	0.184	no
CK-0d	RHSIM_Rhsim06G0204200	0.277	-0.197	-0.474	no
CK-0d	RHSIM_Rhsim06G0241500	1.592	3.024	1.432	high
CK-0d	RHSIM_Rhsim08G0237900	0.732	0.385	-0.348	no
CK-0d	RHSIM_Rhsim06G0068400	-0.410	-1.316	-0.906	no
CK-0d	RHSIM_Rhsim06G0199700	1.637	0.882	-0.756	high
CK-0d	RHSIM_Rhsim08G0156800	2.714	0.656	-2.059	high
CK-0d	RHSIM_Rhsim07G0175700	0.235	1.097	0.862	no
CK-0d	RHSIM_Rhsim07G0127500	0.171	1.824	1.653	no
CK-0d	RHSIM_Rhsim06G0078900	-2.524	-1.582	0.941	low
CK-0d	RHSIM_Rhsim07G0061800	-0.668	-0.617	0.051	no
CK-0d	RHSIM_Rhsim07G0226200	0.451	0.436	-0.015	no
CK-0d	RHSIM_Rhsim06G0232700	-0.835	-1.280	-0.444	no
CK-0d	RHSIM_Rhsim07G0010400	-0.225	-0.937	-0.712	no
CK-0d	RHSIM_Rhsim06G0051000	1.253	-0.827	-2.080	high
CK-0d	RHSIM_Rhsim07G0084600	-1.954	-0.621	1.333	low
CK-0d	RHSIM_Rhsim07G0075300	-0.290	1.399	1.689	no

Group	Gene	PTR (z-score difference)	z(log2 LFQ)	z(log2 FPKM)	extreme-PTR
CK-0d	RHSIM_Rhsim06G0067800	0.699	-0.850	-1.549	no
CK-0d	RHSIM_Rhsim06G0091500	-0.242	1.541	1.784	no
CK-0d	RHSIM_Rhsim06G0034000	-0.931	0.012	0.943	no
CK-0d	RHSIM_Rhsim06G0142100	-0.350	-0.850	-0.501	no
CK-0d	RHSIM_Rhsim06G0000400	-1.278	-1.042	0.236	low
CK-0d	RHSIM_Rhsim07G0190600	-2.506	-2.147	0.359	low
CK-0d	RHSIM_Rhsim06G0012800	0.602	0.125	-0.478	no
CK-0d	RHSIM_Rhsim06G0228900	-0.220	-2.067	-1.847	no
CK-0d	RHSIM_Rhsim06G0127100	0.482	2.428	1.945	no
CK-0d	RHSIM_Rhsim07G0031000	-0.401	-0.389	0.012	no
CK-0d	RHSIM_Rhsim06G0227100	-0.943	-0.550	0.393	no
CK-0d	RHSIM_Rhsim08G0144900	1.863	2.399	0.536	high
CK-0d	RHSIM_Rhsim06G0074800	-0.760	-0.814	-0.053	no
CK-0d	RHSIM_Rhsim08G0057600	0.162	-0.627	-0.789	no
CK-0d	RHSIM_Rhsim07G0238800	-0.582	-1.367	-0.785	no
CK-0d	RHSIM_Rhsim07G0085900	-0.026	-1.367	-1.341	no
CK-0d	RHSIM_Rhsim08G0039600	-1.767	-0.668	1.099	low
CK-0d	RHSIM_Rhsim08G0005300	1.339	1.927	0.588	high
CK-0d	RHSIM_Rhsim07G0100500	0.142	0.118	-0.024	no
CK-0d	RHSIM_Rhsim08G0062500	-0.074	-0.268	-0.194	no
CK-0d	RHSIM_Rhsim06G0004000	0.407	-0.812	-1.218	no
CK-0d	RHSIM_Rhsim06G0208100	1.045	-0.002	-1.047	no
CK-0d	RHSIM_Rhsim07G0168200	-0.311	0.520	0.831	no
CK-0d	RHSIM_Rhsim06G0104800	-0.150	-0.439	-0.289	no
CK-0d	RHSIM_Rhsim07G0136900	0.653	0.318	-0.335	no
CK-0d	RHSIM_Rhsim06G0178500	-1.244	-0.801	0.444	low
CK-0d	RHSIM_Rhsim06G0121900	1.386	2.265	0.879	high
CK-0d	RHSIM_Rhsim06G0035800	-0.113	0.096	0.208	no
CK-0d	RHSIM_Rhsim07G0044900	1.557	1.503	-0.055	high
CK-0d	RHSIM_Rhsim06G0057100	-0.555	1.034	1.589	no
CK-0d	RHSIM_Rhsim07G0028700	0.346	0.107	-0.238	no
CK-0d	RHSIM_Rhsim06G0231500	-0.529	-1.393	-0.863	no

Group	Gene	PTR (z-score difference)	z(log2 LFQ)	z(log2 FPKM)	extreme-PTR
CK-0d	RHSIM_Rhsim07G0076400	-1.129	-0.896	0.233	low
CK-0d	RHSIM_Rhsim06G0219100	0.051	0.163	0.113	no
CK-0d	RHSIM_Rhsim07G0013400	-0.185	0.218	0.403	no
CK-0d	RHSIM_Rhsim07G0162300	0.947	1.498	0.551	no
CK-0d	RHSIM_Rhsim07G0044800	-0.057	0.333	0.390	no
CK-0d	RHSIM_Rhsim07G0214900	-0.060	0.175	0.234	no
CK-0d	RHSIM_Rhsim06G0179200	1.087	0.102	-0.985	no
CK-0d	RHSIM_Rhsim06G0011800	0.406	3.391	2.985	no
CK-0d	RHSIM_Rhsim08G0077200	-0.276	0.265	0.541	no
CK-0d	RHSIM_Rhsim06G0152000	0.330	0.945	0.615	no
CK-0d	RHSIM_Rhsim07G0048800	0.386	0.413	0.027	no
CK-0d	RHSIM_Rhsim06G0140100	1.191	-0.435	-1.627	high
CK-0d	RHSIM_Rhsim06G0166100	1.142	0.490	-0.652	high
CK-0d	RHSIM_Rhsim06G0035100	-0.024	-1.219	-1.195	no
CK-0d	RHSIM_Rhsim06G0232100	-1.529	-2.433	-0.904	low
CK-0d	RHSIM_Rhsim06G0155400	-0.474	1.597	2.071	no
CK-0d	RHSIM_Rhsim07G0103700	0.551	1.582	1.030	no
CK-0d	RHSIM_Rhsim07G0247400	0.902	-0.028	-0.930	no
CK-0d	RHSIM_Rhsim06G0009800	-0.879	-0.762	0.117	no
CK-0d	RHSIM_Rhsim07G0076600	-2.746	-0.012	2.734	low
CK-0d	RHSIM_Rhsim06G0060000	0.684	-0.823	-1.507	no
CK-0d	RHSIM_Rhsim07G0103800	-0.484	0.667	1.151	no
CK-0d	RHSIM_Rhsim06G0017900	-0.322	-0.247	0.075	no
CK-0d	RHSIM_Rhsim06G0224000	-1.143	0.960	2.103	low
CK-0d	RHSIM_Rhsim06G0101600	0.491	0.564	0.073	no
CK-0d	RHSIM_Rhsim08G0020700	-0.472	0.649	1.120	no
CK-0d	RHSIM_Rhsim06G0005000	0.870	-0.479	-1.348	no
CK-0d	RHSIM_Rhsim07G0018000	1.503	0.821	-0.681	high
CK-0d	RHSIM_Rhsim08G0100900	-0.663	-0.875	-0.212	no
CK-0d	RHSIM_Rhsim06G0243400	-0.237	-0.566	-0.330	no
CK-0d	RHSIM_Rhsim06G0061600	0.539	1.517	0.978	no
CK-0d	RHSIM_Rhsim06G0204400	0.028	-0.996	-1.024	no

Group	Gene	PTR (z-score difference)	z(log2 LFQ)	z(log2 FPKM)	extreme-PTR
CK-0d	RHSIM_Rhsim06G0026000	-0.215	-1.466	-1.251	no
CK-0d	RHSIM_Rhsim06G0137100	0.401	0.922	0.521	no
CK-0d	RHSIM_Rhsim07G0003800	1.082	0.413	-0.670	no
CK-0d	RHSIM_Rhsim06G0013600	-1.117	-0.102	1.015	low
CK-0d	RHSIM_Rhsim06G0129800	0.326	-0.589	-0.915	no
CK-0d	RHSIM_Rhsim07G0033100	2.399	0.587	-1.812	high
CK-0d	RHSIM_Rhsim07G0257000	0.077	1.286	1.209	no
CK-0d	RHSIM_Rhsim06G0135500	-0.945	-0.846	0.098	no
CK-0d	RHSIM_Rhsim07G0139800	-0.134	0.696	0.829	no
CK-0d	RHSIM_Rhsim08G0220400	0.477	0.166	-0.310	no
CK-0d	RHSIM_Rhsim07G0018700	1.081	2.017	0.936	no
CK-0d	RHSIM_Rhsim06G0224100	-0.444	-0.382	0.062	no
CK-0d	RHSIM_Rhsim07G0079200	-0.135	-1.052	-0.916	no
CK-0d	RHSIM_Rhsim08G0093000	-2.030	-1.070	0.960	low
CK-0d	RHSIM_Rhsim07G0013600	0.116	1.616	1.500	no
CK-0d	RHSIM_Rhsim07G0161400	0.657	-0.089	-0.746	no
CK-0d	RHSIM_Rhsim06G0229000	-0.632	-0.664	-0.032	no
CK-0d	RHSIM_Rhsim07G0212200	-0.868	-0.826	0.042	no
CK-0d	RHSIM_Rhsim06G0240700	3.164	0.894	-2.270	high
CK-0d	RHSIM_Rhsim07G0166500	-0.433	0.868	1.301	no
CK-0d	RHSIM_Rhsim07G0243700	-1.109	-0.188	0.921	low
CK-0d	RHSIM_Rhsim06G0173600	-0.421	-1.270	-0.849	no
CK-0d	RHSIM_Rhsim06G0016500	2.441	0.177	-2.264	high
CK-0d	RHSIM_Rhsim06G0196300	-0.488	1.584	2.072	no
CK-0d	RHSIM_Rhsim06G0240900	1.579	-0.150	-1.729	high
CK-0d	RHSIM_Rhsim05G0002300	-0.012	0.489	0.501	no
CK-0d	RHSIM_Rhsim06G0055000	-2.644	-2.561	0.083	low
CK-0d	RHSIM_Rhsim07G0079900	0.252	1.089	0.837	no
CK-0d	RHSIM_Rhsim06G0196500	-0.403	0.058	0.461	no
CK-0d	RHSIM_Rhsim07G0258500	-0.226	0.107	0.333	no
CK-0d	RHSIM_Rhsim06G0164500	0.429	1.120	0.691	no
CK-0d	RHSIM_Rhsim06G0040100	1.719	0.818	-0.901	high

Group	Gene	PTR (z-score difference)	z(log2 LFQ)	z(log2 FPKM)	extreme-PTR
CK-0d	RHSIM_Rhsim06G0040800	0.095	-0.288	-0.383	no
CK-0d	RHSIM_Rhsim06G0114600	-0.343	0.303	0.646	no
CK-0d	RHSIM_Rhsim07G0257400	1.076	1.073	-0.003	no
CK-0d	RHSIM_Rhsim06G0032500	-0.537	-0.869	-0.333	no
CK-0d	RHSIM_Rhsim06G0021200	0.533	-0.122	-0.655	no
CK-0d	RHSIM_Rhsim06G0091400	-1.077	-0.924	0.153	low
CK-0d	RHSIM_Rhsim06G0231200	0.188	-0.829	-1.017	no
CK-0d	RHSIM_Rhsim06G0085000	0.322	0.113	-0.209	no
CK-0d	RHSIM_Rhsim06G0012500	2.741	0.502	-2.239	high
CK-0d	RHSIM_Rhsim07G0224300	-0.680	-0.460	0.220	no
CK-0d	RHSIM_Rhsim07G0248700	0.125	-0.590	-0.715	no
CK-0d	RHSIM_Rhsim06G0178700	-0.369	-0.467	-0.097	no
CK-0d	RHSIM_Rhsim06G0207600	-0.341	-0.129	0.212	no
CK-0d	RHSIM_Rhsim07G0211600	0.357	-0.270	-0.627	no
CK-0d	RHSIM_Rhsim05G0076900	-0.303	-0.156	0.147	no
CK-0d	RHSIM_Rhsim06G0178100	-0.495	-0.568	-0.073	no
CK-0d	RHSIM_Rhsim06G0009500	0.131	0.015	-0.116	no
CK-0d	RHSIM_Rhsim06G0216300	-2.665	-1.782	0.882	low
CK-0d	RHSIM_Rhsim05G0232900	0.644	2.009	1.366	no
CK-0d	RHSIM_Rhsim06G0008000	-0.757	-0.933	-0.176	no
CK-0d	RHSIM_Rhsim06G0174400	0.286	-0.388	-0.675	no
CK-0d	RHSIM_Rhsim06G0069300	-0.484	-0.062	0.422	no
CK-0d	RHSIM_Rhsim06G0223700	0.051	-0.155	-0.206	no
CK-0d	RHSIM_Rhsim06G0000600	0.340	-0.801	-1.141	no
CK-0d	RHSIM_Rhsim06G0146800	1.719	-0.061	-1.780	high
CK-0d	RHSIM_Rhsim06G0238600	1.460	1.314	-0.146	high
CK-0d	RHSIM_Rhsim06G0211200	-1.132	-1.006	0.126	low
CK-0d	RHSIM_Rhsim07G0154200	-1.132	-1.966	-0.834	low
CK-0d	RHSIM_Rhsim06G0225600	-0.314	-1.258	-0.944	no
CK-0d	RHSIM_Rhsim07G0150900	0.232	-0.461	-0.694	no
CK-0d	RHSIM_Rhsim06G0175900	0.200	0.318	0.117	no
CK-0d	RHSIM_Rhsim07G0064800	1.757	0.111	-1.646	high

Group	Gene	PTR (z-score difference)	z(log2 LFQ)	z(log2 FPKM)	extreme-PTR
CK-0d	RHSIM_Rhsim05G0005000	1.687	0.404	-1.283	high
CK-0d	RHSIM_Rhsim06G0073900	1.080	1.908	0.828	no
CK-0d	RHSIM_Rhsim06G0056800	-1.544	-1.458	0.085	low
CK-0d	RHSIM_Rhsim07G0115200	-0.458	-0.873	-0.415	no
CK-0d	RHSIM_Rhsim07G0029000	0.197	0.493	0.297	no
CK-0d	RHSIM_Rhsim06G0197600	0.844	0.815	-0.029	no
CK-0d	RHSIM_Rhsim06G0236900	0.425	-0.298	-0.722	no
CK-0d	RHSIM_Rhsim06G0184100	-0.930	0.265	1.195	no
CK-0d	RHSIM_Rhsim07G0250200	-1.298	-0.363	0.935	low
CK-0d	RHSIM_Rhsim06G0224200	0.881	0.482	-0.398	no
CK-0d	RHSIM_Rhsim06G0075100	-3.954	-1.120	2.834	low
CK-0d	RHSIM_Rhsim05G0126500	0.539	0.442	-0.096	no
CK-0d	RHSIM_Rhsim06G0035600	0.192	-0.642	-0.834	no
CK-0d	RHSIM_Rhsim06G0027600	0.237	-0.348	-0.585	no
CK-0d	RHSIM_Rhsim06G0241000	1.629	1.396	-0.232	high
CK-0d	RHSIM_Rhsim06G0006500	1.274	0.162	-1.112	high
CK-0d	RHSIM_Rhsim06G0207700	2.053	0.219	-1.834	high
CK-0d	RHSIM_Rhsim05G0045000	-1.914	-0.723	1.191	low
CK-0d	RHSIM_Rhsim07G0084000	0.929	0.186	-0.743	no
CK-0d	RHSIM_Rhsim05G0048300	0.562	-0.759	-1.320	no
CK-0d	RHSIM_Rhsim06G0008400	-0.650	-0.109	0.542	no
CK-0d	RHSIM_Rhsim05G0168000	0.402	-0.124	-0.527	no
CK-0d	RHSIM_Rhsim07G0027800	0.519	1.038	0.519	no
CK-0d	RHSIM_Rhsim06G0197200	-2.408	-2.052	0.356	low
CK-0d	RHSIM_Rhsim07G0009400	-0.683	-1.419	-0.735	no
CK-0d	RHSIM_Rhsim05G0132400	1.505	2.271	0.766	high
CK-0d	RHSIM_Rhsim07G0022200	-0.663	-1.198	-0.535	no
CK-0d	RHSIM_Rhsim06G0172900	-0.770	-1.451	-0.681	no
CK-0d	RHSIM_Rhsim05G0073200	1.712	0.697	-1.016	high
CK-0d	RHSIM_Rhsim07G0038600	-0.612	0.151	0.762	no
CK-0d	RHSIM_Rhsim06G0052100	2.211	1.090	-1.121	high
CK-0d	RHSIM_Rhsim06G0023100	-0.330	-1.248	-0.917	no

Group	Gene	PTR (z-score difference)	z(log2 LFQ)	z(log2 FPKM)	extreme-PTR
CK-0d	RHSIM_Rhsim06G0214200	-0.479	-0.766	-0.286	no
CK-0d	RHSIM_Rhsim07G0121200	-2.085	-1.389	0.696	low
CK-0d	RHSIM_Rhsim06G0054400	0.524	1.257	0.732	no
CK-0d	RHSIM_Rhsim05G0018800	-0.497	0.020	0.517	no
CK-0d	RHSIM_Rhsim06G0129900	-0.672	1.014	1.686	no
CK-0d	RHSIM_Rhsim07G0008000	0.196	-0.531	-0.727	no
CK-0d	RHSIM_Rhsim06G0186500	-0.095	0.007	0.102	no
CK-0d	RHSIM_Rhsim05G0115300	1.975	1.929	-0.047	high
CK-0d	RHSIM_Rhsim06G0244600	-1.606	-1.468	0.138	low
CK-0d	RHSIM_Rhsim05G0044300	-0.088	1.579	1.667	no
CK-0d	RHSIM_Rhsim07G0174700	0.822	-0.397	-1.220	no
CK-0d	RHSIM_Rhsim05G0060300	-0.499	-0.847	-0.348	no
CK-0d	RHSIM_Rhsim07G0211400	-0.585	-0.452	0.133	no
CK-0d	RHSIM_Rhsim06G0029700	-0.442	-0.382	0.060	no
CK-0d	RHSIM_Rhsim05G0233100	0.344	-0.031	-0.375	no
CK-0d	RHSIM_Rhsim06G0021700	0.582	1.500	0.918	no
CK-0d	RHSIM_Rhsim05G0060600	-0.564	0.017	0.581	no
CK-0d	RHSIM_Rhsim06G0031100	0.172	-1.410	-1.581	no
CK-0d	RHSIM_Rhsim06G0191800	0.071	1.047	0.976	no
CK-0d	RHSIM_Rhsim07G0119900	-0.378	0.391	0.770	no
CK-0d	RHSIM_Rhsim05G0060400	-0.107	0.445	0.552	no
CK-0d	RHSIM_Rhsim06G0183300	-0.408	0.155	0.563	no
CK-0d	RHSIM_Rhsim06G0018700	-0.268	-0.960	-0.692	no
CK-0d	RHSIM_Rhsim07G0240500	0.431	-0.091	-0.522	no
CK-0d	RHSIM_Rhsim05G0187800	-0.191	1.171	1.362	no
CK-0d	RHSIM_Rhsim06G0046300	0.456	1.153	0.697	no
CK-0d	RHSIM_Rhsim05G0208000	-0.545	-0.335	0.210	no
CK-0d	RHSIM_Rhsim06G0070900	-0.215	-0.714	-0.499	no
CK-0d	RHSIM_Rhsim06G0018400	-0.673	-0.419	0.254	no
CK-0d	RHSIM_Rhsim07G0154100	-0.996	0.454	1.450	no
CK-0d	RHSIM_Rhsim06G0118100	-0.313	-0.635	-0.322	no
CK-0d	RHSIM_Rhsim06G0200600	0.600	0.022	-0.578	no

Group	Gene	PTR (z-score difference)	z(log2 LFQ)	z(log2 FPKM)	extreme-PTR
CK-0d	RHSIM_Rhsim05G0013200	-0.521	-1.705	-1.185	no
CK-0d	RHSIM_Rhsim06G0051800	1.811	-0.060	-1.871	high
CK-0d	RHSIM_Rhsim06G0097000	-0.902	-0.342	0.560	no
CK-0d	RHSIM_Rhsim06G0069400	-0.367	0.070	0.437	no
CK-0d	RHSIM_Rhsim05G0022700	1.011	1.062	0.051	no
CK-0d	RHSIM_Rhsim06G0004600	0.517	3.131	2.614	no
CK-0d	RHSIM_Rhsim06G0213400	0.514	-1.021	-1.535	no
CK-0d	RHSIM_Rhsim06G0166800	-0.633	-0.117	0.515	no
CK-0d	RHSIM_Rhsim06G0048000	1.311	1.144	-0.167	high
CK-0d	RHSIM_Rhsim05G0185900	0.056	-0.027	-0.083	no
CK-0d	RHSIM_Rhsim06G0121700	0.203	0.400	0.197	no
CK-0d	RHSIM_Rhsim06G0238500	-0.182	-0.365	-0.182	no
CK-0d	RHSIM_Rhsim05G0003900	0.540	-0.412	-0.951	no
CK-0d	RHSIM_Rhsim06G0216400	-0.693	0.016	0.708	no
CK-0d	RHSIM_Rhsim05G0216700	-2.325	-1.180	1.145	low
CK-0d	RHSIM_Rhsim06G0218400	0.462	0.651	0.188	no
CK-0d	RHSIM_Rhsim06G0136000	0.709	1.649	0.939	no
CK-0d	RHSIM_Rhsim05G0070300	-0.182	0.337	0.519	no
CK-0d	RHSIM_Rhsim06G0004300	0.948	1.184	0.236	no
CK-0d	RHSIM_Rhsim06G0175300	0.487	-0.832	-1.319	no
CK-0d	RHSIM_Rhsim05G0222900	0.846	0.309	-0.537	no
CK-0d	RHSIM_Rhsim06G0137300	0.432	0.873	0.441	no
CK-0d	RHSIM_Rhsim05G0195200	-0.862	-1.323	-0.462	no
CK-0d	RHSIM_Rhsim06G0147000	-1.084	-1.435	-0.351	low
CK-0d	RHSIM_Rhsim05G0104500	-1.828	-0.223	1.605	low
CK-0d	RHSIM_Rhsim05G0088300	-0.226	-0.632	-0.405	no
CK-0d	RHSIM_Rhsim05G0122300	-0.505	-1.119	-0.614	no
CK-0d	RHSIM_Rhsim06G0063800	0.844	-0.820	-1.664	no
CK-0d	RHSIM_Rhsim07G0104300	0.568	0.078	-0.490	no
CK-0d	RHSIM_Rhsim06G0213200	1.052	0.084	-0.968	no
CK-0d	RHSIM_Rhsim06G0116100	0.345	-0.075	-0.420	no
CK-0d	RHSIM_Rhsim06G0201300	-0.030	1.732	1.762	no

Group	Gene	PTR (z-score difference)	z(log2 LFQ)	z(log2 FPKM)	extreme-PTR
CK-0d	RHSIM_Rhsim06G0153100	0.533	2.083	1.550	no
CK-0d	RHSIM_Rhsim05G0177500	1.212	-0.523	-1.735	high
CK-0d	RHSIM_Rhsim06G0129300	1.269	1.260	-0.009	high
CK-0d	RHSIM_Rhsim06G0232400	-2.725	-1.682	1.043	low
CK-0d	RHSIM_Rhsim05G0172800	0.021	-1.189	-1.210	no
CK-0d	RHSIM_Rhsim06G0211100	0.630	-0.440	-1.070	no
CK-0d	RHSIM_Rhsim06G0054200	0.902	-1.082	-1.984	no
CK-0d	RHSIM_Rhsim06G0165500	-1.040	-0.917	0.123	low
CK-0d	RHSIM_Rhsim05G0197900	2.075	1.366	-0.709	high
CK-0d	RHSIM_Rhsim06G0076700	-0.595	0.538	1.134	no
CK-0d	RHSIM_Rhsim06G0224400	-0.628	-0.840	-0.212	no
CK-0d	RHSIM_Rhsim06G0210200	-0.353	0.107	0.460	no
CK-0d	RHSIM_Rhsim06G0212800	1.191	0.661	-0.530	high
CK-0d	RHSIM_Rhsim06G0092900	-0.759	-1.087	-0.328	no
CK-0d	RHSIM_Rhsim05G0142400	-0.718	-0.812	-0.094	no
CK-0d	RHSIM_Rhsim06G0239000	-1.107	0.009	1.116	low
CK-0d	RHSIM_Rhsim06G0223600	-0.103	-0.895	-0.792	no
CK-0d	RHSIM_Rhsim05G0049000	1.534	0.191	-1.343	high
CK-0d	RHSIM_Rhsim06G0157900	-1.759	-0.614	1.145	low
CK-0d	RHSIM_Rhsim06G0062200	-0.710	-0.057	0.652	no
CK-0d	RHSIM_Rhsim05G0229900	-0.821	-0.576	0.245	no
CK-0d	RHSIM_Rhsim05G0029300	0.738	1.262	0.524	no
CK-0d	RHSIM_Rhsim05G0112500	-0.173	0.481	0.654	no
CK-0d	RHSIM_Rhsim05G0095600	0.784	-0.070	-0.854	no
CK-0d	RHSIM_Rhsim06G0234000	0.183	-0.373	-0.556	no
CK-0d	RHSIM_Rhsim05G0013100	-0.901	-0.397	0.504	no
CK-0d	RHSIM_Rhsim05G0016600	-1.284	-1.208	0.077	low
CK-0d	RHSIM_Rhsim05G0073600	0.428	-1.229	-1.657	no
CK-0d	RHSIM_Rhsim06G0065300	-0.090	-1.139	-1.049	no
CK-0d	RHSIM_Rhsim05G0053200	0.942	0.716	-0.226	no
CK-0d	RHSIM_Rhsim05G0188100	-0.733	0.487	1.220	no
CK-0d	RHSIM_Rhsim05G0132800	0.461	0.520	0.059	no

Group	Gene	PTR (z-score difference)	z(log2 LFQ)	z(log2 FPKM)	extreme-PTR
CK-0d	RHSIM_Rhsim05G0032600	0.658	1.183	0.525	no
CK-0d	RHSIM_Rhsim07G0091100	0.726	1.207	0.481	no
CK-0d	RHSIM_Rhsim06G0243100	-1.393	-0.640	0.753	low
CK-0d	RHSIM_Rhsim05G0009900	0.609	-0.231	-0.839	no
CK-0d	RHSIM_Rhsim05G0056900	-0.481	0.997	1.478	no
CK-0d	RHSIM_Rhsim05G0230200	0.141	0.184	0.043	no
CK-0d	RHSIM_Rhsim06G0103100	-1.011	-0.982	0.030	no
CK-0d	RHSIM_Rhsim06G0086200	-0.347	0.518	0.865	no
CK-0d	RHSIM_Rhsim05G0167800	0.167	-0.437	-0.604	no
CK-0d	RHSIM_Rhsim05G0092800	0.486	-0.738	-1.224	no
CK-0d	RHSIM_Rhsim05G0005700	0.449	-0.052	-0.501	no
CK-0d	RHSIM_Rhsim06G0099200	-0.064	0.012	0.076	no
CK-0d	RHSIM_Rhsim06G0212400	-2.094	0.463	2.557	low
CK-0d	RHSIM_Rhsim06G0166500	-0.419	0.364	0.784	no
CK-0d	RHSIM_Rhsim06G0128200	-0.342	0.303	0.645	no
CK-0d	RHSIM_Rhsim05G0148100	-0.762	-0.318	0.444	no
CK-0d	RHSIM_Rhsim06G0166300	-0.010	-0.512	-0.502	no
CK-0d	RHSIM_Rhsim06G0067500	0.987	0.863	-0.124	no
CK-0d	RHSIM_Rhsim05G0229000	-1.213	-0.052	1.162	low
CK-0d	RHSIM_Rhsim06G0198500	0.438	0.432	-0.006	no
CK-0d	RHSIM_Rhsim05G0102000	-0.429	-1.026	-0.597	no
CK-0d	RHSIM_Rhsim06G0022900	2.000	1.224	-0.776	high
CK-0d	RHSIM_Rhsim05G0079000	-1.102	0.577	1.679	low
CK-0d	RHSIM_Rhsim05G0035300	-0.826	-1.832	-1.007	no
CK-0d	RHSIM_Rhsim05G0184700	0.116	-0.569	-0.685	no
CK-0d	RHSIM_Rhsim06G0009700	2.269	0.833	-1.436	high
CK-0d	RHSIM_Rhsim07G0188500	-0.798	-1.047	-0.249	no
CK-0d	RHSIM_Rhsim05G0014500	-0.793	0.448	1.241	no
CK-0d	RHSIM_Rhsim05G0004300	-0.049	2.105	2.154	no
CK-0d	RHSIM_Rhsim06G0202700	0.527	-0.199	-0.725	no
CK-0d	RHSIM_Rhsim06G0148500	1.774	2.904	1.130	high
CK-0d	RHSIM_Rhsim05G0206700	-2.199	-0.925	1.274	low

Group	Gene	PTR (z-score difference)	z(log2 LFQ)	z(log2 FPKM)	extreme-PTR
CK-0d	RHSIM_Rhsim05G0142600	0.937	-0.707	-1.644	no
CK-0d	RHSIM_Rhsim05G0079100	-1.405	-1.269	0.137	low
CK-0d	RHSIM_Rhsim05G0220000	-0.497	-0.341	0.156	no
CK-0d	RHSIM_Rhsim05G0039500	0.098	1.319	1.221	no
CK-0d	RHSIM_Rhsim05G0056600	-0.693	-0.459	0.234	no
CK-0d	RHSIM_Rhsim05G0193900	0.390	2.139	1.749	no
CK-0d	RHSIM_Rhsim05G0074500	0.580	0.333	-0.247	no
CK-0d	RHSIM_Rhsim07G0191000	-1.161	-0.915	0.246	low
CK-0d	RHSIM_Rhsim06G0032600	0.316	0.214	-0.102	no
CK-0d	RHSIM_Rhsim07G0222600	-0.458	-1.278	-0.819	no
CK-0d	RHSIM_Rhsim06G0131600	-0.729	-0.529	0.200	no
CK-0d	RHSIM_Rhsim05G0055600	0.306	0.330	0.023	no
CK-0d	RHSIM_Rhsim06G0230600	-1.314	-0.992	0.322	low
CK-0d	RHSIM_Rhsim07G0246000	1.634	-0.023	-1.657	high
CK-0d	RHSIM_Rhsim05G0165600	0.724	1.059	0.335	no
CK-0d	RHSIM_Rhsim06G0211900	1.267	-0.208	-1.475	high
CK-0d	RHSIM_Rhsim06G0048700	-0.748	-1.182	-0.435	no
CK-0d	RHSIM_Rhsim05G0094900	-0.542	-1.294	-0.752	no
CK-0d	RHSIM_Rhsim06G0031000	1.608	0.496	-1.112	high
CK-0d	RHSIM_Rhsim05G0170000	0.992	0.188	-0.805	no
CK-0d	RHSIM_Rhsim06G0206300	1.398	1.413	0.015	high
CK-0d	RHSIM_Rhsim05G0114200	-1.590	-1.473	0.116	low
CK-0d	RHSIM_Rhsim06G0239600	0.706	2.042	1.336	no
CK-0d	RHSIM_Rhsim06G0090800	-1.288	-0.652	0.636	low
CK-0d	RHSIM_Rhsim06G0087600	-0.497	-0.232	0.265	no
CK-0d	RHSIM_Rhsim05G0172300	-2.052	-1.530	0.522	low
CK-0d	RHSIM_Rhsim06G0179300	0.429	-0.946	-1.375	no
CK-0d	RHSIM_Rhsim07G0095500	-0.942	-1.028	-0.086	no
CK-0d	RHSIM_Rhsim05G0195100	-0.062	-0.677	-0.615	no
CK-0d	RHSIM_Rhsim05G0107000	0.775	0.210	-0.565	no
CK-0d	RHSIM_Rhsim06G0206000	-0.808	-0.128	0.680	no
CK-0d	RHSIM_Rhsim06G0103300	0.545	3.549	3.004	no

Group	Gene	PTR (z-score difference)	z(log2 LFQ)	z(log2 FPKM)	extreme-PTR
CK-0d	RHSIM_Rhsim05G0075800	2.132	1.354	-0.777	high
CK-0d	RHSIM_Rhsim05G0175300	-0.262	0.952	1.214	no
CK-0d	RHSIM_Rhsim07G0020100	-2.428	-1.754	0.674	low
CK-0d	RHSIM_Rhsim05G0076200	0.867	-0.026	-0.893	no
CK-0d	RHSIM_Rhsim06G0232500	1.615	0.690	-0.925	high
CK-0d	RHSIM_Rhsim05G0016500	-0.537	-1.089	-0.552	no
CK-0d	RHSIM_Rhsim06G0141900	-0.607	-1.094	-0.487	no
CK-0d	RHSIM_Rhsim05G0038200	0.202	1.174	0.971	no
CK-0d	RHSIM_Rhsim05G0048000	-0.319	-1.750	-1.431	no
CK-0d	RHSIM_Rhsim06G0208300	-1.685	-0.262	1.423	low
CK-0d	RHSIM_Rhsim06G0008500	0.002	1.801	1.799	no
CK-0d	RHSIM_Rhsim05G0101600	-1.103	-0.533	0.570	low
CK-0d	RHSIM_Rhsim05G0148700	0.368	0.227	-0.141	no
CK-0d	RHSIM_Rhsim06G0003100	-0.871	-1.548	-0.677	no
CK-0d	RHSIM_Rhsim05G0056500	-0.892	1.385	2.277	no
CK-0d	RHSIM_Rhsim05G0114100	-0.467	-0.922	-0.455	no
CK-0d	RHSIM_Rhsim06G0006600	-0.085	1.405	1.489	no
CK-0d	RHSIM_Rhsim06G0027100	-0.723	1.075	1.798	no
CK-0d	RHSIM_Rhsim05G0081000	-1.949	-2.079	-0.129	low
CK-0d	RHSIM_Rhsim05G0081600	-1.998	-0.118	1.880	low
CK-0d	RHSIM_Rhsim06G0170000	0.562	-0.888	-1.450	no
CK-0d	RHSIM_Rhsim05G0007700	-0.066	-0.823	-0.757	no
CK-0d	RHSIM_Rhsim05G0142500	-1.457	-0.773	0.684	low
CK-0d	RHSIM_Rhsim05G0015000	-1.047	0.499	1.546	low
CK-0d	RHSIM_Rhsim05G0040400	-0.306	-0.251	0.055	no
CK-0d	RHSIM_Rhsim06G0184400	0.231	-0.172	-0.403	no
CK-0d	RHSIM_Rhsim05G0034500	-0.183	-0.936	-0.753	no
CK-0d	RHSIM_Rhsim05G0125700	1.860	1.034	-0.826	high
CK-0d	RHSIM_Rhsim05G0234300	-2.985	0.047	3.032	low
CK-0d	RHSIM_Rhsim05G0054800	0.427	-1.014	-1.441	no
CK-0d	RHSIM_Rhsim06G0176900	0.719	0.163	-0.556	no
CK-0d	RHSIM_Rhsim07G0136800	0.346	-0.279	-0.625	no

Group	Gene	PTR (z-score difference)	z(log2 LFQ)	z(log2 FPKM)	extreme-PTR
CK-0d	RHSIM_Rhsim05G0228100	-0.616	0.068	0.684	no
CK-0d	RHSIM_Rhsim06G0062000	0.237	0.150	-0.087	no
CK-0d	RHSIM_Rhsim05G0203100	-0.506	-0.262	0.243	no
CK-0d	RHSIM_Rhsim05G0094000	-0.591	-1.099	-0.508	no
CK-0d	RHSIM_Rhsim05G0178300	0.245	0.112	-0.132	no
CK-0d	RHSIM_Rhsim05G0160100	-0.132	0.606	0.738	no
CK-0d	RHSIM_Rhsim06G0089200	-0.775	-0.896	-0.121	no
CK-0d	RHSIM_Rhsim06G0022200	0.801	0.384	-0.417	no
CK-0d	RHSIM_Rhsim05G0048900	-2.185	-1.456	0.729	low
CK-0d	RHSIM_Rhsim05G0022400	0.591	0.489	-0.102	no
CK-0d	RHSIM_Rhsim05G0167700	0.135	-0.576	-0.711	no
CK-0d	RHSIM_Rhsim05G0203300	-1.005	-0.631	0.373	no
CK-0d	RHSIM_Rhsim05G0013700	-0.188	-1.305	-1.118	no
CK-0d	RHSIM_Rhsim05G0082400	3.294	1.825	-1.469	high
CK-0d	RHSIM_Rhsim05G0214500	0.623	1.458	0.835	no
CK-0d	RHSIM_Rhsim04G0249900	0.281	-0.293	-0.574	no
CK-0d	RHSIM_Rhsim05G0141700	0.831	0.227	-0.604	no
CK-0d	RHSIM_Rhsim07G0093500	0.842	0.053	-0.789	no
CK-0d	RHSIM_Rhsim06G0039400	-2.339	0.829	3.168	low
CK-0d	RHSIM_Rhsim04G0061200	1.864	0.308	-1.556	high
CK-0d	RHSIM_Rhsim05G0057000	0.178	-0.086	-0.264	no
CK-0d	RHSIM_Rhsim06G0052200	1.847	0.228	-1.619	high
CK-0d	RHSIM_Rhsim05G0035400	-0.141	-0.171	-0.030	no
CK-0d	RHSIM_Rhsim04G0207200	-0.109	-0.376	-0.267	no
CK-0d	RHSIM_Rhsim06G0183900	0.607	0.832	0.225	no
CK-0d	RHSIM_Rhsim05G0179700	0.749	1.372	0.623	no
CK-0d	RHSIM_Rhsim05G0055000	0.059	-0.231	-0.290	no
CK-0d	RHSIM_Rhsim05G0001900	-1.686	-1.522	0.164	low
CK-0d	RHSIM_Rhsim06G0234700	-1.252	-0.012	1.240	low
CK-0d	RHSIM_Rhsim04G0179500	-1.214	-1.376	-0.163	low
CK-0d	RHSIM_Rhsim05G0141900	0.310	0.345	0.035	no
CK-0d	RHSIM_Rhsim05G0165000	0.890	-0.430	-1.320	no

Group	Gene	PTR (z-score difference)	z(log2 LFQ)	z(log2 FPKM)	extreme-PTR
CK-0d	RHSIM_Rhsim05G0169000	-0.483	-0.703	-0.220	no
CK-0d	RHSIM_Rhsim06G0060300	0.447	0.490	0.043	no
CK-0d	RHSIM_Rhsim04G0078200	2.905	0.614	-2.292	high
CK-0d	RHSIM_Rhsim06G0030800	0.813	1.546	0.733	no
CK-0d	RHSIM_Rhsim05G0178900	-0.492	0.277	0.768	no
CK-0d	RHSIM_Rhsim06G0139200	2.179	2.683	0.503	high
CK-0d	RHSIM_Rhsim05G0001300	0.614	-0.238	-0.852	no
CK-0d	RHSIM_Rhsim05G0119100	-0.760	-0.719	0.041	no
CK-0d	RHSIM_Rhsim05G0233200	0.659	0.880	0.221	no
CK-0d	RHSIM_Rhsim05G0141500	-3.312	-0.508	2.804	low
CK-0d	RHSIM_Rhsim06G0114100	-0.154	0.063	0.217	no
CK-0d	RHSIM_Rhsim07G0044200	0.097	-0.858	-0.955	no
CK-0d	RHSIM_Rhsim06G0071600	0.997	0.051	-0.947	no
CK-0d	RHSIM_Rhsim05G0067900	-0.705	-1.496	-0.791	no
CK-0d	RHSIM_Rhsim05G0161500	-0.251	-0.714	-0.463	no
CK-0d	RHSIM_Rhsim07G0149700	0.240	-0.411	-0.651	no
CK-0d	RHSIM_Rhsim05G0203500	0.724	1.247	0.523	no
CK-0d	RHSIM_Rhsim06G0047300	-0.760	0.515	1.275	no
CK-0d	RHSIM_Rhsim05G0028200	1.538	0.253	-1.284	high
CK-0d	RHSIM_Rhsim04G0153900	1.037	0.564	-0.473	no
CK-0d	RHSIM_Rhsim05G0122100	-2.270	-1.060	1.209	low
CK-0d	RHSIM_Rhsim07G0213300	0.825	0.760	-0.065	no
CK-0d	RHSIM_Rhsim05G0221100	0.626	-0.819	-1.445	no
CK-0d	RHSIM_Rhsim06G0238700	-1.291	-1.536	-0.245	low
CK-0d	RHSIM_Rhsim04G0180900	-0.754	0.856	1.611	no
CK-0d	RHSIM_Rhsim07G0102700	0.815	1.021	0.206	no
CK-0d	RHSIM_Rhsim04G0213100	1.380	1.768	0.388	high
CK-0d	RHSIM_Rhsim05G0228600	0.255	1.457	1.202	no
CK-0d	RHSIM_Rhsim05G0018700	1.060	1.064	0.004	no
CK-0d	RHSIM_Rhsim06G0203000	-0.405	0.319	0.724	no
CK-0d	RHSIM_Rhsim06G0096300	0.053	0.295	0.241	no
CK-0d	RHSIM_Rhsim05G0219700	-1.071	-0.858	0.212	low

Group	Gene	PTR (z-score difference)	z(log2 LFQ)	z(log2 FPKM)	extreme-PTR
CK-0d	RHSIM_Rhsim06G0187600	1.537	0.339	-1.198	high
CK-0d	RHSIM_Rhsim05G0115900	0.207	0.400	0.193	no
CK-0d	RHSIM_Rhsim05G0168400	-0.858	-0.932	-0.073	no
CK-0d	RHSIM_Rhsim05G0093000	0.094	-0.119	-0.212	no
CK-0d	RHSIM_Rhsim05G0141400	-1.405	-1.246	0.158	low
CK-0d	RHSIM_Rhsim05G0216400	-0.487	0.414	0.901	no
CK-0d	RHSIM_Rhsim04G0117500	-0.502	-1.645	-1.144	no
CK-0d	RHSIM_Rhsim05G0148400	-0.613	-0.923	-0.310	no
CK-0d	RHSIM_Rhsim06G0086800	-1.707	-1.609	0.098	low
CK-0d	RHSIM_Rhsim06G0176800	1.842	-0.167	-2.009	high
CK-0d	RHSIM_Rhsim07G0176500	-0.255	-0.040	0.215	no
CK-0d	RHSIM_Rhsim05G0107700	-1.027	-0.372	0.655	low
CK-0d	RHSIM_Rhsim05G0068100	1.297	1.534	0.238	high
CK-0d	RHSIM_Rhsim05G0146600	-1.706	-1.802	-0.096	low
CK-0d	RHSIM_Rhsim04G0058800	0.168	-0.510	-0.678	no
CK-0d	RHSIM_Rhsim05G0070600	0.919	-0.089	-1.008	no
CK-0d	RHSIM_Rhsim05G0104400	-0.659	0.492	1.152	no
CK-0d	RHSIM_Rhsim05G0047800	0.596	-0.643	-1.239	no
CK-0d	RHSIM_Rhsim05G0012600	-3.442	-1.658	1.784	low
CK-0d	RHSIM_Rhsim06G0244700	0.342	-0.153	-0.495	no
CK-0d	RHSIM_Rhsim06G0022400	0.863	-0.697	-1.560	no
CK-0d	RHSIM_Rhsim07G0057400	0.277	-0.178	-0.455	no
CK-0d	RHSIM_Rhsim05G0070100	-1.966	-0.310	1.656	low
CK-0d	RHSIM_Rhsim05G0169700	-0.011	3.205	3.216	no
CK-0d	RHSIM_Rhsim04G0131900	0.981	1.201	0.219	no
CK-0d	RHSIM_Rhsim06G0047100	0.590	-0.561	-1.152	no
CK-0d	RHSIM_Rhsim05G0166700	0.837	1.066	0.229	no
CK-0d	RHSIM_Rhsim05G0223800	0.459	2.165	1.707	no
CK-0d	RHSIM_Rhsim05G0020400	-1.669	-0.706	0.962	low
CK-0d	RHSIM_Rhsim05G0196900	1.284	0.824	-0.460	high
CK-0d	RHSIM_Rhsim06G0078800	0.178	1.717	1.540	no
CK-0d	RHSIM_Rhsim05G0190800	-1.674	0.003	1.677	low

Group	Gene	PTR (z-score difference)	z(log2 LFQ)	z(log2 FPKM)	extreme-PTR
CK-0d	RHSIM_Rhsim05G0069700	0.483	0.309	-0.174	no
CK-0d	RHSIM_Rhsim05G0074000	-1.339	2.142	3.481	low
CK-0d	RHSIM_Rhsim05G0150000	-1.031	-0.530	0.501	low
CK-0d	RHSIM_Rhsim05G0096900	0.085	0.482	0.396	no
CK-0d	RHSIM_Rhsim06G0005300	-0.060	-0.066	-0.006	no
CK-0d	RHSIM_Rhsim05G0101100	-0.083	-1.177	-1.093	no
CK-0d	RHSIM_Rhsim05G0170400	0.185	0.905	0.720	no
CK-0d	RHSIM_Rhsim05G0073500	-0.808	-0.035	0.773	no
CK-0d	RHSIM_Rhsim06G0220300	-0.106	-0.013	0.093	no
CK-0d	RHSIM_Rhsim05G0182300	-0.437	-0.488	-0.051	no
CK-0d	RHSIM_Rhsim07G0058500	0.540	0.404	-0.136	no
CK-0d	RHSIM_Rhsim04G0175300	-1.332	-0.574	0.758	low
CK-0d	RHSIM_Rhsim05G0062700	1.450	-0.132	-1.581	high
CK-0d	RHSIM_Rhsim05G0075900	-1.106	0.185	1.291	low
CK-0d	RHSIM_Rhsim05G0103400	1.422	1.129	-0.294	high
CK-0d	RHSIM_Rhsim04G0099500	-1.825	-1.289	0.536	low
CK-0d	RHSIM_Rhsim05G0159700	-1.437	-0.386	1.052	low
CK-0d	RHSIM_Rhsim05G0085900	0.643	0.119	-0.523	no
CK-0d	RHSIM_Rhsim05G0020500	-0.222	-1.220	-0.998	no
CK-0d	RHSIM_Rhsim05G0177100	0.500	0.779	0.279	no
CK-0d	RHSIM_Rhsim05G0180300	0.913	-1.064	-1.977	no
CK-0d	RHSIM_Rhsim04G0126100	-0.628	-0.912	-0.284	no
CK-0d	RHSIM_Rhsim06G0049900	0.020	-0.325	-0.346	no
CK-0d	RHSIM_Rhsim05G0080100	-0.406	0.209	0.615	no
CK-0d	RHSIM_Rhsim06G0002600	-0.085	-0.833	-0.749	no
CK-0d	RHSIM_Rhsim05G0143500	-1.722	-0.846	0.876	low
CK-0d	RHSIM_Rhsim05G0074600	-0.608	-0.006	0.601	no
CK-0d	RHSIM_Rhsim06G0142000	-1.014	-0.985	0.028	no
CK-0d	RHSIM_Rhsim05G0040900	-1.285	-1.986	-0.701	low
CK-0d	RHSIM_Rhsim05G0005100	1.101	-0.444	-1.545	no
CK-0d	RHSIM_Rhsim05G0214100	0.643	1.421	0.778	no
CK-0d	RHSIM_Rhsim05G0222100	0.333	-0.964	-1.297	no

Group	Gene	PTR (z-score difference)	z(log2 LFQ)	z(log2 FPKM)	extreme-PTR
CK-0d	RHSIM_Rhsim07G0247000	0.551	0.426	-0.126	no
CK-0d	RHSIM_Rhsim06G0082000	-0.229	-0.221	0.009	no
CK-0d	RHSIM_Rhsim05G0187300	0.135	0.568	0.434	no
CK-0d	RHSIM_Rhsim05G0143000	1.734	1.752	0.018	high
CK-0d	RHSIM_Rhsim05G0063700	2.021	0.935	-1.087	high
CK-0d	RHSIM_Rhsim04G0095000	1.861	-0.178	-2.039	high
CK-0d	RHSIM_Rhsim04G0132500	0.298	0.991	0.693	no
CK-0d	RHSIM_Rhsim05G0072000	0.527	-0.156	-0.683	no
CK-0d	RHSIM_Rhsim04G0011300	0.014	-0.306	-0.319	no
CK-0d	RHSIM_Rhsim05G0110900	1.153	0.779	-0.374	high
CK-0d	RHSIM_Rhsim05G0106700	-0.117	0.173	0.290	no
CK-0d	RHSIM_Rhsim04G0028000	1.741	1.266	-0.475	high
CK-0d	RHSIM_Rhsim06G0054600	-0.682	-0.904	-0.223	no
CK-0d	RHSIM_Rhsim05G0029400	-0.089	-0.250	-0.162	no
CK-0d	RHSIM_Rhsim04G0009500	-0.715	-1.717	-1.002	no
CK-0d	RHSIM_Rhsim05G0203000	-0.907	0.645	1.551	no
CK-0d	RHSIM_Rhsim06G0047000	0.143	-0.203	-0.347	no
CK-0d	RHSIM_Rhsim05G0113900	1.021	0.648	-0.373	no
CK-0d	RHSIM_Rhsim05G0012900	0.593	0.282	-0.311	no
CK-0d	RHSIM_Rhsim04G0014200	0.837	-0.200	-1.036	no
CK-0d	RHSIM_Rhsim06G0017600	-0.907	0.034	0.941	no
CK-0d	RHSIM_Rhsim05G0086100	-2.193	-1.957	0.236	low
CK-0d	RHSIM_Rhsim04G0002900	0.195	-0.295	-0.490	no
CK-0d	RHSIM_Rhsim05G0068700	-1.430	-0.017	1.413	low
CK-0d	RHSIM_Rhsim05G0233600	0.666	0.049	-0.617	no
CK-0d	RHSIM_Rhsim06G0023700	-0.533	-1.091	-0.559	no
CK-0d	RHSIM_Rhsim04G0241700	-1.165	-0.037	1.128	low
CK-0d	RHSIM_Rhsim04G0106600	0.186	1.024	0.838	no
CK-0d	RHSIM_Rhsim04G0190600	-1.174	0.664	1.838	low
CK-0d	RHSIM_Rhsim06G0199000	0.400	-0.897	-1.297	no
CK-0d	RHSIM_Rhsim05G0027700	-1.224	-0.559	0.665	low
CK-0d	RHSIM_Rhsim04G0148800	-0.810	-1.002	-0.192	no

Group	Gene	PTR (z-score difference)	z(log2 LFQ)	z(log2 FPKM)	extreme-PTR
CK-0d	RHSIM_Rhsim07G0039400	0.451	1.128	0.677	no
CK-0d	RHSIM_Rhsim04G0058200	0.346	-0.044	-0.390	no
CK-0d	RHSIM_Rhsim04G0126600	-0.658	-0.474	0.183	no
CK-0d	RHSIM_Rhsim05G0216600	-1.099	-0.771	0.328	low
CK-0d	RHSIM_Rhsim05G0084000	-0.686	-1.315	-0.630	no
CK-0d	RHSIM_Rhsim05G0030000	0.814	-0.120	-0.934	no
CK-0d	RHSIM_Rhsim04G0196900	-0.068	0.065	0.134	no
CK-0d	RHSIM_Rhsim07G0245300	1.170	-0.022	-1.192	high
CK-0d	RHSIM_Rhsim04G0170600	-1.467	-0.811	0.655	low
CK-0d	RHSIM_Rhsim04G0216600	1.369	0.567	-0.802	high
CK-0d	RHSIM_Rhsim05G0067400	1.718	1.364	-0.355	high
CK-0d	RHSIM_Rhsim04G0074000	-0.415	-0.297	0.119	no
CK-0d	RHSIM_Rhsim05G0075200	-0.780	-1.232	-0.452	no
CK-0d	RHSIM_Rhsim05G0145700	1.016	0.622	-0.394	no
CK-0d	RHSIM_Rhsim05G0175100	-0.926	-1.466	-0.539	no
CK-0d	RHSIM_Rhsim04G0205900	-2.298	-0.186	2.112	low
CK-0d	RHSIM_Rhsim05G0135800	0.112	0.670	0.558	no
CK-0d	RHSIM_Rhsim05G0232100	-0.561	-0.032	0.528	no
CK-0d	RHSIM_Rhsim05G0074800	-0.498	-0.345	0.153	no
CK-0d	RHSIM_Rhsim05G0023600	1.864	0.782	-1.082	high
CK-0d	RHSIM_Rhsim06G0064800	-0.276	0.670	0.947	no
CK-0d	RHSIM_Rhsim05G0044600	1.208	0.780	-0.429	high
CK-0d	RHSIM_Rhsim05G0192400	-0.221	-1.072	-0.852	no
CK-0d	RHSIM_Rhsim05G0095400	1.029	1.338	0.310	no
CK-0d	RHSIM_Rhsim05G0194900	0.205	1.390	1.184	no
CK-0d	RHSIM_Rhsim04G0012800	-0.236	0.585	0.820	no
CK-0d	RHSIM_Rhsim04G0034900	-2.078	-1.767	0.311	low
CK-0d	RHSIM_Rhsim05G0078300	0.738	-0.107	-0.845	no
CK-0d	RHSIM_Rhsim05G0000900	-0.427	-0.307	0.120	no
CK-0d	RHSIM_Rhsim04G0014400	2.159	0.184	-1.975	high
CK-0d	RHSIM_Rhsim05G0227300	0.749	-0.761	-1.510	no
CK-0d	RHSIM_Rhsim04G0015900	-1.367	-0.390	0.977	low

Group	Gene	PTR (z-score difference)	z(log2 LFQ)	z(log2 FPKM)	extreme-PTR
CK-0d	RHSIM_Rhsim05G0164500	0.106	1.747	1.642	no
CK-0d	RHSIM_Rhsim04G0016200	0.391	0.674	0.283	no
CK-0d	RHSIM_Rhsim07G0101200	0.171	0.953	0.782	no
CK-0d	RHSIM_Rhsim04G0073400	-1.972	-2.168	-0.196	low
CK-0d	RHSIM_Rhsim05G0152100	0.452	-0.221	-0.673	no
CK-0d	RHSIM_Rhsim04G0029500	-0.204	1.245	1.449	no
CK-0d	RHSIM_Rhsim05G0044900	0.350	-0.560	-0.910	no
CK-0d	RHSIM_Rhsim04G0099700	-0.481	-0.300	0.181	no
CK-0d	RHSIM_Rhsim05G0202600	-0.844	-1.059	-0.215	no
CK-0d	RHSIM_Rhsim05G0133100	0.418	0.228	-0.190	no
CK-0d	RHSIM_Rhsim04G0003800	-0.180	-0.485	-0.304	no
CK-0d	RHSIM_Rhsim05G0210500	0.675	-0.771	-1.446	no
CK-0d	RHSIM_Rhsim07G0175300	0.730	0.902	0.172	no
CK-0d	RHSIM_Rhsim04G0087500	-0.938	-0.795	0.143	no
CK-0d	RHSIM_Rhsim05G0199700	-0.071	0.026	0.097	no
CK-0d	RHSIM_Rhsim05G0165700	0.032	1.449	1.417	no
CK-0d	RHSIM_Rhsim05G0139600	1.518	-0.195	-1.713	high
CK-0d	RHSIM_Rhsim04G0145800	-1.876	-0.567	1.309	low
CK-0d	RHSIM_Rhsim05G0063100	-1.075	-0.093	0.982	low
CK-0d	RHSIM_Rhsim04G0199100	1.109	1.966	0.856	no
CK-0d	RHSIM_Rhsim04G0033100	-0.331	2.284	2.615	no
CK-0d	RHSIM_Rhsim04G0169600	0.641	0.994	0.353	no
CK-0d	RHSIM_Rhsim05G0067700	-1.907	-0.998	0.909	low
CK-0d	RHSIM_Rhsim05G0159600	1.178	1.347	0.169	high
CK-0d	RHSIM_Rhsim04G0013600	-1.433	0.165	1.599	low
CK-0d	RHSIM_Rhsim05G0022000	-0.887	-1.944	-1.057	no
CK-0d	RHSIM_Rhsim04G0124900	-1.352	0.187	1.539	low
CK-0d	RHSIM_Rhsim05G0175000	-0.571	-0.513	0.058	no
CK-0d	RHSIM_Rhsim05G0133000	-0.055	-2.151	-2.096	no
CK-0d	RHSIM_Rhsim05G0121700	-0.849	-0.212	0.637	no
CK-0d	RHSIM_Rhsim04G0100900	-0.655	0.004	0.660	no
CK-0d	RHSIM_Rhsim07G0134900	0.343	2.836	2.492	no

Group	Gene	PTR (z-score difference)	z(log2 LFQ)	z(log2 FPKM)	extreme-PTR
CK-0d	RHSIM_Rhsim05G0072400	-0.143	-1.013	-0.869	no
CK-0d	RHSIM_Rhsim04G0114200	0.542	-0.623	-1.165	no
CK-0d	RHSIM_Rhsim04G0119400	1.929	1.844	-0.085	high
CK-0d	RHSIM_Rhsim04G0065900	1.067	0.410	-0.658	no
CK-0d	RHSIM_Rhsim04G0134300	0.027	1.060	1.033	no
CK-0d	RHSIM_Rhsim05G0148800	-1.155	-1.954	-0.800	low
CK-0d	RHSIM_Rhsim07G0101600	0.586	-0.339	-0.925	no
CK-0d	RHSIM_Rhsim05G0212900	2.164	3.941	1.777	high
CK-0d	RHSIM_Rhsim07G0167800	0.071	-0.958	-1.028	no
CK-0d	RHSIM_Rhsim05G0084700	-0.689	-0.657	0.033	no
CK-0d	RHSIM_Rhsim04G0150100	-1.149	-1.216	-0.067	low
CK-0d	RHSIM_Rhsim05G0111000	-0.550	-0.601	-0.051	no
CK-0d	RHSIM_Rhsim04G0245000	-1.315	-1.766	-0.451	low
CK-0d	RHSIM_Rhsim05G0060100	-1.188	-0.660	0.528	low
CK-0d	RHSIM_Rhsim04G0009400	0.242	-0.315	-0.557	no
CK-0d	RHSIM_Rhsim05G0083700	-0.524	-0.812	-0.287	no
CK-0d	RHSIM_Rhsim05G0229700	0.191	-0.258	-0.449	no
CK-0d	RHSIM_Rhsim04G0014300	1.581	0.709	-0.873	high
CK-0d	RHSIM_Rhsim05G0053600	1.535	-0.359	-1.895	high
CK-0d	RHSIM_Rhsim05G0095000	0.176	0.240	0.064	no
CK-0d	RHSIM_Rhsim04G0096600	0.643	-0.582	-1.225	no
CK-0d	RHSIM_Rhsim05G0090100	0.251	-0.326	-0.577	no
CK-0d	RHSIM_Rhsim05G0161400	-1.170	-2.182	-1.012	low
CK-0d	RHSIM_Rhsim07G0059400	0.329	0.624	0.295	no
CK-0d	RHSIM_Rhsim04G0023700	0.383	1.307	0.924	no
CK-0d	RHSIM_Rhsim05G0216800	-0.787	-0.172	0.615	no
CK-0d	RHSIM_Rhsim05G0101800	-0.988	-0.311	0.677	no
CK-0d	RHSIM_Rhsim04G0213000	0.292	-0.933	-1.225	no
CK-0d	RHSIM_Rhsim05G0039000	2.040	0.254	-1.786	high
CK-0d	RHSIM_Rhsim05G0192200	0.837	1.284	0.446	no
CK-0d	RHSIM_Rhsim07G0008100	-1.836	-0.535	1.301	low
CK-0d	RHSIM_Rhsim05G0180600	0.621	-0.114	-0.735	no

Group	Gene	PTR (z-score difference)	z(log2 LFQ)	z(log2 FPKM)	extreme-PTR
CK-0d	RHSIM_Rhsim04G0202900	-0.409	0.087	0.496	no
CK-0d	RHSIM_Rhsim04G0084300	1.164	-0.520	-1.684	high
CK-0d	RHSIM_Rhsim04G0184400	0.822	0.782	-0.040	no
CK-0d	RHSIM_Rhsim07G0010700	0.139	0.104	-0.034	no
CK-0d	RHSIM_Rhsim04G0228600	0.791	-0.445	-1.236	no
CK-0d	RHSIM_Rhsim05G0062300	0.194	-0.917	-1.111	no
CK-0d	RHSIM_Rhsim07G0005600	-0.991	-1.264	-0.274	no
CK-0d	RHSIM_Rhsim04G0248100	0.293	0.389	0.096	no
CK-0d	RHSIM_Rhsim07G0031200	-0.089	0.598	0.687	no
CK-0d	RHSIM_Rhsim05G0105400	-0.711	0.024	0.736	no
CK-0d	RHSIM_Rhsim04G0112300	1.814	-0.229	-2.043	high
CK-0d	RHSIM_Rhsim04G0216400	1.035	-0.114	-1.149	no
CK-0d	RHSIM_Rhsim07G0142500	-0.499	-0.440	0.059	no
CK-0d	RHSIM_Rhsim04G0243000	-0.796	-1.422	-0.626	no
CK-0d	RHSIM_Rhsim04G0011600	-1.502	-0.943	0.559	low
CK-0d	RHSIM_Rhsim05G0106400	0.301	-0.606	-0.906	no
CK-0d	RHSIM_Rhsim04G0126000	-1.106	-0.759	0.347	low
CK-0d	RHSIM_Rhsim04G0118000	-0.342	-0.295	0.047	no
CK-0d	RHSIM_Rhsim04G0012200	0.376	0.318	-0.057	no
CK-0d	RHSIM_Rhsim04G0010300	0.365	2.427	2.062	no
CK-0d	RHSIM_Rhsim05G0160800	1.236	0.616	-0.620	high
CK-0d	RHSIM_Rhsim04G0010500	-0.720	-1.359	-0.639	no
CK-0d	RHSIM_Rhsim04G0157000	2.857	0.853	-2.004	high
CK-0d	RHSIM_Rhsim05G0068400	-0.615	-1.079	-0.464	no
CK-0d	RHSIM_Rhsim04G0203200	-0.145	0.893	1.039	no
CK-0d	RHSIM_Rhsim07G0252600	0.247	0.665	0.418	no
CK-0d	RHSIM_Rhsim04G0125900	0.275	-1.694	-1.969	no
CK-0d	RHSIM_Rhsim05G0110800	2.847	0.623	-2.224	high
CK-0d	RHSIM_Rhsim05G0159300	0.766	1.718	0.953	no
CK-0d	RHSIM_Rhsim04G0216900	0.141	-0.729	-0.869	no
CK-0d	RHSIM_Rhsim04G0052800	0.370	0.202	-0.168	no
CK-0d	RHSIM_Rhsim04G0225000	0.166	0.847	0.680	no

Group	Gene	PTR (z-score difference)	z(log2 LFQ)	z(log2 FPKM)	extreme-PTR
CK-0d	RHSIM_Rhsim04G0199900	0.573	1.256	0.683	no
CK-0d	RHSIM_Rhsim05G0131900	0.687	0.372	-0.315	no
CK-0d	RHSIM_Rhsim07G0126100	-0.902	-0.525	0.377	no
CK-0d	RHSIM_Rhsim05G0124800	-0.477	-0.279	0.198	no
CK-0d	RHSIM_Rhsim04G0004200	-1.481	-1.481	0.000	low
CK-0d	RHSIM_Rhsim04G0180700	0.176	-0.305	-0.481	no
CK-0d	RHSIM_Rhsim06G0198800	-0.360	-0.869	-0.509	no
CK-0d	RHSIM_Rhsim04G0202000	-0.413	-0.169	0.244	no
CK-0d	RHSIM_Rhsim04G0052300	0.539	2.762	2.223	no
CK-0d	RHSIM_Rhsim04G0006600	0.436	0.267	-0.169	no
CK-0d	RHSIM_Rhsim06G0033800	-0.591	-0.954	-0.362	no
CK-0d	RHSIM_Rhsim05G0167200	0.935	1.560	0.625	no
CK-0d	RHSIM_Rhsim04G0123300	-0.986	-1.362	-0.376	no
CK-0d	RHSIM_Rhsim04G0220300	0.563	-0.373	-0.935	no
CK-0d	RHSIM_Rhsim05G0201000	-0.239	0.113	0.352	no
CK-0d	RHSIM_Rhsim04G0055900	1.013	-0.353	-1.366	no
CK-0d	RHSIM_Rhsim04G0174200	-0.754	0.375	1.129	no
CK-0d	RHSIM_Rhsim06G0087100	-0.325	-0.443	-0.118	no
CK-0d	RHSIM_Rhsim04G0016600	0.284	0.603	0.319	no
CK-0d	RHSIM_Rhsim04G0175900	1.221	1.076	-0.145	high
CK-0d	RHSIM_Rhsim04G0203300	0.263	0.051	-0.212	no
CK-0d	RHSIM_Rhsim05G0100900	-1.516	0.118	1.633	low
CK-0d	RHSIM_Rhsim04G0186500	-0.031	-1.525	-1.494	no
CK-0d	RHSIM_Rhsim04G0026100	1.236	1.837	0.601	high
CK-0d	RHSIM_Rhsim05G0225500	-0.405	-0.661	-0.256	no
CK-0d	RHSIM_Rhsim06G0006400	-2.523	-0.720	1.803	low
CK-0d	RHSIM_Rhsim05G0031400	0.420	0.264	-0.156	no
CK-0d	RHSIM_Rhsim04G0013400	0.377	-0.356	-0.734	no
CK-0d	RHSIM_Rhsim04G0157800	-2.847	-0.527	2.320	low
CK-0d	RHSIM_Rhsim04G0008200	-0.190	0.393	0.582	no
CK-0d	RHSIM_Rhsim04G0019600	0.388	-0.707	-1.095	no
CK-0d	RHSIM_Rhsim05G0122700	-0.281	-1.093	-0.812	no

Group	Gene	PTR (z-score difference)	z(log2 LFQ)	z(log2 FPKM)	extreme-PTR
CK-0d	RHSIM_Rhsim04G0168600	0.392	0.080	-0.313	no
CK-0d	RHSIM_Rhsim04G0083700	0.576	-0.633	-1.209	no
CK-0d	RHSIM_Rhsim05G0047300	-0.384	-1.560	-1.176	no
CK-0d	RHSIM_Rhsim05G0174900	0.275	1.314	1.038	no
CK-0d	RHSIM_Rhsim04G0006900	-0.590	-0.340	0.250	no
CK-0d	RHSIM_Rhsim04G0167600	-0.439	-0.540	-0.101	no
CK-0d	RHSIM_Rhsim04G0070300	-0.580	0.869	1.449	no
CK-0d	RHSIM_Rhsim04G0154200	-0.414	-1.200	-0.786	no
CK-0d	RHSIM_Rhsim04G0215500	0.251	1.080	0.829	no
CK-0d	RHSIM_Rhsim06G0237200	-1.822	0.658	2.480	low
CK-0d	RHSIM_Rhsim04G0173400	2.267	1.190	-1.077	high
CK-0d	RHSIM_Rhsim04G0075900	2.111	1.511	-0.600	high
CK-0d	RHSIM_Rhsim04G0074900	-0.309	-0.770	-0.461	no
CK-0d	RHSIM_Rhsim04G0126400	-0.233	0.808	1.041	no
CK-0d	RHSIM_Rhsim04G0125700	-2.826	-1.787	1.039	low
CK-0d	RHSIM_Rhsim04G0202300	3.555	2.843	-0.712	high
CK-0d	RHSIM_Rhsim04G0023900	-0.815	0.641	1.457	no
CK-0d	RHSIM_Rhsim05G0172200	0.200	-0.885	-1.085	no
CK-0d	RHSIM_Rhsim04G0093600	-1.074	-0.846	0.228	low
CK-0d	RHSIM_Rhsim04G0230600	1.004	2.774	1.770	no
CK-0d	RHSIM_Rhsim04G0136300	-0.525	0.255	0.780	no
CK-0d	RHSIM_Rhsim04G0030800	1.127	0.355	-0.772	high
CK-0d	RHSIM_Rhsim05G0219800	0.647	-0.836	-1.482	no
CK-0d	RHSIM_Rhsim05G0217200	-1.159	-1.232	-0.073	low
CK-0d	RHSIM_Rhsim04G0207400	0.824	0.144	-0.679	no
CK-0d	RHSIM_Rhsim05G0095100	0.203	1.544	1.342	no
CK-0d	RHSIM_Rhsim03G0030800	2.636	0.751	-1.885	high
CK-0d	RHSIM_Rhsim05G0163700	-0.806	-0.536	0.270	no
CK-0d	RHSIM_Rhsim04G0018100	1.442	0.270	-1.172	high
CK-0d	RHSIM_Rhsim05G0211100	0.347	0.690	0.343	no
CK-0d	RHSIM_Rhsim04G0195100	0.550	1.056	0.506	no
CK-0d	RHSIM_Rhsim04G0156300	-2.011	1.531	3.542	low

Group	Gene	PTR (z-score difference)	z(log2 LFQ)	z(log2 FPKM)	extreme-PTR
CK-0d	RHSIM_Rhsim04G0030700	-0.041	0.361	0.402	no
CK-0d	RHSIM_Rhsim04G0108400	-2.607	0.248	2.855	low
CK-0d	RHSIM_Rhsim04G0217800	-1.008	-0.601	0.407	no
CK-0d	RHSIM_Rhsim04G0195700	-0.618	-0.887	-0.270	no
CK-0d	RHSIM_Rhsim04G0000700	-1.189	-0.417	0.772	low
CK-0d	RHSIM_Rhsim04G0117700	-0.082	-0.361	-0.278	no
CK-0d	RHSIM_Rhsim03G0235300	-0.446	0.426	0.872	no
CK-0d	RHSIM_Rhsim05G0139500	0.209	0.666	0.457	no
CK-0d	RHSIM_Rhsim04G0114100	0.636	1.044	0.408	no
CK-0d	RHSIM_Rhsim04G0009900	-1.320	-0.542	0.778	low
CK-0d	RHSIM_Rhsim04G0084500	-0.420	0.640	1.060	no
CK-0d	RHSIM_Rhsim06G0164200	-0.330	1.575	1.905	no
CK-0d	RHSIM_Rhsim05G0075700	0.986	0.784	-0.201	no
CK-0d	RHSIM_Rhsim05G0086800	-1.316	-0.223	1.093	low
CK-0d	RHSIM_Rhsim04G0104400	-0.882	0.174	1.056	no
CK-0d	RHSIM_Rhsim04G0130500	-0.447	0.466	0.913	no
CK-0d	RHSIM_Rhsim04G0018200	0.098	0.249	0.152	no
CK-0d	RHSIM_Rhsim05G0082100	-0.035	0.454	0.489	no
CK-0d	RHSIM_Rhsim04G0224400	0.139	0.066	-0.073	no
CK-0d	RHSIM_Rhsim04G0232100	0.652	0.448	-0.204	no
CK-0d	RHSIM_Rhsim05G0187700	0.473	1.306	0.833	no
CK-0d	RHSIM_Rhsim03G0241400	-0.777	-0.376	0.401	no
CK-0d	RHSIM_Rhsim04G0194700	0.952	0.995	0.043	no
CK-0d	RHSIM_Rhsim05G0017300	-1.488	-0.954	0.533	low
CK-0d	RHSIM_Rhsim04G0240200	-3.823	-1.393	2.430	low
CK-0d	RHSIM_Rhsim04G0063000	0.556	-0.201	-0.757	no
CK-0d	RHSIM_Rhsim05G0104700	0.356	1.012	0.656	no
CK-0d	RHSIM_Rhsim04G0239000	0.502	1.173	0.671	no
CK-0d	RHSIM_Rhsim04G0170500	0.193	0.127	-0.066	no
CK-0d	RHSIM_Rhsim05G0150300	0.666	-0.535	-1.201	no
CK-0d	RHSIM_Rhsim04G0030100	1.023	1.406	0.382	no
CK-0d	RHSIM_Rhsim04G0200900	-0.548	-1.282	-0.734	no

Group	Gene	PTR (z-score difference)	z(log2 LFQ)	z(log2 FPKM)	extreme-PTR
CK-0d	RHSIM_Rhsim04G0083600	-0.830	-0.996	-0.166	no
CK-0d	RHSIM_Rhsim04G0221400	-1.091	-0.741	0.350	low
CK-0d	RHSIM_Rhsim05G0205100	-0.183	-1.482	-1.299	no
CK-0d	RHSIM_Rhsim04G0122900	1.501	0.828	-0.672	high
CK-0d	RHSIM_Rhsim05G0067100	-0.699	0.661	1.361	no
CK-0d	RHSIM_Rhsim03G0221100	0.917	0.728	-0.189	no
CK-0d	RHSIM_Rhsim04G0011100	0.722	0.998	0.276	no
CK-0d	RHSIM_Rhsim04G0104600	1.095	1.017	-0.078	no
CK-0d	RHSIM_Rhsim04G0158800	0.801	-0.260	-1.061	no
CK-0d	RHSIM_Rhsim04G0177100	0.275	-0.173	-0.447	no
CK-0d	RHSIM_Rhsim04G0050100	-2.365	-0.664	1.701	low
CK-0d	RHSIM_Rhsim03G0145800	0.782	-0.118	-0.899	no
CK-0d	RHSIM_Rhsim04G0235200	0.796	1.868	1.072	no
CK-0d	RHSIM_Rhsim04G0247200	0.374	-0.875	-1.249	no
CK-0d	RHSIM_Rhsim04G0171800	-1.667	-1.729	-0.061	low
CK-0d	RHSIM_Rhsim04G0038600	1.676	4.457	2.781	high
CK-0d	RHSIM_Rhsim04G0103300	-0.208	-0.127	0.081	no
CK-0d	RHSIM_Rhsim04G0144400	-0.031	0.860	0.891	no
CK-0d	RHSIM_Rhsim04G0162900	-1.455	-0.723	0.732	low
CK-0d	RHSIM_Rhsim06G0035400	-1.611	-1.076	0.535	low
CK-0d	RHSIM_Rhsim04G0006200	1.609	3.876	2.267	high
CK-0d	RHSIM_Rhsim04G0219100	0.907	0.680	-0.227	no
CK-0d	RHSIM_Rhsim03G0171900	0.387	0.587	0.200	no
CK-0d	RHSIM_Rhsim06G0232900	0.406	0.055	-0.350	no
CK-0d	RHSIM_Rhsim04G0248000	0.474	0.134	-0.340	no
CK-0d	RHSIM_Rhsim04G0084900	-0.012	-0.029	-0.017	no
CK-0d	RHSIM_Rhsim04G0164200	-0.062	-1.653	-1.591	no
CK-0d	RHSIM_Rhsim04G0224800	0.475	-0.068	-0.543	no
CK-0d	RHSIM_Rhsim03G0219900	1.001	0.897	-0.105	no
CK-0d	RHSIM_Rhsim04G0110400	0.528	0.899	0.371	no
CK-0d	RHSIM_Rhsim04G0235000	-0.754	-0.693	0.061	no
CK-0d	RHSIM_Rhsim03G0220500	0.220	-1.334	-1.555	no

Group	Gene	PTR (z-score difference)	z(log2 LFQ)	z(log2 FPKM)	extreme-PTR
CK-0d	RHSIM_Rhsim04G0196400	0.121	-1.511	-1.632	no
CK-0d	RHSIM_Rhsim04G0015300	0.322	1.073	0.751	no
CK-0d	RHSIM_Rhsim04G0241800	0.102	0.751	0.649	no
CK-0d	RHSIM_Rhsim04G0131000	-0.571	-0.569	0.002	no
CK-0d	RHSIM_Rhsim06G0169700	-0.237	-1.333	-1.095	no
CK-0d	RHSIM_Rhsim05G0108600	-0.049	0.579	0.628	no
CK-0d	RHSIM_Rhsim04G0173800	-1.160	-0.647	0.513	low
CK-0d	RHSIM_Rhsim04G0092500	1.622	1.372	-0.250	high
CK-0d	RHSIM_Rhsim04G0037800	-1.138	-0.730	0.408	low
CK-0d	RHSIM_Rhsim04G0031900	-0.853	0.191	1.045	no
CK-0d	RHSIM_Rhsim03G0057400	-1.328	-1.131	0.198	low
CK-0d	RHSIM_Rhsim04G0043200	-0.234	0.026	0.260	no
CK-0d	RHSIM_Rhsim04G0204600	0.107	-0.780	-0.888	no
CK-0d	RHSIM_Rhsim04G0106500	-2.024	-0.015	2.009	low
CK-0d	RHSIM_Rhsim04G0130700	-0.496	0.279	0.775	no
CK-0d	RHSIM_Rhsim06G0229600	1.220	0.999	-0.220	high
CK-0d	RHSIM_Rhsim04G0100100	0.296	-0.385	-0.681	no
CK-0d	RHSIM_Rhsim04G0084000	-0.872	0.046	0.919	no
CK-0d	RHSIM_Rhsim03G0221500	0.683	-0.929	-1.612	no
CK-0d	RHSIM_Rhsim03G0225700	-0.139	0.481	0.620	no
CK-0d	RHSIM_Rhsim06G0202900	2.021	0.669	-1.352	high
CK-0d	RHSIM_Rhsim04G0197100	1.556	1.880	0.324	high
CK-0d	RHSIM_Rhsim03G0001300	-1.443	-0.561	0.882	low
CK-0d	RHSIM_Rhsim04G0167300	0.745	2.152	1.407	no
CK-0d	RHSIM_Rhsim05G0163500	0.066	0.340	0.274	no
CK-0d	RHSIM_Rhsim04G0198400	-0.671	-1.474	-0.803	no
CK-0d	RHSIM_Rhsim04G0056600	0.034	-0.235	-0.268	no
CK-0d	RHSIM_Rhsim04G0244400	0.928	1.210	0.281	no
CK-0d	RHSIM_Rhsim04G0153200	-0.215	0.922	1.137	no
CK-0d	RHSIM_Rhsim04G0023000	-0.541	0.258	0.799	no
CK-0d	RHSIM_Rhsim03G0083600	1.609	1.627	0.018	high
CK-0d	RHSIM_Rhsim06G0000500	-0.138	1.052	1.190	no

Group	Gene	PTR (z-score difference)	z(log2 LFQ)	z(log2 FPKM)	extreme-PTR
CK-0d	RHSIM_Rhsim04G0172400	-0.383	-0.157	0.226	no
CK-0d	RHSIM_Rhsim04G0032200	-0.402	-0.455	-0.053	no
CK-0d	RHSIM_Rhsim03G0154000	0.543	0.562	0.019	no
CK-0d	RHSIM_Rhsim04G0027200	-1.594	0.865	2.459	low
CK-0d	RHSIM_Rhsim04G0245300	1.152	0.151	-1.002	high
CK-0d	RHSIM_Rhsim04G0072600	-1.133	-1.228	-0.094	low
CK-0d	RHSIM_Rhsim03G0168400	2.556	1.348	-1.208	high
CK-0d	RHSIM_Rhsim03G0075300	0.254	0.627	0.373	no
CK-0d	RHSIM_Rhsim04G0173300	-0.744	0.255	0.999	no
CK-0d	RHSIM_Rhsim04G0219500	-0.309	-0.881	-0.572	no
CK-0d	RHSIM_Rhsim04G0180500	-0.160	-0.098	0.062	no
CK-0d	RHSIM_Rhsim05G0181000	-0.502	0.437	0.939	no
CK-0d	RHSIM_Rhsim04G0096800	1.640	2.275	0.635	high
CK-0d	RHSIM_Rhsim04G0095900	0.220	0.696	0.475	no
CK-0d	RHSIM_Rhsim03G0154300	0.728	-0.484	-1.213	no
CK-0d	RHSIM_Rhsim06G0030600	-0.786	-1.202	-0.416	no
CK-0d	RHSIM_Rhsim04G0038700	0.653	-0.170	-0.823	no
CK-0d	RHSIM_Rhsim04G0001800	0.143	0.756	0.614	no
CK-0d	RHSIM_Rhsim05G0062800	0.316	2.793	2.477	no
CK-0d	RHSIM_Rhsim03G0111900	0.333	1.999	1.666	no
CK-0d	RHSIM_Rhsim05G0133600	0.764	0.653	-0.111	no
CK-0d	RHSIM_Rhsim03G0204000	-1.081	-1.421	-0.340	low
CK-0d	RHSIM_Rhsim04G0162800	-1.396	-0.923	0.472	low
CK-0d	RHSIM_Rhsim04G0087300	0.799	0.002	-0.797	no
CK-0d	RHSIM_Rhsim06G0239100	-0.023	1.693	1.715	no
CK-0d	RHSIM_Rhsim04G0196800	-0.122	1.133	1.255	no
CK-0d	RHSIM_Rhsim03G0044900	-0.037	0.082	0.119	no
CK-0d	RHSIM_Rhsim03G0179500	-0.379	1.336	1.715	no
CK-0d	RHSIM_Rhsim04G0185400	0.260	-0.819	-1.079	no
CK-0d	RHSIM_Rhsim04G0205700	2.090	1.756	-0.334	high
CK-0d	RHSIM_Rhsim04G0020500	2.818	2.088	-0.730	high
CK-0d	RHSIM_Rhsim03G0234000	-0.789	-1.269	-0.481	no

Group	Gene	PTR (z-score difference)	z(log2 LFQ)	z(log2 FPKM)	extreme-PTR
CK-0d	RHSIM_Rhsim04G0233600	0.194	-0.254	-0.448	no
CK-0d	RHSIM_Rhsim03G0019300	-1.216	1.657	2.873	low
CK-0d	RHSIM_Rhsim04G0162300	-0.716	0.478	1.195	no
CK-0d	RHSIM_Rhsim03G0226300	-0.079	0.182	0.261	no
CK-0d	RHSIM_Rhsim04G0218700	-0.910	-0.729	0.181	no
CK-0d	RHSIM_Rhsim03G0155100	0.065	-1.058	-1.123	no
CK-0d	RHSIM_Rhsim04G0177600	-1.241	-0.722	0.520	low
CK-0d	RHSIM_Rhsim03G0254300	0.292	0.953	0.661	no
CK-0d	RHSIM_Rhsim03G0001500	0.464	0.591	0.128	no
CK-0d	RHSIM_Rhsim03G0166500	0.126	-0.238	-0.364	no
CK-0d	RHSIM_Rhsim03G0065700	1.089	-0.180	-1.268	no
CK-0d	RHSIM_Rhsim04G0228500	-0.776	-1.022	-0.246	no
CK-0d	RHSIM_Rhsim03G0253300	-0.214	-0.398	-0.184	no
CK-0d	RHSIM_Rhsim04G0003700	1.477	-0.597	-2.074	high
CK-0d	RHSIM_Rhsim03G0180700	0.449	1.319	0.870	no
CK-0d	RHSIM_Rhsim04G0189900	1.127	0.542	-0.585	high
CK-0d	RHSIM_Rhsim03G0237900	1.949	0.741	-1.208	high
CK-0d	RHSIM_Rhsim04G0233400	-0.569	-0.837	-0.268	no
CK-0d	RHSIM_Rhsim04G0003500	-0.553	-0.260	0.293	no
CK-0d	RHSIM_Rhsim03G0223300	0.200	-0.287	-0.487	no
CK-0d	RHSIM_Rhsim04G0134100	0.356	-0.887	-1.244	no
CK-0d	RHSIM_Rhsim03G0010800	1.074	-0.286	-1.361	no
CK-0d	RHSIM_Rhsim03G0063700	-0.383	0.378	0.761	no
CK-0d	RHSIM_Rhsim04G0030200	-0.300	-1.095	-0.795	no
CK-0d	RHSIM_Rhsim03G0068700	-0.804	1.192	1.995	no
CK-0d	RHSIM_Rhsim04G0158400	-0.471	-0.905	-0.434	no
CK-0d	RHSIM_Rhsim06G0242500	-0.241	-1.189	-0.948	no
CK-0d	RHSIM_Rhsim04G0139400	0.578	-0.716	-1.294	no
CK-0d	RHSIM_Rhsim04G0190700	-0.938	-1.284	-0.346	no
CK-0d	RHSIM_Rhsim04G0230400	1.153	1.449	0.296	high
CK-0d	RHSIM_Rhsim03G0085200	-0.069	-0.688	-0.619	no
CK-0d	RHSIM_Rhsim03G0228800	-0.481	-1.219	-0.738	no

Group	Gene	PTR (z-score difference)	z(log2 LFQ)	z(log2 FPKM)	extreme-PTR
CK-0d	RHSIM_Rhsim04G0120900	-0.877	-0.543	0.334	no
CK-0d	RHSIM_Rhsim04G0155300	-1.081	1.565	2.646	low
CK-0d	RHSIM_Rhsim03G0121700	-0.306	-0.444	-0.138	no
CK-0d	RHSIM_Rhsim04G0004400	0.333	0.590	0.256	no
CK-0d	RHSIM_Rhsim03G0129000	-0.373	0.894	1.266	no
CK-0d	RHSIM_Rhsim04G0130800	-1.456	-0.994	0.462	low
CK-0d	RHSIM_Rhsim06G0082800	-0.750	-0.720	0.030	no
CK-0d	RHSIM_Rhsim03G0235100	-0.239	1.062	1.301	no
CK-0d	RHSIM_Rhsim04G0138700	0.141	0.462	0.321	no
CK-0d	RHSIM_Rhsim04G0003600	0.500	1.279	0.779	no
CK-0d	RHSIM_Rhsim06G0166200	-0.237	0.103	0.340	no
CK-0d	RHSIM_Rhsim03G0198500	0.299	-0.481	-0.780	no
CK-0d	RHSIM_Rhsim03G0000200	0.854	1.047	0.193	no
CK-0d	RHSIM_Rhsim04G0128400	-0.832	-0.604	0.228	no
CK-0d	RHSIM_Rhsim04G0225100	0.560	-0.232	-0.792	no
CK-0d	RHSIM_Rhsim03G0096300	-0.805	0.028	0.834	no
CK-0d	RHSIM_Rhsim04G0235600	1.135	-1.042	-2.178	high
CK-0d	RHSIM_Rhsim03G0234800	-0.275	-0.236	0.039	no
CK-0d	RHSIM_Rhsim03G0066700	0.872	3.468	2.596	no
CK-0d	RHSIM_Rhsim04G0241100	1.330	1.233	-0.096	high
CK-0d	RHSIM_Rhsim04G0084800	-1.048	-0.717	0.331	low
CK-0d	RHSIM_Rhsim04G0021800	0.144	0.423	0.279	no
CK-0d	RHSIM_Rhsim03G0239700	-1.882	-1.832	0.051	low
CK-0d	RHSIM_Rhsim03G0073700	0.609	-0.516	-1.126	no
CK-0d	RHSIM_Rhsim03G0146900	-0.043	-0.624	-0.580	no
CK-0d	RHSIM_Rhsim04G0094400	0.067	-0.758	-0.826	no
CK-0d	RHSIM_Rhsim03G0164200	0.548	0.566	0.018	no
CK-0d	RHSIM_Rhsim03G0006300	-2.288	-1.661	0.628	low
CK-0d	RHSIM_Rhsim06G0174600	-0.157	0.301	0.458	no
CK-0d	RHSIM_Rhsim04G0039400	-0.739	-1.791	-1.052	no
CK-0d	RHSIM_Rhsim06G0073200	-3.363	0.022	3.384	low
CK-0d	RHSIM_Rhsim03G0188200	0.541	-0.172	-0.713	no

Group	Gene	PTR (z-score difference)	z(log2 LFQ)	z(log2 FPKM)	extreme-PTR
CK-0d	RHSIM_Rhsim04G0001500	0.875	0.237	-0.638	no
CK-0d	RHSIM_Rhsim06G0036100	0.535	0.310	-0.225	no
CK-0d	RHSIM_Rhsim04G0129300	0.423	0.801	0.378	no
CK-0d	RHSIM_Rhsim06G0177200	0.165	-0.511	-0.676	no
CK-0d	RHSIM_Rhsim03G0019000	-0.899	0.756	1.655	no
CK-0d	RHSIM_Rhsim03G0122000	0.306	-0.035	-0.341	no
CK-0d	RHSIM_Rhsim03G0229700	-0.705	-0.066	0.639	no
CK-0d	RHSIM_Rhsim06G0153200	-1.076	0.341	1.418	low
CK-0d	RHSIM_Rhsim03G0020300	1.161	-0.103	-1.265	high
CK-0d	RHSIM_Rhsim03G0172000	0.100	-0.978	-1.078	no
CK-0d	RHSIM_Rhsim03G0041200	0.868	0.200	-0.668	no
CK-0d	RHSIM_Rhsim03G0222600	-1.775	-0.855	0.920	low
CK-0d	RHSIM_Rhsim06G0001600	0.237	-0.194	-0.431	no
CK-0d	RHSIM_Rhsim03G0155200	0.932	1.095	0.163	no
CK-0d	RHSIM_Rhsim03G0173300	1.033	0.354	-0.679	no
CK-0d	RHSIM_Rhsim03G0218100	-0.869	-1.375	-0.507	no
CK-0d	RHSIM_Rhsim03G0270100	0.199	-0.918	-1.117	no
CK-0d	RHSIM_Rhsim06G0087400	-0.992	-1.044	-0.053	no
CK-0d	RHSIM_Rhsim03G0212700	-0.049	0.878	0.927	no
CK-0d	RHSIM_Rhsim03G0230000	-0.530	-0.144	0.386	no
CK-0d	RHSIM_Rhsim03G0009200	1.159	1.792	0.632	high
CK-0d	RHSIM_Rhsim03G0050300	-0.126	0.137	0.263	no
CK-0d	RHSIM_Rhsim03G0075800	0.923	1.316	0.393	no
CK-0d	RHSIM_Rhsim06G0129100	-0.429	0.313	0.742	no
CK-0d	RHSIM_Rhsim04G0165300	0.369	0.618	0.249	no
CK-0d	RHSIM_Rhsim03G0143600	-1.637	-1.720	-0.083	low
CK-0d	RHSIM_Rhsim04G0139800	0.561	0.236	-0.325	no
CK-0d	RHSIM_Rhsim03G0177300	0.431	0.328	-0.104	no
CK-0d	RHSIM_Rhsim04G0201800	0.262	0.115	-0.146	no
CK-0d	RHSIM_Rhsim06G0130400	0.425	0.616	0.190	no
CK-0d	RHSIM_Rhsim04G0168700	-0.393	0.367	0.760	no
CK-0d	RHSIM_Rhsim06G0048400	-0.204	-0.260	-0.056	no

Group	Gene	PTR (z-score difference)	z(log2 LFQ)	z(log2 FPKM)	extreme-PTR
CK-0d	RHSIM_Rhsim04G0167800	1.442	0.949	-0.492	high
CK-0d	RHSIM_Rhsim03G0248200	-0.290	-0.987	-0.697	no
CK-0d	RHSIM_Rhsim03G0201100	-0.429	0.085	0.514	no
CK-0d	RHSIM_Rhsim03G0150700	0.016	-0.203	-0.219	no
CK-0d	RHSIM_Rhsim04G0110900	-2.115	-1.997	0.117	low
CK-0d	RHSIM_Rhsim04G0181600	1.371	-0.234	-1.605	high
CK-0d	RHSIM_Rhsim03G0265800	1.403	0.369	-1.034	high
CK-0d	RHSIM_Rhsim04G0014800	-2.133	-1.359	0.774	low
CK-0d	RHSIM_Rhsim03G0004000	1.083	-0.146	-1.228	no
CK-0d	RHSIM_Rhsim04G0173000	1.258	1.940	0.682	high
CK-0d	RHSIM_Rhsim04G0091700	0.464	1.362	0.898	no
CK-0d	RHSIM_Rhsim06G0166000	-0.927	-1.404	-0.477	no
CK-0d	RHSIM_Rhsim03G0151400	0.812	1.761	0.949	no
CK-0d	RHSIM_Rhsim03G0061300	0.487	-1.420	-1.907	no
CK-0d	RHSIM_Rhsim04G0081600	0.851	-0.179	-1.029	no
CK-0d	RHSIM_Rhsim04G0038100	-0.835	-0.648	0.187	no
CK-0d	RHSIM_Rhsim04G0105700	0.549	-0.225	-0.774	no
CK-0d	RHSIM_Rhsim03G0196600	0.044	-0.553	-0.598	no
CK-0d	RHSIM_Rhsim03G0235600	-0.480	-0.170	0.310	no
CK-0d	RHSIM_Rhsim03G0252000	-0.336	0.169	0.504	no
CK-0d	RHSIM_Rhsim04G0197500	0.113	0.225	0.112	no
CK-0d	RHSIM_Rhsim06G0018600	0.486	0.684	0.198	no
CK-0d	RHSIM_Rhsim03G0125700	0.707	0.416	-0.291	no
CK-0d	RHSIM_Rhsim03G0001600	-0.717	-0.311	0.406	no
CK-0d	RHSIM_Rhsim04G0170700	1.830	0.382	-1.449	high
CK-0d	RHSIM_Rhsim03G0093200	-1.416	-0.793	0.622	low
CK-0d	RHSIM_Rhsim03G0248000	-0.048	0.656	0.704	no
CK-0d	RHSIM_Rhsim03G0153100	1.000	-0.601	-1.601	no
CK-0d	RHSIM_Rhsim03G0138600	0.225	0.550	0.325	no
CK-0d	RHSIM_Rhsim04G0008100	0.045	1.248	1.203	no
CK-0d	RHSIM_Rhsim03G0090600	-0.185	-0.409	-0.225	no
CK-0d	RHSIM_Rhsim03G0161600	-0.526	-1.396	-0.870	no

Group	Gene	PTR (z-score difference)	z(log2 LFQ)	z(log2 FPKM)	extreme-PTR
CK-0d	RHSIM_Rhsim03G0264700	-0.857	-0.708	0.149	no
CK-0d	RHSIM_Rhsim03G0078800	0.482	-0.060	-0.542	no
CK-0d	RHSIM_Rhsim05G0113400	1.799	3.055	1.256	high
CK-0d	RHSIM_Rhsim03G0211600	-0.370	-1.337	-0.967	no
CK-0d	RHSIM_Rhsim03G0115800	-0.125	-0.459	-0.334	no
CK-0d	RHSIM_Rhsim05G0090500	-1.112	-0.053	1.059	low
CK-0d	RHSIM_Rhsim03G0249400	0.045	0.540	0.496	no
CK-0d	RHSIM_Rhsim04G0086600	-1.075	-0.978	0.098	low
CK-0d	RHSIM_Rhsim04G0169700	-1.027	-0.191	0.836	low
CK-0d	RHSIM_Rhsim03G0245200	-1.390	-0.337	1.053	low
CK-0d	RHSIM_Rhsim03G0009900	0.412	-0.270	-0.682	no
CK-0d	RHSIM_Rhsim04G0157200	-0.274	-0.612	-0.338	no
CK-0d	RHSIM_Rhsim04G0214000	-0.524	-1.258	-0.734	no
CK-0d	RHSIM_Rhsim03G0219300	0.000	-0.803	-0.803	no
CK-0d	RHSIM_Rhsim03G0159800	-0.536	0.437	0.973	no
CK-0d	RHSIM_Rhsim03G0256600	-0.030	-0.579	-0.549	no
CK-0d	RHSIM_Rhsim03G0180900	-2.287	0.300	2.587	low
CK-0d	RHSIM_Rhsim03G0099600	1.193	0.454	-0.739	high
CK-0d	RHSIM_Rhsim03G0240200	0.281	-0.705	-0.985	no
CK-0d	RHSIM_Rhsim03G0131800	-0.790	0.284	1.074	no
CK-0d	RHSIM_Rhsim03G0110300	1.030	0.680	-0.351	no
CK-0d	RHSIM_Rhsim04G0176900	0.941	1.544	0.604	no
CK-0d	RHSIM_Rhsim03G0100800	0.299	-0.321	-0.619	no
CK-0d	RHSIM_Rhsim03G0069400	0.543	0.063	-0.480	no
CK-0d	RHSIM_Rhsim03G0105700	-0.862	-0.756	0.106	no
CK-0d	RHSIM_Rhsim03G0027100	0.065	-0.551	-0.616	no
CK-0d	RHSIM_Rhsim03G0074700	-1.576	-1.308	0.268	low
CK-0d	RHSIM_Rhsim03G0136400	0.045	0.164	0.118	no
CK-0d	RHSIM_Rhsim04G0147900	0.020	0.149	0.130	no
CK-0d	RHSIM_Rhsim03G0075100	0.811	-0.350	-1.161	no
CK-0d	RHSIM_Rhsim03G0141300	-0.198	-0.016	0.181	no
CK-0d	RHSIM_Rhsim03G0262500	-1.235	-0.498	0.737	low

Group	Gene	PTR (z-score difference)	z(log2 LFQ)	z(log2 FPKM)	extreme-PTR
CK-0d	RHSIM_Rhsim04G0151800	1.021	0.373	-0.647	no
CK-0d	RHSIM_Rhsim04G0068400	-0.737	0.474	1.211	no
CK-0d	RHSIM_Rhsim03G0052700	0.011	-0.527	-0.538	no
CK-0d	RHSIM_Rhsim03G0134500	1.181	1.338	0.157	high
CK-0d	RHSIM_Rhsim03G0021400	0.858	0.344	-0.514	no
CK-0d	RHSIM_Rhsim03G0225200	0.062	0.465	0.403	no
CK-0d	RHSIM_Rhsim04G0071500	0.031	0.015	-0.016	no
CK-0d	RHSIM_Rhsim03G0244600	-0.153	0.099	0.253	no
CK-0d	RHSIM_Rhsim05G0115700	-0.140	0.517	0.657	no
CK-0d	RHSIM_Rhsim03G0099500	1.084	-0.994	-2.077	no
CK-0d	RHSIM_Rhsim03G0254100	1.474	1.572	0.098	high
CK-0d	RHSIM_Rhsim04G0081400	0.537	0.385	-0.152	no
CK-0d	RHSIM_Rhsim04G0166700	0.810	0.752	-0.058	no
CK-0d	RHSIM_Rhsim03G0121200	0.480	-0.014	-0.494	no
CK-0d	RHSIM_Rhsim03G0235000	-1.018	0.688	1.706	no
CK-0d	RHSIM_Rhsim03G0146700	-0.849	0.501	1.350	no
CK-0d	RHSIM_Rhsim03G0146600	-0.000	-0.538	-0.538	no
CK-0d	RHSIM_Rhsim03G0073400	0.209	-0.580	-0.789	no
CK-0d	RHSIM_Rhsim04G0206300	2.055	2.705	0.650	high
CK-0d	RHSIM_Rhsim03G0140300	-0.773	-1.761	-0.987	no
CK-0d	RHSIM_Rhsim03G0080300	-1.403	-0.331	1.072	low
CK-0d	RHSIM_Rhsim05G0116200	0.295	-0.163	-0.457	no
CK-0d	RHSIM_Rhsim03G0144700	0.258	-0.231	-0.489	no
CK-0d	RHSIM_Rhsim04G0066500	0.287	-0.421	-0.707	no
CK-0d	RHSIM_Rhsim03G0061100	1.016	-0.605	-1.621	no
CK-0d	RHSIM_Rhsim04G0182600	-1.495	1.166	2.661	low
CK-0d	RHSIM_Rhsim05G0206900	-1.203	-0.319	0.885	low
CK-0d	RHSIM_Rhsim03G0240400	-1.895	-1.263	0.633	low
CK-0d	RHSIM_Rhsim03G0200600	-0.251	-0.229	0.022	no
CK-0d	RHSIM_Rhsim04G0209400	0.319	2.131	1.812	no
CK-0d	RHSIM_Rhsim03G0223200	-0.051	0.032	0.084	no
CK-0d	RHSIM_Rhsim03G0237400	0.324	1.847	1.523	no

Group	Gene	PTR (z-score difference)	z(log2 LFQ)	z(log2 FPKM)	extreme-PTR
CK-0d	RHSIM_Rhsim04G0012000	-0.749	-0.413	0.336	no
CK-0d	RHSIM_Rhsim03G0179100	-0.737	-0.963	-0.226	no
CK-0d	RHSIM_Rhsim03G0187200	0.824	0.781	-0.043	no
CK-0d	RHSIM_Rhsim04G0160900	0.563	-1.583	-2.146	no
CK-0d	RHSIM_Rhsim03G0058100	-0.186	-0.955	-0.769	no
CK-0d	RHSIM_Rhsim03G0244700	1.393	2.009	0.616	high
CK-0d	RHSIM_Rhsim03G0256500	0.367	1.788	1.421	no
CK-0d	RHSIM_Rhsim03G0214800	-0.994	1.300	2.295	no
CK-0d	RHSIM_Rhsim03G0034500	-0.195	-1.275	-1.080	no
CK-0d	RHSIM_Rhsim03G0240800	0.563	0.887	0.324	no
CK-0d	RHSIM_Rhsim05G0193600	0.294	-0.366	-0.660	no
CK-0d	RHSIM_Rhsim04G0086300	0.569	-0.355	-0.924	no
CK-0d	RHSIM_Rhsim05G0216200	-1.128	-0.656	0.472	low
CK-0d	RHSIM_Rhsim03G0258800	0.366	-0.427	-0.793	no
CK-0d	RHSIM_Rhsim03G0231700	-1.133	-1.505	-0.372	low
CK-0d	RHSIM_Rhsim03G0035400	1.508	-0.498	-2.006	high
CK-0d	RHSIM_Rhsim04G0232000	0.552	0.791	0.240	no
CK-0d	RHSIM_Rhsim05G0067500	0.423	0.763	0.340	no
CK-0d	RHSIM_Rhsim03G0150600	1.185	-0.657	-1.842	high
CK-0d	RHSIM_Rhsim04G0179900	0.202	-0.625	-0.827	no
CK-0d	RHSIM_Rhsim03G0062200	1.313	0.082	-1.230	high
CK-0d	RHSIM_Rhsim04G0075400	-0.866	-1.266	-0.400	no
CK-0d	RHSIM_Rhsim03G0092400	0.956	0.973	0.017	no
CK-0d	RHSIM_Rhsim03G0236300	-0.026	-1.055	-1.029	no
CK-0d	RHSIM_Rhsim03G0264300	0.977	0.483	-0.494	no
CK-0d	RHSIM_Rhsim03G0262700	1.555	1.375	-0.180	high
CK-0d	RHSIM_Rhsim03G0154600	-0.711	-0.886	-0.176	no
CK-0d	RHSIM_Rhsim03G0073300	0.546	-0.902	-1.448	no
CK-0d	RHSIM_Rhsim03G0208100	0.433	0.757	0.324	no
CK-0d	RHSIM_Rhsim05G0101900	-0.930	-0.933	-0.003	no
CK-0d	RHSIM_Rhsim03G0230900	0.266	0.028	-0.237	no
CK-0d	RHSIM_Rhsim04G0197400	-0.880	0.639	1.519	no

Group	Gene	PTR (z-score difference)	z(log2 LFQ)	z(log2 FPKM)	extreme-PTR
CK-0d	RHSIM_Rhsim05G0042100	0.574	0.068	-0.505	no
CK-0d	RHSIM_Rhsim04G0204300	-0.922	-1.188	-0.267	no
CK-0d	RHSIM_Rhsim03G0078700	1.133	-0.132	-1.265	high
CK-0d	RHSIM_Rhsim03G0093900	-0.847	-0.730	0.116	no
CK-0d	RHSIM_Rhsim03G0234500	0.931	1.564	0.633	no
CK-0d	RHSIM_Rhsim05G0098000	-0.257	0.348	0.605	no
CK-0d	RHSIM_Rhsim03G0265300	1.639	0.885	-0.755	high
CK-0d	RHSIM_Rhsim03G0192800	0.424	-0.120	-0.544	no
CK-0d	RHSIM_Rhsim03G0018400	-1.778	-0.824	0.954	low
CK-0d	RHSIM_Rhsim04G0171200	1.014	2.289	1.275	no
CK-0d	RHSIM_Rhsim03G0246200	-1.183	-1.331	-0.148	low
CK-0d	RHSIM_Rhsim04G0024300	0.437	-0.527	-0.965	no
CK-0d	RHSIM_Rhsim03G0176200	1.580	1.052	-0.528	high
CK-0d	RHSIM_Rhsim03G0155500	0.473	-0.076	-0.549	no
CK-0d	RHSIM_Rhsim05G0018500	-0.100	-1.098	-0.999	no
CK-0d	RHSIM_Rhsim03G0192400	0.327	0.854	0.527	no
CK-0d	RHSIM_Rhsim03G0110600	0.299	0.011	-0.288	no
CK-0d	RHSIM_Rhsim05G0214700	-0.952	0.260	1.212	no
CK-0d	RHSIM_Rhsim03G0063000	-0.786	0.475	1.260	no
CK-0d	RHSIM_Rhsim03G0141400	-4.593	-0.923	3.670	low
CK-0d	RHSIM_Rhsim03G0187900	-0.272	-0.946	-0.674	no
CK-0d	RHSIM_Rhsim03G0176300	-0.019	-0.705	-0.686	no
CK-0d	RHSIM_Rhsim03G0145100	0.908	-0.041	-0.950	no
CK-0d	RHSIM_Rhsim03G0208900	-0.469	-0.278	0.191	no
CK-0d	RHSIM_Rhsim03G0119400	0.685	0.941	0.256	no
CK-0d	RHSIM_Rhsim03G0093800	-2.444	-0.664	1.781	low
CK-0d	RHSIM_Rhsim02G0061100	0.156	-0.382	-0.537	no
CK-0d	RHSIM_Rhsim04G0144000	1.120	0.195	-0.925	high
CK-0d	RHSIM_Rhsim03G0059400	1.164	-0.347	-1.511	high
CK-0d	RHSIM_Rhsim03G0123500	-1.868	-1.738	0.131	low
CK-0d	RHSIM_Rhsim03G0083800	-1.838	-1.418	0.419	low
CK-0d	RHSIM_Rhsim03G0101200	-1.295	-1.148	0.146	low

Group	Gene	PTR (z-score difference)	z(log2 LFQ)	z(log2 FPKM)	extreme-PTR
CK-0d	RHSIM_Rhsim03G0188500	0.088	0.661	0.574	no
CK-0d	RHSIM_Rhsim02G0160800	0.016	-0.283	-0.299	no
CK-0d	RHSIM_Rhsim03G0118600	1.072	-0.139	-1.211	no
CK-0d	RHSIM_Rhsim03G0089900	-0.214	-0.601	-0.387	no
CK-0d	RHSIM_Rhsim03G0029800	0.219	0.612	0.393	no
CK-0d	RHSIM_Rhsim03G0088700	0.388	-0.405	-0.792	no
CK-0d	RHSIM_Rhsim04G0145600	-0.663	0.202	0.866	no
CK-0d	RHSIM_Rhsim02G0022400	1.718	0.265	-1.453	high
CK-0d	RHSIM_Rhsim03G0270000	0.035	0.026	-0.009	no
CK-0d	RHSIM_Rhsim03G0049600	0.408	-0.322	-0.729	no
CK-0d	RHSIM_Rhsim03G0130200	-0.360	-0.049	0.311	no
CK-0d	RHSIM_Rhsim05G0108800	0.088	-0.415	-0.503	no
CK-0d	RHSIM_Rhsim03G0086200	2.997	1.662	-1.334	high
CK-0d	RHSIM_Rhsim03G0195300	1.243	-0.296	-1.539	high
CK-0d	RHSIM_Rhsim03G0229600	-0.826	-0.028	0.798	no
CK-0d	RHSIM_Rhsim03G0207200	-0.144	-1.209	-1.066	no
CK-0d	RHSIM_Rhsim03G0231100	-0.115	-0.314	-0.199	no
CK-0d	RHSIM_Rhsim03G0212200	0.428	-0.616	-1.044	no
CK-0d	RHSIM_Rhsim02G0105800	-0.765	-0.803	-0.038	no
CK-0d	RHSIM_Rhsim03G0081700	-0.104	0.116	0.220	no
CK-0d	RHSIM_Rhsim04G0207900	0.576	0.456	-0.120	no
CK-0d	RHSIM_Rhsim03G0090700	0.100	-0.672	-0.772	no
CK-0d	RHSIM_Rhsim04G0168900	-1.097	-1.660	-0.564	low
CK-0d	RHSIM_Rhsim03G0261200	1.571	0.443	-1.128	high
CK-0d	RHSIM_Rhsim03G0075200	-1.385	-0.819	0.566	low
CK-0d	RHSIM_Rhsim03G0231500	-0.106	-1.012	-0.906	no
CK-0d	RHSIM_Rhsim04G0224600	-0.235	0.346	0.582	no
CK-0d	RHSIM_Rhsim02G0015800	0.017	-0.063	-0.080	no
CK-0d	RHSIM_Rhsim02G0060700	-0.909	-1.096	-0.187	no
CK-0d	RHSIM_Rhsim02G0108100	1.037	-0.052	-1.089	no
CK-0d	RHSIM_Rhsim02G0001700	0.072	-0.582	-0.655	no
CK-0d	RHSIM_Rhsim02G0159000	-1.344	-0.730	0.614	low

Group	Gene	PTR (z-score difference)	z(log2 LFQ)	z(log2 FPKM)	extreme-PTR
CK-0d	RHSIM_Rhsim04G0249800	1.150	-1.010	-2.160	high
CK-0d	RHSIM_Rhsim03G0122500	-0.042	0.264	0.306	no
CK-0d	RHSIM_Rhsim05G0165500	0.192	-0.289	-0.481	no
CK-0d	RHSIM_Rhsim03G0085600	1.511	2.099	0.589	high
CK-0d	RHSIM_Rhsim03G0018000	-3.434	-1.611	1.823	low
CK-0d	RHSIM_Rhsim04G0009800	0.780	2.570	1.790	no
CK-0d	RHSIM_Rhsim03G0083300	-1.065	-1.654	-0.589	low
CK-0d	RHSIM_Rhsim03G0219000	-1.119	-0.325	0.794	low
CK-0d	RHSIM_Rhsim03G0238600	1.091	-0.002	-1.093	no
CK-0d	RHSIM_Rhsim02G0120400	-2.461	-1.697	0.764	low
CK-0d	RHSIM_Rhsim03G0196000	2.563	0.612	-1.951	high
CK-0d	RHSIM_Rhsim02G0182400	0.053	-0.546	-0.599	no
CK-0d	RHSIM_Rhsim03G0061800	0.819	0.863	0.044	no
CK-0d	RHSIM_Rhsim03G0234600	-0.259	-1.034	-0.775	no
CK-0d	RHSIM_Rhsim05G0227600	-1.625	-0.514	1.111	low
CK-0d	RHSIM_Rhsim04G0197800	0.390	-1.516	-1.906	no
CK-0d	RHSIM_Rhsim03G0193700	2.057	0.349	-1.708	high
CK-0d	RHSIM_Rhsim03G0225300	-0.837	-0.279	0.558	no
CK-0d	RHSIM_Rhsim03G0267400	-1.544	-1.529	0.014	low
CK-0d	RHSIM_Rhsim04G0226400	-0.079	-1.189	-1.110	no
CK-0d	RHSIM_Rhsim03G0208500	0.321	-0.580	-0.901	no
CK-0d	RHSIM_Rhsim02G0011800	-0.802	-0.114	0.688	no
CK-0d	RHSIM_Rhsim02G0230000	0.511	0.821	0.310	no
CK-0d	RHSIM_Rhsim05G0158900	3.443	1.261	-2.182	high
CK-0d	RHSIM_Rhsim03G0074600	-0.763	-0.564	0.199	no
CK-0d	RHSIM_Rhsim03G0012100	1.547	-0.110	-1.657	high
CK-0d	RHSIM_Rhsim03G0189800	-1.120	0.073	1.193	low
CK-0d	RHSIM_Rhsim03G0082000	1.597	2.793	1.196	high
CK-0d	RHSIM_Rhsim03G0175300	0.040	-0.538	-0.579	no
CK-0d	RHSIM_Rhsim03G0225900	0.350	-0.878	-1.227	no
CK-0d	RHSIM_Rhsim03G0049800	0.219	-0.423	-0.642	no
CK-0d	RHSIM_Rhsim03G0168800	-1.313	0.393	1.706	low

Group	Gene	PTR (z-score difference)	z(log2 LFQ)	z(log2 FPKM)	extreme-PTR
CK-0d	RHSIM_Rhsim03G0200100	0.605	0.008	-0.597	no
CK-0d	RHSIM_Rhsim03G0143400	0.369	-0.690	-1.059	no
CK-0d	RHSIM_Rhsim03G0267700	-0.163	0.681	0.844	no
CK-0d	RHSIM_Rhsim05G0214900	0.334	-0.051	-0.385	no
CK-0d	RHSIM_Rhsim03G0012300	1.059	0.373	-0.686	no
CK-0d	RHSIM_Rhsim03G0152000	0.128	1.230	1.102	no
CK-0d	RHSIM_Rhsim02G0137600	-2.120	-0.453	1.667	low
CK-0d	RHSIM_Rhsim03G0077300	-0.287	-1.183	-0.895	no
CK-0d	RHSIM_Rhsim05G0192800	-0.036	0.353	0.390	no
CK-0d	RHSIM_Rhsim03G0128200	1.073	1.129	0.056	no
CK-0d	RHSIM_Rhsim02G0041500	1.387	3.128	1.742	high
CK-0d	RHSIM_Rhsim03G0052900	-2.155	1.774	3.930	low
CK-0d	RHSIM_Rhsim02G0200900	0.652	0.612	-0.040	no
CK-0d	RHSIM_Rhsim03G0225000	-0.692	-0.535	0.156	no
CK-0d	RHSIM_Rhsim05G0146200	-0.049	0.156	0.206	no
CK-0d	RHSIM_Rhsim03G0159900	-1.161	0.014	1.175	low
CK-0d	RHSIM_Rhsim03G0036100	-1.029	-0.961	0.068	low
CK-0d	RHSIM_Rhsim03G0049500	0.980	0.555	-0.425	no
CK-0d	RHSIM_Rhsim03G0246500	-0.024	-0.281	-0.258	no
CK-0d	RHSIM_Rhsim05G0188400	-1.705	-0.733	0.972	low
CK-0d	RHSIM_Rhsim03G0194300	0.620	-0.652	-1.272	no
CK-0d	RHSIM_Rhsim02G0173300	0.338	-0.680	-1.018	no
CK-0d	RHSIM_Rhsim05G0073400	0.412	1.307	0.895	no
CK-0d	RHSIM_Rhsim03G0238900	0.362	1.680	1.318	no
CK-0d	RHSIM_Rhsim03G0058600	0.253	-0.062	-0.315	no
CK-0d	RHSIM_Rhsim03G0236600	-0.773	-1.010	-0.237	no
CK-0d	RHSIM_Rhsim03G0078200	0.787	-1.248	-2.035	no
CK-0d	RHSIM_Rhsim03G0098400	0.881	1.712	0.831	no
CK-0d	RHSIM_Rhsim03G0058700	0.252	0.125	-0.127	no
CK-0d	RHSIM_Rhsim03G0170100	-1.122	0.014	1.136	low
CK-0d	RHSIM_Rhsim03G0044600	0.153	-0.189	-0.342	no
CK-0d	RHSIM_Rhsim02G0251600	0.542	0.092	-0.450	no

Group	Gene	PTR (z-score difference)	z(log2 LFQ)	z(log2 FPKM)	extreme-PTR
CK-0d	RHSIM_Rhsim02G0203500	0.633	-0.090	-0.723	no
CK-0d	RHSIM_Rhsim03G0216000	-0.478	-0.353	0.125	no
CK-0d	RHSIM_Rhsim03G0087900	-0.315	-0.455	-0.139	no
CK-0d	RHSIM_Rhsim03G0222700	0.045	-0.010	-0.056	no
CK-0d	RHSIM_Rhsim05G0040800	0.374	-0.355	-0.729	no
CK-0d	RHSIM_Rhsim02G0024500	1.083	-1.054	-2.136	no
CK-0d	RHSIM_Rhsim03G0134000	-0.429	-0.123	0.305	no
CK-0d	RHSIM_Rhsim03G0218000	-0.690	-0.410	0.279	no
CK-0d	RHSIM_Rhsim03G0261800	0.941	0.506	-0.436	no
CK-0d	RHSIM_Rhsim05G0087000	-0.411	1.074	1.485	no
CK-0d	RHSIM_Rhsim03G0022100	1.600	0.396	-1.204	high
CK-0d	RHSIM_Rhsim03G0218800	1.201	-0.028	-1.229	high
CK-0d	RHSIM_Rhsim03G0221200	2.078	0.378	-1.700	high
CK-0d	RHSIM_Rhsim02G0083700	-0.132	-0.700	-0.568	no
CK-0d	RHSIM_Rhsim03G0150900	-1.657	-0.650	1.007	low
CK-0d	RHSIM_Rhsim03G0217000	-1.508	-0.098	1.410	low
CK-0d	RHSIM_Rhsim03G0255200	-1.411	-0.484	0.927	low
CK-0d	RHSIM_Rhsim03G0172900	-1.203	1.034	2.237	low
CK-0d	RHSIM_Rhsim03G0136600	1.238	1.915	0.677	high
CK-0d	RHSIM_Rhsim05G0200700	0.429	0.027	-0.402	no
CK-0d	RHSIM_Rhsim03G0143900	0.601	-0.901	-1.502	no
CK-0d	RHSIM_Rhsim03G0078900	-0.619	-0.910	-0.290	no
CK-0d	RHSIM_Rhsim05G0119800	0.576	0.369	-0.207	no
CK-0d	RHSIM_Rhsim02G0212300	1.086	2.125	1.039	no
CK-0d	RHSIM_Rhsim02G0132300	-0.373	1.482	1.855	no
CK-0d	RHSIM_Rhsim03G0216300	-0.719	1.443	2.163	no
CK-0d	RHSIM_Rhsim02G0157500	-1.371	-1.102	0.269	low
CK-0d	RHSIM_Rhsim03G0127200	-1.305	-0.822	0.482	low
CK-0d	RHSIM_Rhsim03G0031000	0.257	-0.632	-0.889	no
CK-0d	RHSIM_Rhsim03G0172800	-0.255	-0.358	-0.104	no
CK-0d	RHSIM_Rhsim03G0149000	1.896	1.257	-0.638	high
CK-0d	RHSIM_Rhsim02G0105600	-1.085	0.003	1.088	low

Group	Gene	PTR (z-score difference)	z(log2 LFQ)	z(log2 FPKM)	extreme-PTR
CK-0d	RHSIM_Rhsim03G0208700	-0.134	-0.202	-0.068	no
CK-0d	RHSIM_Rhsim02G0251800	1.354	0.814	-0.540	high
CK-0d	RHSIM_Rhsim02G0007300	0.159	0.610	0.451	no
CK-0d	RHSIM_Rhsim05G0049100	-0.972	-1.653	-0.682	no
CK-0d	RHSIM_Rhsim02G0243400	-1.491	-1.414	0.077	low
CK-0d	RHSIM_Rhsim03G0158800	-0.903	-0.344	0.559	no
CK-0d	RHSIM_Rhsim02G0056700	1.137	1.205	0.068	high
CK-0d	RHSIM_Rhsim05G0228200	-0.385	-0.784	-0.399	no
CK-0d	RHSIM_Rhsim02G0179000	-0.739	-1.374	-0.635	no
CK-0d	RHSIM_Rhsim02G0220800	0.928	-0.342	-1.270	no
CK-0d	RHSIM_Rhsim03G0174400	0.662	1.763	1.101	no
CK-0d	RHSIM_Rhsim03G0114800	-1.376	-0.278	1.098	low
CK-0d	RHSIM_Rhsim03G0266500	0.736	0.678	-0.059	no
CK-0d	RHSIM_Rhsim03G0092100	1.133	0.687	-0.446	high
CK-0d	RHSIM_Rhsim02G0180400	-0.202	-1.333	-1.131	no
CK-0d	RHSIM_Rhsim03G0145000	0.898	0.244	-0.653	no
CK-0d	RHSIM_Rhsim02G0199800	0.165	0.355	0.190	no
CK-0d	RHSIM_Rhsim03G0169200	0.704	0.311	-0.393	no
CK-0d	RHSIM_Rhsim04G0217900	-1.920	-1.172	0.748	low
CK-0d	RHSIM_Rhsim03G0159700	-0.591	0.349	0.940	no
CK-0d	RHSIM_Rhsim02G0146400	-0.638	0.229	0.867	no
CK-0d	RHSIM_Rhsim02G0090300	-1.420	-0.344	1.077	low
CK-0d	RHSIM_Rhsim03G0163000	-0.700	-0.420	0.280	no
CK-0d	RHSIM_Rhsim02G0229000	-1.234	-0.215	1.019	low
CK-0d	RHSIM_Rhsim04G0032000	1.849	4.248	2.399	high
CK-0d	RHSIM_Rhsim03G0262600	0.106	0.138	0.032	no
CK-0d	RHSIM_Rhsim02G0044500	-1.257	-0.733	0.524	low
CK-0d	RHSIM_Rhsim04G0167100	1.651	2.620	0.969	high
CK-0d	RHSIM_Rhsim02G0059100	-0.491	1.496	1.986	no
CK-0d	RHSIM_Rhsim02G0084900	-0.303	0.038	0.341	no
CK-0d	RHSIM_Rhsim02G0042700	-1.554	0.480	2.034	low
CK-0d	RHSIM_Rhsim03G0228600	-2.065	0.010	2.075	low

Group	Gene	PTR (z-score difference)	z(log2 LFQ)	z(log2 FPKM)	extreme-PTR
CK-0d	RHSIM_Rhsim02G0082300	-0.554	-1.469	-0.915	no
CK-0d	RHSIM_Rhsim03G0170400	1.665	-0.650	-2.315	high
CK-0d	RHSIM_Rhsim02G0036100	0.524	0.007	-0.517	no
CK-0d	RHSIM_Rhsim04G0038900	-0.840	-0.608	0.232	no
CK-0d	RHSIM_Rhsim02G0103000	-0.712	-0.606	0.106	no
CK-0d	RHSIM_Rhsim02G0244000	-2.861	-2.224	0.638	low
CK-0d	RHSIM_Rhsim02G0014100	0.248	0.336	0.088	no
CK-0d	RHSIM_Rhsim03G0127300	0.144	1.478	1.334	no
CK-0d	RHSIM_Rhsim03G0078300	1.285	2.606	1.322	high
CK-0d	RHSIM_Rhsim02G0220000	2.712	1.377	-1.335	high
CK-0d	RHSIM_Rhsim03G0189100	-0.203	-1.332	-1.130	no
CK-0d	RHSIM_Rhsim02G0136400	0.898	2.059	1.162	no
CK-0d	RHSIM_Rhsim03G0057300	0.125	-0.857	-0.982	no
CK-0d	RHSIM_Rhsim02G0012500	-0.523	-1.361	-0.838	no
CK-0d	RHSIM_Rhsim02G0241700	1.324	0.670	-0.653	high
CK-0d	RHSIM_Rhsim03G0046400	-0.499	-0.767	-0.268	no
CK-0d	RHSIM_Rhsim03G0258700	0.024	-1.002	-1.026	no
CK-0d	RHSIM_Rhsim03G0265400	0.364	0.967	0.603	no
CK-0d	RHSIM_Rhsim02G0086200	1.828	0.029	-1.799	high
CK-0d	RHSIM_Rhsim02G0255200	1.271	-0.629	-1.900	high
CK-0d	RHSIM_Rhsim02G0254500	0.948	1.541	0.593	no
CK-0d	RHSIM_Rhsim04G0232600	-0.423	-1.144	-0.721	no
CK-0d	RHSIM_Rhsim02G0173200	-1.075	-0.582	0.492	low
CK-0d	RHSIM_Rhsim03G0116200	1.352	0.528	-0.824	high
CK-0d	RHSIM_Rhsim03G0234900	0.613	-0.500	-1.112	no
CK-0d	RHSIM_Rhsim02G0060400	-1.011	-1.172	-0.161	no
CK-0d	RHSIM_Rhsim03G0151900	-0.798	-0.590	0.208	no
CK-0d	RHSIM_Rhsim02G0140300	0.083	1.398	1.315	no
CK-0d	RHSIM_Rhsim03G0000600	0.615	0.499	-0.117	no
CK-0d	RHSIM_Rhsim02G0216000	-0.908	-1.209	-0.301	no
CK-0d	RHSIM_Rhsim02G0213000	0.717	-1.010	-1.727	no
CK-0d	RHSIM_Rhsim04G0247900	-1.096	-0.245	0.851	low

Group	Gene	PTR (z-score difference)	z(log2 LFQ)	z(log2 FPKM)	extreme-PTR
CK-0d	RHSIM_Rhsim02G0097500	-0.898	-1.937	-1.039	no
CK-0d	RHSIM_Rhsim03G0002000	0.135	0.071	-0.064	no
CK-0d	RHSIM_Rhsim02G0239800	-0.120	-1.392	-1.272	no
CK-0d	RHSIM_Rhsim02G0008500	-0.822	2.122	2.944	no
CK-0d	RHSIM_Rhsim02G0009100	0.370	1.033	0.663	no
CK-0d	RHSIM_Rhsim02G0019700	0.474	-0.387	-0.862	no
CK-0d	RHSIM_Rhsim02G0255500	0.988	0.757	-0.231	no
CK-0d	RHSIM_Rhsim03G0232800	-0.054	-0.596	-0.543	no
CK-0d	RHSIM_Rhsim02G0023300	1.221	0.389	-0.832	high
CK-0d	RHSIM_Rhsim03G0264100	1.609	1.421	-0.188	high
CK-0d	RHSIM_Rhsim02G0043700	0.242	0.074	-0.168	no
CK-0d	RHSIM_Rhsim03G0259400	-0.369	0.877	1.246	no
CK-0d	RHSIM_Rhsim02G0088500	0.663	0.218	-0.444	no
CK-0d	RHSIM_Rhsim04G0016400	0.826	1.117	0.291	no
CK-0d	RHSIM_Rhsim02G0137400	-0.613	-1.503	-0.890	no
CK-0d	RHSIM_Rhsim02G0006600	0.310	-0.264	-0.574	no
CK-0d	RHSIM_Rhsim02G0013600	0.474	-0.470	-0.944	no
CK-0d	RHSIM_Rhsim03G0137100	-1.425	-2.032	-0.607	low
CK-0d	RHSIM_Rhsim04G0172100	-2.431	-1.496	0.935	low
CK-0d	RHSIM_Rhsim02G0254400	-0.655	0.211	0.866	no
CK-0d	RHSIM_Rhsim02G0053400	-0.287	0.797	1.084	no
CK-0d	RHSIM_Rhsim02G0081400	3.302	1.199	-2.103	high
CK-0d	RHSIM_Rhsim03G0136800	0.198	-0.726	-0.924	no
CK-0d	RHSIM_Rhsim02G0068600	3.851	1.725	-2.126	high
CK-0d	RHSIM_Rhsim03G0124200	1.430	0.073	-1.357	high
CK-0d	RHSIM_Rhsim03G0141800	-1.246	-0.138	1.108	low
CK-0d	RHSIM_Rhsim03G0010600	-0.742	-0.757	-0.015	no
CK-0d	RHSIM_Rhsim03G0120800	1.407	1.366	-0.041	high
CK-0d	RHSIM_Rhsim02G0244400	0.589	1.426	0.838	no
CK-0d	RHSIM_Rhsim02G0014600	-0.528	-0.419	0.109	no
CK-0d	RHSIM_Rhsim04G0161900	1.722	1.048	-0.674	high
CK-0d	RHSIM_Rhsim02G0023100	2.920	1.270	-1.650	high

Group	Gene	PTR (z-score difference)	z(log2 LFQ)	z(log2 FPKM)	extreme-PTR
CK-0d	RHSIM_Rhsim03G0011600	0.017	-0.666	-0.683	no
CK-0d	RHSIM_Rhsim02G0066700	1.139	-0.325	-1.463	high
CK-0d	RHSIM_Rhsim03G0075600	-0.733	-0.477	0.257	no
CK-0d	RHSIM_Rhsim02G0089200	-0.553	-0.542	0.011	no
CK-0d	RHSIM_Rhsim04G0239100	0.496	-0.282	-0.777	no
CK-0d	RHSIM_Rhsim02G0216100	-0.507	-0.952	-0.444	no
CK-0d	RHSIM_Rhsim02G0126300	1.106	1.176	0.069	no
CK-0d	RHSIM_Rhsim02G0025700	0.459	0.158	-0.301	no
CK-0d	RHSIM_Rhsim04G0138800	-2.024	-1.469	0.556	low
CK-0d	RHSIM_Rhsim03G0201600	0.196	-0.789	-0.985	no
CK-0d	RHSIM_Rhsim02G0106500	0.862	-0.866	-1.728	no
CK-0d	RHSIM_Rhsim04G0002700	0.877	0.597	-0.280	no
CK-0d	RHSIM_Rhsim03G0150500	1.629	-0.006	-1.635	high
CK-0d	RHSIM_Rhsim02G0181700	-0.438	-0.313	0.126	no
CK-0d	RHSIM_Rhsim04G0106800	0.003	-0.442	-0.445	no
CK-0d	RHSIM_Rhsim02G0138500	0.291	0.145	-0.146	no
CK-0d	RHSIM_Rhsim02G0070200	-0.078	-0.395	-0.317	no
CK-0d	RHSIM_Rhsim02G0119300	-0.164	0.613	0.776	no
CK-0d	RHSIM_Rhsim02G0111900	0.453	0.681	0.228	no
CK-0d	RHSIM_Rhsim02G0254100	1.087	0.054	-1.033	no
CK-0d	RHSIM_Rhsim03G0209200	0.219	0.329	0.109	no
CK-0d	RHSIM_Rhsim03G0089600	0.839	0.026	-0.813	no
CK-0d	RHSIM_Rhsim02G0215900	-0.142	-0.176	-0.033	no
CK-0d	RHSIM_Rhsim02G0001500	0.887	2.195	1.308	no
CK-0d	RHSIM_Rhsim03G0258200	0.381	1.243	0.862	no
CK-0d	RHSIM_Rhsim02G0233100	0.795	1.839	1.044	no
CK-0d	RHSIM_Rhsim02G0154700	1.231	0.693	-0.538	high
CK-0d	RHSIM_Rhsim02G0244500	-2.506	-2.273	0.233	low
CK-0d	RHSIM_Rhsim02G0214700	2.830	0.793	-2.037	high
CK-0d	RHSIM_Rhsim03G0050100	1.367	1.367	-0.000	high
CK-0d	RHSIM_Rhsim02G0214000	0.034	-0.800	-0.834	no
CK-0d	RHSIM_Rhsim02G0086000	-1.238	-2.030	-0.792	low

Group	Gene	PTR (z-score difference)	z(log2 LFQ)	z(log2 FPKM)	extreme-PTR
CK-0d	RHSIM_Rhsim02G0062100	0.339	1.673	1.334	no
CK-0d	RHSIM_Rhsim04G0216800	-1.122	0.693	1.815	low
CK-0d	RHSIM_Rhsim03G0179200	-1.084	-0.906	0.178	low
CK-0d	RHSIM_Rhsim03G0171200	-1.668	-1.577	0.090	low
CK-0d	RHSIM_Rhsim02G0135000	-0.171	1.736	1.907	no
CK-0d	RHSIM_Rhsim02G0083600	-0.749	-0.787	-0.039	no
CK-0d	RHSIM_Rhsim02G0201500	-1.128	-0.232	0.896	low
CK-0d	RHSIM_Rhsim02G0172900	-0.402	-0.785	-0.383	no
CK-0d	RHSIM_Rhsim02G0037100	-1.732	-0.994	0.738	low
CK-0d	RHSIM_Rhsim02G0154900	0.022	0.545	0.523	no
CK-0d	RHSIM_Rhsim03G0123600	-0.804	-0.311	0.493	no
CK-0d	RHSIM_Rhsim02G0179400	0.319	0.350	0.031	no
CK-0d	RHSIM_Rhsim02G0223200	0.285	-0.870	-1.155	no
CK-0d	RHSIM_Rhsim02G0062200	-0.059	-0.285	-0.227	no
CK-0d	RHSIM_Rhsim02G0141000	-0.039	-0.078	-0.038	no
CK-0d	RHSIM_Rhsim03G0214000	-0.315	-0.430	-0.115	no
CK-0d	RHSIM_Rhsim04G0087600	0.897	1.028	0.131	no
CK-0d	RHSIM_Rhsim02G0244300	-1.108	-1.576	-0.468	low
CK-0d	RHSIM_Rhsim02G0000100	0.224	0.176	-0.048	no
CK-0d	RHSIM_Rhsim04G0150200	-1.792	-1.200	0.592	low
CK-0d	RHSIM_Rhsim02G0181200	0.270	0.272	0.002	no
CK-0d	RHSIM_Rhsim02G0231700	-0.388	0.037	0.425	no
CK-0d	RHSIM_Rhsim02G0217900	1.524	0.574	-0.950	high
CK-0d	RHSIM_Rhsim02G0127500	-1.116	-0.515	0.600	low
CK-0d	RHSIM_Rhsim04G0036300	1.973	-0.324	-2.297	high
CK-0d	RHSIM_Rhsim03G0035800	0.621	0.310	-0.311	no
CK-0d	RHSIM_Rhsim02G0056400	-0.440	1.674	2.115	no
CK-0d	RHSIM_Rhsim02G0169200	0.510	-0.376	-0.886	no
CK-0d	RHSIM_Rhsim02G0008900	0.215	-1.119	-1.334	no
CK-0d	RHSIM_Rhsim03G0257200	0.055	0.754	0.699	no
CK-0d	RHSIM_Rhsim02G0140600	0.146	-0.084	-0.230	no
CK-0d	RHSIM_Rhsim02G0187100	1.190	1.546	0.357	high

Group	Gene	PTR (z-score difference)	z(log2 LFQ)	z(log2 FPKM)	extreme-PTR
CK-0d	RHSIM_Rhsim02G0041100	1.951	-0.261	-2.211	high
CK-0d	RHSIM_Rhsim02G0068000	0.291	0.147	-0.144	no
CK-0d	RHSIM_Rhsim02G0239600	0.979	1.064	0.085	no
CK-0d	RHSIM_Rhsim02G0252100	0.391	-1.027	-1.418	no
CK-0d	RHSIM_Rhsim02G0149100	-1.209	0.052	1.261	low
CK-0d	RHSIM_Rhsim04G0022600	0.713	1.339	0.626	no
CK-0d	RHSIM_Rhsim02G0106400	0.394	2.256	1.862	no
CK-0d	RHSIM_Rhsim02G0031400	-0.925	-0.213	0.712	no
CK-0d	RHSIM_Rhsim03G0228400	3.022	2.041	-0.981	high
CK-0d	RHSIM_Rhsim02G0176900	-0.367	-0.385	-0.018	no
CK-0d	RHSIM_Rhsim03G0192600	0.217	1.141	0.923	no
CK-0d	RHSIM_Rhsim02G0144300	-0.302	0.043	0.345	no
CK-0d	RHSIM_Rhsim03G0179700	-1.429	0.045	1.474	low
CK-0d	RHSIM_Rhsim04G0011900	0.596	0.202	-0.394	no
CK-0d	RHSIM_Rhsim02G0099500	-0.190	0.240	0.430	no
CK-0d	RHSIM_Rhsim02G0162600	0.293	1.065	0.772	no
CK-0d	RHSIM_Rhsim02G0055200	-0.994	-0.500	0.494	no
CK-0d	RHSIM_Rhsim02G0104500	-0.067	-0.053	0.013	no
CK-0d	RHSIM_Rhsim03G0224600	0.593	1.497	0.905	no
CK-0d	RHSIM_Rhsim02G0113900	3.121	1.323	-1.798	high
CK-0d	RHSIM_Rhsim01G0236100	-0.705	-0.243	0.463	no
CK-0d	RHSIM_Rhsim04G0247100	0.490	1.370	0.881	no
CK-0d	RHSIM_Rhsim03G0192700	0.941	0.664	-0.277	no
CK-0d	RHSIM_Rhsim01G0001000	0.310	1.313	1.003	no
CK-0d	RHSIM_Rhsim02G0202100	-0.388	2.458	2.846	no
CK-0d	RHSIM_Rhsim02G0164900	-0.665	-1.487	-0.823	no
CK-0d	RHSIM_Rhsim02G0067100	1.088	1.243	0.155	no
CK-0d	RHSIM_Rhsim02G0169800	0.789	0.011	-0.778	no
CK-0d	RHSIM_Rhsim02G0201600	-0.680	-0.498	0.182	no
CK-0d	RHSIM_Rhsim02G0036900	0.207	0.647	0.440	no
CK-0d	RHSIM_Rhsim02G0198800	-0.652	-0.631	0.021	no
CK-0d	RHSIM_Rhsim01G0219200	0.872	0.479	-0.393	no

Group	Gene	PTR (z-score difference)	z(log2 LFQ)	z(log2 FPKM)	extreme-PTR
CK-0d	RHSIM_Rhsim03G0217400	-0.273	1.597	1.870	no
CK-0d	RHSIM_Rhsim01G0232300	-0.014	-0.905	-0.891	no
CK-0d	RHSIM_Rhsim02G0218200	1.487	1.487	-0.000	high
CK-0d	RHSIM_Rhsim01G0254500	-0.978	-0.651	0.327	no
CK-0d	RHSIM_Rhsim02G0069700	-0.847	-1.845	-0.998	no
CK-0d	RHSIM_Rhsim02G0076300	-0.620	-0.024	0.595	no
CK-0d	RHSIM_Rhsim03G0028600	1.072	0.997	-0.075	no
CK-0d	RHSIM_Rhsim02G0035500	-2.260	0.072	2.332	low
CK-0d	RHSIM_Rhsim02G0035300	0.403	-0.099	-0.502	no
CK-0d	RHSIM_Rhsim02G0041900	-0.863	-0.382	0.481	no
CK-0d	RHSIM_Rhsim02G0178300	0.078	-0.595	-0.674	no
CK-0d	RHSIM_Rhsim02G0104000	-0.155	0.288	0.442	no
CK-0d	RHSIM_Rhsim04G0234100	0.903	0.835	-0.068	no
CK-0d	RHSIM_Rhsim03G0135900	-0.269	1.297	1.566	no
CK-0d	RHSIM_Rhsim02G0183000	-0.230	-0.760	-0.529	no
CK-0d	RHSIM_Rhsim02G0177000	0.394	-0.252	-0.646	no
CK-0d	RHSIM_Rhsim02G0176200	1.553	0.308	-1.245	high
CK-0d	RHSIM_Rhsim01G0186900	1.173	0.202	-0.971	high
CK-0d	RHSIM_Rhsim02G0146500	-1.368	-0.801	0.566	low
CK-0d	RHSIM_Rhsim04G0013700	0.868	0.629	-0.239	no
CK-0d	RHSIM_Rhsim01G0245400	-0.309	1.239	1.548	no
CK-0d	RHSIM_Rhsim02G0020900	1.002	0.223	-0.778	no
CK-0d	RHSIM_Rhsim02G0145500	0.968	0.613	-0.355	no
CK-0d	RHSIM_Rhsim02G0251900	-0.421	-0.958	-0.538	no
CK-0d	RHSIM_Rhsim02G0123300	-1.283	-1.226	0.056	low
CK-0d	RHSIM_Rhsim02G0029200	0.804	-1.375	-2.179	no
CK-0d	RHSIM_Rhsim02G0115100	1.233	4.559	3.326	high
CK-0d	RHSIM_Rhsim02G0171500	0.357	0.653	0.296	no
CK-0d	RHSIM_Rhsim02G0070300	1.492	0.461	-1.031	high
CK-0d	RHSIM_Rhsim04G0239600	-0.518	-0.600	-0.082	no
CK-0d	RHSIM_Rhsim01G0225400	-1.252	-1.031	0.221	low
CK-0d	RHSIM_Rhsim02G0014200	0.841	0.054	-0.786	no

Group	Gene	PTR (z-score difference)	z(log2 LFQ)	z(log2 FPKM)	extreme-PTR
CK-0d	RHSIM_Rhsim02G0165800	-0.008	0.364	0.372	no
CK-0d	RHSIM_Rhsim02G0027900	-3.127	-1.246	1.881	low
CK-0d	RHSIM_Rhsim02G0076900	-0.549	-1.399	-0.850	no
CK-0d	RHSIM_Rhsim02G0120700	-1.120	-0.367	0.753	low
CK-0d	RHSIM_Rhsim02G0160300	-1.955	-0.598	1.358	low
CK-0d	RHSIM_Rhsim02G0251500	-3.090	-2.635	0.456	low
CK-0d	RHSIM_Rhsim02G0115500	-0.982	-0.681	0.301	no
CK-0d	RHSIM_Rhsim02G0220400	2.202	1.372	-0.830	high
CK-0d	RHSIM_Rhsim02G0071700	0.685	0.163	-0.522	no
CK-0d	RHSIM_Rhsim02G0159900	0.066	-0.385	-0.452	no
CK-0d	RHSIM_Rhsim02G0165700	-2.085	-0.780	1.306	low
CK-0d	RHSIM_Rhsim04G0100800	-1.520	0.074	1.593	low
CK-0d	RHSIM_Rhsim02G0162800	0.216	-0.800	-1.016	no
CK-0d	RHSIM_Rhsim02G0037200	-1.826	-0.763	1.063	low
CK-0d	RHSIM_Rhsim02G0127600	-0.565	-0.499	0.066	no
CK-0d	RHSIM_Rhsim02G0200700	-1.005	-1.417	-0.412	no
CK-0d	RHSIM_Rhsim03G0210800	0.557	0.857	0.300	no
CK-0d	RHSIM_Rhsim04G0240100	0.696	0.015	-0.681	no
CK-0d	RHSIM_Rhsim02G0057300	-2.624	-0.991	1.633	low
CK-0d	RHSIM_Rhsim03G0230200	0.400	0.146	-0.254	no
CK-0d	RHSIM_Rhsim01G0160900	1.348	1.027	-0.321	high
CK-0d	RHSIM_Rhsim01G0235400	0.390	1.358	0.967	no
CK-0d	RHSIM_Rhsim02G0188300	1.145	0.661	-0.484	high
CK-0d	RHSIM_Rhsim02G0090500	-0.840	-0.979	-0.139	no
CK-0d	RHSIM_Rhsim02G0027800	-0.543	-0.698	-0.155	no
CK-0d	RHSIM_Rhsim02G0027600	0.742	-0.662	-1.404	no
CK-0d	RHSIM_Rhsim01G0230500	-2.634	-1.695	0.940	low
CK-0d	RHSIM_Rhsim02G0021200	1.342	2.069	0.728	high
CK-0d	RHSIM_Rhsim03G0056900	0.732	-0.874	-1.606	no
CK-0d	RHSIM_Rhsim02G0032400	0.405	1.792	1.387	no
CK-0d	RHSIM_Rhsim01G0112900	-2.105	-1.726	0.379	low
CK-0d	RHSIM_Rhsim02G0160100	-0.311	-0.485	-0.174	no

Group	Gene	PTR (z-score difference)	z(log2 LFQ)	z(log2 FPKM)	extreme-PTR
CK-0d	RHSIM_Rhsim01G0217300	0.861	0.612	-0.250	no
CK-0d	RHSIM_Rhsim01G0171700	-0.688	-0.624	0.064	no
CK-0d	RHSIM_Rhsim01G0145800	0.925	0.837	-0.088	no
CK-0d	RHSIM_Rhsim01G0245700	-0.207	-0.767	-0.560	no
CK-0d	RHSIM_Rhsim01G0142200	-0.810	-1.176	-0.365	no
CK-0d	RHSIM_Rhsim02G0168400	-0.554	-0.695	-0.142	no
CK-0d	RHSIM_Rhsim01G0268000	-0.059	-0.819	-0.759	no
CK-0d	RHSIM_Rhsim04G0227400	-1.207	-0.815	0.392	low
CK-0d	RHSIM_Rhsim02G0234500	-1.211	-1.170	0.042	low
CK-0d	RHSIM_Rhsim01G0249200	-0.370	-1.481	-1.112	no
CK-0d	RHSIM_Rhsim01G0069300	0.816	0.234	-0.582	no
CK-0d	RHSIM_Rhsim03G0130400	-0.122	0.376	0.498	no
CK-0d	RHSIM_Rhsim01G0268500	-0.004	-1.076	-1.072	no
CK-0d	RHSIM_Rhsim01G0197500	0.276	0.071	-0.205	no
CK-0d	RHSIM_Rhsim01G0186400	2.239	2.301	0.062	high
CK-0d	RHSIM_Rhsim02G0082500	0.298	0.425	0.127	no
CK-0d	RHSIM_Rhsim02G0072000	0.726	-0.018	-0.744	no
CK-0d	RHSIM_Rhsim01G0280600	-1.096	-0.774	0.322	low
CK-0d	RHSIM_Rhsim04G0110500	0.501	-1.108	-1.609	no
CK-0d	RHSIM_Rhsim01G0002200	0.178	0.162	-0.016	no
CK-0d	RHSIM_Rhsim02G0088100	0.701	1.190	0.489	no
CK-0d	RHSIM_Rhsim04G0172200	0.293	2.063	1.770	no
CK-0d	RHSIM_Rhsim02G0013700	-0.658	-1.270	-0.612	no
CK-0d	RHSIM_Rhsim01G0293800	0.924	1.749	0.825	no
CK-0d	RHSIM_Rhsim01G0263600	0.647	0.122	-0.525	no
CK-0d	RHSIM_Rhsim04G0224200	-0.493	-0.299	0.194	no
CK-0d	RHSIM_Rhsim01G0245600	-3.080	-1.036	2.043	low
CK-0d	RHSIM_Rhsim02G0203400	0.674	0.604	-0.069	no
CK-0d	RHSIM_Rhsim01G0281900	1.536	-0.269	-1.805	high
CK-0d	RHSIM_Rhsim02G0210900	0.253	0.127	-0.125	no
CK-0d	RHSIM_Rhsim01G0101000	-1.903	-1.417	0.486	low
CK-0d	RHSIM_Rhsim04G0176700	-0.252	0.349	0.601	no

Group	Gene	PTR (z-score difference)	z(log2 LFQ)	z(log2 FPKM)	extreme-PTR
CK-0d	RHSIM_Rhsim02G0062300	1.901	0.913	-0.987	high
CK-0d	RHSIM_Rhsim01G0142500	-0.353	-1.291	-0.938	no
CK-0d	RHSIM_Rhsim02G0061900	-0.327	-1.024	-0.697	no
CK-0d	RHSIM_Rhsim03G0011700	0.654	0.037	-0.617	no
CK-0d	RHSIM_Rhsim01G0218000	0.403	0.315	-0.088	no
CK-0d	RHSIM_Rhsim01G0211400	-0.974	-0.463	0.511	no
CK-0d	RHSIM_Rhsim03G0141500	-0.264	1.752	2.016	no
CK-0d	RHSIM_Rhsim02G0018200	1.699	0.727	-0.972	high
CK-0d	RHSIM_Rhsim02G0243200	2.438	0.152	-2.286	high
CK-0d	RHSIM_Rhsim01G0273700	0.220	-0.228	-0.448	no
CK-0d	RHSIM_Rhsim03G0135800	-0.228	0.067	0.294	no
CK-0d	RHSIM_Rhsim02G0162900	-0.396	0.135	0.531	no
CK-0d	RHSIM_Rhsim01G0207700	0.532	1.316	0.784	no
CK-0d	RHSIM_Rhsim02G0033000	-1.299	-0.004	1.295	low
CK-0d	RHSIM_Rhsim02G0066900	0.712	-1.039	-1.751	no
CK-0d	RHSIM_Rhsim02G0168300	0.394	0.375	-0.018	no
CK-0d	RHSIM_Rhsim04G0000500	0.369	-0.869	-1.237	no
CK-0d	RHSIM_Rhsim02G0169300	-1.410	-0.299	1.111	low
CK-0d	RHSIM_Rhsim02G0099100	-1.365	-1.624	-0.259	low
CK-0d	RHSIM_Rhsim01G0060800	1.106	1.053	-0.053	no
CK-0d	RHSIM_Rhsim02G0087400	1.393	0.667	-0.726	high
CK-0d	RHSIM_Rhsim02G0044000	-0.261	-0.502	-0.241	no
CK-0d	RHSIM_Rhsim01G0290800	-0.536	2.911	3.447	no
CK-0d	RHSIM_Rhsim02G0172400	-0.105	-0.168	-0.063	no
CK-0d	RHSIM_Rhsim02G0023800	2.019	0.566	-1.453	high
CK-0d	RHSIM_Rhsim02G0054300	-0.307	0.110	0.417	no
CK-0d	RHSIM_Rhsim02G0003600	0.056	0.311	0.255	no
CK-0d	RHSIM_Rhsim02G0202300	-0.002	-0.210	-0.208	no
CK-0d	RHSIM_Rhsim04G0128500	0.096	0.059	-0.037	no
CK-0d	RHSIM_Rhsim01G0100400	-0.078	-0.862	-0.784	no
CK-0d	RHSIM_Rhsim02G0106700	-1.222	-1.307	-0.085	low
CK-0d	RHSIM_Rhsim01G0182300	0.178	-0.857	-1.036	no

Group	Gene	PTR (z-score difference)	z(log2 LFQ)	z(log2 FPKM)	extreme-PTR
CK-0d	RHSIM_Rhsim02G0195900	-0.371	-1.186	-0.815	no
CK-0d	RHSIM_Rhsim02G0190100	-0.543	-0.226	0.317	no
CK-0d	RHSIM_Rhsim04G0093900	0.949	0.712	-0.237	no
CK-0d	RHSIM_Rhsim02G0067000	-1.516	-0.196	1.321	low
CK-0d	RHSIM_Rhsim02G0247000	1.511	0.291	-1.220	high
CK-0d	RHSIM_Rhsim02G0017000	-0.763	-0.879	-0.116	no
CK-0d	RHSIM_Rhsim02G0213200	-0.135	-0.959	-0.824	no
CK-0d	RHSIM_Rhsim01G0219000	-0.783	-0.711	0.072	no
CK-0d	RHSIM_Rhsim01G0176200	0.049	-1.122	-1.172	no
CK-0d	RHSIM_Rhsim02G0052200	-0.301	-0.183	0.118	no
CK-0d	RHSIM_Rhsim02G0034800	0.977	-0.141	-1.118	no
CK-0d	RHSIM_Rhsim02G0214900	1.073	0.233	-0.840	no
CK-0d	RHSIM_Rhsim01G0243700	0.011	-0.160	-0.171	no
CK-0d	RHSIM_Rhsim02G0110000	-0.225	0.047	0.273	no
CK-0d	RHSIM_Rhsim02G0180500	-1.102	-0.239	0.863	low
CK-0d	RHSIM_Rhsim04G0204400	-1.035	-0.900	0.135	low
CK-0d	RHSIM_Rhsim02G0198400	1.169	-0.255	-1.424	high
CK-0d	RHSIM_Rhsim01G0102000	1.549	-0.523	-2.072	high
CK-0d	RHSIM_Rhsim01G0096100	-0.262	-0.852	-0.590	no
CK-0d	RHSIM_Rhsim01G0136000	0.347	0.693	0.346	no
CK-0d	RHSIM_Rhsim02G0002800	0.006	-0.128	-0.134	no
CK-0d	RHSIM_Rhsim01G0197200	1.092	1.947	0.855	no
CK-0d	RHSIM_Rhsim01G0090900	0.030	2.871	2.842	no
CK-0d	RHSIM_Rhsim01G0095300	0.529	-0.222	-0.751	no
CK-0d	RHSIM_Rhsim02G0171600	0.324	1.248	0.923	no
CK-0d	RHSIM_Rhsim02G0054100	1.267	0.408	-0.859	high
CK-0d	RHSIM_Rhsim01G0196800	0.868	-0.096	-0.963	no
CK-0d	RHSIM_Rhsim02G0040400	-2.449	-1.987	0.462	low
CK-0d	RHSIM_Rhsim03G0218500	0.438	1.114	0.676	no
CK-0d	RHSIM_Rhsim02G0032200	-1.123	-0.847	0.276	low
CK-0d	RHSIM_Rhsim02G0004400	0.192	1.224	1.033	no
CK-0d	RHSIM_Rhsim02G0009000	0.141	-0.623	-0.764	no

Group	Gene	PTR (z-score difference)	z(log2 LFQ)	z(log2 FPKM)	extreme-PTR
CK-0d	RHSIM_Rhsim02G0179200	1.003	0.814	-0.190	no
CK-0d	RHSIM_Rhsim02G0212500	-0.127	0.194	0.320	no
CK-0d	RHSIM_Rhsim02G0202200	0.519	-0.426	-0.945	no
CK-0d	RHSIM_Rhsim01G0123500	-0.022	-0.755	-0.733	no
CK-0d	RHSIM_Rhsim01G0051700	0.288	-0.026	-0.314	no
CK-0d	RHSIM_Rhsim01G0216700	-0.078	0.300	0.378	no
CK-0d	RHSIM_Rhsim01G0151600	-1.238	-1.650	-0.412	low
CK-0d	RHSIM_Rhsim01G0107900	0.696	0.451	-0.245	no
CK-0d	RHSIM_Rhsim03G0170800	0.326	1.375	1.049	no
CK-0d	RHSIM_Rhsim01G0209500	-0.539	1.351	1.890	no
CK-0d	RHSIM_Rhsim01G0259700	0.923	-0.738	-1.661	no
CK-0d	RHSIM_Rhsim02G0194300	0.199	1.870	1.671	no
CK-0d	RHSIM_Rhsim02G0069300	-0.059	-0.170	-0.111	no
CK-0d	RHSIM_Rhsim01G0293700	0.790	-0.277	-1.067	no
CK-0d	RHSIM_Rhsim03G0144200	0.184	0.072	-0.111	no
CK-0d	RHSIM_Rhsim01G0265100	1.015	0.535	-0.480	no
CK-0d	RHSIM_Rhsim01G0291100	0.672	1.293	0.621	no
CK-0d	RHSIM_Rhsim02G0232700	0.138	0.260	0.122	no
CK-0d	RHSIM_Rhsim01G0250300	1.446	-0.489	-1.935	high
CK-0d	RHSIM_Rhsim02G0228400	-0.085	0.243	0.328	no
CK-0d	RHSIM_Rhsim01G0146900	-0.872	-1.033	-0.161	no
CK-0d	RHSIM_Rhsim01G0124300	0.327	-0.444	-0.771	no
CK-0d	RHSIM_Rhsim01G0067700	-2.892	-1.926	0.966	low
CK-0d	RHSIM_Rhsim01G0119100	0.403	-0.875	-1.277	no
CK-0d	RHSIM_Rhsim02G0095200	0.276	-0.884	-1.161	no
CK-0d	RHSIM_Rhsim02G0223500	0.823	-0.898	-1.721	no
CK-0d	RHSIM_Rhsim01G0292800	0.972	1.061	0.089	no
CK-0d	RHSIM_Rhsim03G0247900	2.312	1.164	-1.148	high
CK-0d	RHSIM_Rhsim01G0282100	-0.810	-1.639	-0.829	no
CK-0d	RHSIM_Rhsim01G0189100	-1.740	-1.468	0.272	low
CK-0d	RHSIM_Rhsim02G0216700	-0.323	-0.540	-0.218	no
CK-0d	RHSIM_Rhsim01G0224200	1.467	0.422	-1.045	high

Group	Gene	PTR (z-score difference)	z(log2 LFQ)	z(log2 FPKM)	extreme-PTR
CK-0d	RHSIM_Rhsim03G0036700	-0.157	0.296	0.453	no
CK-0d	RHSIM_Rhsim01G0191800	-1.486	-0.446	1.040	low
CK-0d	RHSIM_Rhsim01G0210800	-0.263	1.226	1.488	no
CK-0d	RHSIM_Rhsim01G0109600	0.867	-0.652	-1.519	no
CK-0d	RHSIM_Rhsim01G0009900	-2.025	-0.975	1.050	low
CK-0d	RHSIM_Rhsim01G0176300	1.027	-0.572	-1.599	no
CK-0d	RHSIM_Rhsim03G0088500	1.063	-0.113	-1.176	no
CK-0d	RHSIM_Rhsim02G0244800	0.860	0.675	-0.186	no
CK-0d	RHSIM_Rhsim01G0197900	-0.135	-0.330	-0.195	no
CK-0d	RHSIM_Rhsim01G0191100	1.056	2.607	1.551	no
CK-0d	RHSIM_Rhsim02G0205100	-2.072	-1.571	0.501	low
CK-0d	RHSIM_Rhsim01G0201600	-0.464	0.081	0.545	no
CK-0d	RHSIM_Rhsim03G0125000	-1.909	-1.005	0.903	low
CK-0d	RHSIM_Rhsim01G0210200	-1.513	-1.345	0.167	low
CK-0d	RHSIM_Rhsim02G0062000	0.228	1.266	1.038	no
CK-0d	RHSIM_Rhsim01G0255600	0.397	0.107	-0.289	no
CK-0d	RHSIM_Rhsim01G0188400	0.135	0.719	0.584	no
CK-0d	RHSIM_Rhsim03G0142300	2.126	1.109	-1.017	high
CK-0d	RHSIM_Rhsim02G0168200	-0.746	-1.591	-0.846	no
CK-0d	RHSIM_Rhsim01G0143400	-0.241	1.385	1.627	no
CK-0d	RHSIM_Rhsim01G0204700	-0.864	0.032	0.896	no
CK-0d	RHSIM_Rhsim01G0259300	0.681	0.631	-0.050	no
CK-0d	RHSIM_Rhsim01G0091000	-0.385	-0.876	-0.491	no
CK-0d	RHSIM_Rhsim01G0276200	-0.921	-0.702	0.219	no
CK-0d	RHSIM_Rhsim02G0148900	-0.430	-0.313	0.117	no
CK-0d	RHSIM_Rhsim01G0262300	0.052	-0.448	-0.500	no
CK-0d	RHSIM_Rhsim01G0180500	-0.425	-0.616	-0.191	no
CK-0d	RHSIM_Rhsim01G0119800	-0.286	-0.513	-0.227	no
CK-0d	RHSIM_Rhsim01G0010000	0.290	0.488	0.197	no
CK-0d	RHSIM_Rhsim01G0204400	0.226	0.171	-0.055	no
CK-0d	RHSIM_Rhsim01G0190200	0.384	0.460	0.075	no
CK-0d	RHSIM_Rhsim01G0074300	0.007	0.712	0.705	no

Group	Gene	PTR (z-score difference)	z(log2 LFQ)	z(log2 FPKM)	extreme-PTR
CK-0d	RHSIM_Rhsim01G0174600	0.063	-0.082	-0.145	no
CK-0d	RHSIM_Rhsim02G0028600	-1.685	-1.367	0.318	low
CK-0d	RHSIM_Rhsim01G0246700	0.454	-0.013	-0.468	no
CK-0d	RHSIM_Rhsim01G0107800	-2.162	0.023	2.184	low
CK-0d	RHSIM_Rhsim02G0076700	-0.226	-0.312	-0.086	no
CK-0d	RHSIM_Rhsim01G0113600	-0.280	0.529	0.808	no
CK-0d	RHSIM_Rhsim02G0071000	1.085	-0.653	-1.738	no
CK-0d	RHSIM_Rhsim02G0005500	-0.422	-1.580	-1.158	no
CK-0d	RHSIM_Rhsim01G0013000	-2.312	0.006	2.318	low
CK-0d	RHSIM_Rhsim01G0202900	0.611	1.141	0.530	no
CK-0d	RHSIM_Rhsim02G0193400	1.213	1.816	0.603	high
CK-0d	RHSIM_Rhsim01G0198900	-2.469	-1.414	1.055	low
CK-0d	RHSIM_Rhsim01G0233900	0.391	0.606	0.215	no
CK-0d	RHSIM_Rhsim01G0067800	0.150	0.748	0.598	no
CK-0d	RHSIM_Rhsim02G0049400	-0.624	-1.256	-0.632	no
CK-0d	RHSIM_Rhsim01G0137100	1.570	0.149	-1.421	high
CK-0d	RHSIM_Rhsim01G0126900	-0.629	1.281	1.910	no
CK-0d	RHSIM_Rhsim01G0284500	-0.761	-0.071	0.690	no
CK-0d	RHSIM_Rhsim01G0205000	0.305	1.902	1.597	no
CK-0d	RHSIM_Rhsim01G0145200	0.147	0.710	0.564	no
CK-0d	RHSIM_Rhsim02G0040300	-0.710	-0.356	0.354	no
CK-0d	RHSIM_Rhsim01G0005500	-0.681	-0.450	0.231	no
CK-0d	RHSIM_Rhsim01G0237200	0.781	1.007	0.226	no
CK-0d	RHSIM_Rhsim01G0263000	0.217	0.610	0.393	no
CK-0d	RHSIM_Rhsim01G0132300	-0.742	-1.168	-0.426	no
CK-0d	RHSIM_Rhsim01G0257300	-0.500	1.149	1.649	no
CK-0d	RHSIM_Rhsim01G0236600	0.698	-0.797	-1.495	no
CK-0d	RHSIM_Rhsim01G0009500	1.314	-1.013	-2.327	high
CK-0d	RHSIM_Rhsim03G0172200	1.572	0.276	-1.295	high
CK-0d	RHSIM_Rhsim01G0122100	-0.272	0.074	0.346	no
CK-0d	RHSIM_Rhsim02G0204900	0.410	0.037	-0.374	no
CK-0d	RHSIM_Rhsim01G0289700	-0.179	0.447	0.626	no

Group	Gene	PTR (z-score difference)	z(log2 LFQ)	z(log2 FPKM)	extreme-PTR
CK-0d	RHSIM_Rhsim01G0075900	0.407	0.263	-0.144	no
CK-0d	RHSIM_Rhsim03G0210700	-1.751	-0.227	1.523	low
CK-0d	RHSIM_Rhsim01G0136100	0.621	-0.587	-1.208	no
CK-0d	RHSIM_Rhsim02G0076500	-1.313	-0.166	1.147	low
CK-0d	RHSIM_Rhsim02G0021000	-1.311	-1.121	0.190	low
CK-0d	RHSIM_Rhsim03G0074800	-0.421	0.610	1.031	no
CK-0d	RHSIM_Rhsim01G0181100	-1.125	-1.228	-0.103	low
CK-0d	RHSIM_Rhsim01G0062600	-0.312	0.381	0.693	no
CK-0d	RHSIM_Rhsim01G0206500	-0.663	-0.764	-0.102	no
CK-0d	RHSIM_Rhsim01G0285000	-2.642	-2.025	0.617	low
CK-0d	RHSIM_Rhsim01G0185400	0.331	0.670	0.339	no
CK-0d	RHSIM_Rhsim02G0255700	0.090	-1.085	-1.175	no
CK-0d	RHSIM_Rhsim01G0009700	0.179	0.623	0.444	no
CK-0d	RHSIM_Rhsim01G0172000	-1.781	-1.868	-0.087	low
CK-0d	RHSIM_Rhsim01G0272700	-0.423	-0.146	0.277	no
CK-0d	RHSIM_Rhsim01G0054700	0.625	0.621	-0.004	no
CK-0d	RHSIM_Rhsim01G0260300	0.980	1.335	0.355	no
CK-0d	RHSIM_Rhsim02G0114900	-0.056	0.501	0.557	no
CK-0d	RHSIM_Rhsim01G0216300	0.308	0.751	0.443	no
CK-0d	RHSIM_Rhsim02G0231500	0.535	-0.329	-0.864	no
CK-0d	RHSIM_Rhsim01G0201300	0.898	0.754	-0.144	no
CK-0d	RHSIM_Rhsim01G0234600	-0.013	-0.064	-0.050	no
CK-0d	RHSIM_Rhsim03G0250000	-2.087	-0.892	1.195	low
CK-0d	RHSIM_Rhsim03G0251500	-0.074	-0.963	-0.888	no
CK-0d	RHSIM_Rhsim01G0124000	-0.337	-0.744	-0.407	no
CK-0d	RHSIM_Rhsim01G0271700	-0.178	-0.658	-0.480	no
CK-0d	RHSIM_Rhsim02G0010800	-1.364	-0.060	1.304	low
CK-0d	RHSIM_Rhsim01G0042700	-0.604	-1.113	-0.508	no
CK-0d	RHSIM_Rhsim01G0223500	2.128	0.179	-1.948	high
CK-0d	RHSIM_Rhsim01G0203800	1.001	0.766	-0.235	no
CK-0d	RHSIM_Rhsim01G0286700	-0.413	0.043	0.457	no
CK-0d	RHSIM_Rhsim01G0187100	0.380	0.418	0.038	no

Group	Gene	PTR (z-score difference)	z(log2 LFQ)	z(log2 FPKM)	extreme-PTR
CK-0d	RHSIM_Rhsim01G0141800	-0.747	-0.376	0.370	no
CK-0d	RHSIM_Rhsim01G0245800	0.331	0.120	-0.211	no
CK-0d	RHSIM_Rhsim01G0042600	-0.156	-0.445	-0.289	no
CK-0d	RHSIM_Rhsim01G0235600	-0.186	-0.161	0.025	no
CK-0d	RHSIM_Rhsim01G0062000	0.300	0.334	0.034	no
CK-0d	RHSIM_Rhsim03G0059500	-0.938	-1.508	-0.570	no
CK-0d	RHSIM_Rhsim01G0179100	-0.694	-0.354	0.340	no
CK-0d	RHSIM_Rhsim01G0198500	0.345	-0.051	-0.396	no
CK-0d	RHSIM_Rhsim01G0164800	1.201	0.615	-0.585	high
CK-0d	RHSIM_Rhsim01G0293000	-0.647	-0.385	0.262	no
CK-0d	RHSIM_Rhsim01G0060200	-2.358	-2.282	0.076	low
CK-0d	RHSIM_Rhsim01G0009300	-0.657	0.444	1.101	no
CK-0d	RHSIM_Rhsim01G0149900	0.205	-0.558	-0.764	no
CK-0d	RHSIM_Rhsim01G0147300	1.289	0.738	-0.551	high
CK-0d	RHSIM_Rhsim01G0274600	1.086	2.765	1.680	no
CK-0d	RHSIM_Rhsim01G0274400	2.226	2.986	0.760	high
CK-0d	RHSIM_Rhsim03G0226900	0.428	1.435	1.008	no
CK-0d	RHSIM_Rhsim02G0217400	0.149	0.768	0.618	no
CK-0d	RHSIM_Rhsim01G0071400	-1.663	-0.375	1.288	low
CK-0d	RHSIM_Rhsim01G0002300	1.182	0.487	-0.696	high
CK-0d	RHSIM_Rhsim01G0033400	0.540	0.072	-0.469	no
CK-0d	RHSIM_Rhsim01G0269700	1.055	0.197	-0.858	no
CK-0d	RHSIM_Rhsim01G0227900	-0.750	-1.152	-0.402	no
CK-0d	RHSIM_Rhsim01G0254100	-2.935	-0.220	2.714	low
CK-0d	RHSIM_Rhsim01G0074500	-0.972	-1.574	-0.602	no
CK-0d	RHSIM_Rhsim01G0192900	-0.003	-0.735	-0.732	no
CK-0d	RHSIM_Rhsim01G0260900	1.504	0.699	-0.805	high
CK-0d	RHSIM_Rhsim01G0006200	-1.383	-0.988	0.396	low
CK-0d	RHSIM_Rhsim01G0088200	1.122	1.024	-0.098	high
CK-0d	RHSIM_Rhsim01G0285400	0.095	-0.622	-0.718	no
CK-0d	RHSIM_Rhsim01G0155600	-0.200	-0.596	-0.396	no
CK-0d	RHSIM_Rhsim01G0039300	-0.642	-0.270	0.372	no

Group	Gene	PTR (z-score difference)	z(log2 LFQ)	z(log2 FPKM)	extreme-PTR
CK-0d	RHSIM_Rhsim03G0141100	0.166	-0.091	-0.258	no
CK-0d	RHSIM_Rhsim01G0272800	0.071	-0.530	-0.601	no
CK-0d	RHSIM_Rhsim01G0089800	0.689	0.944	0.255	no
CK-0d	RHSIM_Rhsim01G0205100	0.615	0.021	-0.595	no
CK-0d	RHSIM_Rhsim01G0100800	0.568	-0.721	-1.289	no
CK-0d	RHSIM_Rhsim03G0176000	-1.207	-1.093	0.114	low
CK-0d	RHSIM_Rhsim01G0055600	0.364	-0.301	-0.665	no
CK-0d	RHSIM_Rhsim01G0262400	0.612	1.158	0.546	no
CK-0d	RHSIM_Rhsim01G0218100	0.784	0.758	-0.026	no
CK-0d	RHSIM_Rhsim01G0245300	-2.622	-0.055	2.567	low
CK-0d	RHSIM_Rhsim01G0291400	-0.336	0.335	0.671	no
CK-0d	RHSIM_Rhsim01G0049400	-0.494	-0.499	-0.005	no
CK-0d	RHSIM_Rhsim01G0151000	0.172	-0.445	-0.617	no
CK-0d	RHSIM_Rhsim01G0106700	0.881	0.222	-0.659	no
CK-0d	RHSIM_Rhsim01G0196600	-0.931	0.721	1.652	no
CK-0d	RHSIM_Rhsim01G0239900	-0.927	0.053	0.980	no
CK-0d	RHSIM_Rhsim01G0113300	0.890	0.043	-0.847	no
CK-0d	RHSIM_Rhsim01G0009800	0.097	-0.585	-0.683	no
CK-0d	RHSIM_Rhsim01G0283500	0.366	0.399	0.033	no
CK-0d	RHSIM_Rhsim01G0233100	-0.693	-0.507	0.185	no
CK-0d	RHSIM_Rhsim01G0217800	-0.159	-0.363	-0.204	no
CK-0d	RHSIM_Rhsim01G0221600	1.167	3.555	2.388	high
CK-0d	RHSIM_Rhsim01G0028300	-3.634	-0.651	2.983	low
CK-0d	RHSIM_Rhsim02G0020800	-2.272	-0.619	1.653	low
CK-0d	RHSIM_Rhsim01G0058000	1.404	1.022	-0.382	high
CK-0d	RHSIM_Rhsim01G0167000	-0.352	-1.144	-0.792	no
CK-0d	RHSIM_Rhsim01G0187900	-0.776	-1.739	-0.964	no
CK-0d	RHSIM_Rhsim01G0247100	-0.026	-0.373	-0.347	no
CK-0d	RHSIM_Rhsim01G0007800	1.426	0.633	-0.794	high
CK-0d	RHSIM_Rhsim03G0163700	-0.246	-0.280	-0.034	no
CK-0d	RHSIM_Rhsim01G0012100	-0.119	-0.932	-0.813	no
CK-0d	RHSIM_Rhsim01G0242500	-0.951	0.946	1.897	no

Group	Gene	PTR (z-score difference)	z(log2 LFQ)	z(log2 FPKM)	extreme-PTR
CK-0d	RHSIM_Rhsim01G0268900	0.000	-0.327	-0.327	no
CK-0d	RHSIM_Rhsim01G0229100	-0.666	-0.498	0.168	no
CK-0d	RHSIM_Rhsim01G0273800	-1.622	-0.897	0.724	low
CK-0d	RHSIM_Rhsim03G0141200	-1.537	1.294	2.831	low
CK-0d	RHSIM_Rhsim03G0215700	-0.994	-1.122	-0.127	no
CK-0d	RHSIM_Rhsim01G0186700	0.624	-0.166	-0.790	no
CK-0d	RHSIM_Rhsim01G0225700	0.531	0.542	0.011	no
CK-0d	RHSIM_Rhsim03G0149300	0.005	-0.085	-0.091	no
CK-0d	RHSIM_Rhsim01G0071100	1.877	1.575	-0.302	high
CK-0d	RHSIM_Rhsim03G0083700	0.500	-0.281	-0.781	no
CK-0d	RHSIM_Rhsim02G0012600	-0.827	-1.382	-0.556	no
CK-0d	RHSIM_Rhsim01G0117300	-2.907	-1.447	1.460	low
CK-0d	RHSIM_Rhsim01G0176100	-1.692	-0.551	1.141	low
CK-0d	RHSIM_Rhsim02G0158200	-0.064	0.247	0.311	no
CK-0d	RHSIM_Rhsim01G0033200	0.374	0.899	0.525	no
CK-0d	RHSIM_Rhsim01G0089700	0.421	-0.919	-1.340	no
CK-0d	RHSIM_Rhsim01G0245500	-0.435	-0.384	0.051	no
CK-0d	RHSIM_Rhsim01G0073000	2.588	1.323	-1.266	high
CK-0d	RHSIM_Rhsim01G0038600	-1.067	0.193	1.260	low
CK-0d	RHSIM_Rhsim01G0065700	0.359	-0.631	-0.990	no
CK-0d	RHSIM_Rhsim01G0068100	-0.434	-1.081	-0.647	no
CK-0d	RHSIM_Rhsim01G0130400	-0.128	-0.287	-0.159	no
CK-0d	RHSIM_Rhsim01G0196000	-2.618	-1.311	1.307	low
CK-0d	RHSIM_Rhsim03G0040000	0.491	1.804	1.313	no
CK-0d	RHSIM_Rhsim01G0134600	0.312	-0.767	-1.079	no
CK-0d	RHSIM_Rhsim01G0255000	0.410	1.418	1.009	no
CK-0d	RHSIM_Rhsim01G0249300	-0.744	-1.045	-0.301	no
CK-0d	RHSIM_Rhsim03G0197300	0.280	-0.202	-0.482	no
CK-0d	RHSIM_Rhsim01G0118500	-0.255	-1.752	-1.497	no
CK-0d	RHSIM_Rhsim01G0200800	-1.683	-1.596	0.087	low
CK-0d	RHSIM_Rhsim01G0061700	-3.167	-2.244	0.923	low
CK-0d	RHSIM_Rhsim01G0027700	-0.122	0.404	0.525	no

Group	Gene	PTR (z-score difference)	z(log2 LFQ)	z(log2 FPKM)	extreme-PTR
CK-0d	RHSIM_Rhsim01G0026600	-0.668	-1.327	-0.659	no
CK-0d	RHSIM_Rhsim01G0103600	-0.296	-0.445	-0.149	no
CK-0d	RHSIM_Rhsim01G0182400	-0.046	-0.776	-0.730	no
CK-0d	RHSIM_Rhsim01G0289900	-0.154	0.576	0.730	no
CK-0d	RHSIM_Rhsim01G0251300	1.739	1.050	-0.689	high
CK-0d	RHSIM_Rhsim01G0228100	-0.997	-1.150	-0.153	no
CK-0d	RHSIM_Rhsim02G0213300	-0.107	2.129	2.236	no
CK-0d	RHSIM_Rhsim01G0027300	-0.658	0.417	1.075	no
CK-0d	RHSIM_Rhsim01G0208600	-0.788	0.669	1.457	no
CK-0d	RHSIM_Rhsim01G0292900	-0.715	-0.920	-0.205	no
CK-0d	RHSIM_Rhsim01G0178200	-0.163	-0.264	-0.101	no
CK-0d	RHSIM_Rhsim01G0257200	1.198	1.457	0.259	high
CK-0d	RHSIM_Rhsim01G0198000	0.570	0.184	-0.385	no
CK-0d	RHSIM_Rhsim01G0128600	0.093	-0.021	-0.114	no
CK-0d	RHSIM_Rhsim02G0180600	0.598	0.621	0.022	no
CK-0d	RHSIM_Rhsim01G0149400	0.404	-0.985	-1.389	no
CK-0d	RHSIM_Rhsim01G0000400	1.598	1.722	0.124	high
CK-0d	RHSIM_Rhsim01G0184500	1.409	2.007	0.598	high
CK-0d	RHSIM_Rhsim01G0155900	0.025	0.164	0.139	no
CK-0d	RHSIM_Rhsim01G0253200	0.245	-0.226	-0.471	no
CK-0d	RHSIM_Rhsim01G0101300	-0.122	-0.496	-0.374	no
CK-0d	RHSIM_Rhsim01G0033300	2.427	1.697	-0.730	high
CK-0d	RHSIM_Rhsim01G0237100	-0.978	-0.497	0.481	no
CK-0d	RHSIM_Rhsim01G0236300	1.703	-0.318	-2.021	high
CK-0d	RHSIM_Rhsim01G0023500	-1.973	-2.135	-0.162	low
CK-0d	RHSIM_Rhsim01G0116400	0.927	1.076	0.148	no
CK-0d	RHSIM_Rhsim01G0002800	-1.132	-0.549	0.583	low
CK-0d	RHSIM_Rhsim02G0205700	0.428	-0.909	-1.337	no
CK-0d	RHSIM_Rhsim02G0173000	-0.039	0.233	0.271	no
CK-0d	RHSIM_Rhsim03G0004900	0.361	0.847	0.485	no
CK-0d	RHSIM_Rhsim01G0125000	-1.236	-0.423	0.813	low
CK-0d	RHSIM_Rhsim01G0033100	0.589	0.409	-0.179	no

Group	Gene	PTR (z-score difference)	z(log2 LFQ)	z(log2 FPKM)	extreme-PTR
CK-0d	RHSIM_Rhsim01G0002900	0.013	-0.001	-0.014	no
CK-0d	RHSIM_Rhsim02G0037400	1.398	2.564	1.167	high
CK-0d	RHSIM_Rhsim01G0275800	0.798	1.073	0.276	no
CK-0d	RHSIM_Rhsim01G0191600	1.137	1.519	0.382	high
CK-0d	RHSIM_Rhsim01G0125800	0.026	0.765	0.740	no
CK-0d	RHSIM_Rhsim01G0067100	-0.204	-0.779	-0.575	no
CK-0d	RHSIM_Rhsim01G0180300	0.045	1.167	1.122	no
CK-0d	RHSIM_Rhsim03G0045300	1.185	1.751	0.566	high
CK-0d	RHSIM_Rhsim01G0274100	-1.317	-1.189	0.128	low
CK-0d	RHSIM_Rhsim01G0257500	-0.073	-0.084	-0.011	no
CK-0d	RHSIM_Rhsim03G0081900	0.567	1.828	1.261	no
CK-0d	RHSIM_Rhsim01G0027600	0.881	-0.743	-1.624	no
CK-0d	RHSIM_Rhsim01G0120500	0.083	1.443	1.360	no
CK-0d	RHSIM_Rhsim01G0224400	-0.149	-0.460	-0.311	no
CK-0d	RHSIM_Rhsim01G0190500	0.299	0.027	-0.272	no
CK-0d	RHSIM_Rhsim01G0255900	-1.094	-0.771	0.323	low
CK-0d	RHSIM_Rhsim03G0221900	-0.226	0.934	1.161	no
CK-0d	RHSIM_Rhsim01G0268200	0.659	1.933	1.274	no
CK-0d	RHSIM_Rhsim01G0006700	-0.495	0.024	0.519	no
CK-0d	RHSIM_Rhsim01G0231000	-0.153	-0.176	-0.023	no
CK-0d	RHSIM_Rhsim03G0079200	1.960	1.224	-0.736	high
CK-0d	RHSIM_Rhsim01G0212100	-0.349	-0.271	0.078	no
CK-0d	RHSIM_Rhsim01G0137400	0.372	-0.119	-0.491	no
CK-0d	RHSIM_Rhsim01G0194700	-0.315	-0.673	-0.358	no
CK-0d	RHSIM_Rhsim01G0250200	0.536	0.019	-0.516	no
CK-0d	RHSIM_Rhsim01G0198200	0.151	0.327	0.176	no
CK-0d	RHSIM_Rhsim01G0246300	-1.525	-1.470	0.055	low
CK-0d	RHSIM_Rhsim01G0122600	-1.530	-1.059	0.471	low
CK-0d	RHSIM_Rhsim01G0254300	-0.396	-0.660	-0.263	no
CK-0d	RHSIM_Rhsim03G0033400	-2.848	-0.737	2.111	low
CK-0d	RHSIM_Rhsim01G0035300	0.456	-1.036	-1.492	no
CK-0d	RHSIM_Rhsim01G0291000	0.777	1.865	1.089	no

Group	Gene	PTR (z-score difference)	z(log2 LFQ)	z(log2 FPKM)	extreme-PTR
CK-0d	RHSIM_Rhsim01G0200700	-0.426	-0.098	0.328	no
CK-0d	RHSIM_Rhsim01G0125200	-1.657	-0.386	1.272	low
CK-0d	RHSIM_Rhsim01G0145500	0.578	-0.423	-1.001	no
CK-0d	RHSIM_Rhsim01G0165300	-0.092	1.041	1.133	no
CK-0d	RHSIM_Rhsim01G0016000	0.525	1.223	0.699	no
CK-0d	RHSIM_Rhsim01G0244900	0.259	-0.076	-0.335	no
CK-0d	RHSIM_Rhsim03G0145600	1.205	0.124	-1.081	high
CK-0d	RHSIM_Rhsim01G0149300	0.375	0.619	0.244	no
CK-0d	RHSIM_Rhsim01G0259900	0.526	0.747	0.220	no
CK-0d	RHSIM_Rhsim03G0063400	0.152	-0.286	-0.437	no
CK-0d	RHSIM_Rhsim01G0238300	0.755	0.269	-0.487	no
CK-0d	RHSIM_Rhsim01G0163300	1.405	1.404	-0.001	high
CK-0d	RHSIM_Rhsim01G0251200	0.365	-0.235	-0.600	no
CK-0d	RHSIM_Rhsim01G0250600	-0.611	-0.893	-0.282	no
CK-0d	RHSIM_Rhsim01G0287300	-1.102	-0.599	0.503	low
CK-0d	RHSIM_Rhsim01G0284700	-0.424	-0.328	0.096	no
CK-0d	RHSIM_Rhsim01G0264000	-1.639	-0.864	0.775	low
CK-0d	RHSIM_Rhsim01G0218900	0.240	0.925	0.685	no
CK-0d	RHSIM_Rhsim01G0217200	-0.244	1.242	1.486	no
CK-0d	RHSIM_Rhsim01G0066600	-0.391	-0.307	0.084	no
CK-0d	RHSIM_Rhsim01G0000300	0.116	-0.474	-0.590	no
CK-0d	RHSIM_Rhsim01G0041200	1.346	0.019	-1.327	high
CK-0d	RHSIM_Rhsim01G0007100	-0.600	-0.779	-0.179	no
CK-0d	RHSIM_Rhsim01G0097100	-0.598	-0.578	0.019	no
CK-0d	RHSIM_Rhsim01G0276700	1.234	1.104	-0.130	high
CK-0d	RHSIM_Rhsim01G0283700	0.374	0.579	0.205	no
CK-0d	RHSIM_Rhsim01G0247500	-0.236	0.076	0.312	no
CK-0d	RHSIM_Rhsim01G0247300	-1.215	0.348	1.563	low
CK-0d	RHSIM_Rhsim01G0192300	-0.380	-0.750	-0.370	no
CK-0d	RHSIM_Rhsim01G0290400	-0.932	-0.016	0.916	no
CK-0d	RHSIM_Rhsim01G0130900	-2.499	-1.987	0.512	low
CK-0d	RHSIM_Rhsim02G0110500	0.467	-0.328	-0.796	no

Group	Gene	PTR (z-score difference)	z(log2 LFQ)	z(log2 FPKM)	extreme-PTR
CK-0d	RHSIM_Rhsim01G0068700	-0.726	0.723	1.449	no
CK-0d	RHSIM_Rhsim01G0040300	0.852	-0.962	-1.815	no
CK-0d	RHSIM_Rhsim01G0145700	0.753	0.146	-0.608	no
CK-0d	RHSIM_Rhsim01G0106500	1.318	0.556	-0.762	high
CK-0d	RHSIM_Rhsim01G0230600	0.217	0.119	-0.098	no
CK-0d	RHSIM_Rhsim01G0248500	0.411	0.533	0.122	no
CK-0d	RHSIM_Rhsim01G0091800	-0.103	0.593	0.696	no
CK-0d	RHSIM_Rhsim02G0046000	-0.938	-0.510	0.427	no
CK-0d	RHSIM_Rhsim01G0192200	1.047	3.461	2.413	no
CK-0d	RHSIM_Rhsim01G0091400	-0.392	-0.908	-0.516	no
CK-0d	RHSIM_Rhsim02G0216500	-1.234	1.066	2.300	low
CK-0d	RHSIM_Rhsim01G0209300	0.479	0.196	-0.283	no
CK-0d	RHSIM_Rhsim01G0275400	-0.798	-0.742	0.056	no
CK-0d	RHSIM_Rhsim02G0005600	0.156	-0.688	-0.843	no
CK-0d	RHSIM_Rhsim01G0008800	0.021	-0.380	-0.402	no
CK-0d	RHSIM_Rhsim02G0128200	0.901	0.921	0.019	no
CK-0d	RHSIM_Rhsim01G0195300	0.294	0.243	-0.051	no
CK-0d	RHSIM_Rhsim01G0073700	0.214	0.033	-0.181	no
CK-0d	RHSIM_Rhsim01G0144700	-0.911	-0.927	-0.016	no
CK-0d	RHSIM_Rhsim01G0126800	0.506	2.594	2.087	no
CK-0d	RHSIM_Rhsim01G0286600	-1.077	0.290	1.367	low
CK-0d	RHSIM_Rhsim02G0256800	0.407	0.026	-0.381	no
CK-0d	RHSIM_Rhsim01G0224800	-0.005	0.510	0.514	no
CK-0d	RHSIM_Rhsim02G0132700	-1.938	0.525	2.463	low
CK-0d	RHSIM_Rhsim02G0142400	-0.238	0.600	0.838	no
CK-0d	RHSIM_Rhsim02G0238900	0.102	0.055	-0.047	no
CK-0d	RHSIM_Rhsim01G0184800	-0.183	0.749	0.931	no
CK-0d	RHSIM_Rhsim01G0209000	-0.774	-1.213	-0.439	no
CK-0d	RHSIM_Rhsim02G0185100	0.068	-0.942	-1.010	no
CK-0d	RHSIM_Rhsim02G0229900	0.461	0.726	0.265	no
CK-0d	RHSIM_Rhsim01G0246100	-1.203	-0.850	0.353	low
CK-0d	RHSIM_Rhsim01G0184000	-1.136	-0.285	0.851	low

Group	Gene	PTR (z-score difference)	z(log2 LFQ)	z(log2 FPKM)	extreme-PTR
CK-0d	RHSIM_Rhsim02G0099600	0.432	-0.967	-1.399	no
CK-0d	RHSIM_Rhsim01G0023900	0.961	1.144	0.183	no
CK-0d	RHSIM_Rhsim02G0009700	-1.626	-0.662	0.964	low
CK-0d	RHSIM_Rhsim01G0162200	-0.084	-0.397	-0.313	no
CK-0d	RHSIM_Rhsim01G0178400	-3.204	-1.699	1.505	low
CK-0d	RHSIM_Rhsim01G0008400	1.686	0.571	-1.115	high
CK-0d	RHSIM_Rhsim01G0236500	-0.378	1.076	1.454	no
CK-0d	RHSIM_Rhsim01G0262900	-0.867	-0.729	0.139	no
CK-0d	RHSIM_Rhsim02G0151800	-0.718	-0.028	0.690	no
CK-0d	RHSIM_Rhsim02G0003700	1.093	0.106	-0.987	no
CK-0d	RHSIM_Rhsim02G0100800	0.919	0.414	-0.506	no
CK-0d	RHSIM_Rhsim01G0090500	-0.941	-1.336	-0.395	no
CK-0d	RHSIM_Rhsim01G0064300	-1.157	-1.770	-0.613	low
CK-0d	RHSIM_Rhsim02G0253600	-1.184	-0.925	0.259	low
CK-0d	RHSIM_Rhsim02G0031500	0.094	0.293	0.200	no
CK-0d	RHSIM_Rhsim02G0000800	-1.695	-1.267	0.428	low
CK-0d	RHSIM_Rhsim01G0141400	0.336	2.071	1.734	no
CK-0d	RHSIM_Rhsim02G0075300	-1.021	-0.521	0.501	no
CK-0d	RHSIM_Rhsim01G0280500	-0.772	-1.106	-0.334	no
CK-0d	RHSIM_Rhsim02G0223600	-0.375	-0.153	0.223	no
CK-0d	RHSIM_Rhsim02G0088700	0.976	0.164	-0.812	no
CK-0d	RHSIM_Rhsim02G0228900	1.048	0.881	-0.167	no
CK-0d	RHSIM_Rhsim02G0026400	-3.403	0.101	3.504	low
CK-0d	RHSIM_Rhsim02G0054000	0.479	-0.336	-0.815	no
CK-0d	RHSIM_Rhsim02G0018300	-0.821	-0.748	0.073	no
CK-0d	RHSIM_Rhsim01G0218400	0.972	0.891	-0.081	no
CK-0d	RHSIM_Rhsim01G0251500	1.928	1.845	-0.083	high
CK-0d	RHSIM_Rhsim01G0106600	-0.689	0.092	0.781	no
CK-0d	RHSIM_Rhsim01G0181700	-0.527	-0.943	-0.416	no
CK-0d	RHSIM_Rhsim01G0220200	-1.140	-1.343	-0.203	low
CK-0d	RHSIM_Rhsim02G0037500	0.928	1.177	0.249	no
CK-0d	RHSIM_Rhsim01G0171200	0.204	-1.396	-1.600	no

Group	Gene	PTR (z-score difference)	z(log2 LFQ)	z(log2 FPKM)	extreme-PTR
CK-0d	RHSIM_Rhsim01G0124800	0.801	0.544	-0.257	no
CK-0d	RHSIM_Rhsim01G0139200	0.630	0.833	0.203	no
CK-0d	RHSIM_Rhsim01G0282400	-0.058	-1.183	-1.125	no
CK-0d	RHSIM_Rhsim01G0220800	0.738	0.041	-0.697	no
CK-0d	RHSIM_Rhsim01G0284300	0.267	0.560	0.293	no
CK-0d	RHSIM_Rhsim01G0102600	1.000	-0.012	-1.012	no
CK-0d	RHSIM_Rhsim01G0208800	0.361	-0.038	-0.399	no
CK-0d	RHSIM_Rhsim01G0023400	-0.159	0.379	0.537	no
CK-0d	RHSIM_Rhsim01G0207000	-1.766	1.204	2.970	low
CK-0d	RHSIM_Rhsim01G0216900	1.211	0.983	-0.227	high
CK-0d	RHSIM_Rhsim01G0217400	-0.353	-1.487	-1.134	no
CK-0d	RHSIM_Rhsim01G0245900	0.180	1.624	1.444	no
CK-0d	RHSIM_Rhsim01G0159800	-0.117	0.568	0.685	no
CK-0d	RHSIM_Rhsim01G0238400	0.256	-0.170	-0.426	no
CK-0d	RHSIM_Rhsim01G0226500	-0.553	0.865	1.418	no
CK-0d	RHSIM_Rhsim01G0229500	-0.030	-0.168	-0.137	no
CK-0d	RHSIM_Rhsim01G0216000	-0.389	-0.932	-0.543	no
CK-0d	RHSIM_Rhsim01G0274200	-0.700	-0.950	-0.250	no
CK-0d	RHSIM_Rhsim01G0049500	-1.336	-1.246	0.090	low
CK-0d	RHSIM_Rhsim01G0193100	0.632	1.296	0.664	no
CK-0d	RHSIM_Rhsim01G0231600	-1.790	-1.329	0.461	low
CK-0d	RHSIM_Rhsim01G0191000	-0.428	-0.742	-0.314	no
CK-0d	RHSIM_Rhsim01G0033000	1.769	0.591	-1.178	high
CK-0d	RHSIM_Rhsim01G0223000	0.102	0.070	-0.032	no
CK-0d	RHSIM_Rhsim01G0123800	0.445	0.500	0.055	no
CK-0d	RHSIM_Rhsim01G0002500	-0.451	-0.476	-0.025	no
CK-0d	RHSIM_Rhsim01G0148500	0.119	-0.571	-0.690	no
CK-0d	RHSIM_Rhsim01G0068000	-0.549	-1.326	-0.778	no
CK-0d	RHSIM_Rhsim01G0202100	-1.348	0.331	1.679	low
CK-0d	RHSIM_Rhsim01G0130500	-0.609	-1.273	-0.664	no
CK-0d	RHSIM_Rhsim01G0076500	1.307	1.057	-0.249	high
CK-0d	RHSIM_Rhsim01G0233700	-0.913	-0.715	0.198	no

Group	Gene	PTR (z-score difference)	z(log2 LFQ)	z(log2 FPKM)	extreme-PTR
CK-0d	RHSIM_Rhsim01G0189000	0.173	0.212	0.039	no
CK-0d	RHSIM_Rhsim01G0185300	-0.225	0.034	0.259	no
CK-0d	RHSIM_Rhsim01G0131100	-0.742	0.022	0.764	no
CK-0d	RHSIM_Rhsim01G0252400	-0.164	-0.503	-0.339	no
CK-0d	RHSIM_Rhsim01G0060300	-1.630	-0.322	1.308	low
CK-0d	RHSIM_Rhsim01G0006800	0.404	-0.773	-1.177	no
CK-0d	RHSIM_Rhsim01G0287900	-1.503	-0.539	0.965	low
CK-0d	RHSIM_Rhsim01G0071600	-2.121	-0.185	1.936	low
CK-0d	RHSIM_Rhsim01G0185000	-0.464	-1.015	-0.551	no
CK-0d	RHSIM_Rhsim01G0196300	-0.172	0.700	0.871	no
CK-0d	RHSIM_Rhsim01G0138800	1.089	0.320	-0.769	no
CK-0d	RHSIM_Rhsim01G0210400	1.225	1.763	0.537	high
CK-0d	RHSIM_Rhsim01G0007000	0.249	0.500	0.251	no
CK-0d	RHSIM_Rhsim01G0000700	0.831	1.057	0.226	no
CK-0d	RHSIM_RhsimUnG0256000	2.738	0.610	-2.128	high
CK-0d	RHSIM_RhsimUnG0254300	-4.391	-0.829	3.561	low
CK-0d	RHSIM_RhsimUnG0258100	0.367	0.392	0.025	no
CK-0d	RHSIM_RhsimUnG0252100	0.083	-0.909	-0.992	no
CK-0d	RHSIM_RhsimUnG0236700	0.827	-0.164	-0.990	no
CK-0d	RHSIM_RhsimUnG0227500	0.246	1.450	1.204	no
CK-0d	RHSIM_RhsimUnG0171600	-1.056	-1.678	-0.622	low
CK-0d	RHSIM_RhsimUnG0169000	-1.410	-1.391	0.019	low
CK-0d	RHSIM_RhsimUnG0118200	0.496	-0.454	-0.950	no
CK-0d	RHSIM_RhsimUnG0113800	1.722	0.053	-1.669	high
CK-0d	RHSIM_RhsimUnG0065600	-1.586	0.420	2.005	low
CK-0d	RHSIM_RhsimUnG0062200	1.673	0.499	-1.174	high
CK-0d	RHSIM_Rhsim10G0103200	0.122	0.017	-0.105	no
CK-0d	RHSIM_RhsimUnG0057700	1.116	0.539	-0.577	high
CK-0d	RHSIM_RhsimUnG0055300	0.148	0.283	0.135	no
CK-0d	RHSIM_RhsimUnG0039300	1.678	0.913	-0.765	high
CK-0d	RHSIM_RhsimUnG0037800	-1.373	-0.726	0.647	low
CK-0d	RHSIM_RhsimUnG0024000	-1.837	-2.161	-0.323	low

Group	Gene	PTR (z-score difference)	z(log2 LFQ)	z(log2 FPKM)	extreme-PTR
CK-0d	RHSIM_RhsimUnG0003000	-0.519	-0.256	0.263	no
CK-0d	RHSIM_RhsimUnG0258700	0.235	-1.123	-1.358	no
CK-0d	RHSIM_RhsimPtG0005800	3.383	3.932	0.549	high
CK-0d	RHSIM_RhsimUnG0253900	1.265	1.172	-0.093	high
CK-0d	RHSIM_RhsimUnG0250200	0.651	1.517	0.865	no
CK-0d	RHSIM_Rhsim13G0233000	0.179	0.016	-0.162	no
CK-0d	RHSIM_Rhsim13G0008700	3.191	1.629	-1.562	high
CK-0d	RHSIM_Rhsim13G0227700	0.331	-0.641	-0.972	no
CK-0d	RHSIM_Rhsim13G0228800	-0.368	1.132	1.500	no
CK-0d	RHSIM_Rhsim13G0222000	-0.958	-1.192	-0.234	no
CK-0d	RHSIM_Rhsim13G0111300	-0.219	0.797	1.016	no
CK-0d	RHSIM_Rhsim13G0180900	-1.828	-0.821	1.007	low
CK-0d	RHSIM_Rhsim13G0004600	-0.458	-0.127	0.331	no
CK-0d	RHSIM_RhsimUnG0217200	2.295	0.399	-1.897	high
CK-0d	RHSIM_RhsimUnG0212100	-0.663	-1.185	-0.522	no
CK-0d	RHSIM_Rhsim13G0203000	-0.310	-0.989	-0.680	no
CK-0d	RHSIM_RhsimUnG0202600	1.025	-0.679	-1.704	no
CK-0d	RHSIM_Rhsim13G0053600	0.227	0.904	0.677	no
CK-0d	RHSIM_Rhsim13G0103100	-1.378	-0.153	1.225	low
CK-0d	RHSIM_RhsimUnG0248000	-0.071	0.007	0.078	no
CK-0d	RHSIM_Rhsim13G0088200	-0.253	-2.133	-1.880	no
CK-0d	RHSIM_RhsimUnG0250500	0.237	1.572	1.335	no
CK-0d	RHSIM_RhsimUnG0253100	-2.663	-0.172	2.490	low
CK-0d	RHSIM_Rhsim13G0021500	-0.442	-0.885	-0.443	no
CK-0d	RHSIM_RhsimUnG0257700	0.556	2.811	2.254	no
CK-0d	RHSIM_RhsimUnG0251000	-0.787	-1.133	-0.346	no
CK-0d	RHSIM_RhsimUnG0248500	0.016	-0.518	-0.534	no
CK-0d	RHSIM_Rhsim13G0120200	-1.152	-1.608	-0.456	low
CK-0d	RHSIM_RhsimUnG0234700	-0.375	-0.694	-0.319	no
CK-0d	RHSIM_Rhsim13G0146900	-1.172	-1.438	-0.266	low
CK-0d	RHSIM_RhsimUnG0239200	1.314	0.514	-0.801	high
CK-0d	RHSIM_RhsimUnG0240700	0.749	1.034	0.286	no

Group	Gene	PTR (z-score difference)	z(log2 LFQ)	z(log2 FPKM)	extreme-PTR
CK-0d	RHSIM_RhsimUnG0226400	-0.761	-0.286	0.475	no
CK-0d	RHSIM_Rhsim13G0041600	1.104	3.290	2.186	no
CK-0d	RHSIM_RhsimUnG0223000	1.334	-0.594	-1.927	high
CK-0d	RHSIM_RhsimUnG0231000	0.061	-1.649	-1.710	no
CK-0d	RHSIM_Rhsim13G0201800	-2.790	-1.170	1.619	low
CK-0d	RHSIM_RhsimUnG0225800	1.053	1.295	0.242	no
CK-0d	RHSIM_Rhsim13G0232300	1.477	1.431	-0.047	high
CK-0d	RHSIM_Rhsim13G0191000	0.180	-0.543	-0.724	no
CK-0d	RHSIM_RhsimUnG0168300	0.891	0.051	-0.841	no
CK-0d	RHSIM_Rhsim13G0040700	0.289	-1.005	-1.295	no
CK-0d	RHSIM_Rhsim13G0054200	-0.474	-1.493	-1.020	no
CK-0d	RHSIM_RhsimUnG0210400	2.692	1.694	-0.999	high
CK-0d	RHSIM_RhsimUnG0210700	0.340	-0.618	-0.959	no
CK-0d	RHSIM_RhsimUnG0209300	0.277	-1.077	-1.354	no
CK-0d	RHSIM_Rhsim12G0052900	1.154	0.657	-0.497	high
CK-0d	RHSIM_RhsimUnG0192400	0.826	0.102	-0.724	no
CK-0d	RHSIM_Rhsim13G0198900	0.449	-0.956	-1.405	no
CK-0d	RHSIM_Rhsim09G0047000	1.832	0.938	-0.894	high
CK-0d	RHSIM_Rhsim13G0121800	-0.391	-1.069	-0.678	no
CK-0d	RHSIM_RhsimUnG0168800	1.297	2.090	0.793	high
CK-0d	RHSIM_Rhsim06G0025600	0.101	0.510	0.409	no
CK-0d	RHSIM_Rhsim13G0184800	-0.103	-0.679	-0.575	no
CK-0d	RHSIM_RhsimUnG0143500	-1.284	-1.390	-0.106	low
CK-0d	RHSIM_RhsimUnG0092900	0.835	-0.369	-1.204	no
CK-0d	RHSIM_RhsimUnG0087600	1.776	0.393	-1.382	high
CK-0d	RHSIM_RhsimUnG0086600	1.160	0.825	-0.335	high
CK-0d	RHSIM_Rhsim13G0020600	-0.627	-0.906	-0.279	no
CK-0d	RHSIM_RhsimUnG0082400	1.644	0.174	-1.470	high
CK-0d	RHSIM_RhsimUnG0142500	0.296	-0.412	-0.708	no
CK-0d	RHSIM_RhsimUnG0135800	2.067	0.231	-1.836	high
CK-0d	RHSIM_RhsimUnG0073800	-0.730	0.077	0.807	no
CK-0d	RHSIM_RhsimUnG0131400	2.354	0.734	-1.620	high

Group	Gene	PTR (z-score difference)	z(log2 LFQ)	z(log2 FPKM)	extreme-PTR
CK-0d	RHSIM_RhsimUnG0120100	0.334	1.055	0.721	no
CK-0d	RHSIM_RhsimUnG0128700	0.074	-0.601	-0.675	no
CK-0d	RHSIM_RhsimUnG0123600	-0.078	-1.218	-1.140	no
CK-0d	RHSIM_Rhsim13G0099200	0.922	1.331	0.409	no
CK-0d	RHSIM_Rhsim13G0029700	-0.439	-1.052	-0.613	no
CK-0d	RHSIM_RhsimUnG0044400	-0.232	0.533	0.765	no
CK-0d	RHSIM_RhsimUnG0092800	0.978	0.675	-0.303	no
CK-0d	RHSIM_RhsimUnG0088000	0.775	-0.115	-0.889	no
CK-0d	RHSIM_RhsimUnG0106100	1.474	-0.298	-1.772	high
CK-0d	RHSIM_RhsimUnG0100600	-0.474	0.295	0.768	no
CK-0d	RHSIM_Rhsim13G0131200	0.125	0.692	0.567	no
CK-0d	RHSIM_RhsimUnG0097600	0.944	0.983	0.040	no
CK-0d	RHSIM_RhsimUnG0091000	0.212	-1.140	-1.352	no
CK-0d	RHSIM_RhsimUnG0085300	-0.052	0.696	0.747	no
CK-0d	RHSIM_RhsimUnG0070600	0.910	-0.937	-1.846	no
CK-0d	RHSIM_Rhsim13G0082400	-0.666	-0.681	-0.015	no
CK-0d	RHSIM_RhsimUnG0005900	2.368	2.059	-0.309	high
CK-0d	RHSIM_RhsimUnG0066300	0.981	-0.320	-1.301	no
CK-0d	RHSIM_RhsimUnG0075600	0.695	0.509	-0.186	no
CK-0d	RHSIM_RhsimUnG0064700	1.105	1.601	0.497	no
CK-0d	RHSIM_RhsimPtG0004000	4.957	3.450	-1.507	high
CK-0d	RHSIM_Rhsim13G0062300	1.053	1.108	0.055	no
CK-0d	RHSIM_RhsimMtG0002200	0.997	-0.871	-1.868	no
CK-0d	RHSIM_Rhsim13G0073900	0.586	-0.587	-1.173	no
CK-0d	RHSIM_RhsimUnG0055400	0.758	1.757	0.999	no
CK-0d	RHSIM_RhsimUnG0046700	-0.650	-1.397	-0.747	no
CK-0d	RHSIM_RhsimUnG0054900	-0.809	1.113	1.921	no
CK-0d	RHSIM_RhsimUnG0042300	-1.097	0.249	1.346	low
CK-0d	RHSIM_Rhsim13G0061900	-0.968	-1.396	-0.427	no
CK-0d	RHSIM_Rhsim13G0227300	0.705	0.106	-0.599	no
CK-0d	RHSIM_Rhsim12G0181000	0.201	0.636	0.435	no
CK-0d	RHSIM_Rhsim13G0093800	-1.169	-1.261	-0.092	low

Group	Gene	PTR (z-score difference)	z(log2 LFQ)	z(log2 FPKM)	extreme-PTR
CK-0d	RHSIM_Rhsim13G0030700	0.031	0.202	0.171	no
CK-0d	RHSIM_Rhsim13G0076000	0.311	1.020	0.709	no
CK-0d	RHSIM_RhsimUnG0019100	0.397	0.271	-0.125	no
CK-0d	RHSIM_RhsimUnG0020700	0.037	-0.888	-0.925	no
CK-0d	RHSIM_Rhsim13G0064100	0.249	-0.615	-0.864	no
CK-0d	RHSIM_Rhsim13G0163100	-0.135	-0.895	-0.760	no
CK-0d	RHSIM_Rhsim13G0066100	-0.015	0.987	1.002	no
CK-0d	RHSIM_RhsimUnG0016900	0.967	1.198	0.231	no
CK-0d	RHSIM_RhsimUnG0016100	-2.571	-1.868	0.704	low
CK-0d	RHSIM_Rhsim13G0207900	-1.789	-0.858	0.931	low
CK-0d	RHSIM_Rhsim12G0037800	0.029	0.442	0.413	no
CK-0d	RHSIM_Rhsim13G0043900	-0.160	-0.140	0.020	no
CK-0d	RHSIM_Rhsim12G0063500	-0.630	-0.289	0.341	no
CK-0d	RHSIM_Rhsim12G0167800	0.229	0.318	0.089	no
CK-0d	RHSIM_Rhsim13G0040000	1.280	0.691	-0.590	high
CK-0d	RHSIM_Rhsim13G0118300	-0.889	-0.263	0.627	no
CK-0d	RHSIM_Rhsim13G0220600	0.725	1.099	0.373	no
CK-0d	RHSIM_Rhsim13G0065700	0.554	0.552	-0.002	no
CK-0d	RHSIM_Rhsim13G0221500	-0.546	-1.004	-0.458	no
CK-0d	RHSIM_Rhsim12G0039700	0.605	0.174	-0.431	no
CK-0d	RHSIM_Rhsim13G0226500	0.972	1.387	0.415	no
CK-0d	RHSIM_Rhsim13G0016700	-0.161	-0.341	-0.180	no
CK-0d	RHSIM_Rhsim13G0174500	0.922	1.582	0.660	no
CK-0d	RHSIM_Rhsim12G0048800	-0.491	-0.822	-0.332	no
CK-0d	RHSIM_Rhsim13G0185200	2.352	2.628	0.275	high
CK-0d	RHSIM_Rhsim13G0234600	0.265	0.656	0.390	no
CK-0d	RHSIM_Rhsim13G0038800	-0.807	0.898	1.705	no
CK-0d	RHSIM_Rhsim13G0196800	-0.505	-0.877	-0.372	no
CK-0d	RHSIM_Rhsim13G0053200	0.797	0.854	0.056	no
CK-0d	RHSIM_Rhsim12G0172600	1.364	0.980	-0.384	high
CK-0d	RHSIM_Rhsim13G0134800	-1.134	-0.171	0.963	low
CK-0d	RHSIM_Rhsim13G0154000	0.723	2.622	1.899	no

Group	Gene	PTR (z-score difference)	z(log2 LFQ)	z(log2 FPKM)	extreme-PTR
CK-0d	RHSIM_Rhsim13G0038600	-0.420	-1.068	-0.648	no
CK-0d	RHSIM_Rhsim12G0139300	-0.513	-0.464	0.048	no
CK-0d	RHSIM_Rhsim12G0115700	-1.276	-1.492	-0.216	low
CK-0d	RHSIM_Rhsim13G0229900	0.686	1.554	0.867	no
CK-0d	RHSIM_Rhsim13G0078100	-2.243	-1.260	0.983	low
CK-0d	RHSIM_Rhsim13G0113300	-0.449	-0.950	-0.500	no
CK-0d	RHSIM_Rhsim13G0082700	-3.992	0.046	4.037	low
CK-0d	RHSIM_Rhsim13G0126700	-0.805	-0.877	-0.072	no
CK-0d	RHSIM_Rhsim13G0159200	-0.289	-0.649	-0.361	no
CK-0d	RHSIM_Rhsim13G0201100	0.506	-0.349	-0.855	no
CK-0d	RHSIM_Rhsim13G0022100	0.987	1.218	0.231	no
CK-0d	RHSIM_Rhsim13G0199200	0.888	-0.273	-1.161	no
CK-0d	RHSIM_Rhsim13G0171300	1.475	0.210	-1.264	high
CK-0d	RHSIM_Rhsim13G0177900	0.536	0.135	-0.401	no
CK-0d	RHSIM_Rhsim12G0123300	0.145	-0.553	-0.699	no
CK-0d	RHSIM_Rhsim13G0093700	0.331	0.670	0.339	no
CK-0d	RHSIM_Rhsim12G0178200	0.315	-0.463	-0.778	no
CK-0d	RHSIM_Rhsim13G0036600	-1.642	-0.925	0.717	low
CK-0d	RHSIM_Rhsim12G0139900	-0.927	-1.116	-0.189	no
CK-0d	RHSIM_Rhsim13G0006800	0.517	1.413	0.896	no
CK-0d	RHSIM_Rhsim13G0079200	-0.238	-0.614	-0.376	no
CK-0d	RHSIM_Rhsim12G0079000	-0.425	-0.788	-0.362	no
CK-0d	RHSIM_Rhsim13G0092400	-1.221	-1.617	-0.396	low
CK-0d	RHSIM_Rhsim13G0220800	-0.834	-0.428	0.406	no
CK-0d	RHSIM_Rhsim13G0011300	0.034	0.025	-0.009	no
CK-0d	RHSIM_Rhsim13G0212000	2.034	0.158	-1.875	high
CK-0d	RHSIM_Rhsim12G0204800	-0.095	-0.079	0.015	no
CK-0d	RHSIM_Rhsim13G0041900	-0.310	-1.299	-0.989	no
CK-0d	RHSIM_Rhsim13G0232100	0.698	0.943	0.246	no
CK-0d	RHSIM_Rhsim13G0144400	1.891	0.906	-0.985	high
CK-0d	RHSIM_Rhsim13G0054900	0.947	-0.153	-1.100	no
CK-0d	RHSIM_Rhsim13G0124200	-0.548	-0.491	0.056	no

Group	Gene	PTR (z-score difference)	z(log2 LFQ)	z(log2 FPKM)	extreme-PTR
CK-0d	RHSIM_Rhsim13G0021700	-1.725	-1.495	0.230	low
CK-0d	RHSIM_Rhsim12G0107800	1.526	-0.217	-1.743	high
CK-0d	RHSIM_Rhsim13G0057600	1.389	0.128	-1.262	high
CK-0d	RHSIM_Rhsim13G0191700	1.138	0.031	-1.108	high
CK-0d	RHSIM_Rhsim13G0114000	-0.378	-0.587	-0.210	no
CK-0d	RHSIM_Rhsim13G0027900	-0.595	0.119	0.714	no
CK-0d	RHSIM_Rhsim13G0029600	-3.288	-2.001	1.287	low
CK-0d	RHSIM_Rhsim13G0051300	-0.142	1.568	1.710	no
CK-0d	RHSIM_Rhsim13G0219700	1.108	1.283	0.174	no
CK-0d	RHSIM_Rhsim13G0164200	0.676	-0.818	-1.493	no
CK-0d	RHSIM_Rhsim13G0086200	-0.721	0.274	0.995	no
CK-0d	RHSIM_Rhsim12G0005600	-0.208	0.806	1.014	no
CK-0d	RHSIM_Rhsim13G0014300	-1.454	-1.381	0.072	low
CK-0d	RHSIM_Rhsim12G0203000	-1.242	-0.782	0.460	low
CK-0d	RHSIM_Rhsim13G0001400	1.948	0.959	-0.989	high
CK-0d	RHSIM_Rhsim13G0111900	-2.041	-1.122	0.919	low
CK-0d	RHSIM_Rhsim12G0174000	-0.231	1.122	1.352	no
CK-0d	RHSIM_Rhsim13G0161700	-0.163	0.943	1.107	no
CK-0d	RHSIM_Rhsim13G0036800	0.726	1.265	0.539	no
CK-0d	RHSIM_Rhsim13G0197800	2.576	0.765	-1.811	high
CK-0d	RHSIM_Rhsim13G0219900	-1.748	-1.263	0.485	low
CK-0d	RHSIM_Rhsim13G0107300	0.929	0.897	-0.032	no
CK-0d	RHSIM_Rhsim13G0075000	-0.327	-0.638	-0.311	no
CK-0d	RHSIM_Rhsim13G0103500	-0.231	0.057	0.289	no
CK-0d	RHSIM_Rhsim13G0101100	0.668	0.595	-0.073	no
CK-0d	RHSIM_Rhsim13G0015700	0.967	0.742	-0.225	no
CK-0d	RHSIM_Rhsim13G0168400	0.844	1.770	0.926	no
CK-0d	RHSIM_Rhsim12G0116800	1.466	0.469	-0.997	high
CK-0d	RHSIM_Rhsim12G0078100	-0.467	-0.322	0.146	no
CK-0d	RHSIM_Rhsim13G0181700	0.195	0.186	-0.009	no
CK-0d	RHSIM_Rhsim13G0161600	-0.846	-1.745	-0.899	no
CK-0d	RHSIM_Rhsim13G0116300	1.330	1.561	0.231	high

Group	Gene	PTR (z-score difference)	z(log2 LFQ)	z(log2 FPKM)	extreme-PTR
CK-0d	RHSIM_Rhsim13G0102200	0.844	-0.276	-1.120	no
CK-0d	RHSIM_Rhsim12G0172200	1.055	1.271	0.216	no
CK-0d	RHSIM_Rhsim12G0103900	-0.132	-0.162	-0.030	no
CK-0d	RHSIM_Rhsim13G0069000	-1.154	-0.200	0.953	low
CK-0d	RHSIM_Rhsim13G0231100	0.728	-0.395	-1.123	no
CK-0d	RHSIM_Rhsim12G0083200	1.560	0.573	-0.987	high
CK-0d	RHSIM_Rhsim12G0177000	1.268	0.676	-0.592	high
CK-0d	RHSIM_Rhsim13G0217100	0.167	0.544	0.377	no
CK-0d	RHSIM_Rhsim13G0138400	-0.386	-0.555	-0.168	no
CK-0d	RHSIM_Rhsim13G0042600	-0.336	0.260	0.596	no
CK-0d	RHSIM_Rhsim12G0100900	-0.016	0.693	0.709	no
CK-0d	RHSIM_Rhsim13G0025700	1.304	1.310	0.006	high
CK-0d	RHSIM_Rhsim13G0038000	2.053	1.527	-0.526	high
CK-0d	RHSIM_Rhsim13G0079600	-1.165	0.283	1.449	low
CK-0d	RHSIM_Rhsim12G0083000	-0.157	0.149	0.306	no
CK-0d	RHSIM_Rhsim13G0233800	-1.462	-0.451	1.011	low
CK-0d	RHSIM_Rhsim13G0073800	-1.561	-0.918	0.643	low
CK-0d	RHSIM_Rhsim13G0055800	0.372	-0.830	-1.202	no
CK-0d	RHSIM_Rhsim13G0031900	1.168	0.536	-0.632	high
CK-0d	RHSIM_Rhsim13G0027500	-0.281	-0.044	0.237	no
CK-0d	RHSIM_Rhsim12G0028500	0.009	0.446	0.436	no
CK-0d	RHSIM_Rhsim13G0095000	0.029	-1.272	-1.301	no
CK-0d	RHSIM_Rhsim13G0035400	-0.178	-0.304	-0.126	no
CK-0d	RHSIM_Rhsim13G0167700	0.196	1.055	0.859	no
CK-0d	RHSIM_Rhsim13G0129900	0.737	-0.076	-0.813	no
CK-0d	RHSIM_Rhsim13G0223700	-0.547	-1.112	-0.565	no
CK-0d	RHSIM_Rhsim13G0157400	1.462	1.225	-0.237	high
CK-0d	RHSIM_Rhsim13G0177000	0.218	0.278	0.060	no
CK-0d	RHSIM_Rhsim13G0030400	-0.585	-0.983	-0.398	no
CK-0d	RHSIM_Rhsim12G0123700	0.215	-0.187	-0.402	no
CK-0d	RHSIM_Rhsim13G0208300	-0.408	0.106	0.514	no
CK-0d	RHSIM_Rhsim13G0070100	0.119	-0.191	-0.310	no

Group	Gene	PTR (z-score difference)	z(log2 LFQ)	z(log2 FPKM)	extreme-PTR
CK-0d	RHSIM_Rhsim13G0003700	0.974	-0.939	-1.913	no
CK-0d	RHSIM_Rhsim11G0144200	-0.205	-0.774	-0.569	no
CK-0d	RHSIM_Rhsim13G0179800	-0.750	-2.282	-1.533	no
CK-0d	RHSIM_Rhsim13G0114600	-0.833	-0.143	0.689	no
CK-0d	RHSIM_Rhsim13G0028300	0.702	0.850	0.147	no
CK-0d	RHSIM_Rhsim13G0122000	0.436	-1.200	-1.636	no
CK-0d	RHSIM_Rhsim13G0127800	0.828	-0.439	-1.267	no
CK-0d	RHSIM_Rhsim13G0092300	1.548	1.720	0.172	high
CK-0d	RHSIM_Rhsim13G0026300	1.274	1.546	0.272	high
CK-0d	RHSIM_Rhsim13G0233500	0.246	-0.157	-0.403	no
CK-0d	RHSIM_Rhsim13G0091400	0.275	-0.313	-0.588	no
CK-0d	RHSIM_Rhsim13G0120600	0.540	1.106	0.565	no
CK-0d	RHSIM_Rhsim11G0149700	0.342	-0.158	-0.500	no
CK-0d	RHSIM_Rhsim13G0220700	-0.288	-0.713	-0.426	no
CK-0d	RHSIM_Rhsim13G0067500	-0.054	0.623	0.677	no
CK-0d	RHSIM_Rhsim13G0146800	0.304	-0.348	-0.653	no
CK-0d	RHSIM_Rhsim13G0138900	-1.297	-0.746	0.550	low
CK-0d	RHSIM_Rhsim13G0156800	-0.036	-1.099	-1.063	no
CK-0d	RHSIM_Rhsim13G0076600	-0.367	-1.021	-0.654	no
CK-0d	RHSIM_Rhsim13G0036200	-2.180	-0.465	1.715	low
CK-0d	RHSIM_Rhsim11G0025400	-0.135	-1.478	-1.343	no
CK-0d	RHSIM_Rhsim13G0173800	-1.433	-0.182	1.251	low
CK-0d	RHSIM_Rhsim13G0171400	0.181	-0.293	-0.474	no
CK-0d	RHSIM_Rhsim11G0195300	2.305	0.486	-1.819	high
CK-0d	RHSIM_Rhsim13G0129200	1.114	0.886	-0.228	high
CK-0d	RHSIM_Rhsim13G0010200	-0.912	-0.040	0.873	no
CK-0d	RHSIM_Rhsim13G0231600	-1.657	-0.477	1.180	low
CK-0d	RHSIM_Rhsim13G0230400	0.853	0.710	-0.143	no
CK-0d	RHSIM_Rhsim11G0194500	-0.891	-1.128	-0.236	no
CK-0d	RHSIM_Rhsim13G0140100	0.376	0.294	-0.082	no
CK-0d	RHSIM_Rhsim13G0142000	0.322	0.246	-0.075	no
CK-0d	RHSIM_Rhsim12G0016400	-0.072	-0.570	-0.498	no

Group	Gene	PTR (z-score difference)	z(log2 LFQ)	z(log2 FPKM)	extreme-PTR
CK-0d	RHSIM_Rhsim13G0056200	1.120	0.407	-0.712	high
CK-0d	RHSIM_Rhsim12G0018000	0.583	-0.246	-0.829	no
CK-0d	RHSIM_Rhsim12G0121700	0.424	-0.172	-0.595	no
CK-0d	RHSIM_Rhsim12G0020900	2.478	0.863	-1.615	high
CK-0d	RHSIM_Rhsim11G0117400	1.051	1.005	-0.045	no
CK-0d	RHSIM_Rhsim12G0014400	1.168	0.926	-0.241	high
CK-0d	RHSIM_Rhsim11G0029100	1.216	0.305	-0.912	high
CK-0d	RHSIM_Rhsim12G0021600	-0.765	-2.324	-1.558	no
CK-0d	RHSIM_Rhsim11G0036500	-0.259	-0.072	0.187	no
CK-0d	RHSIM_Rhsim12G0088300	-0.622	0.469	1.090	no
CK-0d	RHSIM_Rhsim13G0217400	-0.194	-0.775	-0.581	no
CK-0d	RHSIM_Rhsim11G0008400	0.622	-0.131	-0.753	no
CK-0d	RHSIM_Rhsim11G0097700	-0.294	-1.529	-1.235	no
CK-0d	RHSIM_Rhsim13G0124600	-1.074	-1.645	-0.571	low
CK-0d	RHSIM_Rhsim12G0178500	0.099	0.278	0.178	no
CK-0d	RHSIM_Rhsim12G0026500	-0.008	-0.953	-0.945	no
CK-0d	RHSIM_Rhsim13G0008300	-1.750	0.260	2.010	low
CK-0d	RHSIM_Rhsim12G0157000	-0.637	0.666	1.304	no
CK-0d	RHSIM_Rhsim11G0148300	-1.924	-0.543	1.382	low
CK-0d	RHSIM_Rhsim12G0110500	0.463	-0.845	-1.308	no
CK-0d	RHSIM_Rhsim11G0196100	0.055	-0.999	-1.054	no
CK-0d	RHSIM_Rhsim12G0078800	1.143	1.132	-0.012	high
CK-0d	RHSIM_Rhsim12G0079500	2.464	1.138	-1.326	high
CK-0d	RHSIM_Rhsim12G0008200	1.716	0.208	-1.508	high
CK-0d	RHSIM_Rhsim13G0206000	-0.337	0.202	0.539	no
CK-0d	RHSIM_Rhsim11G0077200	0.939	3.122	2.183	no
CK-0d	RHSIM_Rhsim12G0189500	-0.012	-0.987	-0.975	no
CK-0d	RHSIM_Rhsim12G0131300	0.667	1.308	0.641	no
CK-0d	RHSIM_Rhsim12G0190500	0.553	0.571	0.018	no
CK-0d	RHSIM_Rhsim13G0036100	0.966	0.511	-0.454	no
CK-0d	RHSIM_Rhsim12G0057400	1.096	0.807	-0.289	no
CK-0d	RHSIM_Rhsim12G0137900	0.305	-0.069	-0.374	no

Group	Gene	PTR (z-score difference)	z(log2 LFQ)	z(log2 FPKM)	extreme-PTR
CK-0d	RHSIM_Rhsim11G0190900	-0.991	1.080	2.071	no
CK-0d	RHSIM_Rhsim12G0211600	-1.313	-1.124	0.189	low
CK-0d	RHSIM_Rhsim11G0196600	-1.590	-1.717	-0.127	low
CK-0d	RHSIM_Rhsim13G0126400	-0.448	-0.128	0.320	no
CK-0d	RHSIM_Rhsim12G0014900	0.990	0.797	-0.193	no
CK-0d	RHSIM_Rhsim12G0197900	1.118	1.362	0.244	high
CK-0d	RHSIM_Rhsim11G0059000	-0.504	0.201	0.704	no
CK-0d	RHSIM_Rhsim11G0096600	-1.032	-0.213	0.820	low
CK-0d	RHSIM_Rhsim12G0181300	1.446	0.538	-0.908	high
CK-0d	RHSIM_Rhsim12G0092800	-1.262	-1.890	-0.629	low
CK-0d	RHSIM_Rhsim12G0041000	0.002	-1.151	-1.153	no
CK-0d	RHSIM_Rhsim12G0017100	0.826	1.529	0.702	no
CK-0d	RHSIM_Rhsim12G0013600	0.503	1.035	0.533	no
CK-0d	RHSIM_Rhsim12G0023200	0.171	-0.683	-0.854	no
CK-0d	RHSIM_Rhsim13G0010500	-0.536	-0.386	0.150	no
CK-0d	RHSIM_Rhsim12G0122100	-0.600	-0.370	0.230	no
CK-0d	RHSIM_Rhsim12G0197700	-0.594	-0.637	-0.043	no
CK-0d	RHSIM_Rhsim12G0207900	-0.873	-0.104	0.769	no
CK-0d	RHSIM_Rhsim12G0057700	-2.091	-0.509	1.582	low
CK-0d	RHSIM_Rhsim12G0102000	0.595	0.481	-0.114	no
CK-0d	RHSIM_Rhsim12G0198000	0.757	-0.443	-1.201	no
CK-0d	RHSIM_Rhsim12G0121900	-0.219	-0.647	-0.429	no
CK-0d	RHSIM_Rhsim12G0187600	0.256	-0.118	-0.374	no
CK-0d	RHSIM_Rhsim12G0211700	0.244	0.297	0.053	no
CK-0d	RHSIM_Rhsim12G0116600	1.008	1.089	0.081	no
CK-0d	RHSIM_Rhsim11G0192700	0.692	0.788	0.097	no
CK-0d	RHSIM_Rhsim12G0062100	-1.121	-1.421	-0.300	low
CK-0d	RHSIM_Rhsim12G0165600	-0.963	-0.926	0.038	no
CK-0d	RHSIM_Rhsim12G0085300	-0.372	1.153	1.525	no
CK-0d	RHSIM_Rhsim12G0202100	1.393	2.628	1.235	high
CK-0d	RHSIM_Rhsim12G0181600	0.485	-0.672	-1.158	no
CK-0d	RHSIM_Rhsim12G0023100	0.125	-1.121	-1.246	no

Group	Gene	PTR (z-score difference)	z(log2 LFQ)	z(log2 FPKM)	extreme-PTR
CK-0d	RHSIM_Rhsim12G0187800	-2.122	-0.726	1.396	low
CK-0d	RHSIM_Rhsim12G0002600	-1.444	-0.984	0.460	low
CK-0d	RHSIM_Rhsim12G0214000	0.035	-1.371	-1.406	no
CK-0d	RHSIM_Rhsim12G0194700	-0.088	-0.054	0.034	no
CK-0d	RHSIM_Rhsim12G0014100	0.588	0.889	0.301	no
CK-0d	RHSIM_Rhsim12G0164800	-2.428	-0.984	1.444	low
CK-0d	RHSIM_Rhsim12G0211300	-0.865	-1.124	-0.259	no
CK-0d	RHSIM_Rhsim12G0176100	-0.038	0.069	0.107	no
CK-0d	RHSIM_Rhsim12G0086500	-0.164	-0.214	-0.049	no
CK-0d	RHSIM_Rhsim11G0097500	0.003	0.310	0.307	no
CK-0d	RHSIM_Rhsim12G0008600	1.714	0.620	-1.093	high
CK-0d	RHSIM_Rhsim12G0104500	0.741	-0.277	-1.018	no
CK-0d	RHSIM_Rhsim12G0207200	-0.067	-1.067	-1.001	no
CK-0d	RHSIM_Rhsim12G0145700	0.926	2.091	1.165	no
CK-0d	RHSIM_Rhsim12G0046200	3.307	1.186	-2.122	high
CK-0d	RHSIM_Rhsim12G0047900	0.417	0.039	-0.378	no
CK-0d	RHSIM_Rhsim12G0064600	0.748	0.723	-0.025	no
CK-0d	RHSIM_Rhsim12G0108800	-0.281	-1.209	-0.929	no
CK-0d	RHSIM_Rhsim12G0132500	0.290	0.259	-0.031	no
CK-0d	RHSIM_Rhsim12G0189400	0.123	1.045	0.922	no
CK-0d	RHSIM_Rhsim12G0004200	1.511	1.421	-0.089	high
CK-0d	RHSIM_Rhsim12G0037200	0.706	-0.859	-1.565	no
CK-0d	RHSIM_Rhsim12G0208100	0.214	-0.787	-1.000	no
CK-0d	RHSIM_Rhsim12G0060900	-0.881	-0.627	0.254	no
CK-0d	RHSIM_Rhsim12G0158400	0.750	-0.574	-1.324	no
CK-0d	RHSIM_Rhsim12G0146600	-0.739	-0.478	0.261	no
CK-0d	RHSIM_Rhsim12G0152300	-2.490	-1.430	1.060	low
CK-0d	RHSIM_Rhsim11G0164900	1.325	0.142	-1.183	high
CK-0d	RHSIM_Rhsim12G0005300	1.084	-0.322	-1.406	no
CK-0d	RHSIM_Rhsim12G0167600	-2.822	-2.054	0.768	low
CK-0d	RHSIM_Rhsim12G0035600	-0.900	-1.557	-0.657	no
CK-0d	RHSIM_Rhsim12G0182400	-0.977	-0.806	0.171	no

Group	Gene	PTR (z-score difference)	z(log2 LFQ)	z(log2 FPKM)	extreme-PTR
CK-0d	RHSIM_Rhsim10G0099100	0.382	0.930	0.549	no
CK-0d	RHSIM_Rhsim12G0170000	-1.809	0.508	2.316	low
CK-0d	RHSIM_Rhsim12G0210300	-4.338	-0.613	3.725	low
CK-0d	RHSIM_Rhsim12G0006100	-1.401	-0.220	1.181	low
CK-0d	RHSIM_Rhsim12G0047400	-2.091	-1.857	0.234	low
CK-0d	RHSIM_Rhsim12G0043400	-1.267	-1.359	-0.092	low
CK-0d	RHSIM_Rhsim12G0060700	-1.093	-1.188	-0.095	low
CK-0d	RHSIM_Rhsim10G0148000	1.076	1.099	0.023	no
CK-0d	RHSIM_Rhsim12G0138900	-0.615	-1.280	-0.665	no
CK-0d	RHSIM_Rhsim12G0214700	-0.246	-1.465	-1.219	no
CK-0d	RHSIM_Rhsim12G0100800	0.190	2.067	1.877	no
CK-0d	RHSIM_Rhsim12G0061700	0.752	1.715	0.962	no
CK-0d	RHSIM_Rhsim10G0064500	-0.699	0.336	1.035	no
CK-0d	RHSIM_Rhsim12G0176300	0.748	1.175	0.428	no
CK-0d	RHSIM_Rhsim10G0170100	-1.060	-1.211	-0.151	low
CK-0d	RHSIM_Rhsim12G0189900	0.642	1.524	0.882	no
CK-0d	RHSIM_Rhsim12G0121500	-0.173	-0.593	-0.421	no
CK-0d	RHSIM_Rhsim12G0156100	-0.445	1.571	2.016	no
CK-0d	RHSIM_Rhsim10G0144700	-0.407	-0.388	0.019	no
CK-0d	RHSIM_Rhsim10G0012700	0.519	0.059	-0.460	no
CK-0d	RHSIM_Rhsim12G0143100	-1.159	-1.658	-0.499	low
CK-0d	RHSIM_Rhsim12G0072400	0.031	0.139	0.108	no
CK-0d	RHSIM_Rhsim12G0044800	0.445	0.533	0.088	no
CK-0d	RHSIM_Rhsim12G0172500	-0.861	-0.496	0.365	no
CK-0d	RHSIM_Rhsim12G0046500	0.616	2.860	2.243	no
CK-0d	RHSIM_Rhsim10G0043300	-0.062	-0.151	-0.089	no
CK-0d	RHSIM_Rhsim12G0053800	-0.078	1.820	1.898	no
CK-0d	RHSIM_Rhsim12G0036000	0.200	-1.077	-1.278	no
CK-0d	RHSIM_Rhsim12G0129400	1.584	0.746	-0.839	high
CK-0d	RHSIM_Rhsim12G0090600	-0.018	0.367	0.385	no
CK-0d	RHSIM_Rhsim12G0006300	0.208	-0.381	-0.589	no
CK-0d	RHSIM_Rhsim12G0063200	-0.320	-0.699	-0.379	no

Group	Gene	PTR (z-score difference)	z(log2 LFQ)	z(log2 FPKM)	extreme-PTR
CK-0d	RHSIM_Rhsim12G0136000	-0.177	-0.753	-0.576	no
CK-0d	RHSIM_Rhsim12G0183100	0.237	0.051	-0.185	no
CK-0d	RHSIM_Rhsim12G0124300	0.765	0.667	-0.098	no
CK-0d	RHSIM_Rhsim12G0038400	1.046	0.756	-0.289	no
CK-0d	RHSIM_Rhsim12G0074900	1.078	0.844	-0.234	no
CK-0d	RHSIM_Rhsim12G0180300	-0.104	1.533	1.637	no
CK-0d	RHSIM_Rhsim12G0063300	-1.489	-0.807	0.682	low
CK-0d	RHSIM_Rhsim11G0038100	-0.502	0.660	1.162	no
CK-0d	RHSIM_Rhsim12G0002400	0.650	1.189	0.540	no
CK-0d	RHSIM_Rhsim12G0062200	1.898	0.745	-1.153	high
CK-0d	RHSIM_Rhsim12G0129000	-0.007	0.357	0.364	no
CK-0d	RHSIM_Rhsim12G0214600	0.471	0.012	-0.459	no
CK-0d	RHSIM_Rhsim11G0137400	-0.713	-0.087	0.626	no
CK-0d	RHSIM_Rhsim12G0162600	-0.826	-1.350	-0.524	no
CK-0d	RHSIM_Rhsim12G0023300	0.066	1.783	1.717	no
CK-0d	RHSIM_Rhsim11G0021900	0.461	0.025	-0.436	no
CK-0d	RHSIM_Rhsim12G0208800	-0.996	-1.183	-0.187	no
CK-0d	RHSIM_Rhsim11G0168800	0.561	-0.784	-1.345	no
CK-0d	RHSIM_Rhsim10G0180100	-0.183	1.209	1.393	no
CK-0d	RHSIM_Rhsim11G0186600	-0.875	-0.371	0.504	no
CK-0d	RHSIM_Rhsim12G0200100	-1.779	-0.155	1.623	low
CK-0d	RHSIM_Rhsim12G0062000	0.259	0.002	-0.257	no
CK-0d	RHSIM_Rhsim12G0063700	0.649	0.430	-0.219	no
CK-0d	RHSIM_Rhsim12G0104400	2.622	1.788	-0.834	high
CK-0d	RHSIM_Rhsim12G0096800	-1.585	-1.238	0.346	low
CK-0d	RHSIM_Rhsim11G0040500	-0.009	-0.643	-0.634	no
CK-0d	RHSIM_Rhsim12G0080700	-0.197	-0.455	-0.257	no
CK-0d	RHSIM_Rhsim11G0111300	-0.637	0.971	1.608	no
CK-0d	RHSIM_Rhsim11G0127400	0.119	-0.488	-0.607	no
CK-0d	RHSIM_Rhsim11G0115300	1.059	0.508	-0.551	no
CK-0d	RHSIM_Rhsim12G0148700	1.269	1.197	-0.072	high
CK-0d	RHSIM_Rhsim12G0081400	0.695	0.537	-0.157	no

Group	Gene	PTR (z-score difference)	z(log2 LFQ)	z(log2 FPKM)	extreme-PTR
CK-0d	RHSIM_Rhsim11G0186800	-1.549	-0.298	1.251	low
CK-0d	RHSIM_Rhsim11G0024700	0.124	-0.426	-0.550	no
CK-0d	RHSIM_Rhsim12G0136400	0.382	1.094	0.712	no
CK-0d	RHSIM_Rhsim12G0174100	0.294	0.537	0.243	no
CK-0d	RHSIM_Rhsim11G0012900	0.609	0.737	0.128	no
CK-0d	RHSIM_Rhsim10G0171800	-0.307	-0.238	0.069	no
CK-0d	RHSIM_Rhsim10G0206700	-0.644	-1.022	-0.378	no
CK-0d	RHSIM_Rhsim11G0169800	0.461	-0.724	-1.185	no
CK-0d	RHSIM_Rhsim12G0201100	0.005	-0.520	-0.524	no
CK-0d	RHSIM_Rhsim10G0010400	-0.921	-0.746	0.174	no
CK-0d	RHSIM_Rhsim11G0098800	-1.950	0.389	2.338	low
CK-0d	RHSIM_Rhsim10G0092900	0.007	-0.351	-0.358	no
CK-0d	RHSIM_Rhsim11G0164100	-1.170	-1.830	-0.661	low
CK-0d	RHSIM_Rhsim12G0136300	-1.530	-1.032	0.498	low
CK-0d	RHSIM_Rhsim11G0088800	0.518	0.620	0.102	no
CK-0d	RHSIM_Rhsim11G0150000	-1.129	-0.790	0.339	low
CK-0d	RHSIM_Rhsim11G0067900	-0.123	-1.088	-0.965	no
CK-0d	RHSIM_Rhsim12G0187900	-0.294	-0.596	-0.301	no
CK-0d	RHSIM_Rhsim11G0098700	0.898	3.067	2.169	no
CK-0d	RHSIM_Rhsim11G0032600	0.561	0.616	0.055	no
CK-0d	RHSIM_Rhsim12G0200200	-1.391	-1.200	0.191	low
CK-0d	RHSIM_Rhsim11G0134300	-0.637	-0.750	-0.113	no
CK-0d	RHSIM_Rhsim12G0143000	-0.721	-0.659	0.062	no
CK-0d	RHSIM_Rhsim11G0122100	1.379	-0.441	-1.820	high
CK-0d	RHSIM_Rhsim11G0024800	-0.260	0.278	0.538	no
CK-0d	RHSIM_Rhsim11G0043200	-3.362	-1.038	2.323	low
CK-0d	RHSIM_Rhsim11G0103200	-1.779	-2.108	-0.329	low
CK-0d	RHSIM_Rhsim11G0173000	-1.164	1.232	2.397	low
CK-0d	RHSIM_Rhsim11G0182500	-0.780	-0.429	0.350	no
CK-0d	RHSIM_Rhsim10G0059600	1.155	0.354	-0.802	high
CK-0d	RHSIM_Rhsim11G0024900	-0.358	0.179	0.536	no
CK-0d	RHSIM_Rhsim10G0128400	0.319	0.905	0.586	no

Group	Gene	PTR (z-score difference)	z(log2 LFQ)	z(log2 FPKM)	extreme-PTR
CK-0d	RHSIM_Rhsim10G0055700	0.291	-0.003	-0.295	no
CK-0d	RHSIM_Rhsim11G0187800	-0.762	-1.211	-0.449	no
CK-0d	RHSIM_Rhsim11G0034100	0.675	2.581	1.906	no
CK-0d	RHSIM_Rhsim11G0141700	1.977	1.911	-0.065	high
CK-0d	RHSIM_Rhsim11G0159600	0.833	0.164	-0.669	no
CK-0d	RHSIM_Rhsim11G0066600	-0.608	0.769	1.377	no
CK-0d	RHSIM_Rhsim11G0054300	1.197	0.312	-0.885	high
CK-0d	RHSIM_Rhsim11G0069700	-0.111	-0.715	-0.604	no
CK-0d	RHSIM_Rhsim10G0084900	-0.200	-1.283	-1.082	no
CK-0d	RHSIM_Rhsim11G0070000	0.020	-0.084	-0.103	no
CK-0d	RHSIM_Rhsim10G0164900	-0.995	-0.220	0.775	no
CK-0d	RHSIM_Rhsim11G0070400	-2.577	-0.685	1.892	low
CK-0d	RHSIM_Rhsim11G0074900	-0.404	-0.900	-0.496	no
CK-0d	RHSIM_Rhsim11G0179100	-1.387	-0.580	0.807	low
CK-0d	RHSIM_Rhsim10G0131900	-0.048	-0.253	-0.205	no
CK-0d	RHSIM_Rhsim11G0027400	-0.153	-0.274	-0.120	no
CK-0d	RHSIM_Rhsim11G0063300	0.633	-0.999	-1.632	no
CK-0d	RHSIM_Rhsim11G0037700	0.097	-0.950	-1.047	no
CK-0d	RHSIM_Rhsim10G0063500	0.601	1.274	0.673	no
CK-0d	RHSIM_Rhsim11G0187700	0.083	1.998	1.915	no
CK-0d	RHSIM_Rhsim11G0069600	0.406	-0.221	-0.626	no
CK-0d	RHSIM_Rhsim10G0146500	0.542	-1.140	-1.683	no
CK-0d	RHSIM_Rhsim11G0056500	0.200	0.087	-0.113	no
CK-0d	RHSIM_Rhsim11G0008200	1.531	0.012	-1.519	high
CK-0d	RHSIM_Rhsim11G0159400	1.768	-0.098	-1.866	high
CK-0d	RHSIM_Rhsim11G0044300	-0.224	0.324	0.549	no
CK-0d	RHSIM_Rhsim10G0065100	0.064	0.462	0.398	no
CK-0d	RHSIM_Rhsim11G0178600	0.609	-0.579	-1.188	no
CK-0d	RHSIM_Rhsim11G0159100	-1.841	-1.225	0.616	low
CK-0d	RHSIM_Rhsim10G0017800	-1.487	-1.098	0.389	low
CK-0d	RHSIM_Rhsim11G0137700	0.305	0.281	-0.024	no
CK-0d	RHSIM_Rhsim11G0046100	0.252	0.518	0.266	no

Group	Gene	PTR (z-score difference)	z(log2 LFQ)	z(log2 FPKM)	extreme-PTR
CK-0d	RHSIM_Rhsim11G0001100	-2.630	-2.036	0.593	low
CK-0d	RHSIM_Rhsim11G0187200	-0.289	0.107	0.396	no
CK-0d	RHSIM_Rhsim11G0099300	0.039	-0.796	-0.834	no
CK-0d	RHSIM_Rhsim11G0187000	0.202	0.511	0.309	no
CK-0d	RHSIM_Rhsim11G0092100	-0.509	-0.188	0.321	no
CK-0d	RHSIM_Rhsim09G0008800	0.538	0.533	-0.006	no
CK-0d	RHSIM_Rhsim11G0066000	-0.166	-0.443	-0.277	no
CK-0d	RHSIM_Rhsim11G0183000	-0.806	-0.606	0.200	no
CK-0d	RHSIM_Rhsim09G0103900	-0.109	0.013	0.122	no
CK-0d	RHSIM_Rhsim11G0075300	-1.532	0.188	1.720	low
CK-0d	RHSIM_Rhsim11G0049500	-0.590	-1.231	-0.641	no
CK-0d	RHSIM_Rhsim11G0193400	1.554	0.375	-1.179	high
CK-0d	RHSIM_Rhsim11G0019200	-1.077	-0.902	0.175	low
CK-0d	RHSIM_Rhsim11G0121200	0.287	-0.039	-0.326	no
CK-0d	RHSIM_Rhsim09G0002100	1.360	0.933	-0.426	high
CK-0d	RHSIM_Rhsim11G0160800	1.212	1.740	0.528	high
CK-0d	RHSIM_Rhsim11G0001400	0.040	-0.610	-0.651	no
CK-0d	RHSIM_Rhsim11G0021000	-0.348	-0.104	0.245	no
CK-0d	RHSIM_Rhsim11G0160000	-0.364	-0.442	-0.078	no
CK-0d	RHSIM_Rhsim11G0160500	-0.135	-0.508	-0.373	no
CK-0d	RHSIM_Rhsim11G0038900	0.015	-0.019	-0.034	no
CK-0d	RHSIM_Rhsim11G0052300	-0.686	-1.153	-0.467	no
CK-0d	RHSIM_Rhsim11G0088200	-3.122	-0.432	2.689	low
CK-0d	RHSIM_Rhsim11G0138000	0.290	-1.587	-1.877	no
CK-0d	RHSIM_Rhsim11G0058000	1.607	0.267	-1.340	high
CK-0d	RHSIM_Rhsim11G0148400	0.390	1.236	0.846	no
CK-0d	RHSIM_Rhsim11G0003500	-0.933	-0.650	0.283	no
CK-0d	RHSIM_Rhsim10G0186400	1.585	1.217	-0.368	high
CK-0d	RHSIM_Rhsim11G0142200	-0.752	-1.375	-0.623	no
CK-0d	RHSIM_Rhsim11G0129100	-0.024	-0.691	-0.668	no
CK-0d	RHSIM_Rhsim10G0108500	0.327	-0.484	-0.811	no
CK-0d	RHSIM_Rhsim09G0038500	-0.528	-0.854	-0.326	no

Group	Gene	PTR (z-score difference)	z(log2 LFQ)	z(log2 FPKM)	extreme-PTR
CK-0d	RHSIM_Rhsim10G0009700	-0.691	0.233	0.925	no
CK-0d	RHSIM_Rhsim09G0212500	0.151	0.662	0.512	no
CK-0d	RHSIM_Rhsim11G0192800	-0.895	-0.635	0.260	no
CK-0d	RHSIM_Rhsim11G0055800	0.768	0.312	-0.456	no
CK-0d	RHSIM_Rhsim10G0162600	0.033	-0.086	-0.119	no
CK-0d	RHSIM_Rhsim11G0148600	-0.236	0.120	0.356	no
CK-0d	RHSIM_Rhsim09G0023700	0.310	-0.730	-1.041	no
CK-0d	RHSIM_Rhsim10G0193700	1.822	-0.057	-1.879	high
CK-0d	RHSIM_Rhsim11G0032300	-2.097	-1.639	0.458	low
CK-0d	RHSIM_Rhsim11G0035800	1.050	0.389	-0.661	no
CK-0d	RHSIM_Rhsim11G0027600	0.826	1.757	0.931	no
CK-0d	RHSIM_Rhsim09G0052000	0.717	0.058	-0.658	no
CK-0d	RHSIM_Rhsim11G0179300	-0.457	-0.783	-0.326	no
CK-0d	RHSIM_Rhsim11G0027500	3.134	1.079	-2.055	high
CK-0d	RHSIM_Rhsim11G0046900	-0.938	-1.669	-0.730	no
CK-0d	RHSIM_Rhsim11G0057900	1.086	-0.444	-1.530	no
CK-0d	RHSIM_Rhsim11G0072300	0.709	0.081	-0.628	no
CK-0d	RHSIM_Rhsim11G0170000	-0.497	0.213	0.709	no
CK-0d	RHSIM_Rhsim09G0188200	-0.284	-0.140	0.143	no
CK-0d	RHSIM_Rhsim09G0200200	0.282	0.391	0.109	no
CK-0d	RHSIM_Rhsim10G0062400	-0.069	-0.842	-0.774	no
CK-0d	RHSIM_Rhsim11G0038000	-0.043	0.939	0.983	no
CK-0d	RHSIM_Rhsim11G0047500	0.526	-0.773	-1.299	no
CK-0d	RHSIM_Rhsim11G0054400	-1.136	0.163	1.299	low
CK-0d	RHSIM_Rhsim10G0039600	0.977	0.979	0.002	no
CK-0d	RHSIM_Rhsim09G0000600	1.518	-0.518	-2.037	high
CK-0d	RHSIM_Rhsim11G0100000	0.933	1.229	0.296	no
CK-0d	RHSIM_Rhsim11G0026600	0.006	-0.785	-0.791	no
CK-0d	RHSIM_Rhsim10G0063000	0.429	0.629	0.200	no
CK-0d	RHSIM_Rhsim11G0044500	0.046	-0.185	-0.230	no
CK-0d	RHSIM_Rhsim11G0163700	-2.447	-0.724	1.722	low
CK-0d	RHSIM_Rhsim09G0186800	0.405	1.283	0.878	no

Group	Gene	PTR (z-score difference)	z(log2 LFQ)	z(log2 FPKM)	extreme-PTR
CK-0d	RHSIM_Rhsim09G0133100	-0.955	-1.174	-0.219	no
CK-0d	RHSIM_Rhsim09G0178500	-0.834	0.061	0.895	no
CK-0d	RHSIM_Rhsim10G0085800	0.055	-0.557	-0.612	no
CK-0d	RHSIM_Rhsim09G0192900	-0.578	1.233	1.810	no
CK-0d	RHSIM_Rhsim09G0180100	1.339	2.147	0.808	high
CK-0d	RHSIM_Rhsim11G0037300	-0.984	-1.049	-0.066	no
CK-0d	RHSIM_Rhsim09G0165200	-1.117	-0.756	0.361	low
CK-0d	RHSIM_Rhsim09G0103600	0.193	-1.037	-1.231	no
CK-0d	RHSIM_Rhsim09G0087900	-0.225	-0.126	0.099	no
CK-0d	RHSIM_Rhsim10G0118400	-0.505	-0.420	0.085	no
CK-0d	RHSIM_Rhsim11G0141000	0.791	0.362	-0.428	no
CK-0d	RHSIM_Rhsim09G0185200	0.398	0.894	0.496	no
CK-0d	RHSIM_Rhsim09G0151700	-0.222	2.185	2.408	no
CK-0d	RHSIM_Rhsim11G0182100	1.125	0.415	-0.710	high
CK-0d	RHSIM_Rhsim11G0021500	1.712	1.910	0.198	high
CK-0d	RHSIM_Rhsim11G0169500	0.593	0.013	-0.579	no
CK-0d	RHSIM_Rhsim11G0037600	0.205	-0.125	-0.330	no
CK-0d	RHSIM_Rhsim10G0020800	-0.239	-0.266	-0.027	no
CK-0d	RHSIM_Rhsim11G0111400	-1.272	-0.409	0.863	low
CK-0d	RHSIM_Rhsim10G0014700	0.955	-0.254	-1.209	no
CK-0d	RHSIM_Rhsim11G0052900	1.217	1.644	0.427	high
CK-0d	RHSIM_Rhsim10G0138900	-0.505	-1.143	-0.637	no
CK-0d	RHSIM_Rhsim10G0159500	2.114	0.776	-1.338	high
CK-0d	RHSIM_Rhsim10G0082200	-0.218	0.159	0.377	no
CK-0d	RHSIM_Rhsim10G0183600	-1.357	-0.425	0.931	low
CK-0d	RHSIM_Rhsim10G0169300	0.314	-0.920	-1.234	no
CK-0d	RHSIM_Rhsim10G0173800	1.006	1.260	0.254	no
CK-0d	RHSIM_Rhsim09G0142600	0.313	0.149	-0.164	no
CK-0d	RHSIM_Rhsim10G0195900	0.090	-1.327	-1.417	no
CK-0d	RHSIM_Rhsim10G0067500	-0.124	1.431	1.555	no
CK-0d	RHSIM_Rhsim11G0146300	0.993	1.116	0.123	no
CK-0d	RHSIM_Rhsim11G0047200	-1.309	-1.978	-0.669	low

Group	Gene	PTR (z-score difference)	z(log2 LFQ)	z(log2 FPKM)	extreme-PTR
CK-0d	RHSIM_Rhsim10G0052200	1.096	0.517	-0.579	no
CK-0d	RHSIM_Rhsim09G0129300	-0.268	0.143	0.412	no
CK-0d	RHSIM_Rhsim10G0008900	0.281	0.236	-0.045	no
CK-0d	RHSIM_Rhsim10G0007400	-0.898	-1.181	-0.283	no
CK-0d	RHSIM_Rhsim09G0209100	-1.234	0.082	1.317	low
CK-0d	RHSIM_Rhsim10G0004900	0.065	-0.190	-0.255	no
CK-0d	RHSIM_Rhsim10G0129200	-1.376	-1.501	-0.125	low
CK-0d	RHSIM_Rhsim10G0094900	0.915	-0.187	-1.102	no
CK-0d	RHSIM_Rhsim09G0032600	-0.341	-0.695	-0.354	no
CK-0d	RHSIM_Rhsim10G0104300	-0.693	-0.455	0.238	no
CK-0d	RHSIM_Rhsim10G0171200	-0.316	0.143	0.458	no
CK-0d	RHSIM_Rhsim10G0129100	-1.513	-1.246	0.266	low
CK-0d	RHSIM_Rhsim10G0122800	0.067	-0.866	-0.933	no
CK-0d	RHSIM_Rhsim10G0184100	1.663	0.769	-0.894	high
CK-0d	RHSIM_Rhsim09G0158400	1.016	1.207	0.191	no
CK-0d	RHSIM_Rhsim09G0007900	0.481	1.398	0.917	no
CK-0d	RHSIM_Rhsim10G0157900	0.272	0.294	0.022	no
CK-0d	RHSIM_Rhsim09G0073500	0.830	0.753	-0.076	no
CK-0d	RHSIM_Rhsim10G0010500	1.654	1.756	0.102	high
CK-0d	RHSIM_Rhsim10G0156400	0.736	-1.163	-1.900	no
CK-0d	RHSIM_Rhsim10G0188300	0.402	0.267	-0.134	no
CK-0d	RHSIM_Rhsim10G0137900	1.381	0.780	-0.601	high
CK-0d	RHSIM_Rhsim09G0063200	0.328	0.633	0.305	no
CK-0d	RHSIM_Rhsim10G0210900	-0.162	1.036	1.199	no
CK-0d	RHSIM_Rhsim10G0156900	-0.286	0.305	0.590	no
CK-0d	RHSIM_Rhsim10G0080600	1.120	1.010	-0.110	high
CK-0d	RHSIM_Rhsim10G0191400	-1.847	-2.282	-0.435	low
CK-0d	RHSIM_Rhsim10G0043400	0.332	-0.397	-0.729	no
CK-0d	RHSIM_Rhsim10G0044200	0.785	1.087	0.303	no
CK-0d	RHSIM_Rhsim10G0110700	1.301	0.324	-0.977	high
CK-0d	RHSIM_Rhsim09G0114600	-2.746	-0.499	2.248	low
CK-0d	RHSIM_Rhsim10G0174000	1.969	0.992	-0.977	high

Group	Gene	PTR (z-score difference)	z(log2 LFQ)	z(log2 FPKM)	extreme-PTR
CK-0d	RHSIM_Rhsim10G0172400	0.709	0.557	-0.152	no
CK-0d	RHSIM_Rhsim09G0022800	0.374	1.152	0.778	no
CK-0d	RHSIM_Rhsim10G0115200	0.322	-1.028	-1.350	no
CK-0d	RHSIM_Rhsim10G0205500	0.967	-0.895	-1.863	no
CK-0d	RHSIM_Rhsim09G0059100	-3.407	-1.472	1.935	low
CK-0d	RHSIM_Rhsim10G0133100	-0.684	-0.579	0.104	no
CK-0d	RHSIM_Rhsim10G0151000	0.695	-0.058	-0.753	no
CK-0d	RHSIM_Rhsim10G0182800	0.900	1.054	0.154	no
CK-0d	RHSIM_Rhsim10G0156600	0.110	0.677	0.567	no
CK-0d	RHSIM_Rhsim10G0050900	1.782	0.561	-1.221	high
CK-0d	RHSIM_Rhsim10G0172000	-0.865	-0.254	0.611	no
CK-0d	RHSIM_Rhsim10G0085400	0.580	-0.470	-1.050	no
CK-0d	RHSIM_Rhsim08G0156400	0.569	-0.623	-1.192	no
CK-0d	RHSIM_Rhsim10G0147800	-0.093	-1.096	-1.003	no
CK-0d	RHSIM_Rhsim10G0177400	0.115	-1.059	-1.173	no
CK-0d	RHSIM_Rhsim08G0211300	-1.218	-0.340	0.878	low
CK-0d	RHSIM_Rhsim08G0064400	-0.629	-1.222	-0.593	no
CK-0d	RHSIM_Rhsim10G0203500	-1.184	-1.403	-0.219	low
CK-0d	RHSIM_Rhsim10G0032200	1.081	-0.758	-1.839	no
CK-0d	RHSIM_Rhsim08G0204300	0.039	-1.058	-1.097	no
CK-0d	RHSIM_Rhsim10G0114700	0.793	0.142	-0.652	no
CK-0d	RHSIM_Rhsim10G0093100	-0.023	-1.148	-1.126	no
CK-0d	RHSIM_Rhsim10G0191100	-0.712	0.595	1.307	no
CK-0d	RHSIM_Rhsim10G0197500	-0.400	-0.136	0.264	no
CK-0d	RHSIM_Rhsim10G0144500	-1.048	-0.704	0.343	low
CK-0d	RHSIM_Rhsim10G0013400	-0.594	-2.023	-1.429	no
CK-0d	RHSIM_Rhsim08G0099700	-0.244	0.014	0.259	no
CK-0d	RHSIM_Rhsim10G0212300	-0.802	-0.864	-0.062	no
CK-0d	RHSIM_Rhsim08G0220500	0.779	0.919	0.139	no
CK-0d	RHSIM_Rhsim10G0142000	-0.191	-1.796	-1.605	no
CK-0d	RHSIM_Rhsim08G0251300	-0.043	0.240	0.284	no
CK-0d	RHSIM_Rhsim08G0007600	0.166	-0.927	-1.093	no

Group	Gene	PTR (z-score difference)	z(log2 LFQ)	z(log2 FPKM)	extreme-PTR
CK-0d	RHSIM_Rhsim10G0209300	-0.465	-1.475	-1.010	no
CK-0d	RHSIM_Rhsim10G0166800	0.328	0.701	0.373	no
CK-0d	RHSIM_Rhsim09G0002300	0.785	0.226	-0.559	no
CK-0d	RHSIM_Rhsim10G0083700	-0.116	-0.820	-0.704	no
CK-0d	RHSIM_Rhsim10G0150100	-0.334	-0.794	-0.460	no
CK-0d	RHSIM_Rhsim10G0127500	0.362	0.324	-0.038	no
CK-0d	RHSIM_Rhsim08G0012700	0.759	-0.010	-0.769	no
CK-0d	RHSIM_Rhsim08G0146300	0.441	0.029	-0.411	no
CK-0d	RHSIM_Rhsim10G0173500	-0.161	-0.951	-0.790	no
CK-0d	RHSIM_Rhsim08G0066300	0.939	1.229	0.290	no
CK-0d	RHSIM_Rhsim09G0209900	-0.783	-0.562	0.221	no
CK-0d	RHSIM_Rhsim10G0193100	0.338	0.266	-0.073	no
CK-0d	RHSIM_Rhsim10G0124700	-0.373	0.253	0.625	no
CK-0d	RHSIM_Rhsim10G0124000	-1.926	-0.687	1.238	low
CK-0d	RHSIM_Rhsim09G0032900	0.523	1.730	1.207	no
CK-0d	RHSIM_Rhsim10G0157000	0.678	-0.461	-1.140	no
CK-0d	RHSIM_Rhsim10G0021300	0.103	3.598	3.495	no
CK-0d	RHSIM_Rhsim09G0172500	0.474	0.844	0.370	no
CK-0d	RHSIM_Rhsim09G0069400	-0.581	-0.236	0.345	no
CK-0d	RHSIM_Rhsim08G0053400	-0.582	0.339	0.920	no
CK-0d	RHSIM_Rhsim10G0066000	1.550	1.233	-0.317	high
CK-0d	RHSIM_Rhsim09G0205000	-2.282	-0.957	1.325	low
CK-0d	RHSIM_Rhsim09G0034400	1.084	-0.210	-1.295	no
CK-0d	RHSIM_Rhsim10G0190900	-0.236	0.109	0.346	no
CK-0d	RHSIM_Rhsim09G0007800	0.073	-0.591	-0.664	no
CK-0d	RHSIM_Rhsim09G0205300	0.400	-1.337	-1.737	no
CK-0d	RHSIM_Rhsim10G0112400	0.421	-0.345	-0.766	no
CK-0d	RHSIM_Rhsim08G0092300	-0.572	-1.299	-0.727	no
CK-0d	RHSIM_Rhsim10G0005200	-0.417	-0.964	-0.547	no
CK-0d	RHSIM_Rhsim08G0045300	0.573	0.562	-0.011	no
CK-0d	RHSIM_Rhsim09G0195700	0.526	1.634	1.108	no
CK-0d	RHSIM_Rhsim09G0177100	-0.766	0.762	1.528	no

Group	Gene	PTR (z-score difference)	z(log2 LFQ)	z(log2 FPKM)	extreme-PTR
CK-0d	RHSIM_Rhsim09G0205800	-0.827	-1.483	-0.657	no
CK-0d	RHSIM_Rhsim05G0149300	-0.555	0.625	1.180	no
CK-0d	RHSIM_Rhsim08G0128200	-0.297	-1.043	-0.746	no
CK-0d	RHSIM_Rhsim08G0079900	-0.750	0.137	0.888	no
CK-0d	RHSIM_Rhsim09G0163400	0.140	1.587	1.447	no
CK-0d	RHSIM_Rhsim08G0171900	0.081	0.877	0.795	no
CK-0d	RHSIM_Rhsim08G0225400	0.231	0.415	0.185	no
CK-0d	RHSIM_Rhsim10G0011000	1.019	0.611	-0.408	no
CK-0d	RHSIM_Rhsim10G0003100	0.189	-0.698	-0.887	no
CK-0d	RHSIM_Rhsim08G0161200	-0.284	-1.204	-0.921	no
CK-0d	RHSIM_Rhsim09G0060000	0.405	0.539	0.134	no
CK-0d	RHSIM_Rhsim09G0091000	0.234	-0.128	-0.361	no
CK-0d	RHSIM_Rhsim09G0093400	1.073	0.194	-0.879	no
CK-0d	RHSIM_Rhsim10G0163400	-1.241	0.137	1.378	low
CK-0d	RHSIM_Rhsim08G0176500	-0.708	-0.862	-0.154	no
CK-0d	RHSIM_Rhsim09G0198100	1.366	-0.133	-1.500	high
CK-0d	RHSIM_Rhsim10G0063200	-0.202	0.371	0.573	no
CK-0d	RHSIM_Rhsim09G0007600	0.689	-0.500	-1.189	no
CK-0d	RHSIM_Rhsim09G0211900	-0.309	1.322	1.631	no
CK-0d	RHSIM_Rhsim09G0181300	-0.939	-1.054	-0.114	no
CK-0d	RHSIM_Rhsim10G0067400	0.386	-0.072	-0.458	no
CK-0d	RHSIM_Rhsim08G0112400	-0.517	-0.892	-0.375	no
CK-0d	RHSIM_Rhsim09G0209500	-0.646	-1.936	-1.290	no
CK-0d	RHSIM_Rhsim08G0172700	0.309	-1.409	-1.718	no
CK-0d	RHSIM_Rhsim10G0191500	-0.408	-0.833	-0.425	no
CK-0d	RHSIM_Rhsim09G0009500	-1.783	-0.604	1.179	low
CK-0d	RHSIM_Rhsim09G0091300	-0.795	-0.487	0.308	no
CK-0d	RHSIM_Rhsim08G0188100	-0.528	0.087	0.615	no
CK-0d	RHSIM_Rhsim09G0111200	-0.509	-0.697	-0.188	no
CK-0d	RHSIM_Rhsim08G0242700	-1.513	-1.495	0.018	low
CK-0d	RHSIM_Rhsim10G0000800	1.236	-0.866	-2.102	high
CK-0d	RHSIM_Rhsim09G0135300	-0.702	-1.414	-0.712	no

Group	Gene	PTR (z-score difference)	z(log2 LFQ)	z(log2 FPKM)	extreme-PTR
CK-0d	RHSIM_Rhsim09G0137200	-1.855	-1.199	0.656	low
CK-0d	RHSIM_Rhsim10G0114000	-0.302	1.096	1.398	no
CK-0d	RHSIM_Rhsim08G0042300	-0.598	0.171	0.769	no
CK-0d	RHSIM_Rhsim09G0141800	0.739	0.472	-0.267	no
CK-0d	RHSIM_Rhsim10G0165400	-0.152	-0.258	-0.105	no
CK-0d	RHSIM_Rhsim09G0037400	0.134	0.231	0.096	no
CK-0d	RHSIM_Rhsim10G0166900	-0.526	-0.886	-0.360	no
CK-0d	RHSIM_Rhsim09G0029500	-0.629	-0.553	0.075	no
CK-0d	RHSIM_Rhsim08G0236100	-2.007	-1.620	0.387	low
CK-0d	RHSIM_Rhsim08G0123900	0.509	0.569	0.060	no
CK-0d	RHSIM_Rhsim10G0050000	1.452	0.105	-1.347	high
CK-0d	RHSIM_Rhsim10G0021100	1.238	0.064	-1.175	high
CK-0d	RHSIM_Rhsim09G0107300	0.711	0.186	-0.525	no
CK-0d	RHSIM_Rhsim10G0069600	-1.507	-0.093	1.413	low
CK-0d	RHSIM_Rhsim09G0181500	-0.835	-0.896	-0.061	no
CK-0d	RHSIM_Rhsim09G0176600	-0.464	0.612	1.076	no
CK-0d	RHSIM_Rhsim10G0188000	-0.257	-1.292	-1.034	no
CK-0d	RHSIM_Rhsim09G0126200	-1.032	-1.666	-0.634	low
CK-0d	RHSIM_Rhsim08G0033900	-1.269	-1.127	0.142	low
CK-0d	RHSIM_Rhsim09G0011600	-0.775	-0.376	0.400	no
CK-0d	RHSIM_Rhsim08G0070600	-0.125	-0.107	0.018	no
CK-0d	RHSIM_Rhsim09G0008400	1.074	2.227	1.153	no
CK-0d	RHSIM_Rhsim09G0028000	-0.568	-0.407	0.161	no
CK-0d	RHSIM_Rhsim08G0101600	-1.257	-2.000	-0.743	low
CK-0d	RHSIM_Rhsim09G0212400	-0.773	-0.601	0.171	no
CK-0d	RHSIM_Rhsim08G0079700	-1.119	0.967	2.086	low
CK-0d	RHSIM_Rhsim09G0164000	-0.141	0.269	0.410	no
CK-0d	RHSIM_Rhsim09G0179900	0.320	0.996	0.676	no
CK-0d	RHSIM_Rhsim09G0154200	0.037	-0.232	-0.270	no
CK-0d	RHSIM_Rhsim09G0103500	-0.043	-1.123	-1.081	no
CK-0d	RHSIM_Rhsim08G0147100	1.696	0.912	-0.784	high
CK-0d	RHSIM_Rhsim09G0071200	-2.066	-0.889	1.177	low

Group	Gene	PTR (z-score difference)	z(log2 LFQ)	z(log2 FPKM)	extreme-PTR
CK-0d	RHSIM_Rhsim09G0126000	2.420	0.498	-1.922	high
CK-0d	RHSIM_Rhsim09G0012000	-2.262	-0.732	1.530	low
CK-0d	RHSIM_Rhsim09G0191500	0.112	-0.298	-0.410	no
CK-0d	RHSIM_Rhsim09G0177000	0.398	-0.531	-0.928	no
CK-0d	RHSIM_Rhsim09G0086300	-0.342	-0.183	0.159	no
CK-0d	RHSIM_Rhsim09G0192800	-0.962	-0.404	0.558	no
CK-0d	RHSIM_Rhsim09G0063500	-0.874	-1.600	-0.726	no
CK-0d	RHSIM_Rhsim09G0182600	-0.047	0.645	0.692	no
CK-0d	RHSIM_Rhsim09G0010800	-0.748	-0.957	-0.209	no
CK-0d	RHSIM_Rhsim09G0098400	-2.178	-1.648	0.530	low
CK-0d	RHSIM_Rhsim08G0214300	0.653	1.772	1.119	no
CK-0d	RHSIM_Rhsim09G0099000	-0.917	-1.431	-0.514	no
CK-0d	RHSIM_Rhsim08G0035400	-1.154	-1.590	-0.436	low
CK-0d	RHSIM_Rhsim08G0097500	-1.727	-1.545	0.182	low
CK-0d	RHSIM_Rhsim09G0065700	1.023	-0.582	-1.604	no
CK-0d	RHSIM_Rhsim09G0075600	0.022	-0.830	-0.853	no
CK-0d	RHSIM_Rhsim09G0094200	-2.528	-1.399	1.129	low
CK-0d	RHSIM_Rhsim09G0184500	1.461	0.388	-1.073	high
CK-0d	RHSIM_Rhsim09G0147200	-0.163	1.398	1.561	no
CK-0d	RHSIM_Rhsim08G0145400	0.869	0.263	-0.605	no
CK-0d	RHSIM_Rhsim09G0190300	0.023	0.459	0.435	no
CK-0d	RHSIM_Rhsim09G0193300	0.213	0.287	0.074	no
CK-0d	RHSIM_Rhsim09G0055700	0.934	-0.995	-1.929	no
CK-0d	RHSIM_Rhsim09G0204600	-1.637	-1.073	0.564	low
CK-0d	RHSIM_Rhsim08G0200000	-0.177	0.234	0.410	no
CK-0d	RHSIM_Rhsim09G0069300	1.459	0.617	-0.842	high
CK-0d	RHSIM_Rhsim09G0179400	1.238	0.445	-0.793	high
CK-0d	RHSIM_Rhsim09G0157600	0.006	-0.715	-0.721	no
CK-0d	RHSIM_Rhsim09G0091900	-0.816	-0.277	0.539	no
CK-0d	RHSIM_Rhsim09G0117900	0.835	0.235	-0.600	no
CK-0d	RHSIM_Rhsim09G0131900	-0.716	-0.015	0.702	no
CK-0d	RHSIM_Rhsim07G0198600	0.063	2.360	2.297	no

Group	Gene	PTR (z-score difference)	z(log2 LFQ)	z(log2 FPKM)	extreme-PTR
CK-0d	RHSIM_Rhsim09G0070800	0.418	1.023	0.605	no
CK-0d	RHSIM_Rhsim09G0155700	0.436	-0.330	-0.766	no
CK-0d	RHSIM_Rhsim09G0186700	2.036	0.696	-1.341	high
CK-0d	RHSIM_Rhsim09G0145100	-1.548	-1.391	0.157	low
CK-0d	RHSIM_Rhsim09G0129000	-0.527	-0.685	-0.158	no
CK-0d	RHSIM_Rhsim07G0224400	-0.461	-0.959	-0.498	no
CK-0d	RHSIM_Rhsim09G0137500	-2.580	-0.796	1.784	low
CK-0d	RHSIM_Rhsim09G0171900	0.806	2.757	1.951	no
CK-0d	RHSIM_Rhsim09G0010300	0.188	-0.653	-0.841	no
CK-0d	RHSIM_Rhsim07G0144600	-0.554	-0.125	0.429	no
CK-0d	RHSIM_Rhsim09G0025700	1.614	-0.040	-1.654	high
CK-0d	RHSIM_Rhsim07G0074600	-0.420	-0.692	-0.272	no
CK-0d	RHSIM_Rhsim07G0153100	0.159	0.557	0.398	no
CK-0d	RHSIM_Rhsim08G0119600	-0.169	0.065	0.234	no
CK-0d	RHSIM_Rhsim09G0054700	0.338	0.027	-0.311	no
CK-0d	RHSIM_Rhsim07G0217300	2.699	0.548	-2.151	high
CK-0d	RHSIM_Rhsim09G0123100	-0.587	-0.178	0.410	no
CK-0d	RHSIM_Rhsim09G0080400	-0.251	-0.143	0.108	no
CK-0d	RHSIM_Rhsim09G0138200	-1.316	-1.484	-0.168	low
CK-0d	RHSIM_Rhsim08G0135400	-1.386	-1.631	-0.245	low
CK-0d	RHSIM_Rhsim07G0018300	1.273	0.954	-0.318	high
CK-0d	RHSIM_Rhsim08G0214800	-0.863	-0.227	0.636	no
CK-0d	RHSIM_Rhsim03G0130900	0.794	0.801	0.007	no
CK-0d	RHSIM_Rhsim09G0128900	0.270	0.331	0.062	no
CK-0d	RHSIM_Rhsim09G0054900	-0.934	-0.958	-0.024	no
CK-0d	RHSIM_Rhsim08G0045200	0.473	-0.012	-0.485	no
CK-0d	RHSIM_Rhsim07G0258100	0.314	0.149	-0.165	no
CK-0d	RHSIM_Rhsim07G0246300	-1.297	-1.526	-0.229	low
CK-0d	RHSIM_Rhsim09G0044200	-2.088	-0.187	1.902	low
CK-0d	RHSIM_Rhsim08G0089100	-0.343	0.946	1.289	no
CK-0d	RHSIM_Rhsim07G0061000	-2.021	-1.357	0.664	low
CK-0d	RHSIM_Rhsim08G0213200	-2.229	-1.136	1.093	low

Group	Gene	PTR (z-score difference)	z(log2 LFQ)	z(log2 FPKM)	extreme-PTR
CK-0d	RHSIM_Rhsim07G0068600	0.591	-0.144	-0.735	no
CK-0d	RHSIM_Rhsim07G0243600	1.015	-0.568	-1.583	no
CK-0d	RHSIM_Rhsim08G0223600	0.504	1.719	1.215	no
CK-0d	RHSIM_Rhsim09G0089500	0.482	-0.216	-0.698	no
CK-0d	RHSIM_Rhsim07G0088700	0.369	0.655	0.285	no
CK-0d	RHSIM_Rhsim07G0042900	-1.362	-2.109	-0.747	low
CK-0d	RHSIM_Rhsim09G0033900	-2.400	-1.447	0.954	low
CK-0d	RHSIM_Rhsim09G0142900	-0.231	-0.112	0.118	no
CK-0d	RHSIM_Rhsim07G0200500	0.213	-0.054	-0.267	no
CK-0d	RHSIM_Rhsim09G0113800	0.609	-0.396	-1.004	no
CK-0d	RHSIM_Rhsim09G0212800	1.139	0.096	-1.043	high
CK-0d	RHSIM_Rhsim09G0178400	0.827	1.511	0.684	no
CK-0d	RHSIM_Rhsim08G0111900	0.359	0.687	0.328	no
CK-0d	RHSIM_Rhsim09G0094000	-0.460	-0.115	0.346	no
CK-0d	RHSIM_Rhsim08G0126700	1.990	1.569	-0.422	high
CK-0d	RHSIM_Rhsim07G0192400	1.034	-0.453	-1.486	no
CK-0d	RHSIM_Rhsim08G0021800	2.139	2.234	0.095	high
CK-0d	RHSIM_Rhsim09G0088900	0.623	0.245	-0.379	no
CK-0d	RHSIM_Rhsim08G0042600	-1.428	-0.106	1.322	low
CK-0d	RHSIM_Rhsim09G0091400	0.203	-0.243	-0.447	no
CK-0d	RHSIM_Rhsim08G0092200	-0.038	-0.399	-0.361	no
CK-0d	RHSIM_Rhsim09G0130400	-1.070	0.708	1.778	low
CK-0d	RHSIM_Rhsim09G0120100	0.439	1.887	1.448	no
CK-0d	RHSIM_Rhsim08G0043100	1.740	-0.105	-1.845	high
CK-0d	RHSIM_Rhsim09G0190000	0.266	-1.016	-1.282	no
CK-0d	RHSIM_Rhsim09G0102000	-0.882	-0.293	0.589	no
CK-0d	RHSIM_Rhsim07G0108200	0.413	0.176	-0.237	no
CK-0d	RHSIM_Rhsim08G0118300	-1.410	-1.183	0.227	low
CK-0d	RHSIM_Rhsim09G0101400	-0.368	0.434	0.802	no
CK-0d	RHSIM_Rhsim08G0008800	1.677	1.075	-0.602	high
CK-0d	RHSIM_Rhsim08G0105500	1.141	-0.290	-1.432	high
CK-0d	RHSIM_Rhsim08G0178200	0.412	2.772	2.361	no

Group	Gene	PTR (z-score difference)	z(log2 LFQ)	z(log2 FPKM)	extreme-PTR
CK-0d	RHSIM_Rhsim08G0162700	2.291	0.359	-1.932	high
CK-0d	RHSIM_Rhsim07G0234400	-1.734	0.295	2.030	low
CK-0d	RHSIM_Rhsim08G0212700	0.257	-0.005	-0.262	no
CK-0d	RHSIM_Rhsim08G0244600	0.550	0.280	-0.269	no
CK-0d	RHSIM_Rhsim09G0074900	-0.328	1.245	1.573	no
CK-0d	RHSIM_Rhsim07G0030800	2.315	2.057	-0.258	high
CK-0d	RHSIM_Rhsim09G0001500	0.654	1.412	0.758	no
CK-0d	RHSIM_Rhsim07G0104900	1.426	0.192	-1.234	high
CK-0d	RHSIM_Rhsim08G0192000	-1.491	0.150	1.641	low
CK-0d	RHSIM_Rhsim08G0167700	1.009	-0.635	-1.644	no
CK-0d	RHSIM_Rhsim08G0068800	-0.472	-1.308	-0.835	no
CK-0d	RHSIM_Rhsim07G0234600	-0.101	1.285	1.386	no
CK-0d	RHSIM_Rhsim09G0153200	-0.712	-0.594	0.118	no
CK-0d	RHSIM_Rhsim08G0227900	-0.954	-1.436	-0.481	no
CK-0d	RHSIM_Rhsim09G0093900	-0.074	0.945	1.019	no
CK-0d	RHSIM_Rhsim07G0164900	1.053	-0.469	-1.523	no
CK-0d	RHSIM_Rhsim07G0092500	0.531	0.545	0.013	no
CK-0d	RHSIM_Rhsim08G0083700	-0.383	0.530	0.913	no
CK-0d	RHSIM_Rhsim08G0027200	-1.479	-0.851	0.628	low
CK-0d	RHSIM_Rhsim07G0021100	1.106	0.106	-1.001	no
CK-0d	RHSIM_Rhsim08G0236900	0.040	-0.321	-0.361	no
CK-0d	RHSIM_Rhsim09G0198900	0.178	0.376	0.198	no
CK-0d	RHSIM_Rhsim08G0187300	-0.747	-1.262	-0.514	no
CK-0d	RHSIM_Rhsim08G0212200	-0.368	-0.483	-0.115	no
CK-0d	RHSIM_Rhsim09G0072100	-0.296	-0.974	-0.678	no
CK-0d	RHSIM_Rhsim09G0131200	-0.543	-0.549	-0.006	no
CK-0d	RHSIM_Rhsim08G0118700	0.884	0.310	-0.573	no
CK-0d	RHSIM_Rhsim08G0180000	-0.966	-0.795	0.171	no
CK-0d	RHSIM_Rhsim07G0128400	-4.973	-2.908	2.064	low
CK-0d	RHSIM_Rhsim08G0236700	1.384	0.372	-1.012	high
CK-0d	RHSIM_Rhsim09G0213200	-0.067	-0.027	0.040	no
CK-0d	RHSIM_Rhsim07G0155900	1.544	-0.124	-1.668	high

Group	Gene	PTR (z-score difference)	z(log2 LFQ)	z(log2 FPKM)	extreme-PTR
CK-0d	RHSIM_Rhsim08G0246100	1.930	-0.237	-2.167	high
CK-0d	RHSIM_Rhsim08G0098000	-1.711	-0.047	1.663	low
CK-0d	RHSIM_Rhsim08G0082400	-0.620	-0.064	0.556	no
CK-0d	RHSIM_Rhsim08G0071400	-0.676	1.488	2.164	no
CK-0d	RHSIM_Rhsim08G0249700	1.032	0.864	-0.168	no
CK-0d	RHSIM_Rhsim08G0043900	-1.618	-1.427	0.190	low
CK-0d	RHSIM_Rhsim07G0132900	-1.469	-1.403	0.066	low
CK-0d	RHSIM_Rhsim08G0093700	0.024	-1.471	-1.496	no
CK-0d	RHSIM_Rhsim08G0061200	-0.433	-1.076	-0.643	no
CK-0d	RHSIM_Rhsim07G0128700	-0.613	-1.184	-0.570	no
CK-0d	RHSIM_Rhsim08G0052300	0.613	-0.657	-1.270	no
CK-0d	RHSIM_Rhsim08G0234400	1.225	-0.026	-1.250	high
CK-0d	RHSIM_Rhsim09G0021800	-0.108	0.923	1.032	no
CK-0d	RHSIM_Rhsim09G0176500	0.715	1.501	0.787	no
CK-0d	RHSIM_Rhsim08G0200300	-2.007	-1.369	0.638	low
CK-0d	RHSIM_Rhsim07G0258600	-0.550	-0.742	-0.192	no
CK-0d	RHSIM_Rhsim08G0032500	-1.328	-0.989	0.339	low
CK-0d	RHSIM_Rhsim08G0004300	-1.261	-0.723	0.539	low
CK-0d	RHSIM_Rhsim08G0026800	-0.803	-1.310	-0.507	no
CK-0d	RHSIM_Rhsim08G0240700	-0.775	-0.948	-0.172	no
CK-0d	RHSIM_Rhsim08G0247800	-0.497	-0.309	0.188	no
CK-0d	RHSIM_Rhsim09G0073300	-0.284	0.430	0.713	no
CK-0d	RHSIM_Rhsim09G0145600	0.973	0.008	-0.965	no
CK-0d	RHSIM_Rhsim08G0080700	-1.107	-0.557	0.550	low
CK-0d	RHSIM_Rhsim08G0076400	0.074	-0.219	-0.293	no
CK-0d	RHSIM_Rhsim08G0184900	0.328	-0.808	-1.135	no
CK-0d	RHSIM_Rhsim08G0009400	-0.678	1.560	2.238	no
CK-0d	RHSIM_Rhsim07G0050700	-0.460	-0.086	0.374	no
CK-0d	RHSIM_Rhsim08G0019200	-1.758	-0.656	1.102	low
CK-0d	RHSIM_Rhsim08G0059600	0.833	1.146	0.313	no
CK-0d	RHSIM_Rhsim08G0239600	-1.662	-1.158	0.504	low
CK-0d	RHSIM_Rhsim08G0093100	-0.821	-1.103	-0.282	no

Group	Gene	PTR (z-score difference)	z(log2 LFQ)	z(log2 FPKM)	extreme-PTR
CK-0d	RHSIM_Rhsim08G0128400	0.929	0.550	-0.379	no
CK-0d	RHSIM_Rhsim07G0012200	1.141	-0.116	-1.257	high
CK-0d	RHSIM_Rhsim08G0194600	0.896	1.576	0.680	no
CK-0d	RHSIM_Rhsim08G0033500	-0.546	0.077	0.623	no
CK-0d	RHSIM_Rhsim07G0027400	0.459	0.795	0.336	no
CK-0d	RHSIM_Rhsim07G0153400	0.166	-0.340	-0.506	no
CK-0d	RHSIM_Rhsim08G0164000	0.185	-0.510	-0.695	no
CK-0d	RHSIM_Rhsim07G0002300	-0.060	-1.073	-1.013	no
CK-0d	RHSIM_Rhsim08G0222900	1.494	0.344	-1.151	high
CK-0d	RHSIM_Rhsim08G0198500	-0.557	-0.236	0.322	no
CK-0d	RHSIM_Rhsim08G0218500	0.554	0.624	0.070	no
CK-0d	RHSIM_Rhsim09G0155900	0.643	1.228	0.585	no
CK-0d	RHSIM_Rhsim08G0228500	2.935	0.831	-2.104	high
CK-0d	RHSIM_Rhsim09G0195900	2.358	0.734	-1.625	high
CK-0d	RHSIM_Rhsim08G0029800	-0.140	-0.903	-0.763	no
CK-0d	RHSIM_Rhsim07G0228400	0.387	0.690	0.303	no
CK-0d	RHSIM_Rhsim09G0093200	1.122	1.844	0.722	high
CK-0d	RHSIM_Rhsim07G0068800	-0.665	-1.054	-0.388	no
CK-0d	RHSIM_Rhsim08G0037300	2.130	0.270	-1.860	high
CK-0d	RHSIM_Rhsim07G0011300	-0.604	-0.861	-0.257	no
CK-0d	RHSIM_Rhsim08G0233800	0.798	0.110	-0.688	no
CK-0d	RHSIM_Rhsim08G0211000	1.518	1.576	0.058	high
CK-0d	RHSIM_Rhsim07G0169700	-0.246	-0.518	-0.272	no
CK-0d	RHSIM_Rhsim08G0027300	-0.122	-0.855	-0.734	no
CK-0d	RHSIM_Rhsim07G0078300	0.103	0.325	0.223	no
CK-0d	RHSIM_Rhsim08G0055200	-0.395	0.966	1.361	no
CK-0d	RHSIM_Rhsim07G0091400	-0.780	1.380	2.159	no
CK-0d	RHSIM_Rhsim07G0058300	0.331	-0.449	-0.780	no
CK-0d	RHSIM_Rhsim09G0042400	0.652	-0.454	-1.106	no
CK-0d	RHSIM_Rhsim08G0020900	0.702	2.044	1.341	no
CK-0d	RHSIM_Rhsim08G0103100	1.002	-0.179	-1.181	no
CK-0d	RHSIM_Rhsim08G0232100	-1.265	-0.659	0.605	low

Group	Gene	PTR (z-score difference)	z(log2 LFQ)	z(log2 FPKM)	extreme-PTR
CK-0d	RHSIM_Rhsim09G0030000	-0.085	-0.331	-0.245	no
CK-0d	RHSIM_Rhsim08G0121700	1.633	-0.160	-1.794	high
CK-0d	RHSIM_Rhsim08G0011700	-0.041	0.547	0.588	no
CK-0d	RHSIM_Rhsim08G0147000	-0.156	0.753	0.909	no
CK-0d	RHSIM_Rhsim08G0057700	1.023	1.940	0.917	no
CK-0d	RHSIM_Rhsim08G0199300	-2.712	-1.392	1.320	low
CK-0d	RHSIM_Rhsim07G0016300	1.131	-0.737	-1.868	high
CK-0d	RHSIM_Rhsim08G0083000	-2.570	-1.044	1.525	low
CK-0d	RHSIM_Rhsim08G0252400	1.228	0.521	-0.707	high
CK-0d	RHSIM_Rhsim08G0215600	0.067	0.002	-0.065	no
CK-0d	RHSIM_Rhsim09G0009000	0.027	-0.004	-0.031	no
CK-0d	RHSIM_Rhsim08G0207700	1.156	-0.514	-1.670	high
CK-0d	RHSIM_Rhsim09G0152000	1.176	-0.212	-1.388	high
CK-0d	RHSIM_Rhsim08G0089000	2.115	0.763	-1.352	high
CK-0d	RHSIM_Rhsim07G0118200	0.118	1.169	1.051	no
CK-0d	RHSIM_Rhsim07G0028300	-0.501	-0.222	0.279	no
CK-0d	RHSIM_Rhsim08G0161100	0.825	0.686	-0.139	no
CK-0d	RHSIM_Rhsim08G0087900	-1.080	-0.933	0.148	low
CK-0d	RHSIM_Rhsim09G0164200	0.129	1.659	1.530	no
CK-0d	RHSIM_Rhsim07G0246500	0.446	-0.944	-1.391	no
CK-0d	RHSIM_Rhsim08G0055400	1.691	2.156	0.465	high
CK-0d	RHSIM_Rhsim08G0034500	0.272	-0.840	-1.112	no
CK-0d	RHSIM_Rhsim08G0242000	-0.842	-1.526	-0.685	no
CK-0d	RHSIM_Rhsim08G0073200	0.879	0.887	0.009	no
CK-0d	RHSIM_Rhsim08G0223300	0.565	3.141	2.575	no
CK-0d	RHSIM_Rhsim08G0128700	-1.139	-0.637	0.501	low
CK-0d	RHSIM_Rhsim08G0219800	-1.424	-1.776	-0.352	low
CK-0d	RHSIM_Rhsim09G0135900	0.905	-0.151	-1.056	no
CK-0d	RHSIM_Rhsim08G0106300	1.468	-0.666	-2.134	high
CK-0d	RHSIM_Rhsim08G0027700	0.744	-0.071	-0.815	no
CK-0d	RHSIM_Rhsim08G0010500	-0.960	-0.720	0.241	no
CK-0d	RHSIM_Rhsim08G0049000	-0.056	-0.820	-0.764	no

Group	Gene	PTR (z-score difference)	z(log2 LFQ)	z(log2 FPKM)	extreme-PTR
CK-0d	RHSIM_Rhsim08G0188800	-0.067	-0.727	-0.660	no
CK-0d	RHSIM_Rhsim08G0028200	0.334	-0.128	-0.462	no
CK-0d	RHSIM_Rhsim08G0112900	-0.678	-0.519	0.159	no
CK-0d	RHSIM_Rhsim08G0223100	0.063	1.506	1.444	no
CK-0d	RHSIM_Rhsim07G0214500	2.407	2.394	-0.013	high
CK-0d	RHSIM_Rhsim08G0178000	-0.189	-2.260	-2.071	no
CK-0d	RHSIM_Rhsim07G0067400	-0.856	-1.063	-0.207	no
CK-0d	RHSIM_Rhsim07G0124800	0.628	0.363	-0.265	no
CK-0d	RHSIM_Rhsim08G0227600	-0.049	-1.701	-1.651	no
CK-0d	RHSIM_Rhsim07G0009900	-0.295	-0.856	-0.561	no
CK-0d	RHSIM_Rhsim07G0036400	-0.138	2.052	2.190	no
CK-0d	RHSIM_Rhsim08G0228700	-0.552	-1.413	-0.861	no
CK-0d	RHSIM_Rhsim08G0246500	-1.571	0.078	1.649	low
CK-0d	RHSIM_Rhsim08G0015900	0.214	-0.200	-0.415	no
CK-0d	RHSIM_Rhsim07G0124400	0.099	2.713	2.614	no
CK-0d	RHSIM_Rhsim06G0028000	-0.102	1.652	1.754	no
CK-0d	RHSIM_Rhsim08G0248800	-0.015	0.657	0.672	no
CK-0d	RHSIM_Rhsim08G0225500	1.247	1.336	0.090	high
CK-0d	RHSIM_Rhsim08G0196300	0.751	-1.200	-1.950	no
CK-0d	RHSIM_Rhsim08G0011000	0.126	1.205	1.079	no
CK-0d	RHSIM_Rhsim08G0001700	-0.463	-0.455	0.008	no
CK-0d	RHSIM_Rhsim08G0169400	-0.897	-1.355	-0.459	no
CK-0d	RHSIM_Rhsim08G0087000	1.169	1.295	0.125	high
CK-0d	RHSIM_Rhsim07G0046800	-1.752	0.060	1.812	low
CK-0d	RHSIM_Rhsim08G0221800	0.152	-0.621	-0.772	no
CK-0d	RHSIM_Rhsim08G0173800	-0.472	-0.850	-0.378	no
CK-0d	RHSIM_Rhsim06G0095100	-0.188	-1.356	-1.168	no
CK-0d	RHSIM_Rhsim08G0198100	0.559	1.745	1.186	no
CK-0d	RHSIM_Rhsim07G0215600	-0.728	0.271	1.000	no
CK-0d	RHSIM_Rhsim08G0032400	-0.342	-0.039	0.302	no
CK-0d	RHSIM_Rhsim08G0084500	-1.433	-0.012	1.421	low
CK-0d	RHSIM_Rhsim08G0059500	1.279	1.836	0.557	high

Group	Gene	PTR (z-score difference)	z(log2 LFQ)	z(log2 FPKM)	extreme-PTR
CK-0d	RHSIM_Rhsim08G0052400	0.706	0.610	-0.096	no
CK-0d	RHSIM_Rhsim07G0093000	-0.865	-0.312	0.554	no
CK-0d	RHSIM_Rhsim08G0092600	1.672	1.426	-0.247	high
CK-0d	RHSIM_Rhsim08G0052800	-2.476	-1.143	1.333	low
CK-0d	RHSIM_Rhsim08G0048100	-0.925	-0.840	0.085	no
CK-0d	RHSIM_Rhsim07G0017600	-0.161	0.225	0.386	no
CK-0d	RHSIM_Rhsim07G0044000	-0.205	-0.214	-0.009	no
CK-0d	RHSIM_Rhsim08G0042700	0.717	-0.077	-0.794	no
CK-0d	RHSIM_Rhsim08G0066600	0.314	-0.163	-0.477	no
CK-0d	RHSIM_Rhsim07G0106400	0.653	0.721	0.068	no
CK-0d	RHSIM_Rhsim07G0142700	-0.555	-1.771	-1.216	no
CK-0d	RHSIM_Rhsim06G0005200	-0.802	-1.003	-0.200	no
CK-0d	RHSIM_Rhsim08G0135600	-0.712	-1.661	-0.949	no
CK-0d	RHSIM_Rhsim07G0212500	-0.853	-0.450	0.403	no
CK-0d	RHSIM_Rhsim08G0194200	0.712	0.550	-0.162	no
CK-0d	RHSIM_Rhsim08G0155500	-0.805	-1.451	-0.646	no
CK-0d	RHSIM_Rhsim07G0048500	1.036	-1.175	-2.211	no
CK-0d	RHSIM_Rhsim06G0127700	0.312	0.005	-0.308	no
CK-0d	RHSIM_Rhsim08G0017300	0.935	0.148	-0.787	no
CK-0d	RHSIM_Rhsim08G0195700	-0.985	-1.143	-0.157	no
CK-0d	RHSIM_Rhsim08G0007800	-0.381	0.050	0.431	no
CK-0d	RHSIM_Rhsim08G0104200	0.462	-0.576	-1.038	no
CK-0d	RHSIM_Rhsim06G0233900	0.443	0.204	-0.239	no
CK-0d	RHSIM_Rhsim08G0159800	1.071	0.512	-0.559	no
CK-0d	RHSIM_Rhsim08G0212400	-0.319	-1.265	-0.946	no
CK-0d	RHSIM_Rhsim06G0165900	0.300	0.067	-0.233	no
CK-0d	RHSIM_Rhsim08G0236000	-1.767	-1.215	0.552	low
CK-0d	RHSIM_Rhsim07G0219800	0.025	-0.122	-0.147	no
CK-0d	RHSIM_Rhsim08G0196000	-0.265	1.616	1.882	no
CK-0d	RHSIM_Rhsim06G0091300	-0.154	-1.013	-0.859	no
CK-0d	RHSIM_Rhsim08G0095900	1.336	-0.730	-2.065	high
CK-0d	RHSIM_Rhsim08G0078500	1.103	-0.007	-1.110	no

Group	Gene	PTR (z-score difference)	z(log2 LFQ)	z(log2 FPKM)	extreme-PTR
CK-0d	RHSIM_Rhsim07G0170200	0.528	0.556	0.028	no
CK-0d	RHSIM_Rhsim07G0233400	1.421	1.171	-0.250	high
CK-0d	RHSIM_Rhsim06G0211500	-0.176	-0.732	-0.556	no
CK-0d	RHSIM_Rhsim08G0079500	-0.122	0.965	1.087	no
CK-0d	RHSIM_Rhsim07G0202900	-0.109	-0.249	-0.140	no
CK-0d	RHSIM_Rhsim07G0001500	0.813	0.524	-0.289	no
CK-0d	RHSIM_Rhsim07G0178700	0.221	0.156	-0.065	no
CK-0d	RHSIM_Rhsim07G0170500	-1.716	-1.265	0.451	low
CK-0d	RHSIM_Rhsim06G0181200	-0.176	0.463	0.640	no
CK-0d	RHSIM_Rhsim07G0049800	0.650	1.635	0.985	no
CK-0d	RHSIM_Rhsim07G0066200	0.071	-0.155	-0.227	no
CK-0d	RHSIM_Rhsim08G0072200	0.578	-0.261	-0.839	no
CK-0d	RHSIM_Rhsim07G0131800	0.583	0.402	-0.181	no
CK-0d	RHSIM_Rhsim07G0036500	0.182	0.569	0.387	no
CK-0d	RHSIM_Rhsim07G0028800	1.014	3.595	2.581	no
CK-0d	RHSIM_Rhsim07G0132800	0.604	0.078	-0.526	no
CK-0d	RHSIM_Rhsim07G0081100	0.974	-0.737	-1.712	no
CK-0d	RHSIM_Rhsim08G0235700	0.396	0.082	-0.314	no
CK-0d	RHSIM_Rhsim06G0212600	2.490	1.341	-1.150	high
CK-0d	RHSIM_Rhsim07G0018400	0.881	1.343	0.461	no
CK-0d	RHSIM_Rhsim07G0184200	-1.735	0.674	2.409	low
CK-0d	RHSIM_Rhsim06G0002800	0.663	0.405	-0.257	no
CK-0d	RHSIM_Rhsim07G0099200	1.652	-0.016	-1.668	high
CK-0d	RHSIM_Rhsim07G0258700	-0.098	-0.535	-0.437	no
CK-0d	RHSIM_Rhsim07G0045900	-0.315	-0.951	-0.636	no
CK-0d	RHSIM_Rhsim07G0087900	1.425	-0.464	-1.889	high
CK-0d	RHSIM_Rhsim08G0071000	-1.711	-0.207	1.505	low
CK-0d	RHSIM_Rhsim08G0186700	-1.293	-1.028	0.265	low
CK-0d	RHSIM_Rhsim06G0244300	0.358	0.930	0.572	no
CK-0d	RHSIM_Rhsim08G0167500	0.470	0.926	0.456	no
CK-0d	RHSIM_Rhsim07G0030200	-0.095	-0.530	-0.435	no
CK-0d	RHSIM_Rhsim08G0171100	0.502	0.076	-0.426	no

Group	Gene	PTR (z-score difference)	z(log2 LFQ)	z(log2 FPKM)	extreme-PTR
CK-0d	RHSIM_Rhsim07G0083900	-0.705	0.469	1.175	no
CK-0d	RHSIM_Rhsim08G0226300	-0.626	-1.029	-0.404	no
CK-0d	RHSIM_Rhsim07G0017800	0.072	-1.144	-1.216	no
CK-0d	RHSIM_Rhsim07G0027500	0.037	-0.684	-0.720	no
CK-0d	RHSIM_Rhsim07G0202800	-1.051	-0.303	0.748	low
CK-0d	RHSIM_Rhsim06G0052900	-0.669	0.709	1.378	no
CK-0d	RHSIM_Rhsim08G0118600	0.229	0.218	-0.011	no
CK-0d	RHSIM_Rhsim07G0119300	-0.670	1.062	1.732	no
CK-0d	RHSIM_Rhsim07G0049600	0.007	1.156	1.149	no
CK-0d	RHSIM_Rhsim06G0207400	1.154	0.606	-0.548	high
CK-0d	RHSIM_Rhsim07G0056500	-0.885	-0.113	0.772	no
CK-0d	RHSIM_Rhsim07G0103100	-0.174	0.941	1.115	no
CK-0d	RHSIM_Rhsim07G0171800	0.267	0.293	0.026	no
CK-0d	RHSIM_Rhsim07G0121100	-0.400	-0.011	0.388	no
CK-0d	RHSIM_Rhsim08G0036900	0.371	0.815	0.444	no
CK-0d	RHSIM_Rhsim07G0165200	0.762	-0.936	-1.699	no
CK-0d	RHSIM_Rhsim08G0097800	0.361	1.565	1.204	no
CK-0d	RHSIM_Rhsim07G0149800	0.656	-0.809	-1.465	no
CK-0d	RHSIM_Rhsim06G0077600	0.885	0.628	-0.257	no
CK-0d	RHSIM_Rhsim07G0020500	-0.135	0.125	0.260	no
CK-0d	RHSIM_Rhsim07G0045200	1.053	0.554	-0.499	no
CK-0d	RHSIM_Rhsim06G0218800	-1.506	-1.574	-0.067	low
CK-0d	RHSIM_Rhsim07G0067300	0.511	0.131	-0.380	no
CK-0d	RHSIM_Rhsim07G0188200	-1.866	-0.779	1.088	low
CK-0d	RHSIM_Rhsim07G0245900	-0.677	-0.719	-0.042	no
CK-0d	RHSIM_Rhsim06G0187500	-0.848	-0.502	0.347	no
CK-0d	RHSIM_Rhsim06G0105400	-2.058	-1.495	0.563	low
CK-0d	RHSIM_Rhsim08G0152800	0.830	-0.268	-1.098	no
CK-0d	RHSIM_Rhsim08G0171200	-1.302	-1.097	0.205	low
CK-0d	RHSIM_Rhsim07G0092600	-2.162	-1.019	1.143	low
CK-0d	RHSIM_Rhsim07G0204300	0.344	0.536	0.192	no
CK-0d	RHSIM_Rhsim07G0081900	0.141	0.552	0.410	no

Group	Gene	PTR (z-score difference)	z(log2 LFQ)	z(log2 FPKM)	extreme-PTR
CK-0d	RHSIM_Rhsim08G0138500	0.508	0.398	-0.110	no
CK-0d	RHSIM_Rhsim07G0055600	2.231	1.859	-0.371	high
CK-0d	RHSIM_Rhsim07G0125500	-1.608	-0.868	0.739	low
CK-0d	RHSIM_Rhsim06G0222300	2.590	1.719	-0.872	high
CK-0d	RHSIM_Rhsim07G0214600	-0.992	-1.640	-0.647	no
CK-0d	RHSIM_Rhsim07G0075900	0.754	0.743	-0.010	no
CK-0d	RHSIM_Rhsim07G0074000	0.685	0.198	-0.488	no
CK-0d	RHSIM_Rhsim06G0191200	-0.008	-0.541	-0.533	no
CK-0d	RHSIM_Rhsim08G0101100	-0.484	-1.536	-1.052	no
CK-0d	RHSIM_Rhsim08G0183600	0.389	-0.214	-0.603	no
CK-0d	RHSIM_Rhsim06G0178000	0.633	0.509	-0.124	no
CK-0d	RHSIM_Rhsim07G0179500	0.650	0.200	-0.450	no
CK-0d	RHSIM_Rhsim08G0196600	0.932	0.567	-0.365	no
CK-0d	RHSIM_Rhsim06G0035000	0.401	0.066	-0.335	no
CK-0d	RHSIM_Rhsim07G0059500	1.018	-0.648	-1.666	no
CK-0d	RHSIM_Rhsim07G0097100	3.096	0.785	-2.311	high
CK-0d	RHSIM_Rhsim07G0228600	-0.677	-0.329	0.349	no
CK-0d	RHSIM_Rhsim06G0046200	0.637	-0.193	-0.830	no
CK-0d	RHSIM_Rhsim07G0033000	-0.197	-0.779	-0.582	no
CK-0d	RHSIM_Rhsim06G0167500	-2.014	1.044	3.059	low
CK-0d	RHSIM_Rhsim07G0004400	-0.182	0.467	0.649	no
CK-0d	RHSIM_Rhsim06G0000200	-0.322	-0.600	-0.278	no
CK-0d	RHSIM_Rhsim07G0241100	1.043	-1.284	-2.327	no
CK-0d	RHSIM_Rhsim06G0034400	0.571	1.006	0.435	no
CK-0d	RHSIM_Rhsim08G0242800	1.112	-0.451	-1.563	high
CK-0d	RHSIM_Rhsim07G0178300	3.329	3.292	-0.038	high
CK-0d	RHSIM_Rhsim07G0013700	-0.124	0.235	0.359	no
CK-0d	RHSIM_Rhsim07G0176800	0.176	-1.773	-1.949	no
CK-0d	RHSIM_Rhsim06G0003200	1.231	1.383	0.151	high
CK-0d	RHSIM_Rhsim06G0031700	0.250	-0.153	-0.402	no
CK-0d	RHSIM_Rhsim08G0090800	1.220	-0.431	-1.651	high
CK-0d	RHSIM_Rhsim07G0087100	-0.372	-0.500	-0.128	no

Group	Gene	PTR (z-score difference)	z(log2 LFQ)	z(log2 FPKM)	extreme-PTR
CK-0d	RHSIM_Rhsim08G0222200	0.327	-0.860	-1.188	no
CK-0d	RHSIM_Rhsim07G0169100	0.468	0.816	0.348	no
CK-0d	RHSIM_Rhsim08G0251700	1.393	0.676	-0.717	high
CK-0d	RHSIM_Rhsim07G0047300	-0.012	-0.814	-0.803	no
CK-0d	RHSIM_Rhsim08G0236600	0.833	0.826	-0.007	no
CK-0d	RHSIM_Rhsim07G0110300	-0.194	1.200	1.394	no
CK-0d	RHSIM_Rhsim07G0226700	0.018	-0.511	-0.529	no
CK-0d	RHSIM_Rhsim07G0081200	0.105	0.867	0.762	no
CK-0d	RHSIM_Rhsim06G0193100	0.554	1.304	0.750	no
CK-0d	RHSIM_Rhsim08G0036600	0.242	0.284	0.042	no
CK-0d	RHSIM_Rhsim07G0139000	0.456	1.148	0.692	no
CK-0d	RHSIM_Rhsim07G0137200	-1.105	-1.709	-0.604	low
CK-0d	RHSIM_Rhsim07G0103400	0.590	0.005	-0.585	no
CK-0d	RHSIM_Rhsim07G0139200	-0.309	-0.713	-0.404	no
CK-0d	RHSIM_Rhsim07G0210400	1.182	2.083	0.901	high
CK-0d	RHSIM_Rhsim07G0072200	-0.243	-0.262	-0.019	no
CK-0d	RHSIM_Rhsim06G0070400	-0.283	0.677	0.960	no
CK-0d	RHSIM_Rhsim07G0197900	-0.515	0.841	1.356	no
CK-0d	RHSIM_Rhsim07G0130000	-0.118	-0.110	0.007	no
CK-0d	RHSIM_Rhsim08G0190500	-1.069	0.052	1.121	low
CK-0d	RHSIM_Rhsim07G0236500	-1.054	-0.877	0.178	low
CK-0d	RHSIM_Rhsim07G0110500	-0.893	-0.797	0.096	no
CK-0d	RHSIM_Rhsim06G0205600	-0.648	-0.172	0.476	no
CK-0d	RHSIM_Rhsim08G0240800	-0.402	-0.156	0.247	no
CK-0d	RHSIM_Rhsim07G0035600	-0.674	-0.320	0.354	no
CK-0d	RHSIM_Rhsim06G0237100	-2.920	-1.273	1.647	low
CK-0d	RHSIM_Rhsim07G0249300	1.987	0.977	-1.010	high
CK-0d	RHSIM_Rhsim07G0001600	0.673	0.165	-0.508	no
CK-0d	RHSIM_Rhsim06G0047700	-0.416	1.424	1.841	no
CK-0d	RHSIM_Rhsim06G0242300	0.838	0.938	0.100	no
CK-0d	RHSIM_Rhsim07G0018200	-0.019	0.337	0.356	no
CK-0d	RHSIM_Rhsim07G0178100	0.080	-0.815	-0.894	no

Group	Gene	PTR (z-score difference)	z(log2 LFQ)	z(log2 FPKM)	extreme-PTR
CK-0d	RHSIM_Rhsim05G0060700	-0.533	0.840	1.373	no
CK-0d	RHSIM_Rhsim06G0169300	1.146	1.095	-0.052	high
CK-0d	RHSIM_Rhsim07G0092700	-0.291	1.387	1.677	no
CK-0d	RHSIM_Rhsim07G0072000	-1.305	-0.843	0.462	low
CK-0d	RHSIM_Rhsim07G0046600	-1.430	-0.494	0.937	low
CK-0d	RHSIM_Rhsim07G0059000	1.757	0.889	-0.868	high
CK-0d	RHSIM_Rhsim05G0194700	0.184	0.751	0.567	no
CK-0d	RHSIM_Rhsim05G0122800	0.009	0.529	0.520	no
CK-0d	RHSIM_Rhsim06G0028600	1.013	-0.492	-1.504	no
CK-0d	RHSIM_Rhsim07G0204200	-1.122	-1.099	0.023	low
CK-0d	RHSIM_Rhsim06G0243900	2.223	0.687	-1.536	high
CK-0d	RHSIM_Rhsim07G0237600	-0.019	-0.313	-0.294	no
CK-0d	RHSIM_Rhsim07G0247500	0.353	0.491	0.138	no
CK-0d	RHSIM_Rhsim05G0101000	1.174	0.250	-0.924	high
CK-0d	RHSIM_Rhsim07G0012900	-2.052	-0.645	1.407	low
CK-0d	RHSIM_Rhsim07G0026400	-2.778	0.207	2.985	low
CK-0d	RHSIM_Rhsim07G0125200	0.183	-1.424	-1.607	no
CK-0d	RHSIM_Rhsim07G0117900	0.721	1.073	0.352	no
CK-0d	RHSIM_Rhsim07G0150800	0.063	0.083	0.020	no
CK-0d	RHSIM_Rhsim07G0054900	0.696	-0.139	-0.836	no
CK-0d	RHSIM_Rhsim06G0011300	-2.898	-1.960	0.937	low
CK-0d	RHSIM_Rhsim07G0006300	-2.318	-1.323	0.995	low
CK-0d	RHSIM_Rhsim05G0217100	-0.564	-0.851	-0.287	no
CK-0d	RHSIM_Rhsim07G0100900	-1.930	-0.888	1.041	low
CK-0d	RHSIM_Rhsim07G0210900	0.707	1.085	0.378	no
CK-0d	RHSIM_Rhsim07G0179400	-0.750	-0.948	-0.197	no
CK-0d	RHSIM_Rhsim07G0254300	-0.767	-1.658	-0.890	no
CK-0d	RHSIM_Rhsim07G0082100	1.797	2.283	0.486	high
CK-0d	RHSIM_Rhsim07G0048700	-0.286	-1.951	-1.665	no
CK-0d	RHSIM_Rhsim06G0064500	-0.601	-0.657	-0.055	no
CK-0d	RHSIM_Rhsim07G0083200	0.698	0.568	-0.130	no
CK-0d	RHSIM_Rhsim06G0090400	-0.185	-0.110	0.075	no

Group	Gene	PTR (z-score difference)	z(log2 LFQ)	z(log2 FPKM)	extreme-PTR
CK-0d	RHSIM_Rhsim06G0013400	0.375	0.625	0.250	no
CK-0d	RHSIM_Rhsim05G0134300	0.417	-0.478	-0.895	no
CK-0d	RHSIM_Rhsim06G0033500	0.303	-0.348	-0.651	no
CK-0d	RHSIM_Rhsim05G0088500	1.728	1.580	-0.148	high
CK-0d	RHSIM_Rhsim06G0089300	-2.340	-1.528	0.813	low
CK-0d	RHSIM_Rhsim06G0122800	-0.448	-0.872	-0.424	no
CK-0d	RHSIM_Rhsim07G0091600	1.676	-0.017	-1.693	high
CK-0d	RHSIM_Rhsim07G0129700	-1.183	-0.139	1.044	low
CK-0d	RHSIM_Rhsim06G0072400	0.452	0.425	-0.028	no
CK-0d	RHSIM_Rhsim07G0205000	1.635	2.866	1.232	high
CK-0d	RHSIM_Rhsim05G0061100	0.351	-0.478	-0.829	no
CK-0d	RHSIM_Rhsim05G0111600	-0.175	-0.646	-0.471	no
CK-0d	RHSIM_Rhsim06G0084600	-1.493	-1.007	0.486	low
CK-0d	RHSIM_Rhsim07G0092900	-1.247	0.057	1.304	low
CK-0d	RHSIM_Rhsim06G0151500	-0.861	0.810	1.671	no
CK-0d	RHSIM_Rhsim06G0238800	-0.613	-0.229	0.384	no
CK-0d	RHSIM_Rhsim07G0071400	-1.485	-0.372	1.113	low
CK-0d	RHSIM_Rhsim07G0087700	1.007	1.504	0.497	no
CK-0d	RHSIM_Rhsim05G0120100	-1.390	-1.467	-0.077	low
CK-0d	RHSIM_Rhsim06G0161000	-1.805	-0.660	1.144	low
CK-0d	RHSIM_Rhsim07G0225500	0.554	0.377	-0.177	no
CK-0d	RHSIM_Rhsim05G0232300	0.085	1.066	0.981	no
CK-0d	RHSIM_Rhsim07G0047800	1.180	0.872	-0.308	high
CK-0d	RHSIM_Rhsim06G0205800	-0.618	0.667	1.285	no
CK-0d	RHSIM_Rhsim07G0189000	-0.253	0.991	1.244	no
CK-0d	RHSIM_Rhsim06G0004200	1.467	0.984	-0.484	high
CK-0d	RHSIM_Rhsim07G0013800	-1.594	-0.755	0.839	low
CK-0d	RHSIM_Rhsim06G0098800	0.101	-0.418	-0.519	no
CK-0d	RHSIM_Rhsim06G0074700	-0.245	0.608	0.852	no
CK-0d	RHSIM_Rhsim06G0177400	0.742	-0.371	-1.113	no
CK-0d	RHSIM_Rhsim06G0026100	-3.108	-1.253	1.854	low
CK-0d	RHSIM_Rhsim07G0153500	-1.328	-0.499	0.829	low

Group	Gene	PTR (z-score difference)	z(log2 LFQ)	z(log2 FPKM)	extreme-PTR
CK-0d	RHSIM_Rhsim06G0207300	0.475	-0.351	-0.826	no
CK-0d	RHSIM_Rhsim07G0066700	-0.480	-1.548	-1.068	no
CK-0d	RHSIM_Rhsim05G0165300	-0.198	-0.782	-0.584	no
CK-0d	RHSIM_Rhsim06G0137600	1.144	1.726	0.582	high
CK-0d	RHSIM_Rhsim07G0051300	3.085	2.845	-0.240	high
CK-0d	RHSIM_Rhsim06G0116000	0.525	0.519	-0.006	no
CK-0d	RHSIM_Rhsim07G0258000	-1.122	-0.922	0.201	low
CK-0d	RHSIM_Rhsim07G0074300	0.660	0.097	-0.563	no
CK-0d	RHSIM_Rhsim06G0203600	1.649	0.935	-0.714	high
CK-0d	RHSIM_Rhsim07G0049700	-4.048	-1.342	2.707	low
CK-0d	RHSIM_Rhsim06G0032800	-0.340	-0.037	0.303	no
CK-0d	RHSIM_Rhsim05G0004900	-0.071	1.098	1.169	no
CK-0d	RHSIM_Rhsim06G0205900	1.400	0.277	-1.123	high
CK-0d	RHSIM_Rhsim05G0161600	1.177	-0.214	-1.391	high
CK-0d	RHSIM_Rhsim06G0204300	-0.676	-1.866	-1.190	no
CK-0d	RHSIM_Rhsim06G0223100	0.312	1.010	0.698	no
CK-0d	RHSIM_Rhsim06G0130900	-0.390	-0.594	-0.203	no
CK-0d	RHSIM_Rhsim06G0025000	0.838	-0.246	-1.084	no
CK-0d	RHSIM_Rhsim06G0165200	0.176	-0.518	-0.693	no
CK-0d	RHSIM_Rhsim06G0003000	-0.481	-1.543	-1.062	no
CK-0d	RHSIM_Rhsim07G0203600	-1.043	-0.163	0.880	low
CK-0d	RHSIM_Rhsim06G0027400	0.100	-1.186	-1.286	no
CK-0d	RHSIM_Rhsim06G0069500	-0.832	-1.021	-0.189	no
CK-0d	RHSIM_Rhsim07G0187800	0.060	1.322	1.263	no
CK-0d	RHSIM_Rhsim05G0104300	-0.738	0.084	0.821	no
CK-0d	RHSIM_Rhsim05G0167900	-1.263	-0.162	1.102	low
CK-0d	RHSIM_Rhsim06G0034800	0.100	0.432	0.332	no
CK-0d	RHSIM_Rhsim07G0019300	-0.413	1.033	1.446	no
CK-0d	RHSIM_Rhsim07G0129500	-0.767	-1.510	-0.743	no
CK-0d	RHSIM_Rhsim06G0193700	0.313	-0.553	-0.866	no
CK-0d	RHSIM_Rhsim05G0106800	0.026	0.715	0.689	no
CK-0d	RHSIM_Rhsim07G0101400	-0.549	-0.333	0.215	no

Group	Gene	PTR (z-score difference)	z(log2 LFQ)	z(log2 FPKM)	extreme-PTR
CK-0d	RHSIM_Rhsim06G0243000	2.997	2.257	-0.740	high
CK-0d	RHSIM_Rhsim06G0205300	0.633	1.621	0.988	no
CK-0d	RHSIM_Rhsim06G0167300	-0.869	0.693	1.562	no
CK-0d	RHSIM_Rhsim06G0078100	0.927	0.036	-0.891	no
CK-0d	RHSIM_Rhsim07G0143200	-0.015	0.926	0.940	no
CK-0d	RHSIM_Rhsim06G0126000	0.094	0.490	0.396	no
CK-0d	RHSIM_Rhsim06G0212000	0.654	-0.588	-1.242	no
CK-0d	RHSIM_Rhsim06G0176500	1.078	0.189	-0.889	no
CK-0d	RHSIM_Rhsim06G0191300	0.192	-1.125	-1.317	no
CK-0d	RHSIM_Rhsim06G0226100	-0.209	-0.440	-0.231	no
CK-0d	RHSIM_Rhsim07G0134200	-0.234	-0.242	-0.009	no
CK-0d	RHSIM_Rhsim05G0132600	0.615	1.486	0.871	no
CK-0d	RHSIM_Rhsim06G0006900	1.332	1.050	-0.282	high
CK-0d	RHSIM_Rhsim07G0082500	0.246	1.699	1.453	no
CK-0d	RHSIM_Rhsim06G0201700	-0.198	-0.700	-0.502	no
CK-0d	RHSIM_Rhsim07G0131300	1.773	2.212	0.439	high
CK-0d	RHSIM_Rhsim06G0001500	-0.102	0.273	0.375	no
CK-0d	RHSIM_Rhsim06G0236000	-0.724	-0.871	-0.147	no
CK-0d	RHSIM_Rhsim06G0236100	-1.629	-0.681	0.948	low
CK-0d	RHSIM_Rhsim06G0007900	0.002	-0.754	-0.756	no
CK-0d	RHSIM_Rhsim06G0184600	-0.656	-1.207	-0.551	no
CK-0d	RHSIM_Rhsim07G0061200	-0.781	-0.916	-0.135	no
CK-0d	RHSIM_Rhsim06G0163900	0.714	1.031	0.316	no
CK-0d	RHSIM_Rhsim05G0182800	1.085	0.059	-1.026	no
CK-0d	RHSIM_Rhsim06G0035200	-0.117	-1.441	-1.324	no
CK-0d	RHSIM_Rhsim05G0142700	0.943	0.435	-0.508	no
CK-0d	RHSIM_Rhsim07G0053300	0.191	1.598	1.407	no
CK-0d	RHSIM_Rhsim06G0129400	-2.649	-1.734	0.915	low
CK-0d	RHSIM_Rhsim05G0041300	-0.292	-0.369	-0.076	no
CK-0d	RHSIM_Rhsim06G0026800	-0.134	-1.223	-1.089	no
CK-0d	RHSIM_Rhsim07G0020400	-0.865	-1.367	-0.503	no
CK-0d	RHSIM_Rhsim06G0194400	1.434	1.211	-0.223	high

Group	Gene	PTR (z-score difference)	z(log2 LFQ)	z(log2 FPKM)	extreme-PTR
CK-0d	RHSIM_Rhsim06G0191000	0.683	1.708	1.025	no
CK-0d	RHSIM_Rhsim05G0167300	1.393	1.404	0.011	high
CK-0d	RHSIM_Rhsim06G0226700	0.835	0.925	0.090	no
CK-0d	RHSIM_Rhsim05G0062900	0.118	-1.205	-1.323	no
CK-0d	RHSIM_Rhsim07G0019100	-1.406	-0.598	0.808	low
CK-0d	RHSIM_Rhsim07G0064900	0.454	-0.415	-0.869	no
CK-0d	RHSIM_Rhsim06G0055300	0.903	1.408	0.505	no
CK-0d	RHSIM_Rhsim07G0053700	-0.159	-0.999	-0.840	no
CK-0d	RHSIM_Rhsim06G0065900	-0.364	-0.770	-0.405	no
CK-0d	RHSIM_Rhsim06G0041800	-0.994	-0.157	0.837	no
CK-0d	RHSIM_Rhsim06G0047900	-0.178	0.466	0.644	no
CK-0d	RHSIM_Rhsim07G0090200	0.130	1.372	1.243	no
CK-0d	RHSIM_Rhsim05G0002000	-0.383	-0.987	-0.604	no
CK-0d	RHSIM_Rhsim06G0171700	0.677	-0.431	-1.108	no
CK-0d	RHSIM_Rhsim07G0026500	0.465	0.277	-0.188	no
CK-0d	RHSIM_Rhsim05G0139700	2.552	0.327	-2.225	high
CK-0d	RHSIM_Rhsim06G0159400	0.837	0.368	-0.468	no
CK-0d	RHSIM_Rhsim06G0240800	2.579	0.697	-1.881	high
CK-0d	RHSIM_Rhsim05G0231800	1.772	0.962	-0.810	high
CK-0d	RHSIM_Rhsim06G0049600	-0.130	-0.043	0.087	no
CK-0d	RHSIM_Rhsim05G0143600	-0.498	-0.558	-0.060	no
CK-0d	RHSIM_Rhsim07G0039100	1.718	1.992	0.274	high
CK-0d	RHSIM_Rhsim07G0044600	0.937	-0.539	-1.476	no
CK-0d	RHSIM_Rhsim05G0148200	-0.304	0.264	0.568	no
CK-0d	RHSIM_Rhsim06G0122300	-0.877	-0.155	0.722	no
CK-0d	RHSIM_Rhsim07G0113300	0.652	0.957	0.305	no
CK-0d	RHSIM_Rhsim07G0145600	0.464	0.412	-0.052	no
CK-0d	RHSIM_Rhsim06G0092600	0.166	-0.591	-0.756	no
CK-0d	RHSIM_Rhsim05G0096400	0.040	0.988	0.947	no
CK-0d	RHSIM_Rhsim07G0036300	0.800	0.362	-0.438	no
CK-0d	RHSIM_Rhsim06G0218900	-1.790	-1.286	0.504	low
CK-0d	RHSIM_Rhsim05G0163000	1.259	-0.022	-1.281	high

Group	Gene	PTR (z-score difference)	z(log2 LFQ)	z(log2 FPKM)	extreme-PTR
CK-0d	RHSIM_Rhsim06G0046700	-1.808	-1.027	0.781	low
CK-0d	RHSIM_Rhsim06G0131500	1.207	2.917	1.710	high
CK-0d	RHSIM_Rhsim06G0054800	-1.457	1.193	2.651	low
CK-0d	RHSIM_Rhsim06G0087800	-1.085	-0.521	0.564	low
CK-0d	RHSIM_Rhsim04G0083400	-0.402	0.273	0.674	no
CK-0d	RHSIM_Rhsim06G0002900	-0.116	0.399	0.515	no
CK-0d	RHSIM_Rhsim06G0130100	1.240	1.093	-0.147	high
CK-0d	RHSIM_Rhsim04G0085300	-1.911	-1.128	0.783	low
CK-0d	RHSIM_Rhsim06G0229100	-0.827	-1.111	-0.284	no
CK-0d	RHSIM_Rhsim06G0178400	-0.338	0.225	0.564	no
CK-0d	RHSIM_Rhsim06G0135300	-0.146	0.250	0.397	no
CK-0d	RHSIM_Rhsim04G0169200	0.446	0.085	-0.361	no
CK-0d	RHSIM_Rhsim05G0017200	-1.362	-1.453	-0.091	low
CK-0d	RHSIM_Rhsim05G0185400	0.340	-0.544	-0.884	no
CK-0d	RHSIM_Rhsim04G0010400	1.004	0.433	-0.571	no
CK-0d	RHSIM_Rhsim06G0121800	-0.845	-1.515	-0.670	no
CK-0d	RHSIM_Rhsim05G0030300	0.593	1.200	0.607	no
CK-0d	RHSIM_Rhsim05G0059500	1.025	-0.397	-1.422	no
CK-0d	RHSIM_Rhsim04G0118700	0.782	0.734	-0.048	no
CK-0d	RHSIM_Rhsim05G0139000	-2.213	-0.651	1.562	low
CK-0d	RHSIM_Rhsim06G0068800	0.900	-0.867	-1.768	no
CK-0d	RHSIM_Rhsim06G0099300	0.188	-0.143	-0.331	no
CK-0d	RHSIM_Rhsim05G0103900	1.108	0.941	-0.167	no
CK-0d	RHSIM_Rhsim06G0146500	1.142	1.566	0.424	high
CK-0d	RHSIM_Rhsim06G0057900	-0.993	-0.673	0.320	no
CK-0d	RHSIM_Rhsim05G0089600	-3.859	-1.788	2.071	low
CK-0d	RHSIM_Rhsim05G0013800	0.253	0.296	0.044	no
CK-0d	RHSIM_Rhsim06G0156300	-1.086	-0.180	0.906	low
CK-0d	RHSIM_Rhsim05G0050000	0.561	1.022	0.461	no
CK-0d	RHSIM_Rhsim05G0180000	0.689	-0.893	-1.582	no
CK-0d	RHSIM_Rhsim06G0185200	0.843	1.102	0.258	no
CK-0d	RHSIM_Rhsim05G0020600	-0.517	-0.141	0.376	no

Group	Gene	PTR (z-score difference)	z(log2 LFQ)	z(log2 FPKM)	extreme-PTR
CK-0d	RHSIM_Rhsim04G0213800	0.047	0.863	0.816	no
CK-0d	RHSIM_Rhsim05G0069400	1.044	0.057	-0.986	no
CK-0d	RHSIM_Rhsim05G0093200	-0.638	-0.533	0.105	no
CK-0d	RHSIM_Rhsim05G0207300	0.352	-1.174	-1.526	no
CK-0d	RHSIM_Rhsim05G0035000	1.140	1.379	0.239	high
CK-0d	RHSIM_Rhsim04G0032800	-0.615	1.776	2.391	no
CK-0d	RHSIM_Rhsim06G0020600	-1.559	-1.963	-0.404	low
CK-0d	RHSIM_Rhsim05G0118000	-0.025	-0.362	-0.337	no
CK-0d	RHSIM_Rhsim06G0235200	-0.195	-1.103	-0.908	no
CK-0d	RHSIM_Rhsim05G0064500	0.430	0.971	0.542	no
CK-0d	RHSIM_Rhsim06G0064300	-0.691	-1.274	-0.584	no
CK-0d	RHSIM_Rhsim05G0012000	-0.256	1.774	2.031	no
CK-0d	RHSIM_Rhsim06G0066600	1.414	-0.304	-1.718	high
CK-0d	RHSIM_Rhsim05G0022300	0.942	1.225	0.284	no
CK-0d	RHSIM_Rhsim05G0054100	0.082	-0.566	-0.647	no
CK-0d	RHSIM_Rhsim05G0161200	0.261	-0.050	-0.311	no
CK-0d	RHSIM_Rhsim06G0197400	0.724	1.983	1.260	no
CK-0d	RHSIM_Rhsim05G0070000	0.547	0.919	0.372	no
CK-0d	RHSIM_Rhsim06G0049800	-2.060	0.136	2.196	low
CK-0d	RHSIM_Rhsim04G0091600	-0.918	-0.187	0.731	no
CK-0d	RHSIM_Rhsim06G0073600	0.396	1.162	0.765	no
CK-0d	RHSIM_Rhsim06G0212900	0.467	0.730	0.263	no
CK-0d	RHSIM_Rhsim05G0174500	-3.459	-1.161	2.298	low
CK-0d	RHSIM_Rhsim05G0223200	-0.630	-0.709	-0.079	no
CK-0d	RHSIM_Rhsim05G0012400	0.430	0.745	0.315	no
CK-0d	RHSIM_Rhsim06G0049500	1.380	1.023	-0.357	high
CK-0d	RHSIM_Rhsim05G0000600	1.205	-0.361	-1.566	high
CK-0d	RHSIM_Rhsim06G0169400	-1.475	-1.846	-0.371	low
CK-0d	RHSIM_Rhsim05G0202500	0.322	0.166	-0.156	no
CK-0d	RHSIM_Rhsim06G0086900	-1.373	-0.536	0.838	low
CK-0d	RHSIM_Rhsim05G0049500	0.469	-1.208	-1.677	no
CK-0d	RHSIM_Rhsim04G0105200	1.734	0.586	-1.147	high

Group	Gene	PTR (z-score difference)	z(log2 LFQ)	z(log2 FPKM)	extreme-PTR
CK-0d	RHSIM_Rhsim05G0070900	1.145	1.199	0.054	high
CK-0d	RHSIM_Rhsim04G0246500	2.157	0.558	-1.600	high
CK-0d	RHSIM_Rhsim06G0012100	-0.054	0.440	0.494	no
CK-0d	RHSIM_Rhsim05G0011500	0.083	-0.106	-0.189	no
CK-0d	RHSIM_Rhsim06G0236300	0.269	1.044	0.776	no
CK-0d	RHSIM_Rhsim05G0063200	0.899	1.624	0.725	no
CK-0d	RHSIM_Rhsim05G0180400	-0.001	2.565	2.566	no
CK-0d	RHSIM_Rhsim05G0137700	-1.200	-0.097	1.103	low
CK-0d	RHSIM_Rhsim04G0243100	-0.092	1.398	1.489	no
CK-0d	RHSIM_Rhsim04G0234700	0.915	-0.311	-1.226	no
CK-0d	RHSIM_Rhsim05G0202800	2.120	0.983	-1.137	high
CK-0d	RHSIM_Rhsim05G0027600	1.698	-0.342	-2.040	high
CK-0d	RHSIM_Rhsim05G0163600	1.235	-0.640	-1.875	high
CK-0d	RHSIM_Rhsim06G0037800	-0.390	0.803	1.192	no
CK-0d	RHSIM_Rhsim05G0079600	0.289	-0.106	-0.395	no
CK-0d	RHSIM_Rhsim04G0016100	1.513	0.858	-0.655	high
CK-0d	RHSIM_Rhsim05G0088400	0.629	1.044	0.415	no
CK-0d	RHSIM_Rhsim04G0146600	-0.487	-0.994	-0.507	no
CK-0d	RHSIM_Rhsim06G0161200	-0.148	1.046	1.194	no
CK-0d	RHSIM_Rhsim06G0093300	-0.502	-0.630	-0.127	no
CK-0d	RHSIM_Rhsim06G0011900	1.114	-0.823	-1.937	high
CK-0d	RHSIM_Rhsim06G0060100	-0.395	-0.201	0.194	no
CK-0d	RHSIM_Rhsim06G0047800	0.162	0.985	0.823	no
CK-0d	RHSIM_Rhsim05G0062500	0.415	0.096	-0.320	no
CK-0d	RHSIM_Rhsim06G0186600	-0.093	-0.092	0.001	no
CK-0d	RHSIM_Rhsim06G0237500	-0.138	-0.403	-0.265	no
CK-0d	RHSIM_Rhsim06G0166400	-0.117	-0.586	-0.469	no
CK-0d	RHSIM_Rhsim05G0224600	-0.393	0.025	0.418	no
CK-0d	RHSIM_Rhsim06G0072000	0.019	0.127	0.108	no
CK-0d	RHSIM_Rhsim06G0102100	0.719	1.517	0.798	no
CK-0d	RHSIM_Rhsim04G0102500	1.664	0.830	-0.834	high
CK-0d	RHSIM_Rhsim05G0230900	1.169	1.132	-0.037	high

Group	Gene	PTR (z-score difference)	z(log2 LFQ)	z(log2 FPKM)	extreme-PTR
CK-0d	RHSIM_Rhsim06G0014600	1.697	1.108	-0.589	high
CK-0d	RHSIM_Rhsim04G0118500	-0.024	0.610	0.634	no
CK-0d	RHSIM_Rhsim06G0038600	0.292	2.202	1.910	no
CK-0d	RHSIM_Rhsim06G0077300	-1.172	0.630	1.801	low
CK-0d	RHSIM_Rhsim06G0214800	0.779	1.086	0.307	no
CK-0d	RHSIM_Rhsim05G0169300	-0.608	-1.276	-0.668	no
CK-0d	RHSIM_Rhsim04G0101900	0.136	1.461	1.326	no
CK-0d	RHSIM_Rhsim04G0149800	0.037	-0.294	-0.331	no
CK-0d	RHSIM_Rhsim06G0116800	0.437	0.216	-0.221	no
CK-0d	RHSIM_Rhsim04G0119100	0.676	1.413	0.737	no
CK-0d	RHSIM_Rhsim05G0108700	-0.681	-1.244	-0.563	no
CK-0d	RHSIM_Rhsim04G0186900	-0.107	-0.578	-0.471	no
CK-0d	RHSIM_Rhsim06G0076100	1.017	1.495	0.478	no
CK-0d	RHSIM_Rhsim05G0200500	-1.551	0.355	1.906	low
CK-0d	RHSIM_Rhsim06G0007500	-0.408	-0.945	-0.537	no
CK-0d	RHSIM_Rhsim05G0114400	1.190	0.193	-0.997	high
CK-0d	RHSIM_Rhsim06G0110000	-0.090	0.222	0.311	no
CK-0d	RHSIM_Rhsim04G0179200	-1.860	-2.190	-0.330	low
CK-0d	RHSIM_Rhsim05G0101300	-0.442	-1.011	-0.569	no
CK-0d	RHSIM_Rhsim06G0057200	0.262	-0.111	-0.373	no
CK-0d	RHSIM_Rhsim05G0031300	-1.112	0.280	1.392	low
CK-0d	RHSIM_Rhsim04G0126900	1.111	1.337	0.226	high
CK-0d	RHSIM_Rhsim06G0206900	0.115	0.953	0.838	no
CK-0d	RHSIM_Rhsim04G0250200	0.092	-0.982	-1.074	no
CK-0d	RHSIM_Rhsim06G0116700	0.299	0.609	0.310	no
CK-0d	RHSIM_Rhsim05G0205700	0.144	-0.348	-0.492	no
CK-0d	RHSIM_Rhsim04G0130200	1.528	0.680	-0.848	high
CK-0d	RHSIM_Rhsim06G0196800	0.372	-0.699	-1.072	no
CK-0d	RHSIM_Rhsim04G0108100	-0.616	-0.168	0.448	no
CK-0d	RHSIM_Rhsim05G0087800	0.883	0.536	-0.346	no
CK-0d	RHSIM_Rhsim04G0070700	-0.455	0.320	0.775	no
CK-0d	RHSIM_Rhsim05G0029900	-1.458	-0.049	1.409	low

Group	Gene	PTR (z-score difference)	z(log2 LFQ)	z(log2 FPKM)	extreme-PTR
CK-0d	RHSIM_Rhsim04G0174900	-1.295	0.545	1.841	low
CK-0d	RHSIM_Rhsim05G0147900	-0.806	-1.263	-0.457	no
CK-0d	RHSIM_Rhsim04G0221500	-1.662	-1.937	-0.275	low
CK-0d	RHSIM_Rhsim05G0068000	0.664	1.339	0.674	no
CK-0d	RHSIM_Rhsim05G0062100	0.186	1.752	1.566	no
CK-0d	RHSIM_Rhsim05G0039700	1.681	1.139	-0.542	high
CK-0d	RHSIM_Rhsim05G0146400	0.752	0.264	-0.488	no
CK-0d	RHSIM_Rhsim06G0225700	0.419	-0.280	-0.699	no
CK-0d	RHSIM_Rhsim04G0060200	1.061	-0.442	-1.504	no
CK-0d	RHSIM_Rhsim05G0208200	-1.026	-0.212	0.814	no
CK-0d	RHSIM_Rhsim06G0018800	0.237	-0.047	-0.284	no
CK-0d	RHSIM_Rhsim05G0137500	0.177	0.218	0.041	no
CK-0d	RHSIM_Rhsim04G0211500	-1.079	-1.027	0.051	low
CK-0d	RHSIM_Rhsim05G0168900	-0.596	-1.402	-0.807	no
CK-0d	RHSIM_Rhsim05G0159800	-1.175	-0.791	0.384	low
CK-0d	RHSIM_Rhsim05G0224000	-1.490	0.624	2.114	low
CK-0d	RHSIM_Rhsim05G0163400	-0.228	-1.337	-1.109	no
CK-0d	RHSIM_Rhsim06G0201000	-0.512	-0.851	-0.340	no
CK-0d	RHSIM_Rhsim04G0011000	-0.747	-1.194	-0.447	no
CK-0d	RHSIM_Rhsim05G0215900	1.413	0.568	-0.845	high
CK-0d	RHSIM_Rhsim06G0021800	0.087	-1.164	-1.252	no
CK-0d	RHSIM_Rhsim05G0193800	-0.618	-0.093	0.525	no
CK-0d	RHSIM_Rhsim04G0021400	-0.758	0.060	0.817	no
CK-0d	RHSIM_Rhsim05G0161100	0.406	1.958	1.552	no
CK-0d	RHSIM_Rhsim05G0176200	-0.569	-0.819	-0.249	no
CK-0d	RHSIM_Rhsim06G0074900	0.888	0.541	-0.347	no
CK-0d	RHSIM_Rhsim06G0230800	0.980	-1.142	-2.122	no
CK-0d	RHSIM_Rhsim06G0061800	-3.174	-1.530	1.644	low
CK-0d	RHSIM_Rhsim04G0236100	0.644	0.084	-0.560	no
CK-0d	RHSIM_Rhsim04G0206400	0.427	3.253	2.825	no
CK-0d	RHSIM_Rhsim06G0071000	-0.272	-0.104	0.167	no
CK-0d	RHSIM_Rhsim05G0231000	0.329	0.301	-0.028	no

Group	Gene	PTR (z-score difference)	z(log2 LFQ)	z(log2 FPKM)	extreme-PTR
CK-0d	RHSIM_Rhsim05G0116000	0.216	0.049	-0.168	no
CK-0d	RHSIM_Rhsim04G0024600	0.587	0.630	0.043	no
CK-0d	RHSIM_Rhsim05G0065200	0.236	-1.667	-1.903	no
CK-0d	RHSIM_Rhsim05G0000700	0.677	-0.366	-1.043	no
CK-0d	RHSIM_Rhsim05G0069500	0.885	0.995	0.110	no
CK-0d	RHSIM_Rhsim03G0222100	1.806	0.236	-1.570	high
CK-0d	RHSIM_Rhsim04G0167900	0.355	-0.996	-1.352	no
CK-0d	RHSIM_Rhsim03G0253400	0.660	-0.210	-0.870	no
CK-0d	RHSIM_Rhsim05G0069300	0.451	0.317	-0.134	no
CK-0d	RHSIM_Rhsim03G0178900	-1.647	-0.951	0.696	low
CK-0d	RHSIM_Rhsim05G0231600	-0.827	-0.605	0.222	no
CK-0d	RHSIM_Rhsim05G0186700	1.156	0.597	-0.558	high
CK-0d	RHSIM_Rhsim03G0262800	-2.483	-0.991	1.493	low
CK-0d	RHSIM_Rhsim05G0011000	-1.382	-0.935	0.447	low
CK-0d	RHSIM_Rhsim04G0102100	0.262	1.349	1.087	no
CK-0d	RHSIM_Rhsim05G0115500	-0.289	-0.374	-0.085	no
CK-0d	RHSIM_Rhsim05G0186400	-0.092	-0.917	-0.826	no
CK-0d	RHSIM_Rhsim05G0231200	-1.003	-0.980	0.023	no
CK-0d	RHSIM_Rhsim04G0201600	0.349	-0.296	-0.645	no
CK-0d	RHSIM_Rhsim05G0136600	1.034	0.643	-0.391	no
CK-0d	RHSIM_Rhsim03G0064000	0.034	-0.341	-0.374	no
CK-0d	RHSIM_Rhsim05G0043400	0.599	-0.110	-0.709	no
CK-0d	RHSIM_Rhsim05G0167500	-0.381	-1.177	-0.796	no
CK-0d	RHSIM_Rhsim05G0049600	0.081	-0.276	-0.357	no
CK-0d	RHSIM_Rhsim05G0049900	-1.866	-0.725	1.142	low
CK-0d	RHSIM_Rhsim05G0203700	1.929	-0.050	-1.979	high
CK-0d	RHSIM_Rhsim04G0132400	0.170	1.026	0.856	no
CK-0d	RHSIM_Rhsim03G0187300	0.377	-0.116	-0.493	no
CK-0d	RHSIM_Rhsim04G0072400	-0.795	-0.991	-0.197	no
CK-0d	RHSIM_Rhsim04G0097900	0.402	-0.272	-0.675	no
CK-0d	RHSIM_Rhsim05G0189500	-1.183	-0.212	0.971	low
CK-0d	RHSIM_Rhsim05G0196300	-0.603	-0.432	0.171	no

Group	Gene	PTR (z-score difference)	z(log2 LFQ)	z(log2 FPKM)	extreme-PTR
CK-0d	RHSIM_Rhsim05G0072800	0.151	1.098	0.947	no
CK-0d	RHSIM_Rhsim04G0099900	-0.905	-1.793	-0.888	no
CK-0d	RHSIM_Rhsim04G0220700	-0.773	-1.598	-0.825	no
CK-0d	RHSIM_Rhsim05G0014300	-0.334	-1.272	-0.938	no
CK-0d	RHSIM_Rhsim04G0075000	-0.245	-0.802	-0.558	no
CK-0d	RHSIM_Rhsim05G0102200	0.219	-0.055	-0.275	no
CK-0d	RHSIM_Rhsim05G0204700	-0.701	-1.086	-0.384	no
CK-0d	RHSIM_Rhsim04G0231300	3.590	3.168	-0.421	high
CK-0d	RHSIM_Rhsim05G0148900	0.231	-0.722	-0.953	no
CK-0d	RHSIM_Rhsim04G0009700	0.885	-0.482	-1.367	no
CK-0d	RHSIM_Rhsim05G0006600	-0.875	-0.252	0.622	no
CK-0d	RHSIM_Rhsim05G0221700	1.343	1.475	0.132	high
CK-0d	RHSIM_Rhsim04G0049800	-0.243	-0.023	0.220	no
CK-0d	RHSIM_Rhsim05G0128900	0.263	0.217	-0.046	no
CK-0d	RHSIM_Rhsim04G0003400	-2.242	-0.435	1.807	low
CK-0d	RHSIM_Rhsim04G0122300	-0.797	1.602	2.399	no
CK-0d	RHSIM_Rhsim05G0067600	1.299	0.850	-0.449	high
CK-0d	RHSIM_Rhsim03G0127100	1.091	1.069	-0.022	no
CK-0d	RHSIM_Rhsim05G0080700	0.920	-0.543	-1.463	no
CK-0d	RHSIM_Rhsim03G0031300	0.746	0.220	-0.527	no
CK-0d	RHSIM_Rhsim05G0144300	0.731	-0.049	-0.780	no
CK-0d	RHSIM_Rhsim05G0184800	-2.019	-0.865	1.154	low
CK-0d	RHSIM_Rhsim04G0151900	0.905	1.547	0.641	no
CK-0d	RHSIM_Rhsim05G0119700	0.226	-0.307	-0.533	no
CK-0d	RHSIM_Rhsim04G0218900	0.393	-0.284	-0.676	no
CK-0d	RHSIM_Rhsim05G0233000	0.090	0.244	0.154	no
CK-0d	RHSIM_Rhsim05G0217500	0.746	1.602	0.856	no
CK-0d	RHSIM_Rhsim04G0162200	1.545	0.673	-0.872	high
CK-0d	RHSIM_Rhsim05G0013300	-0.943	-0.953	-0.010	no
CK-0d	RHSIM_Rhsim05G0177800	0.288	-0.451	-0.738	no
CK-0d	RHSIM_Rhsim04G0136600	1.069	0.636	-0.433	no
CK-0d	RHSIM_Rhsim04G0157300	-0.260	-0.601	-0.341	no

Group	Gene	PTR (z-score difference)	z(log2 LFQ)	z(log2 FPKM)	extreme-PTR
CK-0d	RHSIM_Rhsim03G0064600	-0.200	-0.137	0.063	no
CK-0d	RHSIM_Rhsim05G0126300	-0.238	0.395	0.632	no
CK-0d	RHSIM_Rhsim04G0183700	0.341	-0.678	-1.019	no
CK-0d	RHSIM_Rhsim05G0219500	-0.124	1.027	1.151	no
CK-0d	RHSIM_Rhsim05G0094600	0.939	2.310	1.371	no
CK-0d	RHSIM_Rhsim03G0235900	1.482	1.314	-0.168	high
CK-0d	RHSIM_Rhsim04G0003000	-1.500	-1.026	0.474	low
CK-0d	RHSIM_Rhsim05G0068600	-1.468	-0.887	0.581	low
CK-0d	RHSIM_Rhsim04G0135600	0.551	2.033	1.482	no
CK-0d	RHSIM_Rhsim05G0115100	-1.362	-1.857	-0.494	low
CK-0d	RHSIM_Rhsim05G0103300	2.071	3.010	0.939	high
CK-0d	RHSIM_Rhsim04G0196000	0.424	0.645	0.220	no
CK-0d	RHSIM_Rhsim05G0171100	-0.064	-0.500	-0.435	no
CK-0d	RHSIM_Rhsim05G0092500	1.161	0.274	-0.887	high
CK-0d	RHSIM_Rhsim04G0204500	-0.128	1.716	1.844	no
CK-0d	RHSIM_Rhsim05G0002100	0.216	0.761	0.545	no
CK-0d	RHSIM_Rhsim03G0234400	-0.185	-0.451	-0.266	no
CK-0d	RHSIM_Rhsim04G0026300	-1.399	-2.212	-0.814	low
CK-0d	RHSIM_Rhsim04G0106100	0.731	-0.047	-0.777	no
CK-0d	RHSIM_Rhsim04G0023600	-0.129	0.376	0.505	no
CK-0d	RHSIM_Rhsim04G0208300	0.413	2.094	1.682	no
CK-0d	RHSIM_Rhsim05G0000100	0.066	0.722	0.655	no
CK-0d	RHSIM_Rhsim03G0122800	-0.504	-0.106	0.398	no
CK-0d	RHSIM_Rhsim04G0247000	-0.121	-0.187	-0.066	no
CK-0d	RHSIM_Rhsim03G0222500	0.194	0.637	0.443	no
CK-0d	RHSIM_Rhsim04G0101200	2.400	0.554	-1.846	high
CK-0d	RHSIM_Rhsim04G0225200	0.174	-0.357	-0.531	no
CK-0d	RHSIM_Rhsim04G0237500	1.346	-0.469	-1.815	high
CK-0d	RHSIM_Rhsim04G0021500	-1.847	-1.369	0.478	low
CK-0d	RHSIM_Rhsim05G0048800	0.342	-0.001	-0.343	no
CK-0d	RHSIM_Rhsim03G0233000	-1.344	-1.220	0.123	low
CK-0d	RHSIM_Rhsim05G0064300	-0.324	-0.011	0.313	no

Group	Gene	PTR (z-score difference)	z(log2 LFQ)	z(log2 FPKM)	extreme-PTR
CK-0d	RHSIM_Rhsim03G0160100	1.169	1.780	0.611	high
CK-0d	RHSIM_Rhsim04G0032700	-0.672	1.674	2.346	no
CK-0d	RHSIM_Rhsim04G0057100	1.074	0.492	-0.582	no
CK-0d	RHSIM_Rhsim04G0091800	-0.062	1.854	1.916	no
CK-0d	RHSIM_Rhsim04G0127300	-0.204	0.137	0.341	no
CK-0d	RHSIM_Rhsim04G0247700	1.073	0.844	-0.229	no
CK-0d	RHSIM_Rhsim04G0046600	-0.647	-1.100	-0.453	no
CK-0d	RHSIM_Rhsim04G0010600	0.622	1.188	0.566	no
CK-0d	RHSIM_Rhsim05G0092700	2.024	0.833	-1.192	high
CK-0d	RHSIM_Rhsim03G0218900	0.424	0.508	0.084	no
CK-0d	RHSIM_Rhsim04G0069300	1.241	0.009	-1.233	high
CK-0d	RHSIM_Rhsim05G0110000	1.453	-0.544	-1.997	high
CK-0d	RHSIM_Rhsim04G0113400	-0.378	-0.434	-0.057	no
CK-0d	RHSIM_Rhsim03G0217600	1.194	0.686	-0.508	high
CK-0d	RHSIM_Rhsim05G0075000	-0.069	0.131	0.200	no
CK-0d	RHSIM_Rhsim05G0036500	1.817	-0.210	-2.027	high
CK-0d	RHSIM_Rhsim04G0021000	1.507	-0.597	-2.104	high
CK-0d	RHSIM_Rhsim03G0200000	-0.974	-0.011	0.963	no
CK-0d	RHSIM_Rhsim03G0028900	0.187	0.978	0.791	no
CK-0d	RHSIM_Rhsim05G0034800	-1.726	-1.154	0.572	low
CK-0d	RHSIM_Rhsim04G0229700	-0.811	0.987	1.798	no
CK-0d	RHSIM_Rhsim05G0076800	-1.243	-0.694	0.549	low
CK-0d	RHSIM_Rhsim04G0089100	1.741	-0.385	-2.126	high
CK-0d	RHSIM_Rhsim05G0079900	0.668	0.683	0.016	no
CK-0d	RHSIM_Rhsim04G0156000	0.401	-0.248	-0.650	no
CK-0d	RHSIM_Rhsim05G0148600	-0.242	-0.176	0.066	no
CK-0d	RHSIM_Rhsim04G0247300	-0.339	-1.192	-0.853	no
CK-0d	RHSIM_Rhsim04G0202200	-1.099	-0.791	0.308	low
CK-0d	RHSIM_Rhsim04G0073800	-0.034	-0.442	-0.408	no
CK-0d	RHSIM_Rhsim03G0167100	-0.271	-0.247	0.024	no
CK-0d	RHSIM_Rhsim03G0257100	1.117	1.194	0.077	high
CK-0d	RHSIM_Rhsim04G0070800	-0.001	-0.341	-0.340	no

Group	Gene	PTR (z-score difference)	z(log2 LFQ)	z(log2 FPKM)	extreme-PTR
CK-0d	RHSIM_Rhsim03G0221000	-0.910	-0.631	0.279	no
CK-0d	RHSIM_Rhsim05G0022100	0.536	0.173	-0.362	no
CK-0d	RHSIM_Rhsim04G0023800	-0.722	-0.930	-0.208	no
CK-0d	RHSIM_Rhsim03G0074500	1.218	-0.563	-1.781	high
CK-0d	RHSIM_Rhsim04G0249000	0.690	1.152	0.462	no
CK-0d	RHSIM_Rhsim03G0246100	0.204	-0.255	-0.459	no
CK-0d	RHSIM_Rhsim04G0052100	-3.824	-2.416	1.408	low
CK-0d	RHSIM_Rhsim04G0008300	0.471	0.305	-0.166	no
CK-0d	RHSIM_Rhsim04G0205500	-1.783	-0.201	1.583	low
CK-0d	RHSIM_Rhsim05G0179500	0.674	0.753	0.080	no
CK-0d	RHSIM_Rhsim03G0025100	1.773	0.453	-1.320	high
CK-0d	RHSIM_Rhsim03G0189600	-0.150	-1.072	-0.922	no
CK-0d	RHSIM_Rhsim03G0080200	0.454	-0.938	-1.392	no
CK-0d	RHSIM_Rhsim03G0171100	-1.731	-1.661	0.070	low
CK-0d	RHSIM_Rhsim05G0223400	0.261	-1.520	-1.781	no
CK-0d	RHSIM_Rhsim03G0227400	-0.539	1.509	2.048	no
CK-0d	RHSIM_Rhsim04G0175800	-1.154	-1.315	-0.161	low
CK-0d	RHSIM_Rhsim03G0250200	-1.278	-0.641	0.638	low
CK-0d	RHSIM_Rhsim04G0222400	0.170	0.144	-0.026	no
CK-0d	RHSIM_Rhsim03G0063800	1.830	0.998	-0.831	high
CK-0d	RHSIM_Rhsim04G0205300	-0.306	-0.686	-0.380	no
CK-0d	RHSIM_Rhsim03G0140700	-1.193	-0.725	0.468	low
CK-0d	RHSIM_Rhsim03G0069800	1.023	0.600	-0.424	no
CK-0d	RHSIM_Rhsim03G0000700	1.687	0.515	-1.172	high
CK-0d	RHSIM_Rhsim04G0134200	0.889	0.756	-0.134	no
CK-0d	RHSIM_Rhsim05G0225700	-1.017	1.429	2.446	no
CK-0d	RHSIM_Rhsim03G0229500	1.612	1.657	0.045	high
CK-0d	RHSIM_Rhsim04G0197000	0.735	1.382	0.647	no
CK-0d	RHSIM_Rhsim04G0238000	0.634	-0.434	-1.068	no
CK-0d	RHSIM_Rhsim04G0190900	-1.128	-1.339	-0.211	low
CK-0d	RHSIM_Rhsim03G0153400	-1.399	-0.382	1.017	low
CK-0d	RHSIM_Rhsim04G0024500	-1.267	-0.955	0.313	low

Group	Gene	PTR (z-score difference)	z(log2 LFQ)	z(log2 FPKM)	extreme-PTR
CK-0d	RHSIM_Rhsim04G0078700	2.572	0.726	-1.846	high
CK-0d	RHSIM_Rhsim03G0113100	-0.594	-0.828	-0.234	no
CK-0d	RHSIM_Rhsim04G0015700	2.006	1.637	-0.369	high
CK-0d	RHSIM_Rhsim02G0086400	0.092	-0.594	-0.686	no
CK-0d	RHSIM_Rhsim04G0167200	2.732	2.231	-0.501	high
CK-0d	RHSIM_Rhsim04G0213900	-1.115	-0.877	0.238	low
CK-0d	RHSIM_Rhsim02G0110200	-1.207	-0.758	0.449	low
CK-0d	RHSIM_Rhsim04G0125000	0.252	-0.797	-1.049	no
CK-0d	RHSIM_Rhsim03G0192100	0.335	-0.066	-0.401	no
CK-0d	RHSIM_Rhsim02G0158900	-0.094	-0.825	-0.731	no
CK-0d	RHSIM_Rhsim04G0046500	-0.980	-0.664	0.316	no
CK-0d	RHSIM_Rhsim04G0019500	0.802	2.040	1.238	no
CK-0d	RHSIM_Rhsim04G0220400	-0.942	0.031	0.973	no
CK-0d	RHSIM_Rhsim04G0003900	-0.003	-0.212	-0.208	no
CK-0d	RHSIM_Rhsim03G0093600	0.317	-0.070	-0.387	no
CK-0d	RHSIM_Rhsim04G0048200	-0.246	0.124	0.370	no
CK-0d	RHSIM_Rhsim04G0166900	0.954	1.809	0.855	no
CK-0d	RHSIM_Rhsim04G0235300	1.529	0.381	-1.148	high
CK-0d	RHSIM_Rhsim04G0124400	-1.508	-0.086	1.422	low
CK-0d	RHSIM_Rhsim04G0120400	-0.997	-0.430	0.567	no
CK-0d	RHSIM_Rhsim03G0045900	-0.120	-0.356	-0.237	no
CK-0d	RHSIM_Rhsim04G0015800	-0.098	-0.922	-0.824	no
CK-0d	RHSIM_Rhsim02G0250400	-0.326	-0.441	-0.115	no
CK-0d	RHSIM_Rhsim04G0195000	-0.431	1.080	1.511	no
CK-0d	RHSIM_Rhsim04G0214300	0.190	0.281	0.091	no
CK-0d	RHSIM_Rhsim04G0153100	0.670	-0.177	-0.847	no
CK-0d	RHSIM_Rhsim04G0085200	-1.143	-0.694	0.448	low
CK-0d	RHSIM_Rhsim03G0153200	0.979	-0.549	-1.528	no
CK-0d	RHSIM_Rhsim04G0053900	0.079	-0.888	-0.967	no
CK-0d	RHSIM_Rhsim04G0164100	0.005	0.005	0.000	no
CK-0d	RHSIM_Rhsim04G0201900	0.990	0.744	-0.246	no
CK-0d	RHSIM_Rhsim04G0176300	-0.374	-1.051	-0.677	no

Group	Gene	PTR (z-score difference)	z(log2 LFQ)	z(log2 FPKM)	extreme-PTR
CK-0d	RHSIM_Rhsim04G0167000	0.305	1.799	1.493	no
CK-0d	RHSIM_Rhsim02G0232000	-0.122	0.477	0.599	no
CK-0d	RHSIM_Rhsim04G0149700	-0.113	0.554	0.668	no
CK-0d	RHSIM_Rhsim04G0160100	1.677	1.295	-0.382	high
CK-0d	RHSIM_Rhsim04G0147500	1.507	0.299	-1.208	high
CK-0d	RHSIM_Rhsim04G0214400	-0.245	0.211	0.455	no
CK-0d	RHSIM_Rhsim04G0016500	0.233	-0.959	-1.192	no
CK-0d	RHSIM_Rhsim04G0203600	1.038	1.596	0.558	no
CK-0d	RHSIM_Rhsim04G0231000	0.346	3.023	2.677	no
CK-0d	RHSIM_Rhsim04G0188800	-1.888	-1.564	0.323	low
CK-0d	RHSIM_Rhsim03G0212900	0.829	0.522	-0.306	no
CK-0d	RHSIM_Rhsim04G0212200	0.372	-1.097	-1.469	no
CK-0d	RHSIM_Rhsim04G0228000	1.377	0.107	-1.270	high
CK-0d	RHSIM_Rhsim04G0246200	-0.564	-0.359	0.205	no
CK-0d	RHSIM_Rhsim03G0223700	-0.051	-0.522	-0.471	no
CK-0d	RHSIM_Rhsim04G0026700	-0.731	-0.043	0.688	no
CK-0d	RHSIM_Rhsim03G0055600	1.295	-0.748	-2.042	high
CK-0d	RHSIM_Rhsim04G0088200	0.996	-0.130	-1.126	no
CK-0d	RHSIM_Rhsim04G0083100	-0.222	1.556	1.778	no
CK-0d	RHSIM_Rhsim04G0008600	0.524	0.630	0.105	no
CK-0d	RHSIM_Rhsim02G0248700	1.562	2.047	0.485	high
CK-0d	RHSIM_Rhsim03G0009100	0.592	1.293	0.701	no
CK-0d	RHSIM_Rhsim04G0052900	0.454	-0.338	-0.792	no
CK-0d	RHSIM_Rhsim04G0109400	0.254	-0.071	-0.325	no
CK-0d	RHSIM_Rhsim04G0008000	-0.944	-1.217	-0.273	no
CK-0d	RHSIM_Rhsim02G0173800	0.592	0.259	-0.333	no
CK-0d	RHSIM_Rhsim04G0234400	1.341	-0.869	-2.210	high
CK-0d	RHSIM_Rhsim02G0254600	0.353	-0.772	-1.125	no
CK-0d	RHSIM_Rhsim04G0065800	1.100	1.530	0.431	no
CK-0d	RHSIM_Rhsim02G0000300	0.695	-0.138	-0.832	no
CK-0d	RHSIM_Rhsim03G0093500	-0.084	-0.986	-0.902	no
CK-0d	RHSIM_Rhsim04G0196300	-2.319	0.087	2.405	low

Group	Gene	PTR (z-score difference)	z(log2 LFQ)	z(log2 FPKM)	extreme-PTR
CK-0d	RHSIM_Rhsim03G0224100	-0.159	0.350	0.509	no
CK-0d	RHSIM_Rhsim04G0228200	-1.102	-1.640	-0.538	low
CK-0d	RHSIM_Rhsim03G0069100	0.752	-0.047	-0.800	no
CK-0d	RHSIM_Rhsim02G0199000	-1.394	-2.272	-0.878	low
CK-0d	RHSIM_Rhsim04G0018700	-0.972	-1.248	-0.276	no
CK-0d	RHSIM_Rhsim02G0143100	-2.244	-1.604	0.640	low
CK-0d	RHSIM_Rhsim02G0172200	0.791	1.217	0.427	no
CK-0d	RHSIM_Rhsim03G0247800	-1.121	-1.201	-0.080	low
CK-0d	RHSIM_Rhsim04G0113500	1.005	1.028	0.023	no
CK-0d	RHSIM_Rhsim03G0031100	-0.944	-2.081	-1.137	no
CK-0d	RHSIM_Rhsim03G0141600	0.424	1.329	0.905	no
CK-0d	RHSIM_Rhsim03G0236500	-0.071	0.114	0.185	no
CK-0d	RHSIM_Rhsim04G0164800	0.964	2.254	1.289	no
CK-0d	RHSIM_Rhsim03G0120500	0.265	-0.407	-0.673	no
CK-0d	RHSIM_Rhsim03G0186100	-1.670	-0.614	1.056	low
CK-0d	RHSIM_Rhsim03G0223900	0.068	0.288	0.220	no
CK-0d	RHSIM_Rhsim03G0017900	0.935	0.275	-0.661	no
CK-0d	RHSIM_Rhsim04G0218600	-1.254	-0.662	0.591	low
CK-0d	RHSIM_Rhsim03G0256000	1.095	-0.097	-1.192	no
CK-0d	RHSIM_Rhsim04G0187900	0.074	-1.002	-1.077	no
CK-0d	RHSIM_Rhsim03G0164300	2.068	0.750	-1.318	high
CK-0d	RHSIM_Rhsim04G0111500	-1.184	-0.889	0.295	low
CK-0d	RHSIM_Rhsim03G0214900	0.890	1.573	0.683	no
CK-0d	RHSIM_Rhsim04G0002800	1.141	3.432	2.291	high
CK-0d	RHSIM_Rhsim04G0245400	0.786	-0.503	-1.289	no
CK-0d	RHSIM_Rhsim04G0208400	0.688	1.386	0.698	no
CK-0d	RHSIM_Rhsim04G0229900	-0.262	3.245	3.507	no
CK-0d	RHSIM_Rhsim04G0216700	2.091	1.246	-0.846	high
CK-0d	RHSIM_Rhsim03G0236800	-0.753	-1.228	-0.475	no
CK-0d	RHSIM_Rhsim04G0123000	1.455	0.090	-1.365	high
CK-0d	RHSIM_Rhsim03G0211000	0.554	0.887	0.333	no
CK-0d	RHSIM_Rhsim02G0193100	-1.221	-0.890	0.330	low

Group	Gene	PTR (z-score difference)	z(log2 LFQ)	z(log2 FPKM)	extreme-PTR
CK-0d	RHSIM_Rhsim03G0208400	1.377	-0.259	-1.636	high
CK-0d	RHSIM_Rhsim03G0055400	1.126	-1.124	-2.249	high
CK-0d	RHSIM_Rhsim03G0121600	0.513	-0.169	-0.682	no
CK-0d	RHSIM_Rhsim03G0088200	1.347	0.289	-1.058	high
CK-0d	RHSIM_Rhsim04G0008500	-0.384	-0.570	-0.186	no
CK-0d	RHSIM_Rhsim03G0230800	0.127	-0.279	-0.406	no
CK-0d	RHSIM_Rhsim04G0222300	0.119	-0.309	-0.428	no
CK-0d	RHSIM_Rhsim03G0161300	0.343	-0.291	-0.634	no
CK-0d	RHSIM_Rhsim03G0188000	-0.517	-0.155	0.362	no
CK-0d	RHSIM_Rhsim03G0046200	0.724	0.534	-0.190	no
CK-0d	RHSIM_Rhsim02G0059700	-0.640	-1.033	-0.393	no
CK-0d	RHSIM_Rhsim03G0009300	1.329	-0.038	-1.366	high
CK-0d	RHSIM_Rhsim03G0245100	0.149	-0.191	-0.340	no
CK-0d	RHSIM_Rhsim03G0028400	0.538	-0.878	-1.417	no
CK-0d	RHSIM_Rhsim04G0245900	1.752	0.881	-0.871	high
CK-0d	RHSIM_Rhsim03G0264400	0.694	-0.047	-0.741	no
CK-0d	RHSIM_Rhsim02G0123600	-1.328	-0.737	0.592	low
CK-0d	RHSIM_Rhsim04G0015500	-0.567	0.072	0.639	no
CK-0d	RHSIM_Rhsim04G0028100	0.654	-0.482	-1.136	no
CK-0d	RHSIM_Rhsim04G0249600	1.330	0.193	-1.137	high
CK-0d	RHSIM_Rhsim03G0070200	0.528	0.118	-0.410	no
CK-0d	RHSIM_Rhsim03G0234300	0.863	0.432	-0.430	no
CK-0d	RHSIM_Rhsim03G0121100	0.242	0.258	0.016	no
CK-0d	RHSIM_Rhsim04G0021900	1.250	1.305	0.055	high
CK-0d	RHSIM_Rhsim02G0237500	0.474	0.942	0.468	no
CK-0d	RHSIM_Rhsim04G0015400	-0.641	-0.091	0.550	no
CK-0d	RHSIM_Rhsim04G0074100	-0.620	-0.439	0.181	no
CK-0d	RHSIM_Rhsim03G0163800	-1.383	-0.430	0.953	low
CK-0d	RHSIM_Rhsim03G0241100	-0.219	-1.183	-0.964	no
CK-0d	RHSIM_Rhsim02G0001600	-0.478	-0.857	-0.379	no
CK-0d	RHSIM_Rhsim03G0007100	-0.567	-0.461	0.107	no
CK-0d	RHSIM_Rhsim03G0197200	-0.461	0.090	0.552	no

Group	Gene	PTR (z-score difference)	z(log2 LFQ)	z(log2 FPKM)	extreme-PTR
CK-0d	RHSIM_Rhsim02G0159600	-0.616	1.493	2.108	no
CK-0d	RHSIM_Rhsim03G0018300	-0.227	1.253	1.480	no
CK-0d	RHSIM_Rhsim03G0011100	-0.287	0.789	1.076	no
CK-0d	RHSIM_Rhsim03G0264900	-1.372	-0.618	0.755	low
CK-0d	RHSIM_Rhsim03G0131600	-0.308	0.709	1.017	no
CK-0d	RHSIM_Rhsim03G0215400	-0.630	0.905	1.534	no
CK-0d	RHSIM_Rhsim02G0070700	0.400	0.784	0.384	no
CK-0d	RHSIM_Rhsim02G0132400	0.384	0.141	-0.242	no
CK-0d	RHSIM_Rhsim03G0058000	1.699	0.702	-0.998	high
CK-0d	RHSIM_Rhsim04G0067400	-0.378	-0.547	-0.170	no
CK-0d	RHSIM_Rhsim02G0193600	-0.423	-1.035	-0.612	no
CK-0d	RHSIM_Rhsim03G0063900	-3.404	0.109	3.513	low
CK-0d	RHSIM_Rhsim03G0259500	0.001	-0.827	-0.828	no
CK-0d	RHSIM_Rhsim04G0243300	0.164	0.366	0.203	no
CK-0d	RHSIM_Rhsim02G0252000	0.661	-0.160	-0.821	no
CK-0d	RHSIM_Rhsim03G0165500	-0.923	0.984	1.906	no
CK-0d	RHSIM_Rhsim03G0076200	-0.089	0.057	0.146	no
CK-0d	RHSIM_Rhsim03G0191300	0.708	0.875	0.166	no
CK-0d	RHSIM_Rhsim03G0191800	0.461	0.180	-0.281	no
CK-0d	RHSIM_Rhsim03G0254400	-1.008	-0.479	0.529	no
CK-0d	RHSIM_Rhsim04G0166600	0.015	-0.527	-0.542	no
CK-0d	RHSIM_Rhsim03G0180600	-0.207	1.215	1.422	no
CK-0d	RHSIM_Rhsim02G0135500	0.421	0.011	-0.410	no
CK-0d	RHSIM_Rhsim02G0217800	2.042	0.681	-1.362	high
CK-0d	RHSIM_Rhsim02G0056200	2.100	2.360	0.260	high
CK-0d	RHSIM_Rhsim04G0242100	0.242	-0.443	-0.685	no
CK-0d	RHSIM_Rhsim02G0131800	-0.744	-0.893	-0.149	no
CK-0d	RHSIM_Rhsim03G0165100	1.502	-0.612	-2.114	high
CK-0d	RHSIM_Rhsim03G0245900	-1.594	0.417	2.010	low
CK-0d	RHSIM_Rhsim02G0071100	0.570	1.015	0.444	no
CK-0d	RHSIM_Rhsim03G0073900	1.398	0.370	-1.028	high
CK-0d	RHSIM_Rhsim03G0171300	0.560	1.457	0.897	no

Group	Gene	PTR (z-score difference)	z(log2 LFQ)	z(log2 FPKM)	extreme-PTR
CK-0d	RHSIM_Rhsim03G0231400	-1.242	-1.398	-0.156	low
CK-0d	RHSIM_Rhsim02G0235500	-0.355	0.713	1.068	no
CK-0d	RHSIM_Rhsim03G0067800	-0.128	-0.433	-0.304	no
CK-0d	RHSIM_Rhsim02G0143300	1.682	2.839	1.157	high
CK-0d	RHSIM_Rhsim03G0105000	-0.290	-0.922	-0.632	no
CK-0d	RHSIM_Rhsim04G0083900	0.717	0.731	0.014	no
CK-0d	RHSIM_Rhsim03G0053000	0.376	1.592	1.216	no
CK-0d	RHSIM_Rhsim02G0123500	0.030	-0.684	-0.714	no
CK-0d	RHSIM_Rhsim03G0075500	-0.568	-0.316	0.253	no
CK-0d	RHSIM_Rhsim02G0088400	0.215	1.448	1.233	no
CK-0d	RHSIM_Rhsim04G0007400	0.823	3.403	2.580	no
CK-0d	RHSIM_Rhsim02G0031300	-1.997	-1.362	0.635	low
CK-0d	RHSIM_Rhsim04G0006500	-1.351	-1.064	0.287	low
CK-0d	RHSIM_Rhsim01G0222900	0.564	-0.266	-0.830	no
CK-0d	RHSIM_Rhsim01G0240100	-1.071	-1.183	-0.112	low
CK-0d	RHSIM_Rhsim02G0171900	1.727	1.322	-0.405	high
CK-0d	RHSIM_Rhsim01G0041300	0.414	0.084	-0.329	no
CK-0d	RHSIM_Rhsim04G0051700	1.494	0.084	-1.410	high
CK-0d	RHSIM_Rhsim03G0067500	-0.812	0.934	1.746	no
CK-0d	RHSIM_Rhsim04G0066100	0.947	0.208	-0.739	no
CK-0d	RHSIM_Rhsim02G0066400	-0.806	-0.800	0.006	no
CK-0d	RHSIM_Rhsim02G0018900	0.281	-0.568	-0.849	no
CK-0d	RHSIM_Rhsim01G0178500	-0.908	-0.208	0.701	no
CK-0d	RHSIM_Rhsim04G0181100	1.703	0.848	-0.856	high
CK-0d	RHSIM_Rhsim03G0255900	0.163	1.364	1.202	no
CK-0d	RHSIM_Rhsim03G0243000	-0.429	-0.203	0.226	no
CK-0d	RHSIM_Rhsim03G0186600	0.440	0.344	-0.097	no
CK-0d	RHSIM_Rhsim02G0241900	0.123	-1.426	-1.549	no
CK-0d	RHSIM_Rhsim03G0245400	0.340	-0.279	-0.620	no
CK-0d	RHSIM_Rhsim01G0167600	0.833	1.063	0.230	no
CK-0d	RHSIM_Rhsim03G0225800	1.248	-0.373	-1.622	high
CK-0d	RHSIM_Rhsim02G0133700	-0.995	-0.166	0.829	no

Group	Gene	PTR (z-score difference)	z(log2 LFQ)	z(log2 FPKM)	extreme-PTR
CK-0d	RHSIM_Rhsim02G0014500	-0.198	-1.292	-1.094	no
CK-0d	RHSIM_Rhsim04G0072300	-0.985	-1.853	-0.868	no
CK-0d	RHSIM_Rhsim04G0091400	-1.828	-0.803	1.025	low
CK-0d	RHSIM_Rhsim03G0059700	0.470	0.483	0.013	no
CK-0d	RHSIM_Rhsim04G0085600	-0.412	0.805	1.217	no
CK-0d	RHSIM_Rhsim04G0121700	1.144	1.230	0.086	high
CK-0d	RHSIM_Rhsim02G0253800	0.098	-0.486	-0.585	no
CK-0d	RHSIM_Rhsim02G0043500	-0.453	0.193	0.646	no
CK-0d	RHSIM_Rhsim03G0097500	-0.557	0.195	0.752	no
CK-0d	RHSIM_Rhsim03G0254000	0.679	0.691	0.011	no
CK-0d	RHSIM_Rhsim02G0031900	-1.145	-0.099	1.045	low
CK-0d	RHSIM_Rhsim03G0235200	-0.130	1.067	1.197	no
CK-0d	RHSIM_Rhsim03G0091100	0.044	-0.424	-0.468	no
CK-0d	RHSIM_Rhsim02G0071800	0.117	0.144	0.028	no
CK-0d	RHSIM_Rhsim01G0292400	-0.819	0.010	0.829	no
CK-0d	RHSIM_Rhsim02G0141300	0.321	-0.395	-0.715	no
CK-0d	RHSIM_Rhsim01G0203700	-0.001	-0.063	-0.063	no
CK-0d	RHSIM_Rhsim03G0129800	-0.742	-0.510	0.232	no
CK-0d	RHSIM_Rhsim03G0038900	0.795	0.354	-0.441	no
CK-0d	RHSIM_Rhsim02G0122100	0.009	0.087	0.077	no
CK-0d	RHSIM_Rhsim01G0238000	-3.795	-0.779	3.016	low
CK-0d	RHSIM_Rhsim02G0243000	1.139	0.127	-1.012	high
CK-0d	RHSIM_Rhsim01G0066800	0.105	-0.911	-1.016	no
CK-0d	RHSIM_Rhsim03G0125400	-1.777	-1.752	0.025	low
CK-0d	RHSIM_Rhsim03G0161700	0.527	0.393	-0.135	no
CK-0d	RHSIM_Rhsim02G0100900	2.152	0.632	-1.520	high
CK-0d	RHSIM_Rhsim03G0261300	-0.633	0.044	0.677	no
CK-0d	RHSIM_Rhsim02G0256200	0.159	-0.065	-0.223	no
CK-0d	RHSIM_Rhsim02G0022900	-1.354	-0.949	0.405	low
CK-0d	RHSIM_Rhsim03G0110700	-0.315	1.038	1.353	no
CK-0d	RHSIM_Rhsim01G0141100	-0.369	0.321	0.690	no
CK-0d	RHSIM_Rhsim03G0268200	0.786	0.016	-0.770	no

Group	Gene	PTR (z-score difference)	z(log2 LFQ)	z(log2 FPKM)	extreme-PTR
CK-0d	RHSIM_Rhsim02G0008200	0.109	0.677	0.568	no
CK-0d	RHSIM_Rhsim03G0225600	-1.972	-1.803	0.169	low
CK-0d	RHSIM_Rhsim03G0152900	0.504	0.459	-0.045	no
CK-0d	RHSIM_Rhsim03G0243200	-1.853	-0.374	1.479	low
CK-0d	RHSIM_Rhsim03G0236200	0.119	1.406	1.287	no
CK-0d	RHSIM_Rhsim01G0105900	0.028	0.299	0.270	no
CK-0d	RHSIM_Rhsim03G0212300	0.889	0.586	-0.303	no
CK-0d	RHSIM_Rhsim03G0054000	0.668	-1.129	-1.797	no
CK-0d	RHSIM_Rhsim03G0090500	-0.220	-0.990	-0.769	no
CK-0d	RHSIM_Rhsim01G0147400	1.024	0.656	-0.368	no
CK-0d	RHSIM_Rhsim02G0248400	0.250	1.625	1.375	no
CK-0d	RHSIM_Rhsim03G0257300	-0.802	-0.193	0.609	no
CK-0d	RHSIM_Rhsim02G0128600	-0.615	-0.424	0.191	no
CK-0d	RHSIM_Rhsim03G0219500	0.000	1.458	1.458	no
CK-0d	RHSIM_Rhsim03G0187400	1.175	0.941	-0.234	high
CK-0d	RHSIM_Rhsim03G0040400	1.567	0.979	-0.588	high
CK-0d	RHSIM_Rhsim03G0251700	-0.891	-0.472	0.419	no
CK-0d	RHSIM_Rhsim03G0005600	-0.447	0.094	0.541	no
CK-0d	RHSIM_Rhsim03G0167200	-0.026	-0.411	-0.385	no
CK-0d	RHSIM_Rhsim03G0254200	-0.534	-1.512	-0.978	no
CK-0d	RHSIM_Rhsim03G0136300	0.738	0.252	-0.486	no
CK-0d	RHSIM_Rhsim02G0071400	0.744	-0.843	-1.587	no
CK-0d	RHSIM_Rhsim03G0230100	0.818	0.915	0.097	no
CK-0d	RHSIM_Rhsim03G0176800	-1.212	-1.483	-0.271	low
CK-0d	RHSIM_Rhsim02G0060200	-0.248	-1.161	-0.913	no
CK-0d	RHSIM_Rhsim02G0143200	0.446	-0.281	-0.727	no
CK-0d	RHSIM_Rhsim03G0070300	0.042	0.994	0.953	no
CK-0d	RHSIM_Rhsim03G0117200	-0.310	-0.553	-0.243	no
CK-0d	RHSIM_Rhsim03G0212500	-1.231	0.140	1.371	low
CK-0d	RHSIM_Rhsim02G0194100	0.144	1.448	1.304	no
CK-0d	RHSIM_Rhsim03G0053900	1.621	-0.026	-1.647	high
CK-0d	RHSIM_Rhsim02G0181400	-0.018	0.253	0.272	no

Group	Gene	PTR (z-score difference)	z(log2 LFQ)	z(log2 FPKM)	extreme-PTR
CK-0d	RHSIM_Rhsim02G0107000	-0.724	0.392	1.116	no
CK-0d	RHSIM_Rhsim02G0028200	1.081	-0.037	-1.118	no
CK-0d	RHSIM_Rhsim03G0219400	-0.529	-1.475	-0.947	no
CK-0d	RHSIM_Rhsim03G0027400	0.820	1.060	0.240	no
CK-0d	RHSIM_Rhsim03G0240000	-2.281	-1.644	0.637	low
CK-0d	RHSIM_Rhsim02G0252600	-1.766	-0.793	0.973	low
CK-0d	RHSIM_Rhsim03G0089800	-0.871	-0.166	0.705	no
CK-0d	RHSIM_Rhsim01G0249900	0.033	1.047	1.014	no
CK-0d	RHSIM_Rhsim03G0214200	0.310	-0.553	-0.863	no
CK-0d	RHSIM_Rhsim02G0150100	-1.614	-0.358	1.256	low
CK-0d	RHSIM_Rhsim03G0169600	0.753	-0.368	-1.121	no
CK-0d	RHSIM_Rhsim03G0253100	-2.203	-1.155	1.048	low
CK-0d	RHSIM_Rhsim02G0053700	-0.782	1.148	1.930	no
CK-0d	RHSIM_Rhsim01G0170800	0.389	0.319	-0.070	no
CK-0d	RHSIM_Rhsim03G0254900	0.937	0.875	-0.062	no
CK-0d	RHSIM_Rhsim02G0085700	0.637	-0.350	-0.987	no
CK-0d	RHSIM_Rhsim03G0150200	2.514	0.866	-1.648	high
CK-0d	RHSIM_Rhsim03G0190600	-0.368	-0.716	-0.348	no
CK-0d	RHSIM_Rhsim03G0062900	-0.317	-0.756	-0.439	no
CK-0d	RHSIM_Rhsim03G0049900	0.165	0.306	0.141	no
CK-0d	RHSIM_Rhsim03G0239600	-0.526	-1.630	-1.105	no
CK-0d	RHSIM_Rhsim02G0002000	0.906	1.139	0.233	no
CK-0d	RHSIM_Rhsim01G0183300	0.268	-0.746	-1.015	no
CK-0d	RHSIM_Rhsim01G0001700	-0.793	-1.322	-0.529	no
CK-0d	RHSIM_Rhsim03G0147100	0.018	0.471	0.452	no
CK-0d	RHSIM_Rhsim03G0076700	0.119	-0.125	-0.244	no
CK-0d	RHSIM_Rhsim01G0283600	-1.651	0.491	2.142	low
CK-0d	RHSIM_Rhsim03G0186300	0.655	-1.197	-1.852	no
CK-0d	RHSIM_Rhsim02G0244700	-0.157	-0.231	-0.074	no
CK-0d	RHSIM_Rhsim02G0058700	2.161	0.509	-1.652	high
CK-0d	RHSIM_Rhsim03G0226600	-0.485	0.435	0.920	no
CK-0d	RHSIM_Rhsim01G0123000	-1.795	-1.073	0.721	low

Group	Gene	PTR (z-score difference)	z(log2 LFQ)	z(log2 FPKM)	extreme-PTR
CK-0d	RHSIM_Rhsim03G0003600	-0.307	-0.172	0.135	no
CK-0d	RHSIM_Rhsim02G0005400	1.692	1.592	-0.100	high
CK-0d	RHSIM_Rhsim01G0192600	-0.846	-0.342	0.504	no
CK-0d	RHSIM_Rhsim01G0258200	-0.239	0.403	0.642	no
CK-0d	RHSIM_Rhsim01G0100600	-0.425	-0.042	0.383	no
CK-0d	RHSIM_Rhsim02G0236800	-1.903	-0.950	0.954	low
CK-0d	RHSIM_Rhsim03G0221300	-1.172	-2.042	-0.870	low
CK-0d	RHSIM_Rhsim01G0280000	-0.035	0.214	0.250	no
CK-0d	RHSIM_Rhsim01G0135600	0.493	-0.389	-0.882	no
CK-0d	RHSIM_Rhsim02G0175300	1.128	0.093	-1.035	high
CK-0d	RHSIM_Rhsim03G0094200	0.567	-0.153	-0.720	no
CK-0d	RHSIM_Rhsim02G0195000	-1.144	-1.257	-0.113	low
CK-0d	RHSIM_Rhsim01G0114000	0.953	1.021	0.068	no
CK-0d	RHSIM_Rhsim03G0052800	-2.932	-0.950	1.982	low
CK-0d	RHSIM_Rhsim01G0259100	-1.779	-1.169	0.610	low
CK-0d	RHSIM_Rhsim01G0049700	-0.794	-0.491	0.303	no
CK-0d	RHSIM_Rhsim01G0190400	-0.088	0.393	0.482	no
CK-0d	RHSIM_Rhsim01G0187400	-0.148	-0.971	-0.823	no
CK-0d	RHSIM_Rhsim01G0044000	0.760	1.459	0.699	no
CK-0d	RHSIM_Rhsim03G0153700	0.479	0.400	-0.080	no
CK-0d	RHSIM_Rhsim02G0246500	-0.283	0.415	0.698	no
CK-0d	RHSIM_Rhsim01G0061400	0.340	-0.433	-0.773	no
CK-0d	RHSIM_Rhsim02G0004600	-1.687	-0.207	1.480	low
CK-0d	RHSIM_Rhsim01G0049100	1.138	0.307	-0.832	high
CK-0d	RHSIM_Rhsim02G0180700	-0.784	-0.273	0.511	no
CK-0d	RHSIM_Rhsim01G0222000	0.782	0.466	-0.315	no
CK-0d	RHSIM_Rhsim02G0207700	-0.006	-1.344	-1.338	no
CK-0d	RHSIM_Rhsim03G0227500	1.408	0.305	-1.103	high
CK-0d	RHSIM_Rhsim02G0241000	-1.579	-1.437	0.142	low
CK-0d	RHSIM_Rhsim02G0018500	-0.174	-0.495	-0.322	no
CK-0d	RHSIM_Rhsim02G0115600	-0.858	-0.200	0.658	no
CK-0d	RHSIM_Rhsim02G0070000	0.393	0.563	0.170	no

Group	Gene	PTR (z-score difference)	z(log2 LFQ)	z(log2 FPKM)	extreme-PTR
CK-0d	RHSIM_Rhsim02G0007400	0.137	-0.425	-0.561	no
CK-0d	RHSIM_Rhsim02G0256900	0.586	-0.010	-0.596	no
CK-0d	RHSIM_Rhsim02G0106100	-0.060	-1.253	-1.193	no
CK-0d	RHSIM_Rhsim03G0213000	0.632	0.146	-0.486	no
CK-0d	RHSIM_Rhsim03G0087300	0.713	-0.253	-0.966	no
CK-0d	RHSIM_Rhsim02G0061700	-0.396	1.141	1.537	no
CK-0d	RHSIM_Rhsim02G0234600	-2.408	-1.045	1.363	low
CK-0d	RHSIM_Rhsim03G0148000	-1.337	-0.868	0.469	low
CK-0d	RHSIM_Rhsim01G0169000	-2.556	-1.397	1.159	low
CK-0d	RHSIM_Rhsim01G0232600	-1.040	-0.365	0.675	low
CK-0d	RHSIM_Rhsim02G0084400	-0.311	-0.959	-0.647	no
CK-0d	RHSIM_Rhsim01G0286300	2.104	0.789	-1.315	high
CK-0d	RHSIM_Rhsim03G0247500	-1.856	-0.918	0.938	low
CK-0d	RHSIM_Rhsim02G0217700	-0.150	0.499	0.649	no
CK-0d	RHSIM_Rhsim02G0104800	0.296	0.055	-0.242	no
CK-0d	RHSIM_Rhsim01G0050400	-0.181	-1.285	-1.104	no
CK-0d	RHSIM_Rhsim01G0101100	-0.061	-1.178	-1.117	no
CK-0d	RHSIM_Rhsim01G0278500	-1.039	-1.030	0.009	low
CK-0d	RHSIM_Rhsim02G0214300	1.084	0.979	-0.105	no
CK-0d	RHSIM_Rhsim01G0060000	-0.388	0.303	0.691	no
CK-0d	RHSIM_Rhsim01G0258800	-0.241	0.280	0.521	no
CK-0d	RHSIM_Rhsim01G0114100	-0.717	-0.469	0.248	no
CK-0d	RHSIM_Rhsim01G0258700	1.139	1.013	-0.127	high
CK-0d	RHSIM_Rhsim02G0007600	1.872	0.365	-1.507	high
CK-0d	RHSIM_Rhsim02G0069600	-0.025	0.470	0.495	no
CK-0d	RHSIM_Rhsim01G0127700	0.639	1.231	0.592	no
CK-0d	RHSIM_Rhsim02G0031200	0.635	-0.620	-1.255	no
CK-0d	RHSIM_Rhsim01G0192800	-0.124	0.489	0.613	no
CK-0d	RHSIM_Rhsim03G0004200	-0.868	-0.508	0.360	no
CK-0d	RHSIM_Rhsim01G0240400	0.592	-0.246	-0.838	no
CK-0d	RHSIM_Rhsim02G0068800	0.216	0.152	-0.065	no
CK-0d	RHSIM_Rhsim01G0291700	-0.197	-0.428	-0.230	no

Group	Gene	PTR (z-score difference)	z(log2 LFQ)	z(log2 FPKM)	extreme-PTR
CK-0d	RHSIM_Rhsim01G0131800	2.251	2.225	-0.025	high
CK-0d	RHSIM_Rhsim01G0171300	0.719	2.425	1.706	no
CK-0d	RHSIM_Rhsim02G0210500	-1.112	-0.797	0.315	low
CK-0d	RHSIM_Rhsim01G0230200	-1.207	-0.983	0.224	low
CK-0d	RHSIM_Rhsim02G0028100	-0.257	1.107	1.364	no
CK-0d	RHSIM_Rhsim01G0131300	1.698	0.627	-1.071	high
CK-0d	RHSIM_Rhsim01G0142100	-0.341	-0.515	-0.173	no
CK-0d	RHSIM_Rhsim01G0220300	0.470	-0.801	-1.270	no
CK-0d	RHSIM_Rhsim03G0090000	-0.735	0.261	0.996	no
CK-0d	RHSIM_Rhsim01G0219100	1.373	3.227	1.854	high
CK-0d	RHSIM_Rhsim03G0151200	0.837	1.309	0.472	no
CK-0d	RHSIM_Rhsim01G0290600	0.257	-0.060	-0.317	no
CK-0d	RHSIM_Rhsim01G0102500	-0.328	-0.197	0.131	no
CK-0d	RHSIM_Rhsim01G0278100	-2.625	-1.435	1.189	low
CK-0d	RHSIM_Rhsim02G0099900	-0.250	-0.769	-0.518	no
CK-0d	RHSIM_Rhsim02G0004900	-0.544	-0.852	-0.308	no
CK-0d	RHSIM_Rhsim01G0226900	-1.697	-0.742	0.954	low
CK-0d	RHSIM_Rhsim02G0124600	0.847	-0.070	-0.918	no
CK-0d	RHSIM_Rhsim02G0157100	-1.397	-1.737	-0.341	low
CK-0d	RHSIM_Rhsim01G0135700	0.736	0.571	-0.166	no
CK-0d	RHSIM_Rhsim02G0181000	0.137	0.220	0.083	no
CK-0d	RHSIM_Rhsim02G0096400	0.433	0.292	-0.141	no
CK-0d	RHSIM_Rhsim02G0089800	0.247	0.351	0.104	no
CK-0d	RHSIM_Rhsim02G0169600	0.028	0.597	0.569	no
CK-0d	RHSIM_Rhsim02G0105100	0.395	-0.719	-1.114	no
CK-0d	RHSIM_Rhsim01G0267800	-2.000	-1.474	0.526	low
CK-0d	RHSIM_Rhsim01G0242700	-0.697	-2.265	-1.568	no
CK-0d	RHSIM_Rhsim01G0152000	-1.255	-1.045	0.210	low
CK-0d	RHSIM_Rhsim02G0172600	0.836	0.856	0.020	no
CK-0d	RHSIM_Rhsim02G0147000	1.456	2.409	0.954	high
CK-0d	RHSIM_Rhsim02G0251400	0.456	0.250	-0.206	no
CK-0d	RHSIM_Rhsim02G0251300	1.598	0.838	-0.759	high

Group	Gene	PTR (z-score difference)	z(log2 LFQ)	z(log2 FPKM)	extreme-PTR
CK-0d	RHSIM_Rhsim02G0007900	-1.691	-0.237	1.454	low
CK-0d	RHSIM_Rhsim02G0252500	-0.013	1.516	1.529	no
CK-0d	RHSIM_Rhsim02G0122400	-0.111	-0.655	-0.545	no
CK-0d	RHSIM_Rhsim02G0159400	-0.071	-0.709	-0.638	no
CK-0d	RHSIM_Rhsim02G0088300	0.256	-1.132	-1.388	no
CK-0d	RHSIM_Rhsim02G0083500	-0.256	-0.169	0.086	no
CK-0d	RHSIM_Rhsim02G0205900	-1.976	-0.077	1.899	low
CK-0d	RHSIM_Rhsim02G0208200	-1.177	-1.470	-0.293	low
CK-0d	RHSIM_Rhsim01G0235200	0.362	0.150	-0.212	no
CK-0d	RHSIM_Rhsim01G0243800	0.164	1.053	0.889	no
CK-0d	RHSIM_Rhsim02G0119200	0.768	0.663	-0.105	no
CK-0d	RHSIM_Rhsim01G0234200	0.945	0.437	-0.508	no
CK-0d	RHSIM_Rhsim01G0025200	0.611	1.226	0.615	no
CK-0d	RHSIM_Rhsim01G0054900	-0.250	-0.078	0.172	no
CK-0d	RHSIM_Rhsim01G0232200	0.242	0.963	0.721	no
CK-0d	RHSIM_Rhsim02G0141400	0.355	-1.096	-1.451	no
CK-0d	RHSIM_Rhsim01G0223600	1.237	-0.638	-1.875	high
CK-0d	RHSIM_Rhsim02G0035600	-2.024	-0.776	1.249	low
CK-0d	RHSIM_Rhsim01G0175000	-2.267	-1.528	0.738	low
CK-0d	RHSIM_Rhsim01G0013100	1.116	2.177	1.060	high
CK-0d	RHSIM_Rhsim01G0073100	-0.290	0.067	0.357	no
CK-0d	RHSIM_Rhsim01G0184400	1.461	2.170	0.709	high
CK-0d	RHSIM_Rhsim01G0269200	0.586	1.482	0.896	no
CK-0d	RHSIM_Rhsim02G0213100	-0.053	-0.820	-0.767	no
CK-0d	RHSIM_Rhsim01G0263700	-0.301	-1.021	-0.720	no
CK-0d	RHSIM_Rhsim01G0182600	0.473	-1.036	-1.509	no
CK-0d	RHSIM_Rhsim01G0241200	-0.318	-0.905	-0.587	no
CK-0d	RHSIM_Rhsim01G0291500	-0.093	-0.638	-0.545	no
CK-0d	RHSIM_Rhsim01G0108800	-0.487	1.451	1.938	no
CK-0d	RHSIM_Rhsim01G0001600	-1.175	-0.346	0.829	low
CK-0d	RHSIM_Rhsim01G0147700	-0.451	-0.678	-0.227	no
CK-0d	RHSIM_Rhsim01G0112300	1.625	-0.692	-2.317	high

Group	Gene	PTR (z-score difference)	z(log2 LFQ)	z(log2 FPKM)	extreme-PTR
CK-0d	RHSIM_Rhsim02G0248800	0.123	0.072	-0.051	no
CK-0d	RHSIM_Rhsim01G0243600	0.082	0.540	0.458	no
CK-0d	RHSIM_Rhsim02G0220200	2.699	2.523	-0.176	high
CK-0d	RHSIM_Rhsim01G0185100	0.278	0.712	0.434	no
CK-0d	RHSIM_Rhsim01G0270000	-0.620	0.436	1.056	no
CK-0d	RHSIM_Rhsim02G0127100	-1.744	-1.487	0.257	low
CK-0d	RHSIM_Rhsim01G0089900	-0.151	0.620	0.771	no
CK-0d	RHSIM_Rhsim02G0145900	-0.514	-0.225	0.289	no
CK-0d	RHSIM_Rhsim01G0114500	-1.357	-1.695	-0.338	low
CK-0d	RHSIM_Rhsim02G0250500	0.435	0.929	0.494	no
CK-0d	RHSIM_Rhsim01G0184600	0.334	-0.239	-0.573	no
CK-0d	RHSIM_Rhsim02G0089500	0.852	-0.628	-1.480	no
CK-0d	RHSIM_Rhsim02G0019600	-0.575	-0.595	-0.020	no
CK-0d	RHSIM_Rhsim01G0247800	0.596	3.604	3.007	no
CK-0d	RHSIM_Rhsim02G0217200	-0.314	-0.528	-0.214	no
CK-0d	RHSIM_Rhsim01G0284000	-1.857	-1.558	0.299	low
CK-0d	RHSIM_Rhsim01G0163700	-0.499	-0.532	-0.033	no
CK-0d	RHSIM_Rhsim01G0144200	0.141	-0.094	-0.236	no
CK-0d	RHSIM_Rhsim02G0184100	-0.370	-1.058	-0.687	no
CK-0d	RHSIM_Rhsim01G0001900	0.222	1.149	0.927	no
CK-0d	RHSIM_Rhsim01G0195500	-1.324	-0.675	0.649	low
CK-0d	RHSIM_Rhsim01G0194400	-0.353	-0.883	-0.530	no
CK-0d	RHSIM_Rhsim01G0196700	-1.117	-0.957	0.160	low
CK-0d	RHSIM_Rhsim01G0287600	-2.179	-0.752	1.427	low
CK-0d	RHSIM_Rhsim01G0227500	0.166	2.640	2.474	no
CK-0d	RHSIM_Rhsim02G0215600	-0.389	0.376	0.765	no
CK-0d	RHSIM_Rhsim02G0109900	1.864	0.799	-1.066	high
CK-0d	RHSIM_Rhsim01G0285600	-1.547	-0.828	0.719	low
CK-0d	RHSIM_Rhsim01G0128500	-1.044	-1.510	-0.466	low
CK-0d	RHSIM_Rhsim01G0286500	0.161	-0.231	-0.392	no
CK-0d	RHSIM_Rhsim01G0008000	1.583	0.647	-0.936	high
CK-0d	RHSIM_Rhsim02G0255400	-0.355	-0.445	-0.090	no

Group	Gene	PTR (z-score difference)	z(log2 LFQ)	z(log2 FPKM)	extreme-PTR
CK-0d	RHSIM_Rhsim01G0244300	-0.489	-0.684	-0.194	no
CK-0d	RHSIM_Rhsim01G0196200	-0.816	0.298	1.115	no
CK-0d	RHSIM_Rhsim01G0265400	-2.404	-1.086	1.318	low
CK-0d	RHSIM_Rhsim02G0083400	0.135	-0.365	-0.500	no
CK-0d	RHSIM_Rhsim01G0144600	0.631	-0.207	-0.838	no
CK-0d	RHSIM_Rhsim01G0276900	-0.900	-1.120	-0.220	no
CK-0d	RHSIM_Rhsim02G0105300	-1.391	-0.753	0.637	low
CK-0d	RHSIM_Rhsim01G0186600	-0.205	-1.106	-0.901	no
CK-0d	RHSIM_Rhsim01G0213500	0.564	2.574	2.010	no
CK-0d	RHSIM_Rhsim01G0265200	-0.265	-1.341	-1.076	no
CK-0d	RHSIM_Rhsim02G0180100	-0.321	-0.300	0.020	no
CK-0d	RHSIM_Rhsim01G0206200	-0.661	-0.155	0.506	no
CK-0d	RHSIM_Rhsim01G0249100	1.085	0.442	-0.644	no
CK-0d	RHSIM_Rhsim01G0059500	-0.108	0.285	0.393	no
CK-0d	RHSIM_Rhsim01G0151800	-2.478	-1.194	1.284	low
CK-0d	RHSIM_Rhsim01G0141500	1.445	1.243	-0.202	high
CK-0d	RHSIM_Rhsim01G0134500	0.071	1.148	1.077	no
CK-0d	RHSIM_Rhsim01G0212900	-1.917	-0.638	1.279	low
CK-0d	RHSIM_Rhsim01G0124100	0.549	0.479	-0.070	no
CK-0d	RHSIM_Rhsim02G0251700	-0.862	-0.663	0.199	no
CK-0d	RHSIM_Rhsim01G0077600	0.151	0.757	0.607	no
CK-0d	RHSIM_Rhsim01G0181300	-0.691	2.279	2.970	no
CK-0d	RHSIM_Rhsim01G0205500	1.153	0.424	-0.729	high
CK-0d	RHSIM_Rhsim01G0204800	0.323	-0.165	-0.488	no
CK-0d	RHSIM_Rhsim02G0043000	0.222	2.301	2.078	no
CK-0d	RHSIM_Rhsim01G0121200	-0.004	-0.875	-0.870	no
CK-0d	RHSIM_Rhsim01G0114600	-0.596	0.977	1.574	no
CK-0d	RHSIM_Rhsim01G0261900	0.678	0.128	-0.550	no
CK-0d	RHSIM_Rhsim01G0290900	0.150	-1.055	-1.205	no
CK-0d	RHSIM_Rhsim01G0215700	-0.505	-0.676	-0.171	no
CK-0d	RHSIM_Rhsim01G0246400	0.336	0.258	-0.078	no
CK-0d	RHSIM_Rhsim01G0176500	0.055	1.038	0.982	no

Group	Gene	PTR (z-score difference)	z(log2 LFQ)	z(log2 FPKM)	extreme-PTR
CK-0d	RHSIM_Rhsim01G0023600	0.745	-0.472	-1.216	no
CK-0d	RHSIM_Rhsim01G0128700	0.424	-0.568	-0.992	no
CK-0d	RHSIM_Rhsim01G0226300	-0.939	-1.128	-0.189	no
CK-0d	RHSIM_Rhsim01G0232700	0.140	-1.625	-1.765	no
CK-0d	RHSIM_Rhsim01G0278000	0.297	0.383	0.086	no
CK-0d	RHSIM_Rhsim01G0001400	0.506	0.246	-0.261	no
CK-0d	RHSIM_Rhsim01G0000100	1.185	-0.590	-1.775	high
CK-0d	RHSIM_Rhsim01G0250800	0.222	1.108	0.887	no
CK-0d	RHSIM_Rhsim01G0245100	0.357	1.059	0.702	no
CK-0d	RHSIM_Rhsim01G0244200	1.098	-0.580	-1.678	no
CK-0d	RHSIM_Rhsim01G0244400	-0.800	0.953	1.753	no
CK-0d	RHSIM_Rhsim01G0001500	0.549	-0.201	-0.750	no
CK-0d	RHSIM_Rhsim01G0292000	0.991	0.431	-0.560	no
CK-0d	RHSIM_Rhsim01G0286100	-0.880	-0.695	0.185	no
CK-0d	RHSIM_Rhsim01G0199200	0.579	0.740	0.161	no
CK-0d	RHSIM_Rhsim01G0004300	-2.788	-0.150	2.638	low
CK-0d	RHSIM_Rhsim01G0256500	-1.479	-1.475	0.004	low
CK-0d	RHSIM_Rhsim01G0060100	-0.462	-0.511	-0.049	no
CK-0d	RHSIM_Rhsim01G0187700	2.822	0.874	-1.949	high
CK-0d	RHSIM_Rhsim01G0108000	-1.501	-1.310	0.191	low
CK-0d	RHSIM_Rhsim01G0210600	0.880	-0.152	-1.032	no
CK-0d	RHSIM_Rhsim01G0276100	2.091	0.552	-1.539	high
CK-0d	RHSIM_Rhsim01G0276800	-0.866	-0.361	0.505	no
CK-0d	RHSIM_Rhsim01G0153000	0.318	0.881	0.562	no
CK-0d	RHSIM_Rhsim01G0193400	1.814	-0.411	-2.225	high
CK-0d	RHSIM_Rhsim01G0241700	1.580	1.290	-0.290	high
CK-0d	RHSIM_Rhsim01G0221500	-2.006	1.110	3.116	low
CK-0d	RHSIM_Rhsim01G0189400	0.209	-0.584	-0.793	no
CK-0d	RHSIM_Rhsim01G0000500	0.513	0.785	0.271	no
CK-0d	RHSIM_Rhsim01G0260600	-0.597	-0.838	-0.240	no
CK-0d	RHSIM_Rhsim01G0286200	-5.352	-1.103	4.250	low
CK-0d	RHSIM_Rhsim01G0279700	0.316	-0.756	-1.072	no

Group	Gene	PTR (z-score difference)	z(log2 LFQ)	z(log2 FPKM)	extreme-PTR
CK-0d	RHSIM_Rhsim01G0055700	1.355	0.238	-1.117	high
CK-0d	RHSIM_Rhsim01G0282300	0.216	-0.336	-0.552	no
CK-0d	RHSIM_Rhsim01G0044100	-0.474	0.231	0.704	no
CK-0d	RHSIM_Rhsim01G0021100	0.559	-0.056	-0.614	no
CK-0d	RHSIM_Rhsim01G0287000	0.539	1.319	0.780	no
CK-0d	RHSIM_Rhsim01G0141200	0.722	1.061	0.339	no
CK-0d	RHSIM_Rhsim01G0123600	1.240	-1.029	-2.270	high
CK-0d	RHSIM_Rhsim01G0091700	0.148	-0.624	-0.772	no
CK-0d	RHSIM_Rhsim01G0210100	2.371	0.424	-1.947	high
CK-0d	RHSIM_Rhsim01G0170700	0.446	0.290	-0.156	no
CK-0d	RHSIM_Rhsim01G0259500	0.833	2.138	1.305	no
CK-0d	RHSIM_Rhsim01G0163000	-0.090	-0.834	-0.743	no
CK-40d	RHSIM_RhsimUnG0249100	-1.859	0.577	2.436	low
CK-40d	RHSIM_RhsimUnG0254800	-2.334	-1.010	1.324	low
CK-40d	RHSIM_RhsimUnG0258600	4.876	2.969	-1.907	high
CK-40d	RHSIM_RhsimUnG0249600	-1.514	0.217	1.731	low
CK-40d	RHSIM_RhsimUnG0251500	-0.233	0.040	0.273	no
CK-40d	RHSIM_RhsimUnG0252300	0.367	-0.434	-0.801	no
CK-40d	RHSIM_RhsimUnG0228100	-0.180	-1.548	-1.368	no
CK-40d	RHSIM_RhsimUnG0251200	-1.135	0.534	1.669	no
CK-40d	RHSIM_RhsimUnG0226800	1.127	-0.584	-1.711	no
CK-40d	RHSIM_RhsimUnG0245900	3.193	3.131	-0.062	high
CK-40d	RHSIM_RhsimUnG0241900	2.860	0.819	-2.041	high
CK-40d	RHSIM_RhsimUnG0250300	-2.222	-1.188	1.035	low
CK-40d	RHSIM_RhsimUnG0250600	-0.557	-0.031	0.526	no
CK-40d	RHSIM_RhsimUnG0216900	0.530	1.784	1.254	no
CK-40d	RHSIM_RhsimUnG0239100	1.165	-0.062	-1.227	high
CK-40d	RHSIM_RhsimUnG0233600	-0.817	-2.277	-1.460	no
CK-40d	RHSIM_RhsimUnG0231700	0.105	-0.706	-0.811	no
CK-40d	RHSIM_RhsimUnG0212900	1.788	0.037	-1.751	high
CK-40d	RHSIM_RhsimUnG0231900	0.346	-0.088	-0.434	no
CK-40d	RHSIM_RhsimUnG0209400	3.066	0.911	-2.155	high

Group	Gene	PTR (z-score difference)	z(log2 LFQ)	z(log2 FPKM)	extreme-PTR
CK-40d	RHSIM_RhsimUnG0219900	1.290	-0.925	-2.216	high
CK-40d	RHSIM_RhsimUnG0217500	1.872	0.606	-1.265	high
CK-40d	RHSIM_Rhsim09G0026600	1.561	-0.791	-2.352	high
CK-40d	RHSIM_RhsimUnG0226200	0.097	0.123	0.026	no
CK-40d	RHSIM_RhsimUnG0210000	-1.317	0.113	1.430	low
CK-40d	RHSIM_RhsimUnG0190800	1.777	0.169	-1.608	high
CK-40d	RHSIM_RhsimUnG0217400	-0.184	-0.240	-0.055	no
CK-40d	RHSIM_RhsimUnG0220800	0.118	-0.476	-0.594	no
CK-40d	RHSIM_RhsimUnG0253500	3.965	1.627	-2.338	high
CK-40d	RHSIM_RhsimUnG0212700	-3.148	-1.254	1.894	low
CK-40d	RHSIM_RhsimUnG0248900	0.096	1.169	1.074	no
CK-40d	RHSIM_RhsimUnG0211400	-0.707	1.351	2.058	no
CK-40d	RHSIM_RhsimUnG0209900	1.806	0.479	-1.327	high
CK-40d	RHSIM_RhsimUnG0205800	0.668	1.955	1.287	no
CK-40d	RHSIM_RhsimUnG0219300	-1.051	-1.515	-0.464	no
CK-40d	RHSIM_Rhsim09G0148400	-0.354	-0.474	-0.120	no
CK-40d	RHSIM_RhsimUnG0209800	0.093	-0.478	-0.572	no
CK-40d	RHSIM_RhsimUnG0225600	-0.323	0.024	0.348	no
CK-40d	RHSIM_RhsimUnG0255000	0.068	0.435	0.367	no
CK-40d	RHSIM_RhsimUnG0156400	-0.034	-1.411	-1.377	no
CK-40d	RHSIM_RhsimUnG0205900	0.049	1.354	1.304	no
CK-40d	RHSIM_RhsimUnG0197600	2.125	0.043	-2.082	high
CK-40d	RHSIM_RhsimUnG0206500	-0.595	-1.318	-0.724	no
CK-40d	RHSIM_Rhsim09G0069900	2.652	1.721	-0.932	high
CK-40d	RHSIM_RhsimUnG0169900	1.145	-0.814	-1.959	high
CK-40d	RHSIM_RhsimUnG0208500	-1.788	-2.039	-0.250	low
CK-40d	RHSIM_RhsimUnG0196500	-1.335	-1.819	-0.484	low
CK-40d	RHSIM_RhsimUnG0176900	0.921	-1.036	-1.957	no
CK-40d	RHSIM_RhsimUnG0185400	0.500	-0.247	-0.747	no
CK-40d	RHSIM_RhsimUnG0158200	1.976	1.802	-0.173	high
CK-40d	RHSIM_RhsimUnG0197500	1.682	-0.397	-2.079	high
CK-40d	RHSIM_Rhsim02G0029800	0.845	-0.860	-1.705	no

Group	Gene	PTR (z-score difference)	z(log2 LFQ)	z(log2 FPKM)	extreme-PTR
CK-40d	RHSIM_Rhsim09G0021500	0.680	1.583	0.903	no
CK-40d	RHSIM_RhsimUnG0136600	1.165	0.483	-0.682	high
CK-40d	RHSIM_RhsimUnG0226700	1.355	-0.386	-1.741	high
CK-40d	RHSIM_RhsimUnG0220700	2.224	0.497	-1.727	high
CK-40d	RHSIM_RhsimUnG0126200	1.467	1.056	-0.410	high
CK-40d	RHSIM_RhsimUnG0217000	-1.114	0.256	1.370	no
CK-40d	RHSIM_RhsimUnG0116800	-1.140	-1.693	-0.553	no
CK-40d	RHSIM_RhsimUnG0109300	1.323	1.764	0.440	high
CK-40d	RHSIM_RhsimUnG0122900	2.450	0.077	-2.372	high
CK-40d	RHSIM_RhsimUnG0158600	1.464	0.386	-1.078	high
CK-40d	RHSIM_RhsimUnG0137100	0.903	1.335	0.432	no
CK-40d	RHSIM_RhsimUnG0190300	0.720	-0.336	-1.056	no
CK-40d	RHSIM_RhsimUnG0256800	0.986	0.426	-0.560	no
CK-40d	RHSIM_RhsimUnG0187600	-1.222	-1.563	-0.341	no
CK-40d	RHSIM_RhsimUnG0185700	-0.011	-1.449	-1.438	no
CK-40d	RHSIM_RhsimUnG0180800	-0.892	-0.243	0.649	no
CK-40d	RHSIM_RhsimUnG0124400	1.676	-0.417	-2.093	high
CK-40d	RHSIM_RhsimUnG0254600	-1.437	-0.505	0.932	low
CK-40d	RHSIM_RhsimUnG0242200	-0.448	-0.951	-0.503	no
CK-40d	RHSIM_RhsimUnG0253800	-0.470	-1.066	-0.596	no
CK-40d	RHSIM_RhsimUnG0093800	-0.030	-0.064	-0.034	no
CK-40d	RHSIM_RhsimUnG0257500	1.567	0.800	-0.767	high
CK-40d	RHSIM_RhsimUnG0118900	-0.820	1.228	2.048	no
CK-40d	RHSIM_RhsimUnG0120200	-0.587	-1.340	-0.752	no
CK-40d	RHSIM_RhsimUnG0111200	-1.470	-1.664	-0.194	low
CK-40d	RHSIM_RhsimUnG0092300	0.231	0.899	0.668	no
CK-40d	RHSIM_RhsimUnG0124200	0.102	-0.503	-0.605	no
CK-40d	RHSIM_RhsimUnG0094500	0.343	0.232	-0.111	no
CK-40d	RHSIM_RhsimUnG0059700	-0.191	-0.337	-0.146	no
CK-40d	RHSIM_RhsimUnG0145200	0.141	-0.936	-1.077	no
CK-40d	RHSIM_RhsimUnG0118400	-1.973	-2.020	-0.047	low
CK-40d	RHSIM_RhsimUnG0138800	2.310	1.162	-1.147	high

Group	Gene	PTR (z-score difference)	z(log2 LFQ)	z(log2 FPKM)	extreme-PTR
CK-40d	RHSIM_RhsimUnG0054600	-0.135	-0.887	-0.752	no
CK-40d	RHSIM_RhsimUnG0216100	-2.757	-0.658	2.098	low
CK-40d	RHSIM_RhsimUnG0213100	-0.078	-0.959	-0.881	no
CK-40d	RHSIM_RhsimUnG0112800	0.837	-1.255	-2.092	no
CK-40d	RHSIM_RhsimUnG0111000	-0.785	-0.622	0.162	no
CK-40d	RHSIM_RhsimUnG0205000	1.407	1.404	-0.003	high
CK-40d	RHSIM_RhsimUnG0221600	1.620	1.409	-0.211	high
CK-40d	RHSIM_RhsimUnG0060900	0.062	-0.232	-0.294	no
CK-40d	RHSIM_RhsimUnG0216800	0.144	0.366	0.223	no
CK-40d	RHSIM_RhsimUnG0215100	1.144	-0.266	-1.410	high
CK-40d	RHSIM_RhsimUnG0124800	-0.059	-1.288	-1.229	no
CK-40d	RHSIM_RhsimUnG0098600	1.168	-0.515	-1.682	high
CK-40d	RHSIM_RhsimUnG0055200	0.403	-0.196	-0.600	no
CK-40d	RHSIM_RhsimUnG0096900	0.406	0.040	-0.366	no
CK-40d	RHSIM_RhsimUnG0119200	-0.686	-0.185	0.501	no
CK-40d	RHSIM_RhsimUnG0032200	-0.621	-1.006	-0.385	no
CK-40d	RHSIM_RhsimUnG0119600	-1.081	-0.130	0.951	no
CK-40d	RHSIM_RhsimUnG0087900	1.648	1.536	-0.112	high
CK-40d	RHSIM_RhsimUnG0065700	0.511	-0.179	-0.690	no
CK-40d	RHSIM_RhsimUnG0113700	0.310	-0.681	-0.992	no
CK-40d	RHSIM_RhsimUnG0109400	0.730	1.200	0.470	no
CK-40d	RHSIM_RhsimUnG0080300	0.032	0.970	0.938	no
CK-40d	RHSIM_RhsimUnG0020600	-0.058	-0.258	-0.201	no
CK-40d	RHSIM_RhsimUnG0019600	1.197	0.458	-0.740	high
CK-40d	RHSIM_RhsimUnG0039700	-0.758	-1.265	-0.507	no
CK-40d	RHSIM_RhsimUnG0117900	0.743	0.903	0.160	no
CK-40d	RHSIM_RhsimUnG0021000	0.169	-0.452	-0.621	no
CK-40d	RHSIM_RhsimUnG0055900	-0.670	-0.208	0.462	no
CK-40d	RHSIM_RhsimUnG0098000	0.568	0.050	-0.518	no
CK-40d	RHSIM_RhsimUnG0194300	-0.783	-0.241	0.542	no
CK-40d	RHSIM_RhsimUnG0119800	-0.676	-1.382	-0.706	no
CK-40d	RHSIM_RhsimUnG0094700	0.403	0.607	0.204	no

Group	Gene	PTR (z-score difference)	z(log2 LFQ)	z(log2 FPKM)	extreme-PTR
CK-40d	RHSIM_RhsimUnG0109200	0.656	0.056	-0.600	no
CK-40d	RHSIM_RhsimUnG0107500	0.089	0.309	0.220	no
CK-40d	RHSIM_RhsimUnG0105000	0.692	1.355	0.664	no
CK-40d	RHSIM_RhsimUnG0188600	0.343	-0.837	-1.180	no
CK-40d	RHSIM_RhsimUnG0006400	1.185	-0.314	-1.499	high
CK-40d	RHSIM_RhsimPtG0002900	2.642	0.560	-2.082	high
CK-40d	RHSIM_RhsimUnG0096000	0.344	-0.777	-1.121	no
CK-40d	RHSIM_RhsimUnG0021300	-1.341	-1.119	0.222	low
CK-40d	RHSIM_RhsimUnG0042900	0.034	-0.419	-0.453	no
CK-40d	RHSIM_Rhsim13G0142900	-1.506	-1.551	-0.045	low
CK-40d	RHSIM_RhsimUnG0045900	3.634	1.656	-1.978	high
CK-40d	RHSIM_RhsimUnG0139700	1.548	-0.391	-1.938	high
CK-40d	RHSIM_RhsimUnG0085800	-1.811	-0.872	0.938	low
CK-40d	RHSIM_RhsimUnG0044200	-1.470	-1.276	0.193	low
CK-40d	RHSIM_Rhsim13G0005000	0.718	0.520	-0.198	no
CK-40d	RHSIM_RhsimUnG0021800	1.723	2.097	0.374	high
CK-40d	RHSIM_Rhsim13G0010100	-1.560	-0.789	0.771	low
CK-40d	RHSIM_RhsimUnG0022700	1.064	-0.793	-1.857	no
CK-40d	RHSIM_RhsimUnG0073900	-0.707	-0.322	0.385	no
CK-40d	RHSIM_RhsimUnG0070500	-2.129	-1.141	0.988	low
CK-40d	RHSIM_Rhsim13G0064900	1.653	0.179	-1.474	high
CK-40d	RHSIM_Rhsim11G0060300	0.871	-0.307	-1.178	no
CK-40d	RHSIM_RhsimUnG0027200	0.758	-0.198	-0.956	no
CK-40d	RHSIM_Rhsim13G0234100	-0.713	-0.798	-0.085	no
CK-40d	RHSIM_RhsimUnG0060500	-0.873	-0.237	0.636	no
CK-40d	RHSIM_Rhsim13G0198200	1.140	0.869	-0.271	high
CK-40d	RHSIM_RhsimUnG0014100	-0.019	-0.757	-0.738	no
CK-40d	RHSIM_RhsimMtG0006700	1.493	-0.404	-1.897	high
CK-40d	RHSIM_Rhsim13G0018300	0.508	-0.097	-0.605	no
CK-40d	RHSIM_RhsimUnG0022900	0.573	0.258	-0.315	no
CK-40d	RHSIM_Rhsim05G0087400	0.060	0.566	0.507	no
CK-40d	RHSIM_RhsimUnG0051400	1.330	1.090	-0.240	high

Group	Gene	PTR (z-score difference)	z(log2 LFQ)	z(log2 FPKM)	extreme-PTR
CK-40d	RHSIM_RhsimUnG0059400	-0.357	-0.798	-0.441	no
CK-40d	RHSIM_RhsimUnG0005600	0.010	-0.647	-0.657	no
CK-40d	RHSIM_Rhsim13G0009100	-0.946	1.233	2.179	no
CK-40d	RHSIM_RhsimPtG0000900	4.158	2.210	-1.949	high
CK-40d	RHSIM_RhsimUnG0015500	0.503	0.110	-0.393	no
CK-40d	RHSIM_RhsimUnG0055000	1.206	-0.282	-1.488	high
CK-40d	RHSIM_Rhsim13G0140400	-0.558	-0.779	-0.220	no
CK-40d	RHSIM_Rhsim13G0104300	0.195	0.730	0.536	no
CK-40d	RHSIM_Rhsim13G0149200	-0.318	1.849	2.167	no
CK-40d	RHSIM_RhsimUnG0052000	2.764	0.444	-2.320	high
CK-40d	RHSIM_Rhsim13G0215900	0.333	1.610	1.276	no
CK-40d	RHSIM_Rhsim13G0000400	-0.073	0.454	0.527	no
CK-40d	RHSIM_Rhsim13G0111800	-1.677	0.144	1.822	low
CK-40d	RHSIM_RhsimUnG0008400	0.909	1.715	0.807	no
CK-40d	RHSIM_Rhsim13G0157800	1.561	1.213	-0.348	high
CK-40d	RHSIM_RhsimUnG0043200	-0.108	2.610	2.718	no
CK-40d	RHSIM_RhsimUnG0002100	0.513	0.184	-0.329	no
CK-40d	RHSIM_Rhsim13G0110800	0.423	0.575	0.152	no
CK-40d	RHSIM_Rhsim13G0229200	2.517	0.640	-1.877	high
CK-40d	RHSIM_Rhsim13G0009200	0.759	-0.616	-1.375	no
CK-40d	RHSIM_Rhsim13G0150700	1.208	0.366	-0.842	high
CK-40d	RHSIM_Rhsim13G0112500	1.460	1.531	0.071	high
CK-40d	RHSIM_Rhsim13G0088500	-0.313	-0.403	-0.090	no
CK-40d	RHSIM_Rhsim13G0186600	-0.853	0.228	1.081	no
CK-40d	RHSIM_Rhsim13G0177500	-2.306	-1.158	1.149	low
CK-40d	RHSIM_Rhsim13G0205900	-0.048	0.344	0.393	no
CK-40d	RHSIM_Rhsim13G0156100	-0.285	-0.734	-0.449	no
CK-40d	RHSIM_RhsimUnG0018800	3.606	1.329	-2.277	high
CK-40d	RHSIM_RhsimUnG0023000	-1.287	-0.955	0.333	low
CK-40d	RHSIM_RhsimUnG0094300	1.519	0.823	-0.696	high
CK-40d	RHSIM_RhsimUnG0025700	-0.161	-0.248	-0.087	no
CK-40d	RHSIM_RhsimUnG0254000	2.175	1.374	-0.801	high

Group	Gene	PTR (z-score difference)	z(log2 LFQ)	z(log2 FPKM)	extreme-PTR
CK-40d	RHSIM_Rhsim13G0077700	-0.072	-0.342	-0.270	no
CK-40d	RHSIM_Rhsim13G0088800	-0.171	-0.422	-0.251	no
CK-40d	RHSIM_Rhsim13G0030500	-0.606	-1.300	-0.694	no
CK-40d	RHSIM_RhsimUnG0252800	-0.069	-0.161	-0.091	no
CK-40d	RHSIM_Rhsim13G0201300	-0.040	-0.100	-0.060	no
CK-40d	RHSIM_Rhsim13G0179600	-1.476	-0.712	0.764	low
CK-40d	RHSIM_Rhsim13G0140500	-0.242	-0.286	-0.044	no
CK-40d	RHSIM_Rhsim13G0175200	-0.771	0.676	1.447	no
CK-40d	RHSIM_Rhsim13G0069400	1.165	1.991	0.826	high
CK-40d	RHSIM_Rhsim13G0169400	0.383	0.117	-0.266	no
CK-40d	RHSIM_Rhsim13G0211800	0.863	0.923	0.060	no
CK-40d	RHSIM_RhsimUnG0139600	1.008	0.118	-0.891	no
CK-40d	RHSIM_RhsimUnG0004000	1.354	0.371	-0.984	high
CK-40d	RHSIM_RhsimUnG0248600	0.395	-0.911	-1.306	no
CK-40d	RHSIM_Rhsim13G0014400	-2.036	-1.242	0.795	low
CK-40d	RHSIM_RhsimUnG0006200	0.947	0.752	-0.196	no
CK-40d	RHSIM_Rhsim13G0147900	0.082	0.211	0.129	no
CK-40d	RHSIM_Rhsim13G0127600	-3.342	-2.373	0.969	low
CK-40d	RHSIM_Rhsim13G0091700	-0.969	0.749	1.718	no
CK-40d	RHSIM_RhsimUnG0135600	-0.549	-0.757	-0.208	no
CK-40d	RHSIM_Rhsim13G0140200	-0.203	0.038	0.241	no
CK-40d	RHSIM_Rhsim13G0039100	-0.518	-0.632	-0.114	no
CK-40d	RHSIM_RhsimUnG0133400	-2.849	-0.911	1.938	low
CK-40d	RHSIM_Rhsim13G0012100	0.638	0.789	0.151	no
CK-40d	RHSIM_Rhsim13G0162700	1.750	1.747	-0.004	high
CK-40d	RHSIM_Rhsim13G0201600	-1.506	-0.645	0.861	low
CK-40d	RHSIM_Rhsim13G0170400	-0.782	0.029	0.811	no
CK-40d	RHSIM_Rhsim13G0218000	-1.668	-1.624	0.044	low
CK-40d	RHSIM_RhsimUnG0126900	-1.984	-2.281	-0.298	low
CK-40d	RHSIM_Rhsim13G0024600	1.021	0.073	-0.948	no
CK-40d	RHSIM_Rhsim13G0122800	1.255	2.069	0.814	high
CK-40d	RHSIM_Rhsim13G0115500	0.194	-0.203	-0.397	no

Group	Gene	PTR (z-score difference)	z(log2 LFQ)	z(log2 FPKM)	extreme-PTR
CK-40d	RHSIM_RhsimUnG0120500	-1.887	-1.503	0.385	low
CK-40d	RHSIM_Rhsim13G0030900	3.016	3.584	0.568	high
CK-40d	RHSIM_Rhsim13G0202300	-3.388	-2.159	1.229	low
CK-40d	RHSIM_Rhsim13G0203400	0.727	1.627	0.900	no
CK-40d	RHSIM_Rhsim13G0233700	-1.061	0.303	1.364	no
CK-40d	RHSIM_Rhsim13G0057000	-0.681	-1.064	-0.383	no
CK-40d	RHSIM_Rhsim13G0044900	-0.270	-0.839	-0.569	no
CK-40d	RHSIM_Rhsim13G0155800	-1.108	-0.283	0.825	no
CK-40d	RHSIM_Rhsim13G0060100	-0.784	-0.763	0.021	no
CK-40d	RHSIM_Rhsim13G0062700	0.158	-0.346	-0.505	no
CK-40d	RHSIM_Rhsim13G0140900	-0.576	-0.404	0.172	no
CK-40d	RHSIM_Rhsim13G0111500	-0.426	1.135	1.561	no
CK-40d	RHSIM_Rhsim13G0111400	-0.426	1.191	1.617	no
CK-40d	RHSIM_RhsimUnG0102900	-0.547	-0.442	0.104	no
CK-40d	RHSIM_Rhsim13G0208400	-0.676	-0.471	0.206	no
CK-40d	RHSIM_Rhsim13G0217600	-0.898	-0.363	0.536	no
CK-40d	RHSIM_Rhsim13G0195100	-1.620	-0.825	0.795	low
CK-40d	RHSIM_Rhsim13G0216400	0.828	-0.783	-1.611	no
CK-40d	RHSIM_Rhsim13G0024400	0.425	-0.120	-0.545	no
CK-40d	RHSIM_Rhsim13G0040400	-1.073	-1.056	0.018	no
CK-40d	RHSIM_RhsimUnG0095400	-2.882	-1.808	1.074	low
CK-40d	RHSIM_Rhsim13G0059900	-0.260	-0.052	0.208	no
CK-40d	RHSIM_Rhsim13G0125900	1.484	0.743	-0.741	high
CK-40d	RHSIM_Rhsim13G0121000	-1.570	-0.674	0.896	low
CK-40d	RHSIM_Rhsim13G0189200	-2.615	-1.607	1.007	low
CK-40d	RHSIM_Rhsim13G0013600	2.232	2.023	-0.209	high
CK-40d	RHSIM_Rhsim13G0071000	-0.604	-0.283	0.320	no
CK-40d	RHSIM_RhsimUnG0086000	1.611	0.559	-1.053	high
CK-40d	RHSIM_Rhsim13G0047200	-1.096	-0.610	0.486	no
CK-40d	RHSIM_Rhsim13G0067900	-2.323	-1.217	1.105	low
CK-40d	RHSIM_Rhsim13G0120300	-1.060	-1.020	0.040	no
CK-40d	RHSIM_Rhsim13G0191600	-0.806	-0.052	0.755	no

Group	Gene	PTR (z-score difference)	z(log2 LFQ)	z(log2 FPKM)	extreme-PTR
CK-40d	RHSIM_Rhsim13G0119400	3.314	3.245	-0.069	high
CK-40d	RHSIM_Rhsim13G0174200	1.119	-0.034	-1.153	no
CK-40d	RHSIM_Rhsim13G0205000	0.625	-0.332	-0.956	no
CK-40d	RHSIM_Rhsim13G0191100	-0.975	-0.429	0.546	no
CK-40d	RHSIM_Rhsim13G0146300	-1.772	-1.216	0.557	low
CK-40d	RHSIM_Rhsim13G0163000	0.136	-0.361	-0.497	no
CK-40d	RHSIM_RhsimUnG0079400	-0.181	-0.593	-0.411	no
CK-40d	RHSIM_Rhsim13G0194200	3.135	2.180	-0.955	high
CK-40d	RHSIM_Rhsim13G0052700	-0.300	-0.327	-0.026	no
CK-40d	RHSIM_Rhsim13G0072700	-0.800	0.069	0.869	no
CK-40d	RHSIM_RhsimUnG0206200	-0.854	-1.131	-0.277	no
CK-40d	RHSIM_Rhsim13G0094700	-0.344	0.172	0.516	no
CK-40d	RHSIM_Rhsim13G0201700	-0.402	0.487	0.889	no
CK-40d	RHSIM_Rhsim13G0056300	-1.138	-1.572	-0.434	no
CK-40d	RHSIM_RhsimUnG0046000	1.327	-0.379	-1.706	high
CK-40d	RHSIM_Rhsim13G0169800	0.949	0.993	0.045	no
CK-40d	RHSIM_Rhsim13G0152000	-0.447	0.822	1.270	no
CK-40d	RHSIM_Rhsim13G0011700	1.007	0.996	-0.011	no
CK-40d	RHSIM_Rhsim13G0056400	0.686	0.963	0.277	no
CK-40d	RHSIM_Rhsim13G0098900	-1.487	0.227	1.714	low
CK-40d	RHSIM_RhsimUnG0065400	1.108	2.676	1.568	no
CK-40d	RHSIM_Rhsim13G0206200	1.719	-0.563	-2.282	high
CK-40d	RHSIM_Rhsim13G0226700	-0.281	-0.044	0.237	no
CK-40d	RHSIM_Rhsim13G0061400	-0.713	-1.379	-0.666	no
CK-40d	RHSIM_Rhsim13G0129400	0.649	-0.604	-1.254	no
CK-40d	RHSIM_Rhsim13G0050600	0.031	-0.033	-0.064	no
CK-40d	RHSIM_Rhsim13G0020400	-2.595	-1.977	0.617	low
CK-40d	RHSIM_Rhsim13G0009900	0.040	-1.561	-1.601	no
CK-40d	RHSIM_Rhsim13G0098700	-1.185	0.665	1.849	no
CK-40d	RHSIM_Rhsim13G0198700	-0.747	-0.298	0.449	no
CK-40d	RHSIM_Rhsim13G0057400	-0.480	-0.649	-0.169	no
CK-40d	RHSIM_Rhsim13G0179900	-1.915	-0.992	0.923	low

Group	Gene	PTR (z-score difference)	z(log2 LFQ)	z(log2 FPKM)	extreme-PTR
CK-40d	RHSIM_Rhsim13G0094300	1.890	0.305	-1.584	high
CK-40d	RHSIM_Rhsim13G0035600	-0.429	-0.274	0.155	no
CK-40d	RHSIM_Rhsim13G0234800	0.504	0.774	0.270	no
CK-40d	RHSIM_Rhsim13G0001000	1.349	0.572	-0.777	high
CK-40d	RHSIM_Rhsim13G0221000	-1.243	-1.061	0.182	low
CK-40d	RHSIM_RhsimUnG0053100	0.203	-1.434	-1.637	no
CK-40d	RHSIM_Rhsim13G0078800	-1.718	-1.975	-0.257	low
CK-40d	RHSIM_Rhsim13G0107100	-0.729	0.400	1.129	no
CK-40d	RHSIM_Rhsim13G0024900	0.993	0.450	-0.543	no
CK-40d	RHSIM_Rhsim13G0045300	0.099	1.471	1.372	no
CK-40d	RHSIM_Rhsim13G0037500	0.580	0.232	-0.347	no
CK-40d	RHSIM_Rhsim13G0211100	-0.633	0.070	0.703	no
CK-40d	RHSIM_Rhsim13G0072000	0.529	-0.430	-0.959	no
CK-40d	RHSIM_Rhsim13G0027700	0.099	-0.426	-0.525	no
CK-40d	RHSIM_Rhsim13G0018600	-0.295	0.136	0.431	no
CK-40d	RHSIM_Rhsim13G0127200	3.395	3.926	0.531	high
CK-40d	RHSIM_RhsimUnG0023900	2.300	0.082	-2.218	high
CK-40d	RHSIM_Rhsim13G0231000	-0.023	-0.033	-0.010	no
CK-40d	RHSIM_Rhsim13G0036500	-1.146	-0.417	0.728	no
CK-40d	RHSIM_Rhsim13G0031600	0.624	0.672	0.048	no
CK-40d	RHSIM_Rhsim13G0065500	0.771	1.070	0.298	no
CK-40d	RHSIM_RhsimUnG0021400	-0.959	-0.126	0.833	no
CK-40d	RHSIM_Rhsim13G0015500	1.192	0.151	-1.041	high
CK-40d	RHSIM_Rhsim13G0056900	0.052	0.678	0.627	no
CK-40d	RHSIM_Rhsim13G0115200	0.730	0.670	-0.060	no
CK-40d	RHSIM_RhsimUnG0172300	0.053	-0.259	-0.312	no
CK-40d	RHSIM_Rhsim13G0209000	-1.169	-1.879	-0.709	no
CK-40d	RHSIM_Rhsim13G0121400	-0.990	0.530	1.521	no
CK-40d	RHSIM_Rhsim13G0208500	-1.819	-1.648	0.171	low
CK-40d	RHSIM_Rhsim13G0058000	-0.194	0.828	1.023	no
CK-40d	RHSIM_Rhsim13G0004800	-0.029	-0.311	-0.283	no
CK-40d	RHSIM_Rhsim13G0221900	0.401	-0.595	-0.997	no

Group	Gene	PTR (z-score difference)	z(log2 LFQ)	z(log2 FPKM)	extreme-PTR
CK-40d	RHSIM_Rhsim13G0104500	-0.258	-1.012	-0.754	no
CK-40d	RHSIM_RhsimUnG0033300	1.145	-0.161	-1.306	high
CK-40d	RHSIM_Rhsim13G0129600	-0.433	-0.555	-0.122	no
CK-40d	RHSIM_Rhsim13G0098500	0.360	-1.182	-1.542	no
CK-40d	RHSIM_Rhsim13G0228700	3.047	2.162	-0.886	high
CK-40d	RHSIM_Rhsim13G0152700	3.893	2.739	-1.155	high
CK-40d	RHSIM_Rhsim13G0228600	-0.119	1.630	1.749	no
CK-40d	RHSIM_Rhsim13G0196900	-1.440	-1.049	0.390	low
CK-40d	RHSIM_RhsimUnG0009300	-0.506	0.884	1.390	no
CK-40d	RHSIM_Rhsim13G0017200	0.065	-0.630	-0.695	no
CK-40d	RHSIM_Rhsim13G0000200	-0.509	0.427	0.937	no
CK-40d	RHSIM_Rhsim13G0073600	0.220	-0.768	-0.988	no
CK-40d	RHSIM_Rhsim13G0209100	-0.768	-1.871	-1.103	no
CK-40d	RHSIM_Rhsim13G0056100	-1.046	-1.043	0.003	no
CK-40d	RHSIM_Rhsim13G0056500	0.554	0.482	-0.072	no
CK-40d	RHSIM_RhsimUnG0023200	-0.585	-0.559	0.025	no
CK-40d	RHSIM_Rhsim13G0200400	-0.003	-0.991	-0.988	no
CK-40d	RHSIM_Rhsim13G0127700	0.993	0.235	-0.758	no
CK-40d	RHSIM_Rhsim13G0230700	-0.756	-0.896	-0.140	no
CK-40d	RHSIM_Rhsim13G0046300	-1.696	0.067	1.763	low
CK-40d	RHSIM_Rhsim13G0112600	-0.337	-0.515	-0.179	no
CK-40d	RHSIM_RhsimUnG0015300	2.377	1.086	-1.292	high
CK-40d	RHSIM_Rhsim13G0082500	1.658	2.976	1.317	high
CK-40d	RHSIM_Rhsim13G0200800	-0.308	-0.305	0.003	no
CK-40d	RHSIM_Rhsim13G0094400	0.936	2.500	1.564	no
CK-40d	RHSIM_Rhsim13G0084100	-0.208	2.674	2.881	no
CK-40d	RHSIM_Rhsim13G0011000	-1.604	0.621	2.225	low
CK-40d	RHSIM_Rhsim13G0137100	0.483	0.361	-0.123	no
CK-40d	RHSIM_Rhsim13G0104400	0.159	-0.441	-0.599	no
CK-40d	RHSIM_RhsimUnG0009100	-0.223	0.730	0.953	no
CK-40d	RHSIM_Rhsim13G0065400	-0.753	-0.906	-0.154	no
CK-40d	RHSIM_RhsimUnG0153300	-0.155	-0.609	-0.454	no

Group	Gene	PTR (z-score difference)	z(log2 LFQ)	z(log2 FPKM)	extreme-PTR
CK-40d	RHSIM_Rhsim13G0088300	-0.054	-0.537	-0.483	no
CK-40d	RHSIM_Rhsim13G0035500	-0.483	-0.021	0.462	no
CK-40d	RHSIM_Rhsim13G0225600	0.917	3.466	2.549	no
CK-40d	RHSIM_Rhsim13G0225300	-0.554	0.433	0.987	no
CK-40d	RHSIM_Rhsim13G0210700	-1.638	-0.386	1.252	low
CK-40d	RHSIM_Rhsim13G0075700	-1.139	0.670	1.809	no
CK-40d	RHSIM_Rhsim13G0141400	-1.744	-0.736	1.008	low
CK-40d	RHSIM_Rhsim13G0021200	0.716	0.603	-0.112	no
CK-40d	RHSIM_Rhsim13G0163700	0.702	0.620	-0.081	no
CK-40d	RHSIM_RhsimUnG0150500	2.630	1.363	-1.266	high
CK-40d	RHSIM_Rhsim13G0095400	-1.095	-1.283	-0.188	no
CK-40d	RHSIM_Rhsim13G0209300	-0.991	-0.877	0.114	no
CK-40d	RHSIM_Rhsim13G0104000	1.292	1.763	0.471	high
CK-40d	RHSIM_Rhsim13G0034500	0.095	-0.465	-0.559	no
CK-40d	RHSIM_Rhsim13G0117100	-1.768	-0.953	0.815	low
CK-40d	RHSIM_Rhsim13G0089200	2.233	2.499	0.267	high
CK-40d	RHSIM_Rhsim13G0232600	-0.370	-1.130	-0.760	no
CK-40d	RHSIM_Rhsim13G0105300	1.657	0.342	-1.316	high
CK-40d	RHSIM_Rhsim13G0207700	2.304	1.965	-0.339	high
CK-40d	RHSIM_Rhsim13G0110000	-0.887	0.229	1.116	no
CK-40d	RHSIM_Rhsim12G0210900	1.752	-0.642	-2.394	high
CK-40d	RHSIM_Rhsim13G0128200	-1.162	-0.931	0.231	no
CK-40d	RHSIM_Rhsim13G0201400	-2.781	-1.165	1.615	low
CK-40d	RHSIM_Rhsim13G0153000	1.006	-0.963	-1.969	no
CK-40d	RHSIM_Rhsim13G0203300	-0.374	-0.367	0.007	no
CK-40d	RHSIM_Rhsim12G0044300	-0.850	0.256	1.106	no
CK-40d	RHSIM_Rhsim13G0051800	1.251	0.243	-1.008	high
CK-40d	RHSIM_Rhsim13G0196400	-2.056	-0.682	1.375	low
CK-40d	RHSIM_Rhsim13G0181500	-0.228	-0.843	-0.615	no
CK-40d	RHSIM_Rhsim13G0066200	-0.021	-0.849	-0.829	no
CK-40d	RHSIM_Rhsim13G0160100	0.014	-1.079	-1.093	no
CK-40d	RHSIM_Rhsim13G0093000	0.415	-0.462	-0.877	no

Group	Gene	PTR (z-score difference)	z(log2 LFQ)	z(log2 FPKM)	extreme-PTR
CK-40d	RHSIM_Rhsim12G0190000	0.250	0.063	-0.187	no
CK-40d	RHSIM_Rhsim13G0123900	0.544	2.053	1.509	no
CK-40d	RHSIM_RhsimUnG0144200	-0.150	1.776	1.926	no
CK-40d	RHSIM_Rhsim13G0044300	0.272	-0.196	-0.468	no
CK-40d	RHSIM_Rhsim13G0018200	-0.200	0.325	0.525	no
CK-40d	RHSIM_Rhsim12G0188700	-1.620	-0.094	1.526	low
CK-40d	RHSIM_Rhsim13G0099900	-0.306	-0.620	-0.314	no
CK-40d	RHSIM_Rhsim12G0145400	-0.000	0.148	0.148	no
CK-40d	RHSIM_Rhsim12G0089400	-1.158	-0.836	0.322	no
CK-40d	RHSIM_Rhsim13G0064700	1.661	0.507	-1.155	high
CK-40d	RHSIM_Rhsim13G0002600	-0.837	-0.449	0.388	no
CK-40d	RHSIM_Rhsim12G0136900	-0.177	0.274	0.450	no
CK-40d	RHSIM_Rhsim13G0102500	1.763	0.708	-1.055	high
CK-40d	RHSIM_Rhsim13G0225400	-0.940	0.591	1.530	no
CK-40d	RHSIM_Rhsim13G0018900	-0.890	0.331	1.220	no
CK-40d	RHSIM_Rhsim13G0191300	1.673	-0.517	-2.190	high
CK-40d	RHSIM_Rhsim13G0178100	-0.029	-0.315	-0.286	no
CK-40d	RHSIM_Rhsim13G0077300	1.686	1.237	-0.449	high
CK-40d	RHSIM_Rhsim13G0072100	-0.591	-0.635	-0.044	no
CK-40d	RHSIM_Rhsim13G0227500	1.995	1.236	-0.759	high
CK-40d	RHSIM_Rhsim13G0162300	-1.251	-0.220	1.031	low
CK-40d	RHSIM_Rhsim13G0134900	0.662	0.333	-0.330	no
CK-40d	RHSIM_Rhsim13G0067700	-0.809	1.310	2.119	no
CK-40d	RHSIM_Rhsim12G0057500	0.488	1.959	1.471	no
CK-40d	RHSIM_RhsimUnG0132500	1.544	-0.394	-1.937	high
CK-40d	RHSIM_Rhsim13G0229700	-0.522	0.361	0.883	no
CK-40d	RHSIM_Rhsim13G0170300	0.663	0.143	-0.520	no
CK-40d	RHSIM_Rhsim13G0002500	0.578	0.327	-0.251	no
CK-40d	RHSIM_Rhsim13G0077400	2.090	3.556	1.466	high
CK-40d	RHSIM_Rhsim13G0013500	0.919	0.538	-0.381	no
CK-40d	RHSIM_Rhsim13G0211600	-0.094	-0.458	-0.365	no
CK-40d	RHSIM_Rhsim13G0102300	0.320	-0.484	-0.804	no

Group	Gene	PTR (z-score difference)	z(log2 LFQ)	z(log2 FPKM)	extreme-PTR
CK-40d	RHSIM_Rhsim13G0015800	0.075	-1.214	-1.289	no
CK-40d	RHSIM_Rhsim13G0023600	-0.906	0.465	1.371	no
CK-40d	RHSIM_Rhsim13G0033000	-2.691	-0.585	2.107	low
CK-40d	RHSIM_Rhsim12G0205600	1.810	-0.624	-2.434	high
CK-40d	RHSIM_Rhsim13G0198600	-0.654	-1.050	-0.396	no
CK-40d	RHSIM_Rhsim13G0126100	-1.412	0.700	2.113	low
CK-40d	RHSIM_Rhsim13G0029500	0.716	-0.871	-1.587	no
CK-40d	RHSIM_Rhsim13G0216500	1.657	0.564	-1.093	high
CK-40d	RHSIM_Rhsim13G0115600	0.060	0.050	-0.010	no
CK-40d	RHSIM_Rhsim13G0044100	-0.321	-0.609	-0.287	no
CK-40d	RHSIM_Rhsim13G0073300	-0.098	-0.725	-0.627	no
CK-40d	RHSIM_RhsimUnG0124700	-1.305	-0.769	0.536	low
CK-40d	RHSIM_Rhsim13G0234200	-0.938	-0.826	0.112	no
CK-40d	RHSIM_Rhsim13G0049400	-0.698	0.175	0.873	no
CK-40d	RHSIM_Rhsim13G0155900	-1.657	-1.086	0.570	low
CK-40d	RHSIM_Rhsim13G0065900	-0.374	-0.907	-0.533	no
CK-40d	RHSIM_Rhsim12G0054400	1.130	0.582	-0.548	no
CK-40d	RHSIM_Rhsim13G0125300	1.143	-0.390	-1.534	high
CK-40d	RHSIM_Rhsim13G0013000	-0.517	-0.283	0.234	no
CK-40d	RHSIM_RhsimUnG0118000	-0.578	0.881	1.459	no
CK-40d	RHSIM_Rhsim13G0150000	-0.081	-0.612	-0.531	no
CK-40d	RHSIM_Rhsim13G0100500	2.023	1.785	-0.239	high
CK-40d	RHSIM_Rhsim12G0041400	-0.490	0.264	0.755	no
CK-40d	RHSIM_Rhsim13G0198800	-0.437	-0.316	0.122	no
CK-40d	RHSIM_Rhsim13G0071100	-0.415	0.363	0.778	no
CK-40d	RHSIM_Rhsim13G0151100	-0.264	-0.025	0.238	no
CK-40d	RHSIM_Rhsim13G0000500	-2.651	-2.081	0.570	low
CK-40d	RHSIM_Rhsim13G0028700	-0.092	-1.125	-1.033	no
CK-40d	RHSIM_Rhsim13G0143800	1.332	0.454	-0.878	high
CK-40d	RHSIM_RhsimUnG0113900	-0.116	-1.856	-1.740	no
CK-40d	RHSIM_Rhsim13G0126200	-1.550	-0.850	0.700	low
CK-40d	RHSIM_Rhsim12G0094700	-0.560	-0.528	0.032	no

Group	Gene	PTR (z-score difference)	z(log2 LFQ)	z(log2 FPKM)	extreme-PTR
CK-40d	RHSIM_Rhsim12G0069500	-0.280	-0.738	-0.458	no
CK-40d	RHSIM_Rhsim13G0228400	-0.355	0.293	0.648	no
CK-40d	RHSIM_Rhsim13G0218100	-0.100	-1.216	-1.117	no
CK-40d	RHSIM_Rhsim13G0127300	-0.755	-1.267	-0.512	no
CK-40d	RHSIM_Rhsim13G0026200	-0.635	0.987	1.622	no
CK-40d	RHSIM_Rhsim13G0088000	1.308	-0.885	-2.194	high
CK-40d	RHSIM_Rhsim12G0000600	4.554	4.458	-0.096	high
CK-40d	RHSIM_Rhsim13G0191900	-0.426	-0.348	0.078	no
CK-40d	RHSIM_Rhsim13G0065600	2.010	1.383	-0.627	high
CK-40d	RHSIM_Rhsim13G0180600	0.853	-0.017	-0.869	no
CK-40d	RHSIM_Rhsim12G0124700	0.001	-0.070	-0.072	no
CK-40d	RHSIM_Rhsim12G0173700	0.241	1.782	1.541	no
CK-40d	RHSIM_Rhsim12G0061300	-0.742	-0.574	0.168	no
CK-40d	RHSIM_Rhsim13G0224500	-1.396	-1.608	-0.212	low
CK-40d	RHSIM_Rhsim13G0199800	1.043	-0.004	-1.046	no
CK-40d	RHSIM_Rhsim13G0076400	-0.318	-0.234	0.084	no
CK-40d	RHSIM_Rhsim13G0063200	-2.299	-0.468	1.831	low
CK-40d	RHSIM_Rhsim13G0202500	0.946	-0.034	-0.980	no
CK-40d	RHSIM_Rhsim12G0204500	-1.633	-0.202	1.431	low
CK-40d	RHSIM_Rhsim13G0235300	0.301	0.704	0.403	no
CK-40d	RHSIM_Rhsim13G0085200	1.756	3.100	1.343	high
CK-40d	RHSIM_Rhsim12G0195700	-1.043	0.837	1.880	no
CK-40d	RHSIM_Rhsim13G0096500	2.856	0.586	-2.270	high
CK-40d	RHSIM_Rhsim12G0197300	0.076	0.417	0.340	no
CK-40d	RHSIM_Rhsim12G0060800	-1.463	-1.322	0.141	low
CK-40d	RHSIM_Rhsim12G0130500	0.018	-0.025	-0.043	no
CK-40d	RHSIM_Rhsim13G0058700	-0.693	-1.487	-0.794	no
CK-40d	RHSIM_Rhsim12G0210700	1.700	1.218	-0.482	high
CK-40d	RHSIM_Rhsim13G0121900	-0.319	-0.957	-0.638	no
CK-40d	RHSIM_Rhsim12G0178300	1.559	0.458	-1.102	high
CK-40d	RHSIM_Rhsim13G0000600	-2.215	-0.820	1.395	low
CK-40d	RHSIM_Rhsim13G0128900	-0.891	-1.076	-0.185	no

Group	Gene	PTR (z-score difference)	z(log2 LFQ)	z(log2 FPKM)	extreme-PTR
CK-40d	RHSIM_RhsimUnG0093000	-0.382	-0.460	-0.078	no
CK-40d	RHSIM_Rhsim12G0182600	-0.353	-0.019	0.335	no
CK-40d	RHSIM_Rhsim13G0152200	2.100	0.283	-1.817	high
CK-40d	RHSIM_Rhsim12G0127000	1.099	0.104	-0.995	no
CK-40d	RHSIM_Rhsim13G0138500	-0.193	0.767	0.960	no
CK-40d	RHSIM_Rhsim13G0072300	2.418	-0.009	-2.427	high
CK-40d	RHSIM_Rhsim13G0097900	-0.036	-0.948	-0.913	no
CK-40d	RHSIM_Rhsim12G0143900	0.402	-0.095	-0.497	no
CK-40d	RHSIM_Rhsim13G0154700	0.700	-0.089	-0.789	no
CK-40d	RHSIM_RhsimUnG0089100	-0.817	0.103	0.921	no
CK-40d	RHSIM_Rhsim12G0035800	-0.293	-0.007	0.286	no
CK-40d	RHSIM_Rhsim12G0047600	0.041	0.632	0.591	no
CK-40d	RHSIM_RhsimUnG0088100	0.224	-1.326	-1.551	no
CK-40d	RHSIM_Rhsim13G0200500	0.249	-0.069	-0.318	no
CK-40d	RHSIM_Rhsim13G0016000	-0.442	-0.480	-0.038	no
CK-40d	RHSIM_Rhsim13G0199600	0.989	0.296	-0.693	no
CK-40d	RHSIM_Rhsim12G0112500	-0.489	-0.683	-0.194	no
CK-40d	RHSIM_Rhsim13G0008000	0.934	1.084	0.151	no
CK-40d	RHSIM_Rhsim13G0197500	-1.208	0.063	1.271	no
CK-40d	RHSIM_Rhsim13G0074100	-1.096	0.403	1.499	no
CK-40d	RHSIM_Rhsim12G0077600	0.059	-0.004	-0.063	no
CK-40d	RHSIM_Rhsim12G0116500	-1.191	-0.359	0.832	no
CK-40d	RHSIM_Rhsim13G0040100	-1.895	-0.910	0.985	low
CK-40d	RHSIM_Rhsim13G0075400	0.623	0.365	-0.258	no
CK-40d	RHSIM_Rhsim12G0207400	0.621	-0.036	-0.657	no
CK-40d	RHSIM_Rhsim12G0135500	-1.065	-1.344	-0.279	no
CK-40d	RHSIM_Rhsim12G0035400	0.423	0.185	-0.237	no
CK-40d	RHSIM_Rhsim13G0037600	0.909	-0.051	-0.960	no
CK-40d	RHSIM_Rhsim13G0173900	-0.767	-0.701	0.066	no
CK-40d	RHSIM_Rhsim13G0102400	0.123	-0.221	-0.344	no
CK-40d	RHSIM_Rhsim12G0130100	-0.305	0.077	0.381	no
CK-40d	RHSIM_Rhsim12G0113000	0.733	0.410	-0.323	no

Group	Gene	PTR (z-score difference)	z(log2 LFQ)	z(log2 FPKM)	extreme-PTR
CK-40d	RHSIM_Rhsim12G0013800	-0.491	0.124	0.615	no
CK-40d	RHSIM_Rhsim13G0223300	1.293	0.985	-0.308	high
CK-40d	RHSIM_Rhsim13G0223000	0.171	2.526	2.356	no
CK-40d	RHSIM_Rhsim13G0154600	-2.190	-1.473	0.717	low
CK-40d	RHSIM_RhsimUnG0067300	0.907	-0.954	-1.861	no
CK-40d	RHSIM_Rhsim12G0212100	0.701	0.039	-0.662	no
CK-40d	RHSIM_Rhsim13G0012200	0.577	0.905	0.328	no
CK-40d	RHSIM_Rhsim12G0143400	-0.530	0.927	1.457	no
CK-40d	RHSIM_Rhsim13G0076300	-1.027	-0.258	0.768	no
CK-40d	RHSIM_Rhsim13G0029400	-0.364	-0.269	0.096	no
CK-40d	RHSIM_Rhsim13G0149600	1.157	2.421	1.263	high
CK-40d	RHSIM_Rhsim12G0021800	-0.696	0.067	0.763	no
CK-40d	RHSIM_Rhsim12G0133000	-0.648	-0.767	-0.119	no
CK-40d	RHSIM_Rhsim13G0224600	-0.432	-0.302	0.130	no
CK-40d	RHSIM_Rhsim12G0149400	2.040	1.704	-0.336	high
CK-40d	RHSIM_Rhsim13G0070200	-0.077	-1.033	-0.956	no
CK-40d	RHSIM_Rhsim12G0076200	-0.276	-0.616	-0.340	no
CK-40d	RHSIM_Rhsim12G0157800	1.031	0.416	-0.615	no
CK-40d	RHSIM_Rhsim13G0023900	-0.143	1.192	1.335	no
CK-40d	RHSIM_Rhsim12G0116400	-2.308	-0.542	1.766	low
CK-40d	RHSIM_Rhsim13G0116000	0.134	1.520	1.386	no
CK-40d	RHSIM_Rhsim13G0119800	-0.750	-1.336	-0.586	no
CK-40d	RHSIM_Rhsim12G0163200	0.570	-0.112	-0.682	no
CK-40d	RHSIM_Rhsim12G0193800	0.348	-0.627	-0.975	no
CK-40d	RHSIM_Rhsim12G0214100	-0.954	-0.510	0.444	no
CK-40d	RHSIM_RhsimUnG0053800	1.650	-0.218	-1.869	high
CK-40d	RHSIM_Rhsim12G0015600	2.477	3.771	1.294	high
CK-40d	RHSIM_Rhsim13G0124300	-2.809	-1.372	1.437	low
CK-40d	RHSIM_Rhsim12G0186700	-0.516	-1.018	-0.502	no
CK-40d	RHSIM_Rhsim12G0134700	-1.250	-0.828	0.423	low
CK-40d	RHSIM_Rhsim13G0198500	0.768	1.581	0.813	no
CK-40d	RHSIM_Rhsim12G0026000	-0.718	0.099	0.817	no

Group	Gene	PTR (z-score difference)	z(log2 LFQ)	z(log2 FPKM)	extreme-PTR
CK-40d	RHSIM_Rhsim12G0078900	-0.367	-0.439	-0.072	no
CK-40d	RHSIM_Rhsim13G0037700	0.997	0.219	-0.778	no
CK-40d	RHSIM_Rhsim12G0140200	0.350	0.528	0.178	no
CK-40d	RHSIM_Rhsim12G0137600	-0.170	0.023	0.193	no
CK-40d	RHSIM_Rhsim13G0039600	0.138	-0.169	-0.307	no
CK-40d	RHSIM_Rhsim12G0108100	-1.261	-0.886	0.376	low
CK-40d	RHSIM_Rhsim12G0009900	0.564	-0.114	-0.678	no
CK-40d	RHSIM_Rhsim13G0095800	-0.756	-1.044	-0.288	no
CK-40d	RHSIM_Rhsim12G0144200	-1.787	-2.100	-0.313	low
CK-40d	RHSIM_Rhsim13G0119500	0.627	0.244	-0.383	no
CK-40d	RHSIM_Rhsim13G0031400	1.781	0.359	-1.422	high
CK-40d	RHSIM_Rhsim12G0195800	0.321	0.434	0.113	no
CK-40d	RHSIM_Rhsim12G0152200	0.138	-0.483	-0.622	no
CK-40d	RHSIM_Rhsim12G0170600	-0.469	0.396	0.865	no
CK-40d	RHSIM_Rhsim13G0210400	2.241	1.007	-1.234	high
CK-40d	RHSIM_Rhsim13G0044700	-0.011	-0.654	-0.643	no
CK-40d	RHSIM_Rhsim12G0165900	-0.298	0.260	0.558	no
CK-40d	RHSIM_Rhsim12G0208900	-0.560	-0.734	-0.174	no
CK-40d	RHSIM_Rhsim13G0222300	-0.496	-1.047	-0.551	no
CK-40d	RHSIM_Rhsim13G0077600	0.593	1.124	0.531	no
CK-40d	RHSIM_Rhsim12G0085200	0.011	-1.279	-1.290	no
CK-40d	RHSIM_Rhsim12G0152500	-1.085	-0.908	0.177	no
CK-40d	RHSIM_Rhsim13G0233300	2.707	1.566	-1.141	high
CK-40d	RHSIM_Rhsim12G0182800	1.910	1.961	0.051	high
CK-40d	RHSIM_Rhsim12G0195100	-0.599	-0.868	-0.269	no
CK-40d	RHSIM_Rhsim12G0169500	2.604	1.327	-1.277	high
CK-40d	RHSIM_Rhsim12G0171400	-1.241	-1.336	-0.095	low
CK-40d	RHSIM_Rhsim12G0109000	2.869	1.250	-1.619	high
CK-40d	RHSIM_Rhsim13G0089100	0.186	1.039	0.853	no
CK-40d	RHSIM_Rhsim12G0041100	-1.237	-0.630	0.607	no
CK-40d	RHSIM_Rhsim12G0180700	0.436	0.805	0.369	no
CK-40d	RHSIM_Rhsim12G0155700	3.643	3.488	-0.155	high

Group	Gene	PTR (z-score difference)	z(log2 LFQ)	z(log2 FPKM)	extreme-PTR
CK-40d	RHSIM_Rhsim12G0188800	-1.377	-1.757	-0.380	low
CK-40d	RHSIM_Rhsim12G0140600	0.395	0.700	0.305	no
CK-40d	RHSIM_Rhsim12G0195900	-1.104	-1.164	-0.060	no
CK-40d	RHSIM_Rhsim12G0015500	-2.308	0.603	2.912	low
CK-40d	RHSIM_Rhsim12G0090000	-0.682	0.181	0.863	no
CK-40d	RHSIM_Rhsim12G0169800	-0.132	0.551	0.683	no
CK-40d	RHSIM_Rhsim12G0074100	-0.367	-0.466	-0.099	no
CK-40d	RHSIM_Rhsim12G0190900	0.011	-0.468	-0.480	no
CK-40d	RHSIM_Rhsim13G0230600	0.303	0.123	-0.180	no
CK-40d	RHSIM_Rhsim12G0168400	-1.093	-0.363	0.729	no
CK-40d	RHSIM_Rhsim12G0208200	-1.922	-0.259	1.663	low
CK-40d	RHSIM_Rhsim12G0167500	-1.528	0.267	1.795	low
CK-40d	RHSIM_Rhsim12G0209100	-0.654	-0.539	0.115	no
CK-40d	RHSIM_Rhsim12G0132700	2.243	0.282	-1.961	high
CK-40d	RHSIM_Rhsim12G0175800	0.344	1.473	1.129	no
CK-40d	RHSIM_Rhsim12G0163900	1.530	0.452	-1.078	high
CK-40d	RHSIM_Rhsim12G0149700	0.523	-1.007	-1.529	no
CK-40d	RHSIM_Rhsim12G0212300	0.630	-0.532	-1.162	no
CK-40d	RHSIM_Rhsim12G0197400	0.431	1.427	0.996	no
CK-40d	RHSIM_Rhsim12G0029400	-0.826	-2.040	-1.214	no
CK-40d	RHSIM_Rhsim12G0076500	0.986	0.037	-0.949	no
CK-40d	RHSIM_Rhsim12G0127200	-1.262	-1.040	0.222	low
CK-40d	RHSIM_Rhsim13G0064000	-0.307	-0.534	-0.227	no
CK-40d	RHSIM_Rhsim13G0071700	2.170	1.713	-0.457	high
CK-40d	RHSIM_Rhsim12G0137100	-2.887	-0.955	1.932	low
CK-40d	RHSIM_Rhsim12G0003100	-1.068	-0.287	0.782	no
CK-40d	RHSIM_Rhsim12G0003800	-0.537	-0.509	0.028	no
CK-40d	RHSIM_Rhsim12G0116900	1.709	0.433	-1.277	high
CK-40d	RHSIM_Rhsim12G0013200	-0.066	-1.237	-1.171	no
CK-40d	RHSIM_Rhsim12G0099000	0.325	-0.029	-0.354	no
CK-40d	RHSIM_Rhsim12G0013400	0.071	1.276	1.205	no
CK-40d	RHSIM_Rhsim12G0000200	0.823	-0.952	-1.776	no

Group	Gene	PTR (z-score difference)	z(log2 LFQ)	z(log2 FPKM)	extreme-PTR
CK-40d	RHSIM_Rhsim12G0017500	-1.536	-1.719	-0.183	low
CK-40d	RHSIM_RhsimUnG0019800	-0.661	-0.363	0.298	no
CK-40d	RHSIM_Rhsim12G0208000	1.336	1.967	0.631	high
CK-40d	RHSIM_Rhsim13G0117600	-0.499	-0.394	0.105	no
CK-40d	RHSIM_Rhsim12G0190700	0.428	0.106	-0.322	no
CK-40d	RHSIM_Rhsim12G0003400	0.066	0.592	0.525	no
CK-40d	RHSIM_Rhsim12G0053900	-3.021	-1.141	1.880	low
CK-40d	RHSIM_RhsimUnG0020300	0.133	0.241	0.108	no
CK-40d	RHSIM_Rhsim13G0102900	-0.541	-0.718	-0.177	no
CK-40d	RHSIM_Rhsim12G0192900	-2.806	-1.066	1.740	low
CK-40d	RHSIM_Rhsim12G0151800	-0.088	0.302	0.390	no
CK-40d	RHSIM_Rhsim13G0204000	-0.106	-0.189	-0.083	no
CK-40d	RHSIM_Rhsim12G0129700	-0.189	-1.107	-0.918	no
CK-40d	RHSIM_Rhsim12G0144700	-0.489	0.533	1.022	no
CK-40d	RHSIM_Rhsim12G0164400	-0.912	-0.918	-0.005	no
CK-40d	RHSIM_Rhsim12G0106200	1.817	3.421	1.604	high
CK-40d	RHSIM_Rhsim13G0136300	3.027	1.049	-1.978	high
CK-40d	RHSIM_Rhsim12G0194200	-1.044	0.706	1.750	no
CK-40d	RHSIM_Rhsim12G0004500	-1.094	-1.193	-0.099	no
CK-40d	RHSIM_Rhsim12G0137400	0.128	-0.388	-0.517	no
CK-40d	RHSIM_Rhsim12G0074200	-0.615	-1.381	-0.766	no
CK-40d	RHSIM_Rhsim12G0107100	0.605	1.174	0.569	no
CK-40d	RHSIM_Rhsim12G0087800	-0.417	0.325	0.742	no
CK-40d	RHSIM_Rhsim12G0043300	0.436	0.356	-0.080	no
CK-40d	RHSIM_Rhsim12G0138800	0.863	0.437	-0.426	no
CK-40d	RHSIM_Rhsim12G0191200	1.282	1.231	-0.051	high
CK-40d	RHSIM_Rhsim12G0099400	-0.855	-1.377	-0.521	no
CK-40d	RHSIM_Rhsim12G0146800	0.395	-0.100	-0.495	no
CK-40d	RHSIM_Rhsim12G0025000	-1.331	-1.900	-0.568	low
CK-40d	RHSIM_Rhsim13G0057500	-2.242	0.435	2.677	low
CK-40d	RHSIM_Rhsim12G0071500	-1.427	-1.421	0.006	low
CK-40d	RHSIM_Rhsim12G0013500	2.923	4.481	1.558	high

Group	Gene	PTR (z-score difference)	z(log2 LFQ)	z(log2 FPKM)	extreme-PTR
CK-40d	RHSIM_Rhsim12G0116700	1.009	0.877	-0.132	no
CK-40d	RHSIM_Rhsim13G0228300	0.314	0.243	-0.071	no
CK-40d	RHSIM_Rhsim12G0063600	1.673	0.164	-1.509	high
CK-40d	RHSIM_Rhsim12G0167900	-2.409	-0.764	1.646	low
CK-40d	RHSIM_Rhsim12G0071800	-1.530	-1.079	0.451	low
CK-40d	RHSIM_Rhsim12G0047500	-0.427	0.698	1.125	no
CK-40d	RHSIM_Rhsim12G0124800	-0.672	1.100	1.772	no
CK-40d	RHSIM_Rhsim12G0046600	-1.055	0.036	1.091	no
CK-40d	RHSIM_Rhsim12G0189200	-0.282	0.100	0.383	no
CK-40d	RHSIM_Rhsim12G0182200	0.151	-0.629	-0.780	no
CK-40d	RHSIM_Rhsim12G0190600	0.415	0.238	-0.177	no
CK-40d	RHSIM_Rhsim13G0131000	-1.501	-0.163	1.338	low
CK-40d	RHSIM_Rhsim11G0139400	-1.126	0.076	1.203	no
CK-40d	RHSIM_Rhsim12G0139100	-0.638	0.421	1.059	no
CK-40d	RHSIM_Rhsim12G0127600	-1.205	0.805	2.010	no
CK-40d	RHSIM_Rhsim12G0193000	0.660	0.052	-0.608	no
CK-40d	RHSIM_Rhsim12G0058100	-1.580	0.206	1.785	low
CK-40d	RHSIM_Rhsim12G0004800	2.521	1.349	-1.172	high
CK-40d	RHSIM_Rhsim12G0179700	-0.490	-0.433	0.057	no
CK-40d	RHSIM_Rhsim13G0166600	-0.466	-0.391	0.075	no
CK-40d	RHSIM_Rhsim12G0197200	-0.252	0.088	0.339	no
CK-40d	RHSIM_Rhsim12G0126700	1.317	-0.306	-1.624	high
CK-40d	RHSIM_Rhsim12G0025400	1.059	-0.042	-1.100	no
CK-40d	RHSIM_Rhsim12G0204700	-0.139	0.413	0.552	no
CK-40d	RHSIM_Rhsim13G0047800	-0.858	-0.811	0.047	no
CK-40d	RHSIM_Rhsim12G0199300	-0.998	0.546	1.544	no
CK-40d	RHSIM_Rhsim12G0214300	0.103	-0.023	-0.126	no
CK-40d	RHSIM_Rhsim13G0039000	-3.603	-0.864	2.739	low
CK-40d	RHSIM_Rhsim12G0139200	-1.184	-1.326	-0.142	no
CK-40d	RHSIM_Rhsim12G0162500	-1.293	-1.882	-0.589	low
CK-40d	RHSIM_Rhsim11G0006900	-1.047	-1.188	-0.140	no
CK-40d	RHSIM_Rhsim12G0191400	-0.075	0.690	0.766	no

Group	Gene	PTR (z-score difference)	z(log2 LFQ)	z(log2 FPKM)	extreme-PTR
CK-40d	RHSIM_Rhsim13G0098600	0.426	1.731	1.305	no
CK-40d	RHSIM_Rhsim12G0193700	1.099	0.863	-0.235	no
CK-40d	RHSIM_Rhsim12G0109300	-1.369	-0.835	0.534	low
CK-40d	RHSIM_Rhsim12G0121200	-0.434	-0.459	-0.025	no
CK-40d	RHSIM_Rhsim13G0042200	-0.618	0.896	1.514	no
CK-40d	RHSIM_Rhsim12G0213600	1.702	2.592	0.890	high
CK-40d	RHSIM_Rhsim13G0138000	1.675	1.049	-0.626	high
CK-40d	RHSIM_Rhsim13G0234000	-1.484	-0.828	0.657	low
CK-40d	RHSIM_Rhsim13G0074400	0.752	0.223	-0.529	no
CK-40d	RHSIM_Rhsim13G0100300	-0.218	-1.036	-0.818	no
CK-40d	RHSIM_Rhsim12G0144100	1.084	0.690	-0.394	no
CK-40d	RHSIM_Rhsim12G0070500	2.477	0.984	-1.492	high
CK-40d	RHSIM_Rhsim12G0185700	-2.520	-0.821	1.699	low
CK-40d	RHSIM_Rhsim12G0104600	0.331	0.550	0.219	no
CK-40d	RHSIM_Rhsim12G0075100	0.734	-0.885	-1.619	no
CK-40d	RHSIM_Rhsim13G0081400	-0.939	-0.941	-0.002	no
CK-40d	RHSIM_Rhsim13G0045200	-0.417	0.079	0.495	no
CK-40d	RHSIM_Rhsim12G0206900	0.291	-0.007	-0.298	no
CK-40d	RHSIM_Rhsim13G0009500	1.034	-0.901	-1.935	no
CK-40d	RHSIM_Rhsim12G0144000	-1.404	-0.875	0.530	low
CK-40d	RHSIM_Rhsim12G0004100	1.739	0.713	-1.027	high
CK-40d	RHSIM_Rhsim13G0199500	-1.346	-0.897	0.449	low
CK-40d	RHSIM_Rhsim11G0191500	-0.984	0.718	1.702	no
CK-40d	RHSIM_Rhsim12G0055000	-2.027	-1.372	0.655	low
CK-40d	RHSIM_Rhsim13G0096300	-0.382	-0.761	-0.378	no
CK-40d	RHSIM_Rhsim12G0005700	0.432	-0.786	-1.218	no
CK-40d	RHSIM_Rhsim13G0050700	-1.071	1.240	2.310	no
CK-40d	RHSIM_Rhsim13G0194300	-0.831	1.250	2.080	no
CK-40d	RHSIM_Rhsim12G0027600	-1.034	0.556	1.590	no
CK-40d	RHSIM_Rhsim12G0087900	0.879	0.540	-0.339	no
CK-40d	RHSIM_Rhsim11G0052100	1.534	0.670	-0.864	high
CK-40d	RHSIM_Rhsim13G0026100	0.633	-0.137	-0.770	no

Group	Gene	PTR (z-score difference)	z(log2 LFQ)	z(log2 FPKM)	extreme-PTR
CK-40d	RHSIM_Rhsim12G0048100	0.349	0.715	0.365	no
CK-40d	RHSIM_Rhsim12G0002800	0.349	0.055	-0.293	no
CK-40d	RHSIM_Rhsim12G0038100	-0.627	-0.679	-0.052	no
CK-40d	RHSIM_Rhsim12G0168200	-0.132	-0.602	-0.469	no
CK-40d	RHSIM_Rhsim12G0148300	-0.631	1.032	1.663	no
CK-40d	RHSIM_Rhsim12G0177900	-0.008	1.141	1.149	no
CK-40d	RHSIM_Rhsim13G0174400	1.738	0.268	-1.470	high
CK-40d	RHSIM_Rhsim12G0138000	1.537	0.266	-1.271	high
CK-40d	RHSIM_Rhsim12G0093200	2.544	0.687	-1.856	high
CK-40d	RHSIM_Rhsim12G0025300	-1.787	-0.755	1.032	low
CK-40d	RHSIM_Rhsim12G0113100	-0.851	-0.713	0.138	no
CK-40d	RHSIM_Rhsim13G0148600	-1.864	0.008	1.872	low
CK-40d	RHSIM_Rhsim12G0203300	-0.167	-1.411	-1.244	no
CK-40d	RHSIM_Rhsim13G0163200	0.444	-0.595	-1.039	no
CK-40d	RHSIM_Rhsim12G0126600	0.506	-0.723	-1.229	no
CK-40d	RHSIM_Rhsim12G0205400	-0.489	-0.536	-0.047	no
CK-40d	RHSIM_Rhsim12G0006200	0.736	1.542	0.806	no
CK-40d	RHSIM_Rhsim12G0070300	2.284	0.370	-1.913	high
CK-40d	RHSIM_Rhsim12G0165000	-0.630	-0.687	-0.058	no
CK-40d	RHSIM_Rhsim12G0006800	-0.859	-0.947	-0.088	no
CK-40d	RHSIM_Rhsim12G0172100	-0.633	-0.519	0.114	no
CK-40d	RHSIM_Rhsim12G0110200	1.242	0.528	-0.714	high
CK-40d	RHSIM_Rhsim12G0048500	-1.674	-0.853	0.821	low
CK-40d	RHSIM_Rhsim12G0137200	1.458	0.528	-0.930	high
CK-40d	RHSIM_Rhsim12G0161200	0.817	0.216	-0.601	no
CK-40d	RHSIM_Rhsim12G0003000	0.283	0.469	0.185	no
CK-40d	RHSIM_Rhsim11G0167100	1.080	1.259	0.179	no
CK-40d	RHSIM_Rhsim12G0052000	0.372	1.528	1.157	no
CK-40d	RHSIM_Rhsim12G0198100	-0.589	-1.139	-0.550	no
CK-40d	RHSIM_Rhsim12G0147400	-1.268	-0.173	1.095	low
CK-40d	RHSIM_Rhsim11G0124300	-1.192	-0.122	1.070	no
CK-40d	RHSIM_Rhsim12G0115900	0.199	1.839	1.640	no

Group	Gene	PTR (z-score difference)	z(log2 LFQ)	z(log2 FPKM)	extreme-PTR
CK-40d	RHSIM_Rhsim12G0192800	0.531	-0.923	-1.454	no
CK-40d	RHSIM_Rhsim13G0126800	-0.160	-0.504	-0.344	no
CK-40d	RHSIM_Rhsim12G0171000	1.177	-0.082	-1.260	high
CK-40d	RHSIM_Rhsim11G0045800	-1.061	-0.282	0.779	no
CK-40d	RHSIM_Rhsim12G0192300	0.553	1.081	0.529	no
CK-40d	RHSIM_Rhsim11G0195600	-2.264	-1.049	1.215	low
CK-40d	RHSIM_Rhsim12G0102400	0.360	1.248	0.888	no
CK-40d	RHSIM_Rhsim11G0129600	-0.768	0.423	1.191	no
CK-40d	RHSIM_Rhsim12G0133400	-0.376	2.036	2.412	no
CK-40d	RHSIM_Rhsim12G0043200	-1.883	0.057	1.940	low
CK-40d	RHSIM_Rhsim12G0153900	0.409	0.499	0.089	no
CK-40d	RHSIM_Rhsim12G0015200	-0.548	0.102	0.650	no
CK-40d	RHSIM_Rhsim11G0167300	0.335	1.495	1.160	no
CK-40d	RHSIM_Rhsim11G0127100	-0.758	-0.302	0.456	no
CK-40d	RHSIM_Rhsim12G0138500	0.338	1.227	0.888	no
CK-40d	RHSIM_Rhsim11G0100300	-0.622	1.608	2.230	no
CK-40d	RHSIM_Rhsim11G0188300	-1.653	-0.517	1.136	low
CK-40d	RHSIM_Rhsim12G0099800	2.038	2.461	0.423	high
CK-40d	RHSIM_Rhsim12G0135900	-0.387	-0.459	-0.072	no
CK-40d	RHSIM_Rhsim11G0132400	-2.381	-1.651	0.730	low
CK-40d	RHSIM_Rhsim12G0047700	-0.227	0.531	0.758	no
CK-40d	RHSIM_Rhsim11G0129000	-2.203	-0.051	2.152	low
CK-40d	RHSIM_Rhsim12G0214500	-0.967	-1.730	-0.763	no
CK-40d	RHSIM_Rhsim11G0034700	1.611	1.213	-0.398	high
CK-40d	RHSIM_Rhsim11G0172800	2.672	1.646	-1.025	high
CK-40d	RHSIM_Rhsim12G0017000	1.973	0.772	-1.201	high
CK-40d	RHSIM_Rhsim12G0152700	-0.572	-1.760	-1.188	no
CK-40d	RHSIM_Rhsim12G0054500	-0.529	1.533	2.063	no
CK-40d	RHSIM_Rhsim12G0183500	1.618	1.777	0.159	high
CK-40d	RHSIM_Rhsim11G0011600	-0.073	0.661	0.734	no
CK-40d	RHSIM_Rhsim13G0178500	-1.278	-1.077	0.201	low
CK-40d	RHSIM_Rhsim12G0168600	0.097	0.080	-0.017	no

Group	Gene	PTR (z-score difference)	z(log2 LFQ)	z(log2 FPKM)	extreme-PTR
CK-40d	RHSIM_Rhsim12G0065100	-1.271	-0.429	0.842	low
CK-40d	RHSIM_Rhsim11G0129800	-1.483	-0.762	0.722	low
CK-40d	RHSIM_Rhsim11G0092300	-0.584	-1.047	-0.463	no
CK-40d	RHSIM_Rhsim12G0132900	-0.524	-0.110	0.414	no
CK-40d	RHSIM_Rhsim13G0080100	0.471	-0.232	-0.704	no
CK-40d	RHSIM_Rhsim12G0063100	0.061	0.174	0.113	no
CK-40d	RHSIM_Rhsim12G0188200	-3.260	-1.972	1.288	low
CK-40d	RHSIM_Rhsim12G0050400	-2.074	0.315	2.389	low
CK-40d	RHSIM_Rhsim11G0076700	-3.435	-1.517	1.918	low
CK-40d	RHSIM_Rhsim12G0166300	-0.100	0.312	0.412	no
CK-40d	RHSIM_Rhsim11G0025100	-0.632	-0.525	0.108	no
CK-40d	RHSIM_Rhsim12G0124500	-0.820	-1.173	-0.353	no
CK-40d	RHSIM_Rhsim12G0207300	1.323	0.564	-0.759	high
CK-40d	RHSIM_Rhsim11G0101800	1.378	-0.763	-2.141	high
CK-40d	RHSIM_Rhsim12G0134100	-0.551	-0.786	-0.235	no
CK-40d	RHSIM_Rhsim13G0101800	1.075	-0.816	-1.891	no
CK-40d	RHSIM_Rhsim12G0197600	1.528	0.681	-0.848	high
CK-40d	RHSIM_Rhsim11G0114600	0.790	-0.080	-0.870	no
CK-40d	RHSIM_Rhsim12G0164700	-0.478	0.064	0.542	no
CK-40d	RHSIM_Rhsim12G0089200	-0.290	-0.761	-0.471	no
CK-40d	RHSIM_Rhsim12G0064700	-0.639	-0.574	0.065	no
CK-40d	RHSIM_Rhsim11G0093300	0.058	0.079	0.021	no
CK-40d	RHSIM_Rhsim12G0042000	0.893	-0.470	-1.363	no
CK-40d	RHSIM_Rhsim12G0171100	1.621	1.573	-0.047	high
CK-40d	RHSIM_Rhsim11G0034400	0.343	-0.012	-0.356	no
CK-40d	RHSIM_Rhsim12G0182900	1.295	-0.034	-1.329	high
CK-40d	RHSIM_Rhsim13G0038100	-0.501	-0.485	0.017	no
CK-40d	RHSIM_Rhsim11G0188100	-0.256	1.635	1.891	no
CK-40d	RHSIM_Rhsim12G0096200	-0.276	-0.676	-0.400	no
CK-40d	RHSIM_Rhsim13G0074500	-1.243	-0.649	0.594	low
CK-40d	RHSIM_Rhsim11G0135800	1.141	1.962	0.822	high
CK-40d	RHSIM_Rhsim12G0046400	1.602	1.946	0.343	high

Group	Gene	PTR (z-score difference)	z(log2 LFQ)	z(log2 FPKM)	extreme-PTR
CK-40d	RHSIM_Rhsim11G0001200	-0.103	-0.148	-0.046	no
CK-40d	RHSIM_Rhsim11G0136900	-0.389	1.132	1.521	no
CK-40d	RHSIM_Rhsim12G0065000	2.496	1.517	-0.978	high
CK-40d	RHSIM_Rhsim11G0039200	-1.558	0.270	1.829	low
CK-40d	RHSIM_Rhsim12G0161700	-0.156	-1.912	-1.756	no
CK-40d	RHSIM_Rhsim11G0125200	-0.698	-0.009	0.689	no
CK-40d	RHSIM_Rhsim11G0098400	2.648	1.520	-1.127	high
CK-40d	RHSIM_Rhsim12G0047800	-0.467	-1.060	-0.593	no
CK-40d	RHSIM_Rhsim11G0186300	-0.177	-0.446	-0.269	no
CK-40d	RHSIM_Rhsim11G0072000	1.639	0.235	-1.403	high
CK-40d	RHSIM_Rhsim12G0073200	1.435	0.605	-0.830	high
CK-40d	RHSIM_Rhsim12G0129600	-1.502	-1.414	0.087	low
CK-40d	RHSIM_Rhsim11G0176000	0.439	1.003	0.564	no
CK-40d	RHSIM_Rhsim11G0133600	-0.860	-0.120	0.740	no
CK-40d	RHSIM_Rhsim11G0171300	-0.928	-1.901	-0.973	no
CK-40d	RHSIM_Rhsim12G0130600	0.365	0.683	0.318	no
CK-40d	RHSIM_Rhsim11G0197000	-0.408	0.213	0.622	no
CK-40d	RHSIM_Rhsim11G0113300	0.139	0.567	0.428	no
CK-40d	RHSIM_Rhsim11G0192400	-0.784	1.110	1.894	no
CK-40d	RHSIM_Rhsim12G0016200	-0.629	-0.943	-0.314	no
CK-40d	RHSIM_Rhsim12G0079100	-1.188	-0.015	1.173	no
CK-40d	RHSIM_Rhsim11G0095300	-0.096	-0.648	-0.552	no
CK-40d	RHSIM_Rhsim13G0229400	0.786	-0.520	-1.306	no
CK-40d	RHSIM_Rhsim11G0016500	1.422	0.453	-0.969	high
CK-40d	RHSIM_Rhsim12G0206500	0.802	1.571	0.769	no
CK-40d	RHSIM_Rhsim11G0121500	0.553	1.181	0.627	no
CK-40d	RHSIM_Rhsim11G0069300	-2.902	-1.364	1.538	low
CK-40d	RHSIM_Rhsim13G0197700	0.289	1.239	0.950	no
CK-40d	RHSIM_Rhsim13G0015200	-1.303	-0.822	0.481	low
CK-40d	RHSIM_Rhsim11G0160100	-0.747	-0.812	-0.064	no
CK-40d	RHSIM_Rhsim12G0201500	-0.151	-0.170	-0.019	no
CK-40d	RHSIM_Rhsim11G0177100	0.138	0.817	0.679	no

Group	Gene	PTR (z-score difference)	z(log2 LFQ)	z(log2 FPKM)	extreme-PTR
CK-40d	RHSIM_Rhsim13G0133100	2.599	1.250	-1.349	high
CK-40d	RHSIM_Rhsim11G0161200	-0.683	-0.043	0.640	no
CK-40d	RHSIM_Rhsim12G0128500	0.460	-0.707	-1.167	no
CK-40d	RHSIM_Rhsim11G0121900	-0.613	-0.687	-0.074	no
CK-40d	RHSIM_Rhsim11G0196200	-0.137	-0.770	-0.633	no
CK-40d	RHSIM_Rhsim13G0234500	0.435	0.092	-0.343	no
CK-40d	RHSIM_Rhsim13G0201500	0.589	2.762	2.173	no
CK-40d	RHSIM_Rhsim11G0148100	0.561	0.365	-0.196	no
CK-40d	RHSIM_Rhsim13G0052600	-0.732	-0.908	-0.176	no
CK-40d	RHSIM_Rhsim11G0122800	-0.183	-0.068	0.115	no
CK-40d	RHSIM_Rhsim11G0130100	2.213	1.945	-0.268	high
CK-40d	RHSIM_Rhsim13G0010400	-0.022	-0.443	-0.421	no
CK-40d	RHSIM_Rhsim12G0087000	-2.699	-1.139	1.559	low
CK-40d	RHSIM_Rhsim11G0167000	1.826	1.404	-0.422	high
CK-40d	RHSIM_Rhsim11G0153500	0.094	0.336	0.242	no
CK-40d	RHSIM_Rhsim12G0080800	1.707	0.157	-1.550	high
CK-40d	RHSIM_Rhsim11G0165700	0.003	0.067	0.064	no
CK-40d	RHSIM_Rhsim13G0153900	0.987	-0.096	-1.083	no
CK-40d	RHSIM_Rhsim11G0097900	0.115	-0.700	-0.816	no
CK-40d	RHSIM_Rhsim12G0010700	1.680	1.688	0.008	high
CK-40d	RHSIM_Rhsim11G0099800	0.452	0.052	-0.400	no
CK-40d	RHSIM_Rhsim13G0049600	-1.719	-0.023	1.696	low
CK-40d	RHSIM_Rhsim12G0076700	0.868	-0.323	-1.192	no
CK-40d	RHSIM_Rhsim12G0209000	0.792	0.610	-0.183	no
CK-40d	RHSIM_Rhsim12G0090500	-1.072	-0.387	0.685	no
CK-40d	RHSIM_Rhsim11G0132600	-0.864	-0.638	0.226	no
CK-40d	RHSIM_Rhsim11G0099600	-2.467	-0.643	1.824	low
CK-40d	RHSIM_Rhsim11G0121300	-0.631	1.373	2.004	no
CK-40d	RHSIM_Rhsim11G0123700	-0.574	0.214	0.788	no
CK-40d	RHSIM_Rhsim11G0161300	1.436	0.282	-1.154	high
CK-40d	RHSIM_Rhsim11G0097400	-0.759	-0.954	-0.195	no
CK-40d	RHSIM_Rhsim11G0145600	0.658	1.446	0.788	no

Group	Gene	PTR (z-score difference)	z(log2 LFQ)	z(log2 FPKM)	extreme-PTR
CK-40d	RHSIM_Rhsim11G0045300	1.307	2.166	0.859	high
CK-40d	RHSIM_Rhsim12G0206700	0.345	-0.499	-0.844	no
CK-40d	RHSIM_Rhsim11G0058800	0.620	1.126	0.505	no
CK-40d	RHSIM_Rhsim12G0077400	0.264	-1.330	-1.594	no
CK-40d	RHSIM_Rhsim11G0191300	-0.438	-0.734	-0.296	no
CK-40d	RHSIM_Rhsim12G0215400	-0.708	-0.039	0.669	no
CK-40d	RHSIM_Rhsim11G0142100	0.831	1.211	0.381	no
CK-40d	RHSIM_Rhsim11G0199200	-0.233	-0.212	0.021	no
CK-40d	RHSIM_Rhsim13G0037000	0.164	0.376	0.212	no
CK-40d	RHSIM_Rhsim11G0044200	0.453	1.087	0.634	no
CK-40d	RHSIM_Rhsim12G0025900	-0.738	-0.272	0.466	no
CK-40d	RHSIM_Rhsim12G0200000	0.463	-0.742	-1.205	no
CK-40d	RHSIM_Rhsim11G0142400	1.111	1.115	0.004	no
CK-40d	RHSIM_Rhsim11G0036800	-1.480	-1.411	0.069	low
CK-40d	RHSIM_Rhsim11G0105300	-1.009	-1.229	-0.220	no
CK-40d	RHSIM_Rhsim11G0149600	0.941	-0.018	-0.959	no
CK-40d	RHSIM_Rhsim12G0149800	0.867	1.526	0.659	no
CK-40d	RHSIM_Rhsim11G0053800	0.845	-0.625	-1.470	no
CK-40d	RHSIM_Rhsim11G0197800	0.553	-1.202	-1.755	no
CK-40d	RHSIM_Rhsim11G0006500	-0.647	-0.646	0.001	no
CK-40d	RHSIM_Rhsim12G0003500	1.349	1.801	0.452	high
CK-40d	RHSIM_Rhsim11G0179400	0.853	0.157	-0.697	no
CK-40d	RHSIM_Rhsim11G0008100	2.664	1.516	-1.148	high
CK-40d	RHSIM_Rhsim11G0084200	-0.324	2.462	2.785	no
CK-40d	RHSIM_Rhsim04G0146800	0.601	0.225	-0.377	no
CK-40d	RHSIM_Rhsim11G0166500	-0.770	-0.638	0.132	no
CK-40d	RHSIM_Rhsim13G0114900	-3.075	-1.490	1.586	low
CK-40d	RHSIM_Rhsim11G0036600	-0.628	-1.081	-0.453	no
CK-40d	RHSIM_Rhsim11G0087200	-0.211	0.637	0.848	no
CK-40d	RHSIM_Rhsim12G0157100	-0.908	-1.389	-0.481	no
CK-40d	RHSIM_Rhsim12G0151900	-0.959	1.879	2.838	no
CK-40d	RHSIM_Rhsim11G0088900	0.630	0.576	-0.054	no

Group	Gene	PTR (z-score difference)	z(log2 LFQ)	z(log2 FPKM)	extreme-PTR
CK-40d	RHSIM_Rhsim11G0108000	-1.336	-0.433	0.903	low
CK-40d	RHSIM_Rhsim13G0225000	-0.917	-1.710	-0.792	no
CK-40d	RHSIM_Rhsim11G0168200	0.429	-1.191	-1.620	no
CK-40d	RHSIM_Rhsim11G0003700	0.038	0.827	0.789	no
CK-40d	RHSIM_Rhsim11G0136600	0.855	-0.118	-0.972	no
CK-40d	RHSIM_Rhsim11G0168600	-0.861	-0.844	0.017	no
CK-40d	RHSIM_Rhsim12G0179900	-1.579	-1.810	-0.231	low
CK-40d	RHSIM_Rhsim11G0193300	-1.019	-0.857	0.162	no
CK-40d	RHSIM_Rhsim11G0105100	1.527	1.031	-0.496	high
CK-40d	RHSIM_Rhsim11G0088000	-0.257	-0.492	-0.235	no
CK-40d	RHSIM_Rhsim12G0090900	-1.230	-0.793	0.438	no
CK-40d	RHSIM_Rhsim12G0193400	-1.037	0.819	1.857	no
CK-40d	RHSIM_Rhsim11G0183800	2.528	3.623	1.095	high
CK-40d	RHSIM_Rhsim13G0213200	-0.944	1.552	2.495	no
CK-40d	RHSIM_Rhsim11G0008300	1.316	-0.130	-1.447	high
CK-40d	RHSIM_Rhsim12G0145800	-0.148	0.271	0.420	no
CK-40d	RHSIM_Rhsim13G0229300	1.702	2.434	0.733	high
CK-40d	RHSIM_Rhsim11G0098600	-0.491	0.224	0.715	no
CK-40d	RHSIM_Rhsim11G0180700	0.203	0.252	0.049	no
CK-40d	RHSIM_Rhsim12G0201700	-0.029	1.101	1.130	no
CK-40d	RHSIM_Rhsim13G0029900	1.720	1.087	-0.633	high
CK-40d	RHSIM_Rhsim12G0180500	-0.081	-0.312	-0.231	no
CK-40d	RHSIM_Rhsim11G0065900	-1.603	-0.497	1.106	low
CK-40d	RHSIM_Rhsim11G0159300	0.676	0.699	0.024	no
CK-40d	RHSIM_Rhsim12G0167000	-1.340	-0.947	0.393	low
CK-40d	RHSIM_Rhsim11G0055100	2.022	-0.210	-2.232	high
CK-40d	RHSIM_Rhsim11G0016400	0.525	-1.021	-1.547	no
CK-40d	RHSIM_Rhsim11G0022000	-0.917	-0.407	0.510	no
CK-40d	RHSIM_Rhsim11G0184900	0.377	0.084	-0.293	no
CK-40d	RHSIM_Rhsim11G0063600	1.536	0.758	-0.778	high
CK-40d	RHSIM_Rhsim13G0166900	0.074	-0.977	-1.052	no
CK-40d	RHSIM_Rhsim11G0010400	0.213	-1.075	-1.288	no

Group	Gene	PTR (z-score difference)	z(log2 LFQ)	z(log2 FPKM)	extreme-PTR
CK-40d	RHSIM_Rhsim11G0063800	-1.342	-1.173	0.169	low
CK-40d	RHSIM_Rhsim11G0174300	0.758	-0.080	-0.838	no
CK-40d	RHSIM_Rhsim11G0085900	-0.570	1.558	2.129	no
CK-40d	RHSIM_Rhsim11G0041300	2.135	1.864	-0.271	high
CK-40d	RHSIM_Rhsim12G0209200	-0.342	-0.199	0.143	no
CK-40d	RHSIM_Rhsim12G0134600	1.009	0.405	-0.604	no
CK-40d	RHSIM_Rhsim11G0146000	-1.800	-1.010	0.790	low
CK-40d	RHSIM_Rhsim12G0024700	-0.966	-0.763	0.204	no
CK-40d	RHSIM_Rhsim12G0054800	-1.519	-1.162	0.357	low
CK-40d	RHSIM_Rhsim11G0198200	0.089	0.381	0.292	no
CK-40d	RHSIM_Rhsim11G0099700	-1.091	-1.424	-0.333	no
CK-40d	RHSIM_Rhsim12G0025500	-1.723	-0.954	0.769	low
CK-40d	RHSIM_Rhsim11G0112000	0.047	-0.619	-0.667	no
CK-40d	RHSIM_Rhsim12G0133300	1.123	0.178	-0.945	no
CK-40d	RHSIM_Rhsim12G0173900	-1.163	-1.180	-0.017	no
CK-40d	RHSIM_Rhsim11G0193600	0.468	0.338	-0.129	no
CK-40d	RHSIM_Rhsim12G0016800	0.880	0.131	-0.749	no
CK-40d	RHSIM_Rhsim12G0112100	0.728	1.459	0.731	no
CK-40d	RHSIM_Rhsim11G0042300	1.036	-0.228	-1.263	no
CK-40d	RHSIM_Rhsim12G0212900	-0.466	-0.195	0.271	no
CK-40d	RHSIM_Rhsim12G0144900	-0.288	-0.173	0.114	no
CK-40d	RHSIM_Rhsim11G0090900	1.240	-0.442	-1.682	high
CK-40d	RHSIM_Rhsim11G0196900	-0.703	-0.406	0.298	no
CK-40d	RHSIM_Rhsim11G0157200	0.120	0.310	0.190	no
CK-40d	RHSIM_Rhsim12G0162200	0.218	-0.518	-0.736	no
CK-40d	RHSIM_Rhsim11G0176400	0.248	0.165	-0.083	no
CK-40d	RHSIM_Rhsim11G0036900	0.705	-0.375	-1.080	no
CK-40d	RHSIM_Rhsim11G0177200	-0.197	0.461	0.658	no
CK-40d	RHSIM_Rhsim11G0051900	1.127	-0.252	-1.379	no
CK-40d	RHSIM_Rhsim12G0137000	0.093	0.116	0.023	no
CK-40d	RHSIM_Rhsim11G0045000	-0.019	-0.441	-0.422	no
CK-40d	RHSIM_Rhsim12G0130000	1.448	0.652	-0.796	high

Group	Gene	PTR (z-score difference)	z(log2 LFQ)	z(log2 FPKM)	extreme-PTR
CK-40d	RHSIM_Rhsim11G0122200	0.910	2.529	1.619	no
CK-40d	RHSIM_Rhsim12G0005000	0.435	-0.082	-0.517	no
CK-40d	RHSIM_Rhsim11G0194700	-0.316	0.872	1.188	no
CK-40d	RHSIM_Rhsim12G0003200	-0.544	-0.705	-0.161	no
CK-40d	RHSIM_Rhsim11G0182000	0.318	0.701	0.383	no
CK-40d	RHSIM_Rhsim11G0134200	1.311	0.357	-0.954	high
CK-40d	RHSIM_Rhsim11G0177800	-0.430	1.482	1.912	no
CK-40d	RHSIM_Rhsim11G0046400	0.083	0.060	-0.023	no
CK-40d	RHSIM_Rhsim12G0041700	-1.805	-0.401	1.405	low
CK-40d	RHSIM_Rhsim11G0195000	-0.118	-1.264	-1.146	no
CK-40d	RHSIM_Rhsim11G0054800	-1.065	-1.256	-0.191	no
CK-40d	RHSIM_Rhsim12G0002100	-0.315	-0.917	-0.601	no
CK-40d	RHSIM_Rhsim11G0065700	0.080	0.866	0.786	no
CK-40d	RHSIM_Rhsim11G0167500	1.132	0.984	-0.148	no
CK-40d	RHSIM_Rhsim11G0045700	-0.035	-0.906	-0.871	no
CK-40d	RHSIM_Rhsim11G0118400	-0.308	0.573	0.881	no
CK-40d	RHSIM_Rhsim12G0204600	-0.749	1.538	2.286	no
CK-40d	RHSIM_Rhsim12G0051400	-1.326	-0.518	0.808	low
CK-40d	RHSIM_Rhsim11G0147400	-0.344	-0.588	-0.245	no
CK-40d	RHSIM_Rhsim12G0075500	-0.622	1.836	2.458	no
CK-40d	RHSIM_Rhsim11G0184600	-1.440	-1.637	-0.197	low
CK-40d	RHSIM_Rhsim11G0199100	-0.175	-0.693	-0.519	no
CK-40d	RHSIM_Rhsim11G0053000	-2.267	-1.146	1.121	low
CK-40d	RHSIM_Rhsim11G0020200	1.376	2.037	0.661	high
CK-40d	RHSIM_Rhsim11G0197500	0.347	0.032	-0.316	no
CK-40d	RHSIM_Rhsim11G0131700	-2.491	-1.522	0.969	low
CK-40d	RHSIM_Rhsim11G0121700	-1.121	1.217	2.338	no
CK-40d	RHSIM_Rhsim12G0017200	-0.461	0.625	1.086	no
CK-40d	RHSIM_Rhsim10G0183500	0.663	0.276	-0.386	no
CK-40d	RHSIM_Rhsim12G0037000	-0.088	-1.486	-1.398	no
CK-40d	RHSIM_Rhsim11G0167700	1.732	0.860	-0.872	high
CK-40d	RHSIM_Rhsim10G0007500	-0.796	0.019	0.814	no

Group	Gene	PTR (z-score difference)	z(log2 LFQ)	z(log2 FPKM)	extreme-PTR
CK-40d	RHSIM_Rhsim12G0000100	1.505	0.623	-0.881	high
CK-40d	RHSIM_Rhsim11G0054200	-0.416	-0.969	-0.553	no
CK-40d	RHSIM_Rhsim11G0163600	1.410	0.301	-1.109	high
CK-40d	RHSIM_Rhsim10G0040200	-0.092	0.654	0.745	no
CK-40d	RHSIM_Rhsim11G0195400	0.505	-0.010	-0.514	no
CK-40d	RHSIM_Rhsim11G0156300	2.284	0.835	-1.449	high
CK-40d	RHSIM_Rhsim10G0102500	1.171	0.071	-1.100	high
CK-40d	RHSIM_Rhsim11G0122000	2.170	0.023	-2.147	high
CK-40d	RHSIM_Rhsim12G0102200	-0.466	-0.779	-0.313	no
CK-40d	RHSIM_Rhsim11G0161100	-2.442	-0.735	1.707	low
CK-40d	RHSIM_Rhsim11G0052000	0.708	-0.479	-1.187	no
CK-40d	RHSIM_Rhsim10G0183700	0.206	1.759	1.554	no
CK-40d	RHSIM_Rhsim11G0175900	-0.674	-0.944	-0.270	no
CK-40d	RHSIM_Rhsim10G0204000	0.458	0.387	-0.070	no
CK-40d	RHSIM_Rhsim10G0079900	1.196	1.062	-0.134	high
CK-40d	RHSIM_Rhsim12G0090700	0.390	0.555	0.165	no
CK-40d	RHSIM_Rhsim11G0010500	-0.698	0.374	1.072	no
CK-40d	RHSIM_Rhsim11G0095500	0.215	0.041	-0.175	no
CK-40d	RHSIM_Rhsim10G0133700	3.678	3.228	-0.450	high
CK-40d	RHSIM_Rhsim10G0079400	0.922	-0.091	-1.013	no
CK-40d	RHSIM_Rhsim10G0135700	1.913	1.338	-0.575	high
CK-40d	RHSIM_Rhsim12G0205300	-0.086	-0.119	-0.033	no
CK-40d	RHSIM_Rhsim12G0036900	-1.505	-0.818	0.687	low
CK-40d	RHSIM_Rhsim10G0196000	-0.273	0.814	1.087	no
CK-40d	RHSIM_Rhsim11G0190300	0.015	0.398	0.383	no
CK-40d	RHSIM_Rhsim12G0105500	2.019	1.154	-0.865	high
CK-40d	RHSIM_Rhsim10G0045000	-0.987	0.145	1.132	no
CK-40d	RHSIM_Rhsim12G0185400	-1.215	-0.774	0.440	no
CK-40d	RHSIM_Rhsim10G0210800	1.056	-0.475	-1.531	no
CK-40d	RHSIM_Rhsim12G0110600	-1.912	-0.982	0.930	low
CK-40d	RHSIM_Rhsim12G0080300	0.592	-0.816	-1.408	no
CK-40d	RHSIM_Rhsim10G0121300	0.495	-0.477	-0.972	no

Group	Gene	PTR (z-score difference)	z(log2 LFQ)	z(log2 FPKM)	extreme-PTR
CK-40d	RHSIM_Rhsim11G0008500	-0.264	-0.068	0.195	no
CK-40d	RHSIM_Rhsim10G0069400	-2.692	-2.237	0.455	low
CK-40d	RHSIM_Rhsim12G0168500	-0.238	-0.260	-0.022	no
CK-40d	RHSIM_Rhsim11G0196800	-0.085	-0.177	-0.092	no
CK-40d	RHSIM_Rhsim10G0029000	1.296	-0.143	-1.439	high
CK-40d	RHSIM_Rhsim10G0155900	0.337	-1.123	-1.460	no
CK-40d	RHSIM_Rhsim10G0009900	-0.163	-1.735	-1.571	no
CK-40d	RHSIM_Rhsim10G0164700	-0.516	-0.952	-0.437	no
CK-40d	RHSIM_Rhsim12G0166000	0.782	-0.707	-1.489	no
CK-40d	RHSIM_Rhsim10G0197300	0.596	1.121	0.525	no
CK-40d	RHSIM_Rhsim11G0097800	0.646	0.643	-0.002	no
CK-40d	RHSIM_Rhsim12G0007900	-0.888	0.471	1.358	no
CK-40d	RHSIM_Rhsim10G0158900	-0.513	-2.199	-1.686	no
CK-40d	RHSIM_Rhsim12G0139700	1.300	0.594	-0.706	high
CK-40d	RHSIM_Rhsim10G0171300	-2.261	-1.866	0.395	low
CK-40d	RHSIM_Rhsim12G0159900	-1.079	-1.008	0.071	no
CK-40d	RHSIM_Rhsim11G0167600	1.050	1.573	0.522	no
CK-40d	RHSIM_Rhsim10G0067100	-1.152	-0.590	0.562	no
CK-40d	RHSIM_Rhsim11G0126200	-0.423	-0.460	-0.037	no
CK-40d	RHSIM_Rhsim11G0023700	1.645	0.881	-0.763	high
CK-40d	RHSIM_Rhsim12G0068000	-0.330	0.446	0.776	no
CK-40d	RHSIM_Rhsim11G0198400	1.137	1.136	-0.000	no
CK-40d	RHSIM_Rhsim11G0052800	0.355	-1.394	-1.748	no
CK-40d	RHSIM_Rhsim10G0051200	-1.214	-0.703	0.511	no
CK-40d	RHSIM_Rhsim10G0191900	-0.008	-0.555	-0.546	no
CK-40d	RHSIM_Rhsim10G0026400	2.034	0.796	-1.238	high
CK-40d	RHSIM_Rhsim10G0030500	-0.983	-1.723	-0.740	no
CK-40d	RHSIM_Rhsim10G0005500	-0.527	0.206	0.733	no
CK-40d	RHSIM_Rhsim10G0105200	-0.046	-0.071	-0.025	no
CK-40d	RHSIM_Rhsim12G0114000	-1.133	-0.142	0.991	no
CK-40d	RHSIM_Rhsim10G0005700	0.024	-0.399	-0.423	no
CK-40d	RHSIM_Rhsim10G0117200	2.128	1.158	-0.969	high

Group	Gene	PTR (z-score difference)	z(log2 LFQ)	z(log2 FPKM)	extreme-PTR
CK-40d	RHSIM_Rhsim10G0191600	0.445	-0.929	-1.373	no
CK-40d	RHSIM_Rhsim10G0194400	0.546	0.586	0.040	no
CK-40d	RHSIM_Rhsim11G0053100	1.182	1.378	0.197	high
CK-40d	RHSIM_Rhsim11G0198000	-0.054	-0.399	-0.344	no
CK-40d	RHSIM_Rhsim11G0113700	-0.624	-0.365	0.260	no
CK-40d	RHSIM_Rhsim10G0199000	1.490	0.007	-1.483	high
CK-40d	RHSIM_Rhsim12G0008000	0.203	-0.082	-0.285	no
CK-40d	RHSIM_Rhsim10G0035600	-0.419	-0.417	0.003	no
CK-40d	RHSIM_Rhsim10G0116000	-1.127	-0.823	0.304	no
CK-40d	RHSIM_Rhsim11G0048900	-1.470	-1.203	0.267	low
CK-40d	RHSIM_Rhsim11G0167200	0.718	0.809	0.091	no
CK-40d	RHSIM_Rhsim12G0070400	1.908	0.419	-1.490	high
CK-40d	RHSIM_Rhsim10G0148300	1.335	0.452	-0.883	high
CK-40d	RHSIM_Rhsim10G0099800	-1.138	1.263	2.401	no
CK-40d	RHSIM_Rhsim10G0136800	-0.051	-0.632	-0.581	no
CK-40d	RHSIM_Rhsim12G0115000	-2.162	-1.185	0.977	low
CK-40d	RHSIM_Rhsim10G0186500	1.587	0.781	-0.805	high
CK-40d	RHSIM_Rhsim12G0166200	-0.450	-1.108	-0.658	no
CK-40d	RHSIM_Rhsim11G0135900	3.296	3.240	-0.055	high
CK-40d	RHSIM_Rhsim12G0189800	-0.018	-0.107	-0.089	no
CK-40d	RHSIM_Rhsim10G0108600	-0.065	0.033	0.099	no
CK-40d	RHSIM_Rhsim12G0014000	0.529	0.032	-0.497	no
CK-40d	RHSIM_Rhsim10G0161200	-0.579	-1.238	-0.659	no
CK-40d	RHSIM_Rhsim11G0125700	-1.479	-1.706	-0.227	low
CK-40d	RHSIM_Rhsim10G0192800	-0.520	-0.609	-0.089	no
CK-40d	RHSIM_Rhsim10G0197900	-0.403	0.663	1.066	no
CK-40d	RHSIM_Rhsim12G0136800	-0.547	-1.294	-0.747	no
CK-40d	RHSIM_Rhsim10G0014000	0.034	-0.635	-0.668	no
CK-40d	RHSIM_Rhsim10G0094500	-0.050	-0.368	-0.318	no
CK-40d	RHSIM_Rhsim11G0173400	-0.280	-0.681	-0.401	no
CK-40d	RHSIM_Rhsim12G0150700	0.064	0.742	0.678	no
CK-40d	RHSIM_Rhsim10G0069000	1.351	1.145	-0.206	high

Group	Gene	PTR (z-score difference)	z(log2 LFQ)	z(log2 FPKM)	extreme-PTR
CK-40d	RHSIM_Rhsim11G0135200	0.061	-0.209	-0.270	no
CK-40d	RHSIM_Rhsim10G0019600	-0.203	-0.277	-0.074	no
CK-40d	RHSIM_Rhsim12G0001500	0.843	0.540	-0.303	no
CK-40d	RHSIM_Rhsim11G0106300	1.114	1.323	0.210	no
CK-40d	RHSIM_Rhsim10G0126200	1.888	1.753	-0.135	high
CK-40d	RHSIM_Rhsim11G0074300	-0.703	0.950	1.653	no
CK-40d	RHSIM_Rhsim11G0155300	2.405	0.990	-1.415	high
CK-40d	RHSIM_Rhsim10G0149500	0.921	-0.741	-1.661	no
CK-40d	RHSIM_Rhsim12G0128400	1.202	0.274	-0.928	high
CK-40d	RHSIM_Rhsim12G0213700	1.629	1.103	-0.526	high
CK-40d	RHSIM_Rhsim10G0141300	1.611	3.246	1.635	high
CK-40d	RHSIM_Rhsim10G0115500	-0.322	-1.579	-1.257	no
CK-40d	RHSIM_Rhsim11G0130000	-0.828	-0.823	0.006	no
CK-40d	RHSIM_Rhsim11G0064900	0.537	-0.692	-1.229	no
CK-40d	RHSIM_Rhsim10G0202500	1.706	1.627	-0.080	high
CK-40d	RHSIM_Rhsim10G0183200	-0.776	-0.600	0.176	no
CK-40d	RHSIM_Rhsim11G0192900	0.088	0.781	0.693	no
CK-40d	RHSIM_Rhsim11G0015100	-1.132	-1.143	-0.011	no
CK-40d	RHSIM_Rhsim10G0078300	-0.909	-0.832	0.076	no
CK-40d	RHSIM_Rhsim10G0014400	-1.575	-1.368	0.207	low
CK-40d	RHSIM_Rhsim10G0101200	1.293	1.992	0.699	high
CK-40d	RHSIM_Rhsim10G0211500	-0.401	-1.471	-1.069	no
CK-40d	RHSIM_Rhsim11G0159000	-0.847	1.562	2.409	no
CK-40d	RHSIM_Rhsim11G0099900	-0.425	-0.782	-0.356	no
CK-40d	RHSIM_Rhsim11G0075200	-1.026	-0.359	0.668	no
CK-40d	RHSIM_Rhsim11G0094000	-0.346	1.777	2.123	no
CK-40d	RHSIM_Rhsim10G0156300	1.045	0.539	-0.506	no
CK-40d	RHSIM_Rhsim11G0003600	-0.211	0.094	0.305	no
CK-40d	RHSIM_Rhsim11G0077900	0.950	0.213	-0.737	no
CK-40d	RHSIM_Rhsim10G0167100	3.348	1.726	-1.622	high
CK-40d	RHSIM_Rhsim11G0075500	-2.972	-1.079	1.892	low
CK-40d	RHSIM_Rhsim10G0071100	2.562	0.827	-1.734	high

Group	Gene	PTR (z-score difference)	z(log2 LFQ)	z(log2 FPKM)	extreme-PTR
CK-40d	RHSIM_Rhsim10G0006900	-0.991	-1.643	-0.652	no
CK-40d	RHSIM_Rhsim11G0133300	4.049	1.715	-2.334	high
CK-40d	RHSIM_Rhsim11G0078100	-0.289	1.016	1.305	no
CK-40d	RHSIM_Rhsim11G0121800	0.168	1.733	1.565	no
CK-40d	RHSIM_Rhsim11G0094400	-0.304	0.925	1.229	no
CK-40d	RHSIM_Rhsim11G0093100	-0.166	-0.478	-0.312	no
CK-40d	RHSIM_Rhsim11G0159200	-0.492	-1.101	-0.609	no
CK-40d	RHSIM_Rhsim10G0198000	-0.510	-1.133	-0.622	no
CK-40d	RHSIM_Rhsim10G0211400	1.859	2.350	0.491	high
CK-40d	RHSIM_Rhsim10G0188500	-0.633	0.329	0.962	no
CK-40d	RHSIM_Rhsim11G0138200	-2.384	-1.044	1.340	low
CK-40d	RHSIM_Rhsim10G0172500	0.655	-0.280	-0.935	no
CK-40d	RHSIM_Rhsim11G0117700	-0.382	0.469	0.851	no
CK-40d	RHSIM_Rhsim11G0196300	1.255	1.339	0.084	high
CK-40d	RHSIM_Rhsim11G0019600	0.176	0.128	-0.048	no
CK-40d	RHSIM_Rhsim10G0006300	1.074	0.154	-0.920	no
CK-40d	RHSIM_Rhsim10G0112600	0.327	-0.184	-0.511	no
CK-40d	RHSIM_Rhsim11G0136500	-0.648	-0.529	0.119	no
CK-40d	RHSIM_Rhsim10G0107900	1.767	0.673	-1.094	high
CK-40d	RHSIM_Rhsim11G0090400	-2.270	0.353	2.623	low
CK-40d	RHSIM_Rhsim11G0190500	-0.496	0.140	0.636	no
CK-40d	RHSIM_Rhsim10G0008800	-0.932	-1.047	-0.115	no
CK-40d	RHSIM_Rhsim11G0136000	0.007	0.643	0.636	no
CK-40d	RHSIM_Rhsim10G0014200	0.293	-0.684	-0.977	no
CK-40d	RHSIM_Rhsim11G0141900	-0.554	-0.422	0.131	no
CK-40d	RHSIM_Rhsim11G0131600	-0.621	-0.873	-0.252	no
CK-40d	RHSIM_Rhsim10G0141900	-1.427	-0.257	1.170	low
CK-40d	RHSIM_Rhsim11G0072400	-1.240	-0.818	0.421	low
CK-40d	RHSIM_Rhsim10G0022700	-0.783	0.433	1.216	no
CK-40d	RHSIM_Rhsim11G0008000	-0.319	-0.756	-0.437	no
CK-40d	RHSIM_Rhsim11G0077600	0.503	-0.651	-1.154	no
CK-40d	RHSIM_Rhsim11G0128300	1.155	0.231	-0.924	high

Group	Gene	PTR (z-score difference)	z(log2 LFQ)	z(log2 FPKM)	extreme-PTR
CK-40d	RHSIM_Rhsim10G0068700	-1.748	-2.294	-0.546	low
CK-40d	RHSIM_Rhsim11G0172600	-1.008	-0.801	0.208	no
CK-40d	RHSIM_Rhsim11G0178400	0.628	-0.390	-1.018	no
CK-40d	RHSIM_Rhsim10G0171700	1.380	2.609	1.230	high
CK-40d	RHSIM_Rhsim11G0165400	-0.081	0.042	0.123	no
CK-40d	RHSIM_Rhsim10G0006400	0.470	0.782	0.313	no
CK-40d	RHSIM_Rhsim11G0180400	0.686	-0.255	-0.940	no
CK-40d	RHSIM_Rhsim11G0132900	-1.589	-1.546	0.043	low
CK-40d	RHSIM_Rhsim10G0072100	-1.248	0.423	1.671	low
CK-40d	RHSIM_Rhsim10G0134600	1.635	-0.448	-2.084	high
CK-40d	RHSIM_Rhsim10G0061400	-0.737	-0.183	0.554	no
CK-40d	RHSIM_Rhsim10G0039800	-0.135	1.200	1.335	no
CK-40d	RHSIM_Rhsim10G0151800	0.398	-0.910	-1.307	no
CK-40d	RHSIM_Rhsim10G0127000	-1.542	-1.741	-0.198	low
CK-40d	RHSIM_Rhsim11G0184700	-0.921	-0.105	0.816	no
CK-40d	RHSIM_Rhsim11G0111500	0.120	-0.510	-0.630	no
CK-40d	RHSIM_Rhsim10G0021200	2.930	3.490	0.560	high
CK-40d	RHSIM_Rhsim10G0013900	-0.700	0.408	1.108	no
CK-40d	RHSIM_Rhsim10G0015700	1.315	-0.482	-1.796	high
CK-40d	RHSIM_Rhsim10G0149000	0.287	0.273	-0.014	no
CK-40d	RHSIM_Rhsim10G0179500	-1.087	0.228	1.316	no
CK-40d	RHSIM_Rhsim10G0178400	0.854	3.204	2.350	no
CK-40d	RHSIM_Rhsim10G0177300	0.190	1.385	1.195	no
CK-40d	RHSIM_Rhsim10G0127200	0.947	0.183	-0.764	no
CK-40d	RHSIM_Rhsim10G0019900	0.730	0.931	0.201	no
CK-40d	RHSIM_Rhsim10G0060000	-0.847	-1.733	-0.885	no
CK-40d	RHSIM_Rhsim10G0118700	0.220	-0.557	-0.777	no
CK-40d	RHSIM_Rhsim10G0079500	1.098	0.766	-0.332	no
CK-40d	RHSIM_Rhsim10G0174300	2.722	1.667	-1.056	high
CK-40d	RHSIM_Rhsim10G0046600	1.598	-0.092	-1.690	high
CK-40d	RHSIM_Rhsim10G0104000	-1.227	-0.929	0.298	no
CK-40d	RHSIM_Rhsim10G0078600	0.092	0.035	-0.057	no

Group	Gene	PTR (z-score difference)	z(log2 LFQ)	z(log2 FPKM)	extreme-PTR
CK-40d	RHSIM_Rhsim11G0028300	1.011	1.153	0.143	no
CK-40d	RHSIM_Rhsim10G0174600	-3.523	-1.353	2.170	low
CK-40d	RHSIM_Rhsim11G0045600	-1.268	0.279	1.546	low
CK-40d	RHSIM_Rhsim10G0185600	2.296	0.044	-2.252	high
CK-40d	RHSIM_Rhsim11G0197700	-0.386	-0.982	-0.596	no
CK-40d	RHSIM_Rhsim10G0082000	-0.910	-0.419	0.491	no
CK-40d	RHSIM_Rhsim10G0048900	0.669	1.192	0.523	no
CK-40d	RHSIM_Rhsim10G0022600	1.118	1.133	0.015	no
CK-40d	RHSIM_Rhsim11G0169400	0.141	-0.006	-0.147	no
CK-40d	RHSIM_Rhsim10G0199600	1.064	1.048	-0.016	no
CK-40d	RHSIM_Rhsim10G0175100	-0.339	1.315	1.654	no
CK-40d	RHSIM_Rhsim11G0173300	0.931	0.403	-0.528	no
CK-40d	RHSIM_Rhsim11G0014400	-0.025	0.484	0.509	no
CK-40d	RHSIM_Rhsim11G0071200	-0.477	-0.784	-0.307	no
CK-40d	RHSIM_Rhsim11G0069400	0.752	2.030	1.278	no
CK-40d	RHSIM_Rhsim10G0163500	1.202	1.730	0.528	high
CK-40d	RHSIM_Rhsim11G0031700	-0.414	-0.448	-0.034	no
CK-40d	RHSIM_Rhsim11G0133700	-1.594	-2.120	-0.526	low
CK-40d	RHSIM_Rhsim10G0152000	2.751	1.194	-1.558	high
CK-40d	RHSIM_Rhsim10G0185900	-1.384	-1.087	0.297	low
CK-40d	RHSIM_Rhsim10G0062200	2.417	1.573	-0.844	high
CK-40d	RHSIM_Rhsim11G0184000	-0.209	0.414	0.624	no
CK-40d	RHSIM_Rhsim10G0100700	1.509	-0.899	-2.408	high
CK-40d	RHSIM_Rhsim11G0158600	-0.251	-1.261	-1.010	no
CK-40d	RHSIM_Rhsim11G0077800	2.042	0.282	-1.759	high
CK-40d	RHSIM_Rhsim10G0154300	2.517	0.707	-1.810	high
CK-40d	RHSIM_Rhsim10G0012800	0.391	-0.321	-0.712	no
CK-40d	RHSIM_Rhsim10G0207900	0.443	0.767	0.325	no
CK-40d	RHSIM_Rhsim09G0201000	-0.642	0.315	0.958	no
CK-40d	RHSIM_Rhsim10G0035200	1.440	0.781	-0.659	high
CK-40d	RHSIM_Rhsim10G0043800	1.543	2.222	0.680	high
CK-40d	RHSIM_Rhsim10G0128200	0.350	-0.809	-1.159	no

Group	Gene	PTR (z-score difference)	z(log2 LFQ)	z(log2 FPKM)	extreme-PTR
CK-40d	RHSIM_Rhsim11G0021700	0.320	1.102	0.782	no
CK-40d	RHSIM_Rhsim10G0209100	-0.154	0.108	0.262	no
CK-40d	RHSIM_Rhsim10G0006600	0.933	0.727	-0.206	no
CK-40d	RHSIM_Rhsim11G0113500	1.792	0.788	-1.004	high
CK-40d	RHSIM_Rhsim10G0064900	-0.026	-0.519	-0.493	no
CK-40d	RHSIM_Rhsim10G0202400	-0.950	0.589	1.540	no
CK-40d	RHSIM_Rhsim10G0213600	-1.755	-0.985	0.770	low
CK-40d	RHSIM_Rhsim10G0072600	-0.543	-1.244	-0.701	no
CK-40d	RHSIM_Rhsim09G0032200	0.986	0.006	-0.980	no
CK-40d	RHSIM_Rhsim10G0008600	2.068	0.426	-1.642	high
CK-40d	RHSIM_Rhsim10G0131300	-0.938	-1.964	-1.026	no
CK-40d	RHSIM_Rhsim10G0102800	-0.685	-1.423	-0.738	no
CK-40d	RHSIM_Rhsim11G0098900	-0.232	-0.221	0.011	no
CK-40d	RHSIM_Rhsim11G0100500	-1.239	-0.086	1.153	low
CK-40d	RHSIM_Rhsim10G0138000	1.788	1.488	-0.300	high
CK-40d	RHSIM_Rhsim09G0097600	-0.906	-0.180	0.726	no
CK-40d	RHSIM_Rhsim10G0102700	0.092	-0.511	-0.602	no
CK-40d	RHSIM_Rhsim11G0088600	0.321	-0.154	-0.475	no
CK-40d	RHSIM_Rhsim10G0034800	1.263	-0.092	-1.355	high
CK-40d	RHSIM_Rhsim11G0178900	-0.631	-0.644	-0.013	no
CK-40d	RHSIM_Rhsim10G0030200	-0.353	0.237	0.590	no
CK-40d	RHSIM_Rhsim09G0168000	0.962	-0.809	-1.771	no
CK-40d	RHSIM_Rhsim10G0058700	3.648	1.557	-2.091	high
CK-40d	RHSIM_Rhsim10G0036600	-0.150	0.027	0.178	no
CK-40d	RHSIM_Rhsim11G0030300	-0.584	-0.328	0.256	no
CK-40d	RHSIM_Rhsim10G0109900	-0.866	-0.402	0.464	no
CK-40d	RHSIM_Rhsim11G0134800	-0.765	0.205	0.970	no
CK-40d	RHSIM_Rhsim11G0104600	1.193	-0.744	-1.937	high
CK-40d	RHSIM_Rhsim10G0077000	-1.095	-1.086	0.009	no
CK-40d	RHSIM_Rhsim10G0019400	-1.392	-1.269	0.123	low
CK-40d	RHSIM_Rhsim09G0144400	2.527	0.564	-1.964	high
CK-40d	RHSIM_Rhsim10G0084700	-0.528	-0.550	-0.022	no

Group	Gene	PTR (z-score difference)	z(log2 LFQ)	z(log2 FPKM)	extreme-PTR
CK-40d	RHSIM_Rhsim09G0121900	-0.053	-0.286	-0.233	no
CK-40d	RHSIM_Rhsim09G0169200	-1.475	-0.263	1.213	low
CK-40d	RHSIM_Rhsim11G0184800	0.697	1.386	0.689	no
CK-40d	RHSIM_Rhsim10G0202900	1.514	1.281	-0.233	high
CK-40d	RHSIM_Rhsim10G0138300	-0.575	-0.988	-0.413	no
CK-40d	RHSIM_Rhsim11G0058900	0.176	0.237	0.061	no
CK-40d	RHSIM_Rhsim10G0028000	1.207	1.737	0.529	high
CK-40d	RHSIM_Rhsim09G0123000	-0.415	0.159	0.574	no
CK-40d	RHSIM_Rhsim10G0210700	0.366	-0.641	-1.006	no
CK-40d	RHSIM_Rhsim10G0023700	-0.657	-1.186	-0.530	no
CK-40d	RHSIM_Rhsim10G0156100	-0.773	-0.183	0.590	no
CK-40d	RHSIM_Rhsim09G0140200	-0.361	-0.451	-0.089	no
CK-40d	RHSIM_Rhsim11G0089800	1.847	-0.047	-1.894	high
CK-40d	RHSIM_Rhsim09G0181100	-0.731	-0.862	-0.130	no
CK-40d	RHSIM_Rhsim09G0069500	0.203	0.837	0.634	no
CK-40d	RHSIM_Rhsim10G0209800	0.506	1.562	1.056	no
CK-40d	RHSIM_Rhsim10G0009000	0.571	-0.011	-0.582	no
CK-40d	RHSIM_Rhsim09G0208200	0.142	0.532	0.390	no
CK-40d	RHSIM_Rhsim10G0029200	-0.117	0.233	0.350	no
CK-40d	RHSIM_Rhsim09G0208400	-0.089	1.358	1.447	no
CK-40d	RHSIM_Rhsim10G0180300	-0.146	-0.549	-0.403	no
CK-40d	RHSIM_Rhsim10G0212700	0.969	0.325	-0.644	no
CK-40d	RHSIM_Rhsim10G0185200	-0.921	-1.416	-0.495	no
CK-40d	RHSIM_Rhsim10G0056100	-0.133	0.322	0.455	no
CK-40d	RHSIM_Rhsim10G0176600	2.800	1.852	-0.948	high
CK-40d	RHSIM_Rhsim09G0110200	0.388	-1.111	-1.499	no
CK-40d	RHSIM_Rhsim09G0157700	-0.213	1.322	1.535	no
CK-40d	RHSIM_Rhsim09G0003700	1.775	1.514	-0.261	high
CK-40d	RHSIM_Rhsim10G0189100	-0.809	-0.697	0.111	no
CK-40d	RHSIM_Rhsim10G0011300	-1.304	-1.156	0.148	low
CK-40d	RHSIM_Rhsim09G0208000	-0.320	-0.480	-0.159	no
CK-40d	RHSIM_Rhsim09G0060100	-1.714	-1.307	0.407	low

Group	Gene	PTR (z-score difference)	z(log2 LFQ)	z(log2 FPKM)	extreme-PTR
CK-40d	RHSIM_Rhsim09G0141900	-0.653	0.737	1.389	no
CK-40d	RHSIM_Rhsim11G0099400	-1.170	0.790	1.961	no
CK-40d	RHSIM_Rhsim10G0034700	-1.091	-0.021	1.069	no
CK-40d	RHSIM_Rhsim10G0029400	-1.088	-1.354	-0.266	no
CK-40d	RHSIM_Rhsim10G0005000	-0.318	-0.682	-0.365	no
CK-40d	RHSIM_Rhsim09G0111300	-0.015	-0.464	-0.449	no
CK-40d	RHSIM_Rhsim09G0207500	-0.165	0.124	0.289	no
CK-40d	RHSIM_Rhsim10G0030100	0.534	-1.013	-1.547	no
CK-40d	RHSIM_Rhsim10G0064100	-2.350	-0.810	1.541	low
CK-40d	RHSIM_Rhsim10G0026800	0.399	-0.825	-1.223	no
CK-40d	RHSIM_Rhsim10G0113200	-1.315	0.430	1.745	low
CK-40d	RHSIM_Rhsim09G0018700	0.382	-0.559	-0.941	no
CK-40d	RHSIM_Rhsim10G0067800	0.849	0.363	-0.486	no
CK-40d	RHSIM_Rhsim09G0141100	-0.979	0.257	1.236	no
CK-40d	RHSIM_Rhsim10G0018300	-0.368	0.325	0.693	no
CK-40d	RHSIM_Rhsim09G0004300	-1.704	-0.882	0.822	low
CK-40d	RHSIM_Rhsim10G0015600	0.931	0.568	-0.363	no
CK-40d	RHSIM_Rhsim11G0141800	-1.883	-1.371	0.512	low
CK-40d	RHSIM_Rhsim09G0167500	-1.435	-2.037	-0.602	low
CK-40d	RHSIM_Rhsim09G0187300	0.669	0.181	-0.487	no
CK-40d	RHSIM_Rhsim10G0126900	-1.859	-0.956	0.903	low
CK-40d	RHSIM_Rhsim09G0208500	-0.136	-0.195	-0.059	no
CK-40d	RHSIM_Rhsim11G0137800	1.296	-0.149	-1.445	high
CK-40d	RHSIM_Rhsim09G0005100	0.176	0.637	0.461	no
CK-40d	RHSIM_Rhsim10G0011500	-0.737	-1.171	-0.434	no
CK-40d	RHSIM_Rhsim11G0040600	1.629	0.162	-1.466	high
CK-40d	RHSIM_Rhsim10G0077900	-0.600	-0.650	-0.049	no
CK-40d	RHSIM_Rhsim10G0102900	-0.194	-0.915	-0.721	no
CK-40d	RHSIM_Rhsim09G0168600	-0.423	-0.634	-0.210	no
CK-40d	RHSIM_Rhsim11G0145300	2.430	1.154	-1.276	high
CK-40d	RHSIM_Rhsim10G0065800	-0.663	-0.911	-0.248	no
CK-40d	RHSIM_Rhsim09G0155300	-0.894	-1.118	-0.223	no

Group	Gene	PTR (z-score difference)	z(log2 LFQ)	z(log2 FPKM)	extreme-PTR
CK-40d	RHSIM_Rhsim10G0212500	0.464	-1.537	-2.001	no
CK-40d	RHSIM_Rhsim09G0151400	0.918	0.747	-0.172	no
CK-40d	RHSIM_Rhsim09G0003600	0.839	1.803	0.963	no
CK-40d	RHSIM_Rhsim10G0130500	0.078	-0.781	-0.859	no
CK-40d	RHSIM_Rhsim10G0162800	0.652	0.271	-0.381	no
CK-40d	RHSIM_Rhsim10G0134900	1.888	1.502	-0.386	high
CK-40d	RHSIM_Rhsim09G0043400	-0.252	0.632	0.885	no
CK-40d	RHSIM_Rhsim10G0057900	1.683	1.597	-0.086	high
CK-40d	RHSIM_Rhsim10G0162400	0.604	-0.803	-1.407	no
CK-40d	RHSIM_Rhsim09G0165400	0.105	-0.454	-0.559	no
CK-40d	RHSIM_Rhsim09G0152300	-1.926	-0.803	1.122	low
CK-40d	RHSIM_Rhsim10G0144800	0.940	-0.703	-1.643	no
CK-40d	RHSIM_Rhsim10G0081500	0.078	-0.085	-0.162	no
CK-40d	RHSIM_Rhsim09G0199500	-0.024	1.761	1.785	no
CK-40d	RHSIM_Rhsim10G0043600	-0.281	0.555	0.837	no
CK-40d	RHSIM_Rhsim09G0146200	-0.551	1.443	1.995	no
CK-40d	RHSIM_Rhsim10G0162700	-0.296	-0.695	-0.398	no
CK-40d	RHSIM_Rhsim09G0146500	-1.629	-0.734	0.896	low
CK-40d	RHSIM_Rhsim09G0137600	0.254	0.620	0.365	no
CK-40d	RHSIM_Rhsim10G0165500	-0.080	-0.721	-0.641	no
CK-40d	RHSIM_Rhsim10G0133000	0.430	1.274	0.844	no
CK-40d	RHSIM_Rhsim10G0034600	-1.025	-0.694	0.331	no
CK-40d	RHSIM_Rhsim09G0128000	1.508	0.543	-0.966	high
CK-40d	RHSIM_Rhsim09G0111600	1.033	-0.169	-1.203	no
CK-40d	RHSIM_Rhsim09G0025300	-0.538	1.148	1.686	no
CK-40d	RHSIM_Rhsim11G0091000	-1.022	-0.765	0.257	no
CK-40d	RHSIM_Rhsim09G0136800	0.414	0.188	-0.226	no
CK-40d	RHSIM_Rhsim09G0010200	-0.271	0.573	0.844	no
CK-40d	RHSIM_Rhsim11G0153300	0.087	0.134	0.048	no
CK-40d	RHSIM_Rhsim09G0037000	-1.852	-1.595	0.257	low
CK-40d	RHSIM_Rhsim10G0019000	-1.485	0.699	2.184	low
CK-40d	RHSIM_Rhsim09G0113200	0.429	0.430	0.001	no

Group	Gene	PTR (z-score difference)	z(log2 LFQ)	z(log2 FPKM)	extreme-PTR
CK-40d	RHSIM_Rhsim10G0186700	-0.022	-0.685	-0.663	no
CK-40d	RHSIM_Rhsim10G0167000	-0.653	-1.252	-0.599	no
CK-40d	RHSIM_Rhsim10G0141700	-0.968	-1.099	-0.131	no
CK-40d	RHSIM_Rhsim09G0165600	1.189	0.310	-0.879	high
CK-40d	RHSIM_Rhsim10G0177900	0.441	0.268	-0.174	no
CK-40d	RHSIM_Rhsim11G0183100	-0.794	-1.272	-0.477	no
CK-40d	RHSIM_Rhsim09G0209400	-0.707	0.934	1.641	no
CK-40d	RHSIM_Rhsim10G0000500	0.259	-0.249	-0.508	no
CK-40d	RHSIM_Rhsim09G0067900	1.366	0.958	-0.408	high
CK-40d	RHSIM_Rhsim09G0001400	0.317	1.491	1.174	no
CK-40d	RHSIM_Rhsim09G0140900	-2.307	-0.916	1.391	low
CK-40d	RHSIM_Rhsim09G0197000	-1.608	-1.203	0.405	low
CK-40d	RHSIM_Rhsim09G0129700	-0.200	0.098	0.298	no
CK-40d	RHSIM_Rhsim10G0003500	-0.064	-0.789	-0.726	no
CK-40d	RHSIM_Rhsim10G0040000	2.821	1.417	-1.403	high
CK-40d	RHSIM_Rhsim11G0056000	1.529	-0.400	-1.929	high
CK-40d	RHSIM_Rhsim09G0213100	-0.433	-0.463	-0.030	no
CK-40d	RHSIM_Rhsim10G0145200	-0.135	-0.511	-0.376	no
CK-40d	RHSIM_Rhsim10G0001300	-0.039	-0.204	-0.165	no
CK-40d	RHSIM_Rhsim09G0066100	0.348	-0.763	-1.112	no
CK-40d	RHSIM_Rhsim10G0049300	0.154	-0.468	-0.622	no
CK-40d	RHSIM_Rhsim11G0134900	-0.289	-0.477	-0.188	no
CK-40d	RHSIM_Rhsim09G0165800	0.260	0.160	-0.100	no
CK-40d	RHSIM_Rhsim09G0027800	-0.147	-0.092	0.055	no
CK-40d	RHSIM_Rhsim11G0197300	-0.320	1.148	1.468	no
CK-40d	RHSIM_Rhsim10G0113000	-0.636	-0.413	0.223	no
CK-40d	RHSIM_Rhsim09G0072200	-0.328	-1.581	-1.253	no
CK-40d	RHSIM_Rhsim10G0053700	-0.018	-1.297	-1.279	no
CK-40d	RHSIM_Rhsim09G0107700	1.100	1.792	0.692	no
CK-40d	RHSIM_Rhsim09G0112700	-2.934	-0.539	2.395	low
CK-40d	RHSIM_Rhsim09G0103400	-1.663	-0.110	1.552	low
CK-40d	RHSIM_Rhsim11G0111700	0.036	-0.363	-0.399	no

Group	Gene	PTR (z-score difference)	z(log2 LFQ)	z(log2 FPKM)	extreme-PTR
CK-40d	RHSIM_Rhsim09G0058600	-0.942	-1.322	-0.380	no
CK-40d	RHSIM_Rhsim09G0199000	1.171	0.417	-0.754	high
CK-40d	RHSIM_Rhsim09G0157900	1.922	1.640	-0.282	high
CK-40d	RHSIM_Rhsim11G0060100	0.531	-0.100	-0.631	no
CK-40d	RHSIM_Rhsim10G0105000	-1.285	-1.683	-0.397	low
CK-40d	RHSIM_Rhsim11G0049400	-1.058	0.498	1.556	no
CK-40d	RHSIM_Rhsim09G0061400	0.106	0.990	0.884	no
CK-40d	RHSIM_Rhsim09G0012100	-1.284	-0.352	0.932	low
CK-40d	RHSIM_Rhsim09G0207600	0.262	-0.149	-0.411	no
CK-40d	RHSIM_Rhsim09G0037600	-0.770	0.288	1.058	no
CK-40d	RHSIM_Rhsim09G0195600	0.915	-0.120	-1.035	no
CK-40d	RHSIM_Rhsim10G0020400	0.680	0.317	-0.364	no
CK-40d	RHSIM_Rhsim10G0111500	-2.209	0.842	3.051	low
CK-40d	RHSIM_Rhsim10G0121000	0.431	1.021	0.590	no
CK-40d	RHSIM_Rhsim09G0166500	-0.316	-1.017	-0.702	no
CK-40d	RHSIM_Rhsim10G0006500	-1.296	-1.090	0.206	low
CK-40d	RHSIM_Rhsim09G0199700	-0.669	-0.862	-0.193	no
CK-40d	RHSIM_Rhsim09G0214500	0.924	0.301	-0.623	no
CK-40d	RHSIM_Rhsim10G0044100	0.136	1.118	0.983	no
CK-40d	RHSIM_Rhsim09G0044400	1.443	0.042	-1.401	high
CK-40d	RHSIM_Rhsim09G0179000	-1.378	-0.994	0.384	low
CK-40d	RHSIM_Rhsim09G0213800	-0.623	0.800	1.423	no
CK-40d	RHSIM_Rhsim09G0071800	0.665	-0.308	-0.973	no
CK-40d	RHSIM_Rhsim09G0158600	-1.204	-0.839	0.365	no
CK-40d	RHSIM_Rhsim09G0051700	-0.521	0.459	0.979	no
CK-40d	RHSIM_Rhsim09G0126300	-1.205	-0.634	0.571	no
CK-40d	RHSIM_Rhsim09G0140700	-1.672	1.381	3.053	low
CK-40d	RHSIM_Rhsim09G0064600	2.211	-0.115	-2.326	high
CK-40d	RHSIM_Rhsim10G0183900	-0.270	1.414	1.684	no
CK-40d	RHSIM_Rhsim10G0072700	-0.737	-0.662	0.075	no
CK-40d	RHSIM_Rhsim10G0020900	0.510	-0.318	-0.828	no
CK-40d	RHSIM_Rhsim09G0132000	0.247	1.497	1.250	no

Group	Gene	PTR (z-score difference)	z(log2 LFQ)	z(log2 FPKM)	extreme-PTR
CK-40d	RHSIM_Rhsim10G0176900	-0.817	0.251	1.068	no
CK-40d	RHSIM_Rhsim09G0208100	-0.351	-0.214	0.138	no
CK-40d	RHSIM_Rhsim09G0073200	-0.427	-1.187	-0.760	no
CK-40d	RHSIM_Rhsim09G0140100	1.227	-0.718	-1.945	high
CK-40d	RHSIM_Rhsim10G0180400	-0.943	-0.936	0.007	no
CK-40d	RHSIM_Rhsim10G0080000	2.641	0.961	-1.680	high
CK-40d	RHSIM_Rhsim09G0020400	0.160	-0.304	-0.464	no
CK-40d	RHSIM_Rhsim09G0105400	-0.581	-0.112	0.469	no
CK-40d	RHSIM_Rhsim09G0005500	1.079	0.707	-0.372	no
CK-40d	RHSIM_Rhsim10G0128500	0.652	0.005	-0.647	no
CK-40d	RHSIM_Rhsim10G0192700	-0.985	0.060	1.045	no
CK-40d	RHSIM_Rhsim10G0002200	-1.044	-0.721	0.324	no
CK-40d	RHSIM_Rhsim09G0188800	0.127	0.807	0.679	no
CK-40d	RHSIM_Rhsim10G0002800	-0.155	-0.707	-0.552	no
CK-40d	RHSIM_Rhsim09G0092400	-1.321	-0.847	0.473	low
CK-40d	RHSIM_Rhsim09G0154300	-0.009	-0.566	-0.557	no
CK-40d	RHSIM_Rhsim09G0184000	0.666	0.737	0.071	no
CK-40d	RHSIM_Rhsim09G0195100	0.039	0.306	0.267	no
CK-40d	RHSIM_Rhsim09G0087000	0.609	-0.232	-0.841	no
CK-40d	RHSIM_Rhsim09G0172200	-1.067	-0.559	0.508	no
CK-40d	RHSIM_Rhsim09G0206900	2.607	2.348	-0.259	high
CK-40d	RHSIM_Rhsim10G0041000	-1.124	-0.427	0.697	no
CK-40d	RHSIM_Rhsim09G0037900	-0.821	-1.292	-0.470	no
CK-40d	RHSIM_Rhsim09G0107200	0.458	-0.579	-1.037	no
CK-40d	RHSIM_Rhsim09G0179700	0.647	-1.135	-1.781	no
CK-40d	RHSIM_Rhsim10G0083200	0.435	0.766	0.332	no
CK-40d	RHSIM_Rhsim09G0212900	-1.120	-1.772	-0.652	no
CK-40d	RHSIM_Rhsim09G0096600	-1.715	-0.163	1.552	low
CK-40d	RHSIM_Rhsim09G0109400	0.491	0.477	-0.014	no
CK-40d	RHSIM_Rhsim09G0058800	1.394	0.201	-1.193	high
CK-40d	RHSIM_Rhsim10G0185700	-0.723	-0.060	0.663	no
CK-40d	RHSIM_Rhsim09G0190900	-0.307	-0.826	-0.518	no

Group	Gene	PTR (z-score difference)	z(log2 LFQ)	z(log2 FPKM)	extreme-PTR
CK-40d	RHSIM_Rhsim09G0214300	0.423	-0.314	-0.737	no
CK-40d	RHSIM_Rhsim09G0110100	0.332	0.938	0.606	no
CK-40d	RHSIM_Rhsim09G0043700	1.531	0.276	-1.255	high
CK-40d	RHSIM_Rhsim09G0176800	-0.058	-1.081	-1.023	no
CK-40d	RHSIM_Rhsim07G0015900	-1.036	0.811	1.847	no
CK-40d	RHSIM_Rhsim09G0028200	-1.746	-0.802	0.943	low
CK-40d	RHSIM_Rhsim10G0076800	1.071	1.444	0.373	no
CK-40d	RHSIM_Rhsim10G0021400	2.225	0.909	-1.315	high
CK-40d	RHSIM_Rhsim09G0143800	0.973	-0.279	-1.252	no
CK-40d	RHSIM_Rhsim09G0066000	-0.198	1.240	1.437	no
CK-40d	RHSIM_Rhsim09G0087100	1.946	0.526	-1.420	high
CK-40d	RHSIM_Rhsim09G0058700	-0.653	-1.033	-0.380	no
CK-40d	RHSIM_Rhsim10G0127700	-0.811	-0.379	0.431	no
CK-40d	RHSIM_Rhsim09G0204400	-1.477	-0.864	0.613	low
CK-40d	RHSIM_Rhsim09G0066200	-1.430	-0.666	0.764	low
CK-40d	RHSIM_Rhsim10G0034300	0.656	0.494	-0.163	no
CK-40d	RHSIM_Rhsim09G0211500	-0.799	-0.887	-0.088	no
CK-40d	RHSIM_Rhsim09G0084400	0.353	-0.501	-0.854	no
CK-40d	RHSIM_Rhsim09G0154600	-0.112	-0.197	-0.085	no
CK-40d	RHSIM_Rhsim10G0214200	-0.716	-0.299	0.417	no
CK-40d	RHSIM_Rhsim09G0130100	-0.276	1.604	1.880	no
CK-40d	RHSIM_Rhsim09G0011700	-1.656	0.401	2.057	low
CK-40d	RHSIM_Rhsim09G0103000	-0.873	-0.767	0.106	no
CK-40d	RHSIM_Rhsim10G0186800	-3.337	-1.004	2.333	low
CK-40d	RHSIM_Rhsim09G0146700	-1.619	-1.840	-0.221	low
CK-40d	RHSIM_Rhsim09G0063000	1.093	-0.792	-1.885	no
CK-40d	RHSIM_Rhsim10G0038900	-1.850	-0.591	1.258	low
CK-40d	RHSIM_Rhsim09G0214600	-0.029	-0.301	-0.272	no
CK-40d	RHSIM_Rhsim10G0084800	-1.003	0.410	1.413	no
CK-40d	RHSIM_Rhsim09G0122300	0.751	-0.264	-1.015	no
CK-40d	RHSIM_Rhsim10G0167600	-3.410	-1.121	2.289	low
CK-40d	RHSIM_Rhsim09G0151300	-1.937	0.161	2.097	low

Group	Gene	PTR (z-score difference)	z(log2 LFQ)	z(log2 FPKM)	extreme-PTR
CK-40d	RHSIM_Rhsim09G0012200	1.821	1.074	-0.747	high
CK-40d	RHSIM_Rhsim09G0061700	0.752	-0.438	-1.190	no
CK-40d	RHSIM_Rhsim09G0166000	-0.814	0.505	1.319	no
CK-40d	RHSIM_Rhsim09G0043800	0.919	0.422	-0.497	no
CK-40d	RHSIM_Rhsim10G0185500	0.248	0.707	0.459	no
CK-40d	RHSIM_Rhsim09G0150500	0.558	-0.434	-0.991	no
CK-40d	RHSIM_Rhsim09G0152400	-0.837	0.379	1.217	no
CK-40d	RHSIM_Rhsim10G0163100	-0.284	0.041	0.325	no
CK-40d	RHSIM_Rhsim09G0035000	-0.539	-0.717	-0.178	no
CK-40d	RHSIM_Rhsim09G0117700	-0.347	1.534	1.881	no
CK-40d	RHSIM_Rhsim09G0196800	0.828	-0.619	-1.447	no
CK-40d	RHSIM_Rhsim09G0033400	1.495	0.864	-0.631	high
CK-40d	RHSIM_Rhsim09G0087600	0.402	-1.416	-1.818	no
CK-40d	RHSIM_Rhsim09G0084100	-0.968	0.504	1.472	no
CK-40d	RHSIM_Rhsim09G0090200	0.477	-0.630	-1.106	no
CK-40d	RHSIM_Rhsim09G0094700	0.253	0.318	0.065	no
CK-40d	RHSIM_Rhsim09G0143200	-0.771	1.958	2.728	no
CK-40d	RHSIM_Rhsim10G0125000	-0.410	-0.638	-0.228	no
CK-40d	RHSIM_Rhsim09G0004100	0.472	1.653	1.180	no
CK-40d	RHSIM_Rhsim09G0140500	0.752	-0.624	-1.377	no
CK-40d	RHSIM_Rhsim10G0004100	-0.259	-0.592	-0.333	no
CK-40d	RHSIM_Rhsim09G0196200	-1.794	-0.578	1.216	low
CK-40d	RHSIM_Rhsim09G0127700	-0.994	-0.877	0.117	no
CK-40d	RHSIM_Rhsim09G0065100	-0.912	-0.999	-0.087	no
CK-40d	RHSIM_Rhsim09G0202400	0.357	1.055	0.698	no
CK-40d	RHSIM_Rhsim09G0148900	0.598	0.361	-0.237	no
CK-40d	RHSIM_Rhsim09G0210400	1.213	1.013	-0.199	high
CK-40d	RHSIM_Rhsim09G0011500	-0.906	-0.909	-0.004	no
CK-40d	RHSIM_Rhsim09G0184800	0.813	-0.411	-1.224	no
CK-40d	RHSIM_Rhsim10G0174500	-2.397	0.803	3.200	low
CK-40d	RHSIM_Rhsim09G0105600	-0.207	-0.060	0.147	no
CK-40d	RHSIM_Rhsim10G0004300	-0.689	0.246	0.935	no

Group	Gene	PTR (z-score difference)	z(log2 LFQ)	z(log2 FPKM)	extreme-PTR
CK-40d	RHSIM_Rhsim09G0194000	-2.296	-2.124	0.172	low
CK-40d	RHSIM_Rhsim09G0132100	-0.219	-0.561	-0.342	no
CK-40d	RHSIM_Rhsim09G0078200	-0.469	-0.830	-0.362	no
CK-40d	RHSIM_Rhsim09G0206100	-0.572	-0.421	0.151	no
CK-40d	RHSIM_Rhsim10G0166700	-0.469	0.002	0.471	no
CK-40d	RHSIM_Rhsim09G0049700	1.531	-0.363	-1.894	high
CK-40d	RHSIM_Rhsim08G0034300	-0.817	-0.255	0.562	no
CK-40d	RHSIM_Rhsim09G0030100	-0.308	-0.673	-0.364	no
CK-40d	RHSIM_Rhsim10G0020100	1.059	2.535	1.476	no
CK-40d	RHSIM_Rhsim09G0044600	1.589	0.667	-0.921	high
CK-40d	RHSIM_Rhsim10G0092800	-0.381	-0.156	0.225	no
CK-40d	RHSIM_Rhsim10G0064800	0.862	0.383	-0.480	no
CK-40d	RHSIM_Rhsim09G0034100	-0.060	-0.425	-0.365	no
CK-40d	RHSIM_Rhsim08G0095100	-1.280	-1.785	-0.504	low
CK-40d	RHSIM_Rhsim09G0173200	-1.128	0.512	1.640	no
CK-40d	RHSIM_Rhsim10G0019700	-3.252	-0.958	2.294	low
CK-40d	RHSIM_Rhsim09G0004500	-0.139	-1.155	-1.015	no
CK-40d	RHSIM_Rhsim08G0223900	2.187	2.265	0.078	high
CK-40d	RHSIM_Rhsim08G0096200	-0.332	0.463	0.795	no
CK-40d	RHSIM_Rhsim09G0140000	0.398	0.336	-0.062	no
CK-40d	RHSIM_Rhsim10G0138100	-2.113	-2.112	0.000	low
CK-40d	RHSIM_Rhsim08G0116000	-0.545	-0.423	0.122	no
CK-40d	RHSIM_Rhsim09G0208800	2.231	1.414	-0.817	high
CK-40d	RHSIM_Rhsim08G0081800	0.281	-0.664	-0.945	no
CK-40d	RHSIM_Rhsim08G0039900	0.386	1.898	1.513	no
CK-40d	RHSIM_Rhsim09G0091600	-0.268	-1.325	-1.057	no
CK-40d	RHSIM_Rhsim08G0000800	-1.249	-0.510	0.739	low
CK-40d	RHSIM_Rhsim09G0034800	-1.064	-0.052	1.012	no
CK-40d	RHSIM_Rhsim10G0008400	-0.110	-1.113	-1.002	no
CK-40d	RHSIM_Rhsim09G0154900	-0.158	-0.348	-0.190	no
CK-40d	RHSIM_Rhsim09G0206000	-0.804	-0.702	0.102	no
CK-40d	RHSIM_Rhsim09G0013300	0.690	0.242	-0.448	no

Group	Gene	PTR (z-score difference)	z(log2 LFQ)	z(log2 FPKM)	extreme-PTR
CK-40d	RHSIM_Rhsim10G0104900	0.092	-0.096	-0.189	no
CK-40d	RHSIM_Rhsim08G0244500	1.892	1.554	-0.338	high
CK-40d	RHSIM_Rhsim08G0231100	-0.214	0.115	0.329	no
CK-40d	RHSIM_Rhsim10G0119200	-0.790	-0.431	0.359	no
CK-40d	RHSIM_Rhsim08G0237400	-0.158	-0.582	-0.424	no
CK-40d	RHSIM_Rhsim08G0102500	0.269	-0.876	-1.145	no
CK-40d	RHSIM_Rhsim09G0097800	-0.775	-0.841	-0.066	no
CK-40d	RHSIM_Rhsim08G0128100	0.129	-0.786	-0.915	no
CK-40d	RHSIM_Rhsim10G0016800	1.190	-0.011	-1.201	high
CK-40d	RHSIM_Rhsim09G0049500	-0.340	-1.240	-0.901	no
CK-40d	RHSIM_Rhsim09G0128800	0.892	-0.320	-1.212	no
CK-40d	RHSIM_Rhsim09G0005700	2.027	0.381	-1.645	high
CK-40d	RHSIM_Rhsim09G0081900	-1.044	0.636	1.681	no
CK-40d	RHSIM_Rhsim08G0128800	1.041	0.128	-0.913	no
CK-40d	RHSIM_Rhsim09G0137900	-0.363	-0.781	-0.418	no
CK-40d	RHSIM_Rhsim09G0187600	0.189	-0.692	-0.881	no
CK-40d	RHSIM_Rhsim09G0214000	1.345	0.870	-0.475	high
CK-40d	RHSIM_Rhsim08G0039400	1.010	1.910	0.900	no
CK-40d	RHSIM_Rhsim09G0005000	2.181	-0.038	-2.219	high
CK-40d	RHSIM_Rhsim09G0145300	1.255	0.112	-1.142	high
CK-40d	RHSIM_Rhsim09G0193000	-0.021	0.334	0.356	no
CK-40d	RHSIM_Rhsim09G0203700	-0.635	-0.865	-0.230	no
CK-40d	RHSIM_Rhsim08G0027400	-1.329	-0.701	0.628	low
CK-40d	RHSIM_Rhsim09G0204300	-1.822	-1.392	0.430	low
CK-40d	RHSIM_Rhsim10G0151200	-0.211	-0.542	-0.332	no
CK-40d	RHSIM_Rhsim10G0049800	-0.220	-0.574	-0.354	no
CK-40d	RHSIM_Rhsim09G0129900	-1.657	0.313	1.971	low
CK-40d	RHSIM_Rhsim08G0146000	-0.123	0.102	0.225	no
CK-40d	RHSIM_Rhsim09G0122200	-0.751	-1.093	-0.341	no
CK-40d	RHSIM_Rhsim09G0142800	-1.796	-0.905	0.891	low
CK-40d	RHSIM_Rhsim09G0141500	0.566	0.641	0.075	no
CK-40d	RHSIM_Rhsim08G0115800	-0.962	-0.097	0.864	no

Group	Gene	PTR (z-score difference)	z(log2 LFQ)	z(log2 FPKM)	extreme-PTR
CK-40d	RHSIM_Rhsim09G0177600	-2.606	-1.287	1.320	low
CK-40d	RHSIM_Rhsim09G0153300	-1.170	-0.703	0.467	no
CK-40d	RHSIM_Rhsim08G0250700	-0.481	-0.572	-0.091	no
CK-40d	RHSIM_Rhsim09G0201300	-1.075	-0.801	0.274	no
CK-40d	RHSIM_Rhsim09G0142500	-0.909	0.389	1.299	no
CK-40d	RHSIM_Rhsim09G0074600	-0.094	0.153	0.246	no
CK-40d	RHSIM_Rhsim09G0089600	-0.582	-0.519	0.064	no
CK-40d	RHSIM_Rhsim09G0030700	-0.032	-0.369	-0.338	no
CK-40d	RHSIM_Rhsim08G0043800	-0.157	0.080	0.237	no
CK-40d	RHSIM_Rhsim09G0169300	-0.581	-0.622	-0.041	no
CK-40d	RHSIM_Rhsim09G0127200	0.670	0.187	-0.483	no
CK-40d	RHSIM_Rhsim08G0125200	-0.853	-0.941	-0.088	no
CK-40d	RHSIM_Rhsim08G0191700	-0.043	0.122	0.165	no
CK-40d	RHSIM_Rhsim09G0069100	1.698	2.959	1.262	high
CK-40d	RHSIM_Rhsim08G0067800	1.081	0.567	-0.514	no
CK-40d	RHSIM_Rhsim09G0201100	-0.689	-0.446	0.242	no
CK-40d	RHSIM_Rhsim09G0029200	-2.067	-1.084	0.983	low
CK-40d	RHSIM_Rhsim09G0061500	1.301	0.210	-1.091	high
CK-40d	RHSIM_Rhsim08G0227500	-1.859	-1.353	0.507	low
CK-40d	RHSIM_Rhsim08G0041900	-2.424	-1.914	0.510	low
CK-40d	RHSIM_Rhsim09G0171700	1.306	0.625	-0.681	high
CK-40d	RHSIM_Rhsim08G0183700	0.608	-0.154	-0.761	no
CK-40d	RHSIM_Rhsim09G0028800	0.022	-1.022	-1.044	no
CK-40d	RHSIM_Rhsim09G0179300	-1.249	-1.504	-0.255	low
CK-40d	RHSIM_Rhsim08G0006400	1.989	0.488	-1.501	high
CK-40d	RHSIM_Rhsim09G0039400	0.592	-0.344	-0.935	no
CK-40d	RHSIM_Rhsim09G0145500	-0.692	-0.213	0.479	no
CK-40d	RHSIM_Rhsim10G0145000	-0.098	0.916	1.015	no
CK-40d	RHSIM_Rhsim09G0113100	1.490	-0.070	-1.560	high
CK-40d	RHSIM_Rhsim08G0121400	1.152	0.191	-0.961	high
CK-40d	RHSIM_Rhsim08G0202500	1.211	0.888	-0.324	high
CK-40d	RHSIM_Rhsim08G0084400	-0.284	-0.258	0.026	no

Group	Gene	PTR (z-score difference)	z(log2 LFQ)	z(log2 FPKM)	extreme-PTR
CK-40d	RHSIM_Rhsim08G0167000	0.639	-0.158	-0.796	no
CK-40d	RHSIM_Rhsim09G0023000	0.888	-0.353	-1.241	no
CK-40d	RHSIM_Rhsim08G0224700	0.549	2.335	1.786	no
CK-40d	RHSIM_Rhsim09G0151500	0.779	-0.106	-0.884	no
CK-40d	RHSIM_Rhsim09G0125900	-1.062	-0.668	0.394	no
CK-40d	RHSIM_Rhsim09G0197100	-0.909	0.576	1.485	no
CK-40d	RHSIM_Rhsim09G0117300	0.415	0.244	-0.171	no
CK-40d	RHSIM_Rhsim08G0028900	-1.277	-1.450	-0.173	low
CK-40d	RHSIM_Rhsim10G0212000	-0.061	0.974	1.035	no
CK-40d	RHSIM_Rhsim09G0181400	-0.815	-0.025	0.790	no
CK-40d	RHSIM_Rhsim09G0088400	-0.073	-0.543	-0.470	no
CK-40d	RHSIM_Rhsim08G0214200	-0.494	0.093	0.587	no
CK-40d	RHSIM_Rhsim08G0063900	0.075	0.220	0.145	no
CK-40d	RHSIM_Rhsim09G0082300	-0.972	-1.092	-0.120	no
CK-40d	RHSIM_Rhsim09G0172400	-0.518	0.538	1.056	no
CK-40d	RHSIM_Rhsim08G0055000	-0.035	1.585	1.621	no
CK-40d	RHSIM_Rhsim09G0040800	0.411	-0.825	-1.236	no
CK-40d	RHSIM_Rhsim08G0044300	-0.039	-0.440	-0.401	no
CK-40d	RHSIM_Rhsim08G0006500	1.505	0.550	-0.954	high
CK-40d	RHSIM_Rhsim09G0171600	-1.464	-0.456	1.008	low
CK-40d	RHSIM_Rhsim09G0168800	1.322	0.366	-0.956	high
CK-40d	RHSIM_Rhsim08G0043500	-0.939	-0.602	0.337	no
CK-40d	RHSIM_Rhsim09G0183900	-0.832	-0.424	0.408	no
CK-40d	RHSIM_Rhsim09G0152700	3.603	3.466	-0.137	high
CK-40d	RHSIM_Rhsim08G0182800	0.703	-1.269	-1.972	no
CK-40d	RHSIM_Rhsim09G0046600	0.402	0.168	-0.234	no
CK-40d	RHSIM_Rhsim08G0082200	-1.395	-1.590	-0.195	low
CK-40d	RHSIM_Rhsim08G0014900	0.512	0.428	-0.083	no
CK-40d	RHSIM_Rhsim08G0081400	0.340	-1.095	-1.435	no
CK-40d	RHSIM_Rhsim08G0159000	1.282	0.666	-0.617	high
CK-40d	RHSIM_Rhsim08G0171800	-0.849	-0.247	0.602	no
CK-40d	RHSIM_Rhsim09G0092900	0.297	-1.305	-1.602	no

Group	Gene	PTR (z-score difference)	z(log2 LFQ)	z(log2 FPKM)	extreme-PTR
CK-40d	RHSIM_Rhsim08G0033300	-1.115	-0.464	0.651	no
CK-40d	RHSIM_Rhsim10G0045100	1.104	0.682	-0.421	no
CK-40d	RHSIM_Rhsim09G0077000	-0.529	-0.713	-0.184	no
CK-40d	RHSIM_Rhsim09G0198800	1.752	0.168	-1.584	high
CK-40d	RHSIM_Rhsim08G0196800	0.838	1.554	0.716	no
CK-40d	RHSIM_Rhsim08G0037100	-1.031	-0.589	0.442	no
CK-40d	RHSIM_Rhsim08G0042900	-0.849	0.004	0.853	no
CK-40d	RHSIM_Rhsim08G0181700	0.206	1.589	1.384	no
CK-40d	RHSIM_Rhsim10G0122600	0.681	-0.673	-1.353	no
CK-40d	RHSIM_Rhsim08G0114500	-1.328	-0.259	1.069	low
CK-40d	RHSIM_Rhsim08G0240000	0.480	-0.113	-0.593	no
CK-40d	RHSIM_Rhsim09G0133500	0.469	0.249	-0.220	no
CK-40d	RHSIM_Rhsim08G0100200	-0.098	0.395	0.493	no
CK-40d	RHSIM_Rhsim08G0248700	-0.930	-0.841	0.089	no
CK-40d	RHSIM_Rhsim09G0184200	-2.567	-0.992	1.575	low
CK-40d	RHSIM_Rhsim08G0026000	0.163	-0.128	-0.291	no
CK-40d	RHSIM_Rhsim09G0141000	-0.453	-1.133	-0.681	no
CK-40d	RHSIM_Rhsim08G0088900	-0.277	-1.083	-0.806	no
CK-40d	RHSIM_Rhsim08G0229600	-0.224	-0.192	0.032	no
CK-40d	RHSIM_Rhsim08G0241300	-0.054	-0.140	-0.086	no
CK-40d	RHSIM_Rhsim09G0164300	-0.383	-1.537	-1.154	no
CK-40d	RHSIM_Rhsim09G0145400	-0.138	0.027	0.165	no
CK-40d	RHSIM_Rhsim08G0209300	1.429	0.970	-0.459	high
CK-40d	RHSIM_Rhsim08G0122300	-2.802	-0.603	2.199	low
CK-40d	RHSIM_Rhsim09G0189300	-0.289	0.545	0.834	no
CK-40d	RHSIM_Rhsim08G0054900	2.453	1.755	-0.698	high
CK-40d	RHSIM_Rhsim09G0117800	1.632	0.419	-1.213	high
CK-40d	RHSIM_Rhsim08G0230600	-1.730	0.199	1.929	low
CK-40d	RHSIM_Rhsim09G0000900	-0.185	0.067	0.251	no
CK-40d	RHSIM_Rhsim08G0150200	0.149	-0.487	-0.636	no
CK-40d	RHSIM_Rhsim08G0067900	0.973	0.982	0.008	no
CK-40d	RHSIM_Rhsim10G0129000	1.760	0.162	-1.598	high

Group	Gene	PTR (z-score difference)	z(log2 LFQ)	z(log2 FPKM)	extreme-PTR
CK-40d	RHSIM_Rhsim08G0121500	-0.363	-0.946	-0.583	no
CK-40d	RHSIM_Rhsim08G0040400	0.019	0.769	0.750	no
CK-40d	RHSIM_Rhsim09G0010600	-0.544	0.149	0.692	no
CK-40d	RHSIM_Rhsim10G0125300	-0.456	-0.634	-0.178	no
CK-40d	RHSIM_Rhsim08G0223400	-0.502	1.419	1.921	no
CK-40d	RHSIM_Rhsim08G0103000	1.595	0.846	-0.749	high
CK-40d	RHSIM_Rhsim10G0139000	0.304	-0.299	-0.603	no
CK-40d	RHSIM_Rhsim09G0008600	-0.728	0.832	1.560	no
CK-40d	RHSIM_Rhsim08G0183000	-0.539	0.680	1.219	no
CK-40d	RHSIM_Rhsim08G0215400	1.310	0.426	-0.884	high
CK-40d	RHSIM_Rhsim09G0210600	0.728	1.544	0.815	no
CK-40d	RHSIM_Rhsim08G0020800	-0.319	-0.430	-0.111	no
CK-40d	RHSIM_Rhsim09G0111700	0.643	0.453	-0.191	no
CK-40d	RHSIM_Rhsim08G0188900	-0.638	-0.051	0.587	no
CK-40d	RHSIM_Rhsim10G0078400	-0.067	-0.593	-0.526	no
CK-40d	RHSIM_Rhsim08G0053600	0.494	0.088	-0.406	no
CK-40d	RHSIM_Rhsim09G0099400	-0.638	-0.454	0.184	no
CK-40d	RHSIM_Rhsim09G0106300	0.650	0.587	-0.063	no
CK-40d	RHSIM_Rhsim08G0008500	-0.333	-0.416	-0.082	no
CK-40d	RHSIM_Rhsim10G0211900	0.942	0.563	-0.380	no
CK-40d	RHSIM_Rhsim08G0033600	-0.797	-0.811	-0.014	no
CK-40d	RHSIM_Rhsim09G0142300	0.038	-1.049	-1.086	no
CK-40d	RHSIM_Rhsim09G0008200	-0.862	-0.661	0.201	no
CK-40d	RHSIM_Rhsim08G0083400	-1.122	-0.366	0.756	no
CK-40d	RHSIM_Rhsim09G0138400	0.854	1.044	0.191	no
CK-40d	RHSIM_Rhsim08G0156200	-0.684	-0.513	0.171	no
CK-40d	RHSIM_Rhsim08G0156700	3.213	1.427	-1.786	high
CK-40d	RHSIM_Rhsim08G0153100	0.076	0.991	0.915	no
CK-40d	RHSIM_Rhsim09G0203300	-1.149	-0.569	0.580	no
CK-40d	RHSIM_Rhsim09G0207900	2.567	1.266	-1.301	high
CK-40d	RHSIM_Rhsim08G0208100	0.028	-0.795	-0.823	no
CK-40d	RHSIM_Rhsim08G0034400	-0.576	-0.831	-0.255	no

Group	Gene	PTR (z-score difference)	z(log2 LFQ)	z(log2 FPKM)	extreme-PTR
CK-40d	RHSIM_Rhsim08G0205700	2.902	1.046	-1.856	high
CK-40d	RHSIM_Rhsim09G0155500	0.201	-1.088	-1.289	no
CK-40d	RHSIM_Rhsim08G0145900	-0.197	0.399	0.595	no
CK-40d	RHSIM_Rhsim08G0041500	-1.127	0.830	1.956	no
CK-40d	RHSIM_Rhsim08G0082100	-0.327	-0.247	0.080	no
CK-40d	RHSIM_Rhsim10G0049400	0.175	0.584	0.409	no
CK-40d	RHSIM_Rhsim08G0174000	0.560	0.701	0.142	no
CK-40d	RHSIM_Rhsim10G0104100	-1.721	-1.391	0.331	low
CK-40d	RHSIM_Rhsim09G0056700	0.753	-0.052	-0.806	no
CK-40d	RHSIM_Rhsim09G0069000	0.664	1.582	0.918	no
CK-40d	RHSIM_Rhsim09G0200500	-0.278	-0.068	0.210	no
CK-40d	RHSIM_Rhsim09G0115400	0.216	0.426	0.210	no
CK-40d	RHSIM_Rhsim09G0085500	-0.678	-0.820	-0.142	no
CK-40d	RHSIM_Rhsim08G0069100	0.933	0.087	-0.846	no
CK-40d	RHSIM_Rhsim08G0202300	-3.023	-1.647	1.376	low
CK-40d	RHSIM_Rhsim09G0024000	-1.890	-0.411	1.479	low
CK-40d	RHSIM_Rhsim09G0022900	-0.732	-0.371	0.361	no
CK-40d	RHSIM_Rhsim08G0038100	-0.289	0.268	0.556	no
CK-40d	RHSIM_Rhsim08G0045400	1.529	1.684	0.156	high
CK-40d	RHSIM_Rhsim08G0180500	1.532	3.892	2.360	high
CK-40d	RHSIM_Rhsim09G0128100	0.500	0.806	0.306	no
CK-40d	RHSIM_Rhsim08G0109700	2.353	4.111	1.758	high
CK-40d	RHSIM_Rhsim08G0047900	2.840	1.104	-1.736	high
CK-40d	RHSIM_Rhsim08G0013000	-1.041	-1.311	-0.270	no
CK-40d	RHSIM_Rhsim08G0088400	-0.185	1.264	1.449	no
CK-40d	RHSIM_Rhsim09G0002200	1.267	1.185	-0.082	high
CK-40d	RHSIM_Rhsim08G0030700	0.945	0.493	-0.452	no
CK-40d	RHSIM_Rhsim08G0020500	0.714	-1.281	-1.995	no
CK-40d	RHSIM_Rhsim08G0045800	-1.884	0.670	2.553	low
CK-40d	RHSIM_Rhsim08G0245700	-0.354	1.064	1.418	no
CK-40d	RHSIM_Rhsim09G0114500	-1.492	0.127	1.619	low
CK-40d	RHSIM_Rhsim09G0109300	-3.118	-1.514	1.603	low

Group	Gene	PTR (z-score difference)	z(log2 LFQ)	z(log2 FPKM)	extreme-PTR
CK-40d	RHSIM_Rhsim08G0043600	0.197	0.902	0.705	no
CK-40d	RHSIM_Rhsim09G0090700	1.235	-0.819	-2.053	high
CK-40d	RHSIM_Rhsim09G0069700	-1.686	-1.886	-0.200	low
CK-40d	RHSIM_Rhsim09G0201200	-0.126	0.931	1.057	no
CK-40d	RHSIM_Rhsim08G0195500	-1.630	-0.191	1.438	low
CK-40d	RHSIM_Rhsim08G0008300	-0.311	-0.908	-0.597	no
CK-40d	RHSIM_Rhsim09G0149700	0.821	0.633	-0.188	no
CK-40d	RHSIM_Rhsim09G0067700	-0.246	0.267	0.513	no
CK-40d	RHSIM_Rhsim09G0144000	2.694	1.080	-1.615	high
CK-40d	RHSIM_Rhsim08G0044100	-2.111	-0.950	1.161	low
CK-40d	RHSIM_Rhsim09G0146300	0.382	1.235	0.853	no
CK-40d	RHSIM_Rhsim08G0188400	1.042	1.613	0.571	no
CK-40d	RHSIM_Rhsim08G0096400	-0.970	-1.462	-0.492	no
CK-40d	RHSIM_Rhsim09G0197500	1.569	1.294	-0.274	high
CK-40d	RHSIM_Rhsim09G0168700	-0.125	1.054	1.178	no
CK-40d	RHSIM_Rhsim09G0025000	-0.515	-1.273	-0.759	no
CK-40d	RHSIM_Rhsim08G0037000	-0.325	0.481	0.807	no
CK-40d	RHSIM_Rhsim08G0072300	1.253	0.378	-0.875	high
CK-40d	RHSIM_Rhsim09G0000500	0.022	0.023	0.000	no
CK-40d	RHSIM_Rhsim08G0176100	0.294	-0.954	-1.248	no
CK-40d	RHSIM_Rhsim08G0250400	-0.455	-2.049	-1.594	no
CK-40d	RHSIM_Rhsim09G0052100	1.393	0.469	-0.923	high
CK-40d	RHSIM_Rhsim08G0185000	-1.693	-1.494	0.199	low
CK-40d	RHSIM_Rhsim09G0074800	-0.164	-0.539	-0.376	no
CK-40d	RHSIM_Rhsim09G0032100	0.851	0.177	-0.675	no
CK-40d	RHSIM_Rhsim09G0143900	2.185	1.303	-0.882	high
CK-40d	RHSIM_Rhsim08G0103200	0.950	0.266	-0.683	no
CK-40d	RHSIM_Rhsim08G0114200	-0.366	-0.334	0.032	no
CK-40d	RHSIM_Rhsim08G0012200	-0.497	-0.379	0.117	no
CK-40d	RHSIM_Rhsim08G0086400	0.027	-1.130	-1.157	no
CK-40d	RHSIM_Rhsim09G0100400	-0.244	-0.660	-0.417	no
CK-40d	RHSIM_Rhsim09G0114100	2.500	0.588	-1.912	high

Group	Gene	PTR (z-score difference)	z(log2 LFQ)	z(log2 FPKM)	extreme-PTR
CK-40d	RHSIM_Rhsim09G0109100	-0.130	0.868	0.998	no
CK-40d	RHSIM_Rhsim09G0000200	-0.058	0.288	0.347	no
CK-40d	RHSIM_Rhsim09G0206600	0.277	-0.495	-0.772	no
CK-40d	RHSIM_Rhsim08G0141600	1.443	1.897	0.453	high
CK-40d	RHSIM_Rhsim09G0035900	-2.471	-0.755	1.717	low
CK-40d	RHSIM_Rhsim08G0221100	0.839	-0.518	-1.357	no
CK-40d	RHSIM_Rhsim08G0185200	1.512	-0.095	-1.607	high
CK-40d	RHSIM_Rhsim08G0231900	-0.970	-1.038	-0.068	no
CK-40d	RHSIM_Rhsim08G0002400	0.765	0.501	-0.264	no
CK-40d	RHSIM_Rhsim08G0239200	3.138	1.162	-1.976	high
CK-40d	RHSIM_Rhsim08G0069300	-0.552	0.666	1.218	no
CK-40d	RHSIM_Rhsim08G0131700	-0.594	-1.590	-0.995	no
CK-40d	RHSIM_Rhsim09G0008700	-0.404	-0.240	0.164	no
CK-40d	RHSIM_Rhsim08G0241200	1.974	1.092	-0.882	high
CK-40d	RHSIM_Rhsim08G0167100	0.930	-0.387	-1.317	no
CK-40d	RHSIM_Rhsim08G0186100	-1.486	-1.371	0.116	low
CK-40d	RHSIM_Rhsim09G0000700	-0.601	-0.694	-0.093	no
CK-40d	RHSIM_Rhsim08G0214400	-0.428	0.863	1.292	no
CK-40d	RHSIM_Rhsim08G0230200	-0.710	-0.850	-0.140	no
CK-40d	RHSIM_Rhsim08G0046300	-0.732	-0.847	-0.115	no
CK-40d	RHSIM_Rhsim08G0241900	-1.174	-0.522	0.652	no
CK-40d	RHSIM_Rhsim08G0242600	0.779	0.146	-0.633	no
CK-40d	RHSIM_Rhsim08G0244800	0.977	0.485	-0.492	no
CK-40d	RHSIM_Rhsim08G0046700	0.818	1.262	0.444	no
CK-40d	RHSIM_Rhsim08G0026100	0.019	-0.140	-0.159	no
CK-40d	RHSIM_Rhsim09G0212200	-0.481	-0.553	-0.072	no
CK-40d	RHSIM_Rhsim09G0099700	-0.172	-1.667	-1.495	no
CK-40d	RHSIM_Rhsim09G0171300	-1.253	-1.731	-0.478	low
CK-40d	RHSIM_Rhsim08G0084800	0.933	0.000	-0.933	no
CK-40d	RHSIM_Rhsim09G0119600	-0.502	-0.896	-0.394	no
CK-40d	RHSIM_Rhsim09G0213700	1.723	0.632	-1.091	high
CK-40d	RHSIM_Rhsim09G0188300	-0.968	-0.919	0.049	no

Group	Gene	PTR (z-score difference)	z(log2 LFQ)	z(log2 FPKM)	extreme-PTR
CK-40d	RHSIM_Rhsim08G0127800	-0.192	0.997	1.189	no
CK-40d	RHSIM_Rhsim08G0030100	1.077	0.286	-0.791	no
CK-40d	RHSIM_Rhsim08G0102200	-0.005	-0.087	-0.082	no
CK-40d	RHSIM_Rhsim08G0214000	0.317	1.628	1.311	no
CK-40d	RHSIM_Rhsim08G0030200	-0.415	-0.145	0.270	no
CK-40d	RHSIM_Rhsim09G0185000	0.339	0.011	-0.328	no
CK-40d	RHSIM_Rhsim09G0171800	0.933	0.556	-0.377	no
CK-40d	RHSIM_Rhsim08G0211400	1.911	0.759	-1.152	high
CK-40d	RHSIM_Rhsim09G0064200	-0.407	0.175	0.581	no
CK-40d	RHSIM_Rhsim08G0094100	-0.062	0.444	0.505	no
CK-40d	RHSIM_Rhsim09G0105100	1.116	-0.088	-1.204	no
CK-40d	RHSIM_Rhsim08G0029600	4.401	3.005	-1.396	high
CK-40d	RHSIM_Rhsim09G0111100	0.138	0.164	0.026	no
CK-40d	RHSIM_Rhsim09G0179800	0.954	-1.099	-2.053	no
CK-40d	RHSIM_Rhsim08G0019700	0.990	1.225	0.235	no
CK-40d	RHSIM_Rhsim09G0093800	0.279	-0.659	-0.938	no
CK-40d	RHSIM_Rhsim09G0209300	-1.154	-0.103	1.051	no
CK-40d	RHSIM_Rhsim08G0094000	0.941	1.881	0.940	no
CK-40d	RHSIM_Rhsim08G0018700	0.457	-1.514	-1.971	no
CK-40d	RHSIM_Rhsim08G0173300	0.681	-0.415	-1.096	no
CK-40d	RHSIM_Rhsim09G0164800	0.144	-0.433	-0.577	no
CK-40d	RHSIM_Rhsim08G0195100	-0.966	0.438	1.404	no
CK-40d	RHSIM_Rhsim08G0199200	-2.255	-0.636	1.619	low
CK-40d	RHSIM_Rhsim08G0034000	-1.499	-1.030	0.470	low
CK-40d	RHSIM_Rhsim09G0164900	0.086	-0.697	-0.783	no
CK-40d	RHSIM_Rhsim09G0000100	-2.298	-1.156	1.142	low
CK-40d	RHSIM_Rhsim08G0045600	-0.149	-0.814	-0.665	no
CK-40d	RHSIM_Rhsim08G0025900	0.431	-0.218	-0.649	no
CK-40d	RHSIM_Rhsim08G0174600	-0.434	-0.409	0.024	no
CK-40d	RHSIM_Rhsim09G0142700	0.199	0.094	-0.105	no
CK-40d	RHSIM_Rhsim08G0237100	-1.191	-1.557	-0.366	no
CK-40d	RHSIM_Rhsim08G0032100	-0.480	0.359	0.839	no

Group	Gene	PTR (z-score difference)	z(log2 LFQ)	z(log2 FPKM)	extreme-PTR
CK-40d	RHSIM_Rhsim08G0148900	0.304	0.459	0.155	no
CK-40d	RHSIM_Rhsim08G0246900	-1.060	-0.324	0.736	no
CK-40d	RHSIM_Rhsim07G0073300	-0.175	0.558	0.733	no
CK-40d	RHSIM_Rhsim08G0045900	0.760	1.586	0.826	no
CK-40d	RHSIM_Rhsim08G0075800	-0.469	0.445	0.914	no
CK-40d	RHSIM_Rhsim08G0109800	-0.114	-1.062	-0.948	no
CK-40d	RHSIM_Rhsim07G0082300	1.148	0.805	-0.344	high
CK-40d	RHSIM_Rhsim08G0074500	0.008	-0.262	-0.270	no
CK-40d	RHSIM_Rhsim09G0152100	0.349	0.553	0.205	no
CK-40d	RHSIM_Rhsim08G0203900	1.747	1.173	-0.574	high
CK-40d	RHSIM_Rhsim08G0028600	-0.636	-0.046	0.590	no
CK-40d	RHSIM_Rhsim07G0065200	4.085	2.294	-1.791	high
CK-40d	RHSIM_Rhsim08G0232500	0.668	1.059	0.391	no
CK-40d	RHSIM_Rhsim08G0233200	-0.847	-0.353	0.494	no
CK-40d	RHSIM_Rhsim07G0063000	0.211	0.054	-0.157	no
CK-40d	RHSIM_Rhsim08G0062700	1.230	1.263	0.033	high
CK-40d	RHSIM_Rhsim08G0195300	-0.088	0.794	0.881	no
CK-40d	RHSIM_Rhsim08G0144000	-0.659	-1.168	-0.509	no
CK-40d	RHSIM_Rhsim08G0190900	-0.293	0.505	0.798	no
CK-40d	RHSIM_Rhsim07G0109500	1.992	-0.050	-2.042	high
CK-40d	RHSIM_Rhsim09G0111900	1.332	0.972	-0.360	high
CK-40d	RHSIM_Rhsim07G0077100	0.511	-0.435	-0.946	no
CK-40d	RHSIM_Rhsim09G0003200	0.026	0.204	0.178	no
CK-40d	RHSIM_Rhsim08G0051500	-0.679	-0.660	0.019	no
CK-40d	RHSIM_Rhsim08G0206900	-0.518	-0.669	-0.151	no
CK-40d	RHSIM_Rhsim09G0013500	-0.534	-1.391	-0.857	no
CK-40d	RHSIM_Rhsim09G0004800	1.776	1.158	-0.618	high
CK-40d	RHSIM_Rhsim08G0152700	-0.716	0.175	0.891	no
CK-40d	RHSIM_Rhsim08G0150000	0.973	0.874	-0.099	no
CK-40d	RHSIM_Rhsim09G0192600	-0.304	0.940	1.244	no
CK-40d	RHSIM_Rhsim09G0073000	0.156	0.116	-0.040	no
CK-40d	RHSIM_Rhsim07G0227100	-0.112	-1.822	-1.710	no

Group	Gene	PTR (z-score difference)	z(log2 LFQ)	z(log2 FPKM)	extreme-PTR
CK-40d	RHSIM_Rhsim08G0232800	0.389	0.089	-0.300	no
CK-40d	RHSIM_Rhsim09G0069200	1.549	0.941	-0.607	high
CK-40d	RHSIM_Rhsim08G0250100	0.697	1.498	0.801	no
CK-40d	RHSIM_Rhsim08G0072500	-0.412	-0.033	0.379	no
CK-40d	RHSIM_Rhsim07G0142300	2.884	2.550	-0.334	high
CK-40d	RHSIM_Rhsim08G0125600	-0.090	0.504	0.594	no
CK-40d	RHSIM_Rhsim07G0079800	-0.404	-0.233	0.171	no
CK-40d	RHSIM_Rhsim08G0106000	0.823	-0.742	-1.566	no
CK-40d	RHSIM_Rhsim08G0225600	-0.510	-1.175	-0.666	no
CK-40d	RHSIM_Rhsim07G0095900	-0.799	0.701	1.500	no
CK-40d	RHSIM_Rhsim08G0008000	0.000	-0.618	-0.618	no
CK-40d	RHSIM_Rhsim08G0005000	-0.348	0.086	0.433	no
CK-40d	RHSIM_Rhsim09G0102400	0.262	-0.306	-0.568	no
CK-40d	RHSIM_Rhsim08G0109400	0.151	-0.490	-0.641	no
CK-40d	RHSIM_Rhsim09G0106700	1.277	-0.030	-1.307	high
CK-40d	RHSIM_Rhsim08G0161300	-0.985	-1.296	-0.311	no
CK-40d	RHSIM_Rhsim08G0003400	-0.024	-0.615	-0.590	no
CK-40d	RHSIM_Rhsim09G0138500	-0.639	0.005	0.645	no
CK-40d	RHSIM_Rhsim09G0066700	1.792	0.740	-1.051	high
CK-40d	RHSIM_Rhsim08G0054100	2.070	1.212	-0.858	high
CK-40d	RHSIM_Rhsim07G0212400	-0.155	-0.455	-0.300	no
CK-40d	RHSIM_Rhsim07G0257800	-1.336	0.119	1.454	low
CK-40d	RHSIM_Rhsim07G0038700	1.439	0.997	-0.442	high
CK-40d	RHSIM_Rhsim07G0074200	-0.196	-0.691	-0.495	no
CK-40d	RHSIM_Rhsim08G0079800	-0.764	-0.841	-0.076	no
CK-40d	RHSIM_Rhsim08G0205400	0.112	-0.633	-0.745	no
CK-40d	RHSIM_Rhsim08G0185100	0.620	1.186	0.566	no
CK-40d	RHSIM_Rhsim08G0160300	-0.330	0.346	0.677	no
CK-40d	RHSIM_Rhsim08G0240600	-0.049	-0.885	-0.836	no
CK-40d	RHSIM_Rhsim07G0106500	0.145	-0.336	-0.481	no
CK-40d	RHSIM_Rhsim09G0203200	-1.056	-1.013	0.043	no
CK-40d	RHSIM_Rhsim08G0202600	-0.304	-0.385	-0.080	no

Group	Gene	PTR (z-score difference)	z(log2 LFQ)	z(log2 FPKM)	extreme-PTR
CK-40d	RHSIM_Rhsim07G0011800	0.315	1.059	0.743	no
CK-40d	RHSIM_Rhsim08G0003500	0.705	0.995	0.291	no
CK-40d	RHSIM_Rhsim07G0040800	1.144	-0.977	-2.121	high
CK-40d	RHSIM_Rhsim08G0136400	0.416	0.139	-0.278	no
CK-40d	RHSIM_Rhsim09G0074000	-0.119	-0.904	-0.785	no
CK-40d	RHSIM_Rhsim08G0044500	-0.454	-1.120	-0.666	no
CK-40d	RHSIM_Rhsim09G0190400	-0.780	-0.376	0.404	no
CK-40d	RHSIM_Rhsim08G0207400	-0.042	1.892	1.934	no
CK-40d	RHSIM_Rhsim07G0010300	-0.364	-0.196	0.167	no
CK-40d	RHSIM_Rhsim07G0204900	1.739	-0.107	-1.846	high
CK-40d	RHSIM_Rhsim08G0190700	0.785	0.372	-0.414	no
CK-40d	RHSIM_Rhsim07G0096600	-0.845	-0.638	0.207	no
CK-40d	RHSIM_Rhsim08G0118900	0.742	-1.004	-1.746	no
CK-40d	RHSIM_Rhsim08G0241100	-1.320	-0.808	0.512	low
CK-40d	RHSIM_Rhsim07G0225100	-2.089	-0.609	1.479	low
CK-40d	RHSIM_Rhsim08G0086000	-0.920	-0.419	0.501	no
CK-40d	RHSIM_Rhsim07G0087000	-0.945	-0.565	0.380	no
CK-40d	RHSIM_Rhsim08G0076000	-1.048	-0.722	0.325	no
CK-40d	RHSIM_Rhsim07G0203200	-0.835	-0.259	0.576	no
CK-40d	RHSIM_Rhsim08G0222800	2.422	0.614	-1.808	high
CK-40d	RHSIM_Rhsim09G0132300	-1.628	-0.136	1.492	low
CK-40d	RHSIM_Rhsim08G0071500	-0.427	-0.366	0.061	no
CK-40d	RHSIM_Rhsim07G0062900	-0.103	-1.020	-0.918	no
CK-40d	RHSIM_Rhsim08G0088200	0.700	1.367	0.667	no
CK-40d	RHSIM_Rhsim09G0031700	-0.936	0.132	1.068	no
CK-40d	RHSIM_Rhsim09G0090500	-1.452	-1.233	0.219	low
CK-40d	RHSIM_Rhsim08G0208000	-2.205	-0.746	1.459	low
CK-40d	RHSIM_Rhsim08G0075200	0.093	0.648	0.554	no
CK-40d	RHSIM_Rhsim08G0197500	-0.424	-0.503	-0.078	no
CK-40d	RHSIM_Rhsim07G0077300	-0.308	0.528	0.835	no
CK-40d	RHSIM_Rhsim07G0006700	0.371	-0.445	-0.817	no
CK-40d	RHSIM_Rhsim07G0119700	0.722	1.301	0.580	no

Group	Gene	PTR (z-score difference)	z(log2 LFQ)	z(log2 FPKM)	extreme-PTR
CK-40d	RHSIM_Rhsim07G0100700	-0.049	-0.712	-0.663	no
CK-40d	RHSIM_Rhsim07G0215500	-0.598	-0.350	0.247	no
CK-40d	RHSIM_Rhsim08G0212100	-0.284	-0.973	-0.689	no
CK-40d	RHSIM_Rhsim08G0216500	0.390	0.080	-0.310	no
CK-40d	RHSIM_Rhsim07G0017000	-0.086	0.825	0.912	no
CK-40d	RHSIM_Rhsim09G0003500	-0.274	-0.405	-0.132	no
CK-40d	RHSIM_Rhsim08G0189100	-0.834	-0.016	0.818	no
CK-40d	RHSIM_Rhsim09G0181600	0.320	-0.227	-0.547	no
CK-40d	RHSIM_Rhsim08G0132500	-0.459	0.383	0.842	no
CK-40d	RHSIM_Rhsim09G0182800	0.763	-0.323	-1.086	no
CK-40d	RHSIM_Rhsim07G0123700	-0.889	-0.428	0.461	no
CK-40d	RHSIM_Rhsim08G0199800	-0.207	0.699	0.906	no
CK-40d	RHSIM_Rhsim09G0146100	0.277	-0.634	-0.911	no
CK-40d	RHSIM_Rhsim08G0228400	-0.422	0.506	0.928	no
CK-40d	RHSIM_Rhsim08G0105300	0.673	-1.123	-1.796	no
CK-40d	RHSIM_Rhsim08G0146100	1.506	2.101	0.595	high
CK-40d	RHSIM_Rhsim07G0165600	1.445	1.455	0.010	high
CK-40d	RHSIM_Rhsim09G0211400	-0.044	-0.961	-0.917	no
CK-40d	RHSIM_Rhsim08G0086100	1.229	1.008	-0.220	high
CK-40d	RHSIM_Rhsim09G0084200	-1.558	0.304	1.862	low
CK-40d	RHSIM_Rhsim07G0010500	0.222	-1.141	-1.362	no
CK-40d	RHSIM_Rhsim07G0118600	-1.427	-1.239	0.188	low
CK-40d	RHSIM_Rhsim08G0067700	-1.298	-1.169	0.129	low
CK-40d	RHSIM_Rhsim08G0080100	-0.945	-0.744	0.201	no
CK-40d	RHSIM_Rhsim08G0002700	-0.726	-0.171	0.556	no
CK-40d	RHSIM_Rhsim09G0126400	-1.853	-1.614	0.239	low
CK-40d	RHSIM_Rhsim09G0199100	-1.820	-1.470	0.350	low
CK-40d	RHSIM_Rhsim09G0210100	1.354	0.551	-0.803	high
CK-40d	RHSIM_Rhsim08G0157100	0.266	-0.162	-0.429	no
CK-40d	RHSIM_Rhsim08G0199500	0.064	-0.247	-0.311	no
CK-40d	RHSIM_Rhsim09G0079200	0.426	0.504	0.078	no
CK-40d	RHSIM_Rhsim08G0225700	-0.726	-0.697	0.029	no

Group	Gene	PTR (z-score difference)	z(log2 LFQ)	z(log2 FPKM)	extreme-PTR
CK-40d	RHSIM_Rhsim08G0191000	2.545	0.501	-2.044	high
CK-40d	RHSIM_Rhsim07G0037800	0.099	0.065	-0.034	no
CK-40d	RHSIM_Rhsim08G0113200	-1.592	-0.583	1.009	low
CK-40d	RHSIM_Rhsim08G0187700	0.480	0.680	0.200	no
CK-40d	RHSIM_Rhsim08G0033400	2.822	2.776	-0.046	high
CK-40d	RHSIM_Rhsim08G0081500	0.571	2.312	1.741	no
CK-40d	RHSIM_Rhsim08G0233000	-1.179	-1.758	-0.580	no
CK-40d	RHSIM_Rhsim08G0176300	0.087	-0.629	-0.716	no
CK-40d	RHSIM_Rhsim08G0221200	1.350	1.658	0.308	high
CK-40d	RHSIM_Rhsim07G0115500	0.179	0.090	-0.089	no
CK-40d	RHSIM_Rhsim08G0009100	-2.105	1.168	3.272	low
CK-40d	RHSIM_Rhsim08G0233300	-0.670	0.309	0.979	no
CK-40d	RHSIM_Rhsim07G0204400	0.152	1.518	1.366	no
CK-40d	RHSIM_Rhsim08G0015000	0.080	-0.718	-0.798	no
CK-40d	RHSIM_Rhsim07G0074900	-0.101	-0.447	-0.346	no
CK-40d	RHSIM_Rhsim08G0100700	0.894	0.799	-0.095	no
CK-40d	RHSIM_Rhsim08G0012800	0.004	0.312	0.309	no
CK-40d	RHSIM_Rhsim08G0000900	0.230	-0.115	-0.346	no
CK-40d	RHSIM_Rhsim08G0009800	0.833	-0.208	-1.041	no
CK-40d	RHSIM_Rhsim08G0075700	-2.844	-1.528	1.316	low
CK-40d	RHSIM_Rhsim08G0118800	1.272	-0.963	-2.234	high
CK-40d	RHSIM_Rhsim08G0020200	0.300	-0.306	-0.606	no
CK-40d	RHSIM_Rhsim07G0118400	0.546	-1.037	-1.583	no
CK-40d	RHSIM_Rhsim07G0173200	1.381	0.462	-0.919	high
CK-40d	RHSIM_Rhsim08G0210300	1.467	0.063	-1.404	high
CK-40d	RHSIM_Rhsim07G0124000	2.698	0.552	-2.146	high
CK-40d	RHSIM_Rhsim08G0151900	-0.213	-0.089	0.125	no
CK-40d	RHSIM_Rhsim08G0070300	-0.010	0.443	0.454	no
CK-40d	RHSIM_Rhsim08G0096300	-0.123	-0.383	-0.260	no
CK-40d	RHSIM_Rhsim07G0016900	0.578	-0.435	-1.013	no
CK-40d	RHSIM_Rhsim08G0249800	-1.252	-1.910	-0.658	low
CK-40d	RHSIM_Rhsim07G0131600	-0.008	-0.648	-0.641	no

Group	Gene	PTR (z-score difference)	z(log2 LFQ)	z(log2 FPKM)	extreme-PTR
CK-40d	RHSIM_Rhsim08G0056900	0.563	-0.918	-1.480	no
CK-40d	RHSIM_Rhsim08G0170400	1.676	1.961	0.285	high
CK-40d	RHSIM_Rhsim07G0175400	1.408	0.102	-1.306	high
CK-40d	RHSIM_Rhsim07G0170000	-0.168	0.837	1.004	no
CK-40d	RHSIM_Rhsim07G0057300	0.754	0.063	-0.691	no
CK-40d	RHSIM_Rhsim08G0071700	-0.202	0.655	0.857	no
CK-40d	RHSIM_Rhsim07G0035700	-0.265	-0.721	-0.456	no
CK-40d	RHSIM_Rhsim08G0077000	-0.545	0.254	0.799	no
CK-40d	RHSIM_Rhsim08G0161000	-1.719	-0.333	1.385	low
CK-40d	RHSIM_Rhsim07G0257200	2.274	1.148	-1.126	high
CK-40d	RHSIM_Rhsim07G0024200	0.548	-0.455	-1.003	no
CK-40d	RHSIM_Rhsim08G0088300	1.378	2.070	0.691	high
CK-40d	RHSIM_Rhsim08G0188700	0.596	-0.054	-0.650	no
CK-40d	RHSIM_Rhsim08G0200500	-1.283	0.345	1.628	low
CK-40d	RHSIM_Rhsim08G0157300	0.055	-0.328	-0.383	no
CK-40d	RHSIM_Rhsim07G0017400	-0.165	0.414	0.578	no
CK-40d	RHSIM_Rhsim07G0085300	0.263	1.314	1.051	no
CK-40d	RHSIM_Rhsim08G0216800	-0.776	0.553	1.329	no
CK-40d	RHSIM_Rhsim08G0126200	0.615	0.571	-0.044	no
CK-40d	RHSIM_Rhsim07G0011500	-0.707	1.212	1.919	no
CK-40d	RHSIM_Rhsim08G0222300	-0.887	-0.354	0.533	no
CK-40d	RHSIM_Rhsim07G0216000	-1.471	-0.338	1.132	low
CK-40d	RHSIM_Rhsim08G0245600	0.778	-0.323	-1.100	no
CK-40d	RHSIM_Rhsim08G0153200	1.247	0.952	-0.295	high
CK-40d	RHSIM_Rhsim07G0101700	-0.338	-0.519	-0.180	no
CK-40d	RHSIM_Rhsim08G0248400	2.489	3.270	0.781	high
CK-40d	RHSIM_Rhsim08G0153400	-0.970	-0.605	0.365	no
CK-40d	RHSIM_Rhsim08G0215500	0.229	-1.115	-1.344	no
CK-40d	RHSIM_Rhsim07G0071000	1.162	-1.045	-2.207	high
CK-40d	RHSIM_Rhsim08G0240500	-0.161	0.264	0.424	no
CK-40d	RHSIM_Rhsim07G0004600	-1.776	-1.243	0.534	low
CK-40d	RHSIM_Rhsim08G0157700	-0.894	-0.607	0.287	no

Group	Gene	PTR (z-score difference)	z(log2 LFQ)	z(log2 FPKM)	extreme-PTR
CK-40d	RHSIM_Rhsim07G0053800	-0.010	0.194	0.205	no
CK-40d	RHSIM_Rhsim08G0118400	1.443	-0.299	-1.741	high
CK-40d	RHSIM_Rhsim08G0230900	-0.765	0.058	0.822	no
CK-40d	RHSIM_Rhsim07G0248600	-0.514	-0.467	0.047	no
CK-40d	RHSIM_Rhsim07G0144500	-0.740	-0.241	0.498	no
CK-40d	RHSIM_Rhsim07G0201100	0.094	-1.336	-1.430	no
CK-40d	RHSIM_Rhsim08G0012300	2.497	1.242	-1.255	high
CK-40d	RHSIM_Rhsim07G0023600	-0.340	-0.749	-0.409	no
CK-40d	RHSIM_Rhsim08G0241600	-0.575	-0.536	0.039	no
CK-40d	RHSIM_Rhsim07G0025600	2.132	0.541	-1.590	high
CK-40d	RHSIM_Rhsim08G0123400	-0.743	0.022	0.764	no
CK-40d	RHSIM_Rhsim08G0040600	0.962	-0.317	-1.279	no
CK-40d	RHSIM_Rhsim08G0119200	-0.403	0.339	0.742	no
CK-40d	RHSIM_Rhsim08G0058800	0.247	-0.370	-0.618	no
CK-40d	RHSIM_Rhsim08G0089400	-0.039	-1.168	-1.129	no
CK-40d	RHSIM_Rhsim07G0019000	0.768	0.669	-0.100	no
CK-40d	RHSIM_Rhsim07G0251100	0.367	1.443	1.076	no
CK-40d	RHSIM_Rhsim08G0169300	-0.379	-1.320	-0.942	no
CK-40d	RHSIM_Rhsim08G0093900	-1.554	0.647	2.201	low
CK-40d	RHSIM_Rhsim08G0098500	-0.466	-0.602	-0.135	no
CK-40d	RHSIM_Rhsim07G0044500	1.430	-0.019	-1.448	high
CK-40d	RHSIM_Rhsim08G0187400	0.907	1.633	0.726	no
CK-40d	RHSIM_Rhsim08G0212600	-0.785	-0.298	0.488	no
CK-40d	RHSIM_Rhsim07G0258200	-1.246	-0.737	0.509	low
CK-40d	RHSIM_Rhsim07G0146900	-0.642	-0.898	-0.257	no
CK-40d	RHSIM_Rhsim08G0035600	2.672	1.014	-1.658	high
CK-40d	RHSIM_Rhsim08G0078700	0.979	-0.172	-1.151	no
CK-40d	RHSIM_Rhsim08G0043300	-0.073	0.483	0.556	no
CK-40d	RHSIM_Rhsim08G0184400	0.976	0.209	-0.767	no
CK-40d	RHSIM_Rhsim08G0119000	1.552	1.310	-0.242	high
CK-40d	RHSIM_Rhsim07G0209200	-1.654	-0.866	0.788	low
CK-40d	RHSIM_Rhsim08G0068000	-0.976	0.036	1.013	no

Group	Gene	PTR (z-score difference)	z(log2 LFQ)	z(log2 FPKM)	extreme-PTR
CK-40d	RHSIM_Rhsim07G0250500	0.036	0.648	0.613	no
CK-40d	RHSIM_Rhsim08G0199900	0.879	0.365	-0.514	no
CK-40d	RHSIM_Rhsim07G0184400	0.874	-0.679	-1.553	no
CK-40d	RHSIM_Rhsim08G0207200	-0.907	0.506	1.413	no
CK-40d	RHSIM_Rhsim08G0216200	0.511	-0.139	-0.650	no
CK-40d	RHSIM_Rhsim08G0227300	-2.116	-1.158	0.958	low
CK-40d	RHSIM_Rhsim08G0124200	1.181	0.195	-0.986	high
CK-40d	RHSIM_Rhsim07G0186000	-0.021	-0.222	-0.201	no
CK-40d	RHSIM_Rhsim08G0027500	-0.609	-0.317	0.291	no
CK-40d	RHSIM_Rhsim08G0000300	-0.235	-0.522	-0.287	no
CK-40d	RHSIM_Rhsim08G0155400	-0.160	0.076	0.236	no
CK-40d	RHSIM_Rhsim07G0190000	-2.818	-1.707	1.111	low
CK-40d	RHSIM_Rhsim08G0125000	-0.706	-0.125	0.580	no
CK-40d	RHSIM_Rhsim07G0256400	0.632	0.267	-0.365	no
CK-40d	RHSIM_Rhsim08G0146900	0.160	-0.358	-0.517	no
CK-40d	RHSIM_Rhsim07G0125300	1.292	1.010	-0.281	high
CK-40d	RHSIM_Rhsim08G0198600	2.394	1.289	-1.105	high
CK-40d	RHSIM_Rhsim08G0124400	-0.663	-0.686	-0.023	no
CK-40d	RHSIM_Rhsim08G0106700	0.821	-0.207	-1.029	no
CK-40d	RHSIM_Rhsim08G0095400	-1.288	-1.159	0.129	low
CK-40d	RHSIM_Rhsim07G0102300	-2.534	-1.551	0.983	low
CK-40d	RHSIM_Rhsim08G0189200	-2.251	-0.256	1.996	low
CK-40d	RHSIM_Rhsim08G0198900	-0.081	-0.931	-0.850	no
CK-40d	RHSIM_Rhsim07G0187600	-1.214	0.806	2.020	no
CK-40d	RHSIM_Rhsim08G0038400	-0.511	-1.352	-0.842	no
CK-40d	RHSIM_Rhsim08G0066200	0.111	2.825	2.714	no
CK-40d	RHSIM_Rhsim08G0129000	0.344	1.874	1.530	no
CK-40d	RHSIM_Rhsim08G0076500	0.668	0.472	-0.196	no
CK-40d	RHSIM_Rhsim08G0171400	0.187	-0.364	-0.551	no
CK-40d	RHSIM_Rhsim08G0047100	1.203	0.288	-0.915	high
CK-40d	RHSIM_Rhsim08G0206000	-0.730	-1.175	-0.445	no
CK-40d	RHSIM_Rhsim07G0219200	-0.631	-1.233	-0.602	no

Group	Gene	PTR (z-score difference)	z(log2 LFQ)	z(log2 FPKM)	extreme-PTR
CK-40d	RHSIM_Rhsim08G0227800	-0.312	0.296	0.608	no
CK-40d	RHSIM_Rhsim08G0249600	-0.154	-0.948	-0.794	no
CK-40d	RHSIM_Rhsim07G0251700	2.381	1.889	-0.492	high
CK-40d	RHSIM_Rhsim08G0038900	0.477	0.911	0.434	no
CK-40d	RHSIM_Rhsim08G0096700	-1.294	-0.510	0.783	low
CK-40d	RHSIM_Rhsim07G0067800	0.843	-0.294	-1.137	no
CK-40d	RHSIM_Rhsim08G0006300	1.372	-0.204	-1.576	high
CK-40d	RHSIM_Rhsim07G0215000	-0.549	-1.003	-0.453	no
CK-40d	RHSIM_Rhsim07G0007400	-0.245	-0.871	-0.626	no
CK-40d	RHSIM_Rhsim08G0115600	0.052	0.051	-0.001	no
CK-40d	RHSIM_Rhsim07G0241500	1.221	0.427	-0.794	high
CK-40d	RHSIM_Rhsim08G0005400	-2.622	-1.535	1.087	low
CK-40d	RHSIM_Rhsim07G0208900	-0.804	-0.271	0.533	no
CK-40d	RHSIM_Rhsim08G0044400	0.117	-1.045	-1.162	no
CK-40d	RHSIM_Rhsim08G0018500	1.277	0.444	-0.833	high
CK-40d	RHSIM_Rhsim08G0090300	0.663	0.785	0.122	no
CK-40d	RHSIM_Rhsim07G0108500	-0.877	-0.817	0.059	no
CK-40d	RHSIM_Rhsim07G0063200	-1.081	-1.087	-0.006	no
CK-40d	RHSIM_Rhsim07G0080500	1.704	0.858	-0.846	high
CK-40d	RHSIM_Rhsim08G0074900	0.187	-0.214	-0.401	no
CK-40d	RHSIM_Rhsim08G0010200	-1.546	0.207	1.753	low
CK-40d	RHSIM_Rhsim07G0032500	1.817	0.869	-0.948	high
CK-40d	RHSIM_Rhsim08G0171600	-1.230	3.377	4.607	no
CK-40d	RHSIM_Rhsim07G0096500	1.559	2.089	0.530	high
CK-40d	RHSIM_Rhsim08G0252300	-0.209	-0.692	-0.482	no
CK-40d	RHSIM_Rhsim07G0181400	-0.020	0.232	0.252	no
CK-40d	RHSIM_Rhsim08G0173200	-1.731	-0.159	1.572	low
CK-40d	RHSIM_Rhsim07G0091300	-0.509	-0.124	0.385	no
CK-40d	RHSIM_Rhsim07G0054000	-2.014	-1.426	0.588	low
CK-40d	RHSIM_Rhsim07G0068700	0.572	-0.105	-0.677	no
CK-40d	RHSIM_Rhsim07G0106900	0.262	-1.416	-1.678	no
CK-40d	RHSIM_Rhsim08G0016700	-0.192	1.089	1.281	no

Group	Gene	PTR (z-score difference)	z(log2 LFQ)	z(log2 FPKM)	extreme-PTR
CK-40d	RHSIM_Rhsim07G0080100	-0.264	0.163	0.426	no
CK-40d	RHSIM_Rhsim07G0245700	0.642	-0.067	-0.710	no
CK-40d	RHSIM_Rhsim08G0080000	-0.664	-1.044	-0.381	no
CK-40d	RHSIM_Rhsim08G0140900	-0.764	-0.416	0.348	no
CK-40d	RHSIM_Rhsim08G0046200	-0.176	0.433	0.608	no
CK-40d	RHSIM_Rhsim08G0010700	-0.668	-0.941	-0.272	no
CK-40d	RHSIM_Rhsim08G0116700	1.796	0.198	-1.598	high
CK-40d	RHSIM_Rhsim07G0145700	-0.326	0.565	0.891	no
CK-40d	RHSIM_Rhsim07G0006200	-1.725	1.014	2.738	low
CK-40d	RHSIM_Rhsim07G0013500	-1.122	-1.073	0.050	no
CK-40d	RHSIM_Rhsim07G0053100	-0.077	0.634	0.712	no
CK-40d	RHSIM_Rhsim08G0249200	0.109	-0.407	-0.516	no
CK-40d	RHSIM_Rhsim07G0096000	0.502	0.701	0.199	no
CK-40d	RHSIM_Rhsim08G0245800	-0.722	-0.311	0.411	no
CK-40d	RHSIM_Rhsim08G0019400	-0.393	-1.610	-1.217	no
CK-40d	RHSIM_Rhsim08G0102300	1.041	-0.807	-1.848	no
CK-40d	RHSIM_Rhsim07G0005700	-0.741	-1.118	-0.377	no
CK-40d	RHSIM_Rhsim07G0056700	0.771	-0.146	-0.918	no
CK-40d	RHSIM_Rhsim08G0210500	0.829	0.525	-0.304	no
CK-40d	RHSIM_Rhsim07G0045400	2.560	0.674	-1.886	high
CK-40d	RHSIM_Rhsim07G0188300	0.314	-0.964	-1.278	no
CK-40d	RHSIM_Rhsim08G0037900	0.189	-0.047	-0.236	no
CK-40d	RHSIM_Rhsim07G0023500	-0.199	-0.374	-0.175	no
CK-40d	RHSIM_Rhsim07G0068500	-0.372	0.317	0.689	no
CK-40d	RHSIM_Rhsim07G0117800	-0.647	1.023	1.670	no
CK-40d	RHSIM_Rhsim08G0119100	0.111	0.657	0.546	no
CK-40d	RHSIM_Rhsim07G0186700	-0.414	-0.848	-0.434	no
CK-40d	RHSIM_Rhsim08G0034600	-0.952	0.750	1.703	no
CK-40d	RHSIM_Rhsim07G0083300	-0.391	-0.861	-0.470	no
CK-40d	RHSIM_Rhsim08G0139800	-0.211	-0.701	-0.491	no
CK-40d	RHSIM_Rhsim07G0126200	-0.675	0.057	0.732	no
CK-40d	RHSIM_Rhsim07G0251400	1.647	2.526	0.879	high

Group	Gene	PTR (z-score difference)	z(log2 LFQ)	z(log2 FPKM)	extreme-PTR
CK-40d	RHSIM_Rhsim07G0127400	-1.440	-0.555	0.885	low
CK-40d	RHSIM_Rhsim07G0022300	-0.908	0.090	0.998	no
CK-40d	RHSIM_Rhsim07G0170800	-1.406	-1.199	0.207	low
CK-40d	RHSIM_Rhsim08G0013500	1.535	-0.549	-2.083	high
CK-40d	RHSIM_Rhsim08G0031200	2.136	1.727	-0.408	high
CK-40d	RHSIM_Rhsim07G0071200	0.922	0.184	-0.738	no
CK-40d	RHSIM_Rhsim08G0248600	1.287	-0.425	-1.712	high
CK-40d	RHSIM_Rhsim08G0067400	2.097	0.554	-1.544	high
CK-40d	RHSIM_Rhsim08G0175500	-0.571	-0.721	-0.150	no
CK-40d	RHSIM_Rhsim07G0172400	-0.533	-1.699	-1.166	no
CK-40d	RHSIM_Rhsim08G0249300	-1.123	-0.119	1.005	no
CK-40d	RHSIM_Rhsim07G0181600	-1.421	-0.897	0.525	low
CK-40d	RHSIM_Rhsim07G0202000	1.249	1.129	-0.120	high
CK-40d	RHSIM_Rhsim08G0221900	-0.568	-0.582	-0.014	no
CK-40d	RHSIM_Rhsim07G0040000	0.287	1.559	1.272	no
CK-40d	RHSIM_Rhsim08G0000400	-0.194	-0.021	0.173	no
CK-40d	RHSIM_Rhsim08G0050800	0.121	0.445	0.324	no
CK-40d	RHSIM_Rhsim07G0236600	-0.003	-0.933	-0.930	no
CK-40d	RHSIM_Rhsim07G0219500	-0.684	-0.205	0.479	no
CK-40d	RHSIM_Rhsim07G0059300	-0.034	-0.909	-0.875	no
CK-40d	RHSIM_Rhsim07G0209000	1.061	0.188	-0.873	no
CK-40d	RHSIM_Rhsim08G0126600	-0.856	-0.443	0.413	no
CK-40d	RHSIM_Rhsim07G0163600	0.795	-0.616	-1.411	no
CK-40d	RHSIM_Rhsim07G0173800	0.166	-0.348	-0.513	no
CK-40d	RHSIM_Rhsim07G0239800	-0.180	-0.222	-0.042	no
CK-40d	RHSIM_Rhsim07G0164400	-1.348	-1.467	-0.119	low
CK-40d	RHSIM_Rhsim07G0031300	-0.544	-0.449	0.095	no
CK-40d	RHSIM_Rhsim07G0016500	0.133	0.138	0.004	no
CK-40d	RHSIM_Rhsim07G0250100	3.140	2.148	-0.992	high
CK-40d	RHSIM_Rhsim07G0107500	-2.910	-0.145	2.765	low
CK-40d	RHSIM_Rhsim08G0024600	-0.626	0.600	1.226	no
CK-40d	RHSIM_Rhsim07G0068000	-0.651	-0.879	-0.228	no

Group	Gene	PTR (z-score difference)	z(log2 LFQ)	z(log2 FPKM)	extreme-PTR
CK-40d	RHSIM_Rhsim07G0024600	-0.722	-0.885	-0.163	no
CK-40d	RHSIM_Rhsim07G0038000	0.157	0.354	0.197	no
CK-40d	RHSIM_Rhsim07G0207200	-1.513	-0.382	1.130	low
CK-40d	RHSIM_Rhsim07G0086400	-0.054	-0.660	-0.606	no
CK-40d	RHSIM_Rhsim07G0052400	0.598	-0.802	-1.400	no
CK-40d	RHSIM_Rhsim07G0029300	0.764	-1.077	-1.841	no
CK-40d	RHSIM_Rhsim08G0068500	-1.665	0.389	2.054	low
CK-40d	RHSIM_Rhsim07G0069700	1.687	-0.584	-2.272	high
CK-40d	RHSIM_Rhsim07G0033300	-0.162	-1.203	-1.041	no
CK-40d	RHSIM_Rhsim07G0105400	0.772	-0.116	-0.888	no
CK-40d	RHSIM_Rhsim08G0085100	1.228	1.069	-0.159	high
CK-40d	RHSIM_Rhsim07G0173600	-1.268	-0.146	1.122	low
CK-40d	RHSIM_Rhsim08G0016400	-0.992	-0.983	0.009	no
CK-40d	RHSIM_Rhsim08G0152500	1.896	2.787	0.891	high
CK-40d	RHSIM_Rhsim07G0027900	-1.916	-1.283	0.634	low
CK-40d	RHSIM_Rhsim07G0026600	-1.279	-1.510	-0.231	low
CK-40d	RHSIM_Rhsim07G0055300	0.843	1.664	0.821	no
CK-40d	RHSIM_Rhsim08G0176400	-1.655	1.160	2.815	low
CK-40d	RHSIM_Rhsim08G0232200	-1.015	-1.045	-0.030	no
CK-40d	RHSIM_Rhsim07G0061300	-0.482	-0.388	0.094	no
CK-40d	RHSIM_Rhsim07G0125000	-0.078	-0.041	0.038	no
CK-40d	RHSIM_Rhsim07G0228300	-0.374	-0.506	-0.133	no
CK-40d	RHSIM_Rhsim07G0020700	-1.021	0.024	1.045	no
CK-40d	RHSIM_Rhsim07G0132100	-1.343	-0.688	0.655	low
CK-40d	RHSIM_Rhsim07G0074100	0.068	0.119	0.051	no
CK-40d	RHSIM_Rhsim07G0198900	0.194	-0.673	-0.866	no
CK-40d	RHSIM_Rhsim07G0007900	2.042	3.078	1.036	high
CK-40d	RHSIM_Rhsim07G0243800	-1.320	2.075	3.394	low
CK-40d	RHSIM_Rhsim07G0025200	-0.144	-0.464	-0.319	no
CK-40d	RHSIM_Rhsim07G0143700	-0.812	-0.282	0.530	no
CK-40d	RHSIM_Rhsim08G0094700	-1.020	-0.197	0.823	no
CK-40d	RHSIM_Rhsim07G0214200	-0.564	-1.704	-1.140	no

Group	Gene	PTR (z-score difference)	z(log2 LFQ)	z(log2 FPKM)	extreme-PTR
CK-40d	RHSIM_Rhsim07G0109900	-0.494	-1.224	-0.730	no
CK-40d	RHSIM_Rhsim08G0173100	-1.284	-0.199	1.086	low
CK-40d	RHSIM_Rhsim07G0049200	-0.408	0.572	0.980	no
CK-40d	RHSIM_Rhsim08G0224200	-0.736	-0.798	-0.062	no
CK-40d	RHSIM_Rhsim08G0112300	-0.307	-0.266	0.041	no
CK-40d	RHSIM_Rhsim07G0172200	-0.578	-0.090	0.487	no
CK-40d	RHSIM_Rhsim08G0226500	-0.090	-0.427	-0.337	no
CK-40d	RHSIM_Rhsim07G0215100	-1.577	-0.704	0.873	low
CK-40d	RHSIM_Rhsim07G0082400	-0.690	-0.323	0.366	no
CK-40d	RHSIM_Rhsim07G0106300	-2.130	0.359	2.489	low
CK-40d	RHSIM_Rhsim07G0200600	-2.909	-2.255	0.654	low
CK-40d	RHSIM_Rhsim07G0029400	-0.271	-0.122	0.149	no
CK-40d	RHSIM_Rhsim07G0171600	0.993	0.652	-0.340	no
CK-40d	RHSIM_Rhsim08G0080600	0.843	0.136	-0.708	no
CK-40d	RHSIM_Rhsim06G0201500	0.065	-0.379	-0.445	no
CK-40d	RHSIM_Rhsim08G0006200	1.269	1.574	0.305	high
CK-40d	RHSIM_Rhsim07G0076300	-1.154	-0.689	0.465	no
CK-40d	RHSIM_Rhsim06G0223900	0.548	-0.975	-1.523	no
CK-40d	RHSIM_Rhsim08G0248900	-1.336	-1.592	-0.257	low
CK-40d	RHSIM_Rhsim06G0029100	-1.910	-0.978	0.932	low
CK-40d	RHSIM_Rhsim07G0102800	0.131	0.195	0.063	no
CK-40d	RHSIM_Rhsim08G0031800	-1.608	-0.984	0.624	low
CK-40d	RHSIM_Rhsim07G0054400	-0.546	-0.147	0.399	no
CK-40d	RHSIM_Rhsim07G0252800	-0.794	-0.978	-0.184	no
CK-40d	RHSIM_Rhsim07G0222500	-1.664	-0.975	0.689	low
CK-40d	RHSIM_Rhsim07G0255000	-0.623	0.074	0.698	no
CK-40d	RHSIM_Rhsim06G0046600	1.171	0.054	-1.118	high
CK-40d	RHSIM_Rhsim08G0240200	0.553	0.141	-0.411	no
CK-40d	RHSIM_Rhsim07G0237100	0.194	-0.165	-0.359	no
CK-40d	RHSIM_Rhsim07G0136400	2.249	0.276	-1.973	high
CK-40d	RHSIM_Rhsim07G0048200	-0.698	-1.360	-0.663	no
CK-40d	RHSIM_Rhsim08G0064800	-0.036	1.649	1.684	no

Group	Gene	PTR (z-score difference)	z(log2 LFQ)	z(log2 FPKM)	extreme-PTR
CK-40d	RHSIM_Rhsim07G0045800	2.481	2.704	0.222	high
CK-40d	RHSIM_Rhsim07G0137100	-0.988	0.120	1.108	no
CK-40d	RHSIM_Rhsim06G0084500	-0.190	-0.570	-0.380	no
CK-40d	RHSIM_Rhsim07G0178600	0.161	-0.168	-0.328	no
CK-40d	RHSIM_Rhsim07G0143500	1.325	0.619	-0.706	high
CK-40d	RHSIM_Rhsim06G0122400	-1.933	-0.200	1.733	low
CK-40d	RHSIM_Rhsim07G0022700	-1.180	-1.254	-0.074	no
CK-40d	RHSIM_Rhsim06G0170400	0.479	-0.009	-0.489	no
CK-40d	RHSIM_Rhsim07G0124300	0.870	0.737	-0.133	no
CK-40d	RHSIM_Rhsim07G0006000	-0.689	0.912	1.601	no
CK-40d	RHSIM_Rhsim07G0130900	0.192	0.014	-0.178	no
CK-40d	RHSIM_Rhsim08G0163100	1.102	-0.427	-1.529	no
CK-40d	RHSIM_Rhsim06G0174100	-2.468	-2.012	0.456	low
CK-40d	RHSIM_Rhsim06G0208000	-0.511	-0.568	-0.057	no
CK-40d	RHSIM_Rhsim07G0201800	-0.247	-0.631	-0.383	no
CK-40d	RHSIM_Rhsim06G0046900	1.064	1.359	0.295	no
CK-40d	RHSIM_Rhsim06G0184300	-0.412	0.223	0.635	no
CK-40d	RHSIM_Rhsim07G0190100	-2.578	-0.017	2.561	low
CK-40d	RHSIM_Rhsim07G0205400	-0.095	0.866	0.960	no
CK-40d	RHSIM_Rhsim07G0137000	2.084	1.061	-1.023	high
CK-40d	RHSIM_Rhsim06G0007800	0.098	0.214	0.116	no
CK-40d	RHSIM_Rhsim07G0014000	0.284	-1.315	-1.599	no
CK-40d	RHSIM_Rhsim07G0159800	0.258	-1.469	-1.727	no
CK-40d	RHSIM_Rhsim06G0140600	0.829	-0.133	-0.962	no
CK-40d	RHSIM_Rhsim07G0120800	-2.629	-1.073	1.555	low
CK-40d	RHSIM_Rhsim08G0230400	-0.024	0.579	0.603	no
CK-40d	RHSIM_Rhsim07G0134100	-0.564	-0.598	-0.034	no
CK-40d	RHSIM_Rhsim06G0008600	-1.218	-0.789	0.429	no
CK-40d	RHSIM_Rhsim07G0244700	-0.445	-0.324	0.121	no
CK-40d	RHSIM_Rhsim07G0096100	-2.187	-2.330	-0.143	low
CK-40d	RHSIM_Rhsim07G0068100	-0.777	0.193	0.970	no
CK-40d	RHSIM_Rhsim06G0227300	0.610	1.504	0.893	no

Group	Gene	PTR (z-score difference)	z(log2 LFQ)	z(log2 FPKM)	extreme-PTR
CK-40d	RHSIM_Rhsim06G0231100	0.654	0.691	0.037	no
CK-40d	RHSIM_Rhsim08G0152000	-0.818	-0.997	-0.180	no
CK-40d	RHSIM_Rhsim06G0121500	0.703	-0.029	-0.732	no
CK-40d	RHSIM_Rhsim06G0180800	0.983	-0.645	-1.628	no
CK-40d	RHSIM_Rhsim07G0037100	-0.293	-0.667	-0.374	no
CK-40d	RHSIM_Rhsim07G0075100	-0.844	-0.343	0.501	no
CK-40d	RHSIM_Rhsim07G0243000	0.257	-0.141	-0.398	no
CK-40d	RHSIM_Rhsim06G0009200	0.603	0.160	-0.443	no
CK-40d	RHSIM_Rhsim07G0193800	-0.267	-0.241	0.026	no
CK-40d	RHSIM_Rhsim07G0175800	1.097	0.759	-0.338	no
CK-40d	RHSIM_Rhsim07G0214800	-1.192	-1.013	0.179	no
CK-40d	RHSIM_Rhsim07G0244600	0.816	0.529	-0.288	no
CK-40d	RHSIM_Rhsim07G0011100	-0.735	-1.295	-0.559	no
CK-40d	RHSIM_Rhsim07G0069200	-0.970	-1.052	-0.083	no
CK-40d	RHSIM_Rhsim08G0163500	0.502	0.126	-0.376	no
CK-40d	RHSIM_Rhsim06G0229900	1.054	-0.441	-1.494	no
CK-40d	RHSIM_Rhsim07G0104500	1.864	1.087	-0.777	high
CK-40d	RHSIM_Rhsim07G0136600	-0.886	-0.951	-0.065	no
CK-40d	RHSIM_Rhsim06G0035500	-0.563	-0.301	0.261	no
CK-40d	RHSIM_Rhsim07G0018800	0.730	1.020	0.290	no
CK-40d	RHSIM_Rhsim06G0089000	-0.206	0.783	0.989	no
CK-40d	RHSIM_Rhsim07G0221800	-4.359	-1.823	2.536	low
CK-40d	RHSIM_Rhsim07G0200400	0.559	-0.021	-0.580	no
CK-40d	RHSIM_Rhsim08G0038600	0.819	0.144	-0.675	no
CK-40d	RHSIM_Rhsim07G0183300	0.011	0.634	0.623	no
CK-40d	RHSIM_Rhsim07G0039600	1.144	1.178	0.035	high
CK-40d	RHSIM_Rhsim07G0229800	2.189	0.815	-1.375	high
CK-40d	RHSIM_Rhsim06G0079300	-1.138	-0.206	0.931	no
CK-40d	RHSIM_Rhsim08G0146700	-0.381	-0.850	-0.469	no
CK-40d	RHSIM_Rhsim07G0172500	0.310	0.098	-0.212	no
CK-40d	RHSIM_Rhsim07G0134000	0.107	0.123	0.016	no
CK-40d	RHSIM_Rhsim07G0078200	-0.469	-0.436	0.033	no

Group	Gene	PTR (z-score difference)	z(log2 LFQ)	z(log2 FPKM)	extreme-PTR
CK-40d	RHSIM_Rhsim07G0179200	0.365	0.135	-0.230	no
CK-40d	RHSIM_Rhsim06G0203800	-2.552	-0.613	1.939	low
CK-40d	RHSIM_Rhsim06G0204600	-0.365	0.754	1.119	no
CK-40d	RHSIM_Rhsim06G0229700	-1.075	-0.889	0.186	no
CK-40d	RHSIM_Rhsim06G0003900	-1.109	0.228	1.337	no
CK-40d	RHSIM_Rhsim07G0176900	-1.371	-0.870	0.501	low
CK-40d	RHSIM_Rhsim06G0211400	0.237	0.935	0.699	no
CK-40d	RHSIM_Rhsim07G0093600	-1.904	-0.953	0.950	low
CK-40d	RHSIM_Rhsim07G0086200	-0.077	0.639	0.715	no
CK-40d	RHSIM_Rhsim07G0017700	-0.913	-0.673	0.239	no
CK-40d	RHSIM_Rhsim07G0102000	0.978	0.351	-0.627	no
CK-40d	RHSIM_Rhsim06G0069100	-2.083	0.428	2.511	low
CK-40d	RHSIM_Rhsim07G0122900	0.191	0.506	0.314	no
CK-40d	RHSIM_Rhsim07G0141400	0.043	-0.243	-0.285	no
CK-40d	RHSIM_Rhsim06G0026300	1.604	1.566	-0.038	high
CK-40d	RHSIM_Rhsim08G0011300	-0.109	0.002	0.111	no
CK-40d	RHSIM_Rhsim06G0050800	-0.495	-0.791	-0.296	no
CK-40d	RHSIM_Rhsim06G0013500	-1.845	0.006	1.851	low
CK-40d	RHSIM_Rhsim06G0131700	-0.690	-0.043	0.647	no
CK-40d	RHSIM_Rhsim07G0243900	-0.037	-0.556	-0.518	no
CK-40d	RHSIM_Rhsim06G0030400	-1.199	-1.737	-0.538	no
CK-40d	RHSIM_Rhsim07G0213800	-1.518	-1.109	0.409	low
CK-40d	RHSIM_Rhsim07G0075700	0.253	0.402	0.149	no
CK-40d	RHSIM_Rhsim06G0175500	-1.119	0.093	1.212	no
CK-40d	RHSIM_Rhsim06G0068700	0.878	1.158	0.279	no
CK-40d	RHSIM_Rhsim07G0257700	-0.058	-0.110	-0.052	no
CK-40d	RHSIM_Rhsim06G0032100	-0.257	-0.694	-0.437	no
CK-40d	RHSIM_Rhsim06G0227500	0.618	0.446	-0.171	no
CK-40d	RHSIM_Rhsim06G0082900	0.329	-0.168	-0.497	no
CK-40d	RHSIM_Rhsim07G0144100	-0.500	0.361	0.861	no
CK-40d	RHSIM_Rhsim07G0039900	-0.091	-1.161	-1.069	no
CK-40d	RHSIM_Rhsim06G0027200	-2.332	-2.170	0.163	low

Group	Gene	PTR (z-score difference)	z(log2 LFQ)	z(log2 FPKM)	extreme-PTR
CK-40d	RHSIM_Rhsim07G0252900	-1.715	-1.783	-0.068	low
CK-40d	RHSIM_Rhsim07G0061100	0.915	-0.674	-1.589	no
CK-40d	RHSIM_Rhsim06G0210400	-0.471	0.262	0.733	no
CK-40d	RHSIM_Rhsim06G0050100	-0.201	-1.127	-0.926	no
CK-40d	RHSIM_Rhsim07G0211300	-0.389	-0.245	0.143	no
CK-40d	RHSIM_Rhsim06G0231000	-0.940	-0.632	0.308	no
CK-40d	RHSIM_Rhsim07G0039700	-0.361	0.597	0.958	no
CK-40d	RHSIM_Rhsim07G0139700	-1.812	0.714	2.526	low
CK-40d	RHSIM_Rhsim06G0154000	0.369	-0.469	-0.839	no
CK-40d	RHSIM_Rhsim07G0045700	-0.514	-0.699	-0.185	no
CK-40d	RHSIM_Rhsim06G0162500	-0.082	2.223	2.306	no
CK-40d	RHSIM_Rhsim06G0242100	0.117	-1.282	-1.399	no
CK-40d	RHSIM_Rhsim06G0213100	-0.212	0.428	0.640	no
CK-40d	RHSIM_Rhsim07G0071500	-2.134	-0.170	1.964	low
CK-40d	RHSIM_Rhsim07G0213700	-1.683	-1.453	0.230	low
CK-40d	RHSIM_Rhsim07G0177400	-0.794	-0.530	0.263	no
CK-40d	RHSIM_Rhsim07G0047700	0.956	0.326	-0.630	no
CK-40d	RHSIM_Rhsim07G0201200	0.763	-1.349	-2.112	no
CK-40d	RHSIM_Rhsim06G0219700	0.364	0.468	0.104	no
CK-40d	RHSIM_Rhsim08G0244100	0.161	0.099	-0.062	no
CK-40d	RHSIM_Rhsim07G0253800	-1.966	0.090	2.056	low
CK-40d	RHSIM_Rhsim07G0137800	-1.001	-0.341	0.660	no
CK-40d	RHSIM_Rhsim07G0209300	-0.998	-0.384	0.615	no
CK-40d	RHSIM_Rhsim06G0207200	0.547	2.184	1.637	no
CK-40d	RHSIM_Rhsim07G0175500	-0.507	-1.303	-0.797	no
CK-40d	RHSIM_Rhsim06G0142500	0.419	0.740	0.321	no
CK-40d	RHSIM_Rhsim07G0141900	-0.237	0.583	0.820	no
CK-40d	RHSIM_Rhsim07G0054800	1.953	2.016	0.063	high
CK-40d	RHSIM_Rhsim07G0236700	-0.857	-1.661	-0.804	no
CK-40d	RHSIM_Rhsim07G0179600	2.942	2.922	-0.020	high
CK-40d	RHSIM_Rhsim06G0198600	-1.387	0.836	2.223	low
CK-40d	RHSIM_Rhsim06G0232600	0.332	2.477	2.145	no

Group	Gene	PTR (z-score difference)	z(log2 LFQ)	z(log2 FPKM)	extreme-PTR
CK-40d	RHSIM_Rhsim07G0043400	-0.691	-1.153	-0.462	no
CK-40d	RHSIM_Rhsim07G0018900	0.588	-0.979	-1.567	no
CK-40d	RHSIM_Rhsim07G0028400	-0.604	-0.261	0.344	no
CK-40d	RHSIM_Rhsim07G0072700	0.227	0.362	0.135	no
CK-40d	RHSIM_Rhsim07G0075600	1.874	0.955	-0.919	high
CK-40d	RHSIM_Rhsim07G0155800	-1.341	-0.887	0.453	low
CK-40d	RHSIM_Rhsim08G0001800	1.063	0.685	-0.377	no
CK-40d	RHSIM_Rhsim07G0024100	0.264	-0.428	-0.693	no
CK-40d	RHSIM_Rhsim07G0012700	0.336	-0.543	-0.879	no
CK-40d	RHSIM_Rhsim07G0048100	0.379	1.017	0.638	no
CK-40d	RHSIM_Rhsim07G0227800	0.989	0.007	-0.982	no
CK-40d	RHSIM_Rhsim08G0022300	-1.411	-0.022	1.389	low
CK-40d	RHSIM_Rhsim06G0063500	-1.759	-1.342	0.417	low
CK-40d	RHSIM_Rhsim07G0034100	-0.725	0.670	1.395	no
CK-40d	RHSIM_Rhsim06G0003400	-0.456	-1.491	-1.035	no
CK-40d	RHSIM_Rhsim06G0027900	-0.030	-0.104	-0.074	no
CK-40d	RHSIM_Rhsim07G0161800	-1.979	-1.106	0.873	low
CK-40d	RHSIM_Rhsim08G0049700	1.293	0.973	-0.320	high
CK-40d	RHSIM_Rhsim06G0078300	0.825	1.333	0.508	no
CK-40d	RHSIM_Rhsim06G0204200	0.245	0.482	0.236	no
CK-40d	RHSIM_Rhsim06G0241500	1.979	3.113	1.135	high
CK-40d	RHSIM_Rhsim08G0237900	1.166	0.324	-0.842	high
CK-40d	RHSIM_Rhsim06G0068400	-0.358	-1.133	-0.775	no
CK-40d	RHSIM_Rhsim06G0199700	2.553	0.974	-1.579	high
CK-40d	RHSIM_Rhsim07G0175700	1.016	1.205	0.189	no
CK-40d	RHSIM_Rhsim07G0127500	-0.364	1.777	2.141	no
CK-40d	RHSIM_Rhsim06G0062100	-1.363	-1.807	-0.444	low
CK-40d	RHSIM_Rhsim06G0175200	1.680	1.217	-0.463	high
CK-40d	RHSIM_Rhsim06G0078900	-1.604	-1.429	0.175	low
CK-40d	RHSIM_Rhsim07G0061800	-1.420	-0.964	0.456	low
CK-40d	RHSIM_Rhsim07G0226200	-0.070	0.493	0.563	no
CK-40d	RHSIM_Rhsim06G0232700	0.178	-0.822	-1.000	no

Group	Gene	PTR (z-score difference)	z(log2 LFQ)	z(log2 FPKM)	extreme-PTR
CK-40d	RHSIM_Rhsim07G0010400	-1.402	-0.354	1.049	low
CK-40d	RHSIM_Rhsim07G0084600	-3.213	-0.654	2.559	low
CK-40d	RHSIM_Rhsim07G0075300	0.498	1.671	1.173	no
CK-40d	RHSIM_Rhsim06G0067800	-0.720	-1.380	-0.660	no
CK-40d	RHSIM_Rhsim06G0091500	0.646	1.455	0.809	no
CK-40d	RHSIM_Rhsim06G0034000	-1.106	-0.192	0.914	no
CK-40d	RHSIM_Rhsim06G0142100	0.170	-0.716	-0.886	no
CK-40d	RHSIM_Rhsim06G0000400	-1.920	-0.925	0.995	low
CK-40d	RHSIM_Rhsim06G0012800	0.442	0.221	-0.221	no
CK-40d	RHSIM_Rhsim06G0228900	-0.354	-1.505	-1.151	no
CK-40d	RHSIM_Rhsim06G0127100	1.936	2.412	0.476	high
CK-40d	RHSIM_Rhsim07G0031000	-0.048	-0.547	-0.498	no
CK-40d	RHSIM_Rhsim06G0227100	-0.392	-0.926	-0.534	no
CK-40d	RHSIM_Rhsim08G0144900	2.930	2.227	-0.703	high
CK-40d	RHSIM_Rhsim06G0074800	-0.973	-0.841	0.132	no
CK-40d	RHSIM_Rhsim08G0057600	0.939	-0.873	-1.811	no
CK-40d	RHSIM_Rhsim07G0238800	1.348	-0.964	-2.313	high
CK-40d	RHSIM_Rhsim07G0085900	-1.095	-1.186	-0.091	no
CK-40d	RHSIM_Rhsim08G0039600	-1.302	-0.335	0.967	low
CK-40d	RHSIM_Rhsim08G0005300	1.691	2.094	0.403	high
CK-40d	RHSIM_Rhsim07G0100500	0.290	0.291	0.001	no
CK-40d	RHSIM_Rhsim08G0062500	-0.835	-0.216	0.618	no
CK-40d	RHSIM_Rhsim06G0004000	0.267	-0.554	-0.821	no
CK-40d	RHSIM_Rhsim06G0208100	0.429	-0.092	-0.521	no
CK-40d	RHSIM_Rhsim07G0168200	-0.497	0.475	0.971	no
CK-40d	RHSIM_Rhsim06G0104800	0.269	-0.160	-0.429	no
CK-40d	RHSIM_Rhsim07G0136900	2.266	0.099	-2.167	high
CK-40d	RHSIM_Rhsim06G0178500	0.052	-0.742	-0.794	no
CK-40d	RHSIM_Rhsim06G0121900	2.673	2.063	-0.610	high
CK-40d	RHSIM_Rhsim06G0035800	-0.671	-0.167	0.504	no
CK-40d	RHSIM_Rhsim06G0059900	-1.831	-0.804	1.027	low
CK-40d	RHSIM_Rhsim07G0044900	3.016	1.691	-1.326	high

Group	Gene	PTR (z-score difference)	z(log2 LFQ)	z(log2 FPKM)	extreme-PTR
CK-40d	RHSIM_Rhsim06G0057100	-0.693	1.138	1.831	no
CK-40d	RHSIM_Rhsim07G0028700	-0.937	-0.125	0.812	no
CK-40d	RHSIM_Rhsim06G0231500	-1.359	-1.459	-0.099	low
CK-40d	RHSIM_Rhsim07G0076400	-1.389	-0.624	0.765	low
CK-40d	RHSIM_Rhsim06G0219100	0.174	0.343	0.169	no
CK-40d	RHSIM_Rhsim07G0013400	-1.192	0.379	1.571	no
CK-40d	RHSIM_Rhsim07G0076100	-2.819	-1.081	1.738	low
CK-40d	RHSIM_Rhsim07G0162300	1.155	1.458	0.304	high
CK-40d	RHSIM_Rhsim07G0044800	0.848	0.367	-0.481	no
CK-40d	RHSIM_Rhsim07G0214900	-0.451	0.110	0.560	no
CK-40d	RHSIM_Rhsim06G0179200	0.890	0.256	-0.635	no
CK-40d	RHSIM_Rhsim06G0011800	2.336	3.613	1.277	high
CK-40d	RHSIM_Rhsim08G0077200	-0.535	0.431	0.965	no
CK-40d	RHSIM_Rhsim06G0152000	0.523	0.857	0.334	no
CK-40d	RHSIM_Rhsim07G0048800	-0.262	0.482	0.745	no
CK-40d	RHSIM_Rhsim06G0166100	1.799	-0.085	-1.884	high
CK-40d	RHSIM_Rhsim06G0035100	-0.820	-1.202	-0.382	no
CK-40d	RHSIM_Rhsim06G0232100	-1.508	-2.153	-0.645	low
CK-40d	RHSIM_Rhsim06G0155400	-1.092	1.410	2.503	no
CK-40d	RHSIM_Rhsim07G0103700	1.328	1.441	0.113	high
CK-40d	RHSIM_Rhsim07G0247400	1.224	-0.076	-1.300	high
CK-40d	RHSIM_Rhsim06G0009800	-0.699	-0.691	0.007	no
CK-40d	RHSIM_Rhsim07G0076600	-0.915	0.329	1.244	no
CK-40d	RHSIM_Rhsim06G0060000	0.560	-1.637	-2.196	no
CK-40d	RHSIM_Rhsim07G0103800	-0.558	0.946	1.503	no
CK-40d	RHSIM_Rhsim06G0017900	-0.575	-0.308	0.267	no
CK-40d	RHSIM_Rhsim06G0224000	-1.257	1.061	2.318	low
CK-40d	RHSIM_Rhsim06G0101600	0.458	0.564	0.106	no
CK-40d	RHSIM_Rhsim08G0020700	-0.350	0.521	0.871	no
CK-40d	RHSIM_Rhsim06G0005000	0.746	-0.309	-1.055	no
CK-40d	RHSIM_Rhsim07G0018000	1.665	0.750	-0.915	high
CK-40d	RHSIM_Rhsim08G0100900	-0.753	-0.897	-0.144	no

Group	Gene	PTR (z-score difference)	z(log2 LFQ)	z(log2 FPKM)	extreme-PTR
CK-40d	RHSIM_Rhsim06G0243400	-1.059	-0.248	0.811	no
CK-40d	RHSIM_Rhsim06G0061600	0.767	1.627	0.860	no
CK-40d	RHSIM_Rhsim06G0204400	1.518	0.655	-0.864	high
CK-40d	RHSIM_Rhsim06G0026000	-0.700	-1.260	-0.560	no
CK-40d	RHSIM_Rhsim06G0137100	0.231	1.013	0.783	no
CK-40d	RHSIM_Rhsim07G0003800	0.387	0.161	-0.226	no
CK-40d	RHSIM_Rhsim06G0013600	-2.373	0.104	2.477	low
CK-40d	RHSIM_Rhsim06G0129800	-0.166	-0.614	-0.447	no
CK-40d	RHSIM_Rhsim07G0033100	1.792	0.373	-1.419	high
CK-40d	RHSIM_Rhsim07G0257000	2.718	1.386	-1.332	high
CK-40d	RHSIM_Rhsim06G0135500	-1.490	-0.780	0.711	low
CK-40d	RHSIM_Rhsim07G0139800	1.008	0.435	-0.573	no
CK-40d	RHSIM_Rhsim08G0220400	-0.270	0.096	0.365	no
CK-40d	RHSIM_Rhsim07G0018700	1.929	2.011	0.082	high
CK-40d	RHSIM_Rhsim06G0224100	-0.412	-0.315	0.097	no
CK-40d	RHSIM_Rhsim07G0079200	-0.453	-1.107	-0.654	no
CK-40d	RHSIM_Rhsim08G0093000	-1.703	-0.695	1.008	low
CK-40d	RHSIM_Rhsim07G0013600	0.060	1.887	1.828	no
CK-40d	RHSIM_Rhsim07G0161400	0.692	-0.219	-0.911	no
CK-40d	RHSIM_Rhsim06G0229000	0.126	-0.435	-0.561	no
CK-40d	RHSIM_Rhsim07G0212200	-1.019	-0.752	0.267	no
CK-40d	RHSIM_Rhsim06G0240700	2.210	0.371	-1.839	high
CK-40d	RHSIM_Rhsim07G0166500	-0.247	0.863	1.110	no
CK-40d	RHSIM_Rhsim07G0243700	-0.944	-0.171	0.774	no
CK-40d	RHSIM_Rhsim06G0173600	-0.608	-1.093	-0.485	no
CK-40d	RHSIM_Rhsim06G0196300	2.234	1.435	-0.799	high
CK-40d	RHSIM_Rhsim06G0240900	-0.639	-1.711	-1.072	no
CK-40d	RHSIM_Rhsim05G0002300	-0.013	0.393	0.406	no
CK-40d	RHSIM_Rhsim07G0079900	-0.100	1.345	1.446	no
CK-40d	RHSIM_Rhsim06G0196500	-2.251	0.536	2.787	low
CK-40d	RHSIM_Rhsim07G0258500	-0.499	-0.148	0.351	no
CK-40d	RHSIM_Rhsim06G0164500	1.589	1.311	-0.278	high

Group	Gene	PTR (z-score difference)	z(log2 LFQ)	z(log2 FPKM)	extreme-PTR
CK-40d	RHSIM_Rhsim06G0040100	1.112	0.743	-0.369	no
CK-40d	RHSIM_Rhsim06G0040800	-0.549	-0.492	0.058	no
CK-40d	RHSIM_Rhsim06G0114600	-0.396	-0.146	0.250	no
CK-40d	RHSIM_Rhsim07G0257400	1.121	1.103	-0.018	no
CK-40d	RHSIM_Rhsim06G0032500	-1.005	-0.717	0.288	no
CK-40d	RHSIM_Rhsim06G0175700	-3.973	-0.408	3.565	low
CK-40d	RHSIM_Rhsim06G0021200	0.091	-0.211	-0.301	no
CK-40d	RHSIM_Rhsim06G0091400	-0.802	-1.029	-0.226	no
CK-40d	RHSIM_Rhsim06G0231200	0.076	-0.765	-0.841	no
CK-40d	RHSIM_Rhsim06G0085000	0.895	0.390	-0.504	no
CK-40d	RHSIM_Rhsim06G0012500	2.278	0.104	-2.174	high
CK-40d	RHSIM_Rhsim07G0224300	-1.450	-0.705	0.745	low
CK-40d	RHSIM_Rhsim07G0248700	-1.129	-0.596	0.533	no
CK-40d	RHSIM_Rhsim06G0178700	-0.981	-0.796	0.185	no
CK-40d	RHSIM_Rhsim06G0207600	0.124	-0.222	-0.346	no
CK-40d	RHSIM_Rhsim06G0195200	-3.439	-0.626	2.813	low
CK-40d	RHSIM_Rhsim07G0211600	-0.413	-0.208	0.204	no
CK-40d	RHSIM_Rhsim06G0178100	0.594	-0.601	-1.195	no
CK-40d	RHSIM_Rhsim06G0009500	0.062	-0.021	-0.084	no
CK-40d	RHSIM_Rhsim06G0216300	-1.918	-1.089	0.829	low
CK-40d	RHSIM_Rhsim05G0232900	0.389	2.082	1.693	no
CK-40d	RHSIM_Rhsim06G0008000	1.001	-0.697	-1.698	no
CK-40d	RHSIM_Rhsim06G0174400	-0.150	-0.581	-0.431	no
CK-40d	RHSIM_Rhsim06G0069300	-0.854	-0.114	0.740	no
CK-40d	RHSIM_Rhsim06G0223700	-0.460	-0.266	0.194	no
CK-40d	RHSIM_Rhsim06G0000600	0.146	-0.821	-0.967	no
CK-40d	RHSIM_Rhsim06G0146800	1.884	0.228	-1.656	high
CK-40d	RHSIM_Rhsim06G0238600	1.407	1.132	-0.274	high
CK-40d	RHSIM_Rhsim06G0211200	-1.552	-1.449	0.103	low
CK-40d	RHSIM_Rhsim07G0154200	-1.330	-1.993	-0.664	low
CK-40d	RHSIM_Rhsim06G0225600	-0.641	-0.905	-0.264	no
CK-40d	RHSIM_Rhsim07G0150900	0.126	-0.458	-0.584	no

Group	Gene	PTR (z-score difference)	z(log2 LFQ)	z(log2 FPKM)	extreme-PTR
CK-40d	RHSIM_Rhsim06G0175900	0.687	0.280	-0.407	no
CK-40d	RHSIM_Rhsim05G0005000	1.574	0.452	-1.122	high
CK-40d	RHSIM_Rhsim06G0073900	1.303	1.966	0.663	high
CK-40d	RHSIM_Rhsim07G0115200	-0.624	-0.985	-0.361	no
CK-40d	RHSIM_Rhsim07G0029000	0.966	0.800	-0.166	no
CK-40d	RHSIM_Rhsim06G0197600	0.398	0.844	0.446	no
CK-40d	RHSIM_Rhsim06G0236900	-0.219	-1.235	-1.016	no
CK-40d	RHSIM_Rhsim06G0184100	-1.001	0.066	1.067	no
CK-40d	RHSIM_Rhsim07G0250200	-0.724	-0.384	0.340	no
CK-40d	RHSIM_Rhsim06G0224200	1.258	0.305	-0.953	high
CK-40d	RHSIM_Rhsim06G0075100	-1.212	-0.477	0.734	no
CK-40d	RHSIM_Rhsim05G0126500	0.194	0.343	0.148	no
CK-40d	RHSIM_Rhsim06G0035600	1.015	-0.726	-1.741	no
CK-40d	RHSIM_Rhsim06G0027600	-0.429	-0.377	0.051	no
CK-40d	RHSIM_Rhsim06G0241000	1.405	1.396	-0.009	high
CK-40d	RHSIM_Rhsim06G0006500	1.962	-0.056	-2.018	high
CK-40d	RHSIM_Rhsim06G0207700	1.405	0.034	-1.371	high
CK-40d	RHSIM_Rhsim05G0045000	-1.297	-0.695	0.602	low
CK-40d	RHSIM_Rhsim07G0084000	0.459	0.120	-0.339	no
CK-40d	RHSIM_Rhsim05G0048300	1.474	-0.687	-2.160	high
CK-40d	RHSIM_Rhsim06G0008400	-0.949	-0.082	0.867	no
CK-40d	RHSIM_Rhsim05G0168000	0.332	-0.215	-0.546	no
CK-40d	RHSIM_Rhsim07G0027800	0.199	1.232	1.033	no
CK-40d	RHSIM_Rhsim06G0197200	-1.873	-1.229	0.645	low
CK-40d	RHSIM_Rhsim07G0009400	-1.063	-1.396	-0.334	no
CK-40d	RHSIM_Rhsim05G0132400	1.617	2.096	0.479	high
CK-40d	RHSIM_Rhsim07G0022200	0.421	-1.298	-1.719	no
CK-40d	RHSIM_Rhsim06G0172900	-0.411	-1.451	-1.041	no
CK-40d	RHSIM_Rhsim06G0242800	-1.207	-0.708	0.499	no
CK-40d	RHSIM_Rhsim07G0038600	-0.540	0.144	0.684	no
CK-40d	RHSIM_Rhsim06G0052100	3.104	0.909	-2.195	high
CK-40d	RHSIM_Rhsim06G0023100	-0.118	-1.621	-1.503	no

Group	Gene	PTR (z-score difference)	z(log2 LFQ)	z(log2 FPKM)	extreme-PTR
CK-40d	RHSIM_Rhsim06G0214200	-1.004	-1.052	-0.048	no
CK-40d	RHSIM_Rhsim07G0121200	-2.728	-1.766	0.961	low
CK-40d	RHSIM_Rhsim06G0054400	1.144	1.133	-0.011	high
CK-40d	RHSIM_Rhsim05G0018800	-0.737	-0.171	0.566	no
CK-40d	RHSIM_Rhsim06G0129900	-0.848	0.969	1.817	no
CK-40d	RHSIM_Rhsim07G0008000	-0.770	-0.685	0.085	no
CK-40d	RHSIM_Rhsim06G0186500	-0.945	-0.186	0.759	no
CK-40d	RHSIM_Rhsim05G0115300	1.988	1.826	-0.162	high
CK-40d	RHSIM_Rhsim06G0244600	-1.101	-1.183	-0.082	no
CK-40d	RHSIM_Rhsim05G0044300	0.193	1.442	1.249	no
CK-40d	RHSIM_Rhsim07G0174700	0.435	-0.572	-1.007	no
CK-40d	RHSIM_Rhsim05G0060300	-0.387	-1.040	-0.653	no
CK-40d	RHSIM_Rhsim07G0211400	-0.799	-0.538	0.261	no
CK-40d	RHSIM_Rhsim06G0029700	-0.647	-0.493	0.154	no
CK-40d	RHSIM_Rhsim05G0233100	0.204	-0.077	-0.281	no
CK-40d	RHSIM_Rhsim06G0021700	-0.091	0.797	0.888	no
CK-40d	RHSIM_Rhsim05G0060600	-0.637	-0.021	0.617	no
CK-40d	RHSIM_Rhsim06G0031100	-0.185	-1.583	-1.398	no
CK-40d	RHSIM_Rhsim06G0191800	-0.232	1.098	1.329	no
CK-40d	RHSIM_Rhsim07G0119900	0.025	0.302	0.277	no
CK-40d	RHSIM_Rhsim06G0059400	-1.132	-0.760	0.372	no
CK-40d	RHSIM_Rhsim05G0060400	0.372	0.583	0.211	no
CK-40d	RHSIM_Rhsim06G0183300	0.560	-0.062	-0.622	no
CK-40d	RHSIM_Rhsim06G0018700	-1.043	-1.063	-0.020	no
CK-40d	RHSIM_Rhsim07G0240500	1.211	0.104	-1.107	high
CK-40d	RHSIM_Rhsim05G0187800	-0.203	1.164	1.366	no
CK-40d	RHSIM_Rhsim06G0046300	1.401	1.273	-0.128	high
CK-40d	RHSIM_Rhsim05G0208000	0.010	-0.649	-0.659	no
CK-40d	RHSIM_Rhsim06G0070900	-0.294	-0.749	-0.455	no
CK-40d	RHSIM_Rhsim06G0018400	-0.908	-0.499	0.410	no
CK-40d	RHSIM_Rhsim07G0154100	1.124	0.316	-0.808	no
CK-40d	RHSIM_Rhsim06G0118100	0.048	-0.607	-0.654	no

Group	Gene	PTR (z-score difference)	z(log2 LFQ)	z(log2 FPKM)	extreme-PTR
CK-40d	RHSIM_Rhsim06G0200600	0.144	-0.433	-0.576	no
CK-40d	RHSIM_Rhsim06G0097000	0.688	-0.336	-1.023	no
CK-40d	RHSIM_Rhsim06G0069400	-0.686	0.068	0.754	no
CK-40d	RHSIM_Rhsim05G0022700	1.036	0.934	-0.103	no
CK-40d	RHSIM_Rhsim06G0004600	2.301	3.020	0.719	high
CK-40d	RHSIM_Rhsim06G0213400	0.229	-1.080	-1.309	no
CK-40d	RHSIM_Rhsim06G0166800	-1.833	-0.433	1.399	low
CK-40d	RHSIM_Rhsim06G0048000	1.338	1.240	-0.099	high
CK-40d	RHSIM_Rhsim05G0185900	0.559	0.036	-0.523	no
CK-40d	RHSIM_Rhsim06G0121700	0.074	0.377	0.303	no
CK-40d	RHSIM_Rhsim06G0238500	-0.445	-0.361	0.084	no
CK-40d	RHSIM_Rhsim05G0003900	-0.096	-0.625	-0.529	no
CK-40d	RHSIM_Rhsim06G0216400	-0.670	0.131	0.801	no
CK-40d	RHSIM_Rhsim05G0216700	-1.971	-0.444	1.527	low
CK-40d	RHSIM_Rhsim06G0218400	0.439	0.739	0.300	no
CK-40d	RHSIM_Rhsim06G0136000	0.536	1.770	1.235	no
CK-40d	RHSIM_Rhsim05G0070300	0.600	0.416	-0.184	no
CK-40d	RHSIM_Rhsim06G0004300	0.852	1.215	0.363	no
CK-40d	RHSIM_Rhsim06G0175300	0.492	-0.734	-1.226	no
CK-40d	RHSIM_Rhsim05G0222900	0.628	0.334	-0.294	no
CK-40d	RHSIM_Rhsim06G0137300	0.304	0.821	0.517	no
CK-40d	RHSIM_Rhsim05G0195200	-1.513	-1.264	0.249	low
CK-40d	RHSIM_Rhsim06G0147000	-1.593	-1.480	0.113	low
CK-40d	RHSIM_Rhsim05G0104500	-1.777	-0.121	1.656	low
CK-40d	RHSIM_Rhsim05G0088300	-0.852	-0.814	0.038	no
CK-40d	RHSIM_Rhsim05G0122300	-0.476	-0.979	-0.503	no
CK-40d	RHSIM_Rhsim06G0063800	1.291	-1.141	-2.432	high
CK-40d	RHSIM_Rhsim07G0104300	1.565	0.314	-1.250	high
CK-40d	RHSIM_Rhsim06G0213200	0.947	-0.013	-0.960	no
CK-40d	RHSIM_Rhsim06G0116100	-0.554	-0.013	0.541	no
CK-40d	RHSIM_Rhsim06G0201300	0.809	1.671	0.862	no
CK-40d	RHSIM_Rhsim06G0153100	0.947	2.050	1.103	no

Group	Gene	PTR (z-score difference)	z(log2 LFQ)	z(log2 FPKM)	extreme-PTR
CK-40d	RHSIM_Rhsim05G0177500	1.187	-1.185	-2.372	high
CK-40d	RHSIM_Rhsim06G0129300	0.730	1.363	0.633	no
CK-40d	RHSIM_Rhsim06G0232400	-2.347	-1.099	1.248	low
CK-40d	RHSIM_Rhsim05G0172800	0.150	-1.093	-1.243	no
CK-40d	RHSIM_Rhsim06G0211100	0.015	-0.428	-0.443	no
CK-40d	RHSIM_Rhsim06G0054200	-1.112	-0.991	0.121	no
CK-40d	RHSIM_Rhsim06G0165500	-1.964	-1.176	0.787	low
CK-40d	RHSIM_Rhsim05G0197900	2.204	1.447	-0.757	high
CK-40d	RHSIM_Rhsim06G0076700	-0.899	0.634	1.533	no
CK-40d	RHSIM_Rhsim06G0224400	-0.692	-0.755	-0.063	no
CK-40d	RHSIM_Rhsim06G0210200	-0.601	0.073	0.674	no
CK-40d	RHSIM_Rhsim06G0212800	1.182	0.666	-0.516	high
CK-40d	RHSIM_Rhsim06G0092900	-0.790	-1.219	-0.429	no
CK-40d	RHSIM_Rhsim05G0142400	-2.584	-0.864	1.720	low
CK-40d	RHSIM_Rhsim06G0239000	-1.126	-0.131	0.994	no
CK-40d	RHSIM_Rhsim06G0223600	-0.144	-0.944	-0.800	no
CK-40d	RHSIM_Rhsim05G0049000	-0.422	0.108	0.530	no
CK-40d	RHSIM_Rhsim06G0157900	-1.795	-0.696	1.099	low
CK-40d	RHSIM_Rhsim06G0062200	-0.179	-0.194	-0.014	no
CK-40d	RHSIM_Rhsim05G0229900	-0.723	-0.589	0.135	no
CK-40d	RHSIM_Rhsim05G0029300	0.761	1.436	0.675	no
CK-40d	RHSIM_Rhsim05G0112500	0.249	0.448	0.199	no
CK-40d	RHSIM_Rhsim05G0095600	0.573	-0.015	-0.588	no
CK-40d	RHSIM_Rhsim06G0234000	-0.076	-0.449	-0.373	no
CK-40d	RHSIM_Rhsim05G0013100	-0.279	-0.310	-0.031	no
CK-40d	RHSIM_Rhsim05G0016600	-0.934	-0.709	0.225	no
CK-40d	RHSIM_Rhsim05G0073600	0.815	-1.092	-1.906	no
CK-40d	RHSIM_Rhsim06G0065300	0.160	-1.002	-1.162	no
CK-40d	RHSIM_Rhsim05G0053200	0.659	0.720	0.062	no
CK-40d	RHSIM_Rhsim05G0188100	-0.442	0.499	0.941	no
CK-40d	RHSIM_Rhsim05G0132800	0.272	0.680	0.408	no
CK-40d	RHSIM_Rhsim05G0032600	2.223	1.115	-1.108	high

Group	Gene	PTR (z-score difference)	z(log2 LFQ)	z(log2 FPKM)	extreme-PTR
CK-40d	RHSIM_Rhsim07G0091100	2.373	0.766	-1.607	high
CK-40d	RHSIM_Rhsim06G0243100	-1.687	-0.500	1.187	low
CK-40d	RHSIM_Rhsim05G0009900	2.216	1.437	-0.779	high
CK-40d	RHSIM_Rhsim05G0056900	-1.124	0.512	1.636	no
CK-40d	RHSIM_Rhsim05G0230200	2.144	0.175	-1.969	high
CK-40d	RHSIM_Rhsim06G0103100	-0.779	-0.791	-0.012	no
CK-40d	RHSIM_Rhsim06G0086200	-0.471	0.561	1.031	no
CK-40d	RHSIM_Rhsim05G0167800	-0.618	-0.531	0.088	no
CK-40d	RHSIM_Rhsim05G0092800	0.313	-0.940	-1.253	no
CK-40d	RHSIM_Rhsim05G0005700	0.293	0.069	-0.224	no
CK-40d	RHSIM_Rhsim06G0099200	-0.716	-0.207	0.509	no
CK-40d	RHSIM_Rhsim06G0212400	0.308	0.716	0.408	no
CK-40d	RHSIM_Rhsim06G0166500	-1.084	0.397	1.481	no
CK-40d	RHSIM_Rhsim06G0128200	-0.219	0.264	0.484	no
CK-40d	RHSIM_Rhsim05G0148100	-0.991	-0.291	0.700	no
CK-40d	RHSIM_Rhsim06G0166300	-0.134	-0.633	-0.498	no
CK-40d	RHSIM_Rhsim06G0067500	1.615	0.963	-0.652	high
CK-40d	RHSIM_Rhsim05G0229000	-1.529	0.035	1.565	low
CK-40d	RHSIM_Rhsim06G0198500	-0.247	0.299	0.546	no
CK-40d	RHSIM_Rhsim05G0102000	-0.484	-0.894	-0.411	no
CK-40d	RHSIM_Rhsim06G0022900	2.471	1.088	-1.382	high
CK-40d	RHSIM_Rhsim05G0079000	-0.014	0.703	0.717	no
CK-40d	RHSIM_Rhsim05G0184700	0.127	-0.569	-0.695	no
CK-40d	RHSIM_Rhsim06G0009700	0.357	0.597	0.240	no
CK-40d	RHSIM_Rhsim07G0188500	-0.398	-1.201	-0.803	no
CK-40d	RHSIM_Rhsim05G0014500	-0.477	0.374	0.851	no
CK-40d	RHSIM_Rhsim05G0004300	0.552	2.133	1.581	no
CK-40d	RHSIM_Rhsim06G0202700	0.161	-0.231	-0.392	no
CK-40d	RHSIM_Rhsim06G0148500	1.332	3.187	1.856	high
CK-40d	RHSIM_Rhsim05G0206700	-2.046	-0.145	1.901	low
CK-40d	RHSIM_Rhsim05G0142600	-0.819	-0.664	0.155	no
CK-40d	RHSIM_Rhsim05G0079100	-1.467	-1.008	0.459	low

Group	Gene	PTR (z-score difference)	z(log2 LFQ)	z(log2 FPKM)	extreme-PTR
CK-40d	RHSIM_Rhsim05G0220000	-0.102	-0.280	-0.178	no
CK-40d	RHSIM_Rhsim05G0039500	0.925	1.119	0.194	no
CK-40d	RHSIM_Rhsim05G0056600	-1.672	-0.857	0.814	low
CK-40d	RHSIM_Rhsim05G0193900	1.041	2.083	1.041	no
CK-40d	RHSIM_Rhsim05G0074500	0.526	0.336	-0.189	no
CK-40d	RHSIM_Rhsim07G0191000	-1.074	-0.917	0.156	no
CK-40d	RHSIM_Rhsim06G0032600	0.322	0.402	0.080	no
CK-40d	RHSIM_Rhsim07G0222600	-2.700	-1.733	0.968	low
CK-40d	RHSIM_Rhsim06G0131600	0.164	-0.746	-0.911	no
CK-40d	RHSIM_Rhsim05G0055600	-0.164	0.272	0.436	no
CK-40d	RHSIM_Rhsim06G0230600	-1.684	-0.981	0.704	low
CK-40d	RHSIM_Rhsim07G0246000	1.485	-0.118	-1.603	high
CK-40d	RHSIM_Rhsim05G0165600	1.022	1.136	0.114	no
CK-40d	RHSIM_Rhsim06G0211900	1.463	-0.154	-1.617	high
CK-40d	RHSIM_Rhsim06G0048700	-0.760	-0.977	-0.217	no
CK-40d	RHSIM_Rhsim05G0094900	-1.050	-1.545	-0.495	no
CK-40d	RHSIM_Rhsim06G0031000	1.774	0.455	-1.319	high
CK-40d	RHSIM_Rhsim05G0170000	0.644	0.099	-0.545	no
CK-40d	RHSIM_Rhsim06G0206300	1.767	1.356	-0.411	high
CK-40d	RHSIM_Rhsim05G0114200	-1.241	-1.577	-0.337	low
CK-40d	RHSIM_Rhsim06G0239600	0.453	1.885	1.432	no
CK-40d	RHSIM_Rhsim06G0090800	-1.467	-0.520	0.947	low
CK-40d	RHSIM_Rhsim06G0087600	-0.839	-0.404	0.435	no
CK-40d	RHSIM_Rhsim05G0172300	-1.520	-1.701	-0.182	low
CK-40d	RHSIM_Rhsim06G0179300	0.572	-0.763	-1.335	no
CK-40d	RHSIM_Rhsim07G0095500	-1.563	-1.011	0.553	low
CK-40d	RHSIM_Rhsim05G0195100	-0.517	-0.508	0.010	no
CK-40d	RHSIM_Rhsim05G0107000	0.642	0.229	-0.413	no
CK-40d	RHSIM_Rhsim06G0206000	-0.223	-0.166	0.056	no
CK-40d	RHSIM_Rhsim06G0103300	2.762	3.470	0.708	high
CK-40d	RHSIM_Rhsim05G0075800	2.197	1.449	-0.748	high
CK-40d	RHSIM_Rhsim05G0175300	0.528	0.818	0.289	no

Group	Gene	PTR (z-score difference)	z(log2 LFQ)	z(log2 FPKM)	extreme-PTR
CK-40d	RHSIM_Rhsim07G0020100	-3.055	-2.141	0.914	low
CK-40d	RHSIM_Rhsim05G0076200	1.131	-0.115	-1.246	no
CK-40d	RHSIM_Rhsim06G0232500	0.945	0.652	-0.294	no
CK-40d	RHSIM_Rhsim06G0141900	-0.617	-0.810	-0.193	no
CK-40d	RHSIM_Rhsim05G0038200	0.550	1.083	0.532	no
CK-40d	RHSIM_Rhsim05G0048000	0.045	-0.988	-1.033	no
CK-40d	RHSIM_Rhsim06G0208300	-1.534	-0.183	1.351	low
CK-40d	RHSIM_Rhsim06G0008500	0.469	1.754	1.285	no
CK-40d	RHSIM_Rhsim05G0101600	-0.831	-0.280	0.551	no
CK-40d	RHSIM_Rhsim05G0148700	0.180	0.101	-0.078	no
CK-40d	RHSIM_Rhsim06G0003100	-2.113	-1.923	0.190	low
CK-40d	RHSIM_Rhsim05G0056500	0.935	1.441	0.506	no
CK-40d	RHSIM_Rhsim05G0114100	0.364	-0.661	-1.025	no
CK-40d	RHSIM_Rhsim06G0006600	0.878	1.651	0.772	no
CK-40d	RHSIM_Rhsim05G0078100	-1.862	-1.086	0.776	low
CK-40d	RHSIM_Rhsim06G0027100	-1.202	0.854	2.057	no
CK-40d	RHSIM_Rhsim05G0081000	-1.600	-1.651	-0.051	low
CK-40d	RHSIM_Rhsim05G0081600	-1.927	0.095	2.022	low
CK-40d	RHSIM_Rhsim06G0170000	0.930	-0.564	-1.494	no
CK-40d	RHSIM_Rhsim05G0007700	-1.069	-1.564	-0.495	no
CK-40d	RHSIM_Rhsim05G0142500	-1.343	-0.539	0.804	low
CK-40d	RHSIM_Rhsim05G0015000	-0.503	0.520	1.023	no
CK-40d	RHSIM_Rhsim05G0040400	-0.414	-0.216	0.198	no
CK-40d	RHSIM_Rhsim06G0184400	-0.201	-0.224	-0.022	no
CK-40d	RHSIM_Rhsim05G0034500	-0.133	-0.792	-0.659	no
CK-40d	RHSIM_Rhsim05G0125700	1.375	0.876	-0.499	high
CK-40d	RHSIM_Rhsim05G0234300	-1.800	0.238	2.038	low
CK-40d	RHSIM_Rhsim06G0176900	1.306	0.030	-1.275	high
CK-40d	RHSIM_Rhsim07G0136800	0.335	-0.350	-0.684	no
CK-40d	RHSIM_Rhsim05G0228100	-0.803	0.220	1.023	no
CK-40d	RHSIM_Rhsim06G0062000	0.408	0.033	-0.376	no
CK-40d	RHSIM_Rhsim05G0203100	-0.118	-0.460	-0.342	no

Group	Gene	PTR (z-score difference)	z(log2 LFQ)	z(log2 FPKM)	extreme-PTR
CK-40d	RHSIM_Rhsim05G0094000	-1.442	-1.137	0.305	low
CK-40d	RHSIM_Rhsim05G0178300	-0.101	0.103	0.204	no
CK-40d	RHSIM_Rhsim05G0160100	-0.562	0.406	0.968	no
CK-40d	RHSIM_Rhsim06G0089200	-1.235	-0.909	0.325	no
CK-40d	RHSIM_Rhsim06G0022200	1.227	-0.226	-1.452	high
CK-40d	RHSIM_Rhsim05G0048900	-1.932	-1.550	0.382	low
CK-40d	RHSIM_Rhsim05G0022400	1.205	0.144	-1.060	high
CK-40d	RHSIM_Rhsim05G0167700	1.035	-1.149	-2.185	no
CK-40d	RHSIM_Rhsim05G0203300	-1.167	-0.532	0.635	no
CK-40d	RHSIM_Rhsim05G0013700	-0.796	-1.096	-0.300	no
CK-40d	RHSIM_Rhsim05G0082400	3.995	1.729	-2.266	high
CK-40d	RHSIM_Rhsim05G0214500	0.302	1.484	1.183	no
CK-40d	RHSIM_Rhsim04G0249900	-0.100	-1.023	-0.923	no
CK-40d	RHSIM_Rhsim05G0141700	1.140	0.310	-0.829	high
CK-40d	RHSIM_Rhsim07G0093500	0.970	0.202	-0.769	no
CK-40d	RHSIM_Rhsim06G0039400	0.304	1.648	1.344	no
CK-40d	RHSIM_Rhsim04G0061200	1.822	0.146	-1.676	high
CK-40d	RHSIM_Rhsim05G0057000	0.528	-0.048	-0.576	no
CK-40d	RHSIM_Rhsim06G0052200	2.186	0.016	-2.170	high
CK-40d	RHSIM_Rhsim05G0035400	-0.130	0.013	0.143	no
CK-40d	RHSIM_Rhsim04G0207200	-0.514	-0.488	0.026	no
CK-40d	RHSIM_Rhsim06G0183900	1.420	0.837	-0.584	high
CK-40d	RHSIM_Rhsim05G0179700	0.963	1.499	0.535	no
CK-40d	RHSIM_Rhsim05G0055000	-0.160	-0.151	0.008	no
CK-40d	RHSIM_Rhsim06G0234700	-1.083	0.056	1.139	no
CK-40d	RHSIM_Rhsim04G0179500	-0.650	-1.468	-0.819	no
CK-40d	RHSIM_Rhsim05G0141900	0.037	0.379	0.342	no
CK-40d	RHSIM_Rhsim05G0165000	0.104	-0.673	-0.777	no
CK-40d	RHSIM_Rhsim05G0169000	0.103	-0.932	-1.035	no
CK-40d	RHSIM_Rhsim06G0060300	0.072	0.439	0.367	no
CK-40d	RHSIM_Rhsim06G0030800	0.453	1.669	1.216	no
CK-40d	RHSIM_Rhsim05G0178900	-0.919	0.245	1.165	no

Group	Gene	PTR (z-score difference)	z(log2 LFQ)	z(log2 FPKM)	extreme-PTR
CK-40d	RHSIM_Rhsim06G0139200	2.068	2.578	0.509	high
CK-40d	RHSIM_Rhsim05G0001300	-0.089	-0.640	-0.551	no
CK-40d	RHSIM_Rhsim05G0119100	-0.625	-0.894	-0.268	no
CK-40d	RHSIM_Rhsim05G0233200	-0.041	0.643	0.684	no
CK-40d	RHSIM_Rhsim05G0141500	-1.984	-0.089	1.895	low
CK-40d	RHSIM_Rhsim06G0114100	0.228	0.181	-0.047	no
CK-40d	RHSIM_Rhsim07G0044200	-0.409	-0.915	-0.507	no
CK-40d	RHSIM_Rhsim06G0071600	0.668	-0.072	-0.740	no
CK-40d	RHSIM_Rhsim05G0067900	-1.071	-1.447	-0.376	no
CK-40d	RHSIM_Rhsim05G0161500	-0.984	-0.418	0.566	no
CK-40d	RHSIM_Rhsim07G0149700	-0.015	-0.375	-0.360	no
CK-40d	RHSIM_Rhsim05G0203500	0.244	1.014	0.770	no
CK-40d	RHSIM_Rhsim06G0047300	1.524	0.813	-0.711	high
CK-40d	RHSIM_Rhsim05G0028200	1.784	0.248	-1.536	high
CK-40d	RHSIM_Rhsim04G0153900	1.372	0.639	-0.733	high
CK-40d	RHSIM_Rhsim05G0122100	-1.509	-1.093	0.416	low
CK-40d	RHSIM_Rhsim07G0213300	0.787	0.697	-0.089	no
CK-40d	RHSIM_Rhsim05G0221100	1.781	-0.560	-2.341	high
CK-40d	RHSIM_Rhsim06G0238700	-1.378	-1.686	-0.308	low
CK-40d	RHSIM_Rhsim04G0180900	-1.296	0.646	1.942	low
CK-40d	RHSIM_Rhsim07G0102700	1.437	1.061	-0.376	high
CK-40d	RHSIM_Rhsim04G0213100	2.511	1.532	-0.979	high
CK-40d	RHSIM_Rhsim05G0228600	-0.292	1.392	1.683	no
CK-40d	RHSIM_Rhsim05G0018700	1.294	0.977	-0.317	high
CK-40d	RHSIM_Rhsim06G0203000	0.054	0.224	0.171	no
CK-40d	RHSIM_Rhsim06G0096300	0.244	0.317	0.073	no
CK-40d	RHSIM_Rhsim05G0219700	-0.523	-1.137	-0.614	no
CK-40d	RHSIM_Rhsim06G0187600	2.106	0.126	-1.980	high
CK-40d	RHSIM_Rhsim05G0115900	0.458	0.456	-0.002	no
CK-40d	RHSIM_Rhsim05G0168400	-1.007	-1.128	-0.121	no
CK-40d	RHSIM_Rhsim05G0093000	-0.161	-0.201	-0.039	no
CK-40d	RHSIM_Rhsim05G0216400	-0.688	0.469	1.157	no

Group	Gene	PTR (z-score difference)	z(log2 LFQ)	z(log2 FPKM)	extreme-PTR
CK-40d	RHSIM_Rhsim04G0117500	-0.549	-1.598	-1.049	no
CK-40d	RHSIM_Rhsim05G0148400	-0.960	-1.003	-0.042	no
CK-40d	RHSIM_Rhsim06G0086800	-0.991	-1.627	-0.636	no
CK-40d	RHSIM_Rhsim07G0176500	-0.281	-0.186	0.095	no
CK-40d	RHSIM_Rhsim05G0107700	-1.445	-0.213	1.232	low
CK-40d	RHSIM_Rhsim05G0068100	2.121	1.119	-1.002	high
CK-40d	RHSIM_Rhsim05G0146600	-1.612	-1.655	-0.043	low
CK-40d	RHSIM_Rhsim04G0058800	-0.104	-0.654	-0.551	no
CK-40d	RHSIM_Rhsim05G0070600	0.580	-0.076	-0.656	no
CK-40d	RHSIM_Rhsim05G0104400	-0.883	0.280	1.162	no
CK-40d	RHSIM_Rhsim05G0047800	1.366	-0.639	-2.005	high
CK-40d	RHSIM_Rhsim05G0012600	-2.921	-1.670	1.251	low
CK-40d	RHSIM_Rhsim06G0244700	0.277	-0.312	-0.589	no
CK-40d	RHSIM_Rhsim06G0022400	0.474	-0.669	-1.144	no
CK-40d	RHSIM_Rhsim07G0057400	1.117	-0.361	-1.478	no
CK-40d	RHSIM_Rhsim05G0070100	-2.427	-0.033	2.394	low
CK-40d	RHSIM_Rhsim05G0169700	2.142	3.286	1.144	high
CK-40d	RHSIM_Rhsim04G0131900	1.430	1.116	-0.314	high
CK-40d	RHSIM_Rhsim06G0047100	0.114	-0.635	-0.749	no
CK-40d	RHSIM_Rhsim05G0166700	0.727	1.003	0.277	no
CK-40d	RHSIM_Rhsim05G0223800	-0.065	2.008	2.074	no
CK-40d	RHSIM_Rhsim05G0020400	-1.370	-0.266	1.104	low
CK-40d	RHSIM_Rhsim05G0196900	0.900	1.035	0.135	no
CK-40d	RHSIM_Rhsim06G0078800	-0.645	1.458	2.103	no
CK-40d	RHSIM_Rhsim05G0190800	-2.206	0.255	2.461	low
CK-40d	RHSIM_Rhsim05G0069700	0.140	0.296	0.156	no
CK-40d	RHSIM_Rhsim05G0074000	-0.889	1.905	2.794	no
CK-40d	RHSIM_Rhsim05G0150000	-0.543	-0.473	0.070	no
CK-40d	RHSIM_Rhsim05G0096900	-0.608	1.302	1.910	no
CK-40d	RHSIM_Rhsim06G0005300	-0.296	0.022	0.318	no
CK-40d	RHSIM_Rhsim05G0101100	0.034	-1.324	-1.358	no
CK-40d	RHSIM_Rhsim05G0170400	1.836	1.226	-0.610	high

Group	Gene	PTR (z-score difference)	z(log2 LFQ)	z(log2 FPKM)	extreme-PTR
CK-40d	RHSIM_Rhsim05G0073500	-1.102	-0.274	0.828	no
CK-40d	RHSIM_Rhsim06G0220300	0.899	0.058	-0.841	no
CK-40d	RHSIM_Rhsim05G0182300	-0.326	-0.548	-0.221	no
CK-40d	RHSIM_Rhsim07G0058500	1.051	0.472	-0.579	no
CK-40d	RHSIM_Rhsim04G0175300	-0.376	-0.679	-0.303	no
CK-40d	RHSIM_Rhsim05G0062700	1.701	0.054	-1.648	high
CK-40d	RHSIM_Rhsim05G0075900	-1.392	-0.473	0.919	low
CK-40d	RHSIM_Rhsim05G0103400	1.328	1.300	-0.028	high
CK-40d	RHSIM_Rhsim04G0099500	-2.028	-1.303	0.725	low
CK-40d	RHSIM_Rhsim05G0159700	-1.277	-0.260	1.017	low
CK-40d	RHSIM_Rhsim05G0085900	0.706	0.219	-0.487	no
CK-40d	RHSIM_Rhsim05G0020500	-0.454	-1.480	-1.025	no
CK-40d	RHSIM_Rhsim05G0177100	0.634	0.899	0.265	no
CK-40d	RHSIM_Rhsim05G0180300	0.128	-0.921	-1.049	no
CK-40d	RHSIM_Rhsim04G0126100	-0.979	-0.986	-0.007	no
CK-40d	RHSIM_Rhsim06G0049900	1.002	-0.329	-1.331	no
CK-40d	RHSIM_Rhsim05G0080100	-0.617	0.046	0.663	no
CK-40d	RHSIM_Rhsim06G0002600	-0.925	-0.796	0.129	no
CK-40d	RHSIM_Rhsim05G0201700	1.567	-0.595	-2.162	high
CK-40d	RHSIM_Rhsim05G0143500	-1.271	-0.671	0.600	low
CK-40d	RHSIM_Rhsim05G0074600	0.554	0.318	-0.237	no
CK-40d	RHSIM_Rhsim06G0142000	-1.752	-0.799	0.954	low
CK-40d	RHSIM_Rhsim05G0040900	-1.058	-1.344	-0.285	no
CK-40d	RHSIM_Rhsim05G0005100	1.561	-0.267	-1.828	high
CK-40d	RHSIM_Rhsim05G0214100	1.427	1.183	-0.243	high
CK-40d	RHSIM_Rhsim05G0222100	0.157	-0.857	-1.014	no
CK-40d	RHSIM_Rhsim07G0247000	0.047	0.357	0.310	no
CK-40d	RHSIM_Rhsim06G0082000	-0.499	-0.069	0.430	no
CK-40d	RHSIM_Rhsim05G0187300	-0.437	0.379	0.815	no
CK-40d	RHSIM_Rhsim05G0143000	2.532	1.952	-0.580	high
CK-40d	RHSIM_Rhsim05G0063700	0.406	0.941	0.535	no
CK-40d	RHSIM_Rhsim04G0132500	1.450	0.900	-0.550	high

Group	Gene	PTR (z-score difference)	z(log2 LFQ)	z(log2 FPKM)	extreme-PTR
CK-40d	RHSIM_Rhsim05G0072000	1.904	-0.048	-1.952	high
CK-40d	RHSIM_Rhsim04G0011300	0.197	-0.060	-0.257	no
CK-40d	RHSIM_Rhsim05G0110900	1.369	0.895	-0.474	high
CK-40d	RHSIM_Rhsim05G0106700	-0.347	0.008	0.355	no
CK-40d	RHSIM_Rhsim04G0028000	2.794	1.379	-1.415	high
CK-40d	RHSIM_Rhsim06G0054600	-1.151	-0.761	0.390	no
CK-40d	RHSIM_Rhsim05G0029400	-1.054	-0.169	0.885	no
CK-40d	RHSIM_Rhsim04G0009500	-0.101	-0.962	-0.861	no
CK-40d	RHSIM_Rhsim05G0203000	0.244	0.392	0.148	no
CK-40d	RHSIM_Rhsim06G0047000	1.108	-0.470	-1.578	no
CK-40d	RHSIM_Rhsim05G0113900	0.755	0.627	-0.128	no
CK-40d	RHSIM_Rhsim05G0012900	0.061	0.256	0.195	no
CK-40d	RHSIM_Rhsim04G0014200	0.667	-0.228	-0.895	no
CK-40d	RHSIM_Rhsim06G0017600	1.649	0.362	-1.288	high
CK-40d	RHSIM_Rhsim05G0086100	-1.647	-0.836	0.811	low
CK-40d	RHSIM_Rhsim04G0002900	0.628	-0.146	-0.774	no
CK-40d	RHSIM_Rhsim05G0068700	-1.176	-0.348	0.828	no
CK-40d	RHSIM_Rhsim05G0233600	0.806	0.038	-0.768	no
CK-40d	RHSIM_Rhsim05G0179200	1.073	-0.572	-1.645	no
CK-40d	RHSIM_Rhsim06G0023700	0.103	-0.779	-0.882	no
CK-40d	RHSIM_Rhsim04G0241700	-1.245	-0.089	1.156	low
CK-40d	RHSIM_Rhsim04G0106600	-0.006	0.994	1.001	no
CK-40d	RHSIM_Rhsim04G0190600	-1.415	0.867	2.282	low
CK-40d	RHSIM_Rhsim06G0199000	0.380	-0.595	-0.974	no
CK-40d	RHSIM_Rhsim05G0027700	-1.065	-0.636	0.428	no
CK-40d	RHSIM_Rhsim04G0148800	-0.518	-1.018	-0.500	no
CK-40d	RHSIM_Rhsim07G0039400	-0.397	1.125	1.522	no
CK-40d	RHSIM_Rhsim04G0058200	-0.927	-0.077	0.851	no
CK-40d	RHSIM_Rhsim04G0126600	-0.365	-0.564	-0.199	no
CK-40d	RHSIM_Rhsim05G0216600	-1.225	-0.767	0.458	no
CK-40d	RHSIM_Rhsim05G0084000	-0.866	-1.444	-0.578	no
CK-40d	RHSIM_Rhsim05G0030000	0.633	0.067	-0.565	no

Group	Gene	PTR (z-score difference)	z(log2 LFQ)	z(log2 FPKM)	extreme-PTR
CK-40d	RHSIM_Rhsim04G0196900	-0.495	0.148	0.643	no
CK-40d	RHSIM_Rhsim07G0245300	1.167	0.152	-1.016	high
CK-40d	RHSIM_Rhsim04G0170600	-1.707	-1.118	0.589	low
CK-40d	RHSIM_Rhsim04G0216600	1.055	0.472	-0.583	no
CK-40d	RHSIM_Rhsim05G0067400	1.350	1.241	-0.108	high
CK-40d	RHSIM_Rhsim04G0074000	-0.658	-0.298	0.360	no
CK-40d	RHSIM_Rhsim05G0075200	-1.590	-1.299	0.290	low
CK-40d	RHSIM_Rhsim05G0145700	0.328	0.232	-0.096	no
CK-40d	RHSIM_Rhsim05G0175100	-1.435	-1.797	-0.362	low
CK-40d	RHSIM_Rhsim04G0205900	-1.817	0.165	1.981	low
CK-40d	RHSIM_Rhsim05G0135800	-0.397	0.595	0.993	no
CK-40d	RHSIM_Rhsim05G0232100	-0.909	-0.079	0.831	no
CK-40d	RHSIM_Rhsim05G0074800	-1.304	-0.378	0.926	low
CK-40d	RHSIM_Rhsim05G0096100	-2.602	-2.112	0.490	low
CK-40d	RHSIM_Rhsim06G0064800	0.473	0.455	-0.018	no
CK-40d	RHSIM_Rhsim05G0044600	0.695	0.692	-0.003	no
CK-40d	RHSIM_Rhsim05G0192400	-0.376	-1.359	-0.982	no
CK-40d	RHSIM_Rhsim05G0095400	1.687	1.311	-0.376	high
CK-40d	RHSIM_Rhsim05G0194900	0.735	1.274	0.539	no
CK-40d	RHSIM_Rhsim04G0012800	-0.346	0.520	0.867	no
CK-40d	RHSIM_Rhsim04G0058700	2.492	0.223	-2.269	high
CK-40d	RHSIM_Rhsim05G0078300	0.354	0.009	-0.345	no
CK-40d	RHSIM_Rhsim05G0000900	-0.693	-0.203	0.491	no
CK-40d	RHSIM_Rhsim04G0014400	1.874	0.179	-1.696	high
CK-40d	RHSIM_Rhsim05G0227300	1.133	-0.805	-1.937	no
CK-40d	RHSIM_Rhsim04G0015900	-0.046	-0.513	-0.467	no
CK-40d	RHSIM_Rhsim05G0164500	1.734	1.610	-0.124	high
CK-40d	RHSIM_Rhsim04G0016200	0.234	0.637	0.403	no
CK-40d	RHSIM_Rhsim07G0101200	-0.815	1.010	1.826	no
CK-40d	RHSIM_Rhsim04G0073400	-1.299	-1.311	-0.012	low
CK-40d	RHSIM_Rhsim05G0152100	0.885	-0.271	-1.155	no
CK-40d	RHSIM_Rhsim04G0029500	-0.179	1.287	1.467	no

Group	Gene	PTR (z-score difference)	z(log2 LFQ)	z(log2 FPKM)	extreme-PTR
CK-40d	RHSIM_Rhsim05G0044900	0.453	-0.759	-1.212	no
CK-40d	RHSIM_Rhsim04G0099700	-0.346	-0.153	0.193	no
CK-40d	RHSIM_Rhsim05G0202600	-0.494	-0.917	-0.423	no
CK-40d	RHSIM_Rhsim05G0133100	1.137	0.193	-0.944	no
CK-40d	RHSIM_Rhsim04G0003800	-0.780	-0.565	0.215	no
CK-40d	RHSIM_Rhsim05G0210500	0.635	-0.865	-1.501	no
CK-40d	RHSIM_Rhsim07G0175300	0.440	0.812	0.372	no
CK-40d	RHSIM_Rhsim04G0087500	-1.221	-0.623	0.597	no
CK-40d	RHSIM_Rhsim05G0199700	-0.045	0.179	0.224	no
CK-40d	RHSIM_Rhsim05G0165700	0.675	1.651	0.976	no
CK-40d	RHSIM_Rhsim05G0139600	1.733	-0.110	-1.844	high
CK-40d	RHSIM_Rhsim04G0145800	-0.835	-0.170	0.665	no
CK-40d	RHSIM_Rhsim05G0063100	-1.185	0.115	1.300	no
CK-40d	RHSIM_Rhsim04G0199100	0.707	1.913	1.206	no
CK-40d	RHSIM_Rhsim04G0033100	1.535	2.361	0.826	high
CK-40d	RHSIM_Rhsim04G0169600	0.358	1.122	0.764	no
CK-40d	RHSIM_Rhsim05G0067700	-0.941	-0.834	0.107	no
CK-40d	RHSIM_Rhsim05G0159600	1.977	1.520	-0.457	high
CK-40d	RHSIM_Rhsim04G0013600	-1.106	-0.000	1.106	no
CK-40d	RHSIM_Rhsim04G0124900	-0.681	0.665	1.346	no
CK-40d	RHSIM_Rhsim05G0175000	-0.557	-0.364	0.193	no
CK-40d	RHSIM_Rhsim05G0133000	-1.542	-1.331	0.210	low
CK-40d	RHSIM_Rhsim05G0121700	-1.595	0.075	1.670	low
CK-40d	RHSIM_Rhsim04G0100900	-1.674	0.159	1.832	low
CK-40d	RHSIM_Rhsim07G0134900	2.710	2.804	0.094	high
CK-40d	RHSIM_Rhsim05G0072400	-0.830	-1.189	-0.360	no
CK-40d	RHSIM_Rhsim04G0114200	1.057	-0.780	-1.836	no
CK-40d	RHSIM_Rhsim04G0119400	1.907	1.670	-0.237	high
CK-40d	RHSIM_Rhsim04G0065900	1.016	0.508	-0.507	no
CK-40d	RHSIM_Rhsim04G0134300	0.897	0.708	-0.189	no
CK-40d	RHSIM_Rhsim05G0148800	-1.530	-1.747	-0.217	low
CK-40d	RHSIM_Rhsim07G0101600	0.815	-0.209	-1.024	no

Group	Gene	PTR (z-score difference)	z(log2 LFQ)	z(log2 FPKM)	extreme-PTR
CK-40d	RHSIM_Rhsim05G0212900	4.294	4.053	-0.241	high
CK-40d	RHSIM_Rhsim07G0167800	0.234	-1.042	-1.276	no
CK-40d	RHSIM_Rhsim05G0084700	-1.448	-0.823	0.625	low
CK-40d	RHSIM_Rhsim04G0150100	-1.847	-1.597	0.249	low
CK-40d	RHSIM_Rhsim05G0111000	0.516	-0.892	-1.408	no
CK-40d	RHSIM_Rhsim04G0245000	-1.100	-1.258	-0.158	no
CK-40d	RHSIM_Rhsim04G0009400	0.039	-0.469	-0.508	no
CK-40d	RHSIM_Rhsim05G0083700	-0.588	-0.616	-0.028	no
CK-40d	RHSIM_Rhsim05G0229700	-0.313	-0.265	0.048	no
CK-40d	RHSIM_Rhsim04G0014300	1.525	0.723	-0.802	high
CK-40d	RHSIM_Rhsim05G0053600	-0.416	-0.298	0.117	no
CK-40d	RHSIM_Rhsim05G0095000	-0.278	-0.087	0.191	no
CK-40d	RHSIM_Rhsim04G0096600	0.360	-0.937	-1.297	no
CK-40d	RHSIM_Rhsim05G0090100	0.060	-0.222	-0.282	no
CK-40d	RHSIM_Rhsim05G0161400	-1.229	-1.303	-0.074	no
CK-40d	RHSIM_Rhsim07G0059400	0.166	0.684	0.518	no
CK-40d	RHSIM_Rhsim04G0023700	-1.301	1.710	3.011	low
CK-40d	RHSIM_Rhsim05G0216800	-1.223	-0.262	0.960	no
CK-40d	RHSIM_Rhsim05G0101800	-0.328	-0.480	-0.152	no
CK-40d	RHSIM_Rhsim04G0213000	-0.413	-0.690	-0.277	no
CK-40d	RHSIM_Rhsim05G0039000	2.393	0.229	-2.164	high
CK-40d	RHSIM_Rhsim05G0192200	1.205	1.204	-0.001	high
CK-40d	RHSIM_Rhsim07G0008100	-2.192	-0.382	1.810	low
CK-40d	RHSIM_Rhsim05G0180600	0.420	-0.168	-0.588	no
CK-40d	RHSIM_Rhsim04G0202900	-0.631	-0.312	0.319	no
CK-40d	RHSIM_Rhsim04G0084300	1.664	-0.295	-1.960	high
CK-40d	RHSIM_Rhsim04G0184400	0.802	0.702	-0.100	no
CK-40d	RHSIM_Rhsim07G0010700	-0.196	0.106	0.301	no
CK-40d	RHSIM_Rhsim04G0228600	0.396	-0.494	-0.890	no
CK-40d	RHSIM_Rhsim05G0062300	-0.345	-1.137	-0.792	no
CK-40d	RHSIM_Rhsim07G0005600	-1.571	-1.610	-0.039	low
CK-40d	RHSIM_Rhsim04G0248100	-0.228	0.482	0.710	no

Group	Gene	PTR (z-score difference)	z(log2 LFQ)	z(log2 FPKM)	extreme-PTR
CK-40d	RHSIM_Rhsim07G0031200	-0.268	0.543	0.811	no
CK-40d	RHSIM_Rhsim05G0105400	-1.691	-0.571	1.120	low
CK-40d	RHSIM_Rhsim04G0039600	-0.751	-1.300	-0.549	no
CK-40d	RHSIM_Rhsim04G0112300	0.244	-1.621	-1.865	no
CK-40d	RHSIM_Rhsim04G0249500	-1.511	-1.064	0.447	low
CK-40d	RHSIM_Rhsim04G0216400	0.785	-0.401	-1.186	no
CK-40d	RHSIM_Rhsim05G0208400	3.011	0.865	-2.146	high
CK-40d	RHSIM_Rhsim07G0142500	-0.863	-0.412	0.451	no
CK-40d	RHSIM_Rhsim04G0243000	-1.626	-1.312	0.314	low
CK-40d	RHSIM_Rhsim04G0011600	-1.905	-1.306	0.599	low
CK-40d	RHSIM_Rhsim05G0106400	-0.235	-0.703	-0.468	no
CK-40d	RHSIM_Rhsim04G0126000	-0.525	-0.628	-0.104	no
CK-40d	RHSIM_Rhsim04G0118000	-0.084	-0.211	-0.127	no
CK-40d	RHSIM_Rhsim04G0012200	0.493	0.208	-0.286	no
CK-40d	RHSIM_Rhsim04G0010300	0.255	2.535	2.280	no
CK-40d	RHSIM_Rhsim05G0160800	0.974	0.625	-0.349	no
CK-40d	RHSIM_Rhsim04G0010500	-0.902	-1.041	-0.139	no
CK-40d	RHSIM_Rhsim04G0157000	2.754	1.028	-1.727	high
CK-40d	RHSIM_Rhsim05G0068400	-1.388	-1.017	0.370	low
CK-40d	RHSIM_Rhsim04G0203200	-2.339	0.355	2.694	low
CK-40d	RHSIM_Rhsim07G0252600	0.208	0.677	0.469	no
CK-40d	RHSIM_Rhsim04G0125900	1.090	-1.264	-2.354	no
CK-40d	RHSIM_Rhsim05G0159300	1.718	1.851	0.133	high
CK-40d	RHSIM_Rhsim04G0216900	-0.219	-0.986	-0.767	no
CK-40d	RHSIM_Rhsim04G0052800	-0.377	-0.215	0.162	no
CK-40d	RHSIM_Rhsim04G0225000	-0.738	0.986	1.723	no
CK-40d	RHSIM_Rhsim04G0199900	0.448	1.457	1.010	no
CK-40d	RHSIM_Rhsim05G0131900	0.430	0.428	-0.002	no
CK-40d	RHSIM_Rhsim07G0126100	-0.915	-0.583	0.332	no
CK-40d	RHSIM_Rhsim05G0124800	0.014	-0.250	-0.263	no
CK-40d	RHSIM_Rhsim04G0004200	-1.842	-1.338	0.503	low
CK-40d	RHSIM_Rhsim04G0180700	-0.231	-0.279	-0.049	no

Group	Gene	PTR (z-score difference)	z(log2 LFQ)	z(log2 FPKM)	extreme-PTR
CK-40d	RHSIM_Rhsim06G0198800	-1.724	-0.925	0.799	low
CK-40d	RHSIM_Rhsim04G0202000	-0.677	-0.450	0.227	no
CK-40d	RHSIM_Rhsim04G0052300	0.210	2.884	2.674	no
CK-40d	RHSIM_Rhsim04G0006600	0.039	0.327	0.288	no
CK-40d	RHSIM_Rhsim06G0033800	-1.248	-0.627	0.620	low
CK-40d	RHSIM_Rhsim05G0167200	1.139	1.643	0.504	high
CK-40d	RHSIM_Rhsim04G0123300	-1.069	-1.184	-0.116	no
CK-40d	RHSIM_Rhsim04G0220300	0.149	-0.402	-0.552	no
CK-40d	RHSIM_Rhsim05G0201000	-0.448	0.084	0.531	no
CK-40d	RHSIM_Rhsim04G0055900	1.516	-0.409	-1.925	high
CK-40d	RHSIM_Rhsim04G0174200	-0.509	0.472	0.981	no
CK-40d	RHSIM_Rhsim06G0087100	-1.076	-0.565	0.510	no
CK-40d	RHSIM_Rhsim04G0016600	0.242	0.691	0.450	no
CK-40d	RHSIM_Rhsim04G0175900	2.028	0.908	-1.120	high
CK-40d	RHSIM_Rhsim04G0203300	0.430	0.054	-0.376	no
CK-40d	RHSIM_Rhsim05G0100900	-2.006	-0.005	2.001	low
CK-40d	RHSIM_Rhsim04G0186500	1.082	-1.168	-2.249	no
CK-40d	RHSIM_Rhsim04G0026100	2.280	1.780	-0.500	high
CK-40d	RHSIM_Rhsim05G0225500	-0.636	-0.609	0.027	no
CK-40d	RHSIM_Rhsim06G0006400	-0.744	-0.841	-0.097	no
CK-40d	RHSIM_Rhsim05G0031400	0.480	0.275	-0.206	no
CK-40d	RHSIM_Rhsim04G0013400	-0.039	-0.412	-0.373	no
CK-40d	RHSIM_Rhsim04G0157800	-0.805	0.350	1.156	no
CK-40d	RHSIM_Rhsim04G0008200	0.534	0.339	-0.194	no
CK-40d	RHSIM_Rhsim04G0019600	0.951	-0.647	-1.597	no
CK-40d	RHSIM_Rhsim05G0122700	-1.248	-1.032	0.216	low
CK-40d	RHSIM_Rhsim04G0168600	0.390	0.319	-0.071	no
CK-40d	RHSIM_Rhsim04G0083700	0.376	-0.700	-1.076	no
CK-40d	RHSIM_Rhsim05G0047300	0.951	-1.415	-2.366	no
CK-40d	RHSIM_Rhsim05G0174900	0.729	1.415	0.686	no
CK-40d	RHSIM_Rhsim04G0006900	-0.502	-0.416	0.086	no
CK-40d	RHSIM_Rhsim04G0167600	-0.686	-0.874	-0.187	no

Group	Gene	PTR (z-score difference)	z(log2 LFQ)	z(log2 FPKM)	extreme-PTR
CK-40d	RHSIM_Rhsim04G0070300	-0.057	0.902	0.959	no
CK-40d	RHSIM_Rhsim04G0154200	-0.370	-0.842	-0.472	no
CK-40d	RHSIM_Rhsim04G0215500	0.808	1.753	0.946	no
CK-40d	RHSIM_Rhsim06G0237200	0.117	0.793	0.676	no
CK-40d	RHSIM_Rhsim04G0173400	2.266	1.248	-1.018	high
CK-40d	RHSIM_Rhsim04G0075900	1.590	1.486	-0.105	high
CK-40d	RHSIM_Rhsim04G0074900	-0.072	-0.782	-0.711	no
CK-40d	RHSIM_Rhsim04G0126400	0.674	0.687	0.013	no
CK-40d	RHSIM_Rhsim04G0125700	-1.715	-0.942	0.772	low
CK-40d	RHSIM_Rhsim04G0202300	3.794	2.835	-0.960	high
CK-40d	RHSIM_Rhsim04G0023900	0.301	0.326	0.025	no
CK-40d	RHSIM_Rhsim05G0172200	0.060	-0.909	-0.969	no
CK-40d	RHSIM_Rhsim04G0093600	-1.642	-0.753	0.889	low
CK-40d	RHSIM_Rhsim04G0230600	0.953	2.764	1.811	no
CK-40d	RHSIM_Rhsim04G0136300	-0.952	0.366	1.317	no
CK-40d	RHSIM_Rhsim04G0030800	0.680	0.374	-0.307	no
CK-40d	RHSIM_Rhsim05G0219800	0.398	-0.849	-1.247	no
CK-40d	RHSIM_Rhsim05G0217200	-1.837	-1.472	0.365	low
CK-40d	RHSIM_Rhsim04G0207400	-0.220	0.031	0.251	no
CK-40d	RHSIM_Rhsim05G0095100	0.001	1.196	1.194	no
CK-40d	RHSIM_Rhsim03G0030800	2.580	1.072	-1.508	high
CK-40d	RHSIM_Rhsim05G0163700	-0.321	-0.444	-0.123	no
CK-40d	RHSIM_Rhsim04G0018100	1.222	0.281	-0.941	high
CK-40d	RHSIM_Rhsim05G0211100	0.085	0.735	0.650	no
CK-40d	RHSIM_Rhsim04G0195100	1.410	1.099	-0.310	high
CK-40d	RHSIM_Rhsim04G0156300	-0.706	1.869	2.575	no
CK-40d	RHSIM_Rhsim04G0030700	-0.142	0.419	0.562	no
CK-40d	RHSIM_Rhsim04G0108400	-1.917	0.322	2.239	low
CK-40d	RHSIM_Rhsim04G0217800	-1.050	-0.487	0.563	no
CK-40d	RHSIM_Rhsim04G0195700	-0.879	-0.735	0.144	no
CK-40d	RHSIM_Rhsim04G0000700	0.141	-0.170	-0.311	no
CK-40d	RHSIM_Rhsim04G0117700	0.704	-0.461	-1.166	no

Group	Gene	PTR (z-score difference)	z(log2 LFQ)	z(log2 FPKM)	extreme-PTR
CK-40d	RHSIM_Rhsim03G0235300	-0.511	0.500	1.011	no
CK-40d	RHSIM_Rhsim05G0139500	0.124	0.664	0.540	no
CK-40d	RHSIM_Rhsim04G0114100	0.898	0.919	0.021	no
CK-40d	RHSIM_Rhsim04G0009900	-0.831	-0.537	0.294	no
CK-40d	RHSIM_Rhsim04G0084500	-0.701	0.593	1.294	no
CK-40d	RHSIM_Rhsim06G0164200	-0.198	1.435	1.633	no
CK-40d	RHSIM_Rhsim05G0075700	1.153	0.788	-0.365	high
CK-40d	RHSIM_Rhsim05G0086800	-1.883	0.300	2.183	low
CK-40d	RHSIM_Rhsim04G0104400	0.018	0.315	0.297	no
CK-40d	RHSIM_Rhsim04G0130500	-0.648	0.208	0.856	no
CK-40d	RHSIM_Rhsim04G0018200	0.494	0.160	-0.335	no
CK-40d	RHSIM_Rhsim05G0082100	0.790	-0.167	-0.957	no
CK-40d	RHSIM_Rhsim04G0224400	-0.914	0.108	1.022	no
CK-40d	RHSIM_Rhsim04G0232100	0.214	0.452	0.238	no
CK-40d	RHSIM_Rhsim05G0187700	0.094	1.221	1.127	no
CK-40d	RHSIM_Rhsim03G0241400	-0.589	-0.285	0.304	no
CK-40d	RHSIM_Rhsim04G0194700	1.278	0.955	-0.322	high
CK-40d	RHSIM_Rhsim05G0017300	-1.130	-1.112	0.018	no
CK-40d	RHSIM_Rhsim04G0240200	-2.123	-0.937	1.186	low
CK-40d	RHSIM_Rhsim04G0063000	-0.041	-0.363	-0.322	no
CK-40d	RHSIM_Rhsim05G0104700	0.564	0.825	0.261	no
CK-40d	RHSIM_Rhsim04G0239000	-0.131	1.064	1.195	no
CK-40d	RHSIM_Rhsim04G0170500	0.410	-0.093	-0.504	no
CK-40d	RHSIM_Rhsim05G0150300	0.120	-0.559	-0.678	no
CK-40d	RHSIM_Rhsim04G0030100	1.257	1.325	0.068	high
CK-40d	RHSIM_Rhsim04G0200900	-0.373	-0.894	-0.520	no
CK-40d	RHSIM_Rhsim04G0083600	-1.452	-0.705	0.747	low
CK-40d	RHSIM_Rhsim04G0221400	-2.036	-0.731	1.306	low
CK-40d	RHSIM_Rhsim05G0205100	-0.188	-1.324	-1.136	no
CK-40d	RHSIM_Rhsim04G0122900	0.651	0.643	-0.008	no
CK-40d	RHSIM_Rhsim05G0067100	-1.114	0.268	1.383	no
CK-40d	RHSIM_Rhsim03G0221100	0.685	0.777	0.092	no

Group	Gene	PTR (z-score difference)	z(log2 LFQ)	z(log2 FPKM)	extreme-PTR
CK-40d	RHSIM_Rhsim04G0011100	0.236	1.133	0.897	no
CK-40d	RHSIM_Rhsim04G0104600	2.892	1.039	-1.853	high
CK-40d	RHSIM_Rhsim04G0158800	0.533	-0.188	-0.721	no
CK-40d	RHSIM_Rhsim04G0177100	0.546	-0.157	-0.703	no
CK-40d	RHSIM_Rhsim04G0050100	-2.320	-0.620	1.699	low
CK-40d	RHSIM_Rhsim03G0145800	1.587	-0.106	-1.693	high
CK-40d	RHSIM_Rhsim04G0235200	0.831	1.847	1.016	no
CK-40d	RHSIM_Rhsim04G0247200	0.029	-0.888	-0.917	no
CK-40d	RHSIM_Rhsim04G0038600	4.470	4.658	0.188	high
CK-40d	RHSIM_Rhsim04G0103300	-0.710	-0.097	0.613	no
CK-40d	RHSIM_Rhsim04G0144400	-0.277	0.787	1.064	no
CK-40d	RHSIM_Rhsim04G0162900	-0.894	-1.098	-0.204	no
CK-40d	RHSIM_Rhsim06G0035400	-0.752	-0.529	0.223	no
CK-40d	RHSIM_Rhsim04G0006200	3.202	3.570	0.369	high
CK-40d	RHSIM_Rhsim04G0219100	0.496	0.596	0.100	no
CK-40d	RHSIM_Rhsim03G0171900	0.548	0.636	0.088	no
CK-40d	RHSIM_Rhsim06G0232900	0.109	-0.032	-0.142	no
CK-40d	RHSIM_Rhsim04G0248000	0.051	0.050	-0.000	no
CK-40d	RHSIM_Rhsim04G0084900	-0.139	-0.019	0.120	no
CK-40d	RHSIM_Rhsim04G0022700	-2.798	-0.679	2.119	low
CK-40d	RHSIM_Rhsim04G0164200	-0.607	-1.674	-1.067	no
CK-40d	RHSIM_Rhsim04G0224800	-0.365	0.041	0.406	no
CK-40d	RHSIM_Rhsim03G0219900	1.507	0.816	-0.691	high
CK-40d	RHSIM_Rhsim04G0110400	0.708	0.862	0.154	no
CK-40d	RHSIM_Rhsim04G0235000	-2.252	-0.883	1.369	low
CK-40d	RHSIM_Rhsim03G0220500	-1.541	-1.113	0.427	low
CK-40d	RHSIM_Rhsim04G0196400	0.164	-1.671	-1.835	no
CK-40d	RHSIM_Rhsim04G0015300	-0.278	1.244	1.521	no
CK-40d	RHSIM_Rhsim04G0241800	-0.110	0.985	1.095	no
CK-40d	RHSIM_Rhsim04G0131000	-0.541	-0.324	0.217	no
CK-40d	RHSIM_Rhsim05G0108600	0.052	0.580	0.528	no
CK-40d	RHSIM_Rhsim04G0173800	-1.228	-0.235	0.994	no

Group	Gene	PTR (z-score difference)	z(log2 LFQ)	z(log2 FPKM)	extreme-PTR
CK-40d	RHSIM_Rhsim04G0092500	2.323	1.402	-0.922	high
CK-40d	RHSIM_Rhsim04G0037800	-0.797	-0.766	0.031	no
CK-40d	RHSIM_Rhsim04G0031900	-0.842	0.068	0.910	no
CK-40d	RHSIM_Rhsim03G0057400	-1.474	-1.007	0.467	low
CK-40d	RHSIM_Rhsim04G0043200	0.599	-0.105	-0.704	no
CK-40d	RHSIM_Rhsim04G0204600	0.098	-0.589	-0.687	no
CK-40d	RHSIM_Rhsim04G0106500	-0.815	0.403	1.218	no
CK-40d	RHSIM_Rhsim04G0130700	0.048	0.117	0.069	no
CK-40d	RHSIM_Rhsim06G0229600	2.037	0.674	-1.363	high
CK-40d	RHSIM_Rhsim04G0100100	-0.134	-0.385	-0.250	no
CK-40d	RHSIM_Rhsim04G0084000	-1.019	0.182	1.201	no
CK-40d	RHSIM_Rhsim03G0221500	-0.340	-0.917	-0.577	no
CK-40d	RHSIM_Rhsim03G0225700	0.068	0.414	0.346	no
CK-40d	RHSIM_Rhsim06G0202900	1.718	0.558	-1.160	high
CK-40d	RHSIM_Rhsim04G0197100	1.337	2.061	0.724	high
CK-40d	RHSIM_Rhsim03G0001300	0.112	-0.605	-0.717	no
CK-40d	RHSIM_Rhsim04G0167300	2.714	2.274	-0.441	high
CK-40d	RHSIM_Rhsim05G0163500	0.210	0.353	0.143	no
CK-40d	RHSIM_Rhsim04G0198400	-1.702	-1.676	0.026	low
CK-40d	RHSIM_Rhsim04G0056300	1.055	-0.180	-1.235	no
CK-40d	RHSIM_Rhsim04G0056600	-0.311	-0.290	0.021	no
CK-40d	RHSIM_Rhsim04G0244400	0.284	1.380	1.096	no
CK-40d	RHSIM_Rhsim04G0153200	-0.338	0.841	1.179	no
CK-40d	RHSIM_Rhsim04G0023000	-0.019	-0.109	-0.090	no
CK-40d	RHSIM_Rhsim03G0083600	1.837	1.575	-0.262	high
CK-40d	RHSIM_Rhsim06G0000500	0.878	0.983	0.104	no
CK-40d	RHSIM_Rhsim04G0172400	-0.674	0.023	0.697	no
CK-40d	RHSIM_Rhsim04G0032200	-0.508	-0.418	0.090	no
CK-40d	RHSIM_Rhsim03G0154000	2.845	0.686	-2.159	high
CK-40d	RHSIM_Rhsim04G0027200	-0.640	0.870	1.511	no
CK-40d	RHSIM_Rhsim04G0245300	0.875	-0.119	-0.994	no
CK-40d	RHSIM_Rhsim04G0072600	-0.995	-0.975	0.020	no

Group	Gene	PTR (z-score difference)	z(log2 LFQ)	z(log2 FPKM)	extreme-PTR
CK-40d	RHSIM_Rhsim03G0168400	2.265	1.366	-0.898	high
CK-40d	RHSIM_Rhsim03G0075300	-0.320	0.512	0.833	no
CK-40d	RHSIM_Rhsim04G0173300	-1.064	0.270	1.334	no
CK-40d	RHSIM_Rhsim04G0219500	-0.541	-0.954	-0.413	no
CK-40d	RHSIM_Rhsim04G0180500	0.006	-0.215	-0.221	no
CK-40d	RHSIM_Rhsim05G0181000	-0.853	0.520	1.373	no
CK-40d	RHSIM_Rhsim04G0096800	2.687	2.229	-0.457	high
CK-40d	RHSIM_Rhsim04G0095900	0.717	0.752	0.035	no
CK-40d	RHSIM_Rhsim03G0154300	1.536	-0.516	-2.052	high
CK-40d	RHSIM_Rhsim06G0030600	-1.416	-0.787	0.629	low
CK-40d	RHSIM_Rhsim04G0038700	1.008	-0.183	-1.191	no
CK-40d	RHSIM_Rhsim04G0001800	0.963	0.746	-0.217	no
CK-40d	RHSIM_Rhsim05G0062800	-0.057	2.773	2.830	no
CK-40d	RHSIM_Rhsim03G0105200	0.588	-0.251	-0.838	no
CK-40d	RHSIM_Rhsim03G0111900	1.337	1.952	0.615	high
CK-40d	RHSIM_Rhsim03G0207700	-0.252	-1.278	-1.025	no
CK-40d	RHSIM_Rhsim05G0133600	0.287	0.499	0.212	no
CK-40d	RHSIM_Rhsim03G0204000	-0.544	-1.488	-0.944	no
CK-40d	RHSIM_Rhsim04G0162800	-1.203	-1.095	0.107	no
CK-40d	RHSIM_Rhsim04G0087300	0.488	0.078	-0.410	no
CK-40d	RHSIM_Rhsim06G0239100	-0.769	1.606	2.375	no
CK-40d	RHSIM_Rhsim04G0196800	-0.444	0.956	1.400	no
CK-40d	RHSIM_Rhsim03G0044900	-0.684	-0.003	0.681	no
CK-40d	RHSIM_Rhsim03G0179500	-0.336	1.491	1.827	no
CK-40d	RHSIM_Rhsim04G0185400	-0.676	-0.659	0.016	no
CK-40d	RHSIM_Rhsim04G0205700	2.500	1.593	-0.907	high
CK-40d	RHSIM_Rhsim04G0020500	3.331	2.095	-1.236	high
CK-40d	RHSIM_Rhsim04G0233600	-0.227	-0.176	0.051	no
CK-40d	RHSIM_Rhsim03G0019300	-0.200	1.759	1.959	no
CK-40d	RHSIM_Rhsim04G0162300	-0.502	0.693	1.194	no
CK-40d	RHSIM_Rhsim03G0226300	0.254	-0.009	-0.263	no
CK-40d	RHSIM_Rhsim03G0123300	0.597	-0.989	-1.585	no

Group	Gene	PTR (z-score difference)	z(log2 LFQ)	z(log2 FPKM)	extreme-PTR
CK-40d	RHSIM_Rhsim04G0218700	-0.633	-0.963	-0.329	no
CK-40d	RHSIM_Rhsim03G0155100	0.112	-1.035	-1.147	no
CK-40d	RHSIM_Rhsim04G0177600	-2.938	-1.244	1.694	low
CK-40d	RHSIM_Rhsim03G0254300	0.888	0.698	-0.190	no
CK-40d	RHSIM_Rhsim03G0001500	0.446	0.734	0.287	no
CK-40d	RHSIM_Rhsim03G0166500	0.978	-0.377	-1.355	no
CK-40d	RHSIM_Rhsim03G0065700	1.579	-0.128	-1.707	high
CK-40d	RHSIM_Rhsim04G0228500	-0.580	0.068	0.648	no
CK-40d	RHSIM_Rhsim03G0253300	-0.914	-0.669	0.245	no
CK-40d	RHSIM_Rhsim03G0056500	-1.950	-0.788	1.162	low
CK-40d	RHSIM_Rhsim04G0003700	0.934	-0.844	-1.777	no
CK-40d	RHSIM_Rhsim03G0180700	-0.237	1.231	1.468	no
CK-40d	RHSIM_Rhsim04G0189900	1.724	0.681	-1.043	high
CK-40d	RHSIM_Rhsim04G0233400	-0.933	-0.767	0.166	no
CK-40d	RHSIM_Rhsim04G0003500	-0.820	-0.307	0.513	no
CK-40d	RHSIM_Rhsim03G0223300	0.765	-0.347	-1.112	no
CK-40d	RHSIM_Rhsim04G0134100	0.657	-0.933	-1.591	no
CK-40d	RHSIM_Rhsim03G0010800	0.463	-0.209	-0.672	no
CK-40d	RHSIM_Rhsim03G0063700	0.786	0.288	-0.498	no
CK-40d	RHSIM_Rhsim04G0030200	0.083	-0.899	-0.982	no
CK-40d	RHSIM_Rhsim03G0068700	-1.063	1.304	2.367	no
CK-40d	RHSIM_Rhsim04G0158400	-0.434	-0.955	-0.521	no
CK-40d	RHSIM_Rhsim06G0242500	0.244	-0.689	-0.933	no
CK-40d	RHSIM_Rhsim04G0230400	0.236	1.484	1.248	no
CK-40d	RHSIM_Rhsim03G0085200	-0.156	-0.646	-0.489	no
CK-40d	RHSIM_Rhsim03G0228800	-0.189	-1.163	-0.974	no
CK-40d	RHSIM_Rhsim04G0120900	0.170	-0.574	-0.744	no
CK-40d	RHSIM_Rhsim04G0155300	-1.068	1.598	2.666	no
CK-40d	RHSIM_Rhsim03G0121700	-0.956	-0.745	0.210	no
CK-40d	RHSIM_Rhsim04G0004400	0.444	0.393	-0.051	no
CK-40d	RHSIM_Rhsim03G0129000	-0.490	0.809	1.299	no
CK-40d	RHSIM_Rhsim04G0130800	-2.253	-1.417	0.836	low

Group	Gene	PTR (z-score difference)	z(log2 LFQ)	z(log2 FPKM)	extreme-PTR
CK-40d	RHSIM_Rhsim06G0082800	-0.326	-0.710	-0.385	no
CK-40d	RHSIM_Rhsim03G0235100	-0.378	1.075	1.453	no
CK-40d	RHSIM_Rhsim04G0138700	-0.031	0.410	0.441	no
CK-40d	RHSIM_Rhsim04G0003600	0.480	1.238	0.759	no
CK-40d	RHSIM_Rhsim06G0166200	0.377	0.214	-0.163	no
CK-40d	RHSIM_Rhsim03G0198500	-0.197	-0.885	-0.688	no
CK-40d	RHSIM_Rhsim03G0000200	0.981	1.113	0.131	no
CK-40d	RHSIM_Rhsim04G0128400	-1.500	-1.072	0.428	low
CK-40d	RHSIM_Rhsim04G0225100	0.835	-0.296	-1.131	no
CK-40d	RHSIM_Rhsim03G0096300	-0.975	-0.025	0.950	no
CK-40d	RHSIM_Rhsim04G0235600	0.711	-1.459	-2.171	no
CK-40d	RHSIM_Rhsim03G0234800	0.679	-0.394	-1.073	no
CK-40d	RHSIM_Rhsim03G0066700	1.965	3.478	1.514	high
CK-40d	RHSIM_Rhsim04G0241100	1.596	1.301	-0.295	high
CK-40d	RHSIM_Rhsim04G0084800	-1.602	-0.779	0.822	low
CK-40d	RHSIM_Rhsim04G0021800	-0.131	0.492	0.623	no
CK-40d	RHSIM_Rhsim03G0239700	-2.098	-1.011	1.087	low
CK-40d	RHSIM_Rhsim03G0073700	1.199	-0.905	-2.104	high
CK-40d	RHSIM_Rhsim03G0146900	1.031	-0.541	-1.572	no
CK-40d	RHSIM_Rhsim04G0094400	0.651	-0.504	-1.155	no
CK-40d	RHSIM_Rhsim03G0164200	0.265	0.676	0.411	no
CK-40d	RHSIM_Rhsim03G0006300	-1.765	-0.829	0.936	low
CK-40d	RHSIM_Rhsim06G0174600	-0.055	0.592	0.647	no
CK-40d	RHSIM_Rhsim04G0039400	-0.605	-1.159	-0.554	no
CK-40d	RHSIM_Rhsim06G0073200	-1.210	-0.008	1.202	no
CK-40d	RHSIM_Rhsim03G0188200	-0.160	-0.577	-0.417	no
CK-40d	RHSIM_Rhsim04G0001500	0.634	0.150	-0.484	no
CK-40d	RHSIM_Rhsim06G0036100	0.240	0.239	-0.001	no
CK-40d	RHSIM_Rhsim04G0129300	0.421	0.742	0.321	no
CK-40d	RHSIM_Rhsim06G0177200	0.686	-0.826	-1.512	no
CK-40d	RHSIM_Rhsim03G0019000	-1.314	0.672	1.987	low
CK-40d	RHSIM_Rhsim04G0116200	0.290	-0.909	-1.199	no

Group	Gene	PTR (z-score difference)	z(log2 LFQ)	z(log2 FPKM)	extreme-PTR
CK-40d	RHSIM_Rhsim03G0122000	0.329	0.149	-0.180	no
CK-40d	RHSIM_Rhsim03G0229700	-1.400	-0.469	0.931	low
CK-40d	RHSIM_Rhsim06G0153200	-0.700	0.393	1.093	no
CK-40d	RHSIM_Rhsim03G0020300	1.153	0.317	-0.835	high
CK-40d	RHSIM_Rhsim03G0172000	0.328	-0.810	-1.139	no
CK-40d	RHSIM_Rhsim03G0041200	0.899	0.333	-0.565	no
CK-40d	RHSIM_Rhsim03G0222600	-1.273	-0.647	0.626	low
CK-40d	RHSIM_Rhsim06G0001600	-0.045	-0.171	-0.126	no
CK-40d	RHSIM_Rhsim03G0155200	0.815	0.948	0.133	no
CK-40d	RHSIM_Rhsim03G0173300	0.675	0.365	-0.310	no
CK-40d	RHSIM_Rhsim03G0218100	-0.929	-1.795	-0.866	no
CK-40d	RHSIM_Rhsim03G0270100	1.465	-0.634	-2.099	high
CK-40d	RHSIM_Rhsim06G0087400	-1.451	-0.763	0.688	low
CK-40d	RHSIM_Rhsim03G0212700	-1.159	0.965	2.124	no
CK-40d	RHSIM_Rhsim03G0230000	-0.428	-0.177	0.251	no
CK-40d	RHSIM_Rhsim03G0009200	3.096	1.671	-1.425	high
CK-40d	RHSIM_Rhsim03G0050300	0.701	0.132	-0.569	no
CK-40d	RHSIM_Rhsim03G0075800	2.161	1.280	-0.881	high
CK-40d	RHSIM_Rhsim06G0129100	0.009	0.554	0.544	no
CK-40d	RHSIM_Rhsim04G0165300	0.862	0.476	-0.387	no
CK-40d	RHSIM_Rhsim04G0139800	1.891	0.192	-1.699	high
CK-40d	RHSIM_Rhsim03G0177300	1.941	0.414	-1.527	high
CK-40d	RHSIM_Rhsim04G0201800	0.197	0.159	-0.039	no
CK-40d	RHSIM_Rhsim06G0130400	1.316	0.758	-0.558	high
CK-40d	RHSIM_Rhsim04G0168700	0.134	0.051	-0.083	no
CK-40d	RHSIM_Rhsim06G0048400	0.241	-0.478	-0.720	no
CK-40d	RHSIM_Rhsim04G0167800	2.019	0.934	-1.085	high
CK-40d	RHSIM_Rhsim03G0248200	-0.262	-0.779	-0.517	no
CK-40d	RHSIM_Rhsim03G0201100	-0.551	0.130	0.681	no
CK-40d	RHSIM_Rhsim03G0150700	0.886	-0.519	-1.405	no
CK-40d	RHSIM_Rhsim04G0181600	0.624	-0.460	-1.084	no
CK-40d	RHSIM_Rhsim03G0265800	1.792	0.269	-1.523	high

Group	Gene	PTR (z-score difference)	z(log2 LFQ)	z(log2 FPKM)	extreme-PTR
CK-40d	RHSIM_Rhsim04G0014800	-1.457	-0.937	0.521	low
CK-40d	RHSIM_Rhsim06G0108900	1.823	-0.129	-1.952	high
CK-40d	RHSIM_Rhsim03G0004000	0.314	-0.104	-0.418	no
CK-40d	RHSIM_Rhsim04G0173000	0.348	1.769	1.421	no
CK-40d	RHSIM_Rhsim04G0091700	2.254	1.054	-1.200	high
CK-40d	RHSIM_Rhsim03G0267600	-0.673	-0.786	-0.112	no
CK-40d	RHSIM_Rhsim06G0166000	-1.405	-1.425	-0.020	low
CK-40d	RHSIM_Rhsim03G0151400	1.977	1.831	-0.145	high
CK-40d	RHSIM_Rhsim03G0061300	0.158	-1.734	-1.892	no
CK-40d	RHSIM_Rhsim04G0081600	1.221	0.018	-1.203	high
CK-40d	RHSIM_Rhsim04G0038100	-0.836	-0.489	0.347	no
CK-40d	RHSIM_Rhsim04G0105700	1.086	-0.172	-1.258	no
CK-40d	RHSIM_Rhsim03G0196600	-0.317	-0.502	-0.185	no
CK-40d	RHSIM_Rhsim03G0235600	-1.071	-0.302	0.769	no
CK-40d	RHSIM_Rhsim03G0119300	-1.105	-1.459	-0.354	no
CK-40d	RHSIM_Rhsim03G0252000	0.097	0.082	-0.015	no
CK-40d	RHSIM_Rhsim06G0022700	0.749	-0.687	-1.436	no
CK-40d	RHSIM_Rhsim04G0197500	1.458	0.336	-1.122	high
CK-40d	RHSIM_Rhsim06G0018600	-0.100	0.652	0.752	no
CK-40d	RHSIM_Rhsim03G0125700	0.741	0.465	-0.276	no
CK-40d	RHSIM_Rhsim03G0001600	-1.227	-0.449	0.778	no
CK-40d	RHSIM_Rhsim04G0170700	2.392	0.769	-1.623	high
CK-40d	RHSIM_Rhsim03G0093200	-1.077	-0.810	0.266	no
CK-40d	RHSIM_Rhsim03G0248000	0.766	0.689	-0.077	no
CK-40d	RHSIM_Rhsim03G0153100	1.532	-0.878	-2.410	high
CK-40d	RHSIM_Rhsim03G0138600	-0.214	0.546	0.760	no
CK-40d	RHSIM_Rhsim04G0008100	-0.070	1.310	1.380	no
CK-40d	RHSIM_Rhsim03G0090600	-0.938	-0.367	0.572	no
CK-40d	RHSIM_Rhsim03G0161600	-0.482	-1.144	-0.662	no
CK-40d	RHSIM_Rhsim03G0264700	-1.247	-0.831	0.416	low
CK-40d	RHSIM_Rhsim03G0078800	0.122	0.000	-0.122	no
CK-40d	RHSIM_Rhsim05G0113400	2.309	2.965	0.656	high

Group	Gene	PTR (z-score difference)	z(log2 LFQ)	z(log2 FPKM)	extreme-PTR
CK-40d	RHSIM_Rhsim03G0211600	-0.881	-1.035	-0.154	no
CK-40d	RHSIM_Rhsim03G0115800	-0.706	-0.485	0.221	no
CK-40d	RHSIM_Rhsim05G0090500	-0.147	-0.050	0.097	no
CK-40d	RHSIM_Rhsim03G0249400	0.069	0.745	0.676	no
CK-40d	RHSIM_Rhsim04G0086600	-0.785	-0.946	-0.161	no
CK-40d	RHSIM_Rhsim04G0169700	-1.203	-0.159	1.044	no
CK-40d	RHSIM_Rhsim03G0245200	-1.789	-0.432	1.357	low
CK-40d	RHSIM_Rhsim03G0009900	1.188	-0.560	-1.749	high
CK-40d	RHSIM_Rhsim04G0157200	-0.465	-0.823	-0.358	no
CK-40d	RHSIM_Rhsim04G0214000	-0.933	-1.297	-0.364	no
CK-40d	RHSIM_Rhsim03G0219300	-0.093	-0.657	-0.564	no
CK-40d	RHSIM_Rhsim03G0159800	-1.156	-0.012	1.144	no
CK-40d	RHSIM_Rhsim03G0256600	-0.542	-0.373	0.169	no
CK-40d	RHSIM_Rhsim03G0180900	-1.514	-0.002	1.512	low
CK-40d	RHSIM_Rhsim03G0099600	1.227	0.423	-0.805	high
CK-40d	RHSIM_Rhsim03G0240200	-0.368	-0.815	-0.447	no
CK-40d	RHSIM_Rhsim03G0131800	-0.770	0.152	0.922	no
CK-40d	RHSIM_Rhsim03G0110300	0.665	0.836	0.170	no
CK-40d	RHSIM_Rhsim04G0176900	0.540	1.427	0.887	no
CK-40d	RHSIM_Rhsim03G0100800	-0.101	-0.098	0.003	no
CK-40d	RHSIM_Rhsim03G0069400	2.142	0.001	-2.140	high
CK-40d	RHSIM_Rhsim03G0105700	-0.625	-0.871	-0.246	no
CK-40d	RHSIM_Rhsim03G0027100	-0.216	-0.541	-0.325	no
CK-40d	RHSIM_Rhsim03G0074700	-1.819	-1.956	-0.136	low
CK-40d	RHSIM_Rhsim03G0136400	0.294	0.089	-0.204	no
CK-40d	RHSIM_Rhsim04G0147900	-0.265	0.139	0.404	no
CK-40d	RHSIM_Rhsim03G0075100	0.325	-0.433	-0.757	no
CK-40d	RHSIM_Rhsim04G0101000	-1.772	-0.836	0.936	low
CK-40d	RHSIM_Rhsim03G0141300	-0.437	0.004	0.441	no
CK-40d	RHSIM_Rhsim03G0262500	-2.046	-0.713	1.333	low
CK-40d	RHSIM_Rhsim04G0151800	1.783	0.364	-1.419	high
CK-40d	RHSIM_Rhsim04G0068400	-0.421	0.854	1.275	no

Group	Gene	PTR (z-score difference)	z(log2 LFQ)	z(log2 FPKM)	extreme-PTR
CK-40d	RHSIM_Rhsim03G0052700	-0.547	-0.351	0.196	no
CK-40d	RHSIM_Rhsim03G0134500	2.345	1.509	-0.836	high
CK-40d	RHSIM_Rhsim03G0021400	0.600	0.071	-0.529	no
CK-40d	RHSIM_Rhsim03G0225200	0.094	0.375	0.281	no
CK-40d	RHSIM_Rhsim04G0071500	0.006	0.009	0.002	no
CK-40d	RHSIM_Rhsim03G0244600	0.909	0.102	-0.807	no
CK-40d	RHSIM_Rhsim05G0115700	0.277	0.724	0.447	no
CK-40d	RHSIM_Rhsim03G0099500	0.392	-1.330	-1.722	no
CK-40d	RHSIM_Rhsim03G0254100	2.796	1.484	-1.312	high
CK-40d	RHSIM_Rhsim04G0081400	0.416	0.216	-0.200	no
CK-40d	RHSIM_Rhsim03G0085000	-0.958	-1.257	-0.299	no
CK-40d	RHSIM_Rhsim04G0166700	0.522	0.944	0.421	no
CK-40d	RHSIM_Rhsim03G0121200	0.541	-0.187	-0.728	no
CK-40d	RHSIM_Rhsim03G0235000	-1.464	0.704	2.168	low
CK-40d	RHSIM_Rhsim03G0146700	0.741	0.499	-0.242	no
CK-40d	RHSIM_Rhsim04G0180800	-2.717	-0.596	2.121	low
CK-40d	RHSIM_Rhsim03G0146600	-0.128	-0.424	-0.296	no
CK-40d	RHSIM_Rhsim03G0073400	-0.860	-0.776	0.084	no
CK-40d	RHSIM_Rhsim04G0206300	3.471	2.770	-0.702	high
CK-40d	RHSIM_Rhsim03G0140300	-2.840	-2.161	0.680	low
CK-40d	RHSIM_Rhsim03G0080300	-1.275	-0.700	0.575	low
CK-40d	RHSIM_Rhsim05G0116200	-0.340	-0.139	0.201	no
CK-40d	RHSIM_Rhsim03G0144700	-0.126	-0.291	-0.165	no
CK-40d	RHSIM_Rhsim04G0066500	-1.539	-0.176	1.363	low
CK-40d	RHSIM_Rhsim04G0182600	-1.717	0.997	2.714	low
CK-40d	RHSIM_Rhsim05G0206900	-0.426	-0.155	0.271	no
CK-40d	RHSIM_Rhsim03G0200600	-0.872	-0.242	0.630	no
CK-40d	RHSIM_Rhsim03G0051100	-1.367	-1.523	-0.156	low
CK-40d	RHSIM_Rhsim04G0209400	2.431	2.094	-0.338	high
CK-40d	RHSIM_Rhsim03G0223200	0.235	-0.004	-0.238	no
CK-40d	RHSIM_Rhsim03G0237400	0.469	1.939	1.470	no
CK-40d	RHSIM_Rhsim04G0012000	-0.872	-0.342	0.530	no

Group	Gene	PTR (z-score difference)	z(log2 LFQ)	z(log2 FPKM)	extreme-PTR
CK-40d	RHSIM_Rhsim03G0179100	-0.998	-0.697	0.301	no
CK-40d	RHSIM_Rhsim03G0187200	1.005	0.658	-0.347	no
CK-40d	RHSIM_Rhsim04G0160900	-0.185	-1.920	-1.735	no
CK-40d	RHSIM_Rhsim03G0058100	-1.112	-0.897	0.215	no
CK-40d	RHSIM_Rhsim03G0244700	2.183	2.114	-0.069	high
CK-40d	RHSIM_Rhsim03G0256500	1.411	1.761	0.351	high
CK-40d	RHSIM_Rhsim03G0214800	-1.107	1.575	2.681	no
CK-40d	RHSIM_Rhsim03G0034500	0.563	-0.945	-1.508	no
CK-40d	RHSIM_Rhsim03G0112100	-0.199	0.395	0.594	no
CK-40d	RHSIM_Rhsim03G0240800	0.833	0.819	-0.014	no
CK-40d	RHSIM_Rhsim05G0193600	0.416	-0.533	-0.949	no
CK-40d	RHSIM_Rhsim04G0086300	0.398	-0.449	-0.846	no
CK-40d	RHSIM_Rhsim05G0216200	-1.216	-0.502	0.714	no
CK-40d	RHSIM_Rhsim03G0258800	0.258	-0.345	-0.603	no
CK-40d	RHSIM_Rhsim03G0231700	-1.238	-1.576	-0.338	no
CK-40d	RHSIM_Rhsim03G0035400	0.703	-0.417	-1.121	no
CK-40d	RHSIM_Rhsim04G0232000	0.816	0.863	0.047	no
CK-40d	RHSIM_Rhsim05G0067500	-0.630	0.641	1.271	no
CK-40d	RHSIM_Rhsim03G0150600	0.613	-1.002	-1.616	no
CK-40d	RHSIM_Rhsim04G0179900	0.483	-0.543	-1.026	no
CK-40d	RHSIM_Rhsim03G0062200	0.815	-0.145	-0.960	no
CK-40d	RHSIM_Rhsim04G0075400	-0.680	-0.852	-0.171	no
CK-40d	RHSIM_Rhsim03G0092400	0.451	0.952	0.500	no
CK-40d	RHSIM_Rhsim03G0236300	-1.322	-1.320	0.002	low
CK-40d	RHSIM_Rhsim05G0011200	-1.627	0.432	2.058	low
CK-40d	RHSIM_Rhsim03G0264300	-0.165	0.431	0.596	no
CK-40d	RHSIM_Rhsim05G0032000	-0.758	-1.320	-0.562	no
CK-40d	RHSIM_Rhsim03G0262700	1.979	1.323	-0.655	high
CK-40d	RHSIM_Rhsim03G0154600	-0.119	-0.762	-0.643	no
CK-40d	RHSIM_Rhsim03G0073300	-0.454	-1.116	-0.662	no
CK-40d	RHSIM_Rhsim03G0208100	0.175	0.965	0.790	no
CK-40d	RHSIM_Rhsim05G0101900	-2.135	-0.800	1.335	low

Group	Gene	PTR (z-score difference)	z(log2 LFQ)	z(log2 FPKM)	extreme-PTR
CK-40d	RHSIM_Rhsim03G0230900	-0.202	0.125	0.326	no
CK-40d	RHSIM_Rhsim04G0197400	-0.732	0.566	1.298	no
CK-40d	RHSIM_Rhsim05G0042100	0.108	-0.454	-0.562	no
CK-40d	RHSIM_Rhsim04G0204300	-1.417	-1.556	-0.139	low
CK-40d	RHSIM_Rhsim03G0078700	0.382	-0.223	-0.605	no
CK-40d	RHSIM_Rhsim03G0093900	-1.162	-0.692	0.470	no
CK-40d	RHSIM_Rhsim03G0234500	0.510	1.479	0.969	no
CK-40d	RHSIM_Rhsim05G0098000	0.114	-0.083	-0.198	no
CK-40d	RHSIM_Rhsim03G0265300	1.557	0.870	-0.687	high
CK-40d	RHSIM_Rhsim03G0192800	0.603	0.155	-0.448	no
CK-40d	RHSIM_Rhsim03G0018400	-0.347	-0.547	-0.200	no
CK-40d	RHSIM_Rhsim04G0171200	1.352	2.091	0.739	high
CK-40d	RHSIM_Rhsim04G0024300	0.700	-0.758	-1.458	no
CK-40d	RHSIM_Rhsim03G0176200	2.965	1.061	-1.904	high
CK-40d	RHSIM_Rhsim03G0155500	0.809	-0.302	-1.111	no
CK-40d	RHSIM_Rhsim05G0018500	0.809	-0.925	-1.734	no
CK-40d	RHSIM_Rhsim03G0192400	0.186	1.151	0.965	no
CK-40d	RHSIM_Rhsim03G0110600	0.182	0.148	-0.034	no
CK-40d	RHSIM_Rhsim05G0214700	-1.101	-0.519	0.582	no
CK-40d	RHSIM_Rhsim03G0063000	0.993	0.538	-0.456	no
CK-40d	RHSIM_Rhsim03G0141400	-2.369	-0.384	1.985	low
CK-40d	RHSIM_Rhsim03G0187900	-0.582	-1.591	-1.009	no
CK-40d	RHSIM_Rhsim03G0176300	-0.055	-1.093	-1.039	no
CK-40d	RHSIM_Rhsim03G0145100	0.197	-0.201	-0.399	no
CK-40d	RHSIM_Rhsim03G0208900	-0.594	-0.323	0.271	no
CK-40d	RHSIM_Rhsim03G0119400	0.548	0.973	0.425	no
CK-40d	RHSIM_Rhsim03G0093800	-1.112	-0.735	0.377	no
CK-40d	RHSIM_Rhsim02G0061100	0.092	-0.977	-1.069	no
CK-40d	RHSIM_Rhsim04G0144000	1.915	-0.454	-2.370	high
CK-40d	RHSIM_Rhsim03G0083800	-1.662	-1.132	0.530	low
CK-40d	RHSIM_Rhsim03G0101200	-0.701	-1.512	-0.811	no
CK-40d	RHSIM_Rhsim03G0188500	1.104	0.464	-0.640	no

Group	Gene	PTR (z-score difference)	z(log2 LFQ)	z(log2 FPKM)	extreme-PTR
CK-40d	RHSIM_Rhsim02G0160800	-0.276	-0.379	-0.103	no
CK-40d	RHSIM_Rhsim03G0118600	-1.588	-0.175	1.413	low
CK-40d	RHSIM_Rhsim03G0089900	-1.228	-0.780	0.448	no
CK-40d	RHSIM_Rhsim03G0029800	-0.502	0.342	0.844	no
CK-40d	RHSIM_Rhsim03G0088700	-0.206	-0.301	-0.095	no
CK-40d	RHSIM_Rhsim04G0145600	-0.690	0.375	1.066	no
CK-40d	RHSIM_Rhsim02G0022400	0.782	0.153	-0.629	no
CK-40d	RHSIM_Rhsim03G0270000	-0.639	-0.049	0.590	no
CK-40d	RHSIM_Rhsim03G0049600	0.573	-0.245	-0.818	no
CK-40d	RHSIM_Rhsim03G0130200	0.334	-0.016	-0.350	no
CK-40d	RHSIM_Rhsim05G0108800	0.306	-0.349	-0.656	no
CK-40d	RHSIM_Rhsim03G0086200	2.808	1.666	-1.142	high
CK-40d	RHSIM_Rhsim03G0195300	1.695	-0.596	-2.291	high
CK-40d	RHSIM_Rhsim03G0229600	-1.153	-0.018	1.134	no
CK-40d	RHSIM_Rhsim03G0207200	-0.608	-1.122	-0.514	no
CK-40d	RHSIM_Rhsim03G0231100	-0.037	-0.486	-0.448	no
CK-40d	RHSIM_Rhsim03G0212200	0.402	-0.499	-0.901	no
CK-40d	RHSIM_Rhsim02G0105800	-1.450	-1.285	0.165	low
CK-40d	RHSIM_Rhsim03G0081700	-0.243	0.007	0.250	no
CK-40d	RHSIM_Rhsim04G0207900	0.099	0.618	0.519	no
CK-40d	RHSIM_Rhsim03G0090700	-0.516	-0.786	-0.270	no
CK-40d	RHSIM_Rhsim03G0261200	0.546	0.575	0.029	no
CK-40d	RHSIM_Rhsim03G0075200	-1.506	-0.789	0.717	low
CK-40d	RHSIM_Rhsim03G0231500	-0.198	-1.088	-0.890	no
CK-40d	RHSIM_Rhsim04G0224600	-0.924	0.280	1.204	no
CK-40d	RHSIM_Rhsim02G0015800	-0.049	-0.094	-0.045	no
CK-40d	RHSIM_Rhsim02G0060700	-1.675	-1.351	0.324	low
CK-40d	RHSIM_Rhsim02G0108100	-0.745	-0.293	0.453	no
CK-40d	RHSIM_Rhsim02G0001700	-0.211	-0.619	-0.408	no
CK-40d	RHSIM_Rhsim02G0159000	-1.892	-1.056	0.836	low
CK-40d	RHSIM_Rhsim04G0249800	1.003	-0.812	-1.815	no
CK-40d	RHSIM_Rhsim03G0122500	-0.459	0.130	0.589	no

Group	Gene	PTR (z-score difference)	z(log2 LFQ)	z(log2 FPKM)	extreme-PTR
CK-40d	RHSIM_Rhsim05G0165500	0.154	-0.398	-0.552	no
CK-40d	RHSIM_Rhsim03G0085600	3.493	2.174	-1.319	high
CK-40d	RHSIM_Rhsim03G0018000	-2.597	-0.932	1.665	low
CK-40d	RHSIM_Rhsim04G0009800	3.230	2.685	-0.545	high
CK-40d	RHSIM_Rhsim03G0083300	-1.515	-1.126	0.389	low
CK-40d	RHSIM_Rhsim03G0219000	-0.936	-0.410	0.526	no
CK-40d	RHSIM_Rhsim03G0238600	0.746	0.030	-0.716	no
CK-40d	RHSIM_Rhsim03G0196000	3.252	0.803	-2.450	high
CK-40d	RHSIM_Rhsim02G0182400	-0.269	-0.727	-0.458	no
CK-40d	RHSIM_Rhsim03G0061800	0.945	0.976	0.031	no
CK-40d	RHSIM_Rhsim03G0234600	-0.293	-0.983	-0.689	no
CK-40d	RHSIM_Rhsim05G0227600	-1.581	-0.269	1.312	low
CK-40d	RHSIM_Rhsim04G0197800	0.025	-1.956	-1.981	no
CK-40d	RHSIM_Rhsim03G0193700	1.765	0.414	-1.351	high
CK-40d	RHSIM_Rhsim03G0225300	-0.898	-0.195	0.703	no
CK-40d	RHSIM_Rhsim03G0267400	-1.203	-1.343	-0.140	no
CK-40d	RHSIM_Rhsim04G0226400	-1.220	-1.297	-0.077	no
CK-40d	RHSIM_Rhsim03G0208500	-0.335	-0.590	-0.255	no
CK-40d	RHSIM_Rhsim02G0011800	-0.920	-0.223	0.697	no
CK-40d	RHSIM_Rhsim02G0230000	-1.662	0.640	2.302	low
CK-40d	RHSIM_Rhsim05G0158900	2.636	1.368	-1.268	high
CK-40d	RHSIM_Rhsim03G0074600	-1.029	-0.709	0.320	no
CK-40d	RHSIM_Rhsim03G0012100	0.291	-0.468	-0.759	no
CK-40d	RHSIM_Rhsim03G0189800	-1.670	0.099	1.770	low
CK-40d	RHSIM_Rhsim03G0082000	2.013	2.839	0.825	high
CK-40d	RHSIM_Rhsim05G0079700	-2.592	-1.010	1.582	low
CK-40d	RHSIM_Rhsim03G0175300	0.166	-0.586	-0.752	no
CK-40d	RHSIM_Rhsim03G0225900	-0.135	-1.118	-0.983	no
CK-40d	RHSIM_Rhsim03G0049800	-0.704	-0.555	0.149	no
CK-40d	RHSIM_Rhsim03G0168800	-1.338	0.488	1.825	low
CK-40d	RHSIM_Rhsim03G0200100	-0.353	0.036	0.388	no
CK-40d	RHSIM_Rhsim02G0252200	-1.831	-0.982	0.849	low

Group	Gene	PTR (z-score difference)	z(log2 LFQ)	z(log2 FPKM)	extreme-PTR
CK-40d	RHSIM_Rhsim03G0143400	-0.977	-0.899	0.078	no
CK-40d	RHSIM_Rhsim03G0267700	-0.376	0.470	0.847	no
CK-40d	RHSIM_Rhsim05G0214900	-1.221	0.082	1.303	no
CK-40d	RHSIM_Rhsim03G0012300	0.839	0.193	-0.646	no
CK-40d	RHSIM_Rhsim03G0152000	-0.408	0.709	1.117	no
CK-40d	RHSIM_Rhsim02G0137600	-2.448	-0.443	2.004	low
CK-40d	RHSIM_Rhsim03G0077300	-2.010	-0.939	1.072	low
CK-40d	RHSIM_Rhsim05G0192800	-0.455	0.557	1.012	no
CK-40d	RHSIM_Rhsim03G0128200	2.360	1.138	-1.222	high
CK-40d	RHSIM_Rhsim02G0041500	2.100	3.221	1.121	high
CK-40d	RHSIM_Rhsim03G0052900	-1.852	1.757	3.609	low
CK-40d	RHSIM_Rhsim02G0200900	0.934	0.523	-0.411	no
CK-40d	RHSIM_Rhsim03G0225000	-0.988	-0.660	0.329	no
CK-40d	RHSIM_Rhsim05G0146200	0.381	0.073	-0.308	no
CK-40d	RHSIM_Rhsim02G0105700	-2.174	-1.542	0.632	low
CK-40d	RHSIM_Rhsim03G0159900	-1.424	0.130	1.554	low
CK-40d	RHSIM_Rhsim03G0036100	0.204	-0.461	-0.665	no
CK-40d	RHSIM_Rhsim03G0049500	1.266	0.388	-0.878	high
CK-40d	RHSIM_Rhsim03G0246500	-0.982	-0.565	0.417	no
CK-40d	RHSIM_Rhsim05G0188400	-2.086	-0.457	1.629	low
CK-40d	RHSIM_Rhsim03G0194300	0.690	-0.646	-1.336	no
CK-40d	RHSIM_Rhsim02G0173300	0.027	-0.681	-0.708	no
CK-40d	RHSIM_Rhsim05G0073400	-1.452	0.562	2.014	low
CK-40d	RHSIM_Rhsim03G0238900	1.169	1.672	0.502	high
CK-40d	RHSIM_Rhsim03G0058600	0.497	-0.190	-0.687	no
CK-40d	RHSIM_Rhsim02G0117400	-1.303	-1.300	0.003	low
CK-40d	RHSIM_Rhsim03G0236600	-1.234	-1.005	0.228	no
CK-40d	RHSIM_Rhsim03G0098400	1.482	1.784	0.302	high
CK-40d	RHSIM_Rhsim03G0058700	0.830	-0.074	-0.904	no
CK-40d	RHSIM_Rhsim03G0170100	-1.649	-0.104	1.545	low
CK-40d	RHSIM_Rhsim03G0044600	-0.492	-0.198	0.294	no
CK-40d	RHSIM_Rhsim02G0251600	0.126	0.082	-0.044	no

Group	Gene	PTR (z-score difference)	z(log2 LFQ)	z(log2 FPKM)	extreme-PTR
CK-40d	RHSIM_Rhsim02G0203500	0.328	-0.249	-0.577	no
CK-40d	RHSIM_Rhsim03G0216000	-1.190	0.519	1.709	no
CK-40d	RHSIM_Rhsim03G0087900	-0.964	-0.491	0.473	no
CK-40d	RHSIM_Rhsim03G0222700	1.012	-0.050	-1.062	no
CK-40d	RHSIM_Rhsim05G0040800	0.337	-0.108	-0.445	no
CK-40d	RHSIM_Rhsim03G0134000	0.029	0.043	0.014	no
CK-40d	RHSIM_Rhsim03G0218000	-1.149	-0.491	0.658	no
CK-40d	RHSIM_Rhsim03G0261800	0.369	0.428	0.059	no
CK-40d	RHSIM_Rhsim05G0087000	-0.889	1.085	1.974	no
CK-40d	RHSIM_Rhsim03G0022100	1.860	0.276	-1.584	high
CK-40d	RHSIM_Rhsim03G0265100	-0.860	-1.269	-0.409	no
CK-40d	RHSIM_Rhsim03G0218800	0.612	0.225	-0.387	no
CK-40d	RHSIM_Rhsim03G0221200	1.403	0.063	-1.340	high
CK-40d	RHSIM_Rhsim02G0083700	0.463	-0.752	-1.216	no
CK-40d	RHSIM_Rhsim03G0150900	-2.196	0.206	2.402	low
CK-40d	RHSIM_Rhsim03G0217000	-0.049	0.096	0.145	no
CK-40d	RHSIM_Rhsim03G0255200	-1.914	0.073	1.987	low
CK-40d	RHSIM_Rhsim03G0172900	0.391	0.951	0.560	no
CK-40d	RHSIM_Rhsim03G0136600	1.803	1.846	0.044	high
CK-40d	RHSIM_Rhsim05G0200700	0.381	0.133	-0.249	no
CK-40d	RHSIM_Rhsim03G0143900	-1.391	-1.486	-0.095	low
CK-40d	RHSIM_Rhsim03G0078900	-0.963	-0.987	-0.024	no
CK-40d	RHSIM_Rhsim05G0119800	0.660	0.439	-0.221	no
CK-40d	RHSIM_Rhsim02G0212300	2.205	1.923	-0.282	high
CK-40d	RHSIM_Rhsim02G0132300	0.254	1.463	1.209	no
CK-40d	RHSIM_Rhsim03G0216300	-0.649	1.576	2.225	no
CK-40d	RHSIM_Rhsim02G0157500	-1.049	-1.598	-0.549	no
CK-40d	RHSIM_Rhsim03G0127200	-0.860	-0.976	-0.116	no
CK-40d	RHSIM_Rhsim03G0031000	-0.432	-0.577	-0.145	no
CK-40d	RHSIM_Rhsim03G0172800	-0.664	-0.332	0.332	no
CK-40d	RHSIM_Rhsim03G0149000	1.806	1.327	-0.478	high
CK-40d	RHSIM_Rhsim02G0105600	-0.762	-0.158	0.604	no

Group	Gene	PTR (z-score difference)	z(log2 LFQ)	z(log2 FPKM)	extreme-PTR
CK-40d	RHSIM_Rhsim03G0208700	-0.195	-0.015	0.180	no
CK-40d	RHSIM_Rhsim02G0251800	0.898	0.767	-0.131	no
CK-40d	RHSIM_Rhsim02G0007300	0.379	0.804	0.424	no
CK-40d	RHSIM_Rhsim03G0158800	-0.665	-0.663	0.002	no
CK-40d	RHSIM_Rhsim02G0056700	0.780	1.162	0.383	no
CK-40d	RHSIM_Rhsim05G0228200	-0.443	-0.816	-0.373	no
CK-40d	RHSIM_Rhsim02G0179000	-0.688	-1.403	-0.715	no
CK-40d	RHSIM_Rhsim03G0174400	2.303	1.659	-0.644	high
CK-40d	RHSIM_Rhsim03G0114800	-1.777	-0.357	1.421	low
CK-40d	RHSIM_Rhsim03G0266500	0.509	0.728	0.219	no
CK-40d	RHSIM_Rhsim03G0092100	1.873	0.620	-1.253	high
CK-40d	RHSIM_Rhsim02G0180400	-0.344	-1.366	-1.022	no
CK-40d	RHSIM_Rhsim03G0145000	0.356	0.167	-0.189	no
CK-40d	RHSIM_Rhsim04G0105900	2.150	-0.083	-2.233	high
CK-40d	RHSIM_Rhsim02G0199800	0.354	0.332	-0.022	no
CK-40d	RHSIM_Rhsim03G0169200	0.556	0.077	-0.479	no
CK-40d	RHSIM_Rhsim04G0217900	-1.436	-1.235	0.201	low
CK-40d	RHSIM_Rhsim03G0159700	-0.668	0.368	1.036	no
CK-40d	RHSIM_Rhsim02G0146400	-0.454	0.315	0.768	no
CK-40d	RHSIM_Rhsim02G0090300	-0.959	-0.507	0.451	no
CK-40d	RHSIM_Rhsim03G0034900	-0.215	-1.126	-0.912	no
CK-40d	RHSIM_Rhsim03G0163000	-0.637	-0.248	0.389	no
CK-40d	RHSIM_Rhsim02G0229000	0.389	-0.087	-0.476	no
CK-40d	RHSIM_Rhsim02G0168000	-1.027	-1.313	-0.286	no
CK-40d	RHSIM_Rhsim04G0032000	4.204	4.339	0.135	high
CK-40d	RHSIM_Rhsim03G0262600	-0.339	0.110	0.449	no
CK-40d	RHSIM_Rhsim02G0044500	-0.380	-0.455	-0.074	no
CK-40d	RHSIM_Rhsim04G0167100	2.005	2.214	0.209	high
CK-40d	RHSIM_Rhsim02G0059100	-0.405	1.549	1.953	no
CK-40d	RHSIM_Rhsim02G0084900	-1.595	0.164	1.760	low
CK-40d	RHSIM_Rhsim02G0042700	-1.102	0.627	1.730	no
CK-40d	RHSIM_Rhsim03G0228600	-0.792	0.307	1.099	no

Group	Gene	PTR (z-score difference)	z(log2 LFQ)	z(log2 FPKM)	extreme-PTR
CK-40d	RHSIM_Rhsim02G0082300	-0.274	-1.120	-0.846	no
CK-40d	RHSIM_Rhsim02G0036100	0.125	0.203	0.078	no
CK-40d	RHSIM_Rhsim04G0038900	-0.984	-0.452	0.532	no
CK-40d	RHSIM_Rhsim02G0103000	-0.787	-0.474	0.313	no
CK-40d	RHSIM_Rhsim02G0014100	-0.857	0.134	0.991	no
CK-40d	RHSIM_Rhsim03G0127300	0.875	1.438	0.563	no
CK-40d	RHSIM_Rhsim03G0078300	0.081	2.607	2.526	no
CK-40d	RHSIM_Rhsim02G0136400	2.214	1.974	-0.240	high
CK-40d	RHSIM_Rhsim03G0057300	0.007	-0.782	-0.789	no
CK-40d	RHSIM_Rhsim02G0241700	1.234	0.530	-0.704	high
CK-40d	RHSIM_Rhsim03G0046400	-0.533	-0.610	-0.077	no
CK-40d	RHSIM_Rhsim03G0258700	-0.114	-0.635	-0.521	no
CK-40d	RHSIM_Rhsim03G0265400	-1.058	0.892	1.949	no
CK-40d	RHSIM_Rhsim02G0086200	1.342	0.174	-1.168	high
CK-40d	RHSIM_Rhsim02G0255200	1.835	-0.467	-2.302	high
CK-40d	RHSIM_Rhsim02G0254500	0.999	1.594	0.596	no
CK-40d	RHSIM_Rhsim02G0173200	0.034	-0.829	-0.862	no
CK-40d	RHSIM_Rhsim03G0116200	1.325	0.496	-0.828	high
CK-40d	RHSIM_Rhsim03G0234900	-0.374	-0.613	-0.239	no
CK-40d	RHSIM_Rhsim02G0060400	-1.409	-1.403	0.006	low
CK-40d	RHSIM_Rhsim03G0151900	0.396	-0.315	-0.711	no
CK-40d	RHSIM_Rhsim02G0140300	1.064	1.291	0.227	no
CK-40d	RHSIM_Rhsim03G0000600	0.057	0.473	0.415	no
CK-40d	RHSIM_Rhsim02G0216000	0.094	-1.317	-1.410	no
CK-40d	RHSIM_Rhsim02G0213000	0.513	-0.921	-1.433	no
CK-40d	RHSIM_Rhsim04G0247900	-0.838	-1.074	-0.236	no
CK-40d	RHSIM_Rhsim03G0002000	0.997	-0.258	-1.255	no
CK-40d	RHSIM_Rhsim02G0008500	-0.628	2.170	2.797	no
CK-40d	RHSIM_Rhsim02G0009100	0.933	1.129	0.196	no
CK-40d	RHSIM_Rhsim02G0217600	-1.192	-1.499	-0.307	no
CK-40d	RHSIM_Rhsim02G0019700	-0.366	-0.699	-0.333	no
CK-40d	RHSIM_Rhsim02G0255500	1.086	0.814	-0.272	no

Group	Gene	PTR (z-score difference)	z(log2 LFQ)	z(log2 FPKM)	extreme-PTR
CK-40d	RHSIM_Rhsim03G0232800	0.337	-0.330	-0.667	no
CK-40d	RHSIM_Rhsim02G0023300	0.989	0.494	-0.495	no
CK-40d	RHSIM_Rhsim03G0264100	1.571	1.405	-0.166	high
CK-40d	RHSIM_Rhsim02G0043700	-0.016	0.038	0.053	no
CK-40d	RHSIM_Rhsim03G0259400	0.227	0.588	0.360	no
CK-40d	RHSIM_Rhsim02G0088500	0.504	0.054	-0.450	no
CK-40d	RHSIM_Rhsim04G0016400	1.909	1.124	-0.785	high
CK-40d	RHSIM_Rhsim02G0137400	-0.931	-1.840	-0.909	no
CK-40d	RHSIM_Rhsim02G0006600	0.308	-0.200	-0.507	no
CK-40d	RHSIM_Rhsim02G0013600	-0.483	-0.718	-0.236	no
CK-40d	RHSIM_Rhsim04G0172100	-1.300	-1.946	-0.646	low
CK-40d	RHSIM_Rhsim02G0254400	0.132	0.179	0.046	no
CK-40d	RHSIM_Rhsim02G0053400	-0.433	0.803	1.236	no
CK-40d	RHSIM_Rhsim02G0081400	2.998	0.937	-2.061	high
CK-40d	RHSIM_Rhsim03G0136800	1.437	-0.582	-2.019	high
CK-40d	RHSIM_Rhsim02G0068600	1.733	1.756	0.023	high
CK-40d	RHSIM_Rhsim03G0124200	1.283	0.019	-1.264	high
CK-40d	RHSIM_Rhsim03G0141800	-0.047	-0.155	-0.108	no
CK-40d	RHSIM_Rhsim03G0010600	-1.114	-0.972	0.142	no
CK-40d	RHSIM_Rhsim03G0120800	0.817	1.385	0.568	no
CK-40d	RHSIM_Rhsim02G0244400	1.052	1.464	0.412	no
CK-40d	RHSIM_Rhsim02G0014600	-0.680	-0.578	0.102	no
CK-40d	RHSIM_Rhsim04G0161900	1.522	0.983	-0.539	high
CK-40d	RHSIM_Rhsim02G0023100	1.102	1.116	0.014	no
CK-40d	RHSIM_Rhsim03G0011600	0.113	-0.836	-0.948	no
CK-40d	RHSIM_Rhsim02G0066700	0.511	-0.151	-0.661	no
CK-40d	RHSIM_Rhsim03G0075600	-1.393	-0.830	0.563	low
CK-40d	RHSIM_Rhsim04G0239100	0.887	-0.064	-0.951	no
CK-40d	RHSIM_Rhsim02G0216100	-0.512	-0.490	0.022	no
CK-40d	RHSIM_Rhsim02G0126300	1.332	1.334	0.002	high
CK-40d	RHSIM_Rhsim02G0025700	1.188	0.282	-0.906	high
CK-40d	RHSIM_Rhsim04G0138800	-1.194	-1.035	0.159	no

Group	Gene	PTR (z-score difference)	z(log2 LFQ)	z(log2 FPKM)	extreme-PTR
CK-40d	RHSIM_Rhsim03G0201600	1.757	-0.693	-2.450	high
CK-40d	RHSIM_Rhsim03G0182700	1.147	-0.913	-2.060	high
CK-40d	RHSIM_Rhsim02G0106500	1.571	-0.660	-2.231	high
CK-40d	RHSIM_Rhsim02G0223100	1.386	-0.468	-1.854	high
CK-40d	RHSIM_Rhsim04G0002700	0.918	0.694	-0.224	no
CK-40d	RHSIM_Rhsim03G0150500	1.244	-0.094	-1.339	high
CK-40d	RHSIM_Rhsim02G0181700	-0.403	-0.383	0.020	no
CK-40d	RHSIM_Rhsim04G0106800	0.212	-0.249	-0.461	no
CK-40d	RHSIM_Rhsim02G0138500	0.341	0.105	-0.236	no
CK-40d	RHSIM_Rhsim02G0070200	-0.138	-0.345	-0.207	no
CK-40d	RHSIM_Rhsim02G0119300	-0.315	0.567	0.881	no
CK-40d	RHSIM_Rhsim02G0111900	0.634	0.584	-0.050	no
CK-40d	RHSIM_Rhsim02G0254100	0.964	-0.038	-1.002	no
CK-40d	RHSIM_Rhsim03G0209200	0.477	-0.042	-0.519	no
CK-40d	RHSIM_Rhsim03G0089600	0.638	-0.254	-0.892	no
CK-40d	RHSIM_Rhsim02G0215900	-0.245	-0.098	0.147	no
CK-40d	RHSIM_Rhsim02G0001500	1.536	2.307	0.771	high
CK-40d	RHSIM_Rhsim03G0258200	-0.079	1.264	1.343	no
CK-40d	RHSIM_Rhsim02G0233100	1.436	1.680	0.244	high
CK-40d	RHSIM_Rhsim02G0154700	0.667	0.833	0.166	no
CK-40d	RHSIM_Rhsim03G0050100	2.549	1.143	-1.406	high
CK-40d	RHSIM_Rhsim02G0214000	-0.155	-0.606	-0.451	no
CK-40d	RHSIM_Rhsim02G0062100	0.119	1.539	1.420	no
CK-40d	RHSIM_Rhsim04G0216800	0.720	0.766	0.047	no
CK-40d	RHSIM_Rhsim03G0179200	-2.051	-1.378	0.674	low
CK-40d	RHSIM_Rhsim03G0171200	-2.026	-1.742	0.284	low
CK-40d	RHSIM_Rhsim02G0135000	0.932	1.459	0.527	no
CK-40d	RHSIM_Rhsim02G0083600	-0.143	-0.792	-0.648	no
CK-40d	RHSIM_Rhsim02G0201500	-1.017	-0.321	0.697	no
CK-40d	RHSIM_Rhsim02G0172900	-0.604	-0.827	-0.223	no
CK-40d	RHSIM_Rhsim02G0037100	-0.948	-0.733	0.215	no
CK-40d	RHSIM_Rhsim02G0154900	0.668	0.780	0.112	no

Group	Gene	PTR (z-score difference)	z(log2 LFQ)	z(log2 FPKM)	extreme-PTR
CK-40d	RHSIM_Rhsim03G0123600	-0.852	-0.426	0.426	no
CK-40d	RHSIM_Rhsim02G0179400	2.059	0.231	-1.828	high
CK-40d	RHSIM_Rhsim02G0223200	-1.327	-0.608	0.719	low
CK-40d	RHSIM_Rhsim02G0062200	0.114	-0.115	-0.229	no
CK-40d	RHSIM_Rhsim02G0141000	-0.430	0.038	0.468	no
CK-40d	RHSIM_Rhsim03G0214000	-0.315	-0.327	-0.012	no
CK-40d	RHSIM_Rhsim04G0087600	1.366	1.114	-0.252	high
CK-40d	RHSIM_Rhsim02G0244300	-1.331	-1.053	0.278	low
CK-40d	RHSIM_Rhsim02G0000100	0.455	0.186	-0.269	no
CK-40d	RHSIM_Rhsim02G0181200	0.001	-0.131	-0.131	no
CK-40d	RHSIM_Rhsim02G0231700	0.118	-0.075	-0.192	no
CK-40d	RHSIM_Rhsim02G0217900	0.871	0.152	-0.719	no
CK-40d	RHSIM_Rhsim02G0127500	-1.948	-0.270	1.679	low
CK-40d	RHSIM_Rhsim04G0036300	2.019	-0.294	-2.313	high
CK-40d	RHSIM_Rhsim03G0035800	1.165	0.274	-0.891	high
CK-40d	RHSIM_Rhsim02G0056400	-1.553	2.101	3.654	low
CK-40d	RHSIM_Rhsim02G0169200	1.314	-0.050	-1.364	high
CK-40d	RHSIM_Rhsim02G0008900	-0.301	-1.086	-0.785	no
CK-40d	RHSIM_Rhsim03G0257200	-0.425	0.676	1.101	no
CK-40d	RHSIM_Rhsim02G0140600	0.134	0.095	-0.039	no
CK-40d	RHSIM_Rhsim02G0187100	1.953	1.463	-0.489	high
CK-40d	RHSIM_Rhsim02G0041100	1.483	-0.233	-1.716	high
CK-40d	RHSIM_Rhsim02G0068000	0.375	0.186	-0.189	no
CK-40d	RHSIM_Rhsim02G0239600	0.383	0.919	0.536	no
CK-40d	RHSIM_Rhsim02G0252100	0.782	-1.382	-2.164	no
CK-40d	RHSIM_Rhsim02G0149100	-0.367	0.160	0.527	no
CK-40d	RHSIM_Rhsim04G0022600	1.423	1.189	-0.234	high
CK-40d	RHSIM_Rhsim02G0106400	1.319	2.276	0.957	high
CK-40d	RHSIM_Rhsim02G0031400	-0.703	-0.129	0.573	no
CK-40d	RHSIM_Rhsim02G0176900	-0.289	-0.321	-0.032	no
CK-40d	RHSIM_Rhsim03G0192600	0.590	1.235	0.644	no
CK-40d	RHSIM_Rhsim02G0144300	-1.090	-0.283	0.807	no

Group	Gene	PTR (z-score difference)	z(log2 LFQ)	z(log2 FPKM)	extreme-PTR
CK-40d	RHSIM_Rhsim03G0179700	-2.841	-1.259	1.582	low
CK-40d	RHSIM_Rhsim04G0011900	-0.164	0.389	0.553	no
CK-40d	RHSIM_Rhsim02G0099500	-0.814	0.079	0.893	no
CK-40d	RHSIM_Rhsim02G0162600	0.636	0.915	0.279	no
CK-40d	RHSIM_Rhsim02G0055200	-0.827	-0.357	0.470	no
CK-40d	RHSIM_Rhsim02G0104500	-0.152	0.073	0.225	no
CK-40d	RHSIM_Rhsim03G0224600	0.752	1.451	0.699	no
CK-40d	RHSIM_Rhsim02G0113900	3.796	1.352	-2.443	high
CK-40d	RHSIM_Rhsim01G0236100	-0.899	-0.423	0.476	no
CK-40d	RHSIM_Rhsim04G0247100	0.186	1.321	1.136	no
CK-40d	RHSIM_Rhsim03G0192700	-0.149	0.485	0.634	no
CK-40d	RHSIM_Rhsim01G0001000	0.059	1.147	1.088	no
CK-40d	RHSIM_Rhsim02G0202100	1.138	2.426	1.288	high
CK-40d	RHSIM_Rhsim01G0131400	-1.332	-0.512	0.820	low
CK-40d	RHSIM_Rhsim02G0067100	0.550	1.231	0.681	no
CK-40d	RHSIM_Rhsim02G0169800	0.413	-0.467	-0.880	no
CK-40d	RHSIM_Rhsim02G0228100	-0.216	-1.526	-1.310	no
CK-40d	RHSIM_Rhsim02G0201600	-0.730	-0.666	0.063	no
CK-40d	RHSIM_Rhsim02G0036900	0.453	0.617	0.164	no
CK-40d	RHSIM_Rhsim02G0198800	-0.099	-0.632	-0.533	no
CK-40d	RHSIM_Rhsim01G0219200	0.155	0.530	0.375	no
CK-40d	RHSIM_Rhsim03G0217400	-0.794	1.656	2.450	no
CK-40d	RHSIM_Rhsim01G0232300	-0.363	-0.891	-0.527	no
CK-40d	RHSIM_Rhsim02G0218200	1.840	0.840	-0.999	high
CK-40d	RHSIM_Rhsim01G0254500	-1.050	-0.753	0.296	no
CK-40d	RHSIM_Rhsim02G0069700	-0.735	-1.126	-0.391	no
CK-40d	RHSIM_Rhsim02G0076300	-0.797	0.057	0.854	no
CK-40d	RHSIM_Rhsim03G0028600	1.828	0.929	-0.899	high
CK-40d	RHSIM_Rhsim02G0035500	-0.689	-0.045	0.645	no
CK-40d	RHSIM_Rhsim02G0035300	-0.195	-0.109	0.086	no
CK-40d	RHSIM_Rhsim02G0041900	-0.689	-0.554	0.135	no
CK-40d	RHSIM_Rhsim02G0178300	0.275	-0.821	-1.096	no

Group	Gene	PTR (z-score difference)	z(log2 LFQ)	z(log2 FPKM)	extreme-PTR
CK-40d	RHSIM_Rhsim02G0104000	-0.120	0.284	0.404	no
CK-40d	RHSIM_Rhsim04G0234100	-0.933	0.153	1.086	no
CK-40d	RHSIM_Rhsim03G0135900	0.459	1.192	0.733	no
CK-40d	RHSIM_Rhsim02G0183000	-0.376	-0.686	-0.310	no
CK-40d	RHSIM_Rhsim02G0177000	-0.448	-0.122	0.326	no
CK-40d	RHSIM_Rhsim02G0176200	1.983	0.191	-1.792	high
CK-40d	RHSIM_Rhsim01G0186900	0.619	0.205	-0.414	no
CK-40d	RHSIM_Rhsim02G0146500	-1.294	-0.823	0.471	low
CK-40d	RHSIM_Rhsim04G0013700	0.641	0.484	-0.157	no
CK-40d	RHSIM_Rhsim01G0245400	0.504	1.159	0.655	no
CK-40d	RHSIM_Rhsim02G0020900	0.979	0.263	-0.716	no
CK-40d	RHSIM_Rhsim02G0145500	1.254	0.684	-0.570	high
CK-40d	RHSIM_Rhsim02G0251900	-1.431	-1.016	0.415	low
CK-40d	RHSIM_Rhsim02G0123300	-1.624	-1.096	0.528	low
CK-40d	RHSIM_Rhsim02G0115100	3.065	4.681	1.615	high
CK-40d	RHSIM_Rhsim02G0171500	1.001	0.549	-0.453	no
CK-40d	RHSIM_Rhsim02G0070300	1.180	0.466	-0.714	high
CK-40d	RHSIM_Rhsim04G0239600	-1.719	-0.631	1.087	low
CK-40d	RHSIM_Rhsim01G0225400	-1.437	-1.100	0.337	low
CK-40d	RHSIM_Rhsim02G0014200	-0.577	-0.245	0.332	no
CK-40d	RHSIM_Rhsim02G0165800	-0.385	0.336	0.721	no
CK-40d	RHSIM_Rhsim02G0027900	-1.499	-1.043	0.456	low
CK-40d	RHSIM_Rhsim02G0076900	-0.610	-1.453	-0.843	no
CK-40d	RHSIM_Rhsim02G0120700	-0.766	-0.098	0.667	no
CK-40d	RHSIM_Rhsim02G0160300	-2.220	-0.444	1.776	low
CK-40d	RHSIM_Rhsim02G0115500	-0.643	-0.651	-0.008	no
CK-40d	RHSIM_Rhsim02G0220400	2.365	1.115	-1.250	high
CK-40d	RHSIM_Rhsim02G0071700	0.593	0.031	-0.562	no
CK-40d	RHSIM_Rhsim02G0159900	0.174	-0.430	-0.604	no
CK-40d	RHSIM_Rhsim02G0165700	-1.980	-0.645	1.335	low
CK-40d	RHSIM_Rhsim04G0100800	-0.596	0.031	0.627	no
CK-40d	RHSIM_Rhsim02G0162800	0.236	-0.938	-1.174	no

Group	Gene	PTR (z-score difference)	z(log2 LFQ)	z(log2 FPKM)	extreme-PTR
CK-40d	RHSIM_Rhsim02G0037200	-1.956	-0.814	1.141	low
CK-40d	RHSIM_Rhsim02G0127600	-1.137	-0.892	0.245	no
CK-40d	RHSIM_Rhsim02G0200700	-1.236	-0.700	0.536	no
CK-40d	RHSIM_Rhsim03G0210800	1.141	0.679	-0.462	high
CK-40d	RHSIM_Rhsim04G0240100	0.494	0.077	-0.417	no
CK-40d	RHSIM_Rhsim02G0057300	-2.605	-0.683	1.922	low
CK-40d	RHSIM_Rhsim03G0230200	0.259	0.082	-0.176	no
CK-40d	RHSIM_Rhsim04G0013000	-1.469	-1.895	-0.425	low
CK-40d	RHSIM_Rhsim01G0160900	1.362	0.790	-0.572	high
CK-40d	RHSIM_Rhsim01G0235400	1.446	1.374	-0.072	high
CK-40d	RHSIM_Rhsim02G0188300	2.027	0.447	-1.581	high
CK-40d	RHSIM_Rhsim02G0027800	-0.050	-0.649	-0.599	no
CK-40d	RHSIM_Rhsim02G0027600	0.683	-0.776	-1.460	no
CK-40d	RHSIM_Rhsim01G0230500	-2.111	-0.186	1.925	low
CK-40d	RHSIM_Rhsim02G0021200	3.113	1.935	-1.178	high
CK-40d	RHSIM_Rhsim03G0056900	0.504	-0.895	-1.398	no
CK-40d	RHSIM_Rhsim02G0032400	1.173	1.848	0.674	high
CK-40d	RHSIM_Rhsim01G0112900	-1.871	-1.581	0.290	low
CK-40d	RHSIM_Rhsim02G0160100	-0.960	-0.592	0.368	no
CK-40d	RHSIM_Rhsim02G0067200	0.174	-0.191	-0.365	no
CK-40d	RHSIM_Rhsim01G0217300	0.561	0.595	0.034	no
CK-40d	RHSIM_Rhsim01G0171700	0.033	-1.057	-1.090	no
CK-40d	RHSIM_Rhsim01G0145800	0.792	0.778	-0.015	no
CK-40d	RHSIM_Rhsim01G0245700	-0.921	-0.633	0.288	no
CK-40d	RHSIM_Rhsim01G0142200	-1.239	-1.363	-0.125	low
CK-40d	RHSIM_Rhsim02G0168400	-1.247	-0.614	0.633	low
CK-40d	RHSIM_Rhsim01G0268000	0.028	-1.193	-1.221	no
CK-40d	RHSIM_Rhsim04G0227400	-1.470	-0.761	0.709	low
CK-40d	RHSIM_Rhsim02G0234500	-1.382	-2.093	-0.712	low
CK-40d	RHSIM_Rhsim01G0249200	-0.447	-1.490	-1.043	no
CK-40d	RHSIM_Rhsim01G0069300	0.178	0.207	0.029	no
CK-40d	RHSIM_Rhsim03G0130400	0.072	0.472	0.400	no

Group	Gene	PTR (z-score difference)	z(log2 LFQ)	z(log2 FPKM)	extreme-PTR
CK-40d	RHSIM_Rhsim01G0268500	-0.687	-1.590	-0.903	no
CK-40d	RHSIM_Rhsim01G0197500	0.110	0.141	0.031	no
CK-40d	RHSIM_Rhsim01G0186400	2.793	2.285	-0.508	high
CK-40d	RHSIM_Rhsim02G0082500	0.402	0.420	0.018	no
CK-40d	RHSIM_Rhsim02G0072000	0.014	-0.097	-0.111	no
CK-40d	RHSIM_Rhsim01G0280600	-1.466	-0.807	0.659	low
CK-40d	RHSIM_Rhsim01G0002200	0.135	0.251	0.116	no
CK-40d	RHSIM_Rhsim02G0088100	2.637	1.145	-1.491	high
CK-40d	RHSIM_Rhsim04G0172200	1.110	2.100	0.990	no
CK-40d	RHSIM_Rhsim02G0013700	-1.021	-0.484	0.537	no
CK-40d	RHSIM_Rhsim01G0293800	2.207	1.598	-0.609	high
CK-40d	RHSIM_Rhsim01G0263600	0.362	0.179	-0.183	no
CK-40d	RHSIM_Rhsim04G0224200	-0.994	-0.328	0.667	no
CK-40d	RHSIM_Rhsim02G0076100	-1.297	-0.673	0.624	low
CK-40d	RHSIM_Rhsim01G0245600	-1.590	-1.187	0.403	low
CK-40d	RHSIM_Rhsim02G0203400	0.242	0.569	0.327	no
CK-40d	RHSIM_Rhsim01G0281900	0.992	-0.189	-1.180	no
CK-40d	RHSIM_Rhsim02G0210900	-1.384	0.292	1.676	low
CK-40d	RHSIM_Rhsim01G0101000	-2.171	-1.473	0.698	low
CK-40d	RHSIM_Rhsim04G0176700	0.585	0.259	-0.326	no
CK-40d	RHSIM_Rhsim02G0062300	1.531	0.997	-0.534	high
CK-40d	RHSIM_Rhsim01G0142500	-1.549	-1.089	0.460	low
CK-40d	RHSIM_Rhsim02G0061900	0.100	-0.948	-1.048	no
CK-40d	RHSIM_Rhsim03G0011700	0.723	0.017	-0.706	no
CK-40d	RHSIM_Rhsim01G0218000	0.204	0.390	0.186	no
CK-40d	RHSIM_Rhsim01G0211400	-0.579	-0.517	0.062	no
CK-40d	RHSIM_Rhsim03G0141500	-0.630	1.659	2.289	no
CK-40d	RHSIM_Rhsim02G0018200	1.838	0.903	-0.936	high
CK-40d	RHSIM_Rhsim02G0243200	1.473	0.094	-1.379	high
CK-40d	RHSIM_Rhsim03G0135800	-0.567	-0.074	0.492	no
CK-40d	RHSIM_Rhsim02G0162900	-0.317	0.056	0.373	no
CK-40d	RHSIM_Rhsim01G0207700	1.716	1.122	-0.594	high

Group	Gene	PTR (z-score difference)	z(log2 LFQ)	z(log2 FPKM)	extreme-PTR
CK-40d	RHSIM_Rhsim02G0033000	-0.947	-0.518	0.429	no
CK-40d	RHSIM_Rhsim02G0066900	-0.267	-0.842	-0.575	no
CK-40d	RHSIM_Rhsim02G0168300	0.512	0.490	-0.022	no
CK-40d	RHSIM_Rhsim04G0000500	0.368	-0.963	-1.331	no
CK-40d	RHSIM_Rhsim02G0169300	-0.720	-0.330	0.389	no
CK-40d	RHSIM_Rhsim02G0099100	-1.108	-1.121	-0.013	no
CK-40d	RHSIM_Rhsim01G0060800	0.208	0.775	0.567	no
CK-40d	RHSIM_Rhsim02G0087400	1.173	0.632	-0.541	high
CK-40d	RHSIM_Rhsim02G0044000	-0.097	-0.471	-0.374	no
CK-40d	RHSIM_Rhsim01G0290800	0.423	2.797	2.374	no
CK-40d	RHSIM_Rhsim02G0172400	1.253	-0.252	-1.505	high
CK-40d	RHSIM_Rhsim02G0023800	1.602	0.494	-1.108	high
CK-40d	RHSIM_Rhsim02G0054300	-0.013	0.327	0.339	no
CK-40d	RHSIM_Rhsim02G0003600	-0.162	0.272	0.434	no
CK-40d	RHSIM_Rhsim02G0179100	0.528	-0.223	-0.751	no
CK-40d	RHSIM_Rhsim02G0202300	0.857	0.198	-0.658	no
CK-40d	RHSIM_Rhsim04G0128500	0.108	-0.116	-0.224	no
CK-40d	RHSIM_Rhsim01G0100400	0.397	-1.129	-1.526	no
CK-40d	RHSIM_Rhsim02G0106700	-1.797	-0.936	0.861	low
CK-40d	RHSIM_Rhsim01G0182300	-0.469	-1.193	-0.724	no
CK-40d	RHSIM_Rhsim02G0195900	-0.248	-0.981	-0.734	no
CK-40d	RHSIM_Rhsim02G0190100	-0.221	-0.661	-0.440	no
CK-40d	RHSIM_Rhsim04G0093900	0.105	0.642	0.537	no
CK-40d	RHSIM_Rhsim02G0067000	-1.252	0.649	1.900	low
CK-40d	RHSIM_Rhsim02G0247000	-0.830	-0.183	0.647	no
CK-40d	RHSIM_Rhsim02G0017000	-0.827	-0.895	-0.068	no
CK-40d	RHSIM_Rhsim02G0213200	0.389	-1.069	-1.459	no
CK-40d	RHSIM_Rhsim01G0219000	-0.927	-0.763	0.164	no
CK-40d	RHSIM_Rhsim01G0176200	-0.012	-1.277	-1.265	no
CK-40d	RHSIM_Rhsim02G0052200	-0.248	-0.362	-0.114	no
CK-40d	RHSIM_Rhsim01G0243700	0.832	-0.122	-0.954	no
CK-40d	RHSIM_Rhsim02G0110000	-0.378	-0.111	0.268	no

Group	Gene	PTR (z-score difference)	z(log2 LFQ)	z(log2 FPKM)	extreme-PTR
CK-40d	RHSIM_Rhsim02G0180500	-2.173	0.069	2.242	low
CK-40d	RHSIM_Rhsim04G0204400	-0.365	-0.834	-0.469	no
CK-40d	RHSIM_Rhsim02G0198400	0.498	-0.400	-0.898	no
CK-40d	RHSIM_Rhsim01G0096100	0.497	-1.038	-1.535	no
CK-40d	RHSIM_Rhsim01G0136000	0.384	0.741	0.357	no
CK-40d	RHSIM_Rhsim02G0002800	-0.140	0.176	0.316	no
CK-40d	RHSIM_Rhsim01G0197200	2.570	1.925	-0.645	high
CK-40d	RHSIM_Rhsim01G0090900	0.822	2.848	2.026	no
CK-40d	RHSIM_Rhsim01G0095300	0.130	-0.447	-0.577	no
CK-40d	RHSIM_Rhsim02G0171600	0.405	1.382	0.976	no
CK-40d	RHSIM_Rhsim02G0054100	0.850	0.346	-0.504	no
CK-40d	RHSIM_Rhsim01G0196800	0.528	-0.519	-1.047	no
CK-40d	RHSIM_Rhsim03G0218500	0.471	0.930	0.460	no
CK-40d	RHSIM_Rhsim02G0004400	0.364	1.111	0.747	no
CK-40d	RHSIM_Rhsim02G0009000	0.640	-0.565	-1.205	no
CK-40d	RHSIM_Rhsim02G0179200	2.365	0.409	-1.955	high
CK-40d	RHSIM_Rhsim02G0212500	-0.116	0.227	0.343	no
CK-40d	RHSIM_Rhsim02G0202200	-0.163	-0.331	-0.168	no
CK-40d	RHSIM_Rhsim01G0123500	0.022	-0.791	-0.814	no
CK-40d	RHSIM_Rhsim01G0051700	0.810	-0.031	-0.841	no
CK-40d	RHSIM_Rhsim01G0216700	-0.368	0.458	0.826	no
CK-40d	RHSIM_Rhsim01G0107900	0.916	0.719	-0.197	no
CK-40d	RHSIM_Rhsim03G0170800	-0.043	1.434	1.477	no
CK-40d	RHSIM_Rhsim01G0209500	-0.173	1.366	1.539	no
CK-40d	RHSIM_Rhsim01G0259700	1.088	-0.848	-1.936	no
CK-40d	RHSIM_Rhsim02G0194300	-0.027	1.872	1.898	no
CK-40d	RHSIM_Rhsim02G0069300	0.335	-0.421	-0.757	no
CK-40d	RHSIM_Rhsim01G0293700	0.833	-0.249	-1.081	no
CK-40d	RHSIM_Rhsim03G0144200	-0.142	-0.001	0.140	no
CK-40d	RHSIM_Rhsim01G0265100	0.759	0.473	-0.286	no
CK-40d	RHSIM_Rhsim01G0291100	0.629	1.339	0.710	no
CK-40d	RHSIM_Rhsim02G0232700	0.750	0.237	-0.513	no

Group	Gene	PTR (z-score difference)	z(log2 LFQ)	z(log2 FPKM)	extreme-PTR
CK-40d	RHSIM_Rhsim02G0228400	-0.133	0.257	0.390	no
CK-40d	RHSIM_Rhsim01G0146900	-1.408	-1.427	-0.019	low
CK-40d	RHSIM_Rhsim01G0124300	0.692	-0.139	-0.831	no
CK-40d	RHSIM_Rhsim01G0067700	-2.073	-1.888	0.184	low
CK-40d	RHSIM_Rhsim01G0119100	0.122	-1.108	-1.230	no
CK-40d	RHSIM_Rhsim02G0095200	-0.562	-0.966	-0.404	no
CK-40d	RHSIM_Rhsim02G0223500	0.024	-0.063	-0.087	no
CK-40d	RHSIM_Rhsim01G0292800	0.489	1.067	0.578	no
CK-40d	RHSIM_Rhsim03G0247900	2.414	1.196	-1.218	high
CK-40d	RHSIM_Rhsim01G0282100	-0.439	-1.342	-0.903	no
CK-40d	RHSIM_Rhsim01G0189100	-0.939	-1.340	-0.401	no
CK-40d	RHSIM_Rhsim02G0216700	-0.377	-0.479	-0.102	no
CK-40d	RHSIM_Rhsim01G0224200	0.802	0.355	-0.448	no
CK-40d	RHSIM_Rhsim03G0036700	-0.435	0.176	0.611	no
CK-40d	RHSIM_Rhsim01G0191800	-1.527	-0.143	1.384	low
CK-40d	RHSIM_Rhsim01G0210800	-0.390	1.306	1.696	no
CK-40d	RHSIM_Rhsim01G0109600	-1.029	-0.591	0.438	no
CK-40d	RHSIM_Rhsim01G0009900	-1.790	-0.587	1.203	low
CK-40d	RHSIM_Rhsim01G0176300	0.826	-0.573	-1.400	no
CK-40d	RHSIM_Rhsim03G0088500	0.257	-0.164	-0.421	no
CK-40d	RHSIM_Rhsim02G0244800	-0.605	0.819	1.424	no
CK-40d	RHSIM_Rhsim01G0197900	0.174	-0.195	-0.370	no
CK-40d	RHSIM_Rhsim01G0191100	2.371	2.422	0.051	high
CK-40d	RHSIM_Rhsim02G0205100	-2.254	-1.697	0.557	low
CK-40d	RHSIM_Rhsim01G0201600	-0.161	-0.010	0.151	no
CK-40d	RHSIM_Rhsim01G0085500	0.740	-1.132	-1.873	no
CK-40d	RHSIM_Rhsim03G0125000	-2.036	-1.065	0.972	low
CK-40d	RHSIM_Rhsim01G0210200	-1.289	-1.284	0.005	low
CK-40d	RHSIM_Rhsim02G0062000	0.410	1.172	0.762	no
CK-40d	RHSIM_Rhsim01G0188400	0.774	0.614	-0.160	no
CK-40d	RHSIM_Rhsim03G0142300	1.160	0.700	-0.459	high
CK-40d	RHSIM_Rhsim02G0168200	-0.932	-1.214	-0.282	no

Group	Gene	PTR (z-score difference)	z(log2 LFQ)	z(log2 FPKM)	extreme-PTR
CK-40d	RHSIM_Rhsim01G0143400	0.253	1.395	1.142	no
CK-40d	RHSIM_Rhsim01G0204700	-1.108	-0.130	0.978	no
CK-40d	RHSIM_Rhsim01G0259300	0.893	0.386	-0.507	no
CK-40d	RHSIM_Rhsim01G0091000	-0.536	-0.918	-0.383	no
CK-40d	RHSIM_Rhsim01G0276200	-0.180	-0.548	-0.368	no
CK-40d	RHSIM_Rhsim02G0148900	-0.883	-0.384	0.499	no
CK-40d	RHSIM_Rhsim01G0262300	0.315	-0.457	-0.772	no
CK-40d	RHSIM_Rhsim01G0180500	-0.505	-0.586	-0.082	no
CK-40d	RHSIM_Rhsim01G0119800	-0.656	-0.657	-0.001	no
CK-40d	RHSIM_Rhsim01G0010000	0.217	0.032	-0.185	no
CK-40d	RHSIM_Rhsim01G0204400	-0.600	0.087	0.687	no
CK-40d	RHSIM_Rhsim01G0190200	0.775	0.276	-0.499	no
CK-40d	RHSIM_Rhsim01G0074300	-0.226	0.688	0.914	no
CK-40d	RHSIM_Rhsim01G0174600	-0.555	0.140	0.696	no
CK-40d	RHSIM_Rhsim02G0028600	-1.314	-1.814	-0.500	low
CK-40d	RHSIM_Rhsim01G0246700	0.083	-0.140	-0.223	no
CK-40d	RHSIM_Rhsim01G0107800	-1.284	0.736	2.019	low
CK-40d	RHSIM_Rhsim02G0076700	0.589	-0.432	-1.021	no
CK-40d	RHSIM_Rhsim01G0113600	-1.092	0.534	1.626	no
CK-40d	RHSIM_Rhsim02G0071000	-1.350	-0.236	1.113	low
CK-40d	RHSIM_Rhsim02G0005500	-2.178	-1.526	0.652	low
CK-40d	RHSIM_Rhsim01G0013000	-2.170	-0.058	2.113	low
CK-40d	RHSIM_Rhsim01G0202900	0.181	1.488	1.307	no
CK-40d	RHSIM_Rhsim02G0193400	1.055	1.749	0.694	no
CK-40d	RHSIM_Rhsim01G0198900	-2.060	-0.998	1.062	low
CK-40d	RHSIM_Rhsim01G0233900	-1.061	0.333	1.393	no
CK-40d	RHSIM_Rhsim01G0067800	0.468	0.902	0.433	no
CK-40d	RHSIM_Rhsim02G0049400	-0.956	-1.388	-0.432	no
CK-40d	RHSIM_Rhsim01G0227300	1.268	-0.669	-1.937	high
CK-40d	RHSIM_Rhsim01G0137100	1.803	0.139	-1.663	high
CK-40d	RHSIM_Rhsim01G0126900	0.745	1.104	0.360	no
CK-40d	RHSIM_Rhsim01G0284500	0.126	-0.499	-0.625	no

Group	Gene	PTR (z-score difference)	z(log2 LFQ)	z(log2 FPKM)	extreme-PTR
CK-40d	RHSIM_Rhsim01G0205000	-0.947	1.655	2.602	no
CK-40d	RHSIM_Rhsim01G0145200	-0.135	0.714	0.850	no
CK-40d	RHSIM_Rhsim02G0040300	-1.055	-0.291	0.764	no
CK-40d	RHSIM_Rhsim01G0005500	-0.984	-0.559	0.425	no
CK-40d	RHSIM_Rhsim01G0237200	0.821	1.023	0.202	no
CK-40d	RHSIM_Rhsim02G0049200	-2.457	-2.514	-0.057	low
CK-40d	RHSIM_Rhsim01G0263000	-0.137	0.424	0.561	no
CK-40d	RHSIM_Rhsim01G0132300	-0.638	-0.983	-0.345	no
CK-40d	RHSIM_Rhsim01G0257300	-0.552	1.033	1.585	no
CK-40d	RHSIM_Rhsim01G0236600	0.312	-0.791	-1.103	no
CK-40d	RHSIM_Rhsim01G0009500	0.646	-1.276	-1.922	no
CK-40d	RHSIM_Rhsim03G0172200	1.316	0.272	-1.044	high
CK-40d	RHSIM_Rhsim01G0122100	-0.354	0.240	0.593	no
CK-40d	RHSIM_Rhsim02G0204900	0.790	0.129	-0.661	no
CK-40d	RHSIM_Rhsim01G0289700	-0.608	0.481	1.089	no
CK-40d	RHSIM_Rhsim01G0075900	1.070	0.100	-0.970	no
CK-40d	RHSIM_Rhsim03G0210700	-0.916	-0.414	0.502	no
CK-40d	RHSIM_Rhsim01G0136100	0.501	-0.804	-1.305	no
CK-40d	RHSIM_Rhsim02G0076500	-1.233	-0.351	0.883	no
CK-40d	RHSIM_Rhsim02G0021000	-1.848	-1.927	-0.080	low
CK-40d	RHSIM_Rhsim03G0074800	0.334	0.371	0.037	no
CK-40d	RHSIM_Rhsim01G0181100	-2.358	-1.872	0.486	low
CK-40d	RHSIM_Rhsim01G0062600	-0.190	0.450	0.640	no
CK-40d	RHSIM_Rhsim01G0206500	-1.059	-0.843	0.216	no
CK-40d	RHSIM_Rhsim01G0285000	-2.678	-1.022	1.656	low
CK-40d	RHSIM_Rhsim01G0185400	-0.205	0.853	1.058	no
CK-40d	RHSIM_Rhsim02G0255700	-0.283	-1.595	-1.312	no
CK-40d	RHSIM_Rhsim01G0009700	1.191	0.515	-0.676	high
CK-40d	RHSIM_Rhsim01G0172000	-0.907	-1.432	-0.525	no
CK-40d	RHSIM_Rhsim01G0272700	-0.719	0.125	0.844	no
CK-40d	RHSIM_Rhsim01G0054700	0.346	0.526	0.180	no
CK-40d	RHSIM_Rhsim01G0260300	2.652	1.275	-1.377	high

Group	Gene	PTR (z-score difference)	z(log2 LFQ)	z(log2 FPKM)	extreme-PTR
CK-40d	RHSIM_Rhsim02G0114900	-0.607	0.679	1.286	no
CK-40d	RHSIM_Rhsim01G0216300	0.565	0.706	0.142	no
CK-40d	RHSIM_Rhsim02G0231500	0.019	-0.312	-0.332	no
CK-40d	RHSIM_Rhsim01G0201300	0.652	0.793	0.141	no
CK-40d	RHSIM_Rhsim01G0234600	-0.398	-0.207	0.191	no
CK-40d	RHSIM_Rhsim03G0250000	-1.564	-0.876	0.688	low
CK-40d	RHSIM_Rhsim03G0251500	-0.421	-0.887	-0.466	no
CK-40d	RHSIM_Rhsim01G0208100	1.636	-0.783	-2.418	high
CK-40d	RHSIM_Rhsim01G0124000	0.127	-0.673	-0.801	no
CK-40d	RHSIM_Rhsim01G0271700	-0.943	-0.985	-0.042	no
CK-40d	RHSIM_Rhsim02G0010800	-0.180	-0.101	0.079	no
CK-40d	RHSIM_Rhsim01G0042700	-0.823	-1.068	-0.245	no
CK-40d	RHSIM_Rhsim01G0223500	0.975	-0.011	-0.986	no
CK-40d	RHSIM_Rhsim01G0203800	0.436	0.734	0.299	no
CK-40d	RHSIM_Rhsim01G0286700	-0.994	0.118	1.112	no
CK-40d	RHSIM_Rhsim01G0187100	0.098	0.466	0.368	no
CK-40d	RHSIM_Rhsim01G0141800	-0.506	-0.302	0.204	no
CK-40d	RHSIM_Rhsim01G0245800	-0.008	0.177	0.184	no
CK-40d	RHSIM_Rhsim01G0042600	0.322	0.060	-0.262	no
CK-40d	RHSIM_Rhsim01G0235600	0.011	-0.445	-0.456	no
CK-40d	RHSIM_Rhsim01G0062000	0.491	0.294	-0.197	no
CK-40d	RHSIM_Rhsim01G0179100	-0.769	-0.296	0.473	no
CK-40d	RHSIM_Rhsim01G0198500	-0.061	-0.067	-0.006	no
CK-40d	RHSIM_Rhsim01G0164800	1.465	0.542	-0.923	high
CK-40d	RHSIM_Rhsim01G0293000	-0.538	-0.671	-0.134	no
CK-40d	RHSIM_Rhsim01G0009300	-0.522	0.340	0.862	no
CK-40d	RHSIM_Rhsim01G0149900	-0.454	-0.375	0.078	no
CK-40d	RHSIM_Rhsim01G0147300	0.942	0.687	-0.255	no
CK-40d	RHSIM_Rhsim01G0274600	3.275	2.699	-0.576	high
CK-40d	RHSIM_Rhsim01G0274400	3.411	3.080	-0.330	high
CK-40d	RHSIM_Rhsim03G0226900	0.320	1.555	1.235	no
CK-40d	RHSIM_Rhsim02G0217400	0.349	0.424	0.075	no

Group	Gene	PTR (z-score difference)	z(log2 LFQ)	z(log2 FPKM)	extreme-PTR
CK-40d	RHSIM_Rhsim01G0071400	-0.898	-0.847	0.051	no
CK-40d	RHSIM_Rhsim01G0002300	0.747	0.323	-0.424	no
CK-40d	RHSIM_Rhsim01G0033400	-0.115	-0.119	-0.003	no
CK-40d	RHSIM_Rhsim01G0269700	0.858	0.027	-0.831	no
CK-40d	RHSIM_Rhsim01G0227900	-0.481	-1.179	-0.698	no
CK-40d	RHSIM_Rhsim01G0254100	-0.763	-0.120	0.643	no
CK-40d	RHSIM_Rhsim01G0074500	-1.001	-1.688	-0.687	no
CK-40d	RHSIM_Rhsim01G0192900	0.289	-0.334	-0.623	no
CK-40d	RHSIM_Rhsim01G0260900	1.223	0.607	-0.617	high
CK-40d	RHSIM_Rhsim01G0006200	-1.118	-0.469	0.650	no
CK-40d	RHSIM_Rhsim01G0088200	2.342	0.641	-1.701	high
CK-40d	RHSIM_Rhsim01G0285400	0.179	-0.805	-0.984	no
CK-40d	RHSIM_Rhsim01G0155600	0.506	-0.938	-1.444	no
CK-40d	RHSIM_Rhsim01G0039300	-0.113	-0.411	-0.298	no
CK-40d	RHSIM_Rhsim03G0141100	0.839	-0.007	-0.847	no
CK-40d	RHSIM_Rhsim01G0272800	-0.131	-0.800	-0.669	no
CK-40d	RHSIM_Rhsim01G0089800	0.268	0.960	0.692	no
CK-40d	RHSIM_Rhsim03G0207500	-0.420	-1.521	-1.100	no
CK-40d	RHSIM_Rhsim01G0205100	0.053	0.216	0.163	no
CK-40d	RHSIM_Rhsim01G0100800	0.378	-0.701	-1.079	no
CK-40d	RHSIM_Rhsim03G0176000	-1.444	-0.911	0.533	low
CK-40d	RHSIM_Rhsim01G0055600	0.370	-0.193	-0.563	no
CK-40d	RHSIM_Rhsim01G0262400	0.472	1.198	0.727	no
CK-40d	RHSIM_Rhsim01G0218100	0.655	0.842	0.187	no
CK-40d	RHSIM_Rhsim01G0245300	-1.622	0.040	1.662	low
CK-40d	RHSIM_Rhsim01G0291400	0.421	0.385	-0.036	no
CK-40d	RHSIM_Rhsim01G0049400	-0.486	-0.535	-0.050	no
CK-40d	RHSIM_Rhsim01G0151000	-0.488	-0.590	-0.103	no
CK-40d	RHSIM_Rhsim01G0106700	0.704	0.241	-0.463	no
CK-40d	RHSIM_Rhsim01G0196600	-1.223	0.700	1.923	no
CK-40d	RHSIM_Rhsim01G0239900	-1.018	0.133	1.151	no
CK-40d	RHSIM_Rhsim01G0113300	-0.915	-0.398	0.517	no

Group	Gene	PTR (z-score difference)	z(log2 LFQ)	z(log2 FPKM)	extreme-PTR
CK-40d	RHSIM_Rhsim01G0009800	-0.553	-1.009	-0.456	no
CK-40d	RHSIM_Rhsim01G0283500	0.265	0.494	0.229	no
CK-40d	RHSIM_Rhsim01G0233100	-0.611	-0.396	0.216	no
CK-40d	RHSIM_Rhsim01G0237700	0.338	-1.644	-1.982	no
CK-40d	RHSIM_Rhsim01G0217800	-1.689	-0.415	1.274	low
CK-40d	RHSIM_Rhsim01G0221600	2.107	3.476	1.369	high
CK-40d	RHSIM_Rhsim01G0028300	-1.891	-0.632	1.258	low
CK-40d	RHSIM_Rhsim02G0020800	-1.138	-0.547	0.591	no
CK-40d	RHSIM_Rhsim01G0058000	1.410	1.129	-0.281	high
CK-40d	RHSIM_Rhsim01G0187900	-0.499	-1.440	-0.941	no
CK-40d	RHSIM_Rhsim01G0124600	-2.342	-1.692	0.651	low
CK-40d	RHSIM_Rhsim01G0247100	0.061	-0.463	-0.524	no
CK-40d	RHSIM_Rhsim01G0007800	1.509	0.051	-1.458	high
CK-40d	RHSIM_Rhsim03G0163700	-0.606	-0.649	-0.043	no
CK-40d	RHSIM_Rhsim01G0012100	0.277	-0.657	-0.935	no
CK-40d	RHSIM_Rhsim01G0242500	-1.517	0.885	2.402	low
CK-40d	RHSIM_Rhsim01G0268900	-0.596	-0.472	0.124	no
CK-40d	RHSIM_Rhsim01G0229100	-0.396	-0.383	0.014	no
CK-40d	RHSIM_Rhsim01G0273800	-2.442	-1.249	1.193	low
CK-40d	RHSIM_Rhsim03G0141200	-1.967	1.462	3.429	low
CK-40d	RHSIM_Rhsim03G0215700	-1.363	-0.585	0.778	low
CK-40d	RHSIM_Rhsim01G0186700	0.404	0.016	-0.388	no
CK-40d	RHSIM_Rhsim01G0225700	-0.027	0.581	0.608	no
CK-40d	RHSIM_Rhsim03G0149300	-0.552	0.111	0.664	no
CK-40d	RHSIM_Rhsim01G0071100	3.499	1.462	-2.037	high
CK-40d	RHSIM_Rhsim03G0083700	0.257	-0.372	-0.629	no
CK-40d	RHSIM_Rhsim02G0012600	-1.242	-1.589	-0.347	low
CK-40d	RHSIM_Rhsim01G0117300	-2.261	-1.251	1.010	low
CK-40d	RHSIM_Rhsim01G0176100	-1.634	-0.301	1.332	low
CK-40d	RHSIM_Rhsim02G0158200	-0.607	0.423	1.029	no
CK-40d	RHSIM_Rhsim01G0033200	0.899	1.038	0.139	no
CK-40d	RHSIM_Rhsim01G0245500	-0.278	-0.296	-0.018	no

Group	Gene	PTR (z-score difference)	z(log2 LFQ)	z(log2 FPKM)	extreme-PTR
CK-40d	RHSIM_Rhsim01G0073000	2.618	1.367	-1.252	high
CK-40d	RHSIM_Rhsim01G0038600	-1.556	0.017	1.572	low
CK-40d	RHSIM_Rhsim01G0065700	-0.361	-0.618	-0.257	no
CK-40d	RHSIM_Rhsim01G0068100	0.108	-0.723	-0.831	no
CK-40d	RHSIM_Rhsim01G0130400	-0.632	-0.148	0.484	no
CK-40d	RHSIM_Rhsim01G0196000	-2.700	-1.476	1.223	low
CK-40d	RHSIM_Rhsim03G0040000	2.063	1.824	-0.240	high
CK-40d	RHSIM_Rhsim01G0134600	0.669	-0.852	-1.521	no
CK-40d	RHSIM_Rhsim01G0255000	0.993	1.593	0.601	no
CK-40d	RHSIM_Rhsim01G0249300	-1.618	-1.666	-0.048	low
CK-40d	RHSIM_Rhsim03G0197300	1.497	-0.607	-2.104	high
CK-40d	RHSIM_Rhsim01G0200800	-2.229	-1.815	0.414	low
CK-40d	RHSIM_Rhsim01G0061700	-2.651	-1.254	1.397	low
CK-40d	RHSIM_Rhsim01G0027700	2.146	0.392	-1.754	high
CK-40d	RHSIM_Rhsim01G0026600	0.776	-1.241	-2.017	no
CK-40d	RHSIM_Rhsim01G0103600	-0.299	-0.411	-0.111	no
CK-40d	RHSIM_Rhsim01G0182400	0.420	-0.936	-1.357	no
CK-40d	RHSIM_Rhsim01G0289900	-0.280	0.594	0.875	no
CK-40d	RHSIM_Rhsim01G0251300	2.410	0.925	-1.486	high
CK-40d	RHSIM_Rhsim01G0228100	-0.989	-1.233	-0.245	no
CK-40d	RHSIM_Rhsim02G0213300	-0.755	2.167	2.922	no
CK-40d	RHSIM_Rhsim01G0027300	-0.144	0.194	0.338	no
CK-40d	RHSIM_Rhsim01G0208600	-1.211	0.655	1.865	no
CK-40d	RHSIM_Rhsim01G0292900	-0.548	-1.034	-0.485	no
CK-40d	RHSIM_Rhsim01G0178200	-0.732	-0.315	0.416	no
CK-40d	RHSIM_Rhsim01G0257200	1.193	1.446	0.253	high
CK-40d	RHSIM_Rhsim01G0198000	1.030	0.057	-0.974	no
CK-40d	RHSIM_Rhsim01G0128600	1.285	-0.013	-1.297	high
CK-40d	RHSIM_Rhsim02G0180600	-0.257	0.510	0.767	no
CK-40d	RHSIM_Rhsim01G0149400	0.058	-1.290	-1.348	no
CK-40d	RHSIM_Rhsim01G0000400	1.365	1.774	0.409	high
CK-40d	RHSIM_Rhsim01G0184500	1.576	1.981	0.404	high

Group	Gene	PTR (z-score difference)	z(log2 LFQ)	z(log2 FPKM)	extreme-PTR
CK-40d	RHSIM_Rhsim01G0155900	-0.404	0.069	0.473	no
CK-40d	RHSIM_Rhsim01G0253200	-0.556	-0.304	0.252	no
CK-40d	RHSIM_Rhsim01G0101300	-0.331	-0.554	-0.224	no
CK-40d	RHSIM_Rhsim01G0033300	2.843	1.762	-1.081	high
CK-40d	RHSIM_Rhsim01G0237100	0.017	-0.552	-0.569	no
CK-40d	RHSIM_Rhsim01G0023500	-2.842	-2.557	0.284	low
CK-40d	RHSIM_Rhsim01G0116400	2.287	1.087	-1.200	high
CK-40d	RHSIM_Rhsim01G0002800	-1.658	-0.671	0.987	low
CK-40d	RHSIM_Rhsim02G0205700	0.185	-0.880	-1.065	no
CK-40d	RHSIM_Rhsim02G0173000	0.579	0.380	-0.199	no
CK-40d	RHSIM_Rhsim03G0004900	0.182	1.004	0.822	no
CK-40d	RHSIM_Rhsim01G0125000	-1.485	-0.513	0.972	low
CK-40d	RHSIM_Rhsim01G0033100	0.308	0.321	0.013	no
CK-40d	RHSIM_Rhsim01G0002900	-0.347	-0.087	0.260	no
CK-40d	RHSIM_Rhsim02G0037400	1.922	2.660	0.738	high
CK-40d	RHSIM_Rhsim01G0275800	0.719	0.668	-0.051	no
CK-40d	RHSIM_Rhsim01G0191600	1.454	1.094	-0.360	high
CK-40d	RHSIM_Rhsim01G0125800	-0.140	1.079	1.219	no
CK-40d	RHSIM_Rhsim01G0067100	-0.361	0.258	0.619	no
CK-40d	RHSIM_Rhsim01G0180300	1.686	0.984	-0.702	high
CK-40d	RHSIM_Rhsim03G0045300	1.107	1.678	0.571	no
CK-40d	RHSIM_Rhsim01G0274100	-1.799	-1.335	0.464	low
CK-40d	RHSIM_Rhsim01G0257500	0.370	-0.026	-0.396	no
CK-40d	RHSIM_Rhsim03G0081900	0.582	2.077	1.496	no
CK-40d	RHSIM_Rhsim01G0027600	1.073	-0.601	-1.675	no
CK-40d	RHSIM_Rhsim01G0120500	-0.404	1.424	1.828	no
CK-40d	RHSIM_Rhsim01G0224400	0.324	-0.395	-0.718	no
CK-40d	RHSIM_Rhsim01G0190500	0.232	0.139	-0.093	no
CK-40d	RHSIM_Rhsim01G0255900	-1.083	-0.661	0.422	no
CK-40d	RHSIM_Rhsim03G0221900	0.031	0.882	0.851	no
CK-40d	RHSIM_Rhsim01G0268200	0.777	1.862	1.086	no
CK-40d	RHSIM_Rhsim01G0006700	-0.619	-0.013	0.607	no

Group	Gene	PTR (z-score difference)	z(log2 LFQ)	z(log2 FPKM)	extreme-PTR
CK-40d	RHSIM_Rhsim01G0231000	-0.612	-0.285	0.327	no
CK-40d	RHSIM_Rhsim03G0079200	1.502	1.203	-0.299	high
CK-40d	RHSIM_Rhsim01G0212100	-1.325	0.093	1.418	low
CK-40d	RHSIM_Rhsim01G0137400	0.417	0.025	-0.392	no
CK-40d	RHSIM_Rhsim01G0194700	-0.344	-0.790	-0.446	no
CK-40d	RHSIM_Rhsim01G0250200	0.214	0.218	0.004	no
CK-40d	RHSIM_Rhsim01G0198200	-0.152	0.321	0.473	no
CK-40d	RHSIM_Rhsim01G0246300	-0.267	-0.485	-0.218	no
CK-40d	RHSIM_Rhsim01G0122600	-1.351	-1.271	0.080	low
CK-40d	RHSIM_Rhsim01G0254300	-0.183	-0.720	-0.536	no
CK-40d	RHSIM_Rhsim03G0033400	-2.965	-0.952	2.013	low
CK-40d	RHSIM_Rhsim01G0035300	0.402	-0.737	-1.139	no
CK-40d	RHSIM_Rhsim01G0291000	0.647	1.844	1.197	no
CK-40d	RHSIM_Rhsim01G0200700	-0.871	-0.057	0.814	no
CK-40d	RHSIM_Rhsim01G0125200	-1.945	-0.451	1.495	low
CK-40d	RHSIM_Rhsim01G0145500	0.364	-0.810	-1.174	no
CK-40d	RHSIM_Rhsim01G0165300	-0.652	0.848	1.500	no
CK-40d	RHSIM_Rhsim01G0016000	0.784	1.210	0.426	no
CK-40d	RHSIM_Rhsim01G0244900	-0.020	-0.076	-0.055	no
CK-40d	RHSIM_Rhsim03G0145600	0.994	0.222	-0.772	no
CK-40d	RHSIM_Rhsim01G0149300	-0.477	0.296	0.773	no
CK-40d	RHSIM_Rhsim01G0259900	0.861	0.863	0.002	no
CK-40d	RHSIM_Rhsim03G0063400	-0.399	-0.365	0.034	no
CK-40d	RHSIM_Rhsim01G0238300	0.723	0.354	-0.369	no
CK-40d	RHSIM_Rhsim01G0163300	1.251	1.291	0.040	high
CK-40d	RHSIM_Rhsim01G0251200	0.877	-0.515	-1.393	no
CK-40d	RHSIM_Rhsim01G0250600	-1.652	-0.672	0.980	low
CK-40d	RHSIM_Rhsim01G0287300	-0.794	-0.803	-0.009	no
CK-40d	RHSIM_Rhsim01G0284700	-0.852	-0.177	0.675	no
CK-40d	RHSIM_Rhsim01G0264000	-1.960	-0.806	1.154	low
CK-40d	RHSIM_Rhsim01G0218900	0.081	0.985	0.903	no
CK-40d	RHSIM_Rhsim01G0217200	-0.026	1.153	1.178	no

Group	Gene	PTR (z-score difference)	z(log2 LFQ)	z(log2 FPKM)	extreme-PTR
CK-40d	RHSIM_Rhsim01G0066600	-0.219	-0.278	-0.058	no
CK-40d	RHSIM_Rhsim01G0000300	-0.148	-0.665	-0.517	no
CK-40d	RHSIM_Rhsim01G0041200	1.288	-0.090	-1.378	high
CK-40d	RHSIM_Rhsim01G0007100	0.195	-0.710	-0.906	no
CK-40d	RHSIM_Rhsim01G0097100	-0.637	-1.344	-0.708	no
CK-40d	RHSIM_Rhsim01G0276700	2.283	1.160	-1.123	high
CK-40d	RHSIM_Rhsim03G0267300	-2.510	-0.878	1.631	low
CK-40d	RHSIM_Rhsim01G0283700	1.217	0.455	-0.763	high
CK-40d	RHSIM_Rhsim01G0247500	0.550	0.126	-0.424	no
CK-40d	RHSIM_Rhsim01G0247300	-0.628	0.230	0.858	no
CK-40d	RHSIM_Rhsim01G0192300	-1.934	-0.997	0.937	low
CK-40d	RHSIM_Rhsim01G0290400	-0.628	0.273	0.901	no
CK-40d	RHSIM_Rhsim01G0130900	-2.699	-1.764	0.936	low
CK-40d	RHSIM_Rhsim02G0110500	0.464	-0.372	-0.836	no
CK-40d	RHSIM_Rhsim01G0068700	-1.344	0.630	1.974	low
CK-40d	RHSIM_Rhsim01G0145700	0.743	0.417	-0.326	no
CK-40d	RHSIM_Rhsim01G0106500	1.730	0.226	-1.505	high
CK-40d	RHSIM_Rhsim01G0230600	-0.130	0.161	0.291	no
CK-40d	RHSIM_Rhsim01G0248500	0.544	0.917	0.374	no
CK-40d	RHSIM_Rhsim01G0091800	1.352	0.328	-1.024	high
CK-40d	RHSIM_Rhsim02G0046000	-0.137	-0.565	-0.428	no
CK-40d	RHSIM_Rhsim01G0192200	2.356	3.423	1.068	high
CK-40d	RHSIM_Rhsim01G0091400	-0.955	-1.203	-0.248	no
CK-40d	RHSIM_Rhsim02G0216500	-0.063	1.194	1.258	no
CK-40d	RHSIM_Rhsim01G0209300	-0.078	0.190	0.268	no
CK-40d	RHSIM_Rhsim02G0193300	-0.599	-0.002	0.596	no
CK-40d	RHSIM_Rhsim01G0275400	-0.951	-0.775	0.176	no
CK-40d	RHSIM_Rhsim02G0005600	-0.648	-0.529	0.119	no
CK-40d	RHSIM_Rhsim01G0008800	0.622	-0.182	-0.804	no
CK-40d	RHSIM_Rhsim02G0128200	1.200	0.946	-0.254	high
CK-40d	RHSIM_Rhsim01G0195300	0.378	-0.056	-0.434	no
CK-40d	RHSIM_Rhsim01G0073700	-0.065	-0.450	-0.385	no

Group	Gene	PTR (z-score difference)	z(log2 LFQ)	z(log2 FPKM)	extreme-PTR
CK-40d	RHSIM_Rhsim01G0144700	-1.317	-1.248	0.069	low
CK-40d	RHSIM_Rhsim01G0126800	1.814	2.414	0.600	high
CK-40d	RHSIM_Rhsim01G0286600	-1.638	0.186	1.824	low
CK-40d	RHSIM_Rhsim02G0256800	0.249	-0.003	-0.252	no
CK-40d	RHSIM_Rhsim01G0224800	-0.397	0.469	0.866	no
CK-40d	RHSIM_Rhsim02G0132700	-2.148	0.259	2.408	low
CK-40d	RHSIM_Rhsim02G0142400	-0.275	0.313	0.588	no
CK-40d	RHSIM_Rhsim02G0238900	0.956	0.110	-0.846	no
CK-40d	RHSIM_Rhsim01G0184800	1.105	0.387	-0.718	no
CK-40d	RHSIM_Rhsim01G0209000	-1.093	-1.083	0.011	no
CK-40d	RHSIM_Rhsim02G0185100	-0.258	-0.733	-0.474	no
CK-40d	RHSIM_Rhsim02G0229900	-1.732	0.609	2.342	low
CK-40d	RHSIM_Rhsim01G0246100	-1.180	-0.995	0.185	no
CK-40d	RHSIM_Rhsim01G0184000	-1.078	-0.315	0.763	no
CK-40d	RHSIM_Rhsim02G0099600	-0.850	-1.211	-0.360	no
CK-40d	RHSIM_Rhsim01G0023900	1.001	1.086	0.085	no
CK-40d	RHSIM_Rhsim02G0009700	-1.518	-0.882	0.636	low
CK-40d	RHSIM_Rhsim01G0162200	-0.377	-0.531	-0.153	no
CK-40d	RHSIM_Rhsim01G0178400	-3.281	-1.665	1.616	low
CK-40d	RHSIM_Rhsim01G0008400	1.533	1.019	-0.514	high
CK-40d	RHSIM_Rhsim01G0236500	-0.063	1.197	1.260	no
CK-40d	RHSIM_Rhsim01G0262900	-1.335	-0.990	0.345	low
CK-40d	RHSIM_Rhsim02G0151800	-0.812	-0.148	0.665	no
CK-40d	RHSIM_Rhsim02G0003700	1.156	0.086	-1.070	high
CK-40d	RHSIM_Rhsim02G0100800	0.849	0.371	-0.478	no
CK-40d	RHSIM_Rhsim01G0090500	-0.866	-1.226	-0.361	no
CK-40d	RHSIM_Rhsim01G0064300	-0.896	-1.621	-0.725	no
CK-40d	RHSIM_Rhsim02G0253600	-1.080	-1.085	-0.005	no
CK-40d	RHSIM_Rhsim02G0031500	0.088	0.219	0.131	no
CK-40d	RHSIM_Rhsim02G0000800	-1.325	-1.225	0.100	low
CK-40d	RHSIM_Rhsim01G0141400	0.298	2.275	1.977	no
CK-40d	RHSIM_Rhsim02G0075300	-1.042	-0.393	0.649	no

Group	Gene	PTR (z-score difference)	z(log2 LFQ)	z(log2 FPKM)	extreme-PTR
CK-40d	RHSIM_Rhsim01G0280500	-1.052	-0.948	0.103	no
CK-40d	RHSIM_Rhsim02G0223600	0.134	-0.396	-0.530	no
CK-40d	RHSIM_Rhsim02G0088700	0.790	-0.178	-0.967	no
CK-40d	RHSIM_Rhsim02G0228900	0.895	0.839	-0.055	no
CK-40d	RHSIM_Rhsim02G0026400	-0.779	0.282	1.060	no
CK-40d	RHSIM_Rhsim02G0054000	0.484	-0.619	-1.103	no
CK-40d	RHSIM_Rhsim02G0018300	0.753	-0.733	-1.486	no
CK-40d	RHSIM_Rhsim01G0218400	1.132	1.020	-0.112	no
CK-40d	RHSIM_Rhsim01G0251500	2.515	1.621	-0.894	high
CK-40d	RHSIM_Rhsim01G0106600	-1.150	0.026	1.176	no
CK-40d	RHSIM_Rhsim01G0181700	-1.522	-1.303	0.219	low
CK-40d	RHSIM_Rhsim01G0220200	-1.398	-1.067	0.331	low
CK-40d	RHSIM_Rhsim02G0037500	2.707	1.155	-1.552	high
CK-40d	RHSIM_Rhsim01G0124800	0.078	0.554	0.477	no
CK-40d	RHSIM_Rhsim01G0139200	0.097	0.787	0.690	no
CK-40d	RHSIM_Rhsim01G0282400	-0.107	-1.056	-0.950	no
CK-40d	RHSIM_Rhsim01G0220800	0.679	-0.015	-0.694	no
CK-40d	RHSIM_Rhsim01G0284300	-1.165	0.294	1.459	no
CK-40d	RHSIM_Rhsim01G0102600	0.676	0.007	-0.669	no
CK-40d	RHSIM_Rhsim01G0208800	-0.780	-0.087	0.693	no
CK-40d	RHSIM_Rhsim01G0023400	1.542	0.215	-1.328	high
CK-40d	RHSIM_Rhsim01G0207000	0.226	1.103	0.877	no
CK-40d	RHSIM_Rhsim01G0216900	1.009	1.154	0.145	no
CK-40d	RHSIM_Rhsim01G0217400	0.220	-0.891	-1.111	no
CK-40d	RHSIM_Rhsim01G0245900	1.794	1.740	-0.054	high
CK-40d	RHSIM_Rhsim01G0159800	-0.295	0.492	0.787	no
CK-40d	RHSIM_Rhsim01G0238400	0.442	-0.014	-0.457	no
CK-40d	RHSIM_Rhsim01G0226500	0.349	0.690	0.341	no
CK-40d	RHSIM_Rhsim01G0229500	-0.429	-0.252	0.177	no
CK-40d	RHSIM_Rhsim01G0216000	-0.505	-0.795	-0.289	no
CK-40d	RHSIM_Rhsim01G0274200	-0.578	-0.536	0.043	no
CK-40d	RHSIM_Rhsim01G0049500	-1.477	-1.150	0.326	low

Group	Gene	PTR (z-score difference)	z(log2 LFQ)	z(log2 FPKM)	extreme-PTR
CK-40d	RHSIM_Rhsim01G0193100	2.512	1.518	-0.994	high
CK-40d	RHSIM_Rhsim01G0231600	-1.313	-1.201	0.112	low
CK-40d	RHSIM_Rhsim01G0191000	-0.361	-1.044	-0.683	no
CK-40d	RHSIM_Rhsim01G0033000	1.150	0.407	-0.742	high
CK-40d	RHSIM_Rhsim01G0223000	0.254	0.295	0.041	no
CK-40d	RHSIM_Rhsim01G0123800	0.712	0.630	-0.081	no
CK-40d	RHSIM_Rhsim01G0002500	-0.697	-0.594	0.103	no
CK-40d	RHSIM_Rhsim01G0148500	0.230	-0.384	-0.613	no
CK-40d	RHSIM_Rhsim01G0271600	-2.320	-1.966	0.354	low
CK-40d	RHSIM_Rhsim01G0068000	-0.740	-1.159	-0.419	no
CK-40d	RHSIM_Rhsim01G0202100	-0.282	0.220	0.502	no
CK-40d	RHSIM_Rhsim01G0130500	-0.643	-1.113	-0.470	no
CK-40d	RHSIM_Rhsim01G0076500	1.823	1.183	-0.639	high
CK-40d	RHSIM_Rhsim01G0265500	-3.311	-1.167	2.144	low
CK-40d	RHSIM_Rhsim01G0233700	-2.433	-0.551	1.882	low
CK-40d	RHSIM_Rhsim01G0189000	1.729	0.585	-1.144	high
CK-40d	RHSIM_Rhsim01G0185300	-0.733	0.262	0.995	no
CK-40d	RHSIM_Rhsim01G0131100	-0.862	-0.163	0.698	no
CK-40d	RHSIM_Rhsim01G0252400	-0.796	-0.810	-0.015	no
CK-40d	RHSIM_Rhsim01G0060300	-0.963	-0.343	0.620	no
CK-40d	RHSIM_Rhsim01G0006800	0.721	-0.696	-1.417	no
CK-40d	RHSIM_Rhsim01G0287900	-1.574	-0.797	0.776	low
CK-40d	RHSIM_Rhsim01G0071600	-1.205	-0.072	1.133	no
CK-40d	RHSIM_Rhsim01G0196300	1.128	0.757	-0.372	no
CK-40d	RHSIM_Rhsim01G0138800	1.142	0.377	-0.766	high
CK-40d	RHSIM_Rhsim01G0210400	1.733	1.629	-0.104	high
CK-40d	RHSIM_Rhsim01G0007000	0.650	0.644	-0.006	no
CK-40d	RHSIM_Rhsim01G0000700	1.648	1.063	-0.585	high
CK-40d	RHSIM_RhsimUnG0254300	-1.563	-0.523	1.040	low
CK-40d	RHSIM_RhsimUnG0258100	0.039	0.316	0.277	no
CK-40d	RHSIM_RhsimUnG0252100	0.258	-0.703	-0.961	no
CK-40d	RHSIM_RhsimUnG0236700	0.007	-0.256	-0.264	no

Group	Gene	PTR (z-score difference)	z(log2 LFQ)	z(log2 FPKM)	extreme-PTR
CK-40d	RHSIM_RhsimUnG0227500	0.807	1.260	0.454	no
CK-40d	RHSIM_RhsimUnG0171600	-2.197	-2.102	0.095	low
CK-40d	RHSIM_RhsimUnG0169000	-1.908	-1.371	0.537	low
CK-40d	RHSIM_RhsimUnG0118200	0.122	-0.672	-0.794	no
CK-40d	RHSIM_RhsimUnG0113800	2.323	0.222	-2.101	high
CK-40d	RHSIM_Rhsim11G0108100	-1.654	-0.208	1.446	low
CK-40d	RHSIM_RhsimUnG0065600	-1.032	0.802	1.833	no
CK-40d	RHSIM_RhsimUnG0062200	1.584	0.097	-1.487	high
CK-40d	RHSIM_Rhsim10G0103200	-0.164	0.124	0.288	no
CK-40d	RHSIM_RhsimUnG0057700	3.146	0.745	-2.401	high
CK-40d	RHSIM_RhsimUnG0055300	-0.769	0.309	1.077	no
CK-40d	RHSIM_RhsimUnG0037800	-2.074	-0.800	1.274	low
CK-40d	RHSIM_RhsimUnG0003000	0.076	-0.403	-0.479	no
CK-40d	RHSIM_RhsimUnG0258700	0.659	-0.927	-1.586	no
CK-40d	RHSIM_RhsimPtG0005800	4.185	4.135	-0.050	high
CK-40d	RHSIM_RhsimUnG0253900	0.926	1.393	0.466	no
CK-40d	RHSIM_RhsimUnG0250200	0.667	1.419	0.751	no
CK-40d	RHSIM_Rhsim13G0233000	0.113	-0.416	-0.530	no
CK-40d	RHSIM_Rhsim13G0008700	4.005	1.571	-2.435	high
CK-40d	RHSIM_Rhsim13G0227700	0.597	-0.120	-0.717	no
CK-40d	RHSIM_Rhsim13G0228800	0.675	2.141	1.466	no
CK-40d	RHSIM_Rhsim13G0222000	-1.439	-1.422	0.017	low
CK-40d	RHSIM_Rhsim13G0111300	1.143	0.780	-0.363	high
CK-40d	RHSIM_Rhsim13G0180900	-0.379	-1.141	-0.761	no
CK-40d	RHSIM_Rhsim13G0004600	0.734	0.023	-0.711	no
CK-40d	RHSIM_RhsimUnG0212100	-1.025	-1.284	-0.260	no
CK-40d	RHSIM_Rhsim13G0203000	0.466	-0.938	-1.404	no
CK-40d	RHSIM_RhsimUnG0202600	1.021	-0.463	-1.484	no
CK-40d	RHSIM_Rhsim13G0053600	0.047	1.090	1.043	no
CK-40d	RHSIM_Rhsim13G0103100	1.156	-0.139	-1.295	high
CK-40d	RHSIM_RhsimUnG0248000	0.316	-0.175	-0.491	no
CK-40d	RHSIM_RhsimUnG0250500	-0.247	1.674	1.921	no

Group	Gene	PTR (z-score difference)	z(log2 LFQ)	z(log2 FPKM)	extreme-PTR
CK-40d	RHSIM_RhsimUnG0253100	-0.862	-0.427	0.435	no
CK-40d	RHSIM_Rhsim13G0021500	-0.909	-0.797	0.111	no
CK-40d	RHSIM_RhsimUnG0257700	1.942	2.829	0.887	high
CK-40d	RHSIM_RhsimUnG0251000	-1.280	-1.104	0.175	low
CK-40d	RHSIM_RhsimUnG0248500	-0.006	-0.906	-0.900	no
CK-40d	RHSIM_Rhsim13G0120200	-1.113	-1.260	-0.147	no
CK-40d	RHSIM_RhsimUnG0234700	-0.603	-1.847	-1.244	no
CK-40d	RHSIM_RhsimUnG0239200	1.058	0.529	-0.528	no
CK-40d	RHSIM_RhsimUnG0240700	0.621	0.975	0.354	no
CK-40d	RHSIM_RhsimUnG0226400	-0.578	-0.208	0.369	no
CK-40d	RHSIM_Rhsim13G0041600	2.605	3.299	0.694	high
CK-40d	RHSIM_RhsimUnG0231000	-0.762	-1.997	-1.236	no
CK-40d	RHSIM_Rhsim13G0201800	-2.601	-1.045	1.555	low
CK-40d	RHSIM_RhsimUnG0225800	2.301	0.919	-1.383	high
CK-40d	RHSIM_Rhsim13G0232300	2.426	1.156	-1.270	high
CK-40d	RHSIM_Rhsim13G0191000	-0.785	-0.766	0.019	no
CK-40d	RHSIM_RhsimUnG0168300	0.381	0.049	-0.332	no
CK-40d	RHSIM_Rhsim13G0040700	-0.218	-1.057	-0.839	no
CK-40d	RHSIM_Rhsim13G0054200	-0.922	-1.298	-0.376	no
CK-40d	RHSIM_RhsimUnG0210400	1.996	1.319	-0.676	high
CK-40d	RHSIM_Rhsim13G0014600	-1.727	-1.061	0.666	low
CK-40d	RHSIM_RhsimUnG0210700	-0.374	-0.423	-0.049	no
CK-40d	RHSIM_RhsimUnG0209300	0.003	-1.380	-1.384	no
CK-40d	RHSIM_Rhsim12G0052900	1.270	0.702	-0.567	high
CK-40d	RHSIM_RhsimUnG0192400	1.084	0.020	-1.064	no
CK-40d	RHSIM_Rhsim13G0198900	0.026	-0.846	-0.872	no
CK-40d	RHSIM_Rhsim09G0047000	2.483	1.123	-1.360	high
CK-40d	RHSIM_Rhsim13G0121800	-0.448	-1.386	-0.939	no
CK-40d	RHSIM_RhsimUnG0168800	0.652	2.125	1.472	no
CK-40d	RHSIM_RhsimUnG0116900	-2.190	-1.893	0.297	low
CK-40d	RHSIM_Rhsim06G0025600	0.283	0.554	0.271	no
CK-40d	RHSIM_Rhsim13G0184800	1.505	-0.766	-2.272	high

Group	Gene	PTR (z-score difference)	z(log2 LFQ)	z(log2 FPKM)	extreme-PTR
CK-40d	RHSIM_RhsimUnG0143500	-1.804	-1.711	0.093	low
CK-40d	RHSIM_RhsimUnG0092900	1.042	-0.418	-1.460	no
CK-40d	RHSIM_RhsimUnG0086600	0.561	0.467	-0.094	no
CK-40d	RHSIM_Rhsim13G0020600	-0.018	-0.927	-0.909	no
CK-40d	RHSIM_RhsimUnG0082400	-0.125	0.185	0.311	no
CK-40d	RHSIM_RhsimUnG0142500	0.546	-0.400	-0.946	no
CK-40d	RHSIM_RhsimUnG0135800	1.338	0.170	-1.168	high
CK-40d	RHSIM_RhsimUnG0073800	-1.080	0.095	1.175	no
CK-40d	RHSIM_RhsimUnG0120100	-0.405	1.663	2.068	no
CK-40d	RHSIM_RhsimUnG0128700	0.458	-0.676	-1.134	no
CK-40d	RHSIM_RhsimUnG0123600	0.353	-1.141	-1.494	no
CK-40d	RHSIM_Rhsim13G0099200	1.674	1.413	-0.261	high
CK-40d	RHSIM_Rhsim13G0029700	0.111	-0.954	-1.065	no
CK-40d	RHSIM_RhsimUnG0044400	-0.596	0.622	1.218	no
CK-40d	RHSIM_RhsimUnG0092800	2.507	0.393	-2.114	high
CK-40d	RHSIM_RhsimUnG0088000	1.036	-0.123	-1.159	no
CK-40d	RHSIM_RhsimUnG0100600	0.696	0.408	-0.288	no
CK-40d	RHSIM_Rhsim13G0131200	1.111	0.629	-0.483	no
CK-40d	RHSIM_RhsimUnG0097600	0.843	0.834	-0.009	no
CK-40d	RHSIM_RhsimUnG0085300	-0.555	0.653	1.208	no
CK-40d	RHSIM_RhsimUnG0070600	0.588	-0.966	-1.554	no
CK-40d	RHSIM_Rhsim13G0082400	-0.957	-0.962	-0.005	no
CK-40d	RHSIM_RhsimUnG0005900	4.104	2.075	-2.029	high
CK-40d	RHSIM_RhsimUnG0066300	1.409	-0.298	-1.707	high
CK-40d	RHSIM_RhsimUnG0075600	1.738	0.611	-1.127	high
CK-40d	RHSIM_RhsimUnG0064700	0.589	1.628	1.039	no
CK-40d	RHSIM_RhsimUnG0061900	-0.334	-1.181	-0.847	no
CK-40d	RHSIM_RhsimPtG0004000	5.225	3.461	-1.764	high
CK-40d	RHSIM_Rhsim13G0062300	0.839	1.045	0.206	no
CK-40d	RHSIM_RhsimMtG0002200	0.887	-0.763	-1.650	no
CK-40d	RHSIM_Rhsim13G0073900	0.003	-0.620	-0.623	no
CK-40d	RHSIM_RhsimUnG0055400	0.727	1.828	1.101	no

Group	Gene	PTR (z-score difference)	z(log2 LFQ)	z(log2 FPKM)	extreme-PTR
CK-40d	RHSIM_Rhsim13G0046200	-2.151	-0.860	1.290	low
CK-40d	RHSIM_RhsimUnG0046700	-1.247	-1.089	0.158	low
CK-40d	RHSIM_RhsimUnG0054900	-0.959	0.912	1.871	no
CK-40d	RHSIM_RhsimUnG0042300	-0.884	0.325	1.208	no
CK-40d	RHSIM_Rhsim13G0173600	-1.196	-0.524	0.672	no
CK-40d	RHSIM_Rhsim13G0227300	0.028	0.074	0.047	no
CK-40d	RHSIM_RhsimUnG0042800	0.769	-0.478	-1.247	no
CK-40d	RHSIM_Rhsim12G0181000	0.310	0.761	0.452	no
CK-40d	RHSIM_Rhsim13G0093800	-1.606	-0.879	0.728	low
CK-40d	RHSIM_Rhsim13G0030700	-0.249	0.089	0.338	no
CK-40d	RHSIM_Rhsim13G0076000	-0.546	1.166	1.711	no
CK-40d	RHSIM_RhsimUnG0019100	2.031	0.114	-1.918	high
CK-40d	RHSIM_RhsimUnG0020700	-0.585	-1.041	-0.456	no
CK-40d	RHSIM_Rhsim13G0064100	-0.227	-0.582	-0.355	no
CK-40d	RHSIM_Rhsim13G0163100	-0.386	-0.970	-0.585	no
CK-40d	RHSIM_Rhsim13G0066100	0.272	0.967	0.695	no
CK-40d	RHSIM_RhsimUnG0016900	0.851	1.345	0.495	no
CK-40d	RHSIM_RhsimUnG0016100	-2.494	-1.756	0.739	low
CK-40d	RHSIM_RhsimUnG0004400	1.488	-0.915	-2.403	high
CK-40d	RHSIM_Rhsim13G0207900	-2.268	-0.775	1.494	low
CK-40d	RHSIM_Rhsim12G0037800	0.309	0.120	-0.189	no
CK-40d	RHSIM_Rhsim13G0043900	-0.621	0.085	0.706	no
CK-40d	RHSIM_Rhsim12G0063500	-0.362	-0.553	-0.191	no
CK-40d	RHSIM_Rhsim12G0167800	0.034	0.305	0.271	no
CK-40d	RHSIM_Rhsim13G0040000	0.840	0.690	-0.149	no
CK-40d	RHSIM_Rhsim13G0118300	0.482	-0.209	-0.690	no
CK-40d	RHSIM_Rhsim13G0220600	-0.070	0.960	1.030	no
CK-40d	RHSIM_Rhsim13G0065700	0.198	0.397	0.199	no
CK-40d	RHSIM_Rhsim13G0221500	-0.719	-1.025	-0.306	no
CK-40d	RHSIM_Rhsim12G0039700	0.017	0.222	0.205	no
CK-40d	RHSIM_Rhsim13G0226500	1.679	1.349	-0.330	high
CK-40d	RHSIM_Rhsim13G0016700	0.298	-0.260	-0.558	no

Group	Gene	PTR (z-score difference)	z(log2 LFQ)	z(log2 FPKM)	extreme-PTR
CK-40d	RHSIM_Rhsim13G0174500	0.657	1.720	1.064	no
CK-40d	RHSIM_Rhsim12G0048800	-0.698	-0.986	-0.288	no
CK-40d	RHSIM_Rhsim13G0185200	2.204	2.560	0.355	high
CK-40d	RHSIM_Rhsim13G0234600	-0.137	0.647	0.784	no
CK-40d	RHSIM_Rhsim13G0038800	-0.727	0.675	1.403	no
CK-40d	RHSIM_Rhsim13G0053200	0.300	0.847	0.547	no
CK-40d	RHSIM_Rhsim12G0172600	1.679	1.089	-0.590	high
CK-40d	RHSIM_Rhsim13G0134800	-1.338	-0.110	1.228	low
CK-40d	RHSIM_Rhsim13G0154000	1.161	2.595	1.434	high
CK-40d	RHSIM_Rhsim13G0038600	-0.314	-0.790	-0.476	no
CK-40d	RHSIM_Rhsim12G0139300	-0.282	-0.492	-0.210	no
CK-40d	RHSIM_Rhsim12G0115700	-0.197	-0.850	-0.654	no
CK-40d	RHSIM_Rhsim13G0229900	1.293	2.452	1.159	high
CK-40d	RHSIM_Rhsim13G0078100	-1.774	-1.400	0.374	low
CK-40d	RHSIM_Rhsim13G0113300	-0.529	-0.773	-0.244	no
CK-40d	RHSIM_Rhsim13G0082700	-3.704	-0.121	3.583	low
CK-40d	RHSIM_Rhsim13G0126700	-0.499	-1.128	-0.629	no
CK-40d	RHSIM_Rhsim13G0159200	-0.044	-1.148	-1.104	no
CK-40d	RHSIM_Rhsim13G0201100	0.563	-0.222	-0.785	no
CK-40d	RHSIM_Rhsim13G0022100	0.925	1.340	0.414	no
CK-40d	RHSIM_Rhsim13G0199200	0.283	-0.400	-0.684	no
CK-40d	RHSIM_Rhsim13G0171300	2.403	0.064	-2.338	high
CK-40d	RHSIM_Rhsim13G0177900	0.470	-0.262	-0.732	no
CK-40d	RHSIM_Rhsim12G0123300	-0.108	-0.496	-0.389	no
CK-40d	RHSIM_Rhsim13G0093700	-1.500	0.524	2.024	low
CK-40d	RHSIM_Rhsim12G0178200	0.269	-0.553	-0.822	no
CK-40d	RHSIM_Rhsim13G0036600	-0.981	-0.593	0.387	no
CK-40d	RHSIM_Rhsim12G0139900	-0.756	-0.940	-0.185	no
CK-40d	RHSIM_Rhsim13G0006800	0.369	1.387	1.018	no
CK-40d	RHSIM_Rhsim13G0079200	-0.430	-0.846	-0.416	no
CK-40d	RHSIM_Rhsim12G0079000	-1.319	-0.552	0.766	low
CK-40d	RHSIM_Rhsim13G0092400	-1.781	-1.330	0.451	low

Group	Gene	PTR (z-score difference)	z(log2 LFQ)	z(log2 FPKM)	extreme-PTR
CK-40d	RHSIM_Rhsim13G0220800	-1.443	-0.684	0.760	low
CK-40d	RHSIM_Rhsim13G0011300	-0.629	-0.106	0.523	no
CK-40d	RHSIM_Rhsim13G0212000	2.564	0.221	-2.343	high
CK-40d	RHSIM_Rhsim12G0204800	-0.469	-0.020	0.449	no
CK-40d	RHSIM_Rhsim13G0041900	-0.981	-1.541	-0.560	no
CK-40d	RHSIM_Rhsim13G0232100	0.413	0.929	0.515	no
CK-40d	RHSIM_Rhsim13G0144400	2.072	0.996	-1.075	high
CK-40d	RHSIM_Rhsim13G0054900	1.256	-0.240	-1.497	high
CK-40d	RHSIM_Rhsim13G0124200	-0.727	-0.515	0.212	no
CK-40d	RHSIM_Rhsim13G0021700	-1.322	-1.525	-0.203	low
CK-40d	RHSIM_Rhsim12G0107800	0.770	-0.227	-0.998	no
CK-40d	RHSIM_Rhsim13G0057600	1.092	0.303	-0.789	no
CK-40d	RHSIM_Rhsim13G0191700	1.959	0.172	-1.787	high
CK-40d	RHSIM_Rhsim13G0114000	-0.632	-0.598	0.034	no
CK-40d	RHSIM_Rhsim13G0027900	0.245	-0.081	-0.326	no
CK-40d	RHSIM_Rhsim13G0029600	-1.930	-0.889	1.041	low
CK-40d	RHSIM_Rhsim13G0051300	-0.762	1.288	2.050	no
CK-40d	RHSIM_Rhsim13G0219700	0.894	1.389	0.496	no
CK-40d	RHSIM_Rhsim13G0164200	1.748	-0.518	-2.266	high
CK-40d	RHSIM_Rhsim13G0086200	-0.465	0.304	0.769	no
CK-40d	RHSIM_Rhsim12G0005600	0.339	0.751	0.412	no
CK-40d	RHSIM_Rhsim12G0203000	-1.425	-0.680	0.745	low
CK-40d	RHSIM_Rhsim13G0001400	1.804	0.716	-1.088	high
CK-40d	RHSIM_Rhsim13G0111900	-2.501	-0.272	2.229	low
CK-40d	RHSIM_Rhsim12G0174000	-0.907	1.032	1.939	no
CK-40d	RHSIM_Rhsim13G0161700	0.766	0.890	0.124	no
CK-40d	RHSIM_Rhsim13G0036800	0.632	1.236	0.605	no
CK-40d	RHSIM_Rhsim13G0197800	2.326	1.167	-1.159	high
CK-40d	RHSIM_Rhsim13G0219900	-1.689	-1.506	0.183	low
CK-40d	RHSIM_Rhsim13G0107300	1.292	0.692	-0.600	high
CK-40d	RHSIM_Rhsim13G0075000	0.240	-0.567	-0.807	no
CK-40d	RHSIM_Rhsim13G0103500	0.437	0.045	-0.393	no

Group	Gene	PTR (z-score difference)	z(log2 LFQ)	z(log2 FPKM)	extreme-PTR
CK-40d	RHSIM_Rhsim13G0101100	-0.667	0.392	1.060	no
CK-40d	RHSIM_Rhsim13G0015700	0.756	0.337	-0.419	no
CK-40d	RHSIM_Rhsim13G0168400	2.604	1.940	-0.665	high
CK-40d	RHSIM_Rhsim12G0116800	2.281	1.314	-0.967	high
CK-40d	RHSIM_Rhsim12G0078100	-0.743	-0.495	0.248	no
CK-40d	RHSIM_Rhsim13G0181700	2.454	0.336	-2.118	high
CK-40d	RHSIM_Rhsim13G0077500	1.142	-0.938	-2.081	high
CK-40d	RHSIM_Rhsim13G0161600	0.883	-1.031	-1.914	no
CK-40d	RHSIM_Rhsim13G0116300	2.652	1.341	-1.311	high
CK-40d	RHSIM_Rhsim13G0102200	1.280	-0.517	-1.797	high
CK-40d	RHSIM_Rhsim12G0172200	1.765	1.263	-0.502	high
CK-40d	RHSIM_Rhsim12G0103900	0.183	-0.160	-0.342	no
CK-40d	RHSIM_Rhsim13G0069000	-1.888	-0.082	1.806	low
CK-40d	RHSIM_Rhsim13G0231100	0.623	-0.477	-1.100	no
CK-40d	RHSIM_Rhsim12G0083200	0.706	0.597	-0.109	no
CK-40d	RHSIM_Rhsim12G0177000	2.083	0.436	-1.647	high
CK-40d	RHSIM_Rhsim13G0217100	1.354	0.383	-0.971	high
CK-40d	RHSIM_Rhsim13G0138400	-0.457	-0.728	-0.271	no
CK-40d	RHSIM_Rhsim13G0042600	0.498	0.374	-0.124	no
CK-40d	RHSIM_Rhsim12G0100900	0.103	0.983	0.880	no
CK-40d	RHSIM_Rhsim13G0025700	1.186	1.366	0.179	high
CK-40d	RHSIM_Rhsim13G0038000	1.884	1.641	-0.244	high
CK-40d	RHSIM_Rhsim13G0079600	-0.497	-0.167	0.330	no
CK-40d	RHSIM_Rhsim12G0083000	-0.048	0.263	0.311	no
CK-40d	RHSIM_Rhsim13G0233800	-1.614	-0.239	1.375	low
CK-40d	RHSIM_Rhsim13G0073800	-1.585	-0.937	0.648	low
CK-40d	RHSIM_Rhsim13G0055800	0.702	-0.558	-1.260	no
CK-40d	RHSIM_Rhsim13G0031900	1.128	0.557	-0.571	no
CK-40d	RHSIM_Rhsim13G0027500	-0.206	0.174	0.380	no
CK-40d	RHSIM_Rhsim12G0028500	0.053	0.616	0.563	no
CK-40d	RHSIM_Rhsim13G0095000	-0.382	-1.162	-0.780	no
CK-40d	RHSIM_Rhsim13G0035400	-0.277	-0.269	0.008	no

Group	Gene	PTR (z-score difference)	z(log2 LFQ)	z(log2 FPKM)	extreme-PTR
CK-40d	RHSIM_Rhsim13G0167700	0.334	1.151	0.817	no
CK-40d	RHSIM_Rhsim13G0129900	0.513	-0.129	-0.641	no
CK-40d	RHSIM_Rhsim13G0223700	-0.929	-1.071	-0.142	no
CK-40d	RHSIM_Rhsim13G0018400	-1.216	-0.998	0.217	no
CK-40d	RHSIM_Rhsim13G0157400	1.069	1.110	0.041	no
CK-40d	RHSIM_Rhsim13G0177000	0.637	0.333	-0.304	no
CK-40d	RHSIM_Rhsim13G0030400	-0.621	-1.088	-0.467	no
CK-40d	RHSIM_Rhsim12G0123700	-0.292	-0.268	0.023	no
CK-40d	RHSIM_Rhsim13G0208300	-0.582	0.243	0.825	no
CK-40d	RHSIM_Rhsim13G0070100	-1.310	-0.163	1.146	low
CK-40d	RHSIM_Rhsim13G0003700	-0.708	-1.077	-0.369	no
CK-40d	RHSIM_Rhsim11G0144200	-0.496	-0.577	-0.081	no
CK-40d	RHSIM_Rhsim13G0114600	-0.398	-0.115	0.284	no
CK-40d	RHSIM_Rhsim13G0028300	0.890	0.929	0.039	no
CK-40d	RHSIM_Rhsim13G0122000	0.178	-1.090	-1.268	no
CK-40d	RHSIM_Rhsim13G0127800	1.318	-0.497	-1.815	high
CK-40d	RHSIM_Rhsim13G0092300	1.067	1.804	0.737	no
CK-40d	RHSIM_Rhsim13G0026300	0.662	1.451	0.789	no
CK-40d	RHSIM_Rhsim13G0233500	0.629	-0.358	-0.986	no
CK-40d	RHSIM_Rhsim13G0091400	0.177	-0.367	-0.544	no
CK-40d	RHSIM_Rhsim13G0120600	0.169	0.939	0.770	no
CK-40d	RHSIM_Rhsim11G0149700	-0.324	-0.258	0.066	no
CK-40d	RHSIM_Rhsim13G0220700	-0.346	-0.594	-0.249	no
CK-40d	RHSIM_Rhsim13G0067500	0.575	0.495	-0.081	no
CK-40d	RHSIM_Rhsim13G0146800	0.085	-0.469	-0.554	no
CK-40d	RHSIM_Rhsim13G0138900	-0.066	-0.772	-0.706	no
CK-40d	RHSIM_Rhsim13G0156800	0.692	-1.067	-1.759	no
CK-40d	RHSIM_Rhsim13G0076600	-1.289	-1.293	-0.005	low
CK-40d	RHSIM_Rhsim13G0075300	-1.215	-1.053	0.163	no
CK-40d	RHSIM_Rhsim13G0036200	-1.365	-0.326	1.040	low
CK-40d	RHSIM_Rhsim11G0025400	-0.628	-1.225	-0.597	no
CK-40d	RHSIM_Rhsim13G0173800	-1.337	-0.173	1.164	low

Group	Gene	PTR (z-score difference)	z(log2 LFQ)	z(log2 FPKM)	extreme-PTR
CK-40d	RHSIM_Rhsim13G0171400	1.043	-0.407	-1.450	no
CK-40d	RHSIM_Rhsim13G0129200	0.875	1.010	0.135	no
CK-40d	RHSIM_Rhsim13G0010200	-0.968	-0.003	0.965	no
CK-40d	RHSIM_Rhsim13G0231600	-0.859	0.104	0.963	no
CK-40d	RHSIM_Rhsim13G0230400	2.538	0.511	-2.027	high
CK-40d	RHSIM_Rhsim11G0194500	0.661	-0.838	-1.499	no
CK-40d	RHSIM_Rhsim13G0140100	0.459	0.249	-0.209	no
CK-40d	RHSIM_Rhsim13G0142000	0.118	0.152	0.034	no
CK-40d	RHSIM_Rhsim12G0016400	0.432	-0.980	-1.412	no
CK-40d	RHSIM_Rhsim13G0056200	2.146	0.373	-1.773	high
CK-40d	RHSIM_Rhsim11G0092700	-1.706	-1.715	-0.009	low
CK-40d	RHSIM_Rhsim12G0018000	0.328	-0.188	-0.517	no
CK-40d	RHSIM_Rhsim12G0121700	-0.069	-0.402	-0.333	no
CK-40d	RHSIM_Rhsim12G0020900	2.364	0.949	-1.415	high
CK-40d	RHSIM_Rhsim11G0117400	1.658	0.772	-0.886	high
CK-40d	RHSIM_Rhsim12G0014400	0.043	0.744	0.701	no
CK-40d	RHSIM_Rhsim11G0029100	1.223	0.295	-0.928	high
CK-40d	RHSIM_Rhsim12G0021600	-0.379	-1.725	-1.346	no
CK-40d	RHSIM_Rhsim11G0036500	-0.585	-0.360	0.226	no
CK-40d	RHSIM_Rhsim12G0088300	0.224	0.326	0.102	no
CK-40d	RHSIM_Rhsim13G0217400	-0.161	-0.834	-0.673	no
CK-40d	RHSIM_Rhsim11G0008400	0.535	-0.347	-0.882	no
CK-40d	RHSIM_Rhsim11G0097700	-0.767	-1.437	-0.670	no
CK-40d	RHSIM_Rhsim13G0124600	-1.147	-1.793	-0.646	no
CK-40d	RHSIM_Rhsim12G0178500	0.887	0.241	-0.646	no
CK-40d	RHSIM_Rhsim12G0026500	0.888	-0.950	-1.838	no
CK-40d	RHSIM_Rhsim13G0008300	-2.212	0.063	2.275	low
CK-40d	RHSIM_Rhsim12G0157000	-0.801	0.645	1.446	no
CK-40d	RHSIM_Rhsim11G0148300	-2.432	-0.736	1.696	low
CK-40d	RHSIM_Rhsim12G0110500	-0.687	-1.091	-0.403	no
CK-40d	RHSIM_Rhsim11G0196100	0.674	-0.505	-1.179	no
CK-40d	RHSIM_Rhsim12G0078800	0.426	1.113	0.688	no

Group	Gene	PTR (z-score difference)	z(log2 LFQ)	z(log2 FPKM)	extreme-PTR
CK-40d	RHSIM_Rhsim12G0079500	1.310	1.128	-0.182	high
CK-40d	RHSIM_Rhsim12G0008200	2.047	0.354	-1.693	high
CK-40d	RHSIM_Rhsim13G0206000	-0.501	0.395	0.896	no
CK-40d	RHSIM_Rhsim11G0077200	2.952	3.009	0.058	high
CK-40d	RHSIM_Rhsim12G0189500	-0.472	-0.904	-0.432	no
CK-40d	RHSIM_Rhsim12G0131300	1.201	1.134	-0.067	high
CK-40d	RHSIM_Rhsim12G0190500	0.200	0.458	0.258	no
CK-40d	RHSIM_Rhsim13G0036100	1.337	0.413	-0.925	high
CK-40d	RHSIM_Rhsim12G0057400	0.555	0.833	0.278	no
CK-40d	RHSIM_Rhsim12G0137900	0.533	0.086	-0.447	no
CK-40d	RHSIM_Rhsim11G0190900	-1.272	1.195	2.467	low
CK-40d	RHSIM_Rhsim12G0211600	-0.739	-0.925	-0.186	no
CK-40d	RHSIM_Rhsim11G0196600	-1.088	-1.579	-0.490	no
CK-40d	RHSIM_Rhsim13G0126400	-0.786	-0.232	0.554	no
CK-40d	RHSIM_Rhsim12G0014900	1.622	0.634	-0.988	high
CK-40d	RHSIM_Rhsim12G0197900	0.760	1.457	0.696	no
CK-40d	RHSIM_Rhsim11G0059000	-1.843	0.397	2.240	low
CK-40d	RHSIM_Rhsim11G0096600	-1.289	-0.256	1.033	low
CK-40d	RHSIM_Rhsim12G0181300	0.917	0.401	-0.517	no
CK-40d	RHSIM_Rhsim12G0092800	-0.650	-1.891	-1.241	no
CK-40d	RHSIM_Rhsim12G0041000	0.177	-1.243	-1.420	no
CK-40d	RHSIM_Rhsim12G0017100	0.232	1.430	1.198	no
CK-40d	RHSIM_Rhsim12G0013600	-0.015	0.809	0.824	no
CK-40d	RHSIM_Rhsim12G0023200	-0.390	-1.045	-0.655	no
CK-40d	RHSIM_Rhsim13G0010500	-0.793	-0.600	0.193	no
CK-40d	RHSIM_Rhsim12G0122100	-0.788	-0.540	0.248	no
CK-40d	RHSIM_Rhsim12G0197700	-0.799	-0.620	0.179	no
CK-40d	RHSIM_Rhsim12G0207900	-1.149	-0.218	0.930	no
CK-40d	RHSIM_Rhsim12G0057700	-2.187	-0.191	1.996	low
CK-40d	RHSIM_Rhsim12G0102000	0.027	0.421	0.394	no
CK-40d	RHSIM_Rhsim12G0198000	1.063	-0.332	-1.395	no
CK-40d	RHSIM_Rhsim12G0121900	-1.437	-0.375	1.062	low

Group	Gene	PTR (z-score difference)	z(log2 LFQ)	z(log2 FPKM)	extreme-PTR
CK-40d	RHSIM_Rhsim12G0187600	-0.197	-0.274	-0.077	no
CK-40d	RHSIM_Rhsim12G0211700	-0.277	0.285	0.562	no
CK-40d	RHSIM_Rhsim12G0116600	1.069	2.061	0.991	no
CK-40d	RHSIM_Rhsim11G0192700	0.626	0.605	-0.021	no
CK-40d	RHSIM_Rhsim12G0062100	-1.623	-1.240	0.383	low
CK-40d	RHSIM_Rhsim12G0165600	-0.729	-0.997	-0.268	no
CK-40d	RHSIM_Rhsim12G0085300	-0.694	1.050	1.745	no
CK-40d	RHSIM_Rhsim12G0202100	2.156	2.648	0.492	high
CK-40d	RHSIM_Rhsim12G0181600	-0.104	-0.317	-0.213	no
CK-40d	RHSIM_Rhsim12G0023100	-0.712	-1.441	-0.729	no
CK-40d	RHSIM_Rhsim12G0187800	-1.073	-0.385	0.688	no
CK-40d	RHSIM_Rhsim12G0002600	-0.261	-0.969	-0.708	no
CK-40d	RHSIM_Rhsim12G0214000	-0.228	-1.291	-1.063	no
CK-40d	RHSIM_Rhsim12G0194700	-0.258	0.065	0.324	no
CK-40d	RHSIM_Rhsim12G0014100	0.074	1.084	1.010	no
CK-40d	RHSIM_Rhsim12G0164800	-2.444	-1.067	1.377	low
CK-40d	RHSIM_Rhsim12G0211300	-0.425	-0.949	-0.524	no
CK-40d	RHSIM_Rhsim12G0176100	-0.058	-0.163	-0.105	no
CK-40d	RHSIM_Rhsim12G0086500	-0.167	-0.528	-0.361	no
CK-40d	RHSIM_Rhsim11G0097500	-0.194	0.369	0.563	no
CK-40d	RHSIM_Rhsim12G0008600	2.109	0.744	-1.365	high
CK-40d	RHSIM_Rhsim12G0104500	1.669	0.028	-1.641	high
CK-40d	RHSIM_Rhsim12G0207200	-0.178	-1.035	-0.857	no
CK-40d	RHSIM_Rhsim12G0145700	2.814	1.946	-0.868	high
CK-40d	RHSIM_Rhsim12G0046200	3.516	1.878	-1.638	high
CK-40d	RHSIM_Rhsim12G0047900	-0.050	-0.053	-0.002	no
CK-40d	RHSIM_Rhsim12G0064600	0.500	0.416	-0.083	no
CK-40d	RHSIM_Rhsim12G0108800	-0.226	-0.994	-0.769	no
CK-40d	RHSIM_Rhsim12G0132500	-0.532	-0.076	0.456	no
CK-40d	RHSIM_Rhsim12G0189400	0.670	1.074	0.404	no
CK-40d	RHSIM_Rhsim12G0004200	1.737	1.413	-0.323	high
CK-40d	RHSIM_Rhsim12G0037200	0.465	-0.812	-1.277	no

Group	Gene	PTR (z-score difference)	z(log2 LFQ)	z(log2 FPKM)	extreme-PTR
CK-40d	RHSIM_Rhsim12G0208100	-0.620	-1.157	-0.537	no
CK-40d	RHSIM_Rhsim12G0060900	-0.933	-0.612	0.322	no
CK-40d	RHSIM_Rhsim12G0158400	1.234	-0.545	-1.779	high
CK-40d	RHSIM_Rhsim12G0146600	-0.429	-0.571	-0.142	no
CK-40d	RHSIM_Rhsim12G0152300	-0.989	-1.089	-0.100	no
CK-40d	RHSIM_Rhsim12G0004300	-1.408	-2.297	-0.889	low
CK-40d	RHSIM_Rhsim11G0164900	1.780	0.215	-1.565	high
CK-40d	RHSIM_Rhsim12G0005300	1.588	-0.370	-1.958	high
CK-40d	RHSIM_Rhsim12G0167600	-2.765	-1.468	1.297	low
CK-40d	RHSIM_Rhsim12G0182400	-1.182	-1.336	-0.154	no
CK-40d	RHSIM_Rhsim10G0099100	0.828	1.211	0.383	no
CK-40d	RHSIM_Rhsim12G0170000	-0.167	0.310	0.477	no
CK-40d	RHSIM_Rhsim12G0210300	-0.792	0.588	1.380	no
CK-40d	RHSIM_Rhsim12G0006100	-0.269	-0.228	0.041	no
CK-40d	RHSIM_Rhsim12G0047400	-1.993	-1.283	0.710	low
CK-40d	RHSIM_Rhsim12G0043400	-2.429	-1.490	0.939	low
CK-40d	RHSIM_Rhsim12G0060700	-1.396	-1.087	0.309	low
CK-40d	RHSIM_Rhsim10G0148000	1.667	0.792	-0.876	high
CK-40d	RHSIM_Rhsim12G0138900	-1.236	-1.329	-0.093	no
CK-40d	RHSIM_Rhsim12G0214700	-0.096	-1.011	-0.915	no
CK-40d	RHSIM_Rhsim12G0028300	-1.313	-1.461	-0.148	low
CK-40d	RHSIM_Rhsim12G0100800	-0.278	2.008	2.286	no
CK-40d	RHSIM_Rhsim12G0061700	0.434	1.602	1.168	no
CK-40d	RHSIM_Rhsim10G0064500	-0.531	0.357	0.889	no
CK-40d	RHSIM_Rhsim12G0176300	0.782	1.453	0.671	no
CK-40d	RHSIM_Rhsim10G0170100	-1.395	-1.391	0.004	low
CK-40d	RHSIM_Rhsim12G0189900	0.567	1.402	0.835	no
CK-40d	RHSIM_Rhsim12G0121500	-0.092	-0.486	-0.394	no
CK-40d	RHSIM_Rhsim12G0156100	0.869	1.254	0.385	no
CK-40d	RHSIM_Rhsim10G0144700	-0.337	-0.314	0.023	no
CK-40d	RHSIM_Rhsim12G0187400	-2.884	-1.407	1.477	low
CK-40d	RHSIM_Rhsim10G0012700	0.044	-0.005	-0.049	no

Group	Gene	PTR (z-score difference)	z(log2 LFQ)	z(log2 FPKM)	extreme-PTR
CK-40d	RHSIM_Rhsim12G0143100	-1.477	-1.434	0.043	low
CK-40d	RHSIM_Rhsim12G0072400	-0.042	0.231	0.273	no
CK-40d	RHSIM_Rhsim12G0044800	1.305	2.601	1.296	high
CK-40d	RHSIM_Rhsim12G0172500	-0.085	-0.802	-0.716	no
CK-40d	RHSIM_Rhsim12G0046500	1.117	2.844	1.727	no
CK-40d	RHSIM_Rhsim10G0043300	1.303	-0.283	-1.586	high
CK-40d	RHSIM_Rhsim12G0053800	0.549	2.255	1.706	no
CK-40d	RHSIM_Rhsim12G0036000	0.223	-1.091	-1.314	no
CK-40d	RHSIM_Rhsim12G0129400	1.383	0.935	-0.448	high
CK-40d	RHSIM_Rhsim12G0090600	-0.773	0.139	0.912	no
CK-40d	RHSIM_Rhsim12G0006300	-1.285	-1.309	-0.024	low
CK-40d	RHSIM_Rhsim12G0063200	0.063	-0.520	-0.583	no
CK-40d	RHSIM_Rhsim12G0136000	0.903	-0.864	-1.766	no
CK-40d	RHSIM_Rhsim12G0183100	1.072	0.201	-0.871	no
CK-40d	RHSIM_Rhsim12G0124300	1.423	0.817	-0.606	high
CK-40d	RHSIM_Rhsim12G0038400	1.205	0.821	-0.384	high
CK-40d	RHSIM_Rhsim12G0074900	1.230	0.900	-0.330	high
CK-40d	RHSIM_Rhsim12G0180300	1.272	1.563	0.291	high
CK-40d	RHSIM_Rhsim12G0063300	-1.429	-1.154	0.276	low
CK-40d	RHSIM_Rhsim11G0038100	-0.083	0.720	0.803	no
CK-40d	RHSIM_Rhsim12G0002400	0.400	1.206	0.807	no
CK-40d	RHSIM_Rhsim12G0062200	1.751	0.837	-0.914	high
CK-40d	RHSIM_Rhsim12G0129000	-0.221	0.390	0.611	no
CK-40d	RHSIM_Rhsim12G0214600	0.583	0.132	-0.451	no
CK-40d	RHSIM_Rhsim11G0137400	-0.779	-0.231	0.548	no
CK-40d	RHSIM_Rhsim12G0162600	-1.307	-1.660	-0.354	low
CK-40d	RHSIM_Rhsim12G0037500	1.058	-0.994	-2.052	no
CK-40d	RHSIM_Rhsim12G0023300	0.540	1.715	1.175	no
CK-40d	RHSIM_Rhsim11G0021900	1.344	-0.021	-1.364	high
CK-40d	RHSIM_Rhsim12G0208800	-1.817	-1.539	0.278	low
CK-40d	RHSIM_Rhsim11G0168800	0.128	-1.002	-1.130	no
CK-40d	RHSIM_Rhsim10G0180100	1.513	1.237	-0.276	high

Group	Gene	PTR (z-score difference)	z(log2 LFQ)	z(log2 FPKM)	extreme-PTR
CK-40d	RHSIM_Rhsim11G0186600	-0.355	-0.483	-0.128	no
CK-40d	RHSIM_Rhsim12G0200100	1.221	-0.398	-1.619	high
CK-40d	RHSIM_Rhsim11G0085000	-1.766	-1.146	0.620	low
CK-40d	RHSIM_Rhsim12G0062000	0.423	0.232	-0.191	no
CK-40d	RHSIM_Rhsim12G0063700	0.236	0.340	0.103	no
CK-40d	RHSIM_Rhsim12G0104400	2.029	1.740	-0.290	high
CK-40d	RHSIM_Rhsim12G0096800	-2.717	-1.511	1.206	low
CK-40d	RHSIM_Rhsim11G0040500	-0.341	-0.451	-0.110	no
CK-40d	RHSIM_Rhsim12G0080700	-0.120	-0.419	-0.298	no
CK-40d	RHSIM_Rhsim11G0111300	0.165	0.679	0.514	no
CK-40d	RHSIM_Rhsim11G0127400	-0.398	-0.329	0.069	no
CK-40d	RHSIM_Rhsim11G0115300	1.316	0.441	-0.875	high
CK-40d	RHSIM_Rhsim12G0148700	1.359	1.270	-0.090	high
CK-40d	RHSIM_Rhsim12G0081400	1.376	0.531	-0.845	high
CK-40d	RHSIM_Rhsim11G0186800	-1.001	-0.376	0.625	no
CK-40d	RHSIM_Rhsim11G0024700	0.023	-0.528	-0.551	no
CK-40d	RHSIM_Rhsim12G0136400	-0.075	1.069	1.144	no
CK-40d	RHSIM_Rhsim12G0174100	-0.360	0.598	0.958	no
CK-40d	RHSIM_Rhsim11G0012900	0.506	0.766	0.260	no
CK-40d	RHSIM_Rhsim10G0171800	-0.728	-0.408	0.320	no
CK-40d	RHSIM_Rhsim10G0206700	0.466	-0.483	-0.949	no
CK-40d	RHSIM_Rhsim11G0169800	-1.700	-0.454	1.246	low
CK-40d	RHSIM_Rhsim12G0201100	-1.659	-0.846	0.814	low
CK-40d	RHSIM_Rhsim10G0010400	-0.861	-0.789	0.072	no
CK-40d	RHSIM_Rhsim11G0098800	-1.404	0.517	1.921	low
CK-40d	RHSIM_Rhsim10G0092900	-0.151	-0.360	-0.210	no
CK-40d	RHSIM_Rhsim10G0202800	2.844	1.212	-1.631	high
CK-40d	RHSIM_Rhsim11G0164100	-1.854	-1.648	0.206	low
CK-40d	RHSIM_Rhsim12G0136300	-2.062	-1.028	1.034	low
CK-40d	RHSIM_Rhsim11G0088800	1.285	0.524	-0.761	high
CK-40d	RHSIM_Rhsim11G0150000	-1.251	-0.963	0.288	low
CK-40d	RHSIM_Rhsim11G0067900	-0.569	-1.942	-1.373	no

Group	Gene	PTR (z-score difference)	z(log2 LFQ)	z(log2 FPKM)	extreme-PTR
CK-40d	RHSIM_Rhsim12G0187900	-0.357	-0.526	-0.169	no
CK-40d	RHSIM_Rhsim11G0098700	2.599	2.849	0.251	high
CK-40d	RHSIM_Rhsim11G0032600	0.129	0.587	0.458	no
CK-40d	RHSIM_Rhsim12G0200200	-1.901	-1.216	0.685	low
CK-40d	RHSIM_Rhsim11G0134300	-0.575	-0.687	-0.111	no
CK-40d	RHSIM_Rhsim12G0143000	-1.251	-1.459	-0.208	low
CK-40d	RHSIM_Rhsim11G0122100	1.166	-0.647	-1.814	high
CK-40d	RHSIM_Rhsim11G0024800	-0.189	0.237	0.426	no
CK-40d	RHSIM_Rhsim11G0043200	-2.292	-1.100	1.192	low
CK-40d	RHSIM_Rhsim11G0103200	-1.829	-1.601	0.229	low
CK-40d	RHSIM_Rhsim11G0173000	-1.126	1.166	2.292	no
CK-40d	RHSIM_Rhsim11G0182500	-0.610	-0.192	0.417	no
CK-40d	RHSIM_Rhsim10G0059600	0.727	0.114	-0.613	no
CK-40d	RHSIM_Rhsim11G0151000	-3.338	-1.211	2.127	low
CK-40d	RHSIM_Rhsim11G0024900	-0.025	0.187	0.213	no
CK-40d	RHSIM_Rhsim10G0128400	0.874	0.687	-0.186	no
CK-40d	RHSIM_Rhsim10G0055700	-0.374	-0.078	0.296	no
CK-40d	RHSIM_Rhsim11G0187800	-0.882	-0.969	-0.087	no
CK-40d	RHSIM_Rhsim11G0034100	1.071	2.493	1.422	no
CK-40d	RHSIM_Rhsim11G0141700	1.857	1.910	0.054	high
CK-40d	RHSIM_Rhsim11G0159600	0.469	0.360	-0.109	no
CK-40d	RHSIM_Rhsim11G0066600	-0.330	0.897	1.227	no
CK-40d	RHSIM_Rhsim11G0054300	0.284	0.108	-0.176	no
CK-40d	RHSIM_Rhsim11G0069700	0.417	-0.485	-0.902	no
CK-40d	RHSIM_Rhsim11G0070000	-0.196	-0.017	0.178	no
CK-40d	RHSIM_Rhsim10G0164900	-1.056	-0.161	0.895	no
CK-40d	RHSIM_Rhsim11G0070400	-1.265	-1.201	0.064	low
CK-40d	RHSIM_Rhsim11G0074900	-1.444	-0.869	0.574	low
CK-40d	RHSIM_Rhsim11G0179100	-1.696	-0.163	1.533	low
CK-40d	RHSIM_Rhsim10G0131900	-0.617	-0.178	0.439	no
CK-40d	RHSIM_Rhsim11G0027400	-0.164	-0.219	-0.055	no
CK-40d	RHSIM_Rhsim11G0063300	0.182	-1.027	-1.208	no

Group	Gene	PTR (z-score difference)	z(log2 LFQ)	z(log2 FPKM)	extreme-PTR
CK-40d	RHSIM_Rhsim11G0037700	-0.152	-0.788	-0.636	no
CK-40d	RHSIM_Rhsim10G0063500	0.300	1.299	1.000	no
CK-40d	RHSIM_Rhsim11G0187700	1.588	2.025	0.436	high
CK-40d	RHSIM_Rhsim11G0069600	0.194	-0.189	-0.383	no
CK-40d	RHSIM_Rhsim10G0146500	0.143	-0.781	-0.924	no
CK-40d	RHSIM_Rhsim11G0056500	-0.054	0.011	0.065	no
CK-40d	RHSIM_Rhsim11G0008200	1.613	-0.205	-1.818	high
CK-40d	RHSIM_Rhsim11G0159400	1.512	-0.008	-1.519	high
CK-40d	RHSIM_Rhsim11G0044300	-0.861	0.407	1.269	no
CK-40d	RHSIM_Rhsim10G0065100	0.154	0.542	0.388	no
CK-40d	RHSIM_Rhsim11G0178600	-0.684	-0.485	0.199	no
CK-40d	RHSIM_Rhsim11G0159100	-1.687	-1.315	0.372	low
CK-40d	RHSIM_Rhsim10G0017800	-1.493	-1.160	0.333	low
CK-40d	RHSIM_Rhsim11G0137700	-1.284	-0.133	1.151	low
CK-40d	RHSIM_Rhsim10G0072400	0.014	-1.705	-1.719	no
CK-40d	RHSIM_Rhsim11G0046100	0.690	0.570	-0.120	no
CK-40d	RHSIM_Rhsim11G0001100	-1.905	-1.621	0.284	low
CK-40d	RHSIM_Rhsim11G0187200	-0.782	-0.269	0.514	no
CK-40d	RHSIM_Rhsim11G0099300	-1.127	-0.894	0.234	no
CK-40d	RHSIM_Rhsim11G0187000	-1.050	0.338	1.388	no
CK-40d	RHSIM_Rhsim11G0165500	-2.077	-1.305	0.772	low
CK-40d	RHSIM_Rhsim11G0092100	-0.425	-0.071	0.354	no
CK-40d	RHSIM_Rhsim09G0008800	0.243	0.568	0.325	no
CK-40d	RHSIM_Rhsim11G0066000	-0.263	-0.675	-0.412	no
CK-40d	RHSIM_Rhsim11G0183000	-0.788	-0.471	0.317	no
CK-40d	RHSIM_Rhsim09G0103900	0.239	0.152	-0.087	no
CK-40d	RHSIM_Rhsim11G0075300	-0.828	0.221	1.050	no
CK-40d	RHSIM_Rhsim11G0049500	-1.257	-2.039	-0.782	low
CK-40d	RHSIM_Rhsim11G0193400	1.963	0.417	-1.546	high
CK-40d	RHSIM_Rhsim11G0019200	-1.246	-0.909	0.337	low
CK-40d	RHSIM_Rhsim11G0121200	-0.149	-0.105	0.044	no
CK-40d	RHSIM_Rhsim09G0002100	2.561	1.080	-1.481	high

Group	Gene	PTR (z-score difference)	z(log2 LFQ)	z(log2 FPKM)	extreme-PTR
CK-40d	RHSIM_Rhsim11G0160800	0.963	1.933	0.971	no
CK-40d	RHSIM_Rhsim11G0001400	0.146	-0.639	-0.785	no
CK-40d	RHSIM_Rhsim11G0021000	0.782	-0.170	-0.952	no
CK-40d	RHSIM_Rhsim11G0160000	-1.904	-0.420	1.484	low
CK-40d	RHSIM_Rhsim11G0160500	0.291	-0.403	-0.694	no
CK-40d	RHSIM_Rhsim11G0131900	-1.476	-1.651	-0.175	low
CK-40d	RHSIM_Rhsim11G0038900	0.834	0.219	-0.615	no
CK-40d	RHSIM_Rhsim11G0052300	-1.199	-1.347	-0.149	no
CK-40d	RHSIM_Rhsim11G0088200	-0.602	0.079	0.681	no
CK-40d	RHSIM_Rhsim11G0138000	-2.138	-0.480	1.658	low
CK-40d	RHSIM_Rhsim11G0058000	1.606	0.387	-1.219	high
CK-40d	RHSIM_Rhsim11G0148400	1.750	1.602	-0.148	high
CK-40d	RHSIM_Rhsim11G0003500	-0.525	-0.539	-0.013	no
CK-40d	RHSIM_Rhsim10G0186400	0.068	1.384	1.317	no
CK-40d	RHSIM_Rhsim11G0142200	-1.126	-1.431	-0.305	no
CK-40d	RHSIM_Rhsim11G0129100	-0.763	-0.840	-0.077	no
CK-40d	RHSIM_Rhsim10G0108500	-0.284	-0.568	-0.284	no
CK-40d	RHSIM_Rhsim09G0038500	-2.108	-0.881	1.227	low
CK-40d	RHSIM_Rhsim10G0009700	-0.065	0.392	0.457	no
CK-40d	RHSIM_Rhsim09G0212500	0.132	0.787	0.655	no
CK-40d	RHSIM_Rhsim11G0192800	-1.337	-0.792	0.545	low
CK-40d	RHSIM_Rhsim11G0055800	1.108	-0.035	-1.144	no
CK-40d	RHSIM_Rhsim10G0162600	-0.398	-0.165	0.234	no
CK-40d	RHSIM_Rhsim11G0148600	0.277	0.894	0.617	no
CK-40d	RHSIM_Rhsim09G0023700	0.149	-0.728	-0.877	no
CK-40d	RHSIM_Rhsim10G0193700	1.646	0.020	-1.625	high
CK-40d	RHSIM_Rhsim10G0174900	2.270	1.303	-0.967	high
CK-40d	RHSIM_Rhsim11G0032300	-2.390	-1.859	0.530	low
CK-40d	RHSIM_Rhsim11G0035800	0.498	0.243	-0.256	no
CK-40d	RHSIM_Rhsim11G0027600	2.940	1.489	-1.451	high
CK-40d	RHSIM_Rhsim09G0052000	1.475	0.232	-1.243	high
CK-40d	RHSIM_Rhsim11G0179300	-0.806	-0.485	0.321	no

Group	Gene	PTR (z-score difference)	z(log2 LFQ)	z(log2 FPKM)	extreme-PTR
CK-40d	RHSIM_Rhsim11G0046900	-0.578	-1.333	-0.755	no
CK-40d	RHSIM_Rhsim11G0057900	0.636	-0.609	-1.245	no
CK-40d	RHSIM_Rhsim11G0072300	0.991	0.078	-0.913	no
CK-40d	RHSIM_Rhsim11G0170000	-1.089	-0.147	0.942	no
CK-40d	RHSIM_Rhsim09G0188200	-0.205	0.013	0.218	no
CK-40d	RHSIM_Rhsim09G0200200	0.392	0.290	-0.102	no
CK-40d	RHSIM_Rhsim10G0062400	-0.641	-0.841	-0.199	no
CK-40d	RHSIM_Rhsim11G0038000	0.326	0.878	0.552	no
CK-40d	RHSIM_Rhsim11G0054400	-0.940	0.003	0.944	no
CK-40d	RHSIM_Rhsim10G0039600	1.030	0.741	-0.289	no
CK-40d	RHSIM_Rhsim09G0000600	1.230	-0.700	-1.930	high
CK-40d	RHSIM_Rhsim11G0100000	0.483	1.134	0.651	no
CK-40d	RHSIM_Rhsim11G0026600	-0.550	-0.665	-0.114	no
CK-40d	RHSIM_Rhsim10G0063000	0.608	0.759	0.152	no
CK-40d	RHSIM_Rhsim11G0044500	-0.249	-0.344	-0.095	no
CK-40d	RHSIM_Rhsim11G0163700	-1.047	0.502	1.549	no
CK-40d	RHSIM_Rhsim09G0186800	0.916	1.305	0.390	no
CK-40d	RHSIM_Rhsim09G0133100	-1.519	-1.673	-0.154	low
CK-40d	RHSIM_Rhsim09G0178500	-0.753	-0.356	0.397	no
CK-40d	RHSIM_Rhsim10G0085800	-0.445	-0.911	-0.465	no
CK-40d	RHSIM_Rhsim09G0192900	-0.813	1.194	2.007	no
CK-40d	RHSIM_Rhsim09G0180100	1.916	2.204	0.288	high
CK-40d	RHSIM_Rhsim11G0037300	-1.468	-1.166	0.303	low
CK-40d	RHSIM_Rhsim09G0165200	-1.822	-0.947	0.874	low
CK-40d	RHSIM_Rhsim09G0103600	-0.463	-1.247	-0.784	no
CK-40d	RHSIM_Rhsim11G0034600	-3.183	-2.043	1.140	low
CK-40d	RHSIM_Rhsim09G0087900	-0.219	-0.207	0.011	no
CK-40d	RHSIM_Rhsim10G0118400	-0.701	-0.852	-0.152	no
CK-40d	RHSIM_Rhsim11G0141000	1.071	0.563	-0.507	no
CK-40d	RHSIM_Rhsim09G0185200	0.923	0.975	0.052	no
CK-40d	RHSIM_Rhsim09G0151700	-0.841	2.358	3.199	no
CK-40d	RHSIM_Rhsim11G0182100	1.123	0.457	-0.666	no

Group	Gene	PTR (z-score difference)	z(log2 LFQ)	z(log2 FPKM)	extreme-PTR
CK-40d	RHSIM_Rhsim11G0021500	1.926	1.846	-0.080	high
CK-40d	RHSIM_Rhsim11G0169500	-1.051	-0.054	0.996	no
CK-40d	RHSIM_Rhsim11G0037600	0.020	-0.087	-0.107	no
CK-40d	RHSIM_Rhsim10G0020800	-0.470	-0.601	-0.131	no
CK-40d	RHSIM_Rhsim11G0111400	-0.874	-0.300	0.574	no
CK-40d	RHSIM_Rhsim10G0014700	0.388	-0.134	-0.522	no
CK-40d	RHSIM_Rhsim11G0087000	4.636	2.797	-1.839	high
CK-40d	RHSIM_Rhsim11G0052900	2.274	1.668	-0.606	high
CK-40d	RHSIM_Rhsim10G0138900	-0.219	-0.927	-0.707	no
CK-40d	RHSIM_Rhsim10G0159500	3.119	0.717	-2.402	high
CK-40d	RHSIM_Rhsim10G0082200	-0.881	0.198	1.079	no
CK-40d	RHSIM_Rhsim10G0183600	-1.208	-0.612	0.596	no
CK-40d	RHSIM_Rhsim10G0169300	-0.687	-0.966	-0.279	no
CK-40d	RHSIM_Rhsim10G0173800	2.055	1.274	-0.781	high
CK-40d	RHSIM_Rhsim09G0142600	0.789	0.206	-0.583	no
CK-40d	RHSIM_Rhsim10G0195900	-0.487	-1.851	-1.363	no
CK-40d	RHSIM_Rhsim10G0067500	-0.263	1.524	1.787	no
CK-40d	RHSIM_Rhsim11G0146300	1.072	1.258	0.186	no
CK-40d	RHSIM_Rhsim11G0047200	-1.903	-1.526	0.377	low
CK-40d	RHSIM_Rhsim11G0174400	-1.517	-1.597	-0.080	low
CK-40d	RHSIM_Rhsim10G0052200	0.982	0.809	-0.174	no
CK-40d	RHSIM_Rhsim09G0129300	-0.213	-0.016	0.196	no
CK-40d	RHSIM_Rhsim10G0008900	0.159	0.197	0.039	no
CK-40d	RHSIM_Rhsim10G0007400	-1.655	-0.823	0.832	low
CK-40d	RHSIM_Rhsim09G0209100	-0.898	-0.208	0.690	no
CK-40d	RHSIM_Rhsim10G0004900	0.879	-0.206	-1.085	no
CK-40d	RHSIM_Rhsim10G0129200	-1.822	-1.275	0.548	low
CK-40d	RHSIM_Rhsim10G0094900	0.786	-0.346	-1.132	no
CK-40d	RHSIM_Rhsim09G0032600	-0.394	-0.708	-0.314	no
CK-40d	RHSIM_Rhsim10G0104300	-1.183	-0.322	0.861	no
CK-40d	RHSIM_Rhsim10G0171200	0.169	0.108	-0.061	no
CK-40d	RHSIM_Rhsim10G0068900	-0.366	-0.689	-0.323	no

Group	Gene	PTR (z-score difference)	z(log2 LFQ)	z(log2 FPKM)	extreme-PTR
CK-40d	RHSIM_Rhsim10G0129100	-2.012	-1.032	0.980	low
CK-40d	RHSIM_Rhsim10G0122800	1.258	-0.644	-1.902	high
CK-40d	RHSIM_Rhsim10G0184100	1.658	0.780	-0.878	high
CK-40d	RHSIM_Rhsim09G0158400	1.948	0.959	-0.989	high
CK-40d	RHSIM_Rhsim09G0007900	2.452	1.421	-1.030	high
CK-40d	RHSIM_Rhsim10G0157900	-0.086	0.182	0.268	no
CK-40d	RHSIM_Rhsim09G0073500	0.386	0.665	0.279	no
CK-40d	RHSIM_Rhsim10G0010500	1.078	1.838	0.760	no
CK-40d	RHSIM_Rhsim10G0156400	0.150	-1.186	-1.336	no
CK-40d	RHSIM_Rhsim10G0188300	-0.612	-0.000	0.612	no
CK-40d	RHSIM_Rhsim10G0137900	1.206	0.678	-0.529	high
CK-40d	RHSIM_Rhsim09G0063200	0.734	0.335	-0.399	no
CK-40d	RHSIM_Rhsim10G0210900	-0.351	0.875	1.226	no
CK-40d	RHSIM_Rhsim10G0156900	0.662	0.372	-0.290	no
CK-40d	RHSIM_Rhsim10G0080600	1.277	0.428	-0.849	high
CK-40d	RHSIM_Rhsim10G0191400	-2.535	-2.153	0.382	low
CK-40d	RHSIM_Rhsim10G0043400	1.713	-0.543	-2.256	high
CK-40d	RHSIM_Rhsim10G0044200	2.594	0.673	-1.921	high
CK-40d	RHSIM_Rhsim10G0110700	1.274	0.283	-0.991	high
CK-40d	RHSIM_Rhsim09G0114600	-1.220	-0.494	0.726	no
CK-40d	RHSIM_Rhsim10G0174000	2.882	0.986	-1.896	high
CK-40d	RHSIM_Rhsim10G0172400	0.820	0.453	-0.367	no
CK-40d	RHSIM_Rhsim09G0022800	1.399	1.263	-0.136	high
CK-40d	RHSIM_Rhsim10G0115200	0.273	-1.237	-1.510	no
CK-40d	RHSIM_Rhsim10G0205500	1.356	-0.759	-2.115	high
CK-40d	RHSIM_Rhsim10G0133100	0.473	-0.560	-1.033	no
CK-40d	RHSIM_Rhsim10G0187200	-3.668	-2.002	1.665	low
CK-40d	RHSIM_Rhsim10G0151000	0.812	-0.297	-1.109	no
CK-40d	RHSIM_Rhsim10G0182800	0.418	0.951	0.533	no
CK-40d	RHSIM_Rhsim10G0156600	2.246	0.682	-1.564	high
CK-40d	RHSIM_Rhsim10G0050900	1.967	0.743	-1.224	high
CK-40d	RHSIM_Rhsim10G0172000	-1.330	-0.301	1.029	low

Group	Gene	PTR (z-score difference)	z(log2 LFQ)	z(log2 FPKM)	extreme-PTR
CK-40d	RHSIM_Rhsim10G0085400	-0.117	-0.534	-0.417	no
CK-40d	RHSIM_Rhsim08G0156400	1.415	-0.324	-1.739	high
CK-40d	RHSIM_Rhsim10G0147800	0.122	-1.014	-1.136	no
CK-40d	RHSIM_Rhsim10G0177400	0.174	-1.220	-1.393	no
CK-40d	RHSIM_Rhsim08G0211300	-1.743	-0.767	0.977	low
CK-40d	RHSIM_Rhsim08G0064400	-0.302	-1.283	-0.981	no
CK-40d	RHSIM_Rhsim10G0203500	-0.750	-2.065	-1.315	no
CK-40d	RHSIM_Rhsim10G0032200	0.841	-0.703	-1.544	no
CK-40d	RHSIM_Rhsim08G0204300	-0.723	-1.017	-0.294	no
CK-40d	RHSIM_Rhsim10G0114700	0.895	-0.082	-0.977	no
CK-40d	RHSIM_Rhsim08G0102800	-1.866	-2.003	-0.137	low
CK-40d	RHSIM_Rhsim10G0093100	-0.417	-1.977	-1.561	no
CK-40d	RHSIM_Rhsim10G0191100	-0.085	0.706	0.791	no
CK-40d	RHSIM_Rhsim10G0197500	-0.886	-0.320	0.566	no
CK-40d	RHSIM_Rhsim10G0144500	-1.350	-0.472	0.878	low
CK-40d	RHSIM_Rhsim08G0099700	-0.314	0.055	0.370	no
CK-40d	RHSIM_Rhsim10G0212300	-0.665	-0.814	-0.149	no
CK-40d	RHSIM_Rhsim08G0220500	0.622	0.909	0.286	no
CK-40d	RHSIM_Rhsim08G0251300	0.056	0.342	0.286	no
CK-40d	RHSIM_Rhsim08G0007600	0.130	-0.723	-0.853	no
CK-40d	RHSIM_Rhsim10G0209300	-0.209	-1.066	-0.857	no
CK-40d	RHSIM_Rhsim10G0166800	-0.280	0.784	1.065	no
CK-40d	RHSIM_Rhsim09G0002300	0.618	0.370	-0.247	no
CK-40d	RHSIM_Rhsim10G0083700	-0.812	-1.318	-0.506	no
CK-40d	RHSIM_Rhsim10G0150100	-1.102	-1.241	-0.139	no
CK-40d	RHSIM_Rhsim10G0127500	0.289	0.475	0.186	no
CK-40d	RHSIM_Rhsim08G0012700	0.418	-0.107	-0.525	no
CK-40d	RHSIM_Rhsim08G0146300	0.245	-0.041	-0.285	no
CK-40d	RHSIM_Rhsim10G0173500	-0.840	-0.989	-0.149	no
CK-40d	RHSIM_Rhsim08G0066300	1.044	1.322	0.278	no
CK-40d	RHSIM_Rhsim09G0209900	-0.425	-0.285	0.140	no
CK-40d	RHSIM_Rhsim10G0193100	0.396	-0.036	-0.432	no

Group	Gene	PTR (z-score difference)	z(log2 LFQ)	z(log2 FPKM)	extreme-PTR
CK-40d	RHSIM_Rhsim10G0124700	-0.732	0.081	0.813	no
CK-40d	RHSIM_Rhsim10G0124000	-1.599	-0.414	1.185	low
CK-40d	RHSIM_Rhsim09G0032900	1.959	1.673	-0.286	high
CK-40d	RHSIM_Rhsim10G0021300	2.732	3.427	0.695	high
CK-40d	RHSIM_Rhsim09G0172500	-0.068	0.876	0.944	no
CK-40d	RHSIM_Rhsim09G0069400	-0.696	-0.037	0.659	no
CK-40d	RHSIM_Rhsim08G0053400	0.039	0.285	0.246	no
CK-40d	RHSIM_Rhsim10G0066000	1.095	1.249	0.154	no
CK-40d	RHSIM_Rhsim09G0205000	-1.527	-0.613	0.914	low
CK-40d	RHSIM_Rhsim10G0190900	1.378	-0.115	-1.493	high
CK-40d	RHSIM_Rhsim09G0007800	-0.392	-0.708	-0.316	no
CK-40d	RHSIM_Rhsim09G0205300	1.269	-0.992	-2.261	high
CK-40d	RHSIM_Rhsim10G0112400	-0.103	-0.668	-0.565	no
CK-40d	RHSIM_Rhsim08G0092300	-0.651	-1.611	-0.961	no
CK-40d	RHSIM_Rhsim10G0005200	-0.894	-0.934	-0.040	no
CK-40d	RHSIM_Rhsim08G0045300	0.797	0.633	-0.164	no
CK-40d	RHSIM_Rhsim09G0195700	1.510	1.571	0.061	high
CK-40d	RHSIM_Rhsim09G0177100	0.444	0.838	0.395	no
CK-40d	RHSIM_Rhsim09G0205800	-1.261	-1.554	-0.293	low
CK-40d	RHSIM_Rhsim05G0149300	-1.078	0.503	1.581	no
CK-40d	RHSIM_Rhsim08G0128200	0.469	-1.572	-2.040	no
CK-40d	RHSIM_Rhsim08G0079900	-0.689	0.255	0.944	no
CK-40d	RHSIM_Rhsim09G0163400	-0.533	1.410	1.942	no
CK-40d	RHSIM_Rhsim08G0171900	0.612	0.614	0.002	no
CK-40d	RHSIM_Rhsim08G0225400	1.183	0.462	-0.721	high
CK-40d	RHSIM_Rhsim08G0008700	0.592	-0.781	-1.373	no
CK-40d	RHSIM_Rhsim10G0011000	0.116	0.600	0.483	no
CK-40d	RHSIM_Rhsim10G0003100	-1.186	-1.175	0.011	no
CK-40d	RHSIM_Rhsim08G0161200	-0.666	-1.220	-0.554	no
CK-40d	RHSIM_Rhsim09G0060000	0.188	0.656	0.468	no
CK-40d	RHSIM_Rhsim09G0091000	0.268	-0.292	-0.560	no
CK-40d	RHSIM_Rhsim09G0093400	1.443	0.019	-1.424	high

Group	Gene	PTR (z-score difference)	z(log2 LFQ)	z(log2 FPKM)	extreme-PTR
CK-40d	RHSIM_Rhsim10G0163400	-0.194	0.295	0.489	no
CK-40d	RHSIM_Rhsim08G0176500	-1.255	-0.898	0.357	low
CK-40d	RHSIM_Rhsim10G0063200	-0.406	0.293	0.700	no
CK-40d	RHSIM_Rhsim09G0007600	0.900	-0.478	-1.378	no
CK-40d	RHSIM_Rhsim09G0211900	2.273	1.402	-0.871	high
CK-40d	RHSIM_Rhsim09G0181300	-1.372	-1.035	0.337	low
CK-40d	RHSIM_Rhsim10G0097300	2.084	0.525	-1.559	high
CK-40d	RHSIM_Rhsim10G0067400	0.603	-0.011	-0.614	no
CK-40d	RHSIM_Rhsim08G0112400	-0.235	-0.726	-0.491	no
CK-40d	RHSIM_Rhsim09G0209500	-0.136	-1.753	-1.617	no
CK-40d	RHSIM_Rhsim08G0172700	0.304	-1.209	-1.513	no
CK-40d	RHSIM_Rhsim10G0191500	-0.476	-0.970	-0.494	no
CK-40d	RHSIM_Rhsim09G0009500	-1.107	-0.551	0.556	no
CK-40d	RHSIM_Rhsim09G0091300	-0.979	-0.720	0.259	no
CK-40d	RHSIM_Rhsim08G0188100	-0.744	0.053	0.797	no
CK-40d	RHSIM_Rhsim09G0111200	-0.164	-0.687	-0.524	no
CK-40d	RHSIM_Rhsim08G0242700	-2.175	-1.814	0.361	low
CK-40d	RHSIM_Rhsim10G0000800	0.152	-0.860	-1.012	no
CK-40d	RHSIM_Rhsim09G0137200	-2.284	-1.645	0.639	low
CK-40d	RHSIM_Rhsim10G0114000	-0.117	1.128	1.245	no
CK-40d	RHSIM_Rhsim08G0042300	-0.940	0.133	1.073	no
CK-40d	RHSIM_Rhsim09G0141800	0.763	0.726	-0.037	no
CK-40d	RHSIM_Rhsim10G0165400	-0.465	-0.460	0.005	no
CK-40d	RHSIM_Rhsim09G0037400	-0.272	0.260	0.533	no
CK-40d	RHSIM_Rhsim10G0166900	-0.758	-0.768	-0.010	no
CK-40d	RHSIM_Rhsim09G0029500	-1.036	-0.867	0.169	no
CK-40d	RHSIM_Rhsim08G0123900	0.023	0.513	0.490	no
CK-40d	RHSIM_Rhsim10G0050000	1.075	0.173	-0.902	no
CK-40d	RHSIM_Rhsim10G0021100	1.623	-0.146	-1.769	high
CK-40d	RHSIM_Rhsim09G0107300	0.254	-0.010	-0.265	no
CK-40d	RHSIM_Rhsim10G0069600	0.452	0.309	-0.143	no
CK-40d	RHSIM_Rhsim09G0181500	-1.238	-0.886	0.352	no

Group	Gene	PTR (z-score difference)	z(log2 LFQ)	z(log2 FPKM)	extreme-PTR
CK-40d	RHSIM_Rhsim09G0176600	-1.081	0.481	1.562	no
CK-40d	RHSIM_Rhsim10G0188000	-0.796	-1.230	-0.435	no
CK-40d	RHSIM_Rhsim08G0033900	-1.168	-0.797	0.371	no
CK-40d	RHSIM_Rhsim09G0011600	-0.386	-0.077	0.310	no
CK-40d	RHSIM_Rhsim08G0070600	-0.324	-0.150	0.173	no
CK-40d	RHSIM_Rhsim09G0008400	1.545	2.237	0.693	high
CK-40d	RHSIM_Rhsim09G0028000	0.899	-0.180	-1.079	no
CK-40d	RHSIM_Rhsim08G0101600	-1.625	-2.473	-0.848	low
CK-40d	RHSIM_Rhsim09G0212400	-1.080	-0.791	0.288	no
CK-40d	RHSIM_Rhsim08G0079700	-1.966	1.727	3.693	low
CK-40d	RHSIM_Rhsim09G0164000	-0.169	0.359	0.528	no
CK-40d	RHSIM_Rhsim09G0179900	0.234	1.054	0.820	no
CK-40d	RHSIM_Rhsim09G0154200	0.066	-0.317	-0.383	no
CK-40d	RHSIM_Rhsim09G0103500	-0.488	-1.216	-0.729	no
CK-40d	RHSIM_Rhsim08G0147100	0.730	0.896	0.166	no
CK-40d	RHSIM_Rhsim09G0071200	-2.425	-0.834	1.591	low
CK-40d	RHSIM_Rhsim09G0126000	2.344	0.626	-1.718	high
CK-40d	RHSIM_Rhsim09G0012000	-1.169	-0.786	0.383	no
CK-40d	RHSIM_Rhsim09G0191500	-0.284	-0.215	0.068	no
CK-40d	RHSIM_Rhsim09G0177000	0.574	-1.309	-1.883	no
CK-40d	RHSIM_Rhsim09G0086300	-0.628	-0.132	0.497	no
CK-40d	RHSIM_Rhsim09G0192800	-0.216	-0.641	-0.424	no
CK-40d	RHSIM_Rhsim09G0063500	-0.551	-1.361	-0.810	no
CK-40d	RHSIM_Rhsim09G0182600	0.076	0.758	0.682	no
CK-40d	RHSIM_Rhsim09G0010800	-0.893	-0.731	0.162	no
CK-40d	RHSIM_Rhsim09G0098400	-2.033	-1.474	0.559	low
CK-40d	RHSIM_Rhsim08G0214300	0.060	1.719	1.659	no
CK-40d	RHSIM_Rhsim09G0099000	0.131	-1.185	-1.316	no
CK-40d	RHSIM_Rhsim09G0075600	-0.916	-0.879	0.038	no
CK-40d	RHSIM_Rhsim09G0094200	-2.993	-0.529	2.464	low
CK-40d	RHSIM_Rhsim09G0184500	2.403	0.248	-2.155	high
CK-40d	RHSIM_Rhsim09G0147200	-0.343	1.232	1.574	no

Group	Gene	PTR (z-score difference)	z(log2 LFQ)	z(log2 FPKM)	extreme-PTR
CK-40d	RHSIM_Rhsim08G0145400	0.317	0.156	-0.161	no
CK-40d	RHSIM_Rhsim09G0190300	-0.028	0.410	0.438	no
CK-40d	RHSIM_Rhsim09G0193300	0.175	0.271	0.096	no
CK-40d	RHSIM_Rhsim09G0055700	0.836	-0.929	-1.765	no
CK-40d	RHSIM_Rhsim09G0204600	-0.423	-1.198	-0.775	no
CK-40d	RHSIM_Rhsim08G0200000	-0.502	0.090	0.591	no
CK-40d	RHSIM_Rhsim09G0069300	1.814	0.709	-1.105	high
CK-40d	RHSIM_Rhsim09G0179400	0.837	0.388	-0.449	no
CK-40d	RHSIM_Rhsim09G0157600	0.016	-0.574	-0.590	no
CK-40d	RHSIM_Rhsim09G0091900	-0.650	-0.394	0.256	no
CK-40d	RHSIM_Rhsim09G0131900	-0.721	0.238	0.960	no
CK-40d	RHSIM_Rhsim07G0198600	1.428	2.380	0.953	high
CK-40d	RHSIM_Rhsim09G0070800	0.314	1.118	0.804	no
CK-40d	RHSIM_Rhsim09G0155700	0.008	-0.349	-0.357	no
CK-40d	RHSIM_Rhsim09G0186700	1.390	0.777	-0.612	high
CK-40d	RHSIM_Rhsim09G0145100	-1.876	-1.475	0.401	low
CK-40d	RHSIM_Rhsim09G0129000	-0.977	-0.702	0.276	no
CK-40d	RHSIM_Rhsim07G0224400	-0.712	-0.852	-0.140	no
CK-40d	RHSIM_Rhsim09G0137500	-1.830	-0.225	1.604	low
CK-40d	RHSIM_Rhsim09G0171900	2.594	2.766	0.172	high
CK-40d	RHSIM_Rhsim09G0010300	1.141	0.388	-0.753	high
CK-40d	RHSIM_Rhsim07G0144600	-0.549	-0.070	0.478	no
CK-40d	RHSIM_Rhsim09G0025700	0.593	0.000	-0.593	no
CK-40d	RHSIM_Rhsim07G0074600	-0.347	-1.073	-0.727	no
CK-40d	RHSIM_Rhsim07G0153100	0.103	0.550	0.446	no
CK-40d	RHSIM_Rhsim08G0119600	0.834	-0.099	-0.933	no
CK-40d	RHSIM_Rhsim07G0217300	2.659	0.629	-2.030	high
CK-40d	RHSIM_Rhsim09G0123100	-0.332	-0.161	0.171	no
CK-40d	RHSIM_Rhsim09G0080400	-0.357	-0.121	0.236	no
CK-40d	RHSIM_Rhsim09G0138200	-2.449	-2.337	0.112	low
CK-40d	RHSIM_Rhsim07G0018300	1.414	0.986	-0.428	high
CK-40d	RHSIM_Rhsim08G0214800	-0.020	-0.404	-0.384	no

Group	Gene	PTR (z-score difference)	z(log2 LFQ)	z(log2 FPKM)	extreme-PTR
CK-40d	RHSIM_Rhsim03G0130900	1.215	0.661	-0.555	high
CK-40d	RHSIM_Rhsim09G0128900	0.994	0.319	-0.675	no
CK-40d	RHSIM_Rhsim09G0054900	-1.316	-0.926	0.389	low
CK-40d	RHSIM_Rhsim08G0045200	0.130	-0.074	-0.204	no
CK-40d	RHSIM_Rhsim07G0258100	-0.265	0.160	0.425	no
CK-40d	RHSIM_Rhsim07G0246300	-1.755	-1.570	0.184	low
CK-40d	RHSIM_Rhsim09G0044200	-1.569	-0.081	1.488	low
CK-40d	RHSIM_Rhsim08G0089100	-0.628	0.924	1.552	no
CK-40d	RHSIM_Rhsim07G0061000	-2.508	-1.623	0.885	low
CK-40d	RHSIM_Rhsim08G0213200	-1.874	-1.286	0.588	low
CK-40d	RHSIM_Rhsim07G0068600	0.152	-0.269	-0.420	no
CK-40d	RHSIM_Rhsim07G0243600	1.132	-0.482	-1.614	no
CK-40d	RHSIM_Rhsim08G0223600	1.672	1.666	-0.006	high
CK-40d	RHSIM_Rhsim09G0089500	0.492	-0.194	-0.686	no
CK-40d	RHSIM_Rhsim07G0088700	0.145	0.658	0.513	no
CK-40d	RHSIM_Rhsim07G0042900	-1.026	-1.705	-0.679	no
CK-40d	RHSIM_Rhsim09G0033900	-1.149	-1.694	-0.544	no
CK-40d	RHSIM_Rhsim09G0142900	-0.162	-0.275	-0.113	no
CK-40d	RHSIM_Rhsim07G0200500	0.078	-0.416	-0.494	no
CK-40d	RHSIM_Rhsim09G0113800	0.659	-0.341	-1.000	no
CK-40d	RHSIM_Rhsim09G0212800	0.851	0.119	-0.731	no
CK-40d	RHSIM_Rhsim09G0178400	0.265	1.498	1.233	no
CK-40d	RHSIM_Rhsim08G0111900	0.295	0.644	0.350	no
CK-40d	RHSIM_Rhsim09G0094000	-0.975	0.087	1.063	no
CK-40d	RHSIM_Rhsim08G0126700	1.962	1.611	-0.351	high
CK-40d	RHSIM_Rhsim07G0192400	1.108	-0.289	-1.397	no
CK-40d	RHSIM_Rhsim08G0070900	1.125	-0.815	-1.939	no
CK-40d	RHSIM_Rhsim08G0021800	2.116	2.230	0.114	high
CK-40d	RHSIM_Rhsim09G0088900	-0.371	0.282	0.653	no
CK-40d	RHSIM_Rhsim08G0042600	-0.194	-0.218	-0.024	no
CK-40d	RHSIM_Rhsim09G0091400	-0.874	-0.335	0.539	no
CK-40d	RHSIM_Rhsim08G0092200	0.025	-0.259	-0.285	no

Group	Gene	PTR (z-score difference)	z(log2 LFQ)	z(log2 FPKM)	extreme-PTR
CK-40d	RHSIM_Rhsim09G0130400	-0.875	0.637	1.512	no
CK-40d	RHSIM_Rhsim09G0120100	1.270	1.892	0.622	high
CK-40d	RHSIM_Rhsim08G0043100	1.867	0.446	-1.420	high
CK-40d	RHSIM_Rhsim09G0190000	-0.194	-0.862	-0.667	no
CK-40d	RHSIM_Rhsim09G0102000	-1.264	-0.499	0.765	low
CK-40d	RHSIM_Rhsim07G0108200	0.673	0.296	-0.377	no
CK-40d	RHSIM_Rhsim08G0118300	-0.550	-1.296	-0.746	no
CK-40d	RHSIM_Rhsim09G0101400	-0.196	0.421	0.618	no
CK-40d	RHSIM_Rhsim08G0008800	0.321	0.860	0.539	no
CK-40d	RHSIM_Rhsim08G0105500	-1.356	0.003	1.358	low
CK-40d	RHSIM_Rhsim08G0178200	1.650	2.732	1.082	high
CK-40d	RHSIM_Rhsim08G0162700	0.717	0.183	-0.534	no
CK-40d	RHSIM_Rhsim07G0234400	-1.498	1.420	2.917	low
CK-40d	RHSIM_Rhsim08G0212700	-1.061	-0.083	0.978	no
CK-40d	RHSIM_Rhsim08G0244600	0.556	0.342	-0.214	no
CK-40d	RHSIM_Rhsim09G0074900	-0.290	1.251	1.541	no
CK-40d	RHSIM_Rhsim07G0030800	1.417	2.029	0.612	high
CK-40d	RHSIM_Rhsim09G0001500	0.625	1.408	0.783	no
CK-40d	RHSIM_Rhsim07G0104900	0.720	-0.289	-1.009	no
CK-40d	RHSIM_Rhsim08G0192000	-0.794	0.337	1.131	no
CK-40d	RHSIM_Rhsim08G0167700	0.641	-0.609	-1.250	no
CK-40d	RHSIM_Rhsim08G0068800	-0.319	-1.191	-0.872	no
CK-40d	RHSIM_Rhsim07G0234600	0.737	1.177	0.440	no
CK-40d	RHSIM_Rhsim09G0153200	-1.007	-0.845	0.162	no
CK-40d	RHSIM_Rhsim08G0227900	0.768	-1.450	-2.219	no
CK-40d	RHSIM_Rhsim09G0093900	1.374	0.772	-0.602	high
CK-40d	RHSIM_Rhsim07G0164900	1.787	-0.259	-2.046	high
CK-40d	RHSIM_Rhsim07G0092500	1.098	0.567	-0.531	no
CK-40d	RHSIM_Rhsim08G0083700	-0.046	0.018	0.064	no
CK-40d	RHSIM_Rhsim08G0027200	-1.651	-0.622	1.029	low
CK-40d	RHSIM_Rhsim07G0021100	0.748	0.179	-0.569	no
CK-40d	RHSIM_Rhsim08G0236900	-0.177	-0.302	-0.124	no

Group	Gene	PTR (z-score difference)	z(log2 LFQ)	z(log2 FPKM)	extreme-PTR
CK-40d	RHSIM_Rhsim09G0198900	0.905	0.375	-0.530	no
CK-40d	RHSIM_Rhsim08G0187300	-1.349	-1.299	0.050	low
CK-40d	RHSIM_Rhsim08G0007300	-3.353	-2.001	1.351	low
CK-40d	RHSIM_Rhsim08G0212200	-0.803	-0.554	0.249	no
CK-40d	RHSIM_Rhsim09G0072100	0.551	-1.114	-1.666	no
CK-40d	RHSIM_Rhsim07G0122800	-0.080	-1.574	-1.495	no
CK-40d	RHSIM_Rhsim09G0131200	-0.557	-0.442	0.115	no
CK-40d	RHSIM_Rhsim08G0118700	1.150	0.157	-0.993	high
CK-40d	RHSIM_Rhsim08G0180000	-0.274	-0.552	-0.278	no
CK-40d	RHSIM_Rhsim08G0236700	-0.059	0.081	0.140	no
CK-40d	RHSIM_Rhsim09G0213200	-0.150	-0.154	-0.003	no
CK-40d	RHSIM_Rhsim07G0155900	1.493	-0.216	-1.709	high
CK-40d	RHSIM_Rhsim09G0130300	-2.130	-1.509	0.622	low
CK-40d	RHSIM_Rhsim08G0246100	1.440	-0.095	-1.535	high
CK-40d	RHSIM_Rhsim08G0098000	-0.391	-0.022	0.369	no
CK-40d	RHSIM_Rhsim08G0082400	-1.798	-0.915	0.884	low
CK-40d	RHSIM_Rhsim08G0071400	-0.456	1.632	2.087	no
CK-40d	RHSIM_Rhsim08G0249700	1.302	1.184	-0.118	high
CK-40d	RHSIM_Rhsim08G0043900	-1.669	-1.319	0.350	low
CK-40d	RHSIM_Rhsim07G0132900	-1.532	-1.355	0.177	low
CK-40d	RHSIM_Rhsim08G0061200	0.529	-1.139	-1.668	no
CK-40d	RHSIM_Rhsim07G0128700	-0.718	-0.980	-0.262	no
CK-40d	RHSIM_Rhsim08G0052300	0.628	-0.566	-1.194	no
CK-40d	RHSIM_Rhsim08G0234400	0.794	-0.283	-1.077	no
CK-40d	RHSIM_Rhsim09G0021800	0.636	1.076	0.440	no
CK-40d	RHSIM_Rhsim09G0176500	0.257	1.425	1.168	no
CK-40d	RHSIM_Rhsim08G0200300	-1.864	-1.222	0.642	low
CK-40d	RHSIM_Rhsim07G0258600	-0.726	-0.826	-0.100	no
CK-40d	RHSIM_Rhsim08G0032500	-1.430	-0.956	0.474	low
CK-40d	RHSIM_Rhsim08G0004300	-1.190	-0.745	0.446	no
CK-40d	RHSIM_Rhsim08G0026800	-2.242	-1.673	0.569	low
CK-40d	RHSIM_Rhsim08G0240700	-1.163	-1.311	-0.147	no

Group	Gene	PTR (z-score difference)	z(log2 LFQ)	z(log2 FPKM)	extreme-PTR
CK-40d	RHSIM_Rhsim08G0247800	-0.732	-0.479	0.253	no
CK-40d	RHSIM_Rhsim09G0073300	-0.435	0.490	0.925	no
CK-40d	RHSIM_Rhsim09G0145600	0.565	-0.039	-0.604	no
CK-40d	RHSIM_Rhsim08G0080700	-1.281	-0.753	0.528	low
CK-40d	RHSIM_Rhsim08G0076400	-0.081	-0.307	-0.226	no
CK-40d	RHSIM_Rhsim08G0184900	0.195	-0.721	-0.915	no
CK-40d	RHSIM_Rhsim08G0009400	0.571	1.897	1.325	no
CK-40d	RHSIM_Rhsim07G0050700	-0.389	-0.003	0.386	no
CK-40d	RHSIM_Rhsim08G0019200	-2.170	-0.594	1.576	low
CK-40d	RHSIM_Rhsim08G0059600	1.566	0.994	-0.572	high
CK-40d	RHSIM_Rhsim08G0239600	-1.429	-0.886	0.543	low
CK-40d	RHSIM_Rhsim08G0093100	-1.022	-0.613	0.409	no
CK-40d	RHSIM_Rhsim08G0128400	1.187	0.790	-0.397	high
CK-40d	RHSIM_Rhsim07G0012200	1.720	-0.525	-2.246	high
CK-40d	RHSIM_Rhsim08G0194600	0.985	1.779	0.794	no
CK-40d	RHSIM_Rhsim08G0033500	0.258	-0.033	-0.292	no
CK-40d	RHSIM_Rhsim07G0027400	0.313	0.939	0.626	no
CK-40d	RHSIM_Rhsim07G0153400	-0.023	-0.295	-0.271	no
CK-40d	RHSIM_Rhsim09G0078400	1.842	-0.135	-1.977	high
CK-40d	RHSIM_Rhsim08G0164000	0.799	-0.581	-1.380	no
CK-40d	RHSIM_Rhsim07G0002300	-0.538	-1.267	-0.729	no
CK-40d	RHSIM_Rhsim08G0222900	1.707	0.406	-1.301	high
CK-40d	RHSIM_Rhsim08G0198500	0.855	-0.224	-1.079	no
CK-40d	RHSIM_Rhsim08G0218500	0.307	0.523	0.216	no
CK-40d	RHSIM_Rhsim09G0155900	0.763	1.007	0.244	no
CK-40d	RHSIM_Rhsim09G0195900	2.172	0.736	-1.436	high
CK-40d	RHSIM_Rhsim08G0029800	0.106	-0.993	-1.099	no
CK-40d	RHSIM_Rhsim07G0228400	0.407	0.552	0.145	no
CK-40d	RHSIM_Rhsim09G0093200	1.208	1.932	0.724	high
CK-40d	RHSIM_Rhsim07G0068800	-1.208	-1.164	0.043	no
CK-40d	RHSIM_Rhsim08G0037300	1.483	0.210	-1.274	high
CK-40d	RHSIM_Rhsim07G0011300	-0.811	-1.042	-0.230	no

Group	Gene	PTR (z-score difference)	z(log2 LFQ)	z(log2 FPKM)	extreme-PTR
CK-40d	RHSIM_Rhsim08G0233800	1.124	0.317	-0.807	no
CK-40d	RHSIM_Rhsim08G0211000	1.305	1.715	0.410	high
CK-40d	RHSIM_Rhsim07G0169700	-0.338	-0.612	-0.274	no
CK-40d	RHSIM_Rhsim08G0027300	-0.579	-0.549	0.030	no
CK-40d	RHSIM_Rhsim07G0078300	0.273	0.429	0.157	no
CK-40d	RHSIM_Rhsim08G0055200	-0.292	0.968	1.261	no
CK-40d	RHSIM_Rhsim07G0091400	-1.410	1.423	2.833	low
CK-40d	RHSIM_Rhsim07G0058300	-0.280	-0.552	-0.272	no
CK-40d	RHSIM_Rhsim09G0042400	1.014	-0.566	-1.580	no
CK-40d	RHSIM_Rhsim08G0020900	1.214	2.144	0.930	high
CK-40d	RHSIM_Rhsim08G0103100	1.170	-0.162	-1.332	high
CK-40d	RHSIM_Rhsim08G0232100	-1.848	-0.324	1.524	low
CK-40d	RHSIM_Rhsim09G0030000	1.260	-0.046	-1.306	high
CK-40d	RHSIM_Rhsim08G0121700	0.852	-0.198	-1.050	no
CK-40d	RHSIM_Rhsim08G0011700	1.980	-0.116	-2.096	high
CK-40d	RHSIM_Rhsim08G0147000	-1.048	0.371	1.419	no
CK-40d	RHSIM_Rhsim08G0192700	-0.894	-1.549	-0.655	no
CK-40d	RHSIM_Rhsim08G0057700	3.362	1.749	-1.613	high
CK-40d	RHSIM_Rhsim08G0199300	-2.059	-0.458	1.600	low
CK-40d	RHSIM_Rhsim07G0016300	0.336	-0.881	-1.218	no
CK-40d	RHSIM_Rhsim08G0083000	-1.185	-0.718	0.467	no
CK-40d	RHSIM_Rhsim08G0252400	2.344	0.443	-1.901	high
CK-40d	RHSIM_Rhsim08G0215600	-0.181	0.106	0.287	no
CK-40d	RHSIM_Rhsim09G0009000	-0.018	0.109	0.127	no
CK-40d	RHSIM_Rhsim08G0207700	0.388	-0.548	-0.936	no
CK-40d	RHSIM_Rhsim09G0152000	0.898	-0.701	-1.600	no
CK-40d	RHSIM_Rhsim08G0089000	1.275	0.558	-0.717	high
CK-40d	RHSIM_Rhsim07G0118200	0.632	1.126	0.494	no
CK-40d	RHSIM_Rhsim08G0102900	-0.959	-1.902	-0.943	no
CK-40d	RHSIM_Rhsim07G0028300	-1.896	-1.153	0.742	low
CK-40d	RHSIM_Rhsim08G0161100	0.512	0.688	0.175	no
CK-40d	RHSIM_Rhsim08G0087900	-0.027	-0.964	-0.936	no

Group	Gene	PTR (z-score difference)	z(log2 LFQ)	z(log2 FPKM)	extreme-PTR
CK-40d	RHSIM_Rhsim09G0164200	-0.294	1.624	1.917	no
CK-40d	RHSIM_Rhsim07G0246500	0.825	-1.259	-2.085	no
CK-40d	RHSIM_Rhsim08G0055400	2.588	2.093	-0.495	high
CK-40d	RHSIM_Rhsim08G0034500	0.409	-0.794	-1.203	no
CK-40d	RHSIM_Rhsim08G0242000	-1.205	-1.662	-0.457	no
CK-40d	RHSIM_Rhsim08G0073200	0.422	0.898	0.476	no
CK-40d	RHSIM_Rhsim08G0223300	2.620	3.215	0.595	high
CK-40d	RHSIM_Rhsim08G0128700	-1.276	-0.495	0.781	low
CK-40d	RHSIM_Rhsim08G0219800	-1.552	-1.686	-0.134	low
CK-40d	RHSIM_Rhsim09G0135900	1.510	-0.637	-2.147	high
CK-40d	RHSIM_Rhsim08G0106300	-0.521	-1.112	-0.591	no
CK-40d	RHSIM_Rhsim08G0027700	0.469	-0.108	-0.577	no
CK-40d	RHSIM_Rhsim08G0010500	-0.873	-0.272	0.601	no
CK-40d	RHSIM_Rhsim08G0049000	-0.430	-0.803	-0.373	no
CK-40d	RHSIM_Rhsim08G0188800	-0.784	-0.709	0.075	no
CK-40d	RHSIM_Rhsim08G0028200	0.062	0.020	-0.042	no
CK-40d	RHSIM_Rhsim08G0112900	-0.272	-0.503	-0.231	no
CK-40d	RHSIM_Rhsim08G0223100	-0.793	1.414	2.207	no
CK-40d	RHSIM_Rhsim07G0214500	2.198	2.456	0.258	high
CK-40d	RHSIM_Rhsim07G0067400	-1.336	-1.127	0.209	low
CK-40d	RHSIM_Rhsim07G0124800	0.291	0.243	-0.049	no
CK-40d	RHSIM_Rhsim08G0227600	-0.356	-1.653	-1.297	no
CK-40d	RHSIM_Rhsim07G0009900	-0.957	-0.715	0.242	no
CK-40d	RHSIM_Rhsim07G0036400	-0.227	2.062	2.289	no
CK-40d	RHSIM_Rhsim08G0228700	-0.216	-1.350	-1.134	no
CK-40d	RHSIM_Rhsim08G0246500	-1.422	0.089	1.511	low
CK-40d	RHSIM_Rhsim08G0015900	0.445	-0.337	-0.782	no
CK-40d	RHSIM_Rhsim07G0124400	1.688	2.737	1.049	high
CK-40d	RHSIM_Rhsim06G0028000	-0.507	1.869	2.376	no
CK-40d	RHSIM_Rhsim08G0248800	0.200	0.638	0.438	no
CK-40d	RHSIM_Rhsim08G0225500	2.352	1.183	-1.169	high
CK-40d	RHSIM_Rhsim08G0196300	0.048	-1.502	-1.550	no

Group	Gene	PTR (z-score difference)	z(log2 LFQ)	z(log2 FPKM)	extreme-PTR
CK-40d	RHSIM_Rhsim08G0011000	-1.746	1.690	3.437	low
CK-40d	RHSIM_Rhsim08G0001700	-0.856	-0.801	0.056	no
CK-40d	RHSIM_Rhsim08G0169400	-0.798	-1.216	-0.418	no
CK-40d	RHSIM_Rhsim08G0087000	2.526	1.263	-1.263	high
CK-40d	RHSIM_Rhsim07G0046800	0.295	0.732	0.437	no
CK-40d	RHSIM_Rhsim08G0221800	-0.516	-0.623	-0.107	no
CK-40d	RHSIM_Rhsim08G0173800	-0.213	-0.610	-0.396	no
CK-40d	RHSIM_Rhsim06G0095100	-0.456	-1.076	-0.620	no
CK-40d	RHSIM_Rhsim08G0198100	1.428	1.931	0.503	high
CK-40d	RHSIM_Rhsim07G0215600	-0.183	0.130	0.313	no
CK-40d	RHSIM_Rhsim08G0032400	-0.188	0.172	0.360	no
CK-40d	RHSIM_Rhsim08G0084500	-2.254	-0.003	2.251	low
CK-40d	RHSIM_Rhsim08G0059500	2.171	1.986	-0.185	high
CK-40d	RHSIM_Rhsim08G0052400	1.041	0.469	-0.572	no
CK-40d	RHSIM_Rhsim07G0093000	-1.922	-0.045	1.877	low
CK-40d	RHSIM_Rhsim08G0181000	3.575	1.852	-1.723	high
CK-40d	RHSIM_Rhsim08G0052800	-1.862	-0.897	0.965	low
CK-40d	RHSIM_Rhsim08G0048100	-0.452	-0.274	0.179	no
CK-40d	RHSIM_Rhsim07G0017600	-0.058	-0.022	0.036	no
CK-40d	RHSIM_Rhsim07G0044000	-0.280	-0.179	0.101	no
CK-40d	RHSIM_Rhsim08G0042700	0.595	-0.167	-0.762	no
CK-40d	RHSIM_Rhsim08G0066600	-0.009	-0.162	-0.153	no
CK-40d	RHSIM_Rhsim07G0106400	0.570	0.720	0.150	no
CK-40d	RHSIM_Rhsim07G0142700	0.099	-1.618	-1.717	no
CK-40d	RHSIM_Rhsim06G0005200	-0.613	-0.920	-0.307	no
CK-40d	RHSIM_Rhsim07G0212500	-0.671	-0.527	0.143	no
CK-40d	RHSIM_Rhsim08G0194200	0.537	0.699	0.162	no
CK-40d	RHSIM_Rhsim08G0155500	-1.212	-1.061	0.151	no
CK-40d	RHSIM_Rhsim07G0048500	-0.587	-1.487	-0.900	no
CK-40d	RHSIM_Rhsim06G0127700	0.333	-0.072	-0.405	no
CK-40d	RHSIM_Rhsim08G0017300	0.731	-0.051	-0.781	no
CK-40d	RHSIM_Rhsim08G0195700	-1.236	-0.961	0.275	no

Group	Gene	PTR (z-score difference)	z(log2 LFQ)	z(log2 FPKM)	extreme-PTR
CK-40d	RHSIM_Rhsim08G0007800	0.621	-0.163	-0.784	no
CK-40d	RHSIM_Rhsim08G0104200	0.291	-0.364	-0.655	no
CK-40d	RHSIM_Rhsim06G0233900	0.475	0.311	-0.164	no
CK-40d	RHSIM_Rhsim08G0159800	0.945	0.663	-0.282	no
CK-40d	RHSIM_Rhsim08G0212400	0.165	-0.949	-1.114	no
CK-40d	RHSIM_Rhsim06G0165900	-0.506	0.145	0.651	no
CK-40d	RHSIM_Rhsim08G0236000	-1.496	-1.070	0.426	low
CK-40d	RHSIM_Rhsim07G0219800	-0.436	0.142	0.578	no
CK-40d	RHSIM_Rhsim08G0196000	-0.462	1.630	2.091	no
CK-40d	RHSIM_Rhsim06G0091300	-0.307	-1.033	-0.726	no
CK-40d	RHSIM_Rhsim08G0078500	1.022	-0.128	-1.150	no
CK-40d	RHSIM_Rhsim07G0170200	1.266	0.626	-0.640	high
CK-40d	RHSIM_Rhsim07G0233400	2.097	1.340	-0.757	high
CK-40d	RHSIM_Rhsim06G0211500	-0.727	-1.006	-0.279	no
CK-40d	RHSIM_Rhsim08G0079500	0.559	0.928	0.370	no
CK-40d	RHSIM_Rhsim07G0202900	0.157	-0.313	-0.470	no
CK-40d	RHSIM_Rhsim07G0001500	0.273	0.481	0.208	no
CK-40d	RHSIM_Rhsim07G0178700	0.547	-0.286	-0.833	no
CK-40d	RHSIM_Rhsim07G0170500	-1.746	-1.283	0.463	low
CK-40d	RHSIM_Rhsim06G0181200	2.406	0.606	-1.801	high
CK-40d	RHSIM_Rhsim07G0049800	0.087	1.619	1.532	no
CK-40d	RHSIM_Rhsim07G0066200	-0.371	-0.182	0.189	no
CK-40d	RHSIM_Rhsim08G0072200	0.053	-0.332	-0.385	no
CK-40d	RHSIM_Rhsim07G0131800	0.537	0.646	0.109	no
CK-40d	RHSIM_Rhsim07G0036500	-0.436	0.785	1.221	no
CK-40d	RHSIM_Rhsim07G0028800	2.109	3.420	1.311	high
CK-40d	RHSIM_Rhsim07G0132800	0.150	-0.115	-0.264	no
CK-40d	RHSIM_Rhsim08G0235700	0.107	0.013	-0.094	no
CK-40d	RHSIM_Rhsim06G0212600	2.821	1.913	-0.908	high
CK-40d	RHSIM_Rhsim07G0018400	1.148	1.310	0.162	high
CK-40d	RHSIM_Rhsim07G0184200	0.574	0.644	0.070	no
CK-40d	RHSIM_Rhsim06G0002800	-0.151	0.823	0.974	no

Group	Gene	PTR (z-score difference)	z(log2 LFQ)	z(log2 FPKM)	extreme-PTR
CK-40d	RHSIM_Rhsim07G0099200	0.768	-0.288	-1.056	no
CK-40d	RHSIM_Rhsim07G0258700	-0.086	-0.729	-0.642	no
CK-40d	RHSIM_Rhsim07G0045900	-0.494	-0.912	-0.418	no
CK-40d	RHSIM_Rhsim07G0087900	1.336	-0.066	-1.402	high
CK-40d	RHSIM_Rhsim08G0071000	-2.110	-0.124	1.986	low
CK-40d	RHSIM_Rhsim08G0105200	-1.239	-0.076	1.162	low
CK-40d	RHSIM_Rhsim08G0186700	-1.007	-1.006	0.000	no
CK-40d	RHSIM_Rhsim06G0244300	0.674	1.002	0.328	no
CK-40d	RHSIM_Rhsim07G0033700	1.240	-0.295	-1.536	high
CK-40d	RHSIM_Rhsim08G0167500	0.518	1.032	0.514	no
CK-40d	RHSIM_Rhsim07G0030200	-1.019	-0.748	0.271	no
CK-40d	RHSIM_Rhsim08G0171100	1.086	0.260	-0.827	no
CK-40d	RHSIM_Rhsim07G0083900	-0.380	0.260	0.640	no
CK-40d	RHSIM_Rhsim08G0226300	-0.845	-0.970	-0.125	no
CK-40d	RHSIM_Rhsim07G0017800	-0.623	-1.258	-0.635	no
CK-40d	RHSIM_Rhsim07G0027500	0.258	-0.512	-0.770	no
CK-40d	RHSIM_Rhsim07G0202800	-1.140	-0.192	0.949	no
CK-40d	RHSIM_Rhsim06G0052900	-0.837	0.844	1.681	no
CK-40d	RHSIM_Rhsim08G0118600	-0.040	0.218	0.258	no
CK-40d	RHSIM_Rhsim07G0119300	0.230	0.775	0.545	no
CK-40d	RHSIM_Rhsim07G0049600	-0.184	1.314	1.499	no
CK-40d	RHSIM_Rhsim06G0207400	0.961	0.505	-0.457	no
CK-40d	RHSIM_Rhsim07G0056500	-0.880	-0.001	0.879	no
CK-40d	RHSIM_Rhsim07G0103100	0.235	1.209	0.974	no
CK-40d	RHSIM_Rhsim07G0171800	0.468	0.244	-0.223	no
CK-40d	RHSIM_Rhsim07G0121100	-0.716	-0.056	0.659	no
CK-40d	RHSIM_Rhsim08G0036900	0.475	0.598	0.124	no
CK-40d	RHSIM_Rhsim07G0165200	1.010	-0.974	-1.984	no
CK-40d	RHSIM_Rhsim08G0097800	1.566	1.586	0.020	high
CK-40d	RHSIM_Rhsim06G0077600	0.654	-0.206	-0.860	no
CK-40d	RHSIM_Rhsim07G0020500	-0.333	0.164	0.496	no
CK-40d	RHSIM_Rhsim07G0045200	2.344	0.941	-1.403	high

Group	Gene	PTR (z-score difference)	z(log2 LFQ)	z(log2 FPKM)	extreme-PTR
CK-40d	RHSIM_Rhsim06G0218800	-1.866	-1.343	0.524	low
CK-40d	RHSIM_Rhsim07G0067300	0.843	0.275	-0.568	no
CK-40d	RHSIM_Rhsim07G0188200	-0.749	-0.582	0.166	no
CK-40d	RHSIM_Rhsim07G0228100	-2.822	-1.868	0.954	low
CK-40d	RHSIM_Rhsim07G0245900	-1.176	-0.982	0.194	no
CK-40d	RHSIM_Rhsim06G0187500	-0.459	-0.668	-0.209	no
CK-40d	RHSIM_Rhsim06G0105400	-2.449	-1.754	0.695	low
CK-40d	RHSIM_Rhsim08G0152800	0.797	-0.362	-1.159	no
CK-40d	RHSIM_Rhsim08G0171200	-1.025	-1.238	-0.214	no
CK-40d	RHSIM_Rhsim07G0092600	-2.521	-0.639	1.881	low
CK-40d	RHSIM_Rhsim07G0204300	0.037	0.435	0.398	no
CK-40d	RHSIM_Rhsim07G0081900	0.955	0.674	-0.281	no
CK-40d	RHSIM_Rhsim08G0138500	0.020	0.177	0.158	no
CK-40d	RHSIM_Rhsim07G0055600	1.789	1.904	0.114	high
CK-40d	RHSIM_Rhsim07G0125500	-0.493	-1.129	-0.636	no
CK-40d	RHSIM_Rhsim06G0222300	2.362	1.635	-0.726	high
CK-40d	RHSIM_Rhsim07G0214600	-1.399	-1.432	-0.033	low
CK-40d	RHSIM_Rhsim07G0075900	0.356	0.872	0.516	no
CK-40d	RHSIM_Rhsim07G0074000	0.685	0.436	-0.249	no
CK-40d	RHSIM_Rhsim06G0191200	-0.269	-0.509	-0.239	no
CK-40d	RHSIM_Rhsim08G0101100	-0.600	-1.185	-0.586	no
CK-40d	RHSIM_Rhsim08G0183600	-0.033	-0.091	-0.058	no
CK-40d	RHSIM_Rhsim06G0178000	1.471	0.263	-1.208	high
CK-40d	RHSIM_Rhsim07G0179500	1.272	0.328	-0.944	high
CK-40d	RHSIM_Rhsim08G0196600	0.428	0.458	0.030	no
CK-40d	RHSIM_Rhsim06G0035000	0.346	0.132	-0.214	no
CK-40d	RHSIM_Rhsim07G0059500	1.482	-0.874	-2.356	high
CK-40d	RHSIM_Rhsim07G0228600	-1.067	-0.494	0.574	no
CK-40d	RHSIM_Rhsim06G0046200	0.719	-0.439	-1.158	no
CK-40d	RHSIM_Rhsim07G0033000	-0.165	-0.551	-0.386	no
CK-40d	RHSIM_Rhsim06G0167500	-1.820	1.000	2.820	low
CK-40d	RHSIM_Rhsim07G0004400	0.097	0.283	0.186	no

Group	Gene	PTR (z-score difference)	z(log2 LFQ)	z(log2 FPKM)	extreme-PTR
CK-40d	RHSIM_Rhsim06G0000200	-0.285	-0.417	-0.132	no
CK-40d	RHSIM_Rhsim07G0241100	1.569	-0.700	-2.269	high
CK-40d	RHSIM_Rhsim06G0034400	1.145	1.192	0.047	high
CK-40d	RHSIM_Rhsim06G0138800	0.143	0.556	0.414	no
CK-40d	RHSIM_Rhsim08G0242800	0.720	-0.260	-0.980	no
CK-40d	RHSIM_Rhsim07G0178300	3.119	3.265	0.146	high
CK-40d	RHSIM_Rhsim07G0013700	-0.709	0.266	0.976	no
CK-40d	RHSIM_Rhsim06G0003200	1.775	1.291	-0.485	high
CK-40d	RHSIM_Rhsim06G0031700	0.235	-0.179	-0.414	no
CK-40d	RHSIM_Rhsim08G0090800	0.895	-0.386	-1.281	no
CK-40d	RHSIM_Rhsim07G0028000	-0.665	-0.302	0.363	no
CK-40d	RHSIM_Rhsim07G0087100	-0.174	-0.028	0.147	no
CK-40d	RHSIM_Rhsim08G0222200	-1.078	-0.814	0.264	no
CK-40d	RHSIM_Rhsim07G0169100	0.082	0.797	0.715	no
CK-40d	RHSIM_Rhsim06G0119000	-0.271	-2.143	-1.871	no
CK-40d	RHSIM_Rhsim08G0251700	1.318	0.707	-0.611	high
CK-40d	RHSIM_Rhsim07G0047300	-0.047	-0.858	-0.811	no
CK-40d	RHSIM_Rhsim08G0236600	1.343	0.660	-0.683	high
CK-40d	RHSIM_Rhsim07G0110300	-0.560	1.107	1.666	no
CK-40d	RHSIM_Rhsim07G0226700	-1.429	-0.364	1.065	low
CK-40d	RHSIM_Rhsim07G0081200	0.192	0.020	-0.171	no
CK-40d	RHSIM_Rhsim06G0193100	0.467	1.300	0.833	no
CK-40d	RHSIM_Rhsim08G0036600	1.402	0.055	-1.346	high
CK-40d	RHSIM_Rhsim07G0139000	1.453	0.906	-0.547	high
CK-40d	RHSIM_Rhsim07G0137200	-1.498	-1.767	-0.269	low
CK-40d	RHSIM_Rhsim07G0103400	-0.440	-0.196	0.244	no
CK-40d	RHSIM_Rhsim07G0139200	-0.623	-0.625	-0.001	no
CK-40d	RHSIM_Rhsim07G0210400	0.945	2.131	1.186	no
CK-40d	RHSIM_Rhsim07G0072200	0.132	-0.316	-0.448	no
CK-40d	RHSIM_Rhsim06G0070400	0.994	0.775	-0.219	no
CK-40d	RHSIM_Rhsim07G0197900	-2.082	1.580	3.662	low
CK-40d	RHSIM_Rhsim07G0130000	0.642	-0.557	-1.198	no

Group	Gene	PTR (z-score difference)	z(log2 LFQ)	z(log2 FPKM)	extreme-PTR
CK-40d	RHSIM_Rhsim08G0190500	-0.868	0.053	0.921	no
CK-40d	RHSIM_Rhsim07G0236500	-1.146	-0.819	0.326	no
CK-40d	RHSIM_Rhsim07G0110500	-1.209	-0.877	0.333	no
CK-40d	RHSIM_Rhsim06G0205600	-0.613	-0.174	0.439	no
CK-40d	RHSIM_Rhsim08G0240800	0.263	-0.046	-0.309	no
CK-40d	RHSIM_Rhsim07G0035600	-0.748	-0.171	0.578	no
CK-40d	RHSIM_Rhsim06G0237100	-1.397	-0.939	0.458	low
CK-40d	RHSIM_Rhsim07G0249300	3.000	0.969	-2.030	high
CK-40d	RHSIM_Rhsim06G0047700	-0.123	1.150	1.273	no
CK-40d	RHSIM_Rhsim06G0242300	1.177	0.945	-0.232	high
CK-40d	RHSIM_Rhsim07G0018200	0.443	0.450	0.006	no
CK-40d	RHSIM_Rhsim07G0178100	-0.367	-0.869	-0.502	no
CK-40d	RHSIM_Rhsim05G0060700	-0.069	0.832	0.901	no
CK-40d	RHSIM_Rhsim06G0169300	0.344	1.215	0.872	no
CK-40d	RHSIM_Rhsim07G0092700	1.411	1.518	0.106	high
CK-40d	RHSIM_Rhsim07G0072000	-1.640	-0.732	0.908	low
CK-40d	RHSIM_Rhsim07G0046600	-0.285	0.286	0.571	no
CK-40d	RHSIM_Rhsim07G0059000	1.811	0.987	-0.824	high
CK-40d	RHSIM_Rhsim05G0194700	0.202	0.811	0.610	no
CK-40d	RHSIM_Rhsim05G0122800	0.937	0.479	-0.457	no
CK-40d	RHSIM_Rhsim06G0028600	0.914	-0.358	-1.272	no
CK-40d	RHSIM_Rhsim07G0204200	-1.902	-1.288	0.613	low
CK-40d	RHSIM_Rhsim07G0237600	-0.281	-0.332	-0.051	no
CK-40d	RHSIM_Rhsim07G0247500	0.004	0.215	0.212	no
CK-40d	RHSIM_Rhsim05G0101000	0.536	0.326	-0.210	no
CK-40d	RHSIM_Rhsim07G0012900	-1.840	-0.489	1.351	low
CK-40d	RHSIM_Rhsim07G0026400	-1.597	1.609	3.206	low
CK-40d	RHSIM_Rhsim07G0125200	-0.060	-1.358	-1.297	no
CK-40d	RHSIM_Rhsim07G0117900	0.481	1.145	0.663	no
CK-40d	RHSIM_Rhsim07G0150800	-0.115	0.121	0.236	no
CK-40d	RHSIM_Rhsim07G0054900	1.232	0.044	-1.189	high
CK-40d	RHSIM_Rhsim07G0006300	-2.102	-1.436	0.666	low

Group	Gene	PTR (z-score difference)	z(log2 LFQ)	z(log2 FPKM)	extreme-PTR
CK-40d	RHSIM_Rhsim05G0217100	-0.256	-0.949	-0.693	no
CK-40d	RHSIM_Rhsim07G0100900	-1.260	-0.872	0.388	low
CK-40d	RHSIM_Rhsim07G0210900	0.361	1.159	0.798	no
CK-40d	RHSIM_Rhsim07G0179400	-1.410	-1.081	0.328	low
CK-40d	RHSIM_Rhsim07G0254300	-1.605	-1.857	-0.252	low
CK-40d	RHSIM_Rhsim07G0082100	1.191	2.365	1.174	high
CK-40d	RHSIM_Rhsim07G0048700	-0.888	-2.243	-1.355	no
CK-40d	RHSIM_Rhsim06G0064500	-0.464	-0.562	-0.099	no
CK-40d	RHSIM_Rhsim07G0083200	1.660	0.412	-1.248	high
CK-40d	RHSIM_Rhsim06G0090400	-0.713	-0.265	0.448	no
CK-40d	RHSIM_Rhsim06G0013400	0.259	0.762	0.502	no
CK-40d	RHSIM_Rhsim05G0134300	0.523	-0.300	-0.823	no
CK-40d	RHSIM_Rhsim06G0033500	-0.292	-0.529	-0.237	no
CK-40d	RHSIM_Rhsim05G0088500	0.972	1.474	0.503	no
CK-40d	RHSIM_Rhsim06G0055200	-3.243	-1.366	1.876	low
CK-40d	RHSIM_Rhsim06G0089300	-2.321	-1.896	0.425	low
CK-40d	RHSIM_Rhsim06G0122800	-0.284	-0.610	-0.326	no
CK-40d	RHSIM_Rhsim07G0091600	1.468	0.059	-1.409	high
CK-40d	RHSIM_Rhsim07G0129700	-0.768	0.081	0.849	no
CK-40d	RHSIM_Rhsim06G0072400	0.113	0.432	0.318	no
CK-40d	RHSIM_Rhsim07G0205000	2.523	2.754	0.231	high
CK-40d	RHSIM_Rhsim05G0061100	0.888	-0.656	-1.544	no
CK-40d	RHSIM_Rhsim05G0111600	0.186	-0.714	-0.900	no
CK-40d	RHSIM_Rhsim06G0084600	-1.568	-1.113	0.455	low
CK-40d	RHSIM_Rhsim07G0092900	-1.200	0.195	1.395	no
CK-40d	RHSIM_Rhsim06G0151500	-1.796	0.673	2.469	low
CK-40d	RHSIM_Rhsim06G0238800	-0.745	-0.193	0.552	no
CK-40d	RHSIM_Rhsim05G0011300	-1.495	-1.018	0.477	low
CK-40d	RHSIM_Rhsim07G0071400	-0.272	0.003	0.275	no
CK-40d	RHSIM_Rhsim07G0087700	0.407	1.325	0.917	no
CK-40d	RHSIM_Rhsim05G0120100	-1.825	-1.572	0.253	low
CK-40d	RHSIM_Rhsim06G0161000	-1.171	-1.092	0.078	no

Group	Gene	PTR (z-score difference)	z(log2 LFQ)	z(log2 FPKM)	extreme-PTR
CK-40d	RHSIM_Rhsim07G0225500	0.198	0.033	-0.165	no
CK-40d	RHSIM_Rhsim05G0232300	-0.270	1.033	1.303	no
CK-40d	RHSIM_Rhsim07G0047800	0.889	0.937	0.048	no
CK-40d	RHSIM_Rhsim06G0205800	-0.481	0.774	1.256	no
CK-40d	RHSIM_Rhsim07G0189000	-0.750	0.515	1.265	no
CK-40d	RHSIM_Rhsim06G0004200	1.411	1.021	-0.389	high
CK-40d	RHSIM_Rhsim07G0013800	-1.988	-0.964	1.024	low
CK-40d	RHSIM_Rhsim06G0098800	-0.082	-0.614	-0.532	no
CK-40d	RHSIM_Rhsim06G0074700	-0.507	0.551	1.058	no
CK-40d	RHSIM_Rhsim06G0177400	-0.027	-0.290	-0.263	no
CK-40d	RHSIM_Rhsim06G0026100	-2.256	-0.704	1.552	low
CK-40d	RHSIM_Rhsim07G0153500	-0.902	-0.735	0.167	no
CK-40d	RHSIM_Rhsim06G0207300	0.033	-0.387	-0.420	no
CK-40d	RHSIM_Rhsim07G0066700	-0.520	-1.540	-1.020	no
CK-40d	RHSIM_Rhsim05G0165300	-0.983	-0.831	0.151	no
CK-40d	RHSIM_Rhsim06G0137600	2.433	1.434	-0.999	high
CK-40d	RHSIM_Rhsim07G0051300	3.948	2.763	-1.185	high
CK-40d	RHSIM_Rhsim06G0116000	0.749	0.606	-0.142	no
CK-40d	RHSIM_Rhsim07G0258000	-1.019	-0.889	0.130	no
CK-40d	RHSIM_Rhsim07G0074300	0.111	0.049	-0.061	no
CK-40d	RHSIM_Rhsim06G0203600	1.850	1.065	-0.785	high
CK-40d	RHSIM_Rhsim05G0216500	-2.629	0.373	3.002	low
CK-40d	RHSIM_Rhsim07G0049700	-2.854	-0.175	2.679	low
CK-40d	RHSIM_Rhsim06G0032800	-0.438	0.123	0.561	no
CK-40d	RHSIM_Rhsim05G0004900	2.223	1.052	-1.171	high
CK-40d	RHSIM_Rhsim07G0071800	-1.794	-1.745	0.048	low
CK-40d	RHSIM_Rhsim06G0205900	0.757	0.365	-0.391	no
CK-40d	RHSIM_Rhsim05G0161600	0.589	0.300	-0.289	no
CK-40d	RHSIM_Rhsim06G0204300	-1.091	-1.513	-0.422	no
CK-40d	RHSIM_Rhsim06G0071400	-0.580	-1.522	-0.942	no
CK-40d	RHSIM_Rhsim06G0223100	0.163	0.924	0.760	no
CK-40d	RHSIM_Rhsim06G0130900	0.357	-0.448	-0.805	no

Group	Gene	PTR (z-score difference)	z(log2 LFQ)	z(log2 FPKM)	extreme-PTR
CK-40d	RHSIM_Rhsim06G0025000	0.913	-0.244	-1.157	no
CK-40d	RHSIM_Rhsim06G0165200	-0.110	-0.821	-0.711	no
CK-40d	RHSIM_Rhsim06G0003000	-1.544	-1.634	-0.090	low
CK-40d	RHSIM_Rhsim07G0203600	-1.371	0.370	1.740	low
CK-40d	RHSIM_Rhsim06G0027400	1.000	-0.970	-1.970	no
CK-40d	RHSIM_Rhsim06G0069500	-1.039	-0.785	0.254	no
CK-40d	RHSIM_Rhsim07G0187800	-0.665	0.814	1.479	no
CK-40d	RHSIM_Rhsim05G0104300	-1.093	-0.265	0.828	no
CK-40d	RHSIM_Rhsim05G0167900	-1.137	-0.423	0.714	no
CK-40d	RHSIM_Rhsim06G0034800	-0.363	0.373	0.735	no
CK-40d	RHSIM_Rhsim07G0019300	-0.285	1.939	2.225	no
CK-40d	RHSIM_Rhsim07G0129500	-0.862	-1.065	-0.203	no
CK-40d	RHSIM_Rhsim06G0193700	0.283	-0.565	-0.849	no
CK-40d	RHSIM_Rhsim05G0106800	-0.253	0.791	1.044	no
CK-40d	RHSIM_Rhsim07G0101400	-1.099	-0.604	0.495	no
CK-40d	RHSIM_Rhsim06G0243000	2.305	2.288	-0.018	high
CK-40d	RHSIM_Rhsim06G0205300	0.982	1.841	0.859	no
CK-40d	RHSIM_Rhsim06G0167300	-0.564	0.798	1.362	no
CK-40d	RHSIM_Rhsim06G0078100	0.903	-0.182	-1.085	no
CK-40d	RHSIM_Rhsim07G0143200	0.101	1.017	0.916	no
CK-40d	RHSIM_Rhsim06G0126000	0.423	0.029	-0.394	no
CK-40d	RHSIM_Rhsim06G0212000	0.932	-0.528	-1.460	no
CK-40d	RHSIM_Rhsim06G0176500	1.192	-0.288	-1.481	high
CK-40d	RHSIM_Rhsim06G0191300	0.146	-0.995	-1.140	no
CK-40d	RHSIM_Rhsim06G0226100	-0.467	-0.344	0.123	no
CK-40d	RHSIM_Rhsim07G0134200	-1.342	-0.886	0.456	low
CK-40d	RHSIM_Rhsim05G0132600	0.247	1.555	1.307	no
CK-40d	RHSIM_Rhsim06G0006900	2.446	0.611	-1.835	high
CK-40d	RHSIM_Rhsim07G0082500	0.305	1.455	1.150	no
CK-40d	RHSIM_Rhsim06G0201700	0.313	-0.894	-1.208	no
CK-40d	RHSIM_Rhsim07G0131300	1.498	2.249	0.750	high
CK-40d	RHSIM_Rhsim06G0001500	1.651	0.404	-1.247	high

Group	Gene	PTR (z-score difference)	z(log2 LFQ)	z(log2 FPKM)	extreme-PTR
CK-40d	RHSIM_Rhsim06G0236000	-0.218	-0.955	-0.737	no
CK-40d	RHSIM_Rhsim06G0236100	-0.357	-0.697	-0.340	no
CK-40d	RHSIM_Rhsim06G0007900	-0.324	-0.656	-0.332	no
CK-40d	RHSIM_Rhsim06G0184600	-0.523	-0.904	-0.381	no
CK-40d	RHSIM_Rhsim07G0061200	-1.711	-1.427	0.284	low
CK-40d	RHSIM_Rhsim06G0163900	0.657	0.800	0.143	no
CK-40d	RHSIM_Rhsim05G0182800	0.280	0.109	-0.171	no
CK-40d	RHSIM_Rhsim06G0035200	-0.762	-1.201	-0.439	no
CK-40d	RHSIM_Rhsim05G0142700	0.620	0.460	-0.160	no
CK-40d	RHSIM_Rhsim07G0053300	1.327	1.533	0.206	high
CK-40d	RHSIM_Rhsim06G0129400	-2.515	-1.809	0.706	low
CK-40d	RHSIM_Rhsim05G0041300	-0.407	-0.169	0.238	no
CK-40d	RHSIM_Rhsim06G0026800	-0.443	-1.058	-0.615	no
CK-40d	RHSIM_Rhsim07G0020400	-1.029	-1.350	-0.321	no
CK-40d	RHSIM_Rhsim07G0078500	0.853	-1.362	-2.215	no
CK-40d	RHSIM_Rhsim06G0194400	2.844	0.993	-1.852	high
CK-40d	RHSIM_Rhsim06G0191000	0.514	1.729	1.215	no
CK-40d	RHSIM_Rhsim05G0167300	1.227	1.495	0.267	high
CK-40d	RHSIM_Rhsim06G0226700	0.865	1.061	0.196	no
CK-40d	RHSIM_Rhsim05G0062900	0.421	-1.084	-1.505	no
CK-40d	RHSIM_Rhsim07G0019100	-1.913	-0.983	0.931	low
CK-40d	RHSIM_Rhsim07G0064900	0.549	-0.275	-0.825	no
CK-40d	RHSIM_Rhsim06G0055300	0.554	1.486	0.932	no
CK-40d	RHSIM_Rhsim07G0053700	0.580	-0.432	-1.012	no
CK-40d	RHSIM_Rhsim06G0041800	0.001	-0.173	-0.174	no
CK-40d	RHSIM_Rhsim06G0047900	0.027	0.385	0.357	no
CK-40d	RHSIM_Rhsim07G0090200	-0.481	1.374	1.855	no
CK-40d	RHSIM_Rhsim05G0002000	-0.708	-1.011	-0.303	no
CK-40d	RHSIM_Rhsim06G0171700	1.013	0.563	-0.450	no
CK-40d	RHSIM_Rhsim07G0026500	0.532	0.201	-0.330	no
CK-40d	RHSIM_Rhsim07G0198500	1.226	-0.299	-1.525	high
CK-40d	RHSIM_Rhsim05G0139700	2.488	0.484	-2.004	high

Group	Gene	PTR (z-score difference)	z(log2 LFQ)	z(log2 FPKM)	extreme-PTR
CK-40d	RHSIM_Rhsim06G0159400	0.555	0.439	-0.115	no
CK-40d	RHSIM_Rhsim06G0240800	1.744	0.092	-1.652	high
CK-40d	RHSIM_Rhsim05G0231800	2.166	0.640	-1.525	high
CK-40d	RHSIM_Rhsim06G0049600	0.634	0.004	-0.631	no
CK-40d	RHSIM_Rhsim05G0143600	-0.642	-0.559	0.083	no
CK-40d	RHSIM_Rhsim07G0039100	1.686	2.106	0.420	high
CK-40d	RHSIM_Rhsim07G0044600	2.220	-0.214	-2.434	high
CK-40d	RHSIM_Rhsim05G0148200	0.447	0.146	-0.301	no
CK-40d	RHSIM_Rhsim06G0122300	-1.205	-0.256	0.948	no
CK-40d	RHSIM_Rhsim07G0113300	0.188	0.964	0.776	no
CK-40d	RHSIM_Rhsim07G0145600	0.609	0.303	-0.306	no
CK-40d	RHSIM_Rhsim06G0092600	-0.057	-0.532	-0.474	no
CK-40d	RHSIM_Rhsim05G0096400	0.105	1.185	1.080	no
CK-40d	RHSIM_Rhsim07G0036300	1.113	0.157	-0.955	no
CK-40d	RHSIM_Rhsim06G0218900	-1.311	-1.030	0.281	low
CK-40d	RHSIM_Rhsim05G0163000	0.646	-0.104	-0.751	no
CK-40d	RHSIM_Rhsim06G0046700	-2.419	-1.462	0.957	low
CK-40d	RHSIM_Rhsim06G0131500	1.388	2.900	1.513	high
CK-40d	RHSIM_Rhsim06G0054800	-1.828	1.283	3.111	low
CK-40d	RHSIM_Rhsim06G0087800	-0.891	-0.681	0.210	no
CK-40d	RHSIM_Rhsim04G0083400	-0.526	0.455	0.981	no
CK-40d	RHSIM_Rhsim06G0002900	-0.537	0.406	0.943	no
CK-40d	RHSIM_Rhsim06G0130100	0.943	1.000	0.057	no
CK-40d	RHSIM_Rhsim04G0085300	-2.393	-1.139	1.254	low
CK-40d	RHSIM_Rhsim06G0229100	-1.112	-0.748	0.365	no
CK-40d	RHSIM_Rhsim06G0178400	-0.814	0.341	1.155	no
CK-40d	RHSIM_Rhsim06G0135300	-0.594	0.081	0.676	no
CK-40d	RHSIM_Rhsim04G0169200	-0.859	-0.306	0.552	no
CK-40d	RHSIM_Rhsim05G0017200	-0.935	-0.969	-0.034	no
CK-40d	RHSIM_Rhsim05G0185400	-0.247	-0.587	-0.340	no
CK-40d	RHSIM_Rhsim04G0010400	0.301	0.246	-0.055	no
CK-40d	RHSIM_Rhsim06G0121800	-1.372	-1.749	-0.377	low

Group	Gene	PTR (z-score difference)	z(log2 LFQ)	z(log2 FPKM)	extreme-PTR
CK-40d	RHSIM_Rhsim05G0030300	0.551	1.277	0.726	no
CK-40d	RHSIM_Rhsim05G0059500	1.545	-0.473	-2.018	high
CK-40d	RHSIM_Rhsim04G0118700	0.494	0.152	-0.341	no
CK-40d	RHSIM_Rhsim05G0139000	-1.192	-0.530	0.662	no
CK-40d	RHSIM_Rhsim06G0099300	0.577	-0.285	-0.862	no
CK-40d	RHSIM_Rhsim05G0103900	0.383	0.742	0.359	no
CK-40d	RHSIM_Rhsim06G0146500	1.034	1.704	0.670	no
CK-40d	RHSIM_Rhsim06G0057900	-0.162	-0.477	-0.315	no
CK-40d	RHSIM_Rhsim05G0089600	-1.993	-1.435	0.558	low
CK-40d	RHSIM_Rhsim05G0013800	0.051	0.330	0.279	no
CK-40d	RHSIM_Rhsim06G0156300	-1.147	-0.055	1.092	no
CK-40d	RHSIM_Rhsim05G0050000	1.671	0.798	-0.873	high
CK-40d	RHSIM_Rhsim06G0172700	-3.386	-1.285	2.100	low
CK-40d	RHSIM_Rhsim05G0180000	0.810	-0.745	-1.556	no
CK-40d	RHSIM_Rhsim06G0185200	0.634	1.080	0.446	no
CK-40d	RHSIM_Rhsim05G0020600	-0.288	-0.042	0.246	no
CK-40d	RHSIM_Rhsim04G0213800	0.662	0.754	0.092	no
CK-40d	RHSIM_Rhsim05G0069400	1.027	-0.237	-1.264	no
CK-40d	RHSIM_Rhsim05G0093200	0.609	-0.659	-1.268	no
CK-40d	RHSIM_Rhsim05G0207300	0.181	-1.092	-1.272	no
CK-40d	RHSIM_Rhsim05G0035000	0.690	1.300	0.610	no
CK-40d	RHSIM_Rhsim04G0032800	0.071	2.076	2.005	no
CK-40d	RHSIM_Rhsim06G0020600	-2.432	-0.651	1.782	low
CK-40d	RHSIM_Rhsim05G0118000	-0.041	-0.298	-0.257	no
CK-40d	RHSIM_Rhsim06G0235200	-0.807	-1.274	-0.467	no
CK-40d	RHSIM_Rhsim05G0064500	0.404	1.038	0.633	no
CK-40d	RHSIM_Rhsim05G0012000	-0.626	1.722	2.348	no
CK-40d	RHSIM_Rhsim06G0066600	0.865	-0.633	-1.499	no
CK-40d	RHSIM_Rhsim05G0022300	1.113	0.766	-0.347	no
CK-40d	RHSIM_Rhsim05G0054100	-0.662	-0.510	0.152	no
CK-40d	RHSIM_Rhsim05G0161200	0.224	0.017	-0.206	no
CK-40d	RHSIM_Rhsim06G0197400	1.086	1.894	0.809	no

Group	Gene	PTR (z-score difference)	z(log2 LFQ)	z(log2 FPKM)	extreme-PTR
CK-40d	RHSIM_Rhsim05G0070000	0.208	0.794	0.587	no
CK-40d	RHSIM_Rhsim06G0049800	-0.966	0.244	1.210	no
CK-40d	RHSIM_Rhsim04G0091600	0.151	0.344	0.194	no
CK-40d	RHSIM_Rhsim06G0073600	0.322	0.918	0.596	no
CK-40d	RHSIM_Rhsim06G0212900	1.307	0.611	-0.696	high
CK-40d	RHSIM_Rhsim05G0174500	-3.120	-0.785	2.335	low
CK-40d	RHSIM_Rhsim05G0223200	0.785	-0.987	-1.773	no
CK-40d	RHSIM_Rhsim05G0012400	0.953	0.726	-0.227	no
CK-40d	RHSIM_Rhsim06G0049500	1.020	1.070	0.050	no
CK-40d	RHSIM_Rhsim05G0000600	1.277	-0.370	-1.646	high
CK-40d	RHSIM_Rhsim05G0202500	0.717	-0.072	-0.789	no
CK-40d	RHSIM_Rhsim06G0086900	-1.332	-1.214	0.118	low
CK-40d	RHSIM_Rhsim05G0049500	-0.037	-0.832	-0.795	no
CK-40d	RHSIM_Rhsim05G0070900	0.573	1.181	0.608	no
CK-40d	RHSIM_Rhsim05G0129000	0.138	-1.923	-2.061	no
CK-40d	RHSIM_Rhsim04G0246500	2.548	0.405	-2.143	high
CK-40d	RHSIM_Rhsim06G0012100	-0.336	0.138	0.474	no
CK-40d	RHSIM_Rhsim05G0011500	0.469	1.295	0.826	no
CK-40d	RHSIM_Rhsim06G0236300	-0.482	0.013	0.495	no
CK-40d	RHSIM_Rhsim05G0063200	1.787	1.561	-0.226	high
CK-40d	RHSIM_Rhsim05G0180400	0.894	2.547	1.653	no
CK-40d	RHSIM_Rhsim05G0137700	-0.192	-0.070	0.122	no
CK-40d	RHSIM_Rhsim04G0243100	0.775	1.361	0.586	no
CK-40d	RHSIM_Rhsim04G0234700	-0.104	0.006	0.110	no
CK-40d	RHSIM_Rhsim05G0202800	2.006	0.974	-1.032	high
CK-40d	RHSIM_Rhsim05G0163600	0.162	-0.689	-0.851	no
CK-40d	RHSIM_Rhsim06G0037800	-0.616	0.514	1.130	no
CK-40d	RHSIM_Rhsim05G0079600	0.133	0.013	-0.121	no
CK-40d	RHSIM_Rhsim04G0016100	1.666	0.837	-0.829	high
CK-40d	RHSIM_Rhsim05G0088400	0.723	0.725	0.001	no
CK-40d	RHSIM_Rhsim04G0146600	-0.540	-0.816	-0.276	no
CK-40d	RHSIM_Rhsim06G0161200	0.751	0.870	0.119	no

Group	Gene	PTR (z-score difference)	z(log2 LFQ)	z(log2 FPKM)	extreme-PTR
CK-40d	RHSIM_Rhsim06G0093300	-1.044	-0.801	0.243	no
CK-40d	RHSIM_Rhsim06G0011900	0.438	-0.878	-1.316	no
CK-40d	RHSIM_Rhsim06G0060100	-0.363	-0.215	0.147	no
CK-40d	RHSIM_Rhsim06G0047800	-0.174	0.990	1.163	no
CK-40d	RHSIM_Rhsim05G0062500	0.850	0.195	-0.655	no
CK-40d	RHSIM_Rhsim06G0186600	0.145	-0.171	-0.316	no
CK-40d	RHSIM_Rhsim06G0237500	-0.615	-0.773	-0.158	no
CK-40d	RHSIM_Rhsim06G0166400	-0.729	-1.059	-0.330	no
CK-40d	RHSIM_Rhsim05G0224600	-0.592	-0.052	0.540	no
CK-40d	RHSIM_Rhsim06G0072000	-0.374	0.405	0.779	no
CK-40d	RHSIM_Rhsim06G0102100	0.780	1.515	0.736	no
CK-40d	RHSIM_Rhsim04G0102500	2.496	0.229	-2.268	high
CK-40d	RHSIM_Rhsim05G0230900	1.396	0.999	-0.396	high
CK-40d	RHSIM_Rhsim06G0014600	2.945	0.993	-1.952	high
CK-40d	RHSIM_Rhsim04G0118500	1.083	0.566	-0.517	no
CK-40d	RHSIM_Rhsim06G0038600	-0.085	2.254	2.339	no
CK-40d	RHSIM_Rhsim06G0077300	-0.009	0.515	0.523	no
CK-40d	RHSIM_Rhsim06G0214800	1.606	0.932	-0.674	high
CK-40d	RHSIM_Rhsim05G0169300	-1.074	-1.437	-0.363	no
CK-40d	RHSIM_Rhsim04G0101900	-0.326	1.368	1.694	no
CK-40d	RHSIM_Rhsim04G0149800	0.395	-0.285	-0.680	no
CK-40d	RHSIM_Rhsim06G0116800	-0.111	0.096	0.207	no
CK-40d	RHSIM_Rhsim04G0119100	2.070	1.062	-1.008	high
CK-40d	RHSIM_Rhsim05G0108700	-0.721	-1.194	-0.473	no
CK-40d	RHSIM_Rhsim04G0186900	0.733	-0.393	-1.126	no
CK-40d	RHSIM_Rhsim06G0040500	-1.063	-0.930	0.133	no
CK-40d	RHSIM_Rhsim06G0076100	-0.289	1.682	1.971	no
CK-40d	RHSIM_Rhsim05G0200500	-1.796	0.900	2.696	low
CK-40d	RHSIM_Rhsim06G0007500	-0.314	-1.008	-0.694	no
CK-40d	RHSIM_Rhsim05G0114400	1.337	-0.126	-1.464	high
CK-40d	RHSIM_Rhsim06G0110000	0.632	0.319	-0.313	no
CK-40d	RHSIM_Rhsim04G0179200	-1.372	-1.888	-0.516	low

Group	Gene	PTR (z-score difference)	z(log2 LFQ)	z(log2 FPKM)	extreme-PTR
CK-40d	RHSIM_Rhsim05G0101300	-0.533	-1.112	-0.579	no
CK-40d	RHSIM_Rhsim06G0057200	0.435	-0.046	-0.482	no
CK-40d	RHSIM_Rhsim05G0031300	-1.348	0.485	1.833	low
CK-40d	RHSIM_Rhsim04G0126900	1.265	1.013	-0.252	high
CK-40d	RHSIM_Rhsim06G0206900	-0.162	1.029	1.191	no
CK-40d	RHSIM_Rhsim04G0250200	0.401	-1.167	-1.568	no
CK-40d	RHSIM_Rhsim06G0116700	0.132	0.749	0.617	no
CK-40d	RHSIM_Rhsim05G0205700	-0.529	-0.285	0.244	no
CK-40d	RHSIM_Rhsim04G0130200	0.862	0.401	-0.461	no
CK-40d	RHSIM_Rhsim06G0196800	0.736	-0.581	-1.317	no
CK-40d	RHSIM_Rhsim04G0108100	0.809	-0.314	-1.124	no
CK-40d	RHSIM_Rhsim05G0087800	1.010	0.524	-0.486	no
CK-40d	RHSIM_Rhsim04G0070700	-0.528	0.166	0.694	no
CK-40d	RHSIM_Rhsim05G0029900	-1.574	-0.099	1.475	low
CK-40d	RHSIM_Rhsim04G0174900	-0.787	0.169	0.956	no
CK-40d	RHSIM_Rhsim05G0147900	-1.242	-1.344	-0.102	low
CK-40d	RHSIM_Rhsim04G0221500	-1.966	-1.792	0.173	low
CK-40d	RHSIM_Rhsim05G0068000	0.496	1.381	0.885	no
CK-40d	RHSIM_Rhsim05G0062100	0.392	1.778	1.386	no
CK-40d	RHSIM_Rhsim05G0039700	1.966	1.232	-0.734	high
CK-40d	RHSIM_Rhsim05G0146400	0.221	0.006	-0.215	no
CK-40d	RHSIM_Rhsim06G0225700	-0.102	-0.337	-0.235	no
CK-40d	RHSIM_Rhsim05G0208200	0.755	-0.510	-1.266	no
CK-40d	RHSIM_Rhsim06G0018800	-0.096	-0.233	-0.138	no
CK-40d	RHSIM_Rhsim05G0137500	0.284	0.145	-0.138	no
CK-40d	RHSIM_Rhsim05G0170800	-0.310	-1.102	-0.791	no
CK-40d	RHSIM_Rhsim04G0211500	-1.738	-1.008	0.730	low
CK-40d	RHSIM_Rhsim05G0168900	0.075	-1.219	-1.295	no
CK-40d	RHSIM_Rhsim05G0159800	-1.527	-0.288	1.239	low
CK-40d	RHSIM_Rhsim05G0224000	-1.151	0.818	1.969	no
CK-40d	RHSIM_Rhsim05G0163400	0.162	-1.436	-1.598	no
CK-40d	RHSIM_Rhsim06G0201000	-0.652	-0.801	-0.149	no

Group	Gene	PTR (z-score difference)	z(log2 LFQ)	z(log2 FPKM)	extreme-PTR
CK-40d	RHSIM_Rhsim04G0011000	-1.221	-1.322	-0.101	no
CK-40d	RHSIM_Rhsim06G0021800	0.286	-1.560	-1.845	no
CK-40d	RHSIM_Rhsim05G0193800	-0.577	-0.175	0.402	no
CK-40d	RHSIM_Rhsim04G0021400	-0.763	0.083	0.847	no
CK-40d	RHSIM_Rhsim05G0161100	1.280	1.976	0.697	high
CK-40d	RHSIM_Rhsim05G0176200	0.386	-0.620	-1.006	no
CK-40d	RHSIM_Rhsim06G0074900	1.376	0.630	-0.746	high
CK-40d	RHSIM_Rhsim06G0061800	-2.359	-1.611	0.749	low
CK-40d	RHSIM_Rhsim04G0236100	0.443	-0.080	-0.523	no
CK-40d	RHSIM_Rhsim04G0206400	2.144	3.368	1.224	high
CK-40d	RHSIM_Rhsim06G0071000	-0.479	0.085	0.564	no
CK-40d	RHSIM_Rhsim05G0231000	-0.367	0.349	0.715	no
CK-40d	RHSIM_Rhsim05G0116000	0.618	-0.298	-0.916	no
CK-40d	RHSIM_Rhsim04G0024600	-0.275	0.810	1.086	no
CK-40d	RHSIM_Rhsim05G0000700	0.955	-0.300	-1.256	no
CK-40d	RHSIM_Rhsim05G0069500	1.986	0.874	-1.112	high
CK-40d	RHSIM_Rhsim03G0222100	2.069	0.107	-1.962	high
CK-40d	RHSIM_Rhsim05G0146700	1.338	-0.860	-2.199	high
CK-40d	RHSIM_Rhsim04G0167900	0.205	-1.121	-1.326	no
CK-40d	RHSIM_Rhsim03G0253400	0.037	-0.129	-0.167	no
CK-40d	RHSIM_Rhsim05G0069300	0.778	0.045	-0.733	no
CK-40d	RHSIM_Rhsim03G0178900	-1.720	-1.038	0.682	low
CK-40d	RHSIM_Rhsim05G0231600	-1.054	-0.478	0.575	no
CK-40d	RHSIM_Rhsim05G0186700	0.992	0.775	-0.217	no
CK-40d	RHSIM_Rhsim03G0262800	-1.985	-0.939	1.047	low
CK-40d	RHSIM_Rhsim05G0011000	-0.750	-0.636	0.114	no
CK-40d	RHSIM_Rhsim04G0102100	0.217	1.436	1.218	no
CK-40d	RHSIM_Rhsim05G0115500	-0.277	-0.185	0.092	no
CK-40d	RHSIM_Rhsim05G0186400	0.218	-0.474	-0.692	no
CK-40d	RHSIM_Rhsim05G0231200	-1.185	-1.166	0.019	no
CK-40d	RHSIM_Rhsim04G0201600	0.203	-0.410	-0.612	no
CK-40d	RHSIM_Rhsim03G0064000	-0.304	-0.537	-0.233	no

Group	Gene	PTR (z-score difference)	z(log2 LFQ)	z(log2 FPKM)	extreme-PTR
CK-40d	RHSIM_Rhsim05G0043400	0.527	-0.153	-0.681	no
CK-40d	RHSIM_Rhsim05G0167500	-0.781	-1.369	-0.588	no
CK-40d	RHSIM_Rhsim05G0049600	-1.943	0.446	2.390	low
CK-40d	RHSIM_Rhsim05G0203700	1.763	-0.376	-2.139	high
CK-40d	RHSIM_Rhsim04G0132400	1.207	0.883	-0.323	high
CK-40d	RHSIM_Rhsim03G0187300	0.386	-0.052	-0.438	no
CK-40d	RHSIM_Rhsim04G0072400	-0.669	-0.849	-0.180	no
CK-40d	RHSIM_Rhsim04G0097900	0.056	-0.216	-0.272	no
CK-40d	RHSIM_Rhsim05G0189500	-0.997	-0.039	0.958	no
CK-40d	RHSIM_Rhsim05G0196300	-2.104	-0.163	1.941	low
CK-40d	RHSIM_Rhsim05G0072800	0.092	1.165	1.072	no
CK-40d	RHSIM_Rhsim04G0220700	-0.531	-1.487	-0.955	no
CK-40d	RHSIM_Rhsim05G0014300	-1.279	-1.690	-0.411	low
CK-40d	RHSIM_Rhsim04G0075000	-0.868	-0.800	0.069	no
CK-40d	RHSIM_Rhsim05G0102200	0.731	-0.237	-0.968	no
CK-40d	RHSIM_Rhsim05G0204700	-1.107	-1.113	-0.006	no
CK-40d	RHSIM_Rhsim04G0231300	3.303	3.159	-0.145	high
CK-40d	RHSIM_Rhsim05G0148900	-0.331	-0.597	-0.266	no
CK-40d	RHSIM_Rhsim04G0009700	0.562	-0.300	-0.863	no
CK-40d	RHSIM_Rhsim05G0006600	-0.867	-0.173	0.694	no
CK-40d	RHSIM_Rhsim05G0221700	1.222	1.258	0.035	high
CK-40d	RHSIM_Rhsim04G0049800	-1.374	-0.197	1.177	low
CK-40d	RHSIM_Rhsim05G0128900	-0.008	0.302	0.311	no
CK-40d	RHSIM_Rhsim04G0003400	-1.201	-0.329	0.872	no
CK-40d	RHSIM_Rhsim04G0122300	-0.625	1.365	1.989	no
CK-40d	RHSIM_Rhsim05G0067600	0.894	0.549	-0.345	no
CK-40d	RHSIM_Rhsim03G0127100	2.233	1.171	-1.062	high
CK-40d	RHSIM_Rhsim05G0080700	0.874	-0.512	-1.386	no
CK-40d	RHSIM_Rhsim03G0031300	0.293	0.143	-0.151	no
CK-40d	RHSIM_Rhsim05G0144300	0.005	-0.104	-0.109	no
CK-40d	RHSIM_Rhsim05G0184800	-2.220	-0.637	1.582	low
CK-40d	RHSIM_Rhsim04G0151900	0.847	1.797	0.951	no

Group	Gene	PTR (z-score difference)	z(log2 LFQ)	z(log2 FPKM)	extreme-PTR
CK-40d	RHSIM_Rhsim05G0119700	0.081	-0.314	-0.395	no
CK-40d	RHSIM_Rhsim04G0218900	-0.230	-0.778	-0.548	no
CK-40d	RHSIM_Rhsim05G0233000	0.250	0.185	-0.064	no
CK-40d	RHSIM_Rhsim05G0135700	-1.389	-1.787	-0.398	low
CK-40d	RHSIM_Rhsim05G0217500	0.181	1.577	1.396	no
CK-40d	RHSIM_Rhsim04G0162200	2.252	0.920	-1.332	high
CK-40d	RHSIM_Rhsim05G0013300	-1.261	-0.986	0.275	low
CK-40d	RHSIM_Rhsim05G0177800	1.625	-0.420	-2.045	high
CK-40d	RHSIM_Rhsim04G0136600	0.272	0.509	0.237	no
CK-40d	RHSIM_Rhsim04G0157300	0.076	-0.467	-0.543	no
CK-40d	RHSIM_Rhsim03G0036800	-2.191	-1.860	0.331	low
CK-40d	RHSIM_Rhsim03G0064600	-0.550	-0.206	0.344	no
CK-40d	RHSIM_Rhsim05G0126300	-0.556	0.501	1.057	no
CK-40d	RHSIM_Rhsim04G0183700	-0.077	-0.624	-0.547	no
CK-40d	RHSIM_Rhsim05G0219500	-1.015	1.042	2.057	no
CK-40d	RHSIM_Rhsim05G0094600	1.942	2.295	0.353	high
CK-40d	RHSIM_Rhsim03G0235900	1.685	1.534	-0.151	high
CK-40d	RHSIM_Rhsim04G0003000	-1.411	-0.663	0.748	low
CK-40d	RHSIM_Rhsim05G0068600	-2.347	-1.304	1.043	low
CK-40d	RHSIM_Rhsim04G0135600	-0.283	2.121	2.404	no
CK-40d	RHSIM_Rhsim05G0115100	-0.963	-1.520	-0.556	no
CK-40d	RHSIM_Rhsim05G0103300	2.135	3.498	1.363	high
CK-40d	RHSIM_Rhsim04G0196000	1.145	0.350	-0.795	high
CK-40d	RHSIM_Rhsim05G0171100	-0.847	-0.987	-0.140	no
CK-40d	RHSIM_Rhsim05G0092500	1.641	0.289	-1.352	high
CK-40d	RHSIM_Rhsim04G0204500	-0.555	1.528	2.083	no
CK-40d	RHSIM_Rhsim05G0002100	0.044	0.686	0.642	no
CK-40d	RHSIM_Rhsim03G0234400	-1.442	-0.608	0.834	low
CK-40d	RHSIM_Rhsim04G0026300	-0.615	-1.855	-1.240	no
CK-40d	RHSIM_Rhsim04G0106100	0.941	0.067	-0.874	no
CK-40d	RHSIM_Rhsim04G0023600	-0.063	0.309	0.372	no
CK-40d	RHSIM_Rhsim04G0208300	-1.050	2.409	3.460	no

Group	Gene	PTR (z-score difference)	z(log2 LFQ)	z(log2 FPKM)	extreme-PTR
CK-40d	RHSIM_Rhsim05G0000100	1.513	0.530	-0.983	high
CK-40d	RHSIM_Rhsim03G0122800	-0.821	-0.232	0.589	no
CK-40d	RHSIM_Rhsim04G0247000	-0.695	-0.260	0.435	no
CK-40d	RHSIM_Rhsim03G0222500	-0.138	0.729	0.867	no
CK-40d	RHSIM_Rhsim04G0225200	0.392	-0.581	-0.972	no
CK-40d	RHSIM_Rhsim04G0021500	-2.507	-1.928	0.579	low
CK-40d	RHSIM_Rhsim05G0048800	0.048	0.024	-0.024	no
CK-40d	RHSIM_Rhsim03G0233000	-1.421	-1.012	0.409	low
CK-40d	RHSIM_Rhsim05G0064300	0.495	-0.057	-0.552	no
CK-40d	RHSIM_Rhsim03G0160100	1.011	1.820	0.809	no
CK-40d	RHSIM_Rhsim04G0032700	0.133	1.710	1.577	no
CK-40d	RHSIM_Rhsim04G0091800	-0.312	1.905	2.217	no
CK-40d	RHSIM_Rhsim04G0127300	-0.469	0.130	0.599	no
CK-40d	RHSIM_Rhsim04G0247700	1.049	0.796	-0.253	no
CK-40d	RHSIM_Rhsim04G0046600	-1.096	-1.451	-0.355	no
CK-40d	RHSIM_Rhsim04G0010600	-0.084	1.212	1.296	no
CK-40d	RHSIM_Rhsim05G0092700	2.767	0.737	-2.029	high
CK-40d	RHSIM_Rhsim03G0218900	0.223	0.618	0.395	no
CK-40d	RHSIM_Rhsim04G0069300	0.755	0.203	-0.552	no
CK-40d	RHSIM_Rhsim05G0110000	1.248	-0.538	-1.786	high
CK-40d	RHSIM_Rhsim04G0113400	-1.822	-0.062	1.761	low
CK-40d	RHSIM_Rhsim03G0217600	1.002	0.804	-0.198	no
CK-40d	RHSIM_Rhsim05G0075000	0.839	0.163	-0.677	no
CK-40d	RHSIM_Rhsim03G0200000	-0.320	-0.230	0.089	no
CK-40d	RHSIM_Rhsim03G0028900	0.666	0.941	0.275	no
CK-40d	RHSIM_Rhsim05G0034800	-1.336	-0.837	0.499	low
CK-40d	RHSIM_Rhsim04G0229700	-0.008	0.934	0.942	no
CK-40d	RHSIM_Rhsim05G0076800	-0.142	-0.861	-0.719	no
CK-40d	RHSIM_Rhsim04G0089100	1.343	-0.471	-1.813	high
CK-40d	RHSIM_Rhsim05G0079900	0.972	0.691	-0.280	no
CK-40d	RHSIM_Rhsim04G0156000	0.948	-0.590	-1.538	no
CK-40d	RHSIM_Rhsim05G0148600	1.253	-0.556	-1.809	high

Group	Gene	PTR (z-score difference)	z(log2 LFQ)	z(log2 FPKM)	extreme-PTR
CK-40d	RHSIM_Rhsim04G0247300	-0.146	-0.919	-0.773	no
CK-40d	RHSIM_Rhsim04G0202200	-0.909	-0.555	0.354	no
CK-40d	RHSIM_Rhsim04G0073800	-0.319	-0.831	-0.512	no
CK-40d	RHSIM_Rhsim03G0167100	0.589	-0.426	-1.015	no
CK-40d	RHSIM_Rhsim03G0257100	1.540	1.245	-0.295	high
CK-40d	RHSIM_Rhsim04G0070800	0.216	-0.421	-0.638	no
CK-40d	RHSIM_Rhsim03G0221000	-0.951	-0.694	0.258	no
CK-40d	RHSIM_Rhsim05G0022100	0.049	0.070	0.021	no
CK-40d	RHSIM_Rhsim04G0023800	-2.294	-0.967	1.327	low
CK-40d	RHSIM_Rhsim03G0074500	1.166	-0.515	-1.681	high
CK-40d	RHSIM_Rhsim04G0249000	0.951	0.943	-0.008	no
CK-40d	RHSIM_Rhsim03G0246100	0.229	-0.636	-0.865	no
CK-40d	RHSIM_Rhsim04G0008300	0.154	0.246	0.091	no
CK-40d	RHSIM_Rhsim04G0205500	-0.410	-0.998	-0.589	no
CK-40d	RHSIM_Rhsim05G0179500	-0.414	0.660	1.074	no
CK-40d	RHSIM_Rhsim05G0212800	1.148	-1.255	-2.403	high
CK-40d	RHSIM_Rhsim03G0025100	1.458	0.485	-0.973	high
CK-40d	RHSIM_Rhsim03G0189600	-0.469	-0.775	-0.306	no
CK-40d	RHSIM_Rhsim03G0080200	-0.785	-0.847	-0.062	no
CK-40d	RHSIM_Rhsim03G0171100	-0.720	-1.999	-1.278	no
CK-40d	RHSIM_Rhsim05G0223400	-0.736	-1.739	-1.003	no
CK-40d	RHSIM_Rhsim03G0227400	-1.021	1.532	2.553	no
CK-40d	RHSIM_Rhsim04G0175800	-1.637	-1.758	-0.121	low
CK-40d	RHSIM_Rhsim03G0250200	-1.211	-0.760	0.451	no
CK-40d	RHSIM_Rhsim04G0222400	0.767	-0.029	-0.796	no
CK-40d	RHSIM_Rhsim03G0063800	1.779	0.942	-0.837	high
CK-40d	RHSIM_Rhsim04G0205300	-1.352	-0.675	0.678	low
CK-40d	RHSIM_Rhsim03G0140700	-0.945	-0.344	0.601	no
CK-40d	RHSIM_Rhsim03G0069800	0.645	0.650	0.006	no
CK-40d	RHSIM_Rhsim03G0000700	0.881	0.286	-0.596	no
CK-40d	RHSIM_Rhsim04G0134200	1.759	0.382	-1.377	high
CK-40d	RHSIM_Rhsim05G0225700	-0.229	1.568	1.797	no

Group	Gene	PTR (z-score difference)	z(log2 LFQ)	z(log2 FPKM)	extreme-PTR
CK-40d	RHSIM_Rhsim03G0229500	1.355	1.822	0.466	high
CK-40d	RHSIM_Rhsim04G0197000	0.914	1.606	0.693	no
CK-40d	RHSIM_Rhsim04G0238000	0.478	-0.770	-1.247	no
CK-40d	RHSIM_Rhsim04G0190900	-1.244	-1.468	-0.224	low
CK-40d	RHSIM_Rhsim03G0153400	-2.323	-0.284	2.039	low
CK-40d	RHSIM_Rhsim04G0024500	-1.220	-0.813	0.406	no
CK-40d	RHSIM_Rhsim03G0113100	-0.483	-0.985	-0.502	no
CK-40d	RHSIM_Rhsim04G0015700	2.447	1.498	-0.949	high
CK-40d	RHSIM_Rhsim02G0086400	-1.024	-0.742	0.282	no
CK-40d	RHSIM_Rhsim04G0167200	2.398	2.109	-0.289	high
CK-40d	RHSIM_Rhsim04G0213900	-1.886	-0.699	1.188	low
CK-40d	RHSIM_Rhsim02G0110200	-2.216	-0.789	1.427	low
CK-40d	RHSIM_Rhsim04G0125000	0.061	-0.400	-0.461	no
CK-40d	RHSIM_Rhsim03G0192100	-0.082	0.033	0.115	no
CK-40d	RHSIM_Rhsim02G0158900	-0.847	-1.433	-0.587	no
CK-40d	RHSIM_Rhsim04G0046500	-0.587	-0.951	-0.364	no
CK-40d	RHSIM_Rhsim04G0019500	2.171	2.028	-0.143	high
CK-40d	RHSIM_Rhsim04G0220400	-0.510	-0.175	0.335	no
CK-40d	RHSIM_Rhsim04G0003900	0.657	-0.661	-1.318	no
CK-40d	RHSIM_Rhsim03G0093600	0.465	0.043	-0.423	no
CK-40d	RHSIM_Rhsim04G0048200	-0.449	0.232	0.681	no
CK-40d	RHSIM_Rhsim04G0166900	0.746	1.517	0.771	no
CK-40d	RHSIM_Rhsim04G0235300	1.671	0.229	-1.442	high
CK-40d	RHSIM_Rhsim04G0124400	-1.366	0.100	1.466	low
CK-40d	RHSIM_Rhsim04G0120400	-1.192	-0.472	0.720	no
CK-40d	RHSIM_Rhsim03G0045900	0.708	-0.648	-1.356	no
CK-40d	RHSIM_Rhsim04G0015800	-0.517	-0.823	-0.306	no
CK-40d	RHSIM_Rhsim02G0250400	-0.376	-0.291	0.085	no
CK-40d	RHSIM_Rhsim04G0195000	0.700	0.831	0.131	no
CK-40d	RHSIM_Rhsim04G0214300	0.069	0.441	0.373	no
CK-40d	RHSIM_Rhsim04G0153100	-0.499	-0.073	0.427	no
CK-40d	RHSIM_Rhsim04G0085200	-1.484	-0.578	0.906	low

Group	Gene	PTR (z-score difference)	z(log2 LFQ)	z(log2 FPKM)	extreme-PTR
CK-40d	RHSIM_Rhsim03G0153200	0.161	-0.592	-0.753	no
CK-40d	RHSIM_Rhsim04G0053900	0.401	-1.042	-1.443	no
CK-40d	RHSIM_Rhsim04G0164100	0.024	0.031	0.007	no
CK-40d	RHSIM_Rhsim04G0201900	0.632	0.979	0.347	no
CK-40d	RHSIM_Rhsim03G0144600	-2.504	-1.050	1.454	low
CK-40d	RHSIM_Rhsim04G0176300	-1.383	-1.747	-0.364	low
CK-40d	RHSIM_Rhsim04G0167000	0.577	1.449	0.871	no
CK-40d	RHSIM_Rhsim02G0232000	0.368	0.430	0.062	no
CK-40d	RHSIM_Rhsim04G0149700	-0.297	0.518	0.816	no
CK-40d	RHSIM_Rhsim02G0045600	-1.934	-1.517	0.417	low
CK-40d	RHSIM_Rhsim04G0160100	2.130	0.994	-1.136	high
CK-40d	RHSIM_Rhsim04G0147500	1.590	0.316	-1.274	high
CK-40d	RHSIM_Rhsim04G0214400	-0.195	0.037	0.232	no
CK-40d	RHSIM_Rhsim04G0016500	-0.433	-0.958	-0.525	no
CK-40d	RHSIM_Rhsim04G0203600	1.322	1.709	0.388	high
CK-40d	RHSIM_Rhsim04G0231000	1.062	2.836	1.774	no
CK-40d	RHSIM_Rhsim03G0212900	0.737	0.689	-0.048	no
CK-40d	RHSIM_Rhsim04G0212200	-0.766	-1.199	-0.432	no
CK-40d	RHSIM_Rhsim04G0228000	0.398	-0.155	-0.553	no
CK-40d	RHSIM_Rhsim04G0246200	-0.562	-0.275	0.287	no
CK-40d	RHSIM_Rhsim03G0223700	-0.365	-0.801	-0.436	no
CK-40d	RHSIM_Rhsim04G0026700	-0.323	-0.134	0.189	no
CK-40d	RHSIM_Rhsim03G0055600	0.638	-0.620	-1.258	no
CK-40d	RHSIM_Rhsim04G0088200	0.813	-0.015	-0.828	no
CK-40d	RHSIM_Rhsim04G0139900	-1.117	-1.424	-0.307	no
CK-40d	RHSIM_Rhsim04G0083100	-0.206	1.614	1.821	no
CK-40d	RHSIM_Rhsim04G0008600	0.289	0.355	0.066	no
CK-40d	RHSIM_Rhsim02G0248700	1.007	2.186	1.179	no
CK-40d	RHSIM_Rhsim03G0009100	0.909	1.481	0.572	no
CK-40d	RHSIM_Rhsim04G0052900	2.054	-0.365	-2.419	high
CK-40d	RHSIM_Rhsim04G0109400	0.164	-0.045	-0.209	no
CK-40d	RHSIM_Rhsim04G0008000	-0.789	-1.171	-0.382	no

Group	Gene	PTR (z-score difference)	z(log2 LFQ)	z(log2 FPKM)	extreme-PTR
CK-40d	RHSIM_Rhsim02G0173800	0.843	-0.045	-0.888	no
CK-40d	RHSIM_Rhsim04G0234400	0.804	-0.453	-1.257	no
CK-40d	RHSIM_Rhsim03G0167300	-2.097	-0.178	1.919	low
CK-40d	RHSIM_Rhsim02G0254600	-0.005	-0.689	-0.685	no
CK-40d	RHSIM_Rhsim04G0065800	0.902	1.506	0.604	no
CK-40d	RHSIM_Rhsim02G0000300	0.330	-0.239	-0.569	no
CK-40d	RHSIM_Rhsim03G0093500	-0.795	-1.384	-0.589	no
CK-40d	RHSIM_Rhsim02G0165400	0.182	-0.674	-0.856	no
CK-40d	RHSIM_Rhsim04G0196300	-1.063	0.562	1.626	no
CK-40d	RHSIM_Rhsim03G0224100	-0.047	0.572	0.619	no
CK-40d	RHSIM_Rhsim03G0069100	-0.263	0.016	0.279	no
CK-40d	RHSIM_Rhsim04G0018700	-2.073	-1.441	0.632	low
CK-40d	RHSIM_Rhsim02G0143100	-1.594	-0.769	0.826	low
CK-40d	RHSIM_Rhsim02G0172200	0.628	1.086	0.458	no
CK-40d	RHSIM_Rhsim03G0247800	-1.634	-1.299	0.334	low
CK-40d	RHSIM_Rhsim04G0113500	0.432	1.232	0.801	no
CK-40d	RHSIM_Rhsim03G0031100	-0.659	-1.467	-0.807	no
CK-40d	RHSIM_Rhsim03G0141600	1.799	1.382	-0.417	high
CK-40d	RHSIM_Rhsim03G0236500	-0.562	0.189	0.751	no
CK-40d	RHSIM_Rhsim04G0164800	2.033	2.189	0.156	high
CK-40d	RHSIM_Rhsim03G0120500	0.027	-0.621	-0.648	no
CK-40d	RHSIM_Rhsim03G0186100	0.142	-0.708	-0.850	no
CK-40d	RHSIM_Rhsim03G0223900	1.044	0.332	-0.713	no
CK-40d	RHSIM_Rhsim03G0017900	0.751	0.085	-0.665	no
CK-40d	RHSIM_Rhsim04G0218600	-1.527	-0.823	0.704	low
CK-40d	RHSIM_Rhsim03G0256000	0.208	-0.233	-0.441	no
CK-40d	RHSIM_Rhsim04G0187900	-0.187	-1.133	-0.946	no
CK-40d	RHSIM_Rhsim03G0164300	1.846	0.909	-0.937	high
CK-40d	RHSIM_Rhsim04G0111500	-1.333	-0.955	0.378	low
CK-40d	RHSIM_Rhsim03G0214900	1.378	1.063	-0.315	high
CK-40d	RHSIM_Rhsim04G0002800	3.047	3.385	0.339	high
CK-40d	RHSIM_Rhsim04G0245400	0.123	-0.627	-0.750	no

Group	Gene	PTR (z-score difference)	z(log2 LFQ)	z(log2 FPKM)	extreme-PTR
CK-40d	RHSIM_Rhsim04G0208400	1.243	1.555	0.312	high
CK-40d	RHSIM_Rhsim04G0229900	0.344	3.291	2.946	no
CK-40d	RHSIM_Rhsim04G0216700	0.683	0.978	0.296	no
CK-40d	RHSIM_Rhsim03G0236800	-2.151	-2.048	0.103	low
CK-40d	RHSIM_Rhsim04G0123000	-0.402	1.173	1.574	no
CK-40d	RHSIM_Rhsim03G0211000	0.462	0.888	0.426	no
CK-40d	RHSIM_Rhsim02G0193100	-0.627	-0.888	-0.261	no
CK-40d	RHSIM_Rhsim03G0208400	0.183	-0.732	-0.915	no
CK-40d	RHSIM_Rhsim03G0055400	0.635	-1.324	-1.959	no
CK-40d	RHSIM_Rhsim03G0121600	0.240	-0.241	-0.481	no
CK-40d	RHSIM_Rhsim03G0088200	-1.071	0.094	1.165	no
CK-40d	RHSIM_Rhsim04G0008500	-0.104	-0.509	-0.405	no
CK-40d	RHSIM_Rhsim03G0230800	-0.114	-0.086	0.027	no
CK-40d	RHSIM_Rhsim04G0222300	0.116	-0.143	-0.259	no
CK-40d	RHSIM_Rhsim03G0161300	-0.701	-0.272	0.429	no
CK-40d	RHSIM_Rhsim03G0188000	-0.989	-0.288	0.701	no
CK-40d	RHSIM_Rhsim03G0046200	-0.462	0.445	0.907	no
CK-40d	RHSIM_Rhsim02G0059700	-1.131	-1.061	0.069	no
CK-40d	RHSIM_Rhsim03G0009300	0.652	0.013	-0.639	no
CK-40d	RHSIM_Rhsim03G0245100	0.203	-0.088	-0.290	no
CK-40d	RHSIM_Rhsim03G0028400	-0.198	-1.277	-1.079	no
CK-40d	RHSIM_Rhsim04G0245900	0.637	0.531	-0.106	no
CK-40d	RHSIM_Rhsim03G0264400	0.961	-0.068	-1.029	no
CK-40d	RHSIM_Rhsim02G0123600	-1.680	-0.575	1.105	low
CK-40d	RHSIM_Rhsim04G0015500	-1.429	-0.141	1.288	low
CK-40d	RHSIM_Rhsim04G0028100	1.745	-0.494	-2.240	high
CK-40d	RHSIM_Rhsim04G0249600	1.640	0.059	-1.581	high
CK-40d	RHSIM_Rhsim03G0070200	0.281	0.108	-0.173	no
CK-40d	RHSIM_Rhsim03G0234300	0.720	0.632	-0.088	no
CK-40d	RHSIM_Rhsim03G0121100	0.372	0.259	-0.113	no
CK-40d	RHSIM_Rhsim04G0021900	0.318	1.170	0.852	no
CK-40d	RHSIM_Rhsim02G0237500	1.758	0.855	-0.903	high

Group	Gene	PTR (z-score difference)	z(log2 LFQ)	z(log2 FPKM)	extreme-PTR
CK-40d	RHSIM_Rhsim04G0015400	-1.775	0.114	1.889	low
CK-40d	RHSIM_Rhsim04G0074100	0.379	-0.447	-0.826	no
CK-40d	RHSIM_Rhsim03G0163800	-1.381	-0.611	0.770	low
CK-40d	RHSIM_Rhsim03G0241100	-0.270	-1.112	-0.842	no
CK-40d	RHSIM_Rhsim02G0001600	-0.640	-0.574	0.066	no
CK-40d	RHSIM_Rhsim03G0007100	-0.567	-0.572	-0.005	no
CK-40d	RHSIM_Rhsim03G0197200	0.251	0.004	-0.247	no
CK-40d	RHSIM_Rhsim02G0159600	0.475	1.272	0.797	no
CK-40d	RHSIM_Rhsim03G0018300	1.182	0.817	-0.364	high
CK-40d	RHSIM_Rhsim03G0011100	-0.269	1.069	1.337	no
CK-40d	RHSIM_Rhsim03G0264900	-1.749	-0.795	0.954	low
CK-40d	RHSIM_Rhsim03G0131600	-0.908	1.010	1.918	no
CK-40d	RHSIM_Rhsim03G0215400	0.013	0.992	0.979	no
CK-40d	RHSIM_Rhsim02G0070700	-0.045	0.772	0.817	no
CK-40d	RHSIM_Rhsim02G0132400	-0.929	-0.843	0.087	no
CK-40d	RHSIM_Rhsim03G0058000	0.956	0.521	-0.435	no
CK-40d	RHSIM_Rhsim04G0067400	-0.259	-0.226	0.033	no
CK-40d	RHSIM_Rhsim02G0193600	-0.087	-1.043	-0.956	no
CK-40d	RHSIM_Rhsim03G0088300	1.150	-0.440	-1.590	high
CK-40d	RHSIM_Rhsim03G0063900	-1.185	0.345	1.531	no
CK-40d	RHSIM_Rhsim03G0259500	-0.201	-0.935	-0.734	no
CK-40d	RHSIM_Rhsim04G0243300	0.780	0.544	-0.236	no
CK-40d	RHSIM_Rhsim02G0252000	-0.667	-0.206	0.462	no
CK-40d	RHSIM_Rhsim03G0165500	-1.155	0.973	2.128	no
CK-40d	RHSIM_Rhsim03G0076200	-0.229	-0.051	0.177	no
CK-40d	RHSIM_Rhsim03G0191300	0.492	1.365	0.873	no
CK-40d	RHSIM_Rhsim03G0191800	-0.374	0.246	0.620	no
CK-40d	RHSIM_Rhsim03G0254400	-1.890	-1.085	0.805	low
CK-40d	RHSIM_Rhsim04G0166600	-0.085	-0.676	-0.591	no
CK-40d	RHSIM_Rhsim03G0180600	-0.651	0.942	1.593	no
CK-40d	RHSIM_Rhsim02G0135500	-0.069	0.054	0.123	no
CK-40d	RHSIM_Rhsim02G0217800	1.160	0.239	-0.921	high

Group	Gene	PTR (z-score difference)	z(log2 LFQ)	z(log2 FPKM)	extreme-PTR
CK-40d	RHSIM_Rhsim02G0056200	0.904	2.233	1.330	no
CK-40d	RHSIM_Rhsim04G0242100	0.519	-0.534	-1.052	no
CK-40d	RHSIM_Rhsim02G0131800	-1.050	-0.447	0.603	no
CK-40d	RHSIM_Rhsim03G0165100	1.117	-0.636	-1.752	no
CK-40d	RHSIM_Rhsim03G0245900	-1.294	0.339	1.633	low
CK-40d	RHSIM_Rhsim02G0071100	0.555	1.027	0.472	no
CK-40d	RHSIM_Rhsim03G0073900	0.017	0.054	0.037	no
CK-40d	RHSIM_Rhsim03G0171300	-0.292	1.531	1.823	no
CK-40d	RHSIM_Rhsim03G0231400	-2.022	-1.769	0.253	low
CK-40d	RHSIM_Rhsim02G0235500	-0.237	0.477	0.715	no
CK-40d	RHSIM_Rhsim03G0067800	0.024	-0.660	-0.685	no
CK-40d	RHSIM_Rhsim02G0143300	0.978	2.857	1.879	no
CK-40d	RHSIM_Rhsim03G0105000	-0.497	-0.955	-0.458	no
CK-40d	RHSIM_Rhsim04G0083900	0.903	0.973	0.070	no
CK-40d	RHSIM_Rhsim03G0053000	0.209	1.509	1.300	no
CK-40d	RHSIM_Rhsim02G0123500	-0.001	-0.532	-0.531	no
CK-40d	RHSIM_Rhsim03G0075500	-0.823	-0.325	0.498	no
CK-40d	RHSIM_Rhsim02G0088400	1.985	1.202	-0.783	high
CK-40d	RHSIM_Rhsim04G0007400	1.820	3.445	1.624	high
CK-40d	RHSIM_Rhsim02G0031300	-2.155	-1.719	0.436	low
CK-40d	RHSIM_Rhsim04G0006500	-1.544	-1.218	0.326	low
CK-40d	RHSIM_Rhsim01G0222900	0.689	-0.099	-0.788	no
CK-40d	RHSIM_Rhsim01G0240100	-1.421	-1.131	0.291	low
CK-40d	RHSIM_Rhsim02G0171900	2.216	1.096	-1.120	high
CK-40d	RHSIM_Rhsim01G0041300	3.057	0.641	-2.416	high
CK-40d	RHSIM_Rhsim04G0051700	0.877	0.170	-0.707	no
CK-40d	RHSIM_Rhsim03G0067500	-0.977	0.943	1.920	no
CK-40d	RHSIM_Rhsim04G0066100	1.582	0.250	-1.332	high
CK-40d	RHSIM_Rhsim02G0066400	-0.226	-0.826	-0.600	no
CK-40d	RHSIM_Rhsim02G0018900	0.202	-0.392	-0.594	no
CK-40d	RHSIM_Rhsim01G0178500	-1.121	-0.263	0.858	no
CK-40d	RHSIM_Rhsim04G0181100	-0.619	0.905	1.524	no

Group	Gene	PTR (z-score difference)	z(log2 LFQ)	z(log2 FPKM)	extreme-PTR
CK-40d	RHSIM_Rhsim03G0255900	0.295	1.351	1.055	no
CK-40d	RHSIM_Rhsim03G0243000	-0.840	-0.179	0.661	no
CK-40d	RHSIM_Rhsim03G0186600	-1.442	0.462	1.905	low
CK-40d	RHSIM_Rhsim02G0241900	-0.202	-2.267	-2.065	no
CK-40d	RHSIM_Rhsim03G0245400	0.085	-0.400	-0.485	no
CK-40d	RHSIM_Rhsim01G0167600	0.388	0.880	0.492	no
CK-40d	RHSIM_Rhsim03G0225800	1.029	-0.509	-1.537	no
CK-40d	RHSIM_Rhsim02G0133700	-1.104	-0.175	0.928	no
CK-40d	RHSIM_Rhsim04G0072300	-1.118	-1.093	0.025	no
CK-40d	RHSIM_Rhsim04G0091400	-0.630	-0.631	-0.001	no
CK-40d	RHSIM_Rhsim03G0059700	0.587	0.664	0.076	no
CK-40d	RHSIM_Rhsim04G0085600	0.154	0.854	0.700	no
CK-40d	RHSIM_Rhsim04G0121700	1.753	0.975	-0.778	high
CK-40d	RHSIM_Rhsim02G0253800	-0.615	-0.704	-0.089	no
CK-40d	RHSIM_Rhsim02G0043500	-0.274	0.252	0.525	no
CK-40d	RHSIM_Rhsim03G0097500	-0.757	0.152	0.908	no
CK-40d	RHSIM_Rhsim03G0254000	1.144	0.164	-0.980	high
CK-40d	RHSIM_Rhsim02G0031900	-0.818	0.073	0.890	no
CK-40d	RHSIM_Rhsim03G0235200	-0.322	0.946	1.268	no
CK-40d	RHSIM_Rhsim03G0144500	-2.148	-0.691	1.457	low
CK-40d	RHSIM_Rhsim03G0091100	-0.891	-0.698	0.193	no
CK-40d	RHSIM_Rhsim02G0071800	-0.090	0.065	0.155	no
CK-40d	RHSIM_Rhsim01G0292400	-0.811	-0.204	0.608	no
CK-40d	RHSIM_Rhsim02G0141300	0.456	-0.618	-1.073	no
CK-40d	RHSIM_Rhsim01G0203700	1.427	-0.105	-1.532	high
CK-40d	RHSIM_Rhsim03G0129800	-0.672	-0.441	0.232	no
CK-40d	RHSIM_Rhsim03G0038900	1.011	0.374	-0.638	no
CK-40d	RHSIM_Rhsim02G0122100	0.153	0.145	-0.008	no
CK-40d	RHSIM_Rhsim01G0238000	-1.572	-0.488	1.084	low
CK-40d	RHSIM_Rhsim02G0243000	0.348	0.260	-0.088	no
CK-40d	RHSIM_Rhsim01G0066800	-1.089	-1.064	0.026	no
CK-40d	RHSIM_Rhsim03G0161700	0.270	0.159	-0.111	no

Group	Gene	PTR (z-score difference)	z(log2 LFQ)	z(log2 FPKM)	extreme-PTR
CK-40d	RHSIM_Rhsim02G0100900	2.786	0.711	-2.075	high
CK-40d	RHSIM_Rhsim03G0261300	-0.472	0.112	0.584	no
CK-40d	RHSIM_Rhsim02G0256200	0.618	-0.215	-0.833	no
CK-40d	RHSIM_Rhsim02G0022900	-1.826	-0.942	0.884	low
CK-40d	RHSIM_Rhsim03G0110700	1.329	0.497	-0.832	high
CK-40d	RHSIM_Rhsim01G0141100	-0.593	0.122	0.715	no
CK-40d	RHSIM_Rhsim03G0268200	1.333	-0.035	-1.368	high
CK-40d	RHSIM_Rhsim02G0008200	-0.065	0.708	0.774	no
CK-40d	RHSIM_Rhsim03G0225600	-1.867	-1.641	0.226	low
CK-40d	RHSIM_Rhsim03G0152900	0.091	0.249	0.158	no
CK-40d	RHSIM_Rhsim03G0243200	0.348	-0.461	-0.809	no
CK-40d	RHSIM_Rhsim03G0236200	0.040	1.410	1.370	no
CK-40d	RHSIM_Rhsim01G0105900	-0.339	0.174	0.512	no
CK-40d	RHSIM_Rhsim03G0212300	0.605	0.634	0.029	no
CK-40d	RHSIM_Rhsim03G0090500	-0.665	-1.032	-0.367	no
CK-40d	RHSIM_Rhsim01G0147400	0.947	0.623	-0.324	no
CK-40d	RHSIM_Rhsim02G0248400	1.123	1.624	0.500	no
CK-40d	RHSIM_Rhsim03G0257300	-1.047	-0.237	0.810	no
CK-40d	RHSIM_Rhsim02G0128600	-0.676	-0.396	0.280	no
CK-40d	RHSIM_Rhsim03G0219500	-0.673	1.162	1.835	no
CK-40d	RHSIM_Rhsim03G0187400	1.056	0.833	-0.223	no
CK-40d	RHSIM_Rhsim03G0040400	1.570	1.037	-0.533	high
CK-40d	RHSIM_Rhsim03G0251700	-1.506	-0.675	0.830	low
CK-40d	RHSIM_Rhsim03G0005600	-0.701	0.075	0.777	no
CK-40d	RHSIM_Rhsim03G0167200	-0.264	-0.415	-0.151	no
CK-40d	RHSIM_Rhsim03G0254200	-1.138	-1.743	-0.605	no
CK-40d	RHSIM_Rhsim03G0136300	0.521	0.338	-0.183	no
CK-40d	RHSIM_Rhsim02G0071400	1.322	-0.767	-2.089	high
CK-40d	RHSIM_Rhsim03G0230100	1.436	1.042	-0.394	high
CK-40d	RHSIM_Rhsim03G0176800	-1.505	-1.623	-0.118	low
CK-40d	RHSIM_Rhsim02G0060200	-0.713	-1.124	-0.411	no
CK-40d	RHSIM_Rhsim02G0143200	0.208	-0.192	-0.400	no

Group	Gene	PTR (z-score difference)	z(log2 LFQ)	z(log2 FPKM)	extreme-PTR
CK-40d	RHSIM_Rhsim03G0070300	1.104	0.760	-0.344	no
CK-40d	RHSIM_Rhsim03G0117200	-0.727	-0.693	0.035	no
CK-40d	RHSIM_Rhsim03G0212500	-1.122	-0.084	1.038	no
CK-40d	RHSIM_Rhsim02G0194100	-0.204	1.594	1.798	no
CK-40d	RHSIM_Rhsim02G0181400	-1.439	0.443	1.881	low
CK-40d	RHSIM_Rhsim02G0107000	0.106	0.343	0.237	no
CK-40d	RHSIM_Rhsim02G0028200	0.836	-0.031	-0.867	no
CK-40d	RHSIM_Rhsim03G0219400	-1.092	-1.138	-0.046	no
CK-40d	RHSIM_Rhsim03G0027400	1.174	0.967	-0.207	high
CK-40d	RHSIM_Rhsim03G0240000	-2.143	-1.260	0.883	low
CK-40d	RHSIM_Rhsim02G0252600	-1.745	-0.416	1.329	low
CK-40d	RHSIM_Rhsim03G0089800	-0.671	-0.003	0.668	no
CK-40d	RHSIM_Rhsim01G0249900	-0.427	1.235	1.661	no
CK-40d	RHSIM_Rhsim03G0214200	0.488	-0.476	-0.964	no
CK-40d	RHSIM_Rhsim02G0150100	-0.999	-0.229	0.770	no
CK-40d	RHSIM_Rhsim03G0169600	0.258	-0.451	-0.709	no
CK-40d	RHSIM_Rhsim03G0253100	-1.019	-0.841	0.178	no
CK-40d	RHSIM_Rhsim02G0053700	-0.951	0.811	1.762	no
CK-40d	RHSIM_Rhsim01G0170800	1.349	0.497	-0.852	high
CK-40d	RHSIM_Rhsim03G0254900	1.156	0.603	-0.553	high
CK-40d	RHSIM_Rhsim02G0085700	0.353	-0.556	-0.910	no
CK-40d	RHSIM_Rhsim03G0150200	2.854	0.959	-1.895	high
CK-40d	RHSIM_Rhsim03G0190600	-0.398	-0.467	-0.069	no
CK-40d	RHSIM_Rhsim03G0062900	-0.207	-0.694	-0.487	no
CK-40d	RHSIM_Rhsim03G0049900	-0.677	0.122	0.799	no
CK-40d	RHSIM_Rhsim03G0239600	-1.232	-1.639	-0.406	no
CK-40d	RHSIM_Rhsim02G0002000	1.336	1.054	-0.282	high
CK-40d	RHSIM_Rhsim01G0183300	-0.139	-0.717	-0.578	no
CK-40d	RHSIM_Rhsim01G0001700	-1.255	-1.188	0.066	low
CK-40d	RHSIM_Rhsim03G0147100	0.789	0.392	-0.397	no
CK-40d	RHSIM_Rhsim03G0076700	-0.669	0.385	1.054	no
CK-40d	RHSIM_Rhsim01G0283600	-2.027	0.507	2.534	low

Group	Gene	PTR (z-score difference)	z(log2 LFQ)	z(log2 FPKM)	extreme-PTR
CK-40d	RHSIM_Rhsim03G0186300	0.566	-1.332	-1.898	no
CK-40d	RHSIM_Rhsim02G0244700	0.017	-0.167	-0.184	no
CK-40d	RHSIM_Rhsim02G0058700	1.445	0.415	-1.030	high
CK-40d	RHSIM_Rhsim03G0226600	-0.438	0.523	0.961	no
CK-40d	RHSIM_Rhsim01G0123000	-2.605	-0.863	1.742	low
CK-40d	RHSIM_Rhsim03G0003600	-0.268	-0.318	-0.051	no
CK-40d	RHSIM_Rhsim02G0005400	1.038	1.441	0.404	no
CK-40d	RHSIM_Rhsim01G0192600	-1.130	-0.183	0.946	no
CK-40d	RHSIM_Rhsim01G0258200	-0.634	0.353	0.987	no
CK-40d	RHSIM_Rhsim01G0100600	-0.485	0.059	0.544	no
CK-40d	RHSIM_Rhsim02G0236800	-2.207	-0.892	1.314	low
CK-40d	RHSIM_Rhsim03G0221300	-1.479	-1.424	0.055	low
CK-40d	RHSIM_Rhsim01G0280000	0.447	0.062	-0.385	no
CK-40d	RHSIM_Rhsim01G0135600	0.018	-0.400	-0.418	no
CK-40d	RHSIM_Rhsim02G0175300	1.457	0.116	-1.341	high
CK-40d	RHSIM_Rhsim03G0094200	0.793	0.230	-0.563	no
CK-40d	RHSIM_Rhsim02G0195000	-1.279	-1.256	0.023	low
CK-40d	RHSIM_Rhsim01G0114000	1.339	0.430	-0.910	high
CK-40d	RHSIM_Rhsim03G0052800	-3.332	-1.105	2.227	low
CK-40d	RHSIM_Rhsim01G0259100	-0.665	-0.916	-0.251	no
CK-40d	RHSIM_Rhsim01G0049700	-1.257	-0.780	0.477	low
CK-40d	RHSIM_Rhsim01G0190400	0.204	0.192	-0.012	no
CK-40d	RHSIM_Rhsim01G0187400	0.596	-0.948	-1.544	no
CK-40d	RHSIM_Rhsim01G0044000	1.611	1.660	0.049	high
CK-40d	RHSIM_Rhsim02G0116000	-0.953	-0.458	0.495	no
CK-40d	RHSIM_Rhsim03G0153700	0.392	0.206	-0.186	no
CK-40d	RHSIM_Rhsim02G0246500	-0.144	0.622	0.766	no
CK-40d	RHSIM_Rhsim01G0061400	0.087	-0.537	-0.624	no
CK-40d	RHSIM_Rhsim02G0004600	-1.325	-0.157	1.168	low
CK-40d	RHSIM_Rhsim01G0049100	1.523	0.551	-0.973	high
CK-40d	RHSIM_Rhsim02G0180700	-1.000	-0.342	0.658	no
CK-40d	RHSIM_Rhsim01G0222000	0.185	0.571	0.386	no

Group	Gene	PTR (z-score difference)	z(log2 LFQ)	z(log2 FPKM)	extreme-PTR
CK-40d	RHSIM_Rhsim02G0242100	-3.483	-0.541	2.942	low
CK-40d	RHSIM_Rhsim02G0207700	1.239	-0.549	-1.789	high
CK-40d	RHSIM_Rhsim03G0227500	1.132	0.336	-0.796	no
CK-40d	RHSIM_Rhsim02G0241000	-1.363	-1.427	-0.064	low
CK-40d	RHSIM_Rhsim02G0018500	0.580	-0.504	-1.084	no
CK-40d	RHSIM_Rhsim02G0115600	-1.099	-0.096	1.003	no
CK-40d	RHSIM_Rhsim02G0070000	0.551	0.759	0.208	no
CK-40d	RHSIM_Rhsim02G0007400	-0.293	-0.364	-0.071	no
CK-40d	RHSIM_Rhsim02G0256900	0.574	-0.131	-0.705	no
CK-40d	RHSIM_Rhsim02G0106100	-0.353	-1.012	-0.660	no
CK-40d	RHSIM_Rhsim03G0213000	0.518	0.308	-0.209	no
CK-40d	RHSIM_Rhsim03G0087300	-0.424	-0.440	-0.015	no
CK-40d	RHSIM_Rhsim02G0061700	-0.537	1.282	1.820	no
CK-40d	RHSIM_Rhsim02G0234600	-1.733	-1.043	0.690	low
CK-40d	RHSIM_Rhsim03G0148000	-0.607	-0.729	-0.123	no
CK-40d	RHSIM_Rhsim01G0169000	-2.615	-1.148	1.467	low
CK-40d	RHSIM_Rhsim01G0232600	-1.399	-0.503	0.896	low
CK-40d	RHSIM_Rhsim02G0084400	-0.316	-0.954	-0.638	no
CK-40d	RHSIM_Rhsim01G0286300	1.323	0.436	-0.887	high
CK-40d	RHSIM_Rhsim03G0247500	-0.923	-0.279	0.644	no
CK-40d	RHSIM_Rhsim02G0217700	-0.175	0.629	0.804	no
CK-40d	RHSIM_Rhsim02G0104800	-0.223	-0.020	0.204	no
CK-40d	RHSIM_Rhsim01G0050400	-0.528	-1.386	-0.858	no
CK-40d	RHSIM_Rhsim01G0101100	-0.417	-0.991	-0.574	no
CK-40d	RHSIM_Rhsim01G0278500	-1.287	-1.059	0.228	low
CK-40d	RHSIM_Rhsim02G0214300	0.275	0.675	0.400	no
CK-40d	RHSIM_Rhsim01G0060000	-0.507	0.210	0.717	no
CK-40d	RHSIM_Rhsim01G0258800	0.187	0.178	-0.009	no
CK-40d	RHSIM_Rhsim01G0114100	-1.071	-0.230	0.841	no
CK-40d	RHSIM_Rhsim01G0258700	1.560	0.812	-0.748	high
CK-40d	RHSIM_Rhsim02G0007600	1.971	0.151	-1.820	high
CK-40d	RHSIM_Rhsim02G0069600	-0.265	0.507	0.772	no

Group	Gene	PTR (z-score difference)	z(log2 LFQ)	z(log2 FPKM)	extreme-PTR
CK-40d	RHSIM_Rhsim01G0127700	1.640	1.057	-0.582	high
CK-40d	RHSIM_Rhsim02G0031200	0.269	-0.784	-1.053	no
CK-40d	RHSIM_Rhsim01G0192800	0.036	0.757	0.721	no
CK-40d	RHSIM_Rhsim03G0004200	-1.138	-0.671	0.467	no
CK-40d	RHSIM_Rhsim02G0066800	-3.077	-0.705	2.373	low
CK-40d	RHSIM_Rhsim01G0240400	0.733	-0.165	-0.898	no
CK-40d	RHSIM_Rhsim02G0068800	0.404	0.115	-0.289	no
CK-40d	RHSIM_Rhsim01G0291700	0.602	-0.796	-1.398	no
CK-40d	RHSIM_Rhsim01G0131800	3.685	2.212	-1.474	high
CK-40d	RHSIM_Rhsim01G0171300	2.197	2.385	0.188	high
CK-40d	RHSIM_Rhsim02G0210500	-1.329	-0.915	0.414	low
CK-40d	RHSIM_Rhsim01G0230200	-0.769	-0.786	-0.017	no
CK-40d	RHSIM_Rhsim02G0028100	-0.687	0.980	1.667	no
CK-40d	RHSIM_Rhsim01G0131300	1.594	0.326	-1.268	high
CK-40d	RHSIM_Rhsim01G0142100	-0.466	-0.289	0.177	no
CK-40d	RHSIM_Rhsim01G0220300	0.511	-0.817	-1.328	no
CK-40d	RHSIM_Rhsim03G0090000	-1.350	0.140	1.490	low
CK-40d	RHSIM_Rhsim01G0219100	1.280	3.249	1.969	high
CK-40d	RHSIM_Rhsim03G0151200	0.269	1.256	0.987	no
CK-40d	RHSIM_Rhsim01G0290600	0.223	-0.059	-0.283	no
CK-40d	RHSIM_Rhsim01G0102500	0.033	-0.061	-0.094	no
CK-40d	RHSIM_Rhsim01G0278100	-1.053	-1.056	-0.003	no
CK-40d	RHSIM_Rhsim02G0099900	-0.610	-0.680	-0.070	no
CK-40d	RHSIM_Rhsim02G0004900	-0.439	-0.734	-0.295	no
CK-40d	RHSIM_Rhsim01G0226900	-1.783	-0.638	1.145	low
CK-40d	RHSIM_Rhsim02G0124600	-0.040	-0.068	-0.028	no
CK-40d	RHSIM_Rhsim02G0157100	-1.088	-1.256	-0.169	no
CK-40d	RHSIM_Rhsim01G0135700	0.187	0.614	0.427	no
CK-40d	RHSIM_Rhsim02G0181000	-0.231	0.339	0.570	no
CK-40d	RHSIM_Rhsim02G0096400	-0.364	0.244	0.608	no
CK-40d	RHSIM_Rhsim02G0089800	0.414	0.146	-0.268	no
CK-40d	RHSIM_Rhsim02G0169600	0.768	0.357	-0.411	no

Group	Gene	PTR (z-score difference)	z(log2 LFQ)	z(log2 FPKM)	extreme-PTR
CK-40d	RHSIM_Rhsim02G0105100	0.059	-0.393	-0.451	no
CK-40d	RHSIM_Rhsim01G0267800	-2.193	-1.596	0.596	low
CK-40d	RHSIM_Rhsim01G0242700	-0.933	-2.077	-1.145	no
CK-40d	RHSIM_Rhsim01G0152000	-0.979	-0.821	0.158	no
CK-40d	RHSIM_Rhsim02G0172600	0.588	1.000	0.412	no
CK-40d	RHSIM_Rhsim02G0147000	2.600	2.417	-0.183	high
CK-40d	RHSIM_Rhsim02G0251400	1.010	0.199	-0.811	no
CK-40d	RHSIM_Rhsim02G0251300	1.163	0.879	-0.284	high
CK-40d	RHSIM_Rhsim02G0007900	-1.039	-0.739	0.299	no
CK-40d	RHSIM_Rhsim02G0252500	-0.163	1.533	1.696	no
CK-40d	RHSIM_Rhsim02G0122400	-0.506	-0.948	-0.442	no
CK-40d	RHSIM_Rhsim02G0159400	-1.140	-1.138	0.003	no
CK-40d	RHSIM_Rhsim02G0088300	-0.069	-0.935	-0.866	no
CK-40d	RHSIM_Rhsim02G0083500	-1.001	-0.289	0.712	no
CK-40d	RHSIM_Rhsim02G0205900	-2.382	-0.388	1.994	low
CK-40d	RHSIM_Rhsim02G0208200	-1.436	-1.669	-0.233	low
CK-40d	RHSIM_Rhsim01G0235200	0.908	0.097	-0.811	no
CK-40d	RHSIM_Rhsim01G0243800	0.031	1.076	1.045	no
CK-40d	RHSIM_Rhsim02G0119200	1.448	0.560	-0.888	high
CK-40d	RHSIM_Rhsim01G0234200	0.864	0.593	-0.271	no
CK-40d	RHSIM_Rhsim01G0025200	0.306	1.290	0.984	no
CK-40d	RHSIM_Rhsim01G0054900	-0.743	-0.213	0.530	no
CK-40d	RHSIM_Rhsim01G0232200	0.927	0.998	0.071	no
CK-40d	RHSIM_Rhsim02G0141400	-0.289	-1.456	-1.167	no
CK-40d	RHSIM_Rhsim01G0223600	0.236	-0.869	-1.105	no
CK-40d	RHSIM_Rhsim02G0035600	-3.261	-1.186	2.075	low
CK-40d	RHSIM_Rhsim01G0175000	-2.448	-1.491	0.957	low
CK-40d	RHSIM_Rhsim01G0013100	0.834	1.841	1.007	no
CK-40d	RHSIM_Rhsim01G0073100	-0.498	0.082	0.580	no
CK-40d	RHSIM_Rhsim01G0184400	0.511	1.978	1.467	no
CK-40d	RHSIM_Rhsim01G0269200	1.416	1.726	0.310	high
CK-40d	RHSIM_Rhsim02G0213100	0.034	-0.752	-0.786	no

Group	Gene	PTR (z-score difference)	z(log2 LFQ)	z(log2 FPKM)	extreme-PTR
CK-40d	RHSIM_Rhsim01G0263700	-0.036	-0.840	-0.804	no
CK-40d	RHSIM_Rhsim01G0182600	0.821	-1.178	-1.999	no
CK-40d	RHSIM_Rhsim01G0132700	-1.580	-1.873	-0.293	low
CK-40d	RHSIM_Rhsim01G0241200	-0.387	-0.630	-0.243	no
CK-40d	RHSIM_Rhsim01G0291500	-0.156	-0.313	-0.157	no
CK-40d	RHSIM_Rhsim01G0108800	0.445	1.258	0.813	no
CK-40d	RHSIM_Rhsim01G0001600	-1.657	-0.493	1.165	low
CK-40d	RHSIM_Rhsim01G0147700	-0.496	-0.710	-0.213	no
CK-40d	RHSIM_Rhsim02G0248800	-0.107	-0.056	0.052	no
CK-40d	RHSIM_Rhsim01G0243600	-1.160	0.396	1.556	no
CK-40d	RHSIM_Rhsim02G0220200	3.808	2.576	-1.232	high
CK-40d	RHSIM_Rhsim01G0185100	0.266	0.774	0.508	no
CK-40d	RHSIM_Rhsim01G0270000	-0.628	0.194	0.821	no
CK-40d	RHSIM_Rhsim02G0127100	-2.105	-1.313	0.792	low
CK-40d	RHSIM_Rhsim01G0089900	0.590	0.336	-0.254	no
CK-40d	RHSIM_Rhsim02G0145900	-1.125	-0.809	0.316	no
CK-40d	RHSIM_Rhsim02G0250500	0.497	1.137	0.641	no
CK-40d	RHSIM_Rhsim01G0184600	0.954	-0.557	-1.512	no
CK-40d	RHSIM_Rhsim02G0089500	0.938	-0.894	-1.832	no
CK-40d	RHSIM_Rhsim02G0019600	-1.067	-0.799	0.268	no
CK-40d	RHSIM_Rhsim01G0247800	2.558	3.652	1.094	high
CK-40d	RHSIM_Rhsim02G0217200	-0.688	-0.547	0.141	no
CK-40d	RHSIM_Rhsim01G0284000	-1.340	-0.840	0.500	low
CK-40d	RHSIM_Rhsim01G0163700	-0.739	-0.422	0.316	no
CK-40d	RHSIM_Rhsim01G0144200	0.385	0.052	-0.334	no
CK-40d	RHSIM_Rhsim02G0184100	-1.395	-0.885	0.510	low
CK-40d	RHSIM_Rhsim01G0001900	0.314	1.070	0.756	no
CK-40d	RHSIM_Rhsim01G0195500	-1.347	-0.665	0.681	low
CK-40d	RHSIM_Rhsim01G0194400	0.074	-0.816	-0.890	no
CK-40d	RHSIM_Rhsim01G0196700	-1.307	-0.860	0.447	low
CK-40d	RHSIM_Rhsim01G0287600	-2.992	-0.859	2.133	low
CK-40d	RHSIM_Rhsim01G0227500	0.434	2.464	2.030	no

Group	Gene	PTR (z-score difference)	z(log2 LFQ)	z(log2 FPKM)	extreme-PTR
CK-40d	RHSIM_Rhsim02G0215600	-0.416	0.384	0.799	no
CK-40d	RHSIM_Rhsim02G0109900	2.305	0.607	-1.697	high
CK-40d	RHSIM_Rhsim01G0285600	-0.917	-0.600	0.317	no
CK-40d	RHSIM_Rhsim02G0193200	-3.086	-0.980	2.106	low
CK-40d	RHSIM_Rhsim01G0128500	-0.828	-1.569	-0.741	no
CK-40d	RHSIM_Rhsim01G0286500	0.083	-0.316	-0.400	no
CK-40d	RHSIM_Rhsim01G0008000	1.276	0.746	-0.530	high
CK-40d	RHSIM_Rhsim02G0255400	-0.294	-0.311	-0.017	no
CK-40d	RHSIM_Rhsim01G0244300	-0.451	-1.196	-0.745	no
CK-40d	RHSIM_Rhsim01G0196200	0.199	0.255	0.056	no
CK-40d	RHSIM_Rhsim01G0265400	-1.611	-1.086	0.525	low
CK-40d	RHSIM_Rhsim02G0083400	-0.743	-0.835	-0.091	no
CK-40d	RHSIM_Rhsim01G0144600	1.199	-0.066	-1.266	high
CK-40d	RHSIM_Rhsim01G0276900	-1.178	-1.125	0.053	no
CK-40d	RHSIM_Rhsim02G0105300	0.922	-0.426	-1.348	no
CK-40d	RHSIM_Rhsim01G0186600	-0.153	-1.244	-1.091	no
CK-40d	RHSIM_Rhsim01G0213500	1.311	2.582	1.271	high
CK-40d	RHSIM_Rhsim01G0265200	-0.560	-1.168	-0.608	no
CK-40d	RHSIM_Rhsim02G0180100	-0.136	-0.213	-0.077	no
CK-40d	RHSIM_Rhsim01G0206200	-0.708	-0.145	0.563	no
CK-40d	RHSIM_Rhsim01G0249100	-0.606	0.196	0.803	no
CK-40d	RHSIM_Rhsim01G0059500	-0.424	0.138	0.561	no
CK-40d	RHSIM_Rhsim01G0074200	-2.464	-1.908	0.557	low
CK-40d	RHSIM_Rhsim01G0151800	-2.371	-1.755	0.616	low
CK-40d	RHSIM_Rhsim01G0141500	1.920	1.149	-0.771	high
CK-40d	RHSIM_Rhsim01G0134500	0.337	1.059	0.722	no
CK-40d	RHSIM_Rhsim01G0212900	-1.294	-0.476	0.818	low
CK-40d	RHSIM_Rhsim01G0124100	0.849	0.538	-0.311	no
CK-40d	RHSIM_Rhsim02G0251700	-1.755	-0.697	1.058	low
CK-40d	RHSIM_Rhsim01G0077600	0.313	0.668	0.355	no
CK-40d	RHSIM_Rhsim01G0181300	1.250	2.133	0.883	high
CK-40d	RHSIM_Rhsim01G0205500	1.148	0.485	-0.663	high

Group	Gene	PTR (z-score difference)	z(log2 LFQ)	z(log2 FPKM)	extreme-PTR
CK-40d	RHSIM_Rhsim01G0204800	0.364	-0.047	-0.411	no
CK-40d	RHSIM_Rhsim02G0043000	-0.049	2.154	2.204	no
CK-40d	RHSIM_Rhsim01G0121200	-0.624	-0.804	-0.180	no
CK-40d	RHSIM_Rhsim01G0114600	-0.737	0.788	1.524	no
CK-40d	RHSIM_Rhsim01G0261900	0.257	0.072	-0.185	no
CK-40d	RHSIM_Rhsim01G0290900	0.833	-0.925	-1.759	no
CK-40d	RHSIM_Rhsim01G0215700	-0.783	-0.558	0.224	no
CK-40d	RHSIM_Rhsim01G0246400	-0.906	0.480	1.386	no
CK-40d	RHSIM_Rhsim01G0176500	1.638	0.764	-0.874	high
CK-40d	RHSIM_Rhsim01G0023600	1.026	-0.662	-1.688	no
CK-40d	RHSIM_Rhsim01G0128700	0.061	-0.625	-0.686	no
CK-40d	RHSIM_Rhsim01G0232700	0.153	-1.707	-1.860	no
CK-40d	RHSIM_Rhsim01G0278000	-1.124	0.213	1.338	no
CK-40d	RHSIM_Rhsim01G0001400	1.186	0.146	-1.040	high
CK-40d	RHSIM_Rhsim01G0000100	0.501	-0.862	-1.362	no
CK-40d	RHSIM_Rhsim01G0250800	0.813	1.029	0.216	no
CK-40d	RHSIM_Rhsim01G0245100	0.842	0.933	0.091	no
CK-40d	RHSIM_Rhsim01G0244200	1.686	-0.563	-2.249	high
CK-40d	RHSIM_Rhsim01G0244400	-1.548	0.701	2.248	low
CK-40d	RHSIM_Rhsim01G0001500	1.372	-0.202	-1.574	high
CK-40d	RHSIM_Rhsim01G0292000	0.981	0.422	-0.559	no
CK-40d	RHSIM_Rhsim01G0286100	-0.822	-0.569	0.253	no
CK-40d	RHSIM_Rhsim01G0199200	0.489	0.908	0.419	no
CK-40d	RHSIM_Rhsim01G0004300	-1.351	0.133	1.484	low
CK-40d	RHSIM_Rhsim01G0256500	-1.529	-1.401	0.128	low
CK-40d	RHSIM_Rhsim01G0060100	-0.940	-0.614	0.327	no
CK-40d	RHSIM_Rhsim01G0108000	-1.453	-1.278	0.175	low
CK-40d	RHSIM_Rhsim01G0210600	1.389	-0.101	-1.490	high
CK-40d	RHSIM_Rhsim01G0276100	0.868	0.388	-0.480	no
CK-40d	RHSIM_Rhsim01G0276800	-0.500	-0.127	0.373	no
CK-40d	RHSIM_Rhsim01G0153000	0.384	0.749	0.365	no
CK-40d	RHSIM_Rhsim01G0241700	-0.198	1.108	1.306	no

Group	Gene	PTR (z-score difference)	z(log2 LFQ)	z(log2 FPKM)	extreme-PTR
CK-40d	RHSIM_Rhsim01G0221500	0.402	1.192	0.790	no
CK-40d	RHSIM_Rhsim01G0189400	1.044	-0.826	-1.871	no
CK-40d	RHSIM_Rhsim01G0000500	0.827	0.559	-0.267	no
CK-40d	RHSIM_Rhsim01G0260600	-1.012	-1.074	-0.061	no
CK-40d	RHSIM_Rhsim01G0286200	-3.997	-0.303	3.694	low
CK-40d	RHSIM_Rhsim01G0279700	0.200	-0.738	-0.938	no
CK-40d	RHSIM_Rhsim01G0055700	-0.279	0.188	0.467	no
CK-40d	RHSIM_Rhsim01G0282300	-0.113	-0.538	-0.425	no
CK-40d	RHSIM_Rhsim01G0044100	-0.712	0.363	1.076	no
CK-40d	RHSIM_Rhsim01G0021100	0.432	-0.044	-0.477	no
CK-40d	RHSIM_Rhsim01G0192500	-0.572	-1.552	-0.980	no
CK-40d	RHSIM_Rhsim01G0287000	-0.655	1.596	2.250	no
CK-40d	RHSIM_Rhsim01G0141200	0.566	1.106	0.540	no
CK-40d	RHSIM_Rhsim01G0091700	-0.895	-0.938	-0.044	no
CK-40d	RHSIM_Rhsim01G0210100	2.527	0.343	-2.184	high
CK-40d	RHSIM_Rhsim01G0170700	0.124	0.411	0.287	no
CK-40d	RHSIM_Rhsim01G0259500	1.600	2.121	0.520	high
CK-40d	RHSIM_Rhsim01G0163000	0.472	-0.507	-0.979	no
Treat-0d	RHSIM_RhsimUnG0249100	-3.550	-0.053	3.497	low
Treat-0d	RHSIM_RhsimUnG0254800	-2.848	-0.959	1.889	low
Treat-0d	RHSIM_RhsimUnG0258600	1.419	2.794	1.374	high
Treat-0d	RHSIM_RhsimUnG0249600	-1.541	-0.470	1.072	low
Treat-0d	RHSIM_RhsimUnG0251500	0.336	0.079	-0.257	no
Treat-0d	RHSIM_RhsimUnG0252300	0.631	-0.178	-0.809	no
Treat-0d	RHSIM_RhsimUnG0228100	0.412	-1.344	-1.757	no
Treat-0d	RHSIM_RhsimUnG0251200	-0.533	0.562	1.096	no
Treat-0d	RHSIM_RhsimUnG0226800	0.447	-0.464	-0.911	no
Treat-0d	RHSIM_RhsimUnG0245900	1.484	3.504	2.021	high
Treat-0d	RHSIM_RhsimUnG0241900	1.585	0.753	-0.832	high
Treat-0d	RHSIM_RhsimUnG0250300	-4.240	-1.449	2.792	low
Treat-0d	RHSIM_RhsimUnG0250600	-0.224	-0.098	0.126	no
Treat-0d	RHSIM_RhsimUnG0216900	0.435	1.602	1.167	no

Group	Gene	PTR (z-score difference)	z(log2 LFQ)	z(log2 FPKM)	extreme-PTR
Treat-0d	RHSIM_RhsimUnG0239100	0.870	0.485	-0.385	no
Treat-0d	RHSIM_RhsimUnG0231700	1.630	-0.551	-2.182	high
Treat-0d	RHSIM_RhsimUnG0212900	2.199	0.311	-1.888	high
Treat-0d	RHSIM_RhsimUnG0231900	1.279	-0.142	-1.421	high
Treat-0d	RHSIM_RhsimUnG0209400	1.663	0.752	-0.911	high
Treat-0d	RHSIM_RhsimUnG0228800	1.773	0.312	-1.461	high
Treat-0d	RHSIM_RhsimUnG0219900	0.455	-0.865	-1.320	no
Treat-0d	RHSIM_RhsimUnG0217500	1.411	0.634	-0.778	high
Treat-0d	RHSIM_Rhsim09G0026600	1.309	-1.068	-2.377	high
Treat-0d	RHSIM_RhsimUnG0226200	-0.014	0.122	0.137	no
Treat-0d	RHSIM_RhsimUnG0210000	1.375	0.402	-0.973	high
Treat-0d	RHSIM_RhsimUnG0190800	0.505	0.322	-0.183	no
Treat-0d	RHSIM_RhsimUnG0217400	0.275	-0.257	-0.533	no
Treat-0d	RHSIM_RhsimUnG0220800	0.750	-0.385	-1.135	no
Treat-0d	RHSIM_RhsimUnG0253500	2.683	1.645	-1.037	high
Treat-0d	RHSIM_RhsimUnG0248900	0.680	1.181	0.501	no
Treat-0d	RHSIM_RhsimUnG0211400	-0.540	1.355	1.895	no
Treat-0d	RHSIM_RhsimUnG0209900	1.480	0.527	-0.953	high
Treat-0d	RHSIM_RhsimUnG0205800	0.084	1.749	1.665	no
Treat-0d	RHSIM_RhsimUnG0219300	-0.462	-1.394	-0.932	no
Treat-0d	RHSIM_Rhsim09G0148400	-1.998	-0.693	1.305	low
Treat-0d	RHSIM_RhsimUnG0209800	0.021	-0.588	-0.609	no
Treat-0d	RHSIM_RhsimUnG0225600	-0.515	-0.132	0.383	no
Treat-0d	RHSIM_RhsimUnG0255000	-0.415	0.534	0.949	no
Treat-0d	RHSIM_RhsimUnG0205900	-0.901	0.974	1.876	no
Treat-0d	RHSIM_RhsimUnG0197600	1.451	0.758	-0.693	high
Treat-0d	RHSIM_RhsimUnG0206500	-0.199	-1.039	-0.840	no
Treat-0d	RHSIM_Rhsim09G0069900	1.498	1.752	0.254	high
Treat-0d	RHSIM_RhsimUnG0169900	-0.121	-0.682	-0.561	no
Treat-0d	RHSIM_RhsimUnG0168100	-1.688	-2.217	-0.529	low
Treat-0d	RHSIM_RhsimUnG0185400	-0.091	-0.546	-0.455	no
Treat-0d	RHSIM_RhsimUnG0158200	1.431	1.791	0.360	high

Group	Gene	PTR (z-score difference)	z(log2 LFQ)	z(log2 FPKM)	extreme-PTR
Treat-0d	RHSIM_RhsimUnG0197500	1.141	-0.154	-1.295	high
Treat-0d	RHSIM_Rhsim02G0029800	1.143	-0.709	-1.852	high
Treat-0d	RHSIM_Rhsim09G0021500	1.530	1.644	0.114	high
Treat-0d	RHSIM_RhsimUnG0136600	0.162	0.132	-0.030	no
Treat-0d	RHSIM_RhsimUnG0226700	0.619	-0.246	-0.866	no
Treat-0d	RHSIM_RhsimUnG0220700	2.022	0.946	-1.076	high
Treat-0d	RHSIM_RhsimUnG0126200	1.318	1.070	-0.248	high
Treat-0d	RHSIM_RhsimUnG0217000	0.898	-0.207	-1.105	no
Treat-0d	RHSIM_RhsimUnG0116800	-1.086	-1.365	-0.280	low
Treat-0d	RHSIM_RhsimUnG0109300	0.891	1.537	0.646	no
Treat-0d	RHSIM_RhsimUnG0122900	0.979	0.081	-0.898	no
Treat-0d	RHSIM_RhsimUnG0158600	1.437	0.262	-1.174	high
Treat-0d	RHSIM_RhsimUnG0137100	1.131	0.485	-0.646	high
Treat-0d	RHSIM_RhsimUnG0190300	0.167	-0.277	-0.444	no
Treat-0d	RHSIM_RhsimUnG0256800	0.643	0.228	-0.415	no
Treat-0d	RHSIM_RhsimUnG0185700	0.708	-1.481	-2.189	no
Treat-0d	RHSIM_RhsimUnG0180800	-1.238	0.288	1.527	low
Treat-0d	RHSIM_RhsimUnG0124400	1.878	-0.368	-2.246	high
Treat-0d	RHSIM_RhsimUnG0254600	-1.315	-0.685	0.630	low
Treat-0d	RHSIM_RhsimUnG0242200	-0.585	-0.643	-0.058	no
Treat-0d	RHSIM_RhsimUnG0253800	-0.506	-1.480	-0.973	no
Treat-0d	RHSIM_RhsimUnG0093800	0.083	0.026	-0.057	no
Treat-0d	RHSIM_RhsimUnG0257500	1.879	0.841	-1.038	high
Treat-0d	RHSIM_RhsimUnG0118900	0.023	1.510	1.487	no
Treat-0d	RHSIM_RhsimUnG0120200	-0.502	-1.005	-0.503	no
Treat-0d	RHSIM_RhsimUnG0111200	-0.617	-1.060	-0.443	no
Treat-0d	RHSIM_RhsimUnG0092300	-0.071	0.845	0.916	no
Treat-0d	RHSIM_RhsimUnG0124200	0.339	-0.604	-0.943	no
Treat-0d	RHSIM_RhsimUnG0094500	0.301	0.284	-0.016	no
Treat-0d	RHSIM_RhsimUnG0059700	0.193	-0.083	-0.276	no
Treat-0d	RHSIM_RhsimUnG0145200	0.735	-1.051	-1.786	no
Treat-0d	RHSIM_RhsimUnG0118400	-0.961	-1.883	-0.921	no

Group	Gene	PTR (z-score difference)	z(log2 LFQ)	z(log2 FPKM)	extreme-PTR
Treat-0d	RHSIM_RhsimUnG0138800	2.441	1.216	-1.226	high
Treat-0d	RHSIM_RhsimUnG0054600	-0.732	-0.684	0.048	no
Treat-0d	RHSIM_RhsimUnG0216100	-2.645	-0.192	2.453	low
Treat-0d	RHSIM_RhsimUnG0141400	-0.254	-1.981	-1.727	no
Treat-0d	RHSIM_RhsimUnG0213100	-0.029	-1.170	-1.141	no
Treat-0d	RHSIM_RhsimUnG0112300	0.465	-1.278	-1.743	no
Treat-0d	RHSIM_RhsimUnG0112800	0.784	-0.883	-1.666	no
Treat-0d	RHSIM_RhsimUnG0111000	-0.276	-0.593	-0.317	no
Treat-0d	RHSIM_RhsimUnG0205000	-0.787	1.290	2.077	no
Treat-0d	RHSIM_RhsimUnG0221600	0.456	1.208	0.752	no
Treat-0d	RHSIM_RhsimUnG0060900	-0.456	-0.361	0.095	no
Treat-0d	RHSIM_RhsimUnG0195900	0.551	-1.182	-1.733	no
Treat-0d	RHSIM_RhsimUnG0215100	1.969	-0.061	-2.030	high
Treat-0d	RHSIM_RhsimUnG0124800	0.238	-0.933	-1.171	no
Treat-0d	RHSIM_RhsimUnG0098600	0.813	-0.211	-1.024	no
Treat-0d	RHSIM_RhsimUnG0055200	0.035	0.000	-0.035	no
Treat-0d	RHSIM_RhsimUnG0096900	0.568	0.097	-0.471	no
Treat-0d	RHSIM_RhsimUnG0119200	-0.662	-0.137	0.526	no
Treat-0d	RHSIM_RhsimUnG0032200	-0.732	-0.980	-0.248	no
Treat-0d	RHSIM_RhsimUnG0119600	-1.069	-0.961	0.108	low
Treat-0d	RHSIM_RhsimUnG0087900	1.938	1.603	-0.335	high
Treat-0d	RHSIM_RhsimUnG0206600	1.489	-0.055	-1.544	high
Treat-0d	RHSIM_RhsimUnG0086700	0.940	-1.212	-2.152	no
Treat-0d	RHSIM_RhsimUnG0065700	1.230	0.099	-1.131	high
Treat-0d	RHSIM_RhsimUnG0113700	0.375	-0.337	-0.712	no
Treat-0d	RHSIM_RhsimUnG0109400	0.776	1.315	0.540	no
Treat-0d	RHSIM_RhsimUnG0080300	1.213	0.695	-0.519	high
Treat-0d	RHSIM_RhsimUnG0020600	0.517	-0.339	-0.856	no
Treat-0d	RHSIM_RhsimUnG0019600	0.859	0.645	-0.214	no
Treat-0d	RHSIM_RhsimUnG0039700	-0.501	-1.175	-0.674	no
Treat-0d	RHSIM_RhsimUnG0117900	0.924	0.880	-0.044	no
Treat-0d	RHSIM_RhsimUnG0021000	0.873	-0.500	-1.373	no

Group	Gene	PTR (z-score difference)	z(log2 LFQ)	z(log2 FPKM)	extreme-PTR
Treat-0d	RHSIM_RhsimUnG0055900	-0.737	-0.131	0.606	no
Treat-0d	RHSIM_RhsimUnG0098000	0.712	0.183	-0.529	no
Treat-0d	RHSIM_RhsimUnG0194300	-0.738	-0.235	0.503	no
Treat-0d	RHSIM_RhsimUnG0119800	-0.454	-1.453	-1.000	no
Treat-0d	RHSIM_RhsimUnG0094700	0.427	0.741	0.314	no
Treat-0d	RHSIM_RhsimUnG0067700	1.599	-0.805	-2.404	high
Treat-0d	RHSIM_RhsimUnG0109200	0.597	0.313	-0.284	no
Treat-0d	RHSIM_RhsimUnG0107500	0.453	0.281	-0.172	no
Treat-0d	RHSIM_RhsimUnG0105000	1.158	1.225	0.067	high
Treat-0d	RHSIM_RhsimUnG0188600	-0.015	-0.587	-0.572	no
Treat-0d	RHSIM_RhsimUnG0006400	1.565	-0.180	-1.745	high
Treat-0d	RHSIM_RhsimPtG0002900	2.692	0.830	-1.863	high
Treat-0d	RHSIM_RhsimUnG0096000	1.362	-1.017	-2.379	high
Treat-0d	RHSIM_RhsimUnG0079300	0.333	-1.282	-1.615	no
Treat-0d	RHSIM_RhsimUnG0021300	-1.206	-1.106	0.100	low
Treat-0d	RHSIM_Rhsim13G0142900	-1.045	-1.538	-0.493	low
Treat-0d	RHSIM_RhsimUnG0026400	2.128	0.089	-2.039	high
Treat-0d	RHSIM_RhsimUnG0045900	2.647	1.403	-1.244	high
Treat-0d	RHSIM_RhsimUnG0139700	0.178	-0.366	-0.544	no
Treat-0d	RHSIM_RhsimUnG0085800	-1.126	-0.149	0.977	low
Treat-0d	RHSIM_RhsimUnG0044200	-0.926	-1.262	-0.337	no
Treat-0d	RHSIM_Rhsim13G0005000	0.478	0.739	0.261	no
Treat-0d	RHSIM_RhsimUnG0021800	1.713	2.238	0.526	high
Treat-0d	RHSIM_Rhsim13G0010100	-1.136	-0.505	0.631	low
Treat-0d	RHSIM_RhsimUnG0073900	0.205	0.154	-0.051	no
Treat-0d	RHSIM_RhsimUnG0070500	-0.342	-0.762	-0.420	no
Treat-0d	RHSIM_Rhsim13G0064900	0.821	0.334	-0.486	no
Treat-0d	RHSIM_Rhsim11G0060300	0.824	-0.165	-0.989	no
Treat-0d	RHSIM_RhsimUnG0027200	0.416	-0.248	-0.663	no
Treat-0d	RHSIM_Rhsim13G0234100	0.114	-0.368	-0.482	no
Treat-0d	RHSIM_RhsimUnG0060500	-0.390	-0.278	0.112	no
Treat-0d	RHSIM_Rhsim13G0198200	0.613	0.939	0.326	no

Group	Gene	PTR (z-score difference)	z(log2 LFQ)	z(log2 FPKM)	extreme-PTR
Treat-0d	RHSIM_RhsimUnG0014100	-0.546	-0.456	0.090	no
Treat-0d	RHSIM_RhsimMtG0006700	1.428	-0.230	-1.658	high
Treat-0d	RHSIM_Rhsim13G0018300	0.632	-0.109	-0.741	no
Treat-0d	RHSIM_RhsimUnG0022900	1.476	0.274	-1.201	high
Treat-0d	RHSIM_Rhsim05G0087400	-0.111	0.528	0.639	no
Treat-0d	RHSIM_RhsimUnG0051400	0.956	0.998	0.043	no
Treat-0d	RHSIM_RhsimUnG0059400	0.126	-0.292	-0.418	no
Treat-0d	RHSIM_RhsimUnG0005600	-0.630	-0.797	-0.167	no
Treat-0d	RHSIM_Rhsim13G0009100	-0.376	1.436	1.812	no
Treat-0d	RHSIM_RhsimPtG0000900	3.850	2.198	-1.652	high
Treat-0d	RHSIM_RhsimUnG0015500	0.519	0.063	-0.456	no
Treat-0d	RHSIM_RhsimUnG0055000	0.970	-0.166	-1.135	no
Treat-0d	RHSIM_Rhsim13G0140400	-0.603	-0.746	-0.143	no
Treat-0d	RHSIM_Rhsim13G0104300	0.572	0.799	0.226	no
Treat-0d	RHSIM_Rhsim13G0149200	0.211	2.091	1.880	no
Treat-0d	RHSIM_Rhsim13G0215900	-0.888	1.581	2.469	no
Treat-0d	RHSIM_Rhsim13G0000400	-0.159	0.510	0.669	no
Treat-0d	RHSIM_Rhsim13G0111800	-1.155	0.150	1.305	low
Treat-0d	RHSIM_RhsimUnG0008400	0.372	1.708	1.336	no
Treat-0d	RHSIM_Rhsim13G0157800	1.415	1.463	0.048	high
Treat-0d	RHSIM_RhsimUnG0043200	0.505	2.645	2.140	no
Treat-0d	RHSIM_RhsimUnG0002100	0.010	0.277	0.267	no
Treat-0d	RHSIM_Rhsim13G0110800	0.378	0.781	0.403	no
Treat-0d	RHSIM_Rhsim13G0229200	2.176	0.495	-1.682	high
Treat-0d	RHSIM_Rhsim13G0150700	0.678	0.372	-0.306	no
Treat-0d	RHSIM_Rhsim13G0112500	1.344	1.501	0.157	high
Treat-0d	RHSIM_Rhsim13G0088500	0.631	-0.251	-0.882	no
Treat-0d	RHSIM_Rhsim13G0186600	0.910	0.424	-0.487	no
Treat-0d	RHSIM_Rhsim13G0177500	-2.014	-1.061	0.953	low
Treat-0d	RHSIM_Rhsim13G0205900	0.148	0.390	0.242	no
Treat-0d	RHSIM_Rhsim13G0156100	0.551	-0.502	-1.053	no
Treat-0d	RHSIM_RhsimUnG0018800	2.192	1.264	-0.928	high

Group	Gene	PTR (z-score difference)	z(log2 LFQ)	z(log2 FPKM)	extreme-PTR
Treat-0d	RHSIM_Rhsim13G0099400	-0.642	-1.621	-0.979	no
Treat-0d	RHSIM_RhsimUnG0023000	-1.195	-0.983	0.212	low
Treat-0d	RHSIM_RhsimUnG0094300	0.661	0.689	0.028	no
Treat-0d	RHSIM_RhsimUnG0025700	0.995	-0.070	-1.065	no
Treat-0d	RHSIM_RhsimUnG0254000	0.837	1.466	0.630	no
Treat-0d	RHSIM_Rhsim13G0077700	0.005	-0.488	-0.493	no
Treat-0d	RHSIM_Rhsim13G0088800	-1.146	-0.603	0.543	low
Treat-0d	RHSIM_Rhsim13G0030500	-0.428	-1.626	-1.198	no
Treat-0d	RHSIM_RhsimUnG0252800	0.615	0.035	-0.580	no
Treat-0d	RHSIM_Rhsim13G0201300	0.238	0.144	-0.094	no
Treat-0d	RHSIM_Rhsim13G0179600	-1.185	-0.263	0.922	low
Treat-0d	RHSIM_Rhsim13G0140500	0.589	-0.071	-0.661	no
Treat-0d	RHSIM_Rhsim13G0175200	-0.179	0.573	0.753	no
Treat-0d	RHSIM_Rhsim13G0069400	1.262	1.961	0.699	high
Treat-0d	RHSIM_Rhsim13G0169400	0.250	0.558	0.308	no
Treat-0d	RHSIM_Rhsim13G0211800	0.862	0.821	-0.041	no
Treat-0d	RHSIM_RhsimUnG0139600	0.557	0.217	-0.339	no
Treat-0d	RHSIM_RhsimUnG0004000	1.249	0.234	-1.014	high
Treat-0d	RHSIM_RhsimUnG0248600	0.788	-0.883	-1.672	no
Treat-0d	RHSIM_Rhsim13G0014400	-1.174	-1.174	0.001	low
Treat-0d	RHSIM_RhsimUnG0006200	1.060	0.714	-0.346	no
Treat-0d	RHSIM_Rhsim13G0147900	0.497	0.076	-0.421	no
Treat-0d	RHSIM_Rhsim13G0127600	-2.072	-1.812	0.260	low
Treat-0d	RHSIM_Rhsim13G0091700	-1.079	0.811	1.890	low
Treat-0d	RHSIM_RhsimUnG0135600	0.930	-0.820	-1.749	no
Treat-0d	RHSIM_Rhsim13G0140200	0.089	-0.107	-0.196	no
Treat-0d	RHSIM_Rhsim13G0039100	-0.064	-0.692	-0.628	no
Treat-0d	RHSIM_Rhsim13G0012100	0.823	0.836	0.012	no
Treat-0d	RHSIM_Rhsim13G0162700	0.360	2.390	2.030	no
Treat-0d	RHSIM_Rhsim13G0201600	0.645	-0.711	-1.355	no
Treat-0d	RHSIM_Rhsim13G0170400	-0.745	0.022	0.768	no
Treat-0d	RHSIM_Rhsim13G0218000	-1.306	-1.752	-0.446	low

Group	Gene	PTR (z-score difference)	z(log2 LFQ)	z(log2 FPKM)	extreme-PTR
Treat-0d	RHSIM_Rhsim13G0024600	1.237	0.208	-1.030	high
Treat-0d	RHSIM_Rhsim13G0024200	-2.804	-1.518	1.286	low
Treat-0d	RHSIM_RhsimUnG0077000	1.581	0.328	-1.253	high
Treat-0d	RHSIM_Rhsim13G0122800	1.692	2.086	0.394	high
Treat-0d	RHSIM_Rhsim13G0115500	0.351	-0.099	-0.450	no
Treat-0d	RHSIM_RhsimUnG0237100	0.694	-1.272	-1.966	no
Treat-0d	RHSIM_RhsimUnG0120500	-1.682	-1.496	0.187	low
Treat-0d	RHSIM_Rhsim13G0030900	2.877	3.558	0.681	high
Treat-0d	RHSIM_Rhsim13G0202300	-2.267	-2.364	-0.097	low
Treat-0d	RHSIM_Rhsim13G0203400	0.352	1.643	1.291	no
Treat-0d	RHSIM_Rhsim13G0233700	-0.621	0.352	0.973	no
Treat-0d	RHSIM_Rhsim13G0057000	-1.000	-0.800	0.200	no
Treat-0d	RHSIM_Rhsim13G0044900	-0.017	-1.051	-1.034	no
Treat-0d	RHSIM_Rhsim13G0155800	-1.356	-0.470	0.887	low
Treat-0d	RHSIM_Rhsim13G0060100	-0.529	-0.568	-0.039	no
Treat-0d	RHSIM_Rhsim13G0062700	0.856	-0.319	-1.175	no
Treat-0d	RHSIM_Rhsim13G0140900	-0.006	-0.054	-0.048	no
Treat-0d	RHSIM_Rhsim13G0111500	0.188	1.569	1.381	no
Treat-0d	RHSIM_Rhsim13G0111400	-0.318	0.919	1.237	no
Treat-0d	RHSIM_RhsimUnG0102900	-0.218	-0.502	-0.284	no
Treat-0d	RHSIM_Rhsim13G0208400	-0.208	-0.374	-0.166	no
Treat-0d	RHSIM_Rhsim13G0217600	-0.593	-0.348	0.244	no
Treat-0d	RHSIM_Rhsim13G0195100	-1.343	-1.095	0.249	low
Treat-0d	RHSIM_Rhsim13G0216400	0.100	-0.334	-0.434	no
Treat-0d	RHSIM_Rhsim13G0024400	0.392	0.002	-0.390	no
Treat-0d	RHSIM_Rhsim13G0040400	-0.977	-1.154	-0.176	no
Treat-0d	RHSIM_RhsimUnG0095400	-2.501	-1.588	0.914	low
Treat-0d	RHSIM_Rhsim13G0059900	0.337	0.135	-0.202	no
Treat-0d	RHSIM_Rhsim13G0125900	0.794	0.448	-0.345	no
Treat-0d	RHSIM_Rhsim13G0121000	-0.937	-0.860	0.077	no
Treat-0d	RHSIM_Rhsim13G0189200	-3.572	-2.138	1.434	low
Treat-0d	RHSIM_Rhsim13G0013600	0.650	1.874	1.224	no

Group	Gene	PTR (z-score difference)	z(log2 LFQ)	z(log2 FPKM)	extreme-PTR
Treat-0d	RHSIM_Rhsim13G0071000	-0.402	-0.214	0.188	no
Treat-0d	RHSIM_RhsimUnG0086000	0.064	0.569	0.505	no
Treat-0d	RHSIM_Rhsim13G0047200	-1.849	-0.607	1.242	low
Treat-0d	RHSIM_Rhsim13G0120300	-0.970	-1.364	-0.394	no
Treat-0d	RHSIM_Rhsim13G0191600	-0.459	0.319	0.777	no
Treat-0d	RHSIM_Rhsim13G0119400	0.871	3.071	2.200	no
Treat-0d	RHSIM_Rhsim13G0174200	0.946	0.227	-0.719	no
Treat-0d	RHSIM_Rhsim13G0205000	1.304	-0.053	-1.357	high
Treat-0d	RHSIM_Rhsim13G0191100	-0.995	-0.383	0.612	no
Treat-0d	RHSIM_Rhsim13G0146300	-1.450	-0.878	0.572	low
Treat-0d	RHSIM_Rhsim13G0163000	0.808	-0.218	-1.026	no
Treat-0d	RHSIM_RhsimUnG0079400	-0.866	-0.847	0.019	no
Treat-0d	RHSIM_Rhsim13G0194200	1.597	2.133	0.536	high
Treat-0d	RHSIM_Rhsim13G0052700	-0.450	-0.362	0.089	no
Treat-0d	RHSIM_Rhsim13G0072700	0.063	0.686	0.623	no
Treat-0d	RHSIM_RhsimUnG0206200	-1.304	-1.281	0.023	low
Treat-0d	RHSIM_Rhsim13G0094700	-0.246	0.254	0.500	no
Treat-0d	RHSIM_Rhsim13G0201700	-0.064	0.753	0.817	no
Treat-0d	RHSIM_Rhsim13G0056300	-0.865	-1.639	-0.775	no
Treat-0d	RHSIM_RhsimUnG0046000	0.919	-0.234	-1.153	no
Treat-0d	RHSIM_Rhsim13G0169800	1.002	1.109	0.107	no
Treat-0d	RHSIM_Rhsim13G0152000	0.009	0.622	0.613	no
Treat-0d	RHSIM_Rhsim13G0011700	-0.496	1.114	1.610	no
Treat-0d	RHSIM_Rhsim13G0056400	1.025	0.947	-0.078	no
Treat-0d	RHSIM_RhsimUnG0066200	-2.394	-0.996	1.398	low
Treat-0d	RHSIM_Rhsim13G0098900	-1.773	0.009	1.782	low
Treat-0d	RHSIM_RhsimUnG0065400	1.275	2.487	1.212	high
Treat-0d	RHSIM_Rhsim13G0226700	-0.586	0.208	0.795	no
Treat-0d	RHSIM_Rhsim13G0061400	-0.635	-0.972	-0.337	no
Treat-0d	RHSIM_Rhsim13G0129400	0.379	-0.747	-1.126	no
Treat-0d	RHSIM_Rhsim13G0050600	-0.054	-0.276	-0.222	no
Treat-0d	RHSIM_Rhsim13G0020400	-1.615	-1.139	0.476	low

Group	Gene	PTR (z-score difference)	z(log2 LFQ)	z(log2 FPKM)	extreme-PTR
Treat-0d	RHSIM_Rhsim13G0009900	0.074	-1.452	-1.525	no
Treat-0d	RHSIM_Rhsim13G0098700	-0.600	0.521	1.121	no
Treat-0d	RHSIM_Rhsim13G0198700	-0.723	-0.380	0.343	no
Treat-0d	RHSIM_Rhsim13G0057400	-0.705	-0.483	0.222	no
Treat-0d	RHSIM_Rhsim13G0179900	-1.526	-1.304	0.221	low
Treat-0d	RHSIM_Rhsim13G0094300	0.889	0.407	-0.482	no
Treat-0d	RHSIM_Rhsim13G0035600	-0.573	-0.254	0.319	no
Treat-0d	RHSIM_Rhsim13G0234800	0.657	0.656	-0.001	no
Treat-0d	RHSIM_Rhsim13G0001000	1.299	0.485	-0.814	high
Treat-0d	RHSIM_Rhsim13G0221000	-1.152	-0.715	0.437	low
Treat-0d	RHSIM_RhsimUnG0053100	-0.873	-1.250	-0.377	no
Treat-0d	RHSIM_Rhsim13G0107100	-1.560	0.035	1.595	low
Treat-0d	RHSIM_Rhsim13G0024900	0.965	0.372	-0.593	no
Treat-0d	RHSIM_Rhsim13G0045300	0.808	1.655	0.847	no
Treat-0d	RHSIM_Rhsim13G0037500	0.748	0.240	-0.508	no
Treat-0d	RHSIM_RhsimUnG0048500	-0.164	-1.198	-1.033	no
Treat-0d	RHSIM_Rhsim13G0211100	-0.061	0.245	0.306	no
Treat-0d	RHSIM_Rhsim13G0072000	-0.437	-0.296	0.141	no
Treat-0d	RHSIM_Rhsim13G0027700	-0.468	-0.240	0.228	no
Treat-0d	RHSIM_Rhsim13G0018600	0.178	0.258	0.080	no
Treat-0d	RHSIM_Rhsim13G0127200	1.533	3.847	2.314	high
Treat-0d	RHSIM_RhsimUnG0023900	1.780	-0.034	-1.814	high
Treat-0d	RHSIM_Rhsim13G0231000	0.454	-0.001	-0.454	no
Treat-0d	RHSIM_Rhsim13G0036500	-1.477	-0.355	1.122	low
Treat-0d	RHSIM_Rhsim13G0031600	0.987	0.661	-0.327	no
Treat-0d	RHSIM_Rhsim13G0065500	0.391	1.060	0.669	no
Treat-0d	RHSIM_RhsimUnG0021400	-1.276	-0.345	0.931	low
Treat-0d	RHSIM_Rhsim13G0015500	0.786	0.195	-0.590	no
Treat-0d	RHSIM_Rhsim13G0056900	-0.397	0.740	1.137	no
Treat-0d	RHSIM_Rhsim13G0115200	0.522	0.496	-0.026	no
Treat-0d	RHSIM_RhsimUnG0172300	0.354	-0.091	-0.446	no
Treat-0d	RHSIM_Rhsim13G0209000	-0.557	-1.177	-0.620	no

Group	Gene	PTR (z-score difference)	z(log2 LFQ)	z(log2 FPKM)	extreme-PTR
Treat-0d	RHSIM_Rhsim13G0121400	0.886	0.088	-0.799	no
Treat-0d	RHSIM_Rhsim13G0208500	-3.271	-2.061	1.210	low
Treat-0d	RHSIM_Rhsim13G0058000	-0.044	0.927	0.971	no
Treat-0d	RHSIM_Rhsim13G0224400	1.632	-0.121	-1.753	high
Treat-0d	RHSIM_Rhsim13G0004800	0.350	-0.259	-0.609	no
Treat-0d	RHSIM_Rhsim13G0221900	0.368	-0.394	-0.763	no
Treat-0d	RHSIM_Rhsim13G0104500	0.785	-0.571	-1.356	no
Treat-0d	RHSIM_RhsimUnG0033300	0.224	-0.359	-0.583	no
Treat-0d	RHSIM_Rhsim13G0129600	-0.641	-0.363	0.278	no
Treat-0d	RHSIM_Rhsim13G0098500	-0.106	-0.448	-0.342	no
Treat-0d	RHSIM_Rhsim13G0228700	2.507	1.819	-0.689	high
Treat-0d	RHSIM_Rhsim13G0152700	2.440	2.851	0.411	high
Treat-0d	RHSIM_Rhsim13G0228600	-0.221	1.282	1.503	no
Treat-0d	RHSIM_Rhsim13G0196900	-1.031	-1.184	-0.153	low
Treat-0d	RHSIM_Rhsim13G0116700	-0.692	-1.383	-0.691	no
Treat-0d	RHSIM_RhsimUnG0009300	-0.132	0.692	0.824	no
Treat-0d	RHSIM_Rhsim13G0017200	-0.462	-0.643	-0.181	no
Treat-0d	RHSIM_Rhsim13G0000200	-0.476	0.448	0.924	no
Treat-0d	RHSIM_Rhsim13G0073600	-0.057	-0.621	-0.564	no
Treat-0d	RHSIM_Rhsim13G0209100	-0.640	-2.014	-1.373	no
Treat-0d	RHSIM_Rhsim13G0056100	-1.102	-1.202	-0.100	low
Treat-0d	RHSIM_Rhsim13G0056500	0.419	0.429	0.009	no
Treat-0d	RHSIM_Rhsim13G0109800	-0.549	-1.164	-0.614	no
Treat-0d	RHSIM_RhsimUnG0023200	0.238	-0.856	-1.094	no
Treat-0d	RHSIM_Rhsim13G0200400	0.389	-0.732	-1.121	no
Treat-0d	RHSIM_Rhsim13G0127700	0.673	0.108	-0.564	no
Treat-0d	RHSIM_Rhsim13G0230700	0.671	-0.355	-1.026	no
Treat-0d	RHSIM_Rhsim13G0046300	-0.486	-0.248	0.238	no
Treat-0d	RHSIM_Rhsim13G0112600	-0.125	-0.348	-0.223	no
Treat-0d	RHSIM_RhsimUnG0015300	1.706	1.268	-0.438	high
Treat-0d	RHSIM_Rhsim13G0053000	-1.819	-1.570	0.249	low
Treat-0d	RHSIM_Rhsim13G0082500	0.645	2.929	2.284	no

Group	Gene	PTR (z-score difference)	z(log2 LFQ)	z(log2 FPKM)	extreme-PTR
Treat-0d	RHSIM_Rhsim13G0200800	-0.271	-0.350	-0.080	no
Treat-0d	RHSIM_Rhsim13G0094400	1.355	2.614	1.259	high
Treat-0d	RHSIM_Rhsim13G0084100	-0.319	2.584	2.902	no
Treat-0d	RHSIM_Rhsim13G0011000	-0.705	0.892	1.597	no
Treat-0d	RHSIM_Rhsim13G0137100	1.113	0.339	-0.773	high
Treat-0d	RHSIM_Rhsim13G0104400	0.440	-0.051	-0.491	no
Treat-0d	RHSIM_RhsimUnG0009100	0.481	0.779	0.298	no
Treat-0d	RHSIM_Rhsim13G0065400	-0.029	-0.976	-0.947	no
Treat-0d	RHSIM_RhsimUnG0007400	-1.088	-1.677	-0.589	low
Treat-0d	RHSIM_Rhsim13G0077000	-1.404	-1.460	-0.056	low
Treat-0d	RHSIM_RhsimUnG0153300	-0.540	-0.513	0.026	no
Treat-0d	RHSIM_Rhsim13G0088300	0.059	-0.585	-0.644	no
Treat-0d	RHSIM_Rhsim13G0035500	-0.336	-0.158	0.178	no
Treat-0d	RHSIM_Rhsim13G0225600	1.289	3.321	2.032	high
Treat-0d	RHSIM_Rhsim13G0225300	0.725	0.734	0.009	no
Treat-0d	RHSIM_Rhsim13G0210700	-2.205	-1.069	1.136	low
Treat-0d	RHSIM_Rhsim13G0075700	0.569	2.037	1.468	no
Treat-0d	RHSIM_Rhsim13G0141400	-1.551	-0.386	1.166	low
Treat-0d	RHSIM_Rhsim13G0021200	-0.304	0.480	0.784	no
Treat-0d	RHSIM_Rhsim13G0163700	0.016	0.543	0.527	no
Treat-0d	RHSIM_RhsimUnG0150500	3.401	1.425	-1.976	high
Treat-0d	RHSIM_Rhsim13G0095400	-1.566	-1.630	-0.063	low
Treat-0d	RHSIM_Rhsim13G0209300	-0.666	-0.797	-0.131	no
Treat-0d	RHSIM_Rhsim13G0104000	0.291	1.594	1.303	no
Treat-0d	RHSIM_Rhsim13G0034500	0.569	-0.428	-0.997	no
Treat-0d	RHSIM_Rhsim13G0117100	-0.850	-0.682	0.167	no
Treat-0d	RHSIM_Rhsim13G0089200	2.496	2.075	-0.422	high
Treat-0d	RHSIM_Rhsim13G0232600	-0.038	-0.917	-0.878	no
Treat-0d	RHSIM_Rhsim13G0105300	0.285	0.256	-0.029	no
Treat-0d	RHSIM_Rhsim13G0207700	1.537	1.805	0.268	high
Treat-0d	RHSIM_Rhsim13G0110000	-0.891	0.295	1.186	no
Treat-0d	RHSIM_Rhsim13G0128200	-0.879	-0.675	0.204	no

Group	Gene	PTR (z-score difference)	z(log2 LFQ)	z(log2 FPKM)	extreme-PTR
Treat-0d	RHSIM_Rhsim12G0014500	-0.039	-0.765	-0.726	no
Treat-0d	RHSIM_Rhsim13G0201400	-2.726	-1.589	1.137	low
Treat-0d	RHSIM_Rhsim13G0203300	-0.180	-0.494	-0.314	no
Treat-0d	RHSIM_Rhsim12G0044300	0.047	0.079	0.032	no
Treat-0d	RHSIM_Rhsim13G0051800	0.206	0.027	-0.179	no
Treat-0d	RHSIM_Rhsim13G0196400	-1.962	-0.597	1.365	low
Treat-0d	RHSIM_Rhsim13G0181500	0.051	-0.864	-0.916	no
Treat-0d	RHSIM_Rhsim13G0066200	0.042	-0.705	-0.748	no
Treat-0d	RHSIM_Rhsim13G0160100	0.079	-2.011	-2.090	no
Treat-0d	RHSIM_Rhsim13G0093000	0.154	-0.542	-0.696	no
Treat-0d	RHSIM_Rhsim12G0190000	0.746	0.271	-0.475	no
Treat-0d	RHSIM_Rhsim13G0123900	-0.029	1.983	2.012	no
Treat-0d	RHSIM_RhsimUnG0144200	0.425	1.851	1.426	no
Treat-0d	RHSIM_Rhsim12G0144400	-1.595	-1.350	0.245	low
Treat-0d	RHSIM_Rhsim13G0044300	0.662	-0.371	-1.033	no
Treat-0d	RHSIM_Rhsim13G0018200	0.202	0.409	0.207	no
Treat-0d	RHSIM_Rhsim12G0188700	-1.083	-0.086	0.997	low
Treat-0d	RHSIM_Rhsim13G0099900	-0.739	-0.787	-0.048	no
Treat-0d	RHSIM_Rhsim12G0145400	0.694	0.251	-0.443	no
Treat-0d	RHSIM_Rhsim12G0089400	-0.739	-0.931	-0.191	no
Treat-0d	RHSIM_Rhsim13G0064700	1.115	0.552	-0.562	high
Treat-0d	RHSIM_Rhsim13G0002600	-0.358	-0.283	0.075	no
Treat-0d	RHSIM_Rhsim12G0136900	-0.113	0.221	0.334	no
Treat-0d	RHSIM_Rhsim13G0102500	1.498	0.934	-0.564	high
Treat-0d	RHSIM_Rhsim13G0225400	0.224	0.753	0.530	no
Treat-0d	RHSIM_Rhsim13G0018900	-0.709	0.300	1.009	no
Treat-0d	RHSIM_Rhsim13G0191300	0.892	-0.519	-1.410	no
Treat-0d	RHSIM_Rhsim13G0178100	0.576	-0.323	-0.899	no
Treat-0d	RHSIM_Rhsim13G0077300	0.999	1.089	0.090	no
Treat-0d	RHSIM_Rhsim13G0072100	-0.687	-0.519	0.168	no
Treat-0d	RHSIM_Rhsim13G0227500	1.664	1.019	-0.645	high
Treat-0d	RHSIM_Rhsim13G0162300	-1.056	-0.130	0.926	low

Group	Gene	PTR (z-score difference)	z(log2 LFQ)	z(log2 FPKM)	extreme-PTR
Treat-0d	RHSIM_Rhsim13G0134900	0.545	0.273	-0.272	no
Treat-0d	RHSIM_Rhsim13G0067700	-0.045	1.364	1.409	no
Treat-0d	RHSIM_Rhsim12G0057500	0.511	1.842	1.330	no
Treat-0d	RHSIM_RhsimUnG0132500	1.802	-0.009	-1.811	high
Treat-0d	RHSIM_Rhsim13G0229700	-0.192	0.357	0.549	no
Treat-0d	RHSIM_Rhsim13G0170300	0.467	-0.063	-0.530	no
Treat-0d	RHSIM_Rhsim13G0002500	0.586	0.597	0.011	no
Treat-0d	RHSIM_Rhsim13G0077400	-0.150	3.579	3.728	no
Treat-0d	RHSIM_Rhsim13G0013500	-0.127	0.468	0.595	no
Treat-0d	RHSIM_Rhsim13G0211600	-0.031	-0.691	-0.660	no
Treat-0d	RHSIM_Rhsim13G0102300	0.454	-0.397	-0.850	no
Treat-0d	RHSIM_Rhsim13G0015800	-0.074	-0.749	-0.675	no
Treat-0d	RHSIM_Rhsim13G0023600	0.097	0.463	0.367	no
Treat-0d	RHSIM_Rhsim12G0205600	1.656	-0.214	-1.870	high
Treat-0d	RHSIM_Rhsim13G0198600	0.276	-0.634	-0.911	no
Treat-0d	RHSIM_Rhsim13G0126100	-1.651	0.692	2.343	low
Treat-0d	RHSIM_Rhsim13G0029500	1.447	-0.944	-2.391	high
Treat-0d	RHSIM_Rhsim13G0216500	0.101	0.418	0.317	no
Treat-0d	RHSIM_Rhsim13G0115600	0.145	0.155	0.010	no
Treat-0d	RHSIM_Rhsim13G0044100	-0.190	-0.708	-0.518	no
Treat-0d	RHSIM_Rhsim13G0073300	-0.458	-1.160	-0.702	no
Treat-0d	RHSIM_RhsimUnG0124700	-0.895	-0.640	0.255	no
Treat-0d	RHSIM_Rhsim13G0234200	-0.972	-0.629	0.343	no
Treat-0d	RHSIM_Rhsim13G0049400	-0.318	0.107	0.425	no
Treat-0d	RHSIM_Rhsim13G0155900	-1.033	-1.300	-0.267	low
Treat-0d	RHSIM_Rhsim13G0065900	0.428	-0.722	-1.150	no
Treat-0d	RHSIM_Rhsim12G0054400	1.602	0.451	-1.151	high
Treat-0d	RHSIM_Rhsim13G0125300	1.240	-0.325	-1.565	high
Treat-0d	RHSIM_Rhsim13G0013000	-0.001	-0.244	-0.243	no
Treat-0d	RHSIM_RhsimUnG0118000	0.397	1.328	0.931	no
Treat-0d	RHSIM_Rhsim13G0150000	0.024	-0.428	-0.452	no
Treat-0d	RHSIM_Rhsim13G0100500	0.873	0.735	-0.138	no

Group	Gene	PTR (z-score difference)	z(log2 LFQ)	z(log2 FPKM)	extreme-PTR
Treat-0d	RHSIM_Rhsim12G0041400	-0.178	0.346	0.525	no
Treat-0d	RHSIM_Rhsim13G0198800	-0.445	-0.516	-0.071	no
Treat-0d	RHSIM_Rhsim13G0071100	-0.164	0.310	0.474	no
Treat-0d	RHSIM_Rhsim13G0151100	0.007	0.118	0.111	no
Treat-0d	RHSIM_Rhsim13G0028700	1.225	-0.395	-1.620	high
Treat-0d	RHSIM_Rhsim13G0143800	1.158	0.304	-0.854	high
Treat-0d	RHSIM_RhsimUnG0113900	-0.583	-1.877	-1.294	no
Treat-0d	RHSIM_Rhsim13G0126200	-2.061	-0.881	1.180	low
Treat-0d	RHSIM_Rhsim12G0094700	0.077	-0.384	-0.461	no
Treat-0d	RHSIM_Rhsim12G0069500	0.068	-0.473	-0.540	no
Treat-0d	RHSIM_Rhsim13G0228400	-0.066	0.630	0.696	no
Treat-0d	RHSIM_Rhsim13G0218100	-0.423	-0.828	-0.406	no
Treat-0d	RHSIM_Rhsim13G0127300	-0.104	-1.266	-1.162	no
Treat-0d	RHSIM_Rhsim13G0026200	0.203	1.422	1.219	no
Treat-0d	RHSIM_Rhsim13G0088000	-0.098	-1.195	-1.097	no
Treat-0d	RHSIM_Rhsim12G0000600	2.034	4.390	2.356	high
Treat-0d	RHSIM_Rhsim12G0123000	0.549	-0.286	-0.836	no
Treat-0d	RHSIM_Rhsim13G0191900	0.481	-0.535	-1.017	no
Treat-0d	RHSIM_Rhsim13G0065600	1.376	1.380	0.004	high
Treat-0d	RHSIM_Rhsim13G0180600	0.605	0.006	-0.599	no
Treat-0d	RHSIM_Rhsim12G0124700	0.666	-0.159	-0.825	no
Treat-0d	RHSIM_Rhsim13G0010300	-0.189	-1.806	-1.617	no
Treat-0d	RHSIM_Rhsim12G0173700	0.152	1.678	1.526	no
Treat-0d	RHSIM_Rhsim12G0061300	-0.179	-0.703	-0.523	no
Treat-0d	RHSIM_Rhsim13G0224500	-0.906	-1.233	-0.327	no
Treat-0d	RHSIM_Rhsim13G0199800	1.102	0.044	-1.059	no
Treat-0d	RHSIM_Rhsim13G0076400	-0.306	-0.146	0.160	no
Treat-0d	RHSIM_Rhsim13G0063200	-3.855	-1.118	2.736	low
Treat-0d	RHSIM_Rhsim13G0202500	0.982	0.282	-0.700	no
Treat-0d	RHSIM_Rhsim12G0204500	-1.759	-0.319	1.440	low
Treat-0d	RHSIM_Rhsim13G0235300	0.004	0.621	0.617	no
Treat-0d	RHSIM_Rhsim13G0085200	1.899	2.950	1.051	high

Group	Gene	PTR (z-score difference)	z(log2 LFQ)	z(log2 FPKM)	extreme-PTR
Treat-0d	RHSIM_Rhsim12G0195700	0.446	0.899	0.453	no
Treat-0d	RHSIM_Rhsim12G0197300	0.753	0.511	-0.242	no
Treat-0d	RHSIM_Rhsim12G0060800	-2.263	-1.414	0.848	low
Treat-0d	RHSIM_Rhsim12G0130500	-0.326	-0.052	0.274	no
Treat-0d	RHSIM_Rhsim13G0058700	-0.829	-1.323	-0.494	no
Treat-0d	RHSIM_Rhsim12G0210700	2.318	1.374	-0.944	high
Treat-0d	RHSIM_Rhsim13G0121900	-0.037	-1.101	-1.064	no
Treat-0d	RHSIM_Rhsim12G0178300	1.538	0.468	-1.070	high
Treat-0d	RHSIM_Rhsim13G0000600	-2.038	-0.954	1.085	low
Treat-0d	RHSIM_Rhsim13G0128900	-1.176	-1.534	-0.358	low
Treat-0d	RHSIM_RhsimUnG0093000	-0.339	-0.192	0.146	no
Treat-0d	RHSIM_Rhsim12G0182600	-0.308	0.067	0.375	no
Treat-0d	RHSIM_Rhsim13G0152200	1.870	0.307	-1.562	high
Treat-0d	RHSIM_Rhsim12G0127000	1.289	0.231	-1.058	high
Treat-0d	RHSIM_Rhsim13G0138500	0.032	0.847	0.816	no
Treat-0d	RHSIM_Rhsim13G0072300	1.804	-0.141	-1.945	high
Treat-0d	RHSIM_Rhsim13G0097900	0.805	-0.817	-1.622	no
Treat-0d	RHSIM_Rhsim12G0143900	0.567	-0.078	-0.645	no
Treat-0d	RHSIM_Rhsim13G0154700	0.854	0.038	-0.816	no
Treat-0d	RHSIM_RhsimUnG0089100	-0.991	0.514	1.506	no
Treat-0d	RHSIM_Rhsim12G0035800	0.152	0.109	-0.043	no
Treat-0d	RHSIM_Rhsim13G0017300	-0.484	-1.393	-0.909	no
Treat-0d	RHSIM_Rhsim12G0047600	-0.449	0.676	1.124	no
Treat-0d	RHSIM_RhsimUnG0088100	1.078	-1.198	-2.275	no
Treat-0d	RHSIM_Rhsim13G0200500	0.703	-0.089	-0.792	no
Treat-0d	RHSIM_Rhsim13G0016000	0.176	-0.316	-0.492	no
Treat-0d	RHSIM_Rhsim13G0199600	0.617	0.304	-0.312	no
Treat-0d	RHSIM_Rhsim12G0112500	0.011	-0.749	-0.760	no
Treat-0d	RHSIM_Rhsim13G0008000	2.012	1.001	-1.012	high
Treat-0d	RHSIM_Rhsim13G0197500	-1.487	-0.114	1.372	low
Treat-0d	RHSIM_Rhsim13G0074100	-1.212	0.327	1.539	low
Treat-0d	RHSIM_Rhsim12G0077600	-0.121	-0.082	0.039	no

Group	Gene	PTR (z-score difference)	z(log2 LFQ)	z(log2 FPKM)	extreme-PTR
Treat-0d	RHSIM_Rhsim12G0116500	-0.079	-0.472	-0.393	no
Treat-0d	RHSIM_Rhsim13G0040100	-5.173	-1.867	3.306	low
Treat-0d	RHSIM_Rhsim13G0075400	0.865	0.227	-0.638	no
Treat-0d	RHSIM_Rhsim12G0207400	0.559	-0.131	-0.689	no
Treat-0d	RHSIM_Rhsim12G0135500	-1.010	-1.113	-0.102	no
Treat-0d	RHSIM_Rhsim12G0035400	1.298	0.028	-1.269	high
Treat-0d	RHSIM_Rhsim13G0037600	0.056	-0.150	-0.206	no
Treat-0d	RHSIM_Rhsim13G0173900	-0.201	-0.485	-0.284	no
Treat-0d	RHSIM_Rhsim13G0102400	0.304	-0.262	-0.565	no
Treat-0d	RHSIM_Rhsim12G0130100	0.498	0.820	0.322	no
Treat-0d	RHSIM_Rhsim12G0113000	0.756	1.068	0.312	no
Treat-0d	RHSIM_Rhsim12G0013800	-0.189	0.398	0.588	no
Treat-0d	RHSIM_Rhsim13G0223300	1.760	0.901	-0.859	high
Treat-0d	RHSIM_Rhsim13G0223000	0.555	2.713	2.158	no
Treat-0d	RHSIM_Rhsim13G0154600	-2.552	-0.994	1.558	low
Treat-0d	RHSIM_RhsimUnG0067300	1.412	-0.916	-2.328	high
Treat-0d	RHSIM_Rhsim12G0212100	0.848	-0.071	-0.919	no
Treat-0d	RHSIM_Rhsim13G0012200	-0.155	0.705	0.860	no
Treat-0d	RHSIM_Rhsim12G0136200	-0.903	-1.295	-0.392	no
Treat-0d	RHSIM_Rhsim12G0143400	-0.670	0.747	1.417	no
Treat-0d	RHSIM_Rhsim13G0076300	-0.924	-0.282	0.643	no
Treat-0d	RHSIM_Rhsim13G0029400	0.427	-0.052	-0.479	no
Treat-0d	RHSIM_Rhsim13G0149600	0.637	2.495	1.858	no
Treat-0d	RHSIM_Rhsim12G0021800	-0.356	0.045	0.401	no
Treat-0d	RHSIM_Rhsim12G0133000	-0.696	-0.651	0.045	no
Treat-0d	RHSIM_Rhsim13G0081700	1.608	-0.127	-1.735	high
Treat-0d	RHSIM_Rhsim13G0224600	-0.002	-0.322	-0.320	no
Treat-0d	RHSIM_Rhsim12G0149400	1.311	1.620	0.309	high
Treat-0d	RHSIM_Rhsim13G0070200	-0.110	-1.199	-1.089	no
Treat-0d	RHSIM_Rhsim12G0076200	-1.316	-0.727	0.589	low
Treat-0d	RHSIM_Rhsim12G0157800	-0.548	0.380	0.928	no
Treat-0d	RHSIM_Rhsim13G0023900	-0.387	0.953	1.340	no

Group	Gene	PTR (z-score difference)	z(log2 LFQ)	z(log2 FPKM)	extreme-PTR
Treat-0d	RHSIM_Rhsim12G0116400	-3.445	0.532	3.977	low
Treat-0d	RHSIM_Rhsim13G0116000	1.113	1.507	0.394	high
Treat-0d	RHSIM_Rhsim13G0119800	-0.383	-1.090	-0.707	no
Treat-0d	RHSIM_Rhsim12G0163200	-0.751	-0.388	0.364	no
Treat-0d	RHSIM_Rhsim12G0193800	0.358	-0.453	-0.811	no
Treat-0d	RHSIM_Rhsim12G0214100	-0.704	-0.383	0.322	no
Treat-0d	RHSIM_Rhsim12G0015600	0.766	3.796	3.030	no
Treat-0d	RHSIM_Rhsim13G0124300	-0.654	-1.096	-0.442	no
Treat-0d	RHSIM_Rhsim12G0186700	-0.510	-0.832	-0.322	no
Treat-0d	RHSIM_Rhsim12G0134700	-1.102	-0.778	0.325	low
Treat-0d	RHSIM_Rhsim13G0198500	0.773	1.542	0.769	no
Treat-0d	RHSIM_Rhsim12G0026000	-0.037	0.105	0.142	no
Treat-0d	RHSIM_Rhsim12G0078900	-0.375	-0.540	-0.165	no
Treat-0d	RHSIM_Rhsim13G0037700	1.108	0.439	-0.669	high
Treat-0d	RHSIM_Rhsim12G0140200	0.184	0.697	0.513	no
Treat-0d	RHSIM_Rhsim12G0137600	0.330	0.209	-0.121	no
Treat-0d	RHSIM_Rhsim13G0039600	0.818	-0.073	-0.891	no
Treat-0d	RHSIM_Rhsim12G0108100	-1.215	-0.921	0.294	low
Treat-0d	RHSIM_Rhsim12G0009900	0.975	0.021	-0.954	no
Treat-0d	RHSIM_Rhsim13G0095800	-0.369	-0.628	-0.259	no
Treat-0d	RHSIM_Rhsim12G0144200	-1.276	-1.890	-0.614	low
Treat-0d	RHSIM_Rhsim13G0119500	0.480	0.383	-0.096	no
Treat-0d	RHSIM_Rhsim13G0031400	1.847	0.144	-1.703	high
Treat-0d	RHSIM_Rhsim13G0017500	0.601	-0.499	-1.101	no
Treat-0d	RHSIM_Rhsim12G0195800	0.465	0.453	-0.012	no
Treat-0d	RHSIM_Rhsim12G0152200	0.298	-0.367	-0.665	no
Treat-0d	RHSIM_Rhsim12G0170600	-1.664	0.387	2.052	low
Treat-0d	RHSIM_Rhsim13G0210400	1.365	0.862	-0.503	high
Treat-0d	RHSIM_Rhsim13G0044700	-0.380	-1.148	-0.768	no
Treat-0d	RHSIM_Rhsim12G0165900	-0.393	0.370	0.763	no
Treat-0d	RHSIM_Rhsim12G0208900	-0.063	-0.566	-0.503	no
Treat-0d	RHSIM_Rhsim13G0222300	0.220	-1.034	-1.254	no

Group	Gene	PTR (z-score difference)	z(log2 LFQ)	z(log2 FPKM)	extreme-PTR
Treat-0d	RHSIM_Rhsim13G0077600	0.768	1.132	0.363	no
Treat-0d	RHSIM_Rhsim12G0085200	-0.550	-1.565	-1.016	no
Treat-0d	RHSIM_Rhsim12G0150200	-0.578	-1.634	-1.056	no
Treat-0d	RHSIM_Rhsim12G0152500	-0.175	-0.434	-0.260	no
Treat-0d	RHSIM_Rhsim13G0233300	0.674	1.547	0.874	no
Treat-0d	RHSIM_Rhsim12G0182800	0.439	1.863	1.423	no
Treat-0d	RHSIM_Rhsim12G0195100	-0.235	-0.564	-0.329	no
Treat-0d	RHSIM_Rhsim12G0169500	1.400	1.383	-0.017	high
Treat-0d	RHSIM_Rhsim12G0171400	-1.379	-1.286	0.093	low
Treat-0d	RHSIM_Rhsim12G0109000	3.625	1.263	-2.362	high
Treat-0d	RHSIM_Rhsim13G0089100	0.098	0.931	0.832	no
Treat-0d	RHSIM_Rhsim12G0041100	-0.503	-0.716	-0.213	no
Treat-0d	RHSIM_Rhsim12G0180700	0.634	0.746	0.112	no
Treat-0d	RHSIM_Rhsim12G0155700	1.065	3.344	2.279	no
Treat-0d	RHSIM_Rhsim12G0188800	-0.735	-1.712	-0.976	no
Treat-0d	RHSIM_Rhsim12G0140600	0.645	0.749	0.105	no
Treat-0d	RHSIM_Rhsim12G0195900	-1.710	-1.316	0.394	low
Treat-0d	RHSIM_Rhsim12G0015500	-1.246	0.754	2.000	low
Treat-0d	RHSIM_Rhsim12G0090000	-0.614	0.280	0.894	no
Treat-0d	RHSIM_Rhsim12G0182500	-0.115	-0.936	-0.821	no
Treat-0d	RHSIM_Rhsim12G0169800	-0.223	0.349	0.571	no
Treat-0d	RHSIM_Rhsim12G0074100	-0.020	-0.405	-0.385	no
Treat-0d	RHSIM_Rhsim12G0190900	0.170	-0.465	-0.635	no
Treat-0d	RHSIM_Rhsim12G0124000	-1.727	-2.051	-0.324	low
Treat-0d	RHSIM_Rhsim13G0230600	-0.642	0.061	0.703	no
Treat-0d	RHSIM_Rhsim12G0168400	-0.677	-0.117	0.559	no
Treat-0d	RHSIM_Rhsim12G0208200	-1.797	-0.453	1.344	low
Treat-0d	RHSIM_Rhsim12G0167500	-0.590	0.578	1.168	no
Treat-0d	RHSIM_Rhsim12G0209100	-0.258	-0.239	0.020	no
Treat-0d	RHSIM_Rhsim12G0086400	-0.621	-1.095	-0.474	no
Treat-0d	RHSIM_Rhsim12G0132700	0.917	0.412	-0.505	no
Treat-0d	RHSIM_Rhsim12G0175800	0.484	1.387	0.904	no

Group	Gene	PTR (z-score difference)	z(log2 LFQ)	z(log2 FPKM)	extreme-PTR
Treat-0d	RHSIM_Rhsim12G0163900	1.406	0.450	-0.956	high
Treat-0d	RHSIM_Rhsim12G0149700	0.673	-0.899	-1.572	no
Treat-0d	RHSIM_Rhsim12G0212300	0.540	-0.670	-1.209	no
Treat-0d	RHSIM_Rhsim12G0197400	-0.768	0.804	1.571	no
Treat-0d	RHSIM_Rhsim12G0029400	-0.064	-1.764	-1.700	no
Treat-0d	RHSIM_Rhsim12G0076500	-0.632	0.119	0.751	no
Treat-0d	RHSIM_Rhsim12G0127200	-0.817	-1.279	-0.463	no
Treat-0d	RHSIM_RhsimUnG0027400	1.473	-0.428	-1.901	high
Treat-0d	RHSIM_Rhsim13G0064000	-0.134	-0.531	-0.397	no
Treat-0d	RHSIM_Rhsim13G0071700	-0.071	1.637	1.709	no
Treat-0d	RHSIM_Rhsim12G0137100	-2.641	-1.138	1.502	low
Treat-0d	RHSIM_Rhsim12G0003100	-1.241	-0.315	0.927	low
Treat-0d	RHSIM_Rhsim12G0003800	-0.315	-0.373	-0.058	no
Treat-0d	RHSIM_Rhsim12G0116900	0.593	0.421	-0.172	no
Treat-0d	RHSIM_Rhsim12G0013200	0.701	-1.205	-1.907	no
Treat-0d	RHSIM_Rhsim12G0099000	0.223	-0.143	-0.366	no
Treat-0d	RHSIM_Rhsim12G0013400	0.038	1.366	1.328	no
Treat-0d	RHSIM_Rhsim12G0000200	0.822	-0.619	-1.441	no
Treat-0d	RHSIM_Rhsim12G0017500	-0.800	-1.216	-0.416	no
Treat-0d	RHSIM_RhsimUnG0019800	-0.187	-0.196	-0.009	no
Treat-0d	RHSIM_Rhsim12G0208000	1.772	2.025	0.253	high
Treat-0d	RHSIM_Rhsim13G0117600	0.595	-0.536	-1.131	no
Treat-0d	RHSIM_Rhsim12G0190700	-0.125	0.187	0.312	no
Treat-0d	RHSIM_Rhsim12G0003400	0.520	0.755	0.235	no
Treat-0d	RHSIM_Rhsim12G0053900	-3.710	-1.422	2.288	low
Treat-0d	RHSIM_RhsimUnG0020300	0.422	0.510	0.088	no
Treat-0d	RHSIM_Rhsim13G0102900	0.064	-0.744	-0.808	no
Treat-0d	RHSIM_Rhsim12G0192900	-2.709	-1.063	1.646	low
Treat-0d	RHSIM_Rhsim12G0151800	-0.374	0.557	0.930	no
Treat-0d	RHSIM_Rhsim13G0204000	0.055	-0.246	-0.301	no
Treat-0d	RHSIM_Rhsim12G0129700	0.616	-0.686	-1.302	no
Treat-0d	RHSIM_Rhsim12G0144700	-1.201	0.501	1.702	low

Group	Gene	PTR (z-score difference)	z(log2 LFQ)	z(log2 FPKM)	extreme-PTR
Treat-0d	RHSIM_Rhsim12G0164400	-0.757	-0.793	-0.036	no
Treat-0d	RHSIM_Rhsim12G0106200	1.446	3.406	1.960	high
Treat-0d	RHSIM_Rhsim13G0136300	0.788	1.536	0.748	no
Treat-0d	RHSIM_Rhsim12G0194200	-0.402	0.902	1.304	no
Treat-0d	RHSIM_Rhsim12G0004500	-0.649	-1.202	-0.552	no
Treat-0d	RHSIM_Rhsim12G0137400	0.640	-0.185	-0.826	no
Treat-0d	RHSIM_Rhsim12G0074200	-1.860	-1.401	0.459	low
Treat-0d	RHSIM_Rhsim12G0107100	-0.063	1.250	1.313	no
Treat-0d	RHSIM_Rhsim12G0087800	-0.144	0.599	0.743	no
Treat-0d	RHSIM_Rhsim12G0043300	0.883	0.542	-0.341	no
Treat-0d	RHSIM_Rhsim12G0138800	0.687	0.245	-0.442	no
Treat-0d	RHSIM_Rhsim12G0191200	0.532	1.295	0.762	no
Treat-0d	RHSIM_Rhsim12G0099400	-1.750	-1.080	0.669	low
Treat-0d	RHSIM_Rhsim12G0146800	-0.284	0.111	0.395	no
Treat-0d	RHSIM_Rhsim12G0025000	-0.491	-1.959	-1.468	no
Treat-0d	RHSIM_Rhsim13G0057500	-0.754	1.136	1.890	no
Treat-0d	RHSIM_Rhsim12G0071500	-0.605	-1.059	-0.454	no
Treat-0d	RHSIM_Rhsim12G0013500	1.307	4.235	2.927	high
Treat-0d	RHSIM_Rhsim12G0116700	1.334	0.143	-1.191	high
Treat-0d	RHSIM_Rhsim13G0228300	0.689	0.440	-0.249	no
Treat-0d	RHSIM_Rhsim12G0063600	1.179	0.317	-0.862	high
Treat-0d	RHSIM_Rhsim12G0167900	-2.358	-0.880	1.478	low
Treat-0d	RHSIM_Rhsim12G0071800	-0.545	-0.584	-0.040	no
Treat-0d	RHSIM_Rhsim12G0047500	0.143	0.821	0.678	no
Treat-0d	RHSIM_Rhsim12G0124800	-2.647	-0.066	2.581	low
Treat-0d	RHSIM_Rhsim12G0046600	-0.764	-0.091	0.673	no
Treat-0d	RHSIM_Rhsim11G0178300	-0.323	-2.336	-2.013	no
Treat-0d	RHSIM_Rhsim12G0189200	-0.275	0.133	0.408	no
Treat-0d	RHSIM_Rhsim12G0182200	0.296	-0.643	-0.939	no
Treat-0d	RHSIM_Rhsim12G0190600	0.966	0.208	-0.758	no
Treat-0d	RHSIM_Rhsim13G0131000	-0.792	0.384	1.177	no
Treat-0d	RHSIM_Rhsim11G0139400	0.699	0.147	-0.552	no

Group	Gene	PTR (z-score difference)	z(log2 LFQ)	z(log2 FPKM)	extreme-PTR
Treat-0d	RHSIM_Rhsim12G0139100	-1.821	0.175	1.995	low
Treat-0d	RHSIM_Rhsim12G0127600	-0.568	0.834	1.403	no
Treat-0d	RHSIM_Rhsim12G0193000	0.639	-0.009	-0.648	no
Treat-0d	RHSIM_Rhsim12G0058100	-1.691	-0.059	1.632	low
Treat-0d	RHSIM_Rhsim13G0116200	2.412	0.190	-2.223	high
Treat-0d	RHSIM_Rhsim12G0004800	0.667	1.184	0.516	no
Treat-0d	RHSIM_Rhsim12G0179700	0.608	-0.457	-1.065	no
Treat-0d	RHSIM_Rhsim13G0166600	-0.664	-0.399	0.265	no
Treat-0d	RHSIM_Rhsim12G0197200	-0.046	0.167	0.213	no
Treat-0d	RHSIM_Rhsim12G0137300	-2.450	-1.254	1.196	low
Treat-0d	RHSIM_Rhsim12G0126700	1.134	0.070	-1.064	high
Treat-0d	RHSIM_Rhsim12G0025400	1.195	0.481	-0.714	high
Treat-0d	RHSIM_Rhsim12G0028200	-0.715	-0.657	0.058	no
Treat-0d	RHSIM_Rhsim12G0204700	0.326	0.561	0.236	no
Treat-0d	RHSIM_Rhsim13G0047800	-0.251	-0.679	-0.428	no
Treat-0d	RHSIM_Rhsim12G0199300	0.288	1.154	0.866	no
Treat-0d	RHSIM_Rhsim12G0214300	-0.689	-0.131	0.558	no
Treat-0d	RHSIM_Rhsim13G0039000	-1.430	-0.679	0.750	low
Treat-0d	RHSIM_Rhsim12G0139200	-0.500	-1.415	-0.915	no
Treat-0d	RHSIM_Rhsim12G0162500	-1.096	-1.519	-0.423	low
Treat-0d	RHSIM_Rhsim11G0006900	-0.747	-1.165	-0.419	no
Treat-0d	RHSIM_Rhsim12G0053200	2.000	0.201	-1.799	high
Treat-0d	RHSIM_Rhsim12G0191400	-0.864	0.437	1.302	no
Treat-0d	RHSIM_Rhsim13G0098600	0.278	1.794	1.515	no
Treat-0d	RHSIM_Rhsim12G0193700	1.175	0.874	-0.301	high
Treat-0d	RHSIM_Rhsim12G0109300	-0.889	-0.735	0.154	no
Treat-0d	RHSIM_Rhsim12G0121200	0.044	-0.255	-0.299	no
Treat-0d	RHSIM_Rhsim13G0042200	-0.247	0.988	1.235	no
Treat-0d	RHSIM_Rhsim12G0213600	2.227	2.661	0.434	high
Treat-0d	RHSIM_Rhsim13G0138000	1.727	0.983	-0.744	high
Treat-0d	RHSIM_Rhsim13G0234000	-1.177	-0.910	0.266	low
Treat-0d	RHSIM_Rhsim13G0074400	1.070	0.237	-0.833	no

Group	Gene	PTR (z-score difference)	z(log2 LFQ)	z(log2 FPKM)	extreme-PTR
Treat-0d	RHSIM_Rhsim13G0100300	-0.082	-0.868	-0.786	no
Treat-0d	RHSIM_Rhsim12G0144100	1.200	0.668	-0.531	high
Treat-0d	RHSIM_Rhsim12G0070500	1.736	1.189	-0.547	high
Treat-0d	RHSIM_Rhsim12G0185700	1.174	-0.300	-1.475	high
Treat-0d	RHSIM_Rhsim12G0104600	0.625	0.640	0.015	no
Treat-0d	RHSIM_Rhsim12G0075100	0.547	-1.208	-1.755	no
Treat-0d	RHSIM_Rhsim13G0081400	-0.482	-1.122	-0.640	no
Treat-0d	RHSIM_Rhsim13G0045200	0.158	0.298	0.140	no
Treat-0d	RHSIM_Rhsim12G0085800	-1.870	-1.963	-0.093	low
Treat-0d	RHSIM_Rhsim12G0206900	-0.940	0.124	1.064	no
Treat-0d	RHSIM_Rhsim13G0009500	0.911	-0.644	-1.555	no
Treat-0d	RHSIM_Rhsim12G0070100	-0.083	-0.891	-0.808	no
Treat-0d	RHSIM_Rhsim12G0144000	-1.944	-0.721	1.223	low
Treat-0d	RHSIM_Rhsim12G0004100	0.932	0.757	-0.175	no
Treat-0d	RHSIM_Rhsim13G0199500	0.616	-0.933	-1.550	no
Treat-0d	RHSIM_Rhsim11G0191500	-2.213	0.606	2.818	low
Treat-0d	RHSIM_Rhsim12G0055000	-2.283	-0.795	1.488	low
Treat-0d	RHSIM_Rhsim13G0096300	-0.976	-0.562	0.414	no
Treat-0d	RHSIM_Rhsim12G0005700	-0.400	-0.599	-0.199	no
Treat-0d	RHSIM_Rhsim13G0050700	-0.490	1.629	2.119	no
Treat-0d	RHSIM_Rhsim12G0138700	1.079	-1.267	-2.347	no
Treat-0d	RHSIM_Rhsim13G0194300	0.216	1.723	1.507	no
Treat-0d	RHSIM_Rhsim12G0027600	-0.131	0.747	0.878	no
Treat-0d	RHSIM_Rhsim12G0087900	0.290	0.943	0.653	no
Treat-0d	RHSIM_Rhsim11G0052100	1.185	0.781	-0.403	high
Treat-0d	RHSIM_Rhsim13G0026100	0.046	-0.185	-0.231	no
Treat-0d	RHSIM_Rhsim12G0048100	0.823	0.788	-0.035	no
Treat-0d	RHSIM_Rhsim12G0002800	0.892	0.069	-0.824	no
Treat-0d	RHSIM_Rhsim12G0038100	-0.072	-0.604	-0.532	no
Treat-0d	RHSIM_Rhsim12G0168200	0.475	-0.956	-1.430	no
Treat-0d	RHSIM_Rhsim12G0148300	-0.685	0.829	1.514	no
Treat-0d	RHSIM_Rhsim12G0177900	-0.443	1.056	1.498	no

Group	Gene	PTR (z-score difference)	z(log2 LFQ)	z(log2 FPKM)	extreme-PTR
Treat-0d	RHSIM_Rhsim13G0174400	1.490	0.092	-1.398	high
Treat-0d	RHSIM_Rhsim12G0138000	-0.065	0.292	0.357	no
Treat-0d	RHSIM_Rhsim13G0119700	2.548	0.169	-2.379	high
Treat-0d	RHSIM_Rhsim12G0025300	-0.865	-0.381	0.484	no
Treat-0d	RHSIM_Rhsim12G0113100	-0.533	-0.595	-0.062	no
Treat-0d	RHSIM_Rhsim13G0148600	-1.449	0.042	1.491	low
Treat-0d	RHSIM_Rhsim12G0203300	-0.262	-1.102	-0.840	no
Treat-0d	RHSIM_Rhsim13G0163200	1.120	-0.412	-1.532	high
Treat-0d	RHSIM_Rhsim12G0126600	0.301	-0.613	-0.914	no
Treat-0d	RHSIM_Rhsim12G0205400	-0.414	-0.394	0.020	no
Treat-0d	RHSIM_Rhsim12G0006200	1.092	1.531	0.439	no
Treat-0d	RHSIM_Rhsim12G0070300	1.436	0.622	-0.813	high
Treat-0d	RHSIM_Rhsim12G0165000	-0.247	-0.507	-0.260	no
Treat-0d	RHSIM_Rhsim12G0006800	-0.388	-0.722	-0.334	no
Treat-0d	RHSIM_Rhsim12G0172100	-0.371	-0.674	-0.303	no
Treat-0d	RHSIM_Rhsim12G0110200	0.485	0.753	0.267	no
Treat-0d	RHSIM_Rhsim12G0048500	-1.154	-0.660	0.494	low
Treat-0d	RHSIM_Rhsim12G0137200	1.391	0.623	-0.768	high
Treat-0d	RHSIM_Rhsim12G0161200	0.007	0.223	0.216	no
Treat-0d	RHSIM_Rhsim12G0003000	-0.464	0.496	0.960	no
Treat-0d	RHSIM_Rhsim11G0167100	0.808	1.367	0.559	no
Treat-0d	RHSIM_Rhsim12G0052000	0.699	1.567	0.868	no
Treat-0d	RHSIM_Rhsim12G0198100	-0.689	-1.273	-0.584	no
Treat-0d	RHSIM_Rhsim12G0147400	-0.316	-0.435	-0.119	no
Treat-0d	RHSIM_Rhsim11G0124300	-0.700	0.076	0.777	no
Treat-0d	RHSIM_Rhsim12G0115900	0.427	1.850	1.423	no
Treat-0d	RHSIM_Rhsim12G0192800	0.654	-0.631	-1.285	no
Treat-0d	RHSIM_Rhsim13G0126800	0.005	-0.584	-0.589	no
Treat-0d	RHSIM_Rhsim12G0171000	1.378	-0.079	-1.458	high
Treat-0d	RHSIM_Rhsim11G0045800	-2.189	-0.612	1.576	low
Treat-0d	RHSIM_Rhsim12G0192300	0.782	1.150	0.369	no
Treat-0d	RHSIM_Rhsim11G0195600	-2.137	-1.019	1.118	low

Group	Gene	PTR (z-score difference)	z(log2 LFQ)	z(log2 FPKM)	extreme-PTR
Treat-0d	RHSIM_Rhsim12G0102400	0.174	1.228	1.054	no
Treat-0d	RHSIM_Rhsim11G0129600	-0.345	0.307	0.652	no
Treat-0d	RHSIM_Rhsim12G0133400	-0.111	2.124	2.234	no
Treat-0d	RHSIM_Rhsim12G0043200	-0.965	0.409	1.375	no
Treat-0d	RHSIM_Rhsim12G0153900	-0.093	0.484	0.578	no
Treat-0d	RHSIM_Rhsim12G0015200	-0.472	0.441	0.913	no
Treat-0d	RHSIM_Rhsim11G0167300	1.112	1.569	0.458	high
Treat-0d	RHSIM_Rhsim11G0127100	-0.677	-0.472	0.205	no
Treat-0d	RHSIM_Rhsim12G0138500	0.110	1.535	1.424	no
Treat-0d	RHSIM_Rhsim11G0100300	-1.514	1.333	2.847	low
Treat-0d	RHSIM_Rhsim11G0188300	-1.579	-0.660	0.919	low
Treat-0d	RHSIM_Rhsim12G0099800	-0.630	2.418	3.048	no
Treat-0d	RHSIM_Rhsim12G0135900	-0.105	-0.353	-0.248	no
Treat-0d	RHSIM_Rhsim11G0132400	-1.117	-1.093	0.024	low
Treat-0d	RHSIM_Rhsim12G0047700	0.287	0.554	0.266	no
Treat-0d	RHSIM_Rhsim11G0129000	-0.456	-0.124	0.332	no
Treat-0d	RHSIM_Rhsim12G0214500	-0.901	-1.069	-0.168	no
Treat-0d	RHSIM_Rhsim11G0034700	0.787	1.126	0.339	no
Treat-0d	RHSIM_Rhsim11G0172800	1.278	1.711	0.433	high
Treat-0d	RHSIM_Rhsim12G0017000	1.179	0.658	-0.521	high
Treat-0d	RHSIM_Rhsim12G0152700	-0.178	-1.297	-1.119	no
Treat-0d	RHSIM_Rhsim12G0054500	-0.001	1.623	1.624	no
Treat-0d	RHSIM_Rhsim12G0183500	0.916	1.906	0.990	no
Treat-0d	RHSIM_Rhsim11G0011600	0.355	0.682	0.328	no
Treat-0d	RHSIM_Rhsim13G0178500	-0.899	-1.036	-0.137	no
Treat-0d	RHSIM_Rhsim12G0168600	-0.129	0.184	0.313	no
Treat-0d	RHSIM_Rhsim11G0129800	-1.053	-0.632	0.421	low
Treat-0d	RHSIM_Rhsim11G0092300	-0.712	-1.039	-0.327	no
Treat-0d	RHSIM_Rhsim12G0132900	-0.101	-0.010	0.091	no
Treat-0d	RHSIM_Rhsim13G0080100	-0.248	-0.125	0.124	no
Treat-0d	RHSIM_Rhsim12G0063100	0.160	0.017	-0.142	no
Treat-0d	RHSIM_Rhsim12G0188200	-2.690	-1.667	1.023	low

Group	Gene	PTR (z-score difference)	z(log2 LFQ)	z(log2 FPKM)	extreme-PTR
Treat-0d	RHSIM_Rhsim11G0076700	-1.127	-0.884	0.243	low
Treat-0d	RHSIM_Rhsim12G0166300	0.215	0.189	-0.026	no
Treat-0d	RHSIM_Rhsim11G0025100	0.600	-0.742	-1.341	no
Treat-0d	RHSIM_Rhsim12G0124500	-0.821	-1.574	-0.753	no
Treat-0d	RHSIM_Rhsim12G0207300	0.937	0.675	-0.262	no
Treat-0d	RHSIM_Rhsim11G0101800	-0.052	-0.935	-0.882	no
Treat-0d	RHSIM_Rhsim12G0134100	-0.335	-0.679	-0.344	no
Treat-0d	RHSIM_Rhsim13G0101800	1.275	-1.026	-2.302	high
Treat-0d	RHSIM_Rhsim12G0197600	1.050	0.652	-0.398	no
Treat-0d	RHSIM_Rhsim11G0114600	1.149	0.003	-1.146	high
Treat-0d	RHSIM_Rhsim12G0164700	-0.183	0.277	0.460	no
Treat-0d	RHSIM_Rhsim12G0089200	-0.216	-0.740	-0.524	no
Treat-0d	RHSIM_Rhsim12G0064700	-0.429	-0.458	-0.029	no
Treat-0d	RHSIM_Rhsim12G0042000	0.475	0.095	-0.380	no
Treat-0d	RHSIM_Rhsim12G0171100	0.772	1.634	0.861	no
Treat-0d	RHSIM_Rhsim11G0034400	0.914	0.010	-0.904	no
Treat-0d	RHSIM_Rhsim12G0182900	0.479	0.028	-0.450	no
Treat-0d	RHSIM_Rhsim13G0038100	-0.896	-0.487	0.409	no
Treat-0d	RHSIM_Rhsim11G0188100	-0.049	1.608	1.657	no
Treat-0d	RHSIM_Rhsim12G0096200	-0.833	-0.505	0.327	no
Treat-0d	RHSIM_Rhsim13G0074500	-1.124	-0.731	0.393	low
Treat-0d	RHSIM_Rhsim11G0135800	1.770	2.066	0.296	high
Treat-0d	RHSIM_Rhsim12G0046400	0.229	1.874	1.645	no
Treat-0d	RHSIM_Rhsim11G0001200	0.058	-0.014	-0.071	no
Treat-0d	RHSIM_Rhsim11G0136900	-0.198	1.214	1.412	no
Treat-0d	RHSIM_Rhsim12G0065000	2.166	1.422	-0.744	high
Treat-0d	RHSIM_Rhsim11G0182800	2.120	-0.271	-2.392	high
Treat-0d	RHSIM_Rhsim11G0039200	-2.641	-0.274	2.367	low
Treat-0d	RHSIM_Rhsim12G0161700	0.089	-1.277	-1.367	no
Treat-0d	RHSIM_Rhsim11G0125200	0.343	-0.009	-0.352	no
Treat-0d	RHSIM_Rhsim11G0098400	1.709	1.525	-0.184	high
Treat-0d	RHSIM_Rhsim12G0047800	-0.283	-1.471	-1.187	no

Group	Gene	PTR (z-score difference)	z(log2 LFQ)	z(log2 FPKM)	extreme-PTR
Treat-0d	RHSIM_Rhsim11G0186300	-0.319	-0.049	0.270	no
Treat-0d	RHSIM_Rhsim11G0072000	2.094	0.511	-1.584	high
Treat-0d	RHSIM_Rhsim12G0073200	1.234	0.572	-0.663	high
Treat-0d	RHSIM_Rhsim12G0129600	-1.132	-1.494	-0.362	low
Treat-0d	RHSIM_Rhsim11G0176000	0.852	1.079	0.227	no
Treat-0d	RHSIM_Rhsim11G0133600	-0.791	-0.157	0.634	no
Treat-0d	RHSIM_Rhsim12G0130600	0.086	0.717	0.631	no
Treat-0d	RHSIM_Rhsim11G0197000	-0.549	0.312	0.861	no
Treat-0d	RHSIM_Rhsim11G0113300	0.099	0.072	-0.027	no
Treat-0d	RHSIM_Rhsim11G0192400	-0.419	1.020	1.439	no
Treat-0d	RHSIM_Rhsim12G0016200	-0.057	-0.655	-0.597	no
Treat-0d	RHSIM_Rhsim12G0079100	-0.605	-0.038	0.567	no
Treat-0d	RHSIM_Rhsim11G0095300	0.107	-0.601	-0.708	no
Treat-0d	RHSIM_Rhsim13G0229400	0.410	-0.319	-0.729	no
Treat-0d	RHSIM_Rhsim11G0016500	-0.095	0.549	0.644	no
Treat-0d	RHSIM_Rhsim12G0206500	1.232	1.574	0.341	high
Treat-0d	RHSIM_Rhsim11G0121500	0.793	1.158	0.365	no
Treat-0d	RHSIM_Rhsim13G0197700	0.429	1.244	0.815	no
Treat-0d	RHSIM_Rhsim13G0015200	-0.655	-0.831	-0.176	no
Treat-0d	RHSIM_Rhsim12G0193900	1.103	-0.086	-1.188	no
Treat-0d	RHSIM_Rhsim11G0160100	-0.049	-0.544	-0.495	no
Treat-0d	RHSIM_Rhsim12G0201500	0.228	0.242	0.014	no
Treat-0d	RHSIM_Rhsim11G0177100	-0.090	0.700	0.790	no
Treat-0d	RHSIM_Rhsim11G0161200	-0.590	0.064	0.653	no
Treat-0d	RHSIM_Rhsim12G0128500	-0.087	0.153	0.240	no
Treat-0d	RHSIM_Rhsim11G0121900	-0.653	-1.194	-0.542	no
Treat-0d	RHSIM_Rhsim11G0196200	-0.119	-0.891	-0.772	no
Treat-0d	RHSIM_Rhsim13G0234500	0.554	0.142	-0.413	no
Treat-0d	RHSIM_Rhsim13G0201500	0.372	2.604	2.232	no
Treat-0d	RHSIM_Rhsim11G0148100	-1.815	-0.299	1.515	low
Treat-0d	RHSIM_Rhsim13G0052600	-0.005	-0.971	-0.966	no
Treat-0d	RHSIM_Rhsim11G0122800	-0.491	-0.287	0.204	no

Group	Gene	PTR (z-score difference)	z(log2 LFQ)	z(log2 FPKM)	extreme-PTR
Treat-0d	RHSIM_Rhsim11G0017900	0.726	-0.794	-1.520	no
Treat-0d	RHSIM_Rhsim11G0130100	0.584	1.755	1.171	no
Treat-0d	RHSIM_Rhsim13G0010400	-0.288	-0.487	-0.198	no
Treat-0d	RHSIM_Rhsim12G0087000	-0.634	0.608	1.241	no
Treat-0d	RHSIM_Rhsim11G0167000	0.928	1.528	0.600	no
Treat-0d	RHSIM_Rhsim11G0153500	0.567	0.490	-0.077	no
Treat-0d	RHSIM_Rhsim12G0080800	1.639	0.218	-1.421	high
Treat-0d	RHSIM_Rhsim11G0106600	0.740	-1.045	-1.785	no
Treat-0d	RHSIM_Rhsim11G0165700	-0.678	0.111	0.789	no
Treat-0d	RHSIM_Rhsim13G0153900	0.150	0.108	-0.042	no
Treat-0d	RHSIM_Rhsim11G0036700	0.225	-0.455	-0.680	no
Treat-0d	RHSIM_Rhsim11G0097900	0.228	-0.662	-0.890	no
Treat-0d	RHSIM_Rhsim12G0010700	1.826	1.790	-0.036	high
Treat-0d	RHSIM_Rhsim11G0099800	0.916	0.246	-0.670	no
Treat-0d	RHSIM_Rhsim13G0049600	-2.439	-0.306	2.134	low
Treat-0d	RHSIM_Rhsim12G0076700	0.184	-0.336	-0.520	no
Treat-0d	RHSIM_Rhsim12G0209000	0.252	0.631	0.379	no
Treat-0d	RHSIM_Rhsim12G0090500	-0.683	-0.584	0.099	no
Treat-0d	RHSIM_Rhsim11G0132600	-0.490	-0.449	0.042	no
Treat-0d	RHSIM_Rhsim11G0099600	-1.945	-0.441	1.504	low
Treat-0d	RHSIM_Rhsim11G0121300	-0.287	1.200	1.486	no
Treat-0d	RHSIM_Rhsim11G0123700	-0.067	0.092	0.159	no
Treat-0d	RHSIM_Rhsim11G0161300	1.163	-0.520	-1.684	high
Treat-0d	RHSIM_Rhsim11G0097400	-0.589	-0.870	-0.281	no
Treat-0d	RHSIM_Rhsim11G0145600	-0.740	1.078	1.818	no
Treat-0d	RHSIM_Rhsim11G0045300	2.133	2.262	0.129	high
Treat-0d	RHSIM_Rhsim12G0206700	0.154	-0.185	-0.339	no
Treat-0d	RHSIM_Rhsim12G0088500	-0.597	-1.580	-0.983	no
Treat-0d	RHSIM_Rhsim11G0058800	-0.567	1.162	1.729	no
Treat-0d	RHSIM_Rhsim12G0077400	0.529	-1.273	-1.802	no
Treat-0d	RHSIM_Rhsim12G0125300	-1.184	-0.844	0.340	low
Treat-0d	RHSIM_Rhsim11G0191300	-1.013	-1.161	-0.148	no

Group	Gene	PTR (z-score difference)	z(log2 LFQ)	z(log2 FPKM)	extreme-PTR
Treat-0d	RHSIM_Rhsim12G0215400	-0.364	0.302	0.666	no
Treat-0d	RHSIM_Rhsim11G0142100	1.173	1.430	0.257	high
Treat-0d	RHSIM_Rhsim11G0199200	0.217	-0.178	-0.395	no
Treat-0d	RHSIM_Rhsim13G0037000	0.229	0.503	0.273	no
Treat-0d	RHSIM_Rhsim11G0044200	2.039	1.095	-0.944	high
Treat-0d	RHSIM_Rhsim12G0025900	-0.335	-0.042	0.293	no
Treat-0d	RHSIM_Rhsim12G0200000	0.361	-0.727	-1.088	no
Treat-0d	RHSIM_Rhsim11G0142400	1.423	1.032	-0.391	high
Treat-0d	RHSIM_Rhsim11G0036800	-0.998	-0.795	0.203	no
Treat-0d	RHSIM_Rhsim11G0105300	-0.514	-0.527	-0.013	no
Treat-0d	RHSIM_Rhsim11G0149600	0.890	0.101	-0.790	no
Treat-0d	RHSIM_Rhsim12G0149800	-0.184	1.643	1.826	no
Treat-0d	RHSIM_Rhsim11G0053800	0.991	-0.821	-1.812	no
Treat-0d	RHSIM_Rhsim11G0197800	-1.569	-1.045	0.524	low
Treat-0d	RHSIM_Rhsim11G0006500	-0.137	-0.695	-0.558	no
Treat-0d	RHSIM_Rhsim12G0003500	1.671	1.836	0.165	high
Treat-0d	RHSIM_Rhsim11G0179400	0.200	0.292	0.092	no
Treat-0d	RHSIM_Rhsim11G0008100	2.843	1.277	-1.566	high
Treat-0d	RHSIM_Rhsim11G0084200	-1.368	2.201	3.569	low
Treat-0d	RHSIM_Rhsim04G0146800	-0.061	0.361	0.423	no
Treat-0d	RHSIM_Rhsim11G0166500	-0.774	-0.539	0.236	no
Treat-0d	RHSIM_Rhsim13G0114900	-1.692	-2.059	-0.367	low
Treat-0d	RHSIM_Rhsim11G0036600	0.391	-0.739	-1.131	no
Treat-0d	RHSIM_Rhsim12G0108900	0.045	-2.109	-2.155	no
Treat-0d	RHSIM_Rhsim11G0087200	-0.664	0.584	1.248	no
Treat-0d	RHSIM_Rhsim12G0157100	-1.339	-1.627	-0.289	low
Treat-0d	RHSIM_Rhsim11G0182400	-2.248	-1.882	0.366	low
Treat-0d	RHSIM_Rhsim12G0151900	-0.487	1.910	2.397	no
Treat-0d	RHSIM_Rhsim11G0088900	0.044	0.377	0.333	no
Treat-0d	RHSIM_Rhsim12G0056500	-2.549	-2.035	0.514	low
Treat-0d	RHSIM_Rhsim11G0108000	0.637	-0.470	-1.106	no
Treat-0d	RHSIM_Rhsim13G0225000	-0.343	-1.300	-0.956	no

Group	Gene	PTR (z-score difference)	z(log2 LFQ)	z(log2 FPKM)	extreme-PTR
Treat-0d	RHSIM_Rhsim11G0168200	-0.468	-1.479	-1.011	no
Treat-0d	RHSIM_Rhsim11G0003700	0.036	0.846	0.811	no
Treat-0d	RHSIM_Rhsim11G0136600	0.926	0.137	-0.789	no
Treat-0d	RHSIM_Rhsim11G0168600	-0.345	-0.817	-0.472	no
Treat-0d	RHSIM_Rhsim12G0179900	-0.308	-1.298	-0.990	no
Treat-0d	RHSIM_Rhsim11G0193300	-0.488	-1.167	-0.679	no
Treat-0d	RHSIM_Rhsim11G0105100	0.460	0.924	0.464	no
Treat-0d	RHSIM_Rhsim11G0088000	-0.465	-0.331	0.135	no
Treat-0d	RHSIM_Rhsim12G0090900	-0.819	-0.984	-0.165	no
Treat-0d	RHSIM_Rhsim12G0193400	-1.382	1.292	2.674	low
Treat-0d	RHSIM_Rhsim11G0183800	-0.326	3.745	4.071	no
Treat-0d	RHSIM_Rhsim13G0213200	-1.016	1.477	2.493	no
Treat-0d	RHSIM_Rhsim11G0008300	1.449	-0.018	-1.467	high
Treat-0d	RHSIM_Rhsim12G0145800	0.299	0.189	-0.110	no
Treat-0d	RHSIM_Rhsim13G0229300	0.217	1.881	1.664	no
Treat-0d	RHSIM_Rhsim11G0098600	-0.402	-0.006	0.395	no
Treat-0d	RHSIM_Rhsim11G0180700	0.198	0.395	0.197	no
Treat-0d	RHSIM_Rhsim12G0201700	-0.043	0.980	1.022	no
Treat-0d	RHSIM_Rhsim13G0029900	0.809	1.356	0.547	no
Treat-0d	RHSIM_Rhsim12G0180500	0.567	-0.298	-0.865	no
Treat-0d	RHSIM_Rhsim11G0065900	-0.887	-0.311	0.576	no
Treat-0d	RHSIM_Rhsim11G0159300	0.955	0.853	-0.102	no
Treat-0d	RHSIM_Rhsim12G0167000	-0.808	-0.994	-0.186	no
Treat-0d	RHSIM_Rhsim11G0055100	1.181	-0.147	-1.327	high
Treat-0d	RHSIM_Rhsim11G0016400	-0.343	-1.038	-0.696	no
Treat-0d	RHSIM_Rhsim11G0022000	0.137	0.027	-0.110	no
Treat-0d	RHSIM_Rhsim11G0184900	-0.610	-0.005	0.605	no
Treat-0d	RHSIM_Rhsim11G0063600	1.608	0.789	-0.819	high
Treat-0d	RHSIM_Rhsim13G0166900	0.394	-0.708	-1.102	no
Treat-0d	RHSIM_Rhsim11G0010400	-0.794	-1.277	-0.483	no
Treat-0d	RHSIM_Rhsim11G0063800	-0.778	-1.174	-0.396	no
Treat-0d	RHSIM_Rhsim11G0174300	1.133	-0.030	-1.162	high

Group	Gene	PTR (z-score difference)	z(log2 LFQ)	z(log2 FPKM)	extreme-PTR
Treat-0d	RHSIM_Rhsim11G0085900	0.169	1.853	1.684	no
Treat-0d	RHSIM_Rhsim11G0041300	1.988	1.880	-0.108	high
Treat-0d	RHSIM_Rhsim12G0209200	0.158	0.072	-0.086	no
Treat-0d	RHSIM_Rhsim12G0134600	0.717	0.359	-0.358	no
Treat-0d	RHSIM_Rhsim11G0146000	-1.966	-1.680	0.285	low
Treat-0d	RHSIM_Rhsim12G0024700	-1.142	-0.790	0.352	low
Treat-0d	RHSIM_Rhsim12G0054800	-2.755	-1.476	1.279	low
Treat-0d	RHSIM_Rhsim11G0198200	0.141	0.415	0.274	no
Treat-0d	RHSIM_Rhsim11G0099700	-0.511	-1.037	-0.526	no
Treat-0d	RHSIM_Rhsim12G0025500	-1.211	-0.831	0.379	low
Treat-0d	RHSIM_Rhsim12G0109900	-0.225	-1.390	-1.165	no
Treat-0d	RHSIM_Rhsim11G0112000	0.162	-0.525	-0.687	no
Treat-0d	RHSIM_Rhsim12G0133300	0.813	-0.043	-0.855	no
Treat-0d	RHSIM_Rhsim12G0173900	-1.062	-1.257	-0.195	low
Treat-0d	RHSIM_Rhsim11G0193600	0.899	0.359	-0.540	no
Treat-0d	RHSIM_Rhsim12G0016800	0.346	0.473	0.127	no
Treat-0d	RHSIM_Rhsim12G0112100	-0.767	1.279	2.046	no
Treat-0d	RHSIM_Rhsim11G0042300	0.430	-0.247	-0.677	no
Treat-0d	RHSIM_Rhsim12G0212900	-0.425	-0.272	0.152	no
Treat-0d	RHSIM_Rhsim12G0144900	-0.078	-0.212	-0.134	no
Treat-0d	RHSIM_Rhsim11G0090900	0.688	-0.451	-1.139	no
Treat-0d	RHSIM_Rhsim11G0196900	-0.152	-0.565	-0.414	no
Treat-0d	RHSIM_Rhsim11G0157200	-0.642	0.267	0.909	no
Treat-0d	RHSIM_Rhsim12G0162200	-0.461	-0.243	0.218	no
Treat-0d	RHSIM_Rhsim11G0176400	0.362	-0.087	-0.448	no
Treat-0d	RHSIM_Rhsim11G0036900	0.982	-0.310	-1.293	no
Treat-0d	RHSIM_Rhsim11G0177200	0.135	0.518	0.383	no
Treat-0d	RHSIM_Rhsim11G0051900	1.519	-0.072	-1.591	high
Treat-0d	RHSIM_Rhsim12G0137000	0.335	0.119	-0.216	no
Treat-0d	RHSIM_Rhsim11G0045000	0.522	-0.274	-0.796	no
Treat-0d	RHSIM_Rhsim12G0130000	0.206	0.581	0.375	no
Treat-0d	RHSIM_Rhsim11G0122200	0.907	2.597	1.690	no

Group	Gene	PTR (z-score difference)	z(log2 LFQ)	z(log2 FPKM)	extreme-PTR
Treat-0d	RHSIM_Rhsim12G0005000	0.656	0.088	-0.568	no
Treat-0d	RHSIM_Rhsim11G0114400	0.646	-0.927	-1.573	no
Treat-0d	RHSIM_Rhsim11G0194700	0.087	0.919	0.833	no
Treat-0d	RHSIM_Rhsim12G0003200	-0.179	-0.641	-0.461	no
Treat-0d	RHSIM_Rhsim11G0182000	0.673	0.848	0.175	no
Treat-0d	RHSIM_Rhsim11G0134200	1.114	0.524	-0.590	high
Treat-0d	RHSIM_Rhsim11G0177800	-1.541	1.112	2.653	low
Treat-0d	RHSIM_Rhsim11G0046400	0.433	-0.053	-0.486	no
Treat-0d	RHSIM_Rhsim12G0041700	-2.281	-0.759	1.522	low
Treat-0d	RHSIM_Rhsim11G0195000	0.070	-1.220	-1.290	no
Treat-0d	RHSIM_Rhsim11G0054800	-1.028	-1.424	-0.396	low
Treat-0d	RHSIM_Rhsim12G0002100	-0.213	-0.904	-0.691	no
Treat-0d	RHSIM_Rhsim11G0065700	-0.984	0.955	1.939	no
Treat-0d	RHSIM_Rhsim11G0167500	1.035	1.016	-0.020	no
Treat-0d	RHSIM_Rhsim11G0045700	0.543	-0.568	-1.111	no
Treat-0d	RHSIM_Rhsim11G0118400	0.024	0.494	0.470	no
Treat-0d	RHSIM_Rhsim12G0204600	-0.125	1.873	1.998	no
Treat-0d	RHSIM_Rhsim12G0051400	-1.173	-0.354	0.820	low
Treat-0d	RHSIM_Rhsim11G0147400	-0.763	-0.524	0.239	no
Treat-0d	RHSIM_Rhsim12G0075500	0.551	1.801	1.250	no
Treat-0d	RHSIM_Rhsim11G0184600	-0.810	-1.108	-0.298	no
Treat-0d	RHSIM_Rhsim11G0199100	0.204	-0.451	-0.654	no
Treat-0d	RHSIM_Rhsim11G0053000	-2.128	-1.194	0.934	low
Treat-0d	RHSIM_Rhsim11G0020200	0.951	2.127	1.176	no
Treat-0d	RHSIM_Rhsim11G0197500	0.414	0.134	-0.280	no
Treat-0d	RHSIM_Rhsim11G0121700	1.515	0.907	-0.607	high
Treat-0d	RHSIM_Rhsim12G0017200	-0.473	0.664	1.137	no
Treat-0d	RHSIM_Rhsim10G0183500	0.359	0.389	0.030	no
Treat-0d	RHSIM_Rhsim12G0037000	-0.263	-1.642	-1.379	no
Treat-0d	RHSIM_Rhsim11G0167700	1.714	0.899	-0.815	high
Treat-0d	RHSIM_Rhsim11G0088700	0.713	-0.929	-1.643	no
Treat-0d	RHSIM_Rhsim10G0007500	-0.452	-0.055	0.396	no

Group	Gene	PTR (z-score difference)	z(log2 LFQ)	z(log2 FPKM)	extreme-PTR
Treat-0d	RHSIM_Rhsim12G0000100	1.417	0.727	-0.689	high
Treat-0d	RHSIM_Rhsim11G0054200	-0.425	-0.798	-0.374	no
Treat-0d	RHSIM_Rhsim10G0040200	0.767	0.939	0.172	no
Treat-0d	RHSIM_Rhsim11G0195400	0.045	0.060	0.015	no
Treat-0d	RHSIM_Rhsim12G0193600	-1.969	-2.508	-0.540	low
Treat-0d	RHSIM_Rhsim11G0156300	2.267	0.822	-1.445	high
Treat-0d	RHSIM_Rhsim10G0102500	0.335	0.121	-0.215	no
Treat-0d	RHSIM_Rhsim11G0122000	1.619	0.133	-1.486	high
Treat-0d	RHSIM_Rhsim12G0102200	-0.458	-0.889	-0.431	no
Treat-0d	RHSIM_Rhsim11G0161100	-3.418	-1.854	1.564	low
Treat-0d	RHSIM_Rhsim11G0052000	0.790	-0.393	-1.183	no
Treat-0d	RHSIM_Rhsim10G0183700	0.053	1.825	1.772	no
Treat-0d	RHSIM_Rhsim11G0175900	-0.422	-1.175	-0.754	no
Treat-0d	RHSIM_Rhsim10G0204000	0.606	0.437	-0.169	no
Treat-0d	RHSIM_Rhsim11G0125500	-0.288	-1.132	-0.844	no
Treat-0d	RHSIM_Rhsim11G0173600	-2.300	-1.583	0.717	low
Treat-0d	RHSIM_Rhsim10G0079900	1.000	1.147	0.148	no
Treat-0d	RHSIM_Rhsim12G0090700	0.222	0.992	0.770	no
Treat-0d	RHSIM_Rhsim11G0010500	-0.327	0.419	0.746	no
Treat-0d	RHSIM_Rhsim11G0095500	0.690	0.035	-0.655	no
Treat-0d	RHSIM_Rhsim10G0133700	1.792	3.173	1.381	high
Treat-0d	RHSIM_Rhsim10G0079400	0.846	0.131	-0.716	no
Treat-0d	RHSIM_Rhsim11G0089500	-0.885	-1.254	-0.370	no
Treat-0d	RHSIM_Rhsim10G0135700	2.179	1.190	-0.988	high
Treat-0d	RHSIM_Rhsim12G0205300	-0.142	0.128	0.270	no
Treat-0d	RHSIM_Rhsim12G0036900	1.314	0.315	-1.000	high
Treat-0d	RHSIM_Rhsim10G0196000	-0.918	1.231	2.149	no
Treat-0d	RHSIM_Rhsim11G0190300	0.311	0.399	0.088	no
Treat-0d	RHSIM_Rhsim12G0105500	1.916	1.226	-0.690	high
Treat-0d	RHSIM_Rhsim10G0045000	-0.877	-0.309	0.568	no
Treat-0d	RHSIM_Rhsim12G0185400	-1.515	-0.913	0.602	low
Treat-0d	RHSIM_Rhsim10G0210800	1.656	-0.506	-2.162	high

Group	Gene	PTR (z-score difference)	z(log2 LFQ)	z(log2 FPKM)	extreme-PTR
Treat-0d	RHSIM_Rhsim12G0110600	-1.450	-0.629	0.821	low
Treat-0d	RHSIM_Rhsim10G0121300	0.002	-0.627	-0.629	no
Treat-0d	RHSIM_Rhsim11G0008500	0.207	0.149	-0.058	no
Treat-0d	RHSIM_Rhsim10G0069400	-1.501	-1.591	-0.090	low
Treat-0d	RHSIM_Rhsim12G0168500	0.217	0.027	-0.190	no
Treat-0d	RHSIM_Rhsim11G0196800	0.259	-0.162	-0.421	no
Treat-0d	RHSIM_Rhsim10G0029000	0.577	0.068	-0.509	no
Treat-0d	RHSIM_Rhsim10G0155900	0.792	-1.263	-2.055	no
Treat-0d	RHSIM_Rhsim10G0009900	-0.482	-1.646	-1.164	no
Treat-0d	RHSIM_Rhsim10G0164700	-0.512	-0.861	-0.349	no
Treat-0d	RHSIM_Rhsim12G0166000	-0.634	-0.816	-0.183	no
Treat-0d	RHSIM_Rhsim10G0197300	-0.489	0.853	1.342	no
Treat-0d	RHSIM_Rhsim11G0097800	-0.456	0.782	1.238	no
Treat-0d	RHSIM_Rhsim12G0007900	-0.494	0.515	1.009	no
Treat-0d	RHSIM_Rhsim10G0158900	0.765	-1.495	-2.260	no
Treat-0d	RHSIM_Rhsim12G0139700	1.190	0.393	-0.797	high
Treat-0d	RHSIM_Rhsim10G0171300	-3.262	-2.329	0.933	low
Treat-0d	RHSIM_Rhsim12G0159900	-0.795	-0.901	-0.106	no
Treat-0d	RHSIM_Rhsim11G0167600	0.816	1.491	0.675	no
Treat-0d	RHSIM_Rhsim10G0067100	-0.619	-0.547	0.073	no
Treat-0d	RHSIM_Rhsim11G0126200	-0.118	-0.474	-0.356	no
Treat-0d	RHSIM_Rhsim11G0023700	0.865	0.957	0.092	no
Treat-0d	RHSIM_Rhsim12G0068000	-0.479	0.594	1.073	no
Treat-0d	RHSIM_Rhsim11G0198400	0.944	1.053	0.109	no
Treat-0d	RHSIM_Rhsim11G0052800	-0.048	-1.330	-1.282	no
Treat-0d	RHSIM_Rhsim10G0051200	-1.009	-0.777	0.232	no
Treat-0d	RHSIM_Rhsim10G0191900	0.138	-0.460	-0.597	no
Treat-0d	RHSIM_Rhsim10G0026400	0.880	0.764	-0.116	no
Treat-0d	RHSIM_Rhsim10G0030500	0.277	-1.430	-1.707	no
Treat-0d	RHSIM_Rhsim10G0005500	-0.260	0.141	0.401	no
Treat-0d	RHSIM_Rhsim10G0105200	0.152	0.039	-0.112	no
Treat-0d	RHSIM_Rhsim12G0114000	-0.335	-0.281	0.054	no

Group	Gene	PTR (z-score difference)	z(log2 LFQ)	z(log2 FPKM)	extreme-PTR
Treat-0d	RHSIM_Rhsim10G0005700	0.235	-0.578	-0.813	no
Treat-0d	RHSIM_Rhsim10G0117200	0.161	1.127	0.966	no
Treat-0d	RHSIM_Rhsim10G0191600	0.068	-0.894	-0.962	no
Treat-0d	RHSIM_Rhsim10G0194400	0.858	0.583	-0.275	no
Treat-0d	RHSIM_Rhsim11G0053100	1.180	1.614	0.434	high
Treat-0d	RHSIM_Rhsim11G0198000	0.129	-0.391	-0.519	no
Treat-0d	RHSIM_Rhsim11G0113700	-0.326	0.192	0.517	no
Treat-0d	RHSIM_Rhsim10G0180700	1.003	-0.259	-1.262	no
Treat-0d	RHSIM_Rhsim10G0199000	1.013	-0.051	-1.063	no
Treat-0d	RHSIM_Rhsim12G0008000	0.752	-0.042	-0.794	no
Treat-0d	RHSIM_Rhsim10G0035600	0.084	-0.344	-0.428	no
Treat-0d	RHSIM_Rhsim10G0116000	-0.879	-1.759	-0.880	no
Treat-0d	RHSIM_Rhsim11G0048900	-1.079	-1.321	-0.242	low
Treat-0d	RHSIM_Rhsim11G0167200	1.711	0.831	-0.881	high
Treat-0d	RHSIM_Rhsim12G0070400	1.010	0.801	-0.209	no
Treat-0d	RHSIM_Rhsim10G0148300	0.638	0.532	-0.106	no
Treat-0d	RHSIM_Rhsim10G0099800	-0.446	1.553	1.999	no
Treat-0d	RHSIM_Rhsim10G0136800	-0.716	-0.480	0.236	no
Treat-0d	RHSIM_Rhsim12G0115000	-2.047	-1.308	0.739	low
Treat-0d	RHSIM_Rhsim10G0186500	0.324	0.592	0.269	no
Treat-0d	RHSIM_Rhsim12G0166200	-0.097	-0.934	-0.837	no
Treat-0d	RHSIM_Rhsim11G0135900	1.100	3.032	1.932	no
Treat-0d	RHSIM_Rhsim12G0189800	-0.222	-0.376	-0.154	no
Treat-0d	RHSIM_Rhsim10G0108600	-0.177	-0.113	0.064	no
Treat-0d	RHSIM_Rhsim10G0162300	0.719	-0.583	-1.302	no
Treat-0d	RHSIM_Rhsim12G0014000	0.553	-0.255	-0.808	no
Treat-0d	RHSIM_Rhsim10G0161200	-1.031	-1.313	-0.282	low
Treat-0d	RHSIM_Rhsim11G0125700	-1.483	-1.637	-0.154	low
Treat-0d	RHSIM_Rhsim10G0192800	-0.044	-0.472	-0.428	no
Treat-0d	RHSIM_Rhsim10G0197900	0.396	0.855	0.460	no
Treat-0d	RHSIM_Rhsim12G0136800	-0.656	-1.294	-0.638	no
Treat-0d	RHSIM_Rhsim10G0014000	-0.291	-0.789	-0.498	no

Group	Gene	PTR (z-score difference)	z(log2 LFQ)	z(log2 FPKM)	extreme-PTR
Treat-0d	RHSIM_Rhsim10G0094500	0.340	-0.253	-0.593	no
Treat-0d	RHSIM_Rhsim11G0173400	-2.672	-0.736	1.936	low
Treat-0d	RHSIM_Rhsim12G0150700	0.085	0.742	0.657	no
Treat-0d	RHSIM_Rhsim10G0069000	0.828	1.008	0.180	no
Treat-0d	RHSIM_Rhsim11G0135200	-0.190	-0.429	-0.239	no
Treat-0d	RHSIM_Rhsim10G0019600	-0.786	-0.113	0.673	no
Treat-0d	RHSIM_Rhsim12G0001500	1.255	0.433	-0.822	high
Treat-0d	RHSIM_Rhsim11G0106300	1.054	1.247	0.193	no
Treat-0d	RHSIM_Rhsim10G0126200	1.425	1.622	0.197	high
Treat-0d	RHSIM_Rhsim11G0074300	-0.535	0.770	1.306	no
Treat-0d	RHSIM_Rhsim11G0155300	2.465	1.062	-1.403	high
Treat-0d	RHSIM_Rhsim12G0128400	0.724	0.273	-0.451	no
Treat-0d	RHSIM_Rhsim12G0213700	0.806	1.160	0.354	no
Treat-0d	RHSIM_Rhsim10G0141300	1.373	3.228	1.855	high
Treat-0d	RHSIM_Rhsim10G0115500	-0.572	-1.416	-0.844	no
Treat-0d	RHSIM_Rhsim11G0130000	-0.397	-0.600	-0.204	no
Treat-0d	RHSIM_Rhsim11G0064900	0.295	-0.774	-1.069	no
Treat-0d	RHSIM_Rhsim10G0202500	0.111	1.108	0.998	no
Treat-0d	RHSIM_Rhsim10G0183800	-0.036	-1.434	-1.398	no
Treat-0d	RHSIM_Rhsim10G0183200	-0.646	-0.616	0.030	no
Treat-0d	RHSIM_Rhsim11G0192900	0.283	0.923	0.640	no
Treat-0d	RHSIM_Rhsim11G0015100	-0.809	-1.413	-0.605	no
Treat-0d	RHSIM_Rhsim10G0078300	-0.862	-0.601	0.262	no
Treat-0d	RHSIM_Rhsim11G0164000	1.554	-0.776	-2.330	high
Treat-0d	RHSIM_Rhsim10G0014400	-1.283	-0.839	0.443	low
Treat-0d	RHSIM_Rhsim10G0101200	0.311	1.668	1.357	no
Treat-0d	RHSIM_Rhsim10G0211500	0.327	-1.868	-2.195	no
Treat-0d	RHSIM_Rhsim11G0159000	0.149	1.047	0.898	no
Treat-0d	RHSIM_Rhsim10G0099300	0.320	-0.237	-0.557	no
Treat-0d	RHSIM_Rhsim11G0099900	-0.415	-1.101	-0.686	no
Treat-0d	RHSIM_Rhsim11G0075200	-0.974	-0.388	0.586	no
Treat-0d	RHSIM_Rhsim11G0094000	0.229	1.781	1.552	no

Group	Gene	PTR (z-score difference)	z(log2 LFQ)	z(log2 FPKM)	extreme-PTR
Treat-0d	RHSIM_Rhsim10G0156300	1.181	0.530	-0.651	high
Treat-0d	RHSIM_Rhsim11G0003600	-0.547	0.042	0.589	no
Treat-0d	RHSIM_Rhsim11G0077900	1.238	0.534	-0.704	high
Treat-0d	RHSIM_Rhsim10G0167100	1.766	1.751	-0.016	high
Treat-0d	RHSIM_Rhsim11G0075500	-4.210	-1.080	3.130	low
Treat-0d	RHSIM_Rhsim10G0071100	0.644	1.533	0.888	no
Treat-0d	RHSIM_Rhsim10G0006900	-0.686	-1.704	-1.018	no
Treat-0d	RHSIM_Rhsim11G0133300	3.192	1.531	-1.661	high
Treat-0d	RHSIM_Rhsim11G0078100	1.519	1.158	-0.361	high
Treat-0d	RHSIM_Rhsim11G0121800	0.413	1.784	1.372	no
Treat-0d	RHSIM_Rhsim11G0094400	-0.442	0.901	1.343	no
Treat-0d	RHSIM_Rhsim11G0093100	0.068	-0.393	-0.461	no
Treat-0d	RHSIM_Rhsim11G0159200	-1.420	-0.981	0.440	low
Treat-0d	RHSIM_Rhsim10G0198000	-0.477	-1.211	-0.735	no
Treat-0d	RHSIM_Rhsim10G0211400	0.145	2.267	2.122	no
Treat-0d	RHSIM_Rhsim10G0188500	-1.115	-0.074	1.041	low
Treat-0d	RHSIM_Rhsim10G0172500	0.329	-0.246	-0.575	no
Treat-0d	RHSIM_Rhsim11G0117700	0.222	0.479	0.257	no
Treat-0d	RHSIM_Rhsim11G0196300	0.701	1.430	0.729	no
Treat-0d	RHSIM_Rhsim11G0019600	-0.211	0.138	0.349	no
Treat-0d	RHSIM_Rhsim10G0006300	1.173	0.119	-1.054	high
Treat-0d	RHSIM_Rhsim10G0112600	0.643	-0.458	-1.101	no
Treat-0d	RHSIM_Rhsim11G0136500	-0.554	-0.580	-0.025	no
Treat-0d	RHSIM_Rhsim10G0107900	0.800	0.842	0.042	no
Treat-0d	RHSIM_Rhsim11G0090400	-0.149	0.179	0.327	no
Treat-0d	RHSIM_Rhsim11G0190500	0.037	0.356	0.319	no
Treat-0d	RHSIM_Rhsim10G0008800	-1.601	-1.450	0.151	low
Treat-0d	RHSIM_Rhsim11G0136000	0.266	0.610	0.344	no
Treat-0d	RHSIM_Rhsim10G0014200	0.603	-0.774	-1.377	no
Treat-0d	RHSIM_Rhsim11G0141900	-0.235	-0.066	0.169	no
Treat-0d	RHSIM_Rhsim11G0131600	0.061	-0.857	-0.918	no
Treat-0d	RHSIM_Rhsim10G0141900	0.121	-0.149	-0.270	no

Group	Gene	PTR (z-score difference)	z(log2 LFQ)	z(log2 FPKM)	extreme-PTR
Treat-0d	RHSIM_Rhsim11G0072400	-1.091	-0.810	0.281	low
Treat-0d	RHSIM_Rhsim10G0099600	-1.888	-2.518	-0.630	low
Treat-0d	RHSIM_Rhsim10G0022700	-0.330	0.526	0.856	no
Treat-0d	RHSIM_Rhsim11G0008000	-0.135	-0.768	-0.633	no
Treat-0d	RHSIM_Rhsim11G0077600	-0.204	-0.997	-0.794	no
Treat-0d	RHSIM_Rhsim11G0128300	0.515	0.400	-0.115	no
Treat-0d	RHSIM_Rhsim10G0068700	-1.330	-1.957	-0.627	low
Treat-0d	RHSIM_Rhsim11G0172600	-0.030	-0.585	-0.555	no
Treat-0d	RHSIM_Rhsim10G0171700	1.232	2.560	1.327	high
Treat-0d	RHSIM_Rhsim11G0165400	0.462	-0.131	-0.593	no
Treat-0d	RHSIM_Rhsim10G0006400	-0.015	0.832	0.846	no
Treat-0d	RHSIM_Rhsim11G0180400	0.760	-0.243	-1.003	no
Treat-0d	RHSIM_Rhsim11G0132900	-1.662	-1.220	0.442	low
Treat-0d	RHSIM_Rhsim10G0072100	-0.718	-0.237	0.481	no
Treat-0d	RHSIM_Rhsim10G0134600	1.481	-0.532	-2.013	high
Treat-0d	RHSIM_Rhsim10G0061400	-0.391	0.005	0.396	no
Treat-0d	RHSIM_Rhsim10G0039800	-0.341	1.147	1.488	no
Treat-0d	RHSIM_Rhsim10G0151800	-0.226	-0.677	-0.451	no
Treat-0d	RHSIM_Rhsim10G0127000	-2.035	-1.222	0.812	low
Treat-0d	RHSIM_Rhsim11G0184700	-0.462	-0.073	0.389	no
Treat-0d	RHSIM_Rhsim11G0111500	-0.173	-0.332	-0.159	no
Treat-0d	RHSIM_Rhsim10G0021200	0.462	3.397	2.934	no
Treat-0d	RHSIM_Rhsim10G0193400	1.784	-0.522	-2.306	high
Treat-0d	RHSIM_Rhsim10G0013900	-0.026	0.038	0.063	no
Treat-0d	RHSIM_Rhsim10G0015700	1.734	-0.601	-2.335	high
Treat-0d	RHSIM_Rhsim10G0149000	0.879	0.467	-0.411	no
Treat-0d	RHSIM_Rhsim10G0179500	-1.072	0.609	1.681	low
Treat-0d	RHSIM_Rhsim10G0178400	-0.546	3.113	3.660	no
Treat-0d	RHSIM_Rhsim10G0177300	0.904	1.650	0.746	no
Treat-0d	RHSIM_Rhsim10G0127200	0.503	0.114	-0.389	no
Treat-0d	RHSIM_Rhsim10G0019900	0.279	0.989	0.710	no
Treat-0d	RHSIM_Rhsim10G0060000	-1.035	-1.449	-0.413	low

Group	Gene	PTR (z-score difference)	z(log2 LFQ)	z(log2 FPKM)	extreme-PTR
Treat-0d	RHSIM_Rhsim10G0079500	1.555	0.945	-0.610	high
Treat-0d	RHSIM_Rhsim10G0046600	0.192	0.062	-0.130	no
Treat-0d	RHSIM_Rhsim10G0078600	0.183	-0.001	-0.184	no
Treat-0d	RHSIM_Rhsim11G0028300	1.218	1.195	-0.023	high
Treat-0d	RHSIM_Rhsim10G0174600	-1.754	-1.454	0.300	low
Treat-0d	RHSIM_Rhsim11G0045600	-0.928	0.341	1.269	no
Treat-0d	RHSIM_Rhsim11G0197700	0.046	-0.850	-0.895	no
Treat-0d	RHSIM_Rhsim10G0082000	-0.386	-0.438	-0.052	no
Treat-0d	RHSIM_Rhsim10G0048900	0.457	1.105	0.648	no
Treat-0d	RHSIM_Rhsim10G0022600	1.929	1.454	-0.475	high
Treat-0d	RHSIM_Rhsim11G0169400	-0.802	0.203	1.005	no
Treat-0d	RHSIM_Rhsim10G0199600	1.369	1.061	-0.308	high
Treat-0d	RHSIM_Rhsim10G0175100	1.212	1.156	-0.056	high
Treat-0d	RHSIM_Rhsim11G0173300	0.910	0.455	-0.455	no
Treat-0d	RHSIM_Rhsim11G0014400	0.074	0.618	0.544	no
Treat-0d	RHSIM_Rhsim11G0071200	-0.384	-0.707	-0.323	no
Treat-0d	RHSIM_Rhsim11G0069400	0.749	1.912	1.162	no
Treat-0d	RHSIM_Rhsim10G0163500	0.494	1.630	1.136	no
Treat-0d	RHSIM_Rhsim11G0031700	-0.401	-0.515	-0.114	no
Treat-0d	RHSIM_Rhsim11G0133700	-0.654	-1.594	-0.940	no
Treat-0d	RHSIM_Rhsim10G0152000	1.327	1.214	-0.113	high
Treat-0d	RHSIM_Rhsim10G0185900	-1.639	-1.484	0.156	low
Treat-0d	RHSIM_Rhsim10G0062200	3.130	1.455	-1.675	high
Treat-0d	RHSIM_Rhsim11G0184000	-0.035	0.408	0.443	no
Treat-0d	RHSIM_Rhsim10G0100700	-0.338	-1.219	-0.881	no
Treat-0d	RHSIM_Rhsim11G0158600	-0.411	-1.350	-0.939	no
Treat-0d	RHSIM_Rhsim10G0014300	-1.857	-1.723	0.134	low
Treat-0d	RHSIM_Rhsim11G0077800	1.647	0.579	-1.068	high
Treat-0d	RHSIM_Rhsim10G0154300	2.769	0.813	-1.956	high
Treat-0d	RHSIM_Rhsim10G0012800	0.709	-0.299	-1.008	no
Treat-0d	RHSIM_Rhsim10G0207900	0.334	0.767	0.433	no
Treat-0d	RHSIM_Rhsim09G0201000	0.047	0.205	0.158	no

Group	Gene	PTR (z-score difference)	z(log2 LFQ)	z(log2 FPKM)	extreme-PTR
Treat-0d	RHSIM_Rhsim10G0035200	0.600	0.799	0.198	no
Treat-0d	RHSIM_Rhsim10G0043800	1.393	2.099	0.706	high
Treat-0d	RHSIM_Rhsim10G0128200	0.036	-0.835	-0.871	no
Treat-0d	RHSIM_Rhsim10G0181800	2.117	0.159	-1.958	high
Treat-0d	RHSIM_Rhsim11G0021700	0.444	1.058	0.614	no
Treat-0d	RHSIM_Rhsim10G0209100	0.116	0.114	-0.002	no
Treat-0d	RHSIM_Rhsim10G0006600	1.002	1.029	0.027	no
Treat-0d	RHSIM_Rhsim11G0113500	2.008	1.062	-0.945	high
Treat-0d	RHSIM_Rhsim10G0064900	0.663	-0.386	-1.048	no
Treat-0d	RHSIM_Rhsim10G0202400	-1.008	0.648	1.656	no
Treat-0d	RHSIM_Rhsim10G0213600	-1.274	-0.810	0.464	low
Treat-0d	RHSIM_Rhsim10G0072600	-0.298	-1.290	-0.992	no
Treat-0d	RHSIM_Rhsim09G0032200	0.371	0.007	-0.363	no
Treat-0d	RHSIM_Rhsim10G0008600	1.754	0.309	-1.446	high
Treat-0d	RHSIM_Rhsim10G0131300	-0.631	-1.255	-0.624	no
Treat-0d	RHSIM_Rhsim10G0102800	0.050	-1.012	-1.062	no
Treat-0d	RHSIM_Rhsim11G0098900	-0.063	-0.111	-0.048	no
Treat-0d	RHSIM_Rhsim11G0100500	-1.317	-0.140	1.177	low
Treat-0d	RHSIM_Rhsim10G0138000	1.797	1.449	-0.348	high
Treat-0d	RHSIM_Rhsim09G0097600	0.014	0.053	0.039	no
Treat-0d	RHSIM_Rhsim10G0102700	0.070	-0.311	-0.381	no
Treat-0d	RHSIM_Rhsim11G0088600	0.029	-0.036	-0.065	no
Treat-0d	RHSIM_Rhsim10G0034800	1.698	-0.034	-1.732	high
Treat-0d	RHSIM_Rhsim11G0178900	-0.572	-0.240	0.332	no
Treat-0d	RHSIM_Rhsim10G0030200	-1.373	0.113	1.486	low
Treat-0d	RHSIM_Rhsim09G0168000	0.895	-0.755	-1.650	no
Treat-0d	RHSIM_Rhsim10G0058700	3.391	1.504	-1.887	high
Treat-0d	RHSIM_Rhsim10G0036600	0.055	0.045	-0.010	no
Treat-0d	RHSIM_Rhsim11G0030300	-0.195	-0.336	-0.141	no
Treat-0d	RHSIM_Rhsim10G0109900	-1.911	-0.371	1.539	low
Treat-0d	RHSIM_Rhsim11G0134800	-0.818	0.059	0.877	no
Treat-0d	RHSIM_Rhsim09G0115100	-0.975	-1.414	-0.438	no

Group	Gene	PTR (z-score difference)	z(log2 LFQ)	z(log2 FPKM)	extreme-PTR
Treat-0d	RHSIM_Rhsim11G0104600	1.534	-0.328	-1.861	high
Treat-0d	RHSIM_Rhsim10G0077000	-0.809	-1.072	-0.263	no
Treat-0d	RHSIM_Rhsim09G0202700	-0.190	-1.249	-1.059	no
Treat-0d	RHSIM_Rhsim10G0019400	-0.943	-1.188	-0.244	no
Treat-0d	RHSIM_Rhsim09G0144400	2.283	0.665	-1.618	high
Treat-0d	RHSIM_Rhsim10G0084700	-0.253	-0.773	-0.520	no
Treat-0d	RHSIM_Rhsim09G0121900	-0.487	-0.176	0.311	no
Treat-0d	RHSIM_Rhsim09G0169200	-1.474	-0.414	1.060	low
Treat-0d	RHSIM_Rhsim11G0184800	0.829	1.529	0.701	no
Treat-0d	RHSIM_Rhsim10G0202900	1.209	0.931	-0.279	high
Treat-0d	RHSIM_Rhsim10G0138300	-0.833	-1.194	-0.360	no
Treat-0d	RHSIM_Rhsim11G0058900	0.095	0.329	0.234	no
Treat-0d	RHSIM_Rhsim09G0150400	2.395	0.875	-1.519	high
Treat-0d	RHSIM_Rhsim10G0028000	0.204	1.792	1.588	no
Treat-0d	RHSIM_Rhsim09G0123000	-0.505	0.119	0.625	no
Treat-0d	RHSIM_Rhsim10G0210700	-0.210	-0.575	-0.364	no
Treat-0d	RHSIM_Rhsim10G0023700	-0.429	-1.031	-0.602	no
Treat-0d	RHSIM_Rhsim10G0156100	-0.463	-0.050	0.413	no
Treat-0d	RHSIM_Rhsim09G0140200	0.014	-0.344	-0.358	no
Treat-0d	RHSIM_Rhsim11G0089800	1.264	0.003	-1.261	high
Treat-0d	RHSIM_Rhsim09G0147900	0.943	-0.364	-1.307	no
Treat-0d	RHSIM_Rhsim09G0181100	-0.134	-0.785	-0.651	no
Treat-0d	RHSIM_Rhsim09G0069500	0.065	0.733	0.668	no
Treat-0d	RHSIM_Rhsim10G0209800	0.543	1.456	0.913	no
Treat-0d	RHSIM_Rhsim10G0009000	0.520	0.135	-0.385	no
Treat-0d	RHSIM_Rhsim09G0208200	0.038	0.583	0.545	no
Treat-0d	RHSIM_Rhsim10G0029200	-0.097	0.228	0.326	no
Treat-0d	RHSIM_Rhsim09G0208400	0.253	1.290	1.037	no
Treat-0d	RHSIM_Rhsim10G0180300	0.118	-0.544	-0.662	no
Treat-0d	RHSIM_Rhsim10G0212700	0.790	0.537	-0.253	no
Treat-0d	RHSIM_Rhsim10G0185200	0.252	-1.012	-1.264	no
Treat-0d	RHSIM_Rhsim10G0056100	0.370	0.326	-0.044	no

Group	Gene	PTR (z-score difference)	z(log2 LFQ)	z(log2 FPKM)	extreme-PTR
Treat-0d	RHSIM_Rhsim10G0176600	1.556	1.995	0.438	high
Treat-0d	RHSIM_Rhsim09G0110200	-0.256	-1.148	-0.892	no
Treat-0d	RHSIM_Rhsim09G0157700	0.155	1.409	1.253	no
Treat-0d	RHSIM_Rhsim09G0003700	1.629	1.564	-0.065	high
Treat-0d	RHSIM_Rhsim10G0189100	-0.625	-0.847	-0.221	no
Treat-0d	RHSIM_Rhsim10G0011300	-0.150	-0.580	-0.430	no
Treat-0d	RHSIM_Rhsim09G0208000	0.002	-0.600	-0.602	no
Treat-0d	RHSIM_Rhsim09G0060100	-2.847	-1.725	1.122	low
Treat-0d	RHSIM_Rhsim09G0141900	-0.159	0.820	0.979	no
Treat-0d	RHSIM_Rhsim11G0099400	-0.533	0.425	0.958	no
Treat-0d	RHSIM_Rhsim10G0034700	-0.931	0.018	0.950	no
Treat-0d	RHSIM_Rhsim10G0029400	-0.801	-0.754	0.047	no
Treat-0d	RHSIM_Rhsim10G0005000	-1.630	-0.992	0.638	low
Treat-0d	RHSIM_Rhsim09G0111300	-0.246	-0.387	-0.140	no
Treat-0d	RHSIM_Rhsim09G0207500	-0.920	0.277	1.198	no
Treat-0d	RHSIM_Rhsim10G0030100	0.220	-1.275	-1.495	no
Treat-0d	RHSIM_Rhsim10G0064100	-2.487	-0.922	1.565	low
Treat-0d	RHSIM_Rhsim10G0026800	0.958	-1.154	-2.112	no
Treat-0d	RHSIM_Rhsim10G0113200	-0.760	0.527	1.286	no
Treat-0d	RHSIM_Rhsim09G0018700	-0.095	-1.276	-1.180	no
Treat-0d	RHSIM_Rhsim10G0067800	0.092	0.220	0.128	no
Treat-0d	RHSIM_Rhsim09G0141100	-0.537	0.114	0.650	no
Treat-0d	RHSIM_Rhsim10G0018300	-0.500	0.585	1.085	no
Treat-0d	RHSIM_Rhsim09G0004300	-0.416	-1.147	-0.732	no
Treat-0d	RHSIM_Rhsim10G0015600	1.023	0.587	-0.436	no
Treat-0d	RHSIM_Rhsim11G0141800	-1.599	-1.027	0.573	low
Treat-0d	RHSIM_Rhsim09G0167500	-0.452	-1.073	-0.620	no
Treat-0d	RHSIM_Rhsim09G0187300	0.663	0.282	-0.381	no
Treat-0d	RHSIM_Rhsim10G0126900	-1.221	-1.392	-0.171	low
Treat-0d	RHSIM_Rhsim09G0208500	-0.116	-0.258	-0.142	no
Treat-0d	RHSIM_Rhsim11G0137800	0.568	0.019	-0.550	no
Treat-0d	RHSIM_Rhsim09G0005100	0.807	0.662	-0.145	no

Group	Gene	PTR (z-score difference)	z(log2 LFQ)	z(log2 FPKM)	extreme-PTR
Treat-0d	RHSIM_Rhsim10G0011500	-0.263	-1.065	-0.802	no
Treat-0d	RHSIM_Rhsim11G0040600	0.739	0.104	-0.636	no
Treat-0d	RHSIM_Rhsim10G0077900	-0.325	-0.650	-0.324	no
Treat-0d	RHSIM_Rhsim10G0102900	-0.808	-0.589	0.218	no
Treat-0d	RHSIM_Rhsim09G0168600	-0.033	-0.658	-0.625	no
Treat-0d	RHSIM_Rhsim11G0145300	1.294	1.088	-0.206	high
Treat-0d	RHSIM_Rhsim10G0065800	-0.636	-0.971	-0.334	no
Treat-0d	RHSIM_Rhsim09G0155300	-0.274	-0.985	-0.711	no
Treat-0d	RHSIM_Rhsim10G0212500	0.967	-1.139	-2.106	no
Treat-0d	RHSIM_Rhsim09G0151400	0.835	0.654	-0.181	no
Treat-0d	RHSIM_Rhsim09G0003600	0.462	1.692	1.230	no
Treat-0d	RHSIM_Rhsim10G0130500	0.537	-0.723	-1.259	no
Treat-0d	RHSIM_Rhsim10G0162800	1.460	-0.179	-1.639	high
Treat-0d	RHSIM_Rhsim10G0187700	0.345	-1.420	-1.765	no
Treat-0d	RHSIM_Rhsim10G0134900	0.670	1.428	0.758	no
Treat-0d	RHSIM_Rhsim09G0043400	-0.112	0.505	0.617	no
Treat-0d	RHSIM_Rhsim10G0057900	0.117	1.841	1.724	no
Treat-0d	RHSIM_Rhsim10G0162400	0.620	-0.838	-1.459	no
Treat-0d	RHSIM_Rhsim09G0165400	0.507	-0.388	-0.894	no
Treat-0d	RHSIM_Rhsim09G0039300	-1.326	-1.254	0.072	low
Treat-0d	RHSIM_Rhsim09G0152300	-0.827	-0.695	0.132	no
Treat-0d	RHSIM_Rhsim10G0144800	0.478	-0.778	-1.257	no
Treat-0d	RHSIM_Rhsim10G0081500	0.045	-0.012	-0.057	no
Treat-0d	RHSIM_Rhsim09G0199500	-1.280	1.346	2.626	low
Treat-0d	RHSIM_Rhsim10G0043600	0.386	0.712	0.326	no
Treat-0d	RHSIM_Rhsim09G0087300	-1.748	-1.493	0.254	low
Treat-0d	RHSIM_Rhsim09G0146200	0.249	1.779	1.531	no
Treat-0d	RHSIM_Rhsim10G0162700	-0.381	-0.552	-0.172	no
Treat-0d	RHSIM_Rhsim09G0146500	-1.536	-0.844	0.693	low
Treat-0d	RHSIM_Rhsim09G0137600	0.500	0.686	0.186	no
Treat-0d	RHSIM_Rhsim10G0165500	-0.143	-0.498	-0.356	no
Treat-0d	RHSIM_Rhsim10G0133000	-0.313	0.869	1.182	no

Group	Gene	PTR (z-score difference)	z(log2 LFQ)	z(log2 FPKM)	extreme-PTR
Treat-0d	RHSIM_Rhsim10G0034600	-0.966	-0.838	0.128	no
Treat-0d	RHSIM_Rhsim09G0128000	1.957	0.584	-1.373	high
Treat-0d	RHSIM_Rhsim09G0111600	1.849	0.383	-1.467	high
Treat-0d	RHSIM_Rhsim09G0025300	-0.043	1.317	1.360	no
Treat-0d	RHSIM_Rhsim11G0091000	0.606	-0.824	-1.430	no
Treat-0d	RHSIM_Rhsim09G0136800	0.670	0.232	-0.438	no
Treat-0d	RHSIM_Rhsim09G0010200	-0.390	0.409	0.799	no
Treat-0d	RHSIM_Rhsim11G0153300	0.485	0.295	-0.191	no
Treat-0d	RHSIM_Rhsim09G0037000	-1.608	-1.692	-0.085	low
Treat-0d	RHSIM_Rhsim10G0019000	-0.614	0.889	1.502	no
Treat-0d	RHSIM_Rhsim09G0113200	-1.046	0.375	1.421	low
Treat-0d	RHSIM_Rhsim10G0186700	-0.206	-0.417	-0.211	no
Treat-0d	RHSIM_Rhsim10G0167000	0.649	-1.049	-1.698	no
Treat-0d	RHSIM_Rhsim10G0141700	-0.440	-0.932	-0.492	no
Treat-0d	RHSIM_Rhsim09G0165600	0.728	0.528	-0.200	no
Treat-0d	RHSIM_Rhsim10G0177900	0.635	0.178	-0.457	no
Treat-0d	RHSIM_Rhsim11G0183100	-0.362	-1.200	-0.838	no
Treat-0d	RHSIM_Rhsim09G0209400	-0.026	1.046	1.073	no
Treat-0d	RHSIM_Rhsim10G0000500	1.920	0.471	-1.450	high
Treat-0d	RHSIM_Rhsim09G0067900	0.639	0.963	0.324	no
Treat-0d	RHSIM_Rhsim09G0001400	0.313	1.631	1.318	no
Treat-0d	RHSIM_Rhsim09G0140900	-3.065	-1.515	1.550	low
Treat-0d	RHSIM_Rhsim09G0197000	-2.787	-0.787	2.000	low
Treat-0d	RHSIM_Rhsim09G0129700	-0.054	0.426	0.480	no
Treat-0d	RHSIM_Rhsim10G0003500	-0.865	-0.856	0.009	no
Treat-0d	RHSIM_Rhsim10G0040000	1.953	1.352	-0.601	high
Treat-0d	RHSIM_Rhsim11G0056000	0.630	-0.323	-0.953	no
Treat-0d	RHSIM_Rhsim09G0213100	-0.110	-0.466	-0.356	no
Treat-0d	RHSIM_Rhsim10G0145200	0.365	-0.357	-0.722	no
Treat-0d	RHSIM_Rhsim10G0001300	0.254	-0.182	-0.437	no
Treat-0d	RHSIM_Rhsim09G0066100	0.584	-0.783	-1.367	no
Treat-0d	RHSIM_Rhsim10G0049300	0.035	-0.295	-0.331	no

Group	Gene	PTR (z-score difference)	z(log2 LFQ)	z(log2 FPKM)	extreme-PTR
Treat-0d	RHSIM_Rhsim11G0134900	-0.009	-0.423	-0.414	no
Treat-0d	RHSIM_Rhsim09G0165800	1.020	0.124	-0.896	no
Treat-0d	RHSIM_Rhsim09G0027800	-0.416	0.055	0.471	no
Treat-0d	RHSIM_Rhsim11G0197300	-0.385	1.194	1.580	no
Treat-0d	RHSIM_Rhsim10G0113000	-0.116	-0.584	-0.468	no
Treat-0d	RHSIM_Rhsim09G0072200	-0.019	-1.558	-1.539	no
Treat-0d	RHSIM_Rhsim10G0053700	0.270	-0.967	-1.237	no
Treat-0d	RHSIM_Rhsim09G0107700	0.680	2.047	1.367	no
Treat-0d	RHSIM_Rhsim09G0112700	-1.835	-0.186	1.649	low
Treat-0d	RHSIM_Rhsim09G0103400	-1.366	-0.248	1.118	low
Treat-0d	RHSIM_Rhsim11G0111700	0.270	-0.270	-0.540	no
Treat-0d	RHSIM_Rhsim09G0058600	-0.608	-1.328	-0.720	no
Treat-0d	RHSIM_Rhsim09G0199000	-0.507	0.443	0.950	no
Treat-0d	RHSIM_Rhsim09G0157900	2.403	1.822	-0.582	high
Treat-0d	RHSIM_Rhsim11G0060100	0.485	-0.062	-0.547	no
Treat-0d	RHSIM_Rhsim10G0105000	-1.147	-1.798	-0.652	low
Treat-0d	RHSIM_Rhsim11G0049400	-1.541	0.269	1.810	low
Treat-0d	RHSIM_Rhsim09G0061400	-0.396	1.042	1.439	no
Treat-0d	RHSIM_Rhsim09G0012100	-2.745	-0.623	2.122	low
Treat-0d	RHSIM_Rhsim09G0207600	-0.355	0.088	0.444	no
Treat-0d	RHSIM_Rhsim09G0037600	-0.539	0.310	0.849	no
Treat-0d	RHSIM_Rhsim09G0195600	-0.476	-0.179	0.297	no
Treat-0d	RHSIM_Rhsim10G0020400	0.861	0.346	-0.515	no
Treat-0d	RHSIM_Rhsim10G0111500	-0.878	0.431	1.308	no
Treat-0d	RHSIM_Rhsim10G0121000	0.737	1.029	0.292	no
Treat-0d	RHSIM_Rhsim09G0166500	0.114	-0.913	-1.026	no
Treat-0d	RHSIM_Rhsim10G0006500	-1.074	-0.961	0.113	low
Treat-0d	RHSIM_Rhsim09G0199700	1.012	0.013	-0.999	no
Treat-0d	RHSIM_Rhsim09G0214500	-1.478	-0.050	1.428	low
Treat-0d	RHSIM_Rhsim10G0044100	-0.860	1.237	2.097	no
Treat-0d	RHSIM_Rhsim09G0044400	1.308	0.235	-1.073	high
Treat-0d	RHSIM_Rhsim09G0179000	-1.168	-0.973	0.196	low

Group	Gene	PTR (z-score difference)	z(log2 LFQ)	z(log2 FPKM)	extreme-PTR
Treat-0d	RHSIM_Rhsim09G0213800	-0.738	0.820	1.558	no
Treat-0d	RHSIM_Rhsim09G0071800	-0.195	-0.051	0.143	no
Treat-0d	RHSIM_Rhsim09G0158600	-1.142	-1.059	0.083	low
Treat-0d	RHSIM_Rhsim09G0051700	-0.078	0.420	0.498	no
Treat-0d	RHSIM_Rhsim09G0126300	-0.578	-0.608	-0.030	no
Treat-0d	RHSIM_Rhsim09G0140700	-1.799	1.320	3.118	low
Treat-0d	RHSIM_Rhsim09G0064600	1.115	0.246	-0.869	high
Treat-0d	RHSIM_Rhsim10G0183900	0.554	1.582	1.028	no
Treat-0d	RHSIM_Rhsim10G0072700	-1.190	-0.746	0.444	low
Treat-0d	RHSIM_Rhsim10G0020900	0.500	-0.396	-0.896	no
Treat-0d	RHSIM_Rhsim09G0132000	0.182	1.490	1.308	no
Treat-0d	RHSIM_Rhsim10G0176900	-0.138	0.339	0.477	no
Treat-0d	RHSIM_Rhsim09G0208100	-0.073	-0.159	-0.086	no
Treat-0d	RHSIM_Rhsim09G0073200	0.024	-1.004	-1.028	no
Treat-0d	RHSIM_Rhsim10G0180400	-0.425	-0.819	-0.394	no
Treat-0d	RHSIM_Rhsim10G0080000	2.966	1.006	-1.961	high
Treat-0d	RHSIM_Rhsim09G0020400	0.145	-0.178	-0.323	no
Treat-0d	RHSIM_Rhsim09G0105400	-0.245	-0.112	0.134	no
Treat-0d	RHSIM_Rhsim09G0005500	1.467	0.851	-0.617	high
Treat-0d	RHSIM_Rhsim10G0128500	0.192	0.249	0.056	no
Treat-0d	RHSIM_Rhsim10G0192700	-1.262	0.284	1.546	low
Treat-0d	RHSIM_Rhsim10G0002200	-3.015	-0.701	2.313	low
Treat-0d	RHSIM_Rhsim09G0188800	-0.173	0.886	1.060	no
Treat-0d	RHSIM_Rhsim10G0002800	-0.340	-0.752	-0.413	no
Treat-0d	RHSIM_Rhsim09G0092400	-1.935	-0.938	0.997	low
Treat-0d	RHSIM_Rhsim09G0154300	0.188	-0.455	-0.643	no
Treat-0d	RHSIM_Rhsim09G0184000	0.291	0.720	0.429	no
Treat-0d	RHSIM_Rhsim09G0195100	0.526	0.267	-0.259	no
Treat-0d	RHSIM_Rhsim09G0087000	0.265	-0.380	-0.645	no
Treat-0d	RHSIM_Rhsim09G0172200	-0.971	-0.443	0.528	no
Treat-0d	RHSIM_Rhsim09G0206900	1.651	2.019	0.368	high
Treat-0d	RHSIM_Rhsim10G0041000	-1.103	-0.473	0.630	low

Group	Gene	PTR (z-score difference)	z(log2 LFQ)	z(log2 FPKM)	extreme-PTR
Treat-0d	RHSIM_Rhsim09G0037900	-1.209	-1.459	-0.250	low
Treat-0d	RHSIM_Rhsim09G0107200	0.985	-0.501	-1.485	no
Treat-0d	RHSIM_Rhsim09G0040700	3.213	1.573	-1.639	high
Treat-0d	RHSIM_Rhsim09G0179700	-0.270	-1.597	-1.327	no
Treat-0d	RHSIM_Rhsim10G0083200	-0.294	0.513	0.807	no
Treat-0d	RHSIM_Rhsim09G0212900	-1.808	-0.591	1.217	low
Treat-0d	RHSIM_Rhsim09G0096600	-0.764	-0.438	0.326	no
Treat-0d	RHSIM_Rhsim09G0109400	0.698	0.573	-0.126	no
Treat-0d	RHSIM_Rhsim09G0058800	0.983	0.139	-0.844	no
Treat-0d	RHSIM_Rhsim10G0185700	-0.501	0.212	0.713	no
Treat-0d	RHSIM_Rhsim09G0190900	0.147	-0.592	-0.740	no
Treat-0d	RHSIM_Rhsim09G0199400	-0.686	-1.472	-0.786	no
Treat-0d	RHSIM_Rhsim09G0214300	-0.148	0.092	0.241	no
Treat-0d	RHSIM_Rhsim09G0110100	-0.144	0.917	1.061	no
Treat-0d	RHSIM_Rhsim09G0043700	1.252	0.050	-1.201	high
Treat-0d	RHSIM_Rhsim09G0176800	-0.017	-1.040	-1.023	no
Treat-0d	RHSIM_Rhsim07G0015900	-0.074	1.194	1.267	no
Treat-0d	RHSIM_Rhsim09G0028200	-0.934	-0.599	0.335	no
Treat-0d	RHSIM_Rhsim10G0076800	0.873	1.527	0.654	no
Treat-0d	RHSIM_Rhsim10G0021400	0.996	0.812	-0.183	no
Treat-0d	RHSIM_Rhsim09G0143800	0.367	-0.178	-0.545	no
Treat-0d	RHSIM_Rhsim09G0066000	0.615	1.109	0.494	no
Treat-0d	RHSIM_Rhsim09G0087100	-0.426	0.059	0.486	no
Treat-0d	RHSIM_Rhsim09G0058700	-1.102	-1.451	-0.349	low
Treat-0d	RHSIM_Rhsim10G0127700	-1.304	-0.562	0.743	low
Treat-0d	RHSIM_Rhsim09G0204400	-1.410	-1.044	0.367	low
Treat-0d	RHSIM_Rhsim09G0066200	-1.152	-0.673	0.479	low
Treat-0d	RHSIM_Rhsim10G0034300	0.388	0.514	0.126	no
Treat-0d	RHSIM_Rhsim09G0211500	-0.650	-0.473	0.177	no
Treat-0d	RHSIM_Rhsim09G0084400	0.478	-0.216	-0.695	no
Treat-0d	RHSIM_Rhsim09G0154600	0.256	-0.025	-0.281	no
Treat-0d	RHSIM_Rhsim10G0214200	-0.527	-0.302	0.224	no

Group	Gene	PTR (z-score difference)	z(log2 LFQ)	z(log2 FPKM)	extreme-PTR
Treat-0d	RHSIM_Rhsim09G0130100	0.116	1.594	1.477	no
Treat-0d	RHSIM_Rhsim09G0011700	-2.841	-0.020	2.821	low
Treat-0d	RHSIM_Rhsim09G0103000	-0.538	-0.749	-0.211	no
Treat-0d	RHSIM_Rhsim10G0186800	-3.418	-1.188	2.230	low
Treat-0d	RHSIM_Rhsim09G0146700	-0.986	-1.264	-0.279	no
Treat-0d	RHSIM_Rhsim09G0063000	1.120	-0.443	-1.563	high
Treat-0d	RHSIM_Rhsim09G0214600	-2.550	-0.858	1.692	low
Treat-0d	RHSIM_Rhsim10G0084800	-0.409	0.172	0.582	no
Treat-0d	RHSIM_Rhsim09G0122300	0.430	-0.443	-0.874	no
Treat-0d	RHSIM_Rhsim10G0167600	-2.390	-1.283	1.107	low
Treat-0d	RHSIM_Rhsim09G0151300	-2.129	0.319	2.448	low
Treat-0d	RHSIM_Rhsim09G0012200	0.561	0.977	0.417	no
Treat-0d	RHSIM_Rhsim09G0061700	0.928	-0.585	-1.512	no
Treat-0d	RHSIM_Rhsim09G0166000	-2.521	-0.084	2.437	low
Treat-0d	RHSIM_Rhsim09G0043800	0.763	0.139	-0.625	no
Treat-0d	RHSIM_Rhsim10G0185500	0.534	0.616	0.082	no
Treat-0d	RHSIM_Rhsim09G0150500	0.066	0.172	0.105	no
Treat-0d	RHSIM_Rhsim09G0152400	-0.413	0.585	0.997	no
Treat-0d	RHSIM_Rhsim10G0163100	-0.217	0.141	0.358	no
Treat-0d	RHSIM_Rhsim09G0035000	0.307	-0.552	-0.859	no
Treat-0d	RHSIM_Rhsim09G0117700	0.585	1.611	1.026	no
Treat-0d	RHSIM_Rhsim09G0196800	-0.612	-0.426	0.186	no
Treat-0d	RHSIM_Rhsim09G0033400	1.142	0.859	-0.283	high
Treat-0d	RHSIM_Rhsim09G0087600	-0.527	-1.366	-0.839	no
Treat-0d	RHSIM_Rhsim09G0084100	-0.887	0.763	1.650	no
Treat-0d	RHSIM_Rhsim09G0090200	0.443	-0.544	-0.987	no
Treat-0d	RHSIM_Rhsim09G0094700	-0.122	0.177	0.299	no
Treat-0d	RHSIM_Rhsim09G0143200	-1.073	2.237	3.310	low
Treat-0d	RHSIM_Rhsim10G0125000	-0.403	-0.699	-0.296	no
Treat-0d	RHSIM_Rhsim09G0004100	0.516	1.426	0.909	no
Treat-0d	RHSIM_Rhsim09G0140500	0.421	-0.592	-1.013	no
Treat-0d	RHSIM_Rhsim10G0004100	-0.469	-0.462	0.007	no

Group	Gene	PTR (z-score difference)	z(log2 LFQ)	z(log2 FPKM)	extreme-PTR
Treat-0d	RHSIM_Rhsim09G0196200	-1.151	-0.542	0.609	low
Treat-0d	RHSIM_Rhsim09G0127700	-0.755	-0.747	0.008	no
Treat-0d	RHSIM_Rhsim09G0065100	-0.831	-0.995	-0.164	no
Treat-0d	RHSIM_Rhsim09G0202400	0.284	1.233	0.949	no
Treat-0d	RHSIM_Rhsim09G0148900	0.455	0.184	-0.271	no
Treat-0d	RHSIM_Rhsim09G0210400	1.336	0.842	-0.494	high
Treat-0d	RHSIM_Rhsim09G0011500	-1.168	-1.330	-0.162	low
Treat-0d	RHSIM_Rhsim09G0184800	1.318	-0.739	-2.057	high
Treat-0d	RHSIM_Rhsim10G0174500	-1.492	-0.021	1.471	low
Treat-0d	RHSIM_Rhsim09G0105600	0.181	0.113	-0.069	no
Treat-0d	RHSIM_Rhsim10G0004300	-0.022	0.688	0.710	no
Treat-0d	RHSIM_Rhsim09G0194000	-2.023	-2.562	-0.539	low
Treat-0d	RHSIM_Rhsim09G0132100	0.232	-0.313	-0.545	no
Treat-0d	RHSIM_Rhsim09G0078200	0.024	-0.855	-0.878	no
Treat-0d	RHSIM_Rhsim09G0126100	-0.205	-1.736	-1.531	no
Treat-0d	RHSIM_Rhsim09G0206100	-0.433	-0.439	-0.006	no
Treat-0d	RHSIM_Rhsim10G0166700	-0.671	-0.470	0.202	no
Treat-0d	RHSIM_Rhsim09G0049700	1.022	-0.542	-1.563	no
Treat-0d	RHSIM_Rhsim09G0170000	1.793	-0.036	-1.829	high
Treat-0d	RHSIM_Rhsim08G0034300	-0.642	-0.313	0.328	no
Treat-0d	RHSIM_Rhsim09G0030100	-0.274	-0.426	-0.152	no
Treat-0d	RHSIM_Rhsim10G0020100	0.677	2.537	1.860	no
Treat-0d	RHSIM_Rhsim09G0044600	0.768	0.727	-0.041	no
Treat-0d	RHSIM_Rhsim10G0092800	-0.017	-0.218	-0.201	no
Treat-0d	RHSIM_Rhsim10G0064800	0.916	0.483	-0.433	no
Treat-0d	RHSIM_Rhsim09G0034100	-0.859	-0.048	0.811	no
Treat-0d	RHSIM_Rhsim08G0095100	-0.829	-0.962	-0.133	no
Treat-0d	RHSIM_Rhsim09G0173200	-1.187	0.485	1.672	low
Treat-0d	RHSIM_Rhsim10G0019700	-2.652	-0.623	2.028	low
Treat-0d	RHSIM_Rhsim09G0004500	-0.552	-1.566	-1.014	no
Treat-0d	RHSIM_Rhsim08G0223900	2.671	2.274	-0.397	high
Treat-0d	RHSIM_Rhsim08G0096200	-1.017	0.462	1.479	no

Group	Gene	PTR (z-score difference)	z(log2 LFQ)	z(log2 FPKM)	extreme-PTR
Treat-0d	RHSIM_Rhsim09G0140000	0.610	0.488	-0.121	no
Treat-0d	RHSIM_Rhsim10G0138100	-1.789	-1.739	0.050	low
Treat-0d	RHSIM_Rhsim08G0116000	-0.056	-0.258	-0.203	no
Treat-0d	RHSIM_Rhsim09G0208800	1.377	1.400	0.023	high
Treat-0d	RHSIM_Rhsim08G0081800	-0.294	0.024	0.319	no
Treat-0d	RHSIM_Rhsim08G0039900	0.802	2.221	1.418	no
Treat-0d	RHSIM_Rhsim09G0091600	-0.249	-0.702	-0.453	no
Treat-0d	RHSIM_Rhsim08G0000800	-0.874	-0.375	0.500	no
Treat-0d	RHSIM_Rhsim09G0034800	-1.225	-0.178	1.046	low
Treat-0d	RHSIM_Rhsim10G0008400	-0.497	-0.994	-0.497	no
Treat-0d	RHSIM_Rhsim09G0154900	-0.278	-0.322	-0.044	no
Treat-0d	RHSIM_Rhsim09G0206000	-0.542	-0.533	0.009	no
Treat-0d	RHSIM_Rhsim09G0013300	1.015	0.189	-0.826	no
Treat-0d	RHSIM_Rhsim10G0104900	0.703	-0.006	-0.709	no
Treat-0d	RHSIM_Rhsim08G0244500	1.590	1.461	-0.128	high
Treat-0d	RHSIM_Rhsim08G0231100	0.060	0.337	0.276	no
Treat-0d	RHSIM_Rhsim10G0119200	-1.190	-0.360	0.830	low
Treat-0d	RHSIM_Rhsim08G0237400	1.079	-0.313	-1.392	no
Treat-0d	RHSIM_Rhsim08G0102500	-0.237	-0.588	-0.351	no
Treat-0d	RHSIM_Rhsim09G0097800	0.280	-0.331	-0.611	no
Treat-0d	RHSIM_Rhsim08G0128100	-0.228	-0.942	-0.714	no
Treat-0d	RHSIM_Rhsim10G0016800	1.806	0.065	-1.741	high
Treat-0d	RHSIM_Rhsim09G0049500	-0.559	-1.179	-0.620	no
Treat-0d	RHSIM_Rhsim09G0128800	0.894	-0.293	-1.188	no
Treat-0d	RHSIM_Rhsim09G0005700	0.528	0.072	-0.456	no
Treat-0d	RHSIM_Rhsim09G0081900	-0.917	0.595	1.512	no
Treat-0d	RHSIM_Rhsim08G0128800	1.761	0.166	-1.595	high
Treat-0d	RHSIM_Rhsim09G0137900	-0.872	-0.606	0.266	no
Treat-0d	RHSIM_Rhsim09G0187600	0.191	-0.732	-0.924	no
Treat-0d	RHSIM_Rhsim09G0214000	0.366	0.683	0.317	no
Treat-0d	RHSIM_Rhsim08G0039400	-0.148	1.539	1.686	no
Treat-0d	RHSIM_Rhsim09G0005000	-0.249	0.095	0.344	no

Group	Gene	PTR (z-score difference)	z(log2 LFQ)	z(log2 FPKM)	extreme-PTR
Treat-0d	RHSIM_Rhsim09G0145300	0.641	0.171	-0.471	no
Treat-0d	RHSIM_Rhsim09G0193000	-0.476	0.194	0.670	no
Treat-0d	RHSIM_Rhsim09G0203700	0.291	-0.975	-1.267	no
Treat-0d	RHSIM_Rhsim08G0027400	-1.793	-0.693	1.100	low
Treat-0d	RHSIM_Rhsim09G0204300	-0.713	-0.748	-0.034	no
Treat-0d	RHSIM_Rhsim10G0151200	-0.196	-0.268	-0.071	no
Treat-0d	RHSIM_Rhsim10G0049800	-0.587	-0.451	0.137	no
Treat-0d	RHSIM_Rhsim09G0129900	-1.658	0.352	2.010	low
Treat-0d	RHSIM_Rhsim08G0146000	0.724	0.250	-0.474	no
Treat-0d	RHSIM_Rhsim09G0122200	0.159	-0.429	-0.587	no
Treat-0d	RHSIM_Rhsim09G0142800	-1.881	-0.738	1.144	low
Treat-0d	RHSIM_Rhsim09G0141500	-0.568	0.627	1.195	no
Treat-0d	RHSIM_Rhsim08G0115800	-1.051	-0.025	1.026	low
Treat-0d	RHSIM_Rhsim09G0177600	-4.166	-0.831	3.334	low
Treat-0d	RHSIM_Rhsim08G0113500	-2.291	-2.257	0.034	low
Treat-0d	RHSIM_Rhsim09G0153300	-0.816	-0.279	0.537	no
Treat-0d	RHSIM_Rhsim08G0250700	0.001	-0.267	-0.268	no
Treat-0d	RHSIM_Rhsim09G0201300	-0.689	-0.471	0.218	no
Treat-0d	RHSIM_Rhsim09G0142500	0.113	0.335	0.222	no
Treat-0d	RHSIM_Rhsim09G0074600	0.257	0.478	0.220	no
Treat-0d	RHSIM_Rhsim09G0089600	-0.019	-0.530	-0.512	no
Treat-0d	RHSIM_Rhsim09G0030700	0.362	-0.248	-0.610	no
Treat-0d	RHSIM_Rhsim08G0043800	0.222	0.296	0.074	no
Treat-0d	RHSIM_Rhsim09G0169300	0.132	-0.388	-0.520	no
Treat-0d	RHSIM_Rhsim09G0127200	0.600	-0.054	-0.654	no
Treat-0d	RHSIM_Rhsim08G0125200	-0.242	-0.713	-0.471	no
Treat-0d	RHSIM_Rhsim08G0191700	-0.096	0.069	0.165	no
Treat-0d	RHSIM_Rhsim09G0069100	1.605	2.911	1.306	high
Treat-0d	RHSIM_Rhsim08G0067800	1.296	0.488	-0.808	high
Treat-0d	RHSIM_Rhsim09G0201100	-1.330	-0.564	0.766	low
Treat-0d	RHSIM_Rhsim09G0029200	-1.598	-0.865	0.733	low
Treat-0d	RHSIM_Rhsim09G0061500	0.818	0.197	-0.621	no

Group	Gene	PTR (z-score difference)	z(log2 LFQ)	z(log2 FPKM)	extreme-PTR
Treat-0d	RHSIM_Rhsim08G0227500	-1.351	-1.128	0.223	low
Treat-0d	RHSIM_Rhsim08G0041900	-2.646	-2.108	0.538	low
Treat-0d	RHSIM_Rhsim09G0171700	1.666	0.368	-1.298	high
Treat-0d	RHSIM_Rhsim08G0183700	0.399	-0.050	-0.450	no
Treat-0d	RHSIM_Rhsim09G0028800	0.219	-0.636	-0.855	no
Treat-0d	RHSIM_Rhsim09G0179300	-0.680	-1.452	-0.772	no
Treat-0d	RHSIM_Rhsim08G0006400	2.129	0.654	-1.475	high
Treat-0d	RHSIM_Rhsim09G0039400	0.776	-0.524	-1.300	no
Treat-0d	RHSIM_Rhsim09G0145500	-0.292	-0.084	0.208	no
Treat-0d	RHSIM_Rhsim10G0145000	0.291	1.160	0.870	no
Treat-0d	RHSIM_Rhsim09G0116000	-2.782	-1.511	1.272	low
Treat-0d	RHSIM_Rhsim09G0205100	0.122	-1.674	-1.796	no
Treat-0d	RHSIM_Rhsim09G0178000	-0.290	-1.442	-1.152	no
Treat-0d	RHSIM_Rhsim09G0113100	1.919	-0.008	-1.927	high
Treat-0d	RHSIM_Rhsim08G0121400	0.872	0.103	-0.768	no
Treat-0d	RHSIM_Rhsim08G0202500	1.074	0.780	-0.294	no
Treat-0d	RHSIM_Rhsim08G0084400	0.185	-0.342	-0.527	no
Treat-0d	RHSIM_Rhsim08G0167000	0.052	-0.056	-0.109	no
Treat-0d	RHSIM_Rhsim09G0023000	1.158	-0.424	-1.582	high
Treat-0d	RHSIM_Rhsim08G0224700	0.133	2.357	2.224	no
Treat-0d	RHSIM_Rhsim09G0151500	0.783	-0.069	-0.853	no
Treat-0d	RHSIM_Rhsim09G0126900	-2.284	-1.671	0.613	low
Treat-0d	RHSIM_Rhsim09G0125900	-0.586	-0.621	-0.035	no
Treat-0d	RHSIM_Rhsim09G0197100	-0.878	0.880	1.758	no
Treat-0d	RHSIM_Rhsim09G0117300	0.517	0.027	-0.491	no
Treat-0d	RHSIM_Rhsim08G0028900	-0.558	-1.068	-0.510	no
Treat-0d	RHSIM_Rhsim10G0212000	-1.317	0.816	2.133	low
Treat-0d	RHSIM_Rhsim09G0181400	-0.323	0.019	0.342	no
Treat-0d	RHSIM_Rhsim09G0088400	-0.046	-0.364	-0.318	no
Treat-0d	RHSIM_Rhsim08G0214200	0.394	0.080	-0.313	no
Treat-0d	RHSIM_Rhsim08G0063900	-0.551	0.134	0.686	no
Treat-0d	RHSIM_Rhsim09G0082300	-0.081	-0.735	-0.653	no

Group	Gene	PTR (z-score difference)	z(log2 LFQ)	z(log2 FPKM)	extreme-PTR
Treat-0d	RHSIM_Rhsim09G0172400	-0.237	0.651	0.888	no
Treat-0d	RHSIM_Rhsim08G0055000	0.987	1.757	0.769	no
Treat-0d	RHSIM_Rhsim09G0040800	-0.093	-0.526	-0.432	no
Treat-0d	RHSIM_Rhsim08G0044300	0.615	-0.351	-0.966	no
Treat-0d	RHSIM_Rhsim08G0006500	0.826	0.579	-0.247	no
Treat-0d	RHSIM_Rhsim09G0171600	-0.771	-0.301	0.470	no
Treat-0d	RHSIM_Rhsim09G0168800	1.382	0.275	-1.108	high
Treat-0d	RHSIM_Rhsim08G0043500	-1.245	-1.176	0.068	low
Treat-0d	RHSIM_Rhsim09G0004000	-1.543	-1.389	0.154	low
Treat-0d	RHSIM_Rhsim09G0183900	-0.558	-0.452	0.106	no
Treat-0d	RHSIM_Rhsim08G0005200	-1.215	-1.798	-0.584	low
Treat-0d	RHSIM_Rhsim09G0152700	0.861	3.405	2.544	no
Treat-0d	RHSIM_Rhsim09G0046600	0.328	0.001	-0.327	no
Treat-0d	RHSIM_Rhsim08G0082200	-1.530	-1.836	-0.307	low
Treat-0d	RHSIM_Rhsim08G0014900	0.399	0.722	0.323	no
Treat-0d	RHSIM_Rhsim08G0081400	-1.372	-1.266	0.106	low
Treat-0d	RHSIM_Rhsim08G0159000	1.469	0.621	-0.848	high
Treat-0d	RHSIM_Rhsim08G0171800	-1.244	-0.446	0.798	low
Treat-0d	RHSIM_Rhsim08G0033300	-0.225	-0.389	-0.164	no
Treat-0d	RHSIM_Rhsim10G0045100	0.896	0.625	-0.272	no
Treat-0d	RHSIM_Rhsim09G0077000	-0.142	-0.445	-0.303	no
Treat-0d	RHSIM_Rhsim09G0198800	1.602	0.286	-1.316	high
Treat-0d	RHSIM_Rhsim08G0141300	-1.423	-1.531	-0.108	low
Treat-0d	RHSIM_Rhsim08G0196800	0.482	1.570	1.087	no
Treat-0d	RHSIM_Rhsim08G0037100	-0.985	-0.767	0.218	no
Treat-0d	RHSIM_Rhsim08G0042900	0.255	-0.191	-0.446	no
Treat-0d	RHSIM_Rhsim08G0181700	0.707	1.594	0.887	no
Treat-0d	RHSIM_Rhsim10G0122600	0.510	-0.594	-1.104	no
Treat-0d	RHSIM_Rhsim08G0243400	1.442	-0.323	-1.765	high
Treat-0d	RHSIM_Rhsim08G0114500	-0.428	-0.164	0.264	no
Treat-0d	RHSIM_Rhsim08G0240000	-1.396	0.070	1.466	low
Treat-0d	RHSIM_Rhsim09G0133500	1.014	0.443	-0.571	no

Group	Gene	PTR (z-score difference)	z(log2 LFQ)	z(log2 FPKM)	extreme-PTR
Treat-0d	RHSIM_Rhsim08G0100200	0.439	0.572	0.134	no
Treat-0d	RHSIM_Rhsim08G0248700	-0.176	-0.597	-0.421	no
Treat-0d	RHSIM_Rhsim08G0026000	0.704	-0.010	-0.714	no
Treat-0d	RHSIM_Rhsim09G0141000	-0.613	-0.966	-0.353	no
Treat-0d	RHSIM_Rhsim08G0088900	-0.214	-1.326	-1.113	no
Treat-0d	RHSIM_Rhsim08G0229600	-0.516	0.006	0.522	no
Treat-0d	RHSIM_Rhsim08G0241300	0.391	-0.138	-0.530	no
Treat-0d	RHSIM_Rhsim09G0164300	0.038	-1.571	-1.609	no
Treat-0d	RHSIM_Rhsim09G0145400	-0.441	-0.189	0.252	no
Treat-0d	RHSIM_Rhsim08G0209300	1.179	1.104	-0.075	high
Treat-0d	RHSIM_Rhsim08G0122300	-1.806	-0.289	1.517	low
Treat-0d	RHSIM_Rhsim09G0189300	-0.970	0.347	1.318	no
Treat-0d	RHSIM_Rhsim08G0054900	1.641	1.842	0.202	high
Treat-0d	RHSIM_Rhsim09G0117800	1.044	0.545	-0.499	no
Treat-0d	RHSIM_Rhsim08G0102400	0.664	-1.076	-1.740	no
Treat-0d	RHSIM_Rhsim08G0230600	-3.051	-0.056	2.995	low
Treat-0d	RHSIM_Rhsim09G0000900	0.122	0.095	-0.027	no
Treat-0d	RHSIM_Rhsim08G0150200	0.696	-0.348	-1.045	no
Treat-0d	RHSIM_Rhsim08G0067900	0.865	0.941	0.076	no
Treat-0d	RHSIM_Rhsim10G0129000	0.871	-0.200	-1.071	no
Treat-0d	RHSIM_Rhsim08G0121500	-0.628	-0.907	-0.279	no
Treat-0d	RHSIM_Rhsim08G0040400	0.445	0.752	0.308	no
Treat-0d	RHSIM_Rhsim09G0010600	-0.296	0.127	0.423	no
Treat-0d	RHSIM_Rhsim10G0125300	0.156	-0.568	-0.724	no
Treat-0d	RHSIM_Rhsim08G0223400	-0.579	1.571	2.150	no
Treat-0d	RHSIM_Rhsim08G0103000	0.844	0.791	-0.053	no
Treat-0d	RHSIM_Rhsim10G0139000	0.122	-0.253	-0.375	no
Treat-0d	RHSIM_Rhsim09G0008600	-0.815	0.750	1.566	no
Treat-0d	RHSIM_Rhsim08G0183000	-0.153	0.687	0.840	no
Treat-0d	RHSIM_Rhsim08G0215400	1.691	0.310	-1.382	high
Treat-0d	RHSIM_Rhsim09G0092000	1.374	-0.422	-1.796	high
Treat-0d	RHSIM_Rhsim09G0210600	1.425	1.567	0.143	high

Group	Gene	PTR (z-score difference)	z(log2 LFQ)	z(log2 FPKM)	extreme-PTR
Treat-0d	RHSIM_Rhsim08G0020800	-0.256	-0.340	-0.084	no
Treat-0d	RHSIM_Rhsim09G0111700	0.348	0.590	0.242	no
Treat-0d	RHSIM_Rhsim08G0188900	-0.081	-0.018	0.063	no
Treat-0d	RHSIM_Rhsim10G0078400	0.030	-0.664	-0.694	no
Treat-0d	RHSIM_Rhsim08G0053600	0.340	0.160	-0.180	no
Treat-0d	RHSIM_Rhsim09G0099400	-0.303	-0.311	-0.009	no
Treat-0d	RHSIM_Rhsim09G0106300	0.678	0.631	-0.047	no
Treat-0d	RHSIM_Rhsim08G0008500	-0.390	-0.386	0.004	no
Treat-0d	RHSIM_Rhsim10G0211900	1.583	0.598	-0.985	high
Treat-0d	RHSIM_Rhsim08G0033600	-1.188	-0.338	0.850	low
Treat-0d	RHSIM_Rhsim09G0142300	0.211	-0.963	-1.174	no
Treat-0d	RHSIM_Rhsim09G0008200	-0.422	-0.558	-0.136	no
Treat-0d	RHSIM_Rhsim08G0083400	-1.478	-0.532	0.946	low
Treat-0d	RHSIM_Rhsim09G0138400	0.654	0.953	0.298	no
Treat-0d	RHSIM_Rhsim08G0156200	-0.726	-0.549	0.177	no
Treat-0d	RHSIM_Rhsim08G0156700	1.416	1.008	-0.409	high
Treat-0d	RHSIM_Rhsim08G0153100	-0.512	1.091	1.603	no
Treat-0d	RHSIM_Rhsim09G0203300	-1.354	-0.669	0.685	low
Treat-0d	RHSIM_Rhsim09G0207900	0.599	1.399	0.800	no
Treat-0d	RHSIM_Rhsim08G0208100	0.095	-0.977	-1.073	no
Treat-0d	RHSIM_Rhsim08G0034400	-0.631	-1.094	-0.462	no
Treat-0d	RHSIM_Rhsim08G0205700	1.164	1.136	-0.027	high
Treat-0d	RHSIM_Rhsim09G0155500	-0.197	-1.027	-0.830	no
Treat-0d	RHSIM_Rhsim08G0145900	-0.972	-0.081	0.891	no
Treat-0d	RHSIM_Rhsim08G0041500	-0.328	1.316	1.645	no
Treat-0d	RHSIM_Rhsim08G0082100	-0.017	-0.111	-0.094	no
Treat-0d	RHSIM_Rhsim10G0049400	0.176	0.591	0.415	no
Treat-0d	RHSIM_Rhsim08G0174000	0.538	0.852	0.315	no
Treat-0d	RHSIM_Rhsim10G0104100	-2.809	-1.660	1.149	low
Treat-0d	RHSIM_Rhsim09G0056700	1.252	-0.022	-1.274	high
Treat-0d	RHSIM_Rhsim09G0069000	0.366	1.569	1.203	no
Treat-0d	RHSIM_Rhsim09G0200500	0.429	-0.216	-0.645	no

Group	Gene	PTR (z-score difference)	z(log2 LFQ)	z(log2 FPKM)	extreme-PTR
Treat-0d	RHSIM_Rhsim09G0115400	0.535	0.592	0.057	no
Treat-0d	RHSIM_Rhsim09G0085500	-0.983	-1.107	-0.124	no
Treat-0d	RHSIM_Rhsim08G0069100	-0.152	-0.241	-0.088	no
Treat-0d	RHSIM_Rhsim08G0202300	-3.070	-1.574	1.496	low
Treat-0d	RHSIM_Rhsim09G0024000	-0.930	-0.574	0.356	no
Treat-0d	RHSIM_Rhsim09G0022900	-1.111	-0.525	0.585	low
Treat-0d	RHSIM_Rhsim08G0038100	0.332	0.438	0.106	no
Treat-0d	RHSIM_Rhsim08G0045400	1.543	1.597	0.054	high
Treat-0d	RHSIM_Rhsim08G0180500	0.498	2.685	2.186	no
Treat-0d	RHSIM_Rhsim09G0128100	0.536	0.784	0.248	no
Treat-0d	RHSIM_Rhsim08G0109700	0.582	3.922	3.340	no
Treat-0d	RHSIM_Rhsim08G0047900	3.516	1.168	-2.348	high
Treat-0d	RHSIM_Rhsim08G0013000	0.528	-0.601	-1.129	no
Treat-0d	RHSIM_Rhsim08G0088400	0.457	1.330	0.873	no
Treat-0d	RHSIM_Rhsim09G0002200	0.770	1.171	0.401	no
Treat-0d	RHSIM_Rhsim08G0030700	0.814	0.385	-0.429	no
Treat-0d	RHSIM_Rhsim08G0020500	0.888	-1.447	-2.335	no
Treat-0d	RHSIM_Rhsim08G0045800	-1.645	0.566	2.211	low
Treat-0d	RHSIM_Rhsim08G0245700	0.223	1.184	0.961	no
Treat-0d	RHSIM_Rhsim09G0114500	-2.871	0.282	3.153	low
Treat-0d	RHSIM_Rhsim09G0109300	-2.377	-0.891	1.486	low
Treat-0d	RHSIM_Rhsim08G0043600	-0.234	1.078	1.312	no
Treat-0d	RHSIM_Rhsim09G0090700	0.536	-0.626	-1.162	no
Treat-0d	RHSIM_Rhsim09G0069700	-1.444	-1.716	-0.272	low
Treat-0d	RHSIM_Rhsim09G0201200	-0.026	0.868	0.895	no
Treat-0d	RHSIM_Rhsim08G0195500	-0.557	-0.166	0.391	no
Treat-0d	RHSIM_Rhsim08G0008300	0.588	-0.624	-1.212	no
Treat-0d	RHSIM_Rhsim09G0210000	-0.607	-1.316	-0.708	no
Treat-0d	RHSIM_Rhsim09G0149700	0.921	0.727	-0.194	no
Treat-0d	RHSIM_Rhsim09G0067700	0.250	0.418	0.169	no
Treat-0d	RHSIM_Rhsim09G0144000	1.166	-0.254	-1.420	high
Treat-0d	RHSIM_Rhsim08G0044100	-4.286	-1.302	2.984	low

Group	Gene	PTR (z-score difference)	z(log2 LFQ)	z(log2 FPKM)	extreme-PTR
Treat-0d	RHSIM_Rhsim09G0146300	0.582	1.250	0.668	no
Treat-0d	RHSIM_Rhsim08G0188400	1.389	1.812	0.422	high
Treat-0d	RHSIM_Rhsim08G0096400	-0.524	-1.091	-0.567	no
Treat-0d	RHSIM_Rhsim09G0197500	1.788	1.082	-0.706	high
Treat-0d	RHSIM_Rhsim09G0168700	0.636	1.036	0.400	no
Treat-0d	RHSIM_Rhsim09G0025000	0.011	-0.996	-1.007	no
Treat-0d	RHSIM_Rhsim08G0037000	0.289	0.439	0.149	no
Treat-0d	RHSIM_Rhsim08G0072300	1.214	0.436	-0.778	high
Treat-0d	RHSIM_Rhsim09G0000500	0.523	0.208	-0.315	no
Treat-0d	RHSIM_Rhsim08G0176100	0.388	-0.937	-1.325	no
Treat-0d	RHSIM_Rhsim08G0250400	-1.261	-1.554	-0.293	low
Treat-0d	RHSIM_Rhsim09G0052100	0.838	0.476	-0.362	no
Treat-0d	RHSIM_Rhsim08G0185000	-0.471	-1.053	-0.582	no
Treat-0d	RHSIM_Rhsim09G0074800	0.148	-0.186	-0.335	no
Treat-0d	RHSIM_Rhsim09G0032100	1.110	0.284	-0.825	high
Treat-0d	RHSIM_Rhsim09G0143900	1.007	0.138	-0.869	no
Treat-0d	RHSIM_Rhsim08G0103200	1.343	0.251	-1.092	high
Treat-0d	RHSIM_Rhsim08G0114200	0.068	-0.189	-0.257	no
Treat-0d	RHSIM_Rhsim08G0012200	-0.457	-0.086	0.372	no
Treat-0d	RHSIM_Rhsim08G0086400	-0.914	-1.080	-0.166	no
Treat-0d	RHSIM_Rhsim09G0100400	0.033	-0.485	-0.518	no
Treat-0d	RHSIM_Rhsim09G0114100	0.726	0.457	-0.269	no
Treat-0d	RHSIM_Rhsim09G0109100	-0.126	0.700	0.826	no
Treat-0d	RHSIM_Rhsim09G0000200	0.414	0.581	0.167	no
Treat-0d	RHSIM_Rhsim09G0206600	0.350	-0.574	-0.924	no
Treat-0d	RHSIM_Rhsim08G0141600	1.908	1.823	-0.085	high
Treat-0d	RHSIM_Rhsim08G0221100	-0.127	-0.347	-0.220	no
Treat-0d	RHSIM_Rhsim08G0185200	0.524	-0.040	-0.564	no
Treat-0d	RHSIM_Rhsim08G0231900	-0.662	-0.932	-0.270	no
Treat-0d	RHSIM_Rhsim08G0002400	0.868	0.569	-0.300	no
Treat-0d	RHSIM_Rhsim08G0239200	-0.422	1.123	1.545	no
Treat-0d	RHSIM_Rhsim08G0069300	-0.008	1.106	1.114	no

Group	Gene	PTR (z-score difference)	z(log2 LFQ)	z(log2 FPKM)	extreme-PTR
Treat-0d	RHSIM_Rhsim08G0131700	0.700	-0.740	-1.440	no
Treat-0d	RHSIM_Rhsim09G0008700	0.185	-0.373	-0.559	no
Treat-0d	RHSIM_Rhsim09G0028300	-0.940	-1.580	-0.640	no
Treat-0d	RHSIM_Rhsim08G0241200	1.515	0.839	-0.677	high
Treat-0d	RHSIM_Rhsim08G0167100	0.555	-0.028	-0.583	no
Treat-0d	RHSIM_Rhsim08G0186100	-0.544	-0.635	-0.092	no
Treat-0d	RHSIM_Rhsim09G0000700	-0.518	-0.630	-0.113	no
Treat-0d	RHSIM_Rhsim08G0214400	0.242	0.996	0.755	no
Treat-0d	RHSIM_Rhsim08G0230200	0.013	-0.790	-0.803	no
Treat-0d	RHSIM_Rhsim08G0046300	-0.244	-0.798	-0.555	no
Treat-0d	RHSIM_Rhsim08G0241900	-1.285	-0.741	0.544	low
Treat-0d	RHSIM_Rhsim08G0242600	1.255	0.298	-0.957	high
Treat-0d	RHSIM_Rhsim08G0244800	2.235	0.780	-1.454	high
Treat-0d	RHSIM_Rhsim08G0046700	0.992	1.377	0.386	no
Treat-0d	RHSIM_Rhsim08G0026100	-0.095	-0.180	-0.085	no
Treat-0d	RHSIM_Rhsim09G0212200	-0.731	-0.350	0.380	no
Treat-0d	RHSIM_Rhsim09G0099700	0.844	-1.244	-2.088	no
Treat-0d	RHSIM_Rhsim09G0171300	-0.647	-1.596	-0.949	no
Treat-0d	RHSIM_Rhsim08G0084800	1.331	-0.009	-1.341	high
Treat-0d	RHSIM_Rhsim09G0119600	-0.654	-1.201	-0.547	no
Treat-0d	RHSIM_Rhsim09G0213700	1.090	0.694	-0.396	no
Treat-0d	RHSIM_Rhsim08G0057900	1.270	0.728	-0.542	high
Treat-0d	RHSIM_Rhsim09G0188300	0.571	-0.633	-1.204	no
Treat-0d	RHSIM_Rhsim08G0127800	0.665	1.223	0.558	no
Treat-0d	RHSIM_Rhsim08G0030100	0.600	0.429	-0.172	no
Treat-0d	RHSIM_Rhsim08G0102200	0.350	0.108	-0.241	no
Treat-0d	RHSIM_Rhsim09G0141400	-1.234	-1.705	-0.472	low
Treat-0d	RHSIM_Rhsim08G0214000	0.729	1.714	0.986	no
Treat-0d	RHSIM_Rhsim08G0030200	-0.282	-0.072	0.210	no
Treat-0d	RHSIM_Rhsim09G0185000	1.327	0.249	-1.078	high
Treat-0d	RHSIM_Rhsim09G0171800	1.034	0.804	-0.229	no
Treat-0d	RHSIM_Rhsim08G0211400	0.577	0.776	0.199	no

Group	Gene	PTR (z-score difference)	z(log2 LFQ)	z(log2 FPKM)	extreme-PTR
Treat-0d	RHSIM_Rhsim09G0064200	-1.391	0.267	1.658	low
Treat-0d	RHSIM_Rhsim08G0094100	-1.404	0.431	1.835	low
Treat-0d	RHSIM_Rhsim09G0105100	0.809	-0.075	-0.884	no
Treat-0d	RHSIM_Rhsim08G0029600	3.177	3.150	-0.027	high
Treat-0d	RHSIM_Rhsim09G0111100	-0.562	0.191	0.753	no
Treat-0d	RHSIM_Rhsim09G0179800	0.368	-1.246	-1.615	no
Treat-0d	RHSIM_Rhsim08G0019700	0.562	1.175	0.613	no
Treat-0d	RHSIM_Rhsim09G0093800	0.099	-0.612	-0.712	no
Treat-0d	RHSIM_Rhsim09G0209300	-1.038	-0.244	0.794	low
Treat-0d	RHSIM_Rhsim08G0094000	0.792	1.824	1.033	no
Treat-0d	RHSIM_Rhsim09G0110000	1.638	-0.067	-1.705	high
Treat-0d	RHSIM_Rhsim08G0018700	0.734	-1.279	-2.013	no
Treat-0d	RHSIM_Rhsim08G0173300	0.424	-0.428	-0.852	no
Treat-0d	RHSIM_Rhsim09G0164800	0.160	-0.172	-0.331	no
Treat-0d	RHSIM_Rhsim08G0195100	-0.787	0.600	1.387	no
Treat-0d	RHSIM_Rhsim08G0199200	-2.560	-1.446	1.115	low
Treat-0d	RHSIM_Rhsim08G0034000	-2.165	-1.067	1.098	low
Treat-0d	RHSIM_Rhsim09G0164900	0.001	-0.362	-0.363	no
Treat-0d	RHSIM_Rhsim09G0000100	-1.765	-0.875	0.890	low
Treat-0d	RHSIM_Rhsim08G0045600	0.327	-0.686	-1.013	no
Treat-0d	RHSIM_Rhsim08G0025900	0.907	-0.002	-0.908	no
Treat-0d	RHSIM_Rhsim08G0174600	-0.151	-0.459	-0.307	no
Treat-0d	RHSIM_Rhsim09G0142700	0.162	0.022	-0.141	no
Treat-0d	RHSIM_Rhsim08G0237100	-0.538	-1.239	-0.702	no
Treat-0d	RHSIM_Rhsim08G0032100	-0.177	0.519	0.696	no
Treat-0d	RHSIM_Rhsim07G0239900	-0.564	-0.602	-0.038	no
Treat-0d	RHSIM_Rhsim08G0148900	0.443	0.315	-0.129	no
Treat-0d	RHSIM_Rhsim08G0246900	-0.712	-0.337	0.375	no
Treat-0d	RHSIM_Rhsim09G0103800	0.317	-1.424	-1.741	no
Treat-0d	RHSIM_Rhsim07G0073300	-0.415	0.554	0.969	no
Treat-0d	RHSIM_Rhsim08G0045900	1.296	1.480	0.183	high
Treat-0d	RHSIM_Rhsim08G0075800	-0.545	0.624	1.169	no

Group	Gene	PTR (z-score difference)	z(log2 LFQ)	z(log2 FPKM)	extreme-PTR
Treat-0d	RHSIM_Rhsim08G0109800	-0.187	-1.239	-1.052	no
Treat-0d	RHSIM_Rhsim07G0082300	1.589	0.889	-0.700	high
Treat-0d	RHSIM_Rhsim08G0074500	0.336	-0.235	-0.572	no
Treat-0d	RHSIM_Rhsim09G0152100	0.021	0.691	0.670	no
Treat-0d	RHSIM_Rhsim08G0203900	1.215	1.080	-0.135	high
Treat-0d	RHSIM_Rhsim08G0028600	0.191	0.189	-0.002	no
Treat-0d	RHSIM_Rhsim08G0098800	-3.070	-1.097	1.972	low
Treat-0d	RHSIM_Rhsim07G0065200	3.133	2.215	-0.918	high
Treat-0d	RHSIM_Rhsim08G0232500	0.691	1.000	0.310	no
Treat-0d	RHSIM_Rhsim08G0233200	-0.198	-0.177	0.021	no
Treat-0d	RHSIM_Rhsim07G0063000	-0.091	0.034	0.125	no
Treat-0d	RHSIM_Rhsim08G0062700	0.307	1.200	0.893	no
Treat-0d	RHSIM_Rhsim08G0195300	0.542	0.730	0.189	no
Treat-0d	RHSIM_Rhsim08G0144000	-0.273	-0.884	-0.611	no
Treat-0d	RHSIM_Rhsim08G0190900	0.405	0.602	0.197	no
Treat-0d	RHSIM_Rhsim07G0109500	0.798	0.406	-0.392	no
Treat-0d	RHSIM_Rhsim09G0111900	0.640	0.992	0.352	no
Treat-0d	RHSIM_Rhsim07G0077100	-1.241	-0.185	1.056	low
Treat-0d	RHSIM_Rhsim09G0003200	0.638	0.321	-0.317	no
Treat-0d	RHSIM_Rhsim08G0051500	-0.702	-0.763	-0.062	no
Treat-0d	RHSIM_Rhsim08G0206900	0.203	-0.547	-0.750	no
Treat-0d	RHSIM_Rhsim09G0013500	-0.214	-1.049	-0.834	no
Treat-0d	RHSIM_Rhsim09G0004800	1.104	1.216	0.113	no
Treat-0d	RHSIM_Rhsim08G0152700	-0.954	0.260	1.214	no
Treat-0d	RHSIM_Rhsim08G0150000	0.317	0.708	0.391	no
Treat-0d	RHSIM_Rhsim09G0192600	0.637	0.599	-0.038	no
Treat-0d	RHSIM_Rhsim09G0073000	0.145	0.091	-0.054	no
Treat-0d	RHSIM_Rhsim07G0227100	1.161	-1.065	-2.226	high
Treat-0d	RHSIM_Rhsim08G0232800	0.527	0.147	-0.381	no
Treat-0d	RHSIM_Rhsim09G0069200	1.475	0.878	-0.597	high
Treat-0d	RHSIM_Rhsim08G0250100	0.519	1.474	0.955	no
Treat-0d	RHSIM_Rhsim08G0072500	-0.365	-0.006	0.359	no

Group	Gene	PTR (z-score difference)	z(log2 LFQ)	z(log2 FPKM)	extreme-PTR
Treat-0d	RHSIM_Rhsim07G0142300	0.699	2.599	1.900	no
Treat-0d	RHSIM_Rhsim08G0125600	-0.055	0.409	0.464	no
Treat-0d	RHSIM_Rhsim07G0079800	-0.092	-0.095	-0.003	no
Treat-0d	RHSIM_Rhsim08G0106000	1.542	-0.461	-2.003	high
Treat-0d	RHSIM_Rhsim08G0225600	0.074	-1.092	-1.166	no
Treat-0d	RHSIM_Rhsim07G0095900	-0.205	0.578	0.782	no
Treat-0d	RHSIM_Rhsim08G0008000	0.608	-0.831	-1.439	no
Treat-0d	RHSIM_Rhsim08G0005000	1.619	0.264	-1.355	high
Treat-0d	RHSIM_Rhsim09G0102400	0.326	-0.339	-0.665	no
Treat-0d	RHSIM_Rhsim08G0109400	0.008	-0.260	-0.268	no
Treat-0d	RHSIM_Rhsim09G0106700	0.859	0.449	-0.410	no
Treat-0d	RHSIM_Rhsim08G0161300	-0.841	-1.648	-0.807	no
Treat-0d	RHSIM_Rhsim08G0003400	-0.010	-0.673	-0.662	no
Treat-0d	RHSIM_Rhsim09G0138500	-0.473	0.146	0.620	no
Treat-0d	RHSIM_Rhsim09G0066700	0.615	0.561	-0.054	no
Treat-0d	RHSIM_Rhsim08G0054100	1.114	1.303	0.189	high
Treat-0d	RHSIM_Rhsim07G0212400	0.494	-0.319	-0.812	no
Treat-0d	RHSIM_Rhsim07G0257800	0.382	0.257	-0.125	no
Treat-0d	RHSIM_Rhsim07G0038700	1.599	1.183	-0.416	high
Treat-0d	RHSIM_Rhsim07G0074200	0.593	-0.677	-1.270	no
Treat-0d	RHSIM_Rhsim08G0079800	0.314	-0.792	-1.106	no
Treat-0d	RHSIM_Rhsim08G0205400	-0.163	-0.435	-0.273	no
Treat-0d	RHSIM_Rhsim08G0185100	0.889	1.341	0.453	no
Treat-0d	RHSIM_Rhsim08G0160300	0.570	0.270	-0.299	no
Treat-0d	RHSIM_Rhsim08G0240600	0.183	-0.853	-1.037	no
Treat-0d	RHSIM_Rhsim07G0106500	1.133	-0.207	-1.341	high
Treat-0d	RHSIM_Rhsim09G0203200	-0.075	-1.181	-1.106	no
Treat-0d	RHSIM_Rhsim08G0202600	0.046	-0.497	-0.543	no
Treat-0d	RHSIM_Rhsim07G0011800	0.524	1.028	0.505	no
Treat-0d	RHSIM_Rhsim08G0242400	0.980	-0.013	-0.993	no
Treat-0d	RHSIM_Rhsim08G0003500	0.319	0.791	0.472	no
Treat-0d	RHSIM_Rhsim07G0040800	0.781	-1.275	-2.056	no

Group	Gene	PTR (z-score difference)	z(log2 LFQ)	z(log2 FPKM)	extreme-PTR
Treat-0d	RHSIM_Rhsim08G0136400	0.180	0.228	0.048	no
Treat-0d	RHSIM_Rhsim09G0074000	-0.015	-0.844	-0.829	no
Treat-0d	RHSIM_Rhsim08G0044500	0.103	-0.766	-0.869	no
Treat-0d	RHSIM_Rhsim09G0190400	-0.622	-0.414	0.207	no
Treat-0d	RHSIM_Rhsim08G0207400	0.428	1.991	1.564	no
Treat-0d	RHSIM_Rhsim07G0010300	0.598	-0.039	-0.637	no
Treat-0d	RHSIM_Rhsim07G0204900	1.917	0.116	-1.800	high
Treat-0d	RHSIM_Rhsim08G0190700	0.130	0.050	-0.081	no
Treat-0d	RHSIM_Rhsim07G0096600	-2.175	0.192	2.368	low
Treat-0d	RHSIM_Rhsim08G0118900	-0.953	-0.585	0.368	no
Treat-0d	RHSIM_Rhsim08G0241100	-1.694	-0.849	0.845	low
Treat-0d	RHSIM_Rhsim07G0225100	-1.669	-0.069	1.600	low
Treat-0d	RHSIM_Rhsim08G0086000	-1.610	-0.240	1.370	low
Treat-0d	RHSIM_Rhsim07G0087000	-1.012	-0.610	0.402	no
Treat-0d	RHSIM_Rhsim08G0076000	-0.875	-0.677	0.198	no
Treat-0d	RHSIM_Rhsim07G0203200	-0.665	-0.062	0.604	no
Treat-0d	RHSIM_Rhsim08G0222800	2.169	0.504	-1.666	high
Treat-0d	RHSIM_Rhsim09G0132300	-0.377	-0.111	0.266	no
Treat-0d	RHSIM_Rhsim08G0071500	-0.336	-0.176	0.161	no
Treat-0d	RHSIM_Rhsim07G0062900	-0.545	-0.772	-0.226	no
Treat-0d	RHSIM_Rhsim08G0088200	-0.794	1.327	2.120	no
Treat-0d	RHSIM_Rhsim09G0031700	-0.513	0.124	0.637	no
Treat-0d	RHSIM_Rhsim09G0090500	-0.289	-0.678	-0.390	no
Treat-0d	RHSIM_Rhsim08G0208000	-2.048	-0.648	1.399	low
Treat-0d	RHSIM_Rhsim08G0075200	0.013	0.629	0.616	no
Treat-0d	RHSIM_Rhsim08G0197500	0.208	-0.506	-0.714	no
Treat-0d	RHSIM_Rhsim07G0077300	-0.207	0.394	0.600	no
Treat-0d	RHSIM_Rhsim07G0006700	0.379	-0.675	-1.053	no
Treat-0d	RHSIM_Rhsim08G0209600	-1.332	-1.603	-0.272	low
Treat-0d	RHSIM_Rhsim07G0119700	1.517	1.502	-0.015	high
Treat-0d	RHSIM_Rhsim08G0189500	-0.736	-1.410	-0.674	no
Treat-0d	RHSIM_Rhsim07G0100700	-0.673	-0.941	-0.268	no

Group	Gene	PTR (z-score difference)	z(log2 LFQ)	z(log2 FPKM)	extreme-PTR
Treat-0d	RHSIM_Rhsim07G0215500	-0.249	-0.233	0.016	no
Treat-0d	RHSIM_Rhsim08G0212100	0.174	-1.082	-1.256	no
Treat-0d	RHSIM_Rhsim08G0216500	1.629	0.193	-1.437	high
Treat-0d	RHSIM_Rhsim07G0017000	-0.709	0.772	1.481	no
Treat-0d	RHSIM_Rhsim09G0003500	-0.142	-0.432	-0.290	no
Treat-0d	RHSIM_Rhsim08G0189100	-0.560	0.331	0.891	no
Treat-0d	RHSIM_Rhsim09G0181600	0.190	-0.286	-0.476	no
Treat-0d	RHSIM_Rhsim08G0132500	-0.156	0.654	0.810	no
Treat-0d	RHSIM_Rhsim09G0182800	1.102	-0.432	-1.534	no
Treat-0d	RHSIM_Rhsim07G0123700	-0.751	-0.609	0.142	no
Treat-0d	RHSIM_Rhsim08G0199800	0.431	0.741	0.310	no
Treat-0d	RHSIM_Rhsim09G0146100	0.145	-0.671	-0.816	no
Treat-0d	RHSIM_Rhsim08G0228400	-0.143	0.223	0.366	no
Treat-0d	RHSIM_Rhsim08G0146100	1.255	2.091	0.836	high
Treat-0d	RHSIM_Rhsim07G0165600	0.769	1.444	0.675	no
Treat-0d	RHSIM_Rhsim09G0211400	-0.066	-1.015	-0.949	no
Treat-0d	RHSIM_Rhsim08G0086100	1.476	1.181	-0.295	high
Treat-0d	RHSIM_Rhsim09G0084200	-0.977	0.193	1.171	no
Treat-0d	RHSIM_Rhsim07G0010500	0.379	-1.233	-1.612	no
Treat-0d	RHSIM_Rhsim07G0118600	-0.554	-0.762	-0.208	no
Treat-0d	RHSIM_Rhsim08G0067700	-1.145	-1.019	0.126	low
Treat-0d	RHSIM_Rhsim09G0185500	2.396	0.407	-1.989	high
Treat-0d	RHSIM_Rhsim08G0080100	-1.223	-0.525	0.698	low
Treat-0d	RHSIM_Rhsim08G0002700	-0.652	-0.355	0.296	no
Treat-0d	RHSIM_Rhsim08G0029700	2.584	1.004	-1.580	high
Treat-0d	RHSIM_Rhsim09G0126400	-2.917	-1.705	1.212	low
Treat-0d	RHSIM_Rhsim09G0199100	-1.263	-1.252	0.011	low
Treat-0d	RHSIM_Rhsim09G0210100	0.895	0.680	-0.215	no
Treat-0d	RHSIM_Rhsim08G0157100	-0.221	-0.317	-0.096	no
Treat-0d	RHSIM_Rhsim08G0199500	0.121	-0.122	-0.243	no
Treat-0d	RHSIM_Rhsim09G0079200	-0.017	0.580	0.598	no
Treat-0d	RHSIM_Rhsim08G0225700	-0.767	-0.728	0.039	no

Group	Gene	PTR (z-score difference)	z(log2 LFQ)	z(log2 FPKM)	extreme-PTR
Treat-0d	RHSIM_Rhsim08G0191000	1.956	0.625	-1.331	high
Treat-0d	RHSIM_Rhsim07G0037800	0.470	0.088	-0.382	no
Treat-0d	RHSIM_Rhsim08G0113200	-0.811	-0.702	0.109	no
Treat-0d	RHSIM_Rhsim08G0187700	0.320	0.706	0.386	no
Treat-0d	RHSIM_Rhsim08G0033400	1.234	2.816	1.581	high
Treat-0d	RHSIM_Rhsim08G0135900	1.261	0.585	-0.675	high
Treat-0d	RHSIM_Rhsim08G0081500	0.597	2.221	1.625	no
Treat-0d	RHSIM_Rhsim08G0233000	-0.192	-1.551	-1.359	no
Treat-0d	RHSIM_Rhsim08G0013100	-2.612	-2.142	0.470	low
Treat-0d	RHSIM_Rhsim08G0176300	0.169	-0.684	-0.853	no
Treat-0d	RHSIM_Rhsim08G0221200	1.063	1.774	0.711	no
Treat-0d	RHSIM_Rhsim07G0115500	-0.100	0.171	0.270	no
Treat-0d	RHSIM_Rhsim08G0009100	-1.056	0.774	1.829	low
Treat-0d	RHSIM_Rhsim08G0233300	0.114	0.281	0.167	no
Treat-0d	RHSIM_Rhsim07G0204400	0.309	1.394	1.085	no
Treat-0d	RHSIM_Rhsim08G0015000	-0.118	-0.692	-0.575	no
Treat-0d	RHSIM_Rhsim07G0074900	-0.648	-0.590	0.058	no
Treat-0d	RHSIM_Rhsim08G0100700	1.332	1.020	-0.311	high
Treat-0d	RHSIM_Rhsim08G0012800	0.110	0.280	0.170	no
Treat-0d	RHSIM_Rhsim08G0000900	-0.380	-0.004	0.376	no
Treat-0d	RHSIM_Rhsim08G0009800	1.042	-0.152	-1.193	no
Treat-0d	RHSIM_Rhsim08G0118800	-0.656	-0.667	-0.012	no
Treat-0d	RHSIM_Rhsim08G0243800	-1.551	-1.339	0.212	low
Treat-0d	RHSIM_Rhsim08G0020200	0.366	-0.176	-0.543	no
Treat-0d	RHSIM_Rhsim08G0032000	-0.234	-0.794	-0.560	no
Treat-0d	RHSIM_Rhsim07G0118400	0.258	-1.479	-1.737	no
Treat-0d	RHSIM_Rhsim07G0173200	0.263	0.456	0.194	no
Treat-0d	RHSIM_Rhsim08G0210300	0.977	0.020	-0.957	no
Treat-0d	RHSIM_Rhsim07G0124000	2.210	0.735	-1.475	high
Treat-0d	RHSIM_Rhsim08G0151900	-0.303	-0.518	-0.215	no
Treat-0d	RHSIM_Rhsim08G0070300	0.163	0.683	0.520	no
Treat-0d	RHSIM_Rhsim08G0096300	-0.268	-0.297	-0.029	no

Group	Gene	PTR (z-score difference)	z(log2 LFQ)	z(log2 FPKM)	extreme-PTR
Treat-0d	RHSIM_Rhsim07G0016900	-0.402	-0.643	-0.241	no
Treat-0d	RHSIM_Rhsim08G0249800	-1.077	-1.608	-0.531	low
Treat-0d	RHSIM_Rhsim07G0131600	0.854	-0.534	-1.388	no
Treat-0d	RHSIM_Rhsim08G0056900	-0.276	-0.832	-0.556	no
Treat-0d	RHSIM_Rhsim08G0170400	0.224	2.016	1.792	no
Treat-0d	RHSIM_Rhsim07G0175400	0.121	0.110	-0.012	no
Treat-0d	RHSIM_Rhsim07G0170000	0.242	1.013	0.771	no
Treat-0d	RHSIM_Rhsim07G0057300	0.016	0.221	0.204	no
Treat-0d	RHSIM_Rhsim08G0071700	0.302	0.771	0.469	no
Treat-0d	RHSIM_Rhsim07G0035700	-0.047	-0.782	-0.735	no
Treat-0d	RHSIM_Rhsim08G0077000	-0.510	0.313	0.823	no
Treat-0d	RHSIM_Rhsim08G0161000	-0.196	0.281	0.477	no
Treat-0d	RHSIM_Rhsim07G0257200	1.539	1.044	-0.495	high
Treat-0d	RHSIM_Rhsim07G0024200	0.693	-0.497	-1.189	no
Treat-0d	RHSIM_Rhsim08G0088300	0.017	2.062	2.045	no
Treat-0d	RHSIM_Rhsim08G0188700	0.193	0.280	0.086	no
Treat-0d	RHSIM_Rhsim08G0200500	-1.675	-0.540	1.135	low
Treat-0d	RHSIM_Rhsim08G0157300	0.266	-0.479	-0.745	no
Treat-0d	RHSIM_Rhsim07G0017400	-1.020	0.520	1.540	no
Treat-0d	RHSIM_Rhsim07G0085300	0.259	1.335	1.076	no
Treat-0d	RHSIM_Rhsim08G0216800	-0.910	0.343	1.253	no
Treat-0d	RHSIM_Rhsim08G0126200	0.683	0.668	-0.015	no
Treat-0d	RHSIM_Rhsim07G0011500	-0.488	1.335	1.823	no
Treat-0d	RHSIM_Rhsim08G0222300	-0.807	-0.873	-0.066	no
Treat-0d	RHSIM_Rhsim07G0216000	-1.214	-0.417	0.797	low
Treat-0d	RHSIM_Rhsim08G0245600	1.390	-0.202	-1.592	high
Treat-0d	RHSIM_Rhsim08G0153200	-0.080	0.896	0.976	no
Treat-0d	RHSIM_Rhsim07G0101700	-0.053	-0.597	-0.544	no
Treat-0d	RHSIM_Rhsim08G0248400	2.980	3.421	0.441	high
Treat-0d	RHSIM_Rhsim08G0153400	-0.732	-0.602	0.130	no
Treat-0d	RHSIM_Rhsim08G0215500	0.811	-1.264	-2.075	no
Treat-0d	RHSIM_Rhsim08G0240500	0.513	0.237	-0.275	no

Group	Gene	PTR (z-score difference)	z(log2 LFQ)	z(log2 FPKM)	extreme-PTR
Treat-0d	RHSIM_Rhsim07G0004600	-1.571	-1.361	0.210	low
Treat-0d	RHSIM_Rhsim08G0157700	-1.228	-0.971	0.257	low
Treat-0d	RHSIM_Rhsim07G0053800	0.166	0.245	0.079	no
Treat-0d	RHSIM_Rhsim08G0230900	-0.085	0.286	0.371	no
Treat-0d	RHSIM_Rhsim07G0248600	0.229	-0.584	-0.812	no
Treat-0d	RHSIM_Rhsim07G0144500	-0.602	-0.348	0.254	no
Treat-0d	RHSIM_Rhsim07G0201100	0.587	-1.061	-1.648	no
Treat-0d	RHSIM_Rhsim08G0012300	0.694	1.462	0.767	no
Treat-0d	RHSIM_Rhsim07G0023600	0.332	-0.569	-0.901	no
Treat-0d	RHSIM_Rhsim08G0241600	-0.220	-0.623	-0.402	no
Treat-0d	RHSIM_Rhsim07G0025600	2.004	0.604	-1.400	high
Treat-0d	RHSIM_Rhsim08G0123400	-0.494	0.068	0.563	no
Treat-0d	RHSIM_Rhsim08G0040600	1.518	-0.439	-1.957	high
Treat-0d	RHSIM_Rhsim08G0119200	-1.246	0.880	2.126	low
Treat-0d	RHSIM_Rhsim08G0058800	-1.495	-0.617	0.878	low
Treat-0d	RHSIM_Rhsim08G0089400	-1.528	-0.400	1.129	low
Treat-0d	RHSIM_Rhsim07G0019000	1.196	0.814	-0.381	high
Treat-0d	RHSIM_Rhsim07G0251100	-0.841	1.518	2.359	no
Treat-0d	RHSIM_Rhsim08G0169300	-1.145	-1.225	-0.080	low
Treat-0d	RHSIM_Rhsim08G0093900	-0.675	0.850	1.525	no
Treat-0d	RHSIM_Rhsim08G0098500	-0.196	-0.610	-0.414	no
Treat-0d	RHSIM_Rhsim07G0044500	2.127	-0.282	-2.409	high
Treat-0d	RHSIM_Rhsim08G0187400	0.826	1.679	0.854	no
Treat-0d	RHSIM_Rhsim08G0212600	-0.290	-0.134	0.156	no
Treat-0d	RHSIM_Rhsim07G0258200	-0.648	-1.328	-0.680	no
Treat-0d	RHSIM_Rhsim07G0146900	-0.181	-1.017	-0.836	no
Treat-0d	RHSIM_Rhsim08G0078700	0.488	-0.080	-0.569	no
Treat-0d	RHSIM_Rhsim08G0043300	0.504	0.527	0.024	no
Treat-0d	RHSIM_Rhsim07G0091700	-0.865	-1.776	-0.911	no
Treat-0d	RHSIM_Rhsim08G0184400	0.650	0.123	-0.527	no
Treat-0d	RHSIM_Rhsim08G0119000	0.973	1.226	0.252	no
Treat-0d	RHSIM_Rhsim07G0209200	-3.382	-1.416	1.966	low

Group	Gene	PTR (z-score difference)	z(log2 LFQ)	z(log2 FPKM)	extreme-PTR
Treat-0d	RHSIM_Rhsim08G0068000	-1.031	-0.079	0.952	low
Treat-0d	RHSIM_Rhsim07G0250500	-0.177	0.817	0.995	no
Treat-0d	RHSIM_Rhsim08G0199900	0.265	0.381	0.116	no
Treat-0d	RHSIM_Rhsim07G0188400	-2.130	-1.740	0.389	low
Treat-0d	RHSIM_Rhsim07G0184400	0.675	-0.680	-1.355	no
Treat-0d	RHSIM_Rhsim08G0207200	-0.799	0.462	1.261	no
Treat-0d	RHSIM_Rhsim08G0216200	0.123	-0.131	-0.254	no
Treat-0d	RHSIM_Rhsim08G0227300	-2.987	-1.273	1.714	low
Treat-0d	RHSIM_Rhsim08G0124200	0.979	0.299	-0.680	no
Treat-0d	RHSIM_Rhsim07G0186000	-0.044	-0.166	-0.122	no
Treat-0d	RHSIM_Rhsim08G0027500	-0.547	-0.336	0.211	no
Treat-0d	RHSIM_Rhsim08G0000300	-0.656	-0.674	-0.019	no
Treat-0d	RHSIM_Rhsim08G0155400	-0.656	-0.192	0.464	no
Treat-0d	RHSIM_Rhsim07G0190000	-1.621	-1.667	-0.047	low
Treat-0d	RHSIM_Rhsim08G0125000	-0.312	-0.031	0.281	no
Treat-0d	RHSIM_Rhsim07G0256400	-0.889	0.228	1.117	no
Treat-0d	RHSIM_Rhsim08G0146900	-0.200	0.101	0.301	no
Treat-0d	RHSIM_Rhsim07G0125300	0.569	1.032	0.463	no
Treat-0d	RHSIM_Rhsim08G0041400	-0.278	-1.658	-1.379	no
Treat-0d	RHSIM_Rhsim08G0198600	0.727	1.207	0.480	no
Treat-0d	RHSIM_Rhsim08G0124400	-0.524	-0.716	-0.192	no
Treat-0d	RHSIM_Rhsim08G0106700	1.524	-0.140	-1.664	high
Treat-0d	RHSIM_Rhsim08G0095400	-0.766	-1.045	-0.279	no
Treat-0d	RHSIM_Rhsim07G0102300	-0.626	-0.835	-0.209	no
Treat-0d	RHSIM_Rhsim08G0198900	-2.592	-1.636	0.956	low
Treat-0d	RHSIM_Rhsim07G0187600	-0.824	0.883	1.707	no
Treat-0d	RHSIM_Rhsim08G0038400	-2.286	-0.943	1.343	low
Treat-0d	RHSIM_Rhsim08G0066200	-0.031	2.659	2.691	no
Treat-0d	RHSIM_Rhsim08G0129000	0.687	1.950	1.263	no
Treat-0d	RHSIM_Rhsim08G0076500	0.249	0.756	0.506	no
Treat-0d	RHSIM_Rhsim08G0171400	0.072	-0.360	-0.432	no
Treat-0d	RHSIM_Rhsim08G0047100	1.455	0.393	-1.062	high

Group	Gene	PTR (z-score difference)	z(log2 LFQ)	z(log2 FPKM)	extreme-PTR
Treat-0d	RHSIM_Rhsim08G0206000	-0.239	-1.058	-0.819	no
Treat-0d	RHSIM_Rhsim07G0219200	0.198	-0.935	-1.133	no
Treat-0d	RHSIM_Rhsim08G0227800	-0.559	0.704	1.263	no
Treat-0d	RHSIM_Rhsim08G0249600	0.531	-0.768	-1.299	no
Treat-0d	RHSIM_Rhsim07G0251700	1.263	1.945	0.683	high
Treat-0d	RHSIM_Rhsim08G0038900	0.081	0.867	0.786	no
Treat-0d	RHSIM_Rhsim08G0096700	-0.365	-0.251	0.114	no
Treat-0d	RHSIM_Rhsim07G0067800	-0.082	-0.407	-0.325	no
Treat-0d	RHSIM_Rhsim08G0006300	0.241	-0.142	-0.384	no
Treat-0d	RHSIM_Rhsim07G0215000	-0.109	-0.879	-0.769	no
Treat-0d	RHSIM_Rhsim07G0007400	-0.318	-0.884	-0.566	no
Treat-0d	RHSIM_Rhsim08G0115600	-0.542	0.031	0.573	no
Treat-0d	RHSIM_Rhsim07G0241500	0.930	0.700	-0.230	no
Treat-0d	RHSIM_Rhsim08G0005400	-2.519	-1.155	1.364	low
Treat-0d	RHSIM_Rhsim07G0208900	-0.731	-0.036	0.695	no
Treat-0d	RHSIM_Rhsim08G0044400	0.241	-0.645	-0.886	no
Treat-0d	RHSIM_Rhsim08G0018500	1.669	0.601	-1.067	high
Treat-0d	RHSIM_Rhsim08G0090300	0.227	0.907	0.679	no
Treat-0d	RHSIM_Rhsim07G0052800	0.612	-0.638	-1.250	no
Treat-0d	RHSIM_Rhsim07G0108500	-0.428	-0.802	-0.373	no
Treat-0d	RHSIM_Rhsim07G0063200	-1.757	-1.128	0.629	low
Treat-0d	RHSIM_Rhsim07G0080500	2.172	0.681	-1.491	high
Treat-0d	RHSIM_Rhsim08G0074900	-0.371	-0.364	0.007	no
Treat-0d	RHSIM_Rhsim08G0010200	-0.103	-0.419	-0.315	no
Treat-0d	RHSIM_Rhsim07G0032500	0.449	0.766	0.318	no
Treat-0d	RHSIM_Rhsim08G0171600	-0.762	3.324	4.086	no
Treat-0d	RHSIM_Rhsim07G0096500	0.413	2.069	1.656	no
Treat-0d	RHSIM_Rhsim08G0252300	-0.278	-0.486	-0.208	no
Treat-0d	RHSIM_Rhsim07G0181400	0.317	0.228	-0.089	no
Treat-0d	RHSIM_Rhsim08G0175700	-0.175	-1.185	-1.010	no
Treat-0d	RHSIM_Rhsim08G0173200	-1.720	0.320	2.040	low
Treat-0d	RHSIM_Rhsim07G0091300	-0.537	-0.081	0.456	no

Group	Gene	PTR (z-score difference)	z(log2 LFQ)	z(log2 FPKM)	extreme-PTR
Treat-0d	RHSIM_Rhsim07G0054000	-1.730	-1.055	0.675	low
Treat-0d	RHSIM_Rhsim07G0068700	0.842	-0.111	-0.952	no
Treat-0d	RHSIM_Rhsim08G0054600	0.764	-0.206	-0.970	no
Treat-0d	RHSIM_Rhsim08G0016700	-0.173	1.161	1.335	no
Treat-0d	RHSIM_Rhsim07G0080100	0.164	0.135	-0.029	no
Treat-0d	RHSIM_Rhsim07G0245700	0.598	-0.274	-0.873	no
Treat-0d	RHSIM_Rhsim08G0080000	0.116	-0.818	-0.934	no
Treat-0d	RHSIM_Rhsim08G0140900	-0.780	-0.425	0.354	no
Treat-0d	RHSIM_Rhsim08G0046200	0.393	0.866	0.474	no
Treat-0d	RHSIM_Rhsim08G0010700	0.418	-0.861	-1.279	no
Treat-0d	RHSIM_Rhsim08G0116700	0.993	0.601	-0.392	no
Treat-0d	RHSIM_Rhsim07G0145700	0.021	0.506	0.485	no
Treat-0d	RHSIM_Rhsim07G0006200	-0.497	0.384	0.881	no
Treat-0d	RHSIM_Rhsim07G0013500	-0.873	-1.233	-0.360	no
Treat-0d	RHSIM_Rhsim07G0053100	-0.533	0.632	1.166	no
Treat-0d	RHSIM_Rhsim08G0249200	-1.042	-0.606	0.436	low
Treat-0d	RHSIM_Rhsim07G0096000	-0.503	0.912	1.415	no
Treat-0d	RHSIM_Rhsim08G0245800	-0.128	-0.075	0.053	no
Treat-0d	RHSIM_Rhsim08G0019400	0.381	-1.581	-1.962	no
Treat-0d	RHSIM_Rhsim08G0102300	-0.510	-0.977	-0.467	no
Treat-0d	RHSIM_Rhsim07G0005700	0.067	-1.016	-1.082	no
Treat-0d	RHSIM_Rhsim07G0056700	0.519	-0.180	-0.699	no
Treat-0d	RHSIM_Rhsim08G0210500	0.182	0.622	0.439	no
Treat-0d	RHSIM_Rhsim07G0045400	0.627	0.842	0.216	no
Treat-0d	RHSIM_Rhsim07G0188300	-0.128	-1.041	-0.913	no
Treat-0d	RHSIM_Rhsim08G0037900	0.170	0.122	-0.048	no
Treat-0d	RHSIM_Rhsim07G0023500	-0.192	-0.274	-0.082	no
Treat-0d	RHSIM_Rhsim07G0068500	-0.405	0.312	0.717	no
Treat-0d	RHSIM_Rhsim07G0081300	1.558	0.159	-1.399	high
Treat-0d	RHSIM_Rhsim07G0117800	-0.521	1.092	1.613	no
Treat-0d	RHSIM_Rhsim08G0119100	-0.132	0.615	0.747	no
Treat-0d	RHSIM_Rhsim07G0186700	0.234	-0.594	-0.828	no

Group	Gene	PTR (z-score difference)	z(log2 LFQ)	z(log2 FPKM)	extreme-PTR
Treat-0d	RHSIM_Rhsim08G0034600	-0.652	0.366	1.017	no
Treat-0d	RHSIM_Rhsim07G0083300	-0.264	-0.765	-0.501	no
Treat-0d	RHSIM_Rhsim08G0139800	-0.133	-0.390	-0.257	no
Treat-0d	RHSIM_Rhsim07G0240400	-1.162	-0.623	0.539	low
Treat-0d	RHSIM_Rhsim07G0126200	-0.239	0.225	0.464	no
Treat-0d	RHSIM_Rhsim07G0251400	-0.721	1.690	2.411	no
Treat-0d	RHSIM_Rhsim07G0127400	-1.117	-0.690	0.426	low
Treat-0d	RHSIM_Rhsim07G0022300	-0.675	0.008	0.683	no
Treat-0d	RHSIM_Rhsim07G0094300	-0.351	-1.052	-0.701	no
Treat-0d	RHSIM_Rhsim07G0170800	-1.584	-1.357	0.227	low
Treat-0d	RHSIM_Rhsim08G0013500	1.437	-0.858	-2.295	high
Treat-0d	RHSIM_Rhsim08G0031200	1.705	1.734	0.029	high
Treat-0d	RHSIM_Rhsim07G0071200	1.252	0.210	-1.042	high
Treat-0d	RHSIM_Rhsim08G0248600	0.064	-0.421	-0.486	no
Treat-0d	RHSIM_Rhsim08G0067400	1.062	0.539	-0.524	no
Treat-0d	RHSIM_Rhsim08G0175500	-0.323	-0.697	-0.374	no
Treat-0d	RHSIM_Rhsim08G0028400	-0.477	-1.692	-1.215	no
Treat-0d	RHSIM_Rhsim07G0172400	-0.073	-1.421	-1.348	no
Treat-0d	RHSIM_Rhsim08G0249300	-0.878	0.034	0.912	no
Treat-0d	RHSIM_Rhsim07G0155300	-0.649	-0.578	0.071	no
Treat-0d	RHSIM_Rhsim07G0181600	-1.160	-1.124	0.036	low
Treat-0d	RHSIM_Rhsim07G0202000	0.494	1.080	0.586	no
Treat-0d	RHSIM_Rhsim08G0221900	0.129	-0.482	-0.611	no
Treat-0d	RHSIM_Rhsim07G0040000	0.272	1.515	1.243	no
Treat-0d	RHSIM_Rhsim08G0000400	-0.085	0.075	0.160	no
Treat-0d	RHSIM_Rhsim08G0050800	1.345	0.617	-0.728	high
Treat-0d	RHSIM_Rhsim07G0236600	-0.119	-0.745	-0.626	no
Treat-0d	RHSIM_Rhsim07G0219500	-1.373	0.028	1.401	low
Treat-0d	RHSIM_Rhsim07G0059300	-0.711	-1.019	-0.308	no
Treat-0d	RHSIM_Rhsim07G0209000	1.358	0.289	-1.068	high
Treat-0d	RHSIM_Rhsim08G0126600	-0.340	-0.282	0.059	no
Treat-0d	RHSIM_Rhsim07G0168300	-0.424	-1.516	-1.092	no

Group	Gene	PTR (z-score difference)	z(log2 LFQ)	z(log2 FPKM)	extreme-PTR
Treat-0d	RHSIM_Rhsim07G0163600	0.911	-0.473	-1.384	no
Treat-0d	RHSIM_Rhsim07G0173800	0.118	-0.408	-0.525	no
Treat-0d	RHSIM_Rhsim07G0239800	-0.389	-0.433	-0.044	no
Treat-0d	RHSIM_Rhsim07G0164400	-0.796	-1.279	-0.484	no
Treat-0d	RHSIM_Rhsim07G0031300	-0.865	-0.760	0.105	no
Treat-0d	RHSIM_Rhsim07G0016500	-0.010	0.006	0.016	no
Treat-0d	RHSIM_Rhsim07G0250100	0.354	2.116	1.762	no
Treat-0d	RHSIM_Rhsim07G0107500	-2.143	-1.097	1.046	low
Treat-0d	RHSIM_Rhsim08G0024600	0.522	-0.975	-1.497	no
Treat-0d	RHSIM_Rhsim07G0068000	0.360	-0.793	-1.153	no
Treat-0d	RHSIM_Rhsim07G0024600	-0.669	-1.190	-0.522	no
Treat-0d	RHSIM_Rhsim07G0038000	0.280	0.301	0.021	no
Treat-0d	RHSIM_Rhsim07G0207200	-2.925	-1.744	1.181	low
Treat-0d	RHSIM_Rhsim07G0086400	0.123	-0.760	-0.882	no
Treat-0d	RHSIM_Rhsim07G0052400	-0.507	-0.774	-0.268	no
Treat-0d	RHSIM_Rhsim07G0029300	0.609	-1.070	-1.679	no
Treat-0d	RHSIM_Rhsim08G0068500	-0.955	0.502	1.457	no
Treat-0d	RHSIM_Rhsim07G0069700	0.918	-0.639	-1.557	no
Treat-0d	RHSIM_Rhsim07G0033300	0.001	-1.439	-1.440	no
Treat-0d	RHSIM_Rhsim07G0105400	0.213	-0.036	-0.249	no
Treat-0d	RHSIM_Rhsim08G0085100	1.129	1.044	-0.085	high
Treat-0d	RHSIM_Rhsim07G0173600	-1.230	0.031	1.261	low
Treat-0d	RHSIM_Rhsim08G0016400	-0.769	-1.030	-0.261	no
Treat-0d	RHSIM_Rhsim08G0152500	0.706	2.800	2.094	no
Treat-0d	RHSIM_Rhsim07G0027900	-1.200	-0.755	0.445	low
Treat-0d	RHSIM_Rhsim07G0026600	-0.724	-1.624	-0.900	no
Treat-0d	RHSIM_Rhsim07G0055300	0.602	1.646	1.044	no
Treat-0d	RHSIM_Rhsim08G0176400	-0.872	1.580	2.452	no
Treat-0d	RHSIM_Rhsim08G0232200	-0.212	-1.179	-0.967	no
Treat-0d	RHSIM_Rhsim07G0061300	-0.833	-0.433	0.399	no
Treat-0d	RHSIM_Rhsim07G0125000	0.825	-0.286	-1.111	no
Treat-0d	RHSIM_Rhsim07G0228300	0.803	-0.328	-1.131	no

Group	Gene	PTR (z-score difference)	z(log2 LFQ)	z(log2 FPKM)	extreme-PTR
Treat-0d	RHSIM_Rhsim07G0020700	-0.878	0.284	1.162	no
Treat-0d	RHSIM_Rhsim07G0132100	-1.058	-0.640	0.417	low
Treat-0d	RHSIM_Rhsim07G0074100	-0.425	-0.116	0.309	no
Treat-0d	RHSIM_Rhsim07G0198900	0.571	-0.181	-0.751	no
Treat-0d	RHSIM_Rhsim07G0007900	1.148	3.023	1.874	high
Treat-0d	RHSIM_Rhsim07G0243800	-0.320	1.724	2.044	no
Treat-0d	RHSIM_Rhsim07G0239400	-1.501	-1.706	-0.205	low
Treat-0d	RHSIM_Rhsim07G0025200	0.040	-0.563	-0.603	no
Treat-0d	RHSIM_Rhsim07G0143700	-1.153	-0.249	0.904	low
Treat-0d	RHSIM_Rhsim08G0094700	-0.711	-0.133	0.578	no
Treat-0d	RHSIM_Rhsim07G0214200	1.371	-0.573	-1.944	high
Treat-0d	RHSIM_Rhsim07G0109900	-0.790	-1.558	-0.768	no
Treat-0d	RHSIM_Rhsim08G0173100	-1.074	-0.176	0.899	low
Treat-0d	RHSIM_Rhsim07G0049200	-0.235	0.621	0.856	no
Treat-0d	RHSIM_Rhsim08G0224200	-0.393	-0.920	-0.526	no
Treat-0d	RHSIM_Rhsim08G0112300	-0.548	-0.391	0.157	no
Treat-0d	RHSIM_Rhsim07G0172200	-0.421	-0.056	0.366	no
Treat-0d	RHSIM_Rhsim08G0226500	-0.224	-0.489	-0.265	no
Treat-0d	RHSIM_Rhsim07G0215100	-0.921	-0.325	0.596	no
Treat-0d	RHSIM_Rhsim07G0082400	-0.459	-0.358	0.101	no
Treat-0d	RHSIM_Rhsim07G0106300	-0.389	0.765	1.154	no
Treat-0d	RHSIM_Rhsim07G0200600	-1.540	-1.159	0.381	low
Treat-0d	RHSIM_Rhsim08G0020400	1.534	-0.719	-2.252	high
Treat-0d	RHSIM_Rhsim07G0029400	0.655	0.037	-0.618	no
Treat-0d	RHSIM_Rhsim07G0171600	0.682	0.546	-0.136	no
Treat-0d	RHSIM_Rhsim08G0080600	0.401	0.276	-0.126	no
Treat-0d	RHSIM_Rhsim06G0201500	0.579	-0.298	-0.878	no
Treat-0d	RHSIM_Rhsim08G0006200	1.130	1.558	0.428	high
Treat-0d	RHSIM_Rhsim07G0076300	-1.118	-0.762	0.356	low
Treat-0d	RHSIM_Rhsim06G0223900	-0.426	-1.363	-0.937	no
Treat-0d	RHSIM_Rhsim08G0157000	1.053	-0.691	-1.744	no
Treat-0d	RHSIM_Rhsim08G0248900	-1.589	-1.748	-0.159	low

Group	Gene	PTR (z-score difference)	z(log2 LFQ)	z(log2 FPKM)	extreme-PTR
Treat-0d	RHSIM_Rhsim06G0029100	-1.484	-0.742	0.742	low
Treat-0d	RHSIM_Rhsim07G0102800	0.194	0.143	-0.051	no
Treat-0d	RHSIM_Rhsim08G0031800	-1.007	-0.736	0.271	no
Treat-0d	RHSIM_Rhsim07G0054400	-0.979	-0.187	0.793	no
Treat-0d	RHSIM_Rhsim07G0252800	-1.642	-1.077	0.565	low
Treat-0d	RHSIM_Rhsim07G0222500	-0.235	0.016	0.251	no
Treat-0d	RHSIM_Rhsim07G0255000	-0.402	0.241	0.644	no
Treat-0d	RHSIM_Rhsim06G0046600	1.310	0.213	-1.097	high
Treat-0d	RHSIM_Rhsim08G0240200	0.436	0.173	-0.263	no
Treat-0d	RHSIM_Rhsim07G0237100	0.354	-0.201	-0.555	no
Treat-0d	RHSIM_Rhsim07G0136400	0.996	0.008	-0.988	no
Treat-0d	RHSIM_Rhsim07G0048200	-0.642	-1.210	-0.568	no
Treat-0d	RHSIM_Rhsim08G0064800	0.110	1.689	1.579	no
Treat-0d	RHSIM_Rhsim07G0045800	1.642	2.628	0.986	high
Treat-0d	RHSIM_Rhsim07G0137100	-1.408	0.294	1.703	low
Treat-0d	RHSIM_Rhsim06G0084500	0.001	-0.415	-0.416	no
Treat-0d	RHSIM_Rhsim07G0178600	-0.164	0.065	0.229	no
Treat-0d	RHSIM_Rhsim07G0143500	1.089	0.665	-0.424	no
Treat-0d	RHSIM_Rhsim06G0122400	-1.494	0.177	1.671	low
Treat-0d	RHSIM_Rhsim07G0022700	-0.883	-0.937	-0.055	no
Treat-0d	RHSIM_Rhsim06G0170400	-0.448	-0.300	0.148	no
Treat-0d	RHSIM_Rhsim07G0065100	1.755	-0.589	-2.344	high
Treat-0d	RHSIM_Rhsim07G0124300	0.771	0.580	-0.190	no
Treat-0d	RHSIM_Rhsim07G0006000	-0.481	0.714	1.196	no
Treat-0d	RHSIM_Rhsim07G0130900	0.066	-0.063	-0.129	no
Treat-0d	RHSIM_Rhsim08G0163100	0.298	-0.329	-0.627	no
Treat-0d	RHSIM_Rhsim06G0174100	-2.026	-1.794	0.232	low
Treat-0d	RHSIM_Rhsim06G0208000	-0.086	-0.489	-0.403	no
Treat-0d	RHSIM_Rhsim07G0201800	-0.937	-0.902	0.035	no
Treat-0d	RHSIM_Rhsim06G0046900	2.158	1.315	-0.843	high
Treat-0d	RHSIM_Rhsim06G0184300	0.794	0.370	-0.423	no
Treat-0d	RHSIM_Rhsim07G0190100	-3.208	-1.351	1.857	low

Group	Gene	PTR (z-score difference)	z(log2 LFQ)	z(log2 FPKM)	extreme-PTR
Treat-0d	RHSIM_Rhsim07G0205400	0.679	0.768	0.089	no
Treat-0d	RHSIM_Rhsim07G0137000	1.393	0.901	-0.492	high
Treat-0d	RHSIM_Rhsim06G0007800	-0.331	0.314	0.645	no
Treat-0d	RHSIM_Rhsim07G0014000	-1.229	-0.795	0.434	low
Treat-0d	RHSIM_Rhsim06G0140600	0.929	-0.164	-1.094	no
Treat-0d	RHSIM_Rhsim07G0120800	-1.848	-1.149	0.699	low
Treat-0d	RHSIM_Rhsim08G0230400	-1.255	0.427	1.682	low
Treat-0d	RHSIM_Rhsim07G0134100	-0.802	-0.452	0.350	no
Treat-0d	RHSIM_Rhsim06G0008600	-1.303	-0.773	0.530	low
Treat-0d	RHSIM_Rhsim07G0244700	-0.791	-0.715	0.076	no
Treat-0d	RHSIM_Rhsim07G0096100	-2.756	-1.724	1.032	low
Treat-0d	RHSIM_Rhsim07G0068100	-0.802	0.129	0.931	no
Treat-0d	RHSIM_Rhsim06G0227300	0.045	1.683	1.638	no
Treat-0d	RHSIM_Rhsim06G0231100	0.258	0.680	0.423	no
Treat-0d	RHSIM_Rhsim08G0152000	-0.475	-1.252	-0.777	no
Treat-0d	RHSIM_Rhsim06G0121500	1.037	-0.227	-1.264	no
Treat-0d	RHSIM_Rhsim06G0180800	0.714	-0.703	-1.417	no
Treat-0d	RHSIM_Rhsim07G0037100	-0.299	-0.661	-0.361	no
Treat-0d	RHSIM_Rhsim07G0075100	-0.150	-0.113	0.037	no
Treat-0d	RHSIM_Rhsim07G0243000	0.446	-0.124	-0.570	no
Treat-0d	RHSIM_Rhsim06G0009200	1.042	0.333	-0.710	no
Treat-0d	RHSIM_Rhsim07G0193800	0.058	-0.168	-0.226	no
Treat-0d	RHSIM_Rhsim07G0175800	0.718	0.725	0.008	no
Treat-0d	RHSIM_Rhsim07G0214800	-2.017	-0.904	1.113	low
Treat-0d	RHSIM_Rhsim07G0244600	0.654	0.431	-0.223	no
Treat-0d	RHSIM_Rhsim07G0011100	-0.558	-1.096	-0.538	no
Treat-0d	RHSIM_Rhsim07G0069200	-0.848	-0.916	-0.068	no
Treat-0d	RHSIM_Rhsim08G0163500	0.699	0.033	-0.665	no
Treat-0d	RHSIM_Rhsim06G0229900	0.134	-0.305	-0.439	no
Treat-0d	RHSIM_Rhsim07G0104500	1.686	1.073	-0.613	high
Treat-0d	RHSIM_Rhsim07G0136600	0.093	-0.943	-1.036	no
Treat-0d	RHSIM_Rhsim06G0035500	-0.448	-0.227	0.221	no

Group	Gene	PTR (z-score difference)	z(log2 LFQ)	z(log2 FPKM)	extreme-PTR
Treat-0d	RHSIM_Rhsim07G0018800	0.282	1.101	0.819	no
Treat-0d	RHSIM_Rhsim06G0089000	-0.147	0.640	0.787	no
Treat-0d	RHSIM_Rhsim07G0221800	-3.290	-1.134	2.156	low
Treat-0d	RHSIM_Rhsim07G0200400	0.006	0.140	0.133	no
Treat-0d	RHSIM_Rhsim08G0038600	1.012	0.238	-0.773	no
Treat-0d	RHSIM_Rhsim07G0183300	0.356	0.582	0.226	no
Treat-0d	RHSIM_Rhsim07G0039600	0.492	1.258	0.766	no
Treat-0d	RHSIM_Rhsim07G0229800	2.494	1.053	-1.441	high
Treat-0d	RHSIM_Rhsim06G0079300	-0.619	-0.135	0.484	no
Treat-0d	RHSIM_Rhsim08G0146700	0.478	-0.784	-1.262	no
Treat-0d	RHSIM_Rhsim07G0172500	0.124	-0.014	-0.137	no
Treat-0d	RHSIM_Rhsim06G0198700	-5.365	-2.093	3.273	low
Treat-0d	RHSIM_Rhsim07G0134000	0.439	0.142	-0.297	no
Treat-0d	RHSIM_Rhsim07G0078200	0.305	-0.241	-0.546	no
Treat-0d	RHSIM_Rhsim07G0179200	1.373	-0.166	-1.540	high
Treat-0d	RHSIM_Rhsim06G0203800	-2.340	-0.002	2.337	low
Treat-0d	RHSIM_Rhsim06G0204600	-0.283	0.570	0.853	no
Treat-0d	RHSIM_Rhsim06G0229700	-1.435	-0.303	1.132	low
Treat-0d	RHSIM_Rhsim06G0003900	-0.968	0.224	1.192	no
Treat-0d	RHSIM_Rhsim07G0176900	0.031	-1.030	-1.061	no
Treat-0d	RHSIM_Rhsim06G0211400	-0.437	1.124	1.562	no
Treat-0d	RHSIM_Rhsim07G0093600	-1.799	-0.961	0.838	low
Treat-0d	RHSIM_Rhsim07G0086200	0.004	0.548	0.544	no
Treat-0d	RHSIM_Rhsim07G0017700	-1.191	-1.180	0.010	low
Treat-0d	RHSIM_Rhsim07G0102000	-0.183	0.478	0.661	no
Treat-0d	RHSIM_Rhsim06G0069100	-0.351	0.676	1.027	no
Treat-0d	RHSIM_Rhsim07G0122900	0.541	0.472	-0.069	no
Treat-0d	RHSIM_Rhsim07G0141400	-0.294	0.089	0.383	no
Treat-0d	RHSIM_Rhsim06G0026300	2.128	1.636	-0.492	high
Treat-0d	RHSIM_Rhsim08G0011300	0.117	0.059	-0.058	no
Treat-0d	RHSIM_Rhsim06G0050800	-0.412	-0.741	-0.329	no
Treat-0d	RHSIM_Rhsim06G0013500	-1.264	0.026	1.291	low

Group	Gene	PTR (z-score difference)	z(log2 LFQ)	z(log2 FPKM)	extreme-PTR
Treat-0d	RHSIM_Rhsim06G0131700	-0.770	-0.013	0.756	no
Treat-0d	RHSIM_Rhsim07G0243900	0.049	-0.334	-0.383	no
Treat-0d	RHSIM_Rhsim07G0213800	-1.072	-0.982	0.091	low
Treat-0d	RHSIM_Rhsim07G0075700	0.373	0.550	0.177	no
Treat-0d	RHSIM_Rhsim06G0175500	-0.551	0.081	0.632	no
Treat-0d	RHSIM_Rhsim06G0068700	0.223	1.478	1.256	no
Treat-0d	RHSIM_Rhsim07G0257700	0.043	-0.056	-0.099	no
Treat-0d	RHSIM_Rhsim06G0032100	0.228	-0.740	-0.968	no
Treat-0d	RHSIM_Rhsim06G0227500	1.081	0.622	-0.459	no
Treat-0d	RHSIM_Rhsim06G0077700	-0.731	-1.678	-0.947	no
Treat-0d	RHSIM_Rhsim06G0082900	-0.126	0.008	0.134	no
Treat-0d	RHSIM_Rhsim07G0144100	-0.301	0.374	0.675	no
Treat-0d	RHSIM_Rhsim07G0039900	0.235	-0.947	-1.182	no
Treat-0d	RHSIM_Rhsim07G0239500	2.067	0.768	-1.299	high
Treat-0d	RHSIM_Rhsim06G0027200	-1.360	-1.375	-0.015	low
Treat-0d	RHSIM_Rhsim07G0252900	-0.266	-0.945	-0.678	no
Treat-0d	RHSIM_Rhsim07G0061100	1.733	-0.665	-2.398	high
Treat-0d	RHSIM_Rhsim06G0210400	0.231	0.301	0.070	no
Treat-0d	RHSIM_Rhsim06G0079600	-2.967	-0.499	2.468	low
Treat-0d	RHSIM_Rhsim06G0050100	0.090	-1.141	-1.231	no
Treat-0d	RHSIM_Rhsim07G0211300	-0.265	-0.151	0.114	no
Treat-0d	RHSIM_Rhsim06G0231000	-0.177	-0.388	-0.211	no
Treat-0d	RHSIM_Rhsim07G0039700	-0.328	0.593	0.921	no
Treat-0d	RHSIM_Rhsim07G0139700	-2.645	0.315	2.960	low
Treat-0d	RHSIM_Rhsim07G0016400	-0.367	-1.065	-0.698	no
Treat-0d	RHSIM_Rhsim06G0154000	-0.301	-0.316	-0.015	no
Treat-0d	RHSIM_Rhsim07G0045700	-0.852	-0.730	0.122	no
Treat-0d	RHSIM_Rhsim06G0162500	1.626	2.307	0.682	high
Treat-0d	RHSIM_Rhsim06G0242100	0.371	-1.114	-1.485	no
Treat-0d	RHSIM_Rhsim06G0213100	1.680	-0.275	-1.954	high
Treat-0d	RHSIM_Rhsim07G0071500	-1.265	-0.728	0.536	low
Treat-0d	RHSIM_Rhsim07G0213700	-1.049	-1.169	-0.119	low

Group	Gene	PTR (z-score difference)	z(log2 LFQ)	z(log2 FPKM)	extreme-PTR
Treat-0d	RHSIM_Rhsim07G0030600	1.762	0.347	-1.415	high
Treat-0d	RHSIM_Rhsim07G0177400	-0.517	-0.549	-0.032	no
Treat-0d	RHSIM_Rhsim07G0047700	-0.642	0.230	0.872	no
Treat-0d	RHSIM_Rhsim07G0201200	0.971	-1.377	-2.348	no
Treat-0d	RHSIM_Rhsim06G0219700	0.894	0.426	-0.468	no
Treat-0d	RHSIM_Rhsim08G0244100	0.357	-0.029	-0.385	no
Treat-0d	RHSIM_Rhsim07G0253800	-1.991	-0.058	1.933	low
Treat-0d	RHSIM_Rhsim07G0137800	-1.100	-0.381	0.718	low
Treat-0d	RHSIM_Rhsim07G0209300	0.292	-0.130	-0.422	no
Treat-0d	RHSIM_Rhsim06G0207200	0.499	2.127	1.628	no
Treat-0d	RHSIM_Rhsim07G0175500	-0.307	-1.119	-0.811	no
Treat-0d	RHSIM_Rhsim06G0142500	0.655	0.710	0.055	no
Treat-0d	RHSIM_Rhsim07G0141900	-0.153	0.612	0.766	no
Treat-0d	RHSIM_Rhsim07G0163200	0.076	-1.265	-1.341	no
Treat-0d	RHSIM_Rhsim07G0054800	1.418	1.817	0.399	high
Treat-0d	RHSIM_Rhsim07G0236700	-0.964	-1.348	-0.385	no
Treat-0d	RHSIM_Rhsim07G0179600	1.416	2.675	1.258	high
Treat-0d	RHSIM_Rhsim06G0198600	-3.827	0.704	4.531	low
Treat-0d	RHSIM_Rhsim06G0232600	0.125	2.375	2.251	no
Treat-0d	RHSIM_Rhsim07G0043400	-0.555	-0.885	-0.330	no
Treat-0d	RHSIM_Rhsim07G0018900	0.686	-1.012	-1.698	no
Treat-0d	RHSIM_Rhsim07G0028400	-0.483	-0.328	0.155	no
Treat-0d	RHSIM_Rhsim07G0072700	0.663	0.383	-0.280	no
Treat-0d	RHSIM_Rhsim07G0075600	-0.127	0.997	1.125	no
Treat-0d	RHSIM_Rhsim07G0155800	-1.228	-0.779	0.449	low
Treat-0d	RHSIM_Rhsim07G0039800	1.082	-0.265	-1.346	no
Treat-0d	RHSIM_Rhsim08G0001800	0.590	0.710	0.119	no
Treat-0d	RHSIM_Rhsim07G0024100	0.655	-0.297	-0.952	no
Treat-0d	RHSIM_Rhsim07G0012700	0.601	-0.798	-1.399	no
Treat-0d	RHSIM_Rhsim07G0048100	-0.187	1.057	1.244	no
Treat-0d	RHSIM_Rhsim07G0227800	1.862	0.311	-1.551	high
Treat-0d	RHSIM_Rhsim08G0022300	-1.368	-0.167	1.201	low

Group	Gene	PTR (z-score difference)	z(log2 LFQ)	z(log2 FPKM)	extreme-PTR
Treat-0d	RHSIM_Rhsim06G0063500	-1.257	-1.423	-0.166	low
Treat-0d	RHSIM_Rhsim07G0034100	-0.291	0.537	0.828	no
Treat-0d	RHSIM_Rhsim06G0003400	-0.385	-1.301	-0.916	no
Treat-0d	RHSIM_Rhsim06G0057600	-1.094	-0.795	0.299	low
Treat-0d	RHSIM_Rhsim06G0027900	-0.007	-0.142	-0.135	no
Treat-0d	RHSIM_Rhsim06G0018200	0.785	-0.447	-1.233	no
Treat-0d	RHSIM_Rhsim07G0161800	-1.756	-1.362	0.393	low
Treat-0d	RHSIM_Rhsim07G0257900	-0.513	-0.778	-0.264	no
Treat-0d	RHSIM_Rhsim08G0049700	0.534	1.095	0.561	no
Treat-0d	RHSIM_Rhsim06G0078300	0.607	1.093	0.486	no
Treat-0d	RHSIM_Rhsim06G0204200	0.119	0.024	-0.095	no
Treat-0d	RHSIM_Rhsim06G0241500	1.619	3.057	1.438	high
Treat-0d	RHSIM_Rhsim08G0237900	1.133	0.255	-0.878	high
Treat-0d	RHSIM_Rhsim06G0068400	-0.636	-1.451	-0.816	no
Treat-0d	RHSIM_Rhsim06G0199700	2.075	0.934	-1.140	high
Treat-0d	RHSIM_Rhsim07G0175700	0.417	1.049	0.632	no
Treat-0d	RHSIM_Rhsim07G0127500	0.149	1.812	1.663	no
Treat-0d	RHSIM_Rhsim06G0175200	1.261	0.984	-0.277	high
Treat-0d	RHSIM_Rhsim06G0078900	-2.409	-1.617	0.792	low
Treat-0d	RHSIM_Rhsim07G0061800	-0.559	-0.582	-0.022	no
Treat-0d	RHSIM_Rhsim07G0226200	0.631	0.629	-0.002	no
Treat-0d	RHSIM_Rhsim06G0232700	-0.223	-1.386	-1.163	no
Treat-0d	RHSIM_Rhsim07G0010400	-0.021	-0.737	-0.716	no
Treat-0d	RHSIM_Rhsim07G0084600	-3.032	-0.477	2.556	low
Treat-0d	RHSIM_Rhsim07G0075300	0.034	1.281	1.247	no
Treat-0d	RHSIM_Rhsim06G0067800	0.139	-1.312	-1.451	no
Treat-0d	RHSIM_Rhsim06G0091500	-0.220	1.491	1.711	no
Treat-0d	RHSIM_Rhsim06G0034000	-1.277	0.125	1.402	low
Treat-0d	RHSIM_Rhsim06G0142100	0.068	-0.760	-0.828	no
Treat-0d	RHSIM_Rhsim06G0000400	-1.109	-0.791	0.319	low
Treat-0d	RHSIM_Rhsim07G0190600	-2.147	-1.882	0.265	low
Treat-0d	RHSIM_Rhsim06G0012800	-0.150	0.316	0.466	no

Group	Gene	PTR (z-score difference)	z(log2 LFQ)	z(log2 FPKM)	extreme-PTR
Treat-0d	RHSIM_Rhsim06G0228900	0.047	-1.832	-1.879	no
Treat-0d	RHSIM_Rhsim06G0127100	0.436	2.398	1.962	no
Treat-0d	RHSIM_Rhsim07G0031000	-0.187	-0.359	-0.171	no
Treat-0d	RHSIM_Rhsim06G0227100	-0.811	-0.541	0.270	no
Treat-0d	RHSIM_Rhsim08G0144900	1.913	2.250	0.337	high
Treat-0d	RHSIM_Rhsim06G0074800	-1.075	-0.975	0.101	low
Treat-0d	RHSIM_Rhsim08G0057600	0.236	-0.806	-1.042	no
Treat-0d	RHSIM_Rhsim07G0085900	-1.372	-1.565	-0.192	low
Treat-0d	RHSIM_Rhsim08G0039600	-1.086	-0.582	0.504	low
Treat-0d	RHSIM_Rhsim08G0005300	1.282	2.035	0.753	high
Treat-0d	RHSIM_Rhsim07G0100500	0.367	0.158	-0.209	no
Treat-0d	RHSIM_Rhsim08G0062500	-0.223	-0.258	-0.036	no
Treat-0d	RHSIM_Rhsim06G0004000	0.366	-0.782	-1.148	no
Treat-0d	RHSIM_Rhsim06G0208100	1.781	0.228	-1.553	high
Treat-0d	RHSIM_Rhsim07G0168200	-0.332	0.449	0.782	no
Treat-0d	RHSIM_Rhsim06G0104800	-0.253	-0.527	-0.274	no
Treat-0d	RHSIM_Rhsim07G0136900	1.126	0.443	-0.682	high
Treat-0d	RHSIM_Rhsim06G0178500	-0.773	-0.628	0.145	no
Treat-0d	RHSIM_Rhsim06G0121900	1.428	2.165	0.736	high
Treat-0d	RHSIM_Rhsim06G0035800	-0.054	0.209	0.262	no
Treat-0d	RHSIM_Rhsim06G0059900	-2.640	-0.746	1.895	low
Treat-0d	RHSIM_Rhsim07G0044900	1.554	1.524	-0.030	high
Treat-0d	RHSIM_Rhsim06G0057100	-0.809	0.967	1.776	no
Treat-0d	RHSIM_Rhsim07G0028700	-0.172	0.199	0.371	no
Treat-0d	RHSIM_Rhsim06G0231500	-0.309	-0.956	-0.646	no
Treat-0d	RHSIM_Rhsim07G0076400	-0.929	-0.782	0.147	no
Treat-0d	RHSIM_Rhsim06G0219100	0.260	0.339	0.079	no
Treat-0d	RHSIM_Rhsim07G0013400	-0.241	0.348	0.589	no
Treat-0d	RHSIM_Rhsim07G0162300	0.927	1.535	0.608	no
Treat-0d	RHSIM_Rhsim07G0044800	-0.007	0.339	0.346	no
Treat-0d	RHSIM_Rhsim07G0214900	-0.283	0.276	0.559	no
Treat-0d	RHSIM_Rhsim06G0179200	0.987	0.087	-0.900	no

Group	Gene	PTR (z-score difference)	z(log2 LFQ)	z(log2 FPKM)	extreme-PTR
Treat-0d	RHSIM_Rhsim06G0011800	-0.078	2.856	2.934	no
Treat-0d	RHSIM_Rhsim08G0077200	-0.467	0.227	0.694	no
Treat-0d	RHSIM_Rhsim06G0152000	0.531	0.865	0.334	no
Treat-0d	RHSIM_Rhsim07G0048800	0.702	0.461	-0.241	no
Treat-0d	RHSIM_Rhsim06G0140100	0.548	-0.625	-1.173	no
Treat-0d	RHSIM_Rhsim06G0166100	1.287	0.351	-0.936	high
Treat-0d	RHSIM_Rhsim06G0035100	0.358	-1.048	-1.405	no
Treat-0d	RHSIM_Rhsim06G0232100	-1.092	-2.260	-1.168	low
Treat-0d	RHSIM_Rhsim06G0155400	-0.482	1.604	2.086	no
Treat-0d	RHSIM_Rhsim07G0103700	0.124	1.502	1.378	no
Treat-0d	RHSIM_Rhsim07G0247400	0.776	-0.069	-0.845	no
Treat-0d	RHSIM_Rhsim06G0009800	-0.680	-0.725	-0.045	no
Treat-0d	RHSIM_Rhsim07G0076600	-2.166	-0.202	1.964	low
Treat-0d	RHSIM_Rhsim06G0060000	0.080	-1.013	-1.093	no
Treat-0d	RHSIM_Rhsim07G0103800	-0.542	0.829	1.372	no
Treat-0d	RHSIM_Rhsim06G0017900	-0.149	-0.130	0.019	no
Treat-0d	RHSIM_Rhsim06G0224000	-1.240	0.927	2.167	low
Treat-0d	RHSIM_Rhsim06G0101600	0.500	0.638	0.138	no
Treat-0d	RHSIM_Rhsim08G0020700	-0.372	0.628	1.000	no
Treat-0d	RHSIM_Rhsim06G0005000	0.615	-0.543	-1.158	no
Treat-0d	RHSIM_Rhsim07G0018000	1.686	0.773	-0.913	high
Treat-0d	RHSIM_Rhsim08G0100900	-0.320	-0.786	-0.466	no
Treat-0d	RHSIM_Rhsim06G0243400	-0.883	-0.678	0.205	no
Treat-0d	RHSIM_Rhsim06G0061600	0.659	1.587	0.927	no
Treat-0d	RHSIM_Rhsim06G0204400	-0.113	-0.840	-0.726	no
Treat-0d	RHSIM_Rhsim06G0026000	-0.361	-1.584	-1.223	no
Treat-0d	RHSIM_Rhsim06G0137100	-0.068	0.939	1.007	no
Treat-0d	RHSIM_Rhsim07G0003800	0.819	0.235	-0.584	no
Treat-0d	RHSIM_Rhsim06G0013600	-0.723	-0.010	0.713	no
Treat-0d	RHSIM_Rhsim06G0129800	0.274	-0.634	-0.907	no
Treat-0d	RHSIM_Rhsim07G0033100	2.644	0.569	-2.075	high
Treat-0d	RHSIM_Rhsim07G0257000	0.678	1.260	0.582	no

Group	Gene	PTR (z-score difference)	z(log2 LFQ)	z(log2 FPKM)	extreme-PTR
Treat-0d	RHSIM_Rhsim06G0135500	-0.772	-0.802	-0.030	no
Treat-0d	RHSIM_Rhsim07G0139800	-0.554	0.529	1.084	no
Treat-0d	RHSIM_Rhsim08G0220400	0.420	0.191	-0.228	no
Treat-0d	RHSIM_Rhsim07G0018700	0.897	1.939	1.042	no
Treat-0d	RHSIM_Rhsim06G0224100	-0.379	-0.329	0.051	no
Treat-0d	RHSIM_Rhsim07G0079200	-0.238	-1.113	-0.875	no
Treat-0d	RHSIM_Rhsim08G0093000	-1.471	-0.925	0.546	low
Treat-0d	RHSIM_Rhsim07G0013600	0.594	1.741	1.147	no
Treat-0d	RHSIM_Rhsim07G0161400	0.748	-0.262	-1.010	no
Treat-0d	RHSIM_Rhsim06G0229000	-0.427	-0.407	0.019	no
Treat-0d	RHSIM_Rhsim07G0212200	-0.789	-0.770	0.019	no
Treat-0d	RHSIM_Rhsim06G0240700	3.005	0.791	-2.215	high
Treat-0d	RHSIM_Rhsim07G0166500	-0.452	0.823	1.275	no
Treat-0d	RHSIM_Rhsim07G0243700	-1.088	-0.047	1.041	low
Treat-0d	RHSIM_Rhsim06G0173600	-0.397	-1.144	-0.747	no
Treat-0d	RHSIM_Rhsim06G0196300	0.746	1.321	0.576	no
Treat-0d	RHSIM_Rhsim06G0240900	1.497	-0.214	-1.711	high
Treat-0d	RHSIM_Rhsim05G0002300	0.330	0.565	0.235	no
Treat-0d	RHSIM_Rhsim06G0055000	-2.260	-2.075	0.185	low
Treat-0d	RHSIM_Rhsim07G0079900	-0.297	1.212	1.510	no
Treat-0d	RHSIM_Rhsim06G0196500	-0.106	0.303	0.410	no
Treat-0d	RHSIM_Rhsim07G0258500	-0.373	-0.018	0.355	no
Treat-0d	RHSIM_Rhsim06G0164500	0.246	1.176	0.930	no
Treat-0d	RHSIM_Rhsim06G0040100	1.431	0.930	-0.501	high
Treat-0d	RHSIM_Rhsim06G0040800	-0.075	-0.265	-0.190	no
Treat-0d	RHSIM_Rhsim06G0114600	-0.220	0.345	0.565	no
Treat-0d	RHSIM_Rhsim07G0257400	0.768	1.008	0.240	no
Treat-0d	RHSIM_Rhsim06G0032500	-0.569	-0.681	-0.112	no
Treat-0d	RHSIM_Rhsim06G0175700	-1.047	-1.316	-0.269	low
Treat-0d	RHSIM_Rhsim06G0021200	0.479	-0.036	-0.515	no
Treat-0d	RHSIM_Rhsim06G0091400	-1.172	-1.062	0.110	low
Treat-0d	RHSIM_Rhsim06G0231200	0.148	-0.604	-0.752	no

Group	Gene	PTR (z-score difference)	z(log2 LFQ)	z(log2 FPKM)	extreme-PTR
Treat-0d	RHSIM_Rhsim06G0085000	0.514	-0.075	-0.589	no
Treat-0d	RHSIM_Rhsim07G0224300	-0.611	-0.416	0.195	no
Treat-0d	RHSIM_Rhsim07G0248700	0.240	-0.514	-0.754	no
Treat-0d	RHSIM_Rhsim06G0178700	-0.632	-0.540	0.093	no
Treat-0d	RHSIM_Rhsim06G0207600	0.036	-0.066	-0.102	no
Treat-0d	RHSIM_Rhsim06G0195200	-4.316	-1.350	2.966	low
Treat-0d	RHSIM_Rhsim07G0211600	0.476	-0.096	-0.573	no
Treat-0d	RHSIM_Rhsim05G0076900	-0.409	-0.727	-0.318	no
Treat-0d	RHSIM_Rhsim06G0178100	-0.525	-0.793	-0.268	no
Treat-0d	RHSIM_Rhsim06G0009500	-0.199	0.008	0.207	no
Treat-0d	RHSIM_Rhsim06G0216300	-2.105	-1.297	0.808	low
Treat-0d	RHSIM_Rhsim05G0232900	0.510	2.089	1.579	no
Treat-0d	RHSIM_Rhsim06G0008000	-0.560	-0.786	-0.227	no
Treat-0d	RHSIM_Rhsim06G0174400	0.331	-0.372	-0.703	no
Treat-0d	RHSIM_Rhsim06G0069300	-0.701	-0.123	0.578	no
Treat-0d	RHSIM_Rhsim06G0223700	-0.084	-0.196	-0.111	no
Treat-0d	RHSIM_Rhsim06G0000600	0.460	-0.769	-1.228	no
Treat-0d	RHSIM_Rhsim06G0146800	2.022	0.183	-1.839	high
Treat-0d	RHSIM_Rhsim06G0238600	1.850	1.278	-0.572	high
Treat-0d	RHSIM_Rhsim06G0211200	-1.856	-1.348	0.508	low
Treat-0d	RHSIM_Rhsim06G0225600	-0.595	-1.380	-0.785	no
Treat-0d	RHSIM_Rhsim07G0150900	0.063	-0.564	-0.627	no
Treat-0d	RHSIM_Rhsim06G0175900	0.480	0.292	-0.188	no
Treat-0d	RHSIM_Rhsim07G0064800	2.107	0.292	-1.815	high
Treat-0d	RHSIM_Rhsim05G0005000	1.219	0.443	-0.776	high
Treat-0d	RHSIM_Rhsim06G0073900	0.983	1.944	0.961	no
Treat-0d	RHSIM_Rhsim06G0056800	-1.089	-1.276	-0.187	low
Treat-0d	RHSIM_Rhsim07G0115200	-0.264	-0.927	-0.663	no
Treat-0d	RHSIM_Rhsim07G0029000	0.625	0.694	0.069	no
Treat-0d	RHSIM_Rhsim06G0197600	0.797	0.862	0.066	no
Treat-0d	RHSIM_Rhsim06G0236900	-0.257	-0.509	-0.251	no
Treat-0d	RHSIM_Rhsim06G0184100	-0.851	0.196	1.046	no

Group	Gene	PTR (z-score difference)	z(log2 LFQ)	z(log2 FPKM)	extreme-PTR
Treat-0d	RHSIM_Rhsim07G0250200	-1.346	-0.475	0.872	low
Treat-0d	RHSIM_Rhsim06G0224200	0.925	0.388	-0.537	no
Treat-0d	RHSIM_Rhsim06G0075100	-3.651	-0.848	2.803	low
Treat-0d	RHSIM_Rhsim05G0126500	0.483	0.513	0.030	no
Treat-0d	RHSIM_Rhsim06G0035600	-0.321	-0.784	-0.463	no
Treat-0d	RHSIM_Rhsim06G0027600	0.250	-0.341	-0.590	no
Treat-0d	RHSIM_Rhsim06G0241000	1.574	1.419	-0.155	high
Treat-0d	RHSIM_Rhsim06G0006500	1.348	0.036	-1.312	high
Treat-0d	RHSIM_Rhsim06G0207700	1.569	-0.079	-1.648	high
Treat-0d	RHSIM_Rhsim05G0045000	-1.530	-0.631	0.900	low
Treat-0d	RHSIM_Rhsim07G0084000	0.624	0.072	-0.552	no
Treat-0d	RHSIM_Rhsim05G0048300	0.948	-0.711	-1.659	no
Treat-0d	RHSIM_Rhsim06G0008400	-0.556	-0.078	0.478	no
Treat-0d	RHSIM_Rhsim05G0168000	0.595	-0.121	-0.716	no
Treat-0d	RHSIM_Rhsim07G0027800	0.800	1.238	0.438	no
Treat-0d	RHSIM_Rhsim06G0197200	-1.256	-1.015	0.241	low
Treat-0d	RHSIM_Rhsim07G0009400	-0.512	-1.299	-0.787	no
Treat-0d	RHSIM_Rhsim05G0132400	1.268	2.247	0.978	high
Treat-0d	RHSIM_Rhsim07G0022200	-0.556	-1.648	-1.091	no
Treat-0d	RHSIM_Rhsim06G0172900	-1.076	-1.412	-0.336	low
Treat-0d	RHSIM_Rhsim05G0073200	0.366	1.036	0.670	no
Treat-0d	RHSIM_Rhsim06G0242800	-1.141	-1.688	-0.547	low
Treat-0d	RHSIM_Rhsim07G0038600	-0.258	0.200	0.458	no
Treat-0d	RHSIM_Rhsim06G0052100	1.746	1.138	-0.608	high
Treat-0d	RHSIM_Rhsim06G0023100	-0.547	-1.180	-0.633	no
Treat-0d	RHSIM_Rhsim06G0214200	-0.782	-0.757	0.025	no
Treat-0d	RHSIM_Rhsim07G0121200	-2.218	-1.507	0.712	low
Treat-0d	RHSIM_Rhsim06G0054400	0.986	1.214	0.228	no
Treat-0d	RHSIM_Rhsim05G0018800	-0.812	0.047	0.859	no
Treat-0d	RHSIM_Rhsim06G0129900	-0.619	1.022	1.641	no
Treat-0d	RHSIM_Rhsim07G0008000	0.259	-0.510	-0.769	no
Treat-0d	RHSIM_Rhsim06G0186500	-0.185	-0.086	0.100	no

Group	Gene	PTR (z-score difference)	z(log2 LFQ)	z(log2 FPKM)	extreme-PTR
Treat-0d	RHSIM_Rhsim05G0115300	2.171	2.003	-0.169	high
Treat-0d	RHSIM_Rhsim06G0244600	-1.594	-1.390	0.204	low
Treat-0d	RHSIM_Rhsim05G0044300	-0.218	1.514	1.732	no
Treat-0d	RHSIM_Rhsim07G0174700	0.620	-0.563	-1.184	no
Treat-0d	RHSIM_Rhsim05G0060300	-0.542	-0.934	-0.392	no
Treat-0d	RHSIM_Rhsim07G0211400	-0.460	-0.359	0.101	no
Treat-0d	RHSIM_Rhsim06G0029700	-0.704	-0.277	0.427	no
Treat-0d	RHSIM_Rhsim05G0233100	0.227	-0.126	-0.352	no
Treat-0d	RHSIM_Rhsim06G0021700	0.139	1.603	1.464	no
Treat-0d	RHSIM_Rhsim05G0060600	-0.355	0.135	0.489	no
Treat-0d	RHSIM_Rhsim06G0031100	-0.472	-1.559	-1.087	no
Treat-0d	RHSIM_Rhsim06G0191800	-0.128	1.096	1.225	no
Treat-0d	RHSIM_Rhsim07G0119900	-0.067	0.432	0.499	no
Treat-0d	RHSIM_Rhsim05G0060400	0.047	0.261	0.215	no
Treat-0d	RHSIM_Rhsim06G0183300	-0.455	0.075	0.530	no
Treat-0d	RHSIM_Rhsim06G0018700	0.103	-0.857	-0.960	no
Treat-0d	RHSIM_Rhsim07G0240500	0.643	-0.107	-0.749	no
Treat-0d	RHSIM_Rhsim05G0187800	-0.281	1.222	1.503	no
Treat-0d	RHSIM_Rhsim06G0046300	0.775	1.224	0.449	no
Treat-0d	RHSIM_Rhsim05G0108500	-2.235	-1.715	0.520	low
Treat-0d	RHSIM_Rhsim05G0208000	-0.488	-0.513	-0.025	no
Treat-0d	RHSIM_Rhsim06G0070900	0.228	-0.772	-0.999	no
Treat-0d	RHSIM_Rhsim06G0018400	-0.728	-0.465	0.263	no
Treat-0d	RHSIM_Rhsim07G0154100	-1.023	0.255	1.277	no
Treat-0d	RHSIM_Rhsim06G0118100	-0.074	-0.640	-0.565	no
Treat-0d	RHSIM_Rhsim06G0200600	1.000	-0.084	-1.084	no
Treat-0d	RHSIM_Rhsim06G0051800	1.459	-0.142	-1.600	high
Treat-0d	RHSIM_Rhsim06G0097000	-0.593	-0.267	0.326	no
Treat-0d	RHSIM_Rhsim06G0069400	-0.546	0.035	0.582	no
Treat-0d	RHSIM_Rhsim05G0022700	1.023	1.057	0.034	no
Treat-0d	RHSIM_Rhsim06G0004600	0.620	3.105	2.484	no
Treat-0d	RHSIM_Rhsim06G0213400	0.655	-0.870	-1.525	no

Group	Gene	PTR (z-score difference)	z(log2 LFQ)	z(log2 FPKM)	extreme-PTR
Treat-0d	RHSIM_Rhsim06G0166800	-1.015	-0.057	0.958	no
Treat-0d	RHSIM_Rhsim06G0048000	1.026	1.107	0.081	no
Treat-0d	RHSIM_Rhsim05G0185900	0.698	0.263	-0.435	no
Treat-0d	RHSIM_Rhsim06G0121700	0.264	0.450	0.185	no
Treat-0d	RHSIM_Rhsim06G0238500	0.090	-0.233	-0.323	no
Treat-0d	RHSIM_Rhsim05G0003900	0.233	-0.466	-0.699	no
Treat-0d	RHSIM_Rhsim06G0216400	-0.379	0.147	0.526	no
Treat-0d	RHSIM_Rhsim05G0216700	-2.210	-0.947	1.263	low
Treat-0d	RHSIM_Rhsim06G0218400	0.376	0.506	0.130	no
Treat-0d	RHSIM_Rhsim06G0136000	0.892	1.737	0.845	no
Treat-0d	RHSIM_Rhsim05G0070300	0.126	0.027	-0.099	no
Treat-0d	RHSIM_Rhsim06G0004300	0.962	1.261	0.299	no
Treat-0d	RHSIM_Rhsim06G0175300	0.899	-0.667	-1.566	no
Treat-0d	RHSIM_Rhsim05G0222900	0.881	0.345	-0.535	no
Treat-0d	RHSIM_Rhsim06G0137300	0.410	0.926	0.516	no
Treat-0d	RHSIM_Rhsim05G0195200	-1.191	-1.059	0.131	low
Treat-0d	RHSIM_Rhsim06G0147000	-0.847	-1.323	-0.476	no
Treat-0d	RHSIM_Rhsim05G0104500	-1.136	0.055	1.191	low
Treat-0d	RHSIM_Rhsim05G0088300	-0.161	-0.553	-0.391	no
Treat-0d	RHSIM_Rhsim05G0122300	-0.519	-1.009	-0.490	no
Treat-0d	RHSIM_Rhsim06G0063800	0.200	-1.077	-1.278	no
Treat-0d	RHSIM_Rhsim07G0104300	0.706	0.249	-0.458	no
Treat-0d	RHSIM_Rhsim06G0213200	1.088	0.154	-0.934	no
Treat-0d	RHSIM_Rhsim06G0116100	0.449	0.006	-0.443	no
Treat-0d	RHSIM_Rhsim06G0201300	0.062	1.749	1.687	no
Treat-0d	RHSIM_Rhsim06G0153100	0.191	1.987	1.796	no
Treat-0d	RHSIM_Rhsim05G0177500	1.470	-0.378	-1.847	high
Treat-0d	RHSIM_Rhsim06G0129300	1.272	1.298	0.026	high
Treat-0d	RHSIM_Rhsim06G0232400	-2.067	-0.978	1.088	low
Treat-0d	RHSIM_Rhsim05G0172800	0.097	-1.074	-1.171	no
Treat-0d	RHSIM_Rhsim06G0054200	1.317	-1.044	-2.361	high
Treat-0d	RHSIM_Rhsim06G0165500	-1.351	-0.953	0.398	low

Group	Gene	PTR (z-score difference)	z(log2 LFQ)	z(log2 FPKM)	extreme-PTR
Treat-0d	RHSIM_Rhsim05G0197900	2.086	1.329	-0.757	high
Treat-0d	RHSIM_Rhsim06G0076700	-0.863	0.463	1.326	no
Treat-0d	RHSIM_Rhsim06G0224400	-0.236	-0.643	-0.407	no
Treat-0d	RHSIM_Rhsim06G0210200	-0.709	-0.204	0.506	no
Treat-0d	RHSIM_Rhsim06G0212800	1.139	0.816	-0.323	high
Treat-0d	RHSIM_Rhsim06G0092900	-0.469	-0.853	-0.384	no
Treat-0d	RHSIM_Rhsim05G0142400	-0.671	-0.747	-0.076	no
Treat-0d	RHSIM_Rhsim06G0239000	-0.719	0.018	0.737	no
Treat-0d	RHSIM_Rhsim06G0223600	0.049	-0.747	-0.796	no
Treat-0d	RHSIM_Rhsim05G0049000	1.349	0.280	-1.070	high
Treat-0d	RHSIM_Rhsim06G0157900	-1.692	-0.562	1.130	low
Treat-0d	RHSIM_Rhsim06G0062200	-0.871	-0.175	0.695	no
Treat-0d	RHSIM_Rhsim05G0229900	-0.705	-0.475	0.230	no
Treat-0d	RHSIM_Rhsim05G0029300	0.756	1.332	0.576	no
Treat-0d	RHSIM_Rhsim05G0112500	-0.005	0.599	0.604	no
Treat-0d	RHSIM_Rhsim05G0095600	0.741	-0.046	-0.787	no
Treat-0d	RHSIM_Rhsim06G0234000	0.212	-0.326	-0.538	no
Treat-0d	RHSIM_Rhsim05G0013100	-0.684	-0.423	0.261	no
Treat-0d	RHSIM_Rhsim05G0016600	-0.578	-0.725	-0.147	no
Treat-0d	RHSIM_Rhsim05G0073600	0.711	-0.812	-1.522	no
Treat-0d	RHSIM_Rhsim06G0065300	-0.398	-1.189	-0.792	no
Treat-0d	RHSIM_Rhsim05G0053200	0.338	0.535	0.197	no
Treat-0d	RHSIM_Rhsim05G0188100	-0.399	0.476	0.875	no
Treat-0d	RHSIM_Rhsim05G0132800	0.312	0.447	0.135	no
Treat-0d	RHSIM_Rhsim05G0032600	0.701	1.067	0.366	no
Treat-0d	RHSIM_Rhsim07G0091100	1.840	1.141	-0.699	high
Treat-0d	RHSIM_Rhsim06G0243100	-1.473	-0.498	0.975	low
Treat-0d	RHSIM_Rhsim05G0009900	0.010	0.331	0.321	no
Treat-0d	RHSIM_Rhsim05G0056900	-0.695	0.995	1.690	no
Treat-0d	RHSIM_Rhsim05G0230200	-0.100	0.345	0.445	no
Treat-0d	RHSIM_Rhsim06G0103100	-1.253	-0.892	0.361	low
Treat-0d	RHSIM_Rhsim06G0086200	-0.229	0.603	0.832	no

Group	Gene	PTR (z-score difference)	z(log2 LFQ)	z(log2 FPKM)	extreme-PTR
Treat-0d	RHSIM_Rhsim05G0167800	0.309	-0.589	-0.899	no
Treat-0d	RHSIM_Rhsim05G0092800	0.604	-0.784	-1.388	no
Treat-0d	RHSIM_Rhsim05G0005700	0.230	-0.012	-0.242	no
Treat-0d	RHSIM_Rhsim06G0099200	-0.151	0.048	0.199	no
Treat-0d	RHSIM_Rhsim06G0212400	-1.888	0.541	2.428	low
Treat-0d	RHSIM_Rhsim06G0166500	-0.530	0.470	1.000	no
Treat-0d	RHSIM_Rhsim06G0128200	-0.236	0.251	0.487	no
Treat-0d	RHSIM_Rhsim05G0148100	-0.892	-0.376	0.516	no
Treat-0d	RHSIM_Rhsim06G0166300	0.072	-0.471	-0.543	no
Treat-0d	RHSIM_Rhsim06G0067500	1.380	0.927	-0.452	high
Treat-0d	RHSIM_Rhsim05G0229000	-1.571	-0.155	1.416	low
Treat-0d	RHSIM_Rhsim06G0198500	0.359	0.418	0.059	no
Treat-0d	RHSIM_Rhsim05G0102000	-0.628	-1.020	-0.393	no
Treat-0d	RHSIM_Rhsim06G0022900	1.358	1.164	-0.194	high
Treat-0d	RHSIM_Rhsim05G0079000	-1.330	0.327	1.657	low
Treat-0d	RHSIM_Rhsim05G0184700	-0.244	-0.866	-0.622	no
Treat-0d	RHSIM_Rhsim06G0009700	2.365	0.750	-1.615	high
Treat-0d	RHSIM_Rhsim07G0188500	-0.494	-1.108	-0.614	no
Treat-0d	RHSIM_Rhsim05G0014500	-0.627	0.474	1.101	no
Treat-0d	RHSIM_Rhsim05G0004300	0.130	2.093	1.963	no
Treat-0d	RHSIM_Rhsim06G0202700	0.619	-0.163	-0.783	no
Treat-0d	RHSIM_Rhsim06G0148500	1.566	2.871	1.305	high
Treat-0d	RHSIM_Rhsim05G0206700	-2.079	-0.616	1.463	low
Treat-0d	RHSIM_Rhsim05G0142600	0.081	-0.612	-0.692	no
Treat-0d	RHSIM_Rhsim05G0079100	-1.374	-1.310	0.064	low
Treat-0d	RHSIM_Rhsim05G0220000	-1.181	-0.436	0.745	low
Treat-0d	RHSIM_Rhsim05G0039500	-0.039	1.219	1.258	no
Treat-0d	RHSIM_Rhsim05G0056600	-0.837	-0.352	0.485	no
Treat-0d	RHSIM_Rhsim05G0193900	0.020	2.044	2.024	no
Treat-0d	RHSIM_Rhsim05G0074500	0.299	0.342	0.043	no
Treat-0d	RHSIM_Rhsim07G0191000	-1.067	-0.821	0.246	low
Treat-0d	RHSIM_Rhsim06G0032600	0.340	0.135	-0.205	no

Group	Gene	PTR (z-score difference)	z(log2 LFQ)	z(log2 FPKM)	extreme-PTR
Treat-0d	RHSIM_Rhsim07G0222600	-0.443	-0.959	-0.516	no
Treat-0d	RHSIM_Rhsim06G0131600	-0.818	-0.886	-0.068	no
Treat-0d	RHSIM_Rhsim05G0055600	0.008	0.385	0.377	no
Treat-0d	RHSIM_Rhsim06G0230600	-1.437	-1.143	0.294	low
Treat-0d	RHSIM_Rhsim07G0246000	1.207	-0.042	-1.250	high
Treat-0d	RHSIM_Rhsim05G0165600	0.614	1.087	0.473	no
Treat-0d	RHSIM_Rhsim06G0211900	1.645	-0.103	-1.748	high
Treat-0d	RHSIM_Rhsim06G0048700	-0.291	-0.872	-0.581	no
Treat-0d	RHSIM_Rhsim05G0094900	-0.225	-1.209	-0.984	no
Treat-0d	RHSIM_Rhsim06G0031000	0.078	0.551	0.473	no
Treat-0d	RHSIM_Rhsim05G0170000	1.462	0.233	-1.229	high
Treat-0d	RHSIM_Rhsim06G0206300	1.683	1.414	-0.269	high
Treat-0d	RHSIM_Rhsim05G0114200	-1.623	-1.808	-0.186	low
Treat-0d	RHSIM_Rhsim06G0239600	0.899	2.151	1.253	no
Treat-0d	RHSIM_Rhsim06G0090800	-1.070	-0.617	0.453	low
Treat-0d	RHSIM_Rhsim06G0087600	-0.570	-0.239	0.332	no
Treat-0d	RHSIM_Rhsim05G0172300	-1.870	-1.679	0.191	low
Treat-0d	RHSIM_Rhsim06G0179300	0.357	-0.861	-1.219	no
Treat-0d	RHSIM_Rhsim07G0095500	-0.757	-0.906	-0.149	no
Treat-0d	RHSIM_Rhsim05G0195100	-0.565	-0.584	-0.019	no
Treat-0d	RHSIM_Rhsim05G0107000	0.962	0.324	-0.638	no
Treat-0d	RHSIM_Rhsim06G0206000	-0.451	-0.254	0.197	no
Treat-0d	RHSIM_Rhsim06G0103300	0.447	3.437	2.990	no
Treat-0d	RHSIM_Rhsim05G0075800	2.137	1.347	-0.790	high
Treat-0d	RHSIM_Rhsim05G0175300	0.144	0.945	0.801	no
Treat-0d	RHSIM_Rhsim07G0020100	-2.320	-1.660	0.660	low
Treat-0d	RHSIM_Rhsim05G0076200	0.590	-0.170	-0.761	no
Treat-0d	RHSIM_Rhsim06G0232500	1.494	0.565	-0.930	high
Treat-0d	RHSIM_Rhsim05G0016500	-0.398	-0.946	-0.548	no
Treat-0d	RHSIM_Rhsim06G0141900	-0.506	-0.725	-0.219	no
Treat-0d	RHSIM_Rhsim05G0038200	0.652	1.204	0.552	no
Treat-0d	RHSIM_Rhsim05G0048000	0.304	-1.120	-1.425	no

Group	Gene	PTR (z-score difference)	z(log2 LFQ)	z(log2 FPKM)	extreme-PTR
Treat-0d	RHSIM_Rhsim06G0208300	-1.523	-0.318	1.205	low
Treat-0d	RHSIM_Rhsim06G0008500	-0.176	1.795	1.971	no
Treat-0d	RHSIM_Rhsim05G0101600	-1.055	-0.398	0.657	low
Treat-0d	RHSIM_Rhsim05G0148700	0.421	0.231	-0.191	no
Treat-0d	RHSIM_Rhsim06G0003100	-0.840	-1.456	-0.617	no
Treat-0d	RHSIM_Rhsim05G0056500	-0.883	1.213	2.096	no
Treat-0d	RHSIM_Rhsim05G0114100	0.124	-0.708	-0.832	no
Treat-0d	RHSIM_Rhsim06G0006600	0.336	1.325	0.989	no
Treat-0d	RHSIM_Rhsim05G0078100	-1.911	-1.360	0.551	low
Treat-0d	RHSIM_Rhsim06G0027100	-1.429	1.041	2.470	low
Treat-0d	RHSIM_Rhsim05G0081000	-2.065	-2.144	-0.079	low
Treat-0d	RHSIM_Rhsim05G0081600	-1.625	0.039	1.664	low
Treat-0d	RHSIM_Rhsim06G0170000	1.113	-0.566	-1.679	high
Treat-0d	RHSIM_Rhsim05G0007700	0.078	-0.835	-0.913	no
Treat-0d	RHSIM_Rhsim05G0142500	-1.307	-0.558	0.749	low
Treat-0d	RHSIM_Rhsim05G0015000	-1.158	0.530	1.688	low
Treat-0d	RHSIM_Rhsim05G0040400	0.037	-0.214	-0.251	no
Treat-0d	RHSIM_Rhsim06G0184400	0.308	-0.092	-0.400	no
Treat-0d	RHSIM_Rhsim05G0034500	-0.186	-0.876	-0.690	no
Treat-0d	RHSIM_Rhsim05G0125700	1.063	0.887	-0.176	no
Treat-0d	RHSIM_Rhsim05G0234300	-2.527	0.104	2.631	low
Treat-0d	RHSIM_Rhsim06G0176900	0.488	0.309	-0.180	no
Treat-0d	RHSIM_Rhsim07G0136800	0.472	-0.328	-0.799	no
Treat-0d	RHSIM_Rhsim06G0182000	1.502	-0.663	-2.165	high
Treat-0d	RHSIM_Rhsim05G0228100	-0.435	0.084	0.519	no
Treat-0d	RHSIM_Rhsim06G0062000	0.295	0.229	-0.066	no
Treat-0d	RHSIM_Rhsim05G0203100	-0.126	-0.298	-0.173	no
Treat-0d	RHSIM_Rhsim05G0094000	-0.666	-1.069	-0.403	no
Treat-0d	RHSIM_Rhsim05G0178300	0.341	0.109	-0.232	no
Treat-0d	RHSIM_Rhsim05G0160100	0.050	0.763	0.713	no
Treat-0d	RHSIM_Rhsim06G0089200	-0.950	-1.056	-0.107	no
Treat-0d	RHSIM_Rhsim06G0022200	1.010	0.196	-0.814	no

Group	Gene	PTR (z-score difference)	z(log2 LFQ)	z(log2 FPKM)	extreme-PTR
Treat-0d	RHSIM_Rhsim05G0048900	-2.115	-1.777	0.338	low
Treat-0d	RHSIM_Rhsim05G0022400	0.546	0.281	-0.265	no
Treat-0d	RHSIM_Rhsim05G0167700	-0.519	-1.054	-0.535	no
Treat-0d	RHSIM_Rhsim05G0203300	-0.944	-0.552	0.393	no
Treat-0d	RHSIM_Rhsim05G0013700	0.260	-1.002	-1.262	no
Treat-0d	RHSIM_Rhsim05G0082400	3.205	1.840	-1.365	high
Treat-0d	RHSIM_Rhsim05G0214500	0.777	1.476	0.699	no
Treat-0d	RHSIM_Rhsim04G0249900	0.186	-0.348	-0.533	no
Treat-0d	RHSIM_Rhsim05G0141700	1.012	0.190	-0.822	no
Treat-0d	RHSIM_Rhsim07G0093500	0.586	0.008	-0.578	no
Treat-0d	RHSIM_Rhsim06G0039400	-1.809	0.939	2.748	low
Treat-0d	RHSIM_Rhsim04G0061200	1.394	0.176	-1.218	high
Treat-0d	RHSIM_Rhsim05G0057000	0.402	-0.076	-0.478	no
Treat-0d	RHSIM_Rhsim06G0052200	1.683	0.161	-1.521	high
Treat-0d	RHSIM_Rhsim05G0035400	0.155	-0.044	-0.199	no
Treat-0d	RHSIM_Rhsim04G0207200	-0.438	-0.532	-0.094	no
Treat-0d	RHSIM_Rhsim06G0183900	0.917	0.797	-0.119	no
Treat-0d	RHSIM_Rhsim05G0179700	0.717	1.490	0.773	no
Treat-0d	RHSIM_Rhsim05G0055000	0.285	-0.036	-0.321	no
Treat-0d	RHSIM_Rhsim05G0001900	-2.094	-1.880	0.214	low
Treat-0d	RHSIM_Rhsim06G0234700	-1.263	0.103	1.366	low
Treat-0d	RHSIM_Rhsim04G0179500	-0.848	-1.199	-0.351	no
Treat-0d	RHSIM_Rhsim05G0141900	0.508	0.434	-0.074	no
Treat-0d	RHSIM_Rhsim05G0165000	0.764	-0.471	-1.235	no
Treat-0d	RHSIM_Rhsim05G0169000	-0.231	-0.978	-0.747	no
Treat-0d	RHSIM_Rhsim06G0060300	0.412	0.549	0.136	no
Treat-0d	RHSIM_Rhsim06G0030800	0.592	1.645	1.052	no
Treat-0d	RHSIM_Rhsim05G0178900	-0.347	0.359	0.706	no
Treat-0d	RHSIM_Rhsim06G0139200	2.193	2.761	0.569	high
Treat-0d	RHSIM_Rhsim05G0001300	0.259	-0.098	-0.357	no
Treat-0d	RHSIM_Rhsim05G0119100	-0.655	-0.912	-0.257	no
Treat-0d	RHSIM_Rhsim05G0233200	0.254	0.816	0.562	no

Group	Gene	PTR (z-score difference)	z(log2 LFQ)	z(log2 FPKM)	extreme-PTR
Treat-0d	RHSIM_Rhsim05G0141500	-3.756	-0.736	3.020	low
Treat-0d	RHSIM_Rhsim06G0114100	-0.010	0.143	0.154	no
Treat-0d	RHSIM_Rhsim07G0044200	-0.059	-1.004	-0.945	no
Treat-0d	RHSIM_Rhsim06G0071600	1.291	0.205	-1.085	high
Treat-0d	RHSIM_Rhsim05G0067900	-0.557	-1.280	-0.723	no
Treat-0d	RHSIM_Rhsim05G0161500	0.008	-0.611	-0.619	no
Treat-0d	RHSIM_Rhsim07G0149700	0.206	-0.381	-0.588	no
Treat-0d	RHSIM_Rhsim05G0203500	0.820	1.334	0.514	no
Treat-0d	RHSIM_Rhsim06G0047300	0.072	0.299	0.227	no
Treat-0d	RHSIM_Rhsim05G0028200	1.444	0.099	-1.344	high
Treat-0d	RHSIM_Rhsim04G0153900	1.167	0.633	-0.533	high
Treat-0d	RHSIM_Rhsim05G0122100	-2.134	-1.104	1.030	low
Treat-0d	RHSIM_Rhsim07G0213300	1.017	0.785	-0.232	no
Treat-0d	RHSIM_Rhsim05G0221100	0.617	-0.732	-1.348	no
Treat-0d	RHSIM_Rhsim06G0238700	-1.405	-1.545	-0.140	low
Treat-0d	RHSIM_Rhsim04G0180900	-0.186	0.779	0.965	no
Treat-0d	RHSIM_Rhsim07G0102700	0.739	1.023	0.284	no
Treat-0d	RHSIM_Rhsim04G0213100	1.635	1.710	0.075	high
Treat-0d	RHSIM_Rhsim05G0228600	0.454	1.396	0.942	no
Treat-0d	RHSIM_Rhsim05G0018700	0.902	1.055	0.153	no
Treat-0d	RHSIM_Rhsim06G0203000	-0.811	0.383	1.194	no
Treat-0d	RHSIM_Rhsim06G0096300	-0.684	0.214	0.897	no
Treat-0d	RHSIM_Rhsim05G0219700	-0.646	-0.649	-0.003	no
Treat-0d	RHSIM_Rhsim06G0187600	1.517	0.231	-1.285	high
Treat-0d	RHSIM_Rhsim05G0115900	0.092	0.341	0.249	no
Treat-0d	RHSIM_Rhsim05G0168400	-0.336	-1.047	-0.711	no
Treat-0d	RHSIM_Rhsim05G0093000	0.136	-0.086	-0.222	no
Treat-0d	RHSIM_Rhsim05G0141400	-1.824	-1.812	0.012	low
Treat-0d	RHSIM_Rhsim05G0216400	-0.128	0.616	0.745	no
Treat-0d	RHSIM_Rhsim04G0117500	-0.551	-1.636	-1.085	no
Treat-0d	RHSIM_Rhsim05G0148400	-0.860	-1.019	-0.159	no
Treat-0d	RHSIM_Rhsim06G0086800	-1.157	-1.400	-0.243	low

Group	Gene	PTR (z-score difference)	z(log2 LFQ)	z(log2 FPKM)	extreme-PTR
Treat-0d	RHSIM_Rhsim06G0176800	1.242	-0.580	-1.823	high
Treat-0d	RHSIM_Rhsim07G0176500	-0.053	-0.094	-0.041	no
Treat-0d	RHSIM_Rhsim05G0107700	-1.230	-0.349	0.881	low
Treat-0d	RHSIM_Rhsim05G0068100	1.296	1.349	0.053	high
Treat-0d	RHSIM_Rhsim05G0146600	-1.456	-1.484	-0.028	low
Treat-0d	RHSIM_Rhsim04G0058800	-0.165	-0.621	-0.456	no
Treat-0d	RHSIM_Rhsim05G0070600	0.945	-0.150	-1.095	no
Treat-0d	RHSIM_Rhsim05G0104400	-0.636	0.541	1.177	no
Treat-0d	RHSIM_Rhsim05G0047800	0.539	-0.681	-1.220	no
Treat-0d	RHSIM_Rhsim05G0012600	-3.610	-1.904	1.706	low
Treat-0d	RHSIM_Rhsim06G0244700	0.288	-0.209	-0.497	no
Treat-0d	RHSIM_Rhsim06G0022400	0.743	-0.682	-1.424	no
Treat-0d	RHSIM_Rhsim07G0057400	0.119	-0.239	-0.358	no
Treat-0d	RHSIM_Rhsim05G0070100	-1.870	-0.110	1.760	low
Treat-0d	RHSIM_Rhsim05G0169700	0.557	3.161	2.605	no
Treat-0d	RHSIM_Rhsim04G0131900	0.979	1.101	0.122	no
Treat-0d	RHSIM_Rhsim06G0047100	0.929	-0.629	-1.558	no
Treat-0d	RHSIM_Rhsim05G0166700	1.063	1.013	-0.051	no
Treat-0d	RHSIM_Rhsim05G0223800	0.544	2.261	1.717	no
Treat-0d	RHSIM_Rhsim05G0020400	-1.461	-0.440	1.021	low
Treat-0d	RHSIM_Rhsim05G0196900	1.747	1.121	-0.626	high
Treat-0d	RHSIM_Rhsim06G0078800	0.439	1.915	1.477	no
Treat-0d	RHSIM_Rhsim05G0190800	-1.965	0.111	2.076	low
Treat-0d	RHSIM_Rhsim05G0069700	0.562	0.406	-0.155	no
Treat-0d	RHSIM_Rhsim05G0074000	-0.188	2.089	2.277	no
Treat-0d	RHSIM_Rhsim05G0150000	-0.648	-0.355	0.293	no
Treat-0d	RHSIM_Rhsim05G0096900	0.619	0.573	-0.046	no
Treat-0d	RHSIM_Rhsim06G0005300	0.037	-0.042	-0.079	no
Treat-0d	RHSIM_Rhsim05G0101100	0.146	-1.162	-1.307	no
Treat-0d	RHSIM_Rhsim05G0170400	0.494	0.845	0.351	no
Treat-0d	RHSIM_Rhsim05G0073500	-1.033	-0.054	0.979	low
Treat-0d	RHSIM_Rhsim06G0220300	-0.308	-0.116	0.192	no

Group	Gene	PTR (z-score difference)	z(log2 LFQ)	z(log2 FPKM)	extreme-PTR
Treat-0d	RHSIM_Rhsim05G0182300	-0.371	-0.583	-0.212	no
Treat-0d	RHSIM_Rhsim07G0058500	0.493	0.296	-0.197	no
Treat-0d	RHSIM_Rhsim04G0175300	-0.568	-0.642	-0.074	no
Treat-0d	RHSIM_Rhsim05G0062700	1.016	-0.387	-1.403	no
Treat-0d	RHSIM_Rhsim05G0075900	-1.197	0.180	1.377	low
Treat-0d	RHSIM_Rhsim05G0103400	1.497	1.113	-0.384	high
Treat-0d	RHSIM_Rhsim04G0099500	-1.878	-1.282	0.595	low
Treat-0d	RHSIM_Rhsim05G0159700	-1.165	-0.105	1.060	low
Treat-0d	RHSIM_Rhsim05G0085900	0.913	0.332	-0.580	no
Treat-0d	RHSIM_Rhsim05G0020500	-0.155	-1.064	-0.909	no
Treat-0d	RHSIM_Rhsim05G0177100	0.561	0.790	0.229	no
Treat-0d	RHSIM_Rhsim05G0180300	1.530	-0.785	-2.315	high
Treat-0d	RHSIM_Rhsim04G0126100	-0.349	-0.698	-0.349	no
Treat-0d	RHSIM_Rhsim06G0049900	0.161	-0.319	-0.480	no
Treat-0d	RHSIM_Rhsim05G0080100	-0.382	0.254	0.636	no
Treat-0d	RHSIM_Rhsim06G0002600	-0.395	-0.867	-0.473	no
Treat-0d	RHSIM_Rhsim05G0201700	0.278	-0.545	-0.823	no
Treat-0d	RHSIM_Rhsim05G0143500	-1.636	-0.829	0.807	low
Treat-0d	RHSIM_Rhsim05G0074600	-0.488	0.031	0.520	no
Treat-0d	RHSIM_Rhsim06G0142000	-0.967	-1.142	-0.175	no
Treat-0d	RHSIM_Rhsim05G0040900	-0.971	-1.647	-0.676	no
Treat-0d	RHSIM_Rhsim05G0005100	1.374	-0.217	-1.591	high
Treat-0d	RHSIM_Rhsim05G0214100	0.616	1.391	0.775	no
Treat-0d	RHSIM_Rhsim05G0222100	-0.056	-1.082	-1.026	no
Treat-0d	RHSIM_Rhsim07G0247000	0.406	0.471	0.064	no
Treat-0d	RHSIM_Rhsim06G0082000	-0.060	-0.188	-0.129	no
Treat-0d	RHSIM_Rhsim05G0187300	-0.002	0.543	0.545	no
Treat-0d	RHSIM_Rhsim05G0143000	1.998	1.781	-0.216	high
Treat-0d	RHSIM_Rhsim05G0063700	0.903	0.881	-0.021	no
Treat-0d	RHSIM_Rhsim04G0132500	0.633	0.993	0.361	no
Treat-0d	RHSIM_Rhsim05G0072000	0.388	-0.158	-0.546	no
Treat-0d	RHSIM_Rhsim04G0011300	0.310	-0.123	-0.434	no

Group	Gene	PTR (z-score difference)	z(log2 LFQ)	z(log2 FPKM)	extreme-PTR
Treat-0d	RHSIM_Rhsim05G0110900	1.676	0.812	-0.864	high
Treat-0d	RHSIM_Rhsim05G0106700	-0.258	0.110	0.368	no
Treat-0d	RHSIM_Rhsim04G0028000	2.018	1.205	-0.813	high
Treat-0d	RHSIM_Rhsim06G0054600	-0.923	-0.698	0.225	no
Treat-0d	RHSIM_Rhsim05G0029400	-0.568	-0.453	0.115	no
Treat-0d	RHSIM_Rhsim04G0009500	0.035	-1.050	-1.085	no
Treat-0d	RHSIM_Rhsim05G0203000	-1.186	0.544	1.730	low
Treat-0d	RHSIM_Rhsim06G0047000	0.114	-0.261	-0.375	no
Treat-0d	RHSIM_Rhsim05G0113900	0.947	0.660	-0.287	no
Treat-0d	RHSIM_Rhsim05G0012900	0.598	0.338	-0.260	no
Treat-0d	RHSIM_Rhsim04G0014200	0.751	-0.156	-0.908	no
Treat-0d	RHSIM_Rhsim06G0017600	-0.309	-0.049	0.259	no
Treat-0d	RHSIM_Rhsim05G0086100	-1.767	-1.419	0.347	low
Treat-0d	RHSIM_Rhsim04G0002900	0.487	-0.146	-0.633	no
Treat-0d	RHSIM_Rhsim05G0068700	-1.311	-0.001	1.310	low
Treat-0d	RHSIM_Rhsim05G0233600	0.824	-0.023	-0.846	no
Treat-0d	RHSIM_Rhsim06G0023700	-0.389	-0.845	-0.457	no
Treat-0d	RHSIM_Rhsim04G0241700	-1.068	-0.143	0.925	low
Treat-0d	RHSIM_Rhsim04G0106600	0.145	1.083	0.938	no
Treat-0d	RHSIM_Rhsim04G0190600	-1.216	0.717	1.934	low
Treat-0d	RHSIM_Rhsim06G0199000	1.280	-0.480	-1.760	high
Treat-0d	RHSIM_Rhsim05G0027700	-1.297	-0.517	0.780	low
Treat-0d	RHSIM_Rhsim04G0148800	-0.706	-0.942	-0.236	no
Treat-0d	RHSIM_Rhsim07G0039400	0.376	1.118	0.742	no
Treat-0d	RHSIM_Rhsim04G0058200	0.226	-0.023	-0.249	no
Treat-0d	RHSIM_Rhsim04G0126600	-0.249	-0.524	-0.275	no
Treat-0d	RHSIM_Rhsim05G0216600	-0.986	-0.801	0.185	no
Treat-0d	RHSIM_Rhsim05G0084000	-0.973	-1.655	-0.682	no
Treat-0d	RHSIM_Rhsim05G0030000	0.493	-0.093	-0.586	no
Treat-0d	RHSIM_Rhsim04G0196900	-0.066	0.134	0.201	no
Treat-0d	RHSIM_Rhsim05G0195600	2.626	0.527	-2.098	high
Treat-0d	RHSIM_Rhsim07G0245300	1.437	0.234	-1.204	high

Group	Gene	PTR (z-score difference)	z(log2 LFQ)	z(log2 FPKM)	extreme-PTR
Treat-0d	RHSIM_Rhsim04G0170600	-1.926	-1.202	0.724	low
Treat-0d	RHSIM_Rhsim04G0216600	1.254	0.507	-0.748	high
Treat-0d	RHSIM_Rhsim05G0067400	1.754	1.422	-0.332	high
Treat-0d	RHSIM_Rhsim04G0074000	-0.760	-0.340	0.420	no
Treat-0d	RHSIM_Rhsim05G0075200	-1.513	-1.063	0.451	low
Treat-0d	RHSIM_Rhsim05G0145700	0.710	0.540	-0.170	no
Treat-0d	RHSIM_Rhsim05G0175100	-0.801	-1.317	-0.516	no
Treat-0d	RHSIM_Rhsim04G0205900	-1.660	-0.023	1.637	low
Treat-0d	RHSIM_Rhsim05G0135800	0.347	0.799	0.452	no
Treat-0d	RHSIM_Rhsim05G0232100	-0.667	-0.078	0.589	no
Treat-0d	RHSIM_Rhsim05G0074800	-0.511	-0.390	0.122	no
Treat-0d	RHSIM_Rhsim05G0023600	2.064	0.737	-1.328	high
Treat-0d	RHSIM_Rhsim05G0096100	-2.148	-1.419	0.729	low
Treat-0d	RHSIM_Rhsim06G0064800	-0.168	0.653	0.821	no
Treat-0d	RHSIM_Rhsim05G0044600	0.994	0.808	-0.186	no
Treat-0d	RHSIM_Rhsim05G0192400	-0.357	-1.302	-0.945	no
Treat-0d	RHSIM_Rhsim05G0095400	1.089	1.371	0.282	no
Treat-0d	RHSIM_Rhsim05G0194900	0.048	1.257	1.209	no
Treat-0d	RHSIM_Rhsim04G0012800	-0.408	0.649	1.057	no
Treat-0d	RHSIM_Rhsim04G0034900	-1.548	-1.487	0.061	low
Treat-0d	RHSIM_Rhsim05G0078300	0.527	-0.094	-0.621	no
Treat-0d	RHSIM_Rhsim05G0000900	0.015	-0.044	-0.059	no
Treat-0d	RHSIM_Rhsim04G0014400	2.176	0.204	-1.972	high
Treat-0d	RHSIM_Rhsim05G0227300	0.968	-0.678	-1.646	no
Treat-0d	RHSIM_Rhsim04G0015900	-1.287	-0.491	0.797	low
Treat-0d	RHSIM_Rhsim05G0164500	0.107	1.680	1.573	no
Treat-0d	RHSIM_Rhsim04G0016200	0.304	0.638	0.333	no
Treat-0d	RHSIM_Rhsim07G0101200	0.477	0.905	0.428	no
Treat-0d	RHSIM_Rhsim04G0073400	-0.827	-1.310	-0.484	no
Treat-0d	RHSIM_Rhsim05G0152100	0.131	-0.247	-0.378	no
Treat-0d	RHSIM_Rhsim04G0029500	0.332	1.366	1.034	no
Treat-0d	RHSIM_Rhsim05G0044900	-0.662	-0.777	-0.114	no

Group	Gene	PTR (z-score difference)	z(log2 LFQ)	z(log2 FPKM)	extreme-PTR
Treat-0d	RHSIM_Rhsim04G0099700	-0.579	-0.360	0.219	no
Treat-0d	RHSIM_Rhsim05G0202600	-0.931	-1.081	-0.150	no
Treat-0d	RHSIM_Rhsim05G0133100	0.768	0.301	-0.467	no
Treat-0d	RHSIM_Rhsim04G0003800	0.098	-0.217	-0.315	no
Treat-0d	RHSIM_Rhsim05G0210500	0.492	-0.837	-1.329	no
Treat-0d	RHSIM_Rhsim07G0175300	0.699	0.997	0.298	no
Treat-0d	RHSIM_Rhsim04G0087500	-0.420	-0.456	-0.036	no
Treat-0d	RHSIM_Rhsim05G0199700	0.019	0.123	0.104	no
Treat-0d	RHSIM_Rhsim05G0165700	0.189	1.473	1.284	no
Treat-0d	RHSIM_Rhsim05G0139600	1.244	-0.197	-1.441	high
Treat-0d	RHSIM_Rhsim04G0145800	-1.472	0.097	1.569	low
Treat-0d	RHSIM_Rhsim05G0063100	-1.070	-0.043	1.027	low
Treat-0d	RHSIM_Rhsim04G0199100	1.300	2.039	0.739	high
Treat-0d	RHSIM_Rhsim04G0033100	-0.173	2.107	2.281	no
Treat-0d	RHSIM_Rhsim04G0169600	0.522	1.024	0.502	no
Treat-0d	RHSIM_Rhsim05G0192600	-6.311	-2.273	4.039	low
Treat-0d	RHSIM_Rhsim05G0067700	-2.081	-0.913	1.168	low
Treat-0d	RHSIM_Rhsim05G0159600	1.133	1.398	0.265	high
Treat-0d	RHSIM_Rhsim04G0013600	-1.505	0.358	1.863	low
Treat-0d	RHSIM_Rhsim05G0022000	-0.527	-1.525	-0.999	no
Treat-0d	RHSIM_Rhsim04G0124900	-0.971	0.432	1.404	no
Treat-0d	RHSIM_Rhsim05G0175000	-0.094	-0.396	-0.301	no
Treat-0d	RHSIM_Rhsim05G0133000	0.566	-1.528	-2.094	no
Treat-0d	RHSIM_Rhsim05G0121700	-1.584	-0.141	1.443	low
Treat-0d	RHSIM_Rhsim04G0100900	-0.798	0.622	1.421	no
Treat-0d	RHSIM_Rhsim07G0134900	0.551	2.805	2.254	no
Treat-0d	RHSIM_Rhsim05G0072400	-1.019	-0.815	0.204	no
Treat-0d	RHSIM_Rhsim04G0114200	0.797	-0.602	-1.398	no
Treat-0d	RHSIM_Rhsim04G0119400	1.810	1.741	-0.069	high
Treat-0d	RHSIM_Rhsim05G0082300	0.677	-1.409	-2.087	no
Treat-0d	RHSIM_Rhsim04G0065900	0.938	0.474	-0.464	no
Treat-0d	RHSIM_Rhsim04G0134300	-0.059	0.971	1.029	no

Group	Gene	PTR (z-score difference)	z(log2 LFQ)	z(log2 FPKM)	extreme-PTR
Treat-0d	RHSIM_Rhsim05G0148800	-1.290	-2.078	-0.788	low
Treat-0d	RHSIM_Rhsim07G0101600	0.638	-0.250	-0.888	no
Treat-0d	RHSIM_Rhsim05G0212900	2.478	3.919	1.441	high
Treat-0d	RHSIM_Rhsim07G0167800	0.147	-1.027	-1.174	no
Treat-0d	RHSIM_Rhsim05G0084700	-1.030	-0.742	0.288	low
Treat-0d	RHSIM_Rhsim04G0150100	-1.633	-1.565	0.068	low
Treat-0d	RHSIM_Rhsim05G0111000	0.122	-0.577	-0.699	no
Treat-0d	RHSIM_Rhsim04G0245000	-0.535	-0.882	-0.347	no
Treat-0d	RHSIM_Rhsim04G0009400	0.343	-0.255	-0.598	no
Treat-0d	RHSIM_Rhsim05G0083700	-0.193	-0.668	-0.475	no
Treat-0d	RHSIM_Rhsim05G0229700	0.019	-0.153	-0.171	no
Treat-0d	RHSIM_Rhsim04G0014300	1.519	0.723	-0.796	high
Treat-0d	RHSIM_Rhsim05G0053600	1.845	-0.327	-2.172	high
Treat-0d	RHSIM_Rhsim05G0095000	0.332	0.240	-0.092	no
Treat-0d	RHSIM_Rhsim04G0096600	1.012	-0.390	-1.402	no
Treat-0d	RHSIM_Rhsim05G0090100	-0.060	-0.404	-0.344	no
Treat-0d	RHSIM_Rhsim07G0059400	0.442	0.713	0.272	no
Treat-0d	RHSIM_Rhsim04G0023700	0.723	1.482	0.758	no
Treat-0d	RHSIM_Rhsim05G0216800	-0.959	-0.186	0.773	no
Treat-0d	RHSIM_Rhsim05G0101800	-0.621	-0.352	0.269	no
Treat-0d	RHSIM_Rhsim04G0213000	0.327	-0.762	-1.089	no
Treat-0d	RHSIM_Rhsim05G0039000	2.051	0.266	-1.785	high
Treat-0d	RHSIM_Rhsim05G0192200	0.460	1.193	0.734	no
Treat-0d	RHSIM_Rhsim07G0008100	-2.327	-0.673	1.654	low
Treat-0d	RHSIM_Rhsim05G0180600	0.606	-0.021	-0.627	no
Treat-0d	RHSIM_Rhsim04G0202900	-0.582	0.269	0.851	no
Treat-0d	RHSIM_Rhsim04G0084300	1.535	-0.552	-2.087	high
Treat-0d	RHSIM_Rhsim04G0184400	1.091	0.885	-0.207	no
Treat-0d	RHSIM_Rhsim07G0010700	0.286	0.116	-0.169	no
Treat-0d	RHSIM_Rhsim04G0228600	0.499	-0.571	-1.070	no
Treat-0d	RHSIM_Rhsim05G0062300	0.211	-1.113	-1.323	no
Treat-0d	RHSIM_Rhsim07G0005600	-0.491	-1.084	-0.593	no

Group	Gene	PTR (z-score difference)	z(log2 LFQ)	z(log2 FPKM)	extreme-PTR
Treat-0d	RHSIM_Rhsim04G0248100	0.578	0.538	-0.039	no
Treat-0d	RHSIM_Rhsim07G0031200	-0.090	0.665	0.754	no
Treat-0d	RHSIM_Rhsim05G0105400	-0.482	0.200	0.682	no
Treat-0d	RHSIM_Rhsim04G0039600	-0.372	-1.323	-0.951	no
Treat-0d	RHSIM_Rhsim04G0112300	2.011	0.038	-1.973	high
Treat-0d	RHSIM_Rhsim04G0249500	-2.288	-1.744	0.544	low
Treat-0d	RHSIM_Rhsim04G0216400	1.177	-0.066	-1.243	high
Treat-0d	RHSIM_Rhsim05G0208400	1.403	-0.080	-1.483	high
Treat-0d	RHSIM_Rhsim07G0142500	-0.642	-0.470	0.172	no
Treat-0d	RHSIM_Rhsim04G0243000	-0.551	-1.137	-0.586	no
Treat-0d	RHSIM_Rhsim04G0011600	-1.832	-0.855	0.976	low
Treat-0d	RHSIM_Rhsim05G0106400	0.430	-0.583	-1.013	no
Treat-0d	RHSIM_Rhsim04G0126000	-0.707	-0.562	0.145	no
Treat-0d	RHSIM_Rhsim04G0118000	-0.030	-0.153	-0.124	no
Treat-0d	RHSIM_Rhsim05G0194000	-2.150	-1.359	0.790	low
Treat-0d	RHSIM_Rhsim04G0012200	0.661	0.348	-0.313	no
Treat-0d	RHSIM_Rhsim04G0010300	-0.014	2.487	2.501	no
Treat-0d	RHSIM_Rhsim05G0160800	1.090	0.599	-0.491	no
Treat-0d	RHSIM_Rhsim04G0010500	-0.829	-1.855	-1.026	no
Treat-0d	RHSIM_Rhsim05G0068400	-1.566	-1.111	0.456	low
Treat-0d	RHSIM_Rhsim05G0214400	0.704	-0.871	-1.575	no
Treat-0d	RHSIM_Rhsim04G0203200	-1.226	0.835	2.061	low
Treat-0d	RHSIM_Rhsim07G0252600	-0.528	0.715	1.243	no
Treat-0d	RHSIM_Rhsim04G0125900	0.946	-1.201	-2.147	no
Treat-0d	RHSIM_Rhsim05G0159300	0.830	1.796	0.966	no
Treat-0d	RHSIM_Rhsim04G0216900	0.139	-0.817	-0.955	no
Treat-0d	RHSIM_Rhsim04G0052800	-0.080	0.069	0.148	no
Treat-0d	RHSIM_Rhsim04G0225000	-0.098	0.870	0.969	no
Treat-0d	RHSIM_Rhsim04G0199900	0.444	1.420	0.976	no
Treat-0d	RHSIM_Rhsim05G0131900	1.091	0.521	-0.570	no
Treat-0d	RHSIM_Rhsim07G0126100	-0.898	-0.530	0.369	no
Treat-0d	RHSIM_Rhsim05G0124800	-0.212	-0.263	-0.051	no

Group	Gene	PTR (z-score difference)	z(log2 LFQ)	z(log2 FPKM)	extreme-PTR
Treat-0d	RHSIM_Rhsim04G0004200	-1.764	-1.770	-0.005	low
Treat-0d	RHSIM_Rhsim04G0180700	0.299	-0.130	-0.429	no
Treat-0d	RHSIM_Rhsim06G0198800	-0.478	-0.980	-0.502	no
Treat-0d	RHSIM_Rhsim04G0202000	-0.395	-0.188	0.207	no
Treat-0d	RHSIM_Rhsim04G0052300	0.434	2.831	2.397	no
Treat-0d	RHSIM_Rhsim04G0006600	0.438	0.432	-0.005	no
Treat-0d	RHSIM_Rhsim06G0033800	-0.708	-0.723	-0.015	no
Treat-0d	RHSIM_Rhsim05G0167200	0.860	1.489	0.629	no
Treat-0d	RHSIM_Rhsim04G0123300	-0.559	-1.075	-0.516	no
Treat-0d	RHSIM_Rhsim04G0220300	0.498	-0.285	-0.783	no
Treat-0d	RHSIM_Rhsim05G0201000	-0.350	0.095	0.446	no
Treat-0d	RHSIM_Rhsim04G0055900	1.073	-0.376	-1.449	no
Treat-0d	RHSIM_Rhsim04G0174200	-0.657	0.386	1.043	no
Treat-0d	RHSIM_Rhsim06G0087100	-0.673	-0.509	0.164	no
Treat-0d	RHSIM_Rhsim04G0016600	0.440	0.687	0.246	no
Treat-0d	RHSIM_Rhsim04G0175900	1.284	1.045	-0.239	high
Treat-0d	RHSIM_Rhsim04G0203300	0.342	0.131	-0.210	no
Treat-0d	RHSIM_Rhsim05G0100900	-1.770	-0.014	1.756	low
Treat-0d	RHSIM_Rhsim04G0186500	0.065	-0.657	-0.722	no
Treat-0d	RHSIM_Rhsim04G0026100	1.332	1.912	0.580	high
Treat-0d	RHSIM_Rhsim05G0225500	-0.596	-0.603	-0.007	no
Treat-0d	RHSIM_Rhsim06G0006400	-2.482	-0.764	1.718	low
Treat-0d	RHSIM_Rhsim05G0031400	0.602	0.351	-0.251	no
Treat-0d	RHSIM_Rhsim04G0013400	0.721	-0.273	-0.994	no
Treat-0d	RHSIM_Rhsim04G0157800	-2.206	-0.353	1.853	low
Treat-0d	RHSIM_Rhsim04G0008200	0.073	0.374	0.301	no
Treat-0d	RHSIM_Rhsim04G0019600	0.546	-0.594	-1.140	no
Treat-0d	RHSIM_Rhsim05G0122700	-0.184	-1.014	-0.830	no
Treat-0d	RHSIM_Rhsim04G0168600	0.738	0.240	-0.498	no
Treat-0d	RHSIM_Rhsim04G0083700	0.637	-0.609	-1.246	no
Treat-0d	RHSIM_Rhsim05G0047300	-0.073	-1.521	-1.448	no
Treat-0d	RHSIM_Rhsim05G0174900	0.581	1.378	0.797	no

Group	Gene	PTR (z-score difference)	z(log2 LFQ)	z(log2 FPKM)	extreme-PTR
Treat-0d	RHSIM_Rhsim04G0006900	-1.240	-0.401	0.839	low
Treat-0d	RHSIM_Rhsim04G0167600	-0.352	-0.595	-0.244	no
Treat-0d	RHSIM_Rhsim04G0070300	-0.162	0.954	1.117	no
Treat-0d	RHSIM_Rhsim04G0154200	-0.719	-1.283	-0.565	no
Treat-0d	RHSIM_Rhsim04G0215500	0.061	0.567	0.506	no
Treat-0d	RHSIM_Rhsim06G0237200	-1.071	0.638	1.709	low
Treat-0d	RHSIM_Rhsim04G0173400	1.287	1.211	-0.076	high
Treat-0d	RHSIM_Rhsim04G0075900	2.351	1.598	-0.753	high
Treat-0d	RHSIM_Rhsim04G0074900	-0.160	-0.837	-0.677	no
Treat-0d	RHSIM_Rhsim04G0126400	-0.209	0.661	0.870	no
Treat-0d	RHSIM_Rhsim04G0125700	-2.719	-1.951	0.768	low
Treat-0d	RHSIM_Rhsim04G0202300	3.548	2.676	-0.872	high
Treat-0d	RHSIM_Rhsim04G0023900	-0.840	0.589	1.429	no
Treat-0d	RHSIM_Rhsim05G0172200	-0.013	-1.013	-1.000	no
Treat-0d	RHSIM_Rhsim04G0093600	-0.862	-0.594	0.269	no
Treat-0d	RHSIM_Rhsim04G0230600	0.773	2.876	2.103	no
Treat-0d	RHSIM_Rhsim04G0136300	-0.624	0.200	0.824	no
Treat-0d	RHSIM_Rhsim04G0030800	1.282	0.433	-0.848	high
Treat-0d	RHSIM_Rhsim05G0219800	0.542	-0.825	-1.367	no
Treat-0d	RHSIM_Rhsim05G0217200	-1.339	-1.440	-0.101	low
Treat-0d	RHSIM_Rhsim04G0207400	0.672	0.126	-0.545	no
Treat-0d	RHSIM_Rhsim05G0095100	0.524	1.589	1.065	no
Treat-0d	RHSIM_Rhsim03G0030800	1.881	0.433	-1.448	high
Treat-0d	RHSIM_Rhsim05G0163700	-0.504	-0.454	0.049	no
Treat-0d	RHSIM_Rhsim04G0018100	0.948	0.371	-0.578	no
Treat-0d	RHSIM_Rhsim05G0211100	0.359	0.731	0.372	no
Treat-0d	RHSIM_Rhsim04G0195100	0.628	1.148	0.520	no
Treat-0d	RHSIM_Rhsim04G0156300	-2.042	1.671	3.713	low
Treat-0d	RHSIM_Rhsim04G0030700	0.070	0.562	0.491	no
Treat-0d	RHSIM_Rhsim04G0108400	-2.161	0.418	2.579	low
Treat-0d	RHSIM_Rhsim04G0217800	-0.587	-0.419	0.167	no
Treat-0d	RHSIM_Rhsim06G0053800	-0.937	-2.146	-1.209	no

Group	Gene	PTR (z-score difference)	z(log2 LFQ)	z(log2 FPKM)	extreme-PTR
Treat-0d	RHSIM_Rhsim04G0195700	-0.656	-0.992	-0.336	no
Treat-0d	RHSIM_Rhsim04G0000700	-0.861	-0.240	0.621	no
Treat-0d	RHSIM_Rhsim04G0117700	0.179	-0.524	-0.703	no
Treat-0d	RHSIM_Rhsim03G0235300	-0.586	0.459	1.045	no
Treat-0d	RHSIM_Rhsim05G0139500	0.228	0.649	0.421	no
Treat-0d	RHSIM_Rhsim04G0114100	0.839	1.090	0.251	no
Treat-0d	RHSIM_Rhsim04G0009900	-1.091	-0.498	0.593	low
Treat-0d	RHSIM_Rhsim04G0084500	-0.530	0.750	1.280	no
Treat-0d	RHSIM_Rhsim06G0164200	-0.508	1.465	1.974	no
Treat-0d	RHSIM_Rhsim05G0075700	1.322	0.890	-0.432	high
Treat-0d	RHSIM_Rhsim05G0086800	-1.204	-0.100	1.104	low
Treat-0d	RHSIM_Rhsim04G0104400	-0.331	0.230	0.561	no
Treat-0d	RHSIM_Rhsim04G0130500	-1.149	0.446	1.595	low
Treat-0d	RHSIM_Rhsim04G0018200	0.404	0.245	-0.159	no
Treat-0d	RHSIM_Rhsim05G0082100	-0.331	0.339	0.670	no
Treat-0d	RHSIM_Rhsim04G0224400	0.477	0.037	-0.441	no
Treat-0d	RHSIM_Rhsim04G0232100	0.600	0.450	-0.151	no
Treat-0d	RHSIM_Rhsim05G0187700	0.279	1.217	0.938	no
Treat-0d	RHSIM_Rhsim03G0241400	-0.479	-0.327	0.152	no
Treat-0d	RHSIM_Rhsim04G0194700	1.095	0.961	-0.135	no
Treat-0d	RHSIM_Rhsim05G0017300	-0.939	-0.865	0.074	no
Treat-0d	RHSIM_Rhsim04G0240200	-4.874	-2.399	2.475	low
Treat-0d	RHSIM_Rhsim04G0063000	0.673	-0.196	-0.869	no
Treat-0d	RHSIM_Rhsim05G0104700	0.572	1.054	0.483	no
Treat-0d	RHSIM_Rhsim04G0239000	0.485	1.336	0.851	no
Treat-0d	RHSIM_Rhsim04G0170500	0.418	0.000	-0.417	no
Treat-0d	RHSIM_Rhsim05G0150300	0.904	-0.567	-1.470	no
Treat-0d	RHSIM_Rhsim04G0030100	1.193	1.414	0.221	high
Treat-0d	RHSIM_Rhsim04G0200900	-0.188	-0.869	-0.681	no
Treat-0d	RHSIM_Rhsim04G0083600	-0.779	-0.890	-0.111	no
Treat-0d	RHSIM_Rhsim04G0221400	-0.900	-0.680	0.220	no
Treat-0d	RHSIM_Rhsim05G0205100	0.092	-1.523	-1.615	no

Group	Gene	PTR (z-score difference)	z(log2 LFQ)	z(log2 FPKM)	extreme-PTR
Treat-0d	RHSIM_Rhsim04G0122900	1.143	0.682	-0.460	high
Treat-0d	RHSIM_Rhsim05G0067100	-0.379	0.958	1.337	no
Treat-0d	RHSIM_Rhsim03G0221100	1.164	0.767	-0.397	high
Treat-0d	RHSIM_Rhsim04G0011100	0.359	1.017	0.658	no
Treat-0d	RHSIM_Rhsim04G0104600	0.704	1.061	0.357	no
Treat-0d	RHSIM_Rhsim04G0158800	1.119	-0.042	-1.160	high
Treat-0d	RHSIM_Rhsim04G0177100	0.212	-0.058	-0.270	no
Treat-0d	RHSIM_Rhsim04G0050100	-2.640	-0.507	2.133	low
Treat-0d	RHSIM_Rhsim03G0145800	0.798	-0.134	-0.932	no
Treat-0d	RHSIM_Rhsim04G0235200	0.980	1.872	0.892	no
Treat-0d	RHSIM_Rhsim04G0247200	0.363	-0.958	-1.320	no
Treat-0d	RHSIM_Rhsim04G0171800	-1.463	-1.503	-0.040	low
Treat-0d	RHSIM_Rhsim04G0038600	0.934	4.376	3.442	no
Treat-0d	RHSIM_Rhsim04G0103300	-0.443	-0.004	0.439	no
Treat-0d	RHSIM_Rhsim04G0144400	-0.115	0.977	1.092	no
Treat-0d	RHSIM_Rhsim04G0162900	-1.506	-0.785	0.720	low
Treat-0d	RHSIM_Rhsim06G0035400	-1.123	-0.694	0.429	low
Treat-0d	RHSIM_Rhsim04G0006200	1.874	3.811	1.937	high
Treat-0d	RHSIM_Rhsim04G0219100	0.860	0.634	-0.226	no
Treat-0d	RHSIM_Rhsim03G0171900	0.308	0.556	0.248	no
Treat-0d	RHSIM_Rhsim06G0232900	0.308	0.003	-0.305	no
Treat-0d	RHSIM_Rhsim04G0248000	0.379	0.075	-0.304	no
Treat-0d	RHSIM_Rhsim04G0084900	0.307	0.092	-0.215	no
Treat-0d	RHSIM_Rhsim04G0022700	-1.137	-1.408	-0.271	low
Treat-0d	RHSIM_Rhsim04G0164200	0.169	-1.525	-1.694	no
Treat-0d	RHSIM_Rhsim04G0224800	0.733	0.054	-0.679	no
Treat-0d	RHSIM_Rhsim03G0219900	0.854	0.719	-0.136	no
Treat-0d	RHSIM_Rhsim04G0110400	0.146	0.771	0.625	no
Treat-0d	RHSIM_Rhsim04G0235000	-0.649	-0.634	0.015	no
Treat-0d	RHSIM_Rhsim03G0220500	0.047	-1.204	-1.250	no
Treat-0d	RHSIM_Rhsim04G0196400	1.006	-0.819	-1.824	no
Treat-0d	RHSIM_Rhsim04G0015300	0.079	1.159	1.080	no

Group	Gene	PTR (z-score difference)	z(log2 LFQ)	z(log2 FPKM)	extreme-PTR
Treat-0d	RHSIM_Rhsim04G0241800	0.543	0.943	0.400	no
Treat-0d	RHSIM_Rhsim04G0131000	-0.768	-0.674	0.095	no
Treat-0d	RHSIM_Rhsim06G0169700	-0.332	-0.921	-0.589	no
Treat-0d	RHSIM_Rhsim05G0108600	0.335	0.693	0.358	no
Treat-0d	RHSIM_Rhsim04G0173800	-0.788	-0.347	0.442	no
Treat-0d	RHSIM_Rhsim04G0092500	1.825	1.392	-0.433	high
Treat-0d	RHSIM_Rhsim04G0037800	-1.042	-0.516	0.526	low
Treat-0d	RHSIM_Rhsim04G0031900	-0.930	0.205	1.135	no
Treat-0d	RHSIM_Rhsim03G0057400	-1.171	-0.858	0.312	low
Treat-0d	RHSIM_Rhsim04G0043200	-0.128	0.037	0.165	no
Treat-0d	RHSIM_Rhsim04G0204600	0.175	-0.738	-0.913	no
Treat-0d	RHSIM_Rhsim04G0106500	-1.684	0.164	1.848	low
Treat-0d	RHSIM_Rhsim04G0130700	-0.563	0.111	0.674	no
Treat-0d	RHSIM_Rhsim06G0229600	0.725	0.863	0.138	no
Treat-0d	RHSIM_Rhsim04G0100100	0.274	-0.331	-0.606	no
Treat-0d	RHSIM_Rhsim04G0084000	-0.780	0.114	0.894	no
Treat-0d	RHSIM_Rhsim03G0221500	1.203	-0.854	-2.058	high
Treat-0d	RHSIM_Rhsim03G0225700	-0.490	0.413	0.903	no
Treat-0d	RHSIM_Rhsim06G0202900	1.531	0.686	-0.845	high
Treat-0d	RHSIM_Rhsim04G0197100	1.753	1.929	0.177	high
Treat-0d	RHSIM_Rhsim03G0001300	-1.065	-0.620	0.444	low
Treat-0d	RHSIM_Rhsim04G0167300	0.874	2.036	1.162	no
Treat-0d	RHSIM_Rhsim05G0163500	0.095	0.506	0.411	no
Treat-0d	RHSIM_Rhsim04G0198400	-1.761	-1.932	-0.171	low
Treat-0d	RHSIM_Rhsim04G0056300	0.800	-0.119	-0.919	no
Treat-0d	RHSIM_Rhsim04G0056600	-0.145	-0.216	-0.071	no
Treat-0d	RHSIM_Rhsim04G0244400	0.832	1.385	0.553	no
Treat-0d	RHSIM_Rhsim04G0153200	-0.272	1.005	1.277	no
Treat-0d	RHSIM_Rhsim04G0023000	-0.049	0.216	0.265	no
Treat-0d	RHSIM_Rhsim03G0083600	1.440	1.707	0.267	high
Treat-0d	RHSIM_Rhsim06G0000500	-0.224	0.994	1.217	no
Treat-0d	RHSIM_Rhsim04G0172400	-0.487	-0.102	0.385	no

Group	Gene	PTR (z-score difference)	z(log2 LFQ)	z(log2 FPKM)	extreme-PTR
Treat-0d	RHSIM_Rhsim04G0032200	-0.246	-0.310	-0.064	no
Treat-0d	RHSIM_Rhsim03G0154000	1.026	0.644	-0.382	no
Treat-0d	RHSIM_Rhsim04G0027200	-1.237	0.970	2.206	low
Treat-0d	RHSIM_Rhsim04G0245300	1.187	0.049	-1.138	high
Treat-0d	RHSIM_Rhsim04G0072600	-0.675	-0.912	-0.237	no
Treat-0d	RHSIM_Rhsim03G0168400	2.205	1.204	-1.001	high
Treat-0d	RHSIM_Rhsim03G0075300	0.376	0.715	0.338	no
Treat-0d	RHSIM_Rhsim04G0173300	-0.798	0.288	1.086	no
Treat-0d	RHSIM_Rhsim04G0219500	-0.427	-0.891	-0.464	no
Treat-0d	RHSIM_Rhsim04G0180500	-0.539	-0.217	0.322	no
Treat-0d	RHSIM_Rhsim05G0181000	-0.404	0.487	0.891	no
Treat-0d	RHSIM_Rhsim04G0096800	1.759	2.192	0.433	high
Treat-0d	RHSIM_Rhsim04G0095900	0.857	0.652	-0.206	no
Treat-0d	RHSIM_Rhsim03G0154300	0.699	-0.723	-1.423	no
Treat-0d	RHSIM_Rhsim06G0030600	-0.855	-1.033	-0.179	no
Treat-0d	RHSIM_Rhsim04G0038700	0.701	-0.001	-0.703	no
Treat-0d	RHSIM_Rhsim04G0001800	0.113	0.570	0.456	no
Treat-0d	RHSIM_Rhsim05G0062800	0.316	2.860	2.544	no
Treat-0d	RHSIM_Rhsim03G0111900	0.454	2.045	1.591	no
Treat-0d	RHSIM_Rhsim03G0207700	0.050	-1.444	-1.493	no
Treat-0d	RHSIM_Rhsim05G0133600	0.493	0.626	0.134	no
Treat-0d	RHSIM_Rhsim03G0204000	-0.684	-1.274	-0.589	no
Treat-0d	RHSIM_Rhsim04G0162800	-1.175	-0.804	0.371	low
Treat-0d	RHSIM_Rhsim04G0087300	0.839	0.111	-0.728	no
Treat-0d	RHSIM_Rhsim06G0239100	0.018	1.746	1.728	no
Treat-0d	RHSIM_Rhsim04G0196800	-0.257	1.282	1.539	no
Treat-0d	RHSIM_Rhsim03G0044900	-0.578	-0.124	0.455	no
Treat-0d	RHSIM_Rhsim03G0179500	-0.310	1.355	1.665	no
Treat-0d	RHSIM_Rhsim04G0185400	0.055	-0.616	-0.671	no
Treat-0d	RHSIM_Rhsim04G0205700	1.560	1.409	-0.152	high
Treat-0d	RHSIM_Rhsim04G0020500	2.665	2.034	-0.631	high
Treat-0d	RHSIM_Rhsim03G0234000	-1.261	-1.350	-0.089	low

Group	Gene	PTR (z-score difference)	z(log2 LFQ)	z(log2 FPKM)	extreme-PTR
Treat-0d	RHSIM_Rhsim04G0233600	0.085	-0.091	-0.175	no
Treat-0d	RHSIM_Rhsim03G0019300	-1.093	1.597	2.691	low
Treat-0d	RHSIM_Rhsim04G0162300	-0.869	0.451	1.319	no
Treat-0d	RHSIM_Rhsim03G0226300	-0.459	0.189	0.649	no
Treat-0d	RHSIM_Rhsim04G0218700	-1.028	-1.154	-0.126	low
Treat-0d	RHSIM_Rhsim03G0155100	0.098	-1.423	-1.521	no
Treat-0d	RHSIM_Rhsim04G0177600	-1.611	-0.430	1.181	low
Treat-0d	RHSIM_Rhsim03G0254300	0.110	0.809	0.698	no
Treat-0d	RHSIM_Rhsim03G0001500	0.472	0.704	0.232	no
Treat-0d	RHSIM_Rhsim03G0166500	0.255	-0.447	-0.702	no
Treat-0d	RHSIM_Rhsim03G0065700	0.344	-0.318	-0.662	no
Treat-0d	RHSIM_Rhsim04G0228500	0.170	-0.135	-0.304	no
Treat-0d	RHSIM_Rhsim03G0253300	-0.559	-0.508	0.052	no
Treat-0d	RHSIM_Rhsim04G0003700	1.219	-0.698	-1.917	high
Treat-0d	RHSIM_Rhsim03G0180700	-0.129	1.298	1.427	no
Treat-0d	RHSIM_Rhsim04G0189900	0.968	0.538	-0.430	no
Treat-0d	RHSIM_Rhsim03G0237900	2.492	0.625	-1.867	high
Treat-0d	RHSIM_Rhsim04G0233400	-1.045	-0.949	0.096	low
Treat-0d	RHSIM_Rhsim04G0003500	-0.455	-0.220	0.235	no
Treat-0d	RHSIM_Rhsim03G0223300	0.293	-0.280	-0.573	no
Treat-0d	RHSIM_Rhsim04G0134100	0.661	-0.841	-1.502	no
Treat-0d	RHSIM_Rhsim03G0010800	0.938	-0.333	-1.271	no
Treat-0d	RHSIM_Rhsim03G0063700	0.032	0.330	0.298	no
Treat-0d	RHSIM_Rhsim04G0030200	-0.515	-0.858	-0.343	no
Treat-0d	RHSIM_Rhsim03G0068700	-0.858	1.206	2.064	no
Treat-0d	RHSIM_Rhsim04G0158400	-0.382	-0.930	-0.548	no
Treat-0d	RHSIM_Rhsim06G0242500	0.303	-0.761	-1.064	no
Treat-0d	RHSIM_Rhsim04G0139400	0.685	-0.716	-1.400	no
Treat-0d	RHSIM_Rhsim04G0190700	-0.707	-1.415	-0.708	no
Treat-0d	RHSIM_Rhsim04G0230400	1.374	1.565	0.192	high
Treat-0d	RHSIM_Rhsim03G0124500	0.723	-0.596	-1.319	no
Treat-0d	RHSIM_Rhsim03G0085200	0.160	-0.660	-0.820	no

Group	Gene	PTR (z-score difference)	z(log2 LFQ)	z(log2 FPKM)	extreme-PTR
Treat-0d	RHSIM_Rhsim03G0228800	-0.217	-1.165	-0.948	no
Treat-0d	RHSIM_Rhsim04G0120900	-1.124	-0.822	0.303	low
Treat-0d	RHSIM_Rhsim04G0155300	-1.119	1.595	2.714	low
Treat-0d	RHSIM_Rhsim03G0121700	-0.456	-0.722	-0.267	no
Treat-0d	RHSIM_Rhsim04G0004400	0.577	0.563	-0.013	no
Treat-0d	RHSIM_Rhsim03G0129000	-0.177	1.112	1.289	no
Treat-0d	RHSIM_Rhsim04G0130800	-2.010	-1.357	0.654	low
Treat-0d	RHSIM_Rhsim06G0082800	-0.634	-0.760	-0.127	no
Treat-0d	RHSIM_Rhsim03G0235100	-0.077	1.401	1.478	no
Treat-0d	RHSIM_Rhsim04G0138700	0.086	0.500	0.414	no
Treat-0d	RHSIM_Rhsim04G0003600	0.365	1.280	0.915	no
Treat-0d	RHSIM_Rhsim06G0166200	0.133	0.002	-0.131	no
Treat-0d	RHSIM_Rhsim03G0198500	0.116	-0.545	-0.661	no
Treat-0d	RHSIM_Rhsim03G0000200	0.658	1.089	0.431	no
Treat-0d	RHSIM_Rhsim04G0128400	-0.984	-0.515	0.468	no
Treat-0d	RHSIM_Rhsim04G0225100	0.218	-0.323	-0.542	no
Treat-0d	RHSIM_Rhsim03G0096300	-1.003	0.000	1.003	no
Treat-0d	RHSIM_Rhsim04G0235600	0.952	-1.029	-1.981	no
Treat-0d	RHSIM_Rhsim03G0234800	-0.029	-0.221	-0.192	no
Treat-0d	RHSIM_Rhsim03G0066700	1.052	3.480	2.428	no
Treat-0d	RHSIM_Rhsim04G0241100	1.362	1.278	-0.084	high
Treat-0d	RHSIM_Rhsim04G0084800	-1.268	-1.052	0.216	low
Treat-0d	RHSIM_Rhsim04G0021800	0.032	0.494	0.462	no
Treat-0d	RHSIM_Rhsim03G0239700	-1.763	-1.802	-0.039	low
Treat-0d	RHSIM_Rhsim03G0073700	0.916	-0.897	-1.814	no
Treat-0d	RHSIM_Rhsim03G0146900	-0.409	-0.696	-0.287	no
Treat-0d	RHSIM_Rhsim04G0094400	0.212	-0.778	-0.990	no
Treat-0d	RHSIM_Rhsim03G0164200	0.300	0.650	0.350	no
Treat-0d	RHSIM_Rhsim03G0006300	-1.272	-0.664	0.608	low
Treat-0d	RHSIM_Rhsim06G0174600	0.414	0.415	0.002	no
Treat-0d	RHSIM_Rhsim04G0039400	-0.548	-1.474	-0.926	no
Treat-0d	RHSIM_Rhsim06G0073200	-3.819	-0.408	3.411	low

Group	Gene	PTR (z-score difference)	z(log2 LFQ)	z(log2 FPKM)	extreme-PTR
Treat-0d	RHSIM_Rhsim03G0188200	0.395	-0.258	-0.652	no
Treat-0d	RHSIM_Rhsim04G0001500	0.912	0.259	-0.653	no
Treat-0d	RHSIM_Rhsim06G0036100	-0.200	0.212	0.412	no
Treat-0d	RHSIM_Rhsim04G0129300	0.389	0.860	0.472	no
Treat-0d	RHSIM_Rhsim06G0177200	-0.647	-0.594	0.053	no
Treat-0d	RHSIM_Rhsim03G0019000	-0.787	0.869	1.656	no
Treat-0d	RHSIM_Rhsim04G0116200	1.089	-1.067	-2.156	no
Treat-0d	RHSIM_Rhsim03G0122000	0.369	0.129	-0.240	no
Treat-0d	RHSIM_Rhsim03G0229700	-0.772	-0.126	0.647	no
Treat-0d	RHSIM_Rhsim06G0153200	-0.711	0.395	1.105	no
Treat-0d	RHSIM_Rhsim03G0020300	1.424	0.104	-1.320	high
Treat-0d	RHSIM_Rhsim03G0172000	0.466	-0.751	-1.217	no
Treat-0d	RHSIM_Rhsim03G0041200	0.884	0.115	-0.768	no
Treat-0d	RHSIM_Rhsim03G0222600	-1.719	-0.836	0.884	low
Treat-0d	RHSIM_Rhsim06G0001600	0.584	0.056	-0.528	no
Treat-0d	RHSIM_Rhsim03G0155200	1.072	1.018	-0.054	no
Treat-0d	RHSIM_Rhsim03G0173300	1.041	0.426	-0.614	no
Treat-0d	RHSIM_Rhsim03G0218100	-0.349	-1.339	-0.990	no
Treat-0d	RHSIM_Rhsim03G0270100	-0.067	-0.654	-0.587	no
Treat-0d	RHSIM_Rhsim06G0087400	-1.065	-1.035	0.030	low
Treat-0d	RHSIM_Rhsim03G0212700	-0.352	0.830	1.183	no
Treat-0d	RHSIM_Rhsim03G0230000	-0.670	-0.141	0.529	no
Treat-0d	RHSIM_Rhsim03G0009200	1.654	1.711	0.057	high
Treat-0d	RHSIM_Rhsim03G0050300	0.369	0.049	-0.320	no
Treat-0d	RHSIM_Rhsim03G0075800	1.090	1.289	0.198	no
Treat-0d	RHSIM_Rhsim06G0051900	2.546	0.545	-2.001	high
Treat-0d	RHSIM_Rhsim06G0129100	-0.324	0.400	0.724	no
Treat-0d	RHSIM_Rhsim04G0165300	0.362	0.616	0.255	no
Treat-0d	RHSIM_Rhsim03G0143600	-1.316	-1.521	-0.206	low
Treat-0d	RHSIM_Rhsim04G0139800	0.857	0.228	-0.629	no
Treat-0d	RHSIM_Rhsim03G0177300	1.298	0.398	-0.900	high
Treat-0d	RHSIM_Rhsim04G0201800	0.439	0.001	-0.438	no

Group	Gene	PTR (z-score difference)	z(log2 LFQ)	z(log2 FPKM)	extreme-PTR
Treat-0d	RHSIM_Rhsim06G0130400	0.524	0.695	0.171	no
Treat-0d	RHSIM_Rhsim04G0168700	-0.533	0.253	0.787	no
Treat-0d	RHSIM_Rhsim06G0048400	0.002	-0.276	-0.278	no
Treat-0d	RHSIM_Rhsim04G0167800	1.752	0.813	-0.938	high
Treat-0d	RHSIM_Rhsim03G0248200	-0.157	-0.803	-0.647	no
Treat-0d	RHSIM_Rhsim03G0201100	-0.293	0.113	0.406	no
Treat-0d	RHSIM_Rhsim03G0150700	-0.530	-0.339	0.191	no
Treat-0d	RHSIM_Rhsim04G0181600	1.735	-0.227	-1.962	high
Treat-0d	RHSIM_Rhsim03G0265800	1.630	0.354	-1.276	high
Treat-0d	RHSIM_Rhsim04G0014800	-2.067	-1.365	0.702	low
Treat-0d	RHSIM_Rhsim03G0004000	1.089	-0.159	-1.247	no
Treat-0d	RHSIM_Rhsim04G0173000	1.557	2.010	0.453	high
Treat-0d	RHSIM_Rhsim04G0091700	2.046	1.353	-0.693	high
Treat-0d	RHSIM_Rhsim03G0267600	0.152	-0.992	-1.144	no
Treat-0d	RHSIM_Rhsim03G0151400	0.904	1.981	1.077	no
Treat-0d	RHSIM_Rhsim03G0061300	0.640	-1.444	-2.084	no
Treat-0d	RHSIM_Rhsim04G0081600	0.304	-0.230	-0.534	no
Treat-0d	RHSIM_Rhsim04G0038100	-0.604	-0.503	0.100	no
Treat-0d	RHSIM_Rhsim04G0105700	0.722	-0.086	-0.808	no
Treat-0d	RHSIM_Rhsim03G0196600	0.298	-0.413	-0.711	no
Treat-0d	RHSIM_Rhsim03G0235600	-0.619	-0.156	0.463	no
Treat-0d	RHSIM_Rhsim03G0119300	-0.797	-1.168	-0.371	no
Treat-0d	RHSIM_Rhsim03G0252000	-0.375	0.043	0.418	no
Treat-0d	RHSIM_Rhsim04G0197500	-1.531	0.131	1.662	low
Treat-0d	RHSIM_Rhsim06G0018600	0.564	0.637	0.073	no
Treat-0d	RHSIM_Rhsim03G0125700	0.575	0.359	-0.216	no
Treat-0d	RHSIM_Rhsim03G0001600	-0.823	-0.298	0.525	no
Treat-0d	RHSIM_Rhsim04G0170700	2.169	0.438	-1.731	high
Treat-0d	RHSIM_Rhsim03G0093200	-1.258	-0.937	0.322	low
Treat-0d	RHSIM_Rhsim03G0248000	0.015	0.493	0.478	no
Treat-0d	RHSIM_Rhsim03G0153100	0.814	-0.818	-1.632	no
Treat-0d	RHSIM_Rhsim03G0138600	0.377	0.757	0.379	no

Group	Gene	PTR (z-score difference)	z(log2 LFQ)	z(log2 FPKM)	extreme-PTR
Treat-0d	RHSIM_Rhsim04G0008100	-0.174	1.227	1.400	no
Treat-0d	RHSIM_Rhsim03G0090600	0.197	-0.345	-0.542	no
Treat-0d	RHSIM_Rhsim03G0161600	-0.154	-1.414	-1.260	no
Treat-0d	RHSIM_Rhsim03G0264700	-0.788	-0.726	0.061	no
Treat-0d	RHSIM_Rhsim03G0078800	0.258	-0.027	-0.286	no
Treat-0d	RHSIM_Rhsim05G0113400	1.990	3.043	1.053	high
Treat-0d	RHSIM_Rhsim03G0211600	-0.529	-1.194	-0.665	no
Treat-0d	RHSIM_Rhsim03G0115800	-0.054	-0.464	-0.410	no
Treat-0d	RHSIM_Rhsim05G0090500	-1.073	-0.095	0.978	low
Treat-0d	RHSIM_Rhsim03G0249400	-0.126	0.488	0.615	no
Treat-0d	RHSIM_Rhsim04G0086600	-1.076	-0.986	0.090	low
Treat-0d	RHSIM_Rhsim04G0169700	-1.483	-0.402	1.081	low
Treat-0d	RHSIM_Rhsim03G0245200	-1.453	-0.421	1.033	low
Treat-0d	RHSIM_Rhsim03G0009900	0.136	-0.249	-0.386	no
Treat-0d	RHSIM_Rhsim04G0157200	-0.228	-0.553	-0.325	no
Treat-0d	RHSIM_Rhsim04G0214000	-0.935	-1.371	-0.436	no
Treat-0d	RHSIM_Rhsim03G0219300	0.082	-0.655	-0.737	no
Treat-0d	RHSIM_Rhsim03G0159800	-0.775	0.307	1.082	no
Treat-0d	RHSIM_Rhsim03G0256600	-0.189	-0.328	-0.139	no
Treat-0d	RHSIM_Rhsim03G0180900	-2.001	0.345	2.346	low
Treat-0d	RHSIM_Rhsim03G0099600	0.924	0.504	-0.420	no
Treat-0d	RHSIM_Rhsim03G0240200	0.611	-0.591	-1.202	no
Treat-0d	RHSIM_Rhsim03G0131800	-0.979	0.149	1.128	no
Treat-0d	RHSIM_Rhsim03G0110300	1.110	0.836	-0.274	high
Treat-0d	RHSIM_Rhsim04G0176900	1.099	1.561	0.462	no
Treat-0d	RHSIM_Rhsim03G0100800	0.388	-0.272	-0.661	no
Treat-0d	RHSIM_Rhsim03G0069400	-0.247	-0.055	0.191	no
Treat-0d	RHSIM_Rhsim03G0105700	-0.800	-0.720	0.080	no
Treat-0d	RHSIM_Rhsim03G0027100	0.230	-0.692	-0.922	no
Treat-0d	RHSIM_Rhsim03G0074700	-2.066	-1.738	0.328	low
Treat-0d	RHSIM_Rhsim03G0136400	-0.292	0.172	0.465	no
Treat-0d	RHSIM_Rhsim04G0147900	-0.166	0.144	0.310	no

Group	Gene	PTR (z-score difference)	z(log2 LFQ)	z(log2 FPKM)	extreme-PTR
Treat-0d	RHSIM_Rhsim03G0075100	0.658	-0.360	-1.018	no
Treat-0d	RHSIM_Rhsim04G0101000	0.360	-0.992	-1.351	no
Treat-0d	RHSIM_Rhsim03G0141300	-0.162	0.148	0.311	no
Treat-0d	RHSIM_Rhsim03G0262500	-1.189	-0.639	0.549	low
Treat-0d	RHSIM_Rhsim04G0151800	1.191	0.346	-0.845	high
Treat-0d	RHSIM_Rhsim04G0068400	-0.644	0.485	1.130	no
Treat-0d	RHSIM_Rhsim03G0052700	0.351	-0.405	-0.755	no
Treat-0d	RHSIM_Rhsim03G0134500	1.593	1.240	-0.353	high
Treat-0d	RHSIM_Rhsim03G0021400	0.612	0.335	-0.277	no
Treat-0d	RHSIM_Rhsim03G0225200	0.194	0.543	0.349	no
Treat-0d	RHSIM_Rhsim04G0071500	0.161	0.092	-0.068	no
Treat-0d	RHSIM_Rhsim03G0244600	-0.363	0.219	0.582	no
Treat-0d	RHSIM_Rhsim05G0115700	-0.080	0.572	0.652	no
Treat-0d	RHSIM_Rhsim03G0099500	0.203	-1.399	-1.602	no
Treat-0d	RHSIM_Rhsim03G0254100	1.725	1.543	-0.183	high
Treat-0d	RHSIM_Rhsim04G0081400	-0.333	0.418	0.751	no
Treat-0d	RHSIM_Rhsim03G0085000	-0.300	-1.294	-0.994	no
Treat-0d	RHSIM_Rhsim04G0166700	0.587	0.899	0.312	no
Treat-0d	RHSIM_Rhsim03G0121200	0.579	0.016	-0.564	no
Treat-0d	RHSIM_Rhsim03G0235000	-0.995	0.643	1.638	no
Treat-0d	RHSIM_Rhsim03G0146700	-0.286	0.302	0.588	no
Treat-0d	RHSIM_Rhsim04G0180800	-1.278	-0.652	0.626	low
Treat-0d	RHSIM_Rhsim03G0146600	-0.196	-0.702	-0.505	no
Treat-0d	RHSIM_Rhsim03G0073400	-0.083	-0.539	-0.456	no
Treat-0d	RHSIM_Rhsim04G0206300	2.457	2.748	0.291	high
Treat-0d	RHSIM_Rhsim03G0140300	-0.623	-1.354	-0.731	no
Treat-0d	RHSIM_Rhsim03G0080300	-2.002	-0.527	1.475	low
Treat-0d	RHSIM_Rhsim05G0116200	0.328	-0.057	-0.386	no
Treat-0d	RHSIM_Rhsim03G0144700	0.387	-0.179	-0.566	no
Treat-0d	RHSIM_Rhsim04G0066500	-0.168	-0.371	-0.203	no
Treat-0d	RHSIM_Rhsim03G0061100	-0.062	-0.850	-0.788	no
Treat-0d	RHSIM_Rhsim04G0182600	-1.509	1.129	2.638	low

Group	Gene	PTR (z-score difference)	z(log2 LFQ)	z(log2 FPKM)	extreme-PTR
Treat-0d	RHSIM_Rhsim05G0206900	-1.153	-0.428	0.726	low
Treat-0d	RHSIM_Rhsim03G0240400	-1.832	-1.076	0.756	low
Treat-0d	RHSIM_Rhsim03G0200600	-0.654	-0.187	0.467	no
Treat-0d	RHSIM_Rhsim03G0051100	-0.882	-1.301	-0.419	no
Treat-0d	RHSIM_Rhsim04G0209400	0.563	2.093	1.531	no
Treat-0d	RHSIM_Rhsim03G0223200	-0.207	0.082	0.290	no
Treat-0d	RHSIM_Rhsim03G0237400	0.435	1.913	1.478	no
Treat-0d	RHSIM_Rhsim04G0012000	-0.955	-0.538	0.417	no
Treat-0d	RHSIM_Rhsim03G0179100	-0.630	-0.825	-0.195	no
Treat-0d	RHSIM_Rhsim03G0187200	1.085	0.787	-0.297	no
Treat-0d	RHSIM_Rhsim04G0160900	1.090	-1.012	-2.101	no
Treat-0d	RHSIM_Rhsim03G0058100	-0.053	-0.697	-0.644	no
Treat-0d	RHSIM_Rhsim03G0244700	1.488	2.042	0.554	high
Treat-0d	RHSIM_Rhsim03G0256500	-0.025	1.853	1.878	no
Treat-0d	RHSIM_Rhsim03G0214800	-1.041	1.420	2.461	low
Treat-0d	RHSIM_Rhsim03G0034500	0.098	-1.109	-1.207	no
Treat-0d	RHSIM_Rhsim03G0112100	-0.602	-0.419	0.183	no
Treat-0d	RHSIM_Rhsim03G0240800	0.445	0.784	0.339	no
Treat-0d	RHSIM_Rhsim05G0193600	0.509	-0.397	-0.907	no
Treat-0d	RHSIM_Rhsim04G0086300	0.511	-0.442	-0.953	no
Treat-0d	RHSIM_Rhsim05G0216200	-1.158	-0.649	0.509	low
Treat-0d	RHSIM_Rhsim03G0258800	0.103	-0.510	-0.613	no
Treat-0d	RHSIM_Rhsim03G0231700	-0.826	-1.446	-0.621	no
Treat-0d	RHSIM_Rhsim03G0035400	0.559	-0.722	-1.281	no
Treat-0d	RHSIM_Rhsim04G0232000	0.597	0.880	0.283	no
Treat-0d	RHSIM_Rhsim05G0067500	-0.391	0.690	1.081	no
Treat-0d	RHSIM_Rhsim03G0150600	0.028	-0.697	-0.725	no
Treat-0d	RHSIM_Rhsim04G0179900	0.283	-0.586	-0.868	no
Treat-0d	RHSIM_Rhsim03G0062200	1.229	0.024	-1.204	high
Treat-0d	RHSIM_Rhsim04G0075400	-0.349	-0.816	-0.468	no
Treat-0d	RHSIM_Rhsim03G0092400	0.637	0.888	0.251	no
Treat-0d	RHSIM_Rhsim03G0236300	-0.475	-1.092	-0.617	no

Group	Gene	PTR (z-score difference)	z(log2 LFQ)	z(log2 FPKM)	extreme-PTR
Treat-0d	RHSIM_Rhsim05G0011200	-1.452	-0.813	0.639	low
Treat-0d	RHSIM_Rhsim03G0264300	1.092	0.600	-0.493	no
Treat-0d	RHSIM_Rhsim05G0032000	-1.469	-1.606	-0.136	low
Treat-0d	RHSIM_Rhsim03G0262700	0.722	1.196	0.474	no
Treat-0d	RHSIM_Rhsim03G0154600	-0.803	-0.868	-0.066	no
Treat-0d	RHSIM_Rhsim03G0073300	0.821	-1.060	-1.881	no
Treat-0d	RHSIM_Rhsim03G0208100	0.267	0.909	0.642	no
Treat-0d	RHSIM_Rhsim05G0101900	-1.530	-1.092	0.438	low
Treat-0d	RHSIM_Rhsim03G0230900	0.100	0.080	-0.020	no
Treat-0d	RHSIM_Rhsim04G0197400	-1.170	0.665	1.835	low
Treat-0d	RHSIM_Rhsim05G0042100	0.438	-0.209	-0.647	no
Treat-0d	RHSIM_Rhsim04G0204300	-1.381	-1.523	-0.142	low
Treat-0d	RHSIM_Rhsim03G0078700	1.096	-0.086	-1.181	no
Treat-0d	RHSIM_Rhsim03G0093900	-0.606	-0.598	0.008	no
Treat-0d	RHSIM_Rhsim03G0234500	0.678	1.618	0.940	no
Treat-0d	RHSIM_Rhsim05G0098000	-0.067	0.163	0.230	no
Treat-0d	RHSIM_Rhsim03G0265300	2.067	1.025	-1.042	high
Treat-0d	RHSIM_Rhsim03G0192800	0.684	0.061	-0.623	no
Treat-0d	RHSIM_Rhsim03G0018400	-1.423	-0.895	0.528	low
Treat-0d	RHSIM_Rhsim04G0171200	1.031	2.343	1.312	no
Treat-0d	RHSIM_Rhsim04G0024300	0.522	-0.634	-1.156	no
Treat-0d	RHSIM_Rhsim03G0176200	1.541	0.980	-0.562	high
Treat-0d	RHSIM_Rhsim03G0155500	0.494	-0.095	-0.589	no
Treat-0d	RHSIM_Rhsim05G0018500	0.145	-1.063	-1.208	no
Treat-0d	RHSIM_Rhsim03G0192400	0.297	0.985	0.688	no
Treat-0d	RHSIM_Rhsim03G0110600	0.213	0.096	-0.116	no
Treat-0d	RHSIM_Rhsim05G0214700	-1.596	0.085	1.681	low
Treat-0d	RHSIM_Rhsim03G0063000	-0.943	0.368	1.310	no
Treat-0d	RHSIM_Rhsim03G0141400	-4.755	-1.174	3.582	low
Treat-0d	RHSIM_Rhsim03G0187900	-0.365	-1.179	-0.814	no
Treat-0d	RHSIM_Rhsim03G0176300	-0.873	-0.881	-0.008	no
Treat-0d	RHSIM_Rhsim03G0145100	0.742	-0.089	-0.831	no

Group	Gene	PTR (z-score difference)	z(log2 LFQ)	z(log2 FPKM)	extreme-PTR
Treat-0d	RHSIM_Rhsim03G0208900	-0.247	-0.255	-0.007	no
Treat-0d	RHSIM_Rhsim03G0119400	0.691	0.992	0.301	no
Treat-0d	RHSIM_Rhsim03G0093800	-1.992	-0.662	1.329	low
Treat-0d	RHSIM_Rhsim02G0061100	-0.247	-0.581	-0.335	no
Treat-0d	RHSIM_Rhsim04G0144000	0.959	0.101	-0.859	no
Treat-0d	RHSIM_Rhsim03G0059400	1.037	-0.675	-1.712	no
Treat-0d	RHSIM_Rhsim03G0123500	-1.673	-1.615	0.058	low
Treat-0d	RHSIM_Rhsim03G0083800	-1.456	-0.859	0.597	low
Treat-0d	RHSIM_Rhsim03G0101200	-0.742	-1.089	-0.348	no
Treat-0d	RHSIM_Rhsim03G0188500	-0.098	0.522	0.620	no
Treat-0d	RHSIM_Rhsim02G0160800	-0.057	-0.416	-0.360	no
Treat-0d	RHSIM_Rhsim03G0118600	0.550	-0.103	-0.653	no
Treat-0d	RHSIM_Rhsim03G0089900	-1.166	-0.423	0.742	low
Treat-0d	RHSIM_Rhsim03G0029800	0.066	0.583	0.517	no
Treat-0d	RHSIM_Rhsim03G0088700	0.715	-0.324	-1.039	no
Treat-0d	RHSIM_Rhsim03G0233300	-2.662	-2.485	0.177	low
Treat-0d	RHSIM_Rhsim04G0145600	-0.270	0.351	0.621	no
Treat-0d	RHSIM_Rhsim02G0022400	1.563	0.230	-1.333	high
Treat-0d	RHSIM_Rhsim03G0270000	-0.216	0.053	0.269	no
Treat-0d	RHSIM_Rhsim03G0049600	0.412	-0.193	-0.604	no
Treat-0d	RHSIM_Rhsim03G0130200	-0.045	-0.036	0.009	no
Treat-0d	RHSIM_Rhsim05G0108800	0.583	-0.523	-1.106	no
Treat-0d	RHSIM_Rhsim03G0086200	2.596	1.814	-0.781	high
Treat-0d	RHSIM_Rhsim03G0195300	0.926	-0.421	-1.346	no
Treat-0d	RHSIM_Rhsim03G0229600	-0.940	-0.004	0.937	no
Treat-0d	RHSIM_Rhsim03G0207200	0.185	-0.854	-1.040	no
Treat-0d	RHSIM_Rhsim03G0231100	-0.105	-0.498	-0.393	no
Treat-0d	RHSIM_Rhsim03G0028800	-0.020	-2.100	-2.080	no
Treat-0d	RHSIM_Rhsim03G0212200	0.668	-0.540	-1.209	no
Treat-0d	RHSIM_Rhsim02G0105800	-1.133	-0.815	0.318	low
Treat-0d	RHSIM_Rhsim03G0081700	0.289	0.359	0.069	no
Treat-0d	RHSIM_Rhsim04G0207900	0.499	0.491	-0.008	no

Group	Gene	PTR (z-score difference)	z(log2 LFQ)	z(log2 FPKM)	extreme-PTR
Treat-0d	RHSIM_Rhsim03G0090700	-0.346	-1.051	-0.705	no
Treat-0d	RHSIM_Rhsim04G0168900	-0.709	-1.404	-0.695	no
Treat-0d	RHSIM_Rhsim03G0261200	1.781	0.507	-1.273	high
Treat-0d	RHSIM_Rhsim03G0075200	-1.383	-0.707	0.676	low
Treat-0d	RHSIM_Rhsim03G0231500	0.084	-1.184	-1.268	no
Treat-0d	RHSIM_Rhsim04G0224600	-0.406	0.366	0.772	no
Treat-0d	RHSIM_Rhsim02G0015800	-0.253	-0.197	0.056	no
Treat-0d	RHSIM_Rhsim02G0060700	-0.774	-0.825	-0.051	no
Treat-0d	RHSIM_Rhsim02G0108100	0.205	0.088	-0.117	no
Treat-0d	RHSIM_Rhsim02G0001700	0.140	-0.533	-0.673	no
Treat-0d	RHSIM_Rhsim02G0159000	-1.652	-1.063	0.589	low
Treat-0d	RHSIM_Rhsim04G0249800	1.481	-0.850	-2.331	high
Treat-0d	RHSIM_Rhsim03G0122500	-0.356	0.144	0.500	no
Treat-0d	RHSIM_Rhsim05G0165500	0.343	-0.255	-0.598	no
Treat-0d	RHSIM_Rhsim03G0085600	1.824	2.021	0.197	high
Treat-0d	RHSIM_Rhsim03G0018000	-2.738	-1.551	1.187	low
Treat-0d	RHSIM_Rhsim04G0009800	1.276	2.588	1.312	high
Treat-0d	RHSIM_Rhsim03G0083300	-0.474	-1.049	-0.575	no
Treat-0d	RHSIM_Rhsim03G0219000	-1.125	-0.398	0.727	low
Treat-0d	RHSIM_Rhsim03G0238600	1.015	0.140	-0.875	no
Treat-0d	RHSIM_Rhsim02G0120400	-2.298	-1.261	1.037	low
Treat-0d	RHSIM_Rhsim03G0196000	1.976	0.329	-1.647	high
Treat-0d	RHSIM_Rhsim02G0182400	0.300	-0.498	-0.798	no
Treat-0d	RHSIM_Rhsim03G0061800	0.880	0.941	0.062	no
Treat-0d	RHSIM_Rhsim03G0234600	0.069	-0.765	-0.834	no
Treat-0d	RHSIM_Rhsim05G0227600	-1.505	-0.377	1.128	low
Treat-0d	RHSIM_Rhsim04G0197800	0.110	-1.278	-1.388	no
Treat-0d	RHSIM_Rhsim03G0193700	2.271	0.392	-1.879	high
Treat-0d	RHSIM_Rhsim03G0225300	-0.669	-0.293	0.376	no
Treat-0d	RHSIM_Rhsim03G0267400	-1.269	-1.495	-0.227	low
Treat-0d	RHSIM_Rhsim04G0226400	-0.191	-1.322	-1.131	no
Treat-0d	RHSIM_Rhsim03G0208500	0.315	-0.555	-0.870	no

Group	Gene	PTR (z-score difference)	z(log2 LFQ)	z(log2 FPKM)	extreme-PTR
Treat-0d	RHSIM_Rhsim02G0011800	-0.660	-0.010	0.650	no
Treat-0d	RHSIM_Rhsim02G0230000	0.496	0.818	0.322	no
Treat-0d	RHSIM_Rhsim03G0074600	-0.727	-0.482	0.245	no
Treat-0d	RHSIM_Rhsim03G0012100	-0.978	-0.175	0.803	no
Treat-0d	RHSIM_Rhsim03G0189800	-0.453	0.433	0.886	no
Treat-0d	RHSIM_Rhsim03G0082000	1.582	2.781	1.198	high
Treat-0d	RHSIM_Rhsim05G0079700	-1.084	-1.644	-0.561	low
Treat-0d	RHSIM_Rhsim03G0175300	-0.164	-0.578	-0.414	no
Treat-0d	RHSIM_Rhsim03G0225900	-0.062	-0.855	-0.794	no
Treat-0d	RHSIM_Rhsim03G0049800	0.361	-0.350	-0.710	no
Treat-0d	RHSIM_Rhsim03G0168800	0.113	0.438	0.324	no
Treat-0d	RHSIM_Rhsim03G0200100	0.681	0.049	-0.631	no
Treat-0d	RHSIM_Rhsim02G0252200	-1.606	-1.267	0.338	low
Treat-0d	RHSIM_Rhsim03G0143400	0.546	-0.745	-1.291	no
Treat-0d	RHSIM_Rhsim03G0267700	0.149	0.715	0.566	no
Treat-0d	RHSIM_Rhsim05G0214900	-0.343	0.024	0.367	no
Treat-0d	RHSIM_Rhsim03G0012300	1.287	0.393	-0.894	high
Treat-0d	RHSIM_Rhsim03G0152000	-0.743	1.240	1.983	no
Treat-0d	RHSIM_Rhsim02G0137600	-2.015	-0.306	1.709	low
Treat-0d	RHSIM_Rhsim05G0192800	-0.019	0.350	0.370	no
Treat-0d	RHSIM_Rhsim03G0128200	1.352	1.183	-0.170	high
Treat-0d	RHSIM_Rhsim02G0041500	1.618	3.129	1.511	high
Treat-0d	RHSIM_Rhsim03G0052900	-1.796	1.836	3.633	low
Treat-0d	RHSIM_Rhsim02G0200900	1.311	0.612	-0.699	high
Treat-0d	RHSIM_Rhsim03G0225000	-0.541	-0.460	0.080	no
Treat-0d	RHSIM_Rhsim05G0146200	0.139	0.090	-0.049	no
Treat-0d	RHSIM_Rhsim02G0105700	-1.879	-1.789	0.090	low
Treat-0d	RHSIM_Rhsim03G0159900	-1.015	0.145	1.160	no
Treat-0d	RHSIM_Rhsim03G0036100	-1.333	-1.068	0.265	low
Treat-0d	RHSIM_Rhsim03G0049500	1.426	0.589	-0.838	high
Treat-0d	RHSIM_Rhsim03G0246500	-0.478	-0.259	0.219	no
Treat-0d	RHSIM_Rhsim05G0188400	-1.504	-0.610	0.894	low

Group	Gene	PTR (z-score difference)	z(log2 LFQ)	z(log2 FPKM)	extreme-PTR
Treat-0d	RHSIM_Rhsim03G0194300	0.796	-0.503	-1.299	no
Treat-0d	RHSIM_Rhsim02G0173300	0.464	-0.596	-1.060	no
Treat-0d	RHSIM_Rhsim05G0073400	0.447	2.054	1.606	no
Treat-0d	RHSIM_Rhsim03G0238900	0.491	1.587	1.096	no
Treat-0d	RHSIM_Rhsim03G0058600	-0.942	0.169	1.111	no
Treat-0d	RHSIM_Rhsim03G0236600	-0.497	-0.912	-0.415	no
Treat-0d	RHSIM_Rhsim03G0078200	-1.717	-1.431	0.285	low
Treat-0d	RHSIM_Rhsim03G0098400	0.909	1.651	0.742	no
Treat-0d	RHSIM_Rhsim03G0058700	0.289	-0.012	-0.301	no
Treat-0d	RHSIM_Rhsim03G0170100	-1.037	0.012	1.049	low
Treat-0d	RHSIM_Rhsim03G0044600	0.041	-0.176	-0.217	no
Treat-0d	RHSIM_Rhsim02G0251600	0.539	0.109	-0.430	no
Treat-0d	RHSIM_Rhsim02G0203500	0.957	-0.100	-1.057	no
Treat-0d	RHSIM_Rhsim03G0216000	-0.986	-0.271	0.714	no
Treat-0d	RHSIM_Rhsim03G0087900	-0.324	-0.363	-0.039	no
Treat-0d	RHSIM_Rhsim03G0222700	0.424	0.017	-0.407	no
Treat-0d	RHSIM_Rhsim05G0040800	0.843	-0.195	-1.038	no
Treat-0d	RHSIM_Rhsim02G0024500	1.438	-0.839	-2.276	high
Treat-0d	RHSIM_Rhsim03G0134000	-0.107	-0.080	0.027	no
Treat-0d	RHSIM_Rhsim03G0218000	-0.806	-0.328	0.478	no
Treat-0d	RHSIM_Rhsim03G0261800	0.714	0.548	-0.166	no
Treat-0d	RHSIM_Rhsim05G0087000	-0.349	1.000	1.349	no
Treat-0d	RHSIM_Rhsim03G0022100	1.697	0.470	-1.226	high
Treat-0d	RHSIM_Rhsim03G0218800	1.319	0.147	-1.172	high
Treat-0d	RHSIM_Rhsim03G0221200	0.833	0.373	-0.459	no
Treat-0d	RHSIM_Rhsim02G0083700	0.120	-0.696	-0.817	no
Treat-0d	RHSIM_Rhsim03G0150900	-2.278	-1.057	1.221	low
Treat-0d	RHSIM_Rhsim03G0217000	-1.455	-0.188	1.267	low
Treat-0d	RHSIM_Rhsim03G0255200	-1.058	-0.375	0.682	low
Treat-0d	RHSIM_Rhsim03G0172900	-0.864	0.948	1.812	no
Treat-0d	RHSIM_Rhsim03G0136600	1.807	1.916	0.109	high
Treat-0d	RHSIM_Rhsim05G0200700	0.477	0.079	-0.398	no

Group	Gene	PTR (z-score difference)	z(log2 LFQ)	z(log2 FPKM)	extreme-PTR
Treat-0d	RHSIM_Rhsim03G0143900	0.355	-1.103	-1.457	no
Treat-0d	RHSIM_Rhsim03G0078900	-0.563	-0.910	-0.347	no
Treat-0d	RHSIM_Rhsim05G0119800	0.731	0.409	-0.322	no
Treat-0d	RHSIM_Rhsim02G0212300	0.994	2.079	1.085	no
Treat-0d	RHSIM_Rhsim02G0132300	-0.345	1.404	1.749	no
Treat-0d	RHSIM_Rhsim03G0216300	-0.597	1.451	2.048	no
Treat-0d	RHSIM_Rhsim02G0157500	-1.167	-0.962	0.205	low
Treat-0d	RHSIM_Rhsim03G0127200	-0.683	-0.736	-0.053	no
Treat-0d	RHSIM_Rhsim03G0031000	-0.102	-0.600	-0.498	no
Treat-0d	RHSIM_Rhsim03G0172800	-0.393	-0.179	0.213	no
Treat-0d	RHSIM_Rhsim03G0149000	2.080	1.327	-0.753	high
Treat-0d	RHSIM_Rhsim02G0105600	-0.888	0.044	0.932	no
Treat-0d	RHSIM_Rhsim03G0208700	0.555	-0.138	-0.693	no
Treat-0d	RHSIM_Rhsim02G0251800	1.179	0.790	-0.388	high
Treat-0d	RHSIM_Rhsim02G0007300	0.443	0.654	0.211	no
Treat-0d	RHSIM_Rhsim05G0049100	-0.967	-1.538	-0.571	no
Treat-0d	RHSIM_Rhsim02G0243400	-1.432	-1.432	-0.000	low
Treat-0d	RHSIM_Rhsim03G0158800	-1.261	-0.562	0.699	low
Treat-0d	RHSIM_Rhsim02G0056700	1.057	1.294	0.238	no
Treat-0d	RHSIM_Rhsim05G0228200	0.006	-0.631	-0.637	no
Treat-0d	RHSIM_Rhsim02G0179000	-0.755	-1.280	-0.525	no
Treat-0d	RHSIM_Rhsim02G0220800	0.540	-0.586	-1.126	no
Treat-0d	RHSIM_Rhsim03G0174400	1.043	1.634	0.592	no
Treat-0d	RHSIM_Rhsim03G0114800	-1.190	-0.432	0.758	low
Treat-0d	RHSIM_Rhsim03G0266500	0.690	0.769	0.079	no
Treat-0d	RHSIM_Rhsim03G0092100	1.123	0.409	-0.713	high
Treat-0d	RHSIM_Rhsim02G0180400	-0.276	-1.038	-0.762	no
Treat-0d	RHSIM_Rhsim03G0145000	1.160	0.357	-0.803	high
Treat-0d	RHSIM_Rhsim04G0105900	2.149	0.333	-1.816	high
Treat-0d	RHSIM_Rhsim02G0199800	0.229	0.307	0.077	no
Treat-0d	RHSIM_Rhsim03G0169200	0.823	0.423	-0.400	no
Treat-0d	RHSIM_Rhsim04G0217900	-2.176	-1.305	0.872	low

Group	Gene	PTR (z-score difference)	z(log2 LFQ)	z(log2 FPKM)	extreme-PTR
Treat-0d	RHSIM_Rhsim03G0159700	-0.715	0.282	0.997	no
Treat-0d	RHSIM_Rhsim02G0146400	-0.446	0.193	0.639	no
Treat-0d	RHSIM_Rhsim02G0090300	-1.282	-0.430	0.852	low
Treat-0d	RHSIM_Rhsim03G0163000	-0.689	-0.484	0.205	no
Treat-0d	RHSIM_Rhsim02G0229000	-0.965	-0.238	0.727	no
Treat-0d	RHSIM_Rhsim04G0032000	1.289	4.200	2.911	high
Treat-0d	RHSIM_Rhsim03G0262600	-0.101	0.035	0.135	no
Treat-0d	RHSIM_Rhsim02G0044500	-0.523	-0.454	0.068	no
Treat-0d	RHSIM_Rhsim02G0181100	-2.341	-2.061	0.280	low
Treat-0d	RHSIM_Rhsim04G0167100	1.679	2.543	0.864	high
Treat-0d	RHSIM_Rhsim02G0059100	-0.555	1.537	2.093	no
Treat-0d	RHSIM_Rhsim02G0084900	-0.629	-0.077	0.552	no
Treat-0d	RHSIM_Rhsim02G0042700	-1.361	0.626	1.987	low
Treat-0d	RHSIM_Rhsim03G0228600	-1.418	0.123	1.541	low
Treat-0d	RHSIM_Rhsim02G0082300	-0.098	-0.779	-0.681	no
Treat-0d	RHSIM_Rhsim02G0036100	0.338	0.166	-0.172	no
Treat-0d	RHSIM_Rhsim04G0038900	-0.614	-0.590	0.024	no
Treat-0d	RHSIM_Rhsim02G0103000	-0.341	-0.524	-0.182	no
Treat-0d	RHSIM_Rhsim02G0014100	-0.119	0.329	0.448	no
Treat-0d	RHSIM_Rhsim03G0127300	0.309	1.365	1.056	no
Treat-0d	RHSIM_Rhsim03G0078300	0.998	2.721	1.722	no
Treat-0d	RHSIM_Rhsim02G0220000	2.626	1.294	-1.332	high
Treat-0d	RHSIM_Rhsim03G0189100	0.528	-1.036	-1.564	no
Treat-0d	RHSIM_Rhsim02G0136400	1.112	2.023	0.911	high
Treat-0d	RHSIM_Rhsim03G0057300	-0.204	-0.889	-0.685	no
Treat-0d	RHSIM_Rhsim02G0241700	1.284	0.576	-0.708	high
Treat-0d	RHSIM_Rhsim03G0046400	-0.232	-0.736	-0.504	no
Treat-0d	RHSIM_Rhsim03G0258700	0.230	-0.768	-0.998	no
Treat-0d	RHSIM_Rhsim03G0265400	0.192	0.945	0.753	no
Treat-0d	RHSIM_Rhsim02G0086200	1.734	0.116	-1.618	high
Treat-0d	RHSIM_Rhsim02G0255200	1.295	-0.770	-2.065	high
Treat-0d	RHSIM_Rhsim02G0254500	1.102	1.620	0.518	no

Group	Gene	PTR (z-score difference)	z(log2 LFQ)	z(log2 FPKM)	extreme-PTR
Treat-0d	RHSIM_Rhsim02G0173200	-0.680	-0.541	0.140	no
Treat-0d	RHSIM_Rhsim03G0116200	1.329	0.636	-0.693	high
Treat-0d	RHSIM_Rhsim03G0234900	0.561	-0.475	-1.036	no
Treat-0d	RHSIM_Rhsim02G0060400	-1.744	-1.654	0.089	low
Treat-0d	RHSIM_Rhsim03G0151900	-0.392	-0.395	-0.003	no
Treat-0d	RHSIM_Rhsim02G0140300	0.312	1.313	1.000	no
Treat-0d	RHSIM_Rhsim03G0000600	0.563	0.376	-0.188	no
Treat-0d	RHSIM_Rhsim02G0216000	-0.743	-0.838	-0.095	no
Treat-0d	RHSIM_Rhsim02G0213000	0.547	-1.216	-1.763	no
Treat-0d	RHSIM_Rhsim04G0247900	-1.410	-0.438	0.972	low
Treat-0d	RHSIM_Rhsim02G0097500	-0.739	-2.042	-1.303	no
Treat-0d	RHSIM_Rhsim03G0002000	0.409	-0.031	-0.440	no
Treat-0d	RHSIM_Rhsim02G0239800	-0.864	-1.340	-0.476	no
Treat-0d	RHSIM_Rhsim02G0008500	-0.687	2.149	2.836	no
Treat-0d	RHSIM_Rhsim02G0009100	-0.040	0.873	0.913	no
Treat-0d	RHSIM_Rhsim02G0217600	-1.171	-1.543	-0.372	low
Treat-0d	RHSIM_Rhsim02G0019700	0.123	-0.407	-0.530	no
Treat-0d	RHSIM_Rhsim02G0255500	1.297	0.821	-0.475	high
Treat-0d	RHSIM_Rhsim03G0232800	0.478	-0.478	-0.957	no
Treat-0d	RHSIM_Rhsim02G0023300	1.171	0.676	-0.495	high
Treat-0d	RHSIM_Rhsim03G0264100	1.525	1.424	-0.101	high
Treat-0d	RHSIM_Rhsim02G0043700	0.525	0.083	-0.442	no
Treat-0d	RHSIM_Rhsim03G0259400	-0.143	0.946	1.089	no
Treat-0d	RHSIM_Rhsim02G0088500	0.912	0.349	-0.563	no
Treat-0d	RHSIM_Rhsim04G0016400	1.133	1.066	-0.067	high
Treat-0d	RHSIM_Rhsim02G0137400	-0.858	-1.704	-0.845	no
Treat-0d	RHSIM_Rhsim02G0006600	0.318	-0.229	-0.546	no
Treat-0d	RHSIM_Rhsim02G0013600	0.472	-0.633	-1.105	no
Treat-0d	RHSIM_Rhsim02G0254400	-0.161	0.199	0.360	no
Treat-0d	RHSIM_Rhsim02G0053400	-0.393	0.788	1.182	no
Treat-0d	RHSIM_Rhsim02G0081400	3.341	1.060	-2.281	high
Treat-0d	RHSIM_Rhsim03G0136800	1.258	-0.393	-1.650	high

Group	Gene	PTR (z-score difference)	z(log2 LFQ)	z(log2 FPKM)	extreme-PTR
Treat-0d	RHSIM_Rhsim02G0068600	3.748	1.753	-1.994	high
Treat-0d	RHSIM_Rhsim03G0124200	1.978	0.074	-1.904	high
Treat-0d	RHSIM_Rhsim03G0141800	-1.269	-0.206	1.064	low
Treat-0d	RHSIM_Rhsim03G0010600	-1.267	-0.893	0.374	low
Treat-0d	RHSIM_Rhsim03G0120800	0.957	1.315	0.358	no
Treat-0d	RHSIM_Rhsim02G0244400	0.621	1.427	0.807	no
Treat-0d	RHSIM_Rhsim02G0014600	-0.785	-0.400	0.385	no
Treat-0d	RHSIM_Rhsim04G0161900	1.597	1.062	-0.535	high
Treat-0d	RHSIM_Rhsim02G0023100	1.976	1.417	-0.560	high
Treat-0d	RHSIM_Rhsim03G0011600	-0.125	-0.698	-0.573	no
Treat-0d	RHSIM_Rhsim02G0066700	1.426	-0.140	-1.566	high
Treat-0d	RHSIM_Rhsim03G0075600	-0.856	-0.493	0.363	no
Treat-0d	RHSIM_Rhsim02G0089200	-0.251	-0.684	-0.433	no
Treat-0d	RHSIM_Rhsim04G0239100	0.660	-0.059	-0.718	no
Treat-0d	RHSIM_Rhsim02G0216100	-0.013	-0.581	-0.568	no
Treat-0d	RHSIM_Rhsim02G0126300	0.751	1.271	0.519	no
Treat-0d	RHSIM_Rhsim02G0025700	0.336	0.066	-0.270	no
Treat-0d	RHSIM_Rhsim04G0138800	-1.265	-0.933	0.332	low
Treat-0d	RHSIM_Rhsim03G0201600	0.829	-0.639	-1.468	no
Treat-0d	RHSIM_Rhsim03G0182700	1.384	-0.924	-2.308	high
Treat-0d	RHSIM_Rhsim02G0106500	0.875	-0.692	-1.567	no
Treat-0d	RHSIM_Rhsim04G0002700	0.752	0.560	-0.192	no
Treat-0d	RHSIM_Rhsim03G0150500	0.678	0.137	-0.542	no
Treat-0d	RHSIM_Rhsim02G0181700	-0.239	-0.179	0.060	no
Treat-0d	RHSIM_Rhsim04G0106800	0.230	-0.260	-0.491	no
Treat-0d	RHSIM_Rhsim02G0138500	0.438	0.323	-0.114	no
Treat-0d	RHSIM_Rhsim02G0070200	-0.102	-0.320	-0.218	no
Treat-0d	RHSIM_Rhsim02G0119300	0.103	0.633	0.531	no
Treat-0d	RHSIM_Rhsim02G0111900	0.700	0.645	-0.055	no
Treat-0d	RHSIM_Rhsim02G0254100	0.908	0.049	-0.859	no
Treat-0d	RHSIM_Rhsim03G0209200	0.370	0.271	-0.098	no
Treat-0d	RHSIM_Rhsim03G0089600	1.067	0.125	-0.942	no

Group	Gene	PTR (z-score difference)	z(log2 LFQ)	z(log2 FPKM)	extreme-PTR
Treat-0d	RHSIM_Rhsim02G0215900	-0.282	-0.289	-0.007	no
Treat-0d	RHSIM_Rhsim02G0001500	1.332	2.388	1.056	high
Treat-0d	RHSIM_Rhsim03G0258200	0.613	1.360	0.748	no
Treat-0d	RHSIM_Rhsim02G0233100	1.100	1.849	0.748	no
Treat-0d	RHSIM_Rhsim02G0154700	1.039	0.717	-0.322	no
Treat-0d	RHSIM_Rhsim02G0244500	-1.983	-2.017	-0.034	low
Treat-0d	RHSIM_Rhsim02G0214700	2.890	0.740	-2.151	high
Treat-0d	RHSIM_Rhsim03G0050100	1.688	1.321	-0.367	high
Treat-0d	RHSIM_Rhsim02G0214000	-0.097	-0.825	-0.728	no
Treat-0d	RHSIM_Rhsim02G0086000	-0.999	-1.387	-0.388	no
Treat-0d	RHSIM_Rhsim02G0062100	0.157	1.701	1.543	no
Treat-0d	RHSIM_Rhsim04G0216800	-0.758	0.707	1.465	no
Treat-0d	RHSIM_Rhsim03G0179200	-1.608	-1.280	0.328	low
Treat-0d	RHSIM_Rhsim03G0171200	-0.911	-1.210	-0.299	no
Treat-0d	RHSIM_Rhsim02G0135000	-0.078	1.671	1.749	no
Treat-0d	RHSIM_Rhsim02G0083600	-0.440	-0.979	-0.539	no
Treat-0d	RHSIM_Rhsim02G0201500	-0.855	-0.189	0.666	no
Treat-0d	RHSIM_Rhsim02G0172900	-0.279	-0.702	-0.422	no
Treat-0d	RHSIM_Rhsim02G0037100	-1.377	-0.969	0.408	low
Treat-0d	RHSIM_Rhsim02G0154900	0.036	0.475	0.439	no
Treat-0d	RHSIM_Rhsim03G0123600	-0.773	-0.212	0.562	no
Treat-0d	RHSIM_Rhsim02G0179400	-0.375	0.223	0.597	no
Treat-0d	RHSIM_Rhsim02G0062200	0.119	-0.222	-0.341	no
Treat-0d	RHSIM_Rhsim02G0141000	0.070	0.037	-0.033	no
Treat-0d	RHSIM_Rhsim03G0214000	-0.027	-0.301	-0.275	no
Treat-0d	RHSIM_Rhsim04G0087600	0.677	1.054	0.377	no
Treat-0d	RHSIM_Rhsim02G0244300	-0.843	-1.451	-0.607	no
Treat-0d	RHSIM_Rhsim02G0000100	0.463	0.069	-0.394	no
Treat-0d	RHSIM_Rhsim04G0150200	-1.584	-1.023	0.561	low
Treat-0d	RHSIM_Rhsim02G0181200	0.489	0.339	-0.150	no
Treat-0d	RHSIM_Rhsim02G0231700	0.230	0.147	-0.083	no
Treat-0d	RHSIM_Rhsim02G0217900	0.467	0.547	0.080	no

Group	Gene	PTR (z-score difference)	z(log2 LFQ)	z(log2 FPKM)	extreme-PTR
Treat-0d	RHSIM_Rhsim03G0035800	0.932	0.251	-0.681	no
Treat-0d	RHSIM_Rhsim02G0056400	-0.190	1.832	2.022	no
Treat-0d	RHSIM_Rhsim02G0169200	0.742	-0.216	-0.958	no
Treat-0d	RHSIM_Rhsim02G0008900	-0.078	-1.138	-1.060	no
Treat-0d	RHSIM_Rhsim03G0257200	-0.034	0.752	0.787	no
Treat-0d	RHSIM_Rhsim02G0140600	0.163	-0.064	-0.227	no
Treat-0d	RHSIM_Rhsim02G0187100	1.376	1.430	0.054	high
Treat-0d	RHSIM_Rhsim02G0041100	1.315	-0.561	-1.876	high
Treat-0d	RHSIM_Rhsim02G0068000	0.357	0.395	0.038	no
Treat-0d	RHSIM_Rhsim02G0239600	0.722	1.125	0.403	no
Treat-0d	RHSIM_Rhsim02G0252100	0.427	-1.002	-1.429	no
Treat-0d	RHSIM_Rhsim02G0149100	-0.899	0.037	0.936	no
Treat-0d	RHSIM_Rhsim04G0022600	1.270	1.413	0.142	high
Treat-0d	RHSIM_Rhsim02G0106400	0.062	2.317	2.255	no
Treat-0d	RHSIM_Rhsim02G0031400	-0.959	-0.286	0.673	no
Treat-0d	RHSIM_Rhsim03G0228400	4.397	2.004	-2.393	high
Treat-0d	RHSIM_Rhsim02G0176900	-0.147	-0.431	-0.284	no
Treat-0d	RHSIM_Rhsim03G0192600	0.291	1.159	0.868	no
Treat-0d	RHSIM_Rhsim02G0144300	-0.100	0.130	0.230	no
Treat-0d	RHSIM_Rhsim03G0179700	-1.354	0.239	1.593	low
Treat-0d	RHSIM_Rhsim04G0011900	0.202	0.287	0.085	no
Treat-0d	RHSIM_Rhsim02G0099500	-0.470	0.234	0.704	no
Treat-0d	RHSIM_Rhsim02G0162600	0.220	1.084	0.864	no
Treat-0d	RHSIM_Rhsim02G0055200	-0.653	-0.216	0.436	no
Treat-0d	RHSIM_Rhsim02G0104500	-0.339	-0.132	0.207	no
Treat-0d	RHSIM_Rhsim03G0224600	0.865	1.578	0.713	no
Treat-0d	RHSIM_Rhsim02G0113900	3.042	1.257	-1.785	high
Treat-0d	RHSIM_Rhsim01G0236100	-0.591	-0.308	0.283	no
Treat-0d	RHSIM_Rhsim04G0247100	0.536	1.399	0.862	no
Treat-0d	RHSIM_Rhsim03G0192700	0.246	0.782	0.536	no
Treat-0d	RHSIM_Rhsim01G0001000	0.147	1.412	1.265	no
Treat-0d	RHSIM_Rhsim02G0202100	-0.487	2.364	2.851	no

Group	Gene	PTR (z-score difference)	z(log2 LFQ)	z(log2 FPKM)	extreme-PTR
Treat-0d	RHSIM_Rhsim01G0131400	1.287	-0.589	-1.876	high
Treat-0d	RHSIM_Rhsim02G0164900	-0.602	-1.483	-0.881	no
Treat-0d	RHSIM_Rhsim02G0067100	1.119	1.292	0.173	high
Treat-0d	RHSIM_Rhsim02G0169800	-0.011	-0.007	0.003	no
Treat-0d	RHSIM_Rhsim02G0228100	0.222	-0.981	-1.204	no
Treat-0d	RHSIM_Rhsim02G0201600	-0.674	-0.524	0.150	no
Treat-0d	RHSIM_Rhsim02G0036900	0.222	0.654	0.432	no
Treat-0d	RHSIM_Rhsim02G0198800	-0.571	-0.735	-0.164	no
Treat-0d	RHSIM_Rhsim01G0219200	0.648	0.424	-0.223	no
Treat-0d	RHSIM_Rhsim03G0217400	-0.490	1.615	2.105	no
Treat-0d	RHSIM_Rhsim01G0232300	-0.172	-1.002	-0.830	no
Treat-0d	RHSIM_Rhsim02G0218200	0.889	1.281	0.392	no
Treat-0d	RHSIM_Rhsim01G0254500	-0.875	-0.470	0.405	no
Treat-0d	RHSIM_Rhsim02G0069700	-0.907	-1.889	-0.981	no
Treat-0d	RHSIM_Rhsim02G0076300	-0.561	0.027	0.588	no
Treat-0d	RHSIM_Rhsim03G0028600	1.219	0.940	-0.279	high
Treat-0d	RHSIM_Rhsim02G0035500	-1.872	0.248	2.119	low
Treat-0d	RHSIM_Rhsim02G0035300	-0.202	-0.110	0.092	no
Treat-0d	RHSIM_Rhsim02G0041900	-0.804	-0.303	0.501	no
Treat-0d	RHSIM_Rhsim02G0178300	0.176	-0.638	-0.814	no
Treat-0d	RHSIM_Rhsim02G0104000	-0.569	0.120	0.689	no
Treat-0d	RHSIM_Rhsim04G0234100	0.285	1.306	1.021	no
Treat-0d	RHSIM_Rhsim03G0135900	-0.103	1.286	1.389	no
Treat-0d	RHSIM_Rhsim02G0183000	-0.135	-0.836	-0.701	no
Treat-0d	RHSIM_Rhsim02G0177000	0.156	-0.213	-0.369	no
Treat-0d	RHSIM_Rhsim02G0176200	1.020	0.200	-0.820	no
Treat-0d	RHSIM_Rhsim01G0186900	1.452	0.343	-1.109	high
Treat-0d	RHSIM_Rhsim02G0146500	-1.107	-0.865	0.242	low
Treat-0d	RHSIM_Rhsim04G0013700	0.615	0.665	0.050	no
Treat-0d	RHSIM_Rhsim01G0245400	-0.073	1.138	1.212	no
Treat-0d	RHSIM_Rhsim02G0020900	1.090	0.345	-0.746	no
Treat-0d	RHSIM_Rhsim02G0145500	0.972	0.700	-0.271	no

Group	Gene	PTR (z-score difference)	z(log2 LFQ)	z(log2 FPKM)	extreme-PTR
Treat-0d	RHSIM_Rhsim02G0251900	-0.358	-0.862	-0.503	no
Treat-0d	RHSIM_Rhsim02G0123300	-1.178	-1.023	0.155	low
Treat-0d	RHSIM_Rhsim02G0115100	1.863	4.478	2.615	high
Treat-0d	RHSIM_Rhsim02G0171500	0.336	0.586	0.250	no
Treat-0d	RHSIM_Rhsim02G0070300	1.169	0.664	-0.505	high
Treat-0d	RHSIM_Rhsim04G0239600	-0.677	-0.518	0.159	no
Treat-0d	RHSIM_Rhsim01G0225400	-0.778	-0.828	-0.049	no
Treat-0d	RHSIM_Rhsim02G0014200	0.185	0.035	-0.151	no
Treat-0d	RHSIM_Rhsim02G0165800	-0.129	0.365	0.494	no
Treat-0d	RHSIM_Rhsim02G0027900	-2.161	-0.882	1.279	low
Treat-0d	RHSIM_Rhsim02G0076900	-0.431	-1.721	-1.290	no
Treat-0d	RHSIM_Rhsim02G0120700	-0.685	-0.302	0.383	no
Treat-0d	RHSIM_Rhsim02G0160300	-1.795	-0.518	1.277	low
Treat-0d	RHSIM_Rhsim02G0251500	-1.908	-1.976	-0.068	low
Treat-0d	RHSIM_Rhsim02G0115500	-0.780	-0.575	0.205	no
Treat-0d	RHSIM_Rhsim02G0220400	1.777	1.255	-0.522	high
Treat-0d	RHSIM_Rhsim02G0071700	0.373	-0.039	-0.412	no
Treat-0d	RHSIM_Rhsim02G0159900	0.020	-0.447	-0.466	no
Treat-0d	RHSIM_Rhsim02G0165700	-1.761	-0.773	0.989	low
Treat-0d	RHSIM_Rhsim04G0100800	-1.205	0.160	1.365	low
Treat-0d	RHSIM_Rhsim02G0162800	0.445	-0.715	-1.160	no
Treat-0d	RHSIM_Rhsim02G0037200	-1.903	-0.572	1.331	low
Treat-0d	RHSIM_Rhsim02G0127600	-0.435	-0.437	-0.002	no
Treat-0d	RHSIM_Rhsim02G0200700	-0.599	-0.955	-0.356	no
Treat-0d	RHSIM_Rhsim03G0210800	0.527	0.676	0.149	no
Treat-0d	RHSIM_Rhsim04G0240100	0.685	0.093	-0.592	no
Treat-0d	RHSIM_Rhsim02G0057300	-2.582	-0.880	1.702	low
Treat-0d	RHSIM_Rhsim03G0230200	0.565	0.172	-0.393	no
Treat-0d	RHSIM_Rhsim04G0013000	-0.630	-1.606	-0.975	no
Treat-0d	RHSIM_Rhsim01G0160900	1.261	0.952	-0.308	high
Treat-0d	RHSIM_Rhsim01G0235400	0.602	1.358	0.756	no
Treat-0d	RHSIM_Rhsim02G0188300	1.399	0.503	-0.896	high

Group	Gene	PTR (z-score difference)	z(log2 LFQ)	z(log2 FPKM)	extreme-PTR
Treat-0d	RHSIM_Rhsim02G0027800	-1.097	-0.550	0.547	low
Treat-0d	RHSIM_Rhsim02G0027600	1.089	-0.826	-1.915	no
Treat-0d	RHSIM_Rhsim01G0230500	-2.078	-1.446	0.632	low
Treat-0d	RHSIM_Rhsim02G0021200	1.088	2.061	0.973	no
Treat-0d	RHSIM_Rhsim03G0056900	0.616	-1.008	-1.624	no
Treat-0d	RHSIM_Rhsim02G0032400	0.261	1.793	1.532	no
Treat-0d	RHSIM_Rhsim01G0112900	-2.214	-2.098	0.117	low
Treat-0d	RHSIM_Rhsim02G0160100	0.035	-0.457	-0.492	no
Treat-0d	RHSIM_Rhsim02G0067200	1.573	-0.501	-2.074	high
Treat-0d	RHSIM_Rhsim01G0217300	1.056	0.674	-0.381	no
Treat-0d	RHSIM_Rhsim01G0171700	-0.626	-0.665	-0.039	no
Treat-0d	RHSIM_Rhsim01G0145800	0.847	0.880	0.032	no
Treat-0d	RHSIM_Rhsim01G0245700	-0.530	-0.722	-0.192	no
Treat-0d	RHSIM_Rhsim01G0142200	-0.780	-1.164	-0.384	no
Treat-0d	RHSIM_Rhsim02G0168400	-0.808	-0.695	0.113	no
Treat-0d	RHSIM_Rhsim01G0268000	0.091	-0.621	-0.712	no
Treat-0d	RHSIM_Rhsim04G0227400	-1.378	-0.901	0.477	low
Treat-0d	RHSIM_Rhsim02G0234500	-1.228	-1.119	0.109	low
Treat-0d	RHSIM_Rhsim01G0249200	-0.624	-1.427	-0.802	no
Treat-0d	RHSIM_Rhsim01G0069300	0.466	0.428	-0.038	no
Treat-0d	RHSIM_Rhsim03G0130400	-0.240	0.389	0.629	no
Treat-0d	RHSIM_Rhsim01G0268500	-0.649	-1.582	-0.933	no
Treat-0d	RHSIM_Rhsim01G0197500	0.495	0.167	-0.328	no
Treat-0d	RHSIM_Rhsim01G0186400	2.524	2.352	-0.172	high
Treat-0d	RHSIM_Rhsim02G0082500	-0.768	0.417	1.184	no
Treat-0d	RHSIM_Rhsim02G0072000	0.590	-0.096	-0.686	no
Treat-0d	RHSIM_Rhsim01G0280600	-1.260	-0.885	0.375	low
Treat-0d	RHSIM_Rhsim04G0110500	0.525	-1.140	-1.665	no
Treat-0d	RHSIM_Rhsim01G0002200	0.197	0.230	0.033	no
Treat-0d	RHSIM_Rhsim02G0088100	1.201	1.169	-0.031	high
Treat-0d	RHSIM_Rhsim04G0172200	0.499	1.990	1.491	no
Treat-0d	RHSIM_Rhsim02G0013700	-0.192	-0.638	-0.446	no

Group	Gene	PTR (z-score difference)	z(log2 LFQ)	z(log2 FPKM)	extreme-PTR
Treat-0d	RHSIM_Rhsim01G0293800	0.900	1.550	0.650	no
Treat-0d	RHSIM_Rhsim01G0263600	1.052	0.265	-0.787	no
Treat-0d	RHSIM_Rhsim04G0224200	-0.287	-0.145	0.142	no
Treat-0d	RHSIM_Rhsim02G0076100	-1.960	-1.861	0.100	low
Treat-0d	RHSIM_Rhsim01G0245600	-2.816	-0.861	1.955	low
Treat-0d	RHSIM_Rhsim02G0203400	0.435	0.573	0.137	no
Treat-0d	RHSIM_Rhsim01G0281900	1.040	-0.408	-1.448	no
Treat-0d	RHSIM_Rhsim02G0210900	-0.289	0.289	0.578	no
Treat-0d	RHSIM_Rhsim01G0101000	-2.098	-1.570	0.528	low
Treat-0d	RHSIM_Rhsim04G0176700	-0.172	0.329	0.501	no
Treat-0d	RHSIM_Rhsim02G0062300	1.852	0.927	-0.925	high
Treat-0d	RHSIM_Rhsim01G0142500	-0.083	-0.986	-0.903	no
Treat-0d	RHSIM_Rhsim02G0061900	0.140	-0.860	-1.000	no
Treat-0d	RHSIM_Rhsim03G0011700	0.719	0.083	-0.636	no
Treat-0d	RHSIM_Rhsim01G0218000	0.345	0.312	-0.033	no
Treat-0d	RHSIM_Rhsim01G0211400	-0.868	-0.440	0.428	no
Treat-0d	RHSIM_Rhsim03G0141500	-0.197	1.799	1.996	no
Treat-0d	RHSIM_Rhsim02G0018200	1.736	0.958	-0.777	high
Treat-0d	RHSIM_Rhsim02G0243200	2.487	0.285	-2.202	high
Treat-0d	RHSIM_Rhsim01G0273700	-0.087	-0.140	-0.053	no
Treat-0d	RHSIM_Rhsim03G0135800	-0.415	0.151	0.566	no
Treat-0d	RHSIM_Rhsim02G0162900	-0.228	0.203	0.431	no
Treat-0d	RHSIM_Rhsim01G0207700	0.692	1.269	0.577	no
Treat-0d	RHSIM_Rhsim02G0033000	-0.420	0.276	0.696	no
Treat-0d	RHSIM_Rhsim02G0066900	0.245	-1.129	-1.374	no
Treat-0d	RHSIM_Rhsim02G0168300	0.531	0.359	-0.173	no
Treat-0d	RHSIM_Rhsim04G0000500	0.594	-0.804	-1.397	no
Treat-0d	RHSIM_Rhsim02G0169300	-0.856	0.040	0.896	no
Treat-0d	RHSIM_Rhsim02G0099100	-0.868	-1.176	-0.308	no
Treat-0d	RHSIM_Rhsim01G0060800	1.087	1.034	-0.053	no
Treat-0d	RHSIM_Rhsim02G0087400	1.226	0.619	-0.607	high
Treat-0d	RHSIM_Rhsim02G0044000	-0.203	-0.535	-0.332	no

Group	Gene	PTR (z-score difference)	z(log2 LFQ)	z(log2 FPKM)	extreme-PTR
Treat-0d	RHSIM_Rhsim01G0290800	-0.414	2.774	3.188	no
Treat-0d	RHSIM_Rhsim02G0172400	0.345	-0.126	-0.470	no
Treat-0d	RHSIM_Rhsim02G0023800	2.138	0.533	-1.605	high
Treat-0d	RHSIM_Rhsim02G0054300	0.234	0.186	-0.048	no
Treat-0d	RHSIM_Rhsim02G0003600	0.122	0.332	0.210	no
Treat-0d	RHSIM_Rhsim01G0234400	0.078	-1.517	-1.594	no
Treat-0d	RHSIM_Rhsim02G0202300	0.637	-0.005	-0.642	no
Treat-0d	RHSIM_Rhsim04G0128500	0.311	-0.042	-0.353	no
Treat-0d	RHSIM_Rhsim01G0100400	0.242	-0.788	-1.029	no
Treat-0d	RHSIM_Rhsim02G0106700	-1.354	-1.174	0.180	low
Treat-0d	RHSIM_Rhsim01G0182300	0.156	-0.821	-0.977	no
Treat-0d	RHSIM_Rhsim02G0195900	-0.045	-0.875	-0.831	no
Treat-0d	RHSIM_Rhsim02G0190100	-0.620	-0.389	0.231	no
Treat-0d	RHSIM_Rhsim04G0093900	0.546	0.866	0.320	no
Treat-0d	RHSIM_Rhsim02G0067000	-1.621	-0.295	1.325	low
Treat-0d	RHSIM_Rhsim02G0247000	0.094	-0.015	-0.109	no
Treat-0d	RHSIM_Rhsim02G0017000	-0.452	-0.610	-0.158	no
Treat-0d	RHSIM_Rhsim02G0213200	0.081	-0.772	-0.853	no
Treat-0d	RHSIM_Rhsim01G0219000	-0.954	-0.549	0.405	no
Treat-0d	RHSIM_Rhsim01G0176200	0.397	-1.089	-1.486	no
Treat-0d	RHSIM_Rhsim02G0052200	-0.457	-0.224	0.233	no
Treat-0d	RHSIM_Rhsim02G0034800	-0.031	-0.009	0.022	no
Treat-0d	RHSIM_Rhsim02G0214900	1.588	0.396	-1.192	high
Treat-0d	RHSIM_Rhsim01G0243700	0.400	-0.025	-0.426	no
Treat-0d	RHSIM_Rhsim02G0110000	-0.463	0.162	0.625	no
Treat-0d	RHSIM_Rhsim02G0180500	-1.134	-0.287	0.847	low
Treat-0d	RHSIM_Rhsim04G0204400	-0.779	-0.955	-0.176	no
Treat-0d	RHSIM_Rhsim02G0198400	1.049	-0.285	-1.333	no
Treat-0d	RHSIM_Rhsim01G0102000	1.581	-0.517	-2.098	high
Treat-0d	RHSIM_Rhsim01G0096100	-0.223	-0.967	-0.744	no
Treat-0d	RHSIM_Rhsim01G0136000	0.470	0.778	0.308	no
Treat-0d	RHSIM_Rhsim02G0002800	0.118	-0.031	-0.149	no

Group	Gene	PTR (z-score difference)	z(log2 LFQ)	z(log2 FPKM)	extreme-PTR
Treat-0d	RHSIM_Rhsim01G0197200	1.152	1.915	0.763	high
Treat-0d	RHSIM_Rhsim01G0090900	0.044	3.089	3.044	no
Treat-0d	RHSIM_Rhsim01G0095300	0.344	-0.268	-0.612	no
Treat-0d	RHSIM_Rhsim02G0171600	0.755	1.259	0.505	no
Treat-0d	RHSIM_Rhsim02G0054100	1.219	0.413	-0.807	high
Treat-0d	RHSIM_Rhsim01G0196800	0.614	-0.194	-0.809	no
Treat-0d	RHSIM_Rhsim02G0040400	-1.998	-1.658	0.341	low
Treat-0d	RHSIM_Rhsim03G0218500	0.310	0.983	0.673	no
Treat-0d	RHSIM_Rhsim02G0032200	-0.744	-0.770	-0.026	no
Treat-0d	RHSIM_Rhsim02G0004400	-0.065	1.226	1.292	no
Treat-0d	RHSIM_Rhsim02G0009000	0.160	-0.556	-0.716	no
Treat-0d	RHSIM_Rhsim02G0179200	0.763	0.878	0.116	no
Treat-0d	RHSIM_Rhsim02G0212500	-0.127	0.112	0.239	no
Treat-0d	RHSIM_Rhsim02G0202200	0.438	-0.356	-0.794	no
Treat-0d	RHSIM_Rhsim01G0123500	0.260	-0.702	-0.962	no
Treat-0d	RHSIM_Rhsim01G0051700	0.703	0.041	-0.662	no
Treat-0d	RHSIM_Rhsim01G0216700	-0.214	0.371	0.584	no
Treat-0d	RHSIM_Rhsim01G0151600	-1.029	-1.560	-0.531	low
Treat-0d	RHSIM_Rhsim01G0107900	0.635	0.546	-0.089	no
Treat-0d	RHSIM_Rhsim03G0170800	0.361	1.392	1.032	no
Treat-0d	RHSIM_Rhsim01G0209500	-0.735	1.163	1.898	no
Treat-0d	RHSIM_Rhsim01G0259700	0.333	-0.832	-1.165	no
Treat-0d	RHSIM_Rhsim02G0194300	0.071	1.961	1.891	no
Treat-0d	RHSIM_Rhsim02G0069300	0.127	-0.485	-0.612	no
Treat-0d	RHSIM_Rhsim01G0293700	0.699	-0.223	-0.922	no
Treat-0d	RHSIM_Rhsim03G0144200	0.120	0.033	-0.088	no
Treat-0d	RHSIM_Rhsim01G0265100	1.114	0.609	-0.505	high
Treat-0d	RHSIM_Rhsim01G0291100	0.499	1.347	0.848	no
Treat-0d	RHSIM_Rhsim02G0232700	0.129	0.226	0.097	no
Treat-0d	RHSIM_Rhsim01G0250300	1.046	-0.511	-1.556	no
Treat-0d	RHSIM_Rhsim02G0228400	-0.065	0.222	0.287	no
Treat-0d	RHSIM_Rhsim01G0146900	-0.625	-1.029	-0.404	no

Group	Gene	PTR (z-score difference)	z(log2 LFQ)	z(log2 FPKM)	extreme-PTR
Treat-0d	RHSIM_Rhsim01G0124300	0.371	-0.529	-0.900	no
Treat-0d	RHSIM_Rhsim01G0067700	-2.691	-1.658	1.033	low
Treat-0d	RHSIM_Rhsim01G0281800	-1.212	-1.334	-0.122	low
Treat-0d	RHSIM_Rhsim01G0119100	0.485	-0.880	-1.365	no
Treat-0d	RHSIM_Rhsim02G0095200	0.229	-0.781	-1.010	no
Treat-0d	RHSIM_Rhsim02G0223500	0.800	-0.830	-1.630	no
Treat-0d	RHSIM_Rhsim01G0292800	0.802	1.164	0.361	no
Treat-0d	RHSIM_Rhsim03G0247900	2.735	1.239	-1.497	high
Treat-0d	RHSIM_Rhsim01G0282100	-0.358	-1.289	-0.931	no
Treat-0d	RHSIM_Rhsim01G0189100	-1.270	-1.125	0.146	low
Treat-0d	RHSIM_Rhsim02G0216700	-0.138	-0.553	-0.415	no
Treat-0d	RHSIM_Rhsim01G0224200	1.104	0.432	-0.672	high
Treat-0d	RHSIM_Rhsim03G0036700	-0.085	0.246	0.331	no
Treat-0d	RHSIM_Rhsim01G0191800	-1.899	-0.585	1.315	low
Treat-0d	RHSIM_Rhsim01G0210800	0.188	1.207	1.019	no
Treat-0d	RHSIM_Rhsim01G0109600	-0.239	-0.448	-0.208	no
Treat-0d	RHSIM_Rhsim01G0009900	-1.493	-0.967	0.527	low
Treat-0d	RHSIM_Rhsim01G0176300	0.833	-0.668	-1.501	no
Treat-0d	RHSIM_Rhsim03G0088500	1.127	-0.143	-1.269	high
Treat-0d	RHSIM_Rhsim02G0244800	0.793	0.741	-0.052	no
Treat-0d	RHSIM_Rhsim01G0197900	0.149	-0.184	-0.333	no
Treat-0d	RHSIM_Rhsim01G0191100	1.572	2.623	1.051	high
Treat-0d	RHSIM_Rhsim02G0205100	-1.626	-1.093	0.533	low
Treat-0d	RHSIM_Rhsim01G0201600	-0.151	-0.023	0.128	no
Treat-0d	RHSIM_Rhsim03G0125000	-1.413	-0.809	0.605	low
Treat-0d	RHSIM_Rhsim01G0210200	-1.692	-1.313	0.379	low
Treat-0d	RHSIM_Rhsim02G0062000	0.118	1.256	1.138	no
Treat-0d	RHSIM_Rhsim01G0255600	0.583	-0.182	-0.766	no
Treat-0d	RHSIM_Rhsim01G0188400	0.119	0.749	0.629	no
Treat-0d	RHSIM_Rhsim03G0142300	2.563	1.130	-1.433	high
Treat-0d	RHSIM_Rhsim02G0168200	-0.791	-1.520	-0.728	no
Treat-0d	RHSIM_Rhsim01G0143400	-0.144	1.368	1.513	no

Group	Gene	PTR (z-score difference)	z(log2 LFQ)	z(log2 FPKM)	extreme-PTR
Treat-0d	RHSIM_Rhsim01G0204700	-1.058	-0.047	1.010	low
Treat-0d	RHSIM_Rhsim01G0259300	0.925	0.643	-0.282	no
Treat-0d	RHSIM_Rhsim01G0091000	-0.127	-0.399	-0.272	no
Treat-0d	RHSIM_Rhsim01G0276200	-0.442	-0.699	-0.258	no
Treat-0d	RHSIM_Rhsim02G0148900	-0.359	-0.172	0.188	no
Treat-0d	RHSIM_Rhsim01G0262300	-0.255	-0.573	-0.318	no
Treat-0d	RHSIM_Rhsim01G0180500	-0.760	-0.623	0.137	no
Treat-0d	RHSIM_Rhsim01G0119800	-0.237	-0.422	-0.185	no
Treat-0d	RHSIM_Rhsim01G0010000	-0.013	0.336	0.349	no
Treat-0d	RHSIM_Rhsim01G0204400	0.024	0.217	0.192	no
Treat-0d	RHSIM_Rhsim01G0190200	0.156	0.402	0.246	no
Treat-0d	RHSIM_Rhsim01G0074300	0.078	0.710	0.631	no
Treat-0d	RHSIM_Rhsim01G0174600	0.193	-0.004	-0.197	no
Treat-0d	RHSIM_Rhsim02G0028600	-1.986	-1.627	0.359	low
Treat-0d	RHSIM_Rhsim01G0246700	0.399	-0.045	-0.444	no
Treat-0d	RHSIM_Rhsim01G0107800	-1.695	-0.000	1.694	low
Treat-0d	RHSIM_Rhsim02G0076700	-0.078	-0.188	-0.110	no
Treat-0d	RHSIM_Rhsim01G0113600	-0.376	0.564	0.940	no
Treat-0d	RHSIM_Rhsim02G0071000	0.447	-0.314	-0.761	no
Treat-0d	RHSIM_Rhsim02G0005500	-0.355	-1.348	-0.994	no
Treat-0d	RHSIM_Rhsim01G0013000	-2.318	-0.050	2.268	low
Treat-0d	RHSIM_Rhsim01G0202900	0.567	1.287	0.720	no
Treat-0d	RHSIM_Rhsim02G0193400	1.176	1.890	0.714	high
Treat-0d	RHSIM_Rhsim01G0198900	-2.165	-1.078	1.087	low
Treat-0d	RHSIM_Rhsim01G0233900	0.833	1.745	0.911	no
Treat-0d	RHSIM_Rhsim01G0067800	-0.068	0.592	0.660	no
Treat-0d	RHSIM_Rhsim02G0049400	-0.674	-1.396	-0.722	no
Treat-0d	RHSIM_Rhsim01G0227300	0.829	-0.868	-1.698	no
Treat-0d	RHSIM_Rhsim01G0137100	1.896	0.092	-1.803	high
Treat-0d	RHSIM_Rhsim01G0126900	-0.406	1.225	1.631	no
Treat-0d	RHSIM_Rhsim01G0284500	-1.184	-0.328	0.856	low
Treat-0d	RHSIM_Rhsim01G0205000	0.261	1.989	1.729	no

Group	Gene	PTR (z-score difference)	z(log2 LFQ)	z(log2 FPKM)	extreme-PTR
Treat-0d	RHSIM_Rhsim01G0145200	0.289	0.790	0.501	no
Treat-0d	RHSIM_Rhsim02G0040300	-0.658	-0.359	0.299	no
Treat-0d	RHSIM_Rhsim01G0005500	-0.709	-0.419	0.290	no
Treat-0d	RHSIM_Rhsim01G0237200	1.003	1.130	0.127	no
Treat-0d	RHSIM_Rhsim02G0049200	-2.677	-1.567	1.110	low
Treat-0d	RHSIM_Rhsim01G0263000	0.114	0.504	0.390	no
Treat-0d	RHSIM_Rhsim01G0132300	-0.277	-0.991	-0.714	no
Treat-0d	RHSIM_Rhsim01G0257300	-0.474	1.163	1.637	no
Treat-0d	RHSIM_Rhsim01G0236600	0.744	-0.924	-1.668	no
Treat-0d	RHSIM_Rhsim01G0009500	0.810	-1.369	-2.179	no
Treat-0d	RHSIM_Rhsim03G0172200	1.886	0.274	-1.612	high
Treat-0d	RHSIM_Rhsim01G0122100	-0.066	0.229	0.295	no
Treat-0d	RHSIM_Rhsim02G0204900	0.476	0.115	-0.360	no
Treat-0d	RHSIM_Rhsim01G0289700	-0.390	0.369	0.759	no
Treat-0d	RHSIM_Rhsim01G0075900	-0.005	0.184	0.189	no
Treat-0d	RHSIM_Rhsim03G0210700	-1.601	-0.352	1.249	low
Treat-0d	RHSIM_Rhsim01G0136100	0.570	-0.693	-1.262	no
Treat-0d	RHSIM_Rhsim02G0076500	-1.416	-0.205	1.211	low
Treat-0d	RHSIM_Rhsim02G0021000	-1.669	-1.219	0.450	low
Treat-0d	RHSIM_Rhsim03G0074800	-0.369	0.488	0.858	no
Treat-0d	RHSIM_Rhsim01G0181100	-1.400	-1.201	0.199	low
Treat-0d	RHSIM_Rhsim01G0062600	-0.080	0.334	0.414	no
Treat-0d	RHSIM_Rhsim01G0206500	-0.773	-0.812	-0.039	no
Treat-0d	RHSIM_Rhsim01G0185400	0.281	0.568	0.287	no
Treat-0d	RHSIM_Rhsim02G0255700	0.025	-1.287	-1.311	no
Treat-0d	RHSIM_Rhsim01G0009700	0.534	0.605	0.071	no
Treat-0d	RHSIM_Rhsim01G0172000	-1.848	-1.420	0.428	low
Treat-0d	RHSIM_Rhsim01G0272700	0.074	0.047	-0.027	no
Treat-0d	RHSIM_Rhsim01G0054700	0.698	0.690	-0.009	no
Treat-0d	RHSIM_Rhsim01G0260300	0.833	1.258	0.425	no
Treat-0d	RHSIM_Rhsim02G0114900	0.259	0.384	0.125	no
Treat-0d	RHSIM_Rhsim01G0216300	-0.233	0.940	1.173	no

Group	Gene	PTR (z-score difference)	z(log2 LFQ)	z(log2 FPKM)	extreme-PTR
Treat-0d	RHSIM_Rhsim02G0231500	0.466	-0.354	-0.821	no
Treat-0d	RHSIM_Rhsim01G0201300	0.727	0.678	-0.049	no
Treat-0d	RHSIM_Rhsim01G0234600	-0.157	-0.150	0.007	no
Treat-0d	RHSIM_Rhsim03G0250000	-2.034	-0.730	1.304	low
Treat-0d	RHSIM_Rhsim03G0251500	0.197	-0.976	-1.173	no
Treat-0d	RHSIM_Rhsim01G0208100	0.515	-1.031	-1.546	no
Treat-0d	RHSIM_Rhsim01G0124000	-0.232	-0.768	-0.535	no
Treat-0d	RHSIM_Rhsim01G0271700	-0.218	-0.594	-0.376	no
Treat-0d	RHSIM_Rhsim02G0010800	-1.383	0.022	1.405	low
Treat-0d	RHSIM_Rhsim01G0042700	-0.376	-1.317	-0.941	no
Treat-0d	RHSIM_Rhsim01G0223500	1.604	0.144	-1.460	high
Treat-0d	RHSIM_Rhsim01G0203800	0.895	0.724	-0.171	no
Treat-0d	RHSIM_Rhsim01G0286700	-0.203	0.217	0.421	no
Treat-0d	RHSIM_Rhsim01G0187100	0.270	0.436	0.166	no
Treat-0d	RHSIM_Rhsim01G0141800	-0.362	-0.185	0.178	no
Treat-0d	RHSIM_Rhsim01G0245800	0.684	0.174	-0.510	no
Treat-0d	RHSIM_Rhsim01G0042600	-0.033	-0.856	-0.823	no
Treat-0d	RHSIM_Rhsim01G0235600	-0.507	-0.195	0.312	no
Treat-0d	RHSIM_Rhsim01G0062000	0.628	0.533	-0.095	no
Treat-0d	RHSIM_Rhsim03G0059500	-1.228	-1.847	-0.620	low
Treat-0d	RHSIM_Rhsim01G0179100	-0.450	-0.125	0.325	no
Treat-0d	RHSIM_Rhsim01G0198500	0.534	0.069	-0.466	no
Treat-0d	RHSIM_Rhsim01G0164800	1.254	0.661	-0.593	high
Treat-0d	RHSIM_Rhsim01G0293000	-1.070	-0.522	0.548	low
Treat-0d	RHSIM_Rhsim01G0060200	-2.223	-1.867	0.356	low
Treat-0d	RHSIM_Rhsim01G0009300	-0.392	0.439	0.830	no
Treat-0d	RHSIM_Rhsim01G0149900	0.253	-0.402	-0.655	no
Treat-0d	RHSIM_Rhsim01G0147300	0.966	0.702	-0.264	no
Treat-0d	RHSIM_Rhsim01G0274600	1.396	2.731	1.335	high
Treat-0d	RHSIM_Rhsim01G0274400	2.646	2.956	0.310	high
Treat-0d	RHSIM_Rhsim03G0226900	0.338	1.525	1.187	no
Treat-0d	RHSIM_Rhsim02G0217400	0.355	0.703	0.348	no

Group	Gene	PTR (z-score difference)	z(log2 LFQ)	z(log2 FPKM)	extreme-PTR
Treat-0d	RHSIM_Rhsim01G0071400	-1.677	-0.609	1.068	low
Treat-0d	RHSIM_Rhsim01G0002300	1.208	0.459	-0.749	high
Treat-0d	RHSIM_Rhsim01G0033400	0.339	-0.026	-0.364	no
Treat-0d	RHSIM_Rhsim01G0269700	0.982	0.248	-0.734	no
Treat-0d	RHSIM_Rhsim01G0227900	-0.929	-1.238	-0.309	no
Treat-0d	RHSIM_Rhsim01G0254100	-2.468	-0.272	2.196	low
Treat-0d	RHSIM_Rhsim01G0074500	-1.105	-1.778	-0.673	low
Treat-0d	RHSIM_Rhsim01G0260900	1.342	0.611	-0.731	high
Treat-0d	RHSIM_Rhsim01G0006200	-1.371	-0.809	0.562	low
Treat-0d	RHSIM_Rhsim01G0088200	1.482	0.964	-0.518	high
Treat-0d	RHSIM_Rhsim01G0285400	-0.635	-0.656	-0.021	no
Treat-0d	RHSIM_Rhsim01G0155600	-0.123	-0.806	-0.683	no
Treat-0d	RHSIM_Rhsim01G0039300	-0.509	-0.222	0.287	no
Treat-0d	RHSIM_Rhsim03G0141100	-0.087	-0.028	0.059	no
Treat-0d	RHSIM_Rhsim01G0272800	-0.174	-0.574	-0.399	no
Treat-0d	RHSIM_Rhsim01G0089800	0.760	0.890	0.130	no
Treat-0d	RHSIM_Rhsim03G0207500	0.017	-1.355	-1.372	no
Treat-0d	RHSIM_Rhsim01G0205100	0.444	0.070	-0.374	no
Treat-0d	RHSIM_Rhsim01G0100800	0.619	-0.739	-1.358	no
Treat-0d	RHSIM_Rhsim03G0176000	-1.149	-0.913	0.236	low
Treat-0d	RHSIM_Rhsim01G0055600	0.784	-0.181	-0.964	no
Treat-0d	RHSIM_Rhsim01G0262400	0.574	1.214	0.640	no
Treat-0d	RHSIM_Rhsim01G0218100	0.621	0.767	0.146	no
Treat-0d	RHSIM_Rhsim01G0245300	-2.348	0.084	2.431	low
Treat-0d	RHSIM_Rhsim01G0291400	-0.108	0.335	0.444	no
Treat-0d	RHSIM_Rhsim01G0049400	-0.242	-0.361	-0.119	no
Treat-0d	RHSIM_Rhsim01G0151000	0.010	-0.384	-0.394	no
Treat-0d	RHSIM_Rhsim01G0106700	0.982	0.238	-0.743	no
Treat-0d	RHSIM_Rhsim01G0196600	-0.904	0.796	1.700	no
Treat-0d	RHSIM_Rhsim01G0239900	-0.454	0.180	0.634	no
Treat-0d	RHSIM_Rhsim01G0113300	0.864	0.208	-0.656	no
Treat-0d	RHSIM_Rhsim01G0009800	-0.387	-0.916	-0.529	no

Group	Gene	PTR (z-score difference)	z(log2 LFQ)	z(log2 FPKM)	extreme-PTR
Treat-0d	RHSIM_Rhsim01G0283500	0.255	0.553	0.298	no
Treat-0d	RHSIM_Rhsim01G0233100	-0.851	-0.395	0.456	no
Treat-0d	RHSIM_Rhsim01G0237700	-1.588	-2.149	-0.560	low
Treat-0d	RHSIM_Rhsim01G0217800	-0.149	-0.200	-0.051	no
Treat-0d	RHSIM_Rhsim01G0221600	1.062	3.536	2.473	no
Treat-0d	RHSIM_Rhsim01G0028300	-3.180	-0.767	2.413	low
Treat-0d	RHSIM_Rhsim02G0020800	-2.230	-0.672	1.557	low
Treat-0d	RHSIM_Rhsim01G0058000	1.507	1.129	-0.377	high
Treat-0d	RHSIM_Rhsim01G0167000	-1.152	-1.202	-0.050	low
Treat-0d	RHSIM_Rhsim01G0187900	-0.731	-1.320	-0.589	no
Treat-0d	RHSIM_Rhsim01G0124600	-2.047	-1.297	0.750	low
Treat-0d	RHSIM_Rhsim01G0247100	-0.152	-0.179	-0.027	no
Treat-0d	RHSIM_Rhsim01G0007800	-0.630	0.795	1.425	no
Treat-0d	RHSIM_Rhsim03G0163700	0.028	-0.271	-0.299	no
Treat-0d	RHSIM_Rhsim01G0012100	0.236	-0.892	-1.128	no
Treat-0d	RHSIM_Rhsim01G0242500	-0.939	0.979	1.917	no
Treat-0d	RHSIM_Rhsim01G0268900	0.134	-0.362	-0.496	no
Treat-0d	RHSIM_Rhsim01G0229100	-0.436	-0.277	0.159	no
Treat-0d	RHSIM_Rhsim01G0273800	-1.469	-0.781	0.689	low
Treat-0d	RHSIM_Rhsim03G0141200	-1.410	1.358	2.768	low
Treat-0d	RHSIM_Rhsim03G0215700	-0.561	-0.704	-0.143	no
Treat-0d	RHSIM_Rhsim01G0186700	0.715	0.095	-0.620	no
Treat-0d	RHSIM_Rhsim01G0225700	0.302	0.613	0.311	no
Treat-0d	RHSIM_Rhsim03G0149300	-0.042	-0.126	-0.084	no
Treat-0d	RHSIM_Rhsim01G0071100	2.409	1.502	-0.907	high
Treat-0d	RHSIM_Rhsim03G0083700	0.736	-0.262	-0.998	no
Treat-0d	RHSIM_Rhsim01G0117300	-2.309	-0.894	1.415	low
Treat-0d	RHSIM_Rhsim01G0176100	-0.917	-0.318	0.599	no
Treat-0d	RHSIM_Rhsim02G0158200	-0.876	0.218	1.093	no
Treat-0d	RHSIM_Rhsim01G0033200	0.655	0.952	0.297	no
Treat-0d	RHSIM_Rhsim01G0089700	0.729	-0.791	-1.520	no
Treat-0d	RHSIM_Rhsim01G0245500	-0.515	-0.438	0.077	no

Group	Gene	PTR (z-score difference)	z(log2 LFQ)	z(log2 FPKM)	extreme-PTR
Treat-0d	RHSIM_Rhsim01G0073000	2.559	1.436	-1.124	high
Treat-0d	RHSIM_Rhsim01G0038600	-1.118	0.241	1.359	low
Treat-0d	RHSIM_Rhsim01G0065700	0.385	-0.504	-0.890	no
Treat-0d	RHSIM_Rhsim01G0068100	-0.365	-0.947	-0.583	no
Treat-0d	RHSIM_Rhsim01G0130400	-0.326	-0.231	0.095	no
Treat-0d	RHSIM_Rhsim01G0196000	-3.456	-2.166	1.289	low
Treat-0d	RHSIM_Rhsim03G0040000	0.672	1.804	1.131	no
Treat-0d	RHSIM_Rhsim01G0134600	0.139	-0.835	-0.974	no
Treat-0d	RHSIM_Rhsim01G0255000	0.896	1.378	0.482	no
Treat-0d	RHSIM_Rhsim01G0249300	-0.544	-0.995	-0.450	no
Treat-0d	RHSIM_Rhsim03G0197300	-0.082	-0.488	-0.406	no
Treat-0d	RHSIM_Rhsim01G0118500	-0.343	-1.995	-1.652	no
Treat-0d	RHSIM_Rhsim01G0200800	-1.622	-1.447	0.175	low
Treat-0d	RHSIM_Rhsim01G0061700	-2.102	-1.227	0.875	low
Treat-0d	RHSIM_Rhsim01G0027700	0.155	0.312	0.157	no
Treat-0d	RHSIM_Rhsim01G0026600	-0.232	-1.117	-0.885	no
Treat-0d	RHSIM_Rhsim01G0103600	-0.324	-0.439	-0.115	no
Treat-0d	RHSIM_Rhsim01G0182400	-0.107	-0.957	-0.850	no
Treat-0d	RHSIM_Rhsim01G0289900	-0.513	0.612	1.126	no
Treat-0d	RHSIM_Rhsim01G0251300	1.501	0.862	-0.639	high
Treat-0d	RHSIM_Rhsim01G0228100	-0.995	-1.201	-0.206	no
Treat-0d	RHSIM_Rhsim02G0213300	-0.139	2.234	2.372	no
Treat-0d	RHSIM_Rhsim01G0027300	-0.718	0.247	0.965	no
Treat-0d	RHSIM_Rhsim01G0208600	-0.340	0.932	1.272	no
Treat-0d	RHSIM_Rhsim01G0292900	-0.385	-0.903	-0.519	no
Treat-0d	RHSIM_Rhsim01G0178200	-0.314	-0.322	-0.008	no
Treat-0d	RHSIM_Rhsim01G0257200	0.967	1.324	0.357	no
Treat-0d	RHSIM_Rhsim01G0198000	0.711	0.170	-0.540	no
Treat-0d	RHSIM_Rhsim01G0128600	0.204	-0.036	-0.240	no
Treat-0d	RHSIM_Rhsim02G0180600	0.480	0.649	0.170	no
Treat-0d	RHSIM_Rhsim01G0149400	0.279	-1.170	-1.449	no
Treat-0d	RHSIM_Rhsim01G0000400	1.808	1.845	0.037	high

Group	Gene	PTR (z-score difference)	z(log2 LFQ)	z(log2 FPKM)	extreme-PTR
Treat-0d	RHSIM_Rhsim01G0184500	1.132	2.009	0.876	high
Treat-0d	RHSIM_Rhsim01G0155900	-0.306	0.092	0.398	no
Treat-0d	RHSIM_Rhsim01G0253200	-0.181	-0.339	-0.158	no
Treat-0d	RHSIM_Rhsim01G0101300	-0.060	-0.411	-0.351	no
Treat-0d	RHSIM_Rhsim01G0033300	2.800	1.757	-1.044	high
Treat-0d	RHSIM_Rhsim01G0237100	-0.579	-0.454	0.125	no
Treat-0d	RHSIM_Rhsim01G0116400	1.196	1.246	0.050	high
Treat-0d	RHSIM_Rhsim01G0002800	-1.263	-0.335	0.928	low
Treat-0d	RHSIM_Rhsim02G0205700	0.492	-0.966	-1.459	no
Treat-0d	RHSIM_Rhsim02G0173000	0.079	0.316	0.237	no
Treat-0d	RHSIM_Rhsim03G0004900	0.298	0.991	0.693	no
Treat-0d	RHSIM_Rhsim01G0125000	-1.470	-0.424	1.046	low
Treat-0d	RHSIM_Rhsim01G0033100	0.327	0.266	-0.062	no
Treat-0d	RHSIM_Rhsim01G0002900	0.031	0.057	0.026	no
Treat-0d	RHSIM_Rhsim02G0037400	1.285	2.646	1.361	high
Treat-0d	RHSIM_Rhsim01G0275800	0.964	1.063	0.099	no
Treat-0d	RHSIM_Rhsim01G0191600	0.878	1.486	0.608	no
Treat-0d	RHSIM_Rhsim01G0125800	-0.027	0.887	0.914	no
Treat-0d	RHSIM_Rhsim01G0067100	-0.621	-0.672	-0.050	no
Treat-0d	RHSIM_Rhsim01G0180300	0.437	1.126	0.689	no
Treat-0d	RHSIM_Rhsim03G0045300	1.124	1.741	0.617	high
Treat-0d	RHSIM_Rhsim01G0274100	-1.237	-1.048	0.189	low
Treat-0d	RHSIM_Rhsim01G0257500	0.312	-0.030	-0.342	no
Treat-0d	RHSIM_Rhsim03G0081900	0.442	1.943	1.501	no
Treat-0d	RHSIM_Rhsim01G0027600	1.245	-0.652	-1.897	high
Treat-0d	RHSIM_Rhsim01G0120500	0.141	1.538	1.397	no
Treat-0d	RHSIM_Rhsim01G0224400	0.124	-0.595	-0.719	no
Treat-0d	RHSIM_Rhsim01G0190500	0.385	0.138	-0.247	no
Treat-0d	RHSIM_Rhsim01G0255900	-0.908	-0.627	0.281	no
Treat-0d	RHSIM_Rhsim03G0221900	-0.014	0.909	0.924	no
Treat-0d	RHSIM_Rhsim01G0268200	1.091	2.104	1.013	no
Treat-0d	RHSIM_Rhsim01G0006700	-0.426	0.149	0.575	no

Group	Gene	PTR (z-score difference)	z(log2 LFQ)	z(log2 FPKM)	extreme-PTR
Treat-0d	RHSIM_Rhsim01G0231000	0.121	-0.041	-0.161	no
Treat-0d	RHSIM_Rhsim03G0079200	2.187	1.153	-1.034	high
Treat-0d	RHSIM_Rhsim01G0212100	-0.677	-0.106	0.571	no
Treat-0d	RHSIM_Rhsim01G0137400	0.399	-0.076	-0.476	no
Treat-0d	RHSIM_Rhsim01G0194700	-0.405	-0.638	-0.233	no
Treat-0d	RHSIM_Rhsim01G0250200	0.198	0.012	-0.186	no
Treat-0d	RHSIM_Rhsim01G0198200	0.297	0.397	0.100	no
Treat-0d	RHSIM_Rhsim01G0246300	-0.082	-0.204	-0.122	no
Treat-0d	RHSIM_Rhsim01G0122600	-1.139	-0.770	0.369	low
Treat-0d	RHSIM_Rhsim01G0254300	-0.165	-0.727	-0.563	no
Treat-0d	RHSIM_Rhsim03G0033400	-2.835	-0.664	2.172	low
Treat-0d	RHSIM_Rhsim01G0035300	-0.177	-1.635	-1.458	no
Treat-0d	RHSIM_Rhsim01G0291000	0.673	1.774	1.101	no
Treat-0d	RHSIM_Rhsim01G0200700	-0.386	-0.056	0.329	no
Treat-0d	RHSIM_Rhsim01G0125200	-1.444	-0.180	1.264	low
Treat-0d	RHSIM_Rhsim01G0145500	0.257	-0.786	-1.043	no
Treat-0d	RHSIM_Rhsim01G0165300	0.054	1.163	1.110	no
Treat-0d	RHSIM_Rhsim01G0016000	0.627	1.101	0.474	no
Treat-0d	RHSIM_Rhsim01G0244900	0.433	-0.029	-0.463	no
Treat-0d	RHSIM_Rhsim03G0145600	1.086	0.173	-0.913	no
Treat-0d	RHSIM_Rhsim01G0149300	0.168	0.572	0.405	no
Treat-0d	RHSIM_Rhsim01G0259900	0.361	0.792	0.431	no
Treat-0d	RHSIM_Rhsim03G0063400	-0.026	-0.323	-0.297	no
Treat-0d	RHSIM_Rhsim01G0238300	0.604	0.230	-0.374	no
Treat-0d	RHSIM_Rhsim01G0163300	1.231	1.354	0.123	high
Treat-0d	RHSIM_Rhsim01G0251200	0.259	-0.241	-0.500	no
Treat-0d	RHSIM_Rhsim01G0250600	-0.157	-0.676	-0.519	no
Treat-0d	RHSIM_Rhsim01G0287300	-0.724	-0.558	0.167	no
Treat-0d	RHSIM_Rhsim01G0284700	-0.035	-0.004	0.031	no
Treat-0d	RHSIM_Rhsim01G0264000	-1.507	-0.737	0.770	low
Treat-0d	RHSIM_Rhsim01G0218900	0.115	1.013	0.898	no
Treat-0d	RHSIM_Rhsim01G0217200	-0.180	1.175	1.354	no

Group	Gene	PTR (z-score difference)	z(log2 LFQ)	z(log2 FPKM)	extreme-PTR
Treat-0d	RHSIM_Rhsim01G0066600	-0.260	-0.135	0.126	no
Treat-0d	RHSIM_Rhsim01G0000300	0.052	-0.447	-0.499	no
Treat-0d	RHSIM_Rhsim01G0041200	1.949	-0.069	-2.018	high
Treat-0d	RHSIM_Rhsim01G0007100	-0.456	-0.787	-0.331	no
Treat-0d	RHSIM_Rhsim01G0097100	-1.222	-1.238	-0.016	low
Treat-0d	RHSIM_Rhsim01G0276700	1.154	1.109	-0.045	high
Treat-0d	RHSIM_Rhsim01G0283700	0.605	0.498	-0.107	no
Treat-0d	RHSIM_Rhsim01G0247500	-0.294	0.012	0.306	no
Treat-0d	RHSIM_Rhsim01G0247300	-0.979	0.353	1.332	no
Treat-0d	RHSIM_Rhsim01G0192300	-0.817	-0.995	-0.178	no
Treat-0d	RHSIM_Rhsim01G0290400	-0.645	0.153	0.798	no
Treat-0d	RHSIM_Rhsim01G0130900	-1.954	-1.531	0.423	low
Treat-0d	RHSIM_Rhsim02G0110500	0.317	-0.443	-0.760	no
Treat-0d	RHSIM_Rhsim01G0068700	-1.011	0.741	1.752	no
Treat-0d	RHSIM_Rhsim01G0040300	1.380	-0.875	-2.255	high
Treat-0d	RHSIM_Rhsim01G0145700	0.657	0.241	-0.416	no
Treat-0d	RHSIM_Rhsim01G0106500	1.368	0.496	-0.872	high
Treat-0d	RHSIM_Rhsim01G0230600	0.259	0.223	-0.036	no
Treat-0d	RHSIM_Rhsim01G0248500	0.514	0.593	0.079	no
Treat-0d	RHSIM_Rhsim01G0091800	-0.028	0.488	0.516	no
Treat-0d	RHSIM_Rhsim02G0046000	-0.648	-0.287	0.362	no
Treat-0d	RHSIM_Rhsim01G0192200	1.349	3.510	2.160	high
Treat-0d	RHSIM_Rhsim01G0091400	-0.651	-1.063	-0.412	no
Treat-0d	RHSIM_Rhsim02G0216500	-1.218	1.039	2.256	low
Treat-0d	RHSIM_Rhsim01G0209300	0.775	0.293	-0.481	no
Treat-0d	RHSIM_Rhsim02G0193300	-2.369	-1.993	0.376	low
Treat-0d	RHSIM_Rhsim01G0275400	-0.669	-0.535	0.134	no
Treat-0d	RHSIM_Rhsim02G0005600	-0.487	-0.751	-0.264	no
Treat-0d	RHSIM_Rhsim01G0008800	0.406	-0.254	-0.660	no
Treat-0d	RHSIM_Rhsim02G0128200	0.918	0.917	-0.001	no
Treat-0d	RHSIM_Rhsim01G0195300	0.451	0.221	-0.231	no
Treat-0d	RHSIM_Rhsim01G0073700	-0.090	-0.220	-0.130	no

Group	Gene	PTR (z-score difference)	z(log2 LFQ)	z(log2 FPKM)	extreme-PTR
Treat-0d	RHSIM_Rhsim01G0144700	-1.283	-1.019	0.263	low
Treat-0d	RHSIM_Rhsim01G0126800	0.377	2.457	2.080	no
Treat-0d	RHSIM_Rhsim01G0230900	-1.633	-1.764	-0.130	low
Treat-0d	RHSIM_Rhsim01G0286600	-0.713	0.524	1.237	no
Treat-0d	RHSIM_Rhsim02G0256800	0.550	0.042	-0.508	no
Treat-0d	RHSIM_Rhsim01G0224800	-0.079	0.479	0.558	no
Treat-0d	RHSIM_Rhsim02G0132700	-1.298	0.719	2.017	low
Treat-0d	RHSIM_Rhsim02G0142400	-0.088	0.536	0.624	no
Treat-0d	RHSIM_Rhsim02G0238900	0.335	-0.050	-0.385	no
Treat-0d	RHSIM_Rhsim01G0184800	-0.173	0.630	0.803	no
Treat-0d	RHSIM_Rhsim02G0185100	0.398	-0.804	-1.203	no
Treat-0d	RHSIM_Rhsim02G0229900	0.418	0.683	0.266	no
Treat-0d	RHSIM_Rhsim01G0246100	-1.067	-0.834	0.233	low
Treat-0d	RHSIM_Rhsim01G0184000	-0.907	-0.160	0.748	no
Treat-0d	RHSIM_Rhsim02G0099600	0.240	-1.621	-1.861	no
Treat-0d	RHSIM_Rhsim01G0023900	0.379	1.011	0.632	no
Treat-0d	RHSIM_Rhsim02G0009700	-1.914	-0.999	0.915	low
Treat-0d	RHSIM_Rhsim01G0162200	-0.049	-0.272	-0.223	no
Treat-0d	RHSIM_Rhsim01G0178400	-2.833	-1.706	1.127	low
Treat-0d	RHSIM_Rhsim01G0008400	2.111	0.655	-1.457	high
Treat-0d	RHSIM_Rhsim01G0236500	-0.571	0.966	1.537	no
Treat-0d	RHSIM_Rhsim01G0262900	-0.905	-0.748	0.157	no
Treat-0d	RHSIM_Rhsim02G0151800	-0.627	0.040	0.667	no
Treat-0d	RHSIM_Rhsim02G0003700	0.576	0.310	-0.266	no
Treat-0d	RHSIM_Rhsim02G0100800	1.202	0.386	-0.815	high
Treat-0d	RHSIM_Rhsim01G0090500	-0.628	-1.117	-0.489	no
Treat-0d	RHSIM_Rhsim01G0064300	-0.641	-1.367	-0.727	no
Treat-0d	RHSIM_Rhsim02G0253600	-1.647	-0.897	0.751	low
Treat-0d	RHSIM_Rhsim02G0031500	0.186	0.321	0.135	no
Treat-0d	RHSIM_Rhsim02G0000800	-1.464	-1.180	0.284	low
Treat-0d	RHSIM_Rhsim01G0141400	0.285	2.105	1.821	no
Treat-0d	RHSIM_Rhsim02G0075300	-0.856	-0.484	0.373	no

Group	Gene	PTR (z-score difference)	z(log2 LFQ)	z(log2 FPKM)	extreme-PTR
Treat-0d	RHSIM_Rhsim01G0280500	-0.975	-1.199	-0.224	no
Treat-0d	RHSIM_Rhsim02G0223600	0.131	-0.310	-0.442	no
Treat-0d	RHSIM_Rhsim02G0088700	1.164	0.098	-1.066	high
Treat-0d	RHSIM_Rhsim02G0228900	1.256	0.884	-0.373	high
Treat-0d	RHSIM_Rhsim02G0026400	-2.998	0.143	3.141	low
Treat-0d	RHSIM_Rhsim02G0054000	-0.953	-0.531	0.422	no
Treat-0d	RHSIM_Rhsim02G0018300	-0.666	-0.669	-0.003	no
Treat-0d	RHSIM_Rhsim01G0218400	0.923	0.961	0.039	no
Treat-0d	RHSIM_Rhsim01G0251500	1.897	1.840	-0.057	high
Treat-0d	RHSIM_Rhsim01G0106600	-0.712	0.164	0.876	no
Treat-0d	RHSIM_Rhsim01G0181700	-0.600	-1.189	-0.589	no
Treat-0d	RHSIM_Rhsim01G0220200	-1.441	-1.647	-0.205	low
Treat-0d	RHSIM_Rhsim02G0037500	1.157	1.190	0.033	high
Treat-0d	RHSIM_Rhsim01G0171200	0.019	-1.615	-1.634	no
Treat-0d	RHSIM_Rhsim01G0124800	1.017	0.657	-0.360	no
Treat-0d	RHSIM_Rhsim01G0139200	0.442	0.741	0.299	no
Treat-0d	RHSIM_Rhsim01G0282400	0.332	-0.963	-1.295	no
Treat-0d	RHSIM_Rhsim01G0220800	0.903	0.152	-0.752	no
Treat-0d	RHSIM_Rhsim01G0284300	0.185	0.660	0.474	no
Treat-0d	RHSIM_Rhsim01G0102600	1.113	0.004	-1.109	high
Treat-0d	RHSIM_Rhsim01G0208800	0.328	0.077	-0.251	no
Treat-0d	RHSIM_Rhsim01G0023400	0.085	0.313	0.228	no
Treat-0d	RHSIM_Rhsim01G0207000	-1.224	1.111	2.335	low
Treat-0d	RHSIM_Rhsim01G0216900	1.028	1.018	-0.009	no
Treat-0d	RHSIM_Rhsim01G0245900	0.422	1.653	1.232	no
Treat-0d	RHSIM_Rhsim01G0159800	-0.214	0.625	0.839	no
Treat-0d	RHSIM_Rhsim01G0238400	0.309	-0.014	-0.323	no
Treat-0d	RHSIM_Rhsim01G0226500	-0.449	0.722	1.171	no
Treat-0d	RHSIM_Rhsim01G0229500	-0.357	-0.244	0.113	no
Treat-0d	RHSIM_Rhsim01G0216000	-0.003	-0.812	-0.809	no
Treat-0d	RHSIM_Rhsim01G0274200	-0.568	-0.687	-0.118	no
Treat-0d	RHSIM_Rhsim01G0049500	-0.919	-1.094	-0.175	no

Group	Gene	PTR (z-score difference)	z(log2 LFQ)	z(log2 FPKM)	extreme-PTR
Treat-0d	RHSIM_Rhsim01G0193100	1.297	0.973	-0.323	high
Treat-0d	RHSIM_Rhsim01G0231600	-1.790	-1.307	0.483	low
Treat-0d	RHSIM_Rhsim01G0191000	-0.153	-0.762	-0.609	no
Treat-0d	RHSIM_Rhsim01G0196100	-1.872	-1.717	0.156	low
Treat-0d	RHSIM_Rhsim01G0033000	1.550	0.530	-1.021	high
Treat-0d	RHSIM_Rhsim01G0223000	0.374	0.250	-0.124	no
Treat-0d	RHSIM_Rhsim01G0123800	0.287	0.579	0.291	no
Treat-0d	RHSIM_Rhsim01G0002500	0.061	-0.509	-0.570	no
Treat-0d	RHSIM_Rhsim01G0148500	0.254	-0.513	-0.767	no
Treat-0d	RHSIM_Rhsim01G0271600	-1.654	-1.738	-0.084	low
Treat-0d	RHSIM_Rhsim01G0068000	-0.683	-1.417	-0.734	no
Treat-0d	RHSIM_Rhsim01G0202100	-1.424	0.226	1.650	low
Treat-0d	RHSIM_Rhsim01G0130500	-0.444	-1.182	-0.738	no
Treat-0d	RHSIM_Rhsim01G0076500	1.169	1.178	0.010	high
Treat-0d	RHSIM_Rhsim01G0233700	0.435	0.918	0.484	no
Treat-0d	RHSIM_Rhsim01G0189000	0.404	0.212	-0.192	no
Treat-0d	RHSIM_Rhsim01G0185300	-0.313	0.209	0.522	no
Treat-0d	RHSIM_Rhsim01G0131100	-0.858	-0.149	0.709	no
Treat-0d	RHSIM_Rhsim01G0252400	-0.614	-0.584	0.030	no
Treat-0d	RHSIM_Rhsim01G0060300	-1.147	-0.267	0.881	low
Treat-0d	RHSIM_Rhsim01G0006800	0.774	-0.594	-1.368	no
Treat-0d	RHSIM_Rhsim01G0287900	-1.505	-0.613	0.892	low
Treat-0d	RHSIM_Rhsim01G0071600	-2.170	-0.112	2.057	low
Treat-0d	RHSIM_Rhsim01G0185000	-0.975	-1.476	-0.502	no
Treat-0d	RHSIM_Rhsim01G0196300	0.128	0.869	0.741	no
Treat-0d	RHSIM_Rhsim01G0138800	1.293	0.417	-0.876	high
Treat-0d	RHSIM_Rhsim01G0210400	1.631	1.727	0.095	high
Treat-0d	RHSIM_Rhsim01G0007000	0.343	0.595	0.253	no
Treat-0d	RHSIM_Rhsim01G0000700	1.168	1.020	-0.148	high
Treat-0d	RHSIM_RhsimUnG0254300	-4.234	-0.905	3.329	low
Treat-0d	RHSIM_RhsimUnG0258100	0.125	0.247	0.122	no
Treat-0d	RHSIM_RhsimUnG0252100	0.339	-0.810	-1.149	no

Group	Gene	PTR (z-score difference)	z(log2 LFQ)	z(log2 FPKM)	extreme-PTR
Treat-0d	RHSIM_RhsimUnG0236700	0.606	-0.092	-0.698	no
Treat-0d	RHSIM_RhsimUnG0227500	0.394	1.509	1.115	no
Treat-0d	RHSIM_RhsimUnG0171600	-2.227	-1.450	0.777	low
Treat-0d	RHSIM_RhsimUnG0169000	-1.442	-1.668	-0.226	low
Treat-0d	RHSIM_RhsimUnG0118200	0.473	-0.433	-0.906	no
Treat-0d	RHSIM_RhsimUnG0113800	1.896	0.296	-1.599	high
Treat-0d	RHSIM_Rhsim11G0108100	0.373	-1.210	-1.583	no
Treat-0d	RHSIM_RhsimUnG0065600	-0.805	0.494	1.299	no
Treat-0d	RHSIM_RhsimUnG0062200	1.472	0.348	-1.123	high
Treat-0d	RHSIM_Rhsim10G0103200	0.220	0.056	-0.164	no
Treat-0d	RHSIM_RhsimUnG0057700	1.053	0.757	-0.296	no
Treat-0d	RHSIM_RhsimUnG0055300	-0.342	0.156	0.499	no
Treat-0d	RHSIM_RhsimUnG0039300	2.586	1.074	-1.511	high
Treat-0d	RHSIM_RhsimUnG0037800	-1.458	-0.620	0.838	low
Treat-0d	RHSIM_RhsimUnG0003000	-0.548	-0.424	0.124	no
Treat-0d	RHSIM_RhsimUnG0258700	0.581	-1.047	-1.627	no
Treat-0d	RHSIM_RhsimPtG0005800	3.630	3.876	0.246	high
Treat-0d	RHSIM_RhsimUnG0253900	1.265	1.041	-0.223	high
Treat-0d	RHSIM_RhsimUnG0250200	0.691	1.600	0.908	no
Treat-0d	RHSIM_Rhsim13G0233000	0.185	-0.142	-0.327	no
Treat-0d	RHSIM_Rhsim13G0008700	3.045	1.682	-1.363	high
Treat-0d	RHSIM_Rhsim13G0227700	0.179	-0.669	-0.848	no
Treat-0d	RHSIM_Rhsim13G0228800	-1.062	1.407	2.469	low
Treat-0d	RHSIM_Rhsim13G0222000	-1.248	-1.223	0.025	low
Treat-0d	RHSIM_Rhsim13G0111300	-0.095	0.726	0.821	no
Treat-0d	RHSIM_Rhsim13G0083600	1.882	-0.323	-2.205	high
Treat-0d	RHSIM_Rhsim13G0180900	-1.736	-0.733	1.003	low
Treat-0d	RHSIM_Rhsim13G0004600	-0.368	-0.093	0.275	no
Treat-0d	RHSIM_RhsimUnG0217200	2.679	0.426	-2.253	high
Treat-0d	RHSIM_RhsimUnG0212100	-0.673	-1.180	-0.507	no
Treat-0d	RHSIM_Rhsim13G0203000	-0.159	-1.095	-0.937	no
Treat-0d	RHSIM_RhsimUnG0202600	1.238	-0.435	-1.673	high

Group	Gene	PTR (z-score difference)	z(log2 LFQ)	z(log2 FPKM)	extreme-PTR
Treat-0d	RHSIM_Rhsim13G0053600	-0.039	0.882	0.921	no
Treat-0d	RHSIM_Rhsim13G0103100	-0.358	0.058	0.416	no
Treat-0d	RHSIM_RhsimUnG0248000	-0.151	-0.095	0.056	no
Treat-0d	RHSIM_Rhsim13G0088200	-0.591	-2.293	-1.703	no
Treat-0d	RHSIM_RhsimUnG0250500	0.048	1.490	1.442	no
Treat-0d	RHSIM_RhsimUnG0253100	-2.781	-0.299	2.482	low
Treat-0d	RHSIM_Rhsim13G0021500	-0.317	-0.946	-0.628	no
Treat-0d	RHSIM_RhsimUnG0257700	0.655	2.753	2.098	no
Treat-0d	RHSIM_RhsimUnG0251000	-0.485	-0.722	-0.237	no
Treat-0d	RHSIM_RhsimUnG0248500	-0.180	-0.528	-0.348	no
Treat-0d	RHSIM_Rhsim13G0120200	-0.595	-1.395	-0.800	no
Treat-0d	RHSIM_RhsimUnG0234700	-0.173	-0.862	-0.689	no
Treat-0d	RHSIM_Rhsim13G0146900	-1.222	-1.610	-0.388	low
Treat-0d	RHSIM_RhsimUnG0239200	1.360	0.526	-0.835	high
Treat-0d	RHSIM_RhsimUnG0240700	0.654	1.115	0.461	no
Treat-0d	RHSIM_RhsimUnG0226400	-0.263	-0.231	0.032	no
Treat-0d	RHSIM_Rhsim13G0041600	1.225	3.285	2.059	high
Treat-0d	RHSIM_RhsimUnG0223000	0.707	-0.703	-1.410	no
Treat-0d	RHSIM_RhsimUnG0231000	-0.511	-2.002	-1.491	no
Treat-0d	RHSIM_Rhsim13G0201800	-2.977	-1.278	1.700	low
Treat-0d	RHSIM_RhsimUnG0225800	1.246	1.159	-0.087	high
Treat-0d	RHSIM_Rhsim13G0232300	1.145	1.370	0.225	high
Treat-0d	RHSIM_Rhsim13G0191000	0.187	-0.467	-0.653	no
Treat-0d	RHSIM_RhsimUnG0168300	0.825	0.095	-0.729	no
Treat-0d	RHSIM_Rhsim13G0040700	-0.043	-1.208	-1.164	no
Treat-0d	RHSIM_Rhsim13G0054200	-0.252	-1.411	-1.159	no
Treat-0d	RHSIM_RhsimUnG0210400	1.352	1.729	0.377	high
Treat-0d	RHSIM_RhsimUnG0210700	0.643	-0.410	-1.052	no
Treat-0d	RHSIM_RhsimUnG0209300	0.237	-1.075	-1.312	no
Treat-0d	RHSIM_Rhsim12G0052900	1.398	0.703	-0.695	high
Treat-0d	RHSIM_RhsimUnG0192400	1.585	0.144	-1.441	high
Treat-0d	RHSIM_Rhsim13G0198900	0.270	-0.930	-1.200	no

Group	Gene	PTR (z-score difference)	z(log2 LFQ)	z(log2 FPKM)	extreme-PTR
Treat-0d	RHSIM_RhsimUnG0123300	1.862	0.316	-1.546	high
Treat-0d	RHSIM_Rhsim09G0047000	1.944	1.057	-0.887	high
Treat-0d	RHSIM_Rhsim13G0121800	-0.188	-1.023	-0.835	no
Treat-0d	RHSIM_RhsimUnG0168800	1.116	1.962	0.846	high
Treat-0d	RHSIM_RhsimUnG0116900	-1.636	-1.646	-0.010	low
Treat-0d	RHSIM_Rhsim06G0025600	0.177	0.569	0.391	no
Treat-0d	RHSIM_Rhsim13G0051200	-2.316	-1.706	0.610	low
Treat-0d	RHSIM_Rhsim13G0184800	-0.239	-0.824	-0.585	no
Treat-0d	RHSIM_RhsimUnG0143500	-1.068	-1.451	-0.383	low
Treat-0d	RHSIM_RhsimUnG0092900	0.686	-0.474	-1.160	no
Treat-0d	RHSIM_RhsimUnG0087600	1.706	0.288	-1.418	high
Treat-0d	RHSIM_RhsimUnG0086600	0.181	0.782	0.601	no
Treat-0d	RHSIM_Rhsim13G0020600	-0.741	-1.051	-0.310	no
Treat-0d	RHSIM_RhsimUnG0082400	1.503	0.066	-1.437	high
Treat-0d	RHSIM_RhsimUnG0142500	0.990	-0.456	-1.445	no
Treat-0d	RHSIM_RhsimUnG0135800	2.324	0.207	-2.117	high
Treat-0d	RHSIM_RhsimUnG0073800	-0.736	0.234	0.971	no
Treat-0d	RHSIM_RhsimUnG0131400	2.074	0.714	-1.360	high
Treat-0d	RHSIM_RhsimUnG0120100	0.582	1.258	0.677	no
Treat-0d	RHSIM_RhsimUnG0128700	0.089	-0.565	-0.654	no
Treat-0d	RHSIM_RhsimUnG0123600	-0.213	-1.174	-0.961	no
Treat-0d	RHSIM_Rhsim13G0099200	1.001	1.321	0.321	no
Treat-0d	RHSIM_Rhsim13G0029700	-0.506	-1.305	-0.799	no
Treat-0d	RHSIM_RhsimUnG0044400	-0.257	0.594	0.851	no
Treat-0d	RHSIM_RhsimUnG0092800	0.161	0.549	0.388	no
Treat-0d	RHSIM_RhsimUnG0088000	1.252	-0.135	-1.386	high
Treat-0d	RHSIM_RhsimUnG0106100	0.728	-0.305	-1.033	no
Treat-0d	RHSIM_RhsimUnG0100600	-0.332	0.320	0.652	no
Treat-0d	RHSIM_Rhsim13G0131200	0.122	0.680	0.558	no
Treat-0d	RHSIM_RhsimUnG0097600	0.892	1.076	0.184	no
Treat-0d	RHSIM_RhsimUnG0091000	0.025	-1.453	-1.478	no
Treat-0d	RHSIM_RhsimUnG0085300	0.012	0.696	0.684	no

Group	Gene	PTR (z-score difference)	z(log2 LFQ)	z(log2 FPKM)	extreme-PTR
Treat-0d	RHSIM_RhsimUnG0070600	0.559	-1.071	-1.630	no
Treat-0d	RHSIM_Rhsim13G0082400	-1.062	-0.976	0.087	low
Treat-0d	RHSIM_RhsimUnG0005900	2.592	1.990	-0.602	high
Treat-0d	RHSIM_RhsimUnG0066300	1.067	-0.326	-1.393	no
Treat-0d	RHSIM_RhsimUnG0075600	0.995	0.554	-0.441	no
Treat-0d	RHSIM_RhsimUnG0064700	1.026	1.699	0.673	no
Treat-0d	RHSIM_RhsimPtG0004000	5.347	3.502	-1.845	high
Treat-0d	RHSIM_Rhsim13G0062300	1.545	1.122	-0.423	high
Treat-0d	RHSIM_RhsimMtG0002200	1.376	-0.440	-1.816	high
Treat-0d	RHSIM_Rhsim13G0073900	0.825	-0.489	-1.314	no
Treat-0d	RHSIM_RhsimUnG0055400	0.638	1.754	1.116	no
Treat-0d	RHSIM_RhsimUnG0046700	-0.566	-1.132	-0.566	no
Treat-0d	RHSIM_RhsimUnG0054900	-0.826	0.881	1.707	no
Treat-0d	RHSIM_RhsimUnG0042300	-1.206	0.194	1.400	low
Treat-0d	RHSIM_Rhsim13G0173600	-1.152	-0.761	0.390	low
Treat-0d	RHSIM_Rhsim13G0227300	0.854	0.063	-0.791	no
Treat-0d	RHSIM_RhsimUnG0042800	-0.262	-1.878	-1.616	no
Treat-0d	RHSIM_Rhsim12G0181000	0.289	0.742	0.452	no
Treat-0d	RHSIM_Rhsim13G0093800	-0.552	-0.760	-0.209	no
Treat-0d	RHSIM_Rhsim13G0030700	0.316	0.170	-0.146	no
Treat-0d	RHSIM_Rhsim13G0076000	-0.042	1.047	1.089	no
Treat-0d	RHSIM_RhsimUnG0019100	1.406	0.242	-1.165	high
Treat-0d	RHSIM_RhsimUnG0020700	0.348	-0.854	-1.202	no
Treat-0d	RHSIM_Rhsim13G0064100	0.464	-0.547	-1.011	no
Treat-0d	RHSIM_Rhsim13G0163100	0.488	-0.783	-1.270	no
Treat-0d	RHSIM_Rhsim13G0066100	0.089	1.115	1.026	no
Treat-0d	RHSIM_RhsimUnG0016900	0.940	1.200	0.260	no
Treat-0d	RHSIM_RhsimUnG0016100	-2.238	-1.363	0.874	low
Treat-0d	RHSIM_Rhsim13G0207900	-1.864	-0.741	1.123	low
Treat-0d	RHSIM_Rhsim12G0037800	0.178	0.332	0.154	no
Treat-0d	RHSIM_Rhsim13G0043900	-0.315	-0.154	0.161	no
Treat-0d	RHSIM_Rhsim13G0068500	-0.067	0.087	0.154	no

Group	Gene	PTR (z-score difference)	z(log2 LFQ)	z(log2 FPKM)	extreme-PTR
Treat-0d	RHSIM_Rhsim12G0063500	-0.625	-0.188	0.437	no
Treat-0d	RHSIM_Rhsim12G0167800	0.121	0.254	0.133	no
Treat-0d	RHSIM_Rhsim13G0040000	1.202	0.671	-0.531	high
Treat-0d	RHSIM_Rhsim13G0118300	-0.795	-0.519	0.276	no
Treat-0d	RHSIM_Rhsim13G0220600	0.849	1.147	0.298	no
Treat-0d	RHSIM_Rhsim13G0065700	-0.287	0.490	0.777	no
Treat-0d	RHSIM_Rhsim13G0221500	-0.652	-1.020	-0.368	no
Treat-0d	RHSIM_Rhsim12G0039700	0.633	0.174	-0.459	no
Treat-0d	RHSIM_Rhsim13G0226500	1.539	1.372	-0.168	high
Treat-0d	RHSIM_Rhsim13G0016700	-0.440	-0.446	-0.006	no
Treat-0d	RHSIM_Rhsim13G0174500	0.858	1.727	0.869	no
Treat-0d	RHSIM_Rhsim12G0048800	-0.004	-0.692	-0.688	no
Treat-0d	RHSIM_Rhsim13G0185200	2.506	2.872	0.366	high
Treat-0d	RHSIM_Rhsim13G0234600	-0.053	0.759	0.812	no
Treat-0d	RHSIM_Rhsim13G0038800	-0.651	0.901	1.552	no
Treat-0d	RHSIM_Rhsim13G0196800	-0.209	-0.666	-0.457	no
Treat-0d	RHSIM_Rhsim13G0053200	0.243	0.714	0.471	no
Treat-0d	RHSIM_Rhsim12G0172600	1.373	1.022	-0.351	high
Treat-0d	RHSIM_Rhsim13G0134800	-1.125	-0.257	0.868	low
Treat-0d	RHSIM_Rhsim13G0154000	0.521	2.510	1.989	no
Treat-0d	RHSIM_Rhsim13G0038600	-0.078	-0.875	-0.796	no
Treat-0d	RHSIM_Rhsim12G0139300	-0.325	-0.371	-0.045	no
Treat-0d	RHSIM_Rhsim13G0229900	0.430	1.927	1.497	no
Treat-0d	RHSIM_Rhsim13G0078100	-1.759	-1.102	0.657	low
Treat-0d	RHSIM_Rhsim13G0113300	-0.157	-0.670	-0.513	no
Treat-0d	RHSIM_Rhsim13G0082700	-4.484	-0.279	4.204	low
Treat-0d	RHSIM_Rhsim13G0126700	-0.895	-1.124	-0.229	no
Treat-0d	RHSIM_Rhsim13G0159200	-0.325	-0.861	-0.536	no
Treat-0d	RHSIM_Rhsim13G0201100	0.888	-0.169	-1.057	no
Treat-0d	RHSIM_Rhsim13G0022100	1.425	1.389	-0.036	high
Treat-0d	RHSIM_Rhsim13G0199200	0.668	-0.432	-1.101	no
Treat-0d	RHSIM_Rhsim13G0171300	1.498	0.192	-1.306	high

Group	Gene	PTR (z-score difference)	z(log2 LFQ)	z(log2 FPKM)	extreme-PTR
Treat-0d	RHSIM_Rhsim13G0177900	0.499	0.023	-0.476	no
Treat-0d	RHSIM_Rhsim12G0123300	0.407	-0.427	-0.833	no
Treat-0d	RHSIM_Rhsim13G0093700	-0.283	0.608	0.892	no
Treat-0d	RHSIM_Rhsim12G0178200	0.221	-0.466	-0.686	no
Treat-0d	RHSIM_Rhsim13G0036600	-1.580	-0.908	0.672	low
Treat-0d	RHSIM_Rhsim12G0139900	-0.900	-0.979	-0.079	no
Treat-0d	RHSIM_Rhsim13G0006800	0.490	1.411	0.921	no
Treat-0d	RHSIM_Rhsim13G0079200	-0.432	-0.737	-0.305	no
Treat-0d	RHSIM_Rhsim12G0079000	-0.347	-0.682	-0.335	no
Treat-0d	RHSIM_Rhsim13G0092400	-1.182	-1.531	-0.349	low
Treat-0d	RHSIM_Rhsim13G0220800	-0.942	-0.413	0.530	no
Treat-0d	RHSIM_Rhsim13G0011300	-0.273	-0.034	0.239	no
Treat-0d	RHSIM_Rhsim12G0204800	-0.199	-0.068	0.131	no
Treat-0d	RHSIM_Rhsim13G0041900	-0.230	-1.157	-0.926	no
Treat-0d	RHSIM_Rhsim13G0232100	0.935	1.013	0.078	no
Treat-0d	RHSIM_Rhsim13G0144400	1.850	0.922	-0.928	high
Treat-0d	RHSIM_Rhsim13G0084400	1.352	-0.766	-2.118	high
Treat-0d	RHSIM_Rhsim13G0054900	1.288	-0.151	-1.439	high
Treat-0d	RHSIM_Rhsim13G0124200	-1.488	-0.700	0.788	low
Treat-0d	RHSIM_Rhsim13G0021700	-2.186	-2.028	0.158	low
Treat-0d	RHSIM_Rhsim12G0107800	1.911	-0.319	-2.230	high
Treat-0d	RHSIM_Rhsim13G0057600	1.347	0.050	-1.297	high
Treat-0d	RHSIM_Rhsim13G0191700	1.132	0.001	-1.131	high
Treat-0d	RHSIM_Rhsim13G0114000	-0.330	-0.478	-0.148	no
Treat-0d	RHSIM_Rhsim13G0027900	-0.217	-0.026	0.191	no
Treat-0d	RHSIM_Rhsim13G0029600	-2.093	-0.994	1.098	low
Treat-0d	RHSIM_Rhsim13G0051300	-0.244	1.675	1.919	no
Treat-0d	RHSIM_Rhsim13G0219700	1.028	1.447	0.420	no
Treat-0d	RHSIM_Rhsim13G0164200	1.234	-0.547	-1.781	high
Treat-0d	RHSIM_Rhsim13G0086200	-0.645	0.303	0.948	no
Treat-0d	RHSIM_Rhsim12G0005600	-0.333	0.613	0.946	no
Treat-0d	RHSIM_Rhsim13G0014300	-1.326	-1.267	0.059	low

Group	Gene	PTR (z-score difference)	z(log2 LFQ)	z(log2 FPKM)	extreme-PTR
Treat-0d	RHSIM_Rhsim12G0203000	-1.724	-0.686	1.037	low
Treat-0d	RHSIM_Rhsim13G0001400	1.768	0.801	-0.967	high
Treat-0d	RHSIM_Rhsim13G0111900	-1.986	-0.830	1.156	low
Treat-0d	RHSIM_Rhsim12G0174000	-0.041	1.371	1.412	no
Treat-0d	RHSIM_Rhsim13G0161700	0.235	0.894	0.659	no
Treat-0d	RHSIM_Rhsim13G0036800	0.697	1.284	0.588	no
Treat-0d	RHSIM_Rhsim13G0219900	-2.191	-1.861	0.330	low
Treat-0d	RHSIM_Rhsim13G0107300	0.723	0.880	0.157	no
Treat-0d	RHSIM_Rhsim13G0075000	-0.125	-0.512	-0.387	no
Treat-0d	RHSIM_Rhsim13G0103500	-0.010	0.034	0.044	no
Treat-0d	RHSIM_Rhsim13G0101100	0.010	0.505	0.495	no
Treat-0d	RHSIM_Rhsim13G0015700	1.111	0.646	-0.464	high
Treat-0d	RHSIM_Rhsim13G0168400	0.991	1.797	0.807	no
Treat-0d	RHSIM_Rhsim12G0116800	1.304	0.809	-0.496	high
Treat-0d	RHSIM_Rhsim12G0078100	-0.482	-0.242	0.240	no
Treat-0d	RHSIM_Rhsim13G0181700	-0.039	0.133	0.172	no
Treat-0d	RHSIM_Rhsim13G0161600	-0.266	-1.868	-1.602	no
Treat-0d	RHSIM_Rhsim13G0116300	1.607	1.711	0.105	high
Treat-0d	RHSIM_Rhsim13G0102200	0.028	-0.714	-0.743	no
Treat-0d	RHSIM_Rhsim12G0172200	1.268	1.275	0.006	high
Treat-0d	RHSIM_Rhsim12G0103900	-0.118	-0.086	0.033	no
Treat-0d	RHSIM_Rhsim13G0069000	-1.872	-0.142	1.730	low
Treat-0d	RHSIM_Rhsim13G0231100	0.682	-0.299	-0.981	no
Treat-0d	RHSIM_Rhsim12G0083200	2.005	0.582	-1.424	high
Treat-0d	RHSIM_Rhsim12G0177000	1.108	0.443	-0.664	high
Treat-0d	RHSIM_Rhsim13G0217100	0.640	0.459	-0.181	no
Treat-0d	RHSIM_Rhsim13G0138400	-0.191	-0.422	-0.231	no
Treat-0d	RHSIM_Rhsim13G0042600	-0.283	0.250	0.533	no
Treat-0d	RHSIM_Rhsim12G0100900	-0.151	0.759	0.910	no
Treat-0d	RHSIM_Rhsim13G0025700	1.177	1.186	0.009	high
Treat-0d	RHSIM_Rhsim13G0038000	2.042	1.656	-0.386	high
Treat-0d	RHSIM_Rhsim13G0079600	-1.395	0.099	1.494	low

Group	Gene	PTR (z-score difference)	z(log2 LFQ)	z(log2 FPKM)	extreme-PTR
Treat-0d	RHSIM_Rhsim12G0083000	-0.260	0.220	0.480	no
Treat-0d	RHSIM_Rhsim13G0233800	-1.303	-0.319	0.984	low
Treat-0d	RHSIM_Rhsim13G0073800	-1.470	-0.764	0.706	low
Treat-0d	RHSIM_Rhsim13G0055800	0.603	-0.669	-1.271	no
Treat-0d	RHSIM_Rhsim13G0031900	1.559	0.674	-0.885	high
Treat-0d	RHSIM_Rhsim13G0027500	-0.037	0.066	0.103	no
Treat-0d	RHSIM_Rhsim12G0028500	0.022	0.515	0.493	no
Treat-0d	RHSIM_Rhsim13G0035400	0.110	-0.172	-0.282	no
Treat-0d	RHSIM_Rhsim13G0167700	0.233	1.154	0.922	no
Treat-0d	RHSIM_Rhsim13G0129900	0.649	0.060	-0.590	no
Treat-0d	RHSIM_Rhsim13G0223700	0.224	-0.909	-1.133	no
Treat-0d	RHSIM_Rhsim13G0018400	0.242	-0.536	-0.778	no
Treat-0d	RHSIM_Rhsim13G0157400	1.562	1.199	-0.363	high
Treat-0d	RHSIM_Rhsim13G0177000	0.156	0.330	0.174	no
Treat-0d	RHSIM_Rhsim13G0030400	-0.216	-0.923	-0.707	no
Treat-0d	RHSIM_Rhsim12G0123700	0.292	-0.168	-0.461	no
Treat-0d	RHSIM_Rhsim13G0208300	-0.121	0.225	0.347	no
Treat-0d	RHSIM_Rhsim13G0070100	-0.377	0.049	0.426	no
Treat-0d	RHSIM_Rhsim13G0003700	0.357	-1.197	-1.554	no
Treat-0d	RHSIM_Rhsim11G0144200	0.121	-0.496	-0.617	no
Treat-0d	RHSIM_Rhsim13G0179800	-0.680	-2.357	-1.678	no
Treat-0d	RHSIM_Rhsim13G0114600	-1.072	-0.150	0.921	low
Treat-0d	RHSIM_Rhsim13G0028300	0.720	0.931	0.211	no
Treat-0d	RHSIM_Rhsim13G0122000	1.187	-0.804	-1.991	high
Treat-0d	RHSIM_Rhsim13G0127800	1.023	-0.292	-1.314	no
Treat-0d	RHSIM_Rhsim13G0092300	1.370	1.729	0.359	high
Treat-0d	RHSIM_Rhsim13G0026300	1.327	1.576	0.249	high
Treat-0d	RHSIM_Rhsim13G0233500	-0.093	-0.270	-0.177	no
Treat-0d	RHSIM_Rhsim13G0091400	0.244	-0.327	-0.571	no
Treat-0d	RHSIM_Rhsim13G0120600	0.259	0.935	0.676	no
Treat-0d	RHSIM_Rhsim11G0149700	0.534	-0.232	-0.766	no
Treat-0d	RHSIM_Rhsim13G0220700	-0.257	-0.730	-0.473	no

Group	Gene	PTR (z-score difference)	z(log2 LFQ)	z(log2 FPKM)	extreme-PTR
Treat-0d	RHSIM_Rhsim13G0067500	0.109	0.551	0.443	no
Treat-0d	RHSIM_Rhsim13G0146800	0.325	-0.363	-0.688	no
Treat-0d	RHSIM_Rhsim13G0138900	-0.888	-0.709	0.179	no
Treat-0d	RHSIM_Rhsim13G0156800	0.285	-0.890	-1.175	no
Treat-0d	RHSIM_Rhsim13G0076600	-0.368	-1.199	-0.831	no
Treat-0d	RHSIM_Rhsim13G0075300	-0.664	-1.172	-0.509	no
Treat-0d	RHSIM_Rhsim13G0036200	-2.030	-0.370	1.660	low
Treat-0d	RHSIM_Rhsim11G0025400	-0.040	-1.414	-1.374	no
Treat-0d	RHSIM_Rhsim13G0173800	-1.698	-0.345	1.353	low
Treat-0d	RHSIM_Rhsim13G0171400	-0.006	-0.304	-0.298	no
Treat-0d	RHSIM_Rhsim11G0195300	1.075	0.304	-0.771	no
Treat-0d	RHSIM_Rhsim13G0129200	0.988	0.773	-0.215	no
Treat-0d	RHSIM_Rhsim13G0010200	-0.955	-0.093	0.863	no
Treat-0d	RHSIM_Rhsim13G0231600	-1.019	-0.544	0.476	no
Treat-0d	RHSIM_Rhsim13G0230400	1.451	0.698	-0.753	high
Treat-0d	RHSIM_Rhsim11G0194500	-0.650	-1.213	-0.563	no
Treat-0d	RHSIM_Rhsim13G0140100	-0.109	0.295	0.405	no
Treat-0d	RHSIM_Rhsim13G0142000	0.484	0.172	-0.311	no
Treat-0d	RHSIM_Rhsim12G0016400	-0.283	-0.576	-0.293	no
Treat-0d	RHSIM_Rhsim13G0056200	0.452	0.347	-0.105	no
Treat-0d	RHSIM_Rhsim11G0092700	-4.045	-2.156	1.888	low
Treat-0d	RHSIM_Rhsim12G0018000	0.646	-0.094	-0.740	no
Treat-0d	RHSIM_Rhsim12G0121700	0.216	-0.115	-0.331	no
Treat-0d	RHSIM_Rhsim12G0020900	2.833	0.893	-1.940	high
Treat-0d	RHSIM_Rhsim11G0117400	1.052	1.085	0.033	no
Treat-0d	RHSIM_Rhsim12G0014400	1.328	0.988	-0.340	high
Treat-0d	RHSIM_Rhsim11G0029100	1.256	0.458	-0.798	high
Treat-0d	RHSIM_Rhsim12G0021600	-0.435	-2.278	-1.844	no
Treat-0d	RHSIM_Rhsim11G0036500	-0.770	-0.254	0.516	no
Treat-0d	RHSIM_Rhsim12G0088300	-0.546	0.374	0.920	no
Treat-0d	RHSIM_Rhsim13G0217400	0.053	-0.619	-0.672	no
Treat-0d	RHSIM_Rhsim11G0008400	0.916	-0.105	-1.020	no

Group	Gene	PTR (z-score difference)	z(log2 LFQ)	z(log2 FPKM)	extreme-PTR
Treat-0d	RHSIM_Rhsim11G0097700	0.114	-1.163	-1.277	no
Treat-0d	RHSIM_Rhsim13G0124600	-0.526	-1.445	-0.918	no
Treat-0d	RHSIM_Rhsim12G0178500	-0.069	0.173	0.242	no
Treat-0d	RHSIM_Rhsim12G0026500	0.181	-0.900	-1.081	no
Treat-0d	RHSIM_Rhsim13G0008300	-1.827	0.263	2.090	low
Treat-0d	RHSIM_Rhsim12G0157000	-0.461	0.685	1.146	no
Treat-0d	RHSIM_Rhsim11G0148300	-4.014	-0.940	3.073	low
Treat-0d	RHSIM_Rhsim12G0110500	0.518	-0.952	-1.470	no
Treat-0d	RHSIM_Rhsim11G0196100	-0.211	-0.957	-0.746	no
Treat-0d	RHSIM_Rhsim12G0078800	0.975	1.168	0.193	no
Treat-0d	RHSIM_Rhsim12G0079500	2.180	1.130	-1.050	high
Treat-0d	RHSIM_Rhsim12G0008200	1.825	0.181	-1.644	high
Treat-0d	RHSIM_Rhsim13G0206000	-0.797	0.261	1.057	no
Treat-0d	RHSIM_Rhsim11G0077200	1.033	3.043	2.010	no
Treat-0d	RHSIM_Rhsim12G0189500	0.166	-0.808	-0.974	no
Treat-0d	RHSIM_Rhsim12G0131300	0.952	1.457	0.505	no
Treat-0d	RHSIM_Rhsim12G0190500	0.661	0.700	0.039	no
Treat-0d	RHSIM_Rhsim13G0036100	1.120	0.532	-0.588	high
Treat-0d	RHSIM_Rhsim12G0057400	0.821	0.879	0.057	no
Treat-0d	RHSIM_Rhsim12G0137900	0.488	0.080	-0.408	no
Treat-0d	RHSIM_Rhsim11G0190900	-0.749	1.175	1.924	no
Treat-0d	RHSIM_Rhsim12G0176000	-0.994	-1.699	-0.704	no
Treat-0d	RHSIM_Rhsim12G0211600	-0.904	-0.867	0.037	no
Treat-0d	RHSIM_Rhsim11G0196600	-0.611	-1.336	-0.725	no
Treat-0d	RHSIM_Rhsim13G0126400	-0.554	-0.175	0.379	no
Treat-0d	RHSIM_Rhsim12G0014900	1.252	0.817	-0.435	high
Treat-0d	RHSIM_Rhsim12G0197900	1.008	1.538	0.530	no
Treat-0d	RHSIM_Rhsim11G0059000	-0.556	0.233	0.789	no
Treat-0d	RHSIM_Rhsim11G0096600	-0.779	-0.363	0.417	no
Treat-0d	RHSIM_Rhsim12G0181300	1.251	0.466	-0.785	high
Treat-0d	RHSIM_Rhsim12G0092800	-0.989	-1.717	-0.729	no
Treat-0d	RHSIM_Rhsim12G0041000	0.675	-0.876	-1.552	no

Group	Gene	PTR (z-score difference)	z(log2 LFQ)	z(log2 FPKM)	extreme-PTR
Treat-0d	RHSIM_Rhsim12G0017100	0.665	1.576	0.911	no
Treat-0d	RHSIM_Rhsim12G0013600	0.325	1.092	0.767	no
Treat-0d	RHSIM_Rhsim12G0023200	0.216	-0.869	-1.084	no
Treat-0d	RHSIM_Rhsim13G0010500	-0.050	-0.233	-0.182	no
Treat-0d	RHSIM_Rhsim12G0122100	-0.619	-0.343	0.276	no
Treat-0d	RHSIM_Rhsim12G0197700	-0.486	-0.693	-0.208	no
Treat-0d	RHSIM_Rhsim12G0207900	-0.639	0.020	0.659	no
Treat-0d	RHSIM_Rhsim12G0057700	-1.974	-0.280	1.694	low
Treat-0d	RHSIM_Rhsim12G0102000	0.622	0.533	-0.089	no
Treat-0d	RHSIM_Rhsim12G0198000	0.568	-0.360	-0.928	no
Treat-0d	RHSIM_Rhsim12G0121900	0.309	-0.416	-0.725	no
Treat-0d	RHSIM_Rhsim12G0187600	-0.685	0.016	0.701	no
Treat-0d	RHSIM_Rhsim12G0211700	0.181	0.332	0.152	no
Treat-0d	RHSIM_Rhsim12G0116600	0.269	1.408	1.139	no
Treat-0d	RHSIM_Rhsim11G0192700	0.030	0.846	0.816	no
Treat-0d	RHSIM_Rhsim12G0062100	-0.894	-1.125	-0.232	no
Treat-0d	RHSIM_Rhsim12G0165600	-1.207	-0.890	0.317	low
Treat-0d	RHSIM_Rhsim12G0085300	-0.084	1.148	1.232	no
Treat-0d	RHSIM_Rhsim12G0202100	1.702	2.566	0.864	high
Treat-0d	RHSIM_Rhsim12G0181600	-0.111	-0.932	-0.821	no
Treat-0d	RHSIM_Rhsim12G0023100	0.109	-1.380	-1.490	no
Treat-0d	RHSIM_Rhsim12G0187800	-1.718	-0.406	1.312	low
Treat-0d	RHSIM_Rhsim12G0002600	-1.303	-0.860	0.442	low
Treat-0d	RHSIM_Rhsim12G0214000	1.056	-1.092	-2.147	no
Treat-0d	RHSIM_Rhsim12G0194700	-0.056	-0.026	0.030	no
Treat-0d	RHSIM_Rhsim12G0014100	0.577	0.884	0.308	no
Treat-0d	RHSIM_Rhsim12G0164800	-2.521	-0.716	1.804	low
Treat-0d	RHSIM_Rhsim12G0211300	-0.547	-0.786	-0.239	no
Treat-0d	RHSIM_Rhsim12G0176100	-0.005	-0.039	-0.034	no
Treat-0d	RHSIM_Rhsim12G0086500	-0.229	-0.361	-0.132	no
Treat-0d	RHSIM_Rhsim11G0097500	-0.053	0.370	0.423	no
Treat-0d	RHSIM_Rhsim12G0008600	1.716	0.600	-1.116	high

Group	Gene	PTR (z-score difference)	z(log2 LFQ)	z(log2 FPKM)	extreme-PTR
Treat-0d	RHSIM_Rhsim12G0104500	0.838	-0.135	-0.973	no
Treat-0d	RHSIM_Rhsim12G0207200	0.374	-0.919	-1.294	no
Treat-0d	RHSIM_Rhsim12G0145700	0.682	2.006	1.324	no
Treat-0d	RHSIM_Rhsim12G0047900	0.383	0.119	-0.264	no
Treat-0d	RHSIM_Rhsim12G0064600	0.665	0.611	-0.054	no
Treat-0d	RHSIM_Rhsim12G0108800	-0.453	-1.413	-0.960	no
Treat-0d	RHSIM_Rhsim12G0132500	0.607	0.175	-0.432	no
Treat-0d	RHSIM_Rhsim12G0189400	0.599	1.026	0.427	no
Treat-0d	RHSIM_Rhsim12G0004200	1.606	1.498	-0.108	high
Treat-0d	RHSIM_Rhsim12G0037200	0.847	-0.824	-1.671	no
Treat-0d	RHSIM_Rhsim12G0208100	-0.082	-0.850	-0.768	no
Treat-0d	RHSIM_Rhsim12G0060900	-1.009	-0.472	0.537	no
Treat-0d	RHSIM_Rhsim12G0158400	1.225	-0.367	-1.592	high
Treat-0d	RHSIM_Rhsim12G0146600	-0.772	-0.483	0.289	no
Treat-0d	RHSIM_Rhsim12G0152300	-2.522	-1.696	0.825	low
Treat-0d	RHSIM_Rhsim12G0004300	-0.891	-2.206	-1.315	no
Treat-0d	RHSIM_Rhsim11G0164900	1.470	0.022	-1.448	high
Treat-0d	RHSIM_Rhsim12G0005300	0.541	-0.438	-0.979	no
Treat-0d	RHSIM_Rhsim12G0167600	-1.432	-1.800	-0.368	low
Treat-0d	RHSIM_Rhsim12G0035600	-0.512	-1.181	-0.669	no
Treat-0d	RHSIM_Rhsim12G0182400	-0.938	-0.905	0.033	no
Treat-0d	RHSIM_Rhsim10G0099100	0.374	1.003	0.629	no
Treat-0d	RHSIM_Rhsim12G0170000	-1.671	0.535	2.206	low
Treat-0d	RHSIM_Rhsim12G0210300	-3.784	-0.514	3.270	low
Treat-0d	RHSIM_Rhsim12G0006100	-1.391	-0.078	1.313	low
Treat-0d	RHSIM_Rhsim12G0047400	-1.336	-1.354	-0.018	low
Treat-0d	RHSIM_Rhsim12G0043400	-1.655	-1.328	0.327	low
Treat-0d	RHSIM_Rhsim12G0060700	-0.704	-0.910	-0.206	no
Treat-0d	RHSIM_Rhsim10G0148000	0.944	0.862	-0.082	no
Treat-0d	RHSIM_Rhsim12G0138900	-0.434	-0.990	-0.556	no
Treat-0d	RHSIM_Rhsim12G0214700	-0.436	-1.692	-1.257	no
Treat-0d	RHSIM_Rhsim12G0028300	-1.004	-1.583	-0.579	no

Group	Gene	PTR (z-score difference)	z(log2 LFQ)	z(log2 FPKM)	extreme-PTR
Treat-0d	RHSIM_Rhsim12G0100800	0.341	2.167	1.826	no
Treat-0d	RHSIM_Rhsim12G0061700	0.811	1.781	0.970	no
Treat-0d	RHSIM_Rhsim10G0064500	-0.872	0.346	1.218	no
Treat-0d	RHSIM_Rhsim12G0176300	0.689	1.327	0.637	no
Treat-0d	RHSIM_Rhsim10G0170100	-1.550	-1.036	0.514	low
Treat-0d	RHSIM_Rhsim12G0189900	0.963	1.491	0.528	no
Treat-0d	RHSIM_Rhsim12G0121500	-0.054	-0.408	-0.354	no
Treat-0d	RHSIM_Rhsim12G0156100	-0.166	1.474	1.640	no
Treat-0d	RHSIM_Rhsim10G0144700	-0.136	-0.094	0.042	no
Treat-0d	RHSIM_Rhsim10G0012700	0.546	-0.002	-0.548	no
Treat-0d	RHSIM_Rhsim12G0143100	-0.854	-1.193	-0.338	no
Treat-0d	RHSIM_Rhsim12G0072400	0.094	0.295	0.200	no
Treat-0d	RHSIM_Rhsim12G0044800	1.169	1.285	0.116	high
Treat-0d	RHSIM_Rhsim12G0172500	-0.254	-0.562	-0.308	no
Treat-0d	RHSIM_Rhsim12G0046500	0.681	3.005	2.325	no
Treat-0d	RHSIM_Rhsim10G0043300	0.037	-0.210	-0.248	no
Treat-0d	RHSIM_Rhsim12G0053800	0.244	1.778	1.534	no
Treat-0d	RHSIM_Rhsim12G0036000	0.220	-1.230	-1.450	no
Treat-0d	RHSIM_Rhsim12G0129400	1.661	0.875	-0.787	high
Treat-0d	RHSIM_Rhsim12G0090600	-0.219	0.396	0.615	no
Treat-0d	RHSIM_Rhsim12G0006300	0.644	-0.081	-0.725	no
Treat-0d	RHSIM_Rhsim12G0063200	-0.416	-0.724	-0.308	no
Treat-0d	RHSIM_Rhsim12G0136000	-0.432	-0.906	-0.474	no
Treat-0d	RHSIM_Rhsim12G0183100	0.481	0.127	-0.354	no
Treat-0d	RHSIM_Rhsim12G0124300	1.051	0.615	-0.436	no
Treat-0d	RHSIM_Rhsim12G0038400	1.138	0.860	-0.278	high
Treat-0d	RHSIM_Rhsim12G0074900	1.144	0.942	-0.202	high
Treat-0d	RHSIM_Rhsim12G0180300	0.036	1.474	1.438	no
Treat-0d	RHSIM_Rhsim12G0063300	-1.424	-0.956	0.468	low
Treat-0d	RHSIM_Rhsim11G0038100	-0.830	0.561	1.391	no
Treat-0d	RHSIM_Rhsim12G0002400	0.601	1.236	0.635	no
Treat-0d	RHSIM_Rhsim12G0062200	1.958	0.755	-1.202	high

Group	Gene	PTR (z-score difference)	z(log2 LFQ)	z(log2 FPKM)	extreme-PTR
Treat-0d	RHSIM_Rhsim12G0129000	-0.083	0.387	0.469	no
Treat-0d	RHSIM_Rhsim12G0214600	0.477	0.173	-0.305	no
Treat-0d	RHSIM_Rhsim11G0137400	-0.705	-0.048	0.657	no
Treat-0d	RHSIM_Rhsim12G0162600	-0.784	-1.307	-0.523	no
Treat-0d	RHSIM_Rhsim12G0023300	0.096	1.695	1.599	no
Treat-0d	RHSIM_Rhsim11G0021900	0.466	-0.003	-0.469	no
Treat-0d	RHSIM_Rhsim12G0208800	-1.268	-1.365	-0.097	low
Treat-0d	RHSIM_Rhsim11G0168800	0.375	-0.762	-1.138	no
Treat-0d	RHSIM_Rhsim10G0180100	-0.635	1.028	1.663	no
Treat-0d	RHSIM_Rhsim11G0186600	-0.219	-0.299	-0.081	no
Treat-0d	RHSIM_Rhsim12G0200100	-1.161	-0.267	0.894	low
Treat-0d	RHSIM_Rhsim11G0085000	-3.312	-1.711	1.601	low
Treat-0d	RHSIM_Rhsim12G0062000	0.131	-0.010	-0.141	no
Treat-0d	RHSIM_Rhsim12G0063700	0.846	0.636	-0.210	no
Treat-0d	RHSIM_Rhsim12G0104400	2.192	1.788	-0.404	high
Treat-0d	RHSIM_Rhsim12G0096800	-1.499	-1.265	0.234	low
Treat-0d	RHSIM_Rhsim11G0040500	0.293	-0.442	-0.735	no
Treat-0d	RHSIM_Rhsim12G0080700	0.143	-0.362	-0.505	no
Treat-0d	RHSIM_Rhsim11G0111300	-0.401	0.814	1.215	no
Treat-0d	RHSIM_Rhsim11G0127400	0.213	-0.319	-0.532	no
Treat-0d	RHSIM_Rhsim11G0115300	1.249	0.447	-0.802	high
Treat-0d	RHSIM_Rhsim12G0148700	1.221	1.113	-0.108	high
Treat-0d	RHSIM_Rhsim12G0081400	0.617	0.384	-0.232	no
Treat-0d	RHSIM_Rhsim11G0186800	-0.722	-0.259	0.463	no
Treat-0d	RHSIM_Rhsim11G0024700	-0.100	-0.611	-0.512	no
Treat-0d	RHSIM_Rhsim12G0136400	0.610	1.380	0.770	no
Treat-0d	RHSIM_Rhsim12G0174100	0.323	0.641	0.317	no
Treat-0d	RHSIM_Rhsim11G0012900	0.451	0.686	0.235	no
Treat-0d	RHSIM_Rhsim10G0171800	-0.441	-0.361	0.080	no
Treat-0d	RHSIM_Rhsim10G0206700	-0.884	-1.440	-0.555	no
Treat-0d	RHSIM_Rhsim11G0169800	-1.025	-0.324	0.701	low
Treat-0d	RHSIM_Rhsim12G0201100	-0.433	-0.651	-0.218	no

Group	Gene	PTR (z-score difference)	z(log2 LFQ)	z(log2 FPKM)	extreme-PTR
Treat-0d	RHSIM_Rhsim10G0010400	-0.833	-0.708	0.125	no
Treat-0d	RHSIM_Rhsim11G0098800	-1.939	0.425	2.364	low
Treat-0d	RHSIM_Rhsim10G0092900	0.262	-0.214	-0.476	no
Treat-0d	RHSIM_Rhsim10G0202800	3.012	1.078	-1.934	high
Treat-0d	RHSIM_Rhsim11G0164100	-1.325	-1.639	-0.314	low
Treat-0d	RHSIM_Rhsim12G0136300	-1.151	-1.391	-0.240	low
Treat-0d	RHSIM_Rhsim11G0088800	0.674	0.651	-0.023	no
Treat-0d	RHSIM_Rhsim11G0150000	-1.668	-0.832	0.836	low
Treat-0d	RHSIM_Rhsim11G0067900	-0.308	-1.105	-0.797	no
Treat-0d	RHSIM_Rhsim12G0187900	-0.360	-0.483	-0.123	no
Treat-0d	RHSIM_Rhsim11G0098700	1.030	3.005	1.975	no
Treat-0d	RHSIM_Rhsim11G0032600	0.081	0.543	0.462	no
Treat-0d	RHSIM_Rhsim12G0200200	-1.661	-1.219	0.442	low
Treat-0d	RHSIM_Rhsim11G0134300	-0.565	-0.640	-0.075	no
Treat-0d	RHSIM_Rhsim12G0143000	-0.615	-1.160	-0.545	no
Treat-0d	RHSIM_Rhsim11G0122100	1.662	-0.331	-1.993	high
Treat-0d	RHSIM_Rhsim11G0024800	-0.113	0.333	0.446	no
Treat-0d	RHSIM_Rhsim11G0043200	-4.140	-1.524	2.616	low
Treat-0d	RHSIM_Rhsim11G0103200	-1.687	-2.086	-0.399	low
Treat-0d	RHSIM_Rhsim11G0173000	-0.969	1.174	2.143	no
Treat-0d	RHSIM_Rhsim11G0182500	-0.594	-0.294	0.300	no
Treat-0d	RHSIM_Rhsim10G0059600	1.457	0.434	-1.024	high
Treat-0d	RHSIM_Rhsim11G0024900	-0.305	0.135	0.440	no
Treat-0d	RHSIM_Rhsim10G0128400	0.491	0.842	0.351	no
Treat-0d	RHSIM_Rhsim10G0055700	0.398	-0.089	-0.486	no
Treat-0d	RHSIM_Rhsim11G0187800	-0.541	-1.016	-0.476	no
Treat-0d	RHSIM_Rhsim11G0034100	0.612	2.449	1.838	no
Treat-0d	RHSIM_Rhsim11G0141700	2.039	1.934	-0.105	high
Treat-0d	RHSIM_Rhsim11G0159600	0.076	0.212	0.136	no
Treat-0d	RHSIM_Rhsim11G0066600	-0.861	0.609	1.470	no
Treat-0d	RHSIM_Rhsim11G0054300	0.505	0.318	-0.188	no
Treat-0d	RHSIM_Rhsim11G0069700	0.112	-0.556	-0.669	no

Group	Gene	PTR (z-score difference)	z(log2 LFQ)	z(log2 FPKM)	extreme-PTR
Treat-0d	RHSIM_Rhsim10G0084900	-0.826	-1.729	-0.902	no
Treat-0d	RHSIM_Rhsim11G0070000	-0.120	0.070	0.191	no
Treat-0d	RHSIM_Rhsim10G0164900	-0.744	-0.104	0.640	no
Treat-0d	RHSIM_Rhsim11G0070400	-2.194	-0.472	1.721	low
Treat-0d	RHSIM_Rhsim11G0074900	-1.472	-1.024	0.448	low
Treat-0d	RHSIM_Rhsim11G0179100	-0.879	-0.325	0.554	no
Treat-0d	RHSIM_Rhsim10G0131900	-0.116	-0.222	-0.106	no
Treat-0d	RHSIM_Rhsim11G0027400	-0.063	-0.066	-0.002	no
Treat-0d	RHSIM_Rhsim11G0063300	0.640	-0.966	-1.606	no
Treat-0d	RHSIM_Rhsim11G0037700	0.110	-0.887	-0.997	no
Treat-0d	RHSIM_Rhsim10G0063500	0.796	1.483	0.687	no
Treat-0d	RHSIM_Rhsim11G0187700	0.852	2.006	1.154	no
Treat-0d	RHSIM_Rhsim11G0069600	0.188	-0.244	-0.432	no
Treat-0d	RHSIM_Rhsim10G0146500	0.887	-0.994	-1.882	no
Treat-0d	RHSIM_Rhsim11G0056500	-0.167	0.076	0.243	no
Treat-0d	RHSIM_Rhsim11G0008200	1.908	-0.016	-1.924	high
Treat-0d	RHSIM_Rhsim11G0159400	1.678	0.102	-1.575	high
Treat-0d	RHSIM_Rhsim11G0044300	0.068	0.210	0.143	no
Treat-0d	RHSIM_Rhsim10G0065100	0.356	0.689	0.333	no
Treat-0d	RHSIM_Rhsim11G0178600	0.126	-0.537	-0.663	no
Treat-0d	RHSIM_Rhsim11G0159100	-0.648	-0.879	-0.231	no
Treat-0d	RHSIM_Rhsim10G0017800	-0.925	-0.942	-0.017	no
Treat-0d	RHSIM_Rhsim11G0137700	-0.249	0.288	0.536	no
Treat-0d	RHSIM_Rhsim10G0072400	-0.212	-1.061	-0.849	no
Treat-0d	RHSIM_Rhsim11G0046100	0.425	0.635	0.210	no
Treat-0d	RHSIM_Rhsim11G0001100	-2.228	-1.655	0.574	low
Treat-0d	RHSIM_Rhsim11G0187200	-0.201	0.310	0.511	no
Treat-0d	RHSIM_Rhsim11G0099300	0.122	-0.794	-0.916	no
Treat-0d	RHSIM_Rhsim11G0187000	-0.651	0.584	1.235	no
Treat-0d	RHSIM_Rhsim11G0092100	-0.142	-0.004	0.138	no
Treat-0d	RHSIM_Rhsim09G0008800	0.687	0.577	-0.110	no
Treat-0d	RHSIM_Rhsim11G0066000	-0.295	-0.511	-0.217	no

Group	Gene	PTR (z-score difference)	z(log2 LFQ)	z(log2 FPKM)	extreme-PTR
Treat-0d	RHSIM_Rhsim11G0183000	-0.713	-0.445	0.267	no
Treat-0d	RHSIM_Rhsim09G0103900	0.138	0.038	-0.100	no
Treat-0d	RHSIM_Rhsim11G0075300	-1.360	0.275	1.636	low
Treat-0d	RHSIM_Rhsim11G0049500	-0.730	-1.320	-0.590	no
Treat-0d	RHSIM_Rhsim11G0193400	1.211	0.309	-0.902	high
Treat-0d	RHSIM_Rhsim11G0019200	-0.835	-1.077	-0.242	no
Treat-0d	RHSIM_Rhsim11G0121200	0.442	-0.037	-0.479	no
Treat-0d	RHSIM_Rhsim09G0002100	1.526	0.876	-0.650	high
Treat-0d	RHSIM_Rhsim11G0160800	0.733	1.825	1.091	no
Treat-0d	RHSIM_Rhsim11G0001400	0.131	-0.598	-0.729	no
Treat-0d	RHSIM_Rhsim11G0021000	-0.266	-0.220	0.046	no
Treat-0d	RHSIM_Rhsim11G0160000	-0.789	-0.260	0.529	no
Treat-0d	RHSIM_Rhsim11G0160500	-0.389	-0.423	-0.034	no
Treat-0d	RHSIM_Rhsim11G0131900	-1.594	-1.668	-0.074	low
Treat-0d	RHSIM_Rhsim11G0194100	0.560	-1.820	-2.380	no
Treat-0d	RHSIM_Rhsim11G0038900	-0.897	0.000	0.897	no
Treat-0d	RHSIM_Rhsim11G0052300	-0.155	-1.244	-1.089	no
Treat-0d	RHSIM_Rhsim11G0088200	-2.784	-0.342	2.442	low
Treat-0d	RHSIM_Rhsim11G0138000	-1.932	-0.796	1.135	low
Treat-0d	RHSIM_Rhsim11G0058000	1.806	0.270	-1.535	high
Treat-0d	RHSIM_Rhsim11G0148400	1.130	1.178	0.048	high
Treat-0d	RHSIM_Rhsim11G0003500	-0.821	-0.584	0.236	no
Treat-0d	RHSIM_Rhsim10G0186400	0.710	1.324	0.614	no
Treat-0d	RHSIM_Rhsim11G0142200	-0.386	-1.271	-0.885	no
Treat-0d	RHSIM_Rhsim11G0129100	-0.017	-0.622	-0.606	no
Treat-0d	RHSIM_Rhsim10G0108500	0.006	-0.663	-0.669	no
Treat-0d	RHSIM_Rhsim09G0038500	-0.994	-0.957	0.037	no
Treat-0d	RHSIM_Rhsim10G0009700	-0.848	0.239	1.088	no
Treat-0d	RHSIM_Rhsim09G0212500	0.176	0.669	0.493	no
Treat-0d	RHSIM_Rhsim11G0192800	-1.453	-0.901	0.551	low
Treat-0d	RHSIM_Rhsim11G0055800	-0.111	0.353	0.463	no
Treat-0d	RHSIM_Rhsim10G0162600	-0.063	-0.085	-0.022	no

Group	Gene	PTR (z-score difference)	z(log2 LFQ)	z(log2 FPKM)	extreme-PTR
Treat-0d	RHSIM_Rhsim11G0148600	-0.862	0.493	1.355	no
Treat-0d	RHSIM_Rhsim09G0023700	0.361	-0.564	-0.924	no
Treat-0d	RHSIM_Rhsim10G0193700	2.040	-0.085	-2.124	high
Treat-0d	RHSIM_Rhsim11G0032300	-1.976	-1.561	0.415	low
Treat-0d	RHSIM_Rhsim11G0035800	1.318	0.360	-0.958	high
Treat-0d	RHSIM_Rhsim11G0027600	0.893	1.572	0.679	no
Treat-0d	RHSIM_Rhsim09G0052000	1.143	0.319	-0.823	high
Treat-0d	RHSIM_Rhsim11G0179300	-0.106	-0.674	-0.568	no
Treat-0d	RHSIM_Rhsim11G0027500	2.740	1.011	-1.729	high
Treat-0d	RHSIM_Rhsim11G0046900	-0.522	-1.422	-0.899	no
Treat-0d	RHSIM_Rhsim11G0057900	0.686	-0.392	-1.077	no
Treat-0d	RHSIM_Rhsim11G0072300	0.692	0.076	-0.616	no
Treat-0d	RHSIM_Rhsim11G0170000	0.199	0.247	0.049	no
Treat-0d	RHSIM_Rhsim09G0188200	-0.197	0.007	0.203	no
Treat-0d	RHSIM_Rhsim09G0200200	0.471	0.317	-0.154	no
Treat-0d	RHSIM_Rhsim10G0062400	0.208	-0.750	-0.957	no
Treat-0d	RHSIM_Rhsim11G0038000	-0.263	0.851	1.114	no
Treat-0d	RHSIM_Rhsim11G0047500	0.178	-0.955	-1.133	no
Treat-0d	RHSIM_Rhsim11G0054400	-1.209	-0.010	1.199	low
Treat-0d	RHSIM_Rhsim10G0039600	1.319	1.277	-0.042	high
Treat-0d	RHSIM_Rhsim11G0100000	0.955	1.383	0.428	no
Treat-0d	RHSIM_Rhsim11G0026600	0.359	-0.618	-0.976	no
Treat-0d	RHSIM_Rhsim10G0063000	0.464	0.729	0.265	no
Treat-0d	RHSIM_Rhsim11G0044500	0.070	-0.160	-0.230	no
Treat-0d	RHSIM_Rhsim11G0163700	-2.564	-0.641	1.923	low
Treat-0d	RHSIM_Rhsim09G0186800	0.218	1.329	1.111	no
Treat-0d	RHSIM_Rhsim09G0133100	-1.271	-1.384	-0.113	low
Treat-0d	RHSIM_Rhsim09G0178500	-0.988	-0.035	0.953	no
Treat-0d	RHSIM_Rhsim10G0085800	-0.364	-0.628	-0.264	no
Treat-0d	RHSIM_Rhsim09G0192900	-0.658	1.273	1.931	no
Treat-0d	RHSIM_Rhsim09G0180100	0.943	2.107	1.164	no
Treat-0d	RHSIM_Rhsim11G0037300	-0.894	-1.103	-0.208	no

Group	Gene	PTR (z-score difference)	z(log2 LFQ)	z(log2 FPKM)	extreme-PTR
Treat-0d	RHSIM_Rhsim09G0165200	-1.038	-0.694	0.344	low
Treat-0d	RHSIM_Rhsim09G0103600	0.799	-0.729	-1.528	no
Treat-0d	RHSIM_Rhsim09G0087900	-0.194	-0.214	-0.020	no
Treat-0d	RHSIM_Rhsim10G0118400	-1.538	-0.315	1.223	low
Treat-0d	RHSIM_Rhsim11G0141000	0.892	0.538	-0.354	no
Treat-0d	RHSIM_Rhsim09G0185200	0.048	0.753	0.705	no
Treat-0d	RHSIM_Rhsim09G0151700	-0.501	2.324	2.825	no
Treat-0d	RHSIM_Rhsim11G0182100	1.380	0.382	-0.998	high
Treat-0d	RHSIM_Rhsim11G0021500	1.970	1.839	-0.132	high
Treat-0d	RHSIM_Rhsim11G0169500	-1.511	0.449	1.960	low
Treat-0d	RHSIM_Rhsim11G0037600	0.267	-0.070	-0.337	no
Treat-0d	RHSIM_Rhsim10G0020800	0.159	-0.250	-0.409	no
Treat-0d	RHSIM_Rhsim11G0111400	-0.932	-0.480	0.452	no
Treat-0d	RHSIM_Rhsim10G0014700	0.777	-0.137	-0.915	no
Treat-0d	RHSIM_Rhsim11G0087000	4.721	2.405	-2.316	high
Treat-0d	RHSIM_Rhsim11G0052900	1.426	1.580	0.154	high
Treat-0d	RHSIM_Rhsim10G0138900	-0.661	-1.294	-0.633	no
Treat-0d	RHSIM_Rhsim10G0159500	1.577	0.531	-1.046	high
Treat-0d	RHSIM_Rhsim10G0082200	-0.529	0.385	0.914	no
Treat-0d	RHSIM_Rhsim10G0183600	-1.017	-0.478	0.539	no
Treat-0d	RHSIM_Rhsim10G0169300	0.155	-0.761	-0.916	no
Treat-0d	RHSIM_Rhsim10G0173800	1.054	1.285	0.231	no
Treat-0d	RHSIM_Rhsim09G0142600	0.548	0.154	-0.395	no
Treat-0d	RHSIM_Rhsim10G0195900	0.396	-1.162	-1.558	no
Treat-0d	RHSIM_Rhsim10G0067500	-0.099	1.449	1.548	no
Treat-0d	RHSIM_Rhsim11G0146300	1.058	1.132	0.075	no
Treat-0d	RHSIM_Rhsim11G0047200	-0.831	-1.540	-0.708	no
Treat-0d	RHSIM_Rhsim11G0174400	-1.574	-2.097	-0.522	low
Treat-0d	RHSIM_Rhsim10G0052200	0.754	0.607	-0.147	no
Treat-0d	RHSIM_Rhsim09G0129300	-0.236	0.148	0.384	no
Treat-0d	RHSIM_Rhsim10G0008900	0.318	0.335	0.017	no
Treat-0d	RHSIM_Rhsim10G0007400	-1.016	-0.974	0.042	no

Group	Gene	PTR (z-score difference)	z(log2 LFQ)	z(log2 FPKM)	extreme-PTR
Treat-0d	RHSIM_Rhsim09G0209100	-1.454	-0.059	1.395	low
Treat-0d	RHSIM_Rhsim10G0004900	0.351	-0.241	-0.592	no
Treat-0d	RHSIM_Rhsim10G0129200	-1.535	-1.737	-0.202	low
Treat-0d	RHSIM_Rhsim10G0094900	0.978	-0.101	-1.080	no
Treat-0d	RHSIM_Rhsim09G0032600	-0.459	-0.739	-0.281	no
Treat-0d	RHSIM_Rhsim10G0104300	-0.607	-0.342	0.265	no
Treat-0d	RHSIM_Rhsim10G0171200	0.027	0.163	0.136	no
Treat-0d	RHSIM_Rhsim10G0068900	0.125	-0.772	-0.897	no
Treat-0d	RHSIM_Rhsim10G0129100	-1.576	-1.304	0.272	low
Treat-0d	RHSIM_Rhsim10G0122800	0.773	-0.664	-1.438	no
Treat-0d	RHSIM_Rhsim10G0184100	1.860	0.871	-0.990	high
Treat-0d	RHSIM_Rhsim09G0158400	0.814	1.286	0.472	no
Treat-0d	RHSIM_Rhsim09G0007900	1.403	1.249	-0.154	high
Treat-0d	RHSIM_Rhsim10G0157900	0.392	0.337	-0.055	no
Treat-0d	RHSIM_Rhsim09G0073500	1.161	0.760	-0.401	high
Treat-0d	RHSIM_Rhsim10G0010500	1.687	1.829	0.142	high
Treat-0d	RHSIM_Rhsim10G0156400	0.878	-1.055	-1.934	no
Treat-0d	RHSIM_Rhsim10G0188300	-0.317	0.232	0.549	no
Treat-0d	RHSIM_Rhsim10G0137900	1.185	0.677	-0.508	high
Treat-0d	RHSIM_Rhsim09G0063200	0.482	0.527	0.045	no
Treat-0d	RHSIM_Rhsim10G0210900	-0.409	0.995	1.404	no
Treat-0d	RHSIM_Rhsim10G0156900	-0.603	0.126	0.730	no
Treat-0d	RHSIM_Rhsim10G0080600	0.801	0.870	0.069	no
Treat-0d	RHSIM_Rhsim10G0152400	-0.528	-2.241	-1.713	no
Treat-0d	RHSIM_Rhsim10G0191400	-1.192	-1.895	-0.702	low
Treat-0d	RHSIM_Rhsim10G0043400	0.366	-0.502	-0.868	no
Treat-0d	RHSIM_Rhsim10G0044200	1.226	1.128	-0.098	high
Treat-0d	RHSIM_Rhsim10G0110700	1.367	0.301	-1.066	high
Treat-0d	RHSIM_Rhsim09G0114600	-2.756	-0.667	2.090	low
Treat-0d	RHSIM_Rhsim10G0174000	2.047	1.037	-1.010	high
Treat-0d	RHSIM_Rhsim10G0172400	0.620	0.464	-0.156	no
Treat-0d	RHSIM_Rhsim09G0022800	0.712	1.264	0.552	no

Group	Gene	PTR (z-score difference)	z(log2 LFQ)	z(log2 FPKM)	extreme-PTR
Treat-0d	RHSIM_Rhsim10G0113800	-1.049	-1.524	-0.474	low
Treat-0d	RHSIM_Rhsim10G0115200	0.331	-1.001	-1.332	no
Treat-0d	RHSIM_Rhsim10G0205500	1.457	-0.712	-2.169	high
Treat-0d	RHSIM_Rhsim09G0059100	-3.403	-1.182	2.221	low
Treat-0d	RHSIM_Rhsim10G0133100	-0.483	-0.673	-0.189	no
Treat-0d	RHSIM_Rhsim10G0152800	-1.066	-1.842	-0.775	low
Treat-0d	RHSIM_Rhsim10G0151000	0.924	-0.011	-0.935	no
Treat-0d	RHSIM_Rhsim10G0182800	0.610	1.236	0.627	no
Treat-0d	RHSIM_Rhsim10G0156600	0.645	0.743	0.098	no
Treat-0d	RHSIM_Rhsim10G0050900	1.656	0.643	-1.012	high
Treat-0d	RHSIM_Rhsim10G0172000	-1.089	-0.409	0.680	low
Treat-0d	RHSIM_Rhsim10G0085400	0.419	-0.546	-0.965	no
Treat-0d	RHSIM_Rhsim08G0156400	0.574	-0.552	-1.126	no
Treat-0d	RHSIM_Rhsim10G0147800	0.131	-1.186	-1.317	no
Treat-0d	RHSIM_Rhsim10G0177400	0.106	-1.174	-1.281	no
Treat-0d	RHSIM_Rhsim08G0211300	-1.355	-0.500	0.855	low
Treat-0d	RHSIM_Rhsim08G0064400	-0.153	-1.207	-1.055	no
Treat-0d	RHSIM_Rhsim10G0203500	-0.613	-1.749	-1.136	no
Treat-0d	RHSIM_Rhsim10G0032200	1.154	-0.594	-1.747	high
Treat-0d	RHSIM_Rhsim08G0204300	-0.099	-0.790	-0.691	no
Treat-0d	RHSIM_Rhsim10G0114700	0.978	0.037	-0.942	no
Treat-0d	RHSIM_Rhsim08G0102800	-1.341	-1.962	-0.621	low
Treat-0d	RHSIM_Rhsim10G0093100	0.075	-1.101	-1.175	no
Treat-0d	RHSIM_Rhsim10G0191100	-0.485	0.660	1.145	no
Treat-0d	RHSIM_Rhsim10G0197500	-0.700	-0.164	0.537	no
Treat-0d	RHSIM_Rhsim10G0144500	-0.737	-0.738	-0.002	no
Treat-0d	RHSIM_Rhsim10G0139700	0.315	-1.712	-2.027	no
Treat-0d	RHSIM_Rhsim08G0099700	-0.295	0.142	0.437	no
Treat-0d	RHSIM_Rhsim10G0212300	-0.422	-0.741	-0.318	no
Treat-0d	RHSIM_Rhsim08G0220500	0.941	0.988	0.048	no
Treat-0d	RHSIM_Rhsim10G0142000	-0.091	-1.815	-1.723	no
Treat-0d	RHSIM_Rhsim08G0251300	0.041	0.268	0.228	no

Group	Gene	PTR (z-score difference)	z(log2 LFQ)	z(log2 FPKM)	extreme-PTR
Treat-0d	RHSIM_Rhsim08G0007600	0.870	-0.567	-1.436	no
Treat-0d	RHSIM_Rhsim10G0209300	0.023	-1.081	-1.103	no
Treat-0d	RHSIM_Rhsim10G0166800	0.090	0.686	0.596	no
Treat-0d	RHSIM_Rhsim09G0002300	0.779	0.287	-0.492	no
Treat-0d	RHSIM_Rhsim10G0083700	0.117	-0.947	-1.063	no
Treat-0d	RHSIM_Rhsim10G0150100	-0.651	-0.911	-0.260	no
Treat-0d	RHSIM_Rhsim10G0127500	0.189	0.371	0.182	no
Treat-0d	RHSIM_Rhsim08G0012700	0.566	-0.027	-0.592	no
Treat-0d	RHSIM_Rhsim08G0146300	0.279	0.035	-0.245	no
Treat-0d	RHSIM_Rhsim10G0173500	0.184	-0.951	-1.134	no
Treat-0d	RHSIM_Rhsim08G0066300	0.675	1.224	0.549	no
Treat-0d	RHSIM_Rhsim09G0209900	-0.676	-0.342	0.334	no
Treat-0d	RHSIM_Rhsim10G0193100	0.212	0.116	-0.096	no
Treat-0d	RHSIM_Rhsim10G0124700	-0.238	0.258	0.496	no
Treat-0d	RHSIM_Rhsim10G0124000	-1.928	-0.679	1.249	low
Treat-0d	RHSIM_Rhsim09G0032900	0.979	1.671	0.691	no
Treat-0d	RHSIM_Rhsim10G0021300	0.877	3.648	2.771	no
Treat-0d	RHSIM_Rhsim09G0172500	0.393	0.897	0.504	no
Treat-0d	RHSIM_Rhsim09G0069400	-0.546	-0.201	0.345	no
Treat-0d	RHSIM_Rhsim08G0053400	-0.162	0.347	0.509	no
Treat-0d	RHSIM_Rhsim10G0066000	1.262	1.341	0.079	high
Treat-0d	RHSIM_Rhsim08G0139000	-2.296	-2.066	0.230	low
Treat-0d	RHSIM_Rhsim09G0205000	-2.030	-0.801	1.229	low
Treat-0d	RHSIM_Rhsim09G0034400	1.054	-0.357	-1.411	no
Treat-0d	RHSIM_Rhsim10G0190900	-0.426	0.109	0.535	no
Treat-0d	RHSIM_Rhsim09G0007800	0.143	-0.480	-0.622	no
Treat-0d	RHSIM_Rhsim09G0205300	0.442	-1.101	-1.543	no
Treat-0d	RHSIM_Rhsim10G0112400	0.566	-0.398	-0.964	no
Treat-0d	RHSIM_Rhsim08G0092300	-0.325	-1.251	-0.926	no
Treat-0d	RHSIM_Rhsim10G0005200	-0.451	-0.864	-0.413	no
Treat-0d	RHSIM_Rhsim08G0045300	0.903	0.682	-0.220	no
Treat-0d	RHSIM_Rhsim09G0195700	0.573	1.678	1.105	no

Group	Gene	PTR (z-score difference)	z(log2 LFQ)	z(log2 FPKM)	extreme-PTR
Treat-0d	RHSIM_Rhsim09G0177100	-0.449	0.817	1.265	no
Treat-0d	RHSIM_Rhsim09G0205800	-1.134	-1.657	-0.523	low
Treat-0d	RHSIM_Rhsim05G0149300	-0.528	0.698	1.226	no
Treat-0d	RHSIM_Rhsim08G0128200	-0.237	-1.001	-0.765	no
Treat-0d	RHSIM_Rhsim08G0079900	-0.199	0.272	0.471	no
Treat-0d	RHSIM_Rhsim08G0144800	0.438	-0.858	-1.296	no
Treat-0d	RHSIM_Rhsim09G0163400	0.327	1.640	1.313	no
Treat-0d	RHSIM_Rhsim08G0171900	0.175	0.780	0.604	no
Treat-0d	RHSIM_Rhsim08G0225400	0.308	0.422	0.114	no
Treat-0d	RHSIM_Rhsim08G0208400	-1.229	-1.242	-0.012	low
Treat-0d	RHSIM_Rhsim08G0008700	1.233	-0.769	-2.002	high
Treat-0d	RHSIM_Rhsim10G0011000	0.653	0.653	-0.001	no
Treat-0d	RHSIM_Rhsim10G0003100	-0.063	-0.912	-0.850	no
Treat-0d	RHSIM_Rhsim08G0161200	-0.213	-1.384	-1.171	no
Treat-0d	RHSIM_Rhsim09G0060000	0.441	0.499	0.058	no
Treat-0d	RHSIM_Rhsim09G0091000	0.410	0.014	-0.396	no
Treat-0d	RHSIM_Rhsim09G0093400	1.472	0.190	-1.282	high
Treat-0d	RHSIM_Rhsim10G0163400	-0.473	0.253	0.726	no
Treat-0d	RHSIM_Rhsim08G0176500	-0.286	-0.791	-0.505	no
Treat-0d	RHSIM_Rhsim09G0198100	1.212	-0.059	-1.270	high
Treat-0d	RHSIM_Rhsim10G0063200	0.154	0.452	0.298	no
Treat-0d	RHSIM_Rhsim09G0007600	0.720	-0.390	-1.110	no
Treat-0d	RHSIM_Rhsim09G0211900	-0.023	1.267	1.290	no
Treat-0d	RHSIM_Rhsim09G0181300	-1.338	-1.429	-0.091	low
Treat-0d	RHSIM_Rhsim10G0097300	2.889	0.498	-2.390	high
Treat-0d	RHSIM_Rhsim10G0067400	0.400	-0.126	-0.527	no
Treat-0d	RHSIM_Rhsim08G0112400	-0.725	-1.172	-0.446	no
Treat-0d	RHSIM_Rhsim09G0209500	-0.329	-2.032	-1.703	no
Treat-0d	RHSIM_Rhsim08G0172700	0.667	-0.988	-1.655	no
Treat-0d	RHSIM_Rhsim10G0191500	-0.368	-0.680	-0.312	no
Treat-0d	RHSIM_Rhsim09G0009500	-2.074	-0.541	1.533	low
Treat-0d	RHSIM_Rhsim09G0091300	-0.997	-0.633	0.364	no

Group	Gene	PTR (z-score difference)	z(log2 LFQ)	z(log2 FPKM)	extreme-PTR
Treat-0d	RHSIM_Rhsim08G0188100	0.012	0.331	0.318	no
Treat-0d	RHSIM_Rhsim09G0111200	-0.314	-0.342	-0.028	no
Treat-0d	RHSIM_Rhsim08G0242700	-1.500	-1.503	-0.003	low
Treat-0d	RHSIM_Rhsim10G0000800	1.192	-0.870	-2.062	high
Treat-0d	RHSIM_Rhsim09G0135300	-0.635	-1.538	-0.903	no
Treat-0d	RHSIM_Rhsim09G0137200	-2.268	-1.417	0.851	low
Treat-0d	RHSIM_Rhsim10G0114000	-0.159	1.020	1.179	no
Treat-0d	RHSIM_Rhsim08G0042300	-0.687	0.147	0.834	no
Treat-0d	RHSIM_Rhsim09G0141800	1.029	0.626	-0.403	no
Treat-0d	RHSIM_Rhsim10G0165400	-0.474	-0.192	0.282	no
Treat-0d	RHSIM_Rhsim09G0037400	0.275	0.305	0.031	no
Treat-0d	RHSIM_Rhsim10G0166900	0.006	-0.496	-0.502	no
Treat-0d	RHSIM_Rhsim09G0029500	-0.744	-0.886	-0.142	no
Treat-0d	RHSIM_Rhsim08G0236100	-1.734	-1.364	0.370	low
Treat-0d	RHSIM_Rhsim08G0123900	0.233	0.472	0.239	no
Treat-0d	RHSIM_Rhsim10G0050000	1.399	0.195	-1.204	high
Treat-0d	RHSIM_Rhsim10G0021100	1.386	0.075	-1.312	high
Treat-0d	RHSIM_Rhsim09G0107300	0.631	0.202	-0.429	no
Treat-0d	RHSIM_Rhsim10G0069600	-0.985	-0.166	0.820	no
Treat-0d	RHSIM_Rhsim09G0181500	-1.107	-1.063	0.044	low
Treat-0d	RHSIM_Rhsim09G0176600	-0.690	0.667	1.358	no
Treat-0d	RHSIM_Rhsim10G0188000	-0.634	-1.372	-0.738	no
Treat-0d	RHSIM_Rhsim09G0126200	-1.020	-1.635	-0.615	no
Treat-0d	RHSIM_Rhsim08G0033900	-0.997	-0.931	0.066	no
Treat-0d	RHSIM_Rhsim09G0011600	-0.663	-0.531	0.131	no
Treat-0d	RHSIM_Rhsim08G0070600	0.314	0.029	-0.286	no
Treat-0d	RHSIM_Rhsim09G0008400	1.177	2.219	1.041	high
Treat-0d	RHSIM_Rhsim09G0028000	-0.276	-0.402	-0.125	no
Treat-0d	RHSIM_Rhsim08G0101600	-1.676	-2.477	-0.802	low
Treat-0d	RHSIM_Rhsim09G0212400	-0.666	-0.542	0.124	no
Treat-0d	RHSIM_Rhsim08G0079700	-0.293	1.086	1.379	no
Treat-0d	RHSIM_Rhsim09G0164000	0.059	0.448	0.390	no

Group	Gene	PTR (z-score difference)	z(log2 LFQ)	z(log2 FPKM)	extreme-PTR
Treat-0d	RHSIM_Rhsim09G0179900	0.380	1.090	0.710	no
Treat-0d	RHSIM_Rhsim09G0154200	-0.157	-0.381	-0.224	no
Treat-0d	RHSIM_Rhsim09G0103500	-0.092	-1.027	-0.935	no
Treat-0d	RHSIM_Rhsim08G0147100	1.586	1.182	-0.404	high
Treat-0d	RHSIM_Rhsim09G0071200	-2.282	-0.806	1.476	low
Treat-0d	RHSIM_Rhsim09G0126000	2.687	0.613	-2.073	high
Treat-0d	RHSIM_Rhsim09G0012000	-2.367	-0.898	1.469	low
Treat-0d	RHSIM_Rhsim09G0191500	0.313	-0.020	-0.333	no
Treat-0d	RHSIM_Rhsim09G0177000	0.735	-0.370	-1.105	no
Treat-0d	RHSIM_Rhsim09G0086300	-0.386	-0.185	0.201	no
Treat-0d	RHSIM_Rhsim09G0192800	-1.167	-0.644	0.523	low
Treat-0d	RHSIM_Rhsim09G0063500	-0.649	-1.480	-0.831	no
Treat-0d	RHSIM_Rhsim09G0182600	-0.017	0.597	0.614	no
Treat-0d	RHSIM_Rhsim09G0010800	-1.686	-1.363	0.323	low
Treat-0d	RHSIM_Rhsim09G0098400	-2.090	-1.588	0.502	low
Treat-0d	RHSIM_Rhsim08G0214300	0.799	1.830	1.031	no
Treat-0d	RHSIM_Rhsim09G0099000	-0.671	-1.551	-0.881	no
Treat-0d	RHSIM_Rhsim08G0035400	-1.640	-2.213	-0.574	low
Treat-0d	RHSIM_Rhsim08G0097500	-2.046	-1.602	0.443	low
Treat-0d	RHSIM_Rhsim09G0065700	1.517	-0.357	-1.874	high
Treat-0d	RHSIM_Rhsim09G0075600	-0.201	-0.711	-0.511	no
Treat-0d	RHSIM_Rhsim09G0094200	-1.765	-1.230	0.534	low
Treat-0d	RHSIM_Rhsim09G0184500	0.476	0.006	-0.471	no
Treat-0d	RHSIM_Rhsim09G0147200	-0.246	1.328	1.574	no
Treat-0d	RHSIM_Rhsim08G0145400	0.632	0.097	-0.535	no
Treat-0d	RHSIM_Rhsim09G0190300	-0.189	0.337	0.526	no
Treat-0d	RHSIM_Rhsim08G0056700	-1.068	-2.134	-1.066	low
Treat-0d	RHSIM_Rhsim09G0193300	0.121	0.247	0.126	no
Treat-0d	RHSIM_Rhsim09G0055700	1.008	-0.948	-1.957	no
Treat-0d	RHSIM_Rhsim09G0204600	-1.555	-1.138	0.417	low
Treat-0d	RHSIM_Rhsim08G0200000	-0.513	0.347	0.860	no
Treat-0d	RHSIM_Rhsim09G0069300	1.805	0.777	-1.029	high

Group	Gene	PTR (z-score difference)	z(log2 LFQ)	z(log2 FPKM)	extreme-PTR
Treat-0d	RHSIM_Rhsim09G0179400	1.188	0.497	-0.691	high
Treat-0d	RHSIM_Rhsim09G0157600	0.241	-0.482	-0.722	no
Treat-0d	RHSIM_Rhsim09G0091900	-0.951	-0.435	0.517	no
Treat-0d	RHSIM_Rhsim09G0117900	1.854	0.243	-1.612	high
Treat-0d	RHSIM_Rhsim09G0131900	-0.728	0.202	0.930	no
Treat-0d	RHSIM_Rhsim07G0198600	0.111	2.223	2.112	no
Treat-0d	RHSIM_Rhsim09G0070800	0.417	1.169	0.752	no
Treat-0d	RHSIM_Rhsim09G0155700	0.506	-0.248	-0.754	no
Treat-0d	RHSIM_Rhsim09G0186700	2.147	0.761	-1.386	high
Treat-0d	RHSIM_Rhsim09G0145100	-1.504	-1.088	0.416	low
Treat-0d	RHSIM_Rhsim09G0129000	-0.525	-0.514	0.011	no
Treat-0d	RHSIM_Rhsim07G0224400	-0.240	-0.859	-0.618	no
Treat-0d	RHSIM_Rhsim09G0137500	-2.055	-0.437	1.619	low
Treat-0d	RHSIM_Rhsim09G0171900	0.795	2.722	1.927	no
Treat-0d	RHSIM_Rhsim09G0010300	-0.814	-0.538	0.276	no
Treat-0d	RHSIM_Rhsim07G0144600	-0.521	-0.263	0.258	no
Treat-0d	RHSIM_Rhsim09G0025700	1.724	0.132	-1.592	high
Treat-0d	RHSIM_Rhsim07G0074600	-0.255	-0.812	-0.557	no
Treat-0d	RHSIM_Rhsim07G0153100	0.307	0.703	0.397	no
Treat-0d	RHSIM_Rhsim08G0119600	0.234	-0.064	-0.298	no
Treat-0d	RHSIM_Rhsim09G0054700	0.786	0.157	-0.628	no
Treat-0d	RHSIM_Rhsim09G0123100	-0.524	0.076	0.601	no
Treat-0d	RHSIM_Rhsim09G0080400	-0.371	-0.188	0.183	no
Treat-0d	RHSIM_Rhsim09G0138200	-1.466	-1.330	0.136	low
Treat-0d	RHSIM_Rhsim08G0135400	-1.461	-1.802	-0.341	low
Treat-0d	RHSIM_Rhsim07G0018300	1.248	0.917	-0.331	high
Treat-0d	RHSIM_Rhsim08G0214800	-1.181	-0.460	0.721	low
Treat-0d	RHSIM_Rhsim03G0130900	0.068	0.761	0.693	no
Treat-0d	RHSIM_Rhsim09G0128900	0.397	0.352	-0.045	no
Treat-0d	RHSIM_Rhsim09G0054900	-1.052	-0.865	0.188	low
Treat-0d	RHSIM_Rhsim08G0045200	0.672	0.040	-0.632	no
Treat-0d	RHSIM_Rhsim07G0258100	0.055	0.159	0.104	no

Group	Gene	PTR (z-score difference)	z(log2 LFQ)	z(log2 FPKM)	extreme-PTR
Treat-0d	RHSIM_Rhsim07G0246300	-0.943	-1.386	-0.443	no
Treat-0d	RHSIM_Rhsim09G0044200	-2.523	-0.042	2.481	low
Treat-0d	RHSIM_Rhsim08G0089100	-0.548	0.951	1.498	no
Treat-0d	RHSIM_Rhsim07G0061000	-1.910	-1.235	0.674	low
Treat-0d	RHSIM_Rhsim08G0213200	-2.492	-0.935	1.557	low
Treat-0d	RHSIM_Rhsim07G0068600	0.672	-0.097	-0.769	no
Treat-0d	RHSIM_Rhsim07G0243600	1.110	-0.544	-1.654	high
Treat-0d	RHSIM_Rhsim08G0223600	0.426	1.573	1.147	no
Treat-0d	RHSIM_Rhsim09G0089500	0.800	-0.110	-0.910	no
Treat-0d	RHSIM_Rhsim07G0088700	0.468	0.752	0.284	no
Treat-0d	RHSIM_Rhsim07G0042900	-1.468	-2.261	-0.793	low
Treat-0d	RHSIM_Rhsim09G0033900	-2.988	-2.116	0.872	low
Treat-0d	RHSIM_Rhsim09G0142900	-0.497	-0.214	0.282	no
Treat-0d	RHSIM_Rhsim07G0200500	-0.511	0.180	0.691	no
Treat-0d	RHSIM_Rhsim09G0113800	0.609	-0.282	-0.891	no
Treat-0d	RHSIM_Rhsim09G0212800	1.460	0.195	-1.266	high
Treat-0d	RHSIM_Rhsim09G0178400	0.962	1.629	0.667	no
Treat-0d	RHSIM_Rhsim08G0111900	0.472	0.691	0.219	no
Treat-0d	RHSIM_Rhsim09G0094000	-0.353	0.008	0.361	no
Treat-0d	RHSIM_Rhsim08G0126700	2.204	1.627	-0.577	high
Treat-0d	RHSIM_Rhsim07G0192400	1.257	-0.219	-1.475	high
Treat-0d	RHSIM_Rhsim08G0070900	1.657	-0.653	-2.310	high
Treat-0d	RHSIM_Rhsim08G0021800	2.218	2.303	0.085	high
Treat-0d	RHSIM_Rhsim09G0088900	0.977	0.353	-0.624	no
Treat-0d	RHSIM_Rhsim08G0042600	-1.241	-0.096	1.145	low
Treat-0d	RHSIM_Rhsim09G0091400	-0.114	-0.298	-0.183	no
Treat-0d	RHSIM_Rhsim08G0092200	-0.362	-0.552	-0.190	no
Treat-0d	RHSIM_Rhsim09G0130400	-0.929	0.717	1.646	no
Treat-0d	RHSIM_Rhsim09G0120100	0.690	1.846	1.156	no
Treat-0d	RHSIM_Rhsim08G0043100	1.948	0.161	-1.787	high
Treat-0d	RHSIM_Rhsim09G0190000	0.282	-0.662	-0.944	no
Treat-0d	RHSIM_Rhsim09G0102000	-0.936	-0.343	0.593	no

Group	Gene	PTR (z-score difference)	z(log2 LFQ)	z(log2 FPKM)	extreme-PTR
Treat-0d	RHSIM_Rhsim07G0108200	0.595	0.282	-0.314	no
Treat-0d	RHSIM_Rhsim08G0118300	-1.325	-0.888	0.437	low
Treat-0d	RHSIM_Rhsim09G0101400	-0.211	0.457	0.668	no
Treat-0d	RHSIM_Rhsim08G0008800	1.023	1.130	0.107	no
Treat-0d	RHSIM_Rhsim08G0105500	0.529	-0.210	-0.740	no
Treat-0d	RHSIM_Rhsim08G0178200	0.557	2.753	2.196	no
Treat-0d	RHSIM_Rhsim08G0162700	1.763	0.461	-1.303	high
Treat-0d	RHSIM_Rhsim07G0234400	-0.842	0.719	1.561	no
Treat-0d	RHSIM_Rhsim08G0212700	-0.124	-0.016	0.108	no
Treat-0d	RHSIM_Rhsim08G0244600	0.896	0.458	-0.439	no
Treat-0d	RHSIM_Rhsim09G0074900	0.310	1.500	1.191	no
Treat-0d	RHSIM_Rhsim07G0030800	2.115	2.083	-0.032	high
Treat-0d	RHSIM_Rhsim09G0001500	0.479	1.444	0.965	no
Treat-0d	RHSIM_Rhsim07G0104900	1.007	0.117	-0.890	no
Treat-0d	RHSIM_Rhsim08G0192000	-1.458	0.260	1.718	low
Treat-0d	RHSIM_Rhsim08G0167700	0.660	-0.793	-1.453	no
Treat-0d	RHSIM_Rhsim08G0068800	-0.464	-1.296	-0.832	no
Treat-0d	RHSIM_Rhsim07G0234600	0.117	1.253	1.135	no
Treat-0d	RHSIM_Rhsim09G0153200	-0.308	-0.486	-0.178	no
Treat-0d	RHSIM_Rhsim08G0227900	-0.584	-1.578	-0.995	no
Treat-0d	RHSIM_Rhsim09G0093900	-0.202	0.777	0.979	no
Treat-0d	RHSIM_Rhsim07G0164900	1.132	-0.387	-1.519	high
Treat-0d	RHSIM_Rhsim07G0092500	0.766	0.581	-0.185	no
Treat-0d	RHSIM_Rhsim08G0083700	-0.367	0.440	0.808	no
Treat-0d	RHSIM_Rhsim08G0027200	-1.171	-0.749	0.422	low
Treat-0d	RHSIM_Rhsim07G0021100	1.468	0.100	-1.369	high
Treat-0d	RHSIM_Rhsim08G0236900	-0.020	-0.231	-0.211	no
Treat-0d	RHSIM_Rhsim09G0198900	0.327	0.367	0.040	no
Treat-0d	RHSIM_Rhsim08G0187300	-0.398	-1.061	-0.662	no
Treat-0d	RHSIM_Rhsim08G0007300	-2.056	-1.815	0.241	low
Treat-0d	RHSIM_Rhsim08G0212200	-0.229	-0.445	-0.215	no
Treat-0d	RHSIM_Rhsim09G0072100	-0.801	-1.468	-0.667	no

Group	Gene	PTR (z-score difference)	z(log2 LFQ)	z(log2 FPKM)	extreme-PTR
Treat-0d	RHSIM_Rhsim07G0122800	-0.240	-1.150	-0.910	no
Treat-0d	RHSIM_Rhsim09G0131200	-0.464	-0.367	0.097	no
Treat-0d	RHSIM_Rhsim08G0118700	0.908	0.315	-0.593	no
Treat-0d	RHSIM_Rhsim08G0180000	-0.834	-0.664	0.170	no
Treat-0d	RHSIM_Rhsim07G0128400	-3.936	-2.140	1.796	low
Treat-0d	RHSIM_Rhsim08G0236700	1.417	0.396	-1.021	high
Treat-0d	RHSIM_Rhsim09G0213200	-0.019	-0.091	-0.072	no
Treat-0d	RHSIM_Rhsim07G0155900	1.512	-0.280	-1.792	high
Treat-0d	RHSIM_Rhsim09G0130300	-2.898	-2.353	0.545	low
Treat-0d	RHSIM_Rhsim08G0246100	1.577	-0.243	-1.819	high
Treat-0d	RHSIM_Rhsim08G0098000	-1.074	-0.035	1.039	low
Treat-0d	RHSIM_Rhsim08G0082400	-0.416	0.120	0.536	no
Treat-0d	RHSIM_Rhsim08G0071400	-0.639	1.516	2.155	no
Treat-0d	RHSIM_Rhsim08G0249700	1.118	0.896	-0.222	high
Treat-0d	RHSIM_Rhsim08G0043900	-1.305	-1.388	-0.083	low
Treat-0d	RHSIM_Rhsim07G0132900	-1.257	-1.133	0.124	low
Treat-0d	RHSIM_Rhsim08G0093700	0.101	-1.441	-1.543	no
Treat-0d	RHSIM_Rhsim08G0061200	-0.258	-1.010	-0.752	no
Treat-0d	RHSIM_Rhsim07G0128700	-0.439	-1.047	-0.608	no
Treat-0d	RHSIM_Rhsim08G0052300	1.190	-0.490	-1.680	high
Treat-0d	RHSIM_Rhsim08G0234400	1.372	0.001	-1.371	high
Treat-0d	RHSIM_Rhsim09G0021800	-0.082	0.916	0.998	no
Treat-0d	RHSIM_Rhsim09G0176500	0.546	1.410	0.864	no
Treat-0d	RHSIM_Rhsim08G0200300	-2.386	-1.883	0.502	low
Treat-0d	RHSIM_Rhsim07G0258600	-0.825	-0.827	-0.002	no
Treat-0d	RHSIM_Rhsim08G0032500	-1.322	-0.870	0.452	low
Treat-0d	RHSIM_Rhsim08G0004300	-1.114	-0.563	0.551	low
Treat-0d	RHSIM_Rhsim08G0026800	-0.763	-1.322	-0.558	no
Treat-0d	RHSIM_Rhsim08G0240700	-0.758	-0.877	-0.119	no
Treat-0d	RHSIM_Rhsim08G0247800	-0.401	-0.338	0.063	no
Treat-0d	RHSIM_Rhsim09G0073300	-0.210	0.543	0.753	no
Treat-0d	RHSIM_Rhsim09G0145600	0.886	-0.021	-0.907	no

Group	Gene	PTR (z-score difference)	z(log2 LFQ)	z(log2 FPKM)	extreme-PTR
Treat-0d	RHSIM_Rhsim08G0080700	-1.074	-0.363	0.711	low
Treat-0d	RHSIM_Rhsim08G0076400	0.111	-0.101	-0.212	no
Treat-0d	RHSIM_Rhsim08G0184900	0.410	-0.686	-1.096	no
Treat-0d	RHSIM_Rhsim08G0009400	-0.794	1.554	2.347	no
Treat-0d	RHSIM_Rhsim07G0050700	-0.316	0.001	0.317	no
Treat-0d	RHSIM_Rhsim08G0019200	-1.989	-0.648	1.341	low
Treat-0d	RHSIM_Rhsim08G0059600	0.154	0.721	0.567	no
Treat-0d	RHSIM_Rhsim08G0239600	-1.536	-1.299	0.237	low
Treat-0d	RHSIM_Rhsim08G0093100	-0.862	-0.943	-0.081	no
Treat-0d	RHSIM_Rhsim08G0128400	1.191	0.659	-0.532	high
Treat-0d	RHSIM_Rhsim07G0012200	1.383	-0.145	-1.528	high
Treat-0d	RHSIM_Rhsim08G0194600	0.544	1.785	1.241	no
Treat-0d	RHSIM_Rhsim08G0033500	-0.337	-0.033	0.303	no
Treat-0d	RHSIM_Rhsim07G0027400	0.192	0.893	0.702	no
Treat-0d	RHSIM_Rhsim07G0153400	0.151	-0.271	-0.423	no
Treat-0d	RHSIM_Rhsim09G0078400	1.721	-0.092	-1.813	high
Treat-0d	RHSIM_Rhsim08G0164000	0.482	-0.455	-0.937	no
Treat-0d	RHSIM_Rhsim07G0002300	-0.200	-1.090	-0.890	no
Treat-0d	RHSIM_Rhsim08G0222900	1.454	0.336	-1.119	high
Treat-0d	RHSIM_Rhsim08G0198500	-0.664	-0.336	0.328	no
Treat-0d	RHSIM_Rhsim08G0218500	0.640	0.688	0.047	no
Treat-0d	RHSIM_Rhsim09G0155900	0.609	1.272	0.663	no
Treat-0d	RHSIM_Rhsim08G0228500	2.677	0.615	-2.063	high
Treat-0d	RHSIM_Rhsim09G0195900	2.067	0.636	-1.431	high
Treat-0d	RHSIM_Rhsim08G0029800	-0.212	-1.123	-0.911	no
Treat-0d	RHSIM_Rhsim07G0228400	0.060	0.587	0.526	no
Treat-0d	RHSIM_Rhsim09G0093200	1.070	1.887	0.817	no
Treat-0d	RHSIM_Rhsim07G0068800	-0.585	-1.193	-0.608	no
Treat-0d	RHSIM_Rhsim08G0037300	2.360	0.282	-2.078	high
Treat-0d	RHSIM_Rhsim07G0011300	-0.549	-0.862	-0.313	no
Treat-0d	RHSIM_Rhsim08G0233800	1.360	0.266	-1.093	high
Treat-0d	RHSIM_Rhsim08G0211000	1.022	1.711	0.690	no

Group	Gene	PTR (z-score difference)	z(log2 LFQ)	z(log2 FPKM)	extreme-PTR
Treat-0d	RHSIM_Rhsim07G0169700	-0.199	-0.536	-0.337	no
Treat-0d	RHSIM_Rhsim08G0027300	0.171	-0.808	-0.978	no
Treat-0d	RHSIM_Rhsim07G0078300	0.314	0.377	0.063	no
Treat-0d	RHSIM_Rhsim08G0055200	-0.377	0.796	1.173	no
Treat-0d	RHSIM_Rhsim07G0091400	-1.007	1.259	2.266	no
Treat-0d	RHSIM_Rhsim08G0201400	-1.780	-2.278	-0.498	low
Treat-0d	RHSIM_Rhsim07G0058300	0.204	-0.516	-0.720	no
Treat-0d	RHSIM_Rhsim09G0042400	1.032	-0.343	-1.375	no
Treat-0d	RHSIM_Rhsim08G0020900	1.011	2.078	1.067	no
Treat-0d	RHSIM_Rhsim08G0103100	0.979	-0.229	-1.208	no
Treat-0d	RHSIM_Rhsim08G0232100	-1.422	-0.460	0.962	low
Treat-0d	RHSIM_Rhsim09G0030000	0.476	-0.282	-0.757	no
Treat-0d	RHSIM_Rhsim08G0121700	1.859	-0.108	-1.968	high
Treat-0d	RHSIM_Rhsim08G0011700	-1.820	0.785	2.605	low
Treat-0d	RHSIM_Rhsim08G0147000	-1.287	0.855	2.142	low
Treat-0d	RHSIM_Rhsim08G0192700	-0.590	-1.711	-1.121	no
Treat-0d	RHSIM_Rhsim08G0057700	1.894	1.840	-0.053	high
Treat-0d	RHSIM_Rhsim08G0199300	-2.076	-1.241	0.835	low
Treat-0d	RHSIM_Rhsim07G0016300	1.190	-0.614	-1.805	high
Treat-0d	RHSIM_Rhsim08G0083000	-2.618	-1.109	1.509	low
Treat-0d	RHSIM_Rhsim08G0252400	1.564	0.476	-1.089	high
Treat-0d	RHSIM_Rhsim08G0215600	-0.057	0.002	0.060	no
Treat-0d	RHSIM_Rhsim09G0009000	0.049	-0.076	-0.125	no
Treat-0d	RHSIM_Rhsim08G0207700	1.280	-0.507	-1.787	high
Treat-0d	RHSIM_Rhsim09G0152000	0.466	-0.231	-0.697	no
Treat-0d	RHSIM_Rhsim08G0089000	1.999	0.794	-1.205	high
Treat-0d	RHSIM_Rhsim07G0118200	0.145	1.119	0.973	no
Treat-0d	RHSIM_Rhsim08G0102900	-0.856	-1.386	-0.531	no
Treat-0d	RHSIM_Rhsim07G0028300	-0.320	-0.016	0.304	no
Treat-0d	RHSIM_Rhsim08G0161100	0.783	0.505	-0.278	no
Treat-0d	RHSIM_Rhsim08G0087900	-0.733	-1.111	-0.378	no
Treat-0d	RHSIM_Rhsim09G0164200	0.292	1.765	1.474	no

Group	Gene	PTR (z-score difference)	z(log2 LFQ)	z(log2 FPKM)	extreme-PTR
Treat-0d	RHSIM_Rhsim07G0246500	0.241	-0.952	-1.192	no
Treat-0d	RHSIM_Rhsim08G0055400	2.126	2.011	-0.115	high
Treat-0d	RHSIM_Rhsim08G0034500	0.415	-0.843	-1.258	no
Treat-0d	RHSIM_Rhsim08G0242000	-0.424	-1.261	-0.838	no
Treat-0d	RHSIM_Rhsim08G0073200	1.119	0.990	-0.128	high
Treat-0d	RHSIM_Rhsim08G0223300	0.918	3.072	2.154	no
Treat-0d	RHSIM_Rhsim08G0128700	-1.174	-0.702	0.472	low
Treat-0d	RHSIM_Rhsim08G0219800	-1.459	-1.993	-0.533	low
Treat-0d	RHSIM_Rhsim09G0135900	1.164	-0.111	-1.275	high
Treat-0d	RHSIM_Rhsim08G0106300	0.390	-0.433	-0.824	no
Treat-0d	RHSIM_Rhsim08G0027700	0.690	-0.050	-0.740	no
Treat-0d	RHSIM_Rhsim08G0010500	-0.672	-0.212	0.460	no
Treat-0d	RHSIM_Rhsim08G0049000	-0.218	-0.928	-0.711	no
Treat-0d	RHSIM_Rhsim08G0188800	0.058	-0.615	-0.673	no
Treat-0d	RHSIM_Rhsim08G0028200	0.446	0.049	-0.397	no
Treat-0d	RHSIM_Rhsim08G0112900	-0.456	-0.577	-0.121	no
Treat-0d	RHSIM_Rhsim08G0223100	-0.275	1.565	1.839	no
Treat-0d	RHSIM_Rhsim07G0214500	2.351	2.493	0.142	high
Treat-0d	RHSIM_Rhsim08G0178000	0.691	-1.705	-2.396	no
Treat-0d	RHSIM_Rhsim07G0067400	-0.847	-1.077	-0.230	no
Treat-0d	RHSIM_Rhsim07G0124800	0.315	0.272	-0.043	no
Treat-0d	RHSIM_Rhsim08G0227600	0.329	-1.621	-1.950	no
Treat-0d	RHSIM_Rhsim07G0009900	-0.317	-0.929	-0.612	no
Treat-0d	RHSIM_Rhsim07G0036400	0.143	2.163	2.020	no
Treat-0d	RHSIM_Rhsim08G0228700	-0.299	-1.335	-1.036	no
Treat-0d	RHSIM_Rhsim08G0246500	-1.483	0.171	1.653	low
Treat-0d	RHSIM_Rhsim08G0015900	-0.037	-0.143	-0.106	no
Treat-0d	RHSIM_Rhsim07G0124400	0.178	2.654	2.476	no
Treat-0d	RHSIM_Rhsim06G0028000	-0.154	1.675	1.829	no
Treat-0d	RHSIM_Rhsim08G0248800	0.371	0.601	0.230	no
Treat-0d	RHSIM_Rhsim08G0225500	1.518	1.283	-0.235	high
Treat-0d	RHSIM_Rhsim08G0196300	0.661	-1.194	-1.855	no

Group	Gene	PTR (z-score difference)	z(log2 LFQ)	z(log2 FPKM)	extreme-PTR
Treat-0d	RHSIM_Rhsim08G0011000	0.301	1.370	1.069	no
Treat-0d	RHSIM_Rhsim08G0001700	-0.786	-0.559	0.227	no
Treat-0d	RHSIM_Rhsim08G0169400	-0.684	-1.113	-0.429	no
Treat-0d	RHSIM_Rhsim08G0087000	0.990	1.287	0.297	no
Treat-0d	RHSIM_Rhsim07G0046800	-0.649	0.272	0.921	no
Treat-0d	RHSIM_Rhsim08G0221800	-0.099	-0.615	-0.516	no
Treat-0d	RHSIM_Rhsim08G0173800	-0.125	-0.651	-0.526	no
Treat-0d	RHSIM_Rhsim06G0095100	0.153	-0.930	-1.083	no
Treat-0d	RHSIM_Rhsim08G0198100	0.772	1.828	1.056	no
Treat-0d	RHSIM_Rhsim07G0215600	-0.549	0.298	0.847	no
Treat-0d	RHSIM_Rhsim08G0032400	-0.514	-0.020	0.493	no
Treat-0d	RHSIM_Rhsim08G0084500	-1.233	0.122	1.356	low
Treat-0d	RHSIM_Rhsim08G0059500	0.739	1.627	0.888	no
Treat-0d	RHSIM_Rhsim08G0052400	0.697	0.599	-0.099	no
Treat-0d	RHSIM_Rhsim07G0093000	-1.379	0.142	1.520	low
Treat-0d	RHSIM_Rhsim08G0092600	1.572	1.399	-0.173	high
Treat-0d	RHSIM_Rhsim08G0181000	3.064	1.049	-2.014	high
Treat-0d	RHSIM_Rhsim08G0052800	-1.945	-0.945	1.000	low
Treat-0d	RHSIM_Rhsim08G0048100	-0.739	-0.803	-0.064	no
Treat-0d	RHSIM_Rhsim07G0017600	0.002	0.333	0.331	no
Treat-0d	RHSIM_Rhsim07G0044000	-0.038	-0.127	-0.089	no
Treat-0d	RHSIM_Rhsim08G0042700	0.559	-0.218	-0.777	no
Treat-0d	RHSIM_Rhsim08G0066600	0.315	-0.135	-0.451	no
Treat-0d	RHSIM_Rhsim06G0108700	-1.120	-1.266	-0.146	low
Treat-0d	RHSIM_Rhsim07G0106400	0.232	0.713	0.481	no
Treat-0d	RHSIM_Rhsim07G0142700	-0.081	-1.782	-1.701	no
Treat-0d	RHSIM_Rhsim06G0005200	-0.487	-0.911	-0.424	no
Treat-0d	RHSIM_Rhsim08G0135600	-0.726	-1.906	-1.180	no
Treat-0d	RHSIM_Rhsim07G0212500	-0.555	-0.450	0.105	no
Treat-0d	RHSIM_Rhsim08G0194200	0.709	0.550	-0.159	no
Treat-0d	RHSIM_Rhsim08G0155500	-0.693	-1.131	-0.438	no
Treat-0d	RHSIM_Rhsim06G0127700	0.676	0.108	-0.568	no

Group	Gene	PTR (z-score difference)	z(log2 LFQ)	z(log2 FPKM)	extreme-PTR
Treat-0d	RHSIM_Rhsim08G0017300	1.236	0.280	-0.956	high
Treat-0d	RHSIM_Rhsim08G0195700	-0.811	-1.051	-0.241	no
Treat-0d	RHSIM_Rhsim08G0007800	-0.618	-0.131	0.486	no
Treat-0d	RHSIM_Rhsim08G0104200	0.608	-0.562	-1.170	no
Treat-0d	RHSIM_Rhsim06G0233900	0.480	0.254	-0.226	no
Treat-0d	RHSIM_Rhsim08G0159800	0.970	0.669	-0.302	no
Treat-0d	RHSIM_Rhsim08G0212400	-0.134	-1.036	-0.903	no
Treat-0d	RHSIM_Rhsim06G0165900	0.485	0.078	-0.408	no
Treat-0d	RHSIM_Rhsim08G0236000	-1.540	-1.035	0.504	low
Treat-0d	RHSIM_Rhsim07G0219800	-0.304	-0.401	-0.097	no
Treat-0d	RHSIM_Rhsim08G0196000	-0.123	1.607	1.730	no
Treat-0d	RHSIM_Rhsim06G0091300	0.110	-1.173	-1.283	no
Treat-0d	RHSIM_Rhsim08G0095900	0.616	-0.857	-1.473	no
Treat-0d	RHSIM_Rhsim08G0078500	0.889	-0.085	-0.974	no
Treat-0d	RHSIM_Rhsim07G0170200	0.444	0.471	0.027	no
Treat-0d	RHSIM_Rhsim07G0233400	1.401	1.232	-0.169	high
Treat-0d	RHSIM_Rhsim06G0211500	-0.386	-0.773	-0.387	no
Treat-0d	RHSIM_Rhsim08G0079500	-0.280	0.888	1.167	no
Treat-0d	RHSIM_Rhsim07G0202900	-0.241	-0.369	-0.129	no
Treat-0d	RHSIM_Rhsim07G0001500	0.702	0.501	-0.201	no
Treat-0d	RHSIM_Rhsim07G0178700	0.147	-0.032	-0.179	no
Treat-0d	RHSIM_Rhsim07G0170500	-1.304	-1.085	0.219	low
Treat-0d	RHSIM_Rhsim06G0181200	0.204	0.477	0.273	no
Treat-0d	RHSIM_Rhsim07G0018600	2.099	-0.083	-2.181	high
Treat-0d	RHSIM_Rhsim07G0049800	1.111	1.768	0.656	high
Treat-0d	RHSIM_Rhsim07G0066200	0.315	0.093	-0.221	no
Treat-0d	RHSIM_Rhsim08G0072200	0.671	-0.278	-0.949	no
Treat-0d	RHSIM_Rhsim07G0131800	0.666	0.419	-0.247	no
Treat-0d	RHSIM_Rhsim07G0036500	0.029	0.513	0.483	no
Treat-0d	RHSIM_Rhsim07G0028800	1.427	3.604	2.177	high
Treat-0d	RHSIM_Rhsim07G0132800	0.457	-0.001	-0.458	no
Treat-0d	RHSIM_Rhsim07G0081100	-0.001	-1.157	-1.156	no

Group	Gene	PTR (z-score difference)	z(log2 LFQ)	z(log2 FPKM)	extreme-PTR
Treat-0d	RHSIM_Rhsim08G0235700	0.435	0.152	-0.283	no
Treat-0d	RHSIM_Rhsim06G0212600	2.409	1.421	-0.987	high
Treat-0d	RHSIM_Rhsim07G0018400	0.907	1.295	0.387	no
Treat-0d	RHSIM_Rhsim07G0184200	-1.017	0.913	1.930	no
Treat-0d	RHSIM_Rhsim06G0002800	1.108	0.462	-0.646	high
Treat-0d	RHSIM_Rhsim07G0099200	0.826	-0.165	-0.991	no
Treat-0d	RHSIM_Rhsim07G0258700	-0.167	-0.642	-0.474	no
Treat-0d	RHSIM_Rhsim07G0045900	0.233	-0.659	-0.892	no
Treat-0d	RHSIM_Rhsim07G0087900	1.906	0.024	-1.881	high
Treat-0d	RHSIM_Rhsim08G0071000	-1.591	-0.218	1.373	low
Treat-0d	RHSIM_Rhsim08G0105200	1.390	-0.094	-1.484	high
Treat-0d	RHSIM_Rhsim08G0186700	-1.503	-1.174	0.329	low
Treat-0d	RHSIM_Rhsim06G0244300	0.644	1.008	0.364	no
Treat-0d	RHSIM_Rhsim08G0167500	0.295	0.972	0.677	no
Treat-0d	RHSIM_Rhsim07G0122500	-1.051	-1.913	-0.862	low
Treat-0d	RHSIM_Rhsim07G0030200	-0.437	-0.588	-0.151	no
Treat-0d	RHSIM_Rhsim08G0171100	0.724	0.222	-0.502	no
Treat-0d	RHSIM_Rhsim07G0083900	-0.788	0.449	1.237	no
Treat-0d	RHSIM_Rhsim08G0226300	-0.278	-0.851	-0.572	no
Treat-0d	RHSIM_Rhsim07G0017800	0.166	-1.004	-1.170	no
Treat-0d	RHSIM_Rhsim07G0027500	0.117	-0.616	-0.733	no
Treat-0d	RHSIM_Rhsim07G0202800	-0.737	0.003	0.740	no
Treat-0d	RHSIM_Rhsim06G0052900	-0.348	0.865	1.213	no
Treat-0d	RHSIM_Rhsim08G0118600	-0.088	0.279	0.367	no
Treat-0d	RHSIM_Rhsim07G0119300	-0.638	0.925	1.563	no
Treat-0d	RHSIM_Rhsim07G0049600	-0.244	1.180	1.424	no
Treat-0d	RHSIM_Rhsim06G0207400	1.470	0.886	-0.584	high
Treat-0d	RHSIM_Rhsim07G0056500	-1.027	-0.137	0.890	low
Treat-0d	RHSIM_Rhsim07G0103100	-0.285	1.019	1.304	no
Treat-0d	RHSIM_Rhsim07G0171800	0.495	0.418	-0.077	no
Treat-0d	RHSIM_Rhsim07G0121100	-0.072	0.086	0.158	no
Treat-0d	RHSIM_Rhsim08G0036900	0.214	0.635	0.421	no

Group	Gene	PTR (z-score difference)	z(log2 LFQ)	z(log2 FPKM)	extreme-PTR
Treat-0d	RHSIM_Rhsim07G0165200	0.632	-0.947	-1.579	no
Treat-0d	RHSIM_Rhsim08G0097800	0.568	1.528	0.960	no
Treat-0d	RHSIM_Rhsim06G0077600	-0.271	0.517	0.788	no
Treat-0d	RHSIM_Rhsim07G0020500	-0.222	0.143	0.365	no
Treat-0d	RHSIM_Rhsim07G0045200	0.105	0.540	0.435	no
Treat-0d	RHSIM_Rhsim06G0218800	-1.512	-1.448	0.063	low
Treat-0d	RHSIM_Rhsim07G0067300	0.540	0.168	-0.372	no
Treat-0d	RHSIM_Rhsim07G0188200	-1.715	-0.718	0.997	low
Treat-0d	RHSIM_Rhsim07G0228100	-1.353	-1.303	0.050	low
Treat-0d	RHSIM_Rhsim07G0245900	-0.915	-0.885	0.030	no
Treat-0d	RHSIM_Rhsim06G0187500	-1.170	-0.621	0.549	low
Treat-0d	RHSIM_Rhsim06G0105400	-3.207	-2.513	0.694	low
Treat-0d	RHSIM_Rhsim08G0152800	0.867	-0.270	-1.137	no
Treat-0d	RHSIM_Rhsim08G0171200	-1.143	-1.255	-0.112	low
Treat-0d	RHSIM_Rhsim07G0092600	-1.726	-0.768	0.958	low
Treat-0d	RHSIM_Rhsim07G0204300	0.871	0.656	-0.215	no
Treat-0d	RHSIM_Rhsim07G0081900	0.298	0.533	0.235	no
Treat-0d	RHSIM_Rhsim08G0138500	0.487	0.279	-0.208	no
Treat-0d	RHSIM_Rhsim07G0055600	1.933	1.883	-0.049	high
Treat-0d	RHSIM_Rhsim07G0125500	-1.359	-1.038	0.321	low
Treat-0d	RHSIM_Rhsim06G0222300	2.780	1.756	-1.024	high
Treat-0d	RHSIM_Rhsim07G0214600	-0.595	-1.206	-0.611	no
Treat-0d	RHSIM_Rhsim07G0075900	0.637	0.785	0.148	no
Treat-0d	RHSIM_Rhsim07G0074000	0.849	0.263	-0.586	no
Treat-0d	RHSIM_Rhsim06G0191200	0.504	-0.505	-1.009	no
Treat-0d	RHSIM_Rhsim08G0101100	-0.446	-1.389	-0.943	no
Treat-0d	RHSIM_Rhsim08G0183600	0.779	-0.123	-0.902	no
Treat-0d	RHSIM_Rhsim06G0178000	0.686	0.304	-0.382	no
Treat-0d	RHSIM_Rhsim07G0179500	1.219	0.119	-1.100	high
Treat-0d	RHSIM_Rhsim08G0196600	0.950	0.624	-0.326	no
Treat-0d	RHSIM_Rhsim06G0035000	0.283	0.116	-0.167	no
Treat-0d	RHSIM_Rhsim07G0059500	1.331	-0.538	-1.869	high

Group	Gene	PTR (z-score difference)	z(log2 LFQ)	z(log2 FPKM)	extreme-PTR
Treat-0d	RHSIM_Rhsim07G0097100	3.064	0.711	-2.353	high
Treat-0d	RHSIM_Rhsim07G0228600	-0.652	-0.489	0.163	no
Treat-0d	RHSIM_Rhsim06G0046200	0.962	-0.258	-1.220	no
Treat-0d	RHSIM_Rhsim07G0033000	-0.265	-0.672	-0.407	no
Treat-0d	RHSIM_Rhsim06G0167500	-2.014	1.210	3.224	low
Treat-0d	RHSIM_Rhsim07G0004400	0.181	0.365	0.184	no
Treat-0d	RHSIM_Rhsim06G0000200	-0.234	-0.517	-0.283	no
Treat-0d	RHSIM_Rhsim07G0241100	0.299	-1.437	-1.736	no
Treat-0d	RHSIM_Rhsim06G0034400	0.877	1.148	0.272	no
Treat-0d	RHSIM_Rhsim06G0138800	1.923	0.517	-1.406	high
Treat-0d	RHSIM_Rhsim08G0242800	1.664	-0.385	-2.049	high
Treat-0d	RHSIM_Rhsim07G0178300	3.432	3.291	-0.141	high
Treat-0d	RHSIM_Rhsim07G0013700	-0.273	0.261	0.534	no
Treat-0d	RHSIM_Rhsim07G0176800	0.935	-1.087	-2.023	no
Treat-0d	RHSIM_Rhsim06G0003200	1.213	1.206	-0.007	high
Treat-0d	RHSIM_Rhsim06G0031700	0.220	-0.251	-0.471	no
Treat-0d	RHSIM_Rhsim08G0090800	1.526	-0.419	-1.944	high
Treat-0d	RHSIM_Rhsim07G0028000	-0.487	-0.454	0.033	no
Treat-0d	RHSIM_Rhsim07G0087100	-0.570	-0.275	0.294	no
Treat-0d	RHSIM_Rhsim08G0222200	0.712	-0.931	-1.643	no
Treat-0d	RHSIM_Rhsim07G0169100	0.382	0.906	0.524	no
Treat-0d	RHSIM_Rhsim08G0251700	1.578	0.766	-0.812	high
Treat-0d	RHSIM_Rhsim07G0047300	0.392	-0.726	-1.118	no
Treat-0d	RHSIM_Rhsim08G0236600	0.617	0.858	0.241	no
Treat-0d	RHSIM_Rhsim07G0110300	-0.395	1.232	1.627	no
Treat-0d	RHSIM_Rhsim07G0226700	-0.128	-0.548	-0.420	no
Treat-0d	RHSIM_Rhsim07G0081200	-0.408	0.802	1.210	no
Treat-0d	RHSIM_Rhsim06G0193100	0.582	1.249	0.667	no
Treat-0d	RHSIM_Rhsim08G0036600	0.402	0.355	-0.047	no
Treat-0d	RHSIM_Rhsim07G0139000	0.849	1.031	0.182	no
Treat-0d	RHSIM_Rhsim07G0137200	-1.164	-1.617	-0.452	low
Treat-0d	RHSIM_Rhsim07G0103400	0.180	-0.030	-0.210	no

Group	Gene	PTR (z-score difference)	z(log2 LFQ)	z(log2 FPKM)	extreme-PTR
Treat-0d	RHSIM_Rhsim07G0139200	-0.165	-0.602	-0.437	no
Treat-0d	RHSIM_Rhsim07G0210400	1.086	2.072	0.985	no
Treat-0d	RHSIM_Rhsim07G0072200	0.134	-0.197	-0.331	no
Treat-0d	RHSIM_Rhsim06G0070400	0.024	0.771	0.747	no
Treat-0d	RHSIM_Rhsim07G0197900	-0.578	1.145	1.722	no
Treat-0d	RHSIM_Rhsim07G0130000	-0.791	-0.110	0.681	no
Treat-0d	RHSIM_Rhsim08G0190500	-0.950	0.009	0.960	no
Treat-0d	RHSIM_Rhsim07G0236500	-0.587	-0.690	-0.103	no
Treat-0d	RHSIM_Rhsim07G0110500	-0.674	-0.646	0.028	no
Treat-0d	RHSIM_Rhsim06G0205600	-0.255	0.039	0.295	no
Treat-0d	RHSIM_Rhsim08G0240800	-0.306	-0.260	0.046	no
Treat-0d	RHSIM_Rhsim07G0035600	-0.550	-0.309	0.241	no
Treat-0d	RHSIM_Rhsim06G0237100	-2.473	-1.103	1.370	low
Treat-0d	RHSIM_Rhsim07G0249300	1.307	0.915	-0.392	high
Treat-0d	RHSIM_Rhsim07G0001600	0.282	0.283	0.001	no
Treat-0d	RHSIM_Rhsim06G0047700	0.009	1.444	1.436	no
Treat-0d	RHSIM_Rhsim06G0242300	0.754	0.971	0.217	no
Treat-0d	RHSIM_Rhsim07G0018200	-0.249	0.306	0.554	no
Treat-0d	RHSIM_Rhsim07G0178100	-0.141	-0.802	-0.661	no
Treat-0d	RHSIM_Rhsim05G0060700	-0.348	0.908	1.256	no
Treat-0d	RHSIM_Rhsim06G0169300	1.010	1.178	0.168	no
Treat-0d	RHSIM_Rhsim07G0092700	0.006	1.028	1.022	no
Treat-0d	RHSIM_Rhsim07G0072000	-1.446	-0.828	0.618	low
Treat-0d	RHSIM_Rhsim07G0046600	-0.844	-0.398	0.446	no
Treat-0d	RHSIM_Rhsim07G0059000	1.540	0.823	-0.718	high
Treat-0d	RHSIM_Rhsim05G0194700	-0.050	0.707	0.758	no
Treat-0d	RHSIM_Rhsim05G0122800	0.352	0.456	0.104	no
Treat-0d	RHSIM_Rhsim06G0028600	1.182	-0.501	-1.683	high
Treat-0d	RHSIM_Rhsim07G0204200	-1.881	-1.101	0.781	low
Treat-0d	RHSIM_Rhsim06G0243900	2.371	0.596	-1.775	high
Treat-0d	RHSIM_Rhsim07G0237600	0.330	-0.215	-0.544	no
Treat-0d	RHSIM_Rhsim07G0247500	-0.639	0.805	1.444	no

Group	Gene	PTR (z-score difference)	z(log2 LFQ)	z(log2 FPKM)	extreme-PTR
Treat-0d	RHSIM_Rhsim05G0101000	1.243	0.326	-0.917	high
Treat-0d	RHSIM_Rhsim07G0012900	-2.033	-0.546	1.487	low
Treat-0d	RHSIM_Rhsim07G0026400	-1.868	0.503	2.371	low
Treat-0d	RHSIM_Rhsim07G0125200	-0.091	-1.349	-1.258	no
Treat-0d	RHSIM_Rhsim07G0117900	0.557	1.054	0.497	no
Treat-0d	RHSIM_Rhsim07G0150800	0.000	0.083	0.083	no
Treat-0d	RHSIM_Rhsim07G0054900	0.856	-0.111	-0.967	no
Treat-0d	RHSIM_Rhsim07G0006300	-2.657	-1.357	1.300	low
Treat-0d	RHSIM_Rhsim05G0217100	0.346	-0.897	-1.243	no
Treat-0d	RHSIM_Rhsim07G0100900	-1.975	-1.144	0.831	low
Treat-0d	RHSIM_Rhsim07G0210900	0.628	1.158	0.530	no
Treat-0d	RHSIM_Rhsim07G0179400	-0.809	-0.983	-0.175	no
Treat-0d	RHSIM_Rhsim07G0082100	1.718	2.322	0.604	high
Treat-0d	RHSIM_Rhsim07G0048700	0.361	-1.551	-1.911	no
Treat-0d	RHSIM_Rhsim06G0064500	-0.170	-0.442	-0.272	no
Treat-0d	RHSIM_Rhsim07G0083200	0.332	0.284	-0.048	no
Treat-0d	RHSIM_Rhsim06G0090400	-0.140	-0.109	0.031	no
Treat-0d	RHSIM_Rhsim06G0013400	0.057	0.542	0.485	no
Treat-0d	RHSIM_Rhsim05G0134300	0.404	-0.539	-0.943	no
Treat-0d	RHSIM_Rhsim06G0033500	0.054	-0.468	-0.522	no
Treat-0d	RHSIM_Rhsim05G0088500	1.828	1.648	-0.180	high
Treat-0d	RHSIM_Rhsim06G0089300	-2.590	-1.774	0.816	low
Treat-0d	RHSIM_Rhsim06G0122800	-0.500	-0.840	-0.340	no
Treat-0d	RHSIM_Rhsim07G0091600	1.462	0.107	-1.355	high
Treat-0d	RHSIM_Rhsim07G0129700	-0.777	-0.013	0.764	no
Treat-0d	RHSIM_Rhsim06G0072400	0.305	0.421	0.116	no
Treat-0d	RHSIM_Rhsim07G0205000	1.543	2.717	1.174	high
Treat-0d	RHSIM_Rhsim05G0061100	0.324	-0.534	-0.858	no
Treat-0d	RHSIM_Rhsim05G0111600	-0.114	-0.827	-0.712	no
Treat-0d	RHSIM_Rhsim06G0084600	-1.016	-0.768	0.249	no
Treat-0d	RHSIM_Rhsim07G0092900	-1.063	0.428	1.491	low
Treat-0d	RHSIM_Rhsim06G0151500	-0.641	0.930	1.571	no

Group	Gene	PTR (z-score difference)	z(log2 LFQ)	z(log2 FPKM)	extreme-PTR
Treat-0d	RHSIM_Rhsim07G0250400	-0.858	-1.395	-0.537	no
Treat-0d	RHSIM_Rhsim06G0238800	-0.437	0.028	0.465	no
Treat-0d	RHSIM_Rhsim05G0011300	-0.710	-1.344	-0.635	no
Treat-0d	RHSIM_Rhsim07G0071400	-1.354	-0.152	1.202	low
Treat-0d	RHSIM_Rhsim07G0087700	0.911	1.537	0.626	no
Treat-0d	RHSIM_Rhsim05G0120100	-1.387	-1.274	0.113	low
Treat-0d	RHSIM_Rhsim06G0161000	-1.707	-0.779	0.928	low
Treat-0d	RHSIM_Rhsim07G0225500	0.379	0.343	-0.035	no
Treat-0d	RHSIM_Rhsim05G0232300	0.118	1.069	0.951	no
Treat-0d	RHSIM_Rhsim06G0020700	-0.922	-1.508	-0.585	no
Treat-0d	RHSIM_Rhsim07G0047800	0.995	0.952	-0.043	no
Treat-0d	RHSIM_Rhsim06G0205800	-0.354	0.755	1.108	no
Treat-0d	RHSIM_Rhsim07G0189000	-0.902	0.870	1.772	no
Treat-0d	RHSIM_Rhsim06G0004200	1.228	0.841	-0.387	high
Treat-0d	RHSIM_Rhsim07G0013800	-1.571	-0.738	0.833	low
Treat-0d	RHSIM_Rhsim06G0098800	0.172	-0.378	-0.550	no
Treat-0d	RHSIM_Rhsim06G0074700	-0.487	0.578	1.065	no
Treat-0d	RHSIM_Rhsim06G0177400	1.572	-0.078	-1.650	high
Treat-0d	RHSIM_Rhsim06G0026100	-2.585	-1.182	1.403	low
Treat-0d	RHSIM_Rhsim07G0153500	-0.990	-0.473	0.516	no
Treat-0d	RHSIM_Rhsim06G0207300	0.603	-0.179	-0.782	no
Treat-0d	RHSIM_Rhsim07G0066700	-0.071	-1.155	-1.084	no
Treat-0d	RHSIM_Rhsim05G0165300	-0.682	-0.891	-0.209	no
Treat-0d	RHSIM_Rhsim06G0137600	1.198	1.538	0.340	high
Treat-0d	RHSIM_Rhsim07G0051300	3.748	2.854	-0.894	high
Treat-0d	RHSIM_Rhsim06G0116000	0.603	0.646	0.043	no
Treat-0d	RHSIM_Rhsim07G0258000	-1.019	-0.985	0.034	no
Treat-0d	RHSIM_Rhsim07G0074300	0.625	0.052	-0.573	no
Treat-0d	RHSIM_Rhsim06G0203600	1.580	1.066	-0.514	high
Treat-0d	RHSIM_Rhsim05G0216500	-1.390	-0.853	0.537	low
Treat-0d	RHSIM_Rhsim07G0049700	-3.644	-0.906	2.738	low
Treat-0d	RHSIM_Rhsim06G0032800	-0.082	-0.040	0.043	no

Group	Gene	PTR (z-score difference)	z(log2 LFQ)	z(log2 FPKM)	extreme-PTR
Treat-0d	RHSIM_Rhsim05G0004900	0.209	1.206	0.997	no
Treat-0d	RHSIM_Rhsim06G0205900	1.976	0.344	-1.632	high
Treat-0d	RHSIM_Rhsim05G0161600	1.624	0.015	-1.608	high
Treat-0d	RHSIM_Rhsim06G0204300	-0.443	-1.353	-0.910	no
Treat-0d	RHSIM_Rhsim06G0071400	-1.064	-1.257	-0.194	low
Treat-0d	RHSIM_Rhsim06G0223100	0.290	0.988	0.698	no
Treat-0d	RHSIM_Rhsim06G0130900	-0.430	-0.547	-0.117	no
Treat-0d	RHSIM_Rhsim06G0025000	1.011	-0.165	-1.176	no
Treat-0d	RHSIM_Rhsim06G0165200	-0.505	-0.706	-0.201	no
Treat-0d	RHSIM_Rhsim06G0003000	-0.403	-1.418	-1.015	no
Treat-0d	RHSIM_Rhsim07G0203600	-1.003	-0.495	0.509	no
Treat-0d	RHSIM_Rhsim06G0027400	0.051	-1.105	-1.155	no
Treat-0d	RHSIM_Rhsim06G0069500	-0.791	-0.801	-0.010	no
Treat-0d	RHSIM_Rhsim07G0187800	-0.006	1.268	1.274	no
Treat-0d	RHSIM_Rhsim05G0104300	-0.705	0.141	0.846	no
Treat-0d	RHSIM_Rhsim05G0167900	-1.030	-0.127	0.902	low
Treat-0d	RHSIM_Rhsim06G0034800	-0.303	0.270	0.572	no
Treat-0d	RHSIM_Rhsim07G0019300	-0.624	1.068	1.691	no
Treat-0d	RHSIM_Rhsim07G0129500	-0.536	-1.161	-0.626	no
Treat-0d	RHSIM_Rhsim06G0193700	0.365	-0.421	-0.786	no
Treat-0d	RHSIM_Rhsim05G0106800	-0.459	0.750	1.210	no
Treat-0d	RHSIM_Rhsim07G0101400	-0.922	-0.546	0.375	no
Treat-0d	RHSIM_Rhsim06G0243000	2.756	2.334	-0.422	high
Treat-0d	RHSIM_Rhsim06G0205300	0.573	1.729	1.156	no
Treat-0d	RHSIM_Rhsim06G0167300	-0.912	0.608	1.520	no
Treat-0d	RHSIM_Rhsim06G0078100	0.933	0.032	-0.901	no
Treat-0d	RHSIM_Rhsim07G0143200	-0.028	1.086	1.114	no
Treat-0d	RHSIM_Rhsim06G0126000	0.060	0.199	0.139	no
Treat-0d	RHSIM_Rhsim06G0212000	0.961	-0.412	-1.373	no
Treat-0d	RHSIM_Rhsim06G0176500	0.418	-0.102	-0.520	no
Treat-0d	RHSIM_Rhsim06G0191300	0.246	-1.444	-1.689	no
Treat-0d	RHSIM_Rhsim06G0226100	-0.294	-0.385	-0.091	no

Group	Gene	PTR (z-score difference)	z(log2 LFQ)	z(log2 FPKM)	extreme-PTR
Treat-0d	RHSIM_Rhsim07G0134200	-0.498	-0.282	0.216	no
Treat-0d	RHSIM_Rhsim05G0132600	0.550	1.487	0.937	no
Treat-0d	RHSIM_Rhsim06G0006900	1.201	0.900	-0.302	high
Treat-0d	RHSIM_Rhsim07G0082500	0.331	1.753	1.422	no
Treat-0d	RHSIM_Rhsim06G0201700	-0.102	-0.702	-0.600	no
Treat-0d	RHSIM_Rhsim07G0131300	1.905	2.154	0.248	high
Treat-0d	RHSIM_Rhsim06G0001500	0.059	0.144	0.085	no
Treat-0d	RHSIM_Rhsim06G0236000	-0.911	-1.029	-0.118	no
Treat-0d	RHSIM_Rhsim06G0236100	-1.248	-0.775	0.472	low
Treat-0d	RHSIM_Rhsim06G0007900	0.119	-0.786	-0.904	no
Treat-0d	RHSIM_Rhsim06G0184600	-0.632	-0.846	-0.214	no
Treat-0d	RHSIM_Rhsim07G0061200	-1.436	-1.595	-0.159	low
Treat-0d	RHSIM_Rhsim06G0163900	0.297	0.981	0.684	no
Treat-0d	RHSIM_Rhsim05G0182800	0.694	0.037	-0.656	no
Treat-0d	RHSIM_Rhsim06G0035200	-0.063	-1.249	-1.186	no
Treat-0d	RHSIM_Rhsim05G0142700	0.857	0.459	-0.398	no
Treat-0d	RHSIM_Rhsim07G0053300	0.804	1.489	0.685	no
Treat-0d	RHSIM_Rhsim06G0129400	-2.319	-1.298	1.021	low
Treat-0d	RHSIM_Rhsim05G0041300	-0.616	-0.353	0.263	no
Treat-0d	RHSIM_Rhsim06G0026800	-0.672	-1.615	-0.943	no
Treat-0d	RHSIM_Rhsim07G0020400	-1.105	-1.105	0.001	low
Treat-0d	RHSIM_Rhsim07G0078500	0.453	-0.725	-1.178	no
Treat-0d	RHSIM_Rhsim06G0194400	1.701	1.075	-0.626	high
Treat-0d	RHSIM_Rhsim06G0191000	0.356	1.663	1.307	no
Treat-0d	RHSIM_Rhsim05G0167300	1.455	1.469	0.014	high
Treat-0d	RHSIM_Rhsim06G0226700	0.556	0.870	0.314	no
Treat-0d	RHSIM_Rhsim05G0062900	0.518	-1.054	-1.573	no
Treat-0d	RHSIM_Rhsim07G0019100	-1.152	-0.491	0.661	low
Treat-0d	RHSIM_Rhsim07G0064900	0.467	-0.274	-0.740	no
Treat-0d	RHSIM_Rhsim06G0055300	0.932	1.497	0.565	no
Treat-0d	RHSIM_Rhsim07G0053700	0.251	-0.771	-1.021	no
Treat-0d	RHSIM_Rhsim06G0065900	-1.030	-0.152	0.878	low

Group	Gene	PTR (z-score difference)	z(log2 LFQ)	z(log2 FPKM)	extreme-PTR
Treat-0d	RHSIM_Rhsim06G0041800	-0.996	-0.214	0.782	no
Treat-0d	RHSIM_Rhsim06G0047900	-0.088	0.504	0.592	no
Treat-0d	RHSIM_Rhsim07G0090200	-0.295	1.411	1.705	no
Treat-0d	RHSIM_Rhsim05G0002000	-0.710	-0.900	-0.190	no
Treat-0d	RHSIM_Rhsim06G0171700	-0.419	0.361	0.780	no
Treat-0d	RHSIM_Rhsim07G0026500	0.746	0.318	-0.428	no
Treat-0d	RHSIM_Rhsim07G0225800	0.552	-1.017	-1.569	no
Treat-0d	RHSIM_Rhsim05G0139700	2.456	0.177	-2.278	high
Treat-0d	RHSIM_Rhsim06G0159400	0.652	0.335	-0.317	no
Treat-0d	RHSIM_Rhsim06G0240800	2.347	0.426	-1.921	high
Treat-0d	RHSIM_Rhsim05G0231800	1.560	0.899	-0.661	high
Treat-0d	RHSIM_Rhsim06G0049600	-0.228	-0.111	0.118	no
Treat-0d	RHSIM_Rhsim05G0143600	-0.338	-0.466	-0.129	no
Treat-0d	RHSIM_Rhsim07G0039100	1.561	1.968	0.407	high
Treat-0d	RHSIM_Rhsim07G0044600	0.528	-0.496	-1.024	no
Treat-0d	RHSIM_Rhsim05G0148200	-0.050	0.209	0.259	no
Treat-0d	RHSIM_Rhsim06G0122300	-1.023	-0.188	0.835	low
Treat-0d	RHSIM_Rhsim07G0113300	0.698	1.049	0.351	no
Treat-0d	RHSIM_Rhsim07G0145600	0.529	0.407	-0.123	no
Treat-0d	RHSIM_Rhsim06G0092600	-0.090	-0.611	-0.521	no
Treat-0d	RHSIM_Rhsim05G0096400	0.337	1.171	0.833	no
Treat-0d	RHSIM_Rhsim07G0036300	1.382	0.409	-0.972	high
Treat-0d	RHSIM_Rhsim06G0218900	-1.202	-1.524	-0.322	low
Treat-0d	RHSIM_Rhsim05G0163000	1.414	0.053	-1.362	high
Treat-0d	RHSIM_Rhsim06G0046700	-1.976	-1.199	0.776	low
Treat-0d	RHSIM_Rhsim06G0131500	1.138	2.916	1.778	high
Treat-0d	RHSIM_Rhsim06G0054800	-1.279	1.179	2.458	low
Treat-0d	RHSIM_Rhsim06G0087800	-1.173	-0.488	0.685	low
Treat-0d	RHSIM_Rhsim04G0083400	-0.468	0.272	0.740	no
Treat-0d	RHSIM_Rhsim06G0002900	-0.064	0.362	0.427	no
Treat-0d	RHSIM_Rhsim06G0130100	1.350	1.137	-0.213	high
Treat-0d	RHSIM_Rhsim04G0085300	-1.856	-1.025	0.831	low

Group	Gene	PTR (z-score difference)	z(log2 LFQ)	z(log2 FPKM)	extreme-PTR
Treat-0d	RHSIM_Rhsim06G0229100	-0.329	-0.828	-0.500	no
Treat-0d	RHSIM_Rhsim06G0178400	-0.517	0.431	0.949	no
Treat-0d	RHSIM_Rhsim06G0135300	-0.046	0.165	0.211	no
Treat-0d	RHSIM_Rhsim04G0169200	0.060	0.143	0.082	no
Treat-0d	RHSIM_Rhsim05G0017200	-0.867	-1.290	-0.423	no
Treat-0d	RHSIM_Rhsim05G0185400	0.178	-0.610	-0.789	no
Treat-0d	RHSIM_Rhsim04G0010400	0.738	0.307	-0.431	no
Treat-0d	RHSIM_Rhsim06G0121800	-0.867	-1.643	-0.776	no
Treat-0d	RHSIM_Rhsim05G0030300	0.597	1.293	0.696	no
Treat-0d	RHSIM_Rhsim05G0059500	0.952	-0.375	-1.327	no
Treat-0d	RHSIM_Rhsim04G0118700	0.372	0.453	0.081	no
Treat-0d	RHSIM_Rhsim05G0139000	-1.992	-0.641	1.351	low
Treat-0d	RHSIM_Rhsim06G0068800	-0.192	-0.921	-0.729	no
Treat-0d	RHSIM_Rhsim06G0099300	0.070	-0.185	-0.255	no
Treat-0d	RHSIM_Rhsim05G0103900	1.159	1.007	-0.152	high
Treat-0d	RHSIM_Rhsim06G0146500	1.297	1.667	0.370	high
Treat-0d	RHSIM_Rhsim06G0057900	-0.482	-0.653	-0.171	no
Treat-0d	RHSIM_Rhsim05G0089600	-3.338	-1.359	1.978	low
Treat-0d	RHSIM_Rhsim05G0013800	0.680	0.496	-0.184	no
Treat-0d	RHSIM_Rhsim06G0156300	-0.727	0.121	0.849	no
Treat-0d	RHSIM_Rhsim05G0050000	0.673	0.899	0.226	no
Treat-0d	RHSIM_Rhsim06G0172700	-4.423	-2.030	2.393	low
Treat-0d	RHSIM_Rhsim05G0180000	0.648	-0.894	-1.542	no
Treat-0d	RHSIM_Rhsim06G0185200	0.965	1.195	0.230	no
Treat-0d	RHSIM_Rhsim05G0020600	-0.337	-0.060	0.277	no
Treat-0d	RHSIM_Rhsim04G0213800	-0.051	0.812	0.863	no
Treat-0d	RHSIM_Rhsim05G0069400	1.153	-0.004	-1.157	high
Treat-0d	RHSIM_Rhsim05G0093200	-0.859	-0.551	0.307	no
Treat-0d	RHSIM_Rhsim05G0207300	0.615	-1.179	-1.794	no
Treat-0d	RHSIM_Rhsim05G0035000	1.212	1.451	0.239	high
Treat-0d	RHSIM_Rhsim04G0032800	-0.532	1.787	2.319	no
Treat-0d	RHSIM_Rhsim05G0118000	-0.033	-0.365	-0.331	no

Group	Gene	PTR (z-score difference)	z(log2 LFQ)	z(log2 FPKM)	extreme-PTR
Treat-0d	RHSIM_Rhsim06G0235200	0.284	-0.920	-1.204	no
Treat-0d	RHSIM_Rhsim05G0064500	0.561	1.083	0.522	no
Treat-0d	RHSIM_Rhsim06G0064300	-0.710	-1.264	-0.554	no
Treat-0d	RHSIM_Rhsim05G0012000	-0.208	1.894	2.102	no
Treat-0d	RHSIM_Rhsim06G0066600	1.107	-0.409	-1.516	high
Treat-0d	RHSIM_Rhsim05G0022300	1.014	1.205	0.191	no
Treat-0d	RHSIM_Rhsim05G0054100	0.204	-0.443	-0.647	no
Treat-0d	RHSIM_Rhsim05G0161200	0.236	0.099	-0.137	no
Treat-0d	RHSIM_Rhsim06G0197400	0.650	1.860	1.210	no
Treat-0d	RHSIM_Rhsim05G0070000	0.373	0.889	0.515	no
Treat-0d	RHSIM_Rhsim06G0049800	-1.380	0.116	1.495	low
Treat-0d	RHSIM_Rhsim04G0091600	-0.920	-0.255	0.664	no
Treat-0d	RHSIM_Rhsim06G0073600	0.459	1.116	0.657	no
Treat-0d	RHSIM_Rhsim06G0212900	0.723	0.763	0.039	no
Treat-0d	RHSIM_Rhsim05G0174500	-3.693	-1.239	2.454	low
Treat-0d	RHSIM_Rhsim05G0223200	-0.594	-0.774	-0.181	no
Treat-0d	RHSIM_Rhsim05G0012400	0.790	0.769	-0.021	no
Treat-0d	RHSIM_Rhsim06G0049500	1.203	1.004	-0.198	high
Treat-0d	RHSIM_Rhsim05G0000600	1.277	-0.225	-1.502	high
Treat-0d	RHSIM_Rhsim06G0169400	-0.750	-1.551	-0.801	no
Treat-0d	RHSIM_Rhsim05G0202500	0.376	0.242	-0.134	no
Treat-0d	RHSIM_Rhsim06G0086900	-1.478	-0.835	0.643	low
Treat-0d	RHSIM_Rhsim05G0049500	0.106	-1.050	-1.155	no
Treat-0d	RHSIM_Rhsim04G0105200	1.139	0.595	-0.544	high
Treat-0d	RHSIM_Rhsim05G0070900	0.969	1.292	0.322	no
Treat-0d	RHSIM_Rhsim05G0129000	-0.338	-1.611	-1.273	no
Treat-0d	RHSIM_Rhsim04G0246500	2.354	0.591	-1.764	high
Treat-0d	RHSIM_Rhsim06G0012100	-0.408	0.416	0.824	no
Treat-0d	RHSIM_Rhsim05G0011500	0.673	0.190	-0.483	no
Treat-0d	RHSIM_Rhsim06G0236300	0.003	1.009	1.005	no
Treat-0d	RHSIM_Rhsim05G0063200	1.130	1.559	0.429	high
Treat-0d	RHSIM_Rhsim05G0180400	0.279	2.436	2.157	no

Group	Gene	PTR (z-score difference)	z(log2 LFQ)	z(log2 FPKM)	extreme-PTR
Treat-0d	RHSIM_Rhsim05G0137700	-0.941	0.003	0.944	no
Treat-0d	RHSIM_Rhsim04G0243100	0.036	1.333	1.297	no
Treat-0d	RHSIM_Rhsim04G0234700	0.396	0.177	-0.219	no
Treat-0d	RHSIM_Rhsim05G0202800	2.106	0.934	-1.172	high
Treat-0d	RHSIM_Rhsim05G0027600	1.629	-0.321	-1.951	high
Treat-0d	RHSIM_Rhsim05G0163600	1.309	-0.671	-1.980	high
Treat-0d	RHSIM_Rhsim06G0037800	-0.563	0.682	1.245	no
Treat-0d	RHSIM_Rhsim05G0079600	0.008	-0.195	-0.203	no
Treat-0d	RHSIM_Rhsim04G0016100	1.320	0.809	-0.512	high
Treat-0d	RHSIM_Rhsim05G0088400	0.412	0.884	0.471	no
Treat-0d	RHSIM_Rhsim04G0146600	-0.420	-0.968	-0.548	no
Treat-0d	RHSIM_Rhsim06G0161200	0.345	1.061	0.716	no
Treat-0d	RHSIM_Rhsim06G0093300	-0.930	-1.005	-0.076	no
Treat-0d	RHSIM_Rhsim06G0011900	1.091	-0.739	-1.830	no
Treat-0d	RHSIM_Rhsim06G0060100	-0.380	-0.098	0.283	no
Treat-0d	RHSIM_Rhsim06G0047800	-0.019	1.055	1.074	no
Treat-0d	RHSIM_Rhsim05G0062500	0.272	0.007	-0.265	no
Treat-0d	RHSIM_Rhsim06G0186600	0.022	-0.128	-0.150	no
Treat-0d	RHSIM_Rhsim06G0237500	-0.480	-0.646	-0.166	no
Treat-0d	RHSIM_Rhsim06G0166400	0.049	-0.412	-0.461	no
Treat-0d	RHSIM_Rhsim05G0224600	-0.547	-0.134	0.413	no
Treat-0d	RHSIM_Rhsim06G0072000	0.403	0.382	-0.021	no
Treat-0d	RHSIM_Rhsim06G0102100	0.599	1.509	0.910	no
Treat-0d	RHSIM_Rhsim04G0102500	0.325	0.528	0.204	no
Treat-0d	RHSIM_Rhsim05G0230900	0.731	1.068	0.338	no
Treat-0d	RHSIM_Rhsim06G0014600	2.266	1.047	-1.219	high
Treat-0d	RHSIM_Rhsim04G0118500	0.453	0.625	0.173	no
Treat-0d	RHSIM_Rhsim06G0038600	0.307	2.290	1.983	no
Treat-0d	RHSIM_Rhsim06G0077300	-0.795	0.457	1.252	no
Treat-0d	RHSIM_Rhsim06G0214800	0.751	1.072	0.321	no
Treat-0d	RHSIM_Rhsim05G0169300	-0.806	-1.607	-0.802	no
Treat-0d	RHSIM_Rhsim04G0101900	0.273	1.616	1.343	no

Group	Gene	PTR (z-score difference)	z(log2 LFQ)	z(log2 FPKM)	extreme-PTR
Treat-0d	RHSIM_Rhsim04G0149800	0.054	-0.317	-0.371	no
Treat-0d	RHSIM_Rhsim06G0116800	-0.054	0.066	0.121	no
Treat-0d	RHSIM_Rhsim04G0119100	0.518	1.281	0.763	no
Treat-0d	RHSIM_Rhsim05G0108700	-0.722	-1.310	-0.588	no
Treat-0d	RHSIM_Rhsim04G0186900	-0.418	-0.452	-0.033	no
Treat-0d	RHSIM_Rhsim06G0040500	-0.027	-0.942	-0.915	no
Treat-0d	RHSIM_Rhsim06G0076100	0.771	1.530	0.759	no
Treat-0d	RHSIM_Rhsim05G0200500	-1.623	0.549	2.172	low
Treat-0d	RHSIM_Rhsim06G0007500	-0.031	-0.640	-0.609	no
Treat-0d	RHSIM_Rhsim05G0114400	1.374	0.020	-1.355	high
Treat-0d	RHSIM_Rhsim06G0110000	0.216	0.234	0.017	no
Treat-0d	RHSIM_Rhsim04G0179200	-1.879	-1.978	-0.100	low
Treat-0d	RHSIM_Rhsim05G0101300	-0.307	-0.948	-0.641	no
Treat-0d	RHSIM_Rhsim06G0057200	0.339	-0.094	-0.433	no
Treat-0d	RHSIM_Rhsim05G0031300	-1.117	0.185	1.302	low
Treat-0d	RHSIM_Rhsim04G0126900	1.452	1.280	-0.172	high
Treat-0d	RHSIM_Rhsim06G0206900	-0.064	0.988	1.052	no
Treat-0d	RHSIM_Rhsim04G0250200	0.610	-1.101	-1.711	no
Treat-0d	RHSIM_Rhsim06G0116700	0.261	0.566	0.305	no
Treat-0d	RHSIM_Rhsim05G0205700	0.285	-0.239	-0.524	no
Treat-0d	RHSIM_Rhsim04G0130200	1.318	0.597	-0.722	high
Treat-0d	RHSIM_Rhsim06G0196800	0.193	-0.781	-0.974	no
Treat-0d	RHSIM_Rhsim04G0108100	-0.014	-0.286	-0.272	no
Treat-0d	RHSIM_Rhsim05G0087800	1.044	0.596	-0.448	no
Treat-0d	RHSIM_Rhsim04G0070700	-0.174	0.275	0.448	no
Treat-0d	RHSIM_Rhsim05G0029900	-1.578	-0.058	1.520	low
Treat-0d	RHSIM_Rhsim04G0174900	-1.273	0.352	1.625	low
Treat-0d	RHSIM_Rhsim05G0147900	-0.547	-0.988	-0.441	no
Treat-0d	RHSIM_Rhsim04G0221500	-1.716	-1.994	-0.278	low
Treat-0d	RHSIM_Rhsim05G0068000	0.597	1.387	0.790	no
Treat-0d	RHSIM_Rhsim05G0062100	0.097	1.804	1.707	no
Treat-0d	RHSIM_Rhsim05G0039700	1.553	1.235	-0.319	high

Group	Gene	PTR (z-score difference)	z(log2 LFQ)	z(log2 FPKM)	extreme-PTR
Treat-0d	RHSIM_Rhsim05G0146400	0.635	-0.021	-0.656	no
Treat-0d	RHSIM_Rhsim06G0225700	0.391	-0.282	-0.672	no
Treat-0d	RHSIM_Rhsim04G0060200	0.422	-0.646	-1.068	no
Treat-0d	RHSIM_Rhsim05G0208200	-0.880	-0.369	0.512	no
Treat-0d	RHSIM_Rhsim06G0018800	0.038	-0.200	-0.239	no
Treat-0d	RHSIM_Rhsim05G0137500	0.163	0.167	0.005	no
Treat-0d	RHSIM_Rhsim05G0170800	-0.640	-1.659	-1.019	no
Treat-0d	RHSIM_Rhsim04G0211500	-1.026	-0.844	0.182	low
Treat-0d	RHSIM_Rhsim05G0168900	-0.257	-1.247	-0.990	no
Treat-0d	RHSIM_Rhsim05G0159800	-0.894	-0.512	0.382	no
Treat-0d	RHSIM_Rhsim05G0224000	-0.764	0.638	1.402	no
Treat-0d	RHSIM_Rhsim05G0163400	0.003	-1.241	-1.244	no
Treat-0d	RHSIM_Rhsim06G0201000	-0.407	-0.786	-0.379	no
Treat-0d	RHSIM_Rhsim04G0011000	-0.132	-0.876	-0.744	no
Treat-0d	RHSIM_Rhsim05G0215900	1.973	0.496	-1.477	high
Treat-0d	RHSIM_Rhsim06G0021800	-0.068	-1.224	-1.157	no
Treat-0d	RHSIM_Rhsim05G0193800	-0.441	-0.043	0.399	no
Treat-0d	RHSIM_Rhsim04G0021400	-0.611	0.061	0.672	no
Treat-0d	RHSIM_Rhsim05G0161100	0.644	1.996	1.352	no
Treat-0d	RHSIM_Rhsim05G0176200	0.152	-0.703	-0.855	no
Treat-0d	RHSIM_Rhsim06G0074900	1.066	0.632	-0.434	no
Treat-0d	RHSIM_Rhsim06G0230800	0.585	-1.166	-1.751	no
Treat-0d	RHSIM_Rhsim06G0061800	-3.510	-2.140	1.370	low
Treat-0d	RHSIM_Rhsim04G0236100	0.542	0.177	-0.365	no
Treat-0d	RHSIM_Rhsim04G0206400	0.548	3.247	2.699	no
Treat-0d	RHSIM_Rhsim06G0071000	-0.271	0.004	0.275	no
Treat-0d	RHSIM_Rhsim05G0231000	0.030	0.368	0.338	no
Treat-0d	RHSIM_Rhsim05G0116000	0.307	-0.088	-0.395	no
Treat-0d	RHSIM_Rhsim04G0024600	0.486	0.687	0.201	no
Treat-0d	RHSIM_Rhsim05G0065200	0.681	-1.693	-2.374	no
Treat-0d	RHSIM_Rhsim05G0000700	1.116	-0.234	-1.350	high
Treat-0d	RHSIM_Rhsim05G0069500	1.062	0.818	-0.245	no

Group	Gene	PTR (z-score difference)	z(log2 LFQ)	z(log2 FPKM)	extreme-PTR
Treat-0d	RHSIM_Rhsim03G0222100	1.649	0.030	-1.619	high
Treat-0d	RHSIM_Rhsim04G0167900	0.150	-0.939	-1.089	no
Treat-0d	RHSIM_Rhsim04G0221800	-2.692	-1.773	0.919	low
Treat-0d	RHSIM_Rhsim03G0253400	0.749	-0.109	-0.858	no
Treat-0d	RHSIM_Rhsim05G0069300	-0.123	0.333	0.457	no
Treat-0d	RHSIM_Rhsim03G0178900	-1.571	-1.016	0.555	low
Treat-0d	RHSIM_Rhsim05G0231600	-0.592	-0.432	0.160	no
Treat-0d	RHSIM_Rhsim05G0186700	1.171	0.416	-0.755	high
Treat-0d	RHSIM_Rhsim03G0262800	-2.288	-0.983	1.305	low
Treat-0d	RHSIM_Rhsim05G0011000	-0.857	-0.815	0.042	no
Treat-0d	RHSIM_Rhsim04G0102100	-0.030	1.256	1.286	no
Treat-0d	RHSIM_Rhsim05G0115500	-0.131	-0.279	-0.148	no
Treat-0d	RHSIM_Rhsim05G0186400	0.025	-0.861	-0.886	no
Treat-0d	RHSIM_Rhsim05G0231200	-0.828	-0.736	0.091	no
Treat-0d	RHSIM_Rhsim04G0201600	0.599	-0.299	-0.898	no
Treat-0d	RHSIM_Rhsim05G0136600	1.304	0.504	-0.800	high
Treat-0d	RHSIM_Rhsim03G0064000	-0.040	-0.492	-0.451	no
Treat-0d	RHSIM_Rhsim05G0043400	0.377	-0.095	-0.471	no
Treat-0d	RHSIM_Rhsim05G0167500	-0.756	-1.460	-0.704	no
Treat-0d	RHSIM_Rhsim05G0049600	0.079	0.264	0.185	no
Treat-0d	RHSIM_Rhsim05G0049900	-1.588	-0.720	0.869	low
Treat-0d	RHSIM_Rhsim05G0203700	1.417	0.023	-1.394	high
Treat-0d	RHSIM_Rhsim04G0132400	0.521	1.010	0.490	no
Treat-0d	RHSIM_Rhsim03G0187300	0.463	-0.035	-0.498	no
Treat-0d	RHSIM_Rhsim04G0072400	-0.445	-0.904	-0.459	no
Treat-0d	RHSIM_Rhsim04G0097900	0.303	-0.341	-0.644	no
Treat-0d	RHSIM_Rhsim05G0189500	-1.405	0.052	1.457	low
Treat-0d	RHSIM_Rhsim05G0196300	-0.778	-0.252	0.526	no
Treat-0d	RHSIM_Rhsim05G0072800	0.120	1.188	1.067	no
Treat-0d	RHSIM_Rhsim04G0099900	-0.476	-1.520	-1.044	no
Treat-0d	RHSIM_Rhsim04G0220700	-0.366	-1.309	-0.943	no
Treat-0d	RHSIM_Rhsim05G0014300	-0.315	-0.854	-0.539	no

Group	Gene	PTR (z-score difference)	z(log2 LFQ)	z(log2 FPKM)	extreme-PTR
Treat-0d	RHSIM_Rhsim04G0075000	-0.527	-0.847	-0.320	no
Treat-0d	RHSIM_Rhsim05G0102200	0.120	-0.029	-0.148	no
Treat-0d	RHSIM_Rhsim05G0204700	-0.776	-0.965	-0.189	no
Treat-0d	RHSIM_Rhsim04G0231300	3.871	3.153	-0.718	high
Treat-0d	RHSIM_Rhsim05G0148900	0.530	-0.680	-1.210	no
Treat-0d	RHSIM_Rhsim04G0009700	0.621	-0.504	-1.125	no
Treat-0d	RHSIM_Rhsim05G0006600	-0.721	-0.076	0.645	no
Treat-0d	RHSIM_Rhsim05G0221700	-0.037	1.255	1.292	no
Treat-0d	RHSIM_Rhsim04G0049800	-0.986	-0.202	0.784	no
Treat-0d	RHSIM_Rhsim05G0128900	0.326	0.282	-0.044	no
Treat-0d	RHSIM_Rhsim04G0003400	-1.614	-0.335	1.279	low
Treat-0d	RHSIM_Rhsim04G0122300	-1.229	1.519	2.748	low
Treat-0d	RHSIM_Rhsim05G0067600	1.314	0.814	-0.500	high
Treat-0d	RHSIM_Rhsim03G0127100	1.243	1.279	0.036	high
Treat-0d	RHSIM_Rhsim05G0080700	0.653	-0.583	-1.236	no
Treat-0d	RHSIM_Rhsim03G0031300	1.137	0.245	-0.892	high
Treat-0d	RHSIM_Rhsim05G0144300	0.785	0.011	-0.773	no
Treat-0d	RHSIM_Rhsim05G0184800	-1.300	-0.530	0.771	low
Treat-0d	RHSIM_Rhsim04G0151900	0.617	1.695	1.078	no
Treat-0d	RHSIM_Rhsim05G0119700	0.413	-0.355	-0.768	no
Treat-0d	RHSIM_Rhsim04G0218900	0.335	-0.358	-0.694	no
Treat-0d	RHSIM_Rhsim05G0233000	0.286	0.240	-0.046	no
Treat-0d	RHSIM_Rhsim05G0135700	-0.593	-1.763	-1.169	no
Treat-0d	RHSIM_Rhsim05G0217500	1.008	1.702	0.693	no
Treat-0d	RHSIM_Rhsim04G0162200	1.826	0.794	-1.032	high
Treat-0d	RHSIM_Rhsim05G0013300	-0.935	-0.958	-0.023	no
Treat-0d	RHSIM_Rhsim05G0177800	1.087	-0.408	-1.494	no
Treat-0d	RHSIM_Rhsim04G0136600	0.623	0.757	0.134	no
Treat-0d	RHSIM_Rhsim04G0157300	-0.059	-0.573	-0.514	no
Treat-0d	RHSIM_Rhsim03G0036800	-1.737	-1.737	-0.000	low
Treat-0d	RHSIM_Rhsim03G0064600	-0.454	0.054	0.508	no
Treat-0d	RHSIM_Rhsim05G0126300	-0.135	0.454	0.589	no

Group	Gene	PTR (z-score difference)	z(log2 LFQ)	z(log2 FPKM)	extreme-PTR
Treat-0d	RHSIM_Rhsim04G0183700	0.453	-0.452	-0.905	no
Treat-0d	RHSIM_Rhsim05G0219500	0.201	1.164	0.963	no
Treat-0d	RHSIM_Rhsim05G0094600	0.963	2.303	1.341	no
Treat-0d	RHSIM_Rhsim03G0235900	1.441	1.442	0.001	high
Treat-0d	RHSIM_Rhsim04G0003000	-1.658	-1.205	0.453	low
Treat-0d	RHSIM_Rhsim05G0068600	-1.116	-0.703	0.414	low
Treat-0d	RHSIM_Rhsim04G0135600	0.246	2.130	1.884	no
Treat-0d	RHSIM_Rhsim05G0115100	-0.492	-1.263	-0.771	no
Treat-0d	RHSIM_Rhsim05G0103300	2.420	3.081	0.661	high
Treat-0d	RHSIM_Rhsim04G0196000	0.266	0.681	0.415	no
Treat-0d	RHSIM_Rhsim05G0171100	0.166	-0.527	-0.693	no
Treat-0d	RHSIM_Rhsim05G0092500	0.812	0.058	-0.754	no
Treat-0d	RHSIM_Rhsim04G0204500	-0.082	1.793	1.874	no
Treat-0d	RHSIM_Rhsim05G0002100	0.113	0.779	0.666	no
Treat-0d	RHSIM_Rhsim03G0234400	-0.748	-0.467	0.280	no
Treat-0d	RHSIM_Rhsim04G0026300	-1.058	-1.814	-0.757	low
Treat-0d	RHSIM_Rhsim04G0106100	0.649	-0.112	-0.761	no
Treat-0d	RHSIM_Rhsim04G0023600	-0.105	0.301	0.406	no
Treat-0d	RHSIM_Rhsim04G0208300	0.719	2.186	1.467	no
Treat-0d	RHSIM_Rhsim05G0000100	0.419	0.586	0.167	no
Treat-0d	RHSIM_Rhsim03G0122800	-0.476	-0.065	0.410	no
Treat-0d	RHSIM_Rhsim04G0247000	-0.088	-0.122	-0.034	no
Treat-0d	RHSIM_Rhsim03G0222500	0.157	0.697	0.540	no
Treat-0d	RHSIM_Rhsim04G0101200	1.899	0.438	-1.461	high
Treat-0d	RHSIM_Rhsim04G0225200	0.235	-0.170	-0.405	no
Treat-0d	RHSIM_Rhsim04G0237500	1.082	-0.546	-1.628	no
Treat-0d	RHSIM_Rhsim04G0021500	-1.755	-1.449	0.306	low
Treat-0d	RHSIM_Rhsim05G0048800	0.169	0.077	-0.092	no
Treat-0d	RHSIM_Rhsim03G0233000	-0.936	-1.018	-0.082	no
Treat-0d	RHSIM_Rhsim05G0064300	0.151	0.062	-0.090	no
Treat-0d	RHSIM_Rhsim03G0160100	0.951	1.768	0.816	no
Treat-0d	RHSIM_Rhsim04G0032700	-0.660	1.654	2.314	no

Group	Gene	PTR (z-score difference)	z(log2 LFQ)	z(log2 FPKM)	extreme-PTR
Treat-0d	RHSIM_Rhsim04G0057100	1.016	0.503	-0.513	no
Treat-0d	RHSIM_Rhsim04G0091800	-0.046	1.925	1.971	no
Treat-0d	RHSIM_Rhsim04G0127300	-0.060	0.162	0.222	no
Treat-0d	RHSIM_Rhsim04G0247700	0.848	0.769	-0.079	no
Treat-0d	RHSIM_Rhsim04G0046600	-0.528	-1.028	-0.500	no
Treat-0d	RHSIM_Rhsim04G0010600	0.789	1.249	0.460	no
Treat-0d	RHSIM_Rhsim05G0092700	2.463	0.812	-1.651	high
Treat-0d	RHSIM_Rhsim03G0218900	0.310	0.537	0.226	no
Treat-0d	RHSIM_Rhsim04G0069300	1.017	-0.098	-1.115	no
Treat-0d	RHSIM_Rhsim05G0110000	1.973	-0.366	-2.339	high
Treat-0d	RHSIM_Rhsim04G0113400	0.292	-0.347	-0.639	no
Treat-0d	RHSIM_Rhsim03G0217600	1.242	0.841	-0.401	high
Treat-0d	RHSIM_Rhsim05G0075000	0.061	0.102	0.041	no
Treat-0d	RHSIM_Rhsim05G0036500	1.999	-0.285	-2.284	high
Treat-0d	RHSIM_Rhsim04G0021000	1.372	-0.766	-2.138	high
Treat-0d	RHSIM_Rhsim03G0200000	-0.585	-0.060	0.525	no
Treat-0d	RHSIM_Rhsim03G0028900	0.224	0.994	0.770	no
Treat-0d	RHSIM_Rhsim05G0034800	-1.449	-0.949	0.500	low
Treat-0d	RHSIM_Rhsim04G0229700	-0.687	1.022	1.709	no
Treat-0d	RHSIM_Rhsim05G0076800	-1.103	-0.721	0.382	low
Treat-0d	RHSIM_Rhsim04G0089100	1.886	-0.254	-2.140	high
Treat-0d	RHSIM_Rhsim05G0079900	0.715	0.807	0.092	no
Treat-0d	RHSIM_Rhsim04G0156000	-0.153	-0.378	-0.225	no
Treat-0d	RHSIM_Rhsim05G0148600	-0.816	-0.214	0.602	no
Treat-0d	RHSIM_Rhsim04G0247300	-0.397	-1.075	-0.677	no
Treat-0d	RHSIM_Rhsim04G0202200	-0.599	-0.513	0.086	no
Treat-0d	RHSIM_Rhsim04G0073800	-0.363	-0.750	-0.387	no
Treat-0d	RHSIM_Rhsim03G0167100	-0.092	-0.180	-0.087	no
Treat-0d	RHSIM_Rhsim03G0257100	1.062	1.347	0.285	no
Treat-0d	RHSIM_Rhsim04G0070800	0.526	-0.407	-0.933	no
Treat-0d	RHSIM_Rhsim03G0221000	-0.770	-0.633	0.137	no
Treat-0d	RHSIM_Rhsim05G0022100	0.328	0.137	-0.191	no

Group	Gene	PTR (z-score difference)	z(log2 LFQ)	z(log2 FPKM)	extreme-PTR
Treat-0d	RHSIM_Rhsim04G0023800	-0.465	-0.721	-0.256	no
Treat-0d	RHSIM_Rhsim03G0074500	1.394	-0.580	-1.974	high
Treat-0d	RHSIM_Rhsim05G0114700	-1.343	-1.415	-0.072	low
Treat-0d	RHSIM_Rhsim04G0249000	0.730	1.179	0.449	no
Treat-0d	RHSIM_Rhsim03G0246100	0.006	-0.537	-0.544	no
Treat-0d	RHSIM_Rhsim04G0008300	0.189	0.277	0.088	no
Treat-0d	RHSIM_Rhsim04G0205500	-1.705	-0.206	1.499	low
Treat-0d	RHSIM_Rhsim05G0179500	0.522	0.792	0.270	no
Treat-0d	RHSIM_Rhsim03G0081400	-1.398	-2.058	-0.660	low
Treat-0d	RHSIM_Rhsim03G0025100	1.953	0.540	-1.413	high
Treat-0d	RHSIM_Rhsim03G0189600	-0.207	-1.333	-1.126	no
Treat-0d	RHSIM_Rhsim03G0080200	0.009	-0.765	-0.774	no
Treat-0d	RHSIM_Rhsim03G0171100	-1.317	-1.848	-0.531	low
Treat-0d	RHSIM_Rhsim05G0223400	0.616	-1.136	-1.752	no
Treat-0d	RHSIM_Rhsim03G0227400	-0.762	1.388	2.150	no
Treat-0d	RHSIM_Rhsim04G0175800	-1.601	-1.853	-0.251	low
Treat-0d	RHSIM_Rhsim03G0250200	-0.941	-0.630	0.311	no
Treat-0d	RHSIM_Rhsim04G0222400	0.585	0.103	-0.482	no
Treat-0d	RHSIM_Rhsim03G0063800	1.872	0.984	-0.888	high
Treat-0d	RHSIM_Rhsim04G0205300	-0.164	-0.695	-0.532	no
Treat-0d	RHSIM_Rhsim03G0140700	-1.203	-0.647	0.556	low
Treat-0d	RHSIM_Rhsim03G0069800	1.062	0.695	-0.367	no
Treat-0d	RHSIM_Rhsim03G0000700	1.096	0.518	-0.578	no
Treat-0d	RHSIM_Rhsim04G0134200	0.733	0.617	-0.117	no
Treat-0d	RHSIM_Rhsim05G0225700	-0.952	1.626	2.578	no
Treat-0d	RHSIM_Rhsim03G0229500	1.442	1.651	0.208	high
Treat-0d	RHSIM_Rhsim04G0197000	1.082	1.391	0.310	no
Treat-0d	RHSIM_Rhsim04G0238000	0.641	-0.420	-1.061	no
Treat-0d	RHSIM_Rhsim04G0190900	-0.985	-1.094	-0.109	no
Treat-0d	RHSIM_Rhsim03G0153400	-1.852	-0.361	1.490	low
Treat-0d	RHSIM_Rhsim04G0024500	-0.955	-0.964	-0.009	no
Treat-0d	RHSIM_Rhsim04G0078700	1.805	0.622	-1.182	high

Group	Gene	PTR (z-score difference)	z(log2 LFQ)	z(log2 FPKM)	extreme-PTR
Treat-0d	RHSIM_Rhsim03G0113100	-0.137	-0.625	-0.488	no
Treat-0d	RHSIM_Rhsim04G0015700	1.895	1.604	-0.290	high
Treat-0d	RHSIM_Rhsim02G0086400	-0.469	-0.781	-0.312	no
Treat-0d	RHSIM_Rhsim04G0167200	2.621	2.285	-0.336	high
Treat-0d	RHSIM_Rhsim04G0213900	-1.454	-0.434	1.020	low
Treat-0d	RHSIM_Rhsim02G0110200	-0.762	-0.802	-0.040	no
Treat-0d	RHSIM_Rhsim04G0125000	0.312	-0.707	-1.019	no
Treat-0d	RHSIM_Rhsim03G0192100	0.392	-0.027	-0.419	no
Treat-0d	RHSIM_Rhsim02G0158900	0.080	-0.720	-0.800	no
Treat-0d	RHSIM_Rhsim04G0046500	-0.936	-0.866	0.070	no
Treat-0d	RHSIM_Rhsim04G0019500	0.624	1.970	1.345	no
Treat-0d	RHSIM_Rhsim04G0220400	-0.341	-0.116	0.225	no
Treat-0d	RHSIM_Rhsim04G0003900	0.132	-0.289	-0.421	no
Treat-0d	RHSIM_Rhsim03G0093600	0.667	0.084	-0.583	no
Treat-0d	RHSIM_Rhsim04G0200100	0.684	-0.972	-1.656	no
Treat-0d	RHSIM_Rhsim04G0048200	-0.252	0.075	0.327	no
Treat-0d	RHSIM_Rhsim04G0166900	0.687	1.752	1.065	no
Treat-0d	RHSIM_Rhsim04G0235300	1.771	0.507	-1.263	high
Treat-0d	RHSIM_Rhsim04G0124400	-1.330	0.068	1.399	low
Treat-0d	RHSIM_Rhsim04G0120400	-0.915	-0.427	0.487	no
Treat-0d	RHSIM_Rhsim03G0045900	0.104	-0.523	-0.627	no
Treat-0d	RHSIM_Rhsim04G0015800	-0.053	-0.808	-0.755	no
Treat-0d	RHSIM_Rhsim02G0250400	-0.227	-0.228	-0.001	no
Treat-0d	RHSIM_Rhsim04G0195000	-0.342	0.809	1.151	no
Treat-0d	RHSIM_Rhsim04G0214300	0.002	0.234	0.232	no
Treat-0d	RHSIM_Rhsim04G0153100	0.738	-0.256	-0.994	no
Treat-0d	RHSIM_Rhsim04G0085200	-1.574	-0.640	0.934	low
Treat-0d	RHSIM_Rhsim03G0153200	1.013	-0.704	-1.717	no
Treat-0d	RHSIM_Rhsim04G0053900	0.101	-0.992	-1.093	no
Treat-0d	RHSIM_Rhsim04G0164100	0.079	0.030	-0.049	no
Treat-0d	RHSIM_Rhsim04G0201900	0.737	0.781	0.045	no
Treat-0d	RHSIM_Rhsim03G0144600	-4.882	-2.241	2.641	low

Group	Gene	PTR (z-score difference)	z(log2 LFQ)	z(log2 FPKM)	extreme-PTR
Treat-0d	RHSIM_Rhsim04G0176300	-0.804	-1.236	-0.432	no
Treat-0d	RHSIM_Rhsim04G0167000	0.282	1.750	1.468	no
Treat-0d	RHSIM_Rhsim02G0232000	-0.796	0.470	1.266	no
Treat-0d	RHSIM_Rhsim04G0149700	-0.244	0.663	0.908	no
Treat-0d	RHSIM_Rhsim02G0045600	-0.752	-0.797	-0.045	no
Treat-0d	RHSIM_Rhsim04G0160100	1.714	1.143	-0.572	high
Treat-0d	RHSIM_Rhsim04G0147500	1.573	0.337	-1.236	high
Treat-0d	RHSIM_Rhsim04G0214400	-0.140	0.223	0.364	no
Treat-0d	RHSIM_Rhsim04G0016500	-0.028	-1.176	-1.147	no
Treat-0d	RHSIM_Rhsim04G0203600	0.878	1.660	0.782	no
Treat-0d	RHSIM_Rhsim04G0231000	0.449	2.988	2.539	no
Treat-0d	RHSIM_Rhsim04G0188800	-1.106	-0.996	0.110	low
Treat-0d	RHSIM_Rhsim03G0212900	0.667	0.530	-0.138	no
Treat-0d	RHSIM_Rhsim04G0212200	0.505	-0.952	-1.458	no
Treat-0d	RHSIM_Rhsim04G0228000	1.122	0.119	-1.003	high
Treat-0d	RHSIM_Rhsim04G0246200	-0.222	-0.218	0.004	no
Treat-0d	RHSIM_Rhsim03G0223700	-0.125	-0.704	-0.579	no
Treat-0d	RHSIM_Rhsim04G0026700	-0.561	-0.008	0.553	no
Treat-0d	RHSIM_Rhsim03G0055600	1.370	-0.709	-2.079	high
Treat-0d	RHSIM_Rhsim04G0088200	0.962	-0.167	-1.130	no
Treat-0d	RHSIM_Rhsim04G0139900	-1.546	-1.533	0.014	low
Treat-0d	RHSIM_Rhsim04G0083100	-0.016	1.551	1.567	no
Treat-0d	RHSIM_Rhsim04G0008600	0.005	0.645	0.640	no
Treat-0d	RHSIM_Rhsim02G0248700	1.218	2.079	0.862	high
Treat-0d	RHSIM_Rhsim03G0009100	0.393	1.276	0.883	no
Treat-0d	RHSIM_Rhsim04G0052900	-0.132	-0.368	-0.236	no
Treat-0d	RHSIM_Rhsim04G0109400	0.204	-0.159	-0.363	no
Treat-0d	RHSIM_Rhsim04G0008000	-1.142	-1.374	-0.233	low
Treat-0d	RHSIM_Rhsim02G0173800	0.559	0.272	-0.287	no
Treat-0d	RHSIM_Rhsim04G0234400	1.690	-0.413	-2.102	high
Treat-0d	RHSIM_Rhsim03G0167300	-1.847	-0.902	0.945	low
Treat-0d	RHSIM_Rhsim02G0254600	0.477	-0.769	-1.246	no

Group	Gene	PTR (z-score difference)	z(log2 LFQ)	z(log2 FPKM)	extreme-PTR
Treat-0d	RHSIM_Rhsim04G0065800	0.813	1.578	0.765	no
Treat-0d	RHSIM_Rhsim02G0000300	0.808	-0.081	-0.890	no
Treat-0d	RHSIM_Rhsim03G0093500	-0.116	-0.929	-0.813	no
Treat-0d	RHSIM_Rhsim02G0165400	0.672	-1.194	-1.866	no
Treat-0d	RHSIM_Rhsim04G0196300	-1.508	0.378	1.885	low
Treat-0d	RHSIM_Rhsim03G0224100	-0.084	0.264	0.349	no
Treat-0d	RHSIM_Rhsim04G0228200	-0.332	-1.170	-0.838	no
Treat-0d	RHSIM_Rhsim03G0069100	0.682	-0.013	-0.695	no
Treat-0d	RHSIM_Rhsim02G0199000	-1.215	-1.694	-0.479	low
Treat-0d	RHSIM_Rhsim04G0018700	-0.943	-1.275	-0.332	no
Treat-0d	RHSIM_Rhsim02G0143100	-1.747	-1.280	0.467	low
Treat-0d	RHSIM_Rhsim02G0172200	0.915	1.377	0.462	no
Treat-0d	RHSIM_Rhsim03G0247800	-1.249	-1.309	-0.060	low
Treat-0d	RHSIM_Rhsim04G0113500	1.205	1.179	-0.027	high
Treat-0d	RHSIM_Rhsim03G0031100	-0.376	-1.522	-1.146	no
Treat-0d	RHSIM_Rhsim03G0141600	0.723	1.262	0.539	no
Treat-0d	RHSIM_Rhsim03G0236500	-0.148	0.124	0.272	no
Treat-0d	RHSIM_Rhsim04G0164800	1.115	2.292	1.177	high
Treat-0d	RHSIM_Rhsim03G0120500	0.370	-0.288	-0.658	no
Treat-0d	RHSIM_Rhsim03G0186100	-1.651	-0.686	0.965	low
Treat-0d	RHSIM_Rhsim03G0223900	0.520	0.452	-0.068	no
Treat-0d	RHSIM_Rhsim03G0017900	1.067	0.357	-0.710	no
Treat-0d	RHSIM_Rhsim04G0218600	-1.174	-0.671	0.504	low
Treat-0d	RHSIM_Rhsim03G0256000	0.570	-0.279	-0.849	no
Treat-0d	RHSIM_Rhsim04G0187900	0.101	-1.139	-1.240	no
Treat-0d	RHSIM_Rhsim03G0164300	1.863	0.830	-1.033	high
Treat-0d	RHSIM_Rhsim04G0111500	-1.005	-0.793	0.211	no
Treat-0d	RHSIM_Rhsim03G0214900	-0.971	1.675	2.647	no
Treat-0d	RHSIM_Rhsim04G0002800	1.348	3.387	2.039	high
Treat-0d	RHSIM_Rhsim04G0245400	0.518	-0.651	-1.169	no
Treat-0d	RHSIM_Rhsim04G0208400	0.571	1.385	0.813	no
Treat-0d	RHSIM_Rhsim04G0229900	0.475	3.346	2.870	no

Group	Gene	PTR (z-score difference)	z(log2 LFQ)	z(log2 FPKM)	extreme-PTR
Treat-0d	RHSIM_Rhsim04G0216700	1.192	1.298	0.106	high
Treat-0d	RHSIM_Rhsim03G0236800	-0.989	-1.317	-0.327	no
Treat-0d	RHSIM_Rhsim04G0123000	0.793	-0.104	-0.897	no
Treat-0d	RHSIM_Rhsim03G0211000	0.732	0.900	0.168	no
Treat-0d	RHSIM_Rhsim02G0193100	-0.987	-0.857	0.130	no
Treat-0d	RHSIM_Rhsim03G0208400	0.671	-0.329	-1.000	no
Treat-0d	RHSIM_Rhsim03G0055400	1.338	-0.789	-2.127	high
Treat-0d	RHSIM_Rhsim03G0121600	0.580	-0.175	-0.755	no
Treat-0d	RHSIM_Rhsim03G0088200	0.135	0.473	0.338	no
Treat-0d	RHSIM_Rhsim04G0008500	-0.532	-0.481	0.051	no
Treat-0d	RHSIM_Rhsim03G0230800	0.375	-0.165	-0.541	no
Treat-0d	RHSIM_Rhsim04G0222300	0.180	-0.196	-0.376	no
Treat-0d	RHSIM_Rhsim03G0161300	0.113	-0.247	-0.360	no
Treat-0d	RHSIM_Rhsim03G0188000	-0.756	-0.308	0.448	no
Treat-0d	RHSIM_Rhsim03G0046200	0.470	0.639	0.169	no
Treat-0d	RHSIM_Rhsim02G0059700	-0.819	-1.099	-0.280	no
Treat-0d	RHSIM_Rhsim03G0009300	1.147	0.023	-1.124	high
Treat-0d	RHSIM_Rhsim03G0245100	0.432	-0.083	-0.516	no
Treat-0d	RHSIM_Rhsim03G0028400	0.242	-1.004	-1.246	no
Treat-0d	RHSIM_Rhsim04G0245900	1.749	1.105	-0.644	high
Treat-0d	RHSIM_Rhsim03G0264400	0.494	-0.028	-0.522	no
Treat-0d	RHSIM_Rhsim02G0123600	-0.922	-0.428	0.495	no
Treat-0d	RHSIM_Rhsim04G0015500	-0.240	0.231	0.471	no
Treat-0d	RHSIM_Rhsim04G0028100	0.590	-0.715	-1.305	no
Treat-0d	RHSIM_Rhsim04G0249600	1.345	0.189	-1.156	high
Treat-0d	RHSIM_Rhsim03G0070200	0.342	0.140	-0.202	no
Treat-0d	RHSIM_Rhsim03G0234300	0.995	0.268	-0.727	no
Treat-0d	RHSIM_Rhsim03G0121100	0.243	0.242	-0.001	no
Treat-0d	RHSIM_Rhsim04G0021900	0.693	1.261	0.568	no
Treat-0d	RHSIM_Rhsim02G0237500	0.675	0.794	0.119	no
Treat-0d	RHSIM_Rhsim04G0015400	-0.541	0.084	0.625	no
Treat-0d	RHSIM_Rhsim04G0074100	-0.365	-0.587	-0.222	no

Group	Gene	PTR (z-score difference)	z(log2 LFQ)	z(log2 FPKM)	extreme-PTR
Treat-0d	RHSIM_Rhsim03G0163800	-1.023	-0.441	0.583	low
Treat-0d	RHSIM_Rhsim03G0241100	0.199	-1.003	-1.202	no
Treat-0d	RHSIM_Rhsim02G0001600	-0.463	-0.744	-0.280	no
Treat-0d	RHSIM_Rhsim03G0007100	-0.553	-0.439	0.114	no
Treat-0d	RHSIM_Rhsim03G0197200	-0.313	0.129	0.442	no
Treat-0d	RHSIM_Rhsim02G0159600	-0.493	1.433	1.926	no
Treat-0d	RHSIM_Rhsim03G0018300	-0.249	1.044	1.293	no
Treat-0d	RHSIM_Rhsim03G0011100	-0.651	0.672	1.323	no
Treat-0d	RHSIM_Rhsim03G0264900	-1.678	-0.919	0.760	low
Treat-0d	RHSIM_Rhsim03G0131600	-0.241	0.930	1.172	no
Treat-0d	RHSIM_Rhsim03G0215400	0.079	0.923	0.844	no
Treat-0d	RHSIM_Rhsim02G0070700	-0.158	0.850	1.008	no
Treat-0d	RHSIM_Rhsim02G0132400	0.380	0.555	0.175	no
Treat-0d	RHSIM_Rhsim03G0058000	2.083	0.779	-1.304	high
Treat-0d	RHSIM_Rhsim04G0067400	-0.367	-0.327	0.040	no
Treat-0d	RHSIM_Rhsim02G0193600	-0.730	-0.961	-0.231	no
Treat-0d	RHSIM_Rhsim03G0063900	-3.419	-0.018	3.401	low
Treat-0d	RHSIM_Rhsim03G0259500	0.073	-0.871	-0.944	no
Treat-0d	RHSIM_Rhsim04G0243300	0.196	0.339	0.143	no
Treat-0d	RHSIM_Rhsim02G0252000	0.322	-0.215	-0.537	no
Treat-0d	RHSIM_Rhsim03G0165500	-0.675	1.163	1.838	no
Treat-0d	RHSIM_Rhsim03G0076200	-0.009	0.159	0.168	no
Treat-0d	RHSIM_Rhsim03G0191300	0.926	1.229	0.303	no
Treat-0d	RHSIM_Rhsim03G0191800	0.386	0.038	-0.348	no
Treat-0d	RHSIM_Rhsim03G0254400	-1.541	-0.443	1.098	low
Treat-0d	RHSIM_Rhsim04G0166600	-0.174	-0.497	-0.323	no
Treat-0d	RHSIM_Rhsim03G0180600	-0.457	1.014	1.471	no
Treat-0d	RHSIM_Rhsim02G0135500	0.557	0.133	-0.424	no
Treat-0d	RHSIM_Rhsim02G0217800	0.935	0.558	-0.377	no
Treat-0d	RHSIM_Rhsim02G0056200	1.716	2.423	0.707	high
Treat-0d	RHSIM_Rhsim04G0242100	0.269	-0.565	-0.833	no
Treat-0d	RHSIM_Rhsim02G0131800	-0.617	-1.234	-0.616	no

Group	Gene	PTR (z-score difference)	z(log2 LFQ)	z(log2 FPKM)	extreme-PTR
Treat-0d	RHSIM_Rhsim03G0165100	1.301	-0.844	-2.144	high
Treat-0d	RHSIM_Rhsim03G0245900	-1.565	0.527	2.092	low
Treat-0d	RHSIM_Rhsim02G0071100	0.525	1.003	0.478	no
Treat-0d	RHSIM_Rhsim03G0073900	0.289	0.333	0.044	no
Treat-0d	RHSIM_Rhsim03G0171300	0.178	1.577	1.399	no
Treat-0d	RHSIM_Rhsim03G0231400	-0.671	-0.948	-0.277	no
Treat-0d	RHSIM_Rhsim02G0235500	-0.513	0.559	1.072	no
Treat-0d	RHSIM_Rhsim03G0067800	0.048	-0.580	-0.629	no
Treat-0d	RHSIM_Rhsim02G0143300	1.010	2.695	1.685	no
Treat-0d	RHSIM_Rhsim02G0247300	-1.214	-1.646	-0.432	low
Treat-0d	RHSIM_Rhsim03G0105000	-0.084	-0.839	-0.756	no
Treat-0d	RHSIM_Rhsim04G0083900	0.989	0.772	-0.217	no
Treat-0d	RHSIM_Rhsim03G0053000	0.344	1.635	1.291	no
Treat-0d	RHSIM_Rhsim02G0123500	0.588	-0.491	-1.078	no
Treat-0d	RHSIM_Rhsim03G0075500	-0.646	-0.343	0.303	no
Treat-0d	RHSIM_Rhsim02G0088400	0.313	1.330	1.017	no
Treat-0d	RHSIM_Rhsim04G0007400	0.867	3.367	2.499	no
Treat-0d	RHSIM_Rhsim02G0031300	-1.842	-1.641	0.201	low
Treat-0d	RHSIM_Rhsim04G0006500	-1.133	-0.885	0.248	low
Treat-0d	RHSIM_Rhsim01G0222900	0.802	-0.057	-0.859	no
Treat-0d	RHSIM_Rhsim01G0240100	-0.884	-1.102	-0.218	no
Treat-0d	RHSIM_Rhsim02G0171900	2.084	1.316	-0.768	high
Treat-0d	RHSIM_Rhsim01G0041300	0.910	-0.346	-1.256	no
Treat-0d	RHSIM_Rhsim04G0051700	1.506	0.159	-1.347	high
Treat-0d	RHSIM_Rhsim03G0067500	-0.982	0.964	1.947	no
Treat-0d	RHSIM_Rhsim04G0066100	1.266	0.191	-1.075	high
Treat-0d	RHSIM_Rhsim02G0066400	-0.663	-0.907	-0.244	no
Treat-0d	RHSIM_Rhsim02G0018900	0.325	-0.516	-0.841	no
Treat-0d	RHSIM_Rhsim01G0178500	-1.091	-0.104	0.988	low
Treat-0d	RHSIM_Rhsim04G0181100	1.141	1.087	-0.054	high
Treat-0d	RHSIM_Rhsim03G0255900	0.594	1.386	0.792	no
Treat-0d	RHSIM_Rhsim03G0243000	-0.507	-0.197	0.310	no

Group	Gene	PTR (z-score difference)	z(log2 LFQ)	z(log2 FPKM)	extreme-PTR
Treat-0d	RHSIM_Rhsim03G0186600	0.747	0.389	-0.358	no
Treat-0d	RHSIM_Rhsim02G0241900	0.595	-1.193	-1.787	no
Treat-0d	RHSIM_Rhsim03G0245400	0.436	-0.290	-0.726	no
Treat-0d	RHSIM_Rhsim01G0167600	0.547	1.059	0.512	no
Treat-0d	RHSIM_Rhsim03G0225800	0.281	-0.356	-0.638	no
Treat-0d	RHSIM_Rhsim02G0133700	-1.078	-0.238	0.839	low
Treat-0d	RHSIM_Rhsim02G0014500	0.184	-0.825	-1.009	no
Treat-0d	RHSIM_Rhsim04G0072300	-0.373	-1.188	-0.815	no
Treat-0d	RHSIM_Rhsim04G0091400	-1.438	-0.830	0.608	low
Treat-0d	RHSIM_Rhsim03G0059700	0.403	0.566	0.164	no
Treat-0d	RHSIM_Rhsim04G0085600	-0.617	0.639	1.256	no
Treat-0d	RHSIM_Rhsim04G0121700	1.720	1.167	-0.553	high
Treat-0d	RHSIM_Rhsim02G0253800	-0.403	-0.615	-0.212	no
Treat-0d	RHSIM_Rhsim02G0043500	0.065	0.387	0.322	no
Treat-0d	RHSIM_Rhsim03G0097500	-0.566	0.297	0.863	no
Treat-0d	RHSIM_Rhsim03G0254000	0.045	0.508	0.463	no
Treat-0d	RHSIM_Rhsim02G0031900	-0.845	-0.127	0.718	no
Treat-0d	RHSIM_Rhsim03G0235200	-0.131	1.096	1.228	no
Treat-0d	RHSIM_Rhsim03G0091100	-0.009	-0.371	-0.362	no
Treat-0d	RHSIM_Rhsim02G0071800	0.213	0.275	0.062	no
Treat-0d	RHSIM_Rhsim01G0292400	-1.012	-0.209	0.803	no
Treat-0d	RHSIM_Rhsim02G0141300	0.115	-0.446	-0.561	no
Treat-0d	RHSIM_Rhsim01G0203700	0.659	-0.171	-0.830	no
Treat-0d	RHSIM_Rhsim03G0129800	-0.705	-0.367	0.339	no
Treat-0d	RHSIM_Rhsim03G0038900	0.905	0.383	-0.522	no
Treat-0d	RHSIM_Rhsim02G0122100	0.062	0.063	0.001	no
Treat-0d	RHSIM_Rhsim01G0238000	-3.712	-0.699	3.013	low
Treat-0d	RHSIM_Rhsim02G0243000	1.198	0.262	-0.935	high
Treat-0d	RHSIM_Rhsim01G0066800	0.185	-1.022	-1.207	no
Treat-0d	RHSIM_Rhsim03G0125400	-1.179	-1.319	-0.141	low
Treat-0d	RHSIM_Rhsim03G0161700	0.419	0.379	-0.040	no
Treat-0d	RHSIM_Rhsim01G0112000	-0.721	-1.522	-0.801	no

Group	Gene	PTR (z-score difference)	z(log2 LFQ)	z(log2 FPKM)	extreme-PTR
Treat-0d	RHSIM_Rhsim02G0100900	2.392	0.659	-1.732	high
Treat-0d	RHSIM_Rhsim03G0261300	-0.673	0.067	0.740	no
Treat-0d	RHSIM_Rhsim02G0256200	0.138	-0.149	-0.287	no
Treat-0d	RHSIM_Rhsim02G0022900	-1.052	-0.781	0.271	low
Treat-0d	RHSIM_Rhsim03G0110700	0.463	0.825	0.362	no
Treat-0d	RHSIM_Rhsim01G0141100	-0.351	0.334	0.685	no
Treat-0d	RHSIM_Rhsim03G0268200	0.960	0.095	-0.864	no
Treat-0d	RHSIM_Rhsim02G0008200	0.309	0.801	0.492	no
Treat-0d	RHSIM_Rhsim03G0225600	-1.807	-1.582	0.225	low
Treat-0d	RHSIM_Rhsim03G0152900	0.638	0.586	-0.052	no
Treat-0d	RHSIM_Rhsim03G0243200	-1.808	-0.124	1.684	low
Treat-0d	RHSIM_Rhsim03G0236200	-0.029	1.375	1.404	no
Treat-0d	RHSIM_Rhsim01G0105900	-0.131	0.293	0.423	no
Treat-0d	RHSIM_Rhsim03G0212300	0.782	0.623	-0.159	no
Treat-0d	RHSIM_Rhsim03G0090500	-0.603	-1.161	-0.557	no
Treat-0d	RHSIM_Rhsim01G0147400	0.959	0.559	-0.400	no
Treat-0d	RHSIM_Rhsim02G0248400	0.424	1.485	1.061	no
Treat-0d	RHSIM_Rhsim03G0257300	-0.889	-0.252	0.637	no
Treat-0d	RHSIM_Rhsim02G0128600	-0.409	-0.264	0.145	no
Treat-0d	RHSIM_Rhsim03G0219500	0.429	1.770	1.341	no
Treat-0d	RHSIM_Rhsim03G0187400	1.342	0.863	-0.479	high
Treat-0d	RHSIM_Rhsim03G0040400	0.790	1.077	0.287	no
Treat-0d	RHSIM_Rhsim03G0251700	-0.680	-0.147	0.533	no
Treat-0d	RHSIM_Rhsim03G0005600	-0.576	0.043	0.619	no
Treat-0d	RHSIM_Rhsim03G0167200	-0.242	-0.459	-0.217	no
Treat-0d	RHSIM_Rhsim03G0254200	-1.005	-1.571	-0.565	no
Treat-0d	RHSIM_Rhsim03G0136300	0.588	0.305	-0.283	no
Treat-0d	RHSIM_Rhsim02G0071400	0.583	-0.730	-1.312	no
Treat-0d	RHSIM_Rhsim03G0230100	0.773	0.918	0.145	no
Treat-0d	RHSIM_Rhsim03G0176800	-1.048	-1.247	-0.199	low
Treat-0d	RHSIM_Rhsim02G0060200	-0.297	-1.045	-0.749	no
Treat-0d	RHSIM_Rhsim02G0143200	0.628	-0.205	-0.833	no

Group	Gene	PTR (z-score difference)	z(log2 LFQ)	z(log2 FPKM)	extreme-PTR
Treat-0d	RHSIM_Rhsim03G0070300	-0.158	0.731	0.889	no
Treat-0d	RHSIM_Rhsim03G0117200	-0.584	-0.736	-0.152	no
Treat-0d	RHSIM_Rhsim03G0212500	-0.983	0.241	1.224	no
Treat-0d	RHSIM_Rhsim02G0194100	0.069	1.410	1.342	no
Treat-0d	RHSIM_Rhsim02G0181400	0.006	0.461	0.455	no
Treat-0d	RHSIM_Rhsim02G0107000	-0.812	0.338	1.150	no
Treat-0d	RHSIM_Rhsim02G0028200	0.899	-0.063	-0.962	no
Treat-0d	RHSIM_Rhsim03G0219400	-0.565	-1.557	-0.992	no
Treat-0d	RHSIM_Rhsim03G0027400	0.979	1.036	0.057	no
Treat-0d	RHSIM_Rhsim03G0240000	-1.629	-1.283	0.347	low
Treat-0d	RHSIM_Rhsim02G0252600	-1.484	-0.719	0.765	low
Treat-0d	RHSIM_Rhsim03G0089800	-0.495	0.045	0.540	no
Treat-0d	RHSIM_Rhsim01G0249900	-0.293	1.102	1.395	no
Treat-0d	RHSIM_Rhsim03G0214200	-0.179	-0.691	-0.512	no
Treat-0d	RHSIM_Rhsim02G0150100	-1.238	-0.342	0.896	low
Treat-0d	RHSIM_Rhsim03G0169600	-0.241	-0.781	-0.540	no
Treat-0d	RHSIM_Rhsim03G0253100	-1.630	-0.851	0.778	low
Treat-0d	RHSIM_Rhsim02G0053700	-1.572	1.624	3.196	low
Treat-0d	RHSIM_Rhsim01G0170800	0.553	0.402	-0.151	no
Treat-0d	RHSIM_Rhsim03G0254900	0.819	0.957	0.139	no
Treat-0d	RHSIM_Rhsim02G0085700	0.894	-0.244	-1.137	no
Treat-0d	RHSIM_Rhsim03G0150200	2.114	1.271	-0.843	high
Treat-0d	RHSIM_Rhsim03G0190600	-0.545	-0.723	-0.178	no
Treat-0d	RHSIM_Rhsim03G0062900	-0.352	-0.741	-0.390	no
Treat-0d	RHSIM_Rhsim03G0049900	0.265	0.503	0.237	no
Treat-0d	RHSIM_Rhsim03G0239600	0.232	-1.306	-1.538	no
Treat-0d	RHSIM_Rhsim02G0002000	1.004	1.081	0.077	no
Treat-0d	RHSIM_Rhsim01G0183300	0.352	-0.728	-1.081	no
Treat-0d	RHSIM_Rhsim01G0001700	-0.715	-1.288	-0.573	no
Treat-0d	RHSIM_Rhsim03G0147100	-0.075	0.412	0.486	no
Treat-0d	RHSIM_Rhsim03G0076700	0.370	-0.116	-0.486	no
Treat-0d	RHSIM_Rhsim01G0283600	-1.649	0.557	2.206	low

Group	Gene	PTR (z-score difference)	z(log2 LFQ)	z(log2 FPKM)	extreme-PTR
Treat-0d	RHSIM_Rhsim03G0186300	0.276	-1.689	-1.965	no
Treat-0d	RHSIM_Rhsim02G0244700	0.206	-0.323	-0.530	no
Treat-0d	RHSIM_Rhsim02G0058700	2.066	0.298	-1.768	high
Treat-0d	RHSIM_Rhsim03G0226600	-0.328	0.437	0.765	no
Treat-0d	RHSIM_Rhsim01G0123000	-1.571	-1.105	0.466	low
Treat-0d	RHSIM_Rhsim03G0003600	-0.219	-0.211	0.009	no
Treat-0d	RHSIM_Rhsim02G0005400	0.795	1.486	0.690	no
Treat-0d	RHSIM_Rhsim01G0192600	-0.607	-0.115	0.491	no
Treat-0d	RHSIM_Rhsim01G0258200	-0.258	0.563	0.821	no
Treat-0d	RHSIM_Rhsim01G0100600	-0.545	-0.073	0.471	no
Treat-0d	RHSIM_Rhsim02G0236800	-2.008	-0.786	1.222	low
Treat-0d	RHSIM_Rhsim03G0221300	-0.599	-1.373	-0.774	no
Treat-0d	RHSIM_Rhsim01G0280000	0.527	0.183	-0.344	no
Treat-0d	RHSIM_Rhsim01G0135600	0.838	-0.345	-1.183	no
Treat-0d	RHSIM_Rhsim02G0175300	1.647	0.202	-1.445	high
Treat-0d	RHSIM_Rhsim03G0094200	0.669	0.061	-0.608	no
Treat-0d	RHSIM_Rhsim02G0195000	-0.749	-0.898	-0.149	no
Treat-0d	RHSIM_Rhsim01G0114000	0.287	0.951	0.665	no
Treat-0d	RHSIM_Rhsim03G0052800	-2.960	-1.062	1.897	low
Treat-0d	RHSIM_Rhsim01G0259100	-1.223	-1.249	-0.025	low
Treat-0d	RHSIM_Rhsim01G0049700	-0.828	-0.514	0.314	no
Treat-0d	RHSIM_Rhsim01G0190400	0.236	0.379	0.143	no
Treat-0d	RHSIM_Rhsim01G0187400	-0.073	-1.025	-0.953	no
Treat-0d	RHSIM_Rhsim01G0044000	0.154	1.511	1.357	no
Treat-0d	RHSIM_Rhsim03G0153700	0.513	0.400	-0.113	no
Treat-0d	RHSIM_Rhsim02G0246500	-0.394	0.367	0.761	no
Treat-0d	RHSIM_Rhsim01G0061400	0.461	-0.340	-0.801	no
Treat-0d	RHSIM_Rhsim02G0004600	-1.815	-0.347	1.468	low
Treat-0d	RHSIM_Rhsim01G0049100	1.274	0.343	-0.930	high
Treat-0d	RHSIM_Rhsim02G0180700	-0.964	-0.213	0.751	no
Treat-0d	RHSIM_Rhsim01G0222000	0.727	0.476	-0.250	no
Treat-0d	RHSIM_Rhsim02G0242100	-4.454	-1.625	2.828	low

Group	Gene	PTR (z-score difference)	z(log2 LFQ)	z(log2 FPKM)	extreme-PTR
Treat-0d	RHSIM_Rhsim02G0207700	0.727	-0.715	-1.442	no
Treat-0d	RHSIM_Rhsim03G0227500	1.572	0.470	-1.102	high
Treat-0d	RHSIM_Rhsim02G0241000	-1.247	-1.241	0.006	low
Treat-0d	RHSIM_Rhsim02G0018500	-0.120	-0.410	-0.290	no
Treat-0d	RHSIM_Rhsim02G0115600	-0.875	-0.201	0.674	no
Treat-0d	RHSIM_Rhsim02G0070000	0.253	0.690	0.437	no
Treat-0d	RHSIM_Rhsim02G0007400	0.092	-0.286	-0.378	no
Treat-0d	RHSIM_Rhsim02G0256900	0.815	0.003	-0.812	no
Treat-0d	RHSIM_Rhsim02G0106100	0.138	-1.110	-1.248	no
Treat-0d	RHSIM_Rhsim03G0213000	0.810	0.199	-0.611	no
Treat-0d	RHSIM_Rhsim03G0087300	-0.603	-0.337	0.266	no
Treat-0d	RHSIM_Rhsim02G0061700	-0.078	1.149	1.227	no
Treat-0d	RHSIM_Rhsim02G0234600	-2.238	-0.980	1.258	low
Treat-0d	RHSIM_Rhsim03G0148000	-1.061	-0.972	0.089	low
Treat-0d	RHSIM_Rhsim01G0169000	-2.519	-1.289	1.230	low
Treat-0d	RHSIM_Rhsim01G0232600	-1.096	-0.419	0.677	low
Treat-0d	RHSIM_Rhsim02G0084400	-0.098	-0.696	-0.597	no
Treat-0d	RHSIM_Rhsim01G0286300	1.331	0.736	-0.595	high
Treat-0d	RHSIM_Rhsim03G0247500	-0.845	-0.704	0.141	no
Treat-0d	RHSIM_Rhsim02G0217700	-0.433	0.368	0.800	no
Treat-0d	RHSIM_Rhsim02G0104800	0.280	0.064	-0.216	no
Treat-0d	RHSIM_Rhsim01G0050400	0.281	-0.902	-1.183	no
Treat-0d	RHSIM_Rhsim01G0101100	0.064	-0.868	-0.932	no
Treat-0d	RHSIM_Rhsim01G0278500	-0.930	-0.884	0.046	no
Treat-0d	RHSIM_Rhsim02G0214300	0.658	0.885	0.227	no
Treat-0d	RHSIM_Rhsim01G0060000	-0.048	0.269	0.317	no
Treat-0d	RHSIM_Rhsim01G0258800	-0.361	0.211	0.572	no
Treat-0d	RHSIM_Rhsim01G0114100	-0.908	-0.425	0.482	no
Treat-0d	RHSIM_Rhsim01G0258700	0.976	0.903	-0.074	no
Treat-0d	RHSIM_Rhsim02G0007600	1.802	0.340	-1.462	high
Treat-0d	RHSIM_Rhsim02G0069600	0.156	0.601	0.445	no
Treat-0d	RHSIM_Rhsim01G0127700	0.625	1.254	0.629	no

Group	Gene	PTR (z-score difference)	z(log2 LFQ)	z(log2 FPKM)	extreme-PTR
Treat-0d	RHSIM_Rhsim02G0031200	-0.499	-0.544	-0.046	no
Treat-0d	RHSIM_Rhsim01G0192800	-1.342	0.613	1.955	low
Treat-0d	RHSIM_Rhsim03G0004200	-0.720	-0.406	0.314	no
Treat-0d	RHSIM_Rhsim02G0066800	-1.329	-0.900	0.428	low
Treat-0d	RHSIM_Rhsim01G0240400	0.727	-0.266	-0.993	no
Treat-0d	RHSIM_Rhsim02G0068800	0.171	0.078	-0.093	no
Treat-0d	RHSIM_Rhsim01G0291700	-0.195	-0.786	-0.591	no
Treat-0d	RHSIM_Rhsim01G0131800	2.500	2.374	-0.126	high
Treat-0d	RHSIM_Rhsim01G0171300	0.925	2.367	1.442	no
Treat-0d	RHSIM_Rhsim01G0116600	-1.095	-1.701	-0.606	low
Treat-0d	RHSIM_Rhsim02G0210500	-1.274	-0.888	0.387	low
Treat-0d	RHSIM_Rhsim01G0230200	-0.749	-0.723	0.026	no
Treat-0d	RHSIM_Rhsim02G0028100	-0.224	1.175	1.399	no
Treat-0d	RHSIM_Rhsim01G0131300	1.737	0.526	-1.211	high
Treat-0d	RHSIM_Rhsim01G0142100	-0.289	-0.271	0.018	no
Treat-0d	RHSIM_Rhsim01G0220300	0.933	-0.718	-1.651	no
Treat-0d	RHSIM_Rhsim03G0090000	-0.972	0.401	1.373	no
Treat-0d	RHSIM_Rhsim01G0219100	1.569	3.337	1.768	high
Treat-0d	RHSIM_Rhsim03G0151200	0.919	1.240	0.321	no
Treat-0d	RHSIM_Rhsim01G0290600	0.420	-0.012	-0.432	no
Treat-0d	RHSIM_Rhsim01G0102500	-0.615	-0.124	0.491	no
Treat-0d	RHSIM_Rhsim01G0278100	-2.207	-1.432	0.775	low
Treat-0d	RHSIM_Rhsim02G0099900	-0.310	-0.735	-0.425	no
Treat-0d	RHSIM_Rhsim02G0004900	-0.383	-0.703	-0.320	no
Treat-0d	RHSIM_Rhsim01G0226900	-1.796	-0.879	0.917	low
Treat-0d	RHSIM_Rhsim02G0124600	0.769	-0.015	-0.784	no
Treat-0d	RHSIM_Rhsim02G0157100	-1.014	-1.560	-0.547	no
Treat-0d	RHSIM_Rhsim01G0135700	0.520	0.548	0.028	no
Treat-0d	RHSIM_Rhsim02G0181000	0.078	0.236	0.158	no
Treat-0d	RHSIM_Rhsim02G0096400	0.413	0.333	-0.080	no
Treat-0d	RHSIM_Rhsim02G0089800	0.275	0.210	-0.065	no
Treat-0d	RHSIM_Rhsim02G0169600	0.132	0.593	0.461	no

Group	Gene	PTR (z-score difference)	z(log2 LFQ)	z(log2 FPKM)	extreme-PTR
Treat-0d	RHSIM_Rhsim02G0105100	0.417	-0.445	-0.862	no
Treat-0d	RHSIM_Rhsim01G0267800	-1.881	-1.619	0.263	low
Treat-0d	RHSIM_Rhsim01G0242700	-0.786	-2.114	-1.328	no
Treat-0d	RHSIM_Rhsim01G0152000	-1.177	-0.941	0.236	low
Treat-0d	RHSIM_Rhsim02G0172600	0.881	0.859	-0.022	no
Treat-0d	RHSIM_Rhsim02G0147000	1.682	2.325	0.643	high
Treat-0d	RHSIM_Rhsim02G0251400	0.406	0.028	-0.378	no
Treat-0d	RHSIM_Rhsim02G0251300	1.602	0.926	-0.676	high
Treat-0d	RHSIM_Rhsim01G0232900	-2.160	-1.834	0.326	low
Treat-0d	RHSIM_Rhsim02G0007900	-2.482	-0.368	2.114	low
Treat-0d	RHSIM_Rhsim02G0252500	0.024	1.543	1.519	no
Treat-0d	RHSIM_Rhsim02G0122400	0.012	-0.645	-0.657	no
Treat-0d	RHSIM_Rhsim02G0159400	-0.108	-0.952	-0.843	no
Treat-0d	RHSIM_Rhsim02G0088300	0.275	-1.051	-1.326	no
Treat-0d	RHSIM_Rhsim02G0083500	-0.495	-0.261	0.234	no
Treat-0d	RHSIM_Rhsim02G0205900	-1.744	-0.077	1.667	low
Treat-0d	RHSIM_Rhsim02G0208200	-0.883	-1.271	-0.388	no
Treat-0d	RHSIM_Rhsim01G0235200	0.749	0.225	-0.524	no
Treat-0d	RHSIM_Rhsim01G0243800	0.269	1.136	0.867	no
Treat-0d	RHSIM_Rhsim02G0119200	1.052	0.774	-0.278	no
Treat-0d	RHSIM_Rhsim01G0234200	0.673	0.452	-0.221	no
Treat-0d	RHSIM_Rhsim01G0025200	0.585	1.176	0.590	no
Treat-0d	RHSIM_Rhsim01G0054900	-0.462	-0.190	0.272	no
Treat-0d	RHSIM_Rhsim01G0232200	-0.036	0.964	1.000	no
Treat-0d	RHSIM_Rhsim02G0141400	0.992	-1.077	-2.069	no
Treat-0d	RHSIM_Rhsim01G0223600	1.008	-0.616	-1.624	no
Treat-0d	RHSIM_Rhsim02G0035600	-1.854	-0.685	1.169	low
Treat-0d	RHSIM_Rhsim01G0175000	-2.511	-1.703	0.808	low
Treat-0d	RHSIM_Rhsim01G0013100	0.365	2.271	1.907	no
Treat-0d	RHSIM_Rhsim01G0073100	-0.680	0.007	0.687	no
Treat-0d	RHSIM_Rhsim01G0184400	1.358	2.144	0.785	high
Treat-0d	RHSIM_Rhsim01G0269200	0.847	1.584	0.737	no

Group	Gene	PTR (z-score difference)	z(log2 LFQ)	z(log2 FPKM)	extreme-PTR
Treat-0d	RHSIM_Rhsim02G0213100	0.018	-0.896	-0.915	no
Treat-0d	RHSIM_Rhsim01G0263700	-0.098	-0.760	-0.662	no
Treat-0d	RHSIM_Rhsim01G0182600	0.826	-0.985	-1.811	no
Treat-0d	RHSIM_Rhsim01G0132700	-1.362	-1.938	-0.576	low
Treat-0d	RHSIM_Rhsim01G0241200	0.017	-0.563	-0.580	no
Treat-0d	RHSIM_Rhsim01G0291500	0.140	-0.374	-0.515	no
Treat-0d	RHSIM_Rhsim01G0108800	-0.459	1.377	1.836	no
Treat-0d	RHSIM_Rhsim01G0001600	-1.727	-0.607	1.121	low
Treat-0d	RHSIM_Rhsim01G0147700	-0.436	-0.583	-0.147	no
Treat-0d	RHSIM_Rhsim02G0240500	0.348	-1.701	-2.049	no
Treat-0d	RHSIM_Rhsim02G0248800	0.231	0.123	-0.108	no
Treat-0d	RHSIM_Rhsim01G0243600	-0.474	0.967	1.441	no
Treat-0d	RHSIM_Rhsim02G0220200	1.968	2.340	0.372	high
Treat-0d	RHSIM_Rhsim01G0185100	0.497	0.790	0.293	no
Treat-0d	RHSIM_Rhsim01G0270000	-0.563	0.395	0.958	no
Treat-0d	RHSIM_Rhsim02G0127100	-1.600	-1.625	-0.025	low
Treat-0d	RHSIM_Rhsim01G0089900	0.228	0.626	0.398	no
Treat-0d	RHSIM_Rhsim02G0145900	-0.553	-0.330	0.223	no
Treat-0d	RHSIM_Rhsim02G0250500	0.423	0.997	0.575	no
Treat-0d	RHSIM_Rhsim01G0184600	0.182	-0.386	-0.568	no
Treat-0d	RHSIM_Rhsim02G0089500	1.090	-0.661	-1.751	no
Treat-0d	RHSIM_Rhsim02G0019600	-0.489	-0.523	-0.034	no
Treat-0d	RHSIM_Rhsim01G0247800	0.888	3.592	2.704	no
Treat-0d	RHSIM_Rhsim02G0217200	-0.173	-0.406	-0.232	no
Treat-0d	RHSIM_Rhsim01G0284000	-1.775	-1.525	0.250	low
Treat-0d	RHSIM_Rhsim01G0163700	-0.447	-0.709	-0.262	no
Treat-0d	RHSIM_Rhsim01G0144200	-0.130	-0.054	0.076	no
Treat-0d	RHSIM_Rhsim02G0184100	-0.938	-0.995	-0.057	no
Treat-0d	RHSIM_Rhsim01G0001900	0.158	1.023	0.865	no
Treat-0d	RHSIM_Rhsim01G0195500	-0.421	-0.491	-0.070	no
Treat-0d	RHSIM_Rhsim01G0194400	-0.349	-0.892	-0.542	no
Treat-0d	RHSIM_Rhsim01G0196700	-0.839	-0.718	0.121	no

Group	Gene	PTR (z-score difference)	z(log2 LFQ)	z(log2 FPKM)	extreme-PTR
Treat-0d	RHSIM_Rhsim01G0287600	-1.915	-0.583	1.332	low
Treat-0d	RHSIM_Rhsim01G0227500	-0.054	2.649	2.702	no
Treat-0d	RHSIM_Rhsim01G0247200	-0.854	-1.472	-0.618	no
Treat-0d	RHSIM_Rhsim02G0215600	-0.107	0.488	0.596	no
Treat-0d	RHSIM_Rhsim02G0109900	1.764	0.809	-0.955	high
Treat-0d	RHSIM_Rhsim01G0285600	-1.585	-0.849	0.736	low
Treat-0d	RHSIM_Rhsim01G0097900	-2.854	-1.855	0.999	low
Treat-0d	RHSIM_Rhsim01G0128500	-0.834	-1.469	-0.635	no
Treat-0d	RHSIM_Rhsim01G0286500	0.331	-0.248	-0.579	no
Treat-0d	RHSIM_Rhsim01G0008000	1.384	0.595	-0.789	high
Treat-0d	RHSIM_Rhsim02G0255400	0.041	-0.358	-0.399	no
Treat-0d	RHSIM_Rhsim01G0244300	-1.107	-0.815	0.292	low
Treat-0d	RHSIM_Rhsim01G0196200	-0.394	0.455	0.849	no
Treat-0d	RHSIM_Rhsim01G0265400	-2.279	-1.044	1.235	low
Treat-0d	RHSIM_Rhsim02G0083400	0.141	-0.437	-0.579	no
Treat-0d	RHSIM_Rhsim01G0144600	0.755	-0.145	-0.901	no
Treat-0d	RHSIM_Rhsim01G0276900	-1.085	-1.131	-0.046	low
Treat-0d	RHSIM_Rhsim02G0105300	-0.369	-0.871	-0.502	no
Treat-0d	RHSIM_Rhsim01G0186600	-0.227	-1.226	-0.999	no
Treat-0d	RHSIM_Rhsim01G0213500	0.712	2.500	1.788	no
Treat-0d	RHSIM_Rhsim01G0265200	-0.118	-1.427	-1.309	no
Treat-0d	RHSIM_Rhsim02G0180100	-0.541	-0.180	0.361	no
Treat-0d	RHSIM_Rhsim01G0206200	-0.723	-0.183	0.540	no
Treat-0d	RHSIM_Rhsim01G0249100	0.489	0.281	-0.208	no
Treat-0d	RHSIM_Rhsim01G0059500	-0.319	0.212	0.531	no
Treat-0d	RHSIM_Rhsim01G0151800	-2.209	-1.111	1.098	low
Treat-0d	RHSIM_Rhsim01G0141500	1.496	1.146	-0.350	high
Treat-0d	RHSIM_Rhsim01G0134500	0.475	1.204	0.729	no
Treat-0d	RHSIM_Rhsim01G0212900	-2.305	-0.658	1.648	low
Treat-0d	RHSIM_Rhsim01G0124100	0.634	0.584	-0.050	no
Treat-0d	RHSIM_Rhsim02G0251700	-0.720	-0.684	0.036	no
Treat-0d	RHSIM_Rhsim01G0077600	0.189	0.780	0.591	no

Group	Gene	PTR (z-score difference)	z(log2 LFQ)	z(log2 FPKM)	extreme-PTR
Treat-0d	RHSIM_Rhsim01G0181300	-0.181	2.256	2.437	no
Treat-0d	RHSIM_Rhsim01G0205500	1.091	0.505	-0.586	no
Treat-0d	RHSIM_Rhsim01G0204800	-0.019	0.024	0.043	no
Treat-0d	RHSIM_Rhsim02G0043000	-0.882	2.344	3.226	no
Treat-0d	RHSIM_Rhsim01G0121200	-0.055	-0.735	-0.680	no
Treat-0d	RHSIM_Rhsim01G0114600	-0.403	1.076	1.479	no
Treat-0d	RHSIM_Rhsim01G0261900	0.827	0.154	-0.673	no
Treat-0d	RHSIM_Rhsim01G0290900	0.874	-1.165	-2.038	no
Treat-0d	RHSIM_Rhsim01G0215700	-0.432	-0.615	-0.183	no
Treat-0d	RHSIM_Rhsim01G0246400	0.569	0.328	-0.240	no
Treat-0d	RHSIM_Rhsim01G0176500	0.238	0.919	0.682	no
Treat-0d	RHSIM_Rhsim01G0023600	0.682	-0.492	-1.174	no
Treat-0d	RHSIM_Rhsim01G0128700	0.372	-0.708	-1.080	no
Treat-0d	RHSIM_Rhsim01G0226300	-1.071	-1.276	-0.206	low
Treat-0d	RHSIM_Rhsim01G0232700	0.382	-1.846	-2.228	no
Treat-0d	RHSIM_Rhsim01G0278000	0.488	0.287	-0.201	no
Treat-0d	RHSIM_Rhsim01G0001400	0.969	0.288	-0.680	no
Treat-0d	RHSIM_Rhsim01G0000100	0.915	-0.727	-1.643	no
Treat-0d	RHSIM_Rhsim01G0250800	-0.159	1.103	1.262	no
Treat-0d	RHSIM_Rhsim01G0245100	0.389	1.020	0.630	no
Treat-0d	RHSIM_Rhsim01G0244200	1.054	-0.459	-1.513	no
Treat-0d	RHSIM_Rhsim01G0244400	-0.619	1.248	1.867	no
Treat-0d	RHSIM_Rhsim01G0001500	0.951	-0.185	-1.135	no
Treat-0d	RHSIM_Rhsim01G0292000	1.132	0.426	-0.707	high
Treat-0d	RHSIM_Rhsim01G0286100	-0.703	-0.534	0.169	no
Treat-0d	RHSIM_Rhsim01G0199200	0.468	0.735	0.267	no
Treat-0d	RHSIM_Rhsim01G0004300	-2.057	-0.035	2.022	low
Treat-0d	RHSIM_Rhsim01G0256500	-1.019	-1.221	-0.202	no
Treat-0d	RHSIM_Rhsim01G0060100	-0.273	-0.395	-0.122	no
Treat-0d	RHSIM_Rhsim01G0187700	2.701	0.760	-1.941	high
Treat-0d	RHSIM_Rhsim01G0108000	-1.741	-1.386	0.355	low
Treat-0d	RHSIM_Rhsim01G0210600	1.178	-0.844	-2.022	high

Group	Gene	PTR (z-score difference)	z(log2 LFQ)	z(log2 FPKM)	extreme-PTR
Treat-0d	RHSIM_Rhsim01G0276100	1.683	0.627	-1.056	high
Treat-0d	RHSIM_Rhsim01G0276800	-0.538	-0.200	0.338	no
Treat-0d	RHSIM_Rhsim01G0153000	0.498	0.877	0.379	no
Treat-0d	RHSIM_Rhsim01G0193400	1.621	-0.412	-2.033	high
Treat-0d	RHSIM_Rhsim01G0241700	-0.083	1.421	1.504	no
Treat-0d	RHSIM_Rhsim01G0221500	-1.916	1.096	3.012	low
Treat-0d	RHSIM_Rhsim01G0189400	0.183	-0.577	-0.760	no
Treat-0d	RHSIM_Rhsim01G0000500	0.670	0.723	0.053	no
Treat-0d	RHSIM_Rhsim01G0260600	-0.904	-0.992	-0.088	no
Treat-0d	RHSIM_Rhsim01G0286200	-5.671	-1.511	4.160	low
Treat-0d	RHSIM_Rhsim01G0279700	0.714	-0.786	-1.500	no
Treat-0d	RHSIM_Rhsim01G0055700	1.140	0.278	-0.862	high
Treat-0d	RHSIM_Rhsim01G0282300	0.185	-0.330	-0.514	no
Treat-0d	RHSIM_Rhsim01G0044100	-0.404	0.460	0.864	no
Treat-0d	RHSIM_Rhsim01G0021100	0.676	0.058	-0.618	no
Treat-0d	RHSIM_Rhsim01G0192500	-0.053	-0.922	-0.868	no
Treat-0d	RHSIM_Rhsim01G0287000	0.696	1.558	0.862	no
Treat-0d	RHSIM_Rhsim01G0141200	0.453	1.028	0.575	no
Treat-0d	RHSIM_Rhsim01G0123600	0.737	-1.250	-1.987	no
Treat-0d	RHSIM_Rhsim01G0091700	-0.122	-0.617	-0.495	no
Treat-0d	RHSIM_Rhsim01G0210100	1.960	0.375	-1.585	high
Treat-0d	RHSIM_Rhsim01G0170700	0.704	0.397	-0.307	no
Treat-0d	RHSIM_Rhsim01G0259500	0.829	2.253	1.424	no
Treat-0d	RHSIM_Rhsim01G0163000	0.208	-0.709	-0.917	no
Treat-40d	RHSIM_RhsimUnG0249100	-2.280	0.583	2.863	low
Treat-40d	RHSIM_RhsimUnG0254800	-3.266	-1.012	2.253	low
Treat-40d	RHSIM_RhsimUnG0249600	0.063	-0.941	-1.004	no
Treat-40d	RHSIM_RhsimUnG0251500	-0.038	0.041	0.079	no
Treat-40d	RHSIM_RhsimUnG0252300	0.684	-0.317	-1.001	no
Treat-40d	RHSIM_RhsimUnG0228100	-0.595	-1.323	-0.729	no
Treat-40d	RHSIM_RhsimUnG0251200	-0.867	0.484	1.351	no
Treat-40d	RHSIM_RhsimUnG0226800	0.363	-0.675	-1.038	no

Group	Gene	PTR (z-score difference)	z(log2 LFQ)	z(log2 FPKM)	extreme-PTR
Treat-40d	RHSIM_RhsimUnG0245900	3.788	2.892	-0.896	high
Treat-40d	RHSIM_RhsimUnG0241900	2.344	0.738	-1.606	high
Treat-40d	RHSIM_RhsimUnG0250300	-3.850	-1.204	2.647	low
Treat-40d	RHSIM_RhsimUnG0250600	0.149	0.126	-0.024	no
Treat-40d	RHSIM_RhsimUnG0216900	1.355	1.746	0.391	high
Treat-40d	RHSIM_RhsimUnG0239100	1.247	0.262	-0.985	high
Treat-40d	RHSIM_RhsimUnG0231700	1.250	0.235	-1.015	high
Treat-40d	RHSIM_RhsimUnG0212900	1.982	0.099	-1.883	high
Treat-40d	RHSIM_RhsimUnG0231900	0.785	0.312	-0.474	no
Treat-40d	RHSIM_RhsimUnG0209400	2.413	0.676	-1.737	high
Treat-40d	RHSIM_RhsimUnG0219900	0.583	-1.266	-1.849	no
Treat-40d	RHSIM_RhsimUnG0217500	0.603	0.584	-0.019	no
Treat-40d	RHSIM_Rhsim09G0026600	1.243	-0.864	-2.108	high
Treat-40d	RHSIM_RhsimUnG0226200	-0.052	-0.057	-0.005	no
Treat-40d	RHSIM_RhsimUnG0190800	0.833	0.149	-0.684	no
Treat-40d	RHSIM_RhsimUnG0217400	0.001	-0.100	-0.102	no
Treat-40d	RHSIM_RhsimUnG0220800	0.189	-0.559	-0.748	no
Treat-40d	RHSIM_RhsimUnG0212700	-3.332	-1.480	1.852	low
Treat-40d	RHSIM_RhsimUnG0248900	0.414	1.226	0.813	no
Treat-40d	RHSIM_RhsimUnG0211400	-0.395	1.565	1.960	no
Treat-40d	RHSIM_RhsimUnG0209900	2.769	0.368	-2.401	high
Treat-40d	RHSIM_RhsimUnG0205800	0.267	1.728	1.461	no
Treat-40d	RHSIM_Rhsim09G0148400	-1.088	-0.713	0.375	no
Treat-40d	RHSIM_RhsimUnG0209800	-0.055	-0.528	-0.473	no
Treat-40d	RHSIM_RhsimUnG0225600	-0.444	0.038	0.483	no
Treat-40d	RHSIM_RhsimUnG0255000	-0.124	0.525	0.648	no
Treat-40d	RHSIM_RhsimUnG0156400	0.838	-1.209	-2.047	no
Treat-40d	RHSIM_RhsimUnG0205900	-0.489	1.074	1.563	no
Treat-40d	RHSIM_RhsimUnG0197600	1.855	0.764	-1.090	high
Treat-40d	RHSIM_RhsimUnG0206500	-1.064	-1.000	0.064	no
Treat-40d	RHSIM_Rhsim09G0069900	2.428	1.757	-0.671	high
Treat-40d	RHSIM_RhsimUnG0208500	-2.421	-1.657	0.763	low

Group	Gene	PTR (z-score difference)	z(log2 LFQ)	z(log2 FPKM)	extreme-PTR
Treat-40d	RHSIM_RhsimUnG0196500	-0.861	-1.650	-0.789	no
Treat-40d	RHSIM_RhsimUnG0176900	0.865	-1.183	-2.048	no
Treat-40d	RHSIM_RhsimUnG0185400	0.524	-0.291	-0.815	no
Treat-40d	RHSIM_RhsimUnG0158200	2.131	1.715	-0.417	high
Treat-40d	RHSIM_RhsimUnG0197500	1.706	-0.466	-2.172	high
Treat-40d	RHSIM_Rhsim02G0029800	1.349	-0.423	-1.772	high
Treat-40d	RHSIM_Rhsim09G0021500	1.412	1.549	0.137	high
Treat-40d	RHSIM_RhsimUnG0136600	0.254	0.285	0.032	no
Treat-40d	RHSIM_RhsimUnG0226700	0.566	-0.466	-1.032	no
Treat-40d	RHSIM_RhsimUnG0220700	2.296	0.750	-1.546	high
Treat-40d	RHSIM_RhsimUnG0126200	1.867	1.209	-0.658	high
Treat-40d	RHSIM_RhsimUnG0217000	1.085	0.127	-0.958	no
Treat-40d	RHSIM_RhsimUnG0109300	1.154	1.720	0.565	high
Treat-40d	RHSIM_RhsimUnG0122900	1.368	0.731	-0.637	high
Treat-40d	RHSIM_RhsimUnG0137100	1.527	1.193	-0.334	high
Treat-40d	RHSIM_RhsimUnG0190300	1.029	-0.479	-1.508	no
Treat-40d	RHSIM_RhsimUnG0256800	0.868	0.538	-0.331	no
Treat-40d	RHSIM_RhsimUnG0185700	-0.028	-1.276	-1.248	no
Treat-40d	RHSIM_RhsimUnG0180800	-0.951	0.437	1.389	no
Treat-40d	RHSIM_RhsimUnG0254600	-1.489	-0.470	1.019	low
Treat-40d	RHSIM_RhsimUnG0242200	-0.686	-1.183	-0.497	no
Treat-40d	RHSIM_RhsimUnG0253800	-0.545	-1.228	-0.683	no
Treat-40d	RHSIM_RhsimUnG0093800	-0.149	-0.041	0.108	no
Treat-40d	RHSIM_RhsimUnG0257500	1.644	0.822	-0.822	high
Treat-40d	RHSIM_RhsimUnG0118900	-0.389	1.335	1.724	no
Treat-40d	RHSIM_RhsimUnG0120200	-0.228	-1.179	-0.952	no
Treat-40d	RHSIM_RhsimUnG0092300	0.153	0.962	0.808	no
Treat-40d	RHSIM_RhsimUnG0124200	-0.486	-0.812	-0.326	no
Treat-40d	RHSIM_RhsimUnG0094500	0.538	0.418	-0.120	no
Treat-40d	RHSIM_RhsimUnG0059700	0.171	-0.348	-0.519	no
Treat-40d	RHSIM_RhsimUnG0145200	0.552	-1.113	-1.665	no
Treat-40d	RHSIM_RhsimUnG0118400	-1.786	-1.310	0.476	low

Group	Gene	PTR (z-score difference)	z(log2 LFQ)	z(log2 FPKM)	extreme-PTR
Treat-40d	RHSIM_RhsimUnG0138800	2.296	1.209	-1.087	high
Treat-40d	RHSIM_RhsimUnG0054600	-0.278	-0.905	-0.628	no
Treat-40d	RHSIM_RhsimUnG0216100	-2.702	-0.460	2.243	low
Treat-40d	RHSIM_RhsimUnG0213100	-0.479	-1.260	-0.781	no
Treat-40d	RHSIM_RhsimUnG0112800	0.707	-0.929	-1.636	no
Treat-40d	RHSIM_RhsimUnG0111000	-0.496	-0.571	-0.075	no
Treat-40d	RHSIM_RhsimUnG0205000	-0.670	1.519	2.190	no
Treat-40d	RHSIM_RhsimUnG0221600	2.292	0.926	-1.366	high
Treat-40d	RHSIM_RhsimUnG0141100	0.260	-0.872	-1.132	no
Treat-40d	RHSIM_RhsimUnG0060900	0.295	-0.902	-1.197	no
Treat-40d	RHSIM_RhsimUnG0216800	1.595	0.523	-1.071	high
Treat-40d	RHSIM_RhsimUnG0215100	1.258	-0.191	-1.449	high
Treat-40d	RHSIM_RhsimUnG0124800	0.184	-1.177	-1.360	no
Treat-40d	RHSIM_RhsimUnG0098600	0.948	-0.480	-1.428	no
Treat-40d	RHSIM_RhsimUnG0055200	0.189	-0.080	-0.269	no
Treat-40d	RHSIM_RhsimUnG0096900	0.053	-0.146	-0.198	no
Treat-40d	RHSIM_RhsimUnG0119200	-1.182	-0.471	0.711	low
Treat-40d	RHSIM_RhsimUnG0032200	-0.580	-1.145	-0.564	no
Treat-40d	RHSIM_RhsimUnG0119600	-0.874	-0.177	0.697	no
Treat-40d	RHSIM_RhsimUnG0087900	2.274	1.408	-0.866	high
Treat-40d	RHSIM_RhsimUnG0065700	-0.246	-0.443	-0.197	no
Treat-40d	RHSIM_RhsimUnG0113700	0.112	-0.477	-0.589	no
Treat-40d	RHSIM_RhsimUnG0109400	0.710	1.350	0.640	no
Treat-40d	RHSIM_RhsimUnG0080300	0.686	0.672	-0.014	no
Treat-40d	RHSIM_RhsimUnG0020600	0.048	-0.337	-0.384	no
Treat-40d	RHSIM_RhsimUnG0019600	0.715	0.167	-0.548	no
Treat-40d	RHSIM_RhsimUnG0039700	-0.845	-1.710	-0.865	no
Treat-40d	RHSIM_RhsimUnG0117900	0.507	0.746	0.240	no
Treat-40d	RHSIM_RhsimUnG0021000	0.362	-0.483	-0.845	no
Treat-40d	RHSIM_RhsimUnG0055900	-1.145	-0.093	1.053	low
Treat-40d	RHSIM_RhsimUnG0098000	0.246	-0.111	-0.357	no
Treat-40d	RHSIM_RhsimUnG0194300	-0.385	-0.671	-0.286	no

Group	Gene	PTR (z-score difference)	z(log2 LFQ)	z(log2 FPKM)	extreme-PTR
Treat-40d	RHSIM_RhsimUnG0119800	-0.909	-1.423	-0.514	no
Treat-40d	RHSIM_RhsimUnG0094700	0.990	0.760	-0.230	no
Treat-40d	RHSIM_RhsimUnG0067700	1.199	-1.148	-2.348	high
Treat-40d	RHSIM_RhsimUnG0109200	0.392	-0.189	-0.580	no
Treat-40d	RHSIM_RhsimUnG0107500	0.373	0.304	-0.069	no
Treat-40d	RHSIM_RhsimUnG0105000	0.963	1.309	0.346	no
Treat-40d	RHSIM_RhsimUnG0188600	0.602	-0.853	-1.455	no
Treat-40d	RHSIM_RhsimUnG0006400	1.121	-0.230	-1.350	high
Treat-40d	RHSIM_RhsimPtG0002900	2.721	0.612	-2.109	high
Treat-40d	RHSIM_RhsimUnG0096000	0.514	-1.048	-1.562	no
Treat-40d	RHSIM_RhsimUnG0079300	0.174	-1.102	-1.276	no
Treat-40d	RHSIM_RhsimUnG0021300	-1.627	-1.154	0.473	low
Treat-40d	RHSIM_Rhsim13G0142900	-1.344	-1.558	-0.214	low
Treat-40d	RHSIM_RhsimUnG0045900	2.650	1.426	-1.224	high
Treat-40d	RHSIM_RhsimUnG0139700	0.141	-0.948	-1.090	no
Treat-40d	RHSIM_RhsimUnG0085800	-1.276	-0.603	0.673	low
Treat-40d	RHSIM_RhsimUnG0044200	-1.621	-1.401	0.221	low
Treat-40d	RHSIM_Rhsim13G0005000	0.751	0.707	-0.045	no
Treat-40d	RHSIM_RhsimUnG0021800	1.833	2.124	0.291	high
Treat-40d	RHSIM_Rhsim13G0010100	-1.535	-0.745	0.790	low
Treat-40d	RHSIM_RhsimUnG0022700	0.596	-0.895	-1.491	no
Treat-40d	RHSIM_RhsimUnG0073900	0.695	-0.054	-0.749	no
Treat-40d	RHSIM_RhsimUnG0070500	-0.260	0.249	0.509	no
Treat-40d	RHSIM_Rhsim13G0064900	0.373	0.096	-0.277	no
Treat-40d	RHSIM_Rhsim11G0060300	1.585	-0.032	-1.617	high
Treat-40d	RHSIM_RhsimUnG0027200	0.552	-0.232	-0.783	no
Treat-40d	RHSIM_Rhsim13G0234100	-0.012	-0.497	-0.485	no
Treat-40d	RHSIM_RhsimUnG0060500	-0.308	-0.146	0.162	no
Treat-40d	RHSIM_Rhsim13G0198200	1.130	0.863	-0.267	high
Treat-40d	RHSIM_RhsimUnG0014100	-0.467	-0.886	-0.418	no
Treat-40d	RHSIM_RhsimMtG0006700	1.945	-0.322	-2.267	high
Treat-40d	RHSIM_Rhsim13G0018300	0.057	-0.282	-0.339	no

Group	Gene	PTR (z-score difference)	z(log2 LFQ)	z(log2 FPKM)	extreme-PTR
Treat-40d	RHSIM_RhsimUnG0022900	1.306	0.304	-1.002	high
Treat-40d	RHSIM_Rhsim05G0087400	-0.126	0.501	0.627	no
Treat-40d	RHSIM_RhsimUnG0051400	1.028	1.049	0.021	no
Treat-40d	RHSIM_RhsimUnG0059400	0.125	-0.680	-0.805	no
Treat-40d	RHSIM_RhsimUnG0005600	-0.104	-0.926	-0.822	no
Treat-40d	RHSIM_Rhsim13G0009100	-0.490	1.304	1.794	no
Treat-40d	RHSIM_RhsimPtG0000900	3.686	2.045	-1.641	high
Treat-40d	RHSIM_RhsimUnG0015500	0.635	0.076	-0.559	no
Treat-40d	RHSIM_RhsimUnG0055000	1.205	-0.175	-1.380	high
Treat-40d	RHSIM_Rhsim13G0140400	-0.746	-0.748	-0.002	no
Treat-40d	RHSIM_Rhsim13G0104300	0.411	0.627	0.216	no
Treat-40d	RHSIM_Rhsim13G0149200	0.956	1.042	0.086	no
Treat-40d	RHSIM_Rhsim13G0215900	-0.770	1.402	2.173	no
Treat-40d	RHSIM_Rhsim13G0000400	-0.082	0.333	0.416	no
Treat-40d	RHSIM_Rhsim13G0111800	-1.213	-0.120	1.093	low
Treat-40d	RHSIM_RhsimUnG0008400	0.268	1.625	1.358	no
Treat-40d	RHSIM_Rhsim13G0157800	1.721	1.331	-0.390	high
Treat-40d	RHSIM_RhsimUnG0043200	0.806	2.496	1.691	no
Treat-40d	RHSIM_RhsimUnG0002100	0.095	0.182	0.086	no
Treat-40d	RHSIM_Rhsim13G0110800	0.467	0.600	0.133	no
Treat-40d	RHSIM_Rhsim13G0009200	1.209	-0.207	-1.416	high
Treat-40d	RHSIM_Rhsim13G0150700	0.455	0.264	-0.191	no
Treat-40d	RHSIM_Rhsim13G0112500	1.536	1.461	-0.075	high
Treat-40d	RHSIM_Rhsim13G0088500	-0.378	-0.420	-0.041	no
Treat-40d	RHSIM_Rhsim13G0186600	0.746	0.131	-0.614	no
Treat-40d	RHSIM_Rhsim13G0177500	-2.090	-0.972	1.118	low
Treat-40d	RHSIM_Rhsim13G0205900	0.197	0.269	0.073	no
Treat-40d	RHSIM_Rhsim13G0156100	-0.238	-0.716	-0.477	no
Treat-40d	RHSIM_RhsimUnG0018800	3.002	1.153	-1.849	high
Treat-40d	RHSIM_Rhsim13G0099400	-0.752	-1.722	-0.970	no
Treat-40d	RHSIM_RhsimUnG0023000	-1.472	-1.120	0.352	low
Treat-40d	RHSIM_RhsimUnG0094300	1.104	0.645	-0.458	no

Group	Gene	PTR (z-score difference)	z(log2 LFQ)	z(log2 FPKM)	extreme-PTR
Treat-40d	RHSIM_RhsimUnG0025700	-0.017	-0.162	-0.145	no
Treat-40d	RHSIM_RhsimUnG0254000	1.465	1.433	-0.032	high
Treat-40d	RHSIM_Rhsim13G0077700	-0.336	-0.513	-0.177	no
Treat-40d	RHSIM_Rhsim13G0088800	-0.447	-0.691	-0.243	no
Treat-40d	RHSIM_Rhsim13G0030500	-1.034	-1.394	-0.360	no
Treat-40d	RHSIM_RhsimUnG0252800	-0.369	-0.539	-0.170	no
Treat-40d	RHSIM_Rhsim13G0201300	0.265	0.089	-0.176	no
Treat-40d	RHSIM_Rhsim13G0179600	1.265	-0.476	-1.741	high
Treat-40d	RHSIM_Rhsim13G0140500	-0.195	-0.264	-0.069	no
Treat-40d	RHSIM_Rhsim13G0175200	-0.714	0.618	1.332	no
Treat-40d	RHSIM_Rhsim13G0069400	1.684	1.795	0.112	high
Treat-40d	RHSIM_Rhsim13G0169400	0.382	-0.183	-0.565	no
Treat-40d	RHSIM_Rhsim13G0211800	1.031	0.913	-0.119	no
Treat-40d	RHSIM_RhsimUnG0139600	0.783	-0.044	-0.827	no
Treat-40d	RHSIM_RhsimUnG0004000	1.324	0.376	-0.949	high
Treat-40d	RHSIM_RhsimUnG0248600	0.384	-0.789	-1.173	no
Treat-40d	RHSIM_Rhsim13G0014400	-1.197	-1.457	-0.260	low
Treat-40d	RHSIM_RhsimUnG0006200	0.631	0.892	0.262	no
Treat-40d	RHSIM_Rhsim13G0147900	0.754	0.302	-0.452	no
Treat-40d	RHSIM_Rhsim13G0091700	-1.062	0.834	1.895	no
Treat-40d	RHSIM_RhsimUnG0135600	-0.103	-0.407	-0.303	no
Treat-40d	RHSIM_Rhsim13G0140200	-0.380	-0.087	0.294	no
Treat-40d	RHSIM_Rhsim13G0039100	-0.201	-0.560	-0.359	no
Treat-40d	RHSIM_Rhsim13G0012100	0.413	0.880	0.467	no
Treat-40d	RHSIM_Rhsim13G0162700	1.453	1.931	0.478	high
Treat-40d	RHSIM_Rhsim13G0201600	-1.114	-0.534	0.580	no
Treat-40d	RHSIM_Rhsim13G0170400	-0.866	0.013	0.880	no
Treat-40d	RHSIM_Rhsim13G0218000	-1.929	-1.707	0.222	low
Treat-40d	RHSIM_RhsimUnG0126900	-1.149	-0.891	0.258	low
Treat-40d	RHSIM_Rhsim13G0024600	0.652	0.255	-0.397	no
Treat-40d	RHSIM_Rhsim13G0024200	-2.948	-2.183	0.765	low
Treat-40d	RHSIM_RhsimUnG0077000	2.257	0.355	-1.902	high

Group	Gene	PTR (z-score difference)	z(log2 LFQ)	z(log2 FPKM)	extreme-PTR
Treat-40d	RHSIM_Rhsim13G0122800	0.921	1.964	1.042	no
Treat-40d	RHSIM_Rhsim13G0115500	-0.453	-0.352	0.102	no
Treat-40d	RHSIM_RhsimUnG0120500	-1.792	-1.154	0.637	low
Treat-40d	RHSIM_Rhsim13G0030900	3.300	3.659	0.360	high
Treat-40d	RHSIM_Rhsim13G0202300	-2.658	-1.692	0.966	low
Treat-40d	RHSIM_Rhsim13G0203400	0.940	1.510	0.570	no
Treat-40d	RHSIM_Rhsim13G0233700	-0.754	0.242	0.996	no
Treat-40d	RHSIM_Rhsim13G0057000	-1.039	-0.685	0.354	no
Treat-40d	RHSIM_Rhsim13G0044900	0.278	-0.980	-1.257	no
Treat-40d	RHSIM_Rhsim13G0155800	-1.165	-0.237	0.928	low
Treat-40d	RHSIM_Rhsim13G0060100	-0.587	-0.927	-0.340	no
Treat-40d	RHSIM_Rhsim13G0062700	0.098	-0.325	-0.424	no
Treat-40d	RHSIM_Rhsim13G0140900	0.082	-0.119	-0.202	no
Treat-40d	RHSIM_Rhsim13G0111500	-0.168	1.188	1.356	no
Treat-40d	RHSIM_Rhsim13G0111400	-0.178	1.045	1.222	no
Treat-40d	RHSIM_RhsimUnG0102900	-0.009	-0.473	-0.464	no
Treat-40d	RHSIM_Rhsim13G0208400	-0.724	-0.567	0.157	no
Treat-40d	RHSIM_Rhsim13G0217600	-1.036	-0.192	0.845	no
Treat-40d	RHSIM_Rhsim13G0195100	-1.165	-1.080	0.085	low
Treat-40d	RHSIM_Rhsim13G0216400	0.553	-0.725	-1.278	no
Treat-40d	RHSIM_Rhsim13G0024400	0.285	-0.125	-0.410	no
Treat-40d	RHSIM_Rhsim13G0040400	-1.520	-1.175	0.345	low
Treat-40d	RHSIM_RhsimUnG0095400	-2.928	-1.883	1.044	low
Treat-40d	RHSIM_Rhsim13G0059900	-0.079	0.161	0.241	no
Treat-40d	RHSIM_Rhsim13G0125900	1.228	0.797	-0.431	high
Treat-40d	RHSIM_Rhsim13G0121000	-0.859	-0.564	0.295	no
Treat-40d	RHSIM_Rhsim13G0189200	-3.257	-1.864	1.393	low
Treat-40d	RHSIM_Rhsim13G0013600	1.634	1.907	0.273	high
Treat-40d	RHSIM_Rhsim13G0033100	-1.940	-0.291	1.649	low
Treat-40d	RHSIM_Rhsim13G0071000	-0.078	-0.221	-0.142	no
Treat-40d	RHSIM_RhsimUnG0086000	0.990	0.417	-0.573	no
Treat-40d	RHSIM_Rhsim13G0047200	-1.631	-0.767	0.863	low

Group	Gene	PTR (z-score difference)	z(log2 LFQ)	z(log2 FPKM)	extreme-PTR
Treat-40d	RHSIM_Rhsim13G0067900	-2.754	-0.613	2.141	low
Treat-40d	RHSIM_Rhsim13G0120300	-1.274	-1.257	0.018	low
Treat-40d	RHSIM_Rhsim13G0191600	0.559	0.113	-0.446	no
Treat-40d	RHSIM_Rhsim13G0119400	2.406	3.078	0.671	high
Treat-40d	RHSIM_Rhsim13G0174200	0.461	-0.019	-0.480	no
Treat-40d	RHSIM_Rhsim13G0216600	-1.177	-2.456	-1.279	low
Treat-40d	RHSIM_Rhsim13G0205000	1.073	-0.121	-1.194	no
Treat-40d	RHSIM_Rhsim13G0191100	-1.583	-0.839	0.743	low
Treat-40d	RHSIM_Rhsim13G0146300	-1.704	-0.954	0.750	low
Treat-40d	RHSIM_Rhsim13G0163000	0.488	0.069	-0.419	no
Treat-40d	RHSIM_RhsimUnG0079400	0.255	-0.857	-1.112	no
Treat-40d	RHSIM_Rhsim13G0194200	1.807	1.974	0.167	high
Treat-40d	RHSIM_Rhsim13G0052700	-0.078	-0.071	0.007	no
Treat-40d	RHSIM_Rhsim13G0072700	0.012	0.113	0.101	no
Treat-40d	RHSIM_RhsimUnG0206200	-1.715	-1.783	-0.068	low
Treat-40d	RHSIM_Rhsim13G0094700	-0.263	0.277	0.539	no
Treat-40d	RHSIM_Rhsim13G0201700	-0.243	0.397	0.640	no
Treat-40d	RHSIM_Rhsim13G0056300	-1.022	-1.527	-0.505	no
Treat-40d	RHSIM_RhsimUnG0046000	0.995	-0.299	-1.295	no
Treat-40d	RHSIM_Rhsim13G0169800	1.065	1.092	0.028	no
Treat-40d	RHSIM_Rhsim13G0152000	-1.005	0.996	2.001	no
Treat-40d	RHSIM_Rhsim13G0011700	-0.093	0.923	1.016	no
Treat-40d	RHSIM_Rhsim13G0056400	0.883	0.966	0.083	no
Treat-40d	RHSIM_RhsimUnG0066200	-1.708	-0.648	1.061	low
Treat-40d	RHSIM_Rhsim13G0098900	-1.668	0.675	2.344	low
Treat-40d	RHSIM_RhsimUnG0065400	1.804	2.290	0.485	high
Treat-40d	RHSIM_Rhsim13G0206200	0.951	-0.864	-1.815	no
Treat-40d	RHSIM_Rhsim13G0226700	-0.672	0.036	0.707	no
Treat-40d	RHSIM_Rhsim13G0061400	-0.416	-1.235	-0.819	no
Treat-40d	RHSIM_Rhsim13G0129400	0.516	-0.430	-0.946	no
Treat-40d	RHSIM_Rhsim13G0050600	0.857	-0.238	-1.095	no
Treat-40d	RHSIM_Rhsim13G0020400	-2.065	-1.362	0.704	low

Group	Gene	PTR (z-score difference)	z(log2 LFQ)	z(log2 FPKM)	extreme-PTR
Treat-40d	RHSIM_Rhsim13G0009900	0.141	-1.805	-1.946	no
Treat-40d	RHSIM_Rhsim13G0098700	-0.794	0.754	1.548	no
Treat-40d	RHSIM_Rhsim13G0198700	-0.955	-0.639	0.316	no
Treat-40d	RHSIM_Rhsim13G0057400	-0.520	-0.479	0.041	no
Treat-40d	RHSIM_Rhsim13G0179900	-1.923	-1.306	0.616	low
Treat-40d	RHSIM_Rhsim13G0094300	2.243	0.297	-1.946	high
Treat-40d	RHSIM_Rhsim13G0035600	-0.331	-0.140	0.191	no
Treat-40d	RHSIM_Rhsim13G0234800	0.283	0.663	0.380	no
Treat-40d	RHSIM_Rhsim13G0001000	1.195	0.473	-0.721	high
Treat-40d	RHSIM_Rhsim13G0221000	-0.898	-0.935	-0.037	no
Treat-40d	RHSIM_RhsimUnG0053100	-1.301	-2.244	-0.942	low
Treat-40d	RHSIM_Rhsim13G0107100	-1.165	0.716	1.881	low
Treat-40d	RHSIM_Rhsim13G0024900	0.568	0.454	-0.114	no
Treat-40d	RHSIM_Rhsim13G0045300	0.476	1.573	1.096	no
Treat-40d	RHSIM_Rhsim13G0037500	0.964	0.275	-0.689	no
Treat-40d	RHSIM_RhsimUnG0048500	-0.762	-1.363	-0.601	no
Treat-40d	RHSIM_Rhsim13G0211100	-0.491	-0.062	0.429	no
Treat-40d	RHSIM_Rhsim13G0072000	-0.026	-0.115	-0.089	no
Treat-40d	RHSIM_Rhsim13G0018600	-0.248	0.029	0.278	no
Treat-40d	RHSIM_Rhsim13G0127200	2.590	3.731	1.141	high
Treat-40d	RHSIM_RhsimUnG0023900	1.518	-0.081	-1.599	high
Treat-40d	RHSIM_Rhsim13G0231000	0.495	-0.040	-0.535	no
Treat-40d	RHSIM_Rhsim13G0036500	-2.070	-0.651	1.419	low
Treat-40d	RHSIM_Rhsim13G0031600	0.557	0.533	-0.024	no
Treat-40d	RHSIM_Rhsim13G0065500	1.371	0.960	-0.410	high
Treat-40d	RHSIM_RhsimUnG0021400	-1.028	-0.275	0.753	no
Treat-40d	RHSIM_Rhsim13G0015500	0.735	0.388	-0.347	no
Treat-40d	RHSIM_Rhsim13G0056900	0.135	0.659	0.524	no
Treat-40d	RHSIM_Rhsim13G0115200	0.284	0.624	0.339	no
Treat-40d	RHSIM_RhsimUnG0172300	0.355	-0.174	-0.529	no
Treat-40d	RHSIM_Rhsim13G0209000	-0.474	-1.492	-1.018	no
Treat-40d	RHSIM_Rhsim13G0121400	0.291	0.347	0.056	no

Group	Gene	PTR (z-score difference)	z(log2 LFQ)	z(log2 FPKM)	extreme-PTR
Treat-40d	RHSIM_Rhsim13G0208500	-2.360	-2.088	0.273	low
Treat-40d	RHSIM_Rhsim13G0058000	0.068	0.912	0.844	no
Treat-40d	RHSIM_Rhsim13G0224400	0.904	-0.352	-1.256	no
Treat-40d	RHSIM_Rhsim13G0004800	0.281	-0.417	-0.698	no
Treat-40d	RHSIM_Rhsim13G0221900	0.162	-0.604	-0.765	no
Treat-40d	RHSIM_Rhsim13G0104500	-0.065	-1.093	-1.028	no
Treat-40d	RHSIM_RhsimUnG0033300	0.389	-0.576	-0.965	no
Treat-40d	RHSIM_Rhsim13G0129600	-0.290	-0.461	-0.171	no
Treat-40d	RHSIM_Rhsim13G0098500	0.127	-1.134	-1.261	no
Treat-40d	RHSIM_Rhsim13G0228700	4.171	2.324	-1.847	high
Treat-40d	RHSIM_Rhsim13G0152700	3.660	2.789	-0.871	high
Treat-40d	RHSIM_Rhsim13G0228600	1.816	1.762	-0.054	high
Treat-40d	RHSIM_RhsimUnG0009300	-0.293	0.797	1.090	no
Treat-40d	RHSIM_Rhsim13G0017200	0.425	-0.421	-0.846	no
Treat-40d	RHSIM_Rhsim13G0000200	-0.460	0.320	0.780	no
Treat-40d	RHSIM_Rhsim13G0073600	0.240	-0.530	-0.770	no
Treat-40d	RHSIM_Rhsim13G0209100	-0.866	-1.884	-1.018	no
Treat-40d	RHSIM_Rhsim13G0056100	-1.267	-0.986	0.281	low
Treat-40d	RHSIM_Rhsim13G0056500	0.614	0.513	-0.102	no
Treat-40d	RHSIM_Rhsim13G0109800	-1.482	-2.113	-0.631	low
Treat-40d	RHSIM_RhsimUnG0023200	-0.610	-1.238	-0.627	no
Treat-40d	RHSIM_Rhsim13G0200400	-0.041	-1.026	-0.985	no
Treat-40d	RHSIM_Rhsim13G0127700	1.023	0.327	-0.696	no
Treat-40d	RHSIM_Rhsim13G0230700	0.247	-0.434	-0.681	no
Treat-40d	RHSIM_Rhsim13G0046300	-0.287	0.366	0.653	no
Treat-40d	RHSIM_Rhsim13G0112600	-0.018	-0.378	-0.360	no
Treat-40d	RHSIM_Rhsim13G0193900	-2.242	-1.417	0.825	low
Treat-40d	RHSIM_RhsimUnG0015300	3.157	0.952	-2.206	high
Treat-40d	RHSIM_Rhsim13G0082500	1.000	2.949	1.949	no
Treat-40d	RHSIM_Rhsim13G0200800	-0.466	-0.429	0.037	no
Treat-40d	RHSIM_Rhsim13G0094400	1.399	2.542	1.143	high
Treat-40d	RHSIM_Rhsim13G0084100	-0.102	2.727	2.829	no

Group	Gene	PTR (z-score difference)	z(log2 LFQ)	z(log2 FPKM)	extreme-PTR
Treat-40d	RHSIM_Rhsim13G0011000	-0.549	0.943	1.492	no
Treat-40d	RHSIM_Rhsim13G0137100	-0.262	0.224	0.486	no
Treat-40d	RHSIM_Rhsim13G0104400	0.132	-0.268	-0.400	no
Treat-40d	RHSIM_RhsimUnG0009100	0.675	0.794	0.119	no
Treat-40d	RHSIM_Rhsim13G0065400	-0.552	-0.777	-0.226	no
Treat-40d	RHSIM_RhsimUnG0153300	-1.175	-1.090	0.085	low
Treat-40d	RHSIM_Rhsim13G0088300	0.346	-0.491	-0.837	no
Treat-40d	RHSIM_Rhsim13G0035500	-0.519	-0.239	0.279	no
Treat-40d	RHSIM_Rhsim13G0225600	1.627	3.333	1.707	high
Treat-40d	RHSIM_Rhsim13G0225300	0.212	0.977	0.765	no
Treat-40d	RHSIM_Rhsim13G0210700	-2.128	-0.889	1.239	low
Treat-40d	RHSIM_Rhsim13G0075700	-0.328	1.289	1.616	no
Treat-40d	RHSIM_Rhsim13G0141400	-0.926	-1.148	-0.222	no
Treat-40d	RHSIM_Rhsim13G0021200	0.433	0.315	-0.118	no
Treat-40d	RHSIM_Rhsim13G0163700	0.056	0.394	0.338	no
Treat-40d	RHSIM_RhsimUnG0150500	2.144	1.489	-0.654	high
Treat-40d	RHSIM_Rhsim13G0095400	-0.913	-1.125	-0.212	no
Treat-40d	RHSIM_Rhsim13G0209300	-1.206	-1.129	0.077	low
Treat-40d	RHSIM_Rhsim13G0104000	1.626	1.583	-0.044	high
Treat-40d	RHSIM_Rhsim13G0034500	0.412	-0.482	-0.894	no
Treat-40d	RHSIM_Rhsim13G0117100	-1.312	-0.808	0.504	low
Treat-40d	RHSIM_Rhsim13G0089200	1.400	2.417	1.017	high
Treat-40d	RHSIM_Rhsim13G0232600	-0.739	-1.512	-0.773	no
Treat-40d	RHSIM_Rhsim13G0105300	1.019	0.185	-0.834	no
Treat-40d	RHSIM_Rhsim13G0207700	1.649	1.742	0.093	high
Treat-40d	RHSIM_Rhsim13G0110000	-0.820	0.313	1.133	no
Treat-40d	RHSIM_Rhsim13G0128200	-0.823	-0.660	0.163	no
Treat-40d	RHSIM_Rhsim13G0201400	-2.436	-1.165	1.271	low
Treat-40d	RHSIM_Rhsim13G0153000	1.165	0.873	-0.292	high
Treat-40d	RHSIM_Rhsim13G0203300	-0.066	-0.326	-0.260	no
Treat-40d	RHSIM_Rhsim12G0044300	-0.091	0.254	0.345	no
Treat-40d	RHSIM_Rhsim13G0051800	1.403	-0.141	-1.544	high

Group	Gene	PTR (z-score difference)	z(log2 LFQ)	z(log2 FPKM)	extreme-PTR
Treat-40d	RHSIM_Rhsim13G0196400	-1.779	-1.096	0.683	low
Treat-40d	RHSIM_Rhsim13G0181500	0.055	-0.895	-0.950	no
Treat-40d	RHSIM_Rhsim13G0066200	-0.030	-0.915	-0.885	no
Treat-40d	RHSIM_Rhsim13G0093000	0.527	-0.598	-1.125	no
Treat-40d	RHSIM_Rhsim12G0190000	0.344	0.042	-0.302	no
Treat-40d	RHSIM_Rhsim13G0123900	0.131	2.084	1.953	no
Treat-40d	RHSIM_RhsimUnG0144200	-0.253	1.938	2.192	no
Treat-40d	RHSIM_Rhsim12G0144400	-1.334	-1.006	0.328	low
Treat-40d	RHSIM_Rhsim13G0044300	0.453	-0.248	-0.701	no
Treat-40d	RHSIM_Rhsim13G0018200	-0.077	0.532	0.609	no
Treat-40d	RHSIM_Rhsim12G0188700	-1.016	0.197	1.212	no
Treat-40d	RHSIM_Rhsim13G0099900	-0.587	-0.699	-0.112	no
Treat-40d	RHSIM_Rhsim12G0145400	0.048	0.006	-0.042	no
Treat-40d	RHSIM_Rhsim12G0089400	-1.152	-0.750	0.402	low
Treat-40d	RHSIM_Rhsim13G0064700	1.591	0.435	-1.156	high
Treat-40d	RHSIM_Rhsim13G0002600	-0.721	-0.308	0.412	no
Treat-40d	RHSIM_Rhsim12G0136900	0.012	0.188	0.176	no
Treat-40d	RHSIM_Rhsim13G0102500	2.678	0.572	-2.106	high
Treat-40d	RHSIM_Rhsim13G0225400	-0.425	0.979	1.405	no
Treat-40d	RHSIM_Rhsim13G0018900	-1.190	0.113	1.303	low
Treat-40d	RHSIM_Rhsim13G0191300	1.070	-0.481	-1.551	no
Treat-40d	RHSIM_Rhsim13G0178100	-0.217	-0.503	-0.287	no
Treat-40d	RHSIM_Rhsim13G0031800	-0.678	-1.108	-0.430	no
Treat-40d	RHSIM_Rhsim13G0077300	0.820	1.003	0.183	no
Treat-40d	RHSIM_Rhsim13G0072100	-0.583	-0.516	0.067	no
Treat-40d	RHSIM_Rhsim13G0227500	1.761	0.924	-0.837	high
Treat-40d	RHSIM_Rhsim13G0162300	-0.836	-0.211	0.625	no
Treat-40d	RHSIM_Rhsim13G0134900	0.247	0.069	-0.178	no
Treat-40d	RHSIM_Rhsim13G0067700	-0.515	1.392	1.907	no
Treat-40d	RHSIM_Rhsim12G0057500	0.517	1.888	1.371	no
Treat-40d	RHSIM_RhsimUnG0132500	1.976	-0.272	-2.249	high
Treat-40d	RHSIM_Rhsim13G0229700	-0.830	0.022	0.852	no

Group	Gene	PTR (z-score difference)	z(log2 LFQ)	z(log2 FPKM)	extreme-PTR
Treat-40d	RHSIM_Rhsim13G0170300	0.155	0.009	-0.146	no
Treat-40d	RHSIM_Rhsim13G0002500	0.581	0.184	-0.398	no
Treat-40d	RHSIM_Rhsim13G0077400	0.714	3.460	2.746	no
Treat-40d	RHSIM_Rhsim13G0013500	1.311	0.441	-0.870	high
Treat-40d	RHSIM_Rhsim13G0211600	0.225	-0.597	-0.822	no
Treat-40d	RHSIM_Rhsim13G0102300	0.260	-0.559	-0.819	no
Treat-40d	RHSIM_Rhsim13G0015800	-0.286	-1.170	-0.884	no
Treat-40d	RHSIM_Rhsim13G0023600	0.237	0.386	0.149	no
Treat-40d	RHSIM_Rhsim13G0033000	-0.968	0.198	1.166	no
Treat-40d	RHSIM_Rhsim12G0205600	1.900	-0.458	-2.358	high
Treat-40d	RHSIM_Rhsim13G0198600	-0.232	-0.912	-0.680	no
Treat-40d	RHSIM_Rhsim13G0126100	-0.628	0.700	1.328	no
Treat-40d	RHSIM_Rhsim13G0029500	1.137	-0.861	-1.998	high
Treat-40d	RHSIM_Rhsim13G0216500	0.440	0.327	-0.113	no
Treat-40d	RHSIM_Rhsim13G0115600	0.094	0.313	0.218	no
Treat-40d	RHSIM_Rhsim13G0044100	0.028	-0.687	-0.714	no
Treat-40d	RHSIM_Rhsim13G0073300	-0.486	-0.995	-0.509	no
Treat-40d	RHSIM_RhsimUnG0124700	-1.272	-0.805	0.467	low
Treat-40d	RHSIM_Rhsim13G0234200	-1.045	-0.413	0.632	no
Treat-40d	RHSIM_Rhsim13G0049400	-0.436	0.292	0.728	no
Treat-40d	RHSIM_Rhsim13G0155900	-1.235	-1.408	-0.173	low
Treat-40d	RHSIM_Rhsim13G0065900	-0.256	-0.849	-0.593	no
Treat-40d	RHSIM_Rhsim12G0054400	1.503	0.563	-0.940	high
Treat-40d	RHSIM_Rhsim13G0125300	1.562	-0.303	-1.865	high
Treat-40d	RHSIM_Rhsim13G0013000	-0.558	-0.363	0.195	no
Treat-40d	RHSIM_RhsimUnG0118000	0.123	1.060	0.937	no
Treat-40d	RHSIM_Rhsim13G0150000	-0.270	-0.411	-0.141	no
Treat-40d	RHSIM_Rhsim13G0100500	0.374	0.279	-0.095	no
Treat-40d	RHSIM_Rhsim12G0041400	-0.075	0.270	0.345	no
Treat-40d	RHSIM_Rhsim13G0198800	-0.555	-0.721	-0.166	no
Treat-40d	RHSIM_Rhsim13G0071100	-0.157	0.658	0.815	no
Treat-40d	RHSIM_Rhsim13G0151100	-0.256	0.043	0.299	no

Group	Gene	PTR (z-score difference)	z(log2 LFQ)	z(log2 FPKM)	extreme-PTR
Treat-40d	RHSIM_Rhsim13G0143800	1.467	0.583	-0.884	high
Treat-40d	RHSIM_Rhsim13G0126200	-1.247	-0.749	0.499	low
Treat-40d	RHSIM_Rhsim12G0094700	-0.401	-0.582	-0.181	no
Treat-40d	RHSIM_Rhsim12G0069500	-0.332	-0.746	-0.414	no
Treat-40d	RHSIM_Rhsim13G0228400	-0.204	0.130	0.334	no
Treat-40d	RHSIM_Rhsim13G0127300	-0.785	-1.472	-0.687	no
Treat-40d	RHSIM_Rhsim13G0026200	0.251	1.182	0.931	no
Treat-40d	RHSIM_Rhsim13G0088000	0.634	-0.963	-1.597	no
Treat-40d	RHSIM_Rhsim12G0000600	3.586	4.190	0.605	high
Treat-40d	RHSIM_Rhsim13G0191900	0.240	-0.808	-1.048	no
Treat-40d	RHSIM_Rhsim13G0065600	1.839	1.305	-0.534	high
Treat-40d	RHSIM_Rhsim13G0180600	0.280	-0.294	-0.575	no
Treat-40d	RHSIM_Rhsim12G0124700	0.509	-0.142	-0.651	no
Treat-40d	RHSIM_Rhsim12G0173700	0.438	1.730	1.292	no
Treat-40d	RHSIM_Rhsim12G0061300	-0.433	-0.448	-0.015	no
Treat-40d	RHSIM_Rhsim13G0224500	-0.053	-0.750	-0.698	no
Treat-40d	RHSIM_Rhsim13G0199800	0.928	-0.007	-0.935	no
Treat-40d	RHSIM_Rhsim13G0076400	-0.145	-0.266	-0.120	no
Treat-40d	RHSIM_Rhsim13G0063200	-2.922	-0.734	2.188	low
Treat-40d	RHSIM_Rhsim13G0202500	1.707	0.082	-1.624	high
Treat-40d	RHSIM_Rhsim12G0204500	-2.063	-0.443	1.620	low
Treat-40d	RHSIM_Rhsim13G0235300	0.406	0.623	0.217	no
Treat-40d	RHSIM_Rhsim13G0085200	1.540	3.061	1.521	high
Treat-40d	RHSIM_Rhsim12G0195700	-0.005	1.100	1.105	no
Treat-40d	RHSIM_Rhsim12G0197300	0.487	0.555	0.068	no
Treat-40d	RHSIM_Rhsim12G0130500	-0.299	-0.339	-0.040	no
Treat-40d	RHSIM_Rhsim13G0058700	-0.936	-1.884	-0.948	no
Treat-40d	RHSIM_Rhsim12G0210700	2.285	1.321	-0.964	high
Treat-40d	RHSIM_Rhsim13G0121900	-0.572	-1.373	-0.801	no
Treat-40d	RHSIM_Rhsim12G0178300	1.375	0.535	-0.840	high
Treat-40d	RHSIM_Rhsim13G0000600	-2.498	-1.954	0.545	low
Treat-40d	RHSIM_Rhsim13G0128900	-0.680	-1.047	-0.368	no

Group	Gene	PTR (z-score difference)	z(log2 LFQ)	z(log2 FPKM)	extreme-PTR
Treat-40d	RHSIM_RhsimUnG0093000	-0.431	-0.443	-0.012	no
Treat-40d	RHSIM_Rhsim12G0182600	-0.215	0.074	0.289	no
Treat-40d	RHSIM_Rhsim13G0152200	1.875	0.434	-1.442	high
Treat-40d	RHSIM_Rhsim12G0127000	1.240	0.100	-1.140	high
Treat-40d	RHSIM_Rhsim13G0138500	0.007	0.800	0.793	no
Treat-40d	RHSIM_Rhsim13G0097900	0.943	-0.854	-1.796	no
Treat-40d	RHSIM_Rhsim12G0143900	0.528	-0.118	-0.646	no
Treat-40d	RHSIM_Rhsim13G0154700	0.289	0.001	-0.288	no
Treat-40d	RHSIM_RhsimUnG0089100	0.049	0.469	0.421	no
Treat-40d	RHSIM_Rhsim12G0035800	0.066	0.016	-0.050	no
Treat-40d	RHSIM_Rhsim13G0017300	0.667	-0.810	-1.477	no
Treat-40d	RHSIM_Rhsim12G0047600	-0.055	0.647	0.702	no
Treat-40d	RHSIM_RhsimUnG0088100	0.521	-1.471	-1.992	no
Treat-40d	RHSIM_Rhsim13G0200500	0.328	-0.072	-0.400	no
Treat-40d	RHSIM_Rhsim13G0016000	-0.367	-0.385	-0.018	no
Treat-40d	RHSIM_Rhsim13G0199600	0.625	0.206	-0.419	no
Treat-40d	RHSIM_Rhsim12G0112500	-0.298	-0.835	-0.537	no
Treat-40d	RHSIM_Rhsim13G0008000	1.792	1.120	-0.671	high
Treat-40d	RHSIM_Rhsim13G0197500	-1.561	0.315	1.876	low
Treat-40d	RHSIM_Rhsim13G0074100	-1.007	0.265	1.272	no
Treat-40d	RHSIM_Rhsim12G0077600	0.281	0.053	-0.228	no
Treat-40d	RHSIM_Rhsim12G0116500	-0.291	-0.370	-0.079	no
Treat-40d	RHSIM_Rhsim13G0040100	-3.567	-1.549	2.018	low
Treat-40d	RHSIM_Rhsim13G0075400	1.032	0.253	-0.779	no
Treat-40d	RHSIM_Rhsim12G0207400	0.181	-0.412	-0.594	no
Treat-40d	RHSIM_Rhsim12G0135500	-1.042	-0.874	0.169	no
Treat-40d	RHSIM_Rhsim12G0035400	2.098	0.170	-1.928	high
Treat-40d	RHSIM_Rhsim13G0037600	1.273	-0.543	-1.816	high
Treat-40d	RHSIM_Rhsim13G0173900	-0.810	-0.563	0.247	no
Treat-40d	RHSIM_Rhsim13G0102400	-0.071	-0.190	-0.119	no
Treat-40d	RHSIM_Rhsim12G0130100	-0.397	0.021	0.418	no
Treat-40d	RHSIM_Rhsim12G0113000	0.575	0.801	0.226	no

Group	Gene	PTR (z-score difference)	z(log2 LFQ)	z(log2 FPKM)	extreme-PTR
Treat-40d	RHSIM_Rhsim12G0013800	-0.246	0.274	0.520	no
Treat-40d	RHSIM_Rhsim13G0223300	1.311	0.897	-0.413	high
Treat-40d	RHSIM_Rhsim13G0223000	0.755	2.593	1.838	no
Treat-40d	RHSIM_Rhsim13G0154600	-1.519	-1.248	0.271	low
Treat-40d	RHSIM_RhsimUnG0067300	1.208	-1.154	-2.362	high
Treat-40d	RHSIM_Rhsim12G0212100	0.620	0.027	-0.592	no
Treat-40d	RHSIM_Rhsim13G0012200	0.263	0.642	0.379	no
Treat-40d	RHSIM_Rhsim12G0136200	-1.767	-1.588	0.179	low
Treat-40d	RHSIM_Rhsim12G0143400	-0.882	0.920	1.801	no
Treat-40d	RHSIM_Rhsim13G0076300	-1.270	-0.315	0.954	low
Treat-40d	RHSIM_Rhsim13G0029400	-0.207	-0.129	0.078	no
Treat-40d	RHSIM_Rhsim13G0149600	0.645	2.512	1.867	no
Treat-40d	RHSIM_Rhsim12G0021800	0.268	0.058	-0.211	no
Treat-40d	RHSIM_Rhsim12G0133000	-0.804	-0.845	-0.042	no
Treat-40d	RHSIM_Rhsim13G0224600	-0.103	-0.261	-0.157	no
Treat-40d	RHSIM_Rhsim12G0149400	2.069	1.589	-0.480	high
Treat-40d	RHSIM_Rhsim13G0070200	0.106	-1.588	-1.694	no
Treat-40d	RHSIM_Rhsim12G0076200	-0.481	-0.548	-0.067	no
Treat-40d	RHSIM_Rhsim12G0157800	-0.327	0.548	0.875	no
Treat-40d	RHSIM_Rhsim13G0023900	-0.151	0.964	1.115	no
Treat-40d	RHSIM_Rhsim12G0116400	-3.450	0.430	3.880	low
Treat-40d	RHSIM_Rhsim13G0116000	0.555	1.216	0.661	no
Treat-40d	RHSIM_Rhsim13G0119800	-0.954	-1.324	-0.370	no
Treat-40d	RHSIM_Rhsim12G0163200	0.054	-0.360	-0.414	no
Treat-40d	RHSIM_Rhsim12G0193800	0.186	-0.426	-0.612	no
Treat-40d	RHSIM_Rhsim12G0214100	-0.910	-0.551	0.358	no
Treat-40d	RHSIM_Rhsim12G0015600	1.715	3.749	2.035	high
Treat-40d	RHSIM_Rhsim13G0124300	-1.858	0.241	2.099	low
Treat-40d	RHSIM_Rhsim12G0186700	-0.794	-0.783	0.011	no
Treat-40d	RHSIM_Rhsim12G0134700	-1.457	-0.801	0.656	low
Treat-40d	RHSIM_Rhsim13G0198500	0.954	1.523	0.569	no
Treat-40d	RHSIM_Rhsim12G0026000	-0.235	-0.015	0.220	no

Group	Gene	PTR (z-score difference)	z(log2 LFQ)	z(log2 FPKM)	extreme-PTR
Treat-40d	RHSIM_Rhsim12G0078900	-0.481	-0.302	0.179	no
Treat-40d	RHSIM_Rhsim13G0037700	0.854	-0.020	-0.875	no
Treat-40d	RHSIM_Rhsim12G0140200	0.097	0.642	0.545	no
Treat-40d	RHSIM_Rhsim12G0137600	-0.034	0.022	0.056	no
Treat-40d	RHSIM_Rhsim13G0039600	0.814	-0.365	-1.178	no
Treat-40d	RHSIM_Rhsim12G0108100	-1.305	-0.987	0.318	low
Treat-40d	RHSIM_Rhsim12G0009900	0.365	-0.242	-0.607	no
Treat-40d	RHSIM_Rhsim13G0095800	-0.471	-0.757	-0.286	no
Treat-40d	RHSIM_Rhsim13G0119500	0.291	0.217	-0.074	no
Treat-40d	RHSIM_Rhsim13G0031400	0.280	-0.010	-0.290	no
Treat-40d	RHSIM_Rhsim13G0017500	0.922	-1.354	-2.275	no
Treat-40d	RHSIM_Rhsim12G0195800	0.268	0.392	0.125	no
Treat-40d	RHSIM_Rhsim12G0152200	0.334	-0.465	-0.800	no
Treat-40d	RHSIM_Rhsim12G0170600	-1.369	0.583	1.952	low
Treat-40d	RHSIM_Rhsim13G0210400	1.465	0.885	-0.581	high
Treat-40d	RHSIM_Rhsim13G0044700	0.840	-0.786	-1.625	no
Treat-40d	RHSIM_Rhsim12G0165900	-0.451	0.186	0.637	no
Treat-40d	RHSIM_Rhsim12G0208900	-0.288	-0.550	-0.262	no
Treat-40d	RHSIM_Rhsim13G0222300	-0.436	-0.998	-0.562	no
Treat-40d	RHSIM_Rhsim13G0077600	0.854	1.140	0.285	no
Treat-40d	RHSIM_Rhsim12G0085200	-0.497	-1.363	-0.866	no
Treat-40d	RHSIM_Rhsim12G0150200	-1.551	-2.108	-0.558	low
Treat-40d	RHSIM_Rhsim12G0152500	0.171	0.145	-0.026	no
Treat-40d	RHSIM_Rhsim13G0233300	1.807	1.530	-0.277	high
Treat-40d	RHSIM_Rhsim12G0182800	1.552	1.772	0.220	high
Treat-40d	RHSIM_Rhsim12G0195100	-0.554	-0.592	-0.039	no
Treat-40d	RHSIM_Rhsim12G0169500	1.491	1.245	-0.246	high
Treat-40d	RHSIM_Rhsim12G0171400	-1.380	-1.397	-0.018	low
Treat-40d	RHSIM_Rhsim12G0109000	3.246	1.199	-2.047	high
Treat-40d	RHSIM_Rhsim13G0089100	-0.176	1.201	1.376	no
Treat-40d	RHSIM_Rhsim12G0041100	-0.247	-0.422	-0.175	no
Treat-40d	RHSIM_Rhsim12G0180700	0.337	0.581	0.245	no

Group	Gene	PTR (z-score difference)	z(log2 LFQ)	z(log2 FPKM)	extreme-PTR
Treat-40d	RHSIM_Rhsim12G0155700	2.900	3.194	0.294	high
Treat-40d	RHSIM_Rhsim12G0140600	1.081	0.804	-0.277	no
Treat-40d	RHSIM_Rhsim12G0195900	-1.455	-1.431	0.024	low
Treat-40d	RHSIM_Rhsim12G0015500	-1.737	0.365	2.102	low
Treat-40d	RHSIM_Rhsim12G0090000	-0.850	0.277	1.127	no
Treat-40d	RHSIM_Rhsim12G0182500	-0.322	-0.998	-0.676	no
Treat-40d	RHSIM_Rhsim12G0169800	-0.342	0.465	0.807	no
Treat-40d	RHSIM_Rhsim12G0074100	0.038	-0.253	-0.291	no
Treat-40d	RHSIM_Rhsim12G0190900	0.573	-0.603	-1.176	no
Treat-40d	RHSIM_Rhsim12G0124000	-0.906	-1.874	-0.968	no
Treat-40d	RHSIM_Rhsim13G0230600	0.420	0.137	-0.283	no
Treat-40d	RHSIM_Rhsim12G0168400	-0.644	-0.474	0.170	no
Treat-40d	RHSIM_Rhsim12G0208200	-2.422	-0.493	1.929	low
Treat-40d	RHSIM_Rhsim12G0167500	-0.933	0.392	1.326	no
Treat-40d	RHSIM_Rhsim12G0209100	-0.210	-0.452	-0.242	no
Treat-40d	RHSIM_Rhsim12G0132700	1.450	0.304	-1.147	high
Treat-40d	RHSIM_Rhsim12G0175800	0.450	1.405	0.954	no
Treat-40d	RHSIM_Rhsim12G0163900	1.496	0.402	-1.093	high
Treat-40d	RHSIM_Rhsim12G0149700	0.870	-0.969	-1.840	no
Treat-40d	RHSIM_Rhsim12G0212300	0.666	-0.495	-1.162	no
Treat-40d	RHSIM_Rhsim12G0197400	-0.565	1.200	1.765	no
Treat-40d	RHSIM_Rhsim12G0029400	-0.316	-1.172	-0.857	no
Treat-40d	RHSIM_Rhsim12G0076500	-0.583	-0.740	-0.157	no
Treat-40d	RHSIM_Rhsim12G0127200	-1.449	-1.327	0.121	low
Treat-40d	RHSIM_Rhsim13G0064000	-0.152	-0.499	-0.347	no
Treat-40d	RHSIM_Rhsim13G0071700	1.599	1.363	-0.236	high
Treat-40d	RHSIM_Rhsim12G0137100	-2.435	-0.630	1.805	low
Treat-40d	RHSIM_Rhsim12G0003100	-1.096	-0.079	1.017	no
Treat-40d	RHSIM_Rhsim12G0003800	-0.304	-0.248	0.056	no
Treat-40d	RHSIM_Rhsim12G0116900	1.372	0.302	-1.070	high
Treat-40d	RHSIM_Rhsim12G0013200	0.627	-1.728	-2.355	no
Treat-40d	RHSIM_Rhsim12G0099000	0.102	-0.043	-0.144	no

Group	Gene	PTR (z-score difference)	z(log2 LFQ)	z(log2 FPKM)	extreme-PTR
Treat-40d	RHSIM_Rhsim12G0013400	0.206	1.511	1.304	no
Treat-40d	RHSIM_Rhsim12G0000200	0.752	-0.491	-1.243	no
Treat-40d	RHSIM_Rhsim12G0017500	-0.922	-1.109	-0.187	no
Treat-40d	RHSIM_RhsimUnG0019800	-0.863	-0.335	0.529	no
Treat-40d	RHSIM_Rhsim12G0208000	2.056	2.048	-0.009	high
Treat-40d	RHSIM_Rhsim13G0117600	0.014	-0.530	-0.544	no
Treat-40d	RHSIM_Rhsim12G0190700	-0.090	0.142	0.232	no
Treat-40d	RHSIM_Rhsim12G0003400	0.606	0.643	0.038	no
Treat-40d	RHSIM_Rhsim12G0053900	-2.690	-1.428	1.262	low
Treat-40d	RHSIM_RhsimUnG0020300	0.420	0.334	-0.086	no
Treat-40d	RHSIM_Rhsim13G0102900	-0.685	-0.820	-0.135	no
Treat-40d	RHSIM_Rhsim12G0192900	-2.685	-0.497	2.188	low
Treat-40d	RHSIM_Rhsim12G0151800	-0.122	0.597	0.719	no
Treat-40d	RHSIM_Rhsim13G0204000	-0.023	-0.480	-0.457	no
Treat-40d	RHSIM_Rhsim12G0129700	0.032	-0.942	-0.974	no
Treat-40d	RHSIM_Rhsim12G0144700	-0.367	-0.073	0.295	no
Treat-40d	RHSIM_Rhsim12G0164400	-0.511	-0.886	-0.375	no
Treat-40d	RHSIM_Rhsim12G0106200	1.947	3.382	1.435	high
Treat-40d	RHSIM_Rhsim13G0136300	1.562	1.015	-0.547	high
Treat-40d	RHSIM_Rhsim12G0194200	-0.734	0.833	1.567	no
Treat-40d	RHSIM_Rhsim12G0004500	-0.047	-0.744	-0.697	no
Treat-40d	RHSIM_Rhsim12G0137400	0.294	-0.406	-0.700	no
Treat-40d	RHSIM_Rhsim12G0074200	-0.785	-1.144	-0.358	no
Treat-40d	RHSIM_Rhsim12G0107100	0.233	1.050	0.817	no
Treat-40d	RHSIM_Rhsim12G0087800	-0.006	0.641	0.647	no
Treat-40d	RHSIM_Rhsim12G0043300	0.318	0.246	-0.072	no
Treat-40d	RHSIM_Rhsim12G0138800	1.039	0.176	-0.864	no
Treat-40d	RHSIM_Rhsim12G0191200	0.807	0.987	0.180	no
Treat-40d	RHSIM_Rhsim12G0099400	-1.997	-1.517	0.480	low
Treat-40d	RHSIM_Rhsim12G0146800	-0.370	-0.173	0.196	no
Treat-40d	RHSIM_Rhsim13G0057500	-1.800	0.459	2.259	low
Treat-40d	RHSIM_Rhsim12G0071500	-0.899	-1.671	-0.772	no

Group	Gene	PTR (z-score difference)	z(log2 LFQ)	z(log2 FPKM)	extreme-PTR
Treat-40d	RHSIM_Rhsim12G0013500	2.558	4.165	1.607	high
Treat-40d	RHSIM_Rhsim12G0116700	1.078	0.648	-0.430	no
Treat-40d	RHSIM_Rhsim13G0228300	0.485	0.241	-0.244	no
Treat-40d	RHSIM_Rhsim12G0063600	0.980	-0.051	-1.031	no
Treat-40d	RHSIM_Rhsim12G0167900	-2.398	-0.589	1.809	low
Treat-40d	RHSIM_Rhsim12G0071800	-0.644	-1.023	-0.379	no
Treat-40d	RHSIM_Rhsim12G0047500	0.323	0.790	0.467	no
Treat-40d	RHSIM_Rhsim12G0124800	-1.750	0.123	1.873	low
Treat-40d	RHSIM_Rhsim12G0046600	-0.676	0.026	0.702	no
Treat-40d	RHSIM_Rhsim11G0178300	-1.172	-1.142	0.030	low
Treat-40d	RHSIM_Rhsim12G0189200	-0.261	0.010	0.271	no
Treat-40d	RHSIM_Rhsim12G0182200	-0.045	-0.836	-0.791	no
Treat-40d	RHSIM_Rhsim12G0190600	1.488	-0.020	-1.508	high
Treat-40d	RHSIM_Rhsim13G0131000	-1.071	-0.129	0.941	no
Treat-40d	RHSIM_Rhsim11G0139400	0.485	0.335	-0.149	no
Treat-40d	RHSIM_Rhsim12G0139100	-0.997	-0.139	0.857	no
Treat-40d	RHSIM_Rhsim12G0127600	-0.550	0.798	1.348	no
Treat-40d	RHSIM_RhsimMtG0003200	3.295	1.161	-2.134	high
Treat-40d	RHSIM_Rhsim12G0193000	0.686	-0.135	-0.821	no
Treat-40d	RHSIM_Rhsim12G0058100	-1.340	0.358	1.698	low
Treat-40d	RHSIM_Rhsim13G0116200	1.390	0.041	-1.349	high
Treat-40d	RHSIM_Rhsim12G0004800	1.889	1.190	-0.699	high
Treat-40d	RHSIM_Rhsim12G0179700	0.654	-0.489	-1.143	no
Treat-40d	RHSIM_Rhsim13G0166600	-0.415	-0.133	0.283	no
Treat-40d	RHSIM_Rhsim12G0197200	-0.124	0.214	0.338	no
Treat-40d	RHSIM_Rhsim12G0137300	-2.770	-1.730	1.039	low
Treat-40d	RHSIM_Rhsim12G0126700	0.702	-0.080	-0.782	no
Treat-40d	RHSIM_Rhsim12G0025400	0.470	0.080	-0.390	no
Treat-40d	RHSIM_Rhsim12G0028200	-1.468	-1.290	0.178	low
Treat-40d	RHSIM_Rhsim12G0204700	0.160	0.746	0.586	no
Treat-40d	RHSIM_Rhsim13G0047800	-0.999	-0.907	0.092	no
Treat-40d	RHSIM_Rhsim12G0199300	0.302	0.957	0.655	no

Group	Gene	PTR (z-score difference)	z(log2 LFQ)	z(log2 FPKM)	extreme-PTR
Treat-40d	RHSIM_Rhsim12G0214300	0.063	0.105	0.042	no
Treat-40d	RHSIM_Rhsim13G0039000	-2.845	-1.579	1.265	low
Treat-40d	RHSIM_Rhsim12G0162500	-0.820	-1.498	-0.678	no
Treat-40d	RHSIM_Rhsim11G0006900	-1.132	-1.434	-0.302	no
Treat-40d	RHSIM_Rhsim12G0053200	2.502	0.469	-2.033	high
Treat-40d	RHSIM_Rhsim12G0191400	-0.508	0.464	0.972	no
Treat-40d	RHSIM_Rhsim13G0098600	0.526	1.815	1.290	no
Treat-40d	RHSIM_Rhsim12G0193700	1.457	0.824	-0.633	high
Treat-40d	RHSIM_Rhsim12G0109300	-1.047	-0.568	0.479	no
Treat-40d	RHSIM_Rhsim12G0121200	-0.542	-0.494	0.048	no
Treat-40d	RHSIM_Rhsim13G0042200	-0.528	0.987	1.515	no
Treat-40d	RHSIM_Rhsim12G0213600	1.819	2.524	0.704	high
Treat-40d	RHSIM_Rhsim13G0138000	1.315	0.929	-0.386	high
Treat-40d	RHSIM_Rhsim13G0234000	-0.921	-0.321	0.600	no
Treat-40d	RHSIM_Rhsim13G0074400	0.815	0.290	-0.525	no
Treat-40d	RHSIM_Rhsim13G0100300	-0.411	-0.980	-0.569	no
Treat-40d	RHSIM_Rhsim12G0144100	1.017	0.765	-0.251	no
Treat-40d	RHSIM_Rhsim12G0070500	2.445	0.826	-1.619	high
Treat-40d	RHSIM_Rhsim12G0185700	-1.631	-0.987	0.644	low
Treat-40d	RHSIM_Rhsim12G0104600	1.670	0.248	-1.422	high
Treat-40d	RHSIM_Rhsim12G0075100	1.162	-1.188	-2.350	high
Treat-40d	RHSIM_Rhsim13G0081400	-0.377	-1.225	-0.848	no
Treat-40d	RHSIM_Rhsim13G0045200	0.276	0.212	-0.064	no
Treat-40d	RHSIM_Rhsim12G0206900	-0.649	-0.060	0.589	no
Treat-40d	RHSIM_Rhsim12G0144000	-1.663	-0.230	1.434	low
Treat-40d	RHSIM_Rhsim12G0004100	1.207	0.399	-0.808	high
Treat-40d	RHSIM_Rhsim13G0199500	-1.021	-0.175	0.846	no
Treat-40d	RHSIM_Rhsim11G0191500	-1.819	0.996	2.815	low
Treat-40d	RHSIM_Rhsim12G0055000	-1.907	-0.541	1.366	low
Treat-40d	RHSIM_Rhsim13G0096300	-0.037	-0.618	-0.581	no
Treat-40d	RHSIM_Rhsim13G0050700	-0.635	1.361	1.996	no
Treat-40d	RHSIM_Rhsim13G0194300	0.107	1.559	1.452	no

Group	Gene	PTR (z-score difference)	z(log2 LFQ)	z(log2 FPKM)	extreme-PTR
Treat-40d	RHSIM_Rhsim12G0027600	-0.202	0.542	0.744	no
Treat-40d	RHSIM_Rhsim12G0087900	0.493	1.085	0.592	no
Treat-40d	RHSIM_Rhsim11G0052100	1.005	0.565	-0.440	no
Treat-40d	RHSIM_Rhsim13G0026100	0.406	-0.446	-0.852	no
Treat-40d	RHSIM_Rhsim12G0048100	0.504	0.703	0.199	no
Treat-40d	RHSIM_Rhsim12G0002800	0.648	0.033	-0.615	no
Treat-40d	RHSIM_Rhsim12G0038100	-0.587	-1.004	-0.418	no
Treat-40d	RHSIM_Rhsim12G0168200	-0.028	-0.745	-0.717	no
Treat-40d	RHSIM_Rhsim12G0148300	-0.758	0.915	1.673	no
Treat-40d	RHSIM_Rhsim12G0177900	0.180	1.115	0.934	no
Treat-40d	RHSIM_Rhsim13G0174400	2.069	0.203	-1.867	high
Treat-40d	RHSIM_Rhsim12G0138000	0.419	0.066	-0.353	no
Treat-40d	RHSIM_Rhsim12G0093200	3.168	0.979	-2.190	high
Treat-40d	RHSIM_Rhsim12G0025300	-1.695	-0.984	0.710	low
Treat-40d	RHSIM_Rhsim12G0113100	-1.124	-0.698	0.426	no
Treat-40d	RHSIM_Rhsim13G0148600	-1.684	-0.186	1.498	low
Treat-40d	RHSIM_Rhsim12G0203300	0.062	-1.101	-1.162	no
Treat-40d	RHSIM_Rhsim12G0181400	1.714	-0.634	-2.348	high
Treat-40d	RHSIM_Rhsim13G0163200	0.737	-0.210	-0.947	no
Treat-40d	RHSIM_Rhsim12G0126600	0.567	-0.781	-1.348	no
Treat-40d	RHSIM_Rhsim12G0205400	-0.436	-0.452	-0.016	no
Treat-40d	RHSIM_Rhsim12G0006200	0.105	1.410	1.305	no
Treat-40d	RHSIM_Rhsim12G0165000	-0.301	-0.663	-0.362	no
Treat-40d	RHSIM_Rhsim12G0006800	-0.151	-0.821	-0.670	no
Treat-40d	RHSIM_Rhsim12G0172100	-0.612	-0.569	0.043	no
Treat-40d	RHSIM_Rhsim12G0110200	0.818	0.243	-0.575	no
Treat-40d	RHSIM_Rhsim12G0048500	-0.995	-0.694	0.301	no
Treat-40d	RHSIM_Rhsim12G0137200	1.101	0.540	-0.561	no
Treat-40d	RHSIM_Rhsim12G0161200	0.061	0.385	0.324	no
Treat-40d	RHSIM_Rhsim12G0003000	-0.199	0.296	0.496	no
Treat-40d	RHSIM_Rhsim11G0167100	2.160	1.367	-0.793	high
Treat-40d	RHSIM_Rhsim12G0052000	0.863	1.644	0.781	no

Group	Gene	PTR (z-score difference)	z(log2 LFQ)	z(log2 FPKM)	extreme-PTR
Treat-40d	RHSIM_Rhsim12G0198100	-0.391	-0.925	-0.534	no
Treat-40d	RHSIM_Rhsim12G0147400	-0.025	0.197	0.222	no
Treat-40d	RHSIM_Rhsim11G0124300	-0.785	0.182	0.967	no
Treat-40d	RHSIM_Rhsim12G0115900	0.542	1.774	1.233	no
Treat-40d	RHSIM_Rhsim12G0192800	0.677	-0.882	-1.559	no
Treat-40d	RHSIM_Rhsim13G0126800	-0.330	-0.563	-0.233	no
Treat-40d	RHSIM_Rhsim12G0171000	1.003	-0.122	-1.125	no
Treat-40d	RHSIM_Rhsim11G0045800	-1.414	0.064	1.477	low
Treat-40d	RHSIM_Rhsim12G0192300	0.677	1.204	0.526	no
Treat-40d	RHSIM_Rhsim11G0195600	-2.034	-0.567	1.468	low
Treat-40d	RHSIM_Rhsim12G0102400	0.086	1.250	1.164	no
Treat-40d	RHSIM_Rhsim11G0129600	-0.636	0.522	1.159	no
Treat-40d	RHSIM_Rhsim12G0133400	0.107	2.155	2.049	no
Treat-40d	RHSIM_Rhsim12G0043200	-0.906	0.415	1.321	no
Treat-40d	RHSIM_Rhsim12G0153900	0.028	0.632	0.604	no
Treat-40d	RHSIM_Rhsim12G0015200	-0.623	0.066	0.689	no
Treat-40d	RHSIM_Rhsim11G0167300	2.493	1.416	-1.077	high
Treat-40d	RHSIM_Rhsim11G0127100	-0.571	-0.018	0.554	no
Treat-40d	RHSIM_Rhsim12G0138500	0.511	1.408	0.897	no
Treat-40d	RHSIM_Rhsim11G0100300	-0.826	1.213	2.039	no
Treat-40d	RHSIM_Rhsim11G0188300	-1.535	-0.733	0.802	low
Treat-40d	RHSIM_Rhsim12G0099800	1.050	2.178	1.128	no
Treat-40d	RHSIM_Rhsim12G0135900	0.036	-0.304	-0.340	no
Treat-40d	RHSIM_Rhsim11G0090600	1.233	-0.458	-1.692	high
Treat-40d	RHSIM_Rhsim11G0132400	-1.982	-1.142	0.840	low
Treat-40d	RHSIM_Rhsim12G0047700	-0.435	0.659	1.094	no
Treat-40d	RHSIM_Rhsim11G0129000	-0.670	1.812	2.481	no
Treat-40d	RHSIM_Rhsim12G0214500	-0.412	-1.345	-0.934	no
Treat-40d	RHSIM_Rhsim11G0034700	1.075	1.168	0.093	no
Treat-40d	RHSIM_Rhsim11G0172800	2.637	1.415	-1.222	high
Treat-40d	RHSIM_Rhsim12G0017000	1.345	0.477	-0.867	high
Treat-40d	RHSIM_Rhsim12G0152700	0.061	-1.336	-1.397	no

Group	Gene	PTR (z-score difference)	z(log2 LFQ)	z(log2 FPKM)	extreme-PTR
Treat-40d	RHSIM_Rhsim12G0054500	0.031	1.538	1.508	no
Treat-40d	RHSIM_Rhsim12G0183500	0.984	1.803	0.819	no
Treat-40d	RHSIM_Rhsim11G0011600	0.003	0.542	0.539	no
Treat-40d	RHSIM_Rhsim13G0178500	-1.274	-1.129	0.144	low
Treat-40d	RHSIM_Rhsim12G0168600	-0.157	-0.208	-0.051	no
Treat-40d	RHSIM_Rhsim12G0065100	-1.057	-0.407	0.650	no
Treat-40d	RHSIM_Rhsim11G0129800	-1.526	-0.752	0.774	low
Treat-40d	RHSIM_Rhsim11G0092300	-0.524	-0.851	-0.327	no
Treat-40d	RHSIM_Rhsim12G0132900	0.407	-0.134	-0.541	no
Treat-40d	RHSIM_Rhsim13G0080100	-0.405	-0.220	0.185	no
Treat-40d	RHSIM_Rhsim12G0063100	0.243	0.037	-0.206	no
Treat-40d	RHSIM_Rhsim12G0188200	-2.765	-1.519	1.246	low
Treat-40d	RHSIM_Rhsim12G0050400	0.053	-1.133	-1.186	no
Treat-40d	RHSIM_Rhsim11G0076700	-1.815	0.140	1.955	low
Treat-40d	RHSIM_Rhsim12G0166300	-0.495	0.158	0.653	no
Treat-40d	RHSIM_Rhsim11G0025100	-0.991	-0.554	0.437	no
Treat-40d	RHSIM_Rhsim12G0124500	-0.717	-1.343	-0.626	no
Treat-40d	RHSIM_Rhsim12G0207300	0.889	0.534	-0.355	no
Treat-40d	RHSIM_Rhsim11G0101800	0.942	-0.981	-1.923	no
Treat-40d	RHSIM_Rhsim12G0134100	-0.426	-0.934	-0.508	no
Treat-40d	RHSIM_Rhsim12G0197600	1.387	0.429	-0.959	high
Treat-40d	RHSIM_Rhsim11G0114600	1.384	-0.119	-1.502	high
Treat-40d	RHSIM_Rhsim12G0164700	-0.121	0.267	0.389	no
Treat-40d	RHSIM_Rhsim12G0089200	-0.459	-1.134	-0.676	no
Treat-40d	RHSIM_Rhsim12G0064700	-0.740	-0.543	0.197	no
Treat-40d	RHSIM_Rhsim11G0093300	-0.739	0.093	0.832	no
Treat-40d	RHSIM_Rhsim12G0042000	0.572	-0.347	-0.919	no
Treat-40d	RHSIM_Rhsim12G0171100	1.403	1.520	0.116	high
Treat-40d	RHSIM_Rhsim11G0034400	0.049	-0.011	-0.060	no
Treat-40d	RHSIM_Rhsim12G0182900	0.802	-0.066	-0.867	no
Treat-40d	RHSIM_Rhsim13G0038100	-0.679	-0.231	0.449	no
Treat-40d	RHSIM_Rhsim11G0188100	-0.059	2.146	2.204	no

Group	Gene	PTR (z-score difference)	z(log2 LFQ)	z(log2 FPKM)	extreme-PTR
Treat-40d	RHSIM_Rhsim12G0096200	-0.317	-0.660	-0.343	no
Treat-40d	RHSIM_Rhsim13G0074500	-1.779	-1.159	0.621	low
Treat-40d	RHSIM_Rhsim11G0135800	0.951	1.867	0.916	no
Treat-40d	RHSIM_Rhsim12G0046400	0.577	2.001	1.424	no
Treat-40d	RHSIM_Rhsim11G0001200	0.199	-0.213	-0.412	no
Treat-40d	RHSIM_Rhsim11G0136900	-0.534	1.095	1.630	no
Treat-40d	RHSIM_Rhsim12G0065000	3.280	1.316	-1.963	high
Treat-40d	RHSIM_Rhsim11G0182800	2.007	-0.190	-2.197	high
Treat-40d	RHSIM_Rhsim11G0039200	-1.945	-0.305	1.640	low
Treat-40d	RHSIM_Rhsim12G0161700	-0.342	-1.874	-1.532	no
Treat-40d	RHSIM_Rhsim11G0125200	-0.003	0.152	0.155	no
Treat-40d	RHSIM_Rhsim11G0098400	1.889	1.468	-0.421	high
Treat-40d	RHSIM_Rhsim12G0047800	-0.155	-1.159	-1.005	no
Treat-40d	RHSIM_Rhsim11G0186300	0.747	-0.517	-1.264	no
Treat-40d	RHSIM_Rhsim11G0072000	2.409	0.301	-2.108	high
Treat-40d	RHSIM_Rhsim12G0073200	1.278	0.736	-0.542	high
Treat-40d	RHSIM_Rhsim12G0129600	-1.811	-2.141	-0.330	low
Treat-40d	RHSIM_Rhsim11G0176000	0.740	0.991	0.251	no
Treat-40d	RHSIM_Rhsim11G0133600	-0.844	-0.244	0.600	no
Treat-40d	RHSIM_Rhsim11G0171300	-0.034	-1.175	-1.141	no
Treat-40d	RHSIM_Rhsim12G0130600	-0.054	1.210	1.264	no
Treat-40d	RHSIM_Rhsim11G0197000	-0.620	0.183	0.803	no
Treat-40d	RHSIM_Rhsim11G0113300	0.496	0.661	0.164	no
Treat-40d	RHSIM_Rhsim11G0192400	-0.696	1.155	1.851	no
Treat-40d	RHSIM_Rhsim12G0016200	-0.578	-0.988	-0.410	no
Treat-40d	RHSIM_Rhsim12G0079100	-0.807	0.107	0.914	no
Treat-40d	RHSIM_Rhsim11G0095300	0.214	-0.600	-0.814	no
Treat-40d	RHSIM_Rhsim13G0229400	0.846	-0.511	-1.357	no
Treat-40d	RHSIM_Rhsim11G0016500	0.816	0.397	-0.419	no
Treat-40d	RHSIM_Rhsim12G0206500	1.132	1.617	0.485	high
Treat-40d	RHSIM_Rhsim11G0121500	0.811	1.171	0.360	no
Treat-40d	RHSIM_Rhsim13G0197700	-0.065	1.169	1.234	no

Group	Gene	PTR (z-score difference)	z(log2 LFQ)	z(log2 FPKM)	extreme-PTR
Treat-40d	RHSIM_Rhsim13G0015200	-1.475	-0.958	0.517	low
Treat-40d	RHSIM_Rhsim11G0160100	-0.469	-0.784	-0.315	no
Treat-40d	RHSIM_Rhsim12G0201500	0.052	-0.014	-0.066	no
Treat-40d	RHSIM_Rhsim11G0177100	-0.114	0.898	1.012	no
Treat-40d	RHSIM_Rhsim13G0133100	2.192	1.144	-1.047	high
Treat-40d	RHSIM_Rhsim11G0161200	-0.445	0.160	0.605	no
Treat-40d	RHSIM_Rhsim12G0128500	0.263	-0.323	-0.586	no
Treat-40d	RHSIM_Rhsim11G0121900	-1.151	-1.120	0.031	low
Treat-40d	RHSIM_Rhsim11G0196200	-0.219	-1.311	-1.092	no
Treat-40d	RHSIM_Rhsim13G0234500	0.672	0.066	-0.606	no
Treat-40d	RHSIM_Rhsim13G0201500	0.665	2.713	2.048	no
Treat-40d	RHSIM_Rhsim11G0148100	-1.069	-0.067	1.001	no
Treat-40d	RHSIM_Rhsim13G0052600	-0.553	-1.183	-0.630	no
Treat-40d	RHSIM_Rhsim11G0122800	-0.212	-0.126	0.086	no
Treat-40d	RHSIM_Rhsim11G0130100	1.305	1.876	0.572	high
Treat-40d	RHSIM_Rhsim13G0010400	0.143	-0.369	-0.512	no
Treat-40d	RHSIM_Rhsim12G0087000	-2.175	-0.293	1.882	low
Treat-40d	RHSIM_Rhsim11G0167000	1.232	1.521	0.289	high
Treat-40d	RHSIM_Rhsim11G0153500	0.362	0.570	0.208	no
Treat-40d	RHSIM_Rhsim12G0080800	1.303	-0.008	-1.311	high
Treat-40d	RHSIM_Rhsim11G0165700	-0.516	0.243	0.759	no
Treat-40d	RHSIM_Rhsim13G0153900	0.162	-0.023	-0.186	no
Treat-40d	RHSIM_Rhsim11G0097900	0.470	-0.651	-1.120	no
Treat-40d	RHSIM_Rhsim12G0010700	1.723	1.772	0.049	high
Treat-40d	RHSIM_Rhsim11G0099800	1.150	-0.060	-1.211	high
Treat-40d	RHSIM_Rhsim13G0049600	-2.235	-1.262	0.973	low
Treat-40d	RHSIM_Rhsim12G0076700	0.588	-0.265	-0.853	no
Treat-40d	RHSIM_Rhsim12G0209000	0.313	0.420	0.107	no
Treat-40d	RHSIM_Rhsim12G0090500	-0.535	0.097	0.633	no
Treat-40d	RHSIM_Rhsim11G0132600	-0.549	-0.406	0.143	no
Treat-40d	RHSIM_Rhsim11G0099600	-2.316	-0.746	1.570	low
Treat-40d	RHSIM_Rhsim11G0121300	-0.810	1.339	2.149	no

Group	Gene	PTR (z-score difference)	z(log2 LFQ)	z(log2 FPKM)	extreme-PTR
Treat-40d	RHSIM_Rhsim11G0123700	-0.938	0.677	1.614	no
Treat-40d	RHSIM_Rhsim11G0161300	-0.098	-0.211	-0.113	no
Treat-40d	RHSIM_Rhsim11G0097400	-0.923	-1.128	-0.205	no
Treat-40d	RHSIM_Rhsim11G0145600	-0.164	0.968	1.132	no
Treat-40d	RHSIM_Rhsim11G0045300	2.077	2.231	0.154	high
Treat-40d	RHSIM_Rhsim12G0206700	0.432	-0.406	-0.838	no
Treat-40d	RHSIM_Rhsim11G0058800	-0.113	1.163	1.276	no
Treat-40d	RHSIM_Rhsim12G0077400	1.076	-0.897	-1.973	no
Treat-40d	RHSIM_Rhsim12G0125300	-0.950	-0.861	0.089	no
Treat-40d	RHSIM_Rhsim11G0191300	-1.017	-0.685	0.331	no
Treat-40d	RHSIM_Rhsim12G0215400	-0.329	-0.064	0.265	no
Treat-40d	RHSIM_Rhsim11G0142100	0.776	1.461	0.685	no
Treat-40d	RHSIM_Rhsim11G0199200	0.020	-0.178	-0.198	no
Treat-40d	RHSIM_Rhsim13G0037000	0.386	0.196	-0.190	no
Treat-40d	RHSIM_Rhsim11G0044200	2.339	1.155	-1.184	high
Treat-40d	RHSIM_Rhsim12G0025900	-0.675	-0.568	0.107	no
Treat-40d	RHSIM_Rhsim12G0200000	0.033	-1.058	-1.091	no
Treat-40d	RHSIM_Rhsim11G0142400	0.570	1.190	0.620	no
Treat-40d	RHSIM_Rhsim11G0036800	-0.296	-0.597	-0.302	no
Treat-40d	RHSIM_Rhsim11G0149600	0.744	0.060	-0.684	no
Treat-40d	RHSIM_Rhsim12G0149800	0.454	1.553	1.099	no
Treat-40d	RHSIM_Rhsim11G0197800	-0.352	-1.187	-0.835	no
Treat-40d	RHSIM_Rhsim11G0006500	-0.995	-0.800	0.194	no
Treat-40d	RHSIM_Rhsim12G0003500	1.357	1.858	0.502	high
Treat-40d	RHSIM_Rhsim11G0179400	1.060	0.358	-0.702	no
Treat-40d	RHSIM_Rhsim11G0008100	2.349	1.260	-1.088	high
Treat-40d	RHSIM_Rhsim11G0084200	-1.156	2.471	3.627	low
Treat-40d	RHSIM_Rhsim04G0146800	0.163	0.380	0.218	no
Treat-40d	RHSIM_Rhsim11G0166500	-1.115	-0.963	0.153	no
Treat-40d	RHSIM_Rhsim13G0114900	-1.818	-0.832	0.986	low
Treat-40d	RHSIM_Rhsim11G0036600	-0.209	-0.894	-0.685	no
Treat-40d	RHSIM_Rhsim11G0087200	-0.689	0.714	1.403	no

Group	Gene	PTR (z-score difference)	z(log2 LFQ)	z(log2 FPKM)	extreme-PTR
Treat-40d	RHSIM_Rhsim12G0157100	-0.752	-1.049	-0.297	no
Treat-40d	RHSIM_Rhsim11G0182400	-3.332	-1.621	1.712	low
Treat-40d	RHSIM_Rhsim12G0151900	-0.487	1.914	2.401	no
Treat-40d	RHSIM_Rhsim11G0088900	0.285	0.273	-0.013	no
Treat-40d	RHSIM_Rhsim12G0056500	-2.205	-1.320	0.885	low
Treat-40d	RHSIM_Rhsim11G0108000	-0.478	1.104	1.582	no
Treat-40d	RHSIM_Rhsim13G0225000	0.361	-1.113	-1.474	no
Treat-40d	RHSIM_Rhsim11G0168200	-0.260	-1.183	-0.922	no
Treat-40d	RHSIM_Rhsim11G0003700	-0.194	0.843	1.037	no
Treat-40d	RHSIM_Rhsim11G0136600	0.555	-0.191	-0.746	no
Treat-40d	RHSIM_Rhsim11G0168600	-0.724	-0.816	-0.092	no
Treat-40d	RHSIM_Rhsim12G0179900	-0.782	-1.275	-0.493	no
Treat-40d	RHSIM_Rhsim11G0193300	-0.995	-0.843	0.152	no
Treat-40d	RHSIM_Rhsim11G0105100	0.932	0.725	-0.207	no
Treat-40d	RHSIM_Rhsim11G0088000	-0.283	-0.909	-0.626	no
Treat-40d	RHSIM_Rhsim12G0090900	-1.224	-0.858	0.366	low
Treat-40d	RHSIM_Rhsim12G0193400	-1.004	2.998	4.002	no
Treat-40d	RHSIM_Rhsim11G0183800	1.215	3.666	2.450	high
Treat-40d	RHSIM_Rhsim13G0213200	-1.172	1.694	2.865	low
Treat-40d	RHSIM_Rhsim11G0008300	0.485	0.025	-0.459	no
Treat-40d	RHSIM_Rhsim12G0145800	-0.012	0.300	0.312	no
Treat-40d	RHSIM_Rhsim13G0229300	2.065	1.983	-0.082	high
Treat-40d	RHSIM_Rhsim11G0098600	-0.330	0.125	0.455	no
Treat-40d	RHSIM_Rhsim11G0180700	0.277	0.404	0.127	no
Treat-40d	RHSIM_Rhsim12G0201700	-0.199	0.913	1.112	no
Treat-40d	RHSIM_Rhsim13G0029900	1.218	1.090	-0.128	high
Treat-40d	RHSIM_Rhsim12G0180500	0.057	-0.238	-0.295	no
Treat-40d	RHSIM_Rhsim11G0065900	-1.437	-0.380	1.057	low
Treat-40d	RHSIM_Rhsim11G0159300	1.365	0.850	-0.515	high
Treat-40d	RHSIM_Rhsim12G0167000	-1.074	-0.649	0.424	no
Treat-40d	RHSIM_Rhsim11G0016400	0.614	-0.785	-1.398	no
Treat-40d	RHSIM_Rhsim11G0022000	-0.349	-0.298	0.051	no

Group	Gene	PTR (z-score difference)	z(log2 LFQ)	z(log2 FPKM)	extreme-PTR
Treat-40d	RHSIM_Rhsim11G0184900	0.509	0.148	-0.360	no
Treat-40d	RHSIM_Rhsim11G0063600	1.714	0.640	-1.075	high
Treat-40d	RHSIM_Rhsim13G0166900	0.481	-1.883	-2.364	no
Treat-40d	RHSIM_Rhsim11G0010400	-0.594	-1.373	-0.779	no
Treat-40d	RHSIM_Rhsim11G0174300	0.600	-0.164	-0.763	no
Treat-40d	RHSIM_Rhsim11G0085900	-0.163	1.644	1.807	no
Treat-40d	RHSIM_Rhsim11G0041300	2.370	1.959	-0.410	high
Treat-40d	RHSIM_Rhsim12G0209200	-0.082	-0.055	0.027	no
Treat-40d	RHSIM_Rhsim12G0134600	1.586	0.533	-1.052	high
Treat-40d	RHSIM_Rhsim11G0146000	-1.719	-1.158	0.561	low
Treat-40d	RHSIM_Rhsim12G0024700	-1.099	-0.727	0.372	no
Treat-40d	RHSIM_Rhsim12G0054800	-3.003	-1.851	1.153	low
Treat-40d	RHSIM_Rhsim11G0198200	-0.443	0.240	0.683	no
Treat-40d	RHSIM_Rhsim11G0099700	-0.497	-0.933	-0.435	no
Treat-40d	RHSIM_Rhsim12G0025500	-1.174	-0.722	0.452	low
Treat-40d	RHSIM_Rhsim12G0109900	0.268	-1.836	-2.103	no
Treat-40d	RHSIM_Rhsim11G0112000	-0.234	-0.668	-0.435	no
Treat-40d	RHSIM_Rhsim12G0133300	1.313	0.013	-1.300	high
Treat-40d	RHSIM_Rhsim12G0173900	-0.620	-1.074	-0.455	no
Treat-40d	RHSIM_Rhsim11G0193600	0.465	0.407	-0.058	no
Treat-40d	RHSIM_Rhsim12G0016800	0.891	-0.093	-0.983	no
Treat-40d	RHSIM_Rhsim12G0112100	0.220	1.039	0.819	no
Treat-40d	RHSIM_Rhsim11G0042300	1.076	-0.509	-1.586	no
Treat-40d	RHSIM_Rhsim12G0212900	-0.503	-0.082	0.421	no
Treat-40d	RHSIM_Rhsim12G0144900	-0.105	-0.368	-0.264	no
Treat-40d	RHSIM_Rhsim11G0090900	0.866	-0.754	-1.620	no
Treat-40d	RHSIM_Rhsim11G0196900	-0.801	-0.519	0.282	no
Treat-40d	RHSIM_Rhsim11G0157200	-0.603	0.945	1.547	no
Treat-40d	RHSIM_Rhsim12G0162200	-0.340	0.130	0.471	no
Treat-40d	RHSIM_Rhsim11G0176400	-0.671	-0.185	0.486	no
Treat-40d	RHSIM_Rhsim11G0036900	0.696	-0.080	-0.776	no
Treat-40d	RHSIM_Rhsim11G0177200	0.052	0.440	0.387	no

Group	Gene	PTR (z-score difference)	z(log2 LFQ)	z(log2 FPKM)	extreme-PTR
Treat-40d	RHSIM_Rhsim11G0051900	1.326	-0.097	-1.423	high
Treat-40d	RHSIM_Rhsim12G0137000	0.074	0.182	0.108	no
Treat-40d	RHSIM_Rhsim11G0045000	-0.113	-0.347	-0.234	no
Treat-40d	RHSIM_Rhsim12G0130000	0.629	-0.122	-0.751	no
Treat-40d	RHSIM_Rhsim11G0122200	1.289	2.708	1.419	high
Treat-40d	RHSIM_Rhsim12G0005000	0.714	0.245	-0.469	no
Treat-40d	RHSIM_Rhsim11G0194700	0.434	0.787	0.353	no
Treat-40d	RHSIM_Rhsim12G0003200	-0.267	-0.834	-0.567	no
Treat-40d	RHSIM_Rhsim11G0182000	0.737	0.724	-0.013	no
Treat-40d	RHSIM_Rhsim11G0134200	1.005	0.365	-0.639	no
Treat-40d	RHSIM_Rhsim11G0177800	-1.457	1.205	2.662	low
Treat-40d	RHSIM_Rhsim11G0046400	-0.012	-0.048	-0.036	no
Treat-40d	RHSIM_Rhsim12G0041700	-1.476	-0.253	1.223	low
Treat-40d	RHSIM_Rhsim11G0195000	-0.409	-1.435	-1.026	no
Treat-40d	RHSIM_Rhsim11G0054800	-0.925	-1.236	-0.312	no
Treat-40d	RHSIM_Rhsim12G0002100	-0.422	-0.715	-0.293	no
Treat-40d	RHSIM_Rhsim11G0065700	-0.485	0.932	1.417	no
Treat-40d	RHSIM_Rhsim11G0167500	1.607	1.057	-0.550	high
Treat-40d	RHSIM_Rhsim11G0045700	-0.143	-0.899	-0.755	no
Treat-40d	RHSIM_Rhsim11G0118400	0.038	0.664	0.625	no
Treat-40d	RHSIM_Rhsim12G0204600	-0.067	1.723	1.790	no
Treat-40d	RHSIM_Rhsim12G0051400	-1.051	-0.534	0.517	no
Treat-40d	RHSIM_Rhsim11G0147400	-0.167	-0.625	-0.458	no
Treat-40d	RHSIM_Rhsim12G0075500	-0.152	2.256	2.408	no
Treat-40d	RHSIM_Rhsim11G0184600	-1.287	-1.492	-0.205	low
Treat-40d	RHSIM_Rhsim11G0199100	0.161	-0.767	-0.928	no
Treat-40d	RHSIM_Rhsim11G0053000	-2.535	-1.485	1.050	low
Treat-40d	RHSIM_Rhsim11G0020200	1.301	2.003	0.702	high
Treat-40d	RHSIM_Rhsim11G0197500	0.101	0.024	-0.077	no
Treat-40d	RHSIM_Rhsim11G0121700	-0.083	1.284	1.368	no
Treat-40d	RHSIM_Rhsim12G0017200	-0.652	0.591	1.243	no
Treat-40d	RHSIM_Rhsim10G0183500	0.609	0.423	-0.186	no

Group	Gene	PTR (z-score difference)	z(log2 LFQ)	z(log2 FPKM)	extreme-PTR
Treat-40d	RHSIM_Rhsim11G0167700	2.326	0.972	-1.354	high
Treat-40d	RHSIM_Rhsim11G0088700	0.269	-1.169	-1.438	no
Treat-40d	RHSIM_Rhsim10G0007500	-0.682	0.020	0.702	no
Treat-40d	RHSIM_Rhsim12G0000100	1.043	0.344	-0.699	no
Treat-40d	RHSIM_Rhsim11G0054200	-0.512	-0.915	-0.403	no
Treat-40d	RHSIM_Rhsim11G0163600	0.688	0.278	-0.409	no
Treat-40d	RHSIM_Rhsim10G0040200	0.858	0.818	-0.040	no
Treat-40d	RHSIM_Rhsim11G0195400	1.092	-0.112	-1.204	no
Treat-40d	RHSIM_Rhsim12G0193600	-1.002	-1.224	-0.222	no
Treat-40d	RHSIM_Rhsim11G0156300	2.305	0.826	-1.479	high
Treat-40d	RHSIM_Rhsim10G0102500	0.943	-0.379	-1.322	no
Treat-40d	RHSIM_Rhsim11G0122000	1.166	-0.053	-1.219	high
Treat-40d	RHSIM_Rhsim12G0102200	-0.329	-0.825	-0.496	no
Treat-40d	RHSIM_Rhsim11G0161100	-2.017	0.249	2.266	low
Treat-40d	RHSIM_Rhsim11G0052000	1.182	-0.494	-1.676	high
Treat-40d	RHSIM_Rhsim10G0183700	0.399	1.769	1.370	no
Treat-40d	RHSIM_Rhsim11G0175900	-0.665	-0.836	-0.171	no
Treat-40d	RHSIM_Rhsim10G0204000	0.737	0.106	-0.632	no
Treat-40d	RHSIM_Rhsim11G0125500	-0.308	-0.746	-0.438	no
Treat-40d	RHSIM_Rhsim10G0079900	1.276	1.217	-0.059	high
Treat-40d	RHSIM_Rhsim12G0090700	0.021	0.724	0.703	no
Treat-40d	RHSIM_Rhsim11G0010500	-0.538	0.815	1.353	no
Treat-40d	RHSIM_Rhsim11G0095500	0.553	0.231	-0.321	no
Treat-40d	RHSIM_Rhsim10G0133700	4.340	3.004	-1.335	high
Treat-40d	RHSIM_Rhsim10G0079400	0.733	-0.034	-0.767	no
Treat-40d	RHSIM_Rhsim10G0135700	1.551	1.068	-0.483	high
Treat-40d	RHSIM_Rhsim12G0205300	-0.775	0.027	0.802	no
Treat-40d	RHSIM_Rhsim12G0036900	-0.312	-0.459	-0.148	no
Treat-40d	RHSIM_Rhsim10G0196000	-0.895	0.853	1.748	no
Treat-40d	RHSIM_Rhsim11G0190300	-0.021	0.430	0.451	no
Treat-40d	RHSIM_Rhsim12G0105500	2.275	1.419	-0.856	high
Treat-40d	RHSIM_Rhsim10G0045000	-0.987	0.211	1.198	no

Group	Gene	PTR (z-score difference)	z(log2 LFQ)	z(log2 FPKM)	extreme-PTR
Treat-40d	RHSIM_Rhsim12G0185400	-1.339	-0.534	0.805	low
Treat-40d	RHSIM_Rhsim10G0210800	0.828	-0.662	-1.490	no
Treat-40d	RHSIM_Rhsim12G0110600	-1.255	-0.561	0.694	low
Treat-40d	RHSIM_Rhsim12G0080300	-0.298	-0.746	-0.448	no
Treat-40d	RHSIM_Rhsim10G0121300	-0.319	-0.638	-0.319	no
Treat-40d	RHSIM_Rhsim11G0008500	-0.861	0.148	1.009	no
Treat-40d	RHSIM_Rhsim12G0168500	-0.296	-0.094	0.201	no
Treat-40d	RHSIM_Rhsim11G0196800	0.269	-0.128	-0.397	no
Treat-40d	RHSIM_Rhsim10G0029000	1.167	-0.407	-1.574	high
Treat-40d	RHSIM_Rhsim10G0155900	0.701	-1.183	-1.884	no
Treat-40d	RHSIM_Rhsim10G0009900	-0.577	-0.868	-0.292	no
Treat-40d	RHSIM_Rhsim10G0164700	-0.514	-0.980	-0.466	no
Treat-40d	RHSIM_Rhsim12G0166000	0.191	-1.198	-1.389	no
Treat-40d	RHSIM_Rhsim10G0197300	-0.208	0.606	0.815	no
Treat-40d	RHSIM_Rhsim11G0097800	0.272	0.888	0.616	no
Treat-40d	RHSIM_Rhsim12G0007900	-1.007	0.422	1.429	no
Treat-40d	RHSIM_Rhsim12G0139700	0.830	0.496	-0.334	no
Treat-40d	RHSIM_Rhsim11G0117500	-1.263	-1.174	0.089	low
Treat-40d	RHSIM_Rhsim11G0167600	1.521	1.475	-0.046	high
Treat-40d	RHSIM_Rhsim10G0067100	-0.375	-0.695	-0.321	no
Treat-40d	RHSIM_Rhsim11G0126200	-0.158	-0.462	-0.305	no
Treat-40d	RHSIM_Rhsim11G0023700	1.412	0.926	-0.486	high
Treat-40d	RHSIM_Rhsim12G0068000	-0.710	0.449	1.160	no
Treat-40d	RHSIM_Rhsim11G0198400	0.686	0.992	0.305	no
Treat-40d	RHSIM_Rhsim11G0052800	0.075	-1.331	-1.406	no
Treat-40d	RHSIM_Rhsim10G0051200	-1.171	-0.804	0.367	low
Treat-40d	RHSIM_Rhsim10G0191900	0.417	-0.221	-0.638	no
Treat-40d	RHSIM_Rhsim10G0026400	1.584	0.772	-0.812	high
Treat-40d	RHSIM_Rhsim10G0030500	-0.794	-1.872	-1.078	no
Treat-40d	RHSIM_Rhsim10G0005500	-0.654	-0.109	0.545	no
Treat-40d	RHSIM_Rhsim10G0105200	0.096	-0.082	-0.178	no
Treat-40d	RHSIM_Rhsim12G0114000	-0.279	-0.339	-0.061	no

Group	Gene	PTR (z-score difference)	z(log2 LFQ)	z(log2 FPKM)	extreme-PTR
Treat-40d	RHSIM_Rhsim10G0117200	1.058	0.995	-0.063	no
Treat-40d	RHSIM_Rhsim10G0191600	0.309	-1.058	-1.367	no
Treat-40d	RHSIM_Rhsim10G0194400	0.673	0.503	-0.169	no
Treat-40d	RHSIM_Rhsim11G0053100	0.841	1.304	0.463	no
Treat-40d	RHSIM_Rhsim11G0198000	0.053	-0.689	-0.742	no
Treat-40d	RHSIM_Rhsim10G0199000	1.497	0.102	-1.395	high
Treat-40d	RHSIM_Rhsim12G0008000	0.384	-0.091	-0.475	no
Treat-40d	RHSIM_Rhsim10G0035600	-0.385	-0.372	0.012	no
Treat-40d	RHSIM_Rhsim10G0116000	-1.410	-1.460	-0.050	low
Treat-40d	RHSIM_Rhsim11G0167200	2.474	0.803	-1.671	high
Treat-40d	RHSIM_Rhsim12G0070400	2.209	0.223	-1.986	high
Treat-40d	RHSIM_Rhsim10G0148300	1.291	0.443	-0.848	high
Treat-40d	RHSIM_Rhsim10G0099800	-0.398	1.334	1.732	no
Treat-40d	RHSIM_Rhsim10G0136800	-0.003	-0.587	-0.584	no
Treat-40d	RHSIM_Rhsim12G0115000	-1.805	-1.046	0.759	low
Treat-40d	RHSIM_Rhsim10G0186500	0.551	0.698	0.147	no
Treat-40d	RHSIM_Rhsim12G0166200	0.281	-0.723	-1.003	no
Treat-40d	RHSIM_Rhsim11G0135900	3.252	2.828	-0.423	high
Treat-40d	RHSIM_Rhsim12G0189800	-0.147	-0.379	-0.233	no
Treat-40d	RHSIM_Rhsim10G0108600	-0.022	0.018	0.040	no
Treat-40d	RHSIM_Rhsim10G0162300	0.241	-1.544	-1.785	no
Treat-40d	RHSIM_Rhsim12G0014000	-0.029	-0.569	-0.540	no
Treat-40d	RHSIM_Rhsim11G0125700	-0.759	-1.873	-1.114	no
Treat-40d	RHSIM_Rhsim10G0192800	-0.134	-0.541	-0.408	no
Treat-40d	RHSIM_Rhsim10G0197900	0.292	0.910	0.618	no
Treat-40d	RHSIM_Rhsim12G0136800	-0.705	-1.303	-0.598	no
Treat-40d	RHSIM_Rhsim10G0014000	0.032	-0.974	-1.006	no
Treat-40d	RHSIM_Rhsim10G0094500	-0.226	-0.350	-0.124	no
Treat-40d	RHSIM_Rhsim11G0173400	-1.106	-0.498	0.609	no
Treat-40d	RHSIM_Rhsim12G0150700	0.337	0.692	0.355	no
Treat-40d	RHSIM_Rhsim10G0069000	1.073	1.021	-0.052	no
Treat-40d	RHSIM_Rhsim11G0135200	-0.068	-0.573	-0.505	no

Group	Gene	PTR (z-score difference)	z(log2 LFQ)	z(log2 FPKM)	extreme-PTR
Treat-40d	RHSIM_Rhsim10G0019600	-0.347	0.028	0.375	no
Treat-40d	RHSIM_Rhsim12G0001500	0.985	0.492	-0.492	no
Treat-40d	RHSIM_Rhsim11G0106300	1.439	1.421	-0.018	high
Treat-40d	RHSIM_Rhsim10G0126200	1.334	1.581	0.247	high
Treat-40d	RHSIM_Rhsim11G0074300	-0.600	0.764	1.364	no
Treat-40d	RHSIM_Rhsim11G0155300	2.271	1.053	-1.219	high
Treat-40d	RHSIM_Rhsim10G0149500	0.000	-1.748	-1.748	no
Treat-40d	RHSIM_Rhsim12G0128400	2.113	0.169	-1.943	high
Treat-40d	RHSIM_Rhsim12G0213700	1.222	1.278	0.056	high
Treat-40d	RHSIM_Rhsim10G0141300	1.379	2.952	1.573	high
Treat-40d	RHSIM_Rhsim10G0115500	0.371	-0.966	-1.338	no
Treat-40d	RHSIM_Rhsim11G0130000	-0.293	-0.812	-0.518	no
Treat-40d	RHSIM_Rhsim11G0064900	0.583	-0.806	-1.388	no
Treat-40d	RHSIM_Rhsim10G0202500	3.582	1.398	-2.184	high
Treat-40d	RHSIM_Rhsim10G0183800	-1.140	-1.589	-0.449	low
Treat-40d	RHSIM_Rhsim10G0183200	-0.251	-0.306	-0.055	no
Treat-40d	RHSIM_Rhsim11G0192900	0.219	0.857	0.638	no
Treat-40d	RHSIM_Rhsim11G0015100	-1.160	-1.333	-0.174	low
Treat-40d	RHSIM_Rhsim10G0014400	-1.208	-1.268	-0.059	low
Treat-40d	RHSIM_Rhsim10G0101200	1.141	1.767	0.626	high
Treat-40d	RHSIM_Rhsim10G0211500	0.351	-1.619	-1.970	no
Treat-40d	RHSIM_Rhsim11G0159000	-0.168	1.141	1.309	no
Treat-40d	RHSIM_Rhsim10G0099300	0.787	-0.517	-1.303	no
Treat-40d	RHSIM_Rhsim11G0099900	-0.797	-0.371	0.427	no
Treat-40d	RHSIM_Rhsim11G0075200	-0.709	-0.304	0.405	no
Treat-40d	RHSIM_Rhsim11G0094000	0.198	1.672	1.474	no
Treat-40d	RHSIM_Rhsim10G0156300	1.154	0.682	-0.472	high
Treat-40d	RHSIM_Rhsim11G0003600	-1.209	-0.008	1.201	low
Treat-40d	RHSIM_Rhsim11G0077900	0.083	0.991	0.908	no
Treat-40d	RHSIM_Rhsim10G0167100	3.117	1.776	-1.342	high
Treat-40d	RHSIM_Rhsim11G0075500	-3.472	-0.989	2.483	low
Treat-40d	RHSIM_Rhsim10G0071100	2.375	0.686	-1.690	high

Group	Gene	PTR (z-score difference)	z(log2 LFQ)	z(log2 FPKM)	extreme-PTR
Treat-40d	RHSIM_Rhsim10G0006900	-0.953	-1.920	-0.967	no
Treat-40d	RHSIM_Rhsim11G0078100	2.073	1.261	-0.812	high
Treat-40d	RHSIM_Rhsim11G0121800	0.316	1.754	1.438	no
Treat-40d	RHSIM_Rhsim11G0094400	-0.317	0.936	1.253	no
Treat-40d	RHSIM_Rhsim11G0093100	-0.008	-0.421	-0.412	no
Treat-40d	RHSIM_Rhsim11G0159200	-0.785	-0.682	0.104	no
Treat-40d	RHSIM_Rhsim10G0198000	-1.117	-1.619	-0.503	no
Treat-40d	RHSIM_Rhsim10G0211400	0.818	2.316	1.498	no
Treat-40d	RHSIM_Rhsim10G0188500	-1.113	0.046	1.159	no
Treat-40d	RHSIM_Rhsim10G0172500	-0.276	-0.022	0.254	no
Treat-40d	RHSIM_Rhsim11G0117700	-0.467	0.765	1.232	no
Treat-40d	RHSIM_Rhsim11G0196300	0.134	1.395	1.261	no
Treat-40d	RHSIM_Rhsim11G0019600	0.416	-0.013	-0.430	no
Treat-40d	RHSIM_Rhsim10G0006300	0.945	0.244	-0.700	no
Treat-40d	RHSIM_Rhsim10G0112600	-0.029	-0.262	-0.233	no
Treat-40d	RHSIM_Rhsim11G0136500	-0.724	-0.657	0.066	no
Treat-40d	RHSIM_Rhsim10G0107900	1.215	0.732	-0.483	high
Treat-40d	RHSIM_Rhsim11G0090400	-0.625	0.844	1.469	no
Treat-40d	RHSIM_Rhsim11G0190500	-0.935	0.725	1.660	no
Treat-40d	RHSIM_Rhsim10G0008800	-0.904	-1.079	-0.175	no
Treat-40d	RHSIM_Rhsim11G0136000	0.796	0.855	0.059	no
Treat-40d	RHSIM_Rhsim10G0014200	1.269	-0.972	-2.241	high
Treat-40d	RHSIM_Rhsim11G0141900	0.001	-0.411	-0.412	no
Treat-40d	RHSIM_Rhsim11G0131600	-0.563	-1.011	-0.448	no
Treat-40d	RHSIM_Rhsim10G0141900	-0.804	-0.137	0.667	no
Treat-40d	RHSIM_Rhsim11G0072400	-1.344	-1.088	0.256	low
Treat-40d	RHSIM_Rhsim10G0099600	-2.624	-2.748	-0.125	low
Treat-40d	RHSIM_Rhsim10G0022700	-0.526	0.481	1.008	no
Treat-40d	RHSIM_Rhsim11G0008000	-0.707	-0.952	-0.245	no
Treat-40d	RHSIM_Rhsim11G0077600	-0.476	-1.137	-0.661	no
Treat-40d	RHSIM_Rhsim11G0128300	1.130	0.205	-0.925	high
Treat-40d	RHSIM_Rhsim10G0068700	-0.749	-1.693	-0.945	no

Group	Gene	PTR (z-score difference)	z(log2 LFQ)	z(log2 FPKM)	extreme-PTR
Treat-40d	RHSIM_Rhsim11G0172600	-0.852	-0.972	-0.121	no
Treat-40d	RHSIM_Rhsim10G0171700	1.044	2.297	1.253	no
Treat-40d	RHSIM_Rhsim11G0165400	0.812	-0.406	-1.218	no
Treat-40d	RHSIM_Rhsim10G0006400	-0.012	0.889	0.902	no
Treat-40d	RHSIM_Rhsim11G0180400	1.336	-0.231	-1.567	high
Treat-40d	RHSIM_Rhsim11G0132900	-1.158	-1.234	-0.075	low
Treat-40d	RHSIM_Rhsim10G0072100	-0.636	0.675	1.311	no
Treat-40d	RHSIM_Rhsim10G0134600	1.847	-0.585	-2.432	high
Treat-40d	RHSIM_Rhsim10G0061400	-0.594	-0.108	0.486	no
Treat-40d	RHSIM_Rhsim10G0039800	-0.156	1.007	1.163	no
Treat-40d	RHSIM_Rhsim10G0151800	0.636	-0.788	-1.425	no
Treat-40d	RHSIM_Rhsim10G0127000	-2.074	-1.796	0.278	low
Treat-40d	RHSIM_Rhsim11G0184700	-0.685	0.078	0.763	no
Treat-40d	RHSIM_Rhsim11G0111500	-0.109	-0.336	-0.227	no
Treat-40d	RHSIM_Rhsim10G0021200	2.850	3.188	0.338	high
Treat-40d	RHSIM_Rhsim10G0013900	-1.102	0.380	1.482	no
Treat-40d	RHSIM_Rhsim10G0015700	1.410	-0.545	-1.956	high
Treat-40d	RHSIM_Rhsim10G0149000	0.173	0.019	-0.154	no
Treat-40d	RHSIM_Rhsim10G0179500	-0.630	0.437	1.067	no
Treat-40d	RHSIM_Rhsim10G0178400	-0.113	3.002	3.116	no
Treat-40d	RHSIM_Rhsim10G0177300	0.202	1.424	1.222	no
Treat-40d	RHSIM_Rhsim10G0127200	0.678	0.096	-0.581	no
Treat-40d	RHSIM_Rhsim10G0019900	0.363	0.664	0.300	no
Treat-40d	RHSIM_Rhsim10G0079500	1.509	0.448	-1.061	high
Treat-40d	RHSIM_Rhsim10G0046600	0.874	-0.367	-1.241	no
Treat-40d	RHSIM_Rhsim10G0104000	-1.087	-1.549	-0.462	no
Treat-40d	RHSIM_Rhsim10G0078600	-0.215	0.020	0.234	no
Treat-40d	RHSIM_Rhsim11G0028300	1.210	1.102	-0.107	high
Treat-40d	RHSIM_Rhsim10G0174600	-1.968	-1.266	0.702	low
Treat-40d	RHSIM_Rhsim11G0045600	-1.278	-0.004	1.274	low
Treat-40d	RHSIM_Rhsim10G0185600	1.537	-0.047	-1.584	high
Treat-40d	RHSIM_Rhsim11G0197700	0.059	-0.903	-0.962	no

Group	Gene	PTR (z-score difference)	z(log2 LFQ)	z(log2 FPKM)	extreme-PTR
Treat-40d	RHSIM_Rhsim10G0082000	0.288	-0.144	-0.432	no
Treat-40d	RHSIM_Rhsim10G0048900	0.877	1.398	0.521	no
Treat-40d	RHSIM_Rhsim10G0022600	2.689	1.329	-1.360	high
Treat-40d	RHSIM_Rhsim11G0169400	0.250	0.724	0.474	no
Treat-40d	RHSIM_Rhsim10G0199600	1.540	1.176	-0.365	high
Treat-40d	RHSIM_Rhsim10G0175100	1.180	1.242	0.062	high
Treat-40d	RHSIM_Rhsim11G0173300	0.700	0.460	-0.240	no
Treat-40d	RHSIM_Rhsim11G0014400	0.265	0.480	0.214	no
Treat-40d	RHSIM_Rhsim11G0071200	-0.870	-0.633	0.237	no
Treat-40d	RHSIM_Rhsim11G0069400	1.112	1.909	0.797	high
Treat-40d	RHSIM_Rhsim10G0163500	1.857	1.738	-0.119	high
Treat-40d	RHSIM_Rhsim11G0031700	-0.663	-0.606	0.057	no
Treat-40d	RHSIM_Rhsim11G0133700	-1.358	-1.745	-0.387	low
Treat-40d	RHSIM_Rhsim10G0152000	2.552	0.951	-1.601	high
Treat-40d	RHSIM_Rhsim10G0185900	-1.039	-1.326	-0.286	no
Treat-40d	RHSIM_Rhsim10G0062200	2.772	1.485	-1.287	high
Treat-40d	RHSIM_Rhsim11G0184000	-0.136	0.506	0.641	no
Treat-40d	RHSIM_Rhsim10G0100700	0.931	-0.935	-1.866	no
Treat-40d	RHSIM_Rhsim11G0158600	-0.285	-1.222	-0.937	no
Treat-40d	RHSIM_Rhsim11G0077800	1.203	0.647	-0.556	high
Treat-40d	RHSIM_Rhsim10G0154300	2.928	0.787	-2.142	high
Treat-40d	RHSIM_Rhsim10G0012800	0.334	-0.231	-0.566	no
Treat-40d	RHSIM_Rhsim10G0207900	0.700	0.606	-0.094	no
Treat-40d	RHSIM_Rhsim09G0201000	-0.523	0.254	0.777	no
Treat-40d	RHSIM_Rhsim10G0035200	0.855	0.902	0.046	no
Treat-40d	RHSIM_Rhsim10G0043800	1.396	2.200	0.804	high
Treat-40d	RHSIM_Rhsim10G0128200	-0.080	-0.987	-0.907	no
Treat-40d	RHSIM_Rhsim11G0021700	0.456	1.035	0.578	no
Treat-40d	RHSIM_Rhsim10G0209100	-0.274	0.074	0.348	no
Treat-40d	RHSIM_Rhsim10G0006600	0.458	0.643	0.184	no
Treat-40d	RHSIM_Rhsim10G0064900	0.289	-0.695	-0.985	no
Treat-40d	RHSIM_Rhsim10G0202400	-1.932	1.730	3.663	low

Group	Gene	PTR (z-score difference)	z(log2 LFQ)	z(log2 FPKM)	extreme-PTR
Treat-40d	RHSIM_Rhsim10G0213600	-1.665	-1.218	0.447	low
Treat-40d	RHSIM_Rhsim10G0072600	-0.578	-1.258	-0.680	no
Treat-40d	RHSIM_Rhsim09G0032200	0.589	-0.122	-0.711	no
Treat-40d	RHSIM_Rhsim10G0008600	1.315	0.342	-0.973	high
Treat-40d	RHSIM_Rhsim10G0131300	-0.438	-1.776	-1.338	no
Treat-40d	RHSIM_Rhsim10G0102800	-0.383	-0.883	-0.501	no
Treat-40d	RHSIM_Rhsim11G0098900	0.051	-0.192	-0.243	no
Treat-40d	RHSIM_Rhsim11G0100500	-1.181	0.065	1.246	low
Treat-40d	RHSIM_Rhsim10G0138000	1.102	1.005	-0.097	no
Treat-40d	RHSIM_Rhsim09G0097600	-0.483	-0.225	0.258	no
Treat-40d	RHSIM_Rhsim10G0102700	0.108	-0.224	-0.332	no
Treat-40d	RHSIM_Rhsim11G0088600	-0.007	-0.116	-0.109	no
Treat-40d	RHSIM_Rhsim11G0178900	-0.983	-0.619	0.364	no
Treat-40d	RHSIM_Rhsim10G0030200	-0.380	0.081	0.461	no
Treat-40d	RHSIM_Rhsim09G0168000	0.915	-0.746	-1.661	no
Treat-40d	RHSIM_Rhsim10G0058700	3.214	1.421	-1.793	high
Treat-40d	RHSIM_Rhsim10G0036600	0.237	0.054	-0.183	no
Treat-40d	RHSIM_Rhsim11G0030300	-0.659	-0.216	0.443	no
Treat-40d	RHSIM_Rhsim10G0109900	-0.640	-0.699	-0.059	no
Treat-40d	RHSIM_Rhsim11G0134800	-0.727	-0.224	0.502	no
Treat-40d	RHSIM_Rhsim09G0115100	-1.006	-1.033	-0.026	no
Treat-40d	RHSIM_Rhsim11G0104600	1.804	-0.540	-2.344	high
Treat-40d	RHSIM_Rhsim10G0077000	-0.790	-0.685	0.106	no
Treat-40d	RHSIM_Rhsim09G0202700	-0.044	-0.728	-0.684	no
Treat-40d	RHSIM_Rhsim10G0019400	-1.845	-1.577	0.267	low
Treat-40d	RHSIM_Rhsim09G0144400	1.942	0.534	-1.407	high
Treat-40d	RHSIM_Rhsim10G0084700	-0.573	-0.862	-0.289	no
Treat-40d	RHSIM_Rhsim09G0121900	0.416	-0.348	-0.764	no
Treat-40d	RHSIM_Rhsim09G0169200	-1.602	-0.279	1.323	low
Treat-40d	RHSIM_Rhsim11G0184800	1.158	1.451	0.293	high
Treat-40d	RHSIM_Rhsim10G0202900	-0.813	1.723	2.536	no
Treat-40d	RHSIM_Rhsim10G0138300	-0.624	-0.848	-0.224	no

Group	Gene	PTR (z-score difference)	z(log2 LFQ)	z(log2 FPKM)	extreme-PTR
Treat-40d	RHSIM_Rhsim11G0058900	0.340	0.261	-0.080	no
Treat-40d	RHSIM_Rhsim09G0150400	2.453	0.732	-1.721	high
Treat-40d	RHSIM_Rhsim10G0028000	1.016	1.810	0.795	no
Treat-40d	RHSIM_Rhsim09G0123000	-0.685	-0.140	0.544	no
Treat-40d	RHSIM_Rhsim10G0023700	-0.427	-1.500	-1.073	no
Treat-40d	RHSIM_Rhsim10G0156100	-0.949	-0.151	0.798	no
Treat-40d	RHSIM_Rhsim09G0140200	-0.129	-0.286	-0.157	no
Treat-40d	RHSIM_Rhsim11G0089800	0.676	-0.201	-0.877	no
Treat-40d	RHSIM_Rhsim09G0147900	1.052	-1.254	-2.306	no
Treat-40d	RHSIM_Rhsim09G0181100	0.280	-0.609	-0.889	no
Treat-40d	RHSIM_Rhsim09G0069500	0.058	0.868	0.810	no
Treat-40d	RHSIM_Rhsim10G0209800	0.613	1.499	0.885	no
Treat-40d	RHSIM_Rhsim10G0009000	0.106	-0.091	-0.197	no
Treat-40d	RHSIM_Rhsim09G0208200	0.083	0.523	0.440	no
Treat-40d	RHSIM_Rhsim10G0029200	-0.031	-0.234	-0.203	no
Treat-40d	RHSIM_Rhsim09G0208400	0.134	1.295	1.161	no
Treat-40d	RHSIM_Rhsim10G0180300	-0.118	-0.570	-0.452	no
Treat-40d	RHSIM_Rhsim10G0212700	0.754	0.243	-0.511	no
Treat-40d	RHSIM_Rhsim10G0185200	-1.141	-1.336	-0.196	low
Treat-40d	RHSIM_Rhsim10G0056100	0.246	0.426	0.180	no
Treat-40d	RHSIM_Rhsim10G0176600	1.687	1.826	0.139	high
Treat-40d	RHSIM_Rhsim09G0110200	-0.031	-1.199	-1.169	no
Treat-40d	RHSIM_Rhsim09G0157700	0.265	1.410	1.145	no
Treat-40d	RHSIM_Rhsim11G0030200	2.057	0.477	-1.580	high
Treat-40d	RHSIM_Rhsim09G0003700	1.815	1.386	-0.429	high
Treat-40d	RHSIM_Rhsim10G0189100	-1.014	-0.888	0.125	no
Treat-40d	RHSIM_Rhsim10G0011300	-0.871	-0.769	0.102	no
Treat-40d	RHSIM_Rhsim09G0208000	-0.051	-0.445	-0.394	no
Treat-40d	RHSIM_Rhsim09G0141900	-0.942	0.544	1.486	no
Treat-40d	RHSIM_Rhsim11G0099400	-0.673	0.408	1.081	no
Treat-40d	RHSIM_Rhsim10G0034700	-0.782	0.478	1.260	no
Treat-40d	RHSIM_Rhsim10G0029400	-0.641	-0.725	-0.085	no

Group	Gene	PTR (z-score difference)	z(log2 LFQ)	z(log2 FPKM)	extreme-PTR
Treat-40d	RHSIM_Rhsim10G0005000	-1.106	-0.629	0.477	no
Treat-40d	RHSIM_Rhsim09G0111300	-0.040	-0.496	-0.456	no
Treat-40d	RHSIM_Rhsim09G0207500	0.706	0.350	-0.356	no
Treat-40d	RHSIM_Rhsim10G0064100	-3.175	-1.513	1.663	low
Treat-40d	RHSIM_Rhsim10G0026800	0.094	-1.128	-1.223	no
Treat-40d	RHSIM_Rhsim10G0113200	-1.366	0.590	1.957	low
Treat-40d	RHSIM_Rhsim09G0018700	0.314	-0.883	-1.198	no
Treat-40d	RHSIM_Rhsim10G0067800	0.379	-0.175	-0.553	no
Treat-40d	RHSIM_Rhsim09G0141100	-1.097	0.115	1.212	no
Treat-40d	RHSIM_Rhsim10G0018300	-0.833	1.020	1.854	no
Treat-40d	RHSIM_Rhsim09G0004300	-0.883	-0.518	0.365	no
Treat-40d	RHSIM_Rhsim10G0015600	0.972	0.583	-0.389	no
Treat-40d	RHSIM_Rhsim11G0141800	-1.701	-1.280	0.421	low
Treat-40d	RHSIM_Rhsim09G0167500	-0.311	-1.371	-1.059	no
Treat-40d	RHSIM_Rhsim09G0187300	0.364	0.363	-0.001	no
Treat-40d	RHSIM_Rhsim10G0126900	-1.379	-0.694	0.684	low
Treat-40d	RHSIM_Rhsim09G0208500	-0.703	0.946	1.649	no
Treat-40d	RHSIM_Rhsim11G0137800	0.895	-0.084	-0.979	no
Treat-40d	RHSIM_Rhsim09G0005100	0.563	0.861	0.299	no
Treat-40d	RHSIM_Rhsim10G0011500	-0.609	-1.218	-0.609	no
Treat-40d	RHSIM_Rhsim11G0040600	1.143	0.061	-1.082	high
Treat-40d	RHSIM_Rhsim10G0077900	-0.524	-0.739	-0.216	no
Treat-40d	RHSIM_Rhsim10G0102900	-0.578	-0.710	-0.132	no
Treat-40d	RHSIM_Rhsim09G0168600	-0.447	-1.128	-0.681	no
Treat-40d	RHSIM_Rhsim11G0145300	2.426	1.067	-1.359	high
Treat-40d	RHSIM_Rhsim10G0065800	-0.273	-1.087	-0.814	no
Treat-40d	RHSIM_Rhsim09G0155300	-0.720	-0.877	-0.156	no
Treat-40d	RHSIM_Rhsim10G0212500	0.748	-1.033	-1.781	no
Treat-40d	RHSIM_Rhsim09G0151400	0.815	0.758	-0.057	no
Treat-40d	RHSIM_Rhsim09G0003600	0.607	1.747	1.140	no
Treat-40d	RHSIM_Rhsim10G0130500	-0.364	-0.767	-0.403	no
Treat-40d	RHSIM_Rhsim10G0162800	0.924	0.410	-0.514	no

Group	Gene	PTR (z-score difference)	z(log2 LFQ)	z(log2 FPKM)	extreme-PTR
Treat-40d	RHSIM_Rhsim09G0071300	-0.978	-2.183	-1.205	no
Treat-40d	RHSIM_Rhsim10G0187700	1.423	0.279	-1.145	high
Treat-40d	RHSIM_Rhsim10G0134900	1.111	1.298	0.187	high
Treat-40d	RHSIM_Rhsim09G0043400	-0.370	0.490	0.861	no
Treat-40d	RHSIM_Rhsim10G0057900	0.989	1.346	0.357	no
Treat-40d	RHSIM_Rhsim10G0162400	0.401	-0.943	-1.344	no
Treat-40d	RHSIM_Rhsim09G0165400	0.218	-0.572	-0.790	no
Treat-40d	RHSIM_Rhsim09G0039300	-0.940	-1.036	-0.097	no
Treat-40d	RHSIM_Rhsim09G0152300	-0.859	-1.158	-0.299	no
Treat-40d	RHSIM_Rhsim10G0144800	0.273	-0.763	-1.037	no
Treat-40d	RHSIM_Rhsim10G0081500	0.027	-0.322	-0.348	no
Treat-40d	RHSIM_Rhsim09G0199500	-0.352	1.160	1.512	no
Treat-40d	RHSIM_Rhsim10G0043600	-0.003	0.717	0.719	no
Treat-40d	RHSIM_Rhsim09G0146200	-0.021	1.545	1.566	no
Treat-40d	RHSIM_Rhsim10G0162700	-0.262	-1.093	-0.830	no
Treat-40d	RHSIM_Rhsim09G0146500	-1.711	-0.852	0.859	low
Treat-40d	RHSIM_Rhsim09G0137600	0.164	0.709	0.545	no
Treat-40d	RHSIM_Rhsim10G0165500	0.294	-0.823	-1.117	no
Treat-40d	RHSIM_Rhsim10G0133000	0.225	1.036	0.811	no
Treat-40d	RHSIM_Rhsim10G0034600	-0.163	-0.511	-0.348	no
Treat-40d	RHSIM_Rhsim09G0128000	2.560	0.490	-2.071	high
Treat-40d	RHSIM_Rhsim09G0111600	1.166	0.225	-0.942	high
Treat-40d	RHSIM_Rhsim09G0025300	-0.027	1.412	1.439	no
Treat-40d	RHSIM_Rhsim11G0091000	-0.222	0.645	0.866	no
Treat-40d	RHSIM_Rhsim09G0136800	0.452	0.293	-0.159	no
Treat-40d	RHSIM_Rhsim09G0010200	-0.170	0.446	0.616	no
Treat-40d	RHSIM_Rhsim11G0153300	0.372	0.154	-0.218	no
Treat-40d	RHSIM_Rhsim09G0037000	-1.326	-1.482	-0.156	low
Treat-40d	RHSIM_Rhsim10G0019000	-1.348	1.132	2.480	low
Treat-40d	RHSIM_Rhsim09G0113200	-0.430	0.091	0.521	no
Treat-40d	RHSIM_Rhsim10G0186700	-0.159	-0.499	-0.341	no
Treat-40d	RHSIM_Rhsim10G0167000	0.170	-1.380	-1.550	no

Group	Gene	PTR (z-score difference)	z(log2 LFQ)	z(log2 FPKM)	extreme-PTR
Treat-40d	RHSIM_Rhsim10G0141700	-0.782	-0.947	-0.166	no
Treat-40d	RHSIM_Rhsim09G0165600	1.132	0.302	-0.830	high
Treat-40d	RHSIM_Rhsim10G0177900	1.010	0.128	-0.882	no
Treat-40d	RHSIM_Rhsim11G0183100	-0.895	-1.502	-0.607	no
Treat-40d	RHSIM_Rhsim09G0209400	-0.492	0.895	1.386	no
Treat-40d	RHSIM_Rhsim10G0000500	1.317	-0.528	-1.844	high
Treat-40d	RHSIM_Rhsim09G0067900	0.599	0.874	0.275	no
Treat-40d	RHSIM_Rhsim09G0001400	0.436	1.481	1.044	no
Treat-40d	RHSIM_Rhsim09G0140900	-2.799	-1.245	1.554	low
Treat-40d	RHSIM_Rhsim09G0197000	-2.116	-0.818	1.298	low
Treat-40d	RHSIM_Rhsim09G0129700	-0.267	0.030	0.296	no
Treat-40d	RHSIM_Rhsim10G0003500	-1.323	-1.291	0.032	low
Treat-40d	RHSIM_Rhsim10G0040000	2.180	1.270	-0.911	high
Treat-40d	RHSIM_Rhsim11G0056000	1.366	-0.788	-2.154	high
Treat-40d	RHSIM_Rhsim09G0213100	-0.215	-0.207	0.008	no
Treat-40d	RHSIM_Rhsim10G0145200	0.163	-0.365	-0.527	no
Treat-40d	RHSIM_Rhsim10G0001300	-0.254	-0.327	-0.074	no
Treat-40d	RHSIM_Rhsim09G0066100	-0.027	-0.818	-0.791	no
Treat-40d	RHSIM_Rhsim10G0049300	0.672	-0.803	-1.475	no
Treat-40d	RHSIM_Rhsim11G0134900	-0.704	-0.557	0.147	no
Treat-40d	RHSIM_Rhsim09G0165800	0.223	0.049	-0.174	no
Treat-40d	RHSIM_Rhsim09G0027800	-0.088	-0.247	-0.159	no
Treat-40d	RHSIM_Rhsim11G0197300	-0.544	1.032	1.576	no
Treat-40d	RHSIM_Rhsim10G0113000	-1.363	-1.127	0.237	low
Treat-40d	RHSIM_Rhsim09G0072200	-0.306	-1.494	-1.188	no
Treat-40d	RHSIM_Rhsim10G0053700	0.057	-1.604	-1.661	no
Treat-40d	RHSIM_Rhsim09G0107700	1.010	1.949	0.939	no
Treat-40d	RHSIM_Rhsim09G0112700	-2.480	-0.470	2.010	low
Treat-40d	RHSIM_Rhsim09G0103400	-1.580	-0.613	0.967	low
Treat-40d	RHSIM_Rhsim11G0111700	0.354	-0.355	-0.710	no
Treat-40d	RHSIM_Rhsim09G0109200	-3.141	-0.371	2.770	low
Treat-40d	RHSIM_Rhsim09G0058600	-0.969	-1.198	-0.229	no

Group	Gene	PTR (z-score difference)	z(log2 LFQ)	z(log2 FPKM)	extreme-PTR
Treat-40d	RHSIM_Rhsim09G0199000	-0.178	0.402	0.580	no
Treat-40d	RHSIM_Rhsim09G0157900	2.045	1.706	-0.340	high
Treat-40d	RHSIM_Rhsim11G0060100	0.562	0.004	-0.558	no
Treat-40d	RHSIM_Rhsim11G0049400	-1.086	0.341	1.426	no
Treat-40d	RHSIM_Rhsim09G0061400	0.335	1.250	0.915	no
Treat-40d	RHSIM_Rhsim09G0012100	-3.345	-0.287	3.058	low
Treat-40d	RHSIM_Rhsim09G0207600	-0.058	-0.762	-0.704	no
Treat-40d	RHSIM_Rhsim09G0037600	-0.633	0.351	0.984	no
Treat-40d	RHSIM_Rhsim09G0195600	0.443	-0.290	-0.733	no
Treat-40d	RHSIM_Rhsim10G0020400	0.433	0.515	0.082	no
Treat-40d	RHSIM_Rhsim10G0111500	-1.863	1.032	2.895	low
Treat-40d	RHSIM_Rhsim10G0121000	0.901	1.093	0.192	no
Treat-40d	RHSIM_Rhsim09G0166500	-0.858	-1.643	-0.785	no
Treat-40d	RHSIM_Rhsim10G0006500	-1.310	-0.838	0.472	low
Treat-40d	RHSIM_Rhsim09G0199700	0.827	-0.559	-1.385	no
Treat-40d	RHSIM_Rhsim09G0214500	0.254	0.120	-0.134	no
Treat-40d	RHSIM_Rhsim10G0044100	0.195	1.244	1.050	no
Treat-40d	RHSIM_Rhsim09G0044400	0.376	0.321	-0.056	no
Treat-40d	RHSIM_Rhsim09G0179000	-1.033	-1.147	-0.114	no
Treat-40d	RHSIM_Rhsim09G0213800	-0.452	0.751	1.203	no
Treat-40d	RHSIM_Rhsim09G0071800	0.205	-0.091	-0.296	no
Treat-40d	RHSIM_Rhsim09G0158600	-0.991	-0.829	0.161	no
Treat-40d	RHSIM_Rhsim09G0051700	-0.038	0.631	0.669	no
Treat-40d	RHSIM_Rhsim09G0126300	-1.049	-0.652	0.397	no
Treat-40d	RHSIM_Rhsim09G0140700	-1.357	1.504	2.861	low
Treat-40d	RHSIM_Rhsim09G0064600	1.420	0.002	-1.418	high
Treat-40d	RHSIM_Rhsim10G0183900	0.447	1.579	1.131	no
Treat-40d	RHSIM_Rhsim10G0072700	-0.673	-0.825	-0.152	no
Treat-40d	RHSIM_Rhsim10G0020900	0.389	-0.347	-0.736	no
Treat-40d	RHSIM_Rhsim09G0132000	0.281	1.425	1.143	no
Treat-40d	RHSIM_Rhsim10G0176900	-0.661	0.224	0.885	no
Treat-40d	RHSIM_Rhsim09G0208100	-0.508	-0.320	0.188	no

Group	Gene	PTR (z-score difference)	z(log2 LFQ)	z(log2 FPKM)	extreme-PTR
Treat-40d	RHSIM_Rhsim09G0073200	-0.314	-1.255	-0.942	no
Treat-40d	RHSIM_Rhsim10G0180400	-1.153	-0.897	0.257	low
Treat-40d	RHSIM_Rhsim10G0080000	2.640	1.037	-1.603	high
Treat-40d	RHSIM_Rhsim09G0020400	-0.224	-0.307	-0.083	no
Treat-40d	RHSIM_Rhsim09G0105400	-0.300	0.049	0.349	no
Treat-40d	RHSIM_Rhsim09G0005500	1.787	0.902	-0.885	high
Treat-40d	RHSIM_Rhsim10G0128500	0.502	0.048	-0.454	no
Treat-40d	RHSIM_Rhsim10G0192700	-1.204	0.002	1.206	low
Treat-40d	RHSIM_Rhsim10G0002200	-1.653	0.126	1.779	low
Treat-40d	RHSIM_Rhsim09G0188800	-0.293	0.882	1.175	no
Treat-40d	RHSIM_Rhsim10G0002800	-0.852	-1.017	-0.165	no
Treat-40d	RHSIM_Rhsim09G0092400	-1.154	0.710	1.864	low
Treat-40d	RHSIM_Rhsim09G0154300	-0.145	-0.664	-0.519	no
Treat-40d	RHSIM_Rhsim09G0184000	0.693	0.869	0.176	no
Treat-40d	RHSIM_Rhsim09G0195100	0.194	0.159	-0.034	no
Treat-40d	RHSIM_Rhsim09G0087000	-0.057	-0.595	-0.538	no
Treat-40d	RHSIM_Rhsim09G0172200	-0.678	-0.473	0.206	no
Treat-40d	RHSIM_Rhsim09G0206900	2.133	2.222	0.089	high
Treat-40d	RHSIM_Rhsim10G0041000	-1.335	-0.499	0.836	low
Treat-40d	RHSIM_Rhsim09G0037900	-1.056	-1.281	-0.225	no
Treat-40d	RHSIM_Rhsim09G0107200	0.516	-0.594	-1.110	no
Treat-40d	RHSIM_Rhsim09G0179700	0.105	-1.422	-1.527	no
Treat-40d	RHSIM_Rhsim10G0083200	-0.146	0.470	0.615	no
Treat-40d	RHSIM_Rhsim09G0212900	-1.074	-0.991	0.083	no
Treat-40d	RHSIM_Rhsim09G0096600	-0.685	-0.310	0.374	no
Treat-40d	RHSIM_Rhsim09G0109400	0.740	0.826	0.087	no
Treat-40d	RHSIM_Rhsim09G0058800	0.497	-0.141	-0.639	no
Treat-40d	RHSIM_Rhsim10G0185700	-0.126	-0.106	0.020	no
Treat-40d	RHSIM_Rhsim09G0190900	-0.279	-0.778	-0.499	no
Treat-40d	RHSIM_Rhsim09G0199400	-0.451	-1.404	-0.953	no
Treat-40d	RHSIM_Rhsim09G0214300	0.188	-0.150	-0.338	no
Treat-40d	RHSIM_Rhsim09G0110100	0.277	0.946	0.669	no

Group	Gene	PTR (z-score difference)	z(log2 LFQ)	z(log2 FPKM)	extreme-PTR
Treat-40d	RHSIM_Rhsim09G0043700	1.818	0.034	-1.784	high
Treat-40d	RHSIM_Rhsim09G0176800	-0.258	-1.091	-0.833	no
Treat-40d	RHSIM_Rhsim07G0015900	-0.322	0.914	1.236	no
Treat-40d	RHSIM_Rhsim09G0028200	-1.566	-0.920	0.645	low
Treat-40d	RHSIM_Rhsim10G0076800	1.527	1.313	-0.214	high
Treat-40d	RHSIM_Rhsim10G0021400	1.752	0.864	-0.888	high
Treat-40d	RHSIM_Rhsim09G0143800	0.046	-0.934	-0.980	no
Treat-40d	RHSIM_Rhsim09G0066000	0.015	1.266	1.251	no
Treat-40d	RHSIM_Rhsim09G0058700	-0.808	-2.002	-1.194	no
Treat-40d	RHSIM_Rhsim10G0127700	-0.479	0.013	0.492	no
Treat-40d	RHSIM_Rhsim09G0204400	-1.091	-0.408	0.683	no
Treat-40d	RHSIM_Rhsim09G0066200	-1.164	-0.739	0.426	low
Treat-40d	RHSIM_Rhsim10G0034300	0.527	0.417	-0.110	no
Treat-40d	RHSIM_Rhsim09G0211500	-0.597	-0.746	-0.149	no
Treat-40d	RHSIM_Rhsim09G0084400	0.810	0.011	-0.799	no
Treat-40d	RHSIM_Rhsim09G0154600	0.263	-0.009	-0.271	no
Treat-40d	RHSIM_Rhsim10G0214200	-1.210	-0.387	0.823	low
Treat-40d	RHSIM_Rhsim09G0130100	-0.071	1.622	1.693	no
Treat-40d	RHSIM_Rhsim09G0011700	-2.217	0.210	2.426	low
Treat-40d	RHSIM_Rhsim09G0103000	-1.510	-0.669	0.840	low
Treat-40d	RHSIM_Rhsim10G0186800	-3.139	-0.939	2.200	low
Treat-40d	RHSIM_Rhsim09G0146700	-0.933	-1.086	-0.152	no
Treat-40d	RHSIM_Rhsim09G0063000	1.245	-0.524	-1.769	high
Treat-40d	RHSIM_Rhsim10G0188100	2.087	-0.257	-2.344	high
Treat-40d	RHSIM_Rhsim09G0214600	-0.534	-0.515	0.018	no
Treat-40d	RHSIM_Rhsim10G0084800	0.625	0.117	-0.508	no
Treat-40d	RHSIM_Rhsim09G0122300	0.790	-0.423	-1.213	no
Treat-40d	RHSIM_Rhsim10G0167600	-0.827	-1.446	-0.618	no
Treat-40d	RHSIM_Rhsim09G0151300	-1.995	0.458	2.453	low
Treat-40d	RHSIM_Rhsim09G0012200	0.504	0.750	0.245	no
Treat-40d	RHSIM_Rhsim09G0061700	1.005	-0.360	-1.365	no
Treat-40d	RHSIM_Rhsim09G0166000	-1.706	0.820	2.526	low

Group	Gene	PTR (z-score difference)	z(log2 LFQ)	z(log2 FPKM)	extreme-PTR
Treat-40d	RHSIM_Rhsim09G0043800	1.084	0.192	-0.891	no
Treat-40d	RHSIM_Rhsim10G0185500	0.233	0.618	0.385	no
Treat-40d	RHSIM_Rhsim09G0150500	0.089	0.382	0.293	no
Treat-40d	RHSIM_Rhsim09G0152400	0.020	0.747	0.727	no
Treat-40d	RHSIM_Rhsim10G0163100	-0.366	0.168	0.534	no
Treat-40d	RHSIM_Rhsim09G0035000	-0.439	-0.619	-0.180	no
Treat-40d	RHSIM_Rhsim09G0117700	0.612	1.667	1.055	no
Treat-40d	RHSIM_Rhsim09G0033400	1.231	0.858	-0.373	high
Treat-40d	RHSIM_Rhsim09G0084100	-1.192	0.998	2.190	low
Treat-40d	RHSIM_Rhsim09G0090200	0.729	-0.482	-1.212	no
Treat-40d	RHSIM_Rhsim09G0094700	0.124	0.371	0.246	no
Treat-40d	RHSIM_Rhsim09G0143200	-0.903	2.127	3.030	no
Treat-40d	RHSIM_Rhsim10G0125000	-0.337	-0.645	-0.307	no
Treat-40d	RHSIM_Rhsim09G0004100	0.465	1.618	1.153	no
Treat-40d	RHSIM_Rhsim09G0140500	0.841	-0.714	-1.555	no
Treat-40d	RHSIM_Rhsim10G0004100	-0.542	-0.942	-0.400	no
Treat-40d	RHSIM_Rhsim09G0196200	-1.430	-0.825	0.605	low
Treat-40d	RHSIM_Rhsim09G0127700	-1.307	-0.896	0.411	low
Treat-40d	RHSIM_Rhsim09G0065100	-0.778	-1.031	-0.253	no
Treat-40d	RHSIM_Rhsim09G0202400	0.337	1.052	0.715	no
Treat-40d	RHSIM_Rhsim09G0148900	1.015	0.308	-0.707	no
Treat-40d	RHSIM_Rhsim09G0210400	1.208	0.875	-0.333	high
Treat-40d	RHSIM_Rhsim09G0011500	-0.854	-1.166	-0.312	no
Treat-40d	RHSIM_Rhsim09G0184800	0.297	-0.552	-0.849	no
Treat-40d	RHSIM_Rhsim10G0174500	-1.627	0.356	1.983	low
Treat-40d	RHSIM_Rhsim09G0105600	0.042	0.132	0.090	no
Treat-40d	RHSIM_Rhsim10G0004300	-0.036	0.398	0.435	no
Treat-40d	RHSIM_Rhsim09G0132100	-0.117	-0.246	-0.129	no
Treat-40d	RHSIM_Rhsim09G0078200	-0.821	-0.805	0.016	no
Treat-40d	RHSIM_Rhsim09G0206100	-0.852	-0.526	0.326	no
Treat-40d	RHSIM_Rhsim10G0166700	-0.828	-0.395	0.434	no
Treat-40d	RHSIM_Rhsim09G0049700	0.853	-0.680	-1.533	no

Group	Gene	PTR (z-score difference)	z(log2 LFQ)	z(log2 FPKM)	extreme-PTR
Treat-40d	RHSIM_Rhsim08G0034300	-0.603	-0.066	0.537	no
Treat-40d	RHSIM_Rhsim09G0030100	-0.080	-0.317	-0.237	no
Treat-40d	RHSIM_Rhsim10G0020100	1.372	2.540	1.168	high
Treat-40d	RHSIM_Rhsim09G0044600	0.790	0.423	-0.367	no
Treat-40d	RHSIM_Rhsim10G0092800	-0.261	-0.237	0.024	no
Treat-40d	RHSIM_Rhsim10G0064800	0.440	0.587	0.146	no
Treat-40d	RHSIM_Rhsim09G0034100	0.250	-0.149	-0.400	no
Treat-40d	RHSIM_Rhsim08G0095100	-1.342	-0.493	0.849	low
Treat-40d	RHSIM_Rhsim09G0173200	-1.143	0.569	1.712	low
Treat-40d	RHSIM_Rhsim10G0019700	-3.093	-0.901	2.192	low
Treat-40d	RHSIM_Rhsim09G0004500	-0.152	-1.431	-1.279	no
Treat-40d	RHSIM_Rhsim08G0223900	2.366	2.126	-0.240	high
Treat-40d	RHSIM_Rhsim08G0096200	-0.841	0.656	1.497	no
Treat-40d	RHSIM_Rhsim09G0140000	0.437	0.495	0.059	no
Treat-40d	RHSIM_Rhsim10G0138100	-1.761	-1.541	0.220	low
Treat-40d	RHSIM_Rhsim08G0116000	-0.376	-0.294	0.081	no
Treat-40d	RHSIM_Rhsim09G0208800	1.890	1.330	-0.560	high
Treat-40d	RHSIM_Rhsim08G0039900	0.626	1.995	1.369	no
Treat-40d	RHSIM_Rhsim09G0091600	0.020	-0.892	-0.912	no
Treat-40d	RHSIM_Rhsim08G0000800	-0.956	-0.546	0.410	no
Treat-40d	RHSIM_Rhsim09G0034800	-1.116	-0.043	1.074	no
Treat-40d	RHSIM_Rhsim10G0008400	-0.963	-1.708	-0.745	no
Treat-40d	RHSIM_Rhsim09G0154900	-0.407	-0.590	-0.183	no
Treat-40d	RHSIM_Rhsim09G0206000	-0.908	-0.645	0.263	no
Treat-40d	RHSIM_Rhsim09G0013300	0.587	0.211	-0.376	no
Treat-40d	RHSIM_Rhsim10G0104900	0.555	-0.147	-0.702	no
Treat-40d	RHSIM_Rhsim08G0244500	1.997	1.503	-0.494	high
Treat-40d	RHSIM_Rhsim08G0231100	-0.178	0.351	0.529	no
Treat-40d	RHSIM_Rhsim10G0119200	-1.055	-0.345	0.710	no
Treat-40d	RHSIM_Rhsim08G0237400	-0.090	-0.509	-0.419	no
Treat-40d	RHSIM_Rhsim09G0182100	-2.334	-1.680	0.654	low
Treat-40d	RHSIM_Rhsim08G0102500	0.137	-0.660	-0.798	no

Group	Gene	PTR (z-score difference)	z(log2 LFQ)	z(log2 FPKM)	extreme-PTR
Treat-40d	RHSIM_Rhsim09G0097800	-0.446	-0.364	0.082	no
Treat-40d	RHSIM_Rhsim08G0128100	-0.441	-0.937	-0.496	no
Treat-40d	RHSIM_Rhsim10G0016800	1.434	0.064	-1.370	high
Treat-40d	RHSIM_Rhsim09G0049500	-0.313	-1.054	-0.741	no
Treat-40d	RHSIM_Rhsim09G0128800	0.979	-0.338	-1.317	no
Treat-40d	RHSIM_Rhsim09G0005700	0.817	0.294	-0.523	no
Treat-40d	RHSIM_Rhsim09G0081900	-1.280	0.603	1.883	low
Treat-40d	RHSIM_Rhsim08G0128800	0.414	0.206	-0.207	no
Treat-40d	RHSIM_Rhsim09G0137900	-1.138	-0.822	0.316	no
Treat-40d	RHSIM_Rhsim09G0187600	-0.356	-0.730	-0.373	no
Treat-40d	RHSIM_Rhsim09G0214000	0.817	0.557	-0.260	no
Treat-40d	RHSIM_Rhsim08G0039400	-0.043	1.747	1.790	no
Treat-40d	RHSIM_Rhsim09G0005000	0.938	0.187	-0.751	no
Treat-40d	RHSIM_Rhsim09G0145300	1.128	0.122	-1.005	high
Treat-40d	RHSIM_Rhsim09G0193000	-0.171	0.210	0.381	no
Treat-40d	RHSIM_Rhsim09G0203700	-0.434	-0.873	-0.438	no
Treat-40d	RHSIM_Rhsim08G0027400	-1.156	-0.891	0.266	low
Treat-40d	RHSIM_Rhsim09G0204300	-1.720	-1.793	-0.073	low
Treat-40d	RHSIM_Rhsim10G0151200	-0.653	-0.724	-0.071	no
Treat-40d	RHSIM_Rhsim10G0049800	-0.098	-0.413	-0.315	no
Treat-40d	RHSIM_Rhsim09G0129900	-1.844	0.129	1.973	low
Treat-40d	RHSIM_Rhsim08G0146000	0.418	0.144	-0.274	no
Treat-40d	RHSIM_Rhsim09G0122200	0.272	-0.183	-0.454	no
Treat-40d	RHSIM_Rhsim09G0142800	-2.194	-1.048	1.146	low
Treat-40d	RHSIM_Rhsim09G0141500	-0.033	0.595	0.629	no
Treat-40d	RHSIM_Rhsim08G0115800	-1.331	-0.001	1.330	low
Treat-40d	RHSIM_Rhsim09G0177600	-3.141	-0.890	2.251	low
Treat-40d	RHSIM_Rhsim08G0113500	-2.089	-1.740	0.349	low
Treat-40d	RHSIM_Rhsim09G0153300	-1.295	-0.601	0.693	low
Treat-40d	RHSIM_Rhsim08G0250700	-0.549	-0.364	0.185	no
Treat-40d	RHSIM_Rhsim09G0201300	-0.634	-0.717	-0.083	no
Treat-40d	RHSIM_Rhsim09G0142500	0.062	0.166	0.104	no

Group	Gene	PTR (z-score difference)	z(log2 LFQ)	z(log2 FPKM)	extreme-PTR
Treat-40d	RHSIM_Rhsim09G0074600	0.108	0.426	0.318	no
Treat-40d	RHSIM_Rhsim09G0089600	-0.588	-0.789	-0.201	no
Treat-40d	RHSIM_Rhsim09G0030700	0.460	-0.236	-0.696	no
Treat-40d	RHSIM_Rhsim08G0043800	0.093	0.262	0.169	no
Treat-40d	RHSIM_Rhsim09G0169300	-0.214	-0.507	-0.293	no
Treat-40d	RHSIM_Rhsim09G0127200	0.611	0.249	-0.362	no
Treat-40d	RHSIM_Rhsim08G0125200	-0.439	-0.781	-0.342	no
Treat-40d	RHSIM_Rhsim08G0191700	0.091	0.102	0.012	no
Treat-40d	RHSIM_Rhsim09G0069100	1.909	2.984	1.076	high
Treat-40d	RHSIM_Rhsim08G0067800	1.281	0.455	-0.826	high
Treat-40d	RHSIM_Rhsim09G0201100	-1.212	-0.325	0.887	low
Treat-40d	RHSIM_Rhsim09G0029200	-1.558	-0.551	1.006	low
Treat-40d	RHSIM_Rhsim09G0061500	0.810	0.052	-0.758	no
Treat-40d	RHSIM_Rhsim09G0171700	1.686	0.576	-1.109	high
Treat-40d	RHSIM_Rhsim08G0183700	0.170	-0.242	-0.412	no
Treat-40d	RHSIM_Rhsim09G0179300	-0.982	-1.197	-0.216	no
Treat-40d	RHSIM_Rhsim08G0006400	2.155	0.664	-1.491	high
Treat-40d	RHSIM_Rhsim09G0039400	1.313	0.031	-1.282	high
Treat-40d	RHSIM_Rhsim09G0145500	-0.755	-0.301	0.454	no
Treat-40d	RHSIM_Rhsim10G0145000	-0.056	1.008	1.064	no
Treat-40d	RHSIM_Rhsim09G0205100	-0.810	-1.388	-0.578	no
Treat-40d	RHSIM_Rhsim09G0178000	0.438	-1.578	-2.016	no
Treat-40d	RHSIM_Rhsim09G0113100	1.786	-0.074	-1.861	high
Treat-40d	RHSIM_Rhsim08G0121400	0.900	0.043	-0.856	no
Treat-40d	RHSIM_Rhsim08G0202500	0.681	0.840	0.159	no
Treat-40d	RHSIM_Rhsim08G0084400	-0.347	-0.376	-0.029	no
Treat-40d	RHSIM_Rhsim08G0167000	1.231	-0.356	-1.588	high
Treat-40d	RHSIM_Rhsim09G0023000	0.612	-0.206	-0.818	no
Treat-40d	RHSIM_Rhsim08G0224700	0.074	2.310	2.236	no
Treat-40d	RHSIM_Rhsim09G0151500	0.348	-0.192	-0.540	no
Treat-40d	RHSIM_Rhsim09G0126900	-1.264	-0.406	0.858	low
Treat-40d	RHSIM_Rhsim09G0125900	-0.789	-0.349	0.440	no

Group	Gene	PTR (z-score difference)	z(log2 LFQ)	z(log2 FPKM)	extreme-PTR
Treat-40d	RHSIM_Rhsim09G0197100	-0.608	0.794	1.401	no
Treat-40d	RHSIM_Rhsim09G0117300	0.174	0.216	0.042	no
Treat-40d	RHSIM_Rhsim08G0028900	-0.977	-1.039	-0.062	no
Treat-40d	RHSIM_Rhsim10G0212000	-0.903	0.893	1.797	no
Treat-40d	RHSIM_Rhsim09G0181400	-0.176	-0.057	0.119	no
Treat-40d	RHSIM_Rhsim09G0088400	-0.404	-0.398	0.005	no
Treat-40d	RHSIM_Rhsim08G0214200	-0.025	0.254	0.280	no
Treat-40d	RHSIM_Rhsim08G0063900	-0.175	0.176	0.351	no
Treat-40d	RHSIM_Rhsim09G0082300	-1.428	-1.163	0.265	low
Treat-40d	RHSIM_Rhsim09G0172400	-0.429	0.637	1.066	no
Treat-40d	RHSIM_Rhsim08G0055000	0.447	1.917	1.470	no
Treat-40d	RHSIM_Rhsim09G0040800	0.187	-0.884	-1.071	no
Treat-40d	RHSIM_Rhsim08G0044300	0.316	-0.367	-0.684	no
Treat-40d	RHSIM_Rhsim08G0006500	0.823	0.378	-0.445	no
Treat-40d	RHSIM_Rhsim09G0171600	-1.128	-0.651	0.477	no
Treat-40d	RHSIM_Rhsim09G0168800	1.122	0.259	-0.862	high
Treat-40d	RHSIM_Rhsim08G0043500	-0.806	-0.873	-0.067	no
Treat-40d	RHSIM_Rhsim09G0004000	-1.376	-1.518	-0.142	low
Treat-40d	RHSIM_Rhsim09G0183900	-1.047	-0.271	0.775	no
Treat-40d	RHSIM_Rhsim08G0005200	-2.511	-2.817	-0.306	low
Treat-40d	RHSIM_Rhsim09G0152700	3.021	2.850	-0.171	high
Treat-40d	RHSIM_Rhsim09G0046600	0.499	0.065	-0.434	no
Treat-40d	RHSIM_Rhsim08G0082200	-1.372	-1.653	-0.282	low
Treat-40d	RHSIM_Rhsim08G0014900	0.351	0.479	0.128	no
Treat-40d	RHSIM_Rhsim08G0081400	-1.074	-1.496	-0.423	no
Treat-40d	RHSIM_Rhsim08G0159000	1.196	0.734	-0.462	high
Treat-40d	RHSIM_Rhsim08G0171800	-1.204	-0.391	0.813	low
Treat-40d	RHSIM_Rhsim08G0033300	-1.012	-0.757	0.255	no
Treat-40d	RHSIM_Rhsim10G0045100	0.958	1.036	0.078	no
Treat-40d	RHSIM_Rhsim09G0077000	-0.797	-0.732	0.065	no
Treat-40d	RHSIM_Rhsim09G0198800	2.171	0.075	-2.095	high
Treat-40d	RHSIM_Rhsim08G0141300	0.025	-0.922	-0.947	no

Group	Gene	PTR (z-score difference)	z(log2 LFQ)	z(log2 FPKM)	extreme-PTR
Treat-40d	RHSIM_Rhsim08G0196800	0.942	1.441	0.500	no
Treat-40d	RHSIM_Rhsim08G0037100	-0.871	-0.535	0.336	no
Treat-40d	RHSIM_Rhsim08G0042900	-0.086	0.073	0.159	no
Treat-40d	RHSIM_Rhsim08G0181700	0.723	1.486	0.763	no
Treat-40d	RHSIM_Rhsim10G0122600	0.336	-0.761	-1.097	no
Treat-40d	RHSIM_Rhsim08G0114500	-1.218	-0.369	0.850	low
Treat-40d	RHSIM_Rhsim08G0240000	-0.971	-0.519	0.451	no
Treat-40d	RHSIM_Rhsim09G0133500	0.604	0.202	-0.402	no
Treat-40d	RHSIM_Rhsim08G0100200	0.067	0.502	0.434	no
Treat-40d	RHSIM_Rhsim08G0248700	-0.278	-0.887	-0.609	no
Treat-40d	RHSIM_Rhsim09G0184200	-2.542	-0.876	1.665	low
Treat-40d	RHSIM_Rhsim08G0026000	0.349	0.084	-0.265	no
Treat-40d	RHSIM_Rhsim09G0141000	-0.916	-1.463	-0.547	no
Treat-40d	RHSIM_Rhsim08G0229600	0.836	-0.707	-1.544	no
Treat-40d	RHSIM_Rhsim08G0241300	-0.008	0.005	0.013	no
Treat-40d	RHSIM_Rhsim09G0164300	-0.512	-1.562	-1.050	no
Treat-40d	RHSIM_Rhsim09G0145400	0.003	0.161	0.158	no
Treat-40d	RHSIM_Rhsim08G0209300	1.394	1.050	-0.344	high
Treat-40d	RHSIM_Rhsim08G0122300	-2.045	2.771	4.816	low
Treat-40d	RHSIM_Rhsim09G0189300	-0.880	0.428	1.308	no
Treat-40d	RHSIM_Rhsim08G0054900	2.129	2.023	-0.106	high
Treat-40d	RHSIM_Rhsim09G0117800	0.785	0.347	-0.438	no
Treat-40d	RHSIM_Rhsim08G0102400	0.010	-1.642	-1.652	no
Treat-40d	RHSIM_Rhsim08G0230600	-2.396	-0.103	2.293	low
Treat-40d	RHSIM_Rhsim09G0000900	0.342	0.096	-0.246	no
Treat-40d	RHSIM_Rhsim08G0150200	0.349	-0.402	-0.750	no
Treat-40d	RHSIM_Rhsim08G0067900	1.019	0.966	-0.053	no
Treat-40d	RHSIM_Rhsim10G0129000	1.526	-0.166	-1.692	high
Treat-40d	RHSIM_Rhsim08G0121500	-0.688	-1.195	-0.507	no
Treat-40d	RHSIM_Rhsim08G0040400	0.441	0.745	0.304	no
Treat-40d	RHSIM_Rhsim09G0010600	-0.523	0.097	0.619	no
Treat-40d	RHSIM_Rhsim10G0125300	-0.012	-0.759	-0.747	no

Group	Gene	PTR (z-score difference)	z(log2 LFQ)	z(log2 FPKM)	extreme-PTR
Treat-40d	RHSIM_Rhsim08G0223400	-0.366	1.491	1.857	no
Treat-40d	RHSIM_Rhsim08G0103000	1.743	0.607	-1.135	high
Treat-40d	RHSIM_Rhsim10G0139000	1.280	-0.463	-1.743	high
Treat-40d	RHSIM_Rhsim09G0008600	-1.168	0.807	1.975	low
Treat-40d	RHSIM_Rhsim08G0183000	-0.752	0.859	1.611	no
Treat-40d	RHSIM_Rhsim08G0215400	2.260	0.429	-1.831	high
Treat-40d	RHSIM_Rhsim09G0210600	0.995	1.506	0.511	no
Treat-40d	RHSIM_Rhsim08G0020800	-0.480	-0.191	0.289	no
Treat-40d	RHSIM_Rhsim09G0111700	0.415	0.461	0.045	no
Treat-40d	RHSIM_Rhsim08G0188900	-0.416	-0.150	0.265	no
Treat-40d	RHSIM_Rhsim10G0078400	0.052	-0.977	-1.029	no
Treat-40d	RHSIM_Rhsim08G0053600	-0.302	0.835	1.137	no
Treat-40d	RHSIM_Rhsim09G0099400	-0.402	-0.158	0.244	no
Treat-40d	RHSIM_Rhsim09G0106300	0.418	0.695	0.278	no
Treat-40d	RHSIM_Rhsim08G0008500	-0.412	-0.551	-0.139	no
Treat-40d	RHSIM_Rhsim10G0211900	0.699	0.448	-0.251	no
Treat-40d	RHSIM_Rhsim08G0033600	-0.779	-0.135	0.644	no
Treat-40d	RHSIM_Rhsim09G0142300	1.143	-1.029	-2.172	high
Treat-40d	RHSIM_Rhsim09G0008200	-0.771	-0.666	0.105	no
Treat-40d	RHSIM_Rhsim08G0083400	-1.295	-0.760	0.535	low
Treat-40d	RHSIM_Rhsim09G0138400	1.081	1.149	0.068	no
Treat-40d	RHSIM_Rhsim08G0156200	-0.863	-0.346	0.518	no
Treat-40d	RHSIM_Rhsim08G0153100	-0.251	0.872	1.124	no
Treat-40d	RHSIM_Rhsim09G0203300	-0.896	-0.315	0.581	no
Treat-40d	RHSIM_Rhsim09G0207900	1.451	1.147	-0.304	high
Treat-40d	RHSIM_Rhsim08G0208100	-0.548	-1.260	-0.712	no
Treat-40d	RHSIM_Rhsim08G0034400	-0.406	-0.952	-0.546	no
Treat-40d	RHSIM_Rhsim08G0205700	2.973	0.941	-2.031	high
Treat-40d	RHSIM_Rhsim09G0155500	-0.159	-1.216	-1.057	no
Treat-40d	RHSIM_Rhsim08G0145900	-0.633	0.076	0.709	no
Treat-40d	RHSIM_Rhsim08G0041500	-0.645	0.910	1.555	no
Treat-40d	RHSIM_Rhsim08G0082100	-0.440	-0.222	0.218	no

Group	Gene	PTR (z-score difference)	z(log2 LFQ)	z(log2 FPKM)	extreme-PTR
Treat-40d	RHSIM_Rhsim10G0049400	0.580	0.675	0.094	no
Treat-40d	RHSIM_Rhsim08G0174000	0.880	0.758	-0.122	no
Treat-40d	RHSIM_Rhsim10G0104100	-2.456	-1.757	0.699	low
Treat-40d	RHSIM_Rhsim09G0056700	0.633	0.070	-0.564	no
Treat-40d	RHSIM_Rhsim09G0069000	0.700	1.597	0.897	no
Treat-40d	RHSIM_Rhsim09G0200500	0.110	-0.068	-0.178	no
Treat-40d	RHSIM_Rhsim09G0115400	0.761	0.482	-0.279	no
Treat-40d	RHSIM_Rhsim08G0069100	0.374	-0.178	-0.552	no
Treat-40d	RHSIM_Rhsim08G0202300	-3.367	-1.345	2.021	low
Treat-40d	RHSIM_Rhsim09G0024000	-1.905	0.128	2.033	low
Treat-40d	RHSIM_Rhsim09G0022900	-1.214	-0.231	0.983	low
Treat-40d	RHSIM_Rhsim08G0038100	-0.431	0.250	0.681	no
Treat-40d	RHSIM_Rhsim08G0045400	1.765	1.707	-0.058	high
Treat-40d	RHSIM_Rhsim08G0180500	4.386	3.997	-0.389	high
Treat-40d	RHSIM_Rhsim09G0128100	0.117	0.521	0.404	no
Treat-40d	RHSIM_Rhsim08G0109700	1.342	3.774	2.432	high
Treat-40d	RHSIM_Rhsim08G0047900	3.011	1.131	-1.880	high
Treat-40d	RHSIM_Rhsim08G0013000	-0.457	-0.397	0.060	no
Treat-40d	RHSIM_Rhsim08G0088400	0.289	1.327	1.038	no
Treat-40d	RHSIM_Rhsim09G0002200	1.025	1.143	0.118	no
Treat-40d	RHSIM_Rhsim08G0030700	1.855	0.285	-1.569	high
Treat-40d	RHSIM_Rhsim08G0020500	0.904	-1.273	-2.176	no
Treat-40d	RHSIM_Rhsim08G0045800	-1.710	0.916	2.625	low
Treat-40d	RHSIM_Rhsim08G0245700	-0.292	1.145	1.436	no
Treat-40d	RHSIM_Rhsim09G0114500	-2.492	-0.819	1.673	low
Treat-40d	RHSIM_Rhsim09G0109300	-2.645	0.165	2.810	low
Treat-40d	RHSIM_Rhsim08G0043600	0.268	0.890	0.622	no
Treat-40d	RHSIM_Rhsim09G0090700	1.461	-0.839	-2.300	high
Treat-40d	RHSIM_Rhsim09G0201200	0.168	0.787	0.619	no
Treat-40d	RHSIM_Rhsim08G0195500	-1.207	0.659	1.866	low
Treat-40d	RHSIM_Rhsim08G0008300	-0.068	-1.248	-1.179	no
Treat-40d	RHSIM_Rhsim09G0210000	-0.965	-1.341	-0.376	no

Group	Gene	PTR (z-score difference)	z(log2 LFQ)	z(log2 FPKM)	extreme-PTR
Treat-40d	RHSIM_Rhsim09G0149700	1.082	0.792	-0.291	no
Treat-40d	RHSIM_Rhsim09G0067700	-0.179	0.254	0.432	no
Treat-40d	RHSIM_Rhsim09G0144000	2.902	0.835	-2.067	high
Treat-40d	RHSIM_Rhsim08G0044100	-3.407	-0.932	2.475	low
Treat-40d	RHSIM_Rhsim09G0146300	0.089	1.196	1.107	no
Treat-40d	RHSIM_Rhsim08G0188400	1.283	1.756	0.473	high
Treat-40d	RHSIM_Rhsim08G0096400	-0.742	-1.242	-0.500	no
Treat-40d	RHSIM_Rhsim09G0197500	2.161	1.208	-0.953	high
Treat-40d	RHSIM_Rhsim09G0168700	-0.050	1.157	1.208	no
Treat-40d	RHSIM_Rhsim09G0025000	0.230	-1.235	-1.464	no
Treat-40d	RHSIM_Rhsim08G0037000	0.218	0.595	0.377	no
Treat-40d	RHSIM_Rhsim08G0072300	0.185	0.427	0.242	no
Treat-40d	RHSIM_Rhsim09G0000500	0.268	0.054	-0.214	no
Treat-40d	RHSIM_Rhsim08G0176100	0.479	-0.833	-1.312	no
Treat-40d	RHSIM_Rhsim09G0052100	1.565	0.429	-1.136	high
Treat-40d	RHSIM_Rhsim08G0185000	-1.223	-1.114	0.109	low
Treat-40d	RHSIM_Rhsim09G0074800	0.205	-0.179	-0.384	no
Treat-40d	RHSIM_Rhsim09G0032100	1.227	0.473	-0.754	high
Treat-40d	RHSIM_Rhsim09G0143900	2.764	1.069	-1.695	high
Treat-40d	RHSIM_Rhsim08G0103200	2.583	0.261	-2.323	high
Treat-40d	RHSIM_Rhsim08G0114200	0.255	-0.065	-0.320	no
Treat-40d	RHSIM_Rhsim08G0012200	-0.353	-0.238	0.114	no
Treat-40d	RHSIM_Rhsim08G0086400	-1.043	-1.634	-0.591	no
Treat-40d	RHSIM_Rhsim09G0100400	-0.576	-0.924	-0.348	no
Treat-40d	RHSIM_Rhsim09G0114100	1.535	0.499	-1.036	high
Treat-40d	RHSIM_Rhsim09G0109100	0.242	0.658	0.416	no
Treat-40d	RHSIM_Rhsim09G0000200	0.264	0.365	0.101	no
Treat-40d	RHSIM_Rhsim09G0206600	0.385	-0.535	-0.920	no
Treat-40d	RHSIM_Rhsim08G0141600	1.769	1.688	-0.081	high
Treat-40d	RHSIM_Rhsim08G0221100	0.258	-0.437	-0.695	no
Treat-40d	RHSIM_Rhsim08G0185200	1.275	-0.207	-1.482	high
Treat-40d	RHSIM_Rhsim08G0231900	-0.580	-0.848	-0.268	no

Group	Gene	PTR (z-score difference)	z(log2 LFQ)	z(log2 FPKM)	extreme-PTR
Treat-40d	RHSIM_Rhsim08G0002400	1.061	0.401	-0.659	no
Treat-40d	RHSIM_Rhsim08G0239200	3.156	0.980	-2.176	high
Treat-40d	RHSIM_Rhsim08G0069300	0.275	0.731	0.456	no
Treat-40d	RHSIM_Rhsim08G0131700	0.015	-1.057	-1.072	no
Treat-40d	RHSIM_Rhsim09G0008700	-0.403	-0.071	0.332	no
Treat-40d	RHSIM_Rhsim09G0028300	-1.583	-2.090	-0.507	low
Treat-40d	RHSIM_Rhsim08G0241200	1.377	0.723	-0.654	high
Treat-40d	RHSIM_Rhsim08G0167100	1.638	-0.459	-2.097	high
Treat-40d	RHSIM_Rhsim08G0186100	-1.574	-1.271	0.303	low
Treat-40d	RHSIM_Rhsim09G0000700	-0.771	-0.693	0.077	no
Treat-40d	RHSIM_Rhsim08G0214400	0.033	1.066	1.033	no
Treat-40d	RHSIM_Rhsim08G0230200	-0.937	-1.376	-0.438	no
Treat-40d	RHSIM_Rhsim08G0046300	-0.462	-0.897	-0.435	no
Treat-40d	RHSIM_Rhsim08G0241900	-1.425	-0.961	0.464	low
Treat-40d	RHSIM_Rhsim08G0242600	0.869	0.369	-0.500	no
Treat-40d	RHSIM_Rhsim08G0244800	-0.649	1.028	1.677	no
Treat-40d	RHSIM_Rhsim08G0046700	0.616	1.369	0.752	no
Treat-40d	RHSIM_Rhsim08G0026100	0.162	-0.235	-0.397	no
Treat-40d	RHSIM_Rhsim09G0212200	-1.024	-0.580	0.444	no
Treat-40d	RHSIM_Rhsim09G0099700	0.156	-1.629	-1.785	no
Treat-40d	RHSIM_Rhsim09G0171300	-1.295	-1.677	-0.382	low
Treat-40d	RHSIM_Rhsim08G0084800	1.078	0.018	-1.060	no
Treat-40d	RHSIM_Rhsim09G0119600	-0.550	-0.944	-0.394	no
Treat-40d	RHSIM_Rhsim09G0213700	1.701	0.581	-1.120	high
Treat-40d	RHSIM_Rhsim09G0188300	-0.914	-0.944	-0.031	no
Treat-40d	RHSIM_Rhsim08G0127800	0.523	1.592	1.069	no
Treat-40d	RHSIM_Rhsim08G0030100	0.434	0.173	-0.261	no
Treat-40d	RHSIM_Rhsim08G0102200	0.091	-0.033	-0.124	no
Treat-40d	RHSIM_Rhsim08G0214000	1.001	1.610	0.609	no
Treat-40d	RHSIM_Rhsim08G0030200	-0.557	-0.308	0.249	no
Treat-40d	RHSIM_Rhsim09G0185000	0.494	0.079	-0.414	no
Treat-40d	RHSIM_Rhsim09G0171800	0.575	0.622	0.047	no

Group	Gene	PTR (z-score difference)	z(log2 LFQ)	z(log2 FPKM)	extreme-PTR
Treat-40d	RHSIM_Rhsim08G0211400	1.056	0.524	-0.532	no
Treat-40d	RHSIM_Rhsim09G0064200	-0.960	-0.684	0.276	no
Treat-40d	RHSIM_Rhsim08G0094100	-0.603	0.096	0.699	no
Treat-40d	RHSIM_Rhsim09G0105100	1.061	-0.064	-1.125	no
Treat-40d	RHSIM_Rhsim08G0029600	3.955	2.982	-0.973	high
Treat-40d	RHSIM_Rhsim09G0111100	0.196	0.062	-0.134	no
Treat-40d	RHSIM_Rhsim09G0179800	-0.012	-0.996	-0.983	no
Treat-40d	RHSIM_Rhsim08G0019700	0.671	1.313	0.643	no
Treat-40d	RHSIM_Rhsim09G0093800	-0.338	-0.451	-0.114	no
Treat-40d	RHSIM_Rhsim09G0209300	-1.159	-0.026	1.133	low
Treat-40d	RHSIM_Rhsim08G0094000	0.887	1.823	0.936	no
Treat-40d	RHSIM_Rhsim09G0110000	1.930	-0.393	-2.323	high
Treat-40d	RHSIM_Rhsim08G0018700	0.565	-1.266	-1.830	no
Treat-40d	RHSIM_Rhsim08G0173300	0.538	-0.481	-1.019	no
Treat-40d	RHSIM_Rhsim09G0164800	-0.002	-0.336	-0.334	no
Treat-40d	RHSIM_Rhsim08G0195100	-1.018	0.415	1.434	no
Treat-40d	RHSIM_Rhsim08G0199200	-1.982	-1.351	0.632	low
Treat-40d	RHSIM_Rhsim08G0034000	-1.948	-1.259	0.690	low
Treat-40d	RHSIM_Rhsim09G0164900	0.650	-0.329	-0.979	no
Treat-40d	RHSIM_Rhsim09G0000100	-2.058	-1.081	0.977	low
Treat-40d	RHSIM_Rhsim08G0045600	0.343	-0.687	-1.029	no
Treat-40d	RHSIM_Rhsim08G0025900	0.598	-0.009	-0.607	no
Treat-40d	RHSIM_Rhsim08G0174600	-0.294	-0.289	0.005	no
Treat-40d	RHSIM_Rhsim09G0142700	-0.109	-0.025	0.083	no
Treat-40d	RHSIM_Rhsim08G0237100	-1.471	-1.939	-0.468	low
Treat-40d	RHSIM_Rhsim08G0032100	0.286	0.596	0.310	no
Treat-40d	RHSIM_Rhsim08G0148900	0.709	0.523	-0.186	no
Treat-40d	RHSIM_Rhsim08G0246900	-0.856	-0.448	0.408	no
Treat-40d	RHSIM_Rhsim07G0073300	-0.625	0.752	1.377	no
Treat-40d	RHSIM_Rhsim08G0045900	0.744	1.543	0.798	no
Treat-40d	RHSIM_Rhsim08G0075800	-0.216	0.411	0.627	no
Treat-40d	RHSIM_Rhsim08G0109800	0.247	-0.775	-1.022	no

Group	Gene	PTR (z-score difference)	z(log2 LFQ)	z(log2 FPKM)	extreme-PTR
Treat-40d	RHSIM_Rhsim07G0082300	2.076	0.741	-1.335	high
Treat-40d	RHSIM_Rhsim08G0074500	-0.175	-0.446	-0.271	no
Treat-40d	RHSIM_Rhsim09G0152100	0.719	0.417	-0.301	no
Treat-40d	RHSIM_Rhsim08G0203900	1.186	1.086	-0.100	high
Treat-40d	RHSIM_Rhsim08G0028600	0.348	0.072	-0.276	no
Treat-40d	RHSIM_Rhsim08G0098800	-2.928	-1.159	1.769	low
Treat-40d	RHSIM_Rhsim08G0232500	0.281	0.914	0.634	no
Treat-40d	RHSIM_Rhsim08G0233200	-0.319	-0.229	0.089	no
Treat-40d	RHSIM_Rhsim07G0063000	0.502	0.045	-0.457	no
Treat-40d	RHSIM_Rhsim08G0062700	0.949	1.064	0.115	no
Treat-40d	RHSIM_Rhsim08G0195300	0.546	0.749	0.203	no
Treat-40d	RHSIM_Rhsim08G0190900	0.259	0.524	0.265	no
Treat-40d	RHSIM_Rhsim07G0109500	2.158	-0.006	-2.163	high
Treat-40d	RHSIM_Rhsim09G0111900	0.538	0.836	0.299	no
Treat-40d	RHSIM_Rhsim07G0077100	0.161	-0.876	-1.037	no
Treat-40d	RHSIM_Rhsim09G0003200	0.779	0.231	-0.548	no
Treat-40d	RHSIM_Rhsim08G0051500	-1.024	-0.857	0.167	no
Treat-40d	RHSIM_Rhsim08G0206900	-0.376	-0.783	-0.407	no
Treat-40d	RHSIM_Rhsim09G0013500	-0.054	-1.159	-1.105	no
Treat-40d	RHSIM_Rhsim09G0004800	1.808	1.099	-0.708	high
Treat-40d	RHSIM_Rhsim08G0152700	-0.867	0.276	1.143	no
Treat-40d	RHSIM_Rhsim08G0150000	0.672	0.668	-0.004	no
Treat-40d	RHSIM_Rhsim09G0192600	1.255	0.731	-0.523	high
Treat-40d	RHSIM_Rhsim09G0073000	0.520	-0.021	-0.541	no
Treat-40d	RHSIM_Rhsim07G0227100	1.102	-0.891	-1.993	no
Treat-40d	RHSIM_Rhsim08G0232800	0.366	0.122	-0.244	no
Treat-40d	RHSIM_Rhsim09G0069200	1.675	0.907	-0.768	high
Treat-40d	RHSIM_Rhsim08G0250100	1.073	1.363	0.290	no
Treat-40d	RHSIM_Rhsim08G0072500	-0.623	0.000	0.623	no
Treat-40d	RHSIM_Rhsim07G0142300	1.718	2.539	0.821	high
Treat-40d	RHSIM_Rhsim08G0125600	-0.071	0.560	0.631	no
Treat-40d	RHSIM_Rhsim07G0079800	-0.123	-0.187	-0.064	no

Group	Gene	PTR (z-score difference)	z(log2 LFQ)	z(log2 FPKM)	extreme-PTR
Treat-40d	RHSIM_Rhsim08G0106000	2.049	-0.202	-2.251	high
Treat-40d	RHSIM_Rhsim08G0225600	-0.373	-1.167	-0.794	no
Treat-40d	RHSIM_Rhsim07G0095900	0.062	0.385	0.323	no
Treat-40d	RHSIM_Rhsim08G0008000	0.563	-0.756	-1.319	no
Treat-40d	RHSIM_Rhsim08G0005000	-0.240	-0.165	0.075	no
Treat-40d	RHSIM_Rhsim09G0102400	0.051	-0.448	-0.499	no
Treat-40d	RHSIM_Rhsim08G0109400	0.080	-0.586	-0.666	no
Treat-40d	RHSIM_Rhsim09G0106700	0.877	0.019	-0.857	no
Treat-40d	RHSIM_Rhsim08G0161300	-0.968	-1.439	-0.471	no
Treat-40d	RHSIM_Rhsim08G0003400	0.361	-0.603	-0.964	no
Treat-40d	RHSIM_Rhsim09G0138500	-0.422	0.152	0.575	no
Treat-40d	RHSIM_Rhsim09G0066700	2.092	0.511	-1.580	high
Treat-40d	RHSIM_Rhsim08G0054100	1.827	1.071	-0.755	high
Treat-40d	RHSIM_Rhsim07G0212400	0.339	-0.320	-0.659	no
Treat-40d	RHSIM_Rhsim07G0257800	-0.220	0.306	0.526	no
Treat-40d	RHSIM_Rhsim09G0106600	0.004	-1.325	-1.329	no
Treat-40d	RHSIM_Rhsim07G0038700	1.059	0.947	-0.112	no
Treat-40d	RHSIM_Rhsim07G0074200	-0.128	-0.845	-0.717	no
Treat-40d	RHSIM_Rhsim08G0079800	-0.219	-0.328	-0.109	no
Treat-40d	RHSIM_Rhsim08G0205400	-0.108	-0.641	-0.533	no
Treat-40d	RHSIM_Rhsim08G0185100	0.770	1.233	0.463	no
Treat-40d	RHSIM_Rhsim08G0160300	0.604	0.336	-0.268	no
Treat-40d	RHSIM_Rhsim08G0240600	0.097	-0.877	-0.974	no
Treat-40d	RHSIM_Rhsim07G0106500	0.564	-0.357	-0.921	no
Treat-40d	RHSIM_Rhsim09G0203200	0.161	-0.656	-0.816	no
Treat-40d	RHSIM_Rhsim08G0202600	0.699	-0.411	-1.110	no
Treat-40d	RHSIM_Rhsim07G0011800	0.305	1.189	0.883	no
Treat-40d	RHSIM_Rhsim08G0242400	-1.324	-0.926	0.398	low
Treat-40d	RHSIM_Rhsim08G0003500	0.351	0.780	0.429	no
Treat-40d	RHSIM_Rhsim08G0136400	0.229	-0.057	-0.287	no
Treat-40d	RHSIM_Rhsim09G0074000	0.017	-0.838	-0.854	no
Treat-40d	RHSIM_Rhsim08G0044500	-0.134	-1.045	-0.910	no

Group	Gene	PTR (z-score difference)	z(log2 LFQ)	z(log2 FPKM)	extreme-PTR
Treat-40d	RHSIM_Rhsim09G0190400	-0.632	-0.133	0.499	no
Treat-40d	RHSIM_Rhsim08G0207400	0.321	1.841	1.520	no
Treat-40d	RHSIM_Rhsim07G0010300	1.108	-0.135	-1.243	no
Treat-40d	RHSIM_Rhsim07G0204900	1.763	-0.038	-1.801	high
Treat-40d	RHSIM_Rhsim08G0190700	0.848	-0.179	-1.027	no
Treat-40d	RHSIM_Rhsim07G0096600	-1.162	0.194	1.356	low
Treat-40d	RHSIM_Rhsim08G0118900	-0.141	-0.784	-0.643	no
Treat-40d	RHSIM_Rhsim08G0241100	-1.630	-0.839	0.791	low
Treat-40d	RHSIM_Rhsim07G0225100	-0.619	0.720	1.339	no
Treat-40d	RHSIM_Rhsim08G0086000	-1.852	0.434	2.286	low
Treat-40d	RHSIM_Rhsim07G0087000	-1.133	-0.509	0.624	no
Treat-40d	RHSIM_Rhsim08G0076000	-1.346	-0.638	0.708	low
Treat-40d	RHSIM_Rhsim07G0203200	-0.384	-0.289	0.095	no
Treat-40d	RHSIM_Rhsim08G0222800	2.752	0.424	-2.328	high
Treat-40d	RHSIM_Rhsim09G0132300	-1.541	1.158	2.699	low
Treat-40d	RHSIM_Rhsim08G0071500	-0.398	-0.043	0.355	no
Treat-40d	RHSIM_Rhsim07G0062900	-0.492	-0.996	-0.504	no
Treat-40d	RHSIM_Rhsim08G0088200	0.261	1.220	0.959	no
Treat-40d	RHSIM_Rhsim09G0031700	-0.926	0.232	1.158	no
Treat-40d	RHSIM_Rhsim09G0090500	-0.708	-0.706	0.003	no
Treat-40d	RHSIM_Rhsim08G0208000	-2.124	-0.393	1.731	low
Treat-40d	RHSIM_Rhsim08G0075200	0.648	0.745	0.098	no
Treat-40d	RHSIM_Rhsim08G0197500	-0.038	-0.690	-0.652	no
Treat-40d	RHSIM_Rhsim07G0077300	-0.509	0.323	0.832	no
Treat-40d	RHSIM_Rhsim07G0006700	0.720	-0.627	-1.347	no
Treat-40d	RHSIM_Rhsim08G0209600	-1.193	-1.636	-0.443	low
Treat-40d	RHSIM_Rhsim07G0119700	1.494	1.495	0.001	high
Treat-40d	RHSIM_Rhsim07G0100700	-0.112	-0.692	-0.580	no
Treat-40d	RHSIM_Rhsim07G0215500	-0.197	-0.422	-0.225	no
Treat-40d	RHSIM_Rhsim08G0212100	-0.426	-0.991	-0.566	no
Treat-40d	RHSIM_Rhsim08G0216500	0.539	-0.019	-0.558	no
Treat-40d	RHSIM_Rhsim07G0017000	-0.652	0.700	1.352	no

Group	Gene	PTR (z-score difference)	z(log2 LFQ)	z(log2 FPKM)	extreme-PTR
Treat-40d	RHSIM_Rhsim09G0003500	-0.131	-0.394	-0.263	no
Treat-40d	RHSIM_Rhsim08G0189100	-0.370	0.063	0.434	no
Treat-40d	RHSIM_Rhsim08G0115500	-3.042	-1.103	1.939	low
Treat-40d	RHSIM_Rhsim08G0052700	-0.445	-1.771	-1.325	no
Treat-40d	RHSIM_Rhsim09G0181600	0.166	0.182	0.016	no
Treat-40d	RHSIM_Rhsim08G0132500	-0.346	0.434	0.780	no
Treat-40d	RHSIM_Rhsim09G0182800	0.804	-0.301	-1.105	no
Treat-40d	RHSIM_Rhsim07G0123700	-0.671	-0.497	0.174	no
Treat-40d	RHSIM_Rhsim08G0199800	0.259	0.733	0.474	no
Treat-40d	RHSIM_Rhsim09G0146100	-0.136	-0.546	-0.410	no
Treat-40d	RHSIM_Rhsim08G0228400	-0.382	0.687	1.069	no
Treat-40d	RHSIM_Rhsim08G0146100	1.119	2.091	0.972	high
Treat-40d	RHSIM_Rhsim07G0165600	1.056	1.265	0.209	no
Treat-40d	RHSIM_Rhsim09G0211400	0.053	-0.880	-0.933	no
Treat-40d	RHSIM_Rhsim08G0086100	1.522	1.063	-0.460	high
Treat-40d	RHSIM_Rhsim09G0084200	-1.132	0.566	1.698	no
Treat-40d	RHSIM_Rhsim07G0010500	0.356	-1.020	-1.376	no
Treat-40d	RHSIM_Rhsim07G0118600	-0.861	-0.837	0.024	no
Treat-40d	RHSIM_Rhsim08G0067700	-1.707	-1.517	0.190	low
Treat-40d	RHSIM_Rhsim08G0080100	-1.660	-0.959	0.701	low
Treat-40d	RHSIM_Rhsim08G0002700	-1.086	-0.307	0.778	no
Treat-40d	RHSIM_Rhsim08G0029700	2.918	0.824	-2.093	high
Treat-40d	RHSIM_Rhsim09G0126400	-2.331	-1.649	0.682	low
Treat-40d	RHSIM_Rhsim09G0199100	-1.804	-1.488	0.316	low
Treat-40d	RHSIM_Rhsim09G0210100	0.847	0.668	-0.178	no
Treat-40d	RHSIM_Rhsim08G0157100	-0.077	-0.375	-0.298	no
Treat-40d	RHSIM_Rhsim08G0199500	0.058	-0.168	-0.226	no
Treat-40d	RHSIM_Rhsim09G0079200	-0.138	0.205	0.343	no
Treat-40d	RHSIM_Rhsim08G0225700	-0.764	-0.633	0.131	no
Treat-40d	RHSIM_Rhsim08G0191000	1.938	0.388	-1.550	high
Treat-40d	RHSIM_Rhsim07G0037800	0.467	0.261	-0.206	no
Treat-40d	RHSIM_Rhsim08G0113200	-1.723	-0.780	0.943	low

Group	Gene	PTR (z-score difference)	z(log2 LFQ)	z(log2 FPKM)	extreme-PTR
Treat-40d	RHSIM_Rhsim08G0187700	0.534	0.618	0.084	no
Treat-40d	RHSIM_Rhsim08G0033400	2.658	2.610	-0.048	high
Treat-40d	RHSIM_Rhsim08G0177500	-1.821	-0.972	0.849	low
Treat-40d	RHSIM_Rhsim08G0135900	2.719	0.637	-2.083	high
Treat-40d	RHSIM_Rhsim08G0081500	0.265	2.309	2.044	no
Treat-40d	RHSIM_Rhsim08G0233000	-0.769	-2.086	-1.317	no
Treat-40d	RHSIM_Rhsim08G0013100	-0.427	-0.885	-0.458	no
Treat-40d	RHSIM_Rhsim08G0176300	0.253	-0.580	-0.833	no
Treat-40d	RHSIM_Rhsim08G0221200	1.434	1.748	0.314	high
Treat-40d	RHSIM_Rhsim07G0115500	-0.670	-0.132	0.538	no
Treat-40d	RHSIM_Rhsim08G0009100	-0.327	0.393	0.721	no
Treat-40d	RHSIM_Rhsim08G0233300	-0.187	0.389	0.576	no
Treat-40d	RHSIM_Rhsim07G0204400	0.676	1.393	0.717	no
Treat-40d	RHSIM_Rhsim08G0015000	-0.004	-0.590	-0.586	no
Treat-40d	RHSIM_Rhsim07G0074900	-0.622	-0.513	0.109	no
Treat-40d	RHSIM_Rhsim08G0100700	1.711	0.812	-0.899	high
Treat-40d	RHSIM_Rhsim08G0012800	0.822	0.532	-0.290	no
Treat-40d	RHSIM_Rhsim08G0000900	-0.285	-0.022	0.263	no
Treat-40d	RHSIM_Rhsim08G0009800	0.464	-0.282	-0.747	no
Treat-40d	RHSIM_Rhsim08G0118800	0.128	-0.873	-1.001	no
Treat-40d	RHSIM_Rhsim08G0020200	-0.047	-0.269	-0.223	no
Treat-40d	RHSIM_Rhsim08G0032000	-1.463	0.232	1.695	low
Treat-40d	RHSIM_Rhsim07G0118400	-0.202	-1.652	-1.450	no
Treat-40d	RHSIM_Rhsim07G0173200	1.113	0.409	-0.705	high
Treat-40d	RHSIM_Rhsim08G0210300	2.220	0.174	-2.046	high
Treat-40d	RHSIM_Rhsim07G0124000	2.872	0.786	-2.086	high
Treat-40d	RHSIM_Rhsim08G0151900	-0.689	-0.624	0.066	no
Treat-40d	RHSIM_Rhsim08G0070300	0.723	0.292	-0.431	no
Treat-40d	RHSIM_Rhsim08G0096300	-0.482	-0.273	0.209	no
Treat-40d	RHSIM_Rhsim07G0016900	0.080	-0.595	-0.676	no
Treat-40d	RHSIM_Rhsim08G0249800	-1.000	-1.590	-0.590	no
Treat-40d	RHSIM_Rhsim07G0131600	-0.143	-0.636	-0.492	no

Group	Gene	PTR (z-score difference)	z(log2 LFQ)	z(log2 FPKM)	extreme-PTR
Treat-40d	RHSIM_Rhsim08G0056900	-0.375	-1.282	-0.907	no
Treat-40d	RHSIM_Rhsim08G0170400	1.589	2.036	0.446	high
Treat-40d	RHSIM_Rhsim07G0175400	0.535	0.262	-0.274	no
Treat-40d	RHSIM_Rhsim07G0170000	0.141	0.682	0.541	no
Treat-40d	RHSIM_Rhsim07G0057300	0.565	0.175	-0.389	no
Treat-40d	RHSIM_Rhsim08G0071700	-0.100	0.661	0.760	no
Treat-40d	RHSIM_Rhsim07G0035700	-0.236	-0.442	-0.206	no
Treat-40d	RHSIM_Rhsim07G0172100	1.324	-1.079	-2.403	high
Treat-40d	RHSIM_Rhsim08G0077000	-0.621	0.301	0.922	no
Treat-40d	RHSIM_Rhsim08G0161000	-0.447	0.575	1.022	no
Treat-40d	RHSIM_Rhsim07G0257200	1.890	1.022	-0.868	high
Treat-40d	RHSIM_Rhsim07G0024200	-0.214	-0.982	-0.768	no
Treat-40d	RHSIM_Rhsim08G0088300	1.100	2.008	0.907	no
Treat-40d	RHSIM_Rhsim08G0188700	-0.407	0.029	0.436	no
Treat-40d	RHSIM_Rhsim08G0200500	-2.160	-0.532	1.628	low
Treat-40d	RHSIM_Rhsim08G0157300	-0.247	-0.588	-0.341	no
Treat-40d	RHSIM_Rhsim07G0017400	-1.514	0.685	2.199	low
Treat-40d	RHSIM_Rhsim07G0085300	0.296	1.232	0.936	no
Treat-40d	RHSIM_Rhsim08G0216800	-0.641	0.368	1.010	no
Treat-40d	RHSIM_Rhsim08G0126200	0.546	0.526	-0.020	no
Treat-40d	RHSIM_Rhsim07G0011500	-0.589	1.124	1.713	no
Treat-40d	RHSIM_Rhsim08G0222300	-0.820	-0.580	0.240	no
Treat-40d	RHSIM_Rhsim07G0216000	-1.431	-0.643	0.788	low
Treat-40d	RHSIM_Rhsim08G0245600	1.375	-0.223	-1.597	high
Treat-40d	RHSIM_Rhsim08G0153200	1.291	0.736	-0.555	high
Treat-40d	RHSIM_Rhsim07G0101700	0.415	-0.549	-0.964	no
Treat-40d	RHSIM_Rhsim08G0248400	2.872	3.261	0.389	high
Treat-40d	RHSIM_Rhsim08G0153400	-0.346	-0.418	-0.072	no
Treat-40d	RHSIM_Rhsim08G0215500	0.466	-1.494	-1.960	no
Treat-40d	RHSIM_Rhsim08G0240500	0.542	-0.089	-0.631	no
Treat-40d	RHSIM_Rhsim07G0004600	-2.209	-1.657	0.552	low
Treat-40d	RHSIM_Rhsim08G0157700	-0.578	-0.594	-0.016	no

Group	Gene	PTR (z-score difference)	z(log2 LFQ)	z(log2 FPKM)	extreme-PTR
Treat-40d	RHSIM_Rhsim07G0053800	0.152	0.296	0.143	no
Treat-40d	RHSIM_Rhsim08G0118400	1.969	-0.164	-2.133	high
Treat-40d	RHSIM_Rhsim08G0230900	0.361	0.352	-0.009	no
Treat-40d	RHSIM_Rhsim07G0248600	-0.334	-0.665	-0.332	no
Treat-40d	RHSIM_Rhsim07G0144500	-0.685	-0.344	0.341	no
Treat-40d	RHSIM_Rhsim07G0201100	-0.333	-1.108	-0.775	no
Treat-40d	RHSIM_Rhsim08G0012300	0.884	1.117	0.233	no
Treat-40d	RHSIM_Rhsim07G0023600	0.059	-0.618	-0.678	no
Treat-40d	RHSIM_Rhsim08G0241600	-0.424	-0.775	-0.351	no
Treat-40d	RHSIM_Rhsim07G0025600	2.347	0.612	-1.735	high
Treat-40d	RHSIM_Rhsim08G0123400	-0.427	0.154	0.581	no
Treat-40d	RHSIM_Rhsim08G0040600	0.446	-0.507	-0.953	no
Treat-40d	RHSIM_Rhsim08G0119200	-0.425	0.986	1.410	no
Treat-40d	RHSIM_Rhsim08G0058800	1.059	-0.912	-1.971	no
Treat-40d	RHSIM_Rhsim07G0019000	1.248	0.729	-0.519	high
Treat-40d	RHSIM_Rhsim07G0251100	0.379	1.582	1.203	no
Treat-40d	RHSIM_Rhsim08G0169300	-0.786	-1.493	-0.707	no
Treat-40d	RHSIM_Rhsim08G0093900	-0.672	0.872	1.544	no
Treat-40d	RHSIM_Rhsim08G0098500	-0.335	-0.640	-0.305	no
Treat-40d	RHSIM_Rhsim08G0187400	0.922	1.555	0.632	no
Treat-40d	RHSIM_Rhsim08G0212600	-0.558	-0.283	0.275	no
Treat-40d	RHSIM_Rhsim07G0258200	-1.191	-0.664	0.527	low
Treat-40d	RHSIM_Rhsim07G0146900	-0.632	-0.991	-0.359	no
Treat-40d	RHSIM_Rhsim08G0078700	0.487	-0.211	-0.698	no
Treat-40d	RHSIM_Rhsim08G0043300	0.215	0.574	0.359	no
Treat-40d	RHSIM_Rhsim08G0184400	1.234	0.151	-1.083	high
Treat-40d	RHSIM_Rhsim08G0119000	1.205	1.341	0.136	high
Treat-40d	RHSIM_Rhsim07G0209200	-2.953	-1.326	1.628	low
Treat-40d	RHSIM_Rhsim08G0068000	-0.662	0.067	0.728	no
Treat-40d	RHSIM_Rhsim07G0250500	-0.779	0.573	1.352	no
Treat-40d	RHSIM_Rhsim08G0199900	0.975	0.112	-0.863	no
Treat-40d	RHSIM_Rhsim07G0188400	-3.749	-2.765	0.984	low

Group	Gene	PTR (z-score difference)	z(log2 LFQ)	z(log2 FPKM)	extreme-PTR
Treat-40d	RHSIM_Rhsim07G0184400	-0.004	-1.766	-1.762	no
Treat-40d	RHSIM_Rhsim08G0207200	-0.921	0.345	1.265	no
Treat-40d	RHSIM_Rhsim08G0216200	-0.054	-0.123	-0.069	no
Treat-40d	RHSIM_Rhsim08G0227300	-2.406	-1.088	1.318	low
Treat-40d	RHSIM_Rhsim08G0124200	1.018	-0.019	-1.037	no
Treat-40d	RHSIM_Rhsim07G0186000	-0.255	-0.203	0.052	no
Treat-40d	RHSIM_Rhsim08G0027500	-0.643	-0.296	0.347	no
Treat-40d	RHSIM_Rhsim08G0000300	-0.485	-0.559	-0.075	no
Treat-40d	RHSIM_Rhsim08G0155400	0.067	0.238	0.171	no
Treat-40d	RHSIM_Rhsim07G0190000	-2.006	-0.876	1.130	low
Treat-40d	RHSIM_Rhsim08G0125000	-0.621	-0.271	0.351	no
Treat-40d	RHSIM_Rhsim07G0256400	-0.882	0.012	0.894	no
Treat-40d	RHSIM_Rhsim08G0146900	-0.343	0.079	0.422	no
Treat-40d	RHSIM_Rhsim07G0125300	1.307	1.053	-0.253	high
Treat-40d	RHSIM_Rhsim08G0198600	1.654	1.103	-0.551	high
Treat-40d	RHSIM_Rhsim08G0124400	-1.079	-0.997	0.083	no
Treat-40d	RHSIM_Rhsim08G0106700	1.451	-0.321	-1.772	high
Treat-40d	RHSIM_Rhsim08G0095400	-0.676	-0.878	-0.202	no
Treat-40d	RHSIM_Rhsim08G0022000	-1.426	0.399	1.825	low
Treat-40d	RHSIM_Rhsim07G0102300	-0.101	-0.602	-0.501	no
Treat-40d	RHSIM_Rhsim08G0198900	-1.560	0.024	1.584	low
Treat-40d	RHSIM_Rhsim07G0187600	-0.697	0.518	1.215	no
Treat-40d	RHSIM_Rhsim08G0038400	-1.896	-0.832	1.064	low
Treat-40d	RHSIM_Rhsim08G0066200	-0.129	2.291	2.419	no
Treat-40d	RHSIM_Rhsim08G0129000	0.533	1.820	1.287	no
Treat-40d	RHSIM_Rhsim08G0076500	-0.248	0.447	0.694	no
Treat-40d	RHSIM_Rhsim08G0171400	-0.190	-0.430	-0.240	no
Treat-40d	RHSIM_Rhsim08G0047100	1.621	0.320	-1.302	high
Treat-40d	RHSIM_Rhsim08G0206000	0.478	-0.875	-1.353	no
Treat-40d	RHSIM_Rhsim07G0219200	-0.343	-1.041	-0.698	no
Treat-40d	RHSIM_Rhsim08G0227800	0.579	1.226	0.647	no
Treat-40d	RHSIM_Rhsim08G0249600	-0.166	-1.207	-1.041	no

Group	Gene	PTR (z-score difference)	z(log2 LFQ)	z(log2 FPKM)	extreme-PTR
Treat-40d	RHSIM_Rhsim07G0251700	1.730	1.872	0.142	high
Treat-40d	RHSIM_Rhsim08G0038900	0.587	0.884	0.298	no
Treat-40d	RHSIM_Rhsim08G0096700	-0.527	-0.542	-0.015	no
Treat-40d	RHSIM_Rhsim07G0067800	0.790	-0.131	-0.921	no
Treat-40d	RHSIM_Rhsim08G0006300	0.855	0.190	-0.664	no
Treat-40d	RHSIM_Rhsim07G0075200	-1.334	-1.044	0.290	low
Treat-40d	RHSIM_Rhsim07G0215000	-0.760	-1.048	-0.288	no
Treat-40d	RHSIM_Rhsim07G0007400	-0.354	-1.063	-0.709	no
Treat-40d	RHSIM_Rhsim08G0115600	-0.454	0.143	0.597	no
Treat-40d	RHSIM_Rhsim07G0241500	1.276	0.447	-0.828	high
Treat-40d	RHSIM_Rhsim08G0005400	-2.423	-1.254	1.169	low
Treat-40d	RHSIM_Rhsim07G0208900	-0.984	0.150	1.134	no
Treat-40d	RHSIM_Rhsim08G0044400	0.174	-0.719	-0.893	no
Treat-40d	RHSIM_Rhsim08G0018500	1.239	0.613	-0.626	high
Treat-40d	RHSIM_Rhsim08G0090300	-0.021	1.048	1.069	no
Treat-40d	RHSIM_Rhsim07G0052800	0.648	-0.515	-1.163	no
Treat-40d	RHSIM_Rhsim07G0108500	-1.056	-1.012	0.044	no
Treat-40d	RHSIM_Rhsim07G0063200	-1.085	-1.346	-0.261	no
Treat-40d	RHSIM_Rhsim07G0080500	2.030	1.044	-0.986	high
Treat-40d	RHSIM_Rhsim08G0074900	0.592	-0.399	-0.991	no
Treat-40d	RHSIM_Rhsim08G0010200	-1.876	1.136	3.012	low
Treat-40d	RHSIM_Rhsim07G0032500	1.044	0.681	-0.362	no
Treat-40d	RHSIM_Rhsim08G0171600	0.915	3.421	2.506	no
Treat-40d	RHSIM_Rhsim07G0096500	0.585	1.874	1.290	no
Treat-40d	RHSIM_Rhsim08G0252300	-0.573	-0.830	-0.257	no
Treat-40d	RHSIM_Rhsim07G0181400	0.646	0.055	-0.591	no
Treat-40d	RHSIM_Rhsim08G0173200	-1.678	0.137	1.815	low
Treat-40d	RHSIM_Rhsim07G0091300	-0.248	-0.309	-0.061	no
Treat-40d	RHSIM_Rhsim07G0054000	-1.757	-1.026	0.731	low
Treat-40d	RHSIM_Rhsim07G0068700	0.285	-0.167	-0.451	no
Treat-40d	RHSIM_Rhsim08G0016700	-0.222	0.831	1.053	no
Treat-40d	RHSIM_Rhsim07G0080100	0.083	0.214	0.131	no

Group	Gene	PTR (z-score difference)	z(log2 LFQ)	z(log2 FPKM)	extreme-PTR
Treat-40d	RHSIM_Rhsim07G0245700	0.283	-0.303	-0.586	no
Treat-40d	RHSIM_Rhsim08G0080000	-0.831	-0.281	0.549	no
Treat-40d	RHSIM_Rhsim08G0140900	-0.929	-0.720	0.209	no
Treat-40d	RHSIM_Rhsim08G0046200	-0.118	0.155	0.273	no
Treat-40d	RHSIM_Rhsim08G0010700	-0.746	-0.755	-0.009	no
Treat-40d	RHSIM_Rhsim07G0145700	0.131	0.459	0.328	no
Treat-40d	RHSIM_Rhsim07G0006200	-0.168	0.142	0.311	no
Treat-40d	RHSIM_Rhsim07G0013500	-1.139	-1.509	-0.370	no
Treat-40d	RHSIM_Rhsim07G0053100	-0.200	0.777	0.977	no
Treat-40d	RHSIM_Rhsim08G0249200	-1.297	-0.645	0.652	low
Treat-40d	RHSIM_Rhsim07G0096000	0.834	0.777	-0.057	no
Treat-40d	RHSIM_Rhsim08G0245800	-0.961	-0.339	0.622	no
Treat-40d	RHSIM_Rhsim08G0019400	0.001	-0.995	-0.996	no
Treat-40d	RHSIM_Rhsim08G0102300	0.293	-0.854	-1.147	no
Treat-40d	RHSIM_Rhsim07G0005700	-0.901	-1.221	-0.320	no
Treat-40d	RHSIM_Rhsim07G0056700	0.741	-0.362	-1.103	no
Treat-40d	RHSIM_Rhsim08G0210500	0.539	0.453	-0.086	no
Treat-40d	RHSIM_Rhsim07G0188300	-0.017	-1.294	-1.277	no
Treat-40d	RHSIM_Rhsim08G0037900	0.792	-0.183	-0.975	no
Treat-40d	RHSIM_Rhsim07G0023500	0.771	-0.278	-1.049	no
Treat-40d	RHSIM_Rhsim07G0068500	-0.281	0.458	0.739	no
Treat-40d	RHSIM_Rhsim07G0117800	-0.257	1.043	1.300	no
Treat-40d	RHSIM_Rhsim08G0119100	-0.115	0.622	0.737	no
Treat-40d	RHSIM_Rhsim07G0186700	-0.411	-0.757	-0.346	no
Treat-40d	RHSIM_Rhsim08G0034600	-0.709	0.701	1.410	no
Treat-40d	RHSIM_Rhsim07G0083300	-0.313	-0.983	-0.670	no
Treat-40d	RHSIM_Rhsim08G0139800	-0.427	-0.243	0.184	no
Treat-40d	RHSIM_Rhsim07G0240400	-1.567	-1.050	0.516	low
Treat-40d	RHSIM_Rhsim07G0126200	-0.393	0.095	0.489	no
Treat-40d	RHSIM_Rhsim07G0251400	-0.205	0.709	0.914	no
Treat-40d	RHSIM_Rhsim07G0127400	-1.488	-0.752	0.735	low
Treat-40d	RHSIM_Rhsim07G0022300	-0.617	0.140	0.757	no

Group	Gene	PTR (z-score difference)	z(log2 LFQ)	z(log2 FPKM)	extreme-PTR
Treat-40d	RHSIM_Rhsim07G0094300	-1.091	-0.984	0.108	no
Treat-40d	RHSIM_Rhsim07G0170800	-1.321	-1.343	-0.022	low
Treat-40d	RHSIM_Rhsim08G0031200	2.088	1.719	-0.369	high
Treat-40d	RHSIM_Rhsim07G0071200	1.356	0.129	-1.227	high
Treat-40d	RHSIM_Rhsim08G0067400	1.534	0.441	-1.093	high
Treat-40d	RHSIM_Rhsim08G0175500	-0.541	-0.607	-0.066	no
Treat-40d	RHSIM_Rhsim08G0028400	-0.516	-1.845	-1.329	no
Treat-40d	RHSIM_Rhsim08G0249300	-0.776	0.029	0.806	no
Treat-40d	RHSIM_Rhsim07G0155300	-1.170	-0.697	0.474	low
Treat-40d	RHSIM_Rhsim07G0181600	-1.493	-1.077	0.416	low
Treat-40d	RHSIM_Rhsim07G0202000	0.376	1.482	1.105	no
Treat-40d	RHSIM_Rhsim08G0221900	-0.074	-0.958	-0.884	no
Treat-40d	RHSIM_Rhsim07G0040000	0.098	1.908	1.810	no
Treat-40d	RHSIM_Rhsim08G0000400	-0.362	0.169	0.531	no
Treat-40d	RHSIM_Rhsim08G0050800	1.273	-0.103	-1.376	high
Treat-40d	RHSIM_Rhsim07G0236600	0.331	-1.094	-1.425	no
Treat-40d	RHSIM_Rhsim07G0219500	-1.397	-0.601	0.795	low
Treat-40d	RHSIM_Rhsim07G0059300	-0.181	-1.155	-0.974	no
Treat-40d	RHSIM_Rhsim07G0209000	1.216	0.405	-0.811	high
Treat-40d	RHSIM_Rhsim08G0126600	-0.596	-0.539	0.057	no
Treat-40d	RHSIM_Rhsim07G0163600	0.721	-0.836	-1.558	no
Treat-40d	RHSIM_Rhsim07G0173800	-0.008	-0.414	-0.406	no
Treat-40d	RHSIM_Rhsim07G0239800	-0.603	-0.466	0.137	no
Treat-40d	RHSIM_Rhsim07G0164400	-1.449	-1.529	-0.080	low
Treat-40d	RHSIM_Rhsim07G0031300	-1.157	-0.599	0.558	low
Treat-40d	RHSIM_Rhsim07G0016500	-0.076	-0.051	0.026	no
Treat-40d	RHSIM_Rhsim07G0250100	4.075	1.651	-2.424	high
Treat-40d	RHSIM_Rhsim07G0107500	-1.592	-1.060	0.532	low
Treat-40d	RHSIM_Rhsim08G0024600	0.301	-0.558	-0.859	no
Treat-40d	RHSIM_Rhsim07G0068000	-0.453	-0.744	-0.291	no
Treat-40d	RHSIM_Rhsim07G0024600	-1.119	-1.205	-0.087	no
Treat-40d	RHSIM_Rhsim07G0038000	0.581	0.431	-0.150	no

Group	Gene	PTR (z-score difference)	z(log2 LFQ)	z(log2 FPKM)	extreme-PTR
Treat-40d	RHSIM_Rhsim07G0207200	-1.320	0.137	1.456	low
Treat-40d	RHSIM_Rhsim07G0086400	-0.161	-0.685	-0.524	no
Treat-40d	RHSIM_Rhsim07G0029300	0.339	-1.090	-1.429	no
Treat-40d	RHSIM_Rhsim08G0068500	-0.873	0.557	1.430	no
Treat-40d	RHSIM_Rhsim07G0069700	1.500	-0.874	-2.373	high
Treat-40d	RHSIM_Rhsim07G0033300	-0.440	-0.925	-0.486	no
Treat-40d	RHSIM_Rhsim07G0105400	0.628	-0.095	-0.724	no
Treat-40d	RHSIM_Rhsim08G0085100	0.956	0.907	-0.049	no
Treat-40d	RHSIM_Rhsim07G0173600	-1.937	-0.429	1.508	low
Treat-40d	RHSIM_Rhsim08G0016400	-0.691	-0.813	-0.122	no
Treat-40d	RHSIM_Rhsim08G0152500	1.414	2.835	1.422	high
Treat-40d	RHSIM_Rhsim07G0027900	-1.995	-1.320	0.675	low
Treat-40d	RHSIM_Rhsim07G0026600	-0.811	-1.176	-0.365	no
Treat-40d	RHSIM_Rhsim07G0055300	1.067	1.711	0.645	no
Treat-40d	RHSIM_Rhsim08G0176400	-1.068	1.276	2.344	no
Treat-40d	RHSIM_Rhsim07G0179300	-0.874	-2.265	-1.390	no
Treat-40d	RHSIM_Rhsim08G0232200	-0.134	-0.517	-0.383	no
Treat-40d	RHSIM_Rhsim07G0061300	-0.458	-0.351	0.107	no
Treat-40d	RHSIM_Rhsim07G0125000	-0.263	-0.132	0.131	no
Treat-40d	RHSIM_Rhsim07G0228300	-0.293	-0.349	-0.056	no
Treat-40d	RHSIM_Rhsim07G0020700	-1.083	0.308	1.391	no
Treat-40d	RHSIM_Rhsim07G0132100	-1.225	-0.720	0.505	low
Treat-40d	RHSIM_Rhsim07G0074100	0.248	0.114	-0.135	no
Treat-40d	RHSIM_Rhsim07G0198900	0.270	-0.371	-0.641	no
Treat-40d	RHSIM_Rhsim07G0007900	1.839	2.673	0.834	high
Treat-40d	RHSIM_Rhsim07G0243800	-0.656	2.240	2.896	no
Treat-40d	RHSIM_Rhsim07G0239400	-1.427	-1.280	0.147	low
Treat-40d	RHSIM_Rhsim07G0025200	0.002	-0.737	-0.739	no
Treat-40d	RHSIM_Rhsim07G0143700	-1.374	-0.464	0.911	low
Treat-40d	RHSIM_Rhsim08G0094700	-0.762	-0.262	0.500	no
Treat-40d	RHSIM_Rhsim07G0214200	0.559	-0.920	-1.479	no
Treat-40d	RHSIM_Rhsim07G0109900	-0.430	-1.483	-1.054	no

Group	Gene	PTR (z-score difference)	z(log2 LFQ)	z(log2 FPKM)	extreme-PTR
Treat-40d	RHSIM_Rhsim08G0173100	-0.777	-0.101	0.676	no
Treat-40d	RHSIM_Rhsim07G0049200	-0.299	0.620	0.918	no
Treat-40d	RHSIM_Rhsim08G0224200	-0.554	-0.712	-0.158	no
Treat-40d	RHSIM_Rhsim08G0112300	-0.550	-0.455	0.095	no
Treat-40d	RHSIM_Rhsim07G0172200	-0.701	-0.154	0.547	no
Treat-40d	RHSIM_Rhsim08G0226500	-0.150	-0.379	-0.229	no
Treat-40d	RHSIM_Rhsim07G0215100	-1.146	-0.833	0.313	low
Treat-40d	RHSIM_Rhsim07G0082400	-0.904	-0.454	0.451	no
Treat-40d	RHSIM_Rhsim07G0106300	0.680	0.336	-0.344	no
Treat-40d	RHSIM_Rhsim07G0200600	-1.260	-1.488	-0.227	low
Treat-40d	RHSIM_Rhsim07G0029400	-0.089	0.104	0.192	no
Treat-40d	RHSIM_Rhsim07G0171600	0.194	0.575	0.382	no
Treat-40d	RHSIM_Rhsim08G0080600	0.850	0.895	0.045	no
Treat-40d	RHSIM_Rhsim06G0201500	0.033	-0.337	-0.370	no
Treat-40d	RHSIM_Rhsim08G0006200	1.003	1.504	0.501	no
Treat-40d	RHSIM_Rhsim07G0076300	-1.456	-0.972	0.484	low
Treat-40d	RHSIM_Rhsim06G0223900	-0.568	-1.080	-0.512	no
Treat-40d	RHSIM_Rhsim08G0248900	-1.914	-2.199	-0.285	low
Treat-40d	RHSIM_Rhsim06G0029100	-1.508	-0.744	0.763	low
Treat-40d	RHSIM_Rhsim07G0102800	0.032	0.105	0.073	no
Treat-40d	RHSIM_Rhsim08G0031800	-1.792	-1.012	0.780	low
Treat-40d	RHSIM_Rhsim07G0054400	-0.761	-0.169	0.592	no
Treat-40d	RHSIM_Rhsim07G0252800	0.003	-0.970	-0.973	no
Treat-40d	RHSIM_Rhsim07G0222500	-1.312	-0.520	0.792	low
Treat-40d	RHSIM_Rhsim07G0255000	-0.436	0.105	0.541	no
Treat-40d	RHSIM_Rhsim06G0046600	0.503	0.096	-0.407	no
Treat-40d	RHSIM_Rhsim08G0240200	0.362	0.072	-0.290	no
Treat-40d	RHSIM_Rhsim07G0237100	0.111	-0.622	-0.732	no
Treat-40d	RHSIM_Rhsim07G0136400	1.533	-0.057	-1.590	high
Treat-40d	RHSIM_Rhsim07G0048200	-0.445	-1.068	-0.623	no
Treat-40d	RHSIM_Rhsim08G0064800	-0.491	1.557	2.048	no
Treat-40d	RHSIM_Rhsim07G0045800	1.370	2.705	1.335	high

Group	Gene	PTR (z-score difference)	z(log2 LFQ)	z(log2 FPKM)	extreme-PTR
Treat-40d	RHSIM_Rhsim07G0137100	-1.298	0.926	2.224	low
Treat-40d	RHSIM_Rhsim06G0084500	-0.299	-0.665	-0.365	no
Treat-40d	RHSIM_Rhsim07G0178600	-0.297	0.027	0.323	no
Treat-40d	RHSIM_Rhsim07G0143500	1.316	0.675	-0.641	high
Treat-40d	RHSIM_Rhsim06G0122400	-1.281	0.801	2.082	low
Treat-40d	RHSIM_Rhsim07G0022700	-1.551	-0.867	0.684	low
Treat-40d	RHSIM_Rhsim07G0124300	1.325	0.742	-0.583	high
Treat-40d	RHSIM_Rhsim07G0006000	-0.779	0.819	1.598	no
Treat-40d	RHSIM_Rhsim07G0130900	0.127	-0.142	-0.269	no
Treat-40d	RHSIM_Rhsim08G0163100	1.216	-0.750	-1.966	high
Treat-40d	RHSIM_Rhsim06G0174100	-2.610	-1.998	0.612	low
Treat-40d	RHSIM_Rhsim06G0208000	-0.334	-0.427	-0.093	no
Treat-40d	RHSIM_Rhsim07G0201800	-0.600	-0.379	0.220	no
Treat-40d	RHSIM_Rhsim06G0046900	1.226	1.171	-0.055	high
Treat-40d	RHSIM_Rhsim06G0184300	1.440	0.126	-1.315	high
Treat-40d	RHSIM_Rhsim07G0205400	0.152	0.839	0.686	no
Treat-40d	RHSIM_Rhsim07G0137000	1.162	0.882	-0.279	high
Treat-40d	RHSIM_Rhsim06G0007800	-0.165	0.265	0.430	no
Treat-40d	RHSIM_Rhsim07G0014000	-0.771	-1.042	-0.271	no
Treat-40d	RHSIM_Rhsim06G0140600	0.408	-0.169	-0.577	no
Treat-40d	RHSIM_Rhsim07G0120800	-2.321	-0.971	1.350	low
Treat-40d	RHSIM_Rhsim08G0230400	-1.344	0.374	1.718	low
Treat-40d	RHSIM_Rhsim07G0134100	-0.773	-0.950	-0.177	no
Treat-40d	RHSIM_Rhsim06G0008600	-0.414	-0.816	-0.402	no
Treat-40d	RHSIM_Rhsim07G0244700	-1.408	-1.039	0.369	low
Treat-40d	RHSIM_Rhsim07G0096100	-2.543	-2.383	0.160	low
Treat-40d	RHSIM_Rhsim07G0068100	-0.700	0.080	0.780	no
Treat-40d	RHSIM_Rhsim06G0227300	0.096	1.584	1.488	no
Treat-40d	RHSIM_Rhsim06G0231100	0.553	0.385	-0.168	no
Treat-40d	RHSIM_Rhsim08G0152000	-1.034	-0.900	0.134	no
Treat-40d	RHSIM_Rhsim06G0121500	0.652	-0.114	-0.766	no
Treat-40d	RHSIM_Rhsim06G0180800	1.084	-0.843	-1.927	no

Group	Gene	PTR (z-score difference)	z(log2 LFQ)	z(log2 FPKM)	extreme-PTR
Treat-40d	RHSIM_Rhsim07G0037100	-0.412	-0.220	0.193	no
Treat-40d	RHSIM_Rhsim07G0075100	0.055	-0.062	-0.116	no
Treat-40d	RHSIM_Rhsim07G0243000	0.032	-0.160	-0.191	no
Treat-40d	RHSIM_Rhsim06G0009200	1.585	-0.062	-1.647	high
Treat-40d	RHSIM_Rhsim07G0193800	-0.244	-0.704	-0.461	no
Treat-40d	RHSIM_Rhsim07G0175800	1.100	0.656	-0.444	no
Treat-40d	RHSIM_Rhsim07G0214800	-1.568	-1.154	0.414	low
Treat-40d	RHSIM_Rhsim07G0244600	0.559	0.390	-0.169	no
Treat-40d	RHSIM_Rhsim07G0011100	-0.629	-1.541	-0.912	no
Treat-40d	RHSIM_Rhsim07G0069200	-0.761	-0.830	-0.069	no
Treat-40d	RHSIM_Rhsim08G0163500	0.298	0.146	-0.152	no
Treat-40d	RHSIM_Rhsim06G0229900	0.217	-0.363	-0.580	no
Treat-40d	RHSIM_Rhsim07G0104500	2.005	1.138	-0.867	high
Treat-40d	RHSIM_Rhsim07G0136600	0.165	-0.985	-1.150	no
Treat-40d	RHSIM_Rhsim06G0035500	-0.367	-0.410	-0.043	no
Treat-40d	RHSIM_Rhsim07G0018800	0.863	1.069	0.206	no
Treat-40d	RHSIM_Rhsim06G0089000	-0.065	0.629	0.694	no
Treat-40d	RHSIM_Rhsim07G0221800	-5.183	-2.865	2.318	low
Treat-40d	RHSIM_Rhsim07G0200400	-0.299	-0.344	-0.046	no
Treat-40d	RHSIM_Rhsim08G0038600	0.793	-0.010	-0.803	no
Treat-40d	RHSIM_Rhsim07G0183300	0.239	0.529	0.290	no
Treat-40d	RHSIM_Rhsim07G0039600	1.094	1.260	0.166	no
Treat-40d	RHSIM_Rhsim07G0229800	2.193	1.301	-0.892	high
Treat-40d	RHSIM_Rhsim06G0079300	-0.901	0.312	1.214	no
Treat-40d	RHSIM_Rhsim08G0146700	-0.278	-0.859	-0.581	no
Treat-40d	RHSIM_Rhsim07G0172500	-0.842	-0.636	0.206	no
Treat-40d	RHSIM_Rhsim07G0134000	0.930	0.058	-0.872	no
Treat-40d	RHSIM_Rhsim07G0078200	0.034	-0.383	-0.417	no
Treat-40d	RHSIM_Rhsim07G0179200	0.948	-0.174	-1.122	no
Treat-40d	RHSIM_Rhsim06G0203800	-3.624	-0.532	3.091	low
Treat-40d	RHSIM_Rhsim06G0204600	-0.690	0.693	1.382	no
Treat-40d	RHSIM_Rhsim06G0229700	-2.259	-0.908	1.352	low

Group	Gene	PTR (z-score difference)	z(log2 LFQ)	z(log2 FPKM)	extreme-PTR
Treat-40d	RHSIM_Rhsim06G0003900	-1.173	0.218	1.391	low
Treat-40d	RHSIM_Rhsim07G0176900	-0.586	-0.398	0.188	no
Treat-40d	RHSIM_Rhsim06G0211400	0.095	1.107	1.012	no
Treat-40d	RHSIM_Rhsim07G0093600	-2.075	-0.598	1.477	low
Treat-40d	RHSIM_Rhsim07G0086200	0.090	0.588	0.498	no
Treat-40d	RHSIM_Rhsim07G0017700	-0.614	-0.724	-0.110	no
Treat-40d	RHSIM_Rhsim07G0102000	0.306	0.510	0.204	no
Treat-40d	RHSIM_Rhsim06G0069100	-0.986	0.788	1.774	no
Treat-40d	RHSIM_Rhsim07G0122900	0.053	0.432	0.379	no
Treat-40d	RHSIM_Rhsim07G0141400	-0.329	-0.163	0.166	no
Treat-40d	RHSIM_Rhsim06G0026300	1.735	1.475	-0.260	high
Treat-40d	RHSIM_Rhsim08G0011300	-0.222	-0.289	-0.066	no
Treat-40d	RHSIM_Rhsim06G0050800	-0.476	-0.699	-0.223	no
Treat-40d	RHSIM_Rhsim06G0013500	-1.862	0.099	1.960	low
Treat-40d	RHSIM_Rhsim06G0131700	-0.123	0.063	0.186	no
Treat-40d	RHSIM_Rhsim07G0243900	0.478	-0.344	-0.822	no
Treat-40d	RHSIM_Rhsim06G0030400	-1.077	-1.567	-0.490	no
Treat-40d	RHSIM_Rhsim07G0213800	-1.029	-0.558	0.470	no
Treat-40d	RHSIM_Rhsim07G0075700	0.675	0.404	-0.271	no
Treat-40d	RHSIM_Rhsim06G0175500	-1.476	0.701	2.176	low
Treat-40d	RHSIM_Rhsim06G0068700	-0.024	1.889	1.913	no
Treat-40d	RHSIM_Rhsim07G0257700	0.348	-0.193	-0.541	no
Treat-40d	RHSIM_Rhsim06G0032100	0.412	-0.619	-1.031	no
Treat-40d	RHSIM_Rhsim06G0227500	1.372	0.531	-0.841	high
Treat-40d	RHSIM_Rhsim06G0082900	0.193	-0.234	-0.427	no
Treat-40d	RHSIM_Rhsim07G0144100	-0.344	0.583	0.927	no
Treat-40d	RHSIM_Rhsim07G0039900	0.094	-1.002	-1.095	no
Treat-40d	RHSIM_Rhsim07G0239500	2.851	0.614	-2.237	high
Treat-40d	RHSIM_Rhsim07G0252900	-1.394	-1.864	-0.470	low
Treat-40d	RHSIM_Rhsim07G0061100	0.952	-0.835	-1.786	no
Treat-40d	RHSIM_Rhsim06G0210400	-0.172	0.043	0.215	no
Treat-40d	RHSIM_Rhsim06G0079600	-1.033	-0.820	0.212	no

Group	Gene	PTR (z-score difference)	z(log2 LFQ)	z(log2 FPKM)	extreme-PTR
Treat-40d	RHSIM_Rhsim06G0050100	-0.284	-0.991	-0.707	no
Treat-40d	RHSIM_Rhsim07G0211300	-0.305	-0.252	0.054	no
Treat-40d	RHSIM_Rhsim06G0231000	-0.196	-0.653	-0.456	no
Treat-40d	RHSIM_Rhsim07G0039700	-0.103	0.807	0.910	no
Treat-40d	RHSIM_Rhsim06G0188100	3.316	1.181	-2.135	high
Treat-40d	RHSIM_Rhsim07G0139700	-1.784	1.345	3.129	low
Treat-40d	RHSIM_Rhsim07G0016400	-0.770	-0.969	-0.200	no
Treat-40d	RHSIM_Rhsim06G0154000	-0.174	-0.500	-0.326	no
Treat-40d	RHSIM_Rhsim07G0045700	-0.835	-0.712	0.124	no
Treat-40d	RHSIM_Rhsim06G0162500	0.931	2.415	1.484	no
Treat-40d	RHSIM_Rhsim06G0242100	0.409	-1.490	-1.900	no
Treat-40d	RHSIM_Rhsim06G0213100	2.494	0.276	-2.218	high
Treat-40d	RHSIM_Rhsim07G0071500	0.168	-0.819	-0.988	no
Treat-40d	RHSIM_Rhsim07G0177400	-0.782	-0.592	0.190	no
Treat-40d	RHSIM_Rhsim07G0047700	-0.148	-0.288	-0.140	no
Treat-40d	RHSIM_Rhsim07G0201200	-0.031	-1.112	-1.081	no
Treat-40d	RHSIM_Rhsim06G0219700	0.649	0.514	-0.135	no
Treat-40d	RHSIM_Rhsim08G0244100	0.490	0.090	-0.400	no
Treat-40d	RHSIM_Rhsim07G0253800	-0.962	1.298	2.260	no
Treat-40d	RHSIM_Rhsim07G0137800	-1.010	-0.489	0.521	no
Treat-40d	RHSIM_Rhsim07G0209300	-0.276	-0.215	0.061	no
Treat-40d	RHSIM_Rhsim06G0207200	0.911	1.971	1.060	no
Treat-40d	RHSIM_Rhsim06G0142500	0.776	0.762	-0.014	no
Treat-40d	RHSIM_Rhsim07G0141900	-0.416	0.548	0.964	no
Treat-40d	RHSIM_Rhsim07G0163200	-0.488	-1.341	-0.852	no
Treat-40d	RHSIM_Rhsim07G0054800	1.865	1.822	-0.043	high
Treat-40d	RHSIM_Rhsim07G0236700	-0.458	-1.964	-1.505	no
Treat-40d	RHSIM_Rhsim07G0179600	2.508	2.613	0.105	high
Treat-40d	RHSIM_Rhsim06G0198600	-1.365	1.148	2.513	low
Treat-40d	RHSIM_Rhsim06G0232600	1.002	2.299	1.296	no
Treat-40d	RHSIM_Rhsim07G0043400	-0.750	-1.171	-0.421	no
Treat-40d	RHSIM_Rhsim07G0018900	0.590	-0.804	-1.394	no

Group	Gene	PTR (z-score difference)	z(log2 LFQ)	z(log2 FPKM)	extreme-PTR
Treat-40d	RHSIM_Rhsim07G0028400	-0.531	-0.213	0.318	no
Treat-40d	RHSIM_Rhsim07G0072700	-0.179	0.435	0.614	no
Treat-40d	RHSIM_Rhsim07G0075600	-0.292	1.232	1.524	no
Treat-40d	RHSIM_Rhsim07G0155800	-1.310	-0.744	0.566	low
Treat-40d	RHSIM_Rhsim08G0001800	0.623	0.541	-0.082	no
Treat-40d	RHSIM_Rhsim07G0024100	0.384	-0.356	-0.740	no
Treat-40d	RHSIM_Rhsim07G0012700	0.147	-0.711	-0.858	no
Treat-40d	RHSIM_Rhsim07G0048100	0.612	0.958	0.346	no
Treat-40d	RHSIM_Rhsim07G0227800	1.480	0.177	-1.302	high
Treat-40d	RHSIM_Rhsim08G0022300	-1.527	0.043	1.570	low
Treat-40d	RHSIM_Rhsim06G0063500	-1.141	-1.208	-0.066	low
Treat-40d	RHSIM_Rhsim07G0034100	-0.477	0.474	0.951	no
Treat-40d	RHSIM_Rhsim06G0003400	-0.851	-1.694	-0.843	no
Treat-40d	RHSIM_Rhsim06G0057600	-1.141	-0.226	0.915	low
Treat-40d	RHSIM_Rhsim06G0027900	0.648	0.064	-0.584	no
Treat-40d	RHSIM_Rhsim07G0161800	-1.759	-1.065	0.695	low
Treat-40d	RHSIM_Rhsim07G0257900	-1.481	-1.207	0.274	low
Treat-40d	RHSIM_Rhsim08G0049700	1.093	1.058	-0.035	no
Treat-40d	RHSIM_Rhsim06G0078300	1.000	1.166	0.166	no
Treat-40d	RHSIM_Rhsim06G0204200	0.620	0.308	-0.312	no
Treat-40d	RHSIM_Rhsim06G0241500	2.094	3.063	0.970	high
Treat-40d	RHSIM_Rhsim08G0237900	1.284	0.070	-1.214	high
Treat-40d	RHSIM_Rhsim06G0068400	0.004	-1.154	-1.158	no
Treat-40d	RHSIM_Rhsim06G0199700	2.190	0.993	-1.197	high
Treat-40d	RHSIM_Rhsim07G0175700	0.638	0.705	0.067	no
Treat-40d	RHSIM_Rhsim07G0127500	0.033	1.772	1.739	no
Treat-40d	RHSIM_Rhsim06G0062100	-1.578	-1.423	0.154	low
Treat-40d	RHSIM_Rhsim06G0175200	1.523	1.632	0.109	high
Treat-40d	RHSIM_Rhsim06G0078900	-2.133	-1.101	1.031	low
Treat-40d	RHSIM_Rhsim06G0067300	1.084	-1.273	-2.357	no
Treat-40d	RHSIM_Rhsim07G0061800	-1.014	-0.680	0.334	no
Treat-40d	RHSIM_Rhsim07G0226200	0.274	0.521	0.248	no

Group	Gene	PTR (z-score difference)	z(log2 LFQ)	z(log2 FPKM)	extreme-PTR
Treat-40d	RHSIM_Rhsim06G0232700	-0.798	-1.234	-0.436	no
Treat-40d	RHSIM_Rhsim07G0010400	0.081	-0.429	-0.510	no
Treat-40d	RHSIM_Rhsim07G0084600	-2.771	-0.552	2.219	low
Treat-40d	RHSIM_Rhsim07G0075300	0.241	1.160	0.919	no
Treat-40d	RHSIM_Rhsim06G0067800	-0.582	-1.464	-0.883	no
Treat-40d	RHSIM_Rhsim06G0091500	0.363	1.584	1.221	no
Treat-40d	RHSIM_Rhsim06G0034000	-1.005	-0.023	0.982	no
Treat-40d	RHSIM_Rhsim06G0142100	0.387	-0.691	-1.078	no
Treat-40d	RHSIM_Rhsim06G0000400	-0.945	-0.575	0.370	no
Treat-40d	RHSIM_Rhsim07G0190600	-1.456	-1.627	-0.171	low
Treat-40d	RHSIM_Rhsim06G0012800	-0.126	0.467	0.593	no
Treat-40d	RHSIM_Rhsim06G0228900	-0.369	-2.001	-1.632	no
Treat-40d	RHSIM_Rhsim06G0127100	0.763	2.613	1.851	no
Treat-40d	RHSIM_Rhsim07G0031000	-0.575	-0.671	-0.095	no
Treat-40d	RHSIM_Rhsim06G0227100	-0.817	-0.718	0.100	no
Treat-40d	RHSIM_Rhsim08G0144900	3.141	1.981	-1.160	high
Treat-40d	RHSIM_Rhsim06G0074800	-0.917	-0.908	0.009	no
Treat-40d	RHSIM_Rhsim07G0238800	0.872	-1.340	-2.212	no
Treat-40d	RHSIM_Rhsim07G0085900	0.275	-1.400	-1.675	no
Treat-40d	RHSIM_Rhsim08G0039600	-1.117	-0.359	0.757	no
Treat-40d	RHSIM_Rhsim08G0005300	1.741	2.090	0.349	high
Treat-40d	RHSIM_Rhsim07G0100500	0.721	0.233	-0.488	no
Treat-40d	RHSIM_Rhsim08G0062500	-0.572	-0.154	0.418	no
Treat-40d	RHSIM_Rhsim06G0004000	0.549	-0.434	-0.983	no
Treat-40d	RHSIM_Rhsim06G0208100	1.178	0.048	-1.131	high
Treat-40d	RHSIM_Rhsim07G0168200	-0.517	0.524	1.041	no
Treat-40d	RHSIM_Rhsim07G0043900	-2.988	-1.266	1.722	low
Treat-40d	RHSIM_Rhsim06G0104800	0.370	-0.315	-0.685	no
Treat-40d	RHSIM_Rhsim07G0136900	2.087	-0.082	-2.169	high
Treat-40d	RHSIM_Rhsim06G0178500	-0.321	-0.617	-0.297	no
Treat-40d	RHSIM_Rhsim06G0121900	2.991	2.026	-0.965	high
Treat-40d	RHSIM_Rhsim06G0035800	-0.678	-0.225	0.453	no

Group	Gene	PTR (z-score difference)	z(log2 LFQ)	z(log2 FPKM)	extreme-PTR
Treat-40d	RHSIM_Rhsim06G0059900	-1.707	-0.955	0.753	low
Treat-40d	RHSIM_Rhsim06G0057100	-0.511	0.951	1.461	no
Treat-40d	RHSIM_Rhsim07G0028700	-0.287	0.692	0.979	no
Treat-40d	RHSIM_Rhsim06G0231500	-1.835	-1.896	-0.061	low
Treat-40d	RHSIM_Rhsim07G0076400	-1.517	-0.674	0.842	low
Treat-40d	RHSIM_Rhsim06G0219100	0.309	0.375	0.067	no
Treat-40d	RHSIM_Rhsim07G0013400	-0.359	0.641	0.999	no
Treat-40d	RHSIM_Rhsim07G0162300	1.069	1.632	0.564	no
Treat-40d	RHSIM_Rhsim07G0214900	0.129	0.139	0.010	no
Treat-40d	RHSIM_Rhsim06G0179200	0.944	0.323	-0.622	no
Treat-40d	RHSIM_Rhsim06G0011800	0.449	2.218	1.769	no
Treat-40d	RHSIM_Rhsim08G0077200	-0.588	0.514	1.102	no
Treat-40d	RHSIM_Rhsim06G0152000	1.156	0.874	-0.282	high
Treat-40d	RHSIM_Rhsim07G0048800	0.335	0.388	0.053	no
Treat-40d	RHSIM_Rhsim06G0140100	1.385	-0.843	-2.228	high
Treat-40d	RHSIM_Rhsim06G0166100	0.810	-0.191	-1.002	no
Treat-40d	RHSIM_Rhsim07G0192000	-1.002	-1.231	-0.229	no
Treat-40d	RHSIM_Rhsim06G0035100	-0.492	-0.953	-0.461	no
Treat-40d	RHSIM_Rhsim06G0232100	-1.024	-1.578	-0.554	no
Treat-40d	RHSIM_Rhsim06G0155400	-0.737	1.418	2.155	no
Treat-40d	RHSIM_Rhsim07G0103700	1.006	1.630	0.624	no
Treat-40d	RHSIM_Rhsim07G0247400	0.926	-0.129	-1.054	no
Treat-40d	RHSIM_Rhsim06G0009800	-0.942	-0.793	0.149	no
Treat-40d	RHSIM_Rhsim07G0076600	-2.765	-0.567	2.199	low
Treat-40d	RHSIM_Rhsim06G0060000	1.003	-1.275	-2.277	no
Treat-40d	RHSIM_Rhsim07G0103800	-0.143	0.963	1.106	no
Treat-40d	RHSIM_Rhsim06G0017900	-0.483	-0.180	0.303	no
Treat-40d	RHSIM_Rhsim06G0224000	-1.782	1.081	2.863	low
Treat-40d	RHSIM_Rhsim06G0101600	0.563	0.619	0.057	no
Treat-40d	RHSIM_Rhsim08G0020700	-0.328	0.538	0.866	no
Treat-40d	RHSIM_Rhsim06G0005000	1.672	-0.505	-2.177	high
Treat-40d	RHSIM_Rhsim07G0018000	1.627	0.793	-0.834	high

Group	Gene	PTR (z-score difference)	z(log2 LFQ)	z(log2 FPKM)	extreme-PTR
Treat-40d	RHSIM_Rhsim08G0100900	-0.949	-1.084	-0.135	no
Treat-40d	RHSIM_Rhsim06G0243400	-1.443	-0.564	0.879	low
Treat-40d	RHSIM_Rhsim06G0061600	0.981	1.553	0.572	no
Treat-40d	RHSIM_Rhsim06G0204400	1.044	0.438	-0.606	no
Treat-40d	RHSIM_Rhsim06G0026000	-0.108	-1.135	-1.027	no
Treat-40d	RHSIM_Rhsim06G0137100	-0.303	0.978	1.281	no
Treat-40d	RHSIM_Rhsim07G0003800	0.199	0.318	0.119	no
Treat-40d	RHSIM_Rhsim06G0013600	-1.004	0.065	1.069	no
Treat-40d	RHSIM_Rhsim06G0129800	0.085	-0.670	-0.755	no
Treat-40d	RHSIM_Rhsim07G0033100	1.182	0.415	-0.767	high
Treat-40d	RHSIM_Rhsim07G0257000	3.018	1.280	-1.738	high
Treat-40d	RHSIM_Rhsim06G0135500	-1.275	-1.097	0.178	low
Treat-40d	RHSIM_Rhsim07G0139800	0.474	0.045	-0.428	no
Treat-40d	RHSIM_Rhsim08G0220400	-0.067	0.254	0.321	no
Treat-40d	RHSIM_Rhsim07G0018700	0.988	2.008	1.020	no
Treat-40d	RHSIM_Rhsim06G0224100	-0.321	-0.197	0.123	no
Treat-40d	RHSIM_Rhsim07G0079200	-0.135	-0.801	-0.666	no
Treat-40d	RHSIM_Rhsim08G0093000	-1.395	-0.608	0.786	low
Treat-40d	RHSIM_Rhsim07G0013600	1.123	1.451	0.328	high
Treat-40d	RHSIM_Rhsim07G0161400	0.446	-0.822	-1.268	no
Treat-40d	RHSIM_Rhsim06G0229000	-0.584	-0.589	-0.005	no
Treat-40d	RHSIM_Rhsim07G0212200	-1.032	-0.761	0.270	no
Treat-40d	RHSIM_Rhsim06G0240700	1.908	-0.021	-1.929	high
Treat-40d	RHSIM_Rhsim07G0166500	-0.319	0.902	1.221	no
Treat-40d	RHSIM_Rhsim07G0243700	-0.950	-0.137	0.813	no
Treat-40d	RHSIM_Rhsim06G0173600	-0.094	-1.196	-1.103	no
Treat-40d	RHSIM_Rhsim06G0196300	0.664	0.868	0.204	no
Treat-40d	RHSIM_Rhsim06G0240900	0.438	-1.141	-1.579	no
Treat-40d	RHSIM_Rhsim05G0002300	0.417	0.388	-0.030	no
Treat-40d	RHSIM_Rhsim07G0079900	0.137	1.197	1.060	no
Treat-40d	RHSIM_Rhsim06G0196500	-0.593	1.018	1.611	no
Treat-40d	RHSIM_Rhsim07G0258500	-0.211	-0.048	0.164	no

Group	Gene	PTR (z-score difference)	z(log2 LFQ)	z(log2 FPKM)	extreme-PTR
Treat-40d	RHSIM_Rhsim06G0164500	0.289	1.453	1.164	no
Treat-40d	RHSIM_Rhsim06G0040100	1.448	0.638	-0.810	high
Treat-40d	RHSIM_Rhsim06G0040800	-0.399	-0.513	-0.114	no
Treat-40d	RHSIM_Rhsim06G0114600	0.219	0.017	-0.202	no
Treat-40d	RHSIM_Rhsim07G0257400	0.647	0.950	0.302	no
Treat-40d	RHSIM_Rhsim06G0032500	-0.652	-0.510	0.142	no
Treat-40d	RHSIM_Rhsim06G0175700	-2.444	0.342	2.785	low
Treat-40d	RHSIM_Rhsim06G0021200	0.429	-0.174	-0.603	no
Treat-40d	RHSIM_Rhsim06G0091400	-0.614	-0.967	-0.353	no
Treat-40d	RHSIM_Rhsim06G0231200	0.474	-0.931	-1.406	no
Treat-40d	RHSIM_Rhsim06G0085000	1.448	0.244	-1.205	high
Treat-40d	RHSIM_Rhsim06G0012500	1.681	0.217	-1.463	high
Treat-40d	RHSIM_Rhsim07G0224300	-1.082	-0.298	0.784	no
Treat-40d	RHSIM_Rhsim07G0248700	-0.841	-0.577	0.263	no
Treat-40d	RHSIM_Rhsim06G0178700	-0.485	-0.565	-0.081	no
Treat-40d	RHSIM_Rhsim06G0207600	-0.034	-0.100	-0.066	no
Treat-40d	RHSIM_Rhsim06G0195200	-3.970	-0.537	3.433	low
Treat-40d	RHSIM_Rhsim07G0211600	0.441	-0.262	-0.703	no
Treat-40d	RHSIM_Rhsim05G0076900	-0.138	-0.752	-0.614	no
Treat-40d	RHSIM_Rhsim06G0178100	-0.371	-1.196	-0.825	no
Treat-40d	RHSIM_Rhsim06G0009500	-0.267	-0.246	0.021	no
Treat-40d	RHSIM_Rhsim06G0216300	-1.941	-1.436	0.505	low
Treat-40d	RHSIM_Rhsim05G0232900	0.810	2.098	1.288	no
Treat-40d	RHSIM_Rhsim06G0008000	0.832	-0.321	-1.153	no
Treat-40d	RHSIM_Rhsim06G0174400	-0.122	-0.568	-0.446	no
Treat-40d	RHSIM_Rhsim06G0069300	-0.648	-0.291	0.356	no
Treat-40d	RHSIM_Rhsim06G0223700	-0.740	-0.391	0.348	no
Treat-40d	RHSIM_Rhsim06G0000600	-0.090	-0.711	-0.621	no
Treat-40d	RHSIM_Rhsim06G0146800	1.295	0.440	-0.855	high
Treat-40d	RHSIM_Rhsim06G0238600	1.336	1.217	-0.119	high
Treat-40d	RHSIM_Rhsim06G0211200	-1.700	-1.692	0.007	low
Treat-40d	RHSIM_Rhsim06G0225600	-0.708	-1.050	-0.343	no

Group	Gene	PTR (z-score difference)	z(log2 LFQ)	z(log2 FPKM)	extreme-PTR
Treat-40d	RHSIM_Rhsim07G0150900	0.118	-0.411	-0.529	no
Treat-40d	RHSIM_Rhsim06G0175900	0.754	0.459	-0.294	no
Treat-40d	RHSIM_Rhsim05G0005000	1.144	0.625	-0.519	high
Treat-40d	RHSIM_Rhsim06G0073900	1.489	1.762	0.273	high
Treat-40d	RHSIM_Rhsim06G0056800	-1.900	-1.593	0.307	low
Treat-40d	RHSIM_Rhsim07G0115200	-0.251	-0.848	-0.597	no
Treat-40d	RHSIM_Rhsim07G0029000	0.627	0.915	0.287	no
Treat-40d	RHSIM_Rhsim06G0197600	1.109	0.815	-0.294	no
Treat-40d	RHSIM_Rhsim06G0236900	-0.517	-1.149	-0.633	no
Treat-40d	RHSIM_Rhsim06G0184100	-0.415	0.441	0.856	no
Treat-40d	RHSIM_Rhsim07G0250200	-0.629	-0.239	0.390	no
Treat-40d	RHSIM_Rhsim06G0224200	1.633	0.098	-1.535	high
Treat-40d	RHSIM_Rhsim06G0075100	-2.846	-0.667	2.179	low
Treat-40d	RHSIM_Rhsim05G0126500	-0.012	0.306	0.317	no
Treat-40d	RHSIM_Rhsim06G0035600	-0.653	-0.831	-0.178	no
Treat-40d	RHSIM_Rhsim06G0027600	-0.575	-0.341	0.234	no
Treat-40d	RHSIM_Rhsim06G0241000	1.669	1.316	-0.353	high
Treat-40d	RHSIM_Rhsim06G0006500	1.050	-0.179	-1.229	no
Treat-40d	RHSIM_Rhsim06G0207700	1.527	-0.081	-1.608	high
Treat-40d	RHSIM_Rhsim05G0045000	-1.525	-0.738	0.787	low
Treat-40d	RHSIM_Rhsim07G0084000	0.843	-0.040	-0.883	no
Treat-40d	RHSIM_Rhsim05G0048300	0.629	-0.439	-1.068	no
Treat-40d	RHSIM_Rhsim06G0008400	-0.981	-0.130	0.851	no
Treat-40d	RHSIM_Rhsim05G0168000	0.728	-0.112	-0.839	no
Treat-40d	RHSIM_Rhsim07G0027800	0.316	1.203	0.887	no
Treat-40d	RHSIM_Rhsim06G0197200	-2.216	-1.210	1.006	low
Treat-40d	RHSIM_Rhsim07G0009400	-1.207	-1.518	-0.311	low
Treat-40d	RHSIM_Rhsim05G0132400	1.089	2.049	0.960	no
Treat-40d	RHSIM_Rhsim06G0172900	-0.431	-1.150	-0.719	no
Treat-40d	RHSIM_Rhsim06G0002200	1.320	-0.352	-1.672	high
Treat-40d	RHSIM_Rhsim05G0073200	0.291	2.080	1.790	no
Treat-40d	RHSIM_Rhsim06G0242800	-1.235	-0.941	0.293	low

Group	Gene	PTR (z-score difference)	z(log2 LFQ)	z(log2 FPKM)	extreme-PTR
Treat-40d	RHSIM_Rhsim07G0038600	-0.318	0.043	0.361	no
Treat-40d	RHSIM_Rhsim06G0052100	3.012	0.805	-2.207	high
Treat-40d	RHSIM_Rhsim06G0214200	-0.882	-1.099	-0.217	no
Treat-40d	RHSIM_Rhsim07G0121200	-2.163	-1.411	0.752	low
Treat-40d	RHSIM_Rhsim06G0054400	1.134	1.050	-0.083	high
Treat-40d	RHSIM_Rhsim05G0018800	-0.711	0.001	0.712	no
Treat-40d	RHSIM_Rhsim06G0129900	-0.191	0.996	1.188	no
Treat-40d	RHSIM_Rhsim07G0008000	0.019	-0.615	-0.633	no
Treat-40d	RHSIM_Rhsim06G0186500	-0.797	0.274	1.071	no
Treat-40d	RHSIM_Rhsim05G0115300	2.002	1.954	-0.048	high
Treat-40d	RHSIM_Rhsim06G0244600	-1.219	-1.306	-0.087	low
Treat-40d	RHSIM_Rhsim05G0044300	-0.121	1.302	1.424	no
Treat-40d	RHSIM_Rhsim07G0174700	0.564	-0.521	-1.085	no
Treat-40d	RHSIM_Rhsim05G0060300	-0.116	-1.463	-1.347	no
Treat-40d	RHSIM_Rhsim07G0211400	-0.714	-0.408	0.305	no
Treat-40d	RHSIM_Rhsim06G0029700	-0.361	-0.482	-0.122	no
Treat-40d	RHSIM_Rhsim05G0233100	-0.836	-0.547	0.289	no
Treat-40d	RHSIM_Rhsim06G0021700	-0.196	0.656	0.852	no
Treat-40d	RHSIM_Rhsim05G0060600	-0.781	-0.041	0.740	no
Treat-40d	RHSIM_Rhsim06G0031100	0.527	-1.727	-2.254	no
Treat-40d	RHSIM_Rhsim06G0191800	0.150	1.179	1.029	no
Treat-40d	RHSIM_Rhsim07G0119900	-0.227	0.352	0.579	no
Treat-40d	RHSIM_Rhsim06G0059400	-0.973	-0.247	0.726	no
Treat-40d	RHSIM_Rhsim05G0060400	1.645	0.013	-1.632	high
Treat-40d	RHSIM_Rhsim06G0183300	-0.039	0.072	0.111	no
Treat-40d	RHSIM_Rhsim06G0018700	-0.660	-1.075	-0.415	no
Treat-40d	RHSIM_Rhsim07G0240500	0.940	0.005	-0.935	no
Treat-40d	RHSIM_Rhsim05G0187800	-0.190	1.211	1.401	no
Treat-40d	RHSIM_Rhsim06G0046300	1.128	1.212	0.084	high
Treat-40d	RHSIM_Rhsim05G0208000	0.003	-0.956	-0.960	no
Treat-40d	RHSIM_Rhsim06G0070900	0.433	-0.545	-0.978	no
Treat-40d	RHSIM_Rhsim06G0018400	-1.064	-0.599	0.464	no

Group	Gene	PTR (z-score difference)	z(log2 LFQ)	z(log2 FPKM)	extreme-PTR
Treat-40d	RHSIM_Rhsim07G0154100	0.970	0.415	-0.555	no
Treat-40d	RHSIM_Rhsim06G0118100	0.182	-0.554	-0.736	no
Treat-40d	RHSIM_Rhsim06G0200600	1.899	-0.554	-2.453	high
Treat-40d	RHSIM_Rhsim05G0013200	-0.116	-1.424	-1.307	no
Treat-40d	RHSIM_Rhsim06G0097000	-0.198	-0.311	-0.113	no
Treat-40d	RHSIM_Rhsim06G0069400	-0.453	-0.082	0.370	no
Treat-40d	RHSIM_Rhsim05G0022700	0.613	0.895	0.282	no
Treat-40d	RHSIM_Rhsim06G0004600	1.713	2.981	1.269	high
Treat-40d	RHSIM_Rhsim06G0213400	-0.019	-0.991	-0.972	no
Treat-40d	RHSIM_Rhsim06G0166800	-0.914	0.058	0.971	no
Treat-40d	RHSIM_Rhsim06G0048000	1.467	1.165	-0.302	high
Treat-40d	RHSIM_Rhsim05G0185900	0.096	0.131	0.035	no
Treat-40d	RHSIM_Rhsim06G0121700	0.427	0.465	0.038	no
Treat-40d	RHSIM_Rhsim06G0238500	-0.265	-0.385	-0.120	no
Treat-40d	RHSIM_Rhsim05G0003900	0.079	-0.614	-0.692	no
Treat-40d	RHSIM_Rhsim06G0216400	-0.835	0.171	1.005	no
Treat-40d	RHSIM_Rhsim05G0216700	-1.992	-0.432	1.560	low
Treat-40d	RHSIM_Rhsim06G0218400	0.590	0.642	0.052	no
Treat-40d	RHSIM_Rhsim06G0136000	1.053	1.719	0.666	no
Treat-40d	RHSIM_Rhsim05G0070300	0.463	-0.318	-0.781	no
Treat-40d	RHSIM_Rhsim06G0004300	0.826	1.105	0.279	no
Treat-40d	RHSIM_Rhsim06G0175300	0.385	-0.747	-1.132	no
Treat-40d	RHSIM_Rhsim05G0222900	0.553	0.370	-0.183	no
Treat-40d	RHSIM_Rhsim06G0137300	0.154	0.846	0.693	no
Treat-40d	RHSIM_Rhsim05G0195200	-1.047	-1.514	-0.467	no
Treat-40d	RHSIM_Rhsim06G0147000	-1.096	-1.036	0.060	no
Treat-40d	RHSIM_Rhsim05G0104500	-0.862	-0.058	0.805	no
Treat-40d	RHSIM_Rhsim05G0088300	-0.683	-0.762	-0.078	no
Treat-40d	RHSIM_Rhsim05G0122300	-0.308	-0.951	-0.643	no
Treat-40d	RHSIM_Rhsim06G0063800	0.157	-1.183	-1.340	no
Treat-40d	RHSIM_Rhsim07G0104300	1.545	0.355	-1.190	high
Treat-40d	RHSIM_Rhsim06G0213200	1.535	0.092	-1.443	high

Group	Gene	PTR (z-score difference)	z(log2 LFQ)	z(log2 FPKM)	extreme-PTR
Treat-40d	RHSIM_Rhsim06G0116100	0.284	-0.413	-0.696	no
Treat-40d	RHSIM_Rhsim06G0201300	0.559	1.443	0.884	no
Treat-40d	RHSIM_Rhsim06G0153100	0.669	2.265	1.596	no
Treat-40d	RHSIM_Rhsim05G0177500	0.594	-1.003	-1.598	no
Treat-40d	RHSIM_Rhsim06G0129300	1.025	1.306	0.281	no
Treat-40d	RHSIM_Rhsim06G0232400	-2.555	-1.160	1.395	low
Treat-40d	RHSIM_Rhsim05G0172800	-0.295	-1.332	-1.037	no
Treat-40d	RHSIM_Rhsim06G0211100	-0.811	-1.274	-0.463	no
Treat-40d	RHSIM_Rhsim06G0054200	0.637	-1.038	-1.675	no
Treat-40d	RHSIM_Rhsim06G0165500	-1.419	-1.271	0.148	low
Treat-40d	RHSIM_Rhsim05G0197900	2.936	1.057	-1.880	high
Treat-40d	RHSIM_Rhsim06G0076700	-1.169	0.530	1.698	low
Treat-40d	RHSIM_Rhsim06G0224400	-0.678	-0.688	-0.010	no
Treat-40d	RHSIM_Rhsim06G0210200	-0.903	-0.169	0.734	no
Treat-40d	RHSIM_Rhsim06G0212800	1.970	0.820	-1.150	high
Treat-40d	RHSIM_Rhsim06G0092900	-0.570	-1.012	-0.442	no
Treat-40d	RHSIM_Rhsim05G0142400	-1.620	-0.480	1.141	low
Treat-40d	RHSIM_Rhsim06G0239000	-0.365	0.226	0.590	no
Treat-40d	RHSIM_Rhsim06G0223600	0.477	-0.754	-1.232	no
Treat-40d	RHSIM_Rhsim05G0049000	1.385	0.072	-1.313	high
Treat-40d	RHSIM_Rhsim06G0157900	-1.941	-0.866	1.075	low
Treat-40d	RHSIM_Rhsim06G0062200	-0.149	-0.186	-0.037	no
Treat-40d	RHSIM_Rhsim05G0229900	-0.354	-0.634	-0.280	no
Treat-40d	RHSIM_Rhsim05G0029300	0.909	1.410	0.501	no
Treat-40d	RHSIM_Rhsim05G0112500	-0.029	0.509	0.538	no
Treat-40d	RHSIM_Rhsim05G0095600	0.752	-0.361	-1.113	no
Treat-40d	RHSIM_Rhsim06G0234000	-0.254	-0.444	-0.190	no
Treat-40d	RHSIM_Rhsim05G0013100	-0.239	-0.634	-0.395	no
Treat-40d	RHSIM_Rhsim05G0016600	-1.821	-1.139	0.681	low
Treat-40d	RHSIM_Rhsim05G0073600	1.068	-1.131	-2.199	no
Treat-40d	RHSIM_Rhsim06G0065300	0.236	-1.415	-1.650	no
Treat-40d	RHSIM_Rhsim05G0053200	0.812	0.461	-0.351	no

Group	Gene	PTR (z-score difference)	z(log2 LFQ)	z(log2 FPKM)	extreme-PTR
Treat-40d	RHSIM_Rhsim05G0188100	-0.579	0.254	0.833	no
Treat-40d	RHSIM_Rhsim05G0132800	0.726	0.538	-0.188	no
Treat-40d	RHSIM_Rhsim05G0032600	1.259	1.032	-0.227	high
Treat-40d	RHSIM_Rhsim07G0091100	0.998	0.490	-0.509	no
Treat-40d	RHSIM_Rhsim06G0243100	-1.551	-0.617	0.935	low
Treat-40d	RHSIM_Rhsim05G0056900	-1.401	0.516	1.918	low
Treat-40d	RHSIM_Rhsim05G0230200	1.060	0.769	-0.292	no
Treat-40d	RHSIM_Rhsim06G0103100	-1.726	-0.992	0.734	low
Treat-40d	RHSIM_Rhsim06G0086200	0.155	0.928	0.772	no
Treat-40d	RHSIM_Rhsim05G0167800	-0.027	-0.634	-0.607	no
Treat-40d	RHSIM_Rhsim05G0092800	0.402	-0.698	-1.100	no
Treat-40d	RHSIM_Rhsim05G0005700	0.447	0.044	-0.403	no
Treat-40d	RHSIM_Rhsim06G0099200	-0.657	-0.323	0.334	no
Treat-40d	RHSIM_Rhsim06G0212400	-0.713	0.565	1.278	no
Treat-40d	RHSIM_Rhsim06G0166500	-0.581	0.633	1.214	no
Treat-40d	RHSIM_Rhsim06G0128200	-0.382	0.119	0.501	no
Treat-40d	RHSIM_Rhsim05G0148100	-0.974	-0.158	0.816	no
Treat-40d	RHSIM_Rhsim06G0166300	-0.209	-0.578	-0.370	no
Treat-40d	RHSIM_Rhsim06G0067500	1.725	0.882	-0.843	high
Treat-40d	RHSIM_Rhsim05G0229000	-1.707	-0.211	1.497	low
Treat-40d	RHSIM_Rhsim06G0198500	0.167	0.545	0.378	no
Treat-40d	RHSIM_Rhsim05G0102000	-0.171	-0.898	-0.728	no
Treat-40d	RHSIM_Rhsim06G0022900	2.740	0.938	-1.802	high
Treat-40d	RHSIM_Rhsim05G0079000	-0.037	0.524	0.561	no
Treat-40d	RHSIM_Rhsim05G0184700	-0.217	-0.770	-0.554	no
Treat-40d	RHSIM_Rhsim06G0009700	2.814	0.562	-2.252	high
Treat-40d	RHSIM_Rhsim07G0188500	-0.702	-1.122	-0.420	no
Treat-40d	RHSIM_Rhsim05G0014500	-0.440	0.498	0.937	no
Treat-40d	RHSIM_Rhsim05G0004300	0.709	2.271	1.563	no
Treat-40d	RHSIM_Rhsim06G0202700	0.002	-0.508	-0.510	no
Treat-40d	RHSIM_Rhsim06G0148500	1.065	2.927	1.863	no
Treat-40d	RHSIM_Rhsim05G0206700	-0.185	-0.588	-0.403	no

Group	Gene	PTR (z-score difference)	z(log2 LFQ)	z(log2 FPKM)	extreme-PTR
Treat-40d	RHSIM_Rhsim05G0079100	-1.890	-1.830	0.060	low
Treat-40d	RHSIM_Rhsim05G0220000	0.242	-0.245	-0.487	no
Treat-40d	RHSIM_Rhsim05G0039500	0.232	1.076	0.844	no
Treat-40d	RHSIM_Rhsim05G0056600	-1.409	-0.608	0.801	low
Treat-40d	RHSIM_Rhsim05G0193900	0.541	2.233	1.692	no
Treat-40d	RHSIM_Rhsim05G0074500	0.450	0.305	-0.145	no
Treat-40d	RHSIM_Rhsim07G0191000	-1.351	-1.405	-0.054	low
Treat-40d	RHSIM_Rhsim06G0032600	0.555	0.092	-0.463	no
Treat-40d	RHSIM_Rhsim07G0222600	-1.639	-1.232	0.407	low
Treat-40d	RHSIM_Rhsim06G0131600	-0.219	-1.439	-1.220	no
Treat-40d	RHSIM_Rhsim05G0055600	0.028	0.260	0.232	no
Treat-40d	RHSIM_Rhsim06G0230600	-1.949	-1.315	0.634	low
Treat-40d	RHSIM_Rhsim05G0165600	0.975	1.121	0.145	no
Treat-40d	RHSIM_Rhsim06G0211900	1.308	-0.014	-1.322	high
Treat-40d	RHSIM_Rhsim06G0048700	-0.693	-0.500	0.193	no
Treat-40d	RHSIM_Rhsim05G0094900	-0.922	-1.791	-0.869	no
Treat-40d	RHSIM_Rhsim06G0031000	1.530	1.349	-0.181	high
Treat-40d	RHSIM_Rhsim05G0170000	0.816	0.186	-0.630	no
Treat-40d	RHSIM_Rhsim06G0206300	0.831	0.931	0.100	no
Treat-40d	RHSIM_Rhsim05G0114200	-1.475	-1.516	-0.041	low
Treat-40d	RHSIM_Rhsim06G0239600	0.710	2.024	1.313	no
Treat-40d	RHSIM_Rhsim06G0090800	-1.550	-0.647	0.903	low
Treat-40d	RHSIM_Rhsim06G0087600	-0.901	-0.340	0.560	no
Treat-40d	RHSIM_Rhsim05G0172300	-1.706	-1.620	0.086	low
Treat-40d	RHSIM_Rhsim06G0179300	0.279	-1.024	-1.303	no
Treat-40d	RHSIM_Rhsim07G0095500	-0.654	-0.965	-0.312	no
Treat-40d	RHSIM_Rhsim05G0195100	-0.488	-0.842	-0.353	no
Treat-40d	RHSIM_Rhsim05G0107000	0.403	0.280	-0.123	no
Treat-40d	RHSIM_Rhsim06G0206000	-0.678	-0.489	0.189	no
Treat-40d	RHSIM_Rhsim06G0103300	1.069	3.316	2.247	no
Treat-40d	RHSIM_Rhsim05G0075800	2.538	1.652	-0.887	high
Treat-40d	RHSIM_Rhsim05G0175300	0.292	1.200	0.908	no

Group	Gene	PTR (z-score difference)	z(log2 LFQ)	z(log2 FPKM)	extreme-PTR
Treat-40d	RHSIM_Rhsim07G0020100	-2.586	-1.606	0.980	low
Treat-40d	RHSIM_Rhsim05G0076200	1.004	-0.169	-1.173	no
Treat-40d	RHSIM_Rhsim06G0232500	1.013	0.511	-0.502	no
Treat-40d	RHSIM_Rhsim05G0016500	-1.020	-1.596	-0.576	no
Treat-40d	RHSIM_Rhsim06G0141900	-0.612	-1.091	-0.479	no
Treat-40d	RHSIM_Rhsim05G0038200	0.482	0.980	0.498	no
Treat-40d	RHSIM_Rhsim05G0048000	1.267	-0.860	-2.127	high
Treat-40d	RHSIM_Rhsim06G0208300	-1.536	-0.390	1.146	low
Treat-40d	RHSIM_Rhsim06G0008500	0.021	2.003	1.982	no
Treat-40d	RHSIM_Rhsim05G0101600	-1.611	-0.547	1.064	low
Treat-40d	RHSIM_Rhsim05G0148700	0.121	0.082	-0.039	no
Treat-40d	RHSIM_Rhsim06G0003100	-1.644	-1.666	-0.021	low
Treat-40d	RHSIM_Rhsim05G0056500	-0.510	1.152	1.662	no
Treat-40d	RHSIM_Rhsim05G0114100	-0.036	-0.844	-0.808	no
Treat-40d	RHSIM_Rhsim06G0006600	0.524	1.376	0.852	no
Treat-40d	RHSIM_Rhsim05G0078100	-1.840	-0.639	1.201	low
Treat-40d	RHSIM_Rhsim06G0027100	-1.479	1.040	2.520	low
Treat-40d	RHSIM_Rhsim05G0081000	-2.245	-2.391	-0.146	low
Treat-40d	RHSIM_Rhsim05G0081600	-2.029	-0.190	1.839	low
Treat-40d	RHSIM_Rhsim06G0170000	0.204	-0.894	-1.098	no
Treat-40d	RHSIM_Rhsim05G0007700	-1.073	-1.659	-0.586	no
Treat-40d	RHSIM_Rhsim05G0142500	-1.189	-0.493	0.696	low
Treat-40d	RHSIM_Rhsim05G0015000	-0.910	0.326	1.236	no
Treat-40d	RHSIM_Rhsim05G0040400	0.185	-0.415	-0.600	no
Treat-40d	RHSIM_Rhsim06G0184400	0.102	-0.152	-0.253	no
Treat-40d	RHSIM_Rhsim05G0034500	-0.220	-1.165	-0.944	no
Treat-40d	RHSIM_Rhsim05G0125700	1.539	0.607	-0.932	high
Treat-40d	RHSIM_Rhsim05G0234300	-1.825	0.282	2.107	low
Treat-40d	RHSIM_Rhsim05G0054800	0.982	-1.152	-2.134	no
Treat-40d	RHSIM_Rhsim06G0176900	1.625	0.062	-1.563	high
Treat-40d	RHSIM_Rhsim07G0136800	0.342	-0.115	-0.457	no
Treat-40d	RHSIM_Rhsim05G0228100	-0.761	0.062	0.823	no

Group	Gene	PTR (z-score difference)	z(log2 LFQ)	z(log2 FPKM)	extreme-PTR
Treat-40d	RHSIM_Rhsim06G0062000	0.154	0.041	-0.113	no
Treat-40d	RHSIM_Rhsim05G0203100	-0.183	-0.328	-0.145	no
Treat-40d	RHSIM_Rhsim05G0094000	-1.131	-1.093	0.037	no
Treat-40d	RHSIM_Rhsim05G0178300	0.064	0.120	0.056	no
Treat-40d	RHSIM_Rhsim05G0160100	-0.089	0.540	0.629	no
Treat-40d	RHSIM_Rhsim06G0089200	-1.514	-1.297	0.217	low
Treat-40d	RHSIM_Rhsim06G0022200	0.686	-0.316	-1.002	no
Treat-40d	RHSIM_Rhsim05G0048900	-1.157	-1.541	-0.384	low
Treat-40d	RHSIM_Rhsim05G0022400	0.386	0.107	-0.279	no
Treat-40d	RHSIM_Rhsim05G0167700	0.502	-1.116	-1.618	no
Treat-40d	RHSIM_Rhsim05G0203300	-1.579	-0.725	0.854	low
Treat-40d	RHSIM_Rhsim05G0013700	-0.440	-1.333	-0.893	no
Treat-40d	RHSIM_Rhsim05G0214500	0.828	1.548	0.721	no
Treat-40d	RHSIM_Rhsim04G0249900	-0.239	1.607	1.846	no
Treat-40d	RHSIM_Rhsim05G0141700	1.669	0.222	-1.447	high
Treat-40d	RHSIM_Rhsim07G0093500	0.866	0.235	-0.631	no
Treat-40d	RHSIM_Rhsim06G0039400	-0.717	0.958	1.675	no
Treat-40d	RHSIM_Rhsim04G0061200	1.280	-0.325	-1.605	high
Treat-40d	RHSIM_Rhsim05G0057000	-0.389	0.100	0.489	no
Treat-40d	RHSIM_Rhsim05G0035400	0.121	-0.000	-0.121	no
Treat-40d	RHSIM_Rhsim04G0207200	-0.412	-0.345	0.068	no
Treat-40d	RHSIM_Rhsim06G0183900	1.158	0.945	-0.212	high
Treat-40d	RHSIM_Rhsim05G0179700	1.180	1.597	0.417	high
Treat-40d	RHSIM_Rhsim05G0055000	0.437	0.042	-0.395	no
Treat-40d	RHSIM_Rhsim05G0001900	-1.940	-1.569	0.371	low
Treat-40d	RHSIM_Rhsim06G0234700	-1.666	-0.140	1.526	low
Treat-40d	RHSIM_Rhsim04G0179500	-0.762	-1.656	-0.894	no
Treat-40d	RHSIM_Rhsim05G0141900	0.339	0.614	0.275	no
Treat-40d	RHSIM_Rhsim05G0165000	0.628	-0.594	-1.222	no
Treat-40d	RHSIM_Rhsim05G0169000	0.282	-0.670	-0.952	no
Treat-40d	RHSIM_Rhsim06G0060300	0.142	0.678	0.536	no
Treat-40d	RHSIM_Rhsim06G0030800	1.172	1.713	0.541	high

Group	Gene	PTR (z-score difference)	z(log2 LFQ)	z(log2 FPKM)	extreme-PTR
Treat-40d	RHSIM_Rhsim05G0178900	-0.329	0.569	0.899	no
Treat-40d	RHSIM_Rhsim06G0139200	2.102	2.662	0.560	high
Treat-40d	RHSIM_Rhsim05G0001300	0.204	-0.055	-0.259	no
Treat-40d	RHSIM_Rhsim05G0119100	-1.104	-1.024	0.080	no
Treat-40d	RHSIM_Rhsim05G0233200	0.401	0.393	-0.009	no
Treat-40d	RHSIM_Rhsim05G0141500	-3.045	-0.613	2.432	low
Treat-40d	RHSIM_Rhsim06G0114100	0.029	0.336	0.307	no
Treat-40d	RHSIM_Rhsim07G0044200	-0.207	-0.815	-0.607	no
Treat-40d	RHSIM_Rhsim06G0071600	0.791	0.063	-0.728	no
Treat-40d	RHSIM_Rhsim05G0067900	-0.970	-0.972	-0.002	no
Treat-40d	RHSIM_Rhsim05G0161500	0.486	-0.360	-0.846	no
Treat-40d	RHSIM_Rhsim07G0149700	-0.004	-0.438	-0.434	no
Treat-40d	RHSIM_Rhsim05G0203500	-0.124	1.070	1.194	no
Treat-40d	RHSIM_Rhsim06G0047300	-0.125	-0.065	0.060	no
Treat-40d	RHSIM_Rhsim05G0028200	1.044	0.111	-0.933	no
Treat-40d	RHSIM_Rhsim04G0153900	1.300	0.598	-0.702	high
Treat-40d	RHSIM_Rhsim05G0122100	-1.175	-0.737	0.437	low
Treat-40d	RHSIM_Rhsim07G0213300	0.896	0.656	-0.240	no
Treat-40d	RHSIM_Rhsim05G0221100	1.345	-1.002	-2.347	high
Treat-40d	RHSIM_Rhsim06G0238700	-1.073	-1.549	-0.476	no
Treat-40d	RHSIM_Rhsim04G0180900	-0.370	0.847	1.218	no
Treat-40d	RHSIM_Rhsim07G0102700	1.168	1.211	0.042	high
Treat-40d	RHSIM_Rhsim04G0213100	2.292	1.411	-0.881	high
Treat-40d	RHSIM_Rhsim05G0228600	0.978	0.911	-0.067	no
Treat-40d	RHSIM_Rhsim05G0018700	1.157	1.083	-0.074	high
Treat-40d	RHSIM_Rhsim06G0203000	0.177	0.552	0.375	no
Treat-40d	RHSIM_Rhsim06G0096300	-0.544	0.166	0.710	no
Treat-40d	RHSIM_Rhsim05G0219700	-0.691	-1.317	-0.627	no
Treat-40d	RHSIM_Rhsim06G0187600	1.649	-0.022	-1.671	high
Treat-40d	RHSIM_Rhsim05G0115900	0.026	0.468	0.442	no
Treat-40d	RHSIM_Rhsim05G0168400	-1.229	-1.379	-0.150	low
Treat-40d	RHSIM_Rhsim05G0093000	-0.498	-0.322	0.176	no

Group	Gene	PTR (z-score difference)	z(log2 LFQ)	z(log2 FPKM)	extreme-PTR
Treat-40d	RHSIM_Rhsim05G0141400	-2.013	-1.610	0.403	low
Treat-40d	RHSIM_Rhsim05G0216400	-0.172	0.311	0.483	no
Treat-40d	RHSIM_Rhsim04G0117500	-0.087	-1.394	-1.307	no
Treat-40d	RHSIM_Rhsim05G0148400	-0.722	-0.876	-0.154	no
Treat-40d	RHSIM_Rhsim06G0086800	-1.005	-1.209	-0.205	no
Treat-40d	RHSIM_Rhsim07G0176500	0.172	-0.344	-0.516	no
Treat-40d	RHSIM_Rhsim05G0107700	-1.412	-0.315	1.097	low
Treat-40d	RHSIM_Rhsim05G0068100	1.432	1.144	-0.289	high
Treat-40d	RHSIM_Rhsim05G0146600	-1.403	-1.552	-0.149	low
Treat-40d	RHSIM_Rhsim04G0058800	-0.785	-1.110	-0.325	no
Treat-40d	RHSIM_Rhsim05G0070600	0.722	-0.220	-0.942	no
Treat-40d	RHSIM_Rhsim05G0104400	-0.702	0.808	1.511	no
Treat-40d	RHSIM_Rhsim05G0047800	0.883	-0.789	-1.671	no
Treat-40d	RHSIM_Rhsim05G0012600	-3.609	-1.931	1.678	low
Treat-40d	RHSIM_Rhsim06G0244700	0.651	-0.143	-0.794	no
Treat-40d	RHSIM_Rhsim06G0022400	0.707	-0.781	-1.488	no
Treat-40d	RHSIM_Rhsim07G0057400	0.927	-0.359	-1.286	no
Treat-40d	RHSIM_Rhsim05G0070100	-2.742	-0.219	2.523	low
Treat-40d	RHSIM_Rhsim05G0169700	1.480	3.058	1.578	high
Treat-40d	RHSIM_Rhsim04G0131900	1.251	1.027	-0.224	high
Treat-40d	RHSIM_Rhsim06G0047100	-0.175	-0.613	-0.438	no
Treat-40d	RHSIM_Rhsim05G0166700	0.578	0.979	0.401	no
Treat-40d	RHSIM_Rhsim05G0223800	0.683	2.015	1.333	no
Treat-40d	RHSIM_Rhsim05G0020400	-1.598	-0.224	1.374	low
Treat-40d	RHSIM_Rhsim06G0078800	-0.174	1.730	1.904	no
Treat-40d	RHSIM_Rhsim05G0190800	-1.364	-0.221	1.143	low
Treat-40d	RHSIM_Rhsim05G0069700	0.536	0.317	-0.219	no
Treat-40d	RHSIM_Rhsim05G0074000	-0.615	1.710	2.325	no
Treat-40d	RHSIM_Rhsim05G0150000	-0.977	-0.504	0.472	no
Treat-40d	RHSIM_Rhsim05G0096900	0.299	0.795	0.496	no
Treat-40d	RHSIM_Rhsim06G0005300	-0.230	-0.105	0.126	no
Treat-40d	RHSIM_Rhsim05G0101100	-0.490	-1.127	-0.636	no

Group	Gene	PTR (z-score difference)	z(log2 LFQ)	z(log2 FPKM)	extreme-PTR
Treat-40d	RHSIM_Rhsim05G0170400	0.662	0.796	0.134	no
Treat-40d	RHSIM_Rhsim05G0073500	-1.267	-0.123	1.144	low
Treat-40d	RHSIM_Rhsim06G0220300	-0.268	-0.285	-0.017	no
Treat-40d	RHSIM_Rhsim05G0182300	-0.326	-0.649	-0.322	no
Treat-40d	RHSIM_Rhsim07G0058500	0.954	0.341	-0.613	no
Treat-40d	RHSIM_Rhsim04G0175300	-0.732	-0.847	-0.115	no
Treat-40d	RHSIM_Rhsim05G0062700	2.056	-0.209	-2.264	high
Treat-40d	RHSIM_Rhsim05G0075900	-1.550	-0.153	1.397	low
Treat-40d	RHSIM_Rhsim05G0103400	1.542	1.252	-0.290	high
Treat-40d	RHSIM_Rhsim04G0099500	-1.661	-1.119	0.542	low
Treat-40d	RHSIM_Rhsim05G0159700	-1.369	-0.009	1.359	low
Treat-40d	RHSIM_Rhsim05G0085900	0.614	0.420	-0.194	no
Treat-40d	RHSIM_Rhsim05G0020500	-0.397	-1.172	-0.774	no
Treat-40d	RHSIM_Rhsim05G0177100	0.443	0.770	0.327	no
Treat-40d	RHSIM_Rhsim05G0180300	0.572	-0.903	-1.476	no
Treat-40d	RHSIM_Rhsim04G0126100	-0.573	-0.832	-0.259	no
Treat-40d	RHSIM_Rhsim06G0049900	0.473	-0.546	-1.019	no
Treat-40d	RHSIM_Rhsim05G0080100	-0.149	0.384	0.533	no
Treat-40d	RHSIM_Rhsim06G0002600	-0.865	-1.175	-0.310	no
Treat-40d	RHSIM_Rhsim05G0143500	-1.803	-1.310	0.494	low
Treat-40d	RHSIM_Rhsim05G0074600	0.453	0.238	-0.215	no
Treat-40d	RHSIM_Rhsim06G0142000	-1.543	-0.033	1.510	low
Treat-40d	RHSIM_Rhsim05G0040900	-1.211	-1.534	-0.322	low
Treat-40d	RHSIM_Rhsim05G0214100	0.992	1.044	0.052	no
Treat-40d	RHSIM_Rhsim05G0222100	0.172	-0.777	-0.949	no
Treat-40d	RHSIM_Rhsim07G0247000	0.274	0.363	0.089	no
Treat-40d	RHSIM_Rhsim06G0082000	-0.284	0.052	0.336	no
Treat-40d	RHSIM_Rhsim05G0187300	0.099	0.443	0.344	no
Treat-40d	RHSIM_Rhsim05G0143000	2.241	1.780	-0.461	high
Treat-40d	RHSIM_Rhsim05G0063700	0.518	0.986	0.469	no
Treat-40d	RHSIM_Rhsim04G0132500	0.528	0.949	0.421	no
Treat-40d	RHSIM_Rhsim05G0072000	1.606	-0.444	-2.049	high

Group	Gene	PTR (z-score difference)	z(log2 LFQ)	z(log2 FPKM)	extreme-PTR
Treat-40d	RHSIM_Rhsim04G0011300	0.082	-0.123	-0.206	no
Treat-40d	RHSIM_Rhsim05G0110900	2.514	0.721	-1.793	high
Treat-40d	RHSIM_Rhsim05G0106700	-0.582	0.081	0.664	no
Treat-40d	RHSIM_Rhsim04G0028000	2.721	1.172	-1.549	high
Treat-40d	RHSIM_Rhsim06G0054600	-1.013	-0.375	0.638	no
Treat-40d	RHSIM_Rhsim05G0029400	-1.179	-0.151	1.028	low
Treat-40d	RHSIM_Rhsim04G0009500	-0.580	-1.044	-0.464	no
Treat-40d	RHSIM_Rhsim05G0203000	-0.639	1.023	1.662	no
Treat-40d	RHSIM_Rhsim06G0047000	0.315	-0.349	-0.665	no
Treat-40d	RHSIM_Rhsim05G0113900	1.240	0.598	-0.642	high
Treat-40d	RHSIM_Rhsim05G0012900	0.610	0.110	-0.500	no
Treat-40d	RHSIM_Rhsim04G0014200	0.435	-0.223	-0.658	no
Treat-40d	RHSIM_Rhsim06G0017600	1.375	-0.069	-1.444	high
Treat-40d	RHSIM_Rhsim05G0086100	-1.988	-0.939	1.049	low
Treat-40d	RHSIM_Rhsim04G0002900	0.336	-0.145	-0.481	no
Treat-40d	RHSIM_Rhsim05G0068700	-1.548	-0.471	1.077	low
Treat-40d	RHSIM_Rhsim05G0233600	0.723	-0.079	-0.802	no
Treat-40d	RHSIM_Rhsim05G0179200	1.125	-0.317	-1.442	high
Treat-40d	RHSIM_Rhsim06G0023700	-0.575	-0.913	-0.338	no
Treat-40d	RHSIM_Rhsim04G0241700	-1.498	0.131	1.628	low
Treat-40d	RHSIM_Rhsim04G0106600	0.374	1.105	0.732	no
Treat-40d	RHSIM_Rhsim04G0190600	-1.367	0.828	2.195	low
Treat-40d	RHSIM_Rhsim06G0199000	-0.256	-0.930	-0.674	no
Treat-40d	RHSIM_Rhsim05G0027700	-1.244	-0.548	0.696	low
Treat-40d	RHSIM_Rhsim04G0148800	-0.445	-1.083	-0.638	no
Treat-40d	RHSIM_Rhsim07G0039400	0.336	1.134	0.797	no
Treat-40d	RHSIM_Rhsim04G0058200	-0.109	-0.099	0.010	no
Treat-40d	RHSIM_Rhsim04G0126600	-0.542	-0.530	0.012	no
Treat-40d	RHSIM_Rhsim05G0216600	-1.104	-0.788	0.315	no
Treat-40d	RHSIM_Rhsim05G0084000	-0.828	-1.519	-0.691	no
Treat-40d	RHSIM_Rhsim04G0196900	-0.180	0.247	0.427	no
Treat-40d	RHSIM_Rhsim05G0195600	1.502	0.358	-1.144	high

Group	Gene	PTR (z-score difference)	z(log2 LFQ)	z(log2 FPKM)	extreme-PTR
Treat-40d	RHSIM_Rhsim07G0245300	1.288	0.191	-1.096	high
Treat-40d	RHSIM_Rhsim04G0216600	1.460	0.194	-1.266	high
Treat-40d	RHSIM_Rhsim05G0067400	1.774	1.260	-0.514	high
Treat-40d	RHSIM_Rhsim04G0074000	-0.890	-0.134	0.756	no
Treat-40d	RHSIM_Rhsim05G0075200	-1.138	-1.482	-0.344	no
Treat-40d	RHSIM_Rhsim05G0145700	1.651	-0.139	-1.790	high
Treat-40d	RHSIM_Rhsim05G0175100	-0.710	-1.023	-0.313	no
Treat-40d	RHSIM_Rhsim04G0205900	-1.110	1.115	2.225	no
Treat-40d	RHSIM_Rhsim05G0135800	-0.135	0.621	0.756	no
Treat-40d	RHSIM_Rhsim05G0232100	-1.049	-0.027	1.021	no
Treat-40d	RHSIM_Rhsim05G0074800	-0.965	-0.691	0.274	no
Treat-40d	RHSIM_Rhsim05G0023600	2.348	0.612	-1.736	high
Treat-40d	RHSIM_Rhsim05G0096100	-2.248	-1.367	0.881	low
Treat-40d	RHSIM_Rhsim06G0064800	-0.163	0.074	0.238	no
Treat-40d	RHSIM_Rhsim05G0044600	1.067	0.751	-0.316	no
Treat-40d	RHSIM_Rhsim05G0192400	-0.599	-0.981	-0.382	no
Treat-40d	RHSIM_Rhsim05G0095400	1.859	1.197	-0.662	high
Treat-40d	RHSIM_Rhsim05G0194900	1.437	1.287	-0.150	high
Treat-40d	RHSIM_Rhsim04G0012800	-0.239	0.454	0.692	no
Treat-40d	RHSIM_Rhsim04G0058700	2.513	0.226	-2.287	high
Treat-40d	RHSIM_Rhsim05G0078300	0.728	0.003	-0.725	no
Treat-40d	RHSIM_Rhsim05G0000900	-0.560	-0.241	0.319	no
Treat-40d	RHSIM_Rhsim04G0014400	1.397	-0.065	-1.461	high
Treat-40d	RHSIM_Rhsim05G0227300	0.744	-0.871	-1.615	no
Treat-40d	RHSIM_Rhsim04G0015900	-0.686	-0.566	0.120	no
Treat-40d	RHSIM_Rhsim05G0164500	0.654	1.570	0.916	no
Treat-40d	RHSIM_Rhsim04G0016200	0.307	0.530	0.223	no
Treat-40d	RHSIM_Rhsim07G0101200	-0.678	0.881	1.558	no
Treat-40d	RHSIM_Rhsim04G0073400	-1.313	-1.322	-0.009	low
Treat-40d	RHSIM_Rhsim05G0152100	0.346	-0.364	-0.710	no
Treat-40d	RHSIM_Rhsim04G0029500	0.266	1.207	0.941	no
Treat-40d	RHSIM_Rhsim05G0044900	0.713	-0.618	-1.331	no

Group	Gene	PTR (z-score difference)	z(log2 LFQ)	z(log2 FPKM)	extreme-PTR
Treat-40d	RHSIM_Rhsim04G0099700	-0.785	-0.037	0.748	no
Treat-40d	RHSIM_Rhsim05G0202600	-0.779	-1.127	-0.348	no
Treat-40d	RHSIM_Rhsim05G0133100	1.112	0.373	-0.739	high
Treat-40d	RHSIM_Rhsim04G0003800	-0.220	-0.622	-0.402	no
Treat-40d	RHSIM_Rhsim05G0210500	0.839	-0.976	-1.814	no
Treat-40d	RHSIM_Rhsim07G0175300	0.628	0.883	0.255	no
Treat-40d	RHSIM_Rhsim04G0087500	-0.814	-0.754	0.060	no
Treat-40d	RHSIM_Rhsim05G0199700	-0.211	0.062	0.274	no
Treat-40d	RHSIM_Rhsim05G0165700	0.736	1.205	0.469	no
Treat-40d	RHSIM_Rhsim05G0139600	1.015	-0.362	-1.378	no
Treat-40d	RHSIM_Rhsim04G0145800	-0.492	0.103	0.595	no
Treat-40d	RHSIM_Rhsim05G0063100	-0.887	-0.056	0.831	no
Treat-40d	RHSIM_Rhsim04G0199100	1.259	1.874	0.615	high
Treat-40d	RHSIM_Rhsim04G0033100	0.732	2.180	1.448	no
Treat-40d	RHSIM_Rhsim04G0169600	0.967	0.885	-0.082	no
Treat-40d	RHSIM_Rhsim05G0067700	-1.949	-0.883	1.066	low
Treat-40d	RHSIM_Rhsim05G0159600	1.538	1.346	-0.192	high
Treat-40d	RHSIM_Rhsim04G0013600	-0.600	-0.089	0.510	no
Treat-40d	RHSIM_Rhsim04G0124900	-0.636	0.824	1.460	no
Treat-40d	RHSIM_Rhsim05G0175000	-0.587	-0.564	0.024	no
Treat-40d	RHSIM_Rhsim05G0133000	0.481	-1.750	-2.231	no
Treat-40d	RHSIM_Rhsim05G0121700	-1.364	0.081	1.445	low
Treat-40d	RHSIM_Rhsim04G0100900	-0.426	2.456	2.883	no
Treat-40d	RHSIM_Rhsim07G0134900	1.973	2.757	0.784	high
Treat-40d	RHSIM_Rhsim05G0072400	-0.083	-0.738	-0.655	no
Treat-40d	RHSIM_Rhsim04G0114200	0.892	-1.157	-2.049	no
Treat-40d	RHSIM_Rhsim04G0119400	1.827	1.667	-0.160	high
Treat-40d	RHSIM_Rhsim04G0065900	0.104	0.417	0.313	no
Treat-40d	RHSIM_Rhsim04G0134300	-0.089	0.716	0.805	no
Treat-40d	RHSIM_Rhsim07G0101600	0.574	0.043	-0.531	no
Treat-40d	RHSIM_Rhsim05G0212900	3.239	3.713	0.474	high
Treat-40d	RHSIM_Rhsim07G0167800	-0.410	-0.797	-0.387	no

Group	Gene	PTR (z-score difference)	z(log2 LFQ)	z(log2 FPKM)	extreme-PTR
Treat-40d	RHSIM_Rhsim05G0084700	-1.184	-0.621	0.563	low
Treat-40d	RHSIM_Rhsim04G0150100	-1.386	-1.451	-0.065	low
Treat-40d	RHSIM_Rhsim05G0111000	0.093	-0.794	-0.887	no
Treat-40d	RHSIM_Rhsim04G0245000	-0.567	-0.824	-0.257	no
Treat-40d	RHSIM_Rhsim05G0060100	-1.075	-0.618	0.456	no
Treat-40d	RHSIM_Rhsim04G0009400	-0.066	-0.466	-0.400	no
Treat-40d	RHSIM_Rhsim05G0083700	-0.398	-0.556	-0.158	no
Treat-40d	RHSIM_Rhsim05G0229700	-0.160	-0.301	-0.140	no
Treat-40d	RHSIM_Rhsim04G0014300	1.034	0.553	-0.480	no
Treat-40d	RHSIM_Rhsim05G0053600	1.528	-0.346	-1.874	high
Treat-40d	RHSIM_Rhsim05G0095000	-0.283	0.046	0.329	no
Treat-40d	RHSIM_Rhsim04G0096600	0.718	-0.775	-1.493	no
Treat-40d	RHSIM_Rhsim05G0090100	0.038	-0.096	-0.133	no
Treat-40d	RHSIM_Rhsim05G0161400	0.581	-0.945	-1.526	no
Treat-40d	RHSIM_Rhsim07G0059400	0.337	0.661	0.324	no
Treat-40d	RHSIM_Rhsim04G0023700	-0.151	1.892	2.043	no
Treat-40d	RHSIM_Rhsim05G0216800	-0.971	-0.067	0.904	no
Treat-40d	RHSIM_Rhsim05G0101800	-0.700	-0.270	0.429	no
Treat-40d	RHSIM_Rhsim04G0213000	-0.678	-0.722	-0.044	no
Treat-40d	RHSIM_Rhsim05G0039000	1.836	-0.049	-1.885	high
Treat-40d	RHSIM_Rhsim05G0192200	0.990	0.873	-0.118	no
Treat-40d	RHSIM_Rhsim07G0008100	-2.023	-0.377	1.646	low
Treat-40d	RHSIM_Rhsim05G0180600	0.356	-0.085	-0.440	no
Treat-40d	RHSIM_Rhsim04G0202900	-0.234	-0.392	-0.158	no
Treat-40d	RHSIM_Rhsim04G0084300	1.626	-0.349	-1.974	high
Treat-40d	RHSIM_Rhsim04G0184400	0.869	0.824	-0.045	no
Treat-40d	RHSIM_Rhsim07G0010700	0.019	0.107	0.088	no
Treat-40d	RHSIM_Rhsim04G0228600	-0.040	-0.222	-0.182	no
Treat-40d	RHSIM_Rhsim05G0062300	-0.751	-1.402	-0.651	no
Treat-40d	RHSIM_Rhsim07G0005600	-1.169	-1.224	-0.055	low
Treat-40d	RHSIM_Rhsim04G0248100	-0.474	0.650	1.124	no
Treat-40d	RHSIM_Rhsim07G0031200	-0.210	0.584	0.794	no

Group	Gene	PTR (z-score difference)	z(log2 LFQ)	z(log2 FPKM)	extreme-PTR
Treat-40d	RHSIM_Rhsim05G0105400	-0.872	-0.186	0.686	no
Treat-40d	RHSIM_Rhsim04G0039600	-1.102	-1.761	-0.660	no
Treat-40d	RHSIM_Rhsim04G0112300	1.391	-0.904	-2.295	high
Treat-40d	RHSIM_Rhsim04G0249500	-2.734	-1.698	1.037	low
Treat-40d	RHSIM_Rhsim04G0216400	0.926	-0.322	-1.249	no
Treat-40d	RHSIM_Rhsim07G0142500	-0.547	-0.261	0.286	no
Treat-40d	RHSIM_Rhsim04G0243000	-1.184	-1.144	0.040	low
Treat-40d	RHSIM_Rhsim04G0011600	-2.234	-0.266	1.969	low
Treat-40d	RHSIM_Rhsim05G0106400	0.024	-0.370	-0.394	no
Treat-40d	RHSIM_Rhsim04G0126000	-0.958	-1.209	-0.251	no
Treat-40d	RHSIM_Rhsim04G0118000	-0.610	-0.482	0.129	no
Treat-40d	RHSIM_Rhsim04G0012200	0.282	0.326	0.045	no
Treat-40d	RHSIM_Rhsim04G0010300	0.726	2.413	1.687	no
Treat-40d	RHSIM_Rhsim05G0160800	1.178	0.640	-0.538	high
Treat-40d	RHSIM_Rhsim04G0010500	-1.112	-1.117	-0.005	no
Treat-40d	RHSIM_Rhsim05G0214400	-0.680	1.012	1.692	no
Treat-40d	RHSIM_Rhsim04G0203200	-1.092	1.003	2.095	no
Treat-40d	RHSIM_Rhsim07G0252600	0.488	0.791	0.303	no
Treat-40d	RHSIM_Rhsim04G0125900	0.358	-1.262	-1.620	no
Treat-40d	RHSIM_Rhsim05G0159300	1.156	1.696	0.539	high
Treat-40d	RHSIM_Rhsim04G0216900	-0.055	-0.888	-0.833	no
Treat-40d	RHSIM_Rhsim04G0052800	-0.185	-0.335	-0.150	no
Treat-40d	RHSIM_Rhsim04G0225000	-0.277	0.984	1.262	no
Treat-40d	RHSIM_Rhsim04G0199900	0.521	1.476	0.956	no
Treat-40d	RHSIM_Rhsim05G0131900	0.595	0.571	-0.023	no
Treat-40d	RHSIM_Rhsim07G0126100	-0.862	-0.471	0.391	no
Treat-40d	RHSIM_Rhsim05G0124800	-0.287	-0.160	0.127	no
Treat-40d	RHSIM_Rhsim04G0004200	-1.850	-1.220	0.630	low
Treat-40d	RHSIM_Rhsim04G0180700	1.208	0.153	-1.055	high
Treat-40d	RHSIM_Rhsim06G0198800	-0.768	-0.514	0.254	no
Treat-40d	RHSIM_Rhsim04G0202000	-0.227	-0.479	-0.252	no
Treat-40d	RHSIM_Rhsim04G0052300	1.933	2.698	0.765	high

Group	Gene	PTR (z-score difference)	z(log2 LFQ)	z(log2 FPKM)	extreme-PTR
Treat-40d	RHSIM_Rhsim04G0006600	0.135	0.487	0.352	no
Treat-40d	RHSIM_Rhsim06G0033800	0.600	-1.059	-1.659	no
Treat-40d	RHSIM_Rhsim05G0167200	1.012	1.528	0.517	no
Treat-40d	RHSIM_Rhsim04G0123300	-1.650	-1.178	0.471	low
Treat-40d	RHSIM_Rhsim04G0220300	0.217	-0.217	-0.434	no
Treat-40d	RHSIM_Rhsim05G0201000	0.191	0.057	-0.134	no
Treat-40d	RHSIM_Rhsim04G0055900	1.010	-0.494	-1.504	no
Treat-40d	RHSIM_Rhsim04G0174200	0.147	-0.715	-0.862	no
Treat-40d	RHSIM_Rhsim06G0087100	-0.593	-0.370	0.224	no
Treat-40d	RHSIM_Rhsim04G0016600	0.350	0.861	0.511	no
Treat-40d	RHSIM_Rhsim04G0175900	1.825	0.948	-0.877	high
Treat-40d	RHSIM_Rhsim04G0203300	0.157	0.140	-0.017	no
Treat-40d	RHSIM_Rhsim05G0100900	-2.316	-0.230	2.086	low
Treat-40d	RHSIM_Rhsim04G0026100	1.389	1.678	0.289	high
Treat-40d	RHSIM_Rhsim05G0225500	-0.589	-0.774	-0.184	no
Treat-40d	RHSIM_Rhsim06G0006400	-1.692	-0.812	0.881	low
Treat-40d	RHSIM_Rhsim05G0031400	0.307	0.249	-0.058	no
Treat-40d	RHSIM_Rhsim04G0013400	0.438	-0.105	-0.542	no
Treat-40d	RHSIM_Rhsim04G0157800	-2.059	-0.168	1.891	low
Treat-40d	RHSIM_Rhsim04G0008200	0.115	0.345	0.230	no
Treat-40d	RHSIM_Rhsim04G0019600	0.675	-0.612	-1.287	no
Treat-40d	RHSIM_Rhsim05G0122700	-0.216	-0.905	-0.689	no
Treat-40d	RHSIM_Rhsim04G0168600	0.365	0.278	-0.087	no
Treat-40d	RHSIM_Rhsim04G0083700	0.835	-0.775	-1.610	no
Treat-40d	RHSIM_Rhsim05G0047300	0.199	-1.604	-1.803	no
Treat-40d	RHSIM_Rhsim05G0174900	1.316	0.809	-0.507	high
Treat-40d	RHSIM_Rhsim04G0006900	-0.926	-0.694	0.232	no
Treat-40d	RHSIM_Rhsim04G0167600	-0.304	-0.963	-0.659	no
Treat-40d	RHSIM_Rhsim04G0070300	-0.452	0.802	1.254	no
Treat-40d	RHSIM_Rhsim04G0154200	-0.731	-1.766	-1.034	no
Treat-40d	RHSIM_Rhsim04G0215500	0.036	0.476	0.441	no
Treat-40d	RHSIM_Rhsim06G0237200	-0.541	0.600	1.141	no

Group	Gene	PTR (z-score difference)	z(log2 LFQ)	z(log2 FPKM)	extreme-PTR
Treat-40d	RHSIM_Rhsim04G0173400	1.679	0.990	-0.689	high
Treat-40d	RHSIM_Rhsim04G0075900	2.208	1.511	-0.697	high
Treat-40d	RHSIM_Rhsim04G0074900	-0.041	-0.833	-0.792	no
Treat-40d	RHSIM_Rhsim04G0126400	0.295	0.209	-0.087	no
Treat-40d	RHSIM_Rhsim04G0125700	-2.228	-1.199	1.030	low
Treat-40d	RHSIM_Rhsim04G0202300	3.559	2.490	-1.069	high
Treat-40d	RHSIM_Rhsim04G0023900	-0.581	0.379	0.960	no
Treat-40d	RHSIM_Rhsim05G0172200	0.102	-0.927	-1.028	no
Treat-40d	RHSIM_Rhsim04G0093600	-1.868	-0.617	1.251	low
Treat-40d	RHSIM_Rhsim04G0230600	0.617	2.835	2.218	no
Treat-40d	RHSIM_Rhsim04G0136300	-0.725	0.429	1.154	no
Treat-40d	RHSIM_Rhsim04G0030800	0.837	0.481	-0.356	no
Treat-40d	RHSIM_Rhsim05G0219800	0.739	-0.913	-1.652	no
Treat-40d	RHSIM_Rhsim05G0217200	-1.794	-1.709	0.085	low
Treat-40d	RHSIM_Rhsim04G0207400	0.133	0.336	0.203	no
Treat-40d	RHSIM_Rhsim05G0095100	0.555	1.420	0.865	no
Treat-40d	RHSIM_Rhsim03G0030800	2.325	1.156	-1.169	high
Treat-40d	RHSIM_Rhsim05G0163700	-0.731	-0.399	0.332	no
Treat-40d	RHSIM_Rhsim04G0018100	0.563	0.350	-0.213	no
Treat-40d	RHSIM_Rhsim05G0211100	0.176	0.474	0.298	no
Treat-40d	RHSIM_Rhsim04G0195100	0.915	1.147	0.232	no
Treat-40d	RHSIM_Rhsim04G0156300	-1.042	1.800	2.842	no
Treat-40d	RHSIM_Rhsim04G0030700	-0.169	0.388	0.557	no
Treat-40d	RHSIM_Rhsim04G0108400	-2.290	0.077	2.367	low
Treat-40d	RHSIM_Rhsim04G0217800	-1.443	-0.610	0.834	low
Treat-40d	RHSIM_Rhsim04G0195700	-1.108	-0.846	0.262	no
Treat-40d	RHSIM_Rhsim04G0000700	-0.605	-0.268	0.337	no
Treat-40d	RHSIM_Rhsim04G0117700	0.087	-0.591	-0.678	no
Treat-40d	RHSIM_Rhsim03G0235300	-0.570	0.583	1.153	no
Treat-40d	RHSIM_Rhsim05G0139500	0.184	0.639	0.454	no
Treat-40d	RHSIM_Rhsim04G0114100	0.473	0.995	0.522	no
Treat-40d	RHSIM_Rhsim04G0009900	-0.582	-0.351	0.231	no

Group	Gene	PTR (z-score difference)	z(log2 LFQ)	z(log2 FPKM)	extreme-PTR
Treat-40d	RHSIM_Rhsim04G0084500	-0.708	0.964	1.672	no
Treat-40d	RHSIM_Rhsim06G0164200	-0.809	1.351	2.160	no
Treat-40d	RHSIM_Rhsim05G0075700	1.539	0.740	-0.799	high
Treat-40d	RHSIM_Rhsim05G0086800	-0.870	0.194	1.065	no
Treat-40d	RHSIM_Rhsim04G0104400	0.708	0.382	-0.326	no
Treat-40d	RHSIM_Rhsim04G0130500	-0.645	-0.005	0.640	no
Treat-40d	RHSIM_Rhsim04G0018200	0.274	-0.073	-0.347	no
Treat-40d	RHSIM_Rhsim05G0082100	0.023	0.076	0.054	no
Treat-40d	RHSIM_Rhsim04G0224400	-0.507	-0.084	0.423	no
Treat-40d	RHSIM_Rhsim04G0232100	0.349	0.614	0.265	no
Treat-40d	RHSIM_Rhsim05G0187700	0.351	1.140	0.789	no
Treat-40d	RHSIM_Rhsim03G0241400	-0.924	-0.258	0.665	no
Treat-40d	RHSIM_Rhsim04G0194700	1.402	0.897	-0.505	high
Treat-40d	RHSIM_Rhsim05G0017300	-0.949	-1.100	-0.151	no
Treat-40d	RHSIM_Rhsim04G0240200	-2.800	-1.216	1.584	low
Treat-40d	RHSIM_Rhsim04G0063000	-0.090	-0.399	-0.309	no
Treat-40d	RHSIM_Rhsim05G0104700	0.960	0.986	0.027	no
Treat-40d	RHSIM_Rhsim04G0239000	1.010	1.190	0.180	no
Treat-40d	RHSIM_Rhsim04G0170500	0.631	0.106	-0.525	no
Treat-40d	RHSIM_Rhsim05G0150300	0.234	-0.685	-0.918	no
Treat-40d	RHSIM_Rhsim04G0030100	1.284	1.558	0.274	high
Treat-40d	RHSIM_Rhsim04G0200900	-0.377	-0.878	-0.501	no
Treat-40d	RHSIM_Rhsim04G0083600	-0.181	-0.689	-0.507	no
Treat-40d	RHSIM_Rhsim04G0221400	-1.488	-0.523	0.965	low
Treat-40d	RHSIM_Rhsim05G0205100	-0.280	-1.363	-1.083	no
Treat-40d	RHSIM_Rhsim04G0122900	0.289	0.434	0.145	no
Treat-40d	RHSIM_Rhsim05G0067100	-0.553	0.472	1.025	no
Treat-40d	RHSIM_Rhsim03G0221100	0.738	0.795	0.057	no
Treat-40d	RHSIM_Rhsim04G0011100	0.510	1.099	0.588	no
Treat-40d	RHSIM_Rhsim04G0158800	0.490	0.036	-0.454	no
Treat-40d	RHSIM_Rhsim04G0177100	0.111	-0.234	-0.345	no
Treat-40d	RHSIM_Rhsim04G0050100	-2.661	-0.189	2.472	low

Group	Gene	PTR (z-score difference)	z(log2 LFQ)	z(log2 FPKM)	extreme-PTR
Treat-40d	RHSIM_Rhsim03G0145800	0.890	-0.166	-1.056	no
Treat-40d	RHSIM_Rhsim04G0235200	0.910	2.002	1.093	no
Treat-40d	RHSIM_Rhsim04G0247200	-0.034	-1.091	-1.057	no
Treat-40d	RHSIM_Rhsim04G0038600	3.913	4.328	0.415	high
Treat-40d	RHSIM_Rhsim04G0103300	-0.801	-0.141	0.660	no
Treat-40d	RHSIM_Rhsim04G0144400	1.223	0.787	-0.435	high
Treat-40d	RHSIM_Rhsim04G0162900	-1.774	-0.937	0.837	low
Treat-40d	RHSIM_Rhsim06G0035400	-1.415	-0.715	0.700	low
Treat-40d	RHSIM_Rhsim04G0006200	2.742	3.225	0.483	high
Treat-40d	RHSIM_Rhsim04G0219100	0.651	0.599	-0.053	no
Treat-40d	RHSIM_Rhsim03G0171900	0.665	0.590	-0.075	no
Treat-40d	RHSIM_Rhsim06G0232900	0.409	0.145	-0.264	no
Treat-40d	RHSIM_Rhsim04G0248000	-0.130	0.082	0.212	no
Treat-40d	RHSIM_Rhsim04G0084900	-0.017	0.220	0.238	no
Treat-40d	RHSIM_Rhsim04G0022700	-1.694	-0.189	1.505	low
Treat-40d	RHSIM_Rhsim04G0164200	-1.904	-2.613	-0.709	low
Treat-40d	RHSIM_Rhsim04G0224800	-0.002	0.314	0.316	no
Treat-40d	RHSIM_Rhsim03G0219900	1.284	0.684	-0.600	high
Treat-40d	RHSIM_Rhsim04G0110400	0.491	0.726	0.235	no
Treat-40d	RHSIM_Rhsim04G0235000	-1.066	-0.789	0.276	no
Treat-40d	RHSIM_Rhsim03G0220500	-1.751	-0.667	1.084	low
Treat-40d	RHSIM_Rhsim04G0196400	0.100	-1.341	-1.442	no
Treat-40d	RHSIM_Rhsim04G0015300	0.250	1.228	0.978	no
Treat-40d	RHSIM_Rhsim04G0241800	0.245	1.257	1.013	no
Treat-40d	RHSIM_Rhsim04G0131000	-0.557	-0.339	0.219	no
Treat-40d	RHSIM_Rhsim06G0169700	1.031	-0.858	-1.888	no
Treat-40d	RHSIM_Rhsim05G0108600	-0.020	0.542	0.562	no
Treat-40d	RHSIM_Rhsim04G0173800	-1.042	-0.172	0.870	no
Treat-40d	RHSIM_Rhsim04G0092500	2.267	1.216	-1.050	high
Treat-40d	RHSIM_Rhsim04G0037800	-0.500	-0.669	-0.168	no
Treat-40d	RHSIM_Rhsim04G0031900	-0.860	0.023	0.883	no
Treat-40d	RHSIM_Rhsim03G0057400	-1.435	-0.884	0.550	low

Group	Gene	PTR (z-score difference)	z(log2 LFQ)	z(log2 FPKM)	extreme-PTR
Treat-40d	RHSIM_Rhsim04G0043200	0.197	0.019	-0.177	no
Treat-40d	RHSIM_Rhsim04G0204600	0.555	-0.836	-1.391	no
Treat-40d	RHSIM_Rhsim04G0106500	-1.445	0.352	1.797	low
Treat-40d	RHSIM_Rhsim04G0130700	-0.601	-0.011	0.591	no
Treat-40d	RHSIM_Rhsim06G0229600	1.801	0.829	-0.972	high
Treat-40d	RHSIM_Rhsim04G0100100	0.306	-0.332	-0.638	no
Treat-40d	RHSIM_Rhsim04G0084000	-0.365	-0.055	0.310	no
Treat-40d	RHSIM_Rhsim03G0221500	-0.250	-0.973	-0.723	no
Treat-40d	RHSIM_Rhsim03G0225700	-0.006	0.435	0.442	no
Treat-40d	RHSIM_Rhsim06G0202900	2.409	0.620	-1.790	high
Treat-40d	RHSIM_Rhsim04G0197100	1.400	2.049	0.649	high
Treat-40d	RHSIM_Rhsim03G0001300	-0.225	-1.111	-0.887	no
Treat-40d	RHSIM_Rhsim04G0167300	1.409	2.136	0.727	high
Treat-40d	RHSIM_Rhsim03G0006400	-2.335	-0.117	2.219	low
Treat-40d	RHSIM_Rhsim05G0163500	0.551	0.406	-0.145	no
Treat-40d	RHSIM_Rhsim04G0198400	-1.639	-0.998	0.641	low
Treat-40d	RHSIM_Rhsim04G0056300	0.963	-0.279	-1.241	no
Treat-40d	RHSIM_Rhsim04G0056600	-0.228	-0.550	-0.322	no
Treat-40d	RHSIM_Rhsim04G0244400	0.506	1.476	0.970	no
Treat-40d	RHSIM_Rhsim04G0153200	-0.279	0.900	1.180	no
Treat-40d	RHSIM_Rhsim04G0023000	0.205	-0.132	-0.337	no
Treat-40d	RHSIM_Rhsim03G0083600	1.789	1.607	-0.181	high
Treat-40d	RHSIM_Rhsim06G0000500	0.235	1.188	0.952	no
Treat-40d	RHSIM_Rhsim04G0172400	-0.468	-0.108	0.360	no
Treat-40d	RHSIM_Rhsim04G0032200	-0.921	-0.506	0.415	no
Treat-40d	RHSIM_Rhsim04G0027200	-0.785	0.916	1.701	no
Treat-40d	RHSIM_Rhsim04G0245300	0.746	-0.199	-0.945	no
Treat-40d	RHSIM_Rhsim04G0072600	-1.416	-1.692	-0.276	low
Treat-40d	RHSIM_Rhsim03G0168400	1.643	1.235	-0.408	high
Treat-40d	RHSIM_Rhsim03G0075300	0.243	0.583	0.341	no
Treat-40d	RHSIM_Rhsim04G0173300	-1.095	0.226	1.321	no
Treat-40d	RHSIM_Rhsim04G0219500	-0.609	-0.733	-0.124	no

Group	Gene	PTR (z-score difference)	z(log2 LFQ)	z(log2 FPKM)	extreme-PTR
Treat-40d	RHSIM_Rhsim04G0180500	-0.217	-0.140	0.077	no
Treat-40d	RHSIM_Rhsim05G0181000	-0.053	0.569	0.622	no
Treat-40d	RHSIM_Rhsim04G0096800	2.016	2.148	0.132	high
Treat-40d	RHSIM_Rhsim04G0095900	0.975	0.584	-0.391	no
Treat-40d	RHSIM_Rhsim03G0154300	1.259	-0.812	-2.072	high
Treat-40d	RHSIM_Rhsim06G0030600	-0.801	-0.582	0.219	no
Treat-40d	RHSIM_Rhsim04G0038700	0.617	-0.190	-0.807	no
Treat-40d	RHSIM_Rhsim04G0001800	0.487	0.443	-0.043	no
Treat-40d	RHSIM_Rhsim05G0062800	0.629	2.745	2.116	no
Treat-40d	RHSIM_Rhsim03G0105200	1.421	-0.633	-2.054	high
Treat-40d	RHSIM_Rhsim03G0111900	1.158	2.012	0.854	high
Treat-40d	RHSIM_Rhsim03G0207700	-0.239	-1.383	-1.143	no
Treat-40d	RHSIM_Rhsim05G0133600	0.515	0.571	0.056	no
Treat-40d	RHSIM_Rhsim04G0162800	-1.043	-0.965	0.078	no
Treat-40d	RHSIM_Rhsim04G0087300	0.303	0.229	-0.074	no
Treat-40d	RHSIM_Rhsim06G0239100	0.521	1.652	1.131	no
Treat-40d	RHSIM_Rhsim04G0196800	-0.179	1.623	1.802	no
Treat-40d	RHSIM_Rhsim03G0044900	-0.363	-0.235	0.127	no
Treat-40d	RHSIM_Rhsim03G0179500	-0.363	1.409	1.772	no
Treat-40d	RHSIM_Rhsim04G0185400	0.733	-0.625	-1.359	no
Treat-40d	RHSIM_Rhsim04G0205700	2.785	1.176	-1.609	high
Treat-40d	RHSIM_Rhsim04G0020500	3.275	1.885	-1.390	high
Treat-40d	RHSIM_Rhsim03G0234000	-2.319	-1.330	0.989	low
Treat-40d	RHSIM_Rhsim04G0233600	0.680	-0.259	-0.940	no
Treat-40d	RHSIM_Rhsim03G0019300	-0.767	1.389	2.155	no
Treat-40d	RHSIM_Rhsim04G0162300	-0.878	0.681	1.559	no
Treat-40d	RHSIM_Rhsim03G0226300	0.172	0.123	-0.049	no
Treat-40d	RHSIM_Rhsim04G0218700	-0.971	-0.898	0.073	no
Treat-40d	RHSIM_Rhsim03G0155100	-0.201	-1.152	-0.951	no
Treat-40d	RHSIM_Rhsim04G0177600	-1.289	-1.163	0.126	low
Treat-40d	RHSIM_Rhsim03G0254300	0.132	0.512	0.380	no
Treat-40d	RHSIM_Rhsim03G0001500	0.744	0.778	0.034	no

Group	Gene	PTR (z-score difference)	z(log2 LFQ)	z(log2 FPKM)	extreme-PTR
Treat-40d	RHSIM_Rhsim03G0166500	0.541	-0.607	-1.148	no
Treat-40d	RHSIM_Rhsim03G0065700	0.760	-0.070	-0.830	no
Treat-40d	RHSIM_Rhsim04G0228500	-0.211	0.627	0.837	no
Treat-40d	RHSIM_Rhsim03G0253300	-1.224	-0.579	0.645	low
Treat-40d	RHSIM_Rhsim03G0056500	-2.292	0.194	2.487	low
Treat-40d	RHSIM_Rhsim04G0003700	0.897	-1.214	-2.111	no
Treat-40d	RHSIM_Rhsim03G0180700	-0.051	1.121	1.171	no
Treat-40d	RHSIM_Rhsim04G0189900	1.202	0.620	-0.582	high
Treat-40d	RHSIM_Rhsim04G0233400	-0.876	-1.139	-0.263	no
Treat-40d	RHSIM_Rhsim04G0003500	-0.814	-0.281	0.533	no
Treat-40d	RHSIM_Rhsim03G0223300	1.296	-1.021	-2.317	high
Treat-40d	RHSIM_Rhsim04G0134100	-0.453	-1.602	-1.149	no
Treat-40d	RHSIM_Rhsim03G0010800	0.302	-0.537	-0.839	no
Treat-40d	RHSIM_Rhsim03G0063700	0.332	0.433	0.101	no
Treat-40d	RHSIM_Rhsim04G0030200	-1.021	-0.685	0.335	no
Treat-40d	RHSIM_Rhsim03G0068700	-0.448	1.282	1.729	no
Treat-40d	RHSIM_Rhsim04G0158400	-0.532	-0.909	-0.377	no
Treat-40d	RHSIM_Rhsim04G0139400	1.346	-0.604	-1.950	high
Treat-40d	RHSIM_Rhsim04G0190700	-0.443	-1.244	-0.802	no
Treat-40d	RHSIM_Rhsim04G0140900	0.073	-0.155	-0.228	no
Treat-40d	RHSIM_Rhsim04G0230400	0.950	1.967	1.017	no
Treat-40d	RHSIM_Rhsim03G0085200	-0.534	-0.448	0.087	no
Treat-40d	RHSIM_Rhsim03G0228800	-0.556	-1.224	-0.668	no
Treat-40d	RHSIM_Rhsim04G0120900	-0.366	-0.716	-0.350	no
Treat-40d	RHSIM_Rhsim04G0155300	-0.951	1.636	2.587	no
Treat-40d	RHSIM_Rhsim03G0121700	-0.921	-0.391	0.530	no
Treat-40d	RHSIM_Rhsim04G0004400	0.644	0.680	0.036	no
Treat-40d	RHSIM_Rhsim03G0129000	0.010	1.016	1.006	no
Treat-40d	RHSIM_Rhsim06G0082800	-0.329	-0.727	-0.398	no
Treat-40d	RHSIM_Rhsim03G0235100	-0.289	1.031	1.321	no
Treat-40d	RHSIM_Rhsim04G0138700	-0.255	0.495	0.750	no
Treat-40d	RHSIM_Rhsim04G0003600	0.677	1.188	0.511	no

Group	Gene	PTR (z-score difference)	z(log2 LFQ)	z(log2 FPKM)	extreme-PTR
Treat-40d	RHSIM_Rhsim06G0166200	0.274	0.047	-0.226	no
Treat-40d	RHSIM_Rhsim03G0198500	-0.092	-0.760	-0.668	no
Treat-40d	RHSIM_Rhsim03G0000200	1.116	1.124	0.008	high
Treat-40d	RHSIM_Rhsim04G0128400	-1.432	-0.792	0.639	low
Treat-40d	RHSIM_Rhsim04G0225100	1.101	-0.515	-1.616	no
Treat-40d	RHSIM_Rhsim03G0096300	-0.725	-0.059	0.666	no
Treat-40d	RHSIM_Rhsim03G0234800	0.315	-0.252	-0.567	no
Treat-40d	RHSIM_Rhsim03G0066700	1.418	3.488	2.070	high
Treat-40d	RHSIM_Rhsim04G0241100	1.355	1.359	0.003	high
Treat-40d	RHSIM_Rhsim04G0084800	-1.866	-0.726	1.140	low
Treat-40d	RHSIM_Rhsim04G0021800	-0.221	0.607	0.828	no
Treat-40d	RHSIM_Rhsim03G0239700	-2.569	-0.919	1.649	low
Treat-40d	RHSIM_Rhsim03G0146900	0.494	-0.855	-1.349	no
Treat-40d	RHSIM_Rhsim04G0094400	0.127	-1.080	-1.207	no
Treat-40d	RHSIM_Rhsim03G0164200	0.360	0.584	0.224	no
Treat-40d	RHSIM_Rhsim03G0006300	-1.838	0.963	2.801	low
Treat-40d	RHSIM_Rhsim06G0174600	-0.033	0.483	0.516	no
Treat-40d	RHSIM_Rhsim04G0039400	-0.368	-1.250	-0.881	no
Treat-40d	RHSIM_Rhsim06G0073200	-2.299	-0.188	2.110	low
Treat-40d	RHSIM_Rhsim03G0188200	-0.365	-0.658	-0.293	no
Treat-40d	RHSIM_Rhsim04G0001500	0.841	0.358	-0.483	no
Treat-40d	RHSIM_Rhsim06G0036100	0.537	-0.231	-0.768	no
Treat-40d	RHSIM_Rhsim04G0129300	0.113	0.648	0.535	no
Treat-40d	RHSIM_Rhsim06G0177200	0.364	-0.620	-0.984	no
Treat-40d	RHSIM_Rhsim03G0019000	-1.019	0.627	1.646	no
Treat-40d	RHSIM_Rhsim04G0116200	0.649	-1.115	-1.763	no
Treat-40d	RHSIM_Rhsim03G0122000	0.816	0.211	-0.605	no
Treat-40d	RHSIM_Rhsim03G0229700	-0.541	-0.299	0.242	no
Treat-40d	RHSIM_Rhsim06G0153200	-0.522	0.336	0.858	no
Treat-40d	RHSIM_Rhsim03G0020300	1.856	0.729	-1.127	high
Treat-40d	RHSIM_Rhsim03G0172000	0.199	-0.713	-0.912	no
Treat-40d	RHSIM_Rhsim03G0041200	-0.033	0.204	0.237	no

Group	Gene	PTR (z-score difference)	z(log2 LFQ)	z(log2 FPKM)	extreme-PTR
Treat-40d	RHSIM_Rhsim03G0222600	-1.496	-0.337	1.158	low
Treat-40d	RHSIM_Rhsim06G0001600	-0.198	-0.096	0.102	no
Treat-40d	RHSIM_Rhsim03G0155200	0.693	0.927	0.234	no
Treat-40d	RHSIM_Rhsim03G0173300	0.884	0.351	-0.533	no
Treat-40d	RHSIM_Rhsim03G0218100	-1.919	-2.261	-0.342	low
Treat-40d	RHSIM_Rhsim03G0270100	1.078	-0.733	-1.810	no
Treat-40d	RHSIM_Rhsim06G0087400	-1.532	-1.140	0.392	low
Treat-40d	RHSIM_Rhsim03G0212700	-0.684	1.005	1.689	no
Treat-40d	RHSIM_Rhsim03G0230000	-0.402	-0.248	0.154	no
Treat-40d	RHSIM_Rhsim03G0009200	2.444	1.468	-0.976	high
Treat-40d	RHSIM_Rhsim03G0050300	0.045	0.001	-0.044	no
Treat-40d	RHSIM_Rhsim03G0075800	1.764	1.130	-0.634	high
Treat-40d	RHSIM_Rhsim06G0129100	-0.015	0.283	0.299	no
Treat-40d	RHSIM_Rhsim04G0165300	0.504	0.568	0.065	no
Treat-40d	RHSIM_Rhsim03G0143600	-1.392	-1.754	-0.362	low
Treat-40d	RHSIM_Rhsim04G0139800	1.109	0.080	-1.029	high
Treat-40d	RHSIM_Rhsim03G0177300	2.331	0.307	-2.024	high
Treat-40d	RHSIM_Rhsim04G0201800	0.236	-0.078	-0.314	no
Treat-40d	RHSIM_Rhsim06G0130400	1.465	0.843	-0.621	high
Treat-40d	RHSIM_Rhsim04G0168700	-0.130	0.116	0.246	no
Treat-40d	RHSIM_Rhsim06G0048400	0.178	-0.506	-0.683	no
Treat-40d	RHSIM_Rhsim04G0167800	1.666	0.798	-0.868	high
Treat-40d	RHSIM_Rhsim03G0248200	-0.541	-0.800	-0.259	no
Treat-40d	RHSIM_Rhsim03G0201100	-0.425	0.033	0.458	no
Treat-40d	RHSIM_Rhsim03G0150700	0.249	-0.477	-0.726	no
Treat-40d	RHSIM_Rhsim04G0181600	1.069	-0.343	-1.412	no
Treat-40d	RHSIM_Rhsim03G0265800	2.019	0.235	-1.784	high
Treat-40d	RHSIM_Rhsim04G0014800	-1.483	-1.249	0.234	low
Treat-40d	RHSIM_Rhsim06G0108900	1.398	0.833	-0.565	high
Treat-40d	RHSIM_Rhsim03G0004000	1.804	-0.187	-1.990	high
Treat-40d	RHSIM_Rhsim04G0173000	1.230	1.870	0.639	high
Treat-40d	RHSIM_Rhsim04G0091700	0.752	0.911	0.159	no

Group	Gene	PTR (z-score difference)	z(log2 LFQ)	z(log2 FPKM)	extreme-PTR
Treat-40d	RHSIM_Rhsim03G0267600	-0.082	-0.256	-0.175	no
Treat-40d	RHSIM_Rhsim06G0166000	-0.948	-0.803	0.145	no
Treat-40d	RHSIM_Rhsim03G0151400	1.093	1.903	0.810	no
Treat-40d	RHSIM_Rhsim04G0081600	0.943	-0.348	-1.291	no
Treat-40d	RHSIM_Rhsim04G0038100	-1.043	-0.478	0.566	no
Treat-40d	RHSIM_Rhsim04G0105700	0.548	-0.110	-0.658	no
Treat-40d	RHSIM_Rhsim03G0196600	-0.393	-0.504	-0.111	no
Treat-40d	RHSIM_Rhsim03G0235600	-0.757	-0.328	0.430	no
Treat-40d	RHSIM_Rhsim03G0119300	-1.928	-2.181	-0.253	low
Treat-40d	RHSIM_Rhsim03G0252000	-0.153	-0.094	0.060	no
Treat-40d	RHSIM_Rhsim04G0197500	-0.592	0.259	0.851	no
Treat-40d	RHSIM_Rhsim06G0018600	-0.036	0.809	0.845	no
Treat-40d	RHSIM_Rhsim03G0125700	0.836	0.374	-0.462	no
Treat-40d	RHSIM_Rhsim03G0001600	-1.007	-0.467	0.540	no
Treat-40d	RHSIM_Rhsim04G0170700	2.970	0.587	-2.383	high
Treat-40d	RHSIM_Rhsim03G0093200	-1.234	-1.209	0.025	low
Treat-40d	RHSIM_Rhsim03G0248000	0.088	0.467	0.379	no
Treat-40d	RHSIM_Rhsim03G0153100	1.178	-1.250	-2.429	high
Treat-40d	RHSIM_Rhsim03G0138600	-0.226	0.479	0.706	no
Treat-40d	RHSIM_Rhsim04G0008100	-0.421	1.209	1.631	no
Treat-40d	RHSIM_Rhsim03G0090600	-0.379	-0.162	0.218	no
Treat-40d	RHSIM_Rhsim03G0161600	-0.521	-1.375	-0.854	no
Treat-40d	RHSIM_Rhsim03G0264700	-1.141	-0.653	0.487	low
Treat-40d	RHSIM_Rhsim03G0078800	0.392	0.040	-0.351	no
Treat-40d	RHSIM_Rhsim05G0113400	2.383	3.150	0.768	high
Treat-40d	RHSIM_Rhsim03G0211600	-0.533	-1.193	-0.660	no
Treat-40d	RHSIM_Rhsim03G0115800	-0.602	-0.518	0.084	no
Treat-40d	RHSIM_Rhsim05G0090500	-0.754	-0.048	0.706	no
Treat-40d	RHSIM_Rhsim03G0249400	-0.826	0.703	1.528	no
Treat-40d	RHSIM_Rhsim04G0086600	-0.924	-1.128	-0.205	no
Treat-40d	RHSIM_Rhsim04G0169700	-1.223	0.112	1.335	low
Treat-40d	RHSIM_Rhsim03G0245200	-1.576	-0.130	1.446	low

Group	Gene	PTR (z-score difference)	z(log2 LFQ)	z(log2 FPKM)	extreme-PTR
Treat-40d	RHSIM_Rhsim03G0009900	-0.405	-0.657	-0.252	no
Treat-40d	RHSIM_Rhsim04G0157200	-0.367	-0.779	-0.412	no
Treat-40d	RHSIM_Rhsim04G0214000	-1.732	-1.459	0.272	low
Treat-40d	RHSIM_Rhsim03G0159800	-0.936	0.283	1.219	no
Treat-40d	RHSIM_Rhsim03G0256600	-0.083	-0.374	-0.292	no
Treat-40d	RHSIM_Rhsim03G0180900	-1.105	1.844	2.949	no
Treat-40d	RHSIM_Rhsim03G0099600	0.858	0.506	-0.352	no
Treat-40d	RHSIM_Rhsim03G0240200	-0.020	-0.614	-0.594	no
Treat-40d	RHSIM_Rhsim03G0131800	-0.627	0.297	0.924	no
Treat-40d	RHSIM_Rhsim03G0110300	0.937	0.905	-0.033	no
Treat-40d	RHSIM_Rhsim04G0176900	1.063	1.502	0.439	no
Treat-40d	RHSIM_Rhsim03G0100800	-0.093	0.170	0.263	no
Treat-40d	RHSIM_Rhsim03G0069400	2.258	-0.177	-2.435	high
Treat-40d	RHSIM_Rhsim03G0105700	-0.743	-0.911	-0.168	no
Treat-40d	RHSIM_Rhsim03G0027100	-0.221	-0.572	-0.351	no
Treat-40d	RHSIM_Rhsim03G0074700	-0.232	-1.848	-1.616	no
Treat-40d	RHSIM_Rhsim03G0136400	-0.713	0.475	1.188	no
Treat-40d	RHSIM_Rhsim04G0147900	-0.170	0.090	0.260	no
Treat-40d	RHSIM_Rhsim03G0075100	0.341	-0.292	-0.632	no
Treat-40d	RHSIM_Rhsim04G0101000	-0.952	-0.191	0.761	no
Treat-40d	RHSIM_Rhsim03G0141300	-0.264	0.250	0.514	no
Treat-40d	RHSIM_Rhsim03G0262500	-0.502	-0.759	-0.257	no
Treat-40d	RHSIM_Rhsim04G0151800	1.440	-0.078	-1.518	high
Treat-40d	RHSIM_Rhsim04G0068400	-0.779	0.625	1.404	no
Treat-40d	RHSIM_Rhsim03G0052700	-0.702	1.759	2.460	no
Treat-40d	RHSIM_Rhsim03G0134500	2.117	1.365	-0.752	high
Treat-40d	RHSIM_Rhsim03G0021400	0.640	0.052	-0.589	no
Treat-40d	RHSIM_Rhsim03G0225200	-0.382	0.261	0.643	no
Treat-40d	RHSIM_Rhsim04G0071500	0.451	0.163	-0.288	no
Treat-40d	RHSIM_Rhsim03G0244600	0.751	0.027	-0.724	no
Treat-40d	RHSIM_Rhsim05G0115700	-0.019	0.744	0.763	no
Treat-40d	RHSIM_Rhsim03G0099500	1.149	-1.125	-2.274	high

Group	Gene	PTR (z-score difference)	z(log2 LFQ)	z(log2 FPKM)	extreme-PTR
Treat-40d	RHSIM_Rhsim03G0254100	1.798	1.374	-0.424	high
Treat-40d	RHSIM_Rhsim04G0081400	0.072	0.365	0.293	no
Treat-40d	RHSIM_Rhsim03G0085000	-1.317	-1.814	-0.497	low
Treat-40d	RHSIM_Rhsim04G0166700	0.680	0.974	0.294	no
Treat-40d	RHSIM_Rhsim03G0121200	0.637	-0.342	-0.979	no
Treat-40d	RHSIM_Rhsim03G0235000	-0.887	0.740	1.627	no
Treat-40d	RHSIM_Rhsim03G0146700	-0.069	0.347	0.416	no
Treat-40d	RHSIM_Rhsim04G0180800	-1.163	-0.078	1.085	low
Treat-40d	RHSIM_Rhsim03G0146600	-0.385	-0.676	-0.291	no
Treat-40d	RHSIM_Rhsim03G0073400	-0.351	-0.636	-0.285	no
Treat-40d	RHSIM_Rhsim04G0206300	3.386	2.174	-1.212	high
Treat-40d	RHSIM_Rhsim03G0140300	-1.597	-1.065	0.532	low
Treat-40d	RHSIM_Rhsim03G0080300	-1.543	-0.086	1.458	low
Treat-40d	RHSIM_Rhsim05G0116200	0.282	0.194	-0.088	no
Treat-40d	RHSIM_Rhsim03G0144700	-0.001	-0.136	-0.135	no
Treat-40d	RHSIM_Rhsim04G0066500	0.596	-0.405	-1.002	no
Treat-40d	RHSIM_Rhsim04G0182600	-1.215	1.144	2.359	low
Treat-40d	RHSIM_Rhsim05G0206900	-1.638	-0.176	1.462	low
Treat-40d	RHSIM_Rhsim03G0240400	-2.486	-1.430	1.056	low
Treat-40d	RHSIM_Rhsim03G0200600	-0.700	0.094	0.793	no
Treat-40d	RHSIM_Rhsim03G0051100	-1.022	-1.202	-0.180	no
Treat-40d	RHSIM_Rhsim04G0209400	2.146	2.032	-0.115	high
Treat-40d	RHSIM_Rhsim03G0223200	-0.264	-0.201	0.063	no
Treat-40d	RHSIM_Rhsim03G0237400	0.530	2.043	1.513	no
Treat-40d	RHSIM_Rhsim04G0012000	-1.658	-1.032	0.626	low
Treat-40d	RHSIM_Rhsim03G0179100	-0.938	-0.686	0.252	no
Treat-40d	RHSIM_Rhsim03G0187200	0.697	0.812	0.115	no
Treat-40d	RHSIM_Rhsim04G0160900	0.209	-1.312	-1.521	no
Treat-40d	RHSIM_Rhsim03G0058100	0.018	-0.838	-0.856	no
Treat-40d	RHSIM_Rhsim03G0244700	2.006	2.025	0.020	high
Treat-40d	RHSIM_Rhsim03G0256500	0.647	1.774	1.126	no
Treat-40d	RHSIM_Rhsim03G0214800	-1.074	1.432	2.505	no

Group	Gene	PTR (z-score difference)	z(log2 LFQ)	z(log2 FPKM)	extreme-PTR
Treat-40d	RHSIM_Rhsim03G0112100	0.167	0.332	0.165	no
Treat-40d	RHSIM_Rhsim03G0240800	0.748	0.670	-0.077	no
Treat-40d	RHSIM_Rhsim05G0193600	0.346	-0.378	-0.723	no
Treat-40d	RHSIM_Rhsim04G0086300	0.279	-0.520	-0.799	no
Treat-40d	RHSIM_Rhsim05G0216200	-0.870	-0.526	0.343	no
Treat-40d	RHSIM_Rhsim03G0258800	0.446	-0.355	-0.802	no
Treat-40d	RHSIM_Rhsim03G0231700	-1.204	-1.396	-0.192	low
Treat-40d	RHSIM_Rhsim04G0232000	0.799	0.969	0.170	no
Treat-40d	RHSIM_Rhsim05G0067500	-0.117	0.815	0.932	no
Treat-40d	RHSIM_Rhsim03G0150600	0.330	-1.511	-1.841	no
Treat-40d	RHSIM_Rhsim04G0179900	0.498	-0.468	-0.966	no
Treat-40d	RHSIM_Rhsim03G0062200	0.592	-0.055	-0.647	no
Treat-40d	RHSIM_Rhsim04G0075400	-0.636	-0.888	-0.252	no
Treat-40d	RHSIM_Rhsim03G0092400	0.602	0.904	0.302	no
Treat-40d	RHSIM_Rhsim03G0236300	0.251	-1.044	-1.295	no
Treat-40d	RHSIM_Rhsim05G0011200	1.926	-0.249	-2.175	high
Treat-40d	RHSIM_Rhsim03G0264300	0.684	0.714	0.030	no
Treat-40d	RHSIM_Rhsim05G0032000	-1.079	-1.460	-0.382	no
Treat-40d	RHSIM_Rhsim03G0262700	1.743	1.025	-0.718	high
Treat-40d	RHSIM_Rhsim03G0154600	-0.336	-1.045	-0.709	no
Treat-40d	RHSIM_Rhsim03G0073300	0.989	-0.889	-1.878	no
Treat-40d	RHSIM_Rhsim03G0208100	0.526	0.997	0.471	no
Treat-40d	RHSIM_Rhsim05G0101900	-1.921	-1.039	0.882	low
Treat-40d	RHSIM_Rhsim03G0230900	-0.220	0.160	0.381	no
Treat-40d	RHSIM_Rhsim04G0197400	-1.607	0.397	2.005	low
Treat-40d	RHSIM_Rhsim05G0042100	-0.700	-0.584	0.115	no
Treat-40d	RHSIM_Rhsim04G0204300	-1.189	-1.187	0.002	low
Treat-40d	RHSIM_Rhsim03G0078700	0.283	-0.084	-0.367	no
Treat-40d	RHSIM_Rhsim03G0093900	-1.318	-0.775	0.542	low
Treat-40d	RHSIM_Rhsim03G0234500	0.480	1.523	1.043	no
Treat-40d	RHSIM_Rhsim05G0098000	-0.352	-0.435	-0.083	no
Treat-40d	RHSIM_Rhsim03G0265300	2.019	0.801	-1.219	high

Group	Gene	PTR (z-score difference)	z(log2 LFQ)	z(log2 FPKM)	extreme-PTR
Treat-40d	RHSIM_Rhsim03G0192800	0.557	0.117	-0.439	no
Treat-40d	RHSIM_Rhsim03G0018400	-1.062	-0.767	0.295	no
Treat-40d	RHSIM_Rhsim04G0171200	1.407	1.926	0.519	high
Treat-40d	RHSIM_Rhsim04G0024300	0.347	-0.955	-1.303	no
Treat-40d	RHSIM_Rhsim03G0176200	1.672	0.986	-0.686	high
Treat-40d	RHSIM_Rhsim03G0155500	0.435	-0.036	-0.471	no
Treat-40d	RHSIM_Rhsim05G0018500	0.172	-1.161	-1.333	no
Treat-40d	RHSIM_Rhsim03G0192400	0.341	1.264	0.924	no
Treat-40d	RHSIM_Rhsim03G0110600	-0.042	-0.163	-0.121	no
Treat-40d	RHSIM_Rhsim05G0214700	-1.420	-0.242	1.178	low
Treat-40d	RHSIM_Rhsim03G0063000	0.174	0.315	0.140	no
Treat-40d	RHSIM_Rhsim03G0141400	-3.368	-0.662	2.705	low
Treat-40d	RHSIM_Rhsim03G0187900	-0.875	-2.014	-1.140	no
Treat-40d	RHSIM_Rhsim03G0176300	-1.688	-1.408	0.280	low
Treat-40d	RHSIM_Rhsim03G0145100	0.704	-0.183	-0.887	no
Treat-40d	RHSIM_Rhsim03G0088100	-2.860	-0.987	1.873	low
Treat-40d	RHSIM_Rhsim03G0208900	-0.372	-0.259	0.113	no
Treat-40d	RHSIM_Rhsim03G0119400	0.604	0.870	0.265	no
Treat-40d	RHSIM_Rhsim03G0093800	-1.810	-0.834	0.976	low
Treat-40d	RHSIM_Rhsim02G0061100	-0.010	-1.442	-1.432	no
Treat-40d	RHSIM_Rhsim04G0144000	0.687	-0.419	-1.107	no
Treat-40d	RHSIM_Rhsim03G0083800	-1.725	-1.003	0.721	low
Treat-40d	RHSIM_Rhsim03G0101200	-0.634	-0.998	-0.364	no
Treat-40d	RHSIM_Rhsim03G0188500	-0.081	0.489	0.570	no
Treat-40d	RHSIM_Rhsim02G0160800	-0.154	-0.201	-0.046	no
Treat-40d	RHSIM_Rhsim03G0118600	-0.754	0.689	1.443	no
Treat-40d	RHSIM_Rhsim03G0089900	-1.916	-0.193	1.723	low
Treat-40d	RHSIM_Rhsim03G0029800	-0.075	0.356	0.431	no
Treat-40d	RHSIM_Rhsim03G0088700	-0.199	-0.195	0.004	no
Treat-40d	RHSIM_Rhsim04G0145600	-0.291	0.492	0.783	no
Treat-40d	RHSIM_Rhsim02G0022400	1.497	0.302	-1.195	high
Treat-40d	RHSIM_Rhsim03G0270000	-0.594	-0.100	0.494	no

Group	Gene	PTR (z-score difference)	z(log2 LFQ)	z(log2 FPKM)	extreme-PTR
Treat-40d	RHSIM_Rhsim03G0049600	0.281	-0.111	-0.392	no
Treat-40d	RHSIM_Rhsim03G0130200	-0.142	-0.084	0.058	no
Treat-40d	RHSIM_Rhsim05G0108800	-0.139	-0.682	-0.543	no
Treat-40d	RHSIM_Rhsim03G0086200	3.198	1.621	-1.578	high
Treat-40d	RHSIM_Rhsim03G0229600	-0.781	-0.087	0.694	no
Treat-40d	RHSIM_Rhsim03G0207200	0.078	-0.966	-1.044	no
Treat-40d	RHSIM_Rhsim03G0231100	0.027	-0.639	-0.666	no
Treat-40d	RHSIM_Rhsim03G0028800	0.757	-1.497	-2.254	no
Treat-40d	RHSIM_Rhsim03G0212200	0.497	-0.540	-1.037	no
Treat-40d	RHSIM_Rhsim02G0105800	-1.299	-1.027	0.272	low
Treat-40d	RHSIM_Rhsim03G0081700	-0.174	0.051	0.225	no
Treat-40d	RHSIM_Rhsim04G0207900	0.738	0.547	-0.190	no
Treat-40d	RHSIM_Rhsim03G0090700	0.320	-0.773	-1.093	no
Treat-40d	RHSIM_Rhsim04G0168900	-1.209	-1.182	0.027	low
Treat-40d	RHSIM_Rhsim03G0261200	1.138	0.547	-0.591	high
Treat-40d	RHSIM_Rhsim03G0075200	-1.710	-0.450	1.261	low
Treat-40d	RHSIM_Rhsim03G0231500	-0.234	-1.350	-1.116	no
Treat-40d	RHSIM_Rhsim04G0224600	-0.639	0.648	1.287	no
Treat-40d	RHSIM_Rhsim02G0015800	0.107	-0.198	-0.305	no
Treat-40d	RHSIM_Rhsim02G0060700	-1.042	-0.974	0.068	no
Treat-40d	RHSIM_Rhsim02G0108100	1.050	-0.334	-1.385	no
Treat-40d	RHSIM_Rhsim02G0001700	-0.021	-0.693	-0.672	no
Treat-40d	RHSIM_Rhsim02G0159000	-1.637	-1.089	0.548	low
Treat-40d	RHSIM_Rhsim04G0249800	0.906	-0.882	-1.788	no
Treat-40d	RHSIM_Rhsim03G0122500	-0.242	0.378	0.619	no
Treat-40d	RHSIM_Rhsim05G0165500	-0.196	-0.550	-0.354	no
Treat-40d	RHSIM_Rhsim03G0085600	2.304	1.702	-0.601	high
Treat-40d	RHSIM_Rhsim04G0009800	2.467	2.539	0.071	high
Treat-40d	RHSIM_Rhsim03G0083300	-1.627	-1.085	0.543	low
Treat-40d	RHSIM_Rhsim03G0219000	-0.951	-0.166	0.786	no
Treat-40d	RHSIM_Rhsim03G0238600	1.005	0.011	-0.995	no
Treat-40d	RHSIM_Rhsim02G0120400	-2.501	-1.647	0.854	low

Group	Gene	PTR (z-score difference)	z(log2 LFQ)	z(log2 FPKM)	extreme-PTR
Treat-40d	RHSIM_Rhsim02G0182400	-0.062	-0.665	-0.603	no
Treat-40d	RHSIM_Rhsim03G0061800	1.092	1.224	0.132	no
Treat-40d	RHSIM_Rhsim03G0234600	0.260	-0.729	-0.989	no
Treat-40d	RHSIM_Rhsim05G0227600	-1.224	-0.584	0.640	low
Treat-40d	RHSIM_Rhsim03G0193700	2.461	0.368	-2.093	high
Treat-40d	RHSIM_Rhsim03G0225300	-1.585	-0.350	1.235	low
Treat-40d	RHSIM_Rhsim03G0267400	-0.658	-1.313	-0.655	no
Treat-40d	RHSIM_Rhsim04G0226400	-0.672	-1.660	-0.988	no
Treat-40d	RHSIM_Rhsim03G0208500	-0.148	-0.351	-0.203	no
Treat-40d	RHSIM_Rhsim02G0011800	-1.023	-0.248	0.774	no
Treat-40d	RHSIM_Rhsim02G0230000	0.664	0.766	0.102	no
Treat-40d	RHSIM_Rhsim05G0158900	3.130	1.434	-1.696	high
Treat-40d	RHSIM_Rhsim03G0074600	-1.163	-1.022	0.141	low
Treat-40d	RHSIM_Rhsim03G0012100	-0.613	-0.023	0.590	no
Treat-40d	RHSIM_Rhsim03G0189800	-1.213	0.223	1.436	low
Treat-40d	RHSIM_Rhsim03G0082000	2.295	2.827	0.532	high
Treat-40d	RHSIM_Rhsim05G0079700	-1.457	0.853	2.310	low
Treat-40d	RHSIM_Rhsim03G0175300	0.540	-0.851	-1.391	no
Treat-40d	RHSIM_Rhsim03G0225900	-0.138	-0.581	-0.443	no
Treat-40d	RHSIM_Rhsim03G0049800	-0.421	-0.843	-0.423	no
Treat-40d	RHSIM_Rhsim03G0168800	0.017	-0.046	-0.063	no
Treat-40d	RHSIM_Rhsim03G0200100	0.847	0.269	-0.578	no
Treat-40d	RHSIM_Rhsim02G0252200	-1.856	-0.493	1.364	low
Treat-40d	RHSIM_Rhsim03G0143400	-0.316	-0.870	-0.554	no
Treat-40d	RHSIM_Rhsim03G0267700	-0.793	0.858	1.652	no
Treat-40d	RHSIM_Rhsim05G0214900	-0.866	0.486	1.352	no
Treat-40d	RHSIM_Rhsim03G0012300	0.845	0.126	-0.719	no
Treat-40d	RHSIM_Rhsim03G0152000	-0.452	1.123	1.575	no
Treat-40d	RHSIM_Rhsim02G0137600	-2.718	-0.613	2.104	low
Treat-40d	RHSIM_Rhsim03G0077300	-1.084	-0.636	0.447	no
Treat-40d	RHSIM_Rhsim05G0192800	-0.338	0.298	0.636	no
Treat-40d	RHSIM_Rhsim03G0128200	2.046	1.210	-0.836	high

Group	Gene	PTR (z-score difference)	z(log2 LFQ)	z(log2 FPKM)	extreme-PTR
Treat-40d	RHSIM_Rhsim02G0029300	0.885	-0.552	-1.436	no
Treat-40d	RHSIM_Rhsim02G0041500	2.101	3.150	1.049	high
Treat-40d	RHSIM_Rhsim03G0052900	-1.959	2.067	4.026	low
Treat-40d	RHSIM_Rhsim02G0200900	1.408	0.318	-1.089	high
Treat-40d	RHSIM_Rhsim03G0225000	-1.149	-0.800	0.349	low
Treat-40d	RHSIM_Rhsim05G0146200	0.417	-0.113	-0.530	no
Treat-40d	RHSIM_Rhsim02G0105700	-2.207	-1.204	1.002	low
Treat-40d	RHSIM_Rhsim03G0159900	-1.796	0.034	1.830	low
Treat-40d	RHSIM_Rhsim03G0036100	0.317	-0.662	-0.979	no
Treat-40d	RHSIM_Rhsim03G0049500	1.107	0.468	-0.639	no
Treat-40d	RHSIM_Rhsim03G0246500	-0.372	-0.375	-0.003	no
Treat-40d	RHSIM_Rhsim05G0188400	-1.734	-0.857	0.877	low
Treat-40d	RHSIM_Rhsim03G0035900	0.960	-0.998	-1.959	no
Treat-40d	RHSIM_Rhsim03G0194300	0.894	-0.261	-1.155	no
Treat-40d	RHSIM_Rhsim02G0173300	0.012	-0.673	-0.684	no
Treat-40d	RHSIM_Rhsim05G0073400	-1.162	1.255	2.417	low
Treat-40d	RHSIM_Rhsim03G0238900	0.942	1.729	0.787	no
Treat-40d	RHSIM_Rhsim03G0058600	0.071	0.089	0.018	no
Treat-40d	RHSIM_Rhsim02G0117400	-0.317	-1.205	-0.888	no
Treat-40d	RHSIM_Rhsim03G0236600	-0.540	-1.002	-0.461	no
Treat-40d	RHSIM_Rhsim03G0098400	1.099	1.786	0.687	no
Treat-40d	RHSIM_Rhsim03G0058700	0.592	-0.038	-0.630	no
Treat-40d	RHSIM_Rhsim03G0170100	-1.152	-0.077	1.075	low
Treat-40d	RHSIM_Rhsim03G0044600	-0.440	-0.185	0.256	no
Treat-40d	RHSIM_Rhsim02G0251600	0.639	0.017	-0.622	no
Treat-40d	RHSIM_Rhsim02G0203500	0.189	-0.105	-0.294	no
Treat-40d	RHSIM_Rhsim03G0216000	-0.609	0.028	0.637	no
Treat-40d	RHSIM_Rhsim03G0087900	-0.268	-0.242	0.026	no
Treat-40d	RHSIM_Rhsim03G0222700	0.319	-0.151	-0.470	no
Treat-40d	RHSIM_Rhsim05G0040800	0.664	-0.086	-0.750	no
Treat-40d	RHSIM_Rhsim02G0024500	1.062	-0.807	-1.869	no
Treat-40d	RHSIM_Rhsim03G0134000	0.254	0.053	-0.201	no

Group	Gene	PTR (z-score difference)	z(log2 LFQ)	z(log2 FPKM)	extreme-PTR
Treat-40d	RHSIM_Rhsim03G0218000	-0.726	-0.485	0.241	no
Treat-40d	RHSIM_Rhsim03G0261800	0.937	0.541	-0.396	no
Treat-40d	RHSIM_Rhsim05G0087000	-0.126	1.179	1.305	no
Treat-40d	RHSIM_Rhsim03G0022100	1.632	0.271	-1.362	high
Treat-40d	RHSIM_Rhsim03G0265100	-0.321	-0.659	-0.338	no
Treat-40d	RHSIM_Rhsim03G0218800	2.301	0.083	-2.218	high
Treat-40d	RHSIM_Rhsim03G0221200	0.527	0.021	-0.506	no
Treat-40d	RHSIM_Rhsim02G0083700	0.518	-0.713	-1.231	no
Treat-40d	RHSIM_Rhsim03G0150900	-1.909	-0.166	1.742	low
Treat-40d	RHSIM_Rhsim03G0217000	-0.750	0.025	0.775	no
Treat-40d	RHSIM_Rhsim03G0255200	-0.489	-0.249	0.241	no
Treat-40d	RHSIM_Rhsim03G0172900	-0.607	0.939	1.547	no
Treat-40d	RHSIM_Rhsim03G0136600	1.739	1.803	0.064	high
Treat-40d	RHSIM_Rhsim05G0200700	0.435	-0.039	-0.475	no
Treat-40d	RHSIM_Rhsim03G0143900	-0.857	-1.741	-0.883	no
Treat-40d	RHSIM_Rhsim03G0078900	-1.084	-1.080	0.004	no
Treat-40d	RHSIM_Rhsim05G0119800	0.562	0.301	-0.261	no
Treat-40d	RHSIM_Rhsim02G0212300	1.100	2.090	0.991	no
Treat-40d	RHSIM_Rhsim02G0132300	-0.183	1.514	1.696	no
Treat-40d	RHSIM_Rhsim03G0216300	-0.547	1.413	1.960	no
Treat-40d	RHSIM_Rhsim03G0127200	-0.748	-0.867	-0.119	no
Treat-40d	RHSIM_Rhsim03G0031000	0.209	-0.515	-0.725	no
Treat-40d	RHSIM_Rhsim03G0172800	-0.310	-0.261	0.048	no
Treat-40d	RHSIM_Rhsim03G0149000	1.735	1.255	-0.480	high
Treat-40d	RHSIM_Rhsim02G0105600	-1.295	-0.114	1.181	low
Treat-40d	RHSIM_Rhsim03G0208700	-0.020	0.153	0.173	no
Treat-40d	RHSIM_Rhsim02G0251800	1.142	0.908	-0.234	high
Treat-40d	RHSIM_Rhsim02G0007300	0.555	0.832	0.277	no
Treat-40d	RHSIM_Rhsim05G0049100	-1.341	-2.256	-0.916	low
Treat-40d	RHSIM_Rhsim02G0243400	-2.518	-1.722	0.796	low
Treat-40d	RHSIM_Rhsim03G0158800	-0.716	-1.215	-0.499	no
Treat-40d	RHSIM_Rhsim02G0056700	1.160	1.244	0.084	high

Group	Gene	PTR (z-score difference)	z(log2 LFQ)	z(log2 FPKM)	extreme-PTR
Treat-40d	RHSIM_Rhsim05G0228200	-0.437	-0.908	-0.471	no
Treat-40d	RHSIM_Rhsim02G0179000	-1.523	-1.192	0.331	low
Treat-40d	RHSIM_Rhsim03G0174400	1.282	1.603	0.321	high
Treat-40d	RHSIM_Rhsim03G0114800	-1.600	-0.652	0.947	low
Treat-40d	RHSIM_Rhsim03G0252700	-2.035	-1.868	0.166	low
Treat-40d	RHSIM_Rhsim03G0266500	0.406	0.839	0.433	no
Treat-40d	RHSIM_Rhsim03G0092100	1.638	0.329	-1.309	high
Treat-40d	RHSIM_Rhsim02G0180400	-0.066	-1.104	-1.038	no
Treat-40d	RHSIM_Rhsim03G0145000	0.999	0.092	-0.907	no
Treat-40d	RHSIM_Rhsim04G0105900	2.215	0.283	-1.932	high
Treat-40d	RHSIM_Rhsim02G0199800	0.060	0.439	0.378	no
Treat-40d	RHSIM_Rhsim03G0169200	0.609	0.388	-0.222	no
Treat-40d	RHSIM_Rhsim04G0217900	-2.366	-1.571	0.796	low
Treat-40d	RHSIM_Rhsim03G0159700	-0.798	0.462	1.260	no
Treat-40d	RHSIM_Rhsim02G0146400	-0.407	0.148	0.554	no
Treat-40d	RHSIM_Rhsim02G0090300	-1.312	-0.285	1.027	low
Treat-40d	RHSIM_Rhsim03G0163000	-0.224	-0.101	0.124	no
Treat-40d	RHSIM_Rhsim02G0229000	-0.841	-0.433	0.407	no
Treat-40d	RHSIM_Rhsim02G0168000	-1.756	-1.819	-0.062	low
Treat-40d	RHSIM_Rhsim04G0032000	2.900	4.114	1.214	high
Treat-40d	RHSIM_Rhsim03G0262600	-0.124	-0.092	0.032	no
Treat-40d	RHSIM_Rhsim02G0044500	0.537	-0.452	-0.989	no
Treat-40d	RHSIM_Rhsim04G0167100	1.438	2.372	0.934	high
Treat-40d	RHSIM_Rhsim02G0059100	-0.019	1.402	1.421	no
Treat-40d	RHSIM_Rhsim02G0084900	-0.834	0.239	1.073	no
Treat-40d	RHSIM_Rhsim02G0042700	-1.154	0.554	1.707	low
Treat-40d	RHSIM_Rhsim03G0228600	-0.986	0.125	1.111	no
Treat-40d	RHSIM_Rhsim02G0036100	1.052	0.332	-0.719	no
Treat-40d	RHSIM_Rhsim04G0038900	-0.849	-0.291	0.557	no
Treat-40d	RHSIM_Rhsim02G0103000	-1.115	-0.755	0.360	no
Treat-40d	RHSIM_Rhsim02G0014100	-0.227	0.557	0.784	no
Treat-40d	RHSIM_Rhsim03G0127300	1.523	1.284	-0.238	high

Group	Gene	PTR (z-score difference)	z(log2 LFQ)	z(log2 FPKM)	extreme-PTR
Treat-40d	RHSIM_Rhsim03G0078300	0.644	2.788	2.143	no
Treat-40d	RHSIM_Rhsim02G0136400	1.598	1.963	0.366	high
Treat-40d	RHSIM_Rhsim03G0057300	0.170	-0.894	-1.065	no
Treat-40d	RHSIM_Rhsim02G0241700	1.151	0.835	-0.316	high
Treat-40d	RHSIM_Rhsim03G0046400	-0.354	-0.795	-0.440	no
Treat-40d	RHSIM_Rhsim03G0258700	0.577	-0.556	-1.134	no
Treat-40d	RHSIM_Rhsim03G0265400	-0.690	0.378	1.068	no
Treat-40d	RHSIM_Rhsim02G0086200	0.948	0.120	-0.828	no
Treat-40d	RHSIM_Rhsim02G0254500	1.457	1.574	0.117	high
Treat-40d	RHSIM_Rhsim04G0232600	-0.752	-0.823	-0.071	no
Treat-40d	RHSIM_Rhsim02G0173200	-0.201	-0.458	-0.257	no
Treat-40d	RHSIM_Rhsim03G0116200	1.470	0.496	-0.973	high
Treat-40d	RHSIM_Rhsim03G0234900	0.177	-0.307	-0.484	no
Treat-40d	RHSIM_Rhsim02G0060400	-1.550	-1.688	-0.138	low
Treat-40d	RHSIM_Rhsim03G0151900	0.494	-0.108	-0.603	no
Treat-40d	RHSIM_Rhsim02G0140300	1.082	1.352	0.271	no
Treat-40d	RHSIM_Rhsim03G0000600	0.616	0.622	0.006	no
Treat-40d	RHSIM_Rhsim02G0216000	-0.518	-0.536	-0.018	no
Treat-40d	RHSIM_Rhsim02G0213000	0.102	-1.051	-1.152	no
Treat-40d	RHSIM_Rhsim04G0247900	-0.310	-0.836	-0.525	no
Treat-40d	RHSIM_Rhsim03G0002000	0.524	-0.221	-0.745	no
Treat-40d	RHSIM_Rhsim02G0239800	-0.599	-1.061	-0.462	no
Treat-40d	RHSIM_Rhsim02G0008500	-0.395	2.143	2.538	no
Treat-40d	RHSIM_Rhsim02G0009100	0.539	0.798	0.260	no
Treat-40d	RHSIM_Rhsim02G0019700	-0.216	-0.091	0.125	no
Treat-40d	RHSIM_Rhsim02G0255500	1.378	0.698	-0.679	high
Treat-40d	RHSIM_Rhsim03G0232800	0.026	-0.488	-0.514	no
Treat-40d	RHSIM_Rhsim02G0023300	0.647	0.655	0.007	no
Treat-40d	RHSIM_Rhsim03G0264100	1.708	1.345	-0.363	high
Treat-40d	RHSIM_Rhsim02G0043700	0.273	-0.109	-0.382	no
Treat-40d	RHSIM_Rhsim03G0259400	0.306	0.765	0.458	no
Treat-40d	RHSIM_Rhsim02G0088500	0.419	0.354	-0.065	no

Group	Gene	PTR (z-score difference)	z(log2 LFQ)	z(log2 FPKM)	extreme-PTR
Treat-40d	RHSIM_Rhsim04G0016400	1.288	1.089	-0.199	high
Treat-40d	RHSIM_Rhsim02G0137400	-1.482	-2.071	-0.589	low
Treat-40d	RHSIM_Rhsim02G0006600	-0.350	-0.698	-0.348	no
Treat-40d	RHSIM_Rhsim02G0013600	-0.255	-0.277	-0.022	no
Treat-40d	RHSIM_Rhsim04G0172100	-2.905	-1.639	1.266	low
Treat-40d	RHSIM_Rhsim02G0254400	-0.620	0.252	0.873	no
Treat-40d	RHSIM_Rhsim02G0053400	-0.414	1.050	1.463	no
Treat-40d	RHSIM_Rhsim02G0081400	3.538	1.094	-2.444	high
Treat-40d	RHSIM_Rhsim03G0136800	-0.243	-0.433	-0.191	no
Treat-40d	RHSIM_Rhsim02G0068600	3.069	1.780	-1.289	high
Treat-40d	RHSIM_Rhsim03G0124200	1.437	0.099	-1.338	high
Treat-40d	RHSIM_Rhsim03G0141800	-0.030	-0.102	-0.072	no
Treat-40d	RHSIM_Rhsim03G0010600	-0.446	-0.549	-0.104	no
Treat-40d	RHSIM_Rhsim03G0120800	1.336	1.283	-0.053	high
Treat-40d	RHSIM_Rhsim02G0244400	0.510	1.578	1.068	no
Treat-40d	RHSIM_Rhsim02G0014600	-0.187	-0.350	-0.163	no
Treat-40d	RHSIM_Rhsim04G0161900	1.474	1.001	-0.473	high
Treat-40d	RHSIM_Rhsim02G0023100	1.566	1.474	-0.092	high
Treat-40d	RHSIM_Rhsim03G0011600	-0.936	-0.671	0.265	no
Treat-40d	RHSIM_Rhsim02G0066700	1.853	0.018	-1.835	high
Treat-40d	RHSIM_Rhsim03G0075600	-1.237	-0.556	0.682	low
Treat-40d	RHSIM_Rhsim04G0239100	0.794	-0.455	-1.249	no
Treat-40d	RHSIM_Rhsim02G0216100	-0.740	-0.912	-0.172	no
Treat-40d	RHSIM_Rhsim02G0126300	1.083	1.258	0.175	no
Treat-40d	RHSIM_Rhsim02G0025700	0.239	0.176	-0.063	no
Treat-40d	RHSIM_Rhsim04G0138800	-1.233	-0.679	0.554	low
Treat-40d	RHSIM_Rhsim03G0201600	0.947	-0.866	-1.813	no
Treat-40d	RHSIM_Rhsim03G0182700	0.528	-1.027	-1.554	no
Treat-40d	RHSIM_Rhsim02G0106500	1.742	-0.584	-2.326	high
Treat-40d	RHSIM_Rhsim02G0223100	1.061	-0.693	-1.754	no
Treat-40d	RHSIM_Rhsim04G0002700	0.752	0.736	-0.016	no
Treat-40d	RHSIM_Rhsim03G0150500	1.222	-0.238	-1.460	high

Group	Gene	PTR (z-score difference)	z(log2 LFQ)	z(log2 FPKM)	extreme-PTR
Treat-40d	RHSIM_Rhsim02G0181700	-0.058	-0.237	-0.179	no
Treat-40d	RHSIM_Rhsim04G0106800	0.130	-0.452	-0.583	no
Treat-40d	RHSIM_Rhsim02G0138500	0.659	0.380	-0.278	no
Treat-40d	RHSIM_Rhsim02G0070200	0.243	-0.488	-0.731	no
Treat-40d	RHSIM_Rhsim02G0119300	-0.030	0.230	0.259	no
Treat-40d	RHSIM_Rhsim02G0111900	0.504	0.665	0.161	no
Treat-40d	RHSIM_Rhsim02G0254100	0.753	-0.416	-1.168	no
Treat-40d	RHSIM_Rhsim03G0209200	-0.294	0.193	0.487	no
Treat-40d	RHSIM_Rhsim02G0243100	0.321	-0.997	-1.318	no
Treat-40d	RHSIM_Rhsim03G0089600	0.488	-0.066	-0.553	no
Treat-40d	RHSIM_Rhsim02G0215900	-0.528	-0.302	0.226	no
Treat-40d	RHSIM_Rhsim02G0001500	1.878	2.315	0.437	high
Treat-40d	RHSIM_Rhsim03G0258200	0.337	1.255	0.918	no
Treat-40d	RHSIM_Rhsim02G0233100	1.209	1.763	0.554	high
Treat-40d	RHSIM_Rhsim02G0154700	1.069	0.797	-0.272	no
Treat-40d	RHSIM_Rhsim02G0244500	-1.789	-1.331	0.458	low
Treat-40d	RHSIM_Rhsim03G0050100	1.685	1.143	-0.542	high
Treat-40d	RHSIM_Rhsim02G0214000	-0.704	-0.837	-0.133	no
Treat-40d	RHSIM_Rhsim02G0086000	-1.632	-2.063	-0.431	low
Treat-40d	RHSIM_Rhsim02G0062100	0.526	1.500	0.974	no
Treat-40d	RHSIM_Rhsim04G0216800	-0.852	-0.031	0.821	no
Treat-40d	RHSIM_Rhsim03G0179200	-1.665	-1.021	0.645	low
Treat-40d	RHSIM_Rhsim03G0171200	-1.452	-1.438	0.015	low
Treat-40d	RHSIM_Rhsim02G0135000	0.198	1.367	1.169	no
Treat-40d	RHSIM_Rhsim02G0083600	-0.621	-1.057	-0.435	no
Treat-40d	RHSIM_Rhsim02G0201500	-0.808	-0.532	0.275	no
Treat-40d	RHSIM_Rhsim02G0172900	-0.780	-0.885	-0.104	no
Treat-40d	RHSIM_Rhsim02G0037100	-1.868	-0.767	1.102	low
Treat-40d	RHSIM_Rhsim02G0005200	-0.742	-0.185	0.557	no
Treat-40d	RHSIM_Rhsim02G0154900	0.073	0.598	0.525	no
Treat-40d	RHSIM_Rhsim03G0123600	-0.956	-0.328	0.629	no
Treat-40d	RHSIM_Rhsim02G0179400	0.255	0.565	0.310	no

Group	Gene	PTR (z-score difference)	z(log2 LFQ)	z(log2 FPKM)	extreme-PTR
Treat-40d	RHSIM_Rhsim02G0223200	1.133	-0.642	-1.775	high
Treat-40d	RHSIM_Rhsim02G0062200	0.061	-0.123	-0.184	no
Treat-40d	RHSIM_Rhsim02G0141000	-0.130	-0.081	0.049	no
Treat-40d	RHSIM_Rhsim03G0214000	-0.103	-0.419	-0.315	no
Treat-40d	RHSIM_Rhsim04G0087600	1.595	0.981	-0.613	high
Treat-40d	RHSIM_Rhsim02G0244300	-2.740	-2.309	0.431	low
Treat-40d	RHSIM_Rhsim02G0000100	-0.053	-0.232	-0.179	no
Treat-40d	RHSIM_Rhsim04G0150200	-1.304	-1.504	-0.201	low
Treat-40d	RHSIM_Rhsim02G0181200	0.876	0.252	-0.624	no
Treat-40d	RHSIM_Rhsim02G0231700	-0.022	0.031	0.052	no
Treat-40d	RHSIM_Rhsim02G0217900	0.242	0.830	0.589	no
Treat-40d	RHSIM_Rhsim02G0127500	-1.664	-0.144	1.520	low
Treat-40d	RHSIM_Rhsim03G0035800	1.551	0.333	-1.218	high
Treat-40d	RHSIM_Rhsim02G0056400	-0.182	2.212	2.394	no
Treat-40d	RHSIM_Rhsim02G0169200	0.769	-0.586	-1.356	no
Treat-40d	RHSIM_Rhsim02G0008900	-0.589	-0.909	-0.320	no
Treat-40d	RHSIM_Rhsim03G0257200	-0.102	0.701	0.803	no
Treat-40d	RHSIM_Rhsim02G0140600	0.856	0.085	-0.771	no
Treat-40d	RHSIM_Rhsim02G0187100	2.077	1.429	-0.648	high
Treat-40d	RHSIM_Rhsim02G0041100	1.547	-0.473	-2.021	high
Treat-40d	RHSIM_Rhsim02G0068000	1.322	0.208	-1.114	high
Treat-40d	RHSIM_Rhsim02G0239600	0.784	1.109	0.325	no
Treat-40d	RHSIM_Rhsim02G0252100	0.524	-1.383	-1.907	no
Treat-40d	RHSIM_Rhsim02G0149100	-0.812	0.112	0.924	no
Treat-40d	RHSIM_Rhsim04G0022600	1.305	1.209	-0.096	high
Treat-40d	RHSIM_Rhsim02G0106400	0.039	2.405	2.366	no
Treat-40d	RHSIM_Rhsim02G0031400	-0.664	-0.155	0.510	no
Treat-40d	RHSIM_Rhsim02G0176900	-1.106	-0.594	0.512	no
Treat-40d	RHSIM_Rhsim03G0192600	0.447	1.167	0.720	no
Treat-40d	RHSIM_Rhsim02G0144300	-0.337	0.066	0.404	no
Treat-40d	RHSIM_Rhsim03G0179700	-2.137	-0.462	1.675	low
Treat-40d	RHSIM_Rhsim04G0011900	0.275	0.426	0.151	no

Group	Gene	PTR (z-score difference)	z(log2 LFQ)	z(log2 FPKM)	extreme-PTR
Treat-40d	RHSIM_Rhsim02G0099500	-0.763	0.179	0.942	no
Treat-40d	RHSIM_Rhsim02G0162600	0.216	1.134	0.918	no
Treat-40d	RHSIM_Rhsim02G0055200	-0.872	-0.599	0.273	no
Treat-40d	RHSIM_Rhsim02G0104500	0.048	-0.103	-0.150	no
Treat-40d	RHSIM_Rhsim03G0224600	0.929	1.520	0.591	no
Treat-40d	RHSIM_Rhsim01G0236100	-0.992	0.035	1.026	no
Treat-40d	RHSIM_Rhsim04G0247100	0.335	1.379	1.044	no
Treat-40d	RHSIM_Rhsim03G0192700	0.777	0.660	-0.117	no
Treat-40d	RHSIM_Rhsim01G0001000	0.278	1.288	1.010	no
Treat-40d	RHSIM_Rhsim02G0202100	0.688	2.080	1.392	no
Treat-40d	RHSIM_Rhsim01G0131400	-1.588	-0.457	1.131	low
Treat-40d	RHSIM_Rhsim02G0067100	1.662	1.213	-0.449	high
Treat-40d	RHSIM_Rhsim02G0169800	-0.129	-0.339	-0.211	no
Treat-40d	RHSIM_Rhsim02G0228100	1.318	-0.910	-2.227	high
Treat-40d	RHSIM_Rhsim02G0201600	-0.286	-0.417	-0.131	no
Treat-40d	RHSIM_Rhsim02G0036900	0.840	0.456	-0.384	no
Treat-40d	RHSIM_Rhsim02G0198800	-0.534	-0.696	-0.162	no
Treat-40d	RHSIM_Rhsim01G0219200	0.723	0.432	-0.291	no
Treat-40d	RHSIM_Rhsim03G0217400	-0.203	1.640	1.843	no
Treat-40d	RHSIM_Rhsim01G0232300	-0.279	-1.009	-0.730	no
Treat-40d	RHSIM_Rhsim01G0254500	-1.058	-0.723	0.335	no
Treat-40d	RHSIM_Rhsim02G0069700	-1.413	-1.500	-0.087	low
Treat-40d	RHSIM_Rhsim02G0076300	-0.992	-0.001	0.991	no
Treat-40d	RHSIM_Rhsim03G0028600	1.642	0.727	-0.915	high
Treat-40d	RHSIM_Rhsim02G0035500	-1.908	0.025	1.934	low
Treat-40d	RHSIM_Rhsim02G0035300	0.232	0.271	0.039	no
Treat-40d	RHSIM_Rhsim02G0041900	-0.875	-0.498	0.377	no
Treat-40d	RHSIM_Rhsim02G0178300	0.540	-0.531	-1.071	no
Treat-40d	RHSIM_Rhsim02G0104000	0.188	-0.002	-0.190	no
Treat-40d	RHSIM_Rhsim04G0234100	-0.210	2.423	2.633	no
Treat-40d	RHSIM_Rhsim03G0135900	0.459	1.222	0.763	no
Treat-40d	RHSIM_Rhsim02G0183000	-0.752	-0.832	-0.080	no

Group	Gene	PTR (z-score difference)	z(log2 LFQ)	z(log2 FPKM)	extreme-PTR
Treat-40d	RHSIM_Rhsim02G0177000	-0.256	0.307	0.563	no
Treat-40d	RHSIM_Rhsim02G0176200	1.178	-0.111	-1.288	high
Treat-40d	RHSIM_Rhsim01G0186900	1.057	0.330	-0.727	no
Treat-40d	RHSIM_Rhsim02G0146500	-1.035	-0.871	0.164	no
Treat-40d	RHSIM_Rhsim04G0013700	0.721	0.506	-0.216	no
Treat-40d	RHSIM_Rhsim01G0245400	0.091	0.809	0.718	no
Treat-40d	RHSIM_Rhsim02G0020900	1.105	0.220	-0.885	no
Treat-40d	RHSIM_Rhsim02G0145500	0.815	0.712	-0.103	no
Treat-40d	RHSIM_Rhsim02G0251900	-1.117	-0.978	0.139	no
Treat-40d	RHSIM_Rhsim02G0123300	-0.800	-0.321	0.479	no
Treat-40d	RHSIM_Rhsim02G0029200	-3.339	-0.078	3.262	low
Treat-40d	RHSIM_Rhsim02G0115100	3.414	4.382	0.968	high
Treat-40d	RHSIM_Rhsim02G0171500	0.804	0.501	-0.303	no
Treat-40d	RHSIM_Rhsim02G0070300	-0.001	1.441	1.443	no
Treat-40d	RHSIM_Rhsim04G0239600	-1.328	-0.414	0.914	low
Treat-40d	RHSIM_Rhsim01G0225400	-1.172	-0.965	0.207	low
Treat-40d	RHSIM_Rhsim02G0014200	0.079	0.162	0.083	no
Treat-40d	RHSIM_Rhsim02G0165800	0.058	0.414	0.355	no
Treat-40d	RHSIM_Rhsim02G0027900	-1.781	-0.959	0.822	low
Treat-40d	RHSIM_Rhsim02G0076900	-1.147	-1.725	-0.578	low
Treat-40d	RHSIM_Rhsim02G0120700	-1.189	-0.420	0.770	low
Treat-40d	RHSIM_Rhsim02G0160300	-1.985	-0.072	1.913	low
Treat-40d	RHSIM_Rhsim02G0115500	-1.092	-0.574	0.519	no
Treat-40d	RHSIM_Rhsim02G0071700	0.196	-0.271	-0.468	no
Treat-40d	RHSIM_Rhsim02G0159900	0.275	-0.428	-0.703	no
Treat-40d	RHSIM_Rhsim02G0165700	-2.073	-0.376	1.698	low
Treat-40d	RHSIM_Rhsim04G0100800	-1.097	-0.197	0.901	no
Treat-40d	RHSIM_Rhsim02G0162800	-0.535	-0.822	-0.286	no
Treat-40d	RHSIM_Rhsim02G0037200	-1.892	-0.864	1.029	low
Treat-40d	RHSIM_Rhsim02G0127600	-1.014	-0.621	0.393	no
Treat-40d	RHSIM_Rhsim02G0200700	-0.502	-1.458	-0.956	no
Treat-40d	RHSIM_Rhsim03G0210800	0.499	0.270	-0.229	no

Group	Gene	PTR (z-score difference)	z(log2 LFQ)	z(log2 FPKM)	extreme-PTR
Treat-40d	RHSIM_Rhsim04G0240100	0.562	0.087	-0.475	no
Treat-40d	RHSIM_Rhsim03G0230200	0.308	0.056	-0.251	no
Treat-40d	RHSIM_Rhsim01G0160900	1.419	0.644	-0.775	high
Treat-40d	RHSIM_Rhsim01G0235400	1.487	1.478	-0.009	high
Treat-40d	RHSIM_Rhsim02G0188300	1.173	0.060	-1.113	high
Treat-40d	RHSIM_Rhsim02G0090500	-1.379	-1.303	0.076	low
Treat-40d	RHSIM_Rhsim02G0027800	-0.153	0.185	0.339	no
Treat-40d	RHSIM_Rhsim02G0027600	0.843	-0.793	-1.636	no
Treat-40d	RHSIM_Rhsim02G0021200	2.222	1.938	-0.284	high
Treat-40d	RHSIM_Rhsim03G0056900	0.448	-0.983	-1.431	no
Treat-40d	RHSIM_Rhsim02G0032400	-0.244	1.541	1.785	no
Treat-40d	RHSIM_Rhsim02G0160100	-1.166	-0.341	0.826	low
Treat-40d	RHSIM_Rhsim02G0067200	1.810	-0.388	-2.198	high
Treat-40d	RHSIM_Rhsim01G0217300	0.971	0.733	-0.238	no
Treat-40d	RHSIM_Rhsim01G0171700	-0.584	-0.812	-0.228	no
Treat-40d	RHSIM_Rhsim01G0145800	0.661	0.690	0.028	no
Treat-40d	RHSIM_Rhsim01G0245700	-0.766	-0.768	-0.002	no
Treat-40d	RHSIM_Rhsim01G0142200	-0.373	-0.900	-0.527	no
Treat-40d	RHSIM_Rhsim02G0168400	-1.033	-0.196	0.837	no
Treat-40d	RHSIM_Rhsim01G0268000	-0.520	-1.688	-1.168	no
Treat-40d	RHSIM_Rhsim04G0227400	-1.153	-0.778	0.375	low
Treat-40d	RHSIM_Rhsim02G0234500	0.132	-1.023	-1.155	no
Treat-40d	RHSIM_Rhsim01G0249200	0.728	-1.545	-2.273	no
Treat-40d	RHSIM_Rhsim01G0069300	0.749	0.621	-0.128	no
Treat-40d	RHSIM_Rhsim03G0130400	-0.261	0.463	0.723	no
Treat-40d	RHSIM_Rhsim01G0268500	-0.088	-1.276	-1.188	no
Treat-40d	RHSIM_Rhsim01G0197500	-0.012	0.123	0.136	no
Treat-40d	RHSIM_Rhsim01G0186400	3.536	2.172	-1.364	high
Treat-40d	RHSIM_Rhsim02G0082500	-0.035	0.143	0.178	no
Treat-40d	RHSIM_Rhsim02G0072000	0.324	-0.186	-0.510	no
Treat-40d	RHSIM_Rhsim01G0280600	-1.308	-1.123	0.185	low
Treat-40d	RHSIM_Rhsim01G0002200	0.074	0.200	0.126	no

Group	Gene	PTR (z-score difference)	z(log2 LFQ)	z(log2 FPKM)	extreme-PTR
Treat-40d	RHSIM_Rhsim02G0088100	2.199	1.238	-0.961	high
Treat-40d	RHSIM_Rhsim04G0172200	0.822	1.927	1.104	no
Treat-40d	RHSIM_Rhsim02G0013700	-0.213	0.219	0.432	no
Treat-40d	RHSIM_Rhsim01G0293800	1.772	1.461	-0.310	high
Treat-40d	RHSIM_Rhsim01G0263600	0.313	0.128	-0.185	no
Treat-40d	RHSIM_Rhsim04G0224200	-1.100	-0.281	0.819	no
Treat-40d	RHSIM_Rhsim02G0076100	-1.491	-0.624	0.867	low
Treat-40d	RHSIM_Rhsim01G0245600	-3.037	-1.254	1.783	low
Treat-40d	RHSIM_Rhsim02G0203400	0.224	0.585	0.361	no
Treat-40d	RHSIM_Rhsim01G0281900	0.975	-0.333	-1.309	no
Treat-40d	RHSIM_Rhsim02G0210900	-0.725	0.800	1.525	no
Treat-40d	RHSIM_Rhsim01G0101000	-1.676	-0.755	0.921	low
Treat-40d	RHSIM_Rhsim04G0176700	0.539	0.480	-0.059	no
Treat-40d	RHSIM_Rhsim02G0062300	1.930	1.151	-0.779	high
Treat-40d	RHSIM_Rhsim01G0142500	-0.859	-0.855	0.004	no
Treat-40d	RHSIM_Rhsim02G0061900	-0.151	-0.910	-0.758	no
Treat-40d	RHSIM_Rhsim03G0011700	0.979	-0.147	-1.126	no
Treat-40d	RHSIM_Rhsim01G0218000	0.367	0.323	-0.044	no
Treat-40d	RHSIM_Rhsim01G0211400	-0.731	-0.466	0.265	no
Treat-40d	RHSIM_Rhsim03G0141500	-0.035	1.658	1.693	no
Treat-40d	RHSIM_Rhsim02G0018200	1.673	1.292	-0.380	high
Treat-40d	RHSIM_Rhsim02G0243200	2.425	0.314	-2.111	high
Treat-40d	RHSIM_Rhsim01G0273700	-1.004	1.268	2.272	no
Treat-40d	RHSIM_Rhsim03G0135800	-0.354	0.344	0.698	no
Treat-40d	RHSIM_Rhsim02G0162900	-0.327	-0.111	0.215	no
Treat-40d	RHSIM_Rhsim01G0207700	1.241	1.057	-0.184	high
Treat-40d	RHSIM_Rhsim02G0033000	-0.853	-0.330	0.523	no
Treat-40d	RHSIM_Rhsim02G0066900	0.978	-0.626	-1.604	no
Treat-40d	RHSIM_Rhsim02G0168300	0.658	0.550	-0.108	no
Treat-40d	RHSIM_Rhsim04G0000500	0.008	-1.359	-1.368	no
Treat-40d	RHSIM_Rhsim02G0169300	-0.447	-0.023	0.423	no
Treat-40d	RHSIM_Rhsim02G0099100	-0.999	-0.846	0.153	no

Group	Gene	PTR (z-score difference)	z(log2 LFQ)	z(log2 FPKM)	extreme-PTR
Treat-40d	RHSIM_Rhsim01G0060800	-0.042	1.202	1.245	no
Treat-40d	RHSIM_Rhsim02G0087400	1.144	0.619	-0.525	high
Treat-40d	RHSIM_Rhsim02G0044000	-0.128	-0.590	-0.462	no
Treat-40d	RHSIM_Rhsim01G0290800	0.358	2.813	2.455	no
Treat-40d	RHSIM_Rhsim02G0172400	1.310	-0.491	-1.802	high
Treat-40d	RHSIM_Rhsim02G0023800	1.541	0.390	-1.151	high
Treat-40d	RHSIM_Rhsim02G0054300	-0.028	0.595	0.623	no
Treat-40d	RHSIM_Rhsim02G0003600	0.277	0.370	0.093	no
Treat-40d	RHSIM_Rhsim01G0234400	0.540	-1.299	-1.839	no
Treat-40d	RHSIM_Rhsim02G0202300	1.584	0.083	-1.501	high
Treat-40d	RHSIM_Rhsim04G0128500	0.079	-0.005	-0.085	no
Treat-40d	RHSIM_Rhsim01G0100400	0.467	-0.861	-1.329	no
Treat-40d	RHSIM_Rhsim02G0106700	-1.542	-1.179	0.362	low
Treat-40d	RHSIM_Rhsim01G0182300	0.206	-1.145	-1.352	no
Treat-40d	RHSIM_Rhsim02G0195900	-0.566	-0.930	-0.364	no
Treat-40d	RHSIM_Rhsim02G0190100	0.649	-0.915	-1.564	no
Treat-40d	RHSIM_Rhsim04G0093900	0.670	0.749	0.079	no
Treat-40d	RHSIM_Rhsim02G0067000	-1.230	-0.017	1.214	low
Treat-40d	RHSIM_Rhsim02G0247000	-1.246	-0.421	0.824	low
Treat-40d	RHSIM_Rhsim02G0017000	-0.964	-1.043	-0.079	no
Treat-40d	RHSIM_Rhsim02G0213200	0.153	-1.147	-1.299	no
Treat-40d	RHSIM_Rhsim01G0219000	-0.859	-0.959	-0.100	no
Treat-40d	RHSIM_Rhsim01G0176200	0.117	-1.195	-1.312	no
Treat-40d	RHSIM_Rhsim02G0052200	-0.574	-0.334	0.240	no
Treat-40d	RHSIM_Rhsim02G0034800	0.602	-0.535	-1.138	no
Treat-40d	RHSIM_Rhsim01G0243700	0.579	-0.226	-0.805	no
Treat-40d	RHSIM_Rhsim02G0110000	-0.268	-0.097	0.171	no
Treat-40d	RHSIM_Rhsim02G0180500	-1.889	-0.132	1.757	low
Treat-40d	RHSIM_Rhsim04G0204400	-1.230	-1.121	0.109	low
Treat-40d	RHSIM_Rhsim02G0198400	1.116	-0.475	-1.591	high
Treat-40d	RHSIM_Rhsim01G0096100	-0.280	-1.146	-0.866	no
Treat-40d	RHSIM_Rhsim01G0136000	0.511	0.858	0.347	no

Group	Gene	PTR (z-score difference)	z(log2 LFQ)	z(log2 FPKM)	extreme-PTR
Treat-40d	RHSIM_Rhsim02G0002800	0.096	0.118	0.023	no
Treat-40d	RHSIM_Rhsim01G0197200	1.927	2.156	0.229	high
Treat-40d	RHSIM_Rhsim01G0090900	1.487	2.378	0.891	high
Treat-40d	RHSIM_Rhsim01G0095300	0.642	-0.191	-0.833	no
Treat-40d	RHSIM_Rhsim02G0171600	0.937	1.426	0.489	no
Treat-40d	RHSIM_Rhsim02G0054100	1.257	0.402	-0.855	high
Treat-40d	RHSIM_Rhsim01G0196800	0.558	-0.494	-1.051	no
Treat-40d	RHSIM_Rhsim02G0040400	-2.165	-1.742	0.423	low
Treat-40d	RHSIM_Rhsim03G0218500	0.269	0.794	0.525	no
Treat-40d	RHSIM_Rhsim02G0032200	-0.184	-0.860	-0.676	no
Treat-40d	RHSIM_Rhsim02G0004400	0.573	0.986	0.414	no
Treat-40d	RHSIM_Rhsim02G0009000	0.489	-0.553	-1.042	no
Treat-40d	RHSIM_Rhsim02G0212500	-0.142	0.235	0.377	no
Treat-40d	RHSIM_Rhsim02G0202200	0.645	-0.565	-1.210	no
Treat-40d	RHSIM_Rhsim01G0123500	-0.026	-0.640	-0.614	no
Treat-40d	RHSIM_Rhsim01G0051700	1.101	0.013	-1.089	no
Treat-40d	RHSIM_Rhsim01G0216700	-0.234	0.363	0.596	no
Treat-40d	RHSIM_Rhsim01G0151600	-1.062	-1.516	-0.454	no
Treat-40d	RHSIM_Rhsim01G0107900	0.686	0.705	0.018	no
Treat-40d	RHSIM_Rhsim03G0170800	0.417	1.374	0.957	no
Treat-40d	RHSIM_Rhsim01G0209500	0.132	1.359	1.227	no
Treat-40d	RHSIM_Rhsim01G0259700	0.496	-0.824	-1.319	no
Treat-40d	RHSIM_Rhsim02G0194300	-0.065	2.207	2.272	no
Treat-40d	RHSIM_Rhsim02G0069300	0.883	-0.822	-1.704	no
Treat-40d	RHSIM_Rhsim01G0293700	0.964	-0.147	-1.112	no
Treat-40d	RHSIM_Rhsim03G0144200	-0.329	-0.095	0.234	no
Treat-40d	RHSIM_Rhsim01G0265100	1.050	0.635	-0.414	no
Treat-40d	RHSIM_Rhsim01G0291100	0.426	1.894	1.468	no
Treat-40d	RHSIM_Rhsim02G0232700	0.528	0.007	-0.521	no
Treat-40d	RHSIM_Rhsim02G0228400	-0.322	0.821	1.143	no
Treat-40d	RHSIM_Rhsim01G0146900	-1.386	-1.381	0.006	low
Treat-40d	RHSIM_Rhsim01G0124300	0.855	-0.026	-0.881	no

Group	Gene	PTR (z-score difference)	z(log2 LFQ)	z(log2 FPKM)	extreme-PTR
Treat-40d	RHSIM_Rhsim01G0067700	-2.965	-1.296	1.669	low
Treat-40d	RHSIM_Rhsim01G0119100	0.145	-0.858	-1.003	no
Treat-40d	RHSIM_Rhsim02G0095200	-0.439	-0.992	-0.553	no
Treat-40d	RHSIM_Rhsim02G0223500	1.914	-0.068	-1.982	high
Treat-40d	RHSIM_Rhsim01G0292800	0.660	1.437	0.777	no
Treat-40d	RHSIM_Rhsim03G0247900	2.267	1.184	-1.083	high
Treat-40d	RHSIM_Rhsim01G0282100	-0.707	-1.104	-0.397	no
Treat-40d	RHSIM_Rhsim01G0189100	-1.443	-1.291	0.152	low
Treat-40d	RHSIM_Rhsim02G0216700	-0.518	-0.654	-0.136	no
Treat-40d	RHSIM_Rhsim01G0224200	0.444	0.641	0.197	no
Treat-40d	RHSIM_Rhsim03G0036700	-0.415	0.222	0.637	no
Treat-40d	RHSIM_Rhsim01G0191800	-1.511	0.020	1.532	low
Treat-40d	RHSIM_Rhsim01G0210800	0.178	1.085	0.908	no
Treat-40d	RHSIM_Rhsim01G0109600	-0.119	-0.227	-0.108	no
Treat-40d	RHSIM_Rhsim01G0009900	-1.505	-0.136	1.368	low
Treat-40d	RHSIM_Rhsim01G0176300	0.638	-1.006	-1.644	no
Treat-40d	RHSIM_Rhsim03G0088500	0.268	-0.267	-0.535	no
Treat-40d	RHSIM_Rhsim02G0244800	0.717	1.110	0.393	no
Treat-40d	RHSIM_Rhsim01G0197900	-0.073	-0.247	-0.174	no
Treat-40d	RHSIM_Rhsim01G0191100	1.170	2.861	1.691	high
Treat-40d	RHSIM_Rhsim02G0205100	-2.399	-1.780	0.620	low
Treat-40d	RHSIM_Rhsim01G0201600	-0.167	-0.188	-0.021	no
Treat-40d	RHSIM_Rhsim03G0125000	-2.393	-1.684	0.709	low
Treat-40d	RHSIM_Rhsim01G0210200	-1.440	-1.365	0.074	low
Treat-40d	RHSIM_Rhsim02G0062000	0.571	1.175	0.604	no
Treat-40d	RHSIM_Rhsim01G0255600	0.483	-0.623	-1.106	no
Treat-40d	RHSIM_Rhsim01G0188400	0.126	0.108	-0.017	no
Treat-40d	RHSIM_Rhsim03G0142300	1.482	0.638	-0.844	high
Treat-40d	RHSIM_Rhsim02G0168200	-0.717	-0.781	-0.064	no
Treat-40d	RHSIM_Rhsim01G0143400	0.272	1.528	1.256	no
Treat-40d	RHSIM_Rhsim01G0204700	-1.084	-0.127	0.957	no
Treat-40d	RHSIM_Rhsim01G0259300	0.894	0.468	-0.427	no

Group	Gene	PTR (z-score difference)	z(log2 LFQ)	z(log2 FPKM)	extreme-PTR
Treat-40d	RHSIM_Rhsim01G0091000	-0.307	-0.307	0.001	no
Treat-40d	RHSIM_Rhsim01G0276200	-0.283	-0.382	-0.099	no
Treat-40d	RHSIM_Rhsim02G0148900	-1.056	-0.561	0.495	no
Treat-40d	RHSIM_Rhsim01G0262300	-0.316	-0.527	-0.211	no
Treat-40d	RHSIM_Rhsim01G0180500	-0.829	-0.580	0.249	no
Treat-40d	RHSIM_Rhsim01G0119800	0.189	-0.740	-0.929	no
Treat-40d	RHSIM_Rhsim01G0010000	0.726	-0.001	-0.728	no
Treat-40d	RHSIM_Rhsim01G0204400	-0.492	0.098	0.590	no
Treat-40d	RHSIM_Rhsim01G0190200	0.669	0.246	-0.424	no
Treat-40d	RHSIM_Rhsim01G0074300	-0.148	0.812	0.961	no
Treat-40d	RHSIM_Rhsim01G0174600	-0.676	0.356	1.032	no
Treat-40d	RHSIM_Rhsim01G0246700	0.136	-0.270	-0.406	no
Treat-40d	RHSIM_Rhsim01G0107800	-1.115	-0.046	1.068	no
Treat-40d	RHSIM_Rhsim02G0076700	-0.253	-0.758	-0.505	no
Treat-40d	RHSIM_Rhsim01G0113600	-0.748	0.492	1.239	no
Treat-40d	RHSIM_Rhsim02G0071000	-0.510	0.808	1.317	no
Treat-40d	RHSIM_Rhsim02G0005500	-2.202	-2.072	0.130	low
Treat-40d	RHSIM_Rhsim01G0013000	-1.914	0.757	2.671	low
Treat-40d	RHSIM_Rhsim01G0202900	1.152	1.338	0.186	high
Treat-40d	RHSIM_Rhsim02G0193400	1.138	1.830	0.692	high
Treat-40d	RHSIM_Rhsim01G0198900	-1.974	-1.115	0.860	low
Treat-40d	RHSIM_Rhsim01G0233900	-0.848	0.801	1.649	no
Treat-40d	RHSIM_Rhsim01G0067800	-0.139	0.835	0.974	no
Treat-40d	RHSIM_Rhsim02G0049400	-1.069	-1.495	-0.426	no
Treat-40d	RHSIM_Rhsim01G0137100	1.919	-0.071	-1.990	high
Treat-40d	RHSIM_Rhsim01G0126900	0.124	1.193	1.069	no
Treat-40d	RHSIM_Rhsim01G0015800	-0.455	-1.984	-1.529	no
Treat-40d	RHSIM_Rhsim01G0284500	-1.175	-0.255	0.920	low
Treat-40d	RHSIM_Rhsim01G0205000	0.442	1.784	1.342	no
Treat-40d	RHSIM_Rhsim01G0145200	0.117	0.763	0.646	no
Treat-40d	RHSIM_Rhsim02G0040300	-1.202	-0.349	0.853	low
Treat-40d	RHSIM_Rhsim01G0005500	-0.743	-0.497	0.246	no

Group	Gene	PTR (z-score difference)	z(log2 LFQ)	z(log2 FPKM)	extreme-PTR
Treat-40d	RHSIM_Rhsim01G0237200	1.281	0.999	-0.282	high
Treat-40d	RHSIM_Rhsim02G0049200	-2.223	-0.306	1.917	low
Treat-40d	RHSIM_Rhsim01G0263000	0.125	0.850	0.725	no
Treat-40d	RHSIM_Rhsim01G0132300	-0.518	-1.114	-0.595	no
Treat-40d	RHSIM_Rhsim01G0257300	-0.516	1.254	1.770	no
Treat-40d	RHSIM_Rhsim01G0236600	-0.253	-1.154	-0.901	no
Treat-40d	RHSIM_Rhsim01G0009500	0.638	-1.737	-2.375	no
Treat-40d	RHSIM_Rhsim03G0172200	1.373	0.209	-1.164	high
Treat-40d	RHSIM_Rhsim01G0122100	-0.266	0.320	0.586	no
Treat-40d	RHSIM_Rhsim02G0204900	0.732	0.006	-0.726	no
Treat-40d	RHSIM_Rhsim01G0289700	-0.447	0.292	0.739	no
Treat-40d	RHSIM_Rhsim01G0075900	0.419	0.009	-0.411	no
Treat-40d	RHSIM_Rhsim03G0210700	-1.352	-0.253	1.099	low
Treat-40d	RHSIM_Rhsim01G0136100	0.203	-0.873	-1.075	no
Treat-40d	RHSIM_Rhsim02G0076500	-1.351	-0.291	1.060	low
Treat-40d	RHSIM_Rhsim02G0021000	-1.416	-0.859	0.557	low
Treat-40d	RHSIM_Rhsim03G0074800	-0.297	0.361	0.659	no
Treat-40d	RHSIM_Rhsim01G0181100	-1.581	-0.864	0.716	low
Treat-40d	RHSIM_Rhsim01G0062600	-0.675	0.091	0.766	no
Treat-40d	RHSIM_Rhsim02G0029100	-1.369	0.795	2.164	low
Treat-40d	RHSIM_Rhsim01G0206500	-1.094	-0.381	0.713	no
Treat-40d	RHSIM_Rhsim01G0285000	-2.492	-0.676	1.816	low
Treat-40d	RHSIM_Rhsim01G0185400	-0.458	0.719	1.176	no
Treat-40d	RHSIM_Rhsim02G0255700	-0.066	-1.227	-1.161	no
Treat-40d	RHSIM_Rhsim01G0009700	0.639	0.390	-0.250	no
Treat-40d	RHSIM_Rhsim01G0272700	-0.226	-0.034	0.192	no
Treat-40d	RHSIM_Rhsim01G0054700	0.778	0.678	-0.099	no
Treat-40d	RHSIM_Rhsim01G0260300	1.476	1.244	-0.231	high
Treat-40d	RHSIM_Rhsim02G0114900	0.162	0.357	0.195	no
Treat-40d	RHSIM_Rhsim01G0216300	-0.051	0.586	0.637	no
Treat-40d	RHSIM_Rhsim02G0231500	0.388	-0.182	-0.570	no
Treat-40d	RHSIM_Rhsim01G0201300	0.674	0.679	0.004	no

Group	Gene	PTR (z-score difference)	z(log2 LFQ)	z(log2 FPKM)	extreme-PTR
Treat-40d	RHSIM_Rhsim01G0234600	-0.195	-0.261	-0.066	no
Treat-40d	RHSIM_Rhsim03G0250000	-2.014	-1.096	0.918	low
Treat-40d	RHSIM_Rhsim03G0251500	-0.037	-0.599	-0.562	no
Treat-40d	RHSIM_Rhsim01G0124000	-0.345	-1.205	-0.860	no
Treat-40d	RHSIM_Rhsim01G0271700	-0.549	-0.900	-0.351	no
Treat-40d	RHSIM_Rhsim02G0010800	-1.146	-0.461	0.685	low
Treat-40d	RHSIM_Rhsim01G0042700	-1.195	-1.582	-0.387	low
Treat-40d	RHSIM_Rhsim01G0203800	0.513	0.652	0.139	no
Treat-40d	RHSIM_Rhsim01G0286700	-0.505	0.302	0.807	no
Treat-40d	RHSIM_Rhsim01G0187100	0.305	0.551	0.245	no
Treat-40d	RHSIM_Rhsim01G0141800	-0.923	-0.342	0.581	no
Treat-40d	RHSIM_Rhsim01G0245800	0.425	0.177	-0.248	no
Treat-40d	RHSIM_Rhsim01G0042600	-0.312	-0.202	0.110	no
Treat-40d	RHSIM_Rhsim01G0235600	-0.570	-0.393	0.178	no
Treat-40d	RHSIM_Rhsim01G0062000	0.364	0.230	-0.133	no
Treat-40d	RHSIM_Rhsim01G0179100	-0.607	-0.453	0.154	no
Treat-40d	RHSIM_Rhsim01G0198500	0.921	-0.069	-0.990	no
Treat-40d	RHSIM_Rhsim01G0164800	1.104	0.491	-0.613	no
Treat-40d	RHSIM_Rhsim01G0293000	-1.369	-0.686	0.684	low
Treat-40d	RHSIM_Rhsim01G0060200	-2.711	-1.665	1.046	low
Treat-40d	RHSIM_Rhsim01G0009300	-0.593	0.086	0.680	no
Treat-40d	RHSIM_Rhsim01G0149900	0.086	-0.412	-0.498	no
Treat-40d	RHSIM_Rhsim01G0147300	1.023	0.664	-0.360	no
Treat-40d	RHSIM_Rhsim01G0274600	2.191	2.659	0.468	high
Treat-40d	RHSIM_Rhsim01G0274400	2.973	3.005	0.032	high
Treat-40d	RHSIM_Rhsim03G0226900	0.587	1.541	0.954	no
Treat-40d	RHSIM_Rhsim02G0217400	0.490	0.294	-0.196	no
Treat-40d	RHSIM_Rhsim01G0071400	-1.239	-0.667	0.572	low
Treat-40d	RHSIM_Rhsim01G0002300	0.714	0.189	-0.524	no
Treat-40d	RHSIM_Rhsim01G0033400	-0.209	-0.453	-0.244	no
Treat-40d	RHSIM_Rhsim01G0269700	1.180	0.200	-0.980	high
Treat-40d	RHSIM_Rhsim01G0227900	-0.570	-1.102	-0.532	no

Group	Gene	PTR (z-score difference)	z(log2 LFQ)	z(log2 FPKM)	extreme-PTR
Treat-40d	RHSIM_Rhsim01G0254100	-1.852	-0.611	1.241	low
Treat-40d	RHSIM_Rhsim01G0074500	-1.573	-2.241	-0.668	low
Treat-40d	RHSIM_Rhsim01G0192900	-1.355	-0.588	0.766	low
Treat-40d	RHSIM_Rhsim01G0260900	1.286	0.756	-0.530	high
Treat-40d	RHSIM_Rhsim01G0006200	-0.726	-0.671	0.054	no
Treat-40d	RHSIM_Rhsim01G0088200	2.126	-0.078	-2.204	high
Treat-40d	RHSIM_Rhsim01G0285400	0.123	-0.802	-0.926	no
Treat-40d	RHSIM_Rhsim01G0155600	0.570	-0.782	-1.353	no
Treat-40d	RHSIM_Rhsim01G0039300	-0.868	-0.806	0.062	no
Treat-40d	RHSIM_Rhsim03G0141100	0.738	0.000	-0.738	no
Treat-40d	RHSIM_Rhsim01G0272800	-0.031	-0.861	-0.829	no
Treat-40d	RHSIM_Rhsim01G0089800	1.266	0.766	-0.499	high
Treat-40d	RHSIM_Rhsim01G0205100	0.504	0.104	-0.401	no
Treat-40d	RHSIM_Rhsim01G0100800	0.429	-0.677	-1.106	no
Treat-40d	RHSIM_Rhsim03G0176000	-1.073	-0.652	0.421	no
Treat-40d	RHSIM_Rhsim01G0055600	1.050	-0.128	-1.178	no
Treat-40d	RHSIM_Rhsim01G0262400	0.518	1.056	0.538	no
Treat-40d	RHSIM_Rhsim01G0218100	0.807	0.814	0.007	no
Treat-40d	RHSIM_Rhsim01G0245300	-1.992	0.351	2.343	low
Treat-40d	RHSIM_Rhsim01G0291400	0.108	0.405	0.297	no
Treat-40d	RHSIM_Rhsim01G0049400	-0.557	-0.397	0.160	no
Treat-40d	RHSIM_Rhsim01G0151000	0.070	-0.447	-0.518	no
Treat-40d	RHSIM_Rhsim01G0106700	0.888	0.244	-0.644	no
Treat-40d	RHSIM_Rhsim01G0196600	-0.845	0.625	1.469	no
Treat-40d	RHSIM_Rhsim01G0239900	-0.633	0.366	0.998	no
Treat-40d	RHSIM_Rhsim01G0113300	-0.560	0.098	0.658	no
Treat-40d	RHSIM_Rhsim01G0009800	0.026	-0.712	-0.738	no
Treat-40d	RHSIM_Rhsim01G0283500	0.449	0.279	-0.171	no
Treat-40d	RHSIM_Rhsim01G0233100	-0.336	-0.360	-0.024	no
Treat-40d	RHSIM_Rhsim01G0217800	-0.975	-0.474	0.501	no
Treat-40d	RHSIM_Rhsim01G0221600	1.333	3.255	1.922	high
Treat-40d	RHSIM_Rhsim01G0028300	-2.677	-0.710	1.967	low

Group	Gene	PTR (z-score difference)	z(log2 LFQ)	z(log2 FPKM)	extreme-PTR
Treat-40d	RHSIM_Rhsim02G0020800	-2.003	-0.527	1.476	low
Treat-40d	RHSIM_Rhsim01G0058000	1.584	1.038	-0.545	high
Treat-40d	RHSIM_Rhsim01G0167000	-0.458	-1.676	-1.217	no
Treat-40d	RHSIM_Rhsim01G0187900	-0.015	-1.036	-1.021	no
Treat-40d	RHSIM_Rhsim01G0124600	-1.189	-0.651	0.538	low
Treat-40d	RHSIM_Rhsim01G0247100	-0.335	-0.556	-0.221	no
Treat-40d	RHSIM_Rhsim01G0007800	0.200	0.938	0.738	no
Treat-40d	RHSIM_Rhsim03G0163700	0.479	-0.122	-0.601	no
Treat-40d	RHSIM_Rhsim01G0283900	-1.030	0.645	1.675	no
Treat-40d	RHSIM_Rhsim01G0012100	0.075	-1.089	-1.164	no
Treat-40d	RHSIM_Rhsim01G0242500	-0.972	0.951	1.923	no
Treat-40d	RHSIM_Rhsim01G0268900	-0.064	-0.552	-0.488	no
Treat-40d	RHSIM_Rhsim01G0229100	-0.924	-0.517	0.407	no
Treat-40d	RHSIM_Rhsim01G0273800	-1.092	-1.095	-0.003	no
Treat-40d	RHSIM_Rhsim03G0141200	-1.548	1.363	2.911	low
Treat-40d	RHSIM_Rhsim03G0215700	-1.224	-0.393	0.831	low
Treat-40d	RHSIM_Rhsim01G0186700	0.267	-0.002	-0.269	no
Treat-40d	RHSIM_Rhsim01G0225700	0.047	0.991	0.944	no
Treat-40d	RHSIM_Rhsim03G0149300	-0.174	0.142	0.316	no
Treat-40d	RHSIM_Rhsim01G0071100	1.979	1.237	-0.742	high
Treat-40d	RHSIM_Rhsim03G0083700	0.549	-0.231	-0.781	no
Treat-40d	RHSIM_Rhsim02G0012600	-1.621	-1.350	0.270	low
Treat-40d	RHSIM_Rhsim01G0117300	-2.366	-0.808	1.558	low
Treat-40d	RHSIM_Rhsim01G0176100	-1.640	-0.266	1.374	low
Treat-40d	RHSIM_Rhsim02G0158200	-0.672	0.370	1.041	no
Treat-40d	RHSIM_Rhsim01G0033200	1.209	0.902	-0.307	high
Treat-40d	RHSIM_Rhsim01G0245500	-0.412	-0.402	0.010	no
Treat-40d	RHSIM_Rhsim01G0073000	3.008	1.354	-1.653	high
Treat-40d	RHSIM_Rhsim01G0038600	-1.097	-0.050	1.047	no
Treat-40d	RHSIM_Rhsim01G0065700	-0.031	-0.561	-0.530	no
Treat-40d	RHSIM_Rhsim01G0068100	0.762	-0.542	-1.304	no
Treat-40d	RHSIM_Rhsim01G0130400	-0.316	-0.135	0.181	no

Group	Gene	PTR (z-score difference)	z(log2 LFQ)	z(log2 FPKM)	extreme-PTR
Treat-40d	RHSIM_Rhsim01G0196000	-2.761	-1.713	1.047	low
Treat-40d	RHSIM_Rhsim03G0040000	1.460	1.814	0.353	high
Treat-40d	RHSIM_Rhsim01G0134600	0.320	-0.993	-1.312	no
Treat-40d	RHSIM_Rhsim01G0255000	1.326	1.266	-0.061	high
Treat-40d	RHSIM_Rhsim01G0249300	-0.869	-0.991	-0.122	no
Treat-40d	RHSIM_Rhsim01G0200800	-1.372	-1.115	0.257	low
Treat-40d	RHSIM_Rhsim01G0061700	-1.813	0.777	2.590	low
Treat-40d	RHSIM_Rhsim01G0027700	1.496	-0.048	-1.544	high
Treat-40d	RHSIM_Rhsim01G0026600	-0.038	-1.294	-1.256	no
Treat-40d	RHSIM_Rhsim01G0103600	0.170	-0.235	-0.405	no
Treat-40d	RHSIM_Rhsim01G0182400	-0.269	-0.976	-0.707	no
Treat-40d	RHSIM_Rhsim01G0289900	-0.597	0.615	1.212	no
Treat-40d	RHSIM_Rhsim01G0251300	1.847	0.568	-1.278	high
Treat-40d	RHSIM_Rhsim01G0228100	-1.165	-1.335	-0.171	low
Treat-40d	RHSIM_Rhsim02G0213300	-0.407	2.255	2.663	no
Treat-40d	RHSIM_Rhsim01G0027300	-0.325	0.396	0.722	no
Treat-40d	RHSIM_Rhsim01G0208600	-0.947	0.629	1.575	no
Treat-40d	RHSIM_Rhsim01G0292900	-0.609	-0.618	-0.009	no
Treat-40d	RHSIM_Rhsim01G0178200	-0.634	-0.184	0.450	no
Treat-40d	RHSIM_Rhsim01G0257200	1.096	1.334	0.238	no
Treat-40d	RHSIM_Rhsim01G0198000	1.844	0.104	-1.741	high
Treat-40d	RHSIM_Rhsim01G0128600	1.319	-0.158	-1.476	high
Treat-40d	RHSIM_Rhsim02G0180600	0.570	0.581	0.011	no
Treat-40d	RHSIM_Rhsim01G0149400	0.071	-0.834	-0.905	no
Treat-40d	RHSIM_Rhsim01G0000400	1.661	1.731	0.070	high
Treat-40d	RHSIM_Rhsim01G0184500	1.424	1.821	0.397	high
Treat-40d	RHSIM_Rhsim01G0155900	-0.410	0.138	0.548	no
Treat-40d	RHSIM_Rhsim01G0253200	-0.455	-0.245	0.209	no
Treat-40d	RHSIM_Rhsim01G0101300	-0.053	-0.446	-0.393	no
Treat-40d	RHSIM_Rhsim01G0033300	3.350	1.676	-1.674	high
Treat-40d	RHSIM_Rhsim01G0237100	-0.739	-0.575	0.164	no
Treat-40d	RHSIM_Rhsim01G0236300	1.469	-0.566	-2.035	high

Group	Gene	PTR (z-score difference)	z(log2 LFQ)	z(log2 FPKM)	extreme-PTR
Treat-40d	RHSIM_Rhsim01G0116400	1.983	1.117	-0.866	high
Treat-40d	RHSIM_Rhsim01G0002800	-1.121	-0.173	0.949	no
Treat-40d	RHSIM_Rhsim02G0205700	0.144	-0.826	-0.971	no
Treat-40d	RHSIM_Rhsim02G0173000	0.587	0.515	-0.072	no
Treat-40d	RHSIM_Rhsim03G0004900	0.452	1.105	0.653	no
Treat-40d	RHSIM_Rhsim01G0125000	-1.674	-0.381	1.293	low
Treat-40d	RHSIM_Rhsim01G0033100	0.771	0.170	-0.601	no
Treat-40d	RHSIM_Rhsim01G0002900	-0.297	0.033	0.329	no
Treat-40d	RHSIM_Rhsim02G0037400	1.620	2.687	1.067	high
Treat-40d	RHSIM_Rhsim01G0275800	0.775	0.679	-0.096	no
Treat-40d	RHSIM_Rhsim01G0191600	0.972	1.283	0.311	no
Treat-40d	RHSIM_Rhsim01G0125800	0.012	1.062	1.051	no
Treat-40d	RHSIM_Rhsim01G0067100	-0.109	0.181	0.290	no
Treat-40d	RHSIM_Rhsim01G0180300	0.735	0.937	0.202	no
Treat-40d	RHSIM_Rhsim03G0045300	1.013	1.900	0.887	no
Treat-40d	RHSIM_Rhsim01G0274100	-1.942	-1.625	0.317	low
Treat-40d	RHSIM_Rhsim01G0257500	-0.126	0.054	0.180	no
Treat-40d	RHSIM_Rhsim03G0081900	0.621	2.097	1.476	no
Treat-40d	RHSIM_Rhsim01G0027600	1.009	-0.569	-1.578	no
Treat-40d	RHSIM_Rhsim01G0120500	0.029	1.390	1.361	no
Treat-40d	RHSIM_Rhsim01G0224400	0.357	-0.391	-0.748	no
Treat-40d	RHSIM_Rhsim01G0190500	0.341	0.237	-0.104	no
Treat-40d	RHSIM_Rhsim01G0255900	-1.094	-0.683	0.410	no
Treat-40d	RHSIM_Rhsim03G0221900	0.164	1.047	0.883	no
Treat-40d	RHSIM_Rhsim01G0268200	0.787	1.849	1.061	no
Treat-40d	RHSIM_Rhsim01G0006700	-0.552	-0.125	0.427	no
Treat-40d	RHSIM_Rhsim01G0231000	-0.485	-0.243	0.242	no
Treat-40d	RHSIM_Rhsim03G0079200	2.349	1.335	-1.015	high
Treat-40d	RHSIM_Rhsim01G0212100	-0.906	0.716	1.622	no
Treat-40d	RHSIM_Rhsim01G0137400	0.289	0.068	-0.221	no
Treat-40d	RHSIM_Rhsim01G0194700	-0.142	-0.652	-0.510	no
Treat-40d	RHSIM_Rhsim01G0265800	2.371	0.223	-2.148	high

Group	Gene	PTR (z-score difference)	z(log2 LFQ)	z(log2 FPKM)	extreme-PTR
Treat-40d	RHSIM_Rhsim01G0250200	0.776	0.072	-0.704	no
Treat-40d	RHSIM_Rhsim01G0198200	-0.085	0.419	0.504	no
Treat-40d	RHSIM_Rhsim01G0246300	-0.450	-0.714	-0.265	no
Treat-40d	RHSIM_Rhsim01G0122600	-0.798	-0.622	0.176	no
Treat-40d	RHSIM_Rhsim01G0254300	-0.380	-0.858	-0.478	no
Treat-40d	RHSIM_Rhsim03G0033400	-2.363	-1.659	0.704	low
Treat-40d	RHSIM_Rhsim01G0035300	-0.190	-1.202	-1.012	no
Treat-40d	RHSIM_Rhsim01G0291000	0.750	1.630	0.880	no
Treat-40d	RHSIM_Rhsim01G0200700	-0.636	-0.002	0.634	no
Treat-40d	RHSIM_Rhsim01G0125200	-1.387	-0.296	1.091	low
Treat-40d	RHSIM_Rhsim01G0145500	-0.313	-1.275	-0.962	no
Treat-40d	RHSIM_Rhsim01G0165300	-0.472	0.838	1.310	no
Treat-40d	RHSIM_Rhsim01G0016000	0.843	1.082	0.238	no
Treat-40d	RHSIM_Rhsim01G0244900	0.054	-0.060	-0.114	no
Treat-40d	RHSIM_Rhsim03G0145600	1.456	0.253	-1.202	high
Treat-40d	RHSIM_Rhsim01G0149300	0.329	0.541	0.212	no
Treat-40d	RHSIM_Rhsim01G0259900	0.724	0.951	0.227	no
Treat-40d	RHSIM_Rhsim03G0063400	-0.266	-0.477	-0.211	no
Treat-40d	RHSIM_Rhsim01G0238300	0.392	0.211	-0.181	no
Treat-40d	RHSIM_Rhsim01G0163300	1.036	1.350	0.313	no
Treat-40d	RHSIM_Rhsim01G0251200	0.485	-0.657	-1.142	no
Treat-40d	RHSIM_Rhsim01G0250600	-1.331	-0.570	0.760	low
Treat-40d	RHSIM_Rhsim01G0287300	-1.430	-0.886	0.544	low
Treat-40d	RHSIM_Rhsim01G0284700	-0.637	-0.171	0.465	no
Treat-40d	RHSIM_Rhsim01G0264000	-1.983	-0.819	1.164	low
Treat-40d	RHSIM_Rhsim01G0218900	0.357	1.028	0.672	no
Treat-40d	RHSIM_Rhsim01G0217200	0.689	0.946	0.258	no
Treat-40d	RHSIM_Rhsim01G0066600	-0.185	-0.157	0.028	no
Treat-40d	RHSIM_Rhsim01G0000300	-0.034	-0.569	-0.535	no
Treat-40d	RHSIM_Rhsim01G0041200	1.613	-0.657	-2.270	high
Treat-40d	RHSIM_Rhsim01G0007100	-0.635	-0.693	-0.057	no
Treat-40d	RHSIM_Rhsim01G0276700	1.574	0.932	-0.642	high

Group	Gene	PTR (z-score difference)	z(log2 LFQ)	z(log2 FPKM)	extreme-PTR
Treat-40d	RHSIM_Rhsim03G0267300	-3.568	-1.384	2.184	low
Treat-40d	RHSIM_Rhsim01G0283700	0.998	0.391	-0.607	no
Treat-40d	RHSIM_Rhsim01G0247500	0.315	0.205	-0.110	no
Treat-40d	RHSIM_Rhsim01G0247300	-1.341	-0.311	1.029	low
Treat-40d	RHSIM_Rhsim01G0192300	-1.257	-0.999	0.258	low
Treat-40d	RHSIM_Rhsim01G0290400	-0.227	0.126	0.353	no
Treat-40d	RHSIM_Rhsim01G0130900	-2.425	-1.358	1.067	low
Treat-40d	RHSIM_Rhsim02G0110500	0.628	-0.570	-1.199	no
Treat-40d	RHSIM_Rhsim01G0068700	-1.910	0.688	2.598	low
Treat-40d	RHSIM_Rhsim01G0145700	0.745	0.155	-0.590	no
Treat-40d	RHSIM_Rhsim01G0106500	1.556	0.327	-1.229	high
Treat-40d	RHSIM_Rhsim01G0230600	0.013	0.144	0.131	no
Treat-40d	RHSIM_Rhsim01G0248500	1.224	0.975	-0.249	high
Treat-40d	RHSIM_Rhsim01G0091800	0.286	0.110	-0.177	no
Treat-40d	RHSIM_Rhsim01G0192200	2.054	3.014	0.960	high
Treat-40d	RHSIM_Rhsim01G0091400	-0.718	-1.261	-0.543	no
Treat-40d	RHSIM_Rhsim02G0216500	-1.044	1.237	2.280	no
Treat-40d	RHSIM_Rhsim01G0209300	0.109	0.214	0.105	no
Treat-40d	RHSIM_Rhsim02G0193300	-0.901	-0.143	0.758	no
Treat-40d	RHSIM_Rhsim01G0275400	-1.697	-0.779	0.919	low
Treat-40d	RHSIM_Rhsim02G0005600	-0.617	-0.656	-0.039	no
Treat-40d	RHSIM_Rhsim01G0008800	1.108	-0.358	-1.466	no
Treat-40d	RHSIM_Rhsim02G0128200	0.777	0.941	0.164	no
Treat-40d	RHSIM_Rhsim01G0195300	0.951	-0.052	-1.002	no
Treat-40d	RHSIM_Rhsim01G0073700	0.269	0.011	-0.258	no
Treat-40d	RHSIM_Rhsim01G0144700	-1.334	-0.936	0.398	low
Treat-40d	RHSIM_Rhsim01G0126800	0.792	2.282	1.490	no
Treat-40d	RHSIM_Rhsim01G0230900	-0.568	-1.272	-0.704	no
Treat-40d	RHSIM_Rhsim01G0286600	-0.954	0.390	1.344	no
Treat-40d	RHSIM_Rhsim02G0256800	0.485	0.027	-0.459	no
Treat-40d	RHSIM_Rhsim01G0224800	-0.219	0.459	0.678	no
Treat-40d	RHSIM_Rhsim02G0132700	-1.221	0.574	1.794	low

Group	Gene	PTR (z-score difference)	z(log2 LFQ)	z(log2 FPKM)	extreme-PTR
Treat-40d	RHSIM_Rhsim02G0142400	-0.442	0.411	0.853	no
Treat-40d	RHSIM_Rhsim02G0238900	0.823	0.118	-0.705	no
Treat-40d	RHSIM_Rhsim01G0184800	0.052	0.933	0.881	no
Treat-40d	RHSIM_Rhsim02G0185100	-0.548	-0.909	-0.361	no
Treat-40d	RHSIM_Rhsim02G0229900	0.663	0.745	0.082	no
Treat-40d	RHSIM_Rhsim01G0246100	-1.626	-0.963	0.663	low
Treat-40d	RHSIM_Rhsim01G0184000	-0.728	-0.331	0.397	no
Treat-40d	RHSIM_Rhsim01G0023900	0.290	0.882	0.592	no
Treat-40d	RHSIM_Rhsim01G0162200	-0.128	-0.023	0.105	no
Treat-40d	RHSIM_Rhsim01G0008400	1.481	1.224	-0.257	high
Treat-40d	RHSIM_Rhsim01G0236500	-0.189	1.114	1.303	no
Treat-40d	RHSIM_Rhsim01G0262900	-1.222	-0.672	0.550	low
Treat-40d	RHSIM_Rhsim02G0151800	-1.021	-0.174	0.847	no
Treat-40d	RHSIM_Rhsim02G0003700	0.936	0.090	-0.846	no
Treat-40d	RHSIM_Rhsim02G0100800	0.878	0.378	-0.500	no
Treat-40d	RHSIM_Rhsim01G0090500	-1.681	-1.713	-0.033	low
Treat-40d	RHSIM_Rhsim01G0064300	-0.729	-1.519	-0.790	no
Treat-40d	RHSIM_Rhsim02G0031500	0.338	0.333	-0.005	no
Treat-40d	RHSIM_Rhsim02G0000800	-1.388	-0.840	0.548	low
Treat-40d	RHSIM_Rhsim01G0141400	0.230	2.458	2.229	no
Treat-40d	RHSIM_Rhsim02G0075300	-0.864	-0.658	0.206	no
Treat-40d	RHSIM_Rhsim01G0280500	-1.609	-1.237	0.372	low
Treat-40d	RHSIM_Rhsim02G0223600	-0.214	-0.325	-0.112	no
Treat-40d	RHSIM_Rhsim02G0088700	1.089	-0.533	-1.622	no
Treat-40d	RHSIM_Rhsim02G0228900	0.768	0.597	-0.171	no
Treat-40d	RHSIM_Rhsim02G0026400	-2.062	0.095	2.157	low
Treat-40d	RHSIM_Rhsim02G0054000	-0.560	-0.576	-0.016	no
Treat-40d	RHSIM_Rhsim02G0018300	0.295	-1.128	-1.423	no
Treat-40d	RHSIM_Rhsim01G0218400	1.028	1.015	-0.013	no
Treat-40d	RHSIM_Rhsim01G0251500	1.978	1.301	-0.677	high
Treat-40d	RHSIM_Rhsim01G0106600	-0.979	-0.022	0.957	no
Treat-40d	RHSIM_Rhsim01G0181700	-0.999	-0.689	0.310	no

Group	Gene	PTR (z-score difference)	z(log2 LFQ)	z(log2 FPKM)	extreme-PTR
Treat-40d	RHSIM_Rhsim01G0220200	-0.911	-1.080	-0.169	no
Treat-40d	RHSIM_Rhsim02G0037500	1.046	1.214	0.168	no
Treat-40d	RHSIM_Rhsim01G0124800	0.607	0.638	0.031	no
Treat-40d	RHSIM_Rhsim01G0139200	0.229	0.656	0.427	no
Treat-40d	RHSIM_Rhsim01G0282400	0.044	-0.793	-0.836	no
Treat-40d	RHSIM_Rhsim01G0220800	1.507	0.190	-1.317	high
Treat-40d	RHSIM_Rhsim01G0284300	0.066	0.209	0.143	no
Treat-40d	RHSIM_Rhsim01G0102600	0.454	0.033	-0.421	no
Treat-40d	RHSIM_Rhsim01G0208800	-0.329	0.060	0.389	no
Treat-40d	RHSIM_Rhsim01G0023400	0.295	0.003	-0.292	no
Treat-40d	RHSIM_Rhsim01G0207000	-0.828	0.842	1.670	no
Treat-40d	RHSIM_Rhsim01G0216900	1.415	1.024	-0.391	high
Treat-40d	RHSIM_Rhsim01G0217400	-0.174	-1.323	-1.149	no
Treat-40d	RHSIM_Rhsim01G0245900	1.272	1.571	0.299	high
Treat-40d	RHSIM_Rhsim01G0159800	-0.382	0.520	0.902	no
Treat-40d	RHSIM_Rhsim01G0238400	0.103	0.044	-0.059	no
Treat-40d	RHSIM_Rhsim01G0226500	0.069	0.559	0.490	no
Treat-40d	RHSIM_Rhsim01G0229500	-0.208	-0.103	0.105	no
Treat-40d	RHSIM_Rhsim01G0216000	-0.380	-1.095	-0.716	no
Treat-40d	RHSIM_Rhsim01G0274200	-0.845	-0.569	0.276	no
Treat-40d	RHSIM_Rhsim01G0049500	-1.561	-1.313	0.248	low
Treat-40d	RHSIM_Rhsim01G0193100	1.788	1.330	-0.457	high
Treat-40d	RHSIM_Rhsim01G0231600	-1.878	-1.296	0.582	low
Treat-40d	RHSIM_Rhsim01G0191000	-0.158	-0.622	-0.464	no
Treat-40d	RHSIM_Rhsim01G0033000	1.424	0.391	-1.034	high
Treat-40d	RHSIM_Rhsim01G0223000	0.429	0.325	-0.104	no
Treat-40d	RHSIM_Rhsim01G0123800	0.729	0.612	-0.117	no
Treat-40d	RHSIM_Rhsim01G0002500	-0.362	-0.660	-0.298	no
Treat-40d	RHSIM_Rhsim01G0148500	-0.024	-0.307	-0.283	no
Treat-40d	RHSIM_Rhsim01G0068000	-0.622	-1.221	-0.599	no
Treat-40d	RHSIM_Rhsim01G0202100	-1.301	0.192	1.493	low
Treat-40d	RHSIM_Rhsim01G0130500	-1.024	-1.344	-0.320	no

Group	Gene	PTR (z-score difference)	z(log2 LFQ)	z(log2 FPKM)	extreme-PTR
Treat-40d	RHSIM_Rhsim01G0076500	1.543	1.151	-0.392	high
Treat-40d	RHSIM_Rhsim01G0265500	-3.207	-1.331	1.875	low
Treat-40d	RHSIM_Rhsim01G0233700	-1.937	-0.527	1.410	low
Treat-40d	RHSIM_Rhsim01G0189000	0.764	0.317	-0.447	no
Treat-40d	RHSIM_Rhsim01G0185300	-0.284	-0.008	0.276	no
Treat-40d	RHSIM_Rhsim01G0131100	-0.712	0.019	0.730	no
Treat-40d	RHSIM_Rhsim01G0252400	-0.534	-0.877	-0.342	no
Treat-40d	RHSIM_Rhsim01G0060300	-1.419	-0.231	1.188	low
Treat-40d	RHSIM_Rhsim01G0006800	0.209	-0.742	-0.951	no
Treat-40d	RHSIM_Rhsim01G0287900	-1.770	-1.101	0.669	low
Treat-40d	RHSIM_Rhsim01G0071600	-2.280	1.082	3.361	low
Treat-40d	RHSIM_Rhsim01G0185000	-0.391	-1.220	-0.829	no
Treat-40d	RHSIM_Rhsim01G0196300	0.790	0.661	-0.129	no
Treat-40d	RHSIM_Rhsim01G0138800	1.182	0.344	-0.839	high
Treat-40d	RHSIM_Rhsim01G0210400	2.567	1.639	-0.929	high
Treat-40d	RHSIM_Rhsim01G0007000	0.605	0.622	0.018	no
Treat-40d	RHSIM_Rhsim01G0000700	1.590	0.928	-0.662	high
Treat-40d	RHSIM_RhsimUnG0256000	1.643	0.038	-1.605	high
Treat-40d	RHSIM_RhsimUnG0254300	-3.259	-0.833	2.426	low
Treat-40d	RHSIM_RhsimUnG0258100	-0.067	-0.156	-0.089	no
Treat-40d	RHSIM_RhsimUnG0252100	0.161	-1.007	-1.168	no
Treat-40d	RHSIM_RhsimUnG0236700	0.486	-0.076	-0.562	no
Treat-40d	RHSIM_RhsimUnG0227500	0.529	1.306	0.778	no
Treat-40d	RHSIM_RhsimUnG0171600	-1.386	-1.480	-0.094	low
Treat-40d	RHSIM_RhsimUnG0169000	-1.725	-1.310	0.415	low
Treat-40d	RHSIM_RhsimUnG0118200	0.365	-0.734	-1.099	no
Treat-40d	RHSIM_Rhsim11G0108100	-0.786	0.834	1.620	no
Treat-40d	RHSIM_RhsimUnG0065600	-1.134	0.522	1.657	no
Treat-40d	RHSIM_RhsimUnG0062200	1.735	-0.347	-2.082	high
Treat-40d	RHSIM_Rhsim10G0103200	0.190	0.104	-0.085	no
Treat-40d	RHSIM_RhsimUnG0055300	-0.031	0.372	0.403	no
Treat-40d	RHSIM_RhsimUnG0037800	-1.718	-0.880	0.838	low

Group	Gene	PTR (z-score difference)	z(log2 LFQ)	z(log2 FPKM)	extreme-PTR
Treat-40d	RHSIM_RhsimUnG0024000	-1.469	0.337	1.805	low
Treat-40d	RHSIM_RhsimUnG0003000	0.048	-0.264	-0.312	no
Treat-40d	RHSIM_RhsimUnG0258700	0.103	-1.179	-1.281	no
Treat-40d	RHSIM_RhsimPtG0005800	3.084	3.761	0.677	high
Treat-40d	RHSIM_RhsimUnG0253900	0.918	1.229	0.311	no
Treat-40d	RHSIM_RhsimUnG0250200	0.632	1.601	0.969	no
Treat-40d	RHSIM_Rhsim13G0233000	0.332	-0.182	-0.514	no
Treat-40d	RHSIM_Rhsim13G0008700	3.275	1.502	-1.774	high
Treat-40d	RHSIM_Rhsim13G0227700	0.972	-1.314	-2.286	no
Treat-40d	RHSIM_Rhsim13G0228800	1.279	2.238	0.959	high
Treat-40d	RHSIM_Rhsim13G0222000	-1.450	-1.271	0.178	low
Treat-40d	RHSIM_Rhsim13G0111300	-0.121	0.784	0.906	no
Treat-40d	RHSIM_Rhsim13G0083600	-0.630	-1.260	-0.630	no
Treat-40d	RHSIM_Rhsim13G0180900	-0.806	-1.005	-0.199	no
Treat-40d	RHSIM_Rhsim13G0004600	0.020	-0.108	-0.128	no
Treat-40d	RHSIM_RhsimUnG0212100	-0.729	-0.728	0.001	no
Treat-40d	RHSIM_Rhsim13G0203000	-0.056	-1.002	-0.946	no
Treat-40d	RHSIM_RhsimUnG0202600	1.163	-0.561	-1.724	high
Treat-40d	RHSIM_Rhsim13G0053600	0.385	1.100	0.716	no
Treat-40d	RHSIM_Rhsim13G0103100	-0.214	-0.353	-0.139	no
Treat-40d	RHSIM_RhsimUnG0248000	0.028	-0.026	-0.053	no
Treat-40d	RHSIM_RhsimUnG0250500	0.689	1.639	0.951	no
Treat-40d	RHSIM_RhsimUnG0253100	-1.693	-0.475	1.218	low
Treat-40d	RHSIM_Rhsim13G0021500	-0.636	-0.804	-0.168	no
Treat-40d	RHSIM_RhsimUnG0257700	1.470	2.658	1.189	high
Treat-40d	RHSIM_RhsimUnG0251000	-1.128	-0.704	0.424	no
Treat-40d	RHSIM_RhsimUnG0248500	-1.001	-0.611	0.390	no
Treat-40d	RHSIM_Rhsim13G0120200	-1.539	-1.712	-0.173	low
Treat-40d	RHSIM_RhsimUnG0234700	-1.458	-2.482	-1.024	low
Treat-40d	RHSIM_Rhsim13G0146900	-1.677	-1.821	-0.144	low
Treat-40d	RHSIM_RhsimUnG0239200	1.262	0.554	-0.708	high
Treat-40d	RHSIM_RhsimUnG0240700	0.767	1.065	0.298	no

Group	Gene	PTR (z-score difference)	z(log2 LFQ)	z(log2 FPKM)	extreme-PTR
Treat-40d	RHSIM_RhsimUnG0226400	-0.743	-0.397	0.346	no
Treat-40d	RHSIM_Rhsim13G0041600	2.684	3.166	0.482	high
Treat-40d	RHSIM_RhsimUnG0223000	0.995	-0.869	-1.864	no
Treat-40d	RHSIM_RhsimUnG0231000	-0.284	-1.789	-1.504	no
Treat-40d	RHSIM_Rhsim13G0201800	-2.497	-0.893	1.604	low
Treat-40d	RHSIM_RhsimUnG0225800	1.519	0.795	-0.724	high
Treat-40d	RHSIM_Rhsim13G0232300	1.536	1.072	-0.464	high
Treat-40d	RHSIM_Rhsim13G0191000	-0.515	-0.791	-0.276	no
Treat-40d	RHSIM_RhsimUnG0168300	0.506	-0.016	-0.523	no
Treat-40d	RHSIM_Rhsim13G0040700	-0.400	-0.970	-0.570	no
Treat-40d	RHSIM_Rhsim13G0054200	-0.732	-1.545	-0.813	no
Treat-40d	RHSIM_RhsimUnG0210400	2.621	1.848	-0.774	high
Treat-40d	RHSIM_RhsimUnG0210700	0.146	-0.427	-0.573	no
Treat-40d	RHSIM_RhsimUnG0209300	0.187	-1.048	-1.235	no
Treat-40d	RHSIM_Rhsim12G0052900	1.116	0.741	-0.375	high
Treat-40d	RHSIM_RhsimUnG0192400	1.004	-0.177	-1.181	no
Treat-40d	RHSIM_Rhsim13G0198900	0.256	-0.526	-0.782	no
Treat-40d	RHSIM_RhsimUnG0123300	2.404	0.446	-1.958	high
Treat-40d	RHSIM_Rhsim09G0047000	1.776	1.135	-0.641	high
Treat-40d	RHSIM_Rhsim13G0121800	-0.272	-1.196	-0.925	no
Treat-40d	RHSIM_RhsimUnG0168800	1.018	1.923	0.904	no
Treat-40d	RHSIM_RhsimUnG0116900	-1.997	-1.868	0.128	low
Treat-40d	RHSIM_Rhsim06G0025600	0.104	0.417	0.313	no
Treat-40d	RHSIM_Rhsim13G0051200	-2.109	-1.774	0.335	low
Treat-40d	RHSIM_RhsimUnG0143500	-1.658	-1.293	0.365	low
Treat-40d	RHSIM_RhsimUnG0092900	1.141	-0.637	-1.777	high
Treat-40d	RHSIM_RhsimUnG0086600	1.205	0.535	-0.670	high
Treat-40d	RHSIM_Rhsim13G0020600	-0.380	-1.764	-1.384	no
Treat-40d	RHSIM_RhsimUnG0082400	0.730	0.421	-0.309	no
Treat-40d	RHSIM_RhsimUnG0142500	-0.206	-0.309	-0.103	no
Treat-40d	RHSIM_RhsimUnG0135800	2.075	0.529	-1.546	high
Treat-40d	RHSIM_RhsimUnG0073800	-0.488	0.741	1.229	no

Group	Gene	PTR (z-score difference)	z(log2 LFQ)	z(log2 FPKM)	extreme-PTR
Treat-40d	RHSIM_RhsimUnG0131400	1.254	0.475	-0.778	high
Treat-40d	RHSIM_RhsimUnG0120100	0.533	1.881	1.348	no
Treat-40d	RHSIM_RhsimUnG0128700	0.385	-0.758	-1.144	no
Treat-40d	RHSIM_Rhsim13G0099200	0.853	1.403	0.550	no
Treat-40d	RHSIM_Rhsim13G0029700	-0.542	-1.392	-0.850	no
Treat-40d	RHSIM_RhsimUnG0044400	-0.239	0.615	0.854	no
Treat-40d	RHSIM_RhsimUnG0092800	1.037	0.172	-0.865	no
Treat-40d	RHSIM_RhsimUnG0088000	0.896	-0.370	-1.266	no
Treat-40d	RHSIM_RhsimUnG0100600	-0.007	0.554	0.561	no
Treat-40d	RHSIM_Rhsim13G0131200	0.423	0.536	0.113	no
Treat-40d	RHSIM_RhsimUnG0097600	0.365	1.036	0.671	no
Treat-40d	RHSIM_RhsimUnG0024100	-0.624	-0.864	-0.240	no
Treat-40d	RHSIM_RhsimUnG0085300	-0.450	0.587	1.037	no
Treat-40d	RHSIM_RhsimUnG0070600	0.230	-1.104	-1.334	no
Treat-40d	RHSIM_Rhsim13G0082400	-0.894	-1.675	-0.781	no
Treat-40d	RHSIM_RhsimUnG0005900	3.423	1.923	-1.499	high
Treat-40d	RHSIM_RhsimUnG0066300	1.012	-0.433	-1.446	no
Treat-40d	RHSIM_RhsimUnG0075600	1.350	0.202	-1.148	high
Treat-40d	RHSIM_RhsimUnG0064700	1.030	1.694	0.663	no
Treat-40d	RHSIM_RhsimPtG0004000	5.445	3.368	-2.077	high
Treat-40d	RHSIM_Rhsim13G0062300	0.734	1.004	0.270	no
Treat-40d	RHSIM_Rhsim13G0073900	0.107	-0.807	-0.914	no
Treat-40d	RHSIM_RhsimUnG0055400	0.801	1.765	0.964	no
Treat-40d	RHSIM_Rhsim13G0046200	-0.466	-0.341	0.125	no
Treat-40d	RHSIM_RhsimUnG0046700	0.170	-0.932	-1.102	no
Treat-40d	RHSIM_RhsimUnG0054900	-0.720	0.719	1.439	no
Treat-40d	RHSIM_RhsimUnG0042300	-0.502	0.569	1.071	no
Treat-40d	RHSIM_Rhsim13G0173600	-1.195	-0.693	0.502	low
Treat-40d	RHSIM_Rhsim13G0227300	0.316	-0.010	-0.326	no
Treat-40d	RHSIM_Rhsim12G0181000	0.507	0.743	0.236	no
Treat-40d	RHSIM_Rhsim13G0093800	-1.961	-1.231	0.730	low
Treat-40d	RHSIM_Rhsim13G0030700	0.661	-0.022	-0.683	no

Group	Gene	PTR (z-score difference)	z(log2 LFQ)	z(log2 FPKM)	extreme-PTR
Treat-40d	RHSIM_Rhsim13G0076000	-0.215	1.401	1.616	no
Treat-40d	RHSIM_RhsimUnG0024500	-2.017	-0.852	1.165	low
Treat-40d	RHSIM_RhsimUnG0019100	1.198	-0.074	-1.272	high
Treat-40d	RHSIM_RhsimUnG0020700	-0.143	-1.050	-0.907	no
Treat-40d	RHSIM_Rhsim13G0064100	-0.154	-0.630	-0.476	no
Treat-40d	RHSIM_Rhsim13G0163100	-0.167	-0.801	-0.634	no
Treat-40d	RHSIM_Rhsim13G0066100	-0.059	1.102	1.161	no
Treat-40d	RHSIM_RhsimUnG0016900	0.519	1.307	0.787	no
Treat-40d	RHSIM_RhsimUnG0016100	-2.168	-1.734	0.434	low
Treat-40d	RHSIM_Rhsim13G0207900	-2.036	-0.632	1.404	low
Treat-40d	RHSIM_Rhsim12G0037800	0.192	0.281	0.089	no
Treat-40d	RHSIM_Rhsim13G0043900	0.166	0.079	-0.087	no
Treat-40d	RHSIM_Rhsim12G0063500	-0.658	-0.718	-0.061	no
Treat-40d	RHSIM_Rhsim12G0167800	-0.242	0.037	0.278	no
Treat-40d	RHSIM_Rhsim13G0040000	0.946	0.729	-0.217	no
Treat-40d	RHSIM_Rhsim13G0118300	-0.258	-0.240	0.018	no
Treat-40d	RHSIM_Rhsim13G0220600	0.797	0.966	0.169	no
Treat-40d	RHSIM_Rhsim13G0065700	0.090	0.488	0.398	no
Treat-40d	RHSIM_Rhsim13G0221500	-0.815	-0.957	-0.142	no
Treat-40d	RHSIM_Rhsim12G0039700	0.358	0.251	-0.108	no
Treat-40d	RHSIM_Rhsim13G0226500	1.960	1.383	-0.577	high
Treat-40d	RHSIM_Rhsim13G0016700	-0.048	-0.425	-0.377	no
Treat-40d	RHSIM_Rhsim13G0174500	0.609	1.956	1.347	no
Treat-40d	RHSIM_Rhsim12G0048800	-0.509	-0.804	-0.295	no
Treat-40d	RHSIM_Rhsim13G0185200	2.010	3.292	1.283	high
Treat-40d	RHSIM_Rhsim13G0234600	0.326	0.667	0.341	no
Treat-40d	RHSIM_Rhsim13G0038800	-0.548	0.768	1.316	no
Treat-40d	RHSIM_Rhsim13G0196800	-0.669	-0.953	-0.284	no
Treat-40d	RHSIM_Rhsim13G0053200	0.600	0.617	0.017	no
Treat-40d	RHSIM_Rhsim12G0172600	1.535	1.062	-0.473	high
Treat-40d	RHSIM_Rhsim13G0134800	-1.527	-0.323	1.204	low
Treat-40d	RHSIM_Rhsim13G0154000	1.022	2.249	1.227	no

Group	Gene	PTR (z-score difference)	z(log2 LFQ)	z(log2 FPKM)	extreme-PTR
Treat-40d	RHSIM_Rhsim13G0038600	-0.932	-1.047	-0.115	no
Treat-40d	RHSIM_Rhsim12G0139300	-0.564	-0.596	-0.032	no
Treat-40d	RHSIM_Rhsim13G0229900	3.177	2.573	-0.604	high
Treat-40d	RHSIM_Rhsim13G0078100	-1.687	-0.983	0.704	low
Treat-40d	RHSIM_Rhsim13G0113300	-0.272	-0.495	-0.223	no
Treat-40d	RHSIM_Rhsim13G0082700	-4.130	-0.393	3.737	low
Treat-40d	RHSIM_Rhsim13G0126700	-1.128	-1.464	-0.336	no
Treat-40d	RHSIM_Rhsim13G0159200	0.315	-2.077	-2.392	no
Treat-40d	RHSIM_Rhsim13G0201100	0.416	-0.103	-0.519	no
Treat-40d	RHSIM_Rhsim13G0022100	1.212	1.417	0.205	high
Treat-40d	RHSIM_Rhsim13G0199200	0.622	-0.141	-0.764	no
Treat-40d	RHSIM_Rhsim13G0177900	0.057	-0.377	-0.433	no
Treat-40d	RHSIM_Rhsim12G0123300	0.135	-0.663	-0.797	no
Treat-40d	RHSIM_Rhsim13G0093700	-0.120	0.631	0.751	no
Treat-40d	RHSIM_Rhsim12G0178200	0.586	-0.647	-1.233	no
Treat-40d	RHSIM_Rhsim13G0036600	-1.441	-0.807	0.634	low
Treat-40d	RHSIM_Rhsim12G0139900	-1.105	-0.916	0.189	no
Treat-40d	RHSIM_Rhsim13G0006800	0.336	1.464	1.128	no
Treat-40d	RHSIM_Rhsim13G0079200	-0.571	-0.813	-0.242	no
Treat-40d	RHSIM_Rhsim12G0079000	-1.194	-0.665	0.529	low
Treat-40d	RHSIM_Rhsim13G0092400	-1.429	-1.613	-0.183	low
Treat-40d	RHSIM_Rhsim13G0220800	-1.368	-0.490	0.878	low
Treat-40d	RHSIM_Rhsim13G0011300	-0.067	0.449	0.517	no
Treat-40d	RHSIM_Rhsim13G0212000	2.594	0.520	-2.074	high
Treat-40d	RHSIM_Rhsim12G0204800	-0.067	-0.113	-0.047	no
Treat-40d	RHSIM_Rhsim13G0041900	-0.447	-1.047	-0.600	no
Treat-40d	RHSIM_Rhsim13G0232100	0.517	1.102	0.586	no
Treat-40d	RHSIM_Rhsim13G0144400	1.995	0.951	-1.044	high
Treat-40d	RHSIM_Rhsim13G0054900	1.592	-0.477	-2.068	high
Treat-40d	RHSIM_Rhsim13G0124200	-1.503	0.112	1.615	low
Treat-40d	RHSIM_Rhsim13G0021700	-1.603	-1.440	0.163	low
Treat-40d	RHSIM_Rhsim12G0107800	1.184	-0.503	-1.687	high

Group	Gene	PTR (z-score difference)	z(log2 LFQ)	z(log2 FPKM)	extreme-PTR
Treat-40d	RHSIM_Rhsim13G0057600	0.814	-0.093	-0.907	no
Treat-40d	RHSIM_Rhsim13G0191700	1.862	0.047	-1.814	high
Treat-40d	RHSIM_Rhsim13G0114000	-0.390	-0.686	-0.296	no
Treat-40d	RHSIM_Rhsim13G0027900	0.256	-1.520	-1.776	no
Treat-40d	RHSIM_Rhsim13G0029600	-2.445	-1.178	1.267	low
Treat-40d	RHSIM_Rhsim13G0051300	-0.143	1.492	1.635	no
Treat-40d	RHSIM_Rhsim13G0219700	1.205	1.365	0.159	high
Treat-40d	RHSIM_Rhsim13G0164200	0.698	-1.350	-2.048	no
Treat-40d	RHSIM_Rhsim13G0086200	-0.771	0.213	0.984	no
Treat-40d	RHSIM_Rhsim12G0005600	-0.076	0.700	0.775	no
Treat-40d	RHSIM_Rhsim13G0014300	-1.717	-1.598	0.119	low
Treat-40d	RHSIM_Rhsim12G0203000	-1.654	-0.687	0.967	low
Treat-40d	RHSIM_Rhsim13G0001400	2.109	0.598	-1.511	high
Treat-40d	RHSIM_Rhsim13G0111900	-2.007	-0.272	1.735	low
Treat-40d	RHSIM_Rhsim12G0174000	-0.455	1.137	1.592	no
Treat-40d	RHSIM_Rhsim13G0161700	0.464	0.323	-0.140	no
Treat-40d	RHSIM_Rhsim13G0036800	1.072	1.214	0.142	no
Treat-40d	RHSIM_Rhsim13G0197800	2.056	0.967	-1.088	high
Treat-40d	RHSIM_Rhsim13G0219900	-1.755	-1.673	0.082	low
Treat-40d	RHSIM_Rhsim13G0209700	-0.032	-1.501	-1.469	no
Treat-40d	RHSIM_Rhsim13G0107300	0.907	0.648	-0.259	no
Treat-40d	RHSIM_Rhsim13G0075000	-0.222	-0.747	-0.526	no
Treat-40d	RHSIM_Rhsim13G0103500	0.263	-0.147	-0.409	no
Treat-40d	RHSIM_Rhsim13G0101100	0.040	1.070	1.029	no
Treat-40d	RHSIM_Rhsim13G0015700	0.833	0.441	-0.392	no
Treat-40d	RHSIM_Rhsim13G0168400	2.021	1.661	-0.360	high
Treat-40d	RHSIM_Rhsim12G0116800	1.777	1.022	-0.755	high
Treat-40d	RHSIM_Rhsim12G0078100	-0.476	-0.233	0.243	no
Treat-40d	RHSIM_Rhsim13G0181700	1.644	-0.049	-1.693	high
Treat-40d	RHSIM_Rhsim13G0077500	0.780	-0.945	-1.725	no
Treat-40d	RHSIM_Rhsim13G0116300	1.108	1.570	0.462	no
Treat-40d	RHSIM_Rhsim13G0102200	1.395	-0.590	-1.985	high

Group	Gene	PTR (z-score difference)	z(log2 LFQ)	z(log2 FPKM)	extreme-PTR
Treat-40d	RHSIM_Rhsim12G0172200	1.238	1.297	0.059	high
Treat-40d	RHSIM_Rhsim12G0103900	-0.577	-0.422	0.154	no
Treat-40d	RHSIM_Rhsim13G0069000	-1.294	0.074	1.368	low
Treat-40d	RHSIM_Rhsim13G0231100	0.239	-0.580	-0.818	no
Treat-40d	RHSIM_Rhsim12G0083200	1.416	0.581	-0.835	high
Treat-40d	RHSIM_Rhsim13G0217100	0.136	0.561	0.425	no
Treat-40d	RHSIM_Rhsim13G0138400	0.245	-0.552	-0.798	no
Treat-40d	RHSIM_Rhsim13G0042600	0.331	0.111	-0.220	no
Treat-40d	RHSIM_Rhsim12G0100900	-0.020	0.828	0.848	no
Treat-40d	RHSIM_Rhsim13G0025700	1.153	1.307	0.154	high
Treat-40d	RHSIM_Rhsim13G0038000	2.186	1.678	-0.509	high
Treat-40d	RHSIM_Rhsim13G0079600	-1.878	-0.320	1.558	low
Treat-40d	RHSIM_Rhsim12G0083000	-0.048	0.298	0.346	no
Treat-40d	RHSIM_Rhsim13G0233800	-1.417	-0.443	0.974	low
Treat-40d	RHSIM_Rhsim13G0073800	-1.238	-0.713	0.524	low
Treat-40d	RHSIM_Rhsim13G0055800	0.202	-0.792	-0.994	no
Treat-40d	RHSIM_Rhsim13G0031900	0.880	0.471	-0.409	no
Treat-40d	RHSIM_Rhsim13G0027500	0.134	0.153	0.019	no
Treat-40d	RHSIM_Rhsim12G0028500	-0.084	0.535	0.619	no
Treat-40d	RHSIM_Rhsim13G0095000	-0.549	-1.205	-0.655	no
Treat-40d	RHSIM_Rhsim13G0035400	-0.485	-0.316	0.169	no
Treat-40d	RHSIM_Rhsim13G0167700	0.704	1.373	0.669	no
Treat-40d	RHSIM_Rhsim13G0129900	0.721	-0.087	-0.808	no
Treat-40d	RHSIM_Rhsim13G0223700	0.163	-0.602	-0.765	no
Treat-40d	RHSIM_Rhsim13G0157400	1.153	0.958	-0.195	high
Treat-40d	RHSIM_Rhsim13G0177000	0.514	0.394	-0.120	no
Treat-40d	RHSIM_Rhsim13G0030400	-0.676	-0.988	-0.312	no
Treat-40d	RHSIM_Rhsim12G0123700	-0.062	-0.233	-0.171	no
Treat-40d	RHSIM_Rhsim12G0080000	-1.365	-0.377	0.988	low
Treat-40d	RHSIM_Rhsim13G0208300	-0.448	0.185	0.632	no
Treat-40d	RHSIM_Rhsim13G0070100	-0.824	0.249	1.073	no
Treat-40d	RHSIM_Rhsim13G0003700	0.037	-0.610	-0.646	no

Group	Gene	PTR (z-score difference)	z(log2 LFQ)	z(log2 FPKM)	extreme-PTR
Treat-40d	RHSIM_Rhsim11G0144200	-0.215	-0.694	-0.479	no
Treat-40d	RHSIM_Rhsim13G0179800	-0.509	-2.358	-1.849	no
Treat-40d	RHSIM_Rhsim13G0114600	-0.133	0.149	0.283	no
Treat-40d	RHSIM_Rhsim13G0028300	1.466	0.885	-0.581	high
Treat-40d	RHSIM_Rhsim13G0122000	1.063	-0.825	-1.887	no
Treat-40d	RHSIM_Rhsim13G0127800	0.906	-0.447	-1.352	no
Treat-40d	RHSIM_Rhsim13G0092300	1.356	1.694	0.338	high
Treat-40d	RHSIM_Rhsim13G0026300	1.263	1.496	0.232	high
Treat-40d	RHSIM_Rhsim13G0233500	0.679	-0.924	-1.604	no
Treat-40d	RHSIM_Rhsim13G0091400	0.186	-0.309	-0.495	no
Treat-40d	RHSIM_Rhsim13G0120600	0.352	1.057	0.705	no
Treat-40d	RHSIM_Rhsim11G0149700	0.053	-0.244	-0.297	no
Treat-40d	RHSIM_Rhsim13G0220700	0.608	-0.685	-1.292	no
Treat-40d	RHSIM_Rhsim13G0067500	0.490	0.139	-0.351	no
Treat-40d	RHSIM_Rhsim13G0146800	0.090	-0.547	-0.637	no
Treat-40d	RHSIM_Rhsim13G0138900	-1.110	-0.741	0.369	no
Treat-40d	RHSIM_Rhsim13G0156800	0.902	-1.051	-1.953	no
Treat-40d	RHSIM_Rhsim13G0076600	-1.316	-1.358	-0.042	low
Treat-40d	RHSIM_Rhsim13G0075300	-0.467	-1.285	-0.818	no
Treat-40d	RHSIM_Rhsim13G0036200	-1.781	-0.375	1.406	low
Treat-40d	RHSIM_Rhsim11G0025400	-0.566	-1.425	-0.858	no
Treat-40d	RHSIM_Rhsim13G0173800	-1.513	0.000	1.513	low
Treat-40d	RHSIM_Rhsim13G0171400	1.576	-0.440	-2.016	high
Treat-40d	RHSIM_Rhsim11G0195300	1.585	-0.081	-1.667	high
Treat-40d	RHSIM_Rhsim13G0129200	1.094	1.007	-0.087	no
Treat-40d	RHSIM_Rhsim13G0010200	-1.057	0.250	1.307	no
Treat-40d	RHSIM_Rhsim13G0231600	-0.639	-0.204	0.435	no
Treat-40d	RHSIM_Rhsim11G0194500	0.764	-1.017	-1.781	no
Treat-40d	RHSIM_Rhsim13G0140100	0.217	0.209	-0.007	no
Treat-40d	RHSIM_Rhsim13G0142000	0.536	0.108	-0.428	no
Treat-40d	RHSIM_Rhsim12G0016400	0.939	-1.180	-2.119	no
Treat-40d	RHSIM_Rhsim13G0056200	1.602	-0.019	-1.621	high

Group	Gene	PTR (z-score difference)	z(log2 LFQ)	z(log2 FPKM)	extreme-PTR
Treat-40d	RHSIM_Rhsim11G0092700	-2.658	-1.476	1.182	low
Treat-40d	RHSIM_Rhsim12G0018000	0.759	-0.050	-0.809	no
Treat-40d	RHSIM_Rhsim12G0121700	0.184	-0.470	-0.654	no
Treat-40d	RHSIM_Rhsim12G0020900	1.943	0.719	-1.224	high
Treat-40d	RHSIM_Rhsim11G0117400	1.272	0.916	-0.356	high
Treat-40d	RHSIM_Rhsim12G0014400	1.684	0.459	-1.225	high
Treat-40d	RHSIM_Rhsim11G0029100	1.216	0.537	-0.679	high
Treat-40d	RHSIM_Rhsim12G0021600	-0.691	-2.050	-1.358	no
Treat-40d	RHSIM_Rhsim11G0036500	-0.167	-0.348	-0.181	no
Treat-40d	RHSIM_Rhsim12G0088300	-0.575	-0.024	0.551	no
Treat-40d	RHSIM_Rhsim13G0217400	-0.191	-0.668	-0.478	no
Treat-40d	RHSIM_Rhsim11G0008400	0.086	-0.014	-0.101	no
Treat-40d	RHSIM_Rhsim11G0097700	-0.293	-1.457	-1.164	no
Treat-40d	RHSIM_Rhsim13G0124600	-1.242	-1.767	-0.525	low
Treat-40d	RHSIM_Rhsim12G0178500	0.510	0.207	-0.303	no
Treat-40d	RHSIM_Rhsim12G0026500	1.092	-0.842	-1.934	no
Treat-40d	RHSIM_Rhsim13G0008300	-1.354	0.161	1.515	low
Treat-40d	RHSIM_Rhsim12G0157000	-0.702	0.748	1.449	no
Treat-40d	RHSIM_Rhsim11G0148300	-3.902	0.008	3.910	low
Treat-40d	RHSIM_Rhsim12G0110500	-0.322	-0.897	-0.575	no
Treat-40d	RHSIM_Rhsim11G0196100	0.855	-1.127	-1.982	no
Treat-40d	RHSIM_Rhsim12G0078800	1.047	1.139	0.092	no
Treat-40d	RHSIM_Rhsim12G0079500	2.497	1.440	-1.057	high
Treat-40d	RHSIM_Rhsim12G0008200	2.104	-0.113	-2.217	high
Treat-40d	RHSIM_Rhsim13G0206000	-0.549	0.261	0.810	no
Treat-40d	RHSIM_Rhsim11G0077200	2.097	2.904	0.807	high
Treat-40d	RHSIM_Rhsim12G0189500	-0.600	-0.760	-0.160	no
Treat-40d	RHSIM_Rhsim12G0131300	1.110	1.151	0.041	high
Treat-40d	RHSIM_Rhsim12G0190500	0.130	0.512	0.383	no
Treat-40d	RHSIM_Rhsim13G0036100	0.788	0.242	-0.547	no
Treat-40d	RHSIM_Rhsim12G0080200	-0.898	-0.959	-0.062	no
Treat-40d	RHSIM_Rhsim12G0057400	0.767	1.029	0.262	no

Group	Gene	PTR (z-score difference)	z(log2 LFQ)	z(log2 FPKM)	extreme-PTR
Treat-40d	RHSIM_Rhsim12G0137900	0.572	-0.075	-0.647	no
Treat-40d	RHSIM_Rhsim11G0190900	-0.765	1.154	1.919	no
Treat-40d	RHSIM_Rhsim12G0176000	-0.601	-1.776	-1.174	no
Treat-40d	RHSIM_Rhsim12G0211600	-1.033	-0.954	0.079	no
Treat-40d	RHSIM_Rhsim11G0196600	-1.439	-1.499	-0.059	low
Treat-40d	RHSIM_Rhsim13G0126400	-0.684	-0.250	0.434	no
Treat-40d	RHSIM_Rhsim12G0014900	1.475	0.499	-0.976	high
Treat-40d	RHSIM_Rhsim12G0197900	0.976	1.598	0.622	no
Treat-40d	RHSIM_Rhsim11G0059000	-0.631	0.597	1.229	no
Treat-40d	RHSIM_Rhsim11G0096600	-0.765	-0.304	0.461	no
Treat-40d	RHSIM_Rhsim12G0181300	1.118	0.280	-0.837	high
Treat-40d	RHSIM_Rhsim12G0041000	0.053	-1.231	-1.284	no
Treat-40d	RHSIM_Rhsim12G0017100	0.341	1.697	1.356	no
Treat-40d	RHSIM_Rhsim12G0013600	-0.219	0.514	0.734	no
Treat-40d	RHSIM_Rhsim12G0023200	-0.502	-0.954	-0.452	no
Treat-40d	RHSIM_Rhsim13G0010500	-0.944	-0.656	0.288	no
Treat-40d	RHSIM_Rhsim12G0122100	-0.338	-0.687	-0.349	no
Treat-40d	RHSIM_Rhsim12G0197700	-0.714	-0.631	0.082	no
Treat-40d	RHSIM_Rhsim12G0207900	-1.055	-0.336	0.719	no
Treat-40d	RHSIM_Rhsim12G0057700	-2.318	-0.221	2.097	low
Treat-40d	RHSIM_Rhsim12G0102000	0.245	0.659	0.414	no
Treat-40d	RHSIM_Rhsim12G0198000	1.908	-0.393	-2.301	high
Treat-40d	RHSIM_Rhsim12G0121900	-0.499	-0.415	0.084	no
Treat-40d	RHSIM_Rhsim12G0187600	0.152	-0.459	-0.612	no
Treat-40d	RHSIM_Rhsim12G0211700	-0.111	0.297	0.408	no
Treat-40d	RHSIM_Rhsim12G0116600	1.096	1.764	0.667	no
Treat-40d	RHSIM_Rhsim11G0192700	-0.245	0.605	0.851	no
Treat-40d	RHSIM_Rhsim12G0062100	-1.431	-1.102	0.329	low
Treat-40d	RHSIM_Rhsim12G0165600	-0.656	-0.824	-0.168	no
Treat-40d	RHSIM_Rhsim12G0085300	-0.380	1.003	1.383	no
Treat-40d	RHSIM_Rhsim12G0202100	2.227	2.688	0.461	high
Treat-40d	RHSIM_Rhsim12G0181600	-0.120	-0.432	-0.312	no

Group	Gene	PTR (z-score difference)	z(log2 LFQ)	z(log2 FPKM)	extreme-PTR
Treat-40d	RHSIM_Rhsim12G0023100	-1.026	-1.670	-0.644	no
Treat-40d	RHSIM_Rhsim12G0187800	-1.777	-0.363	1.414	low
Treat-40d	RHSIM_Rhsim12G0002600	-0.467	-0.801	-0.334	no
Treat-40d	RHSIM_Rhsim12G0214000	0.701	-0.547	-1.248	no
Treat-40d	RHSIM_Rhsim12G0022500	1.046	-1.361	-2.407	no
Treat-40d	RHSIM_Rhsim12G0194700	-0.251	-0.038	0.214	no
Treat-40d	RHSIM_Rhsim12G0014100	0.302	0.926	0.624	no
Treat-40d	RHSIM_Rhsim12G0164800	-2.967	-0.254	2.714	low
Treat-40d	RHSIM_Rhsim12G0211300	-0.200	-1.127	-0.927	no
Treat-40d	RHSIM_Rhsim12G0176100	-0.418	-0.111	0.307	no
Treat-40d	RHSIM_Rhsim12G0086500	0.022	-0.652	-0.675	no
Treat-40d	RHSIM_Rhsim11G0097500	-0.172	0.624	0.795	no
Treat-40d	RHSIM_Rhsim12G0008600	2.252	0.470	-1.782	high
Treat-40d	RHSIM_Rhsim12G0104500	0.881	-0.140	-1.021	no
Treat-40d	RHSIM_Rhsim12G0207200	-0.024	-0.681	-0.657	no
Treat-40d	RHSIM_Rhsim12G0145700	1.379	1.856	0.476	high
Treat-40d	RHSIM_Rhsim12G0046200	3.549	1.724	-1.825	high
Treat-40d	RHSIM_Rhsim12G0047900	0.168	0.008	-0.160	no
Treat-40d	RHSIM_Rhsim12G0064600	0.224	0.173	-0.051	no
Treat-40d	RHSIM_Rhsim12G0108800	-0.675	-1.357	-0.681	no
Treat-40d	RHSIM_Rhsim12G0132500	-0.041	0.216	0.257	no
Treat-40d	RHSIM_Rhsim12G0189400	0.556	1.238	0.682	no
Treat-40d	RHSIM_Rhsim12G0004200	1.491	1.389	-0.102	high
Treat-40d	RHSIM_Rhsim12G0037200	0.441	-1.117	-1.558	no
Treat-40d	RHSIM_Rhsim12G0208100	-0.039	-0.685	-0.646	no
Treat-40d	RHSIM_Rhsim12G0060900	-0.922	-0.929	-0.008	no
Treat-40d	RHSIM_Rhsim12G0158400	1.596	-0.520	-2.116	high
Treat-40d	RHSIM_Rhsim12G0146600	-0.507	-0.540	-0.032	no
Treat-40d	RHSIM_Rhsim11G0164900	1.203	-0.382	-1.586	high
Treat-40d	RHSIM_Rhsim12G0005300	1.171	-0.595	-1.766	high
Treat-40d	RHSIM_Rhsim12G0167600	-3.039	-2.008	1.032	low
Treat-40d	RHSIM_Rhsim12G0035600	-0.615	-1.441	-0.826	no

Group	Gene	PTR (z-score difference)	z(log2 LFQ)	z(log2 FPKM)	extreme-PTR
Treat-40d	RHSIM_Rhsim12G0182400	-1.081	-0.917	0.164	no
Treat-40d	RHSIM_Rhsim10G0099100	0.517	1.238	0.721	no
Treat-40d	RHSIM_Rhsim12G0170000	-1.283	-0.298	0.986	low
Treat-40d	RHSIM_Rhsim12G0210300	-2.126	-0.325	1.801	low
Treat-40d	RHSIM_Rhsim12G0006100	-1.243	-0.172	1.071	low
Treat-40d	RHSIM_Rhsim12G0047400	-2.597	-1.907	0.690	low
Treat-40d	RHSIM_Rhsim12G0043400	-1.626	-0.670	0.957	low
Treat-40d	RHSIM_Rhsim12G0060700	-1.445	-1.096	0.349	low
Treat-40d	RHSIM_Rhsim10G0148000	1.016	0.736	-0.280	no
Treat-40d	RHSIM_Rhsim12G0138900	-0.652	-1.013	-0.361	no
Treat-40d	RHSIM_Rhsim12G0028300	-1.299	-1.396	-0.097	low
Treat-40d	RHSIM_Rhsim12G0100800	0.296	1.951	1.655	no
Treat-40d	RHSIM_Rhsim12G0061700	0.654	1.649	0.995	no
Treat-40d	RHSIM_Rhsim10G0064500	-0.608	0.189	0.797	no
Treat-40d	RHSIM_Rhsim12G0176300	1.058	1.474	0.415	no
Treat-40d	RHSIM_Rhsim10G0170100	-0.565	-1.107	-0.542	no
Treat-40d	RHSIM_Rhsim12G0189900	0.436	1.322	0.886	no
Treat-40d	RHSIM_Rhsim12G0121500	-0.454	-0.579	-0.125	no
Treat-40d	RHSIM_Rhsim12G0156100	0.345	1.070	0.725	no
Treat-40d	RHSIM_Rhsim10G0144700	-0.338	-0.425	-0.086	no
Treat-40d	RHSIM_Rhsim12G0187400	-4.062	-1.394	2.668	low
Treat-40d	RHSIM_Rhsim10G0012700	0.347	-0.077	-0.424	no
Treat-40d	RHSIM_Rhsim12G0072400	-0.447	0.006	0.453	no
Treat-40d	RHSIM_Rhsim12G0044800	3.394	2.540	-0.853	high
Treat-40d	RHSIM_Rhsim12G0172500	-0.563	-0.522	0.041	no
Treat-40d	RHSIM_Rhsim12G0046500	1.526	2.760	1.234	high
Treat-40d	RHSIM_Rhsim10G0043300	1.135	-0.597	-1.732	high
Treat-40d	RHSIM_Rhsim12G0053800	2.122	1.634	-0.488	high
Treat-40d	RHSIM_Rhsim12G0036000	-0.093	-1.272	-1.179	no
Treat-40d	RHSIM_Rhsim12G0129400	1.764	0.890	-0.873	high
Treat-40d	RHSIM_Rhsim12G0090600	0.117	0.403	0.285	no
Treat-40d	RHSIM_Rhsim12G0006300	-1.256	-0.988	0.267	low

Group	Gene	PTR (z-score difference)	z(log2 LFQ)	z(log2 FPKM)	extreme-PTR
Treat-40d	RHSIM_Rhsim12G0063200	0.179	-0.492	-0.670	no
Treat-40d	RHSIM_Rhsim12G0136000	-0.391	-1.411	-1.020	no
Treat-40d	RHSIM_Rhsim12G0183100	0.259	-0.078	-0.337	no
Treat-40d	RHSIM_Rhsim12G0124300	1.180	0.822	-0.358	high
Treat-40d	RHSIM_Rhsim12G0038400	1.144	0.908	-0.236	high
Treat-40d	RHSIM_Rhsim12G0074900	0.785	0.769	-0.015	no
Treat-40d	RHSIM_Rhsim12G0180300	0.870	1.500	0.630	no
Treat-40d	RHSIM_Rhsim12G0063300	-1.766	-1.171	0.595	low
Treat-40d	RHSIM_Rhsim11G0038100	-0.672	0.329	1.000	no
Treat-40d	RHSIM_Rhsim12G0002400	0.869	1.256	0.387	no
Treat-40d	RHSIM_Rhsim12G0062200	1.600	0.862	-0.738	high
Treat-40d	RHSIM_Rhsim12G0129000	0.182	0.450	0.268	no
Treat-40d	RHSIM_Rhsim12G0214600	0.446	-0.010	-0.456	no
Treat-40d	RHSIM_Rhsim11G0137400	-0.323	-0.053	0.270	no
Treat-40d	RHSIM_Rhsim12G0162600	-0.637	-1.126	-0.490	no
Treat-40d	RHSIM_Rhsim12G0037500	1.181	-1.199	-2.380	high
Treat-40d	RHSIM_Rhsim12G0023300	0.169	1.756	1.587	no
Treat-40d	RHSIM_Rhsim11G0021900	0.740	0.031	-0.709	no
Treat-40d	RHSIM_Rhsim12G0208800	-1.392	-1.091	0.301	low
Treat-40d	RHSIM_Rhsim11G0168800	-0.100	-0.947	-0.847	no
Treat-40d	RHSIM_Rhsim10G0180100	0.167	1.104	0.937	no
Treat-40d	RHSIM_Rhsim11G0186600	0.056	-0.646	-0.702	no
Treat-40d	RHSIM_Rhsim12G0200100	0.516	-0.772	-1.288	no
Treat-40d	RHSIM_Rhsim11G0085000	-1.499	-1.611	-0.112	low
Treat-40d	RHSIM_Rhsim12G0062000	0.088	0.343	0.255	no
Treat-40d	RHSIM_Rhsim12G0063700	0.851	0.429	-0.421	no
Treat-40d	RHSIM_Rhsim12G0104400	2.904	1.696	-1.208	high
Treat-40d	RHSIM_Rhsim12G0096800	-2.780	-1.831	0.949	low
Treat-40d	RHSIM_Rhsim11G0040500	0.117	-0.382	-0.499	no
Treat-40d	RHSIM_Rhsim12G0080700	0.088	-0.334	-0.422	no
Treat-40d	RHSIM_Rhsim11G0111300	-0.377	1.150	1.527	no
Treat-40d	RHSIM_Rhsim11G0127400	0.069	-0.348	-0.418	no

Group	Gene	PTR (z-score difference)	z(log2 LFQ)	z(log2 FPKM)	extreme-PTR
Treat-40d	RHSIM_Rhsim11G0115300	0.947	0.449	-0.498	no
Treat-40d	RHSIM_Rhsim12G0148700	1.605	1.074	-0.531	high
Treat-40d	RHSIM_Rhsim12G0081400	0.355	0.065	-0.289	no
Treat-40d	RHSIM_Rhsim11G0186800	-0.728	-0.577	0.150	no
Treat-40d	RHSIM_Rhsim11G0024700	-0.111	-0.547	-0.437	no
Treat-40d	RHSIM_Rhsim12G0136400	0.513	1.095	0.582	no
Treat-40d	RHSIM_Rhsim12G0174100	0.181	0.830	0.649	no
Treat-40d	RHSIM_Rhsim11G0012900	0.970	0.657	-0.313	no
Treat-40d	RHSIM_Rhsim10G0171800	-0.373	-0.497	-0.124	no
Treat-40d	RHSIM_Rhsim10G0206700	0.323	-0.599	-0.922	no
Treat-40d	RHSIM_Rhsim11G0169800	-0.771	0.027	0.798	no
Treat-40d	RHSIM_Rhsim12G0201100	-0.296	-0.758	-0.463	no
Treat-40d	RHSIM_Rhsim10G0010400	-0.859	-0.637	0.222	no
Treat-40d	RHSIM_Rhsim11G0098800	-1.643	0.849	2.492	low
Treat-40d	RHSIM_Rhsim10G0092900	-0.095	-0.588	-0.493	no
Treat-40d	RHSIM_Rhsim10G0202800	0.348	1.271	0.923	no
Treat-40d	RHSIM_Rhsim11G0164100	-1.181	-1.713	-0.532	low
Treat-40d	RHSIM_Rhsim12G0136300	-2.665	-1.944	0.720	low
Treat-40d	RHSIM_Rhsim11G0088800	1.007	0.415	-0.592	no
Treat-40d	RHSIM_Rhsim11G0150000	-1.645	-1.180	0.466	low
Treat-40d	RHSIM_Rhsim12G0187900	-0.425	-0.540	-0.115	no
Treat-40d	RHSIM_Rhsim11G0098700	1.819	2.842	1.022	high
Treat-40d	RHSIM_Rhsim11G0032600	0.280	0.590	0.310	no
Treat-40d	RHSIM_Rhsim12G0200200	-1.995	-2.031	-0.036	low
Treat-40d	RHSIM_Rhsim11G0134300	-0.754	-0.443	0.310	no
Treat-40d	RHSIM_Rhsim12G0143000	-0.709	-1.159	-0.450	no
Treat-40d	RHSIM_Rhsim11G0122100	0.874	-0.579	-1.452	no
Treat-40d	RHSIM_Rhsim11G0024800	-0.083	0.490	0.573	no
Treat-40d	RHSIM_Rhsim11G0173000	-0.746	1.108	1.853	no
Treat-40d	RHSIM_Rhsim11G0182500	-0.647	-0.154	0.494	no
Treat-40d	RHSIM_Rhsim10G0059600	1.005	0.352	-0.653	no
Treat-40d	RHSIM_Rhsim11G0151000	-4.112	-1.849	2.263	low

Group	Gene	PTR (z-score difference)	z(log2 LFQ)	z(log2 FPKM)	extreme-PTR
Treat-40d	RHSIM_Rhsim11G0024900	-0.360	0.230	0.590	no
Treat-40d	RHSIM_Rhsim10G0128400	0.770	0.738	-0.032	no
Treat-40d	RHSIM_Rhsim10G0055700	1.106	-0.181	-1.287	no
Treat-40d	RHSIM_Rhsim11G0187800	-1.022	-0.997	0.026	no
Treat-40d	RHSIM_Rhsim11G0034100	0.571	2.304	1.733	no
Treat-40d	RHSIM_Rhsim11G0141700	1.996	2.018	0.022	high
Treat-40d	RHSIM_Rhsim11G0159600	-0.360	0.627	0.987	no
Treat-40d	RHSIM_Rhsim11G0066600	-0.537	0.729	1.266	no
Treat-40d	RHSIM_Rhsim11G0054300	0.682	0.448	-0.234	no
Treat-40d	RHSIM_Rhsim11G0069700	0.023	-0.415	-0.438	no
Treat-40d	RHSIM_Rhsim11G0070000	0.164	-0.055	-0.220	no
Treat-40d	RHSIM_Rhsim10G0164900	-1.095	-0.040	1.056	no
Treat-40d	RHSIM_Rhsim11G0074900	-1.443	-0.368	1.075	low
Treat-40d	RHSIM_Rhsim11G0179100	-1.957	-0.986	0.971	low
Treat-40d	RHSIM_Rhsim10G0131900	-0.645	-0.396	0.249	no
Treat-40d	RHSIM_Rhsim11G0027400	-0.064	-0.248	-0.184	no
Treat-40d	RHSIM_Rhsim11G0063300	0.087	-1.357	-1.445	no
Treat-40d	RHSIM_Rhsim11G0037700	-0.193	-0.819	-0.627	no
Treat-40d	RHSIM_Rhsim10G0063500	1.603	1.354	-0.249	high
Treat-40d	RHSIM_Rhsim11G0187700	1.275	1.912	0.636	high
Treat-40d	RHSIM_Rhsim11G0069600	0.190	-0.219	-0.408	no
Treat-40d	RHSIM_Rhsim10G0146500	0.520	-0.860	-1.381	no
Treat-40d	RHSIM_Rhsim11G0056500	0.244	0.005	-0.239	no
Treat-40d	RHSIM_Rhsim11G0008200	0.987	0.132	-0.854	no
Treat-40d	RHSIM_Rhsim11G0159400	2.286	0.170	-2.116	high
Treat-40d	RHSIM_Rhsim11G0044300	0.370	0.163	-0.207	no
Treat-40d	RHSIM_Rhsim10G0065100	0.591	0.464	-0.127	no
Treat-40d	RHSIM_Rhsim11G0178600	-0.726	0.004	0.730	no
Treat-40d	RHSIM_Rhsim11G0159100	-0.767	-1.140	-0.373	no
Treat-40d	RHSIM_Rhsim10G0017800	-1.058	-0.786	0.272	no
Treat-40d	RHSIM_Rhsim11G0137700	-0.944	-0.291	0.653	no
Treat-40d	RHSIM_Rhsim10G0072400	-1.465	-0.162	1.304	low

Group	Gene	PTR (z-score difference)	z(log2 LFQ)	z(log2 FPKM)	extreme-PTR
Treat-40d	RHSIM_Rhsim11G0046100	0.807	0.653	-0.155	no
Treat-40d	RHSIM_Rhsim11G0001100	-1.890	-1.399	0.491	low
Treat-40d	RHSIM_Rhsim11G0187200	-0.746	-0.087	0.659	no
Treat-40d	RHSIM_Rhsim11G0099300	-0.054	-0.513	-0.459	no
Treat-40d	RHSIM_Rhsim11G0187000	-0.113	1.656	1.769	no
Treat-40d	RHSIM_Rhsim11G0165500	0.006	-1.222	-1.228	no
Treat-40d	RHSIM_Rhsim11G0092100	0.081	0.058	-0.023	no
Treat-40d	RHSIM_Rhsim09G0008800	0.205	0.389	0.183	no
Treat-40d	RHSIM_Rhsim11G0066000	0.197	-0.602	-0.799	no
Treat-40d	RHSIM_Rhsim11G0183000	-0.921	-0.437	0.484	no
Treat-40d	RHSIM_Rhsim09G0103900	0.068	0.090	0.022	no
Treat-40d	RHSIM_Rhsim11G0075300	-1.267	0.456	1.723	low
Treat-40d	RHSIM_Rhsim11G0049500	-0.911	-1.442	-0.530	no
Treat-40d	RHSIM_Rhsim11G0193400	0.611	0.355	-0.256	no
Treat-40d	RHSIM_Rhsim11G0019200	-1.201	-0.908	0.293	low
Treat-40d	RHSIM_Rhsim11G0121200	0.162	-0.522	-0.684	no
Treat-40d	RHSIM_Rhsim11G0160800	0.825	2.095	1.270	no
Treat-40d	RHSIM_Rhsim11G0001400	0.480	-0.661	-1.140	no
Treat-40d	RHSIM_Rhsim11G0021000	0.651	-0.071	-0.722	no
Treat-40d	RHSIM_Rhsim11G0160000	-0.693	-0.008	0.685	no
Treat-40d	RHSIM_Rhsim11G0160500	0.388	-0.495	-0.883	no
Treat-40d	RHSIM_Rhsim11G0131900	-1.180	-1.219	-0.040	low
Treat-40d	RHSIM_Rhsim11G0038900	1.828	-0.261	-2.089	high
Treat-40d	RHSIM_Rhsim11G0052300	-0.979	-1.393	-0.415	no
Treat-40d	RHSIM_Rhsim11G0088200	-2.225	-0.225	2.000	low
Treat-40d	RHSIM_Rhsim11G0138000	-1.614	-1.056	0.558	low
Treat-40d	RHSIM_Rhsim11G0058000	1.494	0.308	-1.186	high
Treat-40d	RHSIM_Rhsim11G0148400	0.328	1.768	1.441	no
Treat-40d	RHSIM_Rhsim11G0003500	-1.169	-0.498	0.672	low
Treat-40d	RHSIM_Rhsim10G0186400	0.288	1.585	1.297	no
Treat-40d	RHSIM_Rhsim11G0142200	-1.346	-0.629	0.718	low
Treat-40d	RHSIM_Rhsim11G0129100	-0.542	-0.896	-0.354	no

Group	Gene	PTR (z-score difference)	z(log2 LFQ)	z(log2 FPKM)	extreme-PTR
Treat-40d	RHSIM_Rhsim10G0108500	-0.602	-0.600	0.001	no
Treat-40d	RHSIM_Rhsim09G0038500	-0.910	-0.799	0.112	no
Treat-40d	RHSIM_Rhsim10G0009700	-0.840	0.343	1.183	no
Treat-40d	RHSIM_Rhsim09G0212500	0.161	0.723	0.563	no
Treat-40d	RHSIM_Rhsim11G0192800	-0.888	-0.683	0.205	no
Treat-40d	RHSIM_Rhsim11G0055800	0.689	-0.266	-0.955	no
Treat-40d	RHSIM_Rhsim10G0162600	-0.362	0.010	0.372	no
Treat-40d	RHSIM_Rhsim11G0148600	-1.557	0.994	2.551	low
Treat-40d	RHSIM_Rhsim09G0023700	-0.547	-0.600	-0.054	no
Treat-40d	RHSIM_Rhsim10G0193700	1.466	-0.295	-1.761	high
Treat-40d	RHSIM_Rhsim10G0174900	3.147	1.055	-2.091	high
Treat-40d	RHSIM_Rhsim11G0032300	-2.182	-1.350	0.831	low
Treat-40d	RHSIM_Rhsim11G0035800	0.733	0.206	-0.527	no
Treat-40d	RHSIM_Rhsim11G0027600	1.286	1.369	0.083	high
Treat-40d	RHSIM_Rhsim09G0052000	1.557	0.155	-1.402	high
Treat-40d	RHSIM_Rhsim11G0179300	0.026	-0.455	-0.482	no
Treat-40d	RHSIM_Rhsim11G0027500	2.503	0.797	-1.706	high
Treat-40d	RHSIM_Rhsim11G0046900	-0.413	-1.043	-0.631	no
Treat-40d	RHSIM_Rhsim11G0057900	0.714	-0.514	-1.228	no
Treat-40d	RHSIM_Rhsim11G0072300	0.442	-0.086	-0.528	no
Treat-40d	RHSIM_Rhsim11G0170000	-0.501	-0.049	0.452	no
Treat-40d	RHSIM_Rhsim09G0188200	-0.895	-0.421	0.474	no
Treat-40d	RHSIM_Rhsim09G0200200	0.335	0.478	0.143	no
Treat-40d	RHSIM_Rhsim10G0062400	-0.368	-0.817	-0.449	no
Treat-40d	RHSIM_Rhsim11G0038000	-0.004	0.872	0.876	no
Treat-40d	RHSIM_Rhsim11G0047500	0.114	-1.177	-1.291	no
Treat-40d	RHSIM_Rhsim11G0054400	-1.451	-0.168	1.283	low
Treat-40d	RHSIM_Rhsim10G0039600	1.126	0.945	-0.181	high
Treat-40d	RHSIM_Rhsim09G0000600	1.641	-0.635	-2.275	high
Treat-40d	RHSIM_Rhsim11G0100000	1.594	1.159	-0.436	high
Treat-40d	RHSIM_Rhsim11G0026600	-0.080	-0.758	-0.679	no
Treat-40d	RHSIM_Rhsim10G0063000	0.985	0.828	-0.158	no

Group	Gene	PTR (z-score difference)	z(log2 LFQ)	z(log2 FPKM)	extreme-PTR
Treat-40d	RHSIM_Rhsim11G0044500	-0.012	-0.476	-0.464	no
Treat-40d	RHSIM_Rhsim11G0163700	-1.986	-0.157	1.830	low
Treat-40d	RHSIM_Rhsim10G0054700	2.017	-0.270	-2.286	high
Treat-40d	RHSIM_Rhsim09G0186800	0.482	1.227	0.745	no
Treat-40d	RHSIM_Rhsim09G0133100	-0.515	-1.223	-0.708	no
Treat-40d	RHSIM_Rhsim09G0178500	-0.637	-0.419	0.218	no
Treat-40d	RHSIM_Rhsim10G0085800	-0.062	-0.626	-0.564	no
Treat-40d	RHSIM_Rhsim09G0192900	-0.452	1.172	1.624	no
Treat-40d	RHSIM_Rhsim09G0180100	1.299	2.297	0.998	high
Treat-40d	RHSIM_Rhsim09G0165200	-1.504	-0.987	0.517	low
Treat-40d	RHSIM_Rhsim09G0103600	0.209	-0.706	-0.915	no
Treat-40d	RHSIM_Rhsim11G0034600	-3.139	-1.391	1.748	low
Treat-40d	RHSIM_Rhsim09G0087900	-0.625	-0.315	0.311	no
Treat-40d	RHSIM_Rhsim10G0118400	-0.976	-0.572	0.404	no
Treat-40d	RHSIM_Rhsim11G0141000	1.271	0.657	-0.613	high
Treat-40d	RHSIM_Rhsim09G0185200	0.814	0.807	-0.007	no
Treat-40d	RHSIM_Rhsim09G0151700	0.539	2.019	1.480	no
Treat-40d	RHSIM_Rhsim11G0182100	1.031	0.430	-0.600	no
Treat-40d	RHSIM_Rhsim11G0021500	1.929	1.761	-0.168	high
Treat-40d	RHSIM_Rhsim11G0169500	-0.402	0.578	0.980	no
Treat-40d	RHSIM_Rhsim11G0037600	-0.307	-0.097	0.211	no
Treat-40d	RHSIM_Rhsim10G0020800	-0.230	-0.422	-0.192	no
Treat-40d	RHSIM_Rhsim11G0111400	-0.856	-0.333	0.523	no
Treat-40d	RHSIM_Rhsim10G0014700	0.531	-0.113	-0.645	no
Treat-40d	RHSIM_Rhsim11G0087000	4.694	3.096	-1.598	high
Treat-40d	RHSIM_Rhsim11G0052900	1.706	1.460	-0.246	high
Treat-40d	RHSIM_Rhsim10G0138900	-0.163	-0.875	-0.712	no
Treat-40d	RHSIM_Rhsim10G0159500	2.506	0.343	-2.163	high
Treat-40d	RHSIM_Rhsim10G0082200	-0.744	0.681	1.425	no
Treat-40d	RHSIM_Rhsim10G0183600	-0.619	-1.243	-0.624	no
Treat-40d	RHSIM_Rhsim10G0169300	-0.509	-0.906	-0.397	no
Treat-40d	RHSIM_Rhsim10G0173800	2.310	1.136	-1.173	high

Group	Gene	PTR (z-score difference)	z(log2 LFQ)	z(log2 FPKM)	extreme-PTR
Treat-40d	RHSIM_Rhsim09G0142600	0.912	0.178	-0.733	no
Treat-40d	RHSIM_Rhsim10G0195900	0.325	-0.962	-1.287	no
Treat-40d	RHSIM_Rhsim10G0067500	0.172	1.501	1.328	no
Treat-40d	RHSIM_Rhsim11G0146300	1.112	1.274	0.163	high
Treat-40d	RHSIM_Rhsim11G0047200	-2.123	-1.779	0.344	low
Treat-40d	RHSIM_Rhsim10G0052200	1.205	0.795	-0.410	high
Treat-40d	RHSIM_Rhsim09G0129300	-0.525	-0.217	0.307	no
Treat-40d	RHSIM_Rhsim10G0008900	0.087	0.258	0.171	no
Treat-40d	RHSIM_Rhsim10G0007400	-1.165	-0.843	0.322	low
Treat-40d	RHSIM_Rhsim09G0209100	-1.121	-0.253	0.868	no
Treat-40d	RHSIM_Rhsim10G0004900	0.224	-0.251	-0.475	no
Treat-40d	RHSIM_Rhsim10G0129200	-0.861	-1.052	-0.191	no
Treat-40d	RHSIM_Rhsim10G0094900	0.702	-0.336	-1.038	no
Treat-40d	RHSIM_Rhsim09G0032600	-0.524	-0.915	-0.390	no
Treat-40d	RHSIM_Rhsim10G0104300	-1.079	-0.422	0.657	no
Treat-40d	RHSIM_Rhsim10G0171200	-0.312	-0.108	0.204	no
Treat-40d	RHSIM_Rhsim10G0068900	-0.669	-1.018	-0.349	no
Treat-40d	RHSIM_Rhsim10G0129100	-1.514	-1.258	0.257	low
Treat-40d	RHSIM_Rhsim10G0122800	0.586	-0.667	-1.253	no
Treat-40d	RHSIM_Rhsim10G0184100	1.788	0.953	-0.836	high
Treat-40d	RHSIM_Rhsim09G0158400	1.045	1.269	0.225	no
Treat-40d	RHSIM_Rhsim09G0007900	1.377	0.979	-0.398	high
Treat-40d	RHSIM_Rhsim10G0157900	-0.105	0.277	0.382	no
Treat-40d	RHSIM_Rhsim09G0073500	0.598	0.518	-0.080	no
Treat-40d	RHSIM_Rhsim10G0010500	1.233	1.751	0.518	high
Treat-40d	RHSIM_Rhsim10G0156400	0.054	-0.895	-0.949	no
Treat-40d	RHSIM_Rhsim10G0188300	-0.571	-0.207	0.364	no
Treat-40d	RHSIM_Rhsim10G0137900	1.271	0.617	-0.655	high
Treat-40d	RHSIM_Rhsim09G0063200	0.294	0.337	0.043	no
Treat-40d	RHSIM_Rhsim10G0210900	-0.156	0.994	1.150	no
Treat-40d	RHSIM_Rhsim10G0156900	-0.215	0.288	0.502	no
Treat-40d	RHSIM_Rhsim10G0080600	0.446	0.076	-0.370	no

Group	Gene	PTR (z-score difference)	z(log2 LFQ)	z(log2 FPKM)	extreme-PTR
Treat-40d	RHSIM_Rhsim10G0152400	-1.749	-2.576	-0.827	low
Treat-40d	RHSIM_Rhsim10G0191400	-2.528	-1.777	0.752	low
Treat-40d	RHSIM_Rhsim10G0044200	0.671	0.808	0.137	no
Treat-40d	RHSIM_Rhsim10G0110700	1.041	0.281	-0.760	no
Treat-40d	RHSIM_Rhsim10G0174000	3.338	0.887	-2.452	high
Treat-40d	RHSIM_Rhsim10G0172400	0.303	0.299	-0.004	no
Treat-40d	RHSIM_Rhsim09G0022800	0.595	1.255	0.661	no
Treat-40d	RHSIM_Rhsim10G0113800	-1.541	-1.432	0.109	low
Treat-40d	RHSIM_Rhsim10G0115200	-0.374	-1.463	-1.089	no
Treat-40d	RHSIM_Rhsim10G0205500	0.417	-0.643	-1.059	no
Treat-40d	RHSIM_Rhsim09G0059100	-3.322	-1.212	2.110	low
Treat-40d	RHSIM_Rhsim10G0133100	0.073	-0.758	-0.831	no
Treat-40d	RHSIM_Rhsim10G0187200	-3.268	-0.815	2.453	low
Treat-40d	RHSIM_Rhsim10G0151000	0.823	-0.149	-0.972	no
Treat-40d	RHSIM_Rhsim10G0182800	0.021	1.199	1.178	no
Treat-40d	RHSIM_Rhsim10G0156600	1.090	0.454	-0.636	no
Treat-40d	RHSIM_Rhsim10G0050900	2.288	0.671	-1.617	high
Treat-40d	RHSIM_Rhsim10G0172000	-1.362	-0.577	0.785	low
Treat-40d	RHSIM_Rhsim10G0085400	0.154	-0.554	-0.708	no
Treat-40d	RHSIM_Rhsim10G0147800	-0.080	-1.026	-0.946	no
Treat-40d	RHSIM_Rhsim10G0177400	0.240	-1.149	-1.388	no
Treat-40d	RHSIM_Rhsim08G0211300	-0.766	-0.931	-0.165	no
Treat-40d	RHSIM_Rhsim10G0203500	-1.123	-2.034	-0.911	no
Treat-40d	RHSIM_Rhsim10G0032200	1.299	-0.702	-2.002	high
Treat-40d	RHSIM_Rhsim08G0204300	-0.578	-1.139	-0.562	no
Treat-40d	RHSIM_Rhsim10G0114700	0.742	-0.008	-0.750	no
Treat-40d	RHSIM_Rhsim08G0102800	-1.457	-1.507	-0.049	low
Treat-40d	RHSIM_Rhsim10G0093100	-0.175	-1.179	-1.004	no
Treat-40d	RHSIM_Rhsim10G0191100	-0.096	0.635	0.731	no
Treat-40d	RHSIM_Rhsim10G0197500	-0.872	-0.210	0.662	no
Treat-40d	RHSIM_Rhsim10G0144500	-1.392	-0.527	0.865	low
Treat-40d	RHSIM_Rhsim08G0099700	-0.661	0.071	0.732	no

Group	Gene	PTR (z-score difference)	z(log2 LFQ)	z(log2 FPKM)	extreme-PTR
Treat-40d	RHSIM_Rhsim10G0212300	-0.753	-0.805	-0.052	no
Treat-40d	RHSIM_Rhsim08G0220500	0.737	0.914	0.177	no
Treat-40d	RHSIM_Rhsim10G0142000	0.001	-1.994	-1.995	no
Treat-40d	RHSIM_Rhsim08G0251300	0.160	0.343	0.182	no
Treat-40d	RHSIM_Rhsim08G0007600	-0.208	-0.710	-0.502	no
Treat-40d	RHSIM_Rhsim10G0209300	-0.696	-1.271	-0.575	no
Treat-40d	RHSIM_Rhsim10G0166800	0.113	0.792	0.680	no
Treat-40d	RHSIM_Rhsim09G0002300	0.703	0.446	-0.258	no
Treat-40d	RHSIM_Rhsim10G0083700	-0.247	-2.086	-1.839	no
Treat-40d	RHSIM_Rhsim10G0150100	-0.543	-0.774	-0.231	no
Treat-40d	RHSIM_Rhsim10G0127500	0.465	0.453	-0.012	no
Treat-40d	RHSIM_Rhsim08G0012700	0.590	-0.031	-0.621	no
Treat-40d	RHSIM_Rhsim08G0146300	0.364	-0.062	-0.425	no
Treat-40d	RHSIM_Rhsim10G0173500	-0.641	-1.001	-0.361	no
Treat-40d	RHSIM_Rhsim08G0066300	0.471	1.336	0.865	no
Treat-40d	RHSIM_Rhsim09G0209900	-0.811	-0.416	0.395	no
Treat-40d	RHSIM_Rhsim10G0193100	0.808	0.043	-0.765	no
Treat-40d	RHSIM_Rhsim10G0124700	-0.622	-0.033	0.589	no
Treat-40d	RHSIM_Rhsim10G0124000	-1.708	-0.651	1.057	low
Treat-40d	RHSIM_Rhsim09G0032900	1.937	1.677	-0.260	high
Treat-40d	RHSIM_Rhsim10G0021300	3.063	3.371	0.308	high
Treat-40d	RHSIM_Rhsim09G0172500	0.554	0.966	0.412	no
Treat-40d	RHSIM_Rhsim09G0069400	-0.444	-0.151	0.292	no
Treat-40d	RHSIM_Rhsim08G0053400	0.445	0.494	0.049	no
Treat-40d	RHSIM_Rhsim10G0066000	0.540	1.369	0.829	no
Treat-40d	RHSIM_Rhsim09G0205000	-1.628	-1.083	0.546	low
Treat-40d	RHSIM_Rhsim10G0190900	0.148	-0.097	-0.245	no
Treat-40d	RHSIM_Rhsim09G0007800	-0.219	-0.612	-0.393	no
Treat-40d	RHSIM_Rhsim09G0205300	1.142	-0.993	-2.135	high
Treat-40d	RHSIM_Rhsim10G0112400	0.062	-0.586	-0.648	no
Treat-40d	RHSIM_Rhsim08G0092300	-0.075	-1.068	-0.993	no
Treat-40d	RHSIM_Rhsim10G0005200	-0.758	-1.154	-0.396	no

Group	Gene	PTR (z-score difference)	z(log2 LFQ)	z(log2 FPKM)	extreme-PTR
Treat-40d	RHSIM_Rhsim08G0045300	0.838	0.732	-0.107	no
Treat-40d	RHSIM_Rhsim09G0195700	1.243	1.365	0.121	high
Treat-40d	RHSIM_Rhsim09G0177100	-0.513	0.698	1.210	no
Treat-40d	RHSIM_Rhsim09G0205800	-1.604	-2.207	-0.603	low
Treat-40d	RHSIM_Rhsim05G0149300	-0.479	0.645	1.123	no
Treat-40d	RHSIM_Rhsim08G0128200	0.293	-1.264	-1.557	no
Treat-40d	RHSIM_Rhsim08G0079900	-0.542	0.013	0.556	no
Treat-40d	RHSIM_Rhsim08G0144800	1.122	-1.329	-2.450	high
Treat-40d	RHSIM_Rhsim09G0163400	0.313	1.485	1.171	no
Treat-40d	RHSIM_Rhsim08G0171900	0.766	0.712	-0.055	no
Treat-40d	RHSIM_Rhsim08G0225400	0.919	0.173	-0.746	no
Treat-40d	RHSIM_Rhsim08G0208400	-1.072	-1.235	-0.163	no
Treat-40d	RHSIM_Rhsim10G0011000	0.754	0.655	-0.099	no
Treat-40d	RHSIM_Rhsim10G0003100	-0.477	-0.777	-0.299	no
Treat-40d	RHSIM_Rhsim08G0161200	-0.914	-1.237	-0.323	no
Treat-40d	RHSIM_Rhsim09G0060000	0.810	0.817	0.007	no
Treat-40d	RHSIM_Rhsim09G0091000	0.053	-0.208	-0.261	no
Treat-40d	RHSIM_Rhsim09G0093400	1.159	0.170	-0.989	high
Treat-40d	RHSIM_Rhsim10G0163400	-0.509	0.166	0.675	no
Treat-40d	RHSIM_Rhsim08G0176500	-1.066	-0.910	0.156	no
Treat-40d	RHSIM_Rhsim09G0198100	1.012	-0.246	-1.258	no
Treat-40d	RHSIM_Rhsim10G0063200	-0.489	0.580	1.068	no
Treat-40d	RHSIM_Rhsim09G0007600	0.932	-0.446	-1.378	no
Treat-40d	RHSIM_Rhsim09G0211900	1.238	1.257	0.019	high
Treat-40d	RHSIM_Rhsim09G0181300	-0.666	-0.857	-0.191	no
Treat-40d	RHSIM_Rhsim10G0097300	2.433	0.636	-1.797	high
Treat-40d	RHSIM_Rhsim10G0067400	0.395	-0.129	-0.524	no
Treat-40d	RHSIM_Rhsim08G0112400	-0.444	-1.009	-0.565	no
Treat-40d	RHSIM_Rhsim08G0172700	0.497	-1.338	-1.835	no
Treat-40d	RHSIM_Rhsim10G0191500	-0.231	-0.878	-0.646	no
Treat-40d	RHSIM_Rhsim10G0146300	0.045	-1.079	-1.124	no
Treat-40d	RHSIM_Rhsim09G0009500	-1.974	-0.403	1.571	low

Group	Gene	PTR (z-score difference)	z(log2 LFQ)	z(log2 FPKM)	extreme-PTR
Treat-40d	RHSIM_Rhsim09G0091300	-1.023	-0.534	0.489	no
Treat-40d	RHSIM_Rhsim08G0188100	-0.189	0.171	0.360	no
Treat-40d	RHSIM_Rhsim09G0111200	-0.141	-0.540	-0.399	no
Treat-40d	RHSIM_Rhsim08G0242700	-1.443	-1.476	-0.033	low
Treat-40d	RHSIM_Rhsim10G0000800	0.850	-0.717	-1.567	no
Treat-40d	RHSIM_Rhsim09G0137200	-2.559	-1.584	0.975	low
Treat-40d	RHSIM_Rhsim10G0114000	-0.394	1.069	1.463	no
Treat-40d	RHSIM_Rhsim08G0042300	-0.412	0.040	0.452	no
Treat-40d	RHSIM_Rhsim09G0141800	0.716	0.647	-0.070	no
Treat-40d	RHSIM_Rhsim10G0165400	0.211	-0.531	-0.742	no
Treat-40d	RHSIM_Rhsim09G0037400	0.080	0.295	0.215	no
Treat-40d	RHSIM_Rhsim10G0166900	-0.529	-0.780	-0.251	no
Treat-40d	RHSIM_Rhsim09G0029500	-1.089	-1.038	0.051	no
Treat-40d	RHSIM_Rhsim08G0236100	-2.171	-1.722	0.449	low
Treat-40d	RHSIM_Rhsim08G0123900	-0.276	0.398	0.674	no
Treat-40d	RHSIM_Rhsim10G0050000	1.048	0.113	-0.935	no
Treat-40d	RHSIM_Rhsim10G0021100	1.428	-0.061	-1.489	high
Treat-40d	RHSIM_Rhsim09G0107300	0.555	0.019	-0.536	no
Treat-40d	RHSIM_Rhsim10G0069600	0.392	-0.031	-0.422	no
Treat-40d	RHSIM_Rhsim09G0181500	-0.961	-0.858	0.103	no
Treat-40d	RHSIM_Rhsim09G0176600	-0.967	0.587	1.554	no
Treat-40d	RHSIM_Rhsim10G0188000	-1.384	-1.977	-0.593	low
Treat-40d	RHSIM_Rhsim09G0126200	-1.225	-1.437	-0.212	low
Treat-40d	RHSIM_Rhsim08G0033900	-1.313	-0.848	0.466	low
Treat-40d	RHSIM_Rhsim09G0011600	-0.630	-0.359	0.271	no
Treat-40d	RHSIM_Rhsim08G0070600	-0.522	-0.184	0.338	no
Treat-40d	RHSIM_Rhsim09G0008400	1.523	2.276	0.753	high
Treat-40d	RHSIM_Rhsim09G0028000	0.297	-0.349	-0.645	no
Treat-40d	RHSIM_Rhsim08G0101600	-0.825	-1.280	-0.455	no
Treat-40d	RHSIM_Rhsim09G0212400	-1.129	-0.820	0.309	no
Treat-40d	RHSIM_Rhsim08G0079700	0.432	1.524	1.092	no
Treat-40d	RHSIM_Rhsim09G0164000	0.072	0.485	0.413	no

Group	Gene	PTR (z-score difference)	z(log2 LFQ)	z(log2 FPKM)	extreme-PTR
Treat-40d	RHSIM_Rhsim09G0179900	0.328	1.056	0.728	no
Treat-40d	RHSIM_Rhsim09G0154200	-0.378	-0.327	0.050	no
Treat-40d	RHSIM_Rhsim09G0103500	-0.266	-1.070	-0.804	no
Treat-40d	RHSIM_Rhsim08G0147100	1.101	0.994	-0.107	no
Treat-40d	RHSIM_Rhsim09G0071200	-1.492	-0.576	0.916	low
Treat-40d	RHSIM_Rhsim09G0126000	2.558	0.687	-1.871	high
Treat-40d	RHSIM_Rhsim09G0012000	-1.341	-1.125	0.217	low
Treat-40d	RHSIM_Rhsim09G0191500	-0.158	-0.717	-0.558	no
Treat-40d	RHSIM_Rhsim09G0177000	0.485	-0.892	-1.378	no
Treat-40d	RHSIM_Rhsim09G0086300	-0.043	-0.032	0.012	no
Treat-40d	RHSIM_Rhsim09G0192800	-0.173	-0.658	-0.485	no
Treat-40d	RHSIM_Rhsim09G0063500	-0.783	-1.663	-0.880	no
Treat-40d	RHSIM_Rhsim09G0182600	-0.033	0.802	0.836	no
Treat-40d	RHSIM_Rhsim09G0010800	-0.123	-0.908	-0.785	no
Treat-40d	RHSIM_Rhsim09G0098400	-1.661	-1.163	0.498	low
Treat-40d	RHSIM_Rhsim08G0214300	0.503	1.837	1.334	no
Treat-40d	RHSIM_Rhsim09G0099000	-0.102	-1.336	-1.234	no
Treat-40d	RHSIM_Rhsim08G0035400	-1.488	-1.788	-0.300	low
Treat-40d	RHSIM_Rhsim08G0097500	-2.068	-1.484	0.584	low
Treat-40d	RHSIM_Rhsim09G0075600	-0.434	-0.700	-0.266	no
Treat-40d	RHSIM_Rhsim09G0094200	-1.139	-0.186	0.953	no
Treat-40d	RHSIM_Rhsim09G0184500	2.031	-0.184	-2.215	high
Treat-40d	RHSIM_Rhsim09G0147200	-0.889	1.011	1.900	no
Treat-40d	RHSIM_Rhsim08G0145400	-0.648	0.058	0.706	no
Treat-40d	RHSIM_Rhsim09G0190300	0.594	0.309	-0.285	no
Treat-40d	RHSIM_Rhsim08G0056700	-0.119	-1.814	-1.694	no
Treat-40d	RHSIM_Rhsim09G0193300	-0.080	0.347	0.427	no
Treat-40d	RHSIM_Rhsim09G0055700	0.689	-0.892	-1.581	no
Treat-40d	RHSIM_Rhsim09G0204600	-1.348	-1.149	0.199	low
Treat-40d	RHSIM_Rhsim08G0200000	-0.315	0.062	0.376	no
Treat-40d	RHSIM_Rhsim09G0069300	1.968	0.491	-1.477	high
Treat-40d	RHSIM_Rhsim09G0179400	0.869	0.440	-0.429	no

Group	Gene	PTR (z-score difference)	z(log2 LFQ)	z(log2 FPKM)	extreme-PTR
Treat-40d	RHSIM_Rhsim09G0157600	0.547	-0.443	-0.990	no
Treat-40d	RHSIM_Rhsim09G0091900	-1.047	-0.300	0.747	no
Treat-40d	RHSIM_Rhsim09G0117900	1.689	0.153	-1.536	high
Treat-40d	RHSIM_Rhsim09G0131900	-0.771	0.346	1.118	no
Treat-40d	RHSIM_Rhsim07G0198600	0.618	1.989	1.372	no
Treat-40d	RHSIM_Rhsim09G0070800	0.157	1.146	0.989	no
Treat-40d	RHSIM_Rhsim09G0155700	0.222	-0.046	-0.268	no
Treat-40d	RHSIM_Rhsim09G0186700	1.898	0.694	-1.204	high
Treat-40d	RHSIM_Rhsim09G0145100	-1.531	-1.165	0.366	low
Treat-40d	RHSIM_Rhsim09G0129000	-0.950	-0.599	0.351	no
Treat-40d	RHSIM_Rhsim07G0224400	-0.402	-0.899	-0.497	no
Treat-40d	RHSIM_Rhsim09G0137500	-1.692	-0.768	0.924	low
Treat-40d	RHSIM_Rhsim09G0171900	1.878	2.777	0.899	high
Treat-40d	RHSIM_Rhsim07G0144600	-0.812	-0.198	0.613	no
Treat-40d	RHSIM_Rhsim09G0025700	0.551	-0.018	-0.569	no
Treat-40d	RHSIM_Rhsim07G0074600	-0.319	-0.825	-0.507	no
Treat-40d	RHSIM_Rhsim07G0153100	0.325	0.636	0.311	no
Treat-40d	RHSIM_Rhsim08G0119600	0.465	0.255	-0.210	no
Treat-40d	RHSIM_Rhsim09G0054700	1.320	-0.034	-1.353	high
Treat-40d	RHSIM_Rhsim07G0217300	2.202	0.627	-1.575	high
Treat-40d	RHSIM_Rhsim09G0123100	-0.877	-0.349	0.528	no
Treat-40d	RHSIM_Rhsim09G0080400	-0.551	-0.127	0.424	no
Treat-40d	RHSIM_Rhsim09G0138200	-1.945	-2.487	-0.542	low
Treat-40d	RHSIM_Rhsim08G0135400	-0.923	-1.305	-0.382	no
Treat-40d	RHSIM_Rhsim07G0018300	1.031	1.018	-0.013	no
Treat-40d	RHSIM_Rhsim08G0214800	-0.804	-0.510	0.294	no
Treat-40d	RHSIM_Rhsim03G0130900	-0.213	0.327	0.540	no
Treat-40d	RHSIM_Rhsim09G0128900	0.800	0.330	-0.471	no
Treat-40d	RHSIM_Rhsim09G0054900	-1.137	-0.677	0.461	no
Treat-40d	RHSIM_Rhsim08G0045200	0.416	-0.036	-0.452	no
Treat-40d	RHSIM_Rhsim07G0258100	0.223	0.300	0.077	no
Treat-40d	RHSIM_Rhsim07G0246300	-1.281	-1.201	0.080	low

Group	Gene	PTR (z-score difference)	z(log2 LFQ)	z(log2 FPKM)	extreme-PTR
Treat-40d	RHSIM_Rhsim09G0044200	-2.306	0.372	2.679	low
Treat-40d	RHSIM_Rhsim08G0089100	-0.424	0.765	1.189	no
Treat-40d	RHSIM_Rhsim08G0213200	-2.033	-1.938	0.095	low
Treat-40d	RHSIM_Rhsim07G0068600	0.454	-0.177	-0.631	no
Treat-40d	RHSIM_Rhsim07G0243600	0.918	-0.237	-1.155	no
Treat-40d	RHSIM_Rhsim08G0223600	1.758	1.474	-0.283	high
Treat-40d	RHSIM_Rhsim09G0089500	0.547	-0.150	-0.697	no
Treat-40d	RHSIM_Rhsim07G0088700	0.413	0.651	0.238	no
Treat-40d	RHSIM_Rhsim07G0042900	-1.096	-1.526	-0.430	no
Treat-40d	RHSIM_Rhsim09G0033900	-1.628	-1.711	-0.083	low
Treat-40d	RHSIM_Rhsim09G0142900	-0.477	0.152	0.629	no
Treat-40d	RHSIM_Rhsim07G0200500	0.154	0.314	0.160	no
Treat-40d	RHSIM_Rhsim09G0113800	0.694	-0.512	-1.206	no
Treat-40d	RHSIM_Rhsim09G0212800	0.994	0.131	-0.863	no
Treat-40d	RHSIM_Rhsim09G0178400	0.684	1.506	0.822	no
Treat-40d	RHSIM_Rhsim08G0111900	0.414	0.783	0.369	no
Treat-40d	RHSIM_Rhsim09G0094000	-0.648	0.063	0.710	no
Treat-40d	RHSIM_Rhsim08G0126700	2.038	1.344	-0.694	high
Treat-40d	RHSIM_Rhsim07G0192400	0.807	-0.397	-1.204	no
Treat-40d	RHSIM_Rhsim08G0021800	2.014	2.195	0.181	high
Treat-40d	RHSIM_Rhsim09G0088900	0.191	0.397	0.206	no
Treat-40d	RHSIM_Rhsim08G0042600	0.119	0.103	-0.016	no
Treat-40d	RHSIM_Rhsim09G0091400	-0.106	-0.265	-0.158	no
Treat-40d	RHSIM_Rhsim08G0092200	-0.041	-0.403	-0.362	no
Treat-40d	RHSIM_Rhsim09G0130400	-0.814	0.626	1.440	no
Treat-40d	RHSIM_Rhsim09G0120100	0.897	2.077	1.180	no
Treat-40d	RHSIM_Rhsim08G0043100	1.354	0.158	-1.197	high
Treat-40d	RHSIM_Rhsim09G0190000	0.629	-0.403	-1.032	no
Treat-40d	RHSIM_Rhsim09G0102000	-1.463	-0.658	0.805	low
Treat-40d	RHSIM_Rhsim07G0108200	0.658	0.287	-0.372	no
Treat-40d	RHSIM_Rhsim08G0118300	-0.781	0.568	1.349	no
Treat-40d	RHSIM_Rhsim09G0101400	-0.204	0.538	0.742	no

Group	Gene	PTR (z-score difference)	z(log2 LFQ)	z(log2 FPKM)	extreme-PTR
Treat-40d	RHSIM_Rhsim08G0008800	1.175	0.952	-0.223	high
Treat-40d	RHSIM_Rhsim08G0105500	0.772	0.211	-0.561	no
Treat-40d	RHSIM_Rhsim08G0178200	1.198	2.857	1.659	high
Treat-40d	RHSIM_Rhsim08G0162700	1.558	0.452	-1.106	high
Treat-40d	RHSIM_Rhsim07G0234400	-0.560	2.412	2.971	no
Treat-40d	RHSIM_Rhsim08G0212700	-0.700	-0.121	0.579	no
Treat-40d	RHSIM_Rhsim08G0244600	0.526	0.421	-0.105	no
Treat-40d	RHSIM_Rhsim09G0074900	-0.042	1.201	1.243	no
Treat-40d	RHSIM_Rhsim07G0030800	1.931	2.298	0.367	high
Treat-40d	RHSIM_Rhsim09G0001500	0.737	1.385	0.648	no
Treat-40d	RHSIM_Rhsim07G0104900	0.792	-0.016	-0.809	no
Treat-40d	RHSIM_Rhsim08G0192000	-0.787	0.572	1.359	no
Treat-40d	RHSIM_Rhsim08G0167700	0.362	-0.616	-0.979	no
Treat-40d	RHSIM_Rhsim08G0068800	-0.685	-0.884	-0.199	no
Treat-40d	RHSIM_Rhsim07G0234600	0.429	1.191	0.761	no
Treat-40d	RHSIM_Rhsim09G0153200	-1.196	-0.911	0.285	low
Treat-40d	RHSIM_Rhsim09G0093900	0.565	0.517	-0.048	no
Treat-40d	RHSIM_Rhsim07G0164900	1.102	-0.082	-1.184	no
Treat-40d	RHSIM_Rhsim07G0092500	0.756	0.626	-0.130	no
Treat-40d	RHSIM_Rhsim08G0083700	-0.263	0.663	0.926	no
Treat-40d	RHSIM_Rhsim08G0027200	-1.611	-0.803	0.808	low
Treat-40d	RHSIM_Rhsim07G0021100	0.550	0.140	-0.409	no
Treat-40d	RHSIM_Rhsim08G0236900	-0.204	-0.165	0.040	no
Treat-40d	RHSIM_Rhsim09G0198900	1.286	0.430	-0.856	high
Treat-40d	RHSIM_Rhsim08G0187300	-0.683	-1.318	-0.635	no
Treat-40d	RHSIM_Rhsim08G0007300	-2.164	-0.996	1.169	low
Treat-40d	RHSIM_Rhsim08G0212200	-0.699	-0.568	0.131	no
Treat-40d	RHSIM_Rhsim09G0072100	0.915	-1.323	-2.238	no
Treat-40d	RHSIM_Rhsim07G0122800	0.359	-1.304	-1.664	no
Treat-40d	RHSIM_Rhsim09G0131200	-0.776	-0.396	0.381	no
Treat-40d	RHSIM_Rhsim08G0118700	1.209	0.237	-0.972	high
Treat-40d	RHSIM_Rhsim08G0180000	-0.054	-0.931	-0.877	no

Group	Gene	PTR (z-score difference)	z(log2 LFQ)	z(log2 FPKM)	extreme-PTR
Treat-40d	RHSIM_Rhsim07G0128400	-4.326	-2.185	2.141	low
Treat-40d	RHSIM_Rhsim08G0236700	0.905	0.275	-0.630	no
Treat-40d	RHSIM_Rhsim09G0213200	-0.348	-0.378	-0.030	no
Treat-40d	RHSIM_Rhsim07G0155900	1.760	-0.164	-1.925	high
Treat-40d	RHSIM_Rhsim09G0130300	-1.882	-1.028	0.854	low
Treat-40d	RHSIM_Rhsim08G0246100	1.449	-0.183	-1.632	high
Treat-40d	RHSIM_Rhsim08G0098000	-1.013	0.014	1.027	no
Treat-40d	RHSIM_Rhsim08G0082400	-0.906	-0.237	0.669	no
Treat-40d	RHSIM_Rhsim08G0071400	-1.056	1.602	2.658	no
Treat-40d	RHSIM_Rhsim08G0249700	1.009	0.836	-0.173	no
Treat-40d	RHSIM_Rhsim08G0043900	-2.175	-2.089	0.086	low
Treat-40d	RHSIM_Rhsim07G0132900	-1.398	-1.129	0.269	low
Treat-40d	RHSIM_Rhsim08G0093700	-0.044	-1.177	-1.132	no
Treat-40d	RHSIM_Rhsim07G0128700	-1.142	-1.300	-0.159	low
Treat-40d	RHSIM_Rhsim08G0052300	0.752	-0.362	-1.114	no
Treat-40d	RHSIM_Rhsim08G0234400	0.468	-0.329	-0.797	no
Treat-40d	RHSIM_Rhsim09G0021800	0.705	0.828	0.123	no
Treat-40d	RHSIM_Rhsim09G0176500	0.404	1.139	0.734	no
Treat-40d	RHSIM_Rhsim08G0200300	-2.016	-1.131	0.885	low
Treat-40d	RHSIM_Rhsim07G0258600	-0.941	-1.269	-0.328	no
Treat-40d	RHSIM_Rhsim08G0032500	-1.272	-0.843	0.429	low
Treat-40d	RHSIM_Rhsim08G0004300	-1.106	-0.898	0.208	no
Treat-40d	RHSIM_Rhsim08G0026800	-1.000	-2.082	-1.082	no
Treat-40d	RHSIM_Rhsim08G0240700	-0.370	-0.925	-0.555	no
Treat-40d	RHSIM_Rhsim08G0247800	-0.648	-0.426	0.221	no
Treat-40d	RHSIM_Rhsim09G0073300	-0.249	0.623	0.872	no
Treat-40d	RHSIM_Rhsim09G0145600	0.645	-0.132	-0.777	no
Treat-40d	RHSIM_Rhsim08G0080700	-1.675	-0.630	1.045	low
Treat-40d	RHSIM_Rhsim08G0076400	-0.218	-0.370	-0.152	no
Treat-40d	RHSIM_Rhsim08G0184900	-0.320	-0.901	-0.581	no
Treat-40d	RHSIM_Rhsim08G0009400	0.070	1.954	1.884	no
Treat-40d	RHSIM_Rhsim07G0050700	-0.683	-0.078	0.605	no

Group	Gene	PTR (z-score difference)	z(log2 LFQ)	z(log2 FPKM)	extreme-PTR
Treat-40d	RHSIM_Rhsim08G0019200	-1.623	-0.440	1.183	low
Treat-40d	RHSIM_Rhsim08G0239600	-1.292	-0.948	0.344	low
Treat-40d	RHSIM_Rhsim08G0093100	-0.731	-0.830	-0.099	no
Treat-40d	RHSIM_Rhsim08G0128400	1.253	0.712	-0.541	high
Treat-40d	RHSIM_Rhsim07G0012200	1.859	-0.409	-2.268	high
Treat-40d	RHSIM_Rhsim08G0194600	0.801	1.821	1.020	no
Treat-40d	RHSIM_Rhsim08G0033500	-0.480	-0.314	0.166	no
Treat-40d	RHSIM_Rhsim07G0027400	0.637	0.971	0.335	no
Treat-40d	RHSIM_Rhsim07G0153400	-0.042	-0.230	-0.188	no
Treat-40d	RHSIM_Rhsim08G0164000	0.464	-0.420	-0.884	no
Treat-40d	RHSIM_Rhsim07G0002300	-0.058	-1.160	-1.103	no
Treat-40d	RHSIM_Rhsim08G0222900	1.832	0.306	-1.526	high
Treat-40d	RHSIM_Rhsim08G0198500	-0.766	-0.678	0.088	no
Treat-40d	RHSIM_Rhsim08G0218500	0.648	0.619	-0.030	no
Treat-40d	RHSIM_Rhsim09G0155900	1.578	1.080	-0.498	high
Treat-40d	RHSIM_Rhsim09G0195900	2.903	0.623	-2.280	high
Treat-40d	RHSIM_Rhsim08G0029800	0.248	-0.933	-1.182	no
Treat-40d	RHSIM_Rhsim07G0228400	0.265	0.964	0.699	no
Treat-40d	RHSIM_Rhsim09G0093200	0.919	2.012	1.093	no
Treat-40d	RHSIM_Rhsim07G0068800	-1.389	-1.479	-0.090	low
Treat-40d	RHSIM_Rhsim08G0037300	1.853	0.373	-1.480	high
Treat-40d	RHSIM_Rhsim07G0011300	-0.740	-0.988	-0.248	no
Treat-40d	RHSIM_Rhsim08G0233800	1.241	0.415	-0.826	high
Treat-40d	RHSIM_Rhsim08G0211000	1.364	1.923	0.559	high
Treat-40d	RHSIM_Rhsim07G0169700	-0.761	-0.604	0.156	no
Treat-40d	RHSIM_Rhsim08G0027300	-0.423	-0.818	-0.396	no
Treat-40d	RHSIM_Rhsim07G0078300	0.007	0.329	0.322	no
Treat-40d	RHSIM_Rhsim08G0055200	0.002	0.805	0.803	no
Treat-40d	RHSIM_Rhsim07G0091400	-0.662	1.676	2.338	no
Treat-40d	RHSIM_Rhsim08G0201400	-2.484	-2.938	-0.454	low
Treat-40d	RHSIM_Rhsim07G0058300	-0.116	-0.717	-0.601	no
Treat-40d	RHSIM_Rhsim09G0042400	1.076	-0.301	-1.377	no

Group	Gene	PTR (z-score difference)	z(log2 LFQ)	z(log2 FPKM)	extreme-PTR
Treat-40d	RHSIM_Rhsim08G0020900	0.995	2.151	1.156	no
Treat-40d	RHSIM_Rhsim08G0103100	1.273	-0.487	-1.760	high
Treat-40d	RHSIM_Rhsim08G0232100	-1.475	-0.191	1.284	low
Treat-40d	RHSIM_Rhsim09G0030000	0.258	-0.308	-0.566	no
Treat-40d	RHSIM_Rhsim08G0121700	0.710	-0.404	-1.113	no
Treat-40d	RHSIM_Rhsim08G0011700	-2.013	-1.906	0.107	low
Treat-40d	RHSIM_Rhsim08G0147000	-1.183	0.735	1.918	low
Treat-40d	RHSIM_Rhsim08G0199300	-2.246	-0.880	1.366	low
Treat-40d	RHSIM_Rhsim09G0209200	-0.828	-1.711	-0.883	no
Treat-40d	RHSIM_Rhsim07G0016300	0.946	-0.785	-1.731	no
Treat-40d	RHSIM_Rhsim08G0083000	-1.976	-1.258	0.718	low
Treat-40d	RHSIM_Rhsim08G0252400	1.584	0.546	-1.037	high
Treat-40d	RHSIM_Rhsim08G0215600	0.490	0.148	-0.342	no
Treat-40d	RHSIM_Rhsim09G0009000	0.270	0.077	-0.193	no
Treat-40d	RHSIM_Rhsim08G0207700	0.442	-0.443	-0.884	no
Treat-40d	RHSIM_Rhsim09G0152000	1.136	0.133	-1.003	high
Treat-40d	RHSIM_Rhsim08G0089000	2.439	0.716	-1.723	high
Treat-40d	RHSIM_Rhsim07G0118200	0.460	1.233	0.773	no
Treat-40d	RHSIM_Rhsim08G0102900	-0.389	-1.334	-0.946	no
Treat-40d	RHSIM_Rhsim07G0028300	-0.619	-0.588	0.031	no
Treat-40d	RHSIM_Rhsim08G0161100	0.482	0.213	-0.269	no
Treat-40d	RHSIM_Rhsim08G0087900	-0.751	-1.420	-0.669	no
Treat-40d	RHSIM_Rhsim09G0164200	0.155	1.678	1.523	no
Treat-40d	RHSIM_Rhsim07G0246500	0.825	-1.230	-2.056	no
Treat-40d	RHSIM_Rhsim08G0055400	1.999	1.953	-0.046	high
Treat-40d	RHSIM_Rhsim08G0034500	0.280	-1.099	-1.379	no
Treat-40d	RHSIM_Rhsim08G0242000	-0.940	-1.328	-0.388	no
Treat-40d	RHSIM_Rhsim08G0073200	0.743	1.005	0.263	no
Treat-40d	RHSIM_Rhsim08G0223300	2.369	3.019	0.650	high
Treat-40d	RHSIM_Rhsim08G0128700	-1.054	-0.688	0.365	no
Treat-40d	RHSIM_Rhsim08G0219800	-1.690	-1.622	0.067	low
Treat-40d	RHSIM_Rhsim08G0106300	-0.129	-0.790	-0.661	no

Group	Gene	PTR (z-score difference)	z(log2 LFQ)	z(log2 FPKM)	extreme-PTR
Treat-40d	RHSIM_Rhsim08G0027700	0.345	-0.175	-0.520	no
Treat-40d	RHSIM_Rhsim08G0010500	-0.801	-0.448	0.353	no
Treat-40d	RHSIM_Rhsim08G0049000	-0.274	-0.975	-0.701	no
Treat-40d	RHSIM_Rhsim08G0188800	-0.702	-0.790	-0.088	no
Treat-40d	RHSIM_Rhsim08G0028200	0.088	0.111	0.023	no
Treat-40d	RHSIM_Rhsim08G0112900	-0.610	-0.426	0.184	no
Treat-40d	RHSIM_Rhsim08G0223100	-0.379	1.571	1.950	no
Treat-40d	RHSIM_Rhsim07G0214500	2.505	2.377	-0.128	high
Treat-40d	RHSIM_Rhsim08G0178000	-0.254	-2.098	-1.845	no
Treat-40d	RHSIM_Rhsim07G0067400	-1.071	-1.129	-0.058	no
Treat-40d	RHSIM_Rhsim07G0124800	0.763	0.377	-0.387	no
Treat-40d	RHSIM_Rhsim07G0009900	-0.098	-0.668	-0.570	no
Treat-40d	RHSIM_Rhsim07G0036400	0.389	1.962	1.573	no
Treat-40d	RHSIM_Rhsim08G0228700	-0.054	-1.007	-0.952	no
Treat-40d	RHSIM_Rhsim08G0246500	-1.012	0.066	1.078	no
Treat-40d	RHSIM_Rhsim08G0015900	1.143	-0.463	-1.606	high
Treat-40d	RHSIM_Rhsim07G0124400	1.278	2.854	1.576	high
Treat-40d	RHSIM_Rhsim06G0028000	-0.358	1.722	2.080	no
Treat-40d	RHSIM_Rhsim08G0248800	-0.213	0.551	0.764	no
Treat-40d	RHSIM_Rhsim08G0225500	2.754	1.003	-1.750	high
Treat-40d	RHSIM_Rhsim08G0196300	0.575	-1.383	-1.957	no
Treat-40d	RHSIM_Rhsim08G0011000	-0.116	2.022	2.138	no
Treat-40d	RHSIM_Rhsim08G0001700	-0.140	-0.668	-0.529	no
Treat-40d	RHSIM_Rhsim08G0169400	-1.119	-1.181	-0.063	no
Treat-40d	RHSIM_Rhsim08G0234500	-2.067	-0.687	1.380	low
Treat-40d	RHSIM_Rhsim08G0087000	2.102	1.273	-0.829	high
Treat-40d	RHSIM_Rhsim07G0046800	-0.045	0.798	0.842	no
Treat-40d	RHSIM_Rhsim08G0221800	-0.350	-0.767	-0.417	no
Treat-40d	RHSIM_Rhsim08G0173800	-0.508	-0.646	-0.138	no
Treat-40d	RHSIM_Rhsim06G0095100	-0.190	-1.118	-0.928	no
Treat-40d	RHSIM_Rhsim08G0198100	1.271	1.840	0.569	high
Treat-40d	RHSIM_Rhsim07G0215600	0.090	0.236	0.146	no

Group	Gene	PTR (z-score difference)	z(log2 LFQ)	z(log2 FPKM)	extreme-PTR
Treat-40d	RHSIM_Rhsim08G0032400	0.283	0.070	-0.213	no
Treat-40d	RHSIM_Rhsim08G0084500	-1.348	0.278	1.626	low
Treat-40d	RHSIM_Rhsim08G0052400	0.767	0.522	-0.245	no
Treat-40d	RHSIM_Rhsim07G0093000	-2.718	1.868	4.585	low
Treat-40d	RHSIM_Rhsim08G0092600	2.171	1.515	-0.657	high
Treat-40d	RHSIM_Rhsim08G0052800	-1.718	-0.848	0.870	low
Treat-40d	RHSIM_Rhsim08G0048100	-0.490	-0.393	0.097	no
Treat-40d	RHSIM_Rhsim07G0017600	0.126	0.170	0.044	no
Treat-40d	RHSIM_Rhsim07G0044000	-0.159	-0.084	0.075	no
Treat-40d	RHSIM_Rhsim08G0042700	0.651	-0.302	-0.954	no
Treat-40d	RHSIM_Rhsim08G0066600	0.475	-0.152	-0.626	no
Treat-40d	RHSIM_Rhsim06G0108700	-0.644	-1.574	-0.931	no
Treat-40d	RHSIM_Rhsim07G0106400	0.413	0.834	0.421	no
Treat-40d	RHSIM_Rhsim06G0005200	-0.816	-0.875	-0.059	no
Treat-40d	RHSIM_Rhsim07G0212500	-1.018	-0.727	0.290	no
Treat-40d	RHSIM_Rhsim08G0194200	0.738	0.660	-0.078	no
Treat-40d	RHSIM_Rhsim08G0155500	-1.439	-1.229	0.209	low
Treat-40d	RHSIM_Rhsim06G0127700	0.283	-0.168	-0.451	no
Treat-40d	RHSIM_Rhsim08G0017300	1.260	0.237	-1.024	high
Treat-40d	RHSIM_Rhsim08G0195700	-1.171	-0.849	0.323	low
Treat-40d	RHSIM_Rhsim08G0007800	-0.518	-0.664	-0.146	no
Treat-40d	RHSIM_Rhsim08G0104200	0.815	-0.571	-1.386	no
Treat-40d	RHSIM_Rhsim06G0233900	0.601	0.108	-0.493	no
Treat-40d	RHSIM_Rhsim08G0159800	1.035	0.436	-0.599	no
Treat-40d	RHSIM_Rhsim08G0212400	-0.177	-1.139	-0.962	no
Treat-40d	RHSIM_Rhsim06G0165900	-0.250	0.047	0.297	no
Treat-40d	RHSIM_Rhsim08G0236000	-2.263	-1.445	0.818	low
Treat-40d	RHSIM_Rhsim07G0219800	-0.361	-0.441	-0.080	no
Treat-40d	RHSIM_Rhsim08G0196000	-0.236	1.533	1.769	no
Treat-40d	RHSIM_Rhsim06G0091300	-0.029	-0.866	-0.836	no
Treat-40d	RHSIM_Rhsim08G0095900	0.406	-1.225	-1.631	no
Treat-40d	RHSIM_Rhsim08G0078500	1.414	-0.181	-1.595	high

Group	Gene	PTR (z-score difference)	z(log2 LFQ)	z(log2 FPKM)	extreme-PTR
Treat-40d	RHSIM_Rhsim07G0170200	0.855	0.555	-0.299	no
Treat-40d	RHSIM_Rhsim07G0233400	1.468	1.345	-0.123	high
Treat-40d	RHSIM_Rhsim06G0211500	-0.609	-1.145	-0.536	no
Treat-40d	RHSIM_Rhsim08G0079500	-0.472	0.637	1.108	no
Treat-40d	RHSIM_Rhsim07G0202900	0.179	-0.414	-0.594	no
Treat-40d	RHSIM_Rhsim07G0001500	1.085	0.430	-0.656	no
Treat-40d	RHSIM_Rhsim07G0178700	-0.304	-0.178	0.126	no
Treat-40d	RHSIM_Rhsim07G0170500	-1.823	-1.291	0.531	low
Treat-40d	RHSIM_Rhsim06G0181200	0.591	-0.147	-0.739	no
Treat-40d	RHSIM_Rhsim07G0018600	1.263	-1.058	-2.321	high
Treat-40d	RHSIM_Rhsim07G0049800	0.586	1.764	1.178	no
Treat-40d	RHSIM_Rhsim07G0066200	-0.111	0.066	0.177	no
Treat-40d	RHSIM_Rhsim08G0072200	0.037	-0.150	-0.187	no
Treat-40d	RHSIM_Rhsim07G0131800	0.823	0.578	-0.245	no
Treat-40d	RHSIM_Rhsim07G0036500	-0.144	0.782	0.926	no
Treat-40d	RHSIM_Rhsim07G0028800	1.887	3.339	1.452	high
Treat-40d	RHSIM_Rhsim07G0132800	0.257	-0.031	-0.288	no
Treat-40d	RHSIM_Rhsim08G0235700	0.407	0.212	-0.195	no
Treat-40d	RHSIM_Rhsim06G0212600	2.940	1.817	-1.123	high
Treat-40d	RHSIM_Rhsim07G0018400	1.083	1.319	0.235	no
Treat-40d	RHSIM_Rhsim07G0184200	0.269	0.709	0.439	no
Treat-40d	RHSIM_Rhsim06G0002800	0.904	0.576	-0.328	no
Treat-40d	RHSIM_Rhsim07G0099200	0.915	-0.237	-1.152	no
Treat-40d	RHSIM_Rhsim07G0258700	-0.224	-0.740	-0.516	no
Treat-40d	RHSIM_Rhsim07G0045900	-0.392	-1.107	-0.715	no
Treat-40d	RHSIM_Rhsim07G0087900	1.430	-0.114	-1.545	high
Treat-40d	RHSIM_Rhsim08G0071000	-1.130	0.388	1.518	no
Treat-40d	RHSIM_Rhsim08G0105200	-0.237	0.638	0.876	no
Treat-40d	RHSIM_Rhsim08G0186700	-1.082	-0.924	0.158	no
Treat-40d	RHSIM_Rhsim06G0244300	0.502	1.119	0.617	no
Treat-40d	RHSIM_Rhsim07G0033700	1.029	0.037	-0.991	no
Treat-40d	RHSIM_Rhsim08G0167500	0.301	0.980	0.679	no

Group	Gene	PTR (z-score difference)	z(log2 LFQ)	z(log2 FPKM)	extreme-PTR
Treat-40d	RHSIM_Rhsim07G0122500	-1.273	-1.657	-0.384	low
Treat-40d	RHSIM_Rhsim07G0030200	-0.704	-0.636	0.068	no
Treat-40d	RHSIM_Rhsim08G0171100	1.257	0.358	-0.899	high
Treat-40d	RHSIM_Rhsim07G0083900	-0.916	0.033	0.949	no
Treat-40d	RHSIM_Rhsim08G0226300	-0.455	-0.894	-0.439	no
Treat-40d	RHSIM_Rhsim07G0017800	-0.196	-0.985	-0.789	no
Treat-40d	RHSIM_Rhsim07G0027500	-0.050	-0.427	-0.377	no
Treat-40d	RHSIM_Rhsim07G0202800	-0.872	-0.128	0.744	no
Treat-40d	RHSIM_Rhsim06G0052900	-0.264	0.850	1.114	no
Treat-40d	RHSIM_Rhsim08G0118600	-0.315	0.122	0.436	no
Treat-40d	RHSIM_Rhsim07G0119300	-0.035	0.633	0.668	no
Treat-40d	RHSIM_Rhsim07G0049600	-0.269	1.288	1.557	no
Treat-40d	RHSIM_Rhsim06G0207400	2.224	0.120	-2.104	high
Treat-40d	RHSIM_Rhsim07G0056500	-1.095	-0.137	0.958	no
Treat-40d	RHSIM_Rhsim07G0103100	0.271	1.135	0.864	no
Treat-40d	RHSIM_Rhsim07G0171800	-0.209	0.341	0.550	no
Treat-40d	RHSIM_Rhsim07G0121100	-0.564	-0.002	0.561	no
Treat-40d	RHSIM_Rhsim08G0036900	0.290	0.632	0.341	no
Treat-40d	RHSIM_Rhsim07G0165200	0.622	-0.710	-1.332	no
Treat-40d	RHSIM_Rhsim08G0097800	2.321	1.541	-0.780	high
Treat-40d	RHSIM_Rhsim06G0077600	0.058	0.533	0.475	no
Treat-40d	RHSIM_Rhsim07G0020500	-0.300	0.235	0.535	no
Treat-40d	RHSIM_Rhsim06G0218800	-1.725	-1.349	0.376	low
Treat-40d	RHSIM_Rhsim07G0067300	0.914	0.233	-0.682	no
Treat-40d	RHSIM_Rhsim07G0188200	-1.095	-0.512	0.583	no
Treat-40d	RHSIM_Rhsim07G0228100	-1.900	-0.701	1.198	low
Treat-40d	RHSIM_Rhsim07G0245900	-0.938	-0.661	0.277	no
Treat-40d	RHSIM_Rhsim06G0187500	-0.694	-0.543	0.151	no
Treat-40d	RHSIM_Rhsim06G0105400	-2.733	-2.215	0.518	low
Treat-40d	RHSIM_Rhsim08G0152800	0.570	-0.329	-0.898	no
Treat-40d	RHSIM_Rhsim08G0171200	-1.196	-0.966	0.230	low
Treat-40d	RHSIM_Rhsim07G0092600	-1.874	-0.070	1.804	low

Group	Gene	PTR (z-score difference)	z(log2 LFQ)	z(log2 FPKM)	extreme-PTR
Treat-40d	RHSIM_Rhsim07G0204300	0.746	0.704	-0.042	no
Treat-40d	RHSIM_Rhsim07G0081900	0.945	0.524	-0.421	no
Treat-40d	RHSIM_Rhsim08G0138500	0.107	-0.136	-0.242	no
Treat-40d	RHSIM_Rhsim07G0055600	2.250	1.841	-0.410	high
Treat-40d	RHSIM_Rhsim07G0125500	-0.965	-1.087	-0.122	no
Treat-40d	RHSIM_Rhsim06G0222300	2.543	1.696	-0.848	high
Treat-40d	RHSIM_Rhsim07G0214600	-1.155	-1.365	-0.210	low
Treat-40d	RHSIM_Rhsim07G0075900	0.696	0.884	0.188	no
Treat-40d	RHSIM_Rhsim07G0074000	0.744	0.450	-0.293	no
Treat-40d	RHSIM_Rhsim06G0191200	-0.559	-1.023	-0.464	no
Treat-40d	RHSIM_Rhsim08G0101100	-0.300	-1.166	-0.866	no
Treat-40d	RHSIM_Rhsim08G0183600	0.229	-0.225	-0.453	no
Treat-40d	RHSIM_Rhsim06G0178000	0.872	0.353	-0.519	no
Treat-40d	RHSIM_Rhsim07G0179500	0.952	0.165	-0.787	no
Treat-40d	RHSIM_Rhsim08G0196600	0.816	0.468	-0.349	no
Treat-40d	RHSIM_Rhsim06G0035000	0.416	0.214	-0.202	no
Treat-40d	RHSIM_Rhsim07G0059500	1.703	-0.753	-2.455	high
Treat-40d	RHSIM_Rhsim07G0228600	-1.465	-0.997	0.468	low
Treat-40d	RHSIM_Rhsim06G0046200	0.775	-0.493	-1.268	no
Treat-40d	RHSIM_Rhsim07G0033000	-0.385	-0.938	-0.553	no
Treat-40d	RHSIM_Rhsim06G0167500	-1.040	0.625	1.665	no
Treat-40d	RHSIM_Rhsim07G0004400	-0.237	0.289	0.525	no
Treat-40d	RHSIM_Rhsim06G0000200	-0.349	-0.605	-0.256	no
Treat-40d	RHSIM_Rhsim06G0034400	0.773	1.151	0.378	no
Treat-40d	RHSIM_Rhsim06G0138800	0.336	0.500	0.164	no
Treat-40d	RHSIM_Rhsim08G0242800	1.187	-0.378	-1.565	high
Treat-40d	RHSIM_Rhsim07G0178300	2.878	3.157	0.278	high
Treat-40d	RHSIM_Rhsim07G0013700	-0.802	0.454	1.256	no
Treat-40d	RHSIM_Rhsim06G0003200	1.894	1.110	-0.785	high
Treat-40d	RHSIM_Rhsim06G0031700	0.633	-0.141	-0.774	no
Treat-40d	RHSIM_Rhsim08G0090800	0.973	-0.472	-1.445	no
Treat-40d	RHSIM_Rhsim07G0028000	-1.412	-1.138	0.274	low

Group	Gene	PTR (z-score difference)	z(log2 LFQ)	z(log2 FPKM)	extreme-PTR
Treat-40d	RHSIM_Rhsim07G0087100	-0.142	-0.258	-0.115	no
Treat-40d	RHSIM_Rhsim08G0222200	-0.179	-1.201	-1.022	no
Treat-40d	RHSIM_Rhsim07G0169100	-0.168	0.860	1.028	no
Treat-40d	RHSIM_Rhsim08G0251700	1.339	0.763	-0.576	high
Treat-40d	RHSIM_Rhsim07G0047300	-0.294	-0.832	-0.537	no
Treat-40d	RHSIM_Rhsim08G0236600	1.334	0.510	-0.825	high
Treat-40d	RHSIM_Rhsim07G0110300	0.032	1.066	1.034	no
Treat-40d	RHSIM_Rhsim07G0226700	0.515	-0.273	-0.787	no
Treat-40d	RHSIM_Rhsim07G0081200	0.071	-0.108	-0.179	no
Treat-40d	RHSIM_Rhsim06G0193100	0.688	1.200	0.512	no
Treat-40d	RHSIM_Rhsim08G0036600	0.976	-0.277	-1.254	no
Treat-40d	RHSIM_Rhsim07G0139000	0.650	0.859	0.209	no
Treat-40d	RHSIM_Rhsim07G0137200	-1.190	-1.341	-0.151	low
Treat-40d	RHSIM_Rhsim07G0103400	-1.007	-0.304	0.703	no
Treat-40d	RHSIM_Rhsim07G0139200	-0.343	-0.586	-0.243	no
Treat-40d	RHSIM_Rhsim07G0210400	0.852	1.983	1.131	no
Treat-40d	RHSIM_Rhsim07G0072200	-0.209	-0.263	-0.054	no
Treat-40d	RHSIM_Rhsim06G0070400	1.188	0.633	-0.556	high
Treat-40d	RHSIM_Rhsim07G0197900	-0.655	2.309	2.963	no
Treat-40d	RHSIM_Rhsim07G0130000	0.443	-0.504	-0.947	no
Treat-40d	RHSIM_Rhsim08G0190500	-0.365	0.029	0.395	no
Treat-40d	RHSIM_Rhsim07G0236500	-1.072	-0.728	0.344	no
Treat-40d	RHSIM_Rhsim07G0110500	-1.181	-0.974	0.207	low
Treat-40d	RHSIM_Rhsim06G0205600	-0.579	-0.200	0.379	no
Treat-40d	RHSIM_Rhsim08G0240800	0.304	-0.353	-0.656	no
Treat-40d	RHSIM_Rhsim07G0035600	-0.965	-0.140	0.825	no
Treat-40d	RHSIM_Rhsim06G0237100	-2.626	-1.460	1.167	low
Treat-40d	RHSIM_Rhsim07G0249300	2.264	0.843	-1.421	high
Treat-40d	RHSIM_Rhsim07G0001600	1.540	0.340	-1.199	high
Treat-40d	RHSIM_Rhsim06G0047700	0.019	1.369	1.350	no
Treat-40d	RHSIM_Rhsim06G0242300	1.372	1.008	-0.364	high
Treat-40d	RHSIM_Rhsim07G0018200	-0.096	0.467	0.563	no

Group	Gene	PTR (z-score difference)	z(log2 LFQ)	z(log2 FPKM)	extreme-PTR
Treat-40d	RHSIM_Rhsim07G0178100	-0.418	-1.034	-0.616	no
Treat-40d	RHSIM_Rhsim05G0060700	-0.300	0.893	1.193	no
Treat-40d	RHSIM_Rhsim06G0169300	0.955	1.136	0.181	no
Treat-40d	RHSIM_Rhsim07G0092700	1.248	1.186	-0.062	high
Treat-40d	RHSIM_Rhsim07G0072000	-1.580	-0.818	0.762	low
Treat-40d	RHSIM_Rhsim07G0046600	0.264	0.298	0.034	no
Treat-40d	RHSIM_Rhsim07G0059000	2.502	0.722	-1.779	high
Treat-40d	RHSIM_Rhsim05G0194700	0.388	0.805	0.417	no
Treat-40d	RHSIM_Rhsim05G0122800	0.334	0.418	0.085	no
Treat-40d	RHSIM_Rhsim06G0028600	0.540	-0.552	-1.092	no
Treat-40d	RHSIM_Rhsim07G0204200	-1.388	-0.477	0.911	low
Treat-40d	RHSIM_Rhsim06G0243900	2.454	0.567	-1.887	high
Treat-40d	RHSIM_Rhsim07G0237600	0.026	-0.324	-0.350	no
Treat-40d	RHSIM_Rhsim07G0247500	-0.047	0.970	1.018	no
Treat-40d	RHSIM_Rhsim05G0101000	0.606	0.435	-0.171	no
Treat-40d	RHSIM_Rhsim07G0012900	-1.508	-0.383	1.125	low
Treat-40d	RHSIM_Rhsim07G0026400	0.736	1.089	0.353	no
Treat-40d	RHSIM_Rhsim07G0125200	-0.090	-1.870	-1.780	no
Treat-40d	RHSIM_Rhsim07G0117900	0.626	1.192	0.566	no
Treat-40d	RHSIM_Rhsim07G0150800	-0.203	0.236	0.439	no
Treat-40d	RHSIM_Rhsim07G0054900	1.172	0.035	-1.137	high
Treat-40d	RHSIM_Rhsim07G0006300	-2.487	-1.866	0.620	low
Treat-40d	RHSIM_Rhsim05G0217100	-0.502	-1.137	-0.635	no
Treat-40d	RHSIM_Rhsim07G0100900	-1.702	-1.051	0.651	low
Treat-40d	RHSIM_Rhsim07G0210900	0.279	1.171	0.892	no
Treat-40d	RHSIM_Rhsim07G0179400	-1.401	-1.184	0.217	low
Treat-40d	RHSIM_Rhsim07G0082100	1.246	2.435	1.188	high
Treat-40d	RHSIM_Rhsim07G0048700	-0.636	-1.531	-0.895	no
Treat-40d	RHSIM_Rhsim06G0064500	-0.803	-0.648	0.155	no
Treat-40d	RHSIM_Rhsim07G0083200	-0.101	0.118	0.218	no
Treat-40d	RHSIM_Rhsim06G0090400	-0.388	-0.138	0.250	no
Treat-40d	RHSIM_Rhsim06G0013400	0.395	0.484	0.089	no

Group	Gene	PTR (z-score difference)	z(log2 LFQ)	z(log2 FPKM)	extreme-PTR
Treat-40d	RHSIM_Rhsim05G0134300	0.506	-0.203	-0.709	no
Treat-40d	RHSIM_Rhsim06G0033500	0.039	0.079	0.040	no
Treat-40d	RHSIM_Rhsim05G0088500	2.067	1.557	-0.510	high
Treat-40d	RHSIM_Rhsim06G0055200	-2.355	-1.162	1.193	low
Treat-40d	RHSIM_Rhsim06G0122800	-0.997	-0.944	0.053	no
Treat-40d	RHSIM_Rhsim07G0091600	1.755	0.178	-1.577	high
Treat-40d	RHSIM_Rhsim07G0129700	-0.145	-0.005	0.140	no
Treat-40d	RHSIM_Rhsim06G0072400	0.428	0.444	0.015	no
Treat-40d	RHSIM_Rhsim07G0205000	2.036	2.564	0.528	high
Treat-40d	RHSIM_Rhsim05G0061100	1.545	-0.627	-2.172	high
Treat-40d	RHSIM_Rhsim05G0111600	0.050	-0.726	-0.776	no
Treat-40d	RHSIM_Rhsim06G0084600	-1.142	-0.899	0.244	low
Treat-40d	RHSIM_Rhsim07G0092900	-1.165	1.119	2.284	low
Treat-40d	RHSIM_Rhsim06G0151500	-0.966	0.874	1.841	no
Treat-40d	RHSIM_Rhsim07G0250400	-1.581	-1.971	-0.390	low
Treat-40d	RHSIM_Rhsim06G0238800	-0.578	-0.181	0.397	no
Treat-40d	RHSIM_Rhsim07G0071400	0.342	0.018	-0.325	no
Treat-40d	RHSIM_Rhsim07G0087700	0.892	1.441	0.549	no
Treat-40d	RHSIM_Rhsim05G0120100	-1.046	-1.001	0.045	no
Treat-40d	RHSIM_Rhsim06G0161000	-0.479	-1.305	-0.826	no
Treat-40d	RHSIM_Rhsim07G0225500	0.382	0.039	-0.343	no
Treat-40d	RHSIM_Rhsim05G0232300	-0.515	0.819	1.334	no
Treat-40d	RHSIM_Rhsim07G0047800	0.902	0.995	0.094	no
Treat-40d	RHSIM_Rhsim06G0205800	-0.243	0.781	1.024	no
Treat-40d	RHSIM_Rhsim07G0189000	-1.002	0.732	1.734	no
Treat-40d	RHSIM_Rhsim06G0004200	1.951	0.915	-1.036	high
Treat-40d	RHSIM_Rhsim07G0013800	-2.222	-0.663	1.558	low
Treat-40d	RHSIM_Rhsim06G0098800	-0.092	-0.774	-0.682	no
Treat-40d	RHSIM_Rhsim06G0074700	-0.561	0.666	1.227	no
Treat-40d	RHSIM_Rhsim06G0177400	0.442	-0.572	-1.014	no
Treat-40d	RHSIM_Rhsim06G0026100	-2.705	-1.093	1.612	low
Treat-40d	RHSIM_Rhsim07G0153500	-0.805	-0.493	0.312	no

Group	Gene	PTR (z-score difference)	z(log2 LFQ)	z(log2 FPKM)	extreme-PTR
Treat-40d	RHSIM_Rhsim06G0207300	-0.138	-0.449	-0.311	no
Treat-40d	RHSIM_Rhsim07G0066700	-0.984	-1.996	-1.012	no
Treat-40d	RHSIM_Rhsim05G0165300	-1.367	-0.330	1.037	low
Treat-40d	RHSIM_Rhsim06G0137600	1.747	1.341	-0.405	high
Treat-40d	RHSIM_Rhsim06G0116000	1.149	0.402	-0.747	high
Treat-40d	RHSIM_Rhsim07G0258000	-1.262	-0.902	0.360	low
Treat-40d	RHSIM_Rhsim07G0074300	0.583	0.094	-0.489	no
Treat-40d	RHSIM_Rhsim06G0203600	1.068	1.086	0.017	no
Treat-40d	RHSIM_Rhsim07G0049700	-2.996	-0.097	2.899	low
Treat-40d	RHSIM_Rhsim06G0032800	-0.293	0.074	0.367	no
Treat-40d	RHSIM_Rhsim05G0004900	1.015	0.957	-0.058	no
Treat-40d	RHSIM_Rhsim07G0071800	-1.638	-1.870	-0.232	low
Treat-40d	RHSIM_Rhsim06G0205900	1.045	0.487	-0.559	no
Treat-40d	RHSIM_Rhsim05G0161600	2.056	0.336	-1.720	high
Treat-40d	RHSIM_Rhsim06G0204300	-1.261	-1.207	0.054	low
Treat-40d	RHSIM_Rhsim06G0071400	-0.402	-1.014	-0.612	no
Treat-40d	RHSIM_Rhsim06G0223100	-0.248	0.858	1.106	no
Treat-40d	RHSIM_Rhsim06G0130900	0.097	-0.311	-0.407	no
Treat-40d	RHSIM_Rhsim06G0025000	1.255	-0.400	-1.655	high
Treat-40d	RHSIM_Rhsim06G0165200	0.126	-0.607	-0.733	no
Treat-40d	RHSIM_Rhsim07G0203600	-0.409	0.162	0.571	no
Treat-40d	RHSIM_Rhsim06G0027400	0.026	-1.423	-1.449	no
Treat-40d	RHSIM_Rhsim06G0069500	-0.907	-0.554	0.353	no
Treat-40d	RHSIM_Rhsim07G0187800	0.023	1.152	1.129	no
Treat-40d	RHSIM_Rhsim05G0104300	-0.822	0.272	1.095	no
Treat-40d	RHSIM_Rhsim05G0167900	-0.868	-0.448	0.421	no
Treat-40d	RHSIM_Rhsim06G0034800	-0.085	0.327	0.412	no
Treat-40d	RHSIM_Rhsim07G0019300	0.488	1.835	1.347	no
Treat-40d	RHSIM_Rhsim07G0129500	-0.871	-1.177	-0.307	no
Treat-40d	RHSIM_Rhsim06G0193700	0.377	-0.627	-1.004	no
Treat-40d	RHSIM_Rhsim05G0106800	-0.468	0.751	1.219	no
Treat-40d	RHSIM_Rhsim07G0101400	-1.105	-0.649	0.456	no

Group	Gene	PTR (z-score difference)	z(log2 LFQ)	z(log2 FPKM)	extreme-PTR
Treat-40d	RHSIM_Rhsim06G0243000	2.239	2.456	0.217	high
Treat-40d	RHSIM_Rhsim06G0205300	1.146	1.774	0.628	high
Treat-40d	RHSIM_Rhsim06G0167300	-1.025	0.757	1.783	no
Treat-40d	RHSIM_Rhsim06G0078100	0.944	-0.052	-0.996	no
Treat-40d	RHSIM_Rhsim07G0143200	0.066	1.173	1.108	no
Treat-40d	RHSIM_Rhsim06G0126000	0.401	-0.011	-0.412	no
Treat-40d	RHSIM_Rhsim06G0212000	0.817	-0.825	-1.642	no
Treat-40d	RHSIM_Rhsim06G0176500	1.850	-0.273	-2.123	high
Treat-40d	RHSIM_Rhsim06G0226100	-0.564	-0.463	0.101	no
Treat-40d	RHSIM_Rhsim07G0134200	-0.789	-0.617	0.172	no
Treat-40d	RHSIM_Rhsim05G0132600	0.478	1.475	0.997	no
Treat-40d	RHSIM_Rhsim06G0006900	0.766	0.910	0.144	no
Treat-40d	RHSIM_Rhsim07G0082500	0.041	1.596	1.555	no
Treat-40d	RHSIM_Rhsim06G0201700	0.033	-0.817	-0.850	no
Treat-40d	RHSIM_Rhsim07G0131300	2.123	2.337	0.214	high
Treat-40d	RHSIM_Rhsim06G0001500	0.620	-0.158	-0.779	no
Treat-40d	RHSIM_Rhsim06G0236000	-0.365	-1.562	-1.197	no
Treat-40d	RHSIM_Rhsim06G0236100	-0.800	-1.114	-0.315	no
Treat-40d	RHSIM_Rhsim06G0007900	0.067	-0.826	-0.893	no
Treat-40d	RHSIM_Rhsim06G0184600	-0.497	-1.638	-1.141	no
Treat-40d	RHSIM_Rhsim07G0061200	-1.209	-1.182	0.028	low
Treat-40d	RHSIM_Rhsim06G0163900	0.535	0.968	0.432	no
Treat-40d	RHSIM_Rhsim05G0182800	0.603	-0.071	-0.674	no
Treat-40d	RHSIM_Rhsim06G0035200	-0.984	-1.519	-0.535	no
Treat-40d	RHSIM_Rhsim05G0142700	0.851	0.460	-0.391	no
Treat-40d	RHSIM_Rhsim07G0053300	1.251	1.535	0.284	high
Treat-40d	RHSIM_Rhsim05G0041300	-0.492	-0.283	0.209	no
Treat-40d	RHSIM_Rhsim06G0026800	0.176	-1.273	-1.449	no
Treat-40d	RHSIM_Rhsim07G0020400	-0.661	-1.320	-0.660	no
Treat-40d	RHSIM_Rhsim07G0078500	0.004	-1.401	-1.405	no
Treat-40d	RHSIM_Rhsim06G0194400	2.418	0.908	-1.509	high
Treat-40d	RHSIM_Rhsim06G0191000	0.099	1.772	1.674	no

Group	Gene	PTR (z-score difference)	z(log2 LFQ)	z(log2 FPKM)	extreme-PTR
Treat-40d	RHSIM_Rhsim05G0167300	1.139	1.328	0.189	high
Treat-40d	RHSIM_Rhsim06G0226700	0.964	0.994	0.029	no
Treat-40d	RHSIM_Rhsim05G0062900	-0.117	-0.964	-0.847	no
Treat-40d	RHSIM_Rhsim07G0019100	-1.624	-0.380	1.244	low
Treat-40d	RHSIM_Rhsim07G0064900	0.330	-0.494	-0.824	no
Treat-40d	RHSIM_Rhsim06G0055300	0.982	1.614	0.632	no
Treat-40d	RHSIM_Rhsim07G0053700	0.186	-0.311	-0.497	no
Treat-40d	RHSIM_Rhsim06G0065900	-0.549	-0.354	0.195	no
Treat-40d	RHSIM_Rhsim06G0041800	-0.742	-0.539	0.204	no
Treat-40d	RHSIM_Rhsim06G0047900	0.139	0.521	0.382	no
Treat-40d	RHSIM_Rhsim07G0090200	-0.714	1.218	1.933	no
Treat-40d	RHSIM_Rhsim05G0002000	-0.891	-0.801	0.090	no
Treat-40d	RHSIM_Rhsim06G0171700	0.538	0.592	0.054	no
Treat-40d	RHSIM_Rhsim07G0026500	0.748	0.179	-0.569	no
Treat-40d	RHSIM_Rhsim07G0198500	0.895	-0.776	-1.671	no
Treat-40d	RHSIM_Rhsim05G0139700	1.473	0.441	-1.033	high
Treat-40d	RHSIM_Rhsim06G0159400	0.549	0.176	-0.373	no
Treat-40d	RHSIM_Rhsim06G0240800	1.779	-0.051	-1.830	high
Treat-40d	RHSIM_Rhsim05G0231800	0.446	0.733	0.286	no
Treat-40d	RHSIM_Rhsim06G0049600	1.282	-0.258	-1.541	high
Treat-40d	RHSIM_Rhsim05G0143600	-0.405	-0.774	-0.369	no
Treat-40d	RHSIM_Rhsim07G0039100	1.897	2.031	0.134	high
Treat-40d	RHSIM_Rhsim05G0148200	-0.256	0.185	0.441	no
Treat-40d	RHSIM_Rhsim06G0122300	-1.373	-0.280	1.093	low
Treat-40d	RHSIM_Rhsim07G0113300	0.418	0.894	0.476	no
Treat-40d	RHSIM_Rhsim07G0145600	0.465	0.228	-0.237	no
Treat-40d	RHSIM_Rhsim06G0092600	0.367	-0.511	-0.877	no
Treat-40d	RHSIM_Rhsim05G0096400	0.151	1.297	1.146	no
Treat-40d	RHSIM_Rhsim07G0036300	0.830	0.262	-0.568	no
Treat-40d	RHSIM_Rhsim06G0218900	-1.143	-1.725	-0.582	low
Treat-40d	RHSIM_Rhsim05G0163000	0.694	0.046	-0.648	no
Treat-40d	RHSIM_Rhsim06G0046700	-1.538	-1.161	0.377	low

Group	Gene	PTR (z-score difference)	z(log2 LFQ)	z(log2 FPKM)	extreme-PTR
Treat-40d	RHSIM_Rhsim06G0131500	1.505	2.835	1.330	high
Treat-40d	RHSIM_Rhsim06G0054800	-1.563	1.208	2.772	low
Treat-40d	RHSIM_Rhsim06G0087800	-0.763	-1.000	-0.237	no
Treat-40d	RHSIM_Rhsim04G0083400	0.595	0.329	-0.266	no
Treat-40d	RHSIM_Rhsim06G0002900	0.092	0.245	0.153	no
Treat-40d	RHSIM_Rhsim06G0130100	0.087	1.078	0.991	no
Treat-40d	RHSIM_Rhsim04G0085300	-1.936	-1.087	0.849	low
Treat-40d	RHSIM_Rhsim06G0229100	-1.185	-0.724	0.461	low
Treat-40d	RHSIM_Rhsim06G0178400	-0.433	0.373	0.806	no
Treat-40d	RHSIM_Rhsim06G0135300	-0.216	0.242	0.458	no
Treat-40d	RHSIM_Rhsim04G0169200	-0.467	0.001	0.469	no
Treat-40d	RHSIM_Rhsim05G0017200	-1.217	-1.434	-0.217	low
Treat-40d	RHSIM_Rhsim05G0185400	-0.259	-0.166	0.093	no
Treat-40d	RHSIM_Rhsim04G0010400	0.328	0.336	0.008	no
Treat-40d	RHSIM_Rhsim06G0121800	-1.113	-1.387	-0.274	no
Treat-40d	RHSIM_Rhsim05G0030300	0.739	1.252	0.513	no
Treat-40d	RHSIM_Rhsim05G0059500	0.897	-0.408	-1.305	no
Treat-40d	RHSIM_Rhsim04G0118700	0.194	0.005	-0.189	no
Treat-40d	RHSIM_Rhsim05G0139000	-1.708	-0.466	1.243	low
Treat-40d	RHSIM_Rhsim06G0099300	0.885	-0.256	-1.142	no
Treat-40d	RHSIM_Rhsim05G0103900	0.644	0.734	0.090	no
Treat-40d	RHSIM_Rhsim06G0146500	0.849	1.811	0.962	no
Treat-40d	RHSIM_Rhsim06G0057900	-0.298	-0.532	-0.233	no
Treat-40d	RHSIM_Rhsim05G0089600	-2.815	-1.229	1.586	low
Treat-40d	RHSIM_Rhsim05G0013800	0.133	0.739	0.606	no
Treat-40d	RHSIM_Rhsim06G0156300	-0.584	0.102	0.686	no
Treat-40d	RHSIM_Rhsim05G0050000	1.565	0.787	-0.778	high
Treat-40d	RHSIM_Rhsim06G0172700	-3.833	-1.391	2.442	low
Treat-40d	RHSIM_Rhsim05G0180000	0.153	-0.758	-0.911	no
Treat-40d	RHSIM_Rhsim06G0185200	0.952	1.117	0.165	no
Treat-40d	RHSIM_Rhsim05G0020600	-0.715	-0.069	0.647	no
Treat-40d	RHSIM_Rhsim04G0213800	0.149	0.630	0.481	no

Group	Gene	PTR (z-score difference)	z(log2 LFQ)	z(log2 FPKM)	extreme-PTR
Treat-40d	RHSIM_Rhsim05G0069400	1.281	-0.114	-1.395	high
Treat-40d	RHSIM_Rhsim05G0093200	-0.119	-0.827	-0.708	no
Treat-40d	RHSIM_Rhsim05G0207300	0.435	-1.377	-1.812	no
Treat-40d	RHSIM_Rhsim05G0035000	0.849	1.332	0.483	no
Treat-40d	RHSIM_Rhsim04G0032800	0.299	1.805	1.506	no
Treat-40d	RHSIM_Rhsim06G0020600	-1.216	-1.034	0.181	low
Treat-40d	RHSIM_Rhsim05G0118000	-0.523	-0.380	0.144	no
Treat-40d	RHSIM_Rhsim06G0235200	-0.628	-1.365	-0.736	no
Treat-40d	RHSIM_Rhsim05G0064500	0.759	1.181	0.422	no
Treat-40d	RHSIM_Rhsim06G0064300	-0.701	-0.998	-0.297	no
Treat-40d	RHSIM_Rhsim05G0012000	-0.156	1.792	1.947	no
Treat-40d	RHSIM_Rhsim06G0066600	1.021	-0.635	-1.656	no
Treat-40d	RHSIM_Rhsim05G0022300	1.118	0.755	-0.363	high
Treat-40d	RHSIM_Rhsim05G0054100	-0.409	-0.513	-0.104	no
Treat-40d	RHSIM_Rhsim05G0161200	0.168	0.046	-0.122	no
Treat-40d	RHSIM_Rhsim06G0197400	1.420	1.734	0.313	high
Treat-40d	RHSIM_Rhsim05G0070000	-0.154	0.717	0.871	no
Treat-40d	RHSIM_Rhsim06G0049800	-1.010	0.161	1.171	no
Treat-40d	RHSIM_Rhsim04G0091600	-0.779	-0.546	0.233	no
Treat-40d	RHSIM_Rhsim06G0073600	0.716	0.755	0.039	no
Treat-40d	RHSIM_Rhsim06G0212900	0.840	0.752	-0.087	no
Treat-40d	RHSIM_Rhsim05G0174500	-3.122	0.560	3.682	low
Treat-40d	RHSIM_Rhsim05G0223200	-0.755	-1.575	-0.820	no
Treat-40d	RHSIM_Rhsim05G0012400	1.728	0.537	-1.191	high
Treat-40d	RHSIM_Rhsim06G0049500	1.029	0.807	-0.222	no
Treat-40d	RHSIM_Rhsim05G0000600	1.283	-0.352	-1.635	high
Treat-40d	RHSIM_Rhsim05G0202500	0.843	-0.077	-0.920	no
Treat-40d	RHSIM_Rhsim06G0086900	-1.115	-0.568	0.547	no
Treat-40d	RHSIM_Rhsim05G0049500	-0.513	0.325	0.838	no
Treat-40d	RHSIM_Rhsim05G0070900	1.050	1.254	0.204	no
Treat-40d	RHSIM_Rhsim04G0246500	2.102	0.334	-1.769	high
Treat-40d	RHSIM_Rhsim06G0012100	0.121	-0.018	-0.139	no

Group	Gene	PTR (z-score difference)	z(log2 LFQ)	z(log2 FPKM)	extreme-PTR
Treat-40d	RHSIM_Rhsim06G0236300	1.480	-0.420	-1.900	high
Treat-40d	RHSIM_Rhsim05G0063200	1.863	1.399	-0.464	high
Treat-40d	RHSIM_Rhsim05G0180400	0.612	2.346	1.734	no
Treat-40d	RHSIM_Rhsim05G0137700	-0.819	0.126	0.945	no
Treat-40d	RHSIM_Rhsim04G0243100	0.377	1.332	0.955	no
Treat-40d	RHSIM_Rhsim04G0234700	0.355	0.304	-0.051	no
Treat-40d	RHSIM_Rhsim05G0202800	1.880	0.700	-1.180	high
Treat-40d	RHSIM_Rhsim05G0163600	0.787	-0.645	-1.432	no
Treat-40d	RHSIM_Rhsim06G0037800	-0.686	0.587	1.273	no
Treat-40d	RHSIM_Rhsim05G0079600	-0.256	-0.288	-0.032	no
Treat-40d	RHSIM_Rhsim04G0016100	1.599	0.861	-0.738	high
Treat-40d	RHSIM_Rhsim05G0088400	0.454	0.684	0.229	no
Treat-40d	RHSIM_Rhsim04G0146600	-0.761	-1.174	-0.413	no
Treat-40d	RHSIM_Rhsim06G0161200	0.643	1.004	0.361	no
Treat-40d	RHSIM_Rhsim06G0093300	-0.991	-0.798	0.193	no
Treat-40d	RHSIM_Rhsim06G0011900	0.218	-1.259	-1.477	no
Treat-40d	RHSIM_Rhsim06G0060100	0.009	-0.146	-0.154	no
Treat-40d	RHSIM_Rhsim06G0047800	0.146	1.028	0.883	no
Treat-40d	RHSIM_Rhsim05G0062500	1.081	0.063	-1.019	no
Treat-40d	RHSIM_Rhsim06G0186600	-0.449	-0.443	0.006	no
Treat-40d	RHSIM_Rhsim06G0237500	-0.018	-0.555	-0.537	no
Treat-40d	RHSIM_Rhsim06G0166400	0.285	-0.866	-1.152	no
Treat-40d	RHSIM_Rhsim05G0224600	-0.798	-0.348	0.450	no
Treat-40d	RHSIM_Rhsim06G0072000	0.044	0.404	0.361	no
Treat-40d	RHSIM_Rhsim06G0102100	0.556	1.558	1.001	no
Treat-40d	RHSIM_Rhsim04G0102500	1.591	0.047	-1.544	high
Treat-40d	RHSIM_Rhsim05G0230900	0.812	0.851	0.039	no
Treat-40d	RHSIM_Rhsim06G0014600	1.204	1.039	-0.165	high
Treat-40d	RHSIM_Rhsim04G0118500	1.016	0.557	-0.459	no
Treat-40d	RHSIM_Rhsim06G0038600	0.285	2.293	2.008	no
Treat-40d	RHSIM_Rhsim06G0077300	-0.259	0.311	0.570	no
Treat-40d	RHSIM_Rhsim06G0214800	0.328	0.807	0.478	no

Group	Gene	PTR (z-score difference)	z(log2 LFQ)	z(log2 FPKM)	extreme-PTR
Treat-40d	RHSIM_Rhsim05G0169300	-1.162	-1.298	-0.136	low
Treat-40d	RHSIM_Rhsim04G0101900	-0.011	1.354	1.365	no
Treat-40d	RHSIM_Rhsim04G0149800	0.311	-0.360	-0.670	no
Treat-40d	RHSIM_Rhsim06G0116800	-0.057	0.014	0.071	no
Treat-40d	RHSIM_Rhsim04G0119100	1.027	0.760	-0.267	no
Treat-40d	RHSIM_Rhsim05G0108700	-1.263	-1.701	-0.438	low
Treat-40d	RHSIM_Rhsim04G0186900	0.804	-0.613	-1.417	no
Treat-40d	RHSIM_Rhsim06G0040500	0.317	-1.138	-1.455	no
Treat-40d	RHSIM_Rhsim06G0076100	1.108	1.717	0.609	no
Treat-40d	RHSIM_Rhsim05G0200500	-1.332	1.723	3.055	low
Treat-40d	RHSIM_Rhsim06G0007500	-0.481	-1.114	-0.633	no
Treat-40d	RHSIM_Rhsim05G0114400	0.877	0.280	-0.597	no
Treat-40d	RHSIM_Rhsim06G0110000	0.500	0.414	-0.086	no
Treat-40d	RHSIM_Rhsim04G0179200	-1.671	-1.987	-0.316	low
Treat-40d	RHSIM_Rhsim05G0101300	-0.596	-0.662	-0.067	no
Treat-40d	RHSIM_Rhsim06G0057200	0.558	0.074	-0.484	no
Treat-40d	RHSIM_Rhsim05G0031300	-0.837	0.379	1.216	no
Treat-40d	RHSIM_Rhsim04G0126900	1.286	1.333	0.047	high
Treat-40d	RHSIM_Rhsim06G0206900	0.225	1.064	0.839	no
Treat-40d	RHSIM_Rhsim04G0250200	0.628	-0.762	-1.390	no
Treat-40d	RHSIM_Rhsim06G0116700	0.453	0.647	0.195	no
Treat-40d	RHSIM_Rhsim05G0205700	-0.316	-0.241	0.075	no
Treat-40d	RHSIM_Rhsim04G0130200	0.775	0.502	-0.273	no
Treat-40d	RHSIM_Rhsim06G0196800	0.683	-0.843	-1.526	no
Treat-40d	RHSIM_Rhsim04G0108100	0.862	-0.238	-1.100	no
Treat-40d	RHSIM_Rhsim05G0087800	1.220	0.558	-0.662	high
Treat-40d	RHSIM_Rhsim04G0070700	-0.544	0.237	0.781	no
Treat-40d	RHSIM_Rhsim05G0029900	-1.274	-0.091	1.183	low
Treat-40d	RHSIM_Rhsim04G0174900	-0.270	0.460	0.730	no
Treat-40d	RHSIM_Rhsim05G0147900	-0.941	-1.125	-0.183	no
Treat-40d	RHSIM_Rhsim04G0221500	-1.486	-1.381	0.105	low
Treat-40d	RHSIM_Rhsim05G0068000	0.658	1.387	0.729	no

Group	Gene	PTR (z-score difference)	z(log2 LFQ)	z(log2 FPKM)	extreme-PTR
Treat-40d	RHSIM_Rhsim05G0062100	0.511	1.600	1.089	no
Treat-40d	RHSIM_Rhsim05G0039700	2.208	1.174	-1.034	high
Treat-40d	RHSIM_Rhsim05G0146400	0.017	-0.090	-0.107	no
Treat-40d	RHSIM_Rhsim06G0225700	0.496	-0.456	-0.951	no
Treat-40d	RHSIM_Rhsim04G0060200	0.623	-1.149	-1.772	no
Treat-40d	RHSIM_Rhsim06G0018800	0.227	-0.544	-0.771	no
Treat-40d	RHSIM_Rhsim05G0137500	0.262	0.159	-0.104	no
Treat-40d	RHSIM_Rhsim05G0170800	-0.291	-1.252	-0.961	no
Treat-40d	RHSIM_Rhsim04G0211500	-1.060	-0.960	0.101	no
Treat-40d	RHSIM_Rhsim05G0159800	-1.044	-0.538	0.507	no
Treat-40d	RHSIM_Rhsim05G0224000	-1.178	0.835	2.013	low
Treat-40d	RHSIM_Rhsim05G0163400	-0.620	-1.420	-0.800	no
Treat-40d	RHSIM_Rhsim06G0201000	-0.382	-0.927	-0.545	no
Treat-40d	RHSIM_Rhsim04G0011000	-1.062	-1.312	-0.250	no
Treat-40d	RHSIM_Rhsim05G0215900	2.216	0.567	-1.649	high
Treat-40d	RHSIM_Rhsim06G0021800	0.083	-1.602	-1.685	no
Treat-40d	RHSIM_Rhsim05G0193800	-0.675	-0.631	0.045	no
Treat-40d	RHSIM_Rhsim04G0021400	-0.637	-0.024	0.613	no
Treat-40d	RHSIM_Rhsim05G0161100	1.664	1.994	0.330	high
Treat-40d	RHSIM_Rhsim05G0176200	-0.035	-0.625	-0.590	no
Treat-40d	RHSIM_Rhsim06G0074900	0.814	0.573	-0.242	no
Treat-40d	RHSIM_Rhsim06G0230800	0.703	-1.756	-2.458	no
Treat-40d	RHSIM_Rhsim06G0061800	-3.174	-1.547	1.627	low
Treat-40d	RHSIM_Rhsim04G0236100	1.239	-0.054	-1.293	high
Treat-40d	RHSIM_Rhsim04G0206400	2.092	3.192	1.101	high
Treat-40d	RHSIM_Rhsim06G0071000	-0.317	0.022	0.339	no
Treat-40d	RHSIM_Rhsim05G0231000	-0.022	0.583	0.606	no
Treat-40d	RHSIM_Rhsim05G0116000	-0.015	-0.232	-0.217	no
Treat-40d	RHSIM_Rhsim04G0024600	0.594	0.815	0.222	no
Treat-40d	RHSIM_Rhsim05G0000700	1.002	-0.289	-1.291	no
Treat-40d	RHSIM_Rhsim05G0069500	1.166	0.734	-0.432	high
Treat-40d	RHSIM_Rhsim03G0222100	2.187	-0.101	-2.289	high

Group	Gene	PTR (z-score difference)	z(log2 LFQ)	z(log2 FPKM)	extreme-PTR
Treat-40d	RHSIM_Rhsim05G0146700	1.348	-0.450	-1.798	high
Treat-40d	RHSIM_Rhsim04G0167900	0.067	-1.358	-1.426	no
Treat-40d	RHSIM_Rhsim03G0253400	0.176	-0.110	-0.285	no
Treat-40d	RHSIM_Rhsim05G0069300	0.470	0.099	-0.371	no
Treat-40d	RHSIM_Rhsim03G0178900	-1.543	-1.254	0.289	low
Treat-40d	RHSIM_Rhsim05G0231600	-0.894	-0.481	0.413	no
Treat-40d	RHSIM_Rhsim05G0186700	1.391	0.735	-0.656	high
Treat-40d	RHSIM_Rhsim03G0262800	-1.995	-1.045	0.949	low
Treat-40d	RHSIM_Rhsim05G0011000	-0.926	-0.852	0.074	no
Treat-40d	RHSIM_Rhsim04G0102100	0.014	1.193	1.179	no
Treat-40d	RHSIM_Rhsim05G0115500	-0.580	0.021	0.601	no
Treat-40d	RHSIM_Rhsim05G0186400	0.866	-0.243	-1.109	no
Treat-40d	RHSIM_Rhsim05G0231200	-0.966	-0.558	0.407	no
Treat-40d	RHSIM_Rhsim04G0201600	0.383	-0.313	-0.696	no
Treat-40d	RHSIM_Rhsim05G0136600	1.949	0.513	-1.435	high
Treat-40d	RHSIM_Rhsim03G0064000	-0.288	-0.617	-0.328	no
Treat-40d	RHSIM_Rhsim05G0043400	0.231	-0.117	-0.347	no
Treat-40d	RHSIM_Rhsim05G0167500	-0.791	-1.264	-0.473	no
Treat-40d	RHSIM_Rhsim05G0049600	-1.236	1.154	2.390	low
Treat-40d	RHSIM_Rhsim05G0049900	-1.106	-1.205	-0.100	no
Treat-40d	RHSIM_Rhsim05G0203700	0.068	-0.148	-0.215	no
Treat-40d	RHSIM_Rhsim04G0132400	0.391	0.982	0.591	no
Treat-40d	RHSIM_Rhsim03G0187300	0.313	-0.039	-0.352	no
Treat-40d	RHSIM_Rhsim04G0072400	-0.507	-0.708	-0.201	no
Treat-40d	RHSIM_Rhsim04G0097900	0.094	-0.208	-0.302	no
Treat-40d	RHSIM_Rhsim05G0189500	-1.237	-0.082	1.155	low
Treat-40d	RHSIM_Rhsim05G0196300	-0.988	0.398	1.385	no
Treat-40d	RHSIM_Rhsim05G0072800	0.197	1.073	0.876	no
Treat-40d	RHSIM_Rhsim04G0220700	-0.647	-1.272	-0.625	no
Treat-40d	RHSIM_Rhsim05G0014300	-0.429	-0.225	0.204	no
Treat-40d	RHSIM_Rhsim04G0075000	-0.408	-0.490	-0.082	no
Treat-40d	RHSIM_Rhsim05G0102200	-0.024	-0.023	0.001	no

Group	Gene	PTR (z-score difference)	z(log2 LFQ)	z(log2 FPKM)	extreme-PTR
Treat-40d	RHSIM_Rhsim05G0204700	-1.223	-0.874	0.349	low
Treat-40d	RHSIM_Rhsim04G0231300	4.600	3.075	-1.525	high
Treat-40d	RHSIM_Rhsim05G0148900	-0.258	-0.943	-0.685	no
Treat-40d	RHSIM_Rhsim04G0009700	0.559	-0.327	-0.886	no
Treat-40d	RHSIM_Rhsim05G0006600	-0.243	-0.130	0.113	no
Treat-40d	RHSIM_Rhsim05G0221700	0.663	1.328	0.665	no
Treat-40d	RHSIM_Rhsim04G0049800	-0.596	0.125	0.720	no
Treat-40d	RHSIM_Rhsim05G0128900	-0.113	0.425	0.538	no
Treat-40d	RHSIM_Rhsim04G0003400	-1.499	-0.455	1.044	low
Treat-40d	RHSIM_Rhsim04G0122300	-1.004	1.931	2.935	no
Treat-40d	RHSIM_Rhsim05G0067600	1.146	0.486	-0.660	high
Treat-40d	RHSIM_Rhsim03G0127100	1.218	1.321	0.104	high
Treat-40d	RHSIM_Rhsim05G0080700	0.776	-0.423	-1.199	no
Treat-40d	RHSIM_Rhsim03G0031300	1.010	0.490	-0.521	no
Treat-40d	RHSIM_Rhsim05G0144300	0.257	-0.017	-0.274	no
Treat-40d	RHSIM_Rhsim05G0184800	-2.539	-0.801	1.738	low
Treat-40d	RHSIM_Rhsim04G0151900	0.460	1.846	1.387	no
Treat-40d	RHSIM_Rhsim05G0119700	0.185	-0.328	-0.513	no
Treat-40d	RHSIM_Rhsim04G0218900	0.280	-0.693	-0.973	no
Treat-40d	RHSIM_Rhsim05G0233000	0.731	0.029	-0.702	no
Treat-40d	RHSIM_Rhsim05G0135700	-1.815	-2.331	-0.516	low
Treat-40d	RHSIM_Rhsim05G0217500	1.057	1.557	0.499	no
Treat-40d	RHSIM_Rhsim04G0162200	2.172	1.037	-1.136	high
Treat-40d	RHSIM_Rhsim05G0013300	-1.252	-0.661	0.591	low
Treat-40d	RHSIM_Rhsim05G0177800	0.977	-0.404	-1.381	no
Treat-40d	RHSIM_Rhsim04G0136600	0.538	0.624	0.086	no
Treat-40d	RHSIM_Rhsim04G0157300	-0.402	-0.623	-0.221	no
Treat-40d	RHSIM_Rhsim03G0036800	-2.067	-1.657	0.410	low
Treat-40d	RHSIM_Rhsim03G0064600	-1.360	-0.377	0.983	low
Treat-40d	RHSIM_Rhsim05G0126300	-0.147	0.370	0.517	no
Treat-40d	RHSIM_Rhsim04G0183700	0.122	-0.452	-0.574	no
Treat-40d	RHSIM_Rhsim05G0219500	-0.544	0.974	1.518	no

Group	Gene	PTR (z-score difference)	z(log2 LFQ)	z(log2 FPKM)	extreme-PTR
Treat-40d	RHSIM_Rhsim05G0094600	2.005	2.232	0.227	high
Treat-40d	RHSIM_Rhsim03G0235900	1.525	1.520	-0.005	high
Treat-40d	RHSIM_Rhsim04G0003000	-1.365	-1.058	0.307	low
Treat-40d	RHSIM_Rhsim05G0068600	-1.785	-0.954	0.831	low
Treat-40d	RHSIM_Rhsim04G0135600	0.094	2.248	2.153	no
Treat-40d	RHSIM_Rhsim05G0103300	2.350	3.355	1.006	high
Treat-40d	RHSIM_Rhsim04G0196000	1.390	0.320	-1.070	high
Treat-40d	RHSIM_Rhsim05G0171100	-1.131	-1.399	-0.268	no
Treat-40d	RHSIM_Rhsim05G0092500	0.934	-0.229	-1.164	no
Treat-40d	RHSIM_Rhsim04G0204500	0.020	1.640	1.621	no
Treat-40d	RHSIM_Rhsim05G0002100	0.256	0.845	0.589	no
Treat-40d	RHSIM_Rhsim03G0234400	-0.512	-0.720	-0.208	no
Treat-40d	RHSIM_Rhsim04G0106100	0.900	-0.002	-0.902	no
Treat-40d	RHSIM_Rhsim04G0023600	-0.072	0.526	0.599	no
Treat-40d	RHSIM_Rhsim04G0208300	0.860	2.348	1.488	no
Treat-40d	RHSIM_Rhsim05G0000100	0.040	0.227	0.187	no
Treat-40d	RHSIM_Rhsim03G0122800	-0.913	-0.220	0.693	no
Treat-40d	RHSIM_Rhsim04G0247000	-0.721	-0.263	0.459	no
Treat-40d	RHSIM_Rhsim03G0222500	0.169	0.764	0.595	no
Treat-40d	RHSIM_Rhsim04G0225200	0.636	-0.585	-1.221	no
Treat-40d	RHSIM_Rhsim04G0237500	1.327	-0.898	-2.225	high
Treat-40d	RHSIM_Rhsim04G0021500	-2.178	-1.846	0.333	low
Treat-40d	RHSIM_Rhsim05G0048800	0.136	0.075	-0.060	no
Treat-40d	RHSIM_Rhsim03G0233000	-1.418	-0.997	0.422	low
Treat-40d	RHSIM_Rhsim05G0064300	-0.288	-0.168	0.120	no
Treat-40d	RHSIM_Rhsim03G0160100	1.168	1.833	0.665	high
Treat-40d	RHSIM_Rhsim04G0032700	0.324	1.655	1.331	no
Treat-40d	RHSIM_Rhsim04G0057100	1.506	0.044	-1.462	high
Treat-40d	RHSIM_Rhsim04G0091800	0.098	1.891	1.792	no
Treat-40d	RHSIM_Rhsim04G0127300	-0.482	0.109	0.591	no
Treat-40d	RHSIM_Rhsim04G0247700	0.968	0.564	-0.404	no
Treat-40d	RHSIM_Rhsim04G0046600	-0.734	-1.246	-0.512	no

Group	Gene	PTR (z-score difference)	z(log2 LFQ)	z(log2 FPKM)	extreme-PTR
Treat-40d	RHSIM_Rhsim04G0010600	0.730	1.227	0.497	no
Treat-40d	RHSIM_Rhsim05G0092700	1.780	0.718	-1.062	high
Treat-40d	RHSIM_Rhsim03G0218900	0.146	0.422	0.276	no
Treat-40d	RHSIM_Rhsim04G0069300	0.554	0.156	-0.398	no
Treat-40d	RHSIM_Rhsim05G0110000	0.941	-0.891	-1.832	no
Treat-40d	RHSIM_Rhsim04G0113400	0.462	0.083	-0.379	no
Treat-40d	RHSIM_Rhsim03G0217600	1.702	0.828	-0.874	high
Treat-40d	RHSIM_Rhsim05G0075000	0.289	0.186	-0.103	no
Treat-40d	RHSIM_Rhsim03G0200000	-0.552	-0.075	0.477	no
Treat-40d	RHSIM_Rhsim03G0028900	0.240	0.923	0.683	no
Treat-40d	RHSIM_Rhsim05G0034800	-1.601	-0.859	0.741	low
Treat-40d	RHSIM_Rhsim04G0229700	0.088	0.849	0.760	no
Treat-40d	RHSIM_Rhsim05G0076800	-1.047	-1.402	-0.355	no
Treat-40d	RHSIM_Rhsim04G0089100	1.633	-0.220	-1.853	high
Treat-40d	RHSIM_Rhsim05G0079900	1.067	0.789	-0.278	no
Treat-40d	RHSIM_Rhsim04G0156000	0.460	-1.224	-1.684	no
Treat-40d	RHSIM_Rhsim05G0148600	-0.450	-0.149	0.301	no
Treat-40d	RHSIM_Rhsim04G0247300	0.387	-0.844	-1.230	no
Treat-40d	RHSIM_Rhsim04G0202200	-0.645	-0.586	0.060	no
Treat-40d	RHSIM_Rhsim04G0073800	0.151	-0.824	-0.975	no
Treat-40d	RHSIM_Rhsim03G0167100	0.859	-0.458	-1.317	no
Treat-40d	RHSIM_Rhsim03G0257100	1.780	1.280	-0.501	high
Treat-40d	RHSIM_Rhsim04G0070800	0.794	-0.375	-1.169	no
Treat-40d	RHSIM_Rhsim03G0221000	-1.520	-1.178	0.342	low
Treat-40d	RHSIM_Rhsim05G0022100	-0.173	-0.002	0.171	no
Treat-40d	RHSIM_Rhsim04G0023800	-1.316	-0.810	0.505	low
Treat-40d	RHSIM_Rhsim03G0074500	0.261	-0.752	-1.013	no
Treat-40d	RHSIM_Rhsim04G0249000	0.497	1.092	0.594	no
Treat-40d	RHSIM_Rhsim03G0246100	0.797	-0.613	-1.410	no
Treat-40d	RHSIM_Rhsim04G0052100	-2.307	-1.344	0.963	low
Treat-40d	RHSIM_Rhsim04G0008300	-0.001	0.306	0.308	no
Treat-40d	RHSIM_Rhsim04G0205500	-2.450	-1.000	1.451	low

Group	Gene	PTR (z-score difference)	z(log2 LFQ)	z(log2 FPKM)	extreme-PTR
Treat-40d	RHSIM_Rhsim05G0179500	0.616	0.739	0.123	no
Treat-40d	RHSIM_Rhsim03G0025100	1.171	0.582	-0.589	high
Treat-40d	RHSIM_Rhsim03G0189600	-0.082	-0.685	-0.602	no
Treat-40d	RHSIM_Rhsim03G0080200	-0.744	-0.475	0.269	no
Treat-40d	RHSIM_Rhsim03G0171100	0.010	-2.067	-2.078	no
Treat-40d	RHSIM_Rhsim05G0223400	0.048	-1.320	-1.368	no
Treat-40d	RHSIM_Rhsim03G0227400	-0.918	1.182	2.100	no
Treat-40d	RHSIM_Rhsim04G0175800	-1.180	-1.190	-0.009	low
Treat-40d	RHSIM_Rhsim03G0250200	-0.800	-0.670	0.130	no
Treat-40d	RHSIM_Rhsim04G0222400	0.451	0.048	-0.402	no
Treat-40d	RHSIM_Rhsim03G0063800	1.641	0.883	-0.759	high
Treat-40d	RHSIM_Rhsim04G0205300	-0.809	-0.222	0.587	no
Treat-40d	RHSIM_Rhsim03G0140700	-0.924	-0.474	0.450	no
Treat-40d	RHSIM_Rhsim03G0069800	0.926	0.538	-0.388	no
Treat-40d	RHSIM_Rhsim03G0000700	1.836	0.187	-1.649	high
Treat-40d	RHSIM_Rhsim04G0134200	0.852	0.420	-0.433	no
Treat-40d	RHSIM_Rhsim05G0225700	-0.711	1.514	2.224	no
Treat-40d	RHSIM_Rhsim03G0229500	1.691	1.505	-0.187	high
Treat-40d	RHSIM_Rhsim04G0197000	0.827	1.503	0.677	no
Treat-40d	RHSIM_Rhsim04G0238000	0.842	-0.748	-1.590	no
Treat-40d	RHSIM_Rhsim04G0190900	-1.588	-1.706	-0.118	low
Treat-40d	RHSIM_Rhsim03G0153400	-1.969	0.262	2.231	low
Treat-40d	RHSIM_Rhsim04G0024500	-1.713	-0.988	0.725	low
Treat-40d	RHSIM_Rhsim03G0113100	-0.951	-1.337	-0.386	no
Treat-40d	RHSIM_Rhsim04G0015700	1.852	1.469	-0.383	high
Treat-40d	RHSIM_Rhsim02G0086400	-0.184	-0.824	-0.641	no
Treat-40d	RHSIM_Rhsim04G0217300	-2.032	-2.086	-0.055	low
Treat-40d	RHSIM_Rhsim04G0167200	2.672	2.329	-0.343	high
Treat-40d	RHSIM_Rhsim04G0213900	-0.442	-0.543	-0.100	no
Treat-40d	RHSIM_Rhsim02G0110200	-1.406	-0.841	0.565	low
Treat-40d	RHSIM_Rhsim04G0125000	-0.050	-0.661	-0.611	no
Treat-40d	RHSIM_Rhsim03G0192100	0.184	-0.103	-0.287	no

Group	Gene	PTR (z-score difference)	z(log2 LFQ)	z(log2 FPKM)	extreme-PTR
Treat-40d	RHSIM_Rhsim02G0158900	-0.366	-0.789	-0.423	no
Treat-40d	RHSIM_Rhsim03G0068500	-0.261	-1.908	-1.648	no
Treat-40d	RHSIM_Rhsim04G0046500	-0.675	-0.608	0.067	no
Treat-40d	RHSIM_Rhsim04G0019500	1.276	1.577	0.301	high
Treat-40d	RHSIM_Rhsim04G0220400	-0.543	-0.206	0.337	no
Treat-40d	RHSIM_Rhsim04G0003900	0.477	-0.594	-1.070	no
Treat-40d	RHSIM_Rhsim03G0093600	0.499	0.131	-0.367	no
Treat-40d	RHSIM_Rhsim04G0200100	1.307	-0.161	-1.469	high
Treat-40d	RHSIM_Rhsim04G0048200	-0.237	-0.062	0.175	no
Treat-40d	RHSIM_Rhsim04G0166900	0.818	1.694	0.876	no
Treat-40d	RHSIM_Rhsim04G0235300	1.395	-0.041	-1.436	high
Treat-40d	RHSIM_Rhsim04G0124400	-0.972	-0.112	0.860	no
Treat-40d	RHSIM_Rhsim04G0120400	-1.388	-0.525	0.863	low
Treat-40d	RHSIM_Rhsim03G0045900	0.366	-0.632	-0.998	no
Treat-40d	RHSIM_Rhsim04G0015800	-0.610	-0.886	-0.276	no
Treat-40d	RHSIM_Rhsim02G0250400	-0.220	-0.260	-0.040	no
Treat-40d	RHSIM_Rhsim04G0195000	0.632	0.693	0.062	no
Treat-40d	RHSIM_Rhsim04G0214300	0.300	0.318	0.019	no
Treat-40d	RHSIM_Rhsim04G0153100	0.525	0.044	-0.481	no
Treat-40d	RHSIM_Rhsim04G0085200	-1.272	-0.630	0.641	low
Treat-40d	RHSIM_Rhsim03G0153200	0.357	-0.522	-0.879	no
Treat-40d	RHSIM_Rhsim04G0053900	0.920	-1.180	-2.100	no
Treat-40d	RHSIM_Rhsim04G0164100	0.387	0.023	-0.364	no
Treat-40d	RHSIM_Rhsim04G0201900	0.665	0.874	0.209	no
Treat-40d	RHSIM_Rhsim04G0176300	-0.712	-1.067	-0.355	no
Treat-40d	RHSIM_Rhsim04G0167000	0.223	1.658	1.435	no
Treat-40d	RHSIM_Rhsim02G0232000	-1.002	0.171	1.173	no
Treat-40d	RHSIM_Rhsim04G0149700	-0.234	0.541	0.776	no
Treat-40d	RHSIM_Rhsim04G0160100	1.579	0.972	-0.607	high
Treat-40d	RHSIM_Rhsim04G0147500	1.270	-0.039	-1.309	high
Treat-40d	RHSIM_Rhsim04G0214400	0.512	0.386	-0.125	no
Treat-40d	RHSIM_Rhsim04G0016500	-1.334	-2.041	-0.707	low

Group	Gene	PTR (z-score difference)	z(log2 LFQ)	z(log2 FPKM)	extreme-PTR
Treat-40d	RHSIM_Rhsim04G0203600	1.235	1.818	0.583	high
Treat-40d	RHSIM_Rhsim04G0231000	0.504	2.614	2.110	no
Treat-40d	RHSIM_Rhsim04G0188800	-1.498	-1.302	0.196	low
Treat-40d	RHSIM_Rhsim03G0212900	0.766	0.586	-0.180	no
Treat-40d	RHSIM_Rhsim04G0212200	-0.427	-0.956	-0.529	no
Treat-40d	RHSIM_Rhsim04G0228000	1.415	-0.085	-1.500	high
Treat-40d	RHSIM_Rhsim04G0246200	-0.568	-0.188	0.380	no
Treat-40d	RHSIM_Rhsim03G0223700	-0.350	-0.479	-0.129	no
Treat-40d	RHSIM_Rhsim04G0026700	-0.961	-0.327	0.633	no
Treat-40d	RHSIM_Rhsim03G0055600	0.963	-0.796	-1.759	no
Treat-40d	RHSIM_Rhsim04G0088200	0.507	-0.226	-0.733	no
Treat-40d	RHSIM_Rhsim04G0083100	0.386	1.261	0.875	no
Treat-40d	RHSIM_Rhsim04G0008600	0.195	0.286	0.091	no
Treat-40d	RHSIM_Rhsim02G0248700	1.321	2.047	0.726	high
Treat-40d	RHSIM_Rhsim03G0009100	0.650	1.435	0.785	no
Treat-40d	RHSIM_Rhsim04G0109400	0.471	-0.162	-0.633	no
Treat-40d	RHSIM_Rhsim04G0008000	-1.431	-1.492	-0.061	low
Treat-40d	RHSIM_Rhsim02G0173800	0.854	0.050	-0.803	no
Treat-40d	RHSIM_Rhsim04G0234400	1.475	-0.187	-1.662	high
Treat-40d	RHSIM_Rhsim03G0167300	-2.248	-0.453	1.795	low
Treat-40d	RHSIM_Rhsim02G0254600	0.572	-0.693	-1.266	no
Treat-40d	RHSIM_Rhsim04G0243200	-1.171	-0.575	0.595	low
Treat-40d	RHSIM_Rhsim04G0065800	0.875	1.378	0.503	no
Treat-40d	RHSIM_Rhsim02G0000300	-0.064	-0.470	-0.405	no
Treat-40d	RHSIM_Rhsim03G0093500	-0.470	-0.933	-0.463	no
Treat-40d	RHSIM_Rhsim02G0165400	-0.683	-0.978	-0.295	no
Treat-40d	RHSIM_Rhsim04G0196300	0.144	0.548	0.404	no
Treat-40d	RHSIM_Rhsim03G0224100	-0.031	0.351	0.382	no
Treat-40d	RHSIM_Rhsim04G0228200	-1.157	-1.434	-0.277	low
Treat-40d	RHSIM_Rhsim03G0069100	0.134	-0.017	-0.151	no
Treat-40d	RHSIM_Rhsim02G0199000	-1.902	-1.587	0.315	low
Treat-40d	RHSIM_Rhsim04G0018700	-1.206	-0.992	0.214	low

Group	Gene	PTR (z-score difference)	z(log2 LFQ)	z(log2 FPKM)	extreme-PTR
Treat-40d	RHSIM_Rhsim02G0143100	-1.627	-0.731	0.896	low
Treat-40d	RHSIM_Rhsim02G0172200	0.993	1.081	0.088	no
Treat-40d	RHSIM_Rhsim03G0247800	-1.276	-0.991	0.285	low
Treat-40d	RHSIM_Rhsim04G0113500	0.585	1.259	0.674	no
Treat-40d	RHSIM_Rhsim03G0031100	-0.919	-1.705	-0.787	no
Treat-40d	RHSIM_Rhsim03G0141600	0.699	1.116	0.417	no
Treat-40d	RHSIM_Rhsim03G0236500	-0.295	0.334	0.629	no
Treat-40d	RHSIM_Rhsim04G0164800	2.556	2.155	-0.400	high
Treat-40d	RHSIM_Rhsim03G0120500	0.171	-0.308	-0.479	no
Treat-40d	RHSIM_Rhsim03G0186100	-0.744	-0.919	-0.174	no
Treat-40d	RHSIM_Rhsim03G0223900	1.187	0.270	-0.917	high
Treat-40d	RHSIM_Rhsim03G0017900	1.204	0.021	-1.183	high
Treat-40d	RHSIM_Rhsim04G0218600	-1.516	-1.031	0.485	low
Treat-40d	RHSIM_Rhsim03G0256000	0.731	-0.368	-1.099	no
Treat-40d	RHSIM_Rhsim04G0187900	-0.357	-1.213	-0.856	no
Treat-40d	RHSIM_Rhsim03G0164300	1.728	0.878	-0.850	high
Treat-40d	RHSIM_Rhsim04G0111500	-1.623	-1.007	0.615	low
Treat-40d	RHSIM_Rhsim03G0214900	0.761	0.386	-0.375	no
Treat-40d	RHSIM_Rhsim04G0002800	2.460	3.312	0.852	high
Treat-40d	RHSIM_Rhsim04G0245400	0.474	-0.273	-0.747	no
Treat-40d	RHSIM_Rhsim04G0208400	0.755	1.385	0.630	no
Treat-40d	RHSIM_Rhsim04G0229900	0.472	2.934	2.462	no
Treat-40d	RHSIM_Rhsim04G0216700	0.272	0.936	0.664	no
Treat-40d	RHSIM_Rhsim03G0236800	-1.482	-1.280	0.202	low
Treat-40d	RHSIM_Rhsim04G0123000	0.650	1.348	0.698	no
Treat-40d	RHSIM_Rhsim03G0211000	0.532	0.979	0.447	no
Treat-40d	RHSIM_Rhsim02G0193100	-0.979	-0.836	0.143	no
Treat-40d	RHSIM_Rhsim03G0208400	0.816	-0.657	-1.472	no
Treat-40d	RHSIM_Rhsim03G0055400	0.198	-1.046	-1.244	no
Treat-40d	RHSIM_Rhsim03G0121600	-0.244	-0.316	-0.073	no
Treat-40d	RHSIM_Rhsim03G0088200	0.259	1.018	0.758	no
Treat-40d	RHSIM_Rhsim04G0008500	0.029	-0.362	-0.390	no

Group	Gene	PTR (z-score difference)	z(log2 LFQ)	z(log2 FPKM)	extreme-PTR
Treat-40d	RHSIM_Rhsim03G0230800	-0.083	-0.154	-0.071	no
Treat-40d	RHSIM_Rhsim04G0222300	0.173	-0.368	-0.541	no
Treat-40d	RHSIM_Rhsim03G0161300	-0.391	-0.041	0.350	no
Treat-40d	RHSIM_Rhsim03G0188000	-1.240	-0.314	0.926	low
Treat-40d	RHSIM_Rhsim03G0046200	0.490	0.424	-0.066	no
Treat-40d	RHSIM_Rhsim02G0059700	-1.163	-0.864	0.299	low
Treat-40d	RHSIM_Rhsim03G0009300	1.024	0.203	-0.821	no
Treat-40d	RHSIM_Rhsim03G0245100	0.173	-0.038	-0.211	no
Treat-40d	RHSIM_Rhsim03G0028400	0.120	-0.996	-1.116	no
Treat-40d	RHSIM_Rhsim04G0245900	1.403	0.850	-0.553	high
Treat-40d	RHSIM_Rhsim03G0264400	0.923	-0.137	-1.060	no
Treat-40d	RHSIM_Rhsim02G0123600	-1.726	-0.614	1.112	low
Treat-40d	RHSIM_Rhsim04G0015500	-0.698	-0.130	0.568	no
Treat-40d	RHSIM_Rhsim04G0028100	1.632	-0.467	-2.099	high
Treat-40d	RHSIM_Rhsim04G0249600	0.866	0.168	-0.698	no
Treat-40d	RHSIM_Rhsim03G0070200	-0.146	0.090	0.236	no
Treat-40d	RHSIM_Rhsim03G0234300	0.414	0.173	-0.241	no
Treat-40d	RHSIM_Rhsim03G0121100	0.596	0.185	-0.411	no
Treat-40d	RHSIM_Rhsim04G0021900	0.481	1.860	1.378	no
Treat-40d	RHSIM_Rhsim02G0237500	0.883	0.430	-0.453	no
Treat-40d	RHSIM_Rhsim04G0015400	-0.201	-0.581	-0.380	no
Treat-40d	RHSIM_Rhsim04G0074100	-1.054	-1.119	-0.065	no
Treat-40d	RHSIM_Rhsim03G0163800	-0.983	-0.122	0.861	no
Treat-40d	RHSIM_Rhsim03G0241100	-0.600	-1.014	-0.414	no
Treat-40d	RHSIM_Rhsim02G0001600	-0.904	-0.761	0.143	no
Treat-40d	RHSIM_Rhsim03G0007100	-0.463	-0.485	-0.021	no
Treat-40d	RHSIM_Rhsim03G0197200	0.642	0.378	-0.265	no
Treat-40d	RHSIM_Rhsim02G0159600	-0.470	1.049	1.519	no
Treat-40d	RHSIM_Rhsim03G0018300	0.559	1.206	0.647	no
Treat-40d	RHSIM_Rhsim03G0011100	-0.189	0.914	1.103	no
Treat-40d	RHSIM_Rhsim03G0264900	-1.423	-0.665	0.758	low
Treat-40d	RHSIM_Rhsim03G0131600	-0.704	1.293	1.997	no

Group	Gene	PTR (z-score difference)	z(log2 LFQ)	z(log2 FPKM)	extreme-PTR
Treat-40d	RHSIM_Rhsim03G0215400	-0.271	0.996	1.266	no
Treat-40d	RHSIM_Rhsim02G0070700	0.315	0.842	0.526	no
Treat-40d	RHSIM_Rhsim02G0132400	-0.211	3.041	3.253	no
Treat-40d	RHSIM_Rhsim03G0058000	1.415	0.554	-0.861	high
Treat-40d	RHSIM_Rhsim04G0067400	-0.689	-0.173	0.517	no
Treat-40d	RHSIM_Rhsim02G0193600	-1.221	-2.054	-0.833	low
Treat-40d	RHSIM_Rhsim03G0088300	1.708	0.479	-1.229	high
Treat-40d	RHSIM_Rhsim03G0063900	-1.893	0.378	2.271	low
Treat-40d	RHSIM_Rhsim03G0259500	0.171	-1.168	-1.339	no
Treat-40d	RHSIM_Rhsim03G0052600	-2.760	-2.317	0.442	low
Treat-40d	RHSIM_Rhsim04G0243300	0.986	0.347	-0.638	no
Treat-40d	RHSIM_Rhsim02G0252000	-0.437	-0.140	0.296	no
Treat-40d	RHSIM_Rhsim03G0165500	-0.563	1.064	1.627	no
Treat-40d	RHSIM_Rhsim03G0076200	-0.150	-0.210	-0.060	no
Treat-40d	RHSIM_Rhsim03G0191300	0.548	1.386	0.838	no
Treat-40d	RHSIM_Rhsim03G0191800	0.404	-0.051	-0.455	no
Treat-40d	RHSIM_Rhsim03G0254400	-0.882	-0.514	0.368	no
Treat-40d	RHSIM_Rhsim04G0166600	-0.543	-0.599	-0.057	no
Treat-40d	RHSIM_Rhsim03G0180600	-1.061	1.400	2.461	no
Treat-40d	RHSIM_Rhsim02G0135500	-0.384	-0.101	0.283	no
Treat-40d	RHSIM_Rhsim02G0217800	0.747	0.865	0.118	no
Treat-40d	RHSIM_Rhsim02G0056200	1.343	2.550	1.207	high
Treat-40d	RHSIM_Rhsim04G0242100	0.692	-0.341	-1.033	no
Treat-40d	RHSIM_Rhsim02G0131800	0.784	-0.667	-1.451	no
Treat-40d	RHSIM_Rhsim03G0165100	1.046	-0.957	-2.003	no
Treat-40d	RHSIM_Rhsim03G0245900	-0.963	0.564	1.527	no
Treat-40d	RHSIM_Rhsim02G0071100	0.884	0.871	-0.013	no
Treat-40d	RHSIM_Rhsim03G0073900	1.845	0.110	-1.735	high
Treat-40d	RHSIM_Rhsim03G0171300	-0.005	1.981	1.987	no
Treat-40d	RHSIM_Rhsim03G0231400	-1.965	-2.024	-0.059	low
Treat-40d	RHSIM_Rhsim02G0235500	0.264	0.543	0.280	no
Treat-40d	RHSIM_Rhsim03G0067800	-0.340	-0.602	-0.262	no

Group	Gene	PTR (z-score difference)	z(log2 LFQ)	z(log2 FPKM)	extreme-PTR
Treat-40d	RHSIM_Rhsim02G0143300	1.004	2.662	1.658	no
Treat-40d	RHSIM_Rhsim03G0153500	-0.715	-0.851	-0.136	no
Treat-40d	RHSIM_Rhsim03G0105000	-0.503	-0.941	-0.437	no
Treat-40d	RHSIM_Rhsim04G0083900	0.628	1.053	0.425	no
Treat-40d	RHSIM_Rhsim03G0053000	0.748	1.412	0.664	no
Treat-40d	RHSIM_Rhsim02G0123500	0.031	-0.591	-0.623	no
Treat-40d	RHSIM_Rhsim03G0075500	-1.207	-0.358	0.850	low
Treat-40d	RHSIM_Rhsim02G0088400	0.833	1.413	0.579	no
Treat-40d	RHSIM_Rhsim04G0007400	1.688	3.208	1.519	high
Treat-40d	RHSIM_Rhsim04G0006500	-1.690	-1.367	0.323	low
Treat-40d	RHSIM_Rhsim01G0222900	0.639	0.029	-0.610	no
Treat-40d	RHSIM_Rhsim01G0240100	-1.221	-1.721	-0.500	low
Treat-40d	RHSIM_Rhsim02G0171900	1.817	1.189	-0.628	high
Treat-40d	RHSIM_Rhsim04G0051700	0.462	-0.292	-0.754	no
Treat-40d	RHSIM_Rhsim03G0067500	-0.617	0.972	1.589	no
Treat-40d	RHSIM_Rhsim04G0066100	1.179	0.353	-0.826	high
Treat-40d	RHSIM_Rhsim02G0066400	-0.157	-0.747	-0.590	no
Treat-40d	RHSIM_Rhsim02G0018900	0.774	-0.411	-1.185	no
Treat-40d	RHSIM_Rhsim01G0178500	-1.110	-0.068	1.043	no
Treat-40d	RHSIM_Rhsim04G0181100	0.728	1.375	0.647	no
Treat-40d	RHSIM_Rhsim03G0255900	-0.055	1.719	1.774	no
Treat-40d	RHSIM_Rhsim03G0243000	-0.879	-0.390	0.489	no
Treat-40d	RHSIM_Rhsim03G0186600	-0.372	0.744	1.116	no
Treat-40d	RHSIM_Rhsim02G0241900	-0.183	-1.379	-1.196	no
Treat-40d	RHSIM_Rhsim03G0245400	0.030	-0.442	-0.472	no
Treat-40d	RHSIM_Rhsim01G0167600	0.146	0.744	0.598	no
Treat-40d	RHSIM_Rhsim03G0225800	0.026	-0.666	-0.692	no
Treat-40d	RHSIM_Rhsim02G0133700	-0.790	-0.111	0.679	no
Treat-40d	RHSIM_Rhsim04G0072300	-0.409	-1.648	-1.239	no
Treat-40d	RHSIM_Rhsim04G0091400	-1.690	-1.053	0.637	low
Treat-40d	RHSIM_Rhsim03G0059700	0.773	0.625	-0.148	no
Treat-40d	RHSIM_Rhsim04G0085600	-0.286	0.554	0.839	no

Group	Gene	PTR (z-score difference)	z(log2 LFQ)	z(log2 FPKM)	extreme-PTR
Treat-40d	RHSIM_Rhsim04G0121700	1.400	1.021	-0.379	high
Treat-40d	RHSIM_Rhsim02G0253800	0.032	-0.363	-0.395	no
Treat-40d	RHSIM_Rhsim02G0043500	-0.388	0.317	0.705	no
Treat-40d	RHSIM_Rhsim03G0097500	-0.509	0.533	1.042	no
Treat-40d	RHSIM_Rhsim03G0254000	0.304	-0.492	-0.796	no
Treat-40d	RHSIM_Rhsim02G0031900	-0.554	-0.053	0.501	no
Treat-40d	RHSIM_Rhsim03G0235200	0.177	1.333	1.156	no
Treat-40d	RHSIM_Rhsim03G0091100	0.186	-0.616	-0.801	no
Treat-40d	RHSIM_Rhsim02G0071800	-0.167	0.105	0.273	no
Treat-40d	RHSIM_Rhsim01G0292400	-0.713	-0.292	0.421	no
Treat-40d	RHSIM_Rhsim02G0141300	-0.153	-0.490	-0.337	no
Treat-40d	RHSIM_Rhsim01G0203700	0.471	-0.318	-0.789	no
Treat-40d	RHSIM_Rhsim03G0129800	-0.734	-0.391	0.342	no
Treat-40d	RHSIM_Rhsim03G0038900	0.840	0.396	-0.445	no
Treat-40d	RHSIM_Rhsim02G0122100	0.134	0.369	0.235	no
Treat-40d	RHSIM_Rhsim01G0238000	-2.694	0.190	2.884	low
Treat-40d	RHSIM_Rhsim02G0243000	1.392	0.466	-0.926	high
Treat-40d	RHSIM_Rhsim01G0066800	-0.507	-1.206	-0.699	no
Treat-40d	RHSIM_Rhsim03G0125400	-2.511	-1.996	0.515	low
Treat-40d	RHSIM_Rhsim03G0161700	0.215	0.242	0.026	no
Treat-40d	RHSIM_Rhsim01G0112000	-1.331	-1.337	-0.005	low
Treat-40d	RHSIM_Rhsim02G0100900	2.272	0.527	-1.745	high
Treat-40d	RHSIM_Rhsim03G0261300	-0.736	0.018	0.754	no
Treat-40d	RHSIM_Rhsim02G0256200	-0.113	-0.222	-0.109	no
Treat-40d	RHSIM_Rhsim02G0022900	-1.185	-0.712	0.473	low
Treat-40d	RHSIM_Rhsim03G0110700	0.076	0.401	0.325	no
Treat-40d	RHSIM_Rhsim01G0141100	-0.425	0.122	0.547	no
Treat-40d	RHSIM_Rhsim03G0268200	0.995	0.151	-0.844	no
Treat-40d	RHSIM_Rhsim02G0008200	0.203	0.782	0.578	no
Treat-40d	RHSIM_Rhsim03G0225600	-2.292	-2.202	0.090	low
Treat-40d	RHSIM_Rhsim03G0152900	0.282	0.431	0.149	no
Treat-40d	RHSIM_Rhsim03G0243200	0.173	-0.490	-0.663	no

Group	Gene	PTR (z-score difference)	z(log2 LFQ)	z(log2 FPKM)	extreme-PTR
Treat-40d	RHSIM_Rhsim03G0236200	0.139	1.487	1.348	no
Treat-40d	RHSIM_Rhsim01G0105900	0.082	0.388	0.306	no
Treat-40d	RHSIM_Rhsim03G0212300	0.664	0.645	-0.018	no
Treat-40d	RHSIM_Rhsim03G0090500	-0.434	-0.870	-0.437	no
Treat-40d	RHSIM_Rhsim01G0147400	1.198	0.409	-0.790	high
Treat-40d	RHSIM_Rhsim02G0248400	0.981	1.593	0.612	no
Treat-40d	RHSIM_Rhsim03G0257300	-0.945	-0.332	0.613	no
Treat-40d	RHSIM_Rhsim02G0128600	-0.732	-0.386	0.345	no
Treat-40d	RHSIM_Rhsim03G0219500	0.082	1.446	1.364	no
Treat-40d	RHSIM_Rhsim03G0187400	1.064	0.750	-0.314	no
Treat-40d	RHSIM_Rhsim03G0040400	1.322	1.008	-0.314	high
Treat-40d	RHSIM_Rhsim03G0251700	-1.103	-0.507	0.596	no
Treat-40d	RHSIM_Rhsim03G0005600	-0.785	0.156	0.941	no
Treat-40d	RHSIM_Rhsim03G0167200	-0.117	-0.474	-0.357	no
Treat-40d	RHSIM_Rhsim03G0254200	-0.817	-1.374	-0.557	no
Treat-40d	RHSIM_Rhsim03G0136300	0.698	0.324	-0.374	no
Treat-40d	RHSIM_Rhsim02G0071400	-0.162	-1.037	-0.875	no
Treat-40d	RHSIM_Rhsim03G0230100	1.093	0.946	-0.147	no
Treat-40d	RHSIM_Rhsim03G0176800	-0.353	-1.194	-0.842	no
Treat-40d	RHSIM_Rhsim02G0060200	-0.588	-0.948	-0.361	no
Treat-40d	RHSIM_Rhsim02G0143200	0.269	-0.340	-0.609	no
Treat-40d	RHSIM_Rhsim03G0070300	1.237	0.656	-0.581	high
Treat-40d	RHSIM_Rhsim03G0117200	-0.795	-0.504	0.291	no
Treat-40d	RHSIM_Rhsim03G0212500	-0.950	0.291	1.240	no
Treat-40d	RHSIM_Rhsim02G0194100	0.074	1.414	1.341	no
Treat-40d	RHSIM_Rhsim02G0181400	-1.118	0.650	1.768	no
Treat-40d	RHSIM_Rhsim02G0107000	-0.318	0.341	0.659	no
Treat-40d	RHSIM_Rhsim02G0028200	1.028	0.043	-0.985	no
Treat-40d	RHSIM_Rhsim03G0219400	-0.698	-1.147	-0.449	no
Treat-40d	RHSIM_Rhsim03G0027400	0.927	1.029	0.101	no
Treat-40d	RHSIM_Rhsim03G0240000	-2.498	-1.488	1.010	low
Treat-40d	RHSIM_Rhsim02G0252600	-1.361	-0.498	0.863	low

Group	Gene	PTR (z-score difference)	z(log2 LFQ)	z(log2 FPKM)	extreme-PTR
Treat-40d	RHSIM_Rhsim03G0089800	-0.967	0.088	1.054	no
Treat-40d	RHSIM_Rhsim01G0249900	-0.213	1.196	1.409	no
Treat-40d	RHSIM_Rhsim03G0214200	0.433	-0.495	-0.928	no
Treat-40d	RHSIM_Rhsim02G0150100	-1.379	-0.105	1.274	low
Treat-40d	RHSIM_Rhsim03G0169600	0.443	-0.265	-0.708	no
Treat-40d	RHSIM_Rhsim03G0253100	-1.510	-0.722	0.788	low
Treat-40d	RHSIM_Rhsim02G0053700	-0.913	1.630	2.544	no
Treat-40d	RHSIM_Rhsim01G0170800	1.541	0.435	-1.105	high
Treat-40d	RHSIM_Rhsim03G0254900	0.688	0.890	0.202	no
Treat-40d	RHSIM_Rhsim02G0085700	0.460	-0.398	-0.858	no
Treat-40d	RHSIM_Rhsim03G0150200	2.058	1.184	-0.874	high
Treat-40d	RHSIM_Rhsim03G0190600	-0.288	-0.591	-0.303	no
Treat-40d	RHSIM_Rhsim03G0062900	-0.260	-0.981	-0.721	no
Treat-40d	RHSIM_Rhsim03G0049900	-0.717	0.222	0.939	no
Treat-40d	RHSIM_Rhsim03G0239600	-0.890	-1.234	-0.344	no
Treat-40d	RHSIM_Rhsim02G0002000	0.892	0.970	0.078	no
Treat-40d	RHSIM_Rhsim01G0183300	0.170	-0.616	-0.786	no
Treat-40d	RHSIM_Rhsim01G0001700	-1.090	-1.018	0.072	no
Treat-40d	RHSIM_Rhsim03G0147100	0.560	0.260	-0.300	no
Treat-40d	RHSIM_Rhsim03G0076700	-0.083	0.313	0.396	no
Treat-40d	RHSIM_Rhsim01G0283600	-2.123	0.300	2.423	low
Treat-40d	RHSIM_Rhsim02G0244700	-0.202	-0.173	0.029	no
Treat-40d	RHSIM_Rhsim02G0058700	1.109	-0.370	-1.479	high
Treat-40d	RHSIM_Rhsim03G0226600	-0.635	0.369	1.005	no
Treat-40d	RHSIM_Rhsim01G0123000	-1.507	-1.099	0.408	low
Treat-40d	RHSIM_Rhsim03G0003600	-0.664	-0.204	0.460	no
Treat-40d	RHSIM_Rhsim02G0005400	0.944	1.474	0.531	no
Treat-40d	RHSIM_Rhsim01G0192600	-0.876	-0.254	0.622	no
Treat-40d	RHSIM_Rhsim01G0258200	-0.295	0.588	0.883	no
Treat-40d	RHSIM_Rhsim01G0100600	-0.530	0.007	0.537	no
Treat-40d	RHSIM_Rhsim02G0236800	-2.355	-0.471	1.885	low
Treat-40d	RHSIM_Rhsim01G0280000	0.341	0.109	-0.233	no

Group	Gene	PTR (z-score difference)	z(log2 LFQ)	z(log2 FPKM)	extreme-PTR
Treat-40d	RHSIM_Rhsim01G0135600	-0.059	-0.446	-0.387	no
Treat-40d	RHSIM_Rhsim02G0175300	1.242	0.028	-1.214	high
Treat-40d	RHSIM_Rhsim03G0094200	0.562	0.030	-0.532	no
Treat-40d	RHSIM_Rhsim02G0195000	-0.614	-0.792	-0.178	no
Treat-40d	RHSIM_Rhsim01G0114000	-0.075	0.571	0.645	no
Treat-40d	RHSIM_Rhsim03G0052800	-2.772	0.795	3.567	low
Treat-40d	RHSIM_Rhsim01G0259100	-0.967	-1.059	-0.092	no
Treat-40d	RHSIM_Rhsim01G0049700	-1.045	-0.426	0.619	no
Treat-40d	RHSIM_Rhsim01G0190400	-0.051	0.221	0.272	no
Treat-40d	RHSIM_Rhsim01G0187400	0.593	-1.239	-1.831	no
Treat-40d	RHSIM_Rhsim01G0044000	0.669	2.072	1.404	no
Treat-40d	RHSIM_Rhsim03G0153700	0.372	0.333	-0.040	no
Treat-40d	RHSIM_Rhsim02G0246500	-0.314	0.504	0.819	no
Treat-40d	RHSIM_Rhsim01G0061400	-0.011	-0.457	-0.446	no
Treat-40d	RHSIM_Rhsim02G0004600	-1.366	-0.107	1.259	low
Treat-40d	RHSIM_Rhsim01G0049100	2.057	0.560	-1.497	high
Treat-40d	RHSIM_Rhsim02G0180700	-1.380	-0.437	0.943	low
Treat-40d	RHSIM_Rhsim01G0222000	1.394	0.083	-1.311	high
Treat-40d	RHSIM_Rhsim02G0242100	-4.232	-1.138	3.094	low
Treat-40d	RHSIM_Rhsim02G0207700	1.039	-0.540	-1.578	no
Treat-40d	RHSIM_Rhsim03G0227500	1.502	0.375	-1.126	high
Treat-40d	RHSIM_Rhsim02G0241000	-1.314	-1.337	-0.023	low
Treat-40d	RHSIM_Rhsim02G0018500	0.214	-0.705	-0.919	no
Treat-40d	RHSIM_Rhsim02G0115600	-1.284	-0.218	1.066	low
Treat-40d	RHSIM_Rhsim02G0070000	0.929	0.556	-0.373	no
Treat-40d	RHSIM_Rhsim02G0007400	-0.201	0.065	0.266	no
Treat-40d	RHSIM_Rhsim01G0208500	-1.251	1.574	2.825	low
Treat-40d	RHSIM_Rhsim02G0256900	0.710	-0.058	-0.767	no
Treat-40d	RHSIM_Rhsim02G0106100	-0.395	-0.888	-0.493	no
Treat-40d	RHSIM_Rhsim03G0213000	0.787	0.396	-0.392	no
Treat-40d	RHSIM_Rhsim03G0087300	0.507	-0.702	-1.209	no
Treat-40d	RHSIM_Rhsim02G0061700	0.007	1.406	1.399	no

Group	Gene	PTR (z-score difference)	z(log2 LFQ)	z(log2 FPKM)	extreme-PTR
Treat-40d	RHSIM_Rhsim02G0234600	-1.666	-1.600	0.066	low
Treat-40d	RHSIM_Rhsim03G0148000	-0.484	-0.726	-0.243	no
Treat-40d	RHSIM_Rhsim01G0169000	-2.844	-1.505	1.338	low
Treat-40d	RHSIM_Rhsim01G0232600	-1.018	-0.292	0.726	no
Treat-40d	RHSIM_Rhsim02G0084400	-0.385	-0.982	-0.596	no
Treat-40d	RHSIM_Rhsim01G0286300	0.629	0.325	-0.304	no
Treat-40d	RHSIM_Rhsim03G0247500	-1.817	-0.518	1.299	low
Treat-40d	RHSIM_Rhsim02G0217700	-0.413	0.352	0.765	no
Treat-40d	RHSIM_Rhsim02G0104800	0.195	-0.038	-0.233	no
Treat-40d	RHSIM_Rhsim01G0050400	0.381	-1.184	-1.565	no
Treat-40d	RHSIM_Rhsim01G0278500	-1.263	-1.209	0.053	low
Treat-40d	RHSIM_Rhsim02G0214300	0.814	0.415	-0.399	no
Treat-40d	RHSIM_Rhsim01G0060000	-0.891	0.367	1.258	no
Treat-40d	RHSIM_Rhsim01G0258800	0.546	-0.070	-0.616	no
Treat-40d	RHSIM_Rhsim01G0114100	-0.652	-0.193	0.460	no
Treat-40d	RHSIM_Rhsim01G0258700	1.034	0.910	-0.123	no
Treat-40d	RHSIM_Rhsim02G0007600	2.284	0.284	-2.000	high
Treat-40d	RHSIM_Rhsim02G0069600	-0.298	0.652	0.950	no
Treat-40d	RHSIM_Rhsim01G0127700	1.590	1.041	-0.548	high
Treat-40d	RHSIM_Rhsim02G0031200	-0.081	-0.064	0.016	no
Treat-40d	RHSIM_Rhsim01G0192800	-1.750	1.598	3.347	low
Treat-40d	RHSIM_Rhsim03G0004200	-0.850	-0.489	0.361	no
Treat-40d	RHSIM_Rhsim02G0066800	-1.144	-0.850	0.294	low
Treat-40d	RHSIM_Rhsim01G0240400	0.921	-0.283	-1.203	no
Treat-40d	RHSIM_Rhsim02G0068800	0.470	0.332	-0.138	no
Treat-40d	RHSIM_Rhsim01G0291700	-0.731	-1.602	-0.871	no
Treat-40d	RHSIM_Rhsim01G0131800	2.123	2.217	0.094	high
Treat-40d	RHSIM_Rhsim01G0171300	1.205	2.368	1.163	high
Treat-40d	RHSIM_Rhsim01G0116600	-0.892	-1.304	-0.412	no
Treat-40d	RHSIM_Rhsim02G0210500	-0.869	-0.793	0.076	no
Treat-40d	RHSIM_Rhsim01G0230200	-0.852	-0.699	0.153	no
Treat-40d	RHSIM_Rhsim02G0028100	-0.308	1.021	1.329	no

Group	Gene	PTR (z-score difference)	z(log2 LFQ)	z(log2 FPKM)	extreme-PTR
Treat-40d	RHSIM_Rhsim01G0131300	1.485	0.467	-1.017	high
Treat-40d	RHSIM_Rhsim01G0142100	-0.048	-0.288	-0.241	no
Treat-40d	RHSIM_Rhsim01G0220300	0.368	-0.557	-0.926	no
Treat-40d	RHSIM_Rhsim03G0090000	-1.368	0.169	1.537	low
Treat-40d	RHSIM_Rhsim01G0219100	1.074	3.215	2.141	no
Treat-40d	RHSIM_Rhsim03G0151200	-0.015	1.384	1.399	no
Treat-40d	RHSIM_Rhsim01G0290600	0.193	-0.081	-0.274	no
Treat-40d	RHSIM_Rhsim01G0102500	-0.294	-0.195	0.099	no
Treat-40d	RHSIM_Rhsim01G0278100	-1.153	-1.150	0.003	low
Treat-40d	RHSIM_Rhsim02G0099900	-0.698	-0.844	-0.146	no
Treat-40d	RHSIM_Rhsim02G0004900	-0.760	-0.646	0.114	no
Treat-40d	RHSIM_Rhsim01G0226900	-1.566	-0.920	0.647	low
Treat-40d	RHSIM_Rhsim02G0124600	0.530	0.329	-0.200	no
Treat-40d	RHSIM_Rhsim02G0157100	-0.976	-1.382	-0.406	no
Treat-40d	RHSIM_Rhsim01G0135700	-0.134	0.572	0.706	no
Treat-40d	RHSIM_Rhsim02G0181000	-0.048	0.253	0.301	no
Treat-40d	RHSIM_Rhsim02G0096400	0.384	0.307	-0.077	no
Treat-40d	RHSIM_Rhsim02G0089800	0.131	-0.060	-0.191	no
Treat-40d	RHSIM_Rhsim02G0169600	0.113	0.057	-0.056	no
Treat-40d	RHSIM_Rhsim02G0105100	-0.412	-0.576	-0.164	no
Treat-40d	RHSIM_Rhsim01G0267800	-1.524	-1.649	-0.125	low
Treat-40d	RHSIM_Rhsim01G0242700	-0.755	-1.784	-1.029	no
Treat-40d	RHSIM_Rhsim01G0152000	-0.961	-0.988	-0.026	no
Treat-40d	RHSIM_Rhsim02G0172600	0.363	1.079	0.716	no
Treat-40d	RHSIM_Rhsim02G0147000	1.882	2.382	0.500	high
Treat-40d	RHSIM_Rhsim02G0251400	0.672	-0.198	-0.870	no
Treat-40d	RHSIM_Rhsim02G0251300	1.900	0.948	-0.952	high
Treat-40d	RHSIM_Rhsim01G0232900	-1.669	-1.137	0.533	low
Treat-40d	RHSIM_Rhsim02G0007900	-2.113	-0.750	1.363	low
Treat-40d	RHSIM_Rhsim02G0252500	0.159	1.563	1.405	no
Treat-40d	RHSIM_Rhsim02G0122400	-0.028	-0.794	-0.767	no
Treat-40d	RHSIM_Rhsim02G0159400	-1.044	-1.017	0.027	no

Group	Gene	PTR (z-score difference)	z(log2 LFQ)	z(log2 FPKM)	extreme-PTR
Treat-40d	RHSIM_Rhsim02G0088300	-0.077	-0.878	-0.801	no
Treat-40d	RHSIM_Rhsim02G0083500	-0.782	-0.213	0.570	no
Treat-40d	RHSIM_Rhsim02G0205900	-1.852	-0.382	1.471	low
Treat-40d	RHSIM_Rhsim02G0208200	-0.917	-1.364	-0.447	no
Treat-40d	RHSIM_Rhsim01G0235200	0.871	0.235	-0.637	no
Treat-40d	RHSIM_Rhsim01G0243800	0.171	1.159	0.987	no
Treat-40d	RHSIM_Rhsim02G0119200	1.484	0.588	-0.897	high
Treat-40d	RHSIM_Rhsim01G0234200	0.955	0.686	-0.269	no
Treat-40d	RHSIM_Rhsim01G0025200	0.339	1.132	0.793	no
Treat-40d	RHSIM_Rhsim01G0054900	-0.576	-0.135	0.441	no
Treat-40d	RHSIM_Rhsim01G0232200	0.698	0.887	0.189	no
Treat-40d	RHSIM_Rhsim02G0141400	-0.049	-1.119	-1.070	no
Treat-40d	RHSIM_Rhsim02G0035600	-1.518	-0.444	1.074	low
Treat-40d	RHSIM_Rhsim01G0175000	-2.809	-1.271	1.538	low
Treat-40d	RHSIM_Rhsim01G0013100	0.474	2.169	1.696	no
Treat-40d	RHSIM_Rhsim01G0073100	-0.460	-0.016	0.444	no
Treat-40d	RHSIM_Rhsim01G0184400	1.315	2.000	0.685	high
Treat-40d	RHSIM_Rhsim01G0269200	1.055	1.885	0.830	no
Treat-40d	RHSIM_Rhsim02G0213100	0.317	-0.762	-1.079	no
Treat-40d	RHSIM_Rhsim01G0263700	0.205	-1.142	-1.347	no
Treat-40d	RHSIM_Rhsim01G0241200	-0.238	-0.667	-0.428	no
Treat-40d	RHSIM_Rhsim01G0291500	-0.252	-0.265	-0.013	no
Treat-40d	RHSIM_Rhsim01G0108800	-0.354	1.040	1.394	no
Treat-40d	RHSIM_Rhsim01G0001600	-1.575	-0.677	0.899	low
Treat-40d	RHSIM_Rhsim01G0147700	-0.448	-0.499	-0.051	no
Treat-40d	RHSIM_Rhsim01G0112300	-0.616	-1.178	-0.562	no
Treat-40d	RHSIM_Rhsim02G0248800	0.170	0.120	-0.049	no
Treat-40d	RHSIM_Rhsim01G0243600	-0.381	1.111	1.492	no
Treat-40d	RHSIM_Rhsim01G0185100	0.677	0.936	0.260	no
Treat-40d	RHSIM_Rhsim01G0270000	-0.576	0.278	0.854	no
Treat-40d	RHSIM_Rhsim02G0127100	-1.705	-1.365	0.340	low
Treat-40d	RHSIM_Rhsim01G0089900	0.499	0.448	-0.051	no

Group	Gene	PTR (z-score difference)	z(log2 LFQ)	z(log2 FPKM)	extreme-PTR
Treat-40d	RHSIM_Rhsim02G0145900	-0.751	-0.456	0.295	no
Treat-40d	RHSIM_Rhsim02G0250500	0.479	1.047	0.567	no
Treat-40d	RHSIM_Rhsim01G0184600	0.443	-0.494	-0.937	no
Treat-40d	RHSIM_Rhsim02G0089500	0.701	-1.411	-2.111	no
Treat-40d	RHSIM_Rhsim02G0019600	-1.203	-0.697	0.506	low
Treat-40d	RHSIM_Rhsim01G0247800	1.832	3.381	1.549	high
Treat-40d	RHSIM_Rhsim02G0217200	-0.390	-0.296	0.094	no
Treat-40d	RHSIM_Rhsim01G0284000	-1.503	-0.840	0.663	low
Treat-40d	RHSIM_Rhsim01G0073500	-1.235	-1.329	-0.095	low
Treat-40d	RHSIM_Rhsim01G0163700	-0.349	-0.668	-0.319	no
Treat-40d	RHSIM_Rhsim01G0144200	-0.057	-0.012	0.045	no
Treat-40d	RHSIM_Rhsim02G0184100	-0.000	-0.907	-0.907	no
Treat-40d	RHSIM_Rhsim01G0001900	0.235	0.609	0.374	no
Treat-40d	RHSIM_Rhsim01G0195500	-1.249	-0.737	0.512	low
Treat-40d	RHSIM_Rhsim01G0194400	-0.172	-0.838	-0.666	no
Treat-40d	RHSIM_Rhsim01G0196700	-1.112	-0.794	0.318	no
Treat-40d	RHSIM_Rhsim01G0287600	-1.873	-0.585	1.288	low
Treat-40d	RHSIM_Rhsim01G0227500	0.089	2.663	2.574	no
Treat-40d	RHSIM_Rhsim01G0247200	1.030	-0.366	-1.396	no
Treat-40d	RHSIM_Rhsim02G0215600	-0.593	0.293	0.886	no
Treat-40d	RHSIM_Rhsim02G0109900	1.520	0.844	-0.676	high
Treat-40d	RHSIM_Rhsim01G0285600	-1.135	-0.449	0.686	no
Treat-40d	RHSIM_Rhsim02G0193200	-3.567	-0.986	2.580	low
Treat-40d	RHSIM_Rhsim01G0128500	-1.130	-1.701	-0.571	no
Treat-40d	RHSIM_Rhsim01G0286500	0.244	-0.327	-0.571	no
Treat-40d	RHSIM_Rhsim01G0008000	1.646	0.624	-1.022	high
Treat-40d	RHSIM_Rhsim02G0255400	-0.765	-0.436	0.330	no
Treat-40d	RHSIM_Rhsim01G0196200	0.449	0.007	-0.441	no
Treat-40d	RHSIM_Rhsim01G0265400	-1.711	-0.927	0.784	low
Treat-40d	RHSIM_Rhsim02G0083400	-0.372	-0.619	-0.248	no
Treat-40d	RHSIM_Rhsim01G0144600	1.332	-0.192	-1.523	high
Treat-40d	RHSIM_Rhsim01G0276900	-0.698	-0.942	-0.244	no

Group	Gene	PTR (z-score difference)	z(log2 LFQ)	z(log2 FPKM)	extreme-PTR
Treat-40d	RHSIM_Rhsim02G0105300	-0.059	-0.528	-0.469	no
Treat-40d	RHSIM_Rhsim01G0186600	0.488	-1.164	-1.652	no
Treat-40d	RHSIM_Rhsim01G0213500	1.246	2.675	1.429	high
Treat-40d	RHSIM_Rhsim01G0265200	-0.205	-0.986	-0.781	no
Treat-40d	RHSIM_Rhsim02G0180100	-0.789	-0.378	0.411	no
Treat-40d	RHSIM_Rhsim01G0206200	-0.530	-0.020	0.510	no
Treat-40d	RHSIM_Rhsim01G0249100	0.842	-0.116	-0.958	no
Treat-40d	RHSIM_Rhsim01G0059500	-0.204	-0.037	0.167	no
Treat-40d	RHSIM_Rhsim01G0074200	-2.276	-1.410	0.867	low
Treat-40d	RHSIM_Rhsim01G0151800	-1.451	-1.488	-0.037	low
Treat-40d	RHSIM_Rhsim01G0141500	1.875	1.081	-0.793	high
Treat-40d	RHSIM_Rhsim01G0134500	0.128	0.908	0.780	no
Treat-40d	RHSIM_Rhsim01G0212900	-1.486	0.011	1.497	low
Treat-40d	RHSIM_Rhsim01G0124100	0.526	0.525	-0.001	no
Treat-40d	RHSIM_Rhsim02G0251700	0.570	-1.577	-2.147	no
Treat-40d	RHSIM_Rhsim01G0077600	0.094	0.647	0.553	no
Treat-40d	RHSIM_Rhsim01G0181300	0.061	1.786	1.725	no
Treat-40d	RHSIM_Rhsim01G0205500	0.302	0.591	0.290	no
Treat-40d	RHSIM_Rhsim01G0204800	0.046	-0.044	-0.089	no
Treat-40d	RHSIM_Rhsim02G0043000	-0.547	1.968	2.515	no
Treat-40d	RHSIM_Rhsim01G0121200	-0.370	-0.600	-0.230	no
Treat-40d	RHSIM_Rhsim01G0114600	-0.590	0.803	1.393	no
Treat-40d	RHSIM_Rhsim01G0261900	0.645	0.114	-0.531	no
Treat-40d	RHSIM_Rhsim01G0290900	-0.340	-1.253	-0.913	no
Treat-40d	RHSIM_Rhsim01G0215700	-0.555	-0.619	-0.063	no
Treat-40d	RHSIM_Rhsim01G0246400	0.791	0.286	-0.505	no
Treat-40d	RHSIM_Rhsim01G0176500	0.049	0.620	0.571	no
Treat-40d	RHSIM_Rhsim01G0023600	0.771	-0.775	-1.547	no
Treat-40d	RHSIM_Rhsim01G0128700	-0.282	-0.888	-0.606	no
Treat-40d	RHSIM_Rhsim01G0232700	0.168	-1.707	-1.875	no
Treat-40d	RHSIM_Rhsim01G0278000	-0.161	-0.053	0.107	no
Treat-40d	RHSIM_Rhsim01G0001400	0.691	0.099	-0.591	no

Group	Gene	PTR (z-score difference)	z(log2 LFQ)	z(log2 FPKM)	extreme-PTR
Treat-40d	RHSIM_Rhsim01G0000100	0.893	-0.856	-1.749	no
Treat-40d	RHSIM_Rhsim01G0250800	-0.045	1.461	1.506	no
Treat-40d	RHSIM_Rhsim01G0245100	1.159	0.710	-0.449	high
Treat-40d	RHSIM_Rhsim01G0244200	1.602	-0.779	-2.381	high
Treat-40d	RHSIM_Rhsim01G0244400	-0.996	0.951	1.947	no
Treat-40d	RHSIM_Rhsim01G0001500	1.026	-0.163	-1.189	no
Treat-40d	RHSIM_Rhsim01G0292000	0.781	0.373	-0.408	no
Treat-40d	RHSIM_Rhsim01G0286100	-1.456	-0.600	0.856	low
Treat-40d	RHSIM_Rhsim01G0199200	0.587	0.775	0.187	no
Treat-40d	RHSIM_Rhsim01G0124900	-0.691	-0.068	0.623	no
Treat-40d	RHSIM_Rhsim01G0004300	-1.951	-0.064	1.886	low
Treat-40d	RHSIM_Rhsim01G0256500	-1.659	-1.409	0.250	low
Treat-40d	RHSIM_Rhsim01G0060100	-0.480	-0.358	0.122	no
Treat-40d	RHSIM_Rhsim01G0108000	-1.123	-1.062	0.060	no
Treat-40d	RHSIM_Rhsim01G0276100	1.160	0.617	-0.543	high
Treat-40d	RHSIM_Rhsim01G0276800	-0.564	-0.273	0.291	no
Treat-40d	RHSIM_Rhsim01G0153000	0.215	0.831	0.616	no
Treat-40d	RHSIM_Rhsim01G0193400	0.951	-0.564	-1.515	no
Treat-40d	RHSIM_Rhsim01G0241700	-0.510	1.262	1.772	no
Treat-40d	RHSIM_Rhsim01G0221500	-0.344	1.662	2.005	no
Treat-40d	RHSIM_Rhsim01G0189400	0.998	-1.173	-2.170	no
Treat-40d	RHSIM_Rhsim01G0000500	0.442	0.705	0.263	no
Treat-40d	RHSIM_Rhsim01G0260600	-1.152	-1.120	0.032	low
Treat-40d	RHSIM_Rhsim01G0286200	-5.112	-0.703	4.409	low
Treat-40d	RHSIM_Rhsim01G0279700	0.512	-1.083	-1.595	no
Treat-40d	RHSIM_Rhsim01G0055700	0.839	0.274	-0.565	no
Treat-40d	RHSIM_Rhsim01G0282300	-0.322	-0.713	-0.392	no
Treat-40d	RHSIM_Rhsim01G0044100	-0.589	0.136	0.725	no
Treat-40d	RHSIM_Rhsim01G0021100	-0.044	0.068	0.112	no
Treat-40d	RHSIM_Rhsim01G0192500	-0.299	-1.150	-0.851	no
Treat-40d	RHSIM_Rhsim01G0287000	0.288	1.828	1.540	no
Treat-40d	RHSIM_Rhsim01G0141200	1.001	1.048	0.047	no

Group	Gene	PTR (z-score difference)	z(log2 LFQ)	z(log2 FPKM)	extreme-PTR
Treat-40d	RHSIM_Rhsim01G0123600	0.096	-0.687	-0.782	no
Treat-40d	RHSIM_Rhsim01G0091700	-0.790	-0.525	0.265	no
Treat-40d	RHSIM_Rhsim01G0210100	0.396	0.223	-0.173	no
Treat-40d	RHSIM_Rhsim01G0170700	1.299	0.330	-0.970	high
Treat-40d	RHSIM_Rhsim01G0259500	1.453	2.091	0.638	high
Treat-40d	RHSIM_Rhsim01G0163000	-0.058	-0.979	-0.921	no

Comparison	Group	DE & extreme-PTR	DE & non-extreme-PTR	non-DE & extreme-PTR	non-DE & non-extreme-PTR	Odds ratio	P value
CK-40d vs CK-0d	CK-40d	1132	1560	825	3017	2.654	9.91E-71
CK-40d vs CK-0d	CK-0d	892	1854	940	2917	1.493	4.87E-13
Treat-0d vs CK-0d	Treat-0d	291	471	1565	4399	1.737	1.52E-11
Treat-0d vs CK-0d	CK-0d	270	423	1562	4348	1.777	1.37E-11
Treat-40d vs CK-40d	Treat-40d	788	1411	1089	3222	1.652	1.21E-18
Treat-40d vs CK-40d	CK-40d	919	1319	1038	3258	2.187	1.78E-44
Treat-40d vs Treat-0d	Treat-40d	947	1525	930	3108	2.075	4.02E-39
Treat-40d vs Treat-0d	Treat-0d	879	1756	977	3114	1.595	3.57E-17

PTR was computed per gene and group as PTR = z(log_2 LFQ) − z(log_2 FPKM) (within-group z-scores of replicate-mean abundances); genes retained when protein detected (LFQ > 0) in ≥ 2 of 3 replicates and mean FPKM ≥ 1. extreme-PTR: beyond median ± 1 SD of the group PTR distribution (high/low). Second table: Fisher's exact tests (2 × 2) of over-representation of differentially expressed genes (transcript layer: $|log_2FC| \geq 1$ and BH-adjusted P < 0.05; protein layer: FC ≥ 1.5 or ≤ 0.6667 and $P \leq 0.05$) among extreme-PTR genes; universe = genes quantified at both layers in the indicated group.
