# Supplementary material for: Integrated Physiological, Transcriptomic, and Proteomic Analyses Reveal Alkali Stress Response and Tolerance Mechanisms of Rhododendron obtusum ‘Fenzhuanglou’ Seedlings
Source: Plants (Basel). 2026 Oct 6;15(19):3053. doi: 10.3390/plants15193053 (PMC13666605; doi:10.3390/plants15193053)
Supplement: Supplementary file 1 [file plants-15-03053-s001.zip › Supplementary Table S1.pdf]

Supplementary Table S1. Time courses of physiological indicators in leaves of *Rhododendron obtusum* 'Fenzhuanglou' seedlings under alkali stress ($NaHCO_3$:Na_2CO_3 = 9:1 molar ratio, pH 8.94 ± 0.12; CK, 50, 60 and 70 mmol·L^{-1}, applied once every 3 d for 40 d).

Time (d)	Treatment (mmol·L^{-1})	Chl a (mg·g^{-1} FW)	Chl b (mg·g^{-1} FW)	Lutein (mg·g^{-1} FW)	Carotenoid (mg·g^{-1} FW)	Soluble sugar (mg·g^{-1})	Soluble protein (mg·g^{-1})	Proline (μg·g^{-1})	SOD (U·g^{-1})	POD (U·g^{-1})	CAT (U·g^{-1})	MDA (nmol·g^{-1})
0	CK	0.826 a	0.365 a	0.238 a	0.375 a	7.70 ab	2.66 b	127 a	185 a	710 a	85 a	2.2 a
0	50	0.790 a	0.350 a	0.198 a	0.388 a	8.40 a	2.87 ab	118 a	192 a	690 a	87 a	2.2 a
0	60	0.795 a	0.350 a	0.203 a	0.390 a	7.80 ab	2.97 a	128 a	205 a	730 a	80 a	2.4 a
0	70	0.770 a	0.365 a	0.172 a	0.335 a	7.60 b	2.93 a	117 a	200 a	710 a	83 a	2.3 a
10	CK	0.800 a	0.320 a	0.218 ab	0.330 a	8.90 b	2.85 c	126 b	172 d	690 d	95 d	2.8 d
10	50	0.800 a	0.234 a	0.198 ab	0.280 b	8.50 b	3.93 b	173 a	232 c	800 c	180 c	3.6 c
10	60	0.760 a	0.229 a	0.208 ab	0.253 ab	11.40 a	3.76 b	163 a	272 b	840 b	215 b	4.3 b
10	70	0.740 a	0.280 a	0.190 b	0.295 ab	11.60 a	4.40 a	186 a	310 a	920 a	265 a	4.8 a
20	CK	0.830 a	0.316 a	0.275 a	0.350 a	8.70 d	2.70 b	112 d	152 d	615 d	80 d	4.0 d
20	50	0.790 a	0.183 b	0.258 a	0.240 b	10.20 c	4.98 a	198 c	240 c	850 c	245 c	4.9 c
20	60	0.790 a	0.129 bc	0.310 a	0.205 bc	13.80 b	4.83 a	225 b	330 b	930 b	300 b	5.6 b
20	70	0.800 a	0.096 c	0.225 a	0.130 c	18.20 a	5.00 a	230 a	378 a	980 a	345 a	6.4 a
30	CK	0.892 a	0.343 a	0.310 a	0.315 a	8.50 d	3.30 d	113 d	178 d	710 d	70 d	3.6 d
30	50	0.790 ab	0.145 b	0.338 a	0.190 ab	10.80 c	4.85 b	227 c	238 b	820 b	185 b	7.5 c
30	60	0.786 b	0.142 b	0.440 a	0.153 b	17.10 b	6.24 a	259 b	280 a	900 a	225 a	9.4 b
30	70	0.750 b	0.117 b	0.468 a	0.178 b	19.70 a	4.15 c	276 a	202 c	860 c	130 c	11.2 a
40	CK	0.914 a	0.391 a	0.245 a	0.308 a	8.30 c	2.66 c	132 c	168 c	620 a	75 b	3.8 d
40	50	0.695 ab	0.123 b	0.214 a	0.180 b	11.12 b	4.44 ab	232 b	218 a	520 b	105 a	14.5 c
40	60	0.664 b	0.084 b	0.229 a	0.154 b	19.19 a	4.66 a	253 a	195 b	440 c	65 c	19.8 b
40	70	0.658 b	0.088 b	0.240 a	0.144 b	19.67 a	3.22 b	210 b	130 d	360 d	50 d	22.3 a

Values are means of three biological replicates (n = 3). Different lowercase letters within the same column indicate significant differences among treatments at the same time point (one-way ANOVA followed by Duncan's multiple range test, $P < 0.05$).
