# Supplementary material for: Integrated Physiological, Transcriptomic, and Proteomic Analyses Reveal Alkali Stress Response and Tolerance Mechanisms of Rhododendron obtusum ‘Fenzhuanglou’ Seedlings
Source: Plants (Basel). 2026 Oct 6;15(19):3053. doi: 10.3390/plants15193053 (PMC13666605; doi:10.3390/plants15193053)
Supplement: Supplementary file 1 [file plants-15-03053-s001.zip › Supplementary Table S2.pdf]

Supplementary Table S2. Two-way ANOVA (treatment concentration × sampling time) of the eleven physiological indicators (F values; degrees of freedom in parentheses; error df = 40).

Trait	Treatment (df = 3)	Time (df = 4)	Treatment × Time (df = 12)
Chl a	2.9*	0.9 ns	0.6 ns
Chl b	24.0***	20.7***	2.9**
Lutein	1.1 ns	21.2***	1.6 ns
Carotenoid	13.0***	18.9***	1.5 ns
Soluble sugar	310.5***	180.3***	39.0***
Soluble protein	42.3***	30.5***	6.3***
Proline	182.4***	141.4***	23.8***
SOD	127.9***	105.4***	41.5***
POD	34.3***	359.0***	47.7***
CAT	81.4***	167.4***	27.7***
MDA	177.4***	490.0***	53.0***

ns, not significant; * $P < 0.05$; ** $P < 0.01$; *** $P < 0.001$.
