# Supplementary material for: Integrated Physiological, Transcriptomic, and Proteomic Analyses Reveal Alkali Stress Response and Tolerance Mechanisms of Rhododendron obtusum ‘Fenzhuanglou’ Seedlings
Source: Plants (Basel). 2026 Oct 6;15(19):3053. doi: 10.3390/plants15193053 (PMC13666605; doi:10.3390/plants15193053)
Supplement: Supplementary file 1 [file plants-15-03053-s001.zip › Supplementary Table S3.pdf]

**Supplementary Table S3.** Full results of the treatment × time interaction likelihood-ratio test (28,643 genes).

| Gene | LRT statistic | P value | BH-adjusted P | log2FC (interaction) |
|---|---|---|---|---|
| RHSIM_Rhsim04G0172100 | 3514.54 | <1.0E-300 | <1.0E-300 | 4.405 |
| RHSIM_Rhsim07G0003400 | 1583.50 | <1.0E-300 | <1.0E-300 | 1.717 |
| novel.2734 | 2742.52 | <1.0E-300 | <1.0E-300 | -3.238 |
| RHSIM_Rhsim12G0116600 | 1769.08 | <1.0E-300 | <1.0E-300 | -2.615 |
| RHSIM_Rhsim12G0116400 | 2022.88 | <1.0E-300 | <1.0E-300 | 2.857 |
| RHSIM_Rhsim06G0152400 | 1507.95 | <1.0E-300 | <1.0E-300 | -4.947 |
| RHSIM_Rhsim13G0103100 | 2401.94 | <1.0E-300 | <1.0E-300 | 3.724 |
| RHSIM_Rhsim08G0245100 | 1707.47 | <1.0E-300 | <1.0E-300 | 2.459 |
| RHSIM_Rhsim05G0056500 | 1820.82 | <1.0E-300 | <1.0E-300 | 2.518 |
| RHSIM_Rhsim13G0215900 | 2162.10 | <1.0E-300 | <1.0E-300 | 1.816 |
| RHSIM_Rhsim03G0255200 | 2032.81 | <1.0E-300 | <1.0E-300 | -2.857 |
| RHSIM_Rhsim11G0191500 | 3111.62 | <1.0E-300 | <1.0E-300 | 2.581 |
| RHSIM_Rhsim11G0196300 | 1996.62 | <1.0E-300 | <1.0E-300 | 2.846 |
| RHSIM_Rhsim10G0159000 | 1493.95 | <1.0E-300 | <1.0E-300 | -1.914 |
| RHSIM_Rhsim05G0226500 | 2548.74 | <1.0E-300 | <1.0E-300 | 2.602 |
| RHSIM_Rhsim01G0071600 | 3467.92 | <1.0E-300 | <1.0E-300 | 3.949 |
| RHSIM_Rhsim06G0152500 | 3762.86 | <1.0E-300 | <1.0E-300 | -6.260 |
| RHSIM_Rhsim09G0211800 | 1521.00 | <1.0E-300 | <1.0E-300 | 2.682 |
| RHSIM_Rhsim06G0232600 | 2894.86 | <1.0E-300 | <1.0E-300 | -2.389 |
| RHSIM_Rhsim06G0237200 | 2243.62 | <1.0E-300 | <1.0E-300 | 2.328 |
| RHSIM_Rhsim02G0066800 | 3323.45 | <1.0E-300 | <1.0E-300 | -4.764 |
| RHSIM_Rhsim05G0053700 | 1730.54 | <1.0E-300 | <1.0E-300 | -3.611 |
| RHSIM_Rhsim13G0107100 | 3832.74 | <1.0E-300 | <1.0E-300 | 2.844 |
| RHSIM_Rhsim13G0107000 | 1780.68 | <1.0E-300 | <1.0E-300 | 3.071 |
| RHSIM_Rhsim13G0113000 | 2772.96 | <1.0E-300 | <1.0E-300 | 2.473 |
| RHSIM_Rhsim13G0111300 | 1432.39 | <1.0E-300 | <1.0E-300 | 2.763 |
| RHSIM_Rhsim09G0206400 | 4038.60 | <1.0E-300 | <1.0E-300 | 3.563 |
| RHSIM_Rhsim10G0002200 | 2006.88 | <1.0E-300 | <1.0E-300 | 2.481 |
| RHSIM_Rhsim12G0085100 | 1434.74 | <1.0E-300 | <1.0E-300 | 2.155 |
| RHSIM_Rhsim08G0211300 | 1566.90 | <1.0E-300 | <1.0E-300 | -2.118 |
| RHSIM_Rhsim01G0241100 | 4831.77 | <1.0E-300 | <1.0E-300 | 3.102 |

| Gene | LRT statistic | P value | BH-adjusted P | log2FC (interaction) |
|---|---|---|---|---|
| RHSIM_Rhsim13G0116300 | 1484.04 | <1.0E-300 | <1.0E-300 | 3.593 |
| RHSIM_Rhsim06G0236300 | 3339.88 | <1.0E-300 | <1.0E-300 | -4.941 |
| RHSIM_Rhsim06G0227200 | 1475.98 | <1.0E-300 | <1.0E-300 | 2.667 |
| RHSIM_Rhsim02G0230000 | 2819.05 | <1.0E-300 | <1.0E-300 | -4.206 |
| RHSIM_Rhsim02G0229900 | 1630.49 | <1.0E-300 | <1.0E-300 | -4.287 |
| RHSIM_Rhsim04G0174200 | 1570.33 | <1.0E-300 | <1.0E-300 | -3.314 |
| RHSIM_Rhsim08G0122300 | 1606.22 | <1.0E-300 | <1.0E-300 | 3.475 |
| RHSIM_Rhsim05G0004600 | 2508.38 | <1.0E-300 | <1.0E-300 | -7.200 |
| RHSIM_Rhsim05G0004400 | 2048.48 | <1.0E-300 | <1.0E-300 | 3.900 |
| RHSIM_Rhsim07G0140400 | 2912.58 | <1.0E-300 | <1.0E-300 | 3.310 |
| RHSIM_Rhsim09G0177100 | 1435.70 | <1.0E-300 | <1.0E-300 | 2.027 |
| RHSIM_Rhsim07G0017400 | 3594.68 | <1.0E-300 | <1.0E-300 | 3.265 |
| RHSIM_Rhsim05G0142600 | 1715.48 | <1.0E-300 | <1.0E-300 | -7.430 |
| RHSIM_Rhsim02G0106400 | 1800.94 | <1.0E-300 | <1.0E-300 | 1.904 |
| RHSIM_Rhsim13G0080100 | 2433.29 | <1.0E-300 | <1.0E-300 | 2.368 |
| RHSIM_Rhsim13G0079600 | 2382.68 | <1.0E-300 | <1.0E-300 | 2.225 |
| RHSIM_Rhsim13G0077400 | 5471.65 | <1.0E-300 | <1.0E-300 | 2.966 |
| RHSIM_Rhsim01G0205000 | 2872.83 | <1.0E-300 | <1.0E-300 | -2.654 |
| RHSIM_Rhsim01G0202500 | 1960.52 | <1.0E-300 | <1.0E-300 | 3.230 |
| RHSIM_Rhsim12G0144000 | 1653.63 | <1.0E-300 | <1.0E-300 | 2.248 |
| RHSIM_Rhsim07G0142300 | 2710.78 | <1.0E-300 | <1.0E-300 | 2.712 |
| RHSIM_Rhsim13G0229900 | 3501.56 | <1.0E-300 | <1.0E-300 | -4.519 |
| RHSIM_Rhsim03G0267700 | 1543.49 | <1.0E-300 | <1.0E-300 | 2.021 |
| RHSIM_Rhsim13G0075800 | 2225.77 | <1.0E-300 | <1.0E-300 | -4.849 |
| RHSIM_Rhsim01G0206400 | 2477.98 | <1.0E-300 | <1.0E-300 | 5.091 |
| RHSIM_Rhsim04G0091700 | 2615.35 | <1.0E-300 | <1.0E-300 | 5.574 |
| RHSIM_Rhsim13G0217100 | 2027.52 | <1.0E-300 | <1.0E-300 | 3.693 |
| RHSIM_Rhsim11G0169800 | 3337.79 | <1.0E-300 | <1.0E-300 | -4.418 |
| RHSIM_Rhsim02G0108100 | 1733.78 | <1.0E-300 | <1.0E-300 | -5.287 |
| RHSIM_Rhsim08G0110900 | 1632.92 | <1.0E-300 | <1.0E-300 | -6.367 |
| RHSIM_Rhsim03G0262500 | 2026.01 | <1.0E-300 | <1.0E-300 | -2.655 |
| RHSIM_Rhsim02G0088400 | 2443.39 | <1.0E-300 | <1.0E-300 | 2.989 |

| Gene | LRT statistic | P value | BH-adjusted P | log2FC (interaction) |
|---|---|---|---|---|
| RHSIM_Rhsim01G0207000 | 3177.37 | <1.0E-300 | <1.0E-300 | 2.685 |
| RHSIM_Rhsim07G0006200 | 6848.42 | <1.0E-300 | <1.0E-300 | -4.092 |
| RHSIM_Rhsim08G0230400 | 3930.35 | <1.0E-300 | <1.0E-300 | 3.127 |
| RHSIM_Rhsim08G0227800 | 1792.00 | <1.0E-300 | <1.0E-300 | -4.084 |
| RHSIM_Rhsim13G0093700 | 1472.25 | <1.0E-300 | <1.0E-300 | -3.463 |
| novel.2434 | 4059.60 | <1.0E-300 | <1.0E-300 | -2.961 |
| RHSIM_Rhsim13G0225600 | 1440.61 | <1.0E-300 | <1.0E-300 | -1.603 |
| RHSIM_RhsimUnG0254300 | 5652.80 | <1.0E-300 | <1.0E-300 | 3.046 |
| RHSIM_Rhsim03G0259600 | 1984.58 | <1.0E-300 | <1.0E-300 | 2.381 |
| RHSIM_Rhsim12G0137100 | 2703.45 | <1.0E-300 | <1.0E-300 | -2.289 |
| RHSIM_Rhsim03G0265400 | 1679.09 | <1.0E-300 | <1.0E-300 | -1.974 |
| RHSIM_Rhsim10G0167600 | 2735.06 | <1.0E-300 | <1.0E-300 | -5.156 |
| RHSIM_Rhsim05G0230500 | 1799.62 | <1.0E-300 | <1.0E-300 | 3.223 |
| novel.3955 | 1429.97 | <1.0E-300 | <1.0E-300 | -1.902 |
| RHSIM_Rhsim01G0060300 | 1515.74 | <1.0E-300 | <1.0E-300 | 1.864 |
| RHSIM_Rhsim05G0000100 | 2567.06 | <1.0E-300 | <1.0E-300 | 3.137 |
| RHSIM_Rhsim04G0177600 | 2345.18 | <1.0E-300 | <1.0E-300 | -4.221 |
| RHSIM_Rhsim03G0117500 | 1980.29 | <1.0E-300 | <1.0E-300 | 3.167 |
| RHSIM_Rhsim05G0203200 | 1769.45 | <1.0E-300 | <1.0E-300 | 3.102 |
| RHSIM_Rhsim05G0201100 | 1805.28 | <1.0E-300 | <1.0E-300 | 5.397 |
| RHSIM_Rhsim08G0161000 | 1726.31 | <1.0E-300 | <1.0E-300 | -2.725 |
| RHSIM_Rhsim08G0148800 | 1771.12 | <1.0E-300 | <1.0E-300 | 2.781 |
| RHSIM_Rhsim08G0180500 | 11327.79 | <1.0E-300 | <1.0E-300 | -6.683 |
| RHSIM_Rhsim06G0178200 | 1845.52 | <1.0E-300 | <1.0E-300 | 3.330 |
| RHSIM_Rhsim05G0206900 | 1517.12 | <1.0E-300 | <1.0E-300 | 2.537 |
| RHSIM_Rhsim05G0206700 | 3492.30 | <1.0E-300 | <1.0E-300 | -4.706 |
| RHSIM_Rhsim03G0208000 | 3588.36 | <1.0E-300 | <1.0E-300 | -4.798 |
| RHSIM_Rhsim06G0196300 | 2911.17 | <1.0E-300 | <1.0E-300 | 4.728 |
| RHSIM_Rhsim06G0068700 | 1930.19 | <1.0E-300 | <1.0E-300 | 2.628 |
| RHSIM_Rhsim03G0214900 | 3175.61 | <1.0E-300 | <1.0E-300 | -3.787 |
| RHSIM_Rhsim12G0046400 | 1555.67 | <1.0E-300 | <1.0E-300 | 2.410 |
| RHSIM_Rhsim08G0171600 | 13245.94 | <1.0E-300 | <1.0E-300 | -5.415 |

| Gene | LRT statistic | P value | BH-adjusted P | log2FC (interaction) |
|---|---|---|---|---|
| RHSIM_Rhsim06G0212400 | 1568.60 | <1.0E-300 | <1.0E-300 | 1.885 |
| RHSIM_Rhsim04G0144400 | 2109.84 | <1.0E-300 | <1.0E-300 | -3.201 |
| RHSIM_Rhsim05G0190800 | 4974.81 | <1.0E-300 | <1.0E-300 | -3.260 |
| RHSIM_Rhsim10G0044200 | 2161.57 | <1.0E-300 | <1.0E-300 | 4.655 |
| RHSIM_Rhsim10G0055800 | 1508.41 | <1.0E-300 | <1.0E-300 | -9.268 |
| novel.485 | 1803.46 | <1.0E-300 | <1.0E-300 | 2.291 |
| RHSIM_Rhsim03G0081300 | 2842.61 | <1.0E-300 | <1.0E-300 | 4.214 |
| RHSIM_Rhsim01G0023400 | 1451.65 | <1.0E-300 | <1.0E-300 | 2.552 |
| novel.721 | 4783.58 | <1.0E-300 | <1.0E-300 | 3.360 |
| RHSIM_Rhsim01G0275900 | 1973.75 | <1.0E-300 | <1.0E-300 | 3.465 |
| RHSIM_Rhsim01G0004300 | 1877.72 | <1.0E-300 | <1.0E-300 | 1.900 |
| RHSIM_RhsimUnG0065400 | 2620.07 | <1.0E-300 | <1.0E-300 | -2.655 |
| RHSIM_RhsimUnG0062800 | 4279.20 | <1.0E-300 | <1.0E-300 | -4.295 |
| RHSIM_Rhsim11G0105700 | 4477.50 | <1.0E-300 | <1.0E-300 | 4.065 |
| RHSIM_Rhsim11G0098700 | 1444.34 | <1.0E-300 | <1.0E-300 | 1.825 |
| RHSIM_Rhsim02G0032900 | 7497.48 | <1.0E-300 | <1.0E-300 | 5.909 |
| RHSIM_Rhsim01G0028300 | 2777.12 | <1.0E-300 | <1.0E-300 | 2.396 |
| RHSIM_Rhsim06G0029200 | 1490.24 | <1.0E-300 | <1.0E-300 | 2.688 |
| RHSIM_Rhsim11G0044200 | 1451.04 | <1.0E-300 | <1.0E-300 | -3.516 |
| RHSIM_Rhsim11G0052200 | 1494.39 | <1.0E-300 | <1.0E-300 | 3.374 |
| RHSIM_Rhsim02G0132400 | 1986.93 | <1.0E-300 | <1.0E-300 | 5.162 |
| RHSIM_Rhsim07G0103900 | 1931.61 | <1.0E-300 | <1.0E-300 | 3.091 |
| RHSIM_Rhsim07G0093000 | 1444.33 | <1.0E-300 | <1.0E-300 | 3.238 |
| RHSIM_Rhsim03G0073900 | 1494.20 | <1.0E-300 | <1.0E-300 | -5.334 |
| RHSIM_Rhsim06G0047300 | 1978.14 | <1.0E-300 | <1.0E-300 | 3.438 |
| RHSIM_Rhsim06G0167500 | 2816.69 | <1.0E-300 | <1.0E-300 | -2.513 |
| RHSIM_RhsimUnG0250300 | 3371.57 | <1.0E-300 | <1.0E-300 | 3.041 |
| RHSIM_RhsimUnG0249600 | 2043.59 | <1.0E-300 | <1.0E-300 | -4.272 |
| RHSIM_Rhsim13G0141400 | 2977.85 | <1.0E-300 | <1.0E-300 | -3.523 |
| RHSIM_Rhsim13G0138900 | 1754.85 | <1.0E-300 | <1.0E-300 | 2.734 |
| RHSIM_Rhsim13G0149200 | 2463.66 | <1.0E-300 | <1.0E-300 | -3.517 |
| RHSIM_Rhsim01G0221500 | 2797.16 | <1.0E-300 | <1.0E-300 | 2.484 |

| Gene | LRT statistic | P value | BH-adjusted P | log2FC (interaction) |
|---|---|---|---|---|
| RHSIM_Rhsim12G0019500 | 3465.23 | <1.0E-300 | <1.0E-300 | 3.153 |
| RHSIM_Rhsim12G0015500 | 1769.24 | <1.0E-300 | <1.0E-300 | -2.262 |
| RHSIM_Rhsim12G0021700 | 3265.82 | <1.0E-300 | <1.0E-300 | -4.203 |
| RHSIM_Rhsim05G0228600 | 1441.14 | <1.0E-300 | <1.0E-300 | -2.827 |
| RHSIM_Rhsim05G0216500 | 4853.87 | <1.0E-300 | <1.0E-300 | -5.244 |
| RHSIM_Rhsim06G0160500 | 1784.41 | <1.0E-300 | <1.0E-300 | 1.572 |
| RHSIM_Rhsim08G0189200 | 2382.57 | <1.0E-300 | <1.0E-300 | -6.209 |
| RHSIM_Rhsim08G0188700 | 3116.52 | <1.0E-300 | <1.0E-300 | 3.569 |
| RHSIM_Rhsim04G0157800 | 1911.88 | <1.0E-300 | <1.0E-300 | 2.249 |
| RHSIM_Rhsim01G0238000 | 3978.62 | <1.0E-300 | <1.0E-300 | 3.387 |
| RHSIM_Rhsim05G0011500 | 1463.13 | <1.0E-300 | <1.0E-300 | -5.785 |
| RHSIM_Rhsim05G0011200 | 4940.04 | <1.0E-300 | <1.0E-300 | -7.351 |
| RHSIM_Rhsim13G0190900 | 2120.01 | <1.0E-300 | <1.0E-300 | -4.310 |
| RHSIM_Rhsim02G0035500 | 2984.09 | <1.0E-300 | <1.0E-300 | 2.825 |
| RHSIM_Rhsim02G0032200 | 1582.26 | <1.0E-300 | <1.0E-300 | 5.127 |
| RHSIM_Rhsim13G0180800 | 2287.63 | <1.0E-300 | <1.0E-300 | 3.113 |
| RHSIM_Rhsim10G0130000 | 1845.07 | <1.0E-300 | <1.0E-300 | 3.330 |
| RHSIM_RhsimUnG0221200 | 2202.51 | <1.0E-300 | <1.0E-300 | -2.600 |
| RHSIM_Rhsim04G0135300 | 2445.44 | <1.0E-300 | <1.0E-300 | 3.813 |
| RHSIM_Rhsim02G0251700 | 3734.14 | <1.0E-300 | <1.0E-300 | -5.747 |
| RHSIM_Rhsim02G0029100 | 1642.15 | <1.0E-300 | <1.0E-300 | 9.210 |
| RHSIM_RhsimUnG0205000 | 1664.10 | <1.0E-300 | <1.0E-300 | 2.789 |
| RHSIM_Rhsim05G0017000 | 4037.68 | <1.0E-300 | <1.0E-300 | 2.953 |
| RHSIM_Rhsim13G0098900 | 1921.63 | <1.0E-300 | <1.0E-300 | 1.946 |
| RHSIM_Rhsim13G0176400 | 3237.45 | <1.0E-300 | <1.0E-300 | -6.538 |
| RHSIM_Rhsim03G0018300 | 1605.50 | <1.0E-300 | <1.0E-300 | 2.267 |
| novel.3554 | 1775.90 | <1.0E-300 | <1.0E-300 | 2.234 |
| RHSIM_Rhsim02G0239800 | 1551.28 | <1.0E-300 | <1.0E-300 | -3.167 |
| RHSIM_Rhsim12G0082700 | 2115.66 | <1.0E-300 | <1.0E-300 | 2.068 |
| RHSIM_Rhsim12G0014400 | 1857.30 | <1.0E-300 | <1.0E-300 | -3.444 |
| RHSIM_Rhsim03G0180900 | 2147.03 | <1.0E-300 | <1.0E-300 | 3.139 |
| novel.3810 | 1642.36 | <1.0E-300 | <1.0E-300 | -2.236 |

| Gene | LRT statistic | P value | BH-adjusted P | log2FC (interaction) |
|---|---|---|---|---|
| RHSIM_Rhsim08G0023800 | 2492.95 | <1.0E-300 | <1.0E-300 | -4.285 |
| RHSIM_Rhsim09G0112700 | 4652.32 | <1.0E-300 | <1.0E-300 | -3.828 |
| RHSIM_Rhsim06G0103300 | 4010.82 | <1.0E-300 | <1.0E-300 | 2.925 |
| RHSIM_Rhsim10G0215400 | 2633.49 | <1.0E-300 | <1.0E-300 | -4.644 |
| RHSIM_Rhsim03G0144600 | 3166.14 | <1.0E-300 | <1.0E-300 | 2.238 |
| RHSIM_Rhsim01G0184800 | 1778.53 | <1.0E-300 | <1.0E-300 | 3.267 |
| RHSIM_Rhsim07G0084600 | 1662.86 | <1.0E-300 | <1.0E-300 | -2.973 |
| RHSIM_Rhsim09G0116000 | 1604.67 | <1.0E-300 | <1.0E-300 | -4.995 |
| RHSIM_Rhsim12G0187400 | 4705.84 | <1.0E-300 | <1.0E-300 | 2.944 |
| RHSIM_RhsimUnG0120600 | 2713.00 | <1.0E-300 | <1.0E-300 | -4.159 |
| RHSIM_Rhsim12G0206900 | 1539.60 | <1.0E-300 | <1.0E-300 | 2.340 |
| RHSIM_Rhsim10G0212000 | 2634.05 | <1.0E-300 | <1.0E-300 | 2.203 |
| RHSIM_Rhsim10G0211400 | 2378.30 | <1.0E-300 | <1.0E-300 | 2.433 |
| RHSIM_Rhsim04G0205500 | 2223.84 | <1.0E-300 | <1.0E-300 | 4.011 |
| RHSIM_Rhsim03G0078300 | 4165.34 | <1.0E-300 | <1.0E-300 | -1.513 |
| RHSIM_Rhsim03G0110700 | 1967.54 | <1.0E-300 | <1.0E-300 | 4.064 |
| RHSIM_Rhsim03G0109200 | 1502.84 | <1.0E-300 | <1.0E-300 | -3.192 |
| RHSIM_Rhsim11G0111300 | 2576.21 | <1.0E-300 | <1.0E-300 | 2.639 |
| RHSIM_Rhsim03G0084700 | 2852.08 | <1.0E-300 | <1.0E-300 | -4.493 |
| RHSIM_Rhsim08G0009100 | 23200.29 | <1.0E-300 | <1.0E-300 | -7.835 |
| RHSIM_Rhsim04G0015400 | 3256.83 | <1.0E-300 | <1.0E-300 | -4.436 |
| RHSIM_Rhsim04G0066500 | 3447.30 | <1.0E-300 | <1.0E-300 | -5.419 |
| RHSIM_Rhsim10G0202400 | 1615.34 | <1.0E-300 | <1.0E-300 | 2.616 |
| RHSIM_Rhsim09G0012100 | 2450.61 | <1.0E-300 | <1.0E-300 | 3.745 |
| RHSIM_Rhsim09G0019100 | 1666.61 | <1.0E-300 | <1.0E-300 | 3.374 |
| RHSIM_Rhsim13G0039000 | 3162.85 | <1.0E-300 | <1.0E-300 | -2.952 |
| RHSIM_Rhsim06G0126100 | 1492.21 | <1.0E-300 | <1.0E-300 | -4.106 |
| RHSIM_Rhsim08G0011000 | 2978.18 | <1.0E-300 | <1.0E-300 | -2.481 |
| RHSIM_Rhsim11G0077900 | 1919.39 | <1.0E-300 | <1.0E-300 | 4.728 |
| novel.1977 | 1545.63 | <1.0E-300 | <1.0E-300 | 2.130 |
| RHSIM_Rhsim07G0061000 | 2393.33 | <1.0E-300 | <1.0E-300 | -3.644 |
| RHSIM_Rhsim07G0108400 | 1832.68 | <1.0E-300 | <1.0E-300 | 3.151 |

| Gene | LRT statistic | P value | BH-adjusted P | log2FC (interaction) |
|---|---|---|---|---|
| RHSIM_Rhsim13G0036300 | 1774.53 | <1.0E-300 | <1.0E-300 | 2.900 |
| RHSIM_Rhsim13G0040100 | 1879.90 | <1.0E-300 | <1.0E-300 | 1.932 |
| RHSIM_Rhsim04G0068900 | 1482.06 | <1.0E-300 | <1.0E-300 | 2.410 |
| RHSIM_Rhsim07G0083100 | 1861.57 | <1.0E-300 | <1.0E-300 | 2.561 |
| RHSIM_Rhsim13G0023600 | 1784.78 | <1.0E-300 | <1.0E-300 | -2.903 |
| RHSIM_Rhsim06G0076100 | 2611.06 | <1.0E-300 | <1.0E-300 | -3.121 |
| RHSIM_Rhsim04G0029400 | 1641.83 | <1.0E-300 | <1.0E-300 | 3.846 |
| RHSIM_Rhsim08G0079700 | 2106.79 | <1.0E-300 | <1.0E-300 | -3.615 |
| RHSIM_Rhsim04G0203200 | 3714.61 | <1.0E-300 | <1.0E-300 | -3.092 |
| RHSIM_Rhsim13G0000900 | 1546.45 | <1.0E-300 | <1.0E-300 | 2.114 |
| RHSIM_Rhsim13G0006000 | 1916.18 | <1.0E-300 | <1.0E-300 | 2.330 |
| RHSIM_Rhsim01G0171300 | 1563.72 | <1.0E-300 | <1.0E-300 | 2.336 |
| RHSIM_Rhsim07G0076600 | 2719.36 | <1.0E-300 | <1.0E-300 | 3.234 |
| RHSIM_Rhsim06G0077100 | 3638.89 | <1.0E-300 | <1.0E-300 | 3.861 |
| RHSIM_Rhsim07G0071500 | 2764.18 | <1.0E-300 | <1.0E-300 | -5.358 |
| RHSIM_Rhsim06G0075100 | 2780.84 | <1.0E-300 | <1.0E-300 | 2.779 |
| RHSIM_Rhsim06G0081100 | 4707.39 | <1.0E-300 | <1.0E-300 | 3.059 |
| RHSIM_Rhsim11G0039700 | 1617.73 | <1.0E-300 | <1.0E-300 | -6.359 |
| RHSIM_Rhsim11G0132200 | 1640.32 | <1.0E-300 | <1.0E-300 | 2.864 |
| RHSIM_Rhsim11G0138200 | 2053.50 | <1.0E-300 | <1.0E-300 | -4.643 |
| RHSIM_Rhsim11G0138000 | 8772.12 | <1.0E-300 | <1.0E-300 | -7.763 |
| RHSIM_Rhsim03G0139000 | 1513.84 | <1.0E-300 | <1.0E-300 | 3.642 |
| novel.4492 | 3035.78 | <1.0E-300 | <1.0E-300 | 4.071 |
| RHSIM_Rhsim01G0177300 | 1888.12 | <1.0E-300 | <1.0E-300 | -3.224 |
| RHSIM_Rhsim08G0067100 | 5256.81 | <1.0E-300 | <1.0E-300 | -7.998 |
| RHSIM_Rhsim08G0050800 | 3102.28 | <1.0E-300 | <1.0E-300 | -2.815 |
| RHSIM_Rhsim08G0039400 | 9221.22 | <1.0E-300 | <1.0E-300 | 5.400 |
| RHSIM_Rhsim04G0208300 | 5672.45 | <1.0E-300 | <1.0E-300 | -3.361 |
| RHSIM_Rhsim09G0084100 | 2887.93 | <1.0E-300 | <1.0E-300 | 2.692 |
| RHSIM_Rhsim04G0052300 | 2923.19 | <1.0E-300 | <1.0E-300 | -3.948 |
| RHSIM_Rhsim09G0088300 | 1577.83 | <1.0E-300 | <1.0E-300 | 3.467 |
| RHSIM_Rhsim09G0091800 | 2846.14 | <1.0E-300 | <1.0E-300 | -1.704 |

| Gene | LRT statistic | P value | BH-adjusted P | log2FC (interaction) |
|---|---|---|---|---|
| RHSIM_Rhsim06G0013600 | 1554.99 | <1.0E-300 | <1.0E-300 | -2.124 |
| RHSIM_Rhsim11G0088200 | 4252.04 | <1.0E-300 | <1.0E-300 | 2.946 |
| RHSIM_Rhsim11G0087700 | 2734.41 | <1.0E-300 | <1.0E-300 | 2.806 |
| RHSIM_Rhsim11G0078100 | 1793.63 | <1.0E-300 | <1.0E-300 | -4.227 |
| RHSIM_Rhsim03G0088100 | 1631.69 | <1.0E-300 | <1.0E-300 | 2.943 |
| RHSIM_Rhsim07G0075600 | 3104.07 | <1.0E-300 | <1.0E-300 | 4.123 |
| RHSIM_Rhsim07G0081400 | 1559.61 | <1.0E-300 | <1.0E-300 | 1.866 |
| RHSIM_Rhsim07G0080000 | 1993.93 | <1.0E-300 | <1.0E-300 | 2.552 |
| RHSIM_Rhsim07G0079900 | 1537.44 | <1.0E-300 | <1.0E-300 | -2.011 |
| RHSIM_Rhsim09G0117700 | 1777.20 | <1.0E-300 | <1.0E-300 | -2.458 |
| RHSIM_Rhsim11G0169500 | 3360.40 | <1.0E-300 | <1.0E-300 | -4.822 |
| RHSIM_Rhsim05G0073200 | 1651.34 | <1.0E-300 | <1.0E-300 | 5.923 |
| RHSIM_Rhsim09G0151700 | 2601.21 | <1.0E-300 | <1.0E-300 | -4.055 |
| RHSIM_Rhsim09G0155800 | 1866.17 | <1.0E-300 | <1.0E-300 | 2.165 |
| RHSIM_Rhsim12G0154100 | 3150.72 | <1.0E-300 | <1.0E-300 | 3.611 |
| RHSIM_Rhsim12G0162400 | 2740.47 | <1.0E-300 | <1.0E-300 | -5.473 |
| RHSIM_Rhsim04G0010300 | 2559.35 | <1.0E-300 | <1.0E-300 | -1.968 |
| RHSIM_Rhsim05G0133700 | 2063.12 | <1.0E-300 | <1.0E-300 | 3.193 |
| RHSIM_Rhsim11G0167300 | 2249.11 | <1.0E-300 | <1.0E-300 | -5.721 |
| RHSIM_Rhsim07G0096500 | 1648.69 | <1.0E-300 | <1.0E-300 | 1.933 |
| RHSIM_Rhsim07G0107500 | 2998.09 | <1.0E-300 | <1.0E-300 | -3.449 |
| RHSIM_Rhsim09G0153000 | 1678.08 | <1.0E-300 | <1.0E-300 | 2.191 |
| RHSIM_Rhsim07G0106300 | 2426.45 | <1.0E-300 | <1.0E-300 | -5.804 |
| RHSIM_Rhsim04G0057800 | 4004.33 | <1.0E-300 | <1.0E-300 | -8.289 |
| RHSIM_Rhsim02G0186800 | 2117.35 | <1.0E-300 | <1.0E-300 | 2.186 |
| RHSIM_Rhsim12G0197400 | 4255.55 | <1.0E-300 | <1.0E-300 | 3.126 |
| novel.4081 | 2154.01 | <1.0E-300 | <1.0E-300 | -4.182 |
| RHSIM_Rhsim13G0049600 | 1679.46 | <1.0E-300 | <1.0E-300 | -2.206 |
| RHSIM_Rhsim05G0089600 | 1642.83 | <1.0E-300 | <1.0E-300 | 2.107 |
| RHSIM_Rhsim07G0026400 | 8010.26 | <1.0E-300 | <1.0E-300 | -4.249 |
| novel.2147 | 1839.03 | <1.0E-300 | <1.0E-300 | 3.950 |
| RHSIM_Rhsim06G0127100 | 2729.61 | <1.0E-300 | <1.0E-300 | 2.555 |

| Gene | LRT statistic | P value | BH-adjusted P | log2FC (interaction) |
|---|---|---|---|---|
| RHSIM_Rhsim01G0191100 | 4031.77 | <1.0E-300 | <1.0E-300 | 4.026 |
| novel.1388 | 1537.88 | <1.0E-300 | <1.0E-300 | 2.394 |
| RHSIM_Rhsim04G0081300 | 2829.29 | <1.0E-300 | <1.0E-300 | 4.700 |
| RHSIM_Rhsim06G0002300 | 4109.83 | <1.0E-300 | <1.0E-300 | -6.011 |
| RHSIM_Rhsim04G0009300 | 1570.38 | <1.0E-300 | <1.0E-300 | -4.230 |
| RHSIM_Rhsim13G0228600 | 3389.22 | <1.0E-300 | <1.0E-300 | -5.054 |
| RHSIM_Rhsim02G0001900 | 3258.85 | <1.0E-300 | <1.0E-300 | 2.630 |
| RHSIM_Rhsim08G0018400 | 1626.32 | <1.0E-300 | <1.0E-300 | -3.533 |
| RHSIM_Rhsim04G0249900 | 1424.53 | 9.82E-312 | 1.07E-309 | 5.141 |
| RHSIM_Rhsim01G0090900 | 1424.19 | 1.17E-311 | 1.26E-309 | -2.531 |
| RHSIM_Rhsim04G0109200 | 1422.83 | 2.30E-311 | 2.48E-309 | 1.821 |
| RHSIM_Rhsim03G0174400 | 1418.56 | 1.94E-310 | 2.08E-308 | 2.787 |
| RHSIM_Rhsim11G0099400 | 1413.48 | 2.48E-309 | 2.65E-307 | -2.304 |
| RHSIM_Rhsim11G0008700 | 1411.37 | 7.12E-309 | 7.58E-307 | 2.141 |
| RHSIM_Rhsim11G0092700 | 1405.94 | 1.08E-307 | 1.14E-305 | 2.286 |
| RHSIM_Rhsim05G0164500 | 1401.04 | 1.25E-306 | 1.32E-304 | 2.096 |
| RHSIM_Rhsim13G0036500 | 1397.96 | 5.83E-306 | 6.14E-304 | 1.839 |
| RHSIM_Rhsim04G0239000 | 1397.33 | 8.00E-306 | 8.40E-304 | -2.262 |
| RHSIM_Rhsim06G0059900 | 1395.81 | 1.71E-305 | 1.79E-303 | -3.528 |
| RHSIM_Rhsim04G0033100 | 1386.65 | 1.67E-303 | 1.74E-301 | 1.800 |
| RHSIM_RhsimUnG0256700 | 1384.93 | 3.96E-303 | 4.11E-301 | 3.215 |
| RHSIM_Rhsim03G0247500 | 1377.63 | 1.52E-301 | 1.57E-299 | 2.715 |
| RHSIM_Rhsim02G0056400 | 1367.94 | 1.94E-299 | 2.00E-297 | -2.250 |
| RHSIM_Rhsim07G0248800 | 1367.91 | 1.97E-299 | 2.03E-297 | 3.395 |
| RHSIM_Rhsim02G0032400 | 1359.55 | 1.30E-297 | 1.33E-295 | 1.813 |
| RHSIM_Rhsim03G0006300 | 1357.84 | 3.04E-297 | 3.10E-295 | 3.527 |
| RHSIM_Rhsim06G0065100 | 1355.87 | 8.17E-297 | 8.30E-295 | 2.435 |
| RHSIM_Rhsim06G0076000 | 1353.45 | 2.74E-296 | 2.77E-294 | -3.479 |
| RHSIM_Rhsim09G0103000 | 1348.66 | 3.01E-295 | 3.04E-293 | 2.747 |
| RHSIM_Rhsim05G0086200 | 1347.68 | 4.92E-295 | 4.94E-293 | 1.978 |
| RHSIM_Rhsim01G0245600 | 1341.79 | 9.36E-294 | 9.38E-292 | 2.766 |
| RHSIM_Rhsim01G0274600 | 1340.74 | 1.59E-293 | 1.58E-291 | 2.632 |

| Gene | LRT statistic | P value | BH-adjusted P | log2FC (interaction) |
|---|---|---|---|---|
| RHSIM_Rhsim08G0044100 | 1325.49 | 3.26E-290 | 3.24E-288 | 2.666 |
| RHSIM_Rhsim06G0203800 | 1323.92 | 7.15E-290 | 7.08E-288 | 1.877 |
| RHSIM_Rhsim13G0228800 | 1318.76 | 9.47E-289 | 9.36E-287 | -2.790 |
| RHSIM_Rhsim04G0167300 | 1318.21 | 1.25E-288 | 1.23E-286 | 2.672 |
| RHSIM_Rhsim05G0071600 | 1315.95 | 3.87E-288 | 3.79E-286 | -5.248 |
| RHSIM_Rhsim06G0020600 | 1315.75 | 4.27E-288 | 4.17E-286 | -3.738 |
| RHSIM_Rhsim12G0193400 | 1313.02 | 1.68E-287 | 1.63E-285 | 3.517 |
| RHSIM_Rhsim02G0184100 | 1310.61 | 5.58E-287 | 5.42E-285 | -3.866 |
| RHSIM_Rhsim03G0093800 | 1307.21 | 3.07E-286 | 2.97E-284 | 1.976 |
| RHSIM_Rhsim13G0186600 | 1304.08 | 1.47E-285 | 1.41E-283 | -3.309 |
| RHSIM_Rhsim10G0178200 | 1298.78 | 2.08E-284 | 2.00E-282 | 2.220 |
| novel.1656 | 1298.76 | 2.10E-284 | 2.02E-282 | 1.658 |
| RHSIM_Rhsim07G0013600 | 1297.52 | 3.90E-284 | 3.72E-282 | -2.184 |
| RHSIM_Rhsim04G0163900 | 1296.84 | 5.50E-284 | 5.24E-282 | 2.211 |
| novel.1049 | 1284.19 | 3.09E-281 | 2.93E-279 | 2.034 |
| RHSIM_Rhsim03G0088200 | 1283.55 | 4.24E-281 | 4.01E-279 | -3.413 |
| RHSIM_RhsimUnG0024200 | 1283.04 | 5.48E-281 | 5.16E-279 | 9.630 |
| RHSIM_Rhsim03G0248500 | 1267.70 | 1.18E-277 | 1.11E-275 | -4.779 |
| RHSIM_Rhsim02G0253700 | 1267.29 | 1.45E-277 | 1.36E-275 | 2.531 |
| RHSIM_Rhsim02G0232900 | 1267.01 | 1.67E-277 | 1.55E-275 | 2.188 |
| RHSIM_RhsimUnG0085800 | 1264.81 | 5.02E-277 | 4.67E-275 | -2.207 |
| RHSIM_Rhsim09G0120100 | 1259.58 | 6.88E-276 | 6.38E-274 | 1.594 |
| RHSIM_Rhsim07G0010600 | 1257.45 | 1.99E-275 | 1.84E-273 | 2.164 |
| RHSIM_Rhsim13G0191600 | 1257.12 | 2.35E-275 | 2.16E-273 | -2.933 |
| RHSIM_Rhsim02G0254400 | 1252.33 | 2.59E-274 | 2.37E-272 | 2.504 |
| RHSIM_Rhsim01G0273700 | 1251.99 | 3.07E-274 | 2.81E-272 | 4.322 |
| RHSIM_Rhsim12G0054800 | 1250.78 | 5.63E-274 | 5.13E-272 | 2.301 |
| RHSIM_Rhsim01G0249100 | 1250.04 | 8.14E-274 | 7.40E-272 | -4.134 |
| RHSIM_Rhsim06G0150500 | 1247.43 | 3.00E-273 | 2.72E-271 | 2.408 |
| RHSIM_Rhsim12G0028600 | 1242.12 | 4.29E-272 | 3.87E-270 | 1.632 |
| novel.2779 | 1241.55 | 5.70E-272 | 5.13E-270 | 3.620 |
| RHSIM_Rhsim07G0038900 | 1237.49 | 4.35E-271 | 3.90E-269 | -7.451 |

| Gene | LRT statistic | P value | BH-adjusted P | log2FC (interaction) |
|---|---|---|---|---|
| RHSIM_Rhsim02G0026400 | 1232.25 | 5.99E-270 | 5.36E-268 | 2.748 |
| RHSIM_Rhsim11G0059000 | 1229.97 | 1.87E-269 | 1.67E-267 | -2.101 |
| RHSIM_Rhsim06G0014600 | 1227.57 | 6.22E-269 | 5.53E-267 | 4.570 |
| RHSIM_Rhsim05G0022500 | 1226.18 | 1.25E-268 | 1.11E-266 | 2.728 |
| novel.4523 | 1222.51 | 7.83E-268 | 6.92E-266 | -5.461 |
| RHSIM_Rhsim13G0211300 | 1219.00 | 4.52E-267 | 3.99E-265 | 3.853 |
| RHSIM_Rhsim05G0086800 | 1218.76 | 5.10E-267 | 4.48E-265 | -2.156 |
| RHSIM_Rhsim10G0174600 | 1215.29 | 2.90E-266 | 2.54E-264 | -2.786 |
| RHSIM_Rhsim01G0293600 | 1213.31 | 7.81E-266 | 6.82E-264 | 3.135 |
| RHSIM_Rhsim05G0190900 | 1204.91 | 5.22E-264 | 4.55E-262 | -2.030 |
| RHSIM_Rhsim12G0164900 | 1204.26 | 7.23E-264 | 6.28E-262 | -5.482 |
| RHSIM_Rhsim11G0045800 | 1202.15 | 2.08E-263 | 1.80E-261 | 1.922 |
| RHSIM_Rhsim11G0129000 | 1198.20 | 1.50E-262 | 1.29E-260 | -3.522 |
| RHSIM_Rhsim02G0120400 | 1198.13 | 1.55E-262 | 1.34E-260 | 2.191 |
| RHSIM_Rhsim03G0087300 | 1194.61 | 9.06E-262 | 7.77E-260 | -4.563 |
| RHSIM_Rhsim11G0069300 | 1193.68 | 1.44E-261 | 1.23E-259 | -4.416 |
| novel.629 | 1192.11 | 3.17E-261 | 2.70E-259 | 3.959 |
| RHSIM_Rhsim12G0053800 | 1191.55 | 4.19E-261 | 3.56E-259 | -3.446 |
| RHSIM_RhsimUnG0159800 | 1190.59 | 6.77E-261 | 5.74E-259 | 2.433 |
| RHSIM_Rhsim07G0044800 | 1189.46 | 1.19E-260 | 1.01E-258 | -8.243 |
| RHSIM_RhsimUnG0056300 | 1186.42 | 5.45E-260 | 4.59E-258 | 2.456 |
| RHSIM_Rhsim02G0062700 | 1184.18 | 1.67E-259 | 1.40E-257 | 3.662 |
| RHSIM_Rhsim05G0009900 | 1181.33 | 6.97E-259 | 5.84E-257 | -8.079 |
| RHSIM_Rhsim06G0040600 | 1179.24 | 1.98E-258 | 1.65E-256 | -3.198 |
| RHSIM_Rhsim05G0043600 | 1178.04 | 3.61E-258 | 3.01E-256 | 2.294 |
| RHSIM_Rhsim09G0067000 | 1177.07 | 5.87E-258 | 4.88E-256 | -4.691 |
| RHSIM_Rhsim04G0166000 | 1160.94 | 1.88E-254 | 1.56E-252 | -3.491 |
| RHSIM_Rhsim11G0027600 | 1159.74 | 3.44E-254 | 2.84E-252 | 3.388 |
| RHSIM_Rhsim13G0114900 | 1158.87 | 5.31E-254 | 4.37E-252 | -3.814 |
| RHSIM_Rhsim09G0105000 | 1158.53 | 6.29E-254 | 5.16E-252 | 2.128 |
| RHSIM_Rhsim10G0175100 | 1158.27 | 7.14E-254 | 5.84E-252 | -2.756 |
| RHSIM_Rhsim03G0255900 | 1154.12 | 5.72E-253 | 4.66E-251 | 2.105 |

| Gene | LRT statistic | P value | BH-adjusted P | log2FC (interaction) |
|---|---|---|---|---|
| RHSIM_Rhsim09G0091700 | 1154.01 | 6.03E-253 | 4.90E-251 | 2.163 |
| RHSIM_Rhsim12G0121400 | 1153.83 | 6.59E-253 | 5.35E-251 | 1.732 |
| RHSIM_Rhsim02G0205000 | 1147.55 | 1.53E-251 | 1.24E-249 | 2.317 |
| RHSIM_Rhsim01G0123000 | 1145.83 | 3.62E-251 | 2.92E-249 | -2.059 |
| RHSIM_Rhsim09G0104300 | 1143.97 | 9.18E-251 | 7.39E-249 | 3.012 |
| RHSIM_Rhsim12G0082800 | 1143.60 | 1.10E-250 | 8.85E-249 | 3.239 |
| RHSIM_Rhsim09G0209000 | 1138.42 | 1.48E-249 | 1.18E-247 | 1.778 |
| RHSIM_Rhsim03G0056500 | 1132.26 | 3.22E-248 | 2.57E-246 | 2.610 |
| RHSIM_Rhsim12G0009800 | 1126.90 | 4.70E-247 | 3.74E-245 | 2.282 |
| RHSIM_Rhsim06G0078900 | 1123.69 | 2.34E-246 | 1.86E-244 | 1.891 |
| RHSIM_Rhsim08G0075300 | 1122.29 | 4.74E-246 | 3.75E-244 | -2.812 |
| RHSIM_Rhsim02G0037100 | 1119.40 | 2.01E-245 | 1.59E-243 | 2.285 |
| RHSIM_Rhsim04G0168200 | 1118.58 | 3.03E-245 | 2.38E-243 | -7.234 |
| RHSIM_Rhsim08G0229400 | 1116.12 | 1.03E-244 | 8.12E-243 | 3.303 |
| RHSIM_Rhsim13G0151900 | 1115.34 | 1.53E-244 | 1.20E-242 | 1.512 |
| novel.3499 | 1115.04 | 1.78E-244 | 1.39E-242 | 2.541 |
| RHSIM_RhsimUnG0250500 | 1114.73 | 2.08E-244 | 1.62E-242 | -2.052 |
| RHSIM_Rhsim11G0094000 | 1113.93 | 3.09E-244 | 2.40E-242 | -1.755 |
| RHSIM_Rhsim13G0087300 | 1113.86 | 3.21E-244 | 2.49E-242 | -6.031 |
| novel.458 | 1112.64 | 5.90E-244 | 4.56E-242 | 2.478 |
| RHSIM_Rhsim06G0006900 | 1111.34 | 1.13E-243 | 8.74E-242 | 3.783 |
| RHSIM_Rhsim07G0019300 | 1111.00 | 1.34E-243 | 1.03E-241 | -2.144 |
| RHSIM_Rhsim11G0042500 | 1109.86 | 2.37E-243 | 1.82E-241 | 2.143 |
| RHSIM_Rhsim13G0162700 | 1109.60 | 2.71E-243 | 2.07E-241 | -2.370 |
| RHSIM_Rhsim11G0182700 | 1106.31 | 1.41E-242 | 1.07E-240 | 2.034 |
| RHSIM_Rhsim10G0186400 | 1096.26 | 2.15E-240 | 1.63E-238 | -1.911 |
| RHSIM_Rhsim11G0160000 | 1095.87 | 2.60E-240 | 1.97E-238 | -2.671 |
| RHSIM_Rhsim02G0037500 | 1094.18 | 6.07E-240 | 4.59E-238 | 3.672 |
| RHSIM_Rhsim12G0064400 | 1091.23 | 2.66E-239 | 2.01E-237 | -4.832 |
| RHSIM_Rhsim01G0287000 | 1087.37 | 1.84E-238 | 1.38E-236 | -1.531 |
| RHSIM_Rhsim07G0134900 | 1082.02 | 2.67E-237 | 2.00E-235 | 1.759 |
| RHSIM_Rhsim09G0037800 | 1081.11 | 4.21E-237 | 3.15E-235 | 2.006 |

| Gene | LRT statistic | P value | BH-adjusted P | log2FC (interaction) |
|---|---|---|---|---|
| RHSIM_Rhsim08G0012300 | 1077.07 | 3.18E-236 | 2.37E-234 | 3.448 |
| RHSIM_Rhsim03G0136800 | 1075.04 | 8.77E-236 | 6.52E-234 | 4.825 |
| RHSIM_Rhsim13G0067900 | 1074.50 | 1.15E-235 | 8.53E-234 | 2.126 |
| RHSIM_Rhsim03G0215500 | 1072.73 | 2.79E-235 | 2.06E-233 | 3.508 |
| RHSIM_Rhsim05G0226600 | 1069.53 | 1.38E-234 | 1.02E-232 | 2.784 |
| RHSIM_Rhsim02G0107100 | 1069.28 | 1.57E-234 | 1.16E-232 | -4.992 |
| RHSIM_RhsimUnG0125000 | 1068.05 | 2.90E-234 | 2.13E-232 | 4.419 |
| RHSIM_Rhsim07G0120400 | 1066.98 | 4.95E-234 | 3.63E-232 | -5.344 |
| RHSIM_Rhsim07G0004400 | 1066.84 | 5.33E-234 | 3.90E-232 | 1.510 |
| RHSIM_Rhsim13G0179600 | 1065.83 | 8.84E-234 | 6.44E-232 | -4.726 |
| RHSIM_Rhsim05G0004700 | 1064.98 | 1.35E-233 | 9.82E-232 | 1.482 |
| RHSIM_RhsimUnG0136800 | 1064.74 | 1.52E-233 | 1.10E-231 | 3.907 |
| RHSIM_Rhsim01G0202900 | 1061.46 | 7.88E-233 | 5.70E-231 | -2.482 |
| RHSIM_Rhsim01G0246400 | 1058.82 | 2.94E-232 | 2.12E-230 | -3.282 |
| RHSIM_Rhsim12G0212200 | 1057.68 | 5.22E-232 | 3.76E-230 | 2.206 |
| RHSIM_Rhsim13G0193900 | 1054.13 | 3.09E-231 | 2.22E-229 | 2.760 |
| RHSIM_Rhsim11G0114000 | 1053.50 | 4.22E-231 | 3.02E-229 | 3.880 |
| RHSIM_Rhsim08G0115500 | 1053.37 | 4.50E-231 | 3.22E-229 | 6.206 |
| RHSIM_Rhsim08G0066700 | 1051.44 | 1.19E-230 | 8.45E-229 | -5.982 |
| RHSIM_Rhsim07G0074900 | 1049.37 | 3.34E-230 | 2.38E-228 | 1.912 |
| RHSIM_Rhsim12G0104600 | 1047.14 | 1.02E-229 | 7.23E-228 | -2.797 |
| RHSIM_Rhsim08G0098000 | 1042.42 | 1.08E-228 | 7.66E-227 | 2.413 |
| RHSIM_Rhsim08G0186500 | 1039.79 | 4.04E-228 | 2.85E-226 | 1.654 |
| RHSIM_Rhsim06G0175700 | 1037.70 | 1.15E-227 | 8.06E-226 | -4.298 |
| RHSIM_Rhsim08G0119200 | 1036.88 | 1.73E-227 | 1.21E-225 | -1.826 |
| RHSIM_Rhsim09G0007900 | 1032.91 | 1.26E-226 | 8.85E-225 | 3.218 |
| RHSIM_Rhsim03G0192700 | 1030.91 | 3.42E-226 | 2.39E-224 | -2.948 |
| RHSIM_Rhsim02G0159600 | 1029.80 | 5.99E-226 | 4.18E-224 | 1.696 |
| RHSIM_Rhsim01G0176500 | 1026.69 | 2.83E-225 | 1.97E-223 | 3.305 |
| RHSIM_Rhsim07G0062800 | 1025.04 | 6.47E-225 | 4.49E-223 | -2.794 |
| RHSIM_Rhsim13G0154600 | 1023.38 | 1.49E-224 | 1.03E-222 | -2.770 |
| RHSIM_Rhsim06G0163400 | 1021.26 | 4.31E-224 | 2.97E-222 | -4.438 |

| Gene | LRT statistic | P value | BH-adjusted P | log2FC (interaction) |
|---|---|---|---|---|
| RHSIM_Rhsim11G0105900 | 1020.59 | 6.00E-224 | 4.13E-222 | 2.917 |
| RHSIM_Rhsim12G0043200 | 1020.37 | 6.70E-224 | 4.60E-222 | -1.757 |
| RHSIM_Rhsim13G0072700 | 1019.83 | 8.79E-224 | 6.01E-222 | -2.247 |
| RHSIM_Rhsim09G0094200 | 1019.83 | 8.79E-224 | 6.01E-222 | -1.766 |
| RHSIM_Rhsim01G0217200 | 1013.76 | 1.84E-222 | 1.25E-220 | -1.495 |
| RHSIM_Rhsim07G0045200 | 1010.62 | 8.81E-222 | 6.00E-220 | -7.517 |
| novel.3961 | 1009.67 | 1.42E-221 | 9.63E-220 | -3.537 |
| RHSIM_Rhsim11G0039900 | 1008.71 | 2.29E-221 | 1.55E-219 | -2.439 |
| RHSIM_Rhsim11G0058800 | 1007.26 | 4.75E-221 | 3.21E-219 | 2.076 |
| RHSIM_Rhsim08G0078600 | 1006.07 | 8.59E-221 | 5.79E-219 | 2.176 |
| RHSIM_Rhsim11G0038900 | 1003.99 | 2.43E-220 | 1.64E-218 | -4.506 |
| RHSIM_Rhsim08G0109700 | 999.66 | 2.13E-219 | 1.43E-217 | 1.957 |
| novel.2258 | 997.93 | 5.05E-219 | 3.38E-217 | -3.903 |
| RHSIM_Rhsim04G0023700 | 997.42 | 6.54E-219 | 4.36E-217 | -1.560 |
| RHSIM_Rhsim02G0248700 | 995.94 | 1.37E-218 | 9.13E-217 | -1.578 |
| RHSIM_Rhsim07G0007200 | 994.74 | 2.50E-218 | 1.66E-216 | 1.553 |
| RHSIM_Rhsim09G0092400 | 993.22 | 5.35E-218 | 3.55E-216 | 2.507 |
| RHSIM_Rhsim03G0146700 | 988.41 | 5.93E-217 | 3.92E-215 | 2.683 |
| RHSIM_Rhsim08G0214000 | 985.85 | 2.14E-216 | 1.41E-214 | -1.679 |
| RHSIM_Rhsim05G0148600 | 984.27 | 4.72E-216 | 3.11E-214 | 2.996 |
| RHSIM_Rhsim08G0028600 | 984.25 | 4.76E-216 | 3.13E-214 | -3.311 |
| novel.2688 | 983.92 | 5.61E-216 | 3.68E-214 | -1.329 |
| RHSIM_Rhsim08G0109200 | 983.74 | 6.14E-216 | 4.02E-214 | 2.478 |
| RHSIM_Rhsim06G0240400 | 982.66 | 1.06E-215 | 6.89E-214 | 2.121 |
| novel.1957 | 981.89 | 1.55E-215 | 1.01E-213 | 2.696 |
| RHSIM_Rhsim04G0181100 | 979.46 | 5.24E-215 | 3.41E-213 | -3.191 |
| RHSIM_Rhsim03G0177600 | 978.35 | 9.12E-215 | 5.91E-213 | -2.690 |
| RHSIM_Rhsim02G0138600 | 977.57 | 1.35E-214 | 8.71E-213 | 2.823 |
| RHSIM_Rhsim05G0175900 | 971.37 | 3.00E-213 | 1.94E-211 | 3.633 |
| RHSIM_Rhsim07G0046800 | 966.50 | 3.44E-212 | 2.22E-210 | 2.438 |
| RHSIM_Rhsim07G0141600 | 966.35 | 3.71E-212 | 2.38E-210 | 1.732 |
| RHSIM_Rhsim09G0068600 | 966.31 | 3.78E-212 | 2.42E-210 | 2.403 |

| Gene | LRT statistic | P value | BH-adjusted P | log2FC (interaction) |
|---|---|---|---|---|
| RHSIM_Rhsim06G0036400 | 964.49 | 9.41E-212 | 6.02E-210 | -2.289 |
| RHSIM_Rhsim05G0064300 | 962.69 | 2.31E-211 | 1.47E-209 | 2.028 |
| RHSIM_Rhsim07G0250500 | 961.17 | 4.95E-211 | 3.15E-209 | 2.006 |
| RHSIM_Rhsim04G0182700 | 959.51 | 1.13E-210 | 7.20E-209 | -3.008 |
| RHSIM_Rhsim03G0141600 | 959.24 | 1.30E-210 | 8.26E-209 | 2.269 |
| RHSIM_Rhsim04G0147400 | 958.49 | 1.90E-210 | 1.20E-208 | -2.740 |
| RHSIM_Rhsim13G0234200 | 957.44 | 3.20E-210 | 2.02E-208 | 2.334 |
| RHSIM_Rhsim01G0181300 | 955.04 | 1.07E-209 | 6.71E-208 | 2.589 |
| RHSIM_Rhsim08G0105500 | 953.38 | 2.44E-209 | 1.53E-207 | -4.948 |
| RHSIM_Rhsim09G0006400 | 952.73 | 3.39E-209 | 2.12E-207 | -2.963 |
| RHSIM_Rhsim08G0249200 | 950.52 | 1.02E-208 | 6.38E-207 | 2.824 |
| RHSIM_Rhsim02G0049200 | 947.03 | 5.86E-208 | 3.65E-206 | 2.144 |
| RHSIM_Rhsim03G0231600 | 945.00 | 1.62E-207 | 1.01E-205 | 5.055 |
| RHSIM_Rhsim01G0284500 | 939.41 | 2.66E-206 | 1.65E-204 | 2.603 |
| RHSIM_Rhsim10G0018300 | 933.88 | 4.23E-205 | 2.62E-203 | 2.760 |
| RHSIM_Rhsim02G0006000 | 933.12 | 6.20E-205 | 3.83E-203 | 2.209 |
| novel.514 | 931.08 | 1.72E-204 | 1.06E-202 | 3.124 |
| RHSIM_Rhsim01G0246000 | 927.76 | 9.05E-204 | 5.57E-202 | 3.248 |
| RHSIM_Rhsim02G0244000 | 924.10 | 5.65E-203 | 3.47E-201 | -5.088 |
| RHSIM_Rhsim13G0075900 | 923.80 | 6.56E-203 | 4.03E-201 | -3.098 |
| RHSIM_Rhsim12G0037700 | 923.74 | 6.78E-203 | 4.15E-201 | -2.827 |
| RHSIM_Rhsim04G0144800 | 923.10 | 9.33E-203 | 5.70E-201 | 2.372 |
| RHSIM_RhsimUnG0216700 | 920.85 | 2.88E-202 | 1.75E-200 | -2.827 |
| RHSIM_Rhsim08G0016900 | 920.61 | 3.24E-202 | 1.97E-200 | 1.293 |
| RHSIM_Rhsim13G0150700 | 919.48 | 5.72E-202 | 3.47E-200 | 2.419 |
| RHSIM_Rhsim01G0286200 | 916.81 | 2.18E-201 | 1.32E-199 | 1.470 |
| RHSIM_Rhsim09G0177600 | 914.12 | 8.35E-201 | 5.04E-199 | 1.934 |
| RHSIM_Rhsim03G0226700 | 913.19 | 1.33E-200 | 8.03E-199 | 1.632 |
| RHSIM_Rhsim01G0271500 | 911.34 | 3.37E-200 | 2.03E-198 | 2.669 |
| RHSIM_Rhsim02G0193200 | 911.01 | 3.96E-200 | 2.38E-198 | 1.190 |
| RHSIM_Rhsim04G0017200 | 910.80 | 4.41E-200 | 2.65E-198 | 2.336 |
| RHSIM_Rhsim11G0195600 | 910.17 | 6.05E-200 | 3.62E-198 | 1.864 |

| Gene | LRT statistic | P value | BH-adjusted P | log2FC (interaction) |
|---|---|---|---|---|
| RHSIM_Rhsim12G0210300 | 910.08 | 6.31E-200 | 3.77E-198 | 1.646 |
| RHSIM_Rhsim05G0231800 | 906.44 | 3.90E-199 | 2.33E-197 | 3.149 |
| RHSIM_Rhsim11G0093200 | 905.98 | 4.91E-199 | 2.92E-197 | 2.438 |
| RHSIM_Rhsim06G0207200 | 899.44 | 1.30E-197 | 7.71E-196 | -1.571 |
| RHSIM_Rhsim01G0067700 | 898.83 | 1.76E-197 | 1.04E-195 | 2.668 |
| RHSIM_Rhsim04G0103100 | 895.85 | 7.83E-197 | 4.63E-195 | 2.100 |
| RHSIM_Rhsim04G0213900 | 894.83 | 1.31E-196 | 7.70E-195 | -3.916 |
| RHSIM_Rhsim06G0061800 | 888.69 | 2.83E-195 | 1.66E-193 | 2.157 |
| RHSIM_Rhsim06G0234100 | 888.16 | 3.68E-195 | 2.16E-193 | -2.941 |
| RHSIM_Rhsim05G0061700 | 885.30 | 1.54E-194 | 9.02E-193 | -4.216 |
| RHSIM_Rhsim06G0239100 | 885.09 | 1.71E-194 | 9.99E-193 | -2.393 |
| RHSIM_Rhsim07G0102300 | 879.88 | 2.32E-193 | 1.35E-191 | -4.164 |
| RHSIM_Rhsim13G0010900 | 879.11 | 3.41E-193 | 1.98E-191 | 1.685 |
| RHSIM_RhsimUnG0019600 | 878.71 | 4.17E-193 | 2.42E-191 | 2.322 |
| RHSIM_Rhsim11G0169900 | 878.58 | 4.46E-193 | 2.58E-191 | -10.073 |
| RHSIM_Rhsim03G0216000 | 877.29 | 8.50E-193 | 4.92E-191 | -3.152 |
| RHSIM_Rhsim09G0197000 | 876.21 | 1.46E-192 | 8.43E-191 | 1.715 |
| RHSIM_Rhsim08G0026800 | 875.74 | 1.85E-192 | 1.06E-190 | -3.025 |
| RHSIM_Rhsim01G0202100 | 874.99 | 2.68E-192 | 1.54E-190 | 1.918 |
| RHSIM_Rhsim09G0130700 | 874.91 | 2.80E-192 | 1.61E-190 | 2.729 |
| RHSIM_Rhsim05G0075200 | 874.23 | 3.92E-192 | 2.25E-190 | -2.892 |
| RHSIM_Rhsim09G0010300 | 873.25 | 6.43E-192 | 3.67E-190 | -5.953 |
| RHSIM_Rhsim05G0101800 | 871.66 | 1.42E-191 | 8.11E-190 | 1.868 |
| RHSIM_Rhsim03G0253100 | 870.79 | 2.19E-191 | 1.25E-189 | 1.654 |
| RHSIM_Rhsim06G0222100 | 870.74 | 2.25E-191 | 1.28E-189 | 1.254 |
| RHSIM_Rhsim11G0165700 | 870.11 | 3.09E-191 | 1.75E-189 | 1.601 |
| RHSIM_Rhsim11G0003600 | 869.77 | 3.66E-191 | 2.07E-189 | 1.909 |
| RHSIM_Rhsim05G0137700 | 869.06 | 5.23E-191 | 2.96E-189 | 1.849 |
| RHSIM_Rhsim03G0053000 | 867.74 | 1.01E-190 | 5.71E-189 | -1.352 |
| RHSIM_Rhsim07G0091100 | 866.18 | 2.20E-190 | 1.24E-188 | 4.317 |
| RHSIM_Rhsim05G0174500 | 865.66 | 2.87E-190 | 1.61E-188 | 2.208 |
| RHSIM_Rhsim04G0113400 | 863.40 | 8.87E-190 | 4.97E-188 | -2.964 |

| Gene | LRT statistic | P value | BH-adjusted P | log2FC (interaction) |
|---|---|---|---|---|
| RHSIM_Rhsim05G0108400 | 862.96 | 1.11E-189 | 6.20E-188 | 2.403 |
| RHSIM_Rhsim13G0153400 | 862.28 | 1.55E-189 | 8.68E-188 | -2.861 |
| RHSIM_Rhsim07G0005900 | 861.77 | 2.00E-189 | 1.12E-187 | 2.168 |
| RHSIM_Rhsim08G0031900 | 861.62 | 2.17E-189 | 1.21E-187 | 4.279 |
| RHSIM_Rhsim07G0184200 | 860.96 | 3.01E-189 | 1.67E-187 | 1.607 |
| RHSIM_Rhsim01G0264400 | 860.61 | 3.58E-189 | 1.99E-187 | -2.898 |
| RHSIM_Rhsim08G0200500 | 858.89 | 8.49E-189 | 4.69E-187 | 1.171 |
| RHSIM_Rhsim01G0283900 | 858.81 | 8.86E-189 | 4.89E-187 | 8.431 |
| RHSIM_Rhsim11G0113700 | 858.79 | 8.92E-189 | 4.91E-187 | -3.539 |
| RHSIM_Rhsim07G0017000 | 858.54 | 1.01E-188 | 5.57E-187 | 1.385 |
| RHSIM_Rhsim07G0252600 | 858.19 | 1.21E-188 | 6.61E-187 | -1.869 |
| RHSIM_Rhsim04G0023900 | 857.51 | 1.69E-188 | 9.28E-187 | 1.816 |
| RHSIM_Rhsim08G0242200 | 856.58 | 2.70E-188 | 1.48E-186 | 2.707 |
| RHSIM_Rhsim02G0027900 | 856.11 | 3.42E-188 | 1.86E-186 | 1.819 |
| RHSIM_Rhsim13G0072000 | 854.57 | 7.37E-188 | 4.01E-186 | 2.259 |
| RHSIM_Rhsim10G0172700 | 853.78 | 1.10E-187 | 5.95E-186 | 2.114 |
| RHSIM_Rhsim08G0070300 | 852.29 | 2.31E-187 | 1.25E-185 | -2.095 |
| RHSIM_Rhsim07G0014000 | 851.00 | 4.41E-187 | 2.39E-185 | 2.778 |
| RHSIM_Rhsim13G0011000 | 849.62 | 8.79E-187 | 4.75E-185 | -1.677 |
| novel.3051 | 849.28 | 1.04E-186 | 5.61E-185 | 1.305 |
| RHSIM_Rhsim11G0169400 | 848.92 | 1.25E-186 | 6.73E-185 | -3.351 |
| novel.1152 | 847.68 | 2.33E-186 | 1.25E-184 | 2.276 |
| novel.1569 | 846.38 | 4.44E-186 | 2.38E-184 | 3.474 |
| RHSIM_Rhsim08G0032100 | 846.01 | 5.37E-186 | 2.87E-184 | -1.760 |
| RHSIM_Rhsim02G0143300 | 845.16 | 8.22E-186 | 4.39E-184 | -1.437 |
| RHSIM_Rhsim12G0089300 | 844.97 | 9.04E-186 | 4.82E-184 | 2.187 |
| RHSIM_Rhsim09G0166000 | 843.72 | 1.68E-185 | 8.96E-184 | 2.287 |
| RHSIM_Rhsim03G0033400 | 843.21 | 2.18E-185 | 1.16E-183 | -2.592 |
| RHSIM_Rhsim08G0008800 | 842.91 | 2.52E-185 | 1.34E-183 | -2.782 |
| RHSIM_Rhsim02G0020800 | 842.86 | 2.59E-185 | 1.37E-183 | 1.841 |
| RHSIM_Rhsim01G0229600 | 841.95 | 4.08E-185 | 2.16E-183 | 2.511 |
| RHSIM_Rhsim03G0151100 | 841.93 | 4.14E-185 | 2.18E-183 | 3.217 |

| Gene | LRT statistic | P value | BH-adjusted P | log2FC (interaction) |
|---|---|---|---|---|
| RHSIM_Rhsim06G0229700 | 841.64 | 4.76E-185 | 2.51E-183 | 2.479 |
| RHSIM_Rhsim10G0206500 | 840.96 | 6.73E-185 | 3.54E-183 | -1.469 |
| novel.4233 | 840.56 | 8.22E-185 | 4.31E-183 | -8.544 |
| RHSIM_Rhsim10G0185700 | 836.84 | 5.28E-184 | 2.77E-182 | -2.136 |
| RHSIM_Rhsim11G0167000 | 836.47 | 6.36E-184 | 3.32E-182 | 1.661 |
| RHSIM_Rhsim02G0200700 | 830.21 | 1.46E-182 | 7.59E-181 | -2.919 |
| RHSIM_Rhsim05G0194000 | 829.32 | 2.27E-182 | 1.18E-180 | -2.793 |
| RHSIM_Rhsim01G0189600 | 828.68 | 3.14E-182 | 1.63E-180 | 3.650 |
| RHSIM_Rhsim12G0172500 | 826.88 | 7.72E-182 | 4.00E-180 | 2.702 |
| RHSIM_Rhsim06G0101800 | 826.79 | 8.10E-182 | 4.20E-180 | 3.017 |
| novel.4010 | 825.09 | 1.89E-181 | 9.77E-180 | -1.599 |
| RHSIM_Rhsim04G0156700 | 825.01 | 1.97E-181 | 1.02E-179 | 1.812 |
| RHSIM_Rhsim11G0184100 | 824.92 | 2.06E-181 | 1.06E-179 | 2.181 |
| RHSIM_Rhsim02G0229000 | 822.51 | 6.88E-181 | 3.54E-179 | 2.222 |
| RHSIM_Rhsim10G0094800 | 821.24 | 1.30E-180 | 6.68E-179 | -2.617 |
| RHSIM_Rhsim06G0050200 | 821.20 | 1.32E-180 | 6.78E-179 | 2.705 |
| RHSIM_Rhsim02G0212300 | 819.42 | 3.23E-180 | 1.65E-178 | 2.313 |
| RHSIM_Rhsim02G0067100 | 819.29 | 3.46E-180 | 1.76E-178 | -2.171 |
| RHSIM_Rhsim09G0010800 | 817.78 | 7.35E-180 | 3.75E-178 | -2.783 |
| RHSIM_Rhsim02G0012400 | 816.79 | 1.21E-179 | 6.14E-178 | -5.376 |
| RHSIM_Rhsim10G0163400 | 815.08 | 2.85E-179 | 1.45E-177 | 1.572 |
| RHSIM_Rhsim03G0180800 | 813.87 | 5.20E-179 | 2.64E-177 | 4.842 |
| RHSIM_Rhsim03G0215400 | 812.62 | 9.71E-179 | 4.91E-177 | 1.826 |
| RHSIM_Rhsim11G0170100 | 810.84 | 2.37E-178 | 1.20E-176 | 2.126 |
| novel.4105 | 810.77 | 2.45E-178 | 1.24E-176 | 1.256 |
| RHSIM_Rhsim04G0011600 | 810.50 | 2.82E-178 | 1.42E-176 | 1.779 |
| RHSIM_Rhsim06G0097000 | 809.84 | 3.92E-178 | 1.97E-176 | 2.173 |
| RHSIM_Rhsim01G0237100 | 808.45 | 7.86E-178 | 3.94E-176 | 2.058 |
| RHSIM_Rhsim12G0167700 | 808.28 | 8.57E-178 | 4.29E-176 | 2.132 |
| RHSIM_Rhsim02G0105600 | 807.50 | 1.26E-177 | 6.31E-176 | 1.370 |
| RHSIM_Rhsim06G0227300 | 807.39 | 1.34E-177 | 6.67E-176 | 1.667 |
| RHSIM_Rhsim10G0180600 | 806.88 | 1.73E-177 | 8.59E-176 | 3.374 |

| Gene | LRT statistic | P value | BH-adjusted P | log2FC (interaction) |
|---|---|---|---|---|
| RHSIM_Rhsim03G0066700 | 806.49 | 2.09E-177 | 1.04E-175 | 1.350 |
| RHSIM_Rhsim10G0188300 | 806.05 | 2.61E-177 | 1.30E-175 | -1.760 |
| RHSIM_Rhsim04G0059600 | 805.54 | 3.36E-177 | 1.67E-175 | -9.042 |
| RHSIM_Rhsim12G0055000 | 805.12 | 4.15E-177 | 2.05E-175 | 1.520 |
| RHSIM_Rhsim02G0150100 | 803.41 | 9.80E-177 | 4.84E-175 | 1.614 |
| RHSIM_Rhsim12G0015600 | 801.12 | 3.07E-176 | 1.51E-174 | 1.884 |
| RHSIM_Rhsim01G0201100 | 800.57 | 4.06E-176 | 2.00E-174 | -3.374 |
| RHSIM_Rhsim09G0152400 | 800.45 | 4.30E-176 | 2.11E-174 | -1.624 |
| RHSIM_Rhsim07G0190100 | 799.96 | 5.49E-176 | 2.70E-174 | -1.744 |
| RHSIM_Rhsim13G0080300 | 797.84 | 1.59E-175 | 7.78E-174 | 2.092 |
| RHSIM_Rhsim13G0220600 | 795.71 | 4.61E-175 | 2.25E-173 | -1.497 |
| RHSIM_Rhsim08G0228000 | 795.55 | 5.00E-175 | 2.44E-173 | -2.972 |
| RHSIM_Rhsim12G0085700 | 792.77 | 2.02E-174 | 9.82E-173 | -2.218 |
| RHSIM_Rhsim09G0152300 | 791.74 | 3.37E-174 | 1.64E-172 | -3.112 |
| RHSIM_RhsimUnG0192700 | 790.75 | 5.52E-174 | 2.68E-172 | 1.911 |
| RHSIM_Rhsim03G0127100 | 790.75 | 5.55E-174 | 2.69E-172 | 2.102 |
| RHSIM_Rhsim09G0208900 | 789.53 | 1.02E-173 | 4.93E-172 | -4.875 |
| RHSIM_Rhsim04G0144200 | 789.14 | 1.24E-173 | 6.00E-172 | 1.196 |
| RHSIM_Rhsim08G0249100 | 789.09 | 1.27E-173 | 6.14E-172 | 1.579 |
| RHSIM_Rhsim03G0059600 | 787.51 | 2.81E-173 | 1.35E-171 | 2.114 |
| RHSIM_Rhsim03G0040000 | 783.98 | 1.64E-172 | 7.90E-171 | 1.468 |
| RHSIM_Rhsim09G0096600 | 783.58 | 2.01E-172 | 9.62E-171 | -2.478 |
| RHSIM_Rhsim01G0218600 | 783.57 | 2.02E-172 | 9.67E-171 | 1.422 |
| RHSIM_Rhsim08G0198900 | 783.49 | 2.10E-172 | 1.00E-170 | 3.030 |
| RHSIM_Rhsim01G0254100 | 782.03 | 4.35E-172 | 2.08E-170 | 2.105 |
| RHSIM_Rhsim06G0189200 | 780.39 | 9.90E-172 | 4.72E-170 | -7.516 |
| RHSIM_Rhsim06G0193600 | 779.97 | 1.22E-171 | 5.83E-170 | 2.511 |
| RHSIM_Rhsim01G0290100 | 779.79 | 1.33E-171 | 6.34E-170 | 4.534 |
| novel.3956 | 779.77 | 1.35E-171 | 6.39E-170 | -1.871 |
| RHSIM_Rhsim10G0139500 | 779.35 | 1.67E-171 | 7.90E-170 | 2.779 |
| RHSIM_Rhsim05G0161900 | 778.97 | 2.01E-171 | 9.51E-170 | -3.349 |
| RHSIM_Rhsim02G0116000 | 778.91 | 2.07E-171 | 9.79E-170 | 1.800 |

| Gene | LRT statistic | P value | BH-adjusted P | log2FC (interaction) |
|---|---|---|---|---|
| RHSIM_Rhsim01G0231100 | 777.22 | 4.83E-171 | 2.28E-169 | 3.242 |
| RHSIM_Rhsim04G0002800 | 777.19 | 4.91E-171 | 2.31E-169 | 1.448 |
| RHSIM_Rhsim05G0049000 | 776.58 | 6.66E-171 | 3.13E-169 | -4.006 |
| RHSIM_RhsimUnG0254700 | 774.10 | 2.31E-170 | 1.08E-168 | 2.231 |
| RHSIM_Rhsim08G0160100 | 772.98 | 4.05E-170 | 1.90E-168 | -1.613 |
| RHSIM_Rhsim09G0204600 | 771.75 | 7.49E-170 | 3.50E-168 | 2.121 |
| RHSIM_Rhsim13G0000600 | 770.53 | 1.38E-169 | 6.43E-168 | -1.922 |
| RHSIM_Rhsim13G0127600 | 770.49 | 1.41E-169 | 6.55E-168 | -2.560 |
| RHSIM_Rhsim11G0194700 | 770.08 | 1.73E-169 | 8.04E-168 | -2.230 |
| RHSIM_Rhsim01G0125700 | 769.56 | 2.24E-169 | 1.04E-167 | 4.823 |
| RHSIM_Rhsim13G0154400 | 769.06 | 2.88E-169 | 1.34E-167 | -2.533 |
| RHSIM_Rhsim09G0186300 | 768.03 | 4.81E-169 | 2.23E-167 | 2.046 |
| RHSIM_Rhsim07G0139000 | 764.19 | 3.30E-168 | 1.52E-166 | 2.394 |
| RHSIM_Rhsim07G0055800 | 763.91 | 3.79E-168 | 1.75E-166 | -2.342 |
| RHSIM_Rhsim05G0179500 | 762.60 | 7.31E-168 | 3.37E-166 | -2.166 |
| novel.2291 | 762.50 | 7.67E-168 | 3.53E-166 | 2.010 |
| RHSIM_Rhsim03G0046200 | 762.47 | 7.78E-168 | 3.57E-166 | -2.521 |
| novel.4279 | 760.83 | 1.77E-167 | 8.13E-166 | -2.967 |
| RHSIM_Rhsim01G0217800 | 758.94 | 4.56E-167 | 2.09E-165 | -1.765 |
| RHSIM_Rhsim03G0217000 | 757.03 | 1.19E-166 | 5.42E-165 | 1.457 |
| RHSIM_Rhsim06G0131800 | 755.01 | 3.27E-166 | 1.49E-164 | 1.528 |
| RHSIM_RhsimUnG0219800 | 753.16 | 8.24E-166 | 3.75E-164 | -3.243 |
| RHSIM_Rhsim03G0267300 | 751.23 | 2.17E-165 | 9.85E-164 | 1.762 |
| RHSIM_Rhsim06G0188200 | 751.12 | 2.29E-165 | 1.04E-163 | 1.223 |
| novel.4088 | 750.87 | 2.59E-165 | 1.18E-163 | 1.637 |
| RHSIM_Rhsim05G0232900 | 750.81 | 2.68E-165 | 1.21E-163 | -1.186 |
| RHSIM_Rhsim09G0208500 | 750.21 | 3.61E-165 | 1.63E-163 | 2.932 |
| RHSIM_Rhsim12G0152000 | 748.52 | 8.42E-165 | 3.80E-163 | 1.980 |
| RHSIM_Rhsim04G0091400 | 748.41 | 8.91E-165 | 4.01E-163 | 1.987 |
| RHSIM_Rhsim05G0217500 | 748.17 | 1.00E-164 | 4.51E-163 | -1.404 |
| RHSIM_Rhsim04G0184600 | 747.34 | 1.52E-164 | 6.83E-163 | 2.100 |
| RHSIM_Rhsim08G0040300 | 746.02 | 2.94E-164 | 1.32E-162 | 3.846 |

| Gene | LRT statistic | P value | BH-adjusted P | log2FC (interaction) |
|---|---|---|---|---|
| RHSIM_Rhsim04G0135400 | 745.99 | 2.99E-164 | 1.34E-162 | 3.759 |
| RHSIM_Rhsim06G0156900 | 744.88 | 5.20E-164 | 2.32E-162 | -4.962 |
| RHSIM_Rhsim01G0222000 | 742.39 | 1.81E-163 | 8.08E-162 | -3.333 |
| RHSIM_Rhsim03G0266200 | 740.23 | 5.33E-163 | 2.38E-161 | 2.965 |
| RHSIM_Rhsim13G0194200 | 739.34 | 8.36E-163 | 3.72E-161 | 2.732 |
| RHSIM_Rhsim02G0014200 | 738.78 | 1.10E-162 | 4.90E-161 | -1.673 |
| RHSIM_Rhsim07G0096000 | 737.82 | 1.79E-162 | 7.92E-161 | -2.299 |
| RHSIM_Rhsim03G0052700 | 735.15 | 6.79E-162 | 3.00E-160 | 4.653 |
| RHSIM_Rhsim08G0210900 | 734.85 | 7.90E-162 | 3.49E-160 | 2.257 |
| RHSIM_RhsimUnG0219600 | 734.80 | 8.11E-162 | 3.58E-160 | -3.474 |
| RHSIM_Rhsim01G0192800 | 734.03 | 1.19E-161 | 5.25E-160 | 2.405 |
| RHSIM_Rhsim01G0276500 | 733.60 | 1.48E-161 | 6.50E-160 | 2.558 |
| RHSIM_Rhsim13G0098700 | 731.83 | 3.59E-161 | 1.57E-159 | -1.131 |
| RHSIM_Rhsim13G0118300 | 731.83 | 3.59E-161 | 1.57E-159 | 2.005 |
| RHSIM_Rhsim01G0241700 | 731.10 | 5.18E-161 | 2.27E-159 | -2.521 |
| RHSIM_Rhsim02G0179400 | 730.37 | 7.43E-161 | 3.25E-159 | 2.984 |
| RHSIM_Rhsim13G0196400 | 730.27 | 7.84E-161 | 3.42E-159 | -1.832 |
| RHSIM_Rhsim03G0234400 | 729.73 | 1.03E-160 | 4.47E-159 | -3.001 |
| RHSIM_Rhsim01G0250800 | 728.73 | 1.69E-160 | 7.36E-159 | 1.722 |
| RHSIM_Rhsim03G0144000 | 728.48 | 1.92E-160 | 8.35E-159 | 2.379 |
| RHSIM_Rhsim07G0023100 | 727.90 | 2.56E-160 | 1.11E-158 | -2.148 |
| RHSIM_Rhsim13G0045400 | 726.06 | 6.44E-160 | 2.79E-158 | 2.495 |
| RHSIM_Rhsim10G0183900 | 725.88 | 7.04E-160 | 3.05E-158 | -1.441 |
| RHSIM_Rhsim12G0072200 | 725.11 | 1.04E-159 | 4.49E-158 | -6.853 |
| RHSIM_Rhsim07G0209200 | 724.95 | 1.12E-159 | 4.85E-158 | 2.061 |
| RHSIM_Rhsim06G0072900 | 723.97 | 1.83E-159 | 7.90E-158 | 1.805 |
| RHSIM_Rhsim10G0149900 | 723.38 | 2.47E-159 | 1.06E-157 | 3.240 |
| RHSIM_Rhsim11G0075300 | 721.44 | 6.50E-159 | 2.79E-157 | 1.413 |
| RHSIM_Rhsim01G0126900 | 719.76 | 1.51E-158 | 6.46E-157 | 1.865 |
| RHSIM_Rhsim07G0129400 | 717.82 | 3.99E-158 | 1.71E-156 | 1.553 |
| RHSIM_Rhsim05G0145700 | 717.68 | 4.29E-158 | 1.83E-156 | -3.604 |
| RHSIM_Rhsim10G0170600 | 717.55 | 4.58E-158 | 1.95E-156 | 3.105 |

| Gene | LRT statistic | P value | BH-adjusted P | log2FC (interaction) |
|---|---|---|---|---|
| RHSIM_Rhsim01G0243600 | 717.54 | 4.58E-158 | 1.95E-156 | -1.992 |
| RHSIM_Rhsim03G0217100 | 717.31 | 5.14E-158 | 2.19E-156 | -3.160 |
| RHSIM_Rhsim04G0196300 | 716.77 | 6.76E-158 | 2.87E-156 | -1.331 |
| RHSIM_Rhsim05G0078400 | 715.93 | 1.03E-157 | 4.36E-156 | 2.756 |
| RHSIM_Rhsim08G0092900 | 715.06 | 1.59E-157 | 6.72E-156 | 1.454 |
| RHSIM_Rhsim05G0234200 | 715.00 | 1.64E-157 | 6.92E-156 | 2.633 |
| RHSIM_Rhsim07G0018700 | 714.14 | 2.51E-157 | 1.06E-155 | 1.568 |
| RHSIM_Rhsim07G0004100 | 713.93 | 2.80E-157 | 1.18E-155 | 1.341 |
| RHSIM_Rhsim04G0249100 | 713.89 | 2.85E-157 | 1.20E-155 | -1.524 |
| RHSIM_Rhsim02G0249200 | 713.54 | 3.41E-157 | 1.43E-155 | 1.779 |
| RHSIM_Rhsim05G0223800 | 712.89 | 4.70E-157 | 1.98E-155 | -1.442 |
| RHSIM_Rhsim07G0228200 | 712.56 | 5.54E-157 | 2.32E-155 | 6.194 |
| RHSIM_Rhsim02G0007500 | 711.78 | 8.20E-157 | 3.43E-155 | -5.416 |
| RHSIM_Rhsim02G0223600 | 710.44 | 1.61E-156 | 6.72E-155 | 2.044 |
| RHSIM_Rhsim04G0184200 | 708.31 | 4.65E-156 | 1.94E-154 | -2.603 |
| RHSIM_Rhsim06G0237100 | 708.30 | 4.68E-156 | 1.95E-154 | 1.853 |
| RHSIM_Rhsim09G0138900 | 708.08 | 5.22E-156 | 2.17E-154 | 4.552 |
| RHSIM_Rhsim07G0226700 | 707.55 | 6.81E-156 | 2.83E-154 | -3.706 |
| RHSIM_Rhsim07G0014200 | 705.71 | 1.71E-155 | 7.10E-154 | 1.536 |
| RHSIM_Rhsim06G0191500 | 705.12 | 2.30E-155 | 9.53E-154 | 3.345 |
| RHSIM_Rhsim13G0187700 | 703.81 | 4.44E-155 | 1.84E-153 | -9.754 |
| RHSIM_Rhsim02G0084900 | 703.76 | 4.54E-155 | 1.88E-153 | -1.721 |
| RHSIM_Rhsim03G0139800 | 703.70 | 4.69E-155 | 1.93E-153 | -3.147 |
| RHSIM_Rhsim01G0061700 | 703.25 | 5.86E-155 | 2.42E-153 | 2.309 |
| RHSIM_Rhsim12G0039300 | 703.01 | 6.63E-155 | 2.73E-153 | 1.806 |
| RHSIM_Rhsim06G0046400 | 702.04 | 1.08E-154 | 4.43E-153 | 1.593 |
| RHSIM_Rhsim08G0011100 | 701.27 | 1.59E-154 | 6.51E-153 | 1.572 |
| RHSIM_Rhsim03G0190100 | 699.33 | 4.18E-154 | 1.71E-152 | 1.582 |
| RHSIM_Rhsim03G0176400 | 699.08 | 4.74E-154 | 1.94E-152 | 2.680 |
| RHSIM_Rhsim13G0082500 | 699.00 | 4.93E-154 | 2.02E-152 | 1.110 |
| RHSIM_Rhsim13G0194900 | 698.72 | 5.67E-154 | 2.31E-152 | -3.394 |
| RHSIM_RhsimUnG0216300 | 697.46 | 1.07E-153 | 4.35E-152 | -2.475 |

| Gene | LRT statistic | P value | BH-adjusted P | log2FC (interaction) |
|---|---|---|---|---|
| RHSIM_Rhsim12G0136400 | 697.35 | 1.13E-153 | 4.58E-152 | -1.183 |
| RHSIM_Rhsim03G0150900 | 694.34 | 5.09E-153 | 2.07E-151 | -1.681 |
| RHSIM_Rhsim08G0096700 | 692.49 | 1.28E-152 | 5.20E-151 | -2.228 |
| RHSIM_Rhsim07G0009200 | 692.36 | 1.37E-152 | 5.56E-151 | 1.749 |
| RHSIM_Rhsim01G0178400 | 692.13 | 1.54E-152 | 6.23E-151 | -1.838 |
| RHSIM_Rhsim06G0167600 | 692.01 | 1.63E-152 | 6.59E-151 | 1.970 |
| RHSIM_Rhsim02G0135000 | 691.71 | 1.90E-152 | 7.67E-151 | 1.508 |
| novel.831 | 690.34 | 3.77E-152 | 1.52E-150 | -1.290 |
| RHSIM_Rhsim09G0104500 | 689.22 | 6.60E-152 | 2.66E-150 | 2.506 |
| RHSIM_Rhsim12G0145700 | 689.11 | 6.99E-152 | 2.81E-150 | 2.252 |
| RHSIM_Rhsim10G0084800 | 689.10 | 7.02E-152 | 2.82E-150 | -2.674 |
| RHSIM_Rhsim09G0009500 | 687.42 | 1.63E-151 | 6.53E-150 | 1.240 |
| RHSIM_Rhsim08G0000900 | 687.36 | 1.68E-151 | 6.71E-150 | 1.787 |
| RHSIM_Rhsim04G0169600 | 686.69 | 2.35E-151 | 9.38E-150 | -1.882 |
| RHSIM_Rhsim11G0008500 | 685.81 | 3.65E-151 | 1.45E-149 | 1.816 |
| novel.1005 | 684.73 | 6.25E-151 | 2.49E-149 | 1.824 |
| RHSIM_Rhsim07G0045800 | 683.86 | 9.65E-151 | 3.84E-149 | 1.926 |
| RHSIM_Rhsim06G0116100 | 681.54 | 3.08E-150 | 1.23E-148 | -2.301 |
| RHSIM_Rhsim11G0128900 | 679.82 | 7.31E-150 | 2.90E-148 | -3.418 |
| RHSIM_Rhsim12G0146800 | 678.04 | 1.78E-149 | 7.06E-148 | 1.948 |
| RHSIM_Rhsim01G0284300 | 678.04 | 1.78E-149 | 7.06E-148 | -2.836 |
| RHSIM_Rhsim09G0115100 | 677.52 | 2.31E-149 | 9.13E-148 | 2.047 |
| RHSIM_Rhsim10G0037300 | 677.29 | 2.59E-149 | 1.02E-147 | 1.826 |
| RHSIM_Rhsim03G0162200 | 676.99 | 3.02E-149 | 1.19E-147 | 3.364 |
| RHSIM_Rhsim13G0149800 | 674.89 | 8.64E-149 | 3.40E-147 | -1.560 |
| RHSIM_Rhsim07G0225100 | 674.62 | 9.86E-149 | 3.87E-147 | -2.315 |
| RHSIM_Rhsim01G0114000 | 674.36 | 1.12E-148 | 4.41E-147 | 1.816 |
| RHSIM_Rhsim08G0000400 | 672.91 | 2.33E-148 | 9.11E-147 | 1.918 |
| RHSIM_Rhsim06G0061500 | 672.43 | 2.96E-148 | 1.16E-146 | 1.928 |
| RHSIM_Rhsim04G0134300 | 671.73 | 4.19E-148 | 1.64E-146 | 1.884 |
| RHSIM_Rhsim03G0063900 | 669.74 | 1.14E-147 | 4.44E-146 | 1.596 |
| RHSIM_Rhsim06G0207400 | 669.47 | 1.30E-147 | 5.08E-146 | -3.015 |

| Gene | LRT statistic | P value | BH-adjusted P | log2FC (interaction) |
|---|---|---|---|---|
| RHSIM_Rhsim04G0022700 | 668.84 | 1.79E-147 | 6.96E-146 | -2.057 |
| RHSIM_Rhsim03G0210700 | 668.36 | 2.27E-147 | 8.82E-146 | 1.638 |
| RHSIM_Rhsim12G0045800 | 667.70 | 3.16E-147 | 1.23E-145 | 1.793 |
| RHSIM_Rhsim04G0106500 | 667.08 | 4.31E-147 | 1.67E-145 | 1.381 |
| RHSIM_RhsimUnG0120100 | 666.86 | 4.82E-147 | 1.87E-145 | -1.309 |
| RHSIM_Rhsim03G0254400 | 666.55 | 5.63E-147 | 2.18E-145 | -1.899 |
| novel.3449 | 665.42 | 9.91E-147 | 3.83E-145 | 2.590 |
| RHSIM_Rhsim05G0139000 | 665.06 | 1.19E-146 | 4.57E-145 | 1.484 |
| RHSIM_Rhsim12G0195700 | 662.15 | 5.08E-146 | 1.96E-144 | -1.572 |
| RHSIM_Rhsim09G0068500 | 661.96 | 5.60E-146 | 2.15E-144 | 2.793 |
| RHSIM_Rhsim11G0076700 | 661.10 | 8.62E-146 | 3.31E-144 | -3.101 |
| RHSIM_Rhsim09G0192600 | 661.06 | 8.80E-146 | 3.37E-144 | -3.679 |
| RHSIM_Rhsim03G0180700 | 660.67 | 1.07E-145 | 4.09E-144 | -1.626 |
| RHSIM_Rhsim10G0192200 | 660.28 | 1.30E-145 | 4.97E-144 | 1.596 |
| RHSIM_Rhsim09G0155000 | 659.24 | 2.19E-145 | 8.36E-144 | 1.750 |
| RHSIM_Rhsim01G0195400 | 658.73 | 2.82E-145 | 1.08E-143 | -3.432 |
| RHSIM_Rhsim12G0199600 | 658.42 | 3.30E-145 | 1.26E-143 | 2.576 |
| novel.3037 | 657.95 | 4.17E-145 | 1.59E-143 | 5.174 |
| RHSIM_Rhsim13G0058300 | 657.93 | 4.21E-145 | 1.60E-143 | -1.834 |
| RHSIM_Rhsim03G0209200 | 656.53 | 8.48E-145 | 3.22E-143 | 2.291 |
| RHSIM_Rhsim11G0064800 | 656.13 | 1.04E-144 | 3.92E-143 | 1.469 |
| RHSIM_Rhsim08G0009700 | 655.53 | 1.40E-144 | 5.29E-143 | 2.206 |
| RHSIM_Rhsim13G0111900 | 655.21 | 1.65E-144 | 6.22E-143 | -1.411 |
| RHSIM_Rhsim04G0175300 | 654.14 | 2.80E-144 | 1.06E-142 | 1.925 |
| RHSIM_Rhsim08G0178200 | 652.98 | 5.03E-144 | 1.90E-142 | 1.392 |
| RHSIM_Rhsim03G0227400 | 651.68 | 9.63E-144 | 3.62E-142 | -1.077 |
| RHSIM_Rhsim03G0120800 | 651.16 | 1.25E-143 | 4.69E-142 | -1.927 |
| RHSIM_Rhsim07G0091400 | 648.16 | 5.62E-143 | 2.11E-141 | -1.172 |
| novel.3165 | 646.16 | 1.53E-142 | 5.73E-141 | -5.599 |
| RHSIM_Rhsim10G0180100 | 646.05 | 1.61E-142 | 6.04E-141 | 1.787 |
| RHSIM_Rhsim12G0199800 | 645.73 | 1.89E-142 | 7.07E-141 | 1.662 |
| RHSIM_Rhsim01G0004900 | 645.53 | 2.09E-142 | 7.82E-141 | -2.642 |

| Gene | LRT statistic | P value | BH-adjusted P | log2FC (interaction) |
|---|---|---|---|---|
| RHSIM_Rhsim11G0100000 | 645.32 | 2.33E-142 | 8.68E-141 | -2.302 |
| RHSIM_Rhsim10G0063500 | 644.18 | 4.11E-142 | 1.53E-140 | -2.386 |
| RHSIM_Rhsim12G0187600 | 644.04 | 4.41E-142 | 1.64E-140 | -3.021 |
| novel.402 | 644.03 | 4.43E-142 | 1.65E-140 | -2.412 |
| RHSIM_Rhsim09G0012200 | 643.37 | 6.18E-142 | 2.29E-140 | 2.513 |
| RHSIM_Rhsim07G0216900 | 643.14 | 6.93E-142 | 2.57E-140 | 2.549 |
| RHSIM_Rhsim05G0161700 | 640.66 | 2.40E-141 | 8.88E-140 | -2.747 |
| RHSIM_Rhsim06G0184300 | 640.61 | 2.46E-141 | 9.09E-140 | -3.469 |
| RHSIM_Rhsim04G0194000 | 640.35 | 2.81E-141 | 1.04E-139 | 1.698 |
| RHSIM_Rhsim04G0083600 | 639.79 | 3.72E-141 | 1.37E-139 | -2.472 |
| RHSIM_Rhsim03G0136400 | 639.61 | 4.05E-141 | 1.49E-139 | 1.970 |
| RHSIM_Rhsim03G0035500 | 638.48 | 7.17E-141 | 2.64E-139 | -7.842 |
| RHSIM_Rhsim06G0184800 | 638.38 | 7.51E-141 | 2.76E-139 | 1.762 |
| novel.2671 | 638.37 | 7.56E-141 | 2.77E-139 | -9.492 |
| RHSIM_Rhsim06G0185300 | 635.31 | 3.50E-140 | 1.28E-138 | -1.394 |
| RHSIM_Rhsim10G0169600 | 634.31 | 5.79E-140 | 2.12E-138 | -4.136 |
| novel.1891 | 631.40 | 2.48E-139 | 9.07E-138 | -8.183 |
| RHSIM_Rhsim03G0229900 | 630.29 | 4.33E-139 | 1.58E-137 | 2.504 |
| RHSIM_Rhsim05G0015100 | 629.31 | 7.07E-139 | 2.58E-137 | -2.084 |
| RHSIM_Rhsim10G0104800 | 628.64 | 9.88E-139 | 3.59E-137 | -3.499 |
| RHSIM_Rhsim06G0073300 | 627.90 | 1.43E-138 | 5.19E-137 | 2.081 |
| novel.2607 | 627.80 | 1.50E-138 | 5.46E-137 | 4.155 |
| RHSIM_Rhsim06G0164500 | 621.71 | 3.18E-137 | 1.15E-135 | 2.270 |
| RHSIM_Rhsim04G0233600 | 621.04 | 4.44E-137 | 1.61E-135 | -2.378 |
| RHSIM_Rhsim12G0200200 | 620.80 | 5.01E-137 | 1.81E-135 | -1.839 |
| RHSIM_Rhsim11G0187700 | 619.87 | 7.97E-137 | 2.88E-135 | 1.812 |
| RHSIM_Rhsim13G0049700 | 619.42 | 1.00E-136 | 3.61E-135 | -2.087 |
| RHSIM_Rhsim03G0217400 | 618.85 | 1.33E-136 | 4.79E-135 | -1.615 |
| RHSIM_Rhsim01G0207300 | 617.94 | 2.10E-136 | 7.57E-135 | 2.679 |
| RHSIM_Rhsim04G0071100 | 617.11 | 3.18E-136 | 1.14E-134 | 2.391 |
| RHSIM_Rhsim10G0197300 | 617.00 | 3.35E-136 | 1.20E-134 | 1.381 |
| RHSIM_Rhsim07G0246400 | 616.97 | 3.41E-136 | 1.22E-134 | -3.049 |

| Gene | LRT statistic | P value | BH-adjusted P | log2FC (interaction) |
|---|---|---|---|---|
| RHSIM_Rhsim06G0196500 | 616.37 | 4.61E-136 | 1.65E-134 | -2.165 |
| RHSIM_Rhsim10G0025800 | 616.06 | 5.37E-136 | 1.92E-134 | -2.424 |
| RHSIM_Rhsim06G0006400 | 615.86 | 5.96E-136 | 2.13E-134 | 2.014 |
| RHSIM_Rhsim01G0069000 | 615.60 | 6.76E-136 | 2.41E-134 | -2.606 |
| RHSIM_Rhsim05G0148200 | 614.92 | 9.51E-136 | 3.39E-134 | 1.984 |
| RHSIM_Rhsim08G0089400 | 614.73 | 1.05E-135 | 3.72E-134 | -5.671 |
| RHSIM_Rhsim09G0171900 | 613.97 | 1.53E-135 | 5.44E-134 | 1.418 |
| RHSIM_Rhsim02G0147000 | 613.78 | 1.69E-135 | 5.99E-134 | 1.872 |
| RHSIM_Rhsim12G0143500 | 613.71 | 1.75E-135 | 6.20E-134 | -4.144 |
| RHSIM_Rhsim12G0131900 | 611.56 | 5.13E-135 | 1.82E-133 | -2.562 |
| RHSIM_Rhsim02G0165700 | 610.86 | 7.26E-135 | 2.57E-133 | 1.258 |
| RHSIM_Rhsim08G0215200 | 610.51 | 8.67E-135 | 3.06E-133 | -1.488 |
| RHSIM_Rhsim01G0180300 | 609.83 | 1.22E-134 | 4.30E-133 | 2.538 |
| RHSIM_Rhsim03G0180600 | 609.36 | 1.54E-134 | 5.43E-133 | 1.518 |
| RHSIM_Rhsim08G0209800 | 609.03 | 1.82E-134 | 6.41E-133 | -2.549 |
| RHSIM_Rhsim06G0191100 | 608.85 | 1.99E-134 | 7.00E-133 | 2.534 |
| RHSIM_Rhsim08G0059500 | 608.45 | 2.43E-134 | 8.53E-133 | -6.503 |
| RHSIM_Rhsim04G0174800 | 607.54 | 3.84E-134 | 1.35E-132 | 1.365 |
| RHSIM_RhsimUnG0042900 | 607.44 | 4.04E-134 | 1.41E-132 | -5.808 |
| RHSIM_Rhsim02G0251500 | 607.16 | 4.64E-134 | 1.62E-132 | 1.728 |
| RHSIM_Rhsim03G0218800 | 606.37 | 6.90E-134 | 2.41E-132 | -3.542 |
| RHSIM_Rhsim03G0018400 | 605.90 | 8.73E-134 | 3.05E-132 | 1.739 |
| RHSIM_Rhsim10G0117200 | 604.53 | 1.73E-133 | 6.02E-132 | 1.933 |
| novel.4574 | 604.09 | 2.16E-133 | 7.51E-132 | 2.752 |
| RHSIM_Rhsim13G0096300 | 603.96 | 2.30E-133 | 8.00E-132 | -2.284 |
| RHSIM_Rhsim05G0084300 | 603.76 | 2.55E-133 | 8.84E-132 | 2.546 |
| RHSIM_Rhsim05G0224000 | 603.74 | 2.57E-133 | 8.92E-132 | 1.397 |
| RHSIM_Rhsim08G0119600 | 603.38 | 3.08E-133 | 1.07E-131 | 2.372 |
| RHSIM_Rhsim02G0220200 | 602.84 | 4.05E-133 | 1.40E-131 | -5.574 |
| RHSIM_Rhsim04G0016400 | 601.91 | 6.42E-133 | 2.22E-131 | 1.789 |
| RHSIM_Rhsim09G0097400 | 601.78 | 6.86E-133 | 2.37E-131 | 1.459 |
| RHSIM_Rhsim13G0083500 | 601.00 | 1.02E-132 | 3.50E-131 | 1.963 |

| Gene | LRT statistic | P value | BH-adjusted P | log2FC (interaction) |
|---|---|---|---|---|
| RHSIM_Rhsim13G0211700 | 600.35 | 1.40E-132 | 4.83E-131 | 2.182 |
| RHSIM_Rhsim04G0124000 | 599.67 | 1.97E-132 | 6.78E-131 | 1.847 |
| RHSIM_Rhsim12G0157800 | 598.17 | 4.18E-132 | 1.43E-130 | 2.611 |
| RHSIM_Rhsim06G0210400 | 598.16 | 4.21E-132 | 1.45E-130 | -1.924 |
| RHSIM_Rhsim08G0022100 | 597.89 | 4.83E-132 | 1.65E-130 | -2.868 |
| RHSIM_Rhsim10G0055700 | 597.26 | 6.59E-132 | 2.26E-130 | -2.628 |
| RHSIM_Rhsim05G0014900 | 596.94 | 7.75E-132 | 2.65E-130 | 2.023 |
| RHSIM_RhsimUnG0216900 | 595.30 | 1.76E-131 | 6.01E-130 | -1.904 |
| RHSIM_Rhsim12G0024700 | 595.15 | 1.90E-131 | 6.47E-130 | 1.272 |
| RHSIM_Rhsim04G0083400 | 594.66 | 2.43E-131 | 8.29E-130 | -2.475 |
| RHSIM_Rhsim08G0152500 | 593.82 | 3.69E-131 | 1.26E-129 | 1.302 |
| RHSIM_Rhsim03G0176500 | 593.47 | 4.40E-131 | 1.50E-129 | 2.333 |
| RHSIM_Rhsim13G0195000 | 593.41 | 4.54E-131 | 1.54E-129 | -2.375 |
| RHSIM_Rhsim12G0114000 | 591.90 | 9.67E-131 | 3.28E-129 | -1.938 |
| RHSIM_Rhsim13G0223000 | 589.53 | 3.17E-130 | 1.07E-128 | -0.927 |
| RHSIM_Rhsim13G0004700 | 589.37 | 3.43E-130 | 1.16E-128 | -3.406 |
| RHSIM_Rhsim11G0167100 | 588.43 | 5.50E-130 | 1.86E-128 | -2.585 |
| RHSIM_Rhsim01G0284600 | 588.37 | 5.66E-130 | 1.91E-128 | 2.216 |
| RHSIM_Rhsim05G0183000 | 587.72 | 7.86E-130 | 2.65E-128 | -2.764 |
| RHSIM_Rhsim12G0065300 | 586.04 | 1.82E-129 | 6.14E-128 | -3.379 |
| RHSIM_Rhsim08G0071000 | 586.03 | 1.83E-129 | 6.14E-128 | -0.661 |
| RHSIM_Rhsim07G0096200 | 585.68 | 2.19E-129 | 7.34E-128 | -1.839 |
| RHSIM_Rhsim02G0244800 | 585.53 | 2.35E-129 | 7.89E-128 | -2.218 |
| RHSIM_Rhsim11G0031500 | 585.47 | 2.42E-129 | 8.10E-128 | 2.162 |
| RHSIM_Rhsim09G0214200 | 585.46 | 2.44E-129 | 8.15E-128 | 2.933 |
| RHSIM_Rhsim04G0216800 | 585.33 | 2.60E-129 | 8.69E-128 | 2.126 |
| RHSIM_Rhsim13G0174000 | 584.02 | 5.01E-129 | 1.67E-127 | 3.036 |
| RHSIM_Rhsim11G0194000 | 583.46 | 6.64E-129 | 2.21E-127 | 2.052 |
| RHSIM_Rhsim12G0021800 | 583.33 | 7.09E-129 | 2.36E-127 | -2.505 |
| RHSIM_Rhsim01G0245300 | 582.35 | 1.15E-128 | 3.84E-127 | 1.520 |
| RHSIM_Rhsim01G0009900 | 580.53 | 2.88E-128 | 9.57E-127 | 1.275 |
| RHSIM_Rhsim01G0051000 | 579.36 | 5.16E-128 | 1.71E-126 | 2.857 |

| Gene | LRT statistic | P value | BH-adjusted P | log2FC (interaction) |
|---|---|---|---|---|
| RHSIM_Rhsim08G0090300 | 579.08 | 5.95E-128 | 1.97E-126 | 1.893 |
| RHSIM_Rhsim10G0165400 | 578.94 | 6.38E-128 | 2.11E-126 | -2.137 |
| RHSIM_Rhsim08G0160700 | 578.71 | 7.15E-128 | 2.36E-126 | -1.511 |
| novel.1880 | 577.48 | 1.33E-127 | 4.38E-126 | -6.615 |
| RHSIM_Rhsim12G0006100 | 576.84 | 1.83E-127 | 6.04E-126 | 1.695 |
| RHSIM_Rhsim02G0029200 | 576.53 | 2.13E-127 | 7.02E-126 | 13.215 |
| RHSIM_Rhsim01G0287300 | 576.05 | 2.72E-127 | 8.94E-126 | 1.671 |
| RHSIM_Rhsim09G0210500 | 573.91 | 7.93E-127 | 2.61E-125 | 1.170 |
| RHSIM_Rhsim07G0216100 | 573.62 | 9.18E-127 | 3.02E-125 | 2.982 |
| RHSIM_Rhsim04G0012900 | 573.38 | 1.03E-126 | 3.39E-125 | -3.129 |
| RHSIM_Rhsim04G0188600 | 571.60 | 2.52E-126 | 8.26E-125 | -4.299 |
| RHSIM_Rhsim07G0100600 | 571.17 | 3.12E-126 | 1.02E-124 | 1.412 |
| RHSIM_Rhsim06G0048500 | 571.16 | 3.13E-126 | 1.02E-124 | 1.544 |
| RHSIM_Rhsim02G0070300 | 570.93 | 3.53E-126 | 1.15E-124 | 3.073 |
| RHSIM_Rhsim08G0195500 | 570.52 | 4.32E-126 | 1.41E-124 | 1.421 |
| RHSIM_Rhsim03G0068200 | 570.48 | 4.42E-126 | 1.44E-124 | 1.447 |
| RHSIM_Rhsim07G0126400 | 570.36 | 4.69E-126 | 1.53E-124 | 1.997 |
| RHSIM_Rhsim06G0007300 | 570.13 | 5.26E-126 | 1.71E-124 | 1.666 |
| RHSIM_Rhsim11G0099900 | 569.86 | 6.01E-126 | 1.95E-124 | 2.085 |
| RHSIM_Rhsim13G0014400 | 569.52 | 7.15E-126 | 2.32E-124 | -1.919 |
| RHSIM_Rhsim01G0184400 | 569.40 | 7.57E-126 | 2.45E-124 | -1.636 |
| novel.3552 | 569.29 | 8.01E-126 | 2.59E-124 | 3.525 |
| RHSIM_Rhsim08G0248800 | 566.43 | 3.35E-125 | 1.08E-123 | 1.437 |
| RHSIM_Rhsim12G0021000 | 565.58 | 5.13E-125 | 1.66E-123 | 1.578 |
| RHSIM_Rhsim13G0011700 | 565.26 | 6.03E-125 | 1.94E-123 | 2.059 |
| RHSIM_Rhsim09G0064300 | 565.11 | 6.50E-125 | 2.09E-123 | 1.907 |
| RHSIM_Rhsim06G0111800 | 565.01 | 6.85E-125 | 2.21E-123 | -3.555 |
| RHSIM_Rhsim08G0035300 | 564.49 | 8.88E-125 | 2.85E-123 | 1.483 |
| RHSIM_Rhsim09G0103200 | 563.81 | 1.25E-124 | 4.01E-123 | 1.942 |
| RHSIM_Rhsim06G0029800 | 563.80 | 1.25E-124 | 4.01E-123 | -5.534 |
| RHSIM_Rhsim11G0034500 | 562.77 | 2.10E-124 | 6.73E-123 | -2.111 |
| novel.3913 | 561.51 | 3.95E-124 | 1.26E-122 | 1.673 |

| Gene | LRT statistic | P value | BH-adjusted P | log2FC (interaction) |
|---|---|---|---|---|
| RHSIM_Rhsim05G0030000 | 561.14 | 4.75E-124 | 1.52E-122 | -5.398 |
| RHSIM_Rhsim09G0137900 | 560.90 | 5.35E-124 | 1.71E-122 | 2.296 |
| novel.418 | 560.51 | 6.53E-124 | 2.08E-122 | -1.016 |
| RHSIM_Rhsim09G0127700 | 559.20 | 1.26E-123 | 4.00E-122 | 1.278 |
| RHSIM_Rhsim06G0079800 | 559.07 | 1.34E-123 | 4.25E-122 | 1.962 |
| RHSIM_Rhsim08G0016500 | 557.80 | 2.53E-123 | 8.05E-122 | -2.316 |
| RHSIM_Rhsim08G0241000 | 554.88 | 1.09E-122 | 3.47E-121 | 1.044 |
| RHSIM_Rhsim10G0005000 | 554.74 | 1.17E-122 | 3.71E-121 | 2.443 |
| RHSIM_Rhsim05G0170400 | 554.51 | 1.32E-122 | 4.17E-121 | 2.106 |
| RHSIM_Rhsim01G0097900 | 554.05 | 1.66E-122 | 5.25E-121 | 1.583 |
| RHSIM_Rhsim10G0174500 | 554.01 | 1.69E-122 | 5.33E-121 | -1.580 |
| RHSIM_Rhsim06G0009700 | 553.06 | 2.72E-122 | 8.58E-121 | -4.361 |
| RHSIM_Rhsim12G0019600 | 552.95 | 2.87E-122 | 9.06E-121 | 1.898 |
| RHSIM_Rhsim06G0195200 | 552.90 | 2.94E-122 | 9.26E-121 | 1.106 |
| RHSIM_Rhsim01G0067200 | 552.06 | 4.49E-122 | 1.41E-120 | -1.823 |
| RHSIM_Rhsim08G0064800 | 551.67 | 5.45E-122 | 1.71E-120 | 1.104 |
| RHSIM_Rhsim02G0253600 | 549.00 | 2.08E-121 | 6.53E-120 | -1.902 |
| RHSIM_Rhsim11G0093300 | 548.43 | 2.77E-121 | 8.68E-120 | 1.510 |
| RHSIM_Rhsim08G0229600 | 547.78 | 3.83E-121 | 1.20E-119 | -1.891 |
| RHSIM_Rhsim08G0170500 | 546.78 | 6.32E-121 | 1.98E-119 | 1.774 |
| RHSIM_Rhsim12G0106500 | 545.81 | 1.03E-120 | 3.22E-119 | 1.965 |
| RHSIM_RhsimUnG0171600 | 545.37 | 1.28E-120 | 3.99E-119 | -2.990 |
| RHSIM_Rhsim08G0118300 | 544.30 | 2.19E-120 | 6.82E-119 | 3.554 |
| RHSIM_Rhsim09G0189800 | 544.28 | 2.21E-120 | 6.88E-119 | -1.549 |
| RHSIM_Rhsim07G0197900 | 544.02 | 2.52E-120 | 7.84E-119 | -2.063 |
| RHSIM_Rhsim08G0066000 | 543.40 | 3.43E-120 | 1.07E-118 | 2.035 |
| RHSIM_Rhsim13G0043900 | 543.04 | 4.12E-120 | 1.28E-118 | -1.767 |
| RHSIM_Rhsim12G0125800 | 542.77 | 4.72E-120 | 1.46E-118 | 2.271 |
| RHSIM_Rhsim01G0143500 | 542.47 | 5.48E-120 | 1.70E-118 | -1.626 |
| novel.4380 | 542.00 | 6.91E-120 | 2.14E-118 | 2.928 |
| RHSIM_Rhsim04G0024600 | 541.87 | 7.39E-120 | 2.29E-118 | -1.941 |
| RHSIM_Rhsim03G0226500 | 541.78 | 7.72E-120 | 2.39E-118 | -1.641 |

| Gene | LRT statistic | P value | BH-adjusted P | log2FC (interaction) |
|---|---|---|---|---|
| novel.3036 | 541.12 | 1.08E-119 | 3.33E-118 | 5.494 |
| RHSIM_Rhsim12G0109700 | 539.43 | 2.51E-119 | 7.75E-118 | 2.807 |
| RHSIM_Rhsim08G0009200 | 539.22 | 2.78E-119 | 8.57E-118 | -4.144 |
| RHSIM_RhsimUnG0254800 | 537.49 | 6.62E-119 | 2.04E-117 | 1.378 |
| RHSIM_Rhsim01G0249900 | 537.34 | 7.16E-119 | 2.20E-117 | -1.218 |
| RHSIM_Rhsim08G0084500 | 537.22 | 7.60E-119 | 2.33E-117 | -1.086 |
| RHSIM_Rhsim03G0212100 | 536.09 | 1.33E-118 | 4.09E-117 | 2.218 |
| RHSIM_Rhsim04G0114000 | 536.01 | 1.39E-118 | 4.26E-117 | 1.329 |
| RHSIM_Rhsim02G0070700 | 535.51 | 1.78E-118 | 5.46E-117 | -1.731 |
| RHSIM_Rhsim12G0085000 | 534.67 | 2.72E-118 | 8.33E-117 | 1.563 |
| RHSIM_Rhsim09G0115900 | 534.15 | 3.54E-118 | 1.08E-116 | -7.019 |
| RHSIM_Rhsim02G0076000 | 533.79 | 4.23E-118 | 1.29E-116 | -1.908 |
| RHSIM_Rhsim05G0175300 | 533.11 | 5.96E-118 | 1.82E-116 | 1.945 |
| RHSIM_Rhsim10G0189600 | 532.71 | 7.27E-118 | 2.21E-116 | 1.821 |
| RHSIM_Rhsim06G0164400 | 532.56 | 7.83E-118 | 2.38E-116 | -1.961 |
| RHSIM_Rhsim13G0063200 | 532.12 | 9.75E-118 | 2.96E-116 | 0.927 |
| RHSIM_Rhsim09G0211900 | 531.96 | 1.06E-117 | 3.22E-116 | 2.345 |
| RHSIM_Rhsim05G0231500 | 530.73 | 1.96E-117 | 5.93E-116 | 1.580 |
| RHSIM_Rhsim06G0004600 | 530.03 | 2.78E-117 | 8.42E-116 | 1.282 |
| RHSIM_Rhsim03G0241500 | 529.92 | 2.94E-117 | 8.89E-116 | 2.072 |
| RHSIM_Rhsim02G0136400 | 529.42 | 3.78E-117 | 1.14E-115 | 1.623 |
| RHSIM_Rhsim09G0185800 | 528.75 | 5.28E-117 | 1.59E-115 | 2.676 |
| RHSIM_RhsimUnG0100600 | 528.10 | 7.33E-117 | 2.21E-115 | 1.823 |
| novel.2847 | 527.97 | 7.83E-117 | 2.36E-115 | -3.874 |
| RHSIM_Rhsim11G0191200 | 527.54 | 9.71E-117 | 2.92E-115 | -3.104 |
| RHSIM_Rhsim08G0072300 | 526.72 | 1.46E-116 | 4.39E-115 | 2.436 |
| RHSIM_Rhsim08G0033400 | 526.64 | 1.52E-116 | 4.57E-115 | 1.630 |
| RHSIM_Rhsim13G0008300 | 526.54 | 1.60E-116 | 4.80E-115 | -1.606 |
| RHSIM_Rhsim03G0188500 | 525.28 | 3.01E-116 | 9.01E-115 | 2.205 |
| RHSIM_Rhsim07G0095900 | 524.62 | 4.18E-116 | 1.25E-114 | -1.386 |
| RHSIM_Rhsim03G0027200 | 524.29 | 4.94E-116 | 1.48E-114 | -1.861 |
| novel.2722 | 523.97 | 5.81E-116 | 1.73E-114 | -2.232 |

| Gene | LRT statistic | P value | BH-adjusted P | log2FC (interaction) |
|---|---|---|---|---|
| RHSIM_Rhsim01G0174700 | 522.73 | 1.08E-115 | 3.21E-114 | -3.239 |
| RHSIM_Rhsim03G0064200 | 522.44 | 1.25E-115 | 3.71E-114 | -2.753 |
| RHSIM_Rhsim10G0004600 | 522.15 | 1.44E-115 | 4.28E-114 | 1.774 |
| RHSIM_Rhsim09G0044400 | 522.09 | 1.49E-115 | 4.42E-114 | 2.857 |
| RHSIM_Rhsim04G0034900 | 522.07 | 1.50E-115 | 4.46E-114 | -2.005 |
| RHSIM_Rhsim13G0069000 | 521.64 | 1.87E-115 | 5.54E-114 | -2.308 |
| RHSIM_Rhsim13G0137100 | 520.99 | 2.58E-115 | 7.65E-114 | 2.088 |
| RHSIM_Rhsim11G0130100 | 520.78 | 2.86E-115 | 8.46E-114 | 1.971 |
| RHSIM_Rhsim03G0172900 | 520.78 | 2.86E-115 | 8.46E-114 | 2.662 |
| RHSIM_Rhsim08G0013200 | 520.53 | 3.25E-115 | 9.61E-114 | 1.895 |
| RHSIM_RhsimUnG0210000 | 519.85 | 4.57E-115 | 1.35E-113 | -6.638 |
| RHSIM_Rhsim12G0071800 | 519.42 | 5.66E-115 | 1.67E-113 | -1.835 |
| RHSIM_Rhsim01G0125600 | 519.32 | 5.95E-115 | 1.75E-113 | 3.352 |
| RHSIM_Rhsim09G0148400 | 518.32 | 9.81E-115 | 2.89E-113 | 1.437 |
| RHSIM_Rhsim04G0014100 | 517.27 | 1.66E-114 | 4.88E-113 | 1.575 |
| RHSIM_Rhsim13G0227400 | 516.66 | 2.26E-114 | 6.62E-113 | 1.587 |
| RHSIM_Rhsim13G0099100 | 516.66 | 2.26E-114 | 6.62E-113 | 2.034 |
| RHSIM_Rhsim05G0086300 | 516.54 | 2.40E-114 | 7.03E-113 | 1.916 |
| RHSIM_Rhsim09G0022800 | 514.98 | 5.24E-114 | 1.53E-112 | 1.928 |
| RHSIM_Rhsim10G0087200 | 514.36 | 7.14E-114 | 2.09E-112 | 4.298 |
| RHSIM_Rhsim07G0010400 | 514.14 | 7.97E-114 | 2.33E-112 | -2.934 |
| RHSIM_Rhsim03G0174300 | 513.88 | 9.10E-114 | 2.66E-112 | -7.113 |
| RHSIM_Rhsim06G0073200 | 512.87 | 1.50E-113 | 4.39E-112 | 1.660 |
| RHSIM_Rhsim12G0189400 | 511.41 | 3.13E-113 | 9.11E-112 | 1.449 |
| RHSIM_Rhsim06G0166800 | 511.32 | 3.28E-113 | 9.54E-112 | -1.660 |
| RHSIM_Rhsim13G0194300 | 511.05 | 3.75E-113 | 1.09E-111 | -1.307 |
| novel.1918 | 509.83 | 6.91E-113 | 2.01E-111 | -4.463 |
| RHSIM_Rhsim02G0105300 | 506.78 | 3.19E-112 | 9.25E-111 | 3.814 |
| RHSIM_Rhsim01G0264600 | 506.72 | 3.27E-112 | 9.49E-111 | 2.172 |
| RHSIM_Rhsim05G0196300 | 506.54 | 3.58E-112 | 1.04E-110 | -1.751 |
| RHSIM_Rhsim01G0007100 | 506.23 | 4.19E-112 | 1.21E-110 | 1.892 |
| RHSIM_Rhsim06G0231000 | 505.77 | 5.28E-112 | 1.53E-110 | -2.123 |

| Gene | LRT statistic | P value | BH-adjusted P | log2FC (interaction) |
|---|---|---|---|---|
| RHSIM_Rhsim08G0088200 | 505.24 | 6.90E-112 | 1.99E-110 | 1.247 |
| RHSIM_Rhsim03G0249600 | 504.97 | 7.87E-112 | 2.27E-110 | -2.254 |
| novel.2405 | 504.45 | 1.02E-111 | 2.94E-110 | 2.623 |
| RHSIM_Rhsim01G0285200 | 504.45 | 1.02E-111 | 2.94E-110 | -2.457 |
| novel.2430 | 504.38 | 1.06E-111 | 3.05E-110 | -2.270 |
| RHSIM_Rhsim13G0232700 | 503.01 | 2.11E-111 | 6.05E-110 | 2.402 |
| RHSIM_Rhsim08G0048400 | 501.77 | 3.92E-111 | 1.13E-109 | 1.680 |
| RHSIM_Rhsim10G0055000 | 501.73 | 4.00E-111 | 1.15E-109 | 1.390 |
| RHSIM_Rhsim12G0156500 | 501.39 | 4.74E-111 | 1.36E-109 | -1.975 |
| RHSIM_Rhsim06G0129400 | 501.34 | 4.86E-111 | 1.39E-109 | -1.480 |
| novel.4097 | 501.32 | 4.92E-111 | 1.41E-109 | -2.288 |
| RHSIM_Rhsim13G0078100 | 501.15 | 5.33E-111 | 1.52E-109 | 1.230 |
| RHSIM_Rhsim03G0187200 | 501.10 | 5.49E-111 | 1.57E-109 | 1.348 |
| RHSIM_Rhsim01G0237000 | 499.35 | 1.32E-110 | 3.75E-109 | 1.902 |
| RHSIM_Rhsim13G0053200 | 498.22 | 2.32E-110 | 6.60E-109 | -1.788 |
| RHSIM_Rhsim02G0130600 | 497.31 | 3.66E-110 | 1.04E-108 | 1.804 |
| RHSIM_Rhsim13G0127200 | 496.45 | 5.63E-110 | 1.60E-108 | 1.604 |
| RHSIM_Rhsim03G0074900 | 495.99 | 7.09E-110 | 2.01E-108 | -7.711 |
| novel.3290 | 495.78 | 7.87E-110 | 2.23E-108 | -2.318 |
| RHSIM_Rhsim02G0214300 | 495.46 | 9.23E-110 | 2.62E-108 | -2.132 |
| RHSIM_Rhsim09G0067900 | 494.78 | 1.30E-109 | 3.68E-108 | 1.463 |
| RHSIM_Rhsim05G0226900 | 494.68 | 1.36E-109 | 3.86E-108 | -1.881 |
| novel.3044 | 494.65 | 1.39E-109 | 3.91E-108 | -1.906 |
| RHSIM_Rhsim07G0094800 | 494.00 | 1.92E-109 | 5.43E-108 | 3.083 |
| RHSIM_Rhsim01G0259100 | 493.49 | 2.48E-109 | 7.00E-108 | 1.497 |
| RHSIM_Rhsim13G0001500 | 492.41 | 4.27E-109 | 1.20E-107 | 1.233 |
| RHSIM_Rhsim07G0103800 | 491.61 | 6.37E-109 | 1.79E-107 | -1.184 |
| novel.80 | 491.09 | 8.27E-109 | 2.32E-107 | -1.756 |
| RHSIM_Rhsim06G0213100 | 490.85 | 9.29E-109 | 2.61E-107 | -5.433 |
| RHSIM_Rhsim07G0222100 | 490.65 | 1.03E-108 | 2.88E-107 | 1.551 |
| RHSIM_Rhsim08G0032000 | 490.65 | 1.03E-108 | 2.88E-107 | 3.743 |
| novel.3841 | 490.45 | 1.14E-108 | 3.19E-107 | 2.468 |

| Gene | LRT statistic | P value | BH-adjusted P | log2FC (interaction) |
|---|---|---|---|---|
| RHSIM_Rhsim01G0145400 | 490.35 | 1.20E-108 | 3.35E-107 | -8.870 |
| RHSIM_Rhsim08G0024600 | 490.21 | 1.29E-108 | 3.59E-107 | -4.171 |
| RHSIM_Rhsim11G0034700 | 490.11 | 1.35E-108 | 3.76E-107 | 1.414 |
| RHSIM_Rhsim01G0269200 | 489.67 | 1.69E-108 | 4.70E-107 | 1.275 |
| novel.2310 | 488.89 | 2.48E-108 | 6.91E-107 | 2.096 |
| RHSIM_Rhsim07G0137100 | 488.53 | 2.97E-108 | 8.27E-107 | 1.848 |
| RHSIM_Rhsim02G0168900 | 488.37 | 3.23E-108 | 8.98E-107 | -1.154 |
| RHSIM_Rhsim13G0126200 | 487.85 | 4.18E-108 | 1.16E-106 | 1.366 |
| RHSIM_Rhsim12G0170600 | 487.47 | 5.06E-108 | 1.40E-106 | 1.523 |
| RHSIM_Rhsim04G0225000 | 487.38 | 5.29E-108 | 1.47E-106 | -1.439 |
| RHSIM_Rhsim04G0132400 | 487.18 | 5.86E-108 | 1.62E-106 | 2.418 |
| RHSIM_Rhsim12G0191200 | 486.79 | 7.11E-108 | 1.97E-106 | 1.874 |
| RHSIM_Rhsim04G0124400 | 486.67 | 7.54E-108 | 2.08E-106 | -1.114 |
| RHSIM_Rhsim02G0149100 | 486.00 | 1.06E-107 | 2.92E-106 | 1.351 |
| RHSIM_Rhsim10G0041100 | 485.69 | 1.23E-107 | 3.40E-106 | 1.984 |
| RHSIM_Rhsim07G0010300 | 484.47 | 2.28E-107 | 6.27E-106 | -2.975 |
| RHSIM_Rhsim01G0171500 | 484.33 | 2.44E-107 | 6.72E-106 | 1.775 |
| RHSIM_Rhsim04G0132500 | 483.47 | 3.76E-107 | 1.03E-105 | 2.464 |
| RHSIM_Rhsim06G0183300 | 482.98 | 4.80E-107 | 1.32E-105 | 1.456 |
| RHSIM_Rhsim08G0202500 | 482.78 | 5.30E-107 | 1.46E-105 | 1.788 |
| RHSIM_Rhsim07G0039400 | 482.31 | 6.72E-107 | 1.84E-105 | -1.509 |
| RHSIM_Rhsim07G0085900 | 482.04 | 7.68E-107 | 2.10E-105 | -5.135 |
| RHSIM_Rhsim12G0169500 | 481.93 | 8.13E-107 | 2.23E-105 | 2.771 |
| RHSIM_Rhsim08G0030100 | 480.57 | 1.61E-106 | 4.40E-105 | 1.739 |
| RHSIM_Rhsim12G0089100 | 480.51 | 1.66E-106 | 4.53E-105 | -2.523 |
| RHSIM_Rhsim03G0054700 | 480.47 | 1.69E-106 | 4.61E-105 | 1.607 |
| RHSIM_Rhsim06G0030800 | 480.13 | 2.01E-106 | 5.47E-105 | -1.883 |
| RHSIM_Rhsim03G0044900 | 479.61 | 2.60E-106 | 7.07E-105 | -1.681 |
| RHSIM_Rhsim04G0100500 | 479.12 | 3.32E-106 | 9.04E-105 | -1.686 |
| RHSIM_Rhsim11G0117700 | 479.01 | 3.51E-106 | 9.55E-105 | 1.448 |
| novel.3107 | 478.42 | 4.72E-106 | 1.28E-104 | -2.007 |
| RHSIM_Rhsim03G0243700 | 478.27 | 5.08E-106 | 1.38E-104 | 1.370 |

| Gene | LRT statistic | P value | BH-adjusted P | log2FC (interaction) |
|---|---|---|---|---|
| RHSIM_Rhsim13G0082900 | 477.83 | 6.33E-106 | 1.72E-104 | 1.821 |
| RHSIM_Rhsim04G0109300 | 477.04 | 9.41E-106 | 2.55E-104 | -2.340 |
| RHSIM_Rhsim11G0187000 | 476.47 | 1.25E-105 | 3.39E-104 | -1.050 |
| RHSIM_Rhsim10G0203500 | 476.00 | 1.59E-105 | 4.29E-104 | 2.498 |
| RHSIM_Rhsim05G0010400 | 475.89 | 1.68E-105 | 4.53E-104 | 1.971 |
| RHSIM_Rhsim09G0151200 | 475.23 | 2.33E-105 | 6.28E-104 | 2.274 |
| RHSIM_Rhsim05G0174900 | 473.92 | 4.50E-105 | 1.21E-103 | -1.789 |
| RHSIM_Rhsim09G0189300 | 473.90 | 4.55E-105 | 1.23E-103 | 1.439 |
| novel.345 | 473.77 | 4.85E-105 | 1.30E-103 | 1.301 |
| RHSIM_Rhsim08G0203600 | 472.64 | 8.54E-105 | 2.30E-103 | 1.633 |
| RHSIM_Rhsim01G0171400 | 472.38 | 9.71E-105 | 2.61E-103 | -3.436 |
| RHSIM_Rhsim03G0050300 | 472.24 | 1.04E-104 | 2.80E-103 | 2.095 |
| RHSIM_Rhsim01G0060800 | 472.02 | 1.16E-104 | 3.12E-103 | 1.257 |
| RHSIM_Rhsim06G0192600 | 470.65 | 2.31E-104 | 6.19E-103 | -2.183 |
| RHSIM_Rhsim06G0041900 | 470.55 | 2.43E-104 | 6.50E-103 | -2.638 |
| RHSIM_Rhsim13G0069400 | 470.29 | 2.78E-104 | 7.43E-103 | -1.866 |
| RHSIM_RhsimUnG0161800 | 470.07 | 3.09E-104 | 8.26E-103 | 1.979 |
| RHSIM_RhsimUnG0008500 | 468.81 | 5.82E-104 | 1.55E-102 | 2.501 |
| RHSIM_Rhsim12G0126700 | 468.04 | 8.54E-104 | 2.28E-102 | 2.370 |
| novel.1960 | 467.57 | 1.08E-103 | 2.88E-102 | 1.606 |
| RHSIM_Rhsim13G0051300 | 467.57 | 1.08E-103 | 2.88E-102 | -1.199 |
| RHSIM_Rhsim06G0227500 | 466.40 | 1.95E-103 | 5.19E-102 | -1.656 |
| RHSIM_Rhsim04G0167100 | 466.29 | 2.06E-103 | 5.47E-102 | 1.564 |
| RHSIM_Rhsim04G0026100 | 466.28 | 2.06E-103 | 5.48E-102 | 1.540 |
| RHSIM_Rhsim02G0180600 | 466.16 | 2.19E-103 | 5.82E-102 | -1.709 |
| novel.2605 | 465.99 | 2.39E-103 | 6.34E-102 | -3.019 |
| RHSIM_Rhsim12G0203400 | 465.78 | 2.66E-103 | 7.04E-102 | 1.259 |
| RHSIM_Rhsim13G0229300 | 465.60 | 2.91E-103 | 7.70E-102 | -2.040 |
| RHSIM_Rhsim01G0091800 | 465.53 | 3.01E-103 | 7.96E-102 | 1.956 |
| RHSIM_Rhsim01G0276300 | 465.04 | 3.85E-103 | 1.02E-101 | 2.170 |
| RHSIM_Rhsim02G0062100 | 464.91 | 4.11E-103 | 1.08E-101 | -1.246 |
| RHSIM_Rhsim12G0072500 | 464.91 | 4.11E-103 | 1.08E-101 | 1.677 |

| Gene | LRT statistic | P value | BH-adjusted P | log2FC (interaction) |
|---|---|---|---|---|
| RHSIM_Rhsim06G0190500 | 464.89 | 4.14E-103 | 1.09E-101 | -7.125 |
| RHSIM_Rhsim08G0199200 | 464.46 | 5.14E-103 | 1.35E-101 | -1.258 |
| RHSIM_Rhsim03G0236300 | 464.31 | 5.55E-103 | 1.46E-101 | -3.204 |
| RHSIM_Rhsim04G0157500 | 463.16 | 9.89E-103 | 2.60E-101 | 1.798 |
| RHSIM_Rhsim08G0088400 | 463.09 | 1.02E-102 | 2.68E-101 | -1.144 |
| RHSIM_Rhsim12G0013400 | 463.08 | 1.03E-102 | 2.69E-101 | -1.170 |
| RHSIM_Rhsim11G0139400 | 462.38 | 1.46E-102 | 3.82E-101 | -2.647 |
| RHSIM_Rhsim12G0167600 | 461.99 | 1.77E-102 | 4.63E-101 | 1.609 |
| novel.1620 | 461.72 | 2.03E-102 | 5.32E-101 | -4.417 |
| RHSIM_Rhsim02G0035600 | 461.69 | 2.06E-102 | 5.37E-101 | -1.760 |
| RHSIM_Rhsim06G0204600 | 461.65 | 2.10E-102 | 5.48E-101 | 1.116 |
| RHSIM_Rhsim02G0025300 | 461.41 | 2.38E-102 | 6.19E-101 | -1.561 |
| RHSIM_Rhsim11G0044300 | 461.34 | 2.46E-102 | 6.41E-101 | -2.026 |
| RHSIM_Rhsim08G0245800 | 460.70 | 3.39E-102 | 8.82E-101 | 1.742 |
| RHSIM_Rhsim11G0137700 | 460.63 | 3.51E-102 | 9.12E-101 | -2.007 |
| RHSIM_Rhsim06G0073100 | 460.19 | 4.36E-102 | 1.13E-100 | 1.740 |
| novel.1299 | 459.98 | 4.85E-102 | 1.26E-100 | -11.837 |
| RHSIM_Rhsim04G0235000 | 459.19 | 7.20E-102 | 1.87E-100 | -1.993 |
| RHSIM_Rhsim05G0129100 | 458.68 | 9.33E-102 | 2.42E-100 | 1.704 |
| RHSIM_Rhsim12G0135400 | 458.26 | 1.15E-101 | 2.97E-100 | -4.866 |
| RHSIM_Rhsim11G0165600 | 456.67 | 2.55E-101 | 6.60E-100 | 1.928 |
| RHSIM_Rhsim09G0012400 | 456.46 | 2.83E-101 | 7.31E-100 | 2.434 |
| RHSIM_Rhsim13G0045600 | 456.35 | 3.00E-101 | 7.74E-100 | 1.879 |
| RHSIM_Rhsim10G0176600 | 455.71 | 4.12E-101 | 1.06E-99 | 2.556 |
| RHSIM_Rhsim01G0278000 | 455.03 | 5.79E-101 | 1.49E-99 | -1.801 |
| RHSIM_RhsimUnG0070500 | 454.69 | 6.86E-101 | 1.77E-99 | -4.107 |
| RHSIM_Rhsim06G0078200 | 454.36 | 8.10E-101 | 2.08E-99 | 1.197 |
| RHSIM_Rhsim08G0069300 | 454.01 | 9.66E-101 | 2.48E-99 | -1.573 |
| RHSIM_Rhsim11G0153800 | 453.26 | 1.41E-100 | 3.62E-99 | 4.204 |
| RHSIM_Rhsim13G0046300 | 452.88 | 1.70E-100 | 4.36E-99 | -1.806 |
| RHSIM_Rhsim09G0087300 | 450.93 | 4.52E-100 | 1.16E-98 | 1.796 |
| RHSIM_Rhsim03G0091100 | 450.64 | 5.24E-100 | 1.34E-98 | -2.076 |

| Gene | LRT statistic | P value | BH-adjusted P | log2FC (interaction) |
|---|---|---|---|---|
| RHSIM_Rhsim12G0132900 | 450.05 | 7.02E-100 | 1.79E-98 | -2.303 |
| RHSIM_Rhsim02G0059100 | 449.39 | 9.78E-100 | 2.50E-98 | -1.225 |
| RHSIM_Rhsim03G0246900 | 448.73 | 1.37E-99 | 3.49E-98 | 1.482 |
| RHSIM_Rhsim01G0060000 | 447.67 | 2.32E-99 | 5.91E-98 | 1.703 |
| RHSIM_Rhsim05G0049900 | 447.63 | 2.37E-99 | 6.03E-98 | 3.363 |
| RHSIM_Rhsim07G0065500 | 447.05 | 3.16E-99 | 8.06E-98 | 2.128 |
| RHSIM_Rhsim12G0123900 | 446.98 | 3.27E-99 | 8.32E-98 | -3.214 |
| RHSIM_Rhsim04G0003400 | 446.51 | 4.14E-99 | 1.05E-97 | 1.308 |
| RHSIM_Rhsim03G0008800 | 446.47 | 4.23E-99 | 1.07E-97 | -3.932 |
| RHSIM_Rhsim12G0164800 | 446.31 | 4.58E-99 | 1.16E-97 | 1.817 |
| RHSIM_Rhsim13G0189200 | 446.18 | 4.89E-99 | 1.24E-97 | 0.956 |
| novel.2499 | 446.13 | 5.00E-99 | 1.27E-97 | 1.528 |
| novel.2887 | 444.98 | 8.92E-99 | 2.26E-97 | 1.347 |
| RHSIM_Rhsim02G0223500 | 444.47 | 1.15E-98 | 2.91E-97 | -3.736 |
| RHSIM_Rhsim12G0111600 | 444.45 | 1.17E-98 | 2.95E-97 | -1.427 |
| RHSIM_Rhsim07G0250600 | 444.28 | 1.27E-98 | 3.20E-97 | -2.564 |
| RHSIM_Rhsim08G0048300 | 444.26 | 1.28E-98 | 3.23E-97 | 7.453 |
| RHSIM_Rhsim10G0035200 | 444.25 | 1.28E-98 | 3.24E-97 | 2.073 |
| RHSIM_Rhsim07G0216200 | 444.02 | 1.44E-98 | 3.63E-97 | 2.858 |
| RHSIM_Rhsim13G0088900 | 443.99 | 1.46E-98 | 3.68E-97 | -2.365 |
| RHSIM_Rhsim07G0047700 | 443.92 | 1.52E-98 | 3.81E-97 | 1.733 |
| RHSIM_Rhsim04G0169500 | 443.75 | 1.65E-98 | 4.14E-97 | 1.541 |
| RHSIM_Rhsim11G0190600 | 443.48 | 1.89E-98 | 4.74E-97 | 2.549 |
| RHSIM_Rhsim02G0218200 | 442.99 | 2.42E-98 | 6.06E-97 | -3.536 |
| RHSIM_Rhsim01G0230500 | 442.50 | 3.09E-98 | 7.72E-97 | -1.729 |
| RHSIM_Rhsim07G0043900 | 442.29 | 3.43E-98 | 8.58E-97 | 1.632 |
| RHSIM_Rhsim08G0134100 | 441.86 | 4.26E-98 | 1.07E-96 | 1.651 |
| RHSIM_Rhsim05G0011300 | 441.55 | 4.98E-98 | 1.24E-96 | -7.272 |
| RHSIM_Rhsim04G0013600 | 441.42 | 5.32E-98 | 1.33E-96 | -1.621 |
| RHSIM_Rhsim12G0148500 | 441.28 | 5.69E-98 | 1.42E-96 | 1.764 |
| RHSIM_Rhsim13G0175300 | 440.95 | 6.72E-98 | 1.67E-96 | 3.249 |
| RHSIM_RhsimUnG0126900 | 439.28 | 1.56E-97 | 3.87E-96 | 2.280 |

| Gene | LRT statistic | P value | BH-adjusted P | log2FC (interaction) |
|---|---|---|---|---|
| RHSIM_Rhsim07G0214700 | 438.76 | 2.01E-97 | 5.00E-96 | -1.119 |
| RHSIM_Rhsim03G0229400 | 438.09 | 2.82E-97 | 7.00E-96 | -6.845 |
| RHSIM_Rhsim08G0213200 | 437.83 | 3.22E-97 | 7.99E-96 | -1.798 |
| RHSIM_RhsimUnG0065600 | 437.60 | 3.60E-97 | 8.92E-96 | 0.971 |
| RHSIM_Rhsim08G0128800 | 437.51 | 3.78E-97 | 9.36E-96 | 3.310 |
| RHSIM_Rhsim12G0133400 | 437.14 | 4.55E-97 | 1.13E-95 | -1.018 |
| novel.3479 | 436.84 | 5.28E-97 | 1.31E-95 | 1.788 |
| RHSIM_Rhsim01G0247800 | 436.79 | 5.40E-97 | 1.33E-95 | 1.429 |
| RHSIM_Rhsim10G0030200 | 436.34 | 6.76E-97 | 1.67E-95 | -1.383 |
| RHSIM_Rhsim06G0164200 | 435.98 | 8.12E-97 | 2.00E-95 | 0.840 |
| RHSIM_Rhsim11G0155400 | 434.58 | 1.64E-96 | 4.04E-95 | -5.862 |
| RHSIM_Rhsim13G0234600 | 433.95 | 2.24E-96 | 5.53E-95 | -1.636 |
| RHSIM_Rhsim09G0111800 | 433.33 | 3.06E-96 | 7.53E-95 | -1.944 |
| RHSIM_Rhsim05G0087000 | 432.61 | 4.39E-96 | 1.08E-94 | -1.030 |
| RHSIM_Rhsim02G0023100 | 432.13 | 5.60E-96 | 1.38E-94 | -2.258 |
| RHSIM_Rhsim08G0082300 | 431.32 | 8.37E-96 | 2.05E-94 | -2.266 |
| RHSIM_Rhsim04G0119900 | 431.15 | 9.12E-96 | 2.24E-94 | -1.944 |
| RHSIM_Rhsim05G0096900 | 430.82 | 1.08E-95 | 2.64E-94 | -1.864 |
| RHSIM_Rhsim06G0000500 | 430.28 | 1.41E-95 | 3.46E-94 | 1.547 |
| RHSIM_Rhsim10G0158000 | 429.96 | 1.66E-95 | 4.05E-94 | -2.616 |
| RHSIM_Rhsim06G0111700 | 429.87 | 1.73E-95 | 4.23E-94 | -3.081 |
| RHSIM_RhsimUnG0142500 | 429.86 | 1.75E-95 | 4.27E-94 | 2.987 |
| RHSIM_Rhsim11G0034900 | 429.48 | 2.11E-95 | 5.16E-94 | 2.076 |
| RHSIM_Rhsim06G0002000 | 429.43 | 2.16E-95 | 5.27E-94 | -1.294 |
| RHSIM_Rhsim07G0138900 | 429.04 | 2.63E-95 | 6.41E-94 | 2.097 |
| RHSIM_Rhsim04G0130500 | 428.00 | 4.42E-95 | 1.08E-93 | -1.693 |
| RHSIM_Rhsim04G0012200 | 427.92 | 4.61E-95 | 1.12E-93 | 1.103 |
| RHSIM_Rhsim03G0033200 | 427.84 | 4.79E-95 | 1.16E-93 | 1.936 |
| RHSIM_Rhsim11G0075000 | 427.81 | 4.86E-95 | 1.18E-93 | -2.595 |
| novel.122 | 427.80 | 4.90E-95 | 1.19E-93 | 1.744 |
| RHSIM_Rhsim05G0203000 | 427.49 | 5.73E-95 | 1.39E-93 | 2.519 |
| RHSIM_Rhsim08G0250100 | 427.41 | 5.94E-95 | 1.44E-93 | -1.127 |

| Gene | LRT statistic | P value | BH-adjusted P | log2FC (interaction) |
|---|---|---|---|---|
| RHSIM_Rhsim07G0139600 | 427.41 | 5.96E-95 | 1.44E-93 | 2.723 |
| RHSIM_Rhsim08G0195300 | 427.22 | 6.54E-95 | 1.58E-93 | -1.686 |
| novel.3630 | 426.64 | 8.74E-95 | 2.11E-93 | -2.104 |
| RHSIM_Rhsim09G0038500 | 426.38 | 9.95E-95 | 2.40E-93 | -2.811 |
| RHSIM_Rhsim07G0096600 | 425.54 | 1.52E-94 | 3.67E-93 | -2.289 |
| RHSIM_Rhsim04G0174400 | 425.00 | 1.99E-94 | 4.79E-93 | -2.529 |
| RHSIM_Rhsim04G0015300 | 425.00 | 2.00E-94 | 4.80E-93 | -1.666 |
| RHSIM_Rhsim11G0022600 | 424.62 | 2.40E-94 | 5.78E-93 | 2.708 |
| RHSIM_Rhsim08G0058800 | 424.54 | 2.51E-94 | 6.02E-93 | -3.708 |
| RHSIM_Rhsim01G0049800 | 424.08 | 3.15E-94 | 7.57E-93 | -1.965 |
| RHSIM_Rhsim08G0076000 | 423.25 | 4.79E-94 | 1.15E-92 | 1.443 |
| novel.3483 | 423.15 | 5.04E-94 | 1.21E-92 | -2.868 |
| RHSIM_Rhsim13G0114300 | 422.96 | 5.53E-94 | 1.32E-92 | -1.651 |
| novel.2550 | 422.95 | 5.58E-94 | 1.33E-92 | 2.487 |
| novel.1970 | 422.76 | 6.11E-94 | 1.46E-92 | 1.470 |
| RHSIM_Rhsim02G0043000 | 422.44 | 7.17E-94 | 1.71E-92 | -1.596 |
| RHSIM_Rhsim03G0141400 | 422.44 | 7.20E-94 | 1.72E-92 | 1.508 |
| RHSIM_Rhsim04G0117400 | 422.19 | 8.14E-94 | 1.94E-92 | 1.963 |
| RHSIM_Rhsim04G0024200 | 422.17 | 8.21E-94 | 1.96E-92 | -1.950 |
| RHSIM_Rhsim03G0202700 | 421.96 | 9.14E-94 | 2.18E-92 | 2.099 |
| RHSIM_Rhsim05G0169700 | 421.92 | 9.30E-94 | 2.21E-92 | 1.969 |
| RHSIM_Rhsim06G0037300 | 421.32 | 1.26E-93 | 2.99E-92 | 1.991 |
| RHSIM_Rhsim04G0207300 | 421.19 | 1.35E-93 | 3.20E-92 | -2.537 |
| RHSIM_RhsimUnG0221400 | 421.14 | 1.38E-93 | 3.27E-92 | -2.793 |
| RHSIM_Rhsim04G0205900 | 421.05 | 1.44E-93 | 3.42E-92 | 1.324 |
| RHSIM_Rhsim11G0155700 | 421.02 | 1.46E-93 | 3.47E-92 | 2.318 |
| RHSIM_Rhsim13G0040300 | 420.47 | 1.93E-93 | 4.56E-92 | 4.118 |
| RHSIM_Rhsim08G0239700 | 420.37 | 2.03E-93 | 4.80E-92 | -1.089 |
| RHSIM_Rhsim07G0145200 | 420.08 | 2.35E-93 | 5.55E-92 | -1.599 |
| RHSIM_Rhsim08G0055400 | 419.48 | 3.16E-93 | 7.47E-92 | 1.941 |
| RHSIM_Rhsim11G0142200 | 418.67 | 4.75E-93 | 1.12E-91 | 2.412 |
| RHSIM_Rhsim03G0010500 | 417.48 | 8.62E-93 | 2.03E-91 | -3.149 |

| Gene | LRT statistic | P value | BH-adjusted P | log2FC (interaction) |
|---|---|---|---|---|
| novel.2525 | 416.81 | 1.21E-92 | 2.85E-91 | -1.709 |
| RHSIM_Rhsim11G0139900 | 416.63 | 1.32E-92 | 3.10E-91 | 3.522 |
| RHSIM_Rhsim04G0083100 | 415.03 | 2.95E-92 | 6.93E-91 | -1.402 |
| RHSIM_Rhsim03G0063700 | 414.38 | 4.08E-92 | 9.59E-91 | 2.016 |
| novel.417 | 414.04 | 4.83E-92 | 1.13E-90 | 1.432 |
| RHSIM_Rhsim01G0119800 | 414.02 | 4.89E-92 | 1.15E-90 | -1.826 |
| RHSIM_Rhsim02G0056200 | 413.67 | 5.82E-92 | 1.36E-90 | -1.096 |
| RHSIM_Rhsim09G0101000 | 413.25 | 7.18E-92 | 1.68E-90 | -4.856 |
| RHSIM_Rhsim01G0108800 | 413.18 | 7.44E-92 | 1.74E-90 | 1.282 |
| RHSIM_Rhsim02G0022300 | 413.10 | 7.75E-92 | 1.81E-90 | 1.721 |
| RHSIM_Rhsim13G0225400 | 413.03 | 8.02E-92 | 1.87E-90 | -1.641 |
| RHSIM_Rhsim04G0156400 | 412.55 | 1.02E-91 | 2.39E-90 | 1.237 |
| RHSIM_Rhsim10G0165900 | 412.17 | 1.23E-91 | 2.88E-90 | -4.002 |
| RHSIM_Rhsim06G0130100 | 411.88 | 1.43E-91 | 3.32E-90 | 1.871 |
| RHSIM_Rhsim03G0077300 | 411.82 | 1.47E-91 | 3.42E-90 | -2.025 |
| RHSIM_Rhsim09G0111400 | 411.24 | 1.97E-91 | 4.58E-90 | 1.244 |
| RHSIM_Rhsim13G0016400 | 410.92 | 2.31E-91 | 5.36E-90 | 1.172 |
| RHSIM_Rhsim13G0024600 | 410.45 | 2.93E-91 | 6.80E-90 | 2.047 |
| RHSIM_Rhsim06G0006600 | 409.87 | 3.91E-91 | 9.08E-90 | 1.085 |
| RHSIM_Rhsim09G0137500 | 409.78 | 4.09E-91 | 9.49E-90 | -0.988 |
| RHSIM_Rhsim03G0200000 | 409.03 | 5.96E-91 | 1.38E-89 | 1.556 |
| RHSIM_Rhsim10G0156900 | 408.98 | 6.12E-91 | 1.42E-89 | 1.238 |
| RHSIM_RhsimUnG0258800 | 408.93 | 6.26E-91 | 1.45E-89 | 3.877 |
| RHSIM_Rhsim09G0064600 | 408.82 | 6.62E-91 | 1.53E-89 | 3.515 |
| RHSIM_Rhsim01G0122800 | 408.15 | 9.26E-91 | 2.14E-89 | -1.233 |
| RHSIM_Rhsim01G0186500 | 408.11 | 9.45E-91 | 2.18E-89 | 1.773 |
| RHSIM_Rhsim10G0002800 | 407.66 | 1.18E-90 | 2.73E-89 | 2.096 |
| novel.1153 | 407.60 | 1.22E-90 | 2.81E-89 | 5.135 |
| RHSIM_Rhsim10G0198900 | 407.40 | 1.35E-90 | 3.10E-89 | -1.969 |
| RHSIM_Rhsim02G0090300 | 406.84 | 1.79E-90 | 4.12E-89 | 1.500 |
| RHSIM_Rhsim12G0027600 | 406.65 | 1.97E-90 | 4.53E-89 | -1.463 |
| RHSIM_Rhsim02G0122700 | 406.63 | 1.98E-90 | 4.55E-89 | -13.530 |

| Gene | LRT statistic | P value | BH-adjusted P | log2FC (interaction) |
|---|---|---|---|---|
| RHSIM_Rhsim01G0107200 | 406.49 | 2.12E-90 | 4.87E-89 | -2.567 |
| RHSIM_Rhsim06G0130200 | 406.21 | 2.45E-90 | 5.61E-89 | -1.352 |
| RHSIM_Rhsim07G0011200 | 406.20 | 2.46E-90 | 5.64E-89 | -1.626 |
| RHSIM_Rhsim09G0179800 | 406.04 | 2.67E-90 | 6.10E-89 | 2.916 |
| RHSIM_Rhsim04G0123300 | 405.46 | 3.56E-90 | 8.15E-89 | 1.362 |
| RHSIM_Rhsim12G0113100 | 405.27 | 3.92E-90 | 8.96E-89 | 1.468 |
| RHSIM_Rhsim04G0070300 | 405.13 | 4.21E-90 | 9.62E-89 | 1.168 |
| RHSIM_Rhsim06G0211100 | 404.82 | 4.92E-90 | 1.12E-88 | 3.160 |
| RHSIM_Rhsim03G0212700 | 404.58 | 5.56E-90 | 1.27E-88 | -1.335 |
| RHSIM_Rhsim04G0202900 | 403.87 | 7.93E-90 | 1.81E-88 | -1.565 |
| RHSIM_Rhsim13G0033100 | 403.58 | 9.14E-90 | 2.08E-88 | 2.612 |
| RHSIM_Rhsim11G0092600 | 403.49 | 9.55E-90 | 2.17E-88 | 0.893 |
| RHSIM_Rhsim06G0049800 | 403.42 | 9.92E-90 | 2.26E-88 | 1.236 |
| RHSIM_Rhsim09G0071200 | 403.12 | 1.15E-89 | 2.61E-88 | -1.847 |
| RHSIM_Rhsim12G0171500 | 402.49 | 1.58E-89 | 3.58E-88 | 1.712 |
| RHSIM_Rhsim09G0000900 | 402.30 | 1.74E-89 | 3.95E-88 | -1.855 |
| RHSIM_Rhsim11G0090400 | 401.85 | 2.17E-89 | 4.93E-88 | -3.123 |
| RHSIM_Rhsim04G0091800 | 401.81 | 2.22E-89 | 5.02E-88 | -0.930 |
| RHSIM_Rhsim02G0083100 | 401.79 | 2.24E-89 | 5.08E-88 | 2.902 |
| RHSIM_Rhsim03G0067500 | 401.51 | 2.59E-89 | 5.85E-88 | -1.025 |
| RHSIM_Rhsim01G0006600 | 400.99 | 3.36E-89 | 7.59E-88 | 1.837 |
| RHSIM_Rhsim10G0067100 | 400.80 | 3.69E-89 | 8.33E-88 | -1.839 |
| RHSIM_Rhsim13G0081300 | 400.39 | 4.52E-89 | 1.02E-87 | 2.051 |
| RHSIM_Rhsim02G0014400 | 400.12 | 5.19E-89 | 1.17E-87 | 1.598 |
| RHSIM_Rhsim08G0053900 | 399.72 | 6.33E-89 | 1.42E-87 | -2.209 |
| RHSIM_Rhsim01G0247400 | 399.65 | 6.57E-89 | 1.48E-87 | -1.238 |
| RHSIM_Rhsim11G0148600 | 399.62 | 6.66E-89 | 1.50E-87 | 1.747 |
| RHSIM_Rhsim06G0162500 | 399.54 | 6.95E-89 | 1.56E-87 | -0.793 |
| RHSIM_Rhsim12G0061600 | 399.17 | 8.36E-89 | 1.88E-87 | -2.706 |
| RHSIM_Rhsim07G0110300 | 398.85 | 9.79E-89 | 2.20E-87 | -1.644 |
| RHSIM_Rhsim05G0067700 | 398.51 | 1.16E-88 | 2.61E-87 | 1.317 |
| RHSIM_Rhsim13G0060600 | 398.14 | 1.40E-88 | 3.13E-87 | -1.054 |

| Gene | LRT statistic | P value | BH-adjusted P | log2FC (interaction) |
|---|---|---|---|---|
| RHSIM_Rhsim04G0123000 | 397.56 | 1.88E-88 | 4.20E-87 | -2.565 |
| RHSIM_Rhsim08G0200700 | 396.79 | 2.75E-88 | 6.15E-87 | -4.734 |
| RHSIM_Rhsim02G0228800 | 395.48 | 5.30E-88 | 1.18E-86 | 1.455 |
| RHSIM_RhsimUnG0141700 | 395.35 | 5.65E-88 | 1.26E-86 | 1.531 |
| RHSIM_Rhsim01G0013000 | 395.14 | 6.29E-88 | 1.40E-86 | 1.117 |
| RHSIM_Rhsim08G0033500 | 394.97 | 6.85E-88 | 1.53E-86 | 1.467 |
| RHSIM_Rhsim07G0125400 | 393.96 | 1.14E-87 | 2.54E-86 | 3.495 |
| RHSIM_Rhsim04G0009800 | 393.34 | 1.55E-87 | 3.45E-86 | 2.085 |
| RHSIM_Rhsim09G0207500 | 393.33 | 1.56E-87 | 3.47E-86 | -2.295 |
| RHSIM_Rhsim01G0169100 | 393.03 | 1.81E-87 | 4.02E-86 | -3.223 |
| RHSIM_Rhsim12G0150600 | 392.60 | 2.25E-87 | 4.99E-86 | -2.608 |
| RHSIM_Rhsim09G0129500 | 392.44 | 2.44E-87 | 5.41E-86 | 1.491 |
| RHSIM_Rhsim08G0020900 | 391.22 | 4.50E-87 | 9.98E-86 | 0.930 |
| RHSIM_Rhsim05G0003600 | 391.13 | 4.70E-87 | 1.04E-85 | -4.980 |
| RHSIM_Rhsim09G0177400 | 390.91 | 5.25E-87 | 1.16E-85 | 1.319 |
| RHSIM_Rhsim01G0193100 | 390.57 | 6.21E-87 | 1.37E-85 | 2.888 |
| RHSIM_Rhsim10G0013900 | 390.56 | 6.24E-87 | 1.38E-85 | 1.071 |
| RHSIM_Rhsim11G0183800 | 390.45 | 6.62E-87 | 1.46E-85 | 1.578 |
| RHSIM_Rhsim05G0102100 | 390.07 | 7.98E-87 | 1.76E-85 | 1.250 |
| RHSIM_RhsimUnG0058000 | 389.76 | 9.36E-87 | 2.06E-85 | 2.166 |
| RHSIM_Rhsim06G0146400 | 389.68 | 9.73E-87 | 2.14E-85 | 1.819 |
| RHSIM_Rhsim06G0214800 | 389.61 | 1.01E-86 | 2.22E-85 | 2.150 |
| RHSIM_Rhsim09G0203200 | 389.26 | 1.20E-86 | 2.64E-85 | -1.620 |
| RHSIM_Rhsim09G0197400 | 388.97 | 1.38E-86 | 3.04E-85 | 5.666 |
| RHSIM_Rhsim13G0070100 | 388.84 | 1.48E-86 | 3.25E-85 | -1.543 |
| RHSIM_Rhsim13G0230500 | 388.46 | 1.79E-86 | 3.93E-85 | 5.354 |
| RHSIM_Rhsim01G0149300 | 388.46 | 1.79E-86 | 3.93E-85 | -1.371 |
| RHSIM_Rhsim13G0057500 | 388.43 | 1.82E-86 | 3.98E-85 | -0.980 |
| RHSIM_Rhsim13G0077300 | 388.35 | 1.89E-86 | 4.14E-85 | 1.607 |
| RHSIM_Rhsim08G0061400 | 387.76 | 2.55E-86 | 5.57E-85 | -3.401 |
| novel.3337 | 387.63 | 2.71E-86 | 5.93E-85 | 0.928 |
| RHSIM_Rhsim12G0170000 | 387.61 | 2.75E-86 | 6.01E-85 | 1.173 |

| Gene | LRT statistic | P value | BH-adjusted P | log2FC (interaction) |
|---|---|---|---|---|
| RHSIM_RhsimUnG0063400 | 387.38 | 3.08E-86 | 6.73E-85 | 2.087 |
| novel.1890 | 386.37 | 5.10E-86 | 1.11E-84 | -5.478 |
| RHSIM_Rhsim09G0204900 | 386.35 | 5.17E-86 | 1.13E-84 | 1.770 |
| RHSIM_Rhsim04G0207900 | 386.19 | 5.60E-86 | 1.22E-84 | -1.556 |
| RHSIM_Rhsim09G0206900 | 386.07 | 5.94E-86 | 1.29E-84 | 1.758 |
| RHSIM_Rhsim13G0085300 | 385.64 | 7.38E-86 | 1.60E-84 | 1.665 |
| RHSIM_Rhsim05G0233200 | 385.61 | 7.48E-86 | 1.62E-84 | -1.949 |
| RHSIM_Rhsim02G0234200 | 384.98 | 1.03E-85 | 2.23E-84 | -2.252 |
| RHSIM_Rhsim02G0029600 | 384.74 | 1.16E-85 | 2.51E-84 | 3.342 |
| RHSIM_RhsimUnG0223200 | 384.59 | 1.25E-85 | 2.70E-84 | -7.905 |
| RHSIM_Rhsim03G0249400 | 384.44 | 1.35E-85 | 2.92E-84 | 1.364 |
| novel.1905 | 384.38 | 1.39E-85 | 3.00E-84 | -2.447 |
| RHSIM_Rhsim10G0038600 | 383.55 | 2.10E-85 | 4.53E-84 | -2.603 |
| RHSIM_Rhsim07G0137000 | 383.49 | 2.16E-85 | 4.67E-84 | 2.604 |
| RHSIM_Rhsim08G0099900 | 383.11 | 2.62E-85 | 5.66E-84 | 1.466 |
| RHSIM_Rhsim04G0015900 | 382.95 | 2.84E-85 | 6.13E-84 | 1.454 |
| RHSIM_Rhsim04G0192600 | 382.72 | 3.19E-85 | 6.88E-84 | -2.288 |
| RHSIM_RhsimUnG0255000 | 382.68 | 3.25E-85 | 7.00E-84 | 1.474 |
| RHSIM_Rhsim12G0187800 | 382.13 | 4.28E-85 | 9.21E-84 | 1.514 |
| RHSIM_Rhsim08G0196200 | 382.07 | 4.40E-85 | 9.47E-84 | 0.843 |
| RHSIM_Rhsim11G0132800 | 381.72 | 5.26E-85 | 1.13E-83 | 2.230 |
| RHSIM_Rhsim03G0058100 | 381.70 | 5.32E-85 | 1.14E-83 | -2.265 |
| RHSIM_Rhsim12G0052000 | 381.58 | 5.64E-85 | 1.21E-83 | -1.247 |
| RHSIM_Rhsim08G0167800 | 381.56 | 5.69E-85 | 1.22E-83 | -2.925 |
| RHSIM_Rhsim01G0197200 | 381.00 | 7.55E-85 | 1.62E-83 | 1.831 |
| novel.934 | 380.91 | 7.89E-85 | 1.69E-83 | -6.793 |
| RHSIM_Rhsim06G0009900 | 380.77 | 8.45E-85 | 1.81E-83 | 1.471 |
| RHSIM_Rhsim07G0203600 | 380.72 | 8.69E-85 | 1.86E-83 | -1.527 |
| RHSIM_RhsimUnG0020900 | 380.46 | 9.88E-85 | 2.11E-83 | 1.780 |
| RHSIM_Rhsim05G0152300 | 380.10 | 1.19E-84 | 2.53E-83 | 5.316 |
| novel.4282 | 380.03 | 1.23E-84 | 2.62E-83 | -2.359 |
| novel.926 | 379.57 | 1.54E-84 | 3.29E-83 | -4.753 |

| Gene | LRT statistic | P value | BH-adjusted P | log2FC (interaction) |
|---|---|---|---|---|
| RHSIM_Rhsim08G0088300 | 378.77 | 2.31E-84 | 4.92E-83 | 1.147 |
| RHSIM_Rhsim06G0169300 | 378.46 | 2.69E-84 | 5.73E-83 | -1.729 |
| RHSIM_Rhsim08G0068600 | 378.24 | 3.00E-84 | 6.38E-83 | -5.019 |
| novel.3070 | 378.21 | 3.06E-84 | 6.50E-83 | -1.462 |
| novel.4035 | 378.18 | 3.09E-84 | 6.57E-83 | -4.258 |
| RHSIM_Rhsim05G0078500 | 378.04 | 3.33E-84 | 7.07E-83 | 1.548 |
| novel.1876 | 377.45 | 4.46E-84 | 9.46E-83 | -6.578 |
| RHSIM_Rhsim05G0004900 | 376.67 | 6.60E-84 | 1.40E-82 | 2.452 |
| RHSIM_Rhsim02G0243400 | 376.55 | 7.03E-84 | 1.49E-82 | 1.685 |
| RHSIM_Rhsim03G0014300 | 376.29 | 8.00E-84 | 1.69E-82 | -2.190 |
| RHSIM_Rhsim13G0064900 | 376.09 | 8.86E-84 | 1.87E-82 | 2.872 |
| RHSIM_Rhsim02G0133200 | 375.69 | 1.08E-83 | 2.29E-82 | -3.551 |
| RHSIM_Rhsim05G0060400 | 375.38 | 1.26E-83 | 2.66E-82 | -2.828 |
| RHSIM_Rhsim13G0066100 | 375.36 | 1.27E-83 | 2.69E-82 | 0.825 |
| RHSIM_Rhsim03G0261600 | 375.32 | 1.30E-83 | 2.74E-82 | 1.498 |
| RHSIM_Rhsim05G0225600 | 375.30 | 1.31E-83 | 2.77E-82 | -2.925 |
| RHSIM_Rhsim10G0127000 | 374.77 | 1.72E-83 | 3.61E-82 | 1.341 |
| novel.1607 | 374.70 | 1.77E-83 | 3.73E-82 | 0.967 |
| RHSIM_Rhsim01G0071400 | 374.37 | 2.09E-83 | 4.39E-82 | 1.394 |
| RHSIM_Rhsim07G0175400 | 374.20 | 2.28E-83 | 4.80E-82 | 2.009 |
| RHSIM_Rhsim05G0223300 | 373.85 | 2.72E-83 | 5.71E-82 | 2.407 |
| RHSIM_Rhsim05G0122800 | 373.72 | 2.91E-83 | 6.10E-82 | 1.812 |
| RHSIM_Rhsim04G0232900 | 373.48 | 3.27E-83 | 6.86E-82 | -4.611 |
| RHSIM_Rhsim11G0076900 | 373.47 | 3.29E-83 | 6.90E-82 | 1.189 |
| RHSIM_Rhsim10G0014400 | 373.37 | 3.46E-83 | 7.24E-82 | -1.513 |
| RHSIM_Rhsim07G0200400 | 373.28 | 3.61E-83 | 7.54E-82 | 1.248 |
| novel.2436 | 373.04 | 4.08E-83 | 8.53E-82 | 1.969 |
| RHSIM_Rhsim04G0023800 | 372.84 | 4.50E-83 | 9.41E-82 | -1.485 |
| RHSIM_Rhsim12G0147400 | 372.50 | 5.35E-83 | 1.12E-81 | -2.394 |
| RHSIM_Rhsim12G0074200 | 372.40 | 5.61E-83 | 1.17E-81 | -3.114 |
| RHSIM_Rhsim01G0233600 | 371.68 | 8.07E-83 | 1.68E-81 | -1.805 |
| novel.3634 | 371.43 | 9.13E-83 | 1.90E-81 | 2.969 |

| Gene | LRT statistic | P value | BH-adjusted P | log2FC (interaction) |
|---|---|---|---|---|
| RHSIM_Rhsim10G0141400 | 370.50 | 1.46E-82 | 3.03E-81 | -0.941 |
| RHSIM_Rhsim03G0149200 | 370.25 | 1.65E-82 | 3.43E-81 | -1.521 |
| RHSIM_Rhsim11G0037500 | 370.24 | 1.66E-82 | 3.45E-81 | 1.544 |
| RHSIM_Rhsim04G0195600 | 370.13 | 1.75E-82 | 3.63E-81 | 2.187 |
| RHSIM_Rhsim08G0013100 | 369.12 | 2.90E-82 | 6.02E-81 | 1.937 |
| RHSIM_Rhsim02G0020300 | 368.97 | 3.13E-82 | 6.50E-81 | 5.990 |
| RHSIM_Rhsim11G0077200 | 368.55 | 3.88E-82 | 8.04E-81 | 1.749 |
| RHSIM_Rhsim06G0142000 | 368.31 | 4.36E-82 | 9.02E-81 | 1.406 |
| RHSIM_Rhsim03G0197000 | 368.27 | 4.46E-82 | 9.23E-81 | -2.342 |
| RHSIM_Rhsim09G0035900 | 367.99 | 5.13E-82 | 1.06E-80 | -2.118 |
| RHSIM_Rhsim04G0135600 | 367.95 | 5.22E-82 | 1.08E-80 | -1.261 |
| RHSIM_Rhsim08G0244800 | 367.90 | 5.36E-82 | 1.11E-80 | 3.336 |
| RHSIM_Rhsim13G0155900 | 367.73 | 5.85E-82 | 1.21E-80 | -2.178 |
| RHSIM_Rhsim05G0150000 | 367.67 | 6.02E-82 | 1.24E-80 | 1.147 |
| novel.3800 | 367.44 | 6.75E-82 | 1.39E-80 | 1.249 |
| RHSIM_Rhsim06G0026100 | 367.30 | 7.23E-82 | 1.49E-80 | 0.940 |
| RHSIM_Rhsim01G0086000 | 366.63 | 1.01E-81 | 2.08E-80 | 1.362 |
| novel.457 | 366.61 | 1.03E-81 | 2.11E-80 | 2.222 |
| RHSIM_Rhsim13G0143600 | 365.93 | 1.44E-81 | 2.95E-80 | 1.131 |
| RHSIM_Rhsim06G0036100 | 365.80 | 1.54E-81 | 3.16E-80 | -2.625 |
| RHSIM_Rhsim12G0124800 | 365.44 | 1.84E-81 | 3.78E-80 | 0.780 |
| RHSIM_Rhsim05G0032600 | 365.25 | 2.02E-81 | 4.15E-80 | 1.979 |
| RHSIM_Rhsim12G0056800 | 365.13 | 2.15E-81 | 4.40E-80 | -1.684 |
| RHSIM_Rhsim02G0202200 | 365.11 | 2.17E-81 | 4.44E-80 | -2.248 |
| RHSIM_Rhsim03G0175000 | 364.42 | 3.08E-81 | 6.29E-80 | -1.823 |
| RHSIM_Rhsim13G0118100 | 364.32 | 3.23E-81 | 6.60E-80 | -1.928 |
| RHSIM_Rhsim09G0011700 | 364.14 | 3.53E-81 | 7.22E-80 | 1.413 |
| RHSIM_Rhsim03G0218200 | 364.07 | 3.66E-81 | 7.47E-80 | -2.925 |
| RHSIM_Rhsim04G0100800 | 363.90 | 3.99E-81 | 8.13E-80 | 0.939 |
| RHSIM_Rhsim07G0071100 | 363.81 | 4.17E-81 | 8.50E-80 | 3.413 |
| RHSIM_Rhsim06G0157500 | 363.42 | 5.08E-81 | 1.03E-79 | 3.160 |
| RHSIM_Rhsim04G0170900 | 362.72 | 7.22E-81 | 1.47E-79 | 1.952 |

| Gene | LRT statistic | P value | BH-adjusted P | log2FC (interaction) |
|---|---|---|---|---|
| RHSIM_Rhsim01G0230900 | 362.64 | 7.48E-81 | 1.52E-79 | -3.301 |
| RHSIM_Rhsim11G0084200 | 362.08 | 9.91E-81 | 2.02E-79 | 1.352 |
| RHSIM_RhsimUnG0105200 | 361.87 | 1.10E-80 | 2.24E-79 | -1.909 |
| RHSIM_Rhsim10G0189000 | 361.14 | 1.59E-80 | 3.23E-79 | 2.200 |
| RHSIM_Rhsim01G0000500 | 360.48 | 2.22E-80 | 4.50E-79 | 1.410 |
| RHSIM_Rhsim10G0119200 | 360.28 | 2.44E-80 | 4.95E-79 | 1.230 |
| RHSIM_Rhsim12G0138000 | 359.72 | 3.24E-80 | 6.57E-79 | 2.135 |
| RHSIM_Rhsim04G0074100 | 359.71 | 3.26E-80 | 6.60E-79 | 2.204 |
| RHSIM_Rhsim03G0012100 | 359.71 | 3.26E-80 | 6.60E-79 | -2.078 |
| RHSIM_Rhsim13G0044200 | 359.59 | 3.45E-80 | 6.98E-79 | -4.361 |
| RHSIM_Rhsim03G0050100 | 359.57 | 3.49E-80 | 7.06E-79 | 2.334 |
| RHSIM_Rhsim08G0083700 | 359.47 | 3.68E-80 | 7.43E-79 | 1.825 |
| RHSIM_Rhsim06G0057400 | 359.39 | 3.82E-80 | 7.71E-79 | 2.648 |
| RHSIM_Rhsim06G0181200 | 358.69 | 5.42E-80 | 1.09E-78 | 2.723 |
| RHSIM_Rhsim05G0232200 | 357.98 | 7.77E-80 | 1.56E-78 | 2.248 |
| RHSIM_Rhsim12G0078800 | 357.80 | 8.47E-80 | 1.70E-78 | -1.512 |
| RHSIM_Rhsim04G0024500 | 357.61 | 9.32E-80 | 1.87E-78 | 1.195 |
| RHSIM_Rhsim06G0069100 | 357.21 | 1.14E-79 | 2.29E-78 | -0.919 |
| novel.2623 | 357.16 | 1.17E-79 | 2.34E-78 | 1.383 |
| RHSIM_Rhsim07G0194600 | 357.02 | 1.26E-79 | 2.52E-78 | -1.525 |
| RHSIM_Rhsim10G0186500 | 356.58 | 1.57E-79 | 3.14E-78 | 1.843 |
| RHSIM_RhsimUnG0122800 | 356.56 | 1.58E-79 | 3.17E-78 | -1.376 |
| RHSIM_Rhsim01G0249400 | 356.54 | 1.60E-79 | 3.20E-78 | -2.327 |
| RHSIM_Rhsim05G0121700 | 356.09 | 2.00E-79 | 4.00E-78 | -1.963 |
| RHSIM_Rhsim10G0044900 | 355.75 | 2.37E-79 | 4.74E-78 | 1.299 |
| RHSIM_Rhsim03G0254100 | 355.48 | 2.71E-79 | 5.42E-78 | 2.214 |
| RHSIM_Rhsim01G0034400 | 355.28 | 3.01E-79 | 6.01E-78 | -1.210 |
| RHSIM_Rhsim09G0178400 | 354.96 | 3.52E-79 | 7.03E-78 | -0.761 |
| RHSIM_Rhsim06G0012200 | 354.94 | 3.55E-79 | 7.08E-78 | 2.677 |
| RHSIM_Rhsim07G0123400 | 354.43 | 4.60E-79 | 9.18E-78 | 3.399 |
| RHSIM_Rhsim06G0229900 | 353.57 | 7.08E-79 | 1.41E-77 | 2.859 |
| RHSIM_Rhsim12G0136200 | 353.49 | 7.37E-79 | 1.47E-77 | -2.159 |

| Gene | LRT statistic | P value | BH-adjusted P | log2FC (interaction) |
|---|---|---|---|---|
| RHSIM_Rhsim08G0191600 | 353.18 | 8.62E-79 | 1.72E-77 | -4.889 |
| RHSIM_Rhsim07G0053300 | 352.17 | 1.43E-78 | 2.84E-77 | 1.512 |
| RHSIM_Rhsim05G0070900 | 352.00 | 1.56E-78 | 3.09E-77 | -1.278 |
| RHSIM_Rhsim12G0074300 | 351.17 | 2.36E-78 | 4.68E-77 | -3.093 |
| RHSIM_Rhsim08G0001700 | 351.08 | 2.47E-78 | 4.89E-77 | -1.511 |
| RHSIM_Rhsim09G0176900 | 350.95 | 2.63E-78 | 5.22E-77 | 1.929 |
| RHSIM_Rhsim06G0178400 | 350.85 | 2.76E-78 | 5.48E-77 | -1.394 |
| RHSIM_Rhsim08G0039300 | 350.82 | 2.80E-78 | 5.55E-77 | -2.341 |
| RHSIM_Rhsim07G0028800 | 350.25 | 3.74E-78 | 7.39E-77 | 1.018 |
| RHSIM_Rhsim03G0205000 | 349.89 | 4.48E-78 | 8.86E-77 | 2.283 |
| RHSIM_Rhsim12G0035400 | 349.87 | 4.51E-78 | 8.92E-77 | -4.279 |
| RHSIM_Rhsim03G0186100 | 349.82 | 4.64E-78 | 9.16E-77 | 1.459 |
| RHSIM_Rhsim11G0164500 | 349.81 | 4.65E-78 | 9.18E-77 | -1.852 |
| RHSIM_Rhsim08G0240500 | 349.49 | 5.47E-78 | 1.08E-76 | -1.647 |
| RHSIM_Rhsim03G0227300 | 349.23 | 6.23E-78 | 1.23E-76 | -1.671 |
| RHSIM_RhsimUnG0024000 | 349.18 | 6.38E-78 | 1.26E-76 | 3.193 |
| RHSIM_Rhsim10G0114500 | 348.43 | 9.33E-78 | 1.84E-76 | -1.846 |
| RHSIM_Rhsim12G0201100 | 347.41 | 1.55E-77 | 3.05E-76 | -2.987 |
| RHSIM_Rhsim06G0155900 | 346.76 | 2.15E-77 | 4.23E-76 | -7.925 |
| RHSIM_Rhsim06G0199600 | 346.58 | 2.35E-77 | 4.61E-76 | 3.512 |
| RHSIM_Rhsim12G0048700 | 346.41 | 2.57E-77 | 5.03E-76 | -4.290 |
| RHSIM_Rhsim07G0138800 | 345.61 | 3.84E-77 | 7.52E-76 | 1.044 |
| novel.2526 | 345.57 | 3.91E-77 | 7.65E-76 | 1.040 |
| RHSIM_Rhsim01G0288900 | 345.05 | 5.08E-77 | 9.94E-76 | 2.383 |
| RHSIM_Rhsim10G0012300 | 344.57 | 6.46E-77 | 1.26E-75 | 2.186 |
| RHSIM_Rhsim07G0051300 | 344.20 | 7.75E-77 | 1.52E-75 | -8.499 |
| RHSIM_Rhsim08G0087100 | 343.93 | 8.87E-77 | 1.73E-75 | 1.654 |
| RHSIM_Rhsim01G0141800 | 343.82 | 9.38E-77 | 1.83E-75 | 1.067 |
| RHSIM_Rhsim01G0126800 | 343.64 | 1.03E-76 | 2.00E-75 | 1.692 |
| RHSIM_Rhsim08G0230800 | 343.24 | 1.26E-76 | 2.45E-75 | 3.325 |
| RHSIM_Rhsim12G0100800 | 343.23 | 1.26E-76 | 2.46E-75 | -1.119 |
| RHSIM_Rhsim10G0025600 | 343.09 | 1.35E-76 | 2.63E-75 | -3.060 |

| Gene | LRT statistic | P value | BH-adjusted P | log2FC (interaction) |
|---|---|---|---|---|
| RHSIM_Rhsim01G0148700 | 342.73 | 1.62E-76 | 3.16E-75 | -1.382 |
| RHSIM_Rhsim06G0225900 | 342.39 | 1.93E-76 | 3.75E-75 | 1.435 |
| RHSIM_Rhsim02G0046000 | 341.44 | 3.10E-76 | 6.02E-75 | -3.153 |
| RHSIM_Rhsim11G0008300 | 341.43 | 3.12E-76 | 6.05E-75 | 2.343 |
| RHSIM_Rhsim08G0198500 | 340.96 | 3.95E-76 | 7.67E-75 | 2.199 |
| RHSIM_Rhsim06G0178500 | 340.82 | 4.23E-76 | 8.21E-75 | 1.512 |
| RHSIM_Rhsim11G0141200 | 340.76 | 4.35E-76 | 8.43E-75 | 1.040 |
| novel.3106 | 340.74 | 4.40E-76 | 8.52E-75 | 1.168 |
| RHSIM_Rhsim07G0243800 | 340.53 | 4.88E-76 | 9.45E-75 | -1.203 |
| RHSIM_Rhsim11G0036500 | 340.52 | 4.92E-76 | 9.52E-75 | -1.383 |
| RHSIM_Rhsim13G0206000 | 340.34 | 5.38E-76 | 1.04E-74 | -1.152 |
| RHSIM_Rhsim12G0042000 | 340.31 | 5.46E-76 | 1.05E-74 | 1.601 |
| RHSIM_Rhsim06G0035600 | 338.78 | 1.18E-75 | 2.27E-74 | 2.265 |
| RHSIM_Rhsim06G0049700 | 338.51 | 1.35E-75 | 2.60E-74 | 2.336 |
| RHSIM_Rhsim11G0105100 | 338.44 | 1.39E-75 | 2.68E-74 | 1.425 |
| RHSIM_Rhsim05G0057000 | 338.37 | 1.45E-75 | 2.79E-74 | 2.416 |
| novel.2889 | 338.13 | 1.63E-75 | 3.13E-74 | 3.546 |
| RHSIM_Rhsim13G0026300 | 338.09 | 1.66E-75 | 3.20E-74 | -1.017 |
| RHSIM_Rhsim06G0040500 | 337.98 | 1.76E-75 | 3.38E-74 | -3.839 |
| RHSIM_Rhsim11G0069400 | 337.68 | 2.04E-75 | 3.92E-74 | -0.987 |
| RHSIM_Rhsim07G0250100 | 337.48 | 2.25E-75 | 4.33E-74 | -3.181 |
| RHSIM_Rhsim02G0209000 | 336.99 | 2.88E-75 | 5.52E-74 | -2.931 |
| RHSIM_Rhsim06G0033800 | 336.78 | 3.20E-75 | 6.13E-74 | -4.961 |
| RHSIM_Rhsim13G0203100 | 336.26 | 4.17E-75 | 7.99E-74 | -2.394 |
| RHSIM_Rhsim07G0204400 | 336.16 | 4.38E-75 | 8.38E-74 | -1.377 |
| RHSIM_Rhsim09G0163400 | 335.84 | 5.15E-75 | 9.85E-74 | -1.226 |
| RHSIM_Rhsim04G0130400 | 335.73 | 5.43E-75 | 1.04E-73 | 1.582 |
| RHSIM_Rhsim02G0255400 | 335.43 | 6.31E-75 | 1.21E-73 | 1.230 |
| RHSIM_RhsimUnG0197300 | 335.35 | 6.56E-75 | 1.25E-73 | 3.584 |
| RHSIM_Rhsim07G0036300 | 335.01 | 7.79E-75 | 1.49E-73 | 1.744 |
| RHSIM_Rhsim06G0196600 | 335.00 | 7.83E-75 | 1.49E-73 | -2.434 |
| RHSIM_Rhsim01G0273800 | 334.83 | 8.53E-75 | 1.63E-73 | -2.187 |

| Gene | LRT statistic | P value | BH-adjusted P | log2FC (interaction) |
|---|---|---|---|---|
| RHSIM_Rhsim07G0082500 | 334.79 | 8.71E-75 | 1.66E-73 | 0.807 |
| RHSIM_Rhsim03G0252200 | 333.93 | 1.34E-74 | 2.55E-73 | 5.631 |
| RHSIM_Rhsim09G0023600 | 333.65 | 1.54E-74 | 2.94E-73 | 2.650 |
| RHSIM_Rhsim01G0280000 | 333.24 | 1.89E-74 | 3.60E-73 | 1.409 |
| RHSIM_Rhsim11G0161100 | 333.13 | 2.00E-74 | 3.79E-73 | 0.985 |
| RHSIM_Rhsim11G0145600 | 333.04 | 2.09E-74 | 3.97E-73 | 1.089 |
| RHSIM_Rhsim04G0010600 | 332.96 | 2.17E-74 | 4.13E-73 | -1.325 |
| RHSIM_Rhsim09G0179500 | 332.73 | 2.45E-74 | 4.64E-73 | 1.229 |
| RHSIM_Rhsim04G0049800 | 332.30 | 3.03E-74 | 5.75E-73 | -1.933 |
| RHSIM_Rhsim02G0082500 | 332.08 | 3.38E-74 | 6.40E-73 | -1.690 |
| RHSIM_Rhsim06G0012700 | 331.50 | 4.53E-74 | 8.56E-73 | 4.613 |
| RHSIM_Rhsim02G0067000 | 331.31 | 4.98E-74 | 9.42E-73 | -1.326 |
| RHSIM_Rhsim11G0157200 | 331.00 | 5.81E-74 | 1.10E-72 | 1.626 |
| novel.1107 | 330.95 | 5.97E-74 | 1.13E-72 | -3.398 |
| RHSIM_Rhsim03G0074800 | 330.94 | 6.00E-74 | 1.13E-72 | 1.499 |
| RHSIM_Rhsim09G0014300 | 330.89 | 6.14E-74 | 1.16E-72 | 2.436 |
| RHSIM_Rhsim09G0093800 | 330.75 | 6.60E-74 | 1.24E-72 | 1.840 |
| RHSIM_Rhsim02G0228400 | 330.66 | 6.91E-74 | 1.30E-72 | 1.482 |
| RHSIM_Rhsim13G0022300 | 329.99 | 9.67E-74 | 1.82E-72 | 1.122 |
| RHSIM_Rhsim03G0085600 | 329.75 | 1.09E-73 | 2.05E-72 | 2.117 |
| RHSIM_Rhsim13G0118500 | 329.02 | 1.57E-73 | 2.95E-72 | 2.956 |
| RHSIM_Rhsim07G0094300 | 328.87 | 1.70E-73 | 3.19E-72 | 4.054 |
| RHSIM_Rhsim03G0246500 | 328.27 | 2.28E-73 | 4.29E-72 | -1.694 |
| novel.119 | 327.84 | 2.84E-73 | 5.34E-72 | -5.062 |
| novel.2435 | 327.82 | 2.87E-73 | 5.37E-72 | 2.149 |
| RHSIM_Rhsim09G0100300 | 327.75 | 2.97E-73 | 5.56E-72 | 2.076 |
| RHSIM_Rhsim03G0225500 | 327.62 | 3.17E-73 | 5.93E-72 | 2.017 |
| RHSIM_Rhsim08G0098300 | 327.06 | 4.20E-73 | 7.85E-72 | 1.367 |
| novel.2633 | 326.95 | 4.43E-73 | 8.28E-72 | 1.244 |
| RHSIM_Rhsim12G0080300 | 326.64 | 5.18E-73 | 9.67E-72 | 2.157 |
| RHSIM_Rhsim12G0130600 | 326.63 | 5.21E-73 | 9.73E-72 | 1.224 |
| RHSIM_Rhsim01G0089800 | 326.58 | 5.35E-73 | 9.99E-72 | -2.007 |

| Gene | LRT statistic | P value | BH-adjusted P | log2FC (interaction) |
|---|---|---|---|---|
| RHSIM_Rhsim04G0162900 | 326.14 | 6.66E-73 | 1.24E-71 | 1.982 |
| RHSIM_Rhsim13G0130800 | 326.00 | 7.15E-73 | 1.33E-71 | 1.587 |
| RHSIM_Rhsim01G0255500 | 325.92 | 7.45E-73 | 1.39E-71 | 1.335 |
| RHSIM_Rhsim06G0000400 | 325.76 | 8.06E-73 | 1.50E-71 | -1.350 |
| RHSIM_Rhsim04G0236600 | 325.58 | 8.82E-73 | 1.64E-71 | -1.850 |
| RHSIM_Rhsim02G0173200 | 324.78 | 1.32E-72 | 2.45E-71 | 1.822 |
| RHSIM_Rhsim06G0204200 | 324.58 | 1.46E-72 | 2.70E-71 | -1.751 |
| RHSIM_Rhsim05G0101900 | 324.48 | 1.53E-72 | 2.84E-71 | -1.706 |
| RHSIM_Rhsim13G0061200 | 324.33 | 1.65E-72 | 3.07E-71 | 1.093 |
| RHSIM_Rhsim06G0079700 | 324.28 | 1.69E-72 | 3.14E-71 | 2.210 |
| RHSIM_Rhsim01G0006200 | 324.14 | 1.81E-72 | 3.36E-71 | -1.438 |
| RHSIM_RhsimUnG0228600 | 323.85 | 2.10E-72 | 3.90E-71 | -1.300 |
| RHSIM_Rhsim04G0008600 | 323.65 | 2.33E-72 | 4.31E-71 | -0.960 |
| RHSIM_Rhsim12G0114200 | 323.52 | 2.48E-72 | 4.58E-71 | -1.868 |
| RHSIM_Rhsim08G0177500 | 323.23 | 2.86E-72 | 5.29E-71 | 0.991 |
| RHSIM_Rhsim12G0041100 | 323.15 | 2.98E-72 | 5.50E-71 | -1.707 |
| RHSIM_Rhsim06G0161000 | 323.13 | 3.02E-72 | 5.57E-71 | -1.292 |
| RHSIM_Rhsim07G0013400 | 322.74 | 3.67E-72 | 6.77E-71 | -1.455 |
| RHSIM_Rhsim13G0099200 | 322.27 | 4.63E-72 | 8.54E-71 | 1.699 |
| RHSIM_Rhsim07G0095500 | 322.10 | 5.05E-72 | 9.30E-71 | -1.525 |
| RHSIM_Rhsim07G0036400 | 321.88 | 5.65E-72 | 1.04E-70 | -1.053 |
| novel.2514 | 321.84 | 5.75E-72 | 1.06E-70 | -45.610 |
| novel.1963 | 321.41 | 7.15E-72 | 1.31E-70 | 1.025 |
| RHSIM_Rhsim07G0176900 | 321.35 | 7.35E-72 | 1.35E-70 | -2.621 |
| RHSIM_Rhsim12G0213700 | 321.08 | 8.41E-72 | 1.54E-70 | 1.431 |
| RHSIM_Rhsim01G0154600 | 320.86 | 9.39E-72 | 1.72E-70 | -1.802 |
| RHSIM_Rhsim13G0101100 | 320.53 | 1.11E-71 | 2.04E-70 | -1.146 |
| RHSIM_Rhsim06G0001100 | 320.28 | 1.26E-71 | 2.31E-70 | -2.557 |
| RHSIM_Rhsim06G0179000 | 320.20 | 1.31E-71 | 2.40E-70 | 1.612 |
| RHSIM_Rhsim08G0104300 | 320.05 | 1.41E-71 | 2.59E-70 | 1.281 |
| RHSIM_Rhsim12G0047500 | 320.01 | 1.44E-71 | 2.64E-70 | -1.073 |
| RHSIM_Rhsim03G0228600 | 319.85 | 1.56E-71 | 2.86E-70 | 1.019 |

| Gene | LRT statistic | P value | BH-adjusted P | log2FC (interaction) |
|---|---|---|---|---|
| RHSIM_Rhsim08G0094100 | 319.81 | 1.60E-71 | 2.92E-70 | 0.771 |
| RHSIM_Rhsim08G0171200 | 319.63 | 1.75E-71 | 3.19E-70 | 1.433 |
| RHSIM_Rhsim11G0167200 | 319.35 | 2.01E-71 | 3.67E-70 | -5.135 |
| RHSIM_Rhsim11G0049700 | 319.29 | 2.07E-71 | 3.78E-70 | 1.645 |
| RHSIM_Rhsim03G0000500 | 319.12 | 2.25E-71 | 4.10E-70 | 2.205 |
| RHSIM_Rhsim06G0039200 | 318.48 | 3.11E-71 | 5.65E-70 | 0.974 |
| RHSIM_Rhsim09G0199700 | 318.23 | 3.52E-71 | 6.41E-70 | -2.830 |
| novel.1787 | 318.13 | 3.70E-71 | 6.73E-70 | -1.740 |
| RHSIM_Rhsim12G0144700 | 318.04 | 3.87E-71 | 7.03E-70 | -1.217 |
| RHSIM_Rhsim08G0033600 | 317.99 | 3.98E-71 | 7.23E-70 | -2.260 |
| RHSIM_Rhsim08G0030400 | 317.95 | 4.05E-71 | 7.35E-70 | -1.244 |
| RHSIM_Rhsim02G0187600 | 317.33 | 5.52E-71 | 1.00E-69 | 1.086 |
| novel.3576 | 317.31 | 5.57E-71 | 1.01E-69 | -1.394 |
| RHSIM_Rhsim07G0002700 | 316.75 | 7.39E-71 | 1.34E-69 | 2.683 |
| RHSIM_Rhsim02G0213700 | 316.40 | 8.81E-71 | 1.60E-69 | 1.311 |
| RHSIM_Rhsim07G0203200 | 316.14 | 1.00E-70 | 1.82E-69 | -1.230 |
| RHSIM_Rhsim03G0065200 | 315.85 | 1.16E-70 | 2.10E-69 | 2.122 |
| RHSIM_Rhsim09G0196800 | 315.53 | 1.36E-70 | 2.46E-69 | -3.555 |
| RHSIM_Rhsim02G0104000 | 315.50 | 1.39E-70 | 2.50E-69 | -1.578 |
| RHSIM_Rhsim01G0202300 | 315.46 | 1.41E-70 | 2.55E-69 | 1.480 |
| RHSIM_Rhsim01G0055700 | 315.33 | 1.51E-70 | 2.72E-69 | -2.436 |
| RHSIM_Rhsim11G0179600 | 315.02 | 1.76E-70 | 3.18E-69 | 0.989 |
| novel.3960 | 314.83 | 1.94E-70 | 3.49E-69 | -3.419 |
| RHSIM_Rhsim08G0097900 | 314.74 | 2.03E-70 | 3.65E-69 | 1.501 |
| RHSIM_Rhsim07G0178700 | 314.73 | 2.04E-70 | 3.67E-69 | 2.025 |
| RHSIM_Rhsim12G0054500 | 314.70 | 2.06E-70 | 3.71E-69 | -1.085 |
| RHSIM_Rhsim12G0023300 | 313.21 | 4.36E-70 | 7.84E-69 | 0.985 |
| RHSIM_Rhsim12G0198700 | 312.88 | 5.14E-70 | 9.24E-69 | -3.995 |
| RHSIM_Rhsim13G0216500 | 312.74 | 5.52E-70 | 9.91E-69 | 2.278 |
| RHSIM_Rhsim10G0022600 | 312.56 | 6.04E-70 | 1.08E-68 | -2.458 |
| RHSIM_Rhsim06G0235500 | 312.34 | 6.76E-70 | 1.21E-68 | -1.712 |
| novel.216 | 312.06 | 7.76E-70 | 1.39E-68 | -3.533 |

| Gene | LRT statistic | P value | BH-adjusted P | log2FC (interaction) |
|---|---|---|---|---|
| RHSIM_Rhsim03G0176200 | 312.02 | 7.92E-70 | 1.42E-68 | 2.379 |
| RHSIM_Rhsim04G0166300 | 311.70 | 9.30E-70 | 1.66E-68 | 2.488 |
| RHSIM_Rhsim08G0167600 | 311.55 | 1.01E-69 | 1.80E-68 | -3.015 |
| RHSIM_Rhsim04G0060500 | 311.53 | 1.01E-69 | 1.81E-68 | 3.593 |
| RHSIM_Rhsim08G0081000 | 311.36 | 1.11E-69 | 1.97E-68 | 0.722 |
| RHSIM_Rhsim04G0236100 | 311.04 | 1.30E-69 | 2.32E-68 | -1.811 |
| RHSIM_Rhsim06G0073000 | 310.78 | 1.48E-69 | 2.63E-68 | 1.862 |
| RHSIM_Rhsim04G0027200 | 310.72 | 1.52E-69 | 2.72E-68 | 0.818 |
| RHSIM_Rhsim12G0204600 | 310.69 | 1.54E-69 | 2.75E-68 | -0.990 |
| RHSIM_Rhsim04G0087100 | 310.63 | 1.59E-69 | 2.84E-68 | 1.612 |
| RHSIM_Rhsim10G0009000 | 310.42 | 1.77E-69 | 3.14E-68 | 1.725 |
| RHSIM_Rhsim11G0050300 | 310.38 | 1.80E-69 | 3.21E-68 | 1.878 |
| RHSIM_Rhsim05G0067500 | 310.19 | 1.98E-69 | 3.53E-68 | -2.045 |
| RHSIM_Rhsim10G0172500 | 310.06 | 2.11E-69 | 3.75E-68 | 2.226 |
| RHSIM_Rhsim04G0204400 | 310.04 | 2.14E-69 | 3.79E-68 | 1.677 |
| RHSIM_Rhsim13G0065500 | 309.99 | 2.19E-69 | 3.88E-68 | -1.655 |
| RHSIM_Rhsim06G0179400 | 309.68 | 2.57E-69 | 4.55E-68 | 3.951 |
| RHSIM_Rhsim06G0227100 | 309.54 | 2.75E-69 | 4.86E-68 | 1.432 |
| RHSIM_Rhsim07G0129200 | 309.13 | 3.37E-69 | 5.97E-68 | 1.620 |
| RHSIM_Rhsim03G0086500 | 308.97 | 3.66E-69 | 6.47E-68 | -3.254 |
| RHSIM_Rhsim02G0029400 | 308.90 | 3.79E-69 | 6.71E-68 | 9.535 |
| RHSIM_Rhsim13G0163900 | 308.63 | 4.35E-69 | 7.69E-68 | -2.705 |
| RHSIM_Rhsim08G0163400 | 308.31 | 5.09E-69 | 8.99E-68 | 1.944 |
| RHSIM_Rhsim07G0076500 | 308.01 | 5.91E-69 | 1.04E-67 | 1.234 |
| RHSIM_Rhsim06G0039400 | 307.41 | 8.02E-69 | 1.41E-67 | 1.408 |
| RHSIM_Rhsim11G0182000 | 306.71 | 1.13E-68 | 2.00E-67 | -1.046 |
| RHSIM_Rhsim02G0181100 | 306.59 | 1.21E-68 | 2.13E-67 | -1.135 |
| novel.1647 | 306.32 | 1.38E-68 | 2.43E-67 | 1.348 |
| RHSIM_Rhsim01G0170700 | 306.08 | 1.56E-68 | 2.75E-67 | -2.085 |
| RHSIM_Rhsim05G0177800 | 305.91 | 1.70E-68 | 2.99E-67 | 2.697 |
| RHSIM_Rhsim04G0222400 | 305.87 | 1.74E-68 | 3.05E-67 | 1.606 |
| novel.1537 | 305.70 | 1.89E-68 | 3.32E-67 | 5.142 |

| Gene | LRT statistic | P value | BH-adjusted P | log2FC (interaction) |
|---|---|---|---|---|
| RHSIM_Rhsim13G0169900 | 305.65 | 1.94E-68 | 3.40E-67 | 0.920 |
| RHSIM_Rhsim02G0103900 | 305.62 | 1.97E-68 | 3.45E-67 | -1.848 |
| RHSIM_Rhsim08G0168600 | 305.55 | 2.04E-68 | 3.57E-67 | 1.204 |
| RHSIM_Rhsim03G0111700 | 305.22 | 2.40E-68 | 4.21E-67 | 4.063 |
| RHSIM_Rhsim09G0102900 | 304.98 | 2.72E-68 | 4.75E-67 | 2.165 |
| RHSIM_Rhsim11G0180300 | 304.85 | 2.89E-68 | 5.06E-67 | 1.124 |
| RHSIM_Rhsim07G0222600 | 304.82 | 2.93E-68 | 5.12E-67 | -1.646 |
| RHSIM_Rhsim04G0008200 | 304.16 | 4.09E-68 | 7.15E-67 | 1.333 |
| RHSIM_Rhsim03G0144500 | 304.11 | 4.19E-68 | 7.32E-67 | -1.277 |
| RHSIM_Rhsim13G0215800 | 303.70 | 5.14E-68 | 8.98E-67 | 1.720 |
| RHSIM_Rhsim06G0171700 | 303.45 | 5.83E-68 | 1.02E-66 | -2.595 |
| RHSIM_Rhsim02G0033100 | 303.17 | 6.72E-68 | 1.17E-66 | 1.646 |
| RHSIM_Rhsim10G0205500 | 303.15 | 6.78E-68 | 1.18E-66 | 2.579 |
| novel.2595 | 302.56 | 9.13E-68 | 1.59E-66 | 3.267 |
| RHSIM_Rhsim01G0202700 | 302.03 | 1.19E-67 | 2.07E-66 | 3.279 |
| RHSIM_Rhsim07G0015900 | 301.94 | 1.24E-67 | 2.16E-66 | -1.275 |
| RHSIM_Rhsim05G0039500 | 301.90 | 1.27E-67 | 2.21E-66 | 1.157 |
| RHSIM_Rhsim12G0081500 | 301.69 | 1.41E-67 | 2.45E-66 | 1.553 |
| RHSIM_Rhsim05G0060700 | 301.46 | 1.59E-67 | 2.76E-66 | 0.760 |
| RHSIM_Rhsim05G0224700 | 301.38 | 1.65E-67 | 2.86E-66 | -3.144 |
| RHSIM_Rhsim04G0218700 | 301.38 | 1.65E-67 | 2.86E-66 | 1.338 |
| RHSIM_Rhsim10G0195800 | 301.35 | 1.68E-67 | 2.91E-66 | 3.650 |
| RHSIM_Rhsim13G0000500 | 301.16 | 1.84E-67 | 3.18E-66 | -1.178 |
| RHSIM_Rhsim05G0062800 | 300.83 | 2.17E-67 | 3.75E-66 | -1.502 |
| RHSIM_Rhsim03G0151400 | 300.20 | 2.98E-67 | 5.15E-66 | 1.567 |
| RHSIM_Rhsim12G0199300 | 299.97 | 3.34E-67 | 5.78E-66 | -1.823 |
| RHSIM_Rhsim08G0042500 | 299.97 | 3.35E-67 | 5.79E-66 | -1.878 |
| RHSIM_Rhsim12G0008900 | 299.58 | 4.07E-67 | 7.02E-66 | 1.998 |
| RHSIM_Rhsim13G0088400 | 299.46 | 4.32E-67 | 7.45E-66 | -2.337 |
| novel.4069 | 299.21 | 4.89E-67 | 8.44E-66 | -3.980 |
| RHSIM_Rhsim02G0096400 | 299.16 | 5.01E-67 | 8.63E-66 | -1.417 |
| RHSIM_Rhsim02G0210900 | 298.93 | 5.65E-67 | 9.73E-66 | -1.639 |

| Gene | LRT statistic | P value | BH-adjusted P | log2FC (interaction) |
|---|---|---|---|---|
| RHSIM_RhsimUnG0133400 | 298.72 | 6.27E-67 | 1.08E-65 | -4.471 |
| RHSIM_Rhsim12G0215400 | 298.65 | 6.47E-67 | 1.11E-65 | -1.238 |
| RHSIM_Rhsim01G0009700 | 298.20 | 8.12E-67 | 1.40E-65 | 1.518 |
| RHSIM_Rhsim12G0205300 | 298.20 | 8.14E-67 | 1.40E-65 | 1.222 |
| RHSIM_Rhsim05G0188400 | 298.14 | 8.39E-67 | 1.44E-65 | -1.289 |
| RHSIM_Rhsim05G0214000 | 298.10 | 8.56E-67 | 1.47E-65 | 1.482 |
| novel.1982 | 297.90 | 9.47E-67 | 1.62E-65 | -4.218 |
| RHSIM_Rhsim05G0202200 | 297.49 | 1.16E-66 | 1.99E-65 | -4.294 |
| RHSIM_Rhsim01G0124600 | 297.06 | 1.44E-66 | 2.47E-65 | -1.280 |
| RHSIM_Rhsim13G0124200 | 297.01 | 1.48E-66 | 2.53E-65 | 1.256 |
| RHSIM_Rhsim02G0216500 | 296.77 | 1.66E-66 | 2.85E-65 | 1.997 |
| RHSIM_Rhsim09G0089100 | 296.75 | 1.68E-66 | 2.87E-65 | 1.947 |
| RHSIM_Rhsim07G0095300 | 296.63 | 1.79E-66 | 3.05E-65 | 1.626 |
| RHSIM_Rhsim08G0172100 | 296.59 | 1.82E-66 | 3.11E-65 | 1.382 |
| RHSIM_Rhsim04G0205800 | 296.49 | 1.92E-66 | 3.27E-65 | 1.289 |
| RHSIM_Rhsim10G0139200 | 296.42 | 1.99E-66 | 3.39E-65 | 3.205 |
| novel.1177 | 296.10 | 2.33E-66 | 3.97E-65 | 2.363 |
| RHSIM_Rhsim12G0064000 | 295.93 | 2.53E-66 | 4.32E-65 | 1.270 |
| RHSIM_Rhsim05G0074800 | 295.91 | 2.56E-66 | 4.37E-65 | -1.184 |
| RHSIM_Rhsim02G0179000 | 295.89 | 2.59E-66 | 4.41E-65 | 1.765 |
| RHSIM_Rhsim07G0160000 | 295.72 | 2.81E-66 | 4.79E-65 | 2.067 |
| RHSIM_Rhsim04G0241700 | 295.71 | 2.83E-66 | 4.81E-65 | 1.256 |
| novel.2858 | 295.69 | 2.87E-66 | 4.87E-65 | -1.710 |
| RHSIM_Rhsim08G0031800 | 295.56 | 3.06E-66 | 5.19E-65 | 1.224 |
| RHSIM_Rhsim01G0209200 | 295.52 | 3.13E-66 | 5.30E-65 | 2.053 |
| RHSIM_Rhsim12G0066500 | 295.50 | 3.16E-66 | 5.35E-65 | 0.996 |
| RHSIM_RhsimUnG0127800 | 295.29 | 3.49E-66 | 5.92E-65 | 2.460 |
| RHSIM_Rhsim08G0218400 | 295.29 | 3.50E-66 | 5.94E-65 | -1.506 |
| RHSIM_Rhsim05G0084500 | 294.95 | 4.15E-66 | 7.02E-65 | 1.664 |
| RHSIM_Rhsim11G0186300 | 294.82 | 4.43E-66 | 7.49E-65 | -1.728 |
| RHSIM_RhsimUnG0148400 | 294.23 | 5.94E-66 | 1.01E-64 | -1.675 |
| RHSIM_Rhsim11G0118000 | 294.15 | 6.19E-66 | 1.05E-64 | 1.032 |

| Gene | LRT statistic | P value | BH-adjusted P | log2FC (interaction) |
|---|---|---|---|---|
| RHSIM_Rhsim02G0244400 | 294.05 | 6.51E-66 | 1.10E-64 | 1.289 |
| RHSIM_Rhsim09G0117900 | 293.97 | 6.79E-66 | 1.15E-64 | 4.136 |
| RHSIM_RhsimUnG0037800 | 293.70 | 7.79E-66 | 1.31E-64 | -1.197 |
| RHSIM_Rhsim07G0016900 | 293.57 | 8.28E-66 | 1.40E-64 | 2.072 |
| RHSIM_Rhsim06G0020800 | 293.45 | 8.82E-66 | 1.49E-64 | -1.623 |
| RHSIM_RhsimUnG0254000 | 293.38 | 9.12E-66 | 1.54E-64 | 2.007 |
| RHSIM_Rhsim08G0177200 | 293.33 | 9.36E-66 | 1.58E-64 | 2.038 |
| RHSIM_Rhsim13G0020200 | 293.27 | 9.62E-66 | 1.62E-64 | -1.123 |
| RHSIM_Rhsim04G0121700 | 293.22 | 9.88E-66 | 1.66E-64 | 1.963 |
| RHSIM_Rhsim02G0200800 | 292.98 | 1.12E-65 | 1.88E-64 | 1.414 |
| RHSIM_Rhsim07G0044900 | 292.84 | 1.20E-65 | 2.01E-64 | -3.306 |
| RHSIM_Rhsim05G0192600 | 292.68 | 1.30E-65 | 2.18E-64 | 1.186 |
| RHSIM_Rhsim03G0149400 | 292.67 | 1.30E-65 | 2.18E-64 | -3.684 |
| RHSIM_Rhsim01G0280400 | 292.67 | 1.30E-65 | 2.18E-64 | 2.932 |
| RHSIM_Rhsim13G0024200 | 292.60 | 1.35E-65 | 2.26E-64 | -1.412 |
| RHSIM_Rhsim08G0086000 | 292.58 | 1.36E-65 | 2.28E-64 | 3.188 |
| RHSIM_Rhsim10G0000500 | 292.57 | 1.37E-65 | 2.29E-64 | -3.055 |
| RHSIM_Rhsim08G0062300 | 292.48 | 1.43E-65 | 2.40E-64 | -3.373 |
| RHSIM_Rhsim09G0025300 | 292.32 | 1.55E-65 | 2.59E-64 | -1.263 |
| RHSIM_Rhsim06G0129900 | 292.18 | 1.67E-65 | 2.78E-64 | -1.120 |
| RHSIM_Rhsim06G0094500 | 292.02 | 1.81E-65 | 3.01E-64 | -1.509 |
| RHSIM_Rhsim05G0231400 | 291.89 | 1.93E-65 | 3.21E-64 | 1.012 |
| RHSIM_RhsimUnG0125800 | 291.83 | 1.99E-65 | 3.31E-64 | 1.162 |
| RHSIM_Rhsim03G0036200 | 291.40 | 2.46E-65 | 4.10E-64 | -4.871 |
| RHSIM_Rhsim03G0065500 | 291.24 | 2.67E-65 | 4.45E-64 | 1.883 |
| RHSIM_Rhsim06G0008500 | 291.07 | 2.91E-65 | 4.84E-64 | 0.974 |
| RHSIM_Rhsim03G0239700 | 290.94 | 3.10E-65 | 5.15E-64 | 1.205 |
| RHSIM_Rhsim10G0079500 | 290.62 | 3.64E-65 | 6.05E-64 | -1.267 |
| RHSIM_Rhsim08G0090000 | 290.31 | 4.25E-65 | 7.06E-64 | 1.682 |
| RHSIM_Rhsim04G0084000 | 290.16 | 4.59E-65 | 7.63E-64 | -1.639 |
| RHSIM_Rhsim07G0124400 | 290.04 | 4.88E-65 | 8.09E-64 | 1.248 |
| RHSIM_Rhsim06G0156100 | 289.98 | 5.02E-65 | 8.32E-64 | -6.091 |

| Gene | LRT statistic | P value | BH-adjusted P | log2FC (interaction) |
|---|---|---|---|---|
| RHSIM_Rhsim13G0047200 | 289.42 | 6.65E-65 | 1.10E-63 | 1.295 |
| RHSIM_Rhsim06G0154200 | 289.32 | 6.98E-65 | 1.16E-63 | 1.445 |
| RHSIM_Rhsim02G0122200 | 289.22 | 7.35E-65 | 1.22E-63 | 1.023 |
| RHSIM_Rhsim07G0023500 | 288.93 | 8.50E-65 | 1.41E-63 | -2.246 |
| RHSIM_Rhsim04G0081500 | 288.68 | 9.65E-65 | 1.60E-63 | -4.826 |
| RHSIM_Rhsim02G0090100 | 288.68 | 9.66E-65 | 1.60E-63 | -1.469 |
| RHSIM_Rhsim06G0011000 | 288.58 | 1.01E-64 | 1.67E-63 | 1.199 |
| RHSIM_RhsimUnG0024500 | 288.18 | 1.24E-64 | 2.04E-63 | 11.940 |
| RHSIM_Rhsim10G0005400 | 287.82 | 1.49E-64 | 2.45E-63 | 3.281 |
| RHSIM_Rhsim13G0166000 | 287.80 | 1.50E-64 | 2.48E-63 | 4.284 |
| novel.1839 | 287.71 | 1.57E-64 | 2.58E-63 | -0.843 |
| RHSIM_Rhsim09G0024500 | 287.47 | 1.77E-64 | 2.92E-63 | 0.923 |
| RHSIM_Rhsim02G0158200 | 287.17 | 2.06E-64 | 3.38E-63 | -1.465 |
| RHSIM_Rhsim02G0088100 | 286.66 | 2.66E-64 | 4.38E-63 | 2.010 |
| RHSIM_Rhsim10G0154700 | 286.58 | 2.76E-64 | 4.55E-63 | 1.658 |
| novel.1590 | 286.56 | 2.80E-64 | 4.60E-63 | -2.942 |
| RHSIM_Rhsim12G0172700 | 286.53 | 2.83E-64 | 4.65E-63 | 2.894 |
| RHSIM_Rhsim01G0264500 | 286.42 | 3.00E-64 | 4.92E-63 | 2.422 |
| RHSIM_Rhsim02G0220400 | 286.18 | 3.38E-64 | 5.54E-63 | -3.150 |
| RHSIM_Rhsim07G0178600 | 286.16 | 3.41E-64 | 5.59E-63 | 1.687 |
| novel.4342 | 286.06 | 3.58E-64 | 5.87E-63 | 6.039 |
| RHSIM_Rhsim13G0015500 | 285.53 | 4.68E-64 | 7.67E-63 | 1.676 |
| RHSIM_Rhsim05G0090500 | 285.23 | 5.44E-64 | 8.90E-63 | 1.301 |
| RHSIM_RhsimUnG0253100 | 284.99 | 6.14E-64 | 1.00E-62 | 1.496 |
| RHSIM_Rhsim11G0191300 | 284.76 | 6.88E-64 | 1.12E-62 | 1.617 |
| RHSIM_Rhsim01G0185300 | 284.74 | 6.97E-64 | 1.14E-62 | -1.857 |
| RHSIM_Rhsim04G0223000 | 284.42 | 8.15E-64 | 1.33E-62 | 1.206 |
| RHSIM_Rhsim04G0211500 | 284.23 | 8.98E-64 | 1.47E-62 | -1.441 |
| RHSIM_Rhsim13G0176900 | 283.96 | 1.03E-63 | 1.68E-62 | 1.442 |
| RHSIM_Rhsim03G0058600 | 283.95 | 1.04E-63 | 1.69E-62 | -1.344 |
| RHSIM_Rhsim04G0182600 | 283.20 | 1.51E-63 | 2.46E-62 | -0.659 |
| RHSIM_Rhsim02G0093900 | 282.88 | 1.77E-63 | 2.88E-62 | 2.132 |

| Gene | LRT statistic | P value | BH-adjusted P | log2FC (interaction) |
|---|---|---|---|---|
| RHSIM_Rhsim07G0257800 | 282.83 | 1.81E-63 | 2.95E-62 | -1.947 |
| RHSIM_Rhsim01G0125500 | 282.52 | 2.12E-63 | 3.45E-62 | 3.377 |
| RHSIM_Rhsim07G0200600 | 282.28 | 2.39E-63 | 3.88E-62 | -2.053 |
| RHSIM_Rhsim13G0024000 | 282.04 | 2.70E-63 | 4.39E-62 | -2.981 |
| novel.4509 | 282.03 | 2.71E-63 | 4.41E-62 | -8.860 |
| RHSIM_Rhsim13G0094500 | 281.82 | 3.01E-63 | 4.88E-62 | 1.712 |
| RHSIM_Rhsim01G0200900 | 281.55 | 3.45E-63 | 5.59E-62 | -2.735 |
| RHSIM_Rhsim07G0092900 | 281.54 | 3.47E-63 | 5.62E-62 | 1.301 |
| RHSIM_Rhsim04G0172800 | 281.51 | 3.51E-63 | 5.69E-62 | -2.423 |
| RHSIM_Rhsim06G0197700 | 281.42 | 3.68E-63 | 5.97E-62 | 3.402 |
| RHSIM_Rhsim02G0165500 | 281.32 | 3.87E-63 | 6.26E-62 | 2.506 |
| RHSIM_Rhsim07G0020200 | 281.32 | 3.87E-63 | 6.26E-62 | 2.266 |
| RHSIM_Rhsim03G0052800 | 281.02 | 4.50E-63 | 7.27E-62 | 2.647 |
| RHSIM_Rhsim04G0045900 | 280.99 | 4.58E-63 | 7.39E-62 | 5.501 |
| novel.2205 | 280.88 | 4.84E-63 | 7.81E-62 | -1.532 |
| RHSIM_Rhsim08G0232200 | 280.77 | 5.11E-63 | 8.25E-62 | -1.940 |
| RHSIM_RhsimUnG0126300 | 280.61 | 5.53E-63 | 8.91E-62 | 1.434 |
| RHSIM_Rhsim08G0111300 | 280.59 | 5.59E-63 | 9.01E-62 | 1.359 |
| RHSIM_Rhsim06G0036900 | 280.39 | 6.18E-63 | 9.95E-62 | 1.544 |
| RHSIM_Rhsim13G0017800 | 280.37 | 6.22E-63 | 1.00E-61 | 1.545 |
| RHSIM_Rhsim06G0086900 | 280.15 | 6.95E-63 | 1.12E-61 | 1.174 |
| RHSIM_Rhsim03G0218100 | 279.90 | 7.89E-63 | 1.27E-61 | 1.900 |
| RHSIM_Rhsim01G0134500 | 279.37 | 1.03E-62 | 1.65E-61 | 0.755 |
| novel.2335 | 279.37 | 1.03E-62 | 1.65E-61 | 1.537 |
| RHSIM_Rhsim02G0045600 | 279.00 | 1.24E-62 | 1.99E-61 | 1.751 |
| RHSIM_Rhsim12G0107100 | 278.94 | 1.28E-62 | 2.05E-61 | 1.001 |
| RHSIM_Rhsim06G0169800 | 278.71 | 1.43E-62 | 2.30E-61 | 0.835 |
| RHSIM_Rhsim08G0230700 | 278.53 | 1.57E-62 | 2.52E-61 | 2.992 |
| RHSIM_RhsimUnG0086600 | 278.41 | 1.67E-62 | 2.67E-61 | -2.853 |
| RHSIM_Rhsim10G0157400 | 278.26 | 1.80E-62 | 2.88E-61 | 3.536 |
| RHSIM_Rhsim05G0186900 | 278.15 | 1.90E-62 | 3.04E-61 | -4.371 |
| RHSIM_Rhsim12G0015000 | 278.04 | 2.00E-62 | 3.20E-61 | 1.351 |

| Gene | LRT statistic | P value | BH-adjusted P | log2FC (interaction) |
|---|---|---|---|---|
| RHSIM_Rhsim04G0121900 | 277.86 | 2.20E-62 | 3.51E-61 | 1.368 |
| RHSIM_RhsimPtG0005800 | 277.75 | 2.32E-62 | 3.71E-61 | 1.941 |
| RHSIM_Rhsim11G0165500 | 277.74 | 2.34E-62 | 3.73E-61 | -7.043 |
| RHSIM_Rhsim02G0231700 | 277.43 | 2.73E-62 | 4.35E-61 | 1.419 |
| RHSIM_Rhsim09G0190300 | 277.25 | 2.98E-62 | 4.75E-61 | -1.529 |
| RHSIM_Rhsim08G0173100 | 277.04 | 3.32E-62 | 5.29E-61 | -1.237 |
| RHSIM_Rhsim08G0113600 | 277.01 | 3.37E-62 | 5.37E-61 | 1.954 |
| RHSIM_Rhsim03G0081200 | 276.78 | 3.77E-62 | 6.01E-61 | -1.615 |
| RHSIM_Rhsim09G0201200 | 276.43 | 4.49E-62 | 7.15E-61 | -1.153 |
| RHSIM_Rhsim01G0038600 | 276.05 | 5.46E-62 | 8.68E-61 | -1.193 |
| RHSIM_Rhsim12G0107300 | 276.04 | 5.48E-62 | 8.71E-61 | 3.464 |
| RHSIM_Rhsim09G0061400 | 275.92 | 5.83E-62 | 9.25E-61 | -0.907 |
| RHSIM_Rhsim13G0219700 | 275.84 | 6.06E-62 | 9.63E-61 | -1.102 |
| RHSIM_Rhsim01G0255600 | 275.66 | 6.62E-62 | 1.05E-60 | 3.476 |
| RHSIM_Rhsim13G0200900 | 275.58 | 6.90E-62 | 1.09E-60 | 1.867 |
| RHSIM_Rhsim04G0241500 | 275.50 | 7.20E-62 | 1.14E-60 | 1.634 |
| RHSIM_Rhsim05G0012100 | 275.25 | 8.12E-62 | 1.29E-60 | -0.998 |
| RHSIM_RhsimUnG0217100 | 274.84 | 1.00E-61 | 1.58E-60 | -3.088 |
| RHSIM_Rhsim02G0081500 | 274.63 | 1.11E-61 | 1.76E-60 | 1.813 |
| RHSIM_Rhsim03G0246400 | 274.59 | 1.14E-61 | 1.80E-60 | -2.552 |
| RHSIM_Rhsim05G0028800 | 274.46 | 1.21E-61 | 1.91E-60 | -2.141 |
| RHSIM_Rhsim10G0101000 | 274.46 | 1.21E-61 | 1.91E-60 | -2.671 |
| RHSIM_Rhsim07G0144700 | 274.25 | 1.34E-61 | 2.12E-60 | -1.723 |
| RHSIM_Rhsim12G0135000 | 274.21 | 1.37E-61 | 2.17E-60 | 1.229 |
| RHSIM_Rhsim13G0051100 | 274.10 | 1.45E-61 | 2.29E-60 | 1.155 |
| RHSIM_Rhsim13G0119400 | 273.57 | 1.89E-61 | 2.98E-60 | 1.719 |
| RHSIM_Rhsim12G0039900 | 273.50 | 1.96E-61 | 3.09E-60 | -2.685 |
| RHSIM_Rhsim06G0115300 | 273.38 | 2.08E-61 | 3.28E-60 | 2.320 |
| RHSIM_Rhsim03G0178400 | 273.30 | 2.16E-61 | 3.41E-60 | -1.249 |
| RHSIM_Rhsim13G0084100 | 273.17 | 2.31E-61 | 3.64E-60 | -0.712 |
| RHSIM_Rhsim03G0225300 | 272.68 | 2.96E-61 | 4.65E-60 | 1.333 |
| RHSIM_Rhsim08G0026700 | 272.57 | 3.13E-61 | 4.92E-60 | -0.943 |

| Gene | LRT statistic | P value | BH-adjusted P | log2FC (interaction) |
|---|---|---|---|---|
| RHSIM_Rhsim09G0138200 | 272.49 | 3.25E-61 | 5.11E-60 | -1.798 |
| RHSIM_Rhsim07G0226400 | 272.47 | 3.29E-61 | 5.17E-60 | -4.016 |
| RHSIM_Rhsim09G0087100 | 272.25 | 3.66E-61 | 5.74E-60 | 2.112 |
| RHSIM_Rhsim09G0151600 | 272.21 | 3.75E-61 | 5.88E-60 | -2.734 |
| RHSIM_Rhsim10G0169000 | 272.20 | 3.76E-61 | 5.89E-60 | 1.124 |
| RHSIM_Rhsim11G0183200 | 272.11 | 3.94E-61 | 6.17E-60 | 3.411 |
| RHSIM_Rhsim13G0001100 | 271.77 | 4.68E-61 | 7.32E-60 | 1.605 |
| RHSIM_Rhsim07G0085100 | 271.08 | 6.59E-61 | 1.03E-59 | -0.991 |
| novel.1184 | 271.02 | 6.79E-61 | 1.06E-59 | -1.836 |
| RHSIM_Rhsim09G0140700 | 270.51 | 8.77E-61 | 1.37E-59 | -0.763 |
| RHSIM_Rhsim05G0012000 | 270.42 | 9.20E-61 | 1.44E-59 | -0.917 |
| RHSIM_Rhsim03G0233500 | 269.96 | 1.16E-60 | 1.80E-59 | -1.761 |
| RHSIM_Rhsim12G0044700 | 269.89 | 1.20E-60 | 1.87E-59 | -3.910 |
| RHSIM_Rhsim09G0068900 | 269.44 | 1.50E-60 | 2.34E-59 | 1.541 |
| RHSIM_Rhsim09G0113200 | 269.17 | 1.72E-60 | 2.68E-59 | 0.970 |
| RHSIM_Rhsim13G0153900 | 268.99 | 1.89E-60 | 2.94E-59 | 1.439 |
| RHSIM_Rhsim06G0184600 | 268.95 | 1.92E-60 | 2.99E-59 | -2.051 |
| RHSIM_Rhsim01G0212300 | 268.90 | 1.97E-60 | 3.07E-59 | 1.481 |
| RHSIM_Rhsim08G0007300 | 268.65 | 2.23E-60 | 3.47E-59 | -2.264 |
| RHSIM_Rhsim01G0067100 | 268.47 | 2.44E-60 | 3.80E-59 | -1.619 |
| RHSIM_Rhsim12G0051400 | 268.39 | 2.55E-60 | 3.96E-59 | -1.073 |
| RHSIM_Rhsim08G0081400 | 268.37 | 2.57E-60 | 3.99E-59 | 1.977 |
| RHSIM_Rhsim09G0077000 | 268.14 | 2.88E-60 | 4.47E-59 | 1.124 |
| RHSIM_Rhsim07G0068200 | 267.89 | 3.26E-60 | 5.06E-59 | 1.607 |
| RHSIM_Rhsim01G0176100 | 267.80 | 3.42E-60 | 5.30E-59 | 1.078 |
| RHSIM_Rhsim05G0111000 | 267.72 | 3.57E-60 | 5.53E-59 | 2.215 |
| RHSIM_RhsimUnG0205900 | 267.58 | 3.83E-60 | 5.93E-59 | 1.010 |
| RHSIM_Rhsim07G0217300 | 267.52 | 3.94E-60 | 6.10E-59 | 2.005 |
| RHSIM_Rhsim07G0162600 | 267.32 | 4.35E-60 | 6.73E-59 | 1.785 |
| novel.1545 | 266.98 | 5.17E-60 | 7.99E-59 | -3.864 |
| RHSIM_RhsimUnG0253300 | 266.60 | 6.24E-60 | 9.64E-59 | 1.016 |
| RHSIM_Rhsim01G0203700 | 266.45 | 6.75E-60 | 1.04E-58 | 2.851 |

| Gene | LRT statistic | P value | BH-adjusted P | log2FC (interaction) |
|---|---|---|---|---|
| novel.1300 | 266.42 | 6.85E-60 | 1.06E-58 | -4.971 |
| RHSIM_RhsimUnG0251300 | 266.05 | 8.25E-60 | 1.27E-58 | 1.399 |
| RHSIM_Rhsim07G0255100 | 266.05 | 8.26E-60 | 1.27E-58 | 1.186 |
| RHSIM_Rhsim04G0174300 | 265.79 | 9.37E-60 | 1.44E-58 | 2.535 |
| RHSIM_Rhsim10G0174200 | 265.57 | 1.05E-59 | 1.61E-58 | -3.239 |
| RHSIM_Rhsim12G0003000 | 265.33 | 1.18E-59 | 1.82E-58 | 1.141 |
| RHSIM_Rhsim06G0023100 | 265.15 | 1.30E-59 | 2.00E-58 | -2.476 |
| RHSIM_Rhsim10G0171300 | 265.13 | 1.31E-59 | 2.01E-58 | 1.654 |
| RHSIM_Rhsim04G0049500 | 264.83 | 1.52E-59 | 2.34E-58 | -2.176 |
| RHSIM_Rhsim08G0243800 | 264.79 | 1.55E-59 | 2.38E-58 | 1.152 |
| RHSIM_Rhsim07G0234800 | 264.72 | 1.60E-59 | 2.46E-58 | 1.168 |
| RHSIM_Rhsim04G0136700 | 264.36 | 1.92E-59 | 2.95E-58 | -4.720 |
| novel.888 | 264.34 | 1.95E-59 | 2.98E-58 | -1.969 |
| novel.2732 | 264.04 | 2.26E-59 | 3.47E-58 | -1.307 |
| RHSIM_Rhsim09G0058800 | 264.03 | 2.28E-59 | 3.49E-58 | 1.789 |
| RHSIM_Rhsim03G0041200 | 263.94 | 2.38E-59 | 3.65E-58 | 1.701 |
| RHSIM_Rhsim11G0164100 | 263.90 | 2.42E-59 | 3.70E-58 | -2.037 |
| RHSIM_Rhsim07G0010000 | 263.61 | 2.80E-59 | 4.29E-58 | -3.924 |
| RHSIM_Rhsim05G0176200 | 263.24 | 3.37E-59 | 5.16E-58 | 1.931 |
| RHSIM_Rhsim04G0113800 | 263.20 | 3.45E-59 | 5.27E-58 | 1.391 |
| novel.3275 | 263.04 | 3.74E-59 | 5.71E-58 | -1.448 |
| RHSIM_Rhsim07G0034100 | 262.69 | 4.44E-59 | 6.78E-58 | -1.186 |
| RHSIM_RhsimUnG0060400 | 262.62 | 4.61E-59 | 7.03E-58 | -1.781 |
| RHSIM_Rhsim04G0157100 | 262.38 | 5.19E-59 | 7.91E-58 | -1.218 |
| RHSIM_Rhsim07G0081800 | 262.20 | 5.69E-59 | 8.67E-58 | 1.141 |
| RHSIM_Rhsim07G0214900 | 262.14 | 5.85E-59 | 8.92E-58 | -1.646 |
| RHSIM_Rhsim04G0030200 | 262.05 | 6.13E-59 | 9.32E-58 | 1.638 |
| RHSIM_Rhsim07G0004900 | 262.05 | 6.13E-59 | 9.32E-58 | 1.686 |
| RHSIM_Rhsim13G0178800 | 261.90 | 6.60E-59 | 1.00E-57 | -4.752 |
| novel.3736 | 261.86 | 6.76E-59 | 1.03E-57 | -1.424 |
| RHSIM_RhsimUnG0248500 | 261.79 | 6.98E-59 | 1.06E-57 | 2.087 |
| RHSIM_Rhsim06G0147200 | 261.76 | 7.10E-59 | 1.08E-57 | -1.345 |

| Gene | LRT statistic | P value | BH-adjusted P | log2FC (interaction) |
|---|---|---|---|---|
| RHSIM_Rhsim10G0067500 | 261.73 | 7.19E-59 | 1.09E-57 | -0.875 |
| RHSIM_Rhsim08G0034200 | 261.55 | 7.88E-59 | 1.19E-57 | -2.237 |
| RHSIM_Rhsim02G0201200 | 261.53 | 7.96E-59 | 1.21E-57 | 1.057 |
| RHSIM_Rhsim13G0015300 | 261.49 | 8.12E-59 | 1.23E-57 | 0.922 |
| novel.377 | 261.48 | 8.17E-59 | 1.24E-57 | -1.051 |
| novel.1137 | 261.44 | 8.32E-59 | 1.26E-57 | -2.105 |
| RHSIM_Rhsim01G0252400 | 261.37 | 8.62E-59 | 1.30E-57 | -1.310 |
| RHSIM_Rhsim10G0029800 | 261.12 | 9.79E-59 | 1.48E-57 | -3.172 |
| RHSIM_Rhsim01G0171700 | 260.82 | 1.14E-58 | 1.72E-57 | 1.833 |
| RHSIM_Rhsim04G0057100 | 260.67 | 1.23E-58 | 1.85E-57 | 3.076 |
| RHSIM_Rhsim07G0179600 | 260.42 | 1.39E-58 | 2.09E-57 | 1.225 |
| RHSIM_Rhsim06G0175200 | 260.28 | 1.49E-58 | 2.25E-57 | -3.650 |
| RHSIM_Rhsim05G0161500 | 260.25 | 1.51E-58 | 2.28E-57 | -2.374 |
| RHSIM_Rhsim06G0086800 | 260.17 | 1.57E-58 | 2.37E-57 | 1.458 |
| RHSIM_RhsimUnG0217500 | 260.09 | 1.64E-58 | 2.47E-57 | 2.080 |
| RHSIM_Rhsim13G0141500 | 259.99 | 1.72E-58 | 2.59E-57 | -5.100 |
| RHSIM_Rhsim07G0229000 | 259.60 | 2.10E-58 | 3.15E-57 | -3.804 |
| RHSIM_Rhsim11G0173400 | 259.55 | 2.15E-58 | 3.23E-57 | 1.241 |
| RHSIM_Rhsim13G0083300 | 259.34 | 2.39E-58 | 3.59E-57 | -1.536 |
| RHSIM_Rhsim09G0121900 | 258.68 | 3.34E-58 | 5.01E-57 | -1.835 |
| RHSIM_Rhsim13G0199600 | 258.61 | 3.44E-58 | 5.17E-57 | 1.112 |
| RHSIM_Rhsim13G0228200 | 258.61 | 3.45E-58 | 5.18E-57 | 1.290 |
| RHSIM_Rhsim03G0141500 | 258.60 | 3.47E-58 | 5.20E-57 | -1.110 |
| RHSIM_Rhsim12G0210000 | 258.59 | 3.48E-58 | 5.21E-57 | -1.271 |
| RHSIM_Rhsim01G0001400 | 258.23 | 4.16E-58 | 6.24E-57 | 1.645 |
| RHSIM_Rhsim04G0114100 | 258.23 | 4.18E-58 | 6.25E-57 | 1.239 |
| RHSIM_Rhsim10G0202500 | 257.96 | 4.77E-58 | 7.13E-57 | -2.401 |
| RHSIM_Rhsim01G0195500 | 257.84 | 5.08E-58 | 7.60E-57 | 1.019 |
| novel.356 | 257.63 | 5.63E-58 | 8.42E-57 | -1.239 |
| RHSIM_Rhsim13G0233300 | 257.43 | 6.22E-58 | 9.29E-57 | 1.808 |
| RHSIM_Rhsim12G0131800 | 257.03 | 7.61E-58 | 1.14E-56 | 1.353 |
| RHSIM_Rhsim09G0180300 | 257.00 | 7.74E-58 | 1.16E-56 | -2.692 |

| Gene | LRT statistic | P value | BH-adjusted P | log2FC (interaction) |
|---|---|---|---|---|
| RHSIM_Rhsim07G0221000 | 256.75 | 8.78E-58 | 1.31E-56 | 1.242 |
| RHSIM_Rhsim05G0201700 | 255.74 | 1.46E-57 | 2.17E-56 | -5.447 |
| RHSIM_Rhsim06G0161200 | 255.49 | 1.65E-57 | 2.46E-56 | 1.360 |
| RHSIM_Rhsim10G0156600 | 254.90 | 2.22E-57 | 3.30E-56 | 2.667 |
| RHSIM_Rhsim07G0052500 | 254.85 | 2.27E-57 | 3.38E-56 | -1.364 |
| RHSIM_Rhsim08G0015900 | 254.77 | 2.37E-57 | 3.53E-56 | -2.106 |
| RHSIM_Rhsim03G0151200 | 254.54 | 2.65E-57 | 3.95E-56 | 1.041 |
| RHSIM_Rhsim06G0235700 | 254.42 | 2.83E-57 | 4.20E-56 | -1.735 |
| novel.4329 | 254.37 | 2.89E-57 | 4.30E-56 | -4.684 |
| RHSIM_RhsimUnG0194300 | 254.31 | 2.98E-57 | 4.43E-56 | -1.712 |
| RHSIM_Rhsim02G0086400 | 254.30 | 3.00E-57 | 4.46E-56 | -2.448 |
| RHSIM_Rhsim04G0032800 | 254.25 | 3.07E-57 | 4.55E-56 | -0.827 |
| RHSIM_Rhsim13G0075500 | 254.23 | 3.11E-57 | 4.61E-56 | -4.875 |
| novel.1020 | 254.20 | 3.16E-57 | 4.68E-56 | 1.919 |
| RHSIM_Rhsim05G0217100 | 253.74 | 3.98E-57 | 5.90E-56 | 1.916 |
| RHSIM_Rhsim05G0196000 | 253.33 | 4.88E-57 | 7.23E-56 | -1.071 |
| RHSIM_Rhsim10G0009700 | 253.21 | 5.18E-57 | 7.66E-56 | 1.055 |
| RHSIM_Rhsim04G0233400 | 253.06 | 5.58E-57 | 8.25E-56 | -1.489 |
| RHSIM_Rhsim13G0083200 | 252.85 | 6.20E-57 | 9.17E-56 | -0.950 |
| RHSIM_Rhsim08G0032400 | 252.43 | 7.66E-57 | 1.13E-55 | -1.438 |
| RHSIM_Rhsim10G0082000 | 252.37 | 7.90E-57 | 1.17E-55 | -1.768 |
| RHSIM_Rhsim12G0079500 | 252.28 | 8.26E-57 | 1.22E-55 | -2.167 |
| RHSIM_Rhsim12G0121900 | 252.27 | 8.31E-57 | 1.23E-55 | -1.311 |
| RHSIM_Rhsim09G0056100 | 252.10 | 9.07E-57 | 1.34E-55 | 1.681 |
| RHSIM_Rhsim11G0136000 | 252.01 | 9.47E-57 | 1.40E-55 | -1.846 |
| RHSIM_Rhsim08G0094900 | 252.00 | 9.52E-57 | 1.40E-55 | -1.629 |
| RHSIM_Rhsim09G0111100 | 251.85 | 1.03E-56 | 1.51E-55 | -1.482 |
| RHSIM_Rhsim04G0209600 | 251.81 | 1.04E-56 | 1.54E-55 | 1.263 |
| RHSIM_Rhsim11G0070000 | 251.74 | 1.08E-56 | 1.59E-55 | -1.307 |
| RHSIM_Rhsim10G0099300 | 251.54 | 1.20E-56 | 1.77E-55 | 2.730 |
| RHSIM_Rhsim08G0209600 | 251.52 | 1.21E-56 | 1.78E-55 | 1.989 |
| RHSIM_Rhsim12G0190700 | 251.37 | 1.30E-56 | 1.91E-55 | 1.311 |

| Gene | LRT statistic | P value | BH-adjusted P | log2FC (interaction) |
|---|---|---|---|---|
| RHSIM_Rhsim11G0055900 | 251.30 | 1.35E-56 | 1.98E-55 | -4.145 |
| RHSIM_RhsimUnG0055300 | 251.11 | 1.49E-56 | 2.18E-55 | -1.973 |
| RHSIM_Rhsim05G0133200 | 251.11 | 1.49E-56 | 2.18E-55 | -1.023 |
| RHSIM_Rhsim13G0101900 | 251.09 | 1.50E-56 | 2.20E-55 | 1.251 |
| RHSIM_Rhsim09G0178000 | 250.85 | 1.69E-56 | 2.48E-55 | -2.864 |
| novel.2695 | 250.67 | 1.85E-56 | 2.71E-55 | 0.858 |
| RHSIM_Rhsim13G0028500 | 250.19 | 2.36E-56 | 3.45E-55 | -1.285 |
| RHSIM_Rhsim06G0216800 | 249.91 | 2.72E-56 | 3.98E-55 | -2.350 |
| RHSIM_Rhsim01G0235800 | 249.78 | 2.90E-56 | 4.24E-55 | 1.283 |
| RHSIM_Rhsim02G0107700 | 249.41 | 3.50E-56 | 5.11E-55 | -2.335 |
| RHSIM_Rhsim02G0057300 | 249.38 | 3.54E-56 | 5.18E-55 | -1.806 |
| RHSIM_Rhsim11G0065700 | 249.31 | 3.67E-56 | 5.35E-55 | 1.418 |
| RHSIM_Rhsim11G0167600 | 249.06 | 4.16E-56 | 6.06E-55 | -1.782 |
| RHSIM_Rhsim07G0076300 | 249.00 | 4.29E-56 | 6.25E-55 | 1.547 |
| RHSIM_Rhsim01G0107800 | 248.84 | 4.65E-56 | 6.77E-55 | -0.890 |
| RHSIM_Rhsim08G0181000 | 248.78 | 4.79E-56 | 6.98E-55 | -6.430 |
| RHSIM_RhsimUnG0024300 | 248.68 | 5.05E-56 | 7.34E-55 | 10.330 |
| RHSIM_Rhsim08G0228800 | 248.03 | 6.97E-56 | 1.01E-54 | 1.092 |
| novel.4103 | 247.97 | 7.20E-56 | 1.05E-54 | -1.270 |
| RHSIM_Rhsim08G0156700 | 247.25 | 1.03E-55 | 1.50E-54 | -4.325 |
| RHSIM_RhsimUnG0043200 | 246.62 | 1.41E-55 | 2.05E-54 | -1.046 |
| RHSIM_Rhsim10G0003500 | 246.60 | 1.43E-55 | 2.07E-54 | 1.642 |
| RHSIM_Rhsim02G0070000 | 246.39 | 1.59E-55 | 2.31E-54 | -1.596 |
| RHSIM_Rhsim13G0026200 | 246.24 | 1.71E-55 | 2.48E-54 | -1.339 |
| RHSIM_Rhsim07G0234400 | 245.95 | 1.99E-55 | 2.88E-54 | 0.937 |
| RHSIM_Rhsim11G0176000 | 245.28 | 2.78E-55 | 4.03E-54 | -0.862 |
| RHSIM_Rhsim04G0059900 | 245.20 | 2.88E-55 | 4.18E-54 | -2.224 |
| RHSIM_Rhsim11G0165400 | 244.87 | 3.40E-55 | 4.93E-54 | -2.020 |
| RHSIM_Rhsim04G0051800 | 244.78 | 3.57E-55 | 5.16E-54 | -1.622 |
| RHSIM_RhsimUnG0062700 | 244.73 | 3.66E-55 | 5.29E-54 | -2.116 |
| RHSIM_Rhsim10G0099800 | 244.60 | 3.90E-55 | 5.63E-54 | -1.462 |
| RHSIM_RhsimUnG0087900 | 244.43 | 4.26E-55 | 6.15E-54 | -2.083 |

| Gene | LRT statistic | P value | BH-adjusted P | log2FC (interaction) |
|---|---|---|---|---|
| RHSIM_Rhsim03G0230400 | 244.30 | 4.54E-55 | 6.55E-54 | 1.747 |
| novel.13 | 244.26 | 4.62E-55 | 6.67E-54 | 1.045 |
| RHSIM_Rhsim08G0153100 | 244.26 | 4.63E-55 | 6.68E-54 | 0.966 |
| RHSIM_Rhsim12G0104400 | 243.93 | 5.45E-55 | 7.86E-54 | -2.539 |
| RHSIM_Rhsim11G0089600 | 243.90 | 5.54E-55 | 7.98E-54 | -1.116 |
| RHSIM_Rhsim03G0161400 | 243.86 | 5.68E-55 | 8.17E-54 | 1.316 |
| RHSIM_Rhsim10G0102900 | 243.77 | 5.91E-55 | 8.51E-54 | 1.357 |
| RHSIM_Rhsim13G0087900 | 243.70 | 6.13E-55 | 8.82E-54 | -1.689 |
| RHSIM_Rhsim08G0121500 | 243.63 | 6.36E-55 | 9.15E-54 | 1.975 |
| RHSIM_Rhsim12G0126100 | 243.14 | 8.13E-55 | 1.17E-53 | 3.382 |
| RHSIM_Rhsim03G0106200 | 243.08 | 8.39E-55 | 1.20E-53 | -1.894 |
| RHSIM_RhsimUnG0055900 | 242.96 | 8.88E-55 | 1.28E-53 | 0.986 |
| RHSIM_Rhsim05G0227600 | 242.84 | 9.45E-55 | 1.36E-53 | -1.310 |
| RHSIM_Rhsim10G0051100 | 242.63 | 1.05E-54 | 1.51E-53 | -2.495 |
| RHSIM_Rhsim13G0123900 | 242.62 | 1.05E-54 | 1.51E-53 | 0.887 |
| RHSIM_Rhsim10G0134900 | 242.44 | 1.16E-54 | 1.66E-53 | 1.597 |
| RHSIM_Rhsim06G0166500 | 242.36 | 1.21E-54 | 1.73E-53 | -0.931 |
| RHSIM_RhsimUnG0142400 | 242.35 | 1.21E-54 | 1.73E-53 | 4.277 |
| RHSIM_Rhsim07G0038700 | 242.32 | 1.23E-54 | 1.75E-53 | 1.189 |
| RHSIM_Rhsim01G0092000 | 242.28 | 1.25E-54 | 1.79E-53 | 1.528 |
| RHSIM_Rhsim05G0016600 | 242.16 | 1.33E-54 | 1.90E-53 | 1.269 |
| RHSIM_Rhsim08G0111800 | 242.12 | 1.35E-54 | 1.94E-53 | -1.141 |
| RHSIM_Rhsim09G0013200 | 241.92 | 1.50E-54 | 2.14E-53 | -2.187 |
| RHSIM_Rhsim07G0171800 | 241.85 | 1.55E-54 | 2.22E-53 | 1.650 |
| RHSIM_Rhsim08G0225000 | 241.82 | 1.58E-54 | 2.25E-53 | 1.846 |
| RHSIM_Rhsim07G0044500 | 241.78 | 1.61E-54 | 2.30E-53 | -46.628 |
| RHSIM_Rhsim13G0141800 | 241.68 | 1.69E-54 | 2.41E-53 | 1.415 |
| RHSIM_Rhsim02G0240800 | 241.65 | 1.72E-54 | 2.45E-53 | 1.537 |
| RHSIM_Rhsim05G0116000 | 241.62 | 1.74E-54 | 2.48E-53 | 1.757 |
| RHSIM_RhsimUnG0020400 | 241.61 | 1.75E-54 | 2.49E-53 | -1.170 |
| RHSIM_Rhsim01G0226900 | 241.58 | 1.78E-54 | 2.53E-53 | -0.882 |
| RHSIM_Rhsim04G0062100 | 241.48 | 1.87E-54 | 2.66E-53 | 1.446 |

| Gene | LRT statistic | P value | BH-adjusted P | log2FC (interaction) |
|---|---|---|---|---|
| RHSIM_Rhsim09G0003600 | 241.25 | 2.10E-54 | 2.99E-53 | 0.841 |
| RHSIM_Rhsim09G0209600 | 240.89 | 2.51E-54 | 3.57E-53 | 1.504 |
| novel.2226 | 240.48 | 3.08E-54 | 4.38E-53 | -1.116 |
| RHSIM_Rhsim01G0096800 | 240.34 | 3.31E-54 | 4.70E-53 | 1.397 |
| RHSIM_Rhsim02G0213300 | 240.23 | 3.51E-54 | 4.98E-53 | -0.785 |
| RHSIM_Rhsim09G0143800 | 239.92 | 4.09E-54 | 5.79E-53 | 1.333 |
| RHSIM_Rhsim01G0088700 | 239.44 | 5.22E-54 | 7.39E-53 | -7.124 |
| RHSIM_Rhsim07G0013800 | 239.37 | 5.40E-54 | 7.64E-53 | 0.999 |
| RHSIM_Rhsim13G0029600 | 238.91 | 6.78E-54 | 9.60E-53 | 0.767 |
| novel.2155 | 238.88 | 6.88E-54 | 9.74E-53 | 1.211 |
| RHSIM_Rhsim04G0071600 | 238.61 | 7.90E-54 | 1.12E-52 | -5.976 |
| RHSIM_Rhsim04G0220400 | 238.48 | 8.45E-54 | 1.19E-52 | 1.403 |
| RHSIM_Rhsim10G0104100 | 238.16 | 9.93E-54 | 1.40E-52 | -1.169 |
| RHSIM_Rhsim07G0162400 | 238.12 | 1.01E-53 | 1.42E-52 | -6.197 |
| RHSIM_Rhsim02G0142400 | 237.39 | 1.46E-53 | 2.06E-52 | 0.892 |
| RHSIM_Rhsim09G0191200 | 237.15 | 1.65E-53 | 2.32E-52 | -1.255 |
| novel.2148 | 236.98 | 1.79E-53 | 2.52E-52 | 3.721 |
| RHSIM_Rhsim05G0180400 | 236.79 | 1.97E-53 | 2.77E-52 | 0.907 |
| RHSIM_Rhsim02G0180500 | 236.24 | 2.59E-53 | 3.65E-52 | -0.922 |
| RHSIM_Rhsim05G0029900 | 235.83 | 3.20E-53 | 4.50E-52 | -0.776 |
| RHSIM_Rhsim07G0207000 | 235.82 | 3.21E-53 | 4.52E-52 | 1.973 |
| RHSIM_Rhsim12G0106200 | 235.45 | 3.86E-53 | 5.43E-52 | -0.856 |
| RHSIM_Rhsim07G0116700 | 235.42 | 3.92E-53 | 5.51E-52 | 1.370 |
| RHSIM_Rhsim05G0231000 | 235.42 | 3.92E-53 | 5.51E-52 | -0.907 |
| novel.2630 | 235.36 | 4.03E-53 | 5.66E-52 | -6.957 |
| RHSIM_Rhsim02G0105000 | 235.28 | 4.21E-53 | 5.91E-52 | -4.381 |
| RHSIM_Rhsim04G0230000 | 235.16 | 4.46E-53 | 6.25E-52 | 1.814 |
| RHSIM_Rhsim04G0243100 | 235.10 | 4.60E-53 | 6.45E-52 | 1.055 |
| RHSIM_RhsimUnG0204800 | 235.03 | 4.77E-53 | 6.69E-52 | 1.633 |
| RHSIM_Rhsim12G0017400 | 234.72 | 5.56E-53 | 7.79E-52 | 1.890 |
| RHSIM_Rhsim13G0012200 | 234.70 | 5.63E-53 | 7.89E-52 | -1.683 |
| RHSIM_Rhsim11G0106000 | 234.53 | 6.12E-53 | 8.57E-52 | -1.877 |

| Gene | LRT statistic | P value | BH-adjusted P | log2FC (interaction) |
|---|---|---|---|---|
| RHSIM_Rhsim01G0060200 | 234.35 | 6.69E-53 | 9.36E-52 | -0.920 |
| RHSIM_Rhsim12G0156100 | 234.24 | 7.09E-53 | 9.92E-52 | 1.354 |
| RHSIM_Rhsim03G0162100 | 233.97 | 8.10E-53 | 1.13E-51 | 1.357 |
| RHSIM_Rhsim08G0041500 | 233.96 | 8.16E-53 | 1.14E-51 | -0.899 |
| RHSIM_Rhsim03G0127200 | 233.82 | 8.77E-53 | 1.22E-51 | 1.005 |
| RHSIM_Rhsim08G0068500 | 233.76 | 9.03E-53 | 1.26E-51 | -1.096 |
| RHSIM_Rhsim08G0010200 | 233.69 | 9.33E-53 | 1.30E-51 | 1.702 |
| RHSIM_Rhsim05G0138700 | 233.68 | 9.38E-53 | 1.31E-51 | 1.707 |
| RHSIM_Rhsim02G0006300 | 233.63 | 9.65E-53 | 1.34E-51 | -3.582 |
| RHSIM_Rhsim05G0159800 | 233.59 | 9.81E-53 | 1.37E-51 | -1.393 |
| RHSIM_Rhsim05G0195200 | 233.55 | 1.00E-52 | 1.39E-51 | -2.465 |
| RHSIM_Rhsim02G0135200 | 233.54 | 1.01E-52 | 1.40E-51 | -2.699 |
| RHSIM_Rhsim08G0023600 | 233.49 | 1.04E-52 | 1.44E-51 | 1.226 |
| RHSIM_Rhsim06G0137600 | 233.30 | 1.13E-52 | 1.58E-51 | 1.591 |
| RHSIM_Rhsim07G0032400 | 233.18 | 1.21E-52 | 1.68E-51 | 1.374 |
| RHSIM_Rhsim05G0109700 | 233.13 | 1.24E-52 | 1.72E-51 | -1.754 |
| novel.3866 | 233.01 | 1.31E-52 | 1.82E-51 | 1.782 |
| RHSIM_Rhsim10G0104000 | 232.94 | 1.36E-52 | 1.89E-51 | -2.500 |
| RHSIM_Rhsim08G0083400 | 232.83 | 1.44E-52 | 1.99E-51 | 0.788 |
| RHSIM_Rhsim12G0140600 | 232.71 | 1.53E-52 | 2.12E-51 | -1.456 |
| RHSIM_Rhsim09G0091900 | 232.52 | 1.68E-52 | 2.32E-51 | 0.960 |
| RHSIM_Rhsim07G0215400 | 232.48 | 1.72E-52 | 2.38E-51 | -1.387 |
| RHSIM_Rhsim06G0067000 | 232.42 | 1.77E-52 | 2.45E-51 | 1.958 |
| RHSIM_Rhsim05G0075500 | 232.23 | 1.95E-52 | 2.69E-51 | -2.595 |
| RHSIM_Rhsim06G0002900 | 232.17 | 2.00E-52 | 2.77E-51 | -1.335 |
| RHSIM_Rhsim02G0109300 | 231.99 | 2.19E-52 | 3.03E-51 | 1.573 |
| RHSIM_Rhsim13G0078600 | 231.30 | 3.11E-52 | 4.30E-51 | 4.805 |
| RHSIM_Rhsim12G0155100 | 231.22 | 3.23E-52 | 4.46E-51 | -5.325 |
| RHSIM_Rhsim11G0130000 | 231.01 | 3.59E-52 | 4.96E-51 | -1.418 |
| RHSIM_Rhsim13G0027900 | 230.79 | 4.02E-52 | 5.54E-51 | -1.721 |
| RHSIM_Rhsim10G0043000 | 230.73 | 4.13E-52 | 5.69E-51 | -2.169 |
| RHSIM_Rhsim04G0071000 | 230.72 | 4.15E-52 | 5.71E-51 | -2.112 |

| Gene | LRT statistic | P value | BH-adjusted P | log2FC (interaction) |
|---|---|---|---|---|
| RHSIM_Rhsim02G0211200 | 230.71 | 4.18E-52 | 5.76E-51 | -7.185 |
| novel.3014 | 230.39 | 4.91E-52 | 6.76E-51 | 1.451 |
| RHSIM_Rhsim12G0141500 | 230.31 | 5.09E-52 | 7.00E-51 | -1.859 |
| RHSIM_RhsimUnG0205800 | 230.22 | 5.35E-52 | 7.35E-51 | 1.001 |
| novel.2264 | 230.20 | 5.41E-52 | 7.43E-51 | 2.577 |
| RHSIM_Rhsim04G0048200 | 229.91 | 6.24E-52 | 8.58E-51 | -0.881 |
| novel.1445 | 229.64 | 7.15E-52 | 9.82E-51 | 1.902 |
| novel.4580 | 229.54 | 7.50E-52 | 1.03E-50 | 4.247 |
| novel.2594 | 229.32 | 8.39E-52 | 1.15E-50 | 4.242 |
| RHSIM_Rhsim03G0035400 | 229.28 | 8.58E-52 | 1.18E-50 | -4.429 |
| RHSIM_Rhsim06G0002800 | 229.15 | 9.12E-52 | 1.25E-50 | -1.737 |
| RHSIM_Rhsim12G0006800 | 229.01 | 9.81E-52 | 1.34E-50 | -1.600 |
| RHSIM_Rhsim07G0252800 | 228.57 | 1.22E-51 | 1.67E-50 | -1.554 |
| RHSIM_Rhsim10G0011000 | 228.34 | 1.37E-51 | 1.88E-50 | -1.872 |
| RHSIM_Rhsim13G0185200 | 228.13 | 1.53E-51 | 2.09E-50 | 1.566 |
| novel.421 | 228.03 | 1.60E-51 | 2.19E-50 | 1.583 |
| RHSIM_Rhsim05G0201000 | 227.94 | 1.68E-51 | 2.29E-50 | -1.436 |
| RHSIM_Rhsim02G0025700 | 227.91 | 1.71E-51 | 2.33E-50 | 1.536 |
| RHSIM_Rhsim07G0087700 | 227.89 | 1.72E-51 | 2.34E-50 | -0.951 |
| RHSIM_Rhsim05G0188200 | 227.72 | 1.87E-51 | 2.56E-50 | 1.095 |
| novel.78 | 227.68 | 1.91E-51 | 2.61E-50 | 2.949 |
| RHSIM_Rhsim03G0166000 | 227.64 | 1.95E-51 | 2.66E-50 | 1.777 |
| RHSIM_Rhsim10G0139800 | 227.61 | 1.98E-51 | 2.70E-50 | 3.706 |
| novel.933 | 227.60 | 1.99E-51 | 2.71E-50 | -3.204 |
| RHSIM_Rhsim07G0144800 | 227.54 | 2.05E-51 | 2.79E-50 | 1.926 |
| RHSIM_Rhsim04G0136600 | 227.45 | 2.14E-51 | 2.92E-50 | -1.358 |
| RHSIM_Rhsim12G0046500 | 227.32 | 2.30E-51 | 3.12E-50 | -1.094 |
| RHSIM_Rhsim08G0241100 | 227.17 | 2.46E-51 | 3.35E-50 | 0.946 |
| RHSIM_Rhsim02G0241800 | 227.16 | 2.48E-51 | 3.36E-50 | -3.014 |
| RHSIM_Rhsim09G0022900 | 226.89 | 2.84E-51 | 3.85E-50 | 1.307 |
| RHSIM_Rhsim06G0166100 | 226.43 | 3.58E-51 | 4.86E-50 | 2.221 |
| novel.649 | 226.39 | 3.65E-51 | 4.95E-50 | 1.030 |

| Gene | LRT statistic | P value | BH-adjusted P | log2FC (interaction) |
|---|---|---|---|---|
| RHSIM_Rhsim09G0178100 | 226.38 | 3.68E-51 | 4.99E-50 | -2.926 |
| RHSIM_Rhsim07G0069600 | 226.25 | 3.91E-51 | 5.31E-50 | 2.401 |
| RHSIM_RhsimUnG0086000 | 226.24 | 3.93E-51 | 5.33E-50 | 1.588 |
| RHSIM_Rhsim06G0211400 | 226.07 | 4.30E-51 | 5.82E-50 | 0.862 |
| RHSIM_Rhsim12G0197000 | 226.03 | 4.38E-51 | 5.92E-50 | 1.213 |
| RHSIM_Rhsim04G0104300 | 225.99 | 4.46E-51 | 6.03E-50 | -2.397 |
| RHSIM_Rhsim12G0058000 | 225.81 | 4.88E-51 | 6.60E-50 | 1.656 |
| RHSIM_Rhsim04G0085200 | 225.81 | 4.90E-51 | 6.62E-50 | -1.420 |
| RHSIM_Rhsim01G0244400 | 225.74 | 5.07E-51 | 6.85E-50 | -0.811 |
| RHSIM_Rhsim02G0005400 | 225.68 | 5.22E-51 | 7.05E-50 | -1.258 |
| RHSIM_Rhsim01G0044000 | 225.16 | 6.77E-51 | 9.14E-50 | 1.311 |
| RHSIM_Rhsim11G0169200 | 224.89 | 7.75E-51 | 1.05E-49 | -1.236 |
| RHSIM_Rhsim04G0011900 | 224.71 | 8.50E-51 | 1.15E-49 | -1.667 |
| RHSIM_Rhsim08G0154800 | 224.68 | 8.63E-51 | 1.16E-49 | 0.707 |
| RHSIM_Rhsim06G0184500 | 224.58 | 9.07E-51 | 1.22E-49 | -2.151 |
| RHSIM_Rhsim04G0199100 | 224.47 | 9.57E-51 | 1.29E-49 | -0.909 |
| RHSIM_Rhsim06G0035400 | 224.46 | 9.62E-51 | 1.29E-49 | 1.093 |
| RHSIM_Rhsim06G0056800 | 224.44 | 9.71E-51 | 1.31E-49 | 1.773 |
| RHSIM_Rhsim02G0005500 | 224.39 | 9.98E-51 | 1.34E-49 | -1.313 |
| RHSIM_Rhsim04G0227300 | 224.18 | 1.11E-50 | 1.49E-49 | -2.278 |
| RHSIM_Rhsim01G0064400 | 224.15 | 1.13E-50 | 1.51E-49 | -3.147 |
| RHSIM_Rhsim06G0046700 | 223.95 | 1.25E-50 | 1.67E-49 | -1.095 |
| RHSIM_Rhsim13G0149900 | 223.83 | 1.32E-50 | 1.77E-49 | -4.866 |
| RHSIM_RhsimUnG0217000 | 223.81 | 1.33E-50 | 1.79E-49 | -3.836 |
| RHSIM_Rhsim09G0044600 | 223.57 | 1.50E-50 | 2.01E-49 | 1.579 |
| RHSIM_Rhsim02G0000800 | 223.45 | 1.60E-50 | 2.15E-49 | 1.109 |
| RHSIM_Rhsim02G0162800 | 223.40 | 1.64E-50 | 2.19E-49 | 1.951 |
| RHSIM_Rhsim01G0172300 | 223.28 | 1.75E-50 | 2.34E-49 | 1.312 |
| RHSIM_Rhsim05G0088500 | 223.28 | 1.75E-50 | 2.34E-49 | -1.847 |
| RHSIM_RhsimUnG0225800 | 223.12 | 1.89E-50 | 2.52E-49 | 1.882 |
| novel.3689 | 222.94 | 2.06E-50 | 2.76E-49 | 1.841 |
| RHSIM_Rhsim05G0187300 | 222.86 | 2.15E-50 | 2.88E-49 | -1.107 |

| Gene | LRT statistic | P value | BH-adjusted P | log2FC (interaction) |
|---|---|---|---|---|
| RHSIM_Rhsim12G0185700 | 222.85 | 2.17E-50 | 2.89E-49 | -2.011 |
| RHSIM_Rhsim12G0023400 | 222.63 | 2.41E-50 | 3.22E-49 | -1.312 |
| RHSIM_Rhsim03G0091900 | 222.54 | 2.52E-50 | 3.37E-49 | -1.279 |
| RHSIM_Rhsim06G0131200 | 222.36 | 2.77E-50 | 3.69E-49 | 1.243 |
| RHSIM_Rhsim13G0009100 | 221.95 | 3.40E-50 | 4.53E-49 | -0.882 |
| RHSIM_Rhsim07G0092500 | 221.31 | 4.68E-50 | 6.24E-49 | 1.133 |
| RHSIM_Rhsim01G0247300 | 221.31 | 4.68E-50 | 6.24E-49 | 0.748 |
| novel.4462 | 221.31 | 4.68E-50 | 6.24E-49 | -3.052 |
| RHSIM_Rhsim08G0248400 | 221.10 | 5.21E-50 | 6.94E-49 | -0.886 |
| RHSIM_Rhsim12G0090600 | 220.49 | 7.08E-50 | 9.42E-49 | -1.632 |
| RHSIM_Rhsim05G0072400 | 220.29 | 7.83E-50 | 1.04E-48 | -2.556 |
| RHSIM_Rhsim08G0105100 | 220.14 | 8.41E-50 | 1.12E-48 | -5.280 |
| RHSIM_Rhsim12G0159500 | 220.10 | 8.61E-50 | 1.14E-48 | -1.451 |
| RHSIM_Rhsim01G0274400 | 220.06 | 8.77E-50 | 1.16E-48 | 1.535 |
| RHSIM_Rhsim04G0196700 | 220.01 | 9.02E-50 | 1.20E-48 | 0.965 |
| RHSIM_Rhsim05G0214900 | 219.86 | 9.70E-50 | 1.29E-48 | -1.347 |
| RHSIM_Rhsim02G0236600 | 219.58 | 1.12E-49 | 1.48E-48 | 1.665 |
| RHSIM_Rhsim08G0223100 | 219.49 | 1.17E-49 | 1.55E-48 | -1.257 |
| RHSIM_Rhsim11G0198200 | 219.47 | 1.18E-49 | 1.56E-48 | 0.909 |
| RHSIM_Rhsim07G0144300 | 219.39 | 1.23E-49 | 1.63E-48 | 2.292 |
| RHSIM_Rhsim12G0159600 | 219.17 | 1.37E-49 | 1.82E-48 | 1.307 |
| RHSIM_Rhsim05G0046600 | 219.07 | 1.44E-49 | 1.91E-48 | 2.813 |
| RHSIM_Rhsim03G0207800 | 219.03 | 1.47E-49 | 1.94E-48 | -0.953 |
| RHSIM_Rhsim05G0142400 | 219.03 | 1.47E-49 | 1.94E-48 | -1.162 |
| RHSIM_Rhsim06G0164100 | 219.01 | 1.49E-49 | 1.96E-48 | 4.568 |
| RHSIM_Rhsim06G0232700 | 218.89 | 1.58E-49 | 2.09E-48 | 2.422 |
| RHSIM_Rhsim06G0106500 | 218.64 | 1.79E-49 | 2.37E-48 | 1.902 |
| RHSIM_Rhsim04G0126600 | 218.62 | 1.81E-49 | 2.38E-48 | 1.257 |
| RHSIM_Rhsim08G0055100 | 218.57 | 1.85E-49 | 2.44E-48 | -1.386 |
| RHSIM_Rhsim01G0246500 | 218.38 | 2.04E-49 | 2.68E-48 | 1.288 |
| RHSIM_Rhsim02G0036100 | 218.35 | 2.07E-49 | 2.73E-48 | -2.155 |
| RHSIM_Rhsim13G0005300 | 218.07 | 2.38E-49 | 3.14E-48 | 1.686 |

| Gene | LRT statistic | P value | BH-adjusted P | log2FC (interaction) |
|---|---|---|---|---|
| RHSIM_Rhsim12G0140200 | 218.06 | 2.40E-49 | 3.15E-48 | 0.996 |
| RHSIM_Rhsim02G0216000 | 218.00 | 2.47E-49 | 3.25E-48 | 2.251 |
| RHSIM_Rhsim06G0234600 | 217.79 | 2.75E-49 | 3.61E-48 | -1.056 |
| RHSIM_Rhsim07G0022700 | 217.53 | 3.12E-49 | 4.11E-48 | 0.942 |
| RHSIM_RhsimUnG0054600 | 217.31 | 3.49E-49 | 4.58E-48 | 1.200 |
| RHSIM_Rhsim01G0148600 | 217.30 | 3.52E-49 | 4.62E-48 | -1.542 |
| RHSIM_Rhsim12G0000600 | 217.25 | 3.61E-49 | 4.74E-48 | 1.042 |
| RHSIM_RhsimUnG0020200 | 217.16 | 3.77E-49 | 4.94E-48 | -2.628 |
| RHSIM_Rhsim02G0028100 | 217.10 | 3.89E-49 | 5.10E-48 | -0.723 |
| RHSIM_Rhsim12G0087000 | 217.00 | 4.08E-49 | 5.34E-48 | -1.604 |
| RHSIM_Rhsim11G0075500 | 216.99 | 4.10E-49 | 5.37E-48 | 1.013 |
| RHSIM_Rhsim12G0040100 | 216.87 | 4.35E-49 | 5.70E-48 | 1.506 |
| RHSIM_Rhsim07G0181700 | 216.87 | 4.35E-49 | 5.70E-48 | 1.634 |
| RHSIM_Rhsim05G0005600 | 216.80 | 4.52E-49 | 5.91E-48 | 1.087 |
| RHSIM_Rhsim01G0042600 | 216.67 | 4.81E-49 | 6.30E-48 | 1.706 |
| RHSIM_RhsimUnG0153300 | 216.51 | 5.22E-49 | 6.82E-48 | 1.448 |
| RHSIM_Rhsim06G0057600 | 216.39 | 5.54E-49 | 7.24E-48 | -1.360 |
| RHSIM_Rhsim02G0251100 | 216.31 | 5.78E-49 | 7.55E-48 | 1.168 |
| RHSIM_Rhsim01G0258700 | 216.13 | 6.32E-49 | 8.26E-48 | 1.088 |
| RHSIM_Rhsim04G0108300 | 216.12 | 6.34E-49 | 8.28E-48 | -2.378 |
| RHSIM_RhsimUnG0210400 | 215.82 | 7.39E-49 | 9.64E-48 | -2.750 |
| RHSIM_Rhsim12G0113300 | 215.75 | 7.64E-49 | 9.96E-48 | 3.496 |
| RHSIM_Rhsim08G0025000 | 215.73 | 7.70E-49 | 1.00E-47 | 2.496 |
| RHSIM_Rhsim11G0142600 | 215.72 | 7.76E-49 | 1.01E-47 | 1.741 |
| RHSIM_Rhsim04G0234700 | 215.63 | 8.13E-49 | 1.06E-47 | -2.199 |
| RHSIM_Rhsim12G0048500 | 215.57 | 8.35E-49 | 1.09E-47 | -1.378 |
| RHSIM_Rhsim12G0204800 | 215.52 | 8.57E-49 | 1.11E-47 | -1.159 |
| RHSIM_Rhsim10G0084900 | 215.49 | 8.72E-49 | 1.13E-47 | -2.197 |
| RHSIM_Rhsim05G0184800 | 215.46 | 8.87E-49 | 1.15E-47 | 0.990 |
| RHSIM_Rhsim10G0060300 | 215.13 | 1.04E-48 | 1.35E-47 | 1.257 |
| novel.2898 | 215.04 | 1.09E-48 | 1.42E-47 | 1.757 |
| RHSIM_Rhsim13G0121000 | 214.93 | 1.15E-48 | 1.50E-47 | -1.234 |

| Gene | LRT statistic | P value | BH-adjusted P | log2FC (interaction) |
|---|---|---|---|---|
| RHSIM_Rhsim08G0181700 | 214.85 | 1.20E-48 | 1.56E-47 | -0.835 |
| RHSIM_Rhsim01G0076600 | 214.83 | 1.21E-48 | 1.57E-47 | -3.690 |
| RHSIM_Rhsim13G0231500 | 214.82 | 1.22E-48 | 1.58E-47 | 0.938 |
| RHSIM_Rhsim03G0003600 | 214.76 | 1.26E-48 | 1.63E-47 | 1.195 |
| RHSIM_Rhsim01G0196600 | 214.58 | 1.38E-48 | 1.78E-47 | -0.969 |
| RHSIM_Rhsim04G0217900 | 214.57 | 1.38E-48 | 1.79E-47 | 0.884 |
| RHSIM_Rhsim07G0234900 | 214.43 | 1.49E-48 | 1.92E-47 | 1.306 |
| RHSIM_Rhsim12G0037800 | 214.41 | 1.50E-48 | 1.94E-47 | 1.013 |
| RHSIM_Rhsim13G0008000 | 214.38 | 1.52E-48 | 1.96E-47 | -1.523 |
| RHSIM_Rhsim10G0205800 | 214.35 | 1.54E-48 | 1.99E-47 | -2.390 |
| RHSIM_Rhsim10G0126200 | 214.15 | 1.71E-48 | 2.21E-47 | 1.346 |
| RHSIM_Rhsim12G0060800 | 214.09 | 1.76E-48 | 2.27E-47 | 1.196 |
| RHSIM_Rhsim08G0074400 | 213.93 | 1.91E-48 | 2.46E-47 | 1.131 |
| RHSIM_Rhsim11G0165200 | 213.61 | 2.24E-48 | 2.89E-47 | 4.338 |
| RHSIM_Rhsim05G0012900 | 213.34 | 2.56E-48 | 3.30E-47 | -1.407 |
| RHSIM_Rhsim03G0252700 | 213.33 | 2.58E-48 | 3.33E-47 | -1.679 |
| RHSIM_Rhsim12G0189900 | 213.27 | 2.66E-48 | 3.42E-47 | 0.750 |
| RHSIM_Rhsim02G0235400 | 213.25 | 2.69E-48 | 3.46E-47 | 1.660 |
| RHSIM_Rhsim12G0182800 | 213.06 | 2.96E-48 | 3.80E-47 | 0.938 |
| RHSIM_Rhsim03G0223300 | 212.78 | 3.39E-48 | 4.36E-47 | -2.085 |
| RHSIM_Rhsim10G0079800 | 212.62 | 3.68E-48 | 4.74E-47 | 3.287 |
| novel.3065 | 212.62 | 3.69E-48 | 4.74E-47 | 1.002 |
| RHSIM_Rhsim06G0156000 | 212.51 | 3.89E-48 | 4.99E-47 | -3.520 |
| RHSIM_Rhsim06G0208400 | 212.50 | 3.92E-48 | 5.03E-47 | -1.106 |
| RHSIM_Rhsim07G0040500 | 212.46 | 3.98E-48 | 5.11E-47 | -3.886 |
| RHSIM_Rhsim04G0248600 | 212.23 | 4.47E-48 | 5.74E-47 | 5.648 |
| RHSIM_Rhsim11G0008400 | 212.23 | 4.49E-48 | 5.76E-47 | 1.979 |
| RHSIM_Rhsim10G0126900 | 212.19 | 4.57E-48 | 5.86E-47 | 1.054 |
| RHSIM_Rhsim12G0167900 | 212.07 | 4.86E-48 | 6.22E-47 | 0.628 |
| RHSIM_Rhsim07G0188500 | 211.80 | 5.55E-48 | 7.11E-47 | 1.416 |
| RHSIM_Rhsim03G0153000 | 211.70 | 5.84E-48 | 7.47E-47 | 2.881 |
| RHSIM_RhsimUnG0046700 | 211.70 | 5.84E-48 | 7.47E-47 | -2.706 |

| Gene | LRT statistic | P value | BH-adjusted P | log2FC (interaction) |
|---|---|---|---|---|
| RHSIM_Rhsim10G0200200 | 211.68 | 5.92E-48 | 7.57E-47 | -8.091 |
| RHSIM_Rhsim04G0170600 | 211.65 | 5.99E-48 | 7.66E-47 | -1.333 |
| RHSIM_Rhsim01G0216900 | 211.62 | 6.09E-48 | 7.79E-47 | -1.416 |
| RHSIM_Rhsim04G0164000 | 211.54 | 6.32E-48 | 8.07E-47 | 5.706 |
| RHSIM_Rhsim10G0178400 | 211.38 | 6.87E-48 | 8.76E-47 | 0.884 |
| RHSIM_RhsimUnG0118400 | 211.27 | 7.27E-48 | 9.28E-47 | 1.539 |
| RHSIM_Rhsim13G0162300 | 211.18 | 7.60E-48 | 9.69E-47 | -1.203 |
| RHSIM_Rhsim07G0209300 | 211.01 | 8.26E-48 | 1.05E-46 | -1.265 |
| RHSIM_Rhsim13G0163700 | 210.97 | 8.43E-48 | 1.07E-46 | 1.149 |
| RHSIM_Rhsim08G0074600 | 210.72 | 9.58E-48 | 1.22E-46 | 1.248 |
| RHSIM_Rhsim03G0002700 | 210.57 | 1.03E-47 | 1.31E-46 | 1.269 |
| RHSIM_Rhsim01G0275400 | 210.33 | 1.16E-47 | 1.48E-46 | 1.247 |
| RHSIM_Rhsim09G0196600 | 210.19 | 1.25E-47 | 1.59E-46 | -1.724 |
| RHSIM_Rhsim06G0036800 | 210.17 | 1.26E-47 | 1.60E-46 | 2.445 |
| RHSIM_Rhsim12G0162300 | 209.91 | 1.44E-47 | 1.83E-46 | -2.635 |
| RHSIM_Rhsim03G0145000 | 209.73 | 1.57E-47 | 1.99E-46 | -1.070 |
| RHSIM_Rhsim12G0148600 | 209.49 | 1.78E-47 | 2.26E-46 | 3.992 |
| RHSIM_Rhsim13G0110400 | 209.44 | 1.82E-47 | 2.31E-46 | -3.245 |
| RHSIM_Rhsim09G0095000 | 209.37 | 1.88E-47 | 2.39E-46 | -0.864 |
| RHSIM_Rhsim13G0144800 | 209.26 | 1.99E-47 | 2.52E-46 | -1.515 |
| RHSIM_Rhsim11G0159300 | 209.26 | 1.99E-47 | 2.52E-46 | -1.913 |
| RHSIM_Rhsim07G0000500 | 209.23 | 2.02E-47 | 2.56E-46 | 2.287 |
| RHSIM_Rhsim10G0045100 | 209.14 | 2.11E-47 | 2.68E-46 | 1.152 |
| RHSIM_Rhsim03G0260000 | 209.04 | 2.23E-47 | 2.82E-46 | -4.614 |
| novel.1004 | 209.03 | 2.23E-47 | 2.83E-46 | -3.753 |
| novel.551 | 208.82 | 2.48E-47 | 3.14E-46 | -5.406 |
| RHSIM_Rhsim10G0007400 | 208.60 | 2.78E-47 | 3.51E-46 | -1.594 |
| RHSIM_Rhsim10G0025400 | 208.56 | 2.82E-47 | 3.57E-46 | -1.688 |
| RHSIM_Rhsim01G0257500 | 208.39 | 3.08E-47 | 3.89E-46 | 1.712 |
| RHSIM_Rhsim13G0082400 | 208.37 | 3.12E-47 | 3.94E-46 | -1.657 |
| RHSIM_Rhsim05G0022400 | 208.11 | 3.55E-47 | 4.49E-46 | 1.795 |
| RHSIM_Rhsim04G0103200 | 208.00 | 3.75E-47 | 4.73E-46 | 2.209 |

| Gene | LRT statistic | P value | BH-adjusted P | log2FC (interaction) |
|---|---|---|---|---|
| RHSIM_Rhsim11G0122200 | 207.91 | 3.92E-47 | 4.95E-46 | -0.817 |
| RHSIM_Rhsim07G0058100 | 207.80 | 4.15E-47 | 5.22E-46 | -1.082 |
| RHSIM_Rhsim02G0160100 | 207.76 | 4.23E-47 | 5.33E-46 | 1.449 |
| RHSIM_Rhsim05G0185900 | 207.26 | 5.44E-47 | 6.85E-46 | 1.720 |
| novel.2014 | 207.14 | 5.77E-47 | 7.27E-46 | 1.103 |
| RHSIM_Rhsim07G0015100 | 207.02 | 6.14E-47 | 7.72E-46 | 1.085 |
| RHSIM_Rhsim10G0190900 | 206.85 | 6.69E-47 | 8.42E-46 | 2.011 |
| novel.2706 | 206.81 | 6.82E-47 | 8.57E-46 | -1.310 |
| RHSIM_Rhsim04G0085700 | 206.79 | 6.88E-47 | 8.64E-46 | 0.940 |
| RHSIM_Rhsim04G0154400 | 206.74 | 7.07E-47 | 8.88E-46 | 1.013 |
| RHSIM_Rhsim09G0044200 | 206.73 | 7.09E-47 | 8.89E-46 | 1.135 |
| RHSIM_Rhsim04G0058200 | 206.71 | 7.19E-47 | 9.02E-46 | -1.861 |
| novel.249 | 206.67 | 7.32E-47 | 9.18E-46 | -2.339 |
| RHSIM_Rhsim07G0198600 | 206.64 | 7.44E-47 | 9.33E-46 | 1.134 |
| RHSIM_Rhsim07G0083200 | 206.57 | 7.69E-47 | 9.63E-46 | 2.623 |
| novel.172 | 206.41 | 8.33E-47 | 1.04E-45 | -3.235 |
| RHSIM_Rhsim08G0131100 | 206.23 | 9.11E-47 | 1.14E-45 | 4.136 |
| RHSIM_Rhsim04G0104700 | 206.18 | 9.35E-47 | 1.17E-45 | -3.406 |
| novel.382 | 205.69 | 1.20E-46 | 1.50E-45 | -1.674 |
| RHSIM_Rhsim03G0087000 | 205.64 | 1.23E-46 | 1.53E-45 | 1.092 |
| RHSIM_Rhsim07G0091000 | 205.52 | 1.30E-46 | 1.63E-45 | 1.179 |
| RHSIM_Rhsim07G0256500 | 205.32 | 1.45E-46 | 1.81E-45 | 0.798 |
| RHSIM_Rhsim12G0007800 | 205.28 | 1.47E-46 | 1.84E-45 | -3.164 |
| novel.1661 | 205.16 | 1.57E-46 | 1.96E-45 | 4.316 |
| RHSIM_Rhsim01G0236100 | 205.09 | 1.62E-46 | 2.02E-45 | 1.364 |
| RHSIM_RhsimUnG0094700 | 204.79 | 1.88E-46 | 2.35E-45 | -1.246 |
| RHSIM_Rhsim06G0183900 | 204.68 | 1.98E-46 | 2.47E-45 | 1.353 |
| RHSIM_Rhsim11G0095200 | 204.66 | 2.00E-46 | 2.50E-45 | -4.886 |
| RHSIM_Rhsim01G0057900 | 204.61 | 2.06E-46 | 2.57E-45 | 1.204 |
| RHSIM_Rhsim13G0209500 | 204.58 | 2.09E-46 | 2.60E-45 | -2.664 |
| RHSIM_Rhsim04G0135000 | 204.41 | 2.28E-46 | 2.83E-45 | 2.948 |
| RHSIM_Rhsim04G0068700 | 204.37 | 2.32E-46 | 2.89E-45 | -2.165 |

| Gene | LRT statistic | P value | BH-adjusted P | log2FC (interaction) |
|---|---|---|---|---|
| RHSIM_Rhsim13G0206400 | 204.35 | 2.35E-46 | 2.92E-45 | -1.250 |
| RHSIM_Rhsim02G0223200 | 204.33 | 2.37E-46 | 2.95E-45 | -5.097 |
| RHSIM_Rhsim08G0059600 | 204.31 | 2.40E-46 | 2.98E-45 | -45.000 |
| RHSIM_Rhsim12G0111500 | 204.25 | 2.46E-46 | 3.06E-45 | -4.065 |
| RHSIM_Rhsim13G0166900 | 204.24 | 2.48E-46 | 3.08E-45 | -2.809 |
| RHSIM_Rhsim01G0291100 | 204.21 | 2.52E-46 | 3.13E-45 | 0.987 |
| RHSIM_Rhsim01G0280700 | 204.14 | 2.61E-46 | 3.23E-45 | -1.755 |
| RHSIM_Rhsim04G0012800 | 204.04 | 2.75E-46 | 3.40E-45 | -0.786 |
| novel.3962 | 203.99 | 2.81E-46 | 3.49E-45 | -2.452 |
| RHSIM_Rhsim13G0104000 | 203.92 | 2.91E-46 | 3.60E-45 | -1.272 |
| RHSIM_Rhsim01G0046500 | 203.79 | 3.11E-46 | 3.85E-45 | 2.165 |
| RHSIM_Rhsim13G0201500 | 203.45 | 3.70E-46 | 4.57E-45 | -0.726 |
| RHSIM_Rhsim06G0206900 | 203.42 | 3.74E-46 | 4.63E-45 | -1.085 |
| RHSIM_Rhsim12G0053700 | 203.39 | 3.80E-46 | 4.69E-45 | -2.299 |
| RHSIM_Rhsim13G0152000 | 203.38 | 3.83E-46 | 4.73E-45 | 0.706 |
| RHSIM_Rhsim04G0180800 | 203.35 | 3.89E-46 | 4.80E-45 | -1.344 |
| RHSIM_Rhsim07G0020400 | 203.29 | 3.99E-46 | 4.93E-45 | -1.575 |
| RHSIM_RhsimUnG0002100 | 203.25 | 4.09E-46 | 5.04E-45 | 1.108 |
| RHSIM_Rhsim04G0085300 | 202.99 | 4.65E-46 | 5.73E-45 | -0.872 |
| RHSIM_Rhsim03G0021000 | 202.76 | 5.21E-46 | 6.42E-45 | -2.695 |
| RHSIM_Rhsim04G0013500 | 202.57 | 5.74E-46 | 7.07E-45 | -1.825 |
| RHSIM_Rhsim06G0052900 | 202.55 | 5.80E-46 | 7.15E-45 | -0.779 |
| RHSIM_Rhsim09G0154800 | 202.53 | 5.86E-46 | 7.21E-45 | 3.861 |
| RHSIM_Rhsim07G0237000 | 202.49 | 5.98E-46 | 7.37E-45 | 0.899 |
| RHSIM_Rhsim03G0222600 | 201.96 | 7.80E-46 | 9.59E-45 | 1.061 |
| RHSIM_Rhsim08G0203900 | 201.93 | 7.91E-46 | 9.73E-45 | 1.664 |
| RHSIM_Rhsim01G0023700 | 201.93 | 7.91E-46 | 9.73E-45 | 1.621 |
| RHSIM_Rhsim13G0045200 | 201.85 | 8.26E-46 | 1.01E-44 | -1.812 |
| RHSIM_Rhsim09G0158400 | 201.69 | 8.91E-46 | 1.09E-44 | 1.776 |
| RHSIM_Rhsim10G0083200 | 201.58 | 9.43E-46 | 1.16E-44 | 1.105 |
| RHSIM_Rhsim10G0202900 | 201.48 | 9.92E-46 | 1.22E-44 | 2.565 |
| RHSIM_Rhsim01G0088800 | 201.46 | 1.00E-45 | 1.23E-44 | -1.438 |

| Gene | LRT statistic | P value | BH-adjusted P | log2FC (interaction) |
|---|---|---|---|---|
| RHSIM_Rhsim04G0217800 | 201.34 | 1.07E-45 | 1.31E-44 | 0.947 |
| RHSIM_Rhsim07G0087100 | 201.29 | 1.09E-45 | 1.34E-44 | -1.290 |
| RHSIM_Rhsim09G0192900 | 201.22 | 1.13E-45 | 1.39E-44 | -0.973 |
| RHSIM_Rhsim08G0123600 | 200.99 | 1.27E-45 | 1.56E-44 | -1.230 |
| RHSIM_Rhsim12G0175400 | 200.60 | 1.54E-45 | 1.89E-44 | 0.756 |
| RHSIM_Rhsim08G0075800 | 200.60 | 1.54E-45 | 1.89E-44 | -0.792 |
| RHSIM_Rhsim08G0058100 | 200.57 | 1.57E-45 | 1.92E-44 | -4.877 |
| RHSIM_Rhsim03G0247400 | 200.44 | 1.68E-45 | 2.05E-44 | 1.867 |
| RHSIM_Rhsim10G0169900 | 200.29 | 1.81E-45 | 2.21E-44 | -2.259 |
| RHSIM_Rhsim06G0056900 | 200.22 | 1.87E-45 | 2.28E-44 | 4.669 |
| RHSIM_Rhsim08G0103200 | 200.09 | 2.00E-45 | 2.44E-44 | -2.924 |
| RHSIM_Rhsim13G0180000 | 200.09 | 2.00E-45 | 2.44E-44 | -1.027 |
| RHSIM_Rhsim08G0006500 | 200.06 | 2.02E-45 | 2.47E-44 | 1.993 |
| RHSIM_Rhsim11G0025100 | 200.01 | 2.08E-45 | 2.54E-44 | 1.442 |
| RHSIM_RhsimUnG0042800 | 199.99 | 2.10E-45 | 2.56E-44 | -5.748 |
| RHSIM_Rhsim09G0189900 | 199.85 | 2.25E-45 | 2.74E-44 | -2.588 |
| RHSIM_Rhsim05G0063300 | 199.74 | 2.38E-45 | 2.90E-44 | -2.900 |
| RHSIM_Rhsim12G0213600 | 199.44 | 2.77E-45 | 3.37E-44 | -0.826 |
| RHSIM_Rhsim01G0192300 | 199.38 | 2.85E-45 | 3.47E-44 | -1.654 |
| RHSIM_Rhsim07G0223800 | 199.38 | 2.85E-45 | 3.47E-44 | -2.072 |
| RHSIM_Rhsim12G0205000 | 199.35 | 2.90E-45 | 3.53E-44 | 2.672 |
| RHSIM_Rhsim04G0078700 | 199.31 | 2.96E-45 | 3.59E-44 | -5.051 |
| RHSIM_RhsimUnG0219200 | 198.96 | 3.52E-45 | 4.27E-44 | 1.455 |
| RHSIM_Rhsim12G0113000 | 198.96 | 3.53E-45 | 4.28E-44 | 0.811 |
| RHSIM_Rhsim03G0104900 | 198.89 | 3.65E-45 | 4.43E-44 | 1.503 |
| RHSIM_Rhsim10G0076800 | 198.89 | 3.66E-45 | 4.44E-44 | -1.459 |
| RHSIM_Rhsim06G0211700 | 198.84 | 3.74E-45 | 4.53E-44 | 1.174 |
| RHSIM_Rhsim09G0153200 | 198.73 | 3.96E-45 | 4.80E-44 | 0.781 |
| RHSIM_Rhsim13G0179500 | 198.61 | 4.19E-45 | 5.08E-44 | -1.825 |
| RHSIM_Rhsim10G0197200 | 198.50 | 4.45E-45 | 5.38E-44 | -1.240 |
| RHSIM_Rhsim08G0175800 | 198.21 | 5.13E-45 | 6.21E-44 | -1.470 |
| RHSIM_Rhsim05G0062900 | 198.19 | 5.18E-45 | 6.27E-44 | 1.722 |

| Gene | LRT statistic | P value | BH-adjusted P | log2FC (interaction) |
|---|---|---|---|---|
| RHSIM_Rhsim12G0004800 | 198.05 | 5.57E-45 | 6.73E-44 | 1.219 |
| RHSIM_Rhsim08G0127800 | 197.93 | 5.92E-45 | 7.16E-44 | -0.954 |
| RHSIM_Rhsim11G0155100 | 197.90 | 6.00E-45 | 7.25E-44 | 1.521 |
| RHSIM_Rhsim06G0178600 | 197.83 | 6.22E-45 | 7.51E-44 | 1.174 |
| RHSIM_Rhsim08G0147000 | 197.81 | 6.28E-45 | 7.58E-44 | -1.396 |
| RHSIM_Rhsim12G0158100 | 197.39 | 7.73E-45 | 9.34E-44 | 1.147 |
| RHSIM_Rhsim05G0069500 | 197.26 | 8.28E-45 | 9.99E-44 | 1.958 |
| RHSIM_Rhsim03G0084600 | 197.10 | 8.96E-45 | 1.08E-43 | -1.414 |
| RHSIM_Rhsim11G0184700 | 197.07 | 9.12E-45 | 1.10E-43 | 0.938 |
| RHSIM_Rhsim03G0101200 | 196.89 | 9.99E-45 | 1.20E-43 | 1.777 |
| RHSIM_Rhsim10G0107900 | 196.86 | 1.01E-44 | 1.22E-43 | 1.753 |
| RHSIM_Rhsim12G0014100 | 196.85 | 1.02E-44 | 1.22E-43 | -0.758 |
| RHSIM_Rhsim08G0200000 | 196.78 | 1.05E-44 | 1.26E-43 | -1.260 |
| RHSIM_Rhsim03G0229700 | 196.59 | 1.16E-44 | 1.39E-43 | -1.322 |
| RHSIM_Rhsim04G0034500 | 196.30 | 1.34E-44 | 1.61E-43 | -3.484 |
| RHSIM_Rhsim02G0162600 | 196.21 | 1.40E-44 | 1.69E-43 | 1.026 |
| RHSIM_Rhsim05G0063700 | 196.17 | 1.43E-44 | 1.72E-43 | -2.146 |
| RHSIM_Rhsim03G0226900 | 196.12 | 1.47E-44 | 1.76E-43 | -0.884 |
| RHSIM_Rhsim08G0211400 | 196.12 | 1.47E-44 | 1.76E-43 | 1.862 |
| RHSIM_Rhsim03G0141200 | 196.08 | 1.50E-44 | 1.80E-43 | -0.900 |
| RHSIM_Rhsim06G0231700 | 196.06 | 1.52E-44 | 1.82E-43 | 1.084 |
| RHSIM_RhsimUnG0258600 | 195.97 | 1.58E-44 | 1.90E-43 | -3.535 |
| RHSIM_Rhsim08G0035800 | 195.71 | 1.81E-44 | 2.17E-43 | -1.606 |
| RHSIM_Rhsim03G0052900 | 195.60 | 1.91E-44 | 2.29E-43 | 1.297 |
| RHSIM_Rhsim06G0178000 | 195.54 | 1.97E-44 | 2.36E-43 | 1.801 |
| RHSIM_Rhsim02G0043500 | 195.52 | 1.99E-44 | 2.38E-43 | 0.938 |
| RHSIM_Rhsim04G0009100 | 195.48 | 2.03E-44 | 2.43E-43 | -2.274 |
| novel.918 | 195.41 | 2.10E-44 | 2.51E-43 | 2.732 |
| RHSIM_Rhsim01G0088900 | 195.38 | 2.13E-44 | 2.55E-43 | -3.455 |
| RHSIM_Rhsim08G0101500 | 195.03 | 2.53E-44 | 3.03E-43 | -4.318 |
| RHSIM_RhsimUnG0209900 | 194.65 | 3.08E-44 | 3.68E-43 | -3.575 |
| novel.1885 | 194.54 | 3.25E-44 | 3.88E-43 | -46.009 |

| Gene | LRT statistic | P value | BH-adjusted P | log2FC (interaction) |
|---|---|---|---|---|
| RHSIM_Rhsim13G0126100 | 194.37 | 3.54E-44 | 4.22E-43 | -0.652 |
| novel.105 | 194.26 | 3.73E-44 | 4.45E-43 | -2.720 |
| RHSIM_Rhsim07G0029000 | 194.24 | 3.78E-44 | 4.51E-43 | 1.283 |
| novel.4464 | 194.22 | 3.81E-44 | 4.54E-43 | 3.459 |
| novel.1484 | 194.06 | 4.14E-44 | 4.93E-43 | -1.339 |
| RHSIM_Rhsim12G0080000 | 193.97 | 4.32E-44 | 5.14E-43 | 2.099 |
| RHSIM_Rhsim08G0016800 | 193.97 | 4.32E-44 | 5.15E-43 | 1.794 |
| RHSIM_Rhsim05G0146300 | 193.95 | 4.37E-44 | 5.20E-43 | -2.953 |
| RHSIM_Rhsim12G0127600 | 193.94 | 4.38E-44 | 5.21E-43 | -1.233 |
| RHSIM_Rhsim08G0076500 | 193.91 | 4.46E-44 | 5.30E-43 | 1.195 |
| novel.2015 | 193.77 | 4.79E-44 | 5.69E-43 | -1.449 |
| RHSIM_Rhsim12G0025900 | 193.69 | 4.97E-44 | 5.91E-43 | -1.460 |
| RHSIM_Rhsim13G0075700 | 193.62 | 5.15E-44 | 6.11E-43 | -1.966 |
| RHSIM_Rhsim03G0191100 | 193.57 | 5.28E-44 | 6.26E-43 | -1.531 |
| RHSIM_Rhsim05G0012400 | 193.54 | 5.37E-44 | 6.37E-43 | -1.174 |
| RHSIM_Rhsim08G0038100 | 193.47 | 5.57E-44 | 6.60E-43 | 0.821 |
| RHSIM_Rhsim09G0186900 | 193.09 | 6.73E-44 | 7.98E-43 | 1.088 |
| novel.1822 | 192.99 | 7.09E-44 | 8.40E-43 | 1.162 |
| RHSIM_Rhsim09G0172500 | 192.93 | 7.29E-44 | 8.63E-43 | -1.262 |
| RHSIM_Rhsim04G0043200 | 192.91 | 7.36E-44 | 8.71E-43 | 1.184 |
| RHSIM_Rhsim13G0124400 | 192.74 | 8.02E-44 | 9.49E-43 | -2.033 |
| RHSIM_Rhsim05G0197900 | 192.74 | 8.03E-44 | 9.50E-43 | -2.002 |
| RHSIM_Rhsim03G0222700 | 192.73 | 8.07E-44 | 9.54E-43 | 1.793 |
| RHSIM_Rhsim12G0087800 | 192.56 | 8.80E-44 | 1.04E-42 | -0.986 |
| RHSIM_RhsimUnG0158600 | 192.44 | 9.33E-44 | 1.10E-42 | -3.152 |
| RHSIM_Rhsim11G0054300 | 192.30 | 1.00E-43 | 1.18E-42 | -1.419 |
| RHSIM_Rhsim11G0071900 | 192.15 | 1.08E-43 | 1.28E-42 | -2.134 |
| RHSIM_Rhsim05G0137000 | 192.11 | 1.10E-43 | 1.30E-42 | 2.709 |
| RHSIM_Rhsim09G0092100 | 192.10 | 1.10E-43 | 1.30E-42 | 1.606 |
| RHSIM_Rhsim05G0202400 | 191.91 | 1.22E-43 | 1.44E-42 | 0.863 |
| novel.636 | 191.79 | 1.29E-43 | 1.52E-42 | -1.186 |
| RHSIM_Rhsim03G0248000 | 191.74 | 1.33E-43 | 1.56E-42 | 1.283 |

| Gene | LRT statistic | P value | BH-adjusted P | log2FC (interaction) |
|---|---|---|---|---|
| RHSIM_Rhsim07G0028300 | 191.55 | 1.46E-43 | 1.71E-42 | -1.394 |
| novel.932 | 191.52 | 1.48E-43 | 1.75E-42 | -1.626 |
| RHSIM_Rhsim08G0202300 | 191.47 | 1.52E-43 | 1.79E-42 | 0.661 |
| RHSIM_Rhsim13G0013600 | 191.32 | 1.64E-43 | 1.92E-42 | 1.200 |
| RHSIM_Rhsim07G0117400 | 191.15 | 1.78E-43 | 2.09E-42 | 2.057 |
| RHSIM_Rhsim04G0167500 | 190.87 | 2.05E-43 | 2.41E-42 | 4.308 |
| RHSIM_Rhsim05G0216400 | 190.80 | 2.12E-43 | 2.50E-42 | -0.991 |
| RHSIM_Rhsim08G0122900 | 190.63 | 2.32E-43 | 2.72E-42 | 2.079 |
| RHSIM_Rhsim13G0201400 | 190.50 | 2.47E-43 | 2.90E-42 | -0.629 |
| RHSIM_Rhsim08G0105200 | 190.32 | 2.71E-43 | 3.18E-42 | -4.367 |
| RHSIM_Rhsim07G0201600 | 190.18 | 2.91E-43 | 3.41E-42 | 1.269 |
| RHSIM_RhsimUnG0059700 | 190.14 | 2.96E-43 | 3.48E-42 | -1.410 |
| RHSIM_Rhsim01G0289200 | 190.11 | 3.00E-43 | 3.52E-42 | 1.174 |
| RHSIM_RhsimUnG0009200 | 189.99 | 3.19E-43 | 3.74E-42 | 1.242 |
| RHSIM_Rhsim08G0088900 | 189.76 | 3.58E-43 | 4.19E-42 | -2.424 |
| RHSIM_Rhsim05G0042100 | 189.66 | 3.77E-43 | 4.42E-42 | 1.543 |
| RHSIM_Rhsim08G0118900 | 189.63 | 3.84E-43 | 4.49E-42 | 2.607 |
| RHSIM_Rhsim11G0068500 | 189.22 | 4.70E-43 | 5.49E-42 | 3.515 |
| RHSIM_Rhsim03G0208100 | 189.20 | 4.75E-43 | 5.56E-42 | -1.209 |
| RHSIM_Rhsim09G0198700 | 189.04 | 5.16E-43 | 6.02E-42 | -1.022 |
| RHSIM_Rhsim11G0135900 | 188.98 | 5.30E-43 | 6.19E-42 | -1.479 |
| RHSIM_Rhsim06G0047800 | 188.98 | 5.31E-43 | 6.20E-42 | -1.013 |
| RHSIM_Rhsim08G0094800 | 188.96 | 5.37E-43 | 6.27E-42 | 1.568 |
| novel.2265 | 188.95 | 5.39E-43 | 6.28E-42 | 2.415 |
| RHSIM_Rhsim12G0026000 | 188.83 | 5.73E-43 | 6.68E-42 | -1.004 |
| RHSIM_Rhsim09G0163800 | 188.80 | 5.82E-43 | 6.78E-42 | 1.392 |
| RHSIM_Rhsim07G0145300 | 188.69 | 6.13E-43 | 7.15E-42 | -2.464 |
| novel.2278 | 188.52 | 6.70E-43 | 7.80E-42 | 1.133 |
| RHSIM_Rhsim02G0021200 | 188.50 | 6.74E-43 | 7.85E-42 | 1.244 |
| RHSIM_RhsimUnG0240300 | 188.26 | 7.62E-43 | 8.87E-42 | -2.188 |
| RHSIM_Rhsim03G0167300 | 188.16 | 8.00E-43 | 9.31E-42 | -1.155 |
| RHSIM_Rhsim02G0244700 | 188.15 | 8.07E-43 | 9.39E-42 | 1.254 |

| Gene | LRT statistic | P value | BH-adjusted P | log2FC (interaction) |
|---|---|---|---|---|
| RHSIM_Rhsim03G0049500 | 188.13 | 8.12E-43 | 9.44E-42 | 1.240 |
| RHSIM_Rhsim08G0053600 | 188.12 | 8.18E-43 | 9.51E-42 | 1.982 |
| RHSIM_Rhsim09G0108600 | 188.01 | 8.66E-43 | 1.01E-41 | -4.454 |
| RHSIM_Rhsim08G0174300 | 187.98 | 8.79E-43 | 1.02E-41 | 1.444 |
| novel.4297 | 187.97 | 8.83E-43 | 1.02E-41 | 1.639 |
| RHSIM_Rhsim03G0052600 | 187.93 | 8.98E-43 | 1.04E-41 | 3.642 |
| RHSIM_Rhsim06G0055200 | 187.58 | 1.07E-42 | 1.25E-41 | -1.516 |
| RHSIM_Rhsim01G0069300 | 187.54 | 1.10E-42 | 1.27E-41 | -1.323 |
| RHSIM_Rhsim03G0109600 | 187.34 | 1.21E-42 | 1.40E-41 | 0.942 |
| RHSIM_Rhsim05G0187500 | 187.27 | 1.25E-42 | 1.45E-41 | -2.533 |
| RHSIM_Rhsim03G0238900 | 187.24 | 1.27E-42 | 1.47E-41 | 0.952 |
| RHSIM_Rhsim06G0004800 | 187.17 | 1.32E-42 | 1.53E-41 | -7.566 |
| RHSIM_Rhsim02G0007100 | 187.14 | 1.34E-42 | 1.55E-41 | 1.135 |
| RHSIM_Rhsim02G0089600 | 187.06 | 1.39E-42 | 1.61E-41 | -2.089 |
| RHSIM_RhsimUnG0253400 | 186.70 | 1.67E-42 | 1.93E-41 | -0.996 |
| RHSIM_Rhsim12G0132700 | 186.63 | 1.73E-42 | 1.99E-41 | 2.096 |
| RHSIM_Rhsim04G0217500 | 186.39 | 1.96E-42 | 2.26E-41 | 1.114 |
| RHSIM_Rhsim03G0055500 | 186.35 | 1.99E-42 | 2.30E-41 | 2.761 |
| RHSIM_Rhsim01G0192900 | 186.07 | 2.30E-42 | 2.65E-41 | 2.085 |
| RHSIM_Rhsim11G0132100 | 186.04 | 2.32E-42 | 2.68E-41 | 2.233 |
| RHSIM_Rhsim05G0056800 | 185.98 | 2.40E-42 | 2.77E-41 | 1.283 |
| RHSIM_Rhsim13G0115500 | 185.97 | 2.42E-42 | 2.78E-41 | 1.024 |
| RHSIM_Rhsim13G0131700 | 185.90 | 2.50E-42 | 2.88E-41 | 1.132 |
| RHSIM_Rhsim01G0117000 | 185.75 | 2.69E-42 | 3.10E-41 | -6.047 |
| RHSIM_Rhsim06G0188400 | 185.72 | 2.73E-42 | 3.14E-41 | -44.727 |
| RHSIM_Rhsim03G0264300 | 185.52 | 3.03E-42 | 3.48E-41 | -1.085 |
| RHSIM_Rhsim07G0031500 | 185.51 | 3.03E-42 | 3.49E-41 | -2.437 |
| RHSIM_Rhsim12G0023500 | 185.50 | 3.06E-42 | 3.51E-41 | -1.892 |
| RHSIM_Rhsim08G0007800 | 185.40 | 3.21E-42 | 3.69E-41 | 1.114 |
| RHSIM_Rhsim03G0243800 | 185.29 | 3.39E-42 | 3.90E-41 | 0.735 |
| RHSIM_RhsimUnG0111200 | 185.29 | 3.39E-42 | 3.90E-41 | -1.687 |
| RHSIM_Rhsim10G0002100 | 185.26 | 3.44E-42 | 3.95E-41 | -0.962 |

| Gene | LRT statistic | P value | BH-adjusted P | log2FC (interaction) |
|---|---|---|---|---|
| RHSIM_Rhsim04G0200500 | 185.15 | 3.63E-42 | 4.17E-41 | -2.017 |
| RHSIM_Rhsim07G0188200 | 185.07 | 3.79E-42 | 4.35E-41 | 0.959 |
| RHSIM_Rhsim04G0108400 | 185.06 | 3.81E-42 | 4.37E-41 | 0.734 |
| RHSIM_Rhsim13G0025100 | 184.98 | 3.97E-42 | 4.55E-41 | 1.218 |
| RHSIM_Rhsim04G0195100 | 184.94 | 4.05E-42 | 4.64E-41 | 1.000 |
| RHSIM_Rhsim13G0131200 | 184.86 | 4.22E-42 | 4.83E-41 | 1.147 |
| RHSIM_Rhsim06G0006300 | 184.61 | 4.77E-42 | 5.46E-41 | 2.035 |
| RHSIM_Rhsim05G0175800 | 184.58 | 4.85E-42 | 5.55E-41 | 3.281 |
| RHSIM_Rhsim04G0072300 | 184.55 | 4.93E-42 | 5.64E-41 | -2.491 |
| RHSIM_Rhsim06G0040100 | 184.31 | 5.55E-42 | 6.35E-41 | -1.577 |
| RHSIM_Rhsim07G0011000 | 184.31 | 5.56E-42 | 6.35E-41 | -2.448 |
| RHSIM_Rhsim06G0054700 | 184.28 | 5.62E-42 | 6.43E-41 | 0.985 |
| RHSIM_Rhsim03G0093400 | 184.05 | 6.34E-42 | 7.24E-41 | 1.093 |
| RHSIM_Rhsim12G0006200 | 183.90 | 6.82E-42 | 7.78E-41 | 0.840 |
| novel.323 | 183.76 | 7.32E-42 | 8.35E-41 | -1.264 |
| RHSIM_Rhsim06G0050700 | 183.69 | 7.58E-42 | 8.64E-41 | -1.459 |
| novel.4078 | 183.66 | 7.70E-42 | 8.79E-41 | 6.538 |
| RHSIM_Rhsim07G0076200 | 183.38 | 8.87E-42 | 1.01E-40 | -3.680 |
| RHSIM_Rhsim08G0222200 | 183.35 | 8.99E-42 | 1.02E-40 | -1.590 |
| RHSIM_Rhsim11G0099800 | 183.25 | 9.44E-42 | 1.08E-40 | -1.638 |
| RHSIM_Rhsim13G0225000 | 183.20 | 9.70E-42 | 1.10E-40 | -2.665 |
| novel.2536 | 183.13 | 1.01E-41 | 1.14E-40 | -0.935 |
| RHSIM_Rhsim10G0012500 | 183.11 | 1.01E-41 | 1.15E-40 | -1.304 |
| RHSIM_Rhsim02G0043800 | 182.75 | 1.22E-41 | 1.39E-40 | -1.810 |
| RHSIM_Rhsim06G0131700 | 182.69 | 1.25E-41 | 1.42E-40 | -1.309 |
| RHSIM_Rhsim09G0075700 | 182.63 | 1.29E-41 | 1.47E-40 | 1.476 |
| RHSIM_Rhsim01G0285500 | 182.63 | 1.29E-41 | 1.47E-40 | -2.678 |
| RHSIM_Rhsim11G0121400 | 182.61 | 1.30E-41 | 1.48E-40 | 1.065 |
| RHSIM_Rhsim13G0092400 | 182.51 | 1.37E-41 | 1.56E-40 | -1.290 |
| RHSIM_Rhsim07G0119100 | 182.44 | 1.42E-41 | 1.61E-40 | 3.036 |
| RHSIM_Rhsim13G0003800 | 182.41 | 1.44E-41 | 1.64E-40 | -0.979 |
| RHSIM_Rhsim08G0004000 | 182.40 | 1.45E-41 | 1.65E-40 | -3.031 |

| Gene | LRT statistic | P value | BH-adjusted P | log2FC (interaction) |
|---|---|---|---|---|
| novel.1605 | 182.35 | 1.48E-41 | 1.68E-40 | -4.016 |
| RHSIM_Rhsim11G0106300 | 182.30 | 1.52E-41 | 1.72E-40 | -1.230 |
| RHSIM_Rhsim07G0115500 | 182.30 | 1.53E-41 | 1.73E-40 | 1.089 |
| RHSIM_Rhsim11G0170900 | 182.29 | 1.53E-41 | 1.74E-40 | -4.671 |
| RHSIM_Rhsim12G0002200 | 182.11 | 1.68E-41 | 1.90E-40 | 1.187 |
| RHSIM_Rhsim03G0000700 | 182.04 | 1.73E-41 | 1.96E-40 | -3.096 |
| RHSIM_Rhsim05G0020600 | 181.99 | 1.78E-41 | 2.01E-40 | 0.938 |
| RHSIM_Rhsim05G0058600 | 181.87 | 1.89E-41 | 2.14E-40 | 2.043 |
| RHSIM_Rhsim08G0145400 | 181.67 | 2.10E-41 | 2.37E-40 | 1.490 |
| RHSIM_Rhsim01G0262700 | 181.45 | 2.33E-41 | 2.64E-40 | 0.979 |
| RHSIM_Rhsim12G0016400 | 181.43 | 2.36E-41 | 2.66E-40 | -1.695 |
| RHSIM_Rhsim09G0196200 | 181.24 | 2.60E-41 | 2.93E-40 | -0.827 |
| RHSIM_Rhsim08G0077100 | 181.21 | 2.64E-41 | 2.98E-40 | 1.815 |
| RHSIM_Rhsim01G0209500 | 181.19 | 2.66E-41 | 3.00E-40 | -0.615 |
| RHSIM_Rhsim08G0137900 | 181.17 | 2.69E-41 | 3.03E-40 | 1.022 |
| RHSIM_Rhsim04G0070700 | 181.09 | 2.80E-41 | 3.16E-40 | 0.768 |
| RHSIM_Rhsim07G0202000 | 181.08 | 2.82E-41 | 3.18E-40 | 1.233 |
| RHSIM_Rhsim02G0143900 | 181.05 | 2.85E-41 | 3.21E-40 | -1.581 |
| RHSIM_Rhsim11G0003500 | 180.99 | 2.95E-41 | 3.32E-40 | 1.378 |
| RHSIM_Rhsim04G0097700 | 180.97 | 2.98E-41 | 3.36E-40 | -4.065 |
| novel.2096 | 180.93 | 3.04E-41 | 3.41E-40 | 1.138 |
| RHSIM_Rhsim10G0011100 | 180.78 | 3.28E-41 | 3.69E-40 | -1.215 |
| RHSIM_Rhsim01G0033100 | 180.76 | 3.30E-41 | 3.71E-40 | -1.378 |
| RHSIM_Rhsim07G0167800 | 180.72 | 3.38E-41 | 3.80E-40 | 1.952 |
| RHSIM_Rhsim01G0112300 | 180.70 | 3.40E-41 | 3.82E-40 | 49.497 |
| RHSIM_Rhsim12G0008700 | 180.70 | 3.41E-41 | 3.83E-40 | -2.620 |
| RHSIM_Rhsim04G0093900 | 180.65 | 3.49E-41 | 3.92E-40 | -1.910 |
| RHSIM_Rhsim07G0234200 | 180.59 | 3.59E-41 | 4.03E-40 | 1.742 |
| RHSIM_Rhsim05G0193900 | 180.39 | 3.98E-41 | 4.46E-40 | 0.697 |
| novel.2794 | 180.31 | 4.14E-41 | 4.64E-40 | -1.786 |
| RHSIM_Rhsim07G0032600 | 180.30 | 4.16E-41 | 4.66E-40 | -3.903 |
| RHSIM_Rhsim06G0077300 | 180.14 | 4.51E-41 | 5.05E-40 | 1.116 |

| Gene | LRT statistic | P value | BH-adjusted P | log2FC (interaction) |
|---|---|---|---|---|
| RHSIM_Rhsim03G0162000 | 180.05 | 4.72E-41 | 5.28E-40 | 2.431 |
| novel.3917 | 179.97 | 4.92E-41 | 5.50E-40 | 2.722 |
| RHSIM_RhsimUnG0009100 | 179.96 | 4.94E-41 | 5.53E-40 | -1.695 |
| RHSIM_Rhsim12G0161200 | 179.93 | 5.01E-41 | 5.60E-40 | 1.511 |
| RHSIM_Rhsim06G0034400 | 179.67 | 5.71E-41 | 6.38E-40 | 0.927 |
| RHSIM_Rhsim05G0181000 | 179.64 | 5.82E-41 | 6.50E-40 | -1.339 |
| novel.449 | 179.60 | 5.93E-41 | 6.62E-40 | 2.262 |
| RHSIM_Rhsim01G0141200 | 179.55 | 6.09E-41 | 6.80E-40 | -1.375 |
| RHSIM_Rhsim08G0019200 | 179.38 | 6.63E-41 | 7.39E-40 | -1.206 |
| RHSIM_Rhsim01G0131800 | 179.26 | 7.04E-41 | 7.85E-40 | 3.176 |
| RHSIM_Rhsim07G0073300 | 179.20 | 7.26E-41 | 8.10E-40 | 0.978 |
| RHSIM_Rhsim07G0084000 | 179.16 | 7.39E-41 | 8.23E-40 | -1.381 |
| RHSIM_Rhsim10G0118200 | 179.16 | 7.40E-41 | 8.24E-40 | -1.365 |
| RHSIM_Rhsim03G0127800 | 179.08 | 7.71E-41 | 8.59E-40 | 1.444 |
| RHSIM_Rhsim08G0112900 | 178.92 | 8.36E-41 | 9.30E-40 | 1.314 |
| RHSIM_Rhsim03G0200100 | 178.82 | 8.76E-41 | 9.74E-40 | -1.764 |
| RHSIM_Rhsim05G0068400 | 178.60 | 9.82E-41 | 1.09E-39 | -1.388 |
| RHSIM_Rhsim06G0047700 | 178.56 | 9.97E-41 | 1.11E-39 | 0.891 |
| RHSIM_Rhsim08G0041600 | 178.52 | 1.02E-40 | 1.14E-39 | -2.222 |
| RHSIM_Rhsim05G0053200 | 178.48 | 1.04E-40 | 1.16E-39 | -1.570 |
| RHSIM_Rhsim13G0071200 | 178.44 | 1.06E-40 | 1.18E-39 | -1.371 |
| RHSIM_Rhsim13G0184200 | 178.39 | 1.09E-40 | 1.21E-39 | -1.899 |
| RHSIM_Rhsim13G0175200 | 178.36 | 1.10E-40 | 1.23E-39 | -0.884 |
| RHSIM_Rhsim07G0123800 | 178.31 | 1.13E-40 | 1.26E-39 | -1.296 |
| RHSIM_RhsimUnG0073900 | 178.30 | 1.14E-40 | 1.26E-39 | -2.813 |
| RHSIM_RhsimUnG0136600 | 178.26 | 1.16E-40 | 1.29E-39 | 1.681 |
| RHSIM_Rhsim09G0181400 | 178.24 | 1.17E-40 | 1.30E-39 | -1.509 |
| RHSIM_Rhsim03G0009900 | 178.17 | 1.22E-40 | 1.35E-39 | 2.293 |
| novel.41 | 177.85 | 1.43E-40 | 1.58E-39 | 3.131 |
| RHSIM_Rhsim02G0239700 | 177.81 | 1.45E-40 | 1.61E-39 | 1.412 |
| RHSIM_Rhsim03G0176600 | 177.80 | 1.46E-40 | 1.62E-39 | 2.026 |
| RHSIM_Rhsim09G0115400 | 177.78 | 1.48E-40 | 1.63E-39 | -1.746 |

| Gene | LRT statistic | P value | BH-adjusted P | log2FC (interaction) |
|---|---|---|---|---|
| RHSIM_Rhsim02G0165300 | 177.71 | 1.54E-40 | 1.70E-39 | -1.654 |
| RHSIM_Rhsim05G0063100 | 177.64 | 1.59E-40 | 1.75E-39 | -0.987 |
| RHSIM_Rhsim13G0123700 | 177.60 | 1.62E-40 | 1.79E-39 | -1.667 |
| RHSIM_Rhsim04G0167000 | 177.44 | 1.75E-40 | 1.93E-39 | 1.103 |
| RHSIM_Rhsim05G0068100 | 177.12 | 2.06E-40 | 2.28E-39 | 1.703 |
| RHSIM_Rhsim01G0123600 | 177.10 | 2.08E-40 | 2.30E-39 | 3.977 |
| RHSIM_Rhsim05G0115100 | 177.06 | 2.13E-40 | 2.34E-39 | 1.423 |
| novel.4095 | 176.86 | 2.35E-40 | 2.59E-39 | -1.436 |
| RHSIM_Rhsim13G0233700 | 176.85 | 2.36E-40 | 2.60E-39 | -0.673 |
| RHSIM_Rhsim08G0115800 | 176.84 | 2.38E-40 | 2.62E-39 | 0.823 |
| RHSIM_Rhsim08G0217500 | 176.73 | 2.50E-40 | 2.75E-39 | -3.146 |
| RHSIM_Rhsim09G0128100 | 176.60 | 2.68E-40 | 2.94E-39 | 1.105 |
| RHSIM_Rhsim08G0229800 | 176.57 | 2.72E-40 | 2.99E-39 | -3.765 |
| RHSIM_Rhsim03G0068700 | 176.48 | 2.85E-40 | 3.13E-39 | -1.356 |
| RHSIM_Rhsim05G0194900 | 176.46 | 2.87E-40 | 3.15E-39 | -1.347 |
| RHSIM_Rhsim08G0146000 | 176.39 | 2.98E-40 | 3.28E-39 | -1.764 |
| RHSIM_Rhsim11G0098800 | 176.37 | 3.01E-40 | 3.30E-39 | 1.005 |
| RHSIM_Rhsim07G0045400 | 176.34 | 3.06E-40 | 3.36E-39 | -2.555 |
| RHSIM_Rhsim04G0032000 | 176.33 | 3.07E-40 | 3.36E-39 | 1.085 |
| RHSIM_Rhsim08G0079500 | 176.21 | 3.27E-40 | 3.58E-39 | 1.238 |
| novel.2985 | 176.13 | 3.39E-40 | 3.72E-39 | 3.458 |
| RHSIM_Rhsim08G0221300 | 176.08 | 3.49E-40 | 3.82E-39 | 3.231 |
| RHSIM_Rhsim03G0008900 | 176.04 | 3.55E-40 | 3.88E-39 | -4.418 |
| RHSIM_Rhsim07G0019100 | 175.98 | 3.65E-40 | 4.00E-39 | 0.848 |
| novel.2196 | 175.83 | 3.95E-40 | 4.32E-39 | 1.961 |
| RHSIM_Rhsim13G0209400 | 175.77 | 4.07E-40 | 4.45E-39 | -4.344 |
| RHSIM_Rhsim11G0028200 | 175.69 | 4.23E-40 | 4.63E-39 | 3.196 |
| RHSIM_Rhsim02G0169600 | 175.64 | 4.35E-40 | 4.76E-39 | 0.883 |
| RHSIM_Rhsim04G0214200 | 175.63 | 4.36E-40 | 4.76E-39 | 1.372 |
| RHSIM_Rhsim06G0012100 | 175.53 | 4.58E-40 | 5.01E-39 | -1.769 |
| RHSIM_Rhsim09G0068700 | 175.41 | 4.87E-40 | 5.32E-39 | 1.374 |
| RHSIM_Rhsim01G0001900 | 175.22 | 5.35E-40 | 5.85E-39 | -0.611 |

| Gene | LRT statistic | P value | BH-adjusted P | log2FC (interaction) |
|---|---|---|---|---|
| RHSIM_Rhsim02G0237100 | 174.97 | 6.06E-40 | 6.62E-39 | -2.135 |
| novel.1149 | 174.93 | 6.19E-40 | 6.76E-39 | 0.753 |
| RHSIM_Rhsim06G0130600 | 174.91 | 6.26E-40 | 6.83E-39 | 2.585 |
| RHSIM_Rhsim12G0005500 | 174.87 | 6.40E-40 | 6.98E-39 | 0.991 |
| RHSIM_Rhsim01G0289700 | 174.82 | 6.57E-40 | 7.16E-39 | -0.926 |
| RHSIM_Rhsim01G0117300 | 174.80 | 6.62E-40 | 7.21E-39 | 1.107 |
| RHSIM_Rhsim05G0163700 | 174.62 | 7.26E-40 | 7.90E-39 | 1.274 |
| RHSIM_Rhsim02G0022100 | 174.58 | 7.39E-40 | 8.05E-39 | 2.044 |
| RHSIM_Rhsim03G0031000 | 174.56 | 7.46E-40 | 8.12E-39 | -1.829 |
| RHSIM_Rhsim05G0203700 | 174.55 | 7.51E-40 | 8.17E-39 | 2.537 |
| RHSIM_Rhsim01G0221600 | 174.39 | 8.14E-40 | 8.85E-39 | 0.869 |
| RHSIM_Rhsim03G0186600 | 174.34 | 8.36E-40 | 9.09E-39 | -1.026 |
| RHSIM_RhsimUnG0126600 | 174.33 | 8.40E-40 | 9.12E-39 | -3.847 |
| RHSIM_Rhsim08G0230900 | 174.25 | 8.71E-40 | 9.46E-39 | -1.697 |
| RHSIM_Rhsim01G0190900 | 174.16 | 9.14E-40 | 9.93E-39 | -0.865 |
| RHSIM_Rhsim05G0006700 | 174.13 | 9.26E-40 | 1.01E-38 | 1.162 |
| RHSIM_Rhsim06G0206300 | 173.92 | 1.03E-39 | 1.12E-38 | 1.499 |
| novel.4300 | 173.88 | 1.05E-39 | 1.14E-38 | 2.987 |
| RHSIM_Rhsim10G0183600 | 173.87 | 1.05E-39 | 1.14E-38 | -1.553 |
| RHSIM_Rhsim02G0012600 | 173.78 | 1.10E-39 | 1.20E-38 | 1.143 |
| RHSIM_Rhsim06G0239700 | 173.78 | 1.10E-39 | 1.20E-38 | 2.348 |
| RHSIM_Rhsim04G0015500 | 173.75 | 1.12E-39 | 1.22E-38 | -1.056 |
| RHSIM_Rhsim03G0152000 | 173.75 | 1.12E-39 | 1.22E-38 | -0.809 |
| RHSIM_Rhsim02G0200400 | 173.73 | 1.13E-39 | 1.23E-38 | 1.559 |
| RHSIM_Rhsim11G0055800 | 173.68 | 1.16E-39 | 1.25E-38 | -1.353 |
| RHSIM_Rhsim09G0151100 | 173.61 | 1.20E-39 | 1.30E-38 | -0.798 |
| RHSIM_Rhsim03G0171300 | 173.57 | 1.23E-39 | 1.33E-38 | -0.668 |
| RHSIM_Rhsim12G0115900 | 173.49 | 1.28E-39 | 1.38E-38 | -1.121 |
| RHSIM_RhsimUnG0161900 | 173.45 | 1.30E-39 | 1.41E-38 | 3.426 |
| RHSIM_Rhsim02G0068000 | 173.41 | 1.33E-39 | 1.44E-38 | -2.093 |
| RHSIM_RhsimUnG0118000 | 173.37 | 1.36E-39 | 1.47E-38 | -1.057 |
| RHSIM_Rhsim09G0146100 | 173.27 | 1.43E-39 | 1.54E-38 | 1.403 |

| Gene | LRT statistic | P value | BH-adjusted P | log2FC (interaction) |
|---|---|---|---|---|
| RHSIM_Rhsim09G0007300 | 173.26 | 1.44E-39 | 1.55E-38 | 2.554 |
| novel.653 | 173.17 | 1.51E-39 | 1.62E-38 | 2.004 |
| RHSIM_Rhsim11G0016500 | 173.06 | 1.59E-39 | 1.71E-38 | 1.995 |
| RHSIM_Rhsim04G0185400 | 173.04 | 1.60E-39 | 1.73E-38 | -3.342 |
| novel.1025 | 172.89 | 1.73E-39 | 1.87E-38 | 1.029 |
| RHSIM_Rhsim07G0236400 | 172.88 | 1.73E-39 | 1.87E-38 | -3.067 |
| RHSIM_Rhsim12G0195800 | 172.70 | 1.90E-39 | 2.05E-38 | 1.118 |
| RHSIM_Rhsim02G0056700 | 172.70 | 1.91E-39 | 2.05E-38 | -0.887 |
| RHSIM_Rhsim04G0190200 | 172.67 | 1.93E-39 | 2.08E-38 | -0.939 |
| RHSIM_Rhsim07G0056400 | 172.58 | 2.02E-39 | 2.17E-38 | 0.795 |
| RHSIM_Rhsim02G0059400 | 172.57 | 2.03E-39 | 2.18E-38 | -0.720 |
| RHSIM_Rhsim10G0063200 | 172.56 | 2.05E-39 | 2.20E-38 | 1.202 |
| RHSIM_Rhsim08G0118800 | 172.50 | 2.10E-39 | 2.26E-38 | 2.692 |
| novel.2921 | 172.43 | 2.18E-39 | 2.33E-38 | 2.447 |
| RHSIM_Rhsim09G0003800 | 172.38 | 2.24E-39 | 2.40E-38 | -1.100 |
| RHSIM_Rhsim12G0198000 | 172.34 | 2.28E-39 | 2.45E-38 | -2.188 |
| RHSIM_Rhsim13G0161500 | 172.33 | 2.29E-39 | 2.46E-38 | 2.331 |
| RHSIM_RhsimUnG0168200 | 172.33 | 2.29E-39 | 2.46E-38 | 1.207 |
| RHSIM_Rhsim13G0201200 | 172.19 | 2.45E-39 | 2.63E-38 | 1.957 |
| RHSIM_Rhsim06G0138800 | 172.16 | 2.50E-39 | 2.68E-38 | -2.519 |
| RHSIM_Rhsim01G0005200 | 172.00 | 2.70E-39 | 2.89E-38 | -3.873 |
| RHSIM_Rhsim06G0035500 | 171.99 | 2.71E-39 | 2.90E-38 | -1.232 |
| RHSIM_Rhsim06G0078300 | 171.89 | 2.86E-39 | 3.06E-38 | -1.216 |
| RHSIM_Rhsim12G0005600 | 171.89 | 2.87E-39 | 3.06E-38 | 0.811 |
| novel.1207 | 171.83 | 2.95E-39 | 3.15E-38 | 3.070 |
| RHSIM_Rhsim13G0195100 | 171.81 | 2.97E-39 | 3.18E-38 | -1.412 |
| RHSIM_Rhsim04G0209400 | 171.71 | 3.13E-39 | 3.34E-38 | 0.966 |
| RHSIM_Rhsim01G0091900 | 171.55 | 3.39E-39 | 3.62E-38 | 1.226 |
| RHSIM_Rhsim07G0199700 | 171.44 | 3.58E-39 | 3.82E-38 | -1.560 |
| RHSIM_Rhsim08G0081800 | 171.43 | 3.61E-39 | 3.85E-38 | -2.144 |
| RHSIM_Rhsim01G0189100 | 171.25 | 3.95E-39 | 4.22E-38 | 1.284 |
| RHSIM_Rhsim02G0035700 | 171.14 | 4.18E-39 | 4.45E-38 | 1.292 |

| Gene | LRT statistic | P value | BH-adjusted P | log2FC (interaction) |
|---|---|---|---|---|
| RHSIM_Rhsim02G0054300 | 171.11 | 4.24E-39 | 4.52E-38 | 1.397 |
| RHSIM_Rhsim08G0200300 | 170.99 | 4.50E-39 | 4.79E-38 | 0.701 |
| RHSIM_Rhsim09G0005200 | 170.98 | 4.52E-39 | 4.81E-38 | 1.577 |
| RHSIM_Rhsim10G0039800 | 170.92 | 4.67E-39 | 4.97E-38 | -0.883 |
| RHSIM_Rhsim01G0213500 | 170.67 | 5.29E-39 | 5.62E-38 | 0.705 |
| RHSIM_Rhsim06G0143900 | 170.66 | 5.32E-39 | 5.65E-38 | 1.199 |
| RHSIM_Rhsim12G0055100 | 170.61 | 5.44E-39 | 5.78E-38 | -1.664 |
| RHSIM_Rhsim01G0276200 | 170.52 | 5.68E-39 | 6.04E-38 | 1.409 |
| RHSIM_Rhsim08G0250400 | 170.49 | 5.80E-39 | 6.16E-38 | 1.975 |
| RHSIM_Rhsim04G0073600 | 170.45 | 5.89E-39 | 6.26E-38 | 6.307 |
| RHSIM_Rhsim12G0157300 | 170.30 | 6.36E-39 | 6.75E-38 | -1.046 |
| RHSIM_Rhsim06G0046300 | 170.27 | 6.46E-39 | 6.86E-38 | 0.873 |
| RHSIM_Rhsim11G0069800 | 170.26 | 6.49E-39 | 6.88E-38 | -1.791 |
| RHSIM_Rhsim01G0260300 | 170.08 | 7.11E-39 | 7.54E-38 | 2.058 |
| RHSIM_Rhsim09G0086400 | 170.06 | 7.18E-39 | 7.61E-38 | 1.008 |
| RHSIM_Rhsim03G0130200 | 170.00 | 7.38E-39 | 7.83E-38 | 1.340 |
| RHSIM_Rhsim10G0133700 | 169.96 | 7.54E-39 | 7.99E-38 | -1.708 |
| RHSIM_Rhsim06G0055400 | 169.91 | 7.73E-39 | 8.18E-38 | 0.954 |
| RHSIM_Rhsim09G0212900 | 169.82 | 8.09E-39 | 8.56E-38 | -1.254 |
| novel.3308 | 169.82 | 8.12E-39 | 8.59E-38 | -1.478 |
| RHSIM_Rhsim07G0020900 | 169.72 | 8.52E-39 | 9.01E-38 | -1.890 |
| RHSIM_Rhsim01G0245900 | 169.62 | 8.96E-39 | 9.48E-38 | 1.080 |
| RHSIM_Rhsim08G0216700 | 169.56 | 9.23E-39 | 9.75E-38 | -2.024 |
| RHSIM_Rhsim03G0130300 | 169.53 | 9.39E-39 | 9.92E-38 | -1.142 |
| RHSIM_Rhsim03G0085200 | 169.44 | 9.78E-39 | 1.03E-37 | 1.460 |
| RHSIM_Rhsim10G0046800 | 169.37 | 1.01E-38 | 1.07E-37 | -2.231 |
| RHSIM_Rhsim03G0123300 | 169.19 | 1.11E-38 | 1.17E-37 | -5.599 |
| RHSIM_Rhsim09G0207600 | 169.03 | 1.20E-38 | 1.27E-37 | -1.302 |
| RHSIM_Rhsim09G0066900 | 168.80 | 1.35E-38 | 1.43E-37 | 1.232 |
| RHSIM_Rhsim06G0061200 | 168.72 | 1.41E-38 | 1.48E-37 | -4.656 |
| RHSIM_Rhsim13G0229100 | 168.72 | 1.41E-38 | 1.49E-37 | -2.416 |
| RHSIM_Rhsim05G0082100 | 168.68 | 1.44E-38 | 1.51E-37 | 1.580 |

| Gene | LRT statistic | P value | BH-adjusted P | log2FC (interaction) |
|---|---|---|---|---|
| RHSIM_Rhsim11G0025800 | 168.68 | 1.44E-38 | 1.51E-37 | 1.003 |
| novel.3462 | 168.62 | 1.48E-38 | 1.56E-37 | 3.408 |
| RHSIM_Rhsim12G0180300 | 168.49 | 1.58E-38 | 1.66E-37 | 1.014 |
| RHSIM_Rhsim13G0225300 | 168.41 | 1.65E-38 | 1.73E-37 | -1.776 |
| RHSIM_Rhsim08G0147100 | 168.38 | 1.67E-38 | 1.76E-37 | -1.231 |
| RHSIM_Rhsim12G0083500 | 168.26 | 1.78E-38 | 1.87E-37 | 1.359 |
| RHSIM_Rhsim11G0138400 | 168.22 | 1.81E-38 | 1.90E-37 | -4.518 |
| RHSIM_Rhsim01G0212000 | 168.18 | 1.85E-38 | 1.94E-37 | -2.023 |
| RHSIM_Rhsim03G0264500 | 168.17 | 1.85E-38 | 1.95E-37 | -1.295 |
| RHSIM_Rhsim01G0265500 | 168.15 | 1.87E-38 | 1.97E-37 | -1.362 |
| RHSIM_Rhsim09G0018600 | 168.13 | 1.90E-38 | 1.99E-37 | 1.743 |
| RHSIM_Rhsim09G0142800 | 168.09 | 1.94E-38 | 2.03E-37 | 0.693 |
| RHSIM_Rhsim12G0002400 | 167.92 | 2.11E-38 | 2.21E-37 | -0.978 |
| RHSIM_Rhsim10G0181300 | 167.91 | 2.11E-38 | 2.21E-37 | 0.933 |
| RHSIM_Rhsim13G0056800 | 167.89 | 2.14E-38 | 2.24E-37 | 3.827 |
| RHSIM_Rhsim07G0059100 | 167.87 | 2.16E-38 | 2.26E-37 | 1.236 |
| RHSIM_Rhsim02G0227800 | 167.85 | 2.18E-38 | 2.28E-37 | -1.940 |
| RHSIM_Rhsim04G0018900 | 167.84 | 2.19E-38 | 2.30E-37 | 0.690 |
| RHSIM_Rhsim03G0218000 | 167.70 | 2.35E-38 | 2.46E-37 | -1.164 |
| RHSIM_Rhsim11G0180200 | 167.64 | 2.42E-38 | 2.53E-37 | 1.750 |
| RHSIM_Rhsim08G0168700 | 167.61 | 2.46E-38 | 2.57E-37 | -1.581 |
| RHSIM_Rhsim08G0080400 | 167.60 | 2.48E-38 | 2.59E-37 | -3.526 |
| novel.3670 | 167.58 | 2.50E-38 | 2.62E-37 | 2.967 |
| RHSIM_Rhsim09G0091400 | 167.24 | 2.97E-38 | 3.10E-37 | -1.830 |
| RHSIM_Rhsim07G0181400 | 167.16 | 3.08E-38 | 3.22E-37 | -1.945 |
| RHSIM_Rhsim10G0008600 | 166.62 | 4.04E-38 | 4.22E-37 | 2.269 |
| RHSIM_Rhsim02G0076900 | 166.60 | 4.09E-38 | 4.26E-37 | 1.330 |
| novel.436 | 166.58 | 4.13E-38 | 4.30E-37 | -1.392 |
| novel.715 | 166.55 | 4.20E-38 | 4.38E-37 | 3.232 |
| RHSIM_Rhsim03G0011600 | 166.52 | 4.26E-38 | 4.44E-37 | 2.082 |
| RHSIM_Rhsim09G0095100 | 166.48 | 4.34E-38 | 4.52E-37 | 1.735 |
| RHSIM_Rhsim13G0174200 | 166.46 | 4.39E-38 | 4.57E-37 | 1.904 |

| Gene | LRT statistic | P value | BH-adjusted P | log2FC (interaction) |
|---|---|---|---|---|
| RHSIM_Rhsim04G0151700 | 166.43 | 4.45E-38 | 4.63E-37 | -2.397 |
| RHSIM_Rhsim12G0035700 | 166.42 | 4.47E-38 | 4.66E-37 | 1.511 |
| RHSIM_Rhsim11G0077800 | 166.26 | 4.85E-38 | 5.04E-37 | 2.459 |
| RHSIM_Rhsim03G0192300 | 166.17 | 5.07E-38 | 5.27E-37 | -1.887 |
| RHSIM_Rhsim01G0249200 | 166.10 | 5.26E-38 | 5.47E-37 | -2.855 |
| RHSIM_Rhsim12G0088300 | 166.09 | 5.28E-38 | 5.49E-37 | 1.165 |
| RHSIM_Rhsim05G0141400 | 165.98 | 5.60E-38 | 5.81E-37 | 1.065 |
| RHSIM_Rhsim06G0148100 | 165.94 | 5.69E-38 | 5.91E-37 | -2.505 |
| RHSIM_Rhsim01G0172600 | 165.85 | 5.96E-38 | 6.19E-37 | 0.970 |
| RHSIM_Rhsim04G0104600 | 165.83 | 6.03E-38 | 6.26E-37 | -4.149 |
| RHSIM_Rhsim05G0149300 | 165.83 | 6.04E-38 | 6.26E-37 | -0.967 |
| RHSIM_Rhsim06G0244300 | 165.63 | 6.67E-38 | 6.92E-37 | 0.929 |
| RHSIM_Rhsim02G0171900 | 165.62 | 6.70E-38 | 6.94E-37 | 1.622 |
| RHSIM_Rhsim05G0158800 | 165.55 | 6.95E-38 | 7.20E-37 | 2.031 |
| RHSIM_Rhsim12G0057500 | 165.45 | 7.30E-38 | 7.56E-37 | -0.823 |
| RHSIM_Rhsim02G0103400 | 165.42 | 7.39E-38 | 7.65E-37 | 1.336 |
| RHSIM_Rhsim09G0085500 | 165.40 | 7.46E-38 | 7.72E-37 | -1.331 |
| RHSIM_Rhsim08G0179600 | 165.25 | 8.09E-38 | 8.36E-37 | 1.296 |
| RHSIM_Rhsim10G0040200 | 165.22 | 8.19E-38 | 8.47E-37 | -1.411 |
| RHSIM_Rhsim03G0145900 | 165.20 | 8.25E-38 | 8.53E-37 | 1.214 |
| RHSIM_Rhsim07G0251700 | 164.96 | 9.35E-38 | 9.66E-37 | 1.341 |
| RHSIM_Rhsim13G0062300 | 164.86 | 9.80E-38 | 1.01E-36 | 1.011 |
| RHSIM_Rhsim13G0078200 | 164.77 | 1.03E-37 | 1.06E-36 | 1.267 |
| RHSIM_RhsimUnG0187800 | 164.71 | 1.06E-37 | 1.09E-36 | -0.997 |
| RHSIM_Rhsim04G0151300 | 164.68 | 1.07E-37 | 1.11E-36 | -0.771 |
| RHSIM_Rhsim05G0012600 | 164.56 | 1.14E-37 | 1.18E-36 | 0.935 |
| RHSIM_Rhsim05G0234300 | 164.52 | 1.17E-37 | 1.20E-36 | 0.864 |
| RHSIM_Rhsim07G0193500 | 164.40 | 1.24E-37 | 1.27E-36 | 1.754 |
| RHSIM_Rhsim01G0207700 | 164.33 | 1.28E-37 | 1.32E-36 | 1.182 |
| RHSIM_RhsimUnG0112600 | 164.23 | 1.35E-37 | 1.39E-36 | 3.592 |
| RHSIM_Rhsim06G0000700 | 164.15 | 1.40E-37 | 1.45E-36 | 1.933 |
| RHSIM_Rhsim01G0281400 | 164.15 | 1.41E-37 | 1.45E-36 | -3.494 |

| Gene | LRT statistic | P value | BH-adjusted P | log2FC (interaction) |
|---|---|---|---|---|
| RHSIM_Rhsim09G0078600 | 163.88 | 1.61E-37 | 1.65E-36 | 1.370 |
| RHSIM_Rhsim11G0112000 | 163.87 | 1.62E-37 | 1.66E-36 | 1.003 |
| RHSIM_Rhsim03G0212500 | 163.72 | 1.74E-37 | 1.79E-36 | 0.642 |
| RHSIM_Rhsim01G0074200 | 163.69 | 1.76E-37 | 1.81E-36 | 0.899 |
| RHSIM_Rhsim06G0165700 | 163.69 | 1.77E-37 | 1.82E-36 | 1.465 |
| RHSIM_Rhsim01G0088100 | 163.66 | 1.80E-37 | 1.85E-36 | 0.745 |
| RHSIM_Rhsim13G0227700 | 163.61 | 1.84E-37 | 1.89E-36 | -3.207 |
| RHSIM_Rhsim06G0154000 | 163.53 | 1.91E-37 | 1.96E-36 | -1.214 |
| RHSIM_Rhsim06G0006500 | 163.40 | 2.05E-37 | 2.10E-36 | 1.887 |
| RHSIM_Rhsim12G0087900 | 163.34 | 2.11E-37 | 2.16E-36 | 1.149 |
| RHSIM_Rhsim12G0160500 | 163.30 | 2.16E-37 | 2.21E-36 | 0.896 |
| RHSIM_Rhsim04G0086400 | 163.14 | 2.33E-37 | 2.39E-36 | -3.698 |
| RHSIM_Rhsim08G0234000 | 163.13 | 2.34E-37 | 2.40E-36 | 3.394 |
| RHSIM_Rhsim13G0121400 | 163.07 | 2.42E-37 | 2.48E-36 | -1.417 |
| RHSIM_Rhsim12G0161500 | 162.91 | 2.62E-37 | 2.68E-36 | 0.982 |
| RHSIM_Rhsim13G0115200 | 162.69 | 2.93E-37 | 3.00E-36 | 1.130 |
| RHSIM_Rhsim12G0066600 | 162.68 | 2.94E-37 | 3.01E-36 | 0.683 |
| RHSIM_Rhsim08G0212700 | 162.66 | 2.97E-37 | 3.03E-36 | -1.472 |
| RHSIM_Rhsim02G0120700 | 162.56 | 3.11E-37 | 3.18E-36 | 0.875 |
| RHSIM_Rhsim07G0163100 | 162.51 | 3.19E-37 | 3.26E-36 | 1.416 |
| RHSIM_Rhsim09G0196400 | 162.50 | 3.22E-37 | 3.29E-36 | 1.195 |
| RHSIM_Rhsim09G0118400 | 162.37 | 3.43E-37 | 3.50E-36 | 0.933 |
| novel.1159 | 162.33 | 3.51E-37 | 3.58E-36 | 1.025 |
| RHSIM_Rhsim08G0197200 | 162.27 | 3.62E-37 | 3.69E-36 | 1.536 |
| RHSIM_Rhsim03G0136100 | 162.26 | 3.63E-37 | 3.71E-36 | -1.636 |
| RHSIM_Rhsim08G0052500 | 162.22 | 3.71E-37 | 3.78E-36 | 4.123 |
| RHSIM_Rhsim02G0096300 | 162.18 | 3.78E-37 | 3.86E-36 | -0.637 |
| RHSIM_Rhsim06G0223900 | 162.17 | 3.80E-37 | 3.87E-36 | 2.095 |
| RHSIM_Rhsim04G0165300 | 162.08 | 3.98E-37 | 4.05E-36 | 0.844 |
| RHSIM_Rhsim13G0230800 | 162.06 | 4.01E-37 | 4.08E-36 | -1.793 |
| RHSIM_Rhsim02G0066700 | 162.02 | 4.10E-37 | 4.18E-36 | -2.001 |
| RHSIM_Rhsim09G0200700 | 161.95 | 4.25E-37 | 4.32E-36 | -2.136 |

| Gene | LRT statistic | P value | BH-adjusted P | log2FC (interaction) |
|---|---|---|---|---|
| RHSIM_Rhsim01G0125200 | 161.72 | 4.76E-37 | 4.84E-36 | -0.764 |
| RHSIM_Rhsim13G0177400 | 161.67 | 4.89E-37 | 4.98E-36 | -0.837 |
| RHSIM_Rhsim08G0208000 | 161.63 | 4.99E-37 | 5.07E-36 | 0.709 |
| RHSIM_Rhsim01G0240100 | 161.57 | 5.14E-37 | 5.22E-36 | -1.296 |
| RHSIM_Rhsim02G0067300 | 161.57 | 5.15E-37 | 5.23E-36 | -2.274 |
| RHSIM_Rhsim10G0117100 | 161.38 | 5.65E-37 | 5.74E-36 | 1.650 |
| RHSIM_Rhsim11G0163900 | 161.34 | 5.78E-37 | 5.87E-36 | 0.775 |
| RHSIM_Rhsim06G0006100 | 161.31 | 5.84E-37 | 5.92E-36 | 0.885 |
| RHSIM_Rhsim02G0119900 | 161.17 | 6.27E-37 | 6.36E-36 | 1.870 |
| novel.1317 | 161.04 | 6.71E-37 | 6.81E-36 | 0.891 |
| RHSIM_Rhsim12G0103700 | 161.00 | 6.84E-37 | 6.93E-36 | -1.922 |
| RHSIM_Rhsim11G0045300 | 161.00 | 6.86E-37 | 6.95E-36 | -0.818 |
| RHSIM_Rhsim06G0200600 | 160.94 | 7.04E-37 | 7.13E-36 | -2.542 |
| RHSIM_Rhsim07G0072200 | 160.93 | 7.09E-37 | 7.17E-36 | 1.334 |
| RHSIM_Rhsim08G0039700 | 160.92 | 7.14E-37 | 7.23E-36 | 2.039 |
| RHSIM_Rhsim06G0009200 | 160.88 | 7.25E-37 | 7.34E-36 | -2.084 |
| RHSIM_Rhsim07G0234600 | 160.86 | 7.34E-37 | 7.42E-36 | 1.079 |
| RHSIM_Rhsim12G0052600 | 160.80 | 7.55E-37 | 7.63E-36 | 3.030 |
| RHSIM_Rhsim02G0091100 | 160.78 | 7.65E-37 | 7.73E-36 | 1.645 |
| RHSIM_Rhsim02G0237500 | 160.55 | 8.57E-37 | 8.66E-36 | 1.520 |
| RHSIM_Rhsim13G0023000 | 160.55 | 8.60E-37 | 8.68E-36 | -3.010 |
| RHSIM_Rhsim03G0128000 | 160.49 | 8.86E-37 | 8.94E-36 | 0.928 |
| RHSIM_Rhsim13G0091600 | 160.48 | 8.87E-37 | 8.95E-36 | -2.986 |
| RHSIM_Rhsim04G0130700 | 160.27 | 9.88E-37 | 9.97E-36 | 1.169 |
| RHSIM_Rhsim13G0073700 | 160.18 | 1.03E-36 | 1.04E-35 | -0.785 |
| RHSIM_Rhsim08G0216200 | 160.08 | 1.09E-36 | 1.10E-35 | 1.616 |
| RHSIM_Rhsim03G0258200 | 160.03 | 1.12E-36 | 1.12E-35 | -0.605 |
| RHSIM_Rhsim05G0180900 | 160.00 | 1.13E-36 | 1.14E-35 | -6.069 |
| RHSIM_Rhsim05G0233100 | 159.93 | 1.17E-36 | 1.18E-35 | 1.025 |
| RHSIM_Rhsim06G0034000 | 159.92 | 1.18E-36 | 1.18E-35 | -0.745 |
| RHSIM_Rhsim11G0014500 | 159.77 | 1.27E-36 | 1.28E-35 | -0.879 |
| RHSIM_Rhsim06G0214700 | 159.63 | 1.36E-36 | 1.37E-35 | 1.041 |

| Gene | LRT statistic | P value | BH-adjusted P | log2FC (interaction) |
|---|---|---|---|---|
| RHSIM_Rhsim10G0069400 | 159.59 | 1.39E-36 | 1.40E-35 | -1.402 |
| RHSIM_Rhsim06G0011800 | 159.47 | 1.48E-36 | 1.49E-35 | 1.020 |
| RHSIM_Rhsim13G0065700 | 159.43 | 1.51E-36 | 1.52E-35 | -1.095 |
| RHSIM_Rhsim02G0243300 | 159.37 | 1.55E-36 | 1.56E-35 | -3.048 |
| novel.4465 | 159.32 | 1.60E-36 | 1.60E-35 | 3.883 |
| RHSIM_Rhsim07G0190000 | 159.19 | 1.70E-36 | 1.71E-35 | -1.077 |
| RHSIM_Rhsim06G0133700 | 159.12 | 1.76E-36 | 1.77E-35 | -2.323 |
| RHSIM_Rhsim04G0240500 | 159.04 | 1.83E-36 | 1.84E-35 | 0.959 |
| RHSIM_Rhsim01G0250200 | 158.97 | 1.90E-36 | 1.91E-35 | -1.951 |
| RHSIM_Rhsim12G0076500 | 158.95 | 1.92E-36 | 1.92E-35 | 1.546 |
| RHSIM_RhsimUnG0199600 | 158.94 | 1.93E-36 | 1.94E-35 | 2.436 |
| RHSIM_Rhsim12G0071500 | 158.92 | 1.94E-36 | 1.95E-35 | -2.000 |
| RHSIM_Rhsim09G0104200 | 158.78 | 2.09E-36 | 2.09E-35 | 1.153 |
| RHSIM_Rhsim07G0008100 | 158.65 | 2.23E-36 | 2.24E-35 | -0.999 |
| RHSIM_Rhsim07G0257900 | 158.58 | 2.32E-36 | 2.32E-35 | -2.309 |
| RHSIM_Rhsim07G0131600 | 158.51 | 2.40E-36 | 2.40E-35 | 1.334 |
| RHSIM_Rhsim04G0169200 | 158.40 | 2.53E-36 | 2.53E-35 | -1.006 |
| RHSIM_Rhsim08G0105000 | 158.25 | 2.73E-36 | 2.73E-35 | -3.309 |
| RHSIM_Rhsim05G0208800 | 158.17 | 2.84E-36 | 2.84E-35 | -4.123 |
| novel.75 | 158.14 | 2.89E-36 | 2.89E-35 | 0.945 |
| RHSIM_Rhsim06G0048700 | 158.10 | 2.95E-36 | 2.95E-35 | 1.042 |
| RHSIM_Rhsim06G0152600 | 158.03 | 3.04E-36 | 3.04E-35 | -48.871 |
| RHSIM_Rhsim08G0160300 | 157.91 | 3.23E-36 | 3.23E-35 | -1.560 |
| RHSIM_Rhsim11G0017000 | 157.84 | 3.36E-36 | 3.35E-35 | -3.155 |
| RHSIM_Rhsim09G0152100 | 157.83 | 3.38E-36 | 3.37E-35 | -1.188 |
| RHSIM_Rhsim01G0281700 | 157.70 | 3.61E-36 | 3.60E-35 | -3.237 |
| RHSIM_Rhsim05G0102200 | 157.56 | 3.86E-36 | 3.84E-35 | 1.598 |
| RHSIM_Rhsim07G0055600 | 157.53 | 3.92E-36 | 3.90E-35 | -1.593 |
| RHSIM_Rhsim05G0186600 | 157.49 | 4.00E-36 | 3.99E-35 | 2.167 |
| RHSIM_Rhsim10G0165500 | 157.46 | 4.06E-36 | 4.04E-35 | -1.795 |
| RHSIM_Rhsim01G0109600 | 157.42 | 4.14E-36 | 4.12E-35 | -3.494 |
| RHSIM_Rhsim05G0172400 | 157.42 | 4.14E-36 | 4.12E-35 | -1.803 |

| Gene | LRT statistic | P value | BH-adjusted P | log2FC (interaction) |
|---|---|---|---|---|
| RHSIM_Rhsim05G0223000 | 157.41 | 4.17E-36 | 4.15E-35 | 1.186 |
| RHSIM_Rhsim12G0044800 | 157.33 | 4.34E-36 | 4.32E-35 | -4.119 |
| RHSIM_Rhsim11G0148300 | 157.28 | 4.45E-36 | 4.42E-35 | 0.960 |
| RHSIM_Rhsim01G0090700 | 157.28 | 4.45E-36 | 4.42E-35 | -1.597 |
| RHSIM_Rhsim06G0242700 | 157.23 | 4.56E-36 | 4.53E-35 | 0.803 |
| novel.168 | 157.09 | 4.88E-36 | 4.85E-35 | -2.092 |
| RHSIM_Rhsim13G0067700 | 156.82 | 5.59E-36 | 5.55E-35 | -0.725 |
| RHSIM_Rhsim11G0088600 | 156.80 | 5.65E-36 | 5.60E-35 | 1.254 |
| RHSIM_Rhsim12G0063300 | 156.71 | 5.92E-36 | 5.87E-35 | 0.997 |
| RHSIM_Rhsim02G0215800 | 156.68 | 6.03E-36 | 5.97E-35 | 2.354 |
| novel.650 | 156.66 | 6.07E-36 | 6.02E-35 | -2.265 |
| RHSIM_Rhsim05G0053600 | 156.65 | 6.09E-36 | 6.04E-35 | -3.257 |
| RHSIM_Rhsim13G0081400 | 156.61 | 6.24E-36 | 6.18E-35 | -1.862 |
| RHSIM_Rhsim12G0063600 | 156.52 | 6.52E-36 | 6.46E-35 | 1.912 |
| RHSIM_Rhsim06G0073600 | 156.45 | 6.73E-36 | 6.67E-35 | -0.847 |
| RHSIM_Rhsim03G0256500 | 156.45 | 6.74E-36 | 6.67E-35 | 0.603 |
| RHSIM_Rhsim08G0045800 | 156.20 | 7.66E-36 | 7.58E-35 | 0.472 |
| RHSIM_Rhsim01G0206500 | 156.17 | 7.78E-36 | 7.69E-35 | 0.809 |
| RHSIM_Rhsim01G0074400 | 156.06 | 8.21E-36 | 8.11E-35 | 2.826 |
| RHSIM_Rhsim08G0203800 | 156.05 | 8.27E-36 | 8.17E-35 | -2.526 |
| novel.1973 | 156.02 | 8.38E-36 | 8.28E-35 | 1.303 |
| RHSIM_Rhsim07G0003200 | 155.99 | 8.52E-36 | 8.41E-35 | 1.668 |
| RHSIM_Rhsim07G0117700 | 155.89 | 8.97E-36 | 8.85E-35 | -1.031 |
| RHSIM_Rhsim05G0108800 | 155.57 | 1.05E-35 | 1.04E-34 | 1.357 |
| RHSIM_Rhsim02G0107000 | 155.54 | 1.07E-35 | 1.05E-34 | 0.731 |
| RHSIM_Rhsim07G0076100 | 155.46 | 1.11E-35 | 1.10E-34 | -3.043 |
| RHSIM_Rhsim01G0283500 | 155.42 | 1.13E-35 | 1.12E-34 | -1.255 |
| RHSIM_Rhsim08G0180900 | 155.40 | 1.14E-35 | 1.13E-34 | -1.258 |
| RHSIM_Rhsim07G0134000 | 155.06 | 1.36E-35 | 1.34E-34 | -1.418 |
| RHSIM_Rhsim01G0123200 | 155.03 | 1.38E-35 | 1.36E-34 | -1.502 |
| novel.224 | 154.94 | 1.45E-35 | 1.42E-34 | 1.148 |
| RHSIM_Rhsim04G0226400 | 154.75 | 1.59E-35 | 1.57E-34 | -1.684 |

| Gene | LRT statistic | P value | BH-adjusted P | log2FC (interaction) |
|---|---|---|---|---|
| RHSIM_Rhsim06G0243100 | 154.69 | 1.64E-35 | 1.61E-34 | -0.910 |
| RHSIM_Rhsim02G0228100 | 154.66 | 1.66E-35 | 1.63E-34 | -4.786 |
| RHSIM_Rhsim08G0232000 | 154.66 | 1.66E-35 | 1.63E-34 | -1.083 |
| RHSIM_Rhsim13G0100500 | 154.55 | 1.76E-35 | 1.73E-34 | 1.527 |
| RHSIM_Rhsim09G0103400 | 154.54 | 1.77E-35 | 1.74E-34 | -0.852 |
| RHSIM_Rhsim13G0107600 | 154.52 | 1.78E-35 | 1.75E-34 | 2.796 |
| RHSIM_Rhsim02G0127700 | 154.46 | 1.84E-35 | 1.80E-34 | -1.608 |
| RHSIM_Rhsim04G0126900 | 154.42 | 1.87E-35 | 1.84E-34 | 1.317 |
| RHSIM_Rhsim09G0143200 | 154.35 | 1.94E-35 | 1.90E-34 | 0.690 |
| RHSIM_Rhsim10G0179500 | 154.24 | 2.05E-35 | 2.01E-34 | -1.324 |
| RHSIM_Rhsim07G0032300 | 154.24 | 2.06E-35 | 2.01E-34 | -2.109 |
| RHSIM_Rhsim03G0235000 | 153.98 | 2.34E-35 | 2.30E-34 | -0.918 |
| RHSIM_Rhsim01G0287600 | 153.97 | 2.35E-35 | 2.30E-34 | -1.439 |
| RHSIM_Rhsim04G0110900 | 153.95 | 2.38E-35 | 2.33E-34 | -1.449 |
| RHSIM_Rhsim09G0165700 | 153.86 | 2.48E-35 | 2.43E-34 | 0.843 |
| RHSIM_Rhsim09G0155900 | 153.85 | 2.50E-35 | 2.45E-34 | -1.532 |
| RHSIM_Rhsim04G0063600 | 153.79 | 2.57E-35 | 2.52E-34 | 2.668 |
| RHSIM_Rhsim02G0122600 | 153.50 | 2.98E-35 | 2.91E-34 | -14.192 |
| RHSIM_Rhsim13G0006300 | 153.20 | 3.47E-35 | 3.39E-34 | 1.139 |
| RHSIM_Rhsim05G0068700 | 153.19 | 3.48E-35 | 3.40E-34 | 0.651 |
| RHSIM_Rhsim04G0016300 | 153.09 | 3.66E-35 | 3.58E-34 | 1.145 |
| RHSIM_Rhsim01G0237700 | 152.94 | 3.94E-35 | 3.85E-34 | -2.935 |
| RHSIM_Rhsim05G0229900 | 152.94 | 3.95E-35 | 3.86E-34 | -0.752 |
| RHSIM_RhsimUnG0131800 | 152.93 | 3.97E-35 | 3.88E-34 | 3.030 |
| RHSIM_Rhsim03G0009200 | 152.80 | 4.24E-35 | 4.14E-34 | 1.954 |
| RHSIM_Rhsim02G0002000 | 152.70 | 4.45E-35 | 4.34E-34 | 0.976 |
| RHSIM_Rhsim03G0151300 | 152.62 | 4.63E-35 | 4.51E-34 | 2.211 |
| novel.1538 | 152.62 | 4.65E-35 | 4.53E-34 | -0.832 |
| RHSIM_Rhsim03G0123400 | 152.50 | 4.94E-35 | 4.81E-34 | -2.534 |
| RHSIM_Rhsim07G0040000 | 152.49 | 4.95E-35 | 4.82E-34 | 0.764 |
| RHSIM_Rhsim08G0164000 | 152.44 | 5.08E-35 | 4.95E-34 | 1.405 |
| RHSIM_Rhsim04G0168900 | 152.37 | 5.25E-35 | 5.11E-34 | 1.443 |

| Gene | LRT statistic | P value | BH-adjusted P | log2FC (interaction) |
|---|---|---|---|---|
| RHSIM_Rhsim03G0223100 | 152.36 | 5.28E-35 | 5.13E-34 | -1.744 |
| RHSIM_RhsimUnG0213800 | 152.35 | 5.32E-35 | 5.18E-34 | 5.124 |
| RHSIM_Rhsim02G0106700 | 152.08 | 6.08E-35 | 5.91E-34 | -1.450 |
| RHSIM_Rhsim05G0098000 | 152.00 | 6.34E-35 | 6.17E-34 | 0.932 |
| RHSIM_Rhsim11G0136900 | 151.99 | 6.38E-35 | 6.20E-34 | 0.578 |
| RHSIM_Rhsim04G0011100 | 151.97 | 6.43E-35 | 6.24E-34 | -1.307 |
| RHSIM_Rhsim07G0136200 | 151.91 | 6.63E-35 | 6.44E-34 | -1.024 |
| RHSIM_Rhsim12G0207000 | 151.90 | 6.66E-35 | 6.47E-34 | 4.278 |
| RHSIM_Rhsim12G0136600 | 151.76 | 7.14E-35 | 6.92E-34 | 1.942 |
| RHSIM_Rhsim01G0108900 | 151.72 | 7.29E-35 | 7.07E-34 | -3.427 |
| RHSIM_Rhsim02G0067200 | 151.67 | 7.50E-35 | 7.27E-34 | -4.560 |
| RHSIM_Rhsim10G0060400 | 151.50 | 8.17E-35 | 7.92E-34 | 1.402 |
| RHSIM_Rhsim08G0089100 | 151.45 | 8.34E-35 | 8.09E-34 | -1.094 |
| RHSIM_Rhsim07G0208400 | 151.38 | 8.66E-35 | 8.39E-34 | -1.999 |
| RHSIM_Rhsim09G0187300 | 151.29 | 9.05E-35 | 8.76E-34 | 1.404 |
| RHSIM_Rhsim13G0167300 | 151.20 | 9.46E-35 | 9.16E-34 | 2.551 |
| RHSIM_Rhsim09G0107000 | 151.16 | 9.67E-35 | 9.36E-34 | 2.273 |
| RHSIM_Rhsim02G0234600 | 151.15 | 9.74E-35 | 9.42E-34 | -0.973 |
| RHSIM_Rhsim11G0008200 | 151.11 | 9.91E-35 | 9.58E-34 | 2.597 |
| RHSIM_Rhsim03G0092300 | 151.00 | 1.05E-34 | 1.01E-33 | 1.448 |
| RHSIM_Rhsim07G0229500 | 150.96 | 1.07E-34 | 1.04E-33 | -2.185 |
| RHSIM_Rhsim04G0197400 | 150.63 | 1.26E-34 | 1.22E-33 | 0.718 |
| RHSIM_Rhsim01G0008300 | 150.62 | 1.27E-34 | 1.22E-33 | 0.792 |
| RHSIM_Rhsim08G0073000 | 150.59 | 1.29E-34 | 1.24E-33 | -4.103 |
| RHSIM_Rhsim11G0177800 | 150.52 | 1.33E-34 | 1.29E-33 | 1.837 |
| RHSIM_Rhsim05G0045000 | 150.50 | 1.35E-34 | 1.30E-33 | 0.886 |
| novel.2704 | 150.39 | 1.42E-34 | 1.37E-33 | -0.854 |
| RHSIM_Rhsim06G0050300 | 150.36 | 1.44E-34 | 1.39E-33 | -1.366 |
| RHSIM_Rhsim01G0191400 | 150.35 | 1.45E-34 | 1.40E-33 | 3.208 |
| RHSIM_Rhsim06G0210300 | 150.35 | 1.45E-34 | 1.40E-33 | 2.250 |
| RHSIM_Rhsim13G0136100 | 150.32 | 1.48E-34 | 1.42E-33 | 1.974 |
| RHSIM_Rhsim08G0167900 | 150.28 | 1.50E-34 | 1.45E-33 | 2.916 |

| Gene | LRT statistic | P value | BH-adjusted P | log2FC (interaction) |
|---|---|---|---|---|
| RHSIM_Rhsim03G0137600 | 150.22 | 1.55E-34 | 1.49E-33 | -3.862 |
| RHSIM_Rhsim06G0063700 | 150.18 | 1.58E-34 | 1.52E-33 | 0.899 |
| RHSIM_Rhsim11G0098400 | 150.09 | 1.66E-34 | 1.59E-33 | 2.129 |
| RHSIM_Rhsim13G0070200 | 150.00 | 1.73E-34 | 1.67E-33 | -1.847 |
| RHSIM_Rhsim06G0052600 | 149.98 | 1.75E-34 | 1.69E-33 | -2.217 |
| RHSIM_Rhsim04G0248100 | 149.94 | 1.79E-34 | 1.72E-33 | 1.016 |
| RHSIM_Rhsim12G0118800 | 149.85 | 1.87E-34 | 1.79E-33 | 1.197 |
| novel.4505 | 149.82 | 1.90E-34 | 1.82E-33 | 1.269 |
| RHSIM_Rhsim08G0139800 | 149.64 | 2.07E-34 | 1.99E-33 | 1.421 |
| RHSIM_Rhsim06G0087800 | 149.55 | 2.17E-34 | 2.08E-33 | -1.067 |
| RHSIM_Rhsim01G0249600 | 149.42 | 2.33E-34 | 2.23E-33 | -2.175 |
| novel.3253 | 149.37 | 2.38E-34 | 2.28E-33 | 3.677 |
| RHSIM_Rhsim05G0187700 | 149.33 | 2.43E-34 | 2.33E-33 | -0.847 |
| RHSIM_Rhsim01G0001000 | 149.31 | 2.45E-34 | 2.35E-33 | -0.655 |
| RHSIM_Rhsim09G0157700 | 149.28 | 2.49E-34 | 2.39E-33 | -0.876 |
| RHSIM_Rhsim04G0022500 | 149.28 | 2.49E-34 | 2.39E-33 | -0.858 |
| RHSIM_Rhsim08G0126600 | 149.27 | 2.51E-34 | 2.40E-33 | -1.009 |
| RHSIM_RhsimUnG0123600 | 149.22 | 2.57E-34 | 2.45E-33 | -4.213 |
| RHSIM_Rhsim12G0209800 | 149.12 | 2.70E-34 | 2.58E-33 | -1.644 |
| RHSIM_Rhsim07G0028700 | 148.96 | 2.92E-34 | 2.79E-33 | -0.850 |
| RHSIM_Rhsim13G0186800 | 148.96 | 2.93E-34 | 2.80E-33 | -2.475 |
| RHSIM_Rhsim08G0218800 | 148.80 | 3.18E-34 | 3.04E-33 | -2.830 |
| RHSIM_Rhsim01G0007900 | 148.74 | 3.26E-34 | 3.12E-33 | 1.990 |
| novel.2476 | 148.37 | 3.94E-34 | 3.76E-33 | 1.989 |
| RHSIM_Rhsim13G0001800 | 148.37 | 3.95E-34 | 3.77E-33 | -3.840 |
| RHSIM_Rhsim10G0021600 | 148.35 | 3.98E-34 | 3.80E-33 | -1.168 |
| RHSIM_Rhsim05G0191400 | 148.34 | 4.00E-34 | 3.82E-33 | -0.993 |
| RHSIM_Rhsim13G0217800 | 148.31 | 4.05E-34 | 3.86E-33 | 1.297 |
| RHSIM_Rhsim12G0132300 | 148.31 | 4.05E-34 | 3.87E-33 | -7.445 |
| RHSIM_Rhsim10G0065800 | 148.24 | 4.20E-34 | 4.00E-33 | -1.459 |
| RHSIM_Rhsim13G0086500 | 148.19 | 4.31E-34 | 4.10E-33 | -1.456 |
| RHSIM_Rhsim11G0172600 | 148.19 | 4.32E-34 | 4.11E-33 | -0.915 |

| Gene | LRT statistic | P value | BH-adjusted P | log2FC (interaction) |
|---|---|---|---|---|
| RHSIM_Rhsim03G0229500 | 148.17 | 4.36E-34 | 4.15E-33 | -1.539 |
| novel.31 | 148.08 | 4.57E-34 | 4.35E-33 | -0.899 |
| RHSIM_RhsimUnG0120500 | 148.06 | 4.60E-34 | 4.38E-33 | 0.875 |
| RHSIM_Rhsim09G0142000 | 148.03 | 4.67E-34 | 4.45E-33 | -1.276 |
| RHSIM_Rhsim08G0236000 | 148.02 | 4.70E-34 | 4.47E-33 | 0.818 |
| RHSIM_Rhsim12G0063700 | 148.01 | 4.73E-34 | 4.49E-33 | -1.006 |
| RHSIM_Rhsim10G0147800 | 147.99 | 4.77E-34 | 4.54E-33 | 0.960 |
| RHSIM_RhsimUnG0005700 | 147.96 | 4.83E-34 | 4.59E-33 | -2.226 |
| RHSIM_Rhsim05G0230200 | 147.93 | 4.90E-34 | 4.66E-33 | 2.448 |
| RHSIM_Rhsim11G0056500 | 147.83 | 5.16E-34 | 4.89E-33 | -1.245 |
| RHSIM_Rhsim03G0165500 | 147.83 | 5.17E-34 | 4.91E-33 | -0.839 |
| RHSIM_Rhsim07G0119900 | 147.69 | 5.54E-34 | 5.25E-33 | 1.075 |
| RHSIM_Rhsim13G0221900 | 147.63 | 5.71E-34 | 5.42E-33 | 1.330 |
| RHSIM_Rhsim11G0196600 | 147.61 | 5.77E-34 | 5.47E-33 | 1.935 |
| RHSIM_Rhsim06G0087100 | 147.54 | 5.97E-34 | 5.66E-33 | -1.080 |
| RHSIM_Rhsim08G0108400 | 147.48 | 6.17E-34 | 5.85E-33 | -2.335 |
| RHSIM_Rhsim09G0106300 | 147.45 | 6.25E-34 | 5.92E-33 | 0.920 |
| RHSIM_Rhsim12G0176100 | 147.44 | 6.29E-34 | 5.96E-33 | 1.041 |
| RHSIM_Rhsim07G0077100 | 147.40 | 6.42E-34 | 6.08E-33 | -1.528 |
| RHSIM_Rhsim08G0221900 | 147.37 | 6.51E-34 | 6.16E-33 | -1.350 |
| RHSIM_Rhsim07G0001500 | 147.36 | 6.55E-34 | 6.19E-33 | -1.794 |
| RHSIM_Rhsim10G0154000 | 147.24 | 6.95E-34 | 6.58E-33 | 2.066 |
| RHSIM_Rhsim02G0021600 | 147.04 | 7.69E-34 | 7.27E-33 | 1.748 |
| RHSIM_Rhsim11G0087300 | 147.03 | 7.75E-34 | 7.32E-33 | -1.301 |
| RHSIM_Rhsim09G0072500 | 147.02 | 7.76E-34 | 7.33E-33 | 0.965 |
| RHSIM_Rhsim06G0215000 | 147.02 | 7.78E-34 | 7.35E-33 | -3.118 |
| RHSIM_Rhsim12G0060900 | 146.97 | 7.97E-34 | 7.52E-33 | -1.150 |
| RHSIM_Rhsim13G0169700 | 146.89 | 8.27E-34 | 7.81E-33 | -1.527 |
| novel.1101 | 146.89 | 8.31E-34 | 7.84E-33 | -2.064 |
| RHSIM_Rhsim13G0030700 | 146.83 | 8.53E-34 | 8.05E-33 | -1.329 |
| RHSIM_Rhsim04G0108100 | 146.79 | 8.72E-34 | 8.22E-33 | 1.420 |
| RHSIM_Rhsim02G0172200 | 146.77 | 8.80E-34 | 8.29E-33 | -0.769 |

| Gene | LRT statistic | P value | BH-adjusted P | log2FC (interaction) |
|---|---|---|---|---|
| RHSIM_Rhsim13G0180900 | 146.77 | 8.82E-34 | 8.31E-33 | 1.084 |
| RHSIM_Rhsim01G0007600 | 146.60 | 9.58E-34 | 9.02E-33 | -4.429 |
| RHSIM_Rhsim06G0091500 | 146.50 | 1.01E-33 | 9.50E-33 | 0.906 |
| novel.130 | 146.49 | 1.02E-33 | 9.56E-33 | 3.134 |
| RHSIM_Rhsim11G0053800 | 146.39 | 1.07E-33 | 1.00E-32 | -3.023 |
| RHSIM_Rhsim04G0134200 | 146.33 | 1.10E-33 | 1.04E-32 | 1.762 |
| RHSIM_Rhsim03G0151000 | 146.30 | 1.12E-33 | 1.05E-32 | 1.185 |
| RHSIM_Rhsim13G0071100 | 146.26 | 1.14E-33 | 1.07E-32 | 0.840 |
| RHSIM_RhsimUnG0179200 | 146.17 | 1.19E-33 | 1.12E-32 | -2.647 |
| RHSIM_Rhsim06G0082300 | 146.14 | 1.21E-33 | 1.14E-32 | 2.423 |
| RHSIM_Rhsim03G0231300 | 146.06 | 1.26E-33 | 1.18E-32 | -1.414 |
| RHSIM_Rhsim02G0247000 | 146.05 | 1.27E-33 | 1.19E-32 | -1.768 |
| RHSIM_Rhsim11G0034400 | 146.04 | 1.27E-33 | 1.19E-32 | 1.058 |
| RHSIM_Rhsim06G0029000 | 145.99 | 1.31E-33 | 1.23E-32 | 0.878 |
| RHSIM_Rhsim12G0203000 | 145.93 | 1.35E-33 | 1.26E-32 | -0.679 |
| RHSIM_Rhsim06G0179600 | 145.76 | 1.47E-33 | 1.37E-32 | -1.757 |
| RHSIM_Rhsim08G0030700 | 145.74 | 1.48E-33 | 1.39E-32 | -1.868 |
| RHSIM_Rhsim01G0123900 | 145.67 | 1.53E-33 | 1.43E-32 | 1.414 |
| RHSIM_Rhsim05G0203500 | 145.66 | 1.54E-33 | 1.44E-32 | 0.800 |
| RHSIM_Rhsim03G0249700 | 145.63 | 1.56E-33 | 1.46E-32 | -2.290 |
| RHSIM_Rhsim08G0247000 | 145.61 | 1.58E-33 | 1.48E-32 | 1.569 |
| novel.3829 | 145.59 | 1.59E-33 | 1.49E-32 | 2.895 |
| novel.4581 | 145.48 | 1.69E-33 | 1.58E-32 | 5.828 |
| RHSIM_Rhsim07G0214500 | 145.40 | 1.75E-33 | 1.64E-32 | -1.020 |
| RHSIM_Rhsim13G0114800 | 145.36 | 1.79E-33 | 1.68E-32 | -2.474 |
| RHSIM_Rhsim04G0150200 | 145.34 | 1.81E-33 | 1.69E-32 | -0.951 |
| RHSIM_Rhsim08G0065000 | 145.31 | 1.84E-33 | 1.72E-32 | 1.781 |
| RHSIM_Rhsim03G0158800 | 145.24 | 1.91E-33 | 1.78E-32 | -1.198 |
| RHSIM_Rhsim08G0099100 | 145.12 | 2.02E-33 | 1.88E-32 | -1.060 |
| RHSIM_Rhsim01G0012500 | 145.11 | 2.03E-33 | 1.90E-32 | -9.079 |
| RHSIM_Rhsim13G0192600 | 144.86 | 2.30E-33 | 2.15E-32 | -2.833 |
| RHSIM_Rhsim08G0100700 | 144.83 | 2.34E-33 | 2.18E-32 | -1.706 |

| Gene | LRT statistic | P value | BH-adjusted P | log2FC (interaction) |
|---|---|---|---|---|
| RHSIM_Rhsim06G0047100 | 144.82 | 2.35E-33 | 2.19E-32 | 1.351 |
| RHSIM_Rhsim12G0122100 | 144.45 | 2.84E-33 | 2.64E-32 | -1.216 |
| RHSIM_Rhsim02G0004400 | 144.34 | 3.00E-33 | 2.80E-32 | -1.116 |
| RHSIM_Rhsim06G0006200 | 144.29 | 3.07E-33 | 2.86E-32 | 2.322 |
| novel.965 | 144.26 | 3.11E-33 | 2.90E-32 | -1.757 |
| RHSIM_Rhsim03G0065400 | 144.23 | 3.16E-33 | 2.94E-32 | 2.206 |
| RHSIM_Rhsim12G0054200 | 144.22 | 3.17E-33 | 2.95E-32 | -5.519 |
| RHSIM_Rhsim09G0109100 | 144.18 | 3.24E-33 | 3.02E-32 | -1.210 |
| RHSIM_Rhsim10G0077800 | 144.15 | 3.29E-33 | 3.06E-32 | 1.809 |
| RHSIM_Rhsim06G0034800 | 144.05 | 3.46E-33 | 3.22E-32 | -1.068 |
| novel.2431 | 144.03 | 3.51E-33 | 3.26E-32 | -1.195 |
| RHSIM_Rhsim07G0044600 | 143.99 | 3.57E-33 | 3.32E-32 | -4.822 |
| RHSIM_Rhsim12G0213800 | 143.88 | 3.77E-33 | 3.51E-32 | 0.941 |
| RHSIM_Rhsim01G0286100 | 143.80 | 3.93E-33 | 3.65E-32 | 1.157 |
| RHSIM_Rhsim04G0052800 | 143.73 | 4.08E-33 | 3.78E-32 | -1.189 |
| RHSIM_Rhsim06G0041800 | 143.69 | 4.14E-33 | 3.85E-32 | 0.823 |
| RHSIM_Rhsim12G0129900 | 143.69 | 4.15E-33 | 3.85E-32 | -1.355 |
| RHSIM_Rhsim02G0228500 | 143.63 | 4.28E-33 | 3.96E-32 | -2.560 |
| RHSIM_Rhsim04G0018800 | 143.61 | 4.33E-33 | 4.01E-32 | -3.298 |
| RHSIM_Rhsim07G0011800 | 143.56 | 4.44E-33 | 4.11E-32 | 0.766 |
| novel.516 | 143.48 | 4.62E-33 | 4.28E-32 | 3.067 |
| RHSIM_RhsimUnG0178800 | 143.45 | 4.68E-33 | 4.33E-32 | 2.828 |
| RHSIM_Rhsim08G0211000 | 143.43 | 4.73E-33 | 4.38E-32 | -0.916 |
| RHSIM_Rhsim06G0197000 | 143.29 | 5.09E-33 | 4.71E-32 | 1.471 |
| RHSIM_Rhsim07G0125500 | 143.23 | 5.24E-33 | 4.85E-32 | 1.776 |
| RHSIM_Rhsim02G0036900 | 143.20 | 5.32E-33 | 4.92E-32 | -1.014 |
| RHSIM_Rhsim05G0172300 | 143.20 | 5.33E-33 | 4.92E-32 | 1.133 |
| RHSIM_Rhsim07G0007900 | 143.18 | 5.38E-33 | 4.97E-32 | 0.692 |
| RHSIM_Rhsim06G0179100 | 143.04 | 5.77E-33 | 5.33E-32 | 1.868 |
| RHSIM_Rhsim13G0012100 | 143.04 | 5.77E-33 | 5.33E-32 | 0.820 |
| RHSIM_Rhsim04G0046500 | 142.80 | 6.50E-33 | 6.00E-32 | 1.278 |
| RHSIM_Rhsim03G0232100 | 142.72 | 6.77E-33 | 6.25E-32 | -1.565 |

| Gene | LRT statistic | P value | BH-adjusted P | log2FC (interaction) |
|---|---|---|---|---|
| RHSIM_Rhsim08G0227300 | 142.71 | 6.81E-33 | 6.29E-32 | 0.682 |
| RHSIM_Rhsim09G0001400 | 142.68 | 6.91E-33 | 6.38E-32 | -0.697 |
| RHSIM_Rhsim11G0044800 | 142.66 | 6.97E-33 | 6.43E-32 | -3.116 |
| RHSIM_Rhsim11G0154500 | 142.56 | 7.32E-33 | 6.75E-32 | 1.304 |
| RHSIM_Rhsim02G0140000 | 142.39 | 7.99E-33 | 7.36E-32 | 1.831 |
| RHSIM_Rhsim01G0281800 | 142.36 | 8.13E-33 | 7.49E-32 | -2.379 |
| RHSIM_Rhsim02G0131800 | 142.35 | 8.16E-33 | 7.52E-32 | -2.974 |
| RHSIM_Rhsim07G0139700 | 142.34 | 8.19E-33 | 7.54E-32 | 0.870 |
| RHSIM_Rhsim12G0174000 | 142.34 | 8.20E-33 | 7.54E-32 | -0.791 |
| RHSIM_Rhsim09G0208400 | 142.32 | 8.29E-33 | 7.63E-32 | -1.082 |
| RHSIM_Rhsim07G0228900 | 142.14 | 9.05E-33 | 8.33E-32 | -3.878 |
| novel.215 | 142.14 | 9.07E-33 | 8.34E-32 | 1.668 |
| RHSIM_Rhsim09G0151800 | 142.11 | 9.19E-33 | 8.45E-32 | -4.890 |
| RHSIM_Rhsim05G0122700 | 142.10 | 9.26E-33 | 8.51E-32 | -1.672 |
| RHSIM_Rhsim02G0005600 | 142.03 | 9.56E-33 | 8.78E-32 | -1.388 |
| RHSIM_Rhsim01G0067800 | 142.01 | 9.66E-33 | 8.87E-32 | 0.897 |
| RHSIM_Rhsim02G0084200 | 142.01 | 9.69E-33 | 8.90E-32 | 0.787 |
| RHSIM_RhsimUnG0094400 | 142.00 | 9.74E-33 | 8.94E-32 | 3.925 |
| RHSIM_Rhsim08G0233200 | 141.81 | 1.07E-32 | 9.80E-32 | -0.913 |
| RHSIM_Rhsim05G0136600 | 141.71 | 1.12E-32 | 1.03E-31 | 2.862 |
| RHSIM_Rhsim12G0137700 | 141.70 | 1.13E-32 | 1.04E-31 | 1.275 |
| RHSIM_Rhsim01G0151500 | 141.61 | 1.19E-32 | 1.09E-31 | 5.909 |
| RHSIM_Rhsim04G0060400 | 141.52 | 1.24E-32 | 1.14E-31 | 3.433 |
| RHSIM_Rhsim07G0082700 | 141.48 | 1.27E-32 | 1.16E-31 | 2.868 |
| novel.1687 | 141.38 | 1.33E-32 | 1.21E-31 | 1.305 |
| novel.816 | 141.38 | 1.33E-32 | 1.22E-31 | -1.288 |
| RHSIM_Rhsim07G0007600 | 141.30 | 1.38E-32 | 1.27E-31 | 0.915 |
| RHSIM_RhsimUnG0008100 | 141.18 | 1.47E-32 | 1.34E-31 | -2.238 |
| RHSIM_Rhsim08G0182700 | 141.17 | 1.48E-32 | 1.35E-31 | -4.091 |
| RHSIM_RhsimUnG0122900 | 141.11 | 1.52E-32 | 1.39E-31 | 2.405 |
| novel.2966 | 140.91 | 1.69E-32 | 1.54E-31 | 2.881 |
| RHSIM_Rhsim03G0045500 | 140.91 | 1.69E-32 | 1.54E-31 | 1.195 |

| Gene | LRT statistic | P value | BH-adjusted P | log2FC (interaction) |
|---|---|---|---|---|
| RHSIM_Rhsim13G0004600 | 140.84 | 1.75E-32 | 1.60E-31 | 1.216 |
| RHSIM_Rhsim13G0038100 | 140.60 | 1.97E-32 | 1.80E-31 | 1.070 |
| RHSIM_Rhsim13G0064600 | 140.58 | 1.99E-32 | 1.82E-31 | 0.912 |
| RHSIM_Rhsim04G0031300 | 140.56 | 2.01E-32 | 1.83E-31 | 2.139 |
| RHSIM_Rhsim08G0001100 | 140.50 | 2.07E-32 | 1.89E-31 | -2.511 |
| RHSIM_Rhsim11G0090700 | 140.49 | 2.08E-32 | 1.89E-31 | -1.424 |
| RHSIM_Rhsim07G0256400 | 140.48 | 2.09E-32 | 1.90E-31 | 0.780 |
| RHSIM_Rhsim13G0219600 | 140.47 | 2.11E-32 | 1.92E-31 | -3.563 |
| RHSIM_Rhsim06G0093200 | 140.44 | 2.13E-32 | 1.94E-31 | 1.157 |
| RHSIM_Rhsim11G0083400 | 140.41 | 2.16E-32 | 1.97E-31 | 2.925 |
| RHSIM_Rhsim04G0118000 | 140.35 | 2.23E-32 | 2.03E-31 | 0.803 |
| RHSIM_Rhsim09G0075600 | 140.31 | 2.28E-32 | 2.07E-31 | -1.221 |
| RHSIM_Rhsim04G0087600 | 140.31 | 2.28E-32 | 2.07E-31 | -1.140 |
| RHSIM_Rhsim01G0285600 | 140.15 | 2.46E-32 | 2.24E-31 | 0.662 |
| RHSIM_Rhsim02G0176900 | 140.09 | 2.54E-32 | 2.31E-31 | 1.516 |
| novel.1256 | 140.04 | 2.61E-32 | 2.37E-31 | 4.212 |
| RHSIM_Rhsim01G0027300 | 140.02 | 2.64E-32 | 2.40E-31 | 0.930 |
| RHSIM_Rhsim03G0002000 | 139.96 | 2.72E-32 | 2.47E-31 | 1.684 |
| RHSIM_Rhsim10G0187300 | 139.96 | 2.72E-32 | 2.47E-31 | 0.736 |
| RHSIM_Rhsim07G0090200 | 139.83 | 2.90E-32 | 2.63E-31 | -0.750 |
| RHSIM_Rhsim03G0061000 | 139.83 | 2.91E-32 | 2.64E-31 | -45.888 |
| RHSIM_Rhsim06G0022900 | 139.77 | 2.99E-32 | 2.71E-31 | -1.860 |
| RHSIM_Rhsim06G0136000 | 139.64 | 3.19E-32 | 2.89E-31 | -0.910 |
| RHSIM_Rhsim01G0219200 | 139.64 | 3.20E-32 | 2.90E-31 | -1.590 |
| RHSIM_Rhsim11G0176400 | 139.63 | 3.21E-32 | 2.91E-31 | 1.288 |
| RHSIM_Rhsim11G0099300 | 139.43 | 3.54E-32 | 3.21E-31 | -1.158 |
| RHSIM_Rhsim08G0012800 | 139.37 | 3.66E-32 | 3.31E-31 | -0.952 |
| RHSIM_Rhsim12G0128400 | 139.36 | 3.67E-32 | 3.33E-31 | -1.719 |
| RHSIM_Rhsim13G0005200 | 139.31 | 3.77E-32 | 3.41E-31 | 1.377 |
| novel.2846 | 139.30 | 3.78E-32 | 3.42E-31 | -1.512 |
| RHSIM_Rhsim12G0208000 | 139.30 | 3.78E-32 | 3.42E-31 | -0.858 |
| novel.3493 | 139.29 | 3.81E-32 | 3.44E-31 | 3.525 |

| Gene | LRT statistic | P value | BH-adjusted P | log2FC (interaction) |
|---|---|---|---|---|
| RHSIM_Rhsim01G0272500 | 139.27 | 3.85E-32 | 3.48E-31 | -2.411 |
| RHSIM_Rhsim03G0007400 | 139.16 | 4.06E-32 | 3.67E-31 | 1.358 |
| RHSIM_Rhsim04G0205200 | 139.12 | 4.14E-32 | 3.74E-31 | 2.340 |
| RHSIM_RhsimUnG0143500 | 139.12 | 4.16E-32 | 3.75E-31 | 1.030 |
| RHSIM_Rhsim06G0046600 | 139.10 | 4.18E-32 | 3.77E-31 | 2.113 |
| RHSIM_Rhsim06G0218300 | 139.00 | 4.41E-32 | 3.98E-31 | -2.117 |
| RHSIM_Rhsim05G0223200 | 139.00 | 4.41E-32 | 3.98E-31 | 2.015 |
| RHSIM_Rhsim05G0126700 | 138.98 | 4.45E-32 | 4.01E-31 | 1.146 |
| RHSIM_Rhsim07G0047300 | 138.86 | 4.73E-32 | 4.27E-31 | 1.111 |
| RHSIM_Rhsim05G0115500 | 138.81 | 4.84E-32 | 4.37E-31 | 1.071 |
| RHSIM_RhsimUnG0158000 | 138.78 | 4.92E-32 | 4.43E-31 | 1.647 |
| RHSIM_Rhsim05G0114400 | 138.78 | 4.92E-32 | 4.43E-31 | 2.321 |
| RHSIM_Rhsim11G0197500 | 138.73 | 5.04E-32 | 4.54E-31 | 0.932 |
| RHSIM_Rhsim04G0066300 | 138.71 | 5.10E-32 | 4.59E-31 | -3.337 |
| RHSIM_Rhsim07G0131400 | 138.69 | 5.14E-32 | 4.63E-31 | 0.582 |
| RHSIM_Rhsim10G0037200 | 138.56 | 5.51E-32 | 4.96E-31 | 3.070 |
| RHSIM_Rhsim07G0071400 | 138.49 | 5.69E-32 | 5.12E-31 | -1.287 |
| RHSIM_Rhsim07G0082300 | 138.41 | 5.94E-32 | 5.34E-31 | -1.716 |
| RHSIM_Rhsim09G0063200 | 138.41 | 5.94E-32 | 5.34E-31 | 1.324 |
| RHSIM_Rhsim12G0085900 | 138.16 | 6.72E-32 | 6.04E-31 | 1.147 |
| RHSIM_Rhsim06G0165500 | 138.10 | 6.95E-32 | 6.24E-31 | -1.735 |
| RHSIM_Rhsim08G0190500 | 138.06 | 7.06E-32 | 6.35E-31 | -0.693 |
| RHSIM_Rhsim08G0080000 | 137.98 | 7.35E-32 | 6.60E-31 | 1.428 |
| RHSIM_Rhsim04G0106300 | 137.98 | 7.36E-32 | 6.60E-31 | 2.887 |
| novel.2672 | 137.95 | 7.47E-32 | 6.70E-31 | -8.558 |
| RHSIM_Rhsim01G0220900 | 137.95 | 7.49E-32 | 6.72E-31 | 7.226 |
| RHSIM_Rhsim08G0198400 | 137.94 | 7.52E-32 | 6.75E-31 | 2.894 |
| RHSIM_Rhsim01G0089900 | 137.83 | 7.93E-32 | 7.11E-31 | 1.089 |
| novel.4115 | 137.68 | 8.56E-32 | 7.67E-31 | 1.992 |
| RHSIM_Rhsim08G0183200 | 137.63 | 8.76E-32 | 7.85E-31 | 1.983 |
| RHSIM_Rhsim11G0121200 | 137.61 | 8.87E-32 | 7.94E-31 | -1.085 |
| RHSIM_Rhsim05G0185300 | 137.58 | 9.01E-32 | 8.06E-31 | 0.979 |

| Gene | LRT statistic | P value | BH-adjusted P | log2FC (interaction) |
|---|---|---|---|---|
| RHSIM_Rhsim10G0191000 | 137.56 | 9.11E-32 | 8.16E-31 | -1.183 |
| RHSIM_Rhsim04G0249000 | 137.56 | 9.12E-32 | 8.16E-31 | 1.157 |
| RHSIM_Rhsim01G0191600 | 137.49 | 9.43E-32 | 8.43E-31 | 0.849 |
| RHSIM_Rhsim08G0189300 | 137.47 | 9.52E-32 | 8.51E-31 | -1.453 |
| RHSIM_Rhsim08G0019300 | 137.46 | 9.55E-32 | 8.53E-31 | -2.271 |
| RHSIM_Rhsim03G0067000 | 137.45 | 9.62E-32 | 8.59E-31 | -0.938 |
| RHSIM_Rhsim10G0134800 | 137.36 | 1.01E-31 | 8.98E-31 | 1.512 |
| RHSIM_Rhsim07G0137700 | 137.30 | 1.04E-31 | 9.25E-31 | 3.081 |
| RHSIM_Rhsim10G0066900 | 137.06 | 1.17E-31 | 1.04E-30 | 6.119 |
| RHSIM_Rhsim02G0164100 | 137.05 | 1.18E-31 | 1.05E-30 | -2.393 |
| RHSIM_Rhsim10G0040000 | 137.04 | 1.18E-31 | 1.06E-30 | 1.821 |
| RHSIM_Rhsim04G0003600 | 137.02 | 1.19E-31 | 1.06E-30 | -0.728 |
| novel.4160 | 136.99 | 1.21E-31 | 1.08E-30 | 1.545 |
| RHSIM_Rhsim12G0171100 | 136.96 | 1.23E-31 | 1.09E-30 | 0.935 |
| RHSIM_Rhsim03G0241400 | 136.96 | 1.23E-31 | 1.10E-30 | 1.140 |
| RHSIM_Rhsim06G0020700 | 136.95 | 1.24E-31 | 1.10E-30 | -1.758 |
| RHSIM_Rhsim01G0189300 | 136.90 | 1.27E-31 | 1.13E-30 | 1.574 |
| RHSIM_Rhsim01G0205500 | 136.75 | 1.37E-31 | 1.22E-30 | 1.526 |
| RHSIM_Rhsim13G0179000 | 136.44 | 1.60E-31 | 1.42E-30 | -4.313 |
| RHSIM_Rhsim12G0140300 | 136.43 | 1.61E-31 | 1.43E-30 | -4.334 |
| RHSIM_Rhsim03G0148500 | 136.42 | 1.61E-31 | 1.43E-30 | -1.579 |
| RHSIM_Rhsim09G0058700 | 136.41 | 1.62E-31 | 1.44E-30 | -1.640 |
| RHSIM_Rhsim06G0070000 | 136.41 | 1.62E-31 | 1.44E-30 | -1.376 |
| RHSIM_Rhsim09G0037000 | 136.37 | 1.66E-31 | 1.47E-30 | -1.082 |
| RHSIM_Rhsim02G0199800 | 136.36 | 1.66E-31 | 1.48E-30 | 0.962 |
| RHSIM_Rhsim11G0099600 | 136.35 | 1.68E-31 | 1.49E-30 | -0.643 |
| RHSIM_Rhsim12G0036900 | 136.33 | 1.69E-31 | 1.50E-30 | -1.853 |
| RHSIM_Rhsim01G0246100 | 136.17 | 1.83E-31 | 1.63E-30 | 1.124 |
| RHSIM_Rhsim01G0150800 | 136.16 | 1.84E-31 | 1.64E-30 | -1.695 |
| RHSIM_Rhsim05G0060800 | 136.14 | 1.86E-31 | 1.65E-30 | -2.557 |
| RHSIM_Rhsim13G0139700 | 136.07 | 1.92E-31 | 1.71E-30 | -1.785 |
| RHSIM_Rhsim10G0211000 | 136.06 | 1.94E-31 | 1.72E-30 | 1.156 |

| Gene | LRT statistic | P value | BH-adjusted P | log2FC (interaction) |
|---|---|---|---|---|
| RHSIM_Rhsim03G0013800 | 136.04 | 1.96E-31 | 1.73E-30 | -2.591 |
| RHSIM_Rhsim12G0150700 | 136.03 | 1.97E-31 | 1.74E-30 | -1.033 |
| RHSIM_Rhsim06G0007800 | 135.96 | 2.04E-31 | 1.80E-30 | 0.808 |
| RHSIM_Rhsim11G0037000 | 135.95 | 2.05E-31 | 1.81E-30 | 1.719 |
| RHSIM_Rhsim10G0171200 | 135.93 | 2.07E-31 | 1.83E-30 | 1.108 |
| RHSIM_Rhsim08G0193100 | 135.79 | 2.21E-31 | 1.96E-30 | 3.692 |
| RHSIM_Rhsim12G0046600 | 135.73 | 2.29E-31 | 2.02E-30 | -1.087 |
| RHSIM_Rhsim01G0096100 | 135.71 | 2.31E-31 | 2.04E-30 | 1.569 |
| RHSIM_Rhsim13G0230600 | 135.51 | 2.55E-31 | 2.25E-30 | -1.451 |
| RHSIM_Rhsim11G0193400 | 135.51 | 2.55E-31 | 2.25E-30 | 1.919 |
| RHSIM_Rhsim08G0042300 | 135.51 | 2.55E-31 | 2.25E-30 | -1.296 |
| RHSIM_Rhsim13G0149600 | 135.49 | 2.58E-31 | 2.28E-30 | 0.794 |
| RHSIM_Rhsim01G0278100 | 135.48 | 2.60E-31 | 2.29E-30 | 0.799 |
| RHSIM_Rhsim06G0106300 | 135.36 | 2.76E-31 | 2.43E-30 | 1.580 |
| RHSIM_Rhsim06G0185600 | 135.35 | 2.77E-31 | 2.44E-30 | -4.133 |
| RHSIM_Rhsim05G0149600 | 135.29 | 2.85E-31 | 2.51E-30 | -1.022 |
| novel.2150 | 135.19 | 3.00E-31 | 2.65E-30 | 3.028 |
| novel.26 | 135.17 | 3.02E-31 | 2.67E-30 | 1.171 |
| RHSIM_Rhsim07G0187600 | 135.15 | 3.07E-31 | 2.70E-30 | -1.144 |
| RHSIM_Rhsim02G0245700 | 135.06 | 3.21E-31 | 2.83E-30 | 1.869 |
| RHSIM_Rhsim09G0184100 | 134.96 | 3.36E-31 | 2.96E-30 | -0.995 |
| RHSIM_Rhsim04G0087400 | 134.92 | 3.44E-31 | 3.03E-30 | 0.844 |
| RHSIM_Rhsim07G0119700 | 134.90 | 3.47E-31 | 3.06E-30 | -1.210 |
| RHSIM_Rhsim02G0037200 | 134.80 | 3.64E-31 | 3.21E-30 | -0.731 |
| RHSIM_Rhsim02G0233100 | 134.78 | 3.69E-31 | 3.24E-30 | 1.140 |
| RHSIM_Rhsim02G0009800 | 134.74 | 3.76E-31 | 3.30E-30 | 1.831 |
| novel.3936 | 134.74 | 3.76E-31 | 3.30E-30 | 2.689 |
| RHSIM_Rhsim08G0234500 | 134.73 | 3.78E-31 | 3.32E-30 | 0.701 |
| RHSIM_Rhsim04G0106600 | 134.68 | 3.87E-31 | 3.40E-30 | -0.706 |
| RHSIM_Rhsim01G0105600 | 134.63 | 3.98E-31 | 3.50E-30 | -1.160 |
| RHSIM_Rhsim01G0198500 | 134.56 | 4.12E-31 | 3.61E-30 | -1.722 |
| RHSIM_RhsimUnG0065700 | 134.49 | 4.27E-31 | 3.75E-30 | 1.999 |

| Gene | LRT statistic | P value | BH-adjusted P | log2FC (interaction) |
|---|---|---|---|---|
| RHSIM_Rhsim07G0062900 | 134.37 | 4.54E-31 | 3.98E-30 | 1.204 |
| RHSIM_Rhsim09G0084200 | 134.35 | 4.59E-31 | 4.03E-30 | 0.569 |
| RHSIM_Rhsim06G0057900 | 134.31 | 4.68E-31 | 4.10E-30 | 1.086 |
| RHSIM_Rhsim12G0053900 | 134.29 | 4.71E-31 | 4.13E-30 | -0.784 |
| RHSIM_Rhsim10G0019200 | 134.27 | 4.78E-31 | 4.19E-30 | 2.483 |
| RHSIM_Rhsim03G0257200 | 134.19 | 4.96E-31 | 4.34E-30 | -0.743 |
| RHSIM_Rhsim05G0224400 | 134.19 | 4.97E-31 | 4.35E-30 | -1.858 |
| RHSIM_Rhsim04G0139800 | 134.18 | 4.99E-31 | 4.37E-30 | 1.863 |
| RHSIM_Rhsim11G0113500 | 134.13 | 5.12E-31 | 4.48E-30 | -1.229 |
| RHSIM_Rhsim02G0205900 | 134.10 | 5.20E-31 | 4.55E-30 | -0.573 |
| RHSIM_Rhsim07G0215500 | 134.02 | 5.42E-31 | 4.74E-30 | -0.966 |
| RHSIM_Rhsim04G0239200 | 133.87 | 5.84E-31 | 5.11E-30 | -1.909 |
| RHSIM_Rhsim05G0168700 | 133.82 | 5.99E-31 | 5.24E-30 | 2.444 |
| RHSIM_Rhsim05G0058400 | 133.76 | 6.17E-31 | 5.39E-30 | 2.002 |
| RHSIM_Rhsim02G0115500 | 133.57 | 6.77E-31 | 5.92E-30 | 1.172 |
| RHSIM_Rhsim12G0151900 | 133.50 | 7.04E-31 | 6.15E-30 | -0.738 |
| RHSIM_Rhsim03G0253200 | 133.46 | 7.18E-31 | 6.27E-30 | 1.151 |
| RHSIM_Rhsim09G0164200 | 133.42 | 7.33E-31 | 6.40E-30 | -0.663 |
| RHSIM_Rhsim01G0059800 | 133.38 | 7.47E-31 | 6.52E-30 | 1.102 |
| novel.2243 | 133.30 | 7.78E-31 | 6.79E-30 | 1.105 |
| RHSIM_Rhsim05G0136900 | 133.24 | 8.02E-31 | 6.99E-30 | 2.289 |
| RHSIM_RhsimUnG0163000 | 133.22 | 8.11E-31 | 7.07E-30 | -1.617 |
| RHSIM_Rhsim02G0241500 | 133.20 | 8.17E-31 | 7.12E-30 | 1.522 |
| RHSIM_Rhsim06G0138500 | 133.19 | 8.22E-31 | 7.16E-30 | 1.882 |
| RHSIM_Rhsim10G0144800 | 133.19 | 8.22E-31 | 7.16E-30 | 1.681 |
| RHSIM_Rhsim13G0046900 | 133.16 | 8.33E-31 | 7.26E-30 | 1.489 |
| RHSIM_Rhsim02G0131100 | 133.00 | 9.06E-31 | 7.89E-30 | -2.578 |
| RHSIM_Rhsim07G0192200 | 132.99 | 9.08E-31 | 7.90E-30 | 2.193 |
| novel.682 | 132.97 | 9.16E-31 | 7.97E-30 | 2.514 |
| RHSIM_Rhsim02G0166100 | 132.92 | 9.41E-31 | 8.18E-30 | 1.010 |
| RHSIM_RhsimUnG0044400 | 132.74 | 1.03E-30 | 8.96E-30 | -0.860 |
| RHSIM_Rhsim08G0212300 | 132.72 | 1.04E-30 | 9.05E-30 | 0.810 |

| Gene | LRT statistic | P value | BH-adjusted P | log2FC (interaction) |
|---|---|---|---|---|
| RHSIM_Rhsim08G0148300 | 132.63 | 1.09E-30 | 9.46E-30 | 1.447 |
| RHSIM_Rhsim08G0194000 | 132.55 | 1.13E-30 | 9.83E-30 | 0.846 |
| RHSIM_Rhsim11G0111500 | 132.35 | 1.25E-30 | 1.09E-29 | 1.290 |
| RHSIM_Rhsim03G0195600 | 132.22 | 1.34E-30 | 1.16E-29 | 1.278 |
| RHSIM_Rhsim10G0078600 | 132.20 | 1.35E-30 | 1.17E-29 | 0.844 |
| RHSIM_Rhsim04G0220900 | 132.13 | 1.40E-30 | 1.21E-29 | -8.140 |
| novel.1870 | 132.09 | 1.43E-30 | 1.24E-29 | -5.748 |
| RHSIM_Rhsim04G0149300 | 132.08 | 1.44E-30 | 1.25E-29 | 0.841 |
| RHSIM_Rhsim12G0162100 | 131.99 | 1.50E-30 | 1.30E-29 | -1.458 |
| RHSIM_Rhsim13G0129400 | 131.84 | 1.62E-30 | 1.40E-29 | 1.756 |
| RHSIM_Rhsim12G0074600 | 131.81 | 1.65E-30 | 1.43E-29 | -1.746 |
| RHSIM_Rhsim03G0170800 | 131.81 | 1.65E-30 | 1.43E-29 | -0.964 |
| RHSIM_Rhsim13G0150500 | 131.71 | 1.73E-30 | 1.50E-29 | 1.196 |
| novel.3541 | 131.69 | 1.75E-30 | 1.52E-29 | 4.922 |
| RHSIM_Rhsim01G0151800 | 131.66 | 1.77E-30 | 1.54E-29 | -0.887 |
| novel.1210 | 131.58 | 1.85E-30 | 1.60E-29 | 3.879 |
| RHSIM_Rhsim12G0086100 | 131.55 | 1.87E-30 | 1.62E-29 | -1.744 |
| RHSIM_Rhsim03G0034500 | 131.52 | 1.90E-30 | 1.64E-29 | 1.866 |
| RHSIM_Rhsim07G0082200 | 131.51 | 1.91E-30 | 1.65E-29 | 1.055 |
| novel.3607 | 131.41 | 2.01E-30 | 1.74E-29 | -0.779 |
| RHSIM_Rhsim05G0076900 | 131.41 | 2.01E-30 | 1.74E-29 | 1.202 |
| RHSIM_Rhsim13G0229200 | 131.41 | 2.02E-30 | 1.74E-29 | -8.121 |
| RHSIM_Rhsim06G0079600 | 131.37 | 2.06E-30 | 1.77E-29 | 2.123 |
| RHSIM_Rhsim02G0022800 | 131.30 | 2.13E-30 | 1.83E-29 | -0.754 |
| RHSIM_Rhsim12G0104900 | 131.30 | 2.13E-30 | 1.84E-29 | 0.917 |
| RHSIM_Rhsim10G0004900 | 131.28 | 2.14E-30 | 1.85E-29 | 1.792 |
| RHSIM_RhsimUnG0250600 | 131.13 | 2.32E-30 | 2.00E-29 | -1.318 |
| novel.2501 | 131.12 | 2.33E-30 | 2.01E-29 | 1.391 |
| RHSIM_Rhsim07G0003500 | 131.11 | 2.34E-30 | 2.02E-29 | 2.207 |
| RHSIM_Rhsim01G0223500 | 131.07 | 2.39E-30 | 2.06E-29 | -4.844 |
| RHSIM_Rhsim08G0240300 | 131.07 | 2.39E-30 | 2.06E-29 | -0.779 |
| RHSIM_Rhsim06G0205300 | 130.96 | 2.53E-30 | 2.17E-29 | -0.758 |

| Gene | LRT statistic | P value | BH-adjusted P | log2FC (interaction) |
|---|---|---|---|---|
| RHSIM_Rhsim05G0124800 | 130.94 | 2.56E-30 | 2.20E-29 | 1.204 |
| RHSIM_Rhsim03G0196700 | 130.92 | 2.57E-30 | 2.22E-29 | 3.964 |
| RHSIM_Rhsim12G0048600 | 130.87 | 2.64E-30 | 2.27E-29 | -2.602 |
| novel.698 | 130.83 | 2.70E-30 | 2.32E-29 | 1.080 |
| RHSIM_Rhsim02G0253800 | 130.66 | 2.94E-30 | 2.53E-29 | -1.276 |
| RHSIM_Rhsim08G0071400 | 130.64 | 2.96E-30 | 2.54E-29 | 1.064 |
| RHSIM_RhsimUnG0019100 | 130.63 | 2.99E-30 | 2.57E-29 | 3.190 |
| RHSIM_Rhsim04G0118600 | 130.62 | 2.99E-30 | 2.57E-29 | -2.107 |
| RHSIM_Rhsim08G0198600 | 130.46 | 3.24E-30 | 2.79E-29 | 1.889 |
| RHSIM_Rhsim13G0140900 | 130.39 | 3.36E-30 | 2.88E-29 | -1.372 |
| RHSIM_Rhsim10G0166800 | 130.32 | 3.49E-30 | 3.00E-29 | -1.155 |
| RHSIM_Rhsim03G0010600 | 130.22 | 3.67E-30 | 3.15E-29 | -1.197 |
| RHSIM_Rhsim01G0287500 | 130.19 | 3.72E-30 | 3.19E-29 | -0.659 |
| RHSIM_Rhsim03G0163800 | 130.19 | 3.72E-30 | 3.19E-29 | 0.860 |
| RHSIM_Rhsim13G0075300 | 130.17 | 3.77E-30 | 3.23E-29 | -1.945 |
| RHSIM_Rhsim04G0122300 | 130.02 | 4.06E-30 | 3.48E-29 | 1.100 |
| RHSIM_Rhsim06G0110300 | 129.99 | 4.12E-30 | 3.53E-29 | 0.928 |
| RHSIM_Rhsim11G0011700 | 129.94 | 4.22E-30 | 3.62E-29 | -1.282 |
| RHSIM_Rhsim06G0027900 | 129.88 | 4.35E-30 | 3.72E-29 | -1.774 |
| RHSIM_Rhsim12G0127100 | 129.87 | 4.38E-30 | 3.74E-29 | -0.916 |
| RHSIM_Rhsim02G0005300 | 129.86 | 4.41E-30 | 3.77E-29 | -0.919 |
| RHSIM_Rhsim06G0141600 | 129.85 | 4.42E-30 | 3.78E-29 | 3.764 |
| RHSIM_Rhsim05G0132800 | 129.84 | 4.44E-30 | 3.79E-29 | -1.276 |
| RHSIM_Rhsim11G0018800 | 129.77 | 4.61E-30 | 3.94E-29 | 1.081 |
| RHSIM_Rhsim08G0008700 | 129.74 | 4.67E-30 | 3.99E-29 | -4.166 |
| RHSIM_Rhsim12G0056500 | 129.61 | 5.00E-30 | 4.27E-29 | -1.343 |
| RHSIM_Rhsim11G0034200 | 129.59 | 5.04E-30 | 4.30E-29 | -2.996 |
| RHSIM_Rhsim10G0188600 | 129.57 | 5.10E-30 | 4.35E-29 | 0.784 |
| RHSIM_Rhsim03G0206600 | 129.55 | 5.14E-30 | 4.39E-29 | 1.837 |
| RHSIM_Rhsim04G0171300 | 129.51 | 5.24E-30 | 4.47E-29 | 2.867 |
| RHSIM_RhsimUnG0109400 | 129.49 | 5.31E-30 | 4.53E-29 | -0.831 |
| RHSIM_Rhsim03G0188600 | 129.48 | 5.33E-30 | 4.55E-29 | -1.311 |

| Gene | LRT statistic | P value | BH-adjusted P | log2FC (interaction) |
|---|---|---|---|---|
| RHSIM_Rhsim07G0081200 | 129.46 | 5.37E-30 | 4.58E-29 | -0.843 |
| RHSIM_Rhsim08G0195100 | 129.28 | 5.89E-30 | 5.02E-29 | 0.632 |
| novel.852 | 129.22 | 6.06E-30 | 5.16E-29 | 1.648 |
| RHSIM_Rhsim13G0070000 | 129.18 | 6.20E-30 | 5.28E-29 | -2.162 |
| RHSIM_Rhsim05G0116300 | 129.05 | 6.60E-30 | 5.62E-29 | 1.915 |
| RHSIM_Rhsim07G0116600 | 128.99 | 6.81E-30 | 5.80E-29 | 1.109 |
| RHSIM_Rhsim05G0126300 | 128.94 | 6.97E-30 | 5.93E-29 | -0.952 |
| RHSIM_Rhsim04G0060200 | 128.84 | 7.33E-30 | 6.24E-29 | 2.808 |
| RHSIM_Rhsim12G0207300 | 128.77 | 7.60E-30 | 6.47E-29 | 1.345 |
| RHSIM_Rhsim02G0061900 | 128.72 | 7.82E-30 | 6.65E-29 | 1.126 |
| RHSIM_Rhsim08G0227400 | 128.71 | 7.83E-30 | 6.65E-29 | 4.270 |
| RHSIM_Rhsim07G0144400 | 128.60 | 8.29E-30 | 7.05E-29 | 2.065 |
| RHSIM_Rhsim12G0007100 | 128.57 | 8.43E-30 | 7.16E-29 | 3.017 |
| RHSIM_Rhsim12G0138400 | 128.55 | 8.52E-30 | 7.23E-29 | 1.867 |
| RHSIM_RhsimUnG0256400 | 128.54 | 8.57E-30 | 7.27E-29 | 2.072 |
| RHSIM_RhsimUnG0168900 | 128.49 | 8.77E-30 | 7.45E-29 | 1.994 |
| RHSIM_Rhsim03G0093100 | 128.48 | 8.80E-30 | 7.46E-29 | 1.123 |
| RHSIM_Rhsim05G0133000 | 128.47 | 8.86E-30 | 7.51E-29 | -4.580 |
| RHSIM_Rhsim08G0066200 | 128.43 | 9.04E-30 | 7.67E-29 | -0.404 |
| RHSIM_Rhsim02G0252200 | 128.41 | 9.11E-30 | 7.72E-29 | 0.868 |
| novel.4052 | 128.33 | 9.49E-30 | 8.04E-29 | -2.743 |
| RHSIM_Rhsim04G0191700 | 128.33 | 9.52E-30 | 8.06E-29 | -3.165 |
| novel.3555 | 128.26 | 9.84E-30 | 8.33E-29 | 3.869 |
| RHSIM_Rhsim07G0134200 | 128.24 | 9.94E-30 | 8.41E-29 | -0.966 |
| RHSIM_Rhsim05G0226700 | 128.21 | 1.01E-29 | 8.53E-29 | -1.813 |
| RHSIM_Rhsim01G0215900 | 128.21 | 1.01E-29 | 8.54E-29 | -1.086 |
| RHSIM_Rhsim08G0239000 | 128.18 | 1.03E-29 | 8.68E-29 | -4.978 |
| RHSIM_Rhsim07G0031000 | 128.15 | 1.04E-29 | 8.82E-29 | 1.109 |
| RHSIM_Rhsim01G0220300 | 128.14 | 1.04E-29 | 8.82E-29 | 1.486 |
| novel.2571 | 128.13 | 1.05E-29 | 8.88E-29 | -5.869 |
| RHSIM_Rhsim01G0245700 | 128.13 | 1.05E-29 | 8.88E-29 | -1.246 |
| RHSIM_Rhsim07G0256900 | 128.11 | 1.06E-29 | 8.95E-29 | 0.972 |

| Gene | LRT statistic | P value | BH-adjusted P | log2FC (interaction) |
|---|---|---|---|---|
| RHSIM_Rhsim05G0038900 | 128.00 | 1.12E-29 | 9.49E-29 | 0.776 |
| RHSIM_Rhsim11G0196100 | 127.99 | 1.13E-29 | 9.54E-29 | -2.074 |
| novel.2083 | 127.97 | 1.14E-29 | 9.62E-29 | 2.279 |
| RHSIM_Rhsim13G0170800 | 127.96 | 1.15E-29 | 9.67E-29 | -1.949 |
| RHSIM_Rhsim01G0006900 | 127.83 | 1.22E-29 | 1.03E-28 | 1.177 |
| RHSIM_Rhsim07G0238300 | 127.79 | 1.24E-29 | 1.05E-28 | 0.724 |
| RHSIM_Rhsim08G0202600 | 127.55 | 1.41E-29 | 1.19E-28 | -2.006 |
| RHSIM_Rhsim03G0219100 | 127.45 | 1.48E-29 | 1.25E-28 | 1.168 |
| RHSIM_Rhsim04G0138800 | 127.41 | 1.51E-29 | 1.27E-28 | 1.158 |
| RHSIM_Rhsim01G0189000 | 127.30 | 1.59E-29 | 1.34E-28 | 1.765 |
| RHSIM_Rhsim05G0203800 | 127.30 | 1.60E-29 | 1.34E-28 | -3.309 |
| RHSIM_Rhsim03G0081400 | 127.12 | 1.74E-29 | 1.47E-28 | -1.087 |
| RHSIM_Rhsim13G0050600 | 127.08 | 1.78E-29 | 1.50E-28 | -2.172 |
| RHSIM_Rhsim01G0186400 | 126.96 | 1.89E-29 | 1.59E-28 | -1.164 |
| RHSIM_Rhsim04G0181500 | 126.93 | 1.92E-29 | 1.62E-28 | -1.411 |
| RHSIM_Rhsim01G0182400 | 126.91 | 1.94E-29 | 1.63E-28 | 1.465 |
| RHSIM_Rhsim08G0180600 | 126.90 | 1.95E-29 | 1.64E-28 | -1.835 |
| RHSIM_Rhsim10G0191400 | 126.89 | 1.96E-29 | 1.65E-28 | 1.186 |
| RHSIM_Rhsim08G0230600 | 126.76 | 2.09E-29 | 1.76E-28 | 0.816 |
| RHSIM_Rhsim01G0175000 | 126.73 | 2.13E-29 | 1.79E-28 | 0.945 |
| novel.3409 | 126.68 | 2.19E-29 | 1.84E-28 | -5.353 |
| RHSIM_Rhsim03G0041800 | 126.50 | 2.39E-29 | 2.01E-28 | 3.083 |
| RHSIM_Rhsim02G0114900 | 126.46 | 2.44E-29 | 2.04E-28 | -1.252 |
| RHSIM_Rhsim05G0182800 | 126.44 | 2.46E-29 | 2.06E-28 | -1.643 |
| RHSIM_Rhsim01G0026600 | 126.43 | 2.48E-29 | 2.08E-28 | 1.888 |
| RHSIM_Rhsim05G0013800 | 126.42 | 2.49E-29 | 2.08E-28 | 1.035 |
| RHSIM_Rhsim02G0194100 | 126.27 | 2.68E-29 | 2.24E-28 | -0.951 |
| RHSIM_Rhsim12G0116700 | 126.17 | 2.82E-29 | 2.36E-28 | -2.146 |
| RHSIM_Rhsim01G0245400 | 126.11 | 2.91E-29 | 2.44E-28 | 0.748 |
| RHSIM_Rhsim05G0006000 | 126.06 | 2.98E-29 | 2.49E-28 | -1.526 |
| RHSIM_Rhsim09G0171500 | 126.06 | 2.99E-29 | 2.50E-28 | -1.062 |
| RHSIM_Rhsim07G0120700 | 125.88 | 3.26E-29 | 2.73E-28 | 0.977 |

| Gene | LRT statistic | P value | BH-adjusted P | log2FC (interaction) |
|---|---|---|---|---|
| RHSIM_Rhsim08G0194600 | 125.84 | 3.33E-29 | 2.78E-28 | -0.641 |
| RHSIM_Rhsim01G0118100 | 125.84 | 3.33E-29 | 2.78E-28 | 0.694 |
| RHSIM_Rhsim10G0072700 | 125.79 | 3.42E-29 | 2.86E-28 | -0.949 |
| RHSIM_RhsimUnG0044000 | 125.79 | 3.42E-29 | 2.86E-28 | -1.657 |
| novel.1244 | 125.72 | 3.54E-29 | 2.96E-28 | -2.492 |
| RHSIM_Rhsim01G0291400 | 125.68 | 3.62E-29 | 3.02E-28 | 1.056 |
| RHSIM_Rhsim01G0087100 | 125.56 | 3.84E-29 | 3.20E-28 | -2.111 |
| RHSIM_Rhsim01G0234100 | 125.56 | 3.84E-29 | 3.21E-28 | -2.156 |
| RHSIM_Rhsim08G0235100 | 125.14 | 4.74E-29 | 3.95E-28 | 5.025 |
| RHSIM_RhsimUnG0226400 | 125.12 | 4.79E-29 | 4.00E-28 | 0.781 |
| RHSIM_Rhsim09G0083600 | 125.09 | 4.85E-29 | 4.05E-28 | 1.286 |
| novel.4028 | 125.04 | 4.98E-29 | 4.15E-28 | 2.560 |
| RHSIM_Rhsim02G0227700 | 124.99 | 5.12E-29 | 4.27E-28 | -1.544 |
| RHSIM_Rhsim03G0067800 | 124.94 | 5.25E-29 | 4.37E-28 | 1.410 |
| RHSIM_Rhsim13G0194600 | 124.92 | 5.29E-29 | 4.41E-28 | -0.939 |
| RHSIM_Rhsim06G0067100 | 124.89 | 5.38E-29 | 4.48E-28 | -0.997 |
| RHSIM_Rhsim07G0211600 | 124.85 | 5.49E-29 | 4.57E-28 | -1.806 |
| RHSIM_Rhsim13G0022800 | 124.72 | 5.87E-29 | 4.89E-28 | 2.029 |
| novel.2656 | 124.66 | 6.03E-29 | 5.02E-28 | -2.347 |
| RHSIM_Rhsim02G0217600 | 124.56 | 6.35E-29 | 5.28E-28 | 1.218 |
| RHSIM_Rhsim04G0022600 | 124.54 | 6.43E-29 | 5.35E-28 | 1.174 |
| RHSIM_Rhsim09G0140300 | 124.53 | 6.47E-29 | 5.38E-28 | -1.331 |
| RHSIM_Rhsim05G0222200 | 124.39 | 6.91E-29 | 5.75E-28 | 1.106 |
| RHSIM_Rhsim01G0190400 | 124.26 | 7.40E-29 | 6.15E-28 | 1.168 |
| novel.3861 | 124.22 | 7.56E-29 | 6.28E-28 | 5.093 |
| RHSIM_Rhsim02G0068600 | 124.21 | 7.57E-29 | 6.29E-28 | -2.727 |
| RHSIM_RhsimUnG0227500 | 124.21 | 7.58E-29 | 6.30E-28 | 0.774 |
| RHSIM_Rhsim09G0200200 | 124.19 | 7.67E-29 | 6.37E-28 | 0.955 |
| RHSIM_Rhsim04G0098100 | 124.13 | 7.88E-29 | 6.54E-28 | 1.921 |
| RHSIM_Rhsim10G0073500 | 124.12 | 7.94E-29 | 6.59E-28 | -2.983 |
| RHSIM_Rhsim01G0232400 | 123.93 | 8.71E-29 | 7.22E-28 | -1.338 |
| RHSIM_Rhsim01G0259600 | 123.93 | 8.74E-29 | 7.25E-28 | 1.187 |

| Gene | LRT statistic | P value | BH-adjusted P | log2FC (interaction) |
|---|---|---|---|---|
| RHSIM_Rhsim07G0226000 | 123.92 | 8.76E-29 | 7.26E-28 | 1.912 |
| novel.3822 | 123.90 | 8.84E-29 | 7.32E-28 | 1.754 |
| RHSIM_Rhsim06G0224600 | 123.90 | 8.88E-29 | 7.35E-28 | 0.810 |
| RHSIM_Rhsim12G0183500 | 123.88 | 8.95E-29 | 7.42E-28 | 0.950 |
| novel.3222 | 123.78 | 9.42E-29 | 7.80E-28 | 2.251 |
| RHSIM_Rhsim10G0039200 | 123.74 | 9.63E-29 | 7.97E-28 | -1.476 |
| RHSIM_Rhsim09G0086300 | 123.71 | 9.73E-29 | 8.05E-28 | -0.999 |
| RHSIM_Rhsim01G0009200 | 123.46 | 1.10E-28 | 9.13E-28 | -7.495 |
| RHSIM_Rhsim12G0121700 | 123.43 | 1.12E-28 | 9.26E-28 | -1.096 |
| RHSIM_Rhsim13G0050700 | 123.35 | 1.17E-28 | 9.69E-28 | -0.704 |
| RHSIM_Rhsim10G0034500 | 123.27 | 1.22E-28 | 1.00E-27 | -1.992 |
| RHSIM_Rhsim02G0201100 | 123.26 | 1.23E-28 | 1.01E-27 | 2.158 |
| RHSIM_Rhsim05G0113400 | 123.25 | 1.23E-28 | 1.01E-27 | 0.587 |
| RHSIM_Rhsim06G0021700 | 123.25 | 1.23E-28 | 1.02E-27 | -1.099 |
| RHSIM_Rhsim12G0140400 | 123.21 | 1.26E-28 | 1.04E-27 | -3.435 |
| RHSIM_Rhsim04G0125700 | 123.03 | 1.37E-28 | 1.13E-27 | 0.984 |
| RHSIM_Rhsim06G0202900 | 122.96 | 1.43E-28 | 1.18E-27 | -2.138 |
| novel.429 | 122.92 | 1.45E-28 | 1.20E-27 | 0.551 |
| RHSIM_Rhsim06G0037500 | 122.82 | 1.53E-28 | 1.26E-27 | 1.467 |
| RHSIM_Rhsim13G0140200 | 122.81 | 1.54E-28 | 1.27E-27 | 1.049 |
| RHSIM_Rhsim13G0067800 | 122.73 | 1.60E-28 | 1.32E-27 | 1.027 |
| RHSIM_Rhsim01G0081800 | 122.70 | 1.62E-28 | 1.33E-27 | 1.806 |
| novel.66 | 122.65 | 1.66E-28 | 1.37E-27 | 3.909 |
| RHSIM_Rhsim10G0148000 | 122.64 | 1.67E-28 | 1.37E-27 | 1.331 |
| RHSIM_Rhsim06G0146900 | 122.59 | 1.72E-28 | 1.41E-27 | 1.798 |
| RHSIM_Rhsim07G0059000 | 122.57 | 1.73E-28 | 1.42E-27 | -2.076 |
| RHSIM_Rhsim10G0081500 | 122.56 | 1.74E-28 | 1.43E-27 | -0.916 |
| RHSIM_Rhsim09G0061500 | 122.28 | 2.00E-28 | 1.65E-27 | 1.212 |
| RHSIM_Rhsim12G0185400 | 122.25 | 2.04E-28 | 1.67E-27 | 0.891 |
| RHSIM_Rhsim02G0183600 | 122.21 | 2.08E-28 | 1.71E-27 | 1.584 |
| RHSIM_Rhsim08G0240000 | 122.11 | 2.18E-28 | 1.79E-27 | 0.859 |
| RHSIM_Rhsim11G0137100 | 121.83 | 2.51E-28 | 2.06E-27 | -0.977 |

| Gene | LRT statistic | P value | BH-adjusted P | log2FC (interaction) |
|---|---|---|---|---|
| RHSIM_Rhsim03G0168800 | 121.83 | 2.52E-28 | 2.07E-27 | -0.975 |
| novel.2522 | 121.81 | 2.54E-28 | 2.08E-27 | 2.421 |
| novel.2558 | 121.77 | 2.59E-28 | 2.12E-27 | 1.988 |
| RHSIM_Rhsim05G0195100 | 121.75 | 2.62E-28 | 2.15E-27 | -1.810 |
| RHSIM_Rhsim11G0149100 | 121.73 | 2.64E-28 | 2.17E-27 | 1.919 |
| novel.2139 | 121.71 | 2.68E-28 | 2.19E-27 | -1.085 |
| RHSIM_Rhsim09G0125100 | 121.65 | 2.75E-28 | 2.26E-27 | -1.456 |
| RHSIM_Rhsim06G0067200 | 121.61 | 2.82E-28 | 2.31E-27 | -0.697 |
| RHSIM_Rhsim06G0237600 | 121.49 | 2.98E-28 | 2.44E-27 | -2.487 |
| RHSIM_Rhsim03G0125400 | 121.47 | 3.01E-28 | 2.47E-27 | 0.800 |
| RHSIM_Rhsim12G0192400 | 121.35 | 3.21E-28 | 2.63E-27 | -6.202 |
| RHSIM_Rhsim06G0055300 | 121.31 | 3.28E-28 | 2.68E-27 | -0.691 |
| RHSIM_Rhsim03G0172800 | 121.30 | 3.29E-28 | 2.69E-27 | -1.137 |
| RHSIM_Rhsim10G0004500 | 121.27 | 3.34E-28 | 2.73E-27 | 2.890 |
| RHSIM_Rhsim10G0127500 | 121.24 | 3.39E-28 | 2.77E-27 | -0.791 |
| RHSIM_Rhsim11G0089800 | 121.23 | 3.40E-28 | 2.78E-27 | 1.801 |
| RHSIM_Rhsim10G0194000 | 121.13 | 3.58E-28 | 2.92E-27 | 2.333 |
| RHSIM_Rhsim04G0003800 | 121.12 | 3.59E-28 | 2.94E-27 | -1.145 |
| RHSIM_RhsimUnG0232700 | 121.11 | 3.61E-28 | 2.95E-27 | 0.880 |
| RHSIM_Rhsim11G0167500 | 121.10 | 3.63E-28 | 2.97E-27 | -1.733 |
| RHSIM_Rhsim07G0007800 | 120.99 | 3.85E-28 | 3.14E-27 | 2.828 |
| RHSIM_Rhsim10G0185500 | 120.97 | 3.87E-28 | 3.16E-27 | -1.288 |
| RHSIM_Rhsim11G0074900 | 120.97 | 3.89E-28 | 3.17E-27 | -0.851 |
| RHSIM_Rhsim08G0160200 | 120.96 | 3.89E-28 | 3.17E-27 | -1.203 |
| RHSIM_Rhsim06G0197600 | 120.93 | 3.97E-28 | 3.23E-27 | -1.571 |
| RHSIM_Rhsim02G0256200 | 120.91 | 3.99E-28 | 3.25E-27 | 1.496 |
| RHSIM_Rhsim10G0204000 | 120.87 | 4.08E-28 | 3.33E-27 | -1.383 |
| RHSIM_Rhsim05G0219500 | 120.85 | 4.12E-28 | 3.36E-27 | -0.692 |
| RHSIM_Rhsim02G0136600 | 120.80 | 4.23E-28 | 3.45E-27 | 0.870 |
| RHSIM_Rhsim03G0049200 | 120.75 | 4.34E-28 | 3.53E-27 | -2.089 |
| RHSIM_Rhsim13G0171500 | 120.74 | 4.35E-28 | 3.54E-27 | -2.220 |
| RHSIM_Rhsim02G0104500 | 120.68 | 4.49E-28 | 3.65E-27 | -1.074 |

| Gene | LRT statistic | P value | BH-adjusted P | log2FC (interaction) |
|---|---|---|---|---|
| RHSIM_Rhsim03G0027400 | 120.66 | 4.54E-28 | 3.69E-27 | 0.929 |
| RHSIM_Rhsim06G0135500 | 120.63 | 4.59E-28 | 3.74E-27 | -0.778 |
| RHSIM_Rhsim13G0181600 | 120.62 | 4.63E-28 | 3.77E-27 | 0.725 |
| RHSIM_Rhsim08G0159300 | 120.59 | 4.69E-28 | 3.81E-27 | 3.196 |
| RHSIM_Rhsim09G0143900 | 120.50 | 4.91E-28 | 3.99E-27 | -1.892 |
| RHSIM_Rhsim02G0105700 | 120.43 | 5.10E-28 | 4.15E-27 | 0.952 |
| RHSIM_Rhsim07G0188400 | 120.41 | 5.14E-28 | 4.18E-27 | 0.714 |
| RHSIM_Rhsim09G0147200 | 120.37 | 5.25E-28 | 4.26E-27 | 0.564 |
| RHSIM_RhsimUnG0113800 | 120.33 | 5.35E-28 | 4.34E-27 | -2.811 |
| RHSIM_Rhsim13G0224400 | 120.33 | 5.36E-28 | 4.35E-27 | 2.806 |
| RHSIM_Rhsim13G0154700 | 120.30 | 5.43E-28 | 4.40E-27 | 1.176 |
| RHSIM_RhsimUnG0146700 | 120.27 | 5.51E-28 | 4.47E-27 | -2.808 |
| novel.2816 | 120.18 | 5.78E-28 | 4.68E-27 | 1.685 |
| RHSIM_Rhsim06G0079900 | 120.17 | 5.80E-28 | 4.70E-27 | 1.485 |
| RHSIM_RhsimUnG0187000 | 120.14 | 5.89E-28 | 4.77E-27 | 3.215 |
| RHSIM_Rhsim06G0206000 | 120.14 | 5.91E-28 | 4.79E-27 | 1.156 |
| novel.4296 | 119.95 | 6.49E-28 | 5.26E-27 | -1.568 |
| RHSIM_Rhsim13G0054600 | 119.82 | 6.93E-28 | 5.61E-27 | -1.801 |
| RHSIM_Rhsim11G0117300 | 119.80 | 6.99E-28 | 5.66E-27 | 1.144 |
| RHSIM_Rhsim03G0008400 | 119.77 | 7.09E-28 | 5.74E-27 | -5.575 |
| RHSIM_Rhsim08G0046600 | 119.76 | 7.15E-28 | 5.78E-27 | -1.956 |
| RHSIM_Rhsim10G0013600 | 119.68 | 7.44E-28 | 6.02E-27 | -2.208 |
| RHSIM_Rhsim04G0228000 | 119.67 | 7.48E-28 | 6.05E-27 | -2.284 |
| RHSIM_Rhsim07G0212900 | 119.62 | 7.66E-28 | 6.19E-27 | 0.800 |
| RHSIM_Rhsim02G0234100 | 119.62 | 7.68E-28 | 6.21E-27 | 0.719 |
| RHSIM_RhsimUnG0070400 | 119.60 | 7.76E-28 | 6.27E-27 | -3.722 |
| RHSIM_Rhsim10G0019600 | 119.59 | 7.76E-28 | 6.27E-27 | 0.929 |
| novel.1071 | 119.56 | 7.90E-28 | 6.38E-27 | 1.961 |
| RHSIM_Rhsim01G0141700 | 119.53 | 8.02E-28 | 6.48E-27 | 2.854 |
| RHSIM_Rhsim04G0051400 | 119.52 | 8.07E-28 | 6.52E-27 | -2.033 |
| RHSIM_Rhsim03G0029800 | 119.42 | 8.45E-28 | 6.82E-27 | -1.029 |
| RHSIM_Rhsim06G0074900 | 119.41 | 8.54E-28 | 6.89E-27 | 1.122 |

| Gene | LRT statistic | P value | BH-adjusted P | log2FC (interaction) |
|---|---|---|---|---|
| novel.822 | 119.38 | 8.63E-28 | 6.96E-27 | 1.117 |
| RHSIM_Rhsim03G0119900 | 119.35 | 8.78E-28 | 7.08E-27 | -1.748 |
| RHSIM_Rhsim03G0121700 | 119.34 | 8.81E-28 | 7.10E-27 | 0.835 |
| RHSIM_Rhsim01G0226500 | 119.32 | 8.91E-28 | 7.18E-27 | 0.749 |
| RHSIM_Rhsim03G0249100 | 119.31 | 8.97E-28 | 7.23E-27 | 1.560 |
| RHSIM_Rhsim06G0220300 | 119.27 | 9.12E-28 | 7.35E-27 | 1.383 |
| RHSIM_Rhsim03G0040400 | 119.17 | 9.63E-28 | 7.76E-27 | -1.221 |
| RHSIM_Rhsim06G0005100 | 119.16 | 9.64E-28 | 7.76E-27 | -1.939 |
| RHSIM_Rhsim02G0014100 | 119.10 | 9.95E-28 | 8.00E-27 | -1.087 |
| RHSIM_Rhsim04G0010500 | 119.05 | 1.02E-27 | 8.21E-27 | 0.973 |
| RHSIM_Rhsim02G0111400 | 119.02 | 1.03E-27 | 8.32E-27 | 1.270 |
| RHSIM_Rhsim09G0079200 | 118.93 | 1.09E-27 | 8.74E-27 | 0.775 |
| RHSIM_Rhsim10G0187700 | 118.82 | 1.14E-27 | 9.20E-27 | 3.160 |
| RHSIM_Rhsim01G0124500 | 118.78 | 1.17E-27 | 9.39E-27 | 1.136 |
| RHSIM_Rhsim08G0248600 | 118.77 | 1.18E-27 | 9.45E-27 | 1.323 |
| novel.3038 | 118.72 | 1.20E-27 | 9.66E-27 | -1.108 |
| RHSIM_Rhsim09G0169300 | 118.69 | 1.22E-27 | 9.81E-27 | -1.210 |
| RHSIM_Rhsim03G0093700 | 118.68 | 1.23E-27 | 9.85E-27 | 2.414 |
| RHSIM_Rhsim12G0085600 | 118.67 | 1.24E-27 | 9.95E-27 | 0.895 |
| RHSIM_Rhsim09G0060500 | 118.46 | 1.37E-27 | 1.10E-26 | -5.098 |
| RHSIM_Rhsim12G0188800 | 118.43 | 1.39E-27 | 1.12E-26 | 1.246 |
| RHSIM_Rhsim02G0071100 | 118.35 | 1.46E-27 | 1.17E-26 | -0.980 |
| RHSIM_RhsimUnG0168800 | 118.25 | 1.53E-27 | 1.22E-26 | -1.184 |
| RHSIM_Rhsim03G0111400 | 118.24 | 1.53E-27 | 1.23E-26 | -5.725 |
| RHSIM_Rhsim03G0063000 | 118.14 | 1.61E-27 | 1.29E-26 | 1.049 |
| RHSIM_Rhsim03G0164200 | 118.14 | 1.61E-27 | 1.29E-26 | -0.985 |
| RHSIM_Rhsim07G0177900 | 118.11 | 1.64E-27 | 1.31E-26 | 0.794 |
| RHSIM_Rhsim05G0048900 | 118.07 | 1.68E-27 | 1.34E-26 | -0.707 |
| RHSIM_Rhsim05G0184000 | 118.03 | 1.71E-27 | 1.37E-26 | -2.729 |
| RHSIM_Rhsim10G0213600 | 118.00 | 1.74E-27 | 1.39E-26 | -0.888 |
| RHSIM_Rhsim06G0229000 | 117.94 | 1.79E-27 | 1.43E-26 | 0.958 |
| RHSIM_Rhsim03G0115600 | 117.93 | 1.80E-27 | 1.44E-26 | 1.864 |

| Gene | LRT statistic | P value | BH-adjusted P | log2FC (interaction) |
|---|---|---|---|---|
| RHSIM_Rhsim07G0086300 | 117.75 | 1.96E-27 | 1.57E-26 | 5.935 |
| RHSIM_Rhsim02G0034600 | 117.75 | 1.97E-27 | 1.57E-26 | 0.996 |
| RHSIM_Rhsim02G0123200 | 117.74 | 1.97E-27 | 1.58E-26 | 1.606 |
| RHSIM_Rhsim04G0117700 | 117.68 | 2.04E-27 | 1.63E-26 | 1.729 |
| RHSIM_Rhsim03G0208700 | 117.63 | 2.09E-27 | 1.67E-26 | 1.160 |
| RHSIM_Rhsim05G0031300 | 117.61 | 2.11E-27 | 1.68E-26 | -1.013 |
| novel.3035 | 117.50 | 2.23E-27 | 1.78E-26 | -1.069 |
| RHSIM_Rhsim06G0048000 | 117.40 | 2.35E-27 | 1.88E-26 | -0.844 |
| RHSIM_Rhsim01G0240000 | 117.30 | 2.47E-27 | 1.97E-26 | -4.183 |
| RHSIM_Rhsim08G0046200 | 117.26 | 2.52E-27 | 2.01E-26 | -0.888 |
| RHSIM_Rhsim08G0141800 | 117.26 | 2.52E-27 | 2.01E-26 | 1.528 |
| RHSIM_Rhsim02G0033000 | 117.24 | 2.54E-27 | 2.02E-26 | 1.304 |
| RHSIM_Rhsim06G0124000 | 117.24 | 2.55E-27 | 2.03E-26 | 1.140 |
| RHSIM_Rhsim03G0238700 | 117.23 | 2.56E-27 | 2.04E-26 | -4.372 |
| RHSIM_Rhsim01G0218900 | 117.15 | 2.67E-27 | 2.12E-26 | -0.850 |
| RHSIM_Rhsim01G0028200 | 117.12 | 2.70E-27 | 2.15E-26 | 0.839 |
| RHSIM_Rhsim03G0166300 | 117.07 | 2.77E-27 | 2.21E-26 | 1.700 |
| RHSIM_Rhsim02G0154700 | 117.05 | 2.80E-27 | 2.23E-26 | -1.238 |
| novel.2260 | 117.00 | 2.87E-27 | 2.28E-26 | 0.932 |
| RHSIM_Rhsim05G0125700 | 116.88 | 3.05E-27 | 2.42E-26 | -2.030 |
| novel.3330 | 116.82 | 3.14E-27 | 2.49E-26 | 0.668 |
| RHSIM_Rhsim05G0208600 | 116.80 | 3.17E-27 | 2.52E-26 | -4.778 |
| RHSIM_Rhsim08G0199800 | 116.75 | 3.26E-27 | 2.59E-26 | -0.588 |
| RHSIM_Rhsim13G0183800 | 116.73 | 3.30E-27 | 2.62E-26 | 4.113 |
| RHSIM_Rhsim06G0030600 | 116.67 | 3.39E-27 | 2.69E-26 | -1.232 |
| RHSIM_Rhsim03G0073400 | 116.55 | 3.61E-27 | 2.86E-26 | -1.325 |
| RHSIM_Rhsim03G0063100 | 116.52 | 3.66E-27 | 2.90E-26 | 0.865 |
| RHSIM_Rhsim13G0036200 | 116.44 | 3.81E-27 | 3.02E-26 | 0.786 |
| RHSIM_Rhsim07G0030900 | 116.41 | 3.87E-27 | 3.07E-26 | -1.453 |
| RHSIM_Rhsim07G0021000 | 116.36 | 3.96E-27 | 3.14E-26 | -1.859 |
| RHSIM_Rhsim13G0020000 | 116.36 | 3.96E-27 | 3.14E-26 | 2.242 |
| RHSIM_Rhsim07G0020300 | 116.35 | 3.99E-27 | 3.16E-26 | 0.927 |

| Gene | LRT statistic | P value | BH-adjusted P | log2FC (interaction) |
|---|---|---|---|---|
| RHSIM_Rhsim04G0096700 | 116.34 | 4.00E-27 | 3.17E-26 | -1.734 |
| RHSIM_Rhsim06G0001500 | 116.34 | 4.00E-27 | 3.17E-26 | 1.454 |
| RHSIM_Rhsim11G0096200 | 116.17 | 4.36E-27 | 3.45E-26 | 2.006 |
| RHSIM_Rhsim02G0190100 | 116.15 | 4.40E-27 | 3.48E-26 | -1.953 |
| RHSIM_Rhsim09G0094000 | 116.05 | 4.64E-27 | 3.67E-26 | -0.711 |
| RHSIM_Rhsim12G0013800 | 115.98 | 4.81E-27 | 3.81E-26 | -0.916 |
| RHSIM_Rhsim12G0183100 | 115.87 | 5.06E-27 | 4.00E-26 | 1.333 |
| RHSIM_Rhsim13G0116000 | 115.79 | 5.28E-27 | 4.18E-26 | -0.706 |
| RHSIM_Rhsim10G0112500 | 115.78 | 5.31E-27 | 4.20E-26 | -0.993 |
| RHSIM_Rhsim01G0290300 | 115.74 | 5.42E-27 | 4.28E-26 | 1.493 |
| RHSIM_Rhsim08G0086400 | 115.53 | 6.03E-27 | 4.76E-26 | 1.318 |
| RHSIM_Rhsim13G0031500 | 115.50 | 6.13E-27 | 4.84E-26 | 2.134 |
| RHSIM_Rhsim11G0130300 | 115.48 | 6.19E-27 | 4.89E-26 | -1.371 |
| novel.1936 | 115.38 | 6.50E-27 | 5.13E-26 | 3.455 |
| RHSIM_Rhsim07G0225800 | 115.36 | 6.56E-27 | 5.17E-26 | -3.529 |
| RHSIM_Rhsim13G0089200 | 115.36 | 6.56E-27 | 5.18E-26 | 1.996 |
| RHSIM_Rhsim04G0100900 | 115.18 | 7.20E-27 | 5.68E-26 | 0.511 |
| RHSIM_Rhsim07G0153500 | 115.14 | 7.32E-27 | 5.77E-26 | 0.860 |
| RHSIM_RhsimUnG0118600 | 115.03 | 7.75E-27 | 6.11E-26 | 2.648 |
| RHSIM_Rhsim04G0160100 | 115.03 | 7.75E-27 | 6.11E-26 | 1.367 |
| RHSIM_RhsimUnG0244800 | 114.91 | 8.22E-27 | 6.48E-26 | -1.884 |
| RHSIM_Rhsim13G0031800 | 114.84 | 8.51E-27 | 6.70E-26 | 1.165 |
| RHSIM_Rhsim05G0221700 | 114.84 | 8.51E-27 | 6.70E-26 | -0.992 |
| novel.2551 | 114.82 | 8.63E-27 | 6.79E-26 | 2.531 |
| novel.2588 | 114.71 | 9.13E-27 | 7.18E-26 | -1.216 |
| RHSIM_Rhsim10G0039000 | 114.69 | 9.19E-27 | 7.23E-26 | 3.368 |
| RHSIM_Rhsim07G0127300 | 114.69 | 9.21E-27 | 7.25E-26 | 0.685 |
| RHSIM_Rhsim11G0019900 | 114.68 | 9.27E-27 | 7.29E-26 | -0.948 |
| RHSIM_Rhsim08G0241200 | 114.65 | 9.38E-27 | 7.37E-26 | 1.408 |
| novel.4395 | 114.65 | 9.38E-27 | 7.37E-26 | 2.064 |
| RHSIM_Rhsim13G0084000 | 114.56 | 9.82E-27 | 7.72E-26 | -2.920 |
| RHSIM_Rhsim03G0176300 | 114.53 | 9.97E-27 | 7.83E-26 | 1.221 |

| Gene | LRT statistic | P value | BH-adjusted P | log2FC (interaction) |
|---|---|---|---|---|
| RHSIM_Rhsim02G0076700 | 114.53 | 9.98E-27 | 7.83E-26 | 1.036 |
| RHSIM_Rhsim06G0064500 | 114.44 | 1.04E-26 | 8.19E-26 | 0.885 |
| novel.2126 | 114.39 | 1.07E-26 | 8.38E-26 | 2.196 |
| RHSIM_Rhsim10G0113200 | 114.35 | 1.09E-26 | 8.56E-26 | 0.739 |
| RHSIM_Rhsim05G0132600 | 114.33 | 1.10E-26 | 8.66E-26 | -0.727 |
| RHSIM_Rhsim10G0185100 | 114.30 | 1.12E-26 | 8.77E-26 | 0.708 |
| RHSIM_Rhsim06G0049600 | 114.27 | 1.14E-26 | 8.93E-26 | -1.757 |
| RHSIM_Rhsim08G0218300 | 114.25 | 1.15E-26 | 8.98E-26 | -2.407 |
| RHSIM_Rhsim03G0215000 | 114.24 | 1.16E-26 | 9.05E-26 | 1.516 |
| RHSIM_Rhsim05G0094300 | 114.23 | 1.16E-26 | 9.08E-26 | -1.130 |
| novel.1175 | 114.20 | 1.18E-26 | 9.20E-26 | 2.617 |
| RHSIM_Rhsim13G0102500 | 114.20 | 1.18E-26 | 9.20E-26 | -2.176 |
| RHSIM_Rhsim10G0212300 | 114.19 | 1.18E-26 | 9.24E-26 | 0.664 |
| RHSIM_Rhsim11G0165000 | 114.16 | 1.20E-26 | 9.38E-26 | -1.413 |
| RHSIM_Rhsim07G0132400 | 114.00 | 1.30E-26 | 1.02E-25 | -0.911 |
| novel.2017 | 113.97 | 1.32E-26 | 1.03E-25 | 2.726 |
| RHSIM_Rhsim10G0156100 | 113.96 | 1.33E-26 | 1.04E-25 | 0.571 |
| RHSIM_Rhsim12G0136100 | 113.94 | 1.34E-26 | 1.05E-25 | -1.231 |
| RHSIM_Rhsim08G0018500 | 113.91 | 1.36E-26 | 1.07E-25 | 1.040 |
| RHSIM_Rhsim08G0162700 | 113.90 | 1.37E-26 | 1.07E-25 | -2.255 |
| RHSIM_Rhsim09G0123000 | 113.89 | 1.38E-26 | 1.07E-25 | -0.859 |
| RHSIM_Rhsim08G0137000 | 113.85 | 1.40E-26 | 1.09E-25 | 1.531 |
| RHSIM_Rhsim06G0074400 | 113.84 | 1.41E-26 | 1.10E-25 | -1.522 |
| novel.642 | 113.81 | 1.43E-26 | 1.12E-25 | -4.210 |
| RHSIM_Rhsim09G0097600 | 113.78 | 1.45E-26 | 1.13E-25 | -0.948 |
| RHSIM_Rhsim09G0033900 | 113.78 | 1.46E-26 | 1.14E-25 | 1.042 |
| RHSIM_Rhsim08G0245200 | 113.74 | 1.49E-26 | 1.16E-25 | 3.149 |
| RHSIM_Rhsim07G0031100 | 113.71 | 1.51E-26 | 1.18E-25 | 1.810 |
| RHSIM_Rhsim08G0193500 | 113.68 | 1.53E-26 | 1.19E-25 | -2.364 |
| RHSIM_Rhsim10G0141900 | 113.59 | 1.60E-26 | 1.25E-25 | -0.928 |
| RHSIM_Rhsim10G0109500 | 113.45 | 1.72E-26 | 1.34E-25 | -2.210 |
| RHSIM_Rhsim01G0005600 | 113.45 | 1.72E-26 | 1.34E-25 | 2.357 |

| Gene | LRT statistic | P value | BH-adjusted P | log2FC (interaction) |
|---|---|---|---|---|
| RHSIM_Rhsim13G0148700 | 113.42 | 1.75E-26 | 1.36E-25 | 1.206 |
| novel.4000 | 113.40 | 1.76E-26 | 1.37E-25 | -1.261 |
| RHSIM_Rhsim01G0256200 | 113.38 | 1.78E-26 | 1.39E-25 | 2.326 |
| RHSIM_Rhsim03G0245500 | 113.38 | 1.78E-26 | 1.39E-25 | 1.015 |
| RHSIM_Rhsim02G0028000 | 113.36 | 1.80E-26 | 1.40E-25 | 3.128 |
| RHSIM_Rhsim10G0146600 | 113.29 | 1.86E-26 | 1.45E-25 | -3.174 |
| novel.1014 | 113.12 | 2.03E-26 | 1.58E-25 | -1.723 |
| RHSIM_Rhsim13G0185000 | 113.09 | 2.06E-26 | 1.60E-25 | -1.505 |
| RHSIM_Rhsim07G0183300 | 113.08 | 2.07E-26 | 1.61E-25 | -0.733 |
| RHSIM_Rhsim03G0250300 | 113.01 | 2.14E-26 | 1.66E-25 | 1.550 |
| RHSIM_Rhsim06G0057100 | 112.99 | 2.17E-26 | 1.69E-25 | -1.068 |
| RHSIM_Rhsim09G0028200 | 112.94 | 2.23E-26 | 1.73E-25 | -0.615 |
| RHSIM_Rhsim03G0175300 | 112.86 | 2.32E-26 | 1.80E-25 | -1.502 |
| novel.3680 | 112.83 | 2.35E-26 | 1.82E-25 | -4.983 |
| RHSIM_Rhsim01G0106600 | 112.80 | 2.38E-26 | 1.85E-25 | -0.609 |
| RHSIM_Rhsim05G0114200 | 112.75 | 2.44E-26 | 1.89E-25 | 1.125 |
| RHSIM_Rhsim05G0113900 | 112.73 | 2.47E-26 | 1.91E-25 | -1.125 |
| RHSIM_Rhsim07G0169700 | 112.71 | 2.50E-26 | 1.93E-25 | 0.931 |
| RHSIM_Rhsim01G0039400 | 112.69 | 2.52E-26 | 1.95E-25 | 0.901 |
| RHSIM_Rhsim10G0116000 | 112.66 | 2.56E-26 | 1.98E-25 | 1.154 |
| RHSIM_Rhsim03G0219500 | 112.65 | 2.57E-26 | 1.99E-25 | -0.690 |
| RHSIM_Rhsim06G0116000 | 112.62 | 2.62E-26 | 2.02E-25 | -1.233 |
| RHSIM_Rhsim11G0085000 | 112.57 | 2.67E-26 | 2.07E-25 | -1.252 |
| RHSIM_Rhsim03G0217500 | 112.53 | 2.73E-26 | 2.11E-25 | -1.060 |
| RHSIM_Rhsim05G0194700 | 112.52 | 2.75E-26 | 2.12E-25 | -0.728 |
| RHSIM_Rhsim07G0118200 | 112.51 | 2.76E-26 | 2.13E-25 | 0.668 |
| RHSIM_Rhsim03G0098400 | 112.35 | 2.99E-26 | 2.31E-25 | 0.887 |
| RHSIM_Rhsim03G0217600 | 112.34 | 3.01E-26 | 2.33E-25 | -1.462 |
| RHSIM_Rhsim05G0038200 | 112.31 | 3.05E-26 | 2.36E-25 | 0.716 |
| RHSIM_Rhsim08G0161400 | 112.31 | 3.06E-26 | 2.36E-25 | -0.868 |
| RHSIM_Rhsim08G0236700 | 112.26 | 3.13E-26 | 2.42E-25 | -1.433 |
| RHSIM_Rhsim04G0111100 | 112.25 | 3.15E-26 | 2.43E-25 | 4.457 |

| Gene | LRT statistic | P value | BH-adjusted P | log2FC (interaction) |
|---|---|---|---|---|
| RHSIM_Rhsim05G0048100 | 112.24 | 3.16E-26 | 2.44E-25 | 2.928 |
| RHSIM_Rhsim03G0191800 | 112.22 | 3.20E-26 | 2.47E-25 | -1.911 |
| RHSIM_Rhsim04G0135700 | 112.19 | 3.24E-26 | 2.50E-25 | -2.114 |
| RHSIM_Rhsim08G0083100 | 112.14 | 3.33E-26 | 2.56E-25 | 3.554 |
| novel.2379 | 112.08 | 3.43E-26 | 2.65E-25 | -1.051 |
| RHSIM_Rhsim07G0247500 | 112.01 | 3.55E-26 | 2.74E-25 | -0.938 |
| RHSIM_Rhsim11G0052900 | 112.01 | 3.56E-26 | 2.74E-25 | 1.205 |
| novel.1055 | 111.99 | 3.60E-26 | 2.77E-25 | -2.228 |
| novel.462 | 111.94 | 3.68E-26 | 2.83E-25 | -1.318 |
| RHSIM_Rhsim03G0086300 | 111.88 | 3.80E-26 | 2.92E-25 | 3.711 |
| RHSIM_Rhsim01G0119000 | 111.87 | 3.82E-26 | 2.94E-25 | 1.041 |
| RHSIM_Rhsim11G0021100 | 111.86 | 3.84E-26 | 2.95E-25 | -0.664 |
| RHSIM_Rhsim09G0102800 | 111.80 | 3.95E-26 | 3.04E-25 | 0.806 |
| RHSIM_Rhsim12G0081400 | 111.80 | 3.96E-26 | 3.04E-25 | 1.205 |
| RHSIM_Rhsim01G0073200 | 111.76 | 4.03E-26 | 3.10E-25 | 0.957 |
| RHSIM_RhsimUnG0211400 | 111.66 | 4.23E-26 | 3.25E-25 | -1.223 |
| novel.1386 | 111.61 | 4.34E-26 | 3.34E-25 | -1.108 |
| RHSIM_Rhsim08G0233000 | 111.61 | 4.36E-26 | 3.35E-25 | -1.414 |
| RHSIM_RhsimUnG0131400 | 111.57 | 4.44E-26 | 3.41E-25 | 2.790 |
| RHSIM_Rhsim11G0180900 | 111.55 | 4.48E-26 | 3.44E-25 | -0.675 |
| RHSIM_Rhsim10G0209200 | 111.49 | 4.61E-26 | 3.54E-25 | 2.968 |
| RHSIM_Rhsim01G0233100 | 111.35 | 4.95E-26 | 3.80E-25 | -0.961 |
| RHSIM_Rhsim13G0104700 | 111.33 | 5.00E-26 | 3.84E-25 | 1.194 |
| RHSIM_Rhsim08G0232100 | 111.28 | 5.13E-26 | 3.94E-25 | -1.144 |
| RHSIM_Rhsim13G0000700 | 111.25 | 5.22E-26 | 4.00E-25 | 1.260 |
| RHSIM_Rhsim12G0190600 | 111.22 | 5.30E-26 | 4.07E-25 | -2.088 |
| novel.1734 | 111.20 | 5.35E-26 | 4.10E-25 | -3.446 |
| RHSIM_Rhsim03G0081500 | 111.17 | 5.43E-26 | 4.16E-25 | -1.120 |
| RHSIM_Rhsim08G0037900 | 111.16 | 5.46E-26 | 4.18E-25 | -1.847 |
| RHSIM_Rhsim02G0239600 | 111.10 | 5.63E-26 | 4.31E-25 | -1.001 |
| RHSIM_RhsimUnG0159500 | 111.09 | 5.66E-26 | 4.33E-25 | 2.506 |
| RHSIM_Rhsim13G0013500 | 111.05 | 5.78E-26 | 4.42E-25 | -1.361 |

| Gene | LRT statistic | P value | BH-adjusted P | log2FC (interaction) |
|---|---|---|---|---|
| RHSIM_Rhsim02G0132300 | 111.04 | 5.80E-26 | 4.44E-25 | 1.106 |
| RHSIM_Rhsim13G0207700 | 110.85 | 6.37E-26 | 4.87E-25 | 0.986 |
| RHSIM_Rhsim12G0115000 | 110.84 | 6.40E-26 | 4.90E-25 | -0.617 |
| RHSIM_Rhsim03G0104800 | 110.81 | 6.53E-26 | 4.99E-25 | 0.796 |
| RHSIM_Rhsim05G0104400 | 110.80 | 6.55E-26 | 5.00E-25 | 0.594 |
| RHSIM_Rhsim09G0180000 | 110.79 | 6.58E-26 | 5.03E-25 | 0.969 |
| RHSIM_Rhsim06G0192200 | 110.76 | 6.67E-26 | 5.10E-25 | 1.933 |
| RHSIM_Rhsim03G0251700 | 110.75 | 6.71E-26 | 5.12E-25 | -0.669 |
| novel.767 | 110.73 | 6.79E-26 | 5.18E-25 | 0.825 |
| RHSIM_Rhsim02G0234400 | 110.69 | 6.91E-26 | 5.28E-25 | -1.582 |
| RHSIM_Rhsim03G0074700 | 110.63 | 7.12E-26 | 5.44E-25 | -2.867 |
| RHSIM_Rhsim07G0050400 | 110.62 | 7.17E-26 | 5.47E-25 | 0.912 |
| novel.872 | 110.59 | 7.28E-26 | 5.55E-25 | -4.410 |
| RHSIM_Rhsim13G0073000 | 110.55 | 7.43E-26 | 5.67E-25 | -1.753 |
| RHSIM_Rhsim06G0175900 | 110.47 | 7.74E-26 | 5.90E-25 | 0.793 |
| RHSIM_Rhsim10G0214500 | 110.43 | 7.88E-26 | 6.00E-25 | -1.773 |
| RHSIM_Rhsim08G0008200 | 110.42 | 7.94E-26 | 6.05E-25 | 0.759 |
| RHSIM_Rhsim11G0122400 | 110.35 | 8.20E-26 | 6.25E-25 | 1.032 |
| RHSIM_Rhsim08G0223300 | 110.34 | 8.24E-26 | 6.28E-25 | 0.905 |
| RHSIM_Rhsim02G0229200 | 110.19 | 8.92E-26 | 6.79E-25 | 2.134 |
| RHSIM_Rhsim07G0204200 | 110.07 | 9.46E-26 | 7.20E-25 | -0.873 |
| RHSIM_Rhsim08G0062800 | 109.98 | 9.91E-26 | 7.54E-25 | -0.757 |
| RHSIM_Rhsim12G0172200 | 109.97 | 9.94E-26 | 7.56E-25 | 1.464 |
| RHSIM_Rhsim07G0003900 | 109.95 | 1.00E-25 | 7.62E-25 | 1.200 |
| RHSIM_Rhsim05G0119100 | 109.95 | 1.01E-25 | 7.65E-25 | 1.223 |
| RHSIM_Rhsim05G0069700 | 109.89 | 1.04E-25 | 7.89E-25 | -0.740 |
| RHSIM_RhsimUnG0146800 | 109.87 | 1.04E-25 | 7.94E-25 | -4.523 |
| RHSIM_Rhsim07G0159500 | 109.86 | 1.05E-25 | 8.00E-25 | 1.302 |
| novel.106 | 109.80 | 1.08E-25 | 8.21E-25 | -3.781 |
| novel.1601 | 109.78 | 1.09E-25 | 8.30E-25 | 0.666 |
| RHSIM_Rhsim09G0199500 | 109.76 | 1.10E-25 | 8.38E-25 | -0.831 |
| RHSIM_RhsimUnG0212700 | 109.75 | 1.11E-25 | 8.42E-25 | 0.954 |

| Gene | LRT statistic | P value | BH-adjusted P | log2FC (interaction) |
|---|---|---|---|---|
| RHSIM_Rhsim05G0092700 | 109.70 | 1.14E-25 | 8.66E-25 | 2.711 |
| RHSIM_Rhsim06G0212900 | 109.60 | 1.20E-25 | 9.11E-25 | 1.581 |
| novel.645 | 109.58 | 1.21E-25 | 9.16E-25 | 1.505 |
| RHSIM_Rhsim05G0177700 | 109.50 | 1.26E-25 | 9.56E-25 | 3.297 |
| novel.28 | 109.42 | 1.31E-25 | 9.95E-25 | 7.181 |
| novel.1266 | 109.38 | 1.34E-25 | 1.02E-24 | -3.810 |
| RHSIM_Rhsim12G0003400 | 109.32 | 1.38E-25 | 1.04E-24 | -0.873 |
| RHSIM_Rhsim09G0178300 | 109.27 | 1.41E-25 | 1.07E-24 | -4.350 |
| RHSIM_Rhsim08G0055000 | 109.25 | 1.43E-25 | 1.08E-24 | -0.566 |
| RHSIM_Rhsim06G0137200 | 109.17 | 1.49E-25 | 1.13E-24 | -1.671 |
| RHSIM_Rhsim04G0231000 | 109.16 | 1.50E-25 | 1.13E-24 | 0.877 |
| novel.4565 | 109.15 | 1.50E-25 | 1.14E-24 | 1.600 |
| RHSIM_Rhsim02G0070200 | 109.12 | 1.53E-25 | 1.16E-24 | -1.169 |
| RHSIM_Rhsim09G0171600 | 109.11 | 1.54E-25 | 1.16E-24 | -1.016 |
| RHSIM_Rhsim06G0191800 | 109.00 | 1.62E-25 | 1.23E-24 | -1.050 |
| RHSIM_Rhsim03G0208400 | 108.98 | 1.64E-25 | 1.24E-24 | -2.219 |
| RHSIM_Rhsim12G0018600 | 108.98 | 1.64E-25 | 1.24E-24 | 1.126 |
| RHSIM_Rhsim10G0118400 | 108.96 | 1.66E-25 | 1.25E-24 | -1.089 |
| RHSIM_Rhsim10G0021200 | 108.89 | 1.71E-25 | 1.29E-24 | 0.899 |
| novel.3862 | 108.88 | 1.72E-25 | 1.30E-24 | 1.090 |
| RHSIM_Rhsim09G0107700 | 108.88 | 1.73E-25 | 1.30E-24 | 0.823 |
| RHSIM_Rhsim07G0012900 | 108.87 | 1.73E-25 | 1.31E-24 | -0.590 |
| RHSIM_Rhsim06G0064800 | 108.86 | 1.74E-25 | 1.31E-24 | 0.726 |
| novel.1742 | 108.84 | 1.76E-25 | 1.33E-24 | 2.681 |
| RHSIM_Rhsim10G0049000 | 108.84 | 1.76E-25 | 1.33E-24 | 1.263 |
| RHSIM_Rhsim11G0023700 | 108.83 | 1.77E-25 | 1.33E-24 | 1.211 |
| RHSIM_Rhsim12G0172400 | 108.74 | 1.85E-25 | 1.39E-24 | 1.787 |
| RHSIM_Rhsim05G0165700 | 108.74 | 1.85E-25 | 1.39E-24 | -0.715 |
| RHSIM_Rhsim01G0025300 | 108.69 | 1.89E-25 | 1.43E-24 | 0.864 |
| RHSIM_Rhsim04G0234300 | 108.67 | 1.92E-25 | 1.44E-24 | 0.956 |
| RHSIM_Rhsim12G0136900 | 108.67 | 1.92E-25 | 1.44E-24 | -0.686 |
| RHSIM_Rhsim13G0072500 | 108.61 | 1.98E-25 | 1.49E-24 | -2.015 |

| Gene | LRT statistic | P value | BH-adjusted P | log2FC (interaction) |
|---|---|---|---|---|
| RHSIM_Rhsim04G0003000 | 108.59 | 1.99E-25 | 1.50E-24 | -0.802 |
| RHSIM_Rhsim02G0138900 | 108.57 | 2.02E-25 | 1.52E-24 | 2.508 |
| RHSIM_Rhsim06G0029700 | 108.53 | 2.06E-25 | 1.55E-24 | -1.213 |
| RHSIM_Rhsim08G0073600 | 108.49 | 2.09E-25 | 1.57E-24 | 1.757 |
| RHSIM_Rhsim01G0001600 | 108.46 | 2.13E-25 | 1.60E-24 | -1.067 |
| RHSIM_Rhsim01G0259800 | 108.43 | 2.16E-25 | 1.63E-24 | 0.722 |
| RHSIM_Rhsim09G0201100 | 108.36 | 2.25E-25 | 1.69E-24 | 1.206 |
| RHSIM_Rhsim04G0023600 | 108.29 | 2.32E-25 | 1.74E-24 | 0.606 |
| RHSIM_Rhsim08G0189100 | 108.29 | 2.32E-25 | 1.74E-24 | -0.859 |
| RHSIM_Rhsim08G0214800 | 108.26 | 2.36E-25 | 1.77E-24 | 1.130 |
| RHSIM_Rhsim08G0009400 | 108.23 | 2.39E-25 | 1.80E-24 | 0.835 |
| novel.2351 | 108.21 | 2.41E-25 | 1.81E-24 | 1.600 |
| RHSIM_Rhsim04G0218100 | 108.18 | 2.46E-25 | 1.84E-24 | 2.839 |
| RHSIM_Rhsim07G0213000 | 108.16 | 2.48E-25 | 1.86E-24 | -1.475 |
| RHSIM_Rhsim02G0061600 | 108.12 | 2.53E-25 | 1.90E-24 | 0.988 |
| RHSIM_Rhsim06G0008200 | 108.11 | 2.54E-25 | 1.90E-24 | -1.102 |
| RHSIM_Rhsim10G0109900 | 108.03 | 2.64E-25 | 1.98E-24 | -0.825 |
| RHSIM_Rhsim13G0053400 | 108.03 | 2.65E-25 | 1.99E-24 | -2.426 |
| RHSIM_Rhsim12G0134000 | 107.97 | 2.73E-25 | 2.05E-24 | -1.074 |
| RHSIM_Rhsim01G0021100 | 107.89 | 2.84E-25 | 2.13E-24 | 1.117 |
| RHSIM_Rhsim10G0127800 | 107.88 | 2.85E-25 | 2.13E-24 | 1.994 |
| RHSIM_Rhsim05G0229000 | 107.88 | 2.86E-25 | 2.14E-24 | -0.629 |
| RHSIM_Rhsim05G0076800 | 107.84 | 2.91E-25 | 2.18E-24 | 1.012 |
| RHSIM_Rhsim09G0020400 | 107.78 | 3.00E-25 | 2.24E-24 | 0.986 |
| RHSIM_Rhsim13G0092300 | 107.76 | 3.03E-25 | 2.27E-24 | -1.115 |
| RHSIM_RhsimUnG0187600 | 107.76 | 3.04E-25 | 2.27E-24 | -1.703 |
| RHSIM_Rhsim09G0086600 | 107.72 | 3.09E-25 | 2.31E-24 | 0.768 |
| RHSIM_Rhsim05G0203100 | 107.71 | 3.11E-25 | 2.32E-24 | 1.158 |
| RHSIM_Rhsim09G0021500 | 107.70 | 3.12E-25 | 2.33E-24 | -1.354 |
| RHSIM_Rhsim01G0068700 | 107.58 | 3.33E-25 | 2.48E-24 | 0.572 |
| RHSIM_Rhsim01G0088300 | 107.55 | 3.37E-25 | 2.52E-24 | -7.324 |
| RHSIM_Rhsim05G0023400 | 107.50 | 3.46E-25 | 2.58E-24 | -2.077 |

| Gene | LRT statistic | P value | BH-adjusted P | log2FC (interaction) |
|---|---|---|---|---|
| RHSIM_Rhsim06G0218500 | 107.49 | 3.47E-25 | 2.59E-24 | -1.197 |
| RHSIM_Rhsim06G0202500 | 107.37 | 3.70E-25 | 2.76E-24 | 1.267 |
| RHSIM_RhsimPtG0003100 | 107.34 | 3.75E-25 | 2.79E-24 | 2.706 |
| novel.2301 | 107.32 | 3.78E-25 | 2.82E-24 | 3.706 |
| RHSIM_Rhsim05G0070400 | 107.27 | 3.89E-25 | 2.90E-24 | -1.814 |
| RHSIM_Rhsim10G0098600 | 107.22 | 3.98E-25 | 2.96E-24 | -0.946 |
| RHSIM_Rhsim12G0014600 | 107.22 | 3.99E-25 | 2.97E-24 | -1.289 |
| RHSIM_Rhsim10G0137100 | 107.22 | 3.99E-25 | 2.97E-24 | 1.015 |
| RHSIM_Rhsim11G0091000 | 107.17 | 4.09E-25 | 3.04E-24 | -2.541 |
| RHSIM_Rhsim10G0138000 | 107.08 | 4.28E-25 | 3.19E-24 | 0.911 |
| RHSIM_Rhsim06G0057700 | 107.06 | 4.32E-25 | 3.22E-24 | 0.738 |
| RHSIM_Rhsim10G0081700 | 107.00 | 4.45E-25 | 3.31E-24 | -3.287 |
| RHSIM_Rhsim08G0041300 | 107.00 | 4.46E-25 | 3.31E-24 | -0.969 |
| RHSIM_Rhsim10G0205900 | 106.98 | 4.50E-25 | 3.35E-24 | -1.851 |
| RHSIM_Rhsim01G0267100 | 106.98 | 4.51E-25 | 3.35E-24 | 3.263 |
| RHSIM_Rhsim13G0146300 | 106.96 | 4.54E-25 | 3.38E-24 | -0.727 |
| RHSIM_Rhsim08G0207200 | 106.94 | 4.58E-25 | 3.40E-24 | -0.673 |
| RHSIM_Rhsim13G0099000 | 106.93 | 4.60E-25 | 3.42E-24 | 2.172 |
| RHSIM_Rhsim05G0075000 | 106.93 | 4.61E-25 | 3.42E-24 | 1.396 |
| RHSIM_Rhsim02G0066100 | 106.86 | 4.78E-25 | 3.55E-24 | 4.078 |
| RHSIM_Rhsim08G0236800 | 106.81 | 4.90E-25 | 3.63E-24 | 1.100 |
| RHSIM_Rhsim05G0221600 | 106.76 | 5.02E-25 | 3.72E-24 | -2.452 |
| RHSIM_Rhsim09G0142900 | 106.74 | 5.08E-25 | 3.77E-24 | 1.085 |
| RHSIM_Rhsim08G0205900 | 106.71 | 5.15E-25 | 3.82E-24 | 0.468 |
| RHSIM_Rhsim03G0157000 | 106.71 | 5.15E-25 | 3.82E-24 | 2.568 |
| RHSIM_RhsimUnG0248000 | 106.69 | 5.20E-25 | 3.86E-24 | 0.871 |
| RHSIM_Rhsim04G0176700 | 106.69 | 5.21E-25 | 3.86E-24 | 0.698 |
| RHSIM_Rhsim04G0050100 | 106.68 | 5.23E-25 | 3.87E-24 | 0.617 |
| RHSIM_Rhsim03G0196900 | 106.68 | 5.24E-25 | 3.88E-24 | -0.988 |
| RHSIM_Rhsim05G0075900 | 106.60 | 5.46E-25 | 4.04E-24 | 0.728 |
| RHSIM_Rhsim03G0263900 | 106.55 | 5.59E-25 | 4.14E-24 | -5.992 |
| RHSIM_Rhsim13G0060100 | 106.53 | 5.65E-25 | 4.18E-24 | -1.102 |

| Gene | LRT statistic | P value | BH-adjusted P | log2FC (interaction) |
|---|---|---|---|---|
| RHSIM_Rhsim09G0132600 | 106.52 | 5.68E-25 | 4.20E-24 | -2.199 |
| RHSIM_Rhsim12G0189300 | 106.43 | 5.94E-25 | 4.39E-24 | -0.907 |
| RHSIM_Rhsim11G0077600 | 106.42 | 5.97E-25 | 4.41E-24 | 1.709 |
| RHSIM_Rhsim07G0226800 | 106.22 | 6.61E-25 | 4.88E-24 | -2.645 |
| RHSIM_RhsimUnG0194200 | 106.21 | 6.64E-25 | 4.91E-24 | 1.581 |
| RHSIM_Rhsim08G0042900 | 106.16 | 6.81E-25 | 5.03E-24 | -1.200 |
| RHSIM_Rhsim12G0196000 | 106.07 | 7.13E-25 | 5.27E-24 | -2.372 |
| RHSIM_Rhsim01G0191000 | 106.02 | 7.30E-25 | 5.39E-24 | 0.978 |
| novel.4034 | 106.00 | 7.38E-25 | 5.45E-24 | -4.328 |
| RHSIM_Rhsim10G0022700 | 105.97 | 7.48E-25 | 5.52E-24 | -0.825 |
| RHSIM_Rhsim03G0256000 | 105.95 | 7.56E-25 | 5.58E-24 | -1.875 |
| novel.3532 | 105.85 | 7.97E-25 | 5.88E-24 | 3.902 |
| RHSIM_Rhsim13G0126700 | 105.83 | 8.03E-25 | 5.92E-24 | 0.859 |
| RHSIM_Rhsim09G0185100 | 105.78 | 8.24E-25 | 6.08E-24 | 1.079 |
| RHSIM_Rhsim02G0096700 | 105.71 | 8.53E-25 | 6.29E-24 | -0.953 |
| RHSIM_Rhsim01G0034300 | 105.59 | 9.05E-25 | 6.67E-24 | 1.265 |
| RHSIM_Rhsim08G0089000 | 105.59 | 9.09E-25 | 6.70E-24 | -2.156 |
| RHSIM_Rhsim13G0198200 | 105.53 | 9.34E-25 | 6.88E-24 | -1.034 |
| novel.3240 | 105.46 | 9.67E-25 | 7.12E-24 | 0.806 |
| RHSIM_Rhsim13G0104300 | 105.40 | 9.99E-25 | 7.35E-24 | -1.019 |
| RHSIM_RhsimUnG0206500 | 105.35 | 1.02E-24 | 7.52E-24 | 1.116 |
| RHSIM_Rhsim11G0071000 | 105.34 | 1.03E-24 | 7.58E-24 | -1.390 |
| RHSIM_Rhsim04G0099700 | 105.26 | 1.07E-24 | 7.86E-24 | 0.966 |
| RHSIM_Rhsim09G0064900 | 105.11 | 1.15E-24 | 8.49E-24 | -1.701 |
| RHSIM_Rhsim05G0048300 | 105.08 | 1.17E-24 | 8.62E-24 | 2.735 |
| RHSIM_Rhsim12G0099800 | 104.99 | 1.23E-24 | 9.02E-24 | 0.801 |
| RHSIM_Rhsim02G0145200 | 104.98 | 1.23E-24 | 9.05E-24 | -1.290 |
| RHSIM_Rhsim11G0198700 | 104.94 | 1.26E-24 | 9.24E-24 | 0.747 |
| RHSIM_Rhsim08G0173800 | 104.93 | 1.26E-24 | 9.27E-24 | 0.771 |
| RHSIM_Rhsim12G0041700 | 104.91 | 1.28E-24 | 9.40E-24 | -0.533 |
| RHSIM_Rhsim08G0252300 | 104.84 | 1.32E-24 | 9.72E-24 | 1.051 |
| RHSIM_Rhsim01G0042900 | 104.82 | 1.34E-24 | 9.81E-24 | 1.869 |

| Gene | LRT statistic | P value | BH-adjusted P | log2FC (interaction) |
|---|---|---|---|---|
| RHSIM_Rhsim09G0017900 | 104.59 | 1.50E-24 | 1.10E-23 | 1.434 |
| RHSIM_Rhsim10G0069600 | 104.53 | 1.55E-24 | 1.13E-23 | 0.604 |
| RHSIM_Rhsim02G0140300 | 104.45 | 1.61E-24 | 1.18E-23 | 0.674 |
| RHSIM_Rhsim04G0144900 | 104.44 | 1.62E-24 | 1.19E-23 | 1.978 |
| RHSIM_Rhsim01G0193900 | 104.44 | 1.62E-24 | 1.19E-23 | 0.995 |
| RHSIM_Rhsim03G0207200 | 104.36 | 1.68E-24 | 1.23E-23 | -1.043 |
| RHSIM_Rhsim03G0111900 | 104.32 | 1.72E-24 | 1.26E-23 | 0.588 |
| RHSIM_Rhsim09G0066700 | 104.29 | 1.75E-24 | 1.28E-23 | -1.579 |
| RHSIM_Rhsim02G0052300 | 104.26 | 1.78E-24 | 1.30E-23 | 0.583 |
| RHSIM_Rhsim06G0216400 | 104.25 | 1.78E-24 | 1.30E-23 | 0.714 |
| RHSIM_Rhsim01G0284400 | 104.16 | 1.87E-24 | 1.37E-23 | -0.770 |
| RHSIM_Rhsim07G0075100 | 104.04 | 1.98E-24 | 1.45E-23 | -1.345 |
| RHSIM_Rhsim08G0202400 | 104.02 | 2.01E-24 | 1.47E-23 | 1.151 |
| RHSIM_Rhsim05G0210600 | 103.99 | 2.03E-24 | 1.49E-23 | 1.585 |
| RHSIM_Rhsim06G0114100 | 103.99 | 2.04E-24 | 1.49E-23 | 0.784 |
| RHSIM_Rhsim02G0043300 | 103.90 | 2.12E-24 | 1.55E-23 | -1.252 |
| RHSIM_Rhsim07G0067700 | 103.88 | 2.14E-24 | 1.57E-23 | -2.212 |
| RHSIM_RhsimUnG0190300 | 103.82 | 2.22E-24 | 1.62E-23 | -2.336 |
| RHSIM_Rhsim07G0220800 | 103.77 | 2.27E-24 | 1.66E-23 | 0.939 |
| RHSIM_Rhsim05G0116700 | 103.76 | 2.29E-24 | 1.67E-23 | 1.073 |
| RHSIM_Rhsim06G0111400 | 103.75 | 2.29E-24 | 1.67E-23 | -3.650 |
| RHSIM_Rhsim09G0163000 | 103.75 | 2.30E-24 | 1.68E-23 | -2.499 |
| RHSIM_Rhsim03G0254800 | 103.73 | 2.32E-24 | 1.69E-23 | -2.378 |
| RHSIM_Rhsim07G0246000 | 103.63 | 2.44E-24 | 1.78E-23 | -3.754 |
| RHSIM_Rhsim11G0054400 | 103.60 | 2.47E-24 | 1.80E-23 | 0.812 |
| RHSIM_RhsimUnG0016100 | 103.57 | 2.51E-24 | 1.83E-23 | -0.902 |
| novel.273 | 103.56 | 2.52E-24 | 1.84E-23 | 1.161 |
| RHSIM_Rhsim07G0078000 | 103.48 | 2.64E-24 | 1.92E-23 | -1.601 |
| novel.2913 | 103.41 | 2.73E-24 | 1.99E-23 | -0.619 |
| RHSIM_Rhsim08G0163300 | 103.39 | 2.75E-24 | 2.00E-23 | 1.901 |
| RHSIM_Rhsim02G0214200 | 103.32 | 2.85E-24 | 2.08E-23 | -1.726 |
| RHSIM_Rhsim06G0025500 | 103.31 | 2.87E-24 | 2.09E-23 | -1.671 |

| Gene | LRT statistic | P value | BH-adjusted P | log2FC (interaction) |
|---|---|---|---|---|
| RHSIM_Rhsim13G0192900 | 103.28 | 2.90E-24 | 2.11E-23 | -2.367 |
| RHSIM_Rhsim09G0209300 | 103.24 | 2.97E-24 | 2.16E-23 | 0.567 |
| RHSIM_Rhsim04G0026700 | 103.22 | 3.00E-24 | 2.18E-23 | 1.093 |
| RHSIM_Rhsim08G0039600 | 103.17 | 3.07E-24 | 2.23E-23 | 0.708 |
| RHSIM_Rhsim08G0080700 | 103.17 | 3.07E-24 | 2.23E-23 | 0.662 |
| RHSIM_Rhsim03G0005500 | 103.13 | 3.14E-24 | 2.28E-23 | -3.197 |
| RHSIM_Rhsim01G0271700 | 103.09 | 3.19E-24 | 2.32E-23 | -0.780 |
| RHSIM_Rhsim13G0176500 | 103.08 | 3.21E-24 | 2.33E-23 | -7.030 |
| RHSIM_Rhsim03G0234800 | 103.08 | 3.22E-24 | 2.34E-23 | 1.397 |
| RHSIM_Rhsim13G0210600 | 102.86 | 3.59E-24 | 2.61E-23 | 0.793 |
| RHSIM_Rhsim01G0219100 | 102.82 | 3.66E-24 | 2.66E-23 | 0.459 |
| RHSIM_Rhsim12G0112100 | 102.75 | 3.80E-24 | 2.76E-23 | 0.445 |
| RHSIM_Rhsim13G0030900 | 102.75 | 3.81E-24 | 2.76E-23 | -0.795 |
| RHSIM_Rhsim11G0037300 | 102.71 | 3.87E-24 | 2.81E-23 | -0.948 |
| RHSIM_Rhsim08G0252700 | 102.71 | 3.88E-24 | 2.81E-23 | 0.866 |
| RHSIM_Rhsim09G0037500 | 102.68 | 3.94E-24 | 2.86E-23 | 3.398 |
| RHSIM_RhsimUnG0206600 | 102.67 | 3.95E-24 | 2.86E-23 | 2.721 |
| RHSIM_Rhsim05G0071800 | 102.67 | 3.95E-24 | 2.86E-23 | -1.163 |
| RHSIM_Rhsim08G0002800 | 102.57 | 4.17E-24 | 3.02E-23 | -0.561 |
| RHSIM_Rhsim11G0163400 | 102.55 | 4.20E-24 | 3.04E-23 | -0.771 |
| novel.4142 | 102.51 | 4.30E-24 | 3.11E-23 | 1.729 |
| RHSIM_Rhsim06G0103100 | 102.46 | 4.39E-24 | 3.18E-23 | 0.776 |
| novel.2717 | 102.34 | 4.68E-24 | 3.39E-23 | -1.901 |
| RHSIM_Rhsim08G0208400 | 102.32 | 4.73E-24 | 3.42E-23 | -1.057 |
| RHSIM_Rhsim06G0073900 | 102.20 | 5.02E-24 | 3.63E-23 | -0.983 |
| RHSIM_Rhsim07G0121700 | 102.19 | 5.05E-24 | 3.65E-23 | -1.574 |
| RHSIM_Rhsim10G0170300 | 102.12 | 5.23E-24 | 3.78E-23 | -2.104 |
| RHSIM_Rhsim06G0057000 | 102.12 | 5.23E-24 | 3.78E-23 | -2.030 |
| RHSIM_Rhsim10G0004300 | 102.11 | 5.26E-24 | 3.80E-23 | -0.738 |
| RHSIM_Rhsim05G0059800 | 102.10 | 5.28E-24 | 3.81E-23 | 1.948 |
| RHSIM_RhsimUnG0064700 | 102.09 | 5.30E-24 | 3.83E-23 | -1.050 |
| RHSIM_Rhsim06G0207600 | 102.05 | 5.41E-24 | 3.90E-23 | 1.122 |

| Gene | LRT statistic | P value | BH-adjusted P | log2FC (interaction) |
|---|---|---|---|---|
| RHSIM_RhsimUnG0068300 | 102.05 | 5.42E-24 | 3.92E-23 | -4.188 |
| RHSIM_Rhsim12G0200100 | 102.00 | 5.56E-24 | 4.01E-23 | 2.030 |
| RHSIM_Rhsim03G0229600 | 101.97 | 5.63E-24 | 4.06E-23 | -1.106 |
| RHSIM_Rhsim03G0062400 | 101.91 | 5.82E-24 | 4.20E-23 | -0.841 |
| RHSIM_Rhsim13G0054500 | 101.91 | 5.82E-24 | 4.20E-23 | -2.374 |
| RHSIM_RhsimUnG0257700 | 101.90 | 5.85E-24 | 4.22E-23 | 0.861 |
| RHSIM_Rhsim01G0286600 | 101.86 | 5.94E-24 | 4.28E-23 | -0.682 |
| RHSIM_RhsimUnG0007400 | 101.86 | 5.96E-24 | 4.29E-23 | 1.675 |
| novel.484 | 101.83 | 6.04E-24 | 4.35E-23 | -2.988 |
| RHSIM_Rhsim04G0164800 | 101.83 | 6.06E-24 | 4.36E-23 | -0.819 |
| RHSIM_Rhsim09G0211300 | 101.81 | 6.12E-24 | 4.41E-23 | 1.694 |
| RHSIM_Rhsim07G0215100 | 101.81 | 6.12E-24 | 4.41E-23 | -0.565 |
| RHSIM_Rhsim04G0201200 | 101.78 | 6.20E-24 | 4.46E-23 | -4.573 |
| RHSIM_Rhsim05G0168400 | 101.75 | 6.30E-24 | 4.53E-23 | 1.144 |
| RHSIM_Rhsim01G0272700 | 101.69 | 6.50E-24 | 4.67E-23 | -0.671 |
| novel.2480 | 101.64 | 6.67E-24 | 4.80E-23 | -5.372 |
| RHSIM_Rhsim09G0018400 | 101.62 | 6.71E-24 | 4.83E-23 | 1.536 |
| RHSIM_Rhsim08G0071100 | 101.60 | 6.78E-24 | 4.87E-23 | -1.486 |
| RHSIM_Rhsim13G0036800 | 101.52 | 7.09E-24 | 5.10E-23 | -0.966 |
| RHSIM_Rhsim08G0022000 | 101.51 | 7.12E-24 | 5.11E-23 | 3.069 |
| RHSIM_Rhsim01G0280600 | 101.49 | 7.18E-24 | 5.16E-23 | -1.003 |
| RHSIM_Rhsim13G0076000 | 101.48 | 7.20E-24 | 5.17E-23 | -0.918 |
| RHSIM_Rhsim11G0196200 | 101.44 | 7.38E-24 | 5.30E-23 | -1.177 |
| RHSIM_RhsimUnG0007600 | 101.43 | 7.39E-24 | 5.30E-23 | 1.044 |
| RHSIM_Rhsim10G0166700 | 101.42 | 7.45E-24 | 5.35E-23 | 0.667 |
| RHSIM_Rhsim07G0101700 | 101.39 | 7.56E-24 | 5.42E-23 | -1.620 |
| RHSIM_Rhsim08G0234100 | 101.38 | 7.58E-24 | 5.44E-23 | 2.996 |
| RHSIM_Rhsim06G0026400 | 101.38 | 7.59E-24 | 5.44E-23 | -0.986 |
| novel.2761 | 101.35 | 7.72E-24 | 5.53E-23 | 2.826 |
| RHSIM_Rhsim04G0021600 | 101.35 | 7.73E-24 | 5.54E-23 | -1.339 |
| RHSIM_Rhsim05G0018600 | 101.32 | 7.82E-24 | 5.61E-23 | 2.051 |
| RHSIM_Rhsim12G0175800 | 101.31 | 7.85E-24 | 5.62E-23 | -0.671 |

| Gene | LRT statistic | P value | BH-adjusted P | log2FC (interaction) |
|---|---|---|---|---|
| RHSIM_Rhsim09G0187600 | 101.31 | 7.87E-24 | 5.64E-23 | 1.556 |
| RHSIM_Rhsim07G0141400 | 101.24 | 8.16E-24 | 5.84E-23 | 0.837 |
| RHSIM_Rhsim13G0125900 | 101.19 | 8.34E-24 | 5.97E-23 | 0.830 |
| RHSIM_Rhsim12G0029500 | 101.14 | 8.57E-24 | 6.13E-23 | -1.217 |
| RHSIM_Rhsim11G0021900 | 101.10 | 8.77E-24 | 6.27E-23 | 1.313 |
| RHSIM_Rhsim09G0114500 | 101.06 | 8.93E-24 | 6.39E-23 | 0.717 |
| RHSIM_Rhsim01G0241000 | 101.05 | 8.97E-24 | 6.41E-23 | 2.907 |
| RHSIM_Rhsim13G0072100 | 101.03 | 9.08E-24 | 6.49E-23 | 0.795 |
| novel.4393 | 100.94 | 9.46E-24 | 6.76E-23 | 1.160 |
| RHSIM_Rhsim05G0166700 | 100.89 | 9.70E-24 | 6.93E-23 | 0.751 |
| RHSIM_Rhsim08G0073700 | 100.81 | 1.01E-23 | 7.24E-23 | -1.958 |
| RHSIM_Rhsim09G0037700 | 100.69 | 1.08E-23 | 7.68E-23 | 5.886 |
| RHSIM_Rhsim07G0101000 | 100.67 | 1.09E-23 | 7.77E-23 | 0.898 |
| RHSIM_Rhsim01G0293000 | 100.64 | 1.10E-23 | 7.88E-23 | 1.001 |
| RHSIM_Rhsim09G0212200 | 100.63 | 1.11E-23 | 7.91E-23 | 0.970 |
| RHSIM_Rhsim02G0071000 | 100.62 | 1.12E-23 | 7.96E-23 | -1.496 |
| RHSIM_Rhsim07G0006300 | 100.60 | 1.13E-23 | 8.03E-23 | -0.664 |
| RHSIM_Rhsim13G0037000 | 100.50 | 1.19E-23 | 8.45E-23 | -1.212 |
| RHSIM_Rhsim05G0193400 | 100.49 | 1.19E-23 | 8.50E-23 | -2.499 |
| RHSIM_Rhsim11G0117400 | 100.46 | 1.21E-23 | 8.60E-23 | 0.867 |
| RHSIM_Rhsim10G0105000 | 100.43 | 1.22E-23 | 8.73E-23 | -1.165 |
| RHSIM_Rhsim13G0068500 | 100.39 | 1.25E-23 | 8.90E-23 | -1.577 |
| RHSIM_Rhsim10G0006200 | 100.39 | 1.25E-23 | 8.93E-23 | -1.013 |
| RHSIM_Rhsim04G0114600 | 100.34 | 1.28E-23 | 9.13E-23 | 0.513 |
| RHSIM_Rhsim10G0131800 | 100.32 | 1.30E-23 | 9.22E-23 | 1.405 |
| RHSIM_Rhsim13G0016800 | 100.32 | 1.30E-23 | 9.23E-23 | -1.609 |
| RHSIM_Rhsim02G0109900 | 100.24 | 1.35E-23 | 9.60E-23 | 1.735 |
| RHSIM_Rhsim01G0273400 | 100.23 | 1.36E-23 | 9.66E-23 | -1.181 |
| RHSIM_Rhsim01G0008000 | 100.15 | 1.41E-23 | 1.00E-22 | -1.194 |
| RHSIM_Rhsim06G0013400 | 100.15 | 1.41E-23 | 1.01E-22 | -1.228 |
| RHSIM_Rhsim09G0066100 | 100.09 | 1.45E-23 | 1.03E-22 | 1.295 |
| RHSIM_Rhsim09G0184800 | 100.09 | 1.46E-23 | 1.04E-22 | 2.309 |

| Gene | LRT statistic | P value | BH-adjusted P | log2FC (interaction) |
|---|---|---|---|---|
| RHSIM_Rhsim06G0049500 | 100.07 | 1.47E-23 | 1.04E-22 | -0.814 |
| RHSIM_Rhsim10G0156000 | 100.00 | 1.53E-23 | 1.08E-22 | 1.385 |
| RHSIM_Rhsim09G0037300 | 99.98 | 1.54E-23 | 1.09E-22 | 1.541 |
| RHSIM_Rhsim07G0066800 | 99.91 | 1.60E-23 | 1.13E-22 | -2.717 |
| RHSIM_Rhsim10G0164300 | 99.90 | 1.60E-23 | 1.14E-22 | 0.587 |
| RHSIM_Rhsim01G0059500 | 99.90 | 1.60E-23 | 1.14E-22 | -1.008 |
| RHSIM_Rhsim12G0193300 | 99.88 | 1.62E-23 | 1.15E-22 | 9.835 |
| RHSIM_Rhsim06G0156300 | 99.71 | 1.77E-23 | 1.25E-22 | -0.670 |
| RHSIM_Rhsim04G0031200 | 99.66 | 1.81E-23 | 1.28E-22 | 1.095 |
| novel.1024 | 99.62 | 1.84E-23 | 1.31E-22 | -1.373 |
| RHSIM_Rhsim09G0154200 | 99.60 | 1.86E-23 | 1.32E-22 | 0.730 |
| RHSIM_Rhsim06G0071400 | 99.59 | 1.87E-23 | 1.33E-22 | 1.291 |
| novel.1660 | 99.53 | 1.93E-23 | 1.37E-22 | 2.424 |
| RHSIM_Rhsim02G0099600 | 99.49 | 1.98E-23 | 1.40E-22 | -6.832 |
| RHSIM_Rhsim12G0197900 | 99.48 | 1.98E-23 | 1.40E-22 | -0.686 |
| RHSIM_Rhsim08G0136500 | 99.43 | 2.03E-23 | 1.44E-22 | 0.780 |
| RHSIM_Rhsim06G0032100 | 99.39 | 2.07E-23 | 1.46E-22 | -1.208 |
| novel.3987 | 99.39 | 2.07E-23 | 1.47E-22 | 0.849 |
| RHSIM_Rhsim01G0114100 | 99.38 | 2.08E-23 | 1.47E-22 | -1.171 |
| RHSIM_RhsimUnG0251500 | 99.23 | 2.25E-23 | 1.59E-22 | -0.840 |
| RHSIM_Rhsim08G0204700 | 99.21 | 2.27E-23 | 1.61E-22 | 1.500 |
| RHSIM_Rhsim02G0053700 | 99.20 | 2.28E-23 | 1.61E-22 | -0.927 |
| RHSIM_Rhsim10G0028700 | 99.19 | 2.30E-23 | 1.62E-22 | 1.073 |
| RHSIM_Rhsim05G0110100 | 99.17 | 2.32E-23 | 1.64E-22 | -1.109 |
| RHSIM_Rhsim13G0080000 | 99.16 | 2.33E-23 | 1.65E-22 | 1.886 |
| RHSIM_Rhsim06G0182500 | 99.12 | 2.37E-23 | 1.68E-22 | 1.390 |
| RHSIM_Rhsim08G0228900 | 99.09 | 2.41E-23 | 1.70E-22 | 3.590 |
| RHSIM_Rhsim11G0111400 | 98.99 | 2.54E-23 | 1.79E-22 | 0.672 |
| RHSIM_Rhsim02G0040600 | 98.96 | 2.57E-23 | 1.82E-22 | -0.656 |
| RHSIM_Rhsim08G0152200 | 98.94 | 2.61E-23 | 1.84E-22 | 0.789 |
| RHSIM_Rhsim07G0055300 | 98.89 | 2.67E-23 | 1.88E-22 | -0.798 |
| RHSIM_Rhsim13G0097500 | 98.89 | 2.67E-23 | 1.88E-22 | 1.121 |

| Gene | LRT statistic | P value | BH-adjusted P | log2FC (interaction) |
|---|---|---|---|---|
| RHSIM_Rhsim03G0075300 | 98.86 | 2.71E-23 | 1.91E-22 | -0.873 |
| RHSIM_Rhsim09G0184700 | 98.84 | 2.74E-23 | 1.93E-22 | 1.071 |
| RHSIM_Rhsim11G0197800 | 98.80 | 2.79E-23 | 1.97E-22 | 1.666 |
| RHSIM_Rhsim13G0007700 | 98.76 | 2.85E-23 | 2.01E-22 | 1.578 |
| RHSIM_Rhsim13G0074100 | 98.76 | 2.85E-23 | 2.01E-22 | 0.661 |
| RHSIM_Rhsim12G0050400 | 98.75 | 2.86E-23 | 2.01E-22 | -7.400 |
| RHSIM_Rhsim05G0071200 | 98.71 | 2.92E-23 | 2.06E-22 | -1.303 |
| RHSIM_Rhsim04G0175500 | 98.68 | 2.97E-23 | 2.09E-22 | 5.470 |
| RHSIM_Rhsim04G0119800 | 98.65 | 3.01E-23 | 2.12E-22 | -1.753 |
| RHSIM_Rhsim02G0110200 | 98.58 | 3.12E-23 | 2.20E-22 | -0.731 |
| RHSIM_Rhsim12G0153200 | 98.56 | 3.16E-23 | 2.22E-22 | 2.018 |
| RHSIM_Rhsim03G0032700 | 98.50 | 3.25E-23 | 2.28E-22 | 1.789 |
| RHSIM_Rhsim13G0039800 | 98.49 | 3.27E-23 | 2.30E-22 | 2.194 |
| RHSIM_Rhsim02G0007700 | 98.46 | 3.31E-23 | 2.32E-22 | -2.875 |
| RHSIM_Rhsim06G0126200 | 98.29 | 3.62E-23 | 2.54E-22 | -1.030 |
| RHSIM_Rhsim05G0011000 | 98.29 | 3.62E-23 | 2.54E-22 | 0.689 |
| RHSIM_Rhsim05G0165300 | 98.29 | 3.62E-23 | 2.54E-22 | 0.954 |
| RHSIM_Rhsim12G0076700 | 98.23 | 3.72E-23 | 2.61E-22 | 1.516 |
| RHSIM_Rhsim11G0011500 | 98.22 | 3.74E-23 | 2.62E-22 | -1.446 |
| RHSIM_Rhsim07G0124800 | 98.19 | 3.81E-23 | 2.67E-22 | -1.053 |
| novel.1893 | 98.15 | 3.88E-23 | 2.72E-22 | -6.895 |
| RHSIM_Rhsim05G0055600 | 98.10 | 3.98E-23 | 2.79E-22 | -1.053 |
| RHSIM_RhsimUnG0014100 | 98.07 | 4.04E-23 | 2.83E-22 | 0.998 |
| RHSIM_Rhsim01G0179000 | 98.07 | 4.04E-23 | 2.83E-22 | -2.439 |
| RHSIM_Rhsim03G0262600 | 98.03 | 4.13E-23 | 2.89E-22 | -0.988 |
| RHSIM_Rhsim05G0030700 | 98.02 | 4.15E-23 | 2.91E-22 | 1.046 |
| RHSIM_Rhsim04G0032200 | 98.00 | 4.17E-23 | 2.92E-22 | 0.628 |
| RHSIM_Rhsim02G0116500 | 98.00 | 4.19E-23 | 2.93E-22 | 5.501 |
| RHSIM_Rhsim07G0009900 | 97.99 | 4.20E-23 | 2.94E-22 | -1.441 |
| RHSIM_Rhsim11G0131700 | 97.90 | 4.39E-23 | 3.07E-22 | -0.820 |
| RHSIM_Rhsim01G0239400 | 97.88 | 4.44E-23 | 3.11E-22 | -1.276 |
| RHSIM_Rhsim01G0153000 | 97.86 | 4.49E-23 | 3.14E-22 | 0.814 |

| Gene | LRT statistic | P value | BH-adjusted P | log2FC (interaction) |
|---|---|---|---|---|
| RHSIM_Rhsim13G0044700 | 97.85 | 4.50E-23 | 3.15E-22 | -2.051 |
| RHSIM_Rhsim09G0181100 | 97.83 | 4.56E-23 | 3.19E-22 | -1.631 |
| RHSIM_Rhsim13G0147900 | 97.78 | 4.67E-23 | 3.26E-22 | -1.718 |
| RHSIM_Rhsim11G0122000 | 97.77 | 4.70E-23 | 3.28E-22 | 2.021 |
| RHSIM_Rhsim13G0052200 | 97.75 | 4.75E-23 | 3.32E-22 | -4.570 |
| RHSIM_RhsimUnG0256000 | 97.74 | 4.78E-23 | 3.34E-22 | 2.666 |
| RHSIM_Rhsim07G0037100 | 97.71 | 4.85E-23 | 3.39E-22 | 1.149 |
| RHSIM_Rhsim06G0124600 | 97.70 | 4.87E-23 | 3.40E-22 | 2.111 |
| novel.1548 | 97.69 | 4.89E-23 | 3.41E-22 | -1.994 |
| RHSIM_Rhsim09G0199900 | 97.64 | 5.01E-23 | 3.50E-22 | -2.404 |
| RHSIM_Rhsim08G0113100 | 97.59 | 5.15E-23 | 3.59E-22 | -1.211 |
| novel.2098 | 97.55 | 5.25E-23 | 3.66E-22 | 2.175 |
| novel.1226 | 97.51 | 5.37E-23 | 3.74E-22 | -0.706 |
| novel.4001 | 97.42 | 5.61E-23 | 3.91E-22 | 3.121 |
| RHSIM_Rhsim02G0015700 | 97.42 | 5.61E-23 | 3.91E-22 | -1.175 |
| RHSIM_Rhsim08G0162500 | 97.40 | 5.65E-23 | 3.94E-22 | 1.842 |
| novel.4558 | 97.38 | 5.72E-23 | 3.99E-22 | -44.461 |
| RHSIM_Rhsim07G0127500 | 97.33 | 5.86E-23 | 4.08E-22 | -0.801 |
| RHSIM_Rhsim01G0198700 | 97.32 | 5.91E-23 | 4.11E-22 | -1.190 |
| RHSIM_Rhsim06G0036500 | 97.31 | 5.93E-23 | 4.13E-22 | -1.730 |
| novel.4040 | 97.27 | 6.06E-23 | 4.22E-22 | 1.017 |
| RHSIM_Rhsim08G0036000 | 97.21 | 6.25E-23 | 4.35E-22 | 2.691 |
| RHSIM_Rhsim05G0014800 | 97.19 | 6.31E-23 | 4.39E-22 | 1.280 |
| RHSIM_Rhsim09G0093900 | 97.15 | 6.44E-23 | 4.48E-22 | 1.135 |
| RHSIM_Rhsim06G0047500 | 97.13 | 6.50E-23 | 4.52E-22 | 2.065 |
| RHSIM_Rhsim08G0098400 | 97.07 | 6.71E-23 | 4.66E-22 | 0.937 |
| RHSIM_Rhsim12G0174100 | 97.06 | 6.72E-23 | 4.67E-22 | -0.740 |
| RHSIM_Rhsim02G0189300 | 97.06 | 6.74E-23 | 4.68E-22 | -0.787 |
| RHSIM_Rhsim10G0035800 | 97.05 | 6.76E-23 | 4.70E-22 | -6.346 |
| RHSIM_Rhsim12G0155000 | 97.01 | 6.89E-23 | 4.78E-22 | -1.411 |
| RHSIM_Rhsim12G0209100 | 96.93 | 7.20E-23 | 5.00E-22 | -0.795 |
| novel.3717 | 96.92 | 7.23E-23 | 5.02E-22 | 2.840 |

| Gene | LRT statistic | P value | BH-adjusted P | log2FC (interaction) |
|---|---|---|---|---|
| RHSIM_Rhsim08G0123400 | 96.86 | 7.44E-23 | 5.16E-22 | 0.670 |
| RHSIM_Rhsim08G0106300 | 96.83 | 7.55E-23 | 5.24E-22 | -2.605 |
| RHSIM_Rhsim03G0231000 | 96.81 | 7.62E-23 | 5.28E-22 | 2.895 |
| RHSIM_Rhsim05G0028600 | 96.80 | 7.69E-23 | 5.33E-22 | 3.878 |
| RHSIM_Rhsim06G0013500 | 96.77 | 7.78E-23 | 5.39E-22 | 0.498 |
| RHSIM_Rhsim08G0040600 | 96.76 | 7.83E-23 | 5.43E-22 | 1.473 |
| RHSIM_Rhsim08G0086100 | 96.73 | 7.93E-23 | 5.50E-22 | -0.725 |
| RHSIM_RhsimUnG0040900 | 96.72 | 7.99E-23 | 5.53E-22 | 2.246 |
| RHSIM_Rhsim11G0179300 | 96.71 | 8.02E-23 | 5.55E-22 | -1.067 |
| RHSIM_RhsimUnG0179100 | 96.55 | 8.68E-23 | 6.01E-22 | -1.971 |
| RHSIM_Rhsim02G0010800 | 96.48 | 9.01E-23 | 6.24E-22 | 0.961 |
| RHSIM_Rhsim03G0028900 | 96.39 | 9.45E-23 | 6.54E-22 | 0.804 |
| RHSIM_Rhsim08G0242700 | 96.35 | 9.65E-23 | 6.67E-22 | -0.709 |
| RHSIM_Rhsim03G0081900 | 96.32 | 9.78E-23 | 6.76E-22 | -0.510 |
| RHSIM_Rhsim07G0239800 | 96.30 | 9.87E-23 | 6.83E-22 | 0.880 |
| RHSIM_Rhsim06G0173600 | 96.29 | 9.93E-23 | 6.87E-22 | -1.347 |
| RHSIM_Rhsim13G0009300 | 96.26 | 1.01E-22 | 6.96E-22 | -0.783 |
| novel.797 | 96.25 | 1.01E-22 | 6.99E-22 | 1.554 |
| RHSIM_Rhsim10G0034600 | 96.25 | 1.01E-22 | 6.99E-22 | -1.082 |
| RHSIM_Rhsim05G0105400 | 96.22 | 1.03E-22 | 7.10E-22 | -0.733 |
| RHSIM_Rhsim13G0126800 | 96.18 | 1.05E-22 | 7.25E-22 | 0.965 |
| RHSIM_Rhsim07G0079100 | 96.02 | 1.14E-22 | 7.87E-22 | -0.855 |
| RHSIM_Rhsim03G0270000 | 95.96 | 1.17E-22 | 8.07E-22 | -0.713 |
| novel.4044 | 95.89 | 1.22E-22 | 8.40E-22 | 1.710 |
| RHSIM_Rhsim04G0120900 | 95.86 | 1.23E-22 | 8.52E-22 | 0.818 |
| RHSIM_Rhsim03G0001500 | 95.85 | 1.24E-22 | 8.54E-22 | -0.677 |
| RHSIM_Rhsim06G0033900 | 95.77 | 1.29E-22 | 8.91E-22 | -1.023 |
| RHSIM_Rhsim12G0127900 | 95.72 | 1.32E-22 | 9.13E-22 | 1.359 |
| RHSIM_Rhsim01G0096000 | 95.71 | 1.33E-22 | 9.17E-22 | 1.690 |
| RHSIM_Rhsim12G0136300 | 95.70 | 1.34E-22 | 9.21E-22 | 0.778 |
| RHSIM_Rhsim07G0179500 | 95.69 | 1.34E-22 | 9.24E-22 | 1.532 |
| RHSIM_RhsimUnG0168000 | 95.63 | 1.38E-22 | 9.52E-22 | 1.456 |

| Gene | LRT statistic | P value | BH-adjusted P | log2FC (interaction) |
|---|---|---|---|---|
| RHSIM_Rhsim02G0047100 | 95.60 | 1.40E-22 | 9.67E-22 | 1.569 |
| novel.4217 | 95.44 | 1.52E-22 | 1.05E-21 | 2.485 |
| RHSIM_Rhsim05G0042300 | 95.44 | 1.53E-22 | 1.05E-21 | -0.625 |
| RHSIM_Rhsim05G0067100 | 95.42 | 1.54E-22 | 1.06E-21 | -0.645 |
| RHSIM_Rhsim08G0028400 | 95.34 | 1.61E-22 | 1.11E-21 | -2.282 |
| RHSIM_Rhsim06G0157700 | 95.28 | 1.66E-22 | 1.14E-21 | -1.574 |
| RHSIM_Rhsim09G0138700 | 95.24 | 1.69E-22 | 1.16E-21 | 1.150 |
| RHSIM_Rhsim07G0054600 | 95.22 | 1.70E-22 | 1.17E-21 | -0.810 |
| RHSIM_Rhsim06G0026800 | 95.18 | 1.74E-22 | 1.20E-21 | -1.849 |
| RHSIM_Rhsim09G0186200 | 95.14 | 1.78E-22 | 1.22E-21 | -1.704 |
| RHSIM_Rhsim10G0177200 | 94.94 | 1.96E-22 | 1.35E-21 | 1.352 |
| RHSIM_Rhsim10G0008400 | 94.90 | 2.00E-22 | 1.37E-21 | 0.873 |
| RHSIM_Rhsim13G0059800 | 94.86 | 2.04E-22 | 1.40E-21 | -2.959 |
| RHSIM_Rhsim10G0213500 | 94.70 | 2.21E-22 | 1.52E-21 | 0.745 |
| RHSIM_Rhsim03G0080300 | 94.61 | 2.31E-22 | 1.59E-21 | 0.898 |
| RHSIM_Rhsim06G0110000 | 94.61 | 2.32E-22 | 1.59E-21 | 0.982 |
| novel.3840 | 94.61 | 2.32E-22 | 1.59E-21 | -0.626 |
| RHSIM_Rhsim06G0001600 | 94.45 | 2.51E-22 | 1.72E-21 | 0.608 |
| RHSIM_Rhsim09G0032900 | 94.43 | 2.54E-22 | 1.74E-21 | 1.034 |
| RHSIM_Rhsim10G0141300 | 94.38 | 2.60E-22 | 1.78E-21 | -0.466 |
| RHSIM_Rhsim13G0046200 | 94.35 | 2.65E-22 | 1.81E-21 | -0.793 |
| novel.3209 | 94.33 | 2.67E-22 | 1.83E-21 | 1.522 |
| RHSIM_Rhsim13G0094400 | 94.31 | 2.70E-22 | 1.85E-21 | -0.967 |
| RHSIM_Rhsim07G0206000 | 94.29 | 2.73E-22 | 1.87E-21 | 2.032 |
| novel.2974 | 94.28 | 2.73E-22 | 1.87E-21 | -2.647 |
| RHSIM_Rhsim04G0227400 | 94.28 | 2.74E-22 | 1.87E-21 | -0.792 |
| RHSIM_RhsimUnG0158200 | 94.28 | 2.74E-22 | 1.88E-21 | -0.930 |
| RHSIM_Rhsim08G0132200 | 94.27 | 2.76E-22 | 1.89E-21 | -1.581 |
| RHSIM_Rhsim12G0123100 | 94.23 | 2.81E-22 | 1.92E-21 | -1.262 |
| novel.2308 | 94.21 | 2.84E-22 | 1.94E-21 | 1.547 |
| RHSIM_Rhsim04G0083900 | 94.20 | 2.85E-22 | 1.95E-21 | 1.102 |
| RHSIM_Rhsim05G0171800 | 94.19 | 2.86E-22 | 1.96E-21 | 0.730 |

| Gene | LRT statistic | P value | BH-adjusted P | log2FC (interaction) |
|---|---|---|---|---|
| novel.1502 | 94.18 | 2.88E-22 | 1.97E-21 | -2.111 |
| RHSIM_Rhsim06G0165800 | 94.17 | 2.90E-22 | 1.98E-21 | -1.506 |
| RHSIM_Rhsim04G0234000 | 94.14 | 2.94E-22 | 2.01E-21 | 0.712 |
| RHSIM_Rhsim10G0129100 | 94.10 | 2.99E-22 | 2.04E-21 | -1.382 |
| RHSIM_Rhsim12G0016800 | 94.10 | 2.99E-22 | 2.04E-21 | -0.961 |
| RHSIM_Rhsim12G0144300 | 94.06 | 3.06E-22 | 2.09E-21 | 0.621 |
| novel.4194 | 93.98 | 3.18E-22 | 2.17E-21 | -1.752 |
| RHSIM_Rhsim02G0232000 | 93.97 | 3.20E-22 | 2.19E-21 | 0.842 |
| RHSIM_Rhsim10G0195700 | 93.97 | 3.21E-22 | 2.19E-21 | 1.172 |
| RHSIM_Rhsim09G0099500 | 93.94 | 3.25E-22 | 2.21E-21 | -1.875 |
| RHSIM_Rhsim03G0127300 | 93.87 | 3.37E-22 | 2.29E-21 | -0.993 |
| RHSIM_Rhsim01G0231600 | 93.82 | 3.45E-22 | 2.35E-21 | 0.840 |
| RHSIM_Rhsim13G0199300 | 93.76 | 3.56E-22 | 2.43E-21 | 1.229 |
| RHSIM_Rhsim05G0212900 | 93.68 | 3.71E-22 | 2.53E-21 | 1.995 |
| RHSIM_Rhsim02G0140600 | 93.67 | 3.73E-22 | 2.54E-21 | -1.376 |
| RHSIM_Rhsim13G0028300 | 93.66 | 3.74E-22 | 2.55E-21 | -1.277 |
| RHSIM_Rhsim01G0282100 | 93.66 | 3.76E-22 | 2.56E-21 | 1.151 |
| RHSIM_Rhsim07G0088800 | 93.63 | 3.80E-22 | 2.59E-21 | 1.240 |
| RHSIM_Rhsim04G0056300 | 93.59 | 3.89E-22 | 2.65E-21 | -2.658 |
| RHSIM_Rhsim06G0163800 | 93.58 | 3.90E-22 | 2.65E-21 | -3.741 |
| RHSIM_Rhsim03G0044700 | 93.55 | 3.96E-22 | 2.69E-21 | -2.142 |
| RHSIM_Rhsim06G0094000 | 93.55 | 3.97E-22 | 2.70E-21 | 1.635 |
| RHSIM_Rhsim08G0246500 | 93.53 | 4.00E-22 | 2.72E-21 | -0.837 |
| novel.2763 | 93.53 | 4.01E-22 | 2.72E-21 | 2.422 |
| RHSIM_Rhsim06G0155500 | 93.50 | 4.07E-22 | 2.76E-21 | -3.267 |
| RHSIM_Rhsim09G0030000 | 93.46 | 4.14E-22 | 2.81E-21 | 2.387 |
| RHSIM_Rhsim01G0089500 | 93.46 | 4.15E-22 | 2.81E-21 | -2.078 |
| RHSIM_RhsimUnG0251600 | 93.45 | 4.16E-22 | 2.82E-21 | -1.240 |
| RHSIM_Rhsim13G0180100 | 93.43 | 4.20E-22 | 2.85E-21 | -2.566 |
| RHSIM_Rhsim08G0115600 | 93.42 | 4.23E-22 | 2.87E-21 | 1.130 |
| RHSIM_Rhsim12G0109300 | 93.34 | 4.41E-22 | 2.99E-21 | 0.806 |
| RHSIM_Rhsim07G0053700 | 93.29 | 4.51E-22 | 3.06E-21 | 1.325 |

| Gene | LRT statistic | P value | BH-adjusted P | log2FC (interaction) |
|---|---|---|---|---|
| RHSIM_RhsimUnG0188100 | 93.26 | 4.58E-22 | 3.10E-21 | -7.437 |
| novel.3488 | 93.20 | 4.72E-22 | 3.20E-21 | 3.740 |
| RHSIM_Rhsim08G0141600 | 93.15 | 4.84E-22 | 3.28E-21 | -1.148 |
| RHSIM_Rhsim05G0106800 | 93.15 | 4.85E-22 | 3.29E-21 | -0.664 |
| RHSIM_Rhsim13G0068200 | 93.14 | 4.87E-22 | 3.30E-21 | -5.871 |
| RHSIM_Rhsim05G0069600 | 93.10 | 4.97E-22 | 3.37E-21 | 2.369 |
| RHSIM_Rhsim03G0254900 | 93.01 | 5.21E-22 | 3.53E-21 | 1.049 |
| novel.590 | 93.00 | 5.23E-22 | 3.54E-21 | 2.920 |
| RHSIM_Rhsim08G0180000 | 92.91 | 5.47E-22 | 3.71E-21 | -1.108 |
| RHSIM_Rhsim05G0044900 | 92.91 | 5.48E-22 | 3.71E-21 | -1.689 |
| novel.3211 | 92.88 | 5.55E-22 | 3.75E-21 | 2.926 |
| RHSIM_Rhsim01G0229100 | 92.88 | 5.55E-22 | 3.76E-21 | 0.755 |
| RHSIM_Rhsim01G0182000 | 92.84 | 5.66E-22 | 3.83E-21 | -2.931 |
| RHSIM_Rhsim07G0171600 | 92.74 | 5.98E-22 | 4.04E-21 | 1.357 |
| RHSIM_Rhsim04G0130800 | 92.70 | 6.07E-22 | 4.10E-21 | -0.831 |
| RHSIM_Rhsim05G0161600 | 92.70 | 6.08E-22 | 4.11E-21 | -2.287 |
| RHSIM_Rhsim12G0160600 | 92.65 | 6.24E-22 | 4.21E-21 | 1.341 |
| RHSIM_Rhsim13G0121500 | 92.58 | 6.47E-22 | 4.37E-21 | 1.507 |
| RHSIM_Rhsim09G0028000 | 92.55 | 6.57E-22 | 4.43E-21 | 1.384 |
| RHSIM_RhsimUnG0216200 | 92.54 | 6.60E-22 | 4.45E-21 | -1.591 |
| RHSIM_Rhsim01G0181200 | 92.50 | 6.73E-22 | 4.54E-21 | 7.708 |
| RHSIM_Rhsim08G0116700 | 92.47 | 6.83E-22 | 4.61E-21 | -2.483 |
| novel.3571 | 92.40 | 7.08E-22 | 4.77E-21 | 0.436 |
| RHSIM_Rhsim12G0110900 | 92.40 | 7.08E-22 | 4.78E-21 | 1.685 |
| RHSIM_Rhsim01G0142100 | 92.38 | 7.15E-22 | 4.82E-21 | -1.139 |
| RHSIM_Rhsim01G0050800 | 92.38 | 7.16E-22 | 4.83E-21 | -0.763 |
| RHSIM_Rhsim06G0085400 | 92.36 | 7.24E-22 | 4.88E-21 | 1.435 |
| RHSIM_Rhsim04G0096900 | 92.34 | 7.29E-22 | 4.91E-21 | 0.696 |
| RHSIM_Rhsim06G0174200 | 92.34 | 7.29E-22 | 4.91E-21 | -2.358 |
| RHSIM_Rhsim07G0241800 | 92.25 | 7.64E-22 | 5.14E-21 | -1.064 |
| RHSIM_Rhsim13G0180200 | 92.23 | 7.73E-22 | 5.20E-21 | -4.605 |
| RHSIM_Rhsim02G0111700 | 92.21 | 7.78E-22 | 5.24E-21 | -1.308 |

| Gene | LRT statistic | P value | BH-adjusted P | log2FC (interaction) |
|---|---|---|---|---|
| RHSIM_Rhsim12G0092300 | 92.20 | 7.84E-22 | 5.27E-21 | -1.026 |
| novel.3522 | 92.12 | 8.17E-22 | 5.50E-21 | 4.130 |
| RHSIM_Rhsim08G0161700 | 92.11 | 8.21E-22 | 5.52E-21 | -2.030 |
| RHSIM_Rhsim13G0201700 | 92.11 | 8.22E-22 | 5.53E-21 | -0.793 |
| novel.1065 | 92.07 | 8.36E-22 | 5.62E-21 | 0.805 |
| RHSIM_Rhsim11G0034800 | 92.05 | 8.43E-22 | 5.67E-21 | 2.772 |
| RHSIM_Rhsim02G0141300 | 91.89 | 9.15E-22 | 6.15E-21 | 1.108 |
| RHSIM_Rhsim13G0148600 | 91.89 | 9.15E-22 | 6.15E-21 | -0.481 |
| RHSIM_Rhsim07G0243400 | 91.88 | 9.19E-22 | 6.17E-21 | -3.090 |
| RHSIM_Rhsim08G0039900 | 91.88 | 9.22E-22 | 6.19E-21 | -0.519 |
| RHSIM_Rhsim09G0145800 | 91.86 | 9.32E-22 | 6.26E-21 | -0.511 |
| novel.3058 | 91.82 | 9.48E-22 | 6.36E-21 | -1.165 |
| RHSIM_Rhsim06G0169400 | 91.82 | 9.49E-22 | 6.37E-21 | 1.088 |
| novel.1393 | 91.74 | 9.87E-22 | 6.62E-21 | 1.380 |
| RHSIM_Rhsim06G0158200 | 91.74 | 9.90E-22 | 6.64E-21 | 0.836 |
| RHSIM_Rhsim03G0211600 | 91.73 | 9.92E-22 | 6.65E-21 | -1.524 |
| RHSIM_Rhsim01G0220800 | 91.70 | 1.01E-21 | 6.77E-21 | -1.057 |
| RHSIM_Rhsim02G0118800 | 91.66 | 1.03E-21 | 6.89E-21 | -3.540 |
| RHSIM_Rhsim12G0144900 | 91.65 | 1.03E-21 | 6.92E-21 | -0.862 |
| RHSIM_Rhsim01G0204100 | 91.64 | 1.04E-21 | 6.95E-21 | -1.265 |
| RHSIM_Rhsim02G0243000 | 91.63 | 1.05E-21 | 7.01E-21 | -1.726 |
| RHSIM_Rhsim03G0231700 | 91.53 | 1.10E-21 | 7.37E-21 | 0.741 |
| RHSIM_Rhsim07G0075700 | 91.52 | 1.10E-21 | 7.38E-21 | -0.831 |
| novel.2272 | 91.50 | 1.12E-21 | 7.47E-21 | 4.825 |
| RHSIM_Rhsim06G0186900 | 91.47 | 1.13E-21 | 7.58E-21 | 0.893 |
| RHSIM_Rhsim08G0200900 | 91.44 | 1.15E-21 | 7.71E-21 | 1.087 |
| novel.1978 | 91.41 | 1.17E-21 | 7.79E-21 | -1.112 |
| RHSIM_Rhsim03G0075700 | 91.40 | 1.18E-21 | 7.86E-21 | 2.294 |
| RHSIM_Rhsim03G0165400 | 91.39 | 1.18E-21 | 7.89E-21 | 1.316 |
| RHSIM_Rhsim09G0010200 | 91.37 | 1.19E-21 | 7.95E-21 | -0.921 |
| RHSIM_Rhsim04G0046400 | 91.31 | 1.23E-21 | 8.22E-21 | -1.152 |
| RHSIM_RhsimUnG0204200 | 91.30 | 1.23E-21 | 8.23E-21 | -1.514 |

| Gene | LRT statistic | P value | BH-adjusted P | log2FC (interaction) |
|---|---|---|---|---|
| RHSIM_Rhsim12G0202100 | 91.28 | 1.25E-21 | 8.33E-21 | 0.635 |
| RHSIM_Rhsim06G0108600 | 91.27 | 1.25E-21 | 8.35E-21 | -0.828 |
| RHSIM_Rhsim03G0150600 | 91.19 | 1.31E-21 | 8.72E-21 | -2.494 |
| novel.1104 | 91.18 | 1.31E-21 | 8.74E-21 | 2.353 |
| RHSIM_RhsimUnG0082400 | 91.17 | 1.32E-21 | 8.78E-21 | -1.246 |
| RHSIM_Rhsim12G0050700 | 91.15 | 1.33E-21 | 8.87E-21 | -1.145 |
| novel.2315 | 91.15 | 1.33E-21 | 8.89E-21 | -1.792 |
| RHSIM_Rhsim09G0004000 | 91.15 | 1.34E-21 | 8.90E-21 | -0.589 |
| RHSIM_Rhsim13G0184500 | 91.11 | 1.36E-21 | 9.07E-21 | 5.367 |
| novel.1068 | 91.08 | 1.38E-21 | 9.19E-21 | 1.542 |
| RHSIM_Rhsim10G0197900 | 91.08 | 1.38E-21 | 9.21E-21 | -0.671 |
| RHSIM_Rhsim13G0076400 | 91.07 | 1.39E-21 | 9.25E-21 | -0.905 |
| RHSIM_Rhsim13G0122900 | 91.04 | 1.41E-21 | 9.37E-21 | 1.060 |
| RHSIM_Rhsim06G0026900 | 91.01 | 1.43E-21 | 9.51E-21 | -4.254 |
| RHSIM_Rhsim01G0010000 | 91.01 | 1.43E-21 | 9.53E-21 | -1.297 |
| RHSIM_Rhsim01G0005700 | 90.92 | 1.49E-21 | 9.94E-21 | 2.562 |
| RHSIM_Rhsim09G0028800 | 90.92 | 1.50E-21 | 9.97E-21 | -1.942 |
| novel.4298 | 90.85 | 1.55E-21 | 1.03E-20 | -2.545 |
| RHSIM_Rhsim03G0136600 | 90.85 | 1.55E-21 | 1.03E-20 | 1.104 |
| novel.2678 | 90.82 | 1.58E-21 | 1.05E-20 | -1.003 |
| RHSIM_Rhsim09G0071800 | 90.81 | 1.58E-21 | 1.05E-20 | 1.150 |
| RHSIM_Rhsim04G0025200 | 90.80 | 1.59E-21 | 1.06E-20 | -1.009 |
| RHSIM_Rhsim04G0169000 | 90.75 | 1.63E-21 | 1.08E-20 | 0.922 |
| RHSIM_Rhsim11G0112800 | 90.70 | 1.67E-21 | 1.11E-20 | 1.594 |
| RHSIM_Rhsim06G0151500 | 90.64 | 1.72E-21 | 1.14E-20 | -1.027 |
| RHSIM_Rhsim02G0061300 | 90.60 | 1.76E-21 | 1.17E-20 | 2.328 |
| RHSIM_Rhsim07G0046900 | 90.54 | 1.81E-21 | 1.20E-20 | 2.823 |
| RHSIM_Rhsim01G0205400 | 90.54 | 1.82E-21 | 1.21E-20 | 0.973 |
| RHSIM_Rhsim01G0151000 | 90.51 | 1.84E-21 | 1.22E-20 | -1.205 |
| novel.2153 | 90.49 | 1.86E-21 | 1.24E-20 | -2.680 |
| RHSIM_Rhsim13G0102100 | 90.47 | 1.88E-21 | 1.24E-20 | 7.431 |
| novel.3068 | 90.45 | 1.89E-21 | 1.26E-20 | 1.493 |

| Gene | LRT statistic | P value | BH-adjusted P | log2FC (interaction) |
|---|---|---|---|---|
| RHSIM_Rhsim01G0145300 | 90.36 | 1.98E-21 | 1.31E-20 | -0.697 |
| RHSIM_Rhsim05G0041500 | 90.34 | 2.01E-21 | 1.33E-20 | 1.054 |
| RHSIM_Rhsim08G0216800 | 90.33 | 2.02E-21 | 1.34E-20 | -0.685 |
| RHSIM_Rhsim04G0197800 | 90.32 | 2.03E-21 | 1.34E-20 | -3.985 |
| RHSIM_Rhsim10G0171700 | 90.30 | 2.05E-21 | 1.36E-20 | 0.679 |
| RHSIM_Rhsim04G0171200 | 90.29 | 2.06E-21 | 1.36E-20 | -0.490 |
| RHSIM_Rhsim04G0134100 | 90.25 | 2.10E-21 | 1.39E-20 | 1.334 |
| RHSIM_RhsimUnG0066200 | 90.25 | 2.10E-21 | 1.39E-20 | -0.761 |
| RHSIM_Rhsim12G0173800 | 90.23 | 2.12E-21 | 1.40E-20 | -0.690 |
| RHSIM_Rhsim08G0206000 | 90.22 | 2.13E-21 | 1.41E-20 | -1.713 |
| RHSIM_Rhsim05G0194300 | 90.19 | 2.17E-21 | 1.43E-20 | -1.350 |
| novel.2912 | 90.16 | 2.19E-21 | 1.45E-20 | -0.777 |
| RHSIM_Rhsim01G0196100 | 90.15 | 2.21E-21 | 1.46E-20 | 0.691 |
| RHSIM_Rhsim01G0290900 | 90.14 | 2.22E-21 | 1.47E-20 | 3.170 |
| RHSIM_Rhsim07G0035600 | 90.13 | 2.23E-21 | 1.47E-20 | 0.667 |
| RHSIM_Rhsim10G0016900 | 90.12 | 2.25E-21 | 1.48E-20 | 1.227 |
| RHSIM_Rhsim10G0021300 | 90.11 | 2.25E-21 | 1.49E-20 | 0.642 |
| RHSIM_Rhsim04G0208100 | 90.08 | 2.28E-21 | 1.51E-20 | -1.760 |
| RHSIM_Rhsim11G0034100 | 90.07 | 2.29E-21 | 1.52E-20 | 0.701 |
| RHSIM_Rhsim07G0052400 | 90.07 | 2.30E-21 | 1.52E-20 | -1.519 |
| RHSIM_Rhsim12G0070100 | 90.06 | 2.31E-21 | 1.52E-20 | 2.423 |
| RHSIM_RhsimUnG0085400 | 90.03 | 2.35E-21 | 1.55E-20 | -2.009 |
| RHSIM_Rhsim05G0103600 | 90.03 | 2.35E-21 | 1.55E-20 | -3.660 |
| RHSIM_Rhsim07G0205000 | 90.00 | 2.38E-21 | 1.57E-20 | 0.671 |
| RHSIM_Rhsim13G0084200 | 89.99 | 2.40E-21 | 1.58E-20 | 1.427 |
| RHSIM_Rhsim06G0192400 | 89.98 | 2.41E-21 | 1.59E-20 | 4.520 |
| RHSIM_Rhsim13G0081000 | 89.92 | 2.48E-21 | 1.64E-20 | 1.997 |
| RHSIM_Rhsim11G0091700 | 89.92 | 2.48E-21 | 1.64E-20 | -2.902 |
| RHSIM_Rhsim11G0086700 | 89.91 | 2.50E-21 | 1.65E-20 | -1.726 |
| RHSIM_Rhsim11G0111700 | 89.89 | 2.52E-21 | 1.66E-20 | -0.721 |
| RHSIM_Rhsim05G0139200 | 89.87 | 2.54E-21 | 1.67E-20 | 2.069 |
| novel.2515 | 89.85 | 2.57E-21 | 1.69E-20 | 1.399 |

| Gene | LRT statistic | P value | BH-adjusted P | log2FC (interaction) |
|---|---|---|---|---|
| RHSIM_Rhsim05G0118000 | 89.83 | 2.60E-21 | 1.71E-20 | 0.743 |
| RHSIM_Rhsim07G0001600 | 89.81 | 2.63E-21 | 1.73E-20 | 2.159 |
| RHSIM_Rhsim07G0120800 | 89.78 | 2.66E-21 | 1.75E-20 | -0.421 |
| RHSIM_Rhsim07G0200500 | 89.78 | 2.67E-21 | 1.75E-20 | -0.562 |
| RHSIM_RhsimUnG0154700 | 89.74 | 2.72E-21 | 1.79E-20 | 1.675 |
| RHSIM_Rhsim12G0211600 | 89.69 | 2.79E-21 | 1.83E-20 | 0.788 |
| novel.4252 | 89.60 | 2.91E-21 | 1.91E-20 | 1.583 |
| RHSIM_Rhsim03G0078200 | 89.60 | 2.92E-21 | 1.92E-20 | -2.003 |
| RHSIM_Rhsim05G0233700 | 89.59 | 2.93E-21 | 1.93E-20 | 0.867 |
| RHSIM_Rhsim13G0159200 | 89.58 | 2.94E-21 | 1.93E-20 | -2.085 |
| novel.532 | 89.58 | 2.95E-21 | 1.94E-20 | -3.343 |
| RHSIM_Rhsim04G0105700 | 89.53 | 3.02E-21 | 1.99E-20 | 1.206 |
| RHSIM_Rhsim08G0157400 | 89.53 | 3.03E-21 | 1.99E-20 | -1.751 |
| novel.1513 | 89.52 | 3.04E-21 | 1.99E-20 | 1.894 |
| RHSIM_Rhsim06G0047000 | 89.52 | 3.04E-21 | 2.00E-20 | 1.796 |
| RHSIM_Rhsim01G0243900 | 89.45 | 3.15E-21 | 2.06E-20 | -1.661 |
| RHSIM_Rhsim11G0022500 | 89.41 | 3.21E-21 | 2.10E-20 | 2.999 |
| RHSIM_Rhsim02G0193300 | 89.40 | 3.23E-21 | 2.12E-20 | 0.679 |
| RHSIM_Rhsim08G0085900 | 89.40 | 3.23E-21 | 2.12E-20 | -1.099 |
| RHSIM_Rhsim02G0165800 | 89.40 | 3.23E-21 | 2.12E-20 | -0.926 |
| RHSIM_Rhsim08G0102900 | 89.39 | 3.24E-21 | 2.12E-20 | 1.249 |
| RHSIM_Rhsim11G0078700 | 89.29 | 3.41E-21 | 2.23E-20 | -1.671 |
| RHSIM_Rhsim12G0144400 | 89.26 | 3.46E-21 | 2.27E-20 | -0.943 |
| RHSIM_Rhsim07G0048500 | 89.18 | 3.60E-21 | 2.36E-20 | -1.874 |
| RHSIM_RhsimUnG0258100 | 89.17 | 3.62E-21 | 2.37E-20 | -0.874 |
| RHSIM_Rhsim09G0139100 | 89.15 | 3.65E-21 | 2.39E-20 | 5.438 |
| RHSIM_Rhsim04G0129700 | 89.15 | 3.65E-21 | 2.39E-20 | -1.206 |
| RHSIM_Rhsim09G0087900 | 89.12 | 3.71E-21 | 2.43E-20 | 0.781 |
| RHSIM_Rhsim04G0247900 | 89.09 | 3.77E-21 | 2.47E-20 | -0.763 |
| RHSIM_Rhsim08G0068000 | 89.06 | 3.83E-21 | 2.51E-20 | -0.700 |
| RHSIM_Rhsim11G0022300 | 89.02 | 3.90E-21 | 2.55E-20 | 4.626 |
| RHSIM_Rhsim03G0201600 | 89.00 | 3.94E-21 | 2.58E-20 | 2.135 |

| Gene | LRT statistic | P value | BH-adjusted P | log2FC (interaction) |
|---|---|---|---|---|
| novel.384 | 88.99 | 3.96E-21 | 2.59E-20 | -1.004 |
| RHSIM_Rhsim05G0095200 | 88.94 | 4.08E-21 | 2.66E-20 | -1.275 |
| RHSIM_Rhsim04G0000700 | 88.88 | 4.20E-21 | 2.75E-20 | 1.517 |
| RHSIM_Rhsim09G0053000 | 88.84 | 4.28E-21 | 2.79E-20 | -1.730 |
| RHSIM_Rhsim06G0028000 | 88.83 | 4.30E-21 | 2.81E-20 | -0.728 |
| RHSIM_Rhsim07G0197100 | 88.79 | 4.40E-21 | 2.87E-20 | 2.169 |
| RHSIM_Rhsim10G0165100 | 88.73 | 4.53E-21 | 2.95E-20 | 1.347 |
| RHSIM_Rhsim12G0080200 | 88.69 | 4.61E-21 | 3.01E-20 | 1.983 |
| RHSIM_Rhsim11G0192800 | 88.69 | 4.62E-21 | 3.02E-20 | -1.195 |
| RHSIM_Rhsim08G0093100 | 88.67 | 4.66E-21 | 3.04E-20 | -1.339 |
| RHSIM_Rhsim03G0204000 | 88.63 | 4.76E-21 | 3.11E-20 | 1.153 |
| RHSIM_Rhsim10G0061500 | 88.58 | 4.89E-21 | 3.18E-20 | -2.861 |
| RHSIM_Rhsim04G0215800 | 88.57 | 4.90E-21 | 3.19E-20 | 0.491 |
| RHSIM_Rhsim13G0038000 | 88.56 | 4.94E-21 | 3.22E-20 | -0.758 |
| RHSIM_Rhsim07G0192000 | 88.50 | 5.08E-21 | 3.31E-20 | 2.998 |
| RHSIM_Rhsim08G0245500 | 88.43 | 5.27E-21 | 3.43E-20 | 1.398 |
| RHSIM_Rhsim11G0186800 | 88.43 | 5.28E-21 | 3.44E-20 | 0.584 |
| RHSIM_Rhsim03G0160100 | 88.39 | 5.36E-21 | 3.49E-20 | -0.668 |
| RHSIM_Rhsim04G0246900 | 88.38 | 5.40E-21 | 3.51E-20 | 2.194 |
| RHSIM_Rhsim10G0083700 | 88.33 | 5.54E-21 | 3.60E-20 | -1.818 |
| RHSIM_Rhsim09G0141100 | 88.31 | 5.59E-21 | 3.63E-20 | -0.695 |
| RHSIM_Rhsim13G0011300 | 88.27 | 5.71E-21 | 3.71E-20 | -0.492 |
| RHSIM_Rhsim04G0204500 | 88.26 | 5.75E-21 | 3.74E-20 | -0.950 |
| RHSIM_Rhsim02G0052500 | 88.18 | 5.98E-21 | 3.89E-20 | -1.385 |
| RHSIM_Rhsim07G0100900 | 88.17 | 6.00E-21 | 3.90E-20 | 0.890 |
| novel.1694 | 88.13 | 6.12E-21 | 3.97E-20 | 0.717 |
| RHSIM_Rhsim08G0078000 | 88.12 | 6.15E-21 | 4.00E-20 | 0.959 |
| RHSIM_Rhsim04G0081400 | 88.12 | 6.17E-21 | 4.00E-20 | -0.767 |
| novel.3606 | 88.04 | 6.43E-21 | 4.17E-20 | -0.800 |
| RHSIM_Rhsim08G0079200 | 87.98 | 6.61E-21 | 4.29E-20 | -0.874 |
| RHSIM_Rhsim05G0006600 | 87.96 | 6.69E-21 | 4.34E-20 | -1.136 |
| novel.1225 | 87.95 | 6.72E-21 | 4.36E-20 | 1.303 |

| Gene | LRT statistic | P value | BH-adjusted P | log2FC (interaction) |
|---|---|---|---|---|
| RHSIM_Rhsim05G0216200 | 87.91 | 6.86E-21 | 4.45E-20 | -0.773 |
| RHSIM_Rhsim03G0143900 | 87.90 | 6.88E-21 | 4.46E-20 | -1.574 |
| RHSIM_Rhsim07G0124300 | 87.88 | 6.96E-21 | 4.51E-20 | -1.590 |
| RHSIM_Rhsim03G0086200 | 87.87 | 6.99E-21 | 4.53E-20 | -1.853 |
| RHSIM_Rhsim11G0081400 | 87.87 | 7.00E-21 | 4.54E-20 | 2.758 |
| RHSIM_Rhsim04G0019500 | 87.86 | 7.04E-21 | 4.56E-20 | 0.647 |
| RHSIM_Rhsim09G0088400 | 87.75 | 7.43E-21 | 4.81E-20 | 0.814 |
| RHSIM_Rhsim09G0086100 | 87.70 | 7.62E-21 | 4.93E-20 | -1.259 |
| RHSIM_Rhsim13G0038600 | 87.70 | 7.62E-21 | 4.94E-20 | 0.956 |
| RHSIM_Rhsim11G0140600 | 87.61 | 7.98E-21 | 5.17E-20 | -3.554 |
| RHSIM_Rhsim05G0104500 | 87.60 | 8.02E-21 | 5.19E-20 | -0.836 |
| RHSIM_Rhsim01G0292900 | 87.50 | 8.41E-21 | 5.44E-20 | 1.496 |
| novel.2233 | 87.47 | 8.58E-21 | 5.55E-20 | -0.871 |
| RHSIM_Rhsim04G0153100 | 87.45 | 8.64E-21 | 5.59E-20 | -1.443 |
| RHSIM_Rhsim07G0062200 | 87.41 | 8.81E-21 | 5.69E-20 | 2.487 |
| RHSIM_Rhsim04G0120600 | 87.40 | 8.85E-21 | 5.72E-20 | -0.644 |
| RHSIM_Rhsim06G0074100 | 87.37 | 9.01E-21 | 5.82E-20 | 0.762 |
| RHSIM_Rhsim06G0094900 | 87.29 | 9.39E-21 | 6.07E-20 | 0.954 |
| RHSIM_Rhsim12G0087400 | 87.26 | 9.51E-21 | 6.14E-20 | 1.000 |
| RHSIM_Rhsim12G0041400 | 87.18 | 9.91E-21 | 6.40E-20 | -0.668 |
| RHSIM_Rhsim13G0031400 | 87.16 | 9.99E-21 | 6.45E-20 | 1.968 |
| RHSIM_Rhsim07G0008000 | 87.14 | 1.01E-20 | 6.51E-20 | -1.270 |
| RHSIM_Rhsim11G0024600 | 87.09 | 1.04E-20 | 6.71E-20 | -1.050 |
| RHSIM_RhsimUnG0085200 | 87.08 | 1.04E-20 | 6.72E-20 | -1.977 |
| RHSIM_Rhsim11G0195400 | 87.07 | 1.05E-20 | 6.75E-20 | -1.583 |
| RHSIM_Rhsim09G0048200 | 87.01 | 1.08E-20 | 6.95E-20 | 3.247 |
| RHSIM_Rhsim09G0096100 | 87.00 | 1.08E-20 | 6.99E-20 | 1.813 |
| RHSIM_Rhsim06G0031100 | 86.93 | 1.13E-20 | 7.26E-20 | -2.525 |
| RHSIM_Rhsim08G0075200 | 86.91 | 1.13E-20 | 7.31E-20 | -1.172 |
| RHSIM_Rhsim07G0093100 | 86.90 | 1.14E-20 | 7.36E-20 | 2.824 |
| RHSIM_Rhsim01G0291700 | 86.83 | 1.18E-20 | 7.63E-20 | 1.689 |
| RHSIM_Rhsim05G0205900 | 86.78 | 1.21E-20 | 7.82E-20 | -1.438 |

| Gene | LRT statistic | P value | BH-adjusted P | log2FC (interaction) |
|---|---|---|---|---|
| RHSIM_Rhsim06G0054200 | 86.71 | 1.26E-20 | 8.09E-20 | -2.685 |
| RHSIM_Rhsim05G0049600 | 86.59 | 1.33E-20 | 8.58E-20 | -1.065 |
| RHSIM_Rhsim06G0138200 | 86.57 | 1.35E-20 | 8.69E-20 | 1.053 |
| RHSIM_Rhsim05G0132400 | 86.56 | 1.35E-20 | 8.72E-20 | 0.500 |
| RHSIM_Rhsim06G0069300 | 86.56 | 1.36E-20 | 8.73E-20 | -1.023 |
| RHSIM_Rhsim12G0129000 | 86.54 | 1.37E-20 | 8.82E-20 | -0.849 |
| RHSIM_Rhsim13G0203000 | 86.49 | 1.40E-20 | 9.02E-20 | 1.365 |
| RHSIM_Rhsim01G0120900 | 86.47 | 1.42E-20 | 9.12E-20 | -4.521 |
| RHSIM_Rhsim05G0034800 | 86.47 | 1.42E-20 | 9.13E-20 | 0.582 |
| RHSIM_Rhsim09G0149600 | 86.45 | 1.43E-20 | 9.20E-20 | 2.290 |
| RHSIM_Rhsim06G0131500 | 86.45 | 1.43E-20 | 9.22E-20 | -0.489 |
| RHSIM_Rhsim07G0150700 | 86.39 | 1.48E-20 | 9.48E-20 | 1.575 |
| RHSIM_Rhsim02G0139100 | 86.35 | 1.51E-20 | 9.68E-20 | -2.913 |
| RHSIM_Rhsim03G0250900 | 86.29 | 1.56E-20 | 9.99E-20 | -1.450 |
| RHSIM_Rhsim07G0257200 | 86.25 | 1.58E-20 | 1.02E-19 | 1.034 |
| RHSIM_RhsimUnG0168400 | 86.25 | 1.58E-20 | 1.02E-19 | -43.858 |
| RHSIM_Rhsim01G0127000 | 86.25 | 1.59E-20 | 1.02E-19 | 1.819 |
| RHSIM_Rhsim09G0089000 | 86.17 | 1.65E-20 | 1.06E-19 | -0.915 |
| RHSIM_Rhsim05G0130400 | 86.10 | 1.71E-20 | 1.10E-19 | -3.730 |
| RHSIM_Rhsim13G0216700 | 86.10 | 1.71E-20 | 1.10E-19 | -1.915 |
| RHSIM_Rhsim13G0152900 | 86.08 | 1.73E-20 | 1.11E-19 | 2.936 |
| RHSIM_Rhsim09G0023700 | 86.07 | 1.74E-20 | 1.11E-19 | 1.337 |
| RHSIM_Rhsim01G0189500 | 86.05 | 1.75E-20 | 1.12E-19 | -1.226 |
| RHSIM_Rhsim07G0138500 | 86.03 | 1.77E-20 | 1.13E-19 | 0.462 |
| RHSIM_Rhsim11G0137000 | 86.02 | 1.78E-20 | 1.14E-19 | 1.055 |
| RHSIM_RhsimUnG0184000 | 85.99 | 1.81E-20 | 1.16E-19 | -3.183 |
| RHSIM_Rhsim04G0015700 | 85.99 | 1.81E-20 | 1.16E-19 | 0.931 |
| RHSIM_Rhsim11G0125700 | 85.93 | 1.87E-20 | 1.20E-19 | -1.310 |
| RHSIM_Rhsim01G0125300 | 85.92 | 1.87E-20 | 1.20E-19 | 2.108 |
| RHSIM_RhsimUnG0105000 | 85.87 | 1.93E-20 | 1.23E-19 | -0.998 |
| RHSIM_Rhsim08G0043300 | 85.86 | 1.93E-20 | 1.24E-19 | -0.847 |
| RHSIM_Rhsim01G0196300 | 85.84 | 1.95E-20 | 1.25E-19 | 0.713 |

| Gene | LRT statistic | P value | BH-adjusted P | log2FC (interaction) |
|---|---|---|---|---|
| RHSIM_Rhsim07G0142200 | 85.84 | 1.95E-20 | 1.25E-19 | -1.446 |
| RHSIM_Rhsim07G0132000 | 85.83 | 1.96E-20 | 1.25E-19 | -0.656 |
| RHSIM_Rhsim07G0036500 | 85.82 | 1.97E-20 | 1.26E-19 | -0.757 |
| RHSIM_Rhsim09G0145900 | 85.75 | 2.04E-20 | 1.30E-19 | 0.968 |
| RHSIM_RhsimUnG0053100 | 85.67 | 2.12E-20 | 1.35E-19 | 1.550 |
| RHSIM_Rhsim13G0220700 | 85.66 | 2.13E-20 | 1.36E-19 | -1.864 |
| RHSIM_Rhsim08G0176400 | 85.65 | 2.15E-20 | 1.37E-19 | -0.941 |
| RHSIM_Rhsim01G0288700 | 85.64 | 2.16E-20 | 1.38E-19 | -2.298 |
| novel.199 | 85.64 | 2.16E-20 | 1.38E-19 | 2.034 |
| RHSIM_Rhsim05G0181900 | 85.59 | 2.22E-20 | 1.41E-19 | 1.548 |
| RHSIM_Rhsim01G0044100 | 85.58 | 2.22E-20 | 1.42E-19 | -0.968 |
| RHSIM_Rhsim04G0066100 | 85.56 | 2.25E-20 | 1.43E-19 | 1.597 |
| RHSIM_RhsimUnG0220700 | 85.53 | 2.28E-20 | 1.45E-19 | -2.392 |
| RHSIM_Rhsim06G0231200 | 85.48 | 2.34E-20 | 1.49E-19 | -1.547 |
| RHSIM_Rhsim01G0149900 | 85.44 | 2.39E-20 | 1.52E-19 | -1.300 |
| RHSIM_Rhsim11G0173000 | 85.39 | 2.45E-20 | 1.56E-19 | -0.376 |
| RHSIM_Rhsim10G0162200 | 85.38 | 2.46E-20 | 1.57E-19 | -1.020 |
| RHSIM_Rhsim11G0019600 | 85.37 | 2.47E-20 | 1.57E-19 | -0.934 |
| RHSIM_Rhsim07G0016700 | 85.33 | 2.52E-20 | 1.61E-19 | -2.844 |
| RHSIM_Rhsim11G0084100 | 85.29 | 2.58E-20 | 1.64E-19 | 0.934 |
| RHSIM_Rhsim09G0206500 | 85.26 | 2.62E-20 | 1.67E-19 | -1.082 |
| RHSIM_Rhsim07G0022000 | 85.26 | 2.62E-20 | 1.67E-19 | 1.724 |
| RHSIM_Rhsim04G0086900 | 85.22 | 2.66E-20 | 1.69E-19 | -1.408 |
| novel.4582 | 85.17 | 2.74E-20 | 1.74E-19 | 5.134 |
| novel.632 | 85.11 | 2.82E-20 | 1.79E-19 | -6.524 |
| RHSIM_Rhsim01G0289800 | 85.11 | 2.82E-20 | 1.79E-19 | 1.396 |
| RHSIM_Rhsim05G0077900 | 85.06 | 2.89E-20 | 1.84E-19 | -3.314 |
| RHSIM_Rhsim10G0127700 | 85.03 | 2.94E-20 | 1.87E-19 | 0.662 |
| RHSIM_Rhsim04G0052400 | 85.03 | 2.95E-20 | 1.87E-19 | -3.499 |
| RHSIM_Rhsim12G0085500 | 84.98 | 3.02E-20 | 1.92E-19 | 0.829 |
| RHSIM_Rhsim10G0045400 | 84.94 | 3.07E-20 | 1.95E-19 | 1.233 |
| RHSIM_Rhsim13G0131000 | 84.94 | 3.08E-20 | 1.95E-19 | -0.827 |

| Gene | LRT statistic | P value | BH-adjusted P | log2FC (interaction) |
|---|---|---|---|---|
| RHSIM_Rhsim02G0037300 | 84.94 | 3.08E-20 | 1.96E-19 | 1.602 |
| RHSIM_Rhsim09G0214300 | 84.93 | 3.09E-20 | 1.96E-19 | 0.831 |
| RHSIM_Rhsim06G0142500 | 84.86 | 3.20E-20 | 2.03E-19 | -1.018 |
| RHSIM_Rhsim01G0052100 | 84.85 | 3.21E-20 | 2.04E-19 | -1.294 |
| RHSIM_Rhsim04G0065900 | 84.83 | 3.26E-20 | 2.07E-19 | 1.179 |
| RHSIM_Rhsim10G0079700 | 84.78 | 3.33E-20 | 2.11E-19 | 1.019 |
| RHSIM_Rhsim06G0010800 | 84.70 | 3.48E-20 | 2.20E-19 | 2.998 |
| RHSIM_Rhsim10G0129800 | 84.69 | 3.48E-20 | 2.21E-19 | 1.197 |
| RHSIM_Rhsim03G0205700 | 84.66 | 3.55E-20 | 2.25E-19 | 2.335 |
| RHSIM_RhsimUnG0078200 | 84.65 | 3.56E-20 | 2.26E-19 | 0.968 |
| RHSIM_Rhsim06G0178700 | 84.62 | 3.62E-20 | 2.30E-19 | -0.864 |
| RHSIM_Rhsim06G0211200 | 84.61 | 3.63E-20 | 2.30E-19 | -0.897 |
| RHSIM_Rhsim03G0029000 | 84.55 | 3.74E-20 | 2.37E-19 | 0.725 |
| RHSIM_Rhsim07G0221800 | 84.54 | 3.77E-20 | 2.38E-19 | -0.688 |
| RHSIM_Rhsim09G0182000 | 84.52 | 3.81E-20 | 2.41E-19 | 0.794 |
| RHSIM_Rhsim03G0039500 | 84.45 | 3.93E-20 | 2.49E-19 | 0.736 |
| novel.3105 | 84.45 | 3.94E-20 | 2.50E-19 | -2.775 |
| RHSIM_Rhsim07G0034500 | 84.43 | 3.99E-20 | 2.52E-19 | 0.816 |
| novel.1849 | 84.40 | 4.04E-20 | 2.56E-19 | -6.024 |
| RHSIM_Rhsim03G0228000 | 84.40 | 4.05E-20 | 2.56E-19 | 2.971 |
| novel.829 | 84.38 | 4.08E-20 | 2.58E-19 | -0.912 |
| novel.2433 | 84.37 | 4.10E-20 | 2.59E-19 | -1.133 |
| RHSIM_Rhsim08G0142500 | 84.36 | 4.13E-20 | 2.61E-19 | -1.293 |
| RHSIM_Rhsim03G0254300 | 84.35 | 4.14E-20 | 2.61E-19 | 1.009 |
| RHSIM_Rhsim09G0055800 | 84.34 | 4.17E-20 | 2.63E-19 | -1.480 |
| novel.2016 | 84.28 | 4.29E-20 | 2.71E-19 | -4.165 |
| RHSIM_Rhsim02G0119300 | 84.27 | 4.31E-20 | 2.72E-19 | -0.719 |
| RHSIM_Rhsim06G0212800 | 84.27 | 4.32E-20 | 2.73E-19 | -1.574 |
| novel.4441 | 84.20 | 4.48E-20 | 2.82E-19 | 3.075 |
| RHSIM_Rhsim08G0252400 | 84.17 | 4.53E-20 | 2.86E-19 | 2.374 |
| RHSIM_Rhsim01G0267800 | 84.17 | 4.53E-20 | 2.86E-19 | -0.865 |
| RHSIM_Rhsim01G0059400 | 84.16 | 4.56E-20 | 2.88E-19 | 1.055 |

| Gene | LRT statistic | P value | BH-adjusted P | log2FC (interaction) |
|---|---|---|---|---|
| RHSIM_Rhsim08G0070600 | 84.12 | 4.66E-20 | 2.94E-19 | 0.876 |
| RHSIM_Rhsim07G0050700 | 84.08 | 4.75E-20 | 2.99E-19 | 0.513 |
| RHSIM_Rhsim11G0007200 | 84.04 | 4.86E-20 | 3.06E-19 | 1.065 |
| novel.2923 | 84.01 | 4.91E-20 | 3.09E-19 | 1.548 |
| RHSIM_Rhsim11G0126700 | 84.00 | 4.95E-20 | 3.12E-19 | -1.067 |
| RHSIM_Rhsim07G0027400 | 83.98 | 5.01E-20 | 3.15E-19 | -1.244 |
| RHSIM_Rhsim04G0176000 | 83.89 | 5.23E-20 | 3.29E-19 | 0.936 |
| RHSIM_Rhsim04G0244400 | 83.89 | 5.23E-20 | 3.29E-19 | -0.766 |
| RHSIM_Rhsim08G0169500 | 83.88 | 5.25E-20 | 3.30E-19 | -1.339 |
| RHSIM_Rhsim05G0003400 | 83.83 | 5.39E-20 | 3.39E-19 | -1.530 |
| RHSIM_Rhsim08G0250300 | 83.77 | 5.56E-20 | 3.49E-19 | -1.199 |
| RHSIM_Rhsim12G0153600 | 83.77 | 5.56E-20 | 3.50E-19 | -1.228 |
| RHSIM_Rhsim01G0276900 | 83.74 | 5.65E-20 | 3.55E-19 | -0.889 |
| RHSIM_Rhsim01G0020000 | 83.63 | 5.96E-20 | 3.74E-19 | -2.399 |
| RHSIM_Rhsim06G0236100 | 83.56 | 6.18E-20 | 3.89E-19 | 0.948 |
| RHSIM_Rhsim09G0027800 | 83.50 | 6.36E-20 | 3.99E-19 | -1.303 |
| RHSIM_Rhsim12G0023200 | 83.43 | 6.60E-20 | 4.15E-19 | 0.816 |
| novel.1146 | 83.42 | 6.62E-20 | 4.16E-19 | 2.193 |
| RHSIM_Rhsim08G0232300 | 83.42 | 6.63E-20 | 4.16E-19 | -2.425 |
| RHSIM_Rhsim09G0201300 | 83.39 | 6.75E-20 | 4.24E-19 | -1.179 |
| RHSIM_Rhsim12G0043400 | 83.36 | 6.82E-20 | 4.28E-19 | -0.776 |
| RHSIM_Rhsim09G0073000 | 83.36 | 6.82E-20 | 4.28E-19 | -0.927 |
| novel.1089 | 83.34 | 6.92E-20 | 4.34E-19 | 0.787 |
| novel.4257 | 83.30 | 7.04E-20 | 4.42E-19 | -3.779 |
| RHSIM_Rhsim06G0018800 | 83.30 | 7.06E-20 | 4.43E-19 | -1.278 |
| RHSIM_Rhsim09G0188800 | 83.28 | 7.12E-20 | 4.47E-19 | 0.787 |
| RHSIM_Rhsim03G0099800 | 83.27 | 7.14E-20 | 4.48E-19 | 0.702 |
| RHSIM_Rhsim10G0194900 | 83.24 | 7.25E-20 | 4.54E-19 | 3.009 |
| RHSIM_Rhsim08G0192500 | 83.22 | 7.35E-20 | 4.61E-19 | 3.277 |
| RHSIM_Rhsim13G0111800 | 83.22 | 7.36E-20 | 4.61E-19 | -0.681 |
| RHSIM_Rhsim02G0036000 | 83.21 | 7.37E-20 | 4.61E-19 | -0.945 |
| RHSIM_Rhsim12G0209300 | 83.16 | 7.59E-20 | 4.75E-19 | -0.763 |

| Gene | LRT statistic | P value | BH-adjusted P | log2FC (interaction) |
|---|---|---|---|---|
| novel.2158 | 83.15 | 7.59E-20 | 4.75E-19 | 3.195 |
| RHSIM_Rhsim01G0197100 | 83.12 | 7.70E-20 | 4.82E-19 | 0.985 |
| RHSIM_Rhsim02G0020500 | 83.08 | 7.88E-20 | 4.93E-19 | 5.272 |
| RHSIM_Rhsim06G0054400 | 83.05 | 8.02E-20 | 5.01E-19 | 0.816 |
| RHSIM_Rhsim07G0054500 | 83.03 | 8.07E-20 | 5.05E-19 | 0.724 |
| RHSIM_Rhsim09G0089200 | 83.03 | 8.08E-20 | 5.05E-19 | 0.934 |
| RHSIM_Rhsim13G0233500 | 83.01 | 8.18E-20 | 5.11E-19 | -1.564 |
| RHSIM_Rhsim08G0021000 | 83.00 | 8.19E-20 | 5.12E-19 | 0.992 |
| RHSIM_Rhsim03G0176800 | 83.00 | 8.19E-20 | 5.12E-19 | -1.498 |
| RHSIM_Rhsim10G0026200 | 83.00 | 8.22E-20 | 5.14E-19 | 3.912 |
| RHSIM_Rhsim13G0053600 | 82.99 | 8.24E-20 | 5.15E-19 | -1.082 |
| RHSIM_Rhsim05G0165200 | 82.98 | 8.30E-20 | 5.18E-19 | 0.883 |
| RHSIM_Rhsim06G0238900 | 82.95 | 8.41E-20 | 5.25E-19 | -2.260 |
| RHSIM_Rhsim10G0034700 | 82.91 | 8.60E-20 | 5.37E-19 | -0.586 |
| RHSIM_RhsimUnG0137100 | 82.80 | 9.09E-20 | 5.67E-19 | -0.905 |
| RHSIM_Rhsim01G0146800 | 82.80 | 9.09E-20 | 5.67E-19 | 0.867 |
| RHSIM_Rhsim01G0185200 | 82.76 | 9.24E-20 | 5.77E-19 | -1.658 |
| RHSIM_Rhsim02G0149800 | 82.75 | 9.29E-20 | 5.79E-19 | 0.889 |
| RHSIM_Rhsim02G0128200 | 82.73 | 9.43E-20 | 5.88E-19 | 0.828 |
| RHSIM_Rhsim06G0109200 | 82.72 | 9.44E-20 | 5.88E-19 | 0.754 |
| RHSIM_Rhsim05G0192400 | 82.72 | 9.44E-20 | 5.88E-19 | 1.310 |
| RHSIM_Rhsim11G0135100 | 82.66 | 9.74E-20 | 6.07E-19 | 0.706 |
| RHSIM_Rhsim12G0004100 | 82.64 | 9.85E-20 | 6.14E-19 | 1.223 |
| RHSIM_Rhsim08G0138000 | 82.61 | 1.00E-19 | 6.24E-19 | 1.014 |
| RHSIM_Rhsim03G0196000 | 82.59 | 1.01E-19 | 6.30E-19 | -5.462 |
| RHSIM_Rhsim03G0214800 | 82.55 | 1.03E-19 | 6.41E-19 | -0.677 |
| RHSIM_Rhsim03G0129000 | 82.55 | 1.03E-19 | 6.41E-19 | -0.609 |
| RHSIM_Rhsim09G0003200 | 82.53 | 1.04E-19 | 6.49E-19 | -1.282 |
| RHSIM_Rhsim02G0203100 | 82.41 | 1.11E-19 | 6.89E-19 | 4.528 |
| RHSIM_Rhsim02G0021000 | 82.40 | 1.11E-19 | 6.90E-19 | 0.713 |
| RHSIM_Rhsim10G0003000 | 82.39 | 1.12E-19 | 6.95E-19 | 1.307 |
| RHSIM_Rhsim06G0206600 | 82.37 | 1.13E-19 | 7.00E-19 | 2.611 |

| Gene | LRT statistic | P value | BH-adjusted P | log2FC (interaction) |
|---|---|---|---|---|
| novel.1088 | 82.33 | 1.15E-19 | 7.16E-19 | -2.411 |
| RHSIM_RhsimUnG0023300 | 82.32 | 1.15E-19 | 7.18E-19 | -2.223 |
| RHSIM_Rhsim04G0038300 | 82.31 | 1.16E-19 | 7.21E-19 | 0.780 |
| RHSIM_Rhsim01G0262400 | 82.31 | 1.16E-19 | 7.22E-19 | -0.541 |
| RHSIM_Rhsim10G0172400 | 82.31 | 1.16E-19 | 7.22E-19 | 0.694 |
| RHSIM_Rhsim07G0052900 | 82.30 | 1.17E-19 | 7.28E-19 | 1.606 |
| RHSIM_Rhsim06G0152000 | 82.25 | 1.20E-19 | 7.45E-19 | -0.633 |
| RHSIM_Rhsim04G0213100 | 82.21 | 1.22E-19 | 7.58E-19 | 0.795 |
| RHSIM_Rhsim13G0098600 | 82.18 | 1.24E-19 | 7.71E-19 | -0.403 |
| RHSIM_Rhsim09G0142200 | 82.17 | 1.25E-19 | 7.73E-19 | 0.633 |
| RHSIM_Rhsim07G0211700 | 82.16 | 1.26E-19 | 7.79E-19 | -0.782 |
| RHSIM_Rhsim01G0129800 | 82.10 | 1.30E-19 | 8.04E-19 | 2.399 |
| RHSIM_Rhsim02G0104800 | 82.09 | 1.30E-19 | 8.07E-19 | -0.873 |
| RHSIM_Rhsim12G0132800 | 82.09 | 1.30E-19 | 8.07E-19 | 0.852 |
| RHSIM_Rhsim13G0057700 | 82.07 | 1.31E-19 | 8.13E-19 | -2.464 |
| RHSIM_Rhsim06G0041100 | 82.04 | 1.33E-19 | 8.27E-19 | 0.864 |
| RHSIM_Rhsim12G0149000 | 82.01 | 1.36E-19 | 8.39E-19 | 1.078 |
| RHSIM_Rhsim09G0170000 | 82.00 | 1.36E-19 | 8.41E-19 | -2.618 |
| RHSIM_Rhsim03G0019300 | 82.00 | 1.36E-19 | 8.43E-19 | 0.694 |
| RHSIM_Rhsim08G0049700 | 81.97 | 1.38E-19 | 8.55E-19 | 0.793 |
| RHSIM_Rhsim08G0086700 | 81.96 | 1.39E-19 | 8.61E-19 | -2.929 |
| RHSIM_Rhsim13G0195200 | 81.92 | 1.42E-19 | 8.76E-19 | 1.297 |
| RHSIM_Rhsim06G0225700 | 81.87 | 1.45E-19 | 8.99E-19 | -1.404 |
| RHSIM_Rhsim11G0093700 | 81.82 | 1.49E-19 | 9.21E-19 | 4.086 |
| RHSIM_Rhsim07G0231500 | 81.80 | 1.51E-19 | 9.32E-19 | 1.357 |
| RHSIM_Rhsim01G0097800 | 81.79 | 1.51E-19 | 9.34E-19 | 0.823 |
| RHSIM_Rhsim11G0180400 | 81.78 | 1.52E-19 | 9.39E-19 | -1.731 |
| RHSIM_Rhsim08G0072600 | 81.65 | 1.62E-19 | 1.00E-18 | 1.388 |
| RHSIM_Rhsim01G0123500 | 81.64 | 1.63E-19 | 1.01E-18 | 0.816 |
| RHSIM_RhsimUnG0102900 | 81.60 | 1.67E-19 | 1.03E-18 | -0.980 |
| RHSIM_Rhsim07G0045100 | 81.54 | 1.72E-19 | 1.06E-18 | -5.566 |
| RHSIM_Rhsim10G0121300 | 81.52 | 1.73E-19 | 1.07E-18 | 1.720 |

| Gene | LRT statistic | P value | BH-adjusted P | log2FC (interaction) |
|---|---|---|---|---|
| RHSIM_Rhsim04G0117000 | 81.51 | 1.74E-19 | 1.08E-18 | 0.686 |
| novel.1142 | 81.47 | 1.78E-19 | 1.10E-18 | 1.065 |
| novel.665 | 81.47 | 1.78E-19 | 1.10E-18 | -5.836 |
| RHSIM_Rhsim08G0246900 | 81.46 | 1.79E-19 | 1.10E-18 | -0.858 |
| RHSIM_Rhsim03G0225200 | 81.43 | 1.82E-19 | 1.12E-18 | 0.780 |
| RHSIM_Rhsim10G0006600 | 81.41 | 1.83E-19 | 1.13E-18 | 0.868 |
| RHSIM_Rhsim10G0147200 | 81.40 | 1.84E-19 | 1.14E-18 | 2.402 |
| RHSIM_Rhsim12G0098100 | 81.40 | 1.85E-19 | 1.14E-18 | 1.167 |
| RHSIM_RhsimUnG0080300 | 81.40 | 1.85E-19 | 1.14E-18 | -0.834 |
| RHSIM_Rhsim09G0108500 | 81.39 | 1.85E-19 | 1.14E-18 | -1.990 |
| RHSIM_Rhsim11G0129500 | 81.39 | 1.86E-19 | 1.14E-18 | -2.312 |
| novel.1021 | 81.36 | 1.89E-19 | 1.16E-18 | 3.899 |
| RHSIM_RhsimUnG0233400 | 81.29 | 1.94E-19 | 1.20E-18 | -4.754 |
| RHSIM_Rhsim11G0101900 | 81.25 | 1.99E-19 | 1.23E-18 | -1.869 |
| RHSIM_Rhsim10G0181600 | 81.23 | 2.01E-19 | 1.24E-18 | -2.626 |
| RHSIM_Rhsim03G0258700 | 81.19 | 2.05E-19 | 1.26E-18 | -1.202 |
| RHSIM_Rhsim11G0037800 | 81.17 | 2.07E-19 | 1.27E-18 | 1.008 |
| RHSIM_Rhsim08G0075600 | 81.17 | 2.07E-19 | 1.27E-18 | 1.330 |
| RHSIM_Rhsim09G0108400 | 81.15 | 2.10E-19 | 1.29E-18 | 0.989 |
| RHSIM_RhsimUnG0116800 | 81.14 | 2.10E-19 | 1.29E-18 | -1.021 |
| RHSIM_Rhsim09G0213800 | 81.13 | 2.11E-19 | 1.30E-18 | -0.635 |
| RHSIM_Rhsim10G0099900 | 81.00 | 2.26E-19 | 1.39E-18 | 1.401 |
| RHSIM_Rhsim04G0202800 | 80.99 | 2.27E-19 | 1.39E-18 | -1.780 |
| RHSIM_Rhsim05G0044600 | 80.98 | 2.28E-19 | 1.40E-18 | -1.054 |
| RHSIM_Rhsim02G0256000 | 80.96 | 2.31E-19 | 1.42E-18 | 1.450 |
| RHSIM_Rhsim08G0087900 | 80.95 | 2.31E-19 | 1.42E-18 | 1.509 |
| RHSIM_Rhsim05G0009500 | 80.91 | 2.36E-19 | 1.45E-18 | -4.720 |
| RHSIM_Rhsim03G0024900 | 80.91 | 2.36E-19 | 1.45E-18 | -1.744 |
| RHSIM_Rhsim06G0174600 | 80.89 | 2.38E-19 | 1.46E-18 | 0.599 |
| RHSIM_Rhsim07G0134100 | 80.85 | 2.44E-19 | 1.50E-18 | -0.713 |
| RHSIM_Rhsim06G0239800 | 80.84 | 2.45E-19 | 1.50E-18 | 1.286 |
| RHSIM_Rhsim07G0258000 | 80.83 | 2.46E-19 | 1.51E-18 | 0.743 |

| Gene | LRT statistic | P value | BH-adjusted P | log2FC (interaction) |
|---|---|---|---|---|
| novel.400 | 80.82 | 2.47E-19 | 1.52E-18 | 2.344 |
| RHSIM_Rhsim07G0246700 | 80.79 | 2.51E-19 | 1.54E-18 | -2.404 |
| RHSIM_Rhsim08G0245400 | 80.77 | 2.53E-19 | 1.55E-18 | 0.942 |
| RHSIM_Rhsim11G0185700 | 80.76 | 2.54E-19 | 1.56E-18 | -1.757 |
| RHSIM_Rhsim04G0011200 | 80.75 | 2.56E-19 | 1.57E-18 | -4.809 |
| RHSIM_Rhsim11G0036800 | 80.74 | 2.57E-19 | 1.57E-18 | -1.142 |
| RHSIM_Rhsim07G0001200 | 80.70 | 2.63E-19 | 1.61E-18 | -1.171 |
| RHSIM_Rhsim03G0148600 | 80.68 | 2.65E-19 | 1.62E-18 | 3.595 |
| RHSIM_RhsimUnG0249400 | 80.68 | 2.65E-19 | 1.62E-18 | -0.679 |
| RHSIM_Rhsim12G0134600 | 80.63 | 2.72E-19 | 1.66E-18 | -1.249 |
| RHSIM_Rhsim06G0197400 | 80.56 | 2.83E-19 | 1.73E-18 | -0.837 |
| RHSIM_Rhsim09G0150500 | 80.53 | 2.87E-19 | 1.75E-18 | 1.140 |
| RHSIM_Rhsim08G0233300 | 80.45 | 2.98E-19 | 1.82E-18 | -0.528 |
| RHSIM_RhsimUnG0169900 | 80.44 | 3.00E-19 | 1.83E-18 | -2.978 |
| RHSIM_Rhsim01G0142200 | 80.40 | 3.06E-19 | 1.87E-18 | -0.721 |
| RHSIM_Rhsim08G0092500 | 80.37 | 3.10E-19 | 1.89E-18 | 2.275 |
| RHSIM_RhsimUnG0018200 | 80.37 | 3.11E-19 | 1.90E-18 | -1.192 |
| RHSIM_Rhsim08G0006300 | 80.35 | 3.13E-19 | 1.91E-18 | 1.163 |
| RHSIM_Rhsim04G0190300 | 80.33 | 3.16E-19 | 1.93E-18 | -0.961 |
| RHSIM_Rhsim06G0204500 | 80.31 | 3.20E-19 | 1.96E-18 | 1.070 |
| RHSIM_Rhsim10G0007700 | 80.30 | 3.22E-19 | 1.96E-18 | 0.891 |
| novel.1636 | 80.29 | 3.23E-19 | 1.97E-18 | 3.013 |
| RHSIM_Rhsim03G0268200 | 80.29 | 3.23E-19 | 1.97E-18 | 1.183 |
| RHSIM_Rhsim10G0144500 | 80.28 | 3.24E-19 | 1.98E-18 | 0.606 |
| RHSIM_Rhsim05G0101300 | 80.28 | 3.25E-19 | 1.98E-18 | 1.106 |
| RHSIM_Rhsim03G0243600 | 80.27 | 3.26E-19 | 1.99E-18 | 0.995 |
| RHSIM_Rhsim11G0167700 | 80.24 | 3.31E-19 | 2.02E-18 | -2.195 |
| RHSIM_Rhsim01G0066800 | 80.24 | 3.32E-19 | 2.03E-18 | -1.018 |
| RHSIM_RhsimUnG0097600 | 80.23 | 3.34E-19 | 2.03E-18 | 1.014 |
| RHSIM_Rhsim01G0052600 | 80.19 | 3.40E-19 | 2.07E-18 | 2.013 |
| RHSIM_Rhsim12G0171700 | 80.18 | 3.41E-19 | 2.08E-18 | 1.115 |
| RHSIM_Rhsim05G0110900 | 80.10 | 3.55E-19 | 2.16E-18 | -1.551 |

| Gene | LRT statistic | P value | BH-adjusted P | log2FC (interaction) |
|---|---|---|---|---|
| RHSIM_Rhsim03G0113100 | 80.09 | 3.58E-19 | 2.18E-18 | 0.704 |
| RHSIM_Rhsim06G0227700 | 80.04 | 3.66E-19 | 2.23E-18 | 0.913 |
| RHSIM_Rhsim02G0118100 | 80.03 | 3.70E-19 | 2.25E-18 | -0.885 |
| RHSIM_Rhsim07G0233500 | 80.02 | 3.70E-19 | 2.25E-18 | 0.497 |
| RHSIM_Rhsim08G0013300 | 79.99 | 3.77E-19 | 2.29E-18 | 0.638 |
| novel.3788 | 79.88 | 3.97E-19 | 2.42E-18 | -1.572 |
| RHSIM_Rhsim09G0181300 | 79.79 | 4.16E-19 | 2.53E-18 | -1.039 |
| RHSIM_Rhsim13G0231900 | 79.77 | 4.20E-19 | 2.55E-18 | -1.531 |
| RHSIM_Rhsim06G0167300 | 79.77 | 4.21E-19 | 2.56E-18 | 0.856 |
| RHSIM_Rhsim08G0075700 | 79.74 | 4.26E-19 | 2.59E-18 | -0.901 |
| RHSIM_Rhsim07G0103100 | 79.71 | 4.33E-19 | 2.63E-18 | -0.570 |
| RHSIM_Rhsim08G0040700 | 79.70 | 4.37E-19 | 2.65E-18 | 0.753 |
| RHSIM_Rhsim06G0036600 | 79.65 | 4.46E-19 | 2.71E-18 | -1.428 |
| novel.1648 | 79.64 | 4.49E-19 | 2.73E-18 | -1.084 |
| RHSIM_Rhsim03G0222500 | 79.59 | 4.60E-19 | 2.79E-18 | -0.706 |
| RHSIM_Rhsim12G0042200 | 79.54 | 4.73E-19 | 2.87E-18 | -1.339 |
| RHSIM_Rhsim07G0118600 | 79.48 | 4.87E-19 | 2.95E-18 | -1.069 |
| RHSIM_Rhsim08G0234200 | 79.47 | 4.90E-19 | 2.97E-18 | 4.281 |
| RHSIM_Rhsim03G0096300 | 79.46 | 4.92E-19 | 2.99E-18 | -0.860 |
| RHSIM_Rhsim08G0199500 | 79.41 | 5.04E-19 | 3.05E-18 | 0.910 |
| RHSIM_Rhsim08G0134300 | 79.36 | 5.17E-19 | 3.13E-18 | 1.796 |
| RHSIM_Rhsim11G0161300 | 79.35 | 5.20E-19 | 3.15E-18 | 2.016 |
| RHSIM_Rhsim04G0231200 | 79.33 | 5.24E-19 | 3.18E-18 | 0.973 |
| RHSIM_Rhsim09G0195200 | 79.27 | 5.41E-19 | 3.28E-18 | -3.095 |
| RHSIM_Rhsim04G0240200 | 79.26 | 5.45E-19 | 3.30E-18 | 0.661 |
| RHSIM_Rhsim02G0000100 | 79.18 | 5.68E-19 | 3.44E-18 | 0.826 |
| RHSIM_Rhsim06G0052200 | 79.14 | 5.77E-19 | 3.50E-18 | -2.806 |
| RHSIM_Rhsim11G0182400 | 79.13 | 5.81E-19 | 3.52E-18 | 0.557 |
| novel.2486 | 79.11 | 5.88E-19 | 3.56E-18 | -1.078 |
| novel.1053 | 79.10 | 5.89E-19 | 3.57E-18 | 1.283 |
| RHSIM_Rhsim01G0124100 | 79.10 | 5.90E-19 | 3.57E-18 | 0.547 |
| RHSIM_Rhsim10G0038900 | 79.09 | 5.93E-19 | 3.59E-18 | 0.525 |

| Gene | LRT statistic | P value | BH-adjusted P | log2FC (interaction) |
|---|---|---|---|---|
| RHSIM_Rhsim11G0131800 | 79.09 | 5.94E-19 | 3.59E-18 | 3.020 |
| RHSIM_Rhsim08G0175600 | 79.06 | 6.04E-19 | 3.65E-18 | -0.975 |
| RHSIM_Rhsim02G0004000 | 79.04 | 6.09E-19 | 3.68E-18 | 0.949 |
| RHSIM_Rhsim03G0040900 | 78.95 | 6.35E-19 | 3.84E-18 | -42.601 |
| RHSIM_Rhsim01G0113600 | 78.95 | 6.36E-19 | 3.85E-18 | -0.993 |
| RHSIM_Rhsim02G0250700 | 78.80 | 6.86E-19 | 4.15E-18 | 0.913 |
| RHSIM_Rhsim01G0149100 | 78.70 | 7.24E-19 | 4.37E-18 | 1.054 |
| RHSIM_Rhsim09G0158600 | 78.68 | 7.29E-19 | 4.40E-18 | -0.607 |
| RHSIM_Rhsim01G0101600 | 78.67 | 7.33E-19 | 4.43E-18 | 0.876 |
| RHSIM_Rhsim01G0006800 | 78.62 | 7.52E-19 | 4.54E-18 | 1.254 |
| RHSIM_Rhsim10G0019700 | 78.60 | 7.60E-19 | 4.59E-18 | -0.404 |
| RHSIM_Rhsim06G0017600 | 78.52 | 7.92E-19 | 4.78E-18 | 1.034 |
| RHSIM_Rhsim10G0170100 | 78.51 | 7.95E-19 | 4.80E-18 | -2.270 |
| RHSIM_Rhsim10G0100700 | 78.51 | 7.95E-19 | 4.80E-18 | 1.315 |
| RHSIM_Rhsim06G0005000 | 78.49 | 8.05E-19 | 4.85E-18 | -2.456 |
| RHSIM_Rhsim06G0028500 | 78.35 | 8.61E-19 | 5.19E-18 | 0.816 |
| RHSIM_Rhsim13G0105400 | 78.34 | 8.66E-19 | 5.22E-18 | -1.394 |
| RHSIM_Rhsim07G0155600 | 78.34 | 8.67E-19 | 5.23E-18 | 2.661 |
| novel.2018 | 78.33 | 8.70E-19 | 5.24E-18 | -0.716 |
| novel.897 | 78.32 | 8.78E-19 | 5.29E-18 | -1.163 |
| RHSIM_Rhsim05G0120500 | 78.30 | 8.83E-19 | 5.32E-18 | -1.052 |
| RHSIM_Rhsim12G0164700 | 78.28 | 8.95E-19 | 5.39E-18 | -0.570 |
| RHSIM_Rhsim07G0052300 | 78.28 | 8.95E-19 | 5.39E-18 | 1.144 |
| RHSIM_Rhsim08G0029600 | 78.27 | 8.99E-19 | 5.41E-18 | 1.495 |
| RHSIM_Rhsim07G0173400 | 78.20 | 9.30E-19 | 5.60E-18 | 0.727 |
| RHSIM_Rhsim01G0077500 | 78.20 | 9.31E-19 | 5.60E-18 | -0.943 |
| RHSIM_Rhsim07G0063000 | 78.19 | 9.37E-19 | 5.63E-18 | -1.145 |
| RHSIM_Rhsim02G0088500 | 78.15 | 9.54E-19 | 5.74E-18 | 0.955 |
| RHSIM_Rhsim03G0244400 | 78.15 | 9.54E-19 | 5.74E-18 | 1.649 |
| RHSIM_Rhsim09G0051700 | 78.15 | 9.57E-19 | 5.75E-18 | -0.727 |
| RHSIM_Rhsim03G0218700 | 78.15 | 9.57E-19 | 5.75E-18 | -1.248 |
| RHSIM_Rhsim12G0130500 | 78.09 | 9.82E-19 | 5.90E-18 | 0.842 |

| Gene | LRT statistic | P value | BH-adjusted P | log2FC (interaction) |
|---|---|---|---|---|
| RHSIM_Rhsim08G0000500 | 78.09 | 9.86E-19 | 5.93E-18 | -1.341 |
| RHSIM_Rhsim06G0072600 | 78.07 | 9.95E-19 | 5.97E-18 | 0.615 |
| RHSIM_Rhsim13G0231200 | 78.05 | 1.00E-18 | 6.02E-18 | -1.315 |
| RHSIM_Rhsim10G0054600 | 77.99 | 1.03E-18 | 6.21E-18 | -3.674 |
| RHSIM_Rhsim13G0136300 | 77.98 | 1.04E-18 | 6.25E-18 | 1.419 |
| RHSIM_Rhsim04G0038800 | 77.95 | 1.06E-18 | 6.33E-18 | -0.870 |
| RHSIM_Rhsim06G0119000 | 77.95 | 1.06E-18 | 6.34E-18 | -1.917 |
| RHSIM_Rhsim13G0119700 | 77.94 | 1.06E-18 | 6.36E-18 | 2.185 |
| RHSIM_Rhsim09G0106000 | 77.93 | 1.07E-18 | 6.41E-18 | 1.674 |
| RHSIM_Rhsim05G0215900 | 77.78 | 1.15E-18 | 6.89E-18 | 3.128 |
| RHSIM_Rhsim03G0018700 | 77.76 | 1.16E-18 | 6.96E-18 | 1.438 |
| RHSIM_Rhsim13G0092900 | 77.74 | 1.18E-18 | 7.04E-18 | -4.238 |
| novel.756 | 77.72 | 1.19E-18 | 7.10E-18 | 1.770 |
| RHSIM_Rhsim07G0201400 | 77.72 | 1.19E-18 | 7.12E-18 | 0.993 |
| RHSIM_Rhsim05G0018300 | 77.72 | 1.19E-18 | 7.12E-18 | 4.775 |
| RHSIM_Rhsim11G0098600 | 77.70 | 1.20E-18 | 7.18E-18 | -0.931 |
| RHSIM_Rhsim07G0187800 | 77.70 | 1.20E-18 | 7.18E-18 | -0.697 |
| RHSIM_Rhsim07G0066900 | 77.70 | 1.20E-18 | 7.19E-18 | 0.873 |
| RHSIM_Rhsim01G0184000 | 77.67 | 1.22E-18 | 7.30E-18 | -0.504 |
| RHSIM_Rhsim12G0044000 | 77.65 | 1.23E-18 | 7.35E-18 | -0.643 |
| RHSIM_Rhsim08G0055200 | 77.63 | 1.24E-18 | 7.42E-18 | -0.522 |
| RHSIM_Rhsim13G0153100 | 77.61 | 1.26E-18 | 7.51E-18 | 2.621 |
| RHSIM_Rhsim13G0171400 | 77.56 | 1.29E-18 | 7.68E-18 | -1.370 |
| RHSIM_Rhsim13G0040400 | 77.55 | 1.29E-18 | 7.74E-18 | 0.695 |
| RHSIM_Rhsim02G0181000 | 77.51 | 1.32E-18 | 7.88E-18 | -0.655 |
| RHSIM_Rhsim07G0219800 | 77.49 | 1.33E-18 | 7.96E-18 | -1.334 |
| RHSIM_Rhsim05G0095600 | 77.45 | 1.36E-18 | 8.12E-18 | -1.107 |
| novel.2402 | 77.42 | 1.38E-18 | 8.26E-18 | -0.949 |
| RHSIM_Rhsim02G0071400 | 77.39 | 1.41E-18 | 8.40E-18 | 1.795 |
| RHSIM_Rhsim13G0169300 | 77.36 | 1.42E-18 | 8.49E-18 | -1.283 |
| RHSIM_Rhsim04G0180700 | 77.36 | 1.43E-18 | 8.53E-18 | -1.994 |
| RHSIM_Rhsim02G0206900 | 77.31 | 1.46E-18 | 8.72E-18 | -0.951 |

| Gene | LRT statistic | P value | BH-adjusted P | log2FC (interaction) |
|---|---|---|---|---|
| RHSIM_Rhsim07G0257700 | 77.30 | 1.47E-18 | 8.75E-18 | -0.836 |
| novel.4050 | 77.30 | 1.47E-18 | 8.78E-18 | -1.925 |
| RHSIM_Rhsim03G0177500 | 77.29 | 1.47E-18 | 8.79E-18 | 0.844 |
| RHSIM_Rhsim02G0066900 | 77.20 | 1.54E-18 | 9.20E-18 | -2.642 |
| RHSIM_Rhsim02G0129600 | 77.19 | 1.55E-18 | 9.25E-18 | 0.772 |
| RHSIM_Rhsim01G0169200 | 77.16 | 1.57E-18 | 9.39E-18 | -0.826 |
| RHSIM_Rhsim10G0159100 | 77.15 | 1.59E-18 | 9.46E-18 | -1.478 |
| RHSIM_Rhsim08G0051600 | 77.13 | 1.60E-18 | 9.54E-18 | 4.182 |
| novel.3556 | 77.12 | 1.61E-18 | 9.60E-18 | 5.374 |
| novel.2537 | 77.10 | 1.63E-18 | 9.68E-18 | -0.894 |
| RHSIM_Rhsim10G0177300 | 77.09 | 1.63E-18 | 9.70E-18 | 0.578 |
| RHSIM_Rhsim10G0105200 | 77.06 | 1.66E-18 | 9.90E-18 | -0.781 |
| RHSIM_Rhsim03G0122000 | 77.05 | 1.66E-18 | 9.90E-18 | -0.991 |
| RHSIM_Rhsim06G0170400 | 77.04 | 1.68E-18 | 9.99E-18 | -3.841 |
| RHSIM_Rhsim05G0022700 | 77.03 | 1.68E-18 | 1.00E-17 | 0.758 |
| RHSIM_Rhsim11G0084500 | 76.98 | 1.73E-18 | 1.03E-17 | -1.275 |
| RHSIM_Rhsim11G0147000 | 76.97 | 1.73E-18 | 1.03E-17 | 1.530 |
| RHSIM_Rhsim05G0040400 | 76.96 | 1.74E-18 | 1.03E-17 | -0.937 |
| RHSIM_Rhsim03G0076700 | 76.94 | 1.76E-18 | 1.05E-17 | -0.802 |
| RHSIM_Rhsim13G0085900 | 76.91 | 1.79E-18 | 1.06E-17 | 0.980 |
| RHSIM_Rhsim09G0059200 | 76.91 | 1.79E-18 | 1.06E-17 | 0.702 |
| RHSIM_Rhsim09G0107900 | 76.90 | 1.80E-18 | 1.07E-17 | -5.609 |
| RHSIM_Rhsim07G0136600 | 76.89 | 1.81E-18 | 1.08E-17 | -1.955 |
| RHSIM_Rhsim12G0179700 | 76.87 | 1.82E-18 | 1.08E-17 | -2.061 |
| RHSIM_Rhsim06G0178300 | 76.82 | 1.87E-18 | 1.11E-17 | 1.767 |
| RHSIM_Rhsim03G0247900 | 76.80 | 1.89E-18 | 1.12E-17 | 0.921 |
| RHSIM_Rhsim13G0179900 | 76.72 | 1.97E-18 | 1.17E-17 | 0.542 |
| RHSIM_Rhsim07G0154100 | 76.71 | 1.98E-18 | 1.17E-17 | 0.832 |
| RHSIM_Rhsim13G0109500 | 76.69 | 2.00E-18 | 1.18E-17 | -4.641 |
| RHSIM_Rhsim13G0006700 | 76.68 | 2.01E-18 | 1.19E-17 | 1.278 |
| RHSIM_Rhsim13G0021700 | 76.65 | 2.04E-18 | 1.21E-17 | 0.830 |
| RHSIM_Rhsim06G0214200 | 76.65 | 2.05E-18 | 1.21E-17 | -0.900 |

| Gene | LRT statistic | P value | BH-adjusted P | log2FC (interaction) |
|---|---|---|---|---|
| RHSIM_Rhsim07G0048800 | 76.61 | 2.08E-18 | 1.23E-17 | -0.806 |
| RHSIM_Rhsim05G0018200 | 76.61 | 2.08E-18 | 1.23E-17 | 4.833 |
| RHSIM_Rhsim12G0054600 | 76.61 | 2.08E-18 | 1.24E-17 | 2.953 |
| RHSIM_Rhsim02G0181400 | 76.60 | 2.09E-18 | 1.24E-17 | -0.598 |
| RHSIM_Rhsim02G0110000 | 76.60 | 2.10E-18 | 1.24E-17 | -0.842 |
| RHSIM_Rhsim01G0073900 | 76.56 | 2.14E-18 | 1.27E-17 | 0.873 |
| RHSIM_Rhsim10G0009900 | 76.55 | 2.15E-18 | 1.27E-17 | 1.895 |
| RHSIM_RhsimUnG0216100 | 76.51 | 2.19E-18 | 1.30E-17 | 0.479 |
| RHSIM_Rhsim10G0183700 | 76.45 | 2.26E-18 | 1.34E-17 | -0.492 |
| RHSIM_RhsimUnG0189800 | 76.43 | 2.28E-18 | 1.35E-17 | 1.348 |
| RHSIM_Rhsim02G0058900 | 76.43 | 2.28E-18 | 1.35E-17 | -0.915 |
| novel.3887 | 76.43 | 2.28E-18 | 1.35E-17 | -1.656 |
| RHSIM_Rhsim01G0292300 | 76.40 | 2.32E-18 | 1.37E-17 | -2.300 |
| RHSIM_Rhsim06G0029500 | 76.39 | 2.32E-18 | 1.37E-17 | -1.145 |
| novel.3653 | 76.38 | 2.34E-18 | 1.38E-17 | 0.763 |
| RHSIM_Rhsim05G0182200 | 76.36 | 2.36E-18 | 1.39E-17 | -1.873 |
| RHSIM_Rhsim08G0146200 | 76.36 | 2.36E-18 | 1.40E-17 | 0.884 |
| RHSIM_Rhsim10G0018400 | 76.35 | 2.37E-18 | 1.40E-17 | 0.834 |
| RHSIM_RhsimUnG0065500 | 76.35 | 2.38E-18 | 1.41E-17 | -2.243 |
| RHSIM_Rhsim02G0169300 | 76.34 | 2.39E-18 | 1.41E-17 | 0.469 |
| RHSIM_Rhsim06G0157600 | 76.30 | 2.44E-18 | 1.44E-17 | 1.324 |
| RHSIM_Rhsim03G0120900 | 76.27 | 2.48E-18 | 1.46E-17 | 2.002 |
| RHSIM_Rhsim13G0058000 | 76.25 | 2.50E-18 | 1.47E-17 | -0.579 |
| RHSIM_Rhsim10G0128400 | 76.22 | 2.54E-18 | 1.50E-17 | 0.737 |
| RHSIM_Rhsim08G0046700 | 76.21 | 2.55E-18 | 1.50E-17 | -0.874 |
| RHSIM_Rhsim01G0223600 | 76.20 | 2.57E-18 | 1.51E-17 | -3.086 |
| RHSIM_Rhsim10G0044500 | 76.15 | 2.63E-18 | 1.55E-17 | 1.129 |
| RHSIM_Rhsim05G0049500 | 76.04 | 2.78E-18 | 1.64E-17 | 2.091 |
| RHSIM_Rhsim11G0127400 | 76.03 | 2.79E-18 | 1.64E-17 | -1.059 |
| RHSIM_Rhsim03G0164500 | 75.98 | 2.86E-18 | 1.69E-17 | -1.793 |
| RHSIM_Rhsim08G0226800 | 75.97 | 2.88E-18 | 1.70E-17 | 3.158 |
| RHSIM_Rhsim01G0186600 | 75.93 | 2.93E-18 | 1.73E-17 | -0.854 |

| Gene | LRT statistic | P value | BH-adjusted P | log2FC (interaction) |
|---|---|---|---|---|
| RHSIM_Rhsim06G0222600 | 75.92 | 2.95E-18 | 1.74E-17 | -2.796 |
| RHSIM_Rhsim13G0100300 | 75.87 | 3.03E-18 | 1.78E-17 | 0.918 |
| RHSIM_Rhsim03G0058700 | 75.86 | 3.04E-18 | 1.79E-17 | 0.859 |
| RHSIM_Rhsim04G0030100 | 75.81 | 3.13E-18 | 1.84E-17 | 0.690 |
| RHSIM_Rhsim02G0069900 | 75.80 | 3.13E-18 | 1.84E-17 | 3.808 |
| RHSIM_Rhsim03G0054800 | 75.77 | 3.18E-18 | 1.87E-17 | 0.726 |
| RHSIM_Rhsim11G0021700 | 75.73 | 3.25E-18 | 1.91E-17 | -0.922 |
| RHSIM_Rhsim05G0030300 | 75.72 | 3.26E-18 | 1.92E-17 | -0.575 |
| RHSIM_Rhsim13G0003300 | 75.72 | 3.27E-18 | 1.92E-17 | 0.857 |
| RHSIM_Rhsim04G0056900 | 75.69 | 3.32E-18 | 1.95E-17 | -1.405 |
| RHSIM_Rhsim08G0096600 | 75.67 | 3.36E-18 | 1.97E-17 | -1.030 |
| RHSIM_Rhsim08G0077000 | 75.65 | 3.38E-18 | 1.99E-17 | 0.447 |
| RHSIM_Rhsim12G0052300 | 75.63 | 3.42E-18 | 2.01E-17 | 1.067 |
| RHSIM_Rhsim06G0042300 | 75.62 | 3.44E-18 | 2.02E-17 | 0.776 |
| RHSIM_Rhsim01G0049000 | 75.59 | 3.49E-18 | 2.05E-17 | 1.792 |
| RHSIM_Rhsim01G0183000 | 75.58 | 3.51E-18 | 2.06E-17 | 1.024 |
| RHSIM_Rhsim03G0228800 | 75.53 | 3.59E-18 | 2.11E-17 | 0.979 |
| RHSIM_Rhsim09G0214000 | 75.51 | 3.63E-18 | 2.13E-17 | 0.860 |
| RHSIM_Rhsim01G0243300 | 75.48 | 3.69E-18 | 2.16E-17 | -1.971 |
| RHSIM_Rhsim09G0172200 | 75.47 | 3.71E-18 | 2.18E-17 | -0.622 |
| RHSIM_Rhsim04G0172200 | 75.45 | 3.74E-18 | 2.20E-17 | 0.734 |
| RHSIM_Rhsim05G0163500 | 75.44 | 3.76E-18 | 2.21E-17 | -0.803 |
| RHSIM_Rhsim02G0215600 | 75.42 | 3.81E-18 | 2.24E-17 | 0.468 |
| RHSIM_Rhsim10G0045000 | 75.36 | 3.93E-18 | 2.30E-17 | 0.662 |
| RHSIM_Rhsim01G0124800 | 75.33 | 3.98E-18 | 2.33E-17 | -0.657 |
| RHSIM_Rhsim12G0094300 | 75.33 | 3.99E-18 | 2.34E-17 | 1.443 |
| RHSIM_Rhsim13G0017300 | 75.31 | 4.02E-18 | 2.35E-17 | -1.479 |
| RHSIM_Rhsim05G0053500 | 75.29 | 4.07E-18 | 2.38E-17 | -1.182 |
| RHSIM_Rhsim08G0237400 | 75.27 | 4.10E-18 | 2.40E-17 | 0.971 |
| RHSIM_Rhsim09G0164800 | 75.23 | 4.18E-18 | 2.45E-17 | 0.922 |
| RHSIM_Rhsim10G0084500 | 75.23 | 4.19E-18 | 2.45E-17 | 0.726 |
| RHSIM_Rhsim07G0216000 | 75.23 | 4.20E-18 | 2.46E-17 | -0.640 |

| Gene | LRT statistic | P value | BH-adjusted P | log2FC (interaction) |
|---|---|---|---|---|
| novel.4529 | 75.19 | 4.28E-18 | 2.50E-17 | 3.810 |
| RHSIM_Rhsim08G0167000 | 75.17 | 4.31E-18 | 2.52E-17 | -1.279 |
| novel.2978 | 75.16 | 4.33E-18 | 2.53E-17 | 1.083 |
| RHSIM_Rhsim04G0122000 | 75.16 | 4.34E-18 | 2.54E-17 | -1.196 |
| RHSIM_Rhsim10G0192400 | 75.13 | 4.40E-18 | 2.57E-17 | 0.857 |
| RHSIM_Rhsim08G0150700 | 75.13 | 4.41E-18 | 2.58E-17 | 2.312 |
| RHSIM_Rhsim09G0146200 | 75.12 | 4.43E-18 | 2.59E-17 | -0.696 |
| RHSIM_Rhsim01G0208400 | 75.09 | 4.49E-18 | 2.63E-17 | -2.767 |
| RHSIM_Rhsim08G0210500 | 75.09 | 4.51E-18 | 2.63E-17 | 0.798 |
| RHSIM_Rhsim01G0201800 | 75.06 | 4.57E-18 | 2.67E-17 | -1.451 |
| RHSIM_Rhsim08G0091500 | 75.01 | 4.68E-18 | 2.74E-17 | -2.001 |
| RHSIM_Rhsim06G0169500 | 75.00 | 4.70E-18 | 2.74E-17 | 0.718 |
| RHSIM_Rhsim05G0199800 | 74.98 | 4.75E-18 | 2.77E-17 | -2.195 |
| RHSIM_Rhsim10G0049900 | 74.96 | 4.81E-18 | 2.80E-17 | 0.833 |
| RHSIM_Rhsim04G0111500 | 74.96 | 4.81E-18 | 2.81E-17 | 0.596 |
| RHSIM_Rhsim09G0047900 | 74.90 | 4.96E-18 | 2.89E-17 | 0.948 |
| RHSIM_RhsimUnG0027100 | 74.87 | 5.03E-18 | 2.93E-17 | 0.800 |
| RHSIM_Rhsim03G0069100 | 74.82 | 5.15E-18 | 3.00E-17 | -1.012 |
| RHSIM_Rhsim13G0073400 | 74.81 | 5.18E-18 | 3.02E-17 | -1.820 |
| RHSIM_Rhsim11G0199100 | 74.81 | 5.18E-18 | 3.02E-17 | -0.918 |
| RHSIM_Rhsim12G0013500 | 74.78 | 5.25E-18 | 3.06E-17 | 0.636 |
| RHSIM_Rhsim04G0038600 | 74.73 | 5.40E-18 | 3.15E-17 | -0.791 |
| RHSIM_Rhsim01G0220700 | 74.63 | 5.69E-18 | 3.31E-17 | 1.109 |
| RHSIM_Rhsim06G0116800 | 74.60 | 5.76E-18 | 3.35E-17 | -0.906 |
| RHSIM_Rhsim03G0096000 | 74.59 | 5.78E-18 | 3.36E-17 | 2.258 |
| RHSIM_Rhsim04G0243000 | 74.59 | 5.81E-18 | 3.38E-17 | -0.602 |
| novel.2203 | 74.58 | 5.83E-18 | 3.39E-17 | -1.717 |
| RHSIM_Rhsim02G0153500 | 74.57 | 5.85E-18 | 3.41E-17 | 1.464 |
| RHSIM_Rhsim13G0150800 | 74.56 | 5.88E-18 | 3.42E-17 | 1.219 |
| RHSIM_Rhsim01G0261100 | 74.52 | 6.00E-18 | 3.49E-17 | -4.354 |
| RHSIM_Rhsim12G0192600 | 74.52 | 6.01E-18 | 3.50E-17 | 1.645 |
| RHSIM_Rhsim04G0039000 | 74.46 | 6.17E-18 | 3.59E-17 | -2.110 |

| Gene | LRT statistic | P value | BH-adjusted P | log2FC (interaction) |
|---|---|---|---|---|
| RHSIM_Rhsim09G0151900 | 74.45 | 6.23E-18 | 3.62E-17 | -2.211 |
| RHSIM_Rhsim13G0026500 | 74.43 | 6.27E-18 | 3.65E-17 | -0.958 |
| RHSIM_Rhsim09G0108100 | 74.40 | 6.38E-18 | 3.70E-17 | -1.085 |
| RHSIM_Rhsim13G0123000 | 74.34 | 6.58E-18 | 3.82E-17 | 1.094 |
| novel.2471 | 74.33 | 6.62E-18 | 3.84E-17 | -0.849 |
| RHSIM_Rhsim13G0228300 | 74.23 | 6.96E-18 | 4.04E-17 | -0.971 |
| RHSIM_Rhsim03G0146300 | 74.23 | 6.96E-18 | 4.04E-17 | 2.373 |
| RHSIM_Rhsim02G0059200 | 74.19 | 7.11E-18 | 4.13E-17 | -0.924 |
| RHSIM_Rhsim13G0130200 | 74.16 | 7.22E-18 | 4.19E-17 | 5.635 |
| RHSIM_Rhsim04G0054100 | 74.14 | 7.29E-18 | 4.23E-17 | -3.977 |
| novel.4004 | 74.08 | 7.49E-18 | 4.34E-17 | -0.989 |
| RHSIM_Rhsim09G0168400 | 74.06 | 7.57E-18 | 4.39E-17 | 0.624 |
| RHSIM_Rhsim12G0142700 | 74.05 | 7.63E-18 | 4.42E-17 | 1.295 |
| RHSIM_Rhsim08G0183000 | 74.02 | 7.75E-18 | 4.49E-17 | 0.494 |
| RHSIM_Rhsim12G0132000 | 74.01 | 7.76E-18 | 4.50E-17 | 1.439 |
| RHSIM_Rhsim03G0075800 | 73.99 | 7.85E-18 | 4.55E-17 | 0.848 |
| RHSIM_Rhsim02G0248400 | 73.96 | 7.96E-18 | 4.61E-17 | 0.805 |
| RHSIM_RhsimUnG0194100 | 73.92 | 8.13E-18 | 4.71E-17 | -1.139 |
| RHSIM_Rhsim03G0241100 | 73.88 | 8.32E-18 | 4.82E-17 | 1.256 |
| RHSIM_Rhsim02G0007000 | 73.87 | 8.33E-18 | 4.82E-17 | 3.594 |
| RHSIM_Rhsim09G0110900 | 73.85 | 8.44E-18 | 4.88E-17 | 0.951 |
| RHSIM_Rhsim01G0076900 | 73.84 | 8.47E-18 | 4.90E-17 | -3.951 |
| RHSIM_Rhsim12G0155800 | 73.81 | 8.58E-18 | 4.96E-17 | 1.350 |
| RHSIM_Rhsim07G0129700 | 73.81 | 8.60E-18 | 4.98E-17 | -0.811 |
| RHSIM_Rhsim07G0193800 | 73.79 | 8.67E-18 | 5.01E-17 | -0.602 |
| RHSIM_Rhsim13G0143100 | 73.77 | 8.78E-18 | 5.08E-17 | -0.563 |
| RHSIM_Rhsim09G0177200 | 73.73 | 8.97E-18 | 5.19E-17 | 1.730 |
| RHSIM_Rhsim07G0088700 | 73.64 | 9.36E-18 | 5.41E-17 | -0.523 |
| RHSIM_Rhsim12G0191100 | 73.64 | 9.36E-18 | 5.41E-17 | 2.592 |
| RHSIM_Rhsim11G0137400 | 73.64 | 9.36E-18 | 5.41E-17 | -0.586 |
| novel.3333 | 73.61 | 9.50E-18 | 5.49E-17 | -0.653 |
| RHSIM_Rhsim12G0185500 | 73.58 | 9.66E-18 | 5.58E-17 | 1.522 |

| Gene | LRT statistic | P value | BH-adjusted P | log2FC (interaction) |
|---|---|---|---|---|
| RHSIM_Rhsim09G0176500 | 73.56 | 9.77E-18 | 5.64E-17 | -0.975 |
| RHSIM_Rhsim10G0017800 | 73.52 | 9.97E-18 | 5.76E-17 | 0.642 |
| RHSIM_RhsimUnG0150600 | 73.51 | 1.00E-17 | 5.77E-17 | -1.258 |
| RHSIM_Rhsim04G0216600 | 73.48 | 1.02E-17 | 5.88E-17 | -1.386 |
| RHSIM_Rhsim12G0180200 | 73.46 | 1.03E-17 | 5.92E-17 | 1.322 |
| RHSIM_Rhsim13G0010600 | 73.45 | 1.03E-17 | 5.96E-17 | 0.946 |
| RHSIM_Rhsim13G0233800 | 73.36 | 1.08E-17 | 6.23E-17 | -0.724 |
| RHSIM_Rhsim05G0162800 | 73.33 | 1.10E-17 | 6.33E-17 | 0.563 |
| RHSIM_Rhsim08G0236600 | 73.32 | 1.10E-17 | 6.37E-17 | -0.722 |
| RHSIM_Rhsim13G0131100 | 73.31 | 1.11E-17 | 6.39E-17 | -1.105 |
| RHSIM_Rhsim10G0130800 | 73.30 | 1.12E-17 | 6.43E-17 | -3.483 |
| RHSIM_Rhsim03G0075200 | 73.26 | 1.14E-17 | 6.55E-17 | 0.804 |
| RHSIM_Rhsim04G0214000 | 73.24 | 1.15E-17 | 6.61E-17 | 0.634 |
| RHSIM_Rhsim06G0072400 | 73.22 | 1.16E-17 | 6.68E-17 | -0.844 |
| RHSIM_Rhsim03G0198900 | 73.18 | 1.19E-17 | 6.82E-17 | 0.917 |
| novel.3718 | 73.16 | 1.19E-17 | 6.87E-17 | 1.388 |
| RHSIM_Rhsim10G0121100 | 73.09 | 1.24E-17 | 7.13E-17 | 2.142 |
| RHSIM_Rhsim07G0252500 | 73.06 | 1.26E-17 | 7.22E-17 | -1.762 |
| RHSIM_Rhsim03G0261800 | 73.06 | 1.26E-17 | 7.22E-17 | -1.364 |
| RHSIM_Rhsim13G0039500 | 73.06 | 1.26E-17 | 7.23E-17 | -0.938 |
| RHSIM_Rhsim09G0101400 | 73.06 | 1.26E-17 | 7.23E-17 | 0.479 |
| RHSIM_Rhsim05G0013700 | 73.05 | 1.27E-17 | 7.27E-17 | -0.850 |
| RHSIM_Rhsim09G0035700 | 73.04 | 1.27E-17 | 7.32E-17 | -3.337 |
| RHSIM_Rhsim09G0140900 | 72.99 | 1.30E-17 | 7.49E-17 | 0.487 |
| RHSIM_Rhsim06G0015100 | 72.99 | 1.31E-17 | 7.51E-17 | 1.217 |
| RHSIM_Rhsim09G0131400 | 72.91 | 1.36E-17 | 7.79E-17 | 1.057 |
| novel.413 | 72.89 | 1.37E-17 | 7.88E-17 | 0.814 |
| RHSIM_Rhsim06G0201200 | 72.83 | 1.41E-17 | 8.12E-17 | -2.150 |
| RHSIM_Rhsim11G0000900 | 72.83 | 1.41E-17 | 8.12E-17 | -0.633 |
| RHSIM_Rhsim13G0035400 | 72.79 | 1.44E-17 | 8.27E-17 | 0.593 |
| RHSIM_Rhsim07G0254200 | 72.78 | 1.45E-17 | 8.32E-17 | -2.852 |
| RHSIM_Rhsim03G0070000 | 72.69 | 1.52E-17 | 8.71E-17 | 0.787 |

| Gene | LRT statistic | P value | BH-adjusted P | log2FC (interaction) |
|---|---|---|---|---|
| RHSIM_Rhsim05G0047300 | 72.69 | 1.52E-17 | 8.71E-17 | 1.609 |
| RHSIM_Rhsim01G0237500 | 72.69 | 1.52E-17 | 8.72E-17 | -1.336 |
| RHSIM_Rhsim05G0192200 | 72.69 | 1.52E-17 | 8.72E-17 | -0.751 |
| RHSIM_Rhsim04G0130000 | 72.67 | 1.53E-17 | 8.80E-17 | 1.434 |
| novel.530 | 72.66 | 1.54E-17 | 8.81E-17 | 3.237 |
| RHSIM_Rhsim08G0097800 | 72.62 | 1.57E-17 | 8.99E-17 | -1.043 |
| RHSIM_Rhsim03G0257100 | 72.58 | 1.61E-17 | 9.20E-17 | -0.772 |
| RHSIM_Rhsim03G0221900 | 72.55 | 1.63E-17 | 9.35E-17 | 0.496 |
| RHSIM_Rhsim08G0100900 | 72.52 | 1.65E-17 | 9.46E-17 | 0.494 |
| RHSIM_Rhsim05G0094500 | 72.50 | 1.67E-17 | 9.57E-17 | 0.949 |
| RHSIM_Rhsim10G0157800 | 72.48 | 1.69E-17 | 9.65E-17 | 3.870 |
| RHSIM_Rhsim03G0019100 | 72.44 | 1.73E-17 | 9.88E-17 | 1.169 |
| RHSIM_Rhsim05G0161400 | 72.40 | 1.76E-17 | 1.01E-16 | -1.901 |
| RHSIM_Rhsim08G0005000 | 72.40 | 1.76E-17 | 1.01E-16 | -0.844 |
| RHSIM_Rhsim12G0126500 | 72.39 | 1.76E-17 | 1.01E-16 | 0.666 |
| novel.1902 | 72.35 | 1.80E-17 | 1.03E-16 | 1.004 |
| RHSIM_Rhsim08G0070500 | 72.29 | 1.86E-17 | 1.06E-16 | -0.878 |
| RHSIM_Rhsim07G0248500 | 72.18 | 1.96E-17 | 1.12E-16 | -2.210 |
| novel.217 | 72.14 | 2.00E-17 | 1.14E-16 | 1.669 |
| RHSIM_RhsimUnG0205700 | 72.14 | 2.01E-17 | 1.15E-16 | 0.980 |
| RHSIM_Rhsim04G0144000 | 72.12 | 2.03E-17 | 1.16E-16 | 2.290 |
| RHSIM_RhsimUnG0055400 | 72.08 | 2.06E-17 | 1.18E-16 | -0.492 |
| RHSIM_RhsimUnG0137800 | 72.03 | 2.12E-17 | 1.21E-16 | -1.396 |
| RHSIM_Rhsim06G0168700 | 72.02 | 2.12E-17 | 1.21E-16 | -0.807 |
| RHSIM_Rhsim09G0207900 | 72.02 | 2.13E-17 | 1.21E-16 | 1.070 |
| RHSIM_Rhsim08G0094000 | 71.97 | 2.19E-17 | 1.25E-16 | -0.596 |
| RHSIM_Rhsim12G0189000 | 71.92 | 2.24E-17 | 1.28E-16 | 1.327 |
| RHSIM_Rhsim02G0089200 | 71.88 | 2.29E-17 | 1.31E-16 | -2.965 |
| RHSIM_Rhsim05G0088900 | 71.86 | 2.31E-17 | 1.32E-16 | 1.463 |
| RHSIM_Rhsim13G0154000 | 71.85 | 2.32E-17 | 1.32E-16 | -0.571 |
| RHSIM_Rhsim06G0038600 | 71.83 | 2.35E-17 | 1.34E-16 | -0.789 |
| RHSIM_RhsimUnG0248900 | 71.81 | 2.37E-17 | 1.35E-16 | -0.621 |

| Gene | LRT statistic | P value | BH-adjusted P | log2FC (interaction) |
|---|---|---|---|---|
| RHSIM_Rhsim06G0153700 | 71.78 | 2.41E-17 | 1.37E-16 | 1.775 |
| RHSIM_Rhsim06G0167400 | 71.77 | 2.42E-17 | 1.38E-16 | 0.984 |
| RHSIM_Rhsim12G0047700 | 71.74 | 2.45E-17 | 1.40E-16 | 0.790 |
| RHSIM_Rhsim13G0022600 | 71.71 | 2.50E-17 | 1.42E-16 | 0.563 |
| RHSIM_Rhsim09G0088700 | 71.71 | 2.50E-17 | 1.42E-16 | -3.895 |
| RHSIM_Rhsim11G0018300 | 71.70 | 2.50E-17 | 1.42E-16 | 3.328 |
| RHSIM_Rhsim03G0245900 | 71.65 | 2.56E-17 | 1.46E-16 | -0.372 |
| RHSIM_Rhsim12G0013600 | 71.65 | 2.57E-17 | 1.46E-16 | -0.624 |
| RHSIM_RhsimUnG0070300 | 71.60 | 2.63E-17 | 1.50E-16 | -6.851 |
| RHSIM_Rhsim11G0057800 | 71.55 | 2.70E-17 | 1.54E-16 | -0.937 |
| RHSIM_Rhsim10G0171500 | 71.55 | 2.70E-17 | 1.54E-16 | 0.782 |
| RHSIM_Rhsim04G0234100 | 71.55 | 2.70E-17 | 1.54E-16 | 0.839 |
| RHSIM_Rhsim12G0145400 | 71.55 | 2.71E-17 | 1.54E-16 | -0.624 |
| RHSIM_Rhsim08G0086200 | 71.54 | 2.72E-17 | 1.55E-16 | 0.844 |
| novel.2661 | 71.53 | 2.73E-17 | 1.55E-16 | -1.170 |
| RHSIM_Rhsim09G0133500 | 71.51 | 2.76E-17 | 1.57E-16 | -1.237 |
| novel.1443 | 71.48 | 2.79E-17 | 1.59E-16 | -1.506 |
| RHSIM_Rhsim07G0117800 | 71.48 | 2.80E-17 | 1.59E-16 | -0.598 |
| RHSIM_Rhsim13G0124600 | 71.44 | 2.86E-17 | 1.63E-16 | 0.884 |
| RHSIM_Rhsim03G0074400 | 71.40 | 2.91E-17 | 1.65E-16 | 0.831 |
| RHSIM_Rhsim04G0017000 | 71.40 | 2.92E-17 | 1.66E-16 | -1.478 |
| RHSIM_Rhsim05G0208200 | 71.37 | 2.97E-17 | 1.68E-16 | -3.162 |
| RHSIM_Rhsim03G0130900 | 71.36 | 2.98E-17 | 1.69E-16 | 0.780 |
| RHSIM_Rhsim03G0145100 | 71.32 | 3.04E-17 | 1.73E-16 | -1.143 |
| RHSIM_Rhsim09G0002200 | 71.30 | 3.06E-17 | 1.74E-16 | -0.795 |
| RHSIM_Rhsim13G0089300 | 71.29 | 3.09E-17 | 1.75E-16 | 1.936 |
| RHSIM_Rhsim09G0180100 | 71.27 | 3.12E-17 | 1.77E-16 | 0.668 |
| RHSIM_Rhsim08G0057700 | 71.25 | 3.14E-17 | 1.78E-16 | -42.444 |
| RHSIM_Rhsim09G0035100 | 71.22 | 3.20E-17 | 1.81E-16 | 1.566 |
| RHSIM_Rhsim10G0118800 | 71.19 | 3.25E-17 | 1.84E-16 | -1.090 |
| RHSIM_Rhsim07G0054800 | 71.17 | 3.28E-17 | 1.86E-16 | -0.971 |
| RHSIM_Rhsim05G0074000 | 71.16 | 3.29E-17 | 1.86E-16 | 1.351 |

| Gene | LRT statistic | P value | BH-adjusted P | log2FC (interaction) |
|---|---|---|---|---|
| RHSIM_Rhsim13G0217000 | 71.11 | 3.38E-17 | 1.91E-16 | 0.788 |
| RHSIM_Rhsim02G0236800 | 71.04 | 3.50E-17 | 1.98E-16 | 0.547 |
| RHSIM_Rhsim12G0104500 | 71.01 | 3.56E-17 | 2.02E-16 | 1.103 |
| RHSIM_Rhsim11G0032300 | 71.00 | 3.57E-17 | 2.02E-16 | 0.634 |
| novel.3562 | 71.00 | 3.58E-17 | 2.02E-16 | -2.421 |
| RHSIM_Rhsim10G0066600 | 70.98 | 3.60E-17 | 2.04E-16 | -5.146 |
| RHSIM_Rhsim09G0015200 | 70.98 | 3.60E-17 | 2.04E-16 | 2.019 |
| RHSIM_Rhsim08G0187400 | 70.97 | 3.63E-17 | 2.05E-16 | -0.721 |
| RHSIM_Rhsim13G0090200 | 70.87 | 3.82E-17 | 2.16E-16 | -1.049 |
| RHSIM_Rhsim11G0134900 | 70.85 | 3.85E-17 | 2.18E-16 | 0.871 |
| RHSIM_Rhsim03G0128200 | 70.84 | 3.87E-17 | 2.19E-16 | 1.177 |
| RHSIM_Rhsim08G0002200 | 70.80 | 3.95E-17 | 2.23E-16 | -1.444 |
| RHSIM_Rhsim10G0025300 | 70.79 | 3.98E-17 | 2.25E-16 | 0.820 |
| RHSIM_Rhsim11G0128100 | 70.78 | 3.99E-17 | 2.25E-16 | -0.836 |
| RHSIM_RhsimUnG0092800 | 70.68 | 4.19E-17 | 2.37E-16 | 1.081 |
| RHSIM_RhsimUnG0022400 | 70.65 | 4.26E-17 | 2.40E-16 | -5.032 |
| RHSIM_Rhsim13G0045900 | 70.62 | 4.33E-17 | 2.44E-16 | -0.794 |
| RHSIM_Rhsim08G0107900 | 70.61 | 4.34E-17 | 2.45E-16 | -2.061 |
| RHSIM_Rhsim06G0032600 | 70.57 | 4.43E-17 | 2.50E-16 | -0.829 |
| RHSIM_Rhsim11G0071200 | 70.56 | 4.46E-17 | 2.51E-16 | 0.719 |
| novel.1879 | 70.56 | 4.46E-17 | 2.52E-16 | -6.594 |
| RHSIM_Rhsim08G0239400 | 70.49 | 4.62E-17 | 2.61E-16 | -0.991 |
| RHSIM_Rhsim02G0112300 | 70.47 | 4.67E-17 | 2.63E-16 | -0.807 |
| RHSIM_Rhsim10G0055100 | 70.47 | 4.68E-17 | 2.64E-16 | 0.746 |
| RHSIM_Rhsim12G0083100 | 70.45 | 4.72E-17 | 2.66E-16 | 0.662 |
| RHSIM_Rhsim01G0212600 | 70.45 | 4.73E-17 | 2.67E-16 | 0.957 |
| novel.1991 | 70.44 | 4.74E-17 | 2.67E-16 | -2.896 |
| RHSIM_Rhsim05G0037800 | 70.44 | 4.74E-17 | 2.67E-16 | -1.944 |
| RHSIM_Rhsim02G0183500 | 70.43 | 4.76E-17 | 2.68E-16 | 1.370 |
| RHSIM_Rhsim08G0215600 | 70.41 | 4.82E-17 | 2.72E-16 | -1.426 |
| RHSIM_Rhsim07G0170000 | 70.32 | 5.05E-17 | 2.85E-16 | -0.461 |
| RHSIM_Rhsim10G0036600 | 70.31 | 5.07E-17 | 2.85E-16 | -0.965 |

| Gene | LRT statistic | P value | BH-adjusted P | log2FC (interaction) |
|---|---|---|---|---|
| RHSIM_Rhsim07G0011500 | 70.28 | 5.15E-17 | 2.90E-16 | -0.533 |
| RHSIM_Rhsim06G0165200 | 70.23 | 5.28E-17 | 2.97E-16 | -0.954 |
| RHSIM_Rhsim09G0165900 | 70.22 | 5.30E-17 | 2.98E-16 | 1.343 |
| RHSIM_Rhsim03G0099700 | 70.21 | 5.34E-17 | 3.00E-16 | 1.544 |
| RHSIM_RhsimUnG0104800 | 70.21 | 5.34E-17 | 3.00E-16 | 0.992 |
| RHSIM_Rhsim07G0247100 | 70.21 | 5.34E-17 | 3.00E-16 | 0.929 |
| RHSIM_Rhsim04G0203700 | 70.18 | 5.40E-17 | 3.04E-16 | 0.750 |
| RHSIM_Rhsim03G0078800 | 70.17 | 5.43E-17 | 3.05E-16 | -0.911 |
| RHSIM_Rhsim02G0173600 | 70.15 | 5.49E-17 | 3.09E-16 | 1.527 |
| RHSIM_Rhsim02G0134900 | 70.12 | 5.58E-17 | 3.14E-16 | 0.821 |
| RHSIM_Rhsim08G0038800 | 70.03 | 5.84E-17 | 3.28E-16 | 0.589 |
| RHSIM_Rhsim01G0099300 | 69.99 | 5.95E-17 | 3.34E-16 | 1.474 |
| RHSIM_Rhsim12G0194800 | 69.98 | 5.98E-17 | 3.36E-16 | -0.785 |
| novel.3564 | 69.95 | 6.08E-17 | 3.41E-16 | 1.195 |
| RHSIM_Rhsim12G0079100 | 69.95 | 6.09E-17 | 3.42E-16 | -0.811 |
| RHSIM_Rhsim08G0096300 | 69.94 | 6.11E-17 | 3.43E-16 | 0.694 |
| RHSIM_Rhsim11G0071700 | 69.93 | 6.13E-17 | 3.44E-16 | 0.709 |
| RHSIM_Rhsim13G0227500 | 69.92 | 6.16E-17 | 3.46E-16 | 0.958 |
| RHSIM_Rhsim12G0154300 | 69.91 | 6.20E-17 | 3.48E-16 | 0.918 |
| RHSIM_Rhsim09G0115500 | 69.91 | 6.21E-17 | 3.48E-16 | 1.129 |
| RHSIM_Rhsim01G0230200 | 69.89 | 6.26E-17 | 3.51E-16 | 0.692 |
| RHSIM_Rhsim12G0103900 | 69.89 | 6.26E-17 | 3.51E-16 | 0.820 |
| RHSIM_Rhsim08G0117500 | 69.89 | 6.28E-17 | 3.52E-16 | 3.073 |
| RHSIM_Rhsim08G0238400 | 69.88 | 6.30E-17 | 3.53E-16 | -3.203 |
| RHSIM_Rhsim08G0126200 | 69.87 | 6.33E-17 | 3.54E-16 | 0.843 |
| RHSIM_Rhsim13G0068400 | 69.83 | 6.46E-17 | 3.62E-16 | -0.543 |
| RHSIM_Rhsim08G0203500 | 69.82 | 6.48E-17 | 3.63E-16 | 1.469 |
| RHSIM_Rhsim07G0072700 | 69.81 | 6.52E-17 | 3.65E-16 | 0.890 |
| RHSIM_Rhsim08G0039500 | 69.79 | 6.59E-17 | 3.69E-16 | 1.245 |
| novel.231 | 69.77 | 6.66E-17 | 3.73E-16 | 0.702 |
| RHSIM_Rhsim07G0006900 | 69.73 | 6.81E-17 | 3.81E-16 | 1.129 |
| RHSIM_Rhsim12G0151700 | 69.73 | 6.81E-17 | 3.81E-16 | -0.933 |

| Gene | LRT statistic | P value | BH-adjusted P | log2FC (interaction) |
|---|---|---|---|---|
| RHSIM_Rhsim04G0220700 | 69.72 | 6.85E-17 | 3.83E-16 | 0.851 |
| RHSIM_Rhsim01G0039300 | 69.69 | 6.93E-17 | 3.88E-16 | 0.839 |
| RHSIM_Rhsim11G0065600 | 69.64 | 7.11E-17 | 3.98E-16 | 0.592 |
| RHSIM_Rhsim01G0130400 | 69.57 | 7.37E-17 | 4.12E-16 | -1.055 |
| RHSIM_Rhsim09G0171100 | 69.53 | 7.53E-17 | 4.21E-16 | 0.962 |
| RHSIM_Rhsim13G0169500 | 69.53 | 7.53E-17 | 4.21E-16 | -0.699 |
| RHSIM_Rhsim03G0170100 | 69.52 | 7.57E-17 | 4.23E-16 | -0.738 |
| RHSIM_Rhsim04G0102000 | 69.51 | 7.60E-17 | 4.25E-16 | 0.529 |
| RHSIM_Rhsim03G0044500 | 69.48 | 7.71E-17 | 4.31E-16 | -0.842 |
| RHSIM_Rhsim11G0098300 | 69.48 | 7.72E-17 | 4.31E-16 | 1.281 |
| RHSIM_Rhsim10G0197700 | 69.48 | 7.73E-17 | 4.32E-16 | 1.183 |
| novel.4527 | 69.46 | 7.82E-17 | 4.36E-16 | 1.725 |
| RHSIM_Rhsim04G0157400 | 69.43 | 7.91E-17 | 4.41E-16 | 1.618 |
| RHSIM_Rhsim02G0184200 | 69.42 | 7.96E-17 | 4.44E-16 | 0.939 |
| RHSIM_Rhsim08G0120400 | 69.41 | 8.01E-17 | 4.47E-16 | -1.036 |
| RHSIM_Rhsim08G0152100 | 69.39 | 8.08E-17 | 4.51E-16 | 1.466 |
| RHSIM_Rhsim11G0198400 | 69.34 | 8.31E-17 | 4.63E-16 | 0.681 |
| RHSIM_Rhsim07G0187000 | 69.32 | 8.37E-17 | 4.67E-16 | 1.226 |
| RHSIM_Rhsim13G0168400 | 69.28 | 8.55E-17 | 4.77E-16 | 0.828 |
| RHSIM_Rhsim05G0081800 | 69.24 | 8.74E-17 | 4.87E-16 | 2.991 |
| RHSIM_Rhsim05G0220000 | 69.22 | 8.79E-17 | 4.90E-16 | -1.680 |
| RHSIM_Rhsim01G0189800 | 69.21 | 8.86E-17 | 4.93E-16 | 1.772 |
| RHSIM_Rhsim11G0045200 | 69.20 | 8.89E-17 | 4.95E-16 | 1.819 |
| RHSIM_RhsimUnG0062500 | 69.17 | 9.03E-17 | 5.03E-16 | -2.036 |
| RHSIM_Rhsim04G0013000 | 69.17 | 9.05E-17 | 5.04E-16 | -2.890 |
| RHSIM_Rhsim07G0127400 | 69.16 | 9.09E-17 | 5.06E-16 | -0.589 |
| RHSIM_Rhsim12G0073200 | 69.14 | 9.17E-17 | 5.11E-16 | 0.918 |
| RHSIM_Rhsim07G0035700 | 69.12 | 9.26E-17 | 5.15E-16 | 0.975 |
| RHSIM_Rhsim06G0045000 | 69.07 | 9.52E-17 | 5.29E-16 | 1.823 |
| RHSIM_Rhsim11G0087200 | 69.02 | 9.73E-17 | 5.41E-16 | 0.734 |
| RHSIM_Rhsim06G0199700 | 68.99 | 9.88E-17 | 5.50E-16 | 1.467 |
| RHSIM_Rhsim12G0092500 | 68.96 | 1.00E-16 | 5.58E-16 | 0.780 |

| Gene | LRT statistic | P value | BH-adjusted P | log2FC (interaction) |
|---|---|---|---|---|
| RHSIM_Rhsim04G0002900 | 68.96 | 1.01E-16 | 5.59E-16 | 0.830 |
| RHSIM_Rhsim01G0219000 | 68.93 | 1.02E-16 | 5.66E-16 | -1.124 |
| RHSIM_Rhsim09G0177700 | 68.91 | 1.03E-16 | 5.73E-16 | 2.104 |
| RHSIM_Rhsim09G0141000 | 68.89 | 1.04E-16 | 5.78E-16 | 0.964 |
| RHSIM_Rhsim03G0211900 | 68.86 | 1.06E-16 | 5.87E-16 | 1.091 |
| RHSIM_Rhsim06G0082100 | 68.85 | 1.06E-16 | 5.89E-16 | -0.974 |
| RHSIM_Rhsim07G0117100 | 68.79 | 1.10E-16 | 6.08E-16 | 1.813 |
| RHSIM_Rhsim08G0064400 | 68.78 | 1.10E-16 | 6.12E-16 | -1.868 |
| RHSIM_Rhsim12G0148700 | 68.58 | 1.22E-16 | 6.77E-16 | -0.761 |
| RHSIM_Rhsim05G0101600 | 68.56 | 1.23E-16 | 6.82E-16 | 0.791 |
| RHSIM_Rhsim03G0166500 | 68.55 | 1.24E-16 | 6.86E-16 | 1.047 |
| RHSIM_Rhsim13G0165700 | 68.55 | 1.24E-16 | 6.87E-16 | 2.729 |
| RHSIM_RhsimUnG0094900 | 68.54 | 1.24E-16 | 6.89E-16 | 0.810 |
| RHSIM_Rhsim06G0203600 | 68.54 | 1.24E-16 | 6.89E-16 | 1.140 |
| RHSIM_Rhsim09G0078200 | 68.52 | 1.25E-16 | 6.96E-16 | 0.784 |
| RHSIM_Rhsim11G0114700 | 68.49 | 1.28E-16 | 7.08E-16 | 1.034 |
| RHSIM_Rhsim08G0113000 | 68.46 | 1.29E-16 | 7.17E-16 | 0.668 |
| RHSIM_RhsimUnG0035400 | 68.43 | 1.32E-16 | 7.30E-16 | -2.015 |
| novel.1309 | 68.42 | 1.32E-16 | 7.34E-16 | 1.297 |
| RHSIM_Rhsim04G0157300 | 68.41 | 1.33E-16 | 7.37E-16 | 0.935 |
| novel.3778 | 68.40 | 1.34E-16 | 7.41E-16 | -1.024 |
| RHSIM_Rhsim06G0198200 | 68.36 | 1.36E-16 | 7.53E-16 | 2.806 |
| RHSIM_Rhsim10G0030100 | 68.36 | 1.36E-16 | 7.54E-16 | -2.525 |
| RHSIM_Rhsim08G0056800 | 68.33 | 1.38E-16 | 7.65E-16 | 0.563 |
| RHSIM_Rhsim06G0210000 | 68.33 | 1.38E-16 | 7.66E-16 | -0.873 |
| novel.714 | 68.30 | 1.40E-16 | 7.76E-16 | -0.401 |
| RHSIM_Rhsim07G0073600 | 68.29 | 1.41E-16 | 7.81E-16 | 0.999 |
| RHSIM_Rhsim02G0251600 | 68.24 | 1.45E-16 | 8.00E-16 | -1.129 |
| RHSIM_Rhsim05G0204200 | 68.24 | 1.45E-16 | 8.00E-16 | -4.925 |
| RHSIM_Rhsim04G0036000 | 68.23 | 1.46E-16 | 8.05E-16 | -1.345 |
| RHSIM_Rhsim12G0005100 | 68.22 | 1.47E-16 | 8.10E-16 | 2.432 |
| RHSIM_Rhsim05G0052600 | 68.19 | 1.48E-16 | 8.20E-16 | 1.041 |

| Gene | LRT statistic | P value | BH-adjusted P | log2FC (interaction) |
|---|---|---|---|---|
| RHSIM_Rhsim01G0237200 | 68.14 | 1.52E-16 | 8.42E-16 | -0.723 |
| RHSIM_Rhsim07G0203100 | 68.13 | 1.53E-16 | 8.47E-16 | 0.552 |
| RHSIM_Rhsim02G0250900 | 68.09 | 1.56E-16 | 8.62E-16 | 1.114 |
| RHSIM_Rhsim05G0081400 | 68.08 | 1.57E-16 | 8.68E-16 | 1.879 |
| RHSIM_Rhsim09G0193800 | 68.06 | 1.59E-16 | 8.77E-16 | -1.722 |
| RHSIM_Rhsim09G0001200 | 67.99 | 1.65E-16 | 9.09E-16 | -0.725 |
| RHSIM_Rhsim04G0216700 | 67.98 | 1.66E-16 | 9.14E-16 | -1.104 |
| RHSIM_Rhsim06G0037400 | 67.97 | 1.66E-16 | 9.15E-16 | 0.915 |
| RHSIM_Rhsim07G0185300 | 67.95 | 1.68E-16 | 9.24E-16 | 1.364 |
| RHSIM_RhsimUnG0021400 | 67.95 | 1.68E-16 | 9.26E-16 | -0.505 |
| RHSIM_Rhsim04G0097600 | 67.94 | 1.68E-16 | 9.27E-16 | -1.584 |
| RHSIM_Rhsim01G0288000 | 67.93 | 1.69E-16 | 9.34E-16 | 1.762 |
| RHSIM_Rhsim01G0272100 | 67.91 | 1.71E-16 | 9.42E-16 | -1.988 |
| RHSIM_Rhsim05G0163400 | 67.91 | 1.71E-16 | 9.42E-16 | 1.781 |
| RHSIM_Rhsim03G0143200 | 67.87 | 1.74E-16 | 9.61E-16 | 1.125 |
| RHSIM_Rhsim03G0038800 | 67.85 | 1.77E-16 | 9.73E-16 | 0.803 |
| RHSIM_Rhsim03G0032800 | 67.85 | 1.77E-16 | 9.74E-16 | 1.958 |
| RHSIM_Rhsim08G0019700 | 67.84 | 1.77E-16 | 9.77E-16 | 0.583 |
| RHSIM_Rhsim08G0191500 | 67.82 | 1.79E-16 | 9.85E-16 | -0.937 |
| novel.1143 | 67.80 | 1.81E-16 | 9.98E-16 | -1.525 |
| RHSIM_Rhsim07G0085300 | 67.78 | 1.83E-16 | 1.01E-15 | -0.550 |
| RHSIM_Rhsim01G0092200 | 67.77 | 1.83E-16 | 1.01E-15 | 2.163 |
| RHSIM_Rhsim03G0167200 | 67.74 | 1.86E-16 | 1.02E-15 | -0.704 |
| RHSIM_Rhsim03G0118500 | 67.62 | 1.98E-16 | 1.09E-15 | 0.876 |
| RHSIM_Rhsim10G0138300 | 67.56 | 2.04E-16 | 1.12E-15 | 0.952 |
| RHSIM_Rhsim01G0239900 | 67.53 | 2.07E-16 | 1.14E-15 | 0.347 |
| RHSIM_RhsimUnG0222200 | 67.53 | 2.07E-16 | 1.14E-15 | -1.840 |
| RHSIM_RhsimUnG0236700 | 67.52 | 2.08E-16 | 1.14E-15 | -1.111 |
| RHSIM_Rhsim10G0139000 | 67.50 | 2.11E-16 | 1.16E-15 | -1.595 |
| RHSIM_Rhsim08G0131900 | 67.43 | 2.19E-16 | 1.20E-15 | 0.684 |
| RHSIM_Rhsim04G0044600 | 67.42 | 2.19E-16 | 1.20E-15 | -1.311 |
| novel.1613 | 67.42 | 2.20E-16 | 1.21E-15 | 1.695 |

| Gene | LRT statistic | P value | BH-adjusted P | log2FC (interaction) |
|---|---|---|---|---|
| RHSIM_Rhsim02G0220900 | 67.42 | 2.20E-16 | 1.21E-15 | 0.828 |
| RHSIM_Rhsim04G0003200 | 67.40 | 2.21E-16 | 1.22E-15 | 1.266 |
| RHSIM_Rhsim03G0184500 | 67.40 | 2.22E-16 | 1.22E-15 | -2.552 |
| RHSIM_Rhsim06G0148600 | 67.38 | 2.24E-16 | 1.23E-15 | -0.731 |
| RHSIM_Rhsim02G0083600 | 67.36 | 2.27E-16 | 1.24E-15 | 1.344 |
| RHSIM_Rhsim03G0027600 | 67.34 | 2.29E-16 | 1.25E-15 | 1.287 |
| RHSIM_Rhsim02G0008300 | 67.34 | 2.29E-16 | 1.26E-15 | -1.502 |
| RHSIM_Rhsim11G0001200 | 67.33 | 2.30E-16 | 1.26E-15 | -0.722 |
| RHSIM_Rhsim01G0198900 | 67.30 | 2.33E-16 | 1.28E-15 | -0.454 |
| RHSIM_Rhsim06G0059800 | 67.30 | 2.34E-16 | 1.28E-15 | -1.276 |
| RHSIM_Rhsim11G0085900 | 67.29 | 2.34E-16 | 1.28E-15 | -0.357 |
| RHSIM_Rhsim03G0088900 | 67.29 | 2.35E-16 | 1.29E-15 | 0.773 |
| RHSIM_Rhsim09G0166900 | 67.29 | 2.35E-16 | 1.29E-15 | -1.291 |
| RHSIM_Rhsim08G0185100 | 67.26 | 2.38E-16 | 1.30E-15 | -0.625 |
| RHSIM_Rhsim03G0045900 | 67.25 | 2.39E-16 | 1.31E-15 | 1.427 |
| RHSIM_Rhsim01G0130300 | 67.20 | 2.45E-16 | 1.34E-15 | -1.142 |
| RHSIM_Rhsim01G0226200 | 67.19 | 2.46E-16 | 1.35E-15 | 10.093 |
| RHSIM_Rhsim01G0258200 | 67.18 | 2.48E-16 | 1.36E-15 | -0.546 |
| RHSIM_Rhsim08G0040400 | 67.16 | 2.50E-16 | 1.37E-15 | -0.701 |
| RHSIM_Rhsim12G0189200 | 67.13 | 2.54E-16 | 1.39E-15 | -0.460 |
| RHSIM_Rhsim06G0129000 | 67.13 | 2.54E-16 | 1.39E-15 | 1.057 |
| RHSIM_Rhsim10G0195400 | 67.13 | 2.54E-16 | 1.39E-15 | -2.013 |
| RHSIM_Rhsim07G0233400 | 67.10 | 2.58E-16 | 1.41E-15 | 1.048 |
| RHSIM_Rhsim13G0023900 | 67.09 | 2.59E-16 | 1.42E-15 | -1.317 |
| RHSIM_Rhsim12G0029800 | 67.06 | 2.64E-16 | 1.44E-15 | 2.069 |
| RHSIM_Rhsim11G0100500 | 67.03 | 2.67E-16 | 1.46E-15 | 0.613 |
| novel.2133 | 67.02 | 2.68E-16 | 1.47E-15 | 4.246 |
| RHSIM_Rhsim02G0199300 | 67.02 | 2.69E-16 | 1.47E-15 | 1.951 |
| RHSIM_Rhsim10G0095000 | 66.99 | 2.73E-16 | 1.49E-15 | -4.275 |
| novel.807 | 66.95 | 2.78E-16 | 1.52E-15 | 1.521 |
| RHSIM_Rhsim13G0095600 | 66.95 | 2.79E-16 | 1.52E-15 | -1.144 |
| RHSIM_Rhsim04G0218000 | 66.94 | 2.80E-16 | 1.53E-15 | -4.287 |

| Gene | LRT statistic | P value | BH-adjusted P | log2FC (interaction) |
|---|---|---|---|---|
| RHSIM_Rhsim05G0208400 | 66.93 | 2.81E-16 | 1.53E-15 | -7.621 |
| RHSIM_Rhsim04G0186500 | 66.91 | 2.84E-16 | 1.55E-15 | -2.615 |
| novel.4501 | 66.91 | 2.84E-16 | 1.55E-15 | -0.664 |
| RHSIM_Rhsim02G0237200 | 66.90 | 2.85E-16 | 1.56E-15 | -0.627 |
| RHSIM_Rhsim08G0185000 | 66.89 | 2.88E-16 | 1.57E-15 | 0.585 |
| RHSIM_Rhsim03G0169300 | 66.88 | 2.88E-16 | 1.57E-15 | 3.053 |
| RHSIM_Rhsim07G0175100 | 66.88 | 2.89E-16 | 1.57E-15 | 0.775 |
| RHSIM_Rhsim08G0048700 | 66.86 | 2.91E-16 | 1.59E-15 | -2.482 |
| RHSIM_Rhsim09G0207200 | 66.86 | 2.92E-16 | 1.59E-15 | -3.026 |
| RHSIM_Rhsim02G0089700 | 66.85 | 2.92E-16 | 1.59E-15 | 0.817 |
| RHSIM_Rhsim07G0083900 | 66.84 | 2.94E-16 | 1.60E-15 | 0.458 |
| RHSIM_Rhsim07G0000100 | 66.78 | 3.04E-16 | 1.65E-15 | 0.683 |
| RHSIM_Rhsim02G0254800 | 66.77 | 3.05E-16 | 1.66E-15 | -1.716 |
| novel.3008 | 66.70 | 3.17E-16 | 1.72E-15 | -2.235 |
| RHSIM_Rhsim12G0000300 | 66.69 | 3.19E-16 | 1.73E-15 | -0.641 |
| RHSIM_Rhsim06G0241700 | 66.66 | 3.22E-16 | 1.75E-15 | 3.049 |
| RHSIM_Rhsim09G0191500 | 66.66 | 3.22E-16 | 1.75E-15 | -1.338 |
| RHSIM_Rhsim06G0235800 | 66.66 | 3.23E-16 | 1.76E-15 | 0.923 |
| RHSIM_Rhsim12G0038900 | 66.65 | 3.24E-16 | 1.76E-15 | -0.982 |
| RHSIM_Rhsim12G0006500 | 66.64 | 3.26E-16 | 1.77E-15 | -2.648 |
| RHSIM_Rhsim08G0171800 | 66.63 | 3.27E-16 | 1.78E-15 | 0.491 |
| RHSIM_Rhsim10G0121000 | 66.63 | 3.27E-16 | 1.78E-15 | -1.033 |
| RHSIM_Rhsim13G0112600 | 66.63 | 3.28E-16 | 1.78E-15 | -0.782 |
| RHSIM_RhsimUnG0203000 | 66.61 | 3.32E-16 | 1.80E-15 | -2.206 |
| RHSIM_Rhsim07G0082900 | 66.60 | 3.32E-16 | 1.80E-15 | 1.196 |
| RHSIM_Rhsim13G0086300 | 66.59 | 3.35E-16 | 1.82E-15 | -0.906 |
| RHSIM_Rhsim10G0031300 | 66.56 | 3.40E-16 | 1.85E-15 | -1.228 |
| RHSIM_Rhsim10G0130500 | 66.54 | 3.43E-16 | 1.86E-15 | 1.122 |
| RHSIM_Rhsim09G0212100 | 66.52 | 3.47E-16 | 1.88E-15 | 3.089 |
| RHSIM_Rhsim06G0042100 | 66.51 | 3.47E-16 | 1.88E-15 | -1.264 |
| RHSIM_Rhsim05G0084700 | 66.50 | 3.50E-16 | 1.90E-15 | -0.609 |
| RHSIM_Rhsim09G0129800 | 66.50 | 3.50E-16 | 1.90E-15 | -0.811 |

| Gene | LRT statistic | P value | BH-adjusted P | log2FC (interaction) |
|---|---|---|---|---|
| RHSIM_Rhsim13G0173900 | 66.50 | 3.50E-16 | 1.90E-15 | 0.619 |
| RHSIM_Rhsim07G0033100 | 66.49 | 3.52E-16 | 1.91E-15 | 1.735 |
| RHSIM_Rhsim11G0002400 | 66.48 | 3.53E-16 | 1.91E-15 | 1.753 |
| RHSIM_Rhsim07G0002300 | 66.47 | 3.55E-16 | 1.93E-15 | -0.920 |
| RHSIM_Rhsim07G0160900 | 66.47 | 3.56E-16 | 1.93E-15 | 1.334 |
| RHSIM_Rhsim01G0197900 | 66.40 | 3.69E-16 | 2.00E-15 | 0.631 |
| RHSIM_Rhsim06G0156800 | 66.40 | 3.69E-16 | 2.00E-15 | -1.074 |
| RHSIM_Rhsim03G0262900 | 66.37 | 3.74E-16 | 2.02E-15 | -1.118 |
| novel.3783 | 66.33 | 3.82E-16 | 2.07E-15 | 1.013 |
| RHSIM_Rhsim12G0160800 | 66.30 | 3.87E-16 | 2.10E-15 | 4.703 |
| RHSIM_Rhsim03G0169600 | 66.30 | 3.88E-16 | 2.10E-15 | -1.081 |
| RHSIM_Rhsim11G0065800 | 66.27 | 3.93E-16 | 2.13E-15 | 0.859 |
| RHSIM_Rhsim05G0107700 | 66.27 | 3.94E-16 | 2.13E-15 | -0.700 |
| RHSIM_Rhsim10G0135600 | 66.26 | 3.95E-16 | 2.14E-15 | 3.509 |
| RHSIM_Rhsim07G0201100 | 66.23 | 4.01E-16 | 2.17E-15 | 1.867 |
| RHSIM_RhsimUnG0256600 | 66.18 | 4.11E-16 | 2.22E-15 | 2.850 |
| RHSIM_Rhsim13G0198700 | 66.16 | 4.16E-16 | 2.25E-15 | 0.596 |
| novel.278 | 66.14 | 4.20E-16 | 2.27E-15 | -0.928 |
| RHSIM_Rhsim06G0124100 | 66.14 | 4.20E-16 | 2.27E-15 | -0.691 |
| RHSIM_Rhsim01G0273600 | 66.13 | 4.23E-16 | 2.29E-15 | 0.729 |
| RHSIM_Rhsim06G0176400 | 66.12 | 4.25E-16 | 2.30E-15 | 0.961 |
| RHSIM_Rhsim06G0031000 | 66.11 | 4.26E-16 | 2.30E-15 | -0.817 |
| RHSIM_Rhsim08G0045400 | 66.10 | 4.29E-16 | 2.32E-15 | -0.744 |
| novel.2172 | 66.10 | 4.30E-16 | 2.32E-15 | 2.074 |
| RHSIM_Rhsim04G0187700 | 66.09 | 4.31E-16 | 2.33E-15 | -1.247 |
| RHSIM_Rhsim02G0187900 | 66.09 | 4.31E-16 | 2.33E-15 | 0.888 |
| RHSIM_Rhsim09G0069700 | 66.05 | 4.41E-16 | 2.38E-15 | -1.507 |
| RHSIM_Rhsim02G0081900 | 66.03 | 4.43E-16 | 2.39E-15 | 1.412 |
| RHSIM_RhsimUnG0085700 | 66.03 | 4.43E-16 | 2.39E-15 | -0.788 |
| RHSIM_Rhsim06G0077200 | 66.02 | 4.46E-16 | 2.41E-15 | 3.610 |
| RHSIM_RhsimUnG0163700 | 66.01 | 4.49E-16 | 2.42E-15 | -1.527 |
| RHSIM_Rhsim01G0002800 | 65.98 | 4.55E-16 | 2.46E-15 | -0.732 |

| Gene | LRT statistic | P value | BH-adjusted P | log2FC (interaction) |
|---|---|---|---|---|
| RHSIM_Rhsim07G0235100 | 65.97 | 4.58E-16 | 2.47E-15 | 0.748 |
| RHSIM_Rhsim04G0179600 | 65.95 | 4.62E-16 | 2.49E-15 | 1.915 |
| RHSIM_Rhsim07G0126900 | 65.95 | 4.63E-16 | 2.50E-15 | 2.021 |
| RHSIM_Rhsim01G0184500 | 65.94 | 4.64E-16 | 2.50E-15 | -0.543 |
| RHSIM_Rhsim13G0009500 | 65.90 | 4.75E-16 | 2.56E-15 | 1.858 |
| RHSIM_Rhsim06G0194400 | 65.89 | 4.76E-16 | 2.57E-15 | 1.438 |
| RHSIM_Rhsim09G0142300 | 65.80 | 4.98E-16 | 2.68E-15 | -2.266 |
| novel.1874 | 65.80 | 5.00E-16 | 2.69E-15 | -43.862 |
| RHSIM_Rhsim10G0032900 | 65.80 | 5.00E-16 | 2.69E-15 | 1.426 |
| RHSIM_Rhsim08G0224600 | 65.79 | 5.01E-16 | 2.70E-15 | -2.251 |
| novel.252 | 65.74 | 5.15E-16 | 2.77E-15 | 2.211 |
| RHSIM_Rhsim05G0141500 | 65.73 | 5.17E-16 | 2.78E-15 | 0.587 |
| RHSIM_Rhsim10G0140400 | 65.72 | 5.20E-16 | 2.80E-15 | 1.927 |
| RHSIM_Rhsim03G0158600 | 65.72 | 5.21E-16 | 2.80E-15 | 2.094 |
| RHSIM_Rhsim03G0069800 | 65.69 | 5.28E-16 | 2.84E-15 | -0.854 |
| RHSIM_Rhsim06G0017800 | 65.68 | 5.30E-16 | 2.85E-15 | -1.643 |
| RHSIM_Rhsim13G0115600 | 65.67 | 5.33E-16 | 2.87E-15 | 0.734 |
| RHSIM_Rhsim03G0257300 | 65.65 | 5.39E-16 | 2.90E-15 | -0.437 |
| RHSIM_Rhsim08G0223900 | 65.64 | 5.41E-16 | 2.91E-15 | -0.628 |
| RHSIM_Rhsim07G0163200 | 65.63 | 5.44E-16 | 2.92E-15 | 1.354 |
| RHSIM_RhsimUnG0125700 | 65.63 | 5.45E-16 | 2.93E-15 | 0.718 |
| RHSIM_Rhsim13G0056100 | 65.61 | 5.50E-16 | 2.95E-15 | 0.624 |
| RHSIM_Rhsim07G0115700 | 65.61 | 5.50E-16 | 2.95E-15 | 2.846 |
| RHSIM_Rhsim07G0017700 | 65.59 | 5.56E-16 | 2.99E-15 | -0.796 |
| novel.1254 | 65.53 | 5.72E-16 | 3.07E-15 | -1.451 |
| RHSIM_Rhsim03G0000100 | 65.51 | 5.79E-16 | 3.11E-15 | 0.680 |
| RHSIM_Rhsim10G0108800 | 65.51 | 5.79E-16 | 3.11E-15 | -1.307 |
| RHSIM_Rhsim12G0030900 | 65.50 | 5.80E-16 | 3.11E-15 | -1.842 |
| novel.3030 | 65.48 | 5.86E-16 | 3.15E-15 | -4.937 |
| RHSIM_Rhsim11G0132400 | 65.45 | 5.95E-16 | 3.19E-15 | 0.625 |
| RHSIM_Rhsim13G0088300 | 65.45 | 5.96E-16 | 3.20E-15 | -1.034 |
| novel.3663 | 65.43 | 6.01E-16 | 3.22E-15 | 1.167 |

| Gene | LRT statistic | P value | BH-adjusted P | log2FC (interaction) |
|---|---|---|---|---|
| RHSIM_Rhsim09G0166100 | 65.42 | 6.06E-16 | 3.25E-15 | 1.578 |
| RHSIM_Rhsim12G0118700 | 65.41 | 6.08E-16 | 3.26E-15 | -1.920 |
| RHSIM_Rhsim07G0030800 | 65.41 | 6.09E-16 | 3.26E-15 | -0.902 |
| RHSIM_Rhsim05G0123500 | 65.39 | 6.13E-16 | 3.29E-15 | 3.234 |
| RHSIM_Rhsim02G0203500 | 65.38 | 6.19E-16 | 3.32E-15 | 1.158 |
| RHSIM_Rhsim04G0106400 | 65.36 | 6.25E-16 | 3.35E-15 | 1.358 |
| RHSIM_Rhsim02G0172600 | 65.35 | 6.27E-16 | 3.36E-15 | 0.638 |
| RHSIM_Rhsim12G0137200 | 65.35 | 6.27E-16 | 3.36E-15 | 1.203 |
| RHSIM_Rhsim07G0067800 | 65.34 | 6.31E-16 | 3.38E-15 | 1.246 |
| RHSIM_Rhsim13G0154300 | 65.30 | 6.42E-16 | 3.44E-15 | 2.425 |
| RHSIM_Rhsim08G0159000 | 65.29 | 6.46E-16 | 3.45E-15 | 0.898 |
| RHSIM_Rhsim04G0084800 | 65.25 | 6.60E-16 | 3.53E-15 | 0.795 |
| RHSIM_Rhsim11G0018900 | 65.23 | 6.67E-16 | 3.57E-15 | 1.925 |
| novel.4416 | 65.23 | 6.67E-16 | 3.57E-15 | -3.714 |
| RHSIM_Rhsim07G0119200 | 65.21 | 6.72E-16 | 3.59E-15 | 1.012 |
| RHSIM_Rhsim03G0239100 | 65.21 | 6.72E-16 | 3.59E-15 | 0.611 |
| RHSIM_Rhsim12G0188700 | 65.18 | 6.82E-16 | 3.65E-15 | -0.516 |
| RHSIM_RhsimUnG0013500 | 65.13 | 7.02E-16 | 3.75E-15 | 1.509 |
| RHSIM_Rhsim13G0191500 | 65.08 | 7.19E-16 | 3.84E-15 | 0.651 |
| RHSIM_Rhsim01G0053500 | 65.08 | 7.19E-16 | 3.84E-15 | 1.670 |
| novel.2908 | 65.06 | 7.27E-16 | 3.88E-15 | -0.785 |
| RHSIM_Rhsim09G0139300 | 65.05 | 7.30E-16 | 3.89E-15 | 8.244 |
| RHSIM_Rhsim09G0004300 | 65.04 | 7.34E-16 | 3.92E-15 | -1.374 |
| RHSIM_Rhsim11G0069700 | 65.01 | 7.45E-16 | 3.98E-15 | 1.011 |
| RHSIM_Rhsim08G0244500 | 64.99 | 7.52E-16 | 4.01E-15 | -0.702 |
| RHSIM_Rhsim10G0056100 | 64.98 | 7.55E-16 | 4.03E-15 | -0.728 |
| RHSIM_Rhsim13G0105300 | 64.97 | 7.60E-16 | 4.06E-15 | 0.864 |
| RHSIM_Rhsim08G0079000 | 64.97 | 7.61E-16 | 4.06E-15 | 2.767 |
| RHSIM_Rhsim10G0113800 | 64.94 | 7.74E-16 | 4.13E-15 | 0.855 |
| RHSIM_Rhsim02G0179100 | 64.92 | 7.81E-16 | 4.16E-15 | 1.303 |
| RHSIM_Rhsim02G0130900 | 64.85 | 8.08E-16 | 4.31E-15 | -0.699 |
| RHSIM_Rhsim05G0041000 | 64.84 | 8.12E-16 | 4.33E-15 | -1.826 |

| Gene | LRT statistic | P value | BH-adjusted P | log2FC (interaction) |
|---|---|---|---|---|
| RHSIM_Rhsim11G0065900 | 64.83 | 8.17E-16 | 4.35E-15 | 0.448 |
| RHSIM_Rhsim07G0057200 | 64.82 | 8.19E-16 | 4.36E-15 | -0.859 |
| RHSIM_Rhsim10G0186800 | 64.81 | 8.24E-16 | 4.39E-15 | -0.537 |
| RHSIM_Rhsim10G0200500 | 64.80 | 8.30E-16 | 4.42E-15 | -3.720 |
| RHSIM_Rhsim11G0182600 | 64.77 | 8.40E-16 | 4.47E-15 | 1.674 |
| RHSIM_Rhsim03G0019000 | 64.77 | 8.41E-16 | 4.48E-15 | -0.667 |
| RHSIM_Rhsim06G0087400 | 64.77 | 8.42E-16 | 4.48E-15 | -0.728 |
| RHSIM_Rhsim01G0028100 | 64.69 | 8.75E-16 | 4.66E-15 | -1.057 |
| RHSIM_Rhsim02G0076800 | 64.68 | 8.83E-16 | 4.70E-15 | 0.857 |
| RHSIM_Rhsim13G0075000 | 64.64 | 8.98E-16 | 4.77E-15 | 0.690 |
| RHSIM_Rhsim04G0231300 | 64.59 | 9.23E-16 | 4.91E-15 | -2.024 |
| RHSIM_Rhsim02G0003800 | 64.58 | 9.26E-16 | 4.92E-15 | 1.137 |
| RHSIM_RhsimUnG0094800 | 64.58 | 9.27E-16 | 4.93E-15 | 2.316 |
| RHSIM_Rhsim12G0036500 | 64.53 | 9.48E-16 | 5.04E-15 | 5.708 |
| RHSIM_Rhsim05G0193000 | 64.52 | 9.55E-16 | 5.07E-15 | 0.633 |
| RHSIM_Rhsim01G0164700 | 64.50 | 9.65E-16 | 5.13E-15 | -0.546 |
| RHSIM_Rhsim08G0252100 | 64.45 | 9.88E-16 | 5.25E-15 | -0.862 |
| RHSIM_Rhsim06G0134000 | 64.45 | 9.88E-16 | 5.25E-15 | 2.060 |
| RHSIM_Rhsim13G0000800 | 64.42 | 1.00E-15 | 5.33E-15 | -1.425 |
| RHSIM_Rhsim13G0030400 | 64.42 | 1.00E-15 | 5.33E-15 | 0.874 |
| RHSIM_Rhsim07G0130000 | 64.41 | 1.01E-15 | 5.37E-15 | -0.773 |
| RHSIM_Rhsim07G0149800 | 64.40 | 1.01E-15 | 5.38E-15 | 1.378 |
| RHSIM_Rhsim02G0131500 | 64.38 | 1.02E-15 | 5.44E-15 | -0.795 |
| RHSIM_Rhsim05G0057200 | 64.36 | 1.04E-15 | 5.49E-15 | 1.784 |
| RHSIM_Rhsim03G0066400 | 64.33 | 1.05E-15 | 5.58E-15 | -3.306 |
| RHSIM_Rhsim06G0238600 | 64.31 | 1.06E-15 | 5.63E-15 | 1.088 |
| RHSIM_Rhsim10G0006400 | 64.29 | 1.08E-15 | 5.71E-15 | 0.629 |
| novel.1522 | 64.26 | 1.09E-15 | 5.78E-15 | -2.237 |
| RHSIM_Rhsim01G0247000 | 64.25 | 1.09E-15 | 5.79E-15 | 1.773 |
| RHSIM_Rhsim11G0043400 | 64.22 | 1.12E-15 | 5.91E-15 | -1.760 |
| RHSIM_Rhsim08G0232600 | 64.20 | 1.13E-15 | 5.96E-15 | 5.568 |
| RHSIM_Rhsim12G0008400 | 64.16 | 1.15E-15 | 6.08E-15 | -2.139 |

| Gene | LRT statistic | P value | BH-adjusted P | log2FC (interaction) |
|---|---|---|---|---|
| RHSIM_Rhsim04G0166700 | 64.15 | 1.15E-15 | 6.10E-15 | -0.944 |
| RHSIM_Rhsim08G0019400 | 64.14 | 1.16E-15 | 6.13E-15 | 1.162 |
| novel.515 | 64.11 | 1.18E-15 | 6.23E-15 | 3.542 |
| RHSIM_RhsimUnG0179000 | 64.10 | 1.18E-15 | 6.26E-15 | -4.147 |
| RHSIM_Rhsim07G0078200 | 64.09 | 1.19E-15 | 6.29E-15 | -0.742 |
| RHSIM_Rhsim11G0009100 | 64.07 | 1.20E-15 | 6.35E-15 | 1.203 |
| RHSIM_Rhsim06G0186500 | 64.05 | 1.21E-15 | 6.42E-15 | 0.573 |
| RHSIM_Rhsim03G0013600 | 64.04 | 1.22E-15 | 6.44E-15 | -2.305 |
| RHSIM_Rhsim13G0226600 | 63.99 | 1.25E-15 | 6.60E-15 | -3.825 |
| novel.3422 | 63.97 | 1.26E-15 | 6.68E-15 | -0.894 |
| RHSIM_Rhsim02G0251300 | 63.97 | 1.27E-15 | 6.69E-15 | -1.410 |
| RHSIM_Rhsim13G0039400 | 63.95 | 1.28E-15 | 6.74E-15 | -1.369 |
| RHSIM_Rhsim05G0170000 | 63.94 | 1.29E-15 | 6.79E-15 | 0.637 |
| RHSIM_RhsimUnG0249500 | 63.90 | 1.31E-15 | 6.93E-15 | -0.834 |
| RHSIM_Rhsim11G0174000 | 63.89 | 1.32E-15 | 6.96E-15 | -1.257 |
| RHSIM_Rhsim08G0241600 | 63.88 | 1.32E-15 | 6.97E-15 | -1.004 |
| RHSIM_Rhsim03G0256600 | 63.88 | 1.32E-15 | 6.97E-15 | -1.642 |
| RHSIM_Rhsim06G0243400 | 63.88 | 1.32E-15 | 6.99E-15 | -0.894 |
| RHSIM_Rhsim13G0083000 | 63.86 | 1.34E-15 | 7.05E-15 | 0.789 |
| RHSIM_Rhsim12G0144500 | 63.85 | 1.34E-15 | 7.09E-15 | 2.569 |
| RHSIM_Rhsim06G0153100 | 63.80 | 1.38E-15 | 7.27E-15 | 0.452 |
| RHSIM_Rhsim09G0132800 | 63.79 | 1.38E-15 | 7.29E-15 | -5.711 |
| novel.3196 | 63.76 | 1.40E-15 | 7.41E-15 | -2.081 |
| RHSIM_Rhsim01G0006400 | 63.76 | 1.41E-15 | 7.42E-15 | 0.997 |
| RHSIM_Rhsim06G0198800 | 63.74 | 1.42E-15 | 7.49E-15 | -1.046 |
| RHSIM_Rhsim08G0085100 | 63.72 | 1.43E-15 | 7.56E-15 | 0.683 |
| RHSIM_Rhsim03G0224500 | 63.71 | 1.44E-15 | 7.60E-15 | 2.936 |
| RHSIM_Rhsim07G0174400 | 63.71 | 1.44E-15 | 7.61E-15 | -1.189 |
| RHSIM_Rhsim06G0008600 | 63.66 | 1.48E-15 | 7.80E-15 | -0.597 |
| RHSIM_Rhsim04G0173000 | 63.63 | 1.50E-15 | 7.89E-15 | -1.053 |
| novel.4236 | 63.63 | 1.50E-15 | 7.92E-15 | -1.035 |
| RHSIM_Rhsim08G0177600 | 63.62 | 1.51E-15 | 7.95E-15 | 0.768 |

| Gene | LRT statistic | P value | BH-adjusted P | log2FC (interaction) |
|---|---|---|---|---|
| RHSIM_RhsimUnG0215900 | 63.57 | 1.55E-15 | 8.16E-15 | 0.585 |
| RHSIM_Rhsim06G0027200 | 63.55 | 1.56E-15 | 8.23E-15 | -0.890 |
| RHSIM_Rhsim08G0207400 | 63.55 | 1.57E-15 | 8.25E-15 | -0.447 |
| RHSIM_Rhsim05G0164700 | 63.51 | 1.59E-15 | 8.39E-15 | 0.915 |
| RHSIM_Rhsim07G0235800 | 63.48 | 1.62E-15 | 8.50E-15 | -0.782 |
| RHSIM_Rhsim03G0215600 | 63.47 | 1.63E-15 | 8.57E-15 | -1.288 |
| RHSIM_Rhsim11G0169300 | 63.44 | 1.65E-15 | 8.69E-15 | -1.205 |
| RHSIM_Rhsim03G0239200 | 63.44 | 1.66E-15 | 8.71E-15 | -2.040 |
| RHSIM_Rhsim03G0014500 | 63.42 | 1.67E-15 | 8.77E-15 | -2.267 |
| RHSIM_Rhsim12G0202400 | 63.42 | 1.67E-15 | 8.77E-15 | -2.434 |
| RHSIM_Rhsim01G0254000 | 63.40 | 1.69E-15 | 8.87E-15 | -0.741 |
| RHSIM_Rhsim13G0071000 | 63.38 | 1.70E-15 | 8.95E-15 | -1.461 |
| RHSIM_Rhsim06G0122400 | 63.34 | 1.74E-15 | 9.16E-15 | -0.669 |
| RHSIM_Rhsim09G0176800 | 63.33 | 1.75E-15 | 9.20E-15 | 0.936 |
| RHSIM_Rhsim08G0098800 | 63.28 | 1.80E-15 | 9.44E-15 | -0.757 |
| RHSIM_Rhsim07G0101900 | 63.27 | 1.80E-15 | 9.47E-15 | -2.011 |
| RHSIM_RhsimUnG0206900 | 63.25 | 1.82E-15 | 9.54E-15 | -3.442 |
| novel.550 | 63.24 | 1.83E-15 | 9.62E-15 | 1.719 |
| RHSIM_Rhsim08G0203100 | 63.23 | 1.84E-15 | 9.64E-15 | -2.923 |
| RHSIM_Rhsim13G0114000 | 63.15 | 1.91E-15 | 1.00E-14 | -0.742 |
| RHSIM_Rhsim11G0171000 | 63.13 | 1.93E-15 | 1.01E-14 | 0.435 |
| RHSIM_Rhsim01G0293800 | 63.11 | 1.96E-15 | 1.03E-14 | 0.907 |
| RHSIM_Rhsim01G0233700 | 63.06 | 2.01E-15 | 1.05E-14 | -1.454 |
| RHSIM_Rhsim03G0162500 | 63.04 | 2.02E-15 | 1.06E-14 | -0.587 |
| RHSIM_Rhsim12G0070300 | 63.04 | 2.03E-15 | 1.06E-14 | -2.789 |
| RHSIM_Rhsim10G0209800 | 63.03 | 2.04E-15 | 1.07E-14 | -0.624 |
| RHSIM_RhsimUnG0238200 | 63.02 | 2.05E-15 | 1.07E-14 | 1.100 |
| novel.908 | 63.01 | 2.06E-15 | 1.08E-14 | 1.483 |
| RHSIM_Rhsim12G0024400 | 63.01 | 2.06E-15 | 1.08E-14 | -1.757 |
| RHSIM_Rhsim03G0089800 | 63.01 | 2.06E-15 | 1.08E-14 | 1.028 |
| RHSIM_Rhsim07G0205400 | 63.01 | 2.06E-15 | 1.08E-14 | -0.702 |
| RHSIM_Rhsim04G0115600 | 63.00 | 2.06E-15 | 1.08E-14 | -2.755 |

| Gene | LRT statistic | P value | BH-adjusted P | log2FC (interaction) |
|---|---|---|---|---|
| RHSIM_Rhsim07G0040300 | 63.00 | 2.07E-15 | 1.08E-14 | -2.218 |
| RHSIM_Rhsim06G0000600 | 63.00 | 2.07E-15 | 1.08E-14 | 0.821 |
| RHSIM_Rhsim09G0179300 | 62.99 | 2.07E-15 | 1.09E-14 | 0.750 |
| RHSIM_Rhsim08G0057600 | 62.97 | 2.09E-15 | 1.10E-14 | -3.860 |
| RHSIM_Rhsim13G0066900 | 62.97 | 2.10E-15 | 1.10E-14 | 4.091 |
| RHSIM_Rhsim05G0096200 | 62.93 | 2.14E-15 | 1.12E-14 | 0.527 |
| novel.258 | 62.89 | 2.19E-15 | 1.14E-14 | 5.845 |
| RHSIM_Rhsim04G0003900 | 62.85 | 2.23E-15 | 1.16E-14 | 0.890 |
| RHSIM_Rhsim11G0114600 | 62.83 | 2.25E-15 | 1.18E-14 | -1.381 |
| RHSIM_Rhsim07G0102700 | 62.83 | 2.26E-15 | 1.18E-14 | 0.649 |
| RHSIM_Rhsim12G0127500 | 62.81 | 2.28E-15 | 1.19E-14 | -1.080 |
| RHSIM_Rhsim13G0010200 | 62.78 | 2.32E-15 | 1.21E-14 | 0.649 |
| RHSIM_Rhsim03G0000200 | 62.77 | 2.32E-15 | 1.21E-14 | -0.677 |
| RHSIM_RhsimUnG0239300 | 62.71 | 2.40E-15 | 1.25E-14 | -3.564 |
| RHSIM_Rhsim07G0199500 | 62.70 | 2.41E-15 | 1.26E-14 | -1.993 |
| RHSIM_Rhsim08G0044200 | 62.69 | 2.42E-15 | 1.27E-14 | -1.319 |
| RHSIM_Rhsim07G0033300 | 62.69 | 2.43E-15 | 1.27E-14 | 0.922 |
| RHSIM_Rhsim09G0188700 | 62.66 | 2.45E-15 | 1.28E-14 | 1.644 |
| RHSIM_Rhsim10G0063000 | 62.64 | 2.48E-15 | 1.29E-14 | -0.704 |
| RHSIM_Rhsim12G0110600 | 62.62 | 2.51E-15 | 1.31E-14 | -0.640 |
| RHSIM_Rhsim08G0199300 | 62.59 | 2.54E-15 | 1.33E-14 | 0.444 |
| RHSIM_Rhsim08G0201300 | 62.59 | 2.54E-15 | 1.33E-14 | -1.294 |
| RHSIM_Rhsim06G0146200 | 62.58 | 2.56E-15 | 1.34E-14 | 2.206 |
| RHSIM_Rhsim13G0165800 | 62.47 | 2.71E-15 | 1.41E-14 | 2.587 |
| RHSIM_Rhsim12G0158700 | 62.47 | 2.71E-15 | 1.41E-14 | 0.903 |
| RHSIM_Rhsim07G0224100 | 62.43 | 2.76E-15 | 1.44E-14 | 0.805 |
| RHSIM_Rhsim12G0035800 | 62.42 | 2.78E-15 | 1.45E-14 | -0.605 |
| RHSIM_Rhsim06G0004200 | 62.36 | 2.85E-15 | 1.49E-14 | -1.406 |
| RHSIM_Rhsim03G0139300 | 62.36 | 2.86E-15 | 1.49E-14 | 5.507 |
| RHSIM_Rhsim02G0042800 | 62.36 | 2.86E-15 | 1.49E-14 | -0.682 |
| RHSIM_Rhsim01G0044600 | 62.36 | 2.86E-15 | 1.49E-14 | 2.653 |
| RHSIM_Rhsim06G0197200 | 62.30 | 2.94E-15 | 1.53E-14 | 0.886 |

| Gene | LRT statistic | P value | BH-adjusted P | log2FC (interaction) |
|---|---|---|---|---|
| RHSIM_Rhsim04G0197900 | 62.29 | 2.96E-15 | 1.54E-14 | -3.677 |
| novel.4384 | 62.28 | 2.97E-15 | 1.55E-14 | -43.537 |
| RHSIM_RhsimUnG0182800 | 62.26 | 3.01E-15 | 1.57E-14 | -3.380 |
| RHSIM_Rhsim02G0059600 | 62.26 | 3.01E-15 | 1.57E-14 | -0.696 |
| RHSIM_Rhsim10G0065100 | 62.25 | 3.02E-15 | 1.57E-14 | -0.854 |
| RHSIM_Rhsim10G0078400 | 62.23 | 3.06E-15 | 1.59E-14 | -1.723 |
| RHSIM_Rhsim06G0142700 | 62.21 | 3.08E-15 | 1.60E-14 | -0.690 |
| RHSIM_Rhsim01G0270100 | 62.21 | 3.08E-15 | 1.60E-14 | -1.124 |
| RHSIM_Rhsim07G0131100 | 62.19 | 3.11E-15 | 1.62E-14 | -1.976 |
| RHSIM_Rhsim03G0049400 | 62.19 | 3.12E-15 | 1.62E-14 | -2.198 |
| RHSIM_Rhsim06G0225100 | 62.15 | 3.19E-15 | 1.65E-14 | 1.831 |
| RHSIM_Rhsim13G0024900 | 62.12 | 3.23E-15 | 1.68E-14 | 0.907 |
| novel.1369 | 62.12 | 3.24E-15 | 1.68E-14 | -0.873 |
| RHSIM_Rhsim08G0029400 | 62.08 | 3.29E-15 | 1.71E-14 | 1.909 |
| RHSIM_Rhsim01G0251000 | 62.08 | 3.30E-15 | 1.72E-14 | 1.242 |
| RHSIM_Rhsim06G0186000 | 62.06 | 3.33E-15 | 1.73E-14 | -4.173 |
| RHSIM_Rhsim11G0094400 | 62.05 | 3.35E-15 | 1.74E-14 | -0.523 |
| RHSIM_Rhsim03G0145600 | 62.05 | 3.35E-15 | 1.74E-14 | -1.116 |
| RHSIM_Rhsim13G0094000 | 62.04 | 3.36E-15 | 1.74E-14 | -1.295 |
| RHSIM_Rhsim01G0248700 | 62.04 | 3.37E-15 | 1.75E-14 | -1.965 |
| novel.4071 | 62.01 | 3.42E-15 | 1.77E-14 | 0.797 |
| RHSIM_Rhsim01G0212100 | 62.00 | 3.43E-15 | 1.78E-14 | -0.571 |
| novel.2209 | 62.00 | 3.44E-15 | 1.78E-14 | 1.361 |
| RHSIM_Rhsim09G0025000 | 61.97 | 3.49E-15 | 1.81E-14 | -1.806 |
| RHSIM_Rhsim11G0091800 | 61.95 | 3.52E-15 | 1.83E-14 | -1.388 |
| RHSIM_Rhsim07G0226200 | 61.93 | 3.55E-15 | 1.84E-14 | -0.627 |
| RHSIM_Rhsim06G0069400 | 61.92 | 3.58E-15 | 1.85E-14 | -0.998 |
| RHSIM_Rhsim03G0226100 | 61.91 | 3.59E-15 | 1.86E-14 | -1.910 |
| RHSIM_Rhsim08G0108900 | 61.91 | 3.60E-15 | 1.86E-14 | 0.773 |
| RHSIM_Rhsim04G0167800 | 61.90 | 3.62E-15 | 1.88E-14 | 1.264 |
| RHSIM_Rhsim01G0215400 | 61.89 | 3.63E-15 | 1.88E-14 | 0.914 |
| RHSIM_Rhsim11G0134700 | 61.88 | 3.65E-15 | 1.89E-14 | -0.520 |

| Gene | LRT statistic | P value | BH-adjusted P | log2FC (interaction) |
|---|---|---|---|---|
| RHSIM_Rhsim10G0052200 | 61.87 | 3.67E-15 | 1.90E-14 | -1.249 |
| RHSIM_Rhsim10G0203600 | 61.86 | 3.68E-15 | 1.90E-14 | 2.548 |
| RHSIM_Rhsim01G0068200 | 61.85 | 3.71E-15 | 1.92E-14 | -1.873 |
| RHSIM_Rhsim02G0044500 | 61.84 | 3.72E-15 | 1.92E-14 | -0.855 |
| RHSIM_Rhsim07G0016400 | 61.82 | 3.77E-15 | 1.95E-14 | 1.196 |
| RHSIM_Rhsim04G0213800 | 61.79 | 3.82E-15 | 1.98E-14 | 0.647 |
| novel.3640 | 61.78 | 3.83E-15 | 1.98E-14 | -2.896 |
| RHSIM_Rhsim02G0132500 | 61.71 | 3.98E-15 | 2.06E-14 | -2.026 |
| RHSIM_Rhsim04G0112600 | 61.71 | 3.99E-15 | 2.06E-14 | 1.306 |
| RHSIM_Rhsim03G0129400 | 61.69 | 4.03E-15 | 2.08E-14 | 0.741 |
| RHSIM_Rhsim11G0111100 | 61.68 | 4.04E-15 | 2.08E-14 | 0.870 |
| RHSIM_Rhsim12G0108100 | 61.66 | 4.08E-15 | 2.11E-14 | -0.571 |
| RHSIM_Rhsim06G0086200 | 61.65 | 4.10E-15 | 2.12E-14 | -0.440 |
| RHSIM_Rhsim11G0076200 | 61.62 | 4.17E-15 | 2.15E-14 | -2.634 |
| RHSIM_Rhsim04G0110600 | 61.58 | 4.24E-15 | 2.19E-14 | 3.086 |
| RHSIM_Rhsim01G0220200 | 61.58 | 4.25E-15 | 2.19E-14 | -0.940 |
| RHSIM_Rhsim08G0214400 | 61.57 | 4.27E-15 | 2.21E-14 | -0.424 |
| RHSIM_Rhsim11G0012900 | 61.56 | 4.29E-15 | 2.21E-14 | -1.274 |
| RHSIM_Rhsim13G0110800 | 61.55 | 4.31E-15 | 2.22E-14 | -0.597 |
| RHSIM_Rhsim03G0001600 | 61.50 | 4.42E-15 | 2.28E-14 | -0.683 |
| novel.1052 | 61.48 | 4.47E-15 | 2.31E-14 | -1.822 |
| RHSIM_Rhsim03G0069400 | 61.47 | 4.50E-15 | 2.32E-14 | -1.750 |
| RHSIM_Rhsim11G0150000 | 61.45 | 4.55E-15 | 2.34E-14 | -0.604 |
| RHSIM_Rhsim01G0140200 | 61.44 | 4.57E-15 | 2.35E-14 | -1.731 |
| novel.559 | 61.41 | 4.64E-15 | 2.39E-14 | -0.487 |
| RHSIM_Rhsim09G0190000 | 61.40 | 4.66E-15 | 2.40E-14 | -1.312 |
| RHSIM_Rhsim07G0018200 | 61.40 | 4.67E-15 | 2.40E-14 | 0.671 |
| RHSIM_Rhsim01G0135600 | 61.39 | 4.68E-15 | 2.41E-14 | 0.623 |
| RHSIM_Rhsim07G0123700 | 61.37 | 4.73E-15 | 2.44E-14 | -0.829 |
| RHSIM_Rhsim01G0180600 | 61.37 | 4.73E-15 | 2.44E-14 | 1.440 |
| RHSIM_Rhsim07G0110700 | 61.34 | 4.79E-15 | 2.47E-14 | -0.637 |
| RHSIM_Rhsim07G0242500 | 61.34 | 4.80E-15 | 2.47E-14 | -4.024 |

| Gene | LRT statistic | P value | BH-adjusted P | log2FC (interaction) |
|---|---|---|---|---|
| RHSIM_Rhsim05G0141100 | 61.33 | 4.81E-15 | 2.48E-14 | 2.105 |
| RHSIM_Rhsim06G0241000 | 61.33 | 4.81E-15 | 2.48E-14 | -0.790 |
| RHSIM_Rhsim07G0148900 | 61.29 | 4.93E-15 | 2.53E-14 | 0.873 |
| RHSIM_Rhsim08G0182300 | 61.27 | 4.98E-15 | 2.56E-14 | 1.260 |
| RHSIM_Rhsim03G0155800 | 61.22 | 5.10E-15 | 2.62E-14 | 2.408 |
| RHSIM_Rhsim04G0101900 | 61.21 | 5.13E-15 | 2.64E-14 | -0.672 |
| RHSIM_Rhsim06G0073400 | 61.12 | 5.38E-15 | 2.76E-14 | -0.685 |
| RHSIM_Rhsim03G0240000 | 61.10 | 5.43E-15 | 2.79E-14 | 0.774 |
| RHSIM_Rhsim04G0177000 | 61.10 | 5.44E-15 | 2.79E-14 | 1.391 |
| RHSIM_Rhsim13G0031000 | 61.09 | 5.46E-15 | 2.80E-14 | 0.672 |
| RHSIM_RhsimUnG0140000 | 61.09 | 5.46E-15 | 2.81E-14 | 1.176 |
| RHSIM_Rhsim02G0007900 | 61.06 | 5.55E-15 | 2.85E-14 | 0.768 |
| RHSIM_Rhsim01G0129200 | 61.05 | 5.57E-15 | 2.86E-14 | -0.724 |
| RHSIM_Rhsim10G0040300 | 61.04 | 5.61E-15 | 2.88E-14 | 0.740 |
| RHSIM_Rhsim10G0068000 | 60.99 | 5.75E-15 | 2.95E-14 | 1.013 |
| RHSIM_Rhsim07G0003100 | 60.97 | 5.80E-15 | 2.98E-14 | -0.600 |
| RHSIM_Rhsim08G0199900 | 60.97 | 5.80E-15 | 2.98E-14 | -0.816 |
| RHSIM_Rhsim06G0084700 | 60.97 | 5.81E-15 | 2.98E-14 | -0.546 |
| RHSIM_Rhsim03G0155100 | 60.96 | 5.82E-15 | 2.98E-14 | 1.125 |
| RHSIM_Rhsim02G0184300 | 60.92 | 5.95E-15 | 3.05E-14 | -4.829 |
| RHSIM_Rhsim12G0153400 | 60.91 | 5.98E-15 | 3.06E-14 | 0.462 |
| RHSIM_Rhsim04G0145800 | 60.87 | 6.11E-15 | 3.13E-14 | -0.618 |
| RHSIM_Rhsim08G0177800 | 60.86 | 6.13E-15 | 3.14E-14 | 0.540 |
| RHSIM_Rhsim03G0259900 | 60.83 | 6.23E-15 | 3.19E-14 | -4.609 |
| RHSIM_Rhsim03G0262700 | 60.82 | 6.26E-15 | 3.21E-14 | -1.321 |
| RHSIM_Rhsim03G0131700 | 60.81 | 6.29E-15 | 3.22E-14 | 1.402 |
| RHSIM_Rhsim08G0172200 | 60.78 | 6.40E-15 | 3.28E-14 | 1.021 |
| RHSIM_Rhsim09G0125700 | 60.77 | 6.42E-15 | 3.29E-14 | 3.586 |
| RHSIM_Rhsim01G0255300 | 60.63 | 6.88E-15 | 3.52E-14 | 1.175 |
| RHSIM_Rhsim01G0066700 | 60.62 | 6.91E-15 | 3.53E-14 | 1.297 |
| RHSIM_Rhsim11G0071500 | 60.60 | 6.98E-15 | 3.57E-14 | 1.185 |
| RHSIM_Rhsim08G0184500 | 60.59 | 7.02E-15 | 3.59E-14 | 3.363 |

| Gene | LRT statistic | P value | BH-adjusted P | log2FC (interaction) |
|---|---|---|---|---|
| RHSIM_Rhsim01G0100100 | 60.58 | 7.05E-15 | 3.61E-14 | 2.751 |
| novel.800 | 60.58 | 7.07E-15 | 3.62E-14 | 1.683 |
| RHSIM_Rhsim10G0049300 | 60.56 | 7.14E-15 | 3.65E-14 | -1.098 |
| RHSIM_Rhsim05G0003000 | 60.55 | 7.18E-15 | 3.67E-14 | 2.276 |
| RHSIM_Rhsim08G0214300 | 60.55 | 7.19E-15 | 3.68E-14 | -0.473 |
| RHSIM_Rhsim13G0038400 | 60.52 | 7.28E-15 | 3.72E-14 | 0.777 |
| RHSIM_Rhsim07G0092700 | 60.50 | 7.36E-15 | 3.76E-14 | 0.932 |
| RHSIM_Rhsim02G0241700 | 60.48 | 7.45E-15 | 3.80E-14 | 0.842 |
| RHSIM_Rhsim08G0005100 | 60.46 | 7.52E-15 | 3.84E-14 | -1.065 |
| RHSIM_Rhsim02G0014600 | 60.45 | 7.53E-15 | 3.84E-14 | -1.015 |
| RHSIM_Rhsim12G0161900 | 60.40 | 7.74E-15 | 3.95E-14 | -2.782 |
| RHSIM_Rhsim05G0125900 | 60.39 | 7.79E-15 | 3.98E-14 | 1.450 |
| RHSIM_Rhsim08G0240400 | 60.36 | 7.92E-15 | 4.04E-14 | -0.671 |
| novel.3574 | 60.34 | 7.98E-15 | 4.07E-14 | 2.801 |
| RHSIM_Rhsim01G0090500 | 60.29 | 8.20E-15 | 4.18E-14 | 0.793 |
| RHSIM_Rhsim12G0081600 | 60.26 | 8.31E-15 | 4.24E-14 | -0.776 |
| RHSIM_Rhsim07G0073900 | 60.24 | 8.38E-15 | 4.27E-14 | 2.217 |
| novel.386 | 60.23 | 8.46E-15 | 4.31E-14 | -1.105 |
| RHSIM_Rhsim13G0205900 | 60.21 | 8.51E-15 | 4.34E-14 | -0.950 |
| RHSIM_Rhsim13G0161600 | 60.21 | 8.54E-15 | 4.35E-14 | 1.807 |
| RHSIM_Rhsim01G0210100 | 60.19 | 8.60E-15 | 4.38E-14 | 3.143 |
| RHSIM_Rhsim06G0058200 | 60.19 | 8.60E-15 | 4.38E-14 | 1.653 |
| novel.4459 | 60.17 | 8.72E-15 | 4.44E-14 | -2.002 |
| RHSIM_Rhsim12G0192900 | 60.16 | 8.76E-15 | 4.46E-14 | 0.677 |
| novel.329 | 60.14 | 8.83E-15 | 4.50E-14 | -0.734 |
| novel.1579 | 60.12 | 8.91E-15 | 4.54E-14 | 3.726 |
| RHSIM_Rhsim01G0210500 | 60.12 | 8.94E-15 | 4.55E-14 | -1.412 |
| RHSIM_Rhsim02G0090600 | 60.11 | 8.95E-15 | 4.56E-14 | -1.900 |
| RHSIM_Rhsim03G0161000 | 60.11 | 8.99E-15 | 4.58E-14 | -0.709 |
| RHSIM_Rhsim03G0000600 | 60.09 | 9.05E-15 | 4.60E-14 | -0.644 |
| RHSIM_Rhsim06G0089000 | 60.07 | 9.16E-15 | 4.66E-14 | -0.923 |
| RHSIM_Rhsim05G0163300 | 60.04 | 9.30E-15 | 4.73E-14 | 3.599 |

| Gene | LRT statistic | P value | BH-adjusted P | log2FC (interaction) |
|---|---|---|---|---|
| RHSIM_RhsimUnG0184200 | 60.03 | 9.35E-15 | 4.75E-14 | -1.341 |
| RHSIM_Rhsim13G0169000 | 60.00 | 9.49E-15 | 4.82E-14 | 0.807 |
| RHSIM_Rhsim08G0052800 | 60.00 | 9.49E-15 | 4.83E-14 | 0.437 |
| RHSIM_Rhsim04G0070000 | 59.97 | 9.65E-15 | 4.90E-14 | -1.838 |
| RHSIM_Rhsim09G0203600 | 59.96 | 9.69E-15 | 4.93E-14 | -1.064 |
| novel.1732 | 59.94 | 9.76E-15 | 4.96E-14 | 0.713 |
| RHSIM_Rhsim10G0148700 | 59.94 | 9.76E-15 | 4.96E-14 | 1.697 |
| RHSIM_Rhsim09G0078300 | 59.94 | 9.76E-15 | 4.96E-14 | 0.719 |
| novel.1310 | 59.94 | 9.79E-15 | 4.97E-14 | 0.504 |
| RHSIM_Rhsim11G0014400 | 59.93 | 9.80E-15 | 4.98E-14 | -0.613 |
| RHSIM_Rhsim08G0196000 | 59.90 | 9.96E-15 | 5.06E-14 | -0.349 |
| RHSIM_Rhsim03G0004000 | 59.90 | 9.96E-15 | 5.06E-14 | -2.894 |
| RHSIM_Rhsim06G0059300 | 59.90 | 9.98E-15 | 5.07E-14 | -2.036 |
| RHSIM_Rhsim05G0018100 | 59.89 | 1.00E-14 | 5.08E-14 | -2.690 |
| RHSIM_Rhsim05G0169000 | 59.87 | 1.01E-14 | 5.13E-14 | 1.165 |
| RHSIM_Rhsim02G0182300 | 59.86 | 1.02E-14 | 5.17E-14 | 1.156 |
| RHSIM_Rhsim01G0245100 | 59.85 | 1.02E-14 | 5.19E-14 | -0.883 |
| RHSIM_Rhsim02G0035300 | 59.83 | 1.04E-14 | 5.25E-14 | -1.203 |
| RHSIM_RhsimUnG0108500 | 59.82 | 1.04E-14 | 5.26E-14 | -1.833 |
| RHSIM_Rhsim01G0187100 | 59.81 | 1.04E-14 | 5.29E-14 | -0.480 |
| RHSIM_Rhsim04G0100100 | 59.81 | 1.04E-14 | 5.29E-14 | -0.874 |
| RHSIM_Rhsim06G0141900 | 59.79 | 1.06E-14 | 5.36E-14 | -1.047 |
| RHSIM_Rhsim05G0179700 | 59.77 | 1.07E-14 | 5.41E-14 | -0.511 |
| RHSIM_Rhsim03G0221400 | 59.76 | 1.07E-14 | 5.43E-14 | -2.246 |
| RHSIM_Rhsim03G0065100 | 59.72 | 1.09E-14 | 5.53E-14 | 1.179 |
| RHSIM_Rhsim08G0170400 | 59.72 | 1.10E-14 | 5.55E-14 | -0.430 |
| RHSIM_Rhsim12G0017000 | 59.69 | 1.11E-14 | 5.63E-14 | 1.203 |
| RHSIM_Rhsim10G0160200 | 59.66 | 1.12E-14 | 5.69E-14 | -1.665 |
| RHSIM_Rhsim01G0003000 | 59.65 | 1.14E-14 | 5.75E-14 | 0.586 |
| RHSIM_Rhsim11G0134400 | 59.63 | 1.14E-14 | 5.78E-14 | 1.644 |
| RHSIM_Rhsim10G0020800 | 59.63 | 1.14E-14 | 5.79E-14 | 0.600 |
| RHSIM_Rhsim01G0119500 | 59.63 | 1.15E-14 | 5.80E-14 | -1.261 |

| Gene | LRT statistic | P value | BH-adjusted P | log2FC (interaction) |
|---|---|---|---|---|
| RHSIM_Rhsim05G0211100 | 59.63 | 1.15E-14 | 5.80E-14 | -0.724 |
| RHSIM_Rhsim04G0221300 | 59.61 | 1.15E-14 | 5.84E-14 | -1.959 |
| novel.4286 | 59.61 | 1.16E-14 | 5.85E-14 | 2.948 |
| RHSIM_Rhsim01G0049100 | 59.61 | 1.16E-14 | 5.85E-14 | -0.792 |
| RHSIM_Rhsim08G0142400 | 59.60 | 1.16E-14 | 5.87E-14 | -1.006 |
| RHSIM_Rhsim02G0243100 | 59.60 | 1.16E-14 | 5.87E-14 | -2.831 |
| RHSIM_Rhsim05G0169300 | 59.59 | 1.17E-14 | 5.89E-14 | 0.677 |
| RHSIM_Rhsim07G0101100 | 59.59 | 1.17E-14 | 5.91E-14 | -1.210 |
| RHSIM_Rhsim09G0087800 | 59.56 | 1.18E-14 | 5.98E-14 | 0.820 |
| novel.1166 | 59.54 | 1.20E-14 | 6.05E-14 | -3.393 |
| RHSIM_Rhsim04G0198800 | 59.53 | 1.21E-14 | 6.09E-14 | -2.407 |
| novel.3248 | 59.52 | 1.21E-14 | 6.10E-14 | 3.444 |
| RHSIM_Rhsim03G0261500 | 59.52 | 1.21E-14 | 6.11E-14 | 0.873 |
| RHSIM_Rhsim03G0211700 | 59.51 | 1.22E-14 | 6.13E-14 | -1.494 |
| RHSIM_Rhsim12G0062900 | 59.46 | 1.25E-14 | 6.28E-14 | 6.773 |
| RHSIM_Rhsim08G0232400 | 59.42 | 1.28E-14 | 6.44E-14 | -0.671 |
| RHSIM_Rhsim05G0176300 | 59.40 | 1.29E-14 | 6.50E-14 | 3.838 |
| RHSIM_Rhsim11G0178400 | 59.39 | 1.30E-14 | 6.54E-14 | -3.619 |
| RHSIM_Rhsim04G0196000 | 59.36 | 1.31E-14 | 6.62E-14 | -0.866 |
| RHSIM_Rhsim07G0157700 | 59.35 | 1.32E-14 | 6.64E-14 | 0.973 |
| RHSIM_RhsimUnG0255900 | 59.34 | 1.32E-14 | 6.68E-14 | 1.238 |
| RHSIM_Rhsim08G0114100 | 59.31 | 1.35E-14 | 6.79E-14 | -0.793 |
| RHSIM_Rhsim01G0179100 | 59.30 | 1.35E-14 | 6.81E-14 | -0.576 |
| RHSIM_Rhsim07G0093600 | 59.28 | 1.36E-14 | 6.87E-14 | 0.563 |
| RHSIM_Rhsim09G0173400 | 59.28 | 1.37E-14 | 6.88E-14 | -0.881 |
| RHSIM_Rhsim05G0133100 | 59.26 | 1.38E-14 | 6.97E-14 | 0.919 |
| RHSIM_Rhsim11G0124200 | 59.25 | 1.39E-14 | 6.98E-14 | -1.150 |
| RHSIM_RhsimUnG0119100 | 59.24 | 1.40E-14 | 7.02E-14 | 0.995 |
| RHSIM_Rhsim02G0252500 | 59.24 | 1.40E-14 | 7.03E-14 | -0.548 |
| RHSIM_Rhsim09G0000200 | 59.23 | 1.40E-14 | 7.05E-14 | -0.647 |
| novel.3770 | 59.23 | 1.41E-14 | 7.07E-14 | 0.773 |
| RHSIM_Rhsim08G0250700 | 59.22 | 1.41E-14 | 7.08E-14 | 0.550 |

| Gene | LRT statistic | P value | BH-adjusted P | log2FC (interaction) |
|---|---|---|---|---|
| RHSIM_Rhsim02G0249900 | 59.22 | 1.41E-14 | 7.09E-14 | -2.133 |
| RHSIM_Rhsim09G0104400 | 59.20 | 1.43E-14 | 7.18E-14 | 1.634 |
| RHSIM_Rhsim06G0166400 | 59.18 | 1.44E-14 | 7.22E-14 | -1.563 |
| RHSIM_Rhsim04G0166200 | 59.18 | 1.44E-14 | 7.24E-14 | 1.839 |
| RHSIM_Rhsim05G0202300 | 59.17 | 1.45E-14 | 7.26E-14 | -4.503 |
| RHSIM_Rhsim08G0003000 | 59.15 | 1.46E-14 | 7.32E-14 | -0.972 |
| RHSIM_Rhsim12G0025400 | 59.14 | 1.47E-14 | 7.39E-14 | 1.691 |
| RHSIM_Rhsim06G0089600 | 59.12 | 1.48E-14 | 7.45E-14 | 0.718 |
| RHSIM_Rhsim09G0126200 | 59.10 | 1.50E-14 | 7.52E-14 | -0.600 |
| RHSIM_Rhsim01G0120500 | 59.09 | 1.50E-14 | 7.55E-14 | -0.968 |
| RHSIM_Rhsim04G0216000 | 59.08 | 1.52E-14 | 7.61E-14 | -0.802 |
| RHSIM_Rhsim01G0009800 | 59.08 | 1.52E-14 | 7.62E-14 | -0.813 |
| novel.3815 | 59.07 | 1.52E-14 | 7.64E-14 | 0.801 |
| RHSIM_Rhsim11G0026000 | 59.05 | 1.54E-14 | 7.72E-14 | -0.821 |
| RHSIM_Rhsim08G0219700 | 59.04 | 1.54E-14 | 7.75E-14 | -0.746 |
| RHSIM_Rhsim09G0128200 | 59.03 | 1.55E-14 | 7.77E-14 | -3.572 |
| RHSIM_Rhsim05G0133600 | 58.99 | 1.58E-14 | 7.93E-14 | -0.753 |
| RHSIM_Rhsim10G0207900 | 58.99 | 1.58E-14 | 7.94E-14 | -0.987 |
| RHSIM_Rhsim13G0232100 | 58.98 | 1.59E-14 | 7.99E-14 | 0.436 |
| RHSIM_Rhsim09G0079500 | 58.97 | 1.60E-14 | 8.04E-14 | 0.636 |
| RHSIM_Rhsim10G0079400 | 58.97 | 1.60E-14 | 8.04E-14 | 0.731 |
| RHSIM_Rhsim13G0100900 | 58.96 | 1.61E-14 | 8.04E-14 | 1.006 |
| RHSIM_RhsimUnG0142700 | 58.94 | 1.63E-14 | 8.15E-14 | -1.749 |
| RHSIM_Rhsim04G0219400 | 58.93 | 1.63E-14 | 8.16E-14 | 0.739 |
| RHSIM_Rhsim12G0122000 | 58.92 | 1.64E-14 | 8.21E-14 | -1.842 |
| RHSIM_Rhsim03G0257600 | 58.92 | 1.64E-14 | 8.22E-14 | 0.739 |
| RHSIM_Rhsim07G0000300 | 58.91 | 1.65E-14 | 8.28E-14 | 0.628 |
| RHSIM_Rhsim01G0090200 | 58.81 | 1.73E-14 | 8.68E-14 | 1.158 |
| RHSIM_RhsimUnG0234700 | 58.81 | 1.73E-14 | 8.68E-14 | 1.124 |
| novel.2038 | 58.80 | 1.75E-14 | 8.74E-14 | 1.019 |
| RHSIM_Rhsim02G0003700 | 58.79 | 1.75E-14 | 8.76E-14 | -0.917 |
| RHSIM_Rhsim11G0049400 | 58.79 | 1.75E-14 | 8.76E-14 | -0.582 |

| Gene | LRT statistic | P value | BH-adjusted P | log2FC (interaction) |
|---|---|---|---|---|
| RHSIM_Rhsim05G0159700 | 58.79 | 1.76E-14 | 8.78E-14 | 0.617 |
| novel.3585 | 58.78 | 1.76E-14 | 8.81E-14 | 0.681 |
| RHSIM_RhsimPtG0002000 | 58.77 | 1.77E-14 | 8.85E-14 | 1.602 |
| RHSIM_Rhsim12G0128500 | 58.77 | 1.78E-14 | 8.87E-14 | -1.762 |
| RHSIM_Rhsim13G0111400 | 58.74 | 1.80E-14 | 8.98E-14 | -1.133 |
| RHSIM_Rhsim02G0161300 | 58.73 | 1.81E-14 | 9.04E-14 | 0.634 |
| novel.2548 | 58.73 | 1.81E-14 | 9.05E-14 | 2.298 |
| RHSIM_Rhsim10G0000600 | 58.68 | 1.85E-14 | 9.24E-14 | -3.710 |
| novel.1130 | 58.68 | 1.85E-14 | 9.26E-14 | 2.095 |
| novel.2819 | 58.66 | 1.87E-14 | 9.35E-14 | -1.076 |
| RHSIM_RhsimUnG0228400 | 58.66 | 1.87E-14 | 9.35E-14 | -3.588 |
| RHSIM_Rhsim08G0230000 | 58.65 | 1.88E-14 | 9.40E-14 | -1.908 |
| RHSIM_Rhsim12G0029600 | 58.64 | 1.89E-14 | 9.44E-14 | 0.602 |
| RHSIM_Rhsim07G0200100 | 58.61 | 1.92E-14 | 9.57E-14 | 0.655 |
| RHSIM_Rhsim01G0208800 | 58.58 | 1.95E-14 | 9.73E-14 | -0.865 |
| RHSIM_Rhsim09G0176600 | 58.56 | 1.97E-14 | 9.81E-14 | -0.565 |
| RHSIM_Rhsim08G0187300 | 58.53 | 2.01E-14 | 1.00E-13 | -1.010 |
| RHSIM_Rhsim03G0134500 | 58.52 | 2.01E-14 | 1.00E-13 | 1.133 |
| RHSIM_Rhsim12G0137000 | 58.51 | 2.02E-14 | 1.01E-13 | -1.023 |
| RHSIM_Rhsim07G0166700 | 58.51 | 2.02E-14 | 1.01E-13 | 1.827 |
| RHSIM_Rhsim01G0039200 | 58.50 | 2.03E-14 | 1.01E-13 | 1.060 |
| RHSIM_Rhsim08G0036900 | 58.47 | 2.06E-14 | 1.03E-13 | 0.452 |
| RHSIM_Rhsim10G0207400 | 58.47 | 2.06E-14 | 1.03E-13 | -4.125 |
| RHSIM_Rhsim13G0061600 | 58.46 | 2.08E-14 | 1.03E-13 | -1.940 |
| RHSIM_Rhsim04G0095300 | 58.46 | 2.08E-14 | 1.04E-13 | -1.859 |
| RHSIM_Rhsim02G0012300 | 58.46 | 2.08E-14 | 1.04E-13 | 1.001 |
| novel.3121 | 58.41 | 2.13E-14 | 1.06E-13 | 1.165 |
| RHSIM_Rhsim13G0119500 | 58.41 | 2.13E-14 | 1.06E-13 | 0.637 |
| RHSIM_Rhsim13G0032000 | 58.40 | 2.14E-14 | 1.06E-13 | 3.511 |
| RHSIM_Rhsim12G0176300 | 58.40 | 2.14E-14 | 1.07E-13 | -0.878 |
| RHSIM_Rhsim06G0164900 | 58.35 | 2.19E-14 | 1.09E-13 | -1.769 |
| RHSIM_Rhsim03G0256100 | 58.35 | 2.19E-14 | 1.09E-13 | 0.594 |

| Gene | LRT statistic | P value | BH-adjusted P | log2FC (interaction) |
|---|---|---|---|---|
| RHSIM_Rhsim01G0276100 | 58.35 | 2.20E-14 | 1.09E-13 | -1.023 |
| novel.4154 | 58.30 | 2.26E-14 | 1.12E-13 | -1.543 |
| RHSIM_Rhsim12G0028200 | 58.28 | 2.27E-14 | 1.13E-13 | -1.010 |
| RHSIM_Rhsim01G0043100 | 58.28 | 2.27E-14 | 1.13E-13 | 1.168 |
| RHSIM_Rhsim13G0232300 | 58.23 | 2.33E-14 | 1.16E-13 | 1.032 |
| RHSIM_RhsimUnG0250700 | 58.23 | 2.33E-14 | 1.16E-13 | -1.341 |
| RHSIM_Rhsim11G0115300 | 58.21 | 2.36E-14 | 1.17E-13 | 1.183 |
| RHSIM_Rhsim01G0053000 | 58.19 | 2.38E-14 | 1.18E-13 | -5.286 |
| RHSIM_Rhsim05G0205300 | 58.16 | 2.41E-14 | 1.20E-13 | 1.940 |
| novel.722 | 58.15 | 2.43E-14 | 1.21E-13 | 1.613 |
| RHSIM_Rhsim10G0104900 | 58.12 | 2.47E-14 | 1.23E-13 | -0.765 |
| RHSIM_Rhsim11G0019200 | 58.11 | 2.48E-14 | 1.23E-13 | 0.693 |
| RHSIM_Rhsim09G0212300 | 58.09 | 2.50E-14 | 1.24E-13 | -1.137 |
| RHSIM_Rhsim04G0027300 | 58.09 | 2.51E-14 | 1.25E-13 | 0.881 |
| RHSIM_Rhsim08G0219800 | 58.08 | 2.52E-14 | 1.25E-13 | 0.722 |
| novel.4561 | 58.06 | 2.54E-14 | 1.26E-13 | -7.446 |
| RHSIM_Rhsim11G0138500 | 58.06 | 2.54E-14 | 1.26E-13 | -3.898 |
| RHSIM_Rhsim03G0178700 | 58.05 | 2.56E-14 | 1.27E-13 | -1.020 |
| RHSIM_Rhsim09G0156000 | 58.04 | 2.57E-14 | 1.27E-13 | 2.885 |
| RHSIM_Rhsim02G0175000 | 58.04 | 2.57E-14 | 1.28E-13 | 2.100 |
| RHSIM_Rhsim08G0062900 | 58.03 | 2.58E-14 | 1.28E-13 | 0.993 |
| RHSIM_Rhsim11G0133000 | 58.01 | 2.61E-14 | 1.29E-13 | 0.395 |
| RHSIM_Rhsim02G0179200 | 58.00 | 2.62E-14 | 1.30E-13 | -2.465 |
| RHSIM_Rhsim05G0028000 | 57.99 | 2.63E-14 | 1.30E-13 | 2.770 |
| RHSIM_Rhsim07G0073500 | 57.99 | 2.64E-14 | 1.31E-13 | 1.062 |
| novel.4394 | 57.95 | 2.69E-14 | 1.33E-13 | 2.131 |
| RHSIM_Rhsim12G0017600 | 57.94 | 2.71E-14 | 1.34E-13 | -1.188 |
| RHSIM_Rhsim07G0240900 | 57.92 | 2.73E-14 | 1.35E-13 | -1.288 |
| RHSIM_Rhsim07G0227300 | 57.91 | 2.74E-14 | 1.36E-13 | 2.316 |
| RHSIM_Rhsim07G0162200 | 57.89 | 2.77E-14 | 1.37E-13 | 1.788 |
| RHSIM_Rhsim08G0080600 | 57.87 | 2.79E-14 | 1.38E-13 | 0.761 |
| RHSIM_Rhsim10G0171800 | 57.86 | 2.82E-14 | 1.39E-13 | -0.854 |

| Gene | LRT statistic | P value | BH-adjusted P | log2FC (interaction) |
|---|---|---|---|---|
| RHSIM_Rhsim03G0143700 | 57.85 | 2.83E-14 | 1.40E-13 | 1.053 |
| RHSIM_Rhsim08G0075900 | 57.82 | 2.88E-14 | 1.42E-13 | -1.054 |
| RHSIM_Rhsim09G0152800 | 57.79 | 2.91E-14 | 1.44E-13 | -0.985 |
| novel.3759 | 57.78 | 2.92E-14 | 1.45E-13 | -0.754 |
| RHSIM_Rhsim06G0226900 | 57.76 | 2.96E-14 | 1.46E-13 | 2.070 |
| RHSIM_Rhsim07G0180700 | 57.76 | 2.96E-14 | 1.46E-13 | 0.769 |
| RHSIM_Rhsim03G0185600 | 57.76 | 2.96E-14 | 1.46E-13 | 1.048 |
| RHSIM_Rhsim08G0063800 | 57.75 | 2.97E-14 | 1.47E-13 | -2.001 |
| RHSIM_Rhsim10G0164800 | 57.75 | 2.98E-14 | 1.47E-13 | -2.745 |
| RHSIM_Rhsim07G0039100 | 57.75 | 2.98E-14 | 1.47E-13 | -0.790 |
| RHSIM_Rhsim03G0220000 | 57.75 | 2.98E-14 | 1.47E-13 | 1.083 |
| RHSIM_RhsimUnG0216800 | 57.74 | 2.99E-14 | 1.47E-13 | -3.077 |
| RHSIM_Rhsim01G0035100 | 57.73 | 3.00E-14 | 1.48E-13 | 1.007 |
| RHSIM_Rhsim12G0159100 | 57.65 | 3.12E-14 | 1.54E-13 | 0.873 |
| novel.1477 | 57.64 | 3.15E-14 | 1.56E-13 | 0.828 |
| RHSIM_Rhsim13G0117000 | 57.63 | 3.17E-14 | 1.56E-13 | 1.226 |
| RHSIM_Rhsim03G0016500 | 57.63 | 3.17E-14 | 1.56E-13 | 0.886 |
| RHSIM_Rhsim07G0002200 | 57.56 | 3.27E-14 | 1.61E-13 | -1.078 |
| RHSIM_Rhsim12G0122500 | 57.56 | 3.29E-14 | 1.62E-13 | 2.825 |
| RHSIM_Rhsim03G0090700 | 57.55 | 3.30E-14 | 1.62E-13 | -1.682 |
| RHSIM_Rhsim12G0173700 | 57.52 | 3.34E-14 | 1.65E-13 | -0.629 |
| RHSIM_Rhsim03G0070600 | 57.52 | 3.35E-14 | 1.65E-13 | 1.152 |
| RHSIM_Rhsim07G0227700 | 57.51 | 3.36E-14 | 1.66E-13 | 3.549 |
| RHSIM_Rhsim08G0168300 | 57.50 | 3.38E-14 | 1.66E-13 | 0.896 |
| RHSIM_Rhsim08G0144600 | 57.49 | 3.39E-14 | 1.67E-13 | 0.850 |
| novel.1686 | 57.49 | 3.40E-14 | 1.68E-13 | -0.832 |
| RHSIM_Rhsim08G0107500 | 57.45 | 3.46E-14 | 1.70E-13 | -3.372 |
| RHSIM_Rhsim10G0212700 | 57.44 | 3.48E-14 | 1.71E-13 | 0.585 |
| RHSIM_Rhsim09G0132100 | 57.43 | 3.50E-14 | 1.72E-13 | 0.643 |
| RHSIM_Rhsim01G0205100 | 57.42 | 3.52E-14 | 1.73E-13 | -1.489 |
| RHSIM_Rhsim07G0018300 | 57.39 | 3.58E-14 | 1.76E-13 | 0.812 |
| RHSIM_Rhsim08G0206500 | 57.38 | 3.60E-14 | 1.77E-13 | -3.224 |

| Gene | LRT statistic | P value | BH-adjusted P | log2FC (interaction) |
|---|---|---|---|---|
| RHSIM_Rhsim07G0072300 | 57.36 | 3.62E-14 | 1.78E-13 | 2.717 |
| RHSIM_Rhsim03G0201200 | 57.33 | 3.69E-14 | 1.82E-13 | 0.748 |
| RHSIM_Rhsim02G0002500 | 57.32 | 3.70E-14 | 1.82E-13 | 0.541 |
| RHSIM_Rhsim11G0168700 | 57.31 | 3.72E-14 | 1.83E-13 | -0.855 |
| RHSIM_Rhsim11G0136600 | 57.30 | 3.75E-14 | 1.84E-13 | 0.838 |
| RHSIM_RhsimUnG0191900 | 57.29 | 3.76E-14 | 1.85E-13 | 0.755 |
| RHSIM_Rhsim11G0055600 | 57.27 | 3.81E-14 | 1.87E-13 | 2.732 |
| RHSIM_RhsimUnG0239400 | 57.23 | 3.88E-14 | 1.91E-13 | -0.581 |
| novel.553 | 57.22 | 3.89E-14 | 1.91E-13 | -2.242 |
| RHSIM_Rhsim05G0127700 | 57.19 | 3.95E-14 | 1.94E-13 | 1.511 |
| RHSIM_Rhsim12G0075500 | 57.18 | 3.97E-14 | 1.95E-13 | -0.496 |
| RHSIM_Rhsim08G0184700 | 57.18 | 3.97E-14 | 1.95E-13 | 0.660 |
| RHSIM_Rhsim11G0079500 | 57.18 | 3.98E-14 | 1.96E-13 | 1.626 |
| RHSIM_Rhsim12G0050600 | 57.16 | 4.02E-14 | 1.97E-13 | -0.850 |
| RHSIM_Rhsim06G0060100 | 57.11 | 4.11E-14 | 2.02E-13 | -0.739 |
| RHSIM_Rhsim08G0221500 | 57.08 | 4.19E-14 | 2.05E-13 | 0.718 |
| RHSIM_Rhsim08G0196600 | 57.07 | 4.20E-14 | 2.06E-13 | -0.793 |
| RHSIM_Rhsim03G0008500 | 57.06 | 4.24E-14 | 2.08E-13 | -2.396 |
| RHSIM_Rhsim08G0251800 | 57.05 | 4.24E-14 | 2.08E-13 | 1.677 |
| novel.3964 | 57.05 | 4.25E-14 | 2.08E-13 | 1.171 |
| RHSIM_Rhsim08G0116500 | 57.03 | 4.28E-14 | 2.10E-13 | -3.053 |
| RHSIM_Rhsim08G0153400 | 57.03 | 4.30E-14 | 2.11E-13 | -1.339 |
| RHSIM_Rhsim01G0248900 | 57.01 | 4.33E-14 | 2.12E-13 | -1.061 |
| RHSIM_Rhsim13G0203700 | 57.01 | 4.33E-14 | 2.12E-13 | 1.081 |
| RHSIM_Rhsim05G0228900 | 57.01 | 4.35E-14 | 2.13E-13 | -1.419 |
| RHSIM_Rhsim13G0197700 | 57.00 | 4.37E-14 | 2.14E-13 | 0.547 |
| RHSIM_Rhsim01G0038100 | 56.92 | 4.55E-14 | 2.23E-13 | 1.415 |
| RHSIM_Rhsim06G0210900 | 56.89 | 4.61E-14 | 2.26E-13 | 0.653 |
| RHSIM_Rhsim01G0235600 | 56.89 | 4.62E-14 | 2.26E-13 | 0.662 |
| RHSIM_Rhsim08G0068100 | 56.88 | 4.63E-14 | 2.27E-13 | -1.311 |
| RHSIM_Rhsim12G0143300 | 56.87 | 4.66E-14 | 2.28E-13 | -7.292 |
| RHSIM_Rhsim10G0181800 | 56.86 | 4.67E-14 | 2.29E-13 | -42.631 |

| Gene | LRT statistic | P value | BH-adjusted P | log2FC (interaction) |
|---|---|---|---|---|
| RHSIM_Rhsim09G0103100 | 56.86 | 4.69E-14 | 2.29E-13 | 1.866 |
| RHSIM_Rhsim07G0161800 | 56.85 | 4.69E-14 | 2.30E-13 | -0.546 |
| novel.3739 | 56.82 | 4.78E-14 | 2.33E-13 | 2.259 |
| RHSIM_Rhsim02G0171100 | 56.82 | 4.79E-14 | 2.34E-13 | 2.309 |
| RHSIM_RhsimUnG0226000 | 56.80 | 4.83E-14 | 2.36E-13 | 1.218 |
| RHSIM_Rhsim03G0045300 | 56.78 | 4.87E-14 | 2.38E-13 | 0.491 |
| RHSIM_Rhsim08G0192700 | 56.75 | 4.95E-14 | 2.42E-13 | 0.911 |
| RHSIM_Rhsim03G0006000 | 56.75 | 4.95E-14 | 2.42E-13 | 1.795 |
| RHSIM_Rhsim09G0097800 | 56.71 | 5.06E-14 | 2.47E-13 | 0.991 |
| RHSIM_Rhsim12G0153100 | 56.68 | 5.12E-14 | 2.50E-13 | 45.904 |
| RHSIM_Rhsim10G0125300 | 56.68 | 5.13E-14 | 2.50E-13 | -0.767 |
| RHSIM_Rhsim07G0017100 | 56.68 | 5.13E-14 | 2.50E-13 | 1.115 |
| RHSIM_Rhsim07G0207200 | 56.67 | 5.15E-14 | 2.52E-13 | 0.690 |
| RHSIM_RhsimUnG0172700 | 56.66 | 5.18E-14 | 2.53E-13 | 1.181 |
| novel.1506 | 56.66 | 5.18E-14 | 2.53E-13 | 1.480 |
| RHSIM_Rhsim12G0065100 | 56.66 | 5.19E-14 | 2.53E-13 | -0.689 |
| novel.3026 | 56.65 | 5.22E-14 | 2.55E-13 | -1.274 |
| RHSIM_Rhsim02G0072000 | 56.54 | 5.51E-14 | 2.69E-13 | -0.860 |
| RHSIM_Rhsim05G0048000 | 56.52 | 5.57E-14 | 2.72E-13 | -2.039 |
| RHSIM_Rhsim03G0214600 | 56.51 | 5.60E-14 | 2.73E-13 | -0.521 |
| RHSIM_Rhsim02G0010500 | 56.49 | 5.64E-14 | 2.75E-13 | -2.322 |
| RHSIM_Rhsim09G0141400 | 56.49 | 5.66E-14 | 2.76E-13 | -1.042 |
| RHSIM_Rhsim06G0193100 | 56.48 | 5.67E-14 | 2.76E-13 | -0.461 |
| RHSIM_Rhsim02G0062000 | 56.46 | 5.73E-14 | 2.79E-13 | -0.493 |
| RHSIM_Rhsim13G0039600 | 56.45 | 5.78E-14 | 2.82E-13 | -1.252 |
| novel.2252 | 56.43 | 5.82E-14 | 2.83E-13 | 3.955 |
| RHSIM_Rhsim13G0198300 | 56.32 | 6.15E-14 | 3.00E-13 | 1.503 |
| RHSIM_Rhsim13G0142300 | 56.32 | 6.16E-14 | 3.00E-13 | 9.125 |
| RHSIM_Rhsim03G0232900 | 56.30 | 6.21E-14 | 3.02E-13 | 1.457 |
| RHSIM_Rhsim05G0072700 | 56.30 | 6.22E-14 | 3.03E-13 | 2.000 |
| RHSIM_Rhsim05G0110400 | 56.26 | 6.34E-14 | 3.08E-13 | 0.960 |
| RHSIM_Rhsim10G0007500 | 56.26 | 6.36E-14 | 3.09E-13 | -0.651 |

| Gene | LRT statistic | P value | BH-adjusted P | log2FC (interaction) |
|---|---|---|---|---|
| RHSIM_Rhsim04G0205700 | 56.25 | 6.39E-14 | 3.11E-13 | -1.620 |
| RHSIM_Rhsim10G0134700 | 56.23 | 6.45E-14 | 3.14E-13 | 1.328 |
| RHSIM_Rhsim01G0267000 | 56.21 | 6.50E-14 | 3.16E-13 | 0.942 |
| RHSIM_Rhsim07G0238400 | 56.20 | 6.55E-14 | 3.18E-13 | -2.489 |
| RHSIM_Rhsim10G0129200 | 56.20 | 6.55E-14 | 3.18E-13 | -1.250 |
| novel.2638 | 56.15 | 6.71E-14 | 3.26E-13 | -44.479 |
| RHSIM_Rhsim11G0075200 | 56.13 | 6.79E-14 | 3.30E-13 | -0.739 |
| RHSIM_Rhsim01G0211100 | 56.12 | 6.81E-14 | 3.31E-13 | 1.461 |
| RHSIM_Rhsim05G0093600 | 56.08 | 6.97E-14 | 3.38E-13 | -1.507 |
| RHSIM_Rhsim03G0142100 | 56.07 | 6.99E-14 | 3.39E-13 | -0.777 |
| RHSIM_Rhsim03G0236600 | 56.07 | 7.00E-14 | 3.40E-13 | -0.964 |
| RHSIM_Rhsim07G0237100 | 56.06 | 7.04E-14 | 3.42E-13 | -0.833 |
| RHSIM_Rhsim10G0023700 | 56.05 | 7.06E-14 | 3.43E-13 | -1.202 |
| novel.4247 | 56.04 | 7.11E-14 | 3.45E-13 | 2.479 |
| RHSIM_Rhsim08G0065500 | 56.04 | 7.12E-14 | 3.45E-13 | 0.731 |
| RHSIM_Rhsim07G0025200 | 56.04 | 7.12E-14 | 3.45E-13 | -0.920 |
| RHSIM_Rhsim12G0105700 | 56.02 | 7.16E-14 | 3.48E-13 | 2.889 |
| RHSIM_Rhsim02G0075300 | 56.02 | 7.18E-14 | 3.48E-13 | -0.606 |
| RHSIM_Rhsim03G0096800 | 56.01 | 7.21E-14 | 3.50E-13 | 1.699 |
| RHSIM_RhsimUnG0065300 | 55.95 | 7.44E-14 | 3.61E-13 | 1.554 |
| RHSIM_Rhsim07G0094400 | 55.93 | 7.51E-14 | 3.64E-13 | 0.742 |
| RHSIM_Rhsim11G0146000 | 55.87 | 7.75E-14 | 3.76E-13 | 0.458 |
| RHSIM_RhsimUnG0241300 | 55.86 | 7.80E-14 | 3.78E-13 | -0.943 |
| novel.2037 | 55.83 | 7.91E-14 | 3.83E-13 | 2.135 |
| RHSIM_Rhsim13G0175900 | 55.81 | 7.96E-14 | 3.86E-13 | -4.590 |
| RHSIM_Rhsim06G0196200 | 55.80 | 8.00E-14 | 3.88E-13 | -0.671 |
| RHSIM_Rhsim08G0195900 | 55.80 | 8.03E-14 | 3.89E-13 | 1.261 |
| RHSIM_Rhsim06G0002600 | 55.77 | 8.13E-14 | 3.94E-13 | -1.338 |
| RHSIM_Rhsim08G0128100 | 55.77 | 8.13E-14 | 3.94E-13 | 0.872 |
| RHSIM_Rhsim08G0238200 | 55.77 | 8.15E-14 | 3.94E-13 | -6.759 |
| RHSIM_Rhsim08G0234900 | 55.76 | 8.20E-14 | 3.97E-13 | -1.024 |
| RHSIM_Rhsim01G0099900 | 55.72 | 8.37E-14 | 4.05E-13 | 1.409 |

| Gene | LRT statistic | P value | BH-adjusted P | log2FC (interaction) |
|---|---|---|---|---|
| RHSIM_Rhsim01G0228800 | 55.71 | 8.39E-14 | 4.06E-13 | -1.745 |
| RHSIM_Rhsim06G0149400 | 55.70 | 8.43E-14 | 4.08E-13 | -1.035 |
| RHSIM_Rhsim11G0016300 | 55.70 | 8.46E-14 | 4.09E-13 | 0.443 |
| RHSIM_Rhsim10G0187000 | 55.66 | 8.59E-14 | 4.16E-13 | 4.229 |
| RHSIM_Rhsim09G0088800 | 55.66 | 8.63E-14 | 4.17E-13 | -2.518 |
| RHSIM_Rhsim05G0007600 | 55.65 | 8.67E-14 | 4.19E-13 | 1.162 |
| RHSIM_Rhsim02G0018900 | 55.64 | 8.69E-14 | 4.20E-13 | -1.115 |
| RHSIM_Rhsim10G0102300 | 55.64 | 8.70E-14 | 4.20E-13 | 2.811 |
| RHSIM_Rhsim03G0074500 | 55.63 | 8.73E-14 | 4.22E-13 | 1.640 |
| RHSIM_Rhsim06G0183700 | 55.61 | 8.86E-14 | 4.28E-13 | -0.752 |
| RHSIM_Rhsim04G0024400 | 55.58 | 8.99E-14 | 4.34E-13 | -5.378 |
| RHSIM_Rhsim06G0108700 | 55.57 | 9.00E-14 | 4.35E-13 | -1.045 |
| RHSIM_Rhsim05G0108600 | 55.53 | 9.22E-14 | 4.45E-13 | 0.560 |
| RHSIM_Rhsim11G0127100 | 55.52 | 9.27E-14 | 4.47E-13 | 0.588 |
| RHSIM_RhsimUnG0204300 | 55.51 | 9.32E-14 | 4.50E-13 | 2.580 |
| RHSIM_Rhsim01G0050000 | 55.47 | 9.50E-14 | 4.58E-13 | 2.343 |
| RHSIM_RhsimUnG0249000 | 55.44 | 9.64E-14 | 4.65E-13 | 1.712 |
| RHSIM_Rhsim13G0094300 | 55.44 | 9.65E-14 | 4.66E-13 | -1.849 |
| RHSIM_Rhsim03G0144100 | 55.43 | 9.68E-14 | 4.67E-13 | 0.769 |
| RHSIM_Rhsim10G0162700 | 55.43 | 9.69E-14 | 4.68E-13 | -1.258 |
| RHSIM_Rhsim02G0235500 | 55.42 | 9.74E-14 | 4.70E-13 | -0.830 |
| RHSIM_Rhsim13G0036100 | 55.41 | 9.80E-14 | 4.73E-13 | 0.971 |
| RHSIM_Rhsim13G0155300 | 55.40 | 9.82E-14 | 4.74E-13 | 1.397 |
| novel.2041 | 55.40 | 9.85E-14 | 4.75E-13 | -4.688 |
| RHSIM_Rhsim05G0214500 | 55.40 | 9.85E-14 | 4.75E-13 | -0.629 |
| RHSIM_Rhsim08G0243400 | 55.39 | 9.89E-14 | 4.76E-13 | -3.772 |
| RHSIM_Rhsim03G0059500 | 55.38 | 9.91E-14 | 4.78E-13 | -1.472 |
| RHSIM_Rhsim05G0187100 | 55.37 | 9.99E-14 | 4.81E-13 | -0.813 |
| RHSIM_Rhsim08G0251400 | 55.37 | 1.00E-13 | 4.81E-13 | 1.141 |
| RHSIM_Rhsim07G0229900 | 55.37 | 1.00E-13 | 4.81E-13 | -1.446 |
| RHSIM_Rhsim13G0110900 | 55.34 | 1.01E-13 | 4.87E-13 | -0.798 |
| RHSIM_Rhsim11G0105300 | 55.34 | 1.01E-13 | 4.88E-13 | -0.912 |

| Gene | LRT statistic | P value | BH-adjusted P | log2FC (interaction) |
|---|---|---|---|---|
| novel.1487 | 55.34 | 1.01E-13 | 4.88E-13 | -1.259 |
| RHSIM_Rhsim11G0188300 | 55.32 | 1.02E-13 | 4.92E-13 | -0.715 |
| novel.4288 | 55.29 | 1.04E-13 | 5.01E-13 | 2.539 |
| RHSIM_Rhsim03G0027100 | 55.27 | 1.05E-13 | 5.05E-13 | 0.522 |
| novel.1664 | 55.26 | 1.05E-13 | 5.07E-13 | 2.803 |
| RHSIM_Rhsim08G0003500 | 55.24 | 1.07E-13 | 5.14E-13 | 0.731 |
| RHSIM_Rhsim13G0113800 | 55.20 | 1.09E-13 | 5.24E-13 | 2.796 |
| RHSIM_Rhsim09G0030700 | 55.18 | 1.10E-13 | 5.29E-13 | -1.022 |
| RHSIM_Rhsim02G0047700 | 55.17 | 1.11E-13 | 5.32E-13 | -1.656 |
| RHSIM_Rhsim03G0230700 | 55.15 | 1.12E-13 | 5.36E-13 | 1.464 |
| RHSIM_Rhsim09G0183900 | 55.12 | 1.13E-13 | 5.44E-13 | 0.758 |
| RHSIM_Rhsim08G0248700 | 55.12 | 1.14E-13 | 5.46E-13 | -1.105 |
| RHSIM_Rhsim09G0191400 | 55.10 | 1.15E-13 | 5.51E-13 | 0.590 |
| RHSIM_Rhsim12G0071400 | 55.09 | 1.15E-13 | 5.54E-13 | 0.689 |
| novel.2413 | 55.08 | 1.16E-13 | 5.56E-13 | 1.429 |
| RHSIM_Rhsim02G0119500 | 55.06 | 1.17E-13 | 5.63E-13 | -4.355 |
| RHSIM_Rhsim04G0046100 | 55.05 | 1.17E-13 | 5.64E-13 | -6.289 |
| novel.2753 | 55.04 | 1.18E-13 | 5.67E-13 | 1.200 |
| novel.4337 | 55.02 | 1.19E-13 | 5.73E-13 | -2.490 |
| RHSIM_Rhsim06G0053300 | 54.98 | 1.22E-13 | 5.85E-13 | 0.856 |
| RHSIM_Rhsim12G0170700 | 54.97 | 1.22E-13 | 5.87E-13 | 2.416 |
| RHSIM_Rhsim02G0185100 | 54.96 | 1.23E-13 | 5.90E-13 | 0.573 |
| RHSIM_Rhsim03G0012500 | 54.93 | 1.25E-13 | 6.00E-13 | -3.161 |
| RHSIM_Rhsim04G0155300 | 54.90 | 1.27E-13 | 6.07E-13 | -0.312 |
| RHSIM_Rhsim01G0204400 | 54.88 | 1.28E-13 | 6.13E-13 | -0.662 |
| RHSIM_RhsimUnG0100100 | 54.87 | 1.29E-13 | 6.17E-13 | 3.796 |
| RHSIM_Rhsim06G0186100 | 54.86 | 1.29E-13 | 6.20E-13 | 3.037 |
| RHSIM_Rhsim11G0139200 | 54.82 | 1.32E-13 | 6.32E-13 | -3.836 |
| RHSIM_Rhsim03G0265100 | 54.82 | 1.32E-13 | 6.32E-13 | 0.745 |
| novel.3512 | 54.80 | 1.33E-13 | 6.39E-13 | -0.648 |
| novel.552 | 54.80 | 1.34E-13 | 6.40E-13 | -5.023 |
| RHSIM_RhsimUnG0163800 | 54.79 | 1.34E-13 | 6.42E-13 | 0.891 |

| Gene | LRT statistic | P value | BH-adjusted P | log2FC (interaction) |
|---|---|---|---|---|
| RHSIM_Rhsim12G0143000 | 54.78 | 1.35E-13 | 6.44E-13 | 0.690 |
| novel.2001 | 54.78 | 1.35E-13 | 6.47E-13 | -1.357 |
| RHSIM_Rhsim06G0205400 | 54.72 | 1.39E-13 | 6.64E-13 | 1.577 |
| RHSIM_Rhsim03G0041100 | 54.71 | 1.40E-13 | 6.70E-13 | -2.994 |
| RHSIM_Rhsim13G0029900 | 54.70 | 1.40E-13 | 6.70E-13 | 0.894 |
| novel.3071 | 54.70 | 1.41E-13 | 6.72E-13 | 0.892 |
| RHSIM_Rhsim07G0074600 | 54.67 | 1.42E-13 | 6.80E-13 | 0.961 |
| RHSIM_Rhsim13G0213100 | 54.67 | 1.43E-13 | 6.83E-13 | -3.101 |
| RHSIM_Rhsim06G0053100 | 54.66 | 1.43E-13 | 6.85E-13 | -0.712 |
| RHSIM_Rhsim06G0175100 | 54.66 | 1.44E-13 | 6.86E-13 | -1.715 |
| RHSIM_Rhsim12G0149800 | 54.62 | 1.46E-13 | 6.98E-13 | 0.549 |
| RHSIM_Rhsim04G0117900 | 54.62 | 1.46E-13 | 6.98E-13 | -2.551 |
| RHSIM_Rhsim10G0049800 | 54.59 | 1.48E-13 | 7.08E-13 | 0.588 |
| RHSIM_Rhsim13G0003700 | 54.57 | 1.50E-13 | 7.16E-13 | -1.193 |
| RHSIM_RhsimUnG0020300 | 54.52 | 1.54E-13 | 7.36E-13 | -0.544 |
| RHSIM_Rhsim09G0021800 | 54.48 | 1.57E-13 | 7.50E-13 | -0.536 |
| RHSIM_Rhsim06G0003500 | 54.47 | 1.58E-13 | 7.54E-13 | 2.392 |
| RHSIM_Rhsim08G0146400 | 54.47 | 1.58E-13 | 7.55E-13 | 0.644 |
| novel.4037 | 54.46 | 1.59E-13 | 7.58E-13 | -42.959 |
| RHSIM_Rhsim01G0089700 | 54.45 | 1.59E-13 | 7.60E-13 | 1.177 |
| RHSIM_Rhsim03G0056400 | 54.45 | 1.60E-13 | 7.61E-13 | 1.678 |
| novel.4125 | 54.44 | 1.61E-13 | 7.66E-13 | 2.364 |
| RHSIM_Rhsim04G0103300 | 54.42 | 1.62E-13 | 7.71E-13 | -0.598 |
| RHSIM_RhsimUnG0209500 | 54.41 | 1.63E-13 | 7.75E-13 | -0.769 |
| RHSIM_Rhsim07G0126300 | 54.40 | 1.63E-13 | 7.79E-13 | -0.865 |
| novel.2166 | 54.39 | 1.64E-13 | 7.82E-13 | -5.634 |
| RHSIM_Rhsim12G0201400 | 54.39 | 1.64E-13 | 7.83E-13 | 4.389 |
| RHSIM_Rhsim03G0123500 | 54.36 | 1.67E-13 | 7.96E-13 | -0.859 |
| RHSIM_Rhsim05G0115900 | 54.34 | 1.68E-13 | 8.02E-13 | 0.728 |
| RHSIM_Rhsim11G0024900 | 54.33 | 1.69E-13 | 8.06E-13 | 0.890 |
| RHSIM_Rhsim08G0228400 | 54.28 | 1.74E-13 | 8.27E-13 | -0.891 |
| RHSIM_Rhsim06G0068800 | 54.27 | 1.74E-13 | 8.31E-13 | -3.025 |

| Gene | LRT statistic | P value | BH-adjusted P | log2FC (interaction) |
|---|---|---|---|---|
| RHSIM_Rhsim06G0008000 | 54.27 | 1.75E-13 | 8.33E-13 | 1.148 |
| RHSIM_Rhsim09G0069400 | 54.25 | 1.77E-13 | 8.41E-13 | -0.699 |
| RHSIM_Rhsim08G0161100 | 54.23 | 1.78E-13 | 8.48E-13 | -0.583 |
| RHSIM_RhsimUnG0047800 | 54.21 | 1.80E-13 | 8.58E-13 | 2.516 |
| RHSIM_Rhsim12G0129600 | 54.20 | 1.81E-13 | 8.60E-13 | -1.023 |
| RHSIM_Rhsim05G0050000 | 54.20 | 1.81E-13 | 8.62E-13 | 0.637 |
| RHSIM_Rhsim03G0219000 | 54.19 | 1.82E-13 | 8.68E-13 | 0.609 |
| RHSIM_Rhsim04G0246400 | 54.17 | 1.84E-13 | 8.76E-13 | -0.825 |
| novel.586 | 54.16 | 1.85E-13 | 8.79E-13 | -2.032 |
| novel.3057 | 54.16 | 1.85E-13 | 8.79E-13 | 0.641 |
| RHSIM_Rhsim12G0147500 | 54.16 | 1.85E-13 | 8.79E-13 | -1.314 |
| RHSIM_Rhsim08G0196100 | 54.14 | 1.87E-13 | 8.88E-13 | 0.934 |
| RHSIM_Rhsim10G0185900 | 54.12 | 1.88E-13 | 8.94E-13 | -0.852 |
| RHSIM_Rhsim03G0073500 | 54.10 | 1.91E-13 | 9.06E-13 | -1.666 |
| novel.1944 | 54.06 | 1.94E-13 | 9.22E-13 | -3.101 |
| RHSIM_Rhsim09G0122600 | 54.06 | 1.95E-13 | 9.25E-13 | 1.272 |
| RHSIM_Rhsim10G0157000 | 53.97 | 2.03E-13 | 9.66E-13 | 3.122 |
| RHSIM_Rhsim07G0030200 | 53.97 | 2.04E-13 | 9.69E-13 | -0.923 |
| RHSIM_Rhsim13G0203400 | 53.95 | 2.05E-13 | 9.75E-13 | -0.503 |
| RHSIM_Rhsim12G0084400 | 53.92 | 2.09E-13 | 9.92E-13 | -1.309 |
| RHSIM_Rhsim05G0193600 | 53.83 | 2.19E-13 | 1.04E-12 | 0.901 |
| RHSIM_Rhsim06G0043100 | 53.82 | 2.19E-13 | 1.04E-12 | 4.589 |
| RHSIM_Rhsim11G0174300 | 53.82 | 2.20E-13 | 1.04E-12 | 0.649 |
| RHSIM_Rhsim07G0078500 | 53.80 | 2.22E-13 | 1.05E-12 | -2.017 |
| RHSIM_Rhsim07G0008200 | 53.80 | 2.22E-13 | 1.05E-12 | 0.638 |
| RHSIM_RhsimUnG0018600 | 53.76 | 2.26E-13 | 1.07E-12 | -0.819 |
| RHSIM_Rhsim07G0030500 | 53.76 | 2.26E-13 | 1.07E-12 | -1.309 |
| RHSIM_RhsimUnG0218900 | 53.75 | 2.28E-13 | 1.08E-12 | -1.794 |
| RHSIM_Rhsim03G0079600 | 53.74 | 2.29E-13 | 1.08E-12 | 1.518 |
| RHSIM_Rhsim01G0050300 | 53.74 | 2.29E-13 | 1.09E-12 | -2.635 |
| RHSIM_Rhsim05G0130600 | 53.73 | 2.30E-13 | 1.09E-12 | -0.523 |
| novel.2612 | 53.71 | 2.32E-13 | 1.10E-12 | 0.448 |

| Gene | LRT statistic | P value | BH-adjusted P | log2FC (interaction) |
|---|---|---|---|---|
| novel.2960 | 53.68 | 2.36E-13 | 1.12E-12 | -2.546 |
| RHSIM_Rhsim12G0033300 | 53.68 | 2.36E-13 | 1.12E-12 | -2.784 |
| RHSIM_Rhsim07G0145100 | 53.67 | 2.38E-13 | 1.13E-12 | 2.788 |
| RHSIM_Rhsim09G0100800 | 53.64 | 2.40E-13 | 1.14E-12 | 0.574 |
| novel.1878 | 53.63 | 2.42E-13 | 1.15E-12 | -3.299 |
| RHSIM_Rhsim11G0154600 | 53.61 | 2.44E-13 | 1.15E-12 | 1.301 |
| novel.804 | 53.57 | 2.50E-13 | 1.18E-12 | 0.401 |
| RHSIM_Rhsim13G0058400 | 53.56 | 2.51E-13 | 1.19E-12 | 0.983 |
| RHSIM_Rhsim13G0074500 | 53.51 | 2.58E-13 | 1.22E-12 | -0.443 |
| RHSIM_Rhsim08G0030900 | 53.50 | 2.58E-13 | 1.22E-12 | 2.123 |
| RHSIM_RhsimUnG0110400 | 53.48 | 2.62E-13 | 1.24E-12 | 0.846 |
| RHSIM_Rhsim01G0183100 | 53.46 | 2.64E-13 | 1.25E-12 | -1.175 |
| RHSIM_Rhsim02G0243800 | 53.45 | 2.65E-13 | 1.25E-12 | -1.550 |
| RHSIM_Rhsim09G0195800 | 53.42 | 2.70E-13 | 1.27E-12 | -1.277 |
| RHSIM_Rhsim11G0086900 | 53.39 | 2.73E-13 | 1.29E-12 | -0.867 |
| RHSIM_Rhsim06G0186700 | 53.39 | 2.73E-13 | 1.29E-12 | 1.800 |
| RHSIM_Rhsim09G0098900 | 53.36 | 2.78E-13 | 1.31E-12 | -1.361 |
| RHSIM_Rhsim01G0002700 | 53.35 | 2.79E-13 | 1.32E-12 | 1.882 |
| RHSIM_Rhsim01G0242000 | 53.35 | 2.79E-13 | 1.32E-12 | 2.474 |
| RHSIM_Rhsim10G0122800 | 53.30 | 2.86E-13 | 1.35E-12 | 2.198 |
| RHSIM_Rhsim08G0240700 | 53.30 | 2.86E-13 | 1.35E-12 | -0.867 |
| RHSIM_Rhsim07G0028000 | 53.30 | 2.87E-13 | 1.35E-12 | -0.621 |
| RHSIM_Rhsim07G0119300 | 53.29 | 2.88E-13 | 1.36E-12 | 0.556 |
| RHSIM_Rhsim01G0142500 | 53.28 | 2.89E-13 | 1.36E-12 | -0.929 |
| RHSIM_Rhsim06G0214300 | 53.28 | 2.90E-13 | 1.37E-12 | -2.549 |
| RHSIM_Rhsim07G0223400 | 53.25 | 2.93E-13 | 1.38E-12 | -4.540 |
| RHSIM_Rhsim06G0054900 | 53.23 | 2.97E-13 | 1.40E-12 | -0.777 |
| RHSIM_Rhsim06G0102300 | 53.23 | 2.97E-13 | 1.40E-12 | 1.388 |
| RHSIM_Rhsim07G0034600 | 53.20 | 3.02E-13 | 1.42E-12 | 0.591 |
| RHSIM_Rhsim04G0052500 | 53.15 | 3.09E-13 | 1.46E-12 | -4.995 |
| RHSIM_Rhsim11G0147400 | 53.09 | 3.18E-13 | 1.50E-12 | -0.870 |
| RHSIM_Rhsim11G0185000 | 53.03 | 3.29E-13 | 1.55E-12 | -3.029 |

| Gene | LRT statistic | P value | BH-adjusted P | log2FC (interaction) |
|---|---|---|---|---|
| RHSIM_Rhsim06G0215400 | 53.03 | 3.29E-13 | 1.55E-12 | 0.699 |
| RHSIM_Rhsim04G0228400 | 53.01 | 3.32E-13 | 1.56E-12 | -1.040 |
| RHSIM_Rhsim10G0208400 | 53.01 | 3.33E-13 | 1.57E-12 | 1.129 |
| RHSIM_Rhsim06G0053200 | 53.00 | 3.34E-13 | 1.57E-12 | 1.136 |
| RHSIM_Rhsim12G0036800 | 52.99 | 3.35E-13 | 1.58E-12 | -1.739 |
| novel.1019 | 52.99 | 3.36E-13 | 1.58E-12 | -3.221 |
| RHSIM_Rhsim13G0016900 | 52.98 | 3.37E-13 | 1.58E-12 | 4.094 |
| RHSIM_RhsimUnG0141100 | 52.98 | 3.37E-13 | 1.58E-12 | 2.880 |
| RHSIM_Rhsim09G0171800 | 52.94 | 3.43E-13 | 1.61E-12 | 0.735 |
| RHSIM_Rhsim09G0135300 | 52.93 | 3.45E-13 | 1.62E-12 | -2.218 |
| RHSIM_Rhsim06G0058600 | 52.93 | 3.46E-13 | 1.63E-12 | 0.645 |
| novel.3473 | 52.85 | 3.60E-13 | 1.69E-12 | -1.235 |
| RHSIM_Rhsim08G0029700 | 52.82 | 3.65E-13 | 1.72E-12 | 2.342 |
| RHSIM_Rhsim12G0000100 | 52.82 | 3.66E-13 | 1.72E-12 | 0.863 |
| RHSIM_Rhsim07G0177100 | 52.82 | 3.66E-13 | 1.72E-12 | 1.018 |
| RHSIM_Rhsim01G0268400 | 52.81 | 3.68E-13 | 1.73E-12 | 0.475 |
| novel.3452 | 52.80 | 3.69E-13 | 1.73E-12 | 1.840 |
| RHSIM_Rhsim02G0151700 | 52.78 | 3.73E-13 | 1.75E-12 | 0.650 |
| RHSIM_Rhsim07G0215200 | 52.77 | 3.75E-13 | 1.76E-12 | -2.657 |
| RHSIM_Rhsim01G0194500 | 52.76 | 3.76E-13 | 1.77E-12 | -0.717 |
| RHSIM_Rhsim08G0061200 | 52.75 | 3.79E-13 | 1.78E-12 | 1.415 |
| RHSIM_Rhsim13G0086200 | 52.71 | 3.87E-13 | 1.82E-12 | 0.483 |
| RHSIM_Rhsim06G0147300 | 52.70 | 3.88E-13 | 1.82E-12 | -1.509 |
| novel.2149 | 52.65 | 3.98E-13 | 1.87E-12 | 2.607 |
| RHSIM_Rhsim06G0233400 | 52.65 | 3.99E-13 | 1.87E-12 | -2.216 |
| RHSIM_Rhsim07G0170500 | 52.62 | 4.05E-13 | 1.90E-12 | 0.558 |
| RHSIM_Rhsim05G0193500 | 52.58 | 4.13E-13 | 1.94E-12 | -3.672 |
| RHSIM_Rhsim12G0109900 | 52.56 | 4.18E-13 | 1.96E-12 | -1.793 |
| RHSIM_Rhsim10G0099500 | 52.55 | 4.19E-13 | 1.96E-12 | 0.914 |
| RHSIM_Rhsim12G0006300 | 52.51 | 4.29E-13 | 2.01E-12 | 0.793 |
| RHSIM_Rhsim10G0027600 | 52.45 | 4.41E-13 | 2.07E-12 | -1.414 |
| RHSIM_Rhsim11G0098900 | 52.44 | 4.43E-13 | 2.08E-12 | -0.992 |

| Gene | LRT statistic | P value | BH-adjusted P | log2FC (interaction) |
|---|---|---|---|---|
| RHSIM_Rhsim06G0010600 | 52.43 | 4.46E-13 | 2.09E-12 | 1.229 |
| RHSIM_Rhsim13G0140100 | 52.43 | 4.46E-13 | 2.09E-12 | -0.529 |
| RHSIM_Rhsim10G0023600 | 52.43 | 4.46E-13 | 2.09E-12 | 0.944 |
| RHSIM_Rhsim04G0034800 | 52.42 | 4.49E-13 | 2.10E-12 | -1.574 |
| RHSIM_Rhsim11G0018700 | 52.41 | 4.50E-13 | 2.11E-12 | 0.771 |
| RHSIM_Rhsim11G0055300 | 52.40 | 4.54E-13 | 2.12E-12 | 1.180 |
| RHSIM_Rhsim06G0160900 | 52.39 | 4.55E-13 | 2.13E-12 | 1.789 |
| RHSIM_Rhsim01G0067900 | 52.33 | 4.69E-13 | 2.20E-12 | -1.318 |
| RHSIM_Rhsim12G0076200 | 52.31 | 4.74E-13 | 2.22E-12 | 0.637 |
| novel.2786 | 52.29 | 4.78E-13 | 2.24E-12 | 2.309 |
| RHSIM_Rhsim02G0047800 | 52.29 | 4.80E-13 | 2.24E-12 | 0.815 |
| RHSIM_Rhsim05G0163200 | 52.28 | 4.82E-13 | 2.25E-12 | 7.032 |
| RHSIM_Rhsim01G0281000 | 52.26 | 4.85E-13 | 2.27E-12 | -3.068 |
| RHSIM_Rhsim13G0211600 | 52.25 | 4.88E-13 | 2.28E-12 | -1.044 |
| RHSIM_Rhsim11G0072400 | 52.25 | 4.88E-13 | 2.28E-12 | -0.494 |
| RHSIM_Rhsim03G0034900 | 52.23 | 4.95E-13 | 2.31E-12 | -3.582 |
| RHSIM_Rhsim10G0192900 | 52.22 | 4.97E-13 | 2.32E-12 | 0.813 |
| RHSIM_Rhsim10G0202800 | 52.21 | 4.98E-13 | 2.33E-12 | 3.078 |
| RHSIM_Rhsim06G0124300 | 52.20 | 5.01E-13 | 2.34E-12 | -0.817 |
| RHSIM_Rhsim13G0093000 | 52.18 | 5.06E-13 | 2.36E-12 | -1.476 |
| RHSIM_Rhsim02G0217300 | 52.16 | 5.13E-13 | 2.40E-12 | 2.656 |
| RHSIM_Rhsim12G0002600 | 52.14 | 5.17E-13 | 2.42E-12 | 0.749 |
| RHSIM_Rhsim05G0122100 | 52.14 | 5.18E-13 | 2.42E-12 | 0.376 |
| novel.3309 | 52.11 | 5.24E-13 | 2.44E-12 | 2.757 |
| RHSIM_Rhsim04G0214300 | 52.09 | 5.29E-13 | 2.47E-12 | -0.928 |
| RHSIM_Rhsim13G0062500 | 52.08 | 5.34E-13 | 2.49E-12 | 1.107 |
| RHSIM_Rhsim08G0029300 | 52.07 | 5.37E-13 | 2.50E-12 | 2.010 |
| RHSIM_Rhsim01G0027700 | 52.02 | 5.49E-13 | 2.56E-12 | 1.127 |
| RHSIM_Rhsim01G0199200 | 52.01 | 5.51E-13 | 2.57E-12 | -0.643 |
| RHSIM_Rhsim05G0078300 | 52.00 | 5.54E-13 | 2.58E-12 | -1.139 |
| RHSIM_Rhsim04G0065800 | 51.99 | 5.58E-13 | 2.60E-12 | -0.820 |
| RHSIM_Rhsim09G0148900 | 51.97 | 5.63E-13 | 2.62E-12 | -1.077 |

| Gene | LRT statistic | P value | BH-adjusted P | log2FC (interaction) |
|---|---|---|---|---|
| RHSIM_Rhsim03G0040200 | 51.97 | 5.63E-13 | 2.63E-12 | -0.808 |
| RHSIM_Rhsim13G0128500 | 51.96 | 5.65E-13 | 2.64E-12 | 0.729 |
| RHSIM_Rhsim12G0004500 | 51.92 | 5.77E-13 | 2.69E-12 | -1.270 |
| RHSIM_Rhsim10G0157500 | 51.90 | 5.83E-13 | 2.72E-12 | 2.846 |
| novel.2225 | 51.86 | 5.95E-13 | 2.77E-12 | 0.696 |
| RHSIM_Rhsim03G0046500 | 51.84 | 6.01E-13 | 2.80E-12 | -1.208 |
| RHSIM_Rhsim05G0009000 | 51.82 | 6.07E-13 | 2.83E-12 | -2.497 |
| RHSIM_Rhsim13G0070900 | 51.82 | 6.08E-13 | 2.83E-12 | -3.974 |
| novel.159 | 51.81 | 6.12E-13 | 2.85E-12 | 2.532 |
| RHSIM_Rhsim06G0241600 | 51.80 | 6.14E-13 | 2.86E-12 | 0.815 |
| RHSIM_Rhsim08G0224700 | 51.72 | 6.39E-13 | 2.97E-12 | 0.449 |
| RHSIM_Rhsim13G0217400 | 51.72 | 6.39E-13 | 2.98E-12 | 0.545 |
| RHSIM_Rhsim08G0098100 | 51.72 | 6.41E-13 | 2.98E-12 | 2.329 |
| RHSIM_Rhsim03G0254500 | 51.71 | 6.42E-13 | 2.99E-12 | 0.675 |
| novel.2751 | 51.70 | 6.45E-13 | 3.00E-12 | 1.286 |
| RHSIM_Rhsim12G0144100 | 51.70 | 6.46E-13 | 3.00E-12 | 0.723 |
| RHSIM_Rhsim03G0087900 | 51.64 | 6.65E-13 | 3.09E-12 | -1.031 |
| RHSIM_Rhsim01G0225500 | 51.63 | 6.71E-13 | 3.12E-12 | 1.104 |
| RHSIM_Rhsim10G0210600 | 51.62 | 6.72E-13 | 3.12E-12 | -0.934 |
| RHSIM_Rhsim01G0129300 | 51.61 | 6.77E-13 | 3.15E-12 | 4.160 |
| novel.3656 | 51.56 | 6.96E-13 | 3.23E-12 | -0.558 |
| RHSIM_Rhsim07G0055700 | 51.55 | 6.99E-13 | 3.25E-12 | 1.052 |
| RHSIM_Rhsim10G0211500 | 51.53 | 7.06E-13 | 3.28E-12 | -2.167 |
| RHSIM_Rhsim02G0008500 | 51.52 | 7.08E-13 | 3.29E-12 | -0.319 |
| RHSIM_Rhsim07G0222000 | 51.51 | 7.11E-13 | 3.30E-12 | 1.329 |
| RHSIM_Rhsim01G0210800 | 51.51 | 7.12E-13 | 3.31E-12 | -0.620 |
| RHSIM_Rhsim06G0022200 | 51.49 | 7.18E-13 | 3.33E-12 | 1.608 |
| RHSIM_Rhsim02G0060900 | 51.48 | 7.23E-13 | 3.36E-12 | 0.809 |
| novel.1627 | 51.48 | 7.24E-13 | 3.36E-12 | -2.122 |
| RHSIM_Rhsim05G0094200 | 51.48 | 7.25E-13 | 3.36E-12 | 0.363 |
| RHSIM_Rhsim08G0221100 | 51.47 | 7.27E-13 | 3.37E-12 | 0.846 |
| RHSIM_Rhsim08G0076200 | 51.47 | 7.28E-13 | 3.38E-12 | 1.135 |

| Gene | LRT statistic | P value | BH-adjusted P | log2FC (interaction) |
|---|---|---|---|---|
| RHSIM_Rhsim04G0195000 | 51.45 | 7.34E-13 | 3.41E-12 | 0.559 |
| RHSIM_Rhsim09G0000400 | 51.44 | 7.38E-13 | 3.42E-12 | 0.681 |
| novel.1882 | 51.42 | 7.44E-13 | 3.45E-12 | -43.548 |
| RHSIM_Rhsim10G0030800 | 51.41 | 7.50E-13 | 3.48E-12 | -1.201 |
| RHSIM_Rhsim08G0245900 | 51.41 | 7.51E-13 | 3.48E-12 | 0.818 |
| RHSIM_Rhsim12G0191000 | 51.41 | 7.51E-13 | 3.48E-12 | 1.256 |
| RHSIM_Rhsim08G0215400 | 51.40 | 7.52E-13 | 3.48E-12 | -1.766 |
| RHSIM_Rhsim01G0101100 | 51.39 | 7.59E-13 | 3.52E-12 | -1.034 |
| novel.194 | 51.36 | 7.69E-13 | 3.56E-12 | 1.427 |
| RHSIM_Rhsim07G0210500 | 51.35 | 7.71E-13 | 3.57E-12 | -1.258 |
| RHSIM_Rhsim07G0002400 | 51.34 | 7.78E-13 | 3.60E-12 | -2.112 |
| RHSIM_Rhsim07G0001900 | 51.28 | 8.02E-13 | 3.71E-12 | 2.167 |
| novel.4525 | 51.23 | 8.22E-13 | 3.81E-12 | 1.994 |
| RHSIM_Rhsim09G0208800 | 51.18 | 8.42E-13 | 3.90E-12 | 1.007 |
| RHSIM_Rhsim01G0006700 | 51.14 | 8.60E-13 | 3.98E-12 | -0.450 |
| RHSIM_Rhsim09G0105300 | 51.13 | 8.66E-13 | 4.01E-12 | -1.486 |
| RHSIM_Rhsim09G0209200 | 51.12 | 8.69E-13 | 4.02E-12 | 2.236 |
| RHSIM_Rhsim13G0183300 | 51.08 | 8.89E-13 | 4.11E-12 | 2.886 |
| RHSIM_Rhsim06G0243900 | 51.03 | 9.09E-13 | 4.20E-12 | 1.694 |
| RHSIM_Rhsim07G0078600 | 50.98 | 9.31E-13 | 4.30E-12 | -2.007 |
| RHSIM_Rhsim11G0096900 | 50.98 | 9.32E-13 | 4.31E-12 | 2.491 |
| RHSIM_Rhsim05G0189500 | 50.98 | 9.32E-13 | 4.31E-12 | -0.553 |
| RHSIM_Rhsim01G0187000 | 50.98 | 9.33E-13 | 4.31E-12 | -1.340 |
| RHSIM_RhsimUnG0258700 | 50.97 | 9.37E-13 | 4.33E-12 | 1.094 |
| RHSIM_Rhsim04G0237800 | 50.97 | 9.40E-13 | 4.34E-12 | -1.852 |
| RHSIM_Rhsim12G0204400 | 50.95 | 9.50E-13 | 4.39E-12 | 1.037 |
| RHSIM_Rhsim06G0121700 | 50.94 | 9.51E-13 | 4.39E-12 | -0.482 |
| novel.1301 | 50.94 | 9.53E-13 | 4.40E-12 | -9.420 |
| RHSIM_Rhsim08G0001900 | 50.92 | 9.62E-13 | 4.44E-12 | 1.013 |
| RHSIM_Rhsim10G0038500 | 50.87 | 9.85E-13 | 4.55E-12 | 1.255 |
| RHSIM_Rhsim05G0085200 | 50.87 | 9.85E-13 | 4.55E-12 | -3.727 |
| RHSIM_Rhsim13G0031700 | 50.84 | 1.00E-12 | 4.63E-12 | 1.140 |

| Gene | LRT statistic | P value | BH-adjusted P | log2FC (interaction) |
|---|---|---|---|---|
| RHSIM_Rhsim02G0032000 | 50.82 | 1.01E-12 | 4.66E-12 | 0.641 |
| RHSIM_Rhsim12G0190200 | 50.81 | 1.02E-12 | 4.68E-12 | 2.129 |
| RHSIM_Rhsim06G0139500 | 50.81 | 1.02E-12 | 4.68E-12 | -1.391 |
| RHSIM_Rhsim10G0205400 | 50.78 | 1.03E-12 | 4.77E-12 | 1.145 |
| RHSIM_Rhsim04G0004400 | 50.77 | 1.04E-12 | 4.79E-12 | 0.669 |
| RHSIM_RhsimUnG0248800 | 50.77 | 1.04E-12 | 4.79E-12 | 1.023 |
| novel.4522 | 50.75 | 1.05E-12 | 4.84E-12 | 2.648 |
| RHSIM_Rhsim04G0128500 | 50.74 | 1.05E-12 | 4.86E-12 | 0.859 |
| RHSIM_Rhsim12G0159200 | 50.73 | 1.06E-12 | 4.88E-12 | 1.229 |
| RHSIM_Rhsim03G0187500 | 50.71 | 1.07E-12 | 4.93E-12 | -1.020 |
| RHSIM_Rhsim09G0204300 | 50.71 | 1.07E-12 | 4.93E-12 | -0.648 |
| RHSIM_Rhsim08G0121800 | 50.68 | 1.09E-12 | 5.01E-12 | 1.367 |
| RHSIM_Rhsim08G0224100 | 50.68 | 1.09E-12 | 5.02E-12 | 2.113 |
| RHSIM_Rhsim11G0164700 | 50.67 | 1.09E-12 | 5.02E-12 | -1.882 |
| RHSIM_Rhsim09G0033500 | 50.63 | 1.12E-12 | 5.14E-12 | 0.563 |
| RHSIM_Rhsim11G0194100 | 50.62 | 1.12E-12 | 5.15E-12 | 1.714 |
| RHSIM_Rhsim03G0179300 | 50.58 | 1.14E-12 | 5.26E-12 | -1.597 |
| RHSIM_Rhsim05G0041700 | 50.58 | 1.15E-12 | 5.27E-12 | 0.557 |
| RHSIM_Rhsim08G0066600 | 50.56 | 1.16E-12 | 5.33E-12 | -0.933 |
| RHSIM_Rhsim10G0169300 | 50.54 | 1.17E-12 | 5.37E-12 | -0.823 |
| RHSIM_Rhsim13G0162100 | 50.54 | 1.17E-12 | 5.38E-12 | 1.206 |
| RHSIM_Rhsim10G0054400 | 50.53 | 1.17E-12 | 5.39E-12 | 0.648 |
| RHSIM_Rhsim11G0117200 | 50.52 | 1.18E-12 | 5.42E-12 | 1.000 |
| RHSIM_Rhsim01G0283700 | 50.51 | 1.19E-12 | 5.46E-12 | 0.902 |
| RHSIM_Rhsim13G0230200 | 50.49 | 1.20E-12 | 5.51E-12 | 0.828 |
| RHSIM_Rhsim13G0191000 | 50.46 | 1.22E-12 | 5.59E-12 | -0.693 |
| RHSIM_RhsimUnG0258300 | 50.45 | 1.22E-12 | 5.61E-12 | 1.038 |
| RHSIM_Rhsim10G0141500 | 50.45 | 1.22E-12 | 5.62E-12 | -1.161 |
| RHSIM_Rhsim09G0027600 | 50.42 | 1.24E-12 | 5.69E-12 | 2.032 |
| RHSIM_Rhsim04G0008400 | 50.41 | 1.24E-12 | 5.72E-12 | -0.920 |
| RHSIM_Rhsim05G0056400 | 50.40 | 1.25E-12 | 5.74E-12 | -3.808 |
| RHSIM_Rhsim10G0192000 | 50.40 | 1.26E-12 | 5.77E-12 | -2.776 |

| Gene | LRT statistic | P value | BH-adjusted P | log2FC (interaction) |
|---|---|---|---|---|
| novel.3469 | 50.39 | 1.26E-12 | 5.78E-12 | -0.587 |
| RHSIM_Rhsim05G0060300 | 50.39 | 1.26E-12 | 5.79E-12 | -1.211 |
| RHSIM_Rhsim12G0182400 | 50.39 | 1.26E-12 | 5.79E-12 | 0.861 |
| RHSIM_Rhsim01G0139200 | 50.38 | 1.27E-12 | 5.81E-12 | -0.692 |
| novel.2011 | 50.37 | 1.27E-12 | 5.85E-12 | 0.643 |
| RHSIM_RhsimUnG0039300 | 50.34 | 1.29E-12 | 5.92E-12 | -3.999 |
| RHSIM_Rhsim09G0001500 | 50.34 | 1.29E-12 | 5.93E-12 | -0.656 |
| RHSIM_Rhsim01G0240500 | 50.34 | 1.30E-12 | 5.94E-12 | -0.907 |
| RHSIM_Rhsim03G0026900 | 50.32 | 1.30E-12 | 5.97E-12 | 1.267 |
| RHSIM_Rhsim01G0193400 | 50.32 | 1.30E-12 | 5.98E-12 | 2.840 |
| RHSIM_Rhsim07G0113300 | 50.32 | 1.31E-12 | 6.00E-12 | -0.662 |
| RHSIM_Rhsim04G0128900 | 50.32 | 1.31E-12 | 6.00E-12 | 0.731 |
| RHSIM_Rhsim10G0133800 | 50.29 | 1.32E-12 | 6.07E-12 | 0.754 |
| RHSIM_Rhsim06G0184700 | 50.29 | 1.33E-12 | 6.07E-12 | 5.120 |
| RHSIM_Rhsim13G0082600 | 50.23 | 1.37E-12 | 6.26E-12 | 2.560 |
| RHSIM_Rhsim05G0028700 | 50.19 | 1.40E-12 | 6.39E-12 | 4.330 |
| RHSIM_Rhsim11G0148400 | 50.19 | 1.40E-12 | 6.40E-12 | 4.491 |
| RHSIM_Rhsim11G0074400 | 50.16 | 1.41E-12 | 6.47E-12 | -0.905 |
| RHSIM_Rhsim11G0096400 | 50.16 | 1.42E-12 | 6.48E-12 | 0.949 |
| RHSIM_Rhsim09G0054600 | 50.16 | 1.42E-12 | 6.50E-12 | -0.600 |
| RHSIM_Rhsim09G0108800 | 50.13 | 1.44E-12 | 6.60E-12 | -1.253 |
| RHSIM_Rhsim12G0013100 | 50.12 | 1.45E-12 | 6.61E-12 | 1.523 |
| RHSIM_Rhsim10G0063300 | 50.11 | 1.45E-12 | 6.63E-12 | 0.842 |
| RHSIM_Rhsim03G0084800 | 50.09 | 1.47E-12 | 6.70E-12 | 5.183 |
| RHSIM_Rhsim12G0134300 | 50.09 | 1.47E-12 | 6.71E-12 | 2.056 |
| RHSIM_Rhsim06G0070500 | 50.08 | 1.48E-12 | 6.74E-12 | 1.033 |
| RHSIM_Rhsim08G0204800 | 50.08 | 1.48E-12 | 6.76E-12 | 1.834 |
| novel.3102 | 50.06 | 1.49E-12 | 6.81E-12 | 3.898 |
| RHSIM_Rhsim10G0164900 | 50.06 | 1.49E-12 | 6.82E-12 | 0.546 |
| RHSIM_Rhsim01G0106900 | 50.06 | 1.49E-12 | 6.83E-12 | 0.368 |
| RHSIM_Rhsim08G0132100 | 50.05 | 1.50E-12 | 6.84E-12 | 0.844 |
| novel.2399 | 50.04 | 1.50E-12 | 6.86E-12 | 3.024 |

| Gene | LRT statistic | P value | BH-adjusted P | log2FC (interaction) |
|---|---|---|---|---|
| RHSIM_Rhsim03G0179900 | 50.00 | 1.54E-12 | 7.02E-12 | -1.236 |
| RHSIM_Rhsim03G0068000 | 50.00 | 1.54E-12 | 7.03E-12 | -2.037 |
| RHSIM_Rhsim05G0044700 | 49.98 | 1.55E-12 | 7.09E-12 | -1.254 |
| RHSIM_Rhsim03G0189900 | 49.97 | 1.56E-12 | 7.11E-12 | -0.588 |
| RHSIM_RhsimUnG0150400 | 49.95 | 1.58E-12 | 7.21E-12 | 1.078 |
| RHSIM_Rhsim13G0198500 | 49.94 | 1.58E-12 | 7.22E-12 | -0.819 |
| novel.336 | 49.94 | 1.59E-12 | 7.23E-12 | -1.343 |
| RHSIM_Rhsim01G0287400 | 49.93 | 1.59E-12 | 7.25E-12 | -2.392 |
| RHSIM_Rhsim03G0011100 | 49.93 | 1.59E-12 | 7.26E-12 | -0.916 |
| RHSIM_Rhsim13G0069100 | 49.91 | 1.61E-12 | 7.33E-12 | 3.403 |
| RHSIM_Rhsim07G0036100 | 49.91 | 1.61E-12 | 7.34E-12 | -3.936 |
| RHSIM_Rhsim08G0197500 | 49.87 | 1.64E-12 | 7.47E-12 | -0.857 |
| RHSIM_Rhsim10G0003200 | 49.86 | 1.65E-12 | 7.51E-12 | -0.828 |
| RHSIM_Rhsim10G0082200 | 49.86 | 1.65E-12 | 7.54E-12 | -0.378 |
| RHSIM_Rhsim11G0137800 | 49.83 | 1.67E-12 | 7.62E-12 | 1.046 |
| RHSIM_Rhsim11G0134300 | 49.83 | 1.68E-12 | 7.65E-12 | 0.719 |
| RHSIM_Rhsim05G0068000 | 49.82 | 1.68E-12 | 7.67E-12 | -0.526 |
| novel.3708 | 49.79 | 1.71E-12 | 7.77E-12 | 2.715 |
| RHSIM_Rhsim13G0180600 | 49.77 | 1.73E-12 | 7.86E-12 | 0.716 |
| RHSIM_Rhsim09G0005500 | 49.77 | 1.73E-12 | 7.86E-12 | -0.982 |
| RHSIM_Rhsim12G0147100 | 49.77 | 1.73E-12 | 7.88E-12 | 0.759 |
| RHSIM_Rhsim04G0011700 | 49.74 | 1.76E-12 | 8.01E-12 | -1.511 |
| RHSIM_Rhsim08G0093000 | 49.68 | 1.81E-12 | 8.22E-12 | 0.347 |
| RHSIM_Rhsim03G0000400 | 49.66 | 1.83E-12 | 8.31E-12 | 0.994 |
| RHSIM_Rhsim05G0167800 | 49.66 | 1.83E-12 | 8.32E-12 | -0.756 |
| RHSIM_Rhsim11G0172000 | 49.65 | 1.83E-12 | 8.34E-12 | -1.287 |
| RHSIM_Rhsim08G0214200 | 49.64 | 1.85E-12 | 8.42E-12 | -0.916 |
| RHSIM_Rhsim06G0157800 | 49.63 | 1.86E-12 | 8.45E-12 | 3.692 |
| novel.2418 | 49.61 | 1.88E-12 | 8.55E-12 | 4.545 |
| RHSIM_Rhsim09G0043400 | 49.60 | 1.88E-12 | 8.56E-12 | 0.713 |
| RHSIM_Rhsim09G0146600 | 49.59 | 1.89E-12 | 8.60E-12 | 1.393 |
| novel.3615 | 49.57 | 1.91E-12 | 8.68E-12 | -0.939 |

| Gene | LRT statistic | P value | BH-adjusted P | log2FC (interaction) |
|---|---|---|---|---|
| RHSIM_Rhsim02G0227900 | 49.57 | 1.91E-12 | 8.69E-12 | -1.451 |
| RHSIM_Rhsim01G0233900 | 49.56 | 1.93E-12 | 8.76E-12 | -0.850 |
| RHSIM_Rhsim12G0061300 | 49.55 | 1.93E-12 | 8.76E-12 | -0.830 |
| RHSIM_Rhsim07G0034800 | 49.55 | 1.94E-12 | 8.79E-12 | 0.749 |
| RHSIM_Rhsim08G0190700 | 49.55 | 1.94E-12 | 8.80E-12 | -1.093 |
| RHSIM_Rhsim12G0020800 | 49.54 | 1.94E-12 | 8.82E-12 | -2.633 |
| RHSIM_Rhsim12G0024300 | 49.51 | 1.98E-12 | 8.98E-12 | -1.972 |
| RHSIM_Rhsim01G0125800 | 49.50 | 1.98E-12 | 9.01E-12 | -0.663 |
| RHSIM_Rhsim06G0176500 | 49.50 | 1.99E-12 | 9.01E-12 | -1.878 |
| RHSIM_Rhsim09G0212700 | 49.48 | 2.00E-12 | 9.08E-12 | -0.603 |
| RHSIM_Rhsim10G0019400 | 49.48 | 2.00E-12 | 9.09E-12 | 0.549 |
| novel.1800 | 49.46 | 2.02E-12 | 9.16E-12 | 0.820 |
| RHSIM_Rhsim11G0086000 | 49.46 | 2.03E-12 | 9.19E-12 | -1.638 |
| RHSIM_Rhsim05G0229300 | 49.44 | 2.05E-12 | 9.28E-12 | 0.529 |
| RHSIM_Rhsim02G0111900 | 49.44 | 2.05E-12 | 9.28E-12 | 0.928 |
| RHSIM_Rhsim02G0256300 | 49.44 | 2.05E-12 | 9.29E-12 | 1.436 |
| RHSIM_Rhsim10G0006700 | 49.41 | 2.08E-12 | 9.43E-12 | 1.427 |
| RHSIM_Rhsim06G0167800 | 49.39 | 2.10E-12 | 9.52E-12 | 1.265 |
| novel.2696 | 49.38 | 2.11E-12 | 9.55E-12 | -1.232 |
| RHSIM_Rhsim12G0085800 | 49.37 | 2.11E-12 | 9.58E-12 | 0.910 |
| novel.279 | 49.37 | 2.12E-12 | 9.58E-12 | 3.678 |
| RHSIM_Rhsim03G0056200 | 49.37 | 2.12E-12 | 9.61E-12 | 3.296 |
| RHSIM_Rhsim13G0077000 | 49.36 | 2.13E-12 | 9.66E-12 | 0.422 |
| RHSIM_Rhsim07G0236500 | 49.35 | 2.14E-12 | 9.70E-12 | 0.553 |
| RHSIM_Rhsim13G0212900 | 49.34 | 2.15E-12 | 9.74E-12 | 1.539 |
| RHSIM_Rhsim02G0205200 | 49.34 | 2.16E-12 | 9.76E-12 | 0.954 |
| RHSIM_Rhsim07G0031600 | 49.33 | 2.16E-12 | 9.78E-12 | 0.860 |
| novel.4533 | 49.33 | 2.17E-12 | 9.80E-12 | 1.883 |
| RHSIM_Rhsim09G0180500 | 49.30 | 2.20E-12 | 9.96E-12 | 0.426 |
| RHSIM_Rhsim04G0188200 | 49.28 | 2.22E-12 | 1.00E-11 | -1.340 |
| novel.2792 | 49.28 | 2.22E-12 | 1.00E-11 | 2.489 |
| RHSIM_Rhsim02G0241900 | 49.28 | 2.22E-12 | 1.01E-11 | 2.120 |

| Gene | LRT statistic | P value | BH-adjusted P | log2FC (interaction) |
|---|---|---|---|---|
| RHSIM_Rhsim04G0075000 | 49.27 | 2.23E-12 | 1.01E-11 | -0.738 |
| novel.2715 | 49.24 | 2.26E-12 | 1.02E-11 | -1.600 |
| RHSIM_RhsimUnG0184400 | 49.23 | 2.27E-12 | 1.03E-11 | -1.942 |
| novel.4156 | 49.22 | 2.29E-12 | 1.04E-11 | 0.332 |
| RHSIM_Rhsim04G0184100 | 49.20 | 2.32E-12 | 1.05E-11 | -1.197 |
| RHSIM_Rhsim07G0239300 | 49.19 | 2.32E-12 | 1.05E-11 | -1.153 |
| RHSIM_Rhsim02G0082900 | 49.18 | 2.33E-12 | 1.05E-11 | -0.923 |
| RHSIM_RhsimUnG0118700 | 49.18 | 2.34E-12 | 1.06E-11 | 1.011 |
| RHSIM_Rhsim03G0250500 | 49.17 | 2.35E-12 | 1.06E-11 | 1.644 |
| RHSIM_Rhsim09G0100400 | 49.16 | 2.36E-12 | 1.06E-11 | 0.609 |
| RHSIM_Rhsim12G0115600 | 49.16 | 2.36E-12 | 1.07E-11 | -1.124 |
| RHSIM_Rhsim04G0250200 | 49.15 | 2.37E-12 | 1.07E-11 | 1.545 |
| RHSIM_Rhsim04G0135800 | 49.15 | 2.37E-12 | 1.07E-11 | 0.525 |
| RHSIM_RhsimUnG0245900 | 49.11 | 2.41E-12 | 1.09E-11 | -1.729 |
| novel.895 | 49.11 | 2.42E-12 | 1.09E-11 | -1.222 |
| RHSIM_Rhsim06G0234700 | 49.10 | 2.43E-12 | 1.10E-11 | 0.476 |
| RHSIM_RhsimUnG0036900 | 49.09 | 2.45E-12 | 1.10E-11 | -4.353 |
| RHSIM_Rhsim02G0009500 | 49.08 | 2.46E-12 | 1.11E-11 | -1.895 |
| RHSIM_Rhsim02G0141000 | 49.07 | 2.48E-12 | 1.12E-11 | -0.800 |
| RHSIM_Rhsim06G0172100 | 49.05 | 2.50E-12 | 1.12E-11 | -0.966 |
| RHSIM_Rhsim07G0217400 | 49.04 | 2.51E-12 | 1.13E-11 | 1.320 |
| RHSIM_Rhsim05G0079100 | 49.04 | 2.51E-12 | 1.13E-11 | -0.618 |
| RHSIM_Rhsim08G0092100 | 49.02 | 2.53E-12 | 1.14E-11 | 0.541 |
| RHSIM_Rhsim12G0206000 | 48.98 | 2.58E-12 | 1.16E-11 | 43.713 |
| RHSIM_Rhsim09G0165200 | 48.98 | 2.58E-12 | 1.16E-11 | -0.658 |
| RHSIM_Rhsim10G0048200 | 48.97 | 2.60E-12 | 1.17E-11 | 1.840 |
| RHSIM_Rhsim06G0065900 | 48.97 | 2.60E-12 | 1.17E-11 | -0.684 |
| novel.896 | 48.97 | 2.60E-12 | 1.17E-11 | 2.782 |
| RHSIM_Rhsim04G0221000 | 48.93 | 2.65E-12 | 1.19E-11 | -6.921 |
| RHSIM_Rhsim11G0017700 | 48.93 | 2.66E-12 | 1.20E-11 | -1.547 |
| RHSIM_Rhsim12G0057400 | 48.90 | 2.69E-12 | 1.21E-11 | -0.689 |
| RHSIM_Rhsim04G0227200 | 48.90 | 2.70E-12 | 1.21E-11 | -2.012 |

| Gene | LRT statistic | P value | BH-adjusted P | log2FC (interaction) |
|---|---|---|---|---|
| novel.3003 | 48.89 | 2.70E-12 | 1.22E-11 | 3.255 |
| RHSIM_Rhsim06G0023800 | 48.87 | 2.73E-12 | 1.23E-11 | 0.805 |
| RHSIM_RhsimUnG0199700 | 48.86 | 2.74E-12 | 1.23E-11 | 1.417 |
| RHSIM_Rhsim07G0073100 | 48.86 | 2.75E-12 | 1.24E-11 | 1.210 |
| RHSIM_Rhsim04G0118500 | 48.86 | 2.76E-12 | 1.24E-11 | 0.986 |
| RHSIM_Rhsim04G0054300 | 48.84 | 2.77E-12 | 1.25E-11 | -1.311 |
| RHSIM_Rhsim12G0001700 | 48.84 | 2.78E-12 | 1.25E-11 | -1.681 |
| RHSIM_Rhsim03G0247600 | 48.84 | 2.78E-12 | 1.25E-11 | -0.719 |
| RHSIM_Rhsim08G0053200 | 48.82 | 2.80E-12 | 1.26E-11 | -0.364 |
| RHSIM_Rhsim06G0041500 | 48.82 | 2.81E-12 | 1.26E-11 | -2.787 |
| novel.2128 | 48.82 | 2.81E-12 | 1.26E-11 | 1.013 |
| RHSIM_Rhsim08G0143600 | 48.80 | 2.83E-12 | 1.27E-11 | 4.361 |
| RHSIM_Rhsim03G0032600 | 48.80 | 2.83E-12 | 1.27E-11 | 3.533 |
| RHSIM_Rhsim03G0200600 | 48.75 | 2.90E-12 | 1.30E-11 | -0.541 |
| RHSIM_Rhsim01G0006300 | 48.73 | 2.94E-12 | 1.32E-11 | -46.593 |
| RHSIM_Rhsim08G0132600 | 48.72 | 2.95E-12 | 1.32E-11 | 0.739 |
| RHSIM_Rhsim04G0005700 | 48.70 | 2.98E-12 | 1.34E-11 | -2.025 |
| RHSIM_Rhsim09G0075000 | 48.70 | 2.98E-12 | 1.34E-11 | -1.272 |
| RHSIM_Rhsim03G0212600 | 48.67 | 3.03E-12 | 1.36E-11 | 1.016 |
| RHSIM_Rhsim11G0131400 | 48.61 | 3.12E-12 | 1.40E-11 | -1.281 |
| RHSIM_Rhsim04G0018000 | 48.61 | 3.12E-12 | 1.40E-11 | -1.460 |
| RHSIM_Rhsim06G0173800 | 48.61 | 3.12E-12 | 1.40E-11 | 0.745 |
| RHSIM_Rhsim07G0020600 | 48.60 | 3.13E-12 | 1.41E-11 | 0.487 |
| RHSIM_Rhsim01G0169000 | 48.58 | 3.16E-12 | 1.42E-11 | -0.396 |
| RHSIM_Rhsim09G0154100 | 48.58 | 3.17E-12 | 1.42E-11 | -1.380 |
| RHSIM_Rhsim04G0093600 | 48.58 | 3.17E-12 | 1.42E-11 | 0.586 |
| RHSIM_Rhsim03G0092400 | 48.56 | 3.21E-12 | 1.44E-11 | -0.817 |
| RHSIM_Rhsim02G0154900 | 48.54 | 3.24E-12 | 1.45E-11 | 0.936 |
| RHSIM_Rhsim13G0221200 | 48.53 | 3.25E-12 | 1.46E-11 | 1.825 |
| RHSIM_Rhsim07G0074300 | 48.52 | 3.27E-12 | 1.46E-11 | -0.790 |
| RHSIM_Rhsim04G0154200 | 48.51 | 3.29E-12 | 1.47E-11 | -1.472 |
| RHSIM_Rhsim13G0085100 | 48.50 | 3.31E-12 | 1.48E-11 | 1.174 |

| Gene | LRT statistic | P value | BH-adjusted P | log2FC (interaction) |
|---|---|---|---|---|
| RHSIM_RhsimUnG0120700 | 48.47 | 3.36E-12 | 1.50E-11 | -1.296 |
| RHSIM_Rhsim01G0196200 | 48.46 | 3.37E-12 | 1.51E-11 | -0.427 |
| RHSIM_Rhsim05G0226800 | 48.45 | 3.39E-12 | 1.52E-11 | 0.796 |
| RHSIM_Rhsim03G0106700 | 48.44 | 3.40E-12 | 1.52E-11 | 0.778 |
| RHSIM_Rhsim06G0075600 | 48.43 | 3.42E-12 | 1.53E-11 | 0.775 |
| RHSIM_Rhsim13G0235300 | 48.43 | 3.43E-12 | 1.53E-11 | -0.554 |
| novel.3794 | 48.40 | 3.48E-12 | 1.56E-11 | -0.706 |
| RHSIM_Rhsim05G0029300 | 48.39 | 3.50E-12 | 1.56E-11 | -0.437 |
| RHSIM_Rhsim11G0110400 | 48.38 | 3.51E-12 | 1.57E-11 | 2.470 |
| RHSIM_Rhsim13G0105200 | 48.36 | 3.54E-12 | 1.58E-11 | -1.330 |
| RHSIM_Rhsim13G0169400 | 48.36 | 3.55E-12 | 1.59E-11 | -0.794 |
| RHSIM_Rhsim12G0106100 | 48.33 | 3.60E-12 | 1.61E-11 | 1.375 |
| RHSIM_Rhsim01G0210400 | 48.33 | 3.60E-12 | 1.61E-11 | -0.711 |
| RHSIM_Rhsim12G0069900 | 48.32 | 3.63E-12 | 1.62E-11 | -0.793 |
| RHSIM_Rhsim04G0167700 | 48.31 | 3.64E-12 | 1.62E-11 | 1.934 |
| RHSIM_Rhsim08G0141400 | 48.29 | 3.67E-12 | 1.64E-11 | -1.225 |
| RHSIM_Rhsim01G0212900 | 48.28 | 3.69E-12 | 1.65E-11 | 0.579 |
| RHSIM_Rhsim01G0144500 | 48.28 | 3.70E-12 | 1.65E-11 | -1.675 |
| RHSIM_Rhsim02G0212800 | 48.28 | 3.70E-12 | 1.65E-11 | 5.224 |
| novel.2516 | 48.28 | 3.70E-12 | 1.65E-11 | -1.872 |
| RHSIM_Rhsim04G0139900 | 48.28 | 3.70E-12 | 1.65E-11 | 0.708 |
| RHSIM_Rhsim06G0229800 | 48.27 | 3.71E-12 | 1.66E-11 | 1.826 |
| novel.4206 | 48.25 | 3.74E-12 | 1.67E-11 | -0.697 |
| RHSIM_Rhsim09G0056700 | 48.24 | 3.76E-12 | 1.68E-11 | 0.733 |
| RHSIM_Rhsim10G0196000 | 48.23 | 3.79E-12 | 1.69E-11 | 1.603 |
| RHSIM_RhsimUnG0207700 | 48.22 | 3.80E-12 | 1.69E-11 | 0.726 |
| novel.3418 | 48.21 | 3.83E-12 | 1.71E-11 | -1.634 |
| RHSIM_Rhsim10G0127400 | 48.21 | 3.84E-12 | 1.71E-11 | -1.276 |
| novel.2733 | 48.17 | 3.91E-12 | 1.74E-11 | -0.400 |
| RHSIM_Rhsim11G0021000 | 48.15 | 3.95E-12 | 1.76E-11 | 0.822 |
| RHSIM_Rhsim01G0227300 | 48.15 | 3.95E-12 | 1.76E-11 | -2.822 |
| RHSIM_Rhsim10G0172300 | 48.15 | 3.96E-12 | 1.76E-11 | 1.062 |

| Gene | LRT statistic | P value | BH-adjusted P | log2FC (interaction) |
|---|---|---|---|---|
| RHSIM_Rhsim01G0168200 | 48.14 | 3.97E-12 | 1.77E-11 | 0.574 |
| RHSIM_Rhsim08G0101600 | 48.13 | 3.99E-12 | 1.78E-11 | 0.860 |
| RHSIM_Rhsim13G0096800 | 48.12 | 4.00E-12 | 1.78E-11 | -3.424 |
| RHSIM_Rhsim12G0098400 | 48.12 | 4.01E-12 | 1.78E-11 | 0.777 |
| RHSIM_Rhsim08G0100300 | 48.10 | 4.04E-12 | 1.80E-11 | -2.887 |
| RHSIM_Rhsim03G0161300 | 48.08 | 4.08E-12 | 1.82E-11 | -0.680 |
| RHSIM_Rhsim13G0022500 | 48.07 | 4.12E-12 | 1.83E-11 | 0.706 |
| RHSIM_Rhsim05G0041300 | 48.06 | 4.14E-12 | 1.84E-11 | -0.701 |
| novel.2769 | 48.04 | 4.17E-12 | 1.85E-11 | -0.976 |
| RHSIM_Rhsim13G0027700 | 48.04 | 4.18E-12 | 1.86E-11 | 0.474 |
| RHSIM_Rhsim07G0248900 | 48.03 | 4.20E-12 | 1.87E-11 | -4.830 |
| RHSIM_Rhsim04G0221100 | 48.01 | 4.24E-12 | 1.88E-11 | -0.872 |
| novel.1047 | 48.01 | 4.24E-12 | 1.89E-11 | -2.851 |
| RHSIM_Rhsim05G0005700 | 47.97 | 4.32E-12 | 1.92E-11 | -0.825 |
| RHSIM_Rhsim02G0127800 | 47.95 | 4.36E-12 | 1.94E-11 | -0.879 |
| RHSIM_Rhsim12G0134700 | 47.94 | 4.38E-12 | 1.95E-11 | 0.479 |
| RHSIM_Rhsim03G0232600 | 47.91 | 4.45E-12 | 1.98E-11 | -0.997 |
| RHSIM_Rhsim12G0044300 | 47.91 | 4.46E-12 | 1.98E-11 | -0.517 |
| novel.3777 | 47.85 | 4.60E-12 | 2.04E-11 | -5.948 |
| RHSIM_Rhsim04G0032700 | 47.85 | 4.61E-12 | 2.05E-11 | -0.416 |
| novel.2204 | 47.85 | 4.61E-12 | 2.05E-11 | -1.771 |
| RHSIM_Rhsim05G0053900 | 47.84 | 4.62E-12 | 2.05E-11 | -1.013 |
| RHSIM_Rhsim01G0062600 | 47.83 | 4.64E-12 | 2.06E-11 | 0.757 |
| RHSIM_Rhsim07G0075900 | 47.81 | 4.68E-12 | 2.08E-11 | -0.923 |
| RHSIM_Rhsim13G0113400 | 47.81 | 4.69E-12 | 2.08E-11 | 0.480 |
| RHSIM_Rhsim11G0028300 | 47.79 | 4.75E-12 | 2.11E-11 | -0.627 |
| RHSIM_Rhsim13G0126600 | 47.78 | 4.76E-12 | 2.11E-11 | 0.700 |
| RHSIM_Rhsim10G0064500 | 47.77 | 4.79E-12 | 2.12E-11 | -0.522 |
| RHSIM_Rhsim12G0073700 | 47.76 | 4.82E-12 | 2.14E-11 | -1.474 |
| RHSIM_Rhsim09G0208200 | 47.74 | 4.86E-12 | 2.15E-11 | -0.646 |
| RHSIM_Rhsim01G0216200 | 47.73 | 4.89E-12 | 2.17E-11 | -1.416 |
| novel.2569 | 47.73 | 4.90E-12 | 2.17E-11 | -1.011 |

| Gene | LRT statistic | P value | BH-adjusted P | log2FC (interaction) |
|---|---|---|---|---|
| RHSIM_Rhsim06G0140600 | 47.72 | 4.91E-12 | 2.18E-11 | 1.204 |
| RHSIM_Rhsim09G0111700 | 47.72 | 4.92E-12 | 2.18E-11 | 0.721 |
| RHSIM_Rhsim12G0073600 | 47.70 | 4.96E-12 | 2.20E-11 | -6.092 |
| novel.2945 | 47.70 | 4.97E-12 | 2.20E-11 | 2.202 |
| RHSIM_Rhsim13G0106200 | 47.70 | 4.98E-12 | 2.20E-11 | -2.698 |
| RHSIM_Rhsim08G0208300 | 47.69 | 4.99E-12 | 2.21E-11 | -7.907 |
| RHSIM_Rhsim07G0102000 | 47.67 | 5.05E-12 | 2.24E-11 | 0.763 |
| RHSIM_RhsimUnG0028000 | 47.65 | 5.10E-12 | 2.26E-11 | -5.731 |
| RHSIM_Rhsim09G0001700 | 47.59 | 5.25E-12 | 2.33E-11 | -2.305 |
| novel.2059 | 47.57 | 5.30E-12 | 2.34E-11 | 2.282 |
| RHSIM_Rhsim09G0064200 | 47.56 | 5.33E-12 | 2.36E-11 | -0.555 |
| RHSIM_Rhsim08G0136600 | 47.56 | 5.34E-12 | 2.36E-11 | 0.632 |
| RHSIM_Rhsim09G0185700 | 47.54 | 5.38E-12 | 2.38E-11 | 1.512 |
| RHSIM_Rhsim03G0179000 | 47.54 | 5.39E-12 | 2.38E-11 | -2.207 |
| RHSIM_Rhsim02G0183000 | 47.53 | 5.41E-12 | 2.39E-11 | 0.752 |
| RHSIM_Rhsim01G0163700 | 47.52 | 5.45E-12 | 2.41E-11 | -0.769 |
| RHSIM_Rhsim05G0060600 | 47.51 | 5.49E-12 | 2.42E-11 | 0.393 |
| RHSIM_Rhsim01G0265400 | 47.49 | 5.54E-12 | 2.45E-11 | 0.636 |
| RHSIM_Rhsim13G0187200 | 47.48 | 5.55E-12 | 2.45E-11 | -1.331 |
| novel.343 | 47.48 | 5.56E-12 | 2.46E-11 | -1.904 |
| RHSIM_Rhsim06G0009600 | 47.48 | 5.57E-12 | 2.46E-11 | -0.613 |
| RHSIM_Rhsim12G0131300 | 47.47 | 5.59E-12 | 2.47E-11 | 0.465 |
| RHSIM_Rhsim04G0219600 | 47.47 | 5.59E-12 | 2.47E-11 | 1.181 |
| RHSIM_Rhsim05G0141200 | 47.45 | 5.64E-12 | 2.49E-11 | 2.488 |
| RHSIM_Rhsim11G0198100 | 47.45 | 5.65E-12 | 2.49E-11 | 0.632 |
| RHSIM_Rhsim06G0155400 | 47.43 | 5.70E-12 | 2.52E-11 | -0.713 |
| RHSIM_Rhsim07G0227900 | 47.42 | 5.73E-12 | 2.53E-11 | -2.951 |
| RHSIM_Rhsim10G0112800 | 47.42 | 5.74E-12 | 2.53E-11 | -3.683 |
| RHSIM_Rhsim06G0027100 | 47.42 | 5.74E-12 | 2.53E-11 | -0.420 |
| RHSIM_Rhsim12G0177000 | 47.41 | 5.76E-12 | 2.54E-11 | -1.750 |
| RHSIM_Rhsim05G0101100 | 47.40 | 5.80E-12 | 2.56E-11 | 1.761 |
| RHSIM_Rhsim07G0053100 | 47.37 | 5.87E-12 | 2.59E-11 | 0.539 |

| Gene | LRT statistic | P value | BH-adjusted P | log2FC (interaction) |
|---|---|---|---|---|
| RHSIM_Rhsim05G0141600 | 47.36 | 5.90E-12 | 2.60E-11 | 0.601 |
| RHSIM_Rhsim11G0035500 | 47.36 | 5.91E-12 | 2.60E-11 | 0.720 |
| RHSIM_Rhsim07G0150500 | 47.33 | 6.00E-12 | 2.64E-11 | 1.056 |
| RHSIM_Rhsim05G0229700 | 47.31 | 6.05E-12 | 2.67E-11 | -0.886 |
| RHSIM_Rhsim12G0168200 | 47.31 | 6.06E-12 | 2.67E-11 | -1.150 |
| RHSIM_Rhsim13G0223200 | 47.28 | 6.14E-12 | 2.71E-11 | 2.630 |
| RHSIM_Rhsim03G0261200 | 47.28 | 6.17E-12 | 2.72E-11 | -0.900 |
| RHSIM_Rhsim02G0134300 | 47.27 | 6.20E-12 | 2.73E-11 | -2.082 |
| RHSIM_Rhsim03G0092500 | 47.26 | 6.20E-12 | 2.73E-11 | 2.164 |
| RHSIM_Rhsim02G0022900 | 47.24 | 6.29E-12 | 2.77E-11 | -0.532 |
| RHSIM_Rhsim12G0061100 | 47.23 | 6.32E-12 | 2.78E-11 | -2.282 |
| RHSIM_Rhsim11G0127300 | 47.22 | 6.34E-12 | 2.79E-11 | 0.649 |
| RHSIM_Rhsim03G0017700 | 47.22 | 6.36E-12 | 2.80E-11 | -1.334 |
| RHSIM_Rhsim13G0046400 | 47.20 | 6.42E-12 | 2.83E-11 | -1.016 |
| RHSIM_Rhsim13G0204800 | 47.19 | 6.43E-12 | 2.83E-11 | 1.172 |
| RHSIM_Rhsim10G0104200 | 47.14 | 6.60E-12 | 2.90E-11 | -1.447 |
| RHSIM_Rhsim09G0169500 | 47.11 | 6.72E-12 | 2.95E-11 | -0.858 |
| RHSIM_Rhsim12G0008800 | 47.10 | 6.74E-12 | 2.96E-11 | -1.586 |
| RHSIM_Rhsim12G0039200 | 47.08 | 6.80E-12 | 2.99E-11 | 2.735 |
| RHSIM_Rhsim07G0201200 | 47.07 | 6.84E-12 | 3.01E-11 | 2.637 |
| RHSIM_Rhsim03G0102200 | 47.02 | 7.02E-12 | 3.09E-11 | -0.796 |
| RHSIM_Rhsim01G0068600 | 47.02 | 7.04E-12 | 3.09E-11 | 0.816 |
| RHSIM_Rhsim11G0098200 | 47.01 | 7.07E-12 | 3.11E-11 | 2.178 |
| RHSIM_Rhsim11G0159000 | 46.99 | 7.14E-12 | 3.14E-11 | -0.562 |
| novel.3655 | 46.98 | 7.19E-12 | 3.16E-11 | 2.254 |
| novel.4185 | 46.97 | 7.19E-12 | 3.16E-11 | 1.672 |
| RHSIM_Rhsim08G0085800 | 46.97 | 7.19E-12 | 3.16E-11 | -1.080 |
| RHSIM_Rhsim06G0174800 | 46.97 | 7.22E-12 | 3.17E-11 | -0.959 |
| RHSIM_Rhsim10G0051000 | 46.94 | 7.30E-12 | 3.21E-11 | 43.623 |
| novel.1544 | 46.93 | 7.35E-12 | 3.23E-11 | -6.782 |
| RHSIM_Rhsim13G0050000 | 46.91 | 7.43E-12 | 3.26E-11 | 0.568 |
| RHSIM_Rhsim05G0077400 | 46.88 | 7.53E-12 | 3.30E-11 | 0.896 |

| Gene | LRT statistic | P value | BH-adjusted P | log2FC (interaction) |
|---|---|---|---|---|
| RHSIM_Rhsim08G0049000 | 46.88 | 7.53E-12 | 3.30E-11 | -0.717 |
| RHSIM_Rhsim08G0020100 | 46.86 | 7.62E-12 | 3.34E-11 | -2.203 |
| RHSIM_Rhsim03G0038900 | 46.85 | 7.68E-12 | 3.37E-11 | 0.524 |
| RHSIM_Rhsim04G0018700 | 46.82 | 7.80E-12 | 3.42E-11 | -0.696 |
| RHSIM_Rhsim07G0038000 | 46.82 | 7.80E-12 | 3.42E-11 | -0.950 |
| RHSIM_Rhsim13G0036600 | 46.78 | 7.95E-12 | 3.49E-11 | 0.548 |
| RHSIM_Rhsim07G0212500 | 46.77 | 7.99E-12 | 3.50E-11 | 0.840 |
| RHSIM_Rhsim12G0032900 | 46.77 | 7.99E-12 | 3.50E-11 | -1.265 |
| RHSIM_Rhsim07G0006700 | 46.77 | 7.99E-12 | 3.50E-11 | -1.342 |
| RHSIM_Rhsim12G0193900 | 46.76 | 8.04E-12 | 3.52E-11 | 1.754 |
| RHSIM_Rhsim05G0033900 | 46.73 | 8.16E-12 | 3.57E-11 | -1.873 |
| RHSIM_Rhsim07G0176500 | 46.72 | 8.20E-12 | 3.59E-11 | -0.670 |
| RHSIM_Rhsim09G0133100 | 46.70 | 8.29E-12 | 3.63E-11 | -1.231 |
| RHSIM_Rhsim02G0069600 | 46.68 | 8.34E-12 | 3.65E-11 | 0.416 |
| RHSIM_Rhsim01G0016100 | 46.66 | 8.43E-12 | 3.69E-11 | 0.552 |
| novel.2567 | 46.65 | 8.49E-12 | 3.72E-11 | 3.728 |
| novel.2445 | 46.63 | 8.58E-12 | 3.75E-11 | -1.310 |
| RHSIM_Rhsim06G0233800 | 46.62 | 8.61E-12 | 3.77E-11 | -0.873 |
| RHSIM_Rhsim13G0177700 | 46.62 | 8.64E-12 | 3.78E-11 | 0.797 |
| RHSIM_Rhsim05G0049100 | 46.61 | 8.68E-12 | 3.80E-11 | -0.988 |
| RHSIM_Rhsim10G0163100 | 46.58 | 8.80E-12 | 3.85E-11 | 0.438 |
| RHSIM_Rhsim13G0119800 | 46.57 | 8.85E-12 | 3.87E-11 | 0.615 |
| RHSIM_Rhsim11G0184800 | 46.56 | 8.87E-12 | 3.88E-11 | -0.788 |
| RHSIM_Rhsim04G0249600 | 46.54 | 8.96E-12 | 3.92E-11 | 1.718 |
| RHSIM_Rhsim13G0150600 | 46.54 | 8.98E-12 | 3.93E-11 | 0.485 |
| RHSIM_Rhsim06G0230700 | 46.50 | 9.16E-12 | 4.01E-11 | -1.415 |
| RHSIM_Rhsim08G0112800 | 46.48 | 9.26E-12 | 4.05E-11 | -0.670 |
| RHSIM_Rhsim02G0060700 | 46.47 | 9.30E-12 | 4.06E-11 | -0.749 |
| RHSIM_Rhsim09G0207000 | 46.44 | 9.44E-12 | 4.13E-11 | -0.771 |
| RHSIM_Rhsim10G0195200 | 46.43 | 9.52E-12 | 4.16E-11 | 0.857 |
| RHSIM_Rhsim03G0241000 | 46.42 | 9.57E-12 | 4.18E-11 | 0.641 |
| RHSIM_Rhsim03G0052500 | 46.41 | 9.57E-12 | 4.18E-11 | 1.289 |

| Gene | LRT statistic | P value | BH-adjusted P | log2FC (interaction) |
|---|---|---|---|---|
| RHSIM_Rhsim01G0218000 | 46.41 | 9.57E-12 | 4.18E-11 | -0.544 |
| RHSIM_RhsimUnG0246700 | 46.41 | 9.60E-12 | 4.19E-11 | 2.700 |
| RHSIM_Rhsim12G0151000 | 46.40 | 9.63E-12 | 4.20E-11 | 1.464 |
| RHSIM_Rhsim04G0218400 | 46.40 | 9.64E-12 | 4.21E-11 | 1.293 |
| RHSIM_RhsimUnG0199800 | 46.39 | 9.68E-12 | 4.22E-11 | 1.022 |
| RHSIM_Rhsim11G0069900 | 46.39 | 9.68E-12 | 4.22E-11 | -0.550 |
| novel.875 | 46.36 | 9.83E-12 | 4.29E-11 | 3.187 |
| RHSIM_Rhsim08G0231900 | 46.36 | 9.85E-12 | 4.30E-11 | -0.688 |
| RHSIM_Rhsim08G0194700 | 46.36 | 9.86E-12 | 4.30E-11 | -0.541 |
| RHSIM_Rhsim12G0134500 | 46.32 | 1.01E-11 | 4.38E-11 | 1.864 |
| RHSIM_Rhsim06G0005400 | 46.31 | 1.01E-11 | 4.41E-11 | 0.918 |
| RHSIM_Rhsim06G0185800 | 46.28 | 1.02E-11 | 4.46E-11 | -3.630 |
| RHSIM_Rhsim03G0128600 | 46.28 | 1.02E-11 | 4.46E-11 | 2.325 |
| RHSIM_Rhsim06G0236000 | 46.27 | 1.03E-11 | 4.48E-11 | -0.898 |
| RHSIM_RhsimUnG0118900 | 46.26 | 1.04E-11 | 4.51E-11 | -0.448 |
| RHSIM_Rhsim10G0172800 | 46.24 | 1.04E-11 | 4.55E-11 | -0.495 |
| RHSIM_Rhsim08G0198100 | 46.24 | 1.05E-11 | 4.56E-11 | 0.366 |
| RHSIM_Rhsim06G0021200 | 46.23 | 1.05E-11 | 4.58E-11 | -0.829 |
| RHSIM_Rhsim08G0004500 | 46.21 | 1.06E-11 | 4.62E-11 | -0.881 |
| novel.819 | 46.20 | 1.07E-11 | 4.64E-11 | 0.525 |
| RHSIM_Rhsim12G0057300 | 46.20 | 1.07E-11 | 4.65E-11 | 0.735 |
| RHSIM_Rhsim12G0163800 | 46.20 | 1.07E-11 | 4.65E-11 | 0.622 |
| RHSIM_Rhsim03G0233200 | 46.20 | 1.07E-11 | 4.66E-11 | 0.871 |
| novel.2306 | 46.19 | 1.07E-11 | 4.66E-11 | 5.428 |
| RHSIM_Rhsim04G0218900 | 46.19 | 1.07E-11 | 4.67E-11 | -0.767 |
| RHSIM_Rhsim11G0190500 | 46.16 | 1.09E-11 | 4.74E-11 | 0.639 |
| RHSIM_Rhsim13G0071500 | 46.16 | 1.09E-11 | 4.75E-11 | 2.468 |
| RHSIM_Rhsim06G0159400 | 46.16 | 1.09E-11 | 4.75E-11 | -0.771 |
| RHSIM_RhsimUnG0076400 | 46.14 | 1.10E-11 | 4.78E-11 | 1.863 |
| RHSIM_Rhsim04G0239600 | 46.14 | 1.10E-11 | 4.78E-11 | -0.797 |
| RHSIM_Rhsim07G0049300 | 46.12 | 1.11E-11 | 4.82E-11 | 0.647 |
| RHSIM_Rhsim06G0186600 | 46.11 | 1.12E-11 | 4.86E-11 | 0.900 |

| Gene | LRT statistic | P value | BH-adjusted P | log2FC (interaction) |
|---|---|---|---|---|
| RHSIM_Rhsim01G0236800 | 46.10 | 1.12E-11 | 4.87E-11 | -0.698 |
| RHSIM_Rhsim12G0205400 | 46.10 | 1.13E-11 | 4.89E-11 | -0.430 |
| RHSIM_Rhsim03G0224200 | 46.09 | 1.13E-11 | 4.89E-11 | 0.460 |
| RHSIM_Rhsim05G0185400 | 46.07 | 1.14E-11 | 4.96E-11 | 0.632 |
| RHSIM_Rhsim04G0245600 | 46.07 | 1.14E-11 | 4.96E-11 | 1.524 |
| RHSIM_Rhsim09G0028500 | 46.06 | 1.15E-11 | 4.98E-11 | -0.749 |
| RHSIM_Rhsim07G0239500 | 46.06 | 1.15E-11 | 4.98E-11 | 1.320 |
| RHSIM_Rhsim03G0089600 | 46.02 | 1.17E-11 | 5.07E-11 | 0.897 |
| RHSIM_Rhsim11G0093400 | 46.02 | 1.17E-11 | 5.09E-11 | 1.725 |
| RHSIM_Rhsim08G0083200 | 46.01 | 1.18E-11 | 5.11E-11 | 0.651 |
| RHSIM_Rhsim03G0149300 | 46.01 | 1.18E-11 | 5.11E-11 | -0.685 |
| RHSIM_Rhsim09G0002100 | 46.00 | 1.18E-11 | 5.12E-11 | -1.809 |
| RHSIM_Rhsim08G0162600 | 45.98 | 1.20E-11 | 5.19E-11 | 1.749 |
| RHSIM_Rhsim06G0103500 | 45.95 | 1.21E-11 | 5.25E-11 | 1.469 |
| RHSIM_Rhsim01G0292000 | 45.93 | 1.22E-11 | 5.31E-11 | 0.561 |
| RHSIM_Rhsim06G0061600 | 45.92 | 1.23E-11 | 5.33E-11 | -0.459 |
| RHSIM_Rhsim10G0154600 | 45.92 | 1.23E-11 | 5.33E-11 | 1.848 |
| RHSIM_Rhsim07G0214800 | 45.92 | 1.23E-11 | 5.35E-11 | 0.447 |
| RHSIM_Rhsim01G0285300 | 45.91 | 1.24E-11 | 5.36E-11 | -1.256 |
| RHSIM_Rhsim02G0028700 | 45.85 | 1.27E-11 | 5.52E-11 | 45.710 |
| RHSIM_Rhsim01G0215700 | 45.85 | 1.28E-11 | 5.54E-11 | -0.528 |
| RHSIM_Rhsim05G0095000 | 45.84 | 1.28E-11 | 5.56E-11 | 0.545 |
| RHSIM_Rhsim10G0138800 | 45.84 | 1.29E-11 | 5.57E-11 | 2.317 |
| RHSIM_Rhsim12G0092900 | 45.83 | 1.29E-11 | 5.59E-11 | -2.306 |
| RHSIM_Rhsim12G0160400 | 45.82 | 1.29E-11 | 5.61E-11 | -1.694 |
| RHSIM_Rhsim12G0136000 | 45.81 | 1.30E-11 | 5.64E-11 | 1.248 |
| novel.456 | 45.81 | 1.30E-11 | 5.65E-11 | 1.784 |
| RHSIM_RhsimUnG0152400 | 45.80 | 1.31E-11 | 5.66E-11 | 3.527 |
| RHSIM_Rhsim07G0187500 | 45.80 | 1.31E-11 | 5.68E-11 | 0.717 |
| RHSIM_Rhsim02G0110400 | 45.79 | 1.32E-11 | 5.69E-11 | 0.685 |
| RHSIM_Rhsim07G0140900 | 45.76 | 1.34E-11 | 5.79E-11 | 1.618 |
| novel.3754 | 45.74 | 1.35E-11 | 5.83E-11 | -0.794 |

| Gene | LRT statistic | P value | BH-adjusted P | log2FC (interaction) |
|---|---|---|---|---|
| RHSIM_Rhsim10G0044000 | 45.74 | 1.35E-11 | 5.85E-11 | 4.754 |
| RHSIM_Rhsim01G0160500 | 45.71 | 1.37E-11 | 5.93E-11 | 1.282 |
| RHSIM_Rhsim11G0159200 | 45.71 | 1.37E-11 | 5.94E-11 | 0.747 |
| RHSIM_Rhsim09G0197100 | 45.70 | 1.38E-11 | 5.94E-11 | -0.402 |
| RHSIM_Rhsim01G0147300 | 45.69 | 1.38E-11 | 5.97E-11 | -0.735 |
| RHSIM_Rhsim04G0031600 | 45.69 | 1.38E-11 | 5.98E-11 | 0.763 |
| RHSIM_Rhsim03G0169700 | 45.68 | 1.39E-11 | 6.02E-11 | 1.983 |
| RHSIM_RhsimUnG0099100 | 45.68 | 1.39E-11 | 6.02E-11 | 2.948 |
| RHSIM_Rhsim10G0002700 | 45.67 | 1.40E-11 | 6.05E-11 | -2.205 |
| RHSIM_Rhsim11G0198000 | 45.66 | 1.40E-11 | 6.06E-11 | -0.741 |
| novel.3793 | 45.65 | 1.41E-11 | 6.11E-11 | -1.407 |
| RHSIM_Rhsim10G0019900 | 45.64 | 1.42E-11 | 6.12E-11 | 0.521 |
| RHSIM_Rhsim02G0254500 | 45.63 | 1.43E-11 | 6.16E-11 | -0.776 |
| RHSIM_Rhsim10G0162100 | 45.62 | 1.44E-11 | 6.19E-11 | 1.428 |
| RHSIM_Rhsim07G0031800 | 45.60 | 1.45E-11 | 6.26E-11 | -3.341 |
| RHSIM_Rhsim11G0158900 | 45.59 | 1.46E-11 | 6.30E-11 | -0.990 |
| RHSIM_RhsimUnG0250200 | 45.57 | 1.47E-11 | 6.36E-11 | 0.319 |
| RHSIM_Rhsim04G0149000 | 45.56 | 1.48E-11 | 6.38E-11 | 2.362 |
| novel.4051 | 45.55 | 1.49E-11 | 6.42E-11 | -45.008 |
| RHSIM_RhsimUnG0255600 | 45.55 | 1.49E-11 | 6.43E-11 | 1.749 |
| novel.74 | 45.53 | 1.50E-11 | 6.49E-11 | 0.503 |
| RHSIM_Rhsim01G0256500 | 45.52 | 1.51E-11 | 6.50E-11 | 0.608 |
| RHSIM_Rhsim09G0026100 | 45.52 | 1.51E-11 | 6.53E-11 | -3.206 |
| RHSIM_Rhsim01G0118500 | 45.51 | 1.52E-11 | 6.53E-11 | 2.109 |
| RHSIM_Rhsim03G0210800 | 45.51 | 1.52E-11 | 6.54E-11 | 0.738 |
| RHSIM_Rhsim05G0031800 | 45.47 | 1.55E-11 | 6.66E-11 | 1.106 |
| RHSIM_Rhsim09G0093700 | 45.47 | 1.55E-11 | 6.68E-11 | 0.863 |
| RHSIM_Rhsim09G0001600 | 45.45 | 1.57E-11 | 6.75E-11 | -0.383 |
| RHSIM_Rhsim06G0237500 | 45.44 | 1.57E-11 | 6.78E-11 | -0.897 |
| RHSIM_Rhsim04G0179400 | 45.43 | 1.58E-11 | 6.82E-11 | 2.417 |
| RHSIM_Rhsim03G0222800 | 45.42 | 1.59E-11 | 6.85E-11 | 0.401 |
| RHSIM_Rhsim02G0145700 | 45.41 | 1.60E-11 | 6.89E-11 | 1.093 |

| Gene | LRT statistic | P value | BH-adjusted P | log2FC (interaction) |
|---|---|---|---|---|
| RHSIM_Rhsim10G0133000 | 45.39 | 1.61E-11 | 6.94E-11 | -0.512 |
| RHSIM_Rhsim03G0106400 | 45.39 | 1.62E-11 | 6.96E-11 | -0.598 |
| RHSIM_RhsimUnG0237100 | 45.38 | 1.63E-11 | 7.00E-11 | 2.665 |
| RHSIM_Rhsim01G0001500 | 45.34 | 1.66E-11 | 7.13E-11 | 1.472 |
| RHSIM_Rhsim11G0015900 | 45.34 | 1.66E-11 | 7.13E-11 | 1.319 |
| RHSIM_Rhsim09G0018200 | 45.33 | 1.66E-11 | 7.14E-11 | 3.917 |
| RHSIM_Rhsim07G0115600 | 45.33 | 1.67E-11 | 7.17E-11 | 2.428 |
| RHSIM_Rhsim06G0071900 | 45.32 | 1.67E-11 | 7.19E-11 | -0.471 |
| RHSIM_Rhsim10G0012200 | 45.32 | 1.68E-11 | 7.21E-11 | 3.164 |
| RHSIM_Rhsim08G0213500 | 45.31 | 1.68E-11 | 7.23E-11 | 0.845 |
| RHSIM_Rhsim09G0037400 | 45.28 | 1.70E-11 | 7.32E-11 | -0.488 |
| RHSIM_Rhsim02G0022400 | 45.28 | 1.71E-11 | 7.34E-11 | -1.292 |
| RHSIM_Rhsim03G0231200 | 45.24 | 1.74E-11 | 7.47E-11 | -0.902 |
| novel.1039 | 45.23 | 1.75E-11 | 7.52E-11 | 0.483 |
| RHSIM_Rhsim04G0231800 | 45.22 | 1.76E-11 | 7.55E-11 | 1.760 |
| RHSIM_Rhsim12G0170100 | 45.22 | 1.76E-11 | 7.56E-11 | 1.484 |
| RHSIM_Rhsim04G0150100 | 45.21 | 1.77E-11 | 7.59E-11 | -0.843 |
| RHSIM_Rhsim01G0199400 | 45.20 | 1.78E-11 | 7.65E-11 | -0.993 |
| RHSIM_Rhsim11G0033600 | 45.16 | 1.81E-11 | 7.78E-11 | 1.019 |
| RHSIM_Rhsim12G0074400 | 45.16 | 1.82E-11 | 7.79E-11 | -1.802 |
| novel.1813 | 45.16 | 1.82E-11 | 7.79E-11 | -1.999 |
| RHSIM_Rhsim07G0175300 | 45.16 | 1.82E-11 | 7.80E-11 | -0.464 |
| RHSIM_Rhsim03G0018100 | 45.12 | 1.86E-11 | 7.96E-11 | 1.379 |
| RHSIM_Rhsim02G0198800 | 45.11 | 1.86E-11 | 7.98E-11 | 1.049 |
| RHSIM_Rhsim08G0010500 | 45.10 | 1.88E-11 | 8.04E-11 | -0.886 |
| RHSIM_Rhsim11G0179100 | 45.08 | 1.89E-11 | 8.10E-11 | -0.603 |
| RHSIM_Rhsim01G0251100 | 45.07 | 1.91E-11 | 8.17E-11 | -1.826 |
| RHSIM_Rhsim01G0153300 | 45.06 | 1.91E-11 | 8.18E-11 | 0.511 |
| RHSIM_Rhsim01G0269100 | 45.05 | 1.92E-11 | 8.22E-11 | 0.949 |
| RHSIM_Rhsim05G0003200 | 45.02 | 1.95E-11 | 8.35E-11 | 1.934 |
| RHSIM_Rhsim10G0003100 | 44.99 | 1.98E-11 | 8.47E-11 | -0.663 |
| RHSIM_Rhsim06G0058500 | 44.99 | 1.98E-11 | 8.48E-11 | 2.148 |

| Gene | LRT statistic | P value | BH-adjusted P | log2FC (interaction) |
|---|---|---|---|---|
| RHSIM_Rhsim06G0007900 | 44.98 | 1.99E-11 | 8.52E-11 | -0.775 |
| RHSIM_Rhsim09G0060000 | 44.96 | 2.01E-11 | 8.60E-11 | -0.732 |
| RHSIM_Rhsim06G0238800 | 44.95 | 2.02E-11 | 8.64E-11 | -0.449 |
| RHSIM_Rhsim10G0114000 | 44.95 | 2.03E-11 | 8.67E-11 | 0.805 |
| RHSIM_Rhsim08G0044400 | 44.91 | 2.07E-11 | 8.84E-11 | 0.745 |
| RHSIM_Rhsim05G0231200 | 44.90 | 2.07E-11 | 8.87E-11 | 0.599 |
| RHSIM_Rhsim13G0130300 | 44.90 | 2.07E-11 | 8.87E-11 | 2.837 |
| RHSIM_Rhsim05G0017300 | 44.89 | 2.08E-11 | 8.90E-11 | 0.550 |
| RHSIM_Rhsim05G0180100 | 44.89 | 2.09E-11 | 8.93E-11 | -1.225 |
| RHSIM_Rhsim01G0184600 | 44.88 | 2.09E-11 | 8.95E-11 | 1.091 |
| RHSIM_Rhsim09G0184000 | 44.88 | 2.09E-11 | 8.96E-11 | 0.551 |
| RHSIM_Rhsim03G0098900 | 44.88 | 2.10E-11 | 8.97E-11 | 0.960 |
| RHSIM_Rhsim07G0017300 | 44.86 | 2.12E-11 | 9.05E-11 | 1.916 |
| novel.3747 | 44.84 | 2.14E-11 | 9.16E-11 | 43.001 |
| RHSIM_Rhsim03G0190800 | 44.83 | 2.15E-11 | 9.20E-11 | 1.716 |
| RHSIM_Rhsim09G0162900 | 44.82 | 2.16E-11 | 9.23E-11 | -6.361 |
| RHSIM_Rhsim07G0219500 | 44.82 | 2.16E-11 | 9.24E-11 | 0.520 |
| RHSIM_Rhsim03G0018600 | 44.81 | 2.18E-11 | 9.29E-11 | 0.642 |
| RHSIM_Rhsim03G0137800 | 44.79 | 2.19E-11 | 9.37E-11 | -2.038 |
| RHSIM_Rhsim04G0147800 | 44.79 | 2.20E-11 | 9.38E-11 | 0.969 |
| RHSIM_Rhsim01G0257800 | 44.77 | 2.21E-11 | 9.45E-11 | -1.019 |
| RHSIM_Rhsim10G0064000 | 44.75 | 2.24E-11 | 9.55E-11 | -2.773 |
| RHSIM_Rhsim12G0107500 | 44.74 | 2.25E-11 | 9.61E-11 | 0.889 |
| RHSIM_Rhsim03G0106100 | 44.73 | 2.27E-11 | 9.68E-11 | -0.514 |
| RHSIM_Rhsim02G0152200 | 44.72 | 2.28E-11 | 9.71E-11 | -0.516 |
| RHSIM_Rhsim02G0001800 | 44.69 | 2.31E-11 | 9.84E-11 | -1.451 |
| RHSIM_Rhsim12G0024500 | 44.69 | 2.31E-11 | 9.85E-11 | -0.541 |
| RHSIM_Rhsim01G0168500 | 44.68 | 2.32E-11 | 9.88E-11 | -0.652 |
| RHSIM_Rhsim04G0050300 | 44.68 | 2.32E-11 | 9.90E-11 | 0.684 |
| RHSIM_RhsimUnG0095500 | 44.64 | 2.36E-11 | 1.01E-10 | -0.981 |
| RHSIM_Rhsim11G0136400 | 44.64 | 2.36E-11 | 1.01E-10 | 1.151 |
| RHSIM_Rhsim08G0056900 | 44.64 | 2.37E-11 | 1.01E-10 | 1.210 |

| Gene | LRT statistic | P value | BH-adjusted P | log2FC (interaction) |
|---|---|---|---|---|
| RHSIM_Rhsim09G0115700 | 44.62 | 2.39E-11 | 1.02E-10 | 1.237 |
| novel.1447 | 44.60 | 2.42E-11 | 1.03E-10 | 1.822 |
| RHSIM_Rhsim12G0153700 | 44.58 | 2.45E-11 | 1.04E-10 | -0.913 |
| novel.3231 | 44.57 | 2.45E-11 | 1.04E-10 | 0.981 |
| RHSIM_Rhsim08G0122800 | 44.57 | 2.46E-11 | 1.05E-10 | 0.602 |
| RHSIM_Rhsim08G0146300 | 44.57 | 2.46E-11 | 1.05E-10 | -0.573 |
| RHSIM_Rhsim01G0290800 | 44.57 | 2.46E-11 | 1.05E-10 | 0.619 |
| RHSIM_Rhsim12G0124300 | 44.54 | 2.49E-11 | 1.06E-10 | 1.113 |
| RHSIM_Rhsim03G0181000 | 44.52 | 2.52E-11 | 1.07E-10 | -1.532 |
| RHSIM_Rhsim02G0013700 | 44.49 | 2.56E-11 | 1.09E-10 | -0.529 |
| RHSIM_Rhsim03G0256400 | 44.49 | 2.56E-11 | 1.09E-10 | -1.072 |
| novel.1150 | 44.48 | 2.57E-11 | 1.09E-10 | -0.660 |
| RHSIM_Rhsim13G0208400 | 44.48 | 2.57E-11 | 1.09E-10 | 0.397 |
| RHSIM_Rhsim05G0222300 | 44.47 | 2.58E-11 | 1.10E-10 | 2.771 |
| RHSIM_RhsimUnG0256800 | 44.47 | 2.58E-11 | 1.10E-10 | 1.070 |
| RHSIM_Rhsim01G0192600 | 44.45 | 2.61E-11 | 1.11E-10 | -0.602 |
| RHSIM_Rhsim04G0084900 | 44.43 | 2.64E-11 | 1.12E-10 | 0.593 |
| novel.353 | 44.41 | 2.66E-11 | 1.13E-10 | 1.235 |
| RHSIM_Rhsim10G0138500 | 44.41 | 2.67E-11 | 1.13E-10 | -0.546 |
| RHSIM_Rhsim02G0234700 | 44.40 | 2.68E-11 | 1.14E-10 | -3.077 |
| RHSIM_Rhsim02G0097100 | 44.39 | 2.68E-11 | 1.14E-10 | -2.117 |
| RHSIM_Rhsim08G0039200 | 44.38 | 2.70E-11 | 1.15E-10 | 1.038 |
| RHSIM_Rhsim10G0022200 | 44.35 | 2.75E-11 | 1.17E-10 | -0.718 |
| RHSIM_Rhsim08G0096200 | 44.34 | 2.76E-11 | 1.17E-10 | 0.660 |
| RHSIM_Rhsim06G0160600 | 44.33 | 2.78E-11 | 1.18E-10 | -1.058 |
| RHSIM_Rhsim09G0087000 | 44.30 | 2.82E-11 | 1.20E-10 | 0.699 |
| RHSIM_Rhsim05G0003900 | 44.29 | 2.83E-11 | 1.20E-10 | -0.779 |
| RHSIM_Rhsim11G0158700 | 44.29 | 2.84E-11 | 1.21E-10 | 0.825 |
| RHSIM_Rhsim08G0072700 | 44.26 | 2.88E-11 | 1.22E-10 | -0.647 |
| RHSIM_Rhsim07G0055900 | 44.24 | 2.90E-11 | 1.23E-10 | 0.370 |
| RHSIM_Rhsim08G0006600 | 44.22 | 2.93E-11 | 1.24E-10 | -2.013 |
| RHSIM_Rhsim11G0027400 | 44.22 | 2.93E-11 | 1.24E-10 | -0.467 |

| Gene | LRT statistic | P value | BH-adjusted P | log2FC (interaction) |
|---|---|---|---|---|
| RHSIM_Rhsim04G0199900 | 44.22 | 2.93E-11 | 1.24E-10 | -0.667 |
| novel.4 | 44.22 | 2.94E-11 | 1.25E-10 | 1.338 |
| novel.46 | 44.21 | 2.94E-11 | 1.25E-10 | 1.166 |
| RHSIM_Rhsim03G0147900 | 44.21 | 2.95E-11 | 1.25E-10 | -3.458 |
| RHSIM_Rhsim11G0111800 | 44.18 | 2.99E-11 | 1.27E-10 | 0.655 |
| RHSIM_Rhsim02G0050400 | 44.18 | 2.99E-11 | 1.27E-10 | -4.413 |
| RHSIM_Rhsim09G0141600 | 44.18 | 2.99E-11 | 1.27E-10 | -0.812 |
| RHSIM_RhsimUnG0173200 | 44.18 | 2.99E-11 | 1.27E-10 | 2.790 |
| RHSIM_Rhsim01G0272600 | 44.18 | 3.00E-11 | 1.27E-10 | 1.054 |
| RHSIM_Rhsim06G0026000 | 44.17 | 3.01E-11 | 1.28E-10 | -0.932 |
| RHSIM_Rhsim03G0179600 | 44.15 | 3.04E-11 | 1.29E-10 | -0.549 |
| RHSIM_Rhsim11G0160800 | 44.14 | 3.05E-11 | 1.29E-10 | -0.514 |
| RHSIM_Rhsim06G0199400 | 44.14 | 3.06E-11 | 1.30E-10 | 0.453 |
| RHSIM_Rhsim08G0234700 | 44.12 | 3.08E-11 | 1.31E-10 | 0.976 |
| RHSIM_Rhsim10G0010300 | 44.10 | 3.13E-11 | 1.32E-10 | -1.284 |
| RHSIM_Rhsim01G0182300 | 44.07 | 3.17E-11 | 1.34E-10 | -1.281 |
| RHSIM_Rhsim10G0194500 | 44.06 | 3.19E-11 | 1.35E-10 | -0.735 |
| RHSIM_Rhsim11G0106100 | 44.04 | 3.22E-11 | 1.36E-10 | 1.773 |
| RHSIM_Rhsim09G0109300 | 44.03 | 3.24E-11 | 1.37E-10 | 0.401 |
| RHSIM_Rhsim06G0166200 | 44.02 | 3.26E-11 | 1.38E-10 | 0.773 |
| RHSIM_Rhsim10G0015100 | 44.00 | 3.28E-11 | 1.39E-10 | 2.704 |
| RHSIM_Rhsim07G0203900 | 44.00 | 3.29E-11 | 1.39E-10 | 1.742 |
| novel.4166 | 43.97 | 3.33E-11 | 1.41E-10 | -1.189 |
| RHSIM_Rhsim05G0110600 | 43.97 | 3.34E-11 | 1.41E-10 | 0.967 |
| novel.2173 | 43.93 | 3.40E-11 | 1.44E-10 | 2.643 |
| RHSIM_Rhsim08G0125300 | 43.93 | 3.41E-11 | 1.44E-10 | 1.165 |
| RHSIM_Rhsim08G0000800 | 43.91 | 3.44E-11 | 1.45E-10 | -0.406 |
| novel.3561 | 43.90 | 3.46E-11 | 1.46E-10 | 4.062 |
| RHSIM_Rhsim10G0079200 | 43.88 | 3.49E-11 | 1.48E-10 | -2.016 |
| RHSIM_Rhsim09G0140100 | 43.88 | 3.49E-11 | 1.48E-10 | -2.850 |
| RHSIM_Rhsim05G0186000 | 43.87 | 3.51E-11 | 1.48E-10 | 0.550 |
| novel.430 | 43.86 | 3.53E-11 | 1.49E-10 | 0.483 |

| Gene | LRT statistic | P value | BH-adjusted P | log2FC (interaction) |
|---|---|---|---|---|
| RHSIM_Rhsim08G0082600 | 43.85 | 3.54E-11 | 1.49E-10 | 1.233 |
| RHSIM_Rhsim12G0025200 | 43.85 | 3.54E-11 | 1.50E-10 | -1.485 |
| novel.3533 | 43.84 | 3.56E-11 | 1.50E-10 | 0.640 |
| novel.1881 | 43.84 | 3.57E-11 | 1.51E-10 | -43.673 |
| RHSIM_Rhsim04G0007200 | 43.82 | 3.60E-11 | 1.52E-10 | 0.647 |
| RHSIM_Rhsim01G0168400 | 43.82 | 3.60E-11 | 1.52E-10 | 0.704 |
| RHSIM_Rhsim02G0048900 | 43.81 | 3.63E-11 | 1.53E-10 | -0.874 |
| RHSIM_Rhsim01G0235200 | 43.78 | 3.67E-11 | 1.55E-10 | 0.932 |
| RHSIM_Rhsim13G0129900 | 43.78 | 3.68E-11 | 1.55E-10 | -0.723 |
| RHSIM_Rhsim08G0215300 | 43.77 | 3.69E-11 | 1.56E-10 | -42.376 |
| RHSIM_Rhsim03G0262200 | 43.77 | 3.70E-11 | 1.56E-10 | 1.144 |
| novel.2762 | 43.75 | 3.74E-11 | 1.58E-10 | 1.155 |
| RHSIM_Rhsim12G0214500 | 43.75 | 3.74E-11 | 1.58E-10 | -0.970 |
| novel.3727 | 43.75 | 3.74E-11 | 1.58E-10 | 0.741 |
| RHSIM_Rhsim08G0109000 | 43.74 | 3.76E-11 | 1.58E-10 | -6.197 |
| RHSIM_Rhsim05G0233500 | 43.73 | 3.76E-11 | 1.58E-10 | 3.226 |
| RHSIM_Rhsim11G0002200 | 43.73 | 3.76E-11 | 1.59E-10 | 1.522 |
| RHSIM_Rhsim04G0195800 | 43.73 | 3.78E-11 | 1.59E-10 | -1.858 |
| RHSIM_RhsimUnG0086500 | 43.72 | 3.79E-11 | 1.60E-10 | -1.032 |
| RHSIM_Rhsim11G0088000 | 43.72 | 3.80E-11 | 1.60E-10 | -0.667 |
| RHSIM_Rhsim11G0027100 | 43.71 | 3.81E-11 | 1.60E-10 | 3.524 |
| novel.378 | 43.71 | 3.82E-11 | 1.61E-10 | 1.115 |
| novel.3644 | 43.69 | 3.85E-11 | 1.62E-10 | 2.424 |
| RHSIM_Rhsim10G0162400 | 43.68 | 3.87E-11 | 1.63E-10 | 1.046 |
| RHSIM_Rhsim08G0236100 | 43.66 | 3.91E-11 | 1.64E-10 | -0.496 |
| RHSIM_RhsimUnG0033300 | 43.66 | 3.91E-11 | 1.64E-10 | 1.347 |
| RHSIM_Rhsim09G0010000 | 43.63 | 3.97E-11 | 1.67E-10 | -0.947 |
| RHSIM_Rhsim13G0202400 | 43.62 | 3.99E-11 | 1.68E-10 | -0.593 |
| RHSIM_Rhsim12G0142800 | 43.61 | 4.00E-11 | 1.68E-10 | 0.665 |
| novel.2472 | 43.61 | 4.00E-11 | 1.68E-10 | 7.322 |
| RHSIM_Rhsim08G0063200 | 43.58 | 4.07E-11 | 1.71E-10 | -3.647 |
| RHSIM_Rhsim06G0077600 | 43.58 | 4.07E-11 | 1.71E-10 | 0.558 |

| Gene | LRT statistic | P value | BH-adjusted P | log2FC (interaction) |
|---|---|---|---|---|
| RHSIM_Rhsim10G0068300 | 43.58 | 4.07E-11 | 1.71E-10 | -2.075 |
| RHSIM_RhsimUnG0060900 | 43.56 | 4.11E-11 | 1.73E-10 | -1.669 |
| RHSIM_RhsimUnG0082200 | 43.56 | 4.11E-11 | 1.73E-10 | 0.727 |
| RHSIM_Rhsim13G0094900 | 43.55 | 4.13E-11 | 1.74E-10 | 2.052 |
| RHSIM_Rhsim07G0094200 | 43.55 | 4.14E-11 | 1.74E-10 | 4.881 |
| RHSIM_Rhsim06G0008800 | 43.54 | 4.16E-11 | 1.74E-10 | 0.699 |
| RHSIM_RhsimUnG0151000 | 43.50 | 4.23E-11 | 1.78E-10 | -1.447 |
| RHSIM_Rhsim06G0046000 | 43.47 | 4.29E-11 | 1.80E-10 | -1.890 |
| RHSIM_Rhsim02G0120100 | 43.47 | 4.30E-11 | 1.81E-10 | 0.701 |
| RHSIM_Rhsim04G0198500 | 43.47 | 4.31E-11 | 1.81E-10 | -1.299 |
| RHSIM_Rhsim07G0136100 | 43.46 | 4.32E-11 | 1.81E-10 | 1.123 |
| RHSIM_Rhsim13G0226500 | 43.46 | 4.33E-11 | 1.82E-10 | 0.637 |
| RHSIM_Rhsim05G0079700 | 43.45 | 4.35E-11 | 1.82E-10 | -1.495 |
| RHSIM_Rhsim07G0181600 | 43.42 | 4.43E-11 | 1.86E-10 | -0.600 |
| RHSIM_Rhsim01G0005400 | 43.40 | 4.46E-11 | 1.87E-10 | -1.909 |
| RHSIM_RhsimUnG0243600 | 43.40 | 4.47E-11 | 1.87E-10 | 1.270 |
| RHSIM_Rhsim06G0102200 | 43.39 | 4.48E-11 | 1.88E-10 | 1.073 |
| RHSIM_Rhsim02G0120800 | 43.38 | 4.51E-11 | 1.89E-10 | -0.696 |
| RHSIM_Rhsim08G0068800 | 43.35 | 4.57E-11 | 1.92E-10 | 1.268 |
| RHSIM_Rhsim09G0128000 | 43.35 | 4.58E-11 | 1.92E-10 | -2.799 |
| novel.4238 | 43.32 | 4.64E-11 | 1.95E-10 | -1.507 |
| RHSIM_Rhsim01G0187900 | 43.32 | 4.65E-11 | 1.95E-10 | -0.841 |
| RHSIM_Rhsim01G0235000 | 43.32 | 4.65E-11 | 1.95E-10 | -2.297 |
| RHSIM_Rhsim07G0019000 | 43.32 | 4.65E-11 | 1.95E-10 | -0.775 |
| RHSIM_Rhsim07G0141500 | 43.29 | 4.73E-11 | 1.98E-10 | 1.619 |
| RHSIM_Rhsim13G0019300 | 43.25 | 4.83E-11 | 2.02E-10 | 1.189 |
| RHSIM_Rhsim02G0252600 | 43.24 | 4.83E-11 | 2.02E-10 | -0.506 |
| RHSIM_Rhsim02G0027400 | 43.23 | 4.87E-11 | 2.04E-10 | 1.509 |
| RHSIM_Rhsim02G0129800 | 43.21 | 4.92E-11 | 2.06E-10 | 1.152 |
| novel.4017 | 43.20 | 4.95E-11 | 2.07E-10 | 1.851 |
| RHSIM_Rhsim07G0251400 | 43.19 | 4.98E-11 | 2.08E-10 | 0.629 |
| RHSIM_Rhsim13G0026700 | 43.18 | 4.99E-11 | 2.09E-10 | 1.116 |

| Gene | LRT statistic | P value | BH-adjusted P | log2FC (interaction) |
|---|---|---|---|---|
| RHSIM_Rhsim01G0242200 | 43.18 | 5.00E-11 | 2.09E-10 | 1.041 |
| RHSIM_Rhsim13G0053000 | 43.17 | 5.01E-11 | 2.10E-10 | 0.593 |
| RHSIM_Rhsim11G0142500 | 43.15 | 5.08E-11 | 2.12E-10 | -2.619 |
| RHSIM_Rhsim13G0115400 | 43.15 | 5.08E-11 | 2.13E-10 | -1.262 |
| RHSIM_Rhsim11G0049600 | 43.12 | 5.16E-11 | 2.16E-10 | 0.369 |
| RHSIM_Rhsim10G0051900 | 43.11 | 5.17E-11 | 2.16E-10 | 1.093 |
| novel.2271 | 43.11 | 5.18E-11 | 2.16E-10 | -0.484 |
| RHSIM_Rhsim10G0081200 | 43.10 | 5.21E-11 | 2.18E-10 | -1.670 |
| RHSIM_Rhsim03G0104200 | 43.07 | 5.27E-11 | 2.20E-10 | -1.887 |
| RHSIM_Rhsim01G0285400 | 43.07 | 5.27E-11 | 2.20E-10 | -1.188 |
| RHSIM_Rhsim02G0123500 | 43.07 | 5.28E-11 | 2.21E-10 | 0.517 |
| RHSIM_Rhsim10G0003900 | 43.05 | 5.35E-11 | 2.23E-10 | -0.661 |
| RHSIM_Rhsim03G0145800 | 43.04 | 5.35E-11 | 2.24E-10 | 1.277 |
| RHSIM_Rhsim09G0069100 | 43.04 | 5.36E-11 | 2.24E-10 | -0.330 |
| RHSIM_Rhsim07G0034200 | 43.04 | 5.37E-11 | 2.24E-10 | 0.856 |
| novel.3662 | 43.03 | 5.38E-11 | 2.25E-10 | 2.281 |
| RHSIM_Rhsim03G0017900 | 43.03 | 5.40E-11 | 2.25E-10 | -0.872 |
| RHSIM_Rhsim06G0218800 | 43.01 | 5.44E-11 | 2.27E-10 | -0.534 |
| RHSIM_Rhsim08G0146100 | 43.00 | 5.47E-11 | 2.28E-10 | 0.435 |
| RHSIM_Rhsim01G0236300 | 43.00 | 5.48E-11 | 2.29E-10 | 3.201 |
| RHSIM_Rhsim12G0027700 | 43.00 | 5.48E-11 | 2.29E-10 | -1.353 |
| RHSIM_Rhsim04G0203300 | 43.00 | 5.49E-11 | 2.29E-10 | 0.670 |
| RHSIM_Rhsim09G0034300 | 42.99 | 5.51E-11 | 2.30E-10 | -1.450 |
| RHSIM_Rhsim13G0077700 | 42.98 | 5.54E-11 | 2.31E-10 | 0.840 |
| RHSIM_Rhsim11G0192600 | 42.98 | 5.54E-11 | 2.31E-10 | -1.756 |
| RHSIM_Rhsim05G0145100 | 42.98 | 5.54E-11 | 2.31E-10 | 3.258 |
| RHSIM_Rhsim03G0053100 | 42.97 | 5.56E-11 | 2.31E-10 | 0.820 |
| RHSIM_Rhsim12G0211300 | 42.96 | 5.58E-11 | 2.33E-10 | -0.783 |
| RHSIM_Rhsim13G0202500 | 42.95 | 5.62E-11 | 2.34E-10 | -1.103 |
| RHSIM_Rhsim04G0019800 | 42.93 | 5.68E-11 | 2.37E-10 | -0.619 |
| RHSIM_Rhsim04G0113300 | 42.90 | 5.75E-11 | 2.40E-10 | -0.879 |
| RHSIM_Rhsim11G0045600 | 42.90 | 5.76E-11 | 2.40E-10 | -0.477 |

| Gene | LRT statistic | P value | BH-adjusted P | log2FC (interaction) |
|---|---|---|---|---|
| RHSIM_Rhsim01G0242500 | 42.89 | 5.79E-11 | 2.41E-10 | -0.970 |
| novel.3963 | 42.89 | 5.79E-11 | 2.41E-10 | 1.181 |
| RHSIM_Rhsim04G0066900 | 42.89 | 5.80E-11 | 2.41E-10 | 2.723 |
| RHSIM_RhsimUnG0055200 | 42.88 | 5.81E-11 | 2.42E-10 | 0.768 |
| RHSIM_Rhsim03G0018000 | 42.88 | 5.82E-11 | 2.42E-10 | -0.439 |
| RHSIM_Rhsim12G0100900 | 42.87 | 5.84E-11 | 2.43E-10 | -0.449 |
| RHSIM_Rhsim10G0064900 | 42.86 | 5.88E-11 | 2.44E-10 | -1.055 |
| RHSIM_Rhsim05G0014500 | 42.86 | 5.88E-11 | 2.45E-10 | 0.413 |
| RHSIM_Rhsim04G0038100 | 42.86 | 5.89E-11 | 2.45E-10 | 0.565 |
| novel.3018 | 42.85 | 5.90E-11 | 2.45E-10 | -1.139 |
| novel.3381 | 42.83 | 5.97E-11 | 2.48E-10 | -2.074 |
| RHSIM_Rhsim01G0284000 | 42.83 | 5.97E-11 | 2.48E-10 | 0.388 |
| RHSIM_Rhsim05G0055500 | 42.83 | 5.98E-11 | 2.48E-10 | -1.105 |
| RHSIM_Rhsim05G0037300 | 42.81 | 6.02E-11 | 2.50E-10 | 1.682 |
| RHSIM_Rhsim03G0161100 | 42.80 | 6.06E-11 | 2.52E-10 | -0.676 |
| RHSIM_Rhsim10G0177900 | 42.78 | 6.12E-11 | 2.54E-10 | -0.723 |
| RHSIM_Rhsim11G0042100 | 42.78 | 6.14E-11 | 2.55E-10 | -1.649 |
| RHSIM_Rhsim09G0056300 | 42.76 | 6.18E-11 | 2.57E-10 | 2.298 |
| RHSIM_Rhsim05G0082900 | 42.76 | 6.18E-11 | 2.57E-10 | 0.695 |
| RHSIM_Rhsim02G0014300 | 42.76 | 6.19E-11 | 2.57E-10 | 1.703 |
| RHSIM_Rhsim08G0072200 | 42.75 | 6.23E-11 | 2.59E-10 | 0.576 |
| RHSIM_Rhsim10G0034800 | 42.74 | 6.24E-11 | 2.59E-10 | -2.872 |
| RHSIM_Rhsim12G0025300 | 42.74 | 6.25E-11 | 2.60E-10 | -0.525 |
| RHSIM_Rhsim08G0196900 | 42.73 | 6.28E-11 | 2.61E-10 | 2.497 |
| RHSIM_Rhsim13G0042000 | 42.71 | 6.35E-11 | 2.63E-10 | -0.693 |
| RHSIM_Rhsim12G0167500 | 42.71 | 6.36E-11 | 2.64E-10 | -0.904 |
| RHSIM_Rhsim09G0137600 | 42.70 | 6.37E-11 | 2.64E-10 | 0.568 |
| RHSIM_Rhsim06G0108800 | 42.70 | 6.39E-11 | 2.65E-10 | -1.203 |
| RHSIM_Rhsim12G0004200 | 42.67 | 6.48E-11 | 2.68E-10 | 0.455 |
| RHSIM_Rhsim06G0038400 | 42.66 | 6.51E-11 | 2.70E-10 | 1.052 |
| RHSIM_Rhsim13G0179800 | 42.66 | 6.53E-11 | 2.71E-10 | 1.585 |
| RHSIM_Rhsim05G0093100 | 42.65 | 6.56E-11 | 2.72E-10 | -1.041 |

| Gene | LRT statistic | P value | BH-adjusted P | log2FC (interaction) |
|---|---|---|---|---|
| RHSIM_Rhsim04G0006900 | 42.64 | 6.57E-11 | 2.72E-10 | -0.826 |
| RHSIM_Rhsim08G0083300 | 42.63 | 6.61E-11 | 2.74E-10 | -1.750 |
| RHSIM_Rhsim12G0166800 | 42.62 | 6.64E-11 | 2.75E-10 | 2.679 |
| RHSIM_Rhsim09G0211100 | 42.61 | 6.68E-11 | 2.77E-10 | 5.824 |
| RHSIM_RhsimUnG0079300 | 42.57 | 6.82E-11 | 2.82E-10 | 0.740 |
| RHSIM_Rhsim03G0251900 | 42.56 | 6.84E-11 | 2.83E-10 | 1.321 |
| RHSIM_Rhsim08G0008000 | 42.55 | 6.90E-11 | 2.86E-10 | -1.316 |
| RHSIM_Rhsim13G0224500 | 42.55 | 6.91E-11 | 2.86E-10 | -0.815 |
| RHSIM_Rhsim13G0138100 | 42.54 | 6.94E-11 | 2.87E-10 | -0.656 |
| RHSIM_Rhsim13G0190800 | 42.53 | 6.96E-11 | 2.88E-10 | -2.990 |
| novel.4289 | 42.53 | 6.98E-11 | 2.89E-10 | -2.594 |
| RHSIM_Rhsim01G0181600 | 42.52 | 7.00E-11 | 2.90E-10 | -0.621 |
| RHSIM_Rhsim02G0246200 | 42.51 | 7.04E-11 | 2.91E-10 | 1.267 |
| RHSIM_Rhsim05G0148800 | 42.49 | 7.11E-11 | 2.94E-10 | -0.954 |
| RHSIM_Rhsim07G0013500 | 42.48 | 7.16E-11 | 2.96E-10 | -0.414 |
| RHSIM_Rhsim08G0156400 | 42.45 | 7.26E-11 | 3.00E-10 | -2.190 |
| RHSIM_Rhsim05G0112500 | 42.41 | 7.39E-11 | 3.06E-10 | 0.738 |
| novel.3067 | 42.40 | 7.43E-11 | 3.07E-10 | 3.584 |
| RHSIM_RhsimUnG0069000 | 42.40 | 7.45E-11 | 3.08E-10 | -1.543 |
| RHSIM_Rhsim02G0117700 | 42.39 | 7.47E-11 | 3.09E-10 | -0.767 |
| RHSIM_Rhsim03G0007000 | 42.38 | 7.50E-11 | 3.10E-10 | 0.574 |
| RHSIM_Rhsim08G0163100 | 42.36 | 7.60E-11 | 3.14E-10 | -2.083 |
| RHSIM_Rhsim13G0049800 | 42.33 | 7.73E-11 | 3.19E-10 | -1.685 |
| RHSIM_Rhsim04G0068000 | 42.31 | 7.80E-11 | 3.22E-10 | -1.303 |
| RHSIM_Rhsim04G0215200 | 42.29 | 7.88E-11 | 3.25E-10 | 1.123 |
| RHSIM_Rhsim09G0091600 | 42.28 | 7.90E-11 | 3.26E-10 | -2.015 |
| RHSIM_Rhsim07G0191500 | 42.28 | 7.91E-11 | 3.27E-10 | -0.997 |
| novel.1816 | 42.26 | 7.98E-11 | 3.30E-10 | 0.455 |
| novel.3568 | 42.26 | 7.99E-11 | 3.30E-10 | 3.701 |
| RHSIM_Rhsim02G0252000 | 42.25 | 8.02E-11 | 3.31E-10 | -0.863 |
| novel.1417 | 42.25 | 8.03E-11 | 3.31E-10 | 0.946 |
| RHSIM_RhsimUnG0106100 | 42.25 | 8.05E-11 | 3.32E-10 | -1.846 |

| Gene | LRT statistic | P value | BH-adjusted P | log2FC (interaction) |
|---|---|---|---|---|
| RHSIM_Rhsim08G0168800 | 42.20 | 8.23E-11 | 3.39E-10 | -2.245 |
| RHSIM_Rhsim01G0111100 | 42.20 | 8.23E-11 | 3.39E-10 | -3.490 |
| RHSIM_Rhsim07G0050000 | 42.19 | 8.28E-11 | 3.42E-10 | -0.656 |
| RHSIM_Rhsim11G0091200 | 42.18 | 8.31E-11 | 3.43E-10 | 0.846 |
| novel.2045 | 42.18 | 8.33E-11 | 3.43E-10 | 1.371 |
| novel.1577 | 42.17 | 8.38E-11 | 3.45E-10 | -1.613 |
| novel.3472 | 42.14 | 8.48E-11 | 3.49E-10 | 1.018 |
| RHSIM_Rhsim01G0067600 | 42.14 | 8.50E-11 | 3.50E-10 | 0.528 |
| RHSIM_Rhsim01G0074600 | 42.10 | 8.65E-11 | 3.56E-10 | 1.373 |
| RHSIM_Rhsim09G0037900 | 42.09 | 8.70E-11 | 3.59E-10 | 0.626 |
| RHSIM_Rhsim02G0082400 | 42.08 | 8.74E-11 | 3.60E-10 | 0.564 |
| RHSIM_Rhsim12G0037900 | 42.07 | 8.79E-11 | 3.62E-10 | 0.471 |
| RHSIM_Rhsim03G0110400 | 42.07 | 8.81E-11 | 3.63E-10 | -0.689 |
| novel.3542 | 42.06 | 8.84E-11 | 3.64E-10 | 4.621 |
| RHSIM_Rhsim07G0204600 | 42.02 | 9.02E-11 | 3.71E-10 | -0.972 |
| novel.476 | 41.99 | 9.19E-11 | 3.78E-10 | -1.006 |
| RHSIM_Rhsim04G0118400 | 41.96 | 9.29E-11 | 3.82E-10 | -1.708 |
| RHSIM_Rhsim13G0235500 | 41.96 | 9.30E-11 | 3.83E-10 | -1.039 |
| RHSIM_Rhsim13G0115800 | 41.95 | 9.35E-11 | 3.85E-10 | -0.582 |
| novel.3088 | 41.95 | 9.35E-11 | 3.85E-10 | 1.880 |
| RHSIM_Rhsim04G0023500 | 41.95 | 9.37E-11 | 3.85E-10 | 0.500 |
| RHSIM_Rhsim05G0169400 | 41.93 | 9.47E-11 | 3.89E-10 | 0.935 |
| RHSIM_Rhsim07G0058300 | 41.93 | 9.47E-11 | 3.89E-10 | -0.741 |
| novel.614 | 41.92 | 9.49E-11 | 3.90E-10 | 3.278 |
| RHSIM_Rhsim11G0196800 | 41.92 | 9.53E-11 | 3.92E-10 | -0.643 |
| RHSIM_Rhsim02G0244200 | 41.89 | 9.66E-11 | 3.97E-10 | 0.561 |
| RHSIM_Rhsim04G0053900 | 41.88 | 9.69E-11 | 3.98E-10 | -0.970 |
| RHSIM_Rhsim05G0199400 | 41.87 | 9.77E-11 | 4.02E-10 | -1.066 |
| novel.3182 | 41.87 | 9.78E-11 | 4.02E-10 | -0.545 |
| RHSIM_Rhsim08G0066300 | 41.86 | 9.78E-11 | 4.02E-10 | 0.614 |
| RHSIM_Rhsim09G0194700 | 41.86 | 9.82E-11 | 4.03E-10 | 0.759 |
| RHSIM_Rhsim02G0238900 | 41.82 | 1.00E-10 | 4.11E-10 | 0.909 |

| Gene | LRT statistic | P value | BH-adjusted P | log2FC (interaction) |
|---|---|---|---|---|
| RHSIM_Rhsim07G0007500 | 41.80 | 1.01E-10 | 4.15E-10 | 0.969 |
| RHSIM_Rhsim07G0184100 | 41.80 | 1.01E-10 | 4.15E-10 | 1.401 |
| novel.711 | 41.78 | 1.02E-10 | 4.20E-10 | 2.079 |
| RHSIM_Rhsim01G0077200 | 41.76 | 1.03E-10 | 4.23E-10 | 2.324 |
| RHSIM_Rhsim10G0026900 | 41.76 | 1.03E-10 | 4.24E-10 | 1.748 |
| RHSIM_Rhsim13G0221000 | 41.75 | 1.04E-10 | 4.25E-10 | -0.644 |
| RHSIM_Rhsim04G0058000 | 41.71 | 1.06E-10 | 4.34E-10 | -61.721 |
| RHSIM_Rhsim10G0158500 | 41.71 | 1.06E-10 | 4.34E-10 | -4.431 |
| novel.1251 | 41.70 | 1.06E-10 | 4.36E-10 | -1.893 |
| RHSIM_Rhsim07G0047600 | 41.70 | 1.07E-10 | 4.37E-10 | 0.623 |
| RHSIM_Rhsim04G0073000 | 41.69 | 1.07E-10 | 4.39E-10 | -1.017 |
| RHSIM_Rhsim13G0087000 | 41.68 | 1.07E-10 | 4.40E-10 | -1.331 |
| RHSIM_Rhsim13G0113700 | 41.68 | 1.07E-10 | 4.40E-10 | 2.849 |
| RHSIM_Rhsim08G0228100 | 41.68 | 1.08E-10 | 4.41E-10 | -1.810 |
| RHSIM_Rhsim07G0099200 | 41.67 | 1.08E-10 | 4.44E-10 | -1.433 |
| RHSIM_Rhsim12G0192000 | 41.66 | 1.08E-10 | 4.44E-10 | -0.692 |
| RHSIM_Rhsim09G0112800 | 41.65 | 1.09E-10 | 4.46E-10 | -3.323 |
| RHSIM_Rhsim07G0145700 | 41.65 | 1.09E-10 | 4.47E-10 | -0.355 |
| RHSIM_Rhsim03G0085300 | 41.64 | 1.10E-10 | 4.50E-10 | -0.663 |
| RHSIM_Rhsim07G0211000 | 41.63 | 1.10E-10 | 4.51E-10 | 0.900 |
| RHSIM_Rhsim11G0072300 | 41.60 | 1.12E-10 | 4.59E-10 | 0.719 |
| RHSIM_Rhsim08G0172000 | 41.59 | 1.12E-10 | 4.60E-10 | -0.478 |
| RHSIM_Rhsim10G0143900 | 41.58 | 1.13E-10 | 4.63E-10 | 3.172 |
| RHSIM_Rhsim04G0099500 | 41.54 | 1.15E-10 | 4.73E-10 | -0.464 |
| novel.92 | 41.54 | 1.16E-10 | 4.73E-10 | 2.980 |
| RHSIM_Rhsim12G0003200 | 41.54 | 1.16E-10 | 4.73E-10 | -1.042 |
| RHSIM_Rhsim06G0178100 | 41.53 | 1.16E-10 | 4.74E-10 | 1.076 |
| RHSIM_RhsimUnG0057700 | 41.53 | 1.16E-10 | 4.74E-10 | -42.231 |
| RHSIM_Rhsim08G0050900 | 41.53 | 1.16E-10 | 4.75E-10 | -6.374 |
| RHSIM_Rhsim08G0040800 | 41.53 | 1.16E-10 | 4.75E-10 | 1.325 |
| RHSIM_Rhsim02G0026200 | 41.52 | 1.16E-10 | 4.76E-10 | -2.024 |
| RHSIM_RhsimUnG0006300 | 41.52 | 1.17E-10 | 4.76E-10 | 1.097 |

| Gene | LRT statistic | P value | BH-adjusted P | log2FC (interaction) |
|---|---|---|---|---|
| RHSIM_Rhsim06G0218100 | 41.51 | 1.17E-10 | 4.79E-10 | -3.438 |
| RHSIM_Rhsim09G0137100 | 41.51 | 1.17E-10 | 4.79E-10 | 1.128 |
| RHSIM_Rhsim07G0022500 | 41.50 | 1.18E-10 | 4.82E-10 | -1.570 |
| novel.477 | 41.50 | 1.18E-10 | 4.82E-10 | -1.483 |
| RHSIM_Rhsim03G0252300 | 41.49 | 1.18E-10 | 4.83E-10 | -0.964 |
| RHSIM_Rhsim09G0167400 | 41.47 | 1.20E-10 | 4.90E-10 | -0.801 |
| RHSIM_Rhsim01G0027100 | 41.46 | 1.21E-10 | 4.92E-10 | 1.649 |
| RHSIM_Rhsim03G0011700 | 41.45 | 1.21E-10 | 4.94E-10 | -0.743 |
| RHSIM_Rhsim01G0261200 | 41.44 | 1.22E-10 | 4.96E-10 | -0.802 |
| RHSIM_Rhsim13G0230700 | 41.44 | 1.22E-10 | 4.97E-10 | -1.002 |
| RHSIM_Rhsim01G0282900 | 41.43 | 1.22E-10 | 4.99E-10 | 1.715 |
| RHSIM_Rhsim07G0137600 | 41.42 | 1.23E-10 | 5.00E-10 | 1.013 |
| novel.4389 | 41.42 | 1.23E-10 | 5.01E-10 | -2.575 |
| novel.4301 | 41.41 | 1.24E-10 | 5.04E-10 | 1.981 |
| RHSIM_Rhsim09G0169700 | 41.40 | 1.24E-10 | 5.05E-10 | -2.617 |
| RHSIM_Rhsim11G0030200 | 41.36 | 1.27E-10 | 5.18E-10 | 3.399 |
| RHSIM_Rhsim03G0069300 | 41.33 | 1.28E-10 | 5.23E-10 | -0.948 |
| RHSIM_Rhsim10G0014300 | 41.33 | 1.28E-10 | 5.24E-10 | -0.627 |
| novel.3545 | 41.32 | 1.29E-10 | 5.26E-10 | 0.915 |
| RHSIM_Rhsim13G0028700 | 41.32 | 1.30E-10 | 5.28E-10 | 0.527 |
| RHSIM_Rhsim07G0080100 | 41.31 | 1.30E-10 | 5.29E-10 | -0.589 |
| RHSIM_Rhsim09G0003300 | 41.30 | 1.31E-10 | 5.32E-10 | -0.925 |
| RHSIM_Rhsim06G0097900 | 41.28 | 1.32E-10 | 5.37E-10 | 1.502 |
| RHSIM_RhsimUnG0022300 | 41.27 | 1.32E-10 | 5.40E-10 | 1.236 |
| RHSIM_RhsimUnG0168300 | 41.26 | 1.33E-10 | 5.42E-10 | -0.571 |
| RHSIM_Rhsim04G0220500 | 41.25 | 1.34E-10 | 5.46E-10 | -1.607 |
| RHSIM_Rhsim12G0105200 | 41.24 | 1.34E-10 | 5.47E-10 | 4.366 |
| RHSIM_RhsimUnG0100500 | 41.20 | 1.38E-10 | 5.60E-10 | -2.246 |
| novel.1048 | 41.20 | 1.38E-10 | 5.60E-10 | 1.736 |
| RHSIM_Rhsim08G0123700 | 41.19 | 1.38E-10 | 5.61E-10 | -0.812 |
| RHSIM_Rhsim07G0132100 | 41.19 | 1.38E-10 | 5.63E-10 | -0.647 |
| RHSIM_Rhsim08G0241900 | 41.17 | 1.39E-10 | 5.67E-10 | 0.595 |

| Gene | LRT statistic | P value | BH-adjusted P | log2FC (interaction) |
|---|---|---|---|---|
| RHSIM_Rhsim08G0182500 | 41.16 | 1.40E-10 | 5.71E-10 | 2.009 |
| RHSIM_Rhsim02G0145500 | 41.14 | 1.42E-10 | 5.77E-10 | 0.728 |
| RHSIM_Rhsim06G0062800 | 41.12 | 1.43E-10 | 5.81E-10 | -2.956 |
| novel.830 | 41.12 | 1.43E-10 | 5.81E-10 | -5.114 |
| RHSIM_Rhsim04G0059200 | 41.10 | 1.45E-10 | 5.88E-10 | 3.690 |
| RHSIM_Rhsim03G0154900 | 41.09 | 1.46E-10 | 5.92E-10 | -1.178 |
| RHSIM_Rhsim05G0102500 | 41.05 | 1.48E-10 | 6.03E-10 | 1.157 |
| RHSIM_Rhsim06G0100800 | 41.05 | 1.48E-10 | 6.03E-10 | 1.519 |
| RHSIM_Rhsim04G0169400 | 41.05 | 1.49E-10 | 6.04E-10 | 1.153 |
| RHSIM_Rhsim08G0007600 | 41.04 | 1.49E-10 | 6.04E-10 | 1.307 |
| RHSIM_Rhsim02G0204800 | 41.03 | 1.50E-10 | 6.09E-10 | -1.049 |
| RHSIM_Rhsim13G0181200 | 41.03 | 1.50E-10 | 6.10E-10 | 1.591 |
| RHSIM_Rhsim09G0094100 | 41.02 | 1.51E-10 | 6.12E-10 | -0.703 |
| RHSIM_Rhsim12G0194200 | 41.02 | 1.51E-10 | 6.13E-10 | -0.374 |
| RHSIM_Rhsim05G0060200 | 41.01 | 1.51E-10 | 6.15E-10 | 0.549 |
| RHSIM_Rhsim01G0227500 | 41.00 | 1.52E-10 | 6.17E-10 | 0.574 |
| RHSIM_Rhsim13G0127800 | 41.00 | 1.52E-10 | 6.18E-10 | 0.986 |
| RHSIM_Rhsim10G0204900 | 40.97 | 1.55E-10 | 6.27E-10 | -0.660 |
| RHSIM_Rhsim13G0014600 | 40.97 | 1.55E-10 | 6.27E-10 | -0.369 |
| novel.1543 | 40.96 | 1.55E-10 | 6.30E-10 | -6.523 |
| RHSIM_Rhsim06G0223600 | 40.95 | 1.56E-10 | 6.34E-10 | -0.793 |
| RHSIM_Rhsim11G0124400 | 40.95 | 1.56E-10 | 6.35E-10 | 0.717 |
| RHSIM_Rhsim07G0071200 | 40.92 | 1.58E-10 | 6.43E-10 | -1.133 |
| RHSIM_Rhsim01G0209800 | 40.91 | 1.59E-10 | 6.46E-10 | -4.799 |
| RHSIM_Rhsim01G0215300 | 40.90 | 1.61E-10 | 6.51E-10 | -1.787 |
| RHSIM_Rhsim05G0165000 | 40.90 | 1.61E-10 | 6.51E-10 | -0.994 |
| RHSIM_Rhsim03G0139100 | 40.89 | 1.61E-10 | 6.53E-10 | -1.913 |
| RHSIM_Rhsim03G0204200 | 40.88 | 1.62E-10 | 6.55E-10 | 1.728 |
| RHSIM_Rhsim12G0100600 | 40.87 | 1.62E-10 | 6.58E-10 | 3.191 |
| novel.3389 | 40.87 | 1.63E-10 | 6.60E-10 | 1.036 |
| novel.1850 | 40.86 | 1.63E-10 | 6.61E-10 | -43.241 |
| RHSIM_Rhsim12G0105000 | 40.86 | 1.64E-10 | 6.62E-10 | 1.723 |

| Gene | LRT statistic | P value | BH-adjusted P | log2FC (interaction) |
|---|---|---|---|---|
| RHSIM_Rhsim09G0103900 | 40.86 | 1.64E-10 | 6.64E-10 | 0.623 |
| RHSIM_Rhsim06G0131000 | 40.85 | 1.64E-10 | 6.66E-10 | 1.297 |
| RHSIM_Rhsim04G0069500 | 40.83 | 1.66E-10 | 6.73E-10 | -7.772 |
| RHSIM_Rhsim05G0154300 | 40.81 | 1.68E-10 | 6.78E-10 | 1.947 |
| novel.3518 | 40.81 | 1.68E-10 | 6.80E-10 | 0.488 |
| RHSIM_Rhsim05G0150100 | 40.78 | 1.71E-10 | 6.91E-10 | -0.624 |
| novel.2336 | 40.77 | 1.71E-10 | 6.93E-10 | -3.824 |
| RHSIM_Rhsim07G0002600 | 40.76 | 1.72E-10 | 6.98E-10 | 1.091 |
| RHSIM_Rhsim02G0249600 | 40.76 | 1.72E-10 | 6.98E-10 | -1.703 |
| RHSIM_Rhsim11G0160700 | 40.73 | 1.75E-10 | 7.06E-10 | -1.918 |
| RHSIM_Rhsim13G0192800 | 40.71 | 1.76E-10 | 7.13E-10 | -3.038 |
| RHSIM_Rhsim13G0165300 | 40.71 | 1.77E-10 | 7.14E-10 | 1.105 |
| novel.338 | 40.69 | 1.78E-10 | 7.21E-10 | 0.886 |
| RHSIM_Rhsim02G0103000 | 40.68 | 1.80E-10 | 7.26E-10 | 0.625 |
| RHSIM_Rhsim11G0037600 | 40.67 | 1.80E-10 | 7.28E-10 | 0.608 |
| RHSIM_Rhsim08G0016400 | 40.65 | 1.82E-10 | 7.36E-10 | -1.080 |
| RHSIM_Rhsim11G0034300 | 40.64 | 1.83E-10 | 7.39E-10 | -0.919 |
| novel.3001 | 40.63 | 1.84E-10 | 7.45E-10 | 4.697 |
| RHSIM_Rhsim09G0192700 | 40.62 | 1.85E-10 | 7.47E-10 | -1.611 |
| RHSIM_Rhsim07G0144600 | 40.60 | 1.87E-10 | 7.56E-10 | 0.565 |
| RHSIM_Rhsim04G0229900 | 40.54 | 1.93E-10 | 7.78E-10 | 0.252 |
| RHSIM_Rhsim01G0023500 | 40.54 | 1.93E-10 | 7.78E-10 | -0.473 |
| RHSIM_Rhsim13G0133100 | 40.52 | 1.95E-10 | 7.87E-10 | 1.422 |
| RHSIM_Rhsim06G0195000 | 40.51 | 1.95E-10 | 7.88E-10 | 3.028 |
| RHSIM_Rhsim10G0141000 | 40.51 | 1.96E-10 | 7.91E-10 | -2.546 |
| RHSIM_Rhsim12G0203300 | 40.50 | 1.97E-10 | 7.95E-10 | -1.677 |
| RHSIM_Rhsim04G0197500 | 40.49 | 1.97E-10 | 7.96E-10 | 0.823 |
| novel.2136 | 40.49 | 1.98E-10 | 7.98E-10 | -1.848 |
| novel.465 | 40.47 | 1.99E-10 | 8.04E-10 | 3.938 |
| novel.592 | 40.47 | 2.00E-10 | 8.07E-10 | 1.667 |
| RHSIM_Rhsim06G0176300 | 40.42 | 2.05E-10 | 8.26E-10 | 1.971 |
| RHSIM_Rhsim05G0150900 | 40.42 | 2.05E-10 | 8.28E-10 | -0.691 |

| Gene | LRT statistic | P value | BH-adjusted P | log2FC (interaction) |
|---|---|---|---|---|
| RHSIM_Rhsim13G0180300 | 40.41 | 2.06E-10 | 8.30E-10 | 1.692 |
| novel.364 | 40.40 | 2.07E-10 | 8.34E-10 | 3.199 |
| RHSIM_Rhsim07G0078300 | 40.39 | 2.07E-10 | 8.36E-10 | 0.610 |
| RHSIM_Rhsim10G0188800 | 40.39 | 2.08E-10 | 8.38E-10 | 1.138 |
| RHSIM_Rhsim03G0068300 | 40.36 | 2.11E-10 | 8.50E-10 | 1.139 |
| RHSIM_Rhsim06G0163000 | 40.34 | 2.14E-10 | 8.62E-10 | -1.334 |
| RHSIM_Rhsim04G0070600 | 40.32 | 2.15E-10 | 8.67E-10 | -0.548 |
| RHSIM_Rhsim13G0065000 | 40.30 | 2.18E-10 | 8.78E-10 | 0.579 |
| RHSIM_Rhsim08G0094700 | 40.28 | 2.20E-10 | 8.84E-10 | -0.481 |
| RHSIM_Rhsim05G0225700 | 40.28 | 2.20E-10 | 8.86E-10 | 0.538 |
| RHSIM_Rhsim09G0167500 | 40.27 | 2.21E-10 | 8.90E-10 | -0.624 |
| RHSIM_Rhsim10G0128100 | 40.26 | 2.22E-10 | 8.93E-10 | 0.367 |
| RHSIM_Rhsim03G0118600 | 40.26 | 2.22E-10 | 8.94E-10 | -1.030 |
| RHSIM_Rhsim13G0033600 | 40.26 | 2.23E-10 | 8.96E-10 | -5.677 |
| RHSIM_Rhsim01G0087600 | 40.25 | 2.24E-10 | 9.01E-10 | -1.694 |
| novel.420 | 40.25 | 2.24E-10 | 9.01E-10 | 1.058 |
| RHSIM_Rhsim05G0086900 | 40.24 | 2.24E-10 | 9.02E-10 | 0.502 |
| RHSIM_Rhsim09G0162100 | 40.24 | 2.24E-10 | 9.02E-10 | -2.888 |
| RHSIM_Rhsim10G0135700 | 40.24 | 2.25E-10 | 9.03E-10 | 0.590 |
| RHSIM_Rhsim05G0013300 | 40.22 | 2.27E-10 | 9.11E-10 | 0.610 |
| RHSIM_Rhsim10G0178300 | 40.21 | 2.28E-10 | 9.15E-10 | 3.316 |
| RHSIM_Rhsim06G0047600 | 40.20 | 2.29E-10 | 9.21E-10 | -2.953 |
| novel.1895 | 40.19 | 2.30E-10 | 9.26E-10 | -6.626 |
| RHSIM_Rhsim06G0102500 | 40.18 | 2.32E-10 | 9.32E-10 | 1.655 |
| RHSIM_Rhsim05G0054800 | 40.16 | 2.34E-10 | 9.38E-10 | 2.921 |
| RHSIM_Rhsim06G0160800 | 40.13 | 2.37E-10 | 9.53E-10 | -1.464 |
| RHSIM_RhsimUnG0175100 | 40.13 | 2.37E-10 | 9.53E-10 | 0.874 |
| RHSIM_Rhsim02G0203200 | 40.13 | 2.38E-10 | 9.54E-10 | 2.106 |
| novel.4524 | 40.04 | 2.48E-10 | 9.97E-10 | -1.538 |
| RHSIM_Rhsim13G0046700 | 40.04 | 2.49E-10 | 1.00E-09 | -45.106 |
| RHSIM_Rhsim07G0251100 | 40.02 | 2.51E-10 | 1.01E-09 | 0.442 |
| RHSIM_Rhsim03G0144300 | 40.01 | 2.53E-10 | 1.02E-09 | -0.943 |

| Gene | LRT statistic | P value | BH-adjusted P | log2FC (interaction) |
|---|---|---|---|---|
| RHSIM_Rhsim12G0075100 | 40.00 | 2.54E-10 | 1.02E-09 | -1.918 |
| RHSIM_Rhsim13G0183000 | 40.00 | 2.55E-10 | 1.02E-09 | 2.394 |
| novel.3625 | 39.99 | 2.55E-10 | 1.02E-09 | 1.300 |
| RHSIM_Rhsim09G0107300 | 39.98 | 2.56E-10 | 1.03E-09 | -0.688 |
| RHSIM_Rhsim03G0110300 | 39.98 | 2.56E-10 | 1.03E-09 | -0.536 |
| RHSIM_Rhsim09G0156500 | 39.93 | 2.63E-10 | 1.06E-09 | 1.189 |
| RHSIM_Rhsim09G0184200 | 39.92 | 2.65E-10 | 1.06E-09 | 0.567 |
| RHSIM_Rhsim03G0025100 | 39.91 | 2.65E-10 | 1.06E-09 | 0.904 |
| RHSIM_Rhsim10G0208600 | 39.87 | 2.71E-10 | 1.09E-09 | 1.950 |
| RHSIM_Rhsim10G0113000 | 39.87 | 2.71E-10 | 1.09E-09 | 0.636 |
| RHSIM_Rhsim05G0225800 | 39.86 | 2.72E-10 | 1.09E-09 | 1.402 |
| RHSIM_Rhsim13G0008700 | 39.85 | 2.74E-10 | 1.10E-09 | 0.909 |
| RHSIM_Rhsim12G0149600 | 39.84 | 2.75E-10 | 1.10E-09 | 0.617 |
| RHSIM_Rhsim06G0072700 | 39.84 | 2.76E-10 | 1.10E-09 | 1.117 |
| RHSIM_Rhsim04G0214400 | 39.84 | 2.76E-10 | 1.10E-09 | -0.499 |
| RHSIM_Rhsim03G0235600 | 39.83 | 2.77E-10 | 1.11E-09 | -0.937 |
| RHSIM_Rhsim07G0131300 | 39.82 | 2.78E-10 | 1.11E-09 | -0.657 |
| RHSIM_Rhsim06G0122800 | 39.81 | 2.80E-10 | 1.12E-09 | 0.554 |
| RHSIM_Rhsim08G0221800 | 39.81 | 2.80E-10 | 1.12E-09 | -1.062 |
| RHSIM_Rhsim02G0070800 | 39.79 | 2.83E-10 | 1.13E-09 | 0.918 |
| RHSIM_Rhsim01G0224800 | 39.79 | 2.84E-10 | 1.13E-09 | -0.450 |
| RHSIM_Rhsim01G0243700 | 39.78 | 2.84E-10 | 1.14E-09 | 0.781 |
| RHSIM_Rhsim03G0221300 | 39.77 | 2.85E-10 | 1.14E-09 | -1.007 |
| RHSIM_Rhsim05G0001800 | 39.75 | 2.89E-10 | 1.15E-09 | 0.570 |
| RHSIM_Rhsim10G0211800 | 39.72 | 2.93E-10 | 1.17E-09 | 1.317 |
| novel.2657 | 39.71 | 2.95E-10 | 1.18E-09 | -0.424 |
| RHSIM_Rhsim11G0123700 | 39.70 | 2.97E-10 | 1.19E-09 | 0.755 |
| novel.1427 | 39.68 | 2.98E-10 | 1.19E-09 | -44.831 |
| RHSIM_Rhsim11G0069200 | 39.68 | 3.00E-10 | 1.20E-09 | -0.683 |
| RHSIM_RhsimUnG0126100 | 39.64 | 3.05E-10 | 1.22E-09 | 0.567 |
| RHSIM_Rhsim08G0007200 | 39.60 | 3.11E-10 | 1.24E-09 | 2.035 |
| RHSIM_Rhsim10G0179900 | 39.60 | 3.11E-10 | 1.24E-09 | 5.914 |

| Gene | LRT statistic | P value | BH-adjusted P | log2FC (interaction) |
|---|---|---|---|---|
| novel.3417 | 39.60 | 3.12E-10 | 1.25E-09 | -43.599 |
| RHSIM_Rhsim11G0074300 | 39.59 | 3.13E-10 | 1.25E-09 | 0.352 |
| RHSIM_Rhsim01G0136500 | 39.58 | 3.15E-10 | 1.26E-09 | -8.755 |
| RHSIM_Rhsim07G0117600 | 39.56 | 3.18E-10 | 1.27E-09 | -1.132 |
| RHSIM_Rhsim01G0200700 | 39.55 | 3.20E-10 | 1.28E-09 | -0.355 |
| RHSIM_Rhsim08G0195400 | 39.55 | 3.20E-10 | 1.28E-09 | 2.614 |
| RHSIM_Rhsim08G0211100 | 39.55 | 3.20E-10 | 1.28E-09 | 1.625 |
| RHSIM_Rhsim12G0152100 | 39.55 | 3.20E-10 | 1.28E-09 | 0.851 |
| novel.555 | 39.54 | 3.21E-10 | 1.28E-09 | -3.603 |
| novel.2339 | 39.53 | 3.23E-10 | 1.29E-09 | 0.912 |
| RHSIM_Rhsim01G0108000 | 39.51 | 3.26E-10 | 1.30E-09 | -0.523 |
| RHSIM_Rhsim12G0175100 | 39.48 | 3.31E-10 | 1.32E-09 | 1.288 |
| novel.3830 | 39.48 | 3.31E-10 | 1.32E-09 | -0.668 |
| RHSIM_Rhsim04G0132900 | 39.48 | 3.31E-10 | 1.32E-09 | -0.762 |
| RHSIM_Rhsim11G0087600 | 39.47 | 3.32E-10 | 1.33E-09 | -0.686 |
| RHSIM_RhsimUnG0073500 | 39.47 | 3.32E-10 | 1.33E-09 | -1.284 |
| RHSIM_Rhsim08G0091400 | 39.46 | 3.34E-10 | 1.33E-09 | -1.489 |
| RHSIM_Rhsim06G0234300 | 39.46 | 3.35E-10 | 1.33E-09 | 1.107 |
| RHSIM_Rhsim10G0210900 | 39.45 | 3.36E-10 | 1.34E-09 | -0.547 |
| RHSIM_Rhsim13G0218900 | 39.44 | 3.39E-10 | 1.35E-09 | 4.100 |
| RHSIM_Rhsim08G0078900 | 39.43 | 3.39E-10 | 1.35E-09 | -3.721 |
| RHSIM_Rhsim08G0002400 | 39.39 | 3.47E-10 | 1.38E-09 | -0.701 |
| novel.4583 | 39.39 | 3.47E-10 | 1.38E-09 | 1.108 |
| RHSIM_Rhsim08G0178000 | 39.39 | 3.48E-10 | 1.38E-09 | 2.491 |
| novel.4265 | 39.38 | 3.49E-10 | 1.39E-09 | 1.072 |
| RHSIM_Rhsim03G0011000 | 39.36 | 3.53E-10 | 1.41E-09 | 1.984 |
| RHSIM_Rhsim08G0149900 | 39.35 | 3.54E-10 | 1.41E-09 | -1.163 |
| RHSIM_Rhsim12G0039600 | 39.34 | 3.55E-10 | 1.41E-09 | -1.190 |
| RHSIM_Rhsim09G0206000 | 39.34 | 3.56E-10 | 1.42E-09 | 0.787 |
| RHSIM_Rhsim12G0138900 | 39.34 | 3.57E-10 | 1.42E-09 | -0.713 |
| RHSIM_Rhsim08G0078700 | 39.33 | 3.59E-10 | 1.43E-09 | 0.917 |
| RHSIM_RhsimUnG0164800 | 39.30 | 3.64E-10 | 1.45E-09 | -3.039 |

| Gene | LRT statistic | P value | BH-adjusted P | log2FC (interaction) |
|---|---|---|---|---|
| RHSIM_Rhsim12G0090700 | 39.29 | 3.65E-10 | 1.45E-09 | 0.647 |
| RHSIM_Rhsim09G0195900 | 39.28 | 3.67E-10 | 1.46E-09 | -1.943 |
| RHSIM_Rhsim04G0225100 | 39.28 | 3.68E-10 | 1.46E-09 | -1.364 |
| RHSIM_Rhsim07G0011100 | 39.27 | 3.69E-10 | 1.47E-09 | -0.917 |
| RHSIM_Rhsim09G0055600 | 39.26 | 3.71E-10 | 1.48E-09 | -2.348 |
| RHSIM_Rhsim12G0166000 | 39.25 | 3.73E-10 | 1.48E-09 | 0.657 |
| novel.2097 | 39.24 | 3.74E-10 | 1.49E-09 | 2.308 |
| RHSIM_Rhsim09G0133300 | 39.24 | 3.74E-10 | 1.49E-09 | 0.645 |
| RHSIM_Rhsim09G0208300 | 39.23 | 3.77E-10 | 1.50E-09 | -1.159 |
| RHSIM_Rhsim04G0237500 | 39.23 | 3.77E-10 | 1.50E-09 | -1.380 |
| RHSIM_Rhsim01G0288400 | 39.22 | 3.79E-10 | 1.51E-09 | 0.663 |
| RHSIM_Rhsim04G0168700 | 39.19 | 3.84E-10 | 1.52E-09 | 0.572 |
| RHSIM_Rhsim05G0070800 | 39.18 | 3.87E-10 | 1.54E-09 | 2.521 |
| novel.2561 | 39.14 | 3.94E-10 | 1.56E-09 | 1.178 |
| RHSIM_Rhsim03G0160200 | 39.13 | 3.96E-10 | 1.57E-09 | 1.014 |
| RHSIM_Rhsim13G0162200 | 39.13 | 3.96E-10 | 1.57E-09 | -0.455 |
| RHSIM_Rhsim10G0150200 | 39.11 | 4.00E-10 | 1.59E-09 | 1.027 |
| RHSIM_Rhsim13G0229800 | 39.11 | 4.01E-10 | 1.59E-09 | -2.746 |
| RHSIM_Rhsim08G0236500 | 39.10 | 4.03E-10 | 1.60E-09 | 1.334 |
| RHSIM_Rhsim12G0047400 | 39.10 | 4.03E-10 | 1.60E-09 | 0.420 |
| RHSIM_Rhsim13G0140500 | 39.09 | 4.04E-10 | 1.60E-09 | 0.419 |
| RHSIM_Rhsim04G0226700 | 39.09 | 4.05E-10 | 1.61E-09 | -1.612 |
| RHSIM_Rhsim12G0106700 | 39.07 | 4.09E-10 | 1.62E-09 | -2.032 |
| RHSIM_Rhsim09G0094800 | 39.06 | 4.11E-10 | 1.63E-09 | -3.756 |
| novel.1370 | 39.05 | 4.14E-10 | 1.64E-09 | -1.724 |
| RHSIM_Rhsim03G0082800 | 39.05 | 4.14E-10 | 1.64E-09 | -1.493 |
| RHSIM_Rhsim06G0034700 | 39.04 | 4.14E-10 | 1.64E-09 | 2.662 |
| RHSIM_Rhsim13G0004800 | 39.04 | 4.15E-10 | 1.64E-09 | -1.085 |
| RHSIM_Rhsim11G0185100 | 39.03 | 4.16E-10 | 1.65E-09 | -3.638 |
| RHSIM_Rhsim08G0102400 | 39.01 | 4.21E-10 | 1.67E-09 | 1.272 |
| RHSIM_Rhsim08G0002600 | 39.01 | 4.21E-10 | 1.67E-09 | -8.232 |
| RHSIM_Rhsim08G0024200 | 39.01 | 4.22E-10 | 1.67E-09 | 2.337 |

| Gene | LRT statistic | P value | BH-adjusted P | log2FC (interaction) |
|---|---|---|---|---|
| RHSIM_Rhsim03G0170500 | 39.00 | 4.24E-10 | 1.68E-09 | -2.705 |
| RHSIM_Rhsim13G0035700 | 38.99 | 4.26E-10 | 1.69E-09 | -4.269 |
| RHSIM_Rhsim02G0208100 | 38.99 | 4.26E-10 | 1.69E-09 | 1.360 |
| RHSIM_Rhsim10G0001500 | 38.98 | 4.28E-10 | 1.69E-09 | 2.132 |
| RHSIM_Rhsim04G0036400 | 38.96 | 4.34E-10 | 1.72E-09 | 0.690 |
| RHSIM_Rhsim13G0117600 | 38.95 | 4.34E-10 | 1.72E-09 | -0.875 |
| RHSIM_Rhsim13G0179300 | 38.94 | 4.37E-10 | 1.73E-09 | -0.593 |
| RHSIM_Rhsim04G0058600 | 38.93 | 4.40E-10 | 1.74E-09 | -2.388 |
| RHSIM_Rhsim03G0036100 | 38.92 | 4.42E-10 | 1.75E-09 | -0.950 |
| RHSIM_Rhsim05G0206300 | 38.89 | 4.48E-10 | 1.77E-09 | -3.076 |
| RHSIM_Rhsim09G0043900 | 38.89 | 4.48E-10 | 1.77E-09 | -0.882 |
| RHSIM_Rhsim13G0161800 | 38.89 | 4.48E-10 | 1.77E-09 | 1.254 |
| RHSIM_Rhsim05G0160100 | 38.89 | 4.49E-10 | 1.77E-09 | -0.601 |
| RHSIM_Rhsim11G0151000 | 38.89 | 4.49E-10 | 1.78E-09 | 0.576 |
| RHSIM_Rhsim04G0222300 | 38.88 | 4.51E-10 | 1.78E-09 | -0.625 |
| novel.4122 | 38.87 | 4.52E-10 | 1.79E-09 | -0.984 |
| novel.2390 | 38.87 | 4.53E-10 | 1.79E-09 | -1.818 |
| RHSIM_Rhsim11G0002100 | 38.87 | 4.54E-10 | 1.79E-09 | 0.932 |
| RHSIM_Rhsim08G0035600 | 38.86 | 4.54E-10 | 1.79E-09 | -4.159 |
| RHSIM_Rhsim12G0139400 | 38.85 | 4.58E-10 | 1.81E-09 | 2.034 |
| RHSIM_Rhsim01G0003700 | 38.83 | 4.61E-10 | 1.82E-09 | 2.757 |
| RHSIM_Rhsim04G0005600 | 38.83 | 4.62E-10 | 1.82E-09 | -1.870 |
| RHSIM_Rhsim01G0243100 | 38.81 | 4.68E-10 | 1.85E-09 | -0.587 |
| RHSIM_Rhsim09G0111900 | 38.80 | 4.69E-10 | 1.85E-09 | 0.488 |
| RHSIM_Rhsim11G0154900 | 38.79 | 4.72E-10 | 1.87E-09 | 0.812 |
| RHSIM_Rhsim08G0115000 | 38.78 | 4.73E-10 | 1.87E-09 | 1.471 |
| RHSIM_Rhsim12G0015900 | 38.78 | 4.74E-10 | 1.87E-09 | 0.848 |
| RHSIM_Rhsim01G0275300 | 38.78 | 4.74E-10 | 1.87E-09 | 2.079 |
| RHSIM_Rhsim05G0000800 | 38.78 | 4.75E-10 | 1.88E-09 | 0.667 |
| RHSIM_Rhsim07G0083600 | 38.74 | 4.85E-10 | 1.91E-09 | -2.074 |
| RHSIM_Rhsim01G0286700 | 38.73 | 4.86E-10 | 1.92E-09 | -0.524 |
| RHSIM_Rhsim01G0128500 | 38.65 | 5.07E-10 | 2.00E-09 | 0.651 |

| Gene | LRT statistic | P value | BH-adjusted P | log2FC (interaction) |
|---|---|---|---|---|
| RHSIM_Rhsim12G0135500 | 38.65 | 5.08E-10 | 2.00E-09 | 0.674 |
| RHSIM_Rhsim12G0029400 | 38.64 | 5.09E-10 | 2.01E-09 | 1.091 |
| RHSIM_Rhsim10G0003600 | 38.64 | 5.10E-10 | 2.01E-09 | -1.751 |
| novel.2602 | 38.63 | 5.11E-10 | 2.01E-09 | 1.056 |
| RHSIM_Rhsim11G0132000 | 38.61 | 5.17E-10 | 2.04E-09 | 1.368 |
| RHSIM_Rhsim11G0095400 | 38.60 | 5.20E-10 | 2.05E-09 | 0.631 |
| RHSIM_Rhsim08G0071600 | 38.58 | 5.25E-10 | 2.07E-09 | 1.206 |
| RHSIM_Rhsim13G0228000 | 38.57 | 5.29E-10 | 2.08E-09 | 1.541 |
| RHSIM_Rhsim06G0150200 | 38.56 | 5.30E-10 | 2.09E-09 | 1.921 |
| RHSIM_Rhsim03G0025300 | 38.55 | 5.33E-10 | 2.10E-09 | 0.753 |
| RHSIM_Rhsim08G0106700 | 38.55 | 5.34E-10 | 2.10E-09 | -1.784 |
| RHSIM_Rhsim04G0074800 | 38.55 | 5.34E-10 | 2.10E-09 | -0.926 |
| novel.178 | 38.54 | 5.37E-10 | 2.11E-09 | -3.508 |
| RHSIM_Rhsim01G0278400 | 38.52 | 5.43E-10 | 2.14E-09 | -0.443 |
| RHSIM_Rhsim02G0210500 | 38.51 | 5.44E-10 | 2.14E-09 | -0.774 |
| RHSIM_Rhsim11G0172700 | 38.49 | 5.51E-10 | 2.17E-09 | -1.449 |
| RHSIM_RhsimUnG0250900 | 38.48 | 5.52E-10 | 2.17E-09 | 1.952 |
| RHSIM_Rhsim13G0138400 | 38.48 | 5.54E-10 | 2.18E-09 | -0.867 |
| RHSIM_Rhsim12G0037100 | 38.48 | 5.54E-10 | 2.18E-09 | -0.803 |
| RHSIM_Rhsim11G0178300 | 38.48 | 5.54E-10 | 2.18E-09 | -2.329 |
| RHSIM_RhsimUnG0175900 | 38.47 | 5.56E-10 | 2.19E-09 | 6.105 |
| novel.2482 | 38.45 | 5.63E-10 | 2.21E-09 | 1.353 |
| RHSIM_Rhsim09G0156100 | 38.45 | 5.63E-10 | 2.21E-09 | 0.569 |
| novel.754 | 38.41 | 5.72E-10 | 2.25E-09 | 0.640 |
| RHSIM_Rhsim12G0156900 | 38.39 | 5.79E-10 | 2.28E-09 | 2.434 |
| RHSIM_Rhsim09G0098200 | 38.37 | 5.84E-10 | 2.30E-09 | 1.877 |
| RHSIM_Rhsim03G0063600 | 38.35 | 5.91E-10 | 2.32E-09 | 2.251 |
| RHSIM_Rhsim13G0097900 | 38.34 | 5.95E-10 | 2.34E-09 | -1.350 |
| RHSIM_Rhsim09G0056500 | 38.33 | 5.96E-10 | 2.34E-09 | -3.981 |
| novel.2002 | 38.33 | 5.97E-10 | 2.35E-09 | 1.284 |
| RHSIM_Rhsim10G0007100 | 38.33 | 5.99E-10 | 2.35E-09 | -1.463 |
| RHSIM_Rhsim09G0008000 | 38.32 | 6.00E-10 | 2.36E-09 | 1.655 |

| Gene | LRT statistic | P value | BH-adjusted P | log2FC (interaction) |
|---|---|---|---|---|
| RHSIM_Rhsim08G0159800 | 38.32 | 6.01E-10 | 2.36E-09 | -1.080 |
| RHSIM_Rhsim04G0014800 | 38.31 | 6.05E-10 | 2.37E-09 | -0.408 |
| RHSIM_Rhsim12G0038300 | 38.29 | 6.11E-10 | 2.40E-09 | 0.597 |
| RHSIM_Rhsim01G0198600 | 38.28 | 6.13E-10 | 2.40E-09 | 1.819 |
| RHSIM_Rhsim07G0049900 | 38.28 | 6.14E-10 | 2.41E-09 | -1.576 |
| RHSIM_Rhsim04G0025800 | 38.27 | 6.17E-10 | 2.42E-09 | 1.209 |
| RHSIM_Rhsim05G0184400 | 38.25 | 6.22E-10 | 2.44E-09 | -0.633 |
| RHSIM_RhsimUnG0166600 | 38.25 | 6.24E-10 | 2.45E-09 | 1.152 |
| RHSIM_Rhsim02G0023900 | 38.23 | 6.30E-10 | 2.47E-09 | 0.983 |
| RHSIM_Rhsim02G0141400 | 38.22 | 6.31E-10 | 2.48E-09 | 1.363 |
| RHSIM_Rhsim13G0042900 | 38.20 | 6.38E-10 | 2.50E-09 | 0.791 |
| RHSIM_Rhsim03G0218900 | 38.18 | 6.47E-10 | 2.53E-09 | -0.498 |
| RHSIM_Rhsim13G0173800 | 38.17 | 6.48E-10 | 2.54E-09 | 0.448 |
| RHSIM_Rhsim08G0124900 | 38.17 | 6.49E-10 | 2.54E-09 | 0.743 |
| RHSIM_Rhsim09G0071300 | 38.16 | 6.51E-10 | 2.55E-09 | 2.727 |
| RHSIM_Rhsim13G0165900 | 38.15 | 6.55E-10 | 2.57E-09 | 1.543 |
| RHSIM_Rhsim05G0003300 | 38.15 | 6.55E-10 | 2.57E-09 | -1.174 |
| RHSIM_Rhsim04G0104800 | 38.13 | 6.62E-10 | 2.59E-09 | -2.714 |
| RHSIM_Rhsim03G0149800 | 38.12 | 6.64E-10 | 2.60E-09 | -1.064 |
| RHSIM_RhsimUnG0054900 | 38.10 | 6.71E-10 | 2.63E-09 | -0.431 |
| RHSIM_Rhsim08G0034300 | 38.09 | 6.75E-10 | 2.64E-09 | -0.539 |
| RHSIM_RhsimUnG0188500 | 38.08 | 6.81E-10 | 2.66E-09 | -3.484 |
| RHSIM_Rhsim01G0250700 | 38.07 | 6.81E-10 | 2.67E-09 | 1.469 |
| RHSIM_Rhsim05G0063400 | 38.07 | 6.81E-10 | 2.67E-09 | -2.968 |
| RHSIM_Rhsim09G0088200 | 38.07 | 6.81E-10 | 2.67E-09 | 4.426 |
| RHSIM_Rhsim09G0140000 | 38.05 | 6.88E-10 | 2.69E-09 | 0.484 |
| RHSIM_Rhsim09G0205800 | 38.05 | 6.89E-10 | 2.69E-09 | -0.837 |
| RHSIM_Rhsim08G0090200 | 38.04 | 6.94E-10 | 2.71E-09 | 2.141 |
| RHSIM_Rhsim08G0071500 | 38.03 | 6.96E-10 | 2.72E-09 | 0.582 |
| RHSIM_Rhsim09G0082800 | 38.03 | 6.98E-10 | 2.73E-09 | 0.535 |
| RHSIM_Rhsim10G0102200 | 38.02 | 7.01E-10 | 2.74E-09 | 1.005 |
| RHSIM_Rhsim03G0088700 | 38.02 | 7.02E-10 | 2.74E-09 | 0.638 |

| Gene | LRT statistic | P value | BH-adjusted P | log2FC (interaction) |
|---|---|---|---|---|
| RHSIM_Rhsim02G0015800 | 37.99 | 7.10E-10 | 2.77E-09 | -0.738 |
| RHSIM_Rhsim12G0121300 | 37.98 | 7.15E-10 | 2.80E-09 | 2.400 |
| RHSIM_RhsimUnG0043500 | 37.97 | 7.20E-10 | 2.81E-09 | 2.252 |
| RHSIM_Rhsim06G0213600 | 37.96 | 7.22E-10 | 2.82E-09 | 0.752 |
| RHSIM_Rhsim12G0032100 | 37.96 | 7.22E-10 | 2.82E-09 | -2.958 |
| RHSIM_Rhsim04G0195200 | 37.96 | 7.24E-10 | 2.83E-09 | -0.744 |
| RHSIM_Rhsim13G0212000 | 37.93 | 7.32E-10 | 2.86E-09 | 2.228 |
| RHSIM_Rhsim08G0163500 | 37.91 | 7.40E-10 | 2.89E-09 | 0.621 |
| novel.3609 | 37.89 | 7.47E-10 | 2.92E-09 | 2.214 |
| RHSIM_Rhsim02G0251000 | 37.89 | 7.47E-10 | 2.92E-09 | 3.370 |
| RHSIM_Rhsim08G0186400 | 37.88 | 7.51E-10 | 2.93E-09 | 0.594 |
| RHSIM_Rhsim05G0114100 | 37.88 | 7.54E-10 | 2.94E-09 | 1.126 |
| RHSIM_Rhsim07G0101600 | 37.87 | 7.55E-10 | 2.95E-09 | 0.871 |
| RHSIM_Rhsim01G0258800 | 37.85 | 7.62E-10 | 2.97E-09 | -1.233 |
| novel.536 | 37.85 | 7.62E-10 | 2.97E-09 | 1.347 |
| RHSIM_Rhsim10G0173100 | 37.84 | 7.68E-10 | 3.00E-09 | -0.706 |
| RHSIM_Rhsim03G0096900 | 37.84 | 7.68E-10 | 3.00E-09 | 4.143 |
| novel.2513 | 37.83 | 7.70E-10 | 3.00E-09 | -1.967 |
| RHSIM_Rhsim01G0210700 | 37.83 | 7.72E-10 | 3.01E-09 | 0.609 |
| RHSIM_Rhsim05G0004300 | 37.82 | 7.74E-10 | 3.02E-09 | 0.309 |
| novel.1887 | 37.82 | 7.75E-10 | 3.02E-09 | 0.858 |
| RHSIM_Rhsim06G0137500 | 37.80 | 7.83E-10 | 3.05E-09 | -0.439 |
| RHSIM_Rhsim09G0047000 | 37.80 | 7.84E-10 | 3.05E-09 | 1.355 |
| RHSIM_Rhsim06G0168600 | 37.80 | 7.85E-10 | 3.06E-09 | -0.549 |
| RHSIM_Rhsim09G0156200 | 37.79 | 7.88E-10 | 3.07E-09 | -42.622 |
| RHSIM_Rhsim04G0073400 | 37.78 | 7.90E-10 | 3.08E-09 | 0.542 |
| novel.1257 | 37.77 | 7.94E-10 | 3.09E-09 | 4.150 |
| RHSIM_Rhsim10G0029200 | 37.77 | 7.96E-10 | 3.10E-09 | -0.461 |
| RHSIM_Rhsim05G0192800 | 37.75 | 8.03E-10 | 3.13E-09 | -0.684 |
| RHSIM_Rhsim01G0181900 | 37.75 | 8.04E-10 | 3.13E-09 | 1.527 |
| RHSIM_Rhsim02G0026700 | 37.74 | 8.10E-10 | 3.15E-09 | -2.426 |
| RHSIM_Rhsim07G0021100 | 37.73 | 8.12E-10 | 3.16E-09 | 0.995 |

| Gene | LRT statistic | P value | BH-adjusted P | log2FC (interaction) |
|---|---|---|---|---|
| RHSIM_Rhsim12G0191500 | 37.72 | 8.15E-10 | 3.17E-09 | -1.719 |
| RHSIM_Rhsim13G0046100 | 37.72 | 8.15E-10 | 3.17E-09 | -0.773 |
| RHSIM_Rhsim10G0078900 | 37.72 | 8.17E-10 | 3.18E-09 | 1.922 |
| RHSIM_Rhsim13G0034000 | 37.72 | 8.17E-10 | 3.18E-09 | 0.442 |
| novel.806 | 37.71 | 8.19E-10 | 3.19E-09 | -0.849 |
| RHSIM_Rhsim06G0095300 | 37.71 | 8.20E-10 | 3.19E-09 | -0.887 |
| RHSIM_Rhsim11G0129100 | 37.71 | 8.22E-10 | 3.20E-09 | -0.643 |
| RHSIM_RhsimUnG0162000 | 37.70 | 8.26E-10 | 3.21E-09 | 1.871 |
| RHSIM_Rhsim07G0109500 | 37.70 | 8.27E-10 | 3.21E-09 | -1.231 |
| RHSIM_Rhsim01G0269500 | 37.69 | 8.29E-10 | 3.22E-09 | -1.251 |
| RHSIM_Rhsim08G0145800 | 37.69 | 8.30E-10 | 3.23E-09 | -1.157 |
| RHSIM_Rhsim10G0170000 | 37.68 | 8.34E-10 | 3.24E-09 | -2.093 |
| RHSIM_Rhsim11G0044500 | 37.68 | 8.35E-10 | 3.24E-09 | -0.692 |
| RHSIM_Rhsim05G0136200 | 37.67 | 8.36E-10 | 3.25E-09 | 0.979 |
| RHSIM_Rhsim07G0087000 | 37.67 | 8.40E-10 | 3.26E-09 | 0.445 |
| RHSIM_Rhsim02G0157400 | 37.66 | 8.41E-10 | 3.27E-09 | 1.135 |
| RHSIM_Rhsim10G0029700 | 37.66 | 8.44E-10 | 3.28E-09 | 0.889 |
| RHSIM_Rhsim07G0241100 | 37.65 | 8.45E-10 | 3.28E-09 | -2.756 |
| novel.136 | 37.65 | 8.48E-10 | 3.29E-09 | 1.346 |
| RHSIM_Rhsim04G0123500 | 37.64 | 8.50E-10 | 3.30E-09 | 0.683 |
| RHSIM_Rhsim08G0006700 | 37.63 | 8.57E-10 | 3.32E-09 | -3.086 |
| RHSIM_Rhsim06G0065400 | 37.59 | 8.73E-10 | 3.39E-09 | 2.535 |
| RHSIM_Rhsim09G0128900 | 37.57 | 8.83E-10 | 3.43E-09 | 0.597 |
| RHSIM_Rhsim08G0191700 | 37.55 | 8.89E-10 | 3.45E-09 | -0.568 |
| RHSIM_Rhsim07G0009600 | 37.55 | 8.89E-10 | 3.45E-09 | 0.843 |
| RHSIM_Rhsim01G0054700 | 37.55 | 8.90E-10 | 3.45E-09 | -0.523 |
| RHSIM_Rhsim13G0222300 | 37.54 | 8.95E-10 | 3.47E-09 | 0.560 |
| RHSIM_Rhsim03G0061100 | 37.54 | 8.95E-10 | 3.47E-09 | -4.418 |
| novel.3442 | 37.51 | 9.07E-10 | 3.52E-09 | 3.237 |
| RHSIM_Rhsim01G0269000 | 37.51 | 9.08E-10 | 3.52E-09 | 0.758 |
| RHSIM_Rhsim12G0197300 | 37.51 | 9.09E-10 | 3.52E-09 | -0.414 |
| novel.857 | 37.50 | 9.14E-10 | 3.54E-09 | -0.406 |

| Gene | LRT statistic | P value | BH-adjusted P | log2FC (interaction) |
|---|---|---|---|---|
| RHSIM_Rhsim13G0091800 | 37.49 | 9.21E-10 | 3.57E-09 | 1.963 |
| RHSIM_Rhsim04G0195900 | 37.47 | 9.30E-10 | 3.60E-09 | -1.215 |
| RHSIM_Rhsim13G0138000 | 37.45 | 9.36E-10 | 3.63E-09 | 1.019 |
| RHSIM_Rhsim03G0172000 | 37.45 | 9.38E-10 | 3.63E-09 | 0.696 |
| RHSIM_Rhsim05G0215000 | 37.45 | 9.38E-10 | 3.63E-09 | 2.092 |
| RHSIM_RhsimUnG0145600 | 37.44 | 9.43E-10 | 3.65E-09 | 0.669 |
| RHSIM_Rhsim09G0058100 | 37.42 | 9.52E-10 | 3.68E-09 | 1.102 |
| RHSIM_Rhsim06G0062000 | 37.41 | 9.56E-10 | 3.70E-09 | 0.457 |
| RHSIM_Rhsim06G0229300 | 37.41 | 9.59E-10 | 3.71E-09 | -1.165 |
| RHSIM_Rhsim06G0065700 | 37.39 | 9.68E-10 | 3.74E-09 | 0.590 |
| RHSIM_Rhsim06G0138000 | 37.38 | 9.70E-10 | 3.75E-09 | 0.517 |
| novel.3802 | 37.37 | 9.75E-10 | 3.77E-09 | -0.816 |
| RHSIM_Rhsim09G0195100 | 37.35 | 9.85E-10 | 3.81E-09 | -0.704 |
| RHSIM_Rhsim10G0172000 | 37.35 | 9.86E-10 | 3.82E-09 | -0.601 |
| RHSIM_Rhsim03G0197300 | 37.34 | 9.93E-10 | 3.84E-09 | -0.816 |
| RHSIM_Rhsim12G0155200 | 37.34 | 9.93E-10 | 3.84E-09 | -1.829 |
| RHSIM_Rhsim13G0191900 | 37.31 | 1.01E-09 | 3.89E-09 | -2.230 |
| RHSIM_Rhsim04G0122600 | 37.30 | 1.01E-09 | 3.91E-09 | 0.771 |
| novel.131 | 37.30 | 1.01E-09 | 3.92E-09 | 2.527 |
| RHSIM_Rhsim07G0186400 | 37.26 | 1.03E-09 | 3.99E-09 | -1.896 |
| RHSIM_Rhsim12G0063800 | 37.25 | 1.04E-09 | 4.02E-09 | -0.637 |
| RHSIM_Rhsim09G0115000 | 37.23 | 1.05E-09 | 4.06E-09 | -0.884 |
| RHSIM_Rhsim04G0072200 | 37.23 | 1.05E-09 | 4.06E-09 | 1.447 |
| RHSIM_Rhsim01G0257300 | 37.22 | 1.06E-09 | 4.08E-09 | 0.353 |
| RHSIM_Rhsim08G0124400 | 37.20 | 1.07E-09 | 4.12E-09 | 0.600 |
| RHSIM_Rhsim02G0099300 | 37.19 | 1.07E-09 | 4.13E-09 | -0.893 |
| RHSIM_Rhsim09G0076600 | 37.19 | 1.07E-09 | 4.14E-09 | 0.466 |
| RHSIM_Rhsim06G0244100 | 37.16 | 1.09E-09 | 4.20E-09 | 1.465 |
| RHSIM_Rhsim04G0113700 | 37.16 | 1.09E-09 | 4.21E-09 | 7.015 |
| RHSIM_Rhsim01G0254800 | 37.15 | 1.09E-09 | 4.23E-09 | 1.667 |
| RHSIM_Rhsim06G0001900 | 37.13 | 1.11E-09 | 4.27E-09 | 0.269 |
| RHSIM_Rhsim11G0052100 | 37.12 | 1.11E-09 | 4.29E-09 | 0.831 |

| Gene | LRT statistic | P value | BH-adjusted P | log2FC (interaction) |
|---|---|---|---|---|
| RHSIM_Rhsim03G0184700 | 37.12 | 1.11E-09 | 4.29E-09 | 0.904 |
| RHSIM_Rhsim03G0143400 | 37.12 | 1.11E-09 | 4.30E-09 | -0.764 |
| RHSIM_Rhsim08G0161200 | 37.11 | 1.12E-09 | 4.30E-09 | 0.912 |
| RHSIM_RhsimUnG0251200 | 37.11 | 1.12E-09 | 4.31E-09 | -0.277 |
| RHSIM_Rhsim04G0199400 | 37.11 | 1.12E-09 | 4.31E-09 | 0.481 |
| novel.3492 | 37.10 | 1.12E-09 | 4.32E-09 | -0.732 |
| RHSIM_Rhsim09G0023000 | 37.10 | 1.12E-09 | 4.33E-09 | 1.051 |
| RHSIM_Rhsim12G0108000 | 37.09 | 1.13E-09 | 4.35E-09 | 0.961 |
| RHSIM_Rhsim09G0097200 | 37.09 | 1.13E-09 | 4.35E-09 | 0.425 |
| RHSIM_Rhsim03G0132700 | 37.09 | 1.13E-09 | 4.35E-09 | 0.604 |
| novel.631 | 37.08 | 1.13E-09 | 4.36E-09 | -43.910 |
| RHSIM_Rhsim08G0247500 | 37.07 | 1.14E-09 | 4.39E-09 | 2.525 |
| RHSIM_Rhsim04G0239100 | 37.07 | 1.14E-09 | 4.39E-09 | -0.660 |
| RHSIM_Rhsim01G0130900 | 37.07 | 1.14E-09 | 4.39E-09 | 0.399 |
| RHSIM_Rhsim01G0255100 | 37.06 | 1.14E-09 | 4.41E-09 | -1.968 |
| RHSIM_Rhsim06G0105400 | 37.05 | 1.15E-09 | 4.44E-09 | -0.591 |
| RHSIM_Rhsim06G0116500 | 37.03 | 1.16E-09 | 4.47E-09 | 1.531 |
| RHSIM_Rhsim04G0047500 | 37.01 | 1.18E-09 | 4.53E-09 | -2.374 |
| RHSIM_RhsimUnG0258400 | 36.99 | 1.19E-09 | 4.57E-09 | 2.838 |
| RHSIM_Rhsim03G0264100 | 36.97 | 1.20E-09 | 4.62E-09 | -0.530 |
| RHSIM_Rhsim12G0091800 | 36.96 | 1.21E-09 | 4.65E-09 | -5.375 |
| RHSIM_Rhsim07G0190600 | 36.96 | 1.21E-09 | 4.65E-09 | -0.998 |
| RHSIM_Rhsim11G0118500 | 36.95 | 1.21E-09 | 4.67E-09 | -0.544 |
| RHSIM_Rhsim05G0115300 | 36.94 | 1.22E-09 | 4.68E-09 | 0.443 |
| RHSIM_Rhsim04G0186300 | 36.94 | 1.22E-09 | 4.69E-09 | -1.640 |
| RHSIM_Rhsim02G0213600 | 36.93 | 1.22E-09 | 4.71E-09 | 2.817 |
| RHSIM_Rhsim13G0172200 | 36.91 | 1.24E-09 | 4.75E-09 | 0.552 |
| RHSIM_Rhsim02G0116300 | 36.88 | 1.25E-09 | 4.82E-09 | -0.377 |
| RHSIM_Rhsim06G0023700 | 36.88 | 1.26E-09 | 4.82E-09 | 0.842 |
| RHSIM_Rhsim10G0094700 | 36.88 | 1.26E-09 | 4.83E-09 | -0.898 |
| RHSIM_Rhsim02G0167900 | 36.87 | 1.26E-09 | 4.85E-09 | 1.214 |
| RHSIM_Rhsim04G0231900 | 36.87 | 1.26E-09 | 4.85E-09 | -3.416 |

| Gene | LRT statistic | P value | BH-adjusted P | log2FC (interaction) |
|---|---|---|---|---|
| novel.1624 | 36.84 | 1.28E-09 | 4.94E-09 | -0.430 |
| RHSIM_Rhsim13G0201100 | 36.83 | 1.29E-09 | 4.94E-09 | 0.885 |
| RHSIM_Rhsim08G0146700 | 36.83 | 1.29E-09 | 4.94E-09 | 0.516 |
| RHSIM_Rhsim10G0169500 | 36.81 | 1.30E-09 | 4.99E-09 | -5.523 |
| novel.3377 | 36.81 | 1.30E-09 | 5.00E-09 | 2.201 |
| RHSIM_Rhsim07G0126700 | 36.81 | 1.31E-09 | 5.01E-09 | 0.427 |
| RHSIM_Rhsim06G0005200 | 36.80 | 1.31E-09 | 5.04E-09 | 0.882 |
| RHSIM_RhsimUnG0117000 | 36.78 | 1.32E-09 | 5.07E-09 | 1.172 |
| RHSIM_Rhsim03G0109400 | 36.78 | 1.33E-09 | 5.09E-09 | -3.109 |
| RHSIM_Rhsim12G0064200 | 36.77 | 1.33E-09 | 5.11E-09 | -3.833 |
| RHSIM_Rhsim03G0097300 | 36.75 | 1.34E-09 | 5.15E-09 | 1.729 |
| novel.3748 | 36.75 | 1.35E-09 | 5.16E-09 | 42.882 |
| RHSIM_Rhsim11G0161200 | 36.74 | 1.35E-09 | 5.18E-09 | -0.373 |
| RHSIM_Rhsim07G0023900 | 36.74 | 1.35E-09 | 5.18E-09 | -1.116 |
| RHSIM_Rhsim01G0196500 | 36.73 | 1.36E-09 | 5.21E-09 | -0.433 |
| RHSIM_RhsimUnG0095000 | 36.73 | 1.36E-09 | 5.21E-09 | 0.580 |
| novel.2583 | 36.72 | 1.36E-09 | 5.23E-09 | 1.605 |
| RHSIM_Rhsim13G0111500 | 36.71 | 1.37E-09 | 5.24E-09 | -0.341 |
| RHSIM_Rhsim06G0224400 | 36.71 | 1.37E-09 | 5.25E-09 | 0.465 |
| RHSIM_Rhsim06G0096500 | 36.71 | 1.37E-09 | 5.25E-09 | -1.275 |
| novel.407 | 36.71 | 1.37E-09 | 5.25E-09 | 2.052 |
| RHSIM_Rhsim01G0231400 | 36.70 | 1.38E-09 | 5.27E-09 | 1.185 |
| novel.2807 | 36.69 | 1.38E-09 | 5.30E-09 | -1.833 |
| RHSIM_Rhsim06G0007200 | 36.68 | 1.39E-09 | 5.33E-09 | 2.192 |
| novel.1869 | 36.67 | 1.40E-09 | 5.35E-09 | -4.365 |
| RHSIM_Rhsim03G0063400 | 36.66 | 1.41E-09 | 5.40E-09 | -0.727 |
| RHSIM_Rhsim04G0227500 | 36.66 | 1.41E-09 | 5.40E-09 | -0.642 |
| novel.1837 | 36.63 | 1.43E-09 | 5.46E-09 | -2.201 |
| RHSIM_Rhsim02G0001200 | 36.63 | 1.43E-09 | 5.48E-09 | 3.136 |
| RHSIM_Rhsim03G0123900 | 36.60 | 1.45E-09 | 5.56E-09 | 1.696 |
| novel.1642 | 36.58 | 1.46E-09 | 5.60E-09 | -1.084 |
| RHSIM_Rhsim10G0004800 | 36.58 | 1.47E-09 | 5.62E-09 | 0.557 |

| Gene | LRT statistic | P value | BH-adjusted P | log2FC (interaction) |
|---|---|---|---|---|
| RHSIM_Rhsim13G0155000 | 36.56 | 1.48E-09 | 5.67E-09 | 0.591 |
| RHSIM_Rhsim04G0072500 | 36.56 | 1.48E-09 | 5.67E-09 | -1.026 |
| RHSIM_Rhsim12G0193700 | 36.56 | 1.48E-09 | 5.67E-09 | -1.130 |
| RHSIM_Rhsim05G0178900 | 36.55 | 1.49E-09 | 5.69E-09 | -0.398 |
| RHSIM_Rhsim09G0109200 | 36.54 | 1.49E-09 | 5.71E-09 | 0.493 |
| RHSIM_Rhsim08G0083000 | 36.53 | 1.50E-09 | 5.74E-09 | 0.506 |
| RHSIM_Rhsim13G0045300 | 36.53 | 1.50E-09 | 5.75E-09 | -0.466 |
| RHSIM_Rhsim07G0142000 | 36.51 | 1.52E-09 | 5.82E-09 | -0.792 |
| RHSIM_Rhsim08G0123800 | 36.50 | 1.52E-09 | 5.83E-09 | -1.053 |
| RHSIM_Rhsim11G0193300 | 36.48 | 1.54E-09 | 5.89E-09 | -0.876 |
| RHSIM_Rhsim05G0023600 | 36.47 | 1.55E-09 | 5.93E-09 | 2.154 |
| novel.4493 | 36.46 | 1.56E-09 | 5.96E-09 | -0.884 |
| RHSIM_Rhsim07G0212400 | 36.46 | 1.56E-09 | 5.96E-09 | -0.797 |
| RHSIM_Rhsim12G0078400 | 36.45 | 1.56E-09 | 5.97E-09 | 2.024 |
| RHSIM_Rhsim13G0052000 | 36.44 | 1.57E-09 | 6.01E-09 | 0.961 |
| RHSIM_Rhsim07G0006600 | 36.44 | 1.58E-09 | 6.01E-09 | -1.125 |
| RHSIM_Rhsim02G0179300 | 36.43 | 1.58E-09 | 6.03E-09 | 1.735 |
| RHSIM_Rhsim07G0023700 | 36.43 | 1.58E-09 | 6.05E-09 | -0.441 |
| RHSIM_Rhsim05G0036700 | 36.43 | 1.59E-09 | 6.05E-09 | -3.319 |
| RHSIM_Rhsim01G0184300 | 36.42 | 1.59E-09 | 6.06E-09 | 0.445 |
| RHSIM_Rhsim04G0033800 | 36.42 | 1.59E-09 | 6.07E-09 | 1.072 |
| RHSIM_Rhsim11G0164400 | 36.39 | 1.61E-09 | 6.15E-09 | -3.370 |
| RHSIM_Rhsim08G0023000 | 36.39 | 1.62E-09 | 6.17E-09 | -1.525 |
| RHSIM_Rhsim01G0066000 | 36.38 | 1.62E-09 | 6.18E-09 | -2.117 |
| RHSIM_Rhsim04G0138700 | 36.37 | 1.63E-09 | 6.21E-09 | 0.402 |
| RHSIM_Rhsim06G0229100 | 36.37 | 1.63E-09 | 6.21E-09 | 0.579 |
| RHSIM_Rhsim02G0060400 | 36.37 | 1.63E-09 | 6.21E-09 | -0.737 |
| novel.3672 | 36.36 | 1.64E-09 | 6.24E-09 | -2.346 |
| RHSIM_Rhsim01G0290700 | 36.36 | 1.64E-09 | 6.24E-09 | 0.485 |
| RHSIM_Rhsim05G0113500 | 36.36 | 1.64E-09 | 6.25E-09 | 1.544 |
| RHSIM_Rhsim13G0004400 | 36.35 | 1.65E-09 | 6.29E-09 | 0.886 |
| RHSIM_Rhsim01G0012600 | 36.35 | 1.65E-09 | 6.29E-09 | 2.327 |

| Gene | LRT statistic | P value | BH-adjusted P | log2FC (interaction) |
|---|---|---|---|---|
| RHSIM_Rhsim01G0214800 | 36.34 | 1.66E-09 | 6.33E-09 | 3.020 |
| RHSIM_Rhsim01G0284200 | 36.33 | 1.67E-09 | 6.34E-09 | 0.430 |
| novel.1001 | 36.32 | 1.68E-09 | 6.38E-09 | -0.568 |
| RHSIM_Rhsim11G0092100 | 36.30 | 1.69E-09 | 6.45E-09 | -0.371 |
| RHSIM_Rhsim02G0254000 | 36.30 | 1.70E-09 | 6.46E-09 | -1.073 |
| RHSIM_Rhsim11G0095300 | 36.29 | 1.70E-09 | 6.46E-09 | -0.822 |
| RHSIM_Rhsim08G0062500 | 36.29 | 1.70E-09 | 6.48E-09 | -0.683 |
| RHSIM_Rhsim10G0010200 | 36.27 | 1.72E-09 | 6.53E-09 | -0.933 |
| RHSIM_Rhsim06G0030400 | 36.26 | 1.73E-09 | 6.57E-09 | -0.940 |
| RHSIM_Rhsim08G0204300 | 36.26 | 1.73E-09 | 6.57E-09 | -1.269 |
| RHSIM_Rhsim03G0093900 | 36.25 | 1.73E-09 | 6.60E-09 | 0.330 |
| RHSIM_Rhsim07G0103700 | 36.25 | 1.74E-09 | 6.61E-09 | 0.312 |
| RHSIM_Rhsim02G0140700 | 36.23 | 1.75E-09 | 6.65E-09 | 1.029 |
| RHSIM_Rhsim13G0124300 | 36.23 | 1.75E-09 | 6.66E-09 | -0.694 |
| RHSIM_Rhsim01G0112900 | 36.23 | 1.75E-09 | 6.66E-09 | 0.470 |
| RHSIM_Rhsim05G0148000 | 36.22 | 1.76E-09 | 6.70E-09 | -0.633 |
| RHSIM_Rhsim09G0165500 | 36.21 | 1.78E-09 | 6.75E-09 | 1.827 |
| RHSIM_Rhsim04G0105800 | 36.19 | 1.79E-09 | 6.80E-09 | 0.922 |
| RHSIM_Rhsim08G0114000 | 36.18 | 1.80E-09 | 6.84E-09 | 5.433 |
| RHSIM_Rhsim06G0198500 | 36.17 | 1.80E-09 | 6.85E-09 | -0.452 |
| RHSIM_Rhsim09G0092500 | 36.17 | 1.81E-09 | 6.88E-09 | 3.986 |
| RHSIM_Rhsim01G0000400 | 36.16 | 1.82E-09 | 6.90E-09 | -0.482 |
| RHSIM_Rhsim07G0134400 | 36.14 | 1.83E-09 | 6.97E-09 | 1.373 |
| novel.3200 | 36.10 | 1.87E-09 | 7.10E-09 | -3.890 |
| novel.18 | 36.10 | 1.88E-09 | 7.12E-09 | 2.367 |
| novel.2684 | 36.09 | 1.89E-09 | 7.16E-09 | -1.548 |
| RHSIM_Rhsim08G0052300 | 36.08 | 1.89E-09 | 7.19E-09 | 0.931 |
| RHSIM_Rhsim13G0002700 | 36.03 | 1.94E-09 | 7.36E-09 | 1.005 |
| RHSIM_Rhsim11G0097300 | 36.03 | 1.94E-09 | 7.38E-09 | -1.671 |
| novel.538 | 36.02 | 1.95E-09 | 7.40E-09 | -1.344 |
| novel.3622 | 36.00 | 1.97E-09 | 7.48E-09 | -1.313 |
| novel.1501 | 36.00 | 1.97E-09 | 7.49E-09 | -2.337 |

| Gene | LRT statistic | P value | BH-adjusted P | log2FC (interaction) |
|---|---|---|---|---|
| novel.2881 | 35.99 | 1.98E-09 | 7.51E-09 | -6.706 |
| RHSIM_Rhsim01G0078800 | 35.99 | 1.98E-09 | 7.52E-09 | -1.359 |
| RHSIM_RhsimUnG0062200 | 35.99 | 1.98E-09 | 7.52E-09 | -1.199 |
| RHSIM_Rhsim11G0159400 | 35.99 | 1.99E-09 | 7.53E-09 | -1.654 |
| novel.1655 | 35.96 | 2.01E-09 | 7.62E-09 | 1.286 |
| RHSIM_Rhsim08G0184300 | 35.95 | 2.02E-09 | 7.66E-09 | 3.481 |
| RHSIM_Rhsim08G0097500 | 35.95 | 2.02E-09 | 7.66E-09 | -0.756 |
| RHSIM_Rhsim08G0188900 | 35.95 | 2.02E-09 | 7.66E-09 | -0.315 |
| RHSIM_Rhsim08G0199700 | 35.93 | 2.05E-09 | 7.77E-09 | 1.311 |
| RHSIM_Rhsim03G0239600 | 35.92 | 2.06E-09 | 7.79E-09 | 0.925 |
| RHSIM_Rhsim12G0047900 | 35.91 | 2.07E-09 | 7.83E-09 | -0.512 |
| novel.2505 | 35.90 | 2.08E-09 | 7.88E-09 | 2.942 |
| RHSIM_Rhsim04G0111800 | 35.89 | 2.09E-09 | 7.90E-09 | -1.141 |
| novel.3596 | 35.88 | 2.10E-09 | 7.96E-09 | 2.101 |
| RHSIM_Rhsim09G0051200 | 35.84 | 2.14E-09 | 8.10E-09 | 0.827 |
| RHSIM_Rhsim04G0241600 | 35.84 | 2.14E-09 | 8.10E-09 | -1.407 |
| RHSIM_Rhsim12G0090000 | 35.82 | 2.16E-09 | 8.17E-09 | 0.595 |
| RHSIM_Rhsim04G0223700 | 35.81 | 2.17E-09 | 8.21E-09 | 4.740 |
| RHSIM_Rhsim13G0018700 | 35.81 | 2.18E-09 | 8.24E-09 | 1.512 |
| RHSIM_Rhsim05G0062500 | 35.80 | 2.18E-09 | 8.26E-09 | -0.769 |
| novel.3947 | 35.80 | 2.19E-09 | 8.27E-09 | 0.454 |
| novel.2970 | 35.80 | 2.19E-09 | 8.29E-09 | -2.173 |
| RHSIM_Rhsim04G0073900 | 35.79 | 2.19E-09 | 8.29E-09 | -0.936 |
| RHSIM_Rhsim01G0238700 | 35.79 | 2.20E-09 | 8.30E-09 | -0.905 |
| RHSIM_Rhsim06G0206500 | 35.79 | 2.20E-09 | 8.33E-09 | -0.712 |
| novel.1633 | 35.78 | 2.21E-09 | 8.36E-09 | -0.679 |
| RHSIM_Rhsim06G0078800 | 35.75 | 2.24E-09 | 8.47E-09 | -0.287 |
| novel.1009 | 35.75 | 2.25E-09 | 8.49E-09 | 3.351 |
| RHSIM_Rhsim04G0167600 | 35.72 | 2.28E-09 | 8.62E-09 | -0.618 |
| RHSIM_Rhsim09G0081500 | 35.71 | 2.28E-09 | 8.63E-09 | 0.460 |
| RHSIM_Rhsim04G0009500 | 35.70 | 2.30E-09 | 8.71E-09 | 0.906 |
| RHSIM_RhsimUnG0139700 | 35.70 | 2.31E-09 | 8.71E-09 | 1.150 |

| Gene | LRT statistic | P value | BH-adjusted P | log2FC (interaction) |
|---|---|---|---|---|
| RHSIM_Rhsim05G0059500 | 35.69 | 2.31E-09 | 8.72E-09 | 1.197 |
| RHSIM_Rhsim04G0008700 | 35.69 | 2.31E-09 | 8.72E-09 | -1.568 |
| RHSIM_Rhsim13G0221300 | 35.68 | 2.32E-09 | 8.78E-09 | 1.557 |
| RHSIM_Rhsim02G0175400 | 35.68 | 2.33E-09 | 8.79E-09 | 1.099 |
| RHSIM_Rhsim05G0096400 | 35.67 | 2.33E-09 | 8.81E-09 | 0.325 |
| novel.489 | 35.66 | 2.35E-09 | 8.85E-09 | 2.461 |
| RHSIM_Rhsim01G0100400 | 35.66 | 2.35E-09 | 8.88E-09 | 0.855 |
| RHSIM_Rhsim11G0159500 | 35.65 | 2.37E-09 | 8.93E-09 | 0.583 |
| novel.481 | 35.64 | 2.38E-09 | 8.96E-09 | -0.470 |
| RHSIM_Rhsim03G0022400 | 35.63 | 2.39E-09 | 9.02E-09 | -1.155 |
| RHSIM_Rhsim10G0214200 | 35.62 | 2.40E-09 | 9.04E-09 | 0.468 |
| RHSIM_Rhsim07G0178000 | 35.59 | 2.44E-09 | 9.19E-09 | -0.815 |
| RHSIM_Rhsim12G0192500 | 35.57 | 2.46E-09 | 9.28E-09 | 0.637 |
| RHSIM_Rhsim06G0051500 | 35.55 | 2.49E-09 | 9.38E-09 | -4.283 |
| RHSIM_Rhsim11G0001100 | 35.54 | 2.50E-09 | 9.44E-09 | 0.420 |
| RHSIM_Rhsim06G0104600 | 35.53 | 2.51E-09 | 9.47E-09 | 0.796 |
| RHSIM_Rhsim07G0039500 | 35.52 | 2.53E-09 | 9.52E-09 | 0.990 |
| RHSIM_Rhsim03G0105300 | 35.51 | 2.53E-09 | 9.55E-09 | -3.599 |
| RHSIM_Rhsim13G0103000 | 35.51 | 2.54E-09 | 9.56E-09 | 0.758 |
| RHSIM_Rhsim09G0033300 | 35.51 | 2.54E-09 | 9.58E-09 | 2.300 |
| RHSIM_Rhsim01G0198100 | 35.50 | 2.55E-09 | 9.61E-09 | -0.717 |
| RHSIM_Rhsim01G0059300 | 35.50 | 2.56E-09 | 9.62E-09 | -1.935 |
| RHSIM_Rhsim13G0078700 | 35.49 | 2.56E-09 | 9.63E-09 | -0.897 |
| RHSIM_Rhsim12G0079000 | 35.49 | 2.56E-09 | 9.65E-09 | -0.515 |
| RHSIM_Rhsim07G0091700 | 35.48 | 2.58E-09 | 9.72E-09 | 0.919 |
| RHSIM_Rhsim09G0170800 | 35.47 | 2.59E-09 | 9.76E-09 | -1.562 |
| RHSIM_Rhsim09G0193000 | 35.46 | 2.60E-09 | 9.78E-09 | -0.773 |
| RHSIM_Rhsim08G0047100 | 35.46 | 2.60E-09 | 9.78E-09 | -1.655 |
| novel.4562 | 35.46 | 2.60E-09 | 9.79E-09 | 0.960 |
| RHSIM_Rhsim03G0209400 | 35.46 | 2.60E-09 | 9.79E-09 | 6.818 |
| RHSIM_RhsimUnG0015300 | 35.42 | 2.65E-09 | 9.98E-09 | -1.769 |
| RHSIM_Rhsim03G0190500 | 35.42 | 2.66E-09 | 1.00E-08 | 0.971 |

| Gene | LRT statistic | P value | BH-adjusted P | log2FC (interaction) |
|---|---|---|---|---|
| novel.2498 | 35.41 | 2.67E-09 | 1.00E-08 | 1.067 |
| novel.1180 | 35.41 | 2.67E-09 | 1.01E-08 | -42.068 |
| RHSIM_Rhsim09G0060900 | 35.41 | 2.68E-09 | 1.01E-08 | 2.415 |
| RHSIM_Rhsim13G0087100 | 35.40 | 2.68E-09 | 1.01E-08 | 1.510 |
| RHSIM_Rhsim05G0177500 | 35.40 | 2.69E-09 | 1.01E-08 | 1.708 |
| RHSIM_Rhsim11G0039800 | 35.39 | 2.70E-09 | 1.01E-08 | -1.736 |
| RHSIM_Rhsim07G0099100 | 35.39 | 2.70E-09 | 1.01E-08 | -2.158 |
| RHSIM_Rhsim08G0174900 | 35.38 | 2.71E-09 | 1.02E-08 | 0.927 |
| RHSIM_Rhsim01G0235400 | 35.37 | 2.73E-09 | 1.03E-08 | 0.527 |
| RHSIM_Rhsim02G0042000 | 35.37 | 2.73E-09 | 1.03E-08 | -1.192 |
| RHSIM_Rhsim12G0166900 | 35.36 | 2.73E-09 | 1.03E-08 | 2.138 |
| RHSIM_Rhsim08G0049600 | 35.35 | 2.76E-09 | 1.04E-08 | 0.813 |
| RHSIM_Rhsim02G0182100 | 35.33 | 2.79E-09 | 1.05E-08 | -0.943 |
| RHSIM_Rhsim13G0032100 | 35.31 | 2.81E-09 | 1.06E-08 | 1.529 |
| RHSIM_Rhsim13G0190000 | 35.30 | 2.83E-09 | 1.06E-08 | -5.166 |
| RHSIM_Rhsim06G0170100 | 35.30 | 2.83E-09 | 1.06E-08 | 1.005 |
| RHSIM_Rhsim03G0008300 | 35.29 | 2.84E-09 | 1.07E-08 | 1.746 |
| novel.4429 | 35.28 | 2.85E-09 | 1.07E-08 | -0.441 |
| RHSIM_Rhsim13G0088200 | 35.28 | 2.86E-09 | 1.07E-08 | -2.487 |
| RHSIM_Rhsim01G0254300 | 35.25 | 2.89E-09 | 1.09E-08 | 0.683 |
| RHSIM_Rhsim10G0098700 | 35.24 | 2.91E-09 | 1.09E-08 | -0.778 |
| novel.531 | 35.24 | 2.92E-09 | 1.10E-08 | -2.058 |
| RHSIM_Rhsim03G0219300 | 35.22 | 2.94E-09 | 1.10E-08 | -1.466 |
| RHSIM_Rhsim10G0093100 | 35.22 | 2.95E-09 | 1.11E-08 | 1.163 |
| RHSIM_Rhsim10G0055200 | 35.20 | 2.97E-09 | 1.11E-08 | 2.464 |
| RHSIM_Rhsim10G0202200 | 35.20 | 2.98E-09 | 1.12E-08 | 1.635 |
| RHSIM_Rhsim01G0145700 | 35.20 | 2.98E-09 | 1.12E-08 | -0.851 |
| RHSIM_Rhsim07G0188600 | 35.20 | 2.98E-09 | 1.12E-08 | 0.656 |
| RHSIM_Rhsim06G0112600 | 35.19 | 2.99E-09 | 1.12E-08 | -1.958 |
| RHSIM_RhsimUnG0005900 | 35.19 | 3.00E-09 | 1.12E-08 | 1.600 |
| RHSIM_Rhsim06G0192900 | 35.18 | 3.01E-09 | 1.13E-08 | -1.328 |
| RHSIM_Rhsim10G0173200 | 35.17 | 3.03E-09 | 1.13E-08 | -1.711 |

| Gene | LRT statistic | P value | BH-adjusted P | log2FC (interaction) |
|---|---|---|---|---|
| RHSIM_Rhsim05G0214300 | 35.16 | 3.04E-09 | 1.14E-08 | 0.796 |
| RHSIM_Rhsim03G0142500 | 35.16 | 3.04E-09 | 1.14E-08 | -0.673 |
| RHSIM_Rhsim09G0086500 | 35.16 | 3.04E-09 | 1.14E-08 | -0.708 |
| novel.911 | 35.15 | 3.05E-09 | 1.14E-08 | -43.447 |
| RHSIM_Rhsim12G0116500 | 35.15 | 3.06E-09 | 1.15E-08 | -0.611 |
| RHSIM_Rhsim01G0185500 | 35.13 | 3.08E-09 | 1.15E-08 | 0.468 |
| RHSIM_Rhsim10G0001200 | 35.13 | 3.08E-09 | 1.15E-08 | 0.749 |
| novel.2848 | 35.13 | 3.09E-09 | 1.16E-08 | -4.237 |
| RHSIM_Rhsim03G0004900 | 35.12 | 3.09E-09 | 1.16E-08 | -0.714 |
| RHSIM_Rhsim04G0196400 | 35.12 | 3.10E-09 | 1.16E-08 | 1.116 |
| RHSIM_Rhsim03G0227800 | 35.11 | 3.12E-09 | 1.17E-08 | -1.533 |
| RHSIM_Rhsim09G0036900 | 35.10 | 3.13E-09 | 1.17E-08 | -1.492 |
| novel.528 | 35.09 | 3.15E-09 | 1.18E-08 | 0.914 |
| RHSIM_Rhsim03G0123000 | 35.09 | 3.15E-09 | 1.18E-08 | -1.966 |
| RHSIM_Rhsim03G0036300 | 35.08 | 3.16E-09 | 1.18E-08 | -4.608 |
| RHSIM_Rhsim10G0030700 | 35.04 | 3.23E-09 | 1.21E-08 | -1.131 |
| RHSIM_Rhsim12G0085200 | 35.04 | 3.23E-09 | 1.21E-08 | 1.058 |
| RHSIM_Rhsim03G0236500 | 35.03 | 3.25E-09 | 1.21E-08 | -0.408 |
| novel.1505 | 35.02 | 3.26E-09 | 1.22E-08 | -0.721 |
| RHSIM_Rhsim04G0186900 | 35.02 | 3.26E-09 | 1.22E-08 | -1.346 |
| RHSIM_Rhsim09G0182100 | 35.02 | 3.26E-09 | 1.22E-08 | 0.426 |
| RHSIM_Rhsim07G0018100 | 35.02 | 3.27E-09 | 1.22E-08 | 1.134 |
| RHSIM_Rhsim02G0175200 | 35.01 | 3.29E-09 | 1.23E-08 | -1.691 |
| RHSIM_Rhsim06G0234900 | 35.01 | 3.29E-09 | 1.23E-08 | -0.532 |
| RHSIM_Rhsim07G0059400 | 35.00 | 3.29E-09 | 1.23E-08 | -0.328 |
| novel.1402 | 35.00 | 3.30E-09 | 1.23E-08 | -0.698 |
| RHSIM_Rhsim02G0009700 | 35.00 | 3.30E-09 | 1.23E-08 | -0.632 |
| RHSIM_Rhsim07G0226500 | 34.98 | 3.33E-09 | 1.24E-08 | -2.362 |
| RHSIM_Rhsim06G0232100 | 34.98 | 3.34E-09 | 1.25E-08 | 0.669 |
| RHSIM_Rhsim07G0147200 | 34.97 | 3.34E-09 | 1.25E-08 | 1.739 |
| novel.3589 | 34.97 | 3.36E-09 | 1.25E-08 | 0.677 |
| RHSIM_Rhsim06G0223100 | 34.96 | 3.36E-09 | 1.25E-08 | 0.642 |

| Gene | LRT statistic | P value | BH-adjusted P | log2FC (interaction) |
|---|---|---|---|---|
| RHSIM_Rhsim08G0197600 | 34.96 | 3.36E-09 | 1.25E-08 | -1.093 |
| novel.4566 | 34.95 | 3.38E-09 | 1.26E-08 | 0.563 |
| RHSIM_Rhsim09G0061200 | 34.95 | 3.38E-09 | 1.26E-08 | -2.157 |
| novel.2477 | 34.94 | 3.39E-09 | 1.27E-08 | 1.493 |
| RHSIM_Rhsim05G0234400 | 34.94 | 3.40E-09 | 1.27E-08 | 1.618 |
| RHSIM_Rhsim01G0025200 | 34.94 | 3.40E-09 | 1.27E-08 | -0.329 |
| RHSIM_Rhsim04G0184800 | 34.93 | 3.41E-09 | 1.27E-08 | 1.874 |
| RHSIM_Rhsim01G0243500 | 34.92 | 3.43E-09 | 1.28E-08 | 1.048 |
| RHSIM_Rhsim04G0136300 | 34.91 | 3.45E-09 | 1.28E-08 | -0.409 |
| RHSIM_Rhsim03G0121600 | 34.89 | 3.48E-09 | 1.30E-08 | 0.898 |
| RHSIM_Rhsim09G0076300 | 34.89 | 3.49E-09 | 1.30E-08 | 0.547 |
| RHSIM_Rhsim09G0133600 | 34.89 | 3.49E-09 | 1.30E-08 | -0.397 |
| RHSIM_Rhsim05G0062300 | 34.89 | 3.49E-09 | 1.30E-08 | 0.669 |
| RHSIM_Rhsim10G0210700 | 34.89 | 3.50E-09 | 1.30E-08 | -0.813 |
| RHSIM_Rhsim06G0120000 | 34.87 | 3.52E-09 | 1.31E-08 | -1.867 |
| RHSIM_Rhsim03G0176100 | 34.86 | 3.54E-09 | 1.32E-08 | -0.599 |
| novel.2655 | 34.86 | 3.54E-09 | 1.32E-08 | -0.735 |
| RHSIM_Rhsim13G0115300 | 34.85 | 3.56E-09 | 1.32E-08 | -1.134 |
| RHSIM_Rhsim06G0106000 | 34.84 | 3.58E-09 | 1.33E-08 | 3.867 |
| RHSIM_Rhsim03G0115500 | 34.84 | 3.59E-09 | 1.33E-08 | -2.009 |
| novel.4239 | 34.82 | 3.62E-09 | 1.35E-08 | 1.506 |
| RHSIM_Rhsim09G0187400 | 34.81 | 3.63E-09 | 1.35E-08 | 0.917 |
| RHSIM_Rhsim08G0140900 | 34.80 | 3.65E-09 | 1.36E-08 | -0.645 |
| RHSIM_Rhsim09G0040800 | 34.80 | 3.65E-09 | 1.36E-08 | 0.662 |
| RHSIM_Rhsim11G0011600 | 34.77 | 3.71E-09 | 1.38E-08 | -0.523 |
| RHSIM_Rhsim09G0115300 | 34.76 | 3.74E-09 | 1.39E-08 | 0.644 |
| RHSIM_Rhsim09G0123600 | 34.75 | 3.74E-09 | 1.39E-08 | -1.960 |
| RHSIM_Rhsim08G0099000 | 34.74 | 3.77E-09 | 1.40E-08 | -0.713 |
| RHSIM_Rhsim08G0076900 | 34.73 | 3.79E-09 | 1.41E-08 | 0.603 |
| RHSIM_Rhsim04G0109400 | 34.73 | 3.79E-09 | 1.41E-08 | -0.729 |
| novel.2360 | 34.72 | 3.80E-09 | 1.41E-08 | -1.361 |
| RHSIM_Rhsim01G0174600 | 34.71 | 3.82E-09 | 1.42E-08 | 0.710 |

| Gene | LRT statistic | P value | BH-adjusted P | log2FC (interaction) |
|---|---|---|---|---|
| RHSIM_Rhsim03G0190600 | 34.70 | 3.85E-09 | 1.43E-08 | -0.763 |
| novel.2754 | 34.69 | 3.86E-09 | 1.43E-08 | -0.456 |
| RHSIM_Rhsim08G0129000 | 34.69 | 3.87E-09 | 1.44E-08 | -0.458 |
| RHSIM_Rhsim07G0223200 | 34.68 | 3.88E-09 | 1.44E-08 | -0.784 |
| RHSIM_Rhsim10G0094600 | 34.68 | 3.89E-09 | 1.44E-08 | -1.684 |
| RHSIM_Rhsim05G0094000 | 34.67 | 3.90E-09 | 1.45E-08 | -0.715 |
| RHSIM_Rhsim10G0183200 | 34.67 | 3.91E-09 | 1.45E-08 | -0.458 |
| RHSIM_Rhsim10G0096100 | 34.66 | 3.93E-09 | 1.46E-08 | 0.764 |
| novel.2391 | 34.66 | 3.93E-09 | 1.46E-08 | 1.254 |
| RHSIM_Rhsim03G0180200 | 34.65 | 3.94E-09 | 1.46E-08 | -2.771 |
| RHSIM_Rhsim03G0154100 | 34.62 | 4.01E-09 | 1.49E-08 | 1.915 |
| RHSIM_Rhsim03G0261100 | 34.61 | 4.02E-09 | 1.49E-08 | -1.901 |
| RHSIM_RhsimUnG0171500 | 34.61 | 4.04E-09 | 1.50E-08 | -3.827 |
| novel.4080 | 34.60 | 4.04E-09 | 1.50E-08 | -1.820 |
| RHSIM_Rhsim10G0193100 | 34.60 | 4.04E-09 | 1.50E-08 | -0.578 |
| novel.1738 | 34.59 | 4.07E-09 | 1.51E-08 | 5.323 |
| RHSIM_Rhsim02G0253900 | 34.59 | 4.07E-09 | 1.51E-08 | 0.277 |
| RHSIM_Rhsim02G0002300 | 34.59 | 4.08E-09 | 1.51E-08 | 0.502 |
| RHSIM_Rhsim08G0169400 | 34.58 | 4.08E-09 | 1.51E-08 | 0.613 |
| RHSIM_Rhsim11G0038600 | 34.57 | 4.11E-09 | 1.52E-08 | 1.389 |
| RHSIM_Rhsim13G0166500 | 34.57 | 4.12E-09 | 1.53E-08 | 2.183 |
| RHSIM_Rhsim13G0041200 | 34.56 | 4.13E-09 | 1.53E-08 | 0.439 |
| RHSIM_Rhsim09G0200500 | 34.56 | 4.13E-09 | 1.53E-08 | -0.691 |
| RHSIM_Rhsim10G0080600 | 34.56 | 4.13E-09 | 1.53E-08 | 0.582 |
| RHSIM_Rhsim01G0272200 | 34.56 | 4.14E-09 | 1.53E-08 | 0.567 |
| RHSIM_Rhsim01G0213100 | 34.55 | 4.15E-09 | 1.54E-08 | 0.625 |
| novel.3095 | 34.54 | 4.18E-09 | 1.55E-08 | 2.241 |
| RHSIM_Rhsim03G0085800 | 34.54 | 4.18E-09 | 1.55E-08 | 0.643 |
| RHSIM_Rhsim11G0118400 | 34.53 | 4.19E-09 | 1.55E-08 | -0.328 |
| RHSIM_Rhsim07G0186000 | 34.53 | 4.19E-09 | 1.55E-08 | 0.521 |
| RHSIM_Rhsim10G0175300 | 34.53 | 4.21E-09 | 1.56E-08 | -1.523 |
| RHSIM_Rhsim13G0208500 | 34.52 | 4.21E-09 | 1.56E-08 | 0.484 |

| Gene | LRT statistic | P value | BH-adjusted P | log2FC (interaction) |
|---|---|---|---|---|
| novel.4020 | 34.52 | 4.22E-09 | 1.56E-08 | -3.336 |
| RHSIM_Rhsim04G0190600 | 34.52 | 4.23E-09 | 1.56E-08 | -0.374 |
| novel.4362 | 34.51 | 4.23E-09 | 1.56E-08 | 0.759 |
| RHSIM_Rhsim13G0104400 | 34.49 | 4.29E-09 | 1.59E-08 | 0.874 |
| RHSIM_Rhsim03G0090000 | 34.48 | 4.32E-09 | 1.60E-08 | -0.638 |
| RHSIM_Rhsim04G0241100 | 34.47 | 4.33E-09 | 1.60E-08 | 0.545 |
| RHSIM_Rhsim07G0077900 | 34.47 | 4.33E-09 | 1.60E-08 | 0.359 |
| RHSIM_Rhsim01G0196900 | 34.45 | 4.38E-09 | 1.62E-08 | -0.725 |
| novel.3092 | 34.43 | 4.42E-09 | 1.63E-08 | -5.820 |
| RHSIM_Rhsim07G0125200 | 34.43 | 4.42E-09 | 1.63E-08 | -1.545 |
| RHSIM_Rhsim02G0181200 | 34.43 | 4.42E-09 | 1.63E-08 | -0.643 |
| RHSIM_Rhsim11G0059700 | 34.42 | 4.44E-09 | 1.64E-08 | 0.524 |
| RHSIM_Rhsim12G0128900 | 34.42 | 4.44E-09 | 1.64E-08 | 1.284 |
| RHSIM_Rhsim07G0167900 | 34.40 | 4.49E-09 | 1.66E-08 | -0.820 |
| RHSIM_Rhsim03G0210600 | 34.39 | 4.50E-09 | 1.66E-08 | -0.822 |
| RHSIM_Rhsim05G0005800 | 34.35 | 4.59E-09 | 1.70E-08 | 0.878 |
| RHSIM_Rhsim02G0175300 | 34.34 | 4.62E-09 | 1.71E-08 | 1.019 |
| novel.3608 | 34.33 | 4.65E-09 | 1.72E-08 | 4.193 |
| RHSIM_Rhsim10G0060000 | 34.32 | 4.68E-09 | 1.73E-08 | 0.739 |
| RHSIM_Rhsim03G0050000 | 34.31 | 4.70E-09 | 1.73E-08 | -0.728 |
| RHSIM_Rhsim04G0075600 | 34.31 | 4.71E-09 | 1.74E-08 | -0.562 |
| RHSIM_Rhsim10G0007200 | 34.30 | 4.71E-09 | 1.74E-08 | 1.011 |
| RHSIM_Rhsim11G0177400 | 34.30 | 4.72E-09 | 1.74E-08 | 1.299 |
| RHSIM_Rhsim03G0073300 | 34.30 | 4.73E-09 | 1.74E-08 | -1.455 |
| novel.4436 | 34.28 | 4.77E-09 | 1.76E-08 | 2.309 |
| RHSIM_Rhsim01G0290400 | 34.26 | 4.82E-09 | 1.78E-08 | -0.815 |
| novel.4432 | 34.25 | 4.85E-09 | 1.79E-08 | 3.032 |
| RHSIM_Rhsim01G0135900 | 34.25 | 4.86E-09 | 1.79E-08 | -1.118 |
| novel.1709 | 34.24 | 4.86E-09 | 1.79E-08 | 4.520 |
| RHSIM_Rhsim05G0060100 | 34.23 | 4.90E-09 | 1.81E-08 | -0.657 |
| RHSIM_Rhsim05G0048800 | 34.22 | 4.92E-09 | 1.81E-08 | -0.548 |
| RHSIM_Rhsim09G0170400 | 34.22 | 4.93E-09 | 1.81E-08 | 8.614 |

| Gene | LRT statistic | P value | BH-adjusted P | log2FC (interaction) |
|---|---|---|---|---|
| RHSIM_Rhsim12G0181000 | 34.21 | 4.94E-09 | 1.82E-08 | -0.446 |
| RHSIM_Rhsim12G0129400 | 34.21 | 4.96E-09 | 1.83E-08 | -0.888 |
| RHSIM_Rhsim06G0129300 | 34.19 | 5.00E-09 | 1.84E-08 | -0.743 |
| RHSIM_Rhsim06G0190900 | 34.18 | 5.02E-09 | 1.85E-08 | 1.508 |
| RHSIM_Rhsim03G0013000 | 34.18 | 5.02E-09 | 1.85E-08 | -1.489 |
| RHSIM_Rhsim04G0215900 | 34.17 | 5.06E-09 | 1.86E-08 | 0.616 |
| RHSIM_Rhsim08G0250800 | 34.14 | 5.13E-09 | 1.89E-08 | 0.686 |
| RHSIM_Rhsim12G0164400 | 34.14 | 5.13E-09 | 1.89E-08 | -0.728 |
| RHSIM_Rhsim03G0012600 | 34.14 | 5.13E-09 | 1.89E-08 | -2.100 |
| RHSIM_Rhsim11G0033400 | 34.13 | 5.16E-09 | 1.90E-08 | -1.356 |
| RHSIM_Rhsim10G0035900 | 34.13 | 5.16E-09 | 1.90E-08 | 1.515 |
| novel.3558 | 34.11 | 5.20E-09 | 1.91E-08 | 1.353 |
| RHSIM_Rhsim06G0133800 | 34.09 | 5.27E-09 | 1.94E-08 | 1.024 |
| RHSIM_Rhsim08G0076600 | 34.07 | 5.31E-09 | 1.95E-08 | -1.328 |
| RHSIM_Rhsim06G0211500 | 34.07 | 5.31E-09 | 1.95E-08 | -0.797 |
| RHSIM_Rhsim11G0175900 | 34.05 | 5.36E-09 | 1.97E-08 | 0.519 |
| RHSIM_Rhsim13G0112100 | 34.04 | 5.40E-09 | 1.98E-08 | 0.896 |
| RHSIM_Rhsim10G0142800 | 34.04 | 5.41E-09 | 1.99E-08 | -4.008 |
| RHSIM_Rhsim13G0033700 | 34.02 | 5.44E-09 | 2.00E-08 | 0.659 |
| RHSIM_Rhsim11G0190700 | 34.02 | 5.45E-09 | 2.00E-08 | -1.164 |
| RHSIM_Rhsim09G0182600 | 34.01 | 5.48E-09 | 2.01E-08 | 0.423 |
| RHSIM_Rhsim08G0052400 | 34.00 | 5.52E-09 | 2.03E-08 | 0.627 |
| novel.3015 | 33.98 | 5.56E-09 | 2.04E-08 | -3.351 |
| RHSIM_Rhsim12G0095400 | 33.97 | 5.59E-09 | 2.05E-08 | -0.888 |
| RHSIM_Rhsim09G0179000 | 33.97 | 5.60E-09 | 2.06E-08 | -0.805 |
| RHSIM_Rhsim06G0114600 | 33.96 | 5.64E-09 | 2.07E-08 | -0.703 |
| RHSIM_Rhsim04G0059800 | 33.95 | 5.66E-09 | 2.08E-08 | -45.404 |
| RHSIM_Rhsim04G0116400 | 33.91 | 5.77E-09 | 2.12E-08 | -2.173 |
| RHSIM_Rhsim03G0139600 | 33.89 | 5.84E-09 | 2.14E-08 | -0.936 |
| RHSIM_Rhsim09G0119800 | 33.88 | 5.85E-09 | 2.15E-08 | 2.775 |
| RHSIM_Rhsim03G0224700 | 33.88 | 5.87E-09 | 2.15E-08 | -0.650 |
| RHSIM_Rhsim10G0113400 | 33.87 | 5.88E-09 | 2.16E-08 | 1.880 |

| Gene | LRT statistic | P value | BH-adjusted P | log2FC (interaction) |
|---|---|---|---|---|
| RHSIM_Rhsim03G0054400 | 33.87 | 5.88E-09 | 2.16E-08 | -3.285 |
| RHSIM_Rhsim09G0168300 | 33.85 | 5.94E-09 | 2.18E-08 | 0.498 |
| RHSIM_Rhsim11G0109900 | 33.84 | 5.98E-09 | 2.19E-08 | -5.837 |
| novel.778 | 33.84 | 6.00E-09 | 2.20E-08 | -1.067 |
| novel.4556 | 33.82 | 6.05E-09 | 2.22E-08 | 2.736 |
| RHSIM_Rhsim10G0010100 | 33.82 | 6.06E-09 | 2.22E-08 | 1.892 |
| RHSIM_Rhsim04G0015600 | 33.79 | 6.13E-09 | 2.25E-08 | 3.235 |
| RHSIM_Rhsim06G0170000 | 33.79 | 6.14E-09 | 2.25E-08 | 1.191 |
| novel.1433 | 33.79 | 6.15E-09 | 2.25E-08 | -1.585 |
| RHSIM_Rhsim07G0018000 | 33.78 | 6.18E-09 | 2.26E-08 | 0.604 |
| RHSIM_Rhsim03G0216300 | 33.77 | 6.21E-09 | 2.27E-08 | -0.311 |
| RHSIM_Rhsim01G0019600 | 33.75 | 6.26E-09 | 2.29E-08 | 0.953 |
| RHSIM_Rhsim04G0072900 | 33.75 | 6.28E-09 | 2.30E-08 | -2.701 |
| RHSIM_Rhsim01G0174800 | 33.74 | 6.29E-09 | 2.30E-08 | -2.004 |
| RHSIM_Rhsim11G0199000 | 33.74 | 6.31E-09 | 2.31E-08 | 0.600 |
| RHSIM_Rhsim03G0202600 | 33.72 | 6.36E-09 | 2.33E-08 | 1.960 |
| RHSIM_Rhsim01G0211400 | 33.72 | 6.37E-09 | 2.33E-08 | 0.539 |
| RHSIM_Rhsim08G0158500 | 33.71 | 6.40E-09 | 2.34E-08 | 0.779 |
| RHSIM_Rhsim13G0152700 | 33.70 | 6.42E-09 | 2.35E-08 | 0.723 |
| RHSIM_Rhsim12G0187700 | 33.69 | 6.47E-09 | 2.37E-08 | -1.284 |
| RHSIM_RhsimUnG0256100 | 33.68 | 6.51E-09 | 2.38E-08 | 1.102 |
| RHSIM_Rhsim10G0059600 | 33.68 | 6.51E-09 | 2.38E-08 | 0.348 |
| RHSIM_Rhsim07G0022300 | 33.66 | 6.55E-09 | 2.40E-08 | -0.545 |
| RHSIM_Rhsim04G0145900 | 33.66 | 6.56E-09 | 2.40E-08 | 0.931 |
| RHSIM_Rhsim01G0224100 | 33.65 | 6.59E-09 | 2.41E-08 | 2.499 |
| RHSIM_RhsimUnG0091000 | 33.65 | 6.61E-09 | 2.42E-08 | 1.776 |
| RHSIM_Rhsim08G0036300 | 33.63 | 6.67E-09 | 2.44E-08 | -0.685 |
| RHSIM_Rhsim12G0133800 | 33.62 | 6.68E-09 | 2.44E-08 | 2.869 |
| RHSIM_Rhsim03G0220500 | 33.62 | 6.71E-09 | 2.45E-08 | 0.646 |
| novel.1343 | 33.62 | 6.71E-09 | 2.45E-08 | 2.873 |
| RHSIM_Rhsim09G0187800 | 33.61 | 6.72E-09 | 2.46E-08 | 0.800 |
| RHSIM_Rhsim06G0201700 | 33.61 | 6.73E-09 | 2.46E-08 | 0.870 |

| Gene | LRT statistic | P value | BH-adjusted P | log2FC (interaction) |
|---|---|---|---|---|
| RHSIM_Rhsim07G0022200 | 33.61 | 6.74E-09 | 2.46E-08 | -1.374 |
| novel.560 | 33.60 | 6.76E-09 | 2.47E-08 | 1.971 |
| RHSIM_RhsimUnG0221600 | 33.60 | 6.77E-09 | 2.47E-08 | -2.541 |
| RHSIM_Rhsim04G0204700 | 33.59 | 6.81E-09 | 2.49E-08 | 4.667 |
| novel.504 | 33.59 | 6.81E-09 | 2.49E-08 | 2.119 |
| RHSIM_Rhsim07G0096300 | 33.58 | 6.82E-09 | 2.49E-08 | 0.990 |
| RHSIM_Rhsim10G0095600 | 33.58 | 6.82E-09 | 2.49E-08 | 3.347 |
| RHSIM_RhsimUnG0078000 | 33.58 | 6.83E-09 | 2.49E-08 | -1.192 |
| RHSIM_Rhsim10G0094500 | 33.58 | 6.83E-09 | 2.49E-08 | 0.597 |
| RHSIM_Rhsim04G0051300 | 33.57 | 6.89E-09 | 2.51E-08 | -0.840 |
| RHSIM_Rhsim12G0070400 | 33.56 | 6.90E-09 | 2.52E-08 | -2.269 |
| RHSIM_Rhsim05G0084400 | 33.56 | 6.91E-09 | 2.52E-08 | 0.732 |
| RHSIM_Rhsim12G0039400 | 33.56 | 6.92E-09 | 2.52E-08 | 0.813 |
| RHSIM_Rhsim04G0013700 | 33.54 | 6.98E-09 | 2.55E-08 | -0.652 |
| RHSIM_Rhsim03G0102700 | 33.54 | 6.99E-09 | 2.55E-08 | -2.541 |
| RHSIM_Rhsim09G0093200 | 33.53 | 7.01E-09 | 2.55E-08 | 0.504 |
| RHSIM_Rhsim06G0040800 | 33.52 | 7.04E-09 | 2.57E-08 | -0.694 |
| RHSIM_Rhsim01G0275500 | 33.52 | 7.07E-09 | 2.58E-08 | 1.130 |
| novel.2392 | 33.51 | 7.08E-09 | 2.58E-08 | 0.669 |
| RHSIM_Rhsim06G0227800 | 33.51 | 7.08E-09 | 2.58E-08 | 3.057 |
| RHSIM_Rhsim01G0252800 | 33.50 | 7.12E-09 | 2.59E-08 | -1.264 |
| RHSIM_Rhsim11G0022400 | 33.48 | 7.21E-09 | 2.63E-08 | 4.370 |
| RHSIM_Rhsim05G0116200 | 33.47 | 7.23E-09 | 2.63E-08 | -0.684 |
| RHSIM_Rhsim05G0120100 | 33.44 | 7.35E-09 | 2.68E-08 | -0.747 |
| RHSIM_Rhsim01G0288600 | 33.43 | 7.40E-09 | 2.69E-08 | -1.391 |
| RHSIM_Rhsim13G0234500 | 33.43 | 7.40E-09 | 2.70E-08 | -0.677 |
| RHSIM_Rhsim09G0020900 | 33.42 | 7.43E-09 | 2.71E-08 | 1.016 |
| RHSIM_Rhsim12G0048800 | 33.42 | 7.44E-09 | 2.71E-08 | 0.663 |
| RHSIM_Rhsim04G0068400 | 33.42 | 7.44E-09 | 2.71E-08 | 0.379 |
| RHSIM_Rhsim02G0066400 | 33.41 | 7.46E-09 | 2.72E-08 | 0.498 |
| RHSIM_Rhsim13G0030000 | 33.40 | 7.49E-09 | 2.73E-08 | 4.956 |
| RHSIM_Rhsim09G0126900 | 33.40 | 7.51E-09 | 2.73E-08 | 0.568 |

| Gene | LRT statistic | P value | BH-adjusted P | log2FC (interaction) |
|---|---|---|---|---|
| RHSIM_Rhsim02G0194300 | 33.40 | 7.52E-09 | 2.74E-08 | 0.263 |
| novel.2443 | 33.38 | 7.58E-09 | 2.76E-08 | 1.782 |
| RHSIM_Rhsim13G0144000 | 33.37 | 7.63E-09 | 2.78E-08 | 1.634 |
| RHSIM_Rhsim11G0059500 | 33.33 | 7.76E-09 | 2.82E-08 | -0.929 |
| novel.772 | 33.32 | 7.83E-09 | 2.85E-08 | -3.906 |
| RHSIM_Rhsim12G0061500 | 33.31 | 7.86E-09 | 2.86E-08 | -0.470 |
| RHSIM_Rhsim12G0211700 | 33.31 | 7.87E-09 | 2.86E-08 | -0.486 |
| RHSIM_Rhsim01G0263200 | 33.29 | 7.94E-09 | 2.89E-08 | 1.714 |
| RHSIM_RhsimUnG0209400 | 33.29 | 7.96E-09 | 2.89E-08 | 1.496 |
| RHSIM_Rhsim01G0259300 | 33.28 | 7.97E-09 | 2.90E-08 | 0.595 |
| RHSIM_RhsimUnG0126200 | 33.28 | 7.99E-09 | 2.91E-08 | -0.665 |
| RHSIM_Rhsim10G0110700 | 33.27 | 8.02E-09 | 2.92E-08 | 0.613 |
| RHSIM_Rhsim02G0061100 | 33.27 | 8.03E-09 | 2.92E-08 | -1.046 |
| RHSIM_Rhsim10G0118600 | 33.25 | 8.10E-09 | 2.94E-08 | -4.320 |
| RHSIM_Rhsim04G0102100 | 33.23 | 8.19E-09 | 2.97E-08 | -0.465 |
| RHSIM_Rhsim10G0072400 | 33.23 | 8.20E-09 | 2.98E-08 | 2.767 |
| RHSIM_Rhsim08G0171400 | 33.21 | 8.28E-09 | 3.01E-08 | 0.547 |
| RHSIM_Rhsim01G0258300 | 33.21 | 8.29E-09 | 3.01E-08 | -0.830 |
| RHSIM_Rhsim04G0227000 | 33.20 | 8.31E-09 | 3.02E-08 | -3.021 |
| RHSIM_Rhsim05G0234800 | 33.20 | 8.32E-09 | 3.02E-08 | 0.708 |
| novel.2242 | 33.20 | 8.33E-09 | 3.02E-08 | -82.559 |
| RHSIM_Rhsim11G0074100 | 33.19 | 8.37E-09 | 3.04E-08 | 1.382 |
| RHSIM_Rhsim13G0184600 | 33.19 | 8.37E-09 | 3.04E-08 | 42.792 |
| RHSIM_Rhsim06G0185200 | 33.17 | 8.43E-09 | 3.06E-08 | -0.478 |
| RHSIM_Rhsim09G0096900 | 33.17 | 8.43E-09 | 3.06E-08 | -0.774 |
| RHSIM_RhsimUnG0089100 | 33.17 | 8.44E-09 | 3.06E-08 | -0.437 |
| RHSIM_Rhsim12G0002900 | 33.16 | 8.47E-09 | 3.07E-08 | -1.085 |
| RHSIM_Rhsim07G0209100 | 33.14 | 8.56E-09 | 3.11E-08 | 0.672 |
| RHSIM_Rhsim13G0076500 | 33.13 | 8.60E-09 | 3.12E-08 | -3.950 |
| RHSIM_Rhsim05G0039700 | 33.11 | 8.69E-09 | 3.15E-08 | -0.963 |
| RHSIM_Rhsim13G0195600 | 33.10 | 8.75E-09 | 3.17E-08 | -0.727 |
| RHSIM_Rhsim01G0057800 | 33.09 | 8.80E-09 | 3.19E-08 | -1.262 |

| Gene | LRT statistic | P value | BH-adjusted P | log2FC (interaction) |
|---|---|---|---|---|
| RHSIM_Rhsim13G0178600 | 33.07 | 8.88E-09 | 3.22E-08 | -4.635 |
| RHSIM_Rhsim11G0010500 | 33.07 | 8.90E-09 | 3.22E-08 | -0.465 |
| RHSIM_Rhsim05G0193800 | 33.05 | 8.97E-09 | 3.25E-08 | -0.437 |
| RHSIM_Rhsim10G0200000 | 33.04 | 9.04E-09 | 3.28E-08 | -1.766 |
| RHSIM_Rhsim12G0061700 | 33.03 | 9.06E-09 | 3.28E-08 | -0.355 |
| RHSIM_Rhsim07G0068700 | 33.03 | 9.09E-09 | 3.29E-08 | 0.709 |
| RHSIM_Rhsim08G0101800 | 33.01 | 9.15E-09 | 3.31E-08 | 1.225 |
| RHSIM_Rhsim08G0025300 | 33.00 | 9.22E-09 | 3.34E-08 | 0.590 |
| novel.3055 | 33.00 | 9.22E-09 | 3.34E-08 | -0.663 |
| RHSIM_Rhsim11G0185900 | 33.00 | 9.22E-09 | 3.34E-08 | -1.111 |
| RHSIM_Rhsim11G0040600 | 32.97 | 9.35E-09 | 3.38E-08 | 1.161 |
| RHSIM_Rhsim02G0254100 | 32.96 | 9.38E-09 | 3.40E-08 | -0.629 |
| RHSIM_Rhsim04G0201900 | 32.96 | 9.39E-09 | 3.40E-08 | -0.813 |
| RHSIM_Rhsim12G0206500 | 32.96 | 9.41E-09 | 3.40E-08 | -0.386 |
| RHSIM_Rhsim01G0121600 | 32.96 | 9.42E-09 | 3.41E-08 | 0.874 |
| RHSIM_Rhsim06G0192300 | 32.95 | 9.46E-09 | 3.42E-08 | -8.760 |
| RHSIM_Rhsim06G0095800 | 32.93 | 9.57E-09 | 3.46E-08 | -3.530 |
| RHSIM_Rhsim05G0143300 | 32.92 | 9.58E-09 | 3.46E-08 | -0.695 |
| RHSIM_Rhsim01G0224400 | 32.92 | 9.61E-09 | 3.47E-08 | 0.719 |
| RHSIM_Rhsim08G0144000 | 32.92 | 9.62E-09 | 3.48E-08 | 0.621 |
| RHSIM_RhsimUnG0059600 | 32.91 | 9.63E-09 | 3.48E-08 | -1.728 |
| novel.3320 | 32.91 | 9.66E-09 | 3.49E-08 | 1.081 |
| RHSIM_Rhsim06G0240900 | 32.88 | 9.79E-09 | 3.54E-08 | -0.981 |
| RHSIM_Rhsim02G0088700 | 32.87 | 9.84E-09 | 3.56E-08 | -0.737 |
| RHSIM_Rhsim13G0175000 | 32.87 | 9.86E-09 | 3.56E-08 | -0.601 |
| RHSIM_Rhsim01G0230600 | 32.87 | 9.87E-09 | 3.56E-08 | -0.420 |
| RHSIM_Rhsim12G0086300 | 32.84 | 1.00E-08 | 3.62E-08 | -4.127 |
| RHSIM_Rhsim05G0103900 | 32.83 | 1.01E-08 | 3.63E-08 | -0.537 |
| RHSIM_Rhsim07G0165600 | 32.83 | 1.01E-08 | 3.64E-08 | 0.487 |
| RHSIM_Rhsim04G0084500 | 32.82 | 1.01E-08 | 3.64E-08 | 0.278 |
| novel.3808 | 32.82 | 1.01E-08 | 3.65E-08 | -1.150 |
| novel.702 | 32.82 | 1.01E-08 | 3.65E-08 | -2.224 |

| Gene | LRT statistic | P value | BH-adjusted P | log2FC (interaction) |
|---|---|---|---|---|
| RHSIM_Rhsim05G0102000 | 32.82 | 1.01E-08 | 3.65E-08 | -0.967 |
| RHSIM_Rhsim08G0217800 | 32.81 | 1.01E-08 | 3.66E-08 | 1.615 |
| RHSIM_Rhsim03G0005800 | 32.81 | 1.02E-08 | 3.66E-08 | -0.809 |
| RHSIM_Rhsim13G0052300 | 32.81 | 1.02E-08 | 3.67E-08 | -1.094 |
| RHSIM_Rhsim05G0056100 | 32.80 | 1.02E-08 | 3.68E-08 | -0.722 |
| RHSIM_Rhsim10G0069700 | 32.78 | 1.03E-08 | 3.72E-08 | -5.365 |
| RHSIM_Rhsim08G0060400 | 32.77 | 1.04E-08 | 3.74E-08 | -2.357 |
| RHSIM_Rhsim03G0050200 | 32.76 | 1.04E-08 | 3.75E-08 | 0.648 |
| RHSIM_Rhsim11G0032600 | 32.76 | 1.04E-08 | 3.75E-08 | -1.040 |
| novel.802 | 32.74 | 1.06E-08 | 3.81E-08 | 0.939 |
| RHSIM_Rhsim08G0244100 | 32.74 | 1.06E-08 | 3.81E-08 | -0.663 |
| novel.1825 | 32.73 | 1.06E-08 | 3.82E-08 | -0.949 |
| RHSIM_Rhsim02G0099500 | 32.73 | 1.06E-08 | 3.82E-08 | -0.439 |
| RHSIM_Rhsim04G0056600 | 32.72 | 1.06E-08 | 3.83E-08 | -1.018 |
| RHSIM_Rhsim03G0150500 | 32.72 | 1.06E-08 | 3.83E-08 | -2.259 |
| novel.1616 | 32.71 | 1.07E-08 | 3.85E-08 | -0.356 |
| RHSIM_Rhsim13G0109300 | 32.69 | 1.08E-08 | 3.89E-08 | 1.780 |
| RHSIM_Rhsim02G0075800 | 32.68 | 1.09E-08 | 3.92E-08 | -0.668 |
| RHSIM_Rhsim04G0248300 | 32.67 | 1.09E-08 | 3.93E-08 | -0.513 |
| RHSIM_Rhsim08G0249400 | 32.66 | 1.10E-08 | 3.95E-08 | -0.900 |
| RHSIM_RhsimUnG0063500 | 32.65 | 1.10E-08 | 3.97E-08 | 1.002 |
| RHSIM_Rhsim02G0251900 | 32.65 | 1.10E-08 | 3.97E-08 | -0.597 |
| RHSIM_Rhsim05G0055000 | 32.65 | 1.11E-08 | 3.98E-08 | -0.707 |
| novel.1571 | 32.64 | 1.11E-08 | 3.99E-08 | 3.454 |
| RHSIM_Rhsim04G0249500 | 32.64 | 1.11E-08 | 4.00E-08 | 0.556 |
| RHSIM_Rhsim07G0004800 | 32.63 | 1.11E-08 | 4.01E-08 | -1.507 |
| RHSIM_Rhsim12G0155600 | 32.62 | 1.12E-08 | 4.02E-08 | 1.314 |
| RHSIM_Rhsim05G0056600 | 32.62 | 1.12E-08 | 4.04E-08 | -0.507 |
| RHSIM_Rhsim11G0066000 | 32.60 | 1.13E-08 | 4.06E-08 | -0.844 |
| RHSIM_Rhsim12G0171900 | 32.60 | 1.13E-08 | 4.07E-08 | -1.413 |
| RHSIM_Rhsim04G0087500 | 32.60 | 1.13E-08 | 4.07E-08 | -0.680 |
| RHSIM_Rhsim09G0005000 | 32.59 | 1.14E-08 | 4.09E-08 | 1.204 |

| Gene | LRT statistic | P value | BH-adjusted P | log2FC (interaction) |
|---|---|---|---|---|
| RHSIM_Rhsim09G0184600 | 32.59 | 1.14E-08 | 4.09E-08 | 3.606 |
| RHSIM_Rhsim09G0078400 | 32.58 | 1.15E-08 | 4.12E-08 | -2.209 |
| RHSIM_Rhsim07G0155800 | 32.56 | 1.15E-08 | 4.15E-08 | 0.314 |
| RHSIM_Rhsim13G0088000 | 32.55 | 1.16E-08 | 4.17E-08 | 1.345 |
| RHSIM_Rhsim06G0051900 | 32.53 | 1.17E-08 | 4.21E-08 | -3.670 |
| novel.4557 | 32.52 | 1.18E-08 | 4.23E-08 | -42.919 |
| RHSIM_Rhsim08G0152900 | 32.51 | 1.18E-08 | 4.25E-08 | -1.679 |
| RHSIM_Rhsim07G0121200 | 32.51 | 1.19E-08 | 4.26E-08 | -0.440 |
| RHSIM_Rhsim09G0093500 | 32.51 | 1.19E-08 | 4.26E-08 | -1.054 |
| RHSIM_Rhsim01G0239500 | 32.50 | 1.19E-08 | 4.28E-08 | -0.851 |
| RHSIM_Rhsim13G0050100 | 32.50 | 1.19E-08 | 4.29E-08 | 3.092 |
| RHSIM_Rhsim11G0113200 | 32.49 | 1.20E-08 | 4.29E-08 | -4.125 |
| RHSIM_Rhsim13G0218100 | 32.47 | 1.21E-08 | 4.34E-08 | -1.040 |
| RHSIM_Rhsim09G0012500 | 32.47 | 1.21E-08 | 4.35E-08 | 1.414 |
| RHSIM_Rhsim01G0209000 | 32.46 | 1.21E-08 | 4.36E-08 | -1.098 |
| RHSIM_Rhsim04G0190700 | 32.46 | 1.22E-08 | 4.36E-08 | 0.616 |
| RHSIM_Rhsim05G0119900 | 32.46 | 1.22E-08 | 4.38E-08 | -0.636 |
| RHSIM_Rhsim12G0144200 | 32.45 | 1.22E-08 | 4.38E-08 | 0.806 |
| RHSIM_Rhsim08G0054600 | 32.45 | 1.22E-08 | 4.39E-08 | -2.850 |
| RHSIM_Rhsim07G0204900 | 32.45 | 1.22E-08 | 4.39E-08 | 1.214 |
| RHSIM_Rhsim02G0006700 | 32.43 | 1.24E-08 | 4.43E-08 | -1.223 |
| novel.2078 | 32.42 | 1.24E-08 | 4.44E-08 | -3.044 |
| RHSIM_Rhsim03G0034100 | 32.41 | 1.25E-08 | 4.49E-08 | -2.050 |
| RHSIM_Rhsim10G0001800 | 32.40 | 1.25E-08 | 4.49E-08 | 2.018 |
| RHSIM_Rhsim07G0121800 | 32.39 | 1.26E-08 | 4.52E-08 | 1.868 |
| RHSIM_Rhsim04G0027100 | 32.38 | 1.27E-08 | 4.53E-08 | -1.353 |
| RHSIM_Rhsim07G0157100 | 32.36 | 1.28E-08 | 4.59E-08 | 0.950 |
| RHSIM_Rhsim06G0071000 | 32.36 | 1.28E-08 | 4.60E-08 | -0.634 |
| RHSIM_RhsimUnG0136700 | 32.34 | 1.29E-08 | 4.64E-08 | 0.761 |
| RHSIM_Rhsim13G0114600 | 32.32 | 1.30E-08 | 4.67E-08 | -0.438 |
| RHSIM_Rhsim04G0226000 | 32.32 | 1.30E-08 | 4.67E-08 | 0.667 |
| RHSIM_Rhsim02G0031800 | 32.32 | 1.30E-08 | 4.67E-08 | -0.726 |

| Gene | LRT statistic | P value | BH-adjusted P | log2FC (interaction) |
|---|---|---|---|---|
| RHSIM_Rhsim01G0257400 | 32.32 | 1.31E-08 | 4.68E-08 | -1.555 |
| novel.999 | 32.32 | 1.31E-08 | 4.68E-08 | 4.793 |
| RHSIM_Rhsim01G0200600 | 32.31 | 1.31E-08 | 4.69E-08 | -1.466 |
| RHSIM_Rhsim08G0144800 | 32.31 | 1.32E-08 | 4.71E-08 | -1.923 |
| RHSIM_Rhsim01G0004100 | 32.28 | 1.34E-08 | 4.79E-08 | 1.775 |
| RHSIM_Rhsim11G0115200 | 32.27 | 1.34E-08 | 4.79E-08 | 1.482 |
| RHSIM_Rhsim12G0052400 | 32.27 | 1.34E-08 | 4.80E-08 | 0.690 |
| RHSIM_Rhsim08G0074100 | 32.25 | 1.35E-08 | 4.84E-08 | -1.412 |
| RHSIM_Rhsim13G0229700 | 32.22 | 1.37E-08 | 4.91E-08 | 0.450 |
| RHSIM_RhsimUnG0144200 | 32.21 | 1.38E-08 | 4.95E-08 | 0.543 |
| RHSIM_Rhsim13G0001400 | 32.21 | 1.39E-08 | 4.96E-08 | -0.818 |
| RHSIM_Rhsim03G0170900 | 32.21 | 1.39E-08 | 4.96E-08 | 2.409 |
| RHSIM_Rhsim02G0044300 | 32.19 | 1.40E-08 | 4.99E-08 | 1.485 |
| RHSIM_Rhsim04G0132100 | 32.18 | 1.40E-08 | 5.01E-08 | 0.338 |
| RHSIM_Rhsim01G0105900 | 32.17 | 1.42E-08 | 5.06E-08 | -0.681 |
| RHSIM_Rhsim04G0086800 | 32.16 | 1.42E-08 | 5.07E-08 | 2.156 |
| RHSIM_Rhsim10G0015600 | 32.16 | 1.42E-08 | 5.07E-08 | -0.671 |
| RHSIM_Rhsim02G0220000 | 32.14 | 1.43E-08 | 5.11E-08 | -4.174 |
| novel.903 | 32.14 | 1.44E-08 | 5.13E-08 | 0.412 |
| RHSIM_Rhsim04G0078100 | 32.13 | 1.44E-08 | 5.14E-08 | -3.030 |
| novel.3022 | 32.11 | 1.45E-08 | 5.19E-08 | 1.172 |
| RHSIM_Rhsim04G0236700 | 32.10 | 1.46E-08 | 5.22E-08 | -1.696 |
| RHSIM_Rhsim08G0238000 | 32.09 | 1.48E-08 | 5.27E-08 | -0.874 |
| RHSIM_Rhsim01G0221100 | 32.08 | 1.48E-08 | 5.27E-08 | -1.834 |
| RHSIM_Rhsim10G0057900 | 32.08 | 1.48E-08 | 5.29E-08 | 0.601 |
| RHSIM_Rhsim07G0172500 | 32.07 | 1.49E-08 | 5.31E-08 | 0.775 |
| RHSIM_Rhsim13G0040500 | 32.05 | 1.51E-08 | 5.38E-08 | 0.975 |
| RHSIM_RhsimPtG0000800 | 32.04 | 1.51E-08 | 5.38E-08 | 1.131 |
| RHSIM_Rhsim03G0135900 | 32.03 | 1.52E-08 | 5.43E-08 | 0.388 |
| RHSIM_Rhsim03G0225600 | 32.02 | 1.53E-08 | 5.45E-08 | -0.366 |
| novel.3939 | 32.01 | 1.53E-08 | 5.47E-08 | -1.229 |
| novel.2347 | 31.99 | 1.55E-08 | 5.52E-08 | -0.511 |

| Gene | LRT statistic | P value | BH-adjusted P | log2FC (interaction) |
|---|---|---|---|---|
| RHSIM_Rhsim03G0150100 | 31.99 | 1.55E-08 | 5.53E-08 | -4.812 |
| novel.870 | 31.99 | 1.55E-08 | 5.53E-08 | 1.005 |
| RHSIM_Rhsim13G0052100 | 31.97 | 1.56E-08 | 5.57E-08 | -0.838 |
| RHSIM_Rhsim08G0081600 | 31.95 | 1.58E-08 | 5.64E-08 | -2.528 |
| RHSIM_Rhsim04G0098600 | 31.92 | 1.60E-08 | 5.72E-08 | -2.036 |
| RHSIM_Rhsim07G0163700 | 31.91 | 1.62E-08 | 5.76E-08 | 1.872 |
| novel.883 | 31.90 | 1.62E-08 | 5.77E-08 | 0.970 |
| novel.2152 | 31.89 | 1.63E-08 | 5.80E-08 | 1.898 |
| RHSIM_Rhsim01G0291000 | 31.88 | 1.64E-08 | 5.84E-08 | -0.631 |
| RHSIM_Rhsim08G0095900 | 31.87 | 1.64E-08 | 5.86E-08 | 1.500 |
| RHSIM_Rhsim11G0144900 | 31.87 | 1.65E-08 | 5.86E-08 | 1.218 |
| RHSIM_Rhsim10G0131900 | 31.87 | 1.65E-08 | 5.87E-08 | -0.557 |
| RHSIM_Rhsim12G0215100 | 31.86 | 1.66E-08 | 5.89E-08 | 0.749 |
| RHSIM_Rhsim05G0096300 | 31.84 | 1.68E-08 | 5.97E-08 | -0.877 |
| novel.3805 | 31.84 | 1.68E-08 | 5.97E-08 | -2.522 |
| RHSIM_Rhsim10G0071700 | 31.83 | 1.68E-08 | 5.99E-08 | 1.570 |
| RHSIM_Rhsim12G0019200 | 31.81 | 1.70E-08 | 6.05E-08 | -1.592 |
| RHSIM_Rhsim10G0079900 | 31.80 | 1.71E-08 | 6.09E-08 | 0.440 |
| RHSIM_Rhsim06G0143600 | 31.79 | 1.72E-08 | 6.12E-08 | 1.481 |
| RHSIM_Rhsim10G0187200 | 31.77 | 1.74E-08 | 6.17E-08 | 1.364 |
| RHSIM_Rhsim07G0073200 | 31.76 | 1.74E-08 | 6.20E-08 | -1.181 |
| RHSIM_RhsimUnG0090200 | 31.76 | 1.74E-08 | 6.20E-08 | -1.884 |
| novel.1219 | 31.76 | 1.75E-08 | 6.22E-08 | 1.461 |
| RHSIM_Rhsim05G0225500 | 31.75 | 1.75E-08 | 6.24E-08 | -0.869 |
| RHSIM_Rhsim11G0181700 | 31.74 | 1.76E-08 | 6.26E-08 | -1.134 |
| RHSIM_Rhsim11G0079000 | 31.73 | 1.77E-08 | 6.28E-08 | 2.317 |
| RHSIM_Rhsim01G0252300 | 31.72 | 1.78E-08 | 6.33E-08 | 0.797 |
| novel.2071 | 31.70 | 1.79E-08 | 6.38E-08 | 1.518 |
| RHSIM_Rhsim11G0088100 | 31.69 | 1.81E-08 | 6.44E-08 | 1.192 |
| RHSIM_Rhsim08G0051500 | 31.68 | 1.81E-08 | 6.45E-08 | 0.406 |
| RHSIM_Rhsim10G0209300 | 31.68 | 1.81E-08 | 6.45E-08 | 0.713 |
| RHSIM_Rhsim08G0004300 | 31.66 | 1.84E-08 | 6.53E-08 | -0.473 |

| Gene | LRT statistic | P value | BH-adjusted P | log2FC (interaction) |
|---|---|---|---|---|
| RHSIM_Rhsim12G0168700 | 31.65 | 1.84E-08 | 6.55E-08 | 0.328 |
| RHSIM_Rhsim10G0016700 | 31.65 | 1.84E-08 | 6.55E-08 | -0.572 |
| novel.2929 | 31.64 | 1.86E-08 | 6.60E-08 | -1.940 |
| RHSIM_Rhsim08G0252000 | 31.61 | 1.89E-08 | 6.71E-08 | -0.829 |
| RHSIM_Rhsim05G0190200 | 31.60 | 1.89E-08 | 6.71E-08 | 2.102 |
| RHSIM_Rhsim11G0038700 | 31.60 | 1.89E-08 | 6.72E-08 | 3.191 |
| RHSIM_Rhsim07G0091800 | 31.59 | 1.91E-08 | 6.77E-08 | 1.302 |
| novel.1929 | 31.58 | 1.91E-08 | 6.78E-08 | 1.164 |
| RHSIM_Rhsim01G0198400 | 31.58 | 1.91E-08 | 6.78E-08 | 1.949 |
| RHSIM_Rhsim06G0209900 | 31.58 | 1.92E-08 | 6.80E-08 | -1.065 |
| RHSIM_Rhsim08G0011400 | 31.57 | 1.92E-08 | 6.81E-08 | -0.660 |
| RHSIM_RhsimUnG0152500 | 31.57 | 1.92E-08 | 6.81E-08 | 0.789 |
| RHSIM_Rhsim07G0029300 | 31.57 | 1.92E-08 | 6.81E-08 | 0.950 |
| novel.2718 | 31.57 | 1.92E-08 | 6.82E-08 | -0.922 |
| RHSIM_Rhsim11G0179000 | 31.57 | 1.93E-08 | 6.83E-08 | -0.567 |
| RHSIM_Rhsim08G0007000 | 31.57 | 1.93E-08 | 6.83E-08 | 1.841 |
| RHSIM_Rhsim08G0128700 | 31.56 | 1.93E-08 | 6.84E-08 | -0.732 |
| RHSIM_Rhsim08G0218900 | 31.56 | 1.94E-08 | 6.86E-08 | 1.209 |
| RHSIM_Rhsim05G0168300 | 31.55 | 1.95E-08 | 6.90E-08 | 1.149 |
| RHSIM_Rhsim10G0196200 | 31.55 | 1.95E-08 | 6.90E-08 | 2.016 |
| novel.2197 | 31.54 | 1.96E-08 | 6.94E-08 | -0.690 |
| RHSIM_Rhsim07G0146500 | 31.53 | 1.96E-08 | 6.95E-08 | -0.409 |
| RHSIM_Rhsim04G0181400 | 31.52 | 1.97E-08 | 6.98E-08 | -1.392 |
| RHSIM_Rhsim03G0190000 | 31.52 | 1.97E-08 | 6.98E-08 | 1.438 |
| RHSIM_Rhsim03G0206800 | 31.52 | 1.97E-08 | 6.99E-08 | 2.611 |
| RHSIM_Rhsim12G0053200 | 31.51 | 1.99E-08 | 7.04E-08 | 0.984 |
| RHSIM_Rhsim04G0106200 | 31.50 | 1.99E-08 | 7.05E-08 | 3.085 |
| RHSIM_Rhsim05G0229200 | 31.49 | 2.01E-08 | 7.10E-08 | 0.418 |
| RHSIM_Rhsim10G0195300 | 31.48 | 2.02E-08 | 7.14E-08 | 1.215 |
| RHSIM_Rhsim03G0069200 | 31.48 | 2.02E-08 | 7.14E-08 | -0.506 |
| RHSIM_Rhsim02G0069300 | 31.47 | 2.02E-08 | 7.16E-08 | -0.828 |
| RHSIM_Rhsim02G0035400 | 31.47 | 2.02E-08 | 7.16E-08 | -0.596 |

| Gene | LRT statistic | P value | BH-adjusted P | log2FC (interaction) |
|---|---|---|---|---|
| RHSIM_Rhsim08G0217700 | 31.47 | 2.03E-08 | 7.17E-08 | -1.178 |
| RHSIM_Rhsim10G0006800 | 31.47 | 2.03E-08 | 7.17E-08 | 2.171 |
| novel.307 | 31.47 | 2.03E-08 | 7.18E-08 | 0.459 |
| RHSIM_Rhsim12G0121800 | 31.46 | 2.03E-08 | 7.19E-08 | 0.552 |
| RHSIM_Rhsim10G0021400 | 31.44 | 2.06E-08 | 7.27E-08 | 0.746 |
| RHSIM_Rhsim11G0133100 | 31.42 | 2.07E-08 | 7.33E-08 | -1.639 |
| novel.820 | 31.41 | 2.09E-08 | 7.38E-08 | 1.045 |
| RHSIM_Rhsim13G0022200 | 31.41 | 2.09E-08 | 7.39E-08 | -0.394 |
| RHSIM_Rhsim03G0139900 | 31.40 | 2.10E-08 | 7.43E-08 | -0.785 |
| RHSIM_Rhsim02G0168000 | 31.39 | 2.11E-08 | 7.47E-08 | 0.621 |
| RHSIM_Rhsim13G0031200 | 31.39 | 2.12E-08 | 7.48E-08 | -0.542 |
| RHSIM_Rhsim08G0200600 | 31.38 | 2.12E-08 | 7.48E-08 | -0.830 |
| RHSIM_Rhsim13G0231400 | 31.38 | 2.12E-08 | 7.50E-08 | 2.104 |
| RHSIM_Rhsim07G0100200 | 31.37 | 2.13E-08 | 7.54E-08 | -0.715 |
| RHSIM_Rhsim08G0193400 | 31.35 | 2.16E-08 | 7.62E-08 | 0.581 |
| RHSIM_Rhsim06G0108200 | 31.34 | 2.16E-08 | 7.63E-08 | 1.314 |
| RHSIM_Rhsim03G0136000 | 31.32 | 2.19E-08 | 7.74E-08 | 1.453 |
| RHSIM_Rhsim01G0109400 | 31.30 | 2.21E-08 | 7.80E-08 | -2.106 |
| RHSIM_Rhsim07G0043400 | 31.30 | 2.21E-08 | 7.82E-08 | 0.573 |
| RHSIM_Rhsim03G0050900 | 31.29 | 2.22E-08 | 7.83E-08 | 1.527 |
| RHSIM_Rhsim09G0142500 | 31.29 | 2.22E-08 | 7.84E-08 | -0.367 |
| RHSIM_Rhsim09G0189600 | 31.28 | 2.24E-08 | 7.90E-08 | 0.469 |
| RHSIM_Rhsim05G0028500 | 31.28 | 2.24E-08 | 7.90E-08 | -0.476 |
| RHSIM_Rhsim02G0234500 | 31.27 | 2.24E-08 | 7.91E-08 | -0.937 |
| RHSIM_Rhsim05G0143000 | 31.27 | 2.25E-08 | 7.93E-08 | 0.674 |
| RHSIM_Rhsim04G0246200 | 31.26 | 2.25E-08 | 7.94E-08 | 0.548 |
| RHSIM_Rhsim08G0029100 | 31.26 | 2.25E-08 | 7.95E-08 | 1.960 |
| RHSIM_Rhsim08G0234300 | 31.26 | 2.26E-08 | 7.96E-08 | -0.382 |
| RHSIM_RhsimUnG0180500 | 31.25 | 2.27E-08 | 8.00E-08 | 1.044 |
| RHSIM_Rhsim13G0125100 | 31.24 | 2.28E-08 | 8.02E-08 | 1.291 |
| RHSIM_RhsimUnG0145700 | 31.24 | 2.29E-08 | 8.06E-08 | 1.235 |
| RHSIM_Rhsim07G0215000 | 31.23 | 2.29E-08 | 8.07E-08 | 0.445 |

| Gene | LRT statistic | P value | BH-adjusted P | log2FC (interaction) |
|---|---|---|---|---|
| RHSIM_Rhsim04G0155200 | 31.23 | 2.29E-08 | 8.07E-08 | 0.901 |
| RHSIM_Rhsim12G0039700 | 31.23 | 2.29E-08 | 8.08E-08 | -0.539 |
| RHSIM_Rhsim13G0016300 | 31.23 | 2.29E-08 | 8.08E-08 | -0.727 |
| RHSIM_RhsimUnG0103000 | 31.23 | 2.30E-08 | 8.09E-08 | -0.683 |
| RHSIM_Rhsim01G0132900 | 31.22 | 2.30E-08 | 8.10E-08 | 3.358 |
| RHSIM_Rhsim11G0010400 | 31.21 | 2.32E-08 | 8.18E-08 | 0.793 |
| novel.4012 | 31.20 | 2.33E-08 | 8.22E-08 | 1.065 |
| RHSIM_Rhsim04G0177400 | 31.20 | 2.33E-08 | 8.22E-08 | 0.419 |
| RHSIM_RhsimUnG0210700 | 31.19 | 2.34E-08 | 8.22E-08 | -0.815 |
| RHSIM_Rhsim02G0244100 | 31.19 | 2.34E-08 | 8.22E-08 | 0.398 |
| RHSIM_Rhsim06G0172500 | 31.19 | 2.34E-08 | 8.24E-08 | -1.519 |
| novel.1711 | 31.19 | 2.34E-08 | 8.25E-08 | 0.596 |
| RHSIM_Rhsim05G0144200 | 31.18 | 2.35E-08 | 8.26E-08 | 1.034 |
| RHSIM_Rhsim06G0029300 | 31.18 | 2.36E-08 | 8.29E-08 | 3.194 |
| RHSIM_Rhsim04G0108000 | 31.17 | 2.36E-08 | 8.30E-08 | 0.648 |
| RHSIM_Rhsim06G0234800 | 31.17 | 2.36E-08 | 8.31E-08 | -0.573 |
| RHSIM_Rhsim03G0220200 | 31.16 | 2.38E-08 | 8.37E-08 | -1.026 |
| RHSIM_Rhsim06G0097300 | 31.15 | 2.39E-08 | 8.42E-08 | 2.103 |
| RHSIM_Rhsim03G0170700 | 31.11 | 2.44E-08 | 8.57E-08 | -0.572 |
| RHSIM_Rhsim03G0076200 | 31.09 | 2.46E-08 | 8.65E-08 | -0.493 |
| RHSIM_RhsimUnG0044100 | 31.09 | 2.47E-08 | 8.68E-08 | -0.569 |
| RHSIM_Rhsim12G0184600 | 31.08 | 2.48E-08 | 8.71E-08 | 0.583 |
| RHSIM_Rhsim04G0073800 | 31.07 | 2.48E-08 | 8.73E-08 | -0.901 |
| RHSIM_Rhsim08G0107000 | 31.07 | 2.49E-08 | 8.73E-08 | -3.609 |
| RHSIM_Rhsim04G0172400 | 31.07 | 2.49E-08 | 8.74E-08 | -0.939 |
| RHSIM_Rhsim01G0262300 | 31.07 | 2.49E-08 | 8.76E-08 | 0.729 |
| RHSIM_Rhsim09G0051600 | 31.06 | 2.50E-08 | 8.77E-08 | 0.655 |
| RHSIM_Rhsim03G0125700 | 31.06 | 2.50E-08 | 8.78E-08 | -0.489 |
| RHSIM_Rhsim03G0021400 | 31.06 | 2.50E-08 | 8.78E-08 | -0.555 |
| RHSIM_Rhsim05G0044200 | 31.06 | 2.51E-08 | 8.81E-08 | -0.730 |
| RHSIM_Rhsim04G0249200 | 31.04 | 2.53E-08 | 8.87E-08 | 0.600 |
| RHSIM_Rhsim03G0065000 | 31.03 | 2.53E-08 | 8.90E-08 | -4.897 |

| Gene | LRT statistic | P value | BH-adjusted P | log2FC (interaction) |
|---|---|---|---|---|
| RHSIM_Rhsim13G0199500 | 31.02 | 2.56E-08 | 8.98E-08 | 0.672 |
| RHSIM_Rhsim02G0144300 | 31.00 | 2.58E-08 | 9.04E-08 | -0.556 |
| RHSIM_Rhsim08G0003100 | 30.96 | 2.63E-08 | 9.24E-08 | 0.845 |
| RHSIM_Rhsim01G0224500 | 30.96 | 2.64E-08 | 9.25E-08 | -1.886 |
| RHSIM_Rhsim04G0072100 | 30.96 | 2.64E-08 | 9.26E-08 | 1.965 |
| RHSIM_Rhsim12G0149200 | 30.94 | 2.67E-08 | 9.36E-08 | -3.851 |
| RHSIM_Rhsim05G0062700 | 30.93 | 2.67E-08 | 9.36E-08 | -1.477 |
| RHSIM_Rhsim01G0149400 | 30.93 | 2.68E-08 | 9.40E-08 | 0.960 |
| novel.3313 | 30.91 | 2.70E-08 | 9.46E-08 | 1.179 |
| novel.3908 | 30.91 | 2.70E-08 | 9.47E-08 | -0.637 |
| RHSIM_Rhsim09G0171000 | 30.90 | 2.72E-08 | 9.54E-08 | -0.822 |
| RHSIM_Rhsim08G0175000 | 30.90 | 2.72E-08 | 9.54E-08 | 4.384 |
| RHSIM_RhsimUnG0022700 | 30.89 | 2.72E-08 | 9.55E-08 | 1.670 |
| RHSIM_Rhsim08G0093900 | 30.89 | 2.73E-08 | 9.56E-08 | -1.021 |
| novel.2442 | 30.89 | 2.73E-08 | 9.58E-08 | 0.480 |
| RHSIM_Rhsim11G0051300 | 30.86 | 2.78E-08 | 9.73E-08 | 1.126 |
| novel.1894 | 30.86 | 2.78E-08 | 9.73E-08 | -4.947 |
| RHSIM_Rhsim07G0140800 | 30.85 | 2.79E-08 | 9.79E-08 | -1.289 |
| RHSIM_Rhsim09G0163900 | 30.84 | 2.81E-08 | 9.84E-08 | 1.289 |
| RHSIM_Rhsim01G0248500 | 30.82 | 2.82E-08 | 9.89E-08 | -1.083 |
| RHSIM_Rhsim12G0070900 | 30.82 | 2.84E-08 | 9.93E-08 | 0.301 |
| RHSIM_Rhsim12G0014700 | 30.81 | 2.85E-08 | 9.99E-08 | 0.559 |
| RHSIM_Rhsim03G0219400 | 30.80 | 2.85E-08 | 9.99E-08 | -0.683 |
| RHSIM_Rhsim08G0156200 | 30.80 | 2.86E-08 | 1.00E-07 | 0.832 |
| RHSIM_Rhsim01G0292100 | 30.80 | 2.87E-08 | 1.00E-07 | 0.994 |
| RHSIM_RhsimUnG0132500 | 30.77 | 2.90E-08 | 1.02E-07 | -1.818 |
| RHSIM_Rhsim04G0124900 | 30.76 | 2.92E-08 | 1.02E-07 | 0.455 |
| RHSIM_Rhsim03G0243900 | 30.75 | 2.93E-08 | 1.03E-07 | -0.592 |
| RHSIM_Rhsim08G0149800 | 30.74 | 2.96E-08 | 1.03E-07 | 0.862 |
| novel.156 | 30.69 | 3.02E-08 | 1.06E-07 | -3.215 |
| RHSIM_Rhsim06G0165300 | 30.69 | 3.02E-08 | 1.06E-07 | 0.493 |
| RHSIM_RhsimUnG0065200 | 30.69 | 3.03E-08 | 1.06E-07 | 3.627 |

| Gene | LRT statistic | P value | BH-adjusted P | log2FC (interaction) |
|---|---|---|---|---|
| RHSIM_Rhsim11G0125200 | 30.69 | 3.03E-08 | 1.06E-07 | -0.504 |
| RHSIM_Rhsim07G0118800 | 30.68 | 3.05E-08 | 1.06E-07 | -0.807 |
| RHSIM_RhsimUnG0171400 | 30.66 | 3.07E-08 | 1.07E-07 | -2.409 |
| RHSIM_Rhsim03G0155200 | 30.65 | 3.09E-08 | 1.08E-07 | 0.592 |
| RHSIM_Rhsim08G0206100 | 30.63 | 3.12E-08 | 1.09E-07 | 3.726 |
| novel.2992 | 30.62 | 3.14E-08 | 1.10E-07 | -0.413 |
| RHSIM_Rhsim08G0218500 | 30.61 | 3.15E-08 | 1.10E-07 | -0.428 |
| RHSIM_Rhsim03G0254200 | 30.59 | 3.19E-08 | 1.11E-07 | -0.682 |
| novel.2783 | 30.57 | 3.22E-08 | 1.12E-07 | 4.644 |
| novel.2998 | 30.57 | 3.22E-08 | 1.13E-07 | -2.699 |
| RHSIM_Rhsim03G0230000 | 30.55 | 3.25E-08 | 1.13E-07 | -0.459 |
| RHSIM_Rhsim08G0063600 | 30.54 | 3.27E-08 | 1.14E-07 | -2.104 |
| RHSIM_Rhsim10G0064100 | 30.53 | 3.28E-08 | 1.15E-07 | -0.506 |
| RHSIM_Rhsim01G0000700 | 30.52 | 3.30E-08 | 1.15E-07 | 0.571 |
| RHSIM_Rhsim06G0237000 | 30.52 | 3.31E-08 | 1.15E-07 | 0.807 |
| RHSIM_Rhsim04G0095400 | 30.51 | 3.32E-08 | 1.16E-07 | -0.622 |
| RHSIM_Rhsim12G0139000 | 30.48 | 3.38E-08 | 1.18E-07 | 0.471 |
| RHSIM_Rhsim02G0024900 | 30.47 | 3.38E-08 | 1.18E-07 | 0.812 |
| RHSIM_Rhsim04G0204600 | 30.46 | 3.41E-08 | 1.19E-07 | -1.284 |
| RHSIM_Rhsim08G0128200 | 30.46 | 3.42E-08 | 1.19E-07 | 0.973 |
| RHSIM_Rhsim13G0011500 | 30.45 | 3.42E-08 | 1.19E-07 | 0.833 |
| RHSIM_Rhsim03G0243000 | 30.44 | 3.44E-08 | 1.20E-07 | -0.491 |
| RHSIM_Rhsim05G0072900 | 30.43 | 3.45E-08 | 1.20E-07 | 0.962 |
| novel.3610 | 30.43 | 3.45E-08 | 1.20E-07 | -1.345 |
| RHSIM_Rhsim08G0163600 | 30.42 | 3.48E-08 | 1.21E-07 | -0.578 |
| novel.2523 | 30.41 | 3.50E-08 | 1.22E-07 | 0.667 |
| RHSIM_Rhsim13G0190300 | 30.41 | 3.50E-08 | 1.22E-07 | 0.672 |
| RHSIM_Rhsim04G0011400 | 30.40 | 3.51E-08 | 1.22E-07 | -0.485 |
| novel.3478 | 30.39 | 3.52E-08 | 1.23E-07 | 0.500 |
| RHSIM_Rhsim11G0128700 | 30.39 | 3.53E-08 | 1.23E-07 | 2.494 |
| RHSIM_Rhsim13G0234100 | 30.39 | 3.53E-08 | 1.23E-07 | -0.602 |
| RHSIM_Rhsim05G0131900 | 30.39 | 3.54E-08 | 1.23E-07 | 0.438 |

| Gene | LRT statistic | P value | BH-adjusted P | log2FC (interaction) |
|---|---|---|---|---|
| RHSIM_Rhsim03G0226600 | 30.38 | 3.54E-08 | 1.23E-07 | 0.361 |
| RHSIM_Rhsim11G0149600 | 30.38 | 3.55E-08 | 1.24E-07 | 0.761 |
| RHSIM_Rhsim12G0201000 | 30.37 | 3.56E-08 | 1.24E-07 | 42.458 |
| RHSIM_Rhsim03G0235200 | 30.37 | 3.56E-08 | 1.24E-07 | -0.286 |
| RHSIM_Rhsim10G0069000 | 30.37 | 3.57E-08 | 1.24E-07 | 0.431 |
| RHSIM_RhsimUnG0106200 | 30.36 | 3.59E-08 | 1.25E-07 | 0.853 |
| RHSIM_Rhsim02G0087700 | 30.34 | 3.63E-08 | 1.26E-07 | -1.558 |
| novel.3978 | 30.32 | 3.66E-08 | 1.27E-07 | 2.170 |
| RHSIM_Rhsim08G0191400 | 30.31 | 3.67E-08 | 1.28E-07 | 1.819 |
| RHSIM_Rhsim09G0138800 | 30.31 | 3.68E-08 | 1.28E-07 | 2.647 |
| novel.3463 | 30.29 | 3.73E-08 | 1.30E-07 | 2.651 |
| RHSIM_Rhsim03G0212400 | 30.28 | 3.73E-08 | 1.30E-07 | 0.396 |
| RHSIM_Rhsim10G0018500 | 30.27 | 3.76E-08 | 1.31E-07 | 1.041 |
| RHSIM_Rhsim13G0136200 | 30.26 | 3.77E-08 | 1.31E-07 | 2.173 |
| novel.3339 | 30.26 | 3.78E-08 | 1.31E-07 | 1.423 |
| RHSIM_Rhsim08G0164100 | 30.26 | 3.78E-08 | 1.31E-07 | -0.788 |
| RHSIM_Rhsim08G0041900 | 30.25 | 3.81E-08 | 1.32E-07 | -1.165 |
| RHSIM_Rhsim13G0224100 | 30.24 | 3.81E-08 | 1.33E-07 | -0.691 |
| RHSIM_Rhsim10G0153200 | 30.22 | 3.86E-08 | 1.34E-07 | 0.798 |
| RHSIM_Rhsim01G0148500 | 30.22 | 3.87E-08 | 1.34E-07 | 0.774 |
| RHSIM_Rhsim06G0034900 | 30.20 | 3.89E-08 | 1.35E-07 | 0.384 |
| novel.330 | 30.19 | 3.91E-08 | 1.36E-07 | 0.448 |
| RHSIM_Rhsim12G0138500 | 30.18 | 3.93E-08 | 1.37E-07 | 0.499 |
| novel.77 | 30.18 | 3.93E-08 | 1.37E-07 | 1.125 |
| RHSIM_Rhsim04G0184400 | 30.17 | 3.95E-08 | 1.37E-07 | 0.414 |
| RHSIM_Rhsim03G0082200 | 30.16 | 3.97E-08 | 1.38E-07 | 0.772 |
| RHSIM_Rhsim01G0276700 | 30.16 | 3.97E-08 | 1.38E-07 | 0.764 |
| novel.3305 | 30.16 | 3.97E-08 | 1.38E-07 | 0.469 |
| RHSIM_Rhsim13G0010500 | 30.15 | 3.99E-08 | 1.38E-07 | 0.803 |
| RHSIM_Rhsim02G0093200 | 30.14 | 4.01E-08 | 1.39E-07 | 3.730 |
| RHSIM_Rhsim05G0223600 | 30.14 | 4.01E-08 | 1.39E-07 | 1.368 |
| RHSIM_Rhsim08G0034700 | 30.14 | 4.03E-08 | 1.40E-07 | 0.616 |

| Gene | LRT statistic | P value | BH-adjusted P | log2FC (interaction) |
|---|---|---|---|---|
| RHSIM_Rhsim13G0018300 | 30.13 | 4.05E-08 | 1.40E-07 | 0.513 |
| RHSIM_RhsimUnG0105900 | 30.12 | 4.06E-08 | 1.41E-07 | -1.763 |
| RHSIM_Rhsim09G0181500 | 30.12 | 4.06E-08 | 1.41E-07 | -0.672 |
| RHSIM_Rhsim10G0014000 | 30.12 | 4.07E-08 | 1.41E-07 | -0.587 |
| novel.1377 | 30.11 | 4.07E-08 | 1.41E-07 | 3.029 |
| RHSIM_Rhsim06G0029100 | 30.11 | 4.08E-08 | 1.41E-07 | 0.351 |
| RHSIM_Rhsim11G0092800 | 30.11 | 4.08E-08 | 1.41E-07 | 0.905 |
| RHSIM_Rhsim06G0094800 | 30.11 | 4.08E-08 | 1.42E-07 | -1.818 |
| RHSIM_Rhsim02G0042600 | 30.09 | 4.12E-08 | 1.43E-07 | 1.003 |
| RHSIM_Rhsim04G0204900 | 30.09 | 4.13E-08 | 1.43E-07 | 0.566 |
| RHSIM_Rhsim08G0070900 | 30.08 | 4.14E-08 | 1.43E-07 | -2.551 |
| novel.1271 | 30.08 | 4.14E-08 | 1.43E-07 | -0.732 |
| RHSIM_Rhsim04G0076500 | 30.07 | 4.16E-08 | 1.44E-07 | -42.282 |
| novel.428 | 30.06 | 4.19E-08 | 1.45E-07 | 0.406 |
| RHSIM_Rhsim09G0034500 | 30.06 | 4.19E-08 | 1.45E-07 | 3.014 |
| RHSIM_Rhsim10G0169700 | 30.05 | 4.21E-08 | 1.46E-07 | -0.985 |
| RHSIM_Rhsim07G0014100 | 30.05 | 4.22E-08 | 1.46E-07 | -0.914 |
| RHSIM_Rhsim09G0185600 | 30.03 | 4.25E-08 | 1.47E-07 | -1.790 |
| RHSIM_Rhsim01G0293500 | 30.03 | 4.25E-08 | 1.47E-07 | 0.800 |
| RHSIM_Rhsim12G0040600 | 30.03 | 4.26E-08 | 1.48E-07 | -0.559 |
| RHSIM_Rhsim06G0233900 | 30.02 | 4.27E-08 | 1.48E-07 | -0.644 |
| RHSIM_Rhsim08G0211600 | 30.02 | 4.27E-08 | 1.48E-07 | 3.468 |
| RHSIM_Rhsim11G0029800 | 30.02 | 4.28E-08 | 1.48E-07 | -1.479 |
| RHSIM_Rhsim08G0110400 | 30.01 | 4.30E-08 | 1.49E-07 | -0.507 |
| RHSIM_Rhsim11G0045000 | 29.99 | 4.34E-08 | 1.50E-07 | 0.514 |
| RHSIM_Rhsim11G0125300 | 29.97 | 4.38E-08 | 1.52E-07 | 0.745 |
| RHSIM_Rhsim08G0168400 | 29.97 | 4.39E-08 | 1.52E-07 | 0.591 |
| RHSIM_Rhsim08G0074500 | 29.96 | 4.41E-08 | 1.52E-07 | 0.392 |
| RHSIM_RhsimUnG0240700 | 29.95 | 4.43E-08 | 1.53E-07 | -0.439 |
| RHSIM_Rhsim09G0131600 | 29.94 | 4.45E-08 | 1.54E-07 | 0.892 |
| RHSIM_Rhsim09G0045000 | 29.94 | 4.46E-08 | 1.54E-07 | -2.720 |
| RHSIM_Rhsim03G0246100 | 29.94 | 4.46E-08 | 1.54E-07 | -0.843 |

| Gene | LRT statistic | P value | BH-adjusted P | log2FC (interaction) |
|---|---|---|---|---|
| RHSIM_Rhsim10G0008800 | 29.93 | 4.47E-08 | 1.54E-07 | -0.760 |
| RHSIM_RhsimUnG0247200 | 29.92 | 4.49E-08 | 1.55E-07 | -2.161 |
| RHSIM_Rhsim08G0197800 | 29.92 | 4.50E-08 | 1.55E-07 | 3.761 |
| RHSIM_Rhsim04G0196900 | 29.92 | 4.51E-08 | 1.56E-07 | -0.545 |
| RHSIM_Rhsim06G0107700 | 29.91 | 4.52E-08 | 1.56E-07 | -0.658 |
| RHSIM_Rhsim06G0022800 | 29.91 | 4.53E-08 | 1.56E-07 | 0.988 |
| RHSIM_Rhsim08G0046300 | 29.91 | 4.54E-08 | 1.57E-07 | -0.399 |
| RHSIM_Rhsim03G0075500 | 29.90 | 4.54E-08 | 1.57E-07 | 0.555 |
| RHSIM_Rhsim09G0072100 | 29.88 | 4.60E-08 | 1.59E-07 | -1.068 |
| novel.3081 | 29.88 | 4.61E-08 | 1.59E-07 | 1.931 |
| RHSIM_Rhsim10G0145000 | 29.87 | 4.62E-08 | 1.59E-07 | -0.329 |
| RHSIM_Rhsim10G0181400 | 29.87 | 4.63E-08 | 1.60E-07 | -1.239 |
| RHSIM_Rhsim08G0111100 | 29.86 | 4.65E-08 | 1.60E-07 | 1.386 |
| RHSIM_Rhsim11G0105000 | 29.84 | 4.68E-08 | 1.62E-07 | 0.674 |
| RHSIM_Rhsim12G0168300 | 29.81 | 4.76E-08 | 1.64E-07 | -0.834 |
| RHSIM_Rhsim01G0088000 | 29.81 | 4.76E-08 | 1.64E-07 | 0.833 |
| RHSIM_Rhsim02G0073900 | 29.80 | 4.78E-08 | 1.65E-07 | -1.474 |
| RHSIM_RhsimUnG0009900 | 29.79 | 4.82E-08 | 1.66E-07 | -45.483 |
| RHSIM_Rhsim13G0030500 | 29.78 | 4.84E-08 | 1.67E-07 | 0.815 |
| RHSIM_Rhsim09G0185200 | 29.78 | 4.84E-08 | 1.67E-07 | -0.501 |
| RHSIM_Rhsim09G0189500 | 29.78 | 4.85E-08 | 1.67E-07 | 0.530 |
| RHSIM_Rhsim02G0159200 | 29.77 | 4.87E-08 | 1.68E-07 | 2.090 |
| RHSIM_Rhsim07G0224200 | 29.75 | 4.90E-08 | 1.69E-07 | 1.563 |
| RHSIM_Rhsim02G0082000 | 29.75 | 4.91E-08 | 1.69E-07 | -0.554 |
| RHSIM_Rhsim08G0148600 | 29.75 | 4.91E-08 | 1.69E-07 | 2.554 |
| RHSIM_Rhsim01G0203800 | 29.75 | 4.91E-08 | 1.69E-07 | -0.431 |
| RHSIM_Rhsim01G0216700 | 29.75 | 4.93E-08 | 1.70E-07 | -0.824 |
| RHSIM_Rhsim11G0141800 | 29.74 | 4.93E-08 | 1.70E-07 | -0.474 |
| RHSIM_Rhsim07G0202200 | 29.74 | 4.93E-08 | 1.70E-07 | 1.343 |
| RHSIM_Rhsim10G0082800 | 29.74 | 4.94E-08 | 1.70E-07 | 1.499 |
| RHSIM_Rhsim09G0122900 | 29.74 | 4.94E-08 | 1.70E-07 | 1.450 |
| RHSIM_Rhsim01G0004700 | 29.73 | 4.95E-08 | 1.71E-07 | -1.908 |

| Gene | LRT statistic | P value | BH-adjusted P | log2FC (interaction) |
|---|---|---|---|---|
| RHSIM_Rhsim01G0101000 | 29.73 | 4.96E-08 | 1.71E-07 | 0.329 |
| RHSIM_Rhsim03G0180500 | 29.73 | 4.96E-08 | 1.71E-07 | 2.700 |
| RHSIM_RhsimUnG0160300 | 29.73 | 4.97E-08 | 1.71E-07 | -0.533 |
| novel.3635 | 29.69 | 5.06E-08 | 1.74E-07 | 2.359 |
| RHSIM_Rhsim06G0009800 | 29.69 | 5.07E-08 | 1.74E-07 | 0.568 |
| RHSIM_Rhsim08G0136700 | 29.68 | 5.09E-08 | 1.75E-07 | -2.186 |
| RHSIM_Rhsim11G0131900 | 29.68 | 5.11E-08 | 1.76E-07 | 0.524 |
| RHSIM_Rhsim05G0178100 | 29.66 | 5.14E-08 | 1.77E-07 | -1.084 |
| RHSIM_RhsimUnG0042200 | 29.66 | 5.15E-08 | 1.77E-07 | 0.985 |
| RHSIM_Rhsim03G0234000 | 29.65 | 5.16E-08 | 1.78E-07 | 0.532 |
| novel.4045 | 29.64 | 5.20E-08 | 1.79E-07 | 0.700 |
| RHSIM_RhsimUnG0125200 | 29.64 | 5.20E-08 | 1.79E-07 | -3.602 |
| novel.4403 | 29.64 | 5.21E-08 | 1.79E-07 | -3.500 |
| RHSIM_Rhsim07G0119600 | 29.64 | 5.21E-08 | 1.79E-07 | -0.452 |
| RHSIM_Rhsim12G0056000 | 29.64 | 5.21E-08 | 1.79E-07 | 1.384 |
| RHSIM_Rhsim04G0019100 | 29.62 | 5.24E-08 | 1.80E-07 | -4.278 |
| RHSIM_Rhsim04G0031100 | 29.62 | 5.25E-08 | 1.80E-07 | -0.704 |
| RHSIM_Rhsim11G0030300 | 29.62 | 5.26E-08 | 1.81E-07 | 0.499 |
| RHSIM_Rhsim05G0205600 | 29.62 | 5.26E-08 | 1.81E-07 | -0.592 |
| RHSIM_Rhsim07G0070700 | 29.60 | 5.30E-08 | 1.82E-07 | -1.011 |
| RHSIM_Rhsim10G0073100 | 29.60 | 5.32E-08 | 1.83E-07 | 0.755 |
| RHSIM_Rhsim10G0029400 | 29.58 | 5.38E-08 | 1.85E-07 | 0.540 |
| RHSIM_Rhsim10G0180400 | 29.57 | 5.39E-08 | 1.85E-07 | 0.575 |
| RHSIM_Rhsim05G0041600 | 29.56 | 5.41E-08 | 1.86E-07 | 0.820 |
| novel.4471 | 29.55 | 5.46E-08 | 1.87E-07 | 1.170 |
| novel.1553 | 29.54 | 5.49E-08 | 1.88E-07 | 1.713 |
| RHSIM_Rhsim05G0082500 | 29.53 | 5.50E-08 | 1.89E-07 | 4.270 |
| novel.4396 | 29.53 | 5.52E-08 | 1.89E-07 | 2.082 |
| RHSIM_Rhsim05G0143800 | 29.53 | 5.52E-08 | 1.89E-07 | 0.599 |
| RHSIM_Rhsim04G0151400 | 29.52 | 5.53E-08 | 1.90E-07 | 3.368 |
| RHSIM_Rhsim11G0178000 | 29.51 | 5.56E-08 | 1.91E-07 | -2.338 |
| RHSIM_Rhsim11G0088800 | 29.51 | 5.56E-08 | 1.91E-07 | 0.566 |

| Gene | LRT statistic | P value | BH-adjusted P | log2FC (interaction) |
|---|---|---|---|---|
| RHSIM_Rhsim09G0089600 | 29.47 | 5.68E-08 | 1.95E-07 | -0.511 |
| RHSIM_Rhsim12G0185100 | 29.47 | 5.68E-08 | 1.95E-07 | 1.367 |
| RHSIM_Rhsim06G0183200 | 29.45 | 5.75E-08 | 1.97E-07 | -1.323 |
| RHSIM_Rhsim01G0068400 | 29.44 | 5.77E-08 | 1.98E-07 | -0.653 |
| RHSIM_Rhsim10G0006900 | 29.44 | 5.78E-08 | 1.98E-07 | -0.804 |
| RHSIM_Rhsim07G0130100 | 29.43 | 5.78E-08 | 1.98E-07 | 1.831 |
| RHSIM_Rhsim01G0107000 | 29.43 | 5.79E-08 | 1.98E-07 | -1.639 |
| RHSIM_Rhsim11G0121900 | 29.42 | 5.83E-08 | 2.00E-07 | 0.469 |
| RHSIM_Rhsim08G0155600 | 29.42 | 5.84E-08 | 2.00E-07 | -0.598 |
| RHSIM_Rhsim02G0083000 | 29.39 | 5.91E-08 | 2.03E-07 | 0.514 |
| novel.4136 | 29.39 | 5.92E-08 | 2.03E-07 | 1.865 |
| novel.1725 | 29.39 | 5.93E-08 | 2.03E-07 | 7.441 |
| RHSIM_Rhsim07G0141300 | 29.37 | 5.97E-08 | 2.04E-07 | -1.328 |
| RHSIM_Rhsim12G0077600 | 29.37 | 5.99E-08 | 2.05E-07 | -0.726 |
| RHSIM_Rhsim10G0203000 | 29.35 | 6.03E-08 | 2.06E-07 | 1.265 |
| RHSIM_Rhsim07G0089600 | 29.34 | 6.08E-08 | 2.08E-07 | -5.507 |
| RHSIM_Rhsim08G0245700 | 29.33 | 6.09E-08 | 2.08E-07 | -0.327 |
| RHSIM_Rhsim02G0181800 | 29.32 | 6.13E-08 | 2.10E-07 | -1.501 |
| RHSIM_Rhsim10G0160800 | 29.32 | 6.14E-08 | 2.10E-07 | -2.830 |
| RHSIM_Rhsim08G0233800 | 29.30 | 6.19E-08 | 2.12E-07 | 0.735 |
| RHSIM_Rhsim07G0038500 | 29.30 | 6.21E-08 | 2.12E-07 | -0.881 |
| RHSIM_Rhsim04G0247300 | 29.30 | 6.21E-08 | 2.12E-07 | -1.172 |
| RHSIM_Rhsim01G0209100 | 29.29 | 6.22E-08 | 2.13E-07 | 0.709 |
| RHSIM_Rhsim01G0225600 | 29.29 | 6.22E-08 | 2.13E-07 | 1.006 |
| RHSIM_Rhsim03G0192100 | 29.29 | 6.23E-08 | 2.13E-07 | -0.725 |
| RHSIM_Rhsim09G0180600 | 29.29 | 6.24E-08 | 2.13E-07 | 2.012 |
| RHSIM_Rhsim13G0128900 | 29.27 | 6.29E-08 | 2.15E-07 | -0.621 |
| novel.1592 | 29.27 | 6.31E-08 | 2.16E-07 | 4.042 |
| RHSIM_Rhsim12G0127200 | 29.26 | 6.34E-08 | 2.17E-07 | 0.360 |
| RHSIM_Rhsim12G0017700 | 29.26 | 6.34E-08 | 2.17E-07 | 0.793 |
| RHSIM_Rhsim06G0156600 | 29.25 | 6.36E-08 | 2.17E-07 | 2.156 |
| RHSIM_Rhsim05G0171700 | 29.24 | 6.40E-08 | 2.19E-07 | 1.505 |

| Gene | LRT statistic | P value | BH-adjusted P | log2FC (interaction) |
|---|---|---|---|---|
| RHSIM_Rhsim07G0086900 | 29.23 | 6.42E-08 | 2.19E-07 | 0.621 |
| RHSIM_Rhsim08G0214900 | 29.22 | 6.47E-08 | 2.21E-07 | 1.600 |
| RHSIM_Rhsim05G0148400 | 29.21 | 6.49E-08 | 2.22E-07 | -0.497 |
| novel.1462 | 29.20 | 6.54E-08 | 2.23E-07 | 0.516 |
| RHSIM_Rhsim12G0110200 | 29.20 | 6.54E-08 | 2.23E-07 | -0.665 |
| RHSIM_Rhsim03G0121900 | 29.17 | 6.62E-08 | 2.26E-07 | 0.873 |
| RHSIM_Rhsim10G0063400 | 29.17 | 6.63E-08 | 2.26E-07 | 0.877 |
| RHSIM_Rhsim10G0182600 | 29.17 | 6.64E-08 | 2.26E-07 | 1.142 |
| RHSIM_Rhsim11G0125800 | 29.15 | 6.68E-08 | 2.28E-07 | -2.371 |
| RHSIM_Rhsim11G0118300 | 29.15 | 6.70E-08 | 2.29E-07 | 0.941 |
| RHSIM_Rhsim01G0223300 | 29.14 | 6.72E-08 | 2.29E-07 | 0.816 |
| RHSIM_Rhsim05G0061100 | 29.14 | 6.73E-08 | 2.30E-07 | -1.090 |
| RHSIM_Rhsim04G0241900 | 29.14 | 6.74E-08 | 2.30E-07 | -1.112 |
| RHSIM_Rhsim07G0136400 | 29.13 | 6.75E-08 | 2.30E-07 | 1.297 |
| RHSIM_Rhsim07G0039600 | 29.13 | 6.76E-08 | 2.31E-07 | 0.314 |
| RHSIM_Rhsim12G0153900 | 29.13 | 6.77E-08 | 2.31E-07 | 0.437 |
| RHSIM_Rhsim07G0177200 | 29.12 | 6.80E-08 | 2.32E-07 | -0.674 |
| novel.4370 | 29.12 | 6.82E-08 | 2.32E-07 | 3.390 |
| RHSIM_Rhsim06G0009500 | 29.09 | 6.91E-08 | 2.35E-07 | -0.411 |
| RHSIM_Rhsim08G0114200 | 29.09 | 6.93E-08 | 2.36E-07 | -0.671 |
| novel.2885 | 29.08 | 6.93E-08 | 2.36E-07 | 1.579 |
| RHSIM_Rhsim07G0258100 | 29.08 | 6.93E-08 | 2.36E-07 | -1.161 |
| RHSIM_RhsimUnG0043000 | 29.08 | 6.96E-08 | 2.37E-07 | -1.062 |
| RHSIM_Rhsim09G0048800 | 29.07 | 6.98E-08 | 2.38E-07 | 1.333 |
| RHSIM_Rhsim09G0008600 | 29.07 | 6.98E-08 | 2.38E-07 | 0.561 |
| RHSIM_Rhsim05G0092200 | 29.06 | 7.03E-08 | 2.40E-07 | 0.623 |
| RHSIM_Rhsim05G0214600 | 29.05 | 7.06E-08 | 2.40E-07 | 0.828 |
| RHSIM_Rhsim07G0228000 | 29.03 | 7.13E-08 | 2.43E-07 | 1.060 |
| RHSIM_Rhsim12G0181900 | 29.02 | 7.17E-08 | 2.44E-07 | -1.215 |
| RHSIM_Rhsim13G0062000 | 29.01 | 7.19E-08 | 2.45E-07 | -4.459 |
| RHSIM_Rhsim13G0015800 | 29.01 | 7.20E-08 | 2.45E-07 | 0.852 |
| RHSIM_Rhsim07G0015600 | 29.01 | 7.22E-08 | 2.46E-07 | -1.674 |

| Gene | LRT statistic | P value | BH-adjusted P | log2FC (interaction) |
|---|---|---|---|---|
| novel.2702 | 29.00 | 7.23E-08 | 2.46E-07 | -1.642 |
| RHSIM_Rhsim07G0005200 | 29.00 | 7.24E-08 | 2.47E-07 | -0.560 |
| RHSIM_Rhsim04G0120400 | 28.99 | 7.26E-08 | 2.47E-07 | 0.406 |
| RHSIM_Rhsim10G0167300 | 28.98 | 7.30E-08 | 2.48E-07 | 1.450 |
| RHSIM_Rhsim09G0114700 | 28.97 | 7.34E-08 | 2.50E-07 | 3.425 |
| RHSIM_Rhsim06G0162900 | 28.97 | 7.36E-08 | 2.50E-07 | 0.935 |
| RHSIM_Rhsim06G0109400 | 28.95 | 7.43E-08 | 2.53E-07 | -0.960 |
| RHSIM_Rhsim03G0003200 | 28.95 | 7.44E-08 | 2.53E-07 | -0.593 |
| RHSIM_Rhsim03G0258000 | 28.94 | 7.47E-08 | 2.54E-07 | -1.227 |
| RHSIM_Rhsim13G0060500 | 28.93 | 7.49E-08 | 2.55E-07 | 0.511 |
| RHSIM_Rhsim07G0149600 | 28.93 | 7.49E-08 | 2.55E-07 | 0.907 |
| RHSIM_Rhsim05G0029000 | 28.91 | 7.58E-08 | 2.58E-07 | 4.691 |
| RHSIM_Rhsim08G0123500 | 28.90 | 7.60E-08 | 2.58E-07 | 0.844 |
| RHSIM_Rhsim03G0234200 | 28.90 | 7.62E-08 | 2.59E-07 | 1.646 |
| RHSIM_Rhsim08G0150800 | 28.90 | 7.62E-08 | 2.59E-07 | 0.467 |
| RHSIM_Rhsim08G0071700 | 28.89 | 7.66E-08 | 2.60E-07 | -0.437 |
| novel.214 | 28.88 | 7.71E-08 | 2.62E-07 | 1.566 |
| RHSIM_Rhsim02G0254700 | 28.88 | 7.71E-08 | 2.62E-07 | 0.688 |
| RHSIM_Rhsim12G0185300 | 28.85 | 7.83E-08 | 2.66E-07 | -0.353 |
| novel.1105 | 28.83 | 7.89E-08 | 2.68E-07 | 1.236 |
| RHSIM_Rhsim07G0118900 | 28.82 | 7.93E-08 | 2.69E-07 | -2.852 |
| novel.2450 | 28.82 | 7.94E-08 | 2.70E-07 | 1.483 |
| novel.3742 | 28.80 | 8.04E-08 | 2.73E-07 | -1.103 |
| RHSIM_Rhsim11G0041200 | 28.79 | 8.05E-08 | 2.73E-07 | 2.236 |
| RHSIM_Rhsim05G0111600 | 28.79 | 8.08E-08 | 2.74E-07 | 0.700 |
| RHSIM_Rhsim05G0085900 | 28.79 | 8.08E-08 | 2.74E-07 | 0.661 |
| RHSIM_Rhsim13G0184400 | 28.79 | 8.09E-08 | 2.74E-07 | 5.135 |
| RHSIM_Rhsim12G0111000 | 28.79 | 8.09E-08 | 2.74E-07 | 1.467 |
| RHSIM_Rhsim07G0228400 | 28.78 | 8.09E-08 | 2.74E-07 | 0.626 |
| RHSIM_Rhsim10G0173800 | 28.78 | 8.12E-08 | 2.76E-07 | -0.677 |
| RHSIM_Rhsim04G0178100 | 28.77 | 8.16E-08 | 2.77E-07 | -5.937 |
| RHSIM_Rhsim09G0177000 | 28.76 | 8.19E-08 | 2.78E-07 | 1.320 |

| Gene | LRT statistic | P value | BH-adjusted P | log2FC (interaction) |
|---|---|---|---|---|
| RHSIM_Rhsim11G0114800 | 28.76 | 8.20E-08 | 2.78E-07 | -1.300 |
| RHSIM_RhsimUnG0156400 | 28.75 | 8.25E-08 | 2.80E-07 | -1.825 |
| RHSIM_Rhsim09G0205000 | 28.74 | 8.27E-08 | 2.80E-07 | -0.517 |
| RHSIM_Rhsim03G0105200 | 28.74 | 8.28E-08 | 2.81E-07 | -2.042 |
| RHSIM_Rhsim05G0230100 | 28.74 | 8.29E-08 | 2.81E-07 | 3.710 |
| RHSIM_Rhsim12G0134100 | 28.73 | 8.30E-08 | 2.81E-07 | -0.910 |
| RHSIM_Rhsim01G0044300 | 28.73 | 8.30E-08 | 2.81E-07 | 0.750 |
| RHSIM_Rhsim06G0171800 | 28.72 | 8.38E-08 | 2.84E-07 | -1.714 |
| novel.3201 | 28.71 | 8.39E-08 | 2.84E-07 | 4.599 |
| RHSIM_Rhsim04G0038700 | 28.71 | 8.40E-08 | 2.84E-07 | 0.510 |
| RHSIM_Rhsim07G0020700 | 28.71 | 8.40E-08 | 2.85E-07 | 0.382 |
| RHSIM_Rhsim06G0203400 | 28.71 | 8.41E-08 | 2.85E-07 | -0.833 |
| RHSIM_Rhsim05G0072800 | 28.71 | 8.42E-08 | 2.85E-07 | -0.606 |
| RHSIM_Rhsim13G0027100 | 28.69 | 8.49E-08 | 2.87E-07 | -0.842 |
| RHSIM_Rhsim13G0019900 | 28.67 | 8.59E-08 | 2.91E-07 | 0.509 |
| novel.1570 | 28.67 | 8.59E-08 | 2.91E-07 | 3.521 |
| RHSIM_Rhsim08G0023400 | 28.67 | 8.60E-08 | 2.91E-07 | 0.499 |
| novel.59 | 28.66 | 8.61E-08 | 2.91E-07 | 1.243 |
| RHSIM_Rhsim06G0032500 | 28.64 | 8.71E-08 | 2.95E-07 | -0.690 |
| RHSIM_Rhsim03G0238400 | 28.64 | 8.72E-08 | 2.95E-07 | -1.482 |
| RHSIM_Rhsim05G0204100 | 28.63 | 8.74E-08 | 2.96E-07 | -2.315 |
| RHSIM_Rhsim05G0200800 | 28.62 | 8.80E-08 | 2.98E-07 | -0.979 |
| RHSIM_Rhsim04G0184700 | 28.62 | 8.80E-08 | 2.98E-07 | -2.561 |
| RHSIM_Rhsim11G0142100 | 28.61 | 8.84E-08 | 2.99E-07 | -0.312 |
| RHSIM_Rhsim05G0178700 | 28.60 | 8.88E-08 | 3.00E-07 | 1.253 |
| RHSIM_Rhsim08G0008300 | 28.59 | 8.95E-08 | 3.02E-07 | -1.352 |
| RHSIM_Rhsim04G0071700 | 28.57 | 9.05E-08 | 3.06E-07 | -2.982 |
| RHSIM_Rhsim07G0074100 | 28.57 | 9.06E-08 | 3.06E-07 | -0.734 |
| RHSIM_Rhsim06G0244200 | 28.56 | 9.07E-08 | 3.06E-07 | -0.786 |
| RHSIM_Rhsim07G0072000 | 28.56 | 9.07E-08 | 3.06E-07 | -0.577 |
| RHSIM_Rhsim07G0148200 | 28.56 | 9.07E-08 | 3.07E-07 | -0.780 |
| RHSIM_Rhsim08G0136200 | 28.56 | 9.08E-08 | 3.07E-07 | 1.958 |

| Gene | LRT statistic | P value | BH-adjusted P | log2FC (interaction) |
|---|---|---|---|---|
| RHSIM_Rhsim05G0064500 | 28.56 | 9.10E-08 | 3.07E-07 | -0.369 |
| RHSIM_Rhsim13G0178000 | 28.55 | 9.14E-08 | 3.09E-07 | 1.258 |
| RHSIM_RhsimUnG0248100 | 28.54 | 9.17E-08 | 3.10E-07 | 1.326 |
| RHSIM_Rhsim13G0130400 | 28.53 | 9.21E-08 | 3.11E-07 | 2.560 |
| RHSIM_Rhsim06G0116700 | 28.53 | 9.24E-08 | 3.12E-07 | -0.786 |
| RHSIM_Rhsim13G0077500 | 28.48 | 9.45E-08 | 3.19E-07 | 1.575 |
| RHSIM_Rhsim08G0244600 | 28.45 | 9.60E-08 | 3.24E-07 | 0.520 |
| RHSIM_Rhsim02G0008000 | 28.44 | 9.67E-08 | 3.26E-07 | -2.445 |
| RHSIM_Rhsim02G0141900 | 28.43 | 9.70E-08 | 3.27E-07 | 2.552 |
| RHSIM_Rhsim08G0104200 | 28.42 | 9.78E-08 | 3.30E-07 | -1.109 |
| RHSIM_Rhsim06G0001700 | 28.41 | 9.80E-08 | 3.31E-07 | 1.658 |
| RHSIM_Rhsim13G0189800 | 28.41 | 9.81E-08 | 3.31E-07 | -42.130 |
| RHSIM_Rhsim13G0077100 | 28.41 | 9.81E-08 | 3.31E-07 | 0.547 |
| RHSIM_Rhsim07G0158200 | 28.39 | 9.90E-08 | 3.34E-07 | 0.895 |
| novel.434 | 28.38 | 9.96E-08 | 3.36E-07 | 1.684 |
| RHSIM_Rhsim09G0204800 | 28.37 | 1.00E-07 | 3.39E-07 | 2.017 |
| novel.683 | 28.36 | 1.00E-07 | 3.39E-07 | -2.340 |
| RHSIM_Rhsim08G0233900 | 28.36 | 1.01E-07 | 3.39E-07 | 2.201 |
| RHSIM_Rhsim05G0219200 | 28.35 | 1.01E-07 | 3.41E-07 | 0.520 |
| RHSIM_Rhsim05G0036800 | 28.35 | 1.01E-07 | 3.42E-07 | -3.444 |
| RHSIM_Rhsim13G0006600 | 28.35 | 1.01E-07 | 3.42E-07 | -0.559 |
| novel.2726 | 28.34 | 1.02E-07 | 3.43E-07 | -1.469 |
| RHSIM_Rhsim03G0009300 | 28.34 | 1.02E-07 | 3.43E-07 | -0.794 |
| novel.1871 | 28.31 | 1.03E-07 | 3.48E-07 | -6.040 |
| RHSIM_Rhsim12G0173900 | 28.29 | 1.04E-07 | 3.51E-07 | -0.620 |
| RHSIM_Rhsim09G0178500 | 28.29 | 1.04E-07 | 3.52E-07 | -0.450 |
| RHSIM_RhsimUnG0024100 | 28.28 | 1.05E-07 | 3.53E-07 | 4.730 |
| RHSIM_Rhsim11G0077100 | 28.27 | 1.05E-07 | 3.55E-07 | 0.461 |
| RHSIM_Rhsim05G0146100 | 28.26 | 1.06E-07 | 3.57E-07 | 1.310 |
| RHSIM_Rhsim04G0246500 | 28.26 | 1.06E-07 | 3.58E-07 | 1.044 |
| RHSIM_Rhsim02G0083500 | 28.25 | 1.06E-07 | 3.58E-07 | -0.555 |
| RHSIM_Rhsim02G0221000 | 28.25 | 1.07E-07 | 3.59E-07 | 0.634 |

| Gene | LRT statistic | P value | BH-adjusted P | log2FC (interaction) |
|---|---|---|---|---|
| novel.3033 | 28.24 | 1.07E-07 | 3.60E-07 | -0.893 |
| RHSIM_Rhsim05G0203300 | 28.24 | 1.07E-07 | 3.61E-07 | 0.362 |
| RHSIM_Rhsim07G0232100 | 28.23 | 1.08E-07 | 3.62E-07 | 1.628 |
| RHSIM_Rhsim04G0149200 | 28.21 | 1.09E-07 | 3.65E-07 | 0.527 |
| RHSIM_Rhsim05G0058700 | 28.21 | 1.09E-07 | 3.66E-07 | -1.415 |
| RHSIM_Rhsim10G0190700 | 28.21 | 1.09E-07 | 3.67E-07 | -0.510 |
| RHSIM_Rhsim02G0160300 | 28.21 | 1.09E-07 | 3.67E-07 | 0.381 |
| RHSIM_Rhsim10G0167100 | 28.20 | 1.10E-07 | 3.69E-07 | -1.034 |
| RHSIM_Rhsim08G0099700 | 28.19 | 1.10E-07 | 3.69E-07 | 0.341 |
| RHSIM_Rhsim03G0255100 | 28.19 | 1.10E-07 | 3.70E-07 | -2.963 |
| RHSIM_RhsimUnG0180200 | 28.17 | 1.11E-07 | 3.74E-07 | -2.168 |
| RHSIM_Rhsim01G0226400 | 28.16 | 1.12E-07 | 3.75E-07 | 2.294 |
| RHSIM_Rhsim09G0005700 | 28.14 | 1.13E-07 | 3.79E-07 | 1.452 |
| RHSIM_Rhsim13G0044900 | 28.13 | 1.13E-07 | 3.81E-07 | -1.498 |
| RHSIM_Rhsim04G0052600 | 28.13 | 1.14E-07 | 3.82E-07 | -2.136 |
| RHSIM_Rhsim11G0148000 | 28.12 | 1.14E-07 | 3.83E-07 | 4.922 |
| RHSIM_Rhsim05G0143600 | 28.10 | 1.15E-07 | 3.86E-07 | -0.715 |
| RHSIM_Rhsim07G0108000 | 28.09 | 1.16E-07 | 3.89E-07 | 0.754 |
| novel.1282 | 28.08 | 1.16E-07 | 3.90E-07 | -0.410 |
| RHSIM_Rhsim08G0149100 | 28.08 | 1.16E-07 | 3.91E-07 | 0.701 |
| RHSIM_Rhsim07G0012600 | 28.08 | 1.17E-07 | 3.92E-07 | 0.492 |
| RHSIM_Rhsim03G0140300 | 28.08 | 1.17E-07 | 3.92E-07 | -0.787 |
| RHSIM_Rhsim03G0049800 | 28.07 | 1.17E-07 | 3.93E-07 | -0.952 |
| RHSIM_Rhsim05G0062000 | 28.07 | 1.17E-07 | 3.93E-07 | -1.276 |
| novel.3809 | 28.05 | 1.18E-07 | 3.96E-07 | 8.457 |
| RHSIM_Rhsim05G0137800 | 28.05 | 1.18E-07 | 3.97E-07 | 0.502 |
| RHSIM_Rhsim07G0115900 | 28.05 | 1.18E-07 | 3.97E-07 | 0.609 |
| RHSIM_Rhsim01G0050600 | 28.05 | 1.18E-07 | 3.97E-07 | -1.352 |
| RHSIM_Rhsim08G0235500 | 28.04 | 1.19E-07 | 3.98E-07 | 0.499 |
| RHSIM_Rhsim06G0053800 | 28.04 | 1.19E-07 | 3.99E-07 | -0.852 |
| RHSIM_Rhsim03G0014900 | 28.03 | 1.19E-07 | 4.00E-07 | -2.167 |
| novel.813 | 28.02 | 1.20E-07 | 4.02E-07 | -0.483 |

| Gene | LRT statistic | P value | BH-adjusted P | log2FC (interaction) |
|---|---|---|---|---|
| RHSIM_Rhsim11G0198900 | 28.02 | 1.20E-07 | 4.03E-07 | 42.470 |
| RHSIM_RhsimUnG0084800 | 28.01 | 1.21E-07 | 4.04E-07 | -1.830 |
| RHSIM_Rhsim01G0268300 | 27.99 | 1.22E-07 | 4.09E-07 | 0.970 |
| novel.4036 | 27.98 | 1.23E-07 | 4.12E-07 | -42.413 |
| RHSIM_Rhsim12G0080900 | 27.97 | 1.23E-07 | 4.13E-07 | -6.888 |
| RHSIM_Rhsim11G0152400 | 27.96 | 1.24E-07 | 4.14E-07 | -0.639 |
| RHSIM_Rhsim12G0214000 | 27.96 | 1.24E-07 | 4.15E-07 | 1.055 |
| RHSIM_Rhsim09G0145100 | 27.95 | 1.25E-07 | 4.18E-07 | -0.560 |
| RHSIM_Rhsim02G0166600 | 27.94 | 1.25E-07 | 4.18E-07 | 2.177 |
| RHSIM_Rhsim03G0170400 | 27.93 | 1.26E-07 | 4.22E-07 | -3.005 |
| novel.3603 | 27.92 | 1.27E-07 | 4.24E-07 | -1.501 |
| RHSIM_Rhsim08G0109500 | 27.91 | 1.27E-07 | 4.25E-07 | -1.999 |
| RHSIM_Rhsim06G0082600 | 27.91 | 1.27E-07 | 4.26E-07 | 3.948 |
| novel.2138 | 27.90 | 1.27E-07 | 4.27E-07 | -6.214 |
| RHSIM_Rhsim09G0085400 | 27.90 | 1.27E-07 | 4.27E-07 | -0.903 |
| RHSIM_Rhsim02G0112100 | 27.90 | 1.28E-07 | 4.28E-07 | -0.695 |
| novel.3951 | 27.89 | 1.28E-07 | 4.29E-07 | -0.960 |
| RHSIM_Rhsim11G0052300 | 27.88 | 1.29E-07 | 4.32E-07 | 0.666 |
| RHSIM_RhsimUnG0246000 | 27.88 | 1.29E-07 | 4.32E-07 | 1.497 |
| RHSIM_Rhsim02G0216800 | 27.88 | 1.29E-07 | 4.32E-07 | 1.450 |
| RHSIM_Rhsim03G0158700 | 27.87 | 1.30E-07 | 4.34E-07 | 2.569 |
| RHSIM_Rhsim02G0254900 | 27.87 | 1.30E-07 | 4.34E-07 | -3.016 |
| RHSIM_Rhsim06G0032200 | 27.86 | 1.30E-07 | 4.35E-07 | -41.054 |
| RHSIM_Rhsim05G0002300 | 27.85 | 1.31E-07 | 4.37E-07 | -0.325 |
| RHSIM_Rhsim04G0029100 | 27.83 | 1.32E-07 | 4.42E-07 | -4.739 |
| RHSIM_Rhsim11G0052800 | 27.82 | 1.33E-07 | 4.44E-07 | 1.300 |
| RHSIM_Rhsim03G0159900 | 27.82 | 1.33E-07 | 4.44E-07 | 0.527 |
| RHSIM_Rhsim02G0089100 | 27.82 | 1.33E-07 | 4.45E-07 | -2.208 |
| novel.2503 | 27.81 | 1.34E-07 | 4.47E-07 | 1.115 |
| novel.3849 | 27.81 | 1.34E-07 | 4.47E-07 | 0.463 |
| RHSIM_Rhsim11G0074500 | 27.80 | 1.34E-07 | 4.49E-07 | 1.861 |
| RHSIM_Rhsim10G0188000 | 27.78 | 1.36E-07 | 4.54E-07 | -0.849 |

| Gene | LRT statistic | P value | BH-adjusted P | log2FC (interaction) |
|---|---|---|---|---|
| novel.1267 | 27.78 | 1.36E-07 | 4.54E-07 | 1.109 |
| RHSIM_Rhsim01G0195300 | 27.78 | 1.36E-07 | 4.55E-07 | -0.730 |
| RHSIM_Rhsim06G0241500 | 27.77 | 1.37E-07 | 4.57E-07 | -0.333 |
| RHSIM_Rhsim10G0084600 | 27.76 | 1.38E-07 | 4.59E-07 | -1.337 |
| novel.4096 | 27.76 | 1.38E-07 | 4.59E-07 | -2.366 |
| RHSIM_Rhsim13G0015200 | 27.75 | 1.38E-07 | 4.60E-07 | 0.360 |
| RHSIM_RhsimUnG0060600 | 27.75 | 1.38E-07 | 4.61E-07 | 1.676 |
| RHSIM_Rhsim11G0111000 | 27.75 | 1.38E-07 | 4.61E-07 | 0.844 |
| RHSIM_RhsimUnG0023100 | 27.75 | 1.38E-07 | 4.61E-07 | 1.467 |
| RHSIM_Rhsim07G0065200 | 27.74 | 1.38E-07 | 4.62E-07 | -1.939 |
| RHSIM_Rhsim12G0062000 | 27.74 | 1.38E-07 | 4.62E-07 | 0.622 |
| RHSIM_Rhsim11G0141900 | 27.74 | 1.39E-07 | 4.63E-07 | -0.704 |
| RHSIM_Rhsim07G0067400 | 27.73 | 1.39E-07 | 4.65E-07 | -0.467 |
| RHSIM_Rhsim03G0185800 | 27.72 | 1.40E-07 | 4.67E-07 | 0.839 |
| novel.1448 | 27.72 | 1.40E-07 | 4.67E-07 | 1.043 |
| RHSIM_Rhsim13G0204900 | 27.71 | 1.41E-07 | 4.69E-07 | 1.756 |
| RHSIM_Rhsim10G0133100 | 27.71 | 1.41E-07 | 4.70E-07 | 0.943 |
| RHSIM_Rhsim08G0220600 | 27.71 | 1.41E-07 | 4.70E-07 | 2.647 |
| RHSIM_Rhsim04G0007100 | 27.70 | 1.41E-07 | 4.71E-07 | -0.467 |
| RHSIM_Rhsim05G0129500 | 27.70 | 1.42E-07 | 4.72E-07 | 2.282 |
| RHSIM_Rhsim02G0217900 | 27.70 | 1.42E-07 | 4.72E-07 | 0.527 |
| RHSIM_Rhsim05G0233000 | 27.70 | 1.42E-07 | 4.73E-07 | -0.820 |
| RHSIM_Rhsim03G0101000 | 27.69 | 1.42E-07 | 4.73E-07 | 4.031 |
| novel.3321 | 27.69 | 1.43E-07 | 4.75E-07 | 1.972 |
| RHSIM_Rhsim04G0214500 | 27.68 | 1.43E-07 | 4.76E-07 | -0.940 |
| RHSIM_Rhsim03G0004600 | 27.68 | 1.43E-07 | 4.76E-07 | 1.109 |
| RHSIM_Rhsim06G0152300 | 27.68 | 1.43E-07 | 4.78E-07 | 2.588 |
| RHSIM_RhsimUnG0045500 | 27.67 | 1.44E-07 | 4.78E-07 | -1.441 |
| RHSIM_Rhsim03G0198200 | 27.66 | 1.44E-07 | 4.80E-07 | -5.300 |
| novel.3842 | 27.65 | 1.45E-07 | 4.83E-07 | 3.275 |
| RHSIM_Rhsim03G0210900 | 27.65 | 1.45E-07 | 4.84E-07 | -2.958 |
| RHSIM_Rhsim05G0128900 | 27.63 | 1.47E-07 | 4.88E-07 | 0.417 |

| Gene | LRT statistic | P value | BH-adjusted P | log2FC (interaction) |
|---|---|---|---|---|
| novel.4498 | 27.61 | 1.48E-07 | 4.94E-07 | 2.708 |
| RHSIM_Rhsim03G0050400 | 27.61 | 1.49E-07 | 4.94E-07 | 0.834 |
| RHSIM_Rhsim02G0120000 | 27.61 | 1.49E-07 | 4.95E-07 | 0.762 |
| RHSIM_Rhsim07G0247000 | 27.61 | 1.49E-07 | 4.95E-07 | -0.781 |
| RHSIM_Rhsim13G0184000 | 27.60 | 1.49E-07 | 4.96E-07 | -3.971 |
| RHSIM_Rhsim11G0160500 | 27.60 | 1.49E-07 | 4.97E-07 | -0.975 |
| RHSIM_Rhsim08G0077800 | 27.60 | 1.50E-07 | 4.97E-07 | -1.376 |
| RHSIM_Rhsim01G0190800 | 27.59 | 1.50E-07 | 4.98E-07 | 0.835 |
| RHSIM_Rhsim12G0025700 | 27.59 | 1.50E-07 | 4.99E-07 | 0.437 |
| RHSIM_Rhsim07G0163000 | 27.59 | 1.50E-07 | 4.99E-07 | 0.669 |
| RHSIM_Rhsim06G0226700 | 27.59 | 1.50E-07 | 4.99E-07 | -0.730 |
| RHSIM_Rhsim05G0014000 | 27.58 | 1.50E-07 | 5.00E-07 | -0.863 |
| RHSIM_Rhsim13G0122400 | 27.58 | 1.50E-07 | 5.00E-07 | 1.696 |
| RHSIM_Rhsim01G0034600 | 27.58 | 1.51E-07 | 5.01E-07 | -1.816 |
| novel.3179 | 27.58 | 1.51E-07 | 5.01E-07 | -1.365 |
| RHSIM_Rhsim01G0196400 | 27.56 | 1.52E-07 | 5.05E-07 | 0.247 |
| RHSIM_Rhsim07G0136800 | 27.56 | 1.52E-07 | 5.06E-07 | 0.762 |
| RHSIM_Rhsim13G0103600 | 27.56 | 1.52E-07 | 5.06E-07 | 0.560 |
| RHSIM_RhsimUnG0124700 | 27.54 | 1.54E-07 | 5.12E-07 | -0.346 |
| RHSIM_Rhsim04G0015000 | 27.53 | 1.54E-07 | 5.12E-07 | -0.646 |
| RHSIM_Rhsim02G0125900 | 27.53 | 1.55E-07 | 5.14E-07 | 0.464 |
| RHSIM_Rhsim13G0207900 | 27.52 | 1.55E-07 | 5.16E-07 | -0.548 |
| RHSIM_Rhsim11G0162100 | 27.51 | 1.56E-07 | 5.17E-07 | -1.355 |
| RHSIM_Rhsim06G0176900 | 27.51 | 1.56E-07 | 5.19E-07 | -1.209 |
| RHSIM_Rhsim05G0185700 | 27.51 | 1.56E-07 | 5.19E-07 | 0.386 |
| RHSIM_Rhsim11G0096100 | 27.50 | 1.57E-07 | 5.21E-07 | 1.650 |
| RHSIM_Rhsim02G0200600 | 27.49 | 1.58E-07 | 5.23E-07 | -2.577 |
| RHSIM_Rhsim12G0138800 | 27.49 | 1.58E-07 | 5.25E-07 | -1.284 |
| RHSIM_Rhsim10G0024100 | 27.48 | 1.59E-07 | 5.26E-07 | -0.810 |
| RHSIM_Rhsim07G0047400 | 27.48 | 1.59E-07 | 5.27E-07 | 0.890 |
| novel.3881 | 27.47 | 1.59E-07 | 5.28E-07 | -0.364 |
| RHSIM_Rhsim04G0224200 | 27.47 | 1.59E-07 | 5.29E-07 | 0.370 |

| Gene | LRT statistic | P value | BH-adjusted P | log2FC (interaction) |
|---|---|---|---|---|
| RHSIM_Rhsim06G0213200 | 27.47 | 1.60E-07 | 5.30E-07 | -0.965 |
| RHSIM_Rhsim10G0050900 | 27.46 | 1.61E-07 | 5.33E-07 | -1.129 |
| RHSIM_Rhsim03G0229200 | 27.45 | 1.61E-07 | 5.33E-07 | 0.783 |
| RHSIM_Rhsim02G0001400 | 27.45 | 1.61E-07 | 5.33E-07 | 3.397 |
| RHSIM_Rhsim04G0012600 | 27.45 | 1.61E-07 | 5.35E-07 | 1.851 |
| RHSIM_Rhsim12G0023100 | 27.44 | 1.62E-07 | 5.36E-07 | 0.627 |
| RHSIM_Rhsim08G0001800 | 27.43 | 1.63E-07 | 5.39E-07 | 0.421 |
| RHSIM_Rhsim13G0090700 | 27.43 | 1.63E-07 | 5.40E-07 | 2.120 |
| RHSIM_Rhsim06G0089300 | 27.42 | 1.63E-07 | 5.41E-07 | 0.319 |
| RHSIM_Rhsim01G0136600 | 27.42 | 1.64E-07 | 5.42E-07 | 3.953 |
| RHSIM_Rhsim01G0091400 | 27.40 | 1.65E-07 | 5.47E-07 | -0.747 |
| RHSIM_Rhsim03G0057300 | 27.40 | 1.65E-07 | 5.48E-07 | -1.057 |
| RHSIM_Rhsim06G0086500 | 27.40 | 1.66E-07 | 5.49E-07 | 1.506 |
| RHSIM_Rhsim10G0169800 | 27.39 | 1.66E-07 | 5.50E-07 | -2.523 |
| RHSIM_Rhsim08G0227500 | 27.39 | 1.66E-07 | 5.51E-07 | 0.351 |
| RHSIM_Rhsim04G0161100 | 27.38 | 1.67E-07 | 5.53E-07 | -1.190 |
| novel.1457 | 27.36 | 1.69E-07 | 5.60E-07 | 1.406 |
| RHSIM_RhsimUnG0134100 | 27.35 | 1.70E-07 | 5.61E-07 | 0.920 |
| RHSIM_Rhsim12G0101300 | 27.35 | 1.70E-07 | 5.62E-07 | -2.899 |
| RHSIM_Rhsim04G0226600 | 27.35 | 1.70E-07 | 5.63E-07 | 0.523 |
| novel.3467 | 27.34 | 1.71E-07 | 5.65E-07 | -5.817 |
| RHSIM_Rhsim04G0123900 | 27.34 | 1.71E-07 | 5.65E-07 | 5.310 |
| RHSIM_Rhsim02G0206600 | 27.32 | 1.73E-07 | 5.70E-07 | -0.633 |
| RHSIM_Rhsim10G0095800 | 27.31 | 1.73E-07 | 5.73E-07 | 0.936 |
| RHSIM_Rhsim09G0087600 | 27.29 | 1.75E-07 | 5.80E-07 | 0.763 |
| novel.3302 | 27.29 | 1.76E-07 | 5.80E-07 | 2.156 |
| RHSIM_Rhsim01G0049400 | 27.27 | 1.77E-07 | 5.83E-07 | 0.607 |
| RHSIM_Rhsim07G0172700 | 27.27 | 1.77E-07 | 5.84E-07 | 1.037 |
| RHSIM_Rhsim09G0010700 | 27.27 | 1.77E-07 | 5.86E-07 | -1.088 |
| RHSIM_Rhsim01G0052300 | 27.26 | 1.78E-07 | 5.86E-07 | 0.709 |
| RHSIM_Rhsim04G0166600 | 27.22 | 1.81E-07 | 5.99E-07 | 0.596 |
| RHSIM_Rhsim13G0025200 | 27.22 | 1.82E-07 | 6.01E-07 | 0.544 |

| Gene | LRT statistic | P value | BH-adjusted P | log2FC (interaction) |
|---|---|---|---|---|
| RHSIM_Rhsim06G0201600 | 27.21 | 1.82E-07 | 6.02E-07 | -0.783 |
| RHSIM_Rhsim13G0120600 | 27.21 | 1.82E-07 | 6.02E-07 | -0.343 |
| RHSIM_Rhsim11G0181400 | 27.21 | 1.83E-07 | 6.04E-07 | 1.708 |
| RHSIM_Rhsim12G0123300 | 27.19 | 1.85E-07 | 6.09E-07 | -0.517 |
| novel.1989 | 27.18 | 1.85E-07 | 6.11E-07 | -1.094 |
| RHSIM_Rhsim10G0030900 | 27.18 | 1.85E-07 | 6.12E-07 | 1.142 |
| RHSIM_RhsimUnG0151200 | 27.18 | 1.85E-07 | 6.12E-07 | 2.827 |
| RHSIM_Rhsim08G0188000 | 27.17 | 1.86E-07 | 6.15E-07 | 1.038 |
| RHSIM_Rhsim11G0195100 | 27.17 | 1.87E-07 | 6.16E-07 | 0.717 |
| RHSIM_Rhsim13G0051800 | 27.16 | 1.87E-07 | 6.17E-07 | -1.233 |
| RHSIM_Rhsim07G0162300 | 27.15 | 1.88E-07 | 6.21E-07 | 0.378 |
| RHSIM_Rhsim13G0097000 | 27.14 | 1.90E-07 | 6.26E-07 | -0.646 |
| RHSIM_Rhsim06G0058400 | 27.13 | 1.90E-07 | 6.26E-07 | 1.310 |
| RHSIM_Rhsim08G0129700 | 27.11 | 1.92E-07 | 6.33E-07 | 0.977 |
| RHSIM_Rhsim03G0155500 | 27.11 | 1.93E-07 | 6.35E-07 | 1.285 |
| novel.2833 | 27.10 | 1.93E-07 | 6.36E-07 | -0.984 |
| RHSIM_Rhsim08G0106000 | 27.10 | 1.94E-07 | 6.38E-07 | -1.558 |
| RHSIM_Rhsim06G0085200 | 27.09 | 1.94E-07 | 6.39E-07 | -0.918 |
| RHSIM_Rhsim06G0070100 | 27.09 | 1.94E-07 | 6.39E-07 | -0.742 |
| RHSIM_Rhsim08G0085300 | 27.09 | 1.95E-07 | 6.41E-07 | 1.707 |
| RHSIM_Rhsim07G0095100 | 27.08 | 1.95E-07 | 6.43E-07 | -4.121 |
| RHSIM_RhsimUnG0257200 | 27.08 | 1.95E-07 | 6.43E-07 | 0.852 |
| RHSIM_Rhsim03G0085900 | 27.08 | 1.95E-07 | 6.43E-07 | -3.752 |
| RHSIM_Rhsim03G0234900 | 27.07 | 1.96E-07 | 6.45E-07 | -0.608 |
| RHSIM_Rhsim08G0239900 | 27.07 | 1.96E-07 | 6.46E-07 | 4.181 |
| RHSIM_Rhsim05G0090800 | 27.05 | 1.98E-07 | 6.54E-07 | 0.640 |
| RHSIM_Rhsim09G0151500 | 27.04 | 1.99E-07 | 6.56E-07 | 0.576 |
| RHSIM_Rhsim03G0208800 | 27.03 | 2.00E-07 | 6.60E-07 | -0.445 |
| RHSIM_RhsimUnG0019800 | 27.03 | 2.00E-07 | 6.60E-07 | 0.396 |
| RHSIM_Rhsim02G0133700 | 27.03 | 2.01E-07 | 6.60E-07 | -0.497 |
| RHSIM_Rhsim06G0002200 | 27.02 | 2.02E-07 | 6.63E-07 | 3.392 |
| RHSIM_RhsimUnG0117500 | 27.00 | 2.03E-07 | 6.70E-07 | 2.053 |

| Gene | LRT statistic | P value | BH-adjusted P | log2FC (interaction) |
|---|---|---|---|---|
| RHSIM_Rhsim07G0173600 | 27.00 | 2.04E-07 | 6.70E-07 | 0.367 |
| RHSIM_RhsimUnG0187900 | 26.99 | 2.05E-07 | 6.74E-07 | 1.068 |
| RHSIM_Rhsim02G0013600 | 26.98 | 2.05E-07 | 6.75E-07 | 0.697 |
| RHSIM_Rhsim02G0243600 | 26.98 | 2.05E-07 | 6.76E-07 | 2.023 |
| RHSIM_Rhsim12G0008000 | 26.98 | 2.06E-07 | 6.78E-07 | -0.584 |
| RHSIM_Rhsim10G0149400 | 26.96 | 2.07E-07 | 6.82E-07 | -1.776 |
| RHSIM_Rhsim11G0188100 | 26.96 | 2.08E-07 | 6.83E-07 | 0.521 |
| RHSIM_Rhsim01G0089300 | 26.95 | 2.09E-07 | 6.87E-07 | -2.024 |
| RHSIM_Rhsim09G0164500 | 26.95 | 2.09E-07 | 6.88E-07 | -1.053 |
| RHSIM_Rhsim02G0037400 | 26.94 | 2.09E-07 | 6.89E-07 | 0.246 |
| RHSIM_RhsimUnG0093100 | 26.93 | 2.11E-07 | 6.92E-07 | -0.628 |
| RHSIM_Rhsim04G0008000 | 26.93 | 2.11E-07 | 6.93E-07 | 0.529 |
| RHSIM_Rhsim04G0005000 | 26.93 | 2.11E-07 | 6.94E-07 | -2.976 |
| RHSIM_Rhsim05G0164600 | 26.92 | 2.12E-07 | 6.98E-07 | 1.092 |
| RHSIM_Rhsim09G0193300 | 26.91 | 2.13E-07 | 7.01E-07 | 0.525 |
| RHSIM_Rhsim01G0143400 | 26.90 | 2.14E-07 | 7.05E-07 | 0.417 |
| RHSIM_Rhsim05G0139700 | 26.88 | 2.17E-07 | 7.13E-07 | 1.953 |
| RHSIM_Rhsim12G0052200 | 26.87 | 2.18E-07 | 7.16E-07 | -1.123 |
| RHSIM_Rhsim03G0136300 | 26.86 | 2.18E-07 | 7.17E-07 | -0.732 |
| RHSIM_Rhsim06G0071500 | 26.86 | 2.19E-07 | 7.18E-07 | 0.628 |
| RHSIM_Rhsim08G0015000 | 26.85 | 2.20E-07 | 7.23E-07 | 0.869 |
| RHSIM_Rhsim03G0020200 | 26.85 | 2.20E-07 | 7.23E-07 | 0.624 |
| RHSIM_Rhsim01G0234600 | 26.84 | 2.20E-07 | 7.24E-07 | -0.601 |
| RHSIM_Rhsim04G0221800 | 26.84 | 2.21E-07 | 7.27E-07 | 0.421 |
| RHSIM_Rhsim10G0043700 | 26.82 | 2.24E-07 | 7.34E-07 | 0.477 |
| novel.4511 | 26.81 | 2.24E-07 | 7.35E-07 | -3.293 |
| RHSIM_Rhsim11G0070300 | 26.80 | 2.25E-07 | 7.38E-07 | 2.032 |
| RHSIM_Rhsim12G0124700 | 26.80 | 2.26E-07 | 7.40E-07 | -0.593 |
| RHSIM_Rhsim08G0075400 | 26.80 | 2.26E-07 | 7.40E-07 | -1.628 |
| RHSIM_Rhsim07G0170100 | 26.80 | 2.26E-07 | 7.40E-07 | -1.081 |
| RHSIM_Rhsim07G0098000 | 26.79 | 2.26E-07 | 7.42E-07 | -2.523 |
| RHSIM_Rhsim04G0199500 | 26.79 | 2.27E-07 | 7.45E-07 | -0.867 |

| Gene | LRT statistic | P value | BH-adjusted P | log2FC (interaction) |
|---|---|---|---|---|
| RHSIM_Rhsim13G0163100 | 26.78 | 2.28E-07 | 7.46E-07 | 0.873 |
| RHSIM_Rhsim08G0198700 | 26.78 | 2.29E-07 | 7.49E-07 | 1.026 |
| RHSIM_Rhsim08G0013700 | 26.77 | 2.29E-07 | 7.50E-07 | -4.151 |
| RHSIM_Rhsim03G0099500 | 26.77 | 2.29E-07 | 7.50E-07 | -1.903 |
| RHSIM_Rhsim07G0002900 | 26.76 | 2.30E-07 | 7.55E-07 | -0.786 |
| novel.1796 | 26.75 | 2.32E-07 | 7.59E-07 | 0.890 |
| RHSIM_Rhsim04G0023000 | 26.75 | 2.32E-07 | 7.61E-07 | 0.551 |
| RHSIM_RhsimUnG0228100 | 26.73 | 2.34E-07 | 7.67E-07 | 1.234 |
| RHSIM_Rhsim03G0073800 | 26.73 | 2.34E-07 | 7.68E-07 | 1.956 |
| RHSIM_Rhsim06G0072000 | 26.72 | 2.35E-07 | 7.69E-07 | -0.555 |
| novel.1109 | 26.72 | 2.35E-07 | 7.69E-07 | -1.242 |
| RHSIM_Rhsim08G0045300 | 26.71 | 2.37E-07 | 7.75E-07 | 0.507 |
| novel.2179 | 26.71 | 2.37E-07 | 7.75E-07 | -4.731 |
| RHSIM_Rhsim13G0046000 | 26.69 | 2.38E-07 | 7.80E-07 | 0.690 |
| RHSIM_Rhsim04G0023400 | 26.69 | 2.39E-07 | 7.81E-07 | -0.939 |
| RHSIM_Rhsim04G0111200 | 26.69 | 2.39E-07 | 7.82E-07 | 1.945 |
| RHSIM_Rhsim06G0230800 | 26.69 | 2.39E-07 | 7.83E-07 | -1.517 |
| RHSIM_Rhsim06G0020100 | 26.68 | 2.40E-07 | 7.84E-07 | -1.058 |
| novel.304 | 26.68 | 2.40E-07 | 7.85E-07 | 1.107 |
| RHSIM_Rhsim09G0019000 | 26.68 | 2.40E-07 | 7.87E-07 | 2.369 |
| RHSIM_Rhsim05G0175400 | 26.66 | 2.43E-07 | 7.94E-07 | -0.611 |
| RHSIM_Rhsim12G0041000 | 26.64 | 2.46E-07 | 8.03E-07 | 1.019 |
| RHSIM_Rhsim03G0059400 | 26.63 | 2.46E-07 | 8.04E-07 | -1.609 |
| novel.4098 | 26.63 | 2.47E-07 | 8.07E-07 | -4.354 |
| RHSIM_Rhsim07G0245700 | 26.62 | 2.48E-07 | 8.11E-07 | 0.615 |
| RHSIM_Rhsim07G0032500 | 26.60 | 2.51E-07 | 8.19E-07 | 0.776 |
| RHSIM_RhsimUnG0115300 | 26.60 | 2.51E-07 | 8.20E-07 | 1.496 |
| RHSIM_Rhsim10G0179100 | 26.58 | 2.53E-07 | 8.27E-07 | -2.732 |
| novel.2975 | 26.58 | 2.53E-07 | 8.27E-07 | -1.287 |
| RHSIM_Rhsim01G0260100 | 26.57 | 2.54E-07 | 8.30E-07 | -0.580 |
| novel.2979 | 26.55 | 2.57E-07 | 8.39E-07 | 0.835 |
| novel.1722 | 26.53 | 2.59E-07 | 8.46E-07 | -1.368 |

| Gene | LRT statistic | P value | BH-adjusted P | log2FC (interaction) |
|---|---|---|---|---|
| novel.3056 | 26.53 | 2.59E-07 | 8.46E-07 | 0.993 |
| RHSIM_Rhsim07G0183400 | 26.52 | 2.61E-07 | 8.52E-07 | 0.738 |
| RHSIM_Rhsim02G0211800 | 26.50 | 2.63E-07 | 8.59E-07 | -0.886 |
| RHSIM_Rhsim10G0110300 | 26.50 | 2.63E-07 | 8.60E-07 | -0.862 |
| RHSIM_Rhsim08G0149300 | 26.50 | 2.64E-07 | 8.61E-07 | 0.713 |
| RHSIM_Rhsim01G0000800 | 26.49 | 2.64E-07 | 8.63E-07 | -0.658 |
| RHSIM_Rhsim12G0130100 | 26.49 | 2.65E-07 | 8.65E-07 | 0.321 |
| RHSIM_Rhsim01G0206600 | 26.49 | 2.65E-07 | 8.66E-07 | 0.771 |
| RHSIM_RhsimUnG0224700 | 26.48 | 2.66E-07 | 8.67E-07 | -0.605 |
| RHSIM_Rhsim07G0035800 | 26.48 | 2.67E-07 | 8.71E-07 | -0.831 |
| RHSIM_Rhsim01G0247500 | 26.47 | 2.68E-07 | 8.73E-07 | 0.614 |
| novel.21 | 26.47 | 2.68E-07 | 8.75E-07 | -0.707 |
| RHSIM_Rhsim01G0102600 | 26.46 | 2.69E-07 | 8.78E-07 | 0.650 |
| RHSIM_Rhsim08G0230100 | 26.43 | 2.73E-07 | 8.92E-07 | -0.442 |
| novel.1792 | 26.43 | 2.74E-07 | 8.93E-07 | -0.807 |
| RHSIM_Rhsim09G0067700 | 26.41 | 2.77E-07 | 9.02E-07 | -0.396 |
| RHSIM_Rhsim13G0083100 | 26.40 | 2.77E-07 | 9.03E-07 | -1.095 |
| RHSIM_RhsimUnG0105800 | 26.40 | 2.77E-07 | 9.03E-07 | -4.606 |
| RHSIM_Rhsim03G0060100 | 26.40 | 2.78E-07 | 9.05E-07 | 1.436 |
| RHSIM_Rhsim04G0235200 | 26.39 | 2.79E-07 | 9.10E-07 | 0.471 |
| RHSIM_Rhsim02G0080900 | 26.39 | 2.79E-07 | 9.10E-07 | 1.050 |
| RHSIM_Rhsim02G0070600 | 26.39 | 2.79E-07 | 9.11E-07 | -1.801 |
| RHSIM_Rhsim06G0062900 | 26.38 | 2.80E-07 | 9.12E-07 | -1.369 |
| RHSIM_Rhsim07G0039000 | 26.38 | 2.80E-07 | 9.12E-07 | -0.707 |
| novel.1469 | 26.38 | 2.80E-07 | 9.12E-07 | -2.368 |
| novel.1255 | 26.38 | 2.81E-07 | 9.15E-07 | -1.156 |
| RHSIM_Rhsim12G0193500 | 26.38 | 2.81E-07 | 9.15E-07 | -0.703 |
| RHSIM_Rhsim01G0050400 | 26.38 | 2.81E-07 | 9.15E-07 | -1.171 |
| novel.4542 | 26.37 | 2.81E-07 | 9.16E-07 | -4.373 |
| RHSIM_Rhsim01G0277400 | 26.37 | 2.82E-07 | 9.17E-07 | 1.471 |
| novel.3553 | 26.37 | 2.82E-07 | 9.19E-07 | 5.464 |
| RHSIM_Rhsim01G0122400 | 26.36 | 2.83E-07 | 9.21E-07 | -0.969 |

| Gene | LRT statistic | P value | BH-adjusted P | log2FC (interaction) |
|---|---|---|---|---|
| RHSIM_Rhsim13G0197600 | 26.35 | 2.84E-07 | 9.25E-07 | -1.288 |
| RHSIM_Rhsim06G0238700 | 26.35 | 2.84E-07 | 9.26E-07 | -0.514 |
| RHSIM_Rhsim11G0065200 | 26.35 | 2.86E-07 | 9.29E-07 | 0.496 |
| RHSIM_Rhsim10G0174700 | 26.33 | 2.87E-07 | 9.34E-07 | -5.695 |
| RHSIM_RhsimUnG0115200 | 26.33 | 2.87E-07 | 9.35E-07 | -3.538 |
| RHSIM_Rhsim04G0207600 | 26.33 | 2.88E-07 | 9.36E-07 | 1.681 |
| RHSIM_Rhsim09G0186700 | 26.33 | 2.88E-07 | 9.37E-07 | -1.030 |
| RHSIM_Rhsim12G0023600 | 26.32 | 2.89E-07 | 9.39E-07 | 0.350 |
| RHSIM_Rhsim12G0182500 | 26.32 | 2.90E-07 | 9.42E-07 | -0.523 |
| RHSIM_Rhsim04G0156000 | 26.31 | 2.90E-07 | 9.44E-07 | -1.030 |
| novel.1649 | 26.30 | 2.93E-07 | 9.52E-07 | -0.426 |
| RHSIM_RhsimUnG0183700 | 26.29 | 2.93E-07 | 9.53E-07 | 0.748 |
| novel.3582 | 26.29 | 2.93E-07 | 9.53E-07 | 2.190 |
| RHSIM_RhsimUnG0119200 | 26.27 | 2.96E-07 | 9.63E-07 | -0.557 |
| RHSIM_Rhsim02G0127000 | 26.26 | 2.98E-07 | 9.68E-07 | 0.531 |
| RHSIM_Rhsim13G0136500 | 26.25 | 3.00E-07 | 9.75E-07 | -2.784 |
| RHSIM_Rhsim08G0052100 | 26.24 | 3.01E-07 | 9.77E-07 | 4.556 |
| RHSIM_RhsimUnG0248600 | 26.24 | 3.01E-07 | 9.79E-07 | 0.775 |
| RHSIM_Rhsim01G0244300 | 26.24 | 3.02E-07 | 9.81E-07 | -0.642 |
| RHSIM_Rhsim04G0062900 | 26.23 | 3.02E-07 | 9.82E-07 | -2.315 |
| RHSIM_Rhsim09G0002400 | 26.23 | 3.03E-07 | 9.83E-07 | 2.401 |
| RHSIM_Rhsim12G0160100 | 26.23 | 3.03E-07 | 9.85E-07 | -0.435 |
| RHSIM_Rhsim01G0196000 | 26.23 | 3.03E-07 | 9.85E-07 | -0.312 |
| RHSIM_Rhsim07G0056900 | 26.21 | 3.06E-07 | 9.92E-07 | 0.801 |
| RHSIM_Rhsim08G0164200 | 26.19 | 3.10E-07 | 1.00E-06 | -1.500 |
| RHSIM_Rhsim02G0126300 | 26.19 | 3.10E-07 | 1.01E-06 | -0.516 |
| RHSIM_Rhsim13G0190400 | 26.18 | 3.11E-07 | 1.01E-06 | -41.331 |
| RHSIM_Rhsim08G0242400 | 26.18 | 3.11E-07 | 1.01E-06 | -0.949 |
| RHSIM_Rhsim08G0171700 | 26.18 | 3.11E-07 | 1.01E-06 | 0.786 |
| RHSIM_Rhsim02G0214900 | 26.17 | 3.13E-07 | 1.02E-06 | -41.199 |
| RHSIM_Rhsim07G0137800 | 26.16 | 3.15E-07 | 1.02E-06 | -0.428 |
| RHSIM_Rhsim06G0212300 | 26.14 | 3.17E-07 | 1.03E-06 | 0.530 |

| Gene | LRT statistic | P value | BH-adjusted P | log2FC (interaction) |
|---|---|---|---|---|
| RHSIM_Rhsim10G0066000 | 26.14 | 3.17E-07 | 1.03E-06 | 0.515 |
| RHSIM_Rhsim13G0006400 | 26.14 | 3.18E-07 | 1.03E-06 | 0.495 |
| RHSIM_Rhsim01G0201700 | 26.13 | 3.18E-07 | 1.03E-06 | 0.516 |
| RHSIM_Rhsim08G0034800 | 26.08 | 3.27E-07 | 1.06E-06 | 2.573 |
| RHSIM_Rhsim11G0160100 | 26.08 | 3.28E-07 | 1.06E-06 | -0.609 |
| RHSIM_Rhsim05G0016500 | 26.08 | 3.28E-07 | 1.06E-06 | -0.730 |
| RHSIM_Rhsim04G0165500 | 26.06 | 3.31E-07 | 1.07E-06 | 0.418 |
| novel.1900 | 26.06 | 3.31E-07 | 1.07E-06 | -2.575 |
| novel.3066 | 26.06 | 3.31E-07 | 1.07E-06 | 4.046 |
| RHSIM_Rhsim12G0082600 | 26.05 | 3.32E-07 | 1.08E-06 | 0.418 |
| RHSIM_Rhsim06G0191700 | 26.03 | 3.36E-07 | 1.09E-06 | -1.255 |
| RHSIM_RhsimUnG0113600 | 26.02 | 3.37E-07 | 1.09E-06 | -2.739 |
| RHSIM_Rhsim08G0210600 | 26.01 | 3.39E-07 | 1.10E-06 | 0.952 |
| RHSIM_Rhsim11G0044100 | 26.01 | 3.39E-07 | 1.10E-06 | 4.107 |
| RHSIM_Rhsim09G0077700 | 26.01 | 3.40E-07 | 1.10E-06 | 0.663 |
| novel.2363 | 25.99 | 3.43E-07 | 1.11E-06 | 2.328 |
| RHSIM_Rhsim05G0139400 | 25.99 | 3.43E-07 | 1.11E-06 | 0.974 |
| RHSIM_RhsimUnG0119700 | 25.99 | 3.44E-07 | 1.11E-06 | 0.443 |
| RHSIM_Rhsim03G0024800 | 25.99 | 3.44E-07 | 1.11E-06 | -1.716 |
| RHSIM_Rhsim11G0048900 | 25.98 | 3.44E-07 | 1.11E-06 | -0.630 |
| RHSIM_Rhsim05G0018500 | 25.98 | 3.44E-07 | 1.11E-06 | 1.175 |
| novel.4314 | 25.98 | 3.45E-07 | 1.12E-06 | 5.866 |
| RHSIM_Rhsim05G0164400 | 25.98 | 3.45E-07 | 1.12E-06 | 0.977 |
| novel.2105 | 25.97 | 3.46E-07 | 1.12E-06 | -3.780 |
| RHSIM_Rhsim04G0125900 | 25.97 | 3.47E-07 | 1.12E-06 | 1.729 |
| novel.4430 | 25.96 | 3.49E-07 | 1.13E-06 | 2.477 |
| RHSIM_Rhsim05G0128700 | 25.96 | 3.49E-07 | 1.13E-06 | 2.577 |
| RHSIM_RhsimUnG0023500 | 25.94 | 3.52E-07 | 1.14E-06 | -1.937 |
| RHSIM_Rhsim09G0059700 | 25.94 | 3.53E-07 | 1.14E-06 | 0.695 |
| RHSIM_Rhsim03G0039000 | 25.94 | 3.53E-07 | 1.14E-06 | 1.504 |
| RHSIM_Rhsim11G0160300 | 25.92 | 3.55E-07 | 1.15E-06 | -1.788 |
| RHSIM_Rhsim04G0217300 | 25.92 | 3.55E-07 | 1.15E-06 | 0.840 |

| Gene | LRT statistic | P value | BH-adjusted P | log2FC (interaction) |
|---|---|---|---|---|
| RHSIM_Rhsim09G0066800 | 25.92 | 3.56E-07 | 1.15E-06 | 1.021 |
| novel.3 | 25.92 | 3.56E-07 | 1.15E-06 | -0.412 |
| RHSIM_Rhsim01G0114700 | 25.92 | 3.57E-07 | 1.15E-06 | 0.820 |
| RHSIM_Rhsim02G0233400 | 25.90 | 3.59E-07 | 1.16E-06 | -0.810 |
| RHSIM_Rhsim05G0214100 | 25.90 | 3.60E-07 | 1.16E-06 | 0.570 |
| RHSIM_Rhsim03G0064600 | 25.89 | 3.61E-07 | 1.17E-06 | 0.357 |
| RHSIM_Rhsim10G0022300 | 25.89 | 3.61E-07 | 1.17E-06 | -0.668 |
| RHSIM_Rhsim06G0104100 | 25.89 | 3.62E-07 | 1.17E-06 | 1.247 |
| RHSIM_Rhsim12G0094700 | 25.88 | 3.64E-07 | 1.17E-06 | -0.571 |
| novel.4435 | 25.87 | 3.65E-07 | 1.18E-06 | 3.158 |
| RHSIM_Rhsim13G0008600 | 25.87 | 3.66E-07 | 1.18E-06 | -3.345 |
| RHSIM_Rhsim12G0177300 | 25.86 | 3.67E-07 | 1.18E-06 | -6.672 |
| novel.3388 | 25.86 | 3.67E-07 | 1.19E-06 | 5.197 |
| RHSIM_Rhsim04G0067400 | 25.85 | 3.69E-07 | 1.19E-06 | 0.514 |
| RHSIM_Rhsim05G0197800 | 25.85 | 3.70E-07 | 1.19E-06 | -1.259 |
| novel.2791 | 25.84 | 3.70E-07 | 1.19E-06 | 1.264 |
| RHSIM_RhsimUnG0032000 | 25.83 | 3.74E-07 | 1.21E-06 | 0.800 |
| novel.391 | 25.82 | 3.75E-07 | 1.21E-06 | -1.426 |
| RHSIM_Rhsim05G0102700 | 25.80 | 3.78E-07 | 1.22E-06 | -1.042 |
| RHSIM_Rhsim05G0175000 | 25.80 | 3.79E-07 | 1.22E-06 | 0.352 |
| RHSIM_Rhsim10G0104300 | 25.79 | 3.81E-07 | 1.23E-06 | -0.451 |
| RHSIM_Rhsim03G0033800 | 25.78 | 3.83E-07 | 1.24E-06 | -1.433 |
| RHSIM_Rhsim01G0086100 | 25.77 | 3.85E-07 | 1.24E-06 | -0.731 |
| RHSIM_Rhsim08G0246000 | 25.76 | 3.86E-07 | 1.24E-06 | 3.381 |
| RHSIM_Rhsim09G0204500 | 25.76 | 3.86E-07 | 1.24E-06 | 0.708 |
| RHSIM_Rhsim13G0204000 | 25.76 | 3.86E-07 | 1.25E-06 | -0.998 |
| RHSIM_Rhsim11G0181500 | 25.76 | 3.87E-07 | 1.25E-06 | 1.864 |
| RHSIM_RhsimUnG0014200 | 25.75 | 3.88E-07 | 1.25E-06 | 2.440 |
| RHSIM_Rhsim11G0123800 | 25.73 | 3.93E-07 | 1.26E-06 | 1.599 |
| RHSIM_Rhsim06G0211300 | 25.71 | 3.97E-07 | 1.28E-06 | -2.953 |
| RHSIM_Rhsim12G0178500 | 25.71 | 3.97E-07 | 1.28E-06 | 0.542 |
| RHSIM_Rhsim12G0166600 | 25.69 | 4.00E-07 | 1.29E-06 | 0.980 |

| Gene | LRT statistic | P value | BH-adjusted P | log2FC (interaction) |
|---|---|---|---|---|
| RHSIM_Rhsim07G0252700 | 25.66 | 4.06E-07 | 1.31E-06 | 0.795 |
| RHSIM_Rhsim04G0172600 | 25.66 | 4.07E-07 | 1.31E-06 | 0.756 |
| RHSIM_Rhsim03G0176000 | 25.66 | 4.07E-07 | 1.31E-06 | -0.447 |
| novel.4100 | 25.65 | 4.09E-07 | 1.32E-06 | 1.497 |
| RHSIM_Rhsim08G0191000 | 25.65 | 4.10E-07 | 1.32E-06 | 1.223 |
| RHSIM_Rhsim09G0115200 | 25.65 | 4.10E-07 | 1.32E-06 | -0.825 |
| RHSIM_Rhsim03G0242800 | 25.63 | 4.14E-07 | 1.33E-06 | -2.245 |
| RHSIM_Rhsim04G0176900 | 25.63 | 4.14E-07 | 1.33E-06 | -0.589 |
| RHSIM_Rhsim10G0076700 | 25.62 | 4.16E-07 | 1.34E-06 | 1.241 |
| RHSIM_Rhsim10G0195100 | 25.61 | 4.17E-07 | 1.34E-06 | -0.493 |
| novel.2396 | 25.61 | 4.18E-07 | 1.34E-06 | -9.000 |
| novel.3530 | 25.60 | 4.21E-07 | 1.35E-06 | 1.309 |
| RHSIM_Rhsim05G0106700 | 25.58 | 4.24E-07 | 1.36E-06 | 0.424 |
| RHSIM_Rhsim09G0070900 | 25.57 | 4.27E-07 | 1.37E-06 | 0.924 |
| RHSIM_Rhsim01G0061500 | 25.55 | 4.31E-07 | 1.39E-06 | 2.757 |
| RHSIM_Rhsim10G0128500 | 25.54 | 4.33E-07 | 1.39E-06 | 0.743 |
| RHSIM_Rhsim08G0182200 | 25.52 | 4.37E-07 | 1.40E-06 | -0.468 |
| RHSIM_Rhsim09G0086700 | 25.51 | 4.39E-07 | 1.41E-06 | -1.386 |
| novel.3666 | 25.49 | 4.45E-07 | 1.43E-06 | -3.583 |
| RHSIM_Rhsim01G0160800 | 25.49 | 4.45E-07 | 1.43E-06 | 0.882 |
| RHSIM_Rhsim12G0145800 | 25.47 | 4.48E-07 | 1.44E-06 | -0.372 |
| RHSIM_Rhsim08G0190900 | 25.47 | 4.49E-07 | 1.44E-06 | -0.314 |
| RHSIM_Rhsim07G0123600 | 25.47 | 4.49E-07 | 1.44E-06 | 0.959 |
| RHSIM_Rhsim09G0212600 | 25.47 | 4.49E-07 | 1.44E-06 | 0.517 |
| RHSIM_Rhsim07G0252900 | 25.46 | 4.51E-07 | 1.45E-06 | -0.820 |
| RHSIM_RhsimUnG0044200 | 25.46 | 4.51E-07 | 1.45E-06 | 0.347 |
| RHSIM_Rhsim12G0139300 | 25.45 | 4.54E-07 | 1.46E-06 | 0.520 |
| RHSIM_Rhsim07G0069300 | 25.45 | 4.55E-07 | 1.46E-06 | -0.738 |
| RHSIM_RhsimUnG0045900 | 25.44 | 4.57E-07 | 1.47E-06 | 2.122 |
| novel.3753 | 25.43 | 4.58E-07 | 1.47E-06 | 2.672 |
| RHSIM_Rhsim02G0163100 | 25.42 | 4.62E-07 | 1.48E-06 | 0.739 |
| RHSIM_Rhsim06G0201000 | 25.41 | 4.63E-07 | 1.49E-06 | -0.666 |

| Gene | LRT statistic | P value | BH-adjusted P | log2FC (interaction) |
|---|---|---|---|---|
| RHSIM_Rhsim07G0061100 | 25.40 | 4.66E-07 | 1.50E-06 | -1.265 |
| RHSIM_Rhsim04G0202000 | 25.40 | 4.66E-07 | 1.50E-06 | -0.829 |
| RHSIM_Rhsim04G0035400 | 25.40 | 4.66E-07 | 1.50E-06 | 0.494 |
| RHSIM_Rhsim04G0178300 | 25.40 | 4.67E-07 | 1.50E-06 | -5.610 |
| RHSIM_Rhsim08G0091900 | 25.38 | 4.71E-07 | 1.51E-06 | 1.627 |
| RHSIM_Rhsim12G0164200 | 25.37 | 4.74E-07 | 1.52E-06 | -1.793 |
| RHSIM_Rhsim11G0177000 | 25.36 | 4.75E-07 | 1.52E-06 | -1.740 |
| RHSIM_Rhsim05G0142700 | 25.36 | 4.77E-07 | 1.53E-06 | -0.644 |
| RHSIM_Rhsim10G0028000 | 25.35 | 4.78E-07 | 1.53E-06 | 0.637 |
| RHSIM_Rhsim09G0008400 | 25.35 | 4.79E-07 | 1.53E-06 | 0.315 |
| RHSIM_Rhsim07G0009100 | 25.34 | 4.81E-07 | 1.54E-06 | -0.856 |
| novel.3506 | 25.33 | 4.83E-07 | 1.55E-06 | -0.375 |
| RHSIM_Rhsim10G0070900 | 25.32 | 4.85E-07 | 1.55E-06 | -3.875 |
| RHSIM_RhsimUnG0212800 | 25.31 | 4.88E-07 | 1.56E-06 | -1.749 |
| novel.1043 | 25.31 | 4.89E-07 | 1.57E-06 | 0.343 |
| RHSIM_Rhsim01G0254900 | 25.30 | 4.90E-07 | 1.57E-06 | -1.397 |
| RHSIM_Rhsim06G0079500 | 25.30 | 4.91E-07 | 1.57E-06 | -0.452 |
| RHSIM_RhsimUnG0236000 | 25.30 | 4.92E-07 | 1.57E-06 | 1.863 |
| RHSIM_Rhsim07G0223300 | 25.29 | 4.92E-07 | 1.58E-06 | -1.432 |
| RHSIM_Rhsim03G0191300 | 25.29 | 4.92E-07 | 1.58E-06 | -0.339 |
| RHSIM_Rhsim10G0021100 | 25.29 | 4.94E-07 | 1.58E-06 | 0.814 |
| RHSIM_Rhsim06G0194200 | 25.28 | 4.97E-07 | 1.59E-06 | -2.568 |
| RHSIM_Rhsim08G0138500 | 25.27 | 4.98E-07 | 1.59E-06 | -0.574 |
| RHSIM_Rhsim04G0206800 | 25.27 | 4.99E-07 | 1.60E-06 | -42.287 |
| RHSIM_Rhsim01G0068100 | 25.27 | 4.99E-07 | 1.60E-06 | -0.987 |
| RHSIM_Rhsim06G0018200 | 25.26 | 5.00E-07 | 1.60E-06 | -2.425 |
| novel.3259 | 25.26 | 5.02E-07 | 1.61E-06 | -0.320 |
| RHSIM_Rhsim08G0040900 | 25.25 | 5.03E-07 | 1.61E-06 | -0.766 |
| RHSIM_Rhsim04G0140400 | 25.25 | 5.03E-07 | 1.61E-06 | -3.495 |
| RHSIM_Rhsim06G0068400 | 25.25 | 5.03E-07 | 1.61E-06 | -0.884 |
| RHSIM_Rhsim07G0173800 | 25.25 | 5.04E-07 | 1.61E-06 | 0.528 |
| RHSIM_Rhsim06G0090800 | 25.25 | 5.04E-07 | 1.61E-06 | 0.246 |

| Gene | LRT statistic | P value | BH-adjusted P | log2FC (interaction) |
|---|---|---|---|---|
| RHSIM_Rhsim12G0041500 | 25.24 | 5.05E-07 | 1.61E-06 | -1.031 |
| RHSIM_Rhsim02G0001100 | 25.22 | 5.11E-07 | 1.63E-06 | 3.357 |
| novel.2188 | 25.22 | 5.12E-07 | 1.64E-06 | -0.642 |
| novel.2232 | 25.21 | 5.14E-07 | 1.64E-06 | 0.898 |
| RHSIM_Rhsim11G0180700 | 25.19 | 5.19E-07 | 1.66E-06 | 0.474 |
| RHSIM_Rhsim02G0008400 | 25.19 | 5.20E-07 | 1.66E-06 | 2.177 |
| RHSIM_Rhsim07G0165300 | 25.19 | 5.21E-07 | 1.66E-06 | 2.341 |
| RHSIM_Rhsim05G0224200 | 25.18 | 5.22E-07 | 1.67E-06 | 1.134 |
| novel.3732 | 25.18 | 5.23E-07 | 1.67E-06 | -2.562 |
| RHSIM_Rhsim06G0187800 | 25.17 | 5.24E-07 | 1.67E-06 | 0.494 |
| RHSIM_Rhsim13G0163200 | 25.17 | 5.25E-07 | 1.68E-06 | 1.078 |
| RHSIM_Rhsim03G0128800 | 25.17 | 5.26E-07 | 1.68E-06 | 1.236 |
| RHSIM_Rhsim02G0117400 | 25.16 | 5.27E-07 | 1.68E-06 | -1.819 |
| RHSIM_Rhsim08G0041100 | 25.15 | 5.30E-07 | 1.69E-06 | 0.615 |
| RHSIM_Rhsim01G0152000 | 25.15 | 5.30E-07 | 1.69E-06 | -0.397 |
| RHSIM_Rhsim04G0162700 | 25.15 | 5.30E-07 | 1.69E-06 | 0.637 |
| novel.2475 | 25.15 | 5.31E-07 | 1.69E-06 | -0.752 |
| RHSIM_Rhsim02G0084300 | 25.15 | 5.32E-07 | 1.70E-06 | -0.686 |
| RHSIM_Rhsim05G0195500 | 25.14 | 5.32E-07 | 1.70E-06 | 3.569 |
| RHSIM_Rhsim09G0178600 | 25.14 | 5.32E-07 | 1.70E-06 | -1.829 |
| RHSIM_Rhsim11G0153300 | 25.13 | 5.37E-07 | 1.71E-06 | -0.543 |
| RHSIM_Rhsim03G0235700 | 25.12 | 5.40E-07 | 1.72E-06 | -0.293 |
| RHSIM_Rhsim06G0067700 | 25.11 | 5.41E-07 | 1.73E-06 | -2.243 |
| RHSIM_Rhsim08G0197300 | 25.11 | 5.42E-07 | 1.73E-06 | -0.581 |
| RHSIM_Rhsim07G0231400 | 25.11 | 5.42E-07 | 1.73E-06 | -6.163 |
| RHSIM_Rhsim02G0001700 | 25.10 | 5.43E-07 | 1.73E-06 | -0.460 |
| RHSIM_Rhsim06G0058800 | 25.10 | 5.44E-07 | 1.73E-06 | 2.236 |
| RHSIM_Rhsim05G0049700 | 25.10 | 5.45E-07 | 1.74E-06 | 0.685 |
| RHSIM_Rhsim04G0081800 | 25.09 | 5.46E-07 | 1.74E-06 | -0.723 |
| novel.1758 | 25.09 | 5.46E-07 | 1.74E-06 | 4.281 |
| RHSIM_Rhsim03G0001300 | 25.08 | 5.50E-07 | 1.75E-06 | 0.524 |
| RHSIM_Rhsim13G0158800 | 25.07 | 5.53E-07 | 1.76E-06 | 43.952 |

| Gene | LRT statistic | P value | BH-adjusted P | log2FC (interaction) |
|---|---|---|---|---|
| RHSIM_Rhsim13G0075100 | 25.05 | 5.58E-07 | 1.78E-06 | -0.835 |
| RHSIM_Rhsim06G0169600 | 25.05 | 5.60E-07 | 1.78E-06 | -1.917 |
| RHSIM_Rhsim08G0196300 | 25.04 | 5.61E-07 | 1.79E-06 | -0.935 |
| RHSIM_Rhsim10G0004700 | 25.00 | 5.73E-07 | 1.82E-06 | -0.938 |
| RHSIM_Rhsim13G0191200 | 25.00 | 5.73E-07 | 1.83E-06 | -1.358 |
| RHSIM_Rhsim01G0199600 | 25.00 | 5.74E-07 | 1.83E-06 | 1.672 |
| RHSIM_Rhsim08G0245600 | 25.00 | 5.74E-07 | 1.83E-06 | -1.119 |
| RHSIM_Rhsim10G0127200 | 25.00 | 5.74E-07 | 1.83E-06 | 0.621 |
| RHSIM_RhsimUnG0016900 | 25.00 | 5.75E-07 | 1.83E-06 | 0.490 |
| RHSIM_RhsimUnG0138700 | 24.99 | 5.78E-07 | 1.84E-06 | 2.573 |
| novel.876 | 24.98 | 5.80E-07 | 1.85E-06 | 0.914 |
| novel.3537 | 24.95 | 5.88E-07 | 1.87E-06 | 0.530 |
| RHSIM_Rhsim10G0162300 | 24.95 | 5.90E-07 | 1.88E-06 | -1.368 |
| RHSIM_Rhsim10G0151900 | 24.94 | 5.90E-07 | 1.88E-06 | 0.997 |
| RHSIM_Rhsim12G0029000 | 24.94 | 5.93E-07 | 1.89E-06 | 0.752 |
| RHSIM_Rhsim01G0050500 | 24.93 | 5.93E-07 | 1.89E-06 | -1.377 |
| RHSIM_Rhsim09G0106800 | 24.93 | 5.93E-07 | 1.89E-06 | -4.979 |
| RHSIM_Rhsim07G0174300 | 24.93 | 5.94E-07 | 1.89E-06 | 0.988 |
| RHSIM_Rhsim08G0119800 | 24.92 | 5.99E-07 | 1.90E-06 | 0.725 |
| RHSIM_Rhsim02G0081700 | 24.91 | 6.00E-07 | 1.91E-06 | -0.732 |
| RHSIM_Rhsim12G0076900 | 24.91 | 6.00E-07 | 1.91E-06 | 2.851 |
| RHSIM_Rhsim03G0186800 | 24.90 | 6.05E-07 | 1.92E-06 | 0.620 |
| RHSIM_Rhsim05G0216800 | 24.89 | 6.08E-07 | 1.93E-06 | -0.419 |
| RHSIM_Rhsim06G0062400 | 24.89 | 6.08E-07 | 1.93E-06 | -0.614 |
| RHSIM_Rhsim06G0101100 | 24.87 | 6.13E-07 | 1.95E-06 | 1.261 |
| RHSIM_Rhsim05G0006800 | 24.87 | 6.14E-07 | 1.95E-06 | -1.058 |
| RHSIM_RhsimUnG0226700 | 24.87 | 6.14E-07 | 1.95E-06 | 1.074 |
| RHSIM_Rhsim06G0041400 | 24.85 | 6.19E-07 | 1.97E-06 | -0.972 |
| RHSIM_Rhsim02G0193100 | 24.85 | 6.21E-07 | 1.97E-06 | 1.136 |
| RHSIM_Rhsim11G0127500 | 24.84 | 6.22E-07 | 1.97E-06 | 1.318 |
| RHSIM_Rhsim08G0129300 | 24.84 | 6.24E-07 | 1.98E-06 | 0.487 |
| RHSIM_Rhsim01G0268200 | 24.84 | 6.24E-07 | 1.98E-06 | 0.435 |

| Gene | LRT statistic | P value | BH-adjusted P | log2FC (interaction) |
|---|---|---|---|---|
| RHSIM_Rhsim06G0049900 | 24.83 | 6.26E-07 | 1.99E-06 | 0.856 |
| novel.2389 | 24.82 | 6.29E-07 | 2.00E-06 | -0.559 |
| novel.1724 | 24.82 | 6.30E-07 | 2.00E-06 | 1.208 |
| RHSIM_Rhsim13G0144300 | 24.81 | 6.31E-07 | 2.00E-06 | 2.355 |
| RHSIM_Rhsim03G0224800 | 24.81 | 6.33E-07 | 2.01E-06 | 1.040 |
| RHSIM_Rhsim13G0067100 | 24.79 | 6.39E-07 | 2.03E-06 | -0.597 |
| RHSIM_Rhsim07G0244100 | 24.79 | 6.39E-07 | 2.03E-06 | -2.037 |
| RHSIM_Rhsim09G0119200 | 24.78 | 6.42E-07 | 2.04E-06 | 2.647 |
| RHSIM_Rhsim08G0165600 | 24.77 | 6.44E-07 | 2.04E-06 | -4.172 |
| RHSIM_Rhsim02G0019600 | 24.76 | 6.48E-07 | 2.06E-06 | 0.468 |
| RHSIM_Rhsim01G0050900 | 24.76 | 6.48E-07 | 2.06E-06 | 3.105 |
| novel.2808 | 24.75 | 6.52E-07 | 2.07E-06 | 0.938 |
| RHSIM_Rhsim10G0092800 | 24.75 | 6.53E-07 | 2.07E-06 | -0.439 |
| RHSIM_Rhsim03G0011500 | 24.74 | 6.56E-07 | 2.08E-06 | -1.955 |
| RHSIM_Rhsim04G0039400 | 24.73 | 6.61E-07 | 2.09E-06 | -0.850 |
| RHSIM_Rhsim05G0121800 | 24.71 | 6.65E-07 | 2.11E-06 | -0.368 |
| RHSIM_Rhsim01G0234700 | 24.71 | 6.65E-07 | 2.11E-06 | 0.851 |
| novel.342 | 24.71 | 6.67E-07 | 2.11E-06 | -0.519 |
| RHSIM_Rhsim09G0192100 | 24.71 | 6.68E-07 | 2.12E-06 | 1.292 |
| novel.196 | 24.69 | 6.73E-07 | 2.13E-06 | -1.141 |
| novel.1555 | 24.69 | 6.73E-07 | 2.13E-06 | 2.551 |
| RHSIM_Rhsim04G0216300 | 24.69 | 6.73E-07 | 2.13E-06 | -1.106 |
| RHSIM_Rhsim01G0283200 | 24.69 | 6.74E-07 | 2.13E-06 | -0.510 |
| RHSIM_Rhsim07G0142500 | 24.68 | 6.76E-07 | 2.14E-06 | -0.531 |
| RHSIM_Rhsim06G0001000 | 24.68 | 6.76E-07 | 2.14E-06 | 0.341 |
| RHSIM_Rhsim07G0005600 | 24.68 | 6.78E-07 | 2.15E-06 | 0.566 |
| RHSIM_Rhsim09G0200900 | 24.67 | 6.81E-07 | 2.15E-06 | -3.541 |
| RHSIM_Rhsim01G0202600 | 24.67 | 6.81E-07 | 2.16E-06 | -0.909 |
| novel.1775 | 24.67 | 6.82E-07 | 2.16E-06 | 2.793 |
| RHSIM_Rhsim13G0216200 | 24.65 | 6.87E-07 | 2.17E-06 | 1.668 |
| RHSIM_Rhsim12G0136500 | 24.65 | 6.87E-07 | 2.17E-06 | -0.781 |
| RHSIM_Rhsim02G0033200 | 24.65 | 6.89E-07 | 2.18E-06 | 1.275 |

| Gene | LRT statistic | P value | BH-adjusted P | log2FC (interaction) |
|---|---|---|---|---|
| RHSIM_Rhsim03G0046100 | 24.64 | 6.90E-07 | 2.18E-06 | -1.733 |
| RHSIM_Rhsim08G0038600 | 24.64 | 6.91E-07 | 2.18E-06 | -0.575 |
| RHSIM_Rhsim06G0063800 | 24.63 | 6.95E-07 | 2.20E-06 | 1.365 |
| RHSIM_Rhsim09G0106900 | 24.63 | 6.96E-07 | 2.20E-06 | 0.344 |
| RHSIM_Rhsim12G0210100 | 24.62 | 6.98E-07 | 2.21E-06 | 0.637 |
| RHSIM_Rhsim10G0065900 | 24.62 | 6.99E-07 | 2.21E-06 | 0.514 |
| RHSIM_Rhsim02G0042400 | 24.61 | 7.00E-07 | 2.21E-06 | 0.529 |
| RHSIM_Rhsim06G0242300 | 24.61 | 7.03E-07 | 2.22E-06 | -0.465 |
| RHSIM_RhsimUnG0044700 | 24.60 | 7.04E-07 | 2.22E-06 | -0.330 |
| RHSIM_Rhsim11G0173600 | 24.60 | 7.04E-07 | 2.22E-06 | -0.373 |
| novel.1151 | 24.60 | 7.05E-07 | 2.23E-06 | -1.920 |
| RHSIM_Rhsim11G0036600 | 24.58 | 7.12E-07 | 2.25E-06 | -0.668 |
| RHSIM_Rhsim10G0135000 | 24.58 | 7.13E-07 | 2.25E-06 | 2.319 |
| RHSIM_Rhsim09G0001300 | 24.58 | 7.15E-07 | 2.26E-06 | -0.427 |
| RHSIM_Rhsim06G0230500 | 24.57 | 7.17E-07 | 2.26E-06 | 0.501 |
| RHSIM_Rhsim02G0063300 | 24.57 | 7.18E-07 | 2.27E-06 | 1.107 |
| RHSIM_Rhsim05G0072500 | 24.55 | 7.25E-07 | 2.29E-06 | -2.211 |
| RHSIM_Rhsim13G0105600 | 24.55 | 7.25E-07 | 2.29E-06 | -2.484 |
| RHSIM_Rhsim03G0168400 | 24.53 | 7.30E-07 | 2.31E-06 | 0.546 |
| RHSIM_Rhsim10G0036800 | 24.53 | 7.31E-07 | 2.31E-06 | -0.620 |
| RHSIM_Rhsim03G0069500 | 24.53 | 7.31E-07 | 2.31E-06 | -0.785 |
| RHSIM_Rhsim01G0003800 | 24.52 | 7.35E-07 | 2.32E-06 | -0.694 |
| RHSIM_Rhsim12G0018000 | 24.51 | 7.39E-07 | 2.33E-06 | -0.716 |
| novel.2052 | 24.51 | 7.39E-07 | 2.33E-06 | 1.211 |
| RHSIM_Rhsim11G0050900 | 24.50 | 7.42E-07 | 2.34E-06 | -2.685 |
| RHSIM_Rhsim11G0106200 | 24.50 | 7.43E-07 | 2.34E-06 | -0.628 |
| RHSIM_Rhsim09G0172600 | 24.49 | 7.46E-07 | 2.35E-06 | 0.769 |
| RHSIM_Rhsim02G0047900 | 24.48 | 7.52E-07 | 2.37E-06 | 3.806 |
| RHSIM_Rhsim02G0200300 | 24.47 | 7.55E-07 | 2.38E-06 | 0.408 |
| RHSIM_Rhsim01G0269900 | 24.46 | 7.57E-07 | 2.39E-06 | 0.570 |
| RHSIM_Rhsim08G0147300 | 24.46 | 7.59E-07 | 2.39E-06 | 0.485 |
| RHSIM_Rhsim12G0054900 | 24.46 | 7.60E-07 | 2.40E-06 | -1.063 |

| Gene | LRT statistic | P value | BH-adjusted P | log2FC (interaction) |
|---|---|---|---|---|
| RHSIM_Rhsim07G0192100 | 24.44 | 7.65E-07 | 2.41E-06 | 2.653 |
| RHSIM_Rhsim06G0225400 | 24.42 | 7.73E-07 | 2.44E-06 | 4.626 |
| RHSIM_Rhsim12G0182900 | 24.42 | 7.76E-07 | 2.44E-06 | 1.489 |
| RHSIM_Rhsim01G0102700 | 24.40 | 7.82E-07 | 2.46E-06 | -1.552 |
| novel.731 | 24.39 | 7.85E-07 | 2.47E-06 | 1.771 |
| RHSIM_RhsimUnG0254400 | 24.39 | 7.85E-07 | 2.47E-06 | -1.324 |
| RHSIM_Rhsim13G0210800 | 24.39 | 7.86E-07 | 2.48E-06 | -2.567 |
| RHSIM_Rhsim06G0083900 | 24.39 | 7.87E-07 | 2.48E-06 | -0.978 |
| RHSIM_Rhsim03G0233000 | 24.38 | 7.91E-07 | 2.49E-06 | 0.397 |
| RHSIM_Rhsim08G0178100 | 24.36 | 7.97E-07 | 2.51E-06 | 0.593 |
| RHSIM_Rhsim07G0202800 | 24.35 | 8.05E-07 | 2.53E-06 | -0.382 |
| RHSIM_Rhsim07G0236300 | 24.34 | 8.08E-07 | 2.54E-06 | 4.015 |
| RHSIM_RhsimUnG0203600 | 24.34 | 8.08E-07 | 2.54E-06 | -1.877 |
| RHSIM_Rhsim02G0130400 | 24.34 | 8.09E-07 | 2.55E-06 | -1.073 |
| RHSIM_Rhsim09G0161300 | 24.33 | 8.10E-07 | 2.55E-06 | 1.610 |
| novel.4295 | 24.33 | 8.11E-07 | 2.55E-06 | 4.178 |
| RHSIM_Rhsim05G0178500 | 24.33 | 8.14E-07 | 2.56E-06 | -1.258 |
| RHSIM_Rhsim08G0239800 | 24.30 | 8.23E-07 | 2.59E-06 | -3.165 |
| RHSIM_Rhsim11G0097700 | 24.30 | 8.24E-07 | 2.59E-06 | -0.851 |
| RHSIM_Rhsim02G0141800 | 24.29 | 8.27E-07 | 2.60E-06 | 1.408 |
| RHSIM_Rhsim04G0016200 | 24.29 | 8.29E-07 | 2.61E-06 | -0.441 |
| RHSIM_Rhsim07G0052800 | 24.28 | 8.33E-07 | 2.62E-06 | 1.965 |
| RHSIM_Rhsim11G0073400 | 24.27 | 8.37E-07 | 2.63E-06 | -2.769 |
| RHSIM_Rhsim05G0095400 | 24.27 | 8.39E-07 | 2.64E-06 | -0.471 |
| RHSIM_Rhsim03G0176700 | 24.26 | 8.40E-07 | 2.64E-06 | 0.690 |
| RHSIM_Rhsim13G0220900 | 24.26 | 8.41E-07 | 2.64E-06 | 0.559 |
| novel.2919 | 24.26 | 8.41E-07 | 2.64E-06 | 1.711 |
| RHSIM_Rhsim04G0204200 | 24.26 | 8.43E-07 | 2.65E-06 | -0.651 |
| RHSIM_Rhsim06G0077500 | 24.24 | 8.51E-07 | 2.67E-06 | 1.221 |
| RHSIM_Rhsim07G0158100 | 24.23 | 8.54E-07 | 2.68E-06 | 1.227 |
| RHSIM_Rhsim06G0233200 | 24.23 | 8.56E-07 | 2.69E-06 | -1.281 |
| RHSIM_Rhsim07G0067900 | 24.23 | 8.56E-07 | 2.69E-06 | -1.518 |

| Gene | LRT statistic | P value | BH-adjusted P | log2FC (interaction) |
|---|---|---|---|---|
| RHSIM_Rhsim08G0111200 | 24.23 | 8.56E-07 | 2.69E-06 | 0.544 |
| novel.1379 | 24.22 | 8.58E-07 | 2.69E-06 | -1.745 |
| novel.4578 | 24.22 | 8.59E-07 | 2.70E-06 | 1.771 |
| RHSIM_Rhsim12G0207200 | 24.22 | 8.59E-07 | 2.70E-06 | 0.940 |
| RHSIM_Rhsim02G0063500 | 24.21 | 8.63E-07 | 2.71E-06 | -1.411 |
| RHSIM_Rhsim04G0081600 | 24.21 | 8.64E-07 | 2.71E-06 | -1.089 |
| RHSIM_Rhsim09G0066300 | 24.21 | 8.66E-07 | 2.72E-06 | -1.126 |
| RHSIM_Rhsim07G0139500 | 24.20 | 8.68E-07 | 2.72E-06 | 2.579 |
| RHSIM_Rhsim06G0214000 | 24.18 | 8.76E-07 | 2.75E-06 | 1.606 |
| novel.2195 | 24.18 | 8.77E-07 | 2.75E-06 | 0.422 |
| RHSIM_Rhsim04G0021000 | 24.18 | 8.79E-07 | 2.76E-06 | -1.967 |
| RHSIM_Rhsim13G0044100 | 24.18 | 8.79E-07 | 2.76E-06 | -0.720 |
| RHSIM_Rhsim10G0041000 | 24.17 | 8.80E-07 | 2.76E-06 | 0.433 |
| RHSIM_Rhsim11G0121800 | 24.17 | 8.80E-07 | 2.76E-06 | -0.226 |
| novel.3158 | 24.17 | 8.82E-07 | 2.77E-06 | -2.726 |
| RHSIM_Rhsim01G0096900 | 24.17 | 8.84E-07 | 2.77E-06 | 0.641 |
| RHSIM_Rhsim08G0054900 | 24.15 | 8.89E-07 | 2.79E-06 | -0.538 |
| novel.2701 | 24.15 | 8.93E-07 | 2.80E-06 | -2.855 |
| RHSIM_Rhsim02G0004900 | 24.14 | 8.98E-07 | 2.81E-06 | 0.795 |
| RHSIM_Rhsim12G0196200 | 24.12 | 9.05E-07 | 2.84E-06 | -1.456 |
| RHSIM_Rhsim08G0070400 | 24.12 | 9.06E-07 | 2.84E-06 | -1.020 |
| RHSIM_Rhsim11G0034600 | 24.12 | 9.07E-07 | 2.84E-06 | 0.541 |
| RHSIM_RhsimUnG0102600 | 24.11 | 9.08E-07 | 2.84E-06 | -1.702 |
| RHSIM_Rhsim06G0239500 | 24.11 | 9.11E-07 | 2.86E-06 | -0.473 |
| RHSIM_Rhsim04G0231600 | 24.10 | 9.15E-07 | 2.87E-06 | 0.377 |
| RHSIM_Rhsim13G0114700 | 24.09 | 9.20E-07 | 2.88E-06 | 0.293 |
| RHSIM_Rhsim02G0127600 | 24.09 | 9.20E-07 | 2.88E-06 | 0.400 |
| RHSIM_Rhsim08G0236200 | 24.09 | 9.21E-07 | 2.88E-06 | -0.729 |
| RHSIM_Rhsim03G0151900 | 24.08 | 9.24E-07 | 2.89E-06 | 0.616 |
| RHSIM_Rhsim03G0138700 | 24.07 | 9.28E-07 | 2.91E-06 | -0.798 |
| RHSIM_Rhsim02G0143100 | 24.06 | 9.34E-07 | 2.92E-06 | 0.449 |
| RHSIM_Rhsim09G0145000 | 24.05 | 9.37E-07 | 2.93E-06 | -2.445 |

| Gene | LRT statistic | P value | BH-adjusted P | log2FC (interaction) |
|---|---|---|---|---|
| RHSIM_Rhsim03G0234500 | 24.05 | 9.39E-07 | 2.94E-06 | -0.449 |
| novel.435 | 24.04 | 9.42E-07 | 2.95E-06 | 0.652 |
| RHSIM_Rhsim06G0019700 | 24.03 | 9.47E-07 | 2.96E-06 | 0.727 |
| RHSIM_Rhsim07G0236600 | 24.03 | 9.50E-07 | 2.97E-06 | -0.954 |
| RHSIM_Rhsim13G0168800 | 24.01 | 9.57E-07 | 2.99E-06 | -3.513 |
| RHSIM_Rhsim03G0035600 | 24.01 | 9.61E-07 | 3.01E-06 | -0.766 |
| novel.2801 | 24.00 | 9.63E-07 | 3.01E-06 | 2.026 |
| RHSIM_Rhsim12G0087200 | 23.99 | 9.68E-07 | 3.03E-06 | -0.870 |
| novel.36 | 23.98 | 9.75E-07 | 3.05E-06 | -1.414 |
| RHSIM_Rhsim05G0093200 | 23.97 | 9.78E-07 | 3.06E-06 | 0.697 |
| RHSIM_Rhsim13G0083700 | 23.97 | 9.81E-07 | 3.07E-06 | -5.760 |
| RHSIM_Rhsim10G0201100 | 23.96 | 9.85E-07 | 3.08E-06 | -0.911 |
| novel.3292 | 23.95 | 9.89E-07 | 3.09E-06 | -4.599 |
| novel.1349 | 23.94 | 9.93E-07 | 3.10E-06 | 2.172 |
| RHSIM_Rhsim02G0249100 | 23.94 | 9.95E-07 | 3.11E-06 | 1.306 |
| RHSIM_Rhsim02G0218500 | 23.93 | 9.99E-07 | 3.12E-06 | 1.860 |
| RHSIM_Rhsim08G0080500 | 23.92 | 1.01E-06 | 3.14E-06 | 1.580 |
| RHSIM_Rhsim06G0092600 | 23.90 | 1.02E-06 | 3.17E-06 | -1.197 |
| RHSIM_Rhsim13G0227000 | 23.90 | 1.02E-06 | 3.18E-06 | 0.545 |
| RHSIM_Rhsim13G0051200 | 23.89 | 1.02E-06 | 3.19E-06 | -0.372 |
| RHSIM_Rhsim11G0196400 | 23.89 | 1.02E-06 | 3.19E-06 | 0.928 |
| RHSIM_Rhsim09G0070400 | 23.88 | 1.03E-06 | 3.20E-06 | 1.436 |
| RHSIM_Rhsim02G0148300 | 23.88 | 1.03E-06 | 3.21E-06 | 0.435 |
| RHSIM_Rhsim01G0068900 | 23.87 | 1.03E-06 | 3.22E-06 | -0.951 |
| novel.127 | 23.87 | 1.03E-06 | 3.22E-06 | -0.750 |
| RHSIM_Rhsim05G0164800 | 23.86 | 1.03E-06 | 3.23E-06 | 0.753 |
| RHSIM_Rhsim01G0120800 | 23.86 | 1.04E-06 | 3.23E-06 | -3.279 |
| RHSIM_Rhsim07G0100500 | 23.86 | 1.04E-06 | 3.24E-06 | -0.576 |
| RHSIM_Rhsim09G0093400 | 23.85 | 1.04E-06 | 3.25E-06 | 1.608 |
| RHSIM_Rhsim01G0091600 | 23.83 | 1.05E-06 | 3.29E-06 | 1.959 |
| RHSIM_Rhsim06G0185400 | 23.82 | 1.06E-06 | 3.29E-06 | 0.395 |
| RHSIM_Rhsim08G0125500 | 23.82 | 1.06E-06 | 3.29E-06 | 1.772 |

| Gene | LRT statistic | P value | BH-adjusted P | log2FC (interaction) |
|---|---|---|---|---|
| RHSIM_Rhsim04G0175100 | 23.81 | 1.06E-06 | 3.31E-06 | -5.205 |
| RHSIM_Rhsim05G0074500 | 23.80 | 1.07E-06 | 3.34E-06 | -0.457 |
| RHSIM_Rhsim01G0136200 | 23.79 | 1.08E-06 | 3.35E-06 | 1.657 |
| RHSIM_Rhsim10G0158800 | 23.79 | 1.08E-06 | 3.35E-06 | -3.873 |
| RHSIM_Rhsim04G0208900 | 23.78 | 1.08E-06 | 3.37E-06 | 2.382 |
| RHSIM_Rhsim07G0076400 | 23.77 | 1.09E-06 | 3.39E-06 | 0.290 |
| RHSIM_Rhsim12G0034700 | 23.77 | 1.09E-06 | 3.39E-06 | -0.992 |
| RHSIM_Rhsim04G0220600 | 23.77 | 1.09E-06 | 3.39E-06 | -0.556 |
| RHSIM_Rhsim12G0181200 | 23.76 | 1.09E-06 | 3.40E-06 | -0.711 |
| novel.941 | 23.76 | 1.09E-06 | 3.40E-06 | 0.732 |
| RHSIM_Rhsim05G0061900 | 23.74 | 1.10E-06 | 3.43E-06 | -2.765 |
| RHSIM_Rhsim04G0138200 | 23.74 | 1.10E-06 | 3.44E-06 | 1.608 |
| RHSIM_Rhsim09G0037600 | 23.73 | 1.11E-06 | 3.45E-06 | -0.356 |
| RHSIM_Rhsim13G0224300 | 23.73 | 1.11E-06 | 3.45E-06 | 1.025 |
| RHSIM_Rhsim08G0241300 | 23.71 | 1.12E-06 | 3.49E-06 | 0.327 |
| RHSIM_Rhsim09G0146900 | 23.70 | 1.13E-06 | 3.51E-06 | -0.620 |
| RHSIM_Rhsim04G0246100 | 23.69 | 1.13E-06 | 3.52E-06 | 1.584 |
| RHSIM_Rhsim10G0197500 | 23.69 | 1.13E-06 | 3.53E-06 | -0.341 |
| RHSIM_Rhsim03G0129700 | 23.69 | 1.13E-06 | 3.53E-06 | 0.717 |
| RHSIM_RhsimUnG0043100 | 23.68 | 1.14E-06 | 3.54E-06 | -3.787 |
| RHSIM_Rhsim07G0107000 | 23.68 | 1.14E-06 | 3.55E-06 | -1.335 |
| RHSIM_Rhsim09G0043500 | 23.67 | 1.14E-06 | 3.56E-06 | 0.407 |
| RHSIM_Rhsim10G0009400 | 23.67 | 1.14E-06 | 3.56E-06 | -2.588 |
| RHSIM_Rhsim03G0074600 | 23.66 | 1.15E-06 | 3.57E-06 | -0.425 |
| RHSIM_Rhsim01G0152700 | 23.66 | 1.15E-06 | 3.57E-06 | -1.379 |
| RHSIM_Rhsim07G0081100 | 23.66 | 1.15E-06 | 3.58E-06 | -1.510 |
| RHSIM_RhsimUnG0087400 | 23.65 | 1.15E-06 | 3.59E-06 | 0.705 |
| RHSIM_Rhsim10G0202100 | 23.65 | 1.15E-06 | 3.59E-06 | -41.089 |
| novel.3042 | 23.64 | 1.16E-06 | 3.60E-06 | 0.452 |
| RHSIM_Rhsim01G0283600 | 23.64 | 1.16E-06 | 3.62E-06 | -0.363 |
| RHSIM_Rhsim13G0031900 | 23.63 | 1.17E-06 | 3.64E-06 | 0.782 |
| RHSIM_Rhsim07G0250300 | 23.63 | 1.17E-06 | 3.64E-06 | -1.075 |

| Gene | LRT statistic | P value | BH-adjusted P | log2FC (interaction) |
|---|---|---|---|---|
| RHSIM_Rhsim01G0046300 | 23.62 | 1.17E-06 | 3.64E-06 | 0.962 |
| novel.930 | 23.62 | 1.17E-06 | 3.64E-06 | -1.410 |
| novel.1355 | 23.61 | 1.18E-06 | 3.66E-06 | -42.909 |
| RHSIM_Rhsim13G0116200 | 23.60 | 1.18E-06 | 3.68E-06 | 4.104 |
| RHSIM_Rhsim11G0019700 | 23.59 | 1.19E-06 | 3.70E-06 | 1.788 |
| RHSIM_Rhsim07G0140300 | 23.58 | 1.20E-06 | 3.73E-06 | -43.277 |
| RHSIM_Rhsim04G0024700 | 23.57 | 1.20E-06 | 3.73E-06 | 0.446 |
| novel.667 | 23.57 | 1.21E-06 | 3.75E-06 | 2.943 |
| RHSIM_Rhsim01G0210300 | 23.57 | 1.21E-06 | 3.75E-06 | -2.872 |
| RHSIM_Rhsim09G0100900 | 23.56 | 1.21E-06 | 3.76E-06 | -43.457 |
| RHSIM_Rhsim11G0051000 | 23.54 | 1.22E-06 | 3.79E-06 | 1.428 |
| RHSIM_Rhsim06G0216600 | 23.54 | 1.22E-06 | 3.80E-06 | -2.832 |
| RHSIM_Rhsim01G0111600 | 23.54 | 1.22E-06 | 3.80E-06 | -4.379 |
| novel.1073 | 23.53 | 1.23E-06 | 3.81E-06 | -1.109 |
| RHSIM_Rhsim08G0050600 | 23.53 | 1.23E-06 | 3.81E-06 | -0.787 |
| RHSIM_Rhsim04G0055400 | 23.53 | 1.23E-06 | 3.81E-06 | -1.411 |
| RHSIM_Rhsim08G0232900 | 23.53 | 1.23E-06 | 3.81E-06 | 0.641 |
| novel.3505 | 23.53 | 1.23E-06 | 3.82E-06 | 0.469 |
| novel.1198 | 23.52 | 1.23E-06 | 3.83E-06 | -3.022 |
| RHSIM_Rhsim05G0067400 | 23.52 | 1.23E-06 | 3.83E-06 | -0.800 |
| RHSIM_Rhsim11G0096600 | 23.52 | 1.24E-06 | 3.83E-06 | -0.331 |
| RHSIM_Rhsim06G0100900 | 23.52 | 1.24E-06 | 3.83E-06 | 1.327 |
| RHSIM_Rhsim05G0204800 | 23.50 | 1.25E-06 | 3.88E-06 | 2.207 |
| RHSIM_Rhsim08G0138900 | 23.49 | 1.26E-06 | 3.90E-06 | 0.453 |
| RHSIM_Rhsim10G0200100 | 23.49 | 1.26E-06 | 3.90E-06 | -5.785 |
| RHSIM_Rhsim10G0102700 | 23.49 | 1.26E-06 | 3.90E-06 | 0.502 |
| RHSIM_Rhsim05G0189600 | 23.47 | 1.27E-06 | 3.93E-06 | 0.577 |
| RHSIM_Rhsim07G0111300 | 23.47 | 1.27E-06 | 3.93E-06 | 1.685 |
| RHSIM_Rhsim03G0036800 | 23.47 | 1.27E-06 | 3.94E-06 | 0.410 |
| RHSIM_Rhsim09G0030500 | 23.47 | 1.27E-06 | 3.94E-06 | 1.593 |
| RHSIM_Rhsim08G0252600 | 23.47 | 1.27E-06 | 3.94E-06 | 3.059 |
| RHSIM_Rhsim03G0206900 | 23.46 | 1.28E-06 | 3.95E-06 | 1.410 |

| Gene | LRT statistic | P value | BH-adjusted P | log2FC (interaction) |
|---|---|---|---|---|
| RHSIM_Rhsim12G0195100 | 23.46 | 1.28E-06 | 3.96E-06 | -0.635 |
| RHSIM_Rhsim10G0144900 | 23.44 | 1.29E-06 | 4.00E-06 | -0.399 |
| RHSIM_Rhsim10G0028800 | 23.44 | 1.29E-06 | 4.00E-06 | -1.532 |
| RHSIM_Rhsim06G0074800 | 23.43 | 1.29E-06 | 4.01E-06 | -0.520 |
| RHSIM_Rhsim08G0023300 | 23.43 | 1.30E-06 | 4.01E-06 | -0.709 |
| RHSIM_RhsimUnG0167900 | 23.42 | 1.30E-06 | 4.02E-06 | 0.994 |
| RHSIM_Rhsim11G0173300 | 23.41 | 1.31E-06 | 4.05E-06 | 0.436 |
| RHSIM_Rhsim01G0100300 | 23.40 | 1.31E-06 | 4.06E-06 | 2.899 |
| RHSIM_Rhsim04G0158400 | 23.40 | 1.32E-06 | 4.07E-06 | 0.491 |
| novel.1007 | 23.39 | 1.32E-06 | 4.09E-06 | 0.512 |
| RHSIM_Rhsim03G0005100 | 23.39 | 1.32E-06 | 4.09E-06 | -0.570 |
| RHSIM_Rhsim01G0078700 | 23.39 | 1.32E-06 | 4.09E-06 | -6.733 |
| novel.1926 | 23.39 | 1.32E-06 | 4.09E-06 | -1.745 |
| RHSIM_Rhsim01G0229500 | 23.38 | 1.33E-06 | 4.12E-06 | -0.609 |
| RHSIM_Rhsim02G0138300 | 23.37 | 1.34E-06 | 4.14E-06 | -4.383 |
| RHSIM_Rhsim11G0084300 | 23.36 | 1.34E-06 | 4.15E-06 | 0.559 |
| RHSIM_Rhsim12G0166200 | 23.36 | 1.35E-06 | 4.16E-06 | -0.878 |
| novel.2893 | 23.36 | 1.35E-06 | 4.16E-06 | -5.363 |
| RHSIM_RhsimUnG0149600 | 23.34 | 1.36E-06 | 4.19E-06 | -1.686 |
| novel.3252 | 23.34 | 1.36E-06 | 4.19E-06 | 3.534 |
| novel.4005 | 23.34 | 1.36E-06 | 4.19E-06 | -2.234 |
| RHSIM_Rhsim10G0154800 | 23.34 | 1.36E-06 | 4.20E-06 | -0.633 |
| RHSIM_Rhsim08G0224400 | 23.33 | 1.36E-06 | 4.21E-06 | 1.066 |
| RHSIM_Rhsim02G0201900 | 23.33 | 1.37E-06 | 4.22E-06 | -4.582 |
| RHSIM_Rhsim08G0103300 | 23.32 | 1.37E-06 | 4.23E-06 | -1.343 |
| RHSIM_Rhsim03G0096500 | 23.32 | 1.37E-06 | 4.23E-06 | -5.009 |
| RHSIM_Rhsim04G0205000 | 23.32 | 1.37E-06 | 4.23E-06 | 1.645 |
| RHSIM_Rhsim12G0067200 | 23.31 | 1.38E-06 | 4.25E-06 | 1.987 |
| novel.2767 | 23.31 | 1.38E-06 | 4.26E-06 | 3.573 |
| RHSIM_Rhsim09G0201000 | 23.30 | 1.38E-06 | 4.27E-06 | -0.321 |
| RHSIM_Rhsim04G0019000 | 23.30 | 1.39E-06 | 4.28E-06 | 1.342 |
| RHSIM_Rhsim06G0131100 | 23.30 | 1.39E-06 | 4.28E-06 | -5.732 |

| Gene | LRT statistic | P value | BH-adjusted P | log2FC (interaction) |
|---|---|---|---|---|
| RHSIM_Rhsim02G0235000 | 23.29 | 1.39E-06 | 4.30E-06 | 1.560 |
| RHSIM_Rhsim05G0073400 | 23.28 | 1.40E-06 | 4.32E-06 | -0.610 |
| RHSIM_Rhsim07G0121100 | 23.28 | 1.40E-06 | 4.33E-06 | 0.237 |
| RHSIM_Rhsim01G0253200 | 23.28 | 1.40E-06 | 4.33E-06 | -0.669 |
| RHSIM_Rhsim07G0236700 | 23.27 | 1.41E-06 | 4.35E-06 | -1.928 |
| RHSIM_Rhsim03G0050600 | 23.27 | 1.41E-06 | 4.35E-06 | 1.279 |
| RHSIM_Rhsim03G0143600 | 23.27 | 1.41E-06 | 4.35E-06 | -0.644 |
| RHSIM_Rhsim04G0055900 | 23.26 | 1.41E-06 | 4.36E-06 | 0.984 |
| RHSIM_Rhsim03G0211000 | 23.26 | 1.42E-06 | 4.37E-06 | 0.341 |
| RHSIM_Rhsim06G0223200 | 23.26 | 1.42E-06 | 4.37E-06 | 1.400 |
| RHSIM_Rhsim07G0008900 | 23.25 | 1.42E-06 | 4.38E-06 | -1.031 |
| RHSIM_Rhsim03G0115200 | 23.25 | 1.42E-06 | 4.39E-06 | 2.781 |
| RHSIM_Rhsim08G0113200 | 23.25 | 1.42E-06 | 4.39E-06 | 0.339 |
| RHSIM_Rhsim07G0038100 | 23.24 | 1.43E-06 | 4.40E-06 | -1.566 |
| RHSIM_Rhsim04G0005300 | 23.23 | 1.44E-06 | 4.42E-06 | -0.963 |
| RHSIM_Rhsim10G0112900 | 23.23 | 1.44E-06 | 4.44E-06 | 0.974 |
| RHSIM_Rhsim08G0125200 | 23.22 | 1.44E-06 | 4.45E-06 | -0.485 |
| RHSIM_Rhsim10G0012700 | 23.22 | 1.45E-06 | 4.46E-06 | -0.543 |
| RHSIM_Rhsim08G0241800 | 23.21 | 1.45E-06 | 4.47E-06 | -0.581 |
| novel.3090 | 23.21 | 1.45E-06 | 4.47E-06 | 1.180 |
| novel.319 | 23.21 | 1.45E-06 | 4.47E-06 | -0.846 |
| novel.49 | 23.21 | 1.45E-06 | 4.47E-06 | 2.598 |
| RHSIM_Rhsim07G0142400 | 23.20 | 1.46E-06 | 4.49E-06 | -0.410 |
| RHSIM_Rhsim13G0094700 | 23.19 | 1.47E-06 | 4.52E-06 | 0.262 |
| RHSIM_Rhsim01G0181700 | 23.17 | 1.48E-06 | 4.55E-06 | 0.487 |
| RHSIM_Rhsim12G0078200 | 23.17 | 1.48E-06 | 4.57E-06 | 1.683 |
| RHSIM_Rhsim13G0014300 | 23.17 | 1.49E-06 | 4.57E-06 | -0.527 |
| RHSIM_Rhsim03G0197100 | 23.16 | 1.49E-06 | 4.58E-06 | 1.579 |
| RHSIM_Rhsim02G0082200 | 23.15 | 1.50E-06 | 4.61E-06 | -0.575 |
| RHSIM_Rhsim05G0223700 | 23.14 | 1.51E-06 | 4.64E-06 | 1.131 |
| RHSIM_Rhsim05G0190000 | 23.14 | 1.51E-06 | 4.64E-06 | -3.090 |
| RHSIM_RhsimUnG0131200 | 23.14 | 1.51E-06 | 4.64E-06 | 1.228 |

| Gene | LRT statistic | P value | BH-adjusted P | log2FC (interaction) |
|---|---|---|---|---|
| RHSIM_Rhsim01G0256300 | 23.13 | 1.51E-06 | 4.66E-06 | 0.873 |
| RHSIM_Rhsim10G0034300 | 23.12 | 1.52E-06 | 4.69E-06 | -0.481 |
| novel.3221 | 23.10 | 1.54E-06 | 4.72E-06 | -2.181 |
| RHSIM_Rhsim04G0199200 | 23.10 | 1.54E-06 | 4.74E-06 | 1.086 |
| RHSIM_Rhsim13G0166200 | 23.09 | 1.55E-06 | 4.76E-06 | 4.530 |
| RHSIM_Rhsim09G0029600 | 23.07 | 1.56E-06 | 4.81E-06 | -0.435 |
| novel.1925 | 23.07 | 1.57E-06 | 4.81E-06 | -2.360 |
| RHSIM_Rhsim05G0137100 | 23.06 | 1.57E-06 | 4.82E-06 | -1.633 |
| RHSIM_Rhsim11G0191400 | 23.06 | 1.57E-06 | 4.82E-06 | -1.173 |
| RHSIM_RhsimUnG0095400 | 23.06 | 1.57E-06 | 4.82E-06 | -0.437 |
| RHSIM_Rhsim09G0042200 | 23.05 | 1.58E-06 | 4.85E-06 | 1.770 |
| RHSIM_RhsimUnG0062600 | 23.05 | 1.58E-06 | 4.85E-06 | -1.018 |
| RHSIM_Rhsim03G0048700 | 23.04 | 1.58E-06 | 4.86E-06 | -44.458 |
| RHSIM_Rhsim03G0027000 | 23.04 | 1.59E-06 | 4.88E-06 | 1.887 |
| novel.3235 | 23.04 | 1.59E-06 | 4.89E-06 | 1.760 |
| RHSIM_Rhsim01G0039600 | 23.03 | 1.60E-06 | 4.90E-06 | 2.756 |
| RHSIM_Rhsim08G0047400 | 23.02 | 1.60E-06 | 4.91E-06 | -0.905 |
| RHSIM_Rhsim01G0077600 | 23.02 | 1.60E-06 | 4.92E-06 | 0.397 |
| RHSIM_Rhsim05G0219800 | 23.01 | 1.61E-06 | 4.96E-06 | -0.967 |
| RHSIM_RhsimUnG0107700 | 23.01 | 1.61E-06 | 4.96E-06 | 0.907 |
| RHSIM_Rhsim06G0237700 | 22.99 | 1.63E-06 | 5.00E-06 | 0.935 |
| RHSIM_Rhsim06G0065300 | 22.99 | 1.63E-06 | 5.01E-06 | -1.383 |
| RHSIM_Rhsim07G0225500 | 22.98 | 1.64E-06 | 5.03E-06 | -0.601 |
| novel.1845 | 22.97 | 1.64E-06 | 5.04E-06 | 2.072 |
| RHSIM_Rhsim09G0165300 | 22.96 | 1.65E-06 | 5.06E-06 | 2.499 |
| RHSIM_Rhsim09G0091000 | 22.96 | 1.66E-06 | 5.08E-06 | 0.634 |
| RHSIM_Rhsim07G0063200 | 22.95 | 1.66E-06 | 5.10E-06 | 0.342 |
| RHSIM_Rhsim06G0116200 | 22.95 | 1.66E-06 | 5.10E-06 | 0.377 |
| RHSIM_Rhsim09G0214600 | 22.94 | 1.67E-06 | 5.12E-06 | -0.507 |
| RHSIM_Rhsim13G0085600 | 22.93 | 1.68E-06 | 5.16E-06 | 0.535 |
| novel.3601 | 22.92 | 1.69E-06 | 5.18E-06 | 1.884 |
| RHSIM_Rhsim05G0121500 | 22.91 | 1.69E-06 | 5.19E-06 | -0.851 |

| Gene | LRT statistic | P value | BH-adjusted P | log2FC (interaction) |
|---|---|---|---|---|
| RHSIM_Rhsim06G0048600 | 22.91 | 1.70E-06 | 5.20E-06 | -0.444 |
| RHSIM_Rhsim02G0087600 | 22.91 | 1.70E-06 | 5.21E-06 | 0.478 |
| novel.755 | 22.90 | 1.70E-06 | 5.22E-06 | 0.556 |
| RHSIM_Rhsim01G0143100 | 22.90 | 1.71E-06 | 5.24E-06 | -0.526 |
| RHSIM_Rhsim04G0231100 | 22.90 | 1.71E-06 | 5.24E-06 | 3.017 |
| RHSIM_Rhsim01G0238200 | 22.89 | 1.71E-06 | 5.25E-06 | -0.656 |
| RHSIM_Rhsim09G0134200 | 22.89 | 1.71E-06 | 5.25E-06 | 1.954 |
| novel.3331 | 22.89 | 1.72E-06 | 5.26E-06 | -0.688 |
| RHSIM_Rhsim06G0217900 | 22.88 | 1.72E-06 | 5.28E-06 | -3.909 |
| RHSIM_Rhsim08G0046100 | 22.88 | 1.73E-06 | 5.29E-06 | 0.826 |
| RHSIM_Rhsim04G0106700 | 22.87 | 1.73E-06 | 5.31E-06 | 0.578 |
| RHSIM_Rhsim06G0085000 | 22.87 | 1.74E-06 | 5.32E-06 | -0.596 |
| RHSIM_Rhsim09G0102100 | 22.86 | 1.74E-06 | 5.33E-06 | 0.437 |
| RHSIM_Rhsim07G0068000 | 22.85 | 1.75E-06 | 5.36E-06 | 0.407 |
| RHSIM_Rhsim07G0174000 | 22.85 | 1.75E-06 | 5.37E-06 | -0.690 |
| RHSIM_Rhsim04G0067900 | 22.84 | 1.76E-06 | 5.38E-06 | -0.934 |
| RHSIM_Rhsim13G0085200 | 22.84 | 1.76E-06 | 5.39E-06 | 0.473 |
| RHSIM_Rhsim07G0110400 | 22.84 | 1.76E-06 | 5.39E-06 | 0.552 |
| novel.2320 | 22.83 | 1.77E-06 | 5.43E-06 | 1.039 |
| RHSIM_Rhsim01G0269600 | 22.83 | 1.77E-06 | 5.43E-06 | 3.413 |
| RHSIM_Rhsim06G0224000 | 22.82 | 1.77E-06 | 5.43E-06 | 0.876 |
| RHSIM_Rhsim06G0073500 | 22.81 | 1.79E-06 | 5.48E-06 | 1.719 |
| RHSIM_Rhsim13G0170300 | 22.80 | 1.80E-06 | 5.50E-06 | 0.604 |
| RHSIM_Rhsim04G0021200 | 22.80 | 1.80E-06 | 5.50E-06 | -2.227 |
| RHSIM_Rhsim02G0054100 | 22.79 | 1.81E-06 | 5.52E-06 | -0.743 |
| RHSIM_Rhsim02G0216200 | 22.79 | 1.81E-06 | 5.52E-06 | 0.849 |
| RHSIM_Rhsim12G0122400 | 22.79 | 1.81E-06 | 5.53E-06 | 3.407 |
| novel.1527 | 22.79 | 1.81E-06 | 5.53E-06 | -2.373 |
| RHSIM_Rhsim01G0166200 | 22.78 | 1.82E-06 | 5.56E-06 | -2.609 |
| RHSIM_Rhsim12G0030800 | 22.78 | 1.82E-06 | 5.56E-06 | -2.928 |
| RHSIM_Rhsim12G0080800 | 22.77 | 1.82E-06 | 5.57E-06 | 0.825 |
| RHSIM_Rhsim02G0005800 | 22.77 | 1.83E-06 | 5.58E-06 | -0.367 |

| Gene | LRT statistic | P value | BH-adjusted P | log2FC (interaction) |
|---|---|---|---|---|
| RHSIM_Rhsim10G0161200 | 22.75 | 1.85E-06 | 5.65E-06 | -0.818 |
| RHSIM_Rhsim06G0136100 | 22.74 | 1.86E-06 | 5.67E-06 | -0.430 |
| RHSIM_Rhsim08G0129900 | 22.73 | 1.86E-06 | 5.69E-06 | 1.420 |
| novel.3773 | 22.73 | 1.87E-06 | 5.71E-06 | 2.594 |
| RHSIM_Rhsim10G0207600 | 22.72 | 1.87E-06 | 5.72E-06 | -1.248 |
| RHSIM_RhsimUnG0206100 | 22.72 | 1.87E-06 | 5.72E-06 | 0.673 |
| RHSIM_Rhsim03G0259500 | 22.70 | 1.89E-06 | 5.78E-06 | -0.902 |
| RHSIM_Rhsim03G0114800 | 22.70 | 1.89E-06 | 5.78E-06 | -0.271 |
| RHSIM_Rhsim01G0110100 | 22.69 | 1.90E-06 | 5.80E-06 | 1.424 |
| RHSIM_Rhsim10G0034000 | 22.69 | 1.90E-06 | 5.81E-06 | 2.138 |
| novel.1318 | 22.69 | 1.91E-06 | 5.82E-06 | 0.496 |
| RHSIM_Rhsim06G0192100 | 22.68 | 1.91E-06 | 5.82E-06 | -1.137 |
| RHSIM_RhsimUnG0124200 | 22.68 | 1.91E-06 | 5.84E-06 | 0.814 |
| novel.2689 | 22.68 | 1.92E-06 | 5.85E-06 | -1.493 |
| RHSIM_RhsimUnG0108800 | 22.68 | 1.92E-06 | 5.85E-06 | 1.274 |
| RHSIM_Rhsim13G0013200 | 22.67 | 1.92E-06 | 5.87E-06 | 1.567 |
| RHSIM_Rhsim09G0099000 | 22.67 | 1.93E-06 | 5.88E-06 | 0.855 |
| RHSIM_Rhsim03G0179800 | 22.66 | 1.93E-06 | 5.89E-06 | -0.865 |
| RHSIM_Rhsim13G0049400 | 22.65 | 1.94E-06 | 5.92E-06 | -0.350 |
| RHSIM_Rhsim08G0099800 | 22.64 | 1.95E-06 | 5.95E-06 | 0.489 |
| RHSIM_Rhsim02G0139000 | 22.64 | 1.95E-06 | 5.95E-06 | 0.912 |
| RHSIM_Rhsim13G0229400 | 22.64 | 1.96E-06 | 5.96E-06 | -1.641 |
| RHSIM_Rhsim13G0227300 | 22.63 | 1.96E-06 | 5.99E-06 | -0.346 |
| novel.140 | 22.63 | 1.97E-06 | 6.00E-06 | -1.847 |
| novel.195 | 22.62 | 1.97E-06 | 6.01E-06 | 2.392 |
| RHSIM_Rhsim13G0120100 | 22.62 | 1.98E-06 | 6.02E-06 | 0.380 |
| RHSIM_Rhsim01G0179300 | 22.62 | 1.98E-06 | 6.02E-06 | 1.350 |
| RHSIM_Rhsim11G0100300 | 22.61 | 1.98E-06 | 6.04E-06 | -0.266 |
| novel.2766 | 22.61 | 1.98E-06 | 6.04E-06 | 1.912 |
| RHSIM_Rhsim02G0212700 | 22.60 | 1.99E-06 | 6.06E-06 | 0.874 |
| RHSIM_Rhsim02G0241400 | 22.60 | 1.99E-06 | 6.07E-06 | -0.831 |
| RHSIM_Rhsim06G0040400 | 22.60 | 2.00E-06 | 6.08E-06 | 3.291 |

| Gene | LRT statistic | P value | BH-adjusted P | log2FC (interaction) |
|---|---|---|---|---|
| RHSIM_Rhsim01G0063800 | 22.59 | 2.00E-06 | 6.09E-06 | 0.807 |
| RHSIM_Rhsim01G0086600 | 22.58 | 2.01E-06 | 6.13E-06 | -0.541 |
| novel.4063 | 22.58 | 2.01E-06 | 6.13E-06 | 82.962 |
| RHSIM_Rhsim01G0152600 | 22.57 | 2.02E-06 | 6.15E-06 | 0.410 |
| RHSIM_Rhsim13G0120200 | 22.57 | 2.03E-06 | 6.17E-06 | 0.594 |
| RHSIM_Rhsim12G0025800 | 22.56 | 2.03E-06 | 6.19E-06 | 0.781 |
| RHSIM_Rhsim08G0004400 | 22.55 | 2.04E-06 | 6.21E-06 | 0.610 |
| RHSIM_Rhsim08G0140300 | 22.54 | 2.06E-06 | 6.26E-06 | -42.517 |
| RHSIM_Rhsim07G0013300 | 22.54 | 2.06E-06 | 6.27E-06 | -0.459 |
| RHSIM_Rhsim08G0014400 | 22.54 | 2.06E-06 | 6.27E-06 | -1.495 |
| RHSIM_Rhsim09G0041100 | 22.53 | 2.07E-06 | 6.28E-06 | -2.059 |
| RHSIM_Rhsim12G0018700 | 22.52 | 2.08E-06 | 6.32E-06 | -3.734 |
| RHSIM_Rhsim03G0259700 | 22.52 | 2.08E-06 | 6.32E-06 | 2.723 |
| RHSIM_Rhsim10G0007900 | 22.52 | 2.08E-06 | 6.32E-06 | 0.478 |
| RHSIM_Rhsim01G0145100 | 22.51 | 2.09E-06 | 6.34E-06 | 3.144 |
| RHSIM_Rhsim10G0200900 | 22.51 | 2.09E-06 | 6.34E-06 | -2.103 |
| RHSIM_Rhsim08G0037500 | 22.51 | 2.09E-06 | 6.35E-06 | -0.682 |
| RHSIM_Rhsim09G0116600 | 22.51 | 2.09E-06 | 6.36E-06 | 1.262 |
| RHSIM_Rhsim11G0158100 | 22.50 | 2.10E-06 | 6.37E-06 | -1.477 |
| RHSIM_Rhsim06G0124200 | 22.50 | 2.11E-06 | 6.40E-06 | 0.338 |
| RHSIM_RhsimUnG0079800 | 22.49 | 2.11E-06 | 6.40E-06 | 0.718 |
| RHSIM_Rhsim13G0224000 | 22.49 | 2.11E-06 | 6.40E-06 | 0.250 |
| RHSIM_Rhsim07G0117900 | 22.48 | 2.12E-06 | 6.45E-06 | -0.471 |
| RHSIM_Rhsim09G0165100 | 22.47 | 2.13E-06 | 6.47E-06 | 0.658 |
| RHSIM_Rhsim12G0112400 | 22.47 | 2.13E-06 | 6.47E-06 | 0.443 |
| RHSIM_Rhsim07G0095400 | 22.47 | 2.13E-06 | 6.48E-06 | -1.744 |
| novel.3664 | 22.47 | 2.13E-06 | 6.48E-06 | 2.174 |
| RHSIM_Rhsim02G0091500 | 22.46 | 2.14E-06 | 6.51E-06 | 0.501 |
| novel.1463 | 22.45 | 2.15E-06 | 6.54E-06 | 43.197 |
| RHSIM_Rhsim10G0100400 | 22.45 | 2.16E-06 | 6.55E-06 | 1.507 |
| RHSIM_Rhsim11G0026600 | 22.45 | 2.16E-06 | 6.55E-06 | -0.711 |
| RHSIM_Rhsim10G0001400 | 22.44 | 2.17E-06 | 6.59E-06 | 0.346 |

| Gene | LRT statistic | P value | BH-adjusted P | log2FC (interaction) |
|---|---|---|---|---|
| RHSIM_Rhsim05G0115700 | 22.43 | 2.18E-06 | 6.60E-06 | 0.601 |
| RHSIM_Rhsim13G0157300 | 22.43 | 2.18E-06 | 6.61E-06 | 1.811 |
| RHSIM_Rhsim07G0050200 | 22.43 | 2.18E-06 | 6.62E-06 | 2.563 |
| RHSIM_Rhsim08G0028500 | 22.42 | 2.19E-06 | 6.64E-06 | 6.032 |
| RHSIM_Rhsim11G0098000 | 22.42 | 2.19E-06 | 6.64E-06 | 2.139 |
| RHSIM_Rhsim04G0247700 | 22.42 | 2.20E-06 | 6.66E-06 | -0.563 |
| RHSIM_Rhsim10G0115900 | 22.40 | 2.21E-06 | 6.70E-06 | 0.558 |
| RHSIM_Rhsim13G0202000 | 22.40 | 2.21E-06 | 6.70E-06 | -0.376 |
| RHSIM_Rhsim07G0254700 | 22.40 | 2.21E-06 | 6.70E-06 | -0.578 |
| RHSIM_Rhsim03G0143800 | 22.39 | 2.22E-06 | 6.74E-06 | -0.686 |
| novel.1947 | 22.39 | 2.23E-06 | 6.75E-06 | -43.690 |
| RHSIM_Rhsim11G0055200 | 22.39 | 2.23E-06 | 6.76E-06 | 0.989 |
| RHSIM_Rhsim07G0143600 | 22.38 | 2.24E-06 | 6.78E-06 | 0.614 |
| RHSIM_Rhsim13G0171300 | 22.36 | 2.26E-06 | 6.84E-06 | -1.176 |
| RHSIM_Rhsim07G0038300 | 22.34 | 2.28E-06 | 6.91E-06 | -0.375 |
| RHSIM_Rhsim01G0095300 | 22.34 | 2.28E-06 | 6.92E-06 | -0.738 |
| RHSIM_Rhsim05G0030100 | 22.34 | 2.29E-06 | 6.93E-06 | -0.595 |
| RHSIM_Rhsim11G0013300 | 22.31 | 2.31E-06 | 7.01E-06 | 0.478 |
| RHSIM_Rhsim13G0173700 | 22.31 | 2.32E-06 | 7.02E-06 | 6.342 |
| RHSIM_Rhsim13G0221100 | 22.30 | 2.33E-06 | 7.05E-06 | 1.612 |
| RHSIM_Rhsim10G0190000 | 22.30 | 2.34E-06 | 7.07E-06 | 2.398 |
| RHSIM_Rhsim13G0096400 | 22.28 | 2.35E-06 | 7.13E-06 | 0.545 |
| RHSIM_Rhsim03G0204100 | 22.27 | 2.37E-06 | 7.17E-06 | 7.455 |
| RHSIM_Rhsim05G0100400 | 22.27 | 2.37E-06 | 7.17E-06 | 0.731 |
| novel.3973 | 22.27 | 2.37E-06 | 7.18E-06 | 1.782 |
| RHSIM_Rhsim02G0161700 | 22.26 | 2.38E-06 | 7.19E-06 | -1.548 |
| RHSIM_Rhsim13G0150900 | 22.26 | 2.38E-06 | 7.21E-06 | 2.179 |
| RHSIM_Rhsim07G0212100 | 22.25 | 2.40E-06 | 7.25E-06 | 0.660 |
| RHSIM_Rhsim01G0141400 | 22.24 | 2.40E-06 | 7.27E-06 | 0.284 |
| RHSIM_Rhsim07G0083400 | 22.24 | 2.41E-06 | 7.30E-06 | 1.590 |
| RHSIM_Rhsim05G0137900 | 22.22 | 2.43E-06 | 7.34E-06 | 2.086 |
| RHSIM_Rhsim03G0253600 | 22.22 | 2.43E-06 | 7.34E-06 | 0.679 |

| Gene | LRT statistic | P value | BH-adjusted P | log2FC (interaction) |
|---|---|---|---|---|
| RHSIM_Rhsim13G0132200 | 22.22 | 2.43E-06 | 7.35E-06 | 0.498 |
| RHSIM_Rhsim08G0083500 | 22.22 | 2.44E-06 | 7.37E-06 | 0.691 |
| RHSIM_Rhsim08G0034000 | 22.21 | 2.44E-06 | 7.37E-06 | 0.509 |
| RHSIM_Rhsim01G0240900 | 22.21 | 2.44E-06 | 7.38E-06 | -1.198 |
| RHSIM_Rhsim13G0178500 | 22.21 | 2.44E-06 | 7.38E-06 | -0.481 |
| RHSIM_Rhsim13G0231300 | 22.20 | 2.46E-06 | 7.42E-06 | 2.189 |
| RHSIM_Rhsim06G0062500 | 22.20 | 2.46E-06 | 7.43E-06 | 0.983 |
| RHSIM_Rhsim02G0188000 | 22.19 | 2.47E-06 | 7.45E-06 | 43.216 |
| RHSIM_Rhsim05G0070000 | 22.19 | 2.47E-06 | 7.46E-06 | 0.254 |
| novel.4066 | 22.19 | 2.48E-06 | 7.48E-06 | -1.862 |
| RHSIM_Rhsim11G0142300 | 22.18 | 2.48E-06 | 7.48E-06 | 3.824 |
| RHSIM_Rhsim11G0144200 | 22.18 | 2.48E-06 | 7.50E-06 | -0.654 |
| novel.4532 | 22.17 | 2.49E-06 | 7.52E-06 | 4.978 |
| RHSIM_Rhsim13G0181500 | 22.17 | 2.50E-06 | 7.54E-06 | -0.861 |
| RHSIM_Rhsim01G0195900 | 22.16 | 2.50E-06 | 7.56E-06 | -0.420 |
| RHSIM_Rhsim11G0053000 | 22.16 | 2.50E-06 | 7.56E-06 | -0.472 |
| RHSIM_Rhsim01G0076800 | 22.16 | 2.51E-06 | 7.59E-06 | -0.817 |
| RHSIM_Rhsim01G0274100 | 22.16 | 2.51E-06 | 7.59E-06 | -0.396 |
| novel.681 | 22.14 | 2.53E-06 | 7.63E-06 | 1.262 |
| RHSIM_Rhsim03G0226400 | 22.14 | 2.53E-06 | 7.64E-06 | -1.059 |
| RHSIM_Rhsim05G0058800 | 22.14 | 2.53E-06 | 7.64E-06 | -0.792 |
| novel.2906 | 22.14 | 2.53E-06 | 7.65E-06 | 2.847 |
| novel.2066 | 22.14 | 2.53E-06 | 7.65E-06 | 2.953 |
| novel.1945 | 22.13 | 2.55E-06 | 7.68E-06 | -41.809 |
| RHSIM_Rhsim13G0232000 | 22.12 | 2.56E-06 | 7.72E-06 | -0.867 |
| RHSIM_RhsimUnG0188700 | 22.12 | 2.56E-06 | 7.72E-06 | -0.838 |
| RHSIM_Rhsim13G0081800 | 22.12 | 2.56E-06 | 7.73E-06 | 2.613 |
| RHSIM_Rhsim04G0022400 | 22.12 | 2.57E-06 | 7.74E-06 | -1.515 |
| RHSIM_Rhsim07G0015400 | 22.11 | 2.57E-06 | 7.75E-06 | 2.548 |
| RHSIM_Rhsim01G0280200 | 22.11 | 2.58E-06 | 7.76E-06 | -0.297 |
| RHSIM_Rhsim02G0231500 | 22.11 | 2.58E-06 | 7.78E-06 | -0.536 |
| RHSIM_RhsimUnG0238300 | 22.10 | 2.58E-06 | 7.78E-06 | 1.406 |

| Gene | LRT statistic | P value | BH-adjusted P | log2FC (interaction) |
|---|---|---|---|---|
| RHSIM_Rhsim10G0072000 | 22.10 | 2.59E-06 | 7.82E-06 | 1.644 |
| RHSIM_Rhsim01G0221200 | 22.10 | 2.59E-06 | 7.82E-06 | -1.197 |
| RHSIM_Rhsim04G0072400 | 22.09 | 2.60E-06 | 7.82E-06 | 0.456 |
| novel.1090 | 22.08 | 2.61E-06 | 7.87E-06 | 0.371 |
| RHSIM_Rhsim05G0017100 | 22.08 | 2.62E-06 | 7.88E-06 | 0.568 |
| RHSIM_Rhsim05G0105600 | 22.08 | 2.62E-06 | 7.89E-06 | -2.105 |
| RHSIM_Rhsim08G0067000 | 22.08 | 2.62E-06 | 7.89E-06 | 2.697 |
| RHSIM_RhsimUnG0172300 | 22.07 | 2.63E-06 | 7.91E-06 | -0.785 |
| RHSIM_Rhsim10G0075400 | 22.07 | 2.63E-06 | 7.92E-06 | 0.920 |
| RHSIM_Rhsim02G0031300 | 22.07 | 2.63E-06 | 7.92E-06 | -0.500 |
| RHSIM_RhsimUnG0067600 | 22.07 | 2.63E-06 | 7.93E-06 | 1.775 |
| RHSIM_Rhsim01G0033000 | 22.06 | 2.64E-06 | 7.96E-06 | -0.840 |
| RHSIM_Rhsim08G0060500 | 22.05 | 2.65E-06 | 7.98E-06 | -41.864 |
| RHSIM_Rhsim08G0210000 | 22.05 | 2.66E-06 | 8.01E-06 | -0.698 |
| RHSIM_Rhsim13G0184900 | 22.04 | 2.67E-06 | 8.03E-06 | -1.281 |
| RHSIM_Rhsim13G0018900 | 22.04 | 2.67E-06 | 8.04E-06 | 0.575 |
| RHSIM_Rhsim04G0157700 | 22.04 | 2.67E-06 | 8.05E-06 | -0.987 |
| RHSIM_Rhsim06G0029900 | 22.02 | 2.70E-06 | 8.11E-06 | -5.313 |
| RHSIM_Rhsim04G0001800 | 22.02 | 2.70E-06 | 8.14E-06 | 0.639 |
| RHSIM_Rhsim06G0102100 | 22.01 | 2.71E-06 | 8.15E-06 | 0.277 |
| RHSIM_Rhsim02G0070400 | 22.00 | 2.73E-06 | 8.20E-06 | -2.310 |
| RHSIM_Rhsim13G0098500 | 22.00 | 2.73E-06 | 8.20E-06 | 1.281 |
| RHSIM_Rhsim03G0162800 | 21.99 | 2.74E-06 | 8.24E-06 | 0.551 |
| RHSIM_Rhsim05G0028200 | 21.99 | 2.74E-06 | 8.25E-06 | 1.270 |
| RHSIM_Rhsim03G0212900 | 21.98 | 2.76E-06 | 8.28E-06 | -0.565 |
| RHSIM_Rhsim04G0221700 | 21.98 | 2.76E-06 | 8.29E-06 | -2.858 |
| RHSIM_Rhsim10G0174300 | 21.97 | 2.76E-06 | 8.30E-06 | -3.518 |
| RHSIM_RhsimUnG0249100 | 21.97 | 2.76E-06 | 8.31E-06 | 0.319 |
| RHSIM_Rhsim06G0165900 | 21.96 | 2.78E-06 | 8.35E-06 | -0.354 |
| novel.4114 | 21.96 | 2.78E-06 | 8.36E-06 | 1.310 |
| RHSIM_Rhsim05G0078000 | 21.96 | 2.78E-06 | 8.36E-06 | 0.594 |
| novel.405 | 21.96 | 2.79E-06 | 8.37E-06 | -0.570 |

| Gene | LRT statistic | P value | BH-adjusted P | log2FC (interaction) |
|---|---|---|---|---|
| RHSIM_Rhsim01G0109700 | 21.96 | 2.79E-06 | 8.37E-06 | -3.091 |
| RHSIM_Rhsim03G0235500 | 21.95 | 2.80E-06 | 8.40E-06 | 0.337 |
| RHSIM_Rhsim08G0213900 | 21.95 | 2.80E-06 | 8.42E-06 | 0.567 |
| RHSIM_Rhsim03G0119400 | 21.94 | 2.81E-06 | 8.43E-06 | -0.393 |
| RHSIM_RhsimUnG0012600 | 21.94 | 2.82E-06 | 8.46E-06 | 2.064 |
| RHSIM_Rhsim08G0205400 | 21.94 | 2.82E-06 | 8.46E-06 | 0.355 |
| RHSIM_Rhsim06G0088100 | 21.94 | 2.82E-06 | 8.46E-06 | -0.399 |
| RHSIM_Rhsim12G0152500 | 21.93 | 2.82E-06 | 8.47E-06 | -0.881 |
| RHSIM_Rhsim03G0217700 | 21.93 | 2.82E-06 | 8.48E-06 | 0.563 |
| RHSIM_Rhsim02G0146000 | 21.93 | 2.83E-06 | 8.50E-06 | 0.841 |
| RHSIM_Rhsim13G0062700 | 21.92 | 2.84E-06 | 8.52E-06 | 0.379 |
| RHSIM_Rhsim05G0180000 | 21.92 | 2.85E-06 | 8.54E-06 | 1.144 |
| RHSIM_Rhsim13G0233200 | 21.91 | 2.86E-06 | 8.58E-06 | -1.717 |
| RHSIM_Rhsim08G0074900 | 21.91 | 2.86E-06 | 8.59E-06 | -0.827 |
| RHSIM_Rhsim13G0055800 | 21.90 | 2.87E-06 | 8.62E-06 | 0.645 |
| RHSIM_Rhsim13G0053800 | 21.88 | 2.90E-06 | 8.69E-06 | -0.979 |
| RHSIM_Rhsim01G0197500 | 21.88 | 2.90E-06 | 8.70E-06 | 0.423 |
| RHSIM_Rhsim13G0096100 | 21.88 | 2.90E-06 | 8.70E-06 | -0.607 |
| RHSIM_Rhsim09G0051500 | 21.88 | 2.91E-06 | 8.71E-06 | 0.610 |
| novel.1193 | 21.87 | 2.92E-06 | 8.75E-06 | -2.267 |
| RHSIM_RhsimUnG0027200 | 21.87 | 2.92E-06 | 8.75E-06 | 0.670 |
| RHSIM_Rhsim13G0082000 | 21.86 | 2.93E-06 | 8.78E-06 | 2.593 |
| RHSIM_Rhsim08G0204000 | 21.86 | 2.93E-06 | 8.78E-06 | 1.715 |
| RHSIM_Rhsim08G0154600 | 21.86 | 2.93E-06 | 8.78E-06 | 1.367 |
| RHSIM_Rhsim02G0183900 | 21.86 | 2.94E-06 | 8.81E-06 | -6.751 |
| RHSIM_Rhsim10G0140300 | 21.85 | 2.94E-06 | 8.82E-06 | 1.352 |
| novel.978 | 21.85 | 2.94E-06 | 8.82E-06 | -6.610 |
| RHSIM_Rhsim07G0168000 | 21.85 | 2.94E-06 | 8.82E-06 | -0.688 |
| RHSIM_Rhsim09G0164900 | 21.85 | 2.94E-06 | 8.82E-06 | -0.693 |
| RHSIM_Rhsim04G0171100 | 21.85 | 2.95E-06 | 8.82E-06 | 0.946 |
| RHSIM_Rhsim06G0135800 | 21.85 | 2.95E-06 | 8.84E-06 | -0.753 |
| RHSIM_Rhsim01G0134900 | 21.84 | 2.97E-06 | 8.88E-06 | -0.631 |

| Gene | LRT statistic | P value | BH-adjusted P | log2FC (interaction) |
|---|---|---|---|---|
| RHSIM_Rhsim08G0128500 | 21.83 | 2.98E-06 | 8.92E-06 | 10.827 |
| RHSIM_Rhsim11G0181300 | 21.83 | 2.98E-06 | 8.92E-06 | -0.660 |
| RHSIM_Rhsim04G0155800 | 21.83 | 2.98E-06 | 8.92E-06 | -0.740 |
| RHSIM_Rhsim07G0240400 | 21.82 | 2.99E-06 | 8.96E-06 | -0.646 |
| RHSIM_Rhsim01G0100500 | 21.82 | 3.00E-06 | 8.96E-06 | -1.325 |
| RHSIM_Rhsim13G0196800 | 21.82 | 3.00E-06 | 8.97E-06 | -0.400 |
| RHSIM_Rhsim01G0201600 | 21.82 | 3.00E-06 | 8.98E-06 | 0.457 |
| RHSIM_Rhsim09G0045900 | 21.81 | 3.01E-06 | 8.99E-06 | -43.017 |
| RHSIM_Rhsim05G0020400 | 21.81 | 3.01E-06 | 9.01E-06 | 0.385 |
| RHSIM_Rhsim07G0133500 | 21.81 | 3.02E-06 | 9.02E-06 | 4.007 |
| novel.69 | 21.80 | 3.03E-06 | 9.05E-06 | -4.531 |
| RHSIM_Rhsim06G0228800 | 21.80 | 3.03E-06 | 9.05E-06 | -2.428 |
| RHSIM_Rhsim10G0064200 | 21.79 | 3.04E-06 | 9.08E-06 | -0.723 |
| RHSIM_Rhsim10G0012800 | 21.79 | 3.04E-06 | 9.08E-06 | 0.551 |
| RHSIM_Rhsim02G0151800 | 21.79 | 3.04E-06 | 9.09E-06 | 0.377 |
| RHSIM_Rhsim06G0170200 | 21.78 | 3.06E-06 | 9.15E-06 | -0.790 |
| RHSIM_Rhsim12G0002000 | 21.77 | 3.07E-06 | 9.18E-06 | 1.363 |
| RHSIM_Rhsim07G0223700 | 21.77 | 3.08E-06 | 9.20E-06 | -3.375 |
| RHSIM_Rhsim13G0033900 | 21.76 | 3.09E-06 | 9.24E-06 | 5.101 |
| RHSIM_Rhsim11G0129800 | 21.75 | 3.10E-06 | 9.27E-06 | 0.336 |
| RHSIM_Rhsim07G0073400 | 21.75 | 3.11E-06 | 9.29E-06 | 1.093 |
| RHSIM_Rhsim02G0097500 | 21.75 | 3.11E-06 | 9.30E-06 | -1.520 |
| RHSIM_Rhsim02G0097000 | 21.74 | 3.11E-06 | 9.30E-06 | -0.834 |
| RHSIM_Rhsim08G0045000 | 21.74 | 3.13E-06 | 9.34E-06 | 0.644 |
| RHSIM_Rhsim03G0110900 | 21.72 | 3.16E-06 | 9.42E-06 | -0.783 |
| RHSIM_Rhsim04G0017800 | 21.72 | 3.16E-06 | 9.43E-06 | -1.387 |
| RHSIM_Rhsim13G0103500 | 21.72 | 3.16E-06 | 9.43E-06 | 0.433 |
| RHSIM_Rhsim13G0228400 | 21.71 | 3.17E-06 | 9.45E-06 | -0.261 |
| RHSIM_Rhsim03G0169200 | 21.70 | 3.19E-06 | 9.51E-06 | 0.504 |
| RHSIM_Rhsim12G0211800 | 21.70 | 3.19E-06 | 9.52E-06 | -0.955 |
| RHSIM_Rhsim13G0231000 | 21.69 | 3.20E-06 | 9.55E-06 | -0.525 |
| RHSIM_Rhsim06G0000100 | 21.69 | 3.21E-06 | 9.58E-06 | -0.532 |

| Gene | LRT statistic | P value | BH-adjusted P | log2FC (interaction) |
|---|---|---|---|---|
| RHSIM_Rhsim03G0191900 | 21.68 | 3.22E-06 | 9.60E-06 | 0.529 |
| RHSIM_Rhsim03G0142900 | 21.68 | 3.23E-06 | 9.62E-06 | 0.660 |
| RHSIM_Rhsim05G0142300 | 21.67 | 3.24E-06 | 9.66E-06 | -1.126 |
| RHSIM_Rhsim01G0253800 | 21.67 | 3.24E-06 | 9.66E-06 | 5.369 |
| RHSIM_Rhsim12G0105900 | 21.66 | 3.25E-06 | 9.69E-06 | 4.698 |
| RHSIM_Rhsim05G0212700 | 21.66 | 3.25E-06 | 9.71E-06 | -0.817 |
| RHSIM_Rhsim05G0089200 | 21.64 | 3.29E-06 | 9.81E-06 | -1.362 |
| RHSIM_Rhsim03G0205800 | 21.64 | 3.29E-06 | 9.82E-06 | 2.383 |
| RHSIM_Rhsim04G0185200 | 21.63 | 3.30E-06 | 9.84E-06 | -1.094 |
| RHSIM_Rhsim04G0113600 | 21.62 | 3.32E-06 | 9.90E-06 | -0.973 |
| RHSIM_Rhsim13G0209100 | 21.61 | 3.34E-06 | 9.94E-06 | 0.519 |
| novel.2234 | 21.61 | 3.34E-06 | 9.96E-06 | 3.803 |
| RHSIM_RhsimUnG0159900 | 21.61 | 3.34E-06 | 9.96E-06 | 0.832 |
| RHSIM_Rhsim01G0146900 | 21.61 | 3.35E-06 | 9.98E-06 | 0.502 |
| RHSIM_Rhsim09G0168700 | 21.60 | 3.35E-06 | 9.99E-06 | 0.394 |
| RHSIM_Rhsim04G0119500 | 21.59 | 3.38E-06 | 1.01E-05 | 1.257 |
| RHSIM_Rhsim09G0124000 | 21.57 | 3.41E-06 | 1.01E-05 | -1.308 |
| RHSIM_Rhsim08G0197900 | 21.57 | 3.41E-06 | 1.01E-05 | 6.081 |
| RHSIM_Rhsim04G0173400 | 21.56 | 3.42E-06 | 1.02E-05 | -1.248 |
| novel.1456 | 21.56 | 3.42E-06 | 1.02E-05 | 1.394 |
| RHSIM_Rhsim06G0061000 | 21.56 | 3.43E-06 | 1.02E-05 | 1.222 |
| RHSIM_Rhsim04G0227800 | 21.56 | 3.43E-06 | 1.02E-05 | -1.474 |
| RHSIM_Rhsim09G0067800 | 21.56 | 3.43E-06 | 1.02E-05 | 3.065 |
| RHSIM_Rhsim04G0007600 | 21.56 | 3.43E-06 | 1.02E-05 | 0.290 |
| RHSIM_Rhsim04G0180200 | 21.55 | 3.44E-06 | 1.02E-05 | -2.026 |
| novel.974 | 21.55 | 3.44E-06 | 1.03E-05 | 1.022 |
| RHSIM_Rhsim07G0212300 | 21.55 | 3.45E-06 | 1.03E-05 | -0.679 |
| RHSIM_Rhsim13G0131400 | 21.54 | 3.46E-06 | 1.03E-05 | 4.170 |
| RHSIM_Rhsim08G0174400 | 21.53 | 3.48E-06 | 1.03E-05 | -0.427 |
| RHSIM_RhsimUnG0054300 | 21.53 | 3.49E-06 | 1.04E-05 | 1.542 |
| RHSIM_Rhsim11G0177200 | 21.51 | 3.52E-06 | 1.05E-05 | -0.353 |
| RHSIM_Rhsim10G0007300 | 21.51 | 3.53E-06 | 1.05E-05 | -1.221 |

| Gene | LRT statistic | P value | BH-adjusted P | log2FC (interaction) |
|---|---|---|---|---|
| RHSIM_RhsimUnG0107500 | 21.50 | 3.53E-06 | 1.05E-05 | -0.553 |
| RHSIM_Rhsim02G0028900 | 21.50 | 3.54E-06 | 1.05E-05 | 45.715 |
| RHSIM_Rhsim01G0141900 | 21.49 | 3.56E-06 | 1.06E-05 | 0.680 |
| RHSIM_Rhsim04G0237100 | 21.49 | 3.56E-06 | 1.06E-05 | -1.832 |
| novel.2925 | 21.49 | 3.56E-06 | 1.06E-05 | 2.582 |
| RHSIM_RhsimUnG0102800 | 21.47 | 3.59E-06 | 1.07E-05 | -0.636 |
| novel.2285 | 21.47 | 3.59E-06 | 1.07E-05 | -0.502 |
| RHSIM_Rhsim11G0190300 | 21.47 | 3.60E-06 | 1.07E-05 | 0.263 |
| RHSIM_Rhsim11G0108100 | 21.46 | 3.61E-06 | 1.07E-05 | -0.994 |
| novel.2971 | 21.46 | 3.61E-06 | 1.07E-05 | -0.679 |
| RHSIM_Rhsim08G0164300 | 21.46 | 3.62E-06 | 1.07E-05 | 1.301 |
| RHSIM_Rhsim10G0013400 | 21.46 | 3.62E-06 | 1.08E-05 | 1.185 |
| RHSIM_RhsimUnG0180800 | 21.45 | 3.62E-06 | 1.08E-05 | -0.447 |
| RHSIM_Rhsim10G0148600 | 21.45 | 3.63E-06 | 1.08E-05 | 1.459 |
| RHSIM_Rhsim01G0207200 | 21.45 | 3.64E-06 | 1.08E-05 | 0.945 |
| RHSIM_Rhsim10G0123800 | 21.44 | 3.64E-06 | 1.08E-05 | 0.752 |
| RHSIM_Rhsim02G0155000 | 21.44 | 3.65E-06 | 1.08E-05 | 0.677 |
| RHSIM_Rhsim04G0117800 | 21.44 | 3.65E-06 | 1.08E-05 | -1.339 |
| RHSIM_Rhsim06G0092800 | 21.44 | 3.65E-06 | 1.08E-05 | -3.485 |
| RHSIM_Rhsim09G0130900 | 21.44 | 3.66E-06 | 1.09E-05 | 1.383 |
| novel.4049 | 21.43 | 3.67E-06 | 1.09E-05 | 0.457 |
| RHSIM_Rhsim02G0195600 | 21.43 | 3.67E-06 | 1.09E-05 | -1.564 |
| RHSIM_Rhsim03G0254000 | 21.42 | 3.69E-06 | 1.10E-05 | -0.489 |
| RHSIM_Rhsim05G0067900 | 21.42 | 3.70E-06 | 1.10E-05 | 0.572 |
| RHSIM_Rhsim10G0054200 | 21.41 | 3.70E-06 | 1.10E-05 | 1.095 |
| RHSIM_Rhsim01G0131000 | 21.41 | 3.71E-06 | 1.10E-05 | -0.787 |
| RHSIM_Rhsim10G0099100 | 21.41 | 3.71E-06 | 1.10E-05 | 0.481 |
| RHSIM_Rhsim10G0018100 | 21.39 | 3.74E-06 | 1.11E-05 | 0.524 |
| RHSIM_Rhsim07G0117500 | 21.39 | 3.75E-06 | 1.11E-05 | 0.988 |
| RHSIM_Rhsim03G0231400 | 21.39 | 3.76E-06 | 1.11E-05 | -0.370 |
| RHSIM_Rhsim02G0109800 | 21.39 | 3.76E-06 | 1.11E-05 | 0.546 |
| novel.1921 | 21.37 | 3.78E-06 | 1.12E-05 | -2.282 |

| Gene | LRT statistic | P value | BH-adjusted P | log2FC (interaction) |
|---|---|---|---|---|
| RHSIM_Rhsim12G0139700 | 21.37 | 3.78E-06 | 1.12E-05 | 0.596 |
| RHSIM_Rhsim05G0083500 | 21.37 | 3.79E-06 | 1.12E-05 | 2.337 |
| RHSIM_Rhsim02G0003600 | 21.36 | 3.80E-06 | 1.13E-05 | -0.558 |
| RHSIM_Rhsim13G0173500 | 21.36 | 3.81E-06 | 1.13E-05 | -1.538 |
| RHSIM_Rhsim11G0121500 | 21.36 | 3.81E-06 | 1.13E-05 | -0.428 |
| RHSIM_Rhsim13G0102400 | 21.35 | 3.83E-06 | 1.13E-05 | 0.401 |
| RHSIM_Rhsim04G0217100 | 21.35 | 3.83E-06 | 1.13E-05 | 1.035 |
| RHSIM_Rhsim06G0175500 | 21.34 | 3.85E-06 | 1.14E-05 | 0.457 |
| RHSIM_Rhsim01G0272300 | 21.34 | 3.85E-06 | 1.14E-05 | -1.268 |
| RHSIM_Rhsim05G0063000 | 21.34 | 3.85E-06 | 1.14E-05 | 2.604 |
| novel.569 | 21.33 | 3.87E-06 | 1.15E-05 | -2.819 |
| RHSIM_Rhsim09G0108200 | 21.33 | 3.87E-06 | 1.15E-05 | -0.616 |
| RHSIM_Rhsim09G0143300 | 21.33 | 3.87E-06 | 1.15E-05 | 1.555 |
| RHSIM_Rhsim08G0245000 | 21.33 | 3.87E-06 | 1.15E-05 | 0.576 |
| RHSIM_Rhsim02G0142600 | 21.32 | 3.89E-06 | 1.15E-05 | -0.876 |
| RHSIM_Rhsim05G0038400 | 21.31 | 3.90E-06 | 1.16E-05 | 1.329 |
| RHSIM_Rhsim03G0039100 | 21.31 | 3.91E-06 | 1.16E-05 | 2.471 |
| novel.2323 | 21.31 | 3.91E-06 | 1.16E-05 | 2.997 |
| RHSIM_Rhsim02G0075500 | 21.30 | 3.93E-06 | 1.16E-05 | -0.517 |
| RHSIM_Rhsim01G0216300 | 21.30 | 3.93E-06 | 1.16E-05 | -0.436 |
| RHSIM_Rhsim02G0240600 | 21.29 | 3.94E-06 | 1.17E-05 | 1.515 |
| RHSIM_Rhsim13G0101500 | 21.28 | 3.97E-06 | 1.17E-05 | -1.066 |
| RHSIM_Rhsim07G0169900 | 21.28 | 3.97E-06 | 1.17E-05 | 1.956 |
| RHSIM_Rhsim12G0209700 | 21.27 | 3.98E-06 | 1.18E-05 | -0.468 |
| RHSIM_Rhsim06G0033600 | 21.27 | 3.98E-06 | 1.18E-05 | 3.186 |
| RHSIM_Rhsim03G0171900 | 21.27 | 3.99E-06 | 1.18E-05 | -0.399 |
| RHSIM_Rhsim07G0122400 | 21.27 | 3.99E-06 | 1.18E-05 | 1.216 |
| RHSIM_Rhsim02G0018200 | 21.27 | 4.00E-06 | 1.18E-05 | 0.684 |
| RHSIM_Rhsim03G0245100 | 21.26 | 4.01E-06 | 1.18E-05 | 0.477 |
| RHSIM_Rhsim07G0239400 | 21.26 | 4.01E-06 | 1.18E-05 | 0.430 |
| RHSIM_Rhsim02G0144600 | 21.26 | 4.01E-06 | 1.19E-05 | -1.655 |
| RHSIM_Rhsim01G0263700 | 21.26 | 4.02E-06 | 1.19E-05 | -1.117 |

| Gene | LRT statistic | P value | BH-adjusted P | log2FC (interaction) |
|---|---|---|---|---|
| RHSIM_Rhsim11G0142000 | 21.25 | 4.03E-06 | 1.19E-05 | -0.460 |
| RHSIM_Rhsim01G0059900 | 21.24 | 4.05E-06 | 1.20E-05 | -0.431 |
| RHSIM_Rhsim01G0112500 | 21.24 | 4.05E-06 | 1.20E-05 | 2.967 |
| RHSIM_Rhsim05G0191000 | 21.24 | 4.05E-06 | 1.20E-05 | 0.721 |
| RHSIM_Rhsim10G0188500 | 21.24 | 4.06E-06 | 1.20E-05 | 0.449 |
| RHSIM_Rhsim03G0080600 | 21.24 | 4.06E-06 | 1.20E-05 | -2.104 |
| RHSIM_RhsimPtG0005000 | 21.23 | 4.06E-06 | 1.20E-05 | 2.058 |
| RHSIM_Rhsim08G0251900 | 21.22 | 4.10E-06 | 1.21E-05 | -0.626 |
| RHSIM_RhsimUnG0141400 | 21.21 | 4.12E-06 | 1.22E-05 | 1.073 |
| RHSIM_Rhsim04G0096100 | 21.21 | 4.12E-06 | 1.22E-05 | 0.966 |
| RHSIM_RhsimUnG0141600 | 21.20 | 4.13E-06 | 1.22E-05 | 1.452 |
| RHSIM_Rhsim10G0031000 | 21.20 | 4.13E-06 | 1.22E-05 | 42.652 |
| RHSIM_Rhsim02G0079800 | 21.19 | 4.16E-06 | 1.23E-05 | -1.047 |
| RHSIM_Rhsim08G0228700 | 21.17 | 4.20E-06 | 1.24E-05 | 0.696 |
| RHSIM_Rhsim12G0160200 | 21.17 | 4.20E-06 | 1.24E-05 | 0.245 |
| RHSIM_Rhsim01G0180400 | 21.16 | 4.22E-06 | 1.24E-05 | 0.632 |
| novel.2117 | 21.16 | 4.22E-06 | 1.25E-05 | -2.262 |
| RHSIM_Rhsim05G0107000 | 21.15 | 4.24E-06 | 1.25E-05 | 0.681 |
| RHSIM_RhsimUnG0008400 | 21.15 | 4.25E-06 | 1.25E-05 | 0.961 |
| RHSIM_Rhsim07G0170200 | 21.15 | 4.25E-06 | 1.25E-05 | 0.661 |
| RHSIM_Rhsim03G0016200 | 21.15 | 4.25E-06 | 1.25E-05 | -1.895 |
| RHSIM_Rhsim09G0144000 | 21.14 | 4.27E-06 | 1.26E-05 | -1.372 |
| RHSIM_Rhsim10G0100200 | 21.13 | 4.28E-06 | 1.26E-05 | 0.345 |
| RHSIM_Rhsim01G0246200 | 21.12 | 4.31E-06 | 1.27E-05 | 1.124 |
| RHSIM_Rhsim07G0121500 | 21.12 | 4.32E-06 | 1.27E-05 | -6.322 |
| RHSIM_RhsimUnG0160000 | 21.10 | 4.35E-06 | 1.28E-05 | 1.367 |
| RHSIM_Rhsim03G0216100 | 21.10 | 4.36E-06 | 1.28E-05 | -3.993 |
| novel.1582 | 21.09 | 4.37E-06 | 1.29E-05 | 1.164 |
| RHSIM_Rhsim06G0128000 | 21.09 | 4.38E-06 | 1.29E-05 | -0.584 |
| RHSIM_RhsimUnG0166700 | 21.08 | 4.40E-06 | 1.30E-05 | 1.844 |
| RHSIM_Rhsim04G0074300 | 21.08 | 4.40E-06 | 1.30E-05 | 44.230 |
| RHSIM_Rhsim12G0169600 | 21.08 | 4.40E-06 | 1.30E-05 | 0.939 |

| Gene | LRT statistic | P value | BH-adjusted P | log2FC (interaction) |
|---|---|---|---|---|
| RHSIM_Rhsim04G0202100 | 21.08 | 4.41E-06 | 1.30E-05 | 0.335 |
| RHSIM_Rhsim02G0134800 | 21.07 | 4.42E-06 | 1.30E-05 | 0.689 |
| RHSIM_Rhsim13G0042700 | 21.07 | 4.44E-06 | 1.31E-05 | -1.649 |
| RHSIM_Rhsim08G0126700 | 21.06 | 4.46E-06 | 1.31E-05 | -0.349 |
| RHSIM_Rhsim06G0213800 | 21.05 | 4.46E-06 | 1.31E-05 | -0.495 |
| RHSIM_Rhsim02G0016900 | 21.05 | 4.47E-06 | 1.32E-05 | -1.678 |
| RHSIM_Rhsim03G0228200 | 21.04 | 4.50E-06 | 1.33E-05 | -1.260 |
| RHSIM_Rhsim12G0204100 | 21.04 | 4.51E-06 | 1.33E-05 | -3.041 |
| RHSIM_Rhsim09G0009000 | 21.03 | 4.52E-06 | 1.33E-05 | -0.431 |
| RHSIM_Rhsim05G0227300 | 21.02 | 4.54E-06 | 1.34E-05 | 0.890 |
| RHSIM_Rhsim03G0080200 | 21.00 | 4.59E-06 | 1.35E-05 | -0.548 |
| RHSIM_Rhsim02G0180900 | 21.00 | 4.60E-06 | 1.35E-05 | -1.481 |
| RHSIM_Rhsim06G0052100 | 20.99 | 4.62E-06 | 1.36E-05 | -0.962 |
| novel.1551 | 20.99 | 4.62E-06 | 1.36E-05 | 1.660 |
| RHSIM_Rhsim01G0073100 | 20.99 | 4.62E-06 | 1.36E-05 | -0.893 |
| RHSIM_Rhsim11G0155600 | 20.98 | 4.64E-06 | 1.36E-05 | 0.885 |
| RHSIM_Rhsim03G0082000 | 20.98 | 4.64E-06 | 1.36E-05 | -0.561 |
| RHSIM_Rhsim05G0058200 | 20.97 | 4.67E-06 | 1.37E-05 | 1.014 |
| RHSIM_Rhsim11G0044600 | 20.97 | 4.67E-06 | 1.37E-05 | 0.543 |
| RHSIM_Rhsim11G0179900 | 20.97 | 4.67E-06 | 1.37E-05 | -4.169 |
| novel.3779 | 20.96 | 4.69E-06 | 1.38E-05 | 1.489 |
| novel.1933 | 20.96 | 4.70E-06 | 1.38E-05 | 1.653 |
| RHSIM_Rhsim08G0125000 | 20.93 | 4.75E-06 | 1.40E-05 | -0.552 |
| RHSIM_Rhsim12G0029300 | 20.93 | 4.78E-06 | 1.40E-05 | 0.646 |
| RHSIM_Rhsim09G0109700 | 20.92 | 4.80E-06 | 1.41E-05 | 0.364 |
| RHSIM_Rhsim09G0096700 | 20.92 | 4.80E-06 | 1.41E-05 | 0.640 |
| novel.2257 | 20.91 | 4.80E-06 | 1.41E-05 | -1.722 |
| novel.910 | 20.91 | 4.81E-06 | 1.41E-05 | 3.969 |
| RHSIM_Rhsim05G0056700 | 20.91 | 4.82E-06 | 1.42E-05 | -1.517 |
| RHSIM_Rhsim13G0116900 | 20.90 | 4.83E-06 | 1.42E-05 | 1.010 |
| RHSIM_Rhsim13G0211100 | 20.90 | 4.84E-06 | 1.42E-05 | -0.400 |
| novel.4321 | 20.89 | 4.85E-06 | 1.42E-05 | -0.448 |

| Gene | LRT statistic | P value | BH-adjusted P | log2FC (interaction) |
|---|---|---|---|---|
| RHSIM_Rhsim07G0240500 | 20.89 | 4.85E-06 | 1.42E-05 | 0.762 |
| RHSIM_Rhsim09G0172400 | 20.89 | 4.86E-06 | 1.43E-05 | -0.283 |
| RHSIM_Rhsim05G0135800 | 20.89 | 4.87E-06 | 1.43E-05 | -0.261 |
| RHSIM_Rhsim11G0093800 | 20.88 | 4.89E-06 | 1.43E-05 | 0.509 |
| RHSIM_Rhsim02G0221600 | 20.88 | 4.90E-06 | 1.44E-05 | 1.784 |
| RHSIM_Rhsim12G0154200 | 20.88 | 4.90E-06 | 1.44E-05 | 0.988 |
| novel.959 | 20.87 | 4.90E-06 | 1.44E-05 | 2.775 |
| novel.960 | 20.87 | 4.90E-06 | 1.44E-05 | 1.310 |
| RHSIM_Rhsim12G0148800 | 20.87 | 4.91E-06 | 1.44E-05 | 0.778 |
| RHSIM_Rhsim12G0125300 | 20.87 | 4.92E-06 | 1.44E-05 | -0.416 |
| RHSIM_Rhsim01G0167600 | 20.87 | 4.93E-06 | 1.44E-05 | -0.339 |
| RHSIM_Rhsim09G0122700 | 20.86 | 4.93E-06 | 1.44E-05 | -0.591 |
| RHSIM_Rhsim08G0003900 | 20.85 | 4.95E-06 | 1.45E-05 | -0.583 |
| RHSIM_Rhsim11G0056100 | 20.85 | 4.97E-06 | 1.46E-05 | 1.679 |
| RHSIM_Rhsim06G0012500 | 20.84 | 4.98E-06 | 1.46E-05 | 2.010 |
| RHSIM_Rhsim07G0061500 | 20.84 | 4.98E-06 | 1.46E-05 | -1.146 |
| RHSIM_Rhsim08G0081100 | 20.83 | 5.02E-06 | 1.47E-05 | 0.550 |
| RHSIM_Rhsim08G0025500 | 20.83 | 5.03E-06 | 1.47E-05 | 3.167 |
| RHSIM_Rhsim05G0110700 | 20.82 | 5.04E-06 | 1.48E-05 | -1.154 |
| RHSIM_Rhsim08G0148900 | 20.82 | 5.04E-06 | 1.48E-05 | -1.070 |
| RHSIM_Rhsim07G0146400 | 20.82 | 5.04E-06 | 1.48E-05 | 1.528 |
| RHSIM_Rhsim06G0104500 | 20.82 | 5.04E-06 | 1.48E-05 | 3.661 |
| RHSIM_Rhsim09G0000500 | 20.82 | 5.05E-06 | 1.48E-05 | 0.256 |
| RHSIM_RhsimUnG0003000 | 20.82 | 5.05E-06 | 1.48E-05 | 0.586 |
| RHSIM_Rhsim12G0010800 | 20.81 | 5.06E-06 | 1.48E-05 | 1.215 |
| novel.4073 | 20.80 | 5.09E-06 | 1.49E-05 | -2.388 |
| RHSIM_Rhsim07G0046200 | 20.79 | 5.13E-06 | 1.50E-05 | 5.749 |
| RHSIM_Rhsim03G0177700 | 20.79 | 5.14E-06 | 1.50E-05 | 0.363 |
| RHSIM_Rhsim03G0218600 | 20.78 | 5.14E-06 | 1.50E-05 | -0.975 |
| RHSIM_RhsimUnG0212100 | 20.78 | 5.15E-06 | 1.51E-05 | 0.462 |
| RHSIM_Rhsim02G0236300 | 20.78 | 5.15E-06 | 1.51E-05 | 1.233 |
| RHSIM_Rhsim05G0030400 | 20.78 | 5.15E-06 | 1.51E-05 | 1.626 |

| Gene | LRT statistic | P value | BH-adjusted P | log2FC (interaction) |
|---|---|---|---|---|
| RHSIM_Rhsim04G0014500 | 20.77 | 5.17E-06 | 1.51E-05 | 2.901 |
| RHSIM_Rhsim07G0106500 | 20.77 | 5.18E-06 | 1.51E-05 | -1.202 |
| RHSIM_Rhsim03G0155000 | 20.76 | 5.20E-06 | 1.52E-05 | -0.594 |
| RHSIM_Rhsim01G0170600 | 20.76 | 5.20E-06 | 1.52E-05 | 0.279 |
| RHSIM_Rhsim04G0194900 | 20.76 | 5.21E-06 | 1.52E-05 | -0.488 |
| RHSIM_Rhsim06G0203000 | 20.75 | 5.23E-06 | 1.53E-05 | -0.500 |
| RHSIM_Rhsim03G0188100 | 20.75 | 5.23E-06 | 1.53E-05 | -0.485 |
| RHSIM_Rhsim13G0171700 | 20.75 | 5.23E-06 | 1.53E-05 | 0.980 |
| RHSIM_Rhsim04G0102500 | 20.75 | 5.24E-06 | 1.53E-05 | -0.556 |
| RHSIM_Rhsim11G0072000 | 20.74 | 5.25E-06 | 1.53E-05 | -1.356 |
| RHSIM_Rhsim09G0143400 | 20.74 | 5.25E-06 | 1.53E-05 | 4.397 |
| RHSIM_Rhsim12G0195600 | 20.74 | 5.27E-06 | 1.54E-05 | -1.247 |
| RHSIM_Rhsim03G0097200 | 20.73 | 5.28E-06 | 1.54E-05 | -1.736 |
| RHSIM_Rhsim06G0011300 | 20.73 | 5.29E-06 | 1.55E-05 | 0.324 |
| novel.4461 | 20.72 | 5.31E-06 | 1.55E-05 | -0.478 |
| RHSIM_Rhsim05G0077800 | 20.72 | 5.33E-06 | 1.56E-05 | -3.081 |
| RHSIM_Rhsim12G0124000 | 20.71 | 5.35E-06 | 1.56E-05 | -0.877 |
| RHSIM_Rhsim04G0116200 | 20.70 | 5.38E-06 | 1.57E-05 | -1.451 |
| novel.2216 | 20.69 | 5.39E-06 | 1.57E-05 | 0.546 |
| RHSIM_Rhsim05G0075700 | 20.69 | 5.40E-06 | 1.58E-05 | -0.378 |
| RHSIM_Rhsim11G0187600 | 20.69 | 5.40E-06 | 1.58E-05 | -0.380 |
| RHSIM_Rhsim07G0244800 | 20.68 | 5.43E-06 | 1.58E-05 | 0.335 |
| RHSIM_RhsimUnG0099800 | 20.67 | 5.46E-06 | 1.59E-05 | -1.334 |
| RHSIM_Rhsim12G0209000 | 20.67 | 5.46E-06 | 1.59E-05 | 0.370 |
| RHSIM_Rhsim13G0067600 | 20.67 | 5.46E-06 | 1.59E-05 | 4.050 |
| novel.1381 | 20.66 | 5.50E-06 | 1.60E-05 | 1.366 |
| RHSIM_Rhsim03G0244000 | 20.65 | 5.51E-06 | 1.61E-05 | 1.541 |
| RHSIM_Rhsim03G0221100 | 20.65 | 5.51E-06 | 1.61E-05 | 0.316 |
| novel.4025 | 20.65 | 5.53E-06 | 1.61E-05 | 2.279 |
| RHSIM_Rhsim04G0038900 | 20.64 | 5.53E-06 | 1.61E-05 | 0.423 |
| RHSIM_Rhsim09G0155200 | 20.64 | 5.54E-06 | 1.61E-05 | 1.452 |
| RHSIM_Rhsim07G0021900 | 20.63 | 5.57E-06 | 1.63E-05 | 1.858 |

| Gene | LRT statistic | P value | BH-adjusted P | log2FC (interaction) |
|---|---|---|---|---|
| RHSIM_RhsimUnG0248200 | 20.62 | 5.60E-06 | 1.63E-05 | -0.365 |
| novel.335 | 20.62 | 5.60E-06 | 1.63E-05 | 0.662 |
| RHSIM_Rhsim13G0213900 | 20.61 | 5.62E-06 | 1.64E-05 | 0.874 |
| RHSIM_Rhsim13G0056200 | 20.61 | 5.62E-06 | 1.64E-05 | -0.824 |
| RHSIM_Rhsim03G0267400 | 20.60 | 5.67E-06 | 1.65E-05 | -0.511 |
| novel.238 | 20.59 | 5.69E-06 | 1.66E-05 | -0.753 |
| RHSIM_Rhsim01G0288300 | 20.59 | 5.70E-06 | 1.66E-05 | 0.730 |
| RHSIM_Rhsim09G0198800 | 20.59 | 5.70E-06 | 1.66E-05 | -1.130 |
| RHSIM_RhsimUnG0042300 | 20.58 | 5.70E-06 | 1.66E-05 | -0.370 |
| RHSIM_Rhsim04G0087200 | 20.58 | 5.72E-06 | 1.67E-05 | -0.550 |
| novel.118 | 20.58 | 5.73E-06 | 1.67E-05 | -2.527 |
| RHSIM_Rhsim01G0185700 | 20.57 | 5.74E-06 | 1.67E-05 | 1.530 |
| RHSIM_Rhsim11G0148800 | 20.57 | 5.75E-06 | 1.67E-05 | 0.714 |
| RHSIM_Rhsim11G0166600 | 20.56 | 5.77E-06 | 1.68E-05 | -1.678 |
| RHSIM_Rhsim11G0125400 | 20.56 | 5.77E-06 | 1.68E-05 | -0.780 |
| RHSIM_Rhsim06G0146800 | 20.56 | 5.77E-06 | 1.68E-05 | 1.623 |
| RHSIM_Rhsim01G0154800 | 20.56 | 5.79E-06 | 1.68E-05 | 0.793 |
| RHSIM_Rhsim06G0141500 | 20.55 | 5.80E-06 | 1.69E-05 | -0.540 |
| novel.889 | 20.55 | 5.82E-06 | 1.69E-05 | -0.269 |
| RHSIM_RhsimUnG0021300 | 20.55 | 5.82E-06 | 1.69E-05 | 0.519 |
| RHSIM_Rhsim11G0090100 | 20.55 | 5.82E-06 | 1.69E-05 | 0.885 |
| RHSIM_Rhsim02G0231100 | 20.54 | 5.83E-06 | 1.70E-05 | 0.562 |
| RHSIM_Rhsim08G0030000 | 20.54 | 5.85E-06 | 1.70E-05 | 0.429 |
| RHSIM_Rhsim03G0221200 | 20.54 | 5.85E-06 | 1.70E-05 | -0.748 |
| RHSIM_Rhsim10G0044700 | 20.54 | 5.85E-06 | 1.70E-05 | 2.396 |
| RHSIM_Rhsim13G0063800 | 20.53 | 5.89E-06 | 1.71E-05 | 1.777 |
| RHSIM_Rhsim13G0211800 | 20.52 | 5.90E-06 | 1.71E-05 | -0.652 |
| RHSIM_Rhsim07G0075300 | 20.52 | 5.90E-06 | 1.71E-05 | 0.344 |
| RHSIM_Rhsim02G0012700 | 20.52 | 5.91E-06 | 1.72E-05 | -0.534 |
| RHSIM_Rhsim01G0240200 | 20.51 | 5.92E-06 | 1.72E-05 | 1.407 |
| RHSIM_Rhsim13G0174300 | 20.51 | 5.93E-06 | 1.72E-05 | 1.134 |
| RHSIM_Rhsim06G0003100 | 20.51 | 5.94E-06 | 1.73E-05 | -0.521 |

| Gene | LRT statistic | P value | BH-adjusted P | log2FC (interaction) |
|---|---|---|---|---|
| RHSIM_Rhsim11G0104900 | 20.50 | 5.95E-06 | 1.73E-05 | -1.533 |
| RHSIM_Rhsim02G0065000 | 20.49 | 5.98E-06 | 1.74E-05 | 1.183 |
| novel.793 | 20.49 | 5.98E-06 | 1.74E-05 | -2.534 |
| RHSIM_Rhsim01G0044800 | 20.48 | 6.02E-06 | 1.75E-05 | 2.758 |
| RHSIM_Rhsim09G0140800 | 20.48 | 6.03E-06 | 1.75E-05 | -0.609 |
| RHSIM_RhsimUnG0048500 | 20.46 | 6.08E-06 | 1.77E-05 | 0.423 |
| RHSIM_Rhsim13G0098400 | 20.46 | 6.10E-06 | 1.77E-05 | -0.604 |
| RHSIM_Rhsim05G0005100 | 20.45 | 6.11E-06 | 1.77E-05 | -1.336 |
| RHSIM_RhsimUnG0045200 | 20.45 | 6.11E-06 | 1.78E-05 | -0.709 |
| RHSIM_Rhsim01G0223400 | 20.43 | 6.17E-06 | 1.79E-05 | 0.530 |
| RHSIM_Rhsim06G0139400 | 20.43 | 6.19E-06 | 1.80E-05 | -0.496 |
| RHSIM_Rhsim03G0143500 | 20.42 | 6.21E-06 | 1.80E-05 | -1.290 |
| RHSIM_Rhsim13G0192300 | 20.40 | 6.28E-06 | 1.82E-05 | -1.502 |
| RHSIM_Rhsim02G0235600 | 20.40 | 6.29E-06 | 1.82E-05 | -0.747 |
| RHSIM_Rhsim10G0208800 | 20.40 | 6.29E-06 | 1.82E-05 | 1.328 |
| RHSIM_Rhsim03G0221800 | 20.39 | 6.33E-06 | 1.84E-05 | 0.552 |
| RHSIM_Rhsim02G0140400 | 20.38 | 6.35E-06 | 1.84E-05 | 1.234 |
| RHSIM_Rhsim08G0173200 | 20.37 | 6.39E-06 | 1.86E-05 | 0.394 |
| RHSIM_Rhsim04G0058900 | 20.36 | 6.40E-06 | 1.86E-05 | -0.591 |
| RHSIM_Rhsim13G0167700 | 20.36 | 6.41E-06 | 1.86E-05 | -0.402 |
| RHSIM_Rhsim11G0036000 | 20.36 | 6.41E-06 | 1.86E-05 | -0.573 |
| RHSIM_Rhsim07G0056100 | 20.34 | 6.47E-06 | 1.88E-05 | -1.350 |
| novel.1558 | 20.34 | 6.48E-06 | 1.88E-05 | 0.901 |
| novel.3845 | 20.31 | 6.60E-06 | 1.91E-05 | 0.253 |
| RHSIM_Rhsim04G0223900 | 20.29 | 6.64E-06 | 1.93E-05 | 1.945 |
| RHSIM_Rhsim08G0239300 | 20.28 | 6.68E-06 | 1.94E-05 | 3.335 |
| RHSIM_Rhsim04G0175900 | 20.28 | 6.69E-06 | 1.94E-05 | 0.657 |
| RHSIM_Rhsim02G0242700 | 20.27 | 6.71E-06 | 1.95E-05 | -0.758 |
| RHSIM_Rhsim01G0242300 | 20.27 | 6.72E-06 | 1.95E-05 | 1.040 |
| RHSIM_Rhsim08G0041800 | 20.27 | 6.73E-06 | 1.95E-05 | -0.995 |
| RHSIM_RhsimUnG0137700 | 20.27 | 6.74E-06 | 1.95E-05 | 1.404 |
| RHSIM_Rhsim06G0081300 | 20.26 | 6.75E-06 | 1.96E-05 | -0.954 |

| Gene | LRT statistic | P value | BH-adjusted P | log2FC (interaction) |
|---|---|---|---|---|
| RHSIM_Rhsim05G0036100 | 20.26 | 6.76E-06 | 1.96E-05 | -1.765 |
| RHSIM_Rhsim03G0198400 | 20.26 | 6.77E-06 | 1.96E-05 | -0.716 |
| RHSIM_Rhsim12G0002800 | 20.25 | 6.79E-06 | 1.97E-05 | -0.816 |
| RHSIM_Rhsim01G0108100 | 20.25 | 6.80E-06 | 1.97E-05 | 1.534 |
| RHSIM_Rhsim13G0102200 | 20.25 | 6.81E-06 | 1.97E-05 | -1.027 |
| RHSIM_RhsimUnG0185700 | 20.22 | 6.89E-06 | 1.99E-05 | 1.084 |
| RHSIM_Rhsim10G0114700 | 20.22 | 6.91E-06 | 2.00E-05 | 0.984 |
| RHSIM_Rhsim11G0047300 | 20.20 | 6.96E-06 | 2.01E-05 | -0.378 |
| RHSIM_Rhsim03G0068100 | 20.20 | 6.97E-06 | 2.02E-05 | 0.633 |
| RHSIM_Rhsim12G0013200 | 20.20 | 6.99E-06 | 2.02E-05 | -2.085 |
| RHSIM_Rhsim11G0190900 | 20.19 | 7.00E-06 | 2.03E-05 | -0.783 |
| RHSIM_Rhsim05G0122400 | 20.19 | 7.02E-06 | 2.03E-05 | 0.532 |
| RHSIM_Rhsim09G0153600 | 20.19 | 7.03E-06 | 2.03E-05 | -1.145 |
| RHSIM_Rhsim04G0079800 | 20.18 | 7.05E-06 | 2.04E-05 | -3.702 |
| novel.4381 | 20.14 | 7.21E-06 | 2.08E-05 | -2.423 |
| novel.2973 | 20.13 | 7.22E-06 | 2.09E-05 | -4.000 |
| RHSIM_RhsimUnG0098000 | 20.13 | 7.24E-06 | 2.09E-05 | -0.652 |
| RHSIM_Rhsim06G0135700 | 20.12 | 7.26E-06 | 2.10E-05 | -0.620 |
| RHSIM_Rhsim02G0232800 | 20.12 | 7.29E-06 | 2.11E-05 | -0.563 |
| RHSIM_Rhsim11G0089000 | 20.11 | 7.31E-06 | 2.11E-05 | 1.753 |
| RHSIM_Rhsim05G0011100 | 20.10 | 7.37E-06 | 2.13E-05 | 1.763 |
| RHSIM_Rhsim05G0122300 | 20.09 | 7.40E-06 | 2.14E-05 | -0.493 |
| RHSIM_Rhsim07G0138400 | 20.09 | 7.40E-06 | 2.14E-05 | 4.594 |
| RHSIM_Rhsim02G0122100 | 20.08 | 7.41E-06 | 2.14E-05 | 0.593 |
| novel.2284 | 20.08 | 7.42E-06 | 2.14E-05 | -0.391 |
| RHSIM_Rhsim03G0153700 | 20.08 | 7.45E-06 | 2.15E-05 | 0.337 |
| RHSIM_Rhsim13G0119900 | 20.07 | 7.47E-06 | 2.16E-05 | 0.699 |
| RHSIM_Rhsim01G0269800 | 20.07 | 7.47E-06 | 2.16E-05 | -0.666 |
| RHSIM_Rhsim05G0095300 | 20.07 | 7.48E-06 | 2.16E-05 | 0.887 |
| RHSIM_Rhsim04G0159500 | 20.06 | 7.49E-06 | 2.16E-05 | 3.211 |
| novel.3744 | 20.06 | 7.52E-06 | 2.17E-05 | -4.529 |
| RHSIM_Rhsim05G0042000 | 20.06 | 7.52E-06 | 2.17E-05 | 0.728 |

| Gene | LRT statistic | P value | BH-adjusted P | log2FC (interaction) |
|---|---|---|---|---|
| RHSIM_Rhsim08G0098500 | 20.05 | 7.53E-06 | 2.17E-05 | -0.651 |
| RHSIM_Rhsim13G0209000 | 20.05 | 7.56E-06 | 2.18E-05 | -0.490 |
| RHSIM_Rhsim11G0186900 | 20.05 | 7.56E-06 | 2.18E-05 | -1.218 |
| RHSIM_Rhsim10G0005200 | 20.04 | 7.57E-06 | 2.18E-05 | -0.919 |
| RHSIM_Rhsim03G0189700 | 20.03 | 7.62E-06 | 2.20E-05 | 3.599 |
| RHSIM_Rhsim01G0176300 | 20.02 | 7.66E-06 | 2.21E-05 | -0.635 |
| novel.339 | 20.01 | 7.69E-06 | 2.22E-05 | -0.290 |
| RHSIM_Rhsim09G0027900 | 20.00 | 7.74E-06 | 2.23E-05 | 0.663 |
| RHSIM_Rhsim05G0217200 | 20.00 | 7.74E-06 | 2.23E-05 | -0.485 |
| RHSIM_Rhsim02G0044700 | 19.99 | 7.79E-06 | 2.25E-05 | -0.618 |
| RHSIM_Rhsim11G0024800 | 19.98 | 7.82E-06 | 2.26E-05 | 0.446 |
| RHSIM_Rhsim04G0224600 | 19.98 | 7.83E-06 | 2.26E-05 | -0.223 |
| RHSIM_Rhsim01G0291200 | 19.98 | 7.83E-06 | 2.26E-05 | 0.830 |
| RHSIM_Rhsim03G0013100 | 19.97 | 7.87E-06 | 2.27E-05 | -3.916 |
| RHSIM_Rhsim08G0100600 | 19.96 | 7.92E-06 | 2.28E-05 | -0.278 |
| RHSIM_Rhsim09G0204400 | 19.95 | 7.94E-06 | 2.29E-05 | 0.379 |
| RHSIM_Rhsim03G0139500 | 19.95 | 7.95E-06 | 2.29E-05 | -1.396 |
| RHSIM_Rhsim09G0164400 | 19.95 | 7.95E-06 | 2.29E-05 | -1.176 |
| RHSIM_Rhsim06G0032700 | 19.94 | 7.97E-06 | 2.30E-05 | 0.343 |
| RHSIM_Rhsim01G0164800 | 19.94 | 7.98E-06 | 2.30E-05 | 0.612 |
| RHSIM_Rhsim03G0140700 | 19.94 | 7.99E-06 | 2.30E-05 | -0.458 |
| RHSIM_Rhsim07G0205800 | 19.94 | 8.00E-06 | 2.30E-05 | -0.291 |
| RHSIM_Rhsim01G0138900 | 19.94 | 8.00E-06 | 2.30E-05 | 0.361 |
| RHSIM_Rhsim08G0051800 | 19.94 | 8.01E-06 | 2.31E-05 | -1.226 |
| RHSIM_Rhsim04G0049600 | 19.93 | 8.01E-06 | 2.31E-05 | -0.432 |
| RHSIM_Rhsim12G0083200 | 19.93 | 8.03E-06 | 2.31E-05 | -0.545 |
| RHSIM_Rhsim05G0166800 | 19.93 | 8.04E-06 | 2.32E-05 | 5.252 |
| RHSIM_Rhsim09G0111600 | 19.93 | 8.05E-06 | 2.32E-05 | -1.312 |
| RHSIM_Rhsim02G0053400 | 19.92 | 8.07E-06 | 2.32E-05 | 0.226 |
| novel.157 | 19.92 | 8.07E-06 | 2.32E-05 | -3.521 |
| RHSIM_Rhsim07G0081300 | 19.92 | 8.07E-06 | 2.32E-05 | -1.806 |
| RHSIM_Rhsim11G0145100 | 19.92 | 8.09E-06 | 2.33E-05 | 2.207 |

| Gene | LRT statistic | P value | BH-adjusted P | log2FC (interaction) |
|---|---|---|---|---|
| RHSIM_Rhsim09G0007500 | 19.90 | 8.16E-06 | 2.35E-05 | -0.773 |
| RHSIM_Rhsim04G0245300 | 19.88 | 8.24E-06 | 2.37E-05 | 0.357 |
| RHSIM_Rhsim03G0163700 | 19.88 | 8.25E-06 | 2.37E-05 | -0.552 |
| RHSIM_RhsimUnG0223500 | 19.88 | 8.25E-06 | 2.37E-05 | -42.212 |
| RHSIM_Rhsim13G0143500 | 19.88 | 8.27E-06 | 2.38E-05 | 1.645 |
| RHSIM_Rhsim05G0122600 | 19.87 | 8.31E-06 | 2.39E-05 | -1.662 |
| novel.2296 | 19.86 | 8.32E-06 | 2.39E-05 | 1.433 |
| RHSIM_Rhsim04G0055200 | 19.86 | 8.33E-06 | 2.40E-05 | -2.321 |
| RHSIM_Rhsim08G0031100 | 19.85 | 8.36E-06 | 2.40E-05 | -1.947 |
| RHSIM_Rhsim04G0165100 | 19.85 | 8.36E-06 | 2.41E-05 | 0.612 |
| RHSIM_Rhsim01G0123800 | 19.84 | 8.41E-06 | 2.42E-05 | -0.503 |
| RHSIM_Rhsim04G0033000 | 19.84 | 8.42E-06 | 2.42E-05 | -0.683 |
| novel.1100 | 19.84 | 8.42E-06 | 2.42E-05 | -1.101 |
| RHSIM_Rhsim02G0255700 | 19.84 | 8.43E-06 | 2.42E-05 | 0.558 |
| RHSIM_RhsimUnG0213500 | 19.84 | 8.44E-06 | 2.43E-05 | -1.791 |
| RHSIM_Rhsim10G0062600 | 19.83 | 8.45E-06 | 2.43E-05 | 0.300 |
| RHSIM_Rhsim05G0093000 | 19.82 | 8.52E-06 | 2.45E-05 | 0.419 |
| RHSIM_Rhsim10G0036200 | 19.82 | 8.53E-06 | 2.45E-05 | 2.447 |
| novel.3453 | 19.81 | 8.54E-06 | 2.45E-05 | -2.735 |
| RHSIM_Rhsim01G0104100 | 19.81 | 8.57E-06 | 2.46E-05 | 0.749 |
| RHSIM_Rhsim03G0195300 | 19.81 | 8.57E-06 | 2.46E-05 | -1.606 |
| RHSIM_Rhsim12G0171200 | 19.81 | 8.57E-06 | 2.46E-05 | 0.690 |
| RHSIM_Rhsim03G0100800 | 19.80 | 8.61E-06 | 2.47E-05 | 0.563 |
| RHSIM_Rhsim11G0068400 | 19.79 | 8.66E-06 | 2.49E-05 | 2.695 |
| RHSIM_Rhsim11G0020700 | 19.79 | 8.66E-06 | 2.49E-05 | -1.133 |
| RHSIM_Rhsim06G0090400 | 19.78 | 8.67E-06 | 2.49E-05 | -0.302 |
| RHSIM_Rhsim03G0227500 | 19.78 | 8.69E-06 | 2.49E-05 | -0.621 |
| novel.805 | 19.78 | 8.71E-06 | 2.50E-05 | -1.118 |
| RHSIM_Rhsim01G0003200 | 19.77 | 8.75E-06 | 2.51E-05 | -1.258 |
| RHSIM_Rhsim08G0008400 | 19.76 | 8.77E-06 | 2.52E-05 | 0.633 |
| RHSIM_Rhsim10G0108600 | 19.76 | 8.79E-06 | 2.52E-05 | -0.463 |
| RHSIM_Rhsim02G0080000 | 19.75 | 8.81E-06 | 2.53E-05 | -1.152 |

| Gene | LRT statistic | P value | BH-adjusted P | log2FC (interaction) |
|---|---|---|---|---|
| novel.4303 | 19.75 | 8.81E-06 | 2.53E-05 | 1.772 |
| novel.4241 | 19.75 | 8.82E-06 | 2.53E-05 | -0.559 |
| RHSIM_Rhsim09G0089500 | 19.75 | 8.83E-06 | 2.53E-05 | 0.382 |
| novel.3415 | 19.74 | 8.86E-06 | 2.54E-05 | -1.856 |
| RHSIM_Rhsim03G0148000 | 19.74 | 8.89E-06 | 2.55E-05 | 0.496 |
| RHSIM_Rhsim03G0130700 | 19.72 | 8.97E-06 | 2.57E-05 | -1.617 |
| RHSIM_Rhsim03G0150800 | 19.72 | 8.98E-06 | 2.58E-05 | -0.616 |
| RHSIM_Rhsim13G0164100 | 19.70 | 9.04E-06 | 2.59E-05 | 1.240 |
| RHSIM_Rhsim01G0283400 | 19.70 | 9.05E-06 | 2.59E-05 | -1.144 |
| RHSIM_Rhsim07G0225600 | 19.70 | 9.06E-06 | 2.60E-05 | -1.015 |
| RHSIM_Rhsim06G0057800 | 19.70 | 9.06E-06 | 2.60E-05 | 0.713 |
| RHSIM_Rhsim07G0101400 | 19.69 | 9.10E-06 | 2.61E-05 | -0.381 |
| RHSIM_Rhsim08G0241700 | 19.69 | 9.11E-06 | 2.61E-05 | 3.222 |
| RHSIM_Rhsim07G0026700 | 19.68 | 9.14E-06 | 2.62E-05 | -0.319 |
| RHSIM_Rhsim05G0062100 | 19.67 | 9.22E-06 | 2.64E-05 | -0.834 |
| RHSIM_Rhsim13G0207500 | 19.65 | 9.32E-06 | 2.67E-05 | -0.821 |
| RHSIM_Rhsim09G0203300 | 19.64 | 9.34E-06 | 2.67E-05 | -0.382 |
| RHSIM_Rhsim06G0099300 | 19.64 | 9.35E-06 | 2.68E-05 | -0.661 |
| RHSIM_Rhsim07G0131000 | 19.64 | 9.35E-06 | 2.68E-05 | -0.654 |
| novel.1908 | 19.62 | 9.45E-06 | 2.71E-05 | -2.570 |
| RHSIM_RhsimUnG0062000 | 19.61 | 9.48E-06 | 2.71E-05 | -2.350 |
| RHSIM_Rhsim05G0001700 | 19.61 | 9.50E-06 | 2.72E-05 | 1.276 |
| RHSIM_Rhsim02G0056800 | 19.61 | 9.51E-06 | 2.72E-05 | 1.416 |
| novel.2680 | 19.60 | 9.52E-06 | 2.73E-05 | -3.403 |
| RHSIM_Rhsim01G0218800 | 19.60 | 9.53E-06 | 2.73E-05 | 1.723 |
| RHSIM_Rhsim01G0044900 | 19.60 | 9.57E-06 | 2.74E-05 | 1.550 |
| RHSIM_Rhsim03G0007500 | 19.59 | 9.58E-06 | 2.74E-05 | -1.120 |
| RHSIM_Rhsim04G0250100 | 19.59 | 9.60E-06 | 2.75E-05 | 1.218 |
| RHSIM_Rhsim10G0167700 | 19.59 | 9.62E-06 | 2.75E-05 | -1.072 |
| RHSIM_Rhsim08G0246400 | 19.58 | 9.66E-06 | 2.76E-05 | 0.431 |
| RHSIM_Rhsim04G0184000 | 19.58 | 9.66E-06 | 2.76E-05 | -1.906 |
| RHSIM_RhsimUnG0138600 | 19.57 | 9.68E-06 | 2.77E-05 | 2.762 |

| Gene | LRT statistic | P value | BH-adjusted P | log2FC (interaction) |
|---|---|---|---|---|
| novel.975 | 19.57 | 9.71E-06 | 2.78E-05 | -3.629 |
| RHSIM_Rhsim06G0243600 | 19.56 | 9.75E-06 | 2.79E-05 | -4.274 |
| novel.1119 | 19.54 | 9.83E-06 | 2.81E-05 | 2.337 |
| RHSIM_Rhsim09G0178200 | 19.53 | 9.89E-06 | 2.83E-05 | -2.072 |
| RHSIM_Rhsim12G0071900 | 19.53 | 9.90E-06 | 2.83E-05 | 0.618 |
| RHSIM_Rhsim11G0148900 | 19.53 | 9.92E-06 | 2.84E-05 | 0.677 |
| RHSIM_Rhsim01G0147400 | 19.52 | 9.94E-06 | 2.84E-05 | -0.799 |
| RHSIM_Rhsim02G0086500 | 19.52 | 9.95E-06 | 2.84E-05 | -1.128 |
| RHSIM_Rhsim13G0235200 | 19.51 | 9.99E-06 | 2.86E-05 | 1.258 |
| RHSIM_Rhsim01G0009500 | 19.51 | 9.99E-06 | 2.86E-05 | -1.114 |
| RHSIM_Rhsim11G0182100 | 19.50 | 1.01E-05 | 2.87E-05 | 0.677 |
| RHSIM_Rhsim03G0051000 | 19.50 | 1.01E-05 | 2.88E-05 | -1.771 |
| novel.1965 | 19.49 | 1.01E-05 | 2.89E-05 | -6.456 |
| RHSIM_Rhsim07G0028400 | 19.49 | 1.01E-05 | 2.89E-05 | 0.448 |
| RHSIM_Rhsim11G0027000 | 19.48 | 1.01E-05 | 2.90E-05 | -0.826 |
| RHSIM_Rhsim04G0119100 | 19.48 | 1.02E-05 | 2.90E-05 | 1.363 |
| RHSIM_Rhsim05G0159400 | 19.48 | 1.02E-05 | 2.91E-05 | 1.024 |
| RHSIM_Rhsim09G0144300 | 19.48 | 1.02E-05 | 2.91E-05 | 2.853 |
| RHSIM_Rhsim13G0009000 | 19.47 | 1.02E-05 | 2.91E-05 | -0.792 |
| RHSIM_Rhsim03G0028800 | 19.47 | 1.02E-05 | 2.91E-05 | 1.600 |
| RHSIM_Rhsim08G0166900 | 19.47 | 1.02E-05 | 2.91E-05 | 0.443 |
| RHSIM_Rhsim01G0045400 | 19.47 | 1.02E-05 | 2.92E-05 | 0.803 |
| RHSIM_Rhsim06G0021000 | 19.47 | 1.02E-05 | 2.92E-05 | 2.466 |
| RHSIM_Rhsim13G0030600 | 19.47 | 1.02E-05 | 2.92E-05 | 0.475 |
| RHSIM_Rhsim10G0010500 | 19.47 | 1.02E-05 | 2.92E-05 | -0.541 |
| RHSIM_Rhsim05G0031400 | 19.46 | 1.02E-05 | 2.92E-05 | 0.456 |
| RHSIM_Rhsim02G0244900 | 19.46 | 1.03E-05 | 2.93E-05 | 1.546 |
| RHSIM_Rhsim01G0190000 | 19.45 | 1.03E-05 | 2.94E-05 | 3.759 |
| RHSIM_Rhsim11G0177100 | 19.45 | 1.03E-05 | 2.94E-05 | 0.313 |
| RHSIM_Rhsim08G0132300 | 19.45 | 1.03E-05 | 2.95E-05 | 3.466 |
| RHSIM_Rhsim02G0249500 | 19.45 | 1.03E-05 | 2.95E-05 | -1.314 |
| RHSIM_Rhsim10G0014700 | 19.44 | 1.04E-05 | 2.95E-05 | -0.779 |

| Gene | LRT statistic | P value | BH-adjusted P | log2FC (interaction) |
|---|---|---|---|---|
| RHSIM_Rhsim04G0149700 | 19.44 | 1.04E-05 | 2.96E-05 | -0.534 |
| RHSIM_Rhsim04G0013800 | 19.43 | 1.04E-05 | 2.97E-05 | 1.014 |
| RHSIM_Rhsim08G0050700 | 19.43 | 1.04E-05 | 2.97E-05 | -1.306 |
| RHSIM_Rhsim01G0254700 | 19.43 | 1.04E-05 | 2.98E-05 | 1.977 |
| RHSIM_Rhsim06G0033300 | 19.42 | 1.05E-05 | 2.99E-05 | 1.819 |
| RHSIM_Rhsim07G0141700 | 19.41 | 1.05E-05 | 3.00E-05 | -0.774 |
| RHSIM_Rhsim05G0223400 | 19.41 | 1.06E-05 | 3.01E-05 | -0.740 |
| RHSIM_Rhsim10G0175000 | 19.40 | 1.06E-05 | 3.01E-05 | -3.959 |
| RHSIM_Rhsim06G0022600 | 19.40 | 1.06E-05 | 3.02E-05 | -41.546 |
| novel.4423 | 19.40 | 1.06E-05 | 3.02E-05 | 2.233 |
| novel.1173 | 19.40 | 1.06E-05 | 3.02E-05 | 1.916 |
| RHSIM_Rhsim06G0099200 | 19.39 | 1.06E-05 | 3.03E-05 | -0.569 |
| RHSIM_Rhsim11G0080900 | 19.39 | 1.06E-05 | 3.03E-05 | 3.707 |
| RHSIM_Rhsim13G0059300 | 19.38 | 1.07E-05 | 3.05E-05 | -46.000 |
| RHSIM_Rhsim09G0110100 | 19.38 | 1.07E-05 | 3.06E-05 | 0.493 |
| RHSIM_Rhsim12G0106900 | 19.37 | 1.07E-05 | 3.06E-05 | 0.608 |
| RHSIM_Rhsim12G0063100 | 19.37 | 1.07E-05 | 3.06E-05 | -0.685 |
| novel.1061 | 19.37 | 1.08E-05 | 3.07E-05 | -1.079 |
| RHSIM_Rhsim07G0000700 | 19.37 | 1.08E-05 | 3.07E-05 | 0.834 |
| RHSIM_Rhsim10G0048800 | 19.36 | 1.08E-05 | 3.08E-05 | 0.365 |
| RHSIM_Rhsim02G0087100 | 19.35 | 1.09E-05 | 3.09E-05 | 1.466 |
| RHSIM_Rhsim01G0248100 | 19.35 | 1.09E-05 | 3.10E-05 | -0.871 |
| RHSIM_Rhsim12G0208200 | 19.35 | 1.09E-05 | 3.10E-05 | 0.451 |
| RHSIM_Rhsim11G0087400 | 19.35 | 1.09E-05 | 3.10E-05 | -0.409 |
| novel.909 | 19.34 | 1.09E-05 | 3.11E-05 | -0.667 |
| RHSIM_Rhsim06G0027000 | 19.34 | 1.09E-05 | 3.11E-05 | 3.200 |
| RHSIM_Rhsim05G0033800 | 19.34 | 1.10E-05 | 3.11E-05 | 1.594 |
| RHSIM_Rhsim09G0154600 | 19.33 | 1.10E-05 | 3.13E-05 | -0.499 |
| RHSIM_Rhsim03G0210500 | 19.33 | 1.10E-05 | 3.13E-05 | -1.455 |
| novel.3421 | 19.32 | 1.10E-05 | 3.13E-05 | 1.093 |
| RHSIM_Rhsim06G0130900 | 19.32 | 1.10E-05 | 3.14E-05 | 0.596 |
| RHSIM_Rhsim03G0161600 | 19.32 | 1.11E-05 | 3.15E-05 | 0.375 |

| Gene | LRT statistic | P value | BH-adjusted P | log2FC (interaction) |
|---|---|---|---|---|
| RHSIM_Rhsim02G0110500 | 19.31 | 1.11E-05 | 3.15E-05 | -0.745 |
| RHSIM_Rhsim13G0191100 | 19.31 | 1.11E-05 | 3.16E-05 | 0.505 |
| RHSIM_Rhsim01G0120700 | 19.31 | 1.11E-05 | 3.16E-05 | -0.855 |
| RHSIM_Rhsim05G0025500 | 19.31 | 1.11E-05 | 3.16E-05 | 1.110 |
| RHSIM_Rhsim07G0045700 | 19.30 | 1.11E-05 | 3.17E-05 | 0.518 |
| RHSIM_Rhsim08G0043500 | 19.30 | 1.12E-05 | 3.17E-05 | -0.406 |
| RHSIM_Rhsim13G0047300 | 19.29 | 1.12E-05 | 3.18E-05 | -0.248 |
| RHSIM_Rhsim13G0231600 | 19.29 | 1.12E-05 | 3.19E-05 | 0.322 |
| RHSIM_Rhsim05G0186400 | 19.29 | 1.12E-05 | 3.19E-05 | -0.668 |
| RHSIM_Rhsim08G0201400 | 19.28 | 1.13E-05 | 3.21E-05 | -0.631 |
| RHSIM_Rhsim03G0134400 | 19.27 | 1.13E-05 | 3.21E-05 | -1.752 |
| RHSIM_Rhsim12G0139900 | 19.27 | 1.14E-05 | 3.22E-05 | 0.494 |
| RHSIM_Rhsim13G0137200 | 19.26 | 1.14E-05 | 3.23E-05 | 1.321 |
| RHSIM_RhsimUnG0021700 | 19.26 | 1.14E-05 | 3.24E-05 | -0.842 |
| RHSIM_Rhsim07G0019900 | 19.26 | 1.14E-05 | 3.24E-05 | -0.733 |
| RHSIM_RhsimUnG0060000 | 19.26 | 1.14E-05 | 3.24E-05 | 0.362 |
| RHSIM_Rhsim07G0258600 | 19.25 | 1.14E-05 | 3.25E-05 | -0.789 |
| RHSIM_RhsimUnG0185800 | 19.25 | 1.15E-05 | 3.26E-05 | 1.486 |
| RHSIM_Rhsim11G0156600 | 19.24 | 1.15E-05 | 3.27E-05 | -1.619 |
| RHSIM_Rhsim07G0244200 | 19.23 | 1.16E-05 | 3.28E-05 | 1.100 |
| RHSIM_Rhsim04G0162500 | 19.22 | 1.16E-05 | 3.30E-05 | -0.437 |
| RHSIM_Rhsim04G0075900 | 19.22 | 1.16E-05 | 3.30E-05 | -0.817 |
| RHSIM_Rhsim03G0110600 | 19.22 | 1.17E-05 | 3.31E-05 | -0.491 |
| RHSIM_Rhsim07G0010500 | 19.21 | 1.17E-05 | 3.32E-05 | 0.885 |
| RHSIM_Rhsim03G0235100 | 19.20 | 1.18E-05 | 3.33E-05 | -0.594 |
| novel.2178 | 19.20 | 1.18E-05 | 3.34E-05 | -3.723 |
| RHSIM_Rhsim04G0231700 | 19.20 | 1.18E-05 | 3.34E-05 | -0.465 |
| novel.4263 | 19.19 | 1.18E-05 | 3.35E-05 | 1.648 |
| RHSIM_Rhsim09G0068100 | 19.19 | 1.18E-05 | 3.35E-05 | 3.351 |
| RHSIM_Rhsim12G0130700 | 19.18 | 1.19E-05 | 3.37E-05 | 1.735 |
| RHSIM_Rhsim02G0131400 | 19.17 | 1.20E-05 | 3.39E-05 | -5.144 |
| RHSIM_Rhsim11G0021500 | 19.17 | 1.20E-05 | 3.39E-05 | 0.451 |

| Gene | LRT statistic | P value | BH-adjusted P | log2FC (interaction) |
|---|---|---|---|---|
| RHSIM_Rhsim11G0117900 | 19.16 | 1.20E-05 | 3.40E-05 | 0.596 |
| RHSIM_Rhsim04G0188700 | 19.16 | 1.20E-05 | 3.40E-05 | -3.824 |
| RHSIM_Rhsim13G0002400 | 19.16 | 1.20E-05 | 3.41E-05 | 0.863 |
| novel.833 | 19.16 | 1.20E-05 | 3.41E-05 | 2.522 |
| RHSIM_Rhsim01G0289400 | 19.15 | 1.21E-05 | 3.42E-05 | -1.353 |
| RHSIM_Rhsim10G0112600 | 19.15 | 1.21E-05 | 3.43E-05 | 0.694 |
| RHSIM_Rhsim08G0204100 | 19.14 | 1.21E-05 | 3.44E-05 | 2.097 |
| RHSIM_Rhsim03G0065300 | 19.13 | 1.22E-05 | 3.46E-05 | 0.873 |
| RHSIM_Rhsim09G0046600 | 19.12 | 1.23E-05 | 3.47E-05 | -0.557 |
| RHSIM_Rhsim03G0259400 | 19.12 | 1.23E-05 | 3.48E-05 | 0.487 |
| RHSIM_RhsimUnG0201400 | 19.11 | 1.24E-05 | 3.50E-05 | -2.271 |
| RHSIM_Rhsim12G0188300 | 19.11 | 1.24E-05 | 3.50E-05 | -2.085 |
| RHSIM_Rhsim10G0195500 | 19.10 | 1.24E-05 | 3.50E-05 | 1.398 |
| RHSIM_Rhsim07G0193400 | 19.10 | 1.24E-05 | 3.52E-05 | 0.519 |
| RHSIM_Rhsim10G0064800 | 19.09 | 1.24E-05 | 3.52E-05 | 0.458 |
| RHSIM_Rhsim08G0242600 | 19.09 | 1.25E-05 | 3.52E-05 | 0.429 |
| RHSIM_Rhsim03G0056800 | 19.09 | 1.25E-05 | 3.53E-05 | 1.367 |
| RHSIM_Rhsim06G0216300 | 19.09 | 1.25E-05 | 3.53E-05 | -0.473 |
| RHSIM_Rhsim06G0148300 | 19.08 | 1.25E-05 | 3.54E-05 | 1.597 |
| RHSIM_Rhsim11G0040500 | 19.08 | 1.25E-05 | 3.54E-05 | -0.542 |
| RHSIM_Rhsim04G0193400 | 19.08 | 1.25E-05 | 3.54E-05 | 0.341 |
| RHSIM_Rhsim08G0196500 | 19.08 | 1.25E-05 | 3.54E-05 | -1.025 |
| RHSIM_RhsimUnG0008300 | 19.08 | 1.25E-05 | 3.54E-05 | -1.849 |
| RHSIM_Rhsim03G0090900 | 19.08 | 1.26E-05 | 3.55E-05 | -2.404 |
| RHSIM_Rhsim09G0205600 | 19.07 | 1.26E-05 | 3.56E-05 | 1.002 |
| novel.2137 | 19.07 | 1.26E-05 | 3.56E-05 | -0.546 |
| RHSIM_Rhsim04G0019400 | 19.05 | 1.27E-05 | 3.59E-05 | 0.630 |
| RHSIM_Rhsim09G0170100 | 19.04 | 1.28E-05 | 3.61E-05 | -0.606 |
| RHSIM_Rhsim07G0188300 | 19.04 | 1.28E-05 | 3.61E-05 | 0.759 |
| RHSIM_Rhsim13G0162400 | 19.04 | 1.28E-05 | 3.62E-05 | 2.513 |
| RHSIM_Rhsim13G0023700 | 19.04 | 1.28E-05 | 3.62E-05 | 1.703 |
| RHSIM_Rhsim08G0078500 | 19.04 | 1.28E-05 | 3.62E-05 | -1.090 |

| Gene | LRT statistic | P value | BH-adjusted P | log2FC (interaction) |
|---|---|---|---|---|
| RHSIM_Rhsim11G0096800 | 19.04 | 1.28E-05 | 3.62E-05 | 0.457 |
| RHSIM_Rhsim06G0018600 | 19.02 | 1.29E-05 | 3.64E-05 | 0.394 |
| RHSIM_Rhsim01G0272800 | 19.02 | 1.29E-05 | 3.64E-05 | -0.668 |
| RHSIM_Rhsim07G0120100 | 19.01 | 1.30E-05 | 3.67E-05 | 1.389 |
| novel.3801 | 19.01 | 1.30E-05 | 3.68E-05 | -1.779 |
| RHSIM_Rhsim02G0111000 | 19.00 | 1.31E-05 | 3.69E-05 | 2.423 |
| RHSIM_Rhsim08G0125400 | 19.00 | 1.31E-05 | 3.70E-05 | 3.638 |
| RHSIM_Rhsim01G0101800 | 19.00 | 1.31E-05 | 3.70E-05 | 2.441 |
| RHSIM_Rhsim06G0059400 | 18.99 | 1.31E-05 | 3.70E-05 | 0.529 |
| RHSIM_Rhsim12G0115700 | 18.99 | 1.31E-05 | 3.70E-05 | 0.673 |
| RHSIM_Rhsim12G0051300 | 18.99 | 1.31E-05 | 3.71E-05 | 2.124 |
| RHSIM_Rhsim01G0292400 | 18.99 | 1.31E-05 | 3.71E-05 | -0.310 |
| RHSIM_Rhsim13G0144100 | 18.98 | 1.32E-05 | 3.72E-05 | 0.430 |
| RHSIM_Rhsim06G0021300 | 18.98 | 1.32E-05 | 3.73E-05 | 4.300 |
| RHSIM_Rhsim04G0162300 | 18.97 | 1.33E-05 | 3.74E-05 | 0.438 |
| RHSIM_Rhsim04G0242200 | 18.97 | 1.33E-05 | 3.74E-05 | 0.329 |
| RHSIM_Rhsim02G0188100 | 18.97 | 1.33E-05 | 3.75E-05 | 1.435 |
| RHSIM_Rhsim03G0147600 | 18.97 | 1.33E-05 | 3.75E-05 | -0.547 |
| RHSIM_Rhsim07G0157300 | 18.96 | 1.34E-05 | 3.77E-05 | 0.731 |
| RHSIM_Rhsim04G0121000 | 18.94 | 1.35E-05 | 3.80E-05 | 0.982 |
| RHSIM_Rhsim06G0095100 | 18.93 | 1.36E-05 | 3.83E-05 | -0.737 |
| RHSIM_Rhsim06G0038300 | 18.91 | 1.37E-05 | 3.85E-05 | 2.712 |
| novel.3511 | 18.91 | 1.37E-05 | 3.86E-05 | 0.358 |
| RHSIM_Rhsim07G0087600 | 18.90 | 1.38E-05 | 3.89E-05 | -1.784 |
| novel.1779 | 18.90 | 1.38E-05 | 3.89E-05 | 2.051 |
| RHSIM_Rhsim05G0037700 | 18.89 | 1.38E-05 | 3.89E-05 | -4.801 |
| RHSIM_Rhsim13G0035600 | 18.89 | 1.39E-05 | 3.91E-05 | 0.360 |
| RHSIM_Rhsim11G0198300 | 18.88 | 1.39E-05 | 3.91E-05 | 1.030 |
| RHSIM_Rhsim01G0262600 | 18.88 | 1.39E-05 | 3.92E-05 | -0.929 |
| novel.958 | 18.88 | 1.39E-05 | 3.93E-05 | -2.945 |
| RHSIM_Rhsim05G0128100 | 18.88 | 1.39E-05 | 3.93E-05 | 2.217 |
| RHSIM_Rhsim07G0242100 | 18.87 | 1.40E-05 | 3.93E-05 | -0.791 |

| Gene | LRT statistic | P value | BH-adjusted P | log2FC (interaction) |
|---|---|---|---|---|
| RHSIM_Rhsim04G0218200 | 18.87 | 1.40E-05 | 3.93E-05 | 0.759 |
| novel.3271 | 18.87 | 1.40E-05 | 3.94E-05 | 1.969 |
| RHSIM_Rhsim02G0208400 | 18.87 | 1.40E-05 | 3.94E-05 | 1.925 |
| RHSIM_RhsimUnG0251100 | 18.86 | 1.40E-05 | 3.95E-05 | -0.696 |
| novel.1528 | 18.85 | 1.42E-05 | 3.98E-05 | 1.130 |
| RHSIM_Rhsim11G0039200 | 18.84 | 1.42E-05 | 3.99E-05 | -0.237 |
| RHSIM_Rhsim03G0229000 | 18.84 | 1.42E-05 | 3.99E-05 | 2.285 |
| RHSIM_Rhsim08G0241500 | 18.83 | 1.43E-05 | 4.02E-05 | -0.683 |
| RHSIM_Rhsim12G0087100 | 18.83 | 1.43E-05 | 4.02E-05 | -42.066 |
| RHSIM_Rhsim04G0158000 | 18.83 | 1.43E-05 | 4.03E-05 | 1.311 |
| RHSIM_Rhsim03G0073600 | 18.82 | 1.44E-05 | 4.04E-05 | -1.611 |
| RHSIM_Rhsim11G0037100 | 18.82 | 1.44E-05 | 4.04E-05 | -2.205 |
| RHSIM_Rhsim13G0184800 | 18.82 | 1.44E-05 | 4.05E-05 | -0.991 |
| RHSIM_Rhsim12G0195500 | 18.81 | 1.44E-05 | 4.05E-05 | -5.434 |
| RHSIM_Rhsim03G0230100 | 18.81 | 1.44E-05 | 4.05E-05 | 0.384 |
| RHSIM_Rhsim05G0122200 | 18.81 | 1.44E-05 | 4.05E-05 | -1.068 |
| RHSIM_Rhsim12G0174800 | 18.79 | 1.46E-05 | 4.10E-05 | 3.078 |
| RHSIM_RhsimUnG0040800 | 18.79 | 1.46E-05 | 4.10E-05 | -1.400 |
| RHSIM_Rhsim08G0082500 | 18.79 | 1.46E-05 | 4.10E-05 | 0.784 |
| RHSIM_Rhsim03G0130400 | 18.79 | 1.46E-05 | 4.11E-05 | 0.357 |
| RHSIM_Rhsim06G0098200 | 18.78 | 1.47E-05 | 4.12E-05 | 1.080 |
| novel.2142 | 18.78 | 1.47E-05 | 4.12E-05 | -1.587 |
| RHSIM_Rhsim04G0101700 | 18.78 | 1.47E-05 | 4.12E-05 | 1.852 |
| RHSIM_Rhsim08G0236400 | 18.78 | 1.47E-05 | 4.12E-05 | 2.022 |
| RHSIM_Rhsim10G0005500 | 18.78 | 1.47E-05 | 4.13E-05 | -0.624 |
| RHSIM_Rhsim13G0178100 | 18.77 | 1.47E-05 | 4.13E-05 | 0.459 |
| novel.680 | 18.77 | 1.48E-05 | 4.15E-05 | 1.563 |
| novel.1699 | 18.77 | 1.48E-05 | 4.15E-05 | -2.047 |
| RHSIM_Rhsim12G0022200 | 18.76 | 1.48E-05 | 4.16E-05 | -0.576 |
| RHSIM_Rhsim02G0052200 | 18.76 | 1.48E-05 | 4.16E-05 | 0.447 |
| RHSIM_Rhsim11G0015700 | 18.76 | 1.48E-05 | 4.16E-05 | 1.058 |
| RHSIM_Rhsim03G0034300 | 18.75 | 1.49E-05 | 4.18E-05 | -0.718 |

| Gene | LRT statistic | P value | BH-adjusted P | log2FC (interaction) |
|---|---|---|---|---|
| RHSIM_Rhsim02G0215100 | 18.74 | 1.50E-05 | 4.20E-05 | -40.995 |
| RHSIM_Rhsim05G0001300 | 18.74 | 1.50E-05 | 4.21E-05 | -0.376 |
| RHSIM_Rhsim03G0096200 | 18.74 | 1.50E-05 | 4.21E-05 | -2.077 |
| novel.2380 | 18.74 | 1.50E-05 | 4.21E-05 | -1.019 |
| RHSIM_Rhsim10G0119800 | 18.73 | 1.50E-05 | 4.21E-05 | 0.511 |
| RHSIM_Rhsim08G0095000 | 18.73 | 1.50E-05 | 4.22E-05 | -0.981 |
| RHSIM_Rhsim01G0131200 | 18.73 | 1.50E-05 | 4.22E-05 | -0.920 |
| novel.707 | 18.73 | 1.51E-05 | 4.22E-05 | -0.589 |
| RHSIM_Rhsim06G0112200 | 18.73 | 1.51E-05 | 4.23E-05 | -3.485 |
| RHSIM_Rhsim07G0005700 | 18.72 | 1.51E-05 | 4.24E-05 | 0.605 |
| RHSIM_Rhsim08G0107200 | 18.72 | 1.51E-05 | 4.24E-05 | 6.444 |
| RHSIM_Rhsim08G0149400 | 18.72 | 1.51E-05 | 4.24E-05 | 0.953 |
| novel.444 | 18.71 | 1.52E-05 | 4.25E-05 | -1.174 |
| RHSIM_Rhsim05G0038300 | 18.71 | 1.52E-05 | 4.26E-05 | -0.532 |
| RHSIM_Rhsim07G0124600 | 18.70 | 1.53E-05 | 4.29E-05 | -0.488 |
| RHSIM_Rhsim06G0182800 | 18.69 | 1.54E-05 | 4.31E-05 | 5.248 |
| novel.4499 | 18.69 | 1.54E-05 | 4.31E-05 | 1.317 |
| novel.624 | 18.69 | 1.54E-05 | 4.31E-05 | 2.602 |
| RHSIM_Rhsim03G0058400 | 18.69 | 1.54E-05 | 4.32E-05 | 0.683 |
| RHSIM_Rhsim11G0063100 | 18.68 | 1.54E-05 | 4.32E-05 | -1.884 |
| novel.2580 | 18.68 | 1.55E-05 | 4.33E-05 | 1.671 |
| RHSIM_Rhsim03G0221000 | 18.68 | 1.55E-05 | 4.33E-05 | 0.419 |
| RHSIM_Rhsim10G0128200 | 18.67 | 1.55E-05 | 4.35E-05 | 0.798 |
| novel.1262 | 18.66 | 1.56E-05 | 4.37E-05 | 0.871 |
| novel.1280 | 18.66 | 1.57E-05 | 4.38E-05 | -1.512 |
| novel.65 | 18.65 | 1.57E-05 | 4.39E-05 | -4.759 |
| RHSIM_Rhsim04G0158800 | 18.65 | 1.57E-05 | 4.39E-05 | 0.693 |
| RHSIM_RhsimUnG0073700 | 18.65 | 1.57E-05 | 4.40E-05 | -1.887 |
| RHSIM_Rhsim08G0033900 | 18.65 | 1.57E-05 | 4.40E-05 | 0.310 |
| novel.1572 | 18.65 | 1.57E-05 | 4.40E-05 | 3.659 |
| RHSIM_Rhsim07G0258500 | 18.64 | 1.58E-05 | 4.41E-05 | -0.404 |
| RHSIM_Rhsim06G0127800 | 18.64 | 1.58E-05 | 4.42E-05 | 1.332 |

| Gene | LRT statistic | P value | BH-adjusted P | log2FC (interaction) |
|---|---|---|---|---|
| RHSIM_Rhsim09G0147000 | 18.63 | 1.59E-05 | 4.45E-05 | -0.723 |
| RHSIM_Rhsim09G0043300 | 18.62 | 1.59E-05 | 4.45E-05 | 0.419 |
| RHSIM_Rhsim03G0149100 | 18.62 | 1.59E-05 | 4.46E-05 | 0.343 |
| RHSIM_Rhsim08G0003200 | 18.62 | 1.60E-05 | 4.47E-05 | -0.348 |
| novel.302 | 18.61 | 1.60E-05 | 4.47E-05 | -3.104 |
| novel.4531 | 18.60 | 1.61E-05 | 4.50E-05 | -2.203 |
| RHSIM_Rhsim07G0029600 | 18.60 | 1.61E-05 | 4.51E-05 | 1.006 |
| novel.2984 | 18.59 | 1.62E-05 | 4.53E-05 | 1.068 |
| RHSIM_Rhsim02G0086100 | 18.58 | 1.63E-05 | 4.55E-05 | -0.718 |
| RHSIM_Rhsim11G0027500 | 18.57 | 1.63E-05 | 4.57E-05 | 1.196 |
| novel.1082 | 18.57 | 1.63E-05 | 4.57E-05 | 0.711 |
| RHSIM_Rhsim06G0206200 | 18.57 | 1.63E-05 | 4.57E-05 | 1.398 |
| RHSIM_Rhsim06G0113900 | 18.57 | 1.64E-05 | 4.58E-05 | 0.747 |
| RHSIM_Rhsim09G0054700 | 18.56 | 1.64E-05 | 4.59E-05 | 2.979 |
| RHSIM_Rhsim08G0093500 | 18.56 | 1.65E-05 | 4.60E-05 | -0.993 |
| novel.3223 | 18.56 | 1.65E-05 | 4.60E-05 | -0.785 |
| novel.1099 | 18.55 | 1.65E-05 | 4.62E-05 | -0.810 |
| novel.4317 | 18.55 | 1.65E-05 | 4.62E-05 | -0.627 |
| RHSIM_Rhsim02G0247900 | 18.54 | 1.66E-05 | 4.64E-05 | 2.588 |
| RHSIM_Rhsim07G0083300 | 18.54 | 1.66E-05 | 4.64E-05 | -0.675 |
| RHSIM_Rhsim01G0221400 | 18.54 | 1.67E-05 | 4.65E-05 | 0.595 |
| novel.4155 | 18.53 | 1.67E-05 | 4.67E-05 | 2.162 |
| RHSIM_Rhsim05G0234000 | 18.52 | 1.68E-05 | 4.68E-05 | -0.870 |
| RHSIM_Rhsim01G0205800 | 18.52 | 1.68E-05 | 4.68E-05 | -1.529 |
| RHSIM_Rhsim05G0085600 | 18.52 | 1.68E-05 | 4.68E-05 | -1.980 |
| RHSIM_Rhsim13G0089900 | 18.52 | 1.68E-05 | 4.70E-05 | 1.825 |
| RHSIM_Rhsim12G0143700 | 18.50 | 1.70E-05 | 4.74E-05 | 1.551 |
| RHSIM_Rhsim07G0190700 | 18.50 | 1.70E-05 | 4.74E-05 | -0.447 |
| RHSIM_Rhsim04G0214100 | 18.49 | 1.71E-05 | 4.76E-05 | 0.317 |
| RHSIM_Rhsim08G0152700 | 18.49 | 1.71E-05 | 4.77E-05 | 0.333 |
| novel.3626 | 18.48 | 1.72E-05 | 4.78E-05 | 1.361 |
| RHSIM_Rhsim04G0132600 | 18.48 | 1.72E-05 | 4.79E-05 | 0.565 |

| Gene | LRT statistic | P value | BH-adjusted P | log2FC (interaction) |
|---|---|---|---|---|
| RHSIM_Rhsim06G0039300 | 18.48 | 1.72E-05 | 4.80E-05 | 1.019 |
| novel.312 | 18.48 | 1.72E-05 | 4.80E-05 | -0.705 |
| RHSIM_Rhsim04G0026600 | 18.48 | 1.72E-05 | 4.80E-05 | 3.145 |
| novel.3016 | 18.47 | 1.72E-05 | 4.80E-05 | 4.222 |
| RHSIM_Rhsim13G0126400 | 18.47 | 1.73E-05 | 4.82E-05 | -0.347 |
| RHSIM_Rhsim11G0146200 | 18.47 | 1.73E-05 | 4.82E-05 | 0.517 |
| RHSIM_Rhsim12G0096800 | 18.46 | 1.73E-05 | 4.83E-05 | -0.292 |
| RHSIM_Rhsim11G0008000 | 18.46 | 1.74E-05 | 4.84E-05 | 0.473 |
| RHSIM_Rhsim08G0135000 | 18.46 | 1.74E-05 | 4.84E-05 | 0.798 |
| RHSIM_Rhsim05G0214700 | 18.45 | 1.74E-05 | 4.85E-05 | 0.233 |
| RHSIM_Rhsim08G0013000 | 18.44 | 1.75E-05 | 4.88E-05 | -0.760 |
| RHSIM_Rhsim13G0173300 | 18.44 | 1.75E-05 | 4.89E-05 | -0.658 |
| novel.500 | 18.43 | 1.76E-05 | 4.90E-05 | 1.319 |
| RHSIM_Rhsim03G0237000 | 18.43 | 1.77E-05 | 4.92E-05 | 0.674 |
| novel.3565 | 18.42 | 1.77E-05 | 4.94E-05 | -1.498 |
| RHSIM_Rhsim10G0083300 | 18.42 | 1.78E-05 | 4.94E-05 | -1.262 |
| RHSIM_Rhsim11G0037200 | 18.42 | 1.78E-05 | 4.95E-05 | 1.438 |
| RHSIM_Rhsim02G0051200 | 18.41 | 1.78E-05 | 4.95E-05 | -1.121 |
| RHSIM_Rhsim13G0157700 | 18.41 | 1.78E-05 | 4.95E-05 | -1.079 |
| RHSIM_Rhsim05G0044300 | 18.40 | 1.79E-05 | 4.97E-05 | 0.193 |
| RHSIM_Rhsim12G0134800 | 18.40 | 1.79E-05 | 4.98E-05 | -0.599 |
| RHSIM_Rhsim01G0242900 | 18.40 | 1.79E-05 | 4.99E-05 | 4.631 |
| RHSIM_Rhsim01G0082300 | 18.40 | 1.79E-05 | 4.99E-05 | -0.881 |
| novel.333 | 18.40 | 1.80E-05 | 4.99E-05 | -0.264 |
| RHSIM_Rhsim12G0193000 | 18.39 | 1.80E-05 | 5.00E-05 | -0.541 |
| RHSIM_Rhsim07G0110600 | 18.39 | 1.80E-05 | 5.00E-05 | -4.973 |
| RHSIM_Rhsim12G0035000 | 18.39 | 1.80E-05 | 5.00E-05 | -0.877 |
| RHSIM_Rhsim07G0072500 | 18.39 | 1.80E-05 | 5.02E-05 | 6.341 |
| RHSIM_RhsimUnG0219100 | 18.38 | 1.81E-05 | 5.03E-05 | 1.711 |
| RHSIM_Rhsim11G0047500 | 18.37 | 1.82E-05 | 5.05E-05 | -0.739 |
| RHSIM_Rhsim07G0118700 | 18.37 | 1.82E-05 | 5.06E-05 | -1.626 |
| RHSIM_Rhsim04G0188800 | 18.37 | 1.82E-05 | 5.06E-05 | -0.350 |

| Gene | LRT statistic | P value | BH-adjusted P | log2FC (interaction) |
|---|---|---|---|---|
| novel.1737 | 18.37 | 1.82E-05 | 5.06E-05 | 2.815 |
| RHSIM_Rhsim02G0027800 | 18.37 | 1.82E-05 | 5.06E-05 | 0.457 |
| RHSIM_Rhsim12G0043700 | 18.36 | 1.83E-05 | 5.08E-05 | 0.849 |
| RHSIM_Rhsim10G0183300 | 18.36 | 1.83E-05 | 5.09E-05 | 0.396 |
| RHSIM_Rhsim10G0214800 | 18.35 | 1.84E-05 | 5.12E-05 | 0.720 |
| RHSIM_Rhsim02G0206800 | 18.34 | 1.85E-05 | 5.13E-05 | 0.261 |
| novel.1141 | 18.33 | 1.85E-05 | 5.15E-05 | -4.531 |
| novel.3435 | 18.33 | 1.86E-05 | 5.16E-05 | -1.337 |
| RHSIM_Rhsim06G0234000 | 18.32 | 1.87E-05 | 5.18E-05 | 0.312 |
| RHSIM_Rhsim11G0057900 | 18.32 | 1.87E-05 | 5.20E-05 | -0.805 |
| RHSIM_Rhsim13G0200600 | 18.31 | 1.87E-05 | 5.20E-05 | 0.402 |
| RHSIM_Rhsim12G0192300 | 18.31 | 1.88E-05 | 5.22E-05 | -0.330 |
| RHSIM_Rhsim01G0117700 | 18.31 | 1.88E-05 | 5.22E-05 | 2.312 |
| RHSIM_Rhsim03G0203300 | 18.30 | 1.88E-05 | 5.23E-05 | 2.397 |
| RHSIM_Rhsim13G0216600 | 18.30 | 1.89E-05 | 5.24E-05 | -0.706 |
| RHSIM_Rhsim08G0038200 | 18.29 | 1.89E-05 | 5.25E-05 | 0.513 |
| novel.4386 | 18.29 | 1.90E-05 | 5.27E-05 | -0.655 |
| RHSIM_Rhsim05G0176500 | 18.29 | 1.90E-05 | 5.28E-05 | 1.841 |
| RHSIM_Rhsim08G0046000 | 18.28 | 1.90E-05 | 5.28E-05 | 0.514 |
| RHSIM_Rhsim01G0107600 | 18.28 | 1.91E-05 | 5.29E-05 | -0.412 |
| RHSIM_Rhsim09G0028700 | 18.28 | 1.91E-05 | 5.30E-05 | 1.366 |
| RHSIM_Rhsim02G0244300 | 18.27 | 1.91E-05 | 5.31E-05 | 0.535 |
| RHSIM_Rhsim04G0238000 | 18.26 | 1.92E-05 | 5.34E-05 | -0.641 |
| novel.3289 | 18.25 | 1.94E-05 | 5.38E-05 | -43.667 |
| novel.4426 | 18.25 | 1.94E-05 | 5.38E-05 | 1.499 |
| RHSIM_Rhsim13G0001600 | 18.24 | 1.95E-05 | 5.40E-05 | -0.255 |
| RHSIM_Rhsim01G0116400 | 18.24 | 1.95E-05 | 5.40E-05 | 0.824 |
| RHSIM_Rhsim06G0227000 | 18.24 | 1.95E-05 | 5.41E-05 | 0.353 |
| RHSIM_Rhsim06G0209000 | 18.22 | 1.96E-05 | 5.44E-05 | -1.375 |
| RHSIM_Rhsim06G0060900 | 18.22 | 1.97E-05 | 5.45E-05 | 1.720 |
| RHSIM_Rhsim01G0027600 | 18.22 | 1.97E-05 | 5.45E-05 | 0.707 |
| RHSIM_Rhsim10G0140000 | 18.22 | 1.97E-05 | 5.46E-05 | 42.809 |

| Gene | LRT statistic | P value | BH-adjusted P | log2FC (interaction) |
|---|---|---|---|---|
| RHSIM_Rhsim08G0126400 | 18.21 | 1.98E-05 | 5.49E-05 | 2.675 |
| RHSIM_Rhsim07G0056700 | 18.20 | 1.98E-05 | 5.50E-05 | -0.480 |
| novel.2194 | 18.20 | 1.99E-05 | 5.50E-05 | -0.814 |
| RHSIM_Rhsim07G0080400 | 18.19 | 2.00E-05 | 5.53E-05 | -0.551 |
| RHSIM_Rhsim07G0164400 | 18.19 | 2.00E-05 | 5.53E-05 | 0.367 |
| novel.3831 | 18.19 | 2.00E-05 | 5.54E-05 | -1.630 |
| novel.2827 | 18.19 | 2.00E-05 | 5.54E-05 | -4.564 |
| RHSIM_Rhsim13G0142000 | 18.19 | 2.00E-05 | 5.55E-05 | -0.427 |
| novel.499 | 18.18 | 2.01E-05 | 5.55E-05 | 0.738 |
| RHSIM_Rhsim06G0229500 | 18.18 | 2.01E-05 | 5.55E-05 | -0.512 |
| RHSIM_Rhsim06G0019800 | 18.18 | 2.01E-05 | 5.57E-05 | 0.394 |
| RHSIM_Rhsim13G0203800 | 18.18 | 2.01E-05 | 5.58E-05 | -1.727 |
| novel.4313 | 18.17 | 2.02E-05 | 5.59E-05 | 6.733 |
| RHSIM_Rhsim11G0180500 | 18.17 | 2.02E-05 | 5.59E-05 | 1.815 |
| RHSIM_Rhsim03G0067100 | 18.16 | 2.03E-05 | 5.61E-05 | 1.021 |
| RHSIM_Rhsim12G0210600 | 18.16 | 2.03E-05 | 5.63E-05 | 0.893 |
| RHSIM_Rhsim09G0184300 | 18.15 | 2.04E-05 | 5.65E-05 | -0.818 |
| RHSIM_Rhsim09G0111000 | 18.15 | 2.04E-05 | 5.66E-05 | 1.377 |
| RHSIM_RhsimUnG0223300 | 18.14 | 2.05E-05 | 5.66E-05 | -1.830 |
| RHSIM_Rhsim04G0099000 | 18.14 | 2.05E-05 | 5.67E-05 | 1.045 |
| RHSIM_Rhsim01G0165000 | 18.12 | 2.07E-05 | 5.73E-05 | -0.596 |
| RHSIM_Rhsim07G0195500 | 18.12 | 2.07E-05 | 5.73E-05 | 3.046 |
| RHSIM_Rhsim08G0042600 | 18.12 | 2.07E-05 | 5.73E-05 | 0.362 |
| RHSIM_Rhsim07G0197700 | 18.12 | 2.07E-05 | 5.74E-05 | 1.845 |
| RHSIM_Rhsim06G0188700 | 18.12 | 2.08E-05 | 5.74E-05 | -3.579 |
| RHSIM_Rhsim13G0217200 | 18.11 | 2.08E-05 | 5.76E-05 | -1.364 |
| RHSIM_Rhsim03G0121100 | 18.11 | 2.09E-05 | 5.77E-05 | -0.525 |
| RHSIM_Rhsim05G0172500 | 18.10 | 2.09E-05 | 5.78E-05 | 0.663 |
| RHSIM_Rhsim13G0029800 | 18.10 | 2.09E-05 | 5.78E-05 | 1.217 |
| RHSIM_Rhsim07G0165700 | 18.10 | 2.10E-05 | 5.80E-05 | -0.575 |
| RHSIM_Rhsim11G0065100 | 18.10 | 2.10E-05 | 5.81E-05 | 0.368 |
| RHSIM_Rhsim05G0088600 | 18.09 | 2.11E-05 | 5.83E-05 | 0.876 |

| Gene | LRT statistic | P value | BH-adjusted P | log2FC (interaction) |
|---|---|---|---|---|
| RHSIM_Rhsim06G0016400 | 18.09 | 2.11E-05 | 5.83E-05 | 0.499 |
| RHSIM_Rhsim13G0167400 | 18.09 | 2.11E-05 | 5.83E-05 | 1.790 |
| RHSIM_Rhsim09G0167800 | 18.08 | 2.12E-05 | 5.86E-05 | 0.797 |
| novel.4009 | 18.07 | 2.13E-05 | 5.87E-05 | -2.225 |
| RHSIM_Rhsim11G0070200 | 18.06 | 2.14E-05 | 5.90E-05 | 1.914 |
| novel.373 | 18.06 | 2.14E-05 | 5.92E-05 | -3.364 |
| RHSIM_RhsimUnG0119600 | 18.05 | 2.15E-05 | 5.93E-05 | -0.317 |
| RHSIM_Rhsim10G0011500 | 18.05 | 2.15E-05 | 5.93E-05 | -0.436 |
| RHSIM_Rhsim07G0003000 | 18.05 | 2.15E-05 | 5.93E-05 | -0.436 |
| RHSIM_Rhsim09G0213700 | 18.05 | 2.15E-05 | 5.95E-05 | 0.610 |
| RHSIM_Rhsim08G0155100 | 18.04 | 2.16E-05 | 5.97E-05 | 1.754 |
| RHSIM_Rhsim12G0016500 | 18.03 | 2.17E-05 | 6.00E-05 | -0.478 |
| RHSIM_Rhsim04G0186800 | 18.02 | 2.19E-05 | 6.03E-05 | -2.838 |
| RHSIM_Rhsim08G0183400 | 18.02 | 2.19E-05 | 6.04E-05 | 2.542 |
| RHSIM_Rhsim08G0200400 | 18.02 | 2.19E-05 | 6.05E-05 | 0.873 |
| RHSIM_Rhsim10G0212500 | 18.01 | 2.20E-05 | 6.07E-05 | 1.458 |
| RHSIM_Rhsim03G0154500 | 18.01 | 2.20E-05 | 6.07E-05 | 0.748 |
| RHSIM_RhsimUnG0053600 | 17.99 | 2.22E-05 | 6.13E-05 | -4.304 |
| RHSIM_Rhsim12G0123000 | 17.98 | 2.23E-05 | 6.15E-05 | -2.127 |
| RHSIM_Rhsim12G0047600 | 17.98 | 2.23E-05 | 6.16E-05 | -0.246 |
| RHSIM_Rhsim01G0207100 | 17.98 | 2.24E-05 | 6.17E-05 | 2.850 |
| RHSIM_Rhsim06G0236400 | 17.97 | 2.24E-05 | 6.18E-05 | -0.459 |
| novel.2463 | 17.97 | 2.24E-05 | 6.18E-05 | -3.552 |
| novel.1875 | 17.93 | 2.29E-05 | 6.32E-05 | -41.718 |
| RHSIM_Rhsim10G0081800 | 17.93 | 2.29E-05 | 6.32E-05 | 0.307 |
| RHSIM_Rhsim09G0165400 | 17.93 | 2.29E-05 | 6.32E-05 | -0.667 |
| RHSIM_Rhsim01G0200800 | 17.93 | 2.30E-05 | 6.32E-05 | -0.468 |
| RHSIM_Rhsim02G0199000 | 17.93 | 2.30E-05 | 6.32E-05 | 0.744 |
| RHSIM_Rhsim06G0060000 | 17.93 | 2.30E-05 | 6.33E-05 | -0.896 |
| RHSIM_Rhsim02G0194400 | 17.92 | 2.30E-05 | 6.35E-05 | 0.945 |
| RHSIM_Rhsim01G0227200 | 17.92 | 2.31E-05 | 6.35E-05 | -0.566 |
| RHSIM_Rhsim11G0197700 | 17.92 | 2.31E-05 | 6.36E-05 | -0.870 |

| Gene | LRT statistic | P value | BH-adjusted P | log2FC (interaction) |
|---|---|---|---|---|
| RHSIM_Rhsim08G0044000 | 17.92 | 2.31E-05 | 6.36E-05 | 3.930 |
| RHSIM_Rhsim02G0206300 | 17.91 | 2.32E-05 | 6.39E-05 | 1.460 |
| RHSIM_Rhsim07G0242400 | 17.90 | 2.32E-05 | 6.40E-05 | 0.535 |
| RHSIM_Rhsim06G0024700 | 17.90 | 2.32E-05 | 6.40E-05 | -1.405 |
| RHSIM_Rhsim03G0198300 | 17.90 | 2.33E-05 | 6.40E-05 | -1.143 |
| RHSIM_Rhsim13G0027500 | 17.90 | 2.33E-05 | 6.42E-05 | -0.440 |
| RHSIM_Rhsim10G0137400 | 17.90 | 2.33E-05 | 6.42E-05 | 1.831 |
| RHSIM_Rhsim02G0223700 | 17.89 | 2.34E-05 | 6.43E-05 | -0.931 |
| novel.1168 | 17.89 | 2.34E-05 | 6.44E-05 | 0.676 |
| RHSIM_Rhsim01G0210600 | 17.89 | 2.34E-05 | 6.44E-05 | 1.335 |
| RHSIM_Rhsim05G0029400 | 17.89 | 2.34E-05 | 6.45E-05 | -0.272 |
| RHSIM_Rhsim03G0237700 | 17.88 | 2.36E-05 | 6.48E-05 | -1.826 |
| RHSIM_Rhsim11G0173200 | 17.87 | 2.36E-05 | 6.50E-05 | -0.483 |
| novel.3262 | 17.87 | 2.36E-05 | 6.50E-05 | 1.945 |
| novel.1062 | 17.86 | 2.37E-05 | 6.53E-05 | 0.302 |
| RHSIM_Rhsim12G0150500 | 17.86 | 2.38E-05 | 6.54E-05 | -3.979 |
| RHSIM_Rhsim07G0016300 | 17.85 | 2.39E-05 | 6.59E-05 | -1.073 |
| RHSIM_RhsimUnG0135900 | 17.85 | 2.40E-05 | 6.59E-05 | -0.592 |
| RHSIM_Rhsim11G0019800 | 17.84 | 2.40E-05 | 6.59E-05 | -0.700 |
| novel.3199 | 17.84 | 2.40E-05 | 6.60E-05 | -1.691 |
| RHSIM_Rhsim12G0198800 | 17.84 | 2.41E-05 | 6.61E-05 | -0.978 |
| novel.3293 | 17.84 | 2.41E-05 | 6.61E-05 | -2.620 |
| novel.4256 | 17.83 | 2.41E-05 | 6.63E-05 | 4.346 |
| RHSIM_Rhsim12G0006700 | 17.83 | 2.41E-05 | 6.63E-05 | -1.410 |
| RHSIM_Rhsim13G0126000 | 17.83 | 2.41E-05 | 6.63E-05 | -1.455 |
| novel.4368 | 17.83 | 2.42E-05 | 6.64E-05 | 1.802 |
| RHSIM_Rhsim13G0177500 | 17.82 | 2.43E-05 | 6.67E-05 | -0.336 |
| RHSIM_Rhsim12G0165000 | 17.82 | 2.43E-05 | 6.67E-05 | -0.501 |
| RHSIM_Rhsim05G0079400 | 17.82 | 2.43E-05 | 6.67E-05 | -0.325 |
| RHSIM_Rhsim02G0241000 | 17.81 | 2.45E-05 | 6.72E-05 | 0.337 |
| RHSIM_Rhsim04G0108200 | 17.80 | 2.46E-05 | 6.74E-05 | -0.659 |
| RHSIM_Rhsim12G0074900 | 17.78 | 2.48E-05 | 6.80E-05 | 0.541 |

| Gene | LRT statistic | P value | BH-adjusted P | log2FC (interaction) |
|---|---|---|---|---|
| RHSIM_RhsimUnG0030200 | 17.78 | 2.48E-05 | 6.80E-05 | 0.598 |
| RHSIM_Rhsim02G0063700 | 17.78 | 2.48E-05 | 6.81E-05 | 1.341 |
| novel.393 | 17.78 | 2.48E-05 | 6.81E-05 | 4.080 |
| RHSIM_Rhsim11G0057000 | 17.77 | 2.50E-05 | 6.85E-05 | 0.476 |
| RHSIM_Rhsim08G0045600 | 17.77 | 2.50E-05 | 6.86E-05 | -0.813 |
| RHSIM_Rhsim07G0225700 | 17.76 | 2.50E-05 | 6.87E-05 | 1.645 |
| RHSIM_Rhsim11G0012500 | 17.76 | 2.51E-05 | 6.88E-05 | 0.690 |
| RHSIM_Rhsim08G0031600 | 17.75 | 2.52E-05 | 6.90E-05 | 1.712 |
| RHSIM_Rhsim09G0005400 | 17.75 | 2.52E-05 | 6.92E-05 | -0.737 |
| RHSIM_Rhsim12G0121500 | 17.75 | 2.52E-05 | 6.92E-05 | 0.381 |
| RHSIM_Rhsim01G0101300 | 17.75 | 2.52E-05 | 6.92E-05 | -0.361 |
| RHSIM_Rhsim03G0238500 | 17.75 | 2.52E-05 | 6.92E-05 | 1.014 |
| RHSIM_Rhsim11G0074600 | 17.75 | 2.53E-05 | 6.92E-05 | 0.883 |
| RHSIM_Rhsim05G0058500 | 17.75 | 2.53E-05 | 6.93E-05 | -1.492 |
| RHSIM_Rhsim08G0028700 | 17.74 | 2.53E-05 | 6.94E-05 | -0.814 |
| RHSIM_Rhsim04G0170500 | 17.74 | 2.53E-05 | 6.95E-05 | 0.630 |
| RHSIM_Rhsim08G0114500 | 17.74 | 2.54E-05 | 6.95E-05 | -0.259 |
| RHSIM_RhsimUnG0021800 | 17.73 | 2.54E-05 | 6.96E-05 | -0.445 |
| RHSIM_Rhsim13G0177900 | 17.73 | 2.54E-05 | 6.97E-05 | 0.703 |
| RHSIM_Rhsim12G0196900 | 17.73 | 2.55E-05 | 6.99E-05 | 3.209 |
| RHSIM_Rhsim01G0257600 | 17.72 | 2.56E-05 | 7.01E-05 | -0.587 |
| novel.723 | 17.72 | 2.56E-05 | 7.01E-05 | 1.538 |
| RHSIM_Rhsim08G0160600 | 17.72 | 2.56E-05 | 7.02E-05 | -1.746 |
| RHSIM_Rhsim09G0105400 | 17.71 | 2.57E-05 | 7.04E-05 | -0.336 |
| RHSIM_Rhsim11G0192900 | 17.70 | 2.59E-05 | 7.08E-05 | -0.287 |
| RHSIM_Rhsim03G0267000 | 17.69 | 2.59E-05 | 7.11E-05 | 3.854 |
| RHSIM_Rhsim09G0102300 | 17.69 | 2.60E-05 | 7.12E-05 | 4.278 |
| RHSIM_Rhsim12G0100700 | 17.68 | 2.61E-05 | 7.14E-05 | 2.817 |
| RHSIM_Rhsim08G0065200 | 17.68 | 2.61E-05 | 7.16E-05 | 2.050 |
| RHSIM_Rhsim04G0173200 | 17.67 | 2.63E-05 | 7.20E-05 | 1.727 |
| RHSIM_Rhsim11G0141000 | 17.66 | 2.64E-05 | 7.23E-05 | -0.332 |
| RHSIM_Rhsim03G0076400 | 17.66 | 2.64E-05 | 7.23E-05 | -0.781 |

| Gene | LRT statistic | P value | BH-adjusted P | log2FC (interaction) |
|---|---|---|---|---|
| RHSIM_Rhsim05G0159300 | 17.65 | 2.65E-05 | 7.25E-05 | 0.738 |
| RHSIM_Rhsim03G0024700 | 17.65 | 2.65E-05 | 7.27E-05 | -3.169 |
| RHSIM_Rhsim02G0084000 | 17.64 | 2.66E-05 | 7.29E-05 | -1.835 |
| RHSIM_Rhsim08G0099600 | 17.63 | 2.68E-05 | 7.33E-05 | 1.742 |
| RHSIM_Rhsim04G0018300 | 17.63 | 2.69E-05 | 7.35E-05 | -0.477 |
| RHSIM_Rhsim02G0105800 | 17.62 | 2.70E-05 | 7.39E-05 | -0.471 |
| novel.1950 | 17.61 | 2.71E-05 | 7.41E-05 | 1.541 |
| RHSIM_Rhsim09G0200400 | 17.61 | 2.71E-05 | 7.42E-05 | -0.322 |
| RHSIM_Rhsim11G0054800 | 17.60 | 2.72E-05 | 7.45E-05 | -0.390 |
| RHSIM_Rhsim02G0099100 | 17.60 | 2.72E-05 | 7.45E-05 | 0.396 |
| RHSIM_Rhsim07G0193600 | 17.60 | 2.72E-05 | 7.45E-05 | 1.091 |
| RHSIM_Rhsim10G0107800 | 17.60 | 2.72E-05 | 7.45E-05 | 0.588 |
| RHSIM_Rhsim04G0147900 | 17.60 | 2.73E-05 | 7.47E-05 | -0.618 |
| RHSIM_Rhsim01G0156100 | 17.60 | 2.73E-05 | 7.47E-05 | 0.917 |
| RHSIM_Rhsim02G0201500 | 17.59 | 2.73E-05 | 7.47E-05 | -0.362 |
| RHSIM_Rhsim02G0251800 | 17.59 | 2.74E-05 | 7.48E-05 | -0.477 |
| RHSIM_Rhsim08G0063300 | 17.59 | 2.74E-05 | 7.48E-05 | -2.593 |
| RHSIM_Rhsim04G0189900 | 17.59 | 2.74E-05 | 7.49E-05 | 0.586 |
| RHSIM_Rhsim09G0112100 | 17.59 | 2.74E-05 | 7.49E-05 | 1.216 |
| RHSIM_Rhsim12G0131400 | 17.58 | 2.75E-05 | 7.52E-05 | -1.939 |
| RHSIM_Rhsim10G0000800 | 17.58 | 2.76E-05 | 7.53E-05 | -1.120 |
| RHSIM_Rhsim11G0194300 | 17.56 | 2.79E-05 | 7.62E-05 | -1.552 |
| RHSIM_Rhsim07G0212700 | 17.55 | 2.80E-05 | 7.65E-05 | 1.261 |
| novel.3089 | 17.54 | 2.81E-05 | 7.67E-05 | 1.422 |
| RHSIM_Rhsim09G0113800 | 17.54 | 2.82E-05 | 7.70E-05 | -0.587 |
| RHSIM_Rhsim02G0106600 | 17.53 | 2.83E-05 | 7.73E-05 | 3.422 |
| RHSIM_Rhsim01G0005500 | 17.53 | 2.83E-05 | 7.73E-05 | -0.449 |
| RHSIM_Rhsim11G0096700 | 17.52 | 2.84E-05 | 7.75E-05 | -0.735 |
| RHSIM_Rhsim06G0021900 | 17.51 | 2.85E-05 | 7.79E-05 | 0.640 |
| RHSIM_Rhsim03G0140100 | 17.51 | 2.86E-05 | 7.80E-05 | 0.299 |
| RHSIM_Rhsim08G0234400 | 17.51 | 2.86E-05 | 7.80E-05 | 0.759 |
| RHSIM_Rhsim13G0017700 | 17.51 | 2.86E-05 | 7.81E-05 | -0.443 |

| Gene | LRT statistic | P value | BH-adjusted P | log2FC (interaction) |
|---|---|---|---|---|
| RHSIM_Rhsim08G0001400 | 17.51 | 2.86E-05 | 7.82E-05 | -0.372 |
| RHSIM_Rhsim07G0071700 | 17.48 | 2.90E-05 | 7.90E-05 | 0.581 |
| RHSIM_Rhsim05G0172100 | 17.48 | 2.90E-05 | 7.92E-05 | 0.828 |
| RHSIM_Rhsim01G0160900 | 17.48 | 2.91E-05 | 7.93E-05 | -0.393 |
| RHSIM_RhsimUnG0235200 | 17.48 | 2.91E-05 | 7.94E-05 | -3.521 |
| RHSIM_Rhsim07G0170600 | 17.48 | 2.91E-05 | 7.94E-05 | 0.425 |
| RHSIM_Rhsim06G0127700 | 17.47 | 2.92E-05 | 7.97E-05 | 0.406 |
| RHSIM_Rhsim04G0068500 | 17.47 | 2.93E-05 | 7.98E-05 | 1.413 |
| RHSIM_Rhsim11G0083700 | 17.46 | 2.93E-05 | 7.98E-05 | 2.524 |
| RHSIM_Rhsim03G0032400 | 17.46 | 2.93E-05 | 7.98E-05 | 4.331 |
| RHSIM_Rhsim10G0094300 | 17.46 | 2.94E-05 | 8.02E-05 | 0.777 |
| novel.4497 | 17.45 | 2.96E-05 | 8.06E-05 | 2.769 |
| RHSIM_Rhsim07G0169300 | 17.44 | 2.96E-05 | 8.07E-05 | 5.313 |
| RHSIM_Rhsim10G0030400 | 17.44 | 2.96E-05 | 8.08E-05 | -1.551 |
| RHSIM_Rhsim09G0198200 | 17.43 | 2.98E-05 | 8.11E-05 | 2.071 |
| RHSIM_Rhsim10G0085400 | 17.43 | 2.98E-05 | 8.11E-05 | -0.703 |
| novel.190 | 17.43 | 2.98E-05 | 8.11E-05 | 1.283 |
| RHSIM_Rhsim10G0163200 | 17.42 | 2.99E-05 | 8.14E-05 | -0.743 |
| novel.4092 | 17.42 | 3.00E-05 | 8.17E-05 | -0.493 |
| RHSIM_Rhsim10G0051800 | 17.41 | 3.01E-05 | 8.20E-05 | 4.163 |
| RHSIM_Rhsim12G0072000 | 17.41 | 3.02E-05 | 8.22E-05 | 3.889 |
| RHSIM_Rhsim12G0128800 | 17.41 | 3.02E-05 | 8.23E-05 | -0.413 |
| RHSIM_Rhsim07G0207300 | 17.40 | 3.03E-05 | 8.24E-05 | -3.636 |
| RHSIM_Rhsim01G0290500 | 17.40 | 3.03E-05 | 8.26E-05 | 1.778 |
| RHSIM_Rhsim13G0052900 | 17.40 | 3.04E-05 | 8.26E-05 | -0.931 |
| RHSIM_Rhsim08G0196400 | 17.39 | 3.05E-05 | 8.30E-05 | 4.208 |
| RHSIM_RhsimUnG0149500 | 17.38 | 3.06E-05 | 8.32E-05 | -1.332 |
| RHSIM_Rhsim06G0222000 | 17.37 | 3.08E-05 | 8.38E-05 | -1.715 |
| RHSIM_RhsimUnG0022900 | 17.37 | 3.08E-05 | 8.38E-05 | -0.799 |
| RHSIM_Rhsim01G0049500 | 17.37 | 3.08E-05 | 8.39E-05 | 0.343 |
| RHSIM_Rhsim03G0220800 | 17.37 | 3.08E-05 | 8.39E-05 | 1.901 |
| RHSIM_Rhsim04G0101100 | 17.36 | 3.09E-05 | 8.40E-05 | 1.274 |

| Gene | LRT statistic | P value | BH-adjusted P | log2FC (interaction) |
|---|---|---|---|---|
| RHSIM_Rhsim03G0065700 | 17.36 | 3.10E-05 | 8.43E-05 | 0.533 |
| RHSIM_Rhsim02G0024500 | 17.36 | 3.10E-05 | 8.43E-05 | 2.235 |
| RHSIM_Rhsim08G0210300 | 17.34 | 3.12E-05 | 8.48E-05 | -1.533 |
| RHSIM_Rhsim03G0154700 | 17.34 | 3.12E-05 | 8.50E-05 | 0.781 |
| RHSIM_Rhsim12G0180500 | 17.34 | 3.13E-05 | 8.51E-05 | 0.408 |
| RHSIM_Rhsim05G0032000 | 17.33 | 3.13E-05 | 8.52E-05 | -0.661 |
| RHSIM_RhsimUnG0053800 | 17.32 | 3.16E-05 | 8.59E-05 | -1.560 |
| RHSIM_RhsimUnG0058500 | 17.32 | 3.17E-05 | 8.61E-05 | 1.575 |
| RHSIM_RhsimUnG0252400 | 17.32 | 3.17E-05 | 8.61E-05 | 3.088 |
| RHSIM_Rhsim01G0204900 | 17.31 | 3.17E-05 | 8.62E-05 | -1.292 |
| RHSIM_Rhsim08G0077900 | 17.30 | 3.19E-05 | 8.67E-05 | 0.901 |
| RHSIM_Rhsim01G0278600 | 17.29 | 3.21E-05 | 8.73E-05 | 0.932 |
| novel.1140 | 17.28 | 3.23E-05 | 8.79E-05 | -4.523 |
| RHSIM_Rhsim13G0232800 | 17.27 | 3.24E-05 | 8.79E-05 | -1.445 |
| RHSIM_Rhsim10G0142100 | 17.27 | 3.24E-05 | 8.80E-05 | 1.390 |
| RHSIM_Rhsim07G0210400 | 17.26 | 3.25E-05 | 8.84E-05 | -0.280 |
| RHSIM_Rhsim12G0043300 | 17.26 | 3.26E-05 | 8.86E-05 | 0.456 |
| RHSIM_RhsimUnG0034100 | 17.26 | 3.27E-05 | 8.87E-05 | 2.249 |
| novel.1536 | 17.24 | 3.30E-05 | 8.97E-05 | 3.010 |
| RHSIM_Rhsim03G0209700 | 17.23 | 3.31E-05 | 8.98E-05 | -1.033 |
| RHSIM_Rhsim01G0062000 | 17.23 | 3.31E-05 | 8.98E-05 | 0.362 |
| RHSIM_RhsimUnG0183400 | 17.23 | 3.31E-05 | 8.98E-05 | -0.904 |
| RHSIM_Rhsim11G0144300 | 17.23 | 3.31E-05 | 8.99E-05 | 0.423 |
| RHSIM_Rhsim09G0136700 | 17.22 | 3.32E-05 | 9.01E-05 | -0.374 |
| novel.4428 | 17.21 | 3.34E-05 | 9.07E-05 | -0.530 |
| RHSIM_Rhsim06G0097400 | 17.21 | 3.35E-05 | 9.08E-05 | -0.527 |
| RHSIM_Rhsim04G0166800 | 17.21 | 3.35E-05 | 9.10E-05 | -0.464 |
| RHSIM_Rhsim09G0074600 | 17.21 | 3.35E-05 | 9.10E-05 | 0.406 |
| RHSIM_Rhsim06G0088000 | 17.20 | 3.36E-05 | 9.11E-05 | 0.429 |
| RHSIM_Rhsim02G0061500 | 17.20 | 3.36E-05 | 9.11E-05 | -1.522 |
| RHSIM_Rhsim07G0191000 | 17.20 | 3.37E-05 | 9.13E-05 | -0.398 |
| RHSIM_Rhsim06G0208700 | 17.20 | 3.37E-05 | 9.15E-05 | -1.146 |

| Gene | LRT statistic | P value | BH-adjusted P | log2FC (interaction) |
|---|---|---|---|---|
| RHSIM_Rhsim01G0243000 | 17.20 | 3.37E-05 | 9.15E-05 | -1.738 |
| RHSIM_Rhsim13G0028900 | 17.19 | 3.37E-05 | 9.15E-05 | 1.479 |
| RHSIM_Rhsim04G0234200 | 17.19 | 3.38E-05 | 9.16E-05 | -0.313 |
| RHSIM_Rhsim06G0065000 | 17.19 | 3.38E-05 | 9.17E-05 | 0.648 |
| RHSIM_Rhsim09G0191300 | 17.19 | 3.38E-05 | 9.17E-05 | 0.588 |
| RHSIM_Rhsim09G0023900 | 17.19 | 3.39E-05 | 9.19E-05 | 0.978 |
| novel.2118 | 17.19 | 3.39E-05 | 9.19E-05 | -2.510 |
| RHSIM_RhsimUnG0125500 | 17.18 | 3.40E-05 | 9.20E-05 | -1.124 |
| RHSIM_Rhsim01G0205600 | 17.17 | 3.42E-05 | 9.26E-05 | -2.828 |
| RHSIM_RhsimUnG0040000 | 17.16 | 3.43E-05 | 9.30E-05 | 0.615 |
| RHSIM_Rhsim12G0211400 | 17.16 | 3.44E-05 | 9.32E-05 | 1.054 |
| RHSIM_Rhsim06G0208200 | 17.15 | 3.45E-05 | 9.34E-05 | 0.785 |
| RHSIM_Rhsim11G0122100 | 17.15 | 3.46E-05 | 9.37E-05 | 1.032 |
| RHSIM_Rhsim12G0197800 | 17.14 | 3.46E-05 | 9.39E-05 | -1.044 |
| novel.3669 | 17.14 | 3.47E-05 | 9.41E-05 | -4.170 |
| novel.637 | 17.14 | 3.47E-05 | 9.41E-05 | -0.344 |
| RHSIM_Rhsim08G0237000 | 17.13 | 3.49E-05 | 9.44E-05 | -0.879 |
| novel.1197 | 17.13 | 3.49E-05 | 9.46E-05 | -3.818 |
| RHSIM_Rhsim12G0036100 | 17.13 | 3.50E-05 | 9.47E-05 | 0.566 |
| RHSIM_Rhsim05G0014700 | 17.13 | 3.50E-05 | 9.47E-05 | 0.381 |
| RHSIM_Rhsim07G0143000 | 17.12 | 3.51E-05 | 9.49E-05 | 1.332 |
| RHSIM_Rhsim13G0073800 | 17.12 | 3.51E-05 | 9.49E-05 | -0.354 |
| RHSIM_Rhsim05G0013200 | 17.12 | 3.51E-05 | 9.49E-05 | -0.522 |
| RHSIM_RhsimUnG0018800 | 17.12 | 3.51E-05 | 9.49E-05 | 1.067 |
| RHSIM_Rhsim03G0243400 | 17.12 | 3.51E-05 | 9.50E-05 | -0.375 |
| RHSIM_Rhsim01G0013100 | 17.11 | 3.52E-05 | 9.52E-05 | -0.308 |
| RHSIM_Rhsim01G0162900 | 17.11 | 3.54E-05 | 9.57E-05 | 0.338 |
| RHSIM_Rhsim04G0074700 | 17.10 | 3.55E-05 | 9.60E-05 | -0.622 |
| RHSIM_Rhsim07G0014700 | 17.10 | 3.55E-05 | 9.61E-05 | 3.086 |
| RHSIM_Rhsim03G0062900 | 17.09 | 3.56E-05 | 9.64E-05 | -0.526 |
| RHSIM_Rhsim06G0027400 | 17.09 | 3.57E-05 | 9.65E-05 | 0.764 |
| RHSIM_Rhsim09G0188500 | 17.09 | 3.57E-05 | 9.65E-05 | -0.497 |

| Gene | LRT statistic | P value | BH-adjusted P | log2FC (interaction) |
|---|---|---|---|---|
| RHSIM_Rhsim11G0172800 | 17.09 | 3.57E-05 | 9.65E-05 | 0.495 |
| RHSIM_Rhsim02G0049500 | 17.09 | 3.57E-05 | 9.66E-05 | -0.543 |
| RHSIM_Rhsim10G0151000 | 17.08 | 3.58E-05 | 9.68E-05 | 0.608 |
| RHSIM_Rhsim04G0022900 | 17.08 | 3.59E-05 | 9.71E-05 | 3.117 |
| RHSIM_Rhsim04G0031900 | 17.08 | 3.59E-05 | 9.71E-05 | -0.232 |
| RHSIM_Rhsim01G0155600 | 17.07 | 3.60E-05 | 9.73E-05 | 0.738 |
| novel.4141 | 17.07 | 3.60E-05 | 9.74E-05 | 40.749 |
| RHSIM_Rhsim06G0177200 | 17.07 | 3.61E-05 | 9.75E-05 | -0.352 |
| RHSIM_Rhsim12G0004700 | 17.06 | 3.61E-05 | 9.77E-05 | 5.540 |
| RHSIM_Rhsim03G0148700 | 17.06 | 3.62E-05 | 9.78E-05 | -2.488 |
| RHSIM_Rhsim05G0163600 | 17.06 | 3.62E-05 | 9.79E-05 | -0.889 |
| novel.1466 | 17.06 | 3.63E-05 | 9.80E-05 | -0.428 |
| novel.1424 | 17.05 | 3.64E-05 | 9.82E-05 | 1.835 |
| RHSIM_Rhsim01G0220400 | 17.05 | 3.64E-05 | 9.83E-05 | 0.359 |
| RHSIM_Rhsim10G0111700 | 17.04 | 3.66E-05 | 9.90E-05 | 0.748 |
| RHSIM_Rhsim08G0082400 | 17.04 | 3.67E-05 | 9.90E-05 | -0.384 |
| RHSIM_Rhsim03G0030900 | 17.03 | 3.67E-05 | 9.92E-05 | 0.503 |
| RHSIM_RhsimUnG0013600 | 17.03 | 3.68E-05 | 9.92E-05 | 1.507 |
| RHSIM_Rhsim11G0036400 | 17.03 | 3.69E-05 | 9.95E-05 | 1.268 |
| RHSIM_Rhsim06G0077700 | 17.03 | 3.69E-05 | 9.95E-05 | 0.430 |
| RHSIM_Rhsim02G0037600 | 17.03 | 3.69E-05 | 9.95E-05 | 2.235 |
| RHSIM_Rhsim13G0127300 | 17.02 | 3.70E-05 | 9.98E-05 | 0.321 |
| RHSIM_Rhsim08G0232700 | 17.02 | 3.70E-05 | 1.00E-04 | 0.470 |
| novel.2366 | 17.02 | 3.71E-05 | 1.00E-04 | 1.628 |
| RHSIM_Rhsim05G0206100 | 17.01 | 3.72E-05 | 1.00E-04 | -2.119 |
| RHSIM_Rhsim13G0114400 | 17.00 | 3.73E-05 | 1.01E-04 | 2.942 |
| RHSIM_Rhsim11G0063600 | 17.00 | 3.73E-05 | 1.01E-04 | -0.588 |
| RHSIM_Rhsim13G0157200 | 17.00 | 3.73E-05 | 1.01E-04 | 5.632 |
| RHSIM_RhsimUnG0126000 | 17.00 | 3.74E-05 | 1.01E-04 | 5.120 |
| RHSIM_Rhsim07G0139100 | 16.99 | 3.75E-05 | 1.01E-04 | 43.635 |
| novel.3863 | 16.98 | 3.77E-05 | 1.02E-04 | 1.148 |
| RHSIM_RhsimUnG0027300 | 16.98 | 3.77E-05 | 1.02E-04 | -0.475 |

| Gene | LRT statistic | P value | BH-adjusted P | log2FC (interaction) |
|---|---|---|---|---|
| RHSIM_Rhsim07G0154400 | 16.98 | 3.78E-05 | 1.02E-04 | 0.424 |
| RHSIM_Rhsim02G0070500 | 16.98 | 3.78E-05 | 1.02E-04 | -3.067 |
| RHSIM_Rhsim08G0223400 | 16.97 | 3.79E-05 | 1.02E-04 | 0.490 |
| novel.4224 | 16.97 | 3.80E-05 | 1.02E-04 | -0.641 |
| RHSIM_Rhsim08G0179700 | 16.97 | 3.80E-05 | 1.02E-04 | -0.330 |
| RHSIM_Rhsim08G0205700 | 16.96 | 3.82E-05 | 1.03E-04 | 0.745 |
| RHSIM_Rhsim08G0211700 | 16.96 | 3.82E-05 | 1.03E-04 | 2.268 |
| RHSIM_Rhsim08G0038500 | 16.95 | 3.83E-05 | 1.03E-04 | 0.465 |
| RHSIM_Rhsim08G0183100 | 16.95 | 3.83E-05 | 1.03E-04 | 0.534 |
| RHSIM_Rhsim09G0066000 | 16.95 | 3.84E-05 | 1.03E-04 | -0.516 |
| RHSIM_Rhsim13G0020600 | 16.94 | 3.85E-05 | 1.04E-04 | -0.820 |
| RHSIM_Rhsim02G0002800 | 16.94 | 3.85E-05 | 1.04E-04 | -0.526 |
| RHSIM_Rhsim01G0130500 | 16.94 | 3.86E-05 | 1.04E-04 | 0.421 |
| RHSIM_Rhsim04G0114800 | 16.94 | 3.87E-05 | 1.04E-04 | 1.593 |
| RHSIM_Rhsim11G0132500 | 16.93 | 3.87E-05 | 1.04E-04 | -1.879 |
| novel.2022 | 16.93 | 3.87E-05 | 1.04E-04 | 0.281 |
| RHSIM_RhsimUnG0238700 | 16.93 | 3.87E-05 | 1.04E-04 | 4.129 |
| novel.2210 | 16.93 | 3.87E-05 | 1.04E-04 | -0.498 |
| RHSIM_Rhsim04G0080900 | 16.93 | 3.87E-05 | 1.04E-04 | -0.474 |
| RHSIM_Rhsim10G0099200 | 16.92 | 3.90E-05 | 1.05E-04 | -0.777 |
| RHSIM_Rhsim11G0154000 | 16.91 | 3.92E-05 | 1.05E-04 | -4.002 |
| RHSIM_Rhsim01G0272000 | 16.91 | 3.92E-05 | 1.05E-04 | 0.707 |
| RHSIM_Rhsim13G0200700 | 16.91 | 3.92E-05 | 1.06E-04 | 2.242 |
| RHSIM_RhsimUnG0094100 | 16.90 | 3.93E-05 | 1.06E-04 | -3.972 |
| RHSIM_Rhsim10G0154100 | 16.90 | 3.95E-05 | 1.06E-04 | 2.301 |
| novel.1195 | 16.89 | 3.95E-05 | 1.06E-04 | -40.431 |
| novel.2005 | 16.89 | 3.97E-05 | 1.07E-04 | -2.396 |
| RHSIM_Rhsim13G0173600 | 16.88 | 3.99E-05 | 1.07E-04 | 0.280 |
| RHSIM_Rhsim04G0029500 | 16.87 | 4.01E-05 | 1.08E-04 | -0.231 |
| RHSIM_Rhsim04G0175800 | 16.87 | 4.01E-05 | 1.08E-04 | 0.377 |
| RHSIM_Rhsim04G0063100 | 16.86 | 4.02E-05 | 1.08E-04 | 1.195 |
| RHSIM_Rhsim02G0164700 | 16.86 | 4.02E-05 | 1.08E-04 | 1.605 |

| Gene | LRT statistic | P value | BH-adjusted P | log2FC (interaction) |
|---|---|---|---|---|
| RHSIM_Rhsim08G0101100 | 16.86 | 4.02E-05 | 1.08E-04 | -0.728 |
| RHSIM_Rhsim07G0154200 | 16.86 | 4.03E-05 | 1.08E-04 | 0.347 |
| RHSIM_Rhsim10G0041500 | 16.86 | 4.03E-05 | 1.08E-04 | -1.195 |
| RHSIM_Rhsim05G0219700 | 16.86 | 4.03E-05 | 1.08E-04 | 0.397 |
| RHSIM_Rhsim09G0184500 | 16.86 | 4.03E-05 | 1.08E-04 | -1.207 |
| novel.2191 | 16.85 | 4.04E-05 | 1.08E-04 | -0.325 |
| novel.3482 | 16.85 | 4.04E-05 | 1.08E-04 | -1.647 |
| RHSIM_Rhsim09G0120300 | 16.85 | 4.04E-05 | 1.09E-04 | 0.552 |
| RHSIM_Rhsim08G0231100 | 16.85 | 4.05E-05 | 1.09E-04 | 0.397 |
| RHSIM_Rhsim13G0112400 | 16.85 | 4.06E-05 | 1.09E-04 | 0.950 |
| RHSIM_Rhsim10G0163500 | 16.84 | 4.06E-05 | 1.09E-04 | -0.455 |
| RHSIM_Rhsim09G0182500 | 16.84 | 4.07E-05 | 1.09E-04 | -3.251 |
| RHSIM_Rhsim04G0235400 | 16.82 | 4.11E-05 | 1.10E-04 | 3.946 |
| novel.2479 | 16.81 | 4.12E-05 | 1.11E-04 | -0.721 |
| RHSIM_Rhsim06G0203700 | 16.81 | 4.14E-05 | 1.11E-04 | -0.525 |
| RHSIM_Rhsim03G0246300 | 16.81 | 4.14E-05 | 1.11E-04 | 0.407 |
| RHSIM_Rhsim06G0054800 | 16.81 | 4.14E-05 | 1.11E-04 | -0.318 |
| novel.3053 | 16.81 | 4.14E-05 | 1.11E-04 | 2.606 |
| novel.1529 | 16.81 | 4.14E-05 | 1.11E-04 | 0.820 |
| RHSIM_Rhsim09G0115600 | 16.80 | 4.14E-05 | 1.11E-04 | -0.389 |
| novel.2244 | 16.80 | 4.14E-05 | 1.11E-04 | 2.947 |
| RHSIM_Rhsim06G0240100 | 16.80 | 4.15E-05 | 1.11E-04 | 0.428 |
| RHSIM_Rhsim13G0142100 | 16.80 | 4.15E-05 | 1.11E-04 | 2.967 |
| RHSIM_Rhsim06G0201400 | 16.80 | 4.15E-05 | 1.11E-04 | -2.882 |
| RHSIM_Rhsim07G0146800 | 16.80 | 4.15E-05 | 1.11E-04 | -0.348 |
| RHSIM_RhsimUnG0146600 | 16.80 | 4.16E-05 | 1.11E-04 | -2.935 |
| RHSIM_Rhsim04G0193800 | 16.79 | 4.18E-05 | 1.12E-04 | -0.832 |
| RHSIM_RhsimUnG0021100 | 16.79 | 4.18E-05 | 1.12E-04 | -0.489 |
| RHSIM_Rhsim07G0235900 | 16.79 | 4.18E-05 | 1.12E-04 | -3.269 |
| RHSIM_Rhsim02G0189400 | 16.79 | 4.18E-05 | 1.12E-04 | -1.655 |
| RHSIM_Rhsim04G0116000 | 16.79 | 4.19E-05 | 1.12E-04 | -1.759 |
| RHSIM_Rhsim04G0142000 | 16.78 | 4.19E-05 | 1.12E-04 | 1.590 |

| Gene | LRT statistic | P value | BH-adjusted P | log2FC (interaction) |
|---|---|---|---|---|
| RHSIM_Rhsim09G0090300 | 16.78 | 4.19E-05 | 1.12E-04 | -0.465 |
| RHSIM_Rhsim02G0183100 | 16.77 | 4.21E-05 | 1.13E-04 | 2.633 |
| novel.3750 | 16.77 | 4.23E-05 | 1.13E-04 | -2.498 |
| RHSIM_Rhsim13G0216900 | 16.77 | 4.23E-05 | 1.13E-04 | -1.103 |
| RHSIM_Rhsim13G0005000 | 16.76 | 4.24E-05 | 1.14E-04 | 0.266 |
| RHSIM_Rhsim09G0116300 | 16.76 | 4.25E-05 | 1.14E-04 | -1.244 |
| RHSIM_Rhsim13G0159400 | 16.75 | 4.27E-05 | 1.14E-04 | 3.062 |
| RHSIM_Rhsim05G0118600 | 16.74 | 4.29E-05 | 1.15E-04 | 1.060 |
| RHSIM_Rhsim01G0000100 | 16.74 | 4.29E-05 | 1.15E-04 | -0.965 |
| RHSIM_Rhsim07G0040800 | 16.74 | 4.29E-05 | 1.15E-04 | -2.568 |
| RHSIM_Rhsim09G0034800 | 16.73 | 4.30E-05 | 1.15E-04 | 0.243 |
| RHSIM_Rhsim01G0261300 | 16.72 | 4.32E-05 | 1.16E-04 | 0.964 |
| RHSIM_Rhsim01G0033400 | 16.72 | 4.33E-05 | 1.16E-04 | -0.648 |
| RHSIM_RhsimUnG0076300 | 16.72 | 4.34E-05 | 1.16E-04 | 2.428 |
| RHSIM_Rhsim10G0072900 | 16.72 | 4.34E-05 | 1.16E-04 | 0.701 |
| RHSIM_Rhsim05G0200700 | 16.71 | 4.34E-05 | 1.16E-04 | -0.432 |
| novel.1564 | 16.71 | 4.35E-05 | 1.16E-04 | 0.708 |
| RHSIM_Rhsim11G0197600 | 16.71 | 4.35E-05 | 1.16E-04 | 0.646 |
| RHSIM_Rhsim02G0027300 | 16.71 | 4.35E-05 | 1.16E-04 | 1.347 |
| RHSIM_Rhsim07G0100800 | 16.71 | 4.36E-05 | 1.17E-04 | -1.973 |
| RHSIM_Rhsim12G0135600 | 16.71 | 4.36E-05 | 1.17E-04 | 1.269 |
| RHSIM_RhsimUnG0059900 | 16.70 | 4.37E-05 | 1.17E-04 | -1.936 |
| RHSIM_Rhsim10G0166200 | 16.70 | 4.39E-05 | 1.17E-04 | -43.478 |
| RHSIM_Rhsim09G0087200 | 16.69 | 4.39E-05 | 1.17E-04 | 0.888 |
| RHSIM_Rhsim12G0202800 | 16.69 | 4.39E-05 | 1.18E-04 | -0.695 |
| novel.3797 | 16.69 | 4.40E-05 | 1.18E-04 | 0.598 |
| RHSIM_Rhsim12G0139500 | 16.69 | 4.41E-05 | 1.18E-04 | 2.806 |
| RHSIM_Rhsim09G0026700 | 16.68 | 4.42E-05 | 1.18E-04 | 0.883 |
| RHSIM_Rhsim08G0087000 | 16.68 | 4.42E-05 | 1.18E-04 | 0.517 |
| novel.4456 | 16.68 | 4.42E-05 | 1.18E-04 | -1.422 |
| novel.1354 | 16.68 | 4.42E-05 | 1.18E-04 | 2.164 |
| RHSIM_Rhsim06G0093000 | 16.67 | 4.44E-05 | 1.19E-04 | 1.693 |

| Gene | LRT statistic | P value | BH-adjusted P | log2FC (interaction) |
|---|---|---|---|---|
| RHSIM_Rhsim06G0133400 | 16.67 | 4.44E-05 | 1.19E-04 | 1.234 |
| RHSIM_RhsimUnG0256500 | 16.67 | 4.45E-05 | 1.19E-04 | -1.922 |
| novel.2224 | 16.67 | 4.46E-05 | 1.19E-04 | 0.340 |
| novel.2182 | 16.66 | 4.46E-05 | 1.19E-04 | 5.352 |
| RHSIM_Rhsim03G0059700 | 16.65 | 4.49E-05 | 1.20E-04 | -0.707 |
| RHSIM_RhsimUnG0116900 | 16.64 | 4.51E-05 | 1.20E-04 | -0.546 |
| RHSIM_Rhsim13G0150000 | 16.64 | 4.51E-05 | 1.21E-04 | 0.470 |
| RHSIM_Rhsim04G0013900 | 16.63 | 4.55E-05 | 1.21E-04 | 0.324 |
| RHSIM_Rhsim10G0188100 | 16.62 | 4.56E-05 | 1.22E-04 | 3.098 |
| novel.3888 | 16.62 | 4.56E-05 | 1.22E-04 | 1.298 |
| novel.1992 | 16.62 | 4.57E-05 | 1.22E-04 | 40.950 |
| RHSIM_Rhsim02G0041500 | 16.61 | 4.58E-05 | 1.22E-04 | 0.287 |
| RHSIM_Rhsim10G0125500 | 16.61 | 4.59E-05 | 1.22E-04 | 0.583 |
| RHSIM_Rhsim05G0190700 | 16.61 | 4.59E-05 | 1.22E-04 | 2.345 |
| RHSIM_Rhsim07G0224400 | 16.60 | 4.61E-05 | 1.23E-04 | -0.446 |
| RHSIM_Rhsim05G0067600 | 16.60 | 4.62E-05 | 1.23E-04 | -0.490 |
| RHSIM_Rhsim09G0149900 | 16.59 | 4.63E-05 | 1.24E-04 | -1.175 |
| RHSIM_Rhsim05G0197000 | 16.59 | 4.63E-05 | 1.24E-04 | -0.647 |
| RHSIM_Rhsim08G0014000 | 16.59 | 4.64E-05 | 1.24E-04 | -1.480 |
| RHSIM_Rhsim08G0225100 | 16.58 | 4.65E-05 | 1.24E-04 | -0.635 |
| novel.3143 | 16.58 | 4.66E-05 | 1.24E-04 | -2.027 |
| RHSIM_Rhsim01G0106500 | 16.58 | 4.67E-05 | 1.24E-04 | 0.745 |
| RHSIM_Rhsim05G0230700 | 16.58 | 4.67E-05 | 1.25E-04 | -1.022 |
| novel.4254 | 16.57 | 4.68E-05 | 1.25E-04 | 1.312 |
| RHSIM_Rhsim08G0175300 | 16.57 | 4.70E-05 | 1.25E-04 | 1.592 |
| novel.1350 | 16.56 | 4.70E-05 | 1.25E-04 | -3.675 |
| RHSIM_Rhsim07G0027500 | 16.56 | 4.72E-05 | 1.26E-04 | 0.770 |
| RHSIM_Rhsim12G0014900 | 16.56 | 4.72E-05 | 1.26E-04 | 0.498 |
| RHSIM_Rhsim07G0010200 | 16.56 | 4.72E-05 | 1.26E-04 | 1.426 |
| novel.388 | 16.56 | 4.72E-05 | 1.26E-04 | 4.004 |
| RHSIM_Rhsim07G0176100 | 16.55 | 4.75E-05 | 1.27E-04 | 0.404 |
| RHSIM_Rhsim13G0211400 | 16.54 | 4.77E-05 | 1.27E-04 | 0.995 |

| Gene | LRT statistic | P value | BH-adjusted P | log2FC (interaction) |
|---|---|---|---|---|
| RHSIM_Rhsim08G0246600 | 16.54 | 4.78E-05 | 1.27E-04 | 0.493 |
| RHSIM_Rhsim09G0208000 | 16.53 | 4.78E-05 | 1.27E-04 | -0.558 |
| RHSIM_Rhsim02G0240300 | 16.53 | 4.78E-05 | 1.27E-04 | -0.901 |
| novel.3806 | 16.53 | 4.78E-05 | 1.27E-04 | 0.469 |
| RHSIM_Rhsim08G0089200 | 16.53 | 4.79E-05 | 1.27E-04 | 2.384 |
| novel.3005 | 16.53 | 4.79E-05 | 1.28E-04 | 0.374 |
| RHSIM_Rhsim06G0058000 | 16.53 | 4.80E-05 | 1.28E-04 | -0.367 |
| RHSIM_Rhsim02G0005200 | 16.52 | 4.81E-05 | 1.28E-04 | 3.497 |
| RHSIM_Rhsim05G0002100 | 16.52 | 4.81E-05 | 1.28E-04 | -0.334 |
| RHSIM_Rhsim01G0268900 | 16.52 | 4.81E-05 | 1.28E-04 | -0.825 |
| RHSIM_Rhsim04G0050700 | 16.52 | 4.82E-05 | 1.28E-04 | -0.996 |
| RHSIM_Rhsim08G0092200 | 16.51 | 4.83E-05 | 1.29E-04 | -0.461 |
| RHSIM_Rhsim01G0015800 | 16.51 | 4.84E-05 | 1.29E-04 | 1.052 |
| RHSIM_Rhsim06G0021400 | 16.50 | 4.86E-05 | 1.29E-04 | -2.141 |
| RHSIM_Rhsim08G0056700 | 16.50 | 4.87E-05 | 1.29E-04 | -1.024 |
| RHSIM_Rhsim05G0142500 | 16.50 | 4.87E-05 | 1.30E-04 | -0.335 |
| RHSIM_Rhsim02G0235200 | 16.49 | 4.90E-05 | 1.30E-04 | -0.519 |
| novel.2460 | 16.49 | 4.90E-05 | 1.30E-04 | 5.897 |
| RHSIM_Rhsim11G0177300 | 16.48 | 4.92E-05 | 1.31E-04 | -1.098 |
| RHSIM_Rhsim03G0244800 | 16.48 | 4.93E-05 | 1.31E-04 | -0.949 |
| RHSIM_Rhsim06G0071200 | 16.47 | 4.94E-05 | 1.31E-04 | 0.355 |
| RHSIM_Rhsim12G0178200 | 16.47 | 4.94E-05 | 1.31E-04 | -0.932 |
| RHSIM_Rhsim10G0017900 | 16.47 | 4.94E-05 | 1.31E-04 | 1.732 |
| RHSIM_Rhsim07G0088400 | 16.47 | 4.94E-05 | 1.31E-04 | -0.832 |
| RHSIM_Rhsim07G0104100 | 16.47 | 4.95E-05 | 1.32E-04 | -0.657 |
| novel.4031 | 16.47 | 4.95E-05 | 1.32E-04 | -42.032 |
| RHSIM_Rhsim05G0056300 | 16.47 | 4.95E-05 | 1.32E-04 | -3.573 |
| RHSIM_Rhsim05G0013400 | 16.47 | 4.96E-05 | 1.32E-04 | 2.844 |
| RHSIM_Rhsim01G0043700 | 16.46 | 4.96E-05 | 1.32E-04 | 2.500 |
| RHSIM_Rhsim12G0177500 | 16.46 | 4.97E-05 | 1.32E-04 | 0.614 |
| novel.3834 | 16.45 | 4.98E-05 | 1.32E-04 | -0.565 |
| RHSIM_Rhsim04G0076200 | 16.45 | 5.01E-05 | 1.33E-04 | 0.914 |

| Gene | LRT statistic | P value | BH-adjusted P | log2FC (interaction) |
|---|---|---|---|---|
| RHSIM_Rhsim02G0244500 | 16.44 | 5.02E-05 | 1.33E-04 | -0.330 |
| RHSIM_Rhsim12G0158200 | 16.44 | 5.02E-05 | 1.33E-04 | 1.155 |
| RHSIM_Rhsim03G0262300 | 16.44 | 5.02E-05 | 1.33E-04 | 3.127 |
| RHSIM_Rhsim02G0100800 | 16.44 | 5.02E-05 | 1.33E-04 | 0.540 |
| RHSIM_Rhsim01G0113800 | 16.44 | 5.03E-05 | 1.34E-04 | 1.435 |
| RHSIM_Rhsim06G0091300 | 16.43 | 5.04E-05 | 1.34E-04 | 0.598 |
| novel.4333 | 16.43 | 5.05E-05 | 1.34E-04 | 1.585 |
| RHSIM_Rhsim07G0005800 | 16.42 | 5.07E-05 | 1.34E-04 | 0.708 |
| novel.1321 | 16.42 | 5.08E-05 | 1.35E-04 | 0.296 |
| RHSIM_Rhsim05G0069000 | 16.42 | 5.08E-05 | 1.35E-04 | 0.440 |
| RHSIM_Rhsim12G0122200 | 16.41 | 5.10E-05 | 1.35E-04 | -1.756 |
| RHSIM_Rhsim03G0178900 | 16.40 | 5.12E-05 | 1.36E-04 | -0.477 |
| RHSIM_Rhsim01G0121200 | 16.39 | 5.14E-05 | 1.36E-04 | -0.453 |
| RHSIM_Rhsim01G0124400 | 16.39 | 5.15E-05 | 1.37E-04 | -0.409 |
| RHSIM_Rhsim06G0159100 | 16.38 | 5.18E-05 | 1.37E-04 | -1.765 |
| novel.4305 | 16.37 | 5.21E-05 | 1.38E-04 | 1.971 |
| novel.3721 | 16.36 | 5.23E-05 | 1.39E-04 | 4.964 |
| RHSIM_Rhsim07G0213100 | 16.36 | 5.24E-05 | 1.39E-04 | -1.114 |
| RHSIM_Rhsim03G0031400 | 16.36 | 5.25E-05 | 1.39E-04 | -0.852 |
| RHSIM_Rhsim06G0199000 | 16.35 | 5.27E-05 | 1.40E-04 | 1.445 |
| RHSIM_Rhsim03G0055400 | 16.34 | 5.29E-05 | 1.40E-04 | 1.128 |
| RHSIM_Rhsim11G0154800 | 16.34 | 5.29E-05 | 1.40E-04 | 0.821 |
| RHSIM_Rhsim03G0117900 | 16.34 | 5.29E-05 | 1.40E-04 | -0.833 |
| RHSIM_Rhsim02G0176000 | 16.34 | 5.29E-05 | 1.40E-04 | -0.571 |
| RHSIM_Rhsim13G0009200 | 16.34 | 5.30E-05 | 1.40E-04 | -0.567 |
| RHSIM_Rhsim13G0057000 | 16.34 | 5.30E-05 | 1.41E-04 | 0.398 |
| novel.431 | 16.34 | 5.31E-05 | 1.41E-04 | -0.597 |
| RHSIM_Rhsim06G0134700 | 16.33 | 5.31E-05 | 1.41E-04 | 1.172 |
| RHSIM_Rhsim12G0116800 | 16.33 | 5.32E-05 | 1.41E-04 | -0.536 |
| novel.1898 | 16.33 | 5.33E-05 | 1.41E-04 | -4.731 |
| RHSIM_Rhsim06G0071700 | 16.32 | 5.34E-05 | 1.41E-04 | 3.038 |
| RHSIM_Rhsim01G0034500 | 16.32 | 5.35E-05 | 1.42E-04 | -1.359 |

| Gene | LRT statistic | P value | BH-adjusted P | log2FC (interaction) |
|---|---|---|---|---|
| RHSIM_Rhsim01G0122900 | 16.32 | 5.36E-05 | 1.42E-04 | -0.766 |
| RHSIM_Rhsim04G0243300 | 16.31 | 5.37E-05 | 1.42E-04 | -0.639 |
| RHSIM_Rhsim02G0081300 | 16.31 | 5.37E-05 | 1.42E-04 | -1.415 |
| novel.1028 | 16.31 | 5.38E-05 | 1.42E-04 | -5.205 |
| RHSIM_RhsimUnG0121100 | 16.31 | 5.39E-05 | 1.43E-04 | 0.353 |
| RHSIM_Rhsim07G0130900 | 16.30 | 5.39E-05 | 1.43E-04 | -0.481 |
| RHSIM_Rhsim11G0187500 | 16.30 | 5.40E-05 | 1.43E-04 | 0.573 |
| RHSIM_Rhsim06G0051000 | 16.30 | 5.42E-05 | 1.43E-04 | 1.902 |
| RHSIM_Rhsim12G0108700 | 16.29 | 5.43E-05 | 1.44E-04 | -1.296 |
| RHSIM_Rhsim09G0072000 | 16.28 | 5.46E-05 | 1.44E-04 | -0.534 |
| RHSIM_Rhsim08G0186100 | 16.26 | 5.51E-05 | 1.46E-04 | 0.510 |
| RHSIM_RhsimUnG0077100 | 16.26 | 5.51E-05 | 1.46E-04 | 0.734 |
| novel.228 | 16.26 | 5.52E-05 | 1.46E-04 | 3.201 |
| novel.3999 | 16.25 | 5.54E-05 | 1.46E-04 | 0.943 |
| RHSIM_Rhsim08G0136800 | 16.24 | 5.58E-05 | 1.48E-04 | -0.592 |
| RHSIM_Rhsim11G0140900 | 16.24 | 5.58E-05 | 1.48E-04 | 1.171 |
| RHSIM_Rhsim09G0199000 | 16.23 | 5.60E-05 | 1.48E-04 | 0.531 |
| RHSIM_Rhsim09G0032200 | 16.23 | 5.60E-05 | 1.48E-04 | 0.918 |
| novel.3368 | 16.23 | 5.61E-05 | 1.48E-04 | 2.275 |
| RHSIM_Rhsim05G0104300 | 16.22 | 5.63E-05 | 1.49E-04 | 0.447 |
| RHSIM_RhsimUnG0059500 | 16.22 | 5.64E-05 | 1.49E-04 | -1.363 |
| RHSIM_Rhsim03G0173200 | 16.22 | 5.64E-05 | 1.49E-04 | -0.200 |
| RHSIM_RhsimUnG0137900 | 16.22 | 5.64E-05 | 1.49E-04 | 2.026 |
| RHSIM_Rhsim07G0166500 | 16.21 | 5.66E-05 | 1.50E-04 | 0.247 |
| RHSIM_Rhsim09G0007700 | 16.21 | 5.66E-05 | 1.50E-04 | 1.390 |
| RHSIM_Rhsim07G0100700 | 16.21 | 5.67E-05 | 1.50E-04 | -0.461 |
| RHSIM_Rhsim03G0045800 | 16.21 | 5.68E-05 | 1.50E-04 | -1.016 |
| RHSIM_Rhsim03G0110000 | 16.19 | 5.72E-05 | 1.51E-04 | 41.549 |
| RHSIM_Rhsim09G0131700 | 16.19 | 5.73E-05 | 1.51E-04 | 0.436 |
| RHSIM_Rhsim05G0044100 | 16.19 | 5.73E-05 | 1.51E-04 | -1.265 |
| RHSIM_Rhsim05G0092000 | 16.19 | 5.73E-05 | 1.51E-04 | -1.592 |
| RHSIM_Rhsim04G0099400 | 16.18 | 5.76E-05 | 1.52E-04 | 0.886 |

| Gene | LRT statistic | P value | BH-adjusted P | log2FC (interaction) |
|---|---|---|---|---|
| RHSIM_Rhsim13G0211000 | 16.18 | 5.76E-05 | 1.52E-04 | -1.272 |
| RHSIM_Rhsim07G0033400 | 16.18 | 5.77E-05 | 1.52E-04 | 0.333 |
| RHSIM_Rhsim09G0071100 | 16.17 | 5.79E-05 | 1.53E-04 | 1.182 |
| RHSIM_Rhsim06G0043600 | 16.16 | 5.82E-05 | 1.54E-04 | -3.093 |
| RHSIM_Rhsim07G0140100 | 16.16 | 5.84E-05 | 1.54E-04 | 0.334 |
| RHSIM_Rhsim08G0018800 | 16.15 | 5.87E-05 | 1.55E-04 | -0.605 |
| novel.3325 | 16.14 | 5.88E-05 | 1.55E-04 | 1.009 |
| RHSIM_Rhsim03G0191700 | 16.14 | 5.88E-05 | 1.55E-04 | -0.234 |
| RHSIM_Rhsim12G0078700 | 16.14 | 5.88E-05 | 1.55E-04 | 0.955 |
| RHSIM_Rhsim11G0101600 | 16.14 | 5.88E-05 | 1.55E-04 | 2.888 |
| RHSIM_Rhsim02G0066600 | 16.13 | 5.91E-05 | 1.56E-04 | -0.978 |
| RHSIM_Rhsim01G0131300 | 16.13 | 5.91E-05 | 1.56E-04 | 0.755 |
| RHSIM_Rhsim05G0127300 | 16.13 | 5.91E-05 | 1.56E-04 | 0.635 |
| RHSIM_Rhsim12G0211000 | 16.12 | 5.94E-05 | 1.57E-04 | 0.538 |
| RHSIM_Rhsim05G0090200 | 16.11 | 5.96E-05 | 1.57E-04 | 1.207 |
| RHSIM_RhsimUnG0041400 | 16.11 | 5.97E-05 | 1.57E-04 | 41.892 |
| RHSIM_Rhsim02G0090900 | 16.11 | 5.98E-05 | 1.58E-04 | -1.028 |
| RHSIM_Rhsim02G0240700 | 16.11 | 5.98E-05 | 1.58E-04 | -1.002 |
| RHSIM_Rhsim08G0050000 | 16.11 | 5.99E-05 | 1.58E-04 | 0.567 |
| RHSIM_Rhsim07G0006100 | 16.10 | 6.00E-05 | 1.58E-04 | -1.358 |
| novel.2521 | 16.10 | 6.00E-05 | 1.58E-04 | 1.263 |
| RHSIM_Rhsim13G0020400 | 16.10 | 6.01E-05 | 1.58E-04 | 0.275 |
| RHSIM_Rhsim08G0238300 | 16.09 | 6.03E-05 | 1.59E-04 | -7.122 |
| RHSIM_Rhsim04G0104500 | 16.09 | 6.03E-05 | 1.59E-04 | -4.198 |
| RHSIM_Rhsim13G0136600 | 16.09 | 6.03E-05 | 1.59E-04 | 1.454 |
| RHSIM_Rhsim03G0049600 | 16.09 | 6.03E-05 | 1.59E-04 | 0.578 |
| RHSIM_Rhsim13G0189700 | 16.09 | 6.04E-05 | 1.59E-04 | 0.585 |
| RHSIM_Rhsim07G0084500 | 16.09 | 6.05E-05 | 1.59E-04 | -1.158 |
| RHSIM_Rhsim12G0188400 | 16.08 | 6.06E-05 | 1.60E-04 | -0.967 |
| RHSIM_Rhsim07G0176800 | 16.08 | 6.07E-05 | 1.60E-04 | 0.749 |
| RHSIM_Rhsim01G0165300 | 16.08 | 6.08E-05 | 1.60E-04 | -0.333 |
| RHSIM_Rhsim09G0082300 | 16.07 | 6.10E-05 | 1.61E-04 | 0.441 |

| Gene | LRT statistic | P value | BH-adjusted P | log2FC (interaction) |
|---|---|---|---|---|
| RHSIM_Rhsim02G0247800 | 16.07 | 6.11E-05 | 1.61E-04 | 1.049 |
| novel.693 | 16.06 | 6.15E-05 | 1.62E-04 | -2.200 |
| RHSIM_Rhsim11G0047400 | 16.05 | 6.16E-05 | 1.62E-04 | -0.499 |
| RHSIM_Rhsim12G0092400 | 16.05 | 6.18E-05 | 1.63E-04 | 0.578 |
| novel.2667 | 16.04 | 6.21E-05 | 1.63E-04 | 1.999 |
| novel.3883 | 16.04 | 6.21E-05 | 1.63E-04 | 0.402 |
| RHSIM_Rhsim13G0158200 | 16.03 | 6.23E-05 | 1.64E-04 | 0.894 |
| RHSIM_Rhsim08G0102200 | 16.03 | 6.24E-05 | 1.64E-04 | -0.265 |
| RHSIM_Rhsim01G0276400 | 16.03 | 6.24E-05 | 1.64E-04 | 4.070 |
| novel.1162 | 16.02 | 6.27E-05 | 1.65E-04 | 2.573 |
| RHSIM_Rhsim10G0078200 | 16.02 | 6.27E-05 | 1.65E-04 | 0.370 |
| novel.3600 | 16.01 | 6.29E-05 | 1.66E-04 | -1.132 |
| RHSIM_Rhsim05G0206000 | 16.01 | 6.31E-05 | 1.66E-04 | 0.745 |
| RHSIM_RhsimUnG0207200 | 16.00 | 6.33E-05 | 1.66E-04 | 1.284 |
| RHSIM_Rhsim13G0018400 | 16.00 | 6.33E-05 | 1.66E-04 | -0.472 |
| RHSIM_Rhsim01G0068000 | 15.99 | 6.36E-05 | 1.67E-04 | -0.422 |
| RHSIM_Rhsim11G0152500 | 15.99 | 6.36E-05 | 1.67E-04 | 0.531 |
| RHSIM_Rhsim10G0214600 | 15.99 | 6.36E-05 | 1.67E-04 | 0.557 |
| RHSIM_Rhsim04G0121500 | 15.99 | 6.37E-05 | 1.67E-04 | 2.272 |
| novel.1298 | 15.99 | 6.37E-05 | 1.67E-04 | -2.234 |
| RHSIM_RhsimUnG0153700 | 15.98 | 6.40E-05 | 1.68E-04 | 2.266 |
| novel.115 | 15.98 | 6.41E-05 | 1.68E-04 | 1.626 |
| RHSIM_Rhsim04G0235600 | 15.98 | 6.41E-05 | 1.68E-04 | -1.127 |
| RHSIM_Rhsim06G0096600 | 15.98 | 6.41E-05 | 1.68E-04 | -0.749 |
| RHSIM_Rhsim01G0163000 | 15.96 | 6.47E-05 | 1.70E-04 | 0.453 |
| RHSIM_Rhsim01G0232200 | 15.96 | 6.47E-05 | 1.70E-04 | -0.301 |
| RHSIM_Rhsim06G0085500 | 15.95 | 6.49E-05 | 1.71E-04 | 0.937 |
| novel.2930 | 15.95 | 6.50E-05 | 1.71E-04 | 3.929 |
| RHSIM_Rhsim04G0094900 | 15.95 | 6.50E-05 | 1.71E-04 | 1.429 |
| RHSIM_Rhsim11G0115100 | 15.95 | 6.52E-05 | 1.71E-04 | 2.609 |
| RHSIM_Rhsim07G0217700 | 15.94 | 6.53E-05 | 1.71E-04 | -3.402 |
| novel.1144 | 15.94 | 6.55E-05 | 1.72E-04 | -0.704 |

| Gene | LRT statistic | P value | BH-adjusted P | log2FC (interaction) |
|---|---|---|---|---|
| RHSIM_Rhsim09G0153500 | 15.93 | 6.57E-05 | 1.73E-04 | 3.282 |
| RHSIM_Rhsim03G0076600 | 15.92 | 6.60E-05 | 1.73E-04 | 1.957 |
| novel.786 | 15.92 | 6.60E-05 | 1.73E-04 | -0.902 |
| RHSIM_Rhsim07G0005400 | 15.92 | 6.60E-05 | 1.73E-04 | 1.518 |
| RHSIM_Rhsim01G0085600 | 15.92 | 6.62E-05 | 1.74E-04 | 4.457 |
| RHSIM_Rhsim10G0000100 | 15.92 | 6.62E-05 | 1.74E-04 | -0.712 |
| RHSIM_Rhsim01G0139700 | 15.91 | 6.63E-05 | 1.74E-04 | 1.671 |
| novel.2542 | 15.90 | 6.67E-05 | 1.75E-04 | -0.489 |
| RHSIM_Rhsim10G0172600 | 15.90 | 6.69E-05 | 1.75E-04 | -0.676 |
| RHSIM_Rhsim08G0042100 | 15.88 | 6.73E-05 | 1.77E-04 | 2.199 |
| RHSIM_Rhsim09G0039400 | 15.88 | 6.75E-05 | 1.77E-04 | -0.576 |
| RHSIM_Rhsim03G0235400 | 15.88 | 6.76E-05 | 1.77E-04 | 0.533 |
| RHSIM_Rhsim01G0075800 | 15.88 | 6.76E-05 | 1.77E-04 | -0.397 |
| RHSIM_Rhsim09G0140200 | 15.88 | 6.76E-05 | 1.77E-04 | 0.287 |
| RHSIM_Rhsim05G0172700 | 15.88 | 6.77E-05 | 1.77E-04 | -0.324 |
| RHSIM_Rhsim04G0011800 | 15.86 | 6.81E-05 | 1.79E-04 | -0.793 |
| RHSIM_Rhsim01G0191800 | 15.85 | 6.85E-05 | 1.79E-04 | -0.262 |
| RHSIM_RhsimUnG0217600 | 15.85 | 6.85E-05 | 1.80E-04 | -4.130 |
| RHSIM_Rhsim04G0178400 | 15.85 | 6.87E-05 | 1.80E-04 | -2.940 |
| RHSIM_Rhsim07G0091300 | 15.84 | 6.89E-05 | 1.81E-04 | -0.330 |
| RHSIM_Rhsim04G0007300 | 15.84 | 6.89E-05 | 1.81E-04 | -0.438 |
| novel.1840 | 15.83 | 6.92E-05 | 1.81E-04 | -1.198 |
| RHSIM_Rhsim03G0115100 | 15.83 | 6.93E-05 | 1.82E-04 | 1.843 |
| RHSIM_Rhsim13G0075400 | 15.82 | 6.96E-05 | 1.82E-04 | -0.696 |
| RHSIM_Rhsim09G0029900 | 15.82 | 6.98E-05 | 1.83E-04 | 0.257 |
| RHSIM_Rhsim03G0173300 | 15.81 | 7.01E-05 | 1.84E-04 | -0.535 |
| RHSIM_Rhsim01G0033900 | 15.80 | 7.04E-05 | 1.84E-04 | 0.530 |
| novel.1164 | 15.80 | 7.05E-05 | 1.85E-04 | 0.997 |
| RHSIM_Rhsim02G0214800 | 15.79 | 7.06E-05 | 1.85E-04 | -40.647 |
| RHSIM_Rhsim01G0141300 | 15.79 | 7.08E-05 | 1.85E-04 | 1.051 |
| RHSIM_Rhsim02G0137400 | 15.79 | 7.08E-05 | 1.85E-04 | 0.527 |
| RHSIM_RhsimUnG0000900 | 15.79 | 7.08E-05 | 1.85E-04 | 1.760 |

| Gene | LRT statistic | P value | BH-adjusted P | log2FC (interaction) |
|---|---|---|---|---|
| RHSIM_Rhsim09G0199300 | 15.79 | 7.08E-05 | 1.85E-04 | 2.348 |
| RHSIM_Rhsim12G0009100 | 15.78 | 7.11E-05 | 1.86E-04 | 0.421 |
| RHSIM_Rhsim10G0026400 | 15.78 | 7.12E-05 | 1.86E-04 | 1.714 |
| RHSIM_Rhsim07G0248700 | 15.77 | 7.16E-05 | 1.87E-04 | -0.449 |
| RHSIM_Rhsim09G0207100 | 15.77 | 7.17E-05 | 1.88E-04 | 0.453 |
| RHSIM_RhsimUnG0171300 | 15.76 | 7.18E-05 | 1.88E-04 | -2.142 |
| RHSIM_Rhsim03G0026800 | 15.76 | 7.21E-05 | 1.88E-04 | 1.192 |
| RHSIM_Rhsim03G0249500 | 15.76 | 7.21E-05 | 1.88E-04 | -1.320 |
| RHSIM_Rhsim11G0147900 | 15.75 | 7.21E-05 | 1.89E-04 | 4.147 |
| RHSIM_Rhsim03G0094100 | 15.75 | 7.22E-05 | 1.89E-04 | -0.683 |
| RHSIM_Rhsim04G0215600 | 15.75 | 7.23E-05 | 1.89E-04 | 3.645 |
| RHSIM_Rhsim11G0016100 | 15.73 | 7.29E-05 | 1.91E-04 | 0.354 |
| RHSIM_RhsimUnG0192200 | 15.73 | 7.30E-05 | 1.91E-04 | -42.407 |
| RHSIM_Rhsim12G0172100 | 15.73 | 7.31E-05 | 1.91E-04 | -0.372 |
| novel.4248 | 15.72 | 7.34E-05 | 1.92E-04 | 1.042 |
| RHSIM_Rhsim08G0043000 | 15.71 | 7.40E-05 | 1.93E-04 | -0.922 |
| RHSIM_Rhsim06G0106400 | 15.71 | 7.40E-05 | 1.93E-04 | 1.183 |
| novel.2777 | 15.70 | 7.42E-05 | 1.94E-04 | 2.098 |
| novel.4374 | 15.70 | 7.44E-05 | 1.94E-04 | 0.935 |
| RHSIM_Rhsim08G0020200 | 15.69 | 7.46E-05 | 1.95E-04 | -0.683 |
| novel.4530 | 15.69 | 7.46E-05 | 1.95E-04 | 1.966 |
| RHSIM_Rhsim05G0130100 | 15.69 | 7.48E-05 | 1.95E-04 | -1.617 |
| RHSIM_Rhsim08G0246100 | 15.68 | 7.49E-05 | 1.96E-04 | -0.823 |
| RHSIM_RhsimUnG0087700 | 15.68 | 7.50E-05 | 1.96E-04 | 2.647 |
| RHSIM_Rhsim09G0039200 | 15.67 | 7.53E-05 | 1.97E-04 | 1.283 |
| RHSIM_Rhsim08G0107600 | 15.67 | 7.53E-05 | 1.97E-04 | -2.446 |
| RHSIM_Rhsim05G0068200 | 15.67 | 7.55E-05 | 1.97E-04 | -0.645 |
| novel.3897 | 15.67 | 7.56E-05 | 1.97E-04 | 2.224 |
| RHSIM_Rhsim06G0235600 | 15.66 | 7.59E-05 | 1.98E-04 | 0.341 |
| RHSIM_Rhsim09G0190700 | 15.65 | 7.64E-05 | 1.99E-04 | 0.890 |
| RHSIM_Rhsim09G0153100 | 15.64 | 7.67E-05 | 2.00E-04 | -0.882 |
| RHSIM_Rhsim10G0173000 | 15.64 | 7.67E-05 | 2.00E-04 | -0.838 |

| Gene | LRT statistic | P value | BH-adjusted P | log2FC (interaction) |
|---|---|---|---|---|
| RHSIM_Rhsim05G0165500 | 15.64 | 7.68E-05 | 2.00E-04 | 0.595 |
| RHSIM_Rhsim03G0251000 | 15.63 | 7.68E-05 | 2.01E-04 | -0.257 |
| RHSIM_Rhsim04G0018200 | 15.63 | 7.69E-05 | 2.01E-04 | 0.570 |
| RHSIM_Rhsim04G0151600 | 15.62 | 7.75E-05 | 2.02E-04 | 0.371 |
| RHSIM_Rhsim06G0180800 | 15.62 | 7.76E-05 | 2.02E-04 | 0.691 |
| RHSIM_Rhsim05G0160300 | 15.61 | 7.79E-05 | 2.03E-04 | -0.369 |
| RHSIM_RhsimUnG0019200 | 15.61 | 7.79E-05 | 2.03E-04 | 4.037 |
| RHSIM_Rhsim02G0245100 | 15.61 | 7.80E-05 | 2.03E-04 | 1.213 |
| RHSIM_Rhsim07G0040900 | 15.60 | 7.83E-05 | 2.04E-04 | -3.159 |
| RHSIM_Rhsim10G0077900 | 15.60 | 7.83E-05 | 2.04E-04 | -0.468 |
| RHSIM_Rhsim06G0191000 | 15.59 | 7.85E-05 | 2.05E-04 | 0.317 |
| RHSIM_Rhsim09G0034200 | 15.59 | 7.85E-05 | 2.05E-04 | 0.551 |
| novel.3527 | 15.59 | 7.85E-05 | 2.05E-04 | 0.864 |
| RHSIM_Rhsim06G0072500 | 15.59 | 7.86E-05 | 2.05E-04 | -1.181 |
| RHSIM_Rhsim12G0065000 | 15.59 | 7.87E-05 | 2.05E-04 | -1.272 |
| RHSIM_Rhsim12G0213000 | 15.59 | 7.87E-05 | 2.05E-04 | -1.866 |
| RHSIM_Rhsim12G0146500 | 15.59 | 7.87E-05 | 2.05E-04 | 0.365 |
| RHSIM_Rhsim03G0052200 | 15.59 | 7.88E-05 | 2.05E-04 | 0.634 |
| RHSIM_Rhsim02G0094400 | 15.58 | 7.89E-05 | 2.06E-04 | -1.960 |
| RHSIM_Rhsim04G0081000 | 15.58 | 7.90E-05 | 2.06E-04 | 0.244 |
| RHSIM_Rhsim12G0040000 | 15.58 | 7.90E-05 | 2.06E-04 | -0.296 |
| RHSIM_Rhsim07G0055200 | 15.57 | 7.94E-05 | 2.07E-04 | 1.542 |
| RHSIM_Rhsim12G0045300 | 15.57 | 7.96E-05 | 2.07E-04 | 0.958 |
| RHSIM_Rhsim13G0061800 | 15.57 | 7.97E-05 | 2.07E-04 | 0.361 |
| RHSIM_Rhsim06G0066600 | 15.57 | 7.97E-05 | 2.08E-04 | -0.665 |
| RHSIM_Rhsim06G0226800 | 15.56 | 7.99E-05 | 2.08E-04 | -0.554 |
| RHSIM_Rhsim03G0259300 | 15.56 | 8.00E-05 | 2.08E-04 | 1.412 |
| novel.3204 | 15.56 | 8.01E-05 | 2.09E-04 | 1.546 |
| novel.4168 | 15.55 | 8.05E-05 | 2.09E-04 | -0.473 |
| RHSIM_Rhsim08G0000700 | 15.54 | 8.06E-05 | 2.10E-04 | 0.881 |
| RHSIM_Rhsim12G0206400 | 15.54 | 8.08E-05 | 2.10E-04 | -0.602 |
| RHSIM_Rhsim07G0002000 | 15.53 | 8.11E-05 | 2.11E-04 | 2.142 |

| Gene | LRT statistic | P value | BH-adjusted P | log2FC (interaction) |
|---|---|---|---|---|
| novel.3000 | 15.53 | 8.12E-05 | 2.11E-04 | -1.413 |
| RHSIM_Rhsim05G0149900 | 15.53 | 8.13E-05 | 2.11E-04 | -2.353 |
| RHSIM_Rhsim05G0232400 | 15.53 | 8.13E-05 | 2.11E-04 | 0.617 |
| RHSIM_Rhsim07G0040200 | 15.53 | 8.14E-05 | 2.12E-04 | -6.038 |
| RHSIM_Rhsim01G0040600 | 15.52 | 8.14E-05 | 2.12E-04 | 4.211 |
| RHSIM_Rhsim12G0001000 | 15.52 | 8.15E-05 | 2.12E-04 | -0.569 |
| novel.2864 | 15.52 | 8.16E-05 | 2.12E-04 | 5.097 |
| RHSIM_Rhsim07G0129500 | 15.52 | 8.17E-05 | 2.12E-04 | -0.419 |
| RHSIM_Rhsim01G0176900 | 15.51 | 8.20E-05 | 2.13E-04 | -1.378 |
| RHSIM_Rhsim11G0002000 | 15.51 | 8.20E-05 | 2.13E-04 | 1.106 |
| RHSIM_Rhsim11G0003700 | 15.51 | 8.21E-05 | 2.13E-04 | 0.187 |
| RHSIM_Rhsim05G0029700 | 15.51 | 8.22E-05 | 2.14E-04 | -2.338 |
| RHSIM_Rhsim07G0129300 | 15.49 | 8.28E-05 | 2.15E-04 | 2.572 |
| RHSIM_Rhsim11G0045100 | 15.49 | 8.28E-05 | 2.15E-04 | 0.612 |
| RHSIM_Rhsim07G0100400 | 15.49 | 8.29E-05 | 2.15E-04 | -2.299 |
| novel.349 | 15.49 | 8.30E-05 | 2.16E-04 | -0.668 |
| RHSIM_Rhsim08G0082900 | 15.48 | 8.35E-05 | 2.17E-04 | 0.917 |
| RHSIM_Rhsim05G0204600 | 15.48 | 8.35E-05 | 2.17E-04 | -1.049 |
| RHSIM_Rhsim03G0132600 | 15.47 | 8.39E-05 | 2.18E-04 | -0.923 |
| RHSIM_Rhsim10G0167000 | 15.46 | 8.44E-05 | 2.19E-04 | -1.054 |
| novel.4126 | 15.46 | 8.44E-05 | 2.19E-04 | 2.763 |
| RHSIM_Rhsim13G0048000 | 15.46 | 8.44E-05 | 2.19E-04 | 3.013 |
| RHSIM_Rhsim05G0225200 | 15.46 | 8.45E-05 | 2.19E-04 | -1.222 |
| novel.1780 | 15.45 | 8.45E-05 | 2.19E-04 | 0.592 |
| RHSIM_Rhsim04G0196800 | 15.45 | 8.46E-05 | 2.20E-04 | 0.203 |
| RHSIM_Rhsim09G0013500 | 15.45 | 8.47E-05 | 2.20E-04 | -0.688 |
| RHSIM_Rhsim07G0053400 | 15.45 | 8.48E-05 | 2.20E-04 | -1.954 |
| RHSIM_Rhsim02G0000900 | 15.45 | 8.49E-05 | 2.20E-04 | -1.126 |
| RHSIM_Rhsim06G0240200 | 15.44 | 8.51E-05 | 2.21E-04 | 0.997 |
| RHSIM_Rhsim07G0132500 | 15.44 | 8.52E-05 | 2.21E-04 | 0.410 |
| RHSIM_Rhsim04G0222800 | 15.44 | 8.53E-05 | 2.21E-04 | 0.568 |
| novel.997 | 15.43 | 8.56E-05 | 2.22E-04 | -1.429 |

| Gene | LRT statistic | P value | BH-adjusted P | log2FC (interaction) |
|---|---|---|---|---|
| RHSIM_Rhsim10G0011200 | 15.43 | 8.57E-05 | 2.22E-04 | 1.365 |
| RHSIM_Rhsim08G0150000 | 15.43 | 8.58E-05 | 2.22E-04 | 0.404 |
| RHSIM_Rhsim02G0236400 | 15.41 | 8.67E-05 | 2.25E-04 | -4.273 |
| RHSIM_Rhsim06G0002400 | 15.41 | 8.67E-05 | 2.25E-04 | -3.594 |
| RHSIM_Rhsim13G0155800 | 15.41 | 8.67E-05 | 2.25E-04 | 0.287 |
| novel.3560 | 15.41 | 8.67E-05 | 2.25E-04 | 2.765 |
| RHSIM_Rhsim08G0121400 | 15.40 | 8.72E-05 | 2.26E-04 | 0.485 |
| RHSIM_Rhsim12G0045400 | 15.40 | 8.72E-05 | 2.26E-04 | 1.518 |
| RHSIM_Rhsim04G0112200 | 15.39 | 8.72E-05 | 2.26E-04 | -0.901 |
| RHSIM_Rhsim12G0090500 | 15.39 | 8.74E-05 | 2.27E-04 | -0.445 |
| RHSIM_Rhsim10G0161900 | 15.39 | 8.76E-05 | 2.27E-04 | 0.537 |
| RHSIM_Rhsim10G0182500 | 15.38 | 8.79E-05 | 2.28E-04 | 1.446 |
| RHSIM_Rhsim01G0236600 | 15.38 | 8.80E-05 | 2.28E-04 | 0.712 |
| RHSIM_Rhsim05G0216100 | 15.38 | 8.81E-05 | 2.28E-04 | -0.989 |
| RHSIM_Rhsim09G0144400 | 15.38 | 8.81E-05 | 2.28E-04 | 1.393 |
| RHSIM_Rhsim12G0188900 | 15.37 | 8.82E-05 | 2.28E-04 | 1.320 |
| RHSIM_Rhsim02G0238000 | 15.37 | 8.84E-05 | 2.29E-04 | 0.991 |
| RHSIM_Rhsim04G0164100 | 15.37 | 8.84E-05 | 2.29E-04 | -0.601 |
| RHSIM_Rhsim13G0223400 | 15.37 | 8.86E-05 | 2.29E-04 | 1.660 |
| RHSIM_Rhsim06G0136600 | 15.36 | 8.89E-05 | 2.30E-04 | -1.187 |
| RHSIM_Rhsim01G0235900 | 15.36 | 8.89E-05 | 2.30E-04 | 3.304 |
| RHSIM_Rhsim07G0142600 | 15.36 | 8.90E-05 | 2.31E-04 | -0.653 |
| RHSIM_Rhsim06G0004300 | 15.35 | 8.91E-05 | 2.31E-04 | -0.284 |
| novel.905 | 15.35 | 8.93E-05 | 2.31E-04 | 1.472 |
| RHSIM_Rhsim07G0151600 | 15.35 | 8.94E-05 | 2.32E-04 | -42.770 |
| RHSIM_Rhsim12G0035600 | 15.34 | 8.96E-05 | 2.32E-04 | -0.656 |
| RHSIM_Rhsim02G0256400 | 15.34 | 8.97E-05 | 2.32E-04 | 0.658 |
| RHSIM_Rhsim08G0249300 | 15.34 | 8.98E-05 | 2.32E-04 | -0.236 |
| RHSIM_Rhsim01G0108600 | 15.34 | 9.00E-05 | 2.33E-04 | 0.773 |
| RHSIM_Rhsim01G0217500 | 15.33 | 9.04E-05 | 2.34E-04 | -1.012 |
| RHSIM_Rhsim13G0084400 | 15.32 | 9.06E-05 | 2.34E-04 | -1.850 |
| RHSIM_RhsimUnG0060500 | 15.31 | 9.11E-05 | 2.36E-04 | -0.823 |

| Gene | LRT statistic | P value | BH-adjusted P | log2FC (interaction) |
|---|---|---|---|---|
| RHSIM_Rhsim06G0071800 | 15.31 | 9.12E-05 | 2.36E-04 | 0.577 |
| novel.2545 | 15.31 | 9.15E-05 | 2.37E-04 | 3.368 |
| RHSIM_Rhsim05G0018800 | 15.30 | 9.17E-05 | 2.37E-04 | -0.380 |
| RHSIM_Rhsim12G0201700 | 15.30 | 9.19E-05 | 2.38E-04 | -0.290 |
| RHSIM_Rhsim13G0123300 | 15.29 | 9.21E-05 | 2.38E-04 | -1.643 |
| RHSIM_Rhsim02G0032700 | 15.29 | 9.21E-05 | 2.38E-04 | -0.768 |
| novel.929 | 15.29 | 9.21E-05 | 2.38E-04 | 2.081 |
| RHSIM_Rhsim01G0012400 | 15.28 | 9.27E-05 | 2.40E-04 | -3.015 |
| RHSIM_Rhsim09G0170900 | 15.27 | 9.30E-05 | 2.40E-04 | -0.487 |
| RHSIM_Rhsim03G0249000 | 15.26 | 9.36E-05 | 2.42E-04 | 0.798 |
| RHSIM_Rhsim11G0124500 | 15.26 | 9.36E-05 | 2.42E-04 | 0.827 |
| RHSIM_Rhsim13G0059700 | 15.26 | 9.38E-05 | 2.42E-04 | -2.631 |
| RHSIM_Rhsim10G0000700 | 15.26 | 9.39E-05 | 2.43E-04 | -0.482 |
| RHSIM_Rhsim11G0189000 | 15.25 | 9.41E-05 | 2.43E-04 | -1.652 |
| RHSIM_Rhsim05G0016700 | 15.25 | 9.42E-05 | 2.43E-04 | 0.651 |
| novel.1248 | 15.25 | 9.42E-05 | 2.43E-04 | -2.862 |
| RHSIM_Rhsim12G0126300 | 15.25 | 9.43E-05 | 2.44E-04 | -0.763 |
| RHSIM_Rhsim05G0194600 | 15.24 | 9.45E-05 | 2.44E-04 | -0.710 |
| novel.2765 | 15.24 | 9.46E-05 | 2.44E-04 | 1.019 |
| RHSIM_Rhsim03G0223700 | 15.24 | 9.46E-05 | 2.44E-04 | 0.794 |
| RHSIM_Rhsim13G0002600 | 15.23 | 9.50E-05 | 2.45E-04 | 0.257 |
| RHSIM_Rhsim07G0088500 | 15.23 | 9.52E-05 | 2.46E-04 | -41.936 |
| RHSIM_Rhsim11G0124300 | 15.23 | 9.53E-05 | 2.46E-04 | -0.354 |
| RHSIM_Rhsim03G0266000 | 15.23 | 9.53E-05 | 2.46E-04 | -0.575 |
| RHSIM_Rhsim12G0044600 | 15.23 | 9.53E-05 | 2.46E-04 | -0.991 |
| novel.3667 | 15.22 | 9.55E-05 | 2.47E-04 | 1.234 |
| RHSIM_Rhsim11G0026100 | 15.22 | 9.55E-05 | 2.47E-04 | 1.174 |
| RHSIM_Rhsim11G0072600 | 15.22 | 9.56E-05 | 2.47E-04 | 0.497 |
| RHSIM_Rhsim12G0166700 | 15.22 | 9.56E-05 | 2.47E-04 | -2.132 |
| RHSIM_Rhsim01G0107700 | 15.22 | 9.59E-05 | 2.47E-04 | -1.068 |
| RHSIM_Rhsim08G0104400 | 15.21 | 9.60E-05 | 2.48E-04 | 0.435 |
| RHSIM_Rhsim04G0078600 | 15.21 | 9.61E-05 | 2.48E-04 | 1.758 |

| Gene | LRT statistic | P value | BH-adjusted P | log2FC (interaction) |
|---|---|---|---|---|
| RHSIM_Rhsim07G0105500 | 15.21 | 9.63E-05 | 2.48E-04 | -1.678 |
| RHSIM_RhsimUnG0208700 | 15.20 | 9.65E-05 | 2.49E-04 | 1.501 |
| RHSIM_Rhsim01G0218100 | 15.20 | 9.66E-05 | 2.49E-04 | -0.660 |
| RHSIM_Rhsim05G0031700 | 15.20 | 9.66E-05 | 2.49E-04 | 1.901 |
| RHSIM_Rhsim05G0103400 | 15.20 | 9.66E-05 | 2.49E-04 | -0.326 |
| RHSIM_Rhsim05G0029500 | 15.20 | 9.66E-05 | 2.49E-04 | -0.463 |
| RHSIM_Rhsim02G0245400 | 15.19 | 9.71E-05 | 2.50E-04 | 1.670 |
| RHSIM_Rhsim01G0133100 | 15.19 | 9.71E-05 | 2.50E-04 | 2.941 |
| RHSIM_Rhsim13G0235600 | 15.19 | 9.74E-05 | 2.51E-04 | -0.684 |
| RHSIM_Rhsim03G0012300 | 15.18 | 9.76E-05 | 2.52E-04 | 0.261 |
| novel.611 | 15.18 | 9.79E-05 | 2.52E-04 | 0.910 |
| RHSIM_Rhsim10G0052500 | 15.18 | 9.79E-05 | 2.52E-04 | 1.240 |
| RHSIM_Rhsim09G0168800 | 15.18 | 9.79E-05 | 2.52E-04 | 0.526 |
| novel.2714 | 15.17 | 9.83E-05 | 2.53E-04 | -2.048 |
| RHSIM_Rhsim12G0087600 | 15.16 | 9.86E-05 | 2.54E-04 | -0.383 |
| RHSIM_Rhsim09G0034900 | 15.16 | 9.86E-05 | 2.54E-04 | -0.653 |
| RHSIM_Rhsim08G0005900 | 15.16 | 9.87E-05 | 2.54E-04 | -1.018 |
| RHSIM_Rhsim09G0031700 | 15.16 | 9.88E-05 | 2.54E-04 | 0.236 |
| RHSIM_Rhsim11G0194600 | 15.16 | 9.89E-05 | 2.55E-04 | 0.469 |
| RHSIM_Rhsim11G0141600 | 15.16 | 9.90E-05 | 2.55E-04 | 2.217 |
| RHSIM_Rhsim06G0068200 | 15.16 | 9.90E-05 | 2.55E-04 | 0.493 |
| RHSIM_Rhsim03G0188300 | 15.15 | 9.91E-05 | 2.55E-04 | 0.548 |
| RHSIM_Rhsim12G0112800 | 15.15 | 9.92E-05 | 2.55E-04 | -1.553 |
| RHSIM_Rhsim03G0070200 | 15.15 | 9.92E-05 | 2.55E-04 | 0.375 |
| RHSIM_Rhsim07G0255000 | 15.14 | 9.98E-05 | 2.57E-04 | -0.400 |
| RHSIM_Rhsim06G0205900 | 15.14 | 9.99E-05 | 2.57E-04 | 0.651 |
| RHSIM_Rhsim10G0033000 | 15.14 | 9.99E-05 | 2.57E-04 | 1.639 |
| RHSIM_Rhsim12G0060600 | 15.12 | 1.01E-04 | 2.59E-04 | 0.746 |
| RHSIM_Rhsim07G0020800 | 15.11 | 1.01E-04 | 2.61E-04 | -0.459 |
| novel.253 | 15.11 | 1.01E-04 | 2.61E-04 | 42.940 |
| RHSIM_Rhsim13G0112500 | 15.10 | 1.02E-04 | 2.63E-04 | -0.499 |
| RHSIM_Rhsim02G0134600 | 15.10 | 1.02E-04 | 2.63E-04 | 0.930 |

| Gene | LRT statistic | P value | BH-adjusted P | log2FC (interaction) |
|---|---|---|---|---|
| novel.865 | 15.09 | 1.03E-04 | 2.64E-04 | -1.375 |
| RHSIM_Rhsim09G0131200 | 15.08 | 1.03E-04 | 2.65E-04 | 0.301 |
| RHSIM_Rhsim06G0171200 | 15.08 | 1.03E-04 | 2.65E-04 | 0.596 |
| RHSIM_Rhsim01G0090800 | 15.07 | 1.04E-04 | 2.67E-04 | -0.574 |
| RHSIM_Rhsim11G0135800 | 15.06 | 1.04E-04 | 2.67E-04 | 0.518 |
| novel.2693 | 15.06 | 1.04E-04 | 2.67E-04 | -1.975 |
| RHSIM_Rhsim13G0168900 | 15.06 | 1.04E-04 | 2.68E-04 | -0.806 |
| RHSIM_Rhsim02G0062500 | 15.05 | 1.05E-04 | 2.69E-04 | 42.830 |
| novel.3503 | 15.04 | 1.05E-04 | 2.71E-04 | 41.900 |
| RHSIM_Rhsim04G0097800 | 15.03 | 1.06E-04 | 2.71E-04 | 0.942 |
| novel.3682 | 15.03 | 1.06E-04 | 2.72E-04 | 2.912 |
| RHSIM_Rhsim07G0001300 | 15.03 | 1.06E-04 | 2.72E-04 | -43.497 |
| novel.2785 | 15.03 | 1.06E-04 | 2.72E-04 | 2.687 |
| RHSIM_RhsimUnG0244500 | 15.02 | 1.06E-04 | 2.73E-04 | -1.698 |
| RHSIM_Rhsim12G0015700 | 15.01 | 1.07E-04 | 2.75E-04 | -0.816 |
| RHSIM_Rhsim01G0242400 | 15.01 | 1.07E-04 | 2.75E-04 | 0.530 |
| RHSIM_Rhsim01G0212500 | 15.00 | 1.07E-04 | 2.76E-04 | 0.812 |
| RHSIM_Rhsim06G0172000 | 15.00 | 1.07E-04 | 2.76E-04 | -0.923 |
| RHSIM_Rhsim01G0004400 | 15.00 | 1.07E-04 | 2.76E-04 | -3.354 |
| RHSIM_Rhsim13G0225100 | 14.99 | 1.08E-04 | 2.78E-04 | -2.290 |
| RHSIM_Rhsim13G0175100 | 14.99 | 1.08E-04 | 2.78E-04 | 5.074 |
| RHSIM_Rhsim09G0184400 | 14.98 | 1.08E-04 | 2.78E-04 | 1.598 |
| novel.3322 | 14.98 | 1.09E-04 | 2.79E-04 | 4.075 |
| RHSIM_Rhsim03G0145700 | 14.97 | 1.09E-04 | 2.80E-04 | 1.673 |
| RHSIM_Rhsim10G0193200 | 14.97 | 1.09E-04 | 2.80E-04 | 1.504 |
| RHSIM_Rhsim05G0194400 | 14.97 | 1.09E-04 | 2.80E-04 | -0.764 |
| RHSIM_Rhsim13G0037500 | 14.97 | 1.09E-04 | 2.81E-04 | 0.397 |
| RHSIM_Rhsim03G0163000 | 14.97 | 1.09E-04 | 2.81E-04 | -0.370 |
| RHSIM_Rhsim01G0263600 | 14.95 | 1.10E-04 | 2.83E-04 | 0.485 |
| novel.2901 | 14.95 | 1.11E-04 | 2.84E-04 | 0.273 |
| RHSIM_Rhsim09G0146300 | 14.95 | 1.11E-04 | 2.84E-04 | 0.250 |
| RHSIM_Rhsim09G0144800 | 14.94 | 1.11E-04 | 2.84E-04 | -0.815 |

| Gene | LRT statistic | P value | BH-adjusted P | log2FC (interaction) |
|---|---|---|---|---|
| RHSIM_Rhsim05G0034500 | 14.94 | 1.11E-04 | 2.84E-04 | -0.649 |
| RHSIM_Rhsim05G0195300 | 14.94 | 1.11E-04 | 2.84E-04 | -2.846 |
| RHSIM_Rhsim10G0009600 | 14.94 | 1.11E-04 | 2.84E-04 | 1.817 |
| RHSIM_Rhsim09G0122200 | 14.94 | 1.11E-04 | 2.84E-04 | -0.555 |
| novel.314 | 14.94 | 1.11E-04 | 2.84E-04 | -0.670 |
| RHSIM_Rhsim06G0082900 | 14.94 | 1.11E-04 | 2.85E-04 | 0.282 |
| RHSIM_Rhsim08G0228600 | 14.93 | 1.12E-04 | 2.86E-04 | 0.794 |
| RHSIM_RhsimUnG0223600 | 14.93 | 1.12E-04 | 2.86E-04 | -6.564 |
| RHSIM_Rhsim13G0083800 | 14.92 | 1.12E-04 | 2.87E-04 | -1.744 |
| RHSIM_Rhsim06G0244000 | 14.92 | 1.12E-04 | 2.88E-04 | 1.361 |
| novel.1276 | 14.92 | 1.12E-04 | 2.88E-04 | 0.473 |
| RHSIM_Rhsim11G0191800 | 14.92 | 1.12E-04 | 2.88E-04 | 1.845 |
| RHSIM_Rhsim09G0129900 | 14.91 | 1.13E-04 | 2.88E-04 | -0.431 |
| RHSIM_Rhsim10G0110900 | 14.91 | 1.13E-04 | 2.89E-04 | 1.002 |
| novel.171 | 14.91 | 1.13E-04 | 2.89E-04 | 1.493 |
| RHSIM_Rhsim01G0223800 | 14.91 | 1.13E-04 | 2.89E-04 | 0.685 |
| RHSIM_Rhsim09G0147100 | 14.91 | 1.13E-04 | 2.89E-04 | 2.662 |
| RHSIM_Rhsim08G0188100 | 14.91 | 1.13E-04 | 2.90E-04 | -0.278 |
| RHSIM_Rhsim08G0250900 | 14.90 | 1.13E-04 | 2.90E-04 | -0.644 |
| RHSIM_Rhsim01G0068300 | 14.90 | 1.13E-04 | 2.90E-04 | 1.483 |
| RHSIM_Rhsim01G0101500 | 14.89 | 1.14E-04 | 2.91E-04 | 0.496 |
| RHSIM_Rhsim02G0040300 | 14.89 | 1.14E-04 | 2.91E-04 | 0.255 |
| RHSIM_Rhsim04G0169800 | 14.89 | 1.14E-04 | 2.91E-04 | 1.633 |
| RHSIM_Rhsim12G0188600 | 14.89 | 1.14E-04 | 2.91E-04 | -1.125 |
| novel.401 | 14.89 | 1.14E-04 | 2.92E-04 | -3.372 |
| RHSIM_Rhsim04G0100000 | 14.89 | 1.14E-04 | 2.92E-04 | 0.262 |
| novel.874 | 14.89 | 1.14E-04 | 2.92E-04 | -3.651 |
| RHSIM_Rhsim06G0110700 | 14.89 | 1.14E-04 | 2.92E-04 | 1.819 |
| novel.4457 | 14.88 | 1.14E-04 | 2.92E-04 | 3.054 |
| RHSIM_Rhsim08G0187200 | 14.87 | 1.15E-04 | 2.94E-04 | 1.394 |
| RHSIM_Rhsim10G0031500 | 14.86 | 1.16E-04 | 2.96E-04 | 1.217 |
| RHSIM_Rhsim05G0057100 | 14.86 | 1.16E-04 | 2.96E-04 | -0.469 |

| Gene | LRT statistic | P value | BH-adjusted P | log2FC (interaction) |
|---|---|---|---|---|
| RHSIM_Rhsim03G0042100 | 14.85 | 1.16E-04 | 2.97E-04 | 2.154 |
| RHSIM_RhsimUnG0088700 | 14.85 | 1.16E-04 | 2.98E-04 | -1.553 |
| RHSIM_Rhsim02G0034900 | 14.85 | 1.17E-04 | 2.98E-04 | -1.087 |
| RHSIM_Rhsim09G0121400 | 14.84 | 1.17E-04 | 3.00E-04 | -0.954 |
| novel.3344 | 14.84 | 1.17E-04 | 3.00E-04 | 1.669 |
| RHSIM_Rhsim02G0024300 | 14.83 | 1.18E-04 | 3.00E-04 | 0.871 |
| RHSIM_Rhsim03G0040300 | 14.83 | 1.18E-04 | 3.01E-04 | -0.663 |
| RHSIM_Rhsim09G0166700 | 14.83 | 1.18E-04 | 3.01E-04 | -2.042 |
| RHSIM_Rhsim05G0146400 | 14.83 | 1.18E-04 | 3.01E-04 | 0.523 |
| RHSIM_Rhsim07G0022600 | 14.82 | 1.18E-04 | 3.02E-04 | -0.476 |
| RHSIM_Rhsim01G0277700 | 14.82 | 1.19E-04 | 3.03E-04 | 0.885 |
| novel.3989 | 14.82 | 1.19E-04 | 3.03E-04 | 0.462 |
| RHSIM_Rhsim01G0232900 | 14.81 | 1.19E-04 | 3.03E-04 | 0.533 |
| RHSIM_Rhsim07G0250400 | 14.81 | 1.19E-04 | 3.03E-04 | -0.773 |
| RHSIM_Rhsim10G0003400 | 14.81 | 1.19E-04 | 3.03E-04 | -0.628 |
| RHSIM_Rhsim07G0042900 | 14.81 | 1.19E-04 | 3.04E-04 | 0.562 |
| RHSIM_Rhsim06G0020400 | 14.80 | 1.19E-04 | 3.05E-04 | -1.400 |
| RHSIM_Rhsim02G0158900 | 14.80 | 1.19E-04 | 3.05E-04 | 0.441 |
| RHSIM_Rhsim07G0131800 | 14.79 | 1.20E-04 | 3.06E-04 | -0.537 |
| RHSIM_Rhsim08G0222600 | 14.79 | 1.20E-04 | 3.07E-04 | 3.393 |
| RHSIM_Rhsim03G0189000 | 14.78 | 1.21E-04 | 3.08E-04 | -0.512 |
| RHSIM_Rhsim06G0094400 | 14.78 | 1.21E-04 | 3.08E-04 | -1.567 |
| novel.3566 | 14.76 | 1.22E-04 | 3.12E-04 | -42.159 |
| RHSIM_Rhsim13G0214400 | 14.76 | 1.22E-04 | 3.12E-04 | 1.158 |
| RHSIM_Rhsim08G0215500 | 14.76 | 1.22E-04 | 3.12E-04 | -1.083 |
| RHSIM_Rhsim06G0219200 | 14.76 | 1.22E-04 | 3.12E-04 | -0.470 |
| RHSIM_Rhsim02G0089800 | 14.76 | 1.22E-04 | 3.12E-04 | 0.464 |
| RHSIM_Rhsim01G0176800 | 14.75 | 1.23E-04 | 3.14E-04 | -0.934 |
| RHSIM_Rhsim04G0007700 | 14.74 | 1.23E-04 | 3.14E-04 | 0.580 |
| RHSIM_Rhsim02G0247700 | 14.74 | 1.24E-04 | 3.15E-04 | -0.744 |
| RHSIM_Rhsim10G0199600 | 14.74 | 1.24E-04 | 3.15E-04 | -1.364 |
| novel.4328 | 14.74 | 1.24E-04 | 3.15E-04 | 1.612 |

| Gene | LRT statistic | P value | BH-adjusted P | log2FC (interaction) |
|---|---|---|---|---|
| RHSIM_Rhsim12G0208600 | 14.73 | 1.24E-04 | 3.16E-04 | -0.559 |
| RHSIM_RhsimUnG0118200 | 14.73 | 1.24E-04 | 3.17E-04 | -0.638 |
| novel.1183 | 14.72 | 1.25E-04 | 3.17E-04 | 1.264 |
| RHSIM_Rhsim08G0020400 | 14.72 | 1.25E-04 | 3.18E-04 | -2.724 |
| RHSIM_Rhsim09G0008900 | 14.72 | 1.25E-04 | 3.18E-04 | 0.836 |
| RHSIM_Rhsim03G0026500 | 14.72 | 1.25E-04 | 3.19E-04 | 1.411 |
| RHSIM_Rhsim08G0192000 | 14.71 | 1.25E-04 | 3.19E-04 | 0.270 |
| RHSIM_Rhsim07G0049700 | 14.71 | 1.25E-04 | 3.19E-04 | 0.323 |
| novel.2160 | 14.71 | 1.25E-04 | 3.20E-04 | -1.716 |
| RHSIM_Rhsim05G0224300 | 14.70 | 1.26E-04 | 3.21E-04 | 0.819 |
| RHSIM_Rhsim13G0117700 | 14.70 | 1.26E-04 | 3.21E-04 | -0.895 |
| RHSIM_Rhsim06G0067800 | 14.70 | 1.26E-04 | 3.21E-04 | -0.607 |
| RHSIM_Rhsim08G0114900 | 14.69 | 1.27E-04 | 3.23E-04 | -0.376 |
| novel.3611 | 14.69 | 1.27E-04 | 3.23E-04 | 3.017 |
| RHSIM_Rhsim09G0090400 | 14.69 | 1.27E-04 | 3.24E-04 | -0.453 |
| RHSIM_Rhsim01G0141100 | 14.68 | 1.27E-04 | 3.24E-04 | -0.314 |
| RHSIM_Rhsim06G0098000 | 14.68 | 1.27E-04 | 3.24E-04 | 1.006 |
| RHSIM_Rhsim06G0193700 | 14.68 | 1.27E-04 | 3.24E-04 | -0.438 |
| RHSIM_Rhsim07G0003800 | 14.68 | 1.28E-04 | 3.25E-04 | 0.488 |
| RHSIM_Rhsim05G0098200 | 14.67 | 1.28E-04 | 3.25E-04 | -2.013 |
| RHSIM_Rhsim04G0037800 | 14.67 | 1.28E-04 | 3.25E-04 | -0.583 |
| RHSIM_Rhsim08G0217900 | 14.67 | 1.28E-04 | 3.27E-04 | -0.868 |
| RHSIM_Rhsim12G0037600 | 14.67 | 1.28E-04 | 3.27E-04 | 0.765 |
| RHSIM_Rhsim07G0122800 | 14.66 | 1.28E-04 | 3.27E-04 | -0.718 |
| RHSIM_Rhsim02G0255800 | 14.66 | 1.29E-04 | 3.28E-04 | 0.808 |
| RHSIM_Rhsim12G0109600 | 14.66 | 1.29E-04 | 3.28E-04 | 2.298 |
| novel.387 | 14.65 | 1.29E-04 | 3.29E-04 | 1.112 |
| RHSIM_Rhsim01G0236200 | 14.65 | 1.30E-04 | 3.30E-04 | -0.639 |
| RHSIM_RhsimUnG0196500 | 14.64 | 1.30E-04 | 3.31E-04 | -0.421 |
| RHSIM_Rhsim03G0135000 | 14.64 | 1.30E-04 | 3.31E-04 | 2.691 |
| RHSIM_Rhsim08G0045700 | 14.64 | 1.30E-04 | 3.31E-04 | 0.235 |
| RHSIM_Rhsim10G0050200 | 14.64 | 1.30E-04 | 3.31E-04 | 2.105 |

| Gene | LRT statistic | P value | BH-adjusted P | log2FC (interaction) |
|---|---|---|---|---|
| RHSIM_Rhsim12G0025000 | 14.64 | 1.30E-04 | 3.32E-04 | -0.476 |
| RHSIM_RhsimUnG0197800 | 14.62 | 1.32E-04 | 3.35E-04 | -0.978 |
| novel.1615 | 14.61 | 1.32E-04 | 3.35E-04 | 4.753 |
| RHSIM_Rhsim01G0145900 | 14.61 | 1.32E-04 | 3.36E-04 | 1.088 |
| RHSIM_Rhsim12G0005800 | 14.61 | 1.32E-04 | 3.36E-04 | 0.489 |
| RHSIM_Rhsim08G0152800 | 14.60 | 1.33E-04 | 3.38E-04 | 0.580 |
| RHSIM_Rhsim06G0031700 | 14.60 | 1.33E-04 | 3.38E-04 | -0.538 |
| novel.1118 | 14.60 | 1.33E-04 | 3.38E-04 | 4.705 |
| novel.3905 | 14.59 | 1.33E-04 | 3.39E-04 | 1.850 |
| RHSIM_Rhsim13G0029700 | 14.59 | 1.34E-04 | 3.40E-04 | 0.780 |
| RHSIM_Rhsim03G0222200 | 14.58 | 1.35E-04 | 3.42E-04 | 1.810 |
| RHSIM_Rhsim09G0082600 | 14.58 | 1.35E-04 | 3.42E-04 | 2.928 |
| RHSIM_Rhsim02G0127100 | 14.57 | 1.35E-04 | 3.44E-04 | -0.339 |
| RHSIM_Rhsim07G0058900 | 14.57 | 1.35E-04 | 3.44E-04 | -0.649 |
| RHSIM_Rhsim10G0041300 | 14.56 | 1.35E-04 | 3.44E-04 | 0.959 |
| RHSIM_Rhsim13G0167900 | 14.56 | 1.36E-04 | 3.45E-04 | 2.493 |
| RHSIM_Rhsim11G0179200 | 14.56 | 1.36E-04 | 3.45E-04 | -0.448 |
| RHSIM_Rhsim12G0052500 | 14.56 | 1.36E-04 | 3.45E-04 | -0.617 |
| novel.3410 | 14.56 | 1.36E-04 | 3.45E-04 | 6.116 |
| RHSIM_Rhsim07G0199000 | 14.55 | 1.36E-04 | 3.46E-04 | -0.599 |
| RHSIM_Rhsim08G0034600 | 14.55 | 1.36E-04 | 3.46E-04 | 0.322 |
| RHSIM_Rhsim11G0131600 | 14.54 | 1.37E-04 | 3.48E-04 | -0.650 |
| RHSIM_Rhsim07G0182400 | 14.54 | 1.37E-04 | 3.48E-04 | -0.444 |
| RHSIM_Rhsim09G0088600 | 14.54 | 1.38E-04 | 3.49E-04 | -0.555 |
| novel.2502 | 14.52 | 1.39E-04 | 3.52E-04 | 3.078 |
| RHSIM_Rhsim06G0129100 | 14.52 | 1.39E-04 | 3.52E-04 | -0.429 |
| RHSIM_Rhsim07G0095000 | 14.52 | 1.39E-04 | 3.52E-04 | -3.742 |
| RHSIM_Rhsim01G0241600 | 14.52 | 1.39E-04 | 3.52E-04 | -1.564 |
| RHSIM_Rhsim04G0026500 | 14.52 | 1.39E-04 | 3.52E-04 | -0.609 |
| RHSIM_Rhsim07G0247800 | 14.51 | 1.39E-04 | 3.53E-04 | 1.589 |
| RHSIM_Rhsim02G0011800 | 14.50 | 1.40E-04 | 3.55E-04 | 0.206 |
| RHSIM_RhsimUnG0150100 | 14.50 | 1.40E-04 | 3.55E-04 | -0.456 |

| Gene | LRT statistic | P value | BH-adjusted P | log2FC (interaction) |
|---|---|---|---|---|
| RHSIM_Rhsim13G0141200 | 14.50 | 1.40E-04 | 3.56E-04 | -0.559 |
| RHSIM_Rhsim06G0040900 | 14.50 | 1.40E-04 | 3.56E-04 | 0.370 |
| RHSIM_Rhsim08G0220800 | 14.49 | 1.41E-04 | 3.57E-04 | 0.469 |
| RHSIM_Rhsim09G0126300 | 14.49 | 1.41E-04 | 3.57E-04 | -0.574 |
| RHSIM_RhsimUnG0014700 | 14.48 | 1.41E-04 | 3.59E-04 | -5.167 |
| RHSIM_Rhsim04G0124700 | 14.48 | 1.42E-04 | 3.59E-04 | -1.430 |
| RHSIM_Rhsim04G0225200 | 14.48 | 1.42E-04 | 3.59E-04 | -0.693 |
| RHSIM_Rhsim10G0082100 | 14.48 | 1.42E-04 | 3.59E-04 | 0.771 |
| RHSIM_Rhsim07G0072800 | 14.48 | 1.42E-04 | 3.60E-04 | 1.785 |
| RHSIM_Rhsim08G0242900 | 14.47 | 1.42E-04 | 3.61E-04 | -6.893 |
| RHSIM_Rhsim10G0122600 | 14.47 | 1.42E-04 | 3.61E-04 | 0.966 |
| RHSIM_Rhsim06G0121200 | 14.47 | 1.42E-04 | 3.61E-04 | -0.647 |
| RHSIM_Rhsim13G0065800 | 14.47 | 1.42E-04 | 3.61E-04 | -40.880 |
| RHSIM_Rhsim12G0189500 | 14.47 | 1.43E-04 | 3.61E-04 | 0.511 |
| RHSIM_Rhsim07G0019700 | 14.46 | 1.43E-04 | 3.62E-04 | -2.611 |
| RHSIM_Rhsim02G0240500 | 14.46 | 1.43E-04 | 3.62E-04 | -1.526 |
| RHSIM_Rhsim04G0091900 | 14.46 | 1.43E-04 | 3.62E-04 | 42.147 |
| novel.3906 | 14.46 | 1.43E-04 | 3.63E-04 | 3.179 |
| RHSIM_Rhsim13G0101800 | 14.46 | 1.43E-04 | 3.63E-04 | -2.754 |
| RHSIM_RhsimUnG0040700 | 14.45 | 1.44E-04 | 3.63E-04 | -1.853 |
| novel.777 | 14.45 | 1.44E-04 | 3.63E-04 | -0.868 |
| RHSIM_Rhsim11G0139300 | 14.45 | 1.44E-04 | 3.64E-04 | -0.350 |
| RHSIM_Rhsim10G0101200 | 14.45 | 1.44E-04 | 3.64E-04 | -0.219 |
| RHSIM_Rhsim04G0086100 | 14.45 | 1.44E-04 | 3.65E-04 | 1.210 |
| RHSIM_Rhsim02G0053800 | 14.45 | 1.44E-04 | 3.65E-04 | -0.354 |
| RHSIM_Rhsim03G0010700 | 14.45 | 1.44E-04 | 3.65E-04 | -0.894 |
| RHSIM_Rhsim11G0166400 | 14.44 | 1.45E-04 | 3.66E-04 | 0.268 |
| RHSIM_Rhsim05G0160800 | 14.44 | 1.45E-04 | 3.66E-04 | -0.588 |
| RHSIM_Rhsim04G0068300 | 14.44 | 1.45E-04 | 3.67E-04 | -0.634 |
| RHSIM_Rhsim08G0010900 | 14.44 | 1.45E-04 | 3.67E-04 | -0.455 |
| novel.2557 | 14.43 | 1.45E-04 | 3.68E-04 | 1.299 |
| RHSIM_Rhsim06G0048400 | 14.43 | 1.45E-04 | 3.68E-04 | 0.501 |

| Gene | LRT statistic | P value | BH-adjusted P | log2FC (interaction) |
|---|---|---|---|---|
| RHSIM_Rhsim04G0234800 | 14.43 | 1.45E-04 | 3.68E-04 | 0.239 |
| RHSIM_Rhsim07G0026600 | 14.43 | 1.46E-04 | 3.68E-04 | 0.512 |
| novel.3464 | 14.43 | 1.46E-04 | 3.69E-04 | 0.390 |
| RHSIM_Rhsim04G0230100 | 14.42 | 1.47E-04 | 3.70E-04 | 80.319 |
| RHSIM_Rhsim03G0248100 | 14.42 | 1.47E-04 | 3.70E-04 | -1.454 |
| RHSIM_Rhsim04G0153200 | 14.41 | 1.47E-04 | 3.71E-04 | -0.277 |
| RHSIM_Rhsim04G0110700 | 14.41 | 1.47E-04 | 3.72E-04 | -0.318 |
| RHSIM_Rhsim06G0080700 | 14.41 | 1.47E-04 | 3.72E-04 | -0.406 |
| RHSIM_Rhsim13G0155200 | 14.40 | 1.48E-04 | 3.73E-04 | -1.544 |
| novel.1645 | 14.40 | 1.48E-04 | 3.73E-04 | 4.292 |
| novel.2787 | 14.40 | 1.48E-04 | 3.74E-04 | 1.844 |
| RHSIM_Rhsim11G0107000 | 14.39 | 1.48E-04 | 3.75E-04 | -1.288 |
| novel.1341 | 14.39 | 1.49E-04 | 3.75E-04 | -2.540 |
| RHSIM_Rhsim04G0242900 | 14.39 | 1.49E-04 | 3.75E-04 | 0.593 |
| RHSIM_Rhsim08G0101200 | 14.39 | 1.49E-04 | 3.76E-04 | -1.645 |
| RHSIM_Rhsim10G0053900 | 14.36 | 1.51E-04 | 3.81E-04 | 0.474 |
| RHSIM_Rhsim07G0178500 | 14.36 | 1.51E-04 | 3.81E-04 | 0.337 |
| RHSIM_Rhsim08G0141300 | 14.36 | 1.51E-04 | 3.82E-04 | -0.606 |
| RHSIM_Rhsim04G0145100 | 14.36 | 1.51E-04 | 3.82E-04 | -0.651 |
| novel.1389 | 14.35 | 1.52E-04 | 3.82E-04 | 0.823 |
| RHSIM_Rhsim02G0228600 | 14.35 | 1.52E-04 | 3.82E-04 | 0.773 |
| novel.166 | 14.35 | 1.52E-04 | 3.83E-04 | -0.492 |
| RHSIM_Rhsim05G0176800 | 14.35 | 1.52E-04 | 3.84E-04 | 2.680 |
| RHSIM_Rhsim08G0020700 | 14.34 | 1.52E-04 | 3.84E-04 | 0.206 |
| novel.1050 | 14.34 | 1.52E-04 | 3.85E-04 | -1.011 |
| RHSIM_Rhsim04G0018500 | 14.34 | 1.52E-04 | 3.85E-04 | 0.791 |
| RHSIM_Rhsim12G0050300 | 14.34 | 1.52E-04 | 3.85E-04 | -6.735 |
| RHSIM_Rhsim07G0074700 | 14.34 | 1.53E-04 | 3.86E-04 | -0.290 |
| RHSIM_Rhsim12G0208100 | 14.34 | 1.53E-04 | 3.86E-04 | -0.639 |
| RHSIM_Rhsim01G0273100 | 14.34 | 1.53E-04 | 3.86E-04 | -1.390 |
| RHSIM_Rhsim10G0173900 | 14.33 | 1.53E-04 | 3.87E-04 | 1.836 |
| novel.39 | 14.33 | 1.54E-04 | 3.87E-04 | -2.413 |

| Gene | LRT statistic | P value | BH-adjusted P | log2FC (interaction) |
|---|---|---|---|---|
| RHSIM_Rhsim04G0016000 | 14.33 | 1.54E-04 | 3.88E-04 | 0.347 |
| RHSIM_Rhsim01G0198000 | 14.32 | 1.54E-04 | 3.88E-04 | -1.101 |
| novel.441 | 14.32 | 1.54E-04 | 3.89E-04 | -0.942 |
| RHSIM_Rhsim12G0167300 | 14.31 | 1.55E-04 | 3.91E-04 | -1.079 |
| RHSIM_Rhsim13G0116800 | 14.30 | 1.56E-04 | 3.92E-04 | 1.693 |
| RHSIM_Rhsim13G0021200 | 14.30 | 1.56E-04 | 3.92E-04 | -0.355 |
| novel.1093 | 14.30 | 1.56E-04 | 3.92E-04 | 4.399 |
| RHSIM_Rhsim11G0159600 | 14.30 | 1.56E-04 | 3.92E-04 | 0.548 |
| RHSIM_Rhsim10G0072800 | 14.30 | 1.56E-04 | 3.94E-04 | -0.412 |
| novel.2886 | 14.29 | 1.56E-04 | 3.94E-04 | 1.266 |
| RHSIM_Rhsim03G0078000 | 14.29 | 1.57E-04 | 3.95E-04 | -3.178 |
| RHSIM_Rhsim06G0033700 | 14.29 | 1.57E-04 | 3.95E-04 | -0.857 |
| RHSIM_Rhsim04G0239300 | 14.28 | 1.57E-04 | 3.96E-04 | -0.437 |
| novel.200 | 14.28 | 1.58E-04 | 3.97E-04 | 1.491 |
| RHSIM_Rhsim05G0195600 | 14.28 | 1.58E-04 | 3.97E-04 | 1.517 |
| RHSIM_Rhsim03G0002400 | 14.28 | 1.58E-04 | 3.98E-04 | 0.587 |
| novel.3650 | 14.27 | 1.58E-04 | 3.98E-04 | 1.159 |
| RHSIM_Rhsim08G0206900 | 14.27 | 1.58E-04 | 3.98E-04 | -0.507 |
| RHSIM_Rhsim09G0075100 | 14.27 | 1.58E-04 | 3.98E-04 | -0.426 |
| RHSIM_Rhsim03G0255500 | 14.27 | 1.58E-04 | 3.98E-04 | 0.450 |
| RHSIM_Rhsim01G0214100 | 14.27 | 1.58E-04 | 3.99E-04 | -41.800 |
| RHSIM_Rhsim05G0141700 | 14.27 | 1.59E-04 | 3.99E-04 | -0.727 |
| RHSIM_Rhsim08G0021300 | 14.26 | 1.59E-04 | 4.00E-04 | 3.051 |
| RHSIM_Rhsim04G0057600 | 14.26 | 1.59E-04 | 4.01E-04 | -0.846 |
| RHSIM_Rhsim03G0171500 | 14.25 | 1.60E-04 | 4.02E-04 | -3.464 |
| RHSIM_Rhsim11G0073800 | 14.25 | 1.60E-04 | 4.02E-04 | 2.401 |
| RHSIM_Rhsim13G0024300 | 14.25 | 1.60E-04 | 4.02E-04 | 0.551 |
| RHSIM_RhsimUnG0105400 | 14.25 | 1.60E-04 | 4.03E-04 | -3.176 |
| RHSIM_Rhsim10G0014900 | 14.25 | 1.60E-04 | 4.03E-04 | 0.525 |
| RHSIM_Rhsim02G0140100 | 14.25 | 1.60E-04 | 4.03E-04 | 1.672 |
| RHSIM_Rhsim10G0115200 | 14.25 | 1.60E-04 | 4.03E-04 | 0.771 |
| RHSIM_Rhsim08G0245300 | 14.24 | 1.61E-04 | 4.04E-04 | 0.527 |

| Gene | LRT statistic | P value | BH-adjusted P | log2FC (interaction) |
|---|---|---|---|---|
| RHSIM_Rhsim06G0149200 | 14.24 | 1.61E-04 | 4.05E-04 | -0.948 |
| RHSIM_Rhsim13G0025000 | 14.24 | 1.61E-04 | 4.05E-04 | -1.401 |
| RHSIM_Rhsim07G0128600 | 14.23 | 1.61E-04 | 4.06E-04 | -0.425 |
| RHSIM_Rhsim10G0072300 | 14.22 | 1.63E-04 | 4.09E-04 | -0.610 |
| RHSIM_Rhsim11G0077400 | 14.21 | 1.63E-04 | 4.10E-04 | 1.878 |
| RHSIM_Rhsim04G0146800 | 14.21 | 1.63E-04 | 4.11E-04 | 2.251 |
| RHSIM_Rhsim04G0024300 | 14.21 | 1.63E-04 | 4.11E-04 | 0.662 |
| RHSIM_Rhsim09G0106100 | 14.21 | 1.64E-04 | 4.11E-04 | 2.275 |
| RHSIM_Rhsim07G0220000 | 14.21 | 1.64E-04 | 4.12E-04 | 1.839 |
| RHSIM_Rhsim03G0033300 | 14.21 | 1.64E-04 | 4.12E-04 | -0.980 |
| RHSIM_Rhsim08G0222400 | 14.20 | 1.64E-04 | 4.12E-04 | 0.454 |
| RHSIM_Rhsim12G0203900 | 14.20 | 1.64E-04 | 4.12E-04 | -2.948 |
| RHSIM_Rhsim07G0159800 | 14.20 | 1.64E-04 | 4.13E-04 | -2.138 |
| RHSIM_Rhsim08G0174200 | 14.19 | 1.65E-04 | 4.14E-04 | 1.385 |
| RHSIM_Rhsim05G0048200 | 14.19 | 1.65E-04 | 4.14E-04 | 6.161 |
| RHSIM_Rhsim13G0214700 | 14.19 | 1.65E-04 | 4.15E-04 | -1.189 |
| RHSIM_Rhsim09G0032300 | 14.18 | 1.66E-04 | 4.16E-04 | -0.300 |
| RHSIM_Rhsim07G0219300 | 14.18 | 1.66E-04 | 4.17E-04 | 0.469 |
| RHSIM_Rhsim05G0161300 | 14.18 | 1.66E-04 | 4.17E-04 | 0.645 |
| novel.357 | 14.18 | 1.66E-04 | 4.17E-04 | -0.641 |
| novel.4250 | 14.18 | 1.66E-04 | 4.18E-04 | 0.518 |
| RHSIM_Rhsim01G0120200 | 14.17 | 1.67E-04 | 4.18E-04 | 2.965 |
| RHSIM_Rhsim07G0007300 | 14.17 | 1.67E-04 | 4.18E-04 | 2.314 |
| RHSIM_Rhsim07G0221100 | 14.17 | 1.67E-04 | 4.19E-04 | 0.653 |
| RHSIM_Rhsim10G0122100 | 14.17 | 1.67E-04 | 4.19E-04 | 0.883 |
| RHSIM_RhsimUnG0250800 | 14.17 | 1.67E-04 | 4.19E-04 | 1.097 |
| RHSIM_Rhsim03G0196300 | 14.17 | 1.67E-04 | 4.20E-04 | -1.048 |
| novel.533 | 14.16 | 1.68E-04 | 4.20E-04 | 2.579 |
| RHSIM_Rhsim10G0092900 | 14.16 | 1.68E-04 | 4.21E-04 | -0.312 |
| novel.1503 | 14.15 | 1.69E-04 | 4.24E-04 | 2.690 |
| RHSIM_Rhsim06G0171600 | 14.14 | 1.69E-04 | 4.25E-04 | -2.867 |
| novel.2916 | 14.14 | 1.69E-04 | 4.25E-04 | 2.468 |

| Gene | LRT statistic | P value | BH-adjusted P | log2FC (interaction) |
|---|---|---|---|---|
| RHSIM_Rhsim07G0181500 | 14.14 | 1.70E-04 | 4.25E-04 | 1.581 |
| RHSIM_Rhsim12G0040200 | 14.14 | 1.70E-04 | 4.26E-04 | -2.115 |
| RHSIM_Rhsim08G0125100 | 14.14 | 1.70E-04 | 4.26E-04 | -0.412 |
| RHSIM_Rhsim03G0153500 | 14.14 | 1.70E-04 | 4.26E-04 | 0.282 |
| RHSIM_Rhsim01G0038200 | 14.14 | 1.70E-04 | 4.26E-04 | -3.855 |
| RHSIM_Rhsim07G0058400 | 14.13 | 1.71E-04 | 4.28E-04 | -0.374 |
| RHSIM_Rhsim03G0245600 | 14.13 | 1.71E-04 | 4.28E-04 | 0.197 |
| RHSIM_Rhsim09G0190400 | 14.12 | 1.71E-04 | 4.30E-04 | -0.294 |
| RHSIM_Rhsim08G0217600 | 14.12 | 1.72E-04 | 4.30E-04 | -1.387 |
| RHSIM_Rhsim04G0017600 | 14.12 | 1.72E-04 | 4.31E-04 | -1.183 |
| RHSIM_Rhsim12G0201300 | 14.11 | 1.72E-04 | 4.31E-04 | -0.424 |
| novel.2892 | 14.11 | 1.72E-04 | 4.31E-04 | 1.089 |
| RHSIM_Rhsim02G0202300 | 14.11 | 1.72E-04 | 4.31E-04 | -0.747 |
| RHSIM_Rhsim04G0107900 | 14.11 | 1.72E-04 | 4.31E-04 | -0.485 |
| novel.3141 | 14.11 | 1.72E-04 | 4.31E-04 | 2.221 |
| RHSIM_Rhsim08G0248500 | 14.11 | 1.72E-04 | 4.31E-04 | -0.777 |
| RHSIM_Rhsim03G0098700 | 14.10 | 1.73E-04 | 4.34E-04 | 0.921 |
| RHSIM_RhsimUnG0151500 | 14.10 | 1.73E-04 | 4.34E-04 | -42.504 |
| RHSIM_Rhsim10G0134200 | 14.10 | 1.74E-04 | 4.35E-04 | 2.996 |
| RHSIM_Rhsim08G0118600 | 14.09 | 1.74E-04 | 4.35E-04 | -0.381 |
| RHSIM_Rhsim05G0110500 | 14.09 | 1.74E-04 | 4.36E-04 | 4.083 |
| RHSIM_Rhsim12G0174500 | 14.09 | 1.75E-04 | 4.37E-04 | -0.341 |
| RHSIM_Rhsim06G0149600 | 14.08 | 1.75E-04 | 4.37E-04 | 0.863 |
| novel.2877 | 14.08 | 1.75E-04 | 4.38E-04 | 0.603 |
| novel.2231 | 14.08 | 1.76E-04 | 4.39E-04 | -0.540 |
| RHSIM_Rhsim12G0204300 | 14.08 | 1.76E-04 | 4.39E-04 | 0.962 |
| RHSIM_Rhsim05G0064400 | 14.07 | 1.76E-04 | 4.40E-04 | 42.581 |
| RHSIM_Rhsim04G0232800 | 14.07 | 1.76E-04 | 4.40E-04 | -1.187 |
| RHSIM_Rhsim02G0174800 | 14.06 | 1.77E-04 | 4.42E-04 | -0.698 |
| RHSIM_Rhsim12G0189100 | 14.05 | 1.78E-04 | 4.46E-04 | -0.465 |
| RHSIM_Rhsim07G0243600 | 14.04 | 1.79E-04 | 4.47E-04 | 1.028 |
| RHSIM_Rhsim04G0066200 | 14.04 | 1.79E-04 | 4.47E-04 | -2.636 |

| Gene | LRT statistic | P value | BH-adjusted P | log2FC (interaction) |
|---|---|---|---|---|
| RHSIM_RhsimUnG0178900 | 14.04 | 1.79E-04 | 4.47E-04 | -1.500 |
| RHSIM_Rhsim03G0246200 | 14.04 | 1.79E-04 | 4.47E-04 | 0.470 |
| RHSIM_Rhsim08G0174000 | 14.04 | 1.79E-04 | 4.48E-04 | -0.342 |
| RHSIM_Rhsim11G0087500 | 14.04 | 1.79E-04 | 4.48E-04 | 0.968 |
| RHSIM_Rhsim02G0111100 | 14.03 | 1.80E-04 | 4.49E-04 | -0.796 |
| RHSIM_Rhsim05G0196900 | 14.03 | 1.80E-04 | 4.51E-04 | -0.361 |
| novel.126 | 14.02 | 1.80E-04 | 4.51E-04 | 41.306 |
| RHSIM_Rhsim06G0234500 | 14.02 | 1.80E-04 | 4.51E-04 | 1.029 |
| RHSIM_Rhsim07G0080900 | 14.02 | 1.81E-04 | 4.51E-04 | 0.709 |
| RHSIM_Rhsim10G0052400 | 14.02 | 1.81E-04 | 4.52E-04 | -1.087 |
| RHSIM_Rhsim04G0220200 | 14.02 | 1.81E-04 | 4.53E-04 | -0.523 |
| RHSIM_Rhsim10G0061300 | 14.01 | 1.82E-04 | 4.54E-04 | -0.694 |
| RHSIM_RhsimUnG0060100 | 14.01 | 1.82E-04 | 4.55E-04 | 0.805 |
| RHSIM_Rhsim10G0042700 | 14.00 | 1.82E-04 | 4.56E-04 | -1.983 |
| RHSIM_Rhsim12G0089400 | 14.00 | 1.82E-04 | 4.56E-04 | 0.330 |
| RHSIM_Rhsim06G0098500 | 14.00 | 1.83E-04 | 4.56E-04 | 0.399 |
| RHSIM_Rhsim13G0216400 | 13.99 | 1.83E-04 | 4.58E-04 | 0.627 |
| RHSIM_Rhsim04G0112100 | 13.99 | 1.84E-04 | 4.58E-04 | 1.401 |
| novel.2867 | 13.99 | 1.84E-04 | 4.60E-04 | -1.185 |
| RHSIM_Rhsim11G0103500 | 13.99 | 1.84E-04 | 4.60E-04 | 0.716 |
| RHSIM_Rhsim06G0184200 | 13.98 | 1.84E-04 | 4.60E-04 | -1.193 |
| RHSIM_Rhsim03G0083600 | 13.98 | 1.85E-04 | 4.62E-04 | -0.313 |
| RHSIM_Rhsim06G0238400 | 13.98 | 1.85E-04 | 4.62E-04 | -0.261 |
| RHSIM_Rhsim06G0025000 | 13.97 | 1.86E-04 | 4.63E-04 | -0.740 |
| RHSIM_Rhsim02G0114600 | 13.97 | 1.86E-04 | 4.63E-04 | -40.724 |
| RHSIM_Rhsim07G0174500 | 13.97 | 1.86E-04 | 4.64E-04 | -0.531 |
| RHSIM_Rhsim01G0201500 | 13.97 | 1.86E-04 | 4.64E-04 | 0.786 |
| RHSIM_Rhsim03G0036500 | 13.97 | 1.86E-04 | 4.64E-04 | -0.414 |
| novel.2860 | 13.97 | 1.86E-04 | 4.65E-04 | -2.949 |
| RHSIM_Rhsim02G0013900 | 13.95 | 1.87E-04 | 4.67E-04 | 0.398 |
| RHSIM_Rhsim10G0151100 | 13.95 | 1.88E-04 | 4.68E-04 | -0.784 |
| RHSIM_Rhsim12G0204500 | 13.95 | 1.88E-04 | 4.68E-04 | 0.326 |

| Gene | LRT statistic | P value | BH-adjusted P | log2FC (interaction) |
|---|---|---|---|---|
| RHSIM_Rhsim03G0017800 | 13.95 | 1.88E-04 | 4.69E-04 | 1.256 |
| RHSIM_Rhsim04G0068600 | 13.94 | 1.88E-04 | 4.70E-04 | -0.412 |
| novel.2817 | 13.93 | 1.89E-04 | 4.72E-04 | -0.740 |
| RHSIM_Rhsim01G0052500 | 13.93 | 1.90E-04 | 4.73E-04 | 2.504 |
| RHSIM_Rhsim11G0190400 | 13.93 | 1.90E-04 | 4.73E-04 | 1.233 |
| novel.1981 | 13.93 | 1.90E-04 | 4.73E-04 | -0.666 |
| RHSIM_Rhsim02G0135500 | 13.93 | 1.90E-04 | 4.73E-04 | 0.317 |
| RHSIM_Rhsim01G0012200 | 13.93 | 1.90E-04 | 4.74E-04 | 0.725 |
| RHSIM_Rhsim07G0206100 | 13.92 | 1.91E-04 | 4.76E-04 | 0.335 |
| RHSIM_Rhsim12G0140500 | 13.91 | 1.91E-04 | 4.77E-04 | -3.073 |
| RHSIM_Rhsim03G0068500 | 13.91 | 1.92E-04 | 4.77E-04 | 1.241 |
| RHSIM_Rhsim09G0040100 | 13.91 | 1.92E-04 | 4.78E-04 | -2.980 |
| novel.1232 | 13.90 | 1.92E-04 | 4.79E-04 | -0.393 |
| RHSIM_Rhsim11G0160200 | 13.90 | 1.93E-04 | 4.81E-04 | -0.509 |
| RHSIM_Rhsim03G0232800 | 13.90 | 1.93E-04 | 4.81E-04 | 1.068 |
| RHSIM_Rhsim10G0103200 | 13.89 | 1.93E-04 | 4.82E-04 | -0.595 |
| RHSIM_Rhsim13G0234000 | 13.89 | 1.94E-04 | 4.82E-04 | -0.273 |
| RHSIM_Rhsim02G0082600 | 13.89 | 1.94E-04 | 4.83E-04 | -0.316 |
| RHSIM_Rhsim02G0098100 | 13.88 | 1.95E-04 | 4.86E-04 | -0.843 |
| RHSIM_Rhsim05G0234500 | 13.87 | 1.95E-04 | 4.86E-04 | 0.559 |
| RHSIM_Rhsim05G0185000 | 13.87 | 1.96E-04 | 4.88E-04 | -0.371 |
| RHSIM_Rhsim12G0208900 | 13.87 | 1.96E-04 | 4.88E-04 | -0.519 |
| RHSIM_Rhsim12G0003100 | 13.87 | 1.96E-04 | 4.88E-04 | 0.237 |
| RHSIM_Rhsim10G0014200 | 13.86 | 1.97E-04 | 4.89E-04 | -0.630 |
| RHSIM_Rhsim04G0228500 | 13.86 | 1.97E-04 | 4.90E-04 | 0.449 |
| RHSIM_Rhsim12G0112500 | 13.85 | 1.98E-04 | 4.93E-04 | -0.445 |
| RHSIM_Rhsim09G0196700 | 13.84 | 1.99E-04 | 4.95E-04 | -1.585 |
| RHSIM_Rhsim12G0018100 | 13.84 | 1.99E-04 | 4.95E-04 | 3.031 |
| RHSIM_Rhsim10G0002900 | 13.83 | 2.00E-04 | 4.97E-04 | 0.408 |
| RHSIM_Rhsim07G0178300 | 13.83 | 2.00E-04 | 4.97E-04 | 0.441 |
| RHSIM_Rhsim04G0215500 | 13.83 | 2.00E-04 | 4.98E-04 | -0.348 |
| RHSIM_Rhsim07G0143500 | 13.83 | 2.01E-04 | 4.99E-04 | 0.445 |

| Gene | LRT statistic | P value | BH-adjusted P | log2FC (interaction) |
|---|---|---|---|---|
| RHSIM_Rhsim11G0153000 | 13.82 | 2.01E-04 | 5.01E-04 | -0.696 |
| RHSIM_Rhsim05G0121400 | 13.81 | 2.02E-04 | 5.03E-04 | -0.953 |
| RHSIM_RhsimUnG0033200 | 13.81 | 2.02E-04 | 5.03E-04 | -0.403 |
| RHSIM_Rhsim02G0219600 | 13.81 | 2.03E-04 | 5.04E-04 | -41.041 |
| RHSIM_Rhsim12G0214100 | 13.80 | 2.03E-04 | 5.05E-04 | -0.497 |
| RHSIM_Rhsim02G0225000 | 13.80 | 2.04E-04 | 5.06E-04 | 2.004 |
| novel.923 | 13.80 | 2.04E-04 | 5.06E-04 | 1.160 |
| novel.1693 | 13.80 | 2.04E-04 | 5.06E-04 | 1.908 |
| RHSIM_Rhsim01G0289100 | 13.80 | 2.04E-04 | 5.06E-04 | 3.195 |
| RHSIM_Rhsim13G0170400 | 13.80 | 2.04E-04 | 5.06E-04 | 0.197 |
| RHSIM_Rhsim08G0157700 | 13.79 | 2.04E-04 | 5.07E-04 | -0.413 |
| RHSIM_Rhsim02G0202400 | 13.79 | 2.05E-04 | 5.08E-04 | -1.297 |
| RHSIM_Rhsim04G0020500 | 13.79 | 2.05E-04 | 5.08E-04 | -0.452 |
| RHSIM_Rhsim11G0064400 | 13.79 | 2.05E-04 | 5.08E-04 | -0.608 |
| RHSIM_Rhsim10G0008200 | 13.79 | 2.05E-04 | 5.09E-04 | -0.455 |
| novel.3173 | 13.78 | 2.05E-04 | 5.10E-04 | -1.310 |
| RHSIM_Rhsim04G0174700 | 13.78 | 2.06E-04 | 5.10E-04 | 1.806 |
| RHSIM_Rhsim03G0263600 | 13.78 | 2.06E-04 | 5.12E-04 | -0.325 |
| RHSIM_Rhsim10G0156400 | 13.77 | 2.06E-04 | 5.12E-04 | 0.793 |
| RHSIM_Rhsim11G0193800 | 13.77 | 2.07E-04 | 5.13E-04 | 1.136 |
| RHSIM_Rhsim08G0026200 | 13.76 | 2.08E-04 | 5.15E-04 | 0.273 |
| RHSIM_Rhsim03G0119100 | 13.76 | 2.08E-04 | 5.15E-04 | -1.793 |
| RHSIM_Rhsim08G0103500 | 13.76 | 2.08E-04 | 5.16E-04 | -3.796 |
| novel.1811 | 13.75 | 2.08E-04 | 5.17E-04 | 4.374 |
| RHSIM_Rhsim01G0263000 | 13.75 | 2.09E-04 | 5.18E-04 | 0.305 |
| RHSIM_Rhsim03G0197500 | 13.75 | 2.09E-04 | 5.18E-04 | -0.636 |
| RHSIM_Rhsim12G0086500 | 13.75 | 2.09E-04 | 5.18E-04 | -0.429 |
| RHSIM_Rhsim07G0011700 | 13.74 | 2.10E-04 | 5.21E-04 | -0.199 |
| RHSIM_Rhsim12G0018300 | 13.73 | 2.11E-04 | 5.22E-04 | 1.628 |
| novel.4180 | 13.73 | 2.11E-04 | 5.24E-04 | -2.282 |
| RHSIM_RhsimUnG0042100 | 13.73 | 2.11E-04 | 5.24E-04 | 0.693 |
| RHSIM_Rhsim12G0109400 | 13.73 | 2.11E-04 | 5.24E-04 | 1.131 |

| Gene | LRT statistic | P value | BH-adjusted P | log2FC (interaction) |
|---|---|---|---|---|
| RHSIM_Rhsim04G0112500 | 13.73 | 2.11E-04 | 5.24E-04 | 2.190 |
| RHSIM_Rhsim12G0064300 | 13.72 | 2.12E-04 | 5.25E-04 | 0.960 |
| RHSIM_Rhsim01G0155900 | 13.72 | 2.12E-04 | 5.27E-04 | -0.355 |
| RHSIM_Rhsim01G0202000 | 13.72 | 2.13E-04 | 5.27E-04 | 0.622 |
| novel.2326 | 13.72 | 2.13E-04 | 5.27E-04 | 3.284 |
| RHSIM_Rhsim09G0084000 | 13.71 | 2.13E-04 | 5.27E-04 | 1.693 |
| novel.877 | 13.71 | 2.13E-04 | 5.28E-04 | -0.674 |
| RHSIM_Rhsim11G0152600 | 13.71 | 2.13E-04 | 5.29E-04 | 0.734 |
| RHSIM_Rhsim01G0175600 | 13.70 | 2.14E-04 | 5.31E-04 | 0.586 |
| RHSIM_Rhsim03G0009400 | 13.70 | 2.15E-04 | 5.32E-04 | -3.263 |
| novel.3485 | 13.70 | 2.15E-04 | 5.32E-04 | -2.799 |
| RHSIM_Rhsim09G0123100 | 13.69 | 2.15E-04 | 5.33E-04 | 0.307 |
| novel.2937 | 13.69 | 2.15E-04 | 5.33E-04 | -2.240 |
| RHSIM_Rhsim03G0011900 | 13.69 | 2.15E-04 | 5.33E-04 | 1.879 |
| RHSIM_Rhsim04G0052900 | 13.69 | 2.15E-04 | 5.33E-04 | -1.307 |
| RHSIM_Rhsim02G0206500 | 13.69 | 2.15E-04 | 5.34E-04 | 1.337 |
| RHSIM_RhsimUnG0067000 | 13.69 | 2.16E-04 | 5.34E-04 | -43.780 |
| RHSIM_Rhsim09G0129700 | 13.69 | 2.16E-04 | 5.35E-04 | -0.309 |
| RHSIM_Rhsim08G0204200 | 13.69 | 2.16E-04 | 5.35E-04 | 1.042 |
| RHSIM_RhsimUnG0169000 | 13.69 | 2.16E-04 | 5.35E-04 | 0.218 |
| RHSIM_Rhsim04G0193600 | 13.68 | 2.16E-04 | 5.36E-04 | -0.397 |
| novel.3243 | 13.68 | 2.16E-04 | 5.36E-04 | -1.208 |
| RHSIM_Rhsim13G0204100 | 13.68 | 2.16E-04 | 5.36E-04 | -0.359 |
| RHSIM_Rhsim03G0128100 | 13.68 | 2.17E-04 | 5.37E-04 | 2.922 |
| RHSIM_Rhsim01G0201300 | 13.67 | 2.18E-04 | 5.39E-04 | -0.439 |
| RHSIM_RhsimUnG0088300 | 13.67 | 2.18E-04 | 5.40E-04 | -41.811 |
| RHSIM_Rhsim03G0264200 | 13.67 | 2.18E-04 | 5.40E-04 | 1.460 |
| RHSIM_Rhsim07G0025500 | 13.66 | 2.19E-04 | 5.43E-04 | -0.551 |
| RHSIM_Rhsim07G0140200 | 13.65 | 2.20E-04 | 5.44E-04 | 0.445 |
| RHSIM_Rhsim03G0021300 | 13.65 | 2.20E-04 | 5.45E-04 | -2.637 |
| RHSIM_Rhsim02G0090700 | 13.65 | 2.20E-04 | 5.45E-04 | 2.783 |
| RHSIM_Rhsim02G0076300 | 13.65 | 2.21E-04 | 5.46E-04 | 0.258 |

| Gene | LRT statistic | P value | BH-adjusted P | log2FC (interaction) |
|---|---|---|---|---|
| RHSIM_Rhsim03G0199300 | 13.65 | 2.21E-04 | 5.46E-04 | -0.942 |
| novel.837 | 13.64 | 2.21E-04 | 5.46E-04 | -0.598 |
| novel.4107 | 13.64 | 2.21E-04 | 5.47E-04 | 2.376 |
| RHSIM_Rhsim05G0187800 | 13.64 | 2.21E-04 | 5.47E-04 | -0.219 |
| RHSIM_Rhsim06G0046100 | 13.64 | 2.21E-04 | 5.47E-04 | -3.685 |
| RHSIM_Rhsim11G0058200 | 13.64 | 2.21E-04 | 5.47E-04 | -1.255 |
| novel.2101 | 13.64 | 2.22E-04 | 5.48E-04 | 1.811 |
| RHSIM_Rhsim02G0002100 | 13.63 | 2.22E-04 | 5.49E-04 | 1.313 |
| RHSIM_RhsimUnG0111400 | 13.63 | 2.23E-04 | 5.50E-04 | -1.358 |
| RHSIM_Rhsim05G0214200 | 13.63 | 2.23E-04 | 5.50E-04 | -0.870 |
| RHSIM_RhsimUnG0111100 | 13.63 | 2.23E-04 | 5.51E-04 | 2.090 |
| RHSIM_Rhsim12G0080400 | 13.62 | 2.23E-04 | 5.52E-04 | -0.794 |
| RHSIM_Rhsim02G0041300 | 13.62 | 2.24E-04 | 5.53E-04 | 0.748 |
| RHSIM_Rhsim02G0128600 | 13.61 | 2.24E-04 | 5.54E-04 | 0.204 |
| RHSIM_Rhsim09G0013600 | 13.61 | 2.25E-04 | 5.55E-04 | -0.685 |
| RHSIM_Rhsim07G0110800 | 13.61 | 2.25E-04 | 5.55E-04 | 1.175 |
| novel.3504 | 13.61 | 2.25E-04 | 5.56E-04 | 2.470 |
| RHSIM_Rhsim06G0050100 | 13.61 | 2.26E-04 | 5.57E-04 | 0.657 |
| novel.4559 | 13.60 | 2.26E-04 | 5.57E-04 | -42.384 |
| RHSIM_Rhsim02G0096800 | 13.60 | 2.26E-04 | 5.58E-04 | 0.821 |
| novel.1277 | 13.60 | 2.26E-04 | 5.59E-04 | 4.266 |
| RHSIM_Rhsim12G0118600 | 13.59 | 2.27E-04 | 5.61E-04 | 1.433 |
| RHSIM_RhsimUnG0225600 | 13.59 | 2.28E-04 | 5.62E-04 | 0.457 |
| RHSIM_Rhsim02G0000200 | 13.59 | 2.28E-04 | 5.62E-04 | 0.957 |
| RHSIM_Rhsim12G0106800 | 13.59 | 2.28E-04 | 5.62E-04 | -41.098 |
| novel.1952 | 13.58 | 2.28E-04 | 5.64E-04 | -40.178 |
| RHSIM_Rhsim03G0264900 | 13.56 | 2.31E-04 | 5.71E-04 | -0.388 |
| RHSIM_Rhsim08G0188600 | 13.55 | 2.32E-04 | 5.72E-04 | -0.918 |
| RHSIM_Rhsim07G0086200 | 13.55 | 2.32E-04 | 5.72E-04 | -0.200 |
| RHSIM_Rhsim06G0066800 | 13.54 | 2.33E-04 | 5.75E-04 | 0.348 |
| RHSIM_Rhsim12G0102300 | 13.54 | 2.33E-04 | 5.75E-04 | 0.461 |
| RHSIM_Rhsim09G0149700 | 13.54 | 2.33E-04 | 5.76E-04 | -0.472 |

| Gene | LRT statistic | P value | BH-adjusted P | log2FC (interaction) |
|---|---|---|---|---|
| RHSIM_Rhsim03G0220100 | 13.54 | 2.34E-04 | 5.77E-04 | 0.472 |
| RHSIM_Rhsim12G0159900 | 13.54 | 2.34E-04 | 5.77E-04 | 0.300 |
| RHSIM_Rhsim12G0104800 | 13.53 | 2.34E-04 | 5.78E-04 | 0.668 |
| novel.2743 | 13.53 | 2.34E-04 | 5.78E-04 | -1.181 |
| RHSIM_Rhsim09G0157600 | 13.53 | 2.35E-04 | 5.78E-04 | -0.760 |
| RHSIM_Rhsim06G0219900 | 13.53 | 2.35E-04 | 5.79E-04 | 0.529 |
| RHSIM_Rhsim13G0142400 | 13.53 | 2.35E-04 | 5.79E-04 | -0.659 |
| RHSIM_Rhsim04G0095200 | 13.52 | 2.35E-04 | 5.80E-04 | -3.439 |
| novel.30 | 13.52 | 2.36E-04 | 5.81E-04 | 0.827 |
| novel.3013 | 13.51 | 2.37E-04 | 5.84E-04 | -0.993 |
| RHSIM_Rhsim08G0199400 | 13.51 | 2.37E-04 | 5.84E-04 | 0.891 |
| novel.1763 | 13.51 | 2.37E-04 | 5.84E-04 | 0.945 |
| RHSIM_Rhsim03G0192600 | 13.51 | 2.38E-04 | 5.85E-04 | 0.237 |
| RHSIM_Rhsim05G0234100 | 13.51 | 2.38E-04 | 5.85E-04 | -0.376 |
| novel.2813 | 13.51 | 2.38E-04 | 5.86E-04 | 2.164 |
| RHSIM_Rhsim03G0188000 | 13.50 | 2.38E-04 | 5.86E-04 | 0.249 |
| RHSIM_Rhsim08G0048200 | 13.50 | 2.38E-04 | 5.87E-04 | 0.316 |
| RHSIM_Rhsim05G0184900 | 13.50 | 2.39E-04 | 5.88E-04 | 0.353 |
| RHSIM_Rhsim01G0009000 | 13.50 | 2.39E-04 | 5.89E-04 | 3.724 |
| RHSIM_Rhsim12G0157200 | 13.49 | 2.39E-04 | 5.89E-04 | 0.666 |
| RHSIM_Rhsim13G0140400 | 13.49 | 2.39E-04 | 5.90E-04 | 0.471 |
| novel.4113 | 13.49 | 2.40E-04 | 5.91E-04 | 2.714 |
| novel.2473 | 13.49 | 2.40E-04 | 5.91E-04 | 1.705 |
| RHSIM_Rhsim11G0070400 | 13.49 | 2.40E-04 | 5.91E-04 | -0.244 |
| RHSIM_Rhsim09G0001000 | 13.49 | 2.40E-04 | 5.92E-04 | 0.551 |
| RHSIM_RhsimUnG0124300 | 13.48 | 2.41E-04 | 5.94E-04 | 0.709 |
| RHSIM_Rhsim02G0117300 | 13.47 | 2.43E-04 | 5.97E-04 | -1.596 |
| RHSIM_Rhsim04G0177100 | 13.47 | 2.43E-04 | 5.97E-04 | 0.347 |
| RHSIM_Rhsim09G0059000 | 13.46 | 2.44E-04 | 6.00E-04 | 1.406 |
| RHSIM_Rhsim12G0021300 | 13.46 | 2.44E-04 | 6.01E-04 | -1.923 |
| novel.3171 | 13.46 | 2.44E-04 | 6.01E-04 | 1.146 |
| RHSIM_Rhsim04G0162200 | 13.45 | 2.45E-04 | 6.02E-04 | 0.693 |

| Gene | LRT statistic | P value | BH-adjusted P | log2FC (interaction) |
|---|---|---|---|---|
| RHSIM_Rhsim12G0056900 | 13.45 | 2.45E-04 | 6.02E-04 | 1.046 |
| RHSIM_Rhsim04G0015100 | 13.45 | 2.45E-04 | 6.03E-04 | 0.645 |
| RHSIM_Rhsim08G0026300 | 13.45 | 2.46E-04 | 6.04E-04 | 0.337 |
| RHSIM_Rhsim04G0212800 | 13.44 | 2.46E-04 | 6.05E-04 | -0.677 |
| RHSIM_Rhsim03G0058800 | 13.44 | 2.46E-04 | 6.06E-04 | -41.338 |
| RHSIM_Rhsim02G0254600 | 13.43 | 2.47E-04 | 6.08E-04 | -0.857 |
| novel.2603 | 13.43 | 2.48E-04 | 6.09E-04 | 2.641 |
| RHSIM_Rhsim10G0033500 | 13.43 | 2.48E-04 | 6.09E-04 | 1.790 |
| RHSIM_RhsimUnG0134700 | 13.43 | 2.48E-04 | 6.10E-04 | 0.665 |
| RHSIM_Rhsim12G0194300 | 13.42 | 2.48E-04 | 6.10E-04 | -1.287 |
| RHSIM_Rhsim08G0036700 | 13.42 | 2.48E-04 | 6.10E-04 | 0.996 |
| RHSIM_Rhsim05G0059400 | 13.42 | 2.49E-04 | 6.11E-04 | 2.820 |
| RHSIM_RhsimUnG0124100 | 13.42 | 2.49E-04 | 6.11E-04 | 0.356 |
| RHSIM_Rhsim07G0235700 | 13.41 | 2.50E-04 | 6.14E-04 | -0.337 |
| RHSIM_Rhsim08G0103100 | 13.41 | 2.50E-04 | 6.14E-04 | -0.729 |
| RHSIM_Rhsim03G0190200 | 13.41 | 2.50E-04 | 6.14E-04 | 0.623 |
| RHSIM_Rhsim07G0064800 | 13.41 | 2.51E-04 | 6.16E-04 | -1.867 |
| RHSIM_Rhsim08G0094600 | 13.41 | 2.51E-04 | 6.16E-04 | -0.931 |
| novel.1691 | 13.41 | 2.51E-04 | 6.16E-04 | -0.885 |
| RHSIM_Rhsim12G0020900 | 13.40 | 2.51E-04 | 6.16E-04 | 0.983 |
| RHSIM_Rhsim11G0029300 | 13.40 | 2.51E-04 | 6.17E-04 | -2.045 |
| RHSIM_Rhsim13G0161900 | 13.40 | 2.51E-04 | 6.17E-04 | 2.449 |
| RHSIM_Rhsim10G0014800 | 13.40 | 2.52E-04 | 6.18E-04 | 0.751 |
| RHSIM_Rhsim09G0084800 | 13.40 | 2.52E-04 | 6.19E-04 | -0.472 |
| RHSIM_Rhsim10G0168900 | 13.39 | 2.52E-04 | 6.20E-04 | 1.058 |
| RHSIM_Rhsim04G0217600 | 13.39 | 2.53E-04 | 6.20E-04 | 0.931 |
| RHSIM_Rhsim06G0018100 | 13.39 | 2.53E-04 | 6.20E-04 | 0.797 |
| RHSIM_Rhsim05G0021900 | 13.39 | 2.53E-04 | 6.21E-04 | -0.601 |
| RHSIM_Rhsim10G0113300 | 13.39 | 2.53E-04 | 6.21E-04 | 1.487 |
| RHSIM_Rhsim06G0073800 | 13.39 | 2.53E-04 | 6.21E-04 | -0.507 |
| RHSIM_Rhsim11G0126500 | 13.39 | 2.54E-04 | 6.22E-04 | -0.416 |
| novel.1795 | 13.38 | 2.54E-04 | 6.24E-04 | 0.757 |

| Gene | LRT statistic | P value | BH-adjusted P | log2FC (interaction) |
|---|---|---|---|---|
| RHSIM_Rhsim05G0069900 | 13.38 | 2.55E-04 | 6.25E-04 | 2.806 |
| RHSIM_Rhsim07G0147000 | 13.38 | 2.55E-04 | 6.25E-04 | 5.029 |
| RHSIM_RhsimUnG0255500 | 13.37 | 2.55E-04 | 6.26E-04 | 0.939 |
| RHSIM_Rhsim13G0174100 | 13.37 | 2.56E-04 | 6.27E-04 | 0.239 |
| RHSIM_Rhsim06G0064700 | 13.37 | 2.56E-04 | 6.28E-04 | -1.282 |
| RHSIM_Rhsim10G0137700 | 13.37 | 2.56E-04 | 6.28E-04 | -2.667 |
| RHSIM_Rhsim07G0025600 | 13.36 | 2.57E-04 | 6.29E-04 | -1.539 |
| RHSIM_Rhsim04G0045800 | 13.36 | 2.57E-04 | 6.30E-04 | -0.386 |
| RHSIM_Rhsim12G0157000 | 13.36 | 2.57E-04 | 6.30E-04 | 0.278 |
| RHSIM_Rhsim06G0211600 | 13.35 | 2.58E-04 | 6.32E-04 | 0.764 |
| RHSIM_Rhsim03G0089900 | 13.35 | 2.58E-04 | 6.32E-04 | 0.263 |
| RHSIM_Rhsim09G0185000 | 13.35 | 2.59E-04 | 6.33E-04 | -0.869 |
| RHSIM_Rhsim04G0155000 | 13.35 | 2.59E-04 | 6.34E-04 | 2.622 |
| RHSIM_Rhsim03G0270200 | 13.35 | 2.59E-04 | 6.34E-04 | 3.908 |
| RHSIM_Rhsim01G0157600 | 13.34 | 2.59E-04 | 6.35E-04 | 1.040 |
| RHSIM_Rhsim04G0137000 | 13.34 | 2.60E-04 | 6.36E-04 | -0.652 |
| novel.2383 | 13.34 | 2.60E-04 | 6.38E-04 | 1.247 |
| RHSIM_Rhsim05G0205100 | 13.33 | 2.60E-04 | 6.38E-04 | 0.698 |
| RHSIM_Rhsim02G0133300 | 13.33 | 2.62E-04 | 6.41E-04 | 0.249 |
| RHSIM_Rhsim12G0169900 | 13.32 | 2.62E-04 | 6.41E-04 | 1.971 |
| RHSIM_Rhsim02G0196200 | 13.32 | 2.62E-04 | 6.42E-04 | 2.135 |
| novel.3176 | 13.32 | 2.62E-04 | 6.42E-04 | 3.284 |
| novel.3760 | 13.32 | 2.63E-04 | 6.43E-04 | -1.507 |
| RHSIM_Rhsim03G0100100 | 13.32 | 2.63E-04 | 6.43E-04 | 41.528 |
| RHSIM_Rhsim01G0207800 | 13.32 | 2.63E-04 | 6.43E-04 | -0.425 |
| RHSIM_Rhsim06G0128200 | 13.32 | 2.63E-04 | 6.43E-04 | 0.318 |
| RHSIM_Rhsim09G0052300 | 13.31 | 2.64E-04 | 6.46E-04 | -0.434 |
| RHSIM_Rhsim10G0191500 | 13.31 | 2.64E-04 | 6.47E-04 | -0.485 |
| RHSIM_Rhsim10G0188400 | 13.31 | 2.64E-04 | 6.47E-04 | 1.818 |
| novel.2880 | 13.30 | 2.65E-04 | 6.49E-04 | -1.759 |
| RHSIM_RhsimUnG0232100 | 13.30 | 2.66E-04 | 6.50E-04 | 0.855 |
| RHSIM_RhsimUnG0073000 | 13.30 | 2.66E-04 | 6.50E-04 | -0.735 |

| Gene | LRT statistic | P value | BH-adjusted P | log2FC (interaction) |
|---|---|---|---|---|
| RHSIM_Rhsim03G0028600 | 13.29 | 2.66E-04 | 6.51E-04 | 0.372 |
| RHSIM_Rhsim10G0102800 | 13.29 | 2.66E-04 | 6.51E-04 | 0.520 |
| RHSIM_Rhsim09G0077500 | 13.29 | 2.67E-04 | 6.52E-04 | 0.385 |
| novel.3900 | 13.29 | 2.67E-04 | 6.53E-04 | -2.378 |
| RHSIM_Rhsim12G0115200 | 13.29 | 2.67E-04 | 6.54E-04 | 2.271 |
| novel.659 | 13.28 | 2.68E-04 | 6.55E-04 | 1.831 |
| RHSIM_Rhsim01G0033200 | 13.28 | 2.69E-04 | 6.57E-04 | -0.410 |
| RHSIM_Rhsim05G0227000 | 13.27 | 2.69E-04 | 6.57E-04 | -0.404 |
| RHSIM_Rhsim07G0169500 | 13.27 | 2.69E-04 | 6.57E-04 | -0.679 |
| RHSIM_Rhsim06G0241900 | 13.27 | 2.69E-04 | 6.58E-04 | 2.558 |
| novel.1903 | 13.26 | 2.71E-04 | 6.63E-04 | -2.392 |
| RHSIM_Rhsim06G0033500 | 13.25 | 2.73E-04 | 6.66E-04 | 0.279 |
| RHSIM_Rhsim12G0120700 | 13.25 | 2.73E-04 | 6.66E-04 | -1.845 |
| RHSIM_Rhsim11G0093900 | 13.24 | 2.73E-04 | 6.68E-04 | -1.933 |
| RHSIM_Rhsim07G0163300 | 13.24 | 2.74E-04 | 6.68E-04 | -0.579 |
| RHSIM_Rhsim09G0206600 | 13.24 | 2.74E-04 | 6.69E-04 | -0.463 |
| novel.1452 | 13.24 | 2.74E-04 | 6.70E-04 | 2.365 |
| novel.849 | 13.24 | 2.74E-04 | 6.70E-04 | 0.392 |
| RHSIM_Rhsim05G0161100 | 13.23 | 2.75E-04 | 6.72E-04 | -0.310 |
| RHSIM_Rhsim10G0007800 | 13.23 | 2.76E-04 | 6.73E-04 | 0.891 |
| RHSIM_RhsimUnG0142300 | 13.22 | 2.76E-04 | 6.75E-04 | -1.929 |
| RHSIM_Rhsim03G0264800 | 13.22 | 2.76E-04 | 6.75E-04 | -0.325 |
| RHSIM_Rhsim13G0054000 | 13.22 | 2.77E-04 | 6.75E-04 | -0.704 |
| RHSIM_Rhsim07G0107300 | 13.22 | 2.77E-04 | 6.76E-04 | -3.823 |
| RHSIM_Rhsim13G0147000 | 13.22 | 2.77E-04 | 6.76E-04 | -0.734 |
| novel.2093 | 13.22 | 2.77E-04 | 6.76E-04 | 1.681 |
| RHSIM_Rhsim04G0048100 | 13.22 | 2.77E-04 | 6.76E-04 | -0.624 |
| RHSIM_Rhsim05G0030600 | 13.21 | 2.79E-04 | 6.80E-04 | 2.185 |
| RHSIM_Rhsim08G0043200 | 13.21 | 2.79E-04 | 6.81E-04 | 0.606 |
| RHSIM_Rhsim13G0155700 | 13.21 | 2.79E-04 | 6.81E-04 | 0.779 |
| RHSIM_Rhsim12G0119700 | 13.20 | 2.79E-04 | 6.81E-04 | -40.905 |
| novel.1867 | 13.20 | 2.80E-04 | 6.83E-04 | -40.069 |

| Gene | LRT statistic | P value | BH-adjusted P | log2FC (interaction) |
|---|---|---|---|---|
| RHSIM_Rhsim11G0195200 | 13.20 | 2.80E-04 | 6.83E-04 | 1.952 |
| novel.4569 | 13.20 | 2.80E-04 | 6.84E-04 | 0.853 |
| RHSIM_Rhsim02G0095000 | 13.20 | 2.80E-04 | 6.84E-04 | 1.678 |
| RHSIM_Rhsim02G0180400 | 13.19 | 2.81E-04 | 6.85E-04 | -0.716 |
| RHSIM_Rhsim11G0038000 | 13.19 | 2.81E-04 | 6.85E-04 | 0.358 |
| RHSIM_Rhsim05G0004800 | 13.19 | 2.81E-04 | 6.86E-04 | -0.579 |
| RHSIM_Rhsim06G0062300 | 13.19 | 2.82E-04 | 6.87E-04 | -3.281 |
| RHSIM_Rhsim07G0078100 | 13.19 | 2.82E-04 | 6.87E-04 | -2.550 |
| RHSIM_Rhsim01G0004500 | 13.19 | 2.82E-04 | 6.87E-04 | -40.879 |
| novel.4409 | 13.18 | 2.83E-04 | 6.89E-04 | 1.896 |
| RHSIM_Rhsim10G0014600 | 13.18 | 2.83E-04 | 6.89E-04 | 0.582 |
| RHSIM_Rhsim13G0140700 | 13.18 | 2.83E-04 | 6.89E-04 | 0.476 |
| RHSIM_Rhsim10G0037900 | 13.18 | 2.83E-04 | 6.90E-04 | -40.397 |
| RHSIM_Rhsim09G0192300 | 13.18 | 2.83E-04 | 6.90E-04 | -0.461 |
| RHSIM_Rhsim07G0033000 | 13.17 | 2.84E-04 | 6.91E-04 | -0.635 |
| RHSIM_Rhsim02G0142000 | 13.17 | 2.84E-04 | 6.92E-04 | 2.099 |
| RHSIM_RhsimUnG0197900 | 13.17 | 2.84E-04 | 6.93E-04 | -1.774 |
| RHSIM_Rhsim03G0041600 | 13.17 | 2.85E-04 | 6.94E-04 | 1.899 |
| RHSIM_Rhsim12G0137900 | 13.17 | 2.85E-04 | 6.94E-04 | -0.309 |
| RHSIM_Rhsim08G0143800 | 13.16 | 2.85E-04 | 6.95E-04 | -0.909 |
| RHSIM_Rhsim04G0142100 | 13.15 | 2.87E-04 | 6.99E-04 | 2.781 |
| RHSIM_Rhsim06G0189800 | 13.15 | 2.87E-04 | 7.00E-04 | -3.516 |
| RHSIM_Rhsim02G0172900 | 13.15 | 2.88E-04 | 7.01E-04 | 0.296 |
| RHSIM_Rhsim07G0056000 | 13.14 | 2.89E-04 | 7.04E-04 | 0.493 |
| RHSIM_Rhsim07G0082800 | 13.13 | 2.90E-04 | 7.06E-04 | 3.753 |
| RHSIM_Rhsim01G0051900 | 13.13 | 2.90E-04 | 7.07E-04 | 2.434 |
| RHSIM_Rhsim12G0149700 | 13.13 | 2.91E-04 | 7.09E-04 | -0.762 |
| RHSIM_Rhsim05G0144400 | 13.12 | 2.91E-04 | 7.09E-04 | 0.700 |
| novel.1348 | 13.12 | 2.92E-04 | 7.09E-04 | -2.045 |
| RHSIM_Rhsim13G0225200 | 13.12 | 2.92E-04 | 7.10E-04 | -0.603 |
| RHSIM_Rhsim12G0177700 | 13.12 | 2.92E-04 | 7.11E-04 | 0.476 |
| RHSIM_Rhsim09G0111300 | 13.12 | 2.92E-04 | 7.11E-04 | -0.448 |

| Gene | LRT statistic | P value | BH-adjusted P | log2FC (interaction) |
|---|---|---|---|---|
| RHSIM_Rhsim03G0229100 | 13.12 | 2.93E-04 | 7.12E-04 | 3.601 |
| RHSIM_Rhsim08G0141200 | 13.12 | 2.93E-04 | 7.12E-04 | -0.937 |
| RHSIM_Rhsim10G0063100 | 13.12 | 2.93E-04 | 7.12E-04 | 2.177 |
| RHSIM_Rhsim13G0093200 | 13.11 | 2.93E-04 | 7.13E-04 | 1.669 |
| RHSIM_Rhsim10G0135800 | 13.11 | 2.93E-04 | 7.13E-04 | -0.623 |
| RHSIM_Rhsim09G0088900 | 13.11 | 2.93E-04 | 7.13E-04 | -0.395 |
| RHSIM_Rhsim10G0085900 | 13.11 | 2.94E-04 | 7.15E-04 | 2.183 |
| novel.1493 | 13.11 | 2.94E-04 | 7.15E-04 | -2.389 |
| novel.424 | 13.10 | 2.95E-04 | 7.18E-04 | 0.456 |
| RHSIM_Rhsim02G0193400 | 13.10 | 2.96E-04 | 7.18E-04 | -0.224 |
| RHSIM_Rhsim10G0187800 | 13.10 | 2.96E-04 | 7.18E-04 | 1.102 |
| RHSIM_Rhsim08G0109900 | 13.10 | 2.96E-04 | 7.18E-04 | -2.626 |
| RHSIM_Rhsim01G0266300 | 13.09 | 2.96E-04 | 7.20E-04 | 0.513 |
| RHSIM_Rhsim10G0033800 | 13.09 | 2.96E-04 | 7.20E-04 | -4.044 |
| RHSIM_Rhsim06G0099500 | 13.09 | 2.97E-04 | 7.22E-04 | 2.753 |
| RHSIM_Rhsim13G0003200 | 13.08 | 2.98E-04 | 7.24E-04 | -0.436 |
| RHSIM_Rhsim08G0212100 | 13.08 | 2.98E-04 | 7.24E-04 | 0.512 |
| RHSIM_Rhsim07G0122300 | 13.08 | 2.98E-04 | 7.24E-04 | -0.746 |
| RHSIM_Rhsim08G0032500 | 13.07 | 3.00E-04 | 7.28E-04 | -0.303 |
| novel.2261 | 13.07 | 3.00E-04 | 7.29E-04 | -0.615 |
| RHSIM_RhsimUnG0056500 | 13.07 | 3.00E-04 | 7.29E-04 | 0.536 |
| RHSIM_Rhsim12G0118200 | 13.07 | 3.01E-04 | 7.30E-04 | 3.098 |
| novel.1657 | 13.06 | 3.02E-04 | 7.32E-04 | -1.314 |
| RHSIM_Rhsim09G0059100 | 13.06 | 3.02E-04 | 7.33E-04 | 0.183 |
| RHSIM_Rhsim07G0080700 | 13.06 | 3.02E-04 | 7.33E-04 | 1.586 |
| RHSIM_Rhsim04G0186100 | 13.06 | 3.02E-04 | 7.33E-04 | 1.623 |
| RHSIM_Rhsim09G0118100 | 13.06 | 3.02E-04 | 7.33E-04 | 0.550 |
| RHSIM_Rhsim05G0090300 | 13.06 | 3.02E-04 | 7.33E-04 | -1.447 |
| RHSIM_Rhsim07G0072900 | 13.06 | 3.02E-04 | 7.34E-04 | 1.153 |
| RHSIM_Rhsim13G0128200 | 13.05 | 3.03E-04 | 7.36E-04 | -0.448 |
| RHSIM_Rhsim06G0123200 | 13.05 | 3.04E-04 | 7.38E-04 | -1.151 |
| RHSIM_Rhsim02G0040900 | 13.04 | 3.04E-04 | 7.38E-04 | 1.317 |

| Gene | LRT statistic | P value | BH-adjusted P | log2FC (interaction) |
|---|---|---|---|---|
| RHSIM_RhsimUnG0039500 | 13.04 | 3.05E-04 | 7.39E-04 | -1.270 |
| RHSIM_Rhsim07G0253800 | 13.04 | 3.05E-04 | 7.40E-04 | -0.178 |
| RHSIM_Rhsim13G0054100 | 13.04 | 3.05E-04 | 7.40E-04 | 0.797 |
| RHSIM_Rhsim08G0057900 | 13.04 | 3.05E-04 | 7.41E-04 | -40.381 |
| RHSIM_Rhsim06G0055100 | 13.03 | 3.06E-04 | 7.42E-04 | 0.952 |
| RHSIM_RhsimUnG0181300 | 13.03 | 3.06E-04 | 7.42E-04 | 1.423 |
| RHSIM_Rhsim04G0163100 | 13.03 | 3.06E-04 | 7.42E-04 | 0.390 |
| RHSIM_Rhsim02G0113400 | 13.03 | 3.06E-04 | 7.42E-04 | -0.621 |
| novel.1272 | 13.02 | 3.08E-04 | 7.47E-04 | -1.325 |
| RHSIM_Rhsim03G0237300 | 13.02 | 3.08E-04 | 7.47E-04 | 1.085 |
| RHSIM_Rhsim03G0258800 | 13.01 | 3.10E-04 | 7.52E-04 | -0.703 |
| RHSIM_Rhsim08G0099500 | 13.01 | 3.10E-04 | 7.52E-04 | 3.903 |
| RHSIM_Rhsim05G0149700 | 13.00 | 3.11E-04 | 7.53E-04 | -6.974 |
| RHSIM_Rhsim06G0196800 | 13.00 | 3.11E-04 | 7.54E-04 | -0.564 |
| RHSIM_Rhsim03G0110100 | 13.00 | 3.11E-04 | 7.54E-04 | -3.325 |
| RHSIM_Rhsim08G0181300 | 13.00 | 3.12E-04 | 7.55E-04 | -1.508 |
| novel.1791 | 13.00 | 3.12E-04 | 7.56E-04 | 0.786 |
| RHSIM_Rhsim02G0214100 | 12.99 | 3.13E-04 | 7.57E-04 | -0.548 |
| RHSIM_RhsimUnG0012000 | 12.99 | 3.13E-04 | 7.58E-04 | 1.653 |
| RHSIM_Rhsim08G0211500 | 12.99 | 3.13E-04 | 7.59E-04 | 0.635 |
| RHSIM_RhsimUnG0208600 | 12.99 | 3.13E-04 | 7.59E-04 | 3.779 |
| RHSIM_Rhsim12G0000700 | 12.99 | 3.13E-04 | 7.59E-04 | 0.213 |
| RHSIM_Rhsim10G0069800 | 12.98 | 3.14E-04 | 7.61E-04 | -0.621 |
| RHSIM_Rhsim01G0033500 | 12.98 | 3.15E-04 | 7.63E-04 | 1.452 |
| RHSIM_Rhsim02G0119400 | 12.98 | 3.15E-04 | 7.63E-04 | -0.648 |
| RHSIM_Rhsim06G0184400 | 12.97 | 3.16E-04 | 7.65E-04 | -0.444 |
| RHSIM_Rhsim11G0182900 | 12.97 | 3.16E-04 | 7.66E-04 | 43.434 |
| RHSIM_Rhsim01G0214900 | 12.97 | 3.17E-04 | 7.66E-04 | -0.401 |
| novel.1953 | 12.96 | 3.19E-04 | 7.71E-04 | -3.855 |
| RHSIM_Rhsim12G0205700 | 12.96 | 3.19E-04 | 7.71E-04 | 2.003 |
| RHSIM_Rhsim13G0148800 | 12.95 | 3.19E-04 | 7.72E-04 | -1.489 |
| RHSIM_Rhsim11G0108400 | 12.95 | 3.19E-04 | 7.73E-04 | 42.865 |

| Gene | LRT statistic | P value | BH-adjusted P | log2FC (interaction) |
|---|---|---|---|---|
| RHSIM_Rhsim06G0127200 | 12.95 | 3.20E-04 | 7.73E-04 | 0.625 |
| RHSIM_Rhsim06G0228100 | 12.95 | 3.20E-04 | 7.74E-04 | 3.683 |
| RHSIM_Rhsim10G0129000 | 12.95 | 3.20E-04 | 7.75E-04 | 0.786 |
| RHSIM_Rhsim01G0077700 | 12.95 | 3.21E-04 | 7.76E-04 | 42.945 |
| novel.3605 | 12.94 | 3.21E-04 | 7.77E-04 | -80.434 |
| RHSIM_Rhsim08G0207100 | 12.94 | 3.21E-04 | 7.77E-04 | -2.669 |
| RHSIM_Rhsim12G0130800 | 12.94 | 3.22E-04 | 7.78E-04 | -0.289 |
| RHSIM_Rhsim06G0224200 | 12.94 | 3.22E-04 | 7.78E-04 | -0.828 |
| novel.2524 | 12.94 | 3.22E-04 | 7.79E-04 | -0.920 |
| RHSIM_Rhsim01G0157000 | 12.94 | 3.22E-04 | 7.80E-04 | 1.021 |
| RHSIM_Rhsim07G0199300 | 12.93 | 3.23E-04 | 7.80E-04 | -0.708 |
| RHSIM_Rhsim02G0252900 | 12.93 | 3.23E-04 | 7.80E-04 | -1.210 |
| novel.3135 | 12.93 | 3.23E-04 | 7.81E-04 | -3.006 |
| RHSIM_Rhsim05G0044400 | 12.92 | 3.24E-04 | 7.84E-04 | -3.176 |
| RHSIM_Rhsim06G0054600 | 12.92 | 3.25E-04 | 7.84E-04 | -0.382 |
| RHSIM_Rhsim11G0051900 | 12.92 | 3.25E-04 | 7.84E-04 | -0.701 |
| RHSIM_Rhsim03G0115000 | 12.92 | 3.25E-04 | 7.85E-04 | 2.154 |
| novel.90 | 12.92 | 3.25E-04 | 7.85E-04 | -41.012 |
| RHSIM_Rhsim12G0036000 | 12.92 | 3.25E-04 | 7.86E-04 | 0.592 |
| novel.1974 | 12.91 | 3.27E-04 | 7.89E-04 | -1.282 |
| RHSIM_Rhsim04G0014000 | 12.91 | 3.27E-04 | 7.90E-04 | 0.453 |
| RHSIM_Rhsim03G0265300 | 12.91 | 3.28E-04 | 7.91E-04 | -0.450 |
| RHSIM_Rhsim08G0049300 | 12.90 | 3.28E-04 | 7.92E-04 | 0.822 |
| RHSIM_Rhsim09G0172300 | 12.90 | 3.29E-04 | 7.95E-04 | -1.779 |
| RHSIM_Rhsim09G0129100 | 12.89 | 3.30E-04 | 7.97E-04 | -2.502 |
| novel.419 | 12.88 | 3.32E-04 | 8.02E-04 | -0.367 |
| RHSIM_Rhsim04G0156500 | 12.88 | 3.32E-04 | 8.02E-04 | -0.982 |
| RHSIM_Rhsim01G0075500 | 12.88 | 3.33E-04 | 8.03E-04 | -3.631 |
| RHSIM_Rhsim10G0185600 | 12.87 | 3.34E-04 | 8.06E-04 | 1.755 |
| RHSIM_RhsimUnG0061100 | 12.87 | 3.34E-04 | 8.07E-04 | 0.953 |
| RHSIM_Rhsim13G0086900 | 12.86 | 3.35E-04 | 8.08E-04 | 0.275 |
| RHSIM_Rhsim01G0061400 | 12.86 | 3.35E-04 | 8.09E-04 | 0.389 |

| Gene | LRT statistic | P value | BH-adjusted P | log2FC (interaction) |
|---|---|---|---|---|
| RHSIM_Rhsim13G0015700 | 12.86 | 3.36E-04 | 8.10E-04 | 0.499 |
| RHSIM_Rhsim13G0096500 | 12.86 | 3.36E-04 | 8.10E-04 | -3.169 |
| RHSIM_Rhsim01G0119100 | 12.86 | 3.36E-04 | 8.10E-04 | 0.607 |
| RHSIM_Rhsim02G0041200 | 12.86 | 3.36E-04 | 8.11E-04 | 0.739 |
| RHSIM_Rhsim11G0196700 | 12.85 | 3.38E-04 | 8.14E-04 | -0.377 |
| RHSIM_Rhsim07G0129100 | 12.84 | 3.39E-04 | 8.17E-04 | 2.680 |
| RHSIM_Rhsim08G0209300 | 12.84 | 3.39E-04 | 8.17E-04 | 0.398 |
| RHSIM_Rhsim02G0102900 | 12.84 | 3.39E-04 | 8.17E-04 | 0.981 |
| RHSIM_Rhsim13G0197300 | 12.84 | 3.39E-04 | 8.17E-04 | -0.916 |
| RHSIM_Rhsim06G0100100 | 12.83 | 3.40E-04 | 8.20E-04 | 0.960 |
| RHSIM_Rhsim12G0083000 | 12.83 | 3.40E-04 | 8.21E-04 | -0.264 |
| RHSIM_Rhsim02G0118900 | 12.83 | 3.41E-04 | 8.21E-04 | 0.368 |
| RHSIM_Rhsim07G0169100 | 12.83 | 3.41E-04 | 8.22E-04 | 0.246 |
| RHSIM_RhsimUnG0153400 | 12.83 | 3.41E-04 | 8.22E-04 | 2.399 |
| RHSIM_Rhsim09G0131800 | 12.83 | 3.42E-04 | 8.23E-04 | -1.867 |
| novel.1877 | 12.82 | 3.42E-04 | 8.24E-04 | -2.244 |
| RHSIM_Rhsim01G0054500 | 12.82 | 3.42E-04 | 8.24E-04 | -3.560 |
| novel.494 | 12.82 | 3.43E-04 | 8.25E-04 | -1.537 |
| RHSIM_RhsimUnG0075600 | 12.81 | 3.45E-04 | 8.31E-04 | 0.472 |
| RHSIM_Rhsim08G0019600 | 12.81 | 3.45E-04 | 8.32E-04 | -1.651 |
| RHSIM_Rhsim07G0227400 | 12.80 | 3.46E-04 | 8.34E-04 | 1.248 |
| RHSIM_Rhsim02G0256600 | 12.80 | 3.47E-04 | 8.37E-04 | 0.306 |
| RHSIM_Rhsim01G0061800 | 12.79 | 3.48E-04 | 8.37E-04 | -0.559 |
| RHSIM_Rhsim11G0046900 | 12.79 | 3.48E-04 | 8.37E-04 | 0.560 |
| RHSIM_Rhsim08G0189000 | 12.79 | 3.48E-04 | 8.38E-04 | -0.658 |
| RHSIM_Rhsim06G0035200 | 12.79 | 3.49E-04 | 8.39E-04 | -0.440 |
| RHSIM_Rhsim02G0145300 | 12.79 | 3.49E-04 | 8.40E-04 | 0.671 |
| RHSIM_Rhsim05G0076100 | 12.78 | 3.50E-04 | 8.42E-04 | 1.730 |
| RHSIM_RhsimUnG0186300 | 12.78 | 3.50E-04 | 8.43E-04 | -0.648 |
| novel.293 | 12.78 | 3.51E-04 | 8.44E-04 | -0.853 |
| RHSIM_Rhsim04G0185500 | 12.77 | 3.52E-04 | 8.46E-04 | -2.372 |
| RHSIM_Rhsim08G0062200 | 12.75 | 3.56E-04 | 8.56E-04 | -0.693 |

| Gene | LRT statistic | P value | BH-adjusted P | log2FC (interaction) |
|---|---|---|---|---|
| novel.3427 | 12.75 | 3.56E-04 | 8.57E-04 | 1.692 |
| RHSIM_Rhsim03G0263200 | 12.75 | 3.57E-04 | 8.58E-04 | -0.769 |
| RHSIM_Rhsim06G0114400 | 12.74 | 3.58E-04 | 8.61E-04 | 41.089 |
| novel.404 | 12.74 | 3.58E-04 | 8.62E-04 | -1.608 |
| RHSIM_Rhsim02G0185000 | 12.73 | 3.60E-04 | 8.65E-04 | 0.614 |
| RHSIM_Rhsim03G0188700 | 12.73 | 3.60E-04 | 8.66E-04 | 0.893 |
| RHSIM_Rhsim09G0172100 | 12.73 | 3.60E-04 | 8.66E-04 | -3.706 |
| RHSIM_Rhsim07G0014800 | 12.72 | 3.62E-04 | 8.71E-04 | -0.728 |
| RHSIM_Rhsim10G0019500 | 12.71 | 3.64E-04 | 8.75E-04 | -0.320 |
| RHSIM_Rhsim09G0002500 | 12.71 | 3.65E-04 | 8.76E-04 | 1.269 |
| RHSIM_Rhsim07G0211900 | 12.71 | 3.65E-04 | 8.77E-04 | 0.189 |
| RHSIM_Rhsim03G0135700 | 12.70 | 3.65E-04 | 8.78E-04 | -1.312 |
| novel.3270 | 12.70 | 3.66E-04 | 8.79E-04 | -0.446 |
| RHSIM_Rhsim06G0187500 | 12.70 | 3.66E-04 | 8.80E-04 | 0.301 |
| RHSIM_Rhsim05G0088800 | 12.70 | 3.66E-04 | 8.80E-04 | 0.936 |
| RHSIM_Rhsim04G0187900 | 12.70 | 3.66E-04 | 8.81E-04 | 0.481 |
| RHSIM_Rhsim12G0164100 | 12.69 | 3.68E-04 | 8.85E-04 | -0.310 |
| RHSIM_Rhsim10G0075800 | 12.69 | 3.68E-04 | 8.85E-04 | -2.176 |
| RHSIM_Rhsim12G0154400 | 12.68 | 3.69E-04 | 8.86E-04 | 1.737 |
| RHSIM_Rhsim11G0041600 | 12.68 | 3.69E-04 | 8.87E-04 | -0.296 |
| RHSIM_Rhsim01G0238400 | 12.68 | 3.69E-04 | 8.87E-04 | 0.562 |
| RHSIM_Rhsim05G0167900 | 12.68 | 3.70E-04 | 8.88E-04 | -0.184 |
| novel.482 | 12.68 | 3.70E-04 | 8.89E-04 | -1.433 |
| RHSIM_RhsimUnG0150900 | 12.68 | 3.70E-04 | 8.89E-04 | -1.832 |
| RHSIM_Rhsim04G0036200 | 12.67 | 3.71E-04 | 8.90E-04 | -5.121 |
| novel.4054 | 12.67 | 3.71E-04 | 8.90E-04 | -4.554 |
| RHSIM_Rhsim03G0161900 | 12.67 | 3.71E-04 | 8.90E-04 | 2.135 |
| RHSIM_Rhsim07G0061200 | 12.67 | 3.71E-04 | 8.91E-04 | -0.446 |
| RHSIM_Rhsim12G0213200 | 12.67 | 3.72E-04 | 8.92E-04 | -0.270 |
| RHSIM_Rhsim10G0005100 | 12.67 | 3.72E-04 | 8.92E-04 | -1.262 |
| RHSIM_Rhsim12G0139200 | 12.67 | 3.72E-04 | 8.93E-04 | -0.337 |
| novel.511 | 12.67 | 3.72E-04 | 8.93E-04 | 1.334 |

| Gene | LRT statistic | P value | BH-adjusted P | log2FC (interaction) |
|---|---|---|---|---|
| novel.96 | 12.67 | 3.72E-04 | 8.93E-04 | 1.992 |
| RHSIM_Rhsim07G0122100 | 12.67 | 3.72E-04 | 8.94E-04 | -0.263 |
| RHSIM_Rhsim08G0170600 | 12.66 | 3.73E-04 | 8.96E-04 | -0.360 |
| RHSIM_Rhsim10G0157900 | 12.66 | 3.74E-04 | 8.96E-04 | 0.355 |
| RHSIM_Rhsim13G0135800 | 12.66 | 3.74E-04 | 8.97E-04 | 4.450 |
| RHSIM_Rhsim11G0138900 | 12.66 | 3.74E-04 | 8.97E-04 | -41.584 |
| RHSIM_Rhsim12G0030500 | 12.66 | 3.75E-04 | 8.98E-04 | -0.511 |
| RHSIM_Rhsim04G0087300 | 12.65 | 3.75E-04 | 9.00E-04 | 0.506 |
| RHSIM_Rhsim09G0171700 | 12.65 | 3.76E-04 | 9.03E-04 | -0.610 |
| RHSIM_Rhsim05G0010100 | 12.64 | 3.78E-04 | 9.06E-04 | -0.964 |
| RHSIM_Rhsim02G0023000 | 12.64 | 3.78E-04 | 9.07E-04 | -0.348 |
| RHSIM_Rhsim05G0201600 | 12.63 | 3.79E-04 | 9.08E-04 | 2.562 |
| RHSIM_Rhsim03G0237400 | 12.63 | 3.79E-04 | 9.08E-04 | 0.147 |
| novel.1182 | 12.63 | 3.79E-04 | 9.08E-04 | 1.441 |
| RHSIM_Rhsim13G0004000 | 12.63 | 3.79E-04 | 9.09E-04 | -0.550 |
| RHSIM_Rhsim02G0150200 | 12.63 | 3.80E-04 | 9.10E-04 | 5.357 |
| RHSIM_Rhsim11G0044000 | 12.63 | 3.80E-04 | 9.10E-04 | -41.865 |
| RHSIM_Rhsim04G0245700 | 12.63 | 3.80E-04 | 9.11E-04 | 0.498 |
| RHSIM_Rhsim04G0245900 | 12.62 | 3.81E-04 | 9.13E-04 | -1.280 |
| RHSIM_Rhsim05G0234700 | 12.62 | 3.82E-04 | 9.15E-04 | 1.309 |
| RHSIM_Rhsim06G0191200 | 12.62 | 3.82E-04 | 9.16E-04 | 0.468 |
| RHSIM_Rhsim04G0017900 | 12.61 | 3.84E-04 | 9.20E-04 | 1.133 |
| RHSIM_Rhsim10G0193400 | 12.60 | 3.85E-04 | 9.22E-04 | -1.218 |
| novel.924 | 12.60 | 3.85E-04 | 9.23E-04 | -0.374 |
| RHSIM_Rhsim08G0025900 | 12.60 | 3.86E-04 | 9.24E-04 | 0.338 |
| RHSIM_RhsimUnG0094500 | 12.59 | 3.87E-04 | 9.27E-04 | -0.408 |
| RHSIM_Rhsim08G0180700 | 12.59 | 3.87E-04 | 9.27E-04 | -3.669 |
| RHSIM_Rhsim05G0086600 | 12.59 | 3.87E-04 | 9.28E-04 | 3.053 |
| RHSIM_Rhsim08G0208500 | 12.57 | 3.92E-04 | 9.38E-04 | 0.442 |
| RHSIM_Rhsim05G0123600 | 12.57 | 3.92E-04 | 9.38E-04 | 1.410 |
| RHSIM_Rhsim02G0068100 | 12.57 | 3.92E-04 | 9.39E-04 | -3.254 |
| RHSIM_Rhsim04G0219100 | 12.57 | 3.93E-04 | 9.40E-04 | -0.297 |

| Gene | LRT statistic | P value | BH-adjusted P | log2FC (interaction) |
|---|---|---|---|---|
| novel.836 | 12.56 | 3.93E-04 | 9.41E-04 | 0.395 |
| RHSIM_Rhsim06G0042800 | 12.56 | 3.95E-04 | 9.45E-04 | -1.651 |
| RHSIM_Rhsim01G0260400 | 12.55 | 3.95E-04 | 9.46E-04 | 4.416 |
| RHSIM_Rhsim06G0215300 | 12.55 | 3.95E-04 | 9.46E-04 | -2.555 |
| RHSIM_Rhsim08G0084300 | 12.55 | 3.96E-04 | 9.46E-04 | 0.364 |
| RHSIM_Rhsim01G0246800 | 12.55 | 3.96E-04 | 9.47E-04 | -0.386 |
| novel.4458 | 12.55 | 3.96E-04 | 9.47E-04 | -1.943 |
| RHSIM_Rhsim11G0063800 | 12.55 | 3.96E-04 | 9.47E-04 | -0.448 |
| RHSIM_Rhsim04G0221400 | 12.55 | 3.96E-04 | 9.48E-04 | -0.420 |
| RHSIM_Rhsim01G0150000 | 12.55 | 3.97E-04 | 9.49E-04 | 3.002 |
| RHSIM_Rhsim04G0040600 | 12.54 | 3.97E-04 | 9.50E-04 | -2.112 |
| RHSIM_Rhsim13G0009400 | 12.54 | 3.98E-04 | 9.52E-04 | -0.589 |
| novel.1865 | 12.54 | 3.99E-04 | 9.53E-04 | -1.485 |
| RHSIM_Rhsim09G0100200 | 12.53 | 4.00E-04 | 9.56E-04 | -0.348 |
| novel.2982 | 12.53 | 4.01E-04 | 9.58E-04 | 3.814 |
| RHSIM_Rhsim06G0055500 | 12.53 | 4.01E-04 | 9.58E-04 | 5.463 |
| RHSIM_Rhsim03G0196600 | 12.53 | 4.01E-04 | 9.59E-04 | 0.350 |
| novel.4120 | 12.52 | 4.02E-04 | 9.60E-04 | -1.167 |
| RHSIM_Rhsim03G0236200 | 12.52 | 4.02E-04 | 9.60E-04 | -0.275 |
| RHSIM_Rhsim03G0093600 | 12.52 | 4.03E-04 | 9.63E-04 | 0.479 |
| RHSIM_Rhsim08G0005300 | 12.52 | 4.04E-04 | 9.64E-04 | -0.417 |
| RHSIM_Rhsim06G0109300 | 12.51 | 4.04E-04 | 9.65E-04 | -0.828 |
| novel.245 | 12.51 | 4.05E-04 | 9.68E-04 | 0.783 |
| novel.1190 | 12.51 | 4.05E-04 | 9.68E-04 | 1.160 |
| RHSIM_Rhsim08G0069100 | 12.50 | 4.06E-04 | 9.71E-04 | -0.444 |
| RHSIM_RhsimUnG0208500 | 12.50 | 4.07E-04 | 9.72E-04 | 1.554 |
| novel.1818 | 12.50 | 4.07E-04 | 9.72E-04 | -1.118 |
| RHSIM_Rhsim01G0223700 | 12.50 | 4.08E-04 | 9.73E-04 | -2.466 |
| RHSIM_Rhsim12G0064800 | 12.49 | 4.08E-04 | 9.75E-04 | 0.811 |
| novel.3975 | 12.49 | 4.09E-04 | 9.76E-04 | 1.224 |
| RHSIM_Rhsim13G0164200 | 12.48 | 4.12E-04 | 9.83E-04 | 0.984 |
| RHSIM_Rhsim01G0289000 | 12.48 | 4.12E-04 | 9.83E-04 | 3.996 |

| Gene | LRT statistic | P value | BH-adjusted P | log2FC (interaction) |
|---|---|---|---|---|
| novel.2716 | 12.47 | 4.13E-04 | 9.85E-04 | 2.463 |
| RHSIM_Rhsim06G0032000 | 12.47 | 4.13E-04 | 9.86E-04 | 2.141 |
| RHSIM_Rhsim07G0192600 | 12.47 | 4.13E-04 | 9.87E-04 | -0.643 |
| RHSIM_Rhsim09G0186400 | 12.47 | 4.14E-04 | 9.87E-04 | 0.743 |
| RHSIM_Rhsim03G0088300 | 12.47 | 4.14E-04 | 9.88E-04 | -1.780 |
| RHSIM_RhsimUnG0215700 | 12.47 | 4.14E-04 | 9.89E-04 | 2.746 |
| RHSIM_Rhsim03G0016100 | 12.46 | 4.15E-04 | 9.91E-04 | 0.670 |
| RHSIM_Rhsim10G0174000 | 12.46 | 4.16E-04 | 9.91E-04 | -0.942 |
| novel.1678 | 12.46 | 4.16E-04 | 9.91E-04 | 80.372 |
| RHSIM_Rhsim08G0053700 | 12.46 | 4.16E-04 | 9.92E-04 | 0.413 |
| RHSIM_Rhsim13G0201300 | 12.46 | 4.16E-04 | 9.93E-04 | -0.334 |
| novel.2810 | 12.46 | 4.17E-04 | 9.93E-04 | 1.131 |
| RHSIM_Rhsim11G0177500 | 12.46 | 4.17E-04 | 9.94E-04 | 0.578 |
| RHSIM_Rhsim03G0168100 | 12.45 | 4.17E-04 | 9.94E-04 | 0.366 |
| RHSIM_RhsimUnG0212600 | 12.45 | 4.17E-04 | 9.95E-04 | 1.823 |
| RHSIM_Rhsim04G0066700 | 12.45 | 4.17E-04 | 9.95E-04 | -0.572 |
| RHSIM_Rhsim12G0211900 | 12.45 | 4.18E-04 | 9.96E-04 | -0.726 |
| RHSIM_Rhsim05G0188100 | 12.45 | 4.18E-04 | 9.97E-04 | 0.440 |
| RHSIM_Rhsim01G0114500 | 12.45 | 4.19E-04 | 9.98E-04 | -0.396 |
| RHSIM_RhsimUnG0035900 | 12.44 | 4.20E-04 | 0.001 | 0.810 |
| novel.3856 | 12.44 | 4.21E-04 | 0.001 | 1.917 |
| RHSIM_Rhsim10G0010800 | 12.44 | 4.21E-04 | 0.001 | 4.998 |
| RHSIM_Rhsim02G0055200 | 12.44 | 4.21E-04 | 0.001 | -0.272 |
| RHSIM_Rhsim12G0150800 | 12.43 | 4.23E-04 | 0.001 | 1.368 |
| RHSIM_Rhsim05G0218700 | 12.43 | 4.23E-04 | 0.001 | 2.392 |
| RHSIM_Rhsim13G0129600 | 12.42 | 4.24E-04 | 0.001 | -0.299 |
| novel.1035 | 12.42 | 4.24E-04 | 0.001 | 1.731 |
| RHSIM_RhsimUnG0111500 | 12.42 | 4.25E-04 | 0.001 | 0.397 |
| RHSIM_Rhsim06G0121800 | 12.41 | 4.28E-04 | 0.001 | 0.390 |
| novel.3444 | 12.41 | 4.28E-04 | 0.001 | 0.982 |
| novel.2804 | 12.40 | 4.30E-04 | 0.001 | 1.353 |
| novel.3481 | 12.40 | 4.30E-04 | 0.001 | 1.861 |

| Gene | LRT statistic | P value | BH-adjusted P | log2FC (interaction) |
|---|---|---|---|---|
| novel.1658 | 12.39 | 4.30E-04 | 0.001 | -2.836 |
| novel.3933 | 12.39 | 4.32E-04 | 0.001 | -1.329 |
| RHSIM_Rhsim05G0135600 | 12.39 | 4.32E-04 | 0.001 | 0.356 |
| RHSIM_Rhsim04G0066800 | 12.39 | 4.33E-04 | 0.001 | 0.340 |
| RHSIM_Rhsim08G0048600 | 12.38 | 4.33E-04 | 0.001 | 1.426 |
| novel.3147 | 12.38 | 4.33E-04 | 0.001 | 0.645 |
| RHSIM_Rhsim13G0059100 | 12.38 | 4.33E-04 | 0.001 | 1.698 |
| RHSIM_Rhsim11G0030400 | 12.38 | 4.34E-04 | 0.001 | 0.789 |
| RHSIM_Rhsim05G0127500 | 12.38 | 4.34E-04 | 0.001 | 1.261 |
| RHSIM_Rhsim02G0195900 | 12.38 | 4.35E-04 | 0.001 | 0.734 |
| RHSIM_Rhsim01G0224900 | 12.37 | 4.36E-04 | 0.001 | 0.369 |
| novel.3636 | 12.37 | 4.37E-04 | 0.001 | 3.395 |
| novel.473 | 12.37 | 4.37E-04 | 0.001 | 1.646 |
| RHSIM_Rhsim08G0072400 | 12.37 | 4.37E-04 | 0.001 | 1.714 |
| novel.2237 | 12.37 | 4.37E-04 | 0.001 | -0.584 |
| RHSIM_RhsimUnG0139000 | 12.36 | 4.38E-04 | 0.001 | -1.750 |
| RHSIM_Rhsim06G0230400 | 12.36 | 4.38E-04 | 0.001 | -0.509 |
| novel.1131 | 12.36 | 4.38E-04 | 0.001 | 0.310 |
| RHSIM_Rhsim13G0173400 | 12.36 | 4.39E-04 | 0.001 | 0.752 |
| RHSIM_Rhsim05G0123300 | 12.36 | 4.39E-04 | 0.001 | 1.456 |
| RHSIM_Rhsim13G0166700 | 12.36 | 4.40E-04 | 0.001 | 0.969 |
| RHSIM_Rhsim06G0045800 | 12.35 | 4.40E-04 | 0.001 | -0.639 |
| RHSIM_Rhsim02G0214000 | 12.35 | 4.40E-04 | 0.001 | 0.399 |
| RHSIM_Rhsim13G0212800 | 12.35 | 4.41E-04 | 0.001 | 0.458 |
| novel.4438 | 12.35 | 4.41E-04 | 0.001 | 2.642 |
| novel.1748 | 12.34 | 4.42E-04 | 0.001 | 3.128 |
| novel.155 | 12.33 | 4.46E-04 | 0.001 | 1.717 |
| RHSIM_Rhsim09G0028600 | 12.33 | 4.46E-04 | 0.001 | 0.496 |
| RHSIM_RhsimUnG0137500 | 12.32 | 4.47E-04 | 0.001 | 2.112 |
| RHSIM_Rhsim10G0200300 | 12.32 | 4.48E-04 | 0.001 | -41.765 |
| novel.3538 | 12.32 | 4.49E-04 | 0.001 | 0.523 |
| novel.3971 | 12.32 | 4.49E-04 | 0.001 | 1.485 |

| Gene | LRT statistic | P value | BH-adjusted P | log2FC (interaction) |
|---|---|---|---|---|
| RHSIM_Rhsim01G0286400 | 12.30 | 4.52E-04 | 0.001 | -0.861 |
| novel.1245 | 12.30 | 4.53E-04 | 0.001 | -0.833 |
| RHSIM_Rhsim13G0046500 | 12.30 | 4.53E-04 | 0.001 | 5.399 |
| RHSIM_Rhsim02G0245200 | 12.29 | 4.54E-04 | 0.001 | -1.707 |
| RHSIM_Rhsim01G0122600 | 12.29 | 4.56E-04 | 0.001 | 0.377 |
| RHSIM_Rhsim08G0027900 | 12.28 | 4.57E-04 | 0.001 | -0.733 |
| RHSIM_Rhsim11G0182800 | 12.28 | 4.59E-04 | 0.001 | 1.449 |
| RHSIM_Rhsim02G0110300 | 12.28 | 4.59E-04 | 0.001 | 0.467 |
| RHSIM_RhsimUnG0182900 | 12.28 | 4.59E-04 | 0.001 | 1.541 |
| RHSIM_Rhsim07G0018400 | 12.27 | 4.61E-04 | 0.001 | 0.277 |
| RHSIM_Rhsim08G0041000 | 12.27 | 4.61E-04 | 0.001 | 0.825 |
| novel.1951 | 12.26 | 4.62E-04 | 0.001 | 1.817 |
| RHSIM_Rhsim04G0015200 | 12.26 | 4.62E-04 | 0.001 | 0.336 |
| RHSIM_Rhsim12G0143100 | 12.26 | 4.62E-04 | 0.001 | -0.467 |
| novel.4165 | 12.26 | 4.62E-04 | 0.001 | -2.518 |
| RHSIM_Rhsim11G0099100 | 12.26 | 4.63E-04 | 0.001 | 1.036 |
| RHSIM_Rhsim02G0026800 | 12.26 | 4.64E-04 | 0.001 | -2.181 |
| RHSIM_Rhsim01G0270000 | 12.26 | 4.64E-04 | 0.001 | 0.239 |
| RHSIM_Rhsim03G0027900 | 12.25 | 4.64E-04 | 0.001 | 0.971 |
| RHSIM_Rhsim01G0041300 | 12.25 | 4.65E-04 | 0.001 | -1.667 |
| RHSIM_Rhsim04G0096800 | 12.24 | 4.68E-04 | 0.001 | 1.501 |
| RHSIM_Rhsim08G0105300 | 12.24 | 4.68E-04 | 0.001 | -4.185 |
| novel.2314 | 12.24 | 4.68E-04 | 0.001 | 0.912 |
| RHSIM_Rhsim10G0208200 | 12.23 | 4.69E-04 | 0.001 | -0.955 |
| RHSIM_Rhsim11G0072200 | 12.23 | 4.70E-04 | 0.001 | -0.601 |
| RHSIM_Rhsim07G0211800 | 12.23 | 4.71E-04 | 0.001 | -0.237 |
| RHSIM_Rhsim06G0000300 | 12.22 | 4.73E-04 | 0.001 | 0.274 |
| RHSIM_Rhsim07G0030100 | 12.21 | 4.74E-04 | 0.001 | -0.267 |
| RHSIM_Rhsim03G0117600 | 12.21 | 4.75E-04 | 0.001 | 0.553 |
| RHSIM_Rhsim03G0067600 | 12.21 | 4.76E-04 | 0.001 | 0.624 |
| RHSIM_Rhsim01G0004800 | 12.21 | 4.76E-04 | 0.001 | -2.691 |
| RHSIM_Rhsim02G0155900 | 12.21 | 4.76E-04 | 0.001 | 0.627 |

| Gene | LRT statistic | P value | BH-adjusted P | log2FC (interaction) |
|---|---|---|---|---|
| RHSIM_Rhsim06G0186300 | 12.21 | 4.76E-04 | 0.001 | 1.238 |
| RHSIM_Rhsim04G0093500 | 12.20 | 4.79E-04 | 0.001 | 0.388 |
| RHSIM_Rhsim13G0206500 | 12.19 | 4.80E-04 | 0.001 | 0.600 |
| RHSIM_Rhsim08G0246700 | 12.18 | 4.83E-04 | 0.001 | 2.530 |
| RHSIM_Rhsim10G0027900 | 12.18 | 4.84E-04 | 0.001 | -1.012 |
| RHSIM_Rhsim09G0024000 | 12.18 | 4.84E-04 | 0.001 | -0.297 |
| RHSIM_Rhsim03G0079100 | 12.17 | 4.86E-04 | 0.001 | -0.970 |
| RHSIM_Rhsim10G0010000 | 12.16 | 4.88E-04 | 0.001 | -0.978 |
| RHSIM_Rhsim12G0042300 | 12.16 | 4.89E-04 | 0.001 | 1.890 |
| RHSIM_Rhsim07G0022800 | 12.15 | 4.90E-04 | 0.001 | -1.939 |
| RHSIM_Rhsim10G0020200 | 12.15 | 4.90E-04 | 0.001 | 0.698 |
| RHSIM_Rhsim09G0019700 | 12.15 | 4.91E-04 | 0.001 | -0.945 |
| RHSIM_Rhsim09G0071000 | 12.15 | 4.91E-04 | 0.001 | 0.893 |
| RHSIM_Rhsim10G0017100 | 12.15 | 4.92E-04 | 0.001 | -1.564 |
| RHSIM_Rhsim01G0253400 | 12.14 | 4.92E-04 | 0.001 | 42.623 |
| RHSIM_Rhsim07G0214100 | 12.14 | 4.92E-04 | 0.001 | -3.930 |
| RHSIM_Rhsim02G0041900 | 12.14 | 4.93E-04 | 0.001 | 0.415 |
| novel.2874 | 12.14 | 4.93E-04 | 0.001 | 1.152 |
| RHSIM_Rhsim09G0056400 | 12.14 | 4.94E-04 | 0.001 | -1.085 |
| RHSIM_Rhsim12G0053300 | 12.14 | 4.94E-04 | 0.001 | -4.289 |
| RHSIM_Rhsim12G0060700 | 12.14 | 4.94E-04 | 0.001 | 0.272 |
| RHSIM_Rhsim06G0168000 | 12.14 | 4.94E-04 | 0.001 | 0.784 |
| novel.1595 | 12.14 | 4.94E-04 | 0.001 | -1.356 |
| RHSIM_Rhsim06G0080100 | 12.13 | 4.96E-04 | 0.001 | -1.659 |
| novel.4207 | 12.13 | 4.96E-04 | 0.001 | 0.853 |
| RHSIM_Rhsim13G0183900 | 12.13 | 4.97E-04 | 0.001 | 2.264 |
| novel.2092 | 12.13 | 4.97E-04 | 0.001 | 1.379 |
| RHSIM_Rhsim05G0118100 | 12.13 | 4.97E-04 | 0.001 | 0.411 |
| RHSIM_Rhsim06G0124500 | 12.13 | 4.97E-04 | 0.001 | 1.381 |
| RHSIM_Rhsim01G0189400 | 12.12 | 4.98E-04 | 0.001 | -0.589 |
| RHSIM_Rhsim08G0035500 | 12.11 | 5.01E-04 | 0.001 | 0.285 |
| RHSIM_RhsimUnG0169100 | 12.11 | 5.02E-04 | 0.001 | 4.452 |

| Gene | LRT statistic | P value | BH-adjusted P | log2FC (interaction) |
|---|---|---|---|---|
| RHSIM_Rhsim10G0024600 | 12.11 | 5.02E-04 | 0.001 | 1.353 |
| RHSIM_Rhsim03G0186900 | 12.10 | 5.04E-04 | 0.001 | -1.620 |
| RHSIM_Rhsim03G0068600 | 12.10 | 5.04E-04 | 0.001 | -0.389 |
| RHSIM_Rhsim07G0091600 | 12.10 | 5.05E-04 | 0.001 | -0.942 |
| RHSIM_Rhsim01G0058000 | 12.09 | 5.07E-04 | 0.001 | -0.500 |
| RHSIM_Rhsim13G0196600 | 12.09 | 5.07E-04 | 0.001 | -0.793 |
| novel.232 | 12.09 | 5.07E-04 | 0.001 | 1.966 |
| RHSIM_Rhsim09G0141700 | 12.09 | 5.08E-04 | 0.001 | -1.124 |
| RHSIM_Rhsim08G0239500 | 12.08 | 5.08E-04 | 0.001 | 0.680 |
| RHSIM_Rhsim06G0115000 | 12.08 | 5.11E-04 | 0.001 | 2.551 |
| novel.3064 | 12.08 | 5.11E-04 | 0.001 | -0.475 |
| RHSIM_Rhsim04G0007000 | 12.08 | 5.11E-04 | 0.001 | -0.326 |
| RHSIM_Rhsim07G0177300 | 12.07 | 5.11E-04 | 0.001 | 0.487 |
| RHSIM_Rhsim05G0054500 | 12.07 | 5.12E-04 | 0.001 | -0.256 |
| RHSIM_Rhsim02G0040400 | 12.07 | 5.13E-04 | 0.001 | 0.249 |
| RHSIM_Rhsim06G0101700 | 12.07 | 5.13E-04 | 0.001 | 2.352 |
| RHSIM_Rhsim10G0023000 | 12.07 | 5.13E-04 | 0.001 | 0.546 |
| novel.607 | 12.07 | 5.14E-04 | 0.001 | 1.457 |
| novel.2825 | 12.06 | 5.15E-04 | 0.001 | -1.826 |
| RHSIM_Rhsim01G0038400 | 12.06 | 5.15E-04 | 0.001 | -3.107 |
| RHSIM_Rhsim08G0052700 | 12.06 | 5.15E-04 | 0.001 | -0.464 |
| RHSIM_Rhsim01G0202800 | 12.06 | 5.15E-04 | 0.001 | 1.633 |
| RHSIM_Rhsim03G0193700 | 12.06 | 5.16E-04 | 0.001 | -1.062 |
| RHSIM_Rhsim13G0226700 | 12.05 | 5.18E-04 | 0.001 | 0.334 |
| RHSIM_Rhsim12G0147200 | 12.05 | 5.18E-04 | 0.001 | 1.233 |
| RHSIM_Rhsim06G0128900 | 12.05 | 5.18E-04 | 0.001 | -1.361 |
| RHSIM_Rhsim09G0037100 | 12.05 | 5.18E-04 | 0.001 | 2.915 |
| RHSIM_Rhsim13G0008800 | 12.05 | 5.18E-04 | 0.001 | 41.736 |
| novel.306 | 12.05 | 5.19E-04 | 0.001 | 1.236 |
| RHSIM_Rhsim12G0083300 | 12.05 | 5.19E-04 | 0.001 | -0.344 |
| novel.1704 | 12.05 | 5.19E-04 | 0.001 | -2.133 |
| novel.1274 | 12.04 | 5.19E-04 | 0.001 | -5.345 |

| Gene | LRT statistic | P value | BH-adjusted P | log2FC (interaction) |
|---|---|---|---|---|
| novel.4434 | 12.04 | 5.20E-04 | 0.001 | 1.413 |
| RHSIM_Rhsim05G0094700 | 12.04 | 5.21E-04 | 0.001 | -4.494 |
| RHSIM_Rhsim02G0246600 | 12.04 | 5.21E-04 | 0.001 | -0.522 |
| RHSIM_Rhsim02G0217700 | 12.04 | 5.22E-04 | 0.001 | -0.367 |
| novel.3387 | 12.02 | 5.26E-04 | 0.001 | -0.456 |
| novel.3509 | 12.02 | 5.26E-04 | 0.001 | 40.994 |
| RHSIM_Rhsim07G0070400 | 12.01 | 5.28E-04 | 0.001 | 3.396 |
| RHSIM_Rhsim03G0187400 | 12.01 | 5.29E-04 | 0.001 | 0.291 |
| RHSIM_Rhsim09G0042400 | 12.01 | 5.29E-04 | 0.001 | 0.911 |
| RHSIM_Rhsim04G0245500 | 12.01 | 5.30E-04 | 0.001 | -1.003 |
| novel.493 | 12.01 | 5.30E-04 | 0.001 | -1.021 |
| RHSIM_Rhsim12G0127300 | 12.01 | 5.30E-04 | 0.001 | -40.962 |
| RHSIM_RhsimUnG0240500 | 12.00 | 5.31E-04 | 0.001 | 1.681 |
| RHSIM_Rhsim07G0068600 | 12.00 | 5.32E-04 | 0.001 | -0.335 |
| RHSIM_Rhsim10G0177700 | 12.00 | 5.32E-04 | 0.001 | 0.414 |
| RHSIM_Rhsim06G0102900 | 12.00 | 5.32E-04 | 0.001 | -1.707 |
| RHSIM_RhsimUnG0122600 | 12.00 | 5.33E-04 | 0.001 | 1.451 |
| novel.638 | 11.99 | 5.34E-04 | 0.001 | -0.372 |
| RHSIM_Rhsim12G0108500 | 11.99 | 5.35E-04 | 0.001 | -0.383 |
| RHSIM_Rhsim04G0218600 | 11.98 | 5.37E-04 | 0.001 | -0.256 |
| RHSIM_Rhsim11G0007500 | 11.97 | 5.41E-04 | 0.001 | 2.051 |
| novel.3714 | 11.97 | 5.41E-04 | 0.001 | 2.008 |
| novel.3591 | 11.96 | 5.42E-04 | 0.001 | 0.527 |
| RHSIM_Rhsim12G0099900 | 11.96 | 5.43E-04 | 0.001 | 0.630 |
| RHSIM_Rhsim11G0170500 | 11.96 | 5.43E-04 | 0.001 | 2.412 |
| RHSIM_RhsimUnG0227400 | 11.96 | 5.43E-04 | 0.001 | 1.150 |
| RHSIM_Rhsim12G0161000 | 11.96 | 5.44E-04 | 0.001 | -0.472 |
| novel.4029 | 11.95 | 5.47E-04 | 0.001 | -41.216 |
| RHSIM_Rhsim03G0250000 | 11.95 | 5.47E-04 | 0.001 | 0.221 |
| RHSIM_RhsimUnG0206400 | 11.95 | 5.48E-04 | 0.001 | 0.305 |
| RHSIM_Rhsim07G0018800 | 11.94 | 5.48E-04 | 0.001 | -0.220 |
| RHSIM_Rhsim09G0197500 | 11.94 | 5.49E-04 | 0.001 | -0.684 |

| Gene | LRT statistic | P value | BH-adjusted P | log2FC (interaction) |
|---|---|---|---|---|
| RHSIM_Rhsim04G0009400 | 11.94 | 5.51E-04 | 0.001 | 0.283 |
| RHSIM_Rhsim08G0111900 | 11.94 | 5.51E-04 | 0.001 | 0.234 |
| RHSIM_Rhsim05G0080100 | 11.93 | 5.52E-04 | 0.001 | -0.293 |
| RHSIM_Rhsim13G0041000 | 11.93 | 5.53E-04 | 0.001 | -0.783 |
| RHSIM_Rhsim03G0180400 | 11.93 | 5.53E-04 | 0.001 | -0.411 |
| novel.1278 | 11.92 | 5.56E-04 | 0.001 | 2.746 |
| RHSIM_Rhsim02G0195700 | 11.92 | 5.56E-04 | 0.001 | 1.213 |
| RHSIM_Rhsim09G0008200 | 11.91 | 5.57E-04 | 0.001 | -0.391 |
| RHSIM_Rhsim02G0156700 | 11.91 | 5.57E-04 | 0.001 | -2.799 |
| RHSIM_Rhsim09G0110200 | 11.91 | 5.59E-04 | 0.001 | 0.569 |
| RHSIM_Rhsim03G0023300 | 11.91 | 5.59E-04 | 0.001 | 0.742 |
| RHSIM_Rhsim07G0245000 | 11.91 | 5.59E-04 | 0.001 | 0.369 |
| RHSIM_Rhsim08G0197700 | 11.90 | 5.60E-04 | 0.001 | -0.369 |
| RHSIM_Rhsim02G0242500 | 11.90 | 5.61E-04 | 0.001 | -1.685 |
| RHSIM_Rhsim06G0031900 | 11.90 | 5.61E-04 | 0.001 | 1.202 |
| RHSIM_Rhsim08G0002100 | 11.90 | 5.61E-04 | 0.001 | 1.709 |
| RHSIM_Rhsim05G0191600 | 11.90 | 5.62E-04 | 0.001 | 3.346 |
| RHSIM_Rhsim05G0002800 | 11.90 | 5.62E-04 | 0.001 | 4.752 |
| RHSIM_Rhsim04G0202500 | 11.89 | 5.63E-04 | 0.001 | 1.061 |
| RHSIM_Rhsim08G0132000 | 11.89 | 5.64E-04 | 0.001 | -0.356 |
| novel.2592 | 11.89 | 5.66E-04 | 0.001 | -0.936 |
| RHSIM_Rhsim09G0116800 | 11.88 | 5.66E-04 | 0.001 | 1.079 |
| RHSIM_Rhsim05G0137200 | 11.88 | 5.67E-04 | 0.001 | 2.345 |
| RHSIM_Rhsim01G0172000 | 11.88 | 5.68E-04 | 0.001 | -0.340 |
| novel.3843 | 11.88 | 5.68E-04 | 0.001 | 1.071 |
| RHSIM_Rhsim03G0202800 | 11.87 | 5.70E-04 | 0.001 | 0.286 |
| RHSIM_Rhsim04G0008500 | 11.87 | 5.70E-04 | 0.001 | -0.414 |
| RHSIM_RhsimUnG0223400 | 11.87 | 5.71E-04 | 0.001 | -5.010 |
| RHSIM_Rhsim13G0089100 | 11.87 | 5.71E-04 | 0.001 | 0.156 |
| RHSIM_Rhsim06G0150600 | 11.87 | 5.72E-04 | 0.001 | -0.367 |
| RHSIM_Rhsim05G0087600 | 11.87 | 5.72E-04 | 0.001 | 0.234 |
| RHSIM_Rhsim12G0127700 | 11.87 | 5.72E-04 | 0.001 | -1.039 |

| Gene | LRT statistic | P value | BH-adjusted P | log2FC (interaction) |
|---|---|---|---|---|
| RHSIM_Rhsim09G0194200 | 11.86 | 5.73E-04 | 0.001 | 2.273 |
| RHSIM_Rhsim10G0192700 | 11.86 | 5.74E-04 | 0.001 | 0.220 |
| RHSIM_Rhsim08G0176200 | 11.86 | 5.74E-04 | 0.001 | 43.300 |
| RHSIM_Rhsim05G0033700 | 11.86 | 5.74E-04 | 0.001 | 2.758 |
| RHSIM_Rhsim13G0182600 | 11.86 | 5.75E-04 | 0.001 | -39.872 |
| novel.1575 | 11.86 | 5.75E-04 | 0.001 | 0.742 |
| RHSIM_Rhsim10G0060200 | 11.85 | 5.76E-04 | 0.001 | -0.686 |
| RHSIM_Rhsim01G0253300 | 11.85 | 5.77E-04 | 0.001 | 0.459 |
| RHSIM_Rhsim13G0049900 | 11.84 | 5.79E-04 | 0.001 | -0.514 |
| RHSIM_RhsimUnG0113700 | 11.84 | 5.80E-04 | 0.001 | 0.595 |
| RHSIM_Rhsim11G0178100 | 11.84 | 5.80E-04 | 0.001 | -4.129 |
| novel.239 | 11.84 | 5.81E-04 | 0.001 | 0.482 |
| RHSIM_Rhsim07G0227200 | 11.83 | 5.82E-04 | 0.001 | -0.294 |
| RHSIM_Rhsim03G0265500 | 11.83 | 5.84E-04 | 0.001 | -40.694 |
| RHSIM_Rhsim03G0082500 | 11.82 | 5.85E-04 | 0.001 | 0.820 |
| RHSIM_Rhsim01G0121400 | 11.82 | 5.85E-04 | 0.001 | 2.558 |
| RHSIM_Rhsim02G0203400 | 11.82 | 5.86E-04 | 0.001 | -0.331 |
| RHSIM_Rhsim02G0207500 | 11.82 | 5.87E-04 | 0.001 | -1.090 |
| RHSIM_Rhsim07G0133700 | 11.82 | 5.87E-04 | 0.001 | 1.426 |
| RHSIM_Rhsim04G0143900 | 11.82 | 5.87E-04 | 0.001 | 2.301 |
| RHSIM_RhsimPtG0003600 | 11.81 | 5.88E-04 | 0.001 | 0.803 |
| RHSIM_Rhsim10G0073200 | 11.81 | 5.88E-04 | 0.001 | 1.096 |
| RHSIM_Rhsim02G0041700 | 11.81 | 5.88E-04 | 0.001 | 1.150 |
| novel.2600 | 11.81 | 5.89E-04 | 0.001 | 0.293 |
| novel.1713 | 11.81 | 5.90E-04 | 0.001 | -0.586 |
| RHSIM_Rhsim09G0116900 | 11.81 | 5.90E-04 | 0.001 | -1.392 |
| novel.3529 | 11.80 | 5.92E-04 | 0.001 | 0.943 |
| RHSIM_Rhsim13G0122500 | 11.80 | 5.92E-04 | 0.001 | 0.652 |
| RHSIM_Rhsim06G0003600 | 11.80 | 5.93E-04 | 0.001 | -0.526 |
| RHSIM_Rhsim09G0195400 | 11.80 | 5.93E-04 | 0.001 | 0.442 |
| RHSIM_Rhsim04G0071500 | 11.80 | 5.93E-04 | 0.001 | -0.449 |
| RHSIM_RhsimUnG0046000 | 11.79 | 5.95E-04 | 0.001 | 0.899 |

| Gene | LRT statistic | P value | BH-adjusted P | log2FC (interaction) |
|---|---|---|---|---|
| novel.3914 | 11.79 | 5.95E-04 | 0.001 | 1.067 |
| RHSIM_Rhsim04G0105200 | 11.79 | 5.96E-04 | 0.001 | -3.724 |
| RHSIM_Rhsim01G0163200 | 11.79 | 5.96E-04 | 0.001 | 3.629 |
| RHSIM_Rhsim10G0170500 | 11.79 | 5.96E-04 | 0.001 | 1.349 |
| RHSIM_Rhsim10G0061100 | 11.78 | 5.98E-04 | 0.001 | 1.107 |
| RHSIM_Rhsim09G0188200 | 11.78 | 5.99E-04 | 0.001 | 0.360 |
| RHSIM_RhsimUnG0092900 | 11.78 | 5.99E-04 | 0.001 | -0.657 |
| RHSIM_Rhsim07G0191400 | 11.77 | 6.00E-04 | 0.001 | 1.535 |
| novel.2949 | 11.77 | 6.01E-04 | 0.001 | 1.643 |
| RHSIM_Rhsim08G0111500 | 11.77 | 6.02E-04 | 0.001 | 0.319 |
| novel.4345 | 11.76 | 6.05E-04 | 0.001 | 3.023 |
| RHSIM_Rhsim02G0104900 | 11.75 | 6.10E-04 | 0.001 | -0.735 |
| RHSIM_Rhsim03G0243500 | 11.74 | 6.11E-04 | 0.001 | -0.209 |
| RHSIM_RhsimUnG0244400 | 11.74 | 6.11E-04 | 0.001 | 1.827 |
| RHSIM_RhsimUnG0061400 | 11.74 | 6.12E-04 | 0.001 | -0.786 |
| RHSIM_Rhsim03G0179200 | 11.74 | 6.12E-04 | 0.001 | -0.350 |
| RHSIM_Rhsim01G0061100 | 11.74 | 6.13E-04 | 0.001 | 0.393 |
| RHSIM_RhsimUnG0118500 | 11.73 | 6.14E-04 | 0.001 | 1.131 |
| RHSIM_RhsimUnG0028200 | 11.73 | 6.14E-04 | 0.001 | 1.309 |
| RHSIM_Rhsim09G0202400 | 11.72 | 6.18E-04 | 0.001 | -0.246 |
| RHSIM_Rhsim02G0095300 | 11.72 | 6.18E-04 | 0.001 | -1.290 |
| RHSIM_Rhsim02G0132700 | 11.71 | 6.20E-04 | 0.001 | -0.341 |
| novel.1430 | 11.71 | 6.21E-04 | 0.001 | -4.454 |
| RHSIM_Rhsim13G0112300 | 11.71 | 6.21E-04 | 0.001 | -2.624 |
| RHSIM_Rhsim06G0159000 | 11.71 | 6.22E-04 | 0.001 | -0.704 |
| RHSIM_RhsimUnG0048600 | 11.71 | 6.22E-04 | 0.001 | 1.419 |
| RHSIM_Rhsim05G0079800 | 11.71 | 6.23E-04 | 0.001 | 1.201 |
| novel.3746 | 11.71 | 6.23E-04 | 0.001 | 2.822 |
| RHSIM_Rhsim04G0080800 | 11.70 | 6.25E-04 | 0.001 | 2.213 |
| RHSIM_Rhsim07G0081700 | 11.70 | 6.26E-04 | 0.001 | -3.980 |
| RHSIM_Rhsim07G0108300 | 11.69 | 6.28E-04 | 0.001 | -1.265 |
| RHSIM_Rhsim07G0003300 | 11.69 | 6.28E-04 | 0.001 | -4.018 |

| Gene | LRT statistic | P value | BH-adjusted P | log2FC (interaction) |
|---|---|---|---|---|
| RHSIM_Rhsim06G0218200 | 11.69 | 6.29E-04 | 0.001 | -0.949 |
| RHSIM_Rhsim04G0118900 | 11.68 | 6.30E-04 | 0.001 | 0.887 |
| RHSIM_Rhsim07G0070500 | 11.68 | 6.31E-04 | 0.001 | -1.815 |
| RHSIM_Rhsim11G0136200 | 11.68 | 6.33E-04 | 0.001 | 0.487 |
| novel.1449 | 11.67 | 6.33E-04 | 0.001 | -1.470 |
| novel.1215 | 11.67 | 6.36E-04 | 0.001 | -2.652 |
| RHSIM_Rhsim09G0058600 | 11.67 | 6.36E-04 | 0.001 | 0.363 |
| RHSIM_Rhsim13G0088500 | 11.67 | 6.37E-04 | 0.001 | 0.326 |
| RHSIM_Rhsim03G0116200 | 11.66 | 6.37E-04 | 0.001 | -0.509 |
| RHSIM_Rhsim02G0032800 | 11.66 | 6.37E-04 | 0.001 | 0.676 |
| RHSIM_Rhsim13G0197400 | 11.66 | 6.38E-04 | 0.001 | -0.594 |
| RHSIM_Rhsim03G0238600 | 11.65 | 6.42E-04 | 0.001 | -0.920 |
| RHSIM_Rhsim07G0175800 | 11.65 | 6.42E-04 | 0.001 | 0.572 |
| RHSIM_Rhsim12G0046300 | 11.64 | 6.44E-04 | 0.002 | -0.563 |
| RHSIM_Rhsim01G0215200 | 11.64 | 6.45E-04 | 0.002 | -0.560 |
| RHSIM_Rhsim06G0237300 | 11.64 | 6.45E-04 | 0.002 | -0.797 |
| novel.3590 | 11.64 | 6.45E-04 | 0.002 | -1.243 |
| novel.1966 | 11.62 | 6.51E-04 | 0.002 | -1.529 |
| RHSIM_Rhsim02G0076600 | 11.62 | 6.53E-04 | 0.002 | -0.680 |
| RHSIM_Rhsim06G0224700 | 11.62 | 6.53E-04 | 0.002 | -5.414 |
| RHSIM_Rhsim04G0199300 | 11.62 | 6.53E-04 | 0.002 | 1.631 |
| RHSIM_Rhsim04G0086700 | 11.61 | 6.56E-04 | 0.002 | -2.180 |
| novel.3851 | 11.61 | 6.57E-04 | 0.002 | 1.203 |
| RHSIM_Rhsim12G0203500 | 11.60 | 6.59E-04 | 0.002 | -2.246 |
| RHSIM_Rhsim03G0253300 | 11.59 | 6.62E-04 | 0.002 | 0.300 |
| novel.3145 | 11.59 | 6.62E-04 | 0.002 | 2.766 |
| RHSIM_Rhsim13G0194100 | 11.59 | 6.62E-04 | 0.002 | 2.147 |
| RHSIM_Rhsim02G0126900 | 11.59 | 6.65E-04 | 0.002 | 2.272 |
| novel.1969 | 11.58 | 6.66E-04 | 0.002 | 0.571 |
| RHSIM_Rhsim09G0148000 | 11.58 | 6.67E-04 | 0.002 | 1.538 |
| RHSIM_Rhsim10G0029600 | 11.57 | 6.69E-04 | 0.002 | 1.425 |
| RHSIM_Rhsim11G0136500 | 11.57 | 6.69E-04 | 0.002 | -0.264 |

| Gene | LRT statistic | P value | BH-adjusted P | log2FC (interaction) |
|---|---|---|---|---|
| RHSIM_Rhsim08G0171900 | 11.57 | 6.71E-04 | 0.002 | 0.258 |
| RHSIM_Rhsim03G0058500 | 11.57 | 6.72E-04 | 0.002 | 1.024 |
| RHSIM_Rhsim02G0036200 | 11.56 | 6.73E-04 | 0.002 | 1.142 |
| novel.826 | 11.56 | 6.73E-04 | 0.002 | 0.936 |
| RHSIM_Rhsim11G0122800 | 11.56 | 6.74E-04 | 0.002 | -0.402 |
| RHSIM_Rhsim03G0180100 | 11.56 | 6.74E-04 | 0.002 | 0.491 |
| RHSIM_Rhsim08G0142100 | 11.56 | 6.75E-04 | 0.002 | -0.560 |
| RHSIM_Rhsim09G0153300 | 11.56 | 6.75E-04 | 0.002 | 0.311 |
| RHSIM_Rhsim10G0207700 | 11.55 | 6.76E-04 | 0.002 | -0.334 |
| RHSIM_Rhsim03G0162400 | 11.55 | 6.77E-04 | 0.002 | 1.045 |
| RHSIM_Rhsim01G0110000 | 11.55 | 6.77E-04 | 0.002 | 1.091 |
| RHSIM_Rhsim08G0224200 | 11.55 | 6.78E-04 | 0.002 | -0.408 |
| RHSIM_Rhsim12G0133000 | 11.55 | 6.79E-04 | 0.002 | -0.442 |
| RHSIM_Rhsim05G0229600 | 11.54 | 6.81E-04 | 0.002 | 0.160 |
| RHSIM_Rhsim07G0005500 | 11.54 | 6.82E-04 | 0.002 | 0.268 |
| novel.4074 | 11.53 | 6.84E-04 | 0.002 | 3.940 |
| RHSIM_Rhsim13G0091900 | 11.53 | 6.85E-04 | 0.002 | 0.950 |
| RHSIM_Rhsim10G0150100 | 11.53 | 6.85E-04 | 0.002 | -0.553 |
| RHSIM_Rhsim01G0145200 | 11.53 | 6.86E-04 | 0.002 | -0.283 |
| RHSIM_Rhsim09G0101900 | 11.52 | 6.87E-04 | 0.002 | 1.879 |
| RHSIM_RhsimUnG0185500 | 11.52 | 6.87E-04 | 0.002 | -0.418 |
| novel.3515 | 11.52 | 6.89E-04 | 0.002 | -0.618 |
| RHSIM_Rhsim11G0162500 | 11.52 | 6.89E-04 | 0.002 | 2.168 |
| RHSIM_Rhsim01G0103600 | 11.52 | 6.90E-04 | 0.002 | -0.628 |
| RHSIM_Rhsim06G0233500 | 11.51 | 6.91E-04 | 0.002 | -3.117 |
| novel.3575 | 11.50 | 6.95E-04 | 0.002 | -1.385 |
| RHSIM_Rhsim10G0134100 | 11.49 | 6.98E-04 | 0.002 | 1.261 |
| RHSIM_Rhsim13G0040000 | 11.49 | 6.99E-04 | 0.002 | -0.241 |
| RHSIM_Rhsim04G0038200 | 11.49 | 6.99E-04 | 0.002 | 0.694 |
| novel.671 | 11.49 | 7.00E-04 | 0.002 | 2.172 |
| RHSIM_Rhsim08G0153500 | 11.48 | 7.03E-04 | 0.002 | 0.974 |
| RHSIM_RhsimUnG0027400 | 11.48 | 7.03E-04 | 0.002 | -3.610 |

| Gene | LRT statistic | P value | BH-adjusted P | log2FC (interaction) |
|---|---|---|---|---|
| RHSIM_Rhsim08G0250600 | 11.48 | 7.04E-04 | 0.002 | 2.049 |
| RHSIM_Rhsim04G0206500 | 11.48 | 7.05E-04 | 0.002 | -3.815 |
| RHSIM_Rhsim03G0172500 | 11.47 | 7.05E-04 | 0.002 | 3.716 |
| RHSIM_Rhsim05G0120800 | 11.47 | 7.06E-04 | 0.002 | 1.579 |
| RHSIM_Rhsim02G0237900 | 11.47 | 7.08E-04 | 0.002 | -1.669 |
| RHSIM_Rhsim07G0130700 | 11.47 | 7.08E-04 | 0.002 | -0.835 |
| RHSIM_Rhsim02G0103300 | 11.46 | 7.09E-04 | 0.002 | 1.099 |
| RHSIM_Rhsim06G0022400 | 11.46 | 7.09E-04 | 0.002 | -0.892 |
| novel.2944 | 11.46 | 7.11E-04 | 0.002 | 2.733 |
| RHSIM_Rhsim04G0158600 | 11.46 | 7.11E-04 | 0.002 | 2.350 |
| RHSIM_Rhsim06G0137300 | 11.46 | 7.11E-04 | 0.002 | 0.181 |
| RHSIM_Rhsim04G0232500 | 11.46 | 7.11E-04 | 0.002 | -2.399 |
| RHSIM_Rhsim13G0226400 | 11.46 | 7.13E-04 | 0.002 | 0.635 |
| RHSIM_Rhsim07G0046400 | 11.45 | 7.14E-04 | 0.002 | 2.986 |
| RHSIM_Rhsim05G0013900 | 11.45 | 7.15E-04 | 0.002 | -0.962 |
| RHSIM_Rhsim08G0196800 | 11.45 | 7.15E-04 | 0.002 | 0.226 |
| RHSIM_RhsimUnG0030100 | 11.45 | 7.16E-04 | 0.002 | 0.860 |
| RHSIM_RhsimUnG0164600 | 11.45 | 7.16E-04 | 0.002 | -1.304 |
| RHSIM_Rhsim03G0001900 | 11.45 | 7.17E-04 | 0.002 | -0.789 |
| RHSIM_Rhsim10G0137900 | 11.44 | 7.17E-04 | 0.002 | -0.409 |
| RHSIM_Rhsim13G0048200 | 11.44 | 7.17E-04 | 0.002 | -4.598 |
| RHSIM_Rhsim08G0012700 | 11.44 | 7.18E-04 | 0.002 | -0.509 |
| RHSIM_Rhsim09G0049700 | 11.44 | 7.19E-04 | 0.002 | 0.783 |
| novel.1240 | 11.44 | 7.19E-04 | 0.002 | -2.158 |
| RHSIM_Rhsim04G0105300 | 11.44 | 7.20E-04 | 0.002 | 1.030 |
| RHSIM_Rhsim02G0032500 | 11.43 | 7.23E-04 | 0.002 | -0.380 |
| RHSIM_Rhsim11G0180800 | 11.43 | 7.23E-04 | 0.002 | 0.944 |
| RHSIM_Rhsim07G0145400 | 11.43 | 7.24E-04 | 0.002 | -0.286 |
| RHSIM_Rhsim13G0090300 | 11.42 | 7.27E-04 | 0.002 | 1.746 |
| RHSIM_Rhsim05G0027700 | 11.42 | 7.27E-04 | 0.002 | 0.287 |
| RHSIM_Rhsim10G0129900 | 11.42 | 7.28E-04 | 0.002 | 1.027 |
| RHSIM_Rhsim03G0160400 | 11.41 | 7.29E-04 | 0.002 | 1.818 |

| Gene | LRT statistic | P value | BH-adjusted P | log2FC (interaction) |
|---|---|---|---|---|
| novel.1492 | 11.40 | 7.33E-04 | 0.002 | -1.050 |
| novel.2492 | 11.40 | 7.35E-04 | 0.002 | -0.940 |
| RHSIM_Rhsim08G0207500 | 11.40 | 7.36E-04 | 0.002 | 0.458 |
| RHSIM_Rhsim04G0241800 | 11.39 | 7.37E-04 | 0.002 | 0.295 |
| RHSIM_Rhsim09G0057400 | 11.39 | 7.38E-04 | 0.002 | 1.166 |
| RHSIM_Rhsim01G0211200 | 11.39 | 7.38E-04 | 0.002 | 0.281 |
| RHSIM_Rhsim06G0185000 | 11.39 | 7.40E-04 | 0.002 | 0.231 |
| RHSIM_RhsimMtG0002200 | 11.38 | 7.41E-04 | 0.002 | -1.905 |
| RHSIM_Rhsim11G0097800 | 11.38 | 7.43E-04 | 0.002 | 0.429 |
| RHSIM_Rhsim09G0080000 | 11.38 | 7.43E-04 | 0.002 | 0.821 |
| RHSIM_Rhsim09G0100700 | 11.38 | 7.44E-04 | 0.002 | -2.996 |
| RHSIM_Rhsim06G0121900 | 11.37 | 7.46E-04 | 0.002 | -0.374 |
| RHSIM_Rhsim11G0054900 | 11.37 | 7.47E-04 | 0.002 | 0.659 |
| RHSIM_Rhsim09G0029000 | 11.37 | 7.47E-04 | 0.002 | -0.709 |
| RHSIM_Rhsim04G0111000 | 11.37 | 7.47E-04 | 0.002 | -0.412 |
| RHSIM_Rhsim08G0139000 | 11.37 | 7.48E-04 | 0.002 | 0.397 |
| RHSIM_Rhsim01G0145500 | 11.36 | 7.49E-04 | 0.002 | 0.492 |
| RHSIM_Rhsim04G0012100 | 11.36 | 7.50E-04 | 0.002 | 1.402 |
| RHSIM_Rhsim04G0019600 | 11.36 | 7.50E-04 | 0.002 | 0.699 |
| RHSIM_Rhsim13G0111200 | 11.35 | 7.52E-04 | 0.002 | -0.634 |
| RHSIM_Rhsim08G0223200 | 11.35 | 7.54E-04 | 0.002 | 1.721 |
| RHSIM_RhsimUnG0156800 | 11.33 | 7.62E-04 | 0.002 | 0.557 |
| RHSIM_Rhsim12G0107600 | 11.33 | 7.62E-04 | 0.002 | 2.291 |
| RHSIM_Rhsim12G0205500 | 11.33 | 7.63E-04 | 0.002 | 1.834 |
| RHSIM_Rhsim07G0138000 | 11.33 | 7.63E-04 | 0.002 | 0.736 |
| RHSIM_Rhsim09G0080900 | 11.32 | 7.65E-04 | 0.002 | 4.404 |
| novel.2996 | 11.32 | 7.66E-04 | 0.002 | 4.836 |
| RHSIM_Rhsim01G0252900 | 11.32 | 7.67E-04 | 0.002 | -0.486 |
| RHSIM_Rhsim01G0176200 | 11.31 | 7.70E-04 | 0.002 | 0.525 |
| novel.2102 | 11.31 | 7.71E-04 | 0.002 | 2.944 |
| RHSIM_Rhsim05G0143100 | 11.30 | 7.74E-04 | 0.002 | 0.621 |
| RHSIM_Rhsim05G0031100 | 11.30 | 7.75E-04 | 0.002 | 1.423 |

| Gene | LRT statistic | P value | BH-adjusted P | log2FC (interaction) |
|---|---|---|---|---|
| RHSIM_Rhsim06G0165400 | 11.30 | 7.77E-04 | 0.002 | -0.684 |
| RHSIM_Rhsim13G0071600 | 11.29 | 7.77E-04 | 0.002 | 2.417 |
| RHSIM_Rhsim01G0092300 | 11.29 | 7.79E-04 | 0.002 | 2.092 |
| RHSIM_Rhsim13G0041400 | 11.28 | 7.83E-04 | 0.002 | 2.113 |
| RHSIM_Rhsim04G0072600 | 11.27 | 7.88E-04 | 0.002 | -0.287 |
| RHSIM_RhsimUnG0108700 | 11.27 | 7.89E-04 | 0.002 | 0.550 |
| novel.2619 | 11.26 | 7.91E-04 | 0.002 | 0.398 |
| RHSIM_Rhsim01G0025100 | 11.26 | 7.93E-04 | 0.002 | 3.107 |
| RHSIM_Rhsim07G0245100 | 11.25 | 7.95E-04 | 0.002 | 1.614 |
| RHSIM_Rhsim11G0102200 | 11.25 | 7.95E-04 | 0.002 | -0.866 |
| novel.3082 | 11.25 | 7.96E-04 | 0.002 | -0.954 |
| RHSIM_Rhsim02G0209200 | 11.25 | 7.96E-04 | 0.002 | -41.319 |
| RHSIM_Rhsim09G0130800 | 11.24 | 7.99E-04 | 0.002 | -0.303 |
| RHSIM_Rhsim07G0047100 | 11.24 | 7.99E-04 | 0.002 | -0.826 |
| RHSIM_Rhsim08G0167100 | 11.23 | 8.03E-04 | 0.002 | -0.581 |
| RHSIM_Rhsim02G0054400 | 11.23 | 8.04E-04 | 0.002 | 42.954 |
| RHSIM_Rhsim02G0138200 | 11.22 | 8.07E-04 | 0.002 | 0.359 |
| RHSIM_Rhsim02G0088800 | 11.22 | 8.08E-04 | 0.002 | -2.103 |
| RHSIM_Rhsim11G0055100 | 11.22 | 8.08E-04 | 0.002 | -40.253 |
| RHSIM_Rhsim08G0062600 | 11.22 | 8.09E-04 | 0.002 | 1.579 |
| novel.4039 | 11.21 | 8.12E-04 | 0.002 | -1.162 |
| RHSIM_Rhsim03G0214000 | 11.21 | 8.13E-04 | 0.002 | -0.273 |
| RHSIM_Rhsim03G0239000 | 11.21 | 8.13E-04 | 0.002 | 0.418 |
| RHSIM_Rhsim12G0149100 | 11.21 | 8.14E-04 | 0.002 | -3.914 |
| RHSIM_Rhsim07G0078800 | 11.21 | 8.16E-04 | 0.002 | -42.789 |
| RHSIM_Rhsim04G0144300 | 11.20 | 8.16E-04 | 0.002 | 0.660 |
| RHSIM_Rhsim06G0219000 | 11.20 | 8.18E-04 | 0.002 | -0.468 |
| RHSIM_Rhsim07G0019200 | 11.20 | 8.18E-04 | 0.002 | 1.153 |
| RHSIM_RhsimUnG0225000 | 11.19 | 8.20E-04 | 0.002 | -1.548 |
| RHSIM_Rhsim10G0019800 | 11.19 | 8.21E-04 | 0.002 | -3.970 |
| RHSIM_Rhsim13G0044300 | 11.19 | 8.23E-04 | 0.002 | -0.430 |
| RHSIM_Rhsim03G0174700 | 11.19 | 8.23E-04 | 0.002 | -0.929 |

| Gene | LRT statistic | P value | BH-adjusted P | log2FC (interaction) |
|---|---|---|---|---|
| RHSIM_Rhsim08G0193700 | 11.19 | 8.23E-04 | 0.002 | 0.519 |
| RHSIM_Rhsim01G0099400 | 11.19 | 8.24E-04 | 0.002 | 4.205 |
| RHSIM_Rhsim10G0109300 | 11.19 | 8.24E-04 | 0.002 | 0.644 |
| RHSIM_Rhsim11G0145900 | 11.18 | 8.25E-04 | 0.002 | 1.142 |
| novel.3855 | 11.18 | 8.26E-04 | 0.002 | 1.138 |
| novel.3950 | 11.18 | 8.27E-04 | 0.002 | 4.283 |
| RHSIM_Rhsim13G0116600 | 11.18 | 8.27E-04 | 0.002 | 0.605 |
| RHSIM_Rhsim02G0044100 | 11.18 | 8.28E-04 | 0.002 | -0.631 |
| RHSIM_Rhsim07G0244600 | 11.18 | 8.29E-04 | 0.002 | 0.298 |
| RHSIM_Rhsim03G0073100 | 11.17 | 8.31E-04 | 0.002 | -1.624 |
| RHSIM_Rhsim05G0054400 | 11.17 | 8.31E-04 | 0.002 | -0.479 |
| RHSIM_Rhsim01G0119700 | 11.17 | 8.33E-04 | 0.002 | 0.914 |
| RHSIM_Rhsim06G0166900 | 11.16 | 8.34E-04 | 0.002 | -0.829 |
| RHSIM_Rhsim07G0191900 | 11.16 | 8.36E-04 | 0.002 | 4.536 |
| RHSIM_Rhsim02G0004600 | 11.16 | 8.37E-04 | 0.002 | 0.183 |
| RHSIM_Rhsim02G0193600 | 11.15 | 8.40E-04 | 0.002 | -0.468 |
| novel.4018 | 11.15 | 8.40E-04 | 0.002 | 1.125 |
| RHSIM_Rhsim06G0228300 | 11.15 | 8.42E-04 | 0.002 | 2.492 |
| RHSIM_Rhsim08G0186600 | 11.14 | 8.45E-04 | 0.002 | 1.321 |
| RHSIM_Rhsim02G0160200 | 11.13 | 8.48E-04 | 0.002 | 1.044 |
| RHSIM_Rhsim10G0124700 | 11.13 | 8.51E-04 | 0.002 | -0.192 |
| RHSIM_Rhsim10G0036400 | 11.12 | 8.52E-04 | 0.002 | -1.038 |
| novel.3468 | 11.12 | 8.52E-04 | 0.002 | 1.782 |
| RHSIM_Rhsim11G0049000 | 11.11 | 8.58E-04 | 0.002 | 0.371 |
| RHSIM_RhsimUnG0087300 | 11.10 | 8.64E-04 | 0.002 | 0.758 |
| RHSIM_Rhsim04G0077100 | 11.10 | 8.64E-04 | 0.002 | -1.361 |
| RHSIM_Rhsim04G0003700 | 11.10 | 8.65E-04 | 0.002 | -0.888 |
| RHSIM_RhsimUnG0144300 | 11.08 | 8.71E-04 | 0.002 | 42.513 |
| RHSIM_Rhsim08G0095400 | 11.08 | 8.71E-04 | 0.002 | -0.467 |
| RHSIM_Rhsim05G0214400 | 11.08 | 8.72E-04 | 0.002 | 1.105 |
| RHSIM_Rhsim05G0078900 | 11.08 | 8.72E-04 | 0.002 | 1.213 |
| RHSIM_Rhsim07G0040600 | 11.08 | 8.74E-04 | 0.002 | 0.454 |

| Gene | LRT statistic | P value | BH-adjusted P | log2FC (interaction) |
|---|---|---|---|---|
| novel.1279 | 11.08 | 8.74E-04 | 0.002 | -0.639 |
| RHSIM_Rhsim06G0083500 | 11.07 | 8.76E-04 | 0.002 | -2.717 |
| novel.101 | 11.07 | 8.76E-04 | 0.002 | -3.237 |
| RHSIM_Rhsim04G0219500 | 11.07 | 8.77E-04 | 0.002 | 0.344 |
| RHSIM_RhsimUnG0019500 | 11.07 | 8.77E-04 | 0.002 | 3.220 |
| RHSIM_Rhsim12G0199100 | 11.07 | 8.78E-04 | 0.002 | 3.453 |
| RHSIM_Rhsim01G0203900 | 11.07 | 8.78E-04 | 0.002 | -0.374 |
| novel.51 | 11.07 | 8.80E-04 | 0.002 | -1.145 |
| novel.1206 | 11.06 | 8.81E-04 | 0.002 | 1.804 |
| RHSIM_Rhsim07G0251800 | 11.06 | 8.82E-04 | 0.002 | 0.661 |
| RHSIM_Rhsim03G0150700 | 11.06 | 8.82E-04 | 0.002 | 0.538 |
| RHSIM_Rhsim03G0258100 | 11.06 | 8.82E-04 | 0.002 | 1.384 |
| RHSIM_Rhsim06G0115600 | 11.06 | 8.84E-04 | 0.002 | -1.689 |
| RHSIM_RhsimUnG0146900 | 11.05 | 8.85E-04 | 0.002 | -1.246 |
| RHSIM_Rhsim12G0020600 | 11.05 | 8.85E-04 | 0.002 | 2.323 |
| RHSIM_RhsimUnG0203900 | 11.05 | 8.85E-04 | 0.002 | -3.117 |
| RHSIM_Rhsim04G0237600 | 11.05 | 8.86E-04 | 0.002 | -2.398 |
| RHSIM_Rhsim02G0042500 | 11.05 | 8.87E-04 | 0.002 | 0.304 |
| RHSIM_Rhsim09G0133000 | 11.05 | 8.87E-04 | 0.002 | -1.819 |
| RHSIM_Rhsim12G0023900 | 11.05 | 8.89E-04 | 0.002 | -0.912 |
| novel.700 | 11.04 | 8.91E-04 | 0.002 | 0.606 |
| RHSIM_Rhsim03G0066800 | 11.04 | 8.92E-04 | 0.002 | 0.565 |
| novel.3784 | 11.03 | 8.94E-04 | 0.002 | 0.963 |
| novel.2617 | 11.03 | 8.95E-04 | 0.002 | 1.567 |
| RHSIM_Rhsim13G0101200 | 11.03 | 8.97E-04 | 0.002 | 0.963 |
| RHSIM_Rhsim02G0135800 | 11.03 | 8.98E-04 | 0.002 | -0.278 |
| RHSIM_Rhsim12G0024900 | 11.03 | 8.98E-04 | 0.002 | -1.248 |
| RHSIM_Rhsim11G0144600 | 11.03 | 8.99E-04 | 0.002 | -0.624 |
| RHSIM_Rhsim05G0204900 | 11.02 | 9.00E-04 | 0.002 | 1.904 |
| RHSIM_Rhsim09G0110600 | 11.02 | 9.00E-04 | 0.002 | -0.675 |
| RHSIM_Rhsim01G0224200 | 11.02 | 9.03E-04 | 0.002 | 0.520 |
| RHSIM_Rhsim08G0172900 | 11.02 | 9.03E-04 | 0.002 | -0.756 |

| Gene | LRT statistic | P value | BH-adjusted P | log2FC (interaction) |
|---|---|---|---|---|
| RHSIM_Rhsim05G0225900 | 11.01 | 9.05E-04 | 0.002 | -1.318 |
| RHSIM_Rhsim02G0148600 | 11.01 | 9.07E-04 | 0.002 | 1.026 |
| RHSIM_Rhsim08G0178800 | 11.01 | 9.08E-04 | 0.002 | 2.476 |
| RHSIM_Rhsim04G0079600 | 11.01 | 9.08E-04 | 0.002 | -3.595 |
| RHSIM_Rhsim12G0152700 | 11.00 | 9.10E-04 | 0.002 | -0.604 |
| RHSIM_Rhsim01G0103000 | 11.00 | 9.11E-04 | 0.002 | 3.344 |
| RHSIM_Rhsim09G0164000 | 11.00 | 9.13E-04 | 0.002 | -0.186 |
| RHSIM_Rhsim10G0083900 | 10.99 | 9.16E-04 | 0.002 | 4.274 |
| RHSIM_Rhsim01G0278500 | 10.99 | 9.16E-04 | 0.002 | -0.403 |
| RHSIM_Rhsim07G0161200 | 10.99 | 9.18E-04 | 0.002 | 0.910 |
| RHSIM_RhsimUnG0239100 | 10.98 | 9.19E-04 | 0.002 | -1.346 |
| RHSIM_Rhsim10G0189400 | 10.98 | 9.19E-04 | 0.002 | 4.063 |
| RHSIM_Rhsim12G0015200 | 10.98 | 9.21E-04 | 0.002 | -0.218 |
| RHSIM_Rhsim05G0205000 | 10.98 | 9.22E-04 | 0.002 | 1.965 |
| RHSIM_RhsimUnG0169200 | 10.98 | 9.22E-04 | 0.002 | -1.078 |
| RHSIM_Rhsim01G0109900 | 10.98 | 9.23E-04 | 0.002 | -0.597 |
| RHSIM_Rhsim08G0172700 | 10.97 | 9.24E-04 | 0.002 | -0.702 |
| RHSIM_Rhsim06G0213400 | 10.97 | 9.24E-04 | 0.002 | 0.627 |
| RHSIM_Rhsim08G0159500 | 10.97 | 9.24E-04 | 0.002 | 3.612 |
| RHSIM_Rhsim13G0194000 | 10.97 | 9.24E-04 | 0.002 | -0.425 |
| novel.230 | 10.97 | 9.25E-04 | 0.002 | -1.380 |
| RHSIM_RhsimUnG0026100 | 10.97 | 9.26E-04 | 0.002 | 1.556 |
| RHSIM_Rhsim01G0055300 | 10.96 | 9.29E-04 | 0.002 | -2.234 |
| RHSIM_Rhsim04G0009900 | 10.96 | 9.29E-04 | 0.002 | 0.225 |
| RHSIM_Rhsim13G0023500 | 10.96 | 9.32E-04 | 0.002 | -0.382 |
| RHSIM_Rhsim07G0179900 | 10.96 | 9.33E-04 | 0.002 | -1.160 |
| RHSIM_Rhsim08G0208200 | 10.95 | 9.34E-04 | 0.002 | -1.860 |
| RHSIM_RhsimUnG0063900 | 10.95 | 9.36E-04 | 0.002 | 0.558 |
| RHSIM_Rhsim04G0139400 | 10.95 | 9.38E-04 | 0.002 | 1.494 |
| RHSIM_Rhsim13G0099400 | 10.94 | 9.41E-04 | 0.002 | -0.603 |
| RHSIM_Rhsim04G0123100 | 10.93 | 9.44E-04 | 0.002 | -4.218 |
| RHSIM_Rhsim05G0088400 | 10.93 | 9.45E-04 | 0.002 | 0.328 |

| Gene | LRT statistic | P value | BH-adjusted P | log2FC (interaction) |
|---|---|---|---|---|
| RHSIM_Rhsim05G0186100 | 10.93 | 9.45E-04 | 0.002 | -0.461 |
| RHSIM_Rhsim08G0158800 | 10.93 | 9.46E-04 | 0.002 | 1.404 |
| RHSIM_Rhsim05G0081500 | 10.92 | 9.49E-04 | 0.002 | -0.325 |
| RHSIM_Rhsim01G0071100 | 10.92 | 9.51E-04 | 0.002 | 3.607 |
| RHSIM_Rhsim13G0197000 | 10.92 | 9.53E-04 | 0.002 | 1.265 |
| novel.1833 | 10.92 | 9.54E-04 | 0.002 | -0.763 |
| RHSIM_Rhsim13G0142900 | 10.91 | 9.55E-04 | 0.002 | 0.343 |
| RHSIM_Rhsim07G0106000 | 10.91 | 9.55E-04 | 0.002 | -0.783 |
| RHSIM_Rhsim09G0135900 | 10.91 | 9.55E-04 | 0.002 | -0.852 |
| RHSIM_Rhsim04G0247800 | 10.91 | 9.57E-04 | 0.002 | 1.594 |
| RHSIM_Rhsim10G0138400 | 10.91 | 9.58E-04 | 0.002 | 2.938 |
| RHSIM_Rhsim07G0108500 | 10.91 | 9.59E-04 | 0.002 | 0.207 |
| novel.2504 | 10.90 | 9.60E-04 | 0.002 | 1.754 |
| RHSIM_Rhsim02G0166000 | 10.90 | 9.61E-04 | 0.002 | 0.994 |
| RHSIM_Rhsim04G0116900 | 10.90 | 9.61E-04 | 0.002 | -0.393 |
| RHSIM_Rhsim10G0099700 | 10.89 | 9.65E-04 | 0.002 | -0.737 |
| RHSIM_Rhsim01G0286000 | 10.89 | 9.65E-04 | 0.002 | 2.654 |
| RHSIM_Rhsim01G0075900 | 10.89 | 9.66E-04 | 0.002 | 0.439 |
| RHSIM_Rhsim13G0221700 | 10.89 | 9.67E-04 | 0.002 | -2.334 |
| RHSIM_RhsimUnG0035000 | 10.89 | 9.67E-04 | 0.002 | 1.876 |
| RHSIM_Rhsim08G0087700 | 10.89 | 9.67E-04 | 0.002 | -1.784 |
| RHSIM_Rhsim06G0048100 | 10.89 | 9.68E-04 | 0.002 | 1.872 |
| RHSIM_Rhsim08G0074000 | 10.89 | 9.68E-04 | 0.002 | -1.245 |
| novel.3613 | 10.89 | 9.69E-04 | 0.002 | 4.625 |
| novel.977 | 10.89 | 9.69E-04 | 0.002 | 2.472 |
| RHSIM_Rhsim02G0165400 | 10.88 | 9.70E-04 | 0.002 | -0.910 |
| RHSIM_Rhsim10G0215300 | 10.88 | 9.71E-04 | 0.002 | 1.228 |
| RHSIM_Rhsim04G0056200 | 10.88 | 9.71E-04 | 0.002 | -6.202 |
| RHSIM_Rhsim07G0147900 | 10.88 | 9.73E-04 | 0.002 | 1.694 |
| novel.1494 | 10.87 | 9.77E-04 | 0.002 | -1.081 |
| RHSIM_Rhsim01G0049700 | 10.87 | 9.78E-04 | 0.002 | 0.236 |
| novel.507 | 10.87 | 9.79E-04 | 0.002 | -0.722 |

| Gene | LRT statistic | P value | BH-adjusted P | log2FC (interaction) |
|---|---|---|---|---|
| RHSIM_Rhsim04G0085000 | 10.87 | 9.80E-04 | 0.002 | -0.295 |
| novel.1083 | 10.86 | 9.80E-04 | 0.002 | -0.666 |
| RHSIM_Rhsim05G0226400 | 10.86 | 9.84E-04 | 0.002 | 0.228 |
| RHSIM_Rhsim05G0014300 | 10.86 | 9.84E-04 | 0.002 | 0.404 |
| RHSIM_Rhsim11G0188800 | 10.85 | 9.86E-04 | 0.002 | 2.052 |
| novel.2331 | 10.85 | 9.89E-04 | 0.002 | 1.050 |
| RHSIM_Rhsim01G0103900 | 10.85 | 9.90E-04 | 0.002 | -1.735 |
| RHSIM_Rhsim07G0078700 | 10.85 | 9.90E-04 | 0.002 | -2.840 |
| RHSIM_RhsimUnG0070800 | 10.84 | 9.91E-04 | 0.002 | 1.563 |
| RHSIM_Rhsim09G0140500 | 10.84 | 9.92E-04 | 0.002 | -0.683 |
| RHSIM_Rhsim07G0257000 | 10.84 | 9.92E-04 | 0.002 | 0.463 |
| novel.3949 | 10.84 | 9.95E-04 | 0.002 | -2.180 |
| novel.3836 | 10.84 | 9.95E-04 | 0.002 | 5.309 |
| RHSIM_Rhsim06G0111300 | 10.84 | 9.95E-04 | 0.002 | -3.136 |
| RHSIM_RhsimUnG0043300 | 10.84 | 9.95E-04 | 0.002 | -2.449 |
| RHSIM_Rhsim13G0143200 | 10.84 | 9.95E-04 | 0.002 | -1.739 |
| RHSIM_Rhsim04G0075800 | 10.84 | 9.96E-04 | 0.002 | 1.418 |
| RHSIM_Rhsim11G0194200 | 10.84 | 9.96E-04 | 0.002 | 0.920 |
| RHSIM_Rhsim09G0056900 | 10.83 | 9.96E-04 | 0.002 | -2.120 |
| RHSIM_RhsimUnG0004600 | 10.83 | 9.98E-04 | 0.002 | -2.027 |
| RHSIM_Rhsim02G0133400 | 10.83 | 9.98E-04 | 0.002 | 0.264 |
| novel.2924 | 10.83 | 9.99E-04 | 0.002 | 3.019 |
| novel.427 | 10.83 | 0.001 | 0.002 | 0.497 |
| novel.470 | 10.83 | 0.001 | 0.002 | -0.800 |
| RHSIM_Rhsim13G0140000 | 10.82 | 0.001 | 0.002 | 79.481 |
| RHSIM_Rhsim09G0186800 | 10.82 | 0.001 | 0.002 | 0.227 |
| RHSIM_Rhsim11G0132600 | 10.82 | 0.001 | 0.002 | -0.396 |
| RHSIM_Rhsim04G0167900 | 10.82 | 0.001 | 0.002 | -0.671 |
| RHSIM_Rhsim12G0009700 | 10.81 | 0.001 | 0.002 | 2.218 |
| RHSIM_Rhsim03G0124600 | 10.81 | 0.001 | 0.002 | 0.737 |
| RHSIM_Rhsim10G0030600 | 10.80 | 0.001 | 0.002 | -1.778 |
| RHSIM_Rhsim09G0091200 | 10.80 | 0.001 | 0.002 | 1.159 |

| Gene | LRT statistic | P value | BH-adjusted P | log2FC (interaction) |
|---|---|---|---|---|
| RHSIM_Rhsim10G0209600 | 10.80 | 0.001 | 0.002 | -0.451 |
| RHSIM_Rhsim04G0191900 | 10.80 | 0.001 | 0.002 | -2.930 |
| RHSIM_Rhsim01G0118000 | 10.80 | 0.001 | 0.002 | 0.253 |
| RHSIM_RhsimUnG0160400 | 10.80 | 0.001 | 0.002 | -3.095 |
| RHSIM_Rhsim01G0037500 | 10.80 | 0.001 | 0.002 | 1.523 |
| RHSIM_RhsimUnG0254100 | 10.80 | 0.001 | 0.002 | 1.850 |
| RHSIM_Rhsim01G0055600 | 10.79 | 0.001 | 0.002 | -0.588 |
| RHSIM_Rhsim03G0119800 | 10.79 | 0.001 | 0.002 | 1.992 |
| RHSIM_RhsimUnG0232200 | 10.79 | 0.001 | 0.002 | 1.211 |
| RHSIM_RhsimUnG0067100 | 10.79 | 0.001 | 0.002 | -0.863 |
| RHSIM_Rhsim02G0208000 | 10.79 | 0.001 | 0.002 | -0.405 |
| RHSIM_Rhsim05G0054000 | 10.79 | 0.001 | 0.002 | 0.271 |
| RHSIM_Rhsim02G0213200 | 10.78 | 0.001 | 0.002 | 0.372 |
| RHSIM_Rhsim04G0035200 | 10.78 | 0.001 | 0.002 | 1.195 |
| RHSIM_Rhsim11G0040100 | 10.78 | 0.001 | 0.002 | 0.702 |
| RHSIM_Rhsim03G0219600 | 10.78 | 0.001 | 0.002 | 0.514 |
| RHSIM_Rhsim05G0083600 | 10.78 | 0.001 | 0.002 | 1.265 |
| RHSIM_Rhsim03G0118400 | 10.78 | 0.001 | 0.002 | 0.465 |
| RHSIM_Rhsim06G0033100 | 10.77 | 0.001 | 0.002 | 0.437 |
| novel.4330 | 10.77 | 0.001 | 0.002 | -1.655 |
| RHSIM_RhsimUnG0104000 | 10.77 | 0.001 | 0.002 | 0.261 |
| RHSIM_Rhsim06G0118500 | 10.76 | 0.001 | 0.002 | -0.915 |
| RHSIM_Rhsim03G0226300 | 10.76 | 0.001 | 0.002 | -0.313 |
| RHSIM_Rhsim01G0280500 | 10.76 | 0.001 | 0.002 | 0.294 |
| RHSIM_Rhsim04G0216200 | 10.76 | 0.001 | 0.002 | -0.862 |
| novel.1644 | 10.76 | 0.001 | 0.002 | 2.058 |
| RHSIM_Rhsim11G0014300 | 10.76 | 0.001 | 0.002 | 0.900 |
| RHSIM_Rhsim10G0150000 | 10.75 | 0.001 | 0.002 | 1.017 |
| RHSIM_Rhsim01G0273900 | 10.75 | 0.001 | 0.002 | 0.381 |
| novel.3771 | 10.75 | 0.001 | 0.002 | -2.148 |
| RHSIM_Rhsim06G0140400 | 10.75 | 0.001 | 0.002 | 79.598 |
| RHSIM_Rhsim08G0041400 | 10.75 | 0.001 | 0.002 | -0.735 |

| Gene | LRT statistic | P value | BH-adjusted P | log2FC (interaction) |
|---|---|---|---|---|
| RHSIM_Rhsim01G0201900 | 10.75 | 0.001 | 0.002 | 2.704 |
| RHSIM_Rhsim03G0203100 | 10.74 | 0.001 | 0.002 | 0.636 |
| RHSIM_Rhsim11G0022000 | 10.74 | 0.001 | 0.002 | -0.305 |
| RHSIM_Rhsim01G0268500 | 10.74 | 0.001 | 0.002 | -0.788 |
| RHSIM_Rhsim12G0131600 | 10.74 | 0.001 | 0.002 | 1.190 |
| RHSIM_Rhsim11G0101400 | 10.74 | 0.001 | 0.002 | -1.950 |
| novel.625 | 10.73 | 0.001 | 0.002 | 3.975 |
| RHSIM_Rhsim01G0208600 | 10.73 | 0.001 | 0.002 | -0.227 |
| RHSIM_Rhsim12G0154900 | 10.73 | 0.001 | 0.002 | -42.126 |
| RHSIM_Rhsim01G0104200 | 10.73 | 0.001 | 0.002 | 1.310 |
| RHSIM_Rhsim10G0080700 | 10.73 | 0.001 | 0.002 | -0.771 |
| RHSIM_Rhsim03G0245400 | 10.73 | 0.001 | 0.002 | 0.226 |
| RHSIM_Rhsim05G0129000 | 10.72 | 0.001 | 0.002 | -1.404 |
| RHSIM_Rhsim02G0086900 | 10.71 | 0.001 | 0.002 | 0.337 |
| RHSIM_Rhsim11G0097500 | 10.71 | 0.001 | 0.002 | 0.211 |
| RHSIM_Rhsim04G0161700 | 10.70 | 0.001 | 0.002 | 1.443 |
| RHSIM_Rhsim01G0291300 | 10.70 | 0.001 | 0.002 | 1.611 |
| RHSIM_Rhsim11G0192300 | 10.70 | 0.001 | 0.002 | 0.680 |
| RHSIM_Rhsim11G0001600 | 10.70 | 0.001 | 0.002 | 0.670 |
| RHSIM_Rhsim06G0153000 | 10.70 | 0.001 | 0.002 | 3.988 |
| RHSIM_RhsimUnG0167700 | 10.69 | 0.001 | 0.002 | -42.993 |
| novel.1759 | 10.68 | 0.001 | 0.002 | 3.649 |
| novel.4287 | 10.68 | 0.001 | 0.002 | 1.648 |
| RHSIM_Rhsim08G0102800 | 10.68 | 0.001 | 0.002 | -0.648 |
| RHSIM_Rhsim13G0018600 | 10.67 | 0.001 | 0.002 | 0.214 |
| RHSIM_Rhsim13G0201000 | 10.67 | 0.001 | 0.002 | -0.701 |
| RHSIM_Rhsim11G0123600 | 10.67 | 0.001 | 0.002 | 0.346 |
| novel.1335 | 10.67 | 0.001 | 0.002 | -1.255 |
| RHSIM_Rhsim06G0023400 | 10.67 | 0.001 | 0.002 | 0.488 |
| RHSIM_Rhsim11G0126200 | 10.66 | 0.001 | 0.002 | -0.408 |
| RHSIM_Rhsim08G0168500 | 10.66 | 0.001 | 0.002 | -0.575 |
| RHSIM_Rhsim07G0011900 | 10.66 | 0.001 | 0.002 | -1.506 |

| Gene | LRT statistic | P value | BH-adjusted P | log2FC (interaction) |
|---|---|---|---|---|
| RHSIM_Rhsim05G0103300 | 10.66 | 0.001 | 0.002 | -0.170 |
| RHSIM_Rhsim13G0135900 | 10.66 | 0.001 | 0.002 | 2.172 |
| RHSIM_Rhsim06G0007100 | 10.65 | 0.001 | 0.002 | 1.078 |
| RHSIM_Rhsim10G0136100 | 10.65 | 0.001 | 0.002 | 2.119 |
| RHSIM_Rhsim10G0176400 | 10.65 | 0.001 | 0.002 | 1.129 |
| RHSIM_Rhsim07G0197800 | 10.65 | 0.001 | 0.002 | 3.768 |
| novel.498 | 10.65 | 0.001 | 0.002 | -1.177 |
| RHSIM_Rhsim03G0186300 | 10.64 | 0.001 | 0.002 | -1.212 |
| RHSIM_Rhsim05G0017500 | 10.64 | 0.001 | 0.002 | -0.943 |
| RHSIM_Rhsim09G0085200 | 10.64 | 0.001 | 0.002 | 1.030 |
| RHSIM_Rhsim09G0208600 | 10.64 | 0.001 | 0.002 | -0.766 |
| RHSIM_Rhsim08G0146500 | 10.63 | 0.001 | 0.003 | -1.969 |
| RHSIM_RhsimUnG0057600 | 10.63 | 0.001 | 0.003 | -40.331 |
| RHSIM_Rhsim11G0021200 | 10.63 | 0.001 | 0.003 | 0.739 |
| RHSIM_Rhsim04G0177300 | 10.63 | 0.001 | 0.003 | -4.428 |
| RHSIM_Rhsim08G0181600 | 10.63 | 0.001 | 0.003 | -0.510 |
| RHSIM_Rhsim04G0117100 | 10.62 | 0.001 | 0.003 | 0.478 |
| RHSIM_Rhsim02G0231300 | 10.62 | 0.001 | 0.003 | 0.822 |
| novel.344 | 10.62 | 0.001 | 0.003 | -0.874 |
| RHSIM_Rhsim12G0026400 | 10.62 | 0.001 | 0.003 | -1.347 |
| RHSIM_Rhsim07G0153100 | 10.62 | 0.001 | 0.003 | -0.261 |
| novel.2690 | 10.62 | 0.001 | 0.003 | -1.901 |
| RHSIM_Rhsim04G0207500 | 10.62 | 0.001 | 0.003 | -0.696 |
| RHSIM_Rhsim11G0006900 | 10.61 | 0.001 | 0.003 | -0.353 |
| RHSIM_Rhsim07G0124500 | 10.61 | 0.001 | 0.003 | 0.448 |
| RHSIM_Rhsim03G0102000 | 10.61 | 0.001 | 0.003 | -0.327 |
| novel.823 | 10.61 | 0.001 | 0.003 | -0.633 |
| RHSIM_RhsimUnG0052000 | 10.60 | 0.001 | 0.003 | -1.707 |
| RHSIM_Rhsim05G0037400 | 10.60 | 0.001 | 0.003 | -1.143 |
| RHSIM_Rhsim03G0198700 | 10.59 | 0.001 | 0.003 | 0.518 |
| RHSIM_Rhsim04G0049700 | 10.59 | 0.001 | 0.003 | 0.468 |
| RHSIM_Rhsim13G0045500 | 10.59 | 0.001 | 0.003 | 0.459 |

| Gene | LRT statistic | P value | BH-adjusted P | log2FC (interaction) |
|---|---|---|---|---|
| novel.1378 | 10.59 | 0.001 | 0.003 | 0.823 |
| RHSIM_Rhsim07G0069700 | 10.58 | 0.001 | 0.003 | -1.577 |
| novel.2729 | 10.58 | 0.001 | 0.003 | -1.744 |
| RHSIM_Rhsim07G0098200 | 10.58 | 0.001 | 0.003 | 0.579 |
| novel.351 | 10.58 | 0.001 | 0.003 | 0.292 |
| novel.3624 | 10.58 | 0.001 | 0.003 | 42.690 |
| novel.3188 | 10.58 | 0.001 | 0.003 | -1.657 |
| RHSIM_Rhsim08G0082100 | 10.57 | 0.001 | 0.003 | 0.244 |
| novel.1654 | 10.57 | 0.001 | 0.003 | 1.076 |
| RHSIM_Rhsim13G0056400 | 10.57 | 0.001 | 0.003 | -0.296 |
| RHSIM_Rhsim02G0214700 | 10.57 | 0.001 | 0.003 | -40.011 |
| RHSIM_Rhsim09G0069300 | 10.56 | 0.001 | 0.003 | -0.330 |
| RHSIM_Rhsim13G0103900 | 10.56 | 0.001 | 0.003 | -1.465 |
| RHSIM_Rhsim04G0236300 | 10.56 | 0.001 | 0.003 | -0.989 |
| RHSIM_Rhsim10G0173600 | 10.55 | 0.001 | 0.003 | 0.692 |
| novel.1495 | 10.54 | 0.001 | 0.003 | -3.356 |
| novel.1814 | 10.54 | 0.001 | 0.003 | -1.707 |
| RHSIM_RhsimUnG0024800 | 10.54 | 0.001 | 0.003 | 44.487 |
| RHSIM_Rhsim07G0004700 | 10.54 | 0.001 | 0.003 | -0.335 |
| RHSIM_Rhsim05G0135500 | 10.54 | 0.001 | 0.003 | 0.478 |
| novel.2023 | 10.53 | 0.001 | 0.003 | -1.530 |
| RHSIM_Rhsim12G0149400 | 10.53 | 0.001 | 0.003 | 0.371 |
| novel.1323 | 10.53 | 0.001 | 0.003 | 2.114 |
| RHSIM_Rhsim04G0184500 | 10.53 | 0.001 | 0.003 | 1.589 |
| RHSIM_Rhsim01G0091100 | 10.53 | 0.001 | 0.003 | -0.597 |
| RHSIM_Rhsim03G0241300 | 10.52 | 0.001 | 0.003 | 1.077 |
| novel.466 | 10.52 | 0.001 | 0.003 | 0.999 |
| RHSIM_Rhsim06G0095700 | 10.52 | 0.001 | 0.003 | -3.487 |
| RHSIM_Rhsim04G0059400 | 10.52 | 0.001 | 0.003 | -44.346 |
| novel.4245 | 10.51 | 0.001 | 0.003 | -0.351 |
| RHSIM_Rhsim03G0100700 | 10.51 | 0.001 | 0.003 | 2.042 |
| RHSIM_Rhsim02G0034800 | 10.51 | 0.001 | 0.003 | 0.563 |

| Gene | LRT statistic | P value | BH-adjusted P | log2FC (interaction) |
|---|---|---|---|---|
| RHSIM_Rhsim12G0169400 | 10.51 | 0.001 | 0.003 | 4.056 |
| RHSIM_Rhsim13G0107500 | 10.51 | 0.001 | 0.003 | -1.363 |
| novel.2371 | 10.51 | 0.001 | 0.003 | 0.943 |
| RHSIM_Rhsim12G0137600 | 10.50 | 0.001 | 0.003 | -0.230 |
| novel.1 | 10.50 | 0.001 | 0.003 | -0.772 |
| RHSIM_RhsimUnG0145200 | 10.50 | 0.001 | 0.003 | -0.899 |
| RHSIM_Rhsim08G0155700 | 10.50 | 0.001 | 0.003 | -4.938 |
| RHSIM_Rhsim09G0067100 | 10.49 | 0.001 | 0.003 | 0.346 |
| RHSIM_Rhsim12G0098800 | 10.49 | 0.001 | 0.003 | 2.866 |
| RHSIM_RhsimUnG0036700 | 10.49 | 0.001 | 0.003 | -2.510 |
| RHSIM_Rhsim02G0095100 | 10.49 | 0.001 | 0.003 | -0.770 |
| RHSIM_Rhsim07G0053900 | 10.49 | 0.001 | 0.003 | -1.454 |
| RHSIM_Rhsim05G0096700 | 10.48 | 0.001 | 0.003 | -3.225 |
| novel.1322 | 10.48 | 0.001 | 0.003 | 1.609 |
| RHSIM_Rhsim12G0163300 | 10.48 | 0.001 | 0.003 | -0.763 |
| novel.860 | 10.48 | 0.001 | 0.003 | -2.565 |
| novel.1857 | 10.48 | 0.001 | 0.003 | -41.079 |
| RHSIM_Rhsim01G0116500 | 10.47 | 0.001 | 0.003 | -1.849 |
| RHSIM_Rhsim04G0033500 | 10.47 | 0.001 | 0.003 | 0.594 |
| RHSIM_Rhsim06G0195100 | 10.47 | 0.001 | 0.003 | 0.324 |
| novel.2560 | 10.47 | 0.001 | 0.003 | 2.372 |
| RHSIM_Rhsim08G0024900 | 10.47 | 0.001 | 0.003 | 0.825 |
| RHSIM_Rhsim10G0182800 | 10.47 | 0.001 | 0.003 | 0.316 |
| novel.1454 | 10.47 | 0.001 | 0.003 | 0.450 |
| RHSIM_RhsimUnG0197500 | 10.46 | 0.001 | 0.003 | -0.911 |
| RHSIM_Rhsim13G0228700 | 10.46 | 0.001 | 0.003 | -1.391 |
| RHSIM_Rhsim03G0244600 | 10.45 | 0.001 | 0.003 | -0.444 |
| RHSIM_Rhsim09G0126100 | 10.45 | 0.001 | 0.003 | -0.847 |
| RHSIM_Rhsim12G0029700 | 10.45 | 0.001 | 0.003 | -40.664 |
| RHSIM_Rhsim03G0151700 | 10.45 | 0.001 | 0.003 | -2.094 |
| RHSIM_Rhsim12G0008600 | 10.45 | 0.001 | 0.003 | -0.723 |
| RHSIM_Rhsim13G0151100 | 10.45 | 0.001 | 0.003 | 0.273 |

| Gene | LRT statistic | P value | BH-adjusted P | log2FC (interaction) |
|---|---|---|---|---|
| RHSIM_Rhsim04G0173600 | 10.44 | 0.001 | 0.003 | -0.567 |
| RHSIM_Rhsim12G0076800 | 10.44 | 0.001 | 0.003 | 3.139 |
| RHSIM_RhsimUnG0237000 | 10.44 | 0.001 | 0.003 | 1.302 |
| RHSIM_Rhsim10G0136500 | 10.43 | 0.001 | 0.003 | 0.718 |
| RHSIM_Rhsim08G0098900 | 10.42 | 0.001 | 0.003 | -0.720 |
| RHSIM_RhsimUnG0147100 | 10.42 | 0.001 | 0.003 | -1.761 |
| RHSIM_Rhsim05G0173100 | 10.42 | 0.001 | 0.003 | 1.663 |
| RHSIM_Rhsim08G0026100 | 10.42 | 0.001 | 0.003 | -0.396 |
| RHSIM_Rhsim10G0051700 | 10.42 | 0.001 | 0.003 | 0.489 |
| novel.2068 | 10.42 | 0.001 | 0.003 | -0.489 |
| RHSIM_Rhsim10G0073700 | 10.41 | 0.001 | 0.003 | 1.183 |
| RHSIM_Rhsim01G0207900 | 10.41 | 0.001 | 0.003 | -1.207 |
| RHSIM_Rhsim05G0096500 | 10.40 | 0.001 | 0.003 | 0.825 |
| novel.3049 | 10.40 | 0.001 | 0.003 | -0.532 |
| RHSIM_Rhsim07G0221600 | 10.40 | 0.001 | 0.003 | -0.980 |
| novel.474 | 10.39 | 0.001 | 0.003 | 5.522 |
| RHSIM_Rhsim04G0046700 | 10.39 | 0.001 | 0.003 | 0.887 |
| novel.83 | 10.39 | 0.001 | 0.003 | -1.681 |
| RHSIM_Rhsim02G0159400 | 10.38 | 0.001 | 0.003 | 0.419 |
| RHSIM_Rhsim03G0115400 | 10.38 | 0.001 | 0.003 | 1.891 |
| RHSIM_Rhsim08G0136000 | 10.38 | 0.001 | 0.003 | 0.470 |
| RHSIM_Rhsim02G0198400 | 10.38 | 0.001 | 0.003 | -1.442 |
| RHSIM_Rhsim11G0063500 | 10.38 | 0.001 | 0.003 | 5.239 |
| RHSIM_Rhsim01G0116800 | 10.38 | 0.001 | 0.003 | 0.467 |
| novel.3839 | 10.37 | 0.001 | 0.003 | -0.553 |
| RHSIM_Rhsim08G0142600 | 10.37 | 0.001 | 0.003 | 0.506 |
| RHSIM_Rhsim08G0135200 | 10.37 | 0.001 | 0.003 | -0.737 |
| RHSIM_Rhsim11G0181200 | 10.37 | 0.001 | 0.003 | -2.811 |
| RHSIM_Rhsim08G0037400 | 10.37 | 0.001 | 0.003 | 0.580 |
| RHSIM_Rhsim11G0069600 | 10.37 | 0.001 | 0.003 | -0.411 |
| novel.3854 | 10.37 | 0.001 | 0.003 | -4.416 |
| RHSIM_Rhsim03G0134900 | 10.36 | 0.001 | 0.003 | 2.209 |

| Gene | LRT statistic | P value | BH-adjusted P | log2FC (interaction) |
|---|---|---|---|---|
| RHSIM_Rhsim09G0145300 | 10.36 | 0.001 | 0.003 | 0.591 |
| RHSIM_Rhsim03G0057700 | 10.36 | 0.001 | 0.003 | 1.301 |
| novel.948 | 10.36 | 0.001 | 0.003 | -2.131 |
| RHSIM_Rhsim07G0250200 | 10.35 | 0.001 | 0.003 | 0.215 |
| RHSIM_Rhsim13G0189500 | 10.35 | 0.001 | 0.003 | 2.059 |
| RHSIM_Rhsim03G0120600 | 10.35 | 0.001 | 0.003 | -0.398 |
| novel.4482 | 10.35 | 0.001 | 0.003 | 2.396 |
| RHSIM_Rhsim04G0215400 | 10.35 | 0.001 | 0.003 | -0.286 |
| RHSIM_Rhsim06G0124400 | 10.35 | 0.001 | 0.003 | 0.738 |
| novel.846 | 10.35 | 0.001 | 0.003 | 2.788 |
| RHSIM_Rhsim03G0228300 | 10.34 | 0.001 | 0.003 | 0.976 |
| novel.3728 | 10.34 | 0.001 | 0.003 | 1.367 |
| RHSIM_Rhsim01G0240600 | 10.34 | 0.001 | 0.003 | -0.473 |
| RHSIM_Rhsim03G0161200 | 10.34 | 0.001 | 0.003 | -0.519 |
| RHSIM_Rhsim07G0096800 | 10.33 | 0.001 | 0.003 | -0.606 |
| RHSIM_Rhsim03G0130500 | 10.33 | 0.001 | 0.003 | -1.073 |
| RHSIM_Rhsim06G0100500 | 10.33 | 0.001 | 0.003 | 0.256 |
| RHSIM_Rhsim04G0224400 | 10.33 | 0.001 | 0.003 | -0.451 |
| RHSIM_Rhsim03G0092600 | 10.33 | 0.001 | 0.003 | 0.308 |
| RHSIM_Rhsim11G0042300 | 10.33 | 0.001 | 0.003 | -0.708 |
| RHSIM_Rhsim06G0207700 | 10.33 | 0.001 | 0.003 | -0.785 |
| RHSIM_Rhsim01G0216000 | 10.32 | 0.001 | 0.003 | -0.302 |
| novel.3258 | 10.32 | 0.001 | 0.003 | -4.240 |
| RHSIM_Rhsim12G0137400 | 10.31 | 0.001 | 0.003 | -0.447 |
| RHSIM_Rhsim01G0232600 | 10.31 | 0.001 | 0.003 | -0.334 |
| RHSIM_Rhsim11G0107300 | 10.31 | 0.001 | 0.003 | -0.298 |
| RHSIM_Rhsim12G0023000 | 10.30 | 0.001 | 0.003 | -0.705 |
| RHSIM_Rhsim04G0207400 | 10.30 | 0.001 | 0.003 | -0.357 |
| RHSIM_Rhsim03G0254600 | 10.30 | 0.001 | 0.003 | 0.498 |
| RHSIM_Rhsim11G0118600 | 10.29 | 0.001 | 0.003 | 0.487 |
| RHSIM_Rhsim02G0002600 | 10.29 | 0.001 | 0.003 | 3.383 |
| RHSIM_Rhsim03G0224300 | 10.29 | 0.001 | 0.003 | 0.547 |

| Gene | LRT statistic | P value | BH-adjusted P | log2FC (interaction) |
|---|---|---|---|---|
| RHSIM_Rhsim08G0012600 | 10.29 | 0.001 | 0.003 | 0.517 |
| RHSIM_Rhsim07G0253500 | 10.29 | 0.001 | 0.003 | -0.607 |
| RHSIM_Rhsim12G0068200 | 10.29 | 0.001 | 0.003 | 0.733 |
| RHSIM_RhsimUnG0035600 | 10.28 | 0.001 | 0.003 | 0.539 |
| RHSIM_Rhsim09G0200300 | 10.28 | 0.001 | 0.003 | -0.903 |
| RHSIM_Rhsim07G0184000 | 10.28 | 0.001 | 0.003 | 0.820 |
| RHSIM_Rhsim01G0160000 | 10.28 | 0.001 | 0.003 | 0.435 |
| RHSIM_Rhsim01G0115200 | 10.28 | 0.001 | 0.003 | 42.752 |
| RHSIM_Rhsim04G0008900 | 10.28 | 0.001 | 0.003 | 4.309 |
| RHSIM_Rhsim02G0000300 | 10.28 | 0.001 | 0.003 | 0.418 |
| RHSIM_Rhsim06G0001400 | 10.27 | 0.001 | 0.003 | 0.142 |
| novel.936 | 10.27 | 0.001 | 0.003 | 1.720 |
| RHSIM_Rhsim06G0211900 | 10.27 | 0.001 | 0.003 | 1.090 |
| novel.4372 | 10.27 | 0.001 | 0.003 | 0.934 |
| RHSIM_Rhsim11G0049500 | 10.26 | 0.001 | 0.003 | 0.389 |
| RHSIM_Rhsim13G0061400 | 10.26 | 0.001 | 0.003 | -0.577 |
| RHSIM_Rhsim06G0197100 | 10.26 | 0.001 | 0.003 | -0.930 |
| RHSIM_Rhsim06G0067600 | 10.25 | 0.001 | 0.003 | -0.812 |
| RHSIM_Rhsim02G0004100 | 10.25 | 0.001 | 0.003 | 4.295 |
| RHSIM_Rhsim02G0029500 | 10.25 | 0.001 | 0.003 | 2.394 |
| RHSIM_Rhsim04G0044700 | 10.24 | 0.001 | 0.003 | -1.764 |
| RHSIM_Rhsim03G0049100 | 10.24 | 0.001 | 0.003 | -0.590 |
| RHSIM_Rhsim02G0246400 | 10.24 | 0.001 | 0.003 | 1.848 |
| RHSIM_Rhsim10G0021000 | 10.24 | 0.001 | 0.003 | -0.369 |
| RHSIM_Rhsim13G0028200 | 10.24 | 0.001 | 0.003 | -1.101 |
| RHSIM_Rhsim08G0048900 | 10.23 | 0.001 | 0.003 | 0.797 |
| RHSIM_Rhsim09G0179900 | 10.23 | 0.001 | 0.003 | -0.248 |
| RHSIM_Rhsim09G0101600 | 10.23 | 0.001 | 0.003 | -2.550 |
| RHSIM_Rhsim01G0262900 | 10.23 | 0.001 | 0.003 | 0.342 |
| RHSIM_Rhsim08G0113500 | 10.23 | 0.001 | 0.003 | -0.302 |
| RHSIM_Rhsim12G0165600 | 10.23 | 0.001 | 0.003 | -0.334 |
| RHSIM_Rhsim08G0053400 | 10.22 | 0.001 | 0.003 | 0.407 |

| Gene | LRT statistic | P value | BH-adjusted P | log2FC (interaction) |
|---|---|---|---|---|
| novel.981 | 10.21 | 0.001 | 0.003 | 1.894 |
| RHSIM_Rhsim02G0062300 | 10.21 | 0.001 | 0.003 | -0.574 |
| RHSIM_Rhsim09G0000100 | 10.21 | 0.001 | 0.003 | -0.365 |
| RHSIM_RhsimUnG0190100 | 10.21 | 0.001 | 0.003 | 0.926 |
| RHSIM_Rhsim12G0031400 | 10.21 | 0.001 | 0.003 | -0.532 |
| RHSIM_Rhsim08G0026900 | 10.21 | 0.001 | 0.003 | -0.542 |
| RHSIM_Rhsim03G0159700 | 10.20 | 0.001 | 0.003 | 0.301 |
| RHSIM_Rhsim08G0176500 | 10.20 | 0.001 | 0.003 | 0.271 |
| RHSIM_Rhsim02G0187500 | 10.20 | 0.001 | 0.003 | 0.269 |
| RHSIM_Rhsim07G0138200 | 10.20 | 0.001 | 0.003 | -0.933 |
| RHSIM_Rhsim02G0243200 | 10.20 | 0.001 | 0.003 | -1.503 |
| RHSIM_Rhsim09G0152700 | 10.20 | 0.001 | 0.003 | -0.331 |
| RHSIM_Rhsim01G0220100 | 10.20 | 0.001 | 0.003 | -1.053 |
| novel.3316 | 10.19 | 0.001 | 0.003 | 0.317 |
| novel.210 | 10.19 | 0.001 | 0.003 | 40.748 |
| RHSIM_Rhsim01G0068800 | 10.19 | 0.001 | 0.003 | 0.165 |
| RHSIM_Rhsim09G0192200 | 10.19 | 0.001 | 0.003 | -1.548 |
| RHSIM_Rhsim08G0231600 | 10.18 | 0.001 | 0.003 | 0.382 |
| RHSIM_Rhsim09G0117800 | 10.18 | 0.001 | 0.003 | 0.919 |
| novel.3306 | 10.18 | 0.001 | 0.003 | 3.983 |
| RHSIM_Rhsim04G0127500 | 10.18 | 0.001 | 0.003 | 1.663 |
| RHSIM_Rhsim02G0137800 | 10.18 | 0.001 | 0.003 | -0.560 |
| RHSIM_Rhsim06G0187600 | 10.17 | 0.001 | 0.003 | 0.770 |
| RHSIM_Rhsim12G0183200 | 10.17 | 0.001 | 0.003 | 1.559 |
| RHSIM_Rhsim07G0047900 | 10.17 | 0.001 | 0.003 | -0.375 |
| RHSIM_Rhsim05G0106900 | 10.17 | 0.001 | 0.003 | -0.348 |
| RHSIM_Rhsim12G0054400 | 10.16 | 0.001 | 0.003 | -0.593 |
| RHSIM_Rhsim12G0171000 | 10.16 | 0.001 | 0.003 | 0.431 |
| RHSIM_Rhsim08G0095100 | 10.15 | 0.001 | 0.003 | 0.471 |
| RHSIM_Rhsim06G0139100 | 10.15 | 0.001 | 0.003 | 1.277 |
| RHSIM_Rhsim13G0041800 | 10.15 | 0.001 | 0.003 | 0.558 |
| RHSIM_Rhsim11G0044400 | 10.15 | 0.001 | 0.003 | -4.146 |

| Gene | LRT statistic | P value | BH-adjusted P | log2FC (interaction) |
|---|---|---|---|---|
| RHSIM_Rhsim05G0164100 | 10.15 | 0.001 | 0.003 | -3.350 |
| RHSIM_Rhsim04G0137200 | 10.15 | 0.001 | 0.003 | 5.072 |
| novel.1474 | 10.14 | 0.001 | 0.003 | 2.684 |
| novel.2707 | 10.14 | 0.001 | 0.003 | 0.398 |
| RHSIM_Rhsim11G0084400 | 10.14 | 0.001 | 0.003 | -0.759 |
| RHSIM_Rhsim02G0124600 | 10.14 | 0.001 | 0.003 | -0.584 |
| novel.2840 | 10.13 | 0.001 | 0.003 | 1.162 |
| RHSIM_Rhsim09G0098300 | 10.13 | 0.001 | 0.003 | -0.910 |
| RHSIM_Rhsim04G0039800 | 10.13 | 0.001 | 0.003 | -1.107 |
| RHSIM_Rhsim01G0161100 | 10.13 | 0.001 | 0.003 | -1.570 |
| novel.3307 | 10.13 | 0.001 | 0.003 | -0.752 |
| RHSIM_Rhsim11G0006500 | 10.13 | 0.001 | 0.003 | 0.454 |
| RHSIM_Rhsim13G0161700 | 10.12 | 0.001 | 0.003 | 0.353 |
| RHSIM_Rhsim11G0035400 | 10.12 | 0.001 | 0.003 | -1.343 |
| RHSIM_Rhsim07G0126200 | 10.12 | 0.001 | 0.003 | -0.218 |
| RHSIM_Rhsim01G0101200 | 10.12 | 0.001 | 0.003 | 2.513 |
| RHSIM_Rhsim07G0004300 | 10.12 | 0.001 | 0.003 | -0.158 |
| RHSIM_Rhsim09G0141900 | 10.11 | 0.001 | 0.003 | 0.317 |
| RHSIM_Rhsim07G0177500 | 10.11 | 0.001 | 0.003 | 42.420 |
| RHSIM_Rhsim08G0227200 | 10.11 | 0.001 | 0.003 | 0.779 |
| RHSIM_Rhsim04G0239900 | 10.11 | 0.001 | 0.003 | 79.325 |
| RHSIM_Rhsim06G0114000 | 10.11 | 0.001 | 0.003 | 1.120 |
| RHSIM_Rhsim01G0087800 | 10.11 | 0.001 | 0.003 | 3.061 |
| RHSIM_Rhsim10G0137200 | 10.11 | 0.001 | 0.003 | -1.002 |
| RHSIM_Rhsim03G0100600 | 10.11 | 0.001 | 0.003 | 2.323 |
| RHSIM_Rhsim03G0059000 | 10.10 | 0.001 | 0.003 | -0.905 |
| novel.226 | 10.10 | 0.001 | 0.003 | -2.143 |
| RHSIM_Rhsim02G0147500 | 10.10 | 0.001 | 0.003 | 0.295 |
| novel.2256 | 10.10 | 0.001 | 0.003 | 0.415 |
| novel.2790 | 10.10 | 0.001 | 0.003 | 4.156 |
| RHSIM_Rhsim13G0147700 | 10.10 | 0.001 | 0.003 | -0.684 |
| RHSIM_Rhsim02G0053600 | 10.09 | 0.001 | 0.003 | -0.392 |

| Gene | LRT statistic | P value | BH-adjusted P | log2FC (interaction) |
|---|---|---|---|---|
| RHSIM_Rhsim03G0090500 | 10.09 | 0.001 | 0.003 | -0.534 |
| RHSIM_Rhsim02G0256700 | 10.09 | 0.001 | 0.003 | 0.453 |
| RHSIM_Rhsim08G0007100 | 10.09 | 0.001 | 0.003 | -0.715 |
| RHSIM_Rhsim12G0213900 | 10.08 | 0.001 | 0.003 | -0.494 |
| RHSIM_RhsimUnG0166200 | 10.07 | 0.002 | 0.003 | 1.588 |
| RHSIM_Rhsim02G0149000 | 10.07 | 0.002 | 0.003 | 1.271 |
| novel.4392 | 10.07 | 0.002 | 0.003 | 0.803 |
| RHSIM_Rhsim09G0066600 | 10.07 | 0.002 | 0.003 | -0.607 |
| RHSIM_Rhsim08G0183500 | 10.07 | 0.002 | 0.003 | 1.251 |
| RHSIM_Rhsim08G0184400 | 10.07 | 0.002 | 0.003 | -0.518 |
| RHSIM_Rhsim07G0200300 | 10.07 | 0.002 | 0.003 | -0.287 |
| RHSIM_Rhsim08G0213100 | 10.06 | 0.002 | 0.003 | 0.845 |
| RHSIM_Rhsim03G0001100 | 10.05 | 0.002 | 0.003 | 0.505 |
| RHSIM_Rhsim06G0041200 | 10.04 | 0.002 | 0.003 | -0.354 |
| RHSIM_RhsimUnG0073100 | 10.04 | 0.002 | 0.003 | -0.623 |
| novel.501 | 10.04 | 0.002 | 0.003 | 1.362 |
| RHSIM_Rhsim02G0041100 | 10.03 | 0.002 | 0.003 | -1.189 |
| novel.247 | 10.03 | 0.002 | 0.003 | -0.214 |
| novel.4219 | 10.03 | 0.002 | 0.003 | 1.168 |
| RHSIM_Rhsim04G0232600 | 10.02 | 0.002 | 0.003 | -0.346 |
| RHSIM_Rhsim06G0212600 | 10.02 | 0.002 | 0.003 | -0.693 |
| RHSIM_Rhsim01G0091000 | 10.02 | 0.002 | 0.003 | 0.314 |
| RHSIM_Rhsim02G0004800 | 10.02 | 0.002 | 0.003 | 5.250 |
| RHSIM_Rhsim02G0188300 | 10.02 | 0.002 | 0.003 | 1.695 |
| RHSIM_Rhsim03G0050700 | 10.01 | 0.002 | 0.003 | 1.420 |
| RHSIM_Rhsim10G0143300 | 10.01 | 0.002 | 0.003 | -4.255 |
| RHSIM_Rhsim10G0071100 | 10.01 | 0.002 | 0.003 | -1.126 |
| RHSIM_Rhsim13G0159500 | 10.01 | 0.002 | 0.003 | -0.296 |
| novel.1608 | 10.01 | 0.002 | 0.003 | -2.114 |
| RHSIM_Rhsim03G0221500 | 10.01 | 0.002 | 0.003 | 0.555 |
| RHSIM_Rhsim06G0242000 | 10.01 | 0.002 | 0.003 | 5.463 |
| RHSIM_Rhsim02G0236000 | 10.00 | 0.002 | 0.003 | -3.736 |

| Gene | LRT statistic | P value | BH-adjusted P | log2FC (interaction) |
|---|---|---|---|---|
| novel.1542 | 10.00 | 0.002 | 0.003 | 1.408 |
| RHSIM_Rhsim04G0234900 | 10.00 | 0.002 | 0.003 | -0.595 |
| RHSIM_Rhsim09G0090700 | 10.00 | 0.002 | 0.003 | -0.589 |
| RHSIM_RhsimUnG0178200 | 10.00 | 0.002 | 0.003 | 0.847 |
| RHSIM_Rhsim12G0137300 | 10.00 | 0.002 | 0.003 | -0.297 |
| RHSIM_Rhsim02G0041400 | 10.00 | 0.002 | 0.003 | 2.737 |
| RHSIM_Rhsim13G0146500 | 9.99 | 0.002 | 0.003 | 0.686 |
| RHSIM_Rhsim01G0135100 | 9.99 | 0.002 | 0.003 | 0.726 |
| novel.1663 | 9.99 | 0.002 | 0.003 | 1.387 |
| RHSIM_Rhsim01G0122500 | 9.99 | 0.002 | 0.003 | 1.028 |
| RHSIM_Rhsim05G0201300 | 9.98 | 0.002 | 0.003 | -0.834 |
| RHSIM_Rhsim06G0019600 | 9.98 | 0.002 | 0.003 | 1.443 |
| novel.3681 | 9.98 | 0.002 | 0.004 | -4.183 |
| RHSIM_Rhsim08G0240900 | 9.98 | 0.002 | 0.004 | 2.889 |
| RHSIM_Rhsim13G0011400 | 9.98 | 0.002 | 0.004 | 0.211 |
| RHSIM_Rhsim13G0040900 | 9.97 | 0.002 | 0.004 | -0.530 |
| RHSIM_Rhsim11G0162800 | 9.97 | 0.002 | 0.004 | -4.019 |
| RHSIM_Rhsim10G0183800 | 9.97 | 0.002 | 0.004 | 0.523 |
| RHSIM_Rhsim03G0121200 | 9.97 | 0.002 | 0.004 | -0.332 |
| RHSIM_Rhsim01G0222800 | 9.97 | 0.002 | 0.004 | -1.033 |
| RHSIM_Rhsim07G0079800 | 9.96 | 0.002 | 0.004 | -0.257 |
| RHSIM_RhsimUnG0005000 | 9.96 | 0.002 | 0.004 | -1.017 |
| RHSIM_Rhsim02G0215300 | 9.96 | 0.002 | 0.004 | 0.297 |
| RHSIM_Rhsim02G0037000 | 9.96 | 0.002 | 0.004 | -0.531 |
| RHSIM_Rhsim07G0000800 | 9.95 | 0.002 | 0.004 | 0.586 |
| RHSIM_Rhsim12G0040300 | 9.95 | 0.002 | 0.004 | -2.046 |
| novel.453 | 9.95 | 0.002 | 0.004 | 0.816 |
| RHSIM_Rhsim02G0123600 | 9.95 | 0.002 | 0.004 | 0.176 |
| RHSIM_Rhsim04G0152900 | 9.95 | 0.002 | 0.004 | 2.967 |
| novel.1486 | 9.95 | 0.002 | 0.004 | -0.534 |
| novel.4550 | 9.95 | 0.002 | 0.004 | 1.257 |
| novel.2861 | 9.95 | 0.002 | 0.004 | 2.372 |

| Gene | LRT statistic | P value | BH-adjusted P | log2FC (interaction) |
|---|---|---|---|---|
| RHSIM_Rhsim04G0080000 | 9.94 | 0.002 | 0.004 | -40.680 |
| RHSIM_Rhsim10G0213700 | 9.94 | 0.002 | 0.004 | -3.889 |
| RHSIM_Rhsim10G0099600 | 9.94 | 0.002 | 0.004 | 0.352 |
| RHSIM_Rhsim05G0117500 | 9.94 | 0.002 | 0.004 | 0.856 |
| RHSIM_Rhsim03G0067200 | 9.94 | 0.002 | 0.004 | 3.239 |
| RHSIM_RhsimUnG0119800 | 9.94 | 0.002 | 0.004 | 0.364 |
| RHSIM_Rhsim03G0062300 | 9.93 | 0.002 | 0.004 | 1.338 |
| RHSIM_Rhsim07G0049600 | 9.93 | 0.002 | 0.004 | -0.425 |
| RHSIM_Rhsim08G0100400 | 9.92 | 0.002 | 0.004 | 0.259 |
| RHSIM_Rhsim09G0169800 | 9.92 | 0.002 | 0.004 | 1.094 |
| RHSIM_Rhsim04G0095900 | 9.92 | 0.002 | 0.004 | 0.488 |
| RHSIM_Rhsim01G0061000 | 9.92 | 0.002 | 0.004 | -0.452 |
| RHSIM_Rhsim02G0003000 | 9.92 | 0.002 | 0.004 | 0.427 |
| RHSIM_Rhsim06G0218400 | 9.92 | 0.002 | 0.004 | -0.364 |
| RHSIM_Rhsim02G0193700 | 9.92 | 0.002 | 0.004 | 0.243 |
| RHSIM_Rhsim11G0055500 | 9.91 | 0.002 | 0.004 | 1.119 |
| RHSIM_Rhsim09G0044900 | 9.91 | 0.002 | 0.004 | 2.198 |
| RHSIM_Rhsim03G0212300 | 9.90 | 0.002 | 0.004 | -0.366 |
| RHSIM_Rhsim01G0175700 | 9.90 | 0.002 | 0.004 | 0.468 |
| RHSIM_Rhsim06G0037000 | 9.90 | 0.002 | 0.004 | -40.062 |
| RHSIM_Rhsim10G0152800 | 9.90 | 0.002 | 0.004 | -0.611 |
| RHSIM_RhsimUnG0001000 | 9.89 | 0.002 | 0.004 | 1.219 |
| RHSIM_Rhsim07G0068100 | 9.89 | 0.002 | 0.004 | -0.316 |
| RHSIM_Rhsim11G0129900 | 9.89 | 0.002 | 0.004 | -0.908 |
| RHSIM_Rhsim01G0234200 | 9.89 | 0.002 | 0.004 | -0.532 |
| RHSIM_Rhsim06G0100000 | 9.89 | 0.002 | 0.004 | 1.303 |
| novel.838 | 9.89 | 0.002 | 0.004 | -44.057 |
| novel.1265 | 9.88 | 0.002 | 0.004 | -0.397 |
| novel.4047 | 9.88 | 0.002 | 0.004 | 2.130 |
| RHSIM_Rhsim05G0002900 | 9.87 | 0.002 | 0.004 | 0.786 |
| novel.1040 | 9.87 | 0.002 | 0.004 | -0.828 |
| novel.814 | 9.87 | 0.002 | 0.004 | 1.526 |

| Gene | LRT statistic | P value | BH-adjusted P | log2FC (interaction) |
|---|---|---|---|---|
| RHSIM_Rhsim13G0052700 | 9.87 | 0.002 | 0.004 | 0.295 |
| RHSIM_Rhsim04G0119300 | 9.87 | 0.002 | 0.004 | 4.151 |
| RHSIM_Rhsim12G0056300 | 9.86 | 0.002 | 0.004 | -1.281 |
| novel.2868 | 9.85 | 0.002 | 0.004 | -0.591 |
| RHSIM_Rhsim12G0077900 | 9.85 | 0.002 | 0.004 | -0.438 |
| RHSIM_Rhsim03G0173100 | 9.85 | 0.002 | 0.004 | 0.446 |
| RHSIM_Rhsim07G0190200 | 9.85 | 0.002 | 0.004 | -3.652 |
| novel.4192 | 9.85 | 0.002 | 0.004 | 1.439 |
| RHSIM_Rhsim01G0042700 | 9.85 | 0.002 | 0.004 | 0.552 |
| RHSIM_Rhsim02G0006600 | 9.84 | 0.002 | 0.004 | 0.250 |
| RHSIM_Rhsim12G0003800 | 9.84 | 0.002 | 0.004 | 0.255 |
| novel.3400 | 9.84 | 0.002 | 0.004 | -2.251 |
| novel.2487 | 9.84 | 0.002 | 0.004 | 2.117 |
| RHSIM_Rhsim02G0208200 | 9.84 | 0.002 | 0.004 | -0.226 |
| RHSIM_Rhsim10G0062700 | 9.83 | 0.002 | 0.004 | 1.456 |
| RHSIM_Rhsim12G0071600 | 9.83 | 0.002 | 0.004 | -3.939 |
| RHSIM_RhsimUnG0088000 | 9.83 | 0.002 | 0.004 | 0.750 |
| novel.2677 | 9.83 | 0.002 | 0.004 | 1.188 |
| RHSIM_RhsimUnG0208400 | 9.83 | 0.002 | 0.004 | 1.671 |
| RHSIM_Rhsim11G0164000 | 9.83 | 0.002 | 0.004 | -1.290 |
| novel.3678 | 9.83 | 0.002 | 0.004 | -1.077 |
| RHSIM_Rhsim05G0140600 | 9.83 | 0.002 | 0.004 | 3.939 |
| RHSIM_Rhsim12G0092800 | 9.83 | 0.002 | 0.004 | 0.388 |
| RHSIM_Rhsim02G0118600 | 9.82 | 0.002 | 0.004 | -2.612 |
| RHSIM_Rhsim09G0034100 | 9.82 | 0.002 | 0.004 | 0.328 |
| RHSIM_Rhsim08G0232500 | 9.82 | 0.002 | 0.004 | 0.437 |
| RHSIM_Rhsim01G0212700 | 9.81 | 0.002 | 0.004 | 0.611 |
| RHSIM_Rhsim08G0233500 | 9.81 | 0.002 | 0.004 | -0.459 |
| RHSIM_Rhsim03G0147700 | 9.81 | 0.002 | 0.004 | -2.152 |
| RHSIM_Rhsim05G0205700 | 9.80 | 0.002 | 0.004 | -0.269 |
| RHSIM_Rhsim06G0228900 | 9.80 | 0.002 | 0.004 | -0.835 |
| RHSIM_Rhsim01G0136100 | 9.80 | 0.002 | 0.004 | 0.551 |

| Gene | LRT statistic | P value | BH-adjusted P | log2FC (interaction) |
|---|---|---|---|---|
| RHSIM_Rhsim06G0162300 | 9.79 | 0.002 | 0.004 | -0.672 |
| RHSIM_Rhsim11G0023800 | 9.79 | 0.002 | 0.004 | 1.773 |
| RHSIM_Rhsim13G0213200 | 9.79 | 0.002 | 0.004 | 0.204 |
| RHSIM_Rhsim03G0029700 | 9.79 | 0.002 | 0.004 | 1.607 |
| RHSIM_Rhsim05G0144300 | 9.79 | 0.002 | 0.004 | -0.331 |
| RHSIM_Rhsim09G0070300 | 9.78 | 0.002 | 0.004 | 1.247 |
| RHSIM_Rhsim11G0127200 | 9.78 | 0.002 | 0.004 | 1.682 |
| RHSIM_Rhsim04G0153300 | 9.78 | 0.002 | 0.004 | 1.357 |
| RHSIM_RhsimUnG0224400 | 9.78 | 0.002 | 0.004 | 0.617 |
| novel.1637 | 9.77 | 0.002 | 0.004 | 2.530 |
| RHSIM_Rhsim12G0151800 | 9.77 | 0.002 | 0.004 | 0.282 |
| RHSIM_Rhsim05G0148500 | 9.77 | 0.002 | 0.004 | -0.356 |
| RHSIM_Rhsim01G0008800 | 9.77 | 0.002 | 0.004 | -0.756 |
| RHSIM_Rhsim10G0131200 | 9.76 | 0.002 | 0.004 | 0.464 |
| novel.1229 | 9.76 | 0.002 | 0.004 | -3.149 |
| RHSIM_Rhsim05G0088000 | 9.76 | 0.002 | 0.004 | 0.510 |
| RHSIM_Rhsim05G0110000 | 9.76 | 0.002 | 0.004 | 0.574 |
| RHSIM_Rhsim04G0198200 | 9.76 | 0.002 | 0.004 | -1.848 |
| novel.1931 | 9.76 | 0.002 | 0.004 | -1.380 |
| RHSIM_Rhsim08G0111000 | 9.76 | 0.002 | 0.004 | -41.835 |
| RHSIM_Rhsim07G0180800 | 9.76 | 0.002 | 0.004 | -1.801 |
| RHSIM_Rhsim02G0083700 | 9.75 | 0.002 | 0.004 | 0.460 |
| RHSIM_Rhsim01G0006100 | 9.75 | 0.002 | 0.004 | 0.538 |
| RHSIM_Rhsim07G0057600 | 9.75 | 0.002 | 0.004 | -0.430 |
| RHSIM_Rhsim10G0032200 | 9.75 | 0.002 | 0.004 | -1.008 |
| RHSIM_Rhsim01G0276000 | 9.74 | 0.002 | 0.004 | -2.786 |
| RHSIM_Rhsim06G0005300 | 9.74 | 0.002 | 0.004 | -0.234 |
| RHSIM_Rhsim10G0050800 | 9.74 | 0.002 | 0.004 | -1.481 |
| RHSIM_Rhsim01G0292600 | 9.73 | 0.002 | 0.004 | 0.781 |
| RHSIM_Rhsim04G0146900 | 9.73 | 0.002 | 0.004 | 41.289 |
| RHSIM_Rhsim10G0083100 | 9.72 | 0.002 | 0.004 | -0.649 |
| RHSIM_Rhsim13G0068700 | 9.72 | 0.002 | 0.004 | 2.064 |

| Gene | LRT statistic | P value | BH-adjusted P | log2FC (interaction) |
|---|---|---|---|---|
| RHSIM_Rhsim03G0244700 | 9.72 | 0.002 | 0.004 | 0.287 |
| RHSIM_Rhsim01G0162000 | 9.72 | 0.002 | 0.004 | 1.306 |
| RHSIM_Rhsim11G0110100 | 9.72 | 0.002 | 0.004 | -5.276 |
| RHSIM_Rhsim11G0123300 | 9.71 | 0.002 | 0.004 | -4.436 |
| RHSIM_Rhsim08G0088100 | 9.71 | 0.002 | 0.004 | -2.343 |
| novel.3912 | 9.71 | 0.002 | 0.004 | -3.871 |
| RHSIM_Rhsim13G0207100 | 9.71 | 0.002 | 0.004 | -0.620 |
| RHSIM_Rhsim09G0149500 | 9.71 | 0.002 | 0.004 | -0.812 |
| novel.3531 | 9.71 | 0.002 | 0.004 | -1.772 |
| RHSIM_Rhsim10G0178900 | 9.70 | 0.002 | 0.004 | 4.265 |
| RHSIM_Rhsim03G0062800 | 9.70 | 0.002 | 0.004 | -0.485 |
| RHSIM_Rhsim12G0171300 | 9.70 | 0.002 | 0.004 | -0.400 |
| RHSIM_Rhsim09G0082500 | 9.70 | 0.002 | 0.004 | 0.498 |
| RHSIM_Rhsim08G0184800 | 9.70 | 0.002 | 0.004 | 1.527 |
| novel.2865 | 9.70 | 0.002 | 0.004 | -0.736 |
| RHSIM_Rhsim13G0018100 | 9.69 | 0.002 | 0.004 | 0.907 |
| RHSIM_Rhsim12G0207800 | 9.69 | 0.002 | 0.004 | 0.208 |
| novel.1087 | 9.69 | 0.002 | 0.004 | -0.669 |
| novel.666 | 9.69 | 0.002 | 0.004 | -79.014 |
| novel.996 | 9.68 | 0.002 | 0.004 | 0.717 |
| RHSIM_Rhsim01G0140900 | 9.68 | 0.002 | 0.004 | -41.561 |
| RHSIM_Rhsim01G0002500 | 9.67 | 0.002 | 0.004 | 0.269 |
| RHSIM_Rhsim13G0141900 | 9.67 | 0.002 | 0.004 | 3.966 |
| RHSIM_Rhsim03G0265200 | 9.67 | 0.002 | 0.004 | -0.365 |
| RHSIM_Rhsim09G0136600 | 9.66 | 0.002 | 0.004 | -42.164 |
| RHSIM_Rhsim06G0003300 | 9.66 | 0.002 | 0.004 | -0.735 |
| novel.3523 | 9.66 | 0.002 | 0.004 | 0.878 |
| RHSIM_Rhsim09G0151300 | 9.66 | 0.002 | 0.004 | 0.216 |
| RHSIM_Rhsim05G0075300 | 9.65 | 0.002 | 0.004 | -4.175 |
| novel.832 | 9.65 | 0.002 | 0.004 | -0.303 |
| RHSIM_Rhsim12G0052900 | 9.65 | 0.002 | 0.004 | 0.739 |
| RHSIM_RhsimUnG0183300 | 9.65 | 0.002 | 0.004 | 1.028 |

| Gene | LRT statistic | P value | BH-adjusted P | log2FC (interaction) |
|---|---|---|---|---|
| RHSIM_Rhsim13G0193800 | 9.64 | 0.002 | 0.004 | -0.819 |
| RHSIM_Rhsim08G0157000 | 9.64 | 0.002 | 0.004 | -39.754 |
| RHSIM_Rhsim08G0073500 | 9.64 | 0.002 | 0.004 | -1.029 |
| RHSIM_Rhsim03G0225700 | 9.63 | 0.002 | 0.004 | -0.348 |
| RHSIM_Rhsim04G0195700 | 9.63 | 0.002 | 0.004 | 0.345 |
| RHSIM_Rhsim09G0103500 | 9.63 | 0.002 | 0.004 | -0.407 |
| RHSIM_Rhsim11G0174400 | 9.63 | 0.002 | 0.004 | -0.416 |
| RHSIM_Rhsim01G0135800 | 9.63 | 0.002 | 0.004 | 2.198 |
| novel.4291 | 9.63 | 0.002 | 0.004 | 3.201 |
| RHSIM_Rhsim10G0026100 | 9.63 | 0.002 | 0.004 | -0.272 |
| novel.4111 | 9.63 | 0.002 | 0.004 | 2.143 |
| novel.3548 | 9.62 | 0.002 | 0.004 | -3.801 |
| RHSIM_Rhsim04G0157000 | 9.62 | 0.002 | 0.004 | -2.182 |
| RHSIM_Rhsim06G0063500 | 9.62 | 0.002 | 0.004 | -0.417 |
| novel.2012 | 9.62 | 0.002 | 0.004 | 3.342 |
| RHSIM_RhsimUnG0126800 | 9.62 | 0.002 | 0.004 | 0.867 |
| RHSIM_Rhsim03G0201100 | 9.61 | 0.002 | 0.004 | -0.227 |
| novel.1767 | 9.61 | 0.002 | 0.004 | 42.520 |
| RHSIM_Rhsim04G0122800 | 9.61 | 0.002 | 0.004 | 42.145 |
| RHSIM_Rhsim13G0186700 | 9.60 | 0.002 | 0.004 | -0.802 |
| RHSIM_Rhsim12G0130000 | 9.60 | 0.002 | 0.004 | 0.361 |
| RHSIM_Rhsim02G0198900 | 9.60 | 0.002 | 0.004 | -1.216 |
| RHSIM_Rhsim12G0141100 | 9.60 | 0.002 | 0.004 | -0.446 |
| RHSIM_Rhsim13G0146700 | 9.60 | 0.002 | 0.004 | -1.514 |
| novel.112 | 9.60 | 0.002 | 0.004 | -4.137 |
| RHSIM_Rhsim10G0208300 | 9.60 | 0.002 | 0.004 | -0.710 |
| RHSIM_Rhsim09G0083500 | 9.59 | 0.002 | 0.004 | 0.326 |
| novel.2500 | 9.59 | 0.002 | 0.004 | -0.263 |
| RHSIM_Rhsim05G0168500 | 9.59 | 0.002 | 0.004 | 1.021 |
| RHSIM_Rhsim02G0194700 | 9.59 | 0.002 | 0.004 | 1.085 |
| RHSIM_RhsimUnG0221100 | 9.59 | 0.002 | 0.004 | -0.703 |
| RHSIM_Rhsim03G0036700 | 9.59 | 0.002 | 0.004 | 0.266 |

| Gene | LRT statistic | P value | BH-adjusted P | log2FC (interaction) |
|---|---|---|---|---|
| RHSIM_Rhsim13G0164300 | 9.59 | 0.002 | 0.004 | 0.878 |
| RHSIM_Rhsim10G0105600 | 9.59 | 0.002 | 0.004 | 1.996 |
| novel.3901 | 9.58 | 0.002 | 0.004 | 1.670 |
| RHSIM_Rhsim01G0065700 | 9.58 | 0.002 | 0.004 | -0.688 |
| RHSIM_Rhsim04G0017700 | 9.58 | 0.002 | 0.004 | -1.023 |
| RHSIM_Rhsim13G0071900 | 9.58 | 0.002 | 0.004 | 0.443 |
| RHSIM_Rhsim07G0094600 | 9.58 | 0.002 | 0.004 | -0.463 |
| RHSIM_Rhsim08G0122600 | 9.58 | 0.002 | 0.004 | -0.830 |
| RHSIM_Rhsim12G0141300 | 9.57 | 0.002 | 0.004 | 0.714 |
| RHSIM_Rhsim04G0191500 | 9.57 | 0.002 | 0.004 | -40.211 |
| RHSIM_Rhsim11G0173100 | 9.57 | 0.002 | 0.004 | 1.480 |
| RHSIM_Rhsim09G0040900 | 9.57 | 0.002 | 0.004 | 0.265 |
| RHSIM_Rhsim06G0006800 | 9.56 | 0.002 | 0.004 | -0.530 |
| RHSIM_Rhsim02G0049000 | 9.56 | 0.002 | 0.004 | 1.416 |
| RHSIM_Rhsim05G0029200 | 9.56 | 0.002 | 0.004 | -1.229 |
| RHSIM_Rhsim12G0108900 | 9.56 | 0.002 | 0.004 | -0.565 |
| RHSIM_Rhsim13G0104800 | 9.55 | 0.002 | 0.004 | 4.000 |
| RHSIM_Rhsim13G0054400 | 9.55 | 0.002 | 0.004 | 0.398 |
| RHSIM_Rhsim09G0114000 | 9.55 | 0.002 | 0.004 | -0.925 |
| RHSIM_Rhsim04G0206300 | 9.54 | 0.002 | 0.004 | -0.266 |
| RHSIM_Rhsim12G0125100 | 9.54 | 0.002 | 0.004 | 0.500 |
| RHSIM_Rhsim03G0262800 | 9.54 | 0.002 | 0.004 | 0.158 |
| RHSIM_Rhsim09G0206100 | 9.53 | 0.002 | 0.004 | 0.334 |
| novel.4057 | 9.53 | 0.002 | 0.004 | 2.754 |
| RHSIM_Rhsim11G0025400 | 9.53 | 0.002 | 0.004 | -0.434 |
| RHSIM_Rhsim05G0043800 | 9.53 | 0.002 | 0.004 | -0.407 |
| novel.4178 | 9.52 | 0.002 | 0.004 | 0.586 |
| novel.3352 | 9.52 | 0.002 | 0.004 | 0.262 |
| RHSIM_Rhsim01G0124900 | 9.52 | 0.002 | 0.004 | 1.918 |
| novel.1712 | 9.52 | 0.002 | 0.004 | -1.518 |
| novel.3407 | 9.52 | 0.002 | 0.004 | 1.528 |
| RHSIM_Rhsim07G0042700 | 9.51 | 0.002 | 0.004 | 0.950 |

| Gene | LRT statistic | P value | BH-adjusted P | log2FC (interaction) |
|---|---|---|---|---|
| RHSIM_Rhsim12G0113900 | 9.50 | 0.002 | 0.004 | 1.025 |
| RHSIM_Rhsim03G0192400 | 9.50 | 0.002 | 0.004 | -0.390 |
| novel.3894 | 9.50 | 0.002 | 0.004 | -0.802 |
| RHSIM_Rhsim09G0104900 | 9.50 | 0.002 | 0.004 | 2.673 |
| RHSIM_Rhsim07G0082100 | 9.50 | 0.002 | 0.004 | -0.214 |
| RHSIM_Rhsim08G0223800 | 9.50 | 0.002 | 0.004 | 1.418 |
| RHSIM_Rhsim05G0114700 | 9.50 | 0.002 | 0.004 | 0.395 |
| RHSIM_Rhsim03G0131600 | 9.50 | 0.002 | 0.004 | -0.175 |
| RHSIM_Rhsim05G0179600 | 9.50 | 0.002 | 0.004 | -2.911 |
| RHSIM_Rhsim08G0075000 | 9.49 | 0.002 | 0.004 | -1.297 |
| RHSIM_Rhsim13G0167000 | 9.49 | 0.002 | 0.004 | 1.682 |
| RHSIM_Rhsim04G0245000 | 9.49 | 0.002 | 0.004 | -0.384 |
| RHSIM_RhsimUnG0177900 | 9.48 | 0.002 | 0.005 | 1.499 |
| novel.3384 | 9.47 | 0.002 | 0.005 | -3.067 |
| RHSIM_Rhsim01G0227600 | 9.46 | 0.002 | 0.005 | -0.291 |
| RHSIM_Rhsim07G0244700 | 9.46 | 0.002 | 0.005 | 0.235 |
| RHSIM_Rhsim01G0183400 | 9.46 | 0.002 | 0.005 | 43.036 |
| novel.2535 | 9.46 | 0.002 | 0.005 | 0.679 |
| RHSIM_Rhsim01G0161800 | 9.45 | 0.002 | 0.005 | -0.655 |
| RHSIM_Rhsim04G0248500 | 9.45 | 0.002 | 0.005 | -0.330 |
| RHSIM_Rhsim05G0009400 | 9.45 | 0.002 | 0.005 | 1.337 |
| RHSIM_Rhsim01G0035200 | 9.45 | 0.002 | 0.005 | 1.886 |
| RHSIM_Rhsim04G0013100 | 9.45 | 0.002 | 0.005 | 0.646 |
| RHSIM_Rhsim09G0183300 | 9.45 | 0.002 | 0.005 | -0.292 |
| RHSIM_Rhsim07G0027300 | 9.44 | 0.002 | 0.005 | -0.856 |
| RHSIM_Rhsim01G0222100 | 9.44 | 0.002 | 0.005 | -1.967 |
| RHSIM_Rhsim13G0187500 | 9.44 | 0.002 | 0.005 | -0.426 |
| RHSIM_Rhsim10G0068100 | 9.43 | 0.002 | 0.005 | -1.396 |
| RHSIM_Rhsim12G0023800 | 9.43 | 0.002 | 0.005 | 3.828 |
| RHSIM_Rhsim06G0109700 | 9.43 | 0.002 | 0.005 | 40.430 |
| RHSIM_Rhsim02G0068800 | 9.43 | 0.002 | 0.005 | 0.344 |
| RHSIM_RhsimUnG0201100 | 9.42 | 0.002 | 0.005 | -1.121 |

| Gene | LRT statistic | P value | BH-adjusted P | log2FC (interaction) |
|---|---|---|---|---|
| novel.3489 | 9.42 | 0.002 | 0.005 | 3.014 |
| RHSIM_Rhsim02G0242800 | 9.42 | 0.002 | 0.005 | 0.382 |
| novel.1606 | 9.41 | 0.002 | 0.005 | 0.807 |
| RHSIM_Rhsim10G0052700 | 9.41 | 0.002 | 0.005 | 1.981 |
| RHSIM_Rhsim11G0127000 | 9.41 | 0.002 | 0.005 | 1.250 |
| RHSIM_Rhsim03G0266900 | 9.41 | 0.002 | 0.005 | 2.744 |
| RHSIM_Rhsim09G0205700 | 9.40 | 0.002 | 0.005 | 0.498 |
| RHSIM_Rhsim10G0209900 | 9.40 | 0.002 | 0.005 | 42.918 |
| RHSIM_Rhsim11G0038300 | 9.40 | 0.002 | 0.005 | 2.300 |
| RHSIM_Rhsim12G0082100 | 9.40 | 0.002 | 0.005 | -0.849 |
| RHSIM_RhsimUnG0219900 | 9.40 | 0.002 | 0.005 | 0.573 |
| RHSIM_Rhsim10G0044100 | 9.39 | 0.002 | 0.005 | 0.137 |
| novel.1841 | 9.39 | 0.002 | 0.005 | -4.918 |
| RHSIM_Rhsim12G0078300 | 9.39 | 0.002 | 0.005 | 1.274 |
| RHSIM_Rhsim08G0060900 | 9.39 | 0.002 | 0.005 | -39.981 |
| RHSIM_Rhsim01G0100600 | 9.38 | 0.002 | 0.005 | -0.190 |
| RHSIM_RhsimUnG0236500 | 9.38 | 0.002 | 0.005 | 1.656 |
| RHSIM_Rhsim10G0184100 | 9.38 | 0.002 | 0.005 | 0.269 |
| novel.1375 | 9.38 | 0.002 | 0.005 | 0.515 |
| RHSIM_Rhsim10G0019300 | 9.38 | 0.002 | 0.005 | 5.987 |
| RHSIM_Rhsim01G0020900 | 9.38 | 0.002 | 0.005 | -0.301 |
| RHSIM_Rhsim09G0117500 | 9.37 | 0.002 | 0.005 | 0.357 |
| novel.461 | 9.37 | 0.002 | 0.005 | 1.238 |
| novel.1770 | 9.37 | 0.002 | 0.005 | 3.220 |
| RHSIM_Rhsim02G0100900 | 9.37 | 0.002 | 0.005 | 1.048 |
| RHSIM_Rhsim01G0277900 | 9.36 | 0.002 | 0.005 | 0.462 |
| novel.864 | 9.35 | 0.002 | 0.005 | -1.041 |
| RHSIM_Rhsim08G0156600 | 9.35 | 0.002 | 0.005 | -0.432 |
| RHSIM_Rhsim13G0080600 | 9.35 | 0.002 | 0.005 | 0.495 |
| RHSIM_Rhsim01G0036400 | 9.35 | 0.002 | 0.005 | -1.996 |
| RHSIM_Rhsim04G0127400 | 9.34 | 0.002 | 0.005 | 1.095 |
| RHSIM_Rhsim05G0178600 | 9.34 | 0.002 | 0.005 | 0.772 |

| Gene | LRT statistic | P value | BH-adjusted P | log2FC (interaction) |
|---|---|---|---|---|
| RHSIM_Rhsim08G0154500 | 9.34 | 0.002 | 0.005 | -2.090 |
| novel.2803 | 9.34 | 0.002 | 0.005 | 41.594 |
| RHSIM_Rhsim05G0123700 | 9.34 | 0.002 | 0.005 | 1.406 |
| RHSIM_Rhsim01G0243400 | 9.33 | 0.002 | 0.005 | -0.421 |
| RHSIM_Rhsim03G0061900 | 9.33 | 0.002 | 0.005 | 42.868 |
| RHSIM_Rhsim10G0211300 | 9.33 | 0.002 | 0.005 | 1.336 |
| novel.2312 | 9.33 | 0.002 | 0.005 | -0.820 |
| novel.109 | 9.33 | 0.002 | 0.005 | -0.920 |
| RHSIM_Rhsim12G0134900 | 9.33 | 0.002 | 0.005 | 0.830 |
| RHSIM_RhsimUnG0004200 | 9.33 | 0.002 | 0.005 | 0.916 |
| RHSIM_Rhsim01G0060100 | 9.33 | 0.002 | 0.005 | -0.256 |
| RHSIM_RhsimUnG0018400 | 9.32 | 0.002 | 0.005 | -1.634 |
| RHSIM_Rhsim08G0066100 | 9.32 | 0.002 | 0.005 | -0.326 |
| RHSIM_Rhsim07G0118000 | 9.32 | 0.002 | 0.005 | 2.627 |
| RHSIM_Rhsim04G0131900 | 9.32 | 0.002 | 0.005 | 0.356 |
| RHSIM_Rhsim06G0239200 | 9.31 | 0.002 | 0.005 | -0.383 |
| RHSIM_RhsimUnG0016600 | 9.31 | 0.002 | 0.005 | 1.448 |
| RHSIM_Rhsim08G0083900 | 9.31 | 0.002 | 0.005 | -1.235 |
| RHSIM_Rhsim01G0081700 | 9.31 | 0.002 | 0.005 | -93.986 |
| RHSIM_Rhsim03G0053700 | 9.31 | 0.002 | 0.005 | -42.904 |
| novel.98 | 9.31 | 0.002 | 0.005 | 0.987 |
| novel.762 | 9.30 | 0.002 | 0.005 | -1.539 |
| RHSIM_Rhsim04G0228600 | 9.30 | 0.002 | 0.005 | 1.002 |
| RHSIM_Rhsim12G0157900 | 9.30 | 0.002 | 0.005 | 42.263 |
| novel.2544 | 9.30 | 0.002 | 0.005 | -2.894 |
| RHSIM_Rhsim09G0154000 | 9.30 | 0.002 | 0.005 | -3.177 |
| RHSIM_Rhsim05G0114800 | 9.29 | 0.002 | 0.005 | 0.297 |
| RHSIM_Rhsim09G0054900 | 9.29 | 0.002 | 0.005 | -0.275 |
| RHSIM_RhsimUnG0135100 | 9.29 | 0.002 | 0.005 | 2.702 |
| RHSIM_Rhsim07G0013100 | 9.29 | 0.002 | 0.005 | 3.584 |
| novel.2582 | 9.29 | 0.002 | 0.005 | -0.657 |
| RHSIM_Rhsim07G0026100 | 9.29 | 0.002 | 0.005 | -0.573 |

| Gene | LRT statistic | P value | BH-adjusted P | log2FC (interaction) |
|---|---|---|---|---|
| RHSIM_Rhsim01G0151900 | 9.29 | 0.002 | 0.005 | 1.649 |
| RHSIM_Rhsim08G0148000 | 9.28 | 0.002 | 0.005 | 1.507 |
| RHSIM_Rhsim07G0178100 | 9.28 | 0.002 | 0.005 | -0.647 |
| novel.1677 | 9.28 | 0.002 | 0.005 | -1.521 |
| RHSIM_Rhsim01G0035700 | 9.28 | 0.002 | 0.005 | 0.787 |
| RHSIM_RhsimUnG0226100 | 9.28 | 0.002 | 0.005 | 1.594 |
| RHSIM_RhsimUnG0152600 | 9.28 | 0.002 | 0.005 | -0.817 |
| RHSIM_Rhsim08G0188800 | 9.28 | 0.002 | 0.005 | -0.290 |
| RHSIM_Rhsim02G0154300 | 9.27 | 0.002 | 0.005 | -0.548 |
| RHSIM_Rhsim01G0262800 | 9.27 | 0.002 | 0.005 | -0.448 |
| RHSIM_Rhsim10G0194300 | 9.27 | 0.002 | 0.005 | -0.421 |
| RHSIM_Rhsim01G0263900 | 9.27 | 0.002 | 0.005 | -0.563 |
| RHSIM_Rhsim13G0028100 | 9.27 | 0.002 | 0.005 | -0.977 |
| RHSIM_Rhsim01G0167800 | 9.26 | 0.002 | 0.005 | 1.640 |
| RHSIM_Rhsim08G0252200 | 9.26 | 0.002 | 0.005 | 2.160 |
| RHSIM_Rhsim13G0016600 | 9.25 | 0.002 | 0.005 | -0.443 |
| RHSIM_Rhsim02G0218100 | 9.25 | 0.002 | 0.005 | 1.458 |
| RHSIM_Rhsim07G0060900 | 9.25 | 0.002 | 0.005 | -1.316 |
| RHSIM_Rhsim10G0036900 | 9.24 | 0.002 | 0.005 | -3.813 |
| novel.2276 | 9.24 | 0.002 | 0.005 | 2.592 |
| RHSIM_Rhsim05G0199300 | 9.24 | 0.002 | 0.005 | 0.955 |
| novel.1863 | 9.24 | 0.002 | 0.005 | -1.390 |
| RHSIM_RhsimUnG0254900 | 9.24 | 0.002 | 0.005 | 2.336 |
| RHSIM_Rhsim08G0231700 | 9.24 | 0.002 | 0.005 | 0.479 |
| RHSIM_Rhsim07G0074400 | 9.23 | 0.002 | 0.005 | -0.494 |
| novel.818 | 9.23 | 0.002 | 0.005 | 3.755 |
| RHSIM_Rhsim01G0198200 | 9.23 | 0.002 | 0.005 | 0.191 |
| RHSIM_Rhsim11G0155000 | 9.23 | 0.002 | 0.005 | -0.404 |
| RHSIM_Rhsim08G0033800 | 9.23 | 0.002 | 0.005 | -0.522 |
| novel.1329 | 9.23 | 0.002 | 0.005 | -1.466 |
| novel.744 | 9.23 | 0.002 | 0.005 | -41.187 |
| novel.2581 | 9.22 | 0.002 | 0.005 | -0.187 |

| Gene | LRT statistic | P value | BH-adjusted P | log2FC (interaction) |
|---|---|---|---|---|
| RHSIM_Rhsim01G0194400 | 9.22 | 0.002 | 0.005 | 0.467 |
| RHSIM_Rhsim13G0008200 | 9.21 | 0.002 | 0.005 | 0.903 |
| RHSIM_Rhsim04G0242800 | 9.21 | 0.002 | 0.005 | -3.224 |
| RHSIM_Rhsim08G0136400 | 9.21 | 0.002 | 0.005 | 0.431 |
| RHSIM_Rhsim10G0084000 | 9.21 | 0.002 | 0.005 | 42.658 |
| RHSIM_RhsimUnG0044500 | 9.21 | 0.002 | 0.005 | 0.440 |
| RHSIM_Rhsim07G0048700 | 9.20 | 0.002 | 0.005 | 1.332 |
| RHSIM_Rhsim13G0117200 | 9.19 | 0.002 | 0.005 | -1.399 |
| RHSIM_Rhsim03G0257000 | 9.19 | 0.002 | 0.005 | 79.453 |
| RHSIM_Rhsim07G0054000 | 9.19 | 0.002 | 0.005 | -0.256 |
| RHSIM_Rhsim07G0000900 | 9.19 | 0.002 | 0.005 | 0.846 |
| RHSIM_Rhsim01G0003400 | 9.19 | 0.002 | 0.005 | 0.514 |
| novel.1010 | 9.18 | 0.002 | 0.005 | -1.013 |
| RHSIM_Rhsim01G0088400 | 9.18 | 0.002 | 0.005 | -2.494 |
| RHSIM_Rhsim02G0104600 | 9.18 | 0.002 | 0.005 | 2.431 |
| RHSIM_Rhsim11G0192100 | 9.18 | 0.002 | 0.005 | 2.860 |
| RHSIM_Rhsim11G0147200 | 9.18 | 0.002 | 0.005 | -0.763 |
| RHSIM_Rhsim03G0269100 | 9.18 | 0.002 | 0.005 | 1.735 |
| RHSIM_Rhsim05G0215600 | 9.17 | 0.002 | 0.005 | 3.183 |
| RHSIM_Rhsim08G0150400 | 9.17 | 0.002 | 0.005 | 2.991 |
| RHSIM_Rhsim07G0002100 | 9.17 | 0.002 | 0.005 | 1.060 |
| RHSIM_Rhsim05G0035800 | 9.17 | 0.002 | 0.005 | -0.394 |
| RHSIM_Rhsim10G0134400 | 9.17 | 0.002 | 0.005 | -0.383 |
| RHSIM_Rhsim10G0085800 | 9.17 | 0.002 | 0.005 | -0.837 |
| RHSIM_Rhsim01G0213000 | 9.17 | 0.002 | 0.005 | 0.460 |
| RHSIM_Rhsim01G0249500 | 9.17 | 0.002 | 0.005 | 0.427 |
| RHSIM_Rhsim13G0184300 | 9.17 | 0.002 | 0.005 | 41.147 |
| RHSIM_RhsimUnG0234200 | 9.16 | 0.002 | 0.005 | 2.613 |
| novel.4568 | 9.16 | 0.002 | 0.005 | 0.801 |
| RHSIM_Rhsim08G0156100 | 9.16 | 0.002 | 0.005 | -40.799 |
| RHSIM_Rhsim03G0062200 | 9.16 | 0.002 | 0.005 | 0.545 |
| RHSIM_Rhsim10G0193500 | 9.16 | 0.002 | 0.005 | 0.619 |

| Gene | LRT statistic | P value | BH-adjusted P | log2FC (interaction) |
|---|---|---|---|---|
| RHSIM_Rhsim10G0159400 | 9.16 | 0.002 | 0.005 | -2.435 |
| RHSIM_Rhsim11G0173900 | 9.16 | 0.002 | 0.005 | 1.397 |
| RHSIM_Rhsim01G0118800 | 9.16 | 0.002 | 0.005 | -1.831 |
| RHSIM_Rhsim01G0189900 | 9.15 | 0.002 | 0.005 | -1.020 |
| RHSIM_Rhsim04G0197000 | 9.15 | 0.002 | 0.005 | 0.589 |
| RHSIM_Rhsim11G0015100 | 9.15 | 0.002 | 0.005 | -0.433 |
| RHSIM_Rhsim08G0067400 | 9.15 | 0.002 | 0.005 | 0.755 |
| RHSIM_Rhsim11G0020600 | 9.13 | 0.003 | 0.005 | 1.446 |
| RHSIM_Rhsim08G0065600 | 9.13 | 0.003 | 0.005 | 1.605 |
| novel.1116 | 9.13 | 0.003 | 0.005 | 1.128 |
| RHSIM_Rhsim11G0154300 | 9.13 | 0.003 | 0.005 | -0.679 |
| RHSIM_Rhsim08G0197400 | 9.12 | 0.003 | 0.005 | 0.588 |
| RHSIM_Rhsim10G0015000 | 9.12 | 0.003 | 0.005 | 0.646 |
| RHSIM_Rhsim07G0128800 | 9.12 | 0.003 | 0.005 | 2.483 |
| RHSIM_Rhsim08G0171500 | 9.12 | 0.003 | 0.005 | 1.827 |
| novel.2115 | 9.12 | 0.003 | 0.005 | 0.271 |
| RHSIM_Rhsim06G0012400 | 9.12 | 0.003 | 0.005 | -2.421 |
| RHSIM_Rhsim03G0025700 | 9.12 | 0.003 | 0.005 | 0.947 |
| RHSIM_Rhsim03G0007600 | 9.12 | 0.003 | 0.005 | -1.734 |
| RHSIM_Rhsim06G0197300 | 9.11 | 0.003 | 0.005 | -0.344 |
| novel.2900 | 9.11 | 0.003 | 0.005 | 0.927 |
| RHSIM_Rhsim07G0097100 | 9.11 | 0.003 | 0.005 | -3.550 |
| RHSIM_Rhsim02G0061700 | 9.11 | 0.003 | 0.005 | -0.229 |
| RHSIM_Rhsim02G0253400 | 9.10 | 0.003 | 0.005 | -1.453 |
| RHSIM_Rhsim11G0164900 | 9.10 | 0.003 | 0.005 | 0.475 |
| novel.76 | 9.10 | 0.003 | 0.005 | 0.884 |
| novel.1719 | 9.10 | 0.003 | 0.005 | 1.377 |
| novel.2214 | 9.09 | 0.003 | 0.005 | -0.366 |
| RHSIM_Rhsim08G0099300 | 9.09 | 0.003 | 0.005 | 0.510 |
| RHSIM_RhsimUnG0078100 | 9.09 | 0.003 | 0.006 | -2.241 |
| RHSIM_Rhsim12G0121000 | 9.09 | 0.003 | 0.006 | 0.370 |
| RHSIM_Rhsim13G0129200 | 9.08 | 0.003 | 0.006 | -0.441 |

| Gene | LRT statistic | P value | BH-adjusted P | log2FC (interaction) |
|---|---|---|---|---|
| RHSIM_Rhsim03G0215300 | 9.08 | 0.003 | 0.006 | -0.355 |
| RHSIM_Rhsim11G0108300 | 9.08 | 0.003 | 0.006 | -0.763 |
| novel.54 | 9.08 | 0.003 | 0.006 | 2.851 |
| RHSIM_Rhsim06G0118900 | 9.08 | 0.003 | 0.006 | -1.270 |
| RHSIM_RhsimUnG0102400 | 9.08 | 0.003 | 0.006 | 4.696 |
| RHSIM_Rhsim11G0039500 | 9.08 | 0.003 | 0.006 | 2.647 |
| RHSIM_Rhsim11G0187900 | 9.08 | 0.003 | 0.006 | -3.676 |
| novel.1236 | 9.07 | 0.003 | 0.006 | -0.926 |
| RHSIM_Rhsim03G0097800 | 9.07 | 0.003 | 0.006 | -2.591 |
| RHSIM_Rhsim02G0128100 | 9.07 | 0.003 | 0.006 | -0.789 |
| novel.4015 | 9.07 | 0.003 | 0.006 | 0.654 |
| novel.3884 | 9.06 | 0.003 | 0.006 | -0.993 |
| RHSIM_Rhsim10G0078000 | 9.06 | 0.003 | 0.006 | 3.572 |
| RHSIM_Rhsim13G0069900 | 9.06 | 0.003 | 0.006 | -0.310 |
| RHSIM_Rhsim07G0090300 | 9.06 | 0.003 | 0.006 | -0.276 |
| RHSIM_Rhsim13G0209300 | 9.06 | 0.003 | 0.006 | 0.210 |
| RHSIM_Rhsim04G0164200 | 9.05 | 0.003 | 0.006 | 0.868 |
| RHSIM_Rhsim08G0213300 | 9.05 | 0.003 | 0.006 | 0.450 |
| novel.2964 | 9.05 | 0.003 | 0.006 | -2.400 |
| RHSIM_Rhsim05G0185500 | 9.05 | 0.003 | 0.006 | 1.445 |
| RHSIM_Rhsim12G0119800 | 9.05 | 0.003 | 0.006 | -40.231 |
| RHSIM_Rhsim03G0269300 | 9.05 | 0.003 | 0.006 | 1.950 |
| RHSIM_RhsimUnG0231400 | 9.04 | 0.003 | 0.006 | 0.838 |
| RHSIM_Rhsim01G0140000 | 9.04 | 0.003 | 0.006 | 4.284 |
| RHSIM_Rhsim06G0074300 | 9.03 | 0.003 | 0.006 | -2.236 |
| RHSIM_Rhsim02G0024800 | 9.03 | 0.003 | 0.006 | 1.018 |
| RHSIM_Rhsim01G0076700 | 9.03 | 0.003 | 0.006 | -3.644 |
| RHSIM_Rhsim08G0122200 | 9.03 | 0.003 | 0.006 | -42.721 |
| RHSIM_Rhsim09G0139800 | 9.03 | 0.003 | 0.006 | 1.848 |
| RHSIM_Rhsim01G0267900 | 9.03 | 0.003 | 0.006 | 1.436 |
| RHSIM_Rhsim12G0113400 | 9.03 | 0.003 | 0.006 | 2.402 |
| RHSIM_Rhsim01G0164500 | 9.03 | 0.003 | 0.006 | -4.443 |

| Gene | LRT statistic | P value | BH-adjusted P | log2FC (interaction) |
|---|---|---|---|---|
| RHSIM_Rhsim06G0104800 | 9.03 | 0.003 | 0.006 | -0.494 |
| RHSIM_Rhsim06G0007400 | 9.02 | 0.003 | 0.006 | -0.767 |
| novel.2724 | 9.02 | 0.003 | 0.006 | 2.570 |
| RHSIM_Rhsim12G0082300 | 9.02 | 0.003 | 0.006 | 0.348 |
| RHSIM_Rhsim07G0099800 | 9.02 | 0.003 | 0.006 | 3.885 |
| RHSIM_Rhsim08G0154200 | 9.01 | 0.003 | 0.006 | 0.389 |
| novel.1987 | 9.01 | 0.003 | 0.006 | -6.822 |
| RHSIM_Rhsim07G0183700 | 9.01 | 0.003 | 0.006 | 0.521 |
| novel.2839 | 9.01 | 0.003 | 0.006 | 1.607 |
| RHSIM_Rhsim02G0079600 | 9.01 | 0.003 | 0.006 | -2.485 |
| RHSIM_Rhsim09G0185300 | 9.01 | 0.003 | 0.006 | -2.176 |
| RHSIM_Rhsim04G0095500 | 9.01 | 0.003 | 0.006 | -0.346 |
| RHSIM_Rhsim06G0111200 | 9.01 | 0.003 | 0.006 | -2.784 |
| RHSIM_Rhsim12G0042800 | 9.01 | 0.003 | 0.006 | -1.363 |
| RHSIM_RhsimUnG0085300 | 9.01 | 0.003 | 0.006 | -0.224 |
| novel.1115 | 9.00 | 0.003 | 0.006 | -0.722 |
| novel.3393 | 9.00 | 0.003 | 0.006 | -0.431 |
| RHSIM_Rhsim04G0168600 | 9.00 | 0.003 | 0.006 | 0.316 |
| RHSIM_Rhsim07G0168200 | 8.99 | 0.003 | 0.006 | 0.210 |
| RHSIM_Rhsim01G0185400 | 8.99 | 0.003 | 0.006 | 0.301 |
| novel.1041 | 8.99 | 0.003 | 0.006 | 2.518 |
| RHSIM_Rhsim08G0047600 | 8.99 | 0.003 | 0.006 | -0.546 |
| RHSIM_Rhsim08G0009600 | 8.99 | 0.003 | 0.006 | 0.594 |
| novel.3260 | 8.99 | 0.003 | 0.006 | 1.865 |
| RHSIM_Rhsim11G0123000 | 8.99 | 0.003 | 0.006 | 1.373 |
| novel.438 | 8.98 | 0.003 | 0.006 | 0.591 |
| RHSIM_Rhsim03G0200700 | 8.98 | 0.003 | 0.006 | 1.013 |
| RHSIM_RhsimUnG0157300 | 8.98 | 0.003 | 0.006 | 1.379 |
| RHSIM_Rhsim02G0194500 | 8.98 | 0.003 | 0.006 | 0.516 |
| RHSIM_Rhsim03G0253700 | 8.98 | 0.003 | 0.006 | 1.167 |
| RHSIM_Rhsim01G0073600 | 8.98 | 0.003 | 0.006 | 1.889 |
| RHSIM_Rhsim03G0234600 | 8.98 | 0.003 | 0.006 | -0.445 |

| Gene | LRT statistic | P value | BH-adjusted P | log2FC (interaction) |
|---|---|---|---|---|
| RHSIM_RhsimPtG0004700 | 8.97 | 0.003 | 0.006 | 3.353 |
| RHSIM_Rhsim08G0159700 | 8.97 | 0.003 | 0.006 | -41.152 |
| novel.4146 | 8.96 | 0.003 | 0.006 | -2.411 |
| RHSIM_RhsimUnG0143400 | 8.96 | 0.003 | 0.006 | -1.771 |
| RHSIM_Rhsim04G0126100 | 8.96 | 0.003 | 0.006 | -0.353 |
| RHSIM_Rhsim10G0026000 | 8.96 | 0.003 | 0.006 | 0.739 |
| RHSIM_Rhsim04G0071200 | 8.96 | 0.003 | 0.006 | 1.064 |
| novel.1294 | 8.95 | 0.003 | 0.006 | -4.661 |
| RHSIM_Rhsim12G0181600 | 8.95 | 0.003 | 0.006 | -0.833 |
| novel.1286 | 8.95 | 0.003 | 0.006 | -2.717 |
| RHSIM_Rhsim09G0011600 | 8.95 | 0.003 | 0.006 | 0.426 |
| RHSIM_Rhsim11G0141500 | 8.95 | 0.003 | 0.006 | -0.782 |
| novel.2962 | 8.94 | 0.003 | 0.006 | 0.890 |
| RHSIM_Rhsim02G0065100 | 8.94 | 0.003 | 0.006 | 1.207 |
| RHSIM_Rhsim09G0039100 | 8.94 | 0.003 | 0.006 | 0.480 |
| RHSIM_Rhsim01G0204800 | 8.94 | 0.003 | 0.006 | -0.384 |
| RHSIM_Rhsim02G0119000 | 8.94 | 0.003 | 0.006 | -0.521 |
| RHSIM_Rhsim08G0051000 | 8.94 | 0.003 | 0.006 | -3.444 |
| RHSIM_Rhsim05G0125200 | 8.94 | 0.003 | 0.006 | 1.785 |
| RHSIM_Rhsim07G0030400 | 8.94 | 0.003 | 0.006 | 43.679 |
| RHSIM_Rhsim02G0201600 | 8.94 | 0.003 | 0.006 | -0.300 |
| RHSIM_Rhsim07G0000200 | 8.94 | 0.003 | 0.006 | 0.692 |
| RHSIM_Rhsim05G0210500 | 8.94 | 0.003 | 0.006 | -0.789 |
| RHSIM_Rhsim08G0073800 | 8.93 | 0.003 | 0.006 | 0.970 |
| RHSIM_Rhsim07G0225300 | 8.93 | 0.003 | 0.006 | -0.781 |
| RHSIM_Rhsim03G0237900 | 8.93 | 0.003 | 0.006 | 1.354 |
| RHSIM_Rhsim13G0046800 | 8.93 | 0.003 | 0.006 | 2.187 |
| RHSIM_Rhsim04G0171800 | 8.93 | 0.003 | 0.006 | 0.343 |
| novel.537 | 8.91 | 0.003 | 0.006 | 2.637 |
| RHSIM_Rhsim11G0184900 | 8.91 | 0.003 | 0.006 | -0.341 |
| RHSIM_Rhsim07G0005300 | 8.91 | 0.003 | 0.006 | 1.902 |
| RHSIM_Rhsim08G0223600 | 8.91 | 0.003 | 0.006 | -0.379 |

| Gene | LRT statistic | P value | BH-adjusted P | log2FC (interaction) |
|---|---|---|---|---|
| RHSIM_Rhsim04G0246300 | 8.91 | 0.003 | 0.006 | 1.194 |
| RHSIM_Rhsim11G0168200 | 8.91 | 0.003 | 0.006 | 0.624 |
| RHSIM_Rhsim01G0251500 | 8.91 | 0.003 | 0.006 | 0.376 |
| RHSIM_Rhsim10G0006300 | 8.91 | 0.003 | 0.006 | 0.533 |
| RHSIM_Rhsim13G0160900 | 8.90 | 0.003 | 0.006 | 1.336 |
| RHSIM_Rhsim04G0155500 | 8.90 | 0.003 | 0.006 | 0.444 |
| RHSIM_Rhsim04G0248000 | 8.90 | 0.003 | 0.006 | 0.321 |
| RHSIM_Rhsim04G0101200 | 8.90 | 0.003 | 0.006 | -1.509 |
| RHSIM_Rhsim07G0183200 | 8.90 | 0.003 | 0.006 | 0.875 |
| RHSIM_Rhsim03G0079800 | 8.89 | 0.003 | 0.006 | -0.404 |
| RHSIM_Rhsim12G0179900 | 8.89 | 0.003 | 0.006 | -0.387 |
| novel.4198 | 8.89 | 0.003 | 0.006 | 0.793 |
| RHSIM_RhsimUnG0187200 | 8.89 | 0.003 | 0.006 | -39.574 |
| RHSIM_Rhsim04G0155900 | 8.89 | 0.003 | 0.006 | -0.389 |
| RHSIM_Rhsim13G0104600 | 8.88 | 0.003 | 0.006 | 3.651 |
| RHSIM_Rhsim04G0140200 | 8.88 | 0.003 | 0.006 | -2.492 |
| novel.1126 | 8.88 | 0.003 | 0.006 | 1.288 |
| RHSIM_Rhsim06G0107200 | 8.87 | 0.003 | 0.006 | -1.402 |
| RHSIM_Rhsim04G0150600 | 8.87 | 0.003 | 0.006 | 1.225 |
| RHSIM_Rhsim01G0246300 | 8.87 | 0.003 | 0.006 | 0.249 |
| RHSIM_Rhsim08G0001300 | 8.86 | 0.003 | 0.006 | 1.025 |
| RHSIM_Rhsim09G0157100 | 8.86 | 0.003 | 0.006 | 0.906 |
| RHSIM_Rhsim12G0214400 | 8.86 | 0.003 | 0.006 | 3.015 |
| RHSIM_Rhsim11G0168500 | 8.86 | 0.003 | 0.006 | -0.437 |
| RHSIM_Rhsim03G0091000 | 8.86 | 0.003 | 0.006 | -0.434 |
| RHSIM_Rhsim07G0077500 | 8.86 | 0.003 | 0.006 | -40.711 |
| novel.3858 | 8.86 | 0.003 | 0.006 | 0.618 |
| RHSIM_Rhsim06G0198600 | 8.85 | 0.003 | 0.006 | 0.291 |
| RHSIM_Rhsim04G0035500 | 8.85 | 0.003 | 0.006 | 0.838 |
| RHSIM_Rhsim07G0216400 | 8.85 | 0.003 | 0.006 | 2.154 |
| novel.4068 | 8.85 | 0.003 | 0.006 | -0.297 |
| RHSIM_Rhsim07G0170800 | 8.84 | 0.003 | 0.006 | 0.197 |

| Gene | LRT statistic | P value | BH-adjusted P | log2FC (interaction) |
|---|---|---|---|---|
| RHSIM_Rhsim12G0056700 | 8.84 | 0.003 | 0.006 | -1.368 |
| RHSIM_Rhsim02G0035000 | 8.84 | 0.003 | 0.006 | -1.296 |
| RHSIM_Rhsim06G0147900 | 8.84 | 0.003 | 0.006 | 3.742 |
| novel.3946 | 8.84 | 0.003 | 0.006 | 1.982 |
| RHSIM_Rhsim08G0225700 | 8.83 | 0.003 | 0.006 | 0.225 |
| RHSIM_Rhsim06G0161800 | 8.83 | 0.003 | 0.006 | -42.576 |
| RHSIM_Rhsim07G0066600 | 8.83 | 0.003 | 0.006 | -0.765 |
| RHSIM_Rhsim01G0236400 | 8.82 | 0.003 | 0.006 | 0.228 |
| novel.881 | 8.82 | 0.003 | 0.006 | -4.283 |
| novel.33 | 8.82 | 0.003 | 0.006 | 1.047 |
| RHSIM_RhsimUnG0139800 | 8.82 | 0.003 | 0.006 | -1.472 |
| RHSIM_Rhsim01G0054400 | 8.82 | 0.003 | 0.006 | -40.183 |
| novel.4516 | 8.82 | 0.003 | 0.006 | 0.768 |
| RHSIM_Rhsim12G0135900 | 8.82 | 0.003 | 0.006 | -0.322 |
| RHSIM_Rhsim02G0163200 | 8.82 | 0.003 | 0.006 | 1.302 |
| RHSIM_Rhsim02G0213000 | 8.81 | 0.003 | 0.006 | 0.606 |
| RHSIM_Rhsim02G0008200 | 8.81 | 0.003 | 0.006 | -0.238 |
| RHSIM_Rhsim09G0054000 | 8.81 | 0.003 | 0.006 | -0.433 |
| novel.2267 | 8.81 | 0.003 | 0.006 | 2.486 |
| RHSIM_Rhsim09G0031900 | 8.81 | 0.003 | 0.006 | 0.603 |
| RHSIM_Rhsim03G0213400 | 8.81 | 0.003 | 0.006 | 41.866 |
| RHSIM_Rhsim07G0197200 | 8.81 | 0.003 | 0.006 | -0.991 |
| RHSIM_Rhsim13G0183600 | 8.81 | 0.003 | 0.006 | 41.777 |
| RHSIM_Rhsim05G0032500 | 8.81 | 0.003 | 0.006 | -1.100 |
| RHSIM_Rhsim05G0168200 | 8.80 | 0.003 | 0.006 | 41.295 |
| RHSIM_Rhsim08G0162900 | 8.80 | 0.003 | 0.006 | 0.957 |
| RHSIM_Rhsim08G0138100 | 8.80 | 0.003 | 0.006 | 0.713 |
| RHSIM_Rhsim01G0087200 | 8.80 | 0.003 | 0.006 | 0.475 |
| RHSIM_Rhsim02G0256900 | 8.79 | 0.003 | 0.006 | 0.294 |
| RHSIM_Rhsim07G0112400 | 8.79 | 0.003 | 0.006 | -1.939 |
| RHSIM_Rhsim02G0001500 | 8.79 | 0.003 | 0.006 | -0.160 |
| RHSIM_Rhsim10G0069300 | 8.78 | 0.003 | 0.006 | 0.940 |

| Gene | LRT statistic | P value | BH-adjusted P | log2FC (interaction) |
|---|---|---|---|---|
| RHSIM_Rhsim02G0199200 | 8.78 | 0.003 | 0.006 | -0.174 |
| RHSIM_Rhsim09G0075400 | 8.78 | 0.003 | 0.006 | 1.845 |
| RHSIM_RhsimUnG0258900 | 8.78 | 0.003 | 0.006 | 0.347 |
| RHSIM_Rhsim04G0007800 | 8.78 | 0.003 | 0.006 | 42.836 |
| novel.358 | 8.77 | 0.003 | 0.006 | -0.595 |
| RHSIM_Rhsim13G0163500 | 8.77 | 0.003 | 0.006 | -1.961 |
| RHSIM_Rhsim01G0071500 | 8.77 | 0.003 | 0.006 | -0.723 |
| RHSIM_RhsimUnG0210200 | 8.77 | 0.003 | 0.006 | -0.544 |
| RHSIM_Rhsim12G0062100 | 8.77 | 0.003 | 0.006 | -0.240 |
| RHSIM_RhsimUnG0042000 | 8.77 | 0.003 | 0.006 | 0.608 |
| novel.3762 | 8.77 | 0.003 | 0.006 | 1.311 |
| novel.825 | 8.77 | 0.003 | 0.006 | 1.362 |
| RHSIM_Rhsim01G0004000 | 8.77 | 0.003 | 0.006 | -1.564 |
| RHSIM_RhsimUnG0110900 | 8.76 | 0.003 | 0.007 | 1.309 |
| RHSIM_Rhsim07G0019400 | 8.76 | 0.003 | 0.007 | 41.262 |
| novel.746 | 8.76 | 0.003 | 0.007 | 1.444 |
| RHSIM_Rhsim07G0021200 | 8.75 | 0.003 | 0.007 | -2.624 |
| RHSIM_Rhsim09G0145600 | 8.75 | 0.003 | 0.007 | -0.426 |
| RHSIM_Rhsim13G0100200 | 8.75 | 0.003 | 0.007 | -39.848 |
| RHSIM_Rhsim06G0120200 | 8.75 | 0.003 | 0.007 | -2.297 |
| novel.87 | 8.75 | 0.003 | 0.007 | 3.808 |
| RHSIM_Rhsim01G0217000 | 8.75 | 0.003 | 0.007 | -4.611 |
| RHSIM_Rhsim02G0221100 | 8.75 | 0.003 | 0.007 | -0.744 |
| RHSIM_Rhsim06G0208500 | 8.74 | 0.003 | 0.007 | 1.617 |
| RHSIM_Rhsim01G0084900 | 8.74 | 0.003 | 0.007 | -2.144 |
| RHSIM_Rhsim07G0034300 | 8.73 | 0.003 | 0.007 | 0.518 |
| RHSIM_Rhsim04G0191600 | 8.73 | 0.003 | 0.007 | 1.012 |
| RHSIM_Rhsim03G0151800 | 8.73 | 0.003 | 0.007 | 1.162 |
| RHSIM_Rhsim10G0075600 | 8.73 | 0.003 | 0.007 | 1.229 |
| RHSIM_Rhsim09G0090600 | 8.73 | 0.003 | 0.007 | 0.862 |
| RHSIM_Rhsim10G0130900 | 8.73 | 0.003 | 0.007 | -0.858 |
| RHSIM_Rhsim13G0151800 | 8.73 | 0.003 | 0.007 | -4.206 |

| Gene | LRT statistic | P value | BH-adjusted P | log2FC (interaction) |
|---|---|---|---|---|
| RHSIM_Rhsim03G0178600 | 8.73 | 0.003 | 0.007 | 0.711 |
| novel.4127 | 8.72 | 0.003 | 0.007 | 2.610 |
| RHSIM_Rhsim08G0055700 | 8.72 | 0.003 | 0.007 | 0.344 |
| RHSIM_Rhsim06G0116300 | 8.72 | 0.003 | 0.007 | 78.856 |
| RHSIM_Rhsim08G0155800 | 8.72 | 0.003 | 0.007 | -2.741 |
| RHSIM_Rhsim06G0148500 | 8.72 | 0.003 | 0.007 | -0.340 |
| RHSIM_Rhsim09G0040000 | 8.71 | 0.003 | 0.007 | 1.509 |
| novel.3052 | 8.71 | 0.003 | 0.007 | 0.927 |
| RHSIM_Rhsim11G0169100 | 8.71 | 0.003 | 0.007 | -0.940 |
| RHSIM_Rhsim12G0000200 | 8.70 | 0.003 | 0.007 | 0.942 |
| RHSIM_Rhsim03G0160000 | 8.70 | 0.003 | 0.007 | 3.857 |
| RHSIM_Rhsim04G0236200 | 8.70 | 0.003 | 0.007 | -1.770 |
| RHSIM_Rhsim07G0054300 | 8.70 | 0.003 | 0.007 | 40.325 |
| RHSIM_Rhsim02G0061000 | 8.70 | 0.003 | 0.007 | -2.557 |
| RHSIM_Rhsim11G0029400 | 8.69 | 0.003 | 0.007 | 0.567 |
| novel.3374 | 8.69 | 0.003 | 0.007 | 0.550 |
| RHSIM_Rhsim02G0173000 | 8.69 | 0.003 | 0.007 | 0.311 |
| RHSIM_Rhsim06G0063900 | 8.69 | 0.003 | 0.007 | 0.559 |
| RHSIM_Rhsim13G0172700 | 8.69 | 0.003 | 0.007 | -0.642 |
| novel.2120 | 8.69 | 0.003 | 0.007 | 2.039 |
| RHSIM_Rhsim09G0077100 | 8.69 | 0.003 | 0.007 | 3.392 |
| RHSIM_Rhsim12G0036200 | 8.69 | 0.003 | 0.007 | 0.953 |
| RHSIM_Rhsim04G0098200 | 8.69 | 0.003 | 0.007 | 2.506 |
| RHSIM_Rhsim06G0028200 | 8.68 | 0.003 | 0.007 | -0.864 |
| RHSIM_Rhsim01G0040800 | 8.68 | 0.003 | 0.007 | 4.049 |
| novel.1179 | 8.67 | 0.003 | 0.007 | 1.316 |
| RHSIM_RhsimUnG0163100 | 8.67 | 0.003 | 0.007 | -2.576 |
| RHSIM_RhsimUnG0172000 | 8.66 | 0.003 | 0.007 | 0.958 |
| RHSIM_Rhsim10G0148900 | 8.66 | 0.003 | 0.007 | -0.392 |
| RHSIM_Rhsim08G0027300 | 8.66 | 0.003 | 0.007 | -0.351 |
| RHSIM_Rhsim12G0111900 | 8.65 | 0.003 | 0.007 | -0.696 |
| RHSIM_Rhsim09G0162000 | 8.65 | 0.003 | 0.007 | -3.762 |

| Gene | LRT statistic | P value | BH-adjusted P | log2FC (interaction) |
|---|---|---|---|---|
| RHSIM_Rhsim13G0172600 | 8.65 | 0.003 | 0.007 | -0.923 |
| RHSIM_Rhsim03G0018200 | 8.64 | 0.003 | 0.007 | -0.155 |
| RHSIM_Rhsim03G0212800 | 8.64 | 0.003 | 0.007 | 0.250 |
| RHSIM_Rhsim04G0033700 | 8.64 | 0.003 | 0.007 | 1.158 |
| RHSIM_Rhsim10G0039600 | 8.64 | 0.003 | 0.007 | 0.289 |
| RHSIM_Rhsim13G0210300 | 8.64 | 0.003 | 0.007 | 0.906 |
| RHSIM_Rhsim12G0005700 | 8.64 | 0.003 | 0.007 | -0.579 |
| RHSIM_Rhsim08G0194200 | 8.64 | 0.003 | 0.007 | -0.459 |
| RHSIM_RhsimUnG0105300 | 8.63 | 0.003 | 0.007 | -1.414 |
| RHSIM_Rhsim05G0072600 | 8.63 | 0.003 | 0.007 | -1.094 |
| RHSIM_Rhsim03G0086600 | 8.63 | 0.003 | 0.007 | 1.311 |
| RHSIM_RhsimUnG0012200 | 8.63 | 0.003 | 0.007 | 0.443 |
| novel.706 | 8.63 | 0.003 | 0.007 | 0.626 |
| RHSIM_Rhsim07G0035200 | 8.63 | 0.003 | 0.007 | -0.851 |
| RHSIM_Rhsim09G0176400 | 8.63 | 0.003 | 0.007 | 0.916 |
| RHSIM_Rhsim01G0146500 | 8.62 | 0.003 | 0.007 | 1.567 |
| RHSIM_Rhsim06G0159300 | 8.62 | 0.003 | 0.007 | -0.786 |
| RHSIM_Rhsim09G0133400 | 8.62 | 0.003 | 0.007 | -1.108 |
| RHSIM_Rhsim10G0190100 | 8.62 | 0.003 | 0.007 | 2.511 |
| novel.85 | 8.62 | 0.003 | 0.007 | -40.249 |
| RHSIM_Rhsim02G0160600 | 8.62 | 0.003 | 0.007 | 3.168 |
| RHSIM_Rhsim07G0143900 | 8.61 | 0.003 | 0.007 | 1.106 |
| RHSIM_Rhsim08G0089500 | 8.61 | 0.003 | 0.007 | -0.322 |
| novel.1139 | 8.61 | 0.003 | 0.007 | 3.003 |
| RHSIM_Rhsim01G0230300 | 8.61 | 0.003 | 0.007 | 0.212 |
| RHSIM_Rhsim13G0179200 | 8.61 | 0.003 | 0.007 | 41.601 |
| RHSIM_Rhsim01G0242800 | 8.61 | 0.003 | 0.007 | 0.435 |
| RHSIM_Rhsim06G0125800 | 8.61 | 0.003 | 0.007 | -0.487 |
| RHSIM_Rhsim07G0195100 | 8.60 | 0.003 | 0.007 | -0.630 |
| RHSIM_Rhsim02G0040700 | 8.60 | 0.003 | 0.007 | -1.529 |
| RHSIM_Rhsim12G0140800 | 8.60 | 0.003 | 0.007 | 2.136 |
| RHSIM_Rhsim13G0077900 | 8.59 | 0.003 | 0.007 | 0.275 |

| Gene | LRT statistic | P value | BH-adjusted P | log2FC (interaction) |
|---|---|---|---|---|
| RHSIM_Rhsim05G0054100 | 8.59 | 0.003 | 0.007 | -0.485 |
| RHSIM_Rhsim06G0112300 | 8.59 | 0.003 | 0.007 | -2.160 |
| RHSIM_Rhsim02G0171500 | 8.59 | 0.003 | 0.007 | 0.377 |
| RHSIM_Rhsim04G0204000 | 8.58 | 0.003 | 0.007 | 1.041 |
| RHSIM_Rhsim06G0232300 | 8.58 | 0.003 | 0.007 | 2.766 |
| RHSIM_RhsimUnG0227700 | 8.58 | 0.003 | 0.007 | -41.055 |
| RHSIM_Rhsim04G0143100 | 8.58 | 0.003 | 0.007 | 2.101 |
| RHSIM_Rhsim05G0113200 | 8.58 | 0.003 | 0.007 | 0.274 |
| RHSIM_Rhsim03G0046400 | 8.57 | 0.003 | 0.007 | -0.243 |
| RHSIM_Rhsim03G0078700 | 8.57 | 0.003 | 0.007 | 0.292 |
| RHSIM_Rhsim05G0081000 | 8.57 | 0.003 | 0.007 | -0.278 |
| RHSIM_Rhsim04G0228100 | 8.57 | 0.003 | 0.007 | 0.332 |
| RHSIM_Rhsim08G0121000 | 8.57 | 0.003 | 0.007 | 1.073 |
| novel.4479 | 8.57 | 0.003 | 0.007 | 2.256 |
| RHSIM_Rhsim10G0148300 | 8.57 | 0.003 | 0.007 | 0.305 |
| novel.935 | 8.56 | 0.003 | 0.007 | 2.710 |
| RHSIM_Rhsim04G0028000 | 8.56 | 0.003 | 0.007 | 0.406 |
| RHSIM_Rhsim06G0242100 | 8.56 | 0.003 | 0.007 | -0.682 |
| RHSIM_Rhsim12G0128600 | 8.55 | 0.003 | 0.007 | 0.711 |
| RHSIM_RhsimUnG0134200 | 8.55 | 0.003 | 0.007 | -0.836 |
| RHSIM_Rhsim13G0135300 | 8.55 | 0.003 | 0.007 | 5.538 |
| RHSIM_RhsimUnG0148900 | 8.55 | 0.003 | 0.007 | 0.545 |
| RHSIM_Rhsim07G0210100 | 8.55 | 0.003 | 0.007 | 4.604 |
| RHSIM_RhsimUnG0159000 | 8.54 | 0.003 | 0.007 | -0.966 |
| RHSIM_Rhsim07G0211500 | 8.54 | 0.003 | 0.007 | -1.448 |
| RHSIM_Rhsim11G0150200 | 8.54 | 0.003 | 0.007 | 0.686 |
| novel.2933 | 8.54 | 0.003 | 0.007 | -1.885 |
| RHSIM_Rhsim11G0178500 | 8.54 | 0.003 | 0.007 | 0.586 |
| RHSIM_Rhsim06G0160100 | 8.53 | 0.003 | 0.007 | -0.524 |
| RHSIM_Rhsim03G0140600 | 8.53 | 0.003 | 0.007 | 0.637 |
| novel.4490 | 8.53 | 0.004 | 0.007 | -40.828 |
| novel.712 | 8.52 | 0.004 | 0.007 | 1.426 |

| Gene | LRT statistic | P value | BH-adjusted P | log2FC (interaction) |
|---|---|---|---|---|
| RHSIM_Rhsim04G0244600 | 8.52 | 0.004 | 0.007 | 0.343 |
| RHSIM_Rhsim04G0226500 | 8.52 | 0.004 | 0.007 | 0.907 |
| RHSIM_Rhsim01G0170900 | 8.52 | 0.004 | 0.007 | -1.899 |
| RHSIM_Rhsim12G0156600 | 8.52 | 0.004 | 0.007 | 1.712 |
| RHSIM_Rhsim11G0176800 | 8.52 | 0.004 | 0.007 | -0.274 |
| RHSIM_Rhsim11G0153500 | 8.52 | 0.004 | 0.007 | -0.217 |
| RHSIM_Rhsim11G0082700 | 8.52 | 0.004 | 0.007 | -2.057 |
| RHSIM_Rhsim01G0005000 | 8.52 | 0.004 | 0.007 | -0.721 |
| RHSIM_Rhsim04G0011000 | 8.51 | 0.004 | 0.007 | 0.278 |
| RHSIM_Rhsim08G0119000 | 8.51 | 0.004 | 0.007 | 0.287 |
| RHSIM_Rhsim02G0125700 | 8.51 | 0.004 | 0.007 | 1.384 |
| RHSIM_Rhsim06G0163700 | 8.51 | 0.004 | 0.007 | -1.058 |
| RHSIM_Rhsim06G0061400 | 8.51 | 0.004 | 0.007 | -1.722 |
| RHSIM_Rhsim07G0205100 | 8.50 | 0.004 | 0.007 | -1.061 |
| RHSIM_Rhsim09G0069900 | 8.50 | 0.004 | 0.007 | 0.453 |
| RHSIM_Rhsim13G0030200 | 8.50 | 0.004 | 0.007 | -2.191 |
| RHSIM_Rhsim13G0113600 | 8.49 | 0.004 | 0.007 | 4.973 |
| novel.3868 | 8.49 | 0.004 | 0.007 | 1.712 |
| RHSIM_Rhsim05G0216900 | 8.48 | 0.004 | 0.008 | -0.635 |
| RHSIM_RhsimUnG0068800 | 8.48 | 0.004 | 0.008 | -3.885 |
| RHSIM_RhsimUnG0159300 | 8.48 | 0.004 | 0.008 | 0.968 |
| RHSIM_Rhsim07G0124100 | 8.48 | 0.004 | 0.008 | -0.910 |
| RHSIM_Rhsim10G0066500 | 8.47 | 0.004 | 0.008 | 0.380 |
| RHSIM_Rhsim05G0220900 | 8.47 | 0.004 | 0.008 | 0.674 |
| RHSIM_Rhsim04G0156300 | 8.47 | 0.004 | 0.008 | 0.156 |
| RHSIM_Rhsim13G0198600 | 8.47 | 0.004 | 0.008 | -0.298 |
| RHSIM_Rhsim05G0098800 | 8.47 | 0.004 | 0.008 | 42.930 |
| RHSIM_RhsimUnG0222100 | 8.47 | 0.004 | 0.008 | 0.941 |
| RHSIM_Rhsim01G0087500 | 8.46 | 0.004 | 0.008 | 0.258 |
| novel.4061 | 8.46 | 0.004 | 0.008 | -2.006 |
| RHSIM_Rhsim07G0009700 | 8.46 | 0.004 | 0.008 | -2.231 |
| RHSIM_Rhsim03G0002200 | 8.46 | 0.004 | 0.008 | 1.479 |

| Gene | LRT statistic | P value | BH-adjusted P | log2FC (interaction) |
|---|---|---|---|---|
| RHSIM_Rhsim07G0013900 | 8.46 | 0.004 | 0.008 | 2.498 |
| RHSIM_RhsimUnG0132800 | 8.45 | 0.004 | 0.008 | 2.554 |
| RHSIM_Rhsim01G0269300 | 8.45 | 0.004 | 0.008 | -0.898 |
| RHSIM_Rhsim02G0181700 | 8.45 | 0.004 | 0.008 | -0.250 |
| novel.3460 | 8.45 | 0.004 | 0.008 | 42.753 |
| RHSIM_Rhsim13G0115700 | 8.45 | 0.004 | 0.008 | 0.376 |
| RHSIM_Rhsim08G0115100 | 8.45 | 0.004 | 0.008 | 1.659 |
| novel.2654 | 8.45 | 0.004 | 0.008 | -2.230 |
| RHSIM_Rhsim13G0020500 | 8.44 | 0.004 | 0.008 | -2.941 |
| novel.2757 | 8.44 | 0.004 | 0.008 | -40.195 |
| RHSIM_Rhsim01G0222700 | 8.44 | 0.004 | 0.008 | -40.580 |
| RHSIM_Rhsim05G0195000 | 8.44 | 0.004 | 0.008 | -0.457 |
| RHSIM_Rhsim07G0218300 | 8.44 | 0.004 | 0.008 | -0.980 |
| RHSIM_Rhsim04G0241400 | 8.44 | 0.004 | 0.008 | 1.823 |
| RHSIM_Rhsim08G0111600 | 8.43 | 0.004 | 0.008 | 0.470 |
| RHSIM_Rhsim12G0123700 | 8.42 | 0.004 | 0.008 | -0.264 |
| RHSIM_Rhsim04G0078200 | 8.42 | 0.004 | 0.008 | -2.052 |
| novel.3208 | 8.42 | 0.004 | 0.008 | 1.284 |
| novel.4064 | 8.42 | 0.004 | 0.008 | 2.089 |
| novel.4230 | 8.42 | 0.004 | 0.008 | 1.504 |
| RHSIM_Rhsim07G0091900 | 8.42 | 0.004 | 0.008 | 1.136 |
| RHSIM_Rhsim07G0136500 | 8.42 | 0.004 | 0.008 | -0.468 |
| RHSIM_RhsimUnG0206300 | 8.42 | 0.004 | 0.008 | 0.871 |
| RHSIM_Rhsim07G0021700 | 8.42 | 0.004 | 0.008 | 0.330 |
| RHSIM_Rhsim08G0251700 | 8.41 | 0.004 | 0.008 | 0.253 |
| RHSIM_Rhsim13G0004100 | 8.41 | 0.004 | 0.008 | 0.701 |
| RHSIM_Rhsim01G0098900 | 8.41 | 0.004 | 0.008 | 1.075 |
| novel.3628 | 8.41 | 0.004 | 0.008 | -40.783 |
| RHSIM_Rhsim05G0106500 | 8.41 | 0.004 | 0.008 | 2.066 |
| RHSIM_RhsimUnG0155300 | 8.40 | 0.004 | 0.008 | 5.286 |
| RHSIM_Rhsim05G0149800 | 8.40 | 0.004 | 0.008 | -1.119 |
| novel.2713 | 8.40 | 0.004 | 0.008 | -3.867 |

| Gene | LRT statistic | P value | BH-adjusted P | log2FC (interaction) |
|---|---|---|---|---|
| RHSIM_Rhsim03G0240100 | 8.40 | 0.004 | 0.008 | 0.384 |
| novel.2327 | 8.40 | 0.004 | 0.008 | 2.122 |
| RHSIM_Rhsim06G0210100 | 8.39 | 0.004 | 0.008 | -0.520 |
| RHSIM_Rhsim03G0036600 | 8.39 | 0.004 | 0.008 | 0.945 |
| RHSIM_Rhsim01G0238100 | 8.39 | 0.004 | 0.008 | 0.401 |
| novel.1593 | 8.39 | 0.004 | 0.008 | -4.618 |
| RHSIM_Rhsim10G0188900 | 8.39 | 0.004 | 0.008 | 0.481 |
| RHSIM_Rhsim04G0151500 | 8.39 | 0.004 | 0.008 | -3.542 |
| RHSIM_Rhsim06G0193000 | 8.39 | 0.004 | 0.008 | -0.776 |
| RHSIM_Rhsim10G0054900 | 8.39 | 0.004 | 0.008 | -0.711 |
| novel.2299 | 8.39 | 0.004 | 0.008 | -42.624 |
| RHSIM_Rhsim07G0204500 | 8.38 | 0.004 | 0.008 | 5.846 |
| novel.3286 | 8.38 | 0.004 | 0.008 | -4.657 |
| RHSIM_Rhsim10G0001700 | 8.37 | 0.004 | 0.008 | -0.407 |
| RHSIM_Rhsim12G0196800 | 8.37 | 0.004 | 0.008 | 40.280 |
| RHSIM_Rhsim11G0028900 | 8.37 | 0.004 | 0.008 | 0.794 |
| RHSIM_Rhsim12G0080500 | 8.37 | 0.004 | 0.008 | -4.422 |
| RHSIM_Rhsim13G0117400 | 8.36 | 0.004 | 0.008 | 1.644 |
| RHSIM_Rhsim02G0236200 | 8.36 | 0.004 | 0.008 | -3.101 |
| RHSIM_Rhsim03G0196500 | 8.36 | 0.004 | 0.008 | 0.574 |
| novel.1594 | 8.36 | 0.004 | 0.008 | -2.358 |
| RHSIM_Rhsim11G0106600 | 8.35 | 0.004 | 0.008 | 1.377 |
| novel.3916 | 8.35 | 0.004 | 0.008 | 2.407 |
| RHSIM_Rhsim08G0251000 | 8.35 | 0.004 | 0.008 | 0.306 |
| novel.2027 | 8.35 | 0.004 | 0.008 | 2.070 |
| novel.3108 | 8.35 | 0.004 | 0.008 | -1.075 |
| RHSIM_Rhsim01G0054900 | 8.35 | 0.004 | 0.008 | -0.360 |
| RHSIM_Rhsim07G0157600 | 8.35 | 0.004 | 0.008 | 0.776 |
| RHSIM_Rhsim13G0006900 | 8.34 | 0.004 | 0.008 | -0.306 |
| RHSIM_Rhsim03G0084900 | 8.34 | 0.004 | 0.008 | 0.822 |
| RHSIM_Rhsim09G0055300 | 8.34 | 0.004 | 0.008 | 0.626 |
| RHSIM_RhsimUnG0012500 | 8.34 | 0.004 | 0.008 | 0.425 |

| Gene | LRT statistic | P value | BH-adjusted P | log2FC (interaction) |
|---|---|---|---|---|
| RHSIM_Rhsim02G0168700 | 8.34 | 0.004 | 0.008 | -78.856 |
| RHSIM_Rhsim06G0003400 | 8.34 | 0.004 | 0.008 | 0.518 |
| RHSIM_Rhsim08G0250500 | 8.34 | 0.004 | 0.008 | -0.406 |
| RHSIM_Rhsim04G0014300 | 8.33 | 0.004 | 0.008 | 0.469 |
| RHSIM_Rhsim08G0165500 | 8.33 | 0.004 | 0.008 | -2.998 |
| RHSIM_Rhsim07G0109600 | 8.33 | 0.004 | 0.008 | 1.458 |
| RHSIM_Rhsim07G0042500 | 8.33 | 0.004 | 0.008 | 1.679 |
| RHSIM_Rhsim03G0230900 | 8.33 | 0.004 | 0.008 | -0.314 |
| RHSIM_Rhsim05G0228200 | 8.33 | 0.004 | 0.008 | 0.269 |
| novel.570 | 8.32 | 0.004 | 0.008 | 1.639 |
| RHSIM_Rhsim06G0205500 | 8.32 | 0.004 | 0.008 | 1.651 |
| RHSIM_Rhsim06G0166300 | 8.32 | 0.004 | 0.008 | 0.327 |
| RHSIM_Rhsim03G0059100 | 8.32 | 0.004 | 0.008 | -1.607 |
| novel.2081 | 8.32 | 0.004 | 0.008 | 2.869 |
| RHSIM_Rhsim06G0164600 | 8.32 | 0.004 | 0.008 | 0.862 |
| RHSIM_Rhsim11G0138300 | 8.32 | 0.004 | 0.008 | -2.366 |
| novel.3197 | 8.32 | 0.004 | 0.008 | 2.220 |
| novel.1746 | 8.32 | 0.004 | 0.008 | -0.808 |
| RHSIM_Rhsim03G0134700 | 8.31 | 0.004 | 0.008 | 4.979 |
| RHSIM_Rhsim12G0154700 | 8.31 | 0.004 | 0.008 | 0.450 |
| RHSIM_Rhsim12G0008300 | 8.31 | 0.004 | 0.008 | -0.826 |
| RHSIM_RhsimUnG0185000 | 8.31 | 0.004 | 0.008 | 1.988 |
| novel.1270 | 8.31 | 0.004 | 0.008 | -0.582 |
| RHSIM_Rhsim02G0008100 | 8.31 | 0.004 | 0.008 | 6.152 |
| RHSIM_Rhsim02G0213800 | 8.30 | 0.004 | 0.008 | 1.229 |
| RHSIM_RhsimUnG0006000 | 8.30 | 0.004 | 0.008 | 0.790 |
| RHSIM_Rhsim03G0067300 | 8.30 | 0.004 | 0.008 | -3.437 |
| RHSIM_Rhsim06G0230000 | 8.30 | 0.004 | 0.008 | 2.001 |
| RHSIM_Rhsim01G0282400 | 8.30 | 0.004 | 0.008 | 0.544 |
| RHSIM_Rhsim02G0099900 | 8.30 | 0.004 | 0.008 | -0.323 |
| RHSIM_Rhsim02G0127500 | 8.30 | 0.004 | 0.008 | -0.254 |
| RHSIM_Rhsim04G0137400 | 8.30 | 0.004 | 0.008 | 1.529 |

| Gene | LRT statistic | P value | BH-adjusted P | log2FC (interaction) |
|---|---|---|---|---|
| RHSIM_RhsimUnG0187700 | 8.30 | 0.004 | 0.008 | 1.206 |
| RHSIM_Rhsim08G0013500 | 8.30 | 0.004 | 0.008 | -1.707 |
| novel.4357 | 8.29 | 0.004 | 0.008 | -41.902 |
| RHSIM_Rhsim12G0178100 | 8.29 | 0.004 | 0.008 | 5.096 |
| RHSIM_Rhsim13G0006800 | 8.29 | 0.004 | 0.008 | 0.144 |
| RHSIM_Rhsim04G0062300 | 8.29 | 0.004 | 0.008 | -1.701 |
| RHSIM_Rhsim11G0099700 | 8.29 | 0.004 | 0.008 | -0.315 |
| RHSIM_Rhsim05G0176100 | 8.28 | 0.004 | 0.008 | 2.274 |
| RHSIM_Rhsim07G0245500 | 8.28 | 0.004 | 0.008 | -0.241 |
| novel.4143 | 8.28 | 0.004 | 0.008 | 1.076 |
| novel.764 | 8.28 | 0.004 | 0.008 | 0.389 |
| RHSIM_Rhsim07G0170700 | 8.28 | 0.004 | 0.008 | 2.802 |
| RHSIM_Rhsim02G0194200 | 8.28 | 0.004 | 0.008 | 3.835 |
| RHSIM_Rhsim02G0006500 | 8.28 | 0.004 | 0.008 | -0.528 |
| novel.3722 | 8.28 | 0.004 | 0.008 | 1.929 |
| novel.246 | 8.28 | 0.004 | 0.008 | 79.005 |
| novel.2167 | 8.27 | 0.004 | 0.008 | 0.884 |
| RHSIM_Rhsim05G0231700 | 8.27 | 0.004 | 0.008 | 0.514 |
| RHSIM_Rhsim06G0095500 | 8.27 | 0.004 | 0.008 | 0.931 |
| novel.1360 | 8.27 | 0.004 | 0.008 | 1.899 |
| RHSIM_Rhsim07G0010100 | 8.26 | 0.004 | 0.008 | 1.306 |
| RHSIM_Rhsim01G0269700 | 8.26 | 0.004 | 0.008 | -0.505 |
| novel.3046 | 8.26 | 0.004 | 0.008 | 0.145 |
| novel.2956 | 8.26 | 0.004 | 0.008 | -2.700 |
| RHSIM_Rhsim05G0082400 | 8.26 | 0.004 | 0.008 | -1.251 |
| RHSIM_Rhsim07G0154600 | 8.26 | 0.004 | 0.008 | 1.989 |
| RHSIM_Rhsim11G0122900 | 8.26 | 0.004 | 0.008 | -0.238 |
| RHSIM_Rhsim03G0171000 | 8.25 | 0.004 | 0.008 | -0.382 |
| novel.709 | 8.25 | 0.004 | 0.008 | 1.279 |
| RHSIM_Rhsim05G0231900 | 8.25 | 0.004 | 0.008 | 1.543 |
| RHSIM_Rhsim03G0044600 | 8.25 | 0.004 | 0.008 | -0.314 |
| RHSIM_RhsimUnG0021000 | 8.25 | 0.004 | 0.008 | -0.440 |

| Gene | LRT statistic | P value | BH-adjusted P | log2FC (interaction) |
|---|---|---|---|---|
| RHSIM_Rhsim05G0220800 | 8.25 | 0.004 | 0.008 | 2.258 |
| RHSIM_Rhsim11G0146100 | 8.25 | 0.004 | 0.008 | 0.504 |
| RHSIM_Rhsim01G0275800 | 8.25 | 0.004 | 0.008 | 0.247 |
| RHSIM_Rhsim08G0143500 | 8.25 | 0.004 | 0.008 | 0.457 |
| RHSIM_Rhsim10G0167800 | 8.25 | 0.004 | 0.008 | -1.207 |
| RHSIM_Rhsim11G0128300 | 8.24 | 0.004 | 0.008 | 0.357 |
| RHSIM_Rhsim09G0003900 | 8.24 | 0.004 | 0.008 | 0.695 |
| RHSIM_Rhsim11G0186000 | 8.24 | 0.004 | 0.008 | 0.794 |
| RHSIM_Rhsim08G0203200 | 8.24 | 0.004 | 0.008 | 0.480 |
| RHSIM_Rhsim04G0173300 | 8.24 | 0.004 | 0.008 | -0.205 |
| RHSIM_Rhsim10G0153500 | 8.24 | 0.004 | 0.008 | 1.634 |
| RHSIM_Rhsim08G0216500 | 8.23 | 0.004 | 0.009 | 0.417 |
| RHSIM_Rhsim08G0116000 | 8.23 | 0.004 | 0.009 | -0.225 |
| RHSIM_Rhsim10G0074800 | 8.23 | 0.004 | 0.009 | -3.354 |
| novel.2789 | 8.23 | 0.004 | 0.009 | 0.590 |
| novel.2350 | 8.22 | 0.004 | 0.009 | 3.269 |
| RHSIM_Rhsim04G0030000 | 8.22 | 0.004 | 0.009 | 1.408 |
| RHSIM_Rhsim04G0213600 | 8.22 | 0.004 | 0.009 | 2.707 |
| RHSIM_Rhsim03G0248200 | 8.22 | 0.004 | 0.009 | 0.392 |
| RHSIM_Rhsim12G0139800 | 8.22 | 0.004 | 0.009 | 41.906 |
| RHSIM_Rhsim06G0225600 | 8.21 | 0.004 | 0.009 | -0.451 |
| RHSIM_Rhsim07G0128400 | 8.21 | 0.004 | 0.009 | 0.172 |
| RHSIM_Rhsim13G0146400 | 8.21 | 0.004 | 0.009 | 0.321 |
| RHSIM_RhsimUnG0015400 | 8.21 | 0.004 | 0.009 | 0.889 |
| RHSIM_Rhsim08G0144900 | 8.21 | 0.004 | 0.009 | -0.447 |
| RHSIM_Rhsim10G0017600 | 8.21 | 0.004 | 0.009 | 0.990 |
| RHSIM_RhsimUnG0096800 | 8.20 | 0.004 | 0.009 | -0.795 |
| RHSIM_Rhsim13G0078800 | 8.20 | 0.004 | 0.009 | -0.362 |
| RHSIM_Rhsim02G0043900 | 8.20 | 0.004 | 0.009 | 1.662 |
| RHSIM_Rhsim13G0190600 | 8.20 | 0.004 | 0.009 | -39.555 |
| RHSIM_Rhsim01G0232700 | 8.19 | 0.004 | 0.009 | 0.856 |
| RHSIM_Rhsim01G0188400 | 8.19 | 0.004 | 0.009 | 0.191 |

| Gene | LRT statistic | P value | BH-adjusted P | log2FC (interaction) |
|---|---|---|---|---|
| novel.871 | 8.19 | 0.004 | 0.009 | -0.600 |
| RHSIM_Rhsim08G0219400 | 8.19 | 0.004 | 0.009 | -1.082 |
| RHSIM_Rhsim09G0168500 | 8.18 | 0.004 | 0.009 | 3.199 |
| RHSIM_Rhsim06G0029600 | 8.18 | 0.004 | 0.009 | -1.892 |
| RHSIM_Rhsim11G0133400 | 8.18 | 0.004 | 0.009 | 0.351 |
| RHSIM_Rhsim01G0102500 | 8.18 | 0.004 | 0.009 | -0.307 |
| RHSIM_Rhsim07G0161300 | 8.18 | 0.004 | 0.009 | 0.540 |
| RHSIM_Rhsim01G0046800 | 8.18 | 0.004 | 0.009 | 0.953 |
| RHSIM_Rhsim13G0183700 | 8.18 | 0.004 | 0.009 | 2.823 |
| RHSIM_Rhsim10G0083600 | 8.18 | 0.004 | 0.009 | -0.771 |
| RHSIM_Rhsim13G0070400 | 8.18 | 0.004 | 0.009 | 2.110 |
| RHSIM_Rhsim05G0040100 | 8.17 | 0.004 | 0.009 | -0.355 |
| RHSIM_Rhsim03G0111500 | 8.17 | 0.004 | 0.009 | -0.367 |
| RHSIM_Rhsim01G0270700 | 8.17 | 0.004 | 0.009 | 78.891 |
| novel.2770 | 8.17 | 0.004 | 0.009 | -2.245 |
| RHSIM_Rhsim07G0179400 | 8.17 | 0.004 | 0.009 | -0.262 |
| RHSIM_Rhsim10G0079100 | 8.17 | 0.004 | 0.009 | 0.471 |
| RHSIM_Rhsim13G0076200 | 8.17 | 0.004 | 0.009 | 79.360 |
| RHSIM_Rhsim08G0244300 | 8.17 | 0.004 | 0.009 | 0.171 |
| RHSIM_RhsimUnG0148600 | 8.16 | 0.004 | 0.009 | 0.270 |
| RHSIM_Rhsim01G0025900 | 8.16 | 0.004 | 0.009 | -1.658 |
| RHSIM_Rhsim11G0085100 | 8.16 | 0.004 | 0.009 | -39.428 |
| novel.752 | 8.16 | 0.004 | 0.009 | -2.009 |
| RHSIM_Rhsim03G0233400 | 8.16 | 0.004 | 0.009 | -5.437 |
| RHSIM_Rhsim07G0109300 | 8.16 | 0.004 | 0.009 | -1.411 |
| RHSIM_Rhsim06G0048300 | 8.16 | 0.004 | 0.009 | -0.518 |
| novel.3045 | 8.16 | 0.004 | 0.009 | -0.666 |
| novel.2512 | 8.16 | 0.004 | 0.009 | -1.967 |
| RHSIM_Rhsim04G0050400 | 8.15 | 0.004 | 0.009 | -3.586 |
| RHSIM_Rhsim05G0064600 | 8.15 | 0.004 | 0.009 | 41.725 |
| novel.3394 | 8.15 | 0.004 | 0.009 | 4.362 |
| RHSIM_RhsimUnG0234500 | 8.15 | 0.004 | 0.009 | -1.516 |

| Gene | LRT statistic | P value | BH-adjusted P | log2FC (interaction) |
|---|---|---|---|---|
| RHSIM_Rhsim06G0054500 | 8.15 | 0.004 | 0.009 | -0.347 |
| RHSIM_Rhsim06G0037200 | 8.15 | 0.004 | 0.009 | 4.834 |
| RHSIM_Rhsim08G0160400 | 8.14 | 0.004 | 0.009 | -0.769 |
| RHSIM_Rhsim01G0156000 | 8.14 | 0.004 | 0.009 | 4.254 |
| RHSIM_Rhsim01G0225400 | 8.14 | 0.004 | 0.009 | 0.260 |
| RHSIM_Rhsim13G0024800 | 8.14 | 0.004 | 0.009 | 2.639 |
| RHSIM_RhsimUnG0115600 | 8.13 | 0.004 | 0.009 | -3.411 |
| RHSIM_Rhsim05G0037100 | 8.13 | 0.004 | 0.009 | -1.801 |
| RHSIM_Rhsim07G0047500 | 8.13 | 0.004 | 0.009 | 3.118 |
| novel.950 | 8.13 | 0.004 | 0.009 | 40.566 |
| novel.502 | 8.13 | 0.004 | 0.009 | 1.780 |
| RHSIM_Rhsim04G0049100 | 8.13 | 0.004 | 0.009 | 5.325 |
| RHSIM_Rhsim01G0259400 | 8.13 | 0.004 | 0.009 | -0.635 |
| novel.1347 | 8.13 | 0.004 | 0.009 | -2.730 |
| RHSIM_Rhsim01G0281200 | 8.13 | 0.004 | 0.009 | -2.069 |
| RHSIM_Rhsim10G0138100 | 8.12 | 0.004 | 0.009 | 0.411 |
| RHSIM_Rhsim13G0206200 | 8.12 | 0.004 | 0.009 | 1.110 |
| RHSIM_Rhsim07G0248300 | 8.12 | 0.004 | 0.009 | -0.884 |
| novel.2121 | 8.12 | 0.004 | 0.009 | -3.422 |
| RHSIM_Rhsim02G0126600 | 8.12 | 0.004 | 0.009 | 2.699 |
| RHSIM_Rhsim03G0080100 | 8.12 | 0.004 | 0.009 | 1.479 |
| RHSIM_Rhsim13G0112200 | 8.12 | 0.004 | 0.009 | -0.429 |
| RHSIM_Rhsim03G0005200 | 8.12 | 0.004 | 0.009 | 3.523 |
| novel.189 | 8.12 | 0.004 | 0.009 | 1.028 |
| RHSIM_Rhsim03G0196100 | 8.12 | 0.004 | 0.009 | -1.208 |
| RHSIM_Rhsim11G0032200 | 8.12 | 0.004 | 0.009 | -0.976 |
| RHSIM_Rhsim02G0177400 | 8.11 | 0.004 | 0.009 | 1.006 |
| RHSIM_Rhsim02G0071300 | 8.11 | 0.004 | 0.009 | 0.563 |
| RHSIM_Rhsim01G0002600 | 8.11 | 0.004 | 0.009 | 0.299 |
| RHSIM_Rhsim05G0074300 | 8.11 | 0.004 | 0.009 | 0.860 |
| RHSIM_Rhsim13G0028600 | 8.11 | 0.004 | 0.009 | 0.627 |
| novel.1016 | 8.11 | 0.004 | 0.009 | 1.488 |

| Gene | LRT statistic | P value | BH-adjusted P | log2FC (interaction) |
|---|---|---|---|---|
| novel.751 | 8.10 | 0.004 | 0.009 | 1.410 |
| RHSIM_Rhsim09G0146700 | 8.10 | 0.004 | 0.009 | 0.320 |
| RHSIM_Rhsim09G0040700 | 8.10 | 0.004 | 0.009 | -2.000 |
| RHSIM_Rhsim06G0004900 | 8.10 | 0.004 | 0.009 | -0.612 |
| RHSIM_Rhsim11G0030600 | 8.10 | 0.004 | 0.009 | -2.542 |
| RHSIM_Rhsim13G0184700 | 8.10 | 0.004 | 0.009 | 41.229 |
| RHSIM_Rhsim09G0106400 | 8.10 | 0.004 | 0.009 | -0.410 |
| RHSIM_Rhsim03G0011200 | 8.10 | 0.004 | 0.009 | 1.308 |
| RHSIM_Rhsim03G0039900 | 8.09 | 0.004 | 0.009 | -0.354 |
| RHSIM_Rhsim01G0172900 | 8.09 | 0.004 | 0.009 | 1.056 |
| RHSIM_Rhsim04G0160200 | 8.09 | 0.004 | 0.009 | 0.377 |
| RHSIM_Rhsim08G0080100 | 8.09 | 0.004 | 0.009 | 0.296 |
| RHSIM_Rhsim11G0023300 | 8.09 | 0.004 | 0.009 | 2.090 |
| RHSIM_Rhsim05G0031000 | 8.09 | 0.004 | 0.009 | 1.151 |
| RHSIM_Rhsim12G0174200 | 8.09 | 0.004 | 0.009 | 0.731 |
| RHSIM_Rhsim03G0252000 | 8.08 | 0.004 | 0.009 | 0.306 |
| RHSIM_Rhsim11G0021300 | 8.08 | 0.004 | 0.009 | 1.094 |
| RHSIM_Rhsim05G0010800 | 8.08 | 0.004 | 0.009 | -42.328 |
| RHSIM_Rhsim04G0143700 | 8.07 | 0.004 | 0.009 | 1.077 |
| RHSIM_Rhsim02G0054700 | 8.07 | 0.005 | 0.009 | 78.663 |
| RHSIM_Rhsim09G0196900 | 8.06 | 0.005 | 0.009 | -0.394 |
| RHSIM_Rhsim13G0102600 | 8.06 | 0.005 | 0.009 | -2.741 |
| novel.3498 | 8.06 | 0.005 | 0.009 | -0.590 |
| RHSIM_Rhsim02G0203700 | 8.06 | 0.005 | 0.009 | 0.786 |
| novel.2812 | 8.06 | 0.005 | 0.009 | 2.464 |
| novel.4195 | 8.06 | 0.005 | 0.009 | 0.812 |
| RHSIM_Rhsim04G0021400 | 8.05 | 0.005 | 0.009 | -0.177 |
| RHSIM_Rhsim10G0170400 | 8.05 | 0.005 | 0.009 | -2.621 |
| novel.1578 | 8.05 | 0.005 | 0.009 | -1.675 |
| novel.1132 | 8.05 | 0.005 | 0.009 | -0.352 |
| RHSIM_Rhsim09G0033700 | 8.04 | 0.005 | 0.009 | 1.543 |
| RHSIM_Rhsim03G0105800 | 8.04 | 0.005 | 0.009 | 0.790 |

| Gene | LRT statistic | P value | BH-adjusted P | log2FC (interaction) |
|---|---|---|---|---|
| RHSIM_Rhsim06G0129700 | 8.04 | 0.005 | 0.009 | 1.246 |
| RHSIM_Rhsim06G0199100 | 8.04 | 0.005 | 0.009 | 0.439 |
| RHSIM_RhsimMtG0000800 | 8.03 | 0.005 | 0.009 | 1.368 |
| RHSIM_Rhsim12G0048900 | 8.03 | 0.005 | 0.009 | -1.275 |
| RHSIM_Rhsim04G0035600 | 8.03 | 0.005 | 0.009 | 1.452 |
| RHSIM_Rhsim07G0067500 | 8.03 | 0.005 | 0.009 | 3.056 |
| RHSIM_Rhsim10G0133400 | 8.03 | 0.005 | 0.009 | -0.377 |
| novel.3164 | 8.03 | 0.005 | 0.009 | 1.584 |
| novel.4101 | 8.03 | 0.005 | 0.009 | -1.375 |
| RHSIM_Rhsim01G0292700 | 8.02 | 0.005 | 0.009 | -0.399 |
| RHSIM_Rhsim06G0218600 | 8.02 | 0.005 | 0.009 | -3.240 |
| RHSIM_Rhsim13G0116700 | 8.02 | 0.005 | 0.009 | -0.333 |
| RHSIM_Rhsim07G0237700 | 8.02 | 0.005 | 0.009 | -1.871 |
| RHSIM_Rhsim10G0008700 | 8.02 | 0.005 | 0.009 | 2.283 |
| RHSIM_RhsimUnG0048400 | 8.02 | 0.005 | 0.010 | 1.693 |
| RHSIM_RhsimUnG0156700 | 8.02 | 0.005 | 0.010 | -1.155 |
| RHSIM_Rhsim01G0120600 | 8.01 | 0.005 | 0.010 | 2.327 |
| RHSIM_Rhsim09G0137000 | 8.01 | 0.005 | 0.010 | 1.070 |
| RHSIM_Rhsim04G0171700 | 8.01 | 0.005 | 0.010 | 2.464 |
| RHSIM_RhsimUnG0153600 | 8.01 | 0.005 | 0.010 | 1.661 |
| RHSIM_Rhsim07G0019500 | 8.01 | 0.005 | 0.010 | -3.177 |
| RHSIM_RhsimUnG0023900 | 8.01 | 0.005 | 0.010 | 1.243 |
| RHSIM_Rhsim13G0185300 | 8.01 | 0.005 | 0.010 | 42.364 |
| RHSIM_Rhsim02G0186900 | 8.01 | 0.005 | 0.010 | 4.025 |
| RHSIM_Rhsim12G0119600 | 8.01 | 0.005 | 0.010 | -1.841 |
| RHSIM_Rhsim08G0152400 | 8.00 | 0.005 | 0.010 | 1.318 |
| novel.4193 | 8.00 | 0.005 | 0.010 | -2.997 |
| RHSIM_Rhsim10G0198000 | 8.00 | 0.005 | 0.010 | 0.378 |
| RHSIM_Rhsim11G0101000 | 8.00 | 0.005 | 0.010 | 1.083 |
| RHSIM_Rhsim06G0122700 | 8.00 | 0.005 | 0.010 | 0.534 |
| RHSIM_Rhsim08G0008500 | 8.00 | 0.005 | 0.010 | -0.220 |
| RHSIM_Rhsim07G0126600 | 8.00 | 0.005 | 0.010 | -40.801 |

| Gene | LRT statistic | P value | BH-adjusted P | log2FC (interaction) |
|---|---|---|---|---|
| RHSIM_Rhsim07G0223900 | 7.99 | 0.005 | 0.010 | -0.337 |
| novel.543 | 7.99 | 0.005 | 0.010 | -0.540 |
| RHSIM_Rhsim01G0228400 | 7.98 | 0.005 | 0.010 | 0.313 |
| RHSIM_RhsimUnG0085600 | 7.98 | 0.005 | 0.010 | -1.776 |
| RHSIM_Rhsim01G0201200 | 7.98 | 0.005 | 0.010 | -2.540 |
| RHSIM_Rhsim13G0188700 | 7.98 | 0.005 | 0.010 | 1.244 |
| RHSIM_Rhsim10G0171900 | 7.98 | 0.005 | 0.010 | 0.617 |
| RHSIM_Rhsim08G0217200 | 7.98 | 0.005 | 0.010 | 0.908 |
| RHSIM_Rhsim02G0071600 | 7.97 | 0.005 | 0.010 | 1.773 |
| RHSIM_Rhsim13G0035200 | 7.97 | 0.005 | 0.010 | 5.628 |
| RHSIM_Rhsim12G0053500 | 7.97 | 0.005 | 0.010 | -3.154 |
| RHSIM_Rhsim13G0233000 | 7.97 | 0.005 | 0.010 | 0.343 |
| RHSIM_Rhsim01G0222200 | 7.97 | 0.005 | 0.010 | -5.135 |
| RHSIM_Rhsim11G0180100 | 7.96 | 0.005 | 0.010 | 42.593 |
| RHSIM_RhsimUnG0110700 | 7.96 | 0.005 | 0.010 | -0.976 |
| RHSIM_Rhsim13G0146900 | 7.96 | 0.005 | 0.010 | -0.327 |
| RHSIM_Rhsim09G0164300 | 7.96 | 0.005 | 0.010 | 0.340 |
| RHSIM_Rhsim06G0198400 | 7.95 | 0.005 | 0.010 | -0.226 |
| RHSIM_Rhsim07G0163600 | 7.95 | 0.005 | 0.010 | -0.620 |
| RHSIM_Rhsim05G0039400 | 7.95 | 0.005 | 0.010 | -0.699 |
| RHSIM_Rhsim02G0060300 | 7.95 | 0.005 | 0.010 | 0.333 |
| RHSIM_Rhsim04G0178000 | 7.95 | 0.005 | 0.010 | -2.107 |
| novel.1927 | 7.95 | 0.005 | 0.010 | -2.390 |
| RHSIM_Rhsim11G0151500 | 7.95 | 0.005 | 0.010 | -0.555 |
| RHSIM_Rhsim11G0153400 | 7.95 | 0.005 | 0.010 | 2.817 |
| RHSIM_Rhsim09G0001100 | 7.95 | 0.005 | 0.010 | 0.399 |
| RHSIM_Rhsim09G0039500 | 7.95 | 0.005 | 0.010 | -0.712 |
| novel.2382 | 7.95 | 0.005 | 0.010 | 1.078 |
| RHSIM_Rhsim10G0167500 | 7.95 | 0.005 | 0.010 | -2.003 |
| RHSIM_Rhsim03G0140400 | 7.94 | 0.005 | 0.010 | -1.005 |
| RHSIM_Rhsim09G0003700 | 7.94 | 0.005 | 0.010 | -0.372 |
| RHSIM_Rhsim13G0036400 | 7.94 | 0.005 | 0.010 | 0.249 |

| Gene | LRT statistic | P value | BH-adjusted P | log2FC (interaction) |
|---|---|---|---|---|
| RHSIM_Rhsim04G0160300 | 7.93 | 0.005 | 0.010 | 1.231 |
| RHSIM_Rhsim08G0120000 | 7.93 | 0.005 | 0.010 | 0.602 |
| RHSIM_Rhsim06G0229200 | 7.93 | 0.005 | 0.010 | 2.982 |
| novel.1757 | 7.93 | 0.005 | 0.010 | 2.501 |
| RHSIM_Rhsim03G0105900 | 7.93 | 0.005 | 0.010 | -1.980 |
| RHSIM_Rhsim03G0079200 | 7.93 | 0.005 | 0.010 | -0.770 |
| RHSIM_Rhsim12G0188000 | 7.93 | 0.005 | 0.010 | -0.771 |
| RHSIM_Rhsim13G0053100 | 7.93 | 0.005 | 0.010 | -1.755 |
| RHSIM_Rhsim03G0200400 | 7.92 | 0.005 | 0.010 | 1.715 |
| RHSIM_Rhsim04G0004700 | 7.92 | 0.005 | 0.010 | 1.774 |
| RHSIM_Rhsim13G0154800 | 7.92 | 0.005 | 0.010 | -0.411 |
| RHSIM_Rhsim03G0140500 | 7.92 | 0.005 | 0.010 | 0.924 |
| RHSIM_Rhsim02G0242000 | 7.92 | 0.005 | 0.010 | 3.171 |
| RHSIM_Rhsim05G0190300 | 7.91 | 0.005 | 0.010 | 1.244 |
| RHSIM_Rhsim01G0114900 | 7.91 | 0.005 | 0.010 | 6.278 |
| RHSIM_Rhsim12G0070800 | 7.91 | 0.005 | 0.010 | 1.631 |
| RHSIM_Rhsim01G0101900 | 7.91 | 0.005 | 0.010 | -0.400 |
| RHSIM_Rhsim10G0094400 | 7.91 | 0.005 | 0.010 | 0.455 |
| RHSIM_Rhsim11G0157800 | 7.91 | 0.005 | 0.010 | -0.580 |
| RHSIM_Rhsim04G0003100 | 7.90 | 0.005 | 0.010 | 1.757 |
| RHSIM_RhsimUnG0215100 | 7.90 | 0.005 | 0.010 | 0.792 |
| RHSIM_Rhsim03G0124200 | 7.90 | 0.005 | 0.010 | 0.916 |
| RHSIM_Rhsim07G0145000 | 7.90 | 0.005 | 0.010 | -0.590 |
| RHSIM_Rhsim05G0049200 | 7.90 | 0.005 | 0.010 | -0.703 |
| RHSIM_Rhsim11G0132300 | 7.89 | 0.005 | 0.010 | 2.451 |
| RHSIM_Rhsim13G0122800 | 7.89 | 0.005 | 0.010 | 0.244 |
| novel.1117 | 7.89 | 0.005 | 0.010 | 1.546 |
| RHSIM_RhsimUnG0163200 | 7.89 | 0.005 | 0.010 | -2.385 |
| RHSIM_Rhsim03G0082600 | 7.89 | 0.005 | 0.010 | -1.961 |
| RHSIM_Rhsim01G0000600 | 7.89 | 0.005 | 0.010 | 2.440 |
| RHSIM_Rhsim12G0209200 | 7.89 | 0.005 | 0.010 | -0.299 |
| novel.2410 | 7.89 | 0.005 | 0.010 | -2.409 |

| Gene | LRT statistic | P value | BH-adjusted P | log2FC (interaction) |
|---|---|---|---|---|
| RHSIM_Rhsim12G0119400 | 7.88 | 0.005 | 0.010 | -1.182 |
| RHSIM_Rhsim02G0011900 | 7.88 | 0.005 | 0.010 | 0.377 |
| RHSIM_Rhsim10G0117500 | 7.88 | 0.005 | 0.010 | -2.257 |
| RHSIM_Rhsim06G0163500 | 7.88 | 0.005 | 0.010 | -2.242 |
| RHSIM_RhsimUnG0236600 | 7.88 | 0.005 | 0.010 | -0.572 |
| novel.1011 | 7.88 | 0.005 | 0.010 | 0.439 |
| RHSIM_Rhsim12G0123500 | 7.87 | 0.005 | 0.010 | -0.882 |
| novel.2529 | 7.87 | 0.005 | 0.010 | 1.516 |
| RHSIM_Rhsim05G0146600 | 7.87 | 0.005 | 0.010 | -0.328 |
| RHSIM_Rhsim06G0064300 | 7.87 | 0.005 | 0.010 | 0.405 |
| RHSIM_Rhsim01G0281900 | 7.86 | 0.005 | 0.010 | -0.905 |
| RHSIM_Rhsim04G0166900 | 7.86 | 0.005 | 0.010 | -0.208 |
| RHSIM_Rhsim06G0054000 | 7.86 | 0.005 | 0.010 | -0.875 |
| RHSIM_Rhsim07G0229800 | 7.86 | 0.005 | 0.010 | 0.884 |
| novel.3827 | 7.86 | 0.005 | 0.010 | 0.202 |
| RHSIM_Rhsim07G0182000 | 7.85 | 0.005 | 0.010 | 78.663 |
| RHSIM_RhsimUnG0084600 | 7.85 | 0.005 | 0.010 | 1.419 |
| RHSIM_Rhsim06G0022000 | 7.85 | 0.005 | 0.010 | 1.873 |
| RHSIM_Rhsim05G0230600 | 7.85 | 0.005 | 0.010 | 3.755 |
| RHSIM_Rhsim07G0175700 | 7.85 | 0.005 | 0.010 | 0.204 |
| RHSIM_Rhsim03G0169100 | 7.85 | 0.005 | 0.010 | 3.768 |
| novel.2589 | 7.85 | 0.005 | 0.010 | 1.825 |
| novel.2946 | 7.84 | 0.005 | 0.010 | 1.225 |
| RHSIM_Rhsim10G0124000 | 7.84 | 0.005 | 0.010 | -0.273 |
| RHSIM_Rhsim12G0102800 | 7.84 | 0.005 | 0.010 | 1.184 |
| RHSIM_Rhsim04G0127300 | 7.84 | 0.005 | 0.010 | 0.197 |
| RHSIM_Rhsim12G0075600 | 7.83 | 0.005 | 0.010 | -1.918 |
| RHSIM_RhsimUnG0245600 | 7.83 | 0.005 | 0.010 | -3.369 |
| RHSIM_Rhsim12G0116900 | 7.83 | 0.005 | 0.010 | 0.335 |
| RHSIM_Rhsim02G0156800 | 7.83 | 0.005 | 0.010 | 0.281 |
| novel.2220 | 7.83 | 0.005 | 0.010 | 40.604 |
| RHSIM_Rhsim11G0104000 | 7.82 | 0.005 | 0.010 | -1.751 |

| Gene | LRT statistic | P value | BH-adjusted P | log2FC (interaction) |
|---|---|---|---|---|
| RHSIM_Rhsim02G0061800 | 7.82 | 0.005 | 0.010 | 1.785 |
| RHSIM_Rhsim04G0140900 | 7.82 | 0.005 | 0.010 | 43.275 |
| RHSIM_Rhsim07G0160100 | 7.82 | 0.005 | 0.011 | 0.430 |
| RHSIM_Rhsim12G0038400 | 7.82 | 0.005 | 0.011 | 0.262 |
| novel.3761 | 7.82 | 0.005 | 0.011 | -0.474 |
| RHSIM_Rhsim06G0123400 | 7.81 | 0.005 | 0.011 | 0.914 |
| novel.1470 | 7.81 | 0.005 | 0.011 | 0.731 |
| RHSIM_Rhsim06G0176000 | 7.81 | 0.005 | 0.011 | -42.235 |
| RHSIM_Rhsim06G0144200 | 7.81 | 0.005 | 0.011 | 78.836 |
| RHSIM_Rhsim07G0200700 | 7.81 | 0.005 | 0.011 | 1.764 |
| RHSIM_Rhsim05G0218800 | 7.81 | 0.005 | 0.011 | 1.094 |
| novel.4302 | 7.81 | 0.005 | 0.011 | 1.737 |
| novel.2953 | 7.81 | 0.005 | 0.011 | -0.864 |
| RHSIM_Rhsim01G0132800 | 7.80 | 0.005 | 0.011 | -0.341 |
| RHSIM_Rhsim07G0037000 | 7.80 | 0.005 | 0.011 | -3.047 |
| novel.4463 | 7.80 | 0.005 | 0.011 | 3.568 |
| RHSIM_Rhsim12G0003500 | 7.80 | 0.005 | 0.011 | 0.242 |
| RHSIM_Rhsim03G0207600 | 7.80 | 0.005 | 0.011 | 1.003 |
| RHSIM_Rhsim03G0235800 | 7.80 | 0.005 | 0.011 | -1.253 |
| RHSIM_Rhsim10G0213300 | 7.80 | 0.005 | 0.011 | -4.464 |
| RHSIM_Rhsim07G0166400 | 7.79 | 0.005 | 0.011 | 0.415 |
| RHSIM_Rhsim03G0007300 | 7.79 | 0.005 | 0.011 | -0.436 |
| novel.467 | 7.79 | 0.005 | 0.011 | 2.098 |
| RHSIM_Rhsim04G0057400 | 7.78 | 0.005 | 0.011 | 0.540 |
| RHSIM_Rhsim07G0068500 | 7.78 | 0.005 | 0.011 | -0.289 |
| RHSIM_Rhsim09G0011200 | 7.78 | 0.005 | 0.011 | -2.733 |
| RHSIM_RhsimUnG0108300 | 7.77 | 0.005 | 0.011 | -3.235 |
| RHSIM_Rhsim13G0148000 | 7.77 | 0.005 | 0.011 | -2.999 |
| novel.4099 | 7.77 | 0.005 | 0.011 | 3.512 |
| RHSIM_Rhsim08G0084200 | 7.77 | 0.005 | 0.011 | 0.386 |
| RHSIM_Rhsim01G0163300 | 7.77 | 0.005 | 0.011 | 0.278 |
| RHSIM_Rhsim06G0004000 | 7.77 | 0.005 | 0.011 | -0.428 |

| Gene | LRT statistic | P value | BH-adjusted P | log2FC (interaction) |
|---|---|---|---|---|
| RHSIM_Rhsim10G0058000 | 7.77 | 0.005 | 0.011 | 2.168 |
| RHSIM_RhsimUnG0022200 | 7.77 | 0.005 | 0.011 | -1.095 |
| RHSIM_Rhsim04G0010100 | 7.77 | 0.005 | 0.011 | 0.524 |
| novel.520 | 7.77 | 0.005 | 0.011 | 1.461 |
| RHSIM_Rhsim05G0089300 | 7.77 | 0.005 | 0.011 | -1.172 |
| RHSIM_Rhsim04G0016600 | 7.76 | 0.005 | 0.011 | 0.240 |
| novel.11 | 7.76 | 0.005 | 0.011 | 0.943 |
| RHSIM_Rhsim05G0144600 | 7.76 | 0.005 | 0.011 | 1.538 |
| RHSIM_Rhsim04G0225900 | 7.76 | 0.005 | 0.011 | -41.630 |
| RHSIM_RhsimUnG0124600 | 7.76 | 0.005 | 0.011 | 2.361 |
| RHSIM_Rhsim10G0192800 | 7.76 | 0.005 | 0.011 | -0.235 |
| RHSIM_Rhsim02G0243900 | 7.76 | 0.005 | 0.011 | -39.525 |
| RHSIM_Rhsim05G0144000 | 7.76 | 0.005 | 0.011 | -0.708 |
| RHSIM_Rhsim01G0256000 | 7.75 | 0.005 | 0.011 | -0.541 |
| RHSIM_Rhsim09G0080700 | 7.75 | 0.005 | 0.011 | 0.880 |
| RHSIM_Rhsim09G0032600 | 7.75 | 0.005 | 0.011 | -0.280 |
| novel.3284 | 7.74 | 0.005 | 0.011 | -1.929 |
| novel.4444 | 7.74 | 0.005 | 0.011 | 0.664 |
| RHSIM_Rhsim07G0045000 | 7.74 | 0.005 | 0.011 | 1.061 |
| novel.1297 | 7.74 | 0.005 | 0.011 | 2.401 |
| RHSIM_Rhsim08G0207800 | 7.74 | 0.005 | 0.011 | 0.697 |
| RHSIM_Rhsim11G0108000 | 7.73 | 0.005 | 0.011 | -0.872 |
| RHSIM_Rhsim07G0237200 | 7.73 | 0.005 | 0.011 | 1.889 |
| novel.1652 | 7.73 | 0.005 | 0.011 | 1.012 |
| RHSIM_Rhsim11G0164300 | 7.72 | 0.005 | 0.011 | -39.650 |
| RHSIM_Rhsim01G0001200 | 7.72 | 0.005 | 0.011 | -0.417 |
| RHSIM_RhsimUnG0135200 | 7.72 | 0.005 | 0.011 | 4.414 |
| novel.3335 | 7.72 | 0.005 | 0.011 | -1.733 |
| RHSIM_Rhsim01G0196700 | 7.71 | 0.005 | 0.011 | -0.180 |
| RHSIM_RhsimUnG0112300 | 7.71 | 0.005 | 0.011 | 1.167 |
| RHSIM_Rhsim11G0136800 | 7.71 | 0.005 | 0.011 | 2.966 |
| RHSIM_Rhsim05G0183800 | 7.71 | 0.005 | 0.011 | 2.059 |

| Gene | LRT statistic | P value | BH-adjusted P | log2FC (interaction) |
|---|---|---|---|---|
| novel.3992 | 7.71 | 0.005 | 0.011 | -0.829 |
| novel.518 | 7.71 | 0.005 | 0.011 | 1.676 |
| novel.3967 | 7.71 | 0.006 | 0.011 | 2.274 |
| RHSIM_Rhsim10G0163300 | 7.71 | 0.006 | 0.011 | 0.395 |
| RHSIM_Rhsim08G0206600 | 7.70 | 0.006 | 0.011 | 0.622 |
| RHSIM_Rhsim13G0110000 | 7.70 | 0.006 | 0.011 | 0.157 |
| RHSIM_Rhsim06G0035700 | 7.70 | 0.006 | 0.011 | -0.794 |
| RHSIM_Rhsim08G0218000 | 7.70 | 0.006 | 0.011 | -40.095 |
| RHSIM_Rhsim09G0202000 | 7.70 | 0.006 | 0.011 | 1.068 |
| RHSIM_Rhsim05G0232100 | 7.70 | 0.006 | 0.011 | 0.233 |
| RHSIM_Rhsim04G0013300 | 7.70 | 0.006 | 0.011 | 3.222 |
| RHSIM_Rhsim12G0002700 | 7.70 | 0.006 | 0.011 | 2.325 |
| RHSIM_Rhsim10G0190300 | 7.70 | 0.006 | 0.011 | 0.456 |
| RHSIM_Rhsim07G0047800 | 7.69 | 0.006 | 0.011 | -0.409 |
| RHSIM_Rhsim01G0251800 | 7.69 | 0.006 | 0.011 | 0.836 |
| RHSIM_Rhsim07G0173500 | 7.69 | 0.006 | 0.011 | -0.491 |
| RHSIM_Rhsim03G0118700 | 7.69 | 0.006 | 0.011 | 0.314 |
| RHSIM_Rhsim10G0009100 | 7.69 | 0.006 | 0.011 | -40.201 |
| RHSIM_Rhsim03G0142200 | 7.69 | 0.006 | 0.011 | -0.514 |
| RHSIM_Rhsim07G0165200 | 7.69 | 0.006 | 0.011 | 1.033 |
| RHSIM_Rhsim05G0162500 | 7.69 | 0.006 | 0.011 | -1.120 |
| novel.3513 | 7.68 | 0.006 | 0.011 | 42.517 |
| RHSIM_Rhsim03G0259200 | 7.68 | 0.006 | 0.011 | 0.391 |
| novel.2305 | 7.67 | 0.006 | 0.011 | 3.072 |
| RHSIM_Rhsim02G0212500 | 7.67 | 0.006 | 0.011 | 0.212 |
| RHSIM_Rhsim04G0068100 | 7.67 | 0.006 | 0.011 | -0.612 |
| RHSIM_Rhsim11G0155800 | 7.66 | 0.006 | 0.011 | 1.651 |
| RHSIM_Rhsim04G0188300 | 7.66 | 0.006 | 0.011 | -0.395 |
| novel.2353 | 7.66 | 0.006 | 0.011 | 40.972 |
| novel.3207 | 7.66 | 0.006 | 0.011 | 2.013 |
| RHSIM_Rhsim06G0167700 | 7.66 | 0.006 | 0.011 | 2.874 |
| novel.1268 | 7.66 | 0.006 | 0.011 | 1.274 |

| Gene | LRT statistic | P value | BH-adjusted P | log2FC (interaction) |
|---|---|---|---|---|
| novel.4551 | 7.65 | 0.006 | 0.011 | 2.177 |
| RHSIM_Rhsim13G0166600 | 7.65 | 0.006 | 0.011 | 0.203 |
| RHSIM_Rhsim06G0134900 | 7.65 | 0.006 | 0.011 | 1.656 |
| RHSIM_Rhsim11G0008100 | 7.65 | 0.006 | 0.011 | 0.785 |
| RHSIM_Rhsim04G0189500 | 7.64 | 0.006 | 0.011 | 0.687 |
| RHSIM_Rhsim11G0182500 | 7.64 | 0.006 | 0.012 | 0.228 |
| RHSIM_Rhsim05G0059900 | 7.64 | 0.006 | 0.012 | -0.939 |
| RHSIM_Rhsim01G0285100 | 7.64 | 0.006 | 0.012 | 0.314 |
| RHSIM_Rhsim05G0130500 | 7.64 | 0.006 | 0.012 | -1.936 |
| RHSIM_Rhsim09G0051000 | 7.64 | 0.006 | 0.012 | 1.144 |
| novel.2184 | 7.64 | 0.006 | 0.012 | 40.038 |
| RHSIM_Rhsim04G0240600 | 7.64 | 0.006 | 0.012 | -0.287 |
| novel.160 | 7.64 | 0.006 | 0.012 | 0.977 |
| RHSIM_Rhsim13G0019000 | 7.64 | 0.006 | 0.012 | 2.402 |
| RHSIM_Rhsim01G0117800 | 7.64 | 0.006 | 0.012 | 40.861 |
| RHSIM_Rhsim03G0163100 | 7.63 | 0.006 | 0.012 | -0.751 |
| RHSIM_Rhsim06G0051300 | 7.63 | 0.006 | 0.012 | -3.631 |
| RHSIM_Rhsim01G0152800 | 7.63 | 0.006 | 0.012 | 0.379 |
| RHSIM_Rhsim08G0067200 | 7.63 | 0.006 | 0.012 | 0.650 |
| RHSIM_Rhsim08G0113900 | 7.63 | 0.006 | 0.012 | 1.579 |
| RHSIM_Rhsim10G0176800 | 7.62 | 0.006 | 0.012 | -0.783 |
| RHSIM_Rhsim13G0110100 | 7.62 | 0.006 | 0.012 | 0.434 |
| RHSIM_Rhsim03G0147500 | 7.62 | 0.006 | 0.012 | -0.831 |
| RHSIM_Rhsim10G0189500 | 7.61 | 0.006 | 0.012 | 1.645 |
| RHSIM_Rhsim13G0130000 | 7.61 | 0.006 | 0.012 | 42.651 |
| RHSIM_Rhsim09G0166300 | 7.61 | 0.006 | 0.012 | -0.252 |
| novel.2495 | 7.61 | 0.006 | 0.012 | 0.551 |
| RHSIM_Rhsim12G0070000 | 7.60 | 0.006 | 0.012 | 3.301 |
| RHSIM_Rhsim02G0112600 | 7.60 | 0.006 | 0.012 | -0.532 |
| RHSIM_Rhsim01G0077400 | 7.60 | 0.006 | 0.012 | 0.162 |
| RHSIM_RhsimUnG0196000 | 7.60 | 0.006 | 0.012 | 0.661 |
| novel.2035 | 7.60 | 0.006 | 0.012 | 3.845 |

| Gene | LRT statistic | P value | BH-adjusted P | log2FC (interaction) |
|---|---|---|---|---|
| RHSIM_RhsimUnG0253000 | 7.60 | 0.006 | 0.012 | -0.198 |
| RHSIM_Rhsim03G0062100 | 7.59 | 0.006 | 0.012 | 2.492 |
| RHSIM_Rhsim07G0006500 | 7.59 | 0.006 | 0.012 | 1.027 |
| RHSIM_Rhsim01G0230700 | 7.59 | 0.006 | 0.012 | 2.833 |
| RHSIM_Rhsim04G0247100 | 7.59 | 0.006 | 0.012 | -0.155 |
| RHSIM_Rhsim04G0232700 | 7.59 | 0.006 | 0.012 | 2.975 |
| RHSIM_RhsimUnG0015800 | 7.59 | 0.006 | 0.012 | 0.495 |
| RHSIM_Rhsim04G0128700 | 7.59 | 0.006 | 0.012 | 0.897 |
| RHSIM_Rhsim12G0204900 | 7.59 | 0.006 | 0.012 | -0.611 |
| novel.3752 | 7.59 | 0.006 | 0.012 | -41.484 |
| RHSIM_Rhsim06G0000900 | 7.59 | 0.006 | 0.012 | 0.883 |
| RHSIM_Rhsim06G0244700 | 7.59 | 0.006 | 0.012 | -0.384 |
| RHSIM_Rhsim06G0175300 | 7.58 | 0.006 | 0.012 | 0.662 |
| RHSIM_Rhsim07G0106700 | 7.58 | 0.006 | 0.012 | -43.158 |
| RHSIM_Rhsim07G0122500 | 7.58 | 0.006 | 0.012 | -0.425 |
| RHSIM_Rhsim08G0167300 | 7.58 | 0.006 | 0.012 | -0.365 |
| RHSIM_Rhsim11G0149700 | 7.58 | 0.006 | 0.012 | -0.190 |
| RHSIM_Rhsim12G0099700 | 7.58 | 0.006 | 0.012 | 1.117 |
| RHSIM_Rhsim02G0023300 | 7.58 | 0.006 | 0.012 | 0.312 |
| novel.2255 | 7.58 | 0.006 | 0.012 | 2.203 |
| novel.4349 | 7.57 | 0.006 | 0.012 | 2.758 |
| RHSIM_Rhsim07G0127600 | 7.57 | 0.006 | 0.012 | 2.749 |
| RHSIM_Rhsim03G0152500 | 7.57 | 0.006 | 0.012 | 2.447 |
| RHSIM_Rhsim05G0137400 | 7.57 | 0.006 | 0.012 | 1.847 |
| RHSIM_Rhsim13G0092200 | 7.57 | 0.006 | 0.012 | -0.551 |
| RHSIM_Rhsim04G0187400 | 7.56 | 0.006 | 0.012 | 0.413 |
| RHSIM_RhsimUnG0044800 | 7.56 | 0.006 | 0.012 | -0.394 |
| RHSIM_Rhsim08G0080800 | 7.56 | 0.006 | 0.012 | -0.357 |
| RHSIM_Rhsim13G0024400 | 7.56 | 0.006 | 0.012 | 0.288 |
| RHSIM_Rhsim11G0066400 | 7.56 | 0.006 | 0.012 | -1.022 |
| RHSIM_Rhsim05G0044800 | 7.55 | 0.006 | 0.012 | -0.573 |
| RHSIM_Rhsim07G0015300 | 7.55 | 0.006 | 0.012 | 3.131 |

| Gene | LRT statistic | P value | BH-adjusted P | log2FC (interaction) |
|---|---|---|---|---|
| RHSIM_Rhsim05G0069300 | 7.55 | 0.006 | 0.012 | -0.412 |
| RHSIM_Rhsim07G0202900 | 7.55 | 0.006 | 0.012 | -0.246 |
| novel.20 | 7.54 | 0.006 | 0.012 | -2.753 |
| novel.2976 | 7.54 | 0.006 | 0.012 | -0.496 |
| RHSIM_Rhsim12G0168600 | 7.54 | 0.006 | 0.012 | -0.230 |
| RHSIM_Rhsim06G0142100 | 7.54 | 0.006 | 0.012 | 0.267 |
| RHSIM_Rhsim05G0000300 | 7.54 | 0.006 | 0.012 | 0.851 |
| novel.4226 | 7.54 | 0.006 | 0.012 | -2.382 |
| RHSIM_Rhsim03G0224600 | 7.53 | 0.006 | 0.012 | 0.147 |
| RHSIM_Rhsim01G0078600 | 7.53 | 0.006 | 0.012 | 1.529 |
| RHSIM_RhsimUnG0066300 | 7.53 | 0.006 | 0.012 | 0.686 |
| RHSIM_Rhsim04G0141800 | 7.53 | 0.006 | 0.012 | 0.421 |
| RHSIM_Rhsim07G0188900 | 7.52 | 0.006 | 0.012 | 1.095 |
| RHSIM_Rhsim13G0195700 | 7.52 | 0.006 | 0.012 | -1.784 |
| RHSIM_Rhsim09G0126400 | 7.52 | 0.006 | 0.012 | 0.265 |
| novel.3251 | 7.52 | 0.006 | 0.012 | -2.812 |
| novel.2903 | 7.52 | 0.006 | 0.012 | 0.942 |
| RHSIM_Rhsim10G0207800 | 7.52 | 0.006 | 0.012 | 0.963 |
| RHSIM_Rhsim01G0147500 | 7.52 | 0.006 | 0.012 | -0.648 |
| RHSIM_Rhsim12G0202500 | 7.52 | 0.006 | 0.012 | -1.342 |
| RHSIM_Rhsim06G0041600 | 7.51 | 0.006 | 0.012 | 0.399 |
| RHSIM_Rhsim10G0013100 | 7.51 | 0.006 | 0.012 | -0.953 |
| RHSIM_Rhsim03G0120100 | 7.51 | 0.006 | 0.012 | -0.338 |
| RHSIM_Rhsim11G0171900 | 7.51 | 0.006 | 0.012 | 0.619 |
| RHSIM_Rhsim08G0222700 | 7.50 | 0.006 | 0.012 | -0.212 |
| novel.1459 | 7.50 | 0.006 | 0.012 | 0.553 |
| novel.4554 | 7.50 | 0.006 | 0.012 | -1.667 |
| novel.1591 | 7.50 | 0.006 | 0.012 | 41.067 |
| novel.2192 | 7.50 | 0.006 | 0.012 | 0.556 |
| RHSIM_Rhsim09G0015600 | 7.50 | 0.006 | 0.012 | -4.234 |
| RHSIM_Rhsim08G0184900 | 7.50 | 0.006 | 0.012 | 0.566 |
| RHSIM_Rhsim01G0122300 | 7.50 | 0.006 | 0.012 | 78.764 |

| Gene | LRT statistic | P value | BH-adjusted P | log2FC (interaction) |
|---|---|---|---|---|
| RHSIM_Rhsim12G0004900 | 7.50 | 0.006 | 0.012 | 1.153 |
| RHSIM_Rhsim04G0129300 | 7.49 | 0.006 | 0.012 | 0.225 |
| novel.4169 | 7.49 | 0.006 | 0.012 | 4.983 |
| RHSIM_Rhsim08G0223700 | 7.49 | 0.006 | 0.012 | 0.933 |
| RHSIM_Rhsim05G0047600 | 7.49 | 0.006 | 0.012 | -0.955 |
| RHSIM_Rhsim02G0046100 | 7.49 | 0.006 | 0.012 | -0.457 |
| RHSIM_Rhsim05G0210100 | 7.49 | 0.006 | 0.012 | -40.850 |
| novel.4419 | 7.49 | 0.006 | 0.012 | 2.403 |
| RHSIM_Rhsim07G0186200 | 7.49 | 0.006 | 0.012 | 0.793 |
| RHSIM_Rhsim12G0172000 | 7.48 | 0.006 | 0.012 | 0.729 |
| RHSIM_RhsimUnG0032200 | 7.48 | 0.006 | 0.013 | -0.274 |
| RHSIM_Rhsim01G0237400 | 7.48 | 0.006 | 0.013 | 0.503 |
| RHSIM_Rhsim05G0097000 | 7.48 | 0.006 | 0.013 | 2.627 |
| RHSIM_Rhsim02G0226300 | 7.47 | 0.006 | 0.013 | -0.886 |
| novel.2125 | 7.47 | 0.006 | 0.013 | -2.914 |
| RHSIM_Rhsim04G0006300 | 7.47 | 0.006 | 0.013 | -0.252 |
| RHSIM_Rhsim03G0172200 | 7.47 | 0.006 | 0.013 | 0.377 |
| RHSIM_Rhsim07G0090500 | 7.47 | 0.006 | 0.013 | 0.718 |
| novel.308 | 7.47 | 0.006 | 0.013 | -1.546 |
| novel.4085 | 7.47 | 0.006 | 0.013 | 0.481 |
| RHSIM_Rhsim01G0256600 | 7.47 | 0.006 | 0.013 | 2.223 |
| RHSIM_Rhsim09G0108700 | 7.47 | 0.006 | 0.013 | -40.480 |
| RHSIM_Rhsim10G0205100 | 7.47 | 0.006 | 0.013 | 0.455 |
| RHSIM_Rhsim09G0080400 | 7.46 | 0.006 | 0.013 | 0.209 |
| RHSIM_Rhsim09G0196300 | 7.46 | 0.006 | 0.013 | -0.435 |
| RHSIM_Rhsim13G0193700 | 7.46 | 0.006 | 0.013 | 0.414 |
| RHSIM_Rhsim03G0155900 | 7.46 | 0.006 | 0.013 | 2.234 |
| novel.2793 | 7.45 | 0.006 | 0.013 | 2.844 |
| RHSIM_Rhsim13G0056300 | 7.45 | 0.006 | 0.013 | -0.376 |
| novel.2666 | 7.45 | 0.006 | 0.013 | -40.046 |
| novel.2458 | 7.45 | 0.006 | 0.013 | -0.249 |
| RHSIM_Rhsim09G0127100 | 7.45 | 0.006 | 0.013 | 0.482 |

| Gene | LRT statistic | P value | BH-adjusted P | log2FC (interaction) |
|---|---|---|---|---|
| RHSIM_Rhsim08G0227600 | 7.45 | 0.006 | 0.013 | 0.438 |
| novel.4400 | 7.45 | 0.006 | 0.013 | 1.927 |
| novel.3229 | 7.45 | 0.006 | 0.013 | -0.688 |
| RHSIM_Rhsim07G0151400 | 7.44 | 0.006 | 0.013 | 2.636 |
| RHSIM_Rhsim05G0052900 | 7.44 | 0.006 | 0.013 | -1.610 |
| RHSIM_Rhsim13G0061900 | 7.44 | 0.006 | 0.013 | -0.530 |
| RHSIM_RhsimUnG0101800 | 7.43 | 0.006 | 0.013 | -0.815 |
| RHSIM_Rhsim06G0186200 | 7.43 | 0.006 | 0.013 | -2.105 |
| novel.4041 | 7.43 | 0.006 | 0.013 | -1.405 |
| RHSIM_Rhsim12G0193600 | 7.43 | 0.006 | 0.013 | -0.531 |
| RHSIM_Rhsim06G0102400 | 7.43 | 0.006 | 0.013 | 0.646 |
| RHSIM_Rhsim06G0033000 | 7.43 | 0.006 | 0.013 | 3.400 |
| RHSIM_Rhsim12G0133100 | 7.43 | 0.006 | 0.013 | 0.533 |
| RHSIM_RhsimUnG0231000 | 7.42 | 0.006 | 0.013 | -0.888 |
| RHSIM_Rhsim08G0112000 | 7.42 | 0.006 | 0.013 | 0.733 |
| RHSIM_Rhsim13G0099500 | 7.42 | 0.006 | 0.013 | 0.397 |
| RHSIM_Rhsim05G0073500 | 7.42 | 0.006 | 0.013 | 0.195 |
| RHSIM_Rhsim03G0207900 | 7.42 | 0.006 | 0.013 | -0.270 |
| RHSIM_Rhsim02G0250500 | 7.42 | 0.006 | 0.013 | -0.300 |
| novel.2063 | 7.42 | 0.006 | 0.013 | -5.383 |
| RHSIM_Rhsim09G0170700 | 7.41 | 0.006 | 0.013 | 0.534 |
| RHSIM_Rhsim06G0103000 | 7.41 | 0.006 | 0.013 | -0.407 |
| RHSIM_Rhsim08G0246300 | 7.41 | 0.006 | 0.013 | 1.293 |
| RHSIM_Rhsim07G0125000 | 7.41 | 0.006 | 0.013 | 0.313 |
| RHSIM_Rhsim11G0053200 | 7.41 | 0.006 | 0.013 | 40.906 |
| RHSIM_Rhsim04G0216500 | 7.41 | 0.006 | 0.013 | -0.369 |
| RHSIM_Rhsim06G0139000 | 7.41 | 0.007 | 0.013 | 0.329 |
| RHSIM_Rhsim07G0246300 | 7.40 | 0.007 | 0.013 | 0.200 |
| RHSIM_Rhsim03G0123600 | 7.40 | 0.007 | 0.013 | 0.250 |
| RHSIM_Rhsim04G0039900 | 7.40 | 0.007 | 0.013 | -1.158 |
| RHSIM_Rhsim07G0216800 | 7.40 | 0.007 | 0.013 | 0.348 |
| RHSIM_RhsimUnG0179800 | 7.40 | 0.007 | 0.013 | -41.404 |

| Gene | LRT statistic | P value | BH-adjusted P | log2FC (interaction) |
|---|---|---|---|---|
| novel.1670 | 7.39 | 0.007 | 0.013 | 0.859 |
| RHSIM_Rhsim01G0178500 | 7.39 | 0.007 | 0.013 | -0.208 |
| RHSIM_Rhsim03G0138900 | 7.38 | 0.007 | 0.013 | 0.831 |
| RHSIM_Rhsim04G0245800 | 7.38 | 0.007 | 0.013 | 0.747 |
| RHSIM_Rhsim03G0036400 | 7.38 | 0.007 | 0.013 | -3.440 |
| novel.3354 | 7.38 | 0.007 | 0.013 | 2.489 |
| novel.2493 | 7.38 | 0.007 | 0.013 | 1.154 |
| RHSIM_Rhsim04G0201600 | 7.38 | 0.007 | 0.013 | 0.323 |
| RHSIM_Rhsim07G0028900 | 7.38 | 0.007 | 0.013 | 2.450 |
| RHSIM_RhsimPtG0003500 | 7.38 | 0.007 | 0.013 | 0.765 |
| RHSIM_Rhsim10G0061400 | 7.37 | 0.007 | 0.013 | -0.321 |
| RHSIM_Rhsim07G0033800 | 7.37 | 0.007 | 0.013 | 0.193 |
| RHSIM_Rhsim06G0174500 | 7.37 | 0.007 | 0.013 | 0.545 |
| RHSIM_Rhsim09G0091300 | 7.37 | 0.007 | 0.013 | 0.320 |
| RHSIM_Rhsim07G0154500 | 7.37 | 0.007 | 0.013 | 0.311 |
| novel.177 | 7.37 | 0.007 | 0.013 | -1.057 |
| novel.392 | 7.37 | 0.007 | 0.013 | 0.921 |
| RHSIM_Rhsim01G0186900 | 7.36 | 0.007 | 0.013 | -0.332 |
| RHSIM_Rhsim04G0150300 | 7.36 | 0.007 | 0.013 | 1.866 |
| RHSIM_Rhsim12G0018400 | 7.36 | 0.007 | 0.013 | 0.762 |
| RHSIM_Rhsim04G0117600 | 7.36 | 0.007 | 0.013 | -1.034 |
| RHSIM_Rhsim07G0010900 | 7.36 | 0.007 | 0.013 | -0.850 |
| RHSIM_Rhsim02G0110900 | 7.36 | 0.007 | 0.013 | -0.350 |
| RHSIM_Rhsim13G0145500 | 7.35 | 0.007 | 0.013 | 0.771 |
| RHSIM_RhsimUnG0214300 | 7.35 | 0.007 | 0.013 | 1.483 |
| RHSIM_Rhsim13G0014700 | 7.35 | 0.007 | 0.013 | 40.618 |
| RHSIM_Rhsim02G0009100 | 7.35 | 0.007 | 0.013 | -0.347 |
| RHSIM_Rhsim05G0076000 | 7.34 | 0.007 | 0.013 | 0.553 |
| RHSIM_Rhsim09G0193700 | 7.34 | 0.007 | 0.013 | 0.963 |
| RHSIM_Rhsim04G0065400 | 7.34 | 0.007 | 0.013 | -1.355 |
| RHSIM_Rhsim08G0189700 | 7.34 | 0.007 | 0.013 | 0.760 |
| novel.1148 | 7.33 | 0.007 | 0.013 | -0.429 |

| Gene | LRT statistic | P value | BH-adjusted P | log2FC (interaction) |
|---|---|---|---|---|
| RHSIM_Rhsim12G0113700 | 7.33 | 0.007 | 0.013 | 0.413 |
| RHSIM_Rhsim09G0050100 | 7.33 | 0.007 | 0.014 | -0.678 |
| RHSIM_Rhsim10G0161100 | 7.33 | 0.007 | 0.014 | -39.902 |
| RHSIM_Rhsim08G0079400 | 7.33 | 0.007 | 0.014 | 3.773 |
| RHSIM_RhsimUnG0039700 | 7.33 | 0.007 | 0.014 | -0.971 |
| RHSIM_RhsimUnG0185100 | 7.33 | 0.007 | 0.014 | 3.652 |
| RHSIM_Rhsim02G0160800 | 7.33 | 0.007 | 0.014 | 0.216 |
| novel.2349 | 7.32 | 0.007 | 0.014 | 2.354 |
| RHSIM_Rhsim04G0165800 | 7.32 | 0.007 | 0.014 | -0.439 |
| RHSIM_RhsimUnG0098400 | 7.32 | 0.007 | 0.014 | -1.610 |
| RHSIM_Rhsim01G0009600 | 7.32 | 0.007 | 0.014 | 0.173 |
| RHSIM_Rhsim12G0019900 | 7.32 | 0.007 | 0.014 | 1.206 |
| RHSIM_Rhsim02G0063100 | 7.32 | 0.007 | 0.014 | 40.823 |
| RHSIM_RhsimUnG0102000 | 7.32 | 0.007 | 0.014 | 1.075 |
| RHSIM_Rhsim09G0155400 | 7.32 | 0.007 | 0.014 | -0.655 |
| RHSIM_Rhsim02G0229300 | 7.31 | 0.007 | 0.014 | 1.725 |
| RHSIM_RhsimUnG0229100 | 7.31 | 0.007 | 0.014 | -6.078 |
| RHSIM_Rhsim01G0284700 | 7.31 | 0.007 | 0.014 | -0.287 |
| novel.4048 | 7.31 | 0.007 | 0.014 | -2.103 |
| RHSIM_Rhsim13G0125300 | 7.31 | 0.007 | 0.014 | -0.829 |
| RHSIM_Rhsim03G0034400 | 7.31 | 0.007 | 0.014 | -1.109 |
| RHSIM_Rhsim03G0025000 | 7.31 | 0.007 | 0.014 | -0.543 |
| novel.2212 | 7.31 | 0.007 | 0.014 | 2.144 |
| novel.2141 | 7.30 | 0.007 | 0.014 | 1.559 |
| RHSIM_RhsimUnG0105100 | 7.30 | 0.007 | 0.014 | 1.722 |
| RHSIM_Rhsim06G0234200 | 7.30 | 0.007 | 0.014 | -1.477 |
| RHSIM_Rhsim05G0216700 | 7.30 | 0.007 | 0.014 | -0.181 |
| RHSIM_Rhsim07G0189000 | 7.30 | 0.007 | 0.014 | -0.128 |
| RHSIM_Rhsim02G0143800 | 7.30 | 0.007 | 0.014 | -41.248 |
| RHSIM_Rhsim04G0201500 | 7.29 | 0.007 | 0.014 | 41.232 |
| RHSIM_Rhsim04G0134900 | 7.29 | 0.007 | 0.014 | -0.378 |
| RHSIM_Rhsim03G0264700 | 7.29 | 0.007 | 0.014 | 0.291 |

| Gene | LRT statistic | P value | BH-adjusted P | log2FC (interaction) |
|---|---|---|---|---|
| novel.3181 | 7.28 | 0.007 | 0.014 | -0.339 |
| RHSIM_Rhsim01G0112400 | 7.28 | 0.007 | 0.014 | 0.303 |
| RHSIM_Rhsim08G0019800 | 7.28 | 0.007 | 0.014 | 0.667 |
| RHSIM_RhsimUnG0122500 | 7.28 | 0.007 | 0.014 | 1.229 |
| RHSIM_RhsimUnG0208900 | 7.28 | 0.007 | 0.014 | 2.623 |
| RHSIM_Rhsim09G0000300 | 7.28 | 0.007 | 0.014 | 3.275 |
| novel.379 | 7.28 | 0.007 | 0.014 | 2.052 |
| RHSIM_Rhsim07G0177400 | 7.28 | 0.007 | 0.014 | 0.189 |
| RHSIM_Rhsim13G0177300 | 7.27 | 0.007 | 0.014 | -2.765 |
| RHSIM_Rhsim08G0036600 | 7.27 | 0.007 | 0.014 | 0.363 |
| RHSIM_Rhsim01G0056800 | 7.27 | 0.007 | 0.014 | -1.360 |
| RHSIM_Rhsim12G0136700 | 7.27 | 0.007 | 0.014 | 0.271 |
| novel.82 | 7.26 | 0.007 | 0.014 | -2.899 |
| RHSIM_Rhsim08G0195700 | 7.26 | 0.007 | 0.014 | 0.236 |
| RHSIM_Rhsim04G0133000 | 7.26 | 0.007 | 0.014 | 0.617 |
| RHSIM_Rhsim12G0178400 | 7.26 | 0.007 | 0.014 | -0.324 |
| RHSIM_Rhsim04G0161300 | 7.26 | 0.007 | 0.014 | -2.075 |
| novel.2853 | 7.26 | 0.007 | 0.014 | -2.937 |
| RHSIM_RhsimUnG0171000 | 7.26 | 0.007 | 0.014 | 2.079 |
| RHSIM_Rhsim08G0027100 | 7.26 | 0.007 | 0.014 | 2.942 |
| RHSIM_Rhsim06G0155600 | 7.25 | 0.007 | 0.014 | 1.392 |
| RHSIM_Rhsim13G0180500 | 7.25 | 0.007 | 0.014 | 2.600 |
| RHSIM_Rhsim09G0104600 | 7.25 | 0.007 | 0.014 | 0.550 |
| RHSIM_Rhsim04G0142200 | 7.25 | 0.007 | 0.014 | 1.829 |
| RHSIM_RhsimUnG0061900 | 7.25 | 0.007 | 0.014 | 0.622 |
| RHSIM_Rhsim10G0082700 | 7.25 | 0.007 | 0.014 | 0.907 |
| RHSIM_Rhsim07G0179200 | 7.25 | 0.007 | 0.014 | -0.482 |
| novel.3072 | 7.24 | 0.007 | 0.014 | -0.544 |
| RHSIM_Rhsim01G0217300 | 7.24 | 0.007 | 0.014 | -0.265 |
| RHSIM_Rhsim01G0256700 | 7.24 | 0.007 | 0.014 | 3.204 |
| RHSIM_Rhsim02G0027600 | 7.23 | 0.007 | 0.014 | 0.645 |
| RHSIM_Rhsim06G0114200 | 7.23 | 0.007 | 0.014 | 0.331 |

| Gene | LRT statistic | P value | BH-adjusted P | log2FC (interaction) |
|---|---|---|---|---|
| RHSIM_RhsimUnG0114100 | 7.23 | 0.007 | 0.014 | 1.182 |
| RHSIM_Rhsim08G0036800 | 7.23 | 0.007 | 0.014 | 0.571 |
| RHSIM_Rhsim06G0070200 | 7.23 | 0.007 | 0.014 | 2.309 |
| RHSIM_Rhsim01G0173800 | 7.23 | 0.007 | 0.014 | -0.999 |
| RHSIM_Rhsim07G0195400 | 7.23 | 0.007 | 0.014 | 1.398 |
| RHSIM_Rhsim12G0114800 | 7.22 | 0.007 | 0.014 | -0.463 |
| novel.3616 | 7.22 | 0.007 | 0.014 | 0.351 |
| RHSIM_Rhsim08G0176100 | 7.22 | 0.007 | 0.014 | 0.469 |
| novel.3169 | 7.22 | 0.007 | 0.014 | 0.770 |
| RHSIM_Rhsim09G0123700 | 7.22 | 0.007 | 0.014 | -0.520 |
| novel.1293 | 7.22 | 0.007 | 0.014 | 1.761 |
| RHSIM_RhsimUnG0149400 | 7.21 | 0.007 | 0.014 | 0.422 |
| novel.408 | 7.21 | 0.007 | 0.014 | 4.714 |
| RHSIM_Rhsim12G0052100 | 7.21 | 0.007 | 0.014 | 0.218 |
| RHSIM_Rhsim08G0075100 | 7.21 | 0.007 | 0.014 | -0.675 |
| RHSIM_Rhsim07G0088300 | 7.20 | 0.007 | 0.014 | 0.470 |
| novel.3113 | 7.20 | 0.007 | 0.014 | -0.262 |
| RHSIM_Rhsim10G0026300 | 7.20 | 0.007 | 0.014 | 0.842 |
| RHSIM_Rhsim13G0002800 | 7.20 | 0.007 | 0.014 | -4.335 |
| novel.3447 | 7.20 | 0.007 | 0.014 | 1.323 |
| RHSIM_RhsimUnG0144400 | 7.20 | 0.007 | 0.014 | -40.108 |
| RHSIM_Rhsim09G0154900 | 7.20 | 0.007 | 0.014 | -0.308 |
| RHSIM_Rhsim10G0126800 | 7.20 | 0.007 | 0.014 | 0.310 |
| RHSIM_Rhsim03G0223500 | 7.20 | 0.007 | 0.014 | 0.206 |
| RHSIM_Rhsim06G0225300 | 7.19 | 0.007 | 0.014 | 0.439 |
| RHSIM_RhsimUnG0135700 | 7.19 | 0.007 | 0.014 | 0.900 |
| RHSIM_Rhsim12G0026300 | 7.19 | 0.007 | 0.015 | 1.163 |
| RHSIM_Rhsim06G0198300 | 7.19 | 0.007 | 0.015 | -0.456 |
| novel.1628 | 7.19 | 0.007 | 0.015 | -0.212 |
| novel.2248 | 7.19 | 0.007 | 0.015 | 2.923 |
| RHSIM_Rhsim08G0226000 | 7.19 | 0.007 | 0.015 | 1.355 |
| RHSIM_Rhsim07G0057000 | 7.19 | 0.007 | 0.015 | -78.332 |

| Gene | LRT statistic | P value | BH-adjusted P | log2FC (interaction) |
|---|---|---|---|---|
| RHSIM_Rhsim08G0197100 | 7.18 | 0.007 | 0.015 | -0.476 |
| RHSIM_Rhsim12G0142500 | 7.18 | 0.007 | 0.015 | 1.539 |
| RHSIM_Rhsim03G0197400 | 7.18 | 0.007 | 0.015 | -0.511 |
| RHSIM_Rhsim03G0172400 | 7.18 | 0.007 | 0.015 | 2.976 |
| novel.4243 | 7.18 | 0.007 | 0.015 | -1.554 |
| RHSIM_Rhsim07G0230500 | 7.18 | 0.007 | 0.015 | 40.321 |
| RHSIM_RhsimUnG0066800 | 7.17 | 0.007 | 0.015 | 3.829 |
| novel.718 | 7.17 | 0.007 | 0.015 | 4.200 |
| novel.4390 | 7.17 | 0.007 | 0.015 | 1.598 |
| novel.2559 | 7.17 | 0.007 | 0.015 | -2.234 |
| RHSIM_Rhsim04G0001600 | 7.17 | 0.007 | 0.015 | 0.441 |
| novel.2527 | 7.17 | 0.007 | 0.015 | -1.080 |
| novel.798 | 7.17 | 0.007 | 0.015 | 1.351 |
| RHSIM_Rhsim12G0077400 | 7.17 | 0.007 | 0.015 | -0.922 |
| RHSIM_Rhsim03G0083100 | 7.17 | 0.007 | 0.015 | 0.749 |
| novel.3466 | 7.16 | 0.007 | 0.015 | 1.857 |
| RHSIM_Rhsim07G0073700 | 7.16 | 0.007 | 0.015 | -3.525 |
| RHSIM_RhsimMtG0000400 | 7.16 | 0.007 | 0.015 | 2.880 |
| RHSIM_Rhsim09G0187900 | 7.16 | 0.007 | 0.015 | 1.565 |
| RHSIM_Rhsim05G0231600 | 7.15 | 0.007 | 0.015 | -0.203 |
| novel.2760 | 7.15 | 0.007 | 0.015 | -1.043 |
| RHSIM_Rhsim03G0252400 | 7.15 | 0.007 | 0.015 | 1.921 |
| RHSIM_Rhsim11G0120500 | 7.15 | 0.007 | 0.015 | 1.797 |
| RHSIM_Rhsim05G0012300 | 7.15 | 0.008 | 0.015 | -1.544 |
| novel.3567 | 7.15 | 0.008 | 0.015 | -0.778 |
| RHSIM_Rhsim06G0088400 | 7.14 | 0.008 | 0.015 | 0.270 |
| RHSIM_RhsimUnG0069700 | 7.14 | 0.008 | 0.015 | 3.111 |
| novel.3390 | 7.14 | 0.008 | 0.015 | 1.365 |
| RHSIM_RhsimUnG0183000 | 7.14 | 0.008 | 0.015 | 1.788 |
| RHSIM_Rhsim04G0240100 | 7.13 | 0.008 | 0.015 | -0.273 |
| novel.2094 | 7.13 | 0.008 | 0.015 | 2.851 |
| RHSIM_Rhsim03G0124300 | 7.13 | 0.008 | 0.015 | 0.290 |

| Gene | LRT statistic | P value | BH-adjusted P | log2FC (interaction) |
|---|---|---|---|---|
| RHSIM_Rhsim06G0232500 | 7.13 | 0.008 | 0.015 | -0.395 |
| RHSIM_Rhsim02G0216700 | 7.13 | 0.008 | 0.015 | 0.306 |
| RHSIM_RhsimUnG0205100 | 7.12 | 0.008 | 0.015 | 1.522 |
| RHSIM_Rhsim01G0163100 | 7.12 | 0.008 | 0.015 | -1.465 |
| RHSIM_Rhsim13G0052400 | 7.12 | 0.008 | 0.015 | -0.465 |
| RHSIM_Rhsim09G0119700 | 7.12 | 0.008 | 0.015 | 0.279 |
| novel.3871 | 7.12 | 0.008 | 0.015 | 0.352 |
| RHSIM_Rhsim11G0043800 | 7.12 | 0.008 | 0.015 | 0.813 |
| RHSIM_Rhsim06G0110200 | 7.12 | 0.008 | 0.015 | 3.640 |
| RHSIM_Rhsim06G0143700 | 7.11 | 0.008 | 0.015 | 0.165 |
| novel.4278 | 7.11 | 0.008 | 0.015 | -39.414 |
| RHSIM_Rhsim13G0114500 | 7.11 | 0.008 | 0.015 | -0.508 |
| novel.60 | 7.11 | 0.008 | 0.015 | 0.742 |
| RHSIM_Rhsim11G0101800 | 7.11 | 0.008 | 0.015 | 0.511 |
| RHSIM_Rhsim01G0012000 | 7.11 | 0.008 | 0.015 | -0.360 |
| RHSIM_Rhsim07G0129800 | 7.11 | 0.008 | 0.015 | 0.436 |
| RHSIM_Rhsim02G0082300 | 7.10 | 0.008 | 0.015 | -0.429 |
| RHSIM_Rhsim07G0214600 | 7.10 | 0.008 | 0.015 | -0.409 |
| RHSIM_Rhsim10G0050000 | 7.10 | 0.008 | 0.015 | -0.318 |
| RHSIM_Rhsim07G0189300 | 7.10 | 0.008 | 0.015 | 0.586 |
| RHSIM_Rhsim13G0192200 | 7.09 | 0.008 | 0.015 | 78.425 |
| RHSIM_RhsimUnG0088400 | 7.09 | 0.008 | 0.015 | -42.524 |
| RHSIM_Rhsim13G0056600 | 7.09 | 0.008 | 0.015 | 0.917 |
| RHSIM_Rhsim05G0137300 | 7.09 | 0.008 | 0.015 | 1.541 |
| RHSIM_RhsimUnG0080700 | 7.09 | 0.008 | 0.015 | 1.732 |
| RHSIM_Rhsim04G0006400 | 7.09 | 0.008 | 0.015 | 0.385 |
| RHSIM_Rhsim08G0089900 | 7.09 | 0.008 | 0.015 | -0.443 |
| RHSIM_Rhsim05G0062200 | 7.08 | 0.008 | 0.015 | 1.338 |
| RHSIM_Rhsim13G0210400 | 7.08 | 0.008 | 0.015 | 1.353 |
| RHSIM_Rhsim09G0117400 | 7.07 | 0.008 | 0.015 | 3.340 |
| RHSIM_Rhsim13G0121800 | 7.07 | 0.008 | 0.015 | 0.340 |
| RHSIM_Rhsim04G0197300 | 7.07 | 0.008 | 0.015 | 42.917 |

| Gene | LRT statistic | P value | BH-adjusted P | log2FC (interaction) |
|---|---|---|---|---|
| RHSIM_Rhsim02G0006100 | 7.07 | 0.008 | 0.015 | 0.788 |
| RHSIM_Rhsim13G0156100 | 7.07 | 0.008 | 0.015 | 0.420 |
| RHSIM_RhsimUnG0252800 | 7.07 | 0.008 | 0.015 | -0.317 |
| RHSIM_Rhsim08G0133900 | 7.06 | 0.008 | 0.015 | 0.683 |
| RHSIM_RhsimUnG0174800 | 7.06 | 0.008 | 0.015 | 0.487 |
| RHSIM_Rhsim04G0206900 | 7.06 | 0.008 | 0.016 | 0.247 |
| RHSIM_Rhsim05G0191200 | 7.06 | 0.008 | 0.016 | 0.474 |
| novel.250 | 7.06 | 0.008 | 0.016 | -0.798 |
| RHSIM_Rhsim12G0124600 | 7.05 | 0.008 | 0.016 | -1.666 |
| RHSIM_RhsimUnG0087800 | 7.05 | 0.008 | 0.016 | -0.789 |
| RHSIM_Rhsim10G0186600 | 7.05 | 0.008 | 0.016 | 2.459 |
| RHSIM_Rhsim10G0103600 | 7.05 | 0.008 | 0.016 | -0.331 |
| RHSIM_Rhsim06G0142900 | 7.05 | 0.008 | 0.016 | 2.073 |
| RHSIM_Rhsim11G0080300 | 7.05 | 0.008 | 0.016 | 3.275 |
| novel.3821 | 7.05 | 0.008 | 0.016 | -0.462 |
| RHSIM_Rhsim07G0007100 | 7.04 | 0.008 | 0.016 | 0.680 |
| RHSIM_RhsimMtG0003200 | 7.04 | 0.008 | 0.016 | 2.203 |
| RHSIM_Rhsim11G0110200 | 7.04 | 0.008 | 0.016 | -2.118 |
| RHSIM_Rhsim13G0095700 | 7.04 | 0.008 | 0.016 | -0.533 |
| RHSIM_Rhsim13G0058600 | 7.03 | 0.008 | 0.016 | 0.905 |
| RHSIM_Rhsim12G0062700 | 7.03 | 0.008 | 0.016 | 0.436 |
| RHSIM_RhsimUnG0217800 | 7.03 | 0.008 | 0.016 | -0.274 |
| RHSIM_Rhsim01G0197600 | 7.03 | 0.008 | 0.016 | 4.082 |
| RHSIM_RhsimPtG0000900 | 7.03 | 0.008 | 0.016 | 1.291 |
| RHSIM_RhsimUnG0193700 | 7.03 | 0.008 | 0.016 | 2.402 |
| RHSIM_Rhsim11G0176900 | 7.03 | 0.008 | 0.016 | -0.840 |
| RHSIM_Rhsim13G0218200 | 7.03 | 0.008 | 0.016 | 1.530 |
| RHSIM_Rhsim13G0200300 | 7.02 | 0.008 | 0.016 | -1.743 |
| RHSIM_Rhsim09G0007800 | 7.02 | 0.008 | 0.016 | -0.225 |
| RHSIM_Rhsim01G0092700 | 7.02 | 0.008 | 0.016 | 1.269 |
| novel.3652 | 7.02 | 0.008 | 0.016 | 1.175 |
| novel.4364 | 7.02 | 0.008 | 0.016 | -2.168 |

| Gene | LRT statistic | P value | BH-adjusted P | log2FC (interaction) |
|---|---|---|---|---|
| RHSIM_Rhsim07G0186700 | 7.02 | 0.008 | 0.016 | 0.412 |
| RHSIM_Rhsim10G0035300 | 7.01 | 0.008 | 0.016 | 3.164 |
| RHSIM_Rhsim02G0034400 | 7.01 | 0.008 | 0.016 | 0.191 |
| RHSIM_Rhsim06G0064900 | 7.01 | 0.008 | 0.016 | -2.773 |
| RHSIM_Rhsim06G0027600 | 7.01 | 0.008 | 0.016 | 0.348 |
| RHSIM_Rhsim08G0068400 | 7.01 | 0.008 | 0.016 | 1.643 |
| novel.1475 | 7.01 | 0.008 | 0.016 | -2.544 |
| RHSIM_Rhsim04G0210400 | 7.01 | 0.008 | 0.016 | -41.146 |
| RHSIM_Rhsim03G0238000 | 7.01 | 0.008 | 0.016 | -0.895 |
| RHSIM_Rhsim11G0021400 | 7.01 | 0.008 | 0.016 | 0.338 |
| RHSIM_Rhsim07G0054200 | 7.01 | 0.008 | 0.016 | -0.332 |
| RHSIM_Rhsim07G0048600 | 7.00 | 0.008 | 0.016 | -0.305 |
| RHSIM_Rhsim03G0145400 | 7.00 | 0.008 | 0.016 | -3.777 |
| RHSIM_Rhsim12G0098200 | 7.00 | 0.008 | 0.016 | -41.318 |
| RHSIM_Rhsim01G0217600 | 7.00 | 0.008 | 0.016 | 0.226 |
| RHSIM_Rhsim02G0055400 | 7.00 | 0.008 | 0.016 | -0.905 |
| RHSIM_Rhsim07G0184400 | 6.99 | 0.008 | 0.016 | -0.586 |
| novel.633 | 6.99 | 0.008 | 0.016 | 2.871 |
| RHSIM_Rhsim03G0140800 | 6.99 | 0.008 | 0.016 | -78.167 |
| RHSIM_Rhsim10G0199000 | 6.99 | 0.008 | 0.016 | -0.475 |
| RHSIM_Rhsim12G0076600 | 6.99 | 0.008 | 0.016 | 0.334 |
| RHSIM_Rhsim01G0172700 | 6.99 | 0.008 | 0.016 | -2.417 |
| RHSIM_Rhsim13G0081600 | 6.99 | 0.008 | 0.016 | 3.450 |
| novel.3945 | 6.99 | 0.008 | 0.016 | 0.356 |
| RHSIM_Rhsim01G0221800 | 6.99 | 0.008 | 0.016 | 0.210 |
| RHSIM_Rhsim06G0051700 | 6.99 | 0.008 | 0.016 | 1.285 |
| novel.4510 | 6.98 | 0.008 | 0.016 | 1.233 |
| RHSIM_Rhsim12G0214600 | 6.98 | 0.008 | 0.016 | -0.295 |
| RHSIM_Rhsim09G0198100 | 6.98 | 0.008 | 0.016 | 0.565 |
| RHSIM_Rhsim04G0029900 | 6.98 | 0.008 | 0.016 | 0.891 |
| RHSIM_RhsimUnG0117800 | 6.98 | 0.008 | 0.016 | 42.207 |
| RHSIM_Rhsim03G0255700 | 6.97 | 0.008 | 0.016 | 0.947 |

| Gene | LRT statistic | P value | BH-adjusted P | log2FC (interaction) |
|---|---|---|---|---|
| RHSIM_Rhsim05G0080000 | 6.97 | 0.008 | 0.016 | 0.406 |
| RHSIM_Rhsim06G0188100 | 6.97 | 0.008 | 0.016 | 1.584 |
| RHSIM_Rhsim06G0241800 | 6.97 | 0.008 | 0.016 | 1.847 |
| RHSIM_Rhsim02G0017200 | 6.97 | 0.008 | 0.016 | 0.379 |
| RHSIM_Rhsim03G0135100 | 6.97 | 0.008 | 0.016 | 2.733 |
| RHSIM_Rhsim10G0031200 | 6.96 | 0.008 | 0.016 | -1.829 |
| novel.1230 | 6.96 | 0.008 | 0.016 | -1.021 |
| RHSIM_Rhsim12G0207900 | 6.96 | 0.008 | 0.016 | -0.209 |
| novel.3432 | 6.96 | 0.008 | 0.016 | 0.710 |
| RHSIM_Rhsim08G0101300 | 6.96 | 0.008 | 0.016 | -1.359 |
| RHSIM_Rhsim01G0111800 | 6.95 | 0.008 | 0.016 | -3.439 |
| RHSIM_Rhsim09G0047100 | 6.95 | 0.008 | 0.016 | 1.975 |
| novel.580 | 6.95 | 0.008 | 0.016 | -1.166 |
| novel.169 | 6.95 | 0.008 | 0.016 | -2.456 |
| novel.2591 | 6.95 | 0.008 | 0.016 | -1.093 |
| RHSIM_Rhsim11G0095800 | 6.95 | 0.008 | 0.016 | 1.493 |
| RHSIM_Rhsim03G0208600 | 6.95 | 0.008 | 0.016 | -0.708 |
| novel.3224 | 6.95 | 0.008 | 0.016 | 1.962 |
| RHSIM_Rhsim01G0239300 | 6.95 | 0.008 | 0.016 | 1.218 |
| RHSIM_Rhsim04G0246700 | 6.95 | 0.008 | 0.016 | -41.888 |
| RHSIM_Rhsim12G0066800 | 6.94 | 0.008 | 0.016 | -0.718 |
| RHSIM_Rhsim13G0217600 | 6.94 | 0.008 | 0.016 | 0.214 |
| novel.2674 | 6.94 | 0.008 | 0.017 | -4.151 |
| RHSIM_RhsimUnG0079400 | 6.94 | 0.008 | 0.017 | -0.471 |
| RHSIM_Rhsim08G0030800 | 6.94 | 0.008 | 0.017 | 0.255 |
| novel.22 | 6.94 | 0.008 | 0.017 | 0.853 |
| novel.305 | 6.94 | 0.008 | 0.017 | -2.323 |
| RHSIM_Rhsim09G0114200 | 6.94 | 0.008 | 0.017 | 1.108 |
| RHSIM_Rhsim04G0147200 | 6.93 | 0.008 | 0.017 | -1.983 |
| RHSIM_Rhsim02G0250400 | 6.93 | 0.008 | 0.017 | -0.449 |
| RHSIM_Rhsim02G0095200 | 6.93 | 0.008 | 0.017 | -0.567 |
| novel.2424 | 6.93 | 0.008 | 0.017 | 3.025 |

| Gene | LRT statistic | P value | BH-adjusted P | log2FC (interaction) |
|---|---|---|---|---|
| RHSIM_Rhsim04G0143300 | 6.92 | 0.009 | 0.017 | 1.777 |
| RHSIM_Rhsim01G0147000 | 6.92 | 0.009 | 0.017 | 3.019 |
| RHSIM_Rhsim08G0243300 | 6.92 | 0.009 | 0.017 | 0.924 |
| RHSIM_Rhsim01G0211600 | 6.92 | 0.009 | 0.017 | 2.836 |
| novel.3679 | 6.92 | 0.009 | 0.017 | -40.947 |
| RHSIM_Rhsim06G0129800 | 6.92 | 0.009 | 0.017 | -0.584 |
| RHSIM_RhsimUnG0097800 | 6.92 | 0.009 | 0.017 | -3.320 |
| RHSIM_RhsimUnG0194700 | 6.92 | 0.009 | 0.017 | 0.852 |
| RHSIM_RhsimUnG0252200 | 6.92 | 0.009 | 0.017 | 1.995 |
| RHSIM_Rhsim03G0062500 | 6.92 | 0.009 | 0.017 | -0.334 |
| RHSIM_Rhsim13G0003500 | 6.91 | 0.009 | 0.017 | 1.457 |
| RHSIM_Rhsim10G0103500 | 6.91 | 0.009 | 0.017 | 0.995 |
| RHSIM_Rhsim08G0227900 | 6.91 | 0.009 | 0.017 | 0.593 |
| RHSIM_Rhsim01G0040700 | 6.91 | 0.009 | 0.017 | 2.791 |
| RHSIM_Rhsim08G0221200 | 6.91 | 0.009 | 0.017 | -0.172 |
| RHSIM_Rhsim04G0084300 | 6.91 | 0.009 | 0.017 | 0.768 |
| RHSIM_Rhsim08G0190100 | 6.91 | 0.009 | 0.017 | -1.031 |
| RHSIM_Rhsim07G0099300 | 6.90 | 0.009 | 0.017 | 0.659 |
| RHSIM_Rhsim07G0080600 | 6.90 | 0.009 | 0.017 | -1.200 |
| novel.510 | 6.90 | 0.009 | 0.017 | 1.810 |
| RHSIM_RhsimUnG0231700 | 6.90 | 0.009 | 0.017 | -0.863 |
| RHSIM_Rhsim13G0026100 | 6.90 | 0.009 | 0.017 | -0.557 |
| RHSIM_Rhsim12G0163400 | 6.90 | 0.009 | 0.017 | -0.282 |
| RHSIM_Rhsim06G0014400 | 6.90 | 0.009 | 0.017 | 3.577 |
| RHSIM_Rhsim12G0102900 | 6.90 | 0.009 | 0.017 | 1.787 |
| RHSIM_Rhsim03G0122500 | 6.90 | 0.009 | 0.017 | -0.316 |
| RHSIM_Rhsim06G0213900 | 6.90 | 0.009 | 0.017 | -0.700 |
| RHSIM_Rhsim07G0258400 | 6.90 | 0.009 | 0.017 | 0.266 |
| RHSIM_Rhsim08G0075500 | 6.89 | 0.009 | 0.017 | 0.415 |
| novel.2531 | 6.89 | 0.009 | 0.017 | -2.989 |
| RHSIM_Rhsim09G0049500 | 6.89 | 0.009 | 0.017 | 0.408 |
| RHSIM_Rhsim08G0222900 | 6.89 | 0.009 | 0.017 | -0.465 |

| Gene | LRT statistic | P value | BH-adjusted P | log2FC (interaction) |
|---|---|---|---|---|
| RHSIM_Rhsim08G0057200 | 6.89 | 0.009 | 0.017 | -3.529 |
| RHSIM_Rhsim12G0187900 | 6.88 | 0.009 | 0.017 | -0.234 |
| RHSIM_Rhsim13G0054200 | 6.88 | 0.009 | 0.017 | -0.554 |
| RHSIM_Rhsim02G0195000 | 6.88 | 0.009 | 0.017 | -0.315 |
| novel.4129 | 6.88 | 0.009 | 0.017 | -0.635 |
| RHSIM_Rhsim12G0130400 | 6.87 | 0.009 | 0.017 | 1.394 |
| novel.3872 | 6.87 | 0.009 | 0.017 | -2.211 |
| RHSIM_Rhsim12G0069600 | 6.87 | 0.009 | 0.017 | -1.021 |
| RHSIM_Rhsim12G0114100 | 6.87 | 0.009 | 0.017 | -0.321 |
| RHSIM_Rhsim09G0178900 | 6.87 | 0.009 | 0.017 | -0.983 |
| RHSIM_Rhsim09G0202500 | 6.87 | 0.009 | 0.017 | 0.356 |
| RHSIM_Rhsim08G0033300 | 6.87 | 0.009 | 0.017 | -0.476 |
| novel.3318 | 6.87 | 0.009 | 0.017 | 0.605 |
| RHSIM_Rhsim11G0017900 | 6.87 | 0.009 | 0.017 | -0.772 |
| RHSIM_Rhsim08G0107800 | 6.87 | 0.009 | 0.017 | -1.927 |
| RHSIM_RhsimUnG0153800 | 6.87 | 0.009 | 0.017 | -0.399 |
| novel.3074 | 6.87 | 0.009 | 0.017 | -0.537 |
| RHSIM_RhsimUnG0042700 | 6.86 | 0.009 | 0.017 | -3.241 |
| RHSIM_Rhsim05G0138300 | 6.86 | 0.009 | 0.017 | -0.838 |
| RHSIM_Rhsim13G0221400 | 6.86 | 0.009 | 0.017 | 0.638 |
| RHSIM_Rhsim12G0193800 | 6.86 | 0.009 | 0.017 | 0.563 |
| novel.212 | 6.86 | 0.009 | 0.017 | 1.829 |
| RHSIM_Rhsim02G0057900 | 6.85 | 0.009 | 0.017 | 2.514 |
| RHSIM_Rhsim05G0171100 | 6.85 | 0.009 | 0.017 | 0.243 |
| RHSIM_Rhsim02G0069700 | 6.85 | 0.009 | 0.017 | 0.533 |
| RHSIM_RhsimUnG0110000 | 6.85 | 0.009 | 0.017 | -1.368 |
| RHSIM_Rhsim13G0038300 | 6.84 | 0.009 | 0.017 | -4.953 |
| RHSIM_Rhsim10G0084400 | 6.84 | 0.009 | 0.017 | -1.611 |
| RHSIM_Rhsim05G0213200 | 6.84 | 0.009 | 0.017 | -0.792 |
| RHSIM_Rhsim05G0092600 | 6.84 | 0.009 | 0.017 | -2.969 |
| RHSIM_Rhsim01G0025700 | 6.84 | 0.009 | 0.017 | -2.988 |
| RHSIM_Rhsim05G0220500 | 6.83 | 0.009 | 0.017 | -0.487 |

| Gene | LRT statistic | P value | BH-adjusted P | log2FC (interaction) |
|---|---|---|---|---|
| RHSIM_Rhsim05G0071700 | 6.83 | 0.009 | 0.017 | 1.905 |
| RHSIM_Rhsim06G0016500 | 6.83 | 0.009 | 0.017 | 1.600 |
| RHSIM_Rhsim02G0235300 | 6.83 | 0.009 | 0.017 | 1.308 |
| RHSIM_Rhsim08G0024100 | 6.83 | 0.009 | 0.017 | 0.537 |
| novel.609 | 6.83 | 0.009 | 0.017 | 4.587 |
| RHSIM_Rhsim02G0226600 | 6.83 | 0.009 | 0.017 | -2.771 |
| RHSIM_RhsimUnG0031600 | 6.83 | 0.009 | 0.017 | 0.893 |
| RHSIM_Rhsim01G0250500 | 6.83 | 0.009 | 0.018 | 0.252 |
| RHSIM_Rhsim06G0242500 | 6.82 | 0.009 | 0.018 | 0.392 |
| RHSIM_Rhsim06G0231500 | 6.82 | 0.009 | 0.018 | -0.337 |
| RHSIM_Rhsim12G0088500 | 6.82 | 0.009 | 0.018 | 0.370 |
| novel.1324 | 6.82 | 0.009 | 0.018 | 1.485 |
| RHSIM_Rhsim01G0139900 | 6.81 | 0.009 | 0.018 | 0.582 |
| RHSIM_Rhsim01G0025000 | 6.81 | 0.009 | 0.018 | 3.828 |
| novel.1080 | 6.81 | 0.009 | 0.018 | 4.885 |
| novel.4402 | 6.80 | 0.009 | 0.018 | 0.881 |
| novel.3423 | 6.80 | 0.009 | 0.018 | 0.350 |
| novel.4481 | 6.80 | 0.009 | 0.018 | -3.438 |
| RHSIM_Rhsim07G0016500 | 6.80 | 0.009 | 0.018 | -0.424 |
| RHSIM_Rhsim08G0103600 | 6.80 | 0.009 | 0.018 | -3.042 |
| RHSIM_Rhsim05G0073600 | 6.80 | 0.009 | 0.018 | -0.763 |
| RHSIM_Rhsim05G0040500 | 6.79 | 0.009 | 0.018 | 4.743 |
| RHSIM_Rhsim08G0037700 | 6.79 | 0.009 | 0.018 | 1.211 |
| RHSIM_RhsimUnG0020700 | 6.79 | 0.009 | 0.018 | -0.330 |
| RHSIM_Rhsim03G0013700 | 6.79 | 0.009 | 0.018 | 1.157 |
| RHSIM_Rhsim01G0246700 | 6.79 | 0.009 | 0.018 | -0.387 |
| RHSIM_Rhsim04G0243400 | 6.79 | 0.009 | 0.018 | 1.922 |
| RHSIM_Rhsim13G0154500 | 6.78 | 0.009 | 0.018 | -1.088 |
| RHSIM_Rhsim06G0153200 | 6.78 | 0.009 | 0.018 | 0.131 |
| RHSIM_Rhsim12G0024800 | 6.78 | 0.009 | 0.018 | -0.918 |
| RHSIM_Rhsim12G0126600 | 6.78 | 0.009 | 0.018 | -0.502 |
| RHSIM_Rhsim07G0109800 | 6.78 | 0.009 | 0.018 | 1.408 |

| Gene | LRT statistic | P value | BH-adjusted P | log2FC (interaction) |
|---|---|---|---|---|
| novel.3943 | 6.78 | 0.009 | 0.018 | -3.469 |
| RHSIM_Rhsim02G0254300 | 6.78 | 0.009 | 0.018 | 2.709 |
| RHSIM_RhsimUnG0057000 | 6.77 | 0.009 | 0.018 | -2.596 |
| novel.183 | 6.77 | 0.009 | 0.018 | 1.301 |
| RHSIM_Rhsim01G0283000 | 6.77 | 0.009 | 0.018 | 0.556 |
| novel.847 | 6.77 | 0.009 | 0.018 | -1.588 |
| RHSIM_RhsimUnG0006200 | 6.77 | 0.009 | 0.018 | 0.376 |
| novel.422 | 6.77 | 0.009 | 0.018 | 0.640 |
| RHSIM_Rhsim10G0098300 | 6.77 | 0.009 | 0.018 | 3.649 |
| RHSIM_Rhsim11G0052500 | 6.76 | 0.009 | 0.018 | -2.200 |
| RHSIM_Rhsim02G0078000 | 6.76 | 0.009 | 0.018 | 2.721 |
| RHSIM_Rhsim09G0011800 | 6.76 | 0.009 | 0.018 | 0.439 |
| RHSIM_Rhsim01G0062900 | 6.76 | 0.009 | 0.018 | 1.317 |
| RHSIM_Rhsim07G0125300 | 6.76 | 0.009 | 0.018 | 0.219 |
| RHSIM_Rhsim10G0015800 | 6.76 | 0.009 | 0.018 | 0.829 |
| RHSIM_Rhsim02G0130100 | 6.75 | 0.009 | 0.018 | -0.962 |
| RHSIM_Rhsim02G0126500 | 6.75 | 0.009 | 0.018 | 2.408 |
| RHSIM_Rhsim05G0101700 | 6.74 | 0.009 | 0.018 | -0.264 |
| novel.3592 | 6.74 | 0.009 | 0.018 | -2.130 |
| RHSIM_Rhsim04G0017100 | 6.74 | 0.009 | 0.018 | 0.861 |
| RHSIM_Rhsim03G0085500 | 6.74 | 0.009 | 0.018 | 0.259 |
| RHSIM_Rhsim13G0185100 | 6.74 | 0.009 | 0.018 | -0.974 |
| RHSIM_Rhsim06G0035800 | 6.74 | 0.009 | 0.018 | -0.205 |
| RHSIM_Rhsim13G0032600 | 6.73 | 0.009 | 0.018 | -40.691 |
| RHSIM_Rhsim12G0149300 | 6.73 | 0.009 | 0.018 | -1.803 |
| novel.3700 | 6.73 | 0.009 | 0.018 | 2.807 |
| RHSIM_Rhsim09G0064100 | 6.73 | 0.009 | 0.018 | 4.475 |
| RHSIM_Rhsim08G0056500 | 6.73 | 0.010 | 0.018 | 1.511 |
| RHSIM_Rhsim12G0086400 | 6.72 | 0.010 | 0.018 | -0.371 |
| RHSIM_Rhsim13G0226300 | 6.72 | 0.010 | 0.019 | 1.041 |
| RHSIM_Rhsim10G0075900 | 6.72 | 0.010 | 0.019 | 0.920 |
| RHSIM_Rhsim07G0064200 | 6.72 | 0.010 | 0.019 | -1.363 |

| Gene | LRT statistic | P value | BH-adjusted P | log2FC (interaction) |
|---|---|---|---|---|
| RHSIM_Rhsim10G0011400 | 6.72 | 0.010 | 0.019 | -0.361 |
| RHSIM_Rhsim08G0032600 | 6.72 | 0.010 | 0.019 | 0.932 |
| RHSIM_Rhsim12G0045000 | 6.72 | 0.010 | 0.019 | -41.905 |
| RHSIM_RhsimUnG0077200 | 6.71 | 0.010 | 0.019 | 5.399 |
| novel.3699 | 6.71 | 0.010 | 0.019 | -0.401 |
| novel.3767 | 6.71 | 0.010 | 0.019 | -0.692 |
| novel.104 | 6.71 | 0.010 | 0.019 | -0.736 |
| RHSIM_Rhsim13G0230300 | 6.71 | 0.010 | 0.019 | -0.425 |
| novel.154 | 6.71 | 0.010 | 0.019 | 2.658 |
| RHSIM_Rhsim04G0027000 | 6.71 | 0.010 | 0.019 | -0.789 |
| novel.2942 | 6.71 | 0.010 | 0.019 | 2.114 |
| RHSIM_Rhsim12G0214300 | 6.70 | 0.010 | 0.019 | 0.230 |
| RHSIM_Rhsim02G0123300 | 6.70 | 0.010 | 0.019 | -0.290 |
| RHSIM_Rhsim01G0213400 | 6.70 | 0.010 | 0.019 | 1.697 |
| RHSIM_Rhsim13G0072600 | 6.70 | 0.010 | 0.019 | -0.318 |
| novel.4460 | 6.69 | 0.010 | 0.019 | -2.127 |
| RHSIM_Rhsim01G0273500 | 6.69 | 0.010 | 0.019 | 0.359 |
| RHSIM_Rhsim13G0007500 | 6.69 | 0.010 | 0.019 | 3.121 |
| RHSIM_Rhsim07G0195700 | 6.69 | 0.010 | 0.019 | 41.393 |
| novel.1512 | 6.69 | 0.010 | 0.019 | -1.225 |
| RHSIM_Rhsim07G0004500 | 6.69 | 0.010 | 0.019 | 2.937 |
| novel.2003 | 6.69 | 0.010 | 0.019 | -39.967 |
| RHSIM_Rhsim09G0102500 | 6.69 | 0.010 | 0.019 | 0.436 |
| RHSIM_Rhsim03G0157200 | 6.69 | 0.010 | 0.019 | 3.966 |
| RHSIM_Rhsim08G0065900 | 6.69 | 0.010 | 0.019 | 0.398 |
| RHSIM_RhsimUnG0030600 | 6.69 | 0.010 | 0.019 | -1.231 |
| RHSIM_Rhsim08G0243900 | 6.69 | 0.010 | 0.019 | 0.589 |
| novel.1774 | 6.69 | 0.010 | 0.019 | 1.845 |
| RHSIM_Rhsim08G0035200 | 6.68 | 0.010 | 0.019 | 0.240 |
| novel.2275 | 6.68 | 0.010 | 0.019 | 0.191 |
| RHSIM_Rhsim10G0140100 | 6.68 | 0.010 | 0.019 | 2.927 |
| novel.3826 | 6.68 | 0.010 | 0.019 | -0.442 |

| Gene | LRT statistic | P value | BH-adjusted P | log2FC (interaction) |
|---|---|---|---|---|
| novel.894 | 6.68 | 0.010 | 0.019 | -0.605 |
| RHSIM_Rhsim08G0111400 | 6.68 | 0.010 | 0.019 | 0.973 |
| RHSIM_Rhsim10G0142900 | 6.68 | 0.010 | 0.019 | 3.075 |
| RHSIM_Rhsim13G0138500 | 6.68 | 0.010 | 0.019 | -0.246 |
| novel.1483 | 6.68 | 0.010 | 0.019 | 1.817 |
| RHSIM_Rhsim07G0141900 | 6.67 | 0.010 | 0.019 | 0.138 |
| RHSIM_Rhsim07G0109900 | 6.67 | 0.010 | 0.019 | -0.308 |
| RHSIM_Rhsim06G0102800 | 6.67 | 0.010 | 0.019 | -0.416 |
| RHSIM_Rhsim13G0084600 | 6.67 | 0.010 | 0.019 | -1.142 |
| RHSIM_Rhsim11G0181000 | 6.67 | 0.010 | 0.019 | 0.308 |
| RHSIM_Rhsim10G0206700 | 6.67 | 0.010 | 0.019 | 0.397 |
| RHSIM_Rhsim08G0242000 | 6.67 | 0.010 | 0.019 | 0.415 |
| RHSIM_Rhsim08G0016700 | 6.67 | 0.010 | 0.019 | -0.190 |
| RHSIM_Rhsim07G0012000 | 6.66 | 0.010 | 0.019 | -39.058 |
| RHSIM_Rhsim02G0022000 | 6.66 | 0.010 | 0.019 | 2.234 |
| novel.4124 | 6.66 | 0.010 | 0.019 | -0.766 |
| RHSIM_Rhsim03G0143000 | 6.66 | 0.010 | 0.019 | 40.221 |
| RHSIM_Rhsim06G0081900 | 6.66 | 0.010 | 0.019 | 0.592 |
| RHSIM_Rhsim11G0093000 | 6.66 | 0.010 | 0.019 | -0.318 |
| RHSIM_Rhsim10G0050700 | 6.66 | 0.010 | 0.019 | -3.027 |
| RHSIM_Rhsim11G0163700 | 6.66 | 0.010 | 0.019 | 0.132 |
| RHSIM_Rhsim02G0028200 | 6.66 | 0.010 | 0.019 | -0.513 |
| RHSIM_Rhsim04G0061200 | 6.66 | 0.010 | 0.019 | -0.476 |
| RHSIM_Rhsim13G0134900 | 6.66 | 0.010 | 0.019 | 0.393 |
| novel.1112 | 6.66 | 0.010 | 0.019 | -1.466 |
| RHSIM_Rhsim09G0157300 | 6.65 | 0.010 | 0.019 | -1.196 |
| RHSIM_Rhsim03G0025200 | 6.65 | 0.010 | 0.019 | 0.753 |
| novel.3920 | 6.65 | 0.010 | 0.019 | 1.040 |
| RHSIM_Rhsim10G0105700 | 6.65 | 0.010 | 0.019 | 0.238 |
| RHSIM_Rhsim03G0233800 | 6.65 | 0.010 | 0.019 | -2.677 |
| RHSIM_Rhsim06G0108400 | 6.65 | 0.010 | 0.019 | -0.518 |
| novel.4218 | 6.65 | 0.010 | 0.019 | 1.691 |

| Gene | LRT statistic | P value | BH-adjusted P | log2FC (interaction) |
|---|---|---|---|---|
| RHSIM_Rhsim10G0008000 | 6.64 | 0.010 | 0.019 | 1.196 |
| RHSIM_RhsimUnG0090400 | 6.64 | 0.010 | 0.019 | 2.837 |
| RHSIM_Rhsim08G0087600 | 6.64 | 0.010 | 0.019 | -2.798 |
| RHSIM_Rhsim09G0113000 | 6.64 | 0.010 | 0.019 | -0.162 |
| RHSIM_Rhsim02G0046200 | 6.64 | 0.010 | 0.019 | 3.590 |
| RHSIM_Rhsim09G0023200 | 6.64 | 0.010 | 0.019 | -40.604 |
| novel.1888 | 6.63 | 0.010 | 0.019 | 2.221 |
| RHSIM_Rhsim13G0098000 | 6.63 | 0.010 | 0.019 | 0.539 |
| novel.4091 | 6.63 | 0.010 | 0.019 | -1.209 |
| RHSIM_Rhsim04G0202600 | 6.63 | 0.010 | 0.019 | -1.938 |
| RHSIM_Rhsim06G0017200 | 6.63 | 0.010 | 0.019 | 78.161 |
| RHSIM_RhsimUnG0142600 | 6.63 | 0.010 | 0.019 | -0.423 |
| RHSIM_Rhsim08G0098200 | 6.63 | 0.010 | 0.019 | 0.302 |
| novel.354 | 6.62 | 0.010 | 0.019 | 2.451 |
| RHSIM_Rhsim13G0209700 | 6.62 | 0.010 | 0.019 | -3.493 |
| novel.3693 | 6.62 | 0.010 | 0.020 | -40.376 |
| RHSIM_Rhsim05G0156700 | 6.61 | 0.010 | 0.020 | 3.007 |
| RHSIM_Rhsim06G0119900 | 6.61 | 0.010 | 0.020 | -1.244 |
| RHSIM_Rhsim13G0085500 | 6.61 | 0.010 | 0.020 | 0.919 |
| RHSIM_Rhsim03G0251500 | 6.61 | 0.010 | 0.020 | 0.358 |
| RHSIM_Rhsim13G0166800 | 6.60 | 0.010 | 0.020 | 0.386 |
| RHSIM_Rhsim06G0075700 | 6.60 | 0.010 | 0.020 | -0.573 |
| novel.251 | 6.60 | 0.010 | 0.020 | -0.590 |
| novel.1428 | 6.60 | 0.010 | 0.020 | -0.426 |
| RHSIM_Rhsim01G0045100 | 6.60 | 0.010 | 0.020 | 0.569 |
| RHSIM_RhsimUnG0242900 | 6.60 | 0.010 | 0.020 | 1.611 |
| RHSIM_Rhsim04G0242700 | 6.60 | 0.010 | 0.020 | 40.648 |
| RHSIM_RhsimUnG0173100 | 6.60 | 0.010 | 0.020 | 0.843 |
| RHSIM_Rhsim06G0149700 | 6.60 | 0.010 | 0.020 | -0.270 |
| RHSIM_Rhsim04G0199700 | 6.59 | 0.010 | 0.020 | -2.065 |
| RHSIM_Rhsim02G0023400 | 6.59 | 0.010 | 0.020 | -3.269 |
| RHSIM_Rhsim10G0194600 | 6.59 | 0.010 | 0.020 | -1.062 |

| Gene | LRT statistic | P value | BH-adjusted P | log2FC (interaction) |
|---|---|---|---|---|
| RHSIM_Rhsim07G0120300 | 6.59 | 0.010 | 0.020 | 0.849 |
| RHSIM_Rhsim07G0227800 | 6.59 | 0.010 | 0.020 | -0.513 |
| RHSIM_Rhsim01G0227000 | 6.59 | 0.010 | 0.020 | -6.383 |
| RHSIM_Rhsim06G0028600 | 6.58 | 0.010 | 0.020 | 0.689 |
| RHSIM_Rhsim09G0190800 | 6.58 | 0.010 | 0.020 | 0.626 |
| RHSIM_Rhsim10G0069900 | 6.58 | 0.010 | 0.020 | -0.860 |
| RHSIM_Rhsim13G0028400 | 6.58 | 0.010 | 0.020 | -0.336 |
| RHSIM_Rhsim02G0054000 | 6.58 | 0.010 | 0.020 | -0.264 |
| RHSIM_Rhsim02G0234300 | 6.58 | 0.010 | 0.020 | 39.713 |
| RHSIM_Rhsim09G0163700 | 6.58 | 0.010 | 0.020 | 2.054 |
| novel.4543 | 6.58 | 0.010 | 0.020 | 5.292 |
| novel.2897 | 6.58 | 0.010 | 0.020 | 1.016 |
| RHSIM_Rhsim07G0123500 | 6.58 | 0.010 | 0.020 | 39.617 |
| novel.2572 | 6.58 | 0.010 | 0.020 | -2.142 |
| novel.3025 | 6.57 | 0.010 | 0.020 | 1.670 |
| RHSIM_Rhsim08G0016300 | 6.57 | 0.010 | 0.020 | 2.066 |
| novel.1984 | 6.57 | 0.010 | 0.020 | 0.519 |
| RHSIM_Rhsim01G0224300 | 6.57 | 0.010 | 0.020 | -0.447 |
| RHSIM_Rhsim12G0191400 | 6.57 | 0.010 | 0.020 | 0.176 |
| RHSIM_Rhsim06G0046200 | 6.57 | 0.010 | 0.020 | 0.533 |
| RHSIM_Rhsim05G0209800 | 6.57 | 0.010 | 0.020 | -5.198 |
| RHSIM_Rhsim07G0064500 | 6.56 | 0.010 | 0.020 | -0.756 |
| RHSIM_Rhsim11G0054200 | 6.56 | 0.010 | 0.020 | 0.383 |
| RHSIM_Rhsim12G0050500 | 6.56 | 0.010 | 0.020 | -1.582 |
| RHSIM_Rhsim10G0013800 | 6.56 | 0.010 | 0.020 | -0.890 |
| RHSIM_Rhsim13G0110700 | 6.56 | 0.010 | 0.020 | 1.088 |
| RHSIM_Rhsim03G0082300 | 6.56 | 0.010 | 0.020 | 3.209 |
| RHSIM_Rhsim01G0188800 | 6.55 | 0.010 | 0.020 | -0.428 |
| RHSIM_RhsimUnG0117600 | 6.55 | 0.011 | 0.020 | -0.555 |
| RHSIM_Rhsim11G0105800 | 6.55 | 0.011 | 0.020 | 0.814 |
| RHSIM_Rhsim09G0034400 | 6.55 | 0.011 | 0.020 | 1.304 |
| novel.3217 | 6.55 | 0.011 | 0.020 | 1.890 |

| Gene | LRT statistic | P value | BH-adjusted P | log2FC (interaction) |
|---|---|---|---|---|
| RHSIM_Rhsim07G0102800 | 6.54 | 0.011 | 0.020 | -0.433 |
| novel.1342 | 6.54 | 0.011 | 0.020 | 0.699 |
| RHSIM_Rhsim13G0047500 | 6.54 | 0.011 | 0.020 | -0.537 |
| RHSIM_Rhsim07G0012800 | 6.54 | 0.011 | 0.020 | -0.700 |
| RHSIM_Rhsim01G0194700 | 6.54 | 0.011 | 0.020 | -0.352 |
| RHSIM_Rhsim04G0242600 | 6.54 | 0.011 | 0.020 | 0.503 |
| RHSIM_Rhsim10G0163000 | 6.54 | 0.011 | 0.020 | 0.328 |
| RHSIM_Rhsim03G0152100 | 6.53 | 0.011 | 0.020 | 1.633 |
| RHSIM_Rhsim09G0137800 | 6.53 | 0.011 | 0.020 | 6.080 |
| RHSIM_Rhsim01G0165500 | 6.53 | 0.011 | 0.020 | -0.338 |
| RHSIM_Rhsim01G0033300 | 6.53 | 0.011 | 0.020 | -0.503 |
| novel.4023 | 6.53 | 0.011 | 0.020 | 3.392 |
| RHSIM_Rhsim03G0196400 | 6.53 | 0.011 | 0.020 | 0.203 |
| RHSIM_Rhsim05G0145500 | 6.53 | 0.011 | 0.020 | 0.588 |
| RHSIM_Rhsim09G0195500 | 6.52 | 0.011 | 0.020 | -1.221 |
| RHSIM_Rhsim01G0012300 | 6.52 | 0.011 | 0.020 | 5.389 |
| RHSIM_Rhsim10G0187900 | 6.52 | 0.011 | 0.020 | -0.552 |
| RHSIM_Rhsim11G0176200 | 6.52 | 0.011 | 0.020 | -0.872 |
| RHSIM_Rhsim12G0135800 | 6.52 | 0.011 | 0.020 | -41.872 |
| novel.4213 | 6.52 | 0.011 | 0.020 | -0.864 |
| RHSIM_Rhsim02G0171700 | 6.52 | 0.011 | 0.021 | -78.412 |
| RHSIM_Rhsim01G0040300 | 6.52 | 0.011 | 0.021 | -1.536 |
| RHSIM_Rhsim07G0046500 | 6.52 | 0.011 | 0.021 | 3.675 |
| novel.541 | 6.52 | 0.011 | 0.021 | 2.937 |
| novel.163 | 6.52 | 0.011 | 0.021 | -0.927 |
| RHSIM_Rhsim03G0218400 | 6.51 | 0.011 | 0.021 | -0.427 |
| RHSIM_RhsimUnG0184300 | 6.51 | 0.011 | 0.021 | -0.815 |
| RHSIM_Rhsim04G0146600 | 6.51 | 0.011 | 0.021 | -0.177 |
| novel.2622 | 6.50 | 0.011 | 0.021 | -3.774 |
| RHSIM_Rhsim08G0109800 | 6.50 | 0.011 | 0.021 | -0.378 |
| RHSIM_Rhsim08G0216100 | 6.50 | 0.011 | 0.021 | 2.324 |
| RHSIM_Rhsim04G0177800 | 6.50 | 0.011 | 0.021 | 0.751 |

| Gene | LRT statistic | P value | BH-adjusted P | log2FC (interaction) |
|---|---|---|---|---|
| novel.1778 | 6.50 | 0.011 | 0.021 | 0.995 |
| novel.3137 | 6.50 | 0.011 | 0.021 | 1.578 |
| novel.2295 | 6.50 | 0.011 | 0.021 | 2.834 |
| RHSIM_Rhsim10G0064700 | 6.50 | 0.011 | 0.021 | 4.250 |
| RHSIM_Rhsim13G0055200 | 6.50 | 0.011 | 0.021 | 0.526 |
| RHSIM_Rhsim10G0201800 | 6.49 | 0.011 | 0.021 | 0.770 |
| novel.2171 | 6.49 | 0.011 | 0.021 | -4.348 |
| RHSIM_Rhsim13G0223100 | 6.49 | 0.011 | 0.021 | -0.364 |
| RHSIM_RhsimUnG0251700 | 6.49 | 0.011 | 0.021 | -0.296 |
| novel.3440 | 6.49 | 0.011 | 0.021 | -0.785 |
| novel.185 | 6.48 | 0.011 | 0.021 | 1.102 |
| RHSIM_Rhsim10G0175400 | 6.48 | 0.011 | 0.021 | 4.530 |
| RHSIM_RhsimUnG0162600 | 6.48 | 0.011 | 0.021 | 2.717 |
| RHSIM_Rhsim06G0242400 | 6.48 | 0.011 | 0.021 | -0.429 |
| RHSIM_Rhsim03G0006500 | 6.48 | 0.011 | 0.021 | 0.419 |
| novel.1167 | 6.48 | 0.011 | 0.021 | -0.288 |
| RHSIM_Rhsim13G0036000 | 6.48 | 0.011 | 0.021 | 0.328 |
| novel.1415 | 6.48 | 0.011 | 0.021 | 0.486 |
| RHSIM_Rhsim06G0219300 | 6.47 | 0.011 | 0.021 | 2.243 |
| novel.2995 | 6.47 | 0.011 | 0.021 | 1.108 |
| RHSIM_Rhsim05G0079300 | 6.47 | 0.011 | 0.021 | 0.287 |
| RHSIM_Rhsim09G0061100 | 6.47 | 0.011 | 0.021 | 40.987 |
| RHSIM_Rhsim13G0145800 | 6.47 | 0.011 | 0.021 | -1.429 |
| RHSIM_Rhsim12G0080700 | 6.47 | 0.011 | 0.021 | 0.233 |
| RHSIM_RhsimUnG0252700 | 6.47 | 0.011 | 0.021 | 40.318 |
| RHSIM_Rhsim11G0040000 | 6.46 | 0.011 | 0.021 | 2.233 |
| RHSIM_Rhsim09G0202300 | 6.46 | 0.011 | 0.021 | 2.112 |
| RHSIM_Rhsim13G0125700 | 6.46 | 0.011 | 0.021 | -0.175 |
| RHSIM_Rhsim09G0132400 | 6.46 | 0.011 | 0.021 | 1.699 |
| RHSIM_Rhsim07G0229300 | 6.46 | 0.011 | 0.021 | -3.206 |
| RHSIM_Rhsim05G0066900 | 6.45 | 0.011 | 0.021 | 0.512 |
| RHSIM_Rhsim07G0133900 | 6.45 | 0.011 | 0.021 | 3.181 |

| Gene | LRT statistic | P value | BH-adjusted P | log2FC (interaction) |
|---|---|---|---|---|
| RHSIM_Rhsim07G0001700 | 6.45 | 0.011 | 0.021 | -0.327 |
| RHSIM_Rhsim07G0255400 | 6.45 | 0.011 | 0.021 | 0.961 |
| RHSIM_RhsimUnG0167800 | 6.45 | 0.011 | 0.021 | 1.007 |
| RHSIM_Rhsim08G0077400 | 6.45 | 0.011 | 0.021 | -0.239 |
| RHSIM_Rhsim03G0239900 | 6.45 | 0.011 | 0.021 | -0.645 |
| RHSIM_Rhsim07G0124000 | 6.45 | 0.011 | 0.021 | -0.851 |
| RHSIM_Rhsim12G0141600 | 6.44 | 0.011 | 0.021 | -0.455 |
| RHSIM_Rhsim12G0133200 | 6.44 | 0.011 | 0.021 | 1.212 |
| novel.399 | 6.44 | 0.011 | 0.021 | 2.785 |
| RHSIM_Rhsim07G0101200 | 6.44 | 0.011 | 0.021 | 0.127 |
| RHSIM_Rhsim03G0256300 | 6.43 | 0.011 | 0.021 | 2.537 |
| RHSIM_Rhsim11G0009200 | 6.43 | 0.011 | 0.021 | 40.623 |
| novel.1749 | 6.43 | 0.011 | 0.021 | 2.105 |
| RHSIM_Rhsim06G0038500 | 6.43 | 0.011 | 0.022 | -0.297 |
| novel.3109 | 6.43 | 0.011 | 0.022 | -0.610 |
| RHSIM_Rhsim09G0209100 | 6.43 | 0.011 | 0.022 | 0.183 |
| RHSIM_Rhsim04G0059100 | 6.42 | 0.011 | 0.022 | 6.079 |
| RHSIM_Rhsim09G0137200 | 6.42 | 0.011 | 0.022 | 0.257 |
| RHSIM_Rhsim09G0039000 | 6.42 | 0.011 | 0.022 | -2.786 |
| RHSIM_Rhsim06G0226100 | 6.42 | 0.011 | 0.022 | -0.311 |
| RHSIM_Rhsim12G0128200 | 6.42 | 0.011 | 0.022 | 42.485 |
| novel.4222 | 6.42 | 0.011 | 0.022 | 1.431 |
| RHSIM_Rhsim10G0176500 | 6.42 | 0.011 | 0.022 | 2.574 |
| novel.928 | 6.42 | 0.011 | 0.022 | 2.639 |
| novel.2254 | 6.42 | 0.011 | 0.022 | 40.228 |
| RHSIM_Rhsim09G0128400 | 6.41 | 0.011 | 0.022 | 2.008 |
| novel.3998 | 6.41 | 0.011 | 0.022 | 1.896 |
| RHSIM_Rhsim11G0134200 | 6.41 | 0.011 | 0.022 | 0.308 |
| RHSIM_Rhsim08G0108100 | 6.41 | 0.011 | 0.022 | -2.716 |
| RHSIM_Rhsim06G0204300 | 6.41 | 0.011 | 0.022 | 0.363 |
| RHSIM_Rhsim09G0049200 | 6.41 | 0.011 | 0.022 | 2.365 |
| RHSIM_Rhsim09G0145700 | 6.41 | 0.011 | 0.022 | -0.936 |

| Gene | LRT statistic | P value | BH-adjusted P | log2FC (interaction) |
|---|---|---|---|---|
| RHSIM_Rhsim08G0247900 | 6.41 | 0.011 | 0.022 | -0.279 |
| RHSIM_Rhsim01G0207600 | 6.41 | 0.011 | 0.022 | 2.580 |
| RHSIM_Rhsim05G0133800 | 6.40 | 0.011 | 0.022 | 0.576 |
| RHSIM_Rhsim09G0026500 | 6.40 | 0.011 | 0.022 | 0.347 |
| RHSIM_Rhsim13G0228100 | 6.40 | 0.011 | 0.022 | 1.370 |
| RHSIM_Rhsim12G0008100 | 6.40 | 0.011 | 0.022 | -0.538 |
| RHSIM_Rhsim13G0160100 | 6.40 | 0.011 | 0.022 | 0.640 |
| RHSIM_Rhsim13G0063700 | 6.40 | 0.011 | 0.022 | 2.289 |
| RHSIM_Rhsim09G0188900 | 6.40 | 0.011 | 0.022 | -1.798 |
| RHSIM_Rhsim01G0230800 | 6.40 | 0.011 | 0.022 | 1.138 |
| RHSIM_Rhsim09G0147900 | 6.39 | 0.011 | 0.022 | 1.549 |
| RHSIM_Rhsim09G0203500 | 6.39 | 0.011 | 0.022 | -0.372 |
| RHSIM_Rhsim01G0255000 | 6.39 | 0.011 | 0.022 | -0.259 |
| RHSIM_Rhsim12G0048400 | 6.39 | 0.011 | 0.022 | 0.771 |
| novel.3060 | 6.39 | 0.011 | 0.022 | 1.013 |
| RHSIM_Rhsim07G0207400 | 6.39 | 0.011 | 0.022 | -42.519 |
| RHSIM_Rhsim01G0245200 | 6.39 | 0.011 | 0.022 | -0.255 |
| novel.4283 | 6.39 | 0.011 | 0.022 | 39.996 |
| RHSIM_Rhsim07G0033200 | 6.39 | 0.011 | 0.022 | 0.622 |
| RHSIM_Rhsim03G0070500 | 6.39 | 0.011 | 0.022 | -1.757 |
| RHSIM_Rhsim10G0036100 | 6.38 | 0.012 | 0.022 | 0.709 |
| RHSIM_Rhsim13G0182400 | 6.38 | 0.012 | 0.022 | -39.734 |
| novel.3764 | 6.38 | 0.012 | 0.022 | 3.490 |
| novel.651 | 6.38 | 0.012 | 0.022 | -0.957 |
| RHSIM_Rhsim10G0020400 | 6.38 | 0.012 | 0.022 | 0.412 |
| RHSIM_Rhsim06G0017900 | 6.38 | 0.012 | 0.022 | 0.165 |
| RHSIM_Rhsim10G0105500 | 6.38 | 0.012 | 0.022 | 0.437 |
| novel.1169 | 6.38 | 0.012 | 0.022 | -0.676 |
| RHSIM_Rhsim07G0093800 | 6.37 | 0.012 | 0.022 | 2.856 |
| RHSIM_Rhsim10G0211600 | 6.37 | 0.012 | 0.022 | -0.623 |
| RHSIM_Rhsim02G0003400 | 6.37 | 0.012 | 0.022 | 2.689 |
| novel.3078 | 6.37 | 0.012 | 0.022 | 1.299 |

| Gene | LRT statistic | P value | BH-adjusted P | log2FC (interaction) |
|---|---|---|---|---|
| RHSIM_Rhsim01G0261900 | 6.37 | 0.012 | 0.022 | -0.422 |
| RHSIM_Rhsim01G0009300 | 6.37 | 0.012 | 0.022 | 0.155 |
| novel.3820 | 6.36 | 0.012 | 0.022 | 1.314 |
| RHSIM_Rhsim05G0087300 | 6.36 | 0.012 | 0.022 | -1.367 |
| RHSIM_Rhsim08G0023700 | 6.36 | 0.012 | 0.022 | -0.493 |
| RHSIM_Rhsim06G0103600 | 6.36 | 0.012 | 0.022 | -3.594 |
| RHSIM_Rhsim10G0095400 | 6.36 | 0.012 | 0.022 | 1.051 |
| RHSIM_RhsimUnG0249900 | 6.36 | 0.012 | 0.022 | 1.157 |
| novel.3019 | 6.36 | 0.012 | 0.022 | 1.533 |
| RHSIM_Rhsim09G0192400 | 6.35 | 0.012 | 0.022 | -3.249 |
| RHSIM_Rhsim10G0185200 | 6.35 | 0.012 | 0.022 | 0.476 |
| novel.1994 | 6.35 | 0.012 | 0.022 | -1.467 |
| RHSIM_Rhsim01G0093800 | 6.35 | 0.012 | 0.022 | 1.266 |
| RHSIM_Rhsim12G0070700 | 6.34 | 0.012 | 0.022 | 0.620 |
| novel.229 | 6.34 | 0.012 | 0.022 | 1.460 |
| RHSIM_Rhsim01G0185100 | 6.34 | 0.012 | 0.023 | -0.207 |
| RHSIM_Rhsim03G0209000 | 6.34 | 0.012 | 0.023 | 0.696 |
| RHSIM_Rhsim13G0191300 | 6.34 | 0.012 | 0.023 | 0.714 |
| RHSIM_Rhsim09G0073300 | 6.34 | 0.012 | 0.023 | -0.184 |
| RHSIM_Rhsim08G0214600 | 6.34 | 0.012 | 0.023 | 3.097 |
| RHSIM_Rhsim12G0211500 | 6.34 | 0.012 | 0.023 | 0.485 |
| RHSIM_Rhsim10G0191100 | 6.33 | 0.012 | 0.023 | 0.188 |
| novel.2915 | 6.33 | 0.012 | 0.023 | 41.692 |
| RHSIM_Rhsim05G0079200 | 6.33 | 0.012 | 0.023 | 0.939 |
| RHSIM_Rhsim11G0104600 | 6.33 | 0.012 | 0.023 | -0.752 |
| RHSIM_Rhsim10G0052600 | 6.33 | 0.012 | 0.023 | -0.762 |
| RHSIM_Rhsim07G0059600 | 6.33 | 0.012 | 0.023 | 0.400 |
| RHSIM_Rhsim01G0021300 | 6.33 | 0.012 | 0.023 | 5.190 |
| RHSIM_Rhsim04G0212900 | 6.33 | 0.012 | 0.023 | 0.306 |
| novel.3792 | 6.33 | 0.012 | 0.023 | -0.597 |
| RHSIM_Rhsim04G0002300 | 6.32 | 0.012 | 0.023 | 0.378 |
| RHSIM_Rhsim03G0177200 | 6.32 | 0.012 | 0.023 | -0.288 |

| Gene | LRT statistic | P value | BH-adjusted P | log2FC (interaction) |
|---|---|---|---|---|
| RHSIM_Rhsim01G0054300 | 6.32 | 0.012 | 0.023 | -39.459 |
| RHSIM_Rhsim02G0165900 | 6.32 | 0.012 | 0.023 | 2.832 |
| RHSIM_Rhsim12G0057900 | 6.32 | 0.012 | 0.023 | 1.025 |
| RHSIM_Rhsim13G0001700 | 6.32 | 0.012 | 0.023 | 0.265 |
| RHSIM_Rhsim13G0198800 | 6.32 | 0.012 | 0.023 | 0.386 |
| RHSIM_Rhsim03G0074300 | 6.31 | 0.012 | 0.023 | 1.103 |
| novel.1614 | 6.31 | 0.012 | 0.023 | 1.186 |
| RHSIM_Rhsim04G0075700 | 6.31 | 0.012 | 0.023 | 1.782 |
| RHSIM_Rhsim08G0092400 | 6.31 | 0.012 | 0.023 | 0.221 |
| RHSIM_Rhsim11G0180000 | 6.31 | 0.012 | 0.023 | -41.803 |
| RHSIM_Rhsim13G0181700 | 6.31 | 0.012 | 0.023 | 0.495 |
| RHSIM_Rhsim04G0199600 | 6.30 | 0.012 | 0.023 | 0.880 |
| RHSIM_Rhsim06G0193500 | 6.30 | 0.012 | 0.023 | 3.486 |
| RHSIM_RhsimUnG0228800 | 6.30 | 0.012 | 0.023 | -1.491 |
| RHSIM_Rhsim04G0129900 | 6.30 | 0.012 | 0.023 | -0.649 |
| RHSIM_Rhsim13G0193100 | 6.29 | 0.012 | 0.023 | 1.699 |
| RHSIM_Rhsim05G0234600 | 6.29 | 0.012 | 0.023 | 0.315 |
| novel.4422 | 6.29 | 0.012 | 0.023 | 1.176 |
| RHSIM_Rhsim01G0284900 | 6.28 | 0.012 | 0.023 | -0.706 |
| RHSIM_Rhsim08G0222100 | 6.28 | 0.012 | 0.023 | 1.163 |
| RHSIM_Rhsim05G0002200 | 6.28 | 0.012 | 0.023 | 3.447 |
| RHSIM_Rhsim04G0104900 | 6.28 | 0.012 | 0.023 | -1.180 |
| RHSIM_Rhsim03G0245200 | 6.28 | 0.012 | 0.023 | 0.187 |
| RHSIM_Rhsim06G0198700 | 6.28 | 0.012 | 0.023 | -0.950 |
| RHSIM_Rhsim08G0087500 | 6.28 | 0.012 | 0.023 | 6.023 |
| RHSIM_Rhsim06G0100200 | 6.28 | 0.012 | 0.023 | -0.788 |
| RHSIM_Rhsim09G0173200 | 6.27 | 0.012 | 0.023 | -0.167 |
| RHSIM_Rhsim06G0201300 | 6.27 | 0.012 | 0.023 | 0.178 |
| RHSIM_Rhsim12G0032700 | 6.27 | 0.012 | 0.023 | 0.447 |
| RHSIM_Rhsim12G0038200 | 6.27 | 0.012 | 0.023 | 78.272 |
| novel.221 | 6.27 | 0.012 | 0.023 | 0.681 |
| RHSIM_Rhsim01G0255200 | 6.27 | 0.012 | 0.023 | -0.277 |

| Gene | LRT statistic | P value | BH-adjusted P | log2FC (interaction) |
|---|---|---|---|---|
| RHSIM_RhsimUnG0257500 | 6.27 | 0.012 | 0.023 | -0.313 |
| RHSIM_Rhsim12G0001800 | 6.26 | 0.012 | 0.023 | -0.563 |
| RHSIM_Rhsim01G0100700 | 6.26 | 0.012 | 0.023 | 0.526 |
| RHSIM_Rhsim05G0174700 | 6.26 | 0.012 | 0.023 | 0.582 |
| novel.3550 | 6.26 | 0.012 | 0.024 | -2.017 |
| RHSIM_Rhsim13G0027800 | 6.26 | 0.012 | 0.024 | -39.586 |
| novel.2606 | 6.26 | 0.012 | 0.024 | -1.404 |
| RHSIM_Rhsim01G0265800 | 6.26 | 0.012 | 0.024 | 2.554 |
| RHSIM_Rhsim11G0160400 | 6.25 | 0.012 | 0.024 | -0.832 |
| RHSIM_Rhsim11G0022900 | 6.25 | 0.012 | 0.024 | 1.516 |
| RHSIM_Rhsim03G0144700 | 6.25 | 0.012 | 0.024 | 0.199 |
| novel.1798 | 6.25 | 0.012 | 0.024 | -39.804 |
| RHSIM_Rhsim11G0040200 | 6.25 | 0.012 | 0.024 | 0.534 |
| RHSIM_Rhsim06G0063300 | 6.25 | 0.012 | 0.024 | -0.478 |
| RHSIM_Rhsim01G0232300 | 6.25 | 0.012 | 0.024 | -0.486 |
| RHSIM_Rhsim10G0144700 | 6.25 | 0.012 | 0.024 | -0.250 |
| RHSIM_Rhsim01G0024500 | 6.25 | 0.012 | 0.024 | -0.414 |
| RHSIM_Rhsim08G0152000 | 6.25 | 0.012 | 0.024 | 0.230 |
| RHSIM_Rhsim10G0134300 | 6.25 | 0.012 | 0.024 | -0.367 |
| RHSIM_Rhsim02G0255500 | 6.25 | 0.012 | 0.024 | -0.303 |
| RHSIM_Rhsim08G0068300 | 6.25 | 0.012 | 0.024 | -1.322 |
| RHSIM_Rhsim02G0149500 | 6.24 | 0.012 | 0.024 | 2.135 |
| RHSIM_Rhsim11G0171200 | 6.24 | 0.012 | 0.024 | -0.528 |
| RHSIM_Rhsim01G0094400 | 6.24 | 0.013 | 0.024 | -0.460 |
| RHSIM_Rhsim08G0126300 | 6.24 | 0.013 | 0.024 | -0.293 |
| novel.2540 | 6.23 | 0.013 | 0.024 | -0.339 |
| RHSIM_Rhsim08G0122500 | 6.23 | 0.013 | 0.024 | 0.690 |
| RHSIM_Rhsim03G0045700 | 6.23 | 0.013 | 0.024 | 0.745 |
| RHSIM_Rhsim12G0067600 | 6.23 | 0.013 | 0.024 | -4.333 |
| RHSIM_Rhsim11G0043200 | 6.23 | 0.013 | 0.024 | 0.235 |
| novel.3461 | 6.23 | 0.013 | 0.024 | 3.089 |
| RHSIM_Rhsim07G0087200 | 6.22 | 0.013 | 0.024 | -0.398 |

| Gene | LRT statistic | P value | BH-adjusted P | log2FC (interaction) |
|---|---|---|---|---|
| novel.107 | 6.22 | 0.013 | 0.024 | -1.674 |
| RHSIM_Rhsim03G0156900 | 6.22 | 0.013 | 0.024 | 0.196 |
| RHSIM_Rhsim04G0105900 | 6.22 | 0.013 | 0.024 | -0.860 |
| RHSIM_Rhsim02G0079000 | 6.22 | 0.013 | 0.024 | -0.844 |
| RHSIM_Rhsim04G0110800 | 6.22 | 0.013 | 0.024 | -0.319 |
| RHSIM_Rhsim05G0183600 | 6.21 | 0.013 | 0.024 | 1.671 |
| RHSIM_Rhsim01G0122200 | 6.21 | 0.013 | 0.024 | 0.344 |
| novel.4469 | 6.21 | 0.013 | 0.024 | -1.399 |
| novel.3398 | 6.21 | 0.013 | 0.024 | -3.892 |
| novel.2054 | 6.21 | 0.013 | 0.024 | 1.555 |
| RHSIM_Rhsim11G0067400 | 6.21 | 0.013 | 0.024 | 1.001 |
| novel.2648 | 6.21 | 0.013 | 0.024 | 0.890 |
| novel.164 | 6.21 | 0.013 | 0.024 | 2.704 |
| RHSIM_Rhsim04G0227700 | 6.21 | 0.013 | 0.024 | -0.265 |
| RHSIM_Rhsim10G0072100 | 6.21 | 0.013 | 0.024 | 0.576 |
| RHSIM_Rhsim06G0048800 | 6.20 | 0.013 | 0.024 | -2.781 |
| RHSIM_Rhsim09G0006500 | 6.20 | 0.013 | 0.024 | 1.578 |
| RHSIM_Rhsim11G0107400 | 6.20 | 0.013 | 0.024 | 3.301 |
| RHSIM_Rhsim05G0070700 | 6.20 | 0.013 | 0.024 | -0.466 |
| RHSIM_Rhsim11G0087900 | 6.20 | 0.013 | 0.024 | -1.063 |
| RHSIM_Rhsim03G0153400 | 6.20 | 0.013 | 0.024 | -0.556 |
| RHSIM_Rhsim04G0061700 | 6.19 | 0.013 | 0.024 | -0.671 |
| RHSIM_Rhsim12G0185900 | 6.19 | 0.013 | 0.024 | -3.103 |
| novel.3255 | 6.19 | 0.013 | 0.024 | -0.965 |
| RHSIM_Rhsim11G0036700 | 6.19 | 0.013 | 0.024 | 0.245 |
| RHSIM_Rhsim11G0060200 | 6.19 | 0.013 | 0.024 | 2.441 |
| RHSIM_Rhsim03G0010800 | 6.19 | 0.013 | 0.024 | -0.468 |
| RHSIM_Rhsim05G0091600 | 6.19 | 0.013 | 0.024 | -0.306 |
| RHSIM_Rhsim06G0101300 | 6.19 | 0.013 | 0.024 | -5.087 |
| RHSIM_Rhsim11G0042200 | 6.19 | 0.013 | 0.024 | -0.333 |
| RHSIM_Rhsim09G0013900 | 6.19 | 0.013 | 0.024 | 0.632 |
| RHSIM_Rhsim08G0160900 | 6.19 | 0.013 | 0.024 | 5.298 |

| Gene | LRT statistic | P value | BH-adjusted P | log2FC (interaction) |
|---|---|---|---|---|
| RHSIM_Rhsim08G0236300 | 6.18 | 0.013 | 0.024 | 3.718 |
| RHSIM_Rhsim06G0238300 | 6.18 | 0.013 | 0.024 | -0.694 |
| RHSIM_Rhsim12G0173600 | 6.18 | 0.013 | 0.024 | 0.337 |
| RHSIM_Rhsim13G0000300 | 6.18 | 0.013 | 0.024 | 2.101 |
| RHSIM_Rhsim04G0201700 | 6.18 | 0.013 | 0.024 | 0.428 |
| novel.1192 | 6.17 | 0.013 | 0.025 | 3.132 |
| RHSIM_Rhsim06G0064400 | 6.17 | 0.013 | 0.025 | 2.014 |
| RHSIM_Rhsim07G0084300 | 6.17 | 0.013 | 0.025 | 0.420 |
| RHSIM_Rhsim05G0074600 | 6.17 | 0.013 | 0.025 | 0.206 |
| novel.2963 | 6.17 | 0.013 | 0.025 | -2.804 |
| RHSIM_Rhsim13G0139800 | 6.17 | 0.013 | 0.025 | 0.259 |
| RHSIM_Rhsim12G0143900 | 6.16 | 0.013 | 0.025 | -0.255 |
| RHSIM_Rhsim09G0085900 | 6.16 | 0.013 | 0.025 | 1.068 |
| RHSIM_Rhsim02G0011300 | 6.16 | 0.013 | 0.025 | 4.863 |
| RHSIM_Rhsim13G0035300 | 6.16 | 0.013 | 0.025 | -40.540 |
| RHSIM_Rhsim12G0164000 | 6.16 | 0.013 | 0.025 | -0.290 |
| RHSIM_Rhsim01G0213200 | 6.16 | 0.013 | 0.025 | 0.633 |
| RHSIM_RhsimUnG0104100 | 6.16 | 0.013 | 0.025 | 0.604 |
| RHSIM_Rhsim08G0229300 | 6.16 | 0.013 | 0.025 | 1.835 |
| RHSIM_Rhsim04G0167200 | 6.16 | 0.013 | 0.025 | -0.405 |
| RHSIM_Rhsim09G0127600 | 6.16 | 0.013 | 0.025 | -0.389 |
| RHSIM_Rhsim06G0160300 | 6.16 | 0.013 | 0.025 | 1.346 |
| novel.943 | 6.15 | 0.013 | 0.025 | 1.295 |
| RHSIM_Rhsim02G0078600 | 6.15 | 0.013 | 0.025 | -1.259 |
| RHSIM_Rhsim10G0058700 | 6.15 | 0.013 | 0.025 | -0.879 |
| RHSIM_Rhsim08G0155500 | 6.15 | 0.013 | 0.025 | -0.294 |
| RHSIM_Rhsim13G0231100 | 6.15 | 0.013 | 0.025 | 0.275 |
| RHSIM_Rhsim04G0076000 | 6.15 | 0.013 | 0.025 | -0.662 |
| RHSIM_Rhsim12G0193100 | 6.15 | 0.013 | 0.025 | 2.903 |
| RHSIM_Rhsim12G0180400 | 6.15 | 0.013 | 0.025 | 0.334 |
| RHSIM_Rhsim03G0220600 | 6.15 | 0.013 | 0.025 | -0.691 |
| novel.3202 | 6.15 | 0.013 | 0.025 | 1.126 |

| Gene | LRT statistic | P value | BH-adjusted P | log2FC (interaction) |
|---|---|---|---|---|
| RHSIM_Rhsim06G0039700 | 6.14 | 0.013 | 0.025 | -77.697 |
| RHSIM_Rhsim04G0208000 | 6.14 | 0.013 | 0.025 | -42.207 |
| RHSIM_RhsimUnG0242100 | 6.14 | 0.013 | 0.025 | -1.666 |
| RHSIM_Rhsim13G0073900 | 6.14 | 0.013 | 0.025 | -0.277 |
| RHSIM_Rhsim11G0188200 | 6.14 | 0.013 | 0.025 | 0.864 |
| RHSIM_Rhsim01G0291500 | 6.14 | 0.013 | 0.025 | 0.208 |
| RHSIM_RhsimUnG0036100 | 6.14 | 0.013 | 0.025 | -0.996 |
| RHSIM_Rhsim06G0012800 | 6.13 | 0.013 | 0.025 | -0.242 |
| RHSIM_Rhsim10G0072200 | 6.13 | 0.013 | 0.025 | -0.278 |
| RHSIM_Rhsim13G0200800 | 6.13 | 0.013 | 0.025 | 0.222 |
| RHSIM_RhsimUnG0215600 | 6.13 | 0.013 | 0.025 | -0.652 |
| RHSIM_Rhsim03G0066500 | 6.13 | 0.013 | 0.025 | 1.844 |
| RHSIM_Rhsim12G0102200 | 6.13 | 0.013 | 0.025 | -0.301 |
| RHSIM_Rhsim02G0142100 | 6.13 | 0.013 | 0.025 | 1.272 |
| RHSIM_Rhsim07G0231700 | 6.12 | 0.013 | 0.025 | -41.814 |
| novel.3637 | 6.12 | 0.013 | 0.025 | 2.193 |
| RHSIM_Rhsim04G0051100 | 6.12 | 0.013 | 0.025 | -0.579 |
| RHSIM_Rhsim01G0007700 | 6.12 | 0.013 | 0.025 | -2.059 |
| RHSIM_Rhsim13G0132000 | 6.12 | 0.013 | 0.025 | -0.282 |
| RHSIM_Rhsim03G0021100 | 6.11 | 0.013 | 0.025 | 0.614 |
| RHSIM_Rhsim13G0167100 | 6.11 | 0.013 | 0.025 | 1.160 |
| RHSIM_Rhsim12G0196400 | 6.11 | 0.013 | 0.025 | 0.308 |
| RHSIM_Rhsim11G0102700 | 6.11 | 0.013 | 0.025 | 0.615 |
| RHSIM_Rhsim02G0159000 | 6.11 | 0.013 | 0.025 | -0.495 |
| RHSIM_Rhsim12G0124500 | 6.10 | 0.013 | 0.025 | -0.265 |
| RHSIM_Rhsim13G0199800 | 6.10 | 0.013 | 0.025 | 0.336 |
| novel.1387 | 6.10 | 0.014 | 0.025 | -0.414 |
| RHSIM_Rhsim05G0100300 | 6.10 | 0.014 | 0.025 | 0.523 |
| RHSIM_Rhsim10G0012100 | 6.10 | 0.014 | 0.025 | 1.524 |
| RHSIM_Rhsim04G0250000 | 6.10 | 0.014 | 0.026 | -0.272 |
| RHSIM_Rhsim05G0007900 | 6.10 | 0.014 | 0.026 | -2.951 |
| RHSIM_Rhsim03G0014800 | 6.09 | 0.014 | 0.026 | -41.073 |

| Gene | LRT statistic | P value | BH-adjusted P | log2FC (interaction) |
|---|---|---|---|---|
| RHSIM_Rhsim13G0224800 | 6.09 | 0.014 | 0.026 | 1.358 |
| RHSIM_Rhsim08G0165400 | 6.09 | 0.014 | 0.026 | -2.025 |
| novel.1923 | 6.09 | 0.014 | 0.026 | 1.502 |
| RHSIM_Rhsim03G0076000 | 6.09 | 0.014 | 0.026 | -0.766 |
| RHSIM_Rhsim04G0149500 | 6.08 | 0.014 | 0.026 | 0.150 |
| RHSIM_Rhsim02G0062200 | 6.08 | 0.014 | 0.026 | 0.298 |
| novel.287 | 6.08 | 0.014 | 0.026 | -3.286 |
| RHSIM_RhsimUnG0095600 | 6.08 | 0.014 | 0.026 | 40.737 |
| RHSIM_Rhsim03G0266100 | 6.08 | 0.014 | 0.026 | 0.347 |
| RHSIM_Rhsim13G0049200 | 6.08 | 0.014 | 0.026 | 0.458 |
| novel.2528 | 6.08 | 0.014 | 0.026 | 1.212 |
| novel.27 | 6.08 | 0.014 | 0.026 | 2.623 |
| RHSIM_Rhsim11G0061800 | 6.08 | 0.014 | 0.026 | -0.526 |
| RHSIM_Rhsim07G0019600 | 6.07 | 0.014 | 0.026 | -1.397 |
| RHSIM_Rhsim13G0095200 | 6.07 | 0.014 | 0.026 | -4.372 |
| RHSIM_Rhsim10G0168300 | 6.07 | 0.014 | 0.026 | 0.145 |
| novel.4109 | 6.07 | 0.014 | 0.026 | 2.706 |
| RHSIM_Rhsim13G0053500 | 6.07 | 0.014 | 0.026 | -1.734 |
| novel.3276 | 6.07 | 0.014 | 0.026 | -1.406 |
| RHSIM_Rhsim07G0034700 | 6.07 | 0.014 | 0.026 | 1.386 |
| novel.1741 | 6.07 | 0.014 | 0.026 | 1.419 |
| RHSIM_Rhsim05G0106300 | 6.07 | 0.014 | 0.026 | 2.772 |
| RHSIM_Rhsim04G0137100 | 6.07 | 0.014 | 0.026 | -0.603 |
| RHSIM_Rhsim06G0030500 | 6.07 | 0.014 | 0.026 | -0.983 |
| RHSIM_Rhsim07G0036000 | 6.06 | 0.014 | 0.026 | -0.282 |
| RHSIM_Rhsim12G0189800 | 6.06 | 0.014 | 0.026 | 0.354 |
| RHSIM_Rhsim04G0234500 | 6.06 | 0.014 | 0.026 | -0.271 |
| RHSIM_Rhsim06G0174100 | 6.06 | 0.014 | 0.026 | 0.248 |
| RHSIM_Rhsim13G0203300 | 6.06 | 0.014 | 0.026 | -0.340 |
| novel.1031 | 6.06 | 0.014 | 0.026 | 1.240 |
| novel.3617 | 6.06 | 0.014 | 0.026 | 2.449 |
| RHSIM_RhsimUnG0096600 | 6.06 | 0.014 | 0.026 | -0.562 |

| Gene | LRT statistic | P value | BH-adjusted P | log2FC (interaction) |
|---|---|---|---|---|
| RHSIM_Rhsim08G0200100 | 6.06 | 0.014 | 0.026 | 2.121 |
| RHSIM_Rhsim01G0187500 | 6.06 | 0.014 | 0.026 | -1.264 |
| RHSIM_RhsimUnG0214200 | 6.06 | 0.014 | 0.026 | 0.319 |
| RHSIM_Rhsim05G0105900 | 6.06 | 0.014 | 0.026 | -0.852 |
| RHSIM_Rhsim04G0059000 | 6.06 | 0.014 | 0.026 | -0.867 |
| RHSIM_Rhsim05G0050300 | 6.05 | 0.014 | 0.026 | 0.537 |
| RHSIM_Rhsim04G0075100 | 6.05 | 0.014 | 0.026 | -0.681 |
| novel.1145 | 6.05 | 0.014 | 0.026 | 1.550 |
| RHSIM_Rhsim12G0205600 | 6.05 | 0.014 | 0.026 | 0.820 |
| RHSIM_Rhsim08G0056000 | 6.05 | 0.014 | 0.026 | 0.593 |
| RHSIM_Rhsim11G0046400 | 6.05 | 0.014 | 0.026 | -0.329 |
| novel.2631 | 6.05 | 0.014 | 0.026 | 2.526 |
| novel.3083 | 6.05 | 0.014 | 0.026 | 1.362 |
| RHSIM_Rhsim13G0002300 | 6.05 | 0.014 | 0.026 | 0.305 |
| RHSIM_Rhsim07G0097400 | 6.05 | 0.014 | 0.026 | -0.353 |
| RHSIM_Rhsim05G0163100 | 6.05 | 0.014 | 0.026 | 1.876 |
| RHSIM_Rhsim06G0188600 | 6.04 | 0.014 | 0.026 | -41.530 |
| RHSIM_Rhsim13G0222900 | 6.04 | 0.014 | 0.026 | 1.247 |
| RHSIM_Rhsim04G0193500 | 6.04 | 0.014 | 0.026 | 0.273 |
| RHSIM_Rhsim04G0203500 | 6.04 | 0.014 | 0.026 | -2.211 |
| RHSIM_Rhsim09G0076500 | 6.04 | 0.014 | 0.026 | 0.663 |
| novel.759 | 6.04 | 0.014 | 0.026 | 1.991 |
| RHSIM_Rhsim01G0224000 | 6.04 | 0.014 | 0.026 | -0.532 |
| RHSIM_Rhsim05G0134600 | 6.04 | 0.014 | 0.026 | 2.277 |
| RHSIM_Rhsim12G0123200 | 6.04 | 0.014 | 0.026 | 3.005 |
| RHSIM_Rhsim03G0042400 | 6.04 | 0.014 | 0.026 | 2.408 |
| RHSIM_Rhsim05G0176000 | 6.04 | 0.014 | 0.026 | 0.631 |
| novel.4014 | 6.04 | 0.014 | 0.026 | -1.505 |
| RHSIM_Rhsim03G0061200 | 6.03 | 0.014 | 0.026 | -0.613 |
| RHSIM_Rhsim05G0084600 | 6.03 | 0.014 | 0.026 | -39.935 |
| RHSIM_Rhsim10G0082400 | 6.03 | 0.014 | 0.026 | 0.693 |
| RHSIM_Rhsim03G0081700 | 6.03 | 0.014 | 0.026 | 0.230 |

| Gene | LRT statistic | P value | BH-adjusted P | log2FC (interaction) |
|---|---|---|---|---|
| RHSIM_Rhsim07G0058700 | 6.03 | 0.014 | 0.026 | 0.294 |
| RHSIM_Rhsim08G0123100 | 6.03 | 0.014 | 0.026 | 1.182 |
| RHSIM_Rhsim06G0018700 | 6.03 | 0.014 | 0.026 | -0.249 |
| RHSIM_Rhsim07G0251500 | 6.02 | 0.014 | 0.026 | 0.735 |
| RHSIM_Rhsim07G0042200 | 6.02 | 0.014 | 0.026 | -3.753 |
| RHSIM_Rhsim07G0077300 | 6.02 | 0.014 | 0.026 | -0.266 |
| RHSIM_Rhsim05G0123400 | 6.02 | 0.014 | 0.027 | 1.412 |
| RHSIM_RhsimUnG0187300 | 6.02 | 0.014 | 0.027 | 2.595 |
| novel.310 | 6.02 | 0.014 | 0.027 | -2.253 |
| RHSIM_Rhsim10G0056500 | 6.02 | 0.014 | 0.027 | 0.413 |
| RHSIM_Rhsim09G0055500 | 6.02 | 0.014 | 0.027 | 0.448 |
| RHSIM_Rhsim01G0036200 | 6.02 | 0.014 | 0.027 | 0.570 |
| novel.4110 | 6.01 | 0.014 | 0.027 | 1.729 |
| RHSIM_Rhsim13G0074300 | 6.01 | 0.014 | 0.027 | -1.429 |
| RHSIM_Rhsim11G0162600 | 6.01 | 0.014 | 0.027 | -3.277 |
| RHSIM_Rhsim02G0074300 | 6.01 | 0.014 | 0.027 | 0.751 |
| RHSIM_Rhsim11G0039300 | 6.00 | 0.014 | 0.027 | 77.823 |
| RHSIM_Rhsim13G0107300 | 6.00 | 0.014 | 0.027 | 0.304 |
| RHSIM_Rhsim13G0226200 | 6.00 | 0.014 | 0.027 | 0.563 |
| RHSIM_Rhsim07G0208900 | 6.00 | 0.014 | 0.027 | -0.281 |
| novel.3942 | 6.00 | 0.014 | 0.027 | -1.493 |
| RHSIM_Rhsim01G0026100 | 6.00 | 0.014 | 0.027 | -42.025 |
| novel.3256 | 6.00 | 0.014 | 0.027 | -3.560 |
| novel.735 | 6.00 | 0.014 | 0.027 | -2.614 |
| RHSIM_Rhsim06G0086600 | 5.99 | 0.014 | 0.027 | 0.296 |
| RHSIM_Rhsim04G0074200 | 5.99 | 0.014 | 0.027 | 4.353 |
| RHSIM_Rhsim01G0137400 | 5.99 | 0.014 | 0.027 | 0.293 |
| novel.2741 | 5.99 | 0.014 | 0.027 | 2.447 |
| RHSIM_Rhsim02G0164400 | 5.99 | 0.014 | 0.027 | 1.646 |
| RHSIM_Rhsim06G0139900 | 5.99 | 0.014 | 0.027 | 0.799 |
| RHSIM_Rhsim05G0103000 | 5.99 | 0.014 | 0.027 | -1.350 |
| RHSIM_Rhsim06G0078000 | 5.99 | 0.014 | 0.027 | -0.343 |

| Gene | LRT statistic | P value | BH-adjusted P | log2FC (interaction) |
|---|---|---|---|---|
| RHSIM_Rhsim09G0213600 | 5.99 | 0.014 | 0.027 | 1.288 |
| RHSIM_Rhsim01G0235500 | 5.99 | 0.014 | 0.027 | 3.100 |
| RHSIM_Rhsim01G0291800 | 5.98 | 0.014 | 0.027 | -0.833 |
| RHSIM_Rhsim03G0211500 | 5.98 | 0.014 | 0.027 | 0.390 |
| novel.2345 | 5.98 | 0.014 | 0.027 | -1.936 |
| RHSIM_Rhsim04G0118300 | 5.98 | 0.014 | 0.027 | 0.917 |
| RHSIM_Rhsim08G0094300 | 5.98 | 0.014 | 0.027 | -39.895 |
| RHSIM_Rhsim13G0137900 | 5.98 | 0.014 | 0.027 | -0.939 |
| RHSIM_Rhsim06G0027500 | 5.98 | 0.014 | 0.027 | 2.528 |
| RHSIM_Rhsim09G0022600 | 5.98 | 0.014 | 0.027 | -0.839 |
| RHSIM_Rhsim01G0003500 | 5.98 | 0.015 | 0.027 | 0.536 |
| RHSIM_RhsimUnG0198100 | 5.97 | 0.015 | 0.027 | 1.745 |
| RHSIM_Rhsim06G0140700 | 5.97 | 0.015 | 0.027 | -0.836 |
| RHSIM_Rhsim02G0185200 | 5.97 | 0.015 | 0.027 | 0.618 |
| RHSIM_RhsimUnG0253800 | 5.97 | 0.015 | 0.027 | 0.537 |
| RHSIM_Rhsim06G0189600 | 5.97 | 0.015 | 0.027 | -41.885 |
| novel.3140 | 5.97 | 0.015 | 0.027 | 1.355 |
| RHSIM_Rhsim04G0216100 | 5.97 | 0.015 | 0.027 | -1.289 |
| RHSIM_Rhsim04G0137600 | 5.96 | 0.015 | 0.027 | 1.205 |
| RHSIM_Rhsim10G0209100 | 5.96 | 0.015 | 0.027 | 0.155 |
| novel.2917 | 5.96 | 0.015 | 0.027 | -3.580 |
| RHSIM_Rhsim08G0225300 | 5.96 | 0.015 | 0.027 | -40.564 |
| RHSIM_Rhsim07G0184600 | 5.96 | 0.015 | 0.027 | -1.598 |
| RHSIM_Rhsim04G0026400 | 5.96 | 0.015 | 0.027 | -2.391 |
| RHSIM_Rhsim09G0154300 | 5.96 | 0.015 | 0.027 | 0.205 |
| RHSIM_Rhsim06G0241400 | 5.96 | 0.015 | 0.027 | -0.400 |
| novel.2576 | 5.96 | 0.015 | 0.027 | 39.372 |
| novel.730 | 5.96 | 0.015 | 0.027 | 0.897 |
| RHSIM_RhsimUnG0064300 | 5.96 | 0.015 | 0.027 | 2.646 |
| RHSIM_Rhsim09G0064800 | 5.96 | 0.015 | 0.027 | 0.527 |
| novel.4474 | 5.95 | 0.015 | 0.027 | -1.302 |
| novel.4316 | 5.95 | 0.015 | 0.027 | -0.359 |

| Gene | LRT statistic | P value | BH-adjusted P | log2FC (interaction) |
|---|---|---|---|---|
| RHSIM_Rhsim06G0050800 | 5.95 | 0.015 | 0.027 | -0.230 |
| RHSIM_Rhsim08G0088500 | 5.95 | 0.015 | 0.028 | 0.243 |
| RHSIM_Rhsim07G0108600 | 5.95 | 0.015 | 0.028 | 0.183 |
| RHSIM_RhsimUnG0252900 | 5.95 | 0.015 | 0.028 | 0.210 |
| RHSIM_Rhsim05G0068300 | 5.95 | 0.015 | 0.028 | -0.184 |
| RHSIM_Rhsim04G0073200 | 5.94 | 0.015 | 0.028 | 0.581 |
| RHSIM_Rhsim09G0202600 | 5.94 | 0.015 | 0.028 | 1.135 |
| RHSIM_Rhsim13G0100000 | 5.94 | 0.015 | 0.028 | -2.938 |
| novel.2739 | 5.94 | 0.015 | 0.028 | 1.234 |
| novel.4266 | 5.94 | 0.015 | 0.028 | 0.505 |
| RHSIM_Rhsim10G0056000 | 5.94 | 0.015 | 0.028 | -1.000 |
| RHSIM_RhsimUnG0014500 | 5.94 | 0.015 | 0.028 | 0.570 |
| RHSIM_Rhsim03G0091500 | 5.94 | 0.015 | 0.028 | 3.254 |
| RHSIM_Rhsim07G0017800 | 5.93 | 0.015 | 0.028 | -0.379 |
| RHSIM_Rhsim03G0141100 | 5.93 | 0.015 | 0.028 | -0.372 |
| RHSIM_Rhsim11G0103400 | 5.93 | 0.015 | 0.028 | 4.130 |
| novel.2620 | 5.93 | 0.015 | 0.028 | -2.063 |
| novel.1432 | 5.93 | 0.015 | 0.028 | -5.767 |
| novel.2579 | 5.93 | 0.015 | 0.028 | 2.598 |
| RHSIM_Rhsim06G0164300 | 5.93 | 0.015 | 0.028 | 2.267 |
| RHSIM_Rhsim08G0244000 | 5.93 | 0.015 | 0.028 | -3.522 |
| RHSIM_Rhsim07G0118400 | 5.93 | 0.015 | 0.028 | 1.186 |
| RHSIM_Rhsim04G0090800 | 5.93 | 0.015 | 0.028 | 0.540 |
| novel.2199 | 5.92 | 0.015 | 0.028 | 2.720 |
| novel.634 | 5.92 | 0.015 | 0.028 | -1.197 |
| RHSIM_Rhsim01G0288500 | 5.92 | 0.015 | 0.028 | -39.672 |
| RHSIM_Rhsim12G0198100 | 5.92 | 0.015 | 0.028 | -0.631 |
| RHSIM_Rhsim09G0125000 | 5.92 | 0.015 | 0.028 | -3.597 |
| RHSIM_Rhsim13G0088800 | 5.92 | 0.015 | 0.028 | -0.178 |
| novel.298 | 5.92 | 0.015 | 0.028 | -3.017 |
| RHSIM_Rhsim01G0046600 | 5.92 | 0.015 | 0.028 | 1.721 |
| RHSIM_Rhsim03G0228700 | 5.91 | 0.015 | 0.028 | 3.739 |

| Gene | LRT statistic | P value | BH-adjusted P | log2FC (interaction) |
|---|---|---|---|---|
| RHSIM_Rhsim07G0121600 | 5.91 | 0.015 | 0.028 | -2.888 |
| RHSIM_Rhsim01G0028600 | 5.91 | 0.015 | 0.028 | 2.898 |
| RHSIM_Rhsim06G0000200 | 5.91 | 0.015 | 0.028 | -0.225 |
| RHSIM_Rhsim07G0151900 | 5.91 | 0.015 | 0.028 | -2.185 |
| novel.1464 | 5.91 | 0.015 | 0.028 | 0.814 |
| RHSIM_RhsimUnG0109700 | 5.90 | 0.015 | 0.028 | 3.987 |
| RHSIM_Rhsim12G0162200 | 5.90 | 0.015 | 0.028 | -0.311 |
| RHSIM_Rhsim03G0232000 | 5.90 | 0.015 | 0.028 | -0.426 |
| novel.496 | 5.90 | 0.015 | 0.028 | -1.597 |
| novel.1942 | 5.90 | 0.015 | 0.028 | 3.887 |
| RHSIM_Rhsim02G0157500 | 5.89 | 0.015 | 0.028 | 0.354 |
| RHSIM_Rhsim11G0195500 | 5.89 | 0.015 | 0.028 | -0.702 |
| RHSIM_Rhsim12G0190500 | 5.89 | 0.015 | 0.028 | 0.192 |
| RHSIM_Rhsim03G0145500 | 5.89 | 0.015 | 0.028 | -2.572 |
| RHSIM_Rhsim03G0239800 | 5.89 | 0.015 | 0.028 | -2.253 |
| RHSIM_Rhsim02G0044200 | 5.89 | 0.015 | 0.028 | -39.290 |
| RHSIM_Rhsim05G0086100 | 5.89 | 0.015 | 0.028 | 0.224 |
| RHSIM_RhsimUnG0209100 | 5.89 | 0.015 | 0.028 | 0.608 |
| RHSIM_Rhsim07G0043200 | 5.89 | 0.015 | 0.028 | -0.784 |
| novel.1772 | 5.89 | 0.015 | 0.028 | 1.274 |
| novel.1866 | 5.89 | 0.015 | 0.028 | -38.985 |
| RHSIM_Rhsim10G0147500 | 5.89 | 0.015 | 0.028 | 0.289 |
| RHSIM_Rhsim12G0169000 | 5.88 | 0.015 | 0.028 | -77.605 |
| RHSIM_Rhsim02G0057600 | 5.88 | 0.015 | 0.028 | -1.484 |
| RHSIM_Rhsim12G0194700 | 5.88 | 0.015 | 0.028 | -0.205 |
| novel.2417 | 5.88 | 0.015 | 0.028 | 0.316 |
| RHSIM_Rhsim04G0011300 | 5.88 | 0.015 | 0.029 | 0.313 |
| novel.3720 | 5.88 | 0.015 | 0.029 | 3.730 |
| RHSIM_Rhsim08G0209700 | 5.88 | 0.015 | 0.029 | 0.425 |
| RHSIM_Rhsim13G0171100 | 5.88 | 0.015 | 0.029 | -40.038 |
| RHSIM_Rhsim12G0114700 | 5.88 | 0.015 | 0.029 | 0.842 |
| RHSIM_Rhsim13G0079700 | 5.88 | 0.015 | 0.029 | -1.929 |

| Gene | LRT statistic | P value | BH-adjusted P | log2FC (interaction) |
|---|---|---|---|---|
| novel.25 | 5.87 | 0.015 | 0.029 | 1.083 |
| RHSIM_Rhsim04G0099100 | 5.87 | 0.015 | 0.029 | 1.056 |
| RHSIM_Rhsim05G0007400 | 5.87 | 0.015 | 0.029 | -0.864 |
| novel.1859 | 5.87 | 0.015 | 0.029 | -1.129 |
| novel.2888 | 5.87 | 0.015 | 0.029 | 1.052 |
| RHSIM_Rhsim13G0056700 | 5.87 | 0.015 | 0.029 | 0.937 |
| novel.1750 | 5.86 | 0.015 | 0.029 | -0.151 |
| novel.1431 | 5.86 | 0.015 | 0.029 | -40.567 |
| RHSIM_Rhsim03G0236700 | 5.86 | 0.015 | 0.029 | -0.374 |
| RHSIM_Rhsim08G0117300 | 5.86 | 0.015 | 0.029 | -0.599 |
| RHSIM_Rhsim09G0137300 | 5.85 | 0.016 | 0.029 | -2.422 |
| RHSIM_Rhsim02G0207300 | 5.85 | 0.016 | 0.029 | -3.085 |
| novel.3709 | 5.85 | 0.016 | 0.029 | 2.111 |
| RHSIM_Rhsim12G0062200 | 5.85 | 0.016 | 0.029 | 0.425 |
| novel.3938 | 5.85 | 0.016 | 0.029 | 1.551 |
| RHSIM_Rhsim05G0073800 | 5.85 | 0.016 | 0.029 | 0.668 |
| RHSIM_Rhsim08G0126000 | 5.85 | 0.016 | 0.029 | 2.074 |
| RHSIM_Rhsim13G0101400 | 5.85 | 0.016 | 0.029 | -0.223 |
| novel.2185 | 5.85 | 0.016 | 0.029 | -0.603 |
| RHSIM_Rhsim02G0017100 | 5.84 | 0.016 | 0.029 | -0.302 |
| novel.3047 | 5.84 | 0.016 | 0.029 | 2.046 |
| RHSIM_Rhsim09G0078500 | 5.84 | 0.016 | 0.029 | -0.497 |
| RHSIM_Rhsim02G0125800 | 5.84 | 0.016 | 0.029 | 0.906 |
| RHSIM_Rhsim09G0156600 | 5.84 | 0.016 | 0.029 | 0.486 |
| novel.3301 | 5.83 | 0.016 | 0.029 | 2.327 |
| RHSIM_Rhsim07G0169200 | 5.83 | 0.016 | 0.029 | 1.502 |
| RHSIM_Rhsim04G0069700 | 5.83 | 0.016 | 0.029 | -0.947 |
| RHSIM_Rhsim02G0106100 | 5.83 | 0.016 | 0.029 | 0.417 |
| RHSIM_Rhsim13G0154900 | 5.83 | 0.016 | 0.029 | -39.375 |
| RHSIM_Rhsim02G0076200 | 5.83 | 0.016 | 0.029 | 42.155 |
| RHSIM_Rhsim09G0132900 | 5.83 | 0.016 | 0.029 | 0.552 |
| RHSIM_Rhsim04G0189400 | 5.83 | 0.016 | 0.029 | -2.009 |

| Gene | LRT statistic | P value | BH-adjusted P | log2FC (interaction) |
|---|---|---|---|---|
| RHSIM_Rhsim05G0121300 | 5.83 | 0.016 | 0.029 | 0.337 |
| RHSIM_Rhsim12G0077500 | 5.83 | 0.016 | 0.029 | -0.584 |
| RHSIM_Rhsim08G0067800 | 5.83 | 0.016 | 0.029 | -0.436 |
| novel.1334 | 5.83 | 0.016 | 0.029 | 0.582 |
| RHSIM_Rhsim10G0168100 | 5.82 | 0.016 | 0.029 | -0.335 |
| RHSIM_Rhsim01G0285700 | 5.82 | 0.016 | 0.029 | 2.173 |
| RHSIM_RhsimUnG0120300 | 5.82 | 0.016 | 0.029 | 0.935 |
| RHSIM_Rhsim06G0158100 | 5.82 | 0.016 | 0.029 | 0.437 |
| novel.747 | 5.82 | 0.016 | 0.029 | -1.789 |
| RHSIM_Rhsim02G0130300 | 5.82 | 0.016 | 0.029 | -0.429 |
| RHSIM_Rhsim06G0243200 | 5.82 | 0.016 | 0.029 | -0.303 |
| novel.3454 | 5.82 | 0.016 | 0.029 | 3.353 |
| novel.440 | 5.82 | 0.016 | 0.029 | 1.692 |
| RHSIM_Rhsim05G0196500 | 5.81 | 0.016 | 0.029 | 40.903 |
| RHSIM_Rhsim07G0145600 | 5.81 | 0.016 | 0.029 | 0.268 |
| RHSIM_Rhsim07G0200000 | 5.81 | 0.016 | 0.030 | -1.154 |
| RHSIM_Rhsim02G0162000 | 5.81 | 0.016 | 0.030 | -1.946 |
| RHSIM_Rhsim11G0020500 | 5.81 | 0.016 | 0.030 | 2.132 |
| novel.2805 | 5.81 | 0.016 | 0.030 | -0.191 |
| novel.1368 | 5.81 | 0.016 | 0.030 | 0.870 |
| RHSIM_Rhsim01G0290600 | 5.80 | 0.016 | 0.030 | 0.231 |
| RHSIM_Rhsim10G0079600 | 5.80 | 0.016 | 0.030 | 3.302 |
| RHSIM_Rhsim12G0132500 | 5.80 | 0.016 | 0.030 | 0.370 |
| RHSIM_Rhsim08G0229100 | 5.80 | 0.016 | 0.030 | 1.283 |
| RHSIM_Rhsim08G0114600 | 5.79 | 0.016 | 0.030 | 5.203 |
| RHSIM_Rhsim03G0264400 | 5.79 | 0.016 | 0.030 | -0.462 |
| RHSIM_Rhsim04G0168800 | 5.79 | 0.016 | 0.030 | -0.344 |
| RHSIM_Rhsim10G0065400 | 5.79 | 0.016 | 0.030 | 3.718 |
| RHSIM_Rhsim04G0233000 | 5.79 | 0.016 | 0.030 | -40.051 |
| novel.1756 | 5.79 | 0.016 | 0.030 | 0.943 |
| RHSIM_Rhsim02G0152800 | 5.79 | 0.016 | 0.030 | 40.349 |
| RHSIM_Rhsim07G0258300 | 5.78 | 0.016 | 0.030 | 0.423 |

| Gene | LRT statistic | P value | BH-adjusted P | log2FC (interaction) |
|---|---|---|---|---|
| RHSIM_Rhsim11G0188500 | 5.78 | 0.016 | 0.030 | 3.689 |
| RHSIM_Rhsim08G0097200 | 5.78 | 0.016 | 0.030 | -1.358 |
| RHSIM_Rhsim01G0106100 | 5.78 | 0.016 | 0.030 | 2.403 |
| RHSIM_Rhsim05G0167700 | 5.78 | 0.016 | 0.030 | 0.753 |
| RHSIM_Rhsim06G0230100 | 5.78 | 0.016 | 0.030 | 2.936 |
| RHSIM_Rhsim12G0151200 | 5.78 | 0.016 | 0.030 | -41.437 |
| RHSIM_Rhsim07G0031200 | 5.78 | 0.016 | 0.030 | -0.169 |
| RHSIM_Rhsim13G0223600 | 5.78 | 0.016 | 0.030 | -0.591 |
| RHSIM_Rhsim03G0039300 | 5.78 | 0.016 | 0.030 | -0.349 |
| RHSIM_Rhsim02G0031000 | 5.78 | 0.016 | 0.030 | -0.843 |
| RHSIM_Rhsim11G0049200 | 5.78 | 0.016 | 0.030 | 2.282 |
| RHSIM_Rhsim02G0203300 | 5.78 | 0.016 | 0.030 | -0.497 |
| RHSIM_RhsimUnG0161400 | 5.78 | 0.016 | 0.030 | -0.870 |
| RHSIM_Rhsim07G0010700 | 5.78 | 0.016 | 0.030 | -0.155 |
| novel.582 | 5.77 | 0.016 | 0.030 | -0.402 |
| novel.4240 | 5.77 | 0.016 | 0.030 | -0.625 |
| RHSIM_Rhsim08G0232800 | 5.77 | 0.016 | 0.030 | 0.273 |
| RHSIM_Rhsim13G0101000 | 5.77 | 0.016 | 0.030 | 0.700 |
| RHSIM_Rhsim02G0174700 | 5.77 | 0.016 | 0.030 | -0.905 |
| RHSIM_Rhsim01G0102900 | 5.77 | 0.016 | 0.030 | 2.127 |
| RHSIM_Rhsim03G0248800 | 5.77 | 0.016 | 0.030 | -0.802 |
| novel.2941 | 5.77 | 0.016 | 0.030 | -1.454 |
| RHSIM_Rhsim05G0018000 | 5.77 | 0.016 | 0.030 | 0.749 |
| RHSIM_RhsimUnG0001100 | 5.77 | 0.016 | 0.030 | 1.933 |
| RHSIM_RhsimUnG0054800 | 5.76 | 0.016 | 0.030 | 40.754 |
| RHSIM_Rhsim05G0093800 | 5.76 | 0.016 | 0.030 | 0.969 |
| RHSIM_Rhsim03G0005600 | 5.76 | 0.016 | 0.030 | 0.148 |
| RHSIM_Rhsim06G0022700 | 5.76 | 0.016 | 0.030 | -1.116 |
| RHSIM_Rhsim01G0103400 | 5.76 | 0.016 | 0.030 | 2.074 |
| RHSIM_Rhsim02G0250100 | 5.76 | 0.016 | 0.030 | 0.396 |
| RHSIM_Rhsim09G0127400 | 5.76 | 0.016 | 0.030 | 0.855 |
| RHSIM_Rhsim02G0166700 | 5.76 | 0.016 | 0.030 | 40.821 |

| Gene | LRT statistic | P value | BH-adjusted P | log2FC (interaction) |
|---|---|---|---|---|
| RHSIM_Rhsim06G0232900 | 5.76 | 0.016 | 0.030 | -0.315 |
| RHSIM_Rhsim13G0102300 | 5.76 | 0.016 | 0.030 | 0.247 |
| RHSIM_Rhsim07G0136900 | 5.75 | 0.016 | 0.030 | 0.675 |
| RHSIM_Rhsim03G0138600 | 5.75 | 0.016 | 0.030 | -0.215 |
| RHSIM_Rhsim08G0003300 | 5.75 | 0.016 | 0.030 | 0.855 |
| RHSIM_Rhsim08G0134200 | 5.75 | 0.016 | 0.030 | 1.445 |
| RHSIM_Rhsim09G0029500 | 5.75 | 0.016 | 0.030 | 0.183 |
| novel.1640 | 5.75 | 0.016 | 0.030 | 3.424 |
| RHSIM_Rhsim04G0179300 | 5.75 | 0.016 | 0.030 | 1.346 |
| RHSIM_Rhsim09G0213900 | 5.75 | 0.017 | 0.031 | 0.446 |
| RHSIM_Rhsim01G0210200 | 5.75 | 0.017 | 0.031 | -0.266 |
| RHSIM_RhsimUnG0232300 | 5.74 | 0.017 | 0.031 | 0.954 |
| RHSIM_Rhsim10G0122400 | 5.74 | 0.017 | 0.031 | 1.096 |
| novel.2720 | 5.74 | 0.017 | 0.031 | 1.283 |
| RHSIM_Rhsim09G0181800 | 5.74 | 0.017 | 0.031 | -0.263 |
| RHSIM_Rhsim02G0019900 | 5.74 | 0.017 | 0.031 | -0.270 |
| RHSIM_Rhsim03G0263400 | 5.73 | 0.017 | 0.031 | -2.534 |
| RHSIM_RhsimUnG0011000 | 5.73 | 0.017 | 0.031 | -2.830 |
| novel.3029 | 5.73 | 0.017 | 0.031 | 0.330 |
| novel.3705 | 5.73 | 0.017 | 0.031 | 4.080 |
| novel.1076 | 5.73 | 0.017 | 0.031 | 0.657 |
| RHSIM_RhsimUnG0253200 | 5.73 | 0.017 | 0.031 | -0.363 |
| novel.45 | 5.72 | 0.017 | 0.031 | -1.218 |
| RHSIM_Rhsim04G0225600 | 5.72 | 0.017 | 0.031 | 0.928 |
| RHSIM_Rhsim08G0166300 | 5.72 | 0.017 | 0.031 | 1.550 |
| RHSIM_Rhsim10G0120100 | 5.72 | 0.017 | 0.031 | 0.172 |
| RHSIM_Rhsim02G0078100 | 5.72 | 0.017 | 0.031 | -1.243 |
| RHSIM_RhsimUnG0092700 | 5.72 | 0.017 | 0.031 | -5.682 |
| RHSIM_Rhsim02G0132200 | 5.72 | 0.017 | 0.031 | 0.479 |
| novel.248 | 5.72 | 0.017 | 0.031 | 1.544 |
| RHSIM_Rhsim10G0030500 | 5.71 | 0.017 | 0.031 | -0.437 |
| RHSIM_Rhsim05G0127000 | 5.71 | 0.017 | 0.031 | 41.871 |

| Gene | LRT statistic | P value | BH-adjusted P | log2FC (interaction) |
|---|---|---|---|---|
| novel.6 | 5.71 | 0.017 | 0.031 | -0.742 |
| RHSIM_Rhsim06G0226400 | 5.71 | 0.017 | 0.031 | 2.215 |
| RHSIM_Rhsim02G0047400 | 5.71 | 0.017 | 0.031 | -0.710 |
| novel.2262 | 5.71 | 0.017 | 0.031 | -0.394 |
| RHSIM_Rhsim08G0220400 | 5.70 | 0.017 | 0.031 | -0.249 |
| RHSIM_Rhsim07G0196300 | 5.70 | 0.017 | 0.031 | -1.482 |
| RHSIM_Rhsim01G0188500 | 5.70 | 0.017 | 0.031 | -0.985 |
| RHSIM_Rhsim09G0074900 | 5.70 | 0.017 | 0.031 | 0.140 |
| RHSIM_Rhsim09G0069800 | 5.70 | 0.017 | 0.031 | 0.677 |
| RHSIM_Rhsim13G0101700 | 5.70 | 0.017 | 0.031 | -0.229 |
| RHSIM_Rhsim11G0165900 | 5.70 | 0.017 | 0.031 | 2.601 |
| RHSIM_Rhsim03G0134800 | 5.70 | 0.017 | 0.031 | 2.813 |
| RHSIM_Rhsim06G0011900 | 5.69 | 0.017 | 0.031 | -0.494 |
| RHSIM_Rhsim01G0260800 | 5.69 | 0.017 | 0.031 | 0.373 |
| RHSIM_Rhsim03G0193200 | 5.69 | 0.017 | 0.031 | -3.172 |
| RHSIM_Rhsim02G0076500 | 5.69 | 0.017 | 0.031 | 0.203 |
| RHSIM_Rhsim11G0028500 | 5.69 | 0.017 | 0.031 | 40.425 |
| RHSIM_Rhsim04G0224000 | 5.69 | 0.017 | 0.031 | 2.361 |
| RHSIM_Rhsim09G0153900 | 5.69 | 0.017 | 0.031 | 0.584 |
| RHSIM_Rhsim08G0203000 | 5.69 | 0.017 | 0.031 | -0.266 |
| RHSIM_Rhsim01G0008400 | 5.69 | 0.017 | 0.031 | 1.129 |
| RHSIM_Rhsim02G0074400 | 5.69 | 0.017 | 0.031 | -39.456 |
| RHSIM_Rhsim10G0191900 | 5.69 | 0.017 | 0.032 | -0.353 |
| RHSIM_Rhsim02G0256500 | 5.69 | 0.017 | 0.032 | 1.393 |
| RHSIM_Rhsim03G0153800 | 5.68 | 0.017 | 0.032 | 0.189 |
| RHSIM_Rhsim08G0017000 | 5.68 | 0.017 | 0.032 | 4.097 |
| RHSIM_Rhsim12G0164500 | 5.68 | 0.017 | 0.032 | -1.409 |
| RHSIM_Rhsim12G0190900 | 5.68 | 0.017 | 0.032 | -0.400 |
| RHSIM_Rhsim03G0177300 | 5.68 | 0.017 | 0.032 | 0.619 |
| novel.599 | 5.68 | 0.017 | 0.032 | 4.450 |
| novel.547 | 5.68 | 0.017 | 0.032 | 39.550 |
| RHSIM_Rhsim05G0210300 | 5.68 | 0.017 | 0.032 | -1.156 |

| Gene | LRT statistic | P value | BH-adjusted P | log2FC (interaction) |
|---|---|---|---|---|
| RHSIM_Rhsim05G0179200 | 5.68 | 0.017 | 0.032 | 1.134 |
| RHSIM_Rhsim05G0000400 | 5.67 | 0.017 | 0.032 | 2.892 |
| RHSIM_Rhsim09G0046100 | 5.67 | 0.017 | 0.032 | 5.070 |
| RHSIM_Rhsim12G0126200 | 5.67 | 0.017 | 0.032 | 0.565 |
| RHSIM_Rhsim13G0188900 | 5.67 | 0.017 | 0.032 | 2.889 |
| RHSIM_RhsimUnG0008000 | 5.67 | 0.017 | 0.032 | -1.994 |
| RHSIM_Rhsim08G0221700 | 5.67 | 0.017 | 0.032 | -0.660 |
| RHSIM_Rhsim08G0110300 | 5.66 | 0.017 | 0.032 | 0.369 |
| RHSIM_Rhsim02G0002700 | 5.66 | 0.017 | 0.032 | 0.223 |
| RHSIM_Rhsim11G0191600 | 5.66 | 0.017 | 0.032 | -1.988 |
| RHSIM_Rhsim05G0124500 | 5.66 | 0.017 | 0.032 | 0.454 |
| novel.2135 | 5.66 | 0.017 | 0.032 | -0.873 |
| novel.3838 | 5.66 | 0.017 | 0.032 | 1.490 |
| RHSIM_Rhsim02G0106000 | 5.66 | 0.017 | 0.032 | 4.700 |
| RHSIM_Rhsim11G0114400 | 5.66 | 0.017 | 0.032 | -1.570 |
| RHSIM_Rhsim06G0096700 | 5.66 | 0.017 | 0.032 | -4.387 |
| RHSIM_Rhsim03G0255800 | 5.66 | 0.017 | 0.032 | 5.270 |
| RHSIM_Rhsim12G0064500 | 5.66 | 0.017 | 0.032 | -0.283 |
| RHSIM_Rhsim04G0089800 | 5.65 | 0.017 | 0.032 | 0.525 |
| RHSIM_Rhsim12G0157500 | 5.65 | 0.017 | 0.032 | 0.448 |
| RHSIM_Rhsim05G0229100 | 5.65 | 0.017 | 0.032 | 0.242 |
| RHSIM_Rhsim07G0225000 | 5.65 | 0.017 | 0.032 | -3.027 |
| RHSIM_Rhsim01G0038000 | 5.65 | 0.017 | 0.032 | 40.400 |
| RHSIM_Rhsim09G0185900 | 5.64 | 0.018 | 0.032 | 3.173 |
| RHSIM_Rhsim02G0198300 | 5.64 | 0.018 | 0.032 | 1.580 |
| RHSIM_Rhsim01G0108200 | 5.64 | 0.018 | 0.032 | 40.942 |
| RHSIM_Rhsim02G0021100 | 5.64 | 0.018 | 0.032 | -0.286 |
| RHSIM_Rhsim08G0190300 | 5.64 | 0.018 | 0.032 | 1.215 |
| RHSIM_Rhsim02G0114200 | 5.64 | 0.018 | 0.032 | 4.636 |
| RHSIM_Rhsim03G0022100 | 5.64 | 0.018 | 0.032 | 0.479 |
| RHSIM_Rhsim13G0033000 | 5.64 | 0.018 | 0.032 | -0.974 |
| RHSIM_Rhsim02G0241200 | 5.63 | 0.018 | 0.032 | 0.227 |

| Gene | LRT statistic | P value | BH-adjusted P | log2FC (interaction) |
|---|---|---|---|---|
| RHSIM_Rhsim01G0195700 | 5.63 | 0.018 | 0.032 | 1.933 |
| RHSIM_Rhsim13G0145700 | 5.63 | 0.018 | 0.032 | 2.656 |
| RHSIM_Rhsim09G0211400 | 5.63 | 0.018 | 0.032 | -0.441 |
| RHSIM_Rhsim12G0188200 | 5.63 | 0.018 | 0.032 | -0.161 |
| RHSIM_RhsimUnG0003300 | 5.63 | 0.018 | 0.032 | -0.906 |
| RHSIM_Rhsim06G0198900 | 5.63 | 0.018 | 0.032 | 1.279 |
| RHSIM_Rhsim13G0037700 | 5.63 | 0.018 | 0.032 | -0.305 |
| RHSIM_Rhsim08G0021800 | 5.63 | 0.018 | 0.032 | 0.141 |
| RHSIM_RhsimUnG0258200 | 5.63 | 0.018 | 0.032 | 1.602 |
| RHSIM_RhsimUnG0225700 | 5.63 | 0.018 | 0.033 | 2.228 |
| RHSIM_Rhsim02G0187100 | 5.62 | 0.018 | 0.033 | 0.286 |
| novel.2664 | 5.62 | 0.018 | 0.033 | 0.289 |
| RHSIM_Rhsim06G0157400 | 5.62 | 0.018 | 0.033 | -1.281 |
| RHSIM_Rhsim05G0218900 | 5.62 | 0.018 | 0.033 | 0.907 |
| RHSIM_Rhsim05G0221400 | 5.62 | 0.018 | 0.033 | 40.404 |
| RHSIM_Rhsim05G0120200 | 5.62 | 0.018 | 0.033 | 0.502 |
| RHSIM_Rhsim03G0085000 | 5.61 | 0.018 | 0.033 | 0.214 |
| RHSIM_Rhsim06G0069900 | 5.61 | 0.018 | 0.033 | -0.220 |
| novel.3315 | 5.61 | 0.018 | 0.033 | 0.992 |
| RHSIM_Rhsim01G0204700 | 5.61 | 0.018 | 0.033 | -0.264 |
| RHSIM_Rhsim10G0124300 | 5.61 | 0.018 | 0.033 | 0.303 |
| RHSIM_Rhsim01G0166800 | 5.61 | 0.018 | 0.033 | 41.311 |
| RHSIM_Rhsim08G0247800 | 5.61 | 0.018 | 0.033 | 0.170 |
| RHSIM_Rhsim05G0003100 | 5.61 | 0.018 | 0.033 | -1.993 |
| RHSIM_Rhsim07G0083500 | 5.61 | 0.018 | 0.033 | 1.650 |
| RHSIM_Rhsim12G0077800 | 5.61 | 0.018 | 0.033 | 0.582 |
| RHSIM_Rhsim01G0241200 | 5.61 | 0.018 | 0.033 | -0.358 |
| RHSIM_Rhsim09G0051800 | 5.61 | 0.018 | 0.033 | 2.186 |
| RHSIM_Rhsim08G0183600 | 5.60 | 0.018 | 0.033 | -0.191 |
| novel.448 | 5.60 | 0.018 | 0.033 | 1.950 |
| RHSIM_Rhsim09G0123400 | 5.60 | 0.018 | 0.033 | 2.417 |
| RHSIM_Rhsim01G0019900 | 5.59 | 0.018 | 0.033 | -0.571 |

| Gene | LRT statistic | P value | BH-adjusted P | log2FC (interaction) |
|---|---|---|---|---|
| RHSIM_Rhsim07G0210600 | 5.59 | 0.018 | 0.033 | 2.875 |
| RHSIM_Rhsim12G0037200 | 5.59 | 0.018 | 0.033 | -0.321 |
| RHSIM_Rhsim09G0020100 | 5.59 | 0.018 | 0.033 | -0.730 |
| RHSIM_Rhsim01G0003300 | 5.59 | 0.018 | 0.033 | 2.399 |
| RHSIM_Rhsim06G0146500 | 5.59 | 0.018 | 0.033 | 0.643 |
| RHSIM_Rhsim06G0150800 | 5.59 | 0.018 | 0.033 | -1.084 |
| novel.3404 | 5.59 | 0.018 | 0.033 | 3.701 |
| RHSIM_RhsimUnG0015600 | 5.59 | 0.018 | 0.033 | -0.692 |
| RHSIM_Rhsim10G0141200 | 5.59 | 0.018 | 0.033 | -0.906 |
| RHSIM_Rhsim01G0249800 | 5.58 | 0.018 | 0.033 | 0.493 |
| RHSIM_RhsimMtG0001900 | 5.58 | 0.018 | 0.033 | 2.609 |
| RHSIM_Rhsim03G0218300 | 5.58 | 0.018 | 0.033 | -0.348 |
| RHSIM_Rhsim12G0001100 | 5.58 | 0.018 | 0.033 | -1.261 |
| RHSIM_RhsimUnG0170800 | 5.58 | 0.018 | 0.033 | 1.421 |
| RHSIM_Rhsim11G0060300 | 5.58 | 0.018 | 0.033 | -4.479 |
| RHSIM_Rhsim03G0165600 | 5.58 | 0.018 | 0.033 | -0.600 |
| novel.1106 | 5.58 | 0.018 | 0.033 | 77.684 |
| RHSIM_Rhsim04G0085600 | 5.57 | 0.018 | 0.033 | 0.185 |
| RHSIM_Rhsim04G0063000 | 5.57 | 0.018 | 0.033 | 0.232 |
| novel.3528 | 5.57 | 0.018 | 0.033 | 1.032 |
| novel.2768 | 5.57 | 0.018 | 0.033 | 1.298 |
| RHSIM_Rhsim09G0143000 | 5.57 | 0.018 | 0.034 | 0.742 |
| RHSIM_RhsimUnG0043400 | 5.57 | 0.018 | 0.034 | 0.240 |
| RHSIM_Rhsim01G0195600 | 5.57 | 0.018 | 0.034 | 0.471 |
| RHSIM_Rhsim01G0206200 | 5.56 | 0.018 | 0.034 | -0.171 |
| RHSIM_Rhsim10G0010400 | 5.56 | 0.018 | 0.034 | 0.372 |
| RHSIM_Rhsim05G0170900 | 5.56 | 0.018 | 0.034 | -0.359 |
| RHSIM_Rhsim09G0092900 | 5.56 | 0.018 | 0.034 | -2.652 |
| novel.2235 | 5.56 | 0.018 | 0.034 | -0.497 |
| RHSIM_Rhsim01G0010600 | 5.56 | 0.018 | 0.034 | -0.612 |
| RHSIM_Rhsim12G0043900 | 5.56 | 0.018 | 0.034 | 3.886 |
| RHSIM_Rhsim09G0012000 | 5.56 | 0.018 | 0.034 | -0.190 |

| Gene | LRT statistic | P value | BH-adjusted P | log2FC (interaction) |
|---|---|---|---|---|
| novel.689 | 5.56 | 0.018 | 0.034 | 1.757 |
| RHSIM_Rhsim07G0211300 | 5.56 | 0.018 | 0.034 | -0.342 |
| RHSIM_Rhsim06G0125900 | 5.56 | 0.018 | 0.034 | -0.396 |
| RHSIM_Rhsim06G0043300 | 5.56 | 0.018 | 0.034 | 2.269 |
| RHSIM_Rhsim03G0239300 | 5.56 | 0.018 | 0.034 | 2.909 |
| novel.728 | 5.55 | 0.018 | 0.034 | -1.596 |
| novel.1910 | 5.55 | 0.018 | 0.034 | -0.977 |
| RHSIM_Rhsim10G0118000 | 5.55 | 0.018 | 0.034 | 2.455 |
| RHSIM_Rhsim05G0055800 | 5.55 | 0.018 | 0.034 | -6.617 |
| RHSIM_Rhsim07G0029400 | 5.55 | 0.019 | 0.034 | 0.285 |
| RHSIM_Rhsim13G0234800 | 5.55 | 0.019 | 0.034 | 0.280 |
| RHSIM_Rhsim04G0020100 | 5.54 | 0.019 | 0.034 | 1.328 |
| RHSIM_Rhsim13G0124500 | 5.54 | 0.019 | 0.034 | -1.660 |
| novel.4379 | 5.54 | 0.019 | 0.034 | -1.468 |
| RHSIM_Rhsim04G0094300 | 5.53 | 0.019 | 0.034 | 2.193 |
| RHSIM_Rhsim08G0225500 | 5.53 | 0.019 | 0.034 | -0.441 |
| RHSIM_Rhsim08G0215700 | 5.53 | 0.019 | 0.034 | -0.604 |
| novel.4451 | 5.53 | 0.019 | 0.034 | -4.510 |
| novel.3024 | 5.53 | 0.019 | 0.034 | -1.852 |
| RHSIM_Rhsim03G0039200 | 5.52 | 0.019 | 0.034 | 0.989 |
| RHSIM_Rhsim09G0033400 | 5.52 | 0.019 | 0.034 | 0.288 |
| RHSIM_Rhsim07G0127100 | 5.52 | 0.019 | 0.034 | -0.302 |
| RHSIM_Rhsim05G0006900 | 5.52 | 0.019 | 0.034 | 1.456 |
| RHSIM_RhsimUnG0212200 | 5.52 | 0.019 | 0.034 | -0.743 |
| novel.272 | 5.52 | 0.019 | 0.034 | 1.593 |
| RHSIM_Rhsim06G0109000 | 5.51 | 0.019 | 0.035 | 1.521 |
| RHSIM_Rhsim06G0228600 | 5.51 | 0.019 | 0.035 | -2.059 |
| RHSIM_Rhsim11G0155300 | 5.51 | 0.019 | 0.035 | 0.342 |
| RHSIM_Rhsim05G0000200 | 5.51 | 0.019 | 0.035 | 0.477 |
| RHSIM_Rhsim10G0215000 | 5.51 | 0.019 | 0.035 | 0.519 |
| novel.1961 | 5.51 | 0.019 | 0.035 | -2.092 |
| RHSIM_Rhsim01G0146000 | 5.51 | 0.019 | 0.035 | 1.198 |

| Gene | LRT statistic | P value | BH-adjusted P | log2FC (interaction) |
|---|---|---|---|---|
| RHSIM_Rhsim07G0176300 | 5.51 | 0.019 | 0.035 | 1.513 |
| RHSIM_Rhsim03G0225900 | 5.51 | 0.019 | 0.035 | 0.208 |
| RHSIM_Rhsim05G0039900 | 5.50 | 0.019 | 0.035 | 39.453 |
| RHSIM_Rhsim05G0107500 | 5.50 | 0.019 | 0.035 | 0.331 |
| novel.652 | 5.50 | 0.019 | 0.035 | 2.221 |
| RHSIM_Rhsim06G0067500 | 5.50 | 0.019 | 0.035 | 0.268 |
| RHSIM_Rhsim08G0022600 | 5.50 | 0.019 | 0.035 | 0.729 |
| RHSIM_Rhsim05G0035000 | 5.50 | 0.019 | 0.035 | -0.250 |
| RHSIM_Rhsim13G0116100 | 5.50 | 0.019 | 0.035 | -40.686 |
| RHSIM_Rhsim09G0182200 | 5.50 | 0.019 | 0.035 | -0.483 |
| RHSIM_Rhsim04G0219700 | 5.50 | 0.019 | 0.035 | 1.519 |
| RHSIM_Rhsim09G0082400 | 5.50 | 0.019 | 0.035 | 1.821 |
| RHSIM_Rhsim02G0099200 | 5.49 | 0.019 | 0.035 | 1.383 |
| RHSIM_Rhsim13G0058100 | 5.49 | 0.019 | 0.035 | -1.565 |
| RHSIM_Rhsim10G0113700 | 5.49 | 0.019 | 0.035 | -0.222 |
| RHSIM_Rhsim03G0136900 | 5.49 | 0.019 | 0.035 | 2.243 |
| RHSIM_RhsimUnG0121000 | 5.49 | 0.019 | 0.035 | -2.417 |
| RHSIM_Rhsim04G0087700 | 5.49 | 0.019 | 0.035 | 0.489 |
| RHSIM_Rhsim09G0153800 | 5.49 | 0.019 | 0.035 | -0.945 |
| RHSIM_Rhsim03G0063200 | 5.48 | 0.019 | 0.035 | -0.906 |
| RHSIM_Rhsim02G0164800 | 5.48 | 0.019 | 0.035 | -1.637 |
| RHSIM_Rhsim07G0208600 | 5.48 | 0.019 | 0.035 | 0.765 |
| RHSIM_Rhsim10G0215600 | 5.48 | 0.019 | 0.035 | 0.388 |
| RHSIM_Rhsim01G0025500 | 5.48 | 0.019 | 0.035 | -39.393 |
| RHSIM_Rhsim08G0173900 | 5.48 | 0.019 | 0.035 | -39.340 |
| novel.780 | 5.48 | 0.019 | 0.035 | 0.871 |
| RHSIM_Rhsim02G0207600 | 5.48 | 0.019 | 0.035 | -2.228 |
| RHSIM_Rhsim09G0060400 | 5.48 | 0.019 | 0.035 | -0.818 |
| RHSIM_Rhsim02G0025200 | 5.48 | 0.019 | 0.035 | 0.563 |
| RHSIM_Rhsim01G0143700 | 5.48 | 0.019 | 0.035 | 1.215 |
| RHSIM_Rhsim02G0050200 | 5.47 | 0.019 | 0.035 | 4.017 |
| RHSIM_Rhsim13G0175400 | 5.47 | 0.019 | 0.035 | -0.266 |

| Gene | LRT statistic | P value | BH-adjusted P | log2FC (interaction) |
|---|---|---|---|---|
| RHSIM_Rhsim08G0145900 | 5.47 | 0.019 | 0.035 | 0.291 |
| RHSIM_Rhsim03G0105700 | 5.47 | 0.019 | 0.035 | 0.197 |
| RHSIM_Rhsim02G0193800 | 5.47 | 0.019 | 0.035 | 3.007 |
| RHSIM_Rhsim08G0119100 | 5.47 | 0.019 | 0.035 | -0.188 |
| novel.2461 | 5.47 | 0.019 | 0.035 | -0.926 |
| RHSIM_Rhsim08G0005500 | 5.47 | 0.019 | 0.035 | 2.173 |
| RHSIM_RhsimUnG0222600 | 5.46 | 0.019 | 0.035 | -0.396 |
| RHSIM_Rhsim13G0024100 | 5.46 | 0.019 | 0.035 | -1.715 |
| RHSIM_Rhsim05G0119700 | 5.46 | 0.019 | 0.035 | 0.217 |
| RHSIM_Rhsim09G0045600 | 5.46 | 0.019 | 0.035 | -39.415 |
| RHSIM_Rhsim04G0021700 | 5.46 | 0.019 | 0.035 | 1.437 |
| RHSIM_RhsimUnG0082500 | 5.45 | 0.020 | 0.036 | 2.690 |
| RHSIM_Rhsim05G0035700 | 5.45 | 0.020 | 0.036 | -0.944 |
| novel.1975 | 5.45 | 0.020 | 0.036 | -1.697 |
| RHSIM_Rhsim06G0127300 | 5.44 | 0.020 | 0.036 | 1.076 |
| RHSIM_Rhsim05G0163000 | 5.44 | 0.020 | 0.036 | 0.345 |
| RHSIM_Rhsim09G0061300 | 5.44 | 0.020 | 0.036 | 0.243 |
| RHSIM_Rhsim07G0135100 | 5.44 | 0.020 | 0.036 | 2.767 |
| RHSIM_Rhsim06G0232400 | 5.44 | 0.020 | 0.036 | 0.175 |
| RHSIM_Rhsim07G0158800 | 5.44 | 0.020 | 0.036 | 2.033 |
| RHSIM_Rhsim07G0179000 | 5.44 | 0.020 | 0.036 | 0.436 |
| RHSIM_Rhsim09G0209900 | 5.44 | 0.020 | 0.036 | 0.268 |
| RHSIM_Rhsim01G0017500 | 5.44 | 0.020 | 0.036 | 0.582 |
| RHSIM_Rhsim03G0189100 | 5.44 | 0.020 | 0.036 | -1.422 |
| RHSIM_Rhsim03G0199400 | 5.44 | 0.020 | 0.036 | 0.771 |
| RHSIM_Rhsim04G0150500 | 5.44 | 0.020 | 0.036 | 0.602 |
| RHSIM_Rhsim12G0201200 | 5.44 | 0.020 | 0.036 | 1.126 |
| novel.2627 | 5.43 | 0.020 | 0.036 | -1.005 |
| RHSIM_Rhsim05G0192300 | 5.43 | 0.020 | 0.036 | -0.512 |
| RHSIM_Rhsim01G0120300 | 5.43 | 0.020 | 0.036 | -0.485 |
| RHSIM_Rhsim04G0137500 | 5.43 | 0.020 | 0.036 | 1.440 |
| RHSIM_Rhsim08G0054800 | 5.43 | 0.020 | 0.036 | 41.822 |

| Gene | LRT statistic | P value | BH-adjusted P | log2FC (interaction) |
|---|---|---|---|---|
| RHSIM_Rhsim12G0083600 | 5.43 | 0.020 | 0.036 | 0.979 |
| RHSIM_Rhsim07G0228500 | 5.43 | 0.020 | 0.036 | 3.266 |
| novel.2245 | 5.42 | 0.020 | 0.036 | -3.800 |
| RHSIM_Rhsim09G0089400 | 5.42 | 0.020 | 0.036 | 0.428 |
| RHSIM_Rhsim13G0219300 | 5.42 | 0.020 | 0.036 | -41.460 |
| RHSIM_Rhsim01G0241800 | 5.42 | 0.020 | 0.036 | 41.402 |
| RHSIM_Rhsim02G0205700 | 5.42 | 0.020 | 0.036 | 0.410 |
| RHSIM_Rhsim07G0224900 | 5.41 | 0.020 | 0.036 | -2.358 |
| RHSIM_Rhsim04G0104400 | 5.41 | 0.020 | 0.036 | -0.230 |
| RHSIM_Rhsim07G0249200 | 5.41 | 0.020 | 0.036 | -2.845 |
| RHSIM_Rhsim06G0193900 | 5.41 | 0.020 | 0.036 | 2.425 |
| RHSIM_Rhsim08G0063400 | 5.41 | 0.020 | 0.036 | -40.840 |
| novel.1234 | 5.41 | 0.020 | 0.036 | -0.633 |
| RHSIM_Rhsim10G0051400 | 5.40 | 0.020 | 0.037 | 1.669 |
| RHSIM_Rhsim03G0055100 | 5.40 | 0.020 | 0.037 | 1.602 |
| RHSIM_Rhsim08G0192800 | 5.40 | 0.020 | 0.037 | -1.443 |
| RHSIM_Rhsim07G0090100 | 5.40 | 0.020 | 0.037 | 1.194 |
| RHSIM_Rhsim10G0108500 | 5.40 | 0.020 | 0.037 | 0.265 |
| RHSIM_Rhsim11G0153700 | 5.40 | 0.020 | 0.037 | 1.728 |
| RHSIM_RhsimUnG0211500 | 5.40 | 0.020 | 0.037 | 1.717 |
| novel.3833 | 5.40 | 0.020 | 0.037 | 39.671 |
| RHSIM_RhsimUnG0131300 | 5.40 | 0.020 | 0.037 | 2.417 |
| RHSIM_Rhsim06G0104300 | 5.40 | 0.020 | 0.037 | 2.111 |
| novel.4150 | 5.40 | 0.020 | 0.037 | 2.955 |
| RHSIM_Rhsim07G0090400 | 5.39 | 0.020 | 0.037 | -0.390 |
| RHSIM_Rhsim12G0087300 | 5.39 | 0.020 | 0.037 | 0.469 |
| RHSIM_RhsimUnG0169500 | 5.39 | 0.020 | 0.037 | -41.319 |
| RHSIM_Rhsim12G0179800 | 5.39 | 0.020 | 0.037 | -0.474 |
| RHSIM_Rhsim01G0238800 | 5.39 | 0.020 | 0.037 | 40.674 |
| RHSIM_Rhsim06G0007500 | 5.39 | 0.020 | 0.037 | 0.257 |
| RHSIM_Rhsim01G0229900 | 5.39 | 0.020 | 0.037 | 1.248 |
| RHSIM_Rhsim13G0221800 | 5.39 | 0.020 | 0.037 | 1.326 |

| Gene | LRT statistic | P value | BH-adjusted P | log2FC (interaction) |
|---|---|---|---|---|
| RHSIM_Rhsim05G0108100 | 5.39 | 0.020 | 0.037 | 4.637 |
| RHSIM_Rhsim04G0030300 | 5.39 | 0.020 | 0.037 | -0.691 |
| RHSIM_Rhsim13G0072200 | 5.39 | 0.020 | 0.037 | -0.259 |
| RHSIM_Rhsim01G0110900 | 5.39 | 0.020 | 0.037 | -0.286 |
| RHSIM_Rhsim13G0093800 | 5.39 | 0.020 | 0.037 | 0.207 |
| RHSIM_Rhsim04G0037600 | 5.39 | 0.020 | 0.037 | 41.048 |
| RHSIM_Rhsim04G0004200 | 5.38 | 0.020 | 0.037 | 0.240 |
| RHSIM_Rhsim05G0116400 | 5.38 | 0.020 | 0.037 | 2.060 |
| novel.2221 | 5.38 | 0.020 | 0.037 | -0.495 |
| RHSIM_Rhsim05G0127200 | 5.38 | 0.020 | 0.037 | -40.189 |
| RHSIM_Rhsim02G0087300 | 5.38 | 0.020 | 0.037 | -2.652 |
| RHSIM_Rhsim13G0233900 | 5.37 | 0.020 | 0.037 | -40.724 |
| novel.2629 | 5.37 | 0.020 | 0.037 | 0.258 |
| RHSIM_Rhsim03G0016600 | 5.37 | 0.020 | 0.037 | -3.019 |
| novel.954 | 5.37 | 0.020 | 0.037 | -39.047 |
| RHSIM_Rhsim11G0194400 | 5.37 | 0.020 | 0.037 | 0.297 |
| novel.359 | 5.37 | 0.020 | 0.037 | -1.042 |
| novel.517 | 5.37 | 0.021 | 0.037 | -0.715 |
| RHSIM_Rhsim04G0188100 | 5.37 | 0.021 | 0.037 | 1.827 |
| RHSIM_Rhsim03G0199500 | 5.37 | 0.021 | 0.037 | 1.564 |
| RHSIM_Rhsim03G0083300 | 5.37 | 0.021 | 0.037 | 0.250 |
| novel.584 | 5.36 | 0.021 | 0.037 | 2.837 |
| RHSIM_Rhsim05G0079600 | 5.36 | 0.021 | 0.037 | -0.195 |
| RHSIM_Rhsim03G0150300 | 5.36 | 0.021 | 0.037 | -39.257 |
| novel.2058 | 5.36 | 0.021 | 0.037 | 0.963 |
| RHSIM_Rhsim03G0207400 | 5.36 | 0.021 | 0.037 | 40.159 |
| RHSIM_Rhsim04G0110400 | 5.36 | 0.021 | 0.037 | -0.327 |
| RHSIM_RhsimUnG0179500 | 5.36 | 0.021 | 0.037 | -1.710 |
| novel.4201 | 5.36 | 0.021 | 0.037 | 1.255 |
| novel.2485 | 5.36 | 0.021 | 0.037 | 0.609 |
| RHSIM_Rhsim13G0098800 | 5.36 | 0.021 | 0.037 | -0.360 |
| RHSIM_Rhsim09G0125400 | 5.35 | 0.021 | 0.037 | 0.548 |

| Gene | LRT statistic | P value | BH-adjusted P | log2FC (interaction) |
|---|---|---|---|---|
| RHSIM_Rhsim06G0003800 | 5.35 | 0.021 | 0.037 | 41.804 |
| RHSIM_Rhsim07G0094000 | 5.35 | 0.021 | 0.037 | 0.429 |
| RHSIM_Rhsim06G0035000 | 5.35 | 0.021 | 0.037 | -0.295 |
| RHSIM_Rhsim10G0133600 | 5.35 | 0.021 | 0.038 | 0.365 |
| RHSIM_Rhsim01G0248800 | 5.35 | 0.021 | 0.038 | 1.222 |
| novel.1157 | 5.35 | 0.021 | 0.038 | 39.938 |
| RHSIM_Rhsim05G0102800 | 5.35 | 0.021 | 0.038 | -1.218 |
| RHSIM_Rhsim06G0000800 | 5.35 | 0.021 | 0.038 | 1.419 |
| RHSIM_Rhsim01G0287200 | 5.35 | 0.021 | 0.038 | 4.857 |
| RHSIM_Rhsim11G0027300 | 5.35 | 0.021 | 0.038 | 1.253 |
| RHSIM_Rhsim10G0142000 | 5.35 | 0.021 | 0.038 | 1.257 |
| novel.971 | 5.35 | 0.021 | 0.038 | 2.842 |
| novel.3382 | 5.35 | 0.021 | 0.038 | -1.559 |
| RHSIM_Rhsim12G0014200 | 5.34 | 0.021 | 0.038 | 2.579 |
| RHSIM_Rhsim11G0145300 | 5.34 | 0.021 | 0.038 | -0.365 |
| novel.3110 | 5.34 | 0.021 | 0.038 | 2.614 |
| RHSIM_Rhsim06G0215900 | 5.34 | 0.021 | 0.038 | -3.294 |
| RHSIM_Rhsim13G0070800 | 5.34 | 0.021 | 0.038 | -1.007 |
| RHSIM_Rhsim07G0056300 | 5.34 | 0.021 | 0.038 | 39.902 |
| RHSIM_Rhsim05G0020500 | 5.34 | 0.021 | 0.038 | 0.318 |
| novel.3741 | 5.34 | 0.021 | 0.038 | -1.404 |
| novel.3041 | 5.34 | 0.021 | 0.038 | 0.518 |
| novel.2200 | 5.34 | 0.021 | 0.038 | 2.119 |
| RHSIM_Rhsim06G0138100 | 5.34 | 0.021 | 0.038 | -0.693 |
| RHSIM_Rhsim06G0054300 | 5.34 | 0.021 | 0.038 | 2.090 |
| RHSIM_Rhsim09G0071900 | 5.34 | 0.021 | 0.038 | 0.258 |
| RHSIM_Rhsim12G0125700 | 5.33 | 0.021 | 0.038 | -0.170 |
| novel.3477 | 5.33 | 0.021 | 0.038 | -1.248 |
| RHSIM_Rhsim01G0228100 | 5.33 | 0.021 | 0.038 | 0.243 |
| RHSIM_Rhsim08G0073200 | 5.33 | 0.021 | 0.038 | -0.158 |
| RHSIM_Rhsim13G0208800 | 5.33 | 0.021 | 0.038 | 0.565 |
| RHSIM_Rhsim11G0151200 | 5.33 | 0.021 | 0.038 | -0.987 |

| Gene | LRT statistic | P value | BH-adjusted P | log2FC (interaction) |
|---|---|---|---|---|
| RHSIM_Rhsim03G0167100 | 5.33 | 0.021 | 0.038 | -0.345 |
| novel.1554 | 5.33 | 0.021 | 0.038 | 0.896 |
| RHSIM_Rhsim04G0228800 | 5.32 | 0.021 | 0.038 | 0.480 |
| RHSIM_Rhsim13G0145600 | 5.32 | 0.021 | 0.038 | -3.636 |
| RHSIM_Rhsim07G0235000 | 5.32 | 0.021 | 0.038 | 1.414 |
| RHSIM_Rhsim08G0205200 | 5.32 | 0.021 | 0.038 | 1.188 |
| RHSIM_RhsimUnG0211700 | 5.32 | 0.021 | 0.038 | 0.310 |
| RHSIM_Rhsim04G0100300 | 5.32 | 0.021 | 0.038 | 1.273 |
| RHSIM_Rhsim04G0101000 | 5.31 | 0.021 | 0.038 | 0.323 |
| RHSIM_Rhsim02G0169000 | 5.31 | 0.021 | 0.038 | 0.174 |
| RHSIM_Rhsim08G0117400 | 5.31 | 0.021 | 0.038 | -0.862 |
| RHSIM_Rhsim12G0115300 | 5.31 | 0.021 | 0.038 | 1.402 |
| RHSIM_Rhsim03G0021200 | 5.31 | 0.021 | 0.038 | -0.374 |
| RHSIM_Rhsim01G0222900 | 5.31 | 0.021 | 0.038 | 0.391 |
| RHSIM_RhsimUnG0031900 | 5.31 | 0.021 | 0.038 | -39.397 |
| novel.955 | 5.30 | 0.021 | 0.038 | -1.252 |
| novel.2263 | 5.30 | 0.021 | 0.038 | -39.694 |
| novel.58 | 5.30 | 0.021 | 0.038 | 0.821 |
| novel.398 | 5.30 | 0.021 | 0.038 | 2.334 |
| RHSIM_Rhsim08G0057800 | 5.30 | 0.021 | 0.038 | -41.457 |
| RHSIM_Rhsim03G0162900 | 5.30 | 0.021 | 0.039 | -0.392 |
| RHSIM_Rhsim01G0245800 | 5.30 | 0.021 | 0.039 | -0.261 |
| RHSIM_Rhsim07G0149000 | 5.30 | 0.021 | 0.039 | 39.906 |
| RHSIM_Rhsim05G0106400 | 5.30 | 0.021 | 0.039 | 0.346 |
| RHSIM_Rhsim12G0101500 | 5.30 | 0.021 | 0.039 | 0.200 |
| RHSIM_RhsimUnG0197200 | 5.30 | 0.021 | 0.039 | 40.558 |
| novel.3062 | 5.30 | 0.021 | 0.039 | -1.202 |
| RHSIM_Rhsim05G0190400 | 5.30 | 0.021 | 0.039 | 0.350 |
| RHSIM_Rhsim04G0009700 | 5.30 | 0.021 | 0.039 | -0.490 |
| RHSIM_Rhsim02G0130200 | 5.29 | 0.021 | 0.039 | 0.152 |
| novel.3424 | 5.29 | 0.021 | 0.039 | -2.173 |
| RHSIM_Rhsim06G0089200 | 5.29 | 0.021 | 0.039 | -0.240 |

| Gene | LRT statistic | P value | BH-adjusted P | log2FC (interaction) |
|---|---|---|---|---|
| RHSIM_Rhsim10G0065000 | 5.29 | 0.021 | 0.039 | 3.867 |
| novel.1948 | 5.29 | 0.021 | 0.039 | 4.713 |
| RHSIM_Rhsim10G0023500 | 5.29 | 0.021 | 0.039 | -1.634 |
| novel.2073 | 5.29 | 0.021 | 0.039 | 1.341 |
| RHSIM_Rhsim07G0163500 | 5.29 | 0.021 | 0.039 | -0.815 |
| RHSIM_Rhsim09G0181000 | 5.29 | 0.021 | 0.039 | 0.987 |
| RHSIM_Rhsim09G0106600 | 5.28 | 0.022 | 0.039 | 0.561 |
| novel.3172 | 5.28 | 0.022 | 0.039 | 2.257 |
| RHSIM_Rhsim03G0193000 | 5.28 | 0.022 | 0.039 | -2.241 |
| RHSIM_Rhsim09G0090200 | 5.28 | 0.022 | 0.039 | -0.341 |
| novel.604 | 5.28 | 0.022 | 0.039 | -3.080 |
| novel.2772 | 5.28 | 0.022 | 0.039 | 1.296 |
| RHSIM_Rhsim02G0122400 | 5.28 | 0.022 | 0.039 | -0.394 |
| RHSIM_Rhsim09G0000700 | 5.28 | 0.022 | 0.039 | 0.267 |
| RHSIM_Rhsim03G0070300 | 5.28 | 0.022 | 0.039 | -0.304 |
| RHSIM_Rhsim02G0008900 | 5.27 | 0.022 | 0.039 | 0.360 |
| novel.853 | 5.27 | 0.022 | 0.039 | 0.475 |
| RHSIM_Rhsim07G0071900 | 5.27 | 0.022 | 0.039 | 3.243 |
| RHSIM_Rhsim09G0094700 | 5.27 | 0.022 | 0.039 | 0.385 |
| novel.2692 | 5.27 | 0.022 | 0.039 | -1.868 |
| RHSIM_Rhsim04G0084400 | 5.27 | 0.022 | 0.039 | 0.240 |
| RHSIM_Rhsim02G0143000 | 5.27 | 0.022 | 0.039 | -39.251 |
| RHSIM_Rhsim12G0120900 | 5.26 | 0.022 | 0.039 | -40.483 |
| RHSIM_Rhsim13G0037300 | 5.26 | 0.022 | 0.039 | -2.926 |
| RHSIM_Rhsim08G0021200 | 5.26 | 0.022 | 0.039 | -0.457 |
| novel.1490 | 5.26 | 0.022 | 0.039 | -0.404 |
| RHSIM_Rhsim12G0180100 | 5.26 | 0.022 | 0.039 | -0.975 |
| novel.2735 | 5.26 | 0.022 | 0.039 | 0.508 |
| novel.1194 | 5.26 | 0.022 | 0.039 | 1.210 |
| RHSIM_Rhsim13G0223300 | 5.26 | 0.022 | 0.039 | 0.260 |
| RHSIM_Rhsim09G0028100 | 5.26 | 0.022 | 0.039 | -1.222 |
| RHSIM_Rhsim11G0176600 | 5.26 | 0.022 | 0.039 | 41.443 |

| Gene | LRT statistic | P value | BH-adjusted P | log2FC (interaction) |
|---|---|---|---|---|
| RHSIM_Rhsim13G0189300 | 5.25 | 0.022 | 0.039 | -0.472 |
| novel.1703 | 5.25 | 0.022 | 0.039 | 0.575 |
| RHSIM_Rhsim12G0194000 | 5.25 | 0.022 | 0.039 | 4.382 |
| RHSIM_Rhsim13G0121100 | 5.25 | 0.022 | 0.039 | 1.069 |
| RHSIM_Rhsim11G0133700 | 5.25 | 0.022 | 0.039 | 0.334 |
| RHSIM_Rhsim06G0115900 | 5.25 | 0.022 | 0.039 | -1.561 |
| RHSIM_Rhsim12G0088200 | 5.25 | 0.022 | 0.039 | 1.710 |
| RHSIM_Rhsim13G0211500 | 5.25 | 0.022 | 0.040 | 0.888 |
| RHSIM_Rhsim13G0014100 | 5.25 | 0.022 | 0.040 | -3.448 |
| RHSIM_Rhsim11G0006200 | 5.24 | 0.022 | 0.040 | -2.712 |
| RHSIM_Rhsim10G0075300 | 5.24 | 0.022 | 0.040 | -1.132 |
| novel.1420 | 5.24 | 0.022 | 0.040 | 0.556 |
| novel.2293 | 5.23 | 0.022 | 0.040 | -0.954 |
| novel.1202 | 5.23 | 0.022 | 0.040 | -1.090 |
| novel.812 | 5.23 | 0.022 | 0.040 | 0.623 |
| RHSIM_Rhsim07G0043600 | 5.23 | 0.022 | 0.040 | -1.099 |
| RHSIM_Rhsim12G0004400 | 5.23 | 0.022 | 0.040 | -0.778 |
| RHSIM_Rhsim02G0141100 | 5.23 | 0.022 | 0.040 | 0.369 |
| RHSIM_Rhsim07G0092600 | 5.23 | 0.022 | 0.040 | 0.174 |
| RHSIM_Rhsim05G0079000 | 5.23 | 0.022 | 0.040 | -0.251 |
| RHSIM_Rhsim01G0273300 | 5.23 | 0.022 | 0.040 | -0.332 |
| RHSIM_Rhsim11G0072700 | 5.23 | 0.022 | 0.040 | 41.691 |
| RHSIM_Rhsim08G0251600 | 5.23 | 0.022 | 0.040 | 1.606 |
| RHSIM_Rhsim13G0056900 | 5.23 | 0.022 | 0.040 | 0.202 |
| RHSIM_Rhsim04G0190400 | 5.23 | 0.022 | 0.040 | -3.512 |
| RHSIM_Rhsim06G0017700 | 5.23 | 0.022 | 0.040 | -0.342 |
| novel.2615 | 5.23 | 0.022 | 0.040 | 2.606 |
| novel.3192 | 5.22 | 0.022 | 0.040 | -1.151 |
| RHSIM_Rhsim06G0176700 | 5.22 | 0.022 | 0.040 | -0.408 |
| RHSIM_Rhsim07G0022900 | 5.22 | 0.022 | 0.040 | 0.488 |
| RHSIM_Rhsim03G0064800 | 5.22 | 0.022 | 0.040 | 0.278 |
| novel.2781 | 5.22 | 0.022 | 0.040 | 1.544 |

| Gene | LRT statistic | P value | BH-adjusted P | log2FC (interaction) |
|---|---|---|---|---|
| RHSIM_Rhsim12G0021400 | 5.22 | 0.022 | 0.040 | 0.797 |
| novel.4008 | 5.22 | 0.022 | 0.040 | 1.253 |
| RHSIM_Rhsim05G0207300 | 5.22 | 0.022 | 0.040 | -0.491 |
| RHSIM_Rhsim07G0075000 | 5.21 | 0.022 | 0.040 | -0.396 |
| RHSIM_Rhsim09G0139500 | 5.21 | 0.022 | 0.040 | 2.561 |
| RHSIM_Rhsim04G0093400 | 5.21 | 0.022 | 0.040 | -5.646 |
| novel.4570 | 5.21 | 0.022 | 0.040 | 1.503 |
| RHSIM_Rhsim03G0105000 | 5.21 | 0.022 | 0.040 | 0.275 |
| RHSIM_Rhsim04G0046300 | 5.21 | 0.022 | 0.040 | 1.837 |
| novel.2439 | 5.21 | 0.022 | 0.040 | 1.150 |
| RHSIM_Rhsim10G0078300 | 5.21 | 0.022 | 0.040 | -0.246 |
| RHSIM_Rhsim07G0098100 | 5.21 | 0.022 | 0.040 | -1.188 |
| RHSIM_Rhsim04G0187200 | 5.20 | 0.023 | 0.040 | -0.579 |
| RHSIM_Rhsim02G0178500 | 5.20 | 0.023 | 0.040 | -0.703 |
| novel.3180 | 5.20 | 0.023 | 0.041 | 1.410 |
| RHSIM_Rhsim07G0171700 | 5.20 | 0.023 | 0.041 | 40.811 |
| RHSIM_Rhsim05G0143700 | 5.20 | 0.023 | 0.041 | -0.331 |
| RHSIM_Rhsim12G0005000 | 5.20 | 0.023 | 0.041 | -0.331 |
| RHSIM_Rhsim09G0068800 | 5.20 | 0.023 | 0.041 | 1.724 |
| RHSIM_Rhsim13G0017500 | 5.20 | 0.023 | 0.041 | -0.853 |
| RHSIM_Rhsim11G0041800 | 5.20 | 0.023 | 0.041 | -0.694 |
| RHSIM_Rhsim04G0112300 | 5.19 | 0.023 | 0.041 | -0.931 |
| RHSIM_Rhsim03G0164400 | 5.19 | 0.023 | 0.041 | -0.449 |
| RHSIM_Rhsim11G0121700 | 5.19 | 0.023 | 0.041 | 0.186 |
| novel.406 | 5.19 | 0.023 | 0.041 | -0.559 |
| RHSIM_Rhsim06G0068100 | 5.19 | 0.023 | 0.041 | 0.168 |
| novel.1588 | 5.19 | 0.023 | 0.041 | -1.845 |
| RHSIM_Rhsim08G0068200 | 5.19 | 0.023 | 0.041 | 0.711 |
| RHSIM_Rhsim03G0247700 | 5.19 | 0.023 | 0.041 | -0.620 |
| RHSIM_Rhsim07G0063300 | 5.18 | 0.023 | 0.041 | 3.464 |
| RHSIM_Rhsim06G0241100 | 5.18 | 0.023 | 0.041 | -0.569 |
| RHSIM_Rhsim11G0187800 | 5.18 | 0.023 | 0.041 | 0.258 |

| Gene | LRT statistic | P value | BH-adjusted P | log2FC (interaction) |
|---|---|---|---|---|
| RHSIM_Rhsim13G0065200 | 5.18 | 0.023 | 0.041 | 2.388 |
| RHSIM_Rhsim12G0160900 | 5.17 | 0.023 | 0.041 | 0.163 |
| RHSIM_RhsimUnG0099400 | 5.17 | 0.023 | 0.041 | 1.764 |
| RHSIM_Rhsim02G0092800 | 5.17 | 0.023 | 0.041 | 3.952 |
| RHSIM_Rhsim06G0050900 | 5.17 | 0.023 | 0.041 | 0.312 |
| RHSIM_Rhsim04G0027600 | 5.17 | 0.023 | 0.041 | 0.517 |
| RHSIM_Rhsim05G0186700 | 5.17 | 0.023 | 0.041 | -0.446 |
| RHSIM_Rhsim02G0021300 | 5.17 | 0.023 | 0.041 | -0.548 |
| RHSIM_Rhsim12G0047800 | 5.17 | 0.023 | 0.041 | -0.272 |
| RHSIM_Rhsim07G0172100 | 5.17 | 0.023 | 0.041 | 1.073 |
| RHSIM_Rhsim12G0094100 | 5.17 | 0.023 | 0.041 | 1.510 |
| novel.483 | 5.17 | 0.023 | 0.041 | 1.295 |
| RHSIM_Rhsim01G0066200 | 5.17 | 0.023 | 0.041 | -39.478 |
| RHSIM_Rhsim01G0192200 | 5.17 | 0.023 | 0.041 | 0.277 |
| RHSIM_Rhsim03G0171200 | 5.16 | 0.023 | 0.041 | 0.217 |
| RHSIM_Rhsim05G0204700 | 5.16 | 0.023 | 0.041 | 0.301 |
| RHSIM_Rhsim11G0193000 | 5.16 | 0.023 | 0.041 | 0.225 |
| RHSIM_Rhsim05G0120700 | 5.16 | 0.023 | 0.041 | 0.633 |
| RHSIM_Rhsim06G0003200 | 5.16 | 0.023 | 0.041 | -0.262 |
| RHSIM_Rhsim09G0127000 | 5.15 | 0.023 | 0.042 | 3.583 |
| novel.2518 | 5.15 | 0.023 | 0.042 | -0.518 |
| novel.966 | 5.15 | 0.023 | 0.042 | -1.261 |
| RHSIM_Rhsim09G0107800 | 5.15 | 0.023 | 0.042 | -0.210 |
| novel.2563 | 5.15 | 0.023 | 0.042 | 0.948 |
| RHSIM_Rhsim13G0187100 | 5.15 | 0.023 | 0.042 | -0.852 |
| novel.1526 | 5.15 | 0.023 | 0.042 | -2.192 |
| RHSIM_RhsimUnG0048300 | 5.15 | 0.023 | 0.042 | -2.292 |
| RHSIM_Rhsim06G0028400 | 5.15 | 0.023 | 0.042 | -0.296 |
| RHSIM_Rhsim02G0014500 | 5.15 | 0.023 | 0.042 | -0.568 |
| RHSIM_Rhsim07G0171200 | 5.15 | 0.023 | 0.042 | 1.182 |
| novel.2277 | 5.15 | 0.023 | 0.042 | -1.949 |
| RHSIM_Rhsim03G0112100 | 5.15 | 0.023 | 0.042 | -0.168 |

| Gene | LRT statistic | P value | BH-adjusted P | log2FC (interaction) |
|---|---|---|---|---|
| RHSIM_RhsimUnG0176300 | 5.15 | 0.023 | 0.042 | -1.528 |
| RHSIM_Rhsim13G0148400 | 5.15 | 0.023 | 0.042 | -39.961 |
| RHSIM_Rhsim04G0004100 | 5.15 | 0.023 | 0.042 | 0.828 |
| RHSIM_RhsimUnG0026900 | 5.15 | 0.023 | 0.042 | 1.304 |
| RHSIM_Rhsim06G0031500 | 5.15 | 0.023 | 0.042 | 2.945 |
| novel.2764 | 5.15 | 0.023 | 0.042 | 0.365 |
| RHSIM_RhsimUnG0053500 | 5.14 | 0.023 | 0.042 | -1.364 |
| RHSIM_Rhsim12G0084700 | 5.14 | 0.023 | 0.042 | 3.780 |
| novel.2507 | 5.14 | 0.023 | 0.042 | -1.166 |
| RHSIM_Rhsim06G0228500 | 5.14 | 0.023 | 0.042 | -41.554 |
| RHSIM_Rhsim02G0024200 | 5.14 | 0.023 | 0.042 | 0.415 |
| RHSIM_Rhsim04G0119200 | 5.14 | 0.023 | 0.042 | 1.881 |
| novel.4293 | 5.14 | 0.023 | 0.042 | -0.993 |
| RHSIM_Rhsim02G0020900 | 5.14 | 0.023 | 0.042 | -0.376 |
| novel.2922 | 5.14 | 0.023 | 0.042 | 1.753 |
| RHSIM_RhsimUnG0106700 | 5.13 | 0.023 | 0.042 | 1.084 |
| RHSIM_Rhsim04G0026800 | 5.13 | 0.023 | 0.042 | -2.245 |
| RHSIM_Rhsim13G0198100 | 5.13 | 0.023 | 0.042 | 0.340 |
| novel.1858 | 5.13 | 0.024 | 0.042 | 1.303 |
| RHSIM_Rhsim04G0054000 | 5.13 | 0.024 | 0.042 | -1.520 |
| novel.47 | 5.13 | 0.024 | 0.042 | 1.402 |
| RHSIM_Rhsim13G0015400 | 5.13 | 0.024 | 0.042 | 0.662 |
| RHSIM_Rhsim05G0159600 | 5.13 | 0.024 | 0.042 | 0.332 |
| novel.1550 | 5.13 | 0.024 | 0.042 | 2.879 |
| RHSIM_Rhsim09G0125900 | 5.13 | 0.024 | 0.042 | -0.322 |
| RHSIM_Rhsim11G0165300 | 5.13 | 0.024 | 0.042 | 1.836 |
| RHSIM_Rhsim13G0133000 | 5.13 | 0.024 | 0.042 | -0.341 |
| RHSIM_RhsimUnG0181700 | 5.13 | 0.024 | 0.042 | -0.485 |
| RHSIM_Rhsim07G0071000 | 5.12 | 0.024 | 0.042 | -0.663 |
| RHSIM_Rhsim04G0091500 | 5.12 | 0.024 | 0.042 | 0.244 |
| RHSIM_Rhsim04G0234400 | 5.12 | 0.024 | 0.042 | -0.941 |
| novel.3702 | 5.11 | 0.024 | 0.042 | -1.424 |

| Gene | LRT statistic | P value | BH-adjusted P | log2FC (interaction) |
|---|---|---|---|---|
| novel.2809 | 5.11 | 0.024 | 0.042 | 1.336 |
| RHSIM_Rhsim11G0197000 | 5.11 | 0.024 | 0.042 | 0.138 |
| RHSIM_Rhsim11G0011400 | 5.11 | 0.024 | 0.042 | -0.304 |
| novel.442 | 5.11 | 0.024 | 0.042 | 1.000 |
| RHSIM_Rhsim07G0139200 | 5.11 | 0.024 | 0.042 | -0.393 |
| RHSIM_Rhsim05G0077200 | 5.11 | 0.024 | 0.042 | 0.793 |
| novel.1626 | 5.11 | 0.024 | 0.042 | 1.403 |
| RHSIM_Rhsim12G0146200 | 5.11 | 0.024 | 0.043 | 41.640 |
| RHSIM_Rhsim01G0109200 | 5.11 | 0.024 | 0.043 | -1.050 |
| RHSIM_Rhsim08G0105600 | 5.11 | 0.024 | 0.043 | -3.090 |
| RHSIM_RhsimUnG0072500 | 5.11 | 0.024 | 0.043 | 3.317 |
| RHSIM_Rhsim03G0253400 | 5.10 | 0.024 | 0.043 | -0.253 |
| RHSIM_Rhsim11G0046700 | 5.10 | 0.024 | 0.043 | -1.214 |
| novel.1352 | 5.10 | 0.024 | 0.043 | 0.748 |
| RHSIM_Rhsim08G0030200 | 5.10 | 0.024 | 0.043 | 0.214 |
| RHSIM_Rhsim13G0026400 | 5.10 | 0.024 | 0.043 | -1.061 |
| RHSIM_RhsimUnG0065900 | 5.10 | 0.024 | 0.043 | 0.598 |
| RHSIM_Rhsim11G0089200 | 5.10 | 0.024 | 0.043 | -3.625 |
| RHSIM_Rhsim09G0210400 | 5.10 | 0.024 | 0.043 | -0.184 |
| RHSIM_Rhsim02G0235900 | 5.10 | 0.024 | 0.043 | -1.309 |
| RHSIM_Rhsim04G0002700 | 5.09 | 0.024 | 0.043 | 0.226 |
| novel.1258 | 5.09 | 0.024 | 0.043 | -1.812 |
| novel.596 | 5.09 | 0.024 | 0.043 | -1.954 |
| novel.1259 | 5.09 | 0.024 | 0.043 | 1.571 |
| RHSIM_Rhsim05G0089900 | 5.09 | 0.024 | 0.043 | 0.818 |
| RHSIM_Rhsim13G0224600 | 5.09 | 0.024 | 0.043 | -0.231 |
| RHSIM_RhsimUnG0234300 | 5.09 | 0.024 | 0.043 | 2.702 |
| RHSIM_Rhsim03G0032900 | 5.09 | 0.024 | 0.043 | -1.023 |
| novel.2154 | 5.09 | 0.024 | 0.043 | 1.157 |
| RHSIM_Rhsim07G0063900 | 5.08 | 0.024 | 0.043 | -2.667 |
| novel.4382 | 5.08 | 0.024 | 0.043 | 0.841 |
| novel.3569 | 5.08 | 0.024 | 0.043 | 1.200 |

| Gene | LRT statistic | P value | BH-adjusted P | log2FC (interaction) |
|---|---|---|---|---|
| novel.4442 | 5.08 | 0.024 | 0.043 | 2.434 |
| RHSIM_Rhsim09G0052800 | 5.08 | 0.024 | 0.043 | 1.756 |
| RHSIM_Rhsim08G0177900 | 5.08 | 0.024 | 0.043 | -0.547 |
| RHSIM_Rhsim02G0133900 | 5.08 | 0.024 | 0.043 | 0.583 |
| RHSIM_Rhsim02G0019800 | 5.08 | 0.024 | 0.043 | -1.309 |
| RHSIM_Rhsim02G0071800 | 5.08 | 0.024 | 0.043 | 0.151 |
| RHSIM_Rhsim12G0016300 | 5.08 | 0.024 | 0.043 | 0.388 |
| RHSIM_Rhsim12G0209600 | 5.07 | 0.024 | 0.043 | -0.485 |
| RHSIM_Rhsim09G0183500 | 5.07 | 0.024 | 0.043 | 0.906 |
| RHSIM_Rhsim04G0094100 | 5.07 | 0.024 | 0.043 | -3.584 |
| RHSIM_Rhsim13G0093100 | 5.07 | 0.024 | 0.043 | -0.582 |
| RHSIM_Rhsim13G0122700 | 5.06 | 0.024 | 0.044 | -1.492 |
| novel.2334 | 5.06 | 0.024 | 0.044 | 3.438 |
| RHSIM_Rhsim08G0061900 | 5.06 | 0.024 | 0.044 | -39.228 |
| novel.2367 | 5.06 | 0.024 | 0.044 | -1.286 |
| RHSIM_Rhsim08G0049200 | 5.06 | 0.024 | 0.044 | -0.367 |
| novel.1079 | 5.06 | 0.024 | 0.044 | 1.027 |
| RHSIM_Rhsim07G0233900 | 5.06 | 0.024 | 0.044 | -39.721 |
| RHSIM_Rhsim05G0129200 | 5.06 | 0.025 | 0.044 | 0.668 |
| RHSIM_Rhsim09G0003500 | 5.06 | 0.025 | 0.044 | -0.282 |
| RHSIM_RhsimUnG0003800 | 5.06 | 0.025 | 0.044 | -1.530 |
| RHSIM_Rhsim08G0161300 | 5.06 | 0.025 | 0.044 | -0.179 |
| RHSIM_Rhsim09G0006700 | 5.05 | 0.025 | 0.044 | 1.612 |
| RHSIM_Rhsim01G0143200 | 5.05 | 0.025 | 0.044 | 77.724 |
| RHSIM_Rhsim04G0074900 | 5.05 | 0.025 | 0.044 | 0.254 |
| novel.3086 | 5.05 | 0.025 | 0.044 | 0.574 |
| RHSIM_Rhsim03G0270700 | 5.05 | 0.025 | 0.044 | 1.691 |
| novel.89 | 5.05 | 0.025 | 0.044 | -39.509 |
| RHSIM_Rhsim10G0125400 | 5.05 | 0.025 | 0.044 | 2.755 |
| RHSIM_Rhsim02G0029800 | 5.05 | 0.025 | 0.044 | -1.056 |
| RHSIM_Rhsim07G0172200 | 5.05 | 0.025 | 0.044 | 0.170 |
| RHSIM_Rhsim12G0205900 | 5.05 | 0.025 | 0.044 | 3.969 |

| Gene | LRT statistic | P value | BH-adjusted P | log2FC (interaction) |
|---|---|---|---|---|
| novel.1196 | 5.05 | 0.025 | 0.044 | -38.865 |
| RHSIM_Rhsim05G0201900 | 5.04 | 0.025 | 0.044 | -4.913 |
| RHSIM_Rhsim12G0063200 | 5.04 | 0.025 | 0.044 | -0.290 |
| RHSIM_Rhsim03G0036000 | 5.04 | 0.025 | 0.044 | 1.565 |
| RHSIM_Rhsim07G0133200 | 5.04 | 0.025 | 0.044 | -0.701 |
| RHSIM_Rhsim09G0132300 | 5.04 | 0.025 | 0.044 | 0.212 |
| RHSIM_Rhsim02G0123800 | 5.04 | 0.025 | 0.044 | -0.857 |
| RHSIM_Rhsim02G0065800 | 5.04 | 0.025 | 0.044 | 2.408 |
| RHSIM_Rhsim04G0026300 | 5.03 | 0.025 | 0.044 | 0.475 |
| novel.2798 | 5.03 | 0.025 | 0.044 | 0.631 |
| RHSIM_Rhsim02G0207700 | 5.03 | 0.025 | 0.044 | 0.618 |
| RHSIM_Rhsim02G0191900 | 5.03 | 0.025 | 0.044 | 1.781 |
| RHSIM_Rhsim11G0048100 | 5.03 | 0.025 | 0.044 | 0.201 |
| RHSIM_Rhsim04G0242100 | 5.03 | 0.025 | 0.044 | 0.326 |
| novel.3138 | 5.02 | 0.025 | 0.044 | 0.466 |
| RHSIM_Rhsim10G0060800 | 5.02 | 0.025 | 0.044 | -0.638 |
| novel.91 | 5.02 | 0.025 | 0.044 | 1.629 |
| RHSIM_Rhsim11G0170000 | 5.02 | 0.025 | 0.045 | 0.304 |
| RHSIM_Rhsim10G0152400 | 5.02 | 0.025 | 0.045 | 0.548 |
| RHSIM_Rhsim06G0224500 | 5.02 | 0.025 | 0.045 | -2.732 |
| novel.1516 | 5.02 | 0.025 | 0.045 | 1.144 |
| RHSIM_RhsimUnG0035700 | 5.02 | 0.025 | 0.045 | -0.424 |
| RHSIM_Rhsim06G0118100 | 5.02 | 0.025 | 0.045 | 0.318 |
| RHSIM_Rhsim12G0187300 | 5.02 | 0.025 | 0.045 | 1.102 |
| RHSIM_Rhsim04G0229700 | 5.02 | 0.025 | 0.045 | -0.179 |
| RHSIM_Rhsim12G0103200 | 5.02 | 0.025 | 0.045 | 0.629 |
| RHSIM_RhsimMtG0005400 | 5.01 | 0.025 | 0.045 | 2.569 |
| RHSIM_Rhsim12G0200000 | 5.01 | 0.025 | 0.045 | -0.553 |
| novel.2446 | 5.01 | 0.025 | 0.045 | -1.922 |
| RHSIM_Rhsim10G0139700 | 5.01 | 0.025 | 0.045 | 1.073 |
| RHSIM_Rhsim12G0183400 | 5.01 | 0.025 | 0.045 | 0.537 |
| RHSIM_Rhsim03G0012200 | 5.01 | 0.025 | 0.045 | -1.414 |

| Gene | LRT statistic | P value | BH-adjusted P | log2FC (interaction) |
|---|---|---|---|---|
| RHSIM_Rhsim08G0089700 | 5.01 | 0.025 | 0.045 | 1.182 |
| RHSIM_Rhsim06G0231600 | 5.01 | 0.025 | 0.045 | -0.757 |
| RHSIM_Rhsim03G0171100 | 5.01 | 0.025 | 0.045 | -0.331 |
| RHSIM_Rhsim11G0140000 | 5.01 | 0.025 | 0.045 | 0.666 |
| RHSIM_Rhsim01G0143800 | 5.01 | 0.025 | 0.045 | 1.896 |
| novel.380 | 5.00 | 0.025 | 0.045 | -2.301 |
| RHSIM_Rhsim12G0213400 | 5.00 | 0.025 | 0.045 | -0.712 |
| RHSIM_Rhsim01G0094700 | 5.00 | 0.025 | 0.045 | -3.045 |
| RHSIM_Rhsim08G0214100 | 5.00 | 0.025 | 0.045 | -2.597 |
| RHSIM_Rhsim11G0183000 | 5.00 | 0.025 | 0.045 | 0.179 |
| RHSIM_Rhsim13G0109800 | 5.00 | 0.025 | 0.045 | 0.214 |
| novel.884 | 5.00 | 0.025 | 0.045 | 40.634 |
| RHSIM_Rhsim08G0238100 | 5.00 | 0.025 | 0.045 | 0.272 |
| RHSIM_Rhsim12G0030700 | 5.00 | 0.025 | 0.045 | 40.513 |
| RHSIM_Rhsim02G0216600 | 5.00 | 0.025 | 0.045 | 0.285 |
| RHSIM_RhsimUnG0082300 | 5.00 | 0.025 | 0.045 | -0.519 |
| RHSIM_Rhsim04G0006100 | 5.00 | 0.025 | 0.045 | 1.508 |
| RHSIM_Rhsim05G0094900 | 5.00 | 0.025 | 0.045 | -0.269 |
| RHSIM_Rhsim10G0204300 | 5.00 | 0.025 | 0.045 | 3.380 |
| RHSIM_Rhsim11G0078200 | 5.00 | 0.025 | 0.045 | 0.890 |
| novel.598 | 5.00 | 0.025 | 0.045 | 1.718 |
| RHSIM_RhsimUnG0201700 | 5.00 | 0.025 | 0.045 | -2.029 |
| RHSIM_Rhsim03G0029900 | 4.99 | 0.025 | 0.045 | 0.253 |
| RHSIM_RhsimUnG0121900 | 4.99 | 0.025 | 0.045 | -39.342 |
| RHSIM_Rhsim11G0088700 | 4.99 | 0.025 | 0.045 | 0.470 |
| RHSIM_Rhsim12G0069800 | 4.99 | 0.025 | 0.045 | -5.349 |
| RHSIM_Rhsim03G0054100 | 4.99 | 0.026 | 0.045 | -1.352 |
| novel.3719 | 4.99 | 0.026 | 0.045 | 1.462 |
| RHSIM_Rhsim01G0277800 | 4.99 | 0.026 | 0.045 | 0.320 |
| novel.3412 | 4.99 | 0.026 | 0.045 | 3.510 |
| RHSIM_Rhsim06G0177500 | 4.99 | 0.026 | 0.045 | 2.682 |
| novel.2387 | 4.98 | 0.026 | 0.045 | -3.638 |

| Gene | LRT statistic | P value | BH-adjusted P | log2FC (interaction) |
|---|---|---|---|---|
| RHSIM_Rhsim08G0082200 | 4.98 | 0.026 | 0.045 | -0.396 |
| RHSIM_Rhsim10G0139400 | 4.98 | 0.026 | 0.045 | 3.739 |
| RHSIM_Rhsim06G0078500 | 4.98 | 0.026 | 0.045 | 0.351 |
| novel.3790 | 4.98 | 0.026 | 0.045 | 0.765 |
| RHSIM_Rhsim09G0202200 | 4.98 | 0.026 | 0.045 | 0.477 |
| novel.2060 | 4.98 | 0.026 | 0.046 | 0.340 |
| RHSIM_Rhsim01G0212800 | 4.98 | 0.026 | 0.046 | -0.312 |
| RHSIM_Rhsim03G0206000 | 4.97 | 0.026 | 0.046 | -2.019 |
| RHSIM_Rhsim10G0146500 | 4.97 | 0.026 | 0.046 | -0.481 |
| RHSIM_Rhsim05G0173600 | 4.97 | 0.026 | 0.046 | -2.355 |
| RHSIM_Rhsim13G0206100 | 4.97 | 0.026 | 0.046 | -0.356 |
| RHSIM_Rhsim08G0194400 | 4.97 | 0.026 | 0.046 | 5.015 |
| RHSIM_Rhsim05G0187400 | 4.97 | 0.026 | 0.046 | 3.166 |
| RHSIM_Rhsim07G0144000 | 4.97 | 0.026 | 0.046 | 2.637 |
| RHSIM_Rhsim06G0173500 | 4.97 | 0.026 | 0.046 | 41.379 |
| RHSIM_Rhsim03G0094000 | 4.96 | 0.026 | 0.046 | -0.325 |
| novel.2771 | 4.96 | 0.026 | 0.046 | 2.521 |
| RHSIM_Rhsim05G0200400 | 4.96 | 0.026 | 0.046 | -0.317 |
| RHSIM_Rhsim03G0154600 | 4.96 | 0.026 | 0.046 | -0.324 |
| RHSIM_Rhsim04G0125800 | 4.96 | 0.026 | 0.046 | 2.287 |
| novel.1442 | 4.96 | 0.026 | 0.046 | 1.425 |
| novel.2776 | 4.96 | 0.026 | 0.046 | -2.306 |
| RHSIM_Rhsim01G0144600 | 4.96 | 0.026 | 0.046 | -0.350 |
| RHSIM_Rhsim11G0126600 | 4.96 | 0.026 | 0.046 | -0.379 |
| RHSIM_Rhsim12G0174600 | 4.96 | 0.026 | 0.046 | 0.861 |
| RHSIM_Rhsim12G0085300 | 4.95 | 0.026 | 0.046 | -0.150 |
| RHSIM_Rhsim04G0018400 | 4.95 | 0.026 | 0.046 | 0.622 |
| RHSIM_Rhsim05G0210000 | 4.95 | 0.026 | 0.046 | -40.012 |
| RHSIM_Rhsim03G0191500 | 4.95 | 0.026 | 0.046 | 0.258 |
| RHSIM_Rhsim01G0184900 | 4.95 | 0.026 | 0.046 | 0.342 |
| RHSIM_Rhsim01G0261000 | 4.95 | 0.026 | 0.046 | -0.254 |
| RHSIM_Rhsim09G0114900 | 4.95 | 0.026 | 0.046 | 2.611 |

| Gene | LRT statistic | P value | BH-adjusted P | log2FC (interaction) |
|---|---|---|---|---|
| RHSIM_Rhsim03G0031500 | 4.95 | 0.026 | 0.046 | 40.054 |
| novel.341 | 4.95 | 0.026 | 0.046 | 1.315 |
| novel.1059 | 4.95 | 0.026 | 0.046 | -1.530 |
| RHSIM_Rhsim01G0227100 | 4.95 | 0.026 | 0.046 | -2.794 |
| RHSIM_Rhsim12G0151500 | 4.94 | 0.026 | 0.046 | -0.176 |
| RHSIM_Rhsim06G0127500 | 4.94 | 0.026 | 0.046 | 0.879 |
| RHSIM_Rhsim01G0123700 | 4.94 | 0.026 | 0.046 | -0.312 |
| RHSIM_Rhsim04G0220800 | 4.94 | 0.026 | 0.046 | -41.395 |
| RHSIM_Rhsim08G0023500 | 4.94 | 0.026 | 0.046 | 0.880 |
| novel.512 | 4.94 | 0.026 | 0.046 | 1.625 |
| novel.4508 | 4.94 | 0.026 | 0.046 | -0.512 |
| novel.1288 | 4.94 | 0.026 | 0.046 | -1.665 |
| novel.1695 | 4.94 | 0.026 | 0.047 | 41.056 |
| novel.3155 | 4.93 | 0.026 | 0.047 | -1.041 |
| novel.3775 | 4.93 | 0.026 | 0.047 | -0.277 |
| RHSIM_Rhsim13G0109900 | 4.93 | 0.026 | 0.047 | 1.653 |
| RHSIM_Rhsim10G0081900 | 4.93 | 0.026 | 0.047 | -1.388 |
| novel.1328 | 4.93 | 0.026 | 0.047 | 39.789 |
| RHSIM_Rhsim10G0024900 | 4.93 | 0.026 | 0.047 | 1.232 |
| RHSIM_Rhsim05G0044000 | 4.93 | 0.026 | 0.047 | 0.198 |
| RHSIM_Rhsim10G0142600 | 4.92 | 0.026 | 0.047 | -38.837 |
| RHSIM_Rhsim07G0058200 | 4.92 | 0.027 | 0.047 | -0.279 |
| RHSIM_Rhsim04G0166400 | 4.92 | 0.027 | 0.047 | -3.020 |
| RHSIM_RhsimUnG0184700 | 4.92 | 0.027 | 0.047 | -0.922 |
| RHSIM_Rhsim09G0081800 | 4.92 | 0.027 | 0.047 | -0.402 |
| RHSIM_Rhsim01G0174100 | 4.92 | 0.027 | 0.047 | -0.230 |
| RHSIM_Rhsim08G0079800 | 4.91 | 0.027 | 0.047 | -0.250 |
| RHSIM_Rhsim07G0126500 | 4.91 | 0.027 | 0.047 | -0.432 |
| RHSIM_Rhsim11G0192700 | 4.91 | 0.027 | 0.047 | 0.292 |
| RHSIM_Rhsim10G0009500 | 4.91 | 0.027 | 0.047 | 2.056 |
| novel.290 | 4.91 | 0.027 | 0.047 | 0.499 |
| RHSIM_Rhsim09G0129000 | 4.91 | 0.027 | 0.047 | -0.183 |

| Gene | LRT statistic | P value | BH-adjusted P | log2FC (interaction) |
|---|---|---|---|---|
| RHSIM_Rhsim12G0044200 | 4.91 | 0.027 | 0.047 | -2.201 |
| novel.4038 | 4.91 | 0.027 | 0.047 | -39.753 |
| RHSIM_Rhsim09G0081700 | 4.91 | 0.027 | 0.047 | 1.561 |
| RHSIM_Rhsim13G0103700 | 4.90 | 0.027 | 0.047 | -0.667 |
| novel.1892 | 4.90 | 0.027 | 0.047 | -39.202 |
| RHSIM_Rhsim07G0135400 | 4.90 | 0.027 | 0.047 | 40.325 |
| RHSIM_Rhsim02G0146900 | 4.90 | 0.027 | 0.047 | 0.425 |
| novel.1769 | 4.90 | 0.027 | 0.048 | 0.902 |
| RHSIM_Rhsim02G0018300 | 4.89 | 0.027 | 0.048 | 0.278 |
| RHSIM_Rhsim01G0150700 | 4.89 | 0.027 | 0.048 | 1.060 |
| RHSIM_Rhsim02G0016000 | 4.89 | 0.027 | 0.048 | -0.144 |
| novel.1736 | 4.89 | 0.027 | 0.048 | 1.194 |
| novel.4487 | 4.89 | 0.027 | 0.048 | 2.235 |
| RHSIM_Rhsim03G0237200 | 4.89 | 0.027 | 0.048 | 0.664 |
| RHSIM_Rhsim13G0195900 | 4.89 | 0.027 | 0.048 | -40.719 |
| RHSIM_Rhsim02G0059700 | 4.89 | 0.027 | 0.048 | 0.214 |
| RHSIM_Rhsim07G0015800 | 4.89 | 0.027 | 0.048 | -0.286 |
| RHSIM_Rhsim06G0082800 | 4.88 | 0.027 | 0.048 | 0.282 |
| RHSIM_Rhsim04G0065100 | 4.88 | 0.027 | 0.048 | -1.107 |
| RHSIM_Rhsim11G0072500 | 4.88 | 0.027 | 0.048 | -0.395 |
| RHSIM_Rhsim06G0026200 | 4.88 | 0.027 | 0.048 | 1.952 |
| RHSIM_Rhsim10G0213000 | 4.88 | 0.027 | 0.048 | -0.183 |
| RHSIM_Rhsim04G0094800 | 4.88 | 0.027 | 0.048 | 1.193 |
| novel.3931 | 4.88 | 0.027 | 0.048 | 0.194 |
| RHSIM_Rhsim02G0115200 | 4.88 | 0.027 | 0.048 | 0.859 |
| RHSIM_Rhsim07G0090900 | 4.88 | 0.027 | 0.048 | -1.325 |
| RHSIM_Rhsim04G0030600 | 4.88 | 0.027 | 0.048 | -0.631 |
| RHSIM_Rhsim11G0011800 | 4.87 | 0.027 | 0.048 | 1.796 |
| RHSIM_Rhsim02G0123400 | 4.87 | 0.027 | 0.048 | 0.355 |
| RHSIM_Rhsim03G0166100 | 4.87 | 0.027 | 0.048 | -0.370 |
| novel.4043 | 4.87 | 0.027 | 0.048 | 1.536 |
| RHSIM_Rhsim09G0182700 | 4.87 | 0.027 | 0.048 | 0.506 |

| Gene | LRT statistic | P value | BH-adjusted P | log2FC (interaction) |
|---|---|---|---|---|
| RHSIM_Rhsim11G0156200 | 4.87 | 0.027 | 0.048 | 3.783 |
| novel.2660 | 4.87 | 0.027 | 0.048 | 2.514 |
| RHSIM_Rhsim12G0089600 | 4.86 | 0.027 | 0.048 | 40.867 |
| RHSIM_Rhsim12G0156200 | 4.86 | 0.027 | 0.048 | 0.150 |
| novel.244 | 4.86 | 0.027 | 0.048 | 1.191 |
| RHSIM_Rhsim02G0223100 | 4.86 | 0.027 | 0.048 | 0.770 |
| RHSIM_Rhsim01G0121700 | 4.86 | 0.028 | 0.048 | 0.384 |
| RHSIM_Rhsim01G0184200 | 4.86 | 0.028 | 0.048 | -3.673 |
| RHSIM_Rhsim09G0193900 | 4.85 | 0.028 | 0.049 | -0.289 |
| RHSIM_Rhsim06G0121500 | 4.85 | 0.028 | 0.049 | 0.347 |
| RHSIM_Rhsim07G0149700 | 4.85 | 0.028 | 0.049 | -0.252 |
| RHSIM_Rhsim11G0178200 | 4.85 | 0.028 | 0.049 | -41.367 |
| novel.3273 | 4.85 | 0.028 | 0.049 | 1.553 |
| RHSIM_Rhsim12G0168800 | 4.85 | 0.028 | 0.049 | 1.109 |
| RHSIM_Rhsim06G0112800 | 4.85 | 0.028 | 0.049 | -1.427 |
| RHSIM_Rhsim07G0205300 | 4.85 | 0.028 | 0.049 | -5.411 |
| novel.2989 | 4.84 | 0.028 | 0.049 | 1.623 |
| novel.2951 | 4.84 | 0.028 | 0.049 | -2.258 |
| RHSIM_Rhsim11G0135600 | 4.84 | 0.028 | 0.049 | -3.140 |
| RHSIM_Rhsim02G0018500 | 4.84 | 0.028 | 0.049 | 0.277 |
| novel.1472 | 4.84 | 0.028 | 0.049 | 0.828 |
| RHSIM_Rhsim06G0077000 | 4.84 | 0.028 | 0.049 | 1.473 |
| RHSIM_Rhsim07G0239900 | 4.84 | 0.028 | 0.049 | -38.513 |
| novel.1216 | 4.84 | 0.028 | 0.049 | 2.106 |
| RHSIM_Rhsim09G0187500 | 4.84 | 0.028 | 0.049 | -0.178 |
| novel.1619 | 4.84 | 0.028 | 0.049 | 1.349 |
| novel.686 | 4.84 | 0.028 | 0.049 | 1.637 |
| RHSIM_Rhsim12G0078000 | 4.83 | 0.028 | 0.049 | -0.582 |
| RHSIM_Rhsim01G0214600 | 4.83 | 0.028 | 0.049 | 1.239 |
| novel.3740 | 4.83 | 0.028 | 0.049 | -0.646 |
| RHSIM_Rhsim01G0052200 | 4.83 | 0.028 | 0.049 | 0.358 |
| RHSIM_Rhsim11G0058600 | 4.83 | 0.028 | 0.049 | 0.365 |

| Gene | LRT statistic | P value | BH-adjusted P | log2FC (interaction) |
|---|---|---|---|---|
| RHSIM_Rhsim08G0113800 | 4.83 | 0.028 | 0.049 | 1.516 |
| RHSIM_Rhsim01G0248300 | 4.83 | 0.028 | 0.049 | -1.367 |
| RHSIM_Rhsim08G0201200 | 4.83 | 0.028 | 0.049 | -39.826 |
| RHSIM_Rhsim02G0223400 | 4.83 | 0.028 | 0.049 | -1.372 |
| RHSIM_Rhsim05G0222900 | 4.83 | 0.028 | 0.049 | 0.206 |
| novel.2483 | 4.83 | 0.028 | 0.049 | -2.211 |
| RHSIM_Rhsim13G0169800 | 4.82 | 0.028 | 0.049 | -0.185 |
| RHSIM_Rhsim01G0261600 | 4.82 | 0.028 | 0.049 | 0.335 |
| RHSIM_Rhsim08G0099400 | 4.82 | 0.028 | 0.049 | 0.470 |
| RHSIM_Rhsim11G0056000 | 4.82 | 0.028 | 0.049 | -0.626 |
| RHSIM_Rhsim08G0218100 | 4.82 | 0.028 | 0.050 | -0.451 |
| RHSIM_Rhsim04G0071400 | 4.82 | 0.028 | 0.050 | -0.398 |
| RHSIM_Rhsim07G0176600 | 4.82 | 0.028 | 0.050 | 1.439 |
| RHSIM_Rhsim08G0006100 | 4.82 | 0.028 | 0.050 | -0.391 |
| RHSIM_Rhsim09G0199100 | 4.81 | 0.028 | 0.050 | 0.194 |
| RHSIM_Rhsim10G0182200 | 4.81 | 0.028 | 0.050 | -39.401 |
| RHSIM_RhsimUnG0241500 | 4.81 | 0.028 | 0.050 | -2.030 |
| novel.3334 | 4.81 | 0.028 | 0.050 | -0.460 |
| RHSIM_Rhsim06G0073700 | 4.80 | 0.028 | 0.050 | -0.278 |
| novel.3303 | 4.80 | 0.028 | 0.050 | 3.735 |
| RHSIM_Rhsim11G0140800 | 4.80 | 0.028 | 0.050 | -39.625 |
| RHSIM_Rhsim02G0032600 | 4.80 | 0.028 | 0.050 | -0.261 |
| RHSIM_Rhsim11G0150100 | 4.80 | 0.029 | 0.050 | 2.530 |
| novel.2506 | 4.79 | 0.029 | 0.050 | -1.463 |
| RHSIM_Rhsim10G0018600 | 4.79 | 0.029 | 0.050 | 0.933 |
| RHSIM_Rhsim01G0284800 | 4.79 | 0.029 | 0.050 | -0.487 |
| novel.2215 | 4.79 | 0.029 | 0.050 | -2.027 |
| RHSIM_Rhsim08G0092600 | 4.79 | 0.029 | 0.050 | 0.273 |
| novel.2428 | 4.78 | 0.029 | 0.050 | 0.698 |
| RHSIM_Rhsim08G0231500 | 4.78 | 0.029 | 0.050 | -0.301 |
| novel.1325 | 4.78 | 0.029 | 0.050 | -2.012 |
| RHSIM_Rhsim11G0056300 | 4.78 | 0.029 | 0.050 | -0.411 |

| Gene | LRT statistic | P value | BH-adjusted P | log2FC (interaction) |
|---|---|---|---|---|
| RHSIM_Rhsim05G0073300 | 4.78 | 0.029 | 0.050 | 2.141 |
| RHSIM_Rhsim07G0025100 | 4.78 | 0.029 | 0.050 | 2.463 |
| RHSIM_Rhsim02G0108400 | 4.78 | 0.029 | 0.051 | 2.657 |
| RHSIM_Rhsim09G0105500 | 4.78 | 0.029 | 0.051 | -40.005 |
| novel.3803 | 4.78 | 0.029 | 0.051 | 2.330 |
| novel.2552 | 4.77 | 0.029 | 0.051 | 1.172 |
| RHSIM_Rhsim08G0104700 | 4.77 | 0.029 | 0.051 | 1.136 |
| RHSIM_Rhsim08G0244400 | 4.77 | 0.029 | 0.051 | 1.777 |
| RHSIM_RhsimUnG0036000 | 4.77 | 0.029 | 0.051 | 3.524 |
| RHSIM_Rhsim07G0172800 | 4.77 | 0.029 | 0.051 | 39.955 |
| RHSIM_RhsimUnG0159700 | 4.76 | 0.029 | 0.051 | 1.830 |
| RHSIM_Rhsim03G0090100 | 4.76 | 0.029 | 0.051 | 0.347 |
| RHSIM_Rhsim10G0081300 | 4.76 | 0.029 | 0.051 | 3.762 |
| RHSIM_Rhsim11G0126300 | 4.76 | 0.029 | 0.051 | -2.005 |
| RHSIM_Rhsim10G0166900 | 4.76 | 0.029 | 0.051 | -0.188 |
| RHSIM_Rhsim02G0120600 | 4.76 | 0.029 | 0.051 | 0.478 |
| RHSIM_Rhsim01G0024800 | 4.76 | 0.029 | 0.051 | 3.143 |
| RHSIM_Rhsim02G0009900 | 4.76 | 0.029 | 0.051 | 1.245 |
| RHSIM_Rhsim13G0204200 | 4.76 | 0.029 | 0.051 | 0.400 |
| RHSIM_Rhsim10G0109600 | 4.76 | 0.029 | 0.051 | -3.892 |
| novel.1127 | 4.76 | 0.029 | 0.051 | 4.126 |
| novel.2800 | 4.75 | 0.029 | 0.051 | 1.219 |
| RHSIM_Rhsim05G0043400 | 4.75 | 0.029 | 0.051 | 0.187 |
| RHSIM_Rhsim11G0066100 | 4.75 | 0.029 | 0.051 | 0.445 |
| novel.2230 | 4.75 | 0.029 | 0.051 | 0.448 |
| RHSIM_Rhsim13G0007800 | 4.75 | 0.029 | 0.051 | 2.110 |
| RHSIM_Rhsim01G0250300 | 4.75 | 0.029 | 0.051 | -0.995 |
| RHSIM_Rhsim08G0066900 | 4.75 | 0.029 | 0.051 | -1.093 |
| novel.2566 | 4.75 | 0.029 | 0.051 | 2.128 |
| RHSIM_Rhsim10G0211900 | 4.75 | 0.029 | 0.051 | 0.523 |
| novel.2928 | 4.74 | 0.029 | 0.052 | 1.172 |
| RHSIM_RhsimMtG0001000 | 4.74 | 0.029 | 0.052 | 1.696 |

| Gene | LRT statistic | P value | BH-adjusted P | log2FC (interaction) |
|---|---|---|---|---|
| RHSIM_Rhsim13G0143000 | 4.74 | 0.029 | 0.052 | 0.689 |
| RHSIM_Rhsim02G0183700 | 4.74 | 0.030 | 0.052 | 2.524 |
| RHSIM_Rhsim12G0157100 | 4.74 | 0.030 | 0.052 | 0.270 |
| RHSIM_Rhsim02G0007400 | 4.73 | 0.030 | 0.052 | 0.286 |
| RHSIM_Rhsim08G0106400 | 4.73 | 0.030 | 0.052 | 2.053 |
| RHSIM_Rhsim01G0129700 | 4.73 | 0.030 | 0.052 | 2.119 |
| RHSIM_Rhsim06G0059700 | 4.73 | 0.030 | 0.052 | -0.655 |
| RHSIM_Rhsim06G0041000 | 4.73 | 0.030 | 0.052 | 1.068 |
| RHSIM_Rhsim07G0042300 | 4.73 | 0.030 | 0.052 | 2.767 |
| RHSIM_Rhsim05G0052400 | 4.73 | 0.030 | 0.052 | -0.227 |
| RHSIM_Rhsim03G0146400 | 4.73 | 0.030 | 0.052 | 0.447 |
| novel.192 | 4.73 | 0.030 | 0.052 | -38.782 |
| novel.390 | 4.72 | 0.030 | 0.052 | -1.576 |
| RHSIM_Rhsim07G0024100 | 4.72 | 0.030 | 0.052 | 0.177 |
| RHSIM_Rhsim01G0200300 | 4.72 | 0.030 | 0.052 | 3.481 |
| RHSIM_Rhsim13G0035800 | 4.72 | 0.030 | 0.052 | 0.223 |
| novel.3502 | 4.72 | 0.030 | 0.052 | 0.942 |
| RHSIM_RhsimUnG0105500 | 4.72 | 0.030 | 0.052 | -2.943 |
| RHSIM_Rhsim08G0199000 | 4.72 | 0.030 | 0.052 | -0.279 |
| RHSIM_Rhsim01G0006000 | 4.72 | 0.030 | 0.052 | -39.864 |
| RHSIM_Rhsim09G0105600 | 4.72 | 0.030 | 0.052 | -0.176 |
| RHSIM_Rhsim02G0130700 | 4.72 | 0.030 | 0.052 | 0.909 |
| RHSIM_Rhsim11G0074800 | 4.71 | 0.030 | 0.052 | -0.399 |
| RHSIM_Rhsim04G0173500 | 4.71 | 0.030 | 0.052 | 0.716 |
| RHSIM_Rhsim09G0096800 | 4.71 | 0.030 | 0.052 | 0.507 |
| novel.1751 | 4.71 | 0.030 | 0.053 | 40.981 |
| RHSIM_Rhsim07G0048100 | 4.71 | 0.030 | 0.053 | -0.218 |
| novel.2616 | 4.71 | 0.030 | 0.053 | 1.377 |
| RHSIM_Rhsim02G0143600 | 4.70 | 0.030 | 0.053 | 0.349 |
| RHSIM_Rhsim08G0005200 | 4.70 | 0.030 | 0.053 | 0.193 |
| RHSIM_Rhsim11G0009400 | 4.70 | 0.030 | 0.053 | 2.029 |
| RHSIM_RhsimUnG0053700 | 4.70 | 0.030 | 0.053 | -4.072 |

| Gene | LRT statistic | P value | BH-adjusted P | log2FC (interaction) |
|---|---|---|---|---|
| RHSIM_RhsimUnG0244200 | 4.70 | 0.030 | 0.053 | 2.440 |
| novel.1643 | 4.70 | 0.030 | 0.053 | -1.479 |
| RHSIM_Rhsim08G0067600 | 4.70 | 0.030 | 0.053 | 0.284 |
| RHSIM_Rhsim05G0077500 | 4.70 | 0.030 | 0.053 | 2.213 |
| RHSIM_Rhsim04G0217000 | 4.70 | 0.030 | 0.053 | -0.699 |
| RHSIM_RhsimUnG0101400 | 4.70 | 0.030 | 0.053 | 0.592 |
| RHSIM_Rhsim06G0223000 | 4.70 | 0.030 | 0.053 | 41.420 |
| RHSIM_Rhsim07G0079500 | 4.70 | 0.030 | 0.053 | -1.006 |
| RHSIM_Rhsim05G0025100 | 4.70 | 0.030 | 0.053 | 0.805 |
| RHSIM_Rhsim12G0020500 | 4.69 | 0.030 | 0.053 | 2.877 |
| RHSIM_Rhsim07G0064400 | 4.69 | 0.030 | 0.053 | -1.080 |
| RHSIM_Rhsim12G0157400 | 4.69 | 0.030 | 0.053 | 0.305 |
| RHSIM_Rhsim07G0043700 | 4.69 | 0.030 | 0.053 | -0.989 |
| novel.2849 | 4.69 | 0.030 | 0.053 | 0.306 |
| RHSIM_Rhsim06G0082200 | 4.69 | 0.030 | 0.053 | 0.431 |
| RHSIM_Rhsim04G0230400 | 4.69 | 0.030 | 0.053 | -0.259 |
| RHSIM_Rhsim04G0165400 | 4.69 | 0.030 | 0.053 | 0.302 |
| RHSIM_Rhsim05G0228300 | 4.68 | 0.030 | 0.053 | 0.590 |
| RHSIM_Rhsim01G0198300 | 4.68 | 0.030 | 0.053 | 0.662 |
| RHSIM_Rhsim07G0025800 | 4.68 | 0.030 | 0.053 | -0.872 |
| RHSIM_Rhsim07G0061300 | 4.68 | 0.031 | 0.053 | 0.165 |
| RHSIM_Rhsim08G0102500 | 4.68 | 0.031 | 0.053 | 0.316 |
| novel.4019 | 4.67 | 0.031 | 0.053 | -1.600 |
| RHSIM_RhsimUnG0022800 | 4.67 | 0.031 | 0.053 | -0.216 |
| novel.3420 | 4.67 | 0.031 | 0.053 | 1.351 |
| RHSIM_Rhsim12G0055800 | 4.67 | 0.031 | 0.054 | 41.222 |
| RHSIM_Rhsim06G0092300 | 4.67 | 0.031 | 0.054 | 0.932 |
| RHSIM_Rhsim06G0191300 | 4.67 | 0.031 | 0.054 | 0.566 |
| RHSIM_Rhsim07G0153300 | 4.67 | 0.031 | 0.054 | -0.497 |
| RHSIM_RhsimUnG0048800 | 4.67 | 0.031 | 0.054 | 2.209 |
| novel.2411 | 4.67 | 0.031 | 0.054 | -38.898 |
| RHSIM_Rhsim02G0069000 | 4.67 | 0.031 | 0.054 | -1.723 |

| Gene | LRT statistic | P value | BH-adjusted P | log2FC (interaction) |
|---|---|---|---|---|
| RHSIM_Rhsim01G0090600 | 4.67 | 0.031 | 0.054 | -0.304 |
| RHSIM_Rhsim13G0076600 | 4.67 | 0.031 | 0.054 | 0.257 |
| RHSIM_Rhsim03G0147300 | 4.66 | 0.031 | 0.054 | 0.307 |
| novel.3220 | 4.66 | 0.031 | 0.054 | 1.127 |
| RHSIM_Rhsim03G0244900 | 4.66 | 0.031 | 0.054 | 0.537 |
| RHSIM_Rhsim09G0135800 | 4.66 | 0.031 | 0.054 | -3.507 |
| RHSIM_Rhsim09G0177500 | 4.66 | 0.031 | 0.054 | -0.213 |
| RHSIM_Rhsim08G0176300 | 4.66 | 0.031 | 0.054 | 0.295 |
| novel.3536 | 4.66 | 0.031 | 0.054 | 0.937 |
| RHSIM_Rhsim10G0144200 | 4.66 | 0.031 | 0.054 | 0.713 |
| RHSIM_Rhsim03G0204700 | 4.66 | 0.031 | 0.054 | 1.395 |
| RHSIM_Rhsim12G0183300 | 4.66 | 0.031 | 0.054 | 0.475 |
| RHSIM_Rhsim07G0174600 | 4.66 | 0.031 | 0.054 | -1.974 |
| RHSIM_Rhsim12G0161100 | 4.65 | 0.031 | 0.054 | 0.383 |
| RHSIM_Rhsim10G0194700 | 4.65 | 0.031 | 0.054 | 2.110 |
| RHSIM_RhsimUnG0118800 | 4.65 | 0.031 | 0.054 | 0.282 |
| RHSIM_Rhsim11G0115600 | 4.65 | 0.031 | 0.054 | -2.900 |
| RHSIM_Rhsim08G0028800 | 4.65 | 0.031 | 0.054 | 0.818 |
| RHSIM_Rhsim06G0240600 | 4.64 | 0.031 | 0.054 | -0.763 |
| RHSIM_Rhsim04G0227900 | 4.64 | 0.031 | 0.054 | -0.792 |
| RHSIM_Rhsim07G0222300 | 4.64 | 0.031 | 0.054 | 41.344 |
| novel.38 | 4.64 | 0.031 | 0.054 | 2.210 |
| RHSIM_RhsimUnG0170000 | 4.64 | 0.031 | 0.054 | -0.968 |
| RHSIM_Rhsim03G0118000 | 4.64 | 0.031 | 0.054 | -0.667 |
| RHSIM_Rhsim03G0228900 | 4.64 | 0.031 | 0.054 | 0.399 |
| RHSIM_Rhsim03G0195500 | 4.64 | 0.031 | 0.054 | 2.132 |
| RHSIM_RhsimUnG0012300 | 4.64 | 0.031 | 0.054 | 0.866 |
| RHSIM_Rhsim02G0086600 | 4.64 | 0.031 | 0.054 | -2.604 |
| RHSIM_Rhsim08G0045200 | 4.64 | 0.031 | 0.055 | -0.188 |
| RHSIM_Rhsim09G0171300 | 4.64 | 0.031 | 0.055 | 0.255 |
| RHSIM_Rhsim09G0072600 | 4.64 | 0.031 | 0.055 | 0.289 |
| RHSIM_Rhsim06G0010000 | 4.64 | 0.031 | 0.055 | 0.448 |

| Gene | LRT statistic | P value | BH-adjusted P | log2FC (interaction) |
|---|---|---|---|---|
| RHSIM_Rhsim12G0212500 | 4.64 | 0.031 | 0.055 | -0.170 |
| RHSIM_Rhsim09G0085100 | 4.63 | 0.031 | 0.055 | 0.870 |
| novel.1979 | 4.63 | 0.031 | 0.055 | -1.143 |
| RHSIM_Rhsim05G0148700 | 4.63 | 0.031 | 0.055 | 0.164 |
| RHSIM_Rhsim07G0168600 | 4.63 | 0.031 | 0.055 | -3.223 |
| RHSIM_Rhsim08G0089300 | 4.63 | 0.031 | 0.055 | 0.224 |
| novel.2294 | 4.63 | 0.031 | 0.055 | -0.359 |
| RHSIM_RhsimUnG0125100 | 4.63 | 0.031 | 0.055 | -1.998 |
| RHSIM_Rhsim01G0170800 | 4.63 | 0.031 | 0.055 | -0.298 |
| novel.2358 | 4.62 | 0.032 | 0.055 | -0.625 |
| RHSIM_Rhsim01G0000200 | 4.62 | 0.032 | 0.055 | 0.375 |
| RHSIM_Rhsim08G0009800 | 4.62 | 0.032 | 0.055 | 0.430 |
| RHSIM_RhsimUnG0230200 | 4.62 | 0.032 | 0.055 | -40.371 |
| RHSIM_Rhsim03G0040100 | 4.62 | 0.032 | 0.055 | 0.205 |
| RHSIM_Rhsim11G0118800 | 4.62 | 0.032 | 0.055 | -0.402 |
| RHSIM_Rhsim05G0008000 | 4.62 | 0.032 | 0.055 | -4.700 |
| novel.2797 | 4.62 | 0.032 | 0.055 | -42.640 |
| RHSIM_Rhsim06G0177400 | 4.61 | 0.032 | 0.055 | -0.409 |
| novel.815 | 4.61 | 0.032 | 0.055 | -1.996 |
| RHSIM_Rhsim13G0109700 | 4.61 | 0.032 | 0.055 | -40.492 |
| RHSIM_Rhsim03G0196800 | 4.61 | 0.032 | 0.055 | 1.164 |
| RHSIM_RhsimUnG0195900 | 4.61 | 0.032 | 0.055 | 0.687 |
| RHSIM_Rhsim04G0153000 | 4.61 | 0.032 | 0.055 | 1.744 |
| RHSIM_Rhsim08G0006900 | 4.61 | 0.032 | 0.055 | -1.549 |
| RHSIM_Rhsim02G0039500 | 4.61 | 0.032 | 0.055 | -1.107 |
| RHSIM_Rhsim02G0011000 | 4.60 | 0.032 | 0.055 | 0.745 |
| RHSIM_Rhsim08G0083800 | 4.60 | 0.032 | 0.055 | 41.327 |
| RHSIM_Rhsim09G0169900 | 4.60 | 0.032 | 0.056 | -0.456 |
| RHSIM_Rhsim06G0090300 | 4.60 | 0.032 | 0.056 | -0.229 |
| RHSIM_Rhsim07G0041400 | 4.60 | 0.032 | 0.056 | -2.861 |
| RHSIM_Rhsim07G0021500 | 4.60 | 0.032 | 0.056 | 1.134 |
| RHSIM_Rhsim03G0207000 | 4.60 | 0.032 | 0.056 | -3.206 |

| Gene | LRT statistic | P value | BH-adjusted P | log2FC (interaction) |
|---|---|---|---|---|
| RHSIM_Rhsim06G0241300 | 4.60 | 0.032 | 0.056 | -1.297 |
| novel.1308 | 4.60 | 0.032 | 0.056 | -2.023 |
| novel.3116 | 4.60 | 0.032 | 0.056 | 2.796 |
| RHSIM_Rhsim05G0017200 | 4.60 | 0.032 | 0.056 | 0.276 |
| RHSIM_Rhsim06G0092400 | 4.60 | 0.032 | 0.056 | 0.793 |
| RHSIM_Rhsim12G0078900 | 4.60 | 0.032 | 0.056 | -0.274 |
| novel.1828 | 4.59 | 0.032 | 0.056 | 1.634 |
| novel.4575 | 4.59 | 0.032 | 0.056 | 5.350 |
| RHSIM_Rhsim04G0180000 | 4.59 | 0.032 | 0.056 | -39.115 |
| RHSIM_Rhsim03G0032000 | 4.59 | 0.032 | 0.056 | 0.311 |
| RHSIM_Rhsim05G0039000 | 4.59 | 0.032 | 0.056 | 0.550 |
| RHSIM_Rhsim02G0090400 | 4.59 | 0.032 | 0.056 | 3.865 |
| novel.227 | 4.59 | 0.032 | 0.056 | -0.329 |
| RHSIM_Rhsim02G0216900 | 4.59 | 0.032 | 0.056 | 1.606 |
| RHSIM_Rhsim07G0168300 | 4.59 | 0.032 | 0.056 | 0.240 |
| RHSIM_Rhsim05G0019100 | 4.58 | 0.032 | 0.056 | -0.392 |
| RHSIM_Rhsim02G0067700 | 4.58 | 0.032 | 0.056 | -3.578 |
| RHSIM_RhsimUnG0241900 | 4.58 | 0.032 | 0.056 | 0.600 |
| RHSIM_Rhsim01G0162200 | 4.58 | 0.032 | 0.056 | 0.321 |
| RHSIM_Rhsim01G0215800 | 4.58 | 0.032 | 0.056 | -76.976 |
| novel.1698 | 4.58 | 0.032 | 0.056 | 0.761 |
| RHSIM_RhsimUnG0208200 | 4.58 | 0.032 | 0.056 | 2.198 |
| novel.725 | 4.58 | 0.032 | 0.056 | -1.662 |
| novel.2556 | 4.58 | 0.032 | 0.056 | 0.816 |
| RHSIM_Rhsim05G0167000 | 4.58 | 0.032 | 0.056 | 0.758 |
| novel.2742 | 4.57 | 0.032 | 0.056 | 2.767 |
| novel.2670 | 4.57 | 0.033 | 0.056 | -2.664 |
| RHSIM_Rhsim07G0030600 | 4.57 | 0.033 | 0.056 | -2.151 |
| RHSIM_Rhsim09G0019900 | 4.57 | 0.033 | 0.056 | 1.233 |
| RHSIM_Rhsim07G0014600 | 4.57 | 0.033 | 0.057 | 0.345 |
| RHSIM_Rhsim09G0088500 | 4.57 | 0.033 | 0.057 | -0.242 |
| novel.3602 | 4.56 | 0.033 | 0.057 | -1.812 |

| Gene | LRT statistic | P value | BH-adjusted P | log2FC (interaction) |
|---|---|---|---|---|
| RHSIM_Rhsim12G0017100 | 4.56 | 0.033 | 0.057 | -0.115 |
| novel.3593 | 4.56 | 0.033 | 0.057 | 1.521 |
| novel.2355 | 4.56 | 0.033 | 0.057 | 0.310 |
| RHSIM_Rhsim13G0205000 | 4.56 | 0.033 | 0.057 | -0.265 |
| RHSIM_Rhsim06G0068500 | 4.56 | 0.033 | 0.057 | -0.292 |
| RHSIM_RhsimUnG0232000 | 4.56 | 0.033 | 0.057 | 1.061 |
| novel.1788 | 4.55 | 0.033 | 0.057 | 79.202 |
| RHSIM_Rhsim12G0181300 | 4.55 | 0.033 | 0.057 | -0.829 |
| RHSIM_Rhsim08G0056600 | 4.55 | 0.033 | 0.057 | 0.256 |
| novel.3598 | 4.55 | 0.033 | 0.057 | 4.401 |
| RHSIM_Rhsim05G0004100 | 4.55 | 0.033 | 0.057 | -0.319 |
| RHSIM_Rhsim03G0006900 | 4.55 | 0.033 | 0.057 | -77.230 |
| novel.1111 | 4.55 | 0.033 | 0.057 | 2.225 |
| RHSIM_Rhsim05G0090000 | 4.55 | 0.033 | 0.057 | 1.233 |
| RHSIM_RhsimUnG0026800 | 4.55 | 0.033 | 0.057 | 1.938 |
| novel.2464 | 4.55 | 0.033 | 0.057 | 1.550 |
| RHSIM_Rhsim03G0089300 | 4.55 | 0.033 | 0.057 | 0.451 |
| RHSIM_Rhsim12G0087700 | 4.54 | 0.033 | 0.057 | 2.199 |
| RHSIM_Rhsim05G0115600 | 4.54 | 0.033 | 0.057 | 0.256 |
| novel.4234 | 4.54 | 0.033 | 0.057 | -4.699 |
| RHSIM_Rhsim08G0159400 | 4.54 | 0.033 | 0.057 | 40.274 |
| novel.1172 | 4.54 | 0.033 | 0.057 | -0.366 |
| RHSIM_Rhsim11G0083600 | 4.54 | 0.033 | 0.057 | 1.556 |
| RHSIM_Rhsim12G0076100 | 4.54 | 0.033 | 0.057 | -0.275 |
| RHSIM_Rhsim06G0162700 | 4.54 | 0.033 | 0.057 | -0.301 |
| novel.4152 | 4.54 | 0.033 | 0.057 | 0.773 |
| RHSIM_Rhsim03G0053400 | 4.54 | 0.033 | 0.057 | -2.683 |
| RHSIM_Rhsim06G0125300 | 4.54 | 0.033 | 0.057 | 0.383 |
| RHSIM_Rhsim13G0053900 | 4.53 | 0.033 | 0.058 | 40.467 |
| novel.1567 | 4.53 | 0.033 | 0.058 | 4.704 |
| RHSIM_Rhsim03G0234300 | 4.53 | 0.033 | 0.058 | 0.267 |
| RHSIM_Rhsim02G0196500 | 4.53 | 0.033 | 0.058 | -76.823 |

| Gene | LRT statistic | P value | BH-adjusted P | log2FC (interaction) |
|---|---|---|---|---|
| RHSIM_RhsimUnG0246100 | 4.53 | 0.033 | 0.058 | 0.464 |
| RHSIM_Rhsim02G0099700 | 4.52 | 0.033 | 0.058 | -0.451 |
| RHSIM_Rhsim03G0268800 | 4.52 | 0.033 | 0.058 | 0.525 |
| RHSIM_Rhsim02G0146800 | 4.52 | 0.034 | 0.058 | -0.317 |
| novel.3988 | 4.52 | 0.034 | 0.058 | 0.789 |
| novel.2474 | 4.51 | 0.034 | 0.058 | 2.723 |
| RHSIM_Rhsim12G0215300 | 4.51 | 0.034 | 0.058 | -0.355 |
| novel.774 | 4.51 | 0.034 | 0.058 | 2.497 |
| RHSIM_Rhsim12G0109000 | 4.51 | 0.034 | 0.058 | -0.544 |
| novel.3448 | 4.50 | 0.034 | 0.058 | -1.752 |
| RHSIM_Rhsim09G0129400 | 4.50 | 0.034 | 0.059 | 0.449 |
| RHSIM_Rhsim11G0162700 | 4.50 | 0.034 | 0.059 | -0.676 |
| RHSIM_Rhsim08G0204400 | 4.50 | 0.034 | 0.059 | -0.279 |
| RHSIM_Rhsim07G0239200 | 4.50 | 0.034 | 0.059 | -40.059 |
| RHSIM_Rhsim01G0148400 | 4.50 | 0.034 | 0.059 | 0.683 |
| novel.1541 | 4.50 | 0.034 | 0.059 | -1.077 |
| RHSIM_Rhsim02G0178300 | 4.50 | 0.034 | 0.059 | 0.334 |
| RHSIM_Rhsim12G0055400 | 4.49 | 0.034 | 0.059 | 40.216 |
| RHSIM_Rhsim04G0099900 | 4.49 | 0.034 | 0.059 | -0.380 |
| RHSIM_Rhsim12G0008200 | 4.49 | 0.034 | 0.059 | -0.725 |
| RHSIM_Rhsim08G0226100 | 4.49 | 0.034 | 0.059 | 0.946 |
| RHSIM_Rhsim07G0038200 | 4.49 | 0.034 | 0.059 | -1.724 |
| RHSIM_Rhsim01G0106800 | 4.49 | 0.034 | 0.059 | -41.459 |
| RHSIM_Rhsim11G0022800 | 4.49 | 0.034 | 0.059 | 2.040 |
| RHSIM_Rhsim07G0049100 | 4.49 | 0.034 | 0.059 | 1.118 |
| RHSIM_Rhsim04G0175400 | 4.49 | 0.034 | 0.059 | -0.241 |
| RHSIM_Rhsim05G0082000 | 4.49 | 0.034 | 0.059 | 3.014 |
| RHSIM_Rhsim13G0118000 | 4.49 | 0.034 | 0.059 | -0.380 |
| RHSIM_Rhsim09G0055900 | 4.49 | 0.034 | 0.059 | -1.085 |
| RHSIM_Rhsim06G0016600 | 4.49 | 0.034 | 0.059 | -1.890 |
| RHSIM_Rhsim09G0099400 | 4.49 | 0.034 | 0.059 | 0.135 |
| RHSIM_Rhsim01G0044500 | 4.49 | 0.034 | 0.059 | 0.395 |

| Gene | LRT statistic | P value | BH-adjusted P | log2FC (interaction) |
|---|---|---|---|---|
| RHSIM_Rhsim09G0197900 | 4.49 | 0.034 | 0.059 | -0.430 |
| RHSIM_Rhsim09G0074800 | 4.49 | 0.034 | 0.059 | 0.244 |
| RHSIM_Rhsim07G0248200 | 4.48 | 0.034 | 0.059 | 1.381 |
| novel.2496 | 4.48 | 0.034 | 0.059 | 0.380 |
| novel.914 | 4.48 | 0.034 | 0.059 | 1.626 |
| RHSIM_Rhsim03G0153300 | 4.48 | 0.034 | 0.059 | -0.303 |
| RHSIM_Rhsim07G0034400 | 4.48 | 0.034 | 0.059 | -0.261 |
| RHSIM_Rhsim12G0033500 | 4.48 | 0.034 | 0.059 | -1.934 |
| novel.2343 | 4.48 | 0.034 | 0.059 | 0.587 |
| RHSIM_Rhsim06G0175400 | 4.48 | 0.034 | 0.059 | -40.892 |
| RHSIM_Rhsim07G0094700 | 4.48 | 0.034 | 0.059 | -39.253 |
| RHSIM_Rhsim06G0009100 | 4.48 | 0.034 | 0.059 | -0.465 |
| RHSIM_Rhsim11G0119200 | 4.47 | 0.034 | 0.059 | 0.526 |
| novel.3733 | 4.47 | 0.034 | 0.059 | 1.098 |
| novel.2465 | 4.47 | 0.034 | 0.059 | 0.573 |
| RHSIM_Rhsim07G0042800 | 4.47 | 0.034 | 0.059 | -1.507 |
| RHSIM_Rhsim04G0111300 | 4.47 | 0.035 | 0.060 | -1.913 |
| RHSIM_Rhsim08G0235700 | 4.47 | 0.035 | 0.060 | -0.250 |
| RHSIM_Rhsim10G0147100 | 4.47 | 0.035 | 0.060 | 0.541 |
| novel.3471 | 4.46 | 0.035 | 0.060 | -0.130 |
| RHSIM_Rhsim10G0157300 | 4.46 | 0.035 | 0.060 | -0.741 |
| RHSIM_Rhsim13G0073100 | 4.46 | 0.035 | 0.060 | -0.484 |
| RHSIM_Rhsim05G0118900 | 4.46 | 0.035 | 0.060 | 1.065 |
| RHSIM_Rhsim13G0159900 | 4.46 | 0.035 | 0.060 | -1.073 |
| RHSIM_Rhsim07G0243500 | 4.46 | 0.035 | 0.060 | 3.110 |
| novel.3261 | 4.46 | 0.035 | 0.060 | -40.121 |
| RHSIM_Rhsim09G0002700 | 4.46 | 0.035 | 0.060 | -0.888 |
| novel.182 | 4.46 | 0.035 | 0.060 | 2.745 |
| RHSIM_Rhsim11G0029100 | 4.46 | 0.035 | 0.060 | 0.262 |
| novel.719 | 4.45 | 0.035 | 0.060 | -3.642 |
| RHSIM_Rhsim10G0116700 | 4.45 | 0.035 | 0.060 | 2.049 |
| RHSIM_Rhsim08G0035000 | 4.45 | 0.035 | 0.060 | 0.336 |

| Gene | LRT statistic | P value | BH-adjusted P | log2FC (interaction) |
|---|---|---|---|---|
| RHSIM_Rhsim13G0092600 | 4.45 | 0.035 | 0.060 | 2.308 |
| RHSIM_Rhsim13G0017900 | 4.45 | 0.035 | 0.060 | 0.816 |
| novel.3954 | 4.45 | 0.035 | 0.060 | -1.951 |
| RHSIM_Rhsim05G0013100 | 4.45 | 0.035 | 0.060 | -0.218 |
| RHSIM_RhsimUnG0204000 | 4.45 | 0.035 | 0.060 | -2.174 |
| novel.2859 | 4.45 | 0.035 | 0.060 | 0.399 |
| RHSIM_Rhsim02G0165100 | 4.45 | 0.035 | 0.060 | 0.989 |
| novel.3578 | 4.45 | 0.035 | 0.060 | -0.290 |
| RHSIM_Rhsim13G0195300 | 4.45 | 0.035 | 0.060 | -0.231 |
| RHSIM_Rhsim05G0107300 | 4.45 | 0.035 | 0.060 | 5.236 |
| RHSIM_Rhsim03G0184400 | 4.44 | 0.035 | 0.060 | -2.080 |
| RHSIM_Rhsim01G0190200 | 4.44 | 0.035 | 0.060 | -0.170 |
| RHSIM_Rhsim09G0092600 | 4.44 | 0.035 | 0.060 | 1.555 |
| RHSIM_Rhsim10G0028500 | 4.44 | 0.035 | 0.060 | -0.477 |
| RHSIM_Rhsim03G0087100 | 4.44 | 0.035 | 0.060 | 0.211 |
| RHSIM_Rhsim05G0015000 | 4.44 | 0.035 | 0.060 | 0.124 |
| RHSIM_Rhsim04G0200100 | 4.44 | 0.035 | 0.061 | -1.240 |
| RHSIM_Rhsim03G0133600 | 4.44 | 0.035 | 0.061 | -2.678 |
| RHSIM_Rhsim11G0160900 | 4.44 | 0.035 | 0.061 | 1.435 |
| RHSIM_Rhsim07G0066400 | 4.44 | 0.035 | 0.061 | 0.773 |
| novel.4405 | 4.44 | 0.035 | 0.061 | 40.579 |
| RHSIM_RhsimUnG0108100 | 4.43 | 0.035 | 0.061 | 1.256 |
| novel.2046 | 4.43 | 0.035 | 0.061 | 0.246 |
| novel.1002 | 4.43 | 0.035 | 0.061 | -2.594 |
| RHSIM_Rhsim06G0110400 | 4.43 | 0.035 | 0.061 | -0.759 |
| RHSIM_Rhsim02G0171600 | 4.43 | 0.035 | 0.061 | -0.141 |
| RHSIM_Rhsim06G0068900 | 4.43 | 0.035 | 0.061 | 4.335 |
| novel.3216 | 4.43 | 0.035 | 0.061 | -1.449 |
| RHSIM_Rhsim08G0080900 | 4.42 | 0.035 | 0.061 | 0.237 |
| RHSIM_Rhsim07G0141800 | 4.42 | 0.035 | 0.061 | -0.424 |
| RHSIM_Rhsim11G0071100 | 4.42 | 0.035 | 0.061 | 2.771 |
| RHSIM_RhsimUnG0243400 | 4.42 | 0.035 | 0.061 | 1.448 |

| Gene | LRT statistic | P value | BH-adjusted P | log2FC (interaction) |
|---|---|---|---|---|
| RHSIM_Rhsim09G0208700 | 4.42 | 0.035 | 0.061 | -0.987 |
| RHSIM_Rhsim06G0166600 | 4.42 | 0.036 | 0.061 | 0.701 |
| RHSIM_Rhsim01G0188300 | 4.42 | 0.036 | 0.061 | 0.307 |
| RHSIM_Rhsim13G0019400 | 4.42 | 0.036 | 0.061 | -0.452 |
| novel.2835 | 4.42 | 0.036 | 0.061 | 1.744 |
| RHSIM_Rhsim11G0036100 | 4.42 | 0.036 | 0.061 | 0.731 |
| RHSIM_Rhsim06G0187400 | 4.42 | 0.036 | 0.061 | 0.551 |
| RHSIM_Rhsim07G0117000 | 4.42 | 0.036 | 0.061 | 2.676 |
| novel.4046 | 4.41 | 0.036 | 0.061 | 2.852 |
| RHSIM_RhsimUnG0014300 | 4.41 | 0.036 | 0.061 | 1.369 |
| RHSIM_Rhsim08G0217100 | 4.41 | 0.036 | 0.061 | 1.407 |
| RHSIM_Rhsim13G0010100 | 4.41 | 0.036 | 0.061 | -0.229 |
| novel.1600 | 4.41 | 0.036 | 0.061 | -1.625 |
| novel.788 | 4.41 | 0.036 | 0.061 | -2.494 |
| RHSIM_Rhsim10G0125700 | 4.41 | 0.036 | 0.062 | 4.757 |
| RHSIM_Rhsim04G0109800 | 4.41 | 0.036 | 0.062 | 40.090 |
| novel.1422 | 4.41 | 0.036 | 0.062 | 0.437 |
| RHSIM_Rhsim01G0142800 | 4.40 | 0.036 | 0.062 | -40.340 |
| RHSIM_Rhsim11G0063300 | 4.40 | 0.036 | 0.062 | -0.477 |
| RHSIM_Rhsim09G0175300 | 4.40 | 0.036 | 0.062 | -1.059 |
| RHSIM_Rhsim01G0056900 | 4.40 | 0.036 | 0.062 | -1.766 |
| RHSIM_RhsimUnG0103800 | 4.40 | 0.036 | 0.062 | 3.029 |
| RHSIM_RhsimUnG0150500 | 4.40 | 0.036 | 0.062 | 0.757 |
| RHSIM_Rhsim10G0024800 | 4.39 | 0.036 | 0.062 | 1.849 |
| novel.3441 | 4.39 | 0.036 | 0.062 | 0.978 |
| RHSIM_Rhsim06G0224300 | 4.39 | 0.036 | 0.062 | -0.273 |
| novel.863 | 4.39 | 0.036 | 0.062 | 1.394 |
| RHSIM_Rhsim05G0095700 | 4.39 | 0.036 | 0.062 | 2.807 |
| RHSIM_Rhsim03G0053900 | 4.38 | 0.036 | 0.062 | -1.088 |
| RHSIM_Rhsim03G0045200 | 4.38 | 0.036 | 0.062 | 0.814 |
| RHSIM_Rhsim01G0058100 | 4.38 | 0.036 | 0.063 | -1.664 |
| RHSIM_Rhsim05G0197100 | 4.38 | 0.036 | 0.063 | 2.885 |

| Gene | LRT statistic | P value | BH-adjusted P | log2FC (interaction) |
|---|---|---|---|---|
| RHSIM_Rhsim10G0037000 | 4.38 | 0.036 | 0.063 | 0.456 |
| RHSIM_Rhsim05G0005400 | 4.37 | 0.036 | 0.063 | 0.229 |
| RHSIM_RhsimUnG0072300 | 4.37 | 0.036 | 0.063 | -39.406 |
| RHSIM_Rhsim11G0187300 | 4.37 | 0.037 | 0.063 | 2.200 |
| RHSIM_RhsimUnG0256200 | 4.37 | 0.037 | 0.063 | 0.414 |
| RHSIM_Rhsim11G0134500 | 4.37 | 0.037 | 0.063 | -0.571 |
| novel.1359 | 4.37 | 0.037 | 0.063 | -1.280 |
| novel.717 | 4.37 | 0.037 | 0.063 | -0.716 |
| novel.916 | 4.37 | 0.037 | 0.063 | 1.946 |
| RHSIM_Rhsim12G0165900 | 4.37 | 0.037 | 0.063 | -0.291 |
| RHSIM_Rhsim09G0098800 | 4.36 | 0.037 | 0.063 | -2.445 |
| RHSIM_Rhsim07G0258700 | 4.36 | 0.037 | 0.063 | 0.312 |
| novel.4358 | 4.36 | 0.037 | 0.063 | 1.983 |
| RHSIM_Rhsim03G0215100 | 4.36 | 0.037 | 0.063 | 1.693 |
| RHSIM_Rhsim13G0125600 | 4.36 | 0.037 | 0.063 | -1.054 |
| RHSIM_Rhsim05G0076700 | 4.36 | 0.037 | 0.063 | 2.446 |
| RHSIM_Rhsim06G0220200 | 4.36 | 0.037 | 0.063 | 0.157 |
| RHSIM_Rhsim09G0116700 | 4.36 | 0.037 | 0.063 | -0.520 |
| RHSIM_Rhsim09G0198900 | 4.36 | 0.037 | 0.063 | -0.299 |
| RHSIM_RhsimUnG0231200 | 4.35 | 0.037 | 0.063 | 1.854 |
| RHSIM_Rhsim11G0095900 | 4.35 | 0.037 | 0.063 | 1.721 |
| RHSIM_Rhsim03G0061800 | 4.35 | 0.037 | 0.063 | 0.158 |
| RHSIM_Rhsim09G0108000 | 4.35 | 0.037 | 0.063 | -0.379 |
| RHSIM_Rhsim04G0110300 | 4.35 | 0.037 | 0.063 | 1.117 |
| novel.2239 | 4.35 | 0.037 | 0.063 | -0.484 |
| RHSIM_Rhsim05G0134400 | 4.35 | 0.037 | 0.063 | -0.238 |
| novel.4053 | 4.35 | 0.037 | 0.063 | -0.960 |
| RHSIM_Rhsim11G0193600 | 4.35 | 0.037 | 0.063 | 0.178 |
| RHSIM_Rhsim10G0163700 | 4.35 | 0.037 | 0.063 | -4.188 |
| RHSIM_Rhsim12G0051700 | 4.35 | 0.037 | 0.064 | 40.555 |
| RHSIM_Rhsim01G0214200 | 4.35 | 0.037 | 0.064 | 1.149 |
| novel.3737 | 4.35 | 0.037 | 0.064 | 1.041 |

| Gene | LRT statistic | P value | BH-adjusted P | log2FC (interaction) |
|---|---|---|---|---|
| RHSIM_Rhsim03G0205900 | 4.35 | 0.037 | 0.064 | 2.050 |
| RHSIM_Rhsim02G0057000 | 4.34 | 0.037 | 0.064 | 1.078 |
| RHSIM_Rhsim03G0269000 | 4.34 | 0.037 | 0.064 | 0.915 |
| novel.2201 | 4.34 | 0.037 | 0.064 | 1.332 |
| RHSIM_Rhsim08G0158400 | 4.34 | 0.037 | 0.064 | 2.242 |
| RHSIM_Rhsim01G0163900 | 4.34 | 0.037 | 0.064 | 41.144 |
| RHSIM_Rhsim06G0148200 | 4.34 | 0.037 | 0.064 | 3.031 |
| RHSIM_RhsimUnG0251000 | 4.34 | 0.037 | 0.064 | 0.251 |
| RHSIM_Rhsim02G0088900 | 4.34 | 0.037 | 0.064 | -1.699 |
| RHSIM_Rhsim03G0009000 | 4.34 | 0.037 | 0.064 | 2.506 |
| RHSIM_Rhsim08G0009000 | 4.33 | 0.037 | 0.064 | -1.393 |
| RHSIM_Rhsim13G0168200 | 4.33 | 0.037 | 0.064 | 2.572 |
| novel.612 | 4.33 | 0.037 | 0.064 | -38.868 |
| RHSIM_Rhsim03G0137100 | 4.33 | 0.037 | 0.064 | -0.283 |
| novel.2725 | 4.33 | 0.037 | 0.064 | 1.515 |
| RHSIM_Rhsim12G0043600 | 4.33 | 0.037 | 0.064 | -0.202 |
| RHSIM_Rhsim03G0160600 | 4.33 | 0.037 | 0.064 | -0.275 |
| RHSIM_Rhsim13G0181300 | 4.33 | 0.037 | 0.064 | -0.452 |
| RHSIM_Rhsim07G0104900 | 4.33 | 0.037 | 0.064 | -0.257 |
| RHSIM_Rhsim06G0058300 | 4.33 | 0.037 | 0.064 | 0.350 |
| novel.1302 | 4.33 | 0.038 | 0.064 | -0.483 |
| RHSIM_Rhsim02G0016700 | 4.32 | 0.038 | 0.064 | 3.484 |
| RHSIM_Rhsim09G0115800 | 4.32 | 0.038 | 0.064 | -0.792 |
| RHSIM_Rhsim13G0003100 | 4.32 | 0.038 | 0.064 | -0.221 |
| RHSIM_Rhsim01G0145000 | 4.32 | 0.038 | 0.065 | -0.511 |
| novel.2820 | 4.32 | 0.038 | 0.065 | -1.993 |
| RHSIM_Rhsim07G0088600 | 4.32 | 0.038 | 0.065 | 1.401 |
| RHSIM_Rhsim01G0087000 | 4.32 | 0.038 | 0.065 | 1.389 |
| RHSIM_RhsimUnG0125600 | 4.32 | 0.038 | 0.065 | 0.312 |
| RHSIM_Rhsim06G0244400 | 4.31 | 0.038 | 0.065 | 0.324 |
| RHSIM_Rhsim08G0212900 | 4.31 | 0.038 | 0.065 | -0.535 |
| RHSIM_Rhsim07G0220200 | 4.31 | 0.038 | 0.065 | -0.251 |

| Gene | LRT statistic | P value | BH-adjusted P | log2FC (interaction) |
|---|---|---|---|---|
| novel.1465 | 4.31 | 0.038 | 0.065 | -2.335 |
| RHSIM_Rhsim10G0082600 | 4.31 | 0.038 | 0.065 | 0.799 |
| RHSIM_Rhsim13G0083900 | 4.31 | 0.038 | 0.065 | 0.446 |
| RHSIM_Rhsim05G0131800 | 4.31 | 0.038 | 0.065 | 40.463 |
| RHSIM_Rhsim02G0206700 | 4.31 | 0.038 | 0.065 | 2.977 |
| RHSIM_Rhsim10G0068500 | 4.31 | 0.038 | 0.065 | -0.458 |
| RHSIM_Rhsim04G0057200 | 4.31 | 0.038 | 0.065 | 1.025 |
| RHSIM_Rhsim03G0264600 | 4.31 | 0.038 | 0.065 | -0.310 |
| RHSIM_Rhsim10G0146700 | 4.31 | 0.038 | 0.065 | 0.342 |
| RHSIM_RhsimUnG0093000 | 4.30 | 0.038 | 0.065 | 0.241 |
| RHSIM_Rhsim01G0017000 | 4.30 | 0.038 | 0.065 | 39.043 |
| novel.165 | 4.30 | 0.038 | 0.065 | 3.359 |
| RHSIM_Rhsim07G0156000 | 4.30 | 0.038 | 0.065 | 0.994 |
| RHSIM_Rhsim11G0132700 | 4.30 | 0.038 | 0.065 | 1.144 |
| novel.2780 | 4.30 | 0.038 | 0.065 | 0.906 |
| RHSIM_RhsimUnG0018100 | 4.30 | 0.038 | 0.065 | -0.854 |
| RHSIM_Rhsim04G0125100 | 4.30 | 0.038 | 0.065 | 0.719 |
| RHSIM_Rhsim11G0175400 | 4.30 | 0.038 | 0.065 | -4.378 |
| RHSIM_Rhsim04G0240300 | 4.30 | 0.038 | 0.065 | -0.511 |
| RHSIM_Rhsim01G0276800 | 4.30 | 0.038 | 0.065 | 0.154 |
| novel.3520 | 4.30 | 0.038 | 0.065 | 1.120 |
| RHSIM_Rhsim13G0066700 | 4.30 | 0.038 | 0.065 | -0.217 |
| novel.2422 | 4.29 | 0.038 | 0.065 | 0.525 |
| RHSIM_Rhsim02G0094600 | 4.29 | 0.038 | 0.065 | -2.863 |
| RHSIM_Rhsim08G0034900 | 4.29 | 0.038 | 0.066 | -0.507 |
| RHSIM_Rhsim11G0026800 | 4.29 | 0.038 | 0.066 | -0.548 |
| novel.1102 | 4.29 | 0.038 | 0.066 | 0.439 |
| RHSIM_Rhsim02G0069200 | 4.29 | 0.038 | 0.066 | -3.354 |
| RHSIM_Rhsim06G0037800 | 4.29 | 0.038 | 0.066 | 0.158 |
| RHSIM_Rhsim13G0049300 | 4.28 | 0.038 | 0.066 | -39.945 |
| novel.1067 | 4.28 | 0.039 | 0.066 | -1.393 |
| RHSIM_Rhsim07G0204100 | 4.28 | 0.039 | 0.066 | 1.226 |

| Gene | LRT statistic | P value | BH-adjusted P | log2FC (interaction) |
|---|---|---|---|---|
| RHSIM_Rhsim08G0218200 | 4.28 | 0.039 | 0.066 | -1.317 |
| RHSIM_Rhsim02G0007200 | 4.28 | 0.039 | 0.066 | -1.478 |
| RHSIM_Rhsim04G0117500 | 4.28 | 0.039 | 0.066 | -0.589 |
| RHSIM_Rhsim07G0243700 | 4.28 | 0.039 | 0.066 | -0.162 |
| RHSIM_Rhsim02G0175600 | 4.28 | 0.039 | 0.066 | 2.427 |
| RHSIM_Rhsim12G0182200 | 4.27 | 0.039 | 0.066 | 0.325 |
| novel.2621 | 4.27 | 0.039 | 0.066 | 0.551 |
| RHSIM_Rhsim10G0197600 | 4.27 | 0.039 | 0.066 | 40.244 |
| novel.890 | 4.27 | 0.039 | 0.066 | 1.435 |
| RHSIM_Rhsim08G0212600 | 4.27 | 0.039 | 0.066 | -0.099 |
| RHSIM_Rhsim03G0061300 | 4.27 | 0.039 | 0.066 | -0.894 |
| RHSIM_Rhsim07G0023000 | 4.27 | 0.039 | 0.066 | -0.380 |
| novel.7 | 4.27 | 0.039 | 0.066 | 1.852 |
| RHSIM_Rhsim06G0176100 | 4.26 | 0.039 | 0.066 | -0.271 |
| RHSIM_Rhsim04G0143500 | 4.26 | 0.039 | 0.066 | -3.385 |
| novel.1056 | 4.26 | 0.039 | 0.066 | -0.365 |
| RHSIM_Rhsim11G0198500 | 4.26 | 0.039 | 0.066 | 0.793 |
| RHSIM_Rhsim10G0151200 | 4.26 | 0.039 | 0.067 | 0.243 |
| RHSIM_Rhsim05G0167400 | 4.26 | 0.039 | 0.067 | 1.466 |
| RHSIM_Rhsim13G0029400 | 4.26 | 0.039 | 0.067 | 0.152 |
| RHSIM_Rhsim12G0038500 | 4.26 | 0.039 | 0.067 | -41.087 |
| RHSIM_Rhsim01G0056600 | 4.26 | 0.039 | 0.067 | -3.452 |
| RHSIM_RhsimUnG0108000 | 4.26 | 0.039 | 0.067 | 3.330 |
| novel.437 | 4.26 | 0.039 | 0.067 | 1.195 |
| RHSIM_Rhsim05G0232600 | 4.26 | 0.039 | 0.067 | 0.298 |
| RHSIM_Rhsim07G0041200 | 4.26 | 0.039 | 0.067 | -1.960 |
| RHSIM_Rhsim07G0166900 | 4.25 | 0.039 | 0.067 | -0.234 |
| RHSIM_Rhsim12G0190000 | 4.25 | 0.039 | 0.067 | -0.275 |
| RHSIM_Rhsim08G0044300 | 4.25 | 0.039 | 0.067 | -0.396 |
| RHSIM_Rhsim06G0065600 | 4.25 | 0.039 | 0.067 | -0.323 |
| RHSIM_Rhsim11G0068800 | 4.25 | 0.039 | 0.067 | 1.882 |
| RHSIM_Rhsim08G0166500 | 4.25 | 0.039 | 0.067 | -1.200 |

| Gene | LRT statistic | P value | BH-adjusted P | log2FC (interaction) |
|---|---|---|---|---|
| RHSIM_Rhsim03G0155700 | 4.25 | 0.039 | 0.067 | 1.583 |
| RHSIM_Rhsim07G0154700 | 4.24 | 0.039 | 0.067 | 1.240 |
| RHSIM_RhsimUnG0123100 | 4.24 | 0.039 | 0.067 | 1.592 |
| RHSIM_Rhsim05G0160900 | 4.24 | 0.039 | 0.067 | 0.317 |
| RHSIM_Rhsim02G0135700 | 4.24 | 0.039 | 0.067 | -0.947 |
| novel.672 | 4.24 | 0.039 | 0.067 | 1.655 |
| RHSIM_Rhsim11G0123500 | 4.24 | 0.039 | 0.067 | -39.960 |
| RHSIM_Rhsim07G0201800 | 4.24 | 0.039 | 0.067 | 0.246 |
| RHSIM_Rhsim10G0191700 | 4.24 | 0.039 | 0.067 | 0.555 |
| RHSIM_Rhsim11G0051800 | 4.24 | 0.039 | 0.067 | 1.026 |
| RHSIM_Rhsim12G0208300 | 4.24 | 0.040 | 0.067 | -0.764 |
| RHSIM_Rhsim08G0034100 | 4.24 | 0.040 | 0.067 | 1.478 |
| RHSIM_Rhsim09G0044700 | 4.23 | 0.040 | 0.067 | -0.382 |
| RHSIM_Rhsim02G0119200 | 4.23 | 0.040 | 0.067 | 0.332 |
| RHSIM_RhsimUnG0149200 | 4.23 | 0.040 | 0.068 | 0.422 |
| RHSIM_Rhsim03G0002900 | 4.23 | 0.040 | 0.068 | 3.406 |
| RHSIM_Rhsim07G0257400 | 4.23 | 0.040 | 0.068 | 0.144 |
| RHSIM_Rhsim04G0000900 | 4.23 | 0.040 | 0.068 | -4.283 |
| RHSIM_Rhsim06G0112400 | 4.23 | 0.040 | 0.068 | -1.591 |
| novel.591 | 4.23 | 0.040 | 0.068 | 0.749 |
| RHSIM_Rhsim01G0034200 | 4.23 | 0.040 | 0.068 | -0.114 |
| novel.942 | 4.23 | 0.040 | 0.068 | 1.306 |
| RHSIM_RhsimPtG0005300 | 4.23 | 0.040 | 0.068 | 0.458 |
| RHSIM_Rhsim04G0180900 | 4.22 | 0.040 | 0.068 | -0.180 |
| RHSIM_Rhsim12G0023700 | 4.22 | 0.040 | 0.068 | 39.406 |
| RHSIM_Rhsim12G0063400 | 4.22 | 0.040 | 0.068 | 3.403 |
| RHSIM_Rhsim04G0025400 | 4.22 | 0.040 | 0.068 | 1.186 |
| RHSIM_Rhsim03G0223200 | 4.22 | 0.040 | 0.068 | 0.184 |
| RHSIM_Rhsim01G0213900 | 4.22 | 0.040 | 0.068 | 1.467 |
| RHSIM_Rhsim10G0059900 | 4.22 | 0.040 | 0.068 | 0.631 |
| RHSIM_Rhsim11G0028600 | 4.22 | 0.040 | 0.068 | 0.797 |
| RHSIM_Rhsim01G0293700 | 4.22 | 0.040 | 0.068 | -0.315 |

| Gene | LRT statistic | P value | BH-adjusted P | log2FC (interaction) |
|---|---|---|---|---|
| novel.2108 | 4.22 | 0.040 | 0.068 | 39.074 |
| RHSIM_RhsimUnG0250400 | 4.22 | 0.040 | 0.068 | -5.054 |
| RHSIM_Rhsim11G0073200 | 4.22 | 0.040 | 0.068 | -39.170 |
| novel.2938 | 4.21 | 0.040 | 0.068 | -0.554 |
| RHSIM_Rhsim05G0137600 | 4.21 | 0.040 | 0.068 | 2.385 |
| RHSIM_Rhsim02G0202100 | 4.21 | 0.040 | 0.068 | 0.186 |
| novel.1883 | 4.21 | 0.040 | 0.068 | 1.177 |
| RHSIM_Rhsim04G0136900 | 4.21 | 0.040 | 0.068 | 0.788 |
| novel.3769 | 4.21 | 0.040 | 0.068 | 2.046 |
| RHSIM_Rhsim07G0150800 | 4.21 | 0.040 | 0.068 | 0.261 |
| RHSIM_Rhsim08G0034400 | 4.21 | 0.040 | 0.068 | -0.291 |
| RHSIM_Rhsim04G0194400 | 4.21 | 0.040 | 0.068 | 0.283 |
| novel.961 | 4.21 | 0.040 | 0.068 | -1.013 |
| RHSIM_Rhsim11G0133800 | 4.21 | 0.040 | 0.068 | 3.988 |
| RHSIM_Rhsim02G0202800 | 4.21 | 0.040 | 0.068 | -40.284 |
| RHSIM_Rhsim07G0241600 | 4.21 | 0.040 | 0.068 | 0.755 |
| novel.395 | 4.21 | 0.040 | 0.068 | 0.240 |
| RHSIM_Rhsim13G0074600 | 4.21 | 0.040 | 0.069 | -0.593 |
| RHSIM_Rhsim06G0060200 | 4.21 | 0.040 | 0.069 | 39.919 |
| novel.987 | 4.20 | 0.040 | 0.069 | -0.827 |
| RHSIM_Rhsim03G0103800 | 4.20 | 0.040 | 0.069 | 1.032 |
| RHSIM_Rhsim08G0085400 | 4.20 | 0.040 | 0.069 | -0.390 |
| RHSIM_Rhsim06G0126000 | 4.20 | 0.040 | 0.069 | 0.441 |
| RHSIM_Rhsim05G0147900 | 4.20 | 0.040 | 0.069 | -0.189 |
| RHSIM_Rhsim08G0226200 | 4.20 | 0.040 | 0.069 | 79.333 |
| RHSIM_Rhsim04G0091600 | 4.20 | 0.040 | 0.069 | 0.198 |
| RHSIM_Rhsim03G0187300 | 4.20 | 0.040 | 0.069 | 0.176 |
| RHSIM_Rhsim08G0116100 | 4.20 | 0.040 | 0.069 | -0.785 |
| RHSIM_Rhsim03G0081600 | 4.20 | 0.040 | 0.069 | -0.403 |
| RHSIM_Rhsim10G0077000 | 4.20 | 0.040 | 0.069 | 0.259 |
| RHSIM_Rhsim05G0107600 | 4.20 | 0.041 | 0.069 | 0.512 |
| novel.4408 | 4.20 | 0.041 | 0.069 | 2.710 |

| Gene | LRT statistic | P value | BH-adjusted P | log2FC (interaction) |
|---|---|---|---|---|
| RHSIM_Rhsim07G0067200 | 4.20 | 0.041 | 0.069 | 1.999 |
| RHSIM_Rhsim01G0102000 | 4.19 | 0.041 | 0.069 | -2.062 |
| RHSIM_Rhsim12G0102400 | 4.19 | 0.041 | 0.069 | 0.135 |
| novel.2198 | 4.19 | 0.041 | 0.069 | 0.452 |
| RHSIM_RhsimUnG0167000 | 4.19 | 0.041 | 0.069 | -0.488 |
| RHSIM_Rhsim03G0117200 | 4.19 | 0.041 | 0.069 | 0.301 |
| RHSIM_Rhsim11G0193700 | 4.19 | 0.041 | 0.069 | -1.784 |
| RHSIM_Rhsim03G0231800 | 4.19 | 0.041 | 0.069 | 0.205 |
| RHSIM_RhsimUnG0059400 | 4.19 | 0.041 | 0.069 | -1.401 |
| novel.775 | 4.19 | 0.041 | 0.069 | -3.287 |
| RHSIM_Rhsim11G0162400 | 4.19 | 0.041 | 0.069 | -0.953 |
| RHSIM_Rhsim08G0047000 | 4.19 | 0.041 | 0.069 | -0.838 |
| RHSIM_Rhsim08G0157600 | 4.19 | 0.041 | 0.069 | 2.574 |
| RHSIM_Rhsim13G0218000 | 4.19 | 0.041 | 0.069 | 0.233 |
| novel.2710 | 4.18 | 0.041 | 0.069 | -1.934 |
| RHSIM_Rhsim09G0087700 | 4.18 | 0.041 | 0.069 | -1.053 |
| RHSIM_RhsimUnG0133900 | 4.18 | 0.041 | 0.069 | 1.051 |
| RHSIM_Rhsim13G0085400 | 4.18 | 0.041 | 0.069 | 0.346 |
| RHSIM_Rhsim09G0186600 | 4.18 | 0.041 | 0.069 | -1.344 |
| novel.967 | 4.18 | 0.041 | 0.069 | 2.883 |
| novel.2190 | 4.18 | 0.041 | 0.070 | 0.238 |
| RHSIM_Rhsim08G0209400 | 4.17 | 0.041 | 0.070 | 0.699 |
| RHSIM_Rhsim13G0175800 | 4.17 | 0.041 | 0.070 | -1.253 |
| RHSIM_Rhsim05G0187600 | 4.17 | 0.041 | 0.070 | -2.438 |
| RHSIM_Rhsim13G0199700 | 4.17 | 0.041 | 0.070 | 0.365 |
| RHSIM_Rhsim03G0172100 | 4.17 | 0.041 | 0.070 | -1.296 |
| RHSIM_RhsimUnG0139100 | 4.17 | 0.041 | 0.070 | 1.586 |
| RHSIM_Rhsim03G0237500 | 4.17 | 0.041 | 0.070 | 1.536 |
| novel.3853 | 4.17 | 0.041 | 0.070 | -0.739 |
| RHSIM_Rhsim01G0250900 | 4.17 | 0.041 | 0.070 | 2.097 |
| RHSIM_Rhsim05G0203600 | 4.17 | 0.041 | 0.070 | 1.497 |
| RHSIM_Rhsim11G0085400 | 4.17 | 0.041 | 0.070 | 0.507 |

| Gene | LRT statistic | P value | BH-adjusted P | log2FC (interaction) |
|---|---|---|---|---|
| RHSIM_Rhsim13G0217700 | 4.17 | 0.041 | 0.070 | 0.264 |
| RHSIM_Rhsim07G0133800 | 4.17 | 0.041 | 0.070 | 1.370 |
| RHSIM_Rhsim13G0124700 | 4.17 | 0.041 | 0.070 | 0.354 |
| RHSIM_Rhsim07G0081600 | 4.16 | 0.041 | 0.070 | 41.081 |
| RHSIM_RhsimMtG0002000 | 4.16 | 0.041 | 0.070 | 3.264 |
| RHSIM_Rhsim09G0137400 | 4.16 | 0.041 | 0.070 | -0.284 |
| RHSIM_Rhsim10G0113100 | 4.16 | 0.041 | 0.070 | 0.673 |
| RHSIM_Rhsim05G0047000 | 4.16 | 0.041 | 0.070 | -0.926 |
| novel.3816 | 4.16 | 0.041 | 0.070 | 1.136 |
| RHSIM_Rhsim03G0120500 | 4.16 | 0.041 | 0.070 | 0.290 |
| RHSIM_Rhsim12G0182100 | 4.16 | 0.041 | 0.070 | 2.848 |
| RHSIM_Rhsim09G0099600 | 4.16 | 0.041 | 0.070 | 40.513 |
| RHSIM_Rhsim12G0059700 | 4.16 | 0.041 | 0.070 | 1.726 |
| RHSIM_Rhsim08G0187500 | 4.15 | 0.042 | 0.070 | 40.206 |
| RHSIM_Rhsim03G0189300 | 4.15 | 0.042 | 0.070 | 1.636 |
| RHSIM_Rhsim01G0158500 | 4.15 | 0.042 | 0.070 | 0.820 |
| RHSIM_Rhsim06G0222800 | 4.15 | 0.042 | 0.071 | 41.065 |
| RHSIM_Rhsim03G0203200 | 4.15 | 0.042 | 0.071 | 1.266 |
| RHSIM_Rhsim03G0086800 | 4.15 | 0.042 | 0.071 | 0.887 |
| RHSIM_Rhsim11G0092500 | 4.15 | 0.042 | 0.071 | 1.433 |
| RHSIM_Rhsim09G0120000 | 4.15 | 0.042 | 0.071 | 39.704 |
| RHSIM_Rhsim12G0029900 | 4.15 | 0.042 | 0.071 | 0.275 |
| RHSIM_RhsimUnG0091400 | 4.14 | 0.042 | 0.071 | -0.243 |
| RHSIM_Rhsim08G0074300 | 4.14 | 0.042 | 0.071 | 39.599 |
| RHSIM_Rhsim03G0098300 | 4.14 | 0.042 | 0.071 | 0.194 |
| RHSIM_Rhsim04G0221900 | 4.14 | 0.042 | 0.071 | 39.986 |
| RHSIM_Rhsim10G0140600 | 4.14 | 0.042 | 0.071 | -0.404 |
| novel.3353 | 4.14 | 0.042 | 0.071 | 0.407 |
| RHSIM_Rhsim13G0079800 | 4.14 | 0.042 | 0.071 | 2.095 |
| novel.3298 | 4.14 | 0.042 | 0.071 | -0.604 |
| RHSIM_Rhsim05G0221200 | 4.14 | 0.042 | 0.071 | 0.753 |
| RHSIM_Rhsim01G0073500 | 4.13 | 0.042 | 0.071 | -0.367 |

| Gene | LRT statistic | P value | BH-adjusted P | log2FC (interaction) |
|---|---|---|---|---|
| RHSIM_Rhsim13G0024500 | 4.13 | 0.042 | 0.071 | 40.235 |
| RHSIM_Rhsim09G0095400 | 4.13 | 0.042 | 0.071 | 1.171 |
| novel.1896 | 4.13 | 0.042 | 0.071 | -39.679 |
| RHSIM_Rhsim12G0090400 | 4.13 | 0.042 | 0.071 | -0.226 |
| RHSIM_Rhsim02G0012500 | 4.13 | 0.042 | 0.071 | -0.239 |
| RHSIM_Rhsim07G0021400 | 4.13 | 0.042 | 0.071 | 0.653 |
| RHSIM_Rhsim03G0195200 | 4.13 | 0.042 | 0.071 | 0.468 |
| RHSIM_Rhsim03G0096100 | 4.13 | 0.042 | 0.071 | 1.875 |
| novel.4197 | 4.13 | 0.042 | 0.071 | 3.082 |
| RHSIM_Rhsim07G0142900 | 4.13 | 0.042 | 0.071 | 0.482 |
| RHSIM_Rhsim10G0042800 | 4.13 | 0.042 | 0.071 | -0.244 |
| RHSIM_Rhsim05G0035400 | 4.12 | 0.042 | 0.071 | -0.181 |
| RHSIM_RhsimUnG0025800 | 4.12 | 0.042 | 0.072 | -0.292 |
| RHSIM_Rhsim13G0041900 | 4.12 | 0.042 | 0.072 | -0.185 |
| RHSIM_Rhsim03G0206300 | 4.12 | 0.042 | 0.072 | -1.095 |
| RHSIM_Rhsim12G0007000 | 4.12 | 0.042 | 0.072 | -0.334 |
| RHSIM_Rhsim04G0206000 | 4.12 | 0.042 | 0.072 | 0.169 |
| RHSIM_Rhsim02G0237600 | 4.12 | 0.042 | 0.072 | 0.262 |
| novel.2931 | 4.12 | 0.042 | 0.072 | 40.638 |
| RHSIM_Rhsim08G0237800 | 4.12 | 0.042 | 0.072 | 3.225 |
| novel.2280 | 4.12 | 0.042 | 0.072 | 39.244 |
| RHSIM_Rhsim01G0059200 | 4.12 | 0.042 | 0.072 | 1.096 |
| RHSIM_Rhsim01G0127100 | 4.12 | 0.042 | 0.072 | 2.089 |
| novel.3419 | 4.12 | 0.042 | 0.072 | 0.279 |
| RHSIM_Rhsim08G0087300 | 4.11 | 0.043 | 0.072 | 1.943 |
| RHSIM_Rhsim02G0230900 | 4.11 | 0.043 | 0.072 | 1.325 |
| RHSIM_Rhsim08G0054100 | 4.11 | 0.043 | 0.072 | -0.205 |
| novel.4261 | 4.11 | 0.043 | 0.072 | 1.716 |
| RHSIM_Rhsim11G0040700 | 4.11 | 0.043 | 0.072 | 0.467 |
| RHSIM_Rhsim03G0188900 | 4.11 | 0.043 | 0.072 | 0.418 |
| novel.776 | 4.11 | 0.043 | 0.072 | 0.859 |
| RHSIM_RhsimUnG0184100 | 4.11 | 0.043 | 0.072 | -40.293 |

| Gene | LRT statistic | P value | BH-adjusted P | log2FC (interaction) |
|---|---|---|---|---|
| RHSIM_Rhsim01G0257200 | 4.11 | 0.043 | 0.072 | -0.214 |
| RHSIM_Rhsim09G0019200 | 4.11 | 0.043 | 0.072 | 0.917 |
| RHSIM_Rhsim04G0072700 | 4.11 | 0.043 | 0.072 | 0.652 |
| RHSIM_Rhsim07G0063800 | 4.11 | 0.043 | 0.072 | -0.840 |
| RHSIM_Rhsim13G0071400 | 4.11 | 0.043 | 0.072 | -1.134 |
| RHSIM_Rhsim13G0154100 | 4.10 | 0.043 | 0.072 | 0.436 |
| RHSIM_Rhsim03G0131900 | 4.10 | 0.043 | 0.072 | 39.837 |
| RHSIM_Rhsim09G0142700 | 4.10 | 0.043 | 0.072 | 0.268 |
| RHSIM_Rhsim03G0213900 | 4.10 | 0.043 | 0.072 | 1.064 |
| RHSIM_Rhsim06G0140800 | 4.10 | 0.043 | 0.072 | -1.585 |
| RHSIM_Rhsim13G0160700 | 4.10 | 0.043 | 0.072 | -4.510 |
| novel.3150 | 4.10 | 0.043 | 0.072 | 0.731 |
| RHSIM_Rhsim08G0070000 | 4.10 | 0.043 | 0.072 | 0.222 |
| RHSIM_RhsimUnG0025500 | 4.10 | 0.043 | 0.072 | 2.669 |
| RHSIM_Rhsim07G0200800 | 4.10 | 0.043 | 0.072 | -0.241 |
| RHSIM_Rhsim06G0150700 | 4.10 | 0.043 | 0.073 | 2.795 |
| RHSIM_Rhsim04G0111900 | 4.10 | 0.043 | 0.073 | -0.557 |
| RHSIM_Rhsim10G0186700 | 4.09 | 0.043 | 0.073 | -0.267 |
| novel.2782 | 4.09 | 0.043 | 0.073 | -1.569 |
| RHSIM_Rhsim07G0057800 | 4.09 | 0.043 | 0.073 | 1.200 |
| RHSIM_Rhsim03G0102600 | 4.09 | 0.043 | 0.073 | -3.699 |
| RHSIM_Rhsim05G0069200 | 4.09 | 0.043 | 0.073 | -0.479 |
| RHSIM_Rhsim09G0173600 | 4.09 | 0.043 | 0.073 | -1.301 |
| RHSIM_Rhsim04G0123800 | 4.09 | 0.043 | 0.073 | -0.654 |
| RHSIM_Rhsim13G0201900 | 4.09 | 0.043 | 0.073 | 0.439 |
| RHSIM_RhsimUnG0029200 | 4.09 | 0.043 | 0.073 | 0.576 |
| RHSIM_Rhsim02G0184600 | 4.09 | 0.043 | 0.073 | 2.023 |
| RHSIM_Rhsim13G0112700 | 4.09 | 0.043 | 0.073 | -1.942 |
| RHSIM_Rhsim01G0270200 | 4.09 | 0.043 | 0.073 | -0.312 |
| RHSIM_Rhsim12G0006400 | 4.09 | 0.043 | 0.073 | -0.814 |
| RHSIM_Rhsim11G0172200 | 4.08 | 0.043 | 0.073 | -39.192 |
| RHSIM_Rhsim13G0057300 | 4.08 | 0.043 | 0.073 | 1.286 |

| Gene | LRT statistic | P value | BH-adjusted P | log2FC (interaction) |
|---|---|---|---|---|
| RHSIM_Rhsim08G0010600 | 4.08 | 0.043 | 0.073 | -1.369 |
| novel.4212 | 4.08 | 0.043 | 0.073 | -0.967 |
| RHSIM_Rhsim03G0265600 | 4.08 | 0.043 | 0.073 | -0.746 |
| RHSIM_Rhsim10G0068900 | 4.08 | 0.043 | 0.073 | -0.420 |
| RHSIM_RhsimUnG0115000 | 4.08 | 0.043 | 0.073 | 1.275 |
| RHSIM_Rhsim01G0209700 | 4.08 | 0.044 | 0.073 | 1.752 |
| RHSIM_Rhsim12G0053100 | 4.07 | 0.044 | 0.073 | -39.988 |
| RHSIM_Rhsim07G0231300 | 4.07 | 0.044 | 0.073 | -0.605 |
| RHSIM_Rhsim03G0149000 | 4.07 | 0.044 | 0.073 | 0.219 |
| RHSIM_Rhsim03G0004300 | 4.07 | 0.044 | 0.073 | 39.225 |
| RHSIM_Rhsim04G0052100 | 4.07 | 0.044 | 0.073 | 0.110 |
| RHSIM_Rhsim09G0009700 | 4.07 | 0.044 | 0.073 | -1.541 |
| RHSIM_Rhsim06G0070400 | 4.07 | 0.044 | 0.074 | -0.216 |
| RHSIM_Rhsim13G0235700 | 4.07 | 0.044 | 0.074 | -0.293 |
| novel.1993 | 4.07 | 0.044 | 0.074 | 3.325 |
| RHSIM_RhsimUnG0173800 | 4.07 | 0.044 | 0.074 | 0.433 |
| novel.3288 | 4.07 | 0.044 | 0.074 | -5.151 |
| RHSIM_Rhsim13G0234400 | 4.07 | 0.044 | 0.074 | -40.309 |
| RHSIM_Rhsim02G0201300 | 4.07 | 0.044 | 0.074 | -38.505 |
| RHSIM_Rhsim06G0001200 | 4.06 | 0.044 | 0.074 | -3.783 |
| RHSIM_Rhsim05G0013500 | 4.06 | 0.044 | 0.074 | -0.287 |
| RHSIM_Rhsim03G0018500 | 4.06 | 0.044 | 0.074 | 0.356 |
| RHSIM_Rhsim13G0154200 | 4.06 | 0.044 | 0.074 | 0.298 |
| RHSIM_Rhsim01G0180900 | 4.06 | 0.044 | 0.074 | 1.258 |
| RHSIM_Rhsim08G0079600 | 4.06 | 0.044 | 0.074 | -2.286 |
| novel.1012 | 4.06 | 0.044 | 0.074 | -1.338 |
| RHSIM_Rhsim01G0106700 | 4.06 | 0.044 | 0.074 | -0.179 |
| RHSIM_Rhsim11G0152900 | 4.06 | 0.044 | 0.074 | -1.183 |
| RHSIM_Rhsim12G0170400 | 4.06 | 0.044 | 0.074 | -0.276 |
| RHSIM_Rhsim01G0153200 | 4.06 | 0.044 | 0.074 | 1.189 |
| novel.3287 | 4.05 | 0.044 | 0.074 | -3.005 |
| RHSIM_Rhsim08G0210700 | 4.05 | 0.044 | 0.074 | 4.523 |

| Gene | LRT statistic | P value | BH-adjusted P | log2FC (interaction) |
|---|---|---|---|---|
| novel.3658 | 4.05 | 0.044 | 0.074 | 2.054 |
| RHSIM_Rhsim07G0138600 | 4.05 | 0.044 | 0.074 | -0.222 |
| RHSIM_Rhsim10G0128800 | 4.05 | 0.044 | 0.074 | 0.511 |
| RHSIM_Rhsim03G0024300 | 4.04 | 0.044 | 0.075 | -2.418 |
| RHSIM_Rhsim09G0158100 | 4.04 | 0.044 | 0.075 | 3.073 |
| RHSIM_Rhsim13G0131900 | 4.04 | 0.044 | 0.075 | 0.207 |
| RHSIM_Rhsim07G0245200 | 4.04 | 0.044 | 0.075 | 0.317 |
| RHSIM_RhsimUnG0123300 | 4.04 | 0.044 | 0.075 | -0.690 |
| RHSIM_Rhsim12G0052800 | 4.04 | 0.044 | 0.075 | 0.413 |
| RHSIM_Rhsim07G0065300 | 4.04 | 0.044 | 0.075 | 40.319 |
| RHSIM_Rhsim06G0138400 | 4.04 | 0.044 | 0.075 | 1.494 |
| RHSIM_Rhsim01G0265100 | 4.04 | 0.045 | 0.075 | -0.193 |
| novel.2534 | 4.04 | 0.045 | 0.075 | 3.187 |
| RHSIM_Rhsim08G0121300 | 4.04 | 0.045 | 0.075 | -0.436 |
| RHSIM_Rhsim03G0223800 | 4.03 | 0.045 | 0.075 | -0.346 |
| RHSIM_Rhsim10G0196300 | 4.03 | 0.045 | 0.075 | -0.751 |
| RHSIM_Rhsim03G0010300 | 4.03 | 0.045 | 0.075 | 2.756 |
| RHSIM_Rhsim05G0160200 | 4.03 | 0.045 | 0.075 | 0.596 |
| RHSIM_Rhsim02G0011700 | 4.03 | 0.045 | 0.075 | 40.842 |
| RHSIM_Rhsim05G0120000 | 4.03 | 0.045 | 0.075 | 3.950 |
| RHSIM_Rhsim07G0107200 | 4.03 | 0.045 | 0.075 | 0.224 |
| RHSIM_Rhsim04G0153800 | 4.03 | 0.045 | 0.075 | 2.209 |
| RHSIM_Rhsim07G0172400 | 4.03 | 0.045 | 0.075 | -0.537 |
| RHSIM_Rhsim08G0011500 | 4.02 | 0.045 | 0.075 | -41.039 |
| RHSIM_Rhsim06G0109100 | 4.02 | 0.045 | 0.075 | -0.225 |
| RHSIM_Rhsim11G0084000 | 4.02 | 0.045 | 0.075 | 1.691 |
| novel.3877 | 4.02 | 0.045 | 0.075 | 3.303 |
| RHSIM_Rhsim08G0116200 | 4.02 | 0.045 | 0.075 | 0.533 |
| RHSIM_Rhsim07G0181300 | 4.02 | 0.045 | 0.076 | -0.931 |
| RHSIM_Rhsim03G0194300 | 4.02 | 0.045 | 0.076 | 0.408 |
| RHSIM_Rhsim05G0088700 | 4.02 | 0.045 | 0.076 | 3.836 |
| RHSIM_Rhsim01G0275000 | 4.02 | 0.045 | 0.076 | 0.313 |

| Gene | LRT statistic | P value | BH-adjusted P | log2FC (interaction) |
|---|---|---|---|---|
| RHSIM_Rhsim04G0153600 | 4.02 | 0.045 | 0.076 | 1.364 |
| RHSIM_Rhsim09G0136900 | 4.02 | 0.045 | 0.076 | 0.463 |
| RHSIM_Rhsim12G0092100 | 4.02 | 0.045 | 0.076 | 0.788 |
| RHSIM_RhsimUnG0226800 | 4.01 | 0.045 | 0.076 | 0.956 |
| RHSIM_Rhsim06G0132200 | 4.01 | 0.045 | 0.076 | -3.635 |
| RHSIM_RhsimUnG0139500 | 4.01 | 0.045 | 0.076 | 0.351 |
| novel.1218 | 4.01 | 0.045 | 0.076 | 1.466 |
| RHSIM_Rhsim02G0058700 | 4.01 | 0.045 | 0.076 | -0.620 |
| RHSIM_Rhsim10G0210800 | 4.01 | 0.045 | 0.076 | 0.406 |
| RHSIM_Rhsim01G0250000 | 4.01 | 0.045 | 0.076 | 0.653 |
| novel.3396 | 4.01 | 0.045 | 0.076 | 1.525 |
| RHSIM_Rhsim05G0135300 | 4.01 | 0.045 | 0.076 | -0.295 |
| RHSIM_Rhsim06G0017000 | 4.01 | 0.045 | 0.076 | -1.811 |
| RHSIM_Rhsim05G0202500 | 4.01 | 0.045 | 0.076 | -0.277 |
| RHSIM_Rhsim10G0178600 | 4.01 | 0.045 | 0.076 | 0.694 |
| RHSIM_Rhsim13G0160200 | 4.01 | 0.045 | 0.076 | -1.466 |
| RHSIM_Rhsim07G0014400 | 4.01 | 0.045 | 0.076 | -0.789 |
| RHSIM_Rhsim04G0190800 | 4.01 | 0.045 | 0.076 | -0.549 |
| RHSIM_Rhsim09G0206700 | 4.00 | 0.045 | 0.076 | -1.037 |
| novel.4552 | 4.00 | 0.045 | 0.076 | 2.075 |
| RHSIM_Rhsim13G0231800 | 4.00 | 0.045 | 0.076 | -0.262 |
| novel.29 | 4.00 | 0.046 | 0.076 | -39.185 |
| novel.3581 | 4.00 | 0.046 | 0.076 | 0.517 |
| RHSIM_Rhsim03G0201300 | 4.00 | 0.046 | 0.076 | 2.017 |
| RHSIM_Rhsim08G0061500 | 4.00 | 0.046 | 0.077 | -3.478 |
| RHSIM_Rhsim04G0070500 | 3.99 | 0.046 | 0.077 | 1.990 |
| RHSIM_Rhsim07G0217600 | 3.99 | 0.046 | 0.077 | 1.962 |
| RHSIM_Rhsim06G0136400 | 3.99 | 0.046 | 0.077 | 40.008 |
| RHSIM_Rhsim09G0173500 | 3.99 | 0.046 | 0.077 | -0.779 |
| RHSIM_RhsimUnG0109000 | 3.99 | 0.046 | 0.077 | -38.855 |
| RHSIM_Rhsim13G0222000 | 3.99 | 0.046 | 0.077 | -0.186 |
| RHSIM_Rhsim10G0200700 | 3.99 | 0.046 | 0.077 | -1.159 |

| Gene | LRT statistic | P value | BH-adjusted P | log2FC (interaction) |
|---|---|---|---|---|
| RHSIM_Rhsim13G0090600 | 3.98 | 0.046 | 0.077 | -0.920 |
| RHSIM_Rhsim01G0147200 | 3.98 | 0.046 | 0.077 | -1.979 |
| RHSIM_Rhsim07G0094100 | 3.98 | 0.046 | 0.077 | -2.036 |
| RHSIM_RhsimUnG0074300 | 3.98 | 0.046 | 0.077 | 3.203 |
| RHSIM_Rhsim13G0058700 | 3.98 | 0.046 | 0.077 | -0.255 |
| RHSIM_Rhsim07G0046300 | 3.98 | 0.046 | 0.077 | 1.892 |
| RHSIM_Rhsim03G0230300 | 3.98 | 0.046 | 0.077 | 3.952 |
| novel.1560 | 3.98 | 0.046 | 0.077 | -2.427 |
| RHSIM_Rhsim04G0202400 | 3.98 | 0.046 | 0.077 | 1.723 |
| novel.4486 | 3.98 | 0.046 | 0.077 | -1.077 |
| RHSIM_Rhsim05G0233600 | 3.98 | 0.046 | 0.077 | 0.370 |
| RHSIM_Rhsim02G0148900 | 3.98 | 0.046 | 0.077 | -0.151 |
| RHSIM_Rhsim01G0232800 | 3.98 | 0.046 | 0.077 | -0.607 |
| novel.5 | 3.97 | 0.046 | 0.077 | 0.348 |
| RHSIM_Rhsim09G0009900 | 3.97 | 0.046 | 0.077 | -0.835 |
| RHSIM_Rhsim01G0048900 | 3.97 | 0.046 | 0.077 | 0.891 |
| RHSIM_Rhsim02G0007300 | 3.97 | 0.046 | 0.078 | 0.170 |
| RHSIM_Rhsim01G0197400 | 3.97 | 0.046 | 0.078 | -2.037 |
| RHSIM_Rhsim08G0165800 | 3.97 | 0.046 | 0.078 | -2.401 |
| RHSIM_Rhsim12G0110700 | 3.97 | 0.046 | 0.078 | 40.447 |
| novel.2838 | 3.97 | 0.046 | 0.078 | 1.945 |
| RHSIM_Rhsim05G0188300 | 3.97 | 0.046 | 0.078 | 2.327 |
| novel.1701 | 3.97 | 0.046 | 0.078 | 0.656 |
| RHSIM_Rhsim07G0068800 | 3.97 | 0.046 | 0.078 | 0.155 |
| novel.603 | 3.97 | 0.046 | 0.078 | -1.025 |
| RHSIM_Rhsim03G0269900 | 3.97 | 0.046 | 0.078 | 0.444 |
| RHSIM_Rhsim07G0005000 | 3.96 | 0.047 | 0.078 | 0.299 |
| novel.3648 | 3.96 | 0.047 | 0.078 | 0.905 |
| novel.1785 | 3.96 | 0.047 | 0.078 | 0.924 |
| RHSIM_Rhsim06G0212100 | 3.96 | 0.047 | 0.078 | 1.855 |
| novel.3937 | 3.96 | 0.047 | 0.078 | -1.537 |
| RHSIM_Rhsim10G0051300 | 3.95 | 0.047 | 0.078 | 0.679 |

| Gene | LRT statistic | P value | BH-adjusted P | log2FC (interaction) |
|---|---|---|---|---|
| RHSIM_Rhsim02G0185400 | 3.95 | 0.047 | 0.078 | -39.492 |
| RHSIM_RhsimUnG0055600 | 3.95 | 0.047 | 0.078 | -40.112 |
| novel.2333 | 3.95 | 0.047 | 0.078 | 1.248 |
| RHSIM_Rhsim07G0257600 | 3.95 | 0.047 | 0.078 | -3.679 |
| RHSIM_Rhsim05G0180700 | 3.95 | 0.047 | 0.078 | 2.994 |
| novel.3594 | 3.95 | 0.047 | 0.078 | -0.176 |
| RHSIM_RhsimUnG0029700 | 3.95 | 0.047 | 0.079 | 1.534 |
| RHSIM_Rhsim01G0077800 | 3.95 | 0.047 | 0.079 | 1.101 |
| RHSIM_Rhsim09G0087400 | 3.95 | 0.047 | 0.079 | -1.914 |
| RHSIM_Rhsim13G0033800 | 3.94 | 0.047 | 0.079 | 0.303 |
| novel.3050 | 3.94 | 0.047 | 0.079 | -2.539 |
| novel.785 | 3.94 | 0.047 | 0.079 | -0.640 |
| RHSIM_Rhsim03G0109900 | 3.94 | 0.047 | 0.079 | 0.310 |
| RHSIM_Rhsim08G0226300 | 3.94 | 0.047 | 0.079 | -0.282 |
| RHSIM_Rhsim04G0097500 | 3.94 | 0.047 | 0.079 | -1.935 |
| RHSIM_Rhsim09G0005600 | 3.94 | 0.047 | 0.079 | 0.492 |
| RHSIM_Rhsim10G0041700 | 3.94 | 0.047 | 0.079 | -38.601 |
| novel.277 | 3.94 | 0.047 | 0.079 | -1.349 |
| RHSIM_Rhsim02G0026100 | 3.94 | 0.047 | 0.079 | 0.319 |
| novel.3274 | 3.93 | 0.047 | 0.079 | 1.201 |
| RHSIM_Rhsim03G0054900 | 3.93 | 0.047 | 0.079 | -0.651 |
| RHSIM_Rhsim10G0148800 | 3.93 | 0.047 | 0.079 | -0.387 |
| novel.917 | 3.93 | 0.047 | 0.079 | 1.976 |
| RHSIM_Rhsim06G0202600 | 3.93 | 0.047 | 0.079 | 0.776 |
| novel.99 | 3.93 | 0.047 | 0.079 | 1.128 |
| RHSIM_Rhsim09G0092000 | 3.93 | 0.047 | 0.079 | -1.158 |
| RHSIM_Rhsim03G0245000 | 3.93 | 0.047 | 0.079 | 3.159 |
| RHSIM_Rhsim06G0155000 | 3.93 | 0.047 | 0.079 | -1.611 |
| novel.1810 | 3.93 | 0.047 | 0.079 | 1.344 |
| RHSIM_Rhsim05G0182400 | 3.93 | 0.048 | 0.079 | -0.269 |
| RHSIM_Rhsim04G0016700 | 3.93 | 0.048 | 0.079 | 1.560 |
| RHSIM_Rhsim03G0220900 | 3.93 | 0.048 | 0.079 | -0.204 |

| Gene | LRT statistic | P value | BH-adjusted P | log2FC (interaction) |
|---|---|---|---|---|
| RHSIM_Rhsim07G0027700 | 3.93 | 0.048 | 0.079 | -3.020 |
| RHSIM_Rhsim09G0174100 | 3.92 | 0.048 | 0.079 | -3.658 |
| RHSIM_Rhsim11G0197400 | 3.92 | 0.048 | 0.079 | 2.158 |
| RHSIM_Rhsim06G0004100 | 3.92 | 0.048 | 0.080 | -0.984 |
| RHSIM_Rhsim09G0155300 | 3.92 | 0.048 | 0.080 | 0.258 |
| RHSIM_RhsimUnG0058700 | 3.92 | 0.048 | 0.080 | 0.513 |
| RHSIM_Rhsim07G0068300 | 3.92 | 0.048 | 0.080 | 2.520 |
| RHSIM_Rhsim13G0081500 | 3.92 | 0.048 | 0.080 | -0.375 |
| RHSIM_Rhsim10G0081000 | 3.92 | 0.048 | 0.080 | 0.588 |
| RHSIM_Rhsim07G0208200 | 3.92 | 0.048 | 0.080 | -1.810 |
| RHSIM_Rhsim10G0185400 | 3.92 | 0.048 | 0.080 | 3.698 |
| RHSIM_Rhsim12G0207400 | 3.92 | 0.048 | 0.080 | 0.249 |
| RHSIM_Rhsim04G0118200 | 3.92 | 0.048 | 0.080 | 1.450 |
| RHSIM_Rhsim08G0067500 | 3.92 | 0.048 | 0.080 | 1.254 |
| RHSIM_Rhsim09G0190900 | 3.91 | 0.048 | 0.080 | -0.262 |
| RHSIM_Rhsim07G0249400 | 3.91 | 0.048 | 0.080 | -0.193 |
| RHSIM_RhsimUnG0149900 | 3.91 | 0.048 | 0.080 | -0.595 |
| RHSIM_Rhsim13G0043200 | 3.91 | 0.048 | 0.080 | 3.880 |
| RHSIM_Rhsim07G0254000 | 3.91 | 0.048 | 0.080 | 0.282 |
| RHSIM_Rhsim01G0265700 | 3.91 | 0.048 | 0.080 | -2.303 |
| RHSIM_Rhsim04G0030700 | 3.90 | 0.048 | 0.080 | -0.181 |
| RHSIM_RhsimUnG0101700 | 3.90 | 0.048 | 0.080 | 2.188 |
| RHSIM_Rhsim09G0214100 | 3.90 | 0.048 | 0.080 | 0.301 |
| novel.1220 | 3.90 | 0.048 | 0.080 | 3.954 |
| RHSIM_Rhsim10G0108900 | 3.90 | 0.048 | 0.080 | 39.883 |
| RHSIM_Rhsim02G0152700 | 3.90 | 0.048 | 0.080 | 2.245 |
| RHSIM_Rhsim13G0174800 | 3.90 | 0.048 | 0.081 | 39.325 |
| RHSIM_Rhsim11G0048300 | 3.90 | 0.048 | 0.081 | -0.302 |
| RHSIM_Rhsim03G0004200 | 3.90 | 0.048 | 0.081 | -0.119 |
| RHSIM_Rhsim05G0053000 | 3.89 | 0.048 | 0.081 | -0.559 |
| RHSIM_Rhsim05G0142200 | 3.89 | 0.048 | 0.081 | -0.476 |
| RHSIM_Rhsim09G0072300 | 3.89 | 0.049 | 0.081 | 0.300 |

| Gene | LRT statistic | P value | BH-adjusted P | log2FC (interaction) |
|---|---|---|---|---|
| novel.4032 | 3.89 | 0.049 | 0.081 | -39.107 |
| RHSIM_Rhsim13G0041700 | 3.89 | 0.049 | 0.081 | -0.407 |
| RHSIM_Rhsim03G0142800 | 3.89 | 0.049 | 0.081 | -40.290 |
| novel.3715 | 3.89 | 0.049 | 0.081 | -1.691 |
| RHSIM_Rhsim09G0130100 | 3.89 | 0.049 | 0.081 | 0.113 |
| RHSIM_Rhsim06G0128500 | 3.88 | 0.049 | 0.081 | 2.688 |
| RHSIM_Rhsim05G0108500 | 3.88 | 0.049 | 0.081 | 0.206 |
| RHSIM_Rhsim08G0240800 | 3.88 | 0.049 | 0.081 | -0.261 |
| novel.3755 | 3.88 | 0.049 | 0.081 | -39.025 |
| RHSIM_RhsimUnG0255400 | 3.88 | 0.049 | 0.081 | 0.768 |
| RHSIM_Rhsim07G0226100 | 3.88 | 0.049 | 0.081 | -40.753 |
| RHSIM_Rhsim01G0073000 | 3.88 | 0.049 | 0.081 | -1.021 |
| RHSIM_Rhsim03G0232500 | 3.88 | 0.049 | 0.081 | 0.793 |
| RHSIM_RhsimUnG0204600 | 3.88 | 0.049 | 0.081 | -2.249 |
| RHSIM_Rhsim11G0098100 | 3.88 | 0.049 | 0.082 | 1.918 |
| RHSIM_RhsimUnG0020500 | 3.88 | 0.049 | 0.082 | 0.609 |
| RHSIM_Rhsim11G0028000 | 3.87 | 0.049 | 0.082 | 0.445 |
| RHSIM_Rhsim04G0093300 | 3.87 | 0.049 | 0.082 | 1.473 |
| RHSIM_RhsimUnG0244300 | 3.87 | 0.049 | 0.082 | 2.317 |
| RHSIM_RhsimUnG0050800 | 3.87 | 0.049 | 0.082 | -3.149 |
| RHSIM_Rhsim09G0061000 | 3.87 | 0.049 | 0.082 | 1.676 |
| RHSIM_Rhsim10G0147600 | 3.87 | 0.049 | 0.082 | 0.302 |
| RHSIM_Rhsim05G0134300 | 3.87 | 0.049 | 0.082 | 0.311 |
| RHSIM_Rhsim08G0207900 | 3.87 | 0.049 | 0.082 | -0.887 |
| novel.1808 | 3.87 | 0.049 | 0.082 | 0.552 |
| RHSIM_RhsimUnG0086400 | 3.87 | 0.049 | 0.082 | -0.650 |
| RHSIM_Rhsim05G0142900 | 3.87 | 0.049 | 0.082 | -39.144 |
| RHSIM_RhsimUnG0240200 | 3.87 | 0.049 | 0.082 | -1.302 |
| RHSIM_Rhsim03G0092200 | 3.87 | 0.049 | 0.082 | 0.997 |
| novel.3713 | 3.86 | 0.049 | 0.082 | 1.213 |
| novel.2891 | 3.86 | 0.049 | 0.082 | 1.244 |
| RHSIM_Rhsim13G0174400 | 3.86 | 0.049 | 0.082 | -0.557 |

| Gene | LRT statistic | P value | BH-adjusted P | log2FC (interaction) |
|---|---|---|---|---|
| RHSIM_RhsimUnG0054500 | 3.86 | 0.049 | 0.082 | 0.542 |
| novel.1018 | 3.86 | 0.049 | 0.082 | -0.498 |
| RHSIM_Rhsim02G0070900 | 3.86 | 0.049 | 0.082 | 0.792 |
| RHSIM_Rhsim10G0211700 | 3.86 | 0.049 | 0.082 | 0.410 |
| RHSIM_Rhsim05G0102600 | 3.86 | 0.049 | 0.082 | -2.123 |
| RHSIM_Rhsim07G0234700 | 3.86 | 0.050 | 0.082 | 0.462 |
| RHSIM_Rhsim08G0187700 | 3.86 | 0.050 | 0.082 | -0.144 |
| RHSIM_Rhsim05G0102300 | 3.86 | 0.050 | 0.082 | 0.154 |
| RHSIM_Rhsim04G0227100 | 3.85 | 0.050 | 0.082 | -2.639 |
| novel.3573 | 3.85 | 0.050 | 0.083 | -2.569 |
| RHSIM_RhsimUnG0219300 | 3.85 | 0.050 | 0.083 | -0.467 |
| RHSIM_Rhsim13G0024700 | 3.85 | 0.050 | 0.083 | 0.378 |
| RHSIM_Rhsim04G0140500 | 3.85 | 0.050 | 0.083 | -3.115 |
| RHSIM_Rhsim03G0116100 | 3.85 | 0.050 | 0.083 | 2.811 |
| RHSIM_Rhsim02G0145000 | 3.85 | 0.050 | 0.083 | -0.503 |
| RHSIM_Rhsim05G0021800 | 3.85 | 0.050 | 0.083 | 0.561 |
| RHSIM_RhsimUnG0138100 | 3.85 | 0.050 | 0.083 | 39.780 |
| RHSIM_Rhsim07G0143100 | 3.84 | 0.050 | 0.083 | 0.705 |
| RHSIM_Rhsim04G0225300 | 3.84 | 0.050 | 0.083 | 40.333 |
| RHSIM_Rhsim11G0107200 | 3.84 | 0.050 | 0.083 | -1.017 |
| novel.201 | 3.84 | 0.050 | 0.083 | 1.733 |
| RHSIM_Rhsim06G0042700 | 3.84 | 0.050 | 0.083 | -39.906 |
| RHSIM_Rhsim06G0071600 | 3.84 | 0.050 | 0.083 | 0.282 |
| RHSIM_Rhsim12G0021600 | 3.84 | 0.050 | 0.083 | 0.514 |
| RHSIM_Rhsim11G0131100 | 3.84 | 0.050 | 0.083 | 0.551 |
| RHSIM_Rhsim01G0086700 | 3.84 | 0.050 | 0.083 | 2.033 |
| novel.3054 | 3.83 | 0.050 | 0.083 | 2.172 |
| RHSIM_Rhsim08G0084100 | 3.83 | 0.050 | 0.083 | 2.395 |
| RHSIM_Rhsim07G0255600 | 3.83 | 0.050 | 0.083 | 5.335 |
| RHSIM_Rhsim03G0058000 | 3.83 | 0.050 | 0.083 | -0.228 |
| RHSIM_RhsimUnG0082100 | 3.83 | 0.050 | 0.084 | -1.047 |
| RHSIM_Rhsim02G0101000 | 3.83 | 0.050 | 0.084 | -0.488 |

| Gene | LRT statistic | P value | BH-adjusted P | log2FC (interaction) |
|---|---|---|---|---|
| RHSIM_Rhsim07G0037800 | 3.83 | 0.050 | 0.084 | -0.149 |
| RHSIM_Rhsim08G0026000 | 3.83 | 0.050 | 0.084 | 0.259 |
| novel.1646 | 3.83 | 0.050 | 0.084 | -0.750 |
| RHSIM_Rhsim13G0196700 | 3.83 | 0.050 | 0.084 | 0.858 |
| RHSIM_Rhsim12G0041200 | 3.83 | 0.050 | 0.084 | 4.289 |
| RHSIM_Rhsim08G0129800 | 3.83 | 0.050 | 0.084 | 1.397 |
| RHSIM_Rhsim03G0261300 | 3.83 | 0.050 | 0.084 | 0.193 |
| RHSIM_Rhsim01G0074300 | 3.83 | 0.050 | 0.084 | 0.216 |
| RHSIM_Rhsim03G0223400 | 3.83 | 0.050 | 0.084 | -0.397 |
| RHSIM_Rhsim02G0026500 | 3.82 | 0.051 | 0.084 | -0.366 |
| RHSIM_Rhsim03G0214200 | 3.82 | 0.051 | 0.084 | -0.564 |
| RHSIM_Rhsim11G0133200 | 3.82 | 0.051 | 0.084 | 2.636 |
| RHSIM_Rhsim05G0072000 | 3.82 | 0.051 | 0.084 | -0.396 |
| RHSIM_Rhsim01G0069100 | 3.82 | 0.051 | 0.084 | 3.405 |
| RHSIM_Rhsim08G0132500 | 3.82 | 0.051 | 0.084 | 0.138 |
| RHSIM_Rhsim06G0207100 | 3.82 | 0.051 | 0.084 | 39.854 |
| RHSIM_Rhsim06G0204100 | 3.82 | 0.051 | 0.084 | -0.504 |
| RHSIM_Rhsim06G0141700 | 3.82 | 0.051 | 0.084 | -0.296 |
| RHSIM_Rhsim09G0129300 | 3.82 | 0.051 | 0.084 | 0.264 |
| RHSIM_Rhsim03G0187600 | 3.82 | 0.051 | 0.084 | 0.455 |
| RHSIM_Rhsim09G0105800 | 3.81 | 0.051 | 0.084 | -0.478 |
| RHSIM_Rhsim12G0028400 | 3.81 | 0.051 | 0.084 | 1.196 |
| RHSIM_Rhsim02G0011500 | 3.81 | 0.051 | 0.084 | 2.358 |
| RHSIM_Rhsim06G0055700 | 3.81 | 0.051 | 0.084 | -39.227 |
| RHSIM_Rhsim08G0104500 | 3.81 | 0.051 | 0.084 | 0.358 |
| RHSIM_Rhsim05G0070600 | 3.81 | 0.051 | 0.084 | -0.368 |
| RHSIM_Rhsim04G0024900 | 3.81 | 0.051 | 0.084 | 1.865 |
| RHSIM_Rhsim07G0127900 | 3.81 | 0.051 | 0.084 | 1.381 |
| RHSIM_Rhsim01G0049200 | 3.81 | 0.051 | 0.084 | 0.960 |
| RHSIM_RhsimUnG0243300 | 3.81 | 0.051 | 0.084 | 1.062 |
| RHSIM_Rhsim03G0220700 | 3.81 | 0.051 | 0.085 | 1.695 |
| RHSIM_Rhsim13G0041600 | 3.81 | 0.051 | 0.085 | -0.156 |

| Gene | LRT statistic | P value | BH-adjusted P | log2FC (interaction) |
|---|---|---|---|---|
| RHSIM_Rhsim12G0050200 | 3.80 | 0.051 | 0.085 | -0.495 |
| RHSIM_Rhsim08G0185200 | 3.80 | 0.051 | 0.085 | 0.259 |
| RHSIM_Rhsim12G0017900 | 3.80 | 0.051 | 0.085 | -0.999 |
| RHSIM_Rhsim01G0022500 | 3.80 | 0.051 | 0.085 | -40.971 |
| RHSIM_Rhsim01G0135300 | 3.80 | 0.051 | 0.085 | 1.909 |
| RHSIM_Rhsim03G0075400 | 3.80 | 0.051 | 0.085 | 3.024 |
| RHSIM_Rhsim04G0010700 | 3.79 | 0.051 | 0.085 | -0.360 |
| novel.2508 | 3.79 | 0.051 | 0.085 | 0.909 |
| RHSIM_Rhsim04G0232000 | 3.79 | 0.051 | 0.085 | 0.149 |
| RHSIM_Rhsim08G0006200 | 3.79 | 0.051 | 0.085 | -0.211 |
| RHSIM_Rhsim07G0001800 | 3.79 | 0.052 | 0.085 | -1.576 |
| RHSIM_Rhsim11G0160600 | 3.79 | 0.052 | 0.085 | -0.385 |
| novel.1435 | 3.79 | 0.052 | 0.086 | 0.934 |
| RHSIM_Rhsim07G0107100 | 3.79 | 0.052 | 0.086 | -0.507 |
| novel.3203 | 3.79 | 0.052 | 0.086 | 1.643 |
| novel.1064 | 3.79 | 0.052 | 0.086 | 0.496 |
| RHSIM_Rhsim13G0093300 | 3.79 | 0.052 | 0.086 | -0.253 |
| RHSIM_Rhsim01G0037100 | 3.78 | 0.052 | 0.086 | 39.511 |
| RHSIM_Rhsim09G0051400 | 3.78 | 0.052 | 0.086 | 0.212 |
| RHSIM_Rhsim07G0208500 | 3.78 | 0.052 | 0.086 | 0.870 |
| RHSIM_Rhsim03G0133000 | 3.78 | 0.052 | 0.086 | 2.017 |
| RHSIM_Rhsim12G0155900 | 3.78 | 0.052 | 0.086 | 0.447 |
| RHSIM_Rhsim08G0038400 | 3.78 | 0.052 | 0.086 | -0.243 |
| novel.701 | 3.78 | 0.052 | 0.086 | 0.607 |
| RHSIM_Rhsim02G0119100 | 3.78 | 0.052 | 0.086 | -0.349 |
| RHSIM_Rhsim08G0157900 | 3.78 | 0.052 | 0.086 | 0.528 |
| RHSIM_Rhsim02G0049100 | 3.78 | 0.052 | 0.086 | 0.209 |
| RHSIM_Rhsim11G0193500 | 3.78 | 0.052 | 0.086 | 0.407 |
| RHSIM_Rhsim02G0168200 | 3.78 | 0.052 | 0.086 | 0.185 |
| RHSIM_Rhsim06G0168800 | 3.78 | 0.052 | 0.086 | -0.277 |
| RHSIM_Rhsim02G0175800 | 3.77 | 0.052 | 0.086 | 1.126 |
| RHSIM_Rhsim03G0232700 | 3.77 | 0.052 | 0.086 | 2.000 |

| Gene | LRT statistic | P value | BH-adjusted P | log2FC (interaction) |
|---|---|---|---|---|
| novel.2826 | 3.77 | 0.052 | 0.086 | -0.528 |
| RHSIM_Rhsim11G0069500 | 3.77 | 0.052 | 0.086 | -40.071 |
| RHSIM_Rhsim12G0169300 | 3.77 | 0.052 | 0.086 | 1.667 |
| RHSIM_Rhsim05G0115000 | 3.77 | 0.052 | 0.086 | -0.201 |
| RHSIM_Rhsim11G0135500 | 3.77 | 0.052 | 0.086 | -1.555 |
| novel.311 | 3.77 | 0.052 | 0.086 | -0.642 |
| RHSIM_Rhsim03G0173800 | 3.77 | 0.052 | 0.086 | -0.939 |
| RHSIM_Rhsim05G0098600 | 3.77 | 0.052 | 0.086 | 2.413 |
| RHSIM_Rhsim02G0109500 | 3.77 | 0.052 | 0.086 | 1.907 |
| RHSIM_Rhsim05G0069800 | 3.76 | 0.052 | 0.087 | 0.351 |
| RHSIM_Rhsim02G0250000 | 3.76 | 0.052 | 0.087 | -0.159 |
| RHSIM_Rhsim05G0226100 | 3.76 | 0.052 | 0.087 | -2.679 |
| novel.2967 | 3.76 | 0.052 | 0.087 | -1.002 |
| novel.2061 | 3.76 | 0.052 | 0.087 | 0.515 |
| RHSIM_Rhsim08G0030300 | 3.76 | 0.052 | 0.087 | 0.294 |
| novel.3245 | 3.76 | 0.052 | 0.087 | -2.220 |
| RHSIM_Rhsim05G0165600 | 3.76 | 0.052 | 0.087 | -0.199 |
| RHSIM_Rhsim04G0037500 | 3.76 | 0.052 | 0.087 | 0.650 |
| novel.2309 | 3.76 | 0.052 | 0.087 | 0.929 |
| RHSIM_Rhsim03G0255300 | 3.76 | 0.053 | 0.087 | -40.872 |
| RHSIM_Rhsim13G0122000 | 3.76 | 0.053 | 0.087 | -0.487 |
| novel.2935 | 3.76 | 0.053 | 0.087 | 2.210 |
| novel.2251 | 3.76 | 0.053 | 0.087 | -39.625 |
| novel.2438 | 3.76 | 0.053 | 0.087 | -0.476 |
| RHSIM_Rhsim03G0157900 | 3.76 | 0.053 | 0.087 | 1.712 |
| novel.4211 | 3.76 | 0.053 | 0.087 | -1.353 |
| RHSIM_Rhsim06G0107100 | 3.76 | 0.053 | 0.087 | 2.047 |
| RHSIM_Rhsim07G0178800 | 3.75 | 0.053 | 0.087 | 1.840 |
| RHSIM_Rhsim03G0161500 | 3.75 | 0.053 | 0.087 | 1.486 |
| novel.3745 | 3.75 | 0.053 | 0.087 | -2.641 |
| RHSIM_Rhsim02G0102200 | 3.75 | 0.053 | 0.087 | -39.848 |
| RHSIM_Rhsim04G0060800 | 3.75 | 0.053 | 0.087 | 0.948 |

| Gene | LRT statistic | P value | BH-adjusted P | log2FC (interaction) |
|---|---|---|---|---|
| novel.3859 | 3.75 | 0.053 | 0.087 | 0.485 |
| RHSIM_Rhsim07G0222800 | 3.75 | 0.053 | 0.087 | -3.440 |
| novel.326 | 3.75 | 0.053 | 0.087 | 1.576 |
| RHSIM_Rhsim02G0049400 | 3.75 | 0.053 | 0.087 | 0.180 |
| novel.3543 | 3.75 | 0.053 | 0.087 | 1.560 |
| novel.4237 | 3.75 | 0.053 | 0.087 | 1.100 |
| RHSIM_Rhsim10G0036300 | 3.75 | 0.053 | 0.087 | -0.511 |
| novel.1069 | 3.75 | 0.053 | 0.087 | 2.289 |
| RHSIM_Rhsim07G0007400 | 3.75 | 0.053 | 0.087 | -0.330 |
| RHSIM_Rhsim10G0049500 | 3.75 | 0.053 | 0.087 | 3.655 |
| RHSIM_Rhsim06G0190100 | 3.75 | 0.053 | 0.087 | -40.387 |
| novel.1700 | 3.74 | 0.053 | 0.087 | 0.475 |
| RHSIM_Rhsim10G0054500 | 3.74 | 0.053 | 0.087 | -0.751 |
| RHSIM_Rhsim11G0153200 | 3.74 | 0.053 | 0.088 | 0.413 |
| RHSIM_Rhsim03G0033100 | 3.74 | 0.053 | 0.088 | 1.237 |
| RHSIM_Rhsim11G0065000 | 3.74 | 0.053 | 0.088 | 0.147 |
| novel.3696 | 3.74 | 0.053 | 0.088 | 0.737 |
| RHSIM_Rhsim01G0077100 | 3.74 | 0.053 | 0.088 | -2.846 |
| novel.3907 | 3.74 | 0.053 | 0.088 | 0.324 |
| RHSIM_Rhsim11G0175600 | 3.74 | 0.053 | 0.088 | -0.709 |
| RHSIM_Rhsim05G0000700 | 3.74 | 0.053 | 0.088 | 0.522 |
| RHSIM_Rhsim04G0209900 | 3.73 | 0.053 | 0.088 | -0.798 |
| RHSIM_Rhsim09G0186100 | 3.73 | 0.053 | 0.088 | 3.142 |
| RHSIM_RhsimUnG0205300 | 3.73 | 0.053 | 0.088 | 1.656 |
| novel.1356 | 3.73 | 0.053 | 0.088 | 3.034 |
| RHSIM_Rhsim01G0289600 | 3.73 | 0.053 | 0.088 | -1.588 |
| novel.3144 | 3.73 | 0.053 | 0.088 | 0.500 |
| RHSIM_Rhsim03G0182800 | 3.72 | 0.054 | 0.089 | 4.445 |
| RHSIM_Rhsim07G0109700 | 3.72 | 0.054 | 0.089 | 0.291 |
| RHSIM_Rhsim13G0156300 | 3.72 | 0.054 | 0.089 | 39.583 |
| novel.3436 | 3.72 | 0.054 | 0.089 | 3.246 |
| RHSIM_Rhsim12G0041600 | 3.72 | 0.054 | 0.089 | -39.016 |

| Gene | LRT statistic | P value | BH-adjusted P | log2FC (interaction) |
|---|---|---|---|---|
| RHSIM_Rhsim02G0145800 | 3.72 | 0.054 | 0.089 | 3.950 |
| RHSIM_Rhsim05G0168000 | 3.72 | 0.054 | 0.089 | -0.189 |
| RHSIM_Rhsim01G0045000 | 3.72 | 0.054 | 0.089 | 0.586 |
| RHSIM_Rhsim07G0217500 | 3.72 | 0.054 | 0.089 | -0.818 |
| RHSIM_Rhsim07G0000600 | 3.71 | 0.054 | 0.089 | -0.362 |
| RHSIM_Rhsim04G0136800 | 3.71 | 0.054 | 0.089 | 0.692 |
| novel.554 | 3.71 | 0.054 | 0.089 | -2.076 |
| RHSIM_Rhsim10G0136300 | 3.71 | 0.054 | 0.089 | 1.028 |
| RHSIM_Rhsim06G0197900 | 3.71 | 0.054 | 0.089 | -0.243 |
| RHSIM_Rhsim09G0176700 | 3.71 | 0.054 | 0.089 | -0.269 |
| RHSIM_Rhsim02G0077200 | 3.71 | 0.054 | 0.089 | 1.029 |
| RHSIM_Rhsim01G0088600 | 3.71 | 0.054 | 0.089 | 0.644 |
| RHSIM_Rhsim06G0138900 | 3.71 | 0.054 | 0.089 | 2.044 |
| RHSIM_Rhsim13G0205300 | 3.71 | 0.054 | 0.089 | -2.029 |
| novel.664 | 3.70 | 0.054 | 0.089 | 2.645 |
| RHSIM_Rhsim03G0072900 | 3.70 | 0.054 | 0.090 | 1.969 |
| RHSIM_Rhsim13G0055500 | 3.70 | 0.054 | 0.090 | 0.337 |
| RHSIM_Rhsim08G0195800 | 3.70 | 0.054 | 0.090 | 1.315 |
| RHSIM_Rhsim07G0024400 | 3.70 | 0.054 | 0.090 | -0.460 |
| RHSIM_Rhsim11G0196000 | 3.70 | 0.054 | 0.090 | 0.309 |
| novel.3413 | 3.70 | 0.054 | 0.090 | -0.937 |
| novel.269 | 3.70 | 0.054 | 0.090 | -1.117 |
| novel.3131 | 3.70 | 0.054 | 0.090 | 3.432 |
| RHSIM_Rhsim12G0194900 | 3.70 | 0.055 | 0.090 | -0.156 |
| RHSIM_RhsimUnG0015200 | 3.70 | 0.055 | 0.090 | 0.865 |
| novel.301 | 3.70 | 0.055 | 0.090 | -0.491 |
| RHSIM_Rhsim10G0155100 | 3.69 | 0.055 | 0.090 | 0.402 |
| novel.271 | 3.69 | 0.055 | 0.090 | -1.220 |
| RHSIM_Rhsim04G0070900 | 3.69 | 0.055 | 0.090 | -0.275 |
| novel.566 | 3.69 | 0.055 | 0.090 | 1.003 |
| RHSIM_Rhsim11G0074000 | 3.69 | 0.055 | 0.090 | -39.799 |
| novel.3619 | 3.69 | 0.055 | 0.090 | 0.268 |

| Gene | LRT statistic | P value | BH-adjusted P | log2FC (interaction) |
|---|---|---|---|---|
| RHSIM_Rhsim08G0203300 | 3.69 | 0.055 | 0.090 | 3.384 |
| novel.469 | 3.69 | 0.055 | 0.090 | 1.209 |
| RHSIM_Rhsim12G0100200 | 3.69 | 0.055 | 0.090 | 3.520 |
| RHSIM_Rhsim01G0131400 | 3.69 | 0.055 | 0.090 | -0.389 |
| RHSIM_RhsimUnG0183100 | 3.68 | 0.055 | 0.090 | 0.726 |
| RHSIM_Rhsim03G0072700 | 3.68 | 0.055 | 0.090 | -0.939 |
| RHSIM_Rhsim09G0109400 | 3.68 | 0.055 | 0.090 | 0.179 |
| RHSIM_Rhsim07G0177600 | 3.68 | 0.055 | 0.090 | 39.774 |
| RHSIM_RhsimUnG0001200 | 3.68 | 0.055 | 0.090 | 2.712 |
| RHSIM_Rhsim12G0092000 | 3.68 | 0.055 | 0.091 | -0.718 |
| RHSIM_Rhsim02G0019000 | 3.68 | 0.055 | 0.091 | 0.186 |
| RHSIM_Rhsim04G0061000 | 3.68 | 0.055 | 0.091 | -40.433 |
| RHSIM_RhsimUnG0158300 | 3.68 | 0.055 | 0.091 | 2.239 |
| RHSIM_Rhsim06G0219600 | 3.68 | 0.055 | 0.091 | 3.556 |
| RHSIM_Rhsim08G0135100 | 3.68 | 0.055 | 0.091 | 0.560 |
| RHSIM_Rhsim07G0250700 | 3.68 | 0.055 | 0.091 | 4.321 |
| RHSIM_Rhsim02G0078800 | 3.67 | 0.055 | 0.091 | 0.957 |
| RHSIM_Rhsim03G0145200 | 3.67 | 0.055 | 0.091 | -0.674 |
| novel.2890 | 3.67 | 0.055 | 0.091 | -1.750 |
| RHSIM_Rhsim07G0219700 | 3.67 | 0.055 | 0.091 | 0.451 |
| RHSIM_Rhsim09G0141500 | 3.67 | 0.055 | 0.091 | 0.175 |
| novel.4172 | 3.67 | 0.055 | 0.091 | -0.840 |
| novel.3010 | 3.67 | 0.055 | 0.091 | -0.458 |
| RHSIM_RhsimUnG0181100 | 3.67 | 0.055 | 0.091 | -0.479 |
| RHSIM_Rhsim07G0013700 | 3.67 | 0.056 | 0.091 | 0.181 |
| RHSIM_Rhsim07G0115300 | 3.67 | 0.056 | 0.091 | -0.367 |
| RHSIM_Rhsim13G0089800 | 3.66 | 0.056 | 0.091 | -1.069 |
| novel.4397 | 3.66 | 0.056 | 0.091 | 4.827 |
| RHSIM_Rhsim03G0199800 | 3.66 | 0.056 | 0.091 | 1.490 |
| RHSIM_Rhsim13G0157800 | 3.66 | 0.056 | 0.091 | -0.191 |
| RHSIM_Rhsim04G0143600 | 3.66 | 0.056 | 0.091 | 0.960 |
| RHSIM_Rhsim06G0006700 | 3.66 | 0.056 | 0.091 | -39.461 |

| Gene | LRT statistic | P value | BH-adjusted P | log2FC (interaction) |
|---|---|---|---|---|
| RHSIM_Rhsim05G0224100 | 3.66 | 0.056 | 0.091 | 0.979 |
| novel.900 | 3.66 | 0.056 | 0.092 | -0.964 |
| RHSIM_Rhsim06G0005500 | 3.66 | 0.056 | 0.092 | 1.028 |
| RHSIM_Rhsim01G0188900 | 3.66 | 0.056 | 0.092 | 38.954 |
| RHSIM_Rhsim02G0172400 | 3.66 | 0.056 | 0.092 | 0.233 |
| novel.1906 | 3.66 | 0.056 | 0.092 | -2.153 |
| RHSIM_Rhsim04G0244800 | 3.65 | 0.056 | 0.092 | 0.676 |
| RHSIM_Rhsim08G0100100 | 3.65 | 0.056 | 0.092 | -1.330 |
| novel.180 | 3.65 | 0.056 | 0.092 | -1.537 |
| novel.2852 | 3.65 | 0.056 | 0.092 | 39.686 |
| RHSIM_Rhsim06G0201100 | 3.65 | 0.056 | 0.092 | -0.447 |
| RHSIM_Rhsim03G0043000 | 3.65 | 0.056 | 0.092 | 3.787 |
| RHSIM_Rhsim07G0197300 | 3.65 | 0.056 | 0.092 | -0.554 |
| RHSIM_Rhsim02G0180100 | 3.65 | 0.056 | 0.092 | 0.285 |
| RHSIM_Rhsim09G0022400 | 3.65 | 0.056 | 0.092 | -0.843 |
| RHSIM_Rhsim08G0174500 | 3.65 | 0.056 | 0.092 | 1.146 |
| RHSIM_Rhsim02G0031900 | 3.65 | 0.056 | 0.092 | -0.133 |
| RHSIM_Rhsim03G0100500 | 3.65 | 0.056 | 0.092 | 3.691 |
| RHSIM_Rhsim12G0200300 | 3.65 | 0.056 | 0.092 | -0.356 |
| RHSIM_Rhsim11G0133500 | 3.65 | 0.056 | 0.092 | -1.424 |
| novel.270 | 3.65 | 0.056 | 0.092 | 2.489 |
| RHSIM_RhsimUnG0056100 | 3.65 | 0.056 | 0.092 | -1.618 |
| RHSIM_Rhsim07G0116100 | 3.65 | 0.056 | 0.092 | 2.018 |
| RHSIM_RhsimUnG0064400 | 3.64 | 0.056 | 0.092 | 1.408 |
| RHSIM_Rhsim13G0124100 | 3.64 | 0.056 | 0.092 | 0.996 |
| RHSIM_Rhsim01G0234400 | 3.64 | 0.056 | 0.092 | -0.434 |
| novel.893 | 3.64 | 0.056 | 0.092 | -0.782 |
| RHSIM_RhsimUnG0041100 | 3.64 | 0.056 | 0.092 | 3.171 |
| RHSIM_Rhsim01G0086300 | 3.64 | 0.056 | 0.093 | 38.917 |
| RHSIM_Rhsim09G0097300 | 3.64 | 0.056 | 0.093 | 39.835 |
| RHSIM_Rhsim05G0152100 | 3.64 | 0.056 | 0.093 | 0.299 |
| novel.4418 | 3.64 | 0.056 | 0.093 | 1.132 |

| Gene | LRT statistic | P value | BH-adjusted P | log2FC (interaction) |
|---|---|---|---|---|
| RHSIM_Rhsim04G0085500 | 3.64 | 0.056 | 0.093 | -0.544 |
| RHSIM_Rhsim06G0044200 | 3.64 | 0.057 | 0.093 | 4.020 |
| RHSIM_Rhsim10G0188200 | 3.63 | 0.057 | 0.093 | 1.635 |
| novel.583 | 3.63 | 0.057 | 0.093 | -0.764 |
| RHSIM_Rhsim09G0209500 | 3.63 | 0.057 | 0.093 | 0.490 |
| RHSIM_Rhsim06G0217400 | 3.63 | 0.057 | 0.093 | -0.335 |
| RHSIM_Rhsim07G0213800 | 3.63 | 0.057 | 0.093 | 0.340 |
| RHSIM_Rhsim13G0090400 | 3.63 | 0.057 | 0.093 | -1.231 |
| RHSIM_Rhsim08G0131200 | 3.63 | 0.057 | 0.093 | 0.200 |
| novel.1574 | 3.63 | 0.057 | 0.093 | 2.513 |
| novel.3115 | 3.63 | 0.057 | 0.093 | 2.257 |
| RHSIM_RhsimUnG0049200 | 3.63 | 0.057 | 0.093 | 3.517 |
| novel.3232 | 3.63 | 0.057 | 0.093 | -2.605 |
| novel.134 | 3.63 | 0.057 | 0.093 | -0.523 |
| RHSIM_Rhsim01G0019400 | 3.63 | 0.057 | 0.093 | -40.676 |
| novel.2478 | 3.63 | 0.057 | 0.093 | -0.305 |
| RHSIM_Rhsim07G0164900 | 3.63 | 0.057 | 0.093 | 1.635 |
| RHSIM_Rhsim02G0066000 | 3.63 | 0.057 | 0.093 | 0.244 |
| RHSIM_Rhsim07G0058800 | 3.63 | 0.057 | 0.093 | 0.152 |
| RHSIM_Rhsim13G0172100 | 3.63 | 0.057 | 0.093 | -0.953 |
| RHSIM_Rhsim03G0139400 | 3.63 | 0.057 | 0.093 | 40.017 |
| RHSIM_Rhsim11G0035000 | 3.63 | 0.057 | 0.093 | 0.770 |
| RHSIM_RhsimUnG0235600 | 3.63 | 0.057 | 0.093 | 1.405 |
| RHSIM_Rhsim03G0240400 | 3.63 | 0.057 | 0.093 | -0.184 |
| RHSIM_Rhsim12G0182700 | 3.63 | 0.057 | 0.093 | 0.246 |
| RHSIM_RhsimUnG0229400 | 3.62 | 0.057 | 0.093 | 40.262 |
| RHSIM_Rhsim13G0044800 | 3.62 | 0.057 | 0.093 | 0.414 |
| RHSIM_Rhsim13G0233600 | 3.62 | 0.057 | 0.093 | -4.330 |
| novel.1243 | 3.62 | 0.057 | 0.093 | -0.676 |
| RHSIM_Rhsim13G0034500 | 3.62 | 0.057 | 0.093 | -0.261 |
| RHSIM_Rhsim03G0024500 | 3.62 | 0.057 | 0.093 | -3.818 |
| RHSIM_Rhsim07G0040100 | 3.62 | 0.057 | 0.094 | -3.958 |

| Gene | LRT statistic | P value | BH-adjusted P | log2FC (interaction) |
|---|---|---|---|---|
| RHSIM_Rhsim11G0174600 | 3.62 | 0.057 | 0.094 | -0.345 |
| RHSIM_Rhsim01G0132200 | 3.62 | 0.057 | 0.094 | -1.680 |
| RHSIM_Rhsim13G0136800 | 3.62 | 0.057 | 0.094 | -0.259 |
| RHSIM_RhsimUnG0139600 | 3.62 | 0.057 | 0.094 | 0.338 |
| RHSIM_Rhsim11G0156400 | 3.61 | 0.057 | 0.094 | 0.492 |
| novel.2494 | 3.61 | 0.057 | 0.094 | 1.868 |
| novel.4378 | 3.61 | 0.057 | 0.094 | 1.298 |
| RHSIM_Rhsim08G0099200 | 3.61 | 0.057 | 0.094 | 0.227 |
| RHSIM_Rhsim04G0039600 | 3.61 | 0.057 | 0.094 | -0.251 |
| RHSIM_Rhsim05G0200500 | 3.61 | 0.058 | 0.094 | 0.137 |
| RHSIM_Rhsim06G0042900 | 3.61 | 0.058 | 0.094 | 3.003 |
| RHSIM_Rhsim08G0128600 | 3.60 | 0.058 | 0.094 | 0.299 |
| RHSIM_Rhsim04G0229800 | 3.60 | 0.058 | 0.094 | -40.456 |
| novel.2828 | 3.60 | 0.058 | 0.094 | -1.415 |
| novel.573 | 3.60 | 0.058 | 0.095 | 0.776 |
| RHSIM_RhsimUnG0195000 | 3.60 | 0.058 | 0.095 | 0.607 |
| RHSIM_Rhsim06G0051800 | 3.60 | 0.058 | 0.095 | -0.854 |
| RHSIM_Rhsim06G0085800 | 3.60 | 0.058 | 0.095 | 3.492 |
| RHSIM_Rhsim01G0271600 | 3.60 | 0.058 | 0.095 | -0.156 |
| RHSIM_Rhsim07G0129900 | 3.60 | 0.058 | 0.095 | 0.508 |
| novel.3476 | 3.60 | 0.058 | 0.095 | 0.966 |
| novel.4475 | 3.60 | 0.058 | 0.095 | -0.830 |
| RHSIM_Rhsim04G0169900 | 3.59 | 0.058 | 0.095 | -39.470 |
| novel.2 | 3.59 | 0.058 | 0.095 | 1.641 |
| RHSIM_Rhsim08G0042700 | 3.59 | 0.058 | 0.095 | -0.388 |
| RHSIM_Rhsim06G0130500 | 3.59 | 0.058 | 0.095 | -0.364 |
| novel.8 | 3.59 | 0.058 | 0.095 | -4.397 |
| RHSIM_Rhsim09G0054100 | 3.59 | 0.058 | 0.095 | 1.729 |
| RHSIM_Rhsim03G0121000 | 3.59 | 0.058 | 0.095 | 0.955 |
| RHSIM_Rhsim06G0055000 | 3.59 | 0.058 | 0.095 | -0.300 |
| RHSIM_Rhsim08G0183700 | 3.58 | 0.058 | 0.095 | 0.199 |
| RHSIM_Rhsim05G0118200 | 3.58 | 0.058 | 0.095 | 1.025 |

| Gene | LRT statistic | P value | BH-adjusted P | log2FC (interaction) |
|---|---|---|---|---|
| RHSIM_Rhsim05G0232000 | 3.58 | 0.058 | 0.095 | -0.655 |
| RHSIM_Rhsim11G0185800 | 3.58 | 0.058 | 0.095 | -0.513 |
| RHSIM_Rhsim09G0157400 | 3.58 | 0.058 | 0.095 | -1.746 |
| RHSIM_Rhsim13G0059500 | 3.58 | 0.058 | 0.095 | 1.446 |
| RHSIM_Rhsim04G0074000 | 3.58 | 0.058 | 0.095 | 0.169 |
| novel.1674 | 3.58 | 0.058 | 0.095 | -2.623 |
| novel.2943 | 3.58 | 0.058 | 0.095 | 1.455 |
| RHSIM_Rhsim11G0071800 | 3.58 | 0.058 | 0.095 | 1.104 |
| RHSIM_Rhsim11G0033100 | 3.58 | 0.058 | 0.095 | 0.972 |
| novel.334 | 3.58 | 0.058 | 0.095 | -0.426 |
| RHSIM_Rhsim04G0171400 | 3.58 | 0.059 | 0.096 | 2.895 |
| novel.468 | 3.58 | 0.059 | 0.096 | 1.204 |
| RHSIM_Rhsim09G0165600 | 3.58 | 0.059 | 0.096 | -0.223 |
| RHSIM_Rhsim05G0008900 | 3.58 | 0.059 | 0.096 | -2.586 |
| RHSIM_Rhsim08G0011300 | 3.57 | 0.059 | 0.096 | -0.162 |
| novel.1000 | 3.57 | 0.059 | 0.096 | -1.828 |
| RHSIM_Rhsim02G0220300 | 3.57 | 0.059 | 0.096 | 1.348 |
| RHSIM_Rhsim13G0121900 | 3.57 | 0.059 | 0.096 | -0.328 |
| RHSIM_Rhsim11G0148100 | 3.57 | 0.059 | 0.096 | -0.255 |
| RHSIM_RhsimUnG0218800 | 3.57 | 0.059 | 0.096 | -38.877 |
| RHSIM_Rhsim01G0128700 | 3.57 | 0.059 | 0.096 | 0.329 |
| RHSIM_Rhsim08G0162400 | 3.57 | 0.059 | 0.096 | 0.456 |
| RHSIM_Rhsim02G0162900 | 3.57 | 0.059 | 0.096 | -0.114 |
| RHSIM_Rhsim06G0209300 | 3.57 | 0.059 | 0.096 | -39.544 |
| RHSIM_Rhsim01G0108400 | 3.57 | 0.059 | 0.096 | 1.076 |
| RHSIM_Rhsim09G0156800 | 3.56 | 0.059 | 0.096 | 0.901 |
| RHSIM_RhsimUnG0244100 | 3.56 | 0.059 | 0.096 | 2.269 |
| RHSIM_Rhsim04G0046200 | 3.56 | 0.059 | 0.096 | 0.270 |
| RHSIM_Rhsim08G0104000 | 3.56 | 0.059 | 0.096 | 40.796 |
| RHSIM_RhsimUnG0151800 | 3.56 | 0.059 | 0.096 | -38.319 |
| RHSIM_Rhsim07G0183900 | 3.56 | 0.059 | 0.096 | 0.618 |
| novel.2057 | 3.56 | 0.059 | 0.097 | 1.679 |

| Gene | LRT statistic | P value | BH-adjusted P | log2FC (interaction) |
|---|---|---|---|---|
| RHSIM_RhsimMtG0002100 | 3.56 | 0.059 | 0.097 | -1.571 |
| RHSIM_Rhsim10G0019000 | 3.56 | 0.059 | 0.097 | 0.213 |
| RHSIM_Rhsim04G0230900 | 3.56 | 0.059 | 0.097 | 0.475 |
| RHSIM_Rhsim05G0214800 | 3.56 | 0.059 | 0.097 | 0.227 |
| RHSIM_Rhsim07G0247400 | 3.56 | 0.059 | 0.097 | 0.317 |
| RHSIM_Rhsim08G0221600 | 3.56 | 0.059 | 0.097 | -0.993 |
| RHSIM_Rhsim04G0125000 | 3.56 | 0.059 | 0.097 | -0.331 |
| RHSIM_Rhsim01G0244200 | 3.56 | 0.059 | 0.097 | -0.517 |
| RHSIM_Rhsim12G0107900 | 3.55 | 0.059 | 0.097 | 0.429 |
| novel.4229 | 3.55 | 0.059 | 0.097 | -38.791 |
| novel.695 | 3.55 | 0.059 | 0.097 | 1.141 |
| RHSIM_Rhsim07G0215300 | 3.55 | 0.059 | 0.097 | -0.299 |
| RHSIM_Rhsim04G0016500 | 3.55 | 0.060 | 0.097 | -0.415 |
| RHSIM_Rhsim12G0007300 | 3.55 | 0.060 | 0.097 | 3.028 |
| RHSIM_Rhsim07G0096100 | 3.55 | 0.060 | 0.097 | -0.187 |
| novel.3103 | 3.55 | 0.060 | 0.097 | -1.275 |
| RHSIM_RhsimUnG0154000 | 3.55 | 0.060 | 0.097 | -1.684 |
| RHSIM_Rhsim11G0038200 | 3.55 | 0.060 | 0.097 | 0.789 |
| RHSIM_Rhsim03G0154300 | 3.55 | 0.060 | 0.097 | 0.395 |
| RHSIM_Rhsim11G0141400 | 3.54 | 0.060 | 0.097 | 0.332 |
| RHSIM_Rhsim04G0028100 | 3.54 | 0.060 | 0.097 | 0.618 |
| RHSIM_Rhsim02G0020000 | 3.54 | 0.060 | 0.097 | 2.362 |
| RHSIM_Rhsim13G0066200 | 3.54 | 0.060 | 0.098 | -0.294 |
| RHSIM_Rhsim01G0288800 | 3.54 | 0.060 | 0.098 | 0.888 |
| RHSIM_Rhsim08G0136900 | 3.54 | 0.060 | 0.098 | 0.268 |
| novel.3994 | 3.54 | 0.060 | 0.098 | -0.988 |
| novel.1909 | 3.54 | 0.060 | 0.098 | 38.643 |
| RHSIM_Rhsim06G0091600 | 3.54 | 0.060 | 0.098 | -0.264 |
| novel.1768 | 3.54 | 0.060 | 0.098 | 1.929 |
| RHSIM_Rhsim04G0223400 | 3.54 | 0.060 | 0.098 | -0.693 |
| RHSIM_Rhsim06G0136900 | 3.53 | 0.060 | 0.098 | 0.233 |
| RHSIM_Rhsim08G0237900 | 3.53 | 0.060 | 0.098 | 0.308 |

| Gene | LRT statistic | P value | BH-adjusted P | log2FC (interaction) |
|---|---|---|---|---|
| RHSIM_Rhsim07G0014300 | 3.53 | 0.060 | 0.098 | 0.356 |
| novel.3517 | 3.53 | 0.060 | 0.098 | -2.127 |
| RHSIM_Rhsim13G0062400 | 3.53 | 0.060 | 0.098 | 0.542 |
| RHSIM_Rhsim07G0020100 | 3.53 | 0.060 | 0.098 | 0.135 |
| RHSIM_Rhsim04G0006600 | 3.53 | 0.060 | 0.098 | -0.194 |
| RHSIM_Rhsim08G0042000 | 3.52 | 0.060 | 0.098 | -0.585 |
| RHSIM_Rhsim07G0244900 | 3.52 | 0.060 | 0.098 | 0.212 |
| RHSIM_Rhsim05G0112300 | 3.52 | 0.060 | 0.098 | 1.502 |
| RHSIM_Rhsim12G0131700 | 3.52 | 0.061 | 0.098 | -2.263 |
| novel.1413 | 3.52 | 0.061 | 0.099 | -1.311 |
| RHSIM_Rhsim09G0081400 | 3.52 | 0.061 | 0.099 | -38.724 |
| RHSIM_RhsimUnG0237300 | 3.52 | 0.061 | 0.099 | 0.886 |
| RHSIM_Rhsim05G0052800 | 3.52 | 0.061 | 0.099 | -39.357 |
| RHSIM_Rhsim07G0072400 | 3.52 | 0.061 | 0.099 | 0.380 |
| RHSIM_Rhsim12G0206800 | 3.51 | 0.061 | 0.099 | 0.309 |
| RHSIM_Rhsim03G0035000 | 3.51 | 0.061 | 0.099 | -0.489 |
| RHSIM_Rhsim04G0056700 | 3.51 | 0.061 | 0.099 | 40.027 |
| RHSIM_Rhsim04G0224900 | 3.51 | 0.061 | 0.099 | 2.753 |
| RHSIM_Rhsim08G0074200 | 3.51 | 0.061 | 0.099 | -0.381 |
| RHSIM_Rhsim01G0221000 | 3.51 | 0.061 | 0.099 | 1.140 |
| RHSIM_Rhsim13G0009900 | 3.51 | 0.061 | 0.099 | -0.544 |
| RHSIM_Rhsim06G0226000 | 3.51 | 0.061 | 0.099 | 1.898 |
| novel.2409 | 3.51 | 0.061 | 0.099 | 1.661 |
| RHSIM_Rhsim06G0148900 | 3.51 | 0.061 | 0.099 | 39.635 |
| RHSIM_RhsimUnG0237500 | 3.51 | 0.061 | 0.099 | -1.195 |
| RHSIM_Rhsim08G0154300 | 3.50 | 0.061 | 0.099 | 0.303 |
| RHSIM_Rhsim04G0179500 | 3.50 | 0.061 | 0.100 | 0.226 |
| RHSIM_Rhsim06G0080200 | 3.50 | 0.061 | 0.100 | -0.835 |
| RHSIM_Rhsim01G0040100 | 3.50 | 0.061 | 0.100 | 0.895 |
| novel.2186 | 3.50 | 0.061 | 0.100 | -0.251 |
| RHSIM_Rhsim10G0129600 | 3.50 | 0.061 | 0.100 | -0.533 |
| novel.2628 | 3.50 | 0.061 | 0.100 | 1.180 |

| Gene | LRT statistic | P value | BH-adjusted P | log2FC (interaction) |
|---|---|---|---|---|
| novel.2076 | 3.50 | 0.061 | 0.100 | 0.355 |
| RHSIM_Rhsim06G0190800 | 3.50 | 0.061 | 0.100 | 0.818 |
| RHSIM_Rhsim11G0090600 | 3.50 | 0.062 | 0.100 | -2.331 |
| RHSIM_Rhsim12G0215000 | 3.50 | 0.062 | 0.100 | 0.280 |
| RHSIM_Rhsim01G0161900 | 3.49 | 0.062 | 0.100 | 2.246 |
| RHSIM_Rhsim13G0013000 | 3.49 | 0.062 | 0.100 | -0.305 |
| RHSIM_Rhsim01G0041100 | 3.49 | 0.062 | 0.100 | -1.371 |
| novel.3112 | 3.49 | 0.062 | 0.100 | 0.766 |
| RHSIM_Rhsim01G0000300 | 3.49 | 0.062 | 0.100 | -0.201 |
| RHSIM_Rhsim07G0118300 | 3.49 | 0.062 | 0.100 | 3.849 |
| RHSIM_RhsimUnG0009300 | 3.49 | 0.062 | 0.100 | -0.129 |
| RHSIM_Rhsim08G0073300 | 3.49 | 0.062 | 0.100 | 2.844 |
| RHSIM_Rhsim10G0077400 | 3.49 | 0.062 | 0.100 | 3.289 |
| RHSIM_Rhsim02G0086300 | 3.49 | 0.062 | 0.100 | 0.325 |
| RHSIM_Rhsim08G0013400 | 3.48 | 0.062 | 0.101 | 0.257 |
| RHSIM_Rhsim12G0156400 | 3.48 | 0.062 | 0.101 | -0.351 |
| RHSIM_Rhsim07G0189900 | 3.48 | 0.062 | 0.101 | 38.901 |
| RHSIM_Rhsim01G0194000 | 3.48 | 0.062 | 0.101 | 1.801 |
| novel.3265 | 3.48 | 0.062 | 0.101 | -1.066 |
| RHSIM_Rhsim13G0188600 | 3.48 | 0.062 | 0.101 | 2.063 |
| RHSIM_Rhsim08G0186700 | 3.48 | 0.062 | 0.101 | 0.179 |
| RHSIM_Rhsim07G0222500 | 3.48 | 0.062 | 0.101 | -0.192 |
| RHSIM_Rhsim12G0125500 | 3.48 | 0.062 | 0.101 | 2.540 |
| RHSIM_Rhsim06G0090200 | 3.48 | 0.062 | 0.101 | 40.136 |
| RHSIM_Rhsim01G0126000 | 3.48 | 0.062 | 0.101 | 0.187 |
| RHSIM_RhsimUnG0189500 | 3.47 | 0.062 | 0.101 | -2.495 |
| RHSIM_Rhsim08G0222800 | 3.47 | 0.062 | 0.101 | -0.815 |
| RHSIM_Rhsim13G0038800 | 3.47 | 0.062 | 0.101 | 0.112 |
| RHSIM_Rhsim09G0138000 | 3.47 | 0.062 | 0.101 | 0.662 |
| RHSIM_Rhsim09G0195700 | 3.47 | 0.063 | 0.101 | 0.126 |
| RHSIM_Rhsim09G0104800 | 3.47 | 0.063 | 0.101 | 0.915 |
| novel.1669 | 3.47 | 0.063 | 0.101 | -38.652 |

| Gene | LRT statistic | P value | BH-adjusted P | log2FC (interaction) |
|---|---|---|---|---|
| RHSIM_Rhsim12G0057000 | 3.47 | 0.063 | 0.102 | 1.960 |
| RHSIM_Rhsim08G0171300 | 3.47 | 0.063 | 0.102 | 0.861 |
| RHSIM_RhsimUnG0255100 | 3.47 | 0.063 | 0.102 | 0.861 |
| RHSIM_Rhsim10G0205300 | 3.47 | 0.063 | 0.102 | -38.715 |
| novel.4584 | 3.46 | 0.063 | 0.102 | 1.585 |
| novel.1602 | 3.46 | 0.063 | 0.102 | 0.214 |
| RHSIM_Rhsim11G0048200 | 3.46 | 0.063 | 0.102 | 40.289 |
| RHSIM_Rhsim03G0225000 | 3.46 | 0.063 | 0.102 | 0.177 |
| RHSIM_Rhsim02G0081800 | 3.46 | 0.063 | 0.102 | 40.914 |
| RHSIM_Rhsim02G0093800 | 3.46 | 0.063 | 0.102 | -1.107 |
| RHSIM_RhsimUnG0045700 | 3.46 | 0.063 | 0.102 | 2.601 |
| RHSIM_Rhsim02G0022600 | 3.46 | 0.063 | 0.102 | -0.370 |
| RHSIM_Rhsim13G0099800 | 3.46 | 0.063 | 0.102 | -0.657 |
| RHSIM_Rhsim10G0151800 | 3.46 | 0.063 | 0.102 | -0.393 |
| RHSIM_Rhsim11G0165100 | 3.46 | 0.063 | 0.102 | 1.165 |
| RHSIM_Rhsim11G0139700 | 3.46 | 0.063 | 0.102 | -0.401 |
| RHSIM_Rhsim11G0181100 | 3.45 | 0.063 | 0.102 | 1.921 |
| novel.2369 | 3.45 | 0.063 | 0.102 | 0.981 |
| RHSIM_Rhsim04G0161400 | 3.45 | 0.063 | 0.102 | -39.977 |
| RHSIM_Rhsim01G0079700 | 3.45 | 0.063 | 0.102 | 1.918 |
| RHSIM_Rhsim13G0181100 | 3.45 | 0.063 | 0.102 | 39.626 |
| novel.2163 | 3.45 | 0.063 | 0.102 | 4.276 |
| RHSIM_Rhsim01G0126200 | 3.45 | 0.063 | 0.102 | 3.766 |
| RHSIM_Rhsim10G0071800 | 3.45 | 0.063 | 0.102 | 2.876 |
| RHSIM_Rhsim04G0185100 | 3.45 | 0.063 | 0.103 | -1.870 |
| RHSIM_Rhsim05G0113300 | 3.45 | 0.063 | 0.103 | 0.159 |
| RHSIM_Rhsim04G0228200 | 3.45 | 0.063 | 0.103 | 0.187 |
| novel.749 | 3.45 | 0.063 | 0.103 | -1.008 |
| RHSIM_RhsimUnG0119500 | 3.45 | 0.063 | 0.103 | 0.256 |
| RHSIM_Rhsim08G0015400 | 3.44 | 0.063 | 0.103 | -0.723 |
| RHSIM_Rhsim03G0131200 | 3.44 | 0.063 | 0.103 | -39.457 |
| RHSIM_Rhsim07G0036200 | 3.44 | 0.063 | 0.103 | -0.317 |

| Gene | LRT statistic | P value | BH-adjusted P | log2FC (interaction) |
|---|---|---|---|---|
| RHSIM_Rhsim04G0132000 | 3.44 | 0.064 | 0.103 | 0.331 |
| novel.1075 | 3.44 | 0.064 | 0.103 | 3.167 |
| RHSIM_Rhsim11G0184200 | 3.44 | 0.064 | 0.103 | 0.155 |
| RHSIM_Rhsim13G0065300 | 3.44 | 0.064 | 0.103 | -1.015 |
| RHSIM_Rhsim12G0110400 | 3.44 | 0.064 | 0.103 | 0.160 |
| novel.1095 | 3.44 | 0.064 | 0.103 | -2.881 |
| RHSIM_Rhsim10G0197000 | 3.44 | 0.064 | 0.103 | 1.013 |
| novel.1133 | 3.44 | 0.064 | 0.103 | 1.066 |
| RHSIM_Rhsim02G0139900 | 3.44 | 0.064 | 0.103 | 41.022 |
| RHSIM_Rhsim02G0245300 | 3.44 | 0.064 | 0.103 | 0.592 |
| novel.3236 | 3.44 | 0.064 | 0.103 | -0.342 |
| RHSIM_Rhsim09G0150400 | 3.43 | 0.064 | 0.103 | 0.881 |
| novel.4304 | 3.43 | 0.064 | 0.103 | -38.941 |
| RHSIM_Rhsim02G0247300 | 3.43 | 0.064 | 0.103 | 0.252 |
| novel.4538 | 3.43 | 0.064 | 0.104 | -2.477 |
| RHSIM_Rhsim12G0070600 | 3.43 | 0.064 | 0.104 | 0.896 |
| RHSIM_Rhsim10G0190500 | 3.43 | 0.064 | 0.104 | -39.837 |
| RHSIM_Rhsim12G0185600 | 3.43 | 0.064 | 0.104 | 1.486 |
| RHSIM_Rhsim04G0180100 | 3.43 | 0.064 | 0.104 | -38.718 |
| RHSIM_RhsimUnG0175000 | 3.42 | 0.064 | 0.104 | -38.712 |
| novel.3295 | 3.42 | 0.064 | 0.104 | 0.654 |
| RHSIM_Rhsim13G0140600 | 3.42 | 0.064 | 0.104 | 0.290 |
| RHSIM_Rhsim07G0104700 | 3.42 | 0.064 | 0.104 | -0.944 |
| RHSIM_Rhsim10G0010900 | 3.42 | 0.064 | 0.104 | 0.379 |
| RHSIM_Rhsim11G0157100 | 3.42 | 0.064 | 0.104 | 40.553 |
| RHSIM_Rhsim07G0010800 | 3.42 | 0.064 | 0.104 | 0.836 |
| RHSIM_Rhsim10G0018200 | 3.42 | 0.064 | 0.104 | -2.213 |
| RHSIM_Rhsim02G0082800 | 3.42 | 0.065 | 0.104 | 0.165 |
| RHSIM_Rhsim04G0170700 | 3.41 | 0.065 | 0.105 | -0.904 |
| RHSIM_Rhsim01G0262200 | 3.41 | 0.065 | 0.105 | 40.647 |
| RHSIM_Rhsim06G0069500 | 3.41 | 0.065 | 0.105 | -0.159 |
| RHSIM_RhsimUnG0223000 | 3.41 | 0.065 | 0.105 | 0.976 |

| Gene | LRT statistic | P value | BH-adjusted P | log2FC (interaction) |
|---|---|---|---|---|
| RHSIM_Rhsim05G0162000 | 3.41 | 0.065 | 0.105 | -0.932 |
| RHSIM_Rhsim01G0289500 | 3.41 | 0.065 | 0.105 | 0.311 |
| RHSIM_RhsimUnG0115400 | 3.41 | 0.065 | 0.105 | 1.274 |
| RHSIM_Rhsim06G0125200 | 3.41 | 0.065 | 0.105 | -2.184 |
| RHSIM_Rhsim13G0014000 | 3.40 | 0.065 | 0.105 | -38.655 |
| RHSIM_Rhsim07G0102900 | 3.40 | 0.065 | 0.105 | -1.656 |
| RHSIM_Rhsim10G0180300 | 3.40 | 0.065 | 0.105 | -0.169 |
| RHSIM_Rhsim06G0069800 | 3.40 | 0.065 | 0.105 | -0.988 |
| RHSIM_Rhsim03G0125200 | 3.40 | 0.065 | 0.105 | 0.637 |
| RHSIM_Rhsim11G0143900 | 3.40 | 0.065 | 0.105 | -1.055 |
| RHSIM_Rhsim06G0057500 | 3.40 | 0.065 | 0.105 | 1.089 |
| RHSIM_Rhsim02G0153000 | 3.40 | 0.065 | 0.105 | 0.600 |
| RHSIM_Rhsim07G0254800 | 3.40 | 0.065 | 0.105 | 0.245 |
| RHSIM_Rhsim06G0011700 | 3.40 | 0.065 | 0.105 | 39.250 |
| RHSIM_Rhsim10G0002600 | 3.40 | 0.065 | 0.105 | -1.513 |
| RHSIM_Rhsim07G0072100 | 3.40 | 0.065 | 0.106 | -0.296 |
| RHSIM_Rhsim09G0114800 | 3.40 | 0.065 | 0.106 | -0.248 |
| RHSIM_Rhsim13G0170500 | 3.39 | 0.065 | 0.106 | 2.231 |
| RHSIM_RhsimUnG0056400 | 3.39 | 0.065 | 0.106 | 0.601 |
| novel.1507 | 3.39 | 0.065 | 0.106 | -1.381 |
| novel.546 | 3.39 | 0.066 | 0.106 | -0.421 |
| RHSIM_Rhsim08G0168900 | 3.39 | 0.066 | 0.106 | -39.155 |
| RHSIM_Rhsim08G0226400 | 3.39 | 0.066 | 0.106 | 39.792 |
| RHSIM_Rhsim13G0065900 | 3.39 | 0.066 | 0.106 | -0.228 |
| novel.742 | 3.39 | 0.066 | 0.106 | -1.389 |
| RHSIM_RhsimUnG0239700 | 3.39 | 0.066 | 0.106 | -0.956 |
| novel.3186 | 3.39 | 0.066 | 0.106 | 0.593 |
| RHSIM_Rhsim05G0144900 | 3.38 | 0.066 | 0.106 | 40.310 |
| RHSIM_Rhsim03G0075900 | 3.38 | 0.066 | 0.106 | -0.606 |
| RHSIM_Rhsim08G0142200 | 3.38 | 0.066 | 0.106 | 39.256 |
| RHSIM_Rhsim10G0175500 | 3.38 | 0.066 | 0.106 | 2.581 |
| RHSIM_Rhsim04G0068800 | 3.38 | 0.066 | 0.106 | -0.367 |

| Gene | LRT statistic | P value | BH-adjusted P | log2FC (interaction) |
|---|---|---|---|---|
| RHSIM_Rhsim03G0227700 | 3.38 | 0.066 | 0.106 | 0.616 |
| novel.2268 | 3.38 | 0.066 | 0.106 | -38.393 |
| RHSIM_Rhsim01G0279700 | 3.38 | 0.066 | 0.107 | -0.426 |
| RHSIM_Rhsim01G0069800 | 3.37 | 0.066 | 0.107 | 2.701 |
| RHSIM_Rhsim05G0173700 | 3.37 | 0.066 | 0.107 | -1.408 |
| RHSIM_Rhsim11G0025900 | 3.37 | 0.066 | 0.107 | 1.212 |
| novel.4299 | 3.37 | 0.066 | 0.107 | 1.196 |
| RHSIM_RhsimUnG0154900 | 3.37 | 0.066 | 0.107 | 40.104 |
| RHSIM_Rhsim07G0066000 | 3.37 | 0.066 | 0.107 | 2.412 |
| RHSIM_Rhsim08G0092300 | 3.37 | 0.066 | 0.107 | 0.322 |
| RHSIM_Rhsim10G0074300 | 3.37 | 0.066 | 0.107 | 1.339 |
| RHSIM_Rhsim08G0222500 | 3.37 | 0.066 | 0.107 | 1.503 |
| RHSIM_Rhsim04G0154300 | 3.37 | 0.066 | 0.107 | 39.606 |
| RHSIM_Rhsim11G0060100 | 3.37 | 0.066 | 0.107 | -0.345 |
| novel.139 | 3.37 | 0.067 | 0.107 | -1.619 |
| RHSIM_Rhsim02G0088600 | 3.37 | 0.067 | 0.107 | -0.768 |
| RHSIM_Rhsim01G0104900 | 3.37 | 0.067 | 0.107 | -39.628 |
| RHSIM_Rhsim13G0135700 | 3.36 | 0.067 | 0.107 | 40.015 |
| RHSIM_Rhsim10G0098900 | 3.36 | 0.067 | 0.107 | -0.264 |
| novel.291 | 3.36 | 0.067 | 0.107 | -3.431 |
| novel.153 | 3.36 | 0.067 | 0.108 | 0.560 |
| RHSIM_Rhsim12G0184500 | 3.36 | 0.067 | 0.108 | 0.515 |
| RHSIM_RhsimUnG0030400 | 3.36 | 0.067 | 0.108 | 3.948 |
| RHSIM_Rhsim04G0071900 | 3.35 | 0.067 | 0.108 | -38.935 |
| RHSIM_Rhsim13G0214600 | 3.35 | 0.067 | 0.108 | 2.445 |
| RHSIM_Rhsim06G0003900 | 3.35 | 0.067 | 0.108 | -0.181 |
| RHSIM_Rhsim04G0192500 | 3.35 | 0.067 | 0.108 | -2.168 |
| RHSIM_Rhsim13G0061000 | 3.35 | 0.067 | 0.108 | -2.349 |
| RHSIM_Rhsim04G0153700 | 3.35 | 0.067 | 0.108 | 2.159 |
| RHSIM_Rhsim07G0118500 | 3.35 | 0.067 | 0.108 | 1.765 |
| RHSIM_Rhsim10G0076200 | 3.35 | 0.067 | 0.108 | 1.102 |
| novel.3401 | 3.34 | 0.067 | 0.109 | -0.913 |

| Gene | LRT statistic | P value | BH-adjusted P | log2FC (interaction) |
|---|---|---|---|---|
| RHSIM_Rhsim05G0018700 | 3.34 | 0.067 | 0.109 | 0.178 |
| RHSIM_Rhsim02G0228900 | 3.34 | 0.067 | 0.109 | 0.165 |
| RHSIM_Rhsim10G0024400 | 3.34 | 0.068 | 0.109 | -38.702 |
| RHSIM_Rhsim03G0181100 | 3.34 | 0.068 | 0.109 | 1.655 |
| RHSIM_Rhsim09G0132500 | 3.34 | 0.068 | 0.109 | 0.715 |
| RHSIM_Rhsim02G0125300 | 3.34 | 0.068 | 0.109 | -1.676 |
| RHSIM_Rhsim09G0185400 | 3.34 | 0.068 | 0.109 | -38.091 |
| RHSIM_RhsimUnG0213200 | 3.34 | 0.068 | 0.109 | -0.897 |
| RHSIM_Rhsim13G0019700 | 3.34 | 0.068 | 0.109 | 2.646 |
| RHSIM_Rhsim13G0077200 | 3.34 | 0.068 | 0.109 | 39.941 |
| RHSIM_Rhsim06G0160000 | 3.34 | 0.068 | 0.109 | -0.968 |
| RHSIM_Rhsim09G0193400 | 3.34 | 0.068 | 0.109 | -0.232 |
| novel.2955 | 3.34 | 0.068 | 0.109 | -1.007 |
| RHSIM_Rhsim11G0119800 | 3.34 | 0.068 | 0.109 | -2.532 |
| RHSIM_Rhsim11G0169600 | 3.34 | 0.068 | 0.109 | -0.787 |
| RHSIM_Rhsim02G0187700 | 3.34 | 0.068 | 0.109 | 0.843 |
| RHSIM_Rhsim01G0001100 | 3.33 | 0.068 | 0.109 | -0.300 |
| RHSIM_Rhsim11G0166800 | 3.33 | 0.068 | 0.109 | 1.358 |
| RHSIM_Rhsim10G0099400 | 3.33 | 0.068 | 0.109 | 0.755 |
| RHSIM_Rhsim01G0281100 | 3.33 | 0.068 | 0.109 | -2.532 |
| RHSIM_RhsimUnG0036200 | 3.33 | 0.068 | 0.110 | 2.678 |
| RHSIM_Rhsim07G0098300 | 3.33 | 0.068 | 0.110 | -0.542 |
| RHSIM_Rhsim07G0200900 | 3.33 | 0.068 | 0.110 | -0.458 |
| RHSIM_Rhsim08G0121200 | 3.32 | 0.068 | 0.110 | -2.397 |
| novel.2709 | 3.32 | 0.068 | 0.110 | 0.858 |
| RHSIM_Rhsim06G0101600 | 3.32 | 0.068 | 0.110 | -0.219 |
| RHSIM_Rhsim04G0144100 | 3.32 | 0.068 | 0.110 | 0.240 |
| novel.2426 | 3.32 | 0.068 | 0.110 | -38.729 |
| RHSIM_RhsimUnG0241400 | 3.32 | 0.068 | 0.110 | 1.327 |
| novel.3935 | 3.32 | 0.068 | 0.110 | -0.901 |
| RHSIM_Rhsim07G0225900 | 3.32 | 0.069 | 0.110 | -2.889 |
| RHSIM_Rhsim11G0067500 | 3.31 | 0.069 | 0.111 | 1.507 |

| Gene | LRT statistic | P value | BH-adjusted P | log2FC (interaction) |
|---|---|---|---|---|
| RHSIM_Rhsim07G0157200 | 3.31 | 0.069 | 0.111 | 39.775 |
| novel.4134 | 3.31 | 0.069 | 0.111 | 0.888 |
| novel.565 | 3.31 | 0.069 | 0.111 | 0.983 |
| RHSIM_Rhsim04G0096400 | 3.31 | 0.069 | 0.111 | -0.212 |
| RHSIM_Rhsim09G0057700 | 3.31 | 0.069 | 0.111 | 0.624 |
| RHSIM_Rhsim03G0069700 | 3.31 | 0.069 | 0.111 | -0.183 |
| RHSIM_Rhsim05G0209200 | 3.31 | 0.069 | 0.111 | -39.555 |
| RHSIM_Rhsim07G0134700 | 3.31 | 0.069 | 0.111 | 0.140 |
| RHSIM_Rhsim04G0077400 | 3.31 | 0.069 | 0.111 | -38.510 |
| RHSIM_Rhsim04G0175600 | 3.30 | 0.069 | 0.111 | 1.503 |
| RHSIM_Rhsim09G0147800 | 3.30 | 0.069 | 0.111 | 1.973 |
| RHSIM_Rhsim10G0067400 | 3.30 | 0.069 | 0.111 | 0.300 |
| RHSIM_Rhsim06G0154700 | 3.30 | 0.069 | 0.111 | 0.894 |
| novel.409 | 3.30 | 0.069 | 0.111 | 4.058 |
| RHSIM_Rhsim11G0043000 | 3.30 | 0.069 | 0.111 | -0.557 |
| RHSIM_Rhsim03G0174100 | 3.30 | 0.069 | 0.111 | 2.586 |
| RHSIM_Rhsim10G0156300 | 3.30 | 0.069 | 0.111 | 0.206 |
| novel.2330 | 3.30 | 0.069 | 0.111 | -0.308 |
| RHSIM_Rhsim06G0143500 | 3.30 | 0.069 | 0.111 | -2.523 |
| RHSIM_Rhsim04G0114400 | 3.30 | 0.069 | 0.111 | 0.670 |
| RHSIM_Rhsim05G0188900 | 3.30 | 0.069 | 0.111 | -1.347 |
| RHSIM_Rhsim11G0101500 | 3.29 | 0.070 | 0.112 | 39.505 |
| novel.3069 | 3.29 | 0.070 | 0.112 | 0.499 |
| RHSIM_Rhsim04G0129800 | 3.29 | 0.070 | 0.112 | 0.213 |
| novel.142 | 3.29 | 0.070 | 0.112 | 0.684 |
| RHSIM_Rhsim11G0079600 | 3.29 | 0.070 | 0.112 | 1.700 |
| RHSIM_Rhsim06G0203200 | 3.29 | 0.070 | 0.112 | -0.513 |
| RHSIM_Rhsim13G0215300 | 3.29 | 0.070 | 0.112 | 0.411 |
| RHSIM_Rhsim04G0006000 | 3.29 | 0.070 | 0.112 | 0.997 |
| RHSIM_Rhsim13G0178700 | 3.28 | 0.070 | 0.112 | -0.846 |
| RHSIM_Rhsim09G0027000 | 3.28 | 0.070 | 0.112 | -3.073 |
| novel.3157 | 3.28 | 0.070 | 0.113 | -0.633 |

| Gene | LRT statistic | P value | BH-adjusted P | log2FC (interaction) |
|---|---|---|---|---|
| RHSIM_Rhsim02G0030800 | 3.28 | 0.070 | 0.113 | 1.148 |
| RHSIM_Rhsim10G0182400 | 3.27 | 0.070 | 0.113 | 1.368 |
| RHSIM_Rhsim08G0209100 | 3.27 | 0.070 | 0.113 | 39.008 |
| RHSIM_Rhsim09G0114600 | 3.27 | 0.070 | 0.113 | -0.415 |
| RHSIM_Rhsim06G0183500 | 3.27 | 0.070 | 0.113 | -0.624 |
| RHSIM_Rhsim12G0103400 | 3.27 | 0.071 | 0.113 | 0.254 |
| novel.644 | 3.27 | 0.071 | 0.113 | -2.809 |
| RHSIM_Rhsim01G0174300 | 3.27 | 0.071 | 0.113 | 1.289 |
| RHSIM_Rhsim10G0034200 | 3.27 | 0.071 | 0.113 | -3.389 |
| novel.2124 | 3.27 | 0.071 | 0.113 | 1.394 |
| novel.3162 | 3.27 | 0.071 | 0.113 | 0.569 |
| RHSIM_Rhsim07G0121400 | 3.27 | 0.071 | 0.113 | -1.519 |
| RHSIM_Rhsim03G0017100 | 3.26 | 0.071 | 0.113 | -1.371 |
| RHSIM_RhsimUnG0173900 | 3.26 | 0.071 | 0.113 | 1.347 |
| RHSIM_Rhsim01G0280800 | 3.26 | 0.071 | 0.113 | -0.704 |
| RHSIM_Rhsim02G0030600 | 3.26 | 0.071 | 0.114 | 2.151 |
| RHSIM_Rhsim02G0143700 | 3.26 | 0.071 | 0.114 | -39.825 |
| RHSIM_Rhsim11G0174800 | 3.26 | 0.071 | 0.114 | -0.739 |
| RHSIM_Rhsim09G0151400 | 3.26 | 0.071 | 0.114 | 0.183 |
| RHSIM_Rhsim09G0025200 | 3.26 | 0.071 | 0.114 | -0.479 |
| RHSIM_Rhsim13G0068300 | 3.26 | 0.071 | 0.114 | -2.179 |
| RHSIM_Rhsim01G0155400 | 3.26 | 0.071 | 0.114 | 0.238 |
| novel.2954 | 3.26 | 0.071 | 0.114 | 1.111 |
| RHSIM_Rhsim01G0094000 | 3.26 | 0.071 | 0.114 | 1.319 |
| RHSIM_Rhsim09G0154500 | 3.26 | 0.071 | 0.114 | 2.771 |
| RHSIM_Rhsim07G0135200 | 3.26 | 0.071 | 0.114 | 1.953 |
| RHSIM_Rhsim07G0145900 | 3.26 | 0.071 | 0.114 | 0.406 |
| novel.984 | 3.26 | 0.071 | 0.114 | 1.963 |
| RHSIM_Rhsim08G0169200 | 3.25 | 0.071 | 0.114 | 0.125 |
| RHSIM_Rhsim07G0115400 | 3.25 | 0.071 | 0.114 | -0.188 |
| RHSIM_Rhsim01G0004600 | 3.25 | 0.071 | 0.114 | 38.627 |
| novel.3665 | 3.25 | 0.071 | 0.114 | -40.379 |

| Gene | LRT statistic | P value | BH-adjusted P | log2FC (interaction) |
|---|---|---|---|---|
| RHSIM_Rhsim11G0141100 | 3.25 | 0.071 | 0.114 | 0.181 |
| RHSIM_Rhsim10G0024700 | 3.25 | 0.071 | 0.114 | 0.848 |
| RHSIM_Rhsim02G0040800 | 3.25 | 0.071 | 0.114 | 0.484 |
| RHSIM_Rhsim04G0175000 | 3.25 | 0.071 | 0.114 | -38.937 |
| RHSIM_Rhsim06G0066100 | 3.25 | 0.071 | 0.114 | 0.788 |
| RHSIM_Rhsim11G0125600 | 3.25 | 0.071 | 0.114 | 0.333 |
| RHSIM_Rhsim04G0083700 | 3.25 | 0.071 | 0.114 | -0.910 |
| RHSIM_Rhsim01G0267700 | 3.25 | 0.071 | 0.114 | 1.099 |
| RHSIM_RhsimUnG0139200 | 3.25 | 0.071 | 0.114 | -1.575 |
| RHSIM_Rhsim10G0184000 | 3.25 | 0.071 | 0.114 | -0.445 |
| novel.1773 | 3.25 | 0.072 | 0.114 | -0.533 |
| RHSIM_Rhsim05G0025000 | 3.25 | 0.072 | 0.114 | 1.123 |
| RHSIM_Rhsim02G0132100 | 3.25 | 0.072 | 0.115 | 0.441 |
| RHSIM_Rhsim10G0190800 | 3.25 | 0.072 | 0.115 | 0.294 |
| novel.88 | 3.25 | 0.072 | 0.115 | -38.399 |
| RHSIM_Rhsim10G0086200 | 3.25 | 0.072 | 0.115 | -39.849 |
| novel.1263 | 3.24 | 0.072 | 0.115 | -0.572 |
| RHSIM_Rhsim04G0229200 | 3.24 | 0.072 | 0.115 | -0.208 |
| RHSIM_RhsimUnG0207900 | 3.24 | 0.072 | 0.115 | 39.051 |
| RHSIM_Rhsim01G0102200 | 3.24 | 0.072 | 0.115 | 3.956 |
| RHSIM_Rhsim13G0129800 | 3.24 | 0.072 | 0.115 | -0.365 |
| novel.15 | 3.24 | 0.072 | 0.115 | 0.561 |
| RHSIM_Rhsim01G0039100 | 3.24 | 0.072 | 0.115 | 0.707 |
| RHSIM_Rhsim04G0199000 | 3.24 | 0.072 | 0.115 | -4.689 |
| novel.574 | 3.24 | 0.072 | 0.115 | -0.283 |
| RHSIM_Rhsim08G0182800 | 3.24 | 0.072 | 0.115 | -1.302 |
| RHSIM_Rhsim07G0065000 | 3.23 | 0.072 | 0.115 | 0.332 |
| RHSIM_Rhsim13G0184100 | 3.23 | 0.072 | 0.115 | 4.180 |
| RHSIM_RhsimUnG0029100 | 3.23 | 0.072 | 0.115 | -39.860 |
| RHSIM_Rhsim10G0135400 | 3.23 | 0.072 | 0.115 | 38.752 |
| novel.412 | 3.23 | 0.072 | 0.116 | 2.349 |
| RHSIM_Rhsim03G0026600 | 3.23 | 0.072 | 0.116 | 2.461 |

| Gene | LRT statistic | P value | BH-adjusted P | log2FC (interaction) |
|---|---|---|---|---|
| novel.1799 | 3.23 | 0.072 | 0.116 | -38.235 |
| RHSIM_Rhsim08G0058200 | 3.23 | 0.072 | 0.116 | -39.155 |
| RHSIM_Rhsim05G0036500 | 3.23 | 0.072 | 0.116 | 0.879 |
| RHSIM_Rhsim12G0021500 | 3.22 | 0.073 | 0.116 | -2.947 |
| novel.3175 | 3.22 | 0.073 | 0.116 | 1.376 |
| RHSIM_Rhsim06G0226600 | 3.22 | 0.073 | 0.116 | 0.459 |
| novel.2497 | 3.22 | 0.073 | 0.116 | 39.506 |
| RHSIM_Rhsim08G0047500 | 3.22 | 0.073 | 0.116 | 2.038 |
| novel.4573 | 3.22 | 0.073 | 0.116 | -0.529 |
| RHSIM_Rhsim06G0086700 | 3.22 | 0.073 | 0.116 | 1.471 |
| RHSIM_Rhsim03G0222900 | 3.22 | 0.073 | 0.116 | 0.938 |
| RHSIM_Rhsim03G0262000 | 3.22 | 0.073 | 0.116 | -1.152 |
| RHSIM_Rhsim10G0153600 | 3.22 | 0.073 | 0.116 | 0.867 |
| RHSIM_Rhsim08G0064000 | 3.22 | 0.073 | 0.116 | 2.001 |
| novel.3426 | 3.22 | 0.073 | 0.116 | 3.630 |
| RHSIM_RhsimUnG0130800 | 3.22 | 0.073 | 0.116 | 0.371 |
| RHSIM_Rhsim02G0073100 | 3.22 | 0.073 | 0.116 | -1.839 |
| RHSIM_Rhsim07G0063100 | 3.21 | 0.073 | 0.117 | 0.243 |
| RHSIM_Rhsim13G0193300 | 3.21 | 0.073 | 0.117 | -1.786 |
| RHSIM_Rhsim06G0165600 | 3.21 | 0.073 | 0.117 | 1.099 |
| RHSIM_Rhsim08G0060600 | 3.21 | 0.073 | 0.117 | -39.226 |
| novel.2227 | 3.21 | 0.073 | 0.117 | 0.349 |
| RHSIM_Rhsim12G0039000 | 3.21 | 0.073 | 0.117 | 0.558 |
| novel.4118 | 3.21 | 0.073 | 0.117 | 0.724 |
| RHSIM_Rhsim11G0087100 | 3.21 | 0.073 | 0.117 | -0.278 |
| RHSIM_Rhsim07G0139800 | 3.21 | 0.073 | 0.117 | -0.184 |
| RHSIM_Rhsim13G0105000 | 3.21 | 0.073 | 0.117 | 0.270 |
| RHSIM_Rhsim08G0180400 | 3.21 | 0.073 | 0.117 | 3.916 |
| novel.3968 | 3.21 | 0.073 | 0.117 | -0.817 |
| novel.4285 | 3.20 | 0.073 | 0.117 | 39.250 |
| RHSIM_Rhsim11G0064900 | 3.20 | 0.073 | 0.117 | -0.352 |
| RHSIM_Rhsim06G0173900 | 3.20 | 0.073 | 0.117 | -0.161 |

| Gene | LRT statistic | P value | BH-adjusted P | log2FC (interaction) |
|---|---|---|---|---|
| novel.1399 | 3.20 | 0.073 | 0.117 | 0.248 |
| RHSIM_Rhsim10G0076900 | 3.20 | 0.074 | 0.117 | 2.556 |
| RHSIM_Rhsim13G0070600 | 3.20 | 0.074 | 0.117 | 2.165 |
| RHSIM_RhsimMtG0006700 | 3.20 | 0.074 | 0.117 | -0.612 |
| RHSIM_Rhsim10G0062800 | 3.20 | 0.074 | 0.117 | 2.429 |
| RHSIM_Rhsim02G0060200 | 3.20 | 0.074 | 0.117 | -0.211 |
| RHSIM_RhsimUnG0051800 | 3.20 | 0.074 | 0.117 | 3.372 |
| RHSIM_RhsimUnG0013300 | 3.20 | 0.074 | 0.118 | -0.818 |
| novel.3167 | 3.20 | 0.074 | 0.118 | -0.630 |
| novel.3588 | 3.20 | 0.074 | 0.118 | 1.221 |
| RHSIM_Rhsim06G0128800 | 3.19 | 0.074 | 0.118 | 1.213 |
| RHSIM_Rhsim02G0252100 | 3.19 | 0.074 | 0.118 | 0.525 |
| novel.4158 | 3.19 | 0.074 | 0.118 | -1.907 |
| RHSIM_Rhsim05G0168900 | 3.19 | 0.074 | 0.118 | 0.234 |
| RHSIM_Rhsim12G0200500 | 3.19 | 0.074 | 0.118 | 0.783 |
| RHSIM_Rhsim03G0092100 | 3.19 | 0.074 | 0.118 | 0.405 |
| RHSIM_Rhsim04G0200200 | 3.19 | 0.074 | 0.118 | -2.341 |
| RHSIM_RhsimUnG0127100 | 3.19 | 0.074 | 0.118 | 1.055 |
| RHSIM_Rhsim07G0196900 | 3.19 | 0.074 | 0.118 | -1.163 |
| RHSIM_Rhsim05G0111700 | 3.19 | 0.074 | 0.118 | 0.850 |
| RHSIM_Rhsim07G0069400 | 3.19 | 0.074 | 0.118 | -38.726 |
| novel.2425 | 3.19 | 0.074 | 0.118 | 1.260 |
| RHSIM_Rhsim13G0197500 | 3.19 | 0.074 | 0.118 | -0.127 |
| novel.4325 | 3.19 | 0.074 | 0.118 | -1.678 |
| RHSIM_Rhsim05G0130900 | 3.18 | 0.074 | 0.118 | 2.493 |
| RHSIM_Rhsim02G0058800 | 3.18 | 0.074 | 0.119 | -0.213 |
| RHSIM_Rhsim13G0177000 | 3.18 | 0.075 | 0.119 | 0.136 |
| RHSIM_Rhsim05G0094600 | 3.18 | 0.075 | 0.119 | -0.180 |
| RHSIM_Rhsim03G0246600 | 3.18 | 0.075 | 0.119 | 0.267 |
| RHSIM_Rhsim05G0224600 | 3.18 | 0.075 | 0.119 | -0.171 |
| novel.824 | 3.18 | 0.075 | 0.119 | 1.389 |
| RHSIM_Rhsim02G0187400 | 3.18 | 0.075 | 0.119 | 0.543 |

| Gene | LRT statistic | P value | BH-adjusted P | log2FC (interaction) |
|---|---|---|---|---|
| RHSIM_Rhsim03G0187800 | 3.18 | 0.075 | 0.119 | 1.647 |
| RHSIM_Rhsim05G0173800 | 3.18 | 0.075 | 0.119 | 0.903 |
| RHSIM_Rhsim02G0232100 | 3.18 | 0.075 | 0.119 | -2.405 |
| RHSIM_Rhsim10G0018800 | 3.17 | 0.075 | 0.119 | 0.310 |
| RHSIM_Rhsim12G0067700 | 3.17 | 0.075 | 0.119 | -1.634 |
| RHSIM_Rhsim06G0126600 | 3.17 | 0.075 | 0.119 | -1.036 |
| RHSIM_Rhsim12G0022900 | 3.17 | 0.075 | 0.119 | -1.717 |
| RHSIM_Rhsim05G0176900 | 3.17 | 0.075 | 0.119 | 3.018 |
| RHSIM_Rhsim03G0253500 | 3.17 | 0.075 | 0.119 | 0.483 |
| RHSIM_Rhsim06G0155700 | 3.17 | 0.075 | 0.119 | 0.122 |
| RHSIM_Rhsim03G0123200 | 3.17 | 0.075 | 0.119 | 0.431 |
| RHSIM_Rhsim11G0121300 | 3.17 | 0.075 | 0.119 | -0.116 |
| novel.986 | 3.17 | 0.075 | 0.119 | -0.950 |
| RHSIM_Rhsim12G0152600 | 3.17 | 0.075 | 0.119 | -1.821 |
| RHSIM_Rhsim07G0116400 | 3.17 | 0.075 | 0.119 | 0.616 |
| RHSIM_Rhsim05G0071900 | 3.17 | 0.075 | 0.119 | 39.019 |
| RHSIM_Rhsim03G0164300 | 3.17 | 0.075 | 0.120 | -0.355 |
| RHSIM_RhsimUnG0220200 | 3.17 | 0.075 | 0.120 | 1.394 |
| RHSIM_RhsimUnG0174500 | 3.17 | 0.075 | 0.120 | -0.928 |
| RHSIM_Rhsim03G0029600 | 3.16 | 0.075 | 0.120 | 39.959 |
| novel.3257 | 3.16 | 0.075 | 0.120 | -1.319 |
| RHSIM_Rhsim12G0001500 | 3.16 | 0.075 | 0.120 | -0.279 |
| RHSIM_Rhsim05G0221800 | 3.16 | 0.075 | 0.120 | -0.787 |
| RHSIM_Rhsim07G0168400 | 3.16 | 0.075 | 0.120 | -1.640 |
| RHSIM_Rhsim04G0120000 | 3.16 | 0.075 | 0.120 | 1.253 |
| RHSIM_Rhsim10G0182700 | 3.16 | 0.075 | 0.120 | -0.870 |
| RHSIM_Rhsim02G0007600 | 3.16 | 0.075 | 0.120 | -0.390 |
| RHSIM_Rhsim03G0051100 | 3.16 | 0.075 | 0.120 | 0.164 |
| RHSIM_Rhsim13G0208900 | 3.16 | 0.075 | 0.120 | -0.635 |
| RHSIM_Rhsim04G0142900 | 3.16 | 0.075 | 0.120 | 0.329 |
| RHSIM_Rhsim12G0164600 | 3.16 | 0.076 | 0.120 | -2.237 |
| RHSIM_Rhsim04G0050500 | 3.16 | 0.076 | 0.120 | 1.973 |

| Gene | LRT statistic | P value | BH-adjusted P | log2FC (interaction) |
|---|---|---|---|---|
| RHSIM_Rhsim02G0156600 | 3.16 | 0.076 | 0.120 | 39.486 |
| RHSIM_RhsimUnG0115500 | 3.16 | 0.076 | 0.120 | 2.130 |
| novel.1110 | 3.15 | 0.076 | 0.120 | 1.255 |
| RHSIM_Rhsim06G0205600 | 3.15 | 0.076 | 0.120 | 0.226 |
| RHSIM_Rhsim02G0219400 | 3.15 | 0.076 | 0.120 | -38.923 |
| RHSIM_Rhsim06G0053000 | 3.15 | 0.076 | 0.121 | -38.572 |
| RHSIM_Rhsim07G0049200 | 3.15 | 0.076 | 0.121 | -0.123 |
| RHSIM_Rhsim02G0102600 | 3.15 | 0.076 | 0.121 | 1.200 |
| RHSIM_Rhsim13G0215600 | 3.15 | 0.076 | 0.121 | 39.545 |
| RHSIM_Rhsim06G0200700 | 3.15 | 0.076 | 0.121 | -0.510 |
| RHSIM_Rhsim13G0090000 | 3.15 | 0.076 | 0.121 | 0.409 |
| novel.4320 | 3.15 | 0.076 | 0.121 | 2.065 |
| novel.4383 | 3.14 | 0.076 | 0.121 | 0.495 |
| RHSIM_Rhsim05G0162100 | 3.14 | 0.076 | 0.121 | -0.323 |
| RHSIM_Rhsim08G0094400 | 3.14 | 0.076 | 0.121 | -39.671 |
| RHSIM_Rhsim06G0083200 | 3.14 | 0.076 | 0.121 | -0.227 |
| RHSIM_Rhsim06G0205800 | 3.14 | 0.076 | 0.121 | -0.117 |
| RHSIM_Rhsim11G0167400 | 3.14 | 0.076 | 0.121 | -0.867 |
| RHSIM_Rhsim05G0053300 | 3.14 | 0.076 | 0.121 | -1.038 |
| novel.1481 | 3.14 | 0.077 | 0.122 | 0.459 |
| RHSIM_Rhsim05G0102900 | 3.14 | 0.077 | 0.122 | -0.851 |
| novel.4175 | 3.14 | 0.077 | 0.122 | -0.734 |
| RHSIM_Rhsim07G0045300 | 3.13 | 0.077 | 0.122 | -3.172 |
| RHSIM_Rhsim05G0006100 | 3.13 | 0.077 | 0.122 | -2.230 |
| RHSIM_Rhsim13G0102000 | 3.13 | 0.077 | 0.122 | 1.325 |
| RHSIM_Rhsim13G0010300 | 3.13 | 0.077 | 0.122 | 1.314 |
| novel.2712 | 3.13 | 0.077 | 0.122 | 0.318 |
| RHSIM_Rhsim02G0066200 | 3.13 | 0.077 | 0.122 | 0.645 |
| RHSIM_Rhsim03G0052400 | 3.13 | 0.077 | 0.122 | -0.311 |
| RHSIM_Rhsim03G0200200 | 3.13 | 0.077 | 0.122 | 0.147 |
| RHSIM_Rhsim04G0116800 | 3.13 | 0.077 | 0.122 | -0.377 |
| RHSIM_Rhsim12G0119200 | 3.13 | 0.077 | 0.122 | 3.093 |

| Gene | LRT statistic | P value | BH-adjusted P | log2FC (interaction) |
|---|---|---|---|---|
| RHSIM_Rhsim11G0188600 | 3.13 | 0.077 | 0.122 | 1.454 |
| RHSIM_Rhsim03G0079500 | 3.13 | 0.077 | 0.122 | 0.629 |
| RHSIM_Rhsim05G0198000 | 3.13 | 0.077 | 0.122 | -1.604 |
| RHSIM_Rhsim09G0047200 | 3.13 | 0.077 | 0.122 | 0.909 |
| RHSIM_Rhsim13G0090800 | 3.12 | 0.077 | 0.122 | 1.824 |
| RHSIM_Rhsim13G0124000 | 3.12 | 0.077 | 0.122 | -0.352 |
| novel.4310 | 3.12 | 0.077 | 0.123 | 0.656 |
| RHSIM_Rhsim01G0287700 | 3.12 | 0.077 | 0.123 | 0.213 |
| novel.1384 | 3.12 | 0.077 | 0.123 | -1.269 |
| RHSIM_Rhsim11G0003900 | 3.12 | 0.077 | 0.123 | 2.790 |
| RHSIM_Rhsim01G0156700 | 3.12 | 0.077 | 0.123 | 3.206 |
| novel.2950 | 3.12 | 0.077 | 0.123 | 2.068 |
| RHSIM_Rhsim07G0215800 | 3.12 | 0.078 | 0.123 | 0.179 |
| novel.2175 | 3.11 | 0.078 | 0.123 | 1.621 |
| RHSIM_Rhsim08G0163700 | 3.11 | 0.078 | 0.123 | -0.631 |
| RHSIM_Rhsim10G0067800 | 3.11 | 0.078 | 0.123 | 0.292 |
| RHSIM_Rhsim01G0285000 | 3.11 | 0.078 | 0.123 | 0.136 |
| RHSIM_Rhsim05G0209600 | 3.11 | 0.078 | 0.123 | -39.642 |
| RHSIM_Rhsim04G0099200 | 3.11 | 0.078 | 0.123 | -0.403 |
| novel.3043 | 3.11 | 0.078 | 0.123 | -2.463 |
| novel.973 | 3.11 | 0.078 | 0.123 | -0.758 |
| RHSIM_Rhsim03G0110800 | 3.11 | 0.078 | 0.123 | 0.290 |
| RHSIM_Rhsim02G0106300 | 3.11 | 0.078 | 0.123 | 0.491 |
| RHSIM_Rhsim07G0164200 | 3.11 | 0.078 | 0.123 | 2.408 |
| novel.1625 | 3.11 | 0.078 | 0.123 | 0.463 |
| novel.2520 | 3.11 | 0.078 | 0.124 | 0.811 |
| RHSIM_Rhsim05G0095100 | 3.11 | 0.078 | 0.124 | -0.113 |
| novel.661 | 3.11 | 0.078 | 0.124 | -0.091 |
| RHSIM_Rhsim06G0126300 | 3.10 | 0.078 | 0.124 | 0.866 |
| RHSIM_Rhsim07G0056200 | 3.10 | 0.078 | 0.124 | -1.197 |
| RHSIM_Rhsim10G0169400 | 3.10 | 0.078 | 0.124 | 0.722 |
| novel.808 | 3.10 | 0.078 | 0.124 | -0.497 |

| Gene | LRT statistic | P value | BH-adjusted P | log2FC (interaction) |
|---|---|---|---|---|
| RHSIM_RhsimUnG0127500 | 3.10 | 0.078 | 0.124 | 2.074 |
| RHSIM_Rhsim04G0064800 | 3.10 | 0.078 | 0.124 | 2.824 |
| RHSIM_Rhsim04G0062600 | 3.10 | 0.078 | 0.124 | -3.160 |
| RHSIM_Rhsim07G0075200 | 3.10 | 0.078 | 0.124 | -0.455 |
| RHSIM_RhsimUnG0123200 | 3.10 | 0.078 | 0.124 | -2.356 |
| RHSIM_Rhsim12G0064700 | 3.10 | 0.078 | 0.124 | 0.176 |
| novel.620 | 3.10 | 0.079 | 0.124 | -1.276 |
| RHSIM_Rhsim07G0152000 | 3.09 | 0.079 | 0.124 | -3.749 |
| novel.3889 | 3.09 | 0.079 | 0.124 | 0.345 |
| RHSIM_Rhsim03G0067700 | 3.09 | 0.079 | 0.125 | -0.502 |
| novel.526 | 3.09 | 0.079 | 0.125 | -0.350 |
| RHSIM_Rhsim02G0188500 | 3.09 | 0.079 | 0.125 | 0.260 |
| RHSIM_Rhsim13G0099600 | 3.09 | 0.079 | 0.125 | 1.616 |
| RHSIM_RhsimUnG0239200 | 3.09 | 0.079 | 0.125 | -0.282 |
| RHSIM_Rhsim03G0188800 | 3.09 | 0.079 | 0.125 | 0.901 |
| RHSIM_Rhsim13G0157400 | 3.09 | 0.079 | 0.125 | -0.205 |
| novel.1726 | 3.09 | 0.079 | 0.125 | 1.005 |
| novel.3326 | 3.09 | 0.079 | 0.125 | 2.062 |
| novel.3772 | 3.08 | 0.079 | 0.125 | -1.503 |
| RHSIM_Rhsim05G0074100 | 3.08 | 0.079 | 0.125 | -0.593 |
| RHSIM_Rhsim05G0171000 | 3.08 | 0.079 | 0.125 | 4.162 |
| RHSIM_Rhsim12G0178300 | 3.08 | 0.079 | 0.125 | -0.345 |
| RHSIM_Rhsim13G0108200 | 3.08 | 0.079 | 0.125 | -0.673 |
| novel.3328 | 3.08 | 0.079 | 0.125 | -1.245 |
| RHSIM_Rhsim07G0053200 | 3.08 | 0.079 | 0.125 | 2.899 |
| RHSIM_Rhsim02G0228200 | 3.08 | 0.079 | 0.125 | -2.456 |
| novel.292 | 3.08 | 0.079 | 0.125 | 0.585 |
| RHSIM_RhsimUnG0257000 | 3.08 | 0.079 | 0.125 | 1.646 |
| RHSIM_Rhsim07G0078400 | 3.08 | 0.079 | 0.125 | 39.892 |
| RHSIM_Rhsim04G0065200 | 3.08 | 0.079 | 0.126 | -0.559 |
| RHSIM_Rhsim11G0120000 | 3.08 | 0.079 | 0.126 | -0.273 |
| RHSIM_RhsimUnG0210500 | 3.08 | 0.079 | 0.126 | -1.221 |

| Gene | LRT statistic | P value | BH-adjusted P | log2FC (interaction) |
|---|---|---|---|---|
| RHSIM_Rhsim08G0067700 | 3.07 | 0.080 | 0.126 | 0.218 |
| novel.968 | 3.07 | 0.080 | 0.126 | 0.278 |
| RHSIM_Rhsim07G0070200 | 3.07 | 0.080 | 0.126 | -6.758 |
| RHSIM_Rhsim13G0007200 | 3.07 | 0.080 | 0.126 | 1.880 |
| novel.605 | 3.07 | 0.080 | 0.126 | 0.810 |
| RHSIM_Rhsim08G0243700 | 3.07 | 0.080 | 0.126 | 2.105 |
| RHSIM_Rhsim03G0257700 | 3.07 | 0.080 | 0.126 | 1.625 |
| RHSIM_Rhsim09G0210200 | 3.07 | 0.080 | 0.126 | 1.446 |
| RHSIM_Rhsim01G0089400 | 3.07 | 0.080 | 0.126 | -0.259 |
| novel.3508 | 3.07 | 0.080 | 0.126 | 0.510 |
| novel.1990 | 3.07 | 0.080 | 0.126 | -1.525 |
| RHSIM_Rhsim07G0067000 | 3.07 | 0.080 | 0.126 | 1.441 |
| RHSIM_Rhsim04G0208400 | 3.07 | 0.080 | 0.126 | 0.375 |
| RHSIM_Rhsim04G0096300 | 3.06 | 0.080 | 0.126 | -38.605 |
| RHSIM_Rhsim01G0187600 | 3.06 | 0.080 | 0.126 | -1.422 |
| RHSIM_Rhsim04G0223100 | 3.06 | 0.080 | 0.126 | 2.077 |
| RHSIM_Rhsim11G0122300 | 3.06 | 0.080 | 0.127 | -2.242 |
| RHSIM_Rhsim02G0029300 | 3.06 | 0.080 | 0.127 | 2.490 |
| novel.43 | 3.06 | 0.080 | 0.127 | 0.802 |
| RHSIM_Rhsim11G0119500 | 3.06 | 0.080 | 0.127 | 2.110 |
| RHSIM_Rhsim12G0001600 | 3.06 | 0.080 | 0.127 | 0.414 |
| RHSIM_Rhsim01G0080300 | 3.06 | 0.080 | 0.127 | 0.300 |
| RHSIM_Rhsim13G0186400 | 3.06 | 0.080 | 0.127 | 3.300 |
| RHSIM_Rhsim01G0132000 | 3.06 | 0.080 | 0.127 | 0.128 |
| RHSIM_Rhsim05G0111100 | 3.06 | 0.080 | 0.127 | 1.309 |
| RHSIM_Rhsim12G0184700 | 3.06 | 0.080 | 0.127 | 3.208 |
| RHSIM_RhsimUnG0100900 | 3.06 | 0.080 | 0.127 | -39.516 |
| RHSIM_Rhsim10G0023100 | 3.06 | 0.080 | 0.127 | 0.225 |
| novel.1358 | 3.06 | 0.080 | 0.127 | -0.439 |
| RHSIM_Rhsim08G0066800 | 3.06 | 0.080 | 0.127 | 0.870 |
| RHSIM_Rhsim04G0012500 | 3.06 | 0.080 | 0.127 | 0.297 |
| novel.3237 | 3.06 | 0.080 | 0.127 | 0.603 |

| Gene | LRT statistic | P value | BH-adjusted P | log2FC (interaction) |
|---|---|---|---|---|
| novel.2449 | 3.06 | 0.080 | 0.127 | -2.658 |
| RHSIM_Rhsim05G0083800 | 3.06 | 0.080 | 0.127 | 2.408 |
| RHSIM_Rhsim08G0062100 | 3.05 | 0.081 | 0.127 | -2.402 |
| novel.181 | 3.05 | 0.081 | 0.127 | -1.638 |
| novel.284 | 3.05 | 0.081 | 0.127 | 2.259 |
| RHSIM_Rhsim13G0214000 | 3.05 | 0.081 | 0.127 | -0.381 |
| novel.1471 | 3.05 | 0.081 | 0.127 | -2.486 |
| RHSIM_RhsimUnG0149000 | 3.05 | 0.081 | 0.127 | 40.266 |
| RHSIM_Rhsim12G0075800 | 3.05 | 0.081 | 0.127 | 0.947 |
| novel.1161 | 3.05 | 0.081 | 0.127 | -0.889 |
| RHSIM_Rhsim10G0078800 | 3.05 | 0.081 | 0.127 | -39.466 |
| novel.4244 | 3.05 | 0.081 | 0.127 | -0.671 |
| RHSIM_RhsimUnG0166000 | 3.05 | 0.081 | 0.127 | 39.345 |
| RHSIM_Rhsim01G0292200 | 3.05 | 0.081 | 0.127 | 40.361 |
| RHSIM_Rhsim08G0213600 | 3.05 | 0.081 | 0.127 | 0.363 |
| RHSIM_Rhsim11G0058900 | 3.05 | 0.081 | 0.128 | -0.131 |
| RHSIM_Rhsim05G0144700 | 3.05 | 0.081 | 0.128 | 1.571 |
| RHSIM_Rhsim02G0019700 | 3.05 | 0.081 | 0.128 | 0.239 |
| novel.1437 | 3.05 | 0.081 | 0.128 | -0.963 |
| novel.834 | 3.05 | 0.081 | 0.128 | -1.705 |
| novel.4404 | 3.05 | 0.081 | 0.128 | 0.713 |
| RHSIM_Rhsim01G0051200 | 3.05 | 0.081 | 0.128 | 1.377 |
| RHSIM_Rhsim07G0220600 | 3.05 | 0.081 | 0.128 | 1.995 |
| RHSIM_Rhsim03G0227000 | 3.04 | 0.081 | 0.128 | -1.137 |
| RHSIM_Rhsim07G0037200 | 3.04 | 0.081 | 0.128 | 0.320 |
| RHSIM_Rhsim10G0112400 | 3.04 | 0.081 | 0.128 | 0.222 |
| RHSIM_RhsimUnG0117900 | 3.04 | 0.081 | 0.128 | 0.157 |
| RHSIM_Rhsim11G0080000 | 3.04 | 0.081 | 0.128 | 3.277 |
| RHSIM_Rhsim03G0182700 | 3.04 | 0.081 | 0.128 | 0.897 |
| RHSIM_RhsimUnG0229600 | 3.04 | 0.081 | 0.128 | -0.909 |
| novel.4307 | 3.04 | 0.081 | 0.128 | -38.905 |
| RHSIM_Rhsim05G0173300 | 3.04 | 0.081 | 0.128 | 0.767 |

| Gene | LRT statistic | P value | BH-adjusted P | log2FC (interaction) |
|---|---|---|---|---|
| RHSIM_Rhsim09G0168100 | 3.03 | 0.082 | 0.128 | -2.189 |
| RHSIM_Rhsim04G0225800 | 3.03 | 0.082 | 0.129 | 39.193 |
| RHSIM_Rhsim04G0081700 | 3.03 | 0.082 | 0.129 | -0.542 |
| RHSIM_Rhsim03G0186400 | 3.03 | 0.082 | 0.129 | -38.216 |
| RHSIM_Rhsim01G0172100 | 3.03 | 0.082 | 0.129 | 0.259 |
| RHSIM_Rhsim10G0015900 | 3.03 | 0.082 | 0.129 | 1.385 |
| RHSIM_Rhsim01G0013700 | 3.03 | 0.082 | 0.129 | -1.621 |
| RHSIM_Rhsim04G0058400 | 3.02 | 0.082 | 0.129 | 39.375 |
| RHSIM_Rhsim01G0185000 | 3.02 | 0.082 | 0.129 | 0.240 |
| RHSIM_Rhsim08G0014900 | 3.02 | 0.082 | 0.129 | 0.186 |
| RHSIM_Rhsim08G0068700 | 3.02 | 0.082 | 0.129 | -0.158 |
| RHSIM_Rhsim10G0120000 | 3.02 | 0.082 | 0.129 | 1.308 |
| RHSIM_Rhsim05G0222000 | 3.02 | 0.082 | 0.129 | -2.944 |
| RHSIM_Rhsim07G0008500 | 3.02 | 0.082 | 0.129 | -0.625 |
| RHSIM_Rhsim09G0171200 | 3.02 | 0.082 | 0.130 | 1.526 |
| novel.976 | 3.02 | 0.082 | 0.130 | -0.702 |
| RHSIM_Rhsim04G0207200 | 3.02 | 0.082 | 0.130 | -0.248 |
| novel.225 | 3.02 | 0.082 | 0.130 | 40.025 |
| RHSIM_Rhsim05G0178400 | 3.02 | 0.082 | 0.130 | 4.649 |
| RHSIM_Rhsim09G0086900 | 3.02 | 0.082 | 0.130 | 2.625 |
| RHSIM_Rhsim10G0146300 | 3.01 | 0.083 | 0.130 | -0.813 |
| RHSIM_Rhsim06G0098800 | 3.01 | 0.083 | 0.130 | -0.222 |
| novel.4199 | 3.01 | 0.083 | 0.130 | 1.610 |
| RHSIM_Rhsim09G0052900 | 3.01 | 0.083 | 0.130 | 38.859 |
| RHSIM_Rhsim07G0187100 | 3.01 | 0.083 | 0.130 | -2.410 |
| RHSIM_Rhsim05G0090900 | 3.01 | 0.083 | 0.130 | 3.633 |
| RHSIM_Rhsim02G0063400 | 3.01 | 0.083 | 0.130 | -38.354 |
| RHSIM_Rhsim06G0002100 | 3.01 | 0.083 | 0.130 | -1.322 |
| RHSIM_Rhsim08G0050100 | 3.01 | 0.083 | 0.130 | 0.509 |
| RHSIM_Rhsim03G0260100 | 3.01 | 0.083 | 0.130 | -0.402 |
| RHSIM_Rhsim06G0188300 | 3.01 | 0.083 | 0.130 | -2.724 |
| RHSIM_Rhsim07G0098700 | 3.01 | 0.083 | 0.130 | -1.627 |

| Gene | LRT statistic | P value | BH-adjusted P | log2FC (interaction) |
|---|---|---|---|---|
| RHSIM_Rhsim08G0130300 | 3.01 | 0.083 | 0.130 | 2.029 |
| novel.1521 | 3.01 | 0.083 | 0.130 | -2.274 |
| RHSIM_Rhsim01G0183300 | 3.01 | 0.083 | 0.131 | -0.262 |
| RHSIM_Rhsim03G0231500 | 3.00 | 0.083 | 0.131 | 0.265 |
| RHSIM_Rhsim08G0045900 | 3.00 | 0.083 | 0.131 | 0.240 |
| RHSIM_Rhsim12G0037500 | 3.00 | 0.083 | 0.131 | -0.681 |
| RHSIM_Rhsim06G0018000 | 3.00 | 0.083 | 0.131 | 1.147 |
| novel.256 | 3.00 | 0.083 | 0.131 | 0.995 |
| RHSIM_RhsimUnG0121700 | 3.00 | 0.083 | 0.131 | -0.656 |
| RHSIM_Rhsim08G0049900 | 3.00 | 0.083 | 0.131 | -0.319 |
| RHSIM_Rhsim07G0135600 | 3.00 | 0.083 | 0.131 | 2.147 |
| RHSIM_Rhsim01G0076400 | 3.00 | 0.083 | 0.131 | -2.266 |
| RHSIM_Rhsim08G0174700 | 3.00 | 0.083 | 0.131 | -0.377 |
| novel.3577 | 3.00 | 0.083 | 0.131 | -0.253 |
| RHSIM_Rhsim09G0004500 | 3.00 | 0.083 | 0.131 | -0.512 |
| RHSIM_Rhsim09G0153700 | 3.00 | 0.083 | 0.131 | 0.464 |
| RHSIM_RhsimUnG0087600 | 3.00 | 0.083 | 0.131 | 0.800 |
| RHSIM_Rhsim04G0233700 | 3.00 | 0.083 | 0.131 | 2.171 |
| RHSIM_Rhsim09G0146500 | 2.99 | 0.084 | 0.132 | -0.294 |
| RHSIM_Rhsim09G0102400 | 2.99 | 0.084 | 0.132 | 0.221 |
| RHSIM_Rhsim03G0203800 | 2.99 | 0.084 | 0.132 | 1.311 |
| RHSIM_Rhsim09G0105100 | 2.99 | 0.084 | 0.132 | -0.243 |
| RHSIM_Rhsim02G0033500 | 2.99 | 0.084 | 0.132 | -38.759 |
| RHSIM_Rhsim05G0172200 | 2.99 | 0.084 | 0.132 | -0.261 |
| RHSIM_RhsimUnG0101600 | 2.99 | 0.084 | 0.132 | 0.414 |
| RHSIM_Rhsim12G0110100 | 2.99 | 0.084 | 0.132 | -0.379 |
| RHSIM_Rhsim05G0004500 | 2.99 | 0.084 | 0.132 | 4.791 |
| RHSIM_Rhsim01G0132700 | 2.99 | 0.084 | 0.132 | -0.181 |
| RHSIM_Rhsim03G0027700 | 2.99 | 0.084 | 0.132 | -4.615 |
| RHSIM_Rhsim10G0027100 | 2.98 | 0.084 | 0.132 | 2.091 |
| RHSIM_RhsimUnG0054100 | 2.98 | 0.084 | 0.132 | 0.442 |
| RHSIM_RhsimUnG0091100 | 2.98 | 0.084 | 0.132 | -0.463 |

| Gene | LRT statistic | P value | BH-adjusted P | log2FC (interaction) |
|---|---|---|---|---|
| RHSIM_RhsimUnG0125900 | 2.98 | 0.084 | 0.132 | 2.159 |
| RHSIM_Rhsim08G0247600 | 2.98 | 0.084 | 0.133 | 2.169 |
| novel.2223 | 2.98 | 0.084 | 0.133 | 0.759 |
| RHSIM_Rhsim12G0213100 | 2.98 | 0.084 | 0.133 | 39.535 |
| RHSIM_RhsimUnG0220100 | 2.98 | 0.085 | 0.133 | -1.054 |
| novel.506 | 2.98 | 0.085 | 0.133 | 2.121 |
| RHSIM_Rhsim09G0070500 | 2.97 | 0.085 | 0.133 | 38.966 |
| RHSIM_Rhsim12G0199900 | 2.97 | 0.085 | 0.133 | 0.221 |
| RHSIM_Rhsim04G0062800 | 2.97 | 0.085 | 0.133 | -3.680 |
| RHSIM_Rhsim02G0235700 | 2.97 | 0.085 | 0.133 | 0.266 |
| RHSIM_Rhsim03G0061700 | 2.97 | 0.085 | 0.133 | -0.522 |
| RHSIM_Rhsim09G0006200 | 2.97 | 0.085 | 0.133 | -1.402 |
| RHSIM_Rhsim03G0266500 | 2.97 | 0.085 | 0.133 | 0.141 |
| RHSIM_Rhsim09G0212500 | 2.97 | 0.085 | 0.133 | -0.148 |
| RHSIM_Rhsim02G0230100 | 2.97 | 0.085 | 0.133 | -4.463 |
| RHSIM_Rhsim11G0005000 | 2.97 | 0.085 | 0.133 | 0.124 |
| RHSIM_Rhsim08G0130100 | 2.97 | 0.085 | 0.133 | -1.937 |
| RHSIM_Rhsim09G0065100 | 2.97 | 0.085 | 0.133 | -0.341 |
| RHSIM_Rhsim13G0196900 | 2.96 | 0.085 | 0.134 | -0.149 |
| RHSIM_Rhsim05G0148900 | 2.96 | 0.085 | 0.134 | -0.305 |
| RHSIM_Rhsim05G0088300 | 2.96 | 0.085 | 0.134 | -0.245 |
| RHSIM_Rhsim12G0147600 | 2.96 | 0.085 | 0.134 | 1.703 |
| RHSIM_RhsimUnG0125400 | 2.96 | 0.085 | 0.134 | 0.600 |
| novel.2644 | 2.96 | 0.085 | 0.134 | 0.225 |
| novel.3059 | 2.96 | 0.085 | 0.134 | -2.498 |
| RHSIM_RhsimUnG0054700 | 2.96 | 0.085 | 0.134 | -38.635 |
| RHSIM_Rhsim08G0003400 | 2.96 | 0.086 | 0.134 | -0.343 |
| RHSIM_Rhsim03G0232400 | 2.96 | 0.086 | 0.134 | -0.931 |
| RHSIM_Rhsim01G0157500 | 2.95 | 0.086 | 0.134 | 0.655 |
| RHSIM_Rhsim08G0224300 | 2.95 | 0.086 | 0.134 | 0.797 |
| RHSIM_RhsimUnG0015500 | 2.95 | 0.086 | 0.134 | -0.604 |
| RHSIM_Rhsim01G0127400 | 2.95 | 0.086 | 0.134 | 38.803 |

| Gene | LRT statistic | P value | BH-adjusted P | log2FC (interaction) |
|---|---|---|---|---|
| novel.3524 | 2.95 | 0.086 | 0.134 | 1.481 |
| RHSIM_Rhsim07G0057300 | 2.95 | 0.086 | 0.134 | -0.199 |
| novel.1956 | 2.95 | 0.086 | 0.135 | 0.426 |
| RHSIM_Rhsim07G0243000 | 2.95 | 0.086 | 0.135 | 0.319 |
| RHSIM_Rhsim05G0135700 | 2.95 | 0.086 | 0.135 | 0.353 |
| RHSIM_Rhsim11G0171500 | 2.95 | 0.086 | 0.135 | 0.890 |
| RHSIM_Rhsim07G0186900 | 2.95 | 0.086 | 0.135 | 38.674 |
| RHSIM_Rhsim13G0079200 | 2.95 | 0.086 | 0.135 | 0.197 |
| novel.3757 | 2.95 | 0.086 | 0.135 | 0.937 |
| RHSIM_Rhsim08G0074700 | 2.95 | 0.086 | 0.135 | 2.151 |
| RHSIM_Rhsim08G0139700 | 2.95 | 0.086 | 0.135 | 1.350 |
| RHSIM_Rhsim02G0213400 | 2.95 | 0.086 | 0.135 | 1.571 |
| novel.3348 | 2.95 | 0.086 | 0.135 | -1.514 |
| RHSIM_Rhsim04G0209100 | 2.95 | 0.086 | 0.135 | 1.516 |
| RHSIM_Rhsim04G0047000 | 2.94 | 0.086 | 0.135 | -1.298 |
| RHSIM_Rhsim02G0250800 | 2.94 | 0.086 | 0.135 | 1.032 |
| novel.1760 | 2.94 | 0.086 | 0.135 | 39.164 |
| novel.2169 | 2.94 | 0.086 | 0.135 | -38.493 |
| novel.1518 | 2.94 | 0.086 | 0.135 | -38.418 |
| RHSIM_Rhsim03G0199700 | 2.94 | 0.086 | 0.135 | -0.435 |
| novel.1689 | 2.94 | 0.086 | 0.135 | -0.845 |
| RHSIM_Rhsim03G0051200 | 2.94 | 0.086 | 0.135 | -3.261 |
| RHSIM_RhsimPtG0004600 | 2.94 | 0.087 | 0.135 | 0.306 |
| RHSIM_Rhsim01G0066400 | 2.94 | 0.087 | 0.135 | -4.044 |
| novel.110 | 2.94 | 0.087 | 0.136 | -1.784 |
| RHSIM_Rhsim04G0115900 | 2.94 | 0.087 | 0.136 | -1.559 |
| RHSIM_RhsimUnG0120000 | 2.94 | 0.087 | 0.136 | -1.213 |
| RHSIM_Rhsim01G0113900 | 2.93 | 0.087 | 0.136 | 1.110 |
| RHSIM_Rhsim01G0168900 | 2.93 | 0.087 | 0.136 | -0.367 |
| RHSIM_Rhsim05G0053100 | 2.93 | 0.087 | 0.136 | 0.212 |
| RHSIM_Rhsim08G0097600 | 2.93 | 0.087 | 0.136 | 0.827 |
| novel.3357 | 2.93 | 0.087 | 0.136 | 1.603 |

| Gene | LRT statistic | P value | BH-adjusted P | log2FC (interaction) |
|---|---|---|---|---|
| RHSIM_Rhsim04G0005900 | 2.93 | 0.087 | 0.136 | -0.556 |
| RHSIM_Rhsim06G0154100 | 2.93 | 0.087 | 0.136 | -0.240 |
| RHSIM_Rhsim02G0253000 | 2.93 | 0.087 | 0.136 | -2.457 |
| RHSIM_Rhsim03G0024600 | 2.93 | 0.087 | 0.136 | -39.447 |
| RHSIM_Rhsim08G0110000 | 2.92 | 0.087 | 0.137 | -38.585 |
| novel.937 | 2.92 | 0.087 | 0.137 | 0.739 |
| RHSIM_Rhsim02G0233500 | 2.92 | 0.087 | 0.137 | -1.091 |
| RHSIM_Rhsim12G0133500 | 2.92 | 0.087 | 0.137 | -0.532 |
| RHSIM_Rhsim06G0106700 | 2.92 | 0.087 | 0.137 | -39.186 |
| RHSIM_Rhsim02G0236100 | 2.92 | 0.087 | 0.137 | -0.122 |
| RHSIM_Rhsim08G0152600 | 2.92 | 0.087 | 0.137 | -0.348 |
| RHSIM_Rhsim03G0225400 | 2.92 | 0.087 | 0.137 | 0.995 |
| RHSIM_Rhsim02G0195500 | 2.92 | 0.088 | 0.137 | 0.827 |
| RHSIM_Rhsim01G0136700 | 2.92 | 0.088 | 0.137 | 0.477 |
| novel.2927 | 2.92 | 0.088 | 0.137 | 3.166 |
| RHSIM_RhsimUnG0006400 | 2.92 | 0.088 | 0.137 | 1.173 |
| RHSIM_Rhsim03G0221700 | 2.92 | 0.088 | 0.137 | 1.096 |
| RHSIM_Rhsim10G0079000 | 2.92 | 0.088 | 0.137 | 0.402 |
| RHSIM_Rhsim03G0064700 | 2.91 | 0.088 | 0.137 | 0.322 |
| RHSIM_Rhsim05G0170300 | 2.91 | 0.088 | 0.138 | -2.890 |
| RHSIM_Rhsim01G0261700 | 2.91 | 0.088 | 0.138 | 0.299 |
| RHSIM_Rhsim07G0064600 | 2.91 | 0.088 | 0.138 | -0.490 |
| RHSIM_Rhsim09G0133200 | 2.91 | 0.088 | 0.138 | -2.114 |
| RHSIM_Rhsim02G0031500 | 2.91 | 0.088 | 0.138 | -0.134 |
| RHSIM_Rhsim02G0138500 | 2.91 | 0.088 | 0.138 | -0.135 |
| RHSIM_Rhsim06G0043800 | 2.91 | 0.088 | 0.138 | -0.895 |
| RHSIM_Rhsim09G0181200 | 2.91 | 0.088 | 0.138 | 1.697 |
| RHSIM_Rhsim04G0059700 | 2.91 | 0.088 | 0.138 | 1.029 |
| RHSIM_Rhsim11G0079700 | 2.91 | 0.088 | 0.138 | 1.656 |
| novel.3860 | 2.91 | 0.088 | 0.138 | 1.306 |
| RHSIM_Rhsim09G0073800 | 2.91 | 0.088 | 0.138 | 0.413 |
| novel.1962 | 2.90 | 0.088 | 0.138 | 0.517 |

| Gene | LRT statistic | P value | BH-adjusted P | log2FC (interaction) |
|---|---|---|---|---|
| RHSIM_Rhsim04G0113900 | 2.90 | 0.088 | 0.138 | -0.718 |
| RHSIM_Rhsim03G0262100 | 2.90 | 0.088 | 0.138 | 0.285 |
| RHSIM_Rhsim07G0135900 | 2.90 | 0.088 | 0.138 | 0.699 |
| RHSIM_Rhsim12G0079200 | 2.90 | 0.088 | 0.138 | -2.668 |
| RHSIM_Rhsim01G0114600 | 2.90 | 0.088 | 0.138 | -0.087 |
| RHSIM_Rhsim04G0052000 | 2.90 | 0.088 | 0.138 | -0.848 |
| novel.3378 | 2.90 | 0.088 | 0.138 | 0.635 |
| RHSIM_Rhsim07G0158900 | 2.90 | 0.088 | 0.138 | 0.555 |
| RHSIM_Rhsim02G0170800 | 2.90 | 0.089 | 0.138 | 39.064 |
| RHSIM_Rhsim03G0150200 | 2.90 | 0.089 | 0.138 | 0.435 |
| RHSIM_Rhsim06G0007600 | 2.90 | 0.089 | 0.138 | 0.137 |
| RHSIM_Rhsim02G0162700 | 2.90 | 0.089 | 0.139 | 1.406 |
| RHSIM_Rhsim01G0164000 | 2.90 | 0.089 | 0.139 | 0.579 |
| RHSIM_Rhsim13G0218500 | 2.90 | 0.089 | 0.139 | -1.003 |
| RHSIM_Rhsim04G0140600 | 2.90 | 0.089 | 0.139 | 0.965 |
| RHSIM_Rhsim09G0089900 | 2.89 | 0.089 | 0.139 | 0.833 |
| RHSIM_Rhsim02G0182900 | 2.89 | 0.089 | 0.139 | 0.648 |
| RHSIM_Rhsim03G0056600 | 2.89 | 0.089 | 0.139 | -1.371 |
| RHSIM_Rhsim06G0242800 | 2.89 | 0.089 | 0.139 | 0.112 |
| RHSIM_Rhsim02G0181500 | 2.89 | 0.089 | 0.139 | 0.724 |
| RHSIM_Rhsim12G0201800 | 2.89 | 0.089 | 0.139 | -0.382 |
| RHSIM_RhsimPtG0004100 | 2.89 | 0.089 | 0.139 | -0.357 |
| RHSIM_Rhsim13G0010400 | 2.89 | 0.089 | 0.139 | -0.343 |
| RHSIM_Rhsim03G0270100 | 2.89 | 0.089 | 0.139 | -0.426 |
| RHSIM_Rhsim03G0225800 | 2.89 | 0.089 | 0.139 | -0.240 |
| novel.821 | 2.89 | 0.089 | 0.139 | 1.065 |
| RHSIM_Rhsim10G0044800 | 2.89 | 0.089 | 0.139 | 0.637 |
| novel.3324 | 2.89 | 0.089 | 0.139 | -1.031 |
| RHSIM_Rhsim09G0170200 | 2.89 | 0.089 | 0.139 | -1.692 |
| RHSIM_Rhsim09G0131100 | 2.89 | 0.089 | 0.139 | 39.489 |
| RHSIM_Rhsim01G0114400 | 2.88 | 0.090 | 0.140 | -0.408 |
| novel.3459 | 2.88 | 0.090 | 0.140 | 0.630 |

| Gene | LRT statistic | P value | BH-adjusted P | log2FC (interaction) |
|---|---|---|---|---|
| RHSIM_Rhsim08G0096000 | 2.88 | 0.090 | 0.140 | 2.306 |
| RHSIM_Rhsim07G0028200 | 2.88 | 0.090 | 0.140 | -0.205 |
| RHSIM_Rhsim13G0118600 | 2.88 | 0.090 | 0.140 | 2.785 |
| RHSIM_Rhsim01G0070600 | 2.88 | 0.090 | 0.140 | -0.659 |
| RHSIM_Rhsim08G0233700 | 2.88 | 0.090 | 0.140 | 38.650 |
| RHSIM_Rhsim04G0018600 | 2.88 | 0.090 | 0.140 | 0.451 |
| RHSIM_Rhsim13G0199400 | 2.88 | 0.090 | 0.140 | -0.190 |
| RHSIM_Rhsim04G0210100 | 2.88 | 0.090 | 0.140 | 0.778 |
| RHSIM_Rhsim04G0099800 | 2.88 | 0.090 | 0.140 | -0.207 |
| RHSIM_Rhsim08G0021100 | 2.87 | 0.090 | 0.140 | -0.814 |
| RHSIM_Rhsim01G0032800 | 2.87 | 0.090 | 0.140 | 39.879 |
| RHSIM_RhsimUnG0142100 | 2.87 | 0.090 | 0.141 | 1.181 |
| novel.3823 | 2.87 | 0.090 | 0.141 | -37.965 |
| RHSIM_Rhsim13G0136900 | 2.87 | 0.090 | 0.141 | 0.283 |
| novel.4489 | 2.87 | 0.090 | 0.141 | 1.618 |
| RHSIM_Rhsim05G0041800 | 2.87 | 0.090 | 0.141 | 2.535 |
| RHSIM_Rhsim07G0012200 | 2.87 | 0.090 | 0.141 | 0.503 |
| RHSIM_Rhsim06G0239900 | 2.87 | 0.090 | 0.141 | -1.585 |
| novel.4480 | 2.87 | 0.090 | 0.141 | -39.075 |
| RHSIM_RhsimUnG0212900 | 2.86 | 0.091 | 0.141 | -0.625 |
| RHSIM_Rhsim12G0053600 | 2.86 | 0.091 | 0.141 | -0.701 |
| RHSIM_Rhsim07G0238700 | 2.86 | 0.091 | 0.141 | -1.301 |
| novel.3546 | 2.86 | 0.091 | 0.141 | 0.435 |
| RHSIM_Rhsim01G0222600 | 2.86 | 0.091 | 0.142 | -38.540 |
| RHSIM_Rhsim09G0208100 | 2.86 | 0.091 | 0.142 | -0.149 |
| RHSIM_Rhsim05G0065200 | 2.86 | 0.091 | 0.142 | -0.456 |
| RHSIM_Rhsim04G0036700 | 2.86 | 0.091 | 0.142 | -0.587 |
| RHSIM_Rhsim01G0010200 | 2.85 | 0.091 | 0.142 | 0.486 |
| novel.2869 | 2.85 | 0.091 | 0.142 | -2.920 |
| RHSIM_Rhsim04G0240400 | 2.85 | 0.091 | 0.142 | 3.520 |
| RHSIM_Rhsim12G0109500 | 2.85 | 0.091 | 0.142 | 39.266 |
| RHSIM_Rhsim13G0178200 | 2.85 | 0.091 | 0.142 | 0.104 |

| Gene | LRT statistic | P value | BH-adjusted P | log2FC (interaction) |
|---|---|---|---|---|
| RHSIM_Rhsim08G0011700 | 2.85 | 0.091 | 0.142 | 0.392 |
| RHSIM_Rhsim10G0189900 | 2.85 | 0.091 | 0.142 | 38.335 |
| novel.3731 | 2.85 | 0.091 | 0.142 | 0.996 |
| RHSIM_Rhsim10G0081400 | 2.85 | 0.091 | 0.142 | -0.427 |
| novel.3239 | 2.85 | 0.091 | 0.142 | -2.139 |
| novel.2269 | 2.85 | 0.091 | 0.142 | -0.993 |
| RHSIM_Rhsim06G0123600 | 2.85 | 0.091 | 0.142 | 1.288 |
| RHSIM_Rhsim05G0041400 | 2.85 | 0.092 | 0.142 | 0.587 |
| RHSIM_Rhsim09G0142400 | 2.85 | 0.092 | 0.142 | 2.459 |
| RHSIM_Rhsim06G0128300 | 2.85 | 0.092 | 0.142 | -0.605 |
| RHSIM_Rhsim06G0185500 | 2.85 | 0.092 | 0.143 | 0.457 |
| novel.2778 | 2.84 | 0.092 | 0.143 | 2.249 |
| RHSIM_Rhsim10G0103100 | 2.84 | 0.092 | 0.143 | 1.606 |
| RHSIM_Rhsim07G0143800 | 2.84 | 0.092 | 0.143 | -2.047 |
| RHSIM_Rhsim03G0022300 | 2.84 | 0.092 | 0.143 | -0.512 |
| novel.1186 | 2.84 | 0.092 | 0.143 | -1.245 |
| RHSIM_RhsimUnG0089700 | 2.84 | 0.092 | 0.143 | 2.205 |
| RHSIM_Rhsim04G0224300 | 2.84 | 0.092 | 0.143 | 39.693 |
| RHSIM_Rhsim13G0003600 | 2.84 | 0.092 | 0.143 | 0.747 |
| RHSIM_Rhsim07G0140500 | 2.84 | 0.092 | 0.143 | 1.301 |
| novel.2207 | 2.84 | 0.092 | 0.143 | 1.334 |
| RHSIM_Rhsim07G0138300 | 2.84 | 0.092 | 0.143 | 4.129 |
| RHSIM_Rhsim01G0240400 | 2.84 | 0.092 | 0.143 | -0.269 |
| novel.1222 | 2.84 | 0.092 | 0.143 | -1.102 |
| RHSIM_Rhsim05G0091000 | 2.84 | 0.092 | 0.143 | 39.541 |
| RHSIM_Rhsim06G0140000 | 2.84 | 0.092 | 0.143 | 1.743 |
| novel.2553 | 2.84 | 0.092 | 0.143 | 2.568 |
| novel.3371 | 2.83 | 0.092 | 0.143 | 0.577 |
| RHSIM_Rhsim13G0201600 | 2.83 | 0.092 | 0.143 | -0.288 |
| novel.1401 | 2.83 | 0.092 | 0.143 | 2.150 |
| RHSIM_Rhsim07G0133000 | 2.83 | 0.092 | 0.143 | 2.859 |
| RHSIM_Rhsim07G0057500 | 2.83 | 0.092 | 0.143 | 0.551 |

| Gene | LRT statistic | P value | BH-adjusted P | log2FC (interaction) |
|---|---|---|---|---|
| RHSIM_Rhsim11G0128200 | 2.83 | 0.092 | 0.143 | -1.060 |
| novel.4315 | 2.83 | 0.092 | 0.143 | 38.258 |
| RHSIM_RhsimUnG0040200 | 2.83 | 0.092 | 0.144 | -2.773 |
| RHSIM_Rhsim10G0199100 | 2.83 | 0.092 | 0.144 | -0.774 |
| RHSIM_Rhsim11G0178700 | 2.83 | 0.092 | 0.144 | 3.713 |
| RHSIM_Rhsim13G0010700 | 2.83 | 0.092 | 0.144 | 0.608 |
| RHSIM_Rhsim09G0007200 | 2.83 | 0.092 | 0.144 | 0.349 |
| RHSIM_Rhsim02G0031200 | 2.83 | 0.092 | 0.144 | -0.247 |
| RHSIM_Rhsim04G0207100 | 2.83 | 0.092 | 0.144 | 1.928 |
| RHSIM_Rhsim12G0026600 | 2.83 | 0.093 | 0.144 | -37.952 |
| novel.2851 | 2.83 | 0.093 | 0.144 | -1.288 |
| RHSIM_Rhsim09G0196500 | 2.83 | 0.093 | 0.144 | 0.119 |
| RHSIM_RhsimUnG0097000 | 2.83 | 0.093 | 0.144 | -2.823 |
| RHSIM_Rhsim02G0047200 | 2.83 | 0.093 | 0.144 | -0.879 |
| RHSIM_Rhsim05G0119500 | 2.83 | 0.093 | 0.144 | 0.197 |
| RHSIM_Rhsim12G0014800 | 2.83 | 0.093 | 0.144 | -0.305 |
| novel.3539 | 2.83 | 0.093 | 0.144 | 0.868 |
| RHSIM_Rhsim04G0064200 | 2.83 | 0.093 | 0.144 | 1.974 |
| novel.1776 | 2.82 | 0.093 | 0.144 | -1.509 |
| novel.1453 | 2.82 | 0.093 | 0.144 | -1.223 |
| RHSIM_Rhsim01G0098800 | 2.82 | 0.093 | 0.144 | 4.568 |
| RHSIM_Rhsim08G0187100 | 2.82 | 0.093 | 0.144 | -38.331 |
| RHSIM_Rhsim12G0208700 | 2.82 | 0.093 | 0.144 | -1.090 |
| RHSIM_Rhsim10G0043500 | 2.82 | 0.093 | 0.144 | 1.802 |
| RHSIM_Rhsim08G0194500 | 2.82 | 0.093 | 0.144 | 0.533 |
| RHSIM_Rhsim13G0106500 | 2.82 | 0.093 | 0.144 | 0.758 |
| novel.4170 | 2.82 | 0.093 | 0.145 | 1.158 |
| RHSIM_Rhsim02G0142800 | 2.82 | 0.093 | 0.145 | 0.275 |
| novel.4119 | 2.82 | 0.093 | 0.145 | -38.698 |
| RHSIM_Rhsim02G0087200 | 2.82 | 0.093 | 0.145 | -1.566 |
| RHSIM_Rhsim01G0230400 | 2.82 | 0.093 | 0.145 | -0.767 |
| novel.303 | 2.81 | 0.093 | 0.145 | 37.862 |

| Gene | LRT statistic | P value | BH-adjusted P | log2FC (interaction) |
|---|---|---|---|---|
| RHSIM_Rhsim09G0103600 | 2.81 | 0.093 | 0.145 | 0.308 |
| RHSIM_Rhsim04G0123200 | 2.81 | 0.094 | 0.145 | 0.216 |
| RHSIM_Rhsim11G0087800 | 2.81 | 0.094 | 0.145 | -0.354 |
| RHSIM_Rhsim04G0094600 | 2.81 | 0.094 | 0.145 | 2.402 |
| RHSIM_Rhsim11G0047100 | 2.81 | 0.094 | 0.145 | 1.112 |
| RHSIM_Rhsim13G0050500 | 2.81 | 0.094 | 0.145 | 2.953 |
| RHSIM_Rhsim12G0159400 | 2.81 | 0.094 | 0.145 | -0.487 |
| RHSIM_Rhsim03G0234100 | 2.81 | 0.094 | 0.145 | -0.163 |
| novel.946 | 2.81 | 0.094 | 0.145 | 0.797 |
| RHSIM_Rhsim10G0171000 | 2.81 | 0.094 | 0.146 | -2.049 |
| RHSIM_Rhsim11G0137600 | 2.80 | 0.094 | 0.146 | -0.541 |
| RHSIM_Rhsim05G0087500 | 2.80 | 0.094 | 0.146 | 0.479 |
| RHSIM_Rhsim08G0014800 | 2.80 | 0.094 | 0.146 | -38.899 |
| RHSIM_Rhsim02G0033400 | 2.80 | 0.094 | 0.146 | 0.181 |
| RHSIM_Rhsim09G0130600 | 2.80 | 0.094 | 0.146 | 0.560 |
| RHSIM_RhsimUnG0011500 | 2.80 | 0.094 | 0.146 | -0.403 |
| novel.4055 | 2.80 | 0.094 | 0.146 | -1.504 |
| RHSIM_Rhsim04G0036900 | 2.80 | 0.094 | 0.146 | -1.315 |
| RHSIM_Rhsim01G0191900 | 2.80 | 0.094 | 0.146 | -0.426 |
| RHSIM_Rhsim07G0031300 | 2.80 | 0.094 | 0.146 | 0.195 |
| RHSIM_RhsimUnG0112200 | 2.80 | 0.094 | 0.146 | 3.011 |
| novel.2372 | 2.79 | 0.095 | 0.147 | -1.691 |
| RHSIM_Rhsim13G0019800 | 2.79 | 0.095 | 0.147 | 2.105 |
| RHSIM_Rhsim02G0202000 | 2.79 | 0.095 | 0.147 | 0.182 |
| RHSIM_Rhsim01G0162600 | 2.79 | 0.095 | 0.147 | -0.460 |
| RHSIM_Rhsim04G0226100 | 2.79 | 0.095 | 0.147 | 0.314 |
| RHSIM_Rhsim03G0001200 | 2.79 | 0.095 | 0.147 | 1.781 |
| RHSIM_RhsimUnG0106800 | 2.79 | 0.095 | 0.147 | -1.164 |
| RHSIM_RhsimUnG0168100 | 2.79 | 0.095 | 0.147 | 0.221 |
| novel.4002 | 2.79 | 0.095 | 0.147 | -1.926 |
| RHSIM_Rhsim13G0123200 | 2.79 | 0.095 | 0.147 | 1.423 |
| novel.1042 | 2.79 | 0.095 | 0.147 | -0.472 |

| Gene | LRT statistic | P value | BH-adjusted P | log2FC (interaction) |
|---|---|---|---|---|
| RHSIM_Rhsim03G0211400 | 2.79 | 0.095 | 0.147 | -2.321 |
| RHSIM_Rhsim03G0224400 | 2.79 | 0.095 | 0.147 | 1.767 |
| RHSIM_Rhsim03G0031800 | 2.79 | 0.095 | 0.147 | 38.641 |
| RHSIM_Rhsim07G0105600 | 2.79 | 0.095 | 0.147 | -0.937 |
| RHSIM_Rhsim06G0052400 | 2.79 | 0.095 | 0.147 | -38.619 |
| RHSIM_Rhsim13G0085700 | 2.79 | 0.095 | 0.147 | 0.825 |
| RHSIM_Rhsim01G0115800 | 2.79 | 0.095 | 0.147 | 38.942 |
| RHSIM_Rhsim13G0054900 | 2.78 | 0.095 | 0.148 | -0.406 |
| RHSIM_Rhsim13G0034600 | 2.78 | 0.095 | 0.148 | -0.124 |
| RHSIM_Rhsim01G0179200 | 2.78 | 0.095 | 0.148 | 2.119 |
| novel.274 | 2.78 | 0.095 | 0.148 | 0.235 |
| novel.2077 | 2.78 | 0.095 | 0.148 | 1.202 |
| RHSIM_Rhsim11G0043300 | 2.78 | 0.096 | 0.148 | -0.275 |
| RHSIM_Rhsim13G0126300 | 2.78 | 0.096 | 0.148 | 0.928 |
| RHSIM_Rhsim07G0231000 | 2.78 | 0.096 | 0.148 | 2.141 |
| RHSIM_Rhsim02G0135600 | 2.78 | 0.096 | 0.148 | -38.770 |
| novel.2322 | 2.78 | 0.096 | 0.148 | -1.013 |
| RHSIM_Rhsim03G0198500 | 2.78 | 0.096 | 0.148 | -0.179 |
| RHSIM_RhsimUnG0029900 | 2.78 | 0.096 | 0.148 | -1.148 |
| RHSIM_Rhsim07G0069000 | 2.78 | 0.096 | 0.148 | -0.112 |
| RHSIM_RhsimUnG0183500 | 2.78 | 0.096 | 0.148 | -1.828 |
| RHSIM_Rhsim01G0017100 | 2.78 | 0.096 | 0.148 | 38.841 |
| novel.1823 | 2.78 | 0.096 | 0.148 | -1.759 |
| RHSIM_Rhsim07G0179100 | 2.77 | 0.096 | 0.148 | -0.616 |
| RHSIM_RhsimUnG0043600 | 2.77 | 0.096 | 0.148 | -0.633 |
| RHSIM_Rhsim04G0079300 | 2.77 | 0.096 | 0.148 | -0.756 |
| novel.1729 | 2.77 | 0.096 | 0.148 | 1.382 |
| RHSIM_Rhsim11G0067900 | 2.77 | 0.096 | 0.148 | -0.389 |
| RHSIM_Rhsim08G0128000 | 2.77 | 0.096 | 0.148 | 2.364 |
| RHSIM_Rhsim07G0196700 | 2.77 | 0.096 | 0.149 | -2.796 |
| RHSIM_Rhsim08G0215800 | 2.77 | 0.096 | 0.149 | 0.952 |
| RHSIM_Rhsim02G0220100 | 2.77 | 0.096 | 0.149 | -2.351 |

| Gene | LRT statistic | P value | BH-adjusted P | log2FC (interaction) |
|---|---|---|---|---|
| RHSIM_Rhsim04G0014400 | 2.77 | 0.096 | 0.149 | 0.454 |
| RHSIM_Rhsim13G0163600 | 2.77 | 0.096 | 0.149 | -1.672 |
| novel.862 | 2.77 | 0.096 | 0.149 | 1.215 |
| RHSIM_Rhsim07G0221400 | 2.77 | 0.096 | 0.149 | -1.436 |
| RHSIM_Rhsim11G0103100 | 2.76 | 0.096 | 0.149 | -0.656 |
| novel.4190 | 2.76 | 0.096 | 0.149 | -0.161 |
| novel.2565 | 2.76 | 0.097 | 0.149 | 2.381 |
| RHSIM_Rhsim09G0212400 | 2.76 | 0.097 | 0.149 | 0.120 |
| RHSIM_Rhsim03G0248300 | 2.76 | 0.097 | 0.149 | 3.849 |
| RHSIM_Rhsim08G0147200 | 2.76 | 0.097 | 0.149 | -1.374 |
| novel.3869 | 2.76 | 0.097 | 0.149 | 2.891 |
| RHSIM_Rhsim02G0129000 | 2.76 | 0.097 | 0.150 | -1.401 |
| RHSIM_RhsimUnG0210100 | 2.76 | 0.097 | 0.150 | -1.818 |
| RHSIM_Rhsim13G0131300 | 2.76 | 0.097 | 0.150 | 2.231 |
| novel.3254 | 2.75 | 0.097 | 0.150 | -0.472 |
| RHSIM_Rhsim01G0180200 | 2.75 | 0.097 | 0.150 | 0.653 |
| RHSIM_Rhsim06G0172300 | 2.75 | 0.097 | 0.150 | 1.916 |
| novel.841 | 2.75 | 0.097 | 0.150 | 0.661 |
| RHSIM_Rhsim11G0044700 | 2.75 | 0.097 | 0.150 | -38.978 |
| RHSIM_Rhsim08G0155400 | 2.75 | 0.097 | 0.150 | 0.118 |
| RHSIM_RhsimUnG0257600 | 2.75 | 0.097 | 0.150 | -2.254 |
| RHSIM_Rhsim01G0281600 | 2.75 | 0.097 | 0.150 | -78.023 |
| novel.1997 | 2.75 | 0.097 | 0.150 | 0.427 |
| RHSIM_Rhsim06G0081800 | 2.75 | 0.097 | 0.150 | -38.004 |
| RHSIM_Rhsim07G0142700 | 2.75 | 0.097 | 0.150 | -0.892 |
| RHSIM_Rhsim02G0044400 | 2.75 | 0.097 | 0.151 | -37.788 |
| RHSIM_Rhsim11G0107600 | 2.75 | 0.098 | 0.151 | 39.115 |
| RHSIM_Rhsim04G0200000 | 2.74 | 0.098 | 0.151 | -0.367 |
| RHSIM_Rhsim05G0067800 | 2.74 | 0.098 | 0.151 | -0.196 |
| RHSIM_Rhsim12G0163100 | 2.74 | 0.098 | 0.151 | -0.439 |
| RHSIM_Rhsim02G0142700 | 2.74 | 0.098 | 0.151 | -0.984 |
| RHSIM_Rhsim01G0069200 | 2.74 | 0.098 | 0.151 | 1.560 |

| Gene | LRT statistic | P value | BH-adjusted P | log2FC (interaction) |
|---|---|---|---|---|
| novel.3012 | 2.74 | 0.098 | 0.151 | -0.257 |
| RHSIM_RhsimUnG0203100 | 2.74 | 0.098 | 0.151 | 39.271 |
| RHSIM_Rhsim13G0174500 | 2.74 | 0.098 | 0.151 | 0.125 |
| RHSIM_Rhsim11G0156000 | 2.74 | 0.098 | 0.151 | 1.206 |
| novel.1319 | 2.74 | 0.098 | 0.151 | -77.901 |
| RHSIM_Rhsim02G0046400 | 2.74 | 0.098 | 0.151 | -0.566 |
| RHSIM_Rhsim07G0128700 | 2.74 | 0.098 | 0.151 | 0.264 |
| RHSIM_Rhsim07G0135700 | 2.74 | 0.098 | 0.151 | 38.361 |
| RHSIM_Rhsim01G0038700 | 2.74 | 0.098 | 0.151 | -0.862 |
| RHSIM_Rhsim08G0205800 | 2.74 | 0.098 | 0.151 | 0.501 |
| RHSIM_Rhsim06G0115800 | 2.74 | 0.098 | 0.151 | -0.943 |
| novel.2806 | 2.74 | 0.098 | 0.151 | 1.560 |
| RHSIM_Rhsim01G0106400 | 2.74 | 0.098 | 0.151 | 3.288 |
| novel.3983 | 2.73 | 0.098 | 0.151 | -1.925 |
| RHSIM_RhsimUnG0145800 | 2.73 | 0.098 | 0.151 | 1.011 |
| RHSIM_Rhsim08G0130200 | 2.73 | 0.098 | 0.151 | 0.218 |
| RHSIM_Rhsim11G0050500 | 2.73 | 0.098 | 0.152 | 1.025 |
| RHSIM_Rhsim13G0179700 | 2.73 | 0.098 | 0.152 | -0.524 |
| novel.1187 | 2.73 | 0.098 | 0.152 | 39.060 |
| RHSIM_RhsimUnG0249800 | 2.73 | 0.098 | 0.152 | -0.390 |
| RHSIM_Rhsim08G0126500 | 2.73 | 0.098 | 0.152 | 0.323 |
| RHSIM_Rhsim05G0173000 | 2.73 | 0.098 | 0.152 | 39.096 |
| RHSIM_RhsimUnG0250100 | 2.73 | 0.098 | 0.152 | 1.059 |
| RHSIM_Rhsim04G0121400 | 2.73 | 0.098 | 0.152 | 0.265 |
| RHSIM_Rhsim02G0062800 | 2.73 | 0.099 | 0.152 | 0.885 |
| RHSIM_Rhsim05G0191500 | 2.73 | 0.099 | 0.152 | -1.266 |
| RHSIM_Rhsim02G0019400 | 2.73 | 0.099 | 0.152 | 2.133 |
| novel.2241 | 2.73 | 0.099 | 0.152 | -0.611 |
| RHSIM_Rhsim06G0076600 | 2.73 | 0.099 | 0.152 | -39.059 |
| novel.1743 | 2.73 | 0.099 | 0.152 | 2.219 |
| novel.1524 | 2.73 | 0.099 | 0.152 | -0.459 |
| RHSIM_Rhsim03G0187700 | 2.72 | 0.099 | 0.152 | 1.180 |

| Gene | LRT statistic | P value | BH-adjusted P | log2FC (interaction) |
|---|---|---|---|---|
| RHSIM_Rhsim10G0181000 | 2.72 | 0.099 | 0.152 | 2.691 |
| RHSIM_Rhsim13G0039900 | 2.72 | 0.099 | 0.152 | 0.396 |
| RHSIM_Rhsim11G0151400 | 2.72 | 0.099 | 0.152 | -0.182 |
| RHSIM_Rhsim07G0062700 | 2.72 | 0.099 | 0.152 | -0.299 |
| RHSIM_Rhsim12G0170200 | 2.72 | 0.099 | 0.152 | -0.707 |
| RHSIM_Rhsim05G0172000 | 2.72 | 0.099 | 0.153 | 0.220 |
| novel.2683 | 2.72 | 0.099 | 0.153 | -0.443 |
| RHSIM_Rhsim13G0027600 | 2.72 | 0.099 | 0.153 | -0.304 |
| novel.4309 | 2.72 | 0.099 | 0.153 | -1.923 |
| novel.2573 | 2.72 | 0.099 | 0.153 | 0.184 |
| RHSIM_Rhsim10G0099000 | 2.72 | 0.099 | 0.153 | 0.317 |
| RHSIM_Rhsim03G0141700 | 2.72 | 0.099 | 0.153 | -3.405 |
| novel.2697 | 2.72 | 0.099 | 0.153 | -2.090 |
| RHSIM_Rhsim13G0183100 | 2.72 | 0.099 | 0.153 | -38.027 |
| RHSIM_Rhsim05G0184300 | 2.72 | 0.099 | 0.153 | -2.124 |
| RHSIM_Rhsim01G0280100 | 2.72 | 0.099 | 0.153 | -38.516 |
| RHSIM_Rhsim06G0147100 | 2.72 | 0.099 | 0.153 | 1.222 |
| RHSIM_Rhsim03G0081000 | 2.72 | 0.099 | 0.153 | -1.090 |
| novel.2283 | 2.72 | 0.099 | 0.153 | 1.644 |
| novel.1108 | 2.72 | 0.099 | 0.153 | -0.649 |
| RHSIM_Rhsim01G0233500 | 2.72 | 0.099 | 0.153 | -0.696 |
| RHSIM_Rhsim07G0039900 | 2.71 | 0.099 | 0.153 | 0.343 |
| RHSIM_Rhsim03G0230600 | 2.71 | 0.100 | 0.153 | -0.278 |
| novel.763 | 2.71 | 0.100 | 0.153 | 2.142 |
| RHSIM_Rhsim06G0240300 | 2.71 | 0.100 | 0.153 | -38.671 |
| RHSIM_Rhsim03G0078900 | 2.71 | 0.100 | 0.153 | 0.157 |
| RHSIM_Rhsim03G0119600 | 2.71 | 0.100 | 0.153 | 0.544 |
| RHSIM_Rhsim09G0213100 | 2.71 | 0.100 | 0.153 | -0.179 |
| RHSIM_Rhsim07G0222400 | 2.71 | 0.100 | 0.153 | -1.624 |
| RHSIM_Rhsim01G0005100 | 2.71 | 0.100 | 0.153 | -0.915 |
| RHSIM_Rhsim07G0234100 | 2.71 | 0.100 | 0.154 | -0.203 |
| RHSIM_Rhsim08G0010100 | 2.70 | 0.100 | 0.154 | 2.348 |

| Gene | LRT statistic | P value | BH-adjusted P | log2FC (interaction) |
|---|---|---|---|---|
| RHSIM_Rhsim03G0125800 | 2.70 | 0.100 | 0.154 | 1.015 |
| novel.4065 | 2.70 | 0.100 | 0.154 | 1.842 |
| RHSIM_Rhsim05G0092800 | 2.70 | 0.100 | 0.154 | 0.616 |
| RHSIM_Rhsim12G0014500 | 2.70 | 0.100 | 0.154 | -0.414 |
| RHSIM_Rhsim09G0197600 | 2.70 | 0.100 | 0.154 | -0.974 |
| RHSIM_RhsimUnG0207000 | 2.70 | 0.100 | 0.154 | -0.719 |
| novel.3002 | 2.70 | 0.100 | 0.154 | -0.255 |
| RHSIM_Rhsim02G0087500 | 2.70 | 0.100 | 0.154 | -0.296 |
| RHSIM_Rhsim01G0173700 | 2.70 | 0.100 | 0.154 | -0.566 |
| RHSIM_Rhsim08G0050200 | 2.70 | 0.100 | 0.154 | 0.587 |
| RHSIM_Rhsim09G0198600 | 2.70 | 0.100 | 0.154 | -0.255 |
| RHSIM_Rhsim01G0213800 | 2.70 | 0.101 | 0.155 | 0.949 |
| RHSIM_Rhsim06G0015500 | 2.70 | 0.101 | 0.155 | 2.603 |
| novel.548 | 2.70 | 0.101 | 0.155 | 1.183 |
| RHSIM_Rhsim05G0162700 | 2.70 | 0.101 | 0.155 | 0.770 |
| RHSIM_Rhsim05G0191300 | 2.70 | 0.101 | 0.155 | 0.296 |
| RHSIM_Rhsim05G0192700 | 2.69 | 0.101 | 0.155 | 2.188 |
| RHSIM_Rhsim05G0128200 | 2.69 | 0.101 | 0.155 | 0.299 |
| RHSIM_Rhsim13G0117100 | 2.69 | 0.101 | 0.155 | -0.130 |
| novel.858 | 2.69 | 0.101 | 0.155 | 1.560 |
| RHSIM_Rhsim02G0208900 | 2.69 | 0.101 | 0.155 | -2.843 |
| RHSIM_Rhsim10G0003800 | 2.69 | 0.101 | 0.155 | -39.244 |
| RHSIM_Rhsim08G0230300 | 2.69 | 0.101 | 0.155 | 1.526 |
| novel.2119 | 2.69 | 0.101 | 0.155 | 2.305 |
| RHSIM_Rhsim10G0111600 | 2.69 | 0.101 | 0.155 | 1.625 |
| novel.4485 | 2.69 | 0.101 | 0.155 | 1.102 |
| novel.1260 | 2.69 | 0.101 | 0.155 | 1.162 |
| RHSIM_Rhsim08G0072100 | 2.69 | 0.101 | 0.155 | -38.821 |
| RHSIM_Rhsim03G0220300 | 2.69 | 0.101 | 0.155 | -2.565 |
| RHSIM_Rhsim07G0186500 | 2.69 | 0.101 | 0.155 | -39.567 |
| RHSIM_Rhsim13G0132600 | 2.69 | 0.101 | 0.155 | -0.203 |
| novel.2875 | 2.68 | 0.101 | 0.156 | -0.356 |

| Gene | LRT statistic | P value | BH-adjusted P | log2FC (interaction) |
|---|---|---|---|---|
| RHSIM_Rhsim04G0055000 | 2.68 | 0.101 | 0.156 | 40.300 |
| RHSIM_Rhsim10G0067900 | 2.68 | 0.101 | 0.156 | 3.936 |
| novel.4387 | 2.68 | 0.101 | 0.156 | 0.706 |
| RHSIM_Rhsim03G0192900 | 2.68 | 0.101 | 0.156 | -3.373 |
| RHSIM_Rhsim12G0168400 | 2.68 | 0.102 | 0.156 | -0.148 |
| RHSIM_Rhsim03G0137300 | 2.68 | 0.102 | 0.156 | 2.501 |
| RHSIM_RhsimUnG0232900 | 2.68 | 0.102 | 0.156 | 2.264 |
| RHSIM_Rhsim07G0058000 | 2.68 | 0.102 | 0.156 | 0.426 |
| RHSIM_Rhsim12G0212600 | 2.68 | 0.102 | 0.156 | -39.717 |
| RHSIM_Rhsim03G0198800 | 2.68 | 0.102 | 0.156 | -0.256 |
| novel.2070 | 2.68 | 0.102 | 0.156 | -0.931 |
| RHSIM_Rhsim06G0093300 | 2.68 | 0.102 | 0.156 | -0.194 |
| RHSIM_Rhsim09G0072200 | 2.68 | 0.102 | 0.156 | 0.232 |
| RHSIM_Rhsim13G0193600 | 2.68 | 0.102 | 0.156 | -2.111 |
| novel.1581 | 2.67 | 0.102 | 0.156 | 0.845 |
| RHSIM_Rhsim09G0110500 | 2.67 | 0.102 | 0.157 | -4.409 |
| RHSIM_RhsimUnG0100000 | 2.67 | 0.102 | 0.157 | 1.698 |
| novel.3796 | 2.67 | 0.102 | 0.157 | 38.499 |
| RHSIM_Rhsim05G0187000 | 2.67 | 0.102 | 0.157 | -3.523 |
| RHSIM_Rhsim02G0119800 | 2.67 | 0.102 | 0.157 | -0.326 |
| RHSIM_RhsimUnG0039900 | 2.67 | 0.102 | 0.157 | 0.586 |
| RHSIM_Rhsim01G0139000 | 2.67 | 0.102 | 0.157 | 1.898 |
| RHSIM_Rhsim07G0065900 | 2.67 | 0.102 | 0.157 | -1.442 |
| RHSIM_Rhsim05G0123800 | 2.67 | 0.102 | 0.157 | 2.751 |
| RHSIM_Rhsim02G0109100 | 2.67 | 0.103 | 0.157 | -39.830 |
| RHSIM_Rhsim12G0196600 | 2.67 | 0.103 | 0.157 | -0.676 |
| novel.4003 | 2.67 | 0.103 | 0.157 | -1.870 |
| RHSIM_Rhsim03G0138400 | 2.66 | 0.103 | 0.157 | 0.860 |
| RHSIM_Rhsim07G0124700 | 2.66 | 0.103 | 0.157 | -0.383 |
| RHSIM_Rhsim08G0047900 | 2.66 | 0.103 | 0.157 | 0.422 |
| novel.658 | 2.66 | 0.103 | 0.157 | 1.060 |
| novel.1480 | 2.66 | 0.103 | 0.157 | -0.845 |

| Gene | LRT statistic | P value | BH-adjusted P | log2FC (interaction) |
|---|---|---|---|---|
| novel.880 | 2.66 | 0.103 | 0.157 | 1.262 |
| RHSIM_Rhsim06G0160700 | 2.66 | 0.103 | 0.158 | -1.516 |
| RHSIM_Rhsim01G0046000 | 2.66 | 0.103 | 0.158 | 1.731 |
| RHSIM_Rhsim02G0033800 | 2.66 | 0.103 | 0.158 | 3.327 |
| RHSIM_Rhsim01G0272400 | 2.66 | 0.103 | 0.158 | -0.584 |
| RHSIM_Rhsim13G0087400 | 2.66 | 0.103 | 0.158 | -39.763 |
| RHSIM_Rhsim02G0222800 | 2.66 | 0.103 | 0.158 | 39.470 |
| RHSIM_Rhsim02G0089300 | 2.65 | 0.103 | 0.158 | -1.994 |
| RHSIM_Rhsim03G0248400 | 2.65 | 0.103 | 0.158 | -0.399 |
| RHSIM_Rhsim06G0100700 | 2.65 | 0.103 | 0.159 | 0.654 |
| RHSIM_Rhsim13G0190100 | 2.65 | 0.104 | 0.159 | -1.277 |
| novel.3266 | 2.65 | 0.104 | 0.159 | 0.946 |
| RHSIM_Rhsim06G0091400 | 2.65 | 0.104 | 0.159 | -0.152 |
| RHSIM_Rhsim06G0040200 | 2.65 | 0.104 | 0.159 | -1.508 |
| RHSIM_Rhsim13G0226000 | 2.65 | 0.104 | 0.159 | 1.424 |
| RHSIM_Rhsim12G0038700 | 2.64 | 0.104 | 0.159 | 1.317 |
| RHSIM_Rhsim08G0241400 | 2.64 | 0.104 | 0.159 | -0.223 |
| novel.4553 | 2.64 | 0.104 | 0.159 | -2.095 |
| RHSIM_Rhsim04G0137900 | 2.64 | 0.104 | 0.160 | 1.220 |
| RHSIM_Rhsim01G0045300 | 2.64 | 0.104 | 0.160 | -0.432 |
| RHSIM_Rhsim03G0097600 | 2.64 | 0.104 | 0.160 | -3.303 |
| RHSIM_Rhsim11G0191000 | 2.64 | 0.105 | 0.160 | -38.379 |
| RHSIM_Rhsim10G0193900 | 2.63 | 0.105 | 0.160 | 3.897 |
| novel.3627 | 2.63 | 0.105 | 0.160 | -0.938 |
| RHSIM_Rhsim05G0109900 | 2.63 | 0.105 | 0.160 | 0.442 |
| RHSIM_Rhsim08G0001600 | 2.63 | 0.105 | 0.160 | 0.304 |
| RHSIM_Rhsim07G0214300 | 2.63 | 0.105 | 0.160 | -1.227 |
| novel.1224 | 2.63 | 0.105 | 0.160 | -1.468 |
| RHSIM_Rhsim09G0004800 | 2.63 | 0.105 | 0.160 | 0.205 |
| RHSIM_Rhsim01G0181100 | 2.63 | 0.105 | 0.160 | -0.144 |
| RHSIM_Rhsim09G0033100 | 2.63 | 0.105 | 0.160 | -0.615 |
| RHSIM_Rhsim03G0089400 | 2.63 | 0.105 | 0.160 | -3.291 |

| Gene | LRT statistic | P value | BH-adjusted P | log2FC (interaction) |
|---|---|---|---|---|
| RHSIM_Rhsim03G0222300 | 2.63 | 0.105 | 0.160 | 1.976 |
| RHSIM_Rhsim05G0022600 | 2.63 | 0.105 | 0.160 | 0.295 |
| RHSIM_Rhsim08G0182000 | 2.63 | 0.105 | 0.160 | 1.614 |
| RHSIM_Rhsim06G0139700 | 2.63 | 0.105 | 0.160 | 0.870 |
| RHSIM_Rhsim03G0141300 | 2.63 | 0.105 | 0.160 | -0.115 |
| novel.3895 | 2.63 | 0.105 | 0.160 | 0.305 |
| novel.1450 | 2.63 | 0.105 | 0.161 | 1.057 |
| RHSIM_Rhsim12G0196100 | 2.63 | 0.105 | 0.161 | -2.465 |
| RHSIM_Rhsim07G0162800 | 2.63 | 0.105 | 0.161 | -0.281 |
| RHSIM_Rhsim09G0061700 | 2.63 | 0.105 | 0.161 | -0.280 |
| RHSIM_Rhsim13G0017400 | 2.63 | 0.105 | 0.161 | 0.720 |
| RHSIM_Rhsim04G0202200 | 2.63 | 0.105 | 0.161 | -0.142 |
| RHSIM_Rhsim06G0239600 | 2.63 | 0.105 | 0.161 | -0.085 |
| RHSIM_Rhsim08G0193300 | 2.62 | 0.105 | 0.161 | -39.036 |
| RHSIM_Rhsim12G0009200 | 2.62 | 0.105 | 0.161 | 39.123 |
| RHSIM_Rhsim02G0228000 | 2.62 | 0.105 | 0.161 | 1.262 |
| RHSIM_Rhsim07G0175200 | 2.62 | 0.105 | 0.161 | 1.518 |
| RHSIM_Rhsim04G0100700 | 2.62 | 0.105 | 0.161 | -0.907 |
| RHSIM_Rhsim02G0216100 | 2.62 | 0.105 | 0.161 | -0.141 |
| RHSIM_Rhsim10G0062200 | 2.62 | 0.105 | 0.161 | -0.265 |
| RHSIM_Rhsim01G0229400 | 2.62 | 0.106 | 0.161 | 0.275 |
| RHSIM_Rhsim12G0003600 | 2.62 | 0.106 | 0.161 | -3.018 |
| RHSIM_Rhsim04G0170200 | 2.62 | 0.106 | 0.161 | 1.836 |
| RHSIM_Rhsim07G0222900 | 2.62 | 0.106 | 0.161 | -0.936 |
| RHSIM_Rhsim05G0194500 | 2.62 | 0.106 | 0.161 | -3.748 |
| RHSIM_Rhsim10G0196700 | 2.62 | 0.106 | 0.161 | 2.859 |
| RHSIM_RhsimMtG0005600 | 2.62 | 0.106 | 0.161 | 2.584 |
| RHSIM_Rhsim04G0094500 | 2.62 | 0.106 | 0.162 | 0.652 |
| novel.4089 | 2.62 | 0.106 | 0.162 | -0.966 |
| RHSIM_Rhsim03G0222100 | 2.62 | 0.106 | 0.162 | -0.507 |
| RHSIM_Rhsim08G0004200 | 2.61 | 0.106 | 0.162 | -0.210 |
| RHSIM_Rhsim01G0034700 | 2.61 | 0.106 | 0.162 | -1.440 |

| Gene | LRT statistic | P value | BH-adjusted P | log2FC (interaction) |
|---|---|---|---|---|
| RHSIM_Rhsim04G0156800 | 2.61 | 0.106 | 0.162 | -1.724 |
| RHSIM_Rhsim01G0225000 | 2.61 | 0.106 | 0.162 | 0.408 |
| novel.1406 | 2.61 | 0.106 | 0.162 | 0.666 |
| novel.1315 | 2.61 | 0.106 | 0.162 | 0.123 |
| RHSIM_Rhsim03G0164600 | 2.61 | 0.106 | 0.162 | 0.774 |
| RHSIM_Rhsim05G0098400 | 2.61 | 0.106 | 0.162 | 2.593 |
| RHSIM_Rhsim10G0040700 | 2.61 | 0.106 | 0.162 | 1.693 |
| RHSIM_Rhsim13G0073300 | 2.61 | 0.106 | 0.162 | 0.315 |
| RHSIM_Rhsim12G0182600 | 2.61 | 0.106 | 0.163 | -0.184 |
| RHSIM_RhsimUnG0209700 | 2.61 | 0.106 | 0.163 | -0.887 |
| novel.2187 | 2.61 | 0.107 | 0.163 | 0.232 |
| RHSIM_Rhsim01G0117400 | 2.60 | 0.107 | 0.163 | 3.415 |
| RHSIM_Rhsim01G0106300 | 2.60 | 0.107 | 0.163 | 3.590 |
| RHSIM_Rhsim12G0130900 | 2.60 | 0.107 | 0.163 | -2.244 |
| RHSIM_RhsimUnG0209600 | 2.60 | 0.107 | 0.163 | -1.775 |
| RHSIM_Rhsim03G0233300 | 2.60 | 0.107 | 0.163 | -0.250 |
| RHSIM_Rhsim08G0072500 | 2.60 | 0.107 | 0.163 | 0.127 |
| RHSIM_Rhsim08G0085700 | 2.60 | 0.107 | 0.163 | 1.781 |
| RHSIM_Rhsim13G0000100 | 2.60 | 0.107 | 0.163 | 0.211 |
| novel.2217 | 2.60 | 0.107 | 0.163 | 2.696 |
| RHSIM_Rhsim13G0066600 | 2.60 | 0.107 | 0.163 | -0.244 |
| RHSIM_Rhsim04G0160000 | 2.60 | 0.107 | 0.163 | 38.853 |
| RHSIM_Rhsim02G0169800 | 2.60 | 0.107 | 0.163 | -0.195 |
| RHSIM_Rhsim07G0245600 | 2.60 | 0.107 | 0.163 | 1.300 |
| RHSIM_Rhsim03G0163400 | 2.60 | 0.107 | 0.163 | -1.500 |
| RHSIM_Rhsim08G0084400 | 2.60 | 0.107 | 0.163 | 0.182 |
| RHSIM_Rhsim04G0046600 | 2.60 | 0.107 | 0.163 | -0.200 |
| novel.1147 | 2.60 | 0.107 | 0.163 | -0.426 |
| RHSIM_Rhsim06G0147000 | 2.60 | 0.107 | 0.163 | 0.131 |
| RHSIM_Rhsim05G0172800 | 2.60 | 0.107 | 0.163 | 0.337 |
| RHSIM_Rhsim11G0063200 | 2.59 | 0.107 | 0.164 | -0.512 |
| RHSIM_Rhsim13G0069800 | 2.59 | 0.107 | 0.164 | 0.910 |

| Gene | LRT statistic | P value | BH-adjusted P | log2FC (interaction) |
|---|---|---|---|---|
| RHSIM_Rhsim07G0206600 | 2.59 | 0.107 | 0.164 | -0.834 |
| RHSIM_Rhsim06G0219100 | 2.59 | 0.107 | 0.164 | -0.134 |
| novel.4335 | 2.59 | 0.107 | 0.164 | 2.014 |
| RHSIM_Rhsim12G0191700 | 2.59 | 0.108 | 0.164 | 39.805 |
| RHSIM_Rhsim02G0106500 | 2.59 | 0.108 | 0.164 | -0.453 |
| novel.2910 | 2.59 | 0.108 | 0.164 | -37.909 |
| RHSIM_Rhsim11G0187400 | 2.59 | 0.108 | 0.164 | -1.322 |
| RHSIM_Rhsim11G0142700 | 2.59 | 0.108 | 0.164 | 0.570 |
| RHSIM_RhsimUnG0052600 | 2.59 | 0.108 | 0.164 | 0.665 |
| RHSIM_Rhsim10G0111500 | 2.59 | 0.108 | 0.164 | -0.091 |
| RHSIM_Rhsim02G0143400 | 2.59 | 0.108 | 0.164 | -0.430 |
| novel.2610 | 2.59 | 0.108 | 0.164 | 38.554 |
| novel.947 | 2.58 | 0.108 | 0.164 | -3.050 |
| RHSIM_Rhsim09G0026900 | 2.58 | 0.108 | 0.164 | -0.170 |
| RHSIM_Rhsim04G0197200 | 2.58 | 0.108 | 0.165 | 0.130 |
| RHSIM_Rhsim09G0179200 | 2.58 | 0.108 | 0.165 | -0.843 |
| RHSIM_Rhsim07G0057900 | 2.58 | 0.108 | 0.165 | -0.448 |
| novel.4260 | 2.58 | 0.108 | 0.165 | 1.020 |
| novel.3484 | 2.58 | 0.108 | 0.165 | 1.046 |
| RHSIM_Rhsim01G0125000 | 2.58 | 0.108 | 0.165 | 0.155 |
| RHSIM_Rhsim04G0055300 | 2.58 | 0.108 | 0.165 | 1.545 |
| RHSIM_Rhsim11G0026900 | 2.58 | 0.108 | 0.165 | 0.323 |
| RHSIM_Rhsim06G0154900 | 2.58 | 0.108 | 0.165 | -1.041 |
| novel.3075 | 2.58 | 0.108 | 0.165 | 38.845 |
| RHSIM_Rhsim03G0248900 | 2.58 | 0.108 | 0.165 | -0.621 |
| RHSIM_Rhsim07G0088900 | 2.58 | 0.108 | 0.165 | -1.858 |
| RHSIM_Rhsim01G0196800 | 2.58 | 0.108 | 0.165 | -0.274 |
| RHSIM_Rhsim04G0249800 | 2.58 | 0.108 | 0.165 | 0.388 |
| RHSIM_Rhsim05G0179400 | 2.58 | 0.108 | 0.165 | 0.246 |
| novel.4353 | 2.58 | 0.108 | 0.165 | 1.019 |
| RHSIM_Rhsim01G0246600 | 2.58 | 0.109 | 0.165 | -0.511 |
| RHSIM_RhsimUnG0258000 | 2.58 | 0.109 | 0.165 | 0.250 |

| Gene | LRT statistic | P value | BH-adjusted P | log2FC (interaction) |
|---|---|---|---|---|
| RHSIM_Rhsim08G0117600 | 2.57 | 0.109 | 0.165 | -0.300 |
| RHSIM_Rhsim03G0045400 | 2.57 | 0.109 | 0.165 | 0.155 |
| RHSIM_Rhsim11G0095700 | 2.57 | 0.109 | 0.165 | 0.384 |
| RHSIM_Rhsim12G0096200 | 2.57 | 0.109 | 0.165 | 0.242 |
| RHSIM_Rhsim07G0256300 | 2.57 | 0.109 | 0.165 | -1.061 |
| RHSIM_Rhsim06G0183000 | 2.57 | 0.109 | 0.165 | 1.139 |
| RHSIM_Rhsim09G0041300 | 2.57 | 0.109 | 0.165 | 2.169 |
| RHSIM_Rhsim04G0247600 | 2.57 | 0.109 | 0.166 | 0.121 |
| RHSIM_RhsimUnG0196100 | 2.57 | 0.109 | 0.166 | 0.203 |
| RHSIM_Rhsim02G0202500 | 2.57 | 0.109 | 0.166 | 0.167 |
| novel.2407 | 2.57 | 0.109 | 0.166 | -0.195 |
| RHSIM_Rhsim13G0132500 | 2.57 | 0.109 | 0.166 | 39.201 |
| RHSIM_Rhsim07G0236000 | 2.57 | 0.109 | 0.166 | -0.535 |
| RHSIM_Rhsim05G0000500 | 2.57 | 0.109 | 0.166 | 39.157 |
| RHSIM_Rhsim10G0020900 | 2.57 | 0.109 | 0.166 | 0.165 |
| RHSIM_Rhsim01G0263400 | 2.57 | 0.109 | 0.166 | 3.145 |
| novel.3031 | 2.57 | 0.109 | 0.166 | -0.345 |
| RHSIM_Rhsim11G0116600 | 2.57 | 0.109 | 0.166 | -1.475 |
| RHSIM_RhsimUnG0166400 | 2.57 | 0.109 | 0.166 | -2.809 |
| RHSIM_Rhsim11G0098500 | 2.57 | 0.109 | 0.166 | 0.320 |
| RHSIM_Rhsim09G0206800 | 2.56 | 0.109 | 0.166 | 1.461 |
| novel.443 | 2.56 | 0.109 | 0.166 | 2.322 |
| RHSIM_Rhsim06G0122000 | 2.56 | 0.109 | 0.166 | -38.868 |
| RHSIM_Rhsim11G0170600 | 2.56 | 0.109 | 0.166 | -1.146 |
| novel.1057 | 2.56 | 0.109 | 0.166 | -0.145 |
| novel.3206 | 2.56 | 0.110 | 0.167 | 0.764 |
| RHSIM_Rhsim09G0130400 | 2.56 | 0.110 | 0.167 | 0.092 |
| RHSIM_Rhsim04G0090600 | 2.56 | 0.110 | 0.167 | -1.071 |
| RHSIM_Rhsim12G0210500 | 2.56 | 0.110 | 0.167 | -2.186 |
| RHSIM_Rhsim03G0010900 | 2.56 | 0.110 | 0.167 | 1.119 |
| RHSIM_Rhsim01G0113000 | 2.56 | 0.110 | 0.167 | -0.541 |
| novel.3300 | 2.55 | 0.110 | 0.167 | 1.650 |

| Gene | LRT statistic | P value | BH-adjusted P | log2FC (interaction) |
|---|---|---|---|---|
| RHSIM_Rhsim11G0045900 | 2.55 | 0.110 | 0.167 | -0.286 |
| RHSIM_Rhsim11G0161600 | 2.55 | 0.110 | 0.167 | 1.416 |
| RHSIM_Rhsim02G0137600 | 2.55 | 0.110 | 0.167 | 0.081 |
| novel.1478 | 2.55 | 0.110 | 0.167 | -0.713 |
| RHSIM_Rhsim01G0065600 | 2.55 | 0.110 | 0.167 | 2.039 |
| novel.3583 | 2.55 | 0.110 | 0.168 | -1.224 |
| RHSIM_Rhsim12G0146600 | 2.55 | 0.110 | 0.168 | 0.159 |
| RHSIM_Rhsim11G0129400 | 2.55 | 0.111 | 0.168 | 0.211 |
| RHSIM_Rhsim08G0022900 | 2.55 | 0.111 | 0.168 | 2.964 |
| novel.1060 | 2.55 | 0.111 | 0.168 | 1.084 |
| RHSIM_Rhsim10G0025700 | 2.55 | 0.111 | 0.168 | -0.743 |
| RHSIM_Rhsim10G0134500 | 2.55 | 0.111 | 0.168 | 1.596 |
| RHSIM_Rhsim04G0000200 | 2.54 | 0.111 | 0.168 | -0.844 |
| RHSIM_Rhsim09G0055700 | 2.54 | 0.111 | 0.168 | 0.427 |
| RHSIM_Rhsim08G0032900 | 2.54 | 0.111 | 0.168 | -39.663 |
| RHSIM_Rhsim07G0090000 | 2.54 | 0.111 | 0.168 | 0.523 |
| RHSIM_Rhsim13G0007900 | 2.54 | 0.111 | 0.169 | -0.330 |
| RHSIM_Rhsim07G0027800 | 2.54 | 0.111 | 0.169 | -0.136 |
| novel.1755 | 2.54 | 0.111 | 0.169 | 2.022 |
| RHSIM_Rhsim11G0106400 | 2.54 | 0.111 | 0.169 | -1.127 |
| RHSIM_Rhsim01G0291900 | 2.54 | 0.111 | 0.169 | 4.352 |
| RHSIM_Rhsim02G0113100 | 2.54 | 0.111 | 0.169 | 1.685 |
| RHSIM_Rhsim03G0146000 | 2.54 | 0.111 | 0.169 | 0.273 |
| RHSIM_RhsimUnG0180600 | 2.53 | 0.111 | 0.169 | 0.521 |
| RHSIM_Rhsim07G0046700 | 2.53 | 0.111 | 0.169 | 2.899 |
| RHSIM_Rhsim09G0097700 | 2.53 | 0.111 | 0.169 | -0.417 |
| RHSIM_Rhsim11G0072900 | 2.53 | 0.111 | 0.169 | 3.025 |
| novel.2863 | 2.53 | 0.111 | 0.169 | 2.015 |
| RHSIM_Rhsim03G0010400 | 2.53 | 0.111 | 0.169 | 1.115 |
| RHSIM_Rhsim09G0011400 | 2.53 | 0.111 | 0.169 | 38.728 |
| RHSIM_Rhsim02G0021400 | 2.53 | 0.112 | 0.169 | 1.850 |
| RHSIM_Rhsim03G0091400 | 2.53 | 0.112 | 0.169 | 2.530 |

| Gene | LRT statistic | P value | BH-adjusted P | log2FC (interaction) |
|---|---|---|---|---|
| RHSIM_Rhsim07G0112500 | 2.53 | 0.112 | 0.169 | -1.660 |
| RHSIM_Rhsim02G0085700 | 2.53 | 0.112 | 0.169 | 0.381 |
| RHSIM_Rhsim10G0040100 | 2.53 | 0.112 | 0.169 | 0.209 |
| RHSIM_Rhsim12G0017800 | 2.53 | 0.112 | 0.170 | -0.161 |
| RHSIM_Rhsim09G0011300 | 2.53 | 0.112 | 0.170 | 0.633 |
| RHSIM_Rhsim06G0123000 | 2.53 | 0.112 | 0.170 | 3.679 |
| novel.1237 | 2.53 | 0.112 | 0.170 | -1.640 |
| novel.1540 | 2.53 | 0.112 | 0.170 | 0.540 |
| RHSIM_RhsimUnG0138900 | 2.53 | 0.112 | 0.170 | 0.294 |
| RHSIM_Rhsim08G0180200 | 2.53 | 0.112 | 0.170 | 0.644 |
| RHSIM_Rhsim05G0191800 | 2.53 | 0.112 | 0.170 | 2.141 |
| RHSIM_Rhsim12G0103800 | 2.53 | 0.112 | 0.170 | 38.785 |
| RHSIM_Rhsim01G0003900 | 2.52 | 0.112 | 0.170 | 1.141 |
| RHSIM_Rhsim13G0218300 | 2.52 | 0.112 | 0.170 | -37.925 |
| RHSIM_Rhsim10G0054700 | 2.52 | 0.112 | 0.170 | 1.431 |
| RHSIM_Rhsim03G0116300 | 2.52 | 0.112 | 0.170 | 1.038 |
| RHSIM_Rhsim09G0183700 | 2.52 | 0.112 | 0.170 | -1.456 |
| RHSIM_Rhsim10G0155900 | 2.52 | 0.112 | 0.170 | -0.626 |
| novel.1688 | 2.52 | 0.112 | 0.170 | 2.078 |
| RHSIM_Rhsim07G0027200 | 2.52 | 0.113 | 0.171 | 0.170 |
| RHSIM_Rhsim08G0036400 | 2.52 | 0.113 | 0.171 | -0.506 |
| RHSIM_Rhsim02G0076100 | 2.52 | 0.113 | 0.171 | 0.136 |
| RHSIM_Rhsim10G0080900 | 2.52 | 0.113 | 0.171 | -38.808 |
| RHSIM_Rhsim05G0087900 | 2.52 | 0.113 | 0.171 | 0.563 |
| RHSIM_Rhsim06G0175800 | 2.52 | 0.113 | 0.171 | -0.769 |
| RHSIM_Rhsim10G0162800 | 2.52 | 0.113 | 0.171 | 0.343 |
| RHSIM_Rhsim01G0217100 | 2.52 | 0.113 | 0.171 | -2.967 |
| RHSIM_Rhsim01G0246900 | 2.51 | 0.113 | 0.171 | 0.454 |
| RHSIM_Rhsim07G0040400 | 2.51 | 0.113 | 0.171 | 0.361 |
| RHSIM_RhsimUnG0200400 | 2.51 | 0.113 | 0.171 | 0.389 |
| RHSIM_Rhsim07G0104500 | 2.51 | 0.113 | 0.171 | -0.206 |
| RHSIM_Rhsim05G0024600 | 2.51 | 0.113 | 0.171 | -38.675 |

| Gene | LRT statistic | P value | BH-adjusted P | log2FC (interaction) |
|---|---|---|---|---|
| RHSIM_Rhsim13G0115900 | 2.51 | 0.113 | 0.171 | -1.784 |
| RHSIM_Rhsim11G0002600 | 2.50 | 0.114 | 0.172 | 0.862 |
| RHSIM_Rhsim12G0002100 | 2.50 | 0.114 | 0.172 | -0.191 |
| RHSIM_Rhsim07G0225200 | 2.50 | 0.114 | 0.172 | 0.484 |
| RHSIM_Rhsim11G0048400 | 2.50 | 0.114 | 0.172 | 1.805 |
| RHSIM_Rhsim05G0134800 | 2.50 | 0.114 | 0.172 | 1.220 |
| novel.4445 | 2.50 | 0.114 | 0.172 | -2.170 |
| RHSIM_Rhsim06G0161700 | 2.50 | 0.114 | 0.172 | -4.349 |
| RHSIM_Rhsim03G0218500 | 2.50 | 0.114 | 0.172 | 0.125 |
| RHSIM_Rhsim06G0167000 | 2.50 | 0.114 | 0.172 | 0.639 |
| RHSIM_Rhsim08G0029500 | 2.50 | 0.114 | 0.172 | 3.491 |
| RHSIM_Rhsim02G0104100 | 2.50 | 0.114 | 0.172 | -0.235 |
| RHSIM_Rhsim07G0242700 | 2.50 | 0.114 | 0.172 | -0.457 |
| RHSIM_Rhsim10G0065300 | 2.50 | 0.114 | 0.172 | 1.249 |
| RHSIM_Rhsim05G0174600 | 2.50 | 0.114 | 0.173 | -0.647 |
| RHSIM_Rhsim01G0124000 | 2.50 | 0.114 | 0.173 | 0.144 |
| RHSIM_Rhsim13G0107400 | 2.50 | 0.114 | 0.173 | 0.278 |
| RHSIM_Rhsim02G0176600 | 2.50 | 0.114 | 0.173 | 0.486 |
| RHSIM_Rhsim08G0097300 | 2.50 | 0.114 | 0.173 | -39.378 |
| novel.1932 | 2.50 | 0.114 | 0.173 | 0.953 |
| RHSIM_Rhsim01G0135700 | 2.50 | 0.114 | 0.173 | 0.149 |
| RHSIM_Rhsim08G0115300 | 2.49 | 0.114 | 0.173 | 0.647 |
| novel.346 | 2.49 | 0.114 | 0.173 | 2.103 |
| RHSIM_Rhsim04G0064700 | 2.49 | 0.114 | 0.173 | -0.661 |
| novel.1826 | 2.49 | 0.114 | 0.173 | 0.244 |
| RHSIM_Rhsim07G0181800 | 2.49 | 0.115 | 0.173 | 0.649 |
| RHSIM_Rhsim06G0101900 | 2.49 | 0.115 | 0.173 | 3.642 |
| RHSIM_Rhsim04G0159300 | 2.49 | 0.115 | 0.173 | 1.224 |
| RHSIM_Rhsim07G0061900 | 2.49 | 0.115 | 0.173 | -38.572 |
| RHSIM_Rhsim10G0131100 | 2.49 | 0.115 | 0.173 | 38.374 |
| novel.1797 | 2.49 | 0.115 | 0.173 | -37.909 |
| RHSIM_Rhsim09G0026800 | 2.49 | 0.115 | 0.173 | 1.120 |

| Gene | LRT statistic | P value | BH-adjusted P | log2FC (interaction) |
|---|---|---|---|---|
| RHSIM_RhsimUnG0120200 | 2.49 | 0.115 | 0.174 | -0.238 |
| novel.1441 | 2.49 | 0.115 | 0.174 | 1.418 |
| RHSIM_Rhsim07G0060700 | 2.48 | 0.115 | 0.174 | -0.522 |
| RHSIM_RhsimUnG0005400 | 2.48 | 0.115 | 0.174 | -0.615 |
| RHSIM_Rhsim02G0105400 | 2.48 | 0.115 | 0.174 | -38.191 |
| novel.1120 | 2.48 | 0.115 | 0.174 | -1.127 |
| novel.1603 | 2.48 | 0.115 | 0.174 | 3.132 |
| novel.1552 | 2.48 | 0.115 | 0.174 | -0.394 |
| RHSIM_Rhsim09G0168600 | 2.48 | 0.115 | 0.174 | -0.205 |
| RHSIM_Rhsim11G0114500 | 2.48 | 0.115 | 0.174 | -37.789 |
| RHSIM_Rhsim05G0213900 | 2.48 | 0.115 | 0.174 | 38.222 |
| RHSIM_Rhsim03G0116500 | 2.48 | 0.115 | 0.174 | 0.709 |
| RHSIM_Rhsim01G0037700 | 2.48 | 0.116 | 0.175 | -2.048 |
| novel.2652 | 2.48 | 0.116 | 0.175 | -1.771 |
| RHSIM_Rhsim01G0159800 | 2.48 | 0.116 | 0.175 | -0.085 |
| RHSIM_Rhsim02G0073800 | 2.48 | 0.116 | 0.175 | -38.527 |
| RHSIM_Rhsim03G0152200 | 2.48 | 0.116 | 0.175 | -0.330 |
| RHSIM_Rhsim12G0154500 | 2.48 | 0.116 | 0.175 | -0.163 |
| RHSIM_Rhsim03G0237100 | 2.47 | 0.116 | 0.175 | 1.345 |
| RHSIM_Rhsim03G0207500 | 2.47 | 0.116 | 0.175 | 0.307 |
| RHSIM_Rhsim03G0023600 | 2.47 | 0.116 | 0.175 | 2.080 |
| RHSIM_Rhsim10G0045200 | 2.47 | 0.116 | 0.175 | 0.372 |
| RHSIM_Rhsim01G0049300 | 2.47 | 0.116 | 0.175 | -0.716 |
| RHSIM_RhsimUnG0113500 | 2.47 | 0.116 | 0.175 | -3.193 |
| RHSIM_Rhsim11G0036900 | 2.47 | 0.116 | 0.175 | 0.422 |
| RHSIM_RhsimUnG0192400 | 2.47 | 0.116 | 0.175 | 1.150 |
| RHSIM_Rhsim09G0181600 | 2.47 | 0.116 | 0.175 | 0.181 |
| novel.1622 | 2.47 | 0.116 | 0.175 | -38.675 |
| RHSIM_Rhsim08G0227700 | 2.47 | 0.116 | 0.175 | -0.456 |
| novel.3134 | 2.47 | 0.116 | 0.176 | -0.496 |
| RHSIM_Rhsim06G0048900 | 2.47 | 0.116 | 0.176 | -1.300 |
| novel.2034 | 2.47 | 0.116 | 0.176 | 0.350 |

| Gene | LRT statistic | P value | BH-adjusted P | log2FC (interaction) |
|---|---|---|---|---|
| RHSIM_Rhsim09G0086800 | 2.47 | 0.116 | 0.176 | 0.621 |
| RHSIM_Rhsim06G0011400 | 2.47 | 0.116 | 0.176 | -38.328 |
| RHSIM_Rhsim01G0277300 | 2.46 | 0.116 | 0.176 | -0.946 |
| novel.2146 | 2.46 | 0.116 | 0.176 | 2.030 |
| RHSIM_Rhsim04G0163000 | 2.46 | 0.117 | 0.176 | 0.222 |
| RHSIM_RhsimUnG0225100 | 2.46 | 0.117 | 0.176 | 3.155 |
| RHSIM_RhsimUnG0190600 | 2.46 | 0.117 | 0.176 | -0.646 |
| novel.2332 | 2.46 | 0.117 | 0.176 | -1.920 |
| RHSIM_Rhsim13G0004200 | 2.46 | 0.117 | 0.176 | -0.475 |
| RHSIM_Rhsim01G0274200 | 2.46 | 0.117 | 0.176 | 0.188 |
| RHSIM_Rhsim04G0143200 | 2.46 | 0.117 | 0.176 | 0.416 |
| RHSIM_Rhsim07G0211400 | 2.46 | 0.117 | 0.176 | 0.142 |
| RHSIM_Rhsim03G0077400 | 2.46 | 0.117 | 0.176 | -38.128 |
| RHSIM_Rhsim09G0020300 | 2.46 | 0.117 | 0.176 | -2.040 |
| RHSIM_Rhsim06G0166700 | 2.46 | 0.117 | 0.176 | -0.826 |
| RHSIM_Rhsim07G0089900 | 2.46 | 0.117 | 0.176 | 0.727 |
| RHSIM_Rhsim01G0202200 | 2.46 | 0.117 | 0.176 | 2.511 |
| RHSIM_Rhsim11G0011000 | 2.46 | 0.117 | 0.177 | -2.517 |
| RHSIM_Rhsim03G0242100 | 2.45 | 0.117 | 0.177 | 2.121 |
| novel.2113 | 2.45 | 0.117 | 0.177 | -2.801 |
| RHSIM_Rhsim02G0081600 | 2.45 | 0.117 | 0.177 | 0.260 |
| RHSIM_Rhsim05G0010900 | 2.45 | 0.117 | 0.177 | 0.610 |
| RHSIM_Rhsim02G0009000 | 2.45 | 0.117 | 0.177 | 0.232 |
| RHSIM_Rhsim04G0187500 | 2.45 | 0.117 | 0.177 | 0.428 |
| RHSIM_Rhsim09G0040500 | 2.45 | 0.117 | 0.177 | 0.497 |
| RHSIM_Rhsim08G0147800 | 2.45 | 0.117 | 0.177 | -0.468 |
| RHSIM_Rhsim05G0126600 | 2.45 | 0.117 | 0.177 | -0.373 |
| novel.1546 | 2.45 | 0.118 | 0.177 | 0.567 |
| RHSIM_Rhsim03G0270500 | 2.45 | 0.118 | 0.177 | 0.667 |
| novel.1995 | 2.45 | 0.118 | 0.177 | -1.600 |
| RHSIM_Rhsim11G0053400 | 2.45 | 0.118 | 0.177 | -1.556 |
| RHSIM_Rhsim13G0084800 | 2.45 | 0.118 | 0.177 | 38.476 |

| Gene | LRT statistic | P value | BH-adjusted P | log2FC (interaction) |
|---|---|---|---|---|
| RHSIM_Rhsim01G0036000 | 2.45 | 0.118 | 0.178 | -0.229 |
| novel.50 | 2.45 | 0.118 | 0.178 | -0.370 |
| RHSIM_Rhsim01G0136300 | 2.45 | 0.118 | 0.178 | 1.626 |
| novel.3391 | 2.44 | 0.118 | 0.178 | -3.080 |
| novel.2532 | 2.44 | 0.118 | 0.178 | -0.542 |
| RHSIM_Rhsim10G0161800 | 2.44 | 0.118 | 0.178 | -0.395 |
| novel.2597 | 2.44 | 0.118 | 0.178 | -0.268 |
| RHSIM_Rhsim07G0224600 | 2.44 | 0.118 | 0.178 | 41.100 |
| RHSIM_Rhsim06G0050500 | 2.44 | 0.118 | 0.178 | 0.231 |
| RHSIM_Rhsim02G0251400 | 2.44 | 0.118 | 0.178 | 0.223 |
| RHSIM_Rhsim06G0172700 | 2.44 | 0.118 | 0.178 | 0.089 |
| RHSIM_Rhsim03G0263700 | 2.44 | 0.118 | 0.178 | -0.699 |
| RHSIM_Rhsim02G0186700 | 2.44 | 0.118 | 0.178 | -2.848 |
| RHSIM_Rhsim12G0206200 | 2.44 | 0.118 | 0.178 | -37.777 |
| RHSIM_Rhsim01G0071000 | 2.44 | 0.118 | 0.178 | -3.242 |
| RHSIM_Rhsim03G0088800 | 2.44 | 0.118 | 0.178 | 1.268 |
| RHSIM_Rhsim04G0198700 | 2.44 | 0.119 | 0.178 | -1.837 |
| RHSIM_Rhsim04G0010900 | 2.44 | 0.119 | 0.178 | -0.322 |
| novel.1958 | 2.44 | 0.119 | 0.179 | -0.372 |
| novel.1497 | 2.44 | 0.119 | 0.179 | -0.603 |
| RHSIM_Rhsim08G0141900 | 2.44 | 0.119 | 0.179 | 0.471 |
| RHSIM_Rhsim09G0094900 | 2.43 | 0.119 | 0.179 | 2.134 |
| RHSIM_Rhsim10G0144100 | 2.43 | 0.119 | 0.179 | -38.500 |
| RHSIM_Rhsim02G0035900 | 2.43 | 0.119 | 0.179 | -0.368 |
| RHSIM_Rhsim02G0165200 | 2.43 | 0.119 | 0.179 | 2.616 |
| RHSIM_Rhsim04G0248400 | 2.43 | 0.119 | 0.179 | -0.288 |
| RHSIM_Rhsim06G0172800 | 2.43 | 0.119 | 0.179 | 2.626 |
| RHSIM_Rhsim04G0052200 | 2.43 | 0.119 | 0.179 | 0.561 |
| RHSIM_Rhsim01G0066500 | 2.43 | 0.119 | 0.179 | -0.235 |
| RHSIM_Rhsim01G0105700 | 2.43 | 0.119 | 0.179 | -1.008 |
| RHSIM_Rhsim04G0210200 | 2.43 | 0.119 | 0.179 | 0.364 |
| RHSIM_Rhsim09G0060100 | 2.43 | 0.119 | 0.179 | -0.163 |

| Gene | LRT statistic | P value | BH-adjusted P | log2FC (interaction) |
|---|---|---|---|---|
| RHSIM_Rhsim04G0107800 | 2.43 | 0.119 | 0.179 | -37.724 |
| RHSIM_Rhsim11G0063900 | 2.43 | 0.119 | 0.179 | 1.000 |
| RHSIM_Rhsim06G0092200 | 2.43 | 0.119 | 0.179 | -0.255 |
| RHSIM_Rhsim07G0137400 | 2.43 | 0.119 | 0.180 | 1.280 |
| RHSIM_Rhsim13G0197200 | 2.43 | 0.119 | 0.180 | -0.224 |
| novel.3433 | 2.43 | 0.119 | 0.180 | -1.753 |
| RHSIM_Rhsim09G0029200 | 2.42 | 0.120 | 0.180 | -0.100 |
| RHSIM_Rhsim05G0093900 | 2.42 | 0.120 | 0.180 | 0.268 |
| RHSIM_Rhsim08G0096100 | 2.42 | 0.120 | 0.180 | -0.599 |
| RHSIM_RhsimUnG0046300 | 2.42 | 0.120 | 0.180 | -1.902 |
| RHSIM_Rhsim11G0081500 | 2.42 | 0.120 | 0.180 | 38.324 |
| RHSIM_Rhsim02G0171800 | 2.42 | 0.120 | 0.180 | -0.572 |
| novel.2420 | 2.42 | 0.120 | 0.180 | -2.541 |
| RHSIM_Rhsim10G0153900 | 2.42 | 0.120 | 0.180 | 0.566 |
| RHSIM_Rhsim11G0161000 | 2.42 | 0.120 | 0.180 | 0.489 |
| RHSIM_Rhsim12G0033000 | 2.42 | 0.120 | 0.180 | -38.764 |
| RHSIM_Rhsim12G0146700 | 2.42 | 0.120 | 0.180 | 2.871 |
| RHSIM_Rhsim10G0162000 | 2.42 | 0.120 | 0.180 | 0.537 |
| RHSIM_RhsimUnG0132200 | 2.42 | 0.120 | 0.180 | 0.789 |
| RHSIM_RhsimUnG0094000 | 2.42 | 0.120 | 0.180 | -0.584 |
| RHSIM_RhsimUnG0180400 | 2.42 | 0.120 | 0.180 | 0.947 |
| RHSIM_Rhsim11G0009700 | 2.42 | 0.120 | 0.180 | 0.545 |
| novel.4544 | 2.42 | 0.120 | 0.181 | -1.463 |
| RHSIM_Rhsim11G0000800 | 2.41 | 0.120 | 0.181 | 38.749 |
| RHSIM_RhsimUnG0074100 | 2.41 | 0.120 | 0.181 | -1.793 |
| RHSIM_Rhsim05G0192000 | 2.41 | 0.120 | 0.181 | -1.039 |
| RHSIM_Rhsim11G0129700 | 2.41 | 0.120 | 0.181 | 1.824 |
| RHSIM_Rhsim07G0016100 | 2.41 | 0.120 | 0.181 | 0.680 |
| novel.2416 | 2.41 | 0.120 | 0.181 | -38.405 |
| RHSIM_Rhsim09G0114400 | 2.41 | 0.120 | 0.181 | 0.616 |
| RHSIM_RhsimUnG0233300 | 2.41 | 0.120 | 0.181 | 0.226 |
| RHSIM_Rhsim01G0251400 | 2.41 | 0.120 | 0.181 | 2.661 |

| Gene | LRT statistic | P value | BH-adjusted P | log2FC (interaction) |
|---|---|---|---|---|
| novel.1327 | 2.41 | 0.120 | 0.181 | 2.212 |
| RHSIM_Rhsim01G0033800 | 2.41 | 0.120 | 0.181 | -0.193 |
| RHSIM_Rhsim11G0049300 | 2.41 | 0.120 | 0.181 | 1.330 |
| RHSIM_Rhsim06G0018300 | 2.41 | 0.121 | 0.181 | -37.760 |
| RHSIM_Rhsim02G0050700 | 2.41 | 0.121 | 0.181 | 0.302 |
| RHSIM_Rhsim02G0188700 | 2.41 | 0.121 | 0.181 | 1.446 |
| novel.1320 | 2.41 | 0.121 | 0.181 | 1.020 |
| novel.324 | 2.41 | 0.121 | 0.181 | -1.784 |
| novel.2824 | 2.41 | 0.121 | 0.181 | 0.288 |
| RHSIM_Rhsim08G0014100 | 2.41 | 0.121 | 0.181 | -0.730 |
| RHSIM_Rhsim05G0113600 | 2.41 | 0.121 | 0.181 | -0.201 |
| novel.2110 | 2.41 | 0.121 | 0.181 | 0.493 |
| RHSIM_Rhsim10G0209400 | 2.41 | 0.121 | 0.181 | 3.038 |
| RHSIM_Rhsim11G0193200 | 2.40 | 0.121 | 0.182 | 3.635 |
| novel.2575 | 2.40 | 0.121 | 0.182 | -1.825 |
| RHSIM_RhsimUnG0128400 | 2.40 | 0.121 | 0.182 | 0.994 |
| RHSIM_Rhsim07G0039800 | 2.40 | 0.121 | 0.182 | 0.570 |
| RHSIM_RhsimUnG0145500 | 2.40 | 0.121 | 0.182 | 38.766 |
| RHSIM_RhsimUnG0005500 | 2.40 | 0.122 | 0.182 | -0.376 |
| RHSIM_Rhsim04G0146100 | 2.40 | 0.122 | 0.183 | -38.755 |
| RHSIM_Rhsim01G0021200 | 2.40 | 0.122 | 0.183 | -1.631 |
| novel.904 | 2.39 | 0.122 | 0.183 | -2.441 |
| RHSIM_Rhsim01G0008200 | 2.39 | 0.122 | 0.183 | 1.827 |
| RHSIM_Rhsim12G0165800 | 2.39 | 0.122 | 0.183 | -0.161 |
| novel.4276 | 2.39 | 0.122 | 0.183 | -0.554 |
| RHSIM_Rhsim08G0005800 | 2.39 | 0.122 | 0.183 | -1.504 |
| RHSIM_Rhsim05G0040300 | 2.39 | 0.122 | 0.183 | -0.483 |
| novel.261 | 2.39 | 0.122 | 0.183 | -1.634 |
| RHSIM_Rhsim05G0223900 | 2.39 | 0.122 | 0.183 | 4.453 |
| RHSIM_Rhsim06G0144600 | 2.39 | 0.122 | 0.183 | 39.276 |
| RHSIM_Rhsim11G0028700 | 2.39 | 0.122 | 0.183 | 0.463 |
| RHSIM_Rhsim13G0216300 | 2.39 | 0.122 | 0.183 | -38.879 |

| Gene | LRT statistic | P value | BH-adjusted P | log2FC (interaction) |
|---|---|---|---|---|
| novel.4116 | 2.39 | 0.122 | 0.183 | -38.730 |
| RHSIM_Rhsim03G0100300 | 2.39 | 0.122 | 0.183 | 0.564 |
| RHSIM_Rhsim08G0151900 | 2.39 | 0.122 | 0.183 | -0.172 |
| RHSIM_Rhsim04G0140800 | 2.39 | 0.122 | 0.184 | 0.556 |
| RHSIM_Rhsim13G0052600 | 2.39 | 0.122 | 0.184 | 0.327 |
| RHSIM_Rhsim06G0057300 | 2.38 | 0.123 | 0.184 | 1.022 |
| novel.2208 | 2.38 | 0.123 | 0.184 | 0.505 |
| RHSIM_Rhsim02G0128300 | 2.38 | 0.123 | 0.184 | 1.881 |
| RHSIM_Rhsim02G0213100 | 2.38 | 0.123 | 0.184 | -0.254 |
| novel.267 | 2.38 | 0.123 | 0.184 | 38.192 |
| RHSIM_RhsimUnG0245100 | 2.38 | 0.123 | 0.184 | 0.562 |
| RHSIM_Rhsim04G0056000 | 2.38 | 0.123 | 0.184 | -0.126 |
| RHSIM_Rhsim01G0178200 | 2.38 | 0.123 | 0.184 | -0.125 |
| RHSIM_RhsimUnG0150300 | 2.38 | 0.123 | 0.184 | -1.949 |
| RHSIM_Rhsim03G0195000 | 2.38 | 0.123 | 0.184 | -38.643 |
| RHSIM_Rhsim13G0234300 | 2.38 | 0.123 | 0.184 | 0.186 |
| RHSIM_Rhsim03G0051500 | 2.38 | 0.123 | 0.184 | 1.324 |
| RHSIM_RhsimUnG0061500 | 2.38 | 0.123 | 0.185 | 0.731 |
| RHSIM_Rhsim01G0215100 | 2.37 | 0.123 | 0.185 | -1.630 |
| RHSIM_Rhsim10G0113900 | 2.37 | 0.123 | 0.185 | -0.398 |
| RHSIM_Rhsim09G0144100 | 2.37 | 0.123 | 0.185 | 0.704 |
| RHSIM_Rhsim08G0069600 | 2.37 | 0.123 | 0.185 | 0.659 |
| RHSIM_RhsimUnG0132900 | 2.37 | 0.123 | 0.185 | -0.194 |
| RHSIM_Rhsim02G0191400 | 2.37 | 0.123 | 0.185 | -0.687 |
| RHSIM_Rhsim12G0133900 | 2.37 | 0.124 | 0.185 | 0.930 |
| novel.3641 | 2.37 | 0.124 | 0.185 | 0.684 |
| RHSIM_Rhsim05G0045500 | 2.37 | 0.124 | 0.185 | 38.946 |
| RHSIM_Rhsim07G0228600 | 2.37 | 0.124 | 0.185 | 0.145 |
| RHSIM_Rhsim07G0156100 | 2.37 | 0.124 | 0.185 | -0.968 |
| RHSIM_RhsimUnG0073200 | 2.37 | 0.124 | 0.185 | -1.638 |
| RHSIM_Rhsim02G0237700 | 2.37 | 0.124 | 0.185 | 0.398 |
| RHSIM_Rhsim09G0020700 | 2.37 | 0.124 | 0.185 | 0.619 |

| Gene | LRT statistic | P value | BH-adjusted P | log2FC (interaction) |
|---|---|---|---|---|
| RHSIM_Rhsim11G0186700 | 2.37 | 0.124 | 0.186 | -0.714 |
| novel.3332 | 2.37 | 0.124 | 0.186 | 0.424 |
| novel.2530 | 2.37 | 0.124 | 0.186 | -1.599 |
| RHSIM_Rhsim07G0196500 | 2.37 | 0.124 | 0.186 | 2.194 |
| RHSIM_Rhsim07G0021800 | 2.37 | 0.124 | 0.186 | -0.422 |
| RHSIM_Rhsim10G0026800 | 2.37 | 0.124 | 0.186 | 0.472 |
| RHSIM_Rhsim02G0195800 | 2.36 | 0.124 | 0.186 | 0.621 |
| RHSIM_Rhsim08G0175400 | 2.36 | 0.124 | 0.186 | 1.144 |
| RHSIM_Rhsim05G0002500 | 2.36 | 0.124 | 0.186 | 0.251 |
| RHSIM_Rhsim13G0034900 | 2.36 | 0.124 | 0.186 | -1.624 |
| novel.296 | 2.36 | 0.124 | 0.186 | 1.619 |
| RHSIM_Rhsim03G0054600 | 2.36 | 0.124 | 0.186 | -0.251 |
| RHSIM_Rhsim09G0018700 | 2.36 | 0.124 | 0.186 | -0.660 |
| RHSIM_Rhsim06G0004400 | 2.36 | 0.124 | 0.186 | 1.011 |
| RHSIM_Rhsim13G0022700 | 2.36 | 0.125 | 0.186 | 0.093 |
| RHSIM_Rhsim03G0223900 | 2.36 | 0.125 | 0.186 | 0.178 |
| RHSIM_Rhsim07G0108200 | 2.36 | 0.125 | 0.186 | 0.158 |
| RHSIM_RhsimUnG0207800 | 2.36 | 0.125 | 0.187 | 0.693 |
| RHSIM_Rhsim07G0163900 | 2.36 | 0.125 | 0.187 | 0.895 |
| RHSIM_Rhsim12G0006600 | 2.35 | 0.125 | 0.187 | -0.721 |
| RHSIM_Rhsim06G0243000 | 2.35 | 0.125 | 0.187 | -0.158 |
| novel.4354 | 2.35 | 0.125 | 0.187 | 0.495 |
| RHSIM_Rhsim04G0127200 | 2.35 | 0.125 | 0.187 | 38.493 |
| RHSIM_Rhsim11G0101700 | 2.35 | 0.125 | 0.187 | -1.034 |
| novel.1030 | 2.35 | 0.125 | 0.187 | -1.266 |
| RHSIM_Rhsim01G0146100 | 2.35 | 0.125 | 0.187 | 0.461 |
| RHSIM_Rhsim01G0227400 | 2.35 | 0.125 | 0.187 | -0.509 |
| RHSIM_Rhsim06G0236900 | 2.35 | 0.125 | 0.187 | -0.149 |
| RHSIM_Rhsim12G0038100 | 2.35 | 0.125 | 0.187 | -0.175 |
| RHSIM_Rhsim02G0186500 | 2.35 | 0.125 | 0.187 | -2.942 |
| RHSIM_RhsimUnG0230300 | 2.35 | 0.125 | 0.188 | -38.720 |
| RHSIM_Rhsim04G0065500 | 2.35 | 0.126 | 0.188 | -2.730 |

| Gene | LRT statistic | P value | BH-adjusted P | log2FC (interaction) |
|---|---|---|---|---|
| RHSIM_Rhsim07G0050500 | 2.35 | 0.126 | 0.188 | -0.333 |
| RHSIM_Rhsim13G0055600 | 2.35 | 0.126 | 0.188 | 3.141 |
| RHSIM_Rhsim12G0143800 | 2.35 | 0.126 | 0.188 | -37.824 |
| RHSIM_Rhsim02G0178900 | 2.35 | 0.126 | 0.188 | 2.661 |
| RHSIM_Rhsim10G0122500 | 2.35 | 0.126 | 0.188 | 0.129 |
| RHSIM_Rhsim13G0038900 | 2.34 | 0.126 | 0.188 | -0.539 |
| RHSIM_Rhsim02G0115100 | 2.34 | 0.126 | 0.188 | 0.114 |
| novel.4140 | 2.34 | 0.126 | 0.188 | 1.666 |
| RHSIM_RhsimUnG0170200 | 2.34 | 0.126 | 0.188 | -3.872 |
| RHSIM_Rhsim03G0127700 | 2.34 | 0.126 | 0.188 | 2.902 |
| RHSIM_Rhsim13G0210900 | 2.34 | 0.126 | 0.188 | 0.553 |
| RHSIM_Rhsim11G0001300 | 2.34 | 0.126 | 0.188 | -0.501 |
| RHSIM_RhsimUnG0171200 | 2.34 | 0.126 | 0.188 | -0.226 |
| RHSIM_Rhsim06G0133100 | 2.34 | 0.126 | 0.188 | 0.161 |
| novel.3170 | 2.34 | 0.126 | 0.189 | 1.804 |
| RHSIM_Rhsim06G0050400 | 2.34 | 0.126 | 0.189 | 0.242 |
| RHSIM_Rhsim13G0073600 | 2.34 | 0.126 | 0.189 | 0.209 |
| RHSIM_Rhsim09G0141800 | 2.34 | 0.126 | 0.189 | 0.190 |
| RHSIM_RhsimUnG0160200 | 2.34 | 0.126 | 0.189 | -0.486 |
| novel.2274 | 2.33 | 0.127 | 0.189 | 0.947 |
| RHSIM_Rhsim05G0220600 | 2.33 | 0.127 | 0.189 | 0.646 |
| RHSIM_RhsimMtG0005700 | 2.33 | 0.127 | 0.189 | 3.109 |
| novel.3668 | 2.33 | 0.127 | 0.189 | 0.940 |
| RHSIM_Rhsim13G0063100 | 2.33 | 0.127 | 0.189 | -0.216 |
| RHSIM_Rhsim07G0020500 | 2.33 | 0.127 | 0.189 | -0.129 |
| RHSIM_Rhsim05G0212100 | 2.33 | 0.127 | 0.189 | -0.738 |
| RHSIM_RhsimUnG0127200 | 2.33 | 0.127 | 0.189 | 1.104 |
| RHSIM_Rhsim08G0178300 | 2.33 | 0.127 | 0.189 | 0.334 |
| RHSIM_RhsimUnG0038200 | 2.33 | 0.127 | 0.189 | 2.300 |
| RHSIM_Rhsim11G0001400 | 2.33 | 0.127 | 0.189 | -0.513 |
| RHSIM_Rhsim06G0231900 | 2.33 | 0.127 | 0.190 | 39.232 |
| RHSIM_Rhsim08G0124200 | 2.32 | 0.127 | 0.190 | 0.177 |

| Gene | LRT statistic | P value | BH-adjusted P | log2FC (interaction) |
|---|---|---|---|---|
| RHSIM_Rhsim08G0096500 | 2.32 | 0.127 | 0.190 | -0.362 |
| RHSIM_Rhsim10G0131500 | 2.32 | 0.127 | 0.190 | -2.709 |
| RHSIM_Rhsim11G0023600 | 2.32 | 0.127 | 0.190 | -1.195 |
| RHSIM_Rhsim09G0205200 | 2.32 | 0.128 | 0.190 | -0.187 |
| RHSIM_Rhsim04G0147000 | 2.32 | 0.128 | 0.190 | -0.568 |
| RHSIM_Rhsim01G0251300 | 2.32 | 0.128 | 0.190 | 0.318 |
| novel.4112 | 2.32 | 0.128 | 0.191 | 2.069 |
| novel.545 | 2.32 | 0.128 | 0.191 | -1.362 |
| novel.1419 | 2.32 | 0.128 | 0.191 | 0.503 |
| RHSIM_Rhsim05G0048400 | 2.32 | 0.128 | 0.191 | 3.372 |
| RHSIM_Rhsim02G0188600 | 2.32 | 0.128 | 0.191 | 0.206 |
| RHSIM_Rhsim04G0072800 | 2.32 | 0.128 | 0.191 | 2.253 |
| RHSIM_Rhsim12G0139100 | 2.32 | 0.128 | 0.191 | -0.122 |
| novel.1114 | 2.32 | 0.128 | 0.191 | 1.056 |
| RHSIM_Rhsim06G0184100 | 2.32 | 0.128 | 0.191 | -0.135 |
| RHSIM_Rhsim13G0060000 | 2.32 | 0.128 | 0.191 | 1.557 |
| RHSIM_Rhsim05G0066800 | 2.32 | 0.128 | 0.191 | 38.627 |
| RHSIM_Rhsim10G0193800 | 2.32 | 0.128 | 0.191 | -0.953 |
| RHSIM_Rhsim10G0016800 | 2.31 | 0.128 | 0.191 | 0.198 |
| novel.3865 | 2.31 | 0.128 | 0.191 | -1.169 |
| RHSIM_Rhsim01G0288100 | 2.31 | 0.128 | 0.191 | 2.098 |
| novel.3986 | 2.31 | 0.128 | 0.191 | 2.235 |
| RHSIM_Rhsim09G0080500 | 2.31 | 0.129 | 0.192 | -0.774 |
| RHSIM_Rhsim04G0085400 | 2.31 | 0.129 | 0.192 | -0.364 |
| novel.3350 | 2.31 | 0.129 | 0.192 | 1.306 |
| RHSIM_Rhsim08G0035400 | 2.31 | 0.129 | 0.192 | 0.149 |
| RHSIM_Rhsim05G0119800 | 2.31 | 0.129 | 0.192 | 0.138 |
| RHSIM_Rhsim11G0041500 | 2.30 | 0.129 | 0.192 | -0.647 |
| RHSIM_Rhsim05G0139100 | 2.30 | 0.129 | 0.193 | -1.022 |
| RHSIM_Rhsim01G0143300 | 2.30 | 0.129 | 0.193 | -3.682 |
| RHSIM_Rhsim13G0163000 | 2.30 | 0.129 | 0.193 | 0.159 |
| novel.102 | 2.30 | 0.129 | 0.193 | -37.923 |

| Gene | LRT statistic | P value | BH-adjusted P | log2FC (interaction) |
|---|---|---|---|---|
| RHSIM_Rhsim01G0225200 | 2.30 | 0.129 | 0.193 | 0.172 |
| RHSIM_Rhsim12G0135700 | 2.30 | 0.129 | 0.193 | 0.200 |
| novel.3898 | 2.30 | 0.130 | 0.193 | 2.777 |
| RHSIM_Rhsim07G0017600 | 2.30 | 0.130 | 0.193 | 0.118 |
| RHSIM_Rhsim02G0208700 | 2.30 | 0.130 | 0.193 | -1.165 |
| RHSIM_Rhsim05G0118500 | 2.29 | 0.130 | 0.193 | 0.980 |
| novel.3132 | 2.29 | 0.130 | 0.194 | -1.460 |
| RHSIM_Rhsim10G0009200 | 2.29 | 0.130 | 0.194 | 1.633 |
| novel.19 | 2.29 | 0.130 | 0.194 | -2.370 |
| RHSIM_Rhsim02G0167800 | 2.29 | 0.130 | 0.194 | -2.257 |
| RHSIM_Rhsim05G0187200 | 2.29 | 0.130 | 0.194 | 0.862 |
| RHSIM_Rhsim05G0034400 | 2.29 | 0.131 | 0.194 | -1.038 |
| novel.3101 | 2.29 | 0.131 | 0.194 | -0.277 |
| RHSIM_Rhsim08G0031700 | 2.28 | 0.131 | 0.195 | 0.580 |
| RHSIM_Rhsim01G0099500 | 2.28 | 0.131 | 0.195 | 39.082 |
| RHSIM_Rhsim11G0189600 | 2.28 | 0.131 | 0.195 | 3.039 |
| RHSIM_Rhsim09G0033200 | 2.28 | 0.131 | 0.195 | -0.161 |
| RHSIM_Rhsim09G0057300 | 2.28 | 0.131 | 0.195 | 3.050 |
| RHSIM_Rhsim10G0021700 | 2.28 | 0.131 | 0.195 | 2.583 |
| RHSIM_Rhsim05G0161000 | 2.28 | 0.131 | 0.195 | 0.799 |
| novel.522 | 2.28 | 0.131 | 0.195 | -37.865 |
| RHSIM_Rhsim08G0093300 | 2.28 | 0.131 | 0.195 | 0.287 |
| RHSIM_Rhsim09G0204700 | 2.28 | 0.131 | 0.195 | 1.842 |
| RHSIM_Rhsim11G0040400 | 2.28 | 0.131 | 0.195 | 0.213 |
| RHSIM_Rhsim03G0004700 | 2.28 | 0.131 | 0.195 | 1.810 |
| RHSIM_Rhsim12G0210900 | 2.28 | 0.131 | 0.195 | -1.696 |
| RHSIM_Rhsim11G0082600 | 2.28 | 0.131 | 0.195 | 1.477 |
| RHSIM_Rhsim04G0021500 | 2.28 | 0.131 | 0.195 | -0.149 |
| novel.1122 | 2.28 | 0.131 | 0.195 | 1.218 |
| RHSIM_Rhsim05G0103200 | 2.28 | 0.131 | 0.195 | 0.527 |
| RHSIM_Rhsim10G0005900 | 2.28 | 0.131 | 0.195 | 1.891 |
| RHSIM_Rhsim07G0101300 | 2.27 | 0.132 | 0.196 | 0.092 |

| Gene | LRT statistic | P value | BH-adjusted P | log2FC (interaction) |
|---|---|---|---|---|
| RHSIM_Rhsim09G0210100 | 2.27 | 0.132 | 0.196 | 0.175 |
| RHSIM_Rhsim04G0188400 | 2.27 | 0.132 | 0.196 | -0.398 |
| RHSIM_Rhsim04G0233800 | 2.27 | 0.132 | 0.196 | -0.134 |
| RHSIM_Rhsim04G0103800 | 2.27 | 0.132 | 0.196 | 1.318 |
| novel.148 | 2.27 | 0.132 | 0.196 | -2.146 |
| RHSIM_Rhsim01G0182600 | 2.27 | 0.132 | 0.196 | -0.587 |
| RHSIM_Rhsim04G0002000 | 2.27 | 0.132 | 0.196 | -1.550 |
| novel.3516 | 2.27 | 0.132 | 0.196 | 2.055 |
| novel.1414 | 2.27 | 0.132 | 0.196 | 0.435 |
| RHSIM_Rhsim04G0185800 | 2.27 | 0.132 | 0.196 | -1.168 |
| RHSIM_Rhsim03G0253800 | 2.27 | 0.132 | 0.196 | 0.367 |
| RHSIM_Rhsim06G0030300 | 2.27 | 0.132 | 0.196 | -0.874 |
| RHSIM_Rhsim04G0090000 | 2.27 | 0.132 | 0.196 | 0.698 |
| RHSIM_Rhsim01G0169300 | 2.26 | 0.132 | 0.197 | -0.320 |
| RHSIM_Rhsim08G0226700 | 2.26 | 0.132 | 0.197 | -0.698 |
| RHSIM_Rhsim03G0224100 | 2.26 | 0.132 | 0.197 | -0.151 |
| RHSIM_Rhsim03G0165800 | 2.26 | 0.132 | 0.197 | 1.286 |
| RHSIM_Rhsim01G0038800 | 2.26 | 0.132 | 0.197 | 0.580 |
| novel.1331 | 2.26 | 0.132 | 0.197 | 0.715 |
| RHSIM_Rhsim10G0047100 | 2.26 | 0.132 | 0.197 | -2.879 |
| RHSIM_Rhsim09G0194800 | 2.26 | 0.132 | 0.197 | -3.332 |
| RHSIM_Rhsim13G0190500 | 2.26 | 0.133 | 0.197 | -37.700 |
| novel.129 | 2.26 | 0.133 | 0.197 | 2.207 |
| RHSIM_Rhsim13G0222100 | 2.26 | 0.133 | 0.197 | -1.983 |
| RHSIM_Rhsim11G0083500 | 2.26 | 0.133 | 0.197 | -38.337 |
| RHSIM_Rhsim13G0212100 | 2.26 | 0.133 | 0.197 | 3.024 |
| RHSIM_Rhsim04G0235300 | 2.26 | 0.133 | 0.197 | 0.247 |
| novel.1284 | 2.26 | 0.133 | 0.197 | 2.839 |
| RHSIM_Rhsim09G0142600 | 2.26 | 0.133 | 0.197 | 0.163 |
| novel.2837 | 2.26 | 0.133 | 0.197 | 0.392 |
| RHSIM_Rhsim12G0096900 | 2.26 | 0.133 | 0.197 | 0.603 |
| RHSIM_RhsimUnG0046100 | 2.26 | 0.133 | 0.197 | 1.482 |

| Gene | LRT statistic | P value | BH-adjusted P | log2FC (interaction) |
|---|---|---|---|---|
| RHSIM_Rhsim03G0078400 | 2.26 | 0.133 | 0.198 | -0.312 |
| novel.2752 | 2.26 | 0.133 | 0.198 | 0.779 |
| RHSIM_Rhsim08G0069000 | 2.26 | 0.133 | 0.198 | -2.853 |
| RHSIM_Rhsim03G0265700 | 2.25 | 0.133 | 0.198 | -1.958 |
| RHSIM_Rhsim03G0141800 | 2.25 | 0.133 | 0.198 | 0.171 |
| novel.3817 | 2.25 | 0.133 | 0.198 | 1.037 |
| RHSIM_RhsimUnG0141000 | 2.25 | 0.133 | 0.198 | -0.508 |
| RHSIM_Rhsim11G0061600 | 2.25 | 0.133 | 0.198 | 2.717 |
| RHSIM_Rhsim01G0011200 | 2.25 | 0.133 | 0.198 | -0.539 |
| RHSIM_Rhsim12G0108800 | 2.25 | 0.133 | 0.198 | 0.236 |
| RHSIM_Rhsim10G0087400 | 2.25 | 0.134 | 0.198 | -0.785 |
| RHSIM_Rhsim01G0195800 | 2.25 | 0.134 | 0.198 | 0.127 |
| novel.2289 | 2.25 | 0.134 | 0.198 | 0.901 |
| RHSIM_Rhsim07G0001000 | 2.25 | 0.134 | 0.198 | 2.915 |
| RHSIM_Rhsim06G0200900 | 2.25 | 0.134 | 0.198 | -1.932 |
| RHSIM_RhsimUnG0172200 | 2.25 | 0.134 | 0.198 | 3.259 |
| RHSIM_Rhsim06G0200800 | 2.25 | 0.134 | 0.198 | -1.612 |
| RHSIM_Rhsim01G0139100 | 2.25 | 0.134 | 0.198 | 2.351 |
| RHSIM_Rhsim03G0237800 | 2.25 | 0.134 | 0.198 | -1.232 |
| novel.835 | 2.25 | 0.134 | 0.198 | -0.181 |
| RHSIM_Rhsim11G0052000 | 2.25 | 0.134 | 0.199 | 0.304 |
| RHSIM_Rhsim01G0236900 | 2.25 | 0.134 | 0.199 | 38.820 |
| RHSIM_Rhsim13G0106800 | 2.25 | 0.134 | 0.199 | 0.266 |
| RHSIM_Rhsim10G0141700 | 2.24 | 0.134 | 0.199 | 0.179 |
| novel.1697 | 2.24 | 0.134 | 0.199 | -1.244 |
| RHSIM_Rhsim02G0195300 | 2.24 | 0.134 | 0.199 | -38.348 |
| RHSIM_Rhsim05G0177100 | 2.24 | 0.134 | 0.199 | 0.209 |
| RHSIM_Rhsim04G0162800 | 2.24 | 0.134 | 0.199 | 0.133 |
| RHSIM_Rhsim03G0138800 | 2.24 | 0.134 | 0.199 | -3.153 |
| RHSIM_Rhsim11G0149800 | 2.24 | 0.134 | 0.199 | 0.138 |
| RHSIM_Rhsim01G0077300 | 2.24 | 0.134 | 0.199 | 38.554 |
| RHSIM_Rhsim11G0086100 | 2.24 | 0.134 | 0.199 | 1.677 |

| Gene | LRT statistic | P value | BH-adjusted P | log2FC (interaction) |
|---|---|---|---|---|
| RHSIM_Rhsim05G0109000 | 2.24 | 0.134 | 0.199 | 38.107 |
| RHSIM_Rhsim04G0008100 | 2.24 | 0.134 | 0.199 | 0.078 |
| RHSIM_Rhsim09G0129600 | 2.24 | 0.135 | 0.199 | 1.586 |
| RHSIM_Rhsim05G0172900 | 2.24 | 0.135 | 0.199 | 37.986 |
| RHSIM_Rhsim06G0036300 | 2.24 | 0.135 | 0.199 | 1.557 |
| novel.1862 | 2.24 | 0.135 | 0.199 | 0.767 |
| RHSIM_Rhsim06G0022500 | 2.24 | 0.135 | 0.199 | 0.333 |
| RHSIM_Rhsim01G0011700 | 2.24 | 0.135 | 0.199 | -1.666 |
| RHSIM_Rhsim03G0150400 | 2.24 | 0.135 | 0.199 | -1.219 |
| RHSIM_Rhsim03G0267100 | 2.24 | 0.135 | 0.200 | -4.224 |
| novel.2087 | 2.24 | 0.135 | 0.200 | -0.363 |
| novel.2988 | 2.24 | 0.135 | 0.200 | 1.854 |
| RHSIM_Rhsim06G0153900 | 2.24 | 0.135 | 0.200 | -1.192 |
| RHSIM_Rhsim10G0070300 | 2.23 | 0.135 | 0.200 | -38.422 |
| RHSIM_Rhsim05G0012200 | 2.23 | 0.135 | 0.200 | -1.296 |
| RHSIM_Rhsim06G0187700 | 2.23 | 0.135 | 0.200 | -1.318 |
| RHSIM_Rhsim03G0005900 | 2.23 | 0.135 | 0.200 | 2.200 |
| novel.3458 | 2.23 | 0.135 | 0.200 | 0.674 |
| RHSIM_Rhsim11G0166300 | 2.23 | 0.135 | 0.200 | -0.314 |
| RHSIM_Rhsim11G0114200 | 2.23 | 0.135 | 0.200 | 2.658 |
| RHSIM_Rhsim08G0251300 | 2.23 | 0.135 | 0.200 | -0.095 |
| RHSIM_Rhsim05G0063200 | 2.23 | 0.135 | 0.200 | 0.125 |
| RHSIM_Rhsim08G0068900 | 2.23 | 0.135 | 0.200 | -2.186 |
| RHSIM_Rhsim07G0082400 | 2.23 | 0.135 | 0.200 | -0.129 |
| RHSIM_Rhsim06G0176600 | 2.23 | 0.136 | 0.201 | 2.403 |
| RHSIM_Rhsim04G0012300 | 2.23 | 0.136 | 0.201 | 0.784 |
| RHSIM_Rhsim06G0206400 | 2.23 | 0.136 | 0.201 | 0.213 |
| RHSIM_Rhsim07G0028600 | 2.23 | 0.136 | 0.201 | 0.541 |
| RHSIM_Rhsim05G0182100 | 2.23 | 0.136 | 0.201 | 0.127 |
| RHSIM_Rhsim13G0017100 | 2.23 | 0.136 | 0.201 | 1.369 |
| RHSIM_Rhsim05G0126100 | 2.22 | 0.136 | 0.201 | 0.265 |
| novel.995 | 2.22 | 0.136 | 0.201 | -3.011 |

| Gene | LRT statistic | P value | BH-adjusted P | log2FC (interaction) |
|---|---|---|---|---|
| novel.1285 | 2.22 | 0.136 | 0.201 | -1.051 |
| RHSIM_Rhsim07G0054100 | 2.22 | 0.136 | 0.201 | -0.813 |
| RHSIM_Rhsim03G0087600 | 2.22 | 0.136 | 0.201 | 0.704 |
| RHSIM_Rhsim10G0132900 | 2.22 | 0.136 | 0.201 | 1.115 |
| RHSIM_Rhsim01G0278800 | 2.22 | 0.136 | 0.201 | 38.868 |
| RHSIM_Rhsim05G0099000 | 2.22 | 0.136 | 0.201 | -3.028 |
| RHSIM_Rhsim08G0107400 | 2.22 | 0.136 | 0.201 | 1.204 |
| RHSIM_Rhsim01G0289900 | 2.22 | 0.136 | 0.201 | -0.121 |
| RHSIM_Rhsim11G0077000 | 2.22 | 0.136 | 0.202 | 0.155 |
| novel.2596 | 2.22 | 0.136 | 0.202 | -37.316 |
| RHSIM_Rhsim06G0208800 | 2.22 | 0.136 | 0.202 | 0.676 |
| novel.1098 | 2.22 | 0.136 | 0.202 | 1.052 |
| RHSIM_Rhsim09G0163500 | 2.22 | 0.136 | 0.202 | 38.899 |
| RHSIM_Rhsim03G0147100 | 2.22 | 0.136 | 0.202 | 0.128 |
| RHSIM_Rhsim03G0260200 | 2.22 | 0.137 | 0.202 | 0.300 |
| novel.1345 | 2.22 | 0.137 | 0.202 | -1.151 |
| novel.3111 | 2.21 | 0.137 | 0.202 | 0.691 |
| RHSIM_Rhsim05G0132200 | 2.21 | 0.137 | 0.202 | 0.173 |
| RHSIM_Rhsim08G0065100 | 2.21 | 0.137 | 0.202 | -1.266 |
| novel.2388 | 2.21 | 0.137 | 0.202 | 1.486 |
| RHSIM_Rhsim06G0098900 | 2.21 | 0.137 | 0.202 | -0.405 |
| RHSIM_Rhsim07G0092300 | 2.21 | 0.137 | 0.202 | 0.145 |
| RHSIM_Rhsim07G0227100 | 2.21 | 0.137 | 0.202 | -0.407 |
| RHSIM_Rhsim03G0074100 | 2.21 | 0.137 | 0.202 | 0.999 |
| RHSIM_RhsimUnG0025300 | 2.21 | 0.137 | 0.203 | -38.479 |
| RHSIM_Rhsim10G0034900 | 2.21 | 0.137 | 0.203 | 2.131 |
| RHSIM_Rhsim03G0144200 | 2.21 | 0.137 | 0.203 | 0.125 |
| RHSIM_RhsimUnG0088600 | 2.21 | 0.137 | 0.203 | 0.501 |
| RHSIM_Rhsim01G0117100 | 2.21 | 0.137 | 0.203 | -0.334 |
| RHSIM_Rhsim06G0061300 | 2.21 | 0.137 | 0.203 | -0.514 |
| RHSIM_Rhsim02G0075200 | 2.21 | 0.137 | 0.203 | 0.224 |
| novel.2711 | 2.21 | 0.137 | 0.203 | -0.742 |

| Gene | LRT statistic | P value | BH-adjusted P | log2FC (interaction) |
|---|---|---|---|---|
| RHSIM_Rhsim05G0146900 | 2.21 | 0.137 | 0.203 | -3.214 |
| RHSIM_Rhsim01G0117500 | 2.21 | 0.137 | 0.203 | -0.510 |
| RHSIM_Rhsim13G0193000 | 2.21 | 0.138 | 0.203 | 1.632 |
| RHSIM_Rhsim01G0082700 | 2.21 | 0.138 | 0.203 | -0.115 |
| novel.3886 | 2.20 | 0.138 | 0.203 | 0.121 |
| novel.4067 | 2.20 | 0.138 | 0.203 | 0.187 |
| RHSIM_Rhsim13G0036900 | 2.20 | 0.138 | 0.203 | 0.249 |
| novel.1782 | 2.20 | 0.138 | 0.203 | 1.348 |
| RHSIM_Rhsim09G0003100 | 2.20 | 0.138 | 0.203 | 39.049 |
| RHSIM_Rhsim12G0144600 | 2.20 | 0.138 | 0.203 | 3.401 |
| RHSIM_Rhsim11G0036300 | 2.20 | 0.138 | 0.204 | 0.624 |
| RHSIM_Rhsim12G0145100 | 2.20 | 0.138 | 0.204 | -37.859 |
| RHSIM_Rhsim02G0235800 | 2.20 | 0.138 | 0.204 | 3.452 |
| RHSIM_Rhsim05G0135200 | 2.20 | 0.138 | 0.204 | -0.306 |
| RHSIM_Rhsim12G0005300 | 2.20 | 0.138 | 0.204 | -0.420 |
| novel.243 | 2.20 | 0.138 | 0.204 | -1.837 |
| RHSIM_Rhsim04G0046000 | 2.20 | 0.138 | 0.204 | -0.952 |
| RHSIM_Rhsim12G0194500 | 2.20 | 0.138 | 0.204 | -1.544 |
| RHSIM_Rhsim02G0023600 | 2.20 | 0.138 | 0.204 | 0.586 |
| RHSIM_Rhsim12G0006000 | 2.20 | 0.138 | 0.204 | 0.180 |
| RHSIM_Rhsim05G0120400 | 2.20 | 0.138 | 0.204 | 0.182 |
| RHSIM_Rhsim10G0001900 | 2.20 | 0.138 | 0.204 | 4.347 |
| RHSIM_Rhsim08G0173600 | 2.19 | 0.139 | 0.204 | -0.140 |
| RHSIM_Rhsim05G0067000 | 2.19 | 0.139 | 0.204 | 0.197 |
| novel.3660 | 2.19 | 0.139 | 0.204 | -1.368 |
| RHSIM_RhsimUnG0069100 | 2.19 | 0.139 | 0.204 | -38.778 |
| RHSIM_Rhsim02G0046500 | 2.19 | 0.139 | 0.204 | -37.431 |
| RHSIM_Rhsim01G0206000 | 2.19 | 0.139 | 0.205 | -3.006 |
| RHSIM_Rhsim11G0172300 | 2.19 | 0.139 | 0.205 | -1.413 |
| RHSIM_Rhsim09G0155100 | 2.19 | 0.139 | 0.205 | -0.196 |
| RHSIM_Rhsim13G0200200 | 2.19 | 0.139 | 0.205 | 1.638 |
| RHSIM_Rhsim08G0097400 | 2.19 | 0.139 | 0.205 | 0.512 |

| Gene | LRT statistic | P value | BH-adjusted P | log2FC (interaction) |
|---|---|---|---|---|
| RHSIM_Rhsim05G0079900 | 2.19 | 0.139 | 0.205 | -0.136 |
| RHSIM_Rhsim12G0084500 | 2.19 | 0.139 | 0.205 | 0.486 |
| RHSIM_Rhsim13G0034200 | 2.19 | 0.139 | 0.205 | 1.080 |
| RHSIM_Rhsim10G0125600 | 2.19 | 0.139 | 0.205 | 0.365 |
| novel.1720 | 2.19 | 0.139 | 0.205 | 0.591 |
| RHSIM_Rhsim12G0102700 | 2.19 | 0.139 | 0.205 | 0.689 |
| novel.3073 | 2.19 | 0.139 | 0.205 | 0.372 |
| RHSIM_RhsimUnG0095800 | 2.18 | 0.139 | 0.206 | 1.671 |
| RHSIM_Rhsim01G0147700 | 2.18 | 0.139 | 0.206 | 0.156 |
| novel.2290 | 2.18 | 0.139 | 0.206 | -1.337 |
| RHSIM_Rhsim03G0173400 | 2.18 | 0.139 | 0.206 | 0.911 |
| RHSIM_Rhsim07G0122900 | 2.18 | 0.140 | 0.206 | -0.136 |
| RHSIM_Rhsim01G0191500 | 2.18 | 0.140 | 0.206 | -0.313 |
| RHSIM_Rhsim08G0207000 | 2.18 | 0.140 | 0.206 | 0.511 |
| RHSIM_Rhsim04G0151800 | 2.18 | 0.140 | 0.206 | 0.206 |
| RHSIM_Rhsim11G0137200 | 2.18 | 0.140 | 0.206 | -0.283 |
| RHSIM_Rhsim12G0016100 | 2.18 | 0.140 | 0.206 | -0.107 |
| RHSIM_Rhsim01G0171600 | 2.18 | 0.140 | 0.206 | 0.199 |
| novel.2640 | 2.18 | 0.140 | 0.206 | -0.546 |
| RHSIM_Rhsim13G0119600 | 2.18 | 0.140 | 0.206 | -0.258 |
| RHSIM_Rhsim04G0230800 | 2.18 | 0.140 | 0.206 | 2.495 |
| RHSIM_Rhsim05G0153600 | 2.18 | 0.140 | 0.206 | 2.140 |
| RHSIM_Rhsim11G0080500 | 2.18 | 0.140 | 0.206 | 1.601 |
| RHSIM_Rhsim10G0069200 | 2.18 | 0.140 | 0.206 | 2.362 |
| RHSIM_Rhsim03G0214300 | 2.18 | 0.140 | 0.206 | -2.355 |
| RHSIM_Rhsim13G0095400 | 2.18 | 0.140 | 0.207 | -0.154 |
| novel.3429 | 2.18 | 0.140 | 0.207 | 38.782 |
| RHSIM_Rhsim06G0181800 | 2.17 | 0.140 | 0.207 | 2.332 |
| RHSIM_RhsimUnG0213100 | 2.17 | 0.140 | 0.207 | 0.295 |
| RHSIM_Rhsim03G0004800 | 2.17 | 0.140 | 0.207 | 1.841 |
| novel.949 | 2.17 | 0.140 | 0.207 | -1.231 |
| RHSIM_Rhsim07G0213400 | 2.17 | 0.140 | 0.207 | 0.564 |

| Gene | LRT statistic | P value | BH-adjusted P | log2FC (interaction) |
|---|---|---|---|---|
| RHSIM_Rhsim03G0142000 | 2.17 | 0.141 | 0.207 | 0.188 |
| RHSIM_Rhsim03G0134600 | 2.17 | 0.141 | 0.207 | 0.814 |
| RHSIM_Rhsim13G0207600 | 2.17 | 0.141 | 0.207 | 0.379 |
| RHSIM_Rhsim05G0076400 | 2.17 | 0.141 | 0.207 | -0.902 |
| novel.1639 | 2.17 | 0.141 | 0.207 | 1.097 |
| RHSIM_Rhsim09G0168900 | 2.17 | 0.141 | 0.207 | 0.193 |
| novel.4093 | 2.17 | 0.141 | 0.207 | -1.007 |
| RHSIM_Rhsim10G0011800 | 2.17 | 0.141 | 0.207 | 0.263 |
| RHSIM_Rhsim04G0201300 | 2.17 | 0.141 | 0.207 | 0.278 |
| RHSIM_Rhsim11G0097600 | 2.17 | 0.141 | 0.207 | 0.291 |
| RHSIM_Rhsim02G0136700 | 2.17 | 0.141 | 0.207 | -1.237 |
| RHSIM_Rhsim12G0048300 | 2.17 | 0.141 | 0.207 | -0.561 |
| RHSIM_Rhsim01G0161600 | 2.17 | 0.141 | 0.207 | 0.764 |
| RHSIM_Rhsim05G0007800 | 2.17 | 0.141 | 0.207 | -38.031 |
| RHSIM_RhsimUnG0164500 | 2.17 | 0.141 | 0.208 | 1.545 |
| RHSIM_Rhsim11G0068900 | 2.17 | 0.141 | 0.208 | -1.392 |
| RHSIM_Rhsim03G0135800 | 2.17 | 0.141 | 0.208 | -0.134 |
| RHSIM_Rhsim04G0162000 | 2.16 | 0.141 | 0.208 | -1.272 |
| RHSIM_Rhsim06G0148700 | 2.16 | 0.141 | 0.208 | -1.502 |
| RHSIM_Rhsim06G0074700 | 2.16 | 0.141 | 0.208 | -0.095 |
| RHSIM_Rhsim01G0064800 | 2.16 | 0.141 | 0.208 | -0.730 |
| RHSIM_Rhsim03G0131100 | 2.16 | 0.141 | 0.208 | -0.204 |
| novel.1807 | 2.16 | 0.141 | 0.208 | 1.067 |
| RHSIM_Rhsim13G0148100 | 2.16 | 0.141 | 0.208 | 3.108 |
| RHSIM_RhsimUnG0020600 | 2.16 | 0.141 | 0.208 | 0.172 |
| RHSIM_Rhsim06G0217800 | 2.16 | 0.141 | 0.208 | -38.297 |
| RHSIM_Rhsim07G0064100 | 2.16 | 0.141 | 0.208 | -2.215 |
| RHSIM_Rhsim12G0198400 | 2.16 | 0.142 | 0.208 | -1.535 |
| RHSIM_RhsimUnG0181000 | 2.16 | 0.142 | 0.208 | 0.833 |
| RHSIM_Rhsim07G0049800 | 2.16 | 0.142 | 0.208 | -0.075 |
| RHSIM_Rhsim13G0043300 | 2.16 | 0.142 | 0.208 | 38.118 |
| RHSIM_Rhsim09G0164700 | 2.16 | 0.142 | 0.208 | -4.166 |

| Gene | LRT statistic | P value | BH-adjusted P | log2FC (interaction) |
|---|---|---|---|---|
| RHSIM_Rhsim03G0097900 | 2.16 | 0.142 | 0.208 | -0.257 |
| novel.2669 | 2.16 | 0.142 | 0.208 | 0.483 |
| novel.3118 | 2.16 | 0.142 | 0.208 | 1.091 |
| RHSIM_Rhsim09G0042300 | 2.16 | 0.142 | 0.208 | 1.881 |
| RHSIM_Rhsim04G0083300 | 2.16 | 0.142 | 0.209 | 3.043 |
| RHSIM_Rhsim13G0061300 | 2.16 | 0.142 | 0.209 | 1.538 |
| RHSIM_Rhsim09G0004900 | 2.15 | 0.142 | 0.209 | -0.393 |
| RHSIM_Rhsim10G0017000 | 2.15 | 0.142 | 0.209 | -1.273 |
| RHSIM_Rhsim06G0200400 | 2.15 | 0.142 | 0.209 | 1.966 |
| RHSIM_RhsimUnG0063000 | 2.15 | 0.142 | 0.209 | 1.556 |
| novel.1604 | 2.15 | 0.142 | 0.209 | 0.812 |
| RHSIM_Rhsim11G0170400 | 2.15 | 0.143 | 0.209 | 2.217 |
| RHSIM_Rhsim10G0177100 | 2.15 | 0.143 | 0.209 | 1.159 |
| RHSIM_Rhsim13G0040700 | 2.15 | 0.143 | 0.209 | 0.271 |
| novel.4274 | 2.15 | 0.143 | 0.210 | 1.517 |
| RHSIM_Rhsim01G0078500 | 2.15 | 0.143 | 0.210 | 0.320 |
| RHSIM_Rhsim10G0029900 | 2.15 | 0.143 | 0.210 | -3.646 |
| RHSIM_Rhsim05G0097100 | 2.15 | 0.143 | 0.210 | 0.384 |
| RHSIM_Rhsim05G0167600 | 2.15 | 0.143 | 0.210 | 0.184 |
| RHSIM_Rhsim07G0132800 | 2.15 | 0.143 | 0.210 | -0.174 |
| RHSIM_Rhsim04G0025500 | 2.15 | 0.143 | 0.210 | -1.025 |
| RHSIM_Rhsim10G0025200 | 2.14 | 0.143 | 0.210 | 0.565 |
| RHSIM_Rhsim13G0146100 | 2.14 | 0.143 | 0.210 | -1.408 |
| RHSIM_Rhsim05G0173200 | 2.14 | 0.143 | 0.210 | 1.032 |
| RHSIM_Rhsim07G0179300 | 2.14 | 0.143 | 0.210 | 0.358 |
| RHSIM_Rhsim02G0227200 | 2.14 | 0.143 | 0.210 | 1.119 |
| RHSIM_Rhsim01G0250100 | 2.14 | 0.143 | 0.211 | -0.309 |
| RHSIM_Rhsim03G0105400 | 2.14 | 0.143 | 0.211 | 37.972 |
| RHSIM_Rhsim03G0190700 | 2.14 | 0.143 | 0.211 | -0.167 |
| RHSIM_Rhsim03G0015000 | 2.14 | 0.144 | 0.211 | -1.058 |
| RHSIM_Rhsim03G0214500 | 2.14 | 0.144 | 0.211 | -0.967 |
| RHSIM_Rhsim02G0027200 | 2.14 | 0.144 | 0.211 | 2.579 |

| Gene | LRT statistic | P value | BH-adjusted P | log2FC (interaction) |
|---|---|---|---|---|
| novel.2562 | 2.14 | 0.144 | 0.211 | 0.967 |
| RHSIM_Rhsim05G0220300 | 2.14 | 0.144 | 0.211 | -38.264 |
| RHSIM_Rhsim03G0256200 | 2.14 | 0.144 | 0.211 | -37.656 |
| RHSIM_Rhsim13G0172300 | 2.14 | 0.144 | 0.211 | -3.736 |
| novel.2856 | 2.14 | 0.144 | 0.211 | -0.955 |
| novel.3250 | 2.14 | 0.144 | 0.211 | -3.313 |
| RHSIM_Rhsim10G0158900 | 2.14 | 0.144 | 0.211 | 0.819 |
| RHSIM_RhsimUnG0021600 | 2.14 | 0.144 | 0.211 | 0.152 |
| RHSIM_Rhsim08G0123200 | 2.14 | 0.144 | 0.211 | 1.006 |
| novel.2174 | 2.14 | 0.144 | 0.211 | 38.019 |
| novel.4311 | 2.14 | 0.144 | 0.211 | 1.961 |
| RHSIM_Rhsim06G0056000 | 2.14 | 0.144 | 0.211 | 38.089 |
| RHSIM_Rhsim04G0057500 | 2.14 | 0.144 | 0.211 | 1.479 |
| RHSIM_Rhsim01G0097100 | 2.14 | 0.144 | 0.211 | -0.150 |
| RHSIM_Rhsim04G0119700 | 2.14 | 0.144 | 0.211 | -1.710 |
| novel.3572 | 2.14 | 0.144 | 0.211 | 1.505 |
| RHSIM_Rhsim09G0173100 | 2.13 | 0.144 | 0.211 | -0.791 |
| novel.3923 | 2.13 | 0.144 | 0.211 | -2.114 |
| RHSIM_Rhsim03G0260300 | 2.13 | 0.144 | 0.212 | 0.400 |
| RHSIM_Rhsim02G0172300 | 2.13 | 0.145 | 0.212 | 0.352 |
| RHSIM_Rhsim13G0232900 | 2.13 | 0.145 | 0.212 | 1.578 |
| novel.3618 | 2.13 | 0.145 | 0.212 | -0.349 |
| RHSIM_Rhsim04G0192400 | 2.13 | 0.145 | 0.212 | -1.070 |
| RHSIM_Rhsim06G0182000 | 2.12 | 0.145 | 0.212 | 1.015 |
| RHSIM_Rhsim12G0176000 | 2.12 | 0.145 | 0.213 | -0.299 |
| RHSIM_RhsimUnG0009000 | 2.12 | 0.145 | 0.213 | -1.181 |
| RHSIM_Rhsim07G0205200 | 2.12 | 0.145 | 0.213 | -1.535 |
| RHSIM_Rhsim02G0140900 | 2.12 | 0.145 | 0.213 | -38.247 |
| RHSIM_Rhsim04G0205400 | 2.12 | 0.145 | 0.213 | 1.709 |
| RHSIM_Rhsim01G0256100 | 2.12 | 0.146 | 0.213 | -0.227 |
| RHSIM_Rhsim11G0117800 | 2.12 | 0.146 | 0.214 | 1.366 |
| novel.3087 | 2.12 | 0.146 | 0.214 | -0.216 |

| Gene | LRT statistic | P value | BH-adjusted P | log2FC (interaction) |
|---|---|---|---|---|
| RHSIM_Rhsim11G0157000 | 2.12 | 0.146 | 0.214 | 1.264 |
| RHSIM_RhsimUnG0090900 | 2.12 | 0.146 | 0.214 | 2.815 |
| RHSIM_Rhsim02G0126400 | 2.11 | 0.146 | 0.214 | 0.671 |
| RHSIM_RhsimUnG0093800 | 2.11 | 0.146 | 0.214 | 0.141 |
| RHSIM_Rhsim04G0022200 | 2.11 | 0.146 | 0.214 | 3.193 |
| RHSIM_Rhsim03G0039600 | 2.11 | 0.146 | 0.214 | 3.843 |
| RHSIM_Rhsim05G0194800 | 2.11 | 0.146 | 0.214 | -1.301 |
| RHSIM_Rhsim01G0283300 | 2.11 | 0.146 | 0.214 | 0.344 |
| RHSIM_Rhsim05G0009300 | 2.11 | 0.146 | 0.214 | -2.931 |
| novel.10 | 2.11 | 0.146 | 0.214 | 3.157 |
| RHSIM_Rhsim10G0121900 | 2.11 | 0.146 | 0.214 | 38.390 |
| RHSIM_Rhsim06G0087600 | 2.11 | 0.146 | 0.214 | 0.102 |
| RHSIM_Rhsim03G0101100 | 2.11 | 0.146 | 0.214 | -0.113 |
| RHSIM_Rhsim11G0103900 | 2.11 | 0.146 | 0.214 | 39.225 |
| RHSIM_Rhsim07G0180900 | 2.11 | 0.146 | 0.214 | -38.037 |
| RHSIM_Rhsim05G0140100 | 2.11 | 0.146 | 0.214 | -3.449 |
| RHSIM_RhsimUnG0022600 | 2.11 | 0.146 | 0.214 | 0.381 |
| RHSIM_Rhsim06G0042500 | 2.11 | 0.146 | 0.214 | 0.879 |
| RHSIM_Rhsim04G0219000 | 2.11 | 0.146 | 0.214 | 0.329 |
| RHSIM_Rhsim01G0131100 | 2.11 | 0.146 | 0.214 | 0.157 |
| RHSIM_Rhsim11G0071400 | 2.11 | 0.147 | 0.214 | 0.197 |
| RHSIM_Rhsim09G0138400 | 2.11 | 0.147 | 0.214 | -0.193 |
| RHSIM_RhsimUnG0052400 | 2.11 | 0.147 | 0.215 | -38.261 |
| novel.906 | 2.10 | 0.147 | 0.215 | 2.803 |
| novel.839 | 2.10 | 0.147 | 0.215 | -2.625 |
| RHSIM_Rhsim06G0136300 | 2.10 | 0.147 | 0.215 | -4.267 |
| novel.771 | 2.10 | 0.147 | 0.215 | -2.780 |
| novel.4273 | 2.10 | 0.147 | 0.215 | -1.636 |
| RHSIM_Rhsim10G0127600 | 2.10 | 0.147 | 0.215 | 39.106 |
| RHSIM_RhsimUnG0221700 | 2.10 | 0.147 | 0.215 | -0.545 |
| RHSIM_Rhsim11G0171100 | 2.10 | 0.147 | 0.215 | -0.254 |
| RHSIM_Rhsim09G0035500 | 2.10 | 0.147 | 0.216 | 38.164 |

| Gene | LRT statistic | P value | BH-adjusted P | log2FC (interaction) |
|---|---|---|---|---|
| RHSIM_RhsimUnG0241100 | 2.10 | 0.147 | 0.216 | 0.605 |
| RHSIM_Rhsim08G0020600 | 2.10 | 0.147 | 0.216 | 37.816 |
| RHSIM_Rhsim07G0029200 | 2.10 | 0.147 | 0.216 | 2.430 |
| RHSIM_Rhsim06G0038200 | 2.10 | 0.148 | 0.216 | -0.251 |
| RHSIM_Rhsim01G0261800 | 2.10 | 0.148 | 0.216 | -3.168 |
| RHSIM_Rhsim11G0083000 | 2.09 | 0.148 | 0.216 | -0.590 |
| RHSIM_RhsimUnG0080800 | 2.09 | 0.148 | 0.216 | 0.834 |
| novel.4564 | 2.09 | 0.148 | 0.216 | 1.287 |
| RHSIM_Rhsim03G0194700 | 2.09 | 0.148 | 0.216 | -1.768 |
| novel.3957 | 2.09 | 0.148 | 0.217 | -0.247 |
| RHSIM_Rhsim02G0115000 | 2.09 | 0.148 | 0.217 | 1.441 |
| RHSIM_Rhsim04G0042400 | 2.09 | 0.148 | 0.217 | 1.223 |
| novel.3765 | 2.09 | 0.148 | 0.217 | 0.581 |
| RHSIM_Rhsim01G0178600 | 2.09 | 0.148 | 0.217 | -1.235 |
| RHSIM_Rhsim10G0161300 | 2.09 | 0.148 | 0.217 | 0.338 |
| RHSIM_Rhsim10G0080300 | 2.09 | 0.148 | 0.217 | -0.351 |
| RHSIM_Rhsim12G0078100 | 2.09 | 0.148 | 0.217 | -0.186 |
| RHSIM_Rhsim06G0047900 | 2.09 | 0.148 | 0.217 | 0.146 |
| RHSIM_Rhsim01G0023900 | 2.09 | 0.149 | 0.217 | 0.112 |
| RHSIM_Rhsim13G0030100 | 2.09 | 0.149 | 0.217 | -0.350 |
| RHSIM_Rhsim13G0123400 | 2.08 | 0.149 | 0.217 | -38.403 |
| RHSIM_Rhsim09G0119900 | 2.08 | 0.149 | 0.217 | 1.147 |
| RHSIM_Rhsim06G0071300 | 2.08 | 0.149 | 0.218 | -0.233 |
| RHSIM_Rhsim03G0253900 | 2.08 | 0.149 | 0.218 | 0.239 |
| RHSIM_Rhsim10G0109200 | 2.08 | 0.149 | 0.218 | 1.972 |
| RHSIM_Rhsim03G0111000 | 2.08 | 0.149 | 0.218 | -4.155 |
| novel.234 | 2.08 | 0.149 | 0.218 | -1.115 |
| RHSIM_Rhsim07G0209000 | 2.08 | 0.149 | 0.218 | 0.251 |
| novel.125 | 2.08 | 0.149 | 0.218 | 1.892 |
| RHSIM_RhsimUnG0015700 | 2.08 | 0.149 | 0.218 | -0.347 |
| RHSIM_Rhsim12G0208400 | 2.08 | 0.149 | 0.218 | -1.397 |
| RHSIM_Rhsim12G0047100 | 2.08 | 0.149 | 0.218 | -0.334 |

| Gene | LRT statistic | P value | BH-adjusted P | log2FC (interaction) |
|---|---|---|---|---|
| RHSIM_Rhsim13G0005700 | 2.08 | 0.149 | 0.218 | 0.717 |
| RHSIM_Rhsim13G0113500 | 2.08 | 0.149 | 0.218 | 0.201 |
| RHSIM_Rhsim06G0135100 | 2.08 | 0.149 | 0.218 | -1.539 |
| RHSIM_Rhsim06G0009300 | 2.08 | 0.149 | 0.218 | -0.839 |
| novel.854 | 2.08 | 0.149 | 0.218 | 38.157 |
| novel.3791 | 2.08 | 0.149 | 0.218 | -2.098 |
| novel.1576 | 2.08 | 0.149 | 0.218 | 3.507 |
| RHSIM_Rhsim09G0041000 | 2.08 | 0.149 | 0.218 | -0.374 |
| RHSIM_Rhsim13G0055900 | 2.08 | 0.150 | 0.218 | -1.303 |
| RHSIM_Rhsim11G0019400 | 2.08 | 0.150 | 0.218 | 0.347 |
| RHSIM_Rhsim01G0145800 | 2.08 | 0.150 | 0.218 | -0.149 |
| RHSIM_Rhsim05G0096800 | 2.08 | 0.150 | 0.218 | -1.080 |
| RHSIM_Rhsim09G0157500 | 2.08 | 0.150 | 0.218 | -0.952 |
| RHSIM_Rhsim11G0076800 | 2.07 | 0.150 | 0.218 | -2.041 |
| RHSIM_Rhsim03G0260700 | 2.07 | 0.150 | 0.219 | 1.350 |
| RHSIM_Rhsim07G0223500 | 2.07 | 0.150 | 0.219 | -3.574 |
| RHSIM_RhsimUnG0067400 | 2.07 | 0.150 | 0.219 | -1.437 |
| RHSIM_Rhsim01G0286800 | 2.07 | 0.150 | 0.219 | 0.887 |
| RHSIM_Rhsim02G0222900 | 2.07 | 0.150 | 0.219 | 0.482 |
| RHSIM_Rhsim08G0248000 | 2.07 | 0.150 | 0.219 | -0.121 |
| RHSIM_Rhsim01G0095100 | 2.07 | 0.150 | 0.219 | -1.998 |
| RHSIM_Rhsim02G0073300 | 2.07 | 0.150 | 0.219 | 2.669 |
| RHSIM_Rhsim10G0189100 | 2.07 | 0.150 | 0.219 | 0.124 |
| novel.519 | 2.07 | 0.150 | 0.219 | 0.836 |
| novel.3904 | 2.07 | 0.151 | 0.220 | -0.164 |
| RHSIM_RhsimUnG0103100 | 2.07 | 0.151 | 0.220 | 0.201 |
| RHSIM_Rhsim13G0203500 | 2.07 | 0.151 | 0.220 | 0.521 |
| novel.1411 | 2.07 | 0.151 | 0.220 | 1.730 |
| RHSIM_Rhsim02G0173100 | 2.07 | 0.151 | 0.220 | 38.408 |
| novel.3372 | 2.07 | 0.151 | 0.220 | 0.467 |
| RHSIM_Rhsim09G0203700 | 2.07 | 0.151 | 0.220 | -0.214 |
| novel.1954 | 2.07 | 0.151 | 0.220 | 0.114 |

| Gene | LRT statistic | P value | BH-adjusted P | log2FC (interaction) |
|---|---|---|---|---|
| novel.283 | 2.06 | 0.151 | 0.220 | 0.319 |
| RHSIM_Rhsim02G0132000 | 2.06 | 0.151 | 0.220 | -0.500 |
| novel.1561 | 2.06 | 0.151 | 0.220 | -1.527 |
| RHSIM_RhsimUnG0188000 | 2.06 | 0.151 | 0.220 | -0.923 |
| RHSIM_Rhsim07G0186300 | 2.06 | 0.151 | 0.220 | 0.156 |
| novel.4483 | 2.06 | 0.151 | 0.220 | 2.170 |
| RHSIM_Rhsim01G0164400 | 2.06 | 0.151 | 0.220 | -2.524 |
| novel.2099 | 2.06 | 0.151 | 0.220 | 0.718 |
| RHSIM_Rhsim03G0147200 | 2.06 | 0.151 | 0.221 | 0.144 |
| RHSIM_Rhsim04G0152800 | 2.06 | 0.151 | 0.221 | 0.291 |
| RHSIM_Rhsim13G0017200 | 2.06 | 0.151 | 0.221 | -0.236 |
| RHSIM_Rhsim10G0022800 | 2.06 | 0.152 | 0.221 | -0.427 |
| novel.1534 | 2.06 | 0.152 | 0.221 | 1.219 |
| RHSIM_Rhsim04G0066000 | 2.05 | 0.152 | 0.221 | 0.327 |
| RHSIM_Rhsim13G0143800 | 2.05 | 0.152 | 0.221 | -0.181 |
| RHSIM_Rhsim09G0143600 | 2.05 | 0.152 | 0.221 | -2.966 |
| RHSIM_Rhsim04G0212200 | 2.05 | 0.152 | 0.221 | -0.212 |
| RHSIM_Rhsim06G0126700 | 2.05 | 0.152 | 0.221 | -0.936 |
| RHSIM_Rhsim08G0015700 | 2.05 | 0.152 | 0.221 | -1.889 |
| novel.3194 | 2.05 | 0.152 | 0.221 | -2.171 |
| novel.4359 | 2.05 | 0.152 | 0.221 | 1.482 |
| RHSIM_Rhsim07G0210900 | 2.05 | 0.152 | 0.221 | -0.127 |
| RHSIM_Rhsim05G0218300 | 2.05 | 0.152 | 0.222 | 0.441 |
| RHSIM_Rhsim12G0162600 | 2.05 | 0.152 | 0.222 | -0.252 |
| RHSIM_Rhsim12G0043000 | 2.05 | 0.152 | 0.222 | -0.996 |
| RHSIM_Rhsim11G0193100 | 2.05 | 0.152 | 0.222 | 0.185 |
| novel.2645 | 2.05 | 0.152 | 0.222 | 1.896 |
| RHSIM_Rhsim03G0015300 | 2.05 | 0.152 | 0.222 | 4.011 |
| RHSIM_Rhsim06G0052800 | 2.05 | 0.152 | 0.222 | 0.116 |
| RHSIM_Rhsim06G0151600 | 2.05 | 0.152 | 0.222 | -0.819 |
| RHSIM_Rhsim02G0001300 | 2.05 | 0.152 | 0.222 | -0.188 |
| RHSIM_RhsimUnG0065100 | 2.05 | 0.152 | 0.222 | -37.579 |

| Gene | LRT statistic | P value | BH-adjusted P | log2FC (interaction) |
|---|---|---|---|---|
| novel.2993 | 2.05 | 0.153 | 0.222 | 38.153 |
| RHSIM_Rhsim08G0178400 | 2.04 | 0.153 | 0.222 | -38.521 |
| RHSIM_Rhsim08G0070200 | 2.04 | 0.153 | 0.222 | 0.150 |
| RHSIM_Rhsim13G0084900 | 2.04 | 0.153 | 0.223 | 2.563 |
| RHSIM_Rhsim08G0032200 | 2.04 | 0.153 | 0.223 | 38.323 |
| RHSIM_Rhsim04G0031500 | 2.04 | 0.153 | 0.223 | 0.374 |
| RHSIM_Rhsim10G0040800 | 2.04 | 0.153 | 0.223 | -1.258 |
| RHSIM_Rhsim11G0122500 | 2.04 | 0.153 | 0.223 | -0.846 |
| RHSIM_Rhsim11G0174700 | 2.04 | 0.153 | 0.223 | 0.410 |
| RHSIM_Rhsim08G0246800 | 2.04 | 0.153 | 0.223 | 0.105 |
| RHSIM_Rhsim08G0198800 | 2.04 | 0.153 | 0.223 | 0.480 |
| RHSIM_Rhsim01G0089200 | 2.04 | 0.153 | 0.223 | -0.160 |
| RHSIM_Rhsim05G0180800 | 2.04 | 0.153 | 0.223 | -0.637 |
| RHSIM_Rhsim04G0064000 | 2.04 | 0.153 | 0.223 | 0.905 |
| RHSIM_Rhsim02G0174900 | 2.04 | 0.154 | 0.223 | 38.628 |
| RHSIM_Rhsim09G0038300 | 2.04 | 0.154 | 0.223 | 0.419 |
| RHSIM_Rhsim02G0042300 | 2.04 | 0.154 | 0.223 | -2.108 |
| RHSIM_Rhsim02G0143200 | 2.04 | 0.154 | 0.223 | -0.190 |
| RHSIM_Rhsim01G0092900 | 2.03 | 0.154 | 0.224 | 0.630 |
| RHSIM_Rhsim10G0149000 | 2.03 | 0.154 | 0.224 | -0.308 |
| RHSIM_Rhsim06G0065200 | 2.03 | 0.154 | 0.224 | 1.315 |
| RHSIM_Rhsim10G0018000 | 2.03 | 0.154 | 0.224 | -0.344 |
| RHSIM_Rhsim03G0197200 | 2.03 | 0.154 | 0.224 | 0.187 |
| RHSIM_RhsimUnG0160500 | 2.03 | 0.154 | 0.224 | 38.381 |
| RHSIM_Rhsim01G0222300 | 2.03 | 0.154 | 0.224 | -2.000 |
| RHSIM_Rhsim08G0150500 | 2.03 | 0.154 | 0.224 | 1.599 |
| RHSIM_Rhsim01G0048600 | 2.03 | 0.154 | 0.224 | 1.980 |
| RHSIM_Rhsim05G0193700 | 2.03 | 0.154 | 0.224 | 2.552 |
| novel.2008 | 2.03 | 0.154 | 0.224 | 2.042 |
| RHSIM_Rhsim03G0079000 | 2.03 | 0.154 | 0.224 | -0.430 |
| RHSIM_Rhsim08G0043400 | 2.03 | 0.154 | 0.224 | -0.203 |
| RHSIM_Rhsim11G0118100 | 2.03 | 0.154 | 0.224 | 2.897 |

| Gene | LRT statistic | P value | BH-adjusted P | log2FC (interaction) |
|---|---|---|---|---|
| RHSIM_Rhsim06G0017400 | 2.03 | 0.154 | 0.224 | -0.325 |
| RHSIM_RhsimUnG0127700 | 2.03 | 0.154 | 0.224 | -38.399 |
| RHSIM_Rhsim10G0190200 | 2.03 | 0.155 | 0.224 | -0.225 |
| RHSIM_Rhsim11G0195000 | 2.03 | 0.155 | 0.225 | 0.169 |
| novel.2356 | 2.03 | 0.155 | 0.225 | 0.983 |
| RHSIM_Rhsim09G0035000 | 2.02 | 0.155 | 0.225 | 0.252 |
| RHSIM_RhsimUnG0031800 | 2.02 | 0.155 | 0.225 | -0.295 |
| RHSIM_Rhsim12G0127800 | 2.02 | 0.155 | 0.225 | 0.752 |
| RHSIM_Rhsim07G0014500 | 2.02 | 0.155 | 0.225 | 0.448 |
| RHSIM_Rhsim02G0068500 | 2.02 | 0.155 | 0.225 | -3.466 |
| RHSIM_Rhsim08G0167400 | 2.02 | 0.155 | 0.225 | 0.358 |
| novel.280 | 2.02 | 0.155 | 0.225 | 1.114 |
| novel.539 | 2.02 | 0.155 | 0.225 | -0.642 |
| RHSIM_Rhsim06G0060300 | 2.02 | 0.155 | 0.225 | 0.130 |
| RHSIM_RhsimUnG0083500 | 2.02 | 0.155 | 0.225 | 0.433 |
| novel.2316 | 2.02 | 0.155 | 0.225 | -0.423 |
| RHSIM_Rhsim12G0160700 | 2.02 | 0.155 | 0.225 | -0.387 |
| novel.3981 | 2.02 | 0.155 | 0.225 | -1.563 |
| RHSIM_Rhsim11G0162900 | 2.02 | 0.155 | 0.225 | -1.189 |
| novel.1458 | 2.02 | 0.155 | 0.225 | 0.330 |
| novel.769 | 2.02 | 0.155 | 0.226 | -0.644 |
| novel.2872 | 2.02 | 0.155 | 0.226 | 1.455 |
| RHSIM_Rhsim04G0197100 | 2.02 | 0.156 | 0.226 | 0.121 |
| RHSIM_Rhsim07G0174700 | 2.02 | 0.156 | 0.226 | -0.210 |
| RHSIM_Rhsim06G0238500 | 2.02 | 0.156 | 0.226 | -0.125 |
| RHSIM_RhsimMtG0002900 | 2.02 | 0.156 | 0.226 | 1.808 |
| RHSIM_Rhsim07G0106400 | 2.02 | 0.156 | 0.226 | -0.263 |
| RHSIM_Rhsim02G0011400 | 2.02 | 0.156 | 0.226 | 38.461 |
| RHSIM_Rhsim05G0222100 | 2.02 | 0.156 | 0.226 | -0.384 |
| RHSIM_Rhsim10G0041600 | 2.01 | 0.156 | 0.226 | 0.223 |
| RHSIM_Rhsim11G0014900 | 2.01 | 0.156 | 0.226 | 0.490 |
| RHSIM_Rhsim11G0187100 | 2.01 | 0.156 | 0.226 | 1.429 |

| Gene | LRT statistic | P value | BH-adjusted P | log2FC (interaction) |
|---|---|---|---|---|
| RHSIM_Rhsim13G0151700 | 2.01 | 0.156 | 0.226 | -1.483 |
| novel.3540 | 2.01 | 0.156 | 0.226 | 0.810 |
| RHSIM_Rhsim07G0253200 | 2.01 | 0.156 | 0.226 | -38.797 |
| novel.2404 | 2.01 | 0.156 | 0.227 | 0.794 |
| RHSIM_Rhsim02G0243700 | 2.01 | 0.156 | 0.227 | -1.816 |
| RHSIM_Rhsim04G0181900 | 2.01 | 0.156 | 0.227 | 0.246 |
| RHSIM_Rhsim03G0158500 | 2.01 | 0.156 | 0.227 | 2.022 |
| novel.1303 | 2.01 | 0.156 | 0.227 | 0.224 |
| novel.1398 | 2.01 | 0.156 | 0.227 | -2.495 |
| RHSIM_Rhsim01G0169600 | 2.01 | 0.157 | 0.227 | 0.440 |
| novel.318 | 2.01 | 0.157 | 0.227 | -0.212 |
| RHSIM_Rhsim09G0000800 | 2.01 | 0.157 | 0.227 | 0.376 |
| RHSIM_Rhsim05G0100900 | 2.01 | 0.157 | 0.227 | -0.097 |
| RHSIM_Rhsim13G0099900 | 2.01 | 0.157 | 0.227 | 0.155 |
| RHSIM_RhsimUnG0000800 | 2.00 | 0.157 | 0.227 | -37.492 |
| RHSIM_Rhsim03G0099600 | 2.00 | 0.157 | 0.227 | 0.264 |
| RHSIM_Rhsim06G0216500 | 2.00 | 0.157 | 0.228 | -0.197 |
| RHSIM_RhsimUnG0099300 | 2.00 | 0.157 | 0.228 | -0.220 |
| RHSIM_Rhsim12G0165100 | 2.00 | 0.157 | 0.228 | 1.530 |
| RHSIM_Rhsim01G0099600 | 2.00 | 0.157 | 0.228 | 38.109 |
| RHSIM_Rhsim09G0074000 | 2.00 | 0.157 | 0.228 | -0.194 |
| RHSIM_RhsimUnG0076500 | 2.00 | 0.157 | 0.228 | -3.055 |
| RHSIM_Rhsim03G0063800 | 2.00 | 0.157 | 0.228 | 0.268 |
| novel.4352 | 2.00 | 0.157 | 0.228 | 0.860 |
| RHSIM_Rhsim05G0111500 | 2.00 | 0.157 | 0.228 | -0.481 |
| RHSIM_Rhsim09G0144900 | 2.00 | 0.157 | 0.228 | -1.974 |
| RHSIM_RhsimUnG0202600 | 2.00 | 0.157 | 0.228 | -0.482 |
| RHSIM_Rhsim08G0014500 | 2.00 | 0.157 | 0.228 | -0.439 |
| RHSIM_Rhsim07G0246200 | 2.00 | 0.157 | 0.228 | -0.467 |
| RHSIM_Rhsim02G0093100 | 2.00 | 0.157 | 0.228 | 38.297 |
| RHSIM_Rhsim04G0152700 | 2.00 | 0.157 | 0.228 | -0.789 |
| RHSIM_Rhsim03G0246000 | 2.00 | 0.157 | 0.228 | 2.762 |

| Gene | LRT statistic | P value | BH-adjusted P | log2FC (interaction) |
|---|---|---|---|---|
| RHSIM_Rhsim03G0026000 | 2.00 | 0.157 | 0.228 | 0.581 |
| RHSIM_Rhsim03G0025400 | 2.00 | 0.157 | 0.228 | 0.538 |
| RHSIM_Rhsim11G0114900 | 2.00 | 0.158 | 0.228 | 0.137 |
| RHSIM_Rhsim05G0014100 | 2.00 | 0.158 | 0.228 | -1.316 |
| RHSIM_Rhsim09G0123800 | 2.00 | 0.158 | 0.229 | -1.908 |
| RHSIM_RhsimUnG0224900 | 1.99 | 0.158 | 0.229 | -0.797 |
| RHSIM_Rhsim11G0185400 | 1.99 | 0.158 | 0.229 | 0.449 |
| RHSIM_Rhsim01G0128200 | 1.99 | 0.158 | 0.229 | 2.120 |
| RHSIM_Rhsim09G0047800 | 1.99 | 0.158 | 0.229 | -0.332 |
| RHSIM_Rhsim10G0207100 | 1.99 | 0.158 | 0.229 | -0.730 |
| RHSIM_Rhsim10G0142200 | 1.99 | 0.158 | 0.229 | 0.486 |
| RHSIM_RhsimUnG0240800 | 1.99 | 0.158 | 0.229 | 1.266 |
| RHSIM_Rhsim07G0128500 | 1.99 | 0.158 | 0.229 | -39.126 |
| RHSIM_RhsimUnG0042600 | 1.99 | 0.158 | 0.229 | -2.350 |
| RHSIM_RhsimUnG0199500 | 1.99 | 0.158 | 0.229 | 0.243 |
| RHSIM_Rhsim09G0025400 | 1.99 | 0.159 | 0.229 | 0.646 |
| novel.1539 | 1.99 | 0.159 | 0.229 | -3.068 |
| novel.3091 | 1.99 | 0.159 | 0.229 | 2.632 |
| RHSIM_Rhsim12G0196500 | 1.98 | 0.159 | 0.230 | -0.631 |
| RHSIM_Rhsim10G0121800 | 1.98 | 0.159 | 0.230 | 2.574 |
| novel.2578 | 1.98 | 0.159 | 0.230 | 2.602 |
| RHSIM_Rhsim09G0047300 | 1.98 | 0.159 | 0.230 | -0.604 |
| RHSIM_Rhsim08G0006400 | 1.98 | 0.159 | 0.230 | -0.469 |
| RHSIM_Rhsim11G0118700 | 1.98 | 0.159 | 0.230 | -0.256 |
| RHSIM_RhsimUnG0060300 | 1.98 | 0.159 | 0.230 | 0.418 |
| RHSIM_Rhsim13G0062200 | 1.98 | 0.159 | 0.230 | -2.424 |
| RHSIM_Rhsim12G0015400 | 1.98 | 0.159 | 0.231 | 2.368 |
| RHSIM_Rhsim12G0136800 | 1.98 | 0.159 | 0.231 | 0.203 |
| RHSIM_Rhsim11G0197200 | 1.98 | 0.159 | 0.231 | 0.158 |
| RHSIM_Rhsim01G0282800 | 1.98 | 0.159 | 0.231 | 0.678 |
| novel.561 | 1.98 | 0.160 | 0.231 | 1.488 |
| RHSIM_Rhsim13G0094100 | 1.98 | 0.160 | 0.231 | 2.596 |

| Gene | LRT statistic | P value | BH-adjusted P | log2FC (interaction) |
|---|---|---|---|---|
| novel.3647 | 1.98 | 0.160 | 0.231 | 1.919 |
| RHSIM_Rhsim05G0143900 | 1.98 | 0.160 | 0.231 | 0.154 |
| RHSIM_Rhsim13G0077600 | 1.98 | 0.160 | 0.231 | -0.093 |
| novel.4139 | 1.98 | 0.160 | 0.231 | -1.240 |
| RHSIM_Rhsim13G0106300 | 1.98 | 0.160 | 0.231 | 3.111 |
| RHSIM_RhsimUnG0077000 | 1.97 | 0.160 | 0.231 | 1.366 |
| RHSIM_Rhsim03G0180300 | 1.97 | 0.160 | 0.231 | 0.203 |
| RHSIM_Rhsim06G0236200 | 1.97 | 0.160 | 0.231 | -2.858 |
| RHSIM_Rhsim10G0094900 | 1.97 | 0.160 | 0.232 | 0.146 |
| RHSIM_Rhsim12G0126800 | 1.97 | 0.160 | 0.232 | -0.122 |
| novel.563 | 1.97 | 0.160 | 0.232 | -0.952 |
| RHSIM_RhsimUnG0247400 | 1.97 | 0.160 | 0.232 | 3.098 |
| RHSIM_Rhsim12G0089200 | 1.97 | 0.160 | 0.232 | -0.121 |
| novel.1584 | 1.97 | 0.160 | 0.232 | 3.335 |
| RHSIM_Rhsim04G0023200 | 1.97 | 0.160 | 0.232 | -0.630 |
| RHSIM_Rhsim04G0225400 | 1.97 | 0.161 | 0.232 | 37.972 |
| RHSIM_RhsimUnG0035500 | 1.97 | 0.161 | 0.232 | -2.769 |
| RHSIM_Rhsim03G0035900 | 1.97 | 0.161 | 0.232 | 0.830 |
| RHSIM_Rhsim06G0134800 | 1.97 | 0.161 | 0.232 | 0.986 |
| RHSIM_Rhsim02G0192700 | 1.97 | 0.161 | 0.232 | 1.189 |
| RHSIM_Rhsim09G0148300 | 1.97 | 0.161 | 0.232 | 1.094 |
| RHSIM_Rhsim05G0217800 | 1.97 | 0.161 | 0.232 | -0.598 |
| RHSIM_Rhsim13G0117800 | 1.97 | 0.161 | 0.232 | 1.652 |
| RHSIM_Rhsim12G0093600 | 1.97 | 0.161 | 0.233 | -0.438 |
| RHSIM_Rhsim11G0052700 | 1.96 | 0.161 | 0.233 | -0.247 |
| RHSIM_Rhsim09G0152200 | 1.96 | 0.161 | 0.233 | -38.037 |
| RHSIM_Rhsim03G0034600 | 1.96 | 0.161 | 0.233 | 0.146 |
| RHSIM_Rhsim02G0133800 | 1.96 | 0.161 | 0.233 | 37.689 |
| RHSIM_RhsimUnG0191800 | 1.96 | 0.161 | 0.233 | -1.328 |
| RHSIM_Rhsim04G0249400 | 1.96 | 0.161 | 0.233 | -39.000 |
| RHSIM_Rhsim02G0202700 | 1.96 | 0.161 | 0.233 | -37.654 |
| RHSIM_Rhsim10G0170200 | 1.96 | 0.161 | 0.233 | 1.075 |

| Gene | LRT statistic | P value | BH-adjusted P | log2FC (interaction) |
|---|---|---|---|---|
| RHSIM_Rhsim12G0210700 | 1.96 | 0.162 | 0.233 | -1.132 |
| RHSIM_Rhsim13G0139000 | 1.96 | 0.162 | 0.233 | -2.203 |
| RHSIM_Rhsim11G0159900 | 1.96 | 0.162 | 0.233 | -37.287 |
| RHSIM_Rhsim04G0238300 | 1.96 | 0.162 | 0.233 | -1.832 |
| novel.3006 | 1.96 | 0.162 | 0.234 | -1.226 |
| RHSIM_Rhsim05G0103100 | 1.96 | 0.162 | 0.234 | -0.790 |
| RHSIM_Rhsim02G0125600 | 1.96 | 0.162 | 0.234 | 2.099 |
| RHSIM_Rhsim11G0095100 | 1.96 | 0.162 | 0.234 | 2.473 |
| RHSIM_Rhsim06G0219400 | 1.96 | 0.162 | 0.234 | 0.783 |
| RHSIM_Rhsim10G0115300 | 1.95 | 0.162 | 0.234 | -0.188 |
| RHSIM_Rhsim13G0023800 | 1.95 | 0.162 | 0.234 | -2.388 |
| novel.2189 | 1.95 | 0.162 | 0.234 | -1.271 |
| RHSIM_Rhsim03G0028400 | 1.95 | 0.162 | 0.234 | -0.382 |
| RHSIM_Rhsim09G0013300 | 1.95 | 0.162 | 0.234 | 0.326 |
| RHSIM_Rhsim01G0193500 | 1.95 | 0.163 | 0.234 | 0.514 |
| RHSIM_Rhsim10G0154900 | 1.95 | 0.163 | 0.234 | -0.228 |
| RHSIM_Rhsim12G0000400 | 1.95 | 0.163 | 0.235 | 0.388 |
| RHSIM_Rhsim05G0066700 | 1.95 | 0.163 | 0.235 | 0.885 |
| RHSIM_Rhsim12G0121200 | 1.95 | 0.163 | 0.235 | -0.186 |
| novel.1468 | 1.95 | 0.163 | 0.235 | -1.718 |
| novel.3190 | 1.95 | 0.163 | 0.235 | -0.401 |
| novel.4448 | 1.95 | 0.163 | 0.235 | -37.388 |
| RHSIM_Rhsim02G0213500 | 1.95 | 0.163 | 0.235 | 1.076 |
| RHSIM_Rhsim06G0195400 | 1.94 | 0.163 | 0.235 | 1.368 |
| novel.2156 | 1.94 | 0.163 | 0.235 | 1.715 |
| novel.1362 | 1.94 | 0.163 | 0.235 | 1.360 |
| novel.2112 | 1.94 | 0.163 | 0.235 | 2.293 |
| RHSIM_Rhsim01G0244900 | 1.94 | 0.164 | 0.236 | 0.124 |
| RHSIM_Rhsim09G0001800 | 1.94 | 0.164 | 0.236 | 0.596 |
| RHSIM_Rhsim13G0211900 | 1.94 | 0.164 | 0.236 | 0.158 |
| novel.4369 | 1.94 | 0.164 | 0.236 | 1.009 |
| RHSIM_RhsimUnG0039400 | 1.94 | 0.164 | 0.236 | -1.950 |

| Gene | LRT statistic | P value | BH-adjusted P | log2FC (interaction) |
|---|---|---|---|---|
| novel.2088 | 1.94 | 0.164 | 0.236 | -0.346 |
| RHSIM_Rhsim12G0004600 | 1.94 | 0.164 | 0.236 | 0.469 |
| novel.2089 | 1.94 | 0.164 | 0.236 | -1.662 |
| novel.1395 | 1.94 | 0.164 | 0.236 | 1.792 |
| RHSIM_Rhsim03G0254700 | 1.94 | 0.164 | 0.236 | -2.480 |
| RHSIM_Rhsim10G0132700 | 1.94 | 0.164 | 0.237 | 3.070 |
| RHSIM_Rhsim05G0064200 | 1.93 | 0.164 | 0.237 | 1.799 |
| RHSIM_Rhsim03G0098500 | 1.93 | 0.164 | 0.237 | 2.314 |
| RHSIM_Rhsim05G0040700 | 1.93 | 0.164 | 0.237 | -38.544 |
| RHSIM_Rhsim10G0071000 | 1.93 | 0.164 | 0.237 | -1.569 |
| RHSIM_Rhsim10G0102400 | 1.93 | 0.164 | 0.237 | 0.895 |
| RHSIM_Rhsim12G0111800 | 1.93 | 0.165 | 0.237 | -0.101 |
| RHSIM_Rhsim02G0054800 | 1.93 | 0.165 | 0.237 | -38.411 |
| RHSIM_Rhsim06G0076900 | 1.93 | 0.165 | 0.237 | 0.616 |
| RHSIM_Rhsim05G0096100 | 1.93 | 0.165 | 0.238 | 0.166 |
| RHSIM_Rhsim01G0239600 | 1.93 | 0.165 | 0.238 | 38.433 |
| RHSIM_Rhsim03G0105500 | 1.93 | 0.165 | 0.238 | 2.148 |
| RHSIM_Rhsim02G0130800 | 1.93 | 0.165 | 0.238 | 2.772 |
| RHSIM_Rhsim12G0093200 | 1.93 | 0.165 | 0.238 | 0.978 |
| RHSIM_Rhsim07G0024000 | 1.93 | 0.165 | 0.238 | -0.590 |
| RHSIM_Rhsim13G0170700 | 1.92 | 0.165 | 0.238 | 0.365 |
| RHSIM_Rhsim08G0003700 | 1.92 | 0.165 | 0.238 | 2.706 |
| novel.191 | 1.92 | 0.165 | 0.238 | 0.691 |
| RHSIM_Rhsim12G0132600 | 1.92 | 0.165 | 0.238 | -37.508 |
| RHSIM_Rhsim04G0110500 | 1.92 | 0.165 | 0.238 | -0.431 |
| RHSIM_Rhsim06G0125100 | 1.92 | 0.165 | 0.238 | -1.188 |
| RHSIM_Rhsim04G0183700 | 1.92 | 0.165 | 0.238 | -0.260 |
| novel.3735 | 1.92 | 0.165 | 0.238 | 0.517 |
| RHSIM_Rhsim07G0202300 | 1.92 | 0.166 | 0.238 | 2.057 |
| novel.3824 | 1.92 | 0.166 | 0.238 | 1.537 |
| RHSIM_Rhsim10G0119300 | 1.92 | 0.166 | 0.238 | 0.778 |
| RHSIM_Rhsim01G0018300 | 1.92 | 0.166 | 0.238 | 0.811 |

| Gene | LRT statistic | P value | BH-adjusted P | log2FC (interaction) |
|---|---|---|---|---|
| RHSIM_Rhsim01G0197300 | 1.92 | 0.166 | 0.238 | -1.977 |
| RHSIM_RhsimUnG0226200 | 1.92 | 0.166 | 0.238 | -0.099 |
| RHSIM_Rhsim03G0230500 | 1.92 | 0.166 | 0.238 | -0.708 |
| RHSIM_Rhsim12G0093800 | 1.92 | 0.166 | 0.239 | 1.266 |
| RHSIM_Rhsim05G0087400 | 1.92 | 0.166 | 0.239 | 0.138 |
| RHSIM_Rhsim07G0054900 | 1.92 | 0.166 | 0.239 | 0.360 |
| RHSIM_Rhsim07G0049000 | 1.92 | 0.166 | 0.239 | 0.898 |
| RHSIM_Rhsim12G0072100 | 1.92 | 0.166 | 0.239 | 38.385 |
| RHSIM_Rhsim02G0053900 | 1.92 | 0.166 | 0.239 | -1.328 |
| novel.1847 | 1.92 | 0.166 | 0.239 | -0.197 |
| novel.817 | 1.92 | 0.166 | 0.239 | -1.083 |
| RHSIM_Rhsim05G0227800 | 1.92 | 0.166 | 0.239 | -38.539 |
| RHSIM_Rhsim12G0184200 | 1.92 | 0.166 | 0.239 | 1.254 |
| novel.4377 | 1.92 | 0.166 | 0.239 | 0.823 |
| RHSIM_Rhsim09G0191600 | 1.92 | 0.166 | 0.239 | -1.076 |
| RHSIM_RhsimUnG0002000 | 1.92 | 0.166 | 0.239 | 0.587 |
| RHSIM_Rhsim02G0034500 | 1.92 | 0.166 | 0.239 | 3.001 |
| RHSIM_Rhsim05G0182300 | 1.92 | 0.166 | 0.239 | 0.116 |
| RHSIM_Rhsim11G0121600 | 1.91 | 0.166 | 0.239 | 1.583 |
| RHSIM_Rhsim01G0258400 | 1.91 | 0.166 | 0.239 | -0.339 |
| RHSIM_Rhsim08G0051400 | 1.91 | 0.167 | 0.239 | 0.145 |
| RHSIM_Rhsim04G0184300 | 1.91 | 0.167 | 0.239 | 0.414 |
| RHSIM_Rhsim02G0071900 | 1.91 | 0.167 | 0.240 | 0.184 |
| RHSIM_Rhsim10G0024200 | 1.91 | 0.167 | 0.240 | 1.121 |
| RHSIM_Rhsim04G0128300 | 1.91 | 0.167 | 0.240 | 0.339 |
| RHSIM_Rhsim01G0052800 | 1.91 | 0.167 | 0.240 | 0.481 |
| RHSIM_Rhsim01G0002400 | 1.91 | 0.167 | 0.240 | 0.484 |
| RHSIM_Rhsim08G0002000 | 1.91 | 0.167 | 0.240 | -0.817 |
| RHSIM_Rhsim12G0000800 | 1.91 | 0.167 | 0.240 | 0.199 |
| RHSIM_Rhsim10G0056400 | 1.91 | 0.167 | 0.240 | 0.295 |
| RHSIM_Rhsim10G0022900 | 1.91 | 0.167 | 0.240 | -0.517 |
| RHSIM_Rhsim09G0020500 | 1.91 | 0.167 | 0.240 | 38.396 |

| Gene | LRT statistic | P value | BH-adjusted P | log2FC (interaction) |
|---|---|---|---|---|
| RHSIM_Rhsim12G0094200 | 1.91 | 0.167 | 0.240 | -38.009 |
| RHSIM_Rhsim07G0086600 | 1.91 | 0.167 | 0.240 | 37.959 |
| RHSIM_Rhsim08G0101400 | 1.90 | 0.168 | 0.241 | 1.935 |
| RHSIM_Rhsim08G0198000 | 1.90 | 0.168 | 0.241 | -1.648 |
| RHSIM_Rhsim05G0114900 | 1.90 | 0.168 | 0.241 | 2.159 |
| novel.432 | 1.90 | 0.168 | 0.241 | -0.714 |
| novel.1129 | 1.90 | 0.168 | 0.241 | 0.328 |
| novel.3940 | 1.90 | 0.168 | 0.241 | 0.774 |
| novel.332 | 1.90 | 0.168 | 0.241 | 2.773 |
| RHSIM_Rhsim11G0102400 | 1.90 | 0.168 | 0.241 | -38.392 |
| RHSIM_Rhsim11G0032400 | 1.90 | 0.168 | 0.241 | 0.578 |
| RHSIM_Rhsim11G0019500 | 1.90 | 0.168 | 0.241 | -0.355 |
| RHSIM_Rhsim04G0000500 | 1.90 | 0.168 | 0.241 | 0.256 |
| RHSIM_Rhsim08G0226500 | 1.90 | 0.168 | 0.241 | 0.135 |
| novel.3893 | 1.90 | 0.168 | 0.241 | 1.929 |
| RHSIM_Rhsim09G0087500 | 1.90 | 0.168 | 0.242 | -1.828 |
| RHSIM_Rhsim08G0048000 | 1.90 | 0.168 | 0.242 | -0.245 |
| novel.1665 | 1.90 | 0.168 | 0.242 | 0.502 |
| RHSIM_Rhsim03G0236000 | 1.90 | 0.168 | 0.242 | -3.043 |
| RHSIM_Rhsim03G0122800 | 1.90 | 0.168 | 0.242 | 0.161 |
| novel.4388 | 1.90 | 0.168 | 0.242 | -1.855 |
| novel.3241 | 1.90 | 0.169 | 0.242 | -1.026 |
| RHSIM_RhsimUnG0241800 | 1.90 | 0.169 | 0.242 | 0.410 |
| RHSIM_Rhsim11G0009500 | 1.90 | 0.169 | 0.242 | 0.169 |
| RHSIM_Rhsim08G0039800 | 1.89 | 0.169 | 0.242 | -37.603 |
| RHSIM_Rhsim03G0013200 | 1.89 | 0.169 | 0.242 | 0.543 |
| RHSIM_Rhsim08G0073900 | 1.89 | 0.169 | 0.242 | 0.634 |
| RHSIM_Rhsim13G0106600 | 1.89 | 0.169 | 0.242 | -38.069 |
| RHSIM_Rhsim06G0129600 | 1.89 | 0.169 | 0.242 | 0.945 |
| RHSIM_Rhsim01G0165900 | 1.89 | 0.169 | 0.242 | 1.095 |
| RHSIM_Rhsim02G0227500 | 1.89 | 0.169 | 0.242 | -1.105 |
| RHSIM_Rhsim08G0015800 | 1.89 | 0.169 | 0.243 | 1.315 |

| Gene | LRT statistic | P value | BH-adjusted P | log2FC (interaction) |
|---|---|---|---|---|
| RHSIM_Rhsim05G0210700 | 1.89 | 0.169 | 0.243 | 2.141 |
| RHSIM_Rhsim13G0035000 | 1.89 | 0.169 | 0.243 | -0.513 |
| RHSIM_Rhsim08G0051100 | 1.89 | 0.169 | 0.243 | 0.538 |
| RHSIM_Rhsim02G0134400 | 1.89 | 0.169 | 0.243 | -0.882 |
| RHSIM_Rhsim11G0097900 | 1.89 | 0.169 | 0.243 | -0.246 |
| RHSIM_Rhsim06G0019300 | 1.89 | 0.169 | 0.243 | 3.245 |
| RHSIM_Rhsim09G0197200 | 1.89 | 0.169 | 0.243 | 1.209 |
| novel.4294 | 1.89 | 0.169 | 0.243 | -1.133 |
| RHSIM_Rhsim11G0142800 | 1.89 | 0.169 | 0.243 | -1.448 |
| RHSIM_Rhsim09G0161800 | 1.89 | 0.170 | 0.243 | -37.947 |
| RHSIM_Rhsim12G0199400 | 1.89 | 0.170 | 0.243 | 0.211 |
| RHSIM_Rhsim03G0200500 | 1.89 | 0.170 | 0.243 | 2.233 |
| RHSIM_RhsimUnG0178600 | 1.89 | 0.170 | 0.243 | -0.258 |
| RHSIM_Rhsim05G0107100 | 1.89 | 0.170 | 0.243 | 38.310 |
| RHSIM_Rhsim02G0152900 | 1.88 | 0.170 | 0.243 | -0.261 |
| RHSIM_Rhsim07G0109400 | 1.88 | 0.170 | 0.243 | -1.763 |
| RHSIM_Rhsim08G0044700 | 1.88 | 0.170 | 0.243 | 0.481 |
| novel.576 | 1.88 | 0.170 | 0.243 | 0.787 |
| RHSIM_Rhsim07G0174200 | 1.88 | 0.170 | 0.244 | -0.550 |
| novel.3918 | 1.88 | 0.170 | 0.244 | -0.422 |
| RHSIM_Rhsim07G0050600 | 1.88 | 0.170 | 0.244 | -0.244 |
| RHSIM_RhsimUnG0061000 | 1.88 | 0.170 | 0.244 | 1.123 |
| RHSIM_RhsimUnG0114000 | 1.88 | 0.170 | 0.244 | 0.365 |
| RHSIM_Rhsim02G0159300 | 1.88 | 0.170 | 0.244 | -2.428 |
| RHSIM_Rhsim10G0177600 | 1.88 | 0.171 | 0.244 | 38.475 |
| RHSIM_Rhsim03G0154800 | 1.88 | 0.171 | 0.244 | -1.421 |
| RHSIM_Rhsim06G0235400 | 1.88 | 0.171 | 0.245 | -0.955 |
| RHSIM_Rhsim12G0165500 | 1.88 | 0.171 | 0.245 | -0.498 |
| novel.4024 | 1.87 | 0.171 | 0.245 | 1.214 |
| novel.1996 | 1.87 | 0.171 | 0.245 | -1.312 |
| RHSIM_Rhsim01G0274700 | 1.87 | 0.171 | 0.245 | -37.997 |
| novel.3425 | 1.87 | 0.171 | 0.245 | -0.718 |

| Gene | LRT statistic | P value | BH-adjusted P | log2FC (interaction) |
|---|---|---|---|---|
| RHSIM_RhsimUnG0033400 | 1.87 | 0.171 | 0.245 | 1.196 |
| novel.2036 | 1.87 | 0.171 | 0.245 | -0.766 |
| novel.557 | 1.87 | 0.171 | 0.245 | -0.473 |
| RHSIM_Rhsim06G0107800 | 1.87 | 0.172 | 0.246 | 0.637 |
| RHSIM_Rhsim04G0182300 | 1.87 | 0.172 | 0.246 | -1.141 |
| RHSIM_Rhsim02G0129900 | 1.87 | 0.172 | 0.246 | 0.710 |
| RHSIM_Rhsim05G0032300 | 1.87 | 0.172 | 0.246 | 38.434 |
| RHSIM_Rhsim03G0243300 | 1.87 | 0.172 | 0.246 | -0.179 |
| RHSIM_Rhsim13G0210500 | 1.87 | 0.172 | 0.246 | -0.285 |
| RHSIM_Rhsim05G0014600 | 1.87 | 0.172 | 0.246 | 0.090 |
| novel.3612 | 1.87 | 0.172 | 0.246 | 2.337 |
| RHSIM_Rhsim10G0064600 | 1.87 | 0.172 | 0.246 | -0.152 |
| RHSIM_Rhsim02G0178800 | 1.86 | 0.172 | 0.246 | -37.431 |
| novel.2643 | 1.86 | 0.172 | 0.246 | -0.160 |
| RHSIM_Rhsim09G0136500 | 1.86 | 0.172 | 0.246 | -2.707 |
| RHSIM_Rhsim01G0064500 | 1.86 | 0.172 | 0.247 | 2.260 |
| RHSIM_Rhsim01G0205300 | 1.86 | 0.172 | 0.247 | -0.360 |
| RHSIM_RhsimUnG0162700 | 1.86 | 0.172 | 0.247 | -3.429 |
| novel.4135 | 1.86 | 0.172 | 0.247 | -1.441 |
| RHSIM_Rhsim03G0207700 | 1.86 | 0.172 | 0.247 | 0.175 |
| RHSIM_Rhsim11G0129600 | 1.86 | 0.173 | 0.247 | -0.111 |
| RHSIM_Rhsim13G0196300 | 1.86 | 0.173 | 0.247 | -0.953 |
| RHSIM_Rhsim02G0169500 | 1.86 | 0.173 | 0.247 | -0.205 |
| RHSIM_Rhsim08G0213400 | 1.86 | 0.173 | 0.247 | -2.709 |
| novel.2049 | 1.86 | 0.173 | 0.247 | 0.577 |
| RHSIM_Rhsim04G0231400 | 1.86 | 0.173 | 0.247 | -1.275 |
| RHSIM_Rhsim08G0207600 | 1.86 | 0.173 | 0.247 | -1.148 |
| RHSIM_Rhsim01G0235100 | 1.86 | 0.173 | 0.247 | -0.282 |
| novel.1547 | 1.86 | 0.173 | 0.247 | 4.114 |
| RHSIM_Rhsim06G0168900 | 1.86 | 0.173 | 0.247 | 38.015 |
| RHSIM_Rhsim09G0157200 | 1.86 | 0.173 | 0.247 | 0.479 |
| RHSIM_Rhsim03G0137000 | 1.85 | 0.173 | 0.248 | -0.365 |

| Gene | LRT statistic | P value | BH-adjusted P | log2FC (interaction) |
|---|---|---|---|---|
| RHSIM_Rhsim04G0008800 | 1.85 | 0.173 | 0.248 | 0.719 |
| RHSIM_Rhsim13G0176100 | 1.85 | 0.173 | 0.248 | -0.568 |
| novel.2909 | 1.85 | 0.173 | 0.248 | -1.284 |
| RHSIM_Rhsim02G0051700 | 1.85 | 0.173 | 0.248 | -38.535 |
| RHSIM_Rhsim11G0079800 | 1.85 | 0.173 | 0.248 | -1.620 |
| RHSIM_Rhsim01G0027200 | 1.85 | 0.173 | 0.248 | 1.184 |
| novel.3195 | 1.85 | 0.173 | 0.248 | 3.363 |
| RHSIM_Rhsim06G0076800 | 1.85 | 0.174 | 0.248 | -0.960 |
| RHSIM_Rhsim09G0002300 | 1.85 | 0.174 | 0.248 | -0.149 |
| RHSIM_Rhsim03G0157500 | 1.85 | 0.174 | 0.248 | 37.778 |
| RHSIM_Rhsim06G0163900 | 1.85 | 0.174 | 0.248 | -0.142 |
| RHSIM_Rhsim13G0207200 | 1.85 | 0.174 | 0.248 | 0.442 |
| RHSIM_Rhsim09G0114100 | 1.85 | 0.174 | 0.248 | 0.177 |
| novel.369 | 1.85 | 0.174 | 0.248 | 2.476 |
| novel.2632 | 1.85 | 0.174 | 0.248 | 0.623 |
| RHSIM_Rhsim05G0055100 | 1.85 | 0.174 | 0.248 | -38.443 |
| RHSIM_Rhsim12G0038600 | 1.85 | 0.174 | 0.248 | 3.531 |
| RHSIM_Rhsim11G0143400 | 1.85 | 0.174 | 0.248 | -37.899 |
| RHSIM_Rhsim08G0177700 | 1.85 | 0.174 | 0.248 | 0.459 |
| RHSIM_Rhsim13G0187400 | 1.85 | 0.174 | 0.248 | 2.629 |
| RHSIM_Rhsim13G0153200 | 1.85 | 0.174 | 0.249 | 1.080 |
| RHSIM_Rhsim13G0089000 | 1.85 | 0.174 | 0.249 | 4.040 |
| RHSIM_RhsimUnG0029500 | 1.85 | 0.174 | 0.249 | 3.092 |
| RHSIM_Rhsim10G0152000 | 1.85 | 0.174 | 0.249 | 0.210 |
| RHSIM_Rhsim01G0035300 | 1.85 | 0.174 | 0.249 | 0.188 |
| RHSIM_Rhsim09G0197300 | 1.85 | 0.174 | 0.249 | -0.584 |
| RHSIM_Rhsim06G0196400 | 1.85 | 0.174 | 0.249 | -0.124 |
| RHSIM_Rhsim01G0103100 | 1.84 | 0.174 | 0.249 | 2.639 |
| RHSIM_Rhsim03G0068400 | 1.84 | 0.174 | 0.249 | -0.218 |
| RHSIM_Rhsim10G0206300 | 1.84 | 0.174 | 0.249 | -0.112 |
| RHSIM_Rhsim05G0215700 | 1.84 | 0.174 | 0.249 | 0.234 |
| RHSIM_RhsimUnG0140500 | 1.84 | 0.175 | 0.249 | -0.982 |

| Gene | LRT statistic | P value | BH-adjusted P | log2FC (interaction) |
|---|---|---|---|---|
| RHSIM_Rhsim04G0094400 | 1.84 | 0.175 | 0.249 | 0.228 |
| RHSIM_Rhsim06G0166000 | 1.84 | 0.175 | 0.249 | 0.188 |
| RHSIM_Rhsim02G0056600 | 1.84 | 0.175 | 0.249 | 0.210 |
| RHSIM_Rhsim06G0135600 | 1.84 | 0.175 | 0.249 | 37.774 |
| novel.3210 | 1.84 | 0.175 | 0.250 | 1.652 |
| RHSIM_RhsimUnG0142800 | 1.84 | 0.175 | 0.250 | -0.284 |
| RHSIM_Rhsim05G0078100 | 1.84 | 0.175 | 0.250 | 0.116 |
| novel.4184 | 1.84 | 0.175 | 0.250 | 0.588 |
| RHSIM_Rhsim01G0094800 | 1.84 | 0.175 | 0.250 | -37.806 |
| RHSIM_Rhsim13G0031100 | 1.84 | 0.175 | 0.250 | 0.222 |
| RHSIM_Rhsim02G0182200 | 1.84 | 0.175 | 0.250 | -0.198 |
| RHSIM_Rhsim01G0136400 | 1.84 | 0.175 | 0.250 | 37.821 |
| RHSIM_Rhsim09G0194500 | 1.84 | 0.175 | 0.250 | 3.131 |
| RHSIM_Rhsim04G0070200 | 1.84 | 0.176 | 0.250 | 1.540 |
| RHSIM_Rhsim07G0061700 | 1.83 | 0.176 | 0.251 | 1.048 |
| RHSIM_Rhsim02G0057700 | 1.83 | 0.176 | 0.251 | -1.499 |
| RHSIM_Rhsim07G0218200 | 1.83 | 0.176 | 0.251 | 0.231 |
| RHSIM_Rhsim08G0124000 | 1.83 | 0.176 | 0.251 | 1.964 |
| RHSIM_Rhsim01G0292800 | 1.83 | 0.176 | 0.251 | -0.144 |
| RHSIM_Rhsim11G0186500 | 1.83 | 0.176 | 0.251 | -1.803 |
| RHSIM_Rhsim08G0010700 | 1.83 | 0.176 | 0.251 | 0.260 |
| novel.1781 | 1.83 | 0.176 | 0.251 | 0.147 |
| RHSIM_Rhsim04G0014900 | 1.83 | 0.176 | 0.251 | 0.831 |
| RHSIM_Rhsim09G0194000 | 1.83 | 0.176 | 0.251 | -0.115 |
| RHSIM_Rhsim07G0066300 | 1.83 | 0.176 | 0.251 | -0.205 |
| novel.4467 | 1.83 | 0.176 | 0.251 | -0.252 |
| RHSIM_Rhsim04G0105100 | 1.83 | 0.176 | 0.251 | -38.441 |
| RHSIM_Rhsim02G0176500 | 1.83 | 0.176 | 0.251 | -1.136 |
| RHSIM_Rhsim01G0134100 | 1.83 | 0.176 | 0.251 | -1.436 |
| RHSIM_Rhsim02G0195400 | 1.83 | 0.176 | 0.251 | 37.527 |
| RHSIM_Rhsim03G0209100 | 1.83 | 0.176 | 0.251 | 0.230 |
| RHSIM_Rhsim01G0262500 | 1.83 | 0.176 | 0.251 | 3.000 |

| Gene | LRT statistic | P value | BH-adjusted P | log2FC (interaction) |
|---|---|---|---|---|
| RHSIM_Rhsim03G0041500 | 1.83 | 0.176 | 0.251 | 38.078 |
| RHSIM_Rhsim13G0141100 | 1.83 | 0.176 | 0.251 | 1.227 |
| RHSIM_Rhsim02G0074200 | 1.83 | 0.177 | 0.252 | -37.355 |
| RHSIM_Rhsim13G0202200 | 1.82 | 0.177 | 0.252 | -0.124 |
| RHSIM_RhsimUnG0252300 | 1.82 | 0.177 | 0.252 | -0.223 |
| novel.3139 | 1.82 | 0.177 | 0.252 | 1.035 |
| RHSIM_Rhsim02G0028600 | 1.82 | 0.177 | 0.252 | 0.161 |
| RHSIM_Rhsim08G0222300 | 1.82 | 0.177 | 0.252 | -0.161 |
| novel.2466 | 1.82 | 0.177 | 0.252 | -1.616 |
| novel.3688 | 1.82 | 0.177 | 0.252 | -0.443 |
| RHSIM_Rhsim10G0039900 | 1.82 | 0.177 | 0.252 | -2.723 |
| RHSIM_RhsimUnG0111600 | 1.82 | 0.177 | 0.253 | 0.491 |
| novel.2340 | 1.82 | 0.178 | 0.253 | 0.383 |
| novel.1176 | 1.82 | 0.178 | 0.253 | 1.074 |
| RHSIM_Rhsim08G0021900 | 1.82 | 0.178 | 0.253 | -0.187 |
| RHSIM_Rhsim05G0081200 | 1.82 | 0.178 | 0.253 | -2.492 |
| RHSIM_Rhsim03G0206400 | 1.82 | 0.178 | 0.253 | 1.494 |
| RHSIM_RhsimUnG0051900 | 1.82 | 0.178 | 0.253 | -1.902 |
| RHSIM_Rhsim10G0025500 | 1.82 | 0.178 | 0.253 | -0.783 |
| RHSIM_Rhsim10G0028900 | 1.82 | 0.178 | 0.253 | 1.333 |
| RHSIM_Rhsim01G0141500 | 1.81 | 0.178 | 0.253 | 0.250 |
| RHSIM_Rhsim02G0213900 | 1.81 | 0.178 | 0.253 | 0.268 |
| RHSIM_Rhsim09G0149800 | 1.81 | 0.178 | 0.253 | 0.432 |
| RHSIM_RhsimUnG0219700 | 1.81 | 0.178 | 0.253 | 0.788 |
| RHSIM_RhsimUnG0067700 | 1.81 | 0.178 | 0.254 | 2.585 |
| RHSIM_Rhsim13G0138200 | 1.81 | 0.178 | 0.254 | -1.196 |
| novel.2994 | 1.81 | 0.178 | 0.254 | 0.517 |
| RHSIM_RhsimUnG0122300 | 1.81 | 0.178 | 0.254 | 2.519 |
| RHSIM_Rhsim12G0151100 | 1.81 | 0.178 | 0.254 | -37.188 |
| RHSIM_Rhsim07G0251300 | 1.81 | 0.178 | 0.254 | -2.487 |
| RHSIM_Rhsim02G0132800 | 1.81 | 0.178 | 0.254 | 0.218 |
| novel.1476 | 1.81 | 0.178 | 0.254 | 0.762 |

| Gene | LRT statistic | P value | BH-adjusted P | log2FC (interaction) |
|---|---|---|---|---|
| RHSIM_Rhsim10G0152600 | 1.81 | 0.179 | 0.254 | -0.554 |
| RHSIM_Rhsim06G0195800 | 1.81 | 0.179 | 0.254 | -37.588 |
| RHSIM_Rhsim07G0059900 | 1.81 | 0.179 | 0.254 | 1.221 |
| RHSIM_Rhsim13G0213500 | 1.81 | 0.179 | 0.254 | 38.574 |
| RHSIM_Rhsim05G0056900 | 1.81 | 0.179 | 0.254 | 0.112 |
| RHSIM_Rhsim06G0149000 | 1.81 | 0.179 | 0.254 | -0.168 |
| RHSIM_Rhsim06G0011500 | 1.81 | 0.179 | 0.254 | -0.246 |
| RHSIM_Rhsim12G0140700 | 1.81 | 0.179 | 0.255 | 1.778 |
| RHSIM_Rhsim07G0140600 | 1.81 | 0.179 | 0.255 | 0.943 |
| RHSIM_RhsimUnG0039600 | 1.81 | 0.179 | 0.255 | 0.134 |
| RHSIM_RhsimUnG0206000 | 1.81 | 0.179 | 0.255 | -0.105 |
| RHSIM_Rhsim09G0007000 | 1.80 | 0.179 | 0.255 | -0.405 |
| RHSIM_Rhsim01G0234500 | 1.80 | 0.179 | 0.255 | 0.999 |
| novel.4268 | 1.80 | 0.179 | 0.255 | -2.639 |
| RHSIM_Rhsim04G0053500 | 1.80 | 0.179 | 0.255 | -1.766 |
| novel.791 | 1.80 | 0.179 | 0.255 | -0.677 |
| RHSIM_Rhsim02G0234900 | 1.80 | 0.179 | 0.255 | 0.612 |
| novel.1416 | 1.80 | 0.179 | 0.255 | 1.124 |
| RHSIM_Rhsim12G0035500 | 1.80 | 0.179 | 0.255 | 1.675 |
| RHSIM_Rhsim02G0255900 | 1.80 | 0.179 | 0.255 | 0.421 |
| novel.2324 | 1.80 | 0.180 | 0.255 | 0.999 |
| RHSIM_Rhsim06G0233100 | 1.80 | 0.180 | 0.255 | -1.129 |
| RHSIM_Rhsim03G0192200 | 1.80 | 0.180 | 0.255 | -0.174 |
| RHSIM_RhsimUnG0067200 | 1.80 | 0.180 | 0.255 | -0.334 |
| RHSIM_Rhsim04G0088400 | 1.80 | 0.180 | 0.255 | -37.821 |
| novel.727 | 1.80 | 0.180 | 0.255 | 0.923 |
| RHSIM_Rhsim02G0253100 | 1.80 | 0.180 | 0.255 | -37.410 |
| RHSIM_Rhsim08G0028300 | 1.80 | 0.180 | 0.255 | 3.468 |
| RHSIM_Rhsim09G0202100 | 1.80 | 0.180 | 0.256 | 3.071 |
| RHSIM_Rhsim06G0171500 | 1.80 | 0.180 | 0.256 | -2.198 |
| novel.1573 | 1.80 | 0.180 | 0.256 | 2.092 |
| RHSIM_Rhsim05G0233300 | 1.80 | 0.180 | 0.256 | 0.151 |

| Gene | LRT statistic | P value | BH-adjusted P | log2FC (interaction) |
|---|---|---|---|---|
| RHSIM_Rhsim07G0257100 | 1.80 | 0.180 | 0.256 | 2.134 |
| RHSIM_Rhsim10G0049700 | 1.80 | 0.180 | 0.256 | 38.106 |
| RHSIM_Rhsim03G0066100 | 1.79 | 0.181 | 0.257 | -0.647 |
| RHSIM_Rhsim04G0156200 | 1.79 | 0.181 | 0.257 | 0.825 |
| RHSIM_Rhsim04G0230500 | 1.79 | 0.181 | 0.257 | 1.062 |
| RHSIM_Rhsim05G0104700 | 1.79 | 0.181 | 0.257 | -0.113 |
| RHSIM_Rhsim07G0130300 | 1.79 | 0.181 | 0.257 | 3.112 |
| RHSIM_Rhsim12G0048200 | 1.79 | 0.181 | 0.257 | -1.795 |
| RHSIM_Rhsim06G0141100 | 1.79 | 0.181 | 0.257 | -1.564 |
| novel.236 | 1.79 | 0.181 | 0.257 | 1.090 |
| RHSIM_Rhsim04G0219900 | 1.79 | 0.181 | 0.257 | 0.634 |
| RHSIM_Rhsim01G0171200 | 1.79 | 0.181 | 0.257 | 0.635 |
| RHSIM_Rhsim01G0243800 | 1.79 | 0.181 | 0.257 | -0.081 |
| RHSIM_Rhsim03G0175600 | 1.79 | 0.181 | 0.257 | 0.853 |
| RHSIM_RhsimUnG0236100 | 1.79 | 0.181 | 0.257 | -0.464 |
| RHSIM_Rhsim01G0019700 | 1.79 | 0.181 | 0.257 | 1.270 |
| RHSIM_Rhsim12G0126000 | 1.79 | 0.181 | 0.258 | 2.289 |
| RHSIM_RhsimUnG0234100 | 1.79 | 0.181 | 0.258 | 0.092 |
| RHSIM_RhsimUnG0151400 | 1.79 | 0.181 | 0.258 | -37.305 |
| RHSIM_Rhsim13G0158100 | 1.79 | 0.181 | 0.258 | 0.812 |
| RHSIM_Rhsim05G0059300 | 1.78 | 0.182 | 0.258 | -0.613 |
| RHSIM_Rhsim08G0224000 | 1.78 | 0.182 | 0.258 | -0.262 |
| RHSIM_Rhsim13G0213000 | 1.78 | 0.182 | 0.258 | 0.530 |
| RHSIM_Rhsim11G0168300 | 1.78 | 0.182 | 0.258 | 0.899 |
| RHSIM_Rhsim13G0021500 | 1.78 | 0.182 | 0.258 | -0.183 |
| RHSIM_Rhsim07G0135300 | 1.78 | 0.182 | 0.258 | 2.572 |
| RHSIM_Rhsim13G0037600 | 1.78 | 0.182 | 0.258 | 0.431 |
| RHSIM_Rhsim02G0101500 | 1.78 | 0.182 | 0.258 | 2.683 |
| RHSIM_Rhsim13G0095000 | 1.78 | 0.182 | 0.258 | 0.276 |
| RHSIM_Rhsim06G0179300 | 1.78 | 0.182 | 0.258 | -0.213 |
| RHSIM_Rhsim05G0232300 | 1.78 | 0.182 | 0.258 | 0.096 |
| RHSIM_Rhsim10G0201600 | 1.78 | 0.182 | 0.258 | -3.897 |

| Gene | LRT statistic | P value | BH-adjusted P | log2FC (interaction) |
|---|---|---|---|---|
| RHSIM_Rhsim04G0009200 | 1.78 | 0.182 | 0.258 | 38.991 |
| RHSIM_RhsimUnG0079200 | 1.78 | 0.182 | 0.258 | 37.729 |
| RHSIM_Rhsim06G0045400 | 1.78 | 0.182 | 0.258 | 0.635 |
| RHSIM_Rhsim03G0169900 | 1.78 | 0.182 | 0.258 | -2.667 |
| RHSIM_Rhsim11G0087000 | 1.78 | 0.182 | 0.258 | -0.600 |
| RHSIM_Rhsim10G0032800 | 1.78 | 0.182 | 0.259 | 0.745 |
| RHSIM_Rhsim08G0065800 | 1.78 | 0.183 | 0.259 | -0.538 |
| RHSIM_Rhsim11G0042800 | 1.78 | 0.183 | 0.259 | -3.844 |
| RHSIM_Rhsim11G0125500 | 1.78 | 0.183 | 0.259 | -0.281 |
| RHSIM_Rhsim01G0234300 | 1.78 | 0.183 | 0.259 | 3.101 |
| RHSIM_Rhsim02G0189900 | 1.78 | 0.183 | 0.259 | -37.751 |
| RHSIM_Rhsim09G0207400 | 1.78 | 0.183 | 0.259 | -0.366 |
| RHSIM_Rhsim06G0004700 | 1.78 | 0.183 | 0.259 | -0.191 |
| RHSIM_Rhsim06G0065500 | 1.77 | 0.183 | 0.259 | 0.493 |
| RHSIM_Rhsim07G0067100 | 1.77 | 0.183 | 0.259 | -38.237 |
| RHSIM_Rhsim09G0204000 | 1.77 | 0.183 | 0.259 | 0.625 |
| RHSIM_RhsimMtG0002800 | 1.77 | 0.183 | 0.259 | 0.752 |
| RHSIM_Rhsim13G0021800 | 1.77 | 0.183 | 0.259 | 0.092 |
| RHSIM_Rhsim02G0030200 | 1.77 | 0.183 | 0.260 | 0.631 |
| RHSIM_Rhsim08G0037200 | 1.77 | 0.183 | 0.260 | 0.413 |
| RHSIM_Rhsim03G0209600 | 1.77 | 0.183 | 0.260 | 2.394 |
| RHSIM_Rhsim10G0199500 | 1.77 | 0.183 | 0.260 | 2.445 |
| RHSIM_Rhsim11G0168100 | 1.77 | 0.183 | 0.260 | 0.357 |
| novel.2746 | 1.77 | 0.184 | 0.260 | -0.578 |
| RHSIM_Rhsim06G0026700 | 1.77 | 0.184 | 0.260 | -0.546 |
| RHSIM_Rhsim01G0130700 | 1.77 | 0.184 | 0.260 | 2.484 |
| novel.1135 | 1.77 | 0.184 | 0.260 | 0.154 |
| RHSIM_Rhsim07G0154900 | 1.77 | 0.184 | 0.260 | -0.270 |
| RHSIM_Rhsim06G0094300 | 1.77 | 0.184 | 0.260 | -0.400 |
| RHSIM_Rhsim13G0107700 | 1.76 | 0.184 | 0.261 | -0.400 |
| RHSIM_Rhsim03G0260600 | 1.76 | 0.184 | 0.261 | -1.677 |
| RHSIM_Rhsim07G0197600 | 1.76 | 0.184 | 0.261 | 0.367 |

| Gene | LRT statistic | P value | BH-adjusted P | log2FC (interaction) |
|---|---|---|---|---|
| RHSIM_Rhsim12G0009900 | 1.76 | 0.184 | 0.261 | 0.154 |
| RHSIM_Rhsim08G0145600 | 1.76 | 0.184 | 0.261 | 1.429 |
| RHSIM_Rhsim11G0075900 | 1.76 | 0.184 | 0.261 | -2.104 |
| RHSIM_Rhsim07G0240100 | 1.76 | 0.184 | 0.261 | -38.094 |
| RHSIM_Rhsim11G0096300 | 1.76 | 0.184 | 0.261 | 1.837 |
| RHSIM_RhsimUnG0028400 | 1.76 | 0.184 | 0.261 | -0.249 |
| novel.1158 | 1.76 | 0.184 | 0.261 | 1.353 |
| RHSIM_Rhsim04G0027900 | 1.76 | 0.184 | 0.261 | -0.163 |
| RHSIM_Rhsim02G0025400 | 1.76 | 0.185 | 0.261 | 0.469 |
| novel.1829 | 1.76 | 0.185 | 0.261 | -0.428 |
| RHSIM_Rhsim08G0003800 | 1.76 | 0.185 | 0.261 | 0.539 |
| RHSIM_Rhsim09G0043100 | 1.76 | 0.185 | 0.261 | -1.121 |
| RHSIM_Rhsim01G0020100 | 1.76 | 0.185 | 0.261 | 0.752 |
| RHSIM_Rhsim04G0005500 | 1.76 | 0.185 | 0.261 | -2.876 |
| RHSIM_RhsimUnG0124800 | 1.76 | 0.185 | 0.262 | -0.238 |
| RHSIM_Rhsim13G0121200 | 1.76 | 0.185 | 0.262 | 0.466 |
| RHSIM_Rhsim10G0078700 | 1.76 | 0.185 | 0.262 | -0.143 |
| RHSIM_Rhsim06G0074200 | 1.76 | 0.185 | 0.262 | 0.112 |
| RHSIM_Rhsim03G0084000 | 1.76 | 0.185 | 0.262 | 2.315 |
| RHSIM_Rhsim04G0129100 | 1.76 | 0.185 | 0.262 | -0.318 |
| RHSIM_Rhsim08G0081700 | 1.76 | 0.185 | 0.262 | -0.190 |
| RHSIM_Rhsim13G0026900 | 1.76 | 0.185 | 0.262 | -37.846 |
| RHSIM_Rhsim03G0165700 | 1.76 | 0.185 | 0.262 | -1.635 |
| RHSIM_Rhsim13G0094200 | 1.75 | 0.185 | 0.262 | -0.566 |
| RHSIM_Rhsim03G0114500 | 1.75 | 0.185 | 0.262 | -1.847 |
| RHSIM_Rhsim07G0186800 | 1.75 | 0.185 | 0.262 | -0.804 |
| RHSIM_Rhsim07G0132600 | 1.75 | 0.185 | 0.262 | 1.262 |
| RHSIM_RhsimUnG0051200 | 1.75 | 0.185 | 0.262 | 1.072 |
| novel.677 | 1.75 | 0.186 | 0.262 | 38.183 |
| RHSIM_Rhsim05G0222700 | 1.75 | 0.186 | 0.262 | 2.106 |
| RHSIM_Rhsim02G0256800 | 1.75 | 0.186 | 0.262 | -0.149 |
| RHSIM_Rhsim04G0205300 | 1.75 | 0.186 | 0.263 | 0.097 |

| Gene | LRT statistic | P value | BH-adjusted P | log2FC (interaction) |
|---|---|---|---|---|
| novel.4163 | 1.75 | 0.186 | 0.263 | 3.103 |
| RHSIM_Rhsim10G0011600 | 1.75 | 0.186 | 0.263 | -3.125 |
| RHSIM_Rhsim07G0147400 | 1.75 | 0.186 | 0.263 | -0.659 |
| novel.3356 | 1.75 | 0.186 | 0.263 | 1.071 |
| RHSIM_Rhsim03G0223000 | 1.75 | 0.186 | 0.263 | -2.633 |
| RHSIM_Rhsim11G0174100 | 1.75 | 0.186 | 0.263 | 0.147 |
| RHSIM_Rhsim05G0024700 | 1.75 | 0.186 | 0.263 | 37.970 |
| RHSIM_Rhsim10G0213900 | 1.75 | 0.186 | 0.263 | -0.726 |
| RHSIM_RhsimUnG0107400 | 1.75 | 0.186 | 0.263 | -1.342 |
| novel.2134 | 1.75 | 0.186 | 0.263 | 37.200 |
| RHSIM_Rhsim03G0152600 | 1.75 | 0.186 | 0.263 | 0.505 |
| RHSIM_Rhsim11G0045700 | 1.75 | 0.186 | 0.264 | 0.373 |
| RHSIM_Rhsim04G0086200 | 1.75 | 0.186 | 0.264 | -0.152 |
| RHSIM_Rhsim02G0161900 | 1.74 | 0.187 | 0.264 | -0.840 |
| RHSIM_Rhsim05G0005200 | 1.74 | 0.187 | 0.264 | 1.464 |
| RHSIM_Rhsim13G0045100 | 1.74 | 0.187 | 0.264 | -2.542 |
| novel.2412 | 1.74 | 0.187 | 0.264 | 0.634 |
| novel.705 | 1.74 | 0.187 | 0.264 | -0.998 |
| RHSIM_RhsimUnG0004300 | 1.74 | 0.187 | 0.264 | 2.064 |
| novel.1668 | 1.74 | 0.187 | 0.264 | 0.931 |
| RHSIM_Rhsim13G0192500 | 1.74 | 0.187 | 0.264 | 0.907 |
| RHSIM_Rhsim08G0165000 | 1.74 | 0.187 | 0.264 | 0.443 |
| RHSIM_Rhsim06G0127600 | 1.74 | 0.187 | 0.264 | -2.771 |
| RHSIM_Rhsim05G0098500 | 1.74 | 0.187 | 0.264 | 1.534 |
| RHSIM_Rhsim12G0158600 | 1.74 | 0.187 | 0.264 | 0.240 |
| RHSIM_Rhsim04G0243600 | 1.74 | 0.187 | 0.264 | 0.676 |
| RHSIM_Rhsim07G0051800 | 1.74 | 0.187 | 0.264 | 37.612 |
| RHSIM_Rhsim06G0007700 | 1.74 | 0.187 | 0.264 | 0.251 |
| novel.1666 | 1.74 | 0.187 | 0.264 | 0.435 |
| RHSIM_Rhsim09G0195600 | 1.74 | 0.187 | 0.264 | 0.179 |
| RHSIM_RhsimUnG0145100 | 1.74 | 0.187 | 0.264 | 0.840 |
| novel.1959 | 1.74 | 0.187 | 0.265 | -0.425 |

| Gene | LRT statistic | P value | BH-adjusted P | log2FC (interaction) |
|---|---|---|---|---|
| RHSIM_RhsimUnG0017800 | 1.74 | 0.187 | 0.265 | 0.476 |
| RHSIM_Rhsim08G0221000 | 1.74 | 0.188 | 0.265 | -0.839 |
| RHSIM_RhsimUnG0164300 | 1.74 | 0.188 | 0.265 | -0.712 |
| RHSIM_Rhsim10G0111800 | 1.74 | 0.188 | 0.265 | 0.451 |
| RHSIM_Rhsim13G0089400 | 1.74 | 0.188 | 0.265 | 0.551 |
| RHSIM_Rhsim07G0074500 | 1.73 | 0.188 | 0.265 | 0.755 |
| RHSIM_Rhsim03G0012700 | 1.73 | 0.188 | 0.265 | -3.723 |
| RHSIM_Rhsim02G0216300 | 1.73 | 0.188 | 0.265 | -0.228 |
| novel.113 | 1.73 | 0.188 | 0.265 | -37.360 |
| RHSIM_RhsimUnG0047600 | 1.73 | 0.188 | 0.265 | 2.213 |
| RHSIM_Rhsim02G0034700 | 1.73 | 0.188 | 0.265 | -0.509 |
| RHSIM_Rhsim10G0174900 | 1.73 | 0.188 | 0.265 | -1.388 |
| RHSIM_Rhsim09G0144700 | 1.73 | 0.188 | 0.265 | -0.501 |
| RHSIM_Rhsim04G0119400 | 1.73 | 0.188 | 0.265 | 0.118 |
| RHSIM_Rhsim06G0035100 | 1.73 | 0.188 | 0.265 | 0.240 |
| RHSIM_Rhsim12G0183000 | 1.73 | 0.188 | 0.266 | 0.239 |
| RHSIM_Rhsim01G0066600 | 1.73 | 0.188 | 0.266 | 0.083 |
| RHSIM_Rhsim05G0216300 | 1.73 | 0.188 | 0.266 | -0.280 |
| RHSIM_Rhsim07G0122000 | 1.73 | 0.188 | 0.266 | -0.584 |
| RHSIM_Rhsim02G0102100 | 1.73 | 0.188 | 0.266 | -1.519 |
| RHSIM_Rhsim11G0135000 | 1.73 | 0.188 | 0.266 | -1.590 |
| RHSIM_Rhsim01G0288200 | 1.73 | 0.189 | 0.266 | 0.210 |
| RHSIM_Rhsim11G0031700 | 1.73 | 0.189 | 0.266 | -0.591 |
| RHSIM_Rhsim01G0031200 | 1.73 | 0.189 | 0.266 | -1.442 |
| RHSIM_Rhsim06G0070800 | 1.73 | 0.189 | 0.266 | -1.556 |
| RHSIM_Rhsim12G0119300 | 1.73 | 0.189 | 0.266 | 37.940 |
| novel.3712 | 1.73 | 0.189 | 0.267 | 2.080 |
| RHSIM_Rhsim04G0123400 | 1.73 | 0.189 | 0.267 | 0.338 |
| RHSIM_Rhsim02G0164900 | 1.72 | 0.189 | 0.267 | 0.334 |
| RHSIM_Rhsim01G0139500 | 1.72 | 0.189 | 0.267 | 0.183 |
| novel.939 | 1.72 | 0.189 | 0.267 | -1.592 |
| RHSIM_Rhsim06G0087300 | 1.72 | 0.189 | 0.267 | 0.246 |

| Gene | LRT statistic | P value | BH-adjusted P | log2FC (interaction) |
|---|---|---|---|---|
| novel.1919 | 1.72 | 0.189 | 0.267 | 0.180 |
| RHSIM_Rhsim06G0085700 | 1.72 | 0.190 | 0.267 | -0.699 |
| novel.2067 | 1.72 | 0.190 | 0.267 | -1.156 |
| novel.297 | 1.72 | 0.190 | 0.267 | -0.670 |
| RHSIM_Rhsim11G0014700 | 1.72 | 0.190 | 0.267 | -2.031 |
| RHSIM_RhsimUnG0137200 | 1.72 | 0.190 | 0.267 | -2.675 |
| novel.4179 | 1.72 | 0.190 | 0.268 | -36.763 |
| RHSIM_RhsimUnG0080500 | 1.72 | 0.190 | 0.268 | 1.643 |
| RHSIM_Rhsim04G0173800 | 1.72 | 0.190 | 0.268 | -0.113 |
| RHSIM_Rhsim12G0118400 | 1.72 | 0.190 | 0.268 | -0.642 |
| RHSIM_Rhsim06G0081200 | 1.72 | 0.190 | 0.268 | 0.131 |
| RHSIM_Rhsim13G0112900 | 1.72 | 0.190 | 0.268 | -0.906 |
| RHSIM_Rhsim01G0140100 | 1.72 | 0.190 | 0.268 | 0.567 |
| RHSIM_Rhsim07G0155700 | 1.72 | 0.190 | 0.268 | -0.923 |
| RHSIM_Rhsim03G0219900 | 1.71 | 0.190 | 0.268 | 0.250 |
| RHSIM_Rhsim04G0112400 | 1.71 | 0.191 | 0.269 | 38.086 |
| RHSIM_Rhsim05G0167300 | 1.71 | 0.191 | 0.269 | -0.156 |
| novel.331 | 1.71 | 0.191 | 0.269 | 0.282 |
| RHSIM_Rhsim11G0124800 | 1.71 | 0.191 | 0.269 | -0.897 |
| RHSIM_RhsimUnG0096000 | 1.71 | 0.191 | 0.269 | 0.891 |
| RHSIM_Rhsim04G0086300 | 1.71 | 0.191 | 0.269 | 0.153 |
| RHSIM_Rhsim07G0237600 | 1.71 | 0.191 | 0.269 | -0.093 |
| RHSIM_Rhsim09G0045400 | 1.71 | 0.191 | 0.269 | 0.817 |
| novel.1444 | 1.71 | 0.191 | 0.269 | 0.183 |
| novel.237 | 1.71 | 0.191 | 0.269 | 1.141 |
| RHSIM_Rhsim04G0203800 | 1.71 | 0.191 | 0.269 | -37.360 |
| novel.1868 | 1.71 | 0.191 | 0.269 | 0.631 |
| RHSIM_Rhsim11G0159100 | 1.71 | 0.191 | 0.270 | 0.194 |
| RHSIM_RhsimUnG0200100 | 1.71 | 0.192 | 0.270 | 0.569 |
| RHSIM_Rhsim08G0015600 | 1.71 | 0.192 | 0.270 | -1.577 |
| novel.4183 | 1.71 | 0.192 | 0.270 | -1.675 |
| RHSIM_RhsimUnG0217400 | 1.71 | 0.192 | 0.270 | -0.193 |

| Gene | LRT statistic | P value | BH-adjusted P | log2FC (interaction) |
|---|---|---|---|---|
| RHSIM_Rhsim05G0023300 | 1.70 | 0.192 | 0.270 | 38.123 |
| RHSIM_Rhsim13G0153300 | 1.70 | 0.192 | 0.270 | -37.943 |
| RHSIM_Rhsim13G0193400 | 1.70 | 0.192 | 0.270 | 1.586 |
| RHSIM_Rhsim03G0190300 | 1.70 | 0.192 | 0.270 | -1.146 |
| novel.1390 | 1.70 | 0.192 | 0.270 | 0.147 |
| RHSIM_Rhsim07G0081500 | 1.70 | 0.192 | 0.270 | 0.221 |
| RHSIM_Rhsim07G0050100 | 1.70 | 0.192 | 0.270 | 37.874 |
| RHSIM_Rhsim04G0131000 | 1.70 | 0.192 | 0.271 | -0.182 |
| novel.4427 | 1.70 | 0.192 | 0.271 | 1.868 |
| RHSIM_Rhsim09G0021600 | 1.70 | 0.192 | 0.271 | 1.870 |
| RHSIM_Rhsim13G0020300 | 1.70 | 0.193 | 0.271 | 0.226 |
| RHSIM_Rhsim09G0212800 | 1.70 | 0.193 | 0.271 | 0.184 |
| RHSIM_Rhsim04G0203100 | 1.70 | 0.193 | 0.271 | -1.401 |
| novel.2904 | 1.70 | 0.193 | 0.271 | 0.943 |
| novel.3690 | 1.70 | 0.193 | 0.271 | -2.626 |
| RHSIM_Rhsim01G0026500 | 1.70 | 0.193 | 0.271 | -2.020 |
| RHSIM_Rhsim02G0124700 | 1.70 | 0.193 | 0.271 | 1.008 |
| RHSIM_RhsimUnG0190400 | 1.70 | 0.193 | 0.271 | -1.631 |
| RHSIM_Rhsim03G0025600 | 1.70 | 0.193 | 0.271 | 0.575 |
| RHSIM_Rhsim11G0012000 | 1.69 | 0.193 | 0.271 | -0.640 |
| novel.3279 | 1.69 | 0.193 | 0.271 | 1.353 |
| RHSIM_Rhsim06G0217000 | 1.69 | 0.193 | 0.271 | -2.873 |
| novel.264 | 1.69 | 0.193 | 0.271 | 2.210 |
| RHSIM_Rhsim02G0205300 | 1.69 | 0.193 | 0.271 | 0.348 |
| RHSIM_Rhsim01G0081200 | 1.69 | 0.193 | 0.272 | -0.295 |
| RHSIM_Rhsim06G0220500 | 1.69 | 0.193 | 0.272 | -0.944 |
| RHSIM_Rhsim08G0242300 | 1.69 | 0.193 | 0.272 | -0.447 |
| RHSIM_Rhsim09G0010400 | 1.69 | 0.193 | 0.272 | 0.819 |
| RHSIM_Rhsim12G0165700 | 1.69 | 0.193 | 0.272 | -38.668 |
| novel.2368 | 1.69 | 0.193 | 0.272 | 0.291 |
| RHSIM_Rhsim07G0244300 | 1.69 | 0.193 | 0.272 | 1.683 |
| RHSIM_Rhsim13G0235100 | 1.69 | 0.193 | 0.272 | 0.758 |

| Gene | LRT statistic | P value | BH-adjusted P | log2FC (interaction) |
|---|---|---|---|---|
| RHSIM_Rhsim12G0171400 | 1.69 | 0.194 | 0.272 | -0.125 |
| RHSIM_Rhsim08G0091000 | 1.69 | 0.194 | 0.272 | 2.191 |
| RHSIM_Rhsim04G0176300 | 1.69 | 0.194 | 0.272 | -0.427 |
| RHSIM_Rhsim09G0191700 | 1.69 | 0.194 | 0.272 | -1.677 |
| RHSIM_Rhsim10G0049100 | 1.69 | 0.194 | 0.272 | 0.540 |
| RHSIM_RhsimUnG0172400 | 1.69 | 0.194 | 0.273 | -0.266 |
| RHSIM_Rhsim03G0247800 | 1.69 | 0.194 | 0.273 | -0.135 |
| RHSIM_Rhsim13G0071700 | 1.69 | 0.194 | 0.273 | 0.171 |
| novel.1596 | 1.69 | 0.194 | 0.273 | -1.014 |
| RHSIM_Rhsim04G0106800 | 1.69 | 0.194 | 0.273 | -0.141 |
| RHSIM_Rhsim02G0096900 | 1.69 | 0.194 | 0.273 | -0.644 |
| RHSIM_Rhsim03G0240800 | 1.68 | 0.194 | 0.273 | -0.141 |
| RHSIM_Rhsim06G0051400 | 1.68 | 0.194 | 0.273 | 0.181 |
| RHSIM_RhsimUnG0156600 | 1.68 | 0.195 | 0.273 | -2.165 |
| RHSIM_Rhsim13G0096000 | 1.68 | 0.195 | 0.273 | 1.474 |
| RHSIM_Rhsim06G0075800 | 1.68 | 0.195 | 0.273 | -1.501 |
| RHSIM_RhsimUnG0112000 | 1.68 | 0.195 | 0.273 | 1.121 |
| RHSIM_Rhsim08G0091700 | 1.68 | 0.195 | 0.273 | 0.584 |
| novel.368 | 1.68 | 0.195 | 0.273 | 0.577 |
| RHSIM_Rhsim07G0103300 | 1.68 | 0.195 | 0.273 | -37.410 |
| RHSIM_RhsimUnG0195700 | 1.68 | 0.195 | 0.273 | 0.406 |
| novel.2641 | 1.68 | 0.195 | 0.274 | -0.850 |
| RHSIM_Rhsim11G0064000 | 1.68 | 0.195 | 0.274 | -0.200 |
| novel.4547 | 1.68 | 0.195 | 0.274 | -1.412 |
| RHSIM_Rhsim05G0021700 | 1.68 | 0.195 | 0.274 | -0.221 |
| RHSIM_Rhsim09G0108900 | 1.68 | 0.195 | 0.274 | -0.107 |
| novel.3125 | 1.68 | 0.195 | 0.274 | -0.609 |
| RHSIM_Rhsim07G0033500 | 1.68 | 0.195 | 0.274 | 37.586 |
| RHSIM_Rhsim02G0147800 | 1.68 | 0.195 | 0.274 | 37.855 |
| novel.3443 | 1.68 | 0.195 | 0.274 | -1.520 |
| novel.3346 | 1.68 | 0.195 | 0.274 | -0.661 |
| novel.3563 | 1.68 | 0.196 | 0.274 | -1.125 |

| Gene | LRT statistic | P value | BH-adjusted P | log2FC (interaction) |
|---|---|---|---|---|
| RHSIM_Rhsim01G0149700 | 1.68 | 0.196 | 0.274 | 0.130 |
| RHSIM_Rhsim13G0220400 | 1.68 | 0.196 | 0.274 | -1.492 |
| RHSIM_Rhsim08G0129200 | 1.68 | 0.196 | 0.274 | 0.130 |
| novel.593 | 1.67 | 0.196 | 0.274 | 37.282 |
| RHSIM_Rhsim04G0222900 | 1.67 | 0.196 | 0.275 | 0.210 |
| novel.3434 | 1.67 | 0.196 | 0.275 | 0.641 |
| RHSIM_Rhsim02G0022200 | 1.67 | 0.196 | 0.275 | -1.056 |
| RHSIM_Rhsim10G0067300 | 1.67 | 0.196 | 0.275 | -0.223 |
| RHSIM_Rhsim06G0066300 | 1.67 | 0.196 | 0.275 | -0.746 |
| RHSIM_Rhsim03G0233100 | 1.67 | 0.196 | 0.275 | -0.231 |
| novel.3544 | 1.67 | 0.196 | 0.275 | -0.566 |
| RHSIM_Rhsim08G0207700 | 1.67 | 0.196 | 0.275 | 0.331 |
| RHSIM_Rhsim02G0217000 | 1.67 | 0.196 | 0.275 | 0.465 |
| RHSIM_Rhsim09G0091500 | 1.67 | 0.196 | 0.275 | 0.851 |
| RHSIM_Rhsim08G0103900 | 1.67 | 0.196 | 0.275 | 0.820 |
| novel.2253 | 1.67 | 0.196 | 0.275 | 2.968 |
| RHSIM_Rhsim04G0191800 | 1.67 | 0.196 | 0.275 | -1.460 |
| RHSIM_Rhsim09G0214500 | 1.67 | 0.196 | 0.275 | -0.169 |
| RHSIM_Rhsim03G0210000 | 1.67 | 0.197 | 0.276 | 1.686 |
| RHSIM_Rhsim10G0145200 | 1.67 | 0.197 | 0.276 | -0.191 |
| RHSIM_Rhsim08G0188400 | 1.67 | 0.197 | 0.276 | -0.090 |
| novel.2459 | 1.67 | 0.197 | 0.276 | 36.993 |
| novel.1241 | 1.67 | 0.197 | 0.276 | -0.496 |
| RHSIM_Rhsim01G0231000 | 1.67 | 0.197 | 0.276 | 0.091 |
| RHSIM_Rhsim06G0109600 | 1.67 | 0.197 | 0.276 | -2.676 |
| RHSIM_Rhsim03G0077200 | 1.67 | 0.197 | 0.276 | -0.527 |
| RHSIM_Rhsim07G0230000 | 1.67 | 0.197 | 0.276 | -37.220 |
| novel.3007 | 1.67 | 0.197 | 0.276 | 1.373 |
| RHSIM_Rhsim08G0124700 | 1.66 | 0.197 | 0.276 | 0.228 |
| RHSIM_Rhsim07G0004000 | 1.66 | 0.197 | 0.276 | -0.223 |
| novel.2394 | 1.66 | 0.197 | 0.276 | 1.956 |
| RHSIM_Rhsim04G0148800 | 1.66 | 0.197 | 0.276 | -0.171 |

| Gene | LRT statistic | P value | BH-adjusted P | log2FC (interaction) |
|---|---|---|---|---|
| RHSIM_Rhsim07G0242800 | 1.66 | 0.197 | 0.276 | -0.323 |
| RHSIM_Rhsim10G0180200 | 1.66 | 0.197 | 0.276 | -0.201 |
| RHSIM_Rhsim05G0015400 | 1.66 | 0.197 | 0.276 | 1.201 |
| novel.3247 | 1.66 | 0.197 | 0.276 | 0.838 |
| RHSIM_Rhsim03G0028700 | 1.66 | 0.197 | 0.276 | 0.589 |
| RHSIM_Rhsim09G0050000 | 1.66 | 0.197 | 0.276 | -0.399 |
| RHSIM_Rhsim05G0139600 | 1.66 | 0.198 | 0.277 | 0.401 |
| RHSIM_Rhsim12G0133300 | 1.66 | 0.198 | 0.277 | -0.184 |
| novel.1676 | 1.66 | 0.198 | 0.277 | 0.844 |
| RHSIM_Rhsim09G0138600 | 1.66 | 0.198 | 0.277 | -0.715 |
| RHSIM_Rhsim09G0065700 | 1.66 | 0.198 | 0.277 | -1.086 |
| novel.766 | 1.66 | 0.198 | 0.277 | 1.935 |
| RHSIM_Rhsim05G0186300 | 1.66 | 0.198 | 0.277 | 0.568 |
| RHSIM_Rhsim01G0201400 | 1.66 | 0.198 | 0.277 | -0.274 |
| novel.3580 | 1.66 | 0.198 | 0.277 | -0.961 |
| RHSIM_Rhsim12G0119500 | 1.66 | 0.198 | 0.278 | -2.139 |
| RHSIM_Rhsim09G0179700 | 1.65 | 0.198 | 0.278 | -0.266 |
| novel.3189 | 1.65 | 0.198 | 0.278 | 0.176 |
| RHSIM_Rhsim11G0039600 | 1.65 | 0.199 | 0.278 | 1.621 |
| novel.2972 | 1.65 | 0.199 | 0.278 | 2.416 |
| RHSIM_RhsimUnG0240600 | 1.65 | 0.199 | 0.278 | -0.357 |
| RHSIM_Rhsim13G0082800 | 1.65 | 0.199 | 0.278 | -0.215 |
| RHSIM_Rhsim08G0141100 | 1.65 | 0.199 | 0.278 | -0.202 |
| RHSIM_Rhsim05G0022300 | 1.65 | 0.199 | 0.278 | 0.151 |
| RHSIM_Rhsim05G0066300 | 1.65 | 0.199 | 0.278 | 1.639 |
| RHSIM_Rhsim06G0040300 | 1.65 | 0.199 | 0.278 | 2.494 |
| novel.4327 | 1.65 | 0.199 | 0.278 | 0.478 |
| novel.3743 | 1.65 | 0.199 | 0.278 | 1.885 |
| RHSIM_Rhsim04G0090900 | 1.65 | 0.199 | 0.278 | 2.001 |
| RHSIM_Rhsim08G0060000 | 1.65 | 0.199 | 0.278 | -37.531 |
| RHSIM_Rhsim08G0031200 | 1.65 | 0.199 | 0.278 | 0.137 |
| RHSIM_Rhsim06G0144100 | 1.65 | 0.199 | 0.278 | -37.675 |

| Gene | LRT statistic | P value | BH-adjusted P | log2FC (interaction) |
|---|---|---|---|---|
| RHSIM_Rhsim04G0186000 | 1.65 | 0.199 | 0.278 | 0.778 |
| RHSIM_Rhsim07G0213700 | 1.65 | 0.199 | 0.278 | 0.177 |
| RHSIM_Rhsim08G0038000 | 1.65 | 0.199 | 0.278 | -1.365 |
| RHSIM_Rhsim11G0164800 | 1.65 | 0.199 | 0.279 | 1.879 |
| novel.3168 | 1.65 | 0.199 | 0.279 | -0.909 |
| novel.1337 | 1.65 | 0.199 | 0.279 | -1.330 |
| RHSIM_Rhsim08G0244900 | 1.65 | 0.199 | 0.279 | 3.385 |
| RHSIM_Rhsim01G0083500 | 1.65 | 0.199 | 0.279 | -1.274 |
| RHSIM_Rhsim06G0217200 | 1.65 | 0.199 | 0.279 | 37.321 |
| RHSIM_Rhsim11G0157600 | 1.65 | 0.199 | 0.279 | 1.831 |
| RHSIM_Rhsim06G0233700 | 1.65 | 0.199 | 0.279 | -1.447 |
| RHSIM_Rhsim12G0143200 | 1.65 | 0.199 | 0.279 | -4.054 |
| RHSIM_RhsimUnG0231900 | 1.65 | 0.200 | 0.279 | 0.264 |
| RHSIM_Rhsim10G0134600 | 1.65 | 0.200 | 0.279 | -0.569 |
| RHSIM_Rhsim12G0161700 | 1.64 | 0.200 | 0.279 | 0.420 |
| RHSIM_RhsimUnG0119300 | 1.64 | 0.200 | 0.279 | -1.190 |
| RHSIM_Rhsim13G0197800 | 1.64 | 0.200 | 0.279 | -0.265 |
| RHSIM_Rhsim08G0043700 | 1.64 | 0.200 | 0.279 | -0.155 |
| RHSIM_Rhsim08G0167500 | 1.64 | 0.200 | 0.279 | -0.113 |
| RHSIM_Rhsim01G0274900 | 1.64 | 0.200 | 0.279 | -0.316 |
| RHSIM_Rhsim04G0193100 | 1.64 | 0.200 | 0.279 | -37.290 |
| RHSIM_Rhsim10G0074700 | 1.64 | 0.200 | 0.280 | -0.576 |
| novel.3651 | 1.64 | 0.200 | 0.280 | -1.332 |
| RHSIM_Rhsim01G0284100 | 1.64 | 0.200 | 0.280 | -0.091 |
| RHSIM_Rhsim05G0069100 | 1.64 | 0.200 | 0.280 | 2.413 |
| RHSIM_Rhsim09G0034600 | 1.64 | 0.200 | 0.280 | -2.728 |
| RHSIM_Rhsim04G0215700 | 1.64 | 0.201 | 0.280 | -0.303 |
| RHSIM_Rhsim03G0195900 | 1.64 | 0.201 | 0.280 | -37.216 |
| RHSIM_Rhsim10G0164600 | 1.64 | 0.201 | 0.280 | 38.109 |
| RHSIM_Rhsim01G0213600 | 1.64 | 0.201 | 0.281 | 0.859 |
| novel.2855 | 1.63 | 0.201 | 0.281 | -0.229 |
| novel.1586 | 1.63 | 0.201 | 0.281 | 0.956 |

| Gene | LRT statistic | P value | BH-adjusted P | log2FC (interaction) |
|---|---|---|---|---|
| RHSIM_Rhsim05G0052200 | 1.63 | 0.201 | 0.281 | -0.499 |
| novel.12 | 1.63 | 0.201 | 0.281 | 1.070 |
| RHSIM_Rhsim07G0130600 | 1.63 | 0.201 | 0.281 | 0.224 |
| novel.447 | 1.63 | 0.201 | 0.281 | 0.612 |
| RHSIM_Rhsim10G0026700 | 1.63 | 0.201 | 0.281 | 1.371 |
| RHSIM_Rhsim11G0035600 | 1.63 | 0.201 | 0.281 | 1.606 |
| RHSIM_RhsimUnG0183900 | 1.63 | 0.201 | 0.281 | -0.981 |
| RHSIM_Rhsim13G0020800 | 1.63 | 0.202 | 0.281 | -0.578 |
| RHSIM_Rhsim08G0061000 | 1.63 | 0.202 | 0.281 | -36.916 |
| RHSIM_RhsimUnG0097700 | 1.63 | 0.202 | 0.281 | -0.199 |
| RHSIM_Rhsim03G0137200 | 1.63 | 0.202 | 0.282 | 0.426 |
| RHSIM_Rhsim05G0125100 | 1.63 | 0.202 | 0.282 | -37.706 |
| RHSIM_Rhsim07G0186600 | 1.63 | 0.202 | 0.282 | -0.694 |
| RHSIM_RhsimUnG0029600 | 1.63 | 0.202 | 0.282 | -2.445 |
| novel.1249 | 1.63 | 0.202 | 0.282 | 0.617 |
| RHSIM_Rhsim07G0164700 | 1.63 | 0.202 | 0.282 | 0.873 |
| RHSIM_Rhsim03G0093300 | 1.63 | 0.202 | 0.282 | 37.331 |
| novel.2686 | 1.63 | 0.202 | 0.282 | 0.887 |
| RHSIM_Rhsim10G0074400 | 1.62 | 0.202 | 0.282 | 1.401 |
| RHSIM_Rhsim03G0192000 | 1.62 | 0.203 | 0.283 | 0.151 |
| RHSIM_Rhsim05G0105700 | 1.62 | 0.203 | 0.283 | 1.443 |
| novel.1264 | 1.62 | 0.203 | 0.283 | -0.789 |
| novel.3450 | 1.62 | 0.203 | 0.283 | -0.125 |
| RHSIM_Rhsim05G0200600 | 1.62 | 0.203 | 0.283 | 0.191 |
| RHSIM_Rhsim03G0086900 | 1.62 | 0.203 | 0.283 | 1.286 |
| RHSIM_Rhsim12G0210200 | 1.62 | 0.203 | 0.283 | -0.491 |
| novel.381 | 1.62 | 0.203 | 0.284 | -1.654 |
| RHSIM_Rhsim07G0221500 | 1.62 | 0.203 | 0.284 | 0.222 |
| RHSIM_Rhsim07G0037900 | 1.62 | 0.203 | 0.284 | 0.585 |
| novel.3864 | 1.62 | 0.203 | 0.284 | -0.321 |
| RHSIM_Rhsim11G0013400 | 1.62 | 0.204 | 0.284 | 0.168 |
| novel.1659 | 1.62 | 0.204 | 0.284 | 2.201 |

| Gene | LRT statistic | P value | BH-adjusted P | log2FC (interaction) |
|---|---|---|---|---|
| RHSIM_Rhsim10G0040900 | 1.62 | 0.204 | 0.284 | -1.231 |
| RHSIM_Rhsim03G0000900 | 1.62 | 0.204 | 0.284 | 0.782 |
| RHSIM_Rhsim02G0064400 | 1.62 | 0.204 | 0.284 | -0.559 |
| RHSIM_RhsimUnG0154300 | 1.62 | 0.204 | 0.284 | 1.420 |
| RHSIM_Rhsim08G0043100 | 1.62 | 0.204 | 0.284 | 0.325 |
| RHSIM_Rhsim04G0060000 | 1.61 | 0.204 | 0.284 | 37.659 |
| novel.1013 | 1.61 | 0.204 | 0.284 | -1.128 |
| RHSIM_Rhsim01G0241900 | 1.61 | 0.204 | 0.284 | 2.923 |
| RHSIM_Rhsim02G0113600 | 1.61 | 0.204 | 0.284 | -0.355 |
| RHSIM_Rhsim08G0155000 | 1.61 | 0.204 | 0.284 | 2.463 |
| RHSIM_Rhsim01G0021000 | 1.61 | 0.204 | 0.284 | -1.062 |
| RHSIM_RhsimUnG0129200 | 1.61 | 0.204 | 0.284 | 2.832 |
| RHSIM_Rhsim09G0211500 | 1.61 | 0.204 | 0.285 | 0.127 |
| novel.3903 | 1.61 | 0.204 | 0.285 | 1.089 |
| RHSIM_RhsimUnG0051500 | 1.61 | 0.205 | 0.285 | 0.851 |
| RHSIM_Rhsim04G0036600 | 1.61 | 0.205 | 0.286 | -0.427 |
| novel.4549 | 1.61 | 0.205 | 0.286 | 2.988 |
| novel.1930 | 1.61 | 0.205 | 0.286 | 0.287 |
| novel.927 | 1.60 | 0.205 | 0.286 | 2.491 |
| RHSIM_Rhsim11G0043700 | 1.60 | 0.205 | 0.286 | -0.357 |
| RHSIM_Rhsim13G0074800 | 1.60 | 0.205 | 0.286 | 0.305 |
| RHSIM_Rhsim13G0164700 | 1.60 | 0.205 | 0.286 | 1.154 |
| RHSIM_Rhsim01G0072100 | 1.60 | 0.206 | 0.286 | -0.552 |
| RHSIM_Rhsim06G0204000 | 1.60 | 0.206 | 0.286 | -0.198 |
| novel.4221 | 1.60 | 0.206 | 0.286 | 0.557 |
| RHSIM_Rhsim01G0164200 | 1.60 | 0.206 | 0.286 | -3.106 |
| RHSIM_Rhsim05G0065400 | 1.60 | 0.206 | 0.287 | -37.818 |
| RHSIM_Rhsim01G0087900 | 1.60 | 0.206 | 0.287 | -0.276 |
| RHSIM_Rhsim13G0041300 | 1.60 | 0.206 | 0.287 | 2.733 |
| RHSIM_Rhsim03G0100900 | 1.60 | 0.206 | 0.287 | 2.569 |
| novel.1136 | 1.60 | 0.206 | 0.287 | 0.182 |
| novel.1498 | 1.60 | 0.206 | 0.287 | 1.123 |

| Gene | LRT statistic | P value | BH-adjusted P | log2FC (interaction) |
|---|---|---|---|---|
| RHSIM_Rhsim08G0186200 | 1.60 | 0.206 | 0.287 | 2.043 |
| RHSIM_Rhsim12G0072400 | 1.60 | 0.206 | 0.287 | 0.154 |
| RHSIM_Rhsim05G0230900 | 1.60 | 0.206 | 0.287 | 0.123 |
| RHSIM_Rhsim13G0015100 | 1.60 | 0.206 | 0.287 | -0.163 |
| RHSIM_Rhsim08G0078400 | 1.60 | 0.206 | 0.287 | -0.418 |
| RHSIM_Rhsim02G0010300 | 1.60 | 0.206 | 0.287 | -0.721 |
| RHSIM_RhsimUnG0092500 | 1.60 | 0.206 | 0.287 | 0.242 |
| novel.2832 | 1.60 | 0.206 | 0.287 | 1.846 |
| RHSIM_Rhsim06G0219800 | 1.60 | 0.206 | 0.287 | -1.633 |
| novel.2297 | 1.60 | 0.207 | 0.287 | 0.799 |
| novel.1802 | 1.60 | 0.207 | 0.287 | 0.490 |
| RHSIM_Rhsim04G0218500 | 1.59 | 0.207 | 0.288 | 0.879 |
| novel.2738 | 1.59 | 0.207 | 0.288 | 0.551 |
| RHSIM_Rhsim03G0270300 | 1.59 | 0.207 | 0.288 | 2.012 |
| RHSIM_Rhsim04G0127600 | 1.59 | 0.207 | 0.288 | -0.679 |
| novel.1679 | 1.59 | 0.207 | 0.288 | -0.284 |
| RHSIM_Rhsim02G0207900 | 1.59 | 0.207 | 0.288 | 0.614 |
| RHSIM_Rhsim02G0025900 | 1.59 | 0.207 | 0.288 | -0.238 |
| RHSIM_Rhsim03G0073700 | 1.59 | 0.207 | 0.288 | 0.366 |
| novel.1380 | 1.59 | 0.207 | 0.288 | -2.241 |
| RHSIM_Rhsim13G0152200 | 1.59 | 0.207 | 0.288 | 0.381 |
| RHSIM_Rhsim12G0097000 | 1.59 | 0.207 | 0.288 | 0.608 |
| novel.2104 | 1.59 | 0.207 | 0.288 | -2.808 |
| RHSIM_RhsimPtG0002400 | 1.59 | 0.207 | 0.288 | 0.709 |
| RHSIM_Rhsim13G0133700 | 1.59 | 0.207 | 0.288 | -1.800 |
| RHSIM_Rhsim07G0126000 | 1.59 | 0.207 | 0.288 | 1.298 |
| RHSIM_Rhsim12G0091400 | 1.59 | 0.207 | 0.288 | -0.802 |
| RHSIM_Rhsim08G0082000 | 1.59 | 0.207 | 0.288 | -3.532 |
| novel.4173 | 1.59 | 0.208 | 0.288 | -1.994 |
| RHSIM_Rhsim07G0027000 | 1.59 | 0.208 | 0.289 | -0.449 |
| RHSIM_Rhsim01G0118700 | 1.59 | 0.208 | 0.289 | 0.216 |
| RHSIM_Rhsim04G0006800 | 1.59 | 0.208 | 0.289 | -0.306 |

| Gene | LRT statistic | P value | BH-adjusted P | log2FC (interaction) |
|---|---|---|---|---|
| RHSIM_Rhsim04G0027500 | 1.59 | 0.208 | 0.289 | 0.137 |
| RHSIM_Rhsim03G0165300 | 1.59 | 0.208 | 0.289 | 0.256 |
| RHSIM_Rhsim01G0143600 | 1.59 | 0.208 | 0.289 | -37.088 |
| RHSIM_Rhsim05G0109300 | 1.59 | 0.208 | 0.289 | 0.779 |
| RHSIM_Rhsim12G0095900 | 1.59 | 0.208 | 0.289 | 1.104 |
| novel.980 | 1.59 | 0.208 | 0.289 | -0.436 |
| RHSIM_Rhsim12G0078500 | 1.59 | 0.208 | 0.289 | 0.267 |
| novel.488 | 1.59 | 0.208 | 0.289 | 1.178 |
| RHSIM_Rhsim13G0200100 | 1.58 | 0.208 | 0.289 | -0.087 |
| RHSIM_Rhsim13G0055400 | 1.58 | 0.208 | 0.289 | 0.546 |
| RHSIM_Rhsim02G0127200 | 1.58 | 0.208 | 0.289 | -0.498 |
| novel.2635 | 1.58 | 0.208 | 0.289 | 0.092 |
| RHSIM_Rhsim03G0102100 | 1.58 | 0.208 | 0.289 | 1.780 |
| RHSIM_Rhsim12G0068100 | 1.58 | 0.208 | 0.289 | -0.349 |
| novel.2325 | 1.58 | 0.208 | 0.289 | 0.943 |
| RHSIM_Rhsim02G0113000 | 1.58 | 0.209 | 0.290 | 0.936 |
| RHSIM_Rhsim05G0178300 | 1.58 | 0.209 | 0.290 | -0.101 |
| RHSIM_Rhsim06G0138300 | 1.58 | 0.209 | 0.290 | 0.100 |
| RHSIM_Rhsim07G0149100 | 1.58 | 0.209 | 0.290 | 2.591 |
| novel.626 | 1.58 | 0.209 | 0.290 | 2.056 |
| RHSIM_Rhsim07G0140700 | 1.58 | 0.209 | 0.291 | 1.169 |
| RHSIM_Rhsim07G0104200 | 1.58 | 0.209 | 0.291 | 0.568 |
| RHSIM_RhsimUnG0091900 | 1.58 | 0.209 | 0.291 | -1.163 |
| RHSIM_Rhsim11G0007400 | 1.58 | 0.209 | 0.291 | 0.465 |
| RHSIM_Rhsim07G0009400 | 1.57 | 0.210 | 0.291 | 0.135 |
| novel.619 | 1.57 | 0.210 | 0.291 | -1.307 |
| novel.135 | 1.57 | 0.210 | 0.291 | -0.334 |
| RHSIM_Rhsim11G0020200 | 1.57 | 0.210 | 0.291 | 0.100 |
| novel.4187 | 1.57 | 0.210 | 0.291 | -0.420 |
| RHSIM_Rhsim03G0034800 | 1.57 | 0.210 | 0.292 | -0.767 |
| novel.3847 | 1.57 | 0.210 | 0.292 | 0.743 |
| novel.3080 | 1.57 | 0.210 | 0.292 | 1.303 |

| Gene | LRT statistic | P value | BH-adjusted P | log2FC (interaction) |
|---|---|---|---|---|
| novel.1897 | 1.57 | 0.210 | 0.292 | -0.418 |
| RHSIM_Rhsim02G0112000 | 1.57 | 0.210 | 0.292 | 0.170 |
| RHSIM_Rhsim06G0084400 | 1.57 | 0.210 | 0.292 | 1.736 |
| RHSIM_Rhsim02G0180800 | 1.57 | 0.210 | 0.292 | 0.615 |
| RHSIM_RhsimUnG0043800 | 1.57 | 0.211 | 0.292 | -0.184 |
| RHSIM_Rhsim01G0007400 | 1.57 | 0.211 | 0.292 | -2.433 |
| RHSIM_Rhsim12G0101800 | 1.57 | 0.211 | 0.292 | 2.835 |
| RHSIM_Rhsim13G0057600 | 1.57 | 0.211 | 0.292 | -0.153 |
| RHSIM_Rhsim05G0216000 | 1.57 | 0.211 | 0.292 | 0.504 |
| RHSIM_RhsimUnG0256300 | 1.57 | 0.211 | 0.292 | 4.042 |
| RHSIM_Rhsim08G0112600 | 1.57 | 0.211 | 0.293 | 2.291 |
| RHSIM_Rhsim13G0022400 | 1.57 | 0.211 | 0.293 | 0.115 |
| RHSIM_Rhsim13G0034400 | 1.56 | 0.211 | 0.293 | -0.792 |
| novel.2721 | 1.56 | 0.211 | 0.293 | 1.688 |
| RHSIM_Rhsim07G0257300 | 1.56 | 0.211 | 0.293 | -1.081 |
| RHSIM_Rhsim02G0019100 | 1.56 | 0.211 | 0.293 | 0.551 |
| RHSIM_Rhsim12G0186700 | 1.56 | 0.211 | 0.293 | 0.131 |
| RHSIM_Rhsim04G0162400 | 1.56 | 0.211 | 0.293 | -37.152 |
| RHSIM_Rhsim13G0216000 | 1.56 | 0.212 | 0.293 | 0.095 |
| RHSIM_Rhsim05G0173400 | 1.56 | 0.212 | 0.293 | -0.348 |
| RHSIM_Rhsim02G0087800 | 1.56 | 0.212 | 0.294 | 0.099 |
| RHSIM_Rhsim02G0159900 | 1.56 | 0.212 | 0.294 | -0.150 |
| RHSIM_Rhsim02G0198600 | 1.56 | 0.212 | 0.294 | -37.518 |
| RHSIM_Rhsim04G0129500 | 1.56 | 0.212 | 0.294 | 2.134 |
| novel.1710 | 1.56 | 0.212 | 0.294 | 0.791 |
| novel.3345 | 1.56 | 0.212 | 0.294 | -0.388 |
| RHSIM_Rhsim02G0059000 | 1.55 | 0.212 | 0.294 | 0.203 |
| RHSIM_Rhsim01G0261500 | 1.55 | 0.213 | 0.295 | 1.832 |
| RHSIM_Rhsim05G0201500 | 1.55 | 0.213 | 0.295 | -37.152 |
| RHSIM_Rhsim13G0047100 | 1.55 | 0.213 | 0.295 | 0.266 |
| RHSIM_Rhsim04G0092800 | 1.55 | 0.213 | 0.295 | -0.805 |
| RHSIM_Rhsim04G0036300 | 1.55 | 0.213 | 0.295 | 0.477 |

| Gene | LRT statistic | P value | BH-adjusted P | log2FC (interaction) |
|---|---|---|---|---|
| RHSIM_Rhsim09G0069500 | 1.55 | 0.213 | 0.295 | 0.107 |
| RHSIM_Rhsim04G0020700 | 1.55 | 0.213 | 0.295 | -37.266 |
| RHSIM_Rhsim04G0215100 | 1.55 | 0.213 | 0.295 | 37.173 |
| novel.2737 | 1.55 | 0.213 | 0.295 | 0.649 |
| RHSIM_Rhsim09G0150800 | 1.55 | 0.213 | 0.295 | 0.150 |
| novel.403 | 1.55 | 0.213 | 0.295 | -1.527 |
| RHSIM_Rhsim11G0090200 | 1.55 | 0.213 | 0.295 | -1.302 |
| RHSIM_Rhsim03G0127000 | 1.55 | 0.213 | 0.295 | -0.353 |
| RHSIM_Rhsim03G0137500 | 1.55 | 0.213 | 0.295 | -37.918 |
| novel.1653 | 1.55 | 0.213 | 0.295 | 0.134 |
| novel.67 | 1.55 | 0.213 | 0.295 | -1.008 |
| RHSIM_Rhsim10G0015500 | 1.55 | 0.213 | 0.295 | 1.527 |
| RHSIM_RhsimUnG0134500 | 1.55 | 0.213 | 0.295 | 0.347 |
| RHSIM_Rhsim07G0067300 | 1.55 | 0.213 | 0.295 | -0.212 |
| RHSIM_Rhsim08G0017700 | 1.55 | 0.213 | 0.295 | -37.511 |
| RHSIM_Rhsim08G0104800 | 1.55 | 0.213 | 0.295 | -0.378 |
| RHSIM_Rhsim07G0244400 | 1.55 | 0.213 | 0.296 | -0.223 |
| RHSIM_Rhsim08G0149700 | 1.55 | 0.213 | 0.296 | 0.215 |
| novel.1418 | 1.55 | 0.213 | 0.296 | 0.700 |
| novel.740 | 1.55 | 0.214 | 0.296 | 1.393 |
| RHSIM_Rhsim01G0266600 | 1.55 | 0.214 | 0.296 | 2.612 |
| RHSIM_Rhsim13G0060900 | 1.55 | 0.214 | 0.296 | -37.672 |
| RHSIM_Rhsim09G0169200 | 1.55 | 0.214 | 0.296 | -0.102 |
| RHSIM_Rhsim08G0057500 | 1.55 | 0.214 | 0.296 | 0.231 |
| RHSIM_Rhsim01G0048400 | 1.55 | 0.214 | 0.296 | 37.691 |
| RHSIM_Rhsim10G0155200 | 1.55 | 0.214 | 0.296 | -2.341 |
| RHSIM_Rhsim09G0213200 | 1.55 | 0.214 | 0.296 | 0.157 |
| novel.4145 | 1.55 | 0.214 | 0.296 | -0.597 |
| RHSIM_Rhsim08G0062400 | 1.55 | 0.214 | 0.296 | 1.149 |
| RHSIM_Rhsim06G0132900 | 1.54 | 0.214 | 0.296 | 0.935 |
| novel.982 | 1.54 | 0.214 | 0.296 | 0.561 |
| RHSIM_Rhsim07G0135000 | 1.54 | 0.214 | 0.296 | -0.485 |

| Gene | LRT statistic | P value | BH-adjusted P | log2FC (interaction) |
|---|---|---|---|---|
| RHSIM_Rhsim07G0070900 | 1.54 | 0.214 | 0.296 | -1.049 |
| RHSIM_Rhsim07G0044000 | 1.54 | 0.214 | 0.296 | 0.097 |
| novel.2843 | 1.54 | 0.214 | 0.296 | 0.099 |
| novel.2246 | 1.54 | 0.214 | 0.296 | -1.056 |
| RHSIM_Rhsim01G0125400 | 1.54 | 0.214 | 0.296 | 2.675 |
| RHSIM_Rhsim08G0059300 | 1.54 | 0.214 | 0.296 | -37.092 |
| RHSIM_Rhsim02G0055700 | 1.54 | 0.214 | 0.296 | -1.321 |
| novel.4202 | 1.54 | 0.214 | 0.296 | 1.261 |
| RHSIM_Rhsim10G0178700 | 1.54 | 0.214 | 0.297 | 0.098 |
| RHSIM_Rhsim07G0199400 | 1.54 | 0.214 | 0.297 | -0.751 |
| RHSIM_Rhsim04G0045600 | 1.54 | 0.215 | 0.297 | 2.139 |
| novel.1385 | 1.54 | 0.215 | 0.297 | 0.514 |
| RHSIM_Rhsim02G0022700 | 1.54 | 0.215 | 0.297 | -0.093 |
| novel.70 | 1.54 | 0.215 | 0.298 | 0.585 |
| RHSIM_Rhsim09G0004700 | 1.54 | 0.215 | 0.298 | 0.421 |
| novel.144 | 1.54 | 0.215 | 0.298 | 38.207 |
| RHSIM_Rhsim07G0089800 | 1.54 | 0.215 | 0.298 | 0.161 |
| RHSIM_Rhsim09G0212000 | 1.54 | 0.215 | 0.298 | -0.230 |
| RHSIM_Rhsim01G0228300 | 1.54 | 0.215 | 0.298 | 0.235 |
| RHSIM_Rhsim04G0064600 | 1.53 | 0.215 | 0.298 | -1.045 |
| RHSIM_Rhsim08G0138600 | 1.53 | 0.216 | 0.298 | -0.127 |
| RHSIM_Rhsim06G0069600 | 1.53 | 0.216 | 0.298 | 0.938 |
| RHSIM_Rhsim07G0080800 | 1.53 | 0.216 | 0.298 | -37.334 |
| RHSIM_Rhsim01G0199900 | 1.53 | 0.216 | 0.298 | 0.129 |
| RHSIM_Rhsim13G0188800 | 1.53 | 0.216 | 0.298 | -0.368 |
| RHSIM_Rhsim02G0168400 | 1.53 | 0.216 | 0.298 | -0.113 |
| RHSIM_RhsimUnG0076600 | 1.53 | 0.216 | 0.298 | 2.072 |
| novel.856 | 1.53 | 0.216 | 0.298 | -0.591 |
| novel.792 | 1.53 | 0.216 | 0.298 | 0.857 |
| RHSIM_RhsimUnG0121200 | 1.53 | 0.216 | 0.298 | 1.529 |
| novel.3386 | 1.53 | 0.216 | 0.298 | -0.394 |
| RHSIM_Rhsim03G0263800 | 1.53 | 0.216 | 0.298 | 37.541 |

| Gene | LRT statistic | P value | BH-adjusted P | log2FC (interaction) |
|---|---|---|---|---|
| novel.692 | 1.53 | 0.216 | 0.299 | -0.346 |
| RHSIM_Rhsim01G0134600 | 1.53 | 0.216 | 0.299 | 0.221 |
| novel.2300 | 1.53 | 0.217 | 0.299 | 0.951 |
| RHSIM_Rhsim05G0220200 | 1.53 | 0.217 | 0.299 | 1.193 |
| RHSIM_Rhsim12G0157600 | 1.53 | 0.217 | 0.299 | -0.350 |
| RHSIM_Rhsim10G0118300 | 1.53 | 0.217 | 0.299 | -0.751 |
| RHSIM_Rhsim02G0041600 | 1.53 | 0.217 | 0.299 | 0.380 |
| RHSIM_Rhsim11G0176500 | 1.53 | 0.217 | 0.299 | -2.634 |
| RHSIM_Rhsim13G0102900 | 1.53 | 0.217 | 0.299 | 0.221 |
| RHSIM_Rhsim07G0061800 | 1.53 | 0.217 | 0.299 | -0.103 |
| novel.983 | 1.53 | 0.217 | 0.299 | -0.429 |
| RHSIM_Rhsim07G0037600 | 1.53 | 0.217 | 0.299 | 0.238 |
| RHSIM_Rhsim13G0191400 | 1.52 | 0.217 | 0.300 | 37.472 |
| RHSIM_RhsimUnG0063100 | 1.52 | 0.217 | 0.300 | -37.715 |
| RHSIM_Rhsim11G0074200 | 1.52 | 0.217 | 0.300 | -37.639 |
| RHSIM_RhsimUnG0187400 | 1.52 | 0.217 | 0.300 | 0.273 |
| RHSIM_Rhsim09G0211000 | 1.52 | 0.217 | 0.300 | 0.111 |
| RHSIM_Rhsim04G0161200 | 1.52 | 0.218 | 0.300 | 37.314 |
| RHSIM_Rhsim03G0015100 | 1.52 | 0.218 | 0.300 | 0.372 |
| novel.2618 | 1.52 | 0.218 | 0.300 | 0.177 |
| RHSIM_Rhsim13G0235800 | 1.52 | 0.218 | 0.300 | 1.664 |
| RHSIM_Rhsim07G0159400 | 1.52 | 0.218 | 0.301 | -2.005 |
| RHSIM_RhsimUnG0061700 | 1.52 | 0.218 | 0.301 | 1.721 |
| RHSIM_Rhsim06G0143200 | 1.52 | 0.218 | 0.301 | 2.803 |
| RHSIM_Rhsim02G0205800 | 1.52 | 0.218 | 0.301 | 1.278 |
| novel.3697 | 1.52 | 0.218 | 0.301 | 1.245 |
| RHSIM_Rhsim09G0005800 | 1.52 | 0.218 | 0.301 | -0.238 |
| RHSIM_RhsimUnG0192300 | 1.52 | 0.218 | 0.301 | -0.586 |
| RHSIM_Rhsim07G0103400 | 1.52 | 0.218 | 0.301 | 0.147 |
| RHSIM_Rhsim01G0074500 | 1.51 | 0.218 | 0.301 | 0.173 |
| novel.1233 | 1.51 | 0.219 | 0.301 | -1.091 |
| RHSIM_Rhsim09G0155700 | 1.51 | 0.219 | 0.301 | 0.143 |

| Gene | LRT statistic | P value | BH-adjusted P | log2FC (interaction) |
|---|---|---|---|---|
| RHSIM_Rhsim03G0015900 | 1.51 | 0.219 | 0.301 | 0.718 |
| RHSIM_Rhsim07G0069100 | 1.51 | 0.219 | 0.302 | 37.787 |
| RHSIM_Rhsim02G0162200 | 1.51 | 0.219 | 0.302 | 0.768 |
| RHSIM_Rhsim06G0200000 | 1.51 | 0.219 | 0.302 | 37.445 |
| RHSIM_Rhsim01G0047800 | 1.51 | 0.219 | 0.302 | -0.832 |
| RHSIM_RhsimUnG0033900 | 1.51 | 0.219 | 0.302 | 1.360 |
| RHSIM_Rhsim13G0092800 | 1.51 | 0.219 | 0.302 | 1.737 |
| RHSIM_Rhsim07G0016000 | 1.51 | 0.219 | 0.302 | 0.246 |
| RHSIM_Rhsim02G0238300 | 1.51 | 0.219 | 0.302 | 0.246 |
| RHSIM_RhsimUnG0088100 | 1.51 | 0.219 | 0.302 | -0.441 |
| RHSIM_Rhsim04G0242000 | 1.51 | 0.219 | 0.302 | 0.433 |
| RHSIM_Rhsim07G0151300 | 1.51 | 0.219 | 0.302 | 1.057 |
| novel.2682 | 1.51 | 0.219 | 0.302 | -0.108 |
| RHSIM_Rhsim11G0058000 | 1.51 | 0.219 | 0.302 | 0.440 |
| RHSIM_Rhsim10G0212400 | 1.51 | 0.220 | 0.303 | 0.619 |
| RHSIM_Rhsim09G0024900 | 1.51 | 0.220 | 0.303 | -37.247 |
| RHSIM_Rhsim10G0167200 | 1.51 | 0.220 | 0.303 | 0.538 |
| RHSIM_Rhsim11G0089700 | 1.51 | 0.220 | 0.303 | 0.538 |
| RHSIM_Rhsim03G0236400 | 1.51 | 0.220 | 0.303 | -2.266 |
| RHSIM_Rhsim08G0176900 | 1.51 | 0.220 | 0.303 | -37.606 |
| RHSIM_Rhsim12G0098900 | 1.51 | 0.220 | 0.303 | -0.570 |
| RHSIM_Rhsim01G0265000 | 1.51 | 0.220 | 0.303 | -37.679 |
| RHSIM_Rhsim05G0180500 | 1.51 | 0.220 | 0.303 | 2.378 |
| RHSIM_Rhsim06G0200300 | 1.50 | 0.220 | 0.303 | 2.499 |
| RHSIM_Rhsim03G0085100 | 1.50 | 0.220 | 0.303 | 0.166 |
| RHSIM_Rhsim07G0053000 | 1.50 | 0.220 | 0.303 | 0.586 |
| RHSIM_Rhsim05G0125400 | 1.50 | 0.220 | 0.303 | 0.938 |
| RHSIM_Rhsim03G0046700 | 1.50 | 0.220 | 0.303 | -37.899 |
| RHSIM_Rhsim02G0083800 | 1.50 | 0.220 | 0.303 | 1.089 |
| novel.2395 | 1.50 | 0.220 | 0.304 | -0.452 |
| novel.3364 | 1.50 | 0.220 | 0.304 | 1.507 |
| RHSIM_Rhsim03G0166400 | 1.50 | 0.221 | 0.304 | 0.375 |

| Gene | LRT statistic | P value | BH-adjusted P | log2FC (interaction) |
|---|---|---|---|---|
| RHSIM_Rhsim07G0248400 | 1.50 | 0.221 | 0.304 | 1.365 |
| RHSIM_Rhsim11G0060000 | 1.50 | 0.221 | 0.304 | 1.761 |
| novel.2329 | 1.50 | 0.221 | 0.304 | -0.486 |
| RHSIM_Rhsim04G0144600 | 1.50 | 0.221 | 0.304 | 1.544 |
| RHSIM_RhsimUnG0081200 | 1.50 | 0.221 | 0.304 | 0.236 |
| RHSIM_Rhsim07G0120000 | 1.50 | 0.221 | 0.304 | -0.337 |
| RHSIM_Rhsim04G0165900 | 1.50 | 0.221 | 0.304 | 1.582 |
| novel.2384 | 1.50 | 0.221 | 0.305 | -2.275 |
| RHSIM_Rhsim01G0124200 | 1.50 | 0.221 | 0.305 | 3.192 |
| RHSIM_Rhsim03G0087500 | 1.49 | 0.222 | 0.305 | 37.806 |
| RHSIM_Rhsim01G0010400 | 1.49 | 0.222 | 0.305 | 0.263 |
| RHSIM_Rhsim07G0106900 | 1.49 | 0.222 | 0.305 | 0.483 |
| RHSIM_RhsimUnG0008800 | 1.49 | 0.222 | 0.305 | 2.525 |
| RHSIM_Rhsim09G0189400 | 1.49 | 0.222 | 0.305 | 2.468 |
| RHSIM_Rhsim13G0141300 | 1.49 | 0.222 | 0.305 | -0.294 |
| RHSIM_Rhsim09G0135200 | 1.49 | 0.222 | 0.305 | -0.260 |
| RHSIM_Rhsim07G0197500 | 1.49 | 0.222 | 0.305 | 1.684 |
| novel.4262 | 1.49 | 0.222 | 0.305 | 2.027 |
| RHSIM_Rhsim06G0188000 | 1.49 | 0.222 | 0.305 | 37.880 |
| RHSIM_Rhsim07G0178400 | 1.49 | 0.222 | 0.306 | 0.906 |
| RHSIM_Rhsim09G0029100 | 1.49 | 0.222 | 0.306 | 37.555 |
| RHSIM_Rhsim09G0147600 | 1.49 | 0.222 | 0.306 | 0.783 |
| RHSIM_Rhsim05G0070500 | 1.49 | 0.222 | 0.306 | -0.200 |
| novel.1357 | 1.49 | 0.222 | 0.306 | 1.157 |
| RHSIM_Rhsim13G0053300 | 1.49 | 0.222 | 0.306 | -0.219 |
| RHSIM_Rhsim10G0049400 | 1.49 | 0.222 | 0.306 | -0.097 |
| novel.1473 | 1.49 | 0.222 | 0.306 | -0.963 |
| RHSIM_Rhsim03G0159600 | 1.49 | 0.222 | 0.306 | -1.589 |
| RHSIM_Rhsim10G0195900 | 1.49 | 0.222 | 0.306 | 0.435 |
| novel.1884 | 1.49 | 0.223 | 0.306 | 1.573 |
| RHSIM_Rhsim04G0182400 | 1.49 | 0.223 | 0.306 | 1.209 |
| RHSIM_Rhsim03G0043200 | 1.49 | 0.223 | 0.306 | 1.057 |

| Gene | LRT statistic | P value | BH-adjusted P | log2FC (interaction) |
|---|---|---|---|---|
| RHSIM_Rhsim03G0109500 | 1.49 | 0.223 | 0.307 | 1.077 |
| RHSIM_Rhsim11G0092200 | 1.48 | 0.223 | 0.307 | 0.109 |
| RHSIM_Rhsim02G0185600 | 1.48 | 0.223 | 0.307 | 36.963 |
| RHSIM_Rhsim13G0044600 | 1.48 | 0.223 | 0.307 | 0.239 |
| RHSIM_Rhsim12G0044900 | 1.48 | 0.223 | 0.307 | -1.092 |
| novel.3163 | 1.48 | 0.223 | 0.307 | -0.267 |
| RHSIM_Rhsim10G0204600 | 1.48 | 0.223 | 0.307 | -0.310 |
| RHSIM_Rhsim04G0203900 | 1.48 | 0.223 | 0.307 | 0.298 |
| RHSIM_Rhsim10G0096900 | 1.48 | 0.223 | 0.307 | 0.351 |
| RHSIM_Rhsim05G0101200 | 1.48 | 0.223 | 0.307 | 1.247 |
| RHSIM_Rhsim13G0134800 | 1.48 | 0.224 | 0.307 | 0.119 |
| RHSIM_Rhsim13G0206900 | 1.48 | 0.224 | 0.307 | -2.165 |
| RHSIM_Rhsim10G0053100 | 1.48 | 0.224 | 0.307 | -0.173 |
| RHSIM_Rhsim12G0197200 | 1.48 | 0.224 | 0.308 | -0.125 |
| RHSIM_Rhsim03G0179100 | 1.48 | 0.224 | 0.308 | -0.159 |
| novel.544 | 1.48 | 0.224 | 0.308 | -0.480 |
| RHSIM_Rhsim07G0173700 | 1.48 | 0.224 | 0.308 | 0.367 |
| RHSIM_Rhsim08G0095300 | 1.48 | 0.224 | 0.308 | 1.365 |
| RHSIM_Rhsim13G0131500 | 1.48 | 0.224 | 0.308 | -0.114 |
| RHSIM_Rhsim07G0183800 | 1.48 | 0.224 | 0.308 | 0.458 |
| RHSIM_Rhsim05G0001000 | 1.48 | 0.224 | 0.308 | 0.332 |
| RHSIM_Rhsim06G0243500 | 1.48 | 0.224 | 0.308 | 1.217 |
| RHSIM_Rhsim07G0166000 | 1.48 | 0.224 | 0.308 | -37.151 |
| RHSIM_Rhsim06G0244600 | 1.48 | 0.224 | 0.308 | -0.129 |
| RHSIM_Rhsim05G0087800 | 1.48 | 0.224 | 0.308 | -0.133 |
| novel.3474 | 1.48 | 0.224 | 0.308 | -1.675 |
| RHSIM_Rhsim08G0049800 | 1.48 | 0.224 | 0.308 | -37.331 |
| RHSIM_Rhsim02G0000400 | 1.47 | 0.225 | 0.308 | -2.678 |
| RHSIM_Rhsim05G0097400 | 1.47 | 0.225 | 0.309 | 0.873 |
| novel.1820 | 1.47 | 0.225 | 0.309 | -1.659 |
| novel.657 | 1.47 | 0.225 | 0.309 | -0.555 |
| RHSIM_Rhsim02G0008700 | 1.47 | 0.225 | 0.309 | 0.745 |

| Gene | LRT statistic | P value | BH-adjusted P | log2FC (interaction) |
|---|---|---|---|---|
| RHSIM_Rhsim13G0157100 | 1.47 | 0.225 | 0.309 | -0.563 |
| RHSIM_Rhsim12G0212000 | 1.47 | 0.225 | 0.309 | 0.840 |
| RHSIM_Rhsim05G0140900 | 1.47 | 0.225 | 0.309 | 2.127 |
| RHSIM_Rhsim03G0097100 | 1.47 | 0.225 | 0.309 | 0.437 |
| RHSIM_Rhsim12G0073000 | 1.47 | 0.225 | 0.309 | 1.823 |
| RHSIM_Rhsim01G0197700 | 1.47 | 0.225 | 0.309 | 1.893 |
| RHSIM_Rhsim04G0172000 | 1.47 | 0.225 | 0.309 | -0.281 |
| RHSIM_Rhsim03G0093200 | 1.47 | 0.226 | 0.310 | 0.109 |
| novel.979 | 1.47 | 0.226 | 0.310 | 1.121 |
| RHSIM_Rhsim08G0051200 | 1.47 | 0.226 | 0.310 | 0.263 |
| RHSIM_Rhsim13G0177100 | 1.47 | 0.226 | 0.310 | -0.439 |
| RHSIM_Rhsim13G0111100 | 1.47 | 0.226 | 0.310 | 37.055 |
| RHSIM_Rhsim01G0197800 | 1.47 | 0.226 | 0.310 | 3.347 |
| RHSIM_Rhsim04G0213000 | 1.47 | 0.226 | 0.310 | 0.175 |
| RHSIM_Rhsim12G0027500 | 1.47 | 0.226 | 0.310 | -37.780 |
| RHSIM_Rhsim13G0021600 | 1.47 | 0.226 | 0.310 | 1.638 |
| novel.2991 | 1.47 | 0.226 | 0.310 | -1.642 |
| RHSIM_Rhsim12G0044400 | 1.47 | 0.226 | 0.310 | -1.146 |
| RHSIM_Rhsim02G0189800 | 1.47 | 0.226 | 0.310 | -1.602 |
| RHSIM_Rhsim05G0148300 | 1.46 | 0.226 | 0.310 | 0.141 |
| RHSIM_Rhsim06G0207900 | 1.46 | 0.226 | 0.310 | 1.441 |
| RHSIM_Rhsim08G0158600 | 1.46 | 0.226 | 0.310 | 37.494 |
| novel.1998 | 1.46 | 0.226 | 0.310 | 0.924 |
| novel.240 | 1.46 | 0.226 | 0.311 | 1.358 |
| RHSIM_Rhsim05G0136100 | 1.46 | 0.227 | 0.311 | 0.806 |
| RHSIM_Rhsim01G0188100 | 1.46 | 0.227 | 0.311 | -1.051 |
| RHSIM_Rhsim13G0216800 | 1.46 | 0.227 | 0.311 | -0.889 |
| novel.3246 | 1.46 | 0.227 | 0.311 | 0.300 |
| novel.4472 | 1.46 | 0.227 | 0.311 | -0.123 |
| RHSIM_Rhsim01G0259700 | 1.46 | 0.227 | 0.311 | 0.255 |
| RHSIM_Rhsim08G0062700 | 1.46 | 0.227 | 0.311 | 0.135 |
| RHSIM_Rhsim01G0213700 | 1.46 | 0.227 | 0.311 | -2.346 |

| Gene | LRT statistic | P value | BH-adjusted P | log2FC (interaction) |
|---|---|---|---|---|
| RHSIM_Rhsim08G0237100 | 1.46 | 0.227 | 0.311 | 0.129 |
| RHSIM_Rhsim09G0193600 | 1.46 | 0.227 | 0.311 | 0.225 |
| RHSIM_RhsimUnG0021900 | 1.46 | 0.227 | 0.311 | 0.611 |
| novel.2614 | 1.46 | 0.227 | 0.311 | -0.879 |
| RHSIM_Rhsim01G0137500 | 1.46 | 0.227 | 0.312 | -0.255 |
| novel.3198 | 1.46 | 0.227 | 0.312 | -1.254 |
| RHSIM_Rhsim05G0153700 | 1.46 | 0.227 | 0.312 | 0.443 |
| RHSIM_Rhsim05G0109400 | 1.46 | 0.227 | 0.312 | 0.217 |
| novel.3416 | 1.46 | 0.228 | 0.312 | -2.149 |
| RHSIM_RhsimUnG0157600 | 1.46 | 0.228 | 0.312 | 2.317 |
| novel.2555 | 1.45 | 0.228 | 0.312 | 0.352 |
| RHSIM_Rhsim10G0055300 | 1.45 | 0.228 | 0.312 | -1.743 |
| RHSIM_Rhsim05G0138400 | 1.45 | 0.228 | 0.312 | -0.367 |
| RHSIM_Rhsim03G0133900 | 1.45 | 0.228 | 0.312 | -0.451 |
| RHSIM_Rhsim02G0051800 | 1.45 | 0.228 | 0.313 | -1.294 |
| RHSIM_Rhsim13G0117900 | 1.45 | 0.228 | 0.313 | -2.315 |
| RHSIM_RhsimUnG0095700 | 1.45 | 0.228 | 0.313 | 0.320 |
| RHSIM_Rhsim04G0194500 | 1.45 | 0.229 | 0.313 | 0.933 |
| RHSIM_Rhsim04G0097100 | 1.45 | 0.229 | 0.313 | -0.637 |
| RHSIM_Rhsim08G0218700 | 1.45 | 0.229 | 0.313 | 0.187 |
| RHSIM_Rhsim12G0013900 | 1.45 | 0.229 | 0.313 | 0.200 |
| RHSIM_RhsimUnG0123000 | 1.45 | 0.229 | 0.313 | -0.236 |
| novel.679 | 1.45 | 0.229 | 0.313 | 0.186 |
| RHSIM_Rhsim03G0003500 | 1.45 | 0.229 | 0.313 | -0.158 |
| novel.1739 | 1.45 | 0.229 | 0.313 | 0.542 |
| RHSIM_Rhsim13G0178300 | 1.45 | 0.229 | 0.314 | 1.781 |
| novel.2653 | 1.45 | 0.229 | 0.314 | 0.367 |
| RHSIM_Rhsim04G0050600 | 1.45 | 0.229 | 0.314 | 0.123 |
| novel.2386 | 1.45 | 0.229 | 0.314 | -1.212 |
| RHSIM_Rhsim09G0101100 | 1.45 | 0.229 | 0.314 | -0.237 |
| RHSIM_Rhsim01G0241300 | 1.45 | 0.229 | 0.314 | 1.437 |
| RHSIM_Rhsim11G0190200 | 1.44 | 0.229 | 0.314 | -37.736 |

| Gene | LRT statistic | P value | BH-adjusted P | log2FC (interaction) |
|---|---|---|---|---|
| RHSIM_Rhsim13G0095800 | 1.44 | 0.229 | 0.314 | -0.183 |
| RHSIM_RhsimUnG0093600 | 1.44 | 0.229 | 0.314 | -1.824 |
| RHSIM_Rhsim01G0024300 | 1.44 | 0.229 | 0.314 | 0.841 |
| RHSIM_Rhsim10G0080000 | 1.44 | 0.230 | 0.314 | 1.387 |
| RHSIM_Rhsim04G0143800 | 1.44 | 0.230 | 0.314 | 1.569 |
| RHSIM_Rhsim11G0041300 | 1.44 | 0.230 | 0.314 | -0.122 |
| RHSIM_Rhsim04G0145000 | 1.44 | 0.230 | 0.314 | 37.278 |
| RHSIM_Rhsim04G0121200 | 1.44 | 0.230 | 0.314 | -37.181 |
| RHSIM_Rhsim11G0123900 | 1.44 | 0.230 | 0.314 | -0.813 |
| RHSIM_Rhsim10G0119400 | 1.44 | 0.230 | 0.314 | 2.393 |
| RHSIM_Rhsim08G0140700 | 1.44 | 0.230 | 0.314 | -1.721 |
| novel.4398 | 1.44 | 0.230 | 0.314 | -0.869 |
| RHSIM_Rhsim11G0073100 | 1.44 | 0.230 | 0.314 | -0.857 |
| novel.410 | 1.44 | 0.230 | 0.314 | 0.595 |
| novel.855 | 1.44 | 0.230 | 0.314 | 0.488 |
| RHSIM_Rhsim01G0134800 | 1.44 | 0.230 | 0.314 | 1.590 |
| novel.2100 | 1.44 | 0.230 | 0.314 | 0.858 |
| RHSIM_Rhsim04G0054600 | 1.44 | 0.230 | 0.314 | 0.902 |
| RHSIM_Rhsim10G0068200 | 1.44 | 0.230 | 0.314 | 2.817 |
| RHSIM_Rhsim13G0165200 | 1.44 | 0.230 | 0.315 | -0.420 |
| RHSIM_Rhsim09G0201900 | 1.44 | 0.230 | 0.315 | 0.434 |
| RHSIM_Rhsim08G0027600 | 1.44 | 0.230 | 0.315 | -0.298 |
| novel.2533 | 1.44 | 0.230 | 0.315 | -0.582 |
| RHSIM_Rhsim04G0086500 | 1.44 | 0.230 | 0.315 | -0.741 |
| RHSIM_Rhsim12G0070500 | 1.44 | 0.230 | 0.315 | -0.266 |
| RHSIM_Rhsim02G0039800 | 1.44 | 0.230 | 0.315 | -37.658 |
| novel.1508 | 1.44 | 0.230 | 0.315 | 0.537 |
| RHSIM_Rhsim10G0057600 | 1.44 | 0.230 | 0.315 | 2.084 |
| RHSIM_Rhsim07G0121900 | 1.44 | 0.230 | 0.315 | 0.094 |
| RHSIM_Rhsim09G0022100 | 1.44 | 0.230 | 0.315 | 0.699 |
| novel.990 | 1.44 | 0.230 | 0.315 | 1.092 |
| RHSIM_Rhsim06G0222400 | 1.44 | 0.231 | 0.315 | -0.103 |

| Gene | LRT statistic | P value | BH-adjusted P | log2FC (interaction) |
|---|---|---|---|---|
| RHSIM_RhsimUnG0050700 | 1.43 | 0.231 | 0.316 | 0.600 |
| RHSIM_Rhsim08G0070700 | 1.43 | 0.231 | 0.316 | 0.232 |
| RHSIM_RhsimUnG0220000 | 1.43 | 0.231 | 0.316 | -0.207 |
| RHSIM_RhsimUnG0187500 | 1.43 | 0.231 | 0.316 | 0.391 |
| RHSIM_Rhsim01G0166600 | 1.43 | 0.231 | 0.316 | 1.771 |
| RHSIM_Rhsim04G0055700 | 1.43 | 0.231 | 0.316 | -1.488 |
| RHSIM_Rhsim09G0179400 | 1.43 | 0.231 | 0.316 | -0.153 |
| RHSIM_Rhsim12G0169800 | 1.43 | 0.231 | 0.316 | 0.125 |
| RHSIM_RhsimUnG0231300 | 1.43 | 0.231 | 0.316 | -37.044 |
| novel.1672 | 1.43 | 0.231 | 0.316 | 1.125 |
| RHSIM_Rhsim03G0153900 | 1.43 | 0.231 | 0.316 | -0.383 |
| RHSIM_Rhsim09G0047600 | 1.43 | 0.231 | 0.316 | -2.228 |
| RHSIM_RhsimUnG0101900 | 1.43 | 0.232 | 0.316 | 1.120 |
| RHSIM_RhsimUnG0131600 | 1.43 | 0.232 | 0.316 | -1.079 |
| RHSIM_Rhsim01G0221900 | 1.43 | 0.232 | 0.316 | -0.302 |
| RHSIM_RhsimUnG0041900 | 1.43 | 0.232 | 0.317 | 0.677 |
| RHSIM_Rhsim13G0091500 | 1.43 | 0.232 | 0.317 | -0.231 |
| RHSIM_Rhsim01G0259000 | 1.43 | 0.232 | 0.317 | 1.998 |
| novel.2302 | 1.43 | 0.232 | 0.317 | 0.207 |
| RHSIM_Rhsim13G0018000 | 1.43 | 0.232 | 0.317 | -0.394 |
| RHSIM_Rhsim13G0159600 | 1.43 | 0.232 | 0.317 | 1.426 |
| RHSIM_Rhsim06G0177000 | 1.43 | 0.232 | 0.317 | 0.794 |
| RHSIM_Rhsim13G0063600 | 1.43 | 0.232 | 0.317 | -1.745 |
| novel.2222 | 1.43 | 0.232 | 0.317 | -0.481 |
| RHSIM_Rhsim01G0135400 | 1.43 | 0.232 | 0.317 | -37.214 |
| RHSIM_Rhsim12G0058100 | 1.43 | 0.232 | 0.317 | -0.055 |
| RHSIM_Rhsim04G0241300 | 1.43 | 0.232 | 0.317 | -36.894 |
| RHSIM_Rhsim01G0047100 | 1.43 | 0.232 | 0.317 | -1.634 |
| RHSIM_Rhsim06G0196000 | 1.42 | 0.233 | 0.318 | 0.322 |
| RHSIM_Rhsim04G0102200 | 1.42 | 0.233 | 0.318 | -0.534 |
| novel.4355 | 1.42 | 0.233 | 0.318 | 0.367 |
| novel.3032 | 1.42 | 0.233 | 0.318 | -0.678 |

| Gene | LRT statistic | P value | BH-adjusted P | log2FC (interaction) |
|---|---|---|---|---|
| RHSIM_Rhsim12G0064600 | 1.42 | 0.233 | 0.318 | 0.116 |
| RHSIM_Rhsim09G0058500 | 1.42 | 0.233 | 0.318 | -0.095 |
| RHSIM_Rhsim08G0105400 | 1.42 | 0.234 | 0.319 | 37.359 |
| RHSIM_RhsimUnG0230500 | 1.42 | 0.234 | 0.319 | 37.467 |
| RHSIM_Rhsim01G0182200 | 1.42 | 0.234 | 0.319 | 0.144 |
| novel.2926 | 1.42 | 0.234 | 0.319 | 2.016 |
| RHSIM_Rhsim02G0233300 | 1.42 | 0.234 | 0.319 | 0.446 |
| novel.2593 | 1.42 | 0.234 | 0.319 | 1.204 |
| RHSIM_Rhsim13G0195500 | 1.42 | 0.234 | 0.319 | 0.839 |
| RHSIM_Rhsim03G0238800 | 1.42 | 0.234 | 0.319 | -0.105 |
| RHSIM_Rhsim07G0025700 | 1.42 | 0.234 | 0.319 | 0.549 |
| RHSIM_Rhsim13G0229500 | 1.42 | 0.234 | 0.319 | -0.360 |
| novel.2044 | 1.42 | 0.234 | 0.319 | -1.514 |
| novel.4094 | 1.42 | 0.234 | 0.319 | -37.037 |
| novel.616 | 1.42 | 0.234 | 0.319 | 1.051 |
| novel.1363 | 1.42 | 0.234 | 0.319 | -1.549 |
| RHSIM_Rhsim03G0250600 | 1.42 | 0.234 | 0.319 | 0.371 |
| RHSIM_Rhsim01G0233000 | 1.41 | 0.234 | 0.319 | -1.522 |
| novel.4016 | 1.41 | 0.234 | 0.320 | -0.955 |
| RHSIM_Rhsim10G0156200 | 1.41 | 0.234 | 0.320 | -0.136 |
| RHSIM_Rhsim02G0135100 | 1.41 | 0.235 | 0.320 | 37.249 |
| novel.4188 | 1.41 | 0.235 | 0.320 | 1.183 |
| RHSIM_Rhsim08G0209900 | 1.41 | 0.235 | 0.320 | 0.209 |
| RHSIM_Rhsim07G0151500 | 1.41 | 0.235 | 0.320 | -3.150 |
| novel.1914 | 1.41 | 0.235 | 0.320 | 0.304 |
| novel.4246 | 1.41 | 0.235 | 0.320 | -1.118 |
| RHSIM_Rhsim12G0086900 | 1.41 | 0.235 | 0.320 | -0.192 |
| RHSIM_Rhsim05G0060900 | 1.41 | 0.235 | 0.320 | -36.811 |
| RHSIM_Rhsim03G0069600 | 1.41 | 0.235 | 0.320 | 0.197 |
| RHSIM_Rhsim02G0028300 | 1.41 | 0.235 | 0.320 | 37.426 |
| novel.1855 | 1.41 | 0.236 | 0.321 | 0.430 |
| RHSIM_Rhsim05G0097600 | 1.41 | 0.236 | 0.321 | 1.046 |

| Gene | LRT statistic | P value | BH-adjusted P | log2FC (interaction) |
|---|---|---|---|---|
| RHSIM_Rhsim08G0156800 | 1.41 | 0.236 | 0.321 | -1.303 |
| RHSIM_Rhsim10G0158100 | 1.41 | 0.236 | 0.321 | 0.234 |
| RHSIM_Rhsim08G0118000 | 1.41 | 0.236 | 0.321 | -0.722 |
| RHSIM_Rhsim03G0058300 | 1.41 | 0.236 | 0.321 | -0.249 |
| RHSIM_Rhsim02G0156900 | 1.41 | 0.236 | 0.321 | 1.608 |
| RHSIM_Rhsim06G0172200 | 1.41 | 0.236 | 0.321 | -37.306 |
| RHSIM_Rhsim02G0182400 | 1.41 | 0.236 | 0.321 | 0.104 |
| RHSIM_Rhsim11G0083100 | 1.40 | 0.236 | 0.321 | 0.115 |
| RHSIM_Rhsim04G0070100 | 1.40 | 0.236 | 0.321 | -0.136 |
| RHSIM_Rhsim03G0147400 | 1.40 | 0.236 | 0.321 | 0.814 |
| RHSIM_Rhsim04G0159400 | 1.40 | 0.236 | 0.321 | 1.771 |
| RHSIM_Rhsim08G0223000 | 1.40 | 0.236 | 0.322 | 0.396 |
| RHSIM_Rhsim12G0004300 | 1.40 | 0.236 | 0.322 | -0.249 |
| RHSIM_Rhsim12G0150000 | 1.40 | 0.236 | 0.322 | -0.390 |
| RHSIM_RhsimUnG0254200 | 1.40 | 0.236 | 0.322 | -0.183 |
| RHSIM_RhsimUnG0174400 | 1.40 | 0.236 | 0.322 | 1.244 |
| RHSIM_Rhsim10G0215500 | 1.40 | 0.236 | 0.322 | 2.329 |
| RHSIM_Rhsim13G0090100 | 1.40 | 0.237 | 0.322 | 0.873 |
| novel.4267 | 1.40 | 0.237 | 0.322 | -2.026 |
| RHSIM_Rhsim12G0142600 | 1.40 | 0.237 | 0.322 | 1.217 |
| RHSIM_Rhsim10G0153700 | 1.40 | 0.237 | 0.322 | 0.850 |
| RHSIM_Rhsim08G0216600 | 1.40 | 0.237 | 0.322 | 0.113 |
| RHSIM_Rhsim04G0147600 | 1.40 | 0.237 | 0.323 | 1.877 |
| RHSIM_Rhsim05G0092100 | 1.40 | 0.237 | 0.323 | -1.048 |
| RHSIM_Rhsim06G0105800 | 1.40 | 0.237 | 0.323 | 0.856 |
| novel.3291 | 1.40 | 0.237 | 0.323 | -1.453 |
| novel.1972 | 1.40 | 0.237 | 0.323 | -0.922 |
| RHSIM_Rhsim12G0150300 | 1.40 | 0.237 | 0.323 | -0.993 |
| novel.3004 | 1.40 | 0.238 | 0.323 | 0.925 |
| RHSIM_Rhsim09G0019400 | 1.39 | 0.238 | 0.323 | 0.457 |
| RHSIM_Rhsim04G0068200 | 1.39 | 0.238 | 0.323 | -0.502 |
| RHSIM_Rhsim12G0133600 | 1.39 | 0.238 | 0.323 | 0.237 |

| Gene | LRT statistic | P value | BH-adjusted P | log2FC (interaction) |
|---|---|---|---|---|
| RHSIM_Rhsim04G0018100 | 1.39 | 0.238 | 0.323 | 0.259 |
| RHSIM_RhsimUnG0222700 | 1.39 | 0.238 | 0.324 | 1.053 |
| RHSIM_Rhsim01G0205200 | 1.39 | 0.238 | 0.324 | -0.556 |
| RHSIM_Rhsim03G0243200 | 1.39 | 0.238 | 0.324 | -0.080 |
| RHSIM_Rhsim08G0115400 | 1.39 | 0.238 | 0.324 | 0.269 |
| RHSIM_Rhsim03G0261400 | 1.39 | 0.238 | 0.324 | -0.202 |
| RHSIM_Rhsim01G0215600 | 1.39 | 0.238 | 0.324 | -37.747 |
| RHSIM_Rhsim13G0083400 | 1.39 | 0.238 | 0.324 | -2.088 |
| novel.3659 | 1.39 | 0.238 | 0.324 | -0.457 |
| RHSIM_Rhsim13G0061100 | 1.39 | 0.239 | 0.325 | 37.547 |
| RHSIM_Rhsim05G0210400 | 1.39 | 0.239 | 0.325 | -0.293 |
| novel.374 | 1.39 | 0.239 | 0.325 | -2.490 |
| RHSIM_Rhsim09G0160400 | 1.39 | 0.239 | 0.325 | 0.172 |
| RHSIM_Rhsim10G0008900 | 1.39 | 0.239 | 0.325 | 0.130 |
| novel.2218 | 1.39 | 0.239 | 0.325 | -0.660 |
| novel.594 | 1.39 | 0.239 | 0.325 | 0.452 |
| RHSIM_Rhsim13G0194400 | 1.39 | 0.239 | 0.325 | 36.887 |
| novel.316 | 1.39 | 0.239 | 0.325 | -0.595 |
| novel.2282 | 1.39 | 0.239 | 0.325 | 0.528 |
| RHSIM_Rhsim02G0128500 | 1.38 | 0.239 | 0.325 | 0.911 |
| RHSIM_Rhsim10G0173500 | 1.38 | 0.240 | 0.326 | 0.248 |
| RHSIM_RhsimUnG0201900 | 1.38 | 0.240 | 0.326 | -0.445 |
| RHSIM_RhsimUnG0143100 | 1.38 | 0.240 | 0.326 | 1.296 |
| RHSIM_Rhsim01G0200100 | 1.38 | 0.240 | 0.326 | 0.245 |
| RHSIM_Rhsim01G0214500 | 1.38 | 0.240 | 0.326 | 0.927 |
| RHSIM_Rhsim05G0197300 | 1.38 | 0.240 | 0.326 | -0.227 |
| RHSIM_Rhsim06G0115500 | 1.38 | 0.240 | 0.326 | -1.511 |
| novel.1702 | 1.38 | 0.240 | 0.326 | -0.567 |
| RHSIM_Rhsim06G0083300 | 1.38 | 0.240 | 0.326 | -0.417 |
| RHSIM_Rhsim04G0224500 | 1.38 | 0.240 | 0.326 | -0.352 |
| RHSIM_Rhsim10G0199200 | 1.38 | 0.240 | 0.326 | 0.436 |
| novel.1479 | 1.38 | 0.240 | 0.326 | 0.676 |

| Gene | LRT statistic | P value | BH-adjusted P | log2FC (interaction) |
|---|---|---|---|---|
| RHSIM_Rhsim12G0108300 | 1.38 | 0.240 | 0.326 | 1.527 |
| RHSIM_Rhsim04G0102400 | 1.38 | 0.240 | 0.327 | -1.839 |
| RHSIM_Rhsim12G0115500 | 1.38 | 0.240 | 0.327 | -0.262 |
| RHSIM_Rhsim03G0126800 | 1.38 | 0.241 | 0.327 | 37.556 |
| RHSIM_Rhsim04G0032300 | 1.38 | 0.241 | 0.327 | 0.116 |
| RHSIM_Rhsim04G0036100 | 1.38 | 0.241 | 0.327 | -0.146 |
| RHSIM_Rhsim03G0267600 | 1.38 | 0.241 | 0.327 | -0.122 |
| RHSIM_Rhsim04G0113500 | 1.37 | 0.241 | 0.327 | -0.155 |
| RHSIM_Rhsim09G0022700 | 1.37 | 0.241 | 0.327 | 0.936 |
| RHSIM_Rhsim09G0184900 | 1.37 | 0.241 | 0.327 | 1.336 |
| RHSIM_Rhsim06G0104700 | 1.37 | 0.241 | 0.328 | 0.123 |
| RHSIM_Rhsim06G0150000 | 1.37 | 0.242 | 0.328 | 1.278 |
| RHSIM_RhsimUnG0097900 | 1.37 | 0.242 | 0.328 | -37.095 |
| RHSIM_Rhsim08G0043800 | 1.37 | 0.242 | 0.328 | -0.099 |
| RHSIM_Rhsim06G0142200 | 1.37 | 0.242 | 0.328 | -1.357 |
| RHSIM_Rhsim10G0173400 | 1.37 | 0.242 | 0.328 | 2.596 |
| novel.158 | 1.37 | 0.242 | 0.329 | -37.077 |
| novel.2598 | 1.37 | 0.242 | 0.329 | 0.471 |
| RHSIM_Rhsim05G0060000 | 1.37 | 0.242 | 0.329 | 3.127 |
| RHSIM_Rhsim13G0040200 | 1.37 | 0.242 | 0.329 | 3.404 |
| RHSIM_Rhsim03G0192800 | 1.37 | 0.242 | 0.329 | 0.171 |
| RHSIM_Rhsim12G0045700 | 1.37 | 0.242 | 0.329 | -0.670 |
| RHSIM_Rhsim12G0212700 | 1.37 | 0.242 | 0.329 | 37.588 |
| novel.2451 | 1.37 | 0.242 | 0.329 | 0.658 |
| RHSIM_Rhsim01G0189200 | 1.37 | 0.242 | 0.329 | 0.102 |
| RHSIM_Rhsim01G0017900 | 1.37 | 0.243 | 0.329 | 0.999 |
| RHSIM_RhsimUnG0075700 | 1.36 | 0.243 | 0.329 | 1.759 |
| RHSIM_Rhsim04G0246800 | 1.36 | 0.243 | 0.329 | 0.772 |
| RHSIM_Rhsim05G0228100 | 1.36 | 0.243 | 0.329 | -0.082 |
| RHSIM_Rhsim12G0081200 | 1.36 | 0.243 | 0.329 | -0.967 |
| RHSIM_Rhsim07G0013000 | 1.36 | 0.243 | 0.329 | -0.117 |
| RHSIM_Rhsim02G0187000 | 1.36 | 0.243 | 0.329 | -0.154 |

| Gene | LRT statistic | P value | BH-adjusted P | log2FC (interaction) |
|---|---|---|---|---|
| RHSIM_RhsimUnG0172600 | 1.36 | 0.243 | 0.330 | 1.577 |
| RHSIM_Rhsim09G0188000 | 1.36 | 0.243 | 0.330 | -36.643 |
| RHSIM_Rhsim01G0072500 | 1.36 | 0.243 | 0.330 | -37.506 |
| novel.360 | 1.36 | 0.243 | 0.330 | 0.268 |
| RHSIM_Rhsim08G0000300 | 1.36 | 0.243 | 0.330 | 0.113 |
| RHSIM_Rhsim08G0242500 | 1.36 | 0.244 | 0.330 | 0.102 |
| novel.2206 | 1.36 | 0.244 | 0.330 | 0.953 |
| novel.1084 | 1.36 | 0.244 | 0.330 | -2.759 |
| novel.4361 | 1.36 | 0.244 | 0.330 | -3.117 |
| RHSIM_Rhsim09G0005900 | 1.36 | 0.244 | 0.331 | 1.250 |
| RHSIM_Rhsim07G0064900 | 1.36 | 0.244 | 0.331 | -0.232 |
| RHSIM_Rhsim06G0099900 | 1.36 | 0.244 | 0.331 | 0.150 |
| RHSIM_Rhsim13G0189000 | 1.36 | 0.244 | 0.331 | -0.115 |
| RHSIM_Rhsim13G0202100 | 1.35 | 0.244 | 0.331 | -0.155 |
| novel.3683 | 1.35 | 0.245 | 0.331 | 0.730 |
| RHSIM_Rhsim06G0028700 | 1.35 | 0.245 | 0.332 | -0.255 |
| RHSIM_Rhsim08G0103000 | 1.35 | 0.245 | 0.332 | -0.137 |
| novel.787 | 1.35 | 0.245 | 0.332 | -36.883 |
| RHSIM_Rhsim07G0150400 | 1.35 | 0.245 | 0.332 | 0.367 |
| RHSIM_Rhsim06G0218700 | 1.35 | 0.245 | 0.332 | -1.465 |
| RHSIM_Rhsim05G0124000 | 1.35 | 0.245 | 0.332 | -0.134 |
| RHSIM_Rhsim07G0001100 | 1.35 | 0.245 | 0.332 | 0.288 |
| RHSIM_Rhsim11G0064200 | 1.35 | 0.245 | 0.332 | -0.229 |
| novel.1405 | 1.35 | 0.245 | 0.332 | 1.777 |
| novel.3631 | 1.35 | 0.245 | 0.332 | 0.741 |
| RHSIM_Rhsim05G0116800 | 1.35 | 0.245 | 0.332 | -0.717 |
| RHSIM_Rhsim05G0116900 | 1.35 | 0.245 | 0.332 | 0.395 |
| RHSIM_Rhsim05G0159900 | 1.35 | 0.246 | 0.333 | -0.096 |
| RHSIM_Rhsim07G0144100 | 1.35 | 0.246 | 0.333 | -0.103 |
| RHSIM_Rhsim06G0113600 | 1.35 | 0.246 | 0.333 | 0.365 |
| RHSIM_Rhsim10G0117000 | 1.35 | 0.246 | 0.333 | 0.999 |
| RHSIM_Rhsim02G0182500 | 1.35 | 0.246 | 0.333 | -1.556 |

| Gene | LRT statistic | P value | BH-adjusted P | log2FC (interaction) |
|---|---|---|---|---|
| RHSIM_Rhsim03G0227900 | 1.35 | 0.246 | 0.333 | 0.214 |
| novel.2959 | 1.35 | 0.246 | 0.333 | 0.767 |
| novel.1499 | 1.35 | 0.246 | 0.333 | -0.490 |
| RHSIM_Rhsim04G0236500 | 1.35 | 0.246 | 0.333 | -0.691 |
| novel.367 | 1.35 | 0.246 | 0.333 | 0.463 |
| RHSIM_Rhsim01G0242700 | 1.35 | 0.246 | 0.333 | -0.223 |
| RHSIM_Rhsim07G0047000 | 1.34 | 0.246 | 0.333 | -0.367 |
| novel.3587 | 1.34 | 0.246 | 0.334 | 2.566 |
| RHSIM_Rhsim10G0050400 | 1.34 | 0.246 | 0.334 | -2.563 |
| RHSIM_Rhsim02G0051100 | 1.34 | 0.246 | 0.334 | 0.210 |
| RHSIM_RhsimUnG0062400 | 1.34 | 0.247 | 0.334 | 2.085 |
| novel.275 | 1.34 | 0.247 | 0.334 | 0.740 |
| novel.2758 | 1.34 | 0.247 | 0.334 | 1.073 |
| novel.3154 | 1.34 | 0.247 | 0.334 | -0.614 |
| RHSIM_Rhsim10G0017200 | 1.34 | 0.247 | 0.334 | 1.679 |
| RHSIM_Rhsim10G0062400 | 1.34 | 0.247 | 0.334 | -0.127 |
| RHSIM_Rhsim06G0038100 | 1.34 | 0.247 | 0.334 | 1.042 |
| RHSIM_Rhsim09G0053200 | 1.34 | 0.247 | 0.334 | 0.345 |
| RHSIM_RhsimUnG0143800 | 1.34 | 0.247 | 0.334 | 0.808 |
| RHSIM_Rhsim03G0122600 | 1.34 | 0.247 | 0.334 | -0.899 |
| RHSIM_Rhsim13G0118800 | 1.34 | 0.247 | 0.334 | 0.154 |
| RHSIM_Rhsim02G0009600 | 1.34 | 0.247 | 0.334 | 2.366 |
| RHSIM_Rhsim11G0144500 | 1.34 | 0.247 | 0.335 | -3.053 |
| RHSIM_Rhsim08G0202200 | 1.34 | 0.247 | 0.335 | 1.244 |
| RHSIM_Rhsim05G0005000 | 1.34 | 0.247 | 0.335 | 0.192 |
| RHSIM_Rhsim04G0013400 | 1.34 | 0.247 | 0.335 | 0.168 |
| RHSIM_Rhsim06G0215800 | 1.34 | 0.247 | 0.335 | 2.601 |
| RHSIM_Rhsim07G0221300 | 1.34 | 0.248 | 0.335 | 0.391 |
| RHSIM_Rhsim10G0117800 | 1.34 | 0.248 | 0.335 | 0.865 |
| RHSIM_Rhsim02G0185300 | 1.34 | 0.248 | 0.335 | 37.547 |
| RHSIM_Rhsim03G0228400 | 1.34 | 0.248 | 0.335 | 0.954 |
| novel.3457 | 1.34 | 0.248 | 0.335 | 1.244 |

| Gene | LRT statistic | P value | BH-adjusted P | log2FC (interaction) |
|---|---|---|---|---|
| RHSIM_Rhsim02G0190000 | 1.33 | 0.248 | 0.335 | -1.872 |
| RHSIM_Rhsim11G0158600 | 1.33 | 0.248 | 0.336 | 0.330 |
| RHSIM_Rhsim13G0124900 | 1.33 | 0.248 | 0.336 | 1.141 |
| RHSIM_Rhsim05G0117400 | 1.33 | 0.248 | 0.336 | 0.310 |
| RHSIM_Rhsim04G0169700 | 1.33 | 0.249 | 0.336 | 0.073 |
| RHSIM_RhsimUnG0205600 | 1.33 | 0.249 | 0.336 | 0.409 |
| RHSIM_Rhsim02G0045200 | 1.33 | 0.249 | 0.336 | 0.647 |
| novel.2625 | 1.33 | 0.249 | 0.336 | 1.763 |
| RHSIM_Rhsim08G0170200 | 1.33 | 0.249 | 0.336 | 0.431 |
| RHSIM_RhsimUnG0045600 | 1.33 | 0.249 | 0.336 | 1.251 |
| RHSIM_Rhsim04G0010400 | 1.33 | 0.249 | 0.336 | -0.152 |
| RHSIM_Rhsim03G0115800 | 1.33 | 0.249 | 0.336 | -0.124 |
| RHSIM_Rhsim06G0220000 | 1.33 | 0.249 | 0.336 | -0.161 |
| RHSIM_Rhsim10G0014500 | 1.33 | 0.249 | 0.336 | -0.177 |
| RHSIM_Rhsim04G0155700 | 1.33 | 0.249 | 0.336 | 0.258 |
| RHSIM_RhsimUnG0025600 | 1.33 | 0.249 | 0.336 | 37.544 |
| RHSIM_Rhsim13G0086600 | 1.33 | 0.249 | 0.337 | 0.085 |
| RHSIM_Rhsim08G0247200 | 1.33 | 0.249 | 0.337 | 0.080 |
| RHSIM_Rhsim08G0024300 | 1.33 | 0.249 | 0.337 | -1.636 |
| RHSIM_RhsimUnG0233800 | 1.33 | 0.249 | 0.337 | 1.404 |
| RHSIM_Rhsim13G0136000 | 1.33 | 0.249 | 0.337 | -1.826 |
| RHSIM_Rhsim08G0185300 | 1.33 | 0.249 | 0.337 | 3.072 |
| RHSIM_Rhsim05G0174800 | 1.33 | 0.249 | 0.337 | -1.275 |
| RHSIM_Rhsim04G0192800 | 1.33 | 0.250 | 0.337 | -2.694 |
| RHSIM_Rhsim06G0140100 | 1.33 | 0.250 | 0.337 | 0.435 |
| RHSIM_Rhsim13G0215700 | 1.32 | 0.250 | 0.337 | -0.561 |
| RHSIM_RhsimUnG0213000 | 1.32 | 0.250 | 0.337 | 1.417 |
| RHSIM_Rhsim06G0026600 | 1.32 | 0.250 | 0.337 | 0.295 |
| RHSIM_Rhsim04G0219300 | 1.32 | 0.250 | 0.337 | 1.355 |
| RHSIM_Rhsim06G0008400 | 1.32 | 0.250 | 0.338 | 0.076 |
| RHSIM_Rhsim08G0249000 | 1.32 | 0.250 | 0.338 | -0.209 |
| RHSIM_Rhsim09G0174700 | 1.32 | 0.250 | 0.338 | -37.313 |

| Gene | LRT statistic | P value | BH-adjusted P | log2FC (interaction) |
|---|---|---|---|---|
| novel.24 | 1.32 | 0.250 | 0.338 | -1.383 |
| novel.1549 | 1.32 | 0.250 | 0.338 | 1.623 |
| RHSIM_Rhsim11G0073700 | 1.32 | 0.250 | 0.338 | -0.738 |
| RHSIM_Rhsim08G0237600 | 1.32 | 0.250 | 0.338 | -37.239 |
| RHSIM_Rhsim01G0244100 | 1.32 | 0.251 | 0.338 | 0.207 |
| RHSIM_Rhsim05G0004000 | 1.32 | 0.251 | 0.338 | 0.173 |
| novel.3687 | 1.32 | 0.251 | 0.338 | -1.767 |
| RHSIM_Rhsim12G0037000 | 1.32 | 0.251 | 0.338 | 0.181 |
| RHSIM_Rhsim04G0172300 | 1.32 | 0.251 | 0.339 | -0.260 |
| novel.255 | 1.32 | 0.251 | 0.339 | -0.077 |
| RHSIM_Rhsim05G0182500 | 1.32 | 0.251 | 0.339 | -0.966 |
| RHSIM_Rhsim03G0098000 | 1.32 | 0.251 | 0.339 | -0.225 |
| RHSIM_Rhsim12G0141400 | 1.32 | 0.251 | 0.339 | 37.496 |
| RHSIM_Rhsim08G0005600 | 1.32 | 0.251 | 0.339 | -0.476 |
| RHSIM_Rhsim08G0177400 | 1.32 | 0.251 | 0.339 | 2.073 |
| RHSIM_Rhsim02G0247400 | 1.32 | 0.251 | 0.339 | 2.546 |
| RHSIM_Rhsim06G0183600 | 1.32 | 0.251 | 0.339 | -0.576 |
| RHSIM_Rhsim03G0074000 | 1.32 | 0.251 | 0.339 | 2.596 |
| RHSIM_Rhsim11G0118200 | 1.32 | 0.251 | 0.339 | -0.124 |
| RHSIM_RhsimUnG0175500 | 1.32 | 0.251 | 0.339 | 2.456 |
| RHSIM_Rhsim07G0089100 | 1.32 | 0.251 | 0.339 | -0.160 |
| novel.567 | 1.31 | 0.252 | 0.339 | 0.391 |
| RHSIM_RhsimUnG0176700 | 1.31 | 0.252 | 0.339 | -3.746 |
| RHSIM_Rhsim05G0188600 | 1.31 | 0.252 | 0.340 | -37.614 |
| novel.207 | 1.31 | 0.252 | 0.340 | -1.257 |
| RHSIM_Rhsim04G0098700 | 1.31 | 0.252 | 0.340 | 3.168 |
| RHSIM_Rhsim11G0104100 | 1.31 | 0.252 | 0.340 | 1.383 |
| RHSIM_Rhsim06G0029400 | 1.31 | 0.252 | 0.340 | -3.260 |
| RHSIM_RhsimUnG0067300 | 1.31 | 0.252 | 0.340 | -0.852 |
| novel.803 | 1.31 | 0.253 | 0.340 | -0.187 |
| RHSIM_Rhsim05G0110800 | 1.31 | 0.253 | 0.340 | 0.487 |
| RHSIM_Rhsim09G0052200 | 1.31 | 0.253 | 0.340 | -0.145 |

| Gene | LRT statistic | P value | BH-adjusted P | log2FC (interaction) |
|---|---|---|---|---|
| RHSIM_Rhsim13G0194700 | 1.31 | 0.253 | 0.340 | -0.505 |
| RHSIM_Rhsim09G0213500 | 1.31 | 0.253 | 0.340 | 1.547 |
| RHSIM_RhsimUnG0049300 | 1.31 | 0.253 | 0.341 | 0.711 |
| RHSIM_Rhsim06G0169700 | 1.31 | 0.253 | 0.341 | 0.303 |
| novel.1812 | 1.31 | 0.253 | 0.341 | 0.660 |
| RHSIM_Rhsim07G0176200 | 1.31 | 0.253 | 0.341 | 2.086 |
| RHSIM_Rhsim04G0020600 | 1.31 | 0.253 | 0.341 | 37.103 |
| novel.1728 | 1.31 | 0.253 | 0.341 | 1.774 |
| RHSIM_Rhsim01G0209900 | 1.30 | 0.253 | 0.341 | -0.669 |
| RHSIM_Rhsim03G0266300 | 1.30 | 0.253 | 0.341 | 0.457 |
| RHSIM_Rhsim01G0112000 | 1.30 | 0.253 | 0.342 | 0.191 |
| RHSIM_Rhsim03G0049900 | 1.30 | 0.254 | 0.342 | 0.065 |
| novel.4312 | 1.30 | 0.254 | 0.342 | 0.156 |
| RHSIM_Rhsim12G0032800 | 1.30 | 0.254 | 0.342 | 37.586 |
| RHSIM_Rhsim01G0047600 | 1.30 | 0.254 | 0.342 | -1.239 |
| RHSIM_Rhsim03G0240200 | 1.30 | 0.254 | 0.342 | 0.133 |
| novel.1238 | 1.30 | 0.254 | 0.342 | 0.737 |
| RHSIM_Rhsim13G0040800 | 1.30 | 0.254 | 0.342 | -0.166 |
| RHSIM_Rhsim08G0190800 | 1.30 | 0.254 | 0.342 | -0.235 |
| RHSIM_Rhsim04G0242300 | 1.30 | 0.254 | 0.342 | 0.213 |
| novel.9 | 1.30 | 0.254 | 0.342 | -1.324 |
| RHSIM_Rhsim05G0045800 | 1.30 | 0.254 | 0.342 | -3.030 |
| RHSIM_Rhsim12G0112700 | 1.30 | 0.254 | 0.343 | 2.252 |
| RHSIM_Rhsim12G0033800 | 1.30 | 0.254 | 0.343 | -1.050 |
| RHSIM_Rhsim07G0017200 | 1.30 | 0.255 | 0.343 | 0.733 |
| novel.623 | 1.30 | 0.255 | 0.343 | -2.379 |
| RHSIM_Rhsim01G0237800 | 1.30 | 0.255 | 0.343 | -2.062 |
| novel.3366 | 1.30 | 0.255 | 0.343 | 0.664 |
| RHSIM_Rhsim02G0085900 | 1.30 | 0.255 | 0.343 | 0.482 |
| RHSIM_Rhsim08G0212400 | 1.30 | 0.255 | 0.343 | 0.219 |
| RHSIM_Rhsim02G0158800 | 1.30 | 0.255 | 0.343 | 2.235 |
| RHSIM_Rhsim11G0100900 | 1.30 | 0.255 | 0.343 | -0.360 |

| Gene | LRT statistic | P value | BH-adjusted P | log2FC (interaction) |
|---|---|---|---|---|
| RHSIM_Rhsim13G0016000 | 1.30 | 0.255 | 0.343 | 0.140 |
| RHSIM_Rhsim09G0099900 | 1.30 | 0.255 | 0.343 | 0.175 |
| RHSIM_Rhsim01G0113300 | 1.30 | 0.255 | 0.343 | -0.112 |
| RHSIM_Rhsim11G0104700 | 1.29 | 0.255 | 0.343 | 2.463 |
| RHSIM_RhsimUnG0080600 | 1.29 | 0.255 | 0.343 | 1.196 |
| RHSIM_Rhsim11G0066600 | 1.29 | 0.255 | 0.344 | -0.112 |
| novel.2403 | 1.29 | 0.255 | 0.344 | -0.247 |
| novel.3161 | 1.29 | 0.255 | 0.344 | -0.287 |
| RHSIM_Rhsim01G0279800 | 1.29 | 0.255 | 0.344 | 2.036 |
| RHSIM_Rhsim11G0133600 | 1.29 | 0.256 | 0.344 | -0.081 |
| novel.1675 | 1.29 | 0.256 | 0.344 | -0.453 |
| RHSIM_Rhsim03G0205100 | 1.29 | 0.256 | 0.344 | 0.980 |
| RHSIM_Rhsim04G0179900 | 1.29 | 0.256 | 0.344 | 0.201 |
| novel.4326 | 1.29 | 0.256 | 0.344 | 1.364 |
| RHSIM_Rhsim06G0078100 | 1.29 | 0.256 | 0.344 | 0.196 |
| RHSIM_Rhsim03G0194400 | 1.29 | 0.256 | 0.344 | -0.964 |
| RHSIM_Rhsim07G0238600 | 1.29 | 0.256 | 0.344 | -1.006 |
| RHSIM_Rhsim13G0092100 | 1.29 | 0.256 | 0.344 | -1.609 |
| RHSIM_Rhsim11G0152700 | 1.29 | 0.256 | 0.344 | -0.780 |
| RHSIM_Rhsim13G0176300 | 1.29 | 0.256 | 0.344 | 1.924 |
| novel.1103 | 1.29 | 0.256 | 0.344 | -0.588 |
| RHSIM_Rhsim08G0084600 | 1.29 | 0.256 | 0.345 | 0.345 |
| RHSIM_Rhsim03G0206700 | 1.29 | 0.256 | 0.345 | 0.437 |
| RHSIM_Rhsim06G0013200 | 1.29 | 0.256 | 0.345 | -2.272 |
| RHSIM_Rhsim05G0175600 | 1.29 | 0.256 | 0.345 | -0.218 |
| RHSIM_Rhsim01G0041200 | 1.29 | 0.256 | 0.345 | -0.360 |
| RHSIM_Rhsim02G0184500 | 1.29 | 0.256 | 0.345 | 0.343 |
| RHSIM_Rhsim13G0078300 | 1.29 | 0.256 | 0.345 | 1.817 |
| RHSIM_Rhsim03G0157300 | 1.29 | 0.256 | 0.345 | 1.570 |
| RHSIM_Rhsim12G0054700 | 1.29 | 0.257 | 0.345 | 3.198 |
| RHSIM_Rhsim08G0100500 | 1.29 | 0.257 | 0.345 | 0.832 |
| RHSIM_Rhsim10G0017400 | 1.29 | 0.257 | 0.345 | -0.515 |

| Gene | LRT statistic | P value | BH-adjusted P | log2FC (interaction) |
|---|---|---|---|---|
| RHSIM_RhsimUnG0112800 | 1.29 | 0.257 | 0.345 | 0.268 |
| RHSIM_Rhsim05G0089400 | 1.29 | 0.257 | 0.345 | 0.296 |
| RHSIM_Rhsim12G0150900 | 1.29 | 0.257 | 0.345 | 0.263 |
| RHSIM_Rhsim06G0070700 | 1.29 | 0.257 | 0.345 | 0.075 |
| RHSIM_Rhsim05G0088100 | 1.29 | 0.257 | 0.345 | 0.290 |
| RHSIM_Rhsim05G0095900 | 1.29 | 0.257 | 0.345 | -37.408 |
| RHSIM_Rhsim10G0160700 | 1.29 | 0.257 | 0.345 | 0.568 |
| RHSIM_Rhsim02G0115600 | 1.29 | 0.257 | 0.345 | 0.074 |
| novel.952 | 1.29 | 0.257 | 0.345 | 0.981 |
| RHSIM_Rhsim05G0041900 | 1.28 | 0.257 | 0.345 | 0.218 |
| novel.455 | 1.28 | 0.257 | 0.345 | -1.162 |
| novel.4062 | 1.28 | 0.257 | 0.345 | -1.781 |
| RHSIM_Rhsim07G0130200 | 1.28 | 0.257 | 0.345 | -1.114 |
| RHSIM_Rhsim12G0206600 | 1.28 | 0.257 | 0.345 | -0.776 |
| RHSIM_Rhsim01G0091500 | 1.28 | 0.257 | 0.346 | 2.910 |
| RHSIM_RhsimUnG0253500 | 1.28 | 0.257 | 0.346 | -0.417 |
| novel.4292 | 1.28 | 0.258 | 0.346 | -0.890 |
| RHSIM_Rhsim03G0189800 | 1.28 | 0.258 | 0.346 | -0.075 |
| RHSIM_Rhsim12G0163700 | 1.28 | 0.258 | 0.346 | 2.007 |
| RHSIM_Rhsim06G0217300 | 1.28 | 0.258 | 0.346 | 0.159 |
| RHSIM_Rhsim02G0255600 | 1.28 | 0.258 | 0.346 | 1.118 |
| RHSIM_Rhsim10G0128700 | 1.28 | 0.258 | 0.346 | -0.141 |
| RHSIM_Rhsim07G0256700 | 1.28 | 0.258 | 0.346 | -0.157 |
| RHSIM_Rhsim04G0221200 | 1.28 | 0.258 | 0.346 | -1.846 |
| RHSIM_RhsimUnG0000400 | 1.28 | 0.258 | 0.346 | 1.508 |
| novel.1937 | 1.28 | 0.258 | 0.346 | -2.613 |
| RHSIM_Rhsim04G0161900 | 1.28 | 0.258 | 0.347 | -0.132 |
| RHSIM_Rhsim10G0016000 | 1.28 | 0.258 | 0.347 | 0.212 |
| RHSIM_Rhsim08G0119900 | 1.28 | 0.259 | 0.347 | 1.353 |
| RHSIM_RhsimUnG0051300 | 1.28 | 0.259 | 0.347 | 0.266 |
| RHSIM_Rhsim03G0066900 | 1.28 | 0.259 | 0.347 | 0.297 |
| RHSIM_Rhsim09G0067300 | 1.28 | 0.259 | 0.347 | -37.401 |

| Gene | LRT statistic | P value | BH-adjusted P | log2FC (interaction) |
|---|---|---|---|---|
| RHSIM_Rhsim07G0251000 | 1.28 | 0.259 | 0.347 | 2.830 |
| RHSIM_Rhsim10G0087300 | 1.27 | 0.259 | 0.347 | -1.193 |
| RHSIM_Rhsim01G0103700 | 1.27 | 0.259 | 0.347 | 1.714 |
| RHSIM_Rhsim04G0021100 | 1.27 | 0.259 | 0.347 | -0.771 |
| novel.1623 | 1.27 | 0.259 | 0.347 | 0.798 |
| RHSIM_Rhsim07G0173200 | 1.27 | 0.259 | 0.347 | 0.160 |
| RHSIM_RhsimUnG0211800 | 1.27 | 0.259 | 0.348 | 0.288 |
| RHSIM_Rhsim02G0081000 | 1.27 | 0.259 | 0.348 | 37.181 |
| novel.1373 | 1.27 | 0.259 | 0.348 | 0.278 |
| RHSIM_Rhsim07G0019800 | 1.27 | 0.260 | 0.348 | -37.507 |
| RHSIM_Rhsim04G0121800 | 1.27 | 0.260 | 0.348 | -0.463 |
| RHSIM_Rhsim04G0196100 | 1.27 | 0.260 | 0.348 | -0.240 |
| novel.1682 | 1.27 | 0.260 | 0.348 | -0.705 |
| RHSIM_Rhsim06G0208100 | 1.27 | 0.260 | 0.348 | -0.191 |
| RHSIM_Rhsim03G0252100 | 1.27 | 0.260 | 0.348 | 0.339 |
| RHSIM_Rhsim03G0180000 | 1.27 | 0.260 | 0.349 | -0.416 |
| RHSIM_Rhsim09G0130500 | 1.27 | 0.260 | 0.349 | 0.103 |
| RHSIM_Rhsim01G0192500 | 1.27 | 0.260 | 0.349 | 0.188 |
| RHSIM_Rhsim10G0043600 | 1.27 | 0.260 | 0.349 | 0.069 |
| RHSIM_Rhsim11G0042600 | 1.27 | 0.260 | 0.349 | -37.035 |
| novel.4338 | 1.27 | 0.260 | 0.349 | -0.238 |
| RHSIM_Rhsim04G0184900 | 1.27 | 0.260 | 0.349 | -36.492 |
| RHSIM_Rhsim06G0214100 | 1.27 | 0.260 | 0.349 | 1.318 |
| RHSIM_Rhsim06G0213000 | 1.27 | 0.261 | 0.349 | -0.343 |
| RHSIM_Rhsim01G0175800 | 1.27 | 0.261 | 0.349 | -37.071 |
| novel.4496 | 1.27 | 0.261 | 0.349 | -0.365 |
| RHSIM_Rhsim03G0006800 | 1.26 | 0.261 | 0.349 | 2.030 |
| RHSIM_Rhsim05G0170100 | 1.26 | 0.261 | 0.350 | -2.562 |
| novel.1680 | 1.26 | 0.261 | 0.350 | -2.114 |
| RHSIM_Rhsim07G0201000 | 1.26 | 0.261 | 0.350 | 1.035 |
| RHSIM_Rhsim09G0131900 | 1.26 | 0.261 | 0.350 | -0.142 |
| RHSIM_Rhsim02G0033300 | 1.26 | 0.261 | 0.350 | -0.253 |

| Gene | LRT statistic | P value | BH-adjusted P | log2FC (interaction) |
|---|---|---|---|---|
| RHSIM_Rhsim01G0027900 | 1.26 | 0.261 | 0.350 | 36.883 |
| RHSIM_Rhsim02G0232200 | 1.26 | 0.261 | 0.350 | -36.967 |
| novel.2977 | 1.26 | 0.262 | 0.350 | -0.394 |
| RHSIM_Rhsim12G0172600 | 1.26 | 0.262 | 0.351 | 0.160 |
| RHSIM_Rhsim07G0246500 | 1.26 | 0.262 | 0.351 | -0.280 |
| RHSIM_Rhsim03G0090600 | 1.26 | 0.262 | 0.351 | -0.087 |
| RHSIM_Rhsim04G0157200 | 1.26 | 0.262 | 0.351 | -0.119 |
| novel.2818 | 1.26 | 0.262 | 0.351 | -1.123 |
| RHSIM_Rhsim10G0171400 | 1.26 | 0.262 | 0.351 | 1.534 |
| RHSIM_Rhsim13G0152500 | 1.26 | 0.262 | 0.351 | -2.541 |
| novel.4223 | 1.26 | 0.262 | 0.351 | 1.791 |
| RHSIM_Rhsim07G0057400 | 1.26 | 0.262 | 0.351 | 0.196 |
| RHSIM_Rhsim02G0024700 | 1.26 | 0.262 | 0.351 | 0.181 |
| RHSIM_Rhsim12G0026100 | 1.26 | 0.262 | 0.351 | 0.144 |
| RHSIM_RhsimUnG0183800 | 1.26 | 0.262 | 0.351 | -1.639 |
| RHSIM_Rhsim05G0204000 | 1.26 | 0.262 | 0.351 | 37.205 |
| RHSIM_Rhsim04G0095800 | 1.26 | 0.262 | 0.351 | -0.924 |
| RHSIM_Rhsim03G0230200 | 1.26 | 0.263 | 0.352 | 0.118 |
| RHSIM_Rhsim07G0055000 | 1.25 | 0.263 | 0.352 | -37.043 |
| RHSIM_Rhsim04G0116700 | 1.25 | 0.263 | 0.352 | -0.774 |
| RHSIM_Rhsim11G0015000 | 1.25 | 0.263 | 0.352 | 0.484 |
| RHSIM_Rhsim13G0114100 | 1.25 | 0.263 | 0.352 | -1.633 |
| novel.2911 | 1.25 | 0.263 | 0.352 | 0.355 |
| RHSIM_Rhsim07G0087900 | 1.25 | 0.263 | 0.352 | -0.260 |
| RHSIM_Rhsim13G0169100 | 1.25 | 0.263 | 0.352 | -0.353 |
| RHSIM_Rhsim13G0043500 | 1.25 | 0.263 | 0.352 | -1.398 |
| novel.2292 | 1.25 | 0.263 | 0.352 | 0.110 |
| RHSIM_Rhsim01G0286900 | 1.25 | 0.263 | 0.352 | 0.520 |
| RHSIM_Rhsim10G0172200 | 1.25 | 0.263 | 0.352 | 1.437 |
| RHSIM_Rhsim01G0287900 | 1.25 | 0.263 | 0.353 | -0.077 |
| RHSIM_Rhsim12G0115800 | 1.25 | 0.264 | 0.353 | 0.117 |
| novel.2957 | 1.25 | 0.264 | 0.353 | 0.154 |

| Gene | LRT statistic | P value | BH-adjusted P | log2FC (interaction) |
|---|---|---|---|---|
| novel.71 | 1.25 | 0.264 | 0.353 | 1.407 |
| RHSIM_Rhsim03G0013400 | 1.25 | 0.264 | 0.353 | -36.738 |
| RHSIM_Rhsim09G0065200 | 1.25 | 0.264 | 0.353 | 0.629 |
| RHSIM_Rhsim08G0194800 | 1.25 | 0.264 | 0.353 | -37.710 |
| RHSIM_Rhsim11G0163800 | 1.25 | 0.264 | 0.353 | -0.326 |
| novel.4191 | 1.25 | 0.264 | 0.353 | -1.372 |
| RHSIM_Rhsim13G0076800 | 1.25 | 0.264 | 0.353 | 0.527 |
| RHSIM_Rhsim07G0122700 | 1.25 | 0.264 | 0.353 | 37.357 |
| RHSIM_Rhsim08G0063000 | 1.25 | 0.264 | 0.353 | 0.399 |
| RHSIM_Rhsim02G0023200 | 1.25 | 0.264 | 0.353 | -0.134 |
| RHSIM_Rhsim07G0011400 | 1.25 | 0.264 | 0.354 | 0.228 |
| novel.2000 | 1.25 | 0.264 | 0.354 | -0.526 |
| RHSIM_Rhsim07G0223000 | 1.25 | 0.264 | 0.354 | 3.060 |
| novel.1913 | 1.25 | 0.264 | 0.354 | -0.646 |
| RHSIM_Rhsim05G0082200 | 1.24 | 0.265 | 0.354 | 0.542 |
| RHSIM_Rhsim12G0203700 | 1.24 | 0.265 | 0.354 | -0.846 |
| RHSIM_Rhsim07G0199800 | 1.24 | 0.265 | 0.354 | -0.657 |
| RHSIM_Rhsim12G0086600 | 1.24 | 0.265 | 0.354 | -0.528 |
| novel.2095 | 1.24 | 0.265 | 0.354 | -0.683 |
| novel.2509 | 1.24 | 0.265 | 0.354 | -0.610 |
| RHSIM_Rhsim08G0015300 | 1.24 | 0.265 | 0.355 | -0.960 |
| RHSIM_Rhsim07G0144500 | 1.24 | 0.266 | 0.355 | -0.092 |
| RHSIM_Rhsim03G0031900 | 1.24 | 0.266 | 0.355 | 37.058 |
| novel.2470 | 1.24 | 0.266 | 0.355 | 2.095 |
| RHSIM_Rhsim07G0201900 | 1.24 | 0.266 | 0.355 | 1.815 |
| RHSIM_Rhsim11G0146400 | 1.24 | 0.266 | 0.355 | 2.073 |
| RHSIM_Rhsim03G0011800 | 1.24 | 0.266 | 0.355 | -2.341 |
| RHSIM_Rhsim10G0159700 | 1.24 | 0.266 | 0.356 | 0.719 |
| RHSIM_Rhsim03G0024200 | 1.24 | 0.266 | 0.356 | -2.436 |
| RHSIM_Rhsim13G0038500 | 1.24 | 0.266 | 0.356 | 0.141 |
| novel.241 | 1.24 | 0.266 | 0.356 | -0.614 |
| novel.887 | 1.24 | 0.266 | 0.356 | -0.609 |

| Gene | LRT statistic | P value | BH-adjusted P | log2FC (interaction) |
|---|---|---|---|---|
| RHSIM_Rhsim08G0173500 | 1.24 | 0.266 | 0.356 | 1.622 |
| RHSIM_Rhsim02G0197900 | 1.24 | 0.266 | 0.356 | -39.142 |
| RHSIM_Rhsim10G0029000 | 1.23 | 0.266 | 0.356 | 0.248 |
| novel.1922 | 1.23 | 0.267 | 0.356 | 0.960 |
| RHSIM_Rhsim03G0165100 | 1.23 | 0.267 | 0.356 | -0.395 |
| RHSIM_Rhsim08G0174600 | 1.23 | 0.267 | 0.356 | 0.077 |
| RHSIM_Rhsim01G0073700 | 1.23 | 0.267 | 0.357 | 0.159 |
| RHSIM_Rhsim01G0187400 | 1.23 | 0.267 | 0.357 | -0.261 |
| RHSIM_Rhsim03G0269400 | 1.23 | 0.267 | 0.357 | 2.168 |
| novel.4407 | 1.23 | 0.267 | 0.357 | -1.936 |
| RHSIM_Rhsim07G0161400 | 1.23 | 0.268 | 0.357 | -0.161 |
| RHSIM_Rhsim10G0038400 | 1.23 | 0.268 | 0.358 | 0.908 |
| novel.1889 | 1.23 | 0.268 | 0.358 | 2.836 |
| RHSIM_Rhsim04G0003500 | 1.23 | 0.268 | 0.358 | 0.142 |
| RHSIM_Rhsim03G0080800 | 1.23 | 0.268 | 0.358 | -1.676 |
| RHSIM_Rhsim03G0255400 | 1.23 | 0.268 | 0.358 | 0.597 |
| RHSIM_Rhsim05G0027900 | 1.23 | 0.268 | 0.358 | 0.111 |
| novel.1815 | 1.23 | 0.268 | 0.358 | 0.432 |
| RHSIM_Rhsim10G0097400 | 1.23 | 0.268 | 0.358 | -0.623 |
| RHSIM_Rhsim05G0120600 | 1.22 | 0.268 | 0.358 | -0.850 |
| RHSIM_Rhsim07G0247600 | 1.22 | 0.268 | 0.358 | -0.378 |
| RHSIM_Rhsim01G0279200 | 1.22 | 0.268 | 0.358 | 1.089 |
| RHSIM_RhsimUnG0011400 | 1.22 | 0.269 | 0.358 | 0.266 |
| RHSIM_RhsimUnG0135400 | 1.22 | 0.269 | 0.359 | 0.360 |
| RHSIM_Rhsim02G0033600 | 1.22 | 0.269 | 0.359 | 2.130 |
| RHSIM_Rhsim07G0166300 | 1.22 | 0.269 | 0.359 | -2.373 |
| RHSIM_Rhsim13G0164500 | 1.22 | 0.269 | 0.359 | 0.348 |
| RHSIM_Rhsim06G0021600 | 1.22 | 0.269 | 0.359 | 0.302 |
| novel.3495 | 1.22 | 0.269 | 0.359 | 2.271 |
| RHSIM_Rhsim01G0238300 | 1.22 | 0.269 | 0.359 | 0.147 |
| RHSIM_RhsimUnG0004900 | 1.22 | 0.269 | 0.360 | -36.872 |
| RHSIM_Rhsim02G0056300 | 1.22 | 0.270 | 0.360 | -0.772 |

| Gene | LRT statistic | P value | BH-adjusted P | log2FC (interaction) |
|---|---|---|---|---|
| RHSIM_Rhsim07G0198500 | 1.22 | 0.270 | 0.360 | -0.322 |
| RHSIM_Rhsim01G0091200 | 1.22 | 0.270 | 0.360 | -0.683 |
| RHSIM_Rhsim11G0171800 | 1.22 | 0.270 | 0.360 | 0.206 |
| RHSIM_Rhsim11G0084700 | 1.22 | 0.270 | 0.360 | -0.617 |
| RHSIM_Rhsim04G0164400 | 1.22 | 0.270 | 0.360 | 2.601 |
| RHSIM_Rhsim09G0010500 | 1.22 | 0.270 | 0.360 | -0.394 |
| RHSIM_Rhsim07G0240800 | 1.22 | 0.270 | 0.360 | -1.156 |
| novel.608 | 1.22 | 0.270 | 0.360 | 0.776 |
| RHSIM_Rhsim09G0023800 | 1.22 | 0.270 | 0.360 | 0.259 |
| RHSIM_RhsimUnG0044600 | 1.21 | 0.270 | 0.361 | 1.828 |
| novel.4123 | 1.21 | 0.271 | 0.361 | 0.485 |
| RHSIM_Rhsim06G0133200 | 1.21 | 0.271 | 0.361 | 1.221 |
| RHSIM_Rhsim11G0084800 | 1.21 | 0.271 | 0.361 | -1.238 |
| RHSIM_Rhsim09G0166600 | 1.21 | 0.271 | 0.361 | 0.739 |
| novel.3362 | 1.21 | 0.271 | 0.362 | -1.789 |
| RHSIM_Rhsim10G0115700 | 1.21 | 0.271 | 0.362 | -1.393 |
| novel.222 | 1.21 | 0.271 | 0.362 | 1.569 |
| RHSIM_RhsimUnG0014800 | 1.21 | 0.271 | 0.362 | -1.367 |
| RHSIM_Rhsim13G0059600 | 1.21 | 0.271 | 0.362 | 2.295 |
| RHSIM_Rhsim03G0189600 | 1.21 | 0.271 | 0.362 | -0.183 |
| RHSIM_Rhsim10G0162600 | 1.21 | 0.272 | 0.362 | 0.070 |
| RHSIM_Rhsim12G0154000 | 1.21 | 0.272 | 0.362 | 0.529 |
| RHSIM_RhsimUnG0033700 | 1.21 | 0.272 | 0.362 | 1.536 |
| novel.2755 | 1.21 | 0.272 | 0.362 | 0.260 |
| RHSIM_Rhsim10G0177400 | 1.21 | 0.272 | 0.362 | 0.229 |
| RHSIM_Rhsim06G0075300 | 1.21 | 0.272 | 0.362 | -36.915 |
| RHSIM_Rhsim13G0023300 | 1.21 | 0.272 | 0.362 | -36.822 |
| novel.1904 | 1.21 | 0.272 | 0.362 | -1.238 |
| RHSIM_Rhsim05G0170800 | 1.21 | 0.272 | 0.362 | 0.172 |
| RHSIM_Rhsim05G0007200 | 1.21 | 0.272 | 0.363 | -0.125 |
| RHSIM_Rhsim07G0223600 | 1.21 | 0.272 | 0.363 | -0.456 |
| RHSIM_Rhsim01G0084300 | 1.21 | 0.272 | 0.363 | -0.815 |

| Gene | LRT statistic | P value | BH-adjusted P | log2FC (interaction) |
|---|---|---|---|---|
| RHSIM_Rhsim08G0078300 | 1.20 | 0.272 | 0.363 | 0.178 |
| RHSIM_Rhsim07G0007000 | 1.20 | 0.272 | 0.363 | 0.727 |
| RHSIM_Rhsim06G0096800 | 1.20 | 0.273 | 0.363 | 37.332 |
| novel.1762 | 1.20 | 0.273 | 0.363 | 37.487 |
| RHSIM_RhsimUnG0017700 | 1.20 | 0.273 | 0.363 | -0.149 |
| novel.3342 | 1.20 | 0.273 | 0.363 | 37.075 |
| RHSIM_Rhsim06G0100600 | 1.20 | 0.273 | 0.363 | -0.396 |
| RHSIM_Rhsim02G0030000 | 1.20 | 0.273 | 0.363 | -0.117 |
| RHSIM_Rhsim02G0248800 | 1.20 | 0.273 | 0.364 | -0.087 |
| novel.4577 | 1.20 | 0.273 | 0.364 | 2.386 |
| novel.4528 | 1.20 | 0.273 | 0.364 | 0.512 |
| RHSIM_Rhsim12G0051800 | 1.20 | 0.273 | 0.364 | 0.256 |
| RHSIM_Rhsim08G0173300 | 1.20 | 0.273 | 0.364 | -0.256 |
| RHSIM_Rhsim07G0050800 | 1.20 | 0.273 | 0.364 | 1.000 |
| RHSIM_Rhsim06G0075200 | 1.20 | 0.273 | 0.364 | -0.447 |
| novel.3910 | 1.20 | 0.273 | 0.364 | -1.035 |
| RHSIM_Rhsim01G0135000 | 1.20 | 0.273 | 0.364 | -0.389 |
| RHSIM_Rhsim07G0121000 | 1.20 | 0.273 | 0.364 | -0.768 |
| RHSIM_Rhsim13G0060700 | 1.20 | 0.274 | 0.364 | -36.821 |
| RHSIM_Rhsim03G0135200 | 1.20 | 0.274 | 0.364 | 37.224 |
| RHSIM_Rhsim02G0025600 | 1.20 | 0.274 | 0.364 | 1.875 |
| RHSIM_Rhsim08G0138800 | 1.20 | 0.274 | 0.364 | -2.453 |
| RHSIM_Rhsim03G0245700 | 1.20 | 0.274 | 0.364 | -0.222 |
| RHSIM_Rhsim12G0142000 | 1.20 | 0.274 | 0.364 | -36.720 |
| RHSIM_Rhsim07G0124200 | 1.20 | 0.274 | 0.364 | -3.250 |
| RHSIM_Rhsim09G0165800 | 1.20 | 0.274 | 0.365 | -0.153 |
| RHSIM_Rhsim05G0183200 | 1.20 | 0.274 | 0.365 | 0.672 |
| RHSIM_Rhsim03G0161800 | 1.20 | 0.274 | 0.365 | -0.286 |
| RHSIM_Rhsim12G0075200 | 1.20 | 0.274 | 0.365 | 0.393 |
| RHSIM_Rhsim02G0113800 | 1.20 | 0.274 | 0.365 | -0.610 |
| RHSIM_Rhsim02G0239400 | 1.20 | 0.274 | 0.365 | 0.126 |
| RHSIM_Rhsim02G0113900 | 1.20 | 0.274 | 0.365 | -0.289 |

| Gene | LRT statistic | P value | BH-adjusted P | log2FC (interaction) |
|---|---|---|---|---|
| RHSIM_RhsimUnG0142000 | 1.20 | 0.274 | 0.365 | 1.464 |
| RHSIM_Rhsim08G0164400 | 1.20 | 0.274 | 0.365 | 0.257 |
| RHSIM_Rhsim04G0240000 | 1.19 | 0.274 | 0.365 | -0.106 |
| RHSIM_Rhsim07G0068400 | 1.19 | 0.274 | 0.365 | 0.191 |
| RHSIM_Rhsim10G0084700 | 1.19 | 0.274 | 0.365 | -0.127 |
| RHSIM_Rhsim08G0114800 | 1.19 | 0.274 | 0.365 | -0.572 |
| novel.2604 | 1.19 | 0.275 | 0.365 | 36.698 |
| RHSIM_Rhsim12G0048100 | 1.19 | 0.275 | 0.365 | 0.085 |
| RHSIM_Rhsim04G0173100 | 1.19 | 0.275 | 0.365 | 1.804 |
| RHSIM_Rhsim02G0077600 | 1.19 | 0.275 | 0.365 | -0.467 |
| RHSIM_Rhsim13G0147500 | 1.19 | 0.275 | 0.365 | -0.776 |
| RHSIM_Rhsim12G0050900 | 1.19 | 0.275 | 0.365 | 1.789 |
| RHSIM_Rhsim02G0118000 | 1.19 | 0.275 | 0.365 | 0.864 |
| RHSIM_Rhsim01G0065800 | 1.19 | 0.275 | 0.365 | -37.186 |
| RHSIM_Rhsim06G0207300 | 1.19 | 0.275 | 0.365 | 0.122 |
| RHSIM_Rhsim06G0015300 | 1.19 | 0.275 | 0.366 | -0.642 |
| RHSIM_Rhsim04G0225500 | 1.19 | 0.275 | 0.366 | 1.787 |
| RHSIM_Rhsim12G0163900 | 1.19 | 0.275 | 0.366 | 0.147 |
| RHSIM_Rhsim12G0100000 | 1.19 | 0.275 | 0.366 | 1.117 |
| RHSIM_Rhsim05G0058100 | 1.19 | 0.275 | 0.366 | 1.400 |
| RHSIM_RhsimUnG0041000 | 1.19 | 0.275 | 0.366 | 0.229 |
| RHSIM_Rhsim09G0005300 | 1.19 | 0.275 | 0.366 | -1.272 |
| RHSIM_Rhsim04G0019700 | 1.19 | 0.276 | 0.366 | -2.376 |
| RHSIM_Rhsim08G0239600 | 1.19 | 0.276 | 0.366 | 0.122 |
| RHSIM_RhsimUnG0241000 | 1.19 | 0.276 | 0.366 | 1.262 |
| RHSIM_Rhsim02G0117200 | 1.19 | 0.276 | 0.367 | -2.572 |
| RHSIM_Rhsim13G0129000 | 1.19 | 0.276 | 0.367 | -37.093 |
| RHSIM_Rhsim09G0131300 | 1.19 | 0.276 | 0.367 | -0.763 |
| RHSIM_Rhsim05G0183700 | 1.19 | 0.276 | 0.367 | 0.947 |
| RHSIM_Rhsim03G0131800 | 1.18 | 0.276 | 0.367 | -0.114 |
| RHSIM_Rhsim02G0211400 | 1.18 | 0.276 | 0.367 | 1.314 |
| novel.4470 | 1.18 | 0.276 | 0.367 | -1.327 |

| Gene | LRT statistic | P value | BH-adjusted P | log2FC (interaction) |
|---|---|---|---|---|
| RHSIM_Rhsim08G0065400 | 1.18 | 0.276 | 0.367 | 0.463 |
| novel.479 | 1.18 | 0.276 | 0.367 | 0.594 |
| RHSIM_Rhsim02G0119600 | 1.18 | 0.276 | 0.367 | -0.141 |
| RHSIM_Rhsim06G0114900 | 1.18 | 0.277 | 0.367 | 2.527 |
| RHSIM_Rhsim08G0018200 | 1.18 | 0.277 | 0.367 | -1.951 |
| RHSIM_Rhsim06G0068000 | 1.18 | 0.277 | 0.367 | 1.276 |
| RHSIM_RhsimUnG0041500 | 1.18 | 0.277 | 0.367 | -0.898 |
| RHSIM_Rhsim08G0127900 | 1.18 | 0.277 | 0.367 | 1.522 |
| RHSIM_Rhsim04G0211400 | 1.18 | 0.277 | 0.367 | -0.271 |
| novel.3525 | 1.18 | 0.277 | 0.368 | -1.498 |
| RHSIM_Rhsim08G0025600 | 1.18 | 0.277 | 0.368 | 0.567 |
| RHSIM_Rhsim10G0215100 | 1.18 | 0.277 | 0.368 | 0.122 |
| RHSIM_Rhsim01G0109100 | 1.18 | 0.277 | 0.368 | -0.332 |
| RHSIM_Rhsim01G0251700 | 1.18 | 0.277 | 0.368 | -0.205 |
| novel.1744 | 1.18 | 0.277 | 0.368 | -0.413 |
| RHSIM_Rhsim12G0161400 | 1.18 | 0.277 | 0.368 | -36.764 |
| RHSIM_Rhsim01G0045900 | 1.18 | 0.278 | 0.368 | -2.585 |
| RHSIM_Rhsim13G0219900 | 1.18 | 0.278 | 0.368 | 0.100 |
| RHSIM_Rhsim12G0015300 | 1.18 | 0.278 | 0.368 | 0.278 |
| RHSIM_Rhsim08G0205600 | 1.18 | 0.278 | 0.369 | 0.210 |
| RHSIM_Rhsim03G0269700 | 1.18 | 0.278 | 0.369 | 0.189 |
| RHSIM_Rhsim12G0161800 | 1.18 | 0.278 | 0.369 | -0.173 |
| RHSIM_Rhsim13G0134600 | 1.18 | 0.278 | 0.369 | -1.910 |
| novel.1631 | 1.18 | 0.278 | 0.369 | -0.508 |
| RHSIM_Rhsim12G0202700 | 1.18 | 0.278 | 0.369 | -0.061 |
| novel.3408 | 1.18 | 0.278 | 0.369 | 0.203 |
| RHSIM_Rhsim11G0123200 | 1.18 | 0.278 | 0.369 | -0.922 |
| RHSIM_Rhsim12G0056200 | 1.18 | 0.278 | 0.369 | 1.217 |
| novel.4108 | 1.18 | 0.278 | 0.369 | 1.386 |
| RHSIM_Rhsim03G0199000 | 1.18 | 0.278 | 0.369 | -36.605 |
| RHSIM_Rhsim01G0188200 | 1.18 | 0.278 | 0.369 | -1.365 |
| RHSIM_RhsimUnG0135500 | 1.17 | 0.278 | 0.369 | 36.822 |

| Gene | LRT statistic | P value | BH-adjusted P | log2FC (interaction) |
|---|---|---|---|---|
| RHSIM_Rhsim09G0010600 | 1.17 | 0.278 | 0.369 | -0.090 |
| RHSIM_Rhsim07G0126100 | 1.17 | 0.279 | 0.369 | 0.129 |
| RHSIM_Rhsim03G0129100 | 1.17 | 0.279 | 0.369 | -2.533 |
| RHSIM_Rhsim06G0162000 | 1.17 | 0.279 | 0.370 | -0.626 |
| novel.4210 | 1.17 | 0.279 | 0.370 | 1.014 |
| novel.450 | 1.17 | 0.279 | 0.370 | -36.741 |
| RHSIM_Rhsim01G0158600 | 1.17 | 0.279 | 0.370 | -0.217 |
| novel.1485 | 1.17 | 0.279 | 0.370 | 0.205 |
| novel.315 | 1.17 | 0.279 | 0.370 | -1.197 |
| novel.1731 | 1.17 | 0.279 | 0.370 | 1.092 |
| novel.678 | 1.17 | 0.279 | 0.370 | -1.202 |
| novel.3549 | 1.17 | 0.279 | 0.370 | -1.527 |
| RHSIM_Rhsim13G0223800 | 1.17 | 0.279 | 0.370 | 0.056 |
| RHSIM_Rhsim10G0208000 | 1.17 | 0.279 | 0.370 | 0.306 |
| novel.4412 | 1.17 | 0.279 | 0.370 | 1.427 |
| RHSIM_Rhsim11G0196500 | 1.17 | 0.279 | 0.370 | 0.848 |
| RHSIM_Rhsim02G0010200 | 1.17 | 0.279 | 0.370 | 1.636 |
| RHSIM_Rhsim10G0159200 | 1.17 | 0.279 | 0.370 | 1.021 |
| novel.4356 | 1.17 | 0.279 | 0.370 | 0.294 |
| RHSIM_Rhsim04G0125600 | 1.17 | 0.279 | 0.370 | 37.022 |
| novel.4132 | 1.17 | 0.279 | 0.370 | 0.940 |
| RHSIM_Rhsim02G0238800 | 1.17 | 0.280 | 0.370 | 0.239 |
| RHSIM_Rhsim06G0018400 | 1.17 | 0.280 | 0.370 | 0.080 |
| RHSIM_Rhsim03G0188200 | 1.17 | 0.280 | 0.371 | 0.120 |
| novel.915 | 1.17 | 0.280 | 0.371 | 1.517 |
| RHSIM_Rhsim10G0012400 | 1.17 | 0.280 | 0.371 | 0.448 |
| RHSIM_Rhsim07G0158300 | 1.17 | 0.280 | 0.371 | 0.341 |
| RHSIM_Rhsim08G0156000 | 1.17 | 0.280 | 0.371 | -0.622 |
| RHSIM_RhsimUnG0094600 | 1.17 | 0.280 | 0.371 | -0.191 |
| RHSIM_Rhsim09G0018800 | 1.17 | 0.280 | 0.371 | -0.360 |
| RHSIM_RhsimUnG0090500 | 1.17 | 0.280 | 0.371 | 0.212 |
| RHSIM_Rhsim01G0165400 | 1.17 | 0.280 | 0.371 | 1.027 |

| Gene | LRT statistic | P value | BH-adjusted P | log2FC (interaction) |
|---|---|---|---|---|
| RHSIM_Rhsim08G0127200 | 1.17 | 0.280 | 0.371 | -36.990 |
| RHSIM_Rhsim08G0105900 | 1.17 | 0.280 | 0.371 | -1.703 |
| RHSIM_Rhsim06G0216100 | 1.17 | 0.280 | 0.371 | 0.994 |
| RHSIM_Rhsim01G0227900 | 1.17 | 0.280 | 0.371 | 0.143 |
| RHSIM_Rhsim07G0041800 | 1.17 | 0.280 | 0.371 | -0.543 |
| RHSIM_Rhsim06G0063600 | 1.17 | 0.280 | 0.371 | -0.275 |
| RHSIM_Rhsim07G0118100 | 1.16 | 0.280 | 0.371 | 1.311 |
| RHSIM_Rhsim08G0231000 | 1.16 | 0.280 | 0.371 | -0.146 |
| novel.117 | 1.16 | 0.280 | 0.371 | -0.971 |
| novel.3953 | 1.16 | 0.281 | 0.371 | 1.071 |
| novel.4521 | 1.16 | 0.281 | 0.372 | 1.698 |
| RHSIM_RhsimUnG0129100 | 1.16 | 0.281 | 0.372 | -1.237 |
| RHSIM_Rhsim03G0047800 | 1.16 | 0.281 | 0.372 | -36.992 |
| RHSIM_Rhsim05G0063800 | 1.16 | 0.281 | 0.372 | -1.672 |
| RHSIM_Rhsim06G0023300 | 1.16 | 0.281 | 0.372 | -0.216 |
| RHSIM_RhsimUnG0020100 | 1.16 | 0.281 | 0.372 | 1.444 |
| RHSIM_Rhsim03G0110500 | 1.16 | 0.281 | 0.372 | 0.945 |
| novel.799 | 1.16 | 0.281 | 0.372 | 0.934 |
| RHSIM_Rhsim13G0075200 | 1.16 | 0.281 | 0.372 | -1.626 |
| RHSIM_Rhsim01G0086800 | 1.16 | 0.282 | 0.373 | 0.768 |
| RHSIM_Rhsim03G0048000 | 1.16 | 0.282 | 0.373 | -0.348 |
| RHSIM_Rhsim12G0096100 | 1.16 | 0.282 | 0.373 | -1.944 |
| RHSIM_Rhsim02G0043700 | 1.16 | 0.282 | 0.373 | -0.286 |
| RHSIM_Rhsim12G0009400 | 1.16 | 0.282 | 0.373 | -1.819 |
| RHSIM_Rhsim03G0223600 | 1.16 | 0.282 | 0.373 | -0.123 |
| RHSIM_RhsimUnG0159100 | 1.16 | 0.282 | 0.373 | 37.097 |
| novel.34 | 1.16 | 0.282 | 0.373 | 1.962 |
| RHSIM_Rhsim07G0056600 | 1.16 | 0.282 | 0.374 | 0.230 |
| RHSIM_Rhsim09G0007100 | 1.15 | 0.283 | 0.374 | 1.239 |
| RHSIM_Rhsim07G0161000 | 1.15 | 0.283 | 0.374 | 2.480 |
| RHSIM_Rhsim10G0052000 | 1.15 | 0.283 | 0.374 | 1.773 |
| RHSIM_Rhsim07G0239100 | 1.15 | 0.283 | 0.374 | -1.351 |

| Gene | LRT statistic | P value | BH-adjusted P | log2FC (interaction) |
|---|---|---|---|---|
| RHSIM_Rhsim04G0248200 | 1.15 | 0.283 | 0.374 | 0.113 |
| RHSIM_Rhsim08G0119700 | 1.15 | 0.283 | 0.374 | 0.408 |
| novel.4275 | 1.15 | 0.283 | 0.374 | 1.419 |
| RHSIM_Rhsim03G0211800 | 1.15 | 0.283 | 0.374 | 0.479 |
| novel.1217 | 1.15 | 0.283 | 0.374 | 36.833 |
| RHSIM_Rhsim05G0140800 | 1.15 | 0.283 | 0.374 | -36.860 |
| RHSIM_Rhsim06G0215100 | 1.15 | 0.283 | 0.375 | -0.840 |
| RHSIM_Rhsim04G0126000 | 1.15 | 0.283 | 0.375 | 0.110 |
| RHSIM_Rhsim01G0001300 | 1.15 | 0.283 | 0.375 | 0.266 |
| RHSIM_Rhsim01G0081100 | 1.15 | 0.284 | 0.375 | -2.732 |
| RHSIM_Rhsim09G0093000 | 1.15 | 0.284 | 0.375 | 2.064 |
| RHSIM_Rhsim03G0016900 | 1.15 | 0.284 | 0.375 | 0.934 |
| RHSIM_Rhsim12G0074100 | 1.15 | 0.284 | 0.375 | -0.104 |
| RHSIM_Rhsim05G0040900 | 1.15 | 0.284 | 0.375 | -0.118 |
| novel.184 | 1.15 | 0.284 | 0.375 | -36.552 |
| novel.3559 | 1.15 | 0.284 | 0.375 | 0.248 |
| novel.4425 | 1.15 | 0.284 | 0.375 | 2.015 |
| novel.79 | 1.15 | 0.284 | 0.375 | 0.711 |
| novel.1964 | 1.15 | 0.284 | 0.375 | 0.546 |
| novel.4502 | 1.15 | 0.284 | 0.375 | 1.192 |
| RHSIM_Rhsim01G0050100 | 1.15 | 0.284 | 0.376 | 1.441 |
| RHSIM_Rhsim01G0117600 | 1.15 | 0.284 | 0.376 | 37.237 |
| RHSIM_Rhsim08G0071300 | 1.15 | 0.284 | 0.376 | 36.583 |
| RHSIM_Rhsim06G0045500 | 1.15 | 0.284 | 0.376 | 37.179 |
| RHSIM_Rhsim01G0271200 | 1.15 | 0.285 | 0.376 | 36.867 |
| novel.2021 | 1.14 | 0.285 | 0.376 | -0.806 |
| RHSIM_Rhsim08G0054300 | 1.14 | 0.285 | 0.376 | 1.554 |
| novel.1928 | 1.14 | 0.285 | 0.376 | 0.505 |
| novel.3133 | 1.14 | 0.285 | 0.376 | -0.409 |
| RHSIM_Rhsim13G0159700 | 1.14 | 0.285 | 0.376 | 0.811 |
| RHSIM_RhsimUnG0020800 | 1.14 | 0.285 | 0.377 | 0.281 |
| RHSIM_RhsimUnG0251400 | 1.14 | 0.285 | 0.377 | -36.307 |

| Gene | LRT statistic | P value | BH-adjusted P | log2FC (interaction) |
|---|---|---|---|---|
| novel.2228 | 1.14 | 0.286 | 0.377 | 0.445 |
| RHSIM_Rhsim06G0235100 | 1.14 | 0.286 | 0.377 | -1.089 |
| RHSIM_RhsimUnG0209300 | 1.14 | 0.286 | 0.377 | 0.220 |
| novel.114 | 1.14 | 0.286 | 0.377 | 1.712 |
| RHSIM_Rhsim03G0134000 | 1.14 | 0.286 | 0.377 | 0.123 |
| RHSIM_RhsimUnG0013000 | 1.14 | 0.286 | 0.377 | -0.527 |
| novel.2273 | 1.14 | 0.286 | 0.377 | -0.496 |
| RHSIM_Rhsim10G0032000 | 1.14 | 0.286 | 0.377 | 0.412 |
| RHSIM_Rhsim02G0134500 | 1.14 | 0.286 | 0.377 | 1.503 |
| RHSIM_RhsimMtG0007100 | 1.14 | 0.286 | 0.378 | 0.624 |
| RHSIM_Rhsim09G0038600 | 1.14 | 0.286 | 0.378 | 37.004 |
| novel.2668 | 1.14 | 0.286 | 0.378 | 0.839 |
| RHSIM_Rhsim01G0129100 | 1.14 | 0.287 | 0.378 | 0.125 |
| RHSIM_Rhsim09G0151000 | 1.13 | 0.287 | 0.378 | 2.126 |
| RHSIM_Rhsim08G0049400 | 1.13 | 0.287 | 0.378 | -0.168 |
| RHSIM_Rhsim07G0166600 | 1.13 | 0.287 | 0.378 | 1.763 |
| novel.4340 | 1.13 | 0.287 | 0.378 | 0.360 |
| novel.4013 | 1.13 | 0.287 | 0.379 | 0.751 |
| RHSIM_Rhsim11G0112700 | 1.13 | 0.287 | 0.379 | 0.328 |
| novel.748 | 1.13 | 0.287 | 0.379 | 0.346 |
| novel.2646 | 1.13 | 0.287 | 0.379 | 2.241 |
| novel.1834 | 1.13 | 0.287 | 0.379 | 0.827 |
| RHSIM_Rhsim04G0048400 | 1.13 | 0.288 | 0.379 | -37.002 |
| RHSIM_Rhsim01G0088500 | 1.13 | 0.288 | 0.379 | 2.699 |
| RHSIM_Rhsim11G0142900 | 1.13 | 0.288 | 0.379 | -1.140 |
| RHSIM_Rhsim01G0008900 | 1.13 | 0.288 | 0.379 | 0.138 |
| RHSIM_RhsimUnG0121500 | 1.13 | 0.288 | 0.380 | 1.443 |
| novel.1316 | 1.13 | 0.288 | 0.380 | -0.149 |
| RHSIM_Rhsim01G0244600 | 1.13 | 0.288 | 0.380 | -0.143 |
| RHSIM_Rhsim01G0042300 | 1.13 | 0.288 | 0.380 | -36.575 |
| RHSIM_Rhsim07G0208300 | 1.13 | 0.288 | 0.380 | -0.691 |
| RHSIM_Rhsim09G0069000 | 1.13 | 0.288 | 0.380 | 0.091 |

| Gene | LRT statistic | P value | BH-adjusted P | log2FC (interaction) |
|---|---|---|---|---|
| RHSIM_Rhsim01G0018400 | 1.13 | 0.288 | 0.380 | -1.304 |
| RHSIM_Rhsim08G0145500 | 1.13 | 0.288 | 0.380 | 1.147 |
| RHSIM_Rhsim01G0190600 | 1.13 | 0.289 | 0.380 | 1.240 |
| RHSIM_RhsimUnG0098600 | 1.13 | 0.289 | 0.380 | 0.241 |
| RHSIM_Rhsim04G0176200 | 1.13 | 0.289 | 0.380 | 1.997 |
| RHSIM_Rhsim07G0228100 | 1.13 | 0.289 | 0.380 | -0.093 |
| RHSIM_Rhsim07G0196800 | 1.13 | 0.289 | 0.380 | -0.395 |
| novel.945 | 1.13 | 0.289 | 0.380 | 1.633 |
| RHSIM_Rhsim04G0143400 | 1.13 | 0.289 | 0.380 | -0.630 |
| RHSIM_Rhsim08G0003600 | 1.13 | 0.289 | 0.380 | 0.124 |
| RHSIM_Rhsim08G0077300 | 1.12 | 0.289 | 0.381 | 0.211 |
| RHSIM_Rhsim07G0121300 | 1.12 | 0.289 | 0.381 | -0.962 |
| RHSIM_Rhsim04G0146300 | 1.12 | 0.289 | 0.381 | 0.177 |
| RHSIM_Rhsim05G0112200 | 1.12 | 0.289 | 0.381 | 1.489 |
| RHSIM_Rhsim09G0106700 | 1.12 | 0.289 | 0.381 | 0.215 |
| RHSIM_Rhsim09G0044300 | 1.12 | 0.289 | 0.381 | -37.131 |
| RHSIM_RhsimUnG0026500 | 1.12 | 0.289 | 0.381 | 1.537 |
| RHSIM_Rhsim03G0066200 | 1.12 | 0.289 | 0.381 | -1.417 |
| RHSIM_Rhsim01G0060400 | 1.12 | 0.290 | 0.381 | 0.460 |
| RHSIM_Rhsim06G0148800 | 1.12 | 0.290 | 0.382 | -2.163 |
| RHSIM_Rhsim01G0151700 | 1.12 | 0.290 | 0.382 | 2.109 |
| novel.2352 | 1.12 | 0.290 | 0.382 | -1.172 |
| RHSIM_RhsimUnG0060800 | 1.12 | 0.290 | 0.382 | 0.205 |
| RHSIM_Rhsim02G0014000 | 1.12 | 0.290 | 0.382 | 0.217 |
| RHSIM_Rhsim06G0032800 | 1.12 | 0.290 | 0.382 | 0.115 |
| novel.885 | 1.12 | 0.290 | 0.382 | 1.623 |
| RHSIM_Rhsim03G0244100 | 1.12 | 0.290 | 0.382 | 1.274 |
| RHSIM_Rhsim09G0183800 | 1.12 | 0.290 | 0.382 | 1.249 |
| RHSIM_Rhsim12G0158300 | 1.12 | 0.290 | 0.382 | 0.567 |
| RHSIM_Rhsim12G0084300 | 1.12 | 0.291 | 0.382 | 2.097 |
| RHSIM_Rhsim10G0010700 | 1.12 | 0.291 | 0.383 | 2.760 |
| RHSIM_Rhsim06G0179200 | 1.12 | 0.291 | 0.383 | -0.134 |

| Gene | LRT statistic | P value | BH-adjusted P | log2FC (interaction) |
|---|---|---|---|---|
| novel.2899 | 1.12 | 0.291 | 0.383 | -1.022 |
| RHSIM_Rhsim11G0086800 | 1.12 | 0.291 | 0.383 | -0.161 |
| RHSIM_Rhsim04G0012700 | 1.11 | 0.291 | 0.383 | 1.971 |
| RHSIM_Rhsim02G0040500 | 1.11 | 0.291 | 0.383 | 0.252 |
| RHSIM_Rhsim03G0074200 | 1.11 | 0.291 | 0.383 | -0.279 |
| RHSIM_Rhsim11G0099000 | 1.11 | 0.291 | 0.383 | 0.959 |
| RHSIM_Rhsim06G0240700 | 1.11 | 0.291 | 0.384 | -0.259 |
| RHSIM_Rhsim13G0015900 | 1.11 | 0.291 | 0.384 | -0.364 |
| RHSIM_Rhsim09G0044100 | 1.11 | 0.292 | 0.384 | -0.123 |
| RHSIM_Rhsim01G0293100 | 1.11 | 0.292 | 0.384 | -0.232 |
| RHSIM_Rhsim12G0214200 | 1.11 | 0.292 | 0.384 | -0.914 |
| RHSIM_Rhsim13G0173100 | 1.11 | 0.292 | 0.384 | 0.467 |
| RHSIM_Rhsim03G0113000 | 1.11 | 0.292 | 0.384 | 0.161 |
| RHSIM_Rhsim07G0229100 | 1.11 | 0.292 | 0.384 | 36.907 |
| RHSIM_Rhsim06G0223700 | 1.11 | 0.292 | 0.384 | 0.105 |
| RHSIM_Rhsim10G0153000 | 1.11 | 0.292 | 0.384 | -0.455 |
| novel.1113 | 1.11 | 0.292 | 0.385 | 0.608 |
| RHSIM_Rhsim01G0090000 | 1.11 | 0.292 | 0.385 | -0.391 |
| novel.4059 | 1.11 | 0.292 | 0.385 | 0.477 |
| RHSIM_Rhsim07G0131500 | 1.11 | 0.292 | 0.385 | -0.457 |
| RHSIM_Rhsim08G0121700 | 1.11 | 0.292 | 0.385 | 0.214 |
| RHSIM_RhsimUnG0185300 | 1.11 | 0.292 | 0.385 | 2.159 |
| RHSIM_Rhsim05G0169800 | 1.11 | 0.293 | 0.385 | 2.237 |
| novel.3785 | 1.11 | 0.293 | 0.385 | 0.184 |
| RHSIM_Rhsim03G0083200 | 1.11 | 0.293 | 0.385 | 0.460 |
| novel.1296 | 1.11 | 0.293 | 0.385 | 0.506 |
| RHSIM_RhsimUnG0108200 | 1.11 | 0.293 | 0.385 | -2.717 |
| RHSIM_Rhsim02G0255300 | 1.11 | 0.293 | 0.385 | -37.155 |
| RHSIM_Rhsim13G0177600 | 1.11 | 0.293 | 0.385 | 0.709 |
| RHSIM_Rhsim09G0093100 | 1.11 | 0.293 | 0.385 | -36.462 |
| RHSIM_Rhsim06G0067900 | 1.11 | 0.293 | 0.385 | 2.071 |
| RHSIM_Rhsim01G0226300 | 1.11 | 0.293 | 0.385 | -0.110 |

| Gene | LRT statistic | P value | BH-adjusted P | log2FC (interaction) |
|---|---|---|---|---|
| RHSIM_Rhsim04G0207000 | 1.10 | 0.293 | 0.385 | 0.126 |
| RHSIM_Rhsim08G0102600 | 1.10 | 0.293 | 0.386 | 2.125 |
| RHSIM_Rhsim03G0236100 | 1.10 | 0.293 | 0.386 | -36.920 |
| RHSIM_Rhsim09G0136400 | 1.10 | 0.294 | 0.386 | 0.758 |
| novel.4079 | 1.10 | 0.294 | 0.386 | 0.503 |
| RHSIM_Rhsim01G0182900 | 1.10 | 0.294 | 0.386 | -0.207 |
| RHSIM_Rhsim06G0121100 | 1.10 | 0.294 | 0.386 | -2.608 |
| RHSIM_Rhsim10G0086100 | 1.10 | 0.294 | 0.386 | -1.994 |
| RHSIM_Rhsim08G0048100 | 1.10 | 0.294 | 0.386 | 0.125 |
| RHSIM_Rhsim13G0182500 | 1.10 | 0.294 | 0.386 | -36.742 |
| RHSIM_RhsimUnG0013100 | 1.10 | 0.294 | 0.387 | -0.296 |
| novel.3040 | 1.10 | 0.294 | 0.387 | 0.546 |
| RHSIM_Rhsim10G0097300 | 1.10 | 0.294 | 0.387 | -0.397 |
| RHSIM_Rhsim11G0102300 | 1.10 | 0.295 | 0.387 | 0.795 |
| RHSIM_Rhsim09G0077200 | 1.10 | 0.295 | 0.387 | 1.341 |
| RHSIM_Rhsim01G0150400 | 1.10 | 0.295 | 0.387 | 0.267 |
| RHSIM_Rhsim08G0014200 | 1.10 | 0.295 | 0.387 | -1.013 |
| RHSIM_Rhsim03G0057900 | 1.10 | 0.295 | 0.387 | 1.275 |
| RHSIM_Rhsim12G0049500 | 1.10 | 0.295 | 0.387 | -1.317 |
| novel.1058 | 1.10 | 0.295 | 0.387 | 1.631 |
| RHSIM_Rhsim06G0062200 | 1.10 | 0.295 | 0.387 | -0.113 |
| novel.2370 | 1.10 | 0.295 | 0.388 | 1.334 |
| RHSIM_Rhsim05G0104000 | 1.09 | 0.295 | 0.388 | -0.093 |
| RHSIM_Rhsim08G0090500 | 1.09 | 0.295 | 0.388 | 1.742 |
| novel.2132 | 1.09 | 0.295 | 0.388 | -1.430 |
| RHSIM_Rhsim04G0145600 | 1.09 | 0.295 | 0.388 | -0.088 |
| RHSIM_Rhsim08G0005700 | 1.09 | 0.295 | 0.388 | -0.708 |
| RHSIM_Rhsim12G0203600 | 1.09 | 0.295 | 0.388 | -0.601 |
| novel.3604 | 1.09 | 0.296 | 0.388 | 0.860 |
| RHSIM_Rhsim10G0129400 | 1.09 | 0.296 | 0.388 | 0.678 |
| RHSIM_Rhsim03G0213800 | 1.09 | 0.296 | 0.388 | 0.395 |
| RHSIM_Rhsim06G0240800 | 1.09 | 0.296 | 0.388 | -0.236 |

| Gene | LRT statistic | P value | BH-adjusted P | log2FC (interaction) |
|---|---|---|---|---|
| RHSIM_Rhsim01G0172800 | 1.09 | 0.296 | 0.388 | 36.776 |
| novel.4205 | 1.09 | 0.296 | 0.388 | 1.249 |
| novel.1121 | 1.09 | 0.296 | 0.388 | 1.061 |
| novel.886 | 1.09 | 0.296 | 0.388 | -1.343 |
| RHSIM_Rhsim03G0115900 | 1.09 | 0.296 | 0.388 | 0.865 |
| novel.964 | 1.09 | 0.296 | 0.388 | 0.449 |
| RHSIM_Rhsim13G0221500 | 1.09 | 0.296 | 0.388 | 0.145 |
| RHSIM_Rhsim12G0069000 | 1.09 | 0.296 | 0.389 | 0.190 |
| RHSIM_Rhsim13G0171900 | 1.09 | 0.296 | 0.389 | 0.269 |
| RHSIM_Rhsim02G0168100 | 1.09 | 0.296 | 0.389 | -0.237 |
| RHSIM_Rhsim04G0120200 | 1.09 | 0.296 | 0.389 | -0.175 |
| novel.3925 | 1.09 | 0.296 | 0.389 | -0.776 |
| RHSIM_Rhsim04G0187300 | 1.09 | 0.296 | 0.389 | -0.518 |
| RHSIM_Rhsim01G0186200 | 1.09 | 0.297 | 0.389 | 0.173 |
| RHSIM_RhsimUnG0148700 | 1.09 | 0.297 | 0.389 | 2.739 |
| RHSIM_Rhsim11G0156900 | 1.09 | 0.297 | 0.389 | -36.699 |
| RHSIM_Rhsim03G0076900 | 1.09 | 0.297 | 0.389 | -0.107 |
| RHSIM_Rhsim01G0124300 | 1.09 | 0.297 | 0.390 | 0.154 |
| RHSIM_Rhsim11G0125000 | 1.09 | 0.297 | 0.390 | -1.153 |
| RHSIM_Rhsim09G0098100 | 1.09 | 0.297 | 0.390 | -0.056 |
| RHSIM_Rhsim03G0151500 | 1.09 | 0.298 | 0.390 | 1.837 |
| RHSIM_Rhsim01G0194600 | 1.09 | 0.298 | 0.390 | 0.744 |
| novel.1235 | 1.08 | 0.298 | 0.390 | 1.518 |
| RHSIM_Rhsim01G0051700 | 1.08 | 0.298 | 0.390 | 0.199 |
| RHSIM_Rhsim10G0001300 | 1.08 | 0.298 | 0.390 | -0.087 |
| RHSIM_Rhsim06G0201500 | 1.08 | 0.298 | 0.390 | 0.126 |
| RHSIM_Rhsim03G0001400 | 1.08 | 0.298 | 0.390 | 0.286 |
| RHSIM_RhsimUnG0073600 | 1.08 | 0.298 | 0.390 | 0.964 |
| novel.2375 | 1.08 | 0.298 | 0.391 | 0.923 |
| RHSIM_Rhsim03G0267500 | 1.08 | 0.298 | 0.391 | -1.923 |
| RHSIM_Rhsim03G0178200 | 1.08 | 0.298 | 0.391 | -0.097 |
| RHSIM_Rhsim13G0142800 | 1.08 | 0.298 | 0.391 | -0.500 |

| Gene | LRT statistic | P value | BH-adjusted P | log2FC (interaction) |
|---|---|---|---|---|
| RHSIM_RhsimUnG0027600 | 1.08 | 0.298 | 0.391 | -36.989 |
| RHSIM_RhsimUnG0051400 | 1.08 | 0.299 | 0.391 | 0.116 |
| RHSIM_Rhsim02G0078500 | 1.08 | 0.299 | 0.391 | -0.457 |
| RHSIM_Rhsim10G0005300 | 1.08 | 0.299 | 0.392 | 2.433 |
| novel.3470 | 1.08 | 0.299 | 0.392 | -0.425 |
| RHSIM_Rhsim01G0068500 | 1.08 | 0.299 | 0.392 | 0.098 |
| RHSIM_Rhsim02G0171400 | 1.08 | 0.299 | 0.392 | -0.192 |
| novel.1999 | 1.08 | 0.299 | 0.392 | -0.412 |
| RHSIM_Rhsim12G0197700 | 1.08 | 0.299 | 0.392 | 0.128 |
| RHSIM_Rhsim03G0032200 | 1.08 | 0.300 | 0.392 | -0.226 |
| RHSIM_Rhsim01G0104400 | 1.08 | 0.300 | 0.392 | 0.780 |
| RHSIM_Rhsim08G0237200 | 1.08 | 0.300 | 0.392 | -0.792 |
| RHSIM_Rhsim03G0003100 | 1.08 | 0.300 | 0.392 | -36.745 |
| RHSIM_Rhsim13G0152600 | 1.08 | 0.300 | 0.392 | -36.974 |
| RHSIM_Rhsim11G0173700 | 1.08 | 0.300 | 0.393 | -0.346 |
| RHSIM_Rhsim13G0225800 | 1.08 | 0.300 | 0.393 | 0.092 |
| RHSIM_Rhsim09G0019800 | 1.08 | 0.300 | 0.393 | 1.080 |
| RHSIM_Rhsim03G0053500 | 1.07 | 0.300 | 0.393 | -1.593 |
| novel.2650 | 1.07 | 0.300 | 0.393 | -1.502 |
| novel.2539 | 1.07 | 0.300 | 0.393 | -0.161 |
| RHSIM_Rhsim05G0109100 | 1.07 | 0.300 | 0.393 | 1.161 |
| RHSIM_Rhsim02G0080300 | 1.07 | 0.300 | 0.393 | 0.334 |
| novel.840 | 1.07 | 0.300 | 0.393 | 0.379 |
| RHSIM_RhsimUnG0084100 | 1.07 | 0.300 | 0.393 | 0.227 |
| RHSIM_Rhsim07G0097300 | 1.07 | 0.300 | 0.393 | -36.897 |
| RHSIM_Rhsim13G0078000 | 1.07 | 0.300 | 0.393 | 0.219 |
| RHSIM_Rhsim10G0063800 | 1.07 | 0.301 | 0.394 | -2.141 |
| RHSIM_Rhsim05G0075100 | 1.07 | 0.301 | 0.394 | 0.116 |
| RHSIM_Rhsim13G0183500 | 1.07 | 0.301 | 0.394 | -0.650 |
| RHSIM_Rhsim07G0053800 | 1.07 | 0.301 | 0.394 | -0.062 |
| RHSIM_Rhsim04G0022100 | 1.07 | 0.301 | 0.394 | 39.262 |
| RHSIM_Rhsim10G0161700 | 1.07 | 0.301 | 0.394 | 0.215 |

| Gene | LRT statistic | P value | BH-adjusted P | log2FC (interaction) |
|---|---|---|---|---|
| RHSIM_Rhsim10G0074500 | 1.07 | 0.301 | 0.394 | 0.874 |
| RHSIM_Rhsim11G0100200 | 1.07 | 0.301 | 0.394 | -0.154 |
| RHSIM_Rhsim10G0073600 | 1.07 | 0.301 | 0.394 | -0.326 |
| RHSIM_RhsimUnG0013800 | 1.07 | 0.301 | 0.394 | -0.394 |
| RHSIM_Rhsim04G0064100 | 1.07 | 0.301 | 0.394 | -0.935 |
| RHSIM_RhsimUnG0064000 | 1.07 | 0.301 | 0.394 | 36.962 |
| RHSIM_Rhsim01G0289300 | 1.07 | 0.301 | 0.394 | 37.405 |
| RHSIM_Rhsim09G0050900 | 1.07 | 0.301 | 0.394 | 1.113 |
| RHSIM_Rhsim05G0011700 | 1.07 | 0.301 | 0.394 | -1.943 |
| RHSIM_Rhsim04G0017500 | 1.07 | 0.301 | 0.394 | 0.267 |
| RHSIM_Rhsim05G0156600 | 1.07 | 0.302 | 0.395 | -0.990 |
| RHSIM_Rhsim04G0104200 | 1.07 | 0.302 | 0.395 | -36.886 |
| RHSIM_Rhsim10G0041200 | 1.07 | 0.302 | 0.395 | -1.080 |
| RHSIM_Rhsim03G0089100 | 1.07 | 0.302 | 0.395 | 0.889 |
| RHSIM_Rhsim02G0057200 | 1.07 | 0.302 | 0.395 | -0.668 |
| RHSIM_Rhsim13G0200400 | 1.07 | 0.302 | 0.395 | -0.252 |
| RHSIM_Rhsim04G0231500 | 1.07 | 0.302 | 0.395 | 0.103 |
| RHSIM_Rhsim10G0166000 | 1.07 | 0.302 | 0.395 | -1.009 |
| novel.3996 | 1.07 | 0.302 | 0.395 | 0.500 |
| RHSIM_Rhsim10G0188700 | 1.07 | 0.302 | 0.395 | -0.152 |
| RHSIM_Rhsim08G0029800 | 1.06 | 0.302 | 0.395 | 0.134 |
| RHSIM_Rhsim05G0106600 | 1.06 | 0.302 | 0.395 | 0.871 |
| RHSIM_Rhsim08G0240100 | 1.06 | 0.302 | 0.395 | -36.680 |
| novel.2385 | 1.06 | 0.303 | 0.395 | -0.452 |
| RHSIM_RhsimUnG0026400 | 1.06 | 0.303 | 0.396 | 0.493 |
| RHSIM_Rhsim06G0022100 | 1.06 | 0.303 | 0.396 | -0.167 |
| RHSIM_Rhsim06G0094100 | 1.06 | 0.303 | 0.396 | 1.913 |
| RHSIM_Rhsim13G0051700 | 1.06 | 0.303 | 0.396 | 0.097 |
| RHSIM_Rhsim12G0138600 | 1.06 | 0.303 | 0.396 | 0.637 |
| RHSIM_Rhsim06G0083000 | 1.06 | 0.303 | 0.396 | 1.462 |
| RHSIM_Rhsim08G0057000 | 1.06 | 0.303 | 0.396 | 0.567 |
| RHSIM_Rhsim08G0063900 | 1.06 | 0.303 | 0.396 | 0.097 |

| Gene | LRT statistic | P value | BH-adjusted P | log2FC (interaction) |
|---|---|---|---|---|
| novel.1509 | 1.06 | 0.303 | 0.396 | 1.131 |
| novel.1832 | 1.06 | 0.304 | 0.397 | 1.751 |
| RHSIM_Rhsim04G0121300 | 1.06 | 0.304 | 0.397 | 0.530 |
| RHSIM_Rhsim12G0009600 | 1.06 | 0.304 | 0.397 | 1.047 |
| RHSIM_Rhsim03G0251800 | 1.06 | 0.304 | 0.397 | 0.171 |
| RHSIM_Rhsim13G0074700 | 1.06 | 0.304 | 0.397 | 0.252 |
| RHSIM_Rhsim06G0006000 | 1.06 | 0.304 | 0.397 | 2.688 |
| RHSIM_Rhsim07G0170300 | 1.05 | 0.304 | 0.398 | -0.202 |
| RHSIM_Rhsim01G0060500 | 1.05 | 0.305 | 0.398 | 1.327 |
| RHSIM_Rhsim02G0097600 | 1.05 | 0.305 | 0.398 | 0.978 |
| RHSIM_Rhsim10G0078500 | 1.05 | 0.305 | 0.398 | 0.142 |
| RHSIM_Rhsim11G0135700 | 1.05 | 0.305 | 0.398 | 0.976 |
| RHSIM_RhsimUnG0071100 | 1.05 | 0.305 | 0.398 | 0.726 |
| RHSIM_Rhsim02G0052100 | 1.05 | 0.305 | 0.398 | 37.092 |
| RHSIM_Rhsim03G0012900 | 1.05 | 0.305 | 0.398 | 0.571 |
| RHSIM_Rhsim05G0192900 | 1.05 | 0.305 | 0.398 | 0.173 |
| RHSIM_Rhsim09G0117300 | 1.05 | 0.305 | 0.398 | 0.147 |
| novel.789 | 1.05 | 0.305 | 0.398 | -36.590 |
| RHSIM_Rhsim06G0101200 | 1.05 | 0.305 | 0.399 | 1.260 |
| novel.2687 | 1.05 | 0.305 | 0.399 | 0.650 |
| RHSIM_Rhsim07G0110500 | 1.05 | 0.305 | 0.399 | -0.110 |
| novel.3084 | 1.05 | 0.305 | 0.399 | 1.686 |
| RHSIM_Rhsim04G0247200 | 1.05 | 0.306 | 0.399 | -0.126 |
| RHSIM_Rhsim06G0172400 | 1.05 | 0.306 | 0.399 | -0.599 |
| RHSIM_Rhsim05G0073900 | 1.05 | 0.306 | 0.399 | -36.110 |
| RHSIM_Rhsim06G0196100 | 1.05 | 0.306 | 0.399 | 1.994 |
| novel.2303 | 1.05 | 0.306 | 0.399 | -0.857 |
| RHSIM_Rhsim08G0088600 | 1.05 | 0.306 | 0.400 | -1.445 |
| RHSIM_Rhsim13G0121300 | 1.05 | 0.306 | 0.400 | 2.044 |
| RHSIM_Rhsim04G0048700 | 1.05 | 0.306 | 0.400 | 36.864 |
| RHSIM_Rhsim03G0026700 | 1.05 | 0.306 | 0.400 | 0.482 |
| RHSIM_Rhsim01G0268700 | 1.05 | 0.306 | 0.400 | -1.422 |

| Gene | LRT statistic | P value | BH-adjusted P | log2FC (interaction) |
|---|---|---|---|---|
| RHSIM_Rhsim04G0130200 | 1.05 | 0.306 | 0.400 | 0.115 |
| novel.4443 | 1.05 | 0.306 | 0.400 | 1.606 |
| RHSIM_Rhsim09G0018000 | 1.05 | 0.307 | 0.400 | 1.388 |
| RHSIM_Rhsim11G0037900 | 1.05 | 0.307 | 0.400 | 0.214 |
| RHSIM_Rhsim06G0129500 | 1.05 | 0.307 | 0.400 | -0.611 |
| RHSIM_Rhsim12G0072800 | 1.04 | 0.307 | 0.400 | 0.819 |
| RHSIM_Rhsim08G0012200 | 1.04 | 0.307 | 0.400 | 0.128 |
| RHSIM_Rhsim01G0173200 | 1.04 | 0.307 | 0.400 | -0.199 |
| RHSIM_Rhsim08G0028000 | 1.04 | 0.307 | 0.401 | -0.229 |
| RHSIM_Rhsim04G0153900 | 1.04 | 0.307 | 0.401 | 0.184 |
| RHSIM_Rhsim13G0182900 | 1.04 | 0.307 | 0.401 | -36.365 |
| RHSIM_Rhsim11G0175100 | 1.04 | 0.307 | 0.401 | -1.168 |
| RHSIM_Rhsim06G0115400 | 1.04 | 0.307 | 0.401 | -0.398 |
| RHSIM_Rhsim10G0198600 | 1.04 | 0.307 | 0.401 | -1.992 |
| RHSIM_Rhsim03G0247300 | 1.04 | 0.307 | 0.401 | 0.142 |
| RHSIM_Rhsim11G0161500 | 1.04 | 0.307 | 0.401 | 1.197 |
| RHSIM_Rhsim04G0218300 | 1.04 | 0.308 | 0.401 | -0.216 |
| RHSIM_Rhsim04G0001000 | 1.04 | 0.308 | 0.401 | -1.332 |
| RHSIM_Rhsim12G0213300 | 1.04 | 0.308 | 0.401 | -0.257 |
| novel.3142 | 1.04 | 0.308 | 0.401 | 0.385 |
| RHSIM_Rhsim04G0187800 | 1.04 | 0.308 | 0.401 | -0.574 |
| RHSIM_RhsimUnG0037000 | 1.04 | 0.308 | 0.401 | -0.185 |
| RHSIM_Rhsim12G0172300 | 1.04 | 0.308 | 0.401 | -0.908 |
| RHSIM_Rhsim11G0059600 | 1.04 | 0.308 | 0.401 | -0.342 |
| RHSIM_Rhsim02G0058600 | 1.04 | 0.308 | 0.402 | 0.580 |
| novel.1275 | 1.04 | 0.308 | 0.402 | 1.289 |
| RHSIM_Rhsim05G0141800 | 1.04 | 0.308 | 0.402 | 1.925 |
| RHSIM_Rhsim03G0043100 | 1.04 | 0.308 | 0.402 | 1.046 |
| RHSIM_Rhsim10G0111100 | 1.04 | 0.308 | 0.402 | 0.726 |
| novel.2999 | 1.04 | 0.308 | 0.402 | 0.557 |
| RHSIM_Rhsim04G0014200 | 1.04 | 0.308 | 0.402 | 0.210 |
| novel.4555 | 1.04 | 0.308 | 0.402 | 2.215 |

| Gene | LRT statistic | P value | BH-adjusted P | log2FC (interaction) |
|---|---|---|---|---|
| RHSIM_Rhsim09G0036800 | 1.04 | 0.308 | 0.402 | -0.164 |
| novel.1185 | 1.04 | 0.309 | 0.402 | 0.721 |
| RHSIM_Rhsim07G0059200 | 1.04 | 0.309 | 0.402 | -36.298 |
| RHSIM_Rhsim03G0175100 | 1.04 | 0.309 | 0.402 | -0.184 |
| RHSIM_Rhsim10G0069500 | 1.04 | 0.309 | 0.402 | 0.104 |
| RHSIM_Rhsim05G0128300 | 1.04 | 0.309 | 0.402 | -36.769 |
| novel.2821 | 1.03 | 0.309 | 0.403 | 1.480 |
| RHSIM_Rhsim07G0210700 | 1.03 | 0.309 | 0.403 | 0.793 |
| RHSIM_Rhsim10G0068400 | 1.03 | 0.309 | 0.403 | -1.454 |
| RHSIM_Rhsim10G0103700 | 1.03 | 0.309 | 0.403 | 1.962 |
| novel.141 | 1.03 | 0.309 | 0.403 | -0.241 |
| RHSIM_Rhsim13G0018200 | 1.03 | 0.310 | 0.403 | -0.109 |
| RHSIM_Rhsim13G0206800 | 1.03 | 0.310 | 0.403 | 0.305 |
| RHSIM_Rhsim11G0062100 | 1.03 | 0.310 | 0.403 | -36.209 |
| RHSIM_RhsimPtG0005500 | 1.03 | 0.310 | 0.403 | 0.482 |
| novel.743 | 1.03 | 0.310 | 0.403 | 0.746 |
| RHSIM_Rhsim04G0230600 | 1.03 | 0.310 | 0.403 | 0.118 |
| novel.4455 | 1.03 | 0.310 | 0.403 | 1.746 |
| novel.2211 | 1.03 | 0.310 | 0.403 | -1.344 |
| RHSIM_Rhsim02G0159500 | 1.03 | 0.310 | 0.403 | 0.189 |
| RHSIM_RhsimUnG0112900 | 1.03 | 0.310 | 0.403 | -0.415 |
| RHSIM_Rhsim12G0105800 | 1.03 | 0.310 | 0.404 | 0.419 |
| novel.3782 | 1.03 | 0.310 | 0.404 | -0.424 |
| novel.1351 | 1.03 | 0.310 | 0.404 | -1.198 |
| novel.1583 | 1.03 | 0.310 | 0.404 | -0.415 |
| RHSIM_RhsimUnG0016500 | 1.03 | 0.310 | 0.404 | 1.111 |
| RHSIM_Rhsim13G0033300 | 1.03 | 0.311 | 0.404 | 0.547 |
| RHSIM_Rhsim06G0010100 | 1.03 | 0.311 | 0.404 | 0.959 |
| RHSIM_Rhsim06G0213500 | 1.03 | 0.311 | 0.404 | -1.170 |
| novel.3927 | 1.03 | 0.311 | 0.404 | -0.876 |
| RHSIM_Rhsim11G0103200 | 1.03 | 0.311 | 0.404 | -0.136 |
| RHSIM_Rhsim12G0099200 | 1.03 | 0.311 | 0.404 | 0.180 |

| Gene | LRT statistic | P value | BH-adjusted P | log2FC (interaction) |
|---|---|---|---|---|
| RHSIM_Rhsim01G0057600 | 1.03 | 0.311 | 0.404 | -2.201 |
| RHSIM_Rhsim03G0099000 | 1.03 | 0.311 | 0.405 | -0.544 |
| novel.3249 | 1.03 | 0.311 | 0.405 | -0.117 |
| novel.739 | 1.03 | 0.311 | 0.405 | 1.284 |
| RHSIM_Rhsim12G0026700 | 1.03 | 0.311 | 0.405 | -36.545 |
| RHSIM_Rhsim08G0195200 | 1.03 | 0.311 | 0.405 | 0.793 |
| RHSIM_Rhsim08G0184100 | 1.03 | 0.311 | 0.405 | 0.280 |
| RHSIM_Rhsim03G0101400 | 1.03 | 0.311 | 0.405 | -0.302 |
| RHSIM_Rhsim10G0072600 | 1.02 | 0.311 | 0.405 | -0.128 |
| RHSIM_RhsimUnG0066000 | 1.02 | 0.311 | 0.405 | -1.404 |
| RHSIM_Rhsim01G0080800 | 1.02 | 0.312 | 0.405 | 1.200 |
| RHSIM_Rhsim08G0162100 | 1.02 | 0.312 | 0.405 | -36.932 |
| novel.4082 | 1.02 | 0.312 | 0.405 | 0.633 |
| RHSIM_Rhsim08G0158000 | 1.02 | 0.312 | 0.405 | 0.133 |
| RHSIM_Rhsim04G0108500 | 1.02 | 0.312 | 0.406 | -0.401 |
| RHSIM_Rhsim08G0053500 | 1.02 | 0.312 | 0.406 | -0.209 |
| RHSIM_Rhsim01G0119600 | 1.02 | 0.312 | 0.406 | -1.416 |
| novel.2144 | 1.02 | 0.312 | 0.406 | -0.773 |
| novel.2030 | 1.02 | 0.312 | 0.406 | 0.474 |
| RHSIM_Rhsim04G0045300 | 1.02 | 0.312 | 0.406 | -1.889 |
| RHSIM_Rhsim08G0202700 | 1.02 | 0.312 | 0.406 | 1.548 |
| RHSIM_Rhsim09G0132000 | 1.02 | 0.313 | 0.406 | -0.102 |
| novel.3997 | 1.02 | 0.313 | 0.406 | 0.504 |
| RHSIM_Rhsim01G0116600 | 1.02 | 0.313 | 0.406 | 0.128 |
| RHSIM_Rhsim02G0178200 | 1.02 | 0.313 | 0.406 | -1.321 |
| RHSIM_Rhsim04G0126700 | 1.02 | 0.313 | 0.407 | 1.895 |
| RHSIM_Rhsim02G0069100 | 1.02 | 0.313 | 0.407 | 1.274 |
| RHSIM_Rhsim10G0081100 | 1.02 | 0.313 | 0.407 | 0.670 |
| RHSIM_Rhsim09G0200800 | 1.02 | 0.313 | 0.407 | 1.280 |
| RHSIM_Rhsim01G0180700 | 1.02 | 0.313 | 0.407 | -0.352 |
| novel.691 | 1.02 | 0.313 | 0.407 | -1.009 |
| RHSIM_Rhsim01G0092600 | 1.02 | 0.313 | 0.407 | -1.220 |

| Gene | LRT statistic | P value | BH-adjusted P | log2FC (interaction) |
|---|---|---|---|---|
| RHSIM_Rhsim04G0149800 | 1.02 | 0.313 | 0.407 | 0.104 |
| RHSIM_Rhsim10G0194400 | 1.02 | 0.314 | 0.407 | 0.069 |
| RHSIM_Rhsim01G0251200 | 1.01 | 0.314 | 0.408 | 0.310 |
| novel.48 | 1.01 | 0.314 | 0.408 | 1.038 |
| novel.3397 | 1.01 | 0.314 | 0.408 | 0.420 |
| RHSIM_Rhsim10G0157200 | 1.01 | 0.314 | 0.408 | -1.117 |
| RHSIM_Rhsim03G0169800 | 1.01 | 0.314 | 0.408 | 0.474 |
| RHSIM_Rhsim01G0210000 | 1.01 | 0.314 | 0.408 | 1.536 |
| RHSIM_Rhsim08G0137600 | 1.01 | 0.314 | 0.408 | -2.145 |
| RHSIM_Rhsim04G0006200 | 1.01 | 0.314 | 0.408 | 0.834 |
| RHSIM_Rhsim04G0181000 | 1.01 | 0.314 | 0.408 | -0.207 |
| RHSIM_Rhsim10G0150900 | 1.01 | 0.314 | 0.408 | 1.792 |
| RHSIM_Rhsim03G0091600 | 1.01 | 0.314 | 0.408 | 1.101 |
| RHSIM_Rhsim02G0136300 | 1.01 | 0.314 | 0.408 | 0.455 |
| RHSIM_Rhsim06G0128700 | 1.01 | 0.314 | 0.408 | 0.833 |
| RHSIM_Rhsim12G0200800 | 1.01 | 0.315 | 0.408 | 2.897 |
| RHSIM_Rhsim12G0056100 | 1.01 | 0.315 | 0.408 | 1.125 |
| RHSIM_Rhsim10G0119900 | 1.01 | 0.315 | 0.408 | -0.489 |
| RHSIM_Rhsim06G0065800 | 1.01 | 0.315 | 0.409 | -0.777 |
| novel.3629 | 1.01 | 0.315 | 0.409 | -1.054 |
| RHSIM_Rhsim10G0122200 | 1.01 | 0.315 | 0.409 | -2.172 |
| RHSIM_Rhsim01G0024000 | 1.01 | 0.315 | 0.409 | 0.551 |
| RHSIM_RhsimUnG0131900 | 1.01 | 0.315 | 0.409 | 37.079 |
| novel.726 | 1.01 | 0.315 | 0.409 | 36.486 |
| RHSIM_Rhsim08G0164800 | 1.01 | 0.315 | 0.409 | -1.067 |
| novel.1460 | 1.01 | 0.315 | 0.409 | -36.292 |
| RHSIM_RhsimUnG0028800 | 1.01 | 0.316 | 0.409 | 2.529 |
| RHSIM_Rhsim07G0152700 | 1.01 | 0.316 | 0.410 | -0.157 |
| RHSIM_Rhsim12G0036300 | 1.01 | 0.316 | 0.410 | 0.200 |
| RHSIM_Rhsim05G0001900 | 1.01 | 0.316 | 0.410 | 0.076 |
| novel.3355 | 1.01 | 0.316 | 0.410 | 1.146 |
| RHSIM_Rhsim07G0027900 | 1.00 | 0.316 | 0.410 | 0.092 |

| Gene | LRT statistic | P value | BH-adjusted P | log2FC (interaction) |
|---|---|---|---|---|
| RHSIM_Rhsim09G0040200 | 1.00 | 0.316 | 0.410 | 36.635 |
| RHSIM_Rhsim03G0244500 | 1.00 | 0.316 | 0.410 | 0.124 |
| RHSIM_Rhsim11G0156100 | 1.00 | 0.316 | 0.410 | 2.462 |
| RHSIM_Rhsim07G0113000 | 1.00 | 0.316 | 0.410 | -36.552 |
| RHSIM_Rhsim12G0087500 | 1.00 | 0.316 | 0.410 | 0.165 |
| RHSIM_Rhsim08G0077500 | 1.00 | 0.316 | 0.410 | -36.495 |
| RHSIM_Rhsim07G0207900 | 1.00 | 0.317 | 0.411 | -1.923 |
| RHSIM_Rhsim09G0155500 | 1.00 | 0.317 | 0.411 | 0.187 |
| RHSIM_Rhsim07G0195600 | 1.00 | 0.317 | 0.411 | 1.752 |
| novel.426 | 1.00 | 0.317 | 0.411 | 0.115 |
| novel.1174 | 1.00 | 0.317 | 0.411 | 1.115 |
| novel.2681 | 1.00 | 0.317 | 0.411 | -0.626 |
| novel.4131 | 1.00 | 0.317 | 0.411 | -2.209 |
| RHSIM_Rhsim07G0035900 | 1.00 | 0.317 | 0.411 | -0.576 |
| RHSIM_RhsimUnG0165700 | 1.00 | 0.317 | 0.411 | 36.812 |
| RHSIM_Rhsim10G0001600 | 1.00 | 0.317 | 0.411 | 0.143 |
| RHSIM_Rhsim12G0170500 | 1.00 | 0.317 | 0.411 | 2.434 |
| RHSIM_Rhsim03G0153200 | 1.00 | 0.317 | 0.411 | 0.128 |
| RHSIM_Rhsim07G0148000 | 1.00 | 0.317 | 0.411 | 0.425 |
| RHSIM_Rhsim04G0092400 | 1.00 | 0.318 | 0.412 | -0.183 |
| RHSIM_Rhsim07G0128900 | 1.00 | 0.318 | 0.412 | -0.227 |
| RHSIM_Rhsim09G0039700 | 1.00 | 0.318 | 0.412 | 1.914 |
| RHSIM_Rhsim02G0161400 | 1.00 | 0.318 | 0.412 | 2.252 |
| novel.1403 | 1.00 | 0.318 | 0.412 | 0.482 |
| novel.340 | 1.00 | 0.318 | 0.412 | -1.525 |
| RHSIM_RhsimUnG0010900 | 1.00 | 0.318 | 0.412 | -1.467 |
| novel.3269 | 1.00 | 0.318 | 0.412 | 0.703 |
| RHSIM_Rhsim07G0224800 | 1.00 | 0.318 | 0.412 | 1.148 |
| RHSIM_Rhsim13G0108800 | 1.00 | 0.318 | 0.412 | -2.059 |
| novel.4514 | 1.00 | 0.318 | 0.412 | 1.268 |
| RHSIM_Rhsim03G0088600 | 1.00 | 0.318 | 0.412 | -36.905 |
| novel.2983 | 1.00 | 0.318 | 0.412 | -0.323 |

| Gene | LRT statistic | P value | BH-adjusted P | log2FC (interaction) |
|---|---|---|---|---|
| RHSIM_Rhsim06G0137900 | 1.00 | 0.318 | 0.413 | -1.728 |
| RHSIM_RhsimUnG0013700 | 0.99 | 0.319 | 0.413 | 1.018 |
| RHSIM_Rhsim03G0075000 | 0.99 | 0.319 | 0.413 | 0.317 |
| RHSIM_Rhsim13G0133900 | 0.99 | 0.319 | 0.413 | 0.732 |
| RHSIM_Rhsim01G0103500 | 0.99 | 0.319 | 0.413 | 2.002 |
| RHSIM_Rhsim05G0197200 | 0.99 | 0.319 | 0.413 | -0.219 |
| novel.3597 | 0.99 | 0.319 | 0.413 | -1.146 |
| novel.259 | 0.99 | 0.319 | 0.413 | 36.715 |
| RHSIM_Rhsim04G0144500 | 0.99 | 0.319 | 0.413 | 0.781 |
| RHSIM_RhsimUnG0093200 | 0.99 | 0.319 | 0.413 | -0.552 |
| RHSIM_Rhsim10G0124100 | 0.99 | 0.320 | 0.414 | -0.202 |
| novel.750 | 0.99 | 0.320 | 0.414 | 1.157 |
| RHSIM_Rhsim02G0252800 | 0.99 | 0.320 | 0.414 | -1.621 |
| RHSIM_Rhsim08G0160800 | 0.99 | 0.320 | 0.414 | -0.248 |
| novel.4478 | 0.99 | 0.320 | 0.414 | -0.586 |
| RHSIM_Rhsim02G0134000 | 0.99 | 0.320 | 0.414 | 0.585 |
| RHSIM_Rhsim03G0235300 | 0.99 | 0.320 | 0.414 | -0.072 |
| RHSIM_Rhsim05G0137500 | 0.99 | 0.320 | 0.414 | 0.133 |
| RHSIM_RhsimUnG0098800 | 0.99 | 0.320 | 0.414 | 0.677 |
| RHSIM_Rhsim03G0268000 | 0.99 | 0.320 | 0.414 | 0.195 |
| RHSIM_Rhsim10G0043400 | 0.99 | 0.320 | 0.414 | -0.285 |
| RHSIM_Rhsim02G0245000 | 0.99 | 0.320 | 0.415 | -2.697 |
| RHSIM_Rhsim13G0156600 | 0.99 | 0.320 | 0.415 | 0.092 |
| RHSIM_Rhsim01G0113700 | 0.99 | 0.320 | 0.415 | -2.008 |
| RHSIM_Rhsim08G0187000 | 0.99 | 0.320 | 0.415 | 0.829 |
| novel.4242 | 0.99 | 0.320 | 0.415 | 0.397 |
| RHSIM_Rhsim01G0054800 | 0.99 | 0.321 | 0.415 | 1.562 |
| RHSIM_Rhsim11G0016400 | 0.99 | 0.321 | 0.415 | 0.219 |
| RHSIM_Rhsim08G0022500 | 0.99 | 0.321 | 0.415 | 0.692 |
| novel.1440 | 0.99 | 0.321 | 0.415 | -0.929 |
| RHSIM_Rhsim05G0175700 | 0.99 | 0.321 | 0.415 | -0.110 |
| RHSIM_Rhsim11G0058700 | 0.99 | 0.321 | 0.415 | 0.186 |

| Gene | LRT statistic | P value | BH-adjusted P | log2FC (interaction) |
|---|---|---|---|---|
| RHSIM_Rhsim11G0157900 | 0.99 | 0.321 | 0.415 | 0.477 |
| novel.1634 | 0.99 | 0.321 | 0.415 | -0.131 |
| RHSIM_Rhsim13G0031600 | 0.98 | 0.321 | 0.415 | -0.079 |
| novel.3317 | 0.98 | 0.321 | 0.415 | 0.527 |
| RHSIM_Rhsim12G0180000 | 0.98 | 0.321 | 0.416 | -0.482 |
| RHSIM_Rhsim06G0010900 | 0.98 | 0.321 | 0.416 | 1.825 |
| RHSIM_Rhsim07G0240300 | 0.98 | 0.321 | 0.416 | 0.664 |
| novel.782 | 0.98 | 0.321 | 0.416 | -0.369 |
| RHSIM_Rhsim10G0168800 | 0.98 | 0.322 | 0.416 | -0.103 |
| RHSIM_Rhsim08G0229900 | 0.98 | 0.322 | 0.416 | -1.325 |
| RHSIM_Rhsim05G0014400 | 0.98 | 0.322 | 0.416 | 2.142 |
| novel.1696 | 0.98 | 0.322 | 0.416 | 0.583 |
| RHSIM_Rhsim01G0012100 | 0.98 | 0.322 | 0.416 | 0.180 |
| RHSIM_Rhsim10G0103800 | 0.98 | 0.322 | 0.416 | -0.146 |
| RHSIM_Rhsim05G0085100 | 0.98 | 0.322 | 0.416 | -0.150 |
| RHSIM_Rhsim04G0076600 | 0.98 | 0.322 | 0.416 | 0.641 |
| RHSIM_Rhsim12G0051100 | 0.98 | 0.322 | 0.416 | 2.321 |
| novel.1489 | 0.98 | 0.322 | 0.416 | 1.115 |
| RHSIM_Rhsim01G0019500 | 0.98 | 0.322 | 0.416 | 1.925 |
| RHSIM_Rhsim13G0001900 | 0.98 | 0.322 | 0.417 | -1.116 |
| RHSIM_RhsimUnG0122700 | 0.98 | 0.322 | 0.417 | 0.592 |
| novel.2050 | 0.98 | 0.322 | 0.417 | 0.354 |
| RHSIM_Rhsim04G0160800 | 0.98 | 0.323 | 0.417 | -36.384 |
| novel.2328 | 0.98 | 0.323 | 0.417 | -0.270 |
| RHSIM_Rhsim12G0206100 | 0.98 | 0.323 | 0.417 | -1.588 |
| novel.445 | 0.98 | 0.323 | 0.417 | 1.225 |
| RHSIM_Rhsim13G0199000 | 0.98 | 0.323 | 0.417 | 1.580 |
| novel.3267 | 0.98 | 0.323 | 0.417 | 0.202 |
| novel.94 | 0.98 | 0.323 | 0.417 | -0.531 |
| RHSIM_Rhsim06G0039800 | 0.98 | 0.323 | 0.418 | 1.759 |
| RHSIM_Rhsim08G0218600 | 0.98 | 0.323 | 0.418 | -0.180 |
| RHSIM_Rhsim09G0177800 | 0.97 | 0.323 | 0.418 | 1.422 |

| Gene | LRT statistic | P value | BH-adjusted P | log2FC (interaction) |
|---|---|---|---|---|
| RHSIM_Rhsim05G0091400 | 0.97 | 0.323 | 0.418 | 1.182 |
| RHSIM_Rhsim07G0127800 | 0.97 | 0.324 | 0.418 | 0.984 |
| RHSIM_Rhsim02G0206400 | 0.97 | 0.324 | 0.418 | -0.142 |
| RHSIM_Rhsim08G0096400 | 0.97 | 0.324 | 0.418 | 0.091 |
| RHSIM_Rhsim13G0140300 | 0.97 | 0.324 | 0.418 | -0.311 |
| RHSIM_Rhsim04G0245400 | 0.97 | 0.324 | 0.418 | -0.221 |
| RHSIM_Rhsim06G0124900 | 0.97 | 0.324 | 0.418 | 0.910 |
| RHSIM_Rhsim01G0232500 | 0.97 | 0.324 | 0.419 | 0.298 |
| RHSIM_Rhsim07G0069200 | 0.97 | 0.324 | 0.419 | 0.086 |
| novel.2415 | 0.97 | 0.325 | 0.419 | 1.033 |
| novel.4075 | 0.97 | 0.325 | 0.419 | -1.366 |
| RHSIM_Rhsim03G0259800 | 0.97 | 0.325 | 0.419 | -0.394 |
| RHSIM_Rhsim05G0071500 | 0.97 | 0.325 | 0.420 | 2.584 |
| novel.621 | 0.97 | 0.325 | 0.420 | 0.663 |
| RHSIM_Rhsim05G0136800 | 0.97 | 0.325 | 0.420 | 0.396 |
| RHSIM_RhsimUnG0259000 | 0.97 | 0.325 | 0.420 | 0.321 |
| RHSIM_Rhsim01G0208200 | 0.97 | 0.325 | 0.420 | 0.853 |
| RHSIM_Rhsim13G0065400 | 0.97 | 0.325 | 0.420 | 0.158 |
| RHSIM_RhsimUnG0102200 | 0.97 | 0.326 | 0.420 | 0.370 |
| RHSIM_Rhsim12G0188500 | 0.97 | 0.326 | 0.420 | 1.116 |
| RHSIM_Rhsim02G0138700 | 0.97 | 0.326 | 0.420 | 0.607 |
| RHSIM_Rhsim10G0048900 | 0.96 | 0.326 | 0.421 | -0.088 |
| RHSIM_Rhsim08G0124100 | 0.96 | 0.326 | 0.421 | -38.900 |
| RHSIM_Rhsim04G0033200 | 0.96 | 0.326 | 0.421 | 0.400 |
| RHSIM_Rhsim07G0054400 | 0.96 | 0.326 | 0.421 | 0.097 |
| RHSIM_Rhsim06G0202400 | 0.96 | 0.326 | 0.421 | -0.098 |
| RHSIM_RhsimUnG0056200 | 0.96 | 0.326 | 0.421 | 0.281 |
| RHSIM_Rhsim04G0031000 | 0.96 | 0.326 | 0.421 | 0.920 |
| RHSIM_Rhsim09G0124500 | 0.96 | 0.326 | 0.421 | -0.933 |
| RHSIM_Rhsim05G0202700 | 0.96 | 0.326 | 0.421 | -0.262 |
| RHSIM_Rhsim01G0242100 | 0.96 | 0.326 | 0.421 | 0.093 |
| RHSIM_Rhsim05G0130800 | 0.96 | 0.327 | 0.421 | 1.598 |

| Gene | LRT statistic | P value | BH-adjusted P | log2FC (interaction) |
|---|---|---|---|---|
| RHSIM_Rhsim08G0158700 | 0.96 | 0.327 | 0.421 | 1.499 |
| RHSIM_Rhsim01G0211300 | 0.96 | 0.327 | 0.421 | -0.209 |
| RHSIM_Rhsim09G0126500 | 0.96 | 0.327 | 0.421 | -0.172 |
| RHSIM_Rhsim06G0191400 | 0.96 | 0.327 | 0.421 | 0.407 |
| RHSIM_Rhsim04G0093700 | 0.96 | 0.327 | 0.421 | 1.812 |
| RHSIM_Rhsim04G0033400 | 0.96 | 0.327 | 0.421 | -36.471 |
| novel.3130 | 0.96 | 0.327 | 0.422 | 0.822 |
| RHSIM_Rhsim06G0035300 | 0.96 | 0.327 | 0.422 | 2.165 |
| RHSIM_RhsimUnG0242800 | 0.96 | 0.327 | 0.422 | -36.175 |
| RHSIM_Rhsim10G0186200 | 0.96 | 0.328 | 0.422 | 0.156 |
| RHSIM_RhsimUnG0045800 | 0.96 | 0.328 | 0.422 | -0.439 |
| RHSIM_Rhsim05G0180200 | 0.96 | 0.328 | 0.423 | -0.236 |
| RHSIM_Rhsim13G0201800 | 0.96 | 0.328 | 0.423 | -0.077 |
| RHSIM_Rhsim06G0033400 | 0.96 | 0.328 | 0.423 | 0.328 |
| RHSIM_Rhsim13G0079300 | 0.96 | 0.328 | 0.423 | 0.675 |
| RHSIM_Rhsim08G0014600 | 0.96 | 0.328 | 0.423 | 1.972 |
| RHSIM_Rhsim01G0085200 | 0.96 | 0.328 | 0.423 | 0.892 |
| RHSIM_Rhsim03G0111600 | 0.96 | 0.328 | 0.423 | 0.087 |
| RHSIM_Rhsim13G0022100 | 0.96 | 0.328 | 0.423 | 0.097 |
| RHSIM_RhsimUnG0249700 | 0.96 | 0.328 | 0.423 | 0.382 |
| novel.2287 | 0.95 | 0.329 | 0.423 | 1.813 |
| RHSIM_Rhsim07G0092100 | 0.95 | 0.329 | 0.423 | 0.167 |
| novel.3686 | 0.95 | 0.329 | 0.424 | -0.156 |
| novel.3867 | 0.95 | 0.329 | 0.424 | -1.104 |
| RHSIM_Rhsim09G0159100 | 0.95 | 0.329 | 0.424 | -0.287 |
| RHSIM_Rhsim05G0075800 | 0.95 | 0.329 | 0.424 | -0.229 |
| novel.801 | 0.95 | 0.329 | 0.424 | -0.109 |
| RHSIM_Rhsim12G0047300 | 0.95 | 0.329 | 0.424 | 1.142 |
| RHSIM_Rhsim11G0047700 | 0.95 | 0.329 | 0.424 | 0.524 |
| novel.3392 | 0.95 | 0.329 | 0.424 | 1.456 |
| novel.1650 | 0.95 | 0.329 | 0.424 | 1.570 |
| RHSIM_Rhsim03G0170200 | 0.95 | 0.329 | 0.424 | -1.404 |

| Gene | LRT statistic | P value | BH-adjusted P | log2FC (interaction) |
|---|---|---|---|---|
| novel.699 | 0.95 | 0.330 | 0.425 | 0.202 |
| RHSIM_RhsimUnG0018500 | 0.95 | 0.330 | 0.425 | -1.356 |
| novel.3119 | 0.95 | 0.330 | 0.425 | 0.251 |
| RHSIM_Rhsim02G0240000 | 0.95 | 0.330 | 0.425 | -36.445 |
| RHSIM_Rhsim13G0070700 | 0.95 | 0.330 | 0.425 | 1.014 |
| novel.3676 | 0.95 | 0.330 | 0.425 | -0.311 |
| RHSIM_Rhsim09G0065800 | 0.95 | 0.330 | 0.425 | -0.140 |
| RHSIM_Rhsim01G0016500 | 0.95 | 0.330 | 0.425 | -0.771 |
| RHSIM_Rhsim02G0050100 | 0.95 | 0.330 | 0.425 | -0.704 |
| RHSIM_Rhsim06G0125500 | 0.95 | 0.330 | 0.425 | -1.082 |
| novel.3373 | 0.95 | 0.331 | 0.426 | 0.370 |
| RHSIM_Rhsim11G0131500 | 0.95 | 0.331 | 0.426 | 0.215 |
| RHSIM_Rhsim07G0224300 | 0.95 | 0.331 | 0.426 | 0.105 |
| novel.3919 | 0.95 | 0.331 | 0.426 | -0.696 |
| novel.162 | 0.95 | 0.331 | 0.426 | 1.830 |
| novel.639 | 0.95 | 0.331 | 0.426 | -0.794 |
| novel.276 | 0.95 | 0.331 | 0.426 | 0.634 |
| RHSIM_Rhsim06G0057200 | 0.95 | 0.331 | 0.426 | 0.116 |
| novel.2075 | 0.95 | 0.331 | 0.426 | 0.182 |
| RHSIM_Rhsim06G0205200 | 0.94 | 0.331 | 0.426 | 0.206 |
| RHSIM_Rhsim07G0026000 | 0.94 | 0.331 | 0.426 | -0.178 |
| RHSIM_Rhsim02G0217800 | 0.94 | 0.331 | 0.426 | 0.116 |
| novel.1598 | 0.94 | 0.331 | 0.426 | -0.500 |
| RHSIM_Rhsim05G0220100 | 0.94 | 0.331 | 0.426 | 0.341 |
| RHSIM_Rhsim09G0043700 | 0.94 | 0.331 | 0.426 | -0.199 |
| RHSIM_Rhsim11G0189200 | 0.94 | 0.331 | 0.426 | 0.402 |
| RHSIM_Rhsim05G0073000 | 0.94 | 0.332 | 0.427 | 1.066 |
| RHSIM_Rhsim12G0156800 | 0.94 | 0.332 | 0.427 | -1.190 |
| RHSIM_Rhsim01G0129600 | 0.94 | 0.332 | 0.427 | 1.353 |
| RHSIM_Rhsim13G0143400 | 0.94 | 0.332 | 0.427 | 2.007 |
| RHSIM_Rhsim10G0006000 | 0.94 | 0.332 | 0.427 | 0.531 |
| RHSIM_Rhsim05G0197600 | 0.94 | 0.332 | 0.427 | 2.580 |

| Gene | LRT statistic | P value | BH-adjusted P | log2FC (interaction) |
|---|---|---|---|---|
| RHSIM_Rhsim11G0024100 | 0.94 | 0.332 | 0.427 | 2.415 |
| RHSIM_Rhsim07G0022400 | 0.94 | 0.332 | 0.427 | 0.152 |
| RHSIM_Rhsim06G0161100 | 0.94 | 0.332 | 0.427 | -36.373 |
| RHSIM_Rhsim11G0027800 | 0.94 | 0.332 | 0.427 | 0.401 |
| RHSIM_Rhsim08G0127600 | 0.94 | 0.332 | 0.427 | -1.397 |
| RHSIM_Rhsim05G0036600 | 0.94 | 0.332 | 0.427 | -1.132 |
| novel.3148 | 0.94 | 0.333 | 0.428 | -0.198 |
| RHSIM_Rhsim06G0149800 | 0.94 | 0.333 | 0.428 | 0.114 |
| RHSIM_Rhsim07G0022100 | 0.94 | 0.333 | 0.428 | 1.897 |
| RHSIM_Rhsim02G0095700 | 0.94 | 0.333 | 0.428 | 0.347 |
| RHSIM_Rhsim09G0048100 | 0.94 | 0.333 | 0.428 | -2.444 |
| RHSIM_Rhsim03G0044400 | 0.94 | 0.333 | 0.428 | -36.248 |
| RHSIM_Rhsim07G0044200 | 0.94 | 0.333 | 0.428 | -0.200 |
| novel.1366 | 0.94 | 0.333 | 0.428 | -0.215 |
| RHSIM_Rhsim09G0179600 | 0.94 | 0.333 | 0.428 | -2.428 |
| RHSIM_Rhsim09G0039800 | 0.94 | 0.333 | 0.428 | -0.269 |
| RHSIM_Rhsim06G0084800 | 0.94 | 0.333 | 0.428 | 1.374 |
| RHSIM_Rhsim01G0244000 | 0.94 | 0.333 | 0.428 | 0.276 |
| RHSIM_Rhsim04G0005200 | 0.94 | 0.333 | 0.428 | 0.080 |
| RHSIM_Rhsim04G0097900 | 0.94 | 0.333 | 0.428 | -0.121 |
| RHSIM_Rhsim04G0139200 | 0.93 | 0.334 | 0.429 | -0.591 |
| RHSIM_Rhsim12G0177600 | 0.93 | 0.334 | 0.429 | 2.279 |
| novel.2180 | 0.93 | 0.334 | 0.429 | -0.846 |
| RHSIM_Rhsim01G0156800 | 0.93 | 0.334 | 0.429 | 0.523 |
| novel.4539 | 0.93 | 0.334 | 0.429 | 0.444 |
| RHSIM_RhsimUnG0060200 | 0.93 | 0.334 | 0.429 | -0.491 |
| RHSIM_Rhsim03G0214400 | 0.93 | 0.334 | 0.429 | -0.982 |
| RHSIM_Rhsim06G0199300 | 0.93 | 0.334 | 0.429 | 0.088 |
| RHSIM_Rhsim02G0078300 | 0.93 | 0.334 | 0.430 | -0.735 |
| RHSIM_Rhsim09G0052700 | 0.93 | 0.335 | 0.430 | -1.034 |
| RHSIM_Rhsim11G0024700 | 0.93 | 0.335 | 0.430 | 0.149 |
| RHSIM_RhsimUnG0090800 | 0.93 | 0.335 | 0.430 | 3.218 |

| Gene | LRT statistic | P value | BH-adjusted P | log2FC (interaction) |
|---|---|---|---|---|
| novel.446 | 0.93 | 0.335 | 0.430 | 0.177 |
| RHSIM_Rhsim03G0216700 | 0.93 | 0.335 | 0.430 | 1.433 |
| RHSIM_Rhsim03G0250100 | 0.93 | 0.335 | 0.430 | -0.133 |
| RHSIM_Rhsim12G0016200 | 0.93 | 0.335 | 0.430 | -0.083 |
| RHSIM_Rhsim03G0230800 | 0.93 | 0.335 | 0.430 | 0.062 |
| novel.120 | 0.93 | 0.335 | 0.430 | -0.276 |
| RHSIM_Rhsim07G0240200 | 0.93 | 0.335 | 0.430 | 2.296 |
| RHSIM_RhsimUnG0003900 | 0.93 | 0.335 | 0.430 | 0.661 |
| RHSIM_Rhsim04G0189100 | 0.93 | 0.335 | 0.430 | -0.335 |
| novel.521 | 0.93 | 0.335 | 0.430 | 0.325 |
| RHSIM_Rhsim07G0213600 | 0.93 | 0.335 | 0.431 | -0.827 |
| novel.4473 | 0.93 | 0.335 | 0.431 | 0.459 |
| RHSIM_RhsimUnG0080900 | 0.93 | 0.336 | 0.431 | 0.467 |
| RHSIM_Rhsim10G0160300 | 0.93 | 0.336 | 0.431 | -37.176 |
| RHSIM_RhsimUnG0072900 | 0.93 | 0.336 | 0.431 | 1.150 |
| RHSIM_Rhsim13G0210700 | 0.93 | 0.336 | 0.431 | 0.094 |
| novel.2170 | 0.93 | 0.336 | 0.431 | 0.629 |
| RHSIM_Rhsim01G0277100 | 0.93 | 0.336 | 0.431 | -0.598 |
| RHSIM_Rhsim09G0103800 | 0.93 | 0.336 | 0.431 | 0.184 |
| RHSIM_Rhsim08G0139300 | 0.93 | 0.336 | 0.431 | -36.506 |
| novel.2109 | 0.92 | 0.336 | 0.431 | -0.519 |
| RHSIM_Rhsim01G0066100 | 0.92 | 0.336 | 0.431 | -1.058 |
| novel.1511 | 0.92 | 0.336 | 0.432 | 0.632 |
| novel.3491 | 0.92 | 0.337 | 0.432 | 1.575 |
| novel.859 | 0.92 | 0.337 | 0.432 | 1.082 |
| RHSIM_Rhsim05G0072100 | 0.92 | 0.337 | 0.432 | 36.361 |
| RHSIM_Rhsim02G0090500 | 0.92 | 0.337 | 0.432 | -0.129 |
| novel.2004 | 0.92 | 0.337 | 0.432 | -0.396 |
| RHSIM_Rhsim09G0084500 | 0.92 | 0.337 | 0.432 | 0.534 |
| novel.3017 | 0.92 | 0.337 | 0.433 | 0.271 |
| RHSIM_Rhsim06G0148000 | 0.92 | 0.337 | 0.433 | -36.095 |
| RHSIM_Rhsim11G0179400 | 0.92 | 0.337 | 0.433 | -0.115 |

| Gene | LRT statistic | P value | BH-adjusted P | log2FC (interaction) |
|---|---|---|---|---|
| RHSIM_Rhsim13G0153800 | 0.92 | 0.337 | 0.433 | -2.104 |
| RHSIM_Rhsim07G0139400 | 0.92 | 0.337 | 0.433 | 2.741 |
| RHSIM_Rhsim08G0246200 | 0.92 | 0.338 | 0.433 | -0.094 |
| RHSIM_Rhsim09G0001900 | 0.92 | 0.338 | 0.433 | 1.362 |
| RHSIM_Rhsim04G0000400 | 0.92 | 0.338 | 0.433 | -0.113 |
| RHSIM_RhsimUnG0081600 | 0.92 | 0.338 | 0.433 | -1.677 |
| RHSIM_Rhsim04G0150000 | 0.92 | 0.338 | 0.433 | 0.113 |
| RHSIM_Rhsim09G0180700 | 0.92 | 0.338 | 0.434 | -0.648 |
| RHSIM_Rhsim06G0076500 | 0.92 | 0.338 | 0.434 | 36.735 |
| novel.3405 | 0.92 | 0.338 | 0.434 | -1.377 |
| RHSIM_Rhsim04G0223600 | 0.92 | 0.338 | 0.434 | 0.216 |
| RHSIM_Rhsim12G0067300 | 0.92 | 0.338 | 0.434 | 0.928 |
| RHSIM_Rhsim09G0171400 | 0.92 | 0.338 | 0.434 | -1.712 |
| RHSIM_Rhsim08G0249800 | 0.92 | 0.338 | 0.434 | 0.140 |
| RHSIM_Rhsim01G0002300 | 0.92 | 0.339 | 0.434 | -0.087 |
| RHSIM_Rhsim10G0068700 | 0.92 | 0.339 | 0.434 | 0.165 |
| novel.2074 | 0.92 | 0.339 | 0.434 | -0.227 |
| RHSIM_Rhsim12G0117200 | 0.92 | 0.339 | 0.434 | -1.709 |
| novel.4526 | 0.92 | 0.339 | 0.434 | -0.286 |
| RHSIM_Rhsim06G0123800 | 0.92 | 0.339 | 0.434 | 0.364 |
| novel.991 | 0.92 | 0.339 | 0.434 | -0.608 |
| RHSIM_Rhsim09G0035400 | 0.91 | 0.339 | 0.434 | -1.803 |
| RHSIM_Rhsim05G0132300 | 0.91 | 0.339 | 0.434 | -0.186 |
| RHSIM_Rhsim03G0024100 | 0.91 | 0.339 | 0.434 | -2.177 |
| RHSIM_Rhsim12G0151400 | 0.91 | 0.339 | 0.435 | -0.507 |
| RHSIM_Rhsim01G0161500 | 0.91 | 0.339 | 0.435 | 0.127 |
| RHSIM_Rhsim12G0001900 | 0.91 | 0.339 | 0.435 | 0.291 |
| RHSIM_Rhsim05G0138100 | 0.91 | 0.339 | 0.435 | 1.976 |
| RHSIM_Rhsim05G0114000 | 0.91 | 0.340 | 0.435 | 2.032 |
| RHSIM_Rhsim01G0026200 | 0.91 | 0.340 | 0.435 | -36.416 |
| RHSIM_Rhsim10G0193700 | 0.91 | 0.340 | 0.435 | 0.228 |
| RHSIM_Rhsim01G0064100 | 0.91 | 0.340 | 0.435 | 1.262 |

| Gene | LRT statistic | P value | BH-adjusted P | log2FC (interaction) |
|---|---|---|---|---|
| RHSIM_Rhsim04G0223300 | 0.91 | 0.340 | 0.435 | 0.733 |
| RHSIM_Rhsim10G0093300 | 0.91 | 0.340 | 0.435 | -0.821 |
| RHSIM_Rhsim06G0217500 | 0.91 | 0.340 | 0.435 | 0.257 |
| RHSIM_Rhsim02G0075900 | 0.91 | 0.340 | 0.435 | -0.172 |
| RHSIM_Rhsim06G0162400 | 0.91 | 0.340 | 0.436 | -0.685 |
| RHSIM_Rhsim08G0010400 | 0.91 | 0.340 | 0.436 | -0.106 |
| RHSIM_Rhsim13G0031300 | 0.91 | 0.341 | 0.436 | 0.165 |
| RHSIM_Rhsim03G0219700 | 0.91 | 0.341 | 0.436 | -0.527 |
| RHSIM_Rhsim08G0243100 | 0.91 | 0.341 | 0.437 | 0.101 |
| RHSIM_Rhsim05G0161200 | 0.91 | 0.341 | 0.437 | -0.174 |
| RHSIM_Rhsim05G0083700 | 0.91 | 0.341 | 0.437 | 0.105 |
| RHSIM_Rhsim07G0144200 | 0.91 | 0.341 | 0.437 | 1.541 |
| RHSIM_Rhsim08G0175500 | 0.91 | 0.341 | 0.437 | -0.111 |
| RHSIM_Rhsim07G0038400 | 0.91 | 0.341 | 0.437 | -0.087 |
| novel.2033 | 0.91 | 0.341 | 0.437 | -0.958 |
| RHSIM_Rhsim04G0029300 | 0.91 | 0.341 | 0.437 | 0.655 |
| novel.4259 | 0.91 | 0.341 | 0.437 | -1.052 |
| RHSIM_Rhsim11G0010700 | 0.91 | 0.341 | 0.437 | 0.563 |
| RHSIM_Rhsim04G0108900 | 0.90 | 0.342 | 0.437 | -1.302 |
| RHSIM_Rhsim07G0213500 | 0.90 | 0.342 | 0.437 | -0.487 |
| novel.898 | 0.90 | 0.342 | 0.437 | 0.534 |
| RHSIM_Rhsim12G0079300 | 0.90 | 0.342 | 0.437 | -1.700 |
| RHSIM_Rhsim07G0107800 | 0.90 | 0.342 | 0.437 | -1.381 |
| novel.1805 | 0.90 | 0.342 | 0.437 | -1.248 |
| RHSIM_Rhsim05G0040600 | 0.90 | 0.342 | 0.437 | 0.552 |
| novel.1425 | 0.90 | 0.342 | 0.438 | 0.384 |
| RHSIM_Rhsim07G0159100 | 0.90 | 0.342 | 0.438 | 1.823 |
| RHSIM_Rhsim13G0139500 | 0.90 | 0.342 | 0.438 | -0.784 |
| RHSIM_Rhsim02G0243500 | 0.90 | 0.342 | 0.438 | -2.128 |
| RHSIM_Rhsim12G0129500 | 0.90 | 0.342 | 0.438 | -0.194 |
| RHSIM_Rhsim06G0220400 | 0.90 | 0.342 | 0.438 | -0.091 |
| RHSIM_Rhsim13G0213300 | 0.90 | 0.342 | 0.438 | 0.317 |

| Gene | LRT statistic | P value | BH-adjusted P | log2FC (interaction) |
|---|---|---|---|---|
| novel.2457 | 0.90 | 0.343 | 0.438 | 0.826 |
| RHSIM_Rhsim02G0058100 | 0.90 | 0.343 | 0.438 | 0.650 |
| novel.1333 | 0.90 | 0.343 | 0.438 | 0.973 |
| RHSIM_Rhsim06G0099600 | 0.90 | 0.343 | 0.438 | 36.624 |
| novel.3928 | 0.90 | 0.343 | 0.438 | -0.222 |
| RHSIM_Rhsim12G0005400 | 0.90 | 0.343 | 0.439 | 0.433 |
| RHSIM_Rhsim05G0066100 | 0.90 | 0.343 | 0.439 | 2.709 |
| RHSIM_Rhsim06G0122600 | 0.90 | 0.343 | 0.439 | -36.352 |
| RHSIM_RhsimUnG0250000 | 0.90 | 0.344 | 0.439 | -0.434 |
| RHSIM_Rhsim08G0175700 | 0.90 | 0.344 | 0.440 | 0.284 |
| RHSIM_Rhsim04G0188500 | 0.90 | 0.344 | 0.440 | 0.798 |
| novel.1705 | 0.89 | 0.344 | 0.440 | 0.855 |
| RHSIM_Rhsim08G0058700 | 0.89 | 0.345 | 0.440 | 38.620 |
| RHSIM_Rhsim08G0157300 | 0.89 | 0.345 | 0.440 | -0.099 |
| RHSIM_Rhsim02G0200500 | 0.89 | 0.345 | 0.440 | 0.625 |
| RHSIM_Rhsim07G0180200 | 0.89 | 0.345 | 0.441 | 2.464 |
| RHSIM_Rhsim08G0138400 | 0.89 | 0.345 | 0.441 | -1.003 |
| novel.2902 | 0.89 | 0.345 | 0.441 | 0.132 |
| RHSIM_Rhsim09G0169100 | 0.89 | 0.345 | 0.441 | 0.397 |
| RHSIM_Rhsim02G0083400 | 0.89 | 0.346 | 0.442 | -0.155 |
| RHSIM_RhsimUnG0051700 | 0.89 | 0.346 | 0.442 | -1.237 |
| RHSIM_Rhsim03G0048100 | 0.89 | 0.346 | 0.442 | -0.926 |
| novel.1036 | 0.89 | 0.346 | 0.442 | 0.136 |
| novel.527 | 0.89 | 0.346 | 0.442 | 1.497 |
| RHSIM_Rhsim10G0123200 | 0.89 | 0.346 | 0.442 | 1.882 |
| novel.72 | 0.89 | 0.346 | 0.442 | 2.599 |
| RHSIM_Rhsim05G0047900 | 0.89 | 0.346 | 0.442 | 0.926 |
| RHSIM_Rhsim06G0150100 | 0.89 | 0.346 | 0.442 | 0.216 |
| RHSIM_RhsimUnG0096900 | 0.89 | 0.346 | 0.442 | 0.093 |
| RHSIM_Rhsim09G0004600 | 0.89 | 0.346 | 0.442 | 0.645 |
| RHSIM_Rhsim05G0017700 | 0.89 | 0.346 | 0.442 | -0.623 |
| RHSIM_RhsimUnG0093500 | 0.89 | 0.346 | 0.442 | 1.366 |

| Gene | LRT statistic | P value | BH-adjusted P | log2FC (interaction) |
|---|---|---|---|---|
| RHSIM_Rhsim02G0075400 | 0.89 | 0.347 | 0.442 | -0.685 |
| RHSIM_Rhsim08G0109600 | 0.89 | 0.347 | 0.443 | -0.137 |
| novel.2831 | 0.88 | 0.347 | 0.443 | -1.909 |
| RHSIM_Rhsim03G0057500 | 0.88 | 0.347 | 0.443 | 36.793 |
| novel.4512 | 0.88 | 0.347 | 0.443 | 1.872 |
| RHSIM_Rhsim02G0229700 | 0.88 | 0.347 | 0.443 | 0.267 |
| RHSIM_Rhsim02G0204900 | 0.88 | 0.347 | 0.443 | -0.136 |
| RHSIM_Rhsim08G0235800 | 0.88 | 0.347 | 0.443 | 0.136 |
| RHSIM_Rhsim13G0118400 | 0.88 | 0.347 | 0.443 | -0.185 |
| RHSIM_Rhsim01G0159000 | 0.88 | 0.347 | 0.443 | 0.119 |
| RHSIM_Rhsim12G0166100 | 0.88 | 0.348 | 0.444 | 0.327 |
| RHSIM_Rhsim10G0053300 | 0.88 | 0.348 | 0.444 | 0.964 |
| RHSIM_Rhsim07G0032200 | 0.88 | 0.348 | 0.444 | -1.837 |
| RHSIM_Rhsim09G0110300 | 0.88 | 0.348 | 0.444 | -0.176 |
| RHSIM_Rhsim03G0002500 | 0.88 | 0.348 | 0.444 | 36.619 |
| RHSIM_Rhsim06G0172600 | 0.88 | 0.348 | 0.444 | 0.656 |
| RHSIM_Rhsim03G0154000 | 0.88 | 0.348 | 0.444 | -0.276 |
| RHSIM_Rhsim12G0018200 | 0.88 | 0.348 | 0.444 | -1.914 |
| RHSIM_Rhsim11G0050000 | 0.88 | 0.348 | 0.444 | 0.787 |
| RHSIM_RhsimUnG0123900 | 0.88 | 0.348 | 0.444 | -1.588 |
| RHSIM_Rhsim11G0137300 | 0.88 | 0.348 | 0.444 | 0.696 |
| RHSIM_Rhsim09G0031500 | 0.88 | 0.348 | 0.444 | -0.497 |
| RHSIM_Rhsim09G0063400 | 0.88 | 0.348 | 0.444 | 0.155 |
| RHSIM_Rhsim13G0065600 | 0.88 | 0.348 | 0.444 | 0.130 |
| RHSIM_Rhsim05G0027600 | 0.88 | 0.348 | 0.444 | 0.380 |
| RHSIM_Rhsim13G0117500 | 0.88 | 0.348 | 0.444 | 0.173 |
| RHSIM_Rhsim08G0080300 | 0.88 | 0.348 | 0.444 | 1.425 |
| novel.1496 | 0.88 | 0.349 | 0.444 | -0.347 |
| novel.3984 | 0.88 | 0.349 | 0.445 | 0.561 |
| RHSIM_Rhsim08G0179100 | 0.88 | 0.349 | 0.445 | -1.943 |
| RHSIM_Rhsim06G0173700 | 0.88 | 0.349 | 0.445 | -0.824 |
| novel.3465 | 0.88 | 0.349 | 0.445 | 0.925 |

| Gene | LRT statistic | P value | BH-adjusted P | log2FC (interaction) |
|---|---|---|---|---|
| RHSIM_Rhsim10G0044300 | 0.88 | 0.349 | 0.445 | -0.208 |
| novel.1212 | 0.88 | 0.349 | 0.445 | -0.383 |
| RHSIM_Rhsim04G0061500 | 0.88 | 0.349 | 0.445 | 0.691 |
| RHSIM_Rhsim01G0217900 | 0.88 | 0.349 | 0.445 | 0.175 |
| RHSIM_Rhsim07G0052600 | 0.88 | 0.349 | 0.445 | 0.292 |
| novel.2965 | 0.88 | 0.349 | 0.445 | 0.627 |
| RHSIM_Rhsim02G0154200 | 0.88 | 0.349 | 0.445 | 0.822 |
| RHSIM_Rhsim05G0196100 | 0.87 | 0.350 | 0.446 | 36.832 |
| RHSIM_Rhsim02G0215900 | 0.87 | 0.350 | 0.446 | 0.098 |
| RHSIM_Rhsim10G0186100 | 0.87 | 0.350 | 0.446 | -0.222 |
| RHSIM_Rhsim07G0063400 | 0.87 | 0.350 | 0.446 | -36.426 |
| RHSIM_Rhsim04G0051700 | 0.87 | 0.350 | 0.446 | -0.209 |
| RHSIM_Rhsim06G0024600 | 0.87 | 0.350 | 0.446 | 0.306 |
| RHSIM_Rhsim10G0145100 | 0.87 | 0.350 | 0.446 | 0.241 |
| RHSIM_Rhsim08G0044500 | 0.87 | 0.350 | 0.446 | -0.096 |
| RHSIM_Rhsim03G0263300 | 0.87 | 0.350 | 0.446 | 1.660 |
| novel.197 | 0.87 | 0.351 | 0.447 | 1.091 |
| RHSIM_Rhsim07G0230300 | 0.87 | 0.351 | 0.447 | 1.272 |
| RHSIM_Rhsim01G0268600 | 0.87 | 0.351 | 0.447 | 2.427 |
| RHSIM_Rhsim02G0169700 | 0.87 | 0.351 | 0.447 | 0.101 |
| RHSIM_Rhsim13G0064000 | 0.87 | 0.351 | 0.447 | -0.102 |
| novel.4453 | 0.87 | 0.351 | 0.447 | 0.578 |
| RHSIM_Rhsim12G0124200 | 0.87 | 0.351 | 0.448 | -0.107 |
| novel.257 | 0.87 | 0.352 | 0.448 | -36.597 |
| novel.3213 | 0.87 | 0.352 | 0.448 | 0.545 |
| novel.1935 | 0.87 | 0.352 | 0.448 | 0.648 |
| RHSIM_Rhsim10G0212100 | 0.87 | 0.352 | 0.448 | 1.658 |
| RHSIM_Rhsim02G0042900 | 0.87 | 0.352 | 0.448 | 1.600 |
| RHSIM_Rhsim01G0173000 | 0.87 | 0.352 | 0.448 | 2.168 |
| RHSIM_Rhsim01G0189700 | 0.87 | 0.352 | 0.448 | -0.333 |
| RHSIM_Rhsim12G0032300 | 0.87 | 0.352 | 0.449 | 1.363 |
| RHSIM_Rhsim12G0063500 | 0.87 | 0.352 | 0.449 | 0.068 |

| Gene | LRT statistic | P value | BH-adjusted P | log2FC (interaction) |
|---|---|---|---|---|
| RHSIM_Rhsim02G0096500 | 0.86 | 0.352 | 0.449 | 1.402 |
| novel.1854 | 0.86 | 0.352 | 0.449 | 1.541 |
| novel.2259 | 0.86 | 0.353 | 0.449 | -0.138 |
| RHSIM_Rhsim02G0181600 | 0.86 | 0.353 | 0.449 | 0.331 |
| RHSIM_Rhsim02G0253500 | 0.86 | 0.353 | 0.449 | 0.476 |
| novel.2981 | 0.86 | 0.353 | 0.449 | -0.446 |
| RHSIM_Rhsim13G0027200 | 0.86 | 0.353 | 0.449 | 0.948 |
| RHSIM_Rhsim12G0057700 | 0.86 | 0.353 | 0.449 | -0.048 |
| novel.577 | 0.86 | 0.353 | 0.449 | 0.574 |
| RHSIM_Rhsim04G0086600 | 0.86 | 0.353 | 0.450 | -0.066 |
| RHSIM_Rhsim09G0047500 | 0.86 | 0.353 | 0.450 | -0.892 |
| RHSIM_Rhsim05G0184700 | 0.86 | 0.353 | 0.450 | 0.156 |
| RHSIM_Rhsim03G0134100 | 0.86 | 0.353 | 0.450 | 1.025 |
| RHSIM_Rhsim02G0231900 | 0.86 | 0.354 | 0.450 | 0.428 |
| RHSIM_Rhsim11G0055400 | 0.86 | 0.354 | 0.450 | 0.199 |
| novel.1949 | 0.86 | 0.354 | 0.450 | -0.704 |
| novel.2815 | 0.86 | 0.354 | 0.450 | 0.854 |
| novel.2028 | 0.86 | 0.354 | 0.450 | -0.824 |
| RHSIM_Rhsim12G0208800 | 0.86 | 0.354 | 0.450 | -0.136 |
| RHSIM_Rhsim01G0286300 | 0.86 | 0.354 | 0.450 | -0.247 |
| RHSIM_RhsimUnG0148000 | 0.86 | 0.354 | 0.450 | -0.152 |
| novel.2876 | 0.86 | 0.354 | 0.450 | 0.690 |
| RHSIM_Rhsim03G0002100 | 0.86 | 0.354 | 0.451 | 1.648 |
| RHSIM_Rhsim01G0018700 | 0.86 | 0.354 | 0.451 | -2.169 |
| novel.2948 | 0.86 | 0.354 | 0.451 | 0.425 |
| RHSIM_Rhsim05G0150800 | 0.86 | 0.355 | 0.451 | 0.512 |
| RHSIM_Rhsim10G0187600 | 0.86 | 0.355 | 0.451 | 0.867 |
| RHSIM_Rhsim03G0119000 | 0.86 | 0.355 | 0.451 | -1.464 |
| novel.2570 | 0.86 | 0.355 | 0.451 | 0.050 |
| RHSIM_Rhsim04G0053800 | 0.86 | 0.355 | 0.451 | -0.430 |
| RHSIM_Rhsim04G0168400 | 0.86 | 0.355 | 0.451 | -0.464 |
| RHSIM_Rhsim12G0121600 | 0.85 | 0.355 | 0.452 | -0.404 |

| Gene | LRT statistic | P value | BH-adjusted P | log2FC (interaction) |
|---|---|---|---|---|
| RHSIM_RhsimUnG0225500 | 0.85 | 0.355 | 0.452 | 1.497 |
| RHSIM_Rhsim11G0054000 | 0.85 | 0.355 | 0.452 | 0.463 |
| novel.2920 | 0.85 | 0.355 | 0.452 | 0.895 |
| RHSIM_Rhsim07G0056800 | 0.85 | 0.355 | 0.452 | 1.433 |
| RHSIM_Rhsim11G0093500 | 0.85 | 0.355 | 0.452 | -0.153 |
| RHSIM_Rhsim12G0145000 | 0.85 | 0.355 | 0.452 | 1.934 |
| novel.262 | 0.85 | 0.355 | 0.452 | 1.806 |
| RHSIM_Rhsim05G0182600 | 0.85 | 0.355 | 0.452 | 36.416 |
| RHSIM_Rhsim10G0147700 | 0.85 | 0.356 | 0.452 | -0.733 |
| novel.2665 | 0.85 | 0.356 | 0.452 | -1.439 |
| RHSIM_Rhsim02G0111300 | 0.85 | 0.356 | 0.452 | 0.366 |
| RHSIM_Rhsim10G0033100 | 0.85 | 0.356 | 0.452 | -0.627 |
| RHSIM_Rhsim07G0164000 | 0.85 | 0.356 | 0.452 | -1.100 |
| RHSIM_Rhsim06G0001300 | 0.85 | 0.356 | 0.452 | 0.333 |
| RHSIM_RhsimUnG0099600 | 0.85 | 0.356 | 0.452 | -2.775 |
| RHSIM_Rhsim10G0013500 | 0.85 | 0.356 | 0.452 | -0.675 |
| RHSIM_Rhsim09G0053900 | 0.85 | 0.356 | 0.452 | -36.477 |
| novel.2952 | 0.85 | 0.356 | 0.452 | 0.226 |
| novel.1753 | 0.85 | 0.356 | 0.453 | -1.981 |
| RHSIM_Rhsim05G0048500 | 0.85 | 0.356 | 0.453 | 0.136 |
| RHSIM_Rhsim07G0060200 | 0.85 | 0.356 | 0.453 | -0.388 |
| RHSIM_Rhsim07G0201500 | 0.85 | 0.356 | 0.453 | 0.873 |
| RHSIM_Rhsim01G0038500 | 0.85 | 0.356 | 0.453 | -1.392 |
| RHSIM_Rhsim04G0108800 | 0.85 | 0.357 | 0.453 | 0.629 |
| novel.3852 | 0.85 | 0.357 | 0.453 | 0.223 |
| RHSIM_Rhsim07G0011300 | 0.85 | 0.357 | 0.453 | 0.073 |
| novel.2051 | 0.85 | 0.357 | 0.453 | 1.037 |
| RHSIM_Rhsim01G0042800 | 0.85 | 0.357 | 0.453 | 1.590 |
| RHSIM_Rhsim04G0122900 | 0.85 | 0.357 | 0.453 | -0.122 |
| RHSIM_Rhsim13G0176600 | 0.85 | 0.357 | 0.453 | 0.878 |
| RHSIM_RhsimUnG0129300 | 0.85 | 0.357 | 0.454 | -0.922 |
| RHSIM_Rhsim06G0012000 | 0.85 | 0.357 | 0.454 | 0.910 |

| Gene | LRT statistic | P value | BH-adjusted P | log2FC (interaction) |
|---|---|---|---|---|
| RHSIM_Rhsim08G0249500 | 0.85 | 0.357 | 0.454 | -0.925 |
| novel.3097 | 0.85 | 0.357 | 0.454 | -0.491 |
| RHSIM_Rhsim05G0179900 | 0.85 | 0.357 | 0.454 | -1.601 |
| RHSIM_Rhsim01G0104000 | 0.85 | 0.357 | 0.454 | -36.483 |
| RHSIM_Rhsim13G0157000 | 0.85 | 0.358 | 0.454 | 0.465 |
| RHSIM_Rhsim13G0058900 | 0.85 | 0.358 | 0.454 | -0.216 |
| novel.757 | 0.85 | 0.358 | 0.454 | 0.312 |
| novel.4162 | 0.85 | 0.358 | 0.454 | -0.355 |
| RHSIM_RhsimUnG0056700 | 0.85 | 0.358 | 0.454 | 1.438 |
| RHSIM_RhsimUnG0176800 | 0.85 | 0.358 | 0.454 | -1.118 |
| RHSIM_Rhsim12G0187100 | 0.85 | 0.358 | 0.454 | -36.298 |
| RHSIM_Rhsim07G0006400 | 0.84 | 0.358 | 0.454 | -36.370 |
| RHSIM_Rhsim05G0102400 | 0.84 | 0.358 | 0.454 | 0.801 |
| RHSIM_Rhsim04G0146400 | 0.84 | 0.358 | 0.454 | 0.556 |
| RHSIM_Rhsim01G0276600 | 0.84 | 0.358 | 0.454 | 0.159 |
| RHSIM_RhsimUnG0142200 | 0.84 | 0.358 | 0.455 | 2.083 |
| RHSIM_Rhsim13G0037800 | 0.84 | 0.359 | 0.455 | -1.261 |
| RHSIM_Rhsim02G0092000 | 0.84 | 0.359 | 0.455 | 36.437 |
| RHSIM_Rhsim11G0066200 | 0.84 | 0.359 | 0.455 | -1.212 |
| RHSIM_Rhsim07G0131700 | 0.84 | 0.359 | 0.456 | 0.070 |
| novel.1204 | 0.84 | 0.359 | 0.456 | 0.828 |
| novel.4420 | 0.84 | 0.359 | 0.456 | 36.488 |
| novel.3120 | 0.84 | 0.359 | 0.456 | -0.292 |
| RHSIM_Rhsim06G0202700 | 0.84 | 0.359 | 0.456 | -0.108 |
| RHSIM_Rhsim05G0212800 | 0.84 | 0.359 | 0.456 | -0.976 |
| novel.490 | 0.84 | 0.359 | 0.456 | 0.548 |
| novel.940 | 0.84 | 0.359 | 0.456 | -0.360 |
| RHSIM_Rhsim08G0161500 | 0.84 | 0.359 | 0.456 | -0.459 |
| RHSIM_Rhsim03G0017600 | 0.84 | 0.359 | 0.456 | 0.264 |
| novel.2541 | 0.84 | 0.359 | 0.456 | 0.715 |
| RHSIM_Rhsim12G0102500 | 0.84 | 0.359 | 0.456 | 0.578 |
| novel.2086 | 0.84 | 0.359 | 0.456 | -0.153 |

| Gene | LRT statistic | P value | BH-adjusted P | log2FC (interaction) |
|---|---|---|---|---|
| RHSIM_Rhsim09G0188300 | 0.84 | 0.360 | 0.456 | -0.135 |
| RHSIM_Rhsim05G0133400 | 0.84 | 0.360 | 0.456 | 0.245 |
| RHSIM_Rhsim13G0072400 | 0.84 | 0.360 | 0.456 | -0.521 |
| RHSIM_Rhsim07G0137300 | 0.84 | 0.360 | 0.456 | 0.727 |
| RHSIM_Rhsim12G0099000 | 0.84 | 0.360 | 0.456 | 0.112 |
| RHSIM_Rhsim07G0135500 | 0.84 | 0.360 | 0.456 | 1.549 |
| RHSIM_Rhsim05G0050700 | 0.84 | 0.360 | 0.456 | 1.542 |
| RHSIM_Rhsim01G0028000 | 0.84 | 0.360 | 0.456 | -0.142 |
| RHSIM_Rhsim13G0093400 | 0.84 | 0.361 | 0.457 | -36.442 |
| RHSIM_Rhsim11G0169700 | 0.84 | 0.361 | 0.457 | -0.627 |
| novel.3122 | 0.84 | 0.361 | 0.457 | 0.898 |
| RHSIM_Rhsim06G0064100 | 0.84 | 0.361 | 0.457 | -0.563 |
| RHSIM_Rhsim02G0154100 | 0.84 | 0.361 | 0.457 | -0.114 |
| RHSIM_Rhsim03G0266800 | 0.83 | 0.361 | 0.457 | 1.073 |
| RHSIM_Rhsim10G0062300 | 0.83 | 0.361 | 0.457 | 0.832 |
| RHSIM_Rhsim07G0026800 | 0.83 | 0.361 | 0.457 | 0.230 |
| novel.3706 | 0.83 | 0.361 | 0.457 | 0.788 |
| RHSIM_Rhsim01G0194300 | 0.83 | 0.361 | 0.457 | 0.376 |
| RHSIM_Rhsim09G0113100 | 0.83 | 0.361 | 0.458 | -0.280 |
| novel.2440 | 0.83 | 0.361 | 0.458 | -0.671 |
| RHSIM_RhsimUnG0172100 | 0.83 | 0.361 | 0.458 | 0.269 |
| RHSIM_Rhsim06G0189900 | 0.83 | 0.361 | 0.458 | 1.844 |
| RHSIM_Rhsim05G0171900 | 0.83 | 0.361 | 0.458 | -0.111 |
| RHSIM_Rhsim02G0051900 | 0.83 | 0.362 | 0.458 | -0.803 |
| RHSIM_Rhsim11G0168800 | 0.83 | 0.362 | 0.458 | 0.158 |
| RHSIM_Rhsim07G0093500 | 0.83 | 0.362 | 0.458 | -0.131 |
| RHSIM_Rhsim12G0152300 | 0.83 | 0.362 | 0.458 | 0.076 |
| novel.4227 | 0.83 | 0.362 | 0.458 | -1.260 |
| RHSIM_Rhsim01G0094300 | 0.83 | 0.362 | 0.458 | 0.603 |
| RHSIM_Rhsim12G0053000 | 0.83 | 0.362 | 0.458 | 0.337 |
| RHSIM_Rhsim07G0002800 | 0.83 | 0.362 | 0.458 | -1.792 |
| RHSIM_Rhsim12G0197600 | 0.83 | 0.362 | 0.458 | 0.105 |

| Gene | LRT statistic | P value | BH-adjusted P | log2FC (interaction) |
|---|---|---|---|---|
| novel.1673 | 0.83 | 0.362 | 0.458 | -0.696 |
| novel.1831 | 0.83 | 0.362 | 0.458 | -1.694 |
| RHSIM_Rhsim12G0042600 | 0.83 | 0.362 | 0.459 | 0.376 |
| RHSIM_Rhsim02G0077100 | 0.83 | 0.362 | 0.459 | -0.558 |
| novel.708 | 0.83 | 0.362 | 0.459 | -1.071 |
| RHSIM_Rhsim01G0055800 | 0.83 | 0.362 | 0.459 | -0.828 |
| RHSIM_Rhsim09G0127300 | 0.83 | 0.363 | 0.459 | 0.946 |
| RHSIM_Rhsim13G0145200 | 0.83 | 0.363 | 0.459 | 3.139 |
| RHSIM_Rhsim04G0006500 | 0.83 | 0.363 | 0.459 | 0.060 |
| novel.3437 | 0.83 | 0.363 | 0.459 | 38.059 |
| RHSIM_Rhsim13G0047400 | 0.83 | 0.363 | 0.459 | 0.206 |
| RHSIM_Rhsim13G0203600 | 0.83 | 0.363 | 0.459 | -0.384 |
| RHSIM_Rhsim02G0023800 | 0.83 | 0.363 | 0.459 | 0.210 |
| RHSIM_Rhsim05G0068600 | 0.83 | 0.363 | 0.460 | -0.098 |
| RHSIM_Rhsim11G0070500 | 0.83 | 0.363 | 0.460 | -0.102 |
| novel.4258 | 0.83 | 0.363 | 0.460 | 1.786 |
| RHSIM_Rhsim04G0126400 | 0.83 | 0.363 | 0.460 | 0.155 |
| RHSIM_RhsimUnG0124000 | 0.83 | 0.363 | 0.460 | 2.029 |
| RHSIM_RhsimUnG0023000 | 0.83 | 0.363 | 0.460 | 0.071 |
| RHSIM_Rhsim13G0208300 | 0.83 | 0.363 | 0.460 | -0.064 |
| RHSIM_Rhsim03G0046300 | 0.83 | 0.363 | 0.460 | -0.500 |
| RHSIM_RhsimUnG0091800 | 0.83 | 0.363 | 0.460 | 1.072 |
| RHSIM_Rhsim01G0223000 | 0.83 | 0.364 | 0.460 | -0.104 |
| RHSIM_Rhsim13G0170600 | 0.82 | 0.364 | 0.460 | -0.283 |
| RHSIM_Rhsim05G0202800 | 0.82 | 0.364 | 0.460 | -0.200 |
| RHSIM_Rhsim08G0091300 | 0.82 | 0.364 | 0.460 | -0.553 |
| RHSIM_Rhsim08G0189800 | 0.82 | 0.364 | 0.460 | 1.131 |
| RHSIM_Rhsim03G0152800 | 0.82 | 0.364 | 0.460 | 1.416 |
| novel.2691 | 0.82 | 0.364 | 0.460 | -1.402 |
| RHSIM_Rhsim12G0099400 | 0.82 | 0.364 | 0.460 | -0.132 |
| RHSIM_Rhsim10G0068600 | 0.82 | 0.364 | 0.461 | 1.153 |
| novel.2958 | 0.82 | 0.364 | 0.461 | 1.219 |

| Gene | LRT statistic | P value | BH-adjusted P | log2FC (interaction) |
|---|---|---|---|---|
| RHSIM_Rhsim07G0085700 | 0.82 | 0.364 | 0.461 | -0.152 |
| novel.3336 | 0.82 | 0.365 | 0.461 | -0.778 |
| RHSIM_RhsimUnG0111700 | 0.82 | 0.365 | 0.461 | -0.251 |
| RHSIM_Rhsim02G0188800 | 0.82 | 0.365 | 0.461 | 1.741 |
| RHSIM_Rhsim05G0037000 | 0.82 | 0.365 | 0.461 | -0.522 |
| RHSIM_Rhsim07G0012700 | 0.82 | 0.365 | 0.461 | -0.108 |
| RHSIM_Rhsim02G0165000 | 0.82 | 0.365 | 0.461 | 0.678 |
| novel.3151 | 0.82 | 0.365 | 0.461 | -0.543 |
| RHSIM_Rhsim01G0071200 | 0.82 | 0.365 | 0.461 | -0.411 |
| RHSIM_Rhsim13G0092700 | 0.82 | 0.365 | 0.462 | 0.364 |
| RHSIM_Rhsim09G0042900 | 0.82 | 0.365 | 0.462 | -0.684 |
| RHSIM_Rhsim07G0175900 | 0.82 | 0.365 | 0.462 | 1.721 |
| RHSIM_Rhsim11G0039000 | 0.82 | 0.365 | 0.462 | -0.329 |
| novel.549 | 0.82 | 0.365 | 0.462 | 1.037 |
| RHSIM_Rhsim02G0077300 | 0.82 | 0.366 | 0.462 | -0.605 |
| RHSIM_Rhsim09G0121600 | 0.82 | 0.366 | 0.462 | 0.560 |
| RHSIM_Rhsim02G0057400 | 0.82 | 0.366 | 0.462 | -2.210 |
| RHSIM_Rhsim02G0168300 | 0.82 | 0.366 | 0.462 | 0.132 |
| novel.3152 | 0.82 | 0.366 | 0.462 | -0.169 |
| RHSIM_Rhsim10G0098500 | 0.82 | 0.366 | 0.462 | 0.579 |
| novel.220 | 0.82 | 0.366 | 0.462 | 0.242 |
| novel.2107 | 0.82 | 0.366 | 0.462 | -0.120 |
| RHSIM_Rhsim07G0044100 | 0.82 | 0.366 | 0.462 | 1.031 |
| RHSIM_Rhsim06G0161600 | 0.82 | 0.366 | 0.462 | -1.049 |
| RHSIM_RhsimUnG0074400 | 0.82 | 0.366 | 0.463 | -0.838 |
| RHSIM_Rhsim11G0163600 | 0.82 | 0.366 | 0.463 | -0.155 |
| novel.4204 | 0.82 | 0.366 | 0.463 | 0.839 |
| RHSIM_Rhsim01G0157700 | 0.82 | 0.366 | 0.463 | -0.817 |
| RHSIM_Rhsim08G0106900 | 0.82 | 0.366 | 0.463 | -2.112 |
| novel.2452 | 0.82 | 0.367 | 0.463 | -0.092 |
| RHSIM_Rhsim07G0065600 | 0.81 | 0.367 | 0.463 | -1.081 |
| RHSIM_Rhsim01G0155000 | 0.81 | 0.367 | 0.463 | -0.925 |

| Gene | LRT statistic | P value | BH-adjusted P | log2FC (interaction) |
|---|---|---|---|---|
| RHSIM_Rhsim07G0212800 | 0.81 | 0.367 | 0.463 | 0.072 |
| RHSIM_Rhsim07G0065100 | 0.81 | 0.367 | 0.463 | -0.528 |
| RHSIM_RhsimUnG0122100 | 0.81 | 0.367 | 0.463 | 0.148 |
| RHSIM_Rhsim12G0190800 | 0.81 | 0.367 | 0.463 | -0.114 |
| RHSIM_Rhsim12G0069100 | 0.81 | 0.367 | 0.464 | -0.253 |
| RHSIM_Rhsim08G0239200 | 0.81 | 0.367 | 0.464 | -0.274 |
| RHSIM_Rhsim08G0228500 | 0.81 | 0.367 | 0.464 | 0.642 |
| RHSIM_Rhsim07G0111000 | 0.81 | 0.367 | 0.464 | -36.457 |
| RHSIM_Rhsim08G0052000 | 0.81 | 0.367 | 0.464 | -0.468 |
| RHSIM_Rhsim09G0089800 | 0.81 | 0.368 | 0.464 | 0.514 |
| RHSIM_Rhsim01G0002100 | 0.81 | 0.368 | 0.464 | 0.777 |
| RHSIM_Rhsim01G0035400 | 0.81 | 0.368 | 0.464 | 0.075 |
| RHSIM_Rhsim06G0090700 | 0.81 | 0.368 | 0.464 | -0.058 |
| RHSIM_Rhsim03G0091800 | 0.81 | 0.368 | 0.464 | -0.626 |
| RHSIM_Rhsim10G0143000 | 0.81 | 0.368 | 0.464 | 1.988 |
| RHSIM_Rhsim06G0107400 | 0.81 | 0.368 | 0.464 | -0.630 |
| RHSIM_Rhsim09G0130200 | 0.81 | 0.368 | 0.464 | -0.680 |
| RHSIM_RhsimUnG0223700 | 0.81 | 0.368 | 0.464 | -0.721 |
| novel.4399 | 0.81 | 0.368 | 0.464 | -1.372 |
| RHSIM_Rhsim04G0061900 | 0.81 | 0.368 | 0.464 | -1.743 |
| RHSIM_Rhsim01G0148100 | 0.81 | 0.368 | 0.465 | -0.240 |
| RHSIM_Rhsim11G0097100 | 0.81 | 0.368 | 0.465 | -1.502 |
| RHSIM_Rhsim08G0136100 | 0.81 | 0.369 | 0.465 | 0.715 |
| RHSIM_Rhsim02G0133500 | 0.81 | 0.369 | 0.465 | -0.569 |
| novel.475 | 0.81 | 0.369 | 0.465 | 0.254 |
| RHSIM_Rhsim09G0131000 | 0.81 | 0.369 | 0.465 | -0.078 |
| novel.2676 | 0.81 | 0.369 | 0.465 | -0.671 |
| novel.685 | 0.81 | 0.369 | 0.465 | -1.132 |
| RHSIM_Rhsim03G0067400 | 0.81 | 0.369 | 0.465 | -0.134 |
| RHSIM_RhsimUnG0192000 | 0.81 | 0.369 | 0.465 | -1.153 |
| RHSIM_Rhsim06G0177900 | 0.81 | 0.369 | 0.466 | 0.757 |
| RHSIM_Rhsim01G0215500 | 0.81 | 0.369 | 0.466 | -0.761 |

| Gene | LRT statistic | P value | BH-adjusted P | log2FC (interaction) |
|---|---|---|---|---|
| RHSIM_Rhsim06G0233000 | 0.81 | 0.369 | 0.466 | -0.787 |
| novel.1374 | 0.81 | 0.370 | 0.466 | -36.058 |
| RHSIM_Rhsim01G0144200 | 0.81 | 0.370 | 0.466 | 0.132 |
| RHSIM_Rhsim13G0019200 | 0.80 | 0.370 | 0.466 | 0.395 |
| RHSIM_Rhsim08G0017200 | 0.80 | 0.370 | 0.466 | 0.098 |
| RHSIM_Rhsim11G0137900 | 0.80 | 0.370 | 0.466 | 0.817 |
| RHSIM_Rhsim04G0226200 | 0.80 | 0.370 | 0.466 | -1.575 |
| RHSIM_Rhsim07G0056500 | 0.80 | 0.370 | 0.466 | -0.082 |
| novel.3214 | 0.80 | 0.370 | 0.466 | 0.746 |
| RHSIM_Rhsim07G0117300 | 0.80 | 0.370 | 0.466 | 0.146 |
| novel.133 | 0.80 | 0.370 | 0.466 | -0.286 |
| RHSIM_Rhsim08G0101900 | 0.80 | 0.370 | 0.466 | 0.579 |
| RHSIM_Rhsim07G0155900 | 0.80 | 0.370 | 0.467 | -0.158 |
| RHSIM_Rhsim12G0040900 | 0.80 | 0.370 | 0.467 | 0.154 |
| RHSIM_Rhsim12G0084800 | 0.80 | 0.371 | 0.467 | -0.786 |
| RHSIM_Rhsim01G0137300 | 0.80 | 0.371 | 0.467 | 1.550 |
| RHSIM_RhsimUnG0021500 | 0.80 | 0.371 | 0.467 | -0.106 |
| RHSIM_Rhsim07G0074000 | 0.80 | 0.371 | 0.467 | 0.100 |
| RHSIM_Rhsim13G0023400 | 0.80 | 0.371 | 0.467 | 1.441 |
| novel.268 | 0.80 | 0.371 | 0.467 | 0.611 |
| RHSIM_Rhsim06G0091800 | 0.80 | 0.371 | 0.467 | 0.633 |
| RHSIM_Rhsim06G0020000 | 0.80 | 0.371 | 0.468 | -0.091 |
| RHSIM_Rhsim02G0003100 | 0.80 | 0.371 | 0.468 | 0.069 |
| RHSIM_Rhsim07G0226300 | 0.80 | 0.371 | 0.468 | -36.251 |
| RHSIM_Rhsim03G0094200 | 0.80 | 0.371 | 0.468 | -0.140 |
| RHSIM_RhsimUnG0061800 | 0.80 | 0.372 | 0.468 | 0.660 |
| RHSIM_Rhsim07G0008600 | 0.80 | 0.372 | 0.468 | 0.128 |
| novel.4401 | 0.80 | 0.372 | 0.468 | -0.652 |
| RHSIM_Rhsim02G0053500 | 0.80 | 0.372 | 0.468 | -0.107 |
| novel.81 | 0.80 | 0.372 | 0.468 | -0.437 |
| RHSIM_Rhsim08G0073100 | 0.80 | 0.372 | 0.469 | -0.840 |
| RHSIM_RhsimUnG0157700 | 0.80 | 0.372 | 0.469 | -0.637 |

| Gene | LRT statistic | P value | BH-adjusted P | log2FC (interaction) |
|---|---|---|---|---|
| novel.1022 | 0.80 | 0.372 | 0.469 | 1.841 |
| RHSIM_Rhsim07G0159000 | 0.80 | 0.372 | 0.469 | 0.511 |
| RHSIM_Rhsim03G0144800 | 0.79 | 0.373 | 0.469 | 0.166 |
| RHSIM_Rhsim12G0099600 | 0.79 | 0.373 | 0.469 | 0.168 |
| RHSIM_Rhsim13G0091100 | 0.79 | 0.373 | 0.469 | -0.950 |
| RHSIM_Rhsim03G0017500 | 0.79 | 0.373 | 0.469 | -0.656 |
| novel.1273 | 0.79 | 0.373 | 0.469 | -35.897 |
| novel.925 | 0.79 | 0.373 | 0.470 | 0.770 |
| novel.2836 | 0.79 | 0.373 | 0.470 | 0.984 |
| novel.2408 | 0.79 | 0.374 | 0.470 | 0.284 |
| RHSIM_Rhsim06G0127900 | 0.79 | 0.374 | 0.470 | -0.828 |
| RHSIM_Rhsim09G0011500 | 0.79 | 0.374 | 0.470 | 0.168 |
| RHSIM_Rhsim04G0026900 | 0.79 | 0.374 | 0.470 | -0.998 |
| novel.3799 | 0.79 | 0.374 | 0.471 | 0.786 |
| RHSIM_Rhsim13G0139100 | 0.79 | 0.374 | 0.471 | -0.856 |
| RHSIM_Rhsim01G0102300 | 0.79 | 0.374 | 0.471 | -36.321 |
| novel.1916 | 0.79 | 0.374 | 0.471 | 0.248 |
| RHSIM_RhsimUnG0093900 | 0.79 | 0.374 | 0.471 | -0.169 |
| RHSIM_Rhsim13G0138600 | 0.79 | 0.375 | 0.471 | -0.659 |
| novel.4571 | 0.79 | 0.375 | 0.471 | -0.637 |
| novel.2286 | 0.79 | 0.375 | 0.471 | -0.982 |
| RHSIM_Rhsim12G0180800 | 0.79 | 0.375 | 0.471 | 0.180 |
| RHSIM_Rhsim09G0156300 | 0.79 | 0.375 | 0.471 | 0.377 |
| RHSIM_Rhsim04G0089200 | 0.79 | 0.375 | 0.472 | -0.372 |
| novel.266 | 0.79 | 0.375 | 0.472 | 0.793 |
| RHSIM_Rhsim12G0086700 | 0.79 | 0.375 | 0.472 | -0.822 |
| RHSIM_RhsimUnG0161700 | 0.79 | 0.375 | 0.472 | -0.381 |
| RHSIM_Rhsim07G0024200 | 0.79 | 0.375 | 0.472 | 0.183 |
| RHSIM_Rhsim10G0046600 | 0.79 | 0.375 | 0.472 | 0.197 |
| RHSIM_Rhsim07G0170900 | 0.78 | 0.376 | 0.472 | -0.115 |
| RHSIM_Rhsim06G0034600 | 0.78 | 0.376 | 0.473 | 0.647 |
| RHSIM_Rhsim03G0104700 | 0.78 | 0.376 | 0.473 | -0.766 |

| Gene | LRT statistic | P value | BH-adjusted P | log2FC (interaction) |
|---|---|---|---|---|
| RHSIM_Rhsim06G0026300 | 0.78 | 0.376 | 0.473 | -0.081 |
| RHSIM_Rhsim04G0058700 | 0.78 | 0.376 | 0.473 | -0.599 |
| RHSIM_Rhsim13G0020900 | 0.78 | 0.376 | 0.473 | -0.240 |
| RHSIM_Rhsim03G0091300 | 0.78 | 0.376 | 0.473 | -36.226 |
| RHSIM_Rhsim04G0083500 | 0.78 | 0.376 | 0.473 | 36.095 |
| novel.3766 | 0.78 | 0.376 | 0.473 | 1.024 |
| novel.1907 | 0.78 | 0.377 | 0.473 | -0.387 |
| RHSIM_Rhsim10G0135300 | 0.78 | 0.377 | 0.473 | -0.245 |
| RHSIM_Rhsim13G0108000 | 0.78 | 0.377 | 0.473 | -0.339 |
| RHSIM_Rhsim07G0035100 | 0.78 | 0.377 | 0.473 | -0.471 |
| novel.146 | 0.78 | 0.377 | 0.473 | 2.100 |
| RHSIM_Rhsim13G0065100 | 0.78 | 0.377 | 0.473 | -36.187 |
| novel.869 | 0.78 | 0.377 | 0.473 | 0.843 |
| RHSIM_Rhsim02G0084500 | 0.78 | 0.377 | 0.473 | -0.250 |
| novel.1727 | 0.78 | 0.377 | 0.474 | -0.624 |
| novel.174 | 0.78 | 0.377 | 0.474 | -2.098 |
| RHSIM_Rhsim08G0090800 | 0.78 | 0.377 | 0.474 | 0.247 |
| novel.4537 | 0.78 | 0.377 | 0.474 | 0.322 |
| RHSIM_Rhsim11G0113100 | 0.78 | 0.377 | 0.474 | 0.233 |
| RHSIM_Rhsim04G0029600 | 0.78 | 0.377 | 0.474 | 0.895 |
| RHSIM_Rhsim08G0037800 | 0.78 | 0.377 | 0.474 | -0.154 |
| RHSIM_Rhsim03G0139200 | 0.78 | 0.377 | 0.474 | -0.872 |
| RHSIM_Rhsim07G0116000 | 0.78 | 0.377 | 0.474 | -0.421 |
| RHSIM_Rhsim02G0091400 | 0.78 | 0.378 | 0.474 | 2.188 |
| RHSIM_Rhsim10G0166400 | 0.78 | 0.378 | 0.475 | 0.281 |
| RHSIM_Rhsim03G0111800 | 0.78 | 0.378 | 0.475 | 0.176 |
| RHSIM_Rhsim07G0120600 | 0.78 | 0.378 | 0.475 | 0.134 |
| RHSIM_Rhsim02G0055600 | 0.78 | 0.378 | 0.475 | -0.311 |
| RHSIM_Rhsim02G0018600 | 0.77 | 0.379 | 0.476 | -1.527 |
| RHSIM_Rhsim09G0200100 | 0.77 | 0.379 | 0.476 | 1.523 |
| novel.1033 | 0.77 | 0.379 | 0.476 | 0.478 |
| RHSIM_Rhsim07G0098400 | 0.77 | 0.379 | 0.476 | 1.265 |

| Gene | LRT statistic | P value | BH-adjusted P | log2FC (interaction) |
|---|---|---|---|---|
| RHSIM_Rhsim04G0073500 | 0.77 | 0.379 | 0.476 | 0.206 |
| RHSIM_Rhsim05G0229500 | 0.77 | 0.379 | 0.476 | -36.392 |
| RHSIM_Rhsim01G0236500 | 0.77 | 0.380 | 0.476 | -0.087 |
| RHSIM_Rhsim04G0166500 | 0.77 | 0.380 | 0.477 | -36.380 |
| RHSIM_Rhsim10G0207200 | 0.77 | 0.380 | 0.477 | 0.268 |
| RHSIM_Rhsim09G0026200 | 0.77 | 0.380 | 0.477 | -1.200 |
| RHSIM_Rhsim07G0060500 | 0.77 | 0.380 | 0.477 | -0.327 |
| RHSIM_Rhsim05G0212400 | 0.77 | 0.380 | 0.477 | 1.669 |
| RHSIM_Rhsim06G0046500 | 0.77 | 0.380 | 0.477 | -0.218 |
| RHSIM_Rhsim01G0065300 | 0.77 | 0.380 | 0.477 | -36.133 |
| RHSIM_Rhsim02G0016500 | 0.77 | 0.380 | 0.477 | 1.609 |
| RHSIM_Rhsim01G0264700 | 0.77 | 0.381 | 0.477 | 1.511 |
| RHSIM_Rhsim07G0070300 | 0.77 | 0.381 | 0.478 | -0.712 |
| novel.4104 | 0.77 | 0.381 | 0.478 | -1.222 |
| RHSIM_Rhsim09G0124100 | 0.77 | 0.381 | 0.478 | -0.471 |
| RHSIM_Rhsim05G0141900 | 0.77 | 0.381 | 0.478 | 0.069 |
| RHSIM_Rhsim12G0185800 | 0.77 | 0.381 | 0.478 | 1.870 |
| RHSIM_Rhsim08G0192900 | 0.77 | 0.381 | 0.478 | 36.274 |
| novel.4281 | 0.77 | 0.381 | 0.478 | -1.767 |
| RHSIM_Rhsim02G0239500 | 0.77 | 0.381 | 0.478 | 1.620 |
| RHSIM_Rhsim02G0242100 | 0.77 | 0.381 | 0.478 | -0.076 |
| RHSIM_Rhsim01G0191700 | 0.77 | 0.381 | 0.478 | 0.149 |
| novel.648 | 0.77 | 0.381 | 0.478 | 1.846 |
| RHSIM_Rhsim10G0158600 | 0.77 | 0.381 | 0.478 | 0.411 |
| novel.2143 | 0.77 | 0.381 | 0.478 | 1.815 |
| RHSIM_RhsimUnG0207500 | 0.77 | 0.382 | 0.478 | -0.372 |
| RHSIM_Rhsim04G0010800 | 0.77 | 0.382 | 0.479 | 1.621 |
| RHSIM_Rhsim01G0120400 | 0.77 | 0.382 | 0.479 | -2.520 |
| RHSIM_Rhsim08G0092700 | 0.76 | 0.382 | 0.479 | -1.831 |
| RHSIM_Rhsim12G0175200 | 0.76 | 0.382 | 0.479 | -1.354 |
| RHSIM_Rhsim03G0133700 | 0.76 | 0.382 | 0.479 | -1.560 |
| RHSIM_RhsimUnG0098700 | 0.76 | 0.382 | 0.479 | 1.234 |

| Gene | LRT statistic | P value | BH-adjusted P | log2FC (interaction) |
|---|---|---|---|---|
| novel.3534 | 0.76 | 0.382 | 0.479 | -0.788 |
| RHSIM_Rhsim02G0231000 | 0.76 | 0.382 | 0.479 | -0.149 |
| RHSIM_RhsimUnG0216000 | 0.76 | 0.382 | 0.479 | 0.395 |
| RHSIM_RhsimUnG0201800 | 0.76 | 0.383 | 0.480 | -0.648 |
| RHSIM_Rhsim01G0262100 | 0.76 | 0.383 | 0.480 | 0.073 |
| novel.1912 | 0.76 | 0.383 | 0.480 | -0.621 |
| RHSIM_Rhsim13G0100100 | 0.76 | 0.383 | 0.480 | 0.398 |
| novel.4421 | 0.76 | 0.383 | 0.480 | -1.640 |
| RHSIM_Rhsim12G0057600 | 0.76 | 0.383 | 0.480 | 2.616 |
| RHSIM_Rhsim08G0248900 | 0.76 | 0.383 | 0.480 | -0.090 |
| RHSIM_Rhsim13G0222700 | 0.76 | 0.383 | 0.480 | -0.388 |
| RHSIM_Rhsim12G0082400 | 0.76 | 0.384 | 0.481 | 0.392 |
| RHSIM_Rhsim08G0102300 | 0.76 | 0.384 | 0.481 | -0.242 |
| RHSIM_Rhsim07G0111600 | 0.76 | 0.384 | 0.481 | -0.989 |
| RHSIM_Rhsim02G0238100 | 0.76 | 0.384 | 0.481 | 1.299 |
| RHSIM_Rhsim13G0202300 | 0.76 | 0.384 | 0.481 | 0.081 |
| RHSIM_Rhsim08G0047800 | 0.76 | 0.384 | 0.481 | 0.595 |
| RHSIM_Rhsim08G0230500 | 0.76 | 0.384 | 0.481 | 0.131 |
| RHSIM_Rhsim09G0116200 | 0.76 | 0.384 | 0.481 | -0.516 |
| novel.2229 | 0.76 | 0.384 | 0.481 | -0.128 |
| RHSIM_RhsimUnG0004400 | 0.76 | 0.384 | 0.481 | 0.689 |
| RHSIM_Rhsim01G0209600 | 0.76 | 0.384 | 0.481 | 0.144 |
| RHSIM_Rhsim09G0074300 | 0.76 | 0.384 | 0.481 | 1.631 |
| RHSIM_Rhsim13G0040600 | 0.76 | 0.384 | 0.482 | 0.118 |
| RHSIM_Rhsim09G0048700 | 0.75 | 0.385 | 0.482 | -0.079 |
| RHSIM_Rhsim03G0167900 | 0.75 | 0.385 | 0.482 | -0.151 |
| RHSIM_Rhsim10G0204100 | 0.75 | 0.385 | 0.482 | -0.497 |
| novel.2047 | 0.75 | 0.385 | 0.482 | 0.436 |
| RHSIM_Rhsim02G0234000 | 0.75 | 0.385 | 0.482 | 1.074 |
| RHSIM_Rhsim01G0113200 | 0.75 | 0.385 | 0.482 | 0.097 |
| RHSIM_Rhsim01G0033700 | 0.75 | 0.385 | 0.482 | 0.183 |
| novel.1827 | 0.75 | 0.385 | 0.483 | -0.680 |

| Gene | LRT statistic | P value | BH-adjusted P | log2FC (interaction) |
|---|---|---|---|---|
| RHSIM_RhsimUnG0194900 | 0.75 | 0.385 | 0.483 | -0.214 |
| novel.3941 | 0.75 | 0.385 | 0.483 | 1.072 |
| RHSIM_Rhsim08G0120100 | 0.75 | 0.386 | 0.483 | 0.789 |
| RHSIM_Rhsim08G0233400 | 0.75 | 0.386 | 0.483 | 1.576 |
| RHSIM_Rhsim13G0051000 | 0.75 | 0.386 | 0.483 | 0.505 |
| novel.2006 | 0.75 | 0.386 | 0.483 | 0.815 |
| RHSIM_Rhsim06G0122300 | 0.75 | 0.386 | 0.483 | 0.044 |
| RHSIM_Rhsim07G0005100 | 0.75 | 0.386 | 0.483 | 0.111 |
| novel.3455 | 0.75 | 0.386 | 0.483 | -0.546 |
| novel.1939 | 0.75 | 0.386 | 0.483 | -0.626 |
| RHSIM_Rhsim01G0074100 | 0.75 | 0.386 | 0.483 | -1.789 |
| RHSIM_Rhsim02G0086000 | 0.75 | 0.386 | 0.483 | -0.432 |
| RHSIM_Rhsim07G0048200 | 0.75 | 0.386 | 0.484 | -0.117 |
| RHSIM_Rhsim08G0123300 | 0.75 | 0.387 | 0.484 | 0.271 |
| RHSIM_Rhsim05G0139500 | 0.75 | 0.387 | 0.484 | -0.100 |
| RHSIM_Rhsim05G0203900 | 0.75 | 0.387 | 0.484 | -1.238 |
| RHSIM_Rhsim05G0080300 | 0.75 | 0.387 | 0.484 | 0.454 |
| RHSIM_RhsimUnG0239800 | 0.75 | 0.387 | 0.484 | -36.121 |
| RHSIM_Rhsim11G0064500 | 0.75 | 0.387 | 0.484 | 1.974 |
| RHSIM_Rhsim01G0001700 | 0.75 | 0.387 | 0.484 | 0.087 |
| RHSIM_RhsimUnG0181500 | 0.75 | 0.387 | 0.484 | 0.795 |
| RHSIM_Rhsim13G0049500 | 0.75 | 0.387 | 0.484 | 0.389 |
| RHSIM_Rhsim12G0148200 | 0.75 | 0.387 | 0.484 | -0.200 |
| RHSIM_Rhsim07G0015700 | 0.75 | 0.387 | 0.484 | 0.088 |
| RHSIM_Rhsim04G0050200 | 0.75 | 0.387 | 0.484 | -0.122 |
| RHSIM_RhsimUnG0255200 | 0.75 | 0.387 | 0.484 | 0.199 |
| RHSIM_Rhsim10G0015700 | 0.75 | 0.388 | 0.485 | 0.204 |
| RHSIM_Rhsim01G0016700 | 0.75 | 0.388 | 0.485 | 0.312 |
| novel.4033 | 0.75 | 0.388 | 0.485 | -35.885 |
| RHSIM_Rhsim01G0183500 | 0.75 | 0.388 | 0.485 | 0.414 |
| novel.4176 | 0.75 | 0.388 | 0.485 | 0.418 |
| novel.2585 | 0.75 | 0.388 | 0.485 | -0.554 |

| Gene | LRT statistic | P value | BH-adjusted P | log2FC (interaction) |
|---|---|---|---|---|
| RHSIM_Rhsim08G0157200 | 0.75 | 0.388 | 0.485 | -0.159 |
| RHSIM_Rhsim10G0202000 | 0.75 | 0.388 | 0.485 | 0.238 |
| RHSIM_Rhsim07G0043300 | 0.75 | 0.388 | 0.485 | -1.139 |
| RHSIM_Rhsim05G0077000 | 0.75 | 0.388 | 0.485 | -0.205 |
| RHSIM_Rhsim09G0064500 | 0.74 | 0.388 | 0.485 | -35.935 |
| RHSIM_Rhsim02G0189000 | 0.74 | 0.388 | 0.485 | 0.130 |
| RHSIM_Rhsim02G0077400 | 0.74 | 0.388 | 0.485 | -1.561 |
| RHSIM_Rhsim01G0099700 | 0.74 | 0.388 | 0.485 | 1.562 |
| RHSIM_Rhsim10G0049200 | 0.74 | 0.388 | 0.485 | 0.353 |
| RHSIM_RhsimUnG0164200 | 0.74 | 0.388 | 0.485 | 1.185 |
| RHSIM_Rhsim09G0056000 | 0.74 | 0.389 | 0.486 | 0.354 |
| RHSIM_Rhsim11G0191900 | 0.74 | 0.389 | 0.486 | -0.098 |
| RHSIM_Rhsim09G0074700 | 0.74 | 0.389 | 0.486 | 0.245 |
| RHSIM_Rhsim11G0057300 | 0.74 | 0.389 | 0.486 | 2.179 |
| RHSIM_Rhsim06G0153800 | 0.74 | 0.389 | 0.486 | 0.556 |
| RHSIM_Rhsim10G0035600 | 0.74 | 0.389 | 0.487 | 0.163 |
| RHSIM_Rhsim02G0101300 | 0.74 | 0.390 | 0.487 | -1.328 |
| RHSIM_Rhsim05G0046900 | 0.74 | 0.390 | 0.487 | -1.072 |
| RHSIM_Rhsim04G0235100 | 0.74 | 0.390 | 0.487 | 1.903 |
| novel.3414 | 0.74 | 0.390 | 0.488 | 0.839 |
| RHSIM_Rhsim03G0263100 | 0.74 | 0.390 | 0.488 | -36.123 |
| RHSIM_Rhsim02G0242900 | 0.74 | 0.390 | 0.488 | -1.707 |
| RHSIM_Rhsim04G0021300 | 0.74 | 0.390 | 0.488 | -0.362 |
| novel.843 | 0.74 | 0.391 | 0.488 | -36.092 |
| RHSIM_Rhsim08G0097700 | 0.74 | 0.391 | 0.488 | -0.831 |
| RHSIM_Rhsim08G0077700 | 0.74 | 0.391 | 0.488 | -1.323 |
| RHSIM_Rhsim02G0242200 | 0.74 | 0.391 | 0.488 | -0.274 |
| RHSIM_Rhsim06G0041300 | 0.74 | 0.391 | 0.488 | -0.301 |
| RHSIM_Rhsim04G0178600 | 0.74 | 0.391 | 0.488 | -2.307 |
| novel.601 | 0.74 | 0.391 | 0.488 | 1.247 |
| RHSIM_Rhsim08G0130600 | 0.74 | 0.391 | 0.488 | 0.922 |
| novel.2177 | 0.74 | 0.391 | 0.488 | -36.148 |

| Gene | LRT statistic | P value | BH-adjusted P | log2FC (interaction) |
|---|---|---|---|---|
| RHSIM_Rhsim10G0154400 | 0.74 | 0.391 | 0.488 | 0.845 |
| RHSIM_Rhsim10G0040500 | 0.74 | 0.391 | 0.488 | 0.307 |
| novel.3768 | 0.74 | 0.391 | 0.488 | -35.833 |
| novel.1223 | 0.74 | 0.391 | 0.488 | 36.218 |
| RHSIM_Rhsim12G0014000 | 0.74 | 0.391 | 0.488 | -0.084 |
| RHSIM_Rhsim01G0043600 | 0.73 | 0.391 | 0.488 | -36.180 |
| RHSIM_Rhsim02G0225800 | 0.73 | 0.391 | 0.488 | 1.129 |
| RHSIM_Rhsim09G0090500 | 0.73 | 0.391 | 0.488 | -0.149 |
| RHSIM_Rhsim02G0005700 | 0.73 | 0.391 | 0.488 | 0.879 |
| novel.4264 | 0.73 | 0.391 | 0.488 | 0.609 |
| RHSIM_Rhsim05G0018400 | 0.73 | 0.391 | 0.488 | -0.135 |
| RHSIM_Rhsim12G0075700 | 0.73 | 0.392 | 0.489 | 0.675 |
| RHSIM_Rhsim13G0067500 | 0.73 | 0.392 | 0.489 | -0.060 |
| RHSIM_Rhsim04G0126800 | 0.73 | 0.392 | 0.489 | 1.333 |
| RHSIM_Rhsim09G0188400 | 0.73 | 0.392 | 0.489 | -1.842 |
| novel.1171 | 0.73 | 0.392 | 0.489 | 0.540 |
| RHSIM_Rhsim04G0210300 | 0.73 | 0.392 | 0.489 | 1.485 |
| RHSIM_Rhsim13G0187800 | 0.73 | 0.392 | 0.489 | -0.332 |
| RHSIM_Rhsim08G0237500 | 0.73 | 0.392 | 0.490 | -0.208 |
| RHSIM_RhsimUnG0017500 | 0.73 | 0.392 | 0.490 | 1.251 |
| RHSIM_Rhsim09G0209400 | 0.73 | 0.393 | 0.490 | -0.053 |
| RHSIM_Rhsim06G0107300 | 0.73 | 0.393 | 0.490 | 0.196 |
| novel.348 | 0.73 | 0.393 | 0.490 | -0.946 |
| RHSIM_Rhsim11G0117500 | 0.73 | 0.393 | 0.490 | 0.416 |
| RHSIM_Rhsim09G0081000 | 0.73 | 0.393 | 0.490 | 1.737 |
| RHSIM_RhsimUnG0236400 | 0.73 | 0.393 | 0.490 | 0.974 |
| RHSIM_Rhsim01G0260600 | 0.73 | 0.394 | 0.491 | -0.117 |
| RHSIM_Rhsim05G0232800 | 0.73 | 0.394 | 0.491 | -36.067 |
| RHSIM_RhsimUnG0229500 | 0.73 | 0.394 | 0.491 | -1.547 |
| RHSIM_Rhsim02G0075700 | 0.73 | 0.394 | 0.491 | 0.308 |
| RHSIM_Rhsim12G0183900 | 0.73 | 0.394 | 0.491 | 0.619 |
| RHSIM_Rhsim09G0075300 | 0.73 | 0.394 | 0.491 | 0.766 |

| Gene | LRT statistic | P value | BH-adjusted P | log2FC (interaction) |
|---|---|---|---|---|
| novel.670 | 0.73 | 0.394 | 0.492 | 0.664 |
| RHSIM_Rhsim13G0101600 | 0.73 | 0.394 | 0.492 | -0.506 |
| RHSIM_Rhsim01G0045200 | 0.73 | 0.394 | 0.492 | -0.318 |
| novel.1128 | 0.72 | 0.395 | 0.492 | 0.206 |
| RHSIM_Rhsim09G0014000 | 0.72 | 0.395 | 0.492 | 0.642 |
| RHSIM_Rhsim03G0162700 | 0.72 | 0.395 | 0.492 | -1.491 |
| RHSIM_Rhsim07G0242900 | 0.72 | 0.395 | 0.492 | -1.235 |
| RHSIM_Rhsim06G0082500 | 0.72 | 0.395 | 0.492 | -1.153 |
| RHSIM_Rhsim04G0176400 | 0.72 | 0.395 | 0.493 | 2.924 |
| RHSIM_Rhsim13G0034300 | 0.72 | 0.395 | 0.493 | 1.023 |
| novel.729 | 0.72 | 0.396 | 0.493 | 0.216 |
| RHSIM_Rhsim06G0019500 | 0.72 | 0.396 | 0.493 | 0.603 |
| RHSIM_Rhsim01G0182500 | 0.72 | 0.396 | 0.493 | 0.832 |
| RHSIM_Rhsim03G0228500 | 0.72 | 0.396 | 0.493 | 0.839 |
| novel.505 | 0.72 | 0.396 | 0.493 | 1.851 |
| RHSIM_Rhsim09G0157800 | 0.72 | 0.396 | 0.493 | -0.211 |
| RHSIM_RhsimUnG0215200 | 0.72 | 0.396 | 0.493 | 1.039 |
| RHSIM_RhsimUnG0018300 | 0.72 | 0.396 | 0.494 | -0.153 |
| RHSIM_Rhsim10G0060900 | 0.72 | 0.396 | 0.494 | 0.281 |
| RHSIM_Rhsim11G0085800 | 0.72 | 0.396 | 0.494 | -0.065 |
| RHSIM_Rhsim03G0083700 | 0.72 | 0.397 | 0.494 | 0.122 |
| RHSIM_Rhsim12G0020700 | 0.72 | 0.397 | 0.494 | 0.166 |
| novel.40 | 0.72 | 0.397 | 0.494 | 0.400 |
| RHSIM_Rhsim09G0030200 | 0.72 | 0.397 | 0.494 | -0.288 |
| RHSIM_Rhsim01G0128100 | 0.72 | 0.397 | 0.494 | 0.546 |
| RHSIM_Rhsim07G0032900 | 0.72 | 0.397 | 0.494 | -0.220 |
| RHSIM_RhsimUnG0026300 | 0.72 | 0.397 | 0.495 | 1.938 |
| RHSIM_Rhsim06G0169000 | 0.72 | 0.397 | 0.495 | -1.715 |
| RHSIM_Rhsim13G0030800 | 0.72 | 0.397 | 0.495 | 0.350 |
| RHSIM_Rhsim12G0050100 | 0.72 | 0.398 | 0.495 | 0.635 |
| RHSIM_Rhsim02G0086200 | 0.72 | 0.398 | 0.495 | 0.309 |
| RHSIM_Rhsim10G0053500 | 0.72 | 0.398 | 0.495 | 0.852 |

| Gene | LRT statistic | P value | BH-adjusted P | log2FC (interaction) |
|---|---|---|---|---|
| RHSIM_Rhsim08G0216900 | 0.72 | 0.398 | 0.495 | -0.336 |
| RHSIM_Rhsim12G0043500 | 0.72 | 0.398 | 0.495 | -1.588 |
| RHSIM_Rhsim07G0106600 | 0.72 | 0.398 | 0.495 | 0.304 |
| RHSIM_RhsimUnG0190800 | 0.72 | 0.398 | 0.495 | 0.160 |
| RHSIM_Rhsim07G0221700 | 0.72 | 0.398 | 0.495 | 0.072 |
| novel.3704 | 0.72 | 0.398 | 0.495 | 0.567 |
| RHSIM_Rhsim01G0278200 | 0.71 | 0.398 | 0.495 | -0.534 |
| RHSIM_Rhsim05G0077300 | 0.71 | 0.398 | 0.495 | 0.865 |
| RHSIM_Rhsim12G0176200 | 0.71 | 0.398 | 0.495 | -1.343 |
| RHSIM_Rhsim12G0037300 | 0.71 | 0.398 | 0.495 | 0.595 |
| RHSIM_Rhsim04G0186600 | 0.71 | 0.398 | 0.495 | -1.070 |
| novel.3969 | 0.71 | 0.398 | 0.495 | 0.251 |
| RHSIM_Rhsim06G0074600 | 0.71 | 0.398 | 0.495 | -36.188 |
| RHSIM_Rhsim01G0040200 | 0.71 | 0.398 | 0.495 | -0.573 |
| RHSIM_Rhsim09G0169600 | 0.71 | 0.398 | 0.496 | 0.164 |
| RHSIM_Rhsim06G0206100 | 0.71 | 0.398 | 0.496 | 0.626 |
| RHSIM_Rhsim04G0142300 | 0.71 | 0.398 | 0.496 | -0.266 |
| RHSIM_Rhsim02G0050900 | 0.71 | 0.398 | 0.496 | 0.438 |
| RHSIM_Rhsim02G0246300 | 0.71 | 0.398 | 0.496 | 0.151 |
| novel.1915 | 0.71 | 0.398 | 0.496 | -0.428 |
| novel.710 | 0.71 | 0.399 | 0.496 | -0.832 |
| RHSIM_Rhsim03G0207100 | 0.71 | 0.399 | 0.496 | -0.753 |
| RHSIM_Rhsim08G0092000 | 0.71 | 0.399 | 0.496 | 0.323 |
| novel.790 | 0.71 | 0.399 | 0.496 | 0.647 |
| RHSIM_Rhsim01G0208100 | 0.71 | 0.399 | 0.496 | 0.303 |
| RHSIM_Rhsim11G0058400 | 0.71 | 0.399 | 0.496 | -1.115 |
| RHSIM_Rhsim09G0082700 | 0.71 | 0.399 | 0.496 | -1.894 |
| novel.866 | 0.71 | 0.399 | 0.496 | -0.386 |
| novel.1077 | 0.71 | 0.399 | 0.496 | -0.082 |
| RHSIM_Rhsim12G0139600 | 0.71 | 0.399 | 0.496 | -0.222 |
| RHSIM_Rhsim08G0025400 | 0.71 | 0.399 | 0.497 | 1.728 |
| RHSIM_Rhsim08G0108600 | 0.71 | 0.399 | 0.497 | 1.389 |

| Gene | LRT statistic | P value | BH-adjusted P | log2FC (interaction) |
|---|---|---|---|---|
| RHSIM_Rhsim04G0097300 | 0.71 | 0.400 | 0.497 | 1.000 |
| RHSIM_Rhsim09G0195300 | 0.71 | 0.400 | 0.497 | -0.186 |
| novel.3584 | 0.71 | 0.400 | 0.497 | 0.550 |
| RHSIM_Rhsim01G0185800 | 0.71 | 0.400 | 0.497 | 0.792 |
| RHSIM_Rhsim01G0151600 | 0.71 | 0.400 | 0.497 | 0.141 |
| RHSIM_Rhsim05G0087200 | 0.71 | 0.400 | 0.497 | -0.673 |
| RHSIM_Rhsim02G0174300 | 0.71 | 0.400 | 0.497 | 0.096 |
| RHSIM_Rhsim02G0114700 | 0.71 | 0.400 | 0.497 | -1.845 |
| RHSIM_Rhsim07G0023800 | 0.71 | 0.400 | 0.497 | -1.456 |
| RHSIM_Rhsim06G0106200 | 0.71 | 0.400 | 0.497 | -1.845 |
| RHSIM_Rhsim01G0160400 | 0.71 | 0.400 | 0.497 | -36.018 |
| RHSIM_Rhsim07G0255500 | 0.71 | 0.400 | 0.497 | -1.996 |
| RHSIM_RhsimUnG0074700 | 0.71 | 0.401 | 0.498 | 2.114 |
| RHSIM_Rhsim13G0148300 | 0.71 | 0.401 | 0.498 | 0.426 |
| RHSIM_Rhsim02G0029700 | 0.71 | 0.401 | 0.498 | 1.114 |
| novel.3551 | 0.71 | 0.401 | 0.498 | -0.478 |
| RHSIM_RhsimUnG0193600 | 0.70 | 0.401 | 0.499 | 2.129 |
| RHSIM_Rhsim10G0167900 | 0.70 | 0.401 | 0.499 | -0.230 |
| novel.1392 | 0.70 | 0.402 | 0.499 | 0.801 |
| RHSIM_Rhsim08G0120300 | 0.70 | 0.402 | 0.499 | -0.804 |
| novel.3738 | 0.70 | 0.402 | 0.499 | 0.487 |
| RHSIM_Rhsim09G0116400 | 0.70 | 0.402 | 0.499 | 0.694 |
| RHSIM_Rhsim10G0124200 | 0.70 | 0.402 | 0.499 | 0.186 |
| RHSIM_Rhsim06G0042000 | 0.70 | 0.402 | 0.499 | 0.307 |
| novel.2896 | 0.70 | 0.402 | 0.499 | 0.526 |
| RHSIM_Rhsim11G0097400 | 0.70 | 0.402 | 0.499 | 0.109 |
| RHSIM_Rhsim13G0163800 | 0.70 | 0.402 | 0.499 | 2.121 |
| RHSIM_Rhsim13G0214100 | 0.70 | 0.402 | 0.499 | 0.588 |
| RHSIM_Rhsim11G0022700 | 0.70 | 0.402 | 0.499 | 1.318 |
| RHSIM_RhsimUnG0083400 | 0.70 | 0.402 | 0.499 | 2.172 |
| RHSIM_Rhsim07G0255300 | 0.70 | 0.402 | 0.500 | 1.837 |
| RHSIM_Rhsim02G0008600 | 0.70 | 0.403 | 0.500 | -0.185 |

| Gene | LRT statistic | P value | BH-adjusted P | log2FC (interaction) |
|---|---|---|---|---|
| RHSIM_Rhsim13G0147600 | 0.70 | 0.403 | 0.500 | 1.484 |
| RHSIM_Rhsim05G0177000 | 0.70 | 0.403 | 0.500 | -0.672 |
| RHSIM_Rhsim08G0182600 | 0.70 | 0.403 | 0.500 | -1.009 |
| RHSIM_Rhsim07G0012100 | 0.70 | 0.403 | 0.500 | -35.985 |
| RHSIM_Rhsim08G0007900 | 0.70 | 0.403 | 0.500 | 0.375 |
| RHSIM_Rhsim01G0018000 | 0.70 | 0.403 | 0.500 | 0.314 |
| RHSIM_Rhsim07G0258900 | 0.70 | 0.403 | 0.501 | 36.183 |
| RHSIM_Rhsim06G0156200 | 0.70 | 0.403 | 0.501 | -35.747 |
| RHSIM_Rhsim09G0148100 | 0.70 | 0.404 | 0.501 | -0.893 |
| novel.3501 | 0.70 | 0.404 | 0.501 | 0.523 |
| novel.309 | 0.70 | 0.404 | 0.501 | 0.524 |
| RHSIM_Rhsim04G0163800 | 0.70 | 0.404 | 0.501 | 0.576 |
| RHSIM_Rhsim12G0077700 | 0.70 | 0.404 | 0.501 | -0.066 |
| RHSIM_Rhsim13G0199200 | 0.70 | 0.404 | 0.501 | -0.253 |
| RHSIM_Rhsim05G0178200 | 0.70 | 0.404 | 0.502 | -0.598 |
| novel.3694 | 0.70 | 0.404 | 0.502 | 0.581 |
| novel.1609 | 0.69 | 0.405 | 0.502 | -0.878 |
| RHSIM_Rhsim03G0125900 | 0.69 | 0.405 | 0.502 | 36.213 |
| RHSIM_Rhsim05G0086400 | 0.69 | 0.405 | 0.502 | 0.061 |
| RHSIM_Rhsim10G0001100 | 0.69 | 0.405 | 0.502 | 0.425 |
| novel.4203 | 0.69 | 0.405 | 0.502 | 1.056 |
| RHSIM_Rhsim05G0016900 | 0.69 | 0.405 | 0.502 | 0.516 |
| RHSIM_Rhsim02G0094000 | 0.69 | 0.405 | 0.502 | 1.119 |
| RHSIM_Rhsim12G0127000 | 0.69 | 0.405 | 0.502 | -0.162 |
| RHSIM_Rhsim05G0190500 | 0.69 | 0.405 | 0.502 | 1.040 |
| RHSIM_Rhsim12G0138200 | 0.69 | 0.405 | 0.502 | 0.147 |
| RHSIM_Rhsim01G0131700 | 0.69 | 0.405 | 0.502 | 0.552 |
| novel.4500 | 0.69 | 0.405 | 0.503 | 1.445 |
| RHSIM_Rhsim06G0066700 | 0.69 | 0.406 | 0.503 | 1.495 |
| RHSIM_Rhsim13G0091700 | 0.69 | 0.406 | 0.503 | -0.069 |
| RHSIM_Rhsim01G0210900 | 0.69 | 0.406 | 0.503 | 0.227 |
| RHSIM_Rhsim08G0217400 | 0.69 | 0.406 | 0.503 | -0.101 |

| Gene | LRT statistic | P value | BH-adjusted P | log2FC (interaction) |
|---|---|---|---|---|
| novel.3675 | 0.69 | 0.406 | 0.503 | 1.607 |
| RHSIM_Rhsim06G0058100 | 0.69 | 0.406 | 0.503 | 1.590 |
| RHSIM_Rhsim02G0039600 | 0.69 | 0.406 | 0.503 | -0.157 |
| novel.2393 | 0.69 | 0.406 | 0.503 | -0.280 |
| RHSIM_Rhsim07G0247200 | 0.69 | 0.406 | 0.503 | -0.961 |
| RHSIM_Rhsim01G0192400 | 0.69 | 0.406 | 0.503 | 0.171 |
| RHSIM_Rhsim10G0115000 | 0.69 | 0.406 | 0.503 | -2.362 |
| RHSIM_Rhsim01G0266500 | 0.69 | 0.406 | 0.504 | 1.254 |
| RHSIM_RhsimUnG0109500 | 0.69 | 0.406 | 0.504 | 0.292 |
| RHSIM_Rhsim03G0152700 | 0.69 | 0.406 | 0.504 | 0.725 |
| RHSIM_Rhsim03G0216200 | 0.69 | 0.407 | 0.504 | 0.260 |
| novel.2106 | 0.69 | 0.407 | 0.504 | -35.618 |
| RHSIM_Rhsim07G0023600 | 0.69 | 0.407 | 0.504 | -0.143 |
| RHSIM_Rhsim03G0040500 | 0.69 | 0.407 | 0.504 | -0.272 |
| novel.2799 | 0.69 | 0.407 | 0.504 | -35.782 |
| novel.3579 | 0.69 | 0.407 | 0.504 | -0.751 |
| RHSIM_Rhsim12G0138300 | 0.69 | 0.407 | 0.504 | -0.148 |
| RHSIM_Rhsim06G0222900 | 0.69 | 0.407 | 0.504 | -0.179 |
| RHSIM_Rhsim07G0241700 | 0.69 | 0.407 | 0.505 | -0.213 |
| RHSIM_Rhsim11G0107900 | 0.69 | 0.407 | 0.505 | -1.164 |
| RHSIM_Rhsim01G0052400 | 0.69 | 0.408 | 0.505 | 0.092 |
| RHSIM_Rhsim09G0191800 | 0.69 | 0.408 | 0.505 | -35.537 |
| RHSIM_Rhsim01G0002200 | 0.69 | 0.408 | 0.505 | -0.079 |
| novel.1154 | 0.69 | 0.408 | 0.505 | -1.942 |
| RHSIM_Rhsim02G0025100 | 0.69 | 0.408 | 0.505 | 0.425 |
| RHSIM_Rhsim01G0001800 | 0.69 | 0.408 | 0.505 | 0.714 |
| RHSIM_Rhsim13G0011200 | 0.68 | 0.408 | 0.505 | 0.197 |
| RHSIM_Rhsim09G0030100 | 0.68 | 0.408 | 0.505 | -0.098 |
| RHSIM_Rhsim03G0110200 | 0.68 | 0.408 | 0.505 | 0.172 |
| RHSIM_Rhsim10G0153100 | 0.68 | 0.408 | 0.506 | 0.214 |
| RHSIM_Rhsim03G0207300 | 0.68 | 0.408 | 0.506 | -0.969 |
| RHSIM_Rhsim11G0122700 | 0.68 | 0.408 | 0.506 | 0.063 |

| Gene | LRT statistic | P value | BH-adjusted P | log2FC (interaction) |
|---|---|---|---|---|
| RHSIM_Rhsim03G0097000 | 0.68 | 0.408 | 0.506 | -0.189 |
| RHSIM_Rhsim02G0191500 | 0.68 | 0.409 | 0.506 | -1.957 |
| RHSIM_Rhsim08G0175100 | 0.68 | 0.409 | 0.506 | -1.116 |
| RHSIM_Rhsim09G0175700 | 0.68 | 0.409 | 0.506 | 1.841 |
| RHSIM_Rhsim01G0075000 | 0.68 | 0.409 | 0.506 | -0.647 |
| RHSIM_Rhsim09G0156900 | 0.68 | 0.409 | 0.506 | -0.587 |
| novel.1612 | 0.68 | 0.409 | 0.506 | -0.792 |
| novel.1531 | 0.68 | 0.409 | 0.506 | -0.228 |
| RHSIM_Rhsim10G0107500 | 0.68 | 0.409 | 0.506 | -1.389 |
| RHSIM_Rhsim10G0132500 | 0.68 | 0.409 | 0.506 | 0.174 |
| RHSIM_Rhsim01G0265200 | 0.68 | 0.409 | 0.506 | 0.118 |
| novel.617 | 0.68 | 0.409 | 0.506 | -1.131 |
| RHSIM_Rhsim13G0055300 | 0.68 | 0.409 | 0.506 | 0.680 |
| RHSIM_Rhsim07G0110200 | 0.68 | 0.409 | 0.506 | 1.078 |
| RHSIM_Rhsim08G0019100 | 0.68 | 0.409 | 0.506 | 0.741 |
| novel.2055 | 0.68 | 0.409 | 0.506 | 0.372 |
| RHSIM_Rhsim05G0108700 | 0.68 | 0.409 | 0.506 | 0.117 |
| novel.3880 | 0.68 | 0.409 | 0.506 | -0.053 |
| RHSIM_Rhsim10G0214300 | 0.68 | 0.409 | 0.506 | 35.908 |
| RHSIM_Rhsim01G0008100 | 0.68 | 0.409 | 0.506 | 0.782 |
| RHSIM_Rhsim08G0215900 | 0.68 | 0.409 | 0.507 | -1.292 |
| RHSIM_Rhsim02G0133600 | 0.68 | 0.410 | 0.507 | -0.179 |
| RHSIM_RhsimUnG0197600 | 0.68 | 0.410 | 0.507 | -0.268 |
| RHSIM_Rhsim07G0163800 | 0.68 | 0.410 | 0.507 | -0.969 |
| novel.1846 | 0.68 | 0.410 | 0.508 | 0.784 |
| RHSIM_Rhsim04G0023100 | 0.68 | 0.410 | 0.508 | -35.618 |
| RHSIM_Rhsim13G0105100 | 0.68 | 0.410 | 0.508 | -1.765 |
| novel.206 | 0.68 | 0.411 | 0.508 | 0.124 |
| RHSIM_Rhsim13G0004900 | 0.68 | 0.411 | 0.508 | 0.058 |
| RHSIM_RhsimUnG0064800 | 0.68 | 0.411 | 0.508 | -0.122 |
| RHSIM_Rhsim01G0266400 | 0.68 | 0.411 | 0.508 | 2.102 |
| RHSIM_Rhsim01G0171100 | 0.68 | 0.411 | 0.508 | 0.188 |

| Gene | LRT statistic | P value | BH-adjusted P | log2FC (interaction) |
|---|---|---|---|---|
| novel.882 | 0.68 | 0.411 | 0.508 | -1.232 |
| RHSIM_Rhsim08G0176700 | 0.68 | 0.411 | 0.508 | -2.392 |
| RHSIM_Rhsim08G0156300 | 0.68 | 0.411 | 0.508 | -1.283 |
| novel.1835 | 0.68 | 0.411 | 0.508 | -1.721 |
| RHSIM_Rhsim04G0226900 | 0.68 | 0.411 | 0.508 | -1.370 |
| RHSIM_Rhsim13G0125000 | 0.68 | 0.411 | 0.508 | -0.115 |
| RHSIM_RhsimUnG0092200 | 0.68 | 0.411 | 0.508 | -0.343 |
| novel.3128 | 0.68 | 0.411 | 0.508 | 0.300 |
| RHSIM_Rhsim13G0146800 | 0.68 | 0.411 | 0.508 | -0.089 |
| novel.3156 | 0.68 | 0.411 | 0.508 | -0.804 |
| RHSIM_Rhsim09G0002900 | 0.67 | 0.411 | 0.508 | -0.250 |
| RHSIM_Rhsim12G0176600 | 0.67 | 0.412 | 0.509 | -0.491 |
| RHSIM_Rhsim06G0011200 | 0.67 | 0.412 | 0.509 | -35.831 |
| RHSIM_Rhsim06G0226500 | 0.67 | 0.412 | 0.509 | -0.126 |
| RHSIM_Rhsim05G0187900 | 0.67 | 0.412 | 0.509 | 36.225 |
| novel.1189 | 0.67 | 0.412 | 0.509 | -1.008 |
| novel.902 | 0.67 | 0.412 | 0.509 | -0.339 |
| novel.2699 | 0.67 | 0.412 | 0.509 | -0.105 |
| RHSIM_Rhsim09G0027100 | 0.67 | 0.412 | 0.509 | -1.162 |
| RHSIM_RhsimUnG0080200 | 0.67 | 0.412 | 0.509 | 0.233 |
| RHSIM_Rhsim06G0141800 | 0.67 | 0.412 | 0.509 | 0.988 |
| RHSIM_RhsimUnG0087200 | 0.67 | 0.412 | 0.509 | 0.597 |
| RHSIM_RhsimUnG0257300 | 0.67 | 0.412 | 0.509 | 1.961 |
| RHSIM_Rhsim10G0198800 | 0.67 | 0.412 | 0.509 | -0.723 |
| RHSIM_Rhsim12G0181400 | 0.67 | 0.413 | 0.510 | 0.676 |
| RHSIM_Rhsim08G0208100 | 0.67 | 0.413 | 0.510 | 0.095 |
| RHSIM_Rhsim07G0174100 | 0.67 | 0.413 | 0.510 | 0.130 |
| RHSIM_Rhsim07G0160600 | 0.67 | 0.413 | 0.510 | -0.115 |
| novel.1488 | 0.67 | 0.413 | 0.510 | -1.381 |
| novel.4536 | 0.67 | 0.413 | 0.510 | 0.509 |
| RHSIM_Rhsim01G0143000 | 0.67 | 0.413 | 0.510 | -0.097 |
| RHSIM_Rhsim04G0185900 | 0.67 | 0.413 | 0.510 | 35.937 |

| Gene | LRT statistic | P value | BH-adjusted P | log2FC (interaction) |
|---|---|---|---|---|
| RHSIM_Rhsim04G0204100 | 0.67 | 0.413 | 0.510 | -0.503 |
| RHSIM_Rhsim12G0199500 | 0.67 | 0.413 | 0.510 | -0.137 |
| RHSIM_Rhsim02G0125200 | 0.67 | 0.414 | 0.511 | 0.836 |
| novel.3149 | 0.67 | 0.414 | 0.511 | 0.277 |
| RHSIM_Rhsim02G0173300 | 0.67 | 0.414 | 0.511 | 0.132 |
| RHSIM_Rhsim03G0057000 | 0.67 | 0.414 | 0.511 | 1.235 |
| RHSIM_Rhsim07G0203500 | 0.67 | 0.414 | 0.511 | -0.573 |
| RHSIM_Rhsim04G0008300 | 0.67 | 0.414 | 0.511 | -0.076 |
| RHSIM_Rhsim13G0007000 | 0.67 | 0.414 | 0.511 | 0.935 |
| RHSIM_Rhsim09G0019600 | 0.67 | 0.414 | 0.511 | 1.463 |
| RHSIM_Rhsim05G0186200 | 0.67 | 0.414 | 0.511 | -0.643 |
| RHSIM_Rhsim13G0223900 | 0.67 | 0.414 | 0.511 | -0.223 |
| RHSIM_Rhsim11G0031800 | 0.67 | 0.414 | 0.511 | 1.530 |
| novel.3277 | 0.67 | 0.415 | 0.512 | 0.313 |
| RHSIM_Rhsim11G0197300 | 0.67 | 0.415 | 0.512 | -0.053 |
| novel.891 | 0.66 | 0.415 | 0.512 | -35.863 |
| RHSIM_Rhsim01G0249300 | 0.66 | 0.415 | 0.512 | 0.137 |
| RHSIM_Rhsim10G0149500 | 0.66 | 0.415 | 0.512 | -0.357 |
| RHSIM_Rhsim08G0087800 | 0.66 | 0.415 | 0.512 | -35.629 |
| RHSIM_Rhsim02G0099800 | 0.66 | 0.415 | 0.512 | 0.246 |
| novel.1943 | 0.66 | 0.415 | 0.512 | 0.608 |
| novel.1671 | 0.66 | 0.415 | 0.512 | 1.549 |
| RHSIM_Rhsim02G0132600 | 0.66 | 0.416 | 0.513 | -0.140 |
| RHSIM_Rhsim05G0047800 | 0.66 | 0.416 | 0.513 | 0.639 |
| novel.4272 | 0.66 | 0.416 | 0.513 | 0.349 |
| RHSIM_Rhsim07G0046600 | 0.66 | 0.416 | 0.513 | -0.095 |
| RHSIM_Rhsim01G0223200 | 0.66 | 0.416 | 0.513 | 35.957 |
| RHSIM_Rhsim07G0232200 | 0.66 | 0.416 | 0.513 | 1.079 |
| RHSIM_Rhsim04G0248900 | 0.66 | 0.416 | 0.513 | 2.197 |
| RHSIM_Rhsim07G0008700 | 0.66 | 0.416 | 0.513 | -0.243 |
| RHSIM_Rhsim05G0061500 | 0.66 | 0.416 | 0.513 | 0.844 |
| novel.205 | 0.66 | 0.416 | 0.513 | 0.157 |

| Gene | LRT statistic | P value | BH-adjusted P | log2FC (interaction) |
|---|---|---|---|---|
| RHSIM_Rhsim01G0069700 | 0.66 | 0.416 | 0.513 | 0.888 |
| RHSIM_Rhsim07G0059800 | 0.66 | 0.416 | 0.513 | 0.338 |
| RHSIM_Rhsim08G0146600 | 0.66 | 0.416 | 0.513 | -0.805 |
| RHSIM_Rhsim01G0186100 | 0.66 | 0.416 | 0.513 | 0.050 |
| novel.4159 | 0.66 | 0.416 | 0.513 | -0.154 |
| RHSIM_Rhsim11G0005400 | 0.66 | 0.416 | 0.513 | 35.909 |
| RHSIM_Rhsim02G0081400 | 0.66 | 0.417 | 0.513 | -0.358 |
| RHSIM_Rhsim12G0167800 | 0.66 | 0.417 | 0.513 | -0.070 |
| novel.4385 | 0.66 | 0.417 | 0.513 | 1.453 |
| RHSIM_Rhsim09G0070800 | 0.66 | 0.417 | 0.513 | 0.059 |
| novel.4520 | 0.66 | 0.417 | 0.513 | -0.800 |
| RHSIM_Rhsim01G0010700 | 0.66 | 0.417 | 0.514 | -0.123 |
| novel.1434 | 0.66 | 0.417 | 0.514 | 0.182 |
| RHSIM_Rhsim06G0135300 | 0.66 | 0.417 | 0.514 | -0.070 |
| novel.951 | 0.66 | 0.417 | 0.514 | 0.076 |
| RHSIM_Rhsim13G0091300 | 0.66 | 0.417 | 0.514 | 0.994 |
| RHSIM_Rhsim05G0107400 | 0.66 | 0.417 | 0.514 | 1.594 |
| RHSIM_Rhsim12G0120600 | 0.66 | 0.417 | 0.514 | 35.939 |
| RHSIM_Rhsim05G0090100 | 0.66 | 0.417 | 0.514 | -0.159 |
| RHSIM_RhsimUnG0108400 | 0.66 | 0.417 | 0.514 | -0.357 |
| novel.4182 | 0.66 | 0.417 | 0.514 | -0.458 |
| RHSIM_Rhsim03G0114700 | 0.66 | 0.418 | 0.514 | 36.078 |
| RHSIM_Rhsim08G0144400 | 0.66 | 0.418 | 0.514 | 0.099 |
| RHSIM_Rhsim06G0222200 | 0.66 | 0.418 | 0.514 | -1.009 |
| RHSIM_Rhsim10G0159600 | 0.66 | 0.418 | 0.514 | 0.167 |
| RHSIM_Rhsim01G0183700 | 0.66 | 0.418 | 0.514 | 0.114 |
| RHSIM_Rhsim06G0181300 | 0.66 | 0.418 | 0.515 | 35.977 |
| RHSIM_Rhsim03G0215700 | 0.66 | 0.418 | 0.515 | 0.116 |
| RHSIM_Rhsim08G0007500 | 0.66 | 0.418 | 0.515 | -1.537 |
| RHSIM_Rhsim05G0025600 | 0.66 | 0.418 | 0.515 | -1.241 |
| RHSIM_Rhsim09G0084300 | 0.65 | 0.418 | 0.515 | 0.579 |
| novel.3990 | 0.65 | 0.418 | 0.515 | 0.374 |

| Gene | LRT statistic | P value | BH-adjusted P | log2FC (interaction) |
|---|---|---|---|---|
| RHSIM_Rhsim08G0027400 | 0.65 | 0.418 | 0.515 | -0.057 |
| RHSIM_Rhsim01G0273200 | 0.65 | 0.418 | 0.515 | -0.451 |
| RHSIM_Rhsim05G0171500 | 0.65 | 0.419 | 0.515 | -0.643 |
| RHSIM_Rhsim12G0118300 | 0.65 | 0.419 | 0.515 | 35.873 |
| RHSIM_Rhsim01G0002000 | 0.65 | 0.419 | 0.515 | -0.078 |
| novel.193 | 0.65 | 0.419 | 0.516 | -0.406 |
| RHSIM_Rhsim08G0079900 | 0.65 | 0.419 | 0.516 | 0.040 |
| RHSIM_Rhsim03G0133800 | 0.65 | 0.419 | 0.516 | -0.137 |
| RHSIM_Rhsim06G0049200 | 0.65 | 0.419 | 0.516 | -0.250 |
| novel.1585 | 0.65 | 0.419 | 0.516 | -0.303 |
| RHSIM_Rhsim10G0214700 | 0.65 | 0.419 | 0.516 | 1.879 |
| RHSIM_Rhsim02G0017800 | 0.65 | 0.419 | 0.516 | -0.427 |
| RHSIM_Rhsim03G0183300 | 0.65 | 0.419 | 0.516 | 1.171 |
| RHSIM_Rhsim13G0230400 | 0.65 | 0.420 | 0.516 | -0.322 |
| RHSIM_Rhsim01G0221300 | 0.65 | 0.420 | 0.517 | 0.085 |
| novel.1181 | 0.65 | 0.420 | 0.517 | 0.673 |
| novel.1718 | 0.65 | 0.420 | 0.517 | 0.229 |
| RHSIM_Rhsim13G0095500 | 0.65 | 0.420 | 0.517 | -35.523 |
| RHSIM_Rhsim10G0067000 | 0.65 | 0.420 | 0.517 | 0.250 |
| RHSIM_Rhsim06G0085600 | 0.65 | 0.420 | 0.517 | -1.040 |
| RHSIM_Rhsim11G0082200 | 0.65 | 0.420 | 0.517 | 0.678 |
| RHSIM_Rhsim03G0236800 | 0.65 | 0.421 | 0.517 | -0.094 |
| RHSIM_Rhsim03G0030800 | 0.65 | 0.421 | 0.517 | -0.161 |
| RHSIM_Rhsim07G0169600 | 0.65 | 0.421 | 0.518 | -1.329 |
| RHSIM_Rhsim12G0111100 | 0.65 | 0.421 | 0.518 | -0.256 |
| RHSIM_Rhsim10G0176300 | 0.65 | 0.421 | 0.518 | 35.935 |
| novel.994 | 0.65 | 0.421 | 0.518 | -0.867 |
| RHSIM_Rhsim05G0059600 | 0.65 | 0.421 | 0.518 | -0.619 |
| RHSIM_Rhsim04G0128400 | 0.65 | 0.421 | 0.518 | -0.063 |
| RHSIM_Rhsim09G0199600 | 0.65 | 0.421 | 0.518 | 1.015 |
| RHSIM_Rhsim06G0159700 | 0.65 | 0.421 | 0.518 | 0.824 |
| RHSIM_Rhsim02G0017700 | 0.65 | 0.421 | 0.518 | 0.122 |

| Gene | LRT statistic | P value | BH-adjusted P | log2FC (interaction) |
|---|---|---|---|---|
| RHSIM_Rhsim02G0118400 | 0.65 | 0.421 | 0.518 | -0.451 |
| RHSIM_Rhsim12G0114300 | 0.65 | 0.421 | 0.518 | -0.810 |
| RHSIM_Rhsim07G0123000 | 0.65 | 0.421 | 0.518 | 1.559 |
| RHSIM_Rhsim04G0093100 | 0.65 | 0.421 | 0.518 | 0.724 |
| RHSIM_Rhsim09G0057800 | 0.65 | 0.422 | 0.518 | 36.121 |
| RHSIM_Rhsim02G0120500 | 0.65 | 0.422 | 0.518 | -0.477 |
| RHSIM_Rhsim10G0119600 | 0.65 | 0.422 | 0.518 | 0.137 |
| novel.4515 | 0.65 | 0.422 | 0.518 | 0.903 |
| RHSIM_Rhsim05G0041100 | 0.65 | 0.422 | 0.518 | -0.203 |
| RHSIM_RhsimUnG0030800 | 0.65 | 0.422 | 0.518 | -0.329 |
| RHSIM_Rhsim07G0038600 | 0.65 | 0.422 | 0.518 | -0.047 |
| RHSIM_Rhsim02G0250300 | 0.64 | 0.422 | 0.519 | -1.889 |
| RHSIM_Rhsim03G0220400 | 0.64 | 0.422 | 0.519 | 0.355 |
| novel.1824 | 0.64 | 0.422 | 0.519 | -0.100 |
| novel.84 | 0.64 | 0.422 | 0.519 | -1.568 |
| RHSIM_Rhsim08G0210100 | 0.64 | 0.422 | 0.519 | -0.163 |
| RHSIM_RhsimUnG0170300 | 0.64 | 0.422 | 0.519 | 1.045 |
| RHSIM_Rhsim08G0162800 | 0.64 | 0.423 | 0.519 | 0.398 |
| RHSIM_RhsimUnG0246300 | 0.64 | 0.423 | 0.519 | 0.402 |
| RHSIM_Rhsim01G0097200 | 0.64 | 0.423 | 0.519 | 2.549 |
| RHSIM_Rhsim05G0093300 | 0.64 | 0.423 | 0.519 | 1.806 |
| RHSIM_Rhsim03G0123700 | 0.64 | 0.423 | 0.519 | 0.328 |
| RHSIM_Rhsim06G0056400 | 0.64 | 0.423 | 0.519 | -35.689 |
| RHSIM_Rhsim06G0099400 | 0.64 | 0.423 | 0.520 | 0.267 |
| novel.2574 | 0.64 | 0.423 | 0.520 | -1.683 |
| RHSIM_Rhsim04G0101400 | 0.64 | 0.423 | 0.520 | -35.919 |
| RHSIM_Rhsim13G0093500 | 0.64 | 0.423 | 0.520 | -0.449 |
| RHSIM_Rhsim10G0016300 | 0.64 | 0.423 | 0.520 | 0.307 |
| RHSIM_Rhsim02G0104400 | 0.64 | 0.423 | 0.520 | -0.604 |
| RHSIM_Rhsim04G0074400 | 0.64 | 0.423 | 0.520 | 36.100 |
| novel.179 | 0.64 | 0.423 | 0.520 | 1.054 |
| RHSIM_Rhsim04G0217200 | 0.64 | 0.424 | 0.520 | 0.494 |

| Gene | LRT statistic | P value | BH-adjusted P | log2FC (interaction) |
|---|---|---|---|---|
| RHSIM_Rhsim05G0099300 | 0.64 | 0.424 | 0.520 | 0.721 |
| novel.4540 | 0.64 | 0.424 | 0.520 | -1.135 |
| RHSIM_Rhsim02G0055500 | 0.64 | 0.424 | 0.520 | -1.201 |
| RHSIM_Rhsim09G0122100 | 0.64 | 0.424 | 0.520 | -0.303 |
| RHSIM_Rhsim05G0207400 | 0.64 | 0.424 | 0.521 | 0.316 |
| RHSIM_Rhsim03G0199600 | 0.64 | 0.424 | 0.521 | 0.769 |
| novel.3965 | 0.64 | 0.424 | 0.521 | 1.026 |
| RHSIM_Rhsim08G0130800 | 0.64 | 0.424 | 0.521 | -1.190 |
| novel.2072 | 0.64 | 0.424 | 0.521 | 0.614 |
| RHSIM_RhsimUnG0088500 | 0.64 | 0.425 | 0.522 | 35.852 |
| novel.385 | 0.64 | 0.425 | 0.522 | 1.977 |
| RHSIM_Rhsim09G0191100 | 0.64 | 0.425 | 0.522 | -0.177 |
| novel.2019 | 0.64 | 0.425 | 0.522 | -0.732 |
| novel.2987 | 0.64 | 0.425 | 0.522 | 0.609 |
| RHSIM_Rhsim05G0111800 | 0.63 | 0.426 | 0.522 | 0.129 |
| RHSIM_Rhsim11G0163500 | 0.63 | 0.426 | 0.522 | 0.579 |
| RHSIM_Rhsim07G0214200 | 0.63 | 0.426 | 0.523 | 0.177 |
| novel.1138 | 0.63 | 0.426 | 0.523 | 1.080 |
| RHSIM_Rhsim13G0171200 | 0.63 | 0.426 | 0.523 | 0.970 |
| novel.167 | 0.63 | 0.426 | 0.523 | 1.458 |
| RHSIM_Rhsim02G0098500 | 0.63 | 0.426 | 0.523 | -1.994 |
| RHSIM_Rhsim11G0162000 | 0.63 | 0.426 | 0.523 | -1.332 |
| RHSIM_Rhsim05G0120900 | 0.63 | 0.426 | 0.523 | 0.112 |
| RHSIM_RhsimUnG0026700 | 0.63 | 0.426 | 0.523 | 0.398 |
| RHSIM_Rhsim04G0098000 | 0.63 | 0.426 | 0.523 | -0.095 |
| novel.696 | 0.63 | 0.426 | 0.523 | -0.638 |
| RHSIM_Rhsim02G0115400 | 0.63 | 0.426 | 0.523 | -0.250 |
| RHSIM_Rhsim13G0219100 | 0.63 | 0.427 | 0.523 | -1.536 |
| RHSIM_Rhsim05G0124200 | 0.63 | 0.427 | 0.523 | 0.549 |
| RHSIM_Rhsim06G0137400 | 0.63 | 0.427 | 0.523 | 0.700 |
| novel.1955 | 0.63 | 0.427 | 0.523 | 0.403 |
| RHSIM_Rhsim08G0150200 | 0.63 | 0.427 | 0.523 | -0.162 |

| Gene | LRT statistic | P value | BH-adjusted P | log2FC (interaction) |
|---|---|---|---|---|
| RHSIM_Rhsim06G0082400 | 0.63 | 0.427 | 0.523 | 1.362 |
| RHSIM_Rhsim02G0087400 | 0.63 | 0.427 | 0.523 | -0.196 |
| RHSIM_Rhsim03G0057200 | 0.63 | 0.427 | 0.523 | -0.324 |
| RHSIM_Rhsim07G0169400 | 0.63 | 0.427 | 0.523 | -0.561 |
| novel.4331 | 0.63 | 0.427 | 0.523 | -35.535 |
| RHSIM_Rhsim05G0129600 | 0.63 | 0.427 | 0.524 | 0.827 |
| RHSIM_Rhsim09G0065900 | 0.63 | 0.427 | 0.524 | -0.204 |
| RHSIM_Rhsim08G0047300 | 0.63 | 0.427 | 0.524 | 0.898 |
| RHSIM_Rhsim01G0146300 | 0.63 | 0.427 | 0.524 | 0.314 |
| RHSIM_Rhsim06G0079300 | 0.63 | 0.428 | 0.524 | 0.061 |
| RHSIM_Rhsim02G0177000 | 0.63 | 0.428 | 0.524 | -0.087 |
| RHSIM_Rhsim07G0130800 | 0.63 | 0.428 | 0.524 | 0.147 |
| RHSIM_Rhsim08G0011900 | 0.63 | 0.428 | 0.524 | 35.383 |
| RHSIM_Rhsim02G0205100 | 0.63 | 0.428 | 0.525 | 0.048 |
| novel.1786 | 0.63 | 0.428 | 0.525 | 0.581 |
| RHSIM_Rhsim05G0046500 | 0.63 | 0.428 | 0.525 | 1.511 |
| novel.4466 | 0.63 | 0.429 | 0.525 | 0.455 |
| RHSIM_Rhsim07G0127700 | 0.63 | 0.429 | 0.525 | -1.627 |
| RHSIM_Rhsim01G0011600 | 0.63 | 0.429 | 0.525 | -0.375 |
| RHSIM_RhsimUnG0062900 | 0.63 | 0.429 | 0.525 | 0.256 |
| RHSIM_Rhsim04G0221500 | 0.63 | 0.429 | 0.525 | -0.123 |
| RHSIM_Rhsim05G0118800 | 0.63 | 0.429 | 0.525 | 0.884 |
| RHSIM_Rhsim04G0076300 | 0.63 | 0.429 | 0.525 | -1.694 |
| novel.198 | 0.63 | 0.429 | 0.525 | -1.043 |
| novel.2157 | 0.63 | 0.429 | 0.525 | 1.274 |
| novel.3649 | 0.63 | 0.429 | 0.525 | 0.800 |
| RHSIM_Rhsim08G0031500 | 0.63 | 0.429 | 0.525 | 0.206 |
| novel.4332 | 0.63 | 0.429 | 0.525 | 0.162 |
| RHSIM_Rhsim05G0090600 | 0.63 | 0.429 | 0.525 | 2.104 |
| novel.16 | 0.63 | 0.429 | 0.525 | -0.899 |
| novel.2564 | 0.63 | 0.429 | 0.525 | 1.449 |
| novel.4133 | 0.63 | 0.429 | 0.525 | 0.744 |

| Gene | LRT statistic | P value | BH-adjusted P | log2FC (interaction) |
|---|---|---|---|---|
| RHSIM_RhsimUnG0048700 | 0.63 | 0.429 | 0.525 | 0.973 |
| RHSIM_Rhsim04G0207800 | 0.63 | 0.429 | 0.525 | 0.221 |
| RHSIM_Rhsim08G0018900 | 0.62 | 0.430 | 0.526 | 1.974 |
| novel.3882 | 0.62 | 0.430 | 0.526 | -0.543 |
| RHSIM_Rhsim02G0085600 | 0.62 | 0.430 | 0.526 | 0.328 |
| RHSIM_Rhsim04G0037000 | 0.62 | 0.430 | 0.526 | -0.814 |
| RHSIM_Rhsim09G0128800 | 0.62 | 0.430 | 0.526 | 0.137 |
| RHSIM_Rhsim02G0217200 | 0.62 | 0.430 | 0.527 | -0.065 |
| RHSIM_Rhsim09G0109900 | 0.62 | 0.430 | 0.527 | 0.044 |
| RHSIM_Rhsim08G0224800 | 0.62 | 0.430 | 0.527 | 0.112 |
| RHSIM_Rhsim04G0134000 | 0.62 | 0.430 | 0.527 | 0.235 |
| RHSIM_Rhsim04G0089900 | 0.62 | 0.430 | 0.527 | -0.414 |
| RHSIM_Rhsim12G0077200 | 0.62 | 0.431 | 0.527 | 0.251 |
| RHSIM_Rhsim05G0219900 | 0.62 | 0.431 | 0.527 | -1.785 |
| RHSIM_Rhsim07G0256200 | 0.62 | 0.431 | 0.527 | 1.371 |
| RHSIM_RhsimUnG0077600 | 0.62 | 0.431 | 0.528 | 1.028 |
| RHSIM_Rhsim01G0012900 | 0.62 | 0.431 | 0.528 | -0.602 |
| RHSIM_Rhsim09G0054500 | 0.62 | 0.431 | 0.528 | 0.927 |
| RHSIM_Rhsim10G0085600 | 0.62 | 0.431 | 0.528 | 0.890 |
| RHSIM_Rhsim08G0249900 | 0.62 | 0.431 | 0.528 | 0.240 |
| RHSIM_Rhsim09G0058400 | 0.62 | 0.432 | 0.528 | 0.583 |
| RHSIM_Rhsim01G0110200 | 0.62 | 0.432 | 0.528 | 0.099 |
| novel.1461 | 0.62 | 0.432 | 0.528 | 0.424 |
| RHSIM_Rhsim12G0012900 | 0.62 | 0.432 | 0.528 | -0.376 |
| novel.3242 | 0.62 | 0.432 | 0.528 | -0.788 |
| RHSIM_Rhsim01G0105500 | 0.62 | 0.432 | 0.528 | -1.904 |
| RHSIM_Rhsim12G0118000 | 0.62 | 0.432 | 0.528 | 2.119 |
| RHSIM_Rhsim03G0177100 | 0.62 | 0.432 | 0.528 | 1.193 |
| RHSIM_Rhsim10G0199400 | 0.62 | 0.432 | 0.528 | -0.618 |
| RHSIM_Rhsim05G0189700 | 0.62 | 0.432 | 0.529 | -0.275 |
| novel.285 | 0.62 | 0.432 | 0.529 | 0.632 |
| novel.313 | 0.62 | 0.432 | 0.529 | -0.522 |

| Gene | LRT statistic | P value | BH-adjusted P | log2FC (interaction) |
|---|---|---|---|---|
| novel.3724 | 0.62 | 0.432 | 0.529 | -35.652 |
| RHSIM_Rhsim07G0207100 | 0.62 | 0.432 | 0.529 | 1.199 |
| RHSIM_Rhsim03G0076800 | 0.62 | 0.432 | 0.529 | -0.933 |
| RHSIM_Rhsim04G0171600 | 0.62 | 0.433 | 0.529 | -1.449 |
| novel.3117 | 0.62 | 0.433 | 0.529 | 0.182 |
| RHSIM_Rhsim09G0016100 | 0.62 | 0.433 | 0.529 | -0.121 |
| novel.1635 | 0.62 | 0.433 | 0.529 | -0.525 |
| RHSIM_Rhsim09G0083300 | 0.61 | 0.433 | 0.530 | 2.228 |
| RHSIM_Rhsim11G0134600 | 0.61 | 0.433 | 0.530 | 0.201 |
| RHSIM_Rhsim11G0111200 | 0.61 | 0.433 | 0.530 | -0.128 |
| RHSIM_Rhsim05G0157000 | 0.61 | 0.433 | 0.530 | 2.223 |
| RHSIM_Rhsim01G0049600 | 0.61 | 0.433 | 0.530 | 1.732 |
| novel.2468 | 0.61 | 0.433 | 0.530 | 0.333 |
| RHSIM_Rhsim04G0224800 | 0.61 | 0.433 | 0.530 | 0.072 |
| novel.1899 | 0.61 | 0.433 | 0.530 | -37.360 |
| RHSIM_Rhsim10G0070800 | 0.61 | 0.434 | 0.530 | -0.940 |
| novel.1510 | 0.61 | 0.434 | 0.530 | 0.663 |
| RHSIM_Rhsim04G0200800 | 0.61 | 0.434 | 0.530 | -0.384 |
| RHSIM_Rhsim06G0092900 | 0.61 | 0.434 | 0.530 | 0.088 |
| RHSIM_Rhsim05G0008600 | 0.61 | 0.434 | 0.530 | -35.595 |
| RHSIM_Rhsim01G0181500 | 0.61 | 0.434 | 0.530 | 2.155 |
| novel.2694 | 0.61 | 0.434 | 0.530 | -0.691 |
| RHSIM_Rhsim08G0215000 | 0.61 | 0.434 | 0.530 | -1.179 |
| RHSIM_Rhsim10G0154300 | 0.61 | 0.434 | 0.530 | -0.223 |
| novel.3909 | 0.61 | 0.434 | 0.530 | 1.257 |
| RHSIM_Rhsim10G0178100 | 0.61 | 0.434 | 0.530 | -0.680 |
| RHSIM_Rhsim08G0020800 | 0.61 | 0.434 | 0.530 | -0.086 |
| RHSIM_Rhsim05G0096000 | 0.61 | 0.434 | 0.530 | 1.642 |
| RHSIM_Rhsim04G0080700 | 0.61 | 0.434 | 0.530 | 0.538 |
| RHSIM_Rhsim09G0079600 | 0.61 | 0.434 | 0.530 | 1.713 |
| RHSIM_Rhsim07G0114600 | 0.61 | 0.434 | 0.530 | -35.685 |
| RHSIM_Rhsim06G0008900 | 0.61 | 0.434 | 0.530 | -0.173 |

| Gene | LRT statistic | P value | BH-adjusted P | log2FC (interaction) |
|---|---|---|---|---|
| RHSIM_Rhsim06G0097200 | 0.61 | 0.435 | 0.531 | 1.581 |
| RHSIM_Rhsim10G0060600 | 0.61 | 0.435 | 0.531 | -0.091 |
| RHSIM_Rhsim11G0156300 | 0.61 | 0.435 | 0.531 | -0.202 |
| RHSIM_Rhsim05G0149400 | 0.61 | 0.435 | 0.531 | -35.845 |
| RHSIM_Rhsim03G0086700 | 0.61 | 0.435 | 0.531 | 0.678 |
| RHSIM_Rhsim07G0168100 | 0.61 | 0.435 | 0.531 | 0.785 |
| RHSIM_Rhsim09G0098400 | 0.61 | 0.435 | 0.531 | -0.071 |
| RHSIM_Rhsim07G0226900 | 0.61 | 0.435 | 0.531 | 0.352 |
| novel.459 | 0.61 | 0.435 | 0.531 | 1.351 |
| RHSIM_Rhsim03G0056100 | 0.61 | 0.435 | 0.531 | -0.221 |
| RHSIM_Rhsim11G0035900 | 0.61 | 0.435 | 0.531 | 0.100 |
| novel.1597 | 0.61 | 0.436 | 0.532 | -0.877 |
| novel.3445 | 0.61 | 0.436 | 0.532 | 1.947 |
| RHSIM_Rhsim12G0107800 | 0.61 | 0.436 | 0.532 | -0.353 |
| RHSIM_Rhsim03G0266600 | 0.61 | 0.436 | 0.532 | 1.710 |
| RHSIM_Rhsim07G0256800 | 0.61 | 0.436 | 0.532 | 0.391 |
| RHSIM_Rhsim03G0022200 | 0.61 | 0.436 | 0.532 | -1.083 |
| RHSIM_Rhsim08G0031400 | 0.61 | 0.436 | 0.532 | 1.117 |
| RHSIM_Rhsim02G0173700 | 0.61 | 0.436 | 0.532 | -0.132 |
| RHSIM_Rhsim09G0134900 | 0.61 | 0.436 | 0.532 | -1.386 |
| RHSIM_Rhsim12G0068300 | 0.61 | 0.436 | 0.532 | 35.740 |
| novel.3369 | 0.61 | 0.436 | 0.532 | 0.634 |
| RHSIM_Rhsim02G0126200 | 0.61 | 0.436 | 0.532 | -0.555 |
| RHSIM_Rhsim09G0052100 | 0.61 | 0.437 | 0.533 | -0.100 |
| RHSIM_Rhsim05G0148100 | 0.60 | 0.437 | 0.533 | 0.073 |
| RHSIM_Rhsim05G0043000 | 0.60 | 0.437 | 0.533 | -1.786 |
| RHSIM_Rhsim01G0090100 | 0.60 | 0.437 | 0.533 | 0.291 |
| RHSIM_Rhsim10G0066200 | 0.60 | 0.437 | 0.533 | -0.769 |
| RHSIM_Rhsim12G0099300 | 0.60 | 0.437 | 0.533 | -1.715 |
| novel.2587 | 0.60 | 0.437 | 0.534 | 0.482 |
| RHSIM_RhsimUnG0170700 | 0.60 | 0.438 | 0.534 | 0.203 |
| novel.2882 | 0.60 | 0.438 | 0.534 | -1.333 |

| Gene | LRT statistic | P value | BH-adjusted P | log2FC (interaction) |
|---|---|---|---|---|
| RHSIM_Rhsim08G0067900 | 0.60 | 0.438 | 0.534 | -0.069 |
| RHSIM_Rhsim01G0011500 | 0.60 | 0.438 | 0.534 | -0.694 |
| RHSIM_Rhsim02G0207200 | 0.60 | 0.438 | 0.534 | -0.119 |
| RHSIM_Rhsim11G0154200 | 0.60 | 0.438 | 0.534 | -1.079 |
| RHSIM_Rhsim04G0180500 | 0.60 | 0.438 | 0.534 | 0.077 |
| RHSIM_Rhsim02G0148000 | 0.60 | 0.438 | 0.534 | -35.890 |
| RHSIM_Rhsim06G0005600 | 0.60 | 0.438 | 0.534 | -0.090 |
| RHSIM_Rhsim12G0123800 | 0.60 | 0.438 | 0.534 | 1.100 |
| RHSIM_Rhsim01G0129500 | 0.60 | 0.438 | 0.534 | 0.216 |
| RHSIM_RhsimUnG0254600 | 0.60 | 0.438 | 0.534 | 0.082 |
| RHSIM_RhsimUnG0078300 | 0.60 | 0.438 | 0.534 | -0.748 |
| RHSIM_Rhsim06G0133300 | 0.60 | 0.438 | 0.534 | -1.511 |
| RHSIM_Rhsim02G0230400 | 0.60 | 0.438 | 0.534 | -0.274 |
| RHSIM_Rhsim08G0029900 | 0.60 | 0.438 | 0.534 | 1.940 |
| novel.2247 | 0.60 | 0.439 | 0.534 | 0.991 |
| RHSIM_Rhsim01G0171800 | 0.60 | 0.439 | 0.535 | -0.248 |
| RHSIM_Rhsim07G0204300 | 0.60 | 0.439 | 0.535 | -0.074 |
| RHSIM_Rhsim04G0164300 | 0.60 | 0.439 | 0.535 | 0.086 |
| RHSIM_Rhsim03G0197600 | 0.60 | 0.439 | 0.535 | -0.286 |
| novel.1843 | 0.60 | 0.439 | 0.535 | -0.190 |
| RHSIM_Rhsim03G0240300 | 0.60 | 0.439 | 0.535 | -35.730 |
| RHSIM_Rhsim02G0138400 | 0.60 | 0.439 | 0.535 | -0.204 |
| RHSIM_Rhsim11G0012800 | 0.60 | 0.439 | 0.535 | 0.197 |
| RHSIM_Rhsim11G0158000 | 0.60 | 0.440 | 0.536 | 0.437 |
| RHSIM_Rhsim07G0212000 | 0.60 | 0.440 | 0.536 | 0.253 |
| RHSIM_Rhsim02G0169200 | 0.60 | 0.440 | 0.536 | 0.170 |
| RHSIM_Rhsim08G0242800 | 0.60 | 0.440 | 0.536 | -0.193 |
| RHSIM_Rhsim11G0166500 | 0.60 | 0.440 | 0.536 | -0.064 |
| RHSIM_Rhsim06G0130700 | 0.60 | 0.440 | 0.536 | 0.074 |
| RHSIM_Rhsim06G0089800 | 0.60 | 0.440 | 0.536 | -1.235 |
| novel.2116 | 0.60 | 0.440 | 0.536 | 0.873 |
| novel.628 | 0.60 | 0.440 | 0.536 | -0.112 |

| Gene | LRT statistic | P value | BH-adjusted P | log2FC (interaction) |
|---|---|---|---|---|
| RHSIM_Rhsim02G0178600 | 0.60 | 0.440 | 0.536 | 1.303 |
| RHSIM_Rhsim07G0200200 | 0.59 | 0.441 | 0.537 | 1.066 |
| RHSIM_Rhsim11G0062500 | 0.59 | 0.441 | 0.537 | 0.432 |
| RHSIM_RhsimUnG0111000 | 0.59 | 0.441 | 0.537 | -0.095 |
| RHSIM_Rhsim10G0199300 | 0.59 | 0.441 | 0.537 | 0.356 |
| RHSIM_Rhsim07G0104400 | 0.59 | 0.441 | 0.537 | 0.328 |
| RHSIM_Rhsim05G0171600 | 0.59 | 0.441 | 0.537 | 0.816 |
| RHSIM_Rhsim08G0052600 | 0.59 | 0.441 | 0.537 | -0.253 |
| RHSIM_Rhsim10G0146200 | 0.59 | 0.441 | 0.537 | 2.057 |
| RHSIM_Rhsim05G0134700 | 0.59 | 0.441 | 0.537 | 0.279 |
| RHSIM_Rhsim07G0183500 | 0.59 | 0.442 | 0.537 | 0.608 |
| RHSIM_Rhsim13G0135000 | 0.59 | 0.442 | 0.538 | -1.714 |
| RHSIM_Rhsim06G0061700 | 0.59 | 0.442 | 0.538 | 0.483 |
| RHSIM_Rhsim07G0258200 | 0.59 | 0.442 | 0.538 | -0.060 |
| novel.1599 | 0.59 | 0.442 | 0.538 | -0.192 |
| RHSIM_Rhsim12G0082000 | 0.59 | 0.443 | 0.539 | -0.871 |
| RHSIM_Rhsim13G0181400 | 0.59 | 0.443 | 0.539 | -0.188 |
| RHSIM_Rhsim01G0254500 | 0.59 | 0.443 | 0.539 | -0.080 |
| RHSIM_Rhsim11G0175200 | 0.59 | 0.443 | 0.539 | -0.674 |
| RHSIM_RhsimUnG0186900 | 0.59 | 0.443 | 0.539 | 0.854 |
| novel.3360 | 0.59 | 0.443 | 0.539 | 0.249 |
| RHSIM_Rhsim13G0142600 | 0.59 | 0.443 | 0.539 | 0.074 |
| RHSIM_Rhsim09G0026600 | 0.59 | 0.443 | 0.539 | -0.409 |
| novel.3282 | 0.59 | 0.443 | 0.539 | 35.905 |
| RHSIM_Rhsim13G0137700 | 0.59 | 0.443 | 0.539 | 1.614 |
| novel.3707 | 0.59 | 0.443 | 0.539 | 0.745 |
| RHSIM_Rhsim09G0008800 | 0.59 | 0.443 | 0.539 | -0.078 |
| RHSIM_Rhsim09G0172700 | 0.59 | 0.444 | 0.539 | 1.632 |
| RHSIM_Rhsim02G0096600 | 0.59 | 0.444 | 0.540 | 0.157 |
| RHSIM_Rhsim03G0030600 | 0.59 | 0.444 | 0.540 | -0.211 |
| RHSIM_Rhsim04G0235900 | 0.59 | 0.444 | 0.540 | -0.966 |
| RHSIM_Rhsim10G0118700 | 0.59 | 0.444 | 0.540 | 0.199 |

| Gene | LRT statistic | P value | BH-adjusted P | log2FC (interaction) |
|---|---|---|---|---|
| RHSIM_Rhsim02G0237300 | 0.59 | 0.444 | 0.540 | 0.475 |
| RHSIM_Rhsim11G0031000 | 0.59 | 0.444 | 0.540 | -1.507 |
| RHSIM_Rhsim04G0080100 | 0.59 | 0.444 | 0.540 | 0.933 |
| RHSIM_RhsimUnG0022100 | 0.58 | 0.444 | 0.540 | 35.642 |
| RHSIM_Rhsim12G0195900 | 0.58 | 0.444 | 0.540 | -0.101 |
| RHSIM_Rhsim08G0143400 | 0.58 | 0.445 | 0.541 | -0.280 |
| RHSIM_Rhsim07G0103000 | 0.58 | 0.445 | 0.541 | -35.588 |
| RHSIM_Rhsim04G0097400 | 0.58 | 0.445 | 0.541 | -0.344 |
| RHSIM_Rhsim12G0042700 | 0.58 | 0.445 | 0.541 | -0.634 |
| RHSIM_Rhsim07G0023300 | 0.58 | 0.445 | 0.541 | 35.767 |
| novel.600 | 0.58 | 0.445 | 0.541 | 0.657 |
| novel.3932 | 0.58 | 0.445 | 0.541 | -0.077 |
| RHSIM_Rhsim12G0160300 | 0.58 | 0.445 | 0.541 | -0.093 |
| RHSIM_Rhsim01G0251900 | 0.58 | 0.445 | 0.541 | 0.796 |
| novel.2554 | 0.58 | 0.445 | 0.541 | 1.307 |
| RHSIM_Rhsim06G0239000 | 0.58 | 0.445 | 0.541 | -0.060 |
| RHSIM_Rhsim12G0111700 | 0.58 | 0.445 | 0.541 | 0.983 |
| RHSIM_RhsimUnG0197000 | 0.58 | 0.445 | 0.541 | -0.255 |
| RHSIM_RhsimUnG0228300 | 0.58 | 0.445 | 0.541 | -0.291 |
| RHSIM_Rhsim03G0161700 | 0.58 | 0.445 | 0.541 | 0.080 |
| RHSIM_Rhsim10G0049600 | 0.58 | 0.446 | 0.541 | -2.242 |
| RHSIM_Rhsim09G0111200 | 0.58 | 0.446 | 0.541 | -0.058 |
| novel.765 | 0.58 | 0.446 | 0.542 | -0.747 |
| RHSIM_Rhsim04G0131300 | 0.58 | 0.446 | 0.542 | 0.104 |
| RHSIM_Rhsim03G0073200 | 0.58 | 0.446 | 0.542 | -1.765 |
| RHSIM_Rhsim06G0060700 | 0.58 | 0.446 | 0.542 | -1.603 |
| RHSIM_Rhsim13G0100600 | 0.58 | 0.446 | 0.542 | -1.297 |
| RHSIM_Rhsim08G0017100 | 0.58 | 0.446 | 0.542 | 0.162 |
| RHSIM_Rhsim04G0248800 | 0.58 | 0.446 | 0.542 | 0.117 |
| RHSIM_RhsimUnG0166500 | 0.58 | 0.446 | 0.542 | -1.017 |
| RHSIM_Rhsim12G0046200 | 0.58 | 0.446 | 0.542 | 0.410 |
| RHSIM_Rhsim05G0127600 | 0.58 | 0.446 | 0.542 | -35.447 |

| Gene | LRT statistic | P value | BH-adjusted P | log2FC (interaction) |
|---|---|---|---|---|
| RHSIM_Rhsim13G0216100 | 0.58 | 0.446 | 0.542 | 0.842 |
| RHSIM_Rhsim06G0128400 | 0.58 | 0.446 | 0.542 | 35.452 |
| RHSIM_Rhsim10G0121200 | 0.58 | 0.446 | 0.542 | 1.134 |
| novel.2467 | 0.58 | 0.447 | 0.542 | -0.337 |
| novel.2834 | 0.58 | 0.447 | 0.542 | 0.152 |
| RHSIM_RhsimUnG0050500 | 0.58 | 0.447 | 0.543 | 0.471 |
| RHSIM_RhsimUnG0234600 | 0.58 | 0.447 | 0.543 | 0.449 |
| RHSIM_Rhsim03G0092900 | 0.58 | 0.447 | 0.543 | -0.102 |
| novel.4253 | 0.58 | 0.447 | 0.543 | -0.595 |
| RHSIM_Rhsim13G0227800 | 0.58 | 0.447 | 0.543 | 0.979 |
| RHSIM_Rhsim01G0270500 | 0.58 | 0.447 | 0.543 | -0.803 |
| novel.513 | 0.58 | 0.447 | 0.543 | 1.093 |
| RHSIM_Rhsim02G0115800 | 0.58 | 0.448 | 0.543 | -0.155 |
| RHSIM_Rhsim11G0026700 | 0.58 | 0.448 | 0.543 | -1.680 |
| RHSIM_Rhsim09G0063000 | 0.58 | 0.448 | 0.544 | 0.389 |
| RHSIM_Rhsim02G0071500 | 0.58 | 0.448 | 0.544 | 0.108 |
| RHSIM_Rhsim09G0030300 | 0.58 | 0.448 | 0.544 | -0.129 |
| RHSIM_Rhsim01G0293200 | 0.58 | 0.448 | 0.544 | 0.140 |
| RHSIM_Rhsim08G0043600 | 0.57 | 0.448 | 0.544 | 0.071 |
| RHSIM_Rhsim12G0064100 | 0.57 | 0.449 | 0.545 | -0.188 |
| RHSIM_Rhsim03G0127900 | 0.57 | 0.449 | 0.545 | -0.473 |
| RHSIM_Rhsim02G0042100 | 0.57 | 0.449 | 0.545 | 0.265 |
| RHSIM_Rhsim04G0036500 | 0.57 | 0.449 | 0.545 | -0.509 |
| novel.2123 | 0.57 | 0.449 | 0.545 | 0.756 |
| RHSIM_Rhsim09G0063500 | 0.57 | 0.449 | 0.545 | 0.075 |
| RHSIM_Rhsim04G0035900 | 0.57 | 0.449 | 0.545 | 0.291 |
| novel.4106 | 0.57 | 0.449 | 0.545 | -35.688 |
| RHSIM_Rhsim07G0097200 | 0.57 | 0.449 | 0.545 | -1.293 |
| RHSIM_Rhsim12G0156700 | 0.57 | 0.450 | 0.545 | 1.179 |
| RHSIM_RhsimUnG0255300 | 0.57 | 0.450 | 0.545 | -0.663 |
| RHSIM_Rhsim01G0088200 | 0.57 | 0.450 | 0.545 | -0.109 |
| novel.1721 | 0.57 | 0.450 | 0.545 | -0.099 |

| Gene | LRT statistic | P value | BH-adjusted P | log2FC (interaction) |
|---|---|---|---|---|
| RHSIM_Rhsim03G0268600 | 0.57 | 0.450 | 0.546 | 0.647 |
| RHSIM_Rhsim07G0006800 | 0.57 | 0.450 | 0.546 | -1.456 |
| RHSIM_Rhsim07G0120200 | 0.57 | 0.450 | 0.546 | 2.036 |
| RHSIM_Rhsim05G0001500 | 0.57 | 0.450 | 0.546 | -0.475 |
| novel.3879 | 0.57 | 0.450 | 0.546 | 0.916 |
| RHSIM_Rhsim06G0209800 | 0.57 | 0.450 | 0.546 | -0.848 |
| novel.3380 | 0.57 | 0.450 | 0.546 | -0.304 |
| novel.2084 | 0.57 | 0.450 | 0.546 | 0.380 |
| RHSIM_Rhsim04G0088200 | 0.57 | 0.450 | 0.546 | 0.208 |
| RHSIM_Rhsim06G0109800 | 0.57 | 0.450 | 0.546 | 1.308 |
| RHSIM_Rhsim12G0209900 | 0.57 | 0.451 | 0.546 | -0.164 |
| RHSIM_Rhsim03G0146900 | 0.57 | 0.451 | 0.546 | -0.109 |
| novel.503 | 0.57 | 0.451 | 0.546 | -0.796 |
| RHSIM_Rhsim06G0176800 | 0.57 | 0.451 | 0.547 | 0.401 |
| RHSIM_Rhsim04G0034600 | 0.57 | 0.451 | 0.547 | -0.812 |
| RHSIM_Rhsim12G0167000 | 0.57 | 0.451 | 0.547 | -0.073 |
| RHSIM_Rhsim11G0062200 | 0.57 | 0.451 | 0.547 | 0.212 |
| RHSIM_Rhsim01G0279600 | 0.57 | 0.451 | 0.547 | 0.461 |
| novel.758 | 0.57 | 0.451 | 0.547 | -0.912 |
| RHSIM_Rhsim01G0233800 | 0.57 | 0.451 | 0.547 | -0.883 |
| RHSIM_Rhsim11G0172500 | 0.57 | 0.452 | 0.547 | -0.217 |
| RHSIM_Rhsim03G0194100 | 0.57 | 0.452 | 0.547 | -1.105 |
| RHSIM_Rhsim02G0218300 | 0.57 | 0.452 | 0.547 | -35.799 |
| RHSIM_Rhsim09G0135600 | 0.57 | 0.452 | 0.547 | -0.243 |
| novel.781 | 0.57 | 0.452 | 0.547 | -1.219 |
| RHSIM_Rhsim05G0117200 | 0.57 | 0.452 | 0.548 | 1.870 |
| RHSIM_Rhsim12G0103500 | 0.57 | 0.452 | 0.548 | 0.588 |
| novel.575 | 0.57 | 0.452 | 0.548 | -0.515 |
| RHSIM_Rhsim02G0160700 | 0.57 | 0.452 | 0.548 | -35.746 |
| RHSIM_Rhsim13G0021900 | 0.56 | 0.452 | 0.548 | -0.396 |
| novel.235 | 0.56 | 0.453 | 0.548 | 0.107 |
| novel.1391 | 0.56 | 0.453 | 0.548 | 1.787 |

| Gene | LRT statistic | P value | BH-adjusted P | log2FC (interaction) |
|---|---|---|---|---|
| RHSIM_Rhsim08G0225400 | 0.56 | 0.453 | 0.548 | 0.115 |
| RHSIM_RhsimUnG0028600 | 0.56 | 0.453 | 0.548 | 35.626 |
| RHSIM_Rhsim01G0253900 | 0.56 | 0.453 | 0.548 | 1.097 |
| RHSIM_Rhsim12G0124100 | 0.56 | 0.453 | 0.548 | -0.366 |
| novel.2127 | 0.56 | 0.453 | 0.549 | 0.389 |
| RHSIM_Rhsim07G0183100 | 0.56 | 0.453 | 0.549 | 0.521 |
| RHSIM_Rhsim13G0104900 | 0.56 | 0.453 | 0.549 | -1.166 |
| RHSIM_Rhsim05G0138800 | 0.56 | 0.453 | 0.549 | -0.521 |
| RHSIM_Rhsim12G0010700 | 0.56 | 0.453 | 0.549 | -0.079 |
| RHSIM_Rhsim08G0103700 | 0.56 | 0.453 | 0.549 | 0.595 |
| RHSIM_Rhsim03G0173500 | 0.56 | 0.453 | 0.549 | 0.456 |
| RHSIM_Rhsim05G0061800 | 0.56 | 0.453 | 0.549 | -0.152 |
| RHSIM_Rhsim02G0002200 | 0.56 | 0.454 | 0.549 | -35.571 |
| RHSIM_Rhsim05G0095500 | 0.56 | 0.454 | 0.549 | -1.100 |
| RHSIM_Rhsim06G0078700 | 0.56 | 0.454 | 0.549 | -1.711 |
| RHSIM_Rhsim11G0080800 | 0.56 | 0.454 | 0.549 | 1.868 |
| novel.2338 | 0.56 | 0.454 | 0.550 | -0.647 |
| novel.128 | 0.56 | 0.454 | 0.550 | -35.217 |
| RHSIM_Rhsim01G0106200 | 0.56 | 0.454 | 0.550 | -35.572 |
| RHSIM_Rhsim09G0207800 | 0.56 | 0.455 | 0.550 | 0.265 |
| RHSIM_Rhsim08G0016200 | 0.56 | 0.455 | 0.550 | 0.957 |
| novel.32 | 0.56 | 0.455 | 0.550 | 0.613 |
| RHSIM_Rhsim04G0232100 | 0.56 | 0.455 | 0.550 | -0.059 |
| RHSIM_Rhsim13G0194800 | 0.56 | 0.455 | 0.550 | 0.134 |
| RHSIM_Rhsim05G0206600 | 0.56 | 0.455 | 0.550 | -0.313 |
| novel.4341 | 0.56 | 0.455 | 0.551 | 0.277 |
| RHSIM_Rhsim04G0175700 | 0.56 | 0.455 | 0.551 | -1.199 |
| RHSIM_Rhsim09G0166500 | 0.56 | 0.456 | 0.551 | -0.087 |
| RHSIM_Rhsim01G0200500 | 0.56 | 0.456 | 0.551 | 1.494 |
| RHSIM_Rhsim07G0233600 | 0.56 | 0.456 | 0.551 | -0.667 |
| RHSIM_Rhsim10G0071400 | 0.56 | 0.456 | 0.551 | -2.196 |
| novel.4011 | 0.56 | 0.456 | 0.552 | 1.049 |

| Gene | LRT statistic | P value | BH-adjusted P | log2FC (interaction) |
|---|---|---|---|---|
| RHSIM_Rhsim02G0206100 | 0.56 | 0.456 | 0.552 | 1.040 |
| RHSIM_Rhsim09G0086000 | 0.56 | 0.456 | 0.552 | 0.188 |
| RHSIM_Rhsim03G0017400 | 0.56 | 0.456 | 0.552 | -0.161 |
| novel.1313 | 0.55 | 0.456 | 0.552 | 0.412 |
| RHSIM_Rhsim08G0225900 | 0.55 | 0.456 | 0.552 | -0.949 |
| RHSIM_Rhsim10G0046900 | 0.55 | 0.456 | 0.552 | 0.178 |
| novel.2980 | 0.55 | 0.457 | 0.552 | 0.180 |
| RHSIM_Rhsim12G0203100 | 0.55 | 0.457 | 0.552 | 0.053 |
| RHSIM_Rhsim12G0134400 | 0.55 | 0.457 | 0.552 | 0.851 |
| RHSIM_Rhsim01G0094900 | 0.55 | 0.457 | 0.552 | -1.638 |
| RHSIM_Rhsim01G0178700 | 0.55 | 0.457 | 0.552 | -35.740 |
| RHSIM_Rhsim08G0107700 | 0.55 | 0.457 | 0.552 | 1.650 |
| RHSIM_Rhsim13G0165100 | 0.55 | 0.457 | 0.552 | -35.379 |
| RHSIM_Rhsim07G0159300 | 0.55 | 0.457 | 0.553 | 0.075 |
| RHSIM_Rhsim05G0045400 | 0.55 | 0.457 | 0.553 | -1.708 |
| RHSIM_Rhsim12G0212100 | 0.55 | 0.457 | 0.553 | 0.176 |
| RHSIM_Rhsim13G0179100 | 0.55 | 0.457 | 0.553 | -0.946 |
| RHSIM_Rhsim10G0185300 | 0.55 | 0.457 | 0.553 | -35.521 |
| RHSIM_RhsimUnG0013900 | 0.55 | 0.457 | 0.553 | -35.676 |
| novel.2029 | 0.55 | 0.457 | 0.553 | 1.512 |
| RHSIM_Rhsim01G0122100 | 0.55 | 0.458 | 0.553 | 0.069 |
| RHSIM_Rhsim10G0055500 | 0.55 | 0.458 | 0.553 | 0.049 |
| novel.3395 | 0.55 | 0.458 | 0.553 | -1.000 |
| novel.4560 | 0.55 | 0.458 | 0.553 | -0.129 |
| RHSIM_Rhsim09G0027500 | 0.55 | 0.458 | 0.553 | -0.138 |
| novel.170 | 0.55 | 0.458 | 0.554 | -35.602 |
| RHSIM_Rhsim05G0143500 | 0.55 | 0.458 | 0.554 | -0.079 |
| RHSIM_Rhsim02G0078200 | 0.55 | 0.458 | 0.554 | 0.560 |
| novel.1200 | 0.55 | 0.459 | 0.554 | 0.584 |
| RHSIM_Rhsim12G0074700 | 0.55 | 0.459 | 0.554 | 0.174 |
| RHSIM_Rhsim10G0060500 | 0.55 | 0.459 | 0.554 | 0.095 |
| RHSIM_Rhsim03G0208900 | 0.55 | 0.459 | 0.554 | 0.066 |

| Gene | LRT statistic | P value | BH-adjusted P | log2FC (interaction) |
|---|---|---|---|---|
| RHSIM_Rhsim05G0208000 | 0.55 | 0.459 | 0.555 | -0.101 |
| RHSIM_Rhsim08G0067300 | 0.55 | 0.459 | 0.555 | 1.297 |
| RHSIM_Rhsim03G0248700 | 0.55 | 0.459 | 0.555 | -1.700 |
| RHSIM_Rhsim09G0072400 | 0.55 | 0.459 | 0.555 | 0.442 |
| novel.2814 | 0.55 | 0.459 | 0.555 | 0.732 |
| RHSIM_Rhsim13G0235400 | 0.55 | 0.459 | 0.555 | -1.098 |
| RHSIM_Rhsim10G0035000 | 0.55 | 0.459 | 0.555 | -1.013 |
| RHSIM_Rhsim09G0102000 | 0.55 | 0.460 | 0.555 | 0.053 |
| RHSIM_Rhsim02G0234800 | 0.55 | 0.460 | 0.555 | -0.877 |
| novel.4579 | 0.55 | 0.460 | 0.555 | 0.228 |
| RHSIM_Rhsim08G0143900 | 0.55 | 0.460 | 0.555 | 0.086 |
| RHSIM_Rhsim07G0206200 | 0.55 | 0.460 | 0.555 | 35.533 |
| RHSIM_Rhsim13G0082700 | 0.55 | 0.460 | 0.555 | -0.064 |
| RHSIM_Rhsim02G0015200 | 0.55 | 0.460 | 0.555 | -2.077 |
| RHSIM_Rhsim02G0012100 | 0.55 | 0.460 | 0.555 | 1.565 |
| RHSIM_Rhsim03G0249800 | 0.55 | 0.460 | 0.555 | -0.208 |
| RHSIM_Rhsim02G0219200 | 0.55 | 0.460 | 0.555 | -1.744 |
| RHSIM_Rhsim05G0180300 | 0.55 | 0.460 | 0.555 | -0.152 |
| RHSIM_Rhsim01G0287800 | 0.55 | 0.460 | 0.556 | 0.109 |
| RHSIM_Rhsim07G0209500 | 0.54 | 0.461 | 0.556 | -0.954 |
| RHSIM_RhsimUnG0156200 | 0.54 | 0.461 | 0.556 | 0.381 |
| RHSIM_Rhsim05G0041200 | 0.54 | 0.461 | 0.556 | 0.452 |
| RHSIM_Rhsim09G0121500 | 0.54 | 0.461 | 0.556 | -0.861 |
| RHSIM_RhsimUnG0025000 | 0.54 | 0.461 | 0.556 | -0.409 |
| RHSIM_Rhsim11G0127800 | 0.54 | 0.461 | 0.556 | 0.055 |
| novel.3100 | 0.54 | 0.461 | 0.556 | 0.119 |
| RHSIM_Rhsim12G0159800 | 0.54 | 0.461 | 0.556 | 0.136 |
| novel.578 | 0.54 | 0.461 | 0.556 | -0.605 |
| RHSIM_Rhsim04G0092500 | 0.54 | 0.461 | 0.557 | 0.117 |
| RHSIM_Rhsim04G0047300 | 0.54 | 0.461 | 0.557 | 0.152 |
| RHSIM_Rhsim07G0147600 | 0.54 | 0.462 | 0.557 | -0.839 |
| RHSIM_Rhsim04G0032600 | 0.54 | 0.462 | 0.557 | 0.574 |

| Gene | LRT statistic | P value | BH-adjusted P | log2FC (interaction) |
|---|---|---|---|---|
| RHSIM_Rhsim09G0148500 | 0.54 | 0.462 | 0.557 | -1.791 |
| RHSIM_Rhsim09G0205900 | 0.54 | 0.462 | 0.557 | 0.165 |
| RHSIM_Rhsim08G0134400 | 0.54 | 0.462 | 0.558 | 0.346 |
| RHSIM_Rhsim11G0197900 | 0.54 | 0.462 | 0.558 | -1.743 |
| RHSIM_Rhsim02G0218800 | 0.54 | 0.462 | 0.558 | -1.422 |
| RHSIM_Rhsim04G0212000 | 0.54 | 0.462 | 0.558 | 0.055 |
| RHSIM_Rhsim07G0133300 | 0.54 | 0.462 | 0.558 | -1.335 |
| RHSIM_Rhsim03G0209500 | 0.54 | 0.463 | 0.558 | -1.216 |
| RHSIM_Rhsim02G0129700 | 0.54 | 0.463 | 0.558 | -0.235 |
| RHSIM_Rhsim07G0037300 | 0.54 | 0.463 | 0.558 | 0.364 |
| RHSIM_Rhsim03G0039800 | 0.54 | 0.463 | 0.558 | 0.148 |
| RHSIM_Rhsim08G0000200 | 0.54 | 0.463 | 0.558 | 0.089 |
| RHSIM_Rhsim02G0080700 | 0.54 | 0.463 | 0.558 | -0.848 |
| RHSIM_Rhsim05G0129300 | 0.54 | 0.463 | 0.558 | -0.315 |
| RHSIM_Rhsim06G0027800 | 0.54 | 0.463 | 0.558 | -0.166 |
| novel.663 | 0.54 | 0.463 | 0.558 | -0.757 |
| RHSIM_Rhsim13G0123100 | 0.54 | 0.463 | 0.559 | -35.337 |
| RHSIM_Rhsim08G0093400 | 0.54 | 0.464 | 0.559 | -0.830 |
| RHSIM_RhsimUnG0041300 | 0.54 | 0.464 | 0.559 | -0.257 |
| RHSIM_Rhsim08G0159100 | 0.54 | 0.464 | 0.559 | 0.187 |
| RHSIM_Rhsim12G0182300 | 0.54 | 0.464 | 0.559 | 1.172 |
| RHSIM_Rhsim12G0040500 | 0.54 | 0.464 | 0.559 | -0.121 |
| RHSIM_Rhsim12G0141200 | 0.54 | 0.464 | 0.559 | 1.102 |
| RHSIM_Rhsim12G0186500 | 0.54 | 0.464 | 0.559 | 0.104 |
| novel.3639 | 0.54 | 0.464 | 0.559 | 0.111 |
| RHSIM_Rhsim05G0022000 | 0.54 | 0.464 | 0.559 | -0.121 |
| RHSIM_Rhsim03G0146100 | 0.54 | 0.464 | 0.559 | -0.254 |
| RHSIM_Rhsim05G0074400 | 0.54 | 0.464 | 0.560 | -1.344 |
| RHSIM_Rhsim11G0195300 | 0.54 | 0.465 | 0.560 | 0.195 |
| RHSIM_RhsimUnG0038100 | 0.53 | 0.465 | 0.560 | -0.813 |
| RHSIM_Rhsim02G0048300 | 0.53 | 0.465 | 0.560 | 35.485 |
| RHSIM_Rhsim08G0024500 | 0.53 | 0.465 | 0.560 | -0.408 |

| Gene | LRT statistic | P value | BH-adjusted P | log2FC (interaction) |
|---|---|---|---|---|
| RHSIM_Rhsim10G0164700 | 0.53 | 0.465 | 0.560 | -0.096 |
| RHSIM_Rhsim09G0169400 | 0.53 | 0.465 | 0.560 | -2.064 |
| RHSIM_Rhsim10G0054300 | 0.53 | 0.465 | 0.560 | 1.556 |
| RHSIM_Rhsim12G0158500 | 0.53 | 0.465 | 0.560 | -0.292 |
| RHSIM_Rhsim04G0004600 | 0.53 | 0.465 | 0.560 | -0.235 |
| RHSIM_Rhsim02G0209700 | 0.53 | 0.465 | 0.561 | 0.082 |
| RHSIM_Rhsim06G0084500 | 0.53 | 0.465 | 0.561 | 0.111 |
| RHSIM_Rhsim06G0153400 | 0.53 | 0.465 | 0.561 | -0.450 |
| novel.2961 | 0.53 | 0.466 | 0.561 | -0.167 |
| RHSIM_Rhsim07G0025300 | 0.53 | 0.466 | 0.561 | 1.835 |
| RHSIM_Rhsim12G0049900 | 0.53 | 0.466 | 0.561 | -1.677 |
| RHSIM_Rhsim05G0086700 | 0.53 | 0.466 | 0.561 | -0.164 |
| RHSIM_Rhsim08G0147600 | 0.53 | 0.466 | 0.561 | 0.259 |
| RHSIM_Rhsim11G0127900 | 0.53 | 0.466 | 0.561 | -0.466 |
| RHSIM_Rhsim10G0148200 | 0.53 | 0.466 | 0.561 | 0.074 |
| RHSIM_Rhsim13G0039300 | 0.53 | 0.466 | 0.561 | -1.616 |
| RHSIM_Rhsim06G0014700 | 0.53 | 0.466 | 0.561 | 1.923 |
| RHSIM_Rhsim01G0024200 | 0.53 | 0.467 | 0.562 | -0.914 |
| RHSIM_Rhsim02G0192800 | 0.53 | 0.467 | 0.562 | 35.625 |
| RHSIM_Rhsim01G0079800 | 0.53 | 0.467 | 0.562 | 0.658 |
| novel.953 | 0.53 | 0.467 | 0.562 | -0.884 |
| RHSIM_Rhsim05G0030500 | 0.53 | 0.467 | 0.562 | -0.863 |
| novel.3212 | 0.53 | 0.467 | 0.562 | 0.263 |
| RHSIM_Rhsim13G0112800 | 0.53 | 0.467 | 0.562 | 0.812 |
| RHSIM_Rhsim13G0227200 | 0.53 | 0.467 | 0.562 | 0.538 |
| RHSIM_Rhsim01G0244500 | 0.53 | 0.467 | 0.562 | -0.596 |
| RHSIM_RhsimUnG0247300 | 0.53 | 0.467 | 0.562 | -0.896 |
| novel.2441 | 0.53 | 0.467 | 0.563 | 0.392 |
| novel.3730 | 0.53 | 0.468 | 0.563 | 0.137 |
| RHSIM_Rhsim06G0120800 | 0.53 | 0.468 | 0.563 | 1.613 |
| RHSIM_Rhsim13G0109100 | 0.53 | 0.468 | 0.563 | 1.012 |
| novel.1382 | 0.53 | 0.468 | 0.563 | -0.378 |

| Gene | LRT statistic | P value | BH-adjusted P | log2FC (interaction) |
|---|---|---|---|---|
| RHSIM_Rhsim01G0187700 | 0.53 | 0.468 | 0.563 | -0.230 |
| novel.3685 | 0.53 | 0.468 | 0.563 | -1.297 |
| RHSIM_Rhsim01G0034800 | 0.53 | 0.468 | 0.563 | -0.975 |
| RHSIM_Rhsim02G0158700 | 0.53 | 0.468 | 0.563 | -2.086 |
| RHSIM_Rhsim03G0162300 | 0.53 | 0.468 | 0.563 | 0.170 |
| novel.223 | 0.53 | 0.468 | 0.563 | -0.491 |
| RHSIM_Rhsim04G0055600 | 0.53 | 0.468 | 0.563 | -0.246 |
| RHSIM_Rhsim11G0113800 | 0.53 | 0.468 | 0.563 | -35.480 |
| RHSIM_Rhsim03G0237600 | 0.53 | 0.468 | 0.563 | -0.656 |
| RHSIM_Rhsim09G0031800 | 0.53 | 0.468 | 0.563 | 0.121 |
| RHSIM_Rhsim12G0153500 | 0.53 | 0.469 | 0.563 | 0.119 |
| RHSIM_Rhsim09G0006900 | 0.53 | 0.469 | 0.563 | 0.578 |
| RHSIM_Rhsim13G0005600 | 0.53 | 0.469 | 0.564 | 1.203 |
| novel.3811 | 0.53 | 0.469 | 0.564 | -0.245 |
| novel.2114 | 0.52 | 0.469 | 0.564 | -0.826 |
| RHSIM_Rhsim06G0218000 | 0.52 | 0.469 | 0.564 | -0.900 |
| RHSIM_Rhsim10G0085500 | 0.52 | 0.469 | 0.564 | 0.357 |
| novel.4319 | 0.52 | 0.469 | 0.564 | 0.524 |
| RHSIM_RhsimUnG0069500 | 0.52 | 0.469 | 0.564 | -0.227 |
| RHSIM_Rhsim01G0142000 | 0.52 | 0.469 | 0.564 | 0.633 |
| RHSIM_Rhsim06G0181600 | 0.52 | 0.469 | 0.564 | 1.289 |
| RHSIM_Rhsim11G0120300 | 0.52 | 0.469 | 0.564 | 0.572 |
| novel.2042 | 0.52 | 0.469 | 0.564 | 0.599 |
| RHSIM_Rhsim09G0121700 | 0.52 | 0.469 | 0.564 | -0.622 |
| RHSIM_Rhsim09G0038400 | 0.52 | 0.469 | 0.564 | -0.509 |
| RHSIM_Rhsim13G0001000 | 0.52 | 0.469 | 0.564 | 0.127 |
| RHSIM_Rhsim01G0216800 | 0.52 | 0.470 | 0.564 | 0.796 |
| novel.150 | 0.52 | 0.470 | 0.565 | 1.229 |
| RHSIM_Rhsim03G0242700 | 0.52 | 0.470 | 0.565 | 0.181 |
| RHSIM_Rhsim13G0076300 | 0.52 | 0.470 | 0.565 | 0.068 |
| RHSIM_Rhsim02G0030500 | 0.52 | 0.470 | 0.565 | 1.408 |
| RHSIM_Rhsim02G0145900 | 0.52 | 0.470 | 0.565 | 0.088 |

| Gene | LRT statistic | P value | BH-adjusted P | log2FC (interaction) |
|---|---|---|---|---|
| RHSIM_Rhsim13G0217300 | 0.52 | 0.470 | 0.565 | 0.475 |
| RHSIM_Rhsim04G0087000 | 0.52 | 0.470 | 0.565 | 0.133 |
| RHSIM_Rhsim01G0144300 | 0.52 | 0.470 | 0.565 | 0.620 |
| novel.1376 | 0.52 | 0.470 | 0.565 | 0.578 |
| RHSIM_Rhsim09G0123300 | 0.52 | 0.471 | 0.565 | -0.251 |
| RHSIM_RhsimUnG0226500 | 0.52 | 0.471 | 0.565 | 0.953 |
| RHSIM_RhsimUnG0055000 | 0.52 | 0.471 | 0.565 | -0.143 |
| RHSIM_Rhsim06G0215700 | 0.52 | 0.471 | 0.565 | -35.528 |
| RHSIM_Rhsim01G0212400 | 0.52 | 0.471 | 0.565 | -35.505 |
| RHSIM_Rhsim03G0262400 | 0.52 | 0.471 | 0.566 | 0.413 |
| RHSIM_Rhsim02G0121800 | 0.52 | 0.471 | 0.566 | -35.637 |
| novel.2708 | 0.52 | 0.471 | 0.566 | -0.221 |
| RHSIM_Rhsim03G0195100 | 0.52 | 0.471 | 0.566 | -0.916 |
| novel.2373 | 0.52 | 0.471 | 0.566 | -0.723 |
| RHSIM_Rhsim04G0070800 | 0.52 | 0.471 | 0.566 | 0.112 |
| RHSIM_Rhsim11G0019000 | 0.52 | 0.472 | 0.566 | 0.368 |
| RHSIM_RhsimUnG0128800 | 0.52 | 0.472 | 0.567 | -0.322 |
| RHSIM_Rhsim01G0225700 | 0.52 | 0.472 | 0.567 | 0.054 |
| novel.4468 | 0.52 | 0.472 | 0.567 | 0.249 |
| RHSIM_Rhsim10G0065700 | 0.52 | 0.472 | 0.567 | -2.720 |
| RHSIM_Rhsim07G0082600 | 0.52 | 0.472 | 0.567 | 0.301 |
| RHSIM_Rhsim07G0234300 | 0.52 | 0.472 | 0.567 | 0.764 |
| RHSIM_RhsimUnG0082000 | 0.52 | 0.472 | 0.567 | 1.131 |
| RHSIM_Rhsim11G0192000 | 0.52 | 0.472 | 0.567 | -1.537 |
| novel.3934 | 0.52 | 0.473 | 0.567 | -0.215 |
| RHSIM_Rhsim07G0128300 | 0.52 | 0.473 | 0.567 | -0.730 |
| novel.4440 | 0.52 | 0.473 | 0.567 | 0.315 |
| RHSIM_Rhsim10G0000200 | 0.52 | 0.473 | 0.567 | -1.222 |
| RHSIM_RhsimUnG0170100 | 0.52 | 0.473 | 0.568 | -0.595 |
| RHSIM_Rhsim04G0063900 | 0.51 | 0.473 | 0.568 | -0.174 |
| RHSIM_Rhsim06G0145100 | 0.51 | 0.473 | 0.568 | -1.067 |
| RHSIM_Rhsim03G0090800 | 0.51 | 0.473 | 0.568 | -0.486 |

| Gene | LRT statistic | P value | BH-adjusted P | log2FC (interaction) |
|---|---|---|---|---|
| novel.328 | 0.51 | 0.473 | 0.568 | 0.636 |
| RHSIM_Rhsim06G0146300 | 0.51 | 0.473 | 0.568 | -0.888 |
| RHSIM_Rhsim01G0218700 | 0.51 | 0.473 | 0.568 | -1.730 |
| RHSIM_Rhsim13G0212600 | 0.51 | 0.473 | 0.568 | -0.170 |
| RHSIM_Rhsim05G0160700 | 0.51 | 0.473 | 0.568 | 0.589 |
| novel.868 | 0.51 | 0.473 | 0.568 | -1.211 |
| RHSIM_Rhsim04G0238100 | 0.51 | 0.474 | 0.568 | -1.089 |
| RHSIM_Rhsim08G0046400 | 0.51 | 0.474 | 0.568 | 0.245 |
| RHSIM_Rhsim05G0230400 | 0.51 | 0.474 | 0.568 | -0.495 |
| novel.1735 | 0.51 | 0.474 | 0.568 | 0.891 |
| novel.2590 | 0.51 | 0.474 | 0.569 | 0.580 |
| RHSIM_Rhsim12G0142100 | 0.51 | 0.475 | 0.569 | -0.354 |
| novel.3034 | 0.51 | 0.475 | 0.569 | 0.795 |
| RHSIM_Rhsim10G0041900 | 0.51 | 0.475 | 0.570 | 0.217 |
| RHSIM_Rhsim06G0208600 | 0.51 | 0.475 | 0.570 | -1.017 |
| RHSIM_Rhsim03G0148200 | 0.51 | 0.475 | 0.570 | 0.650 |
| RHSIM_Rhsim06G0230600 | 0.51 | 0.475 | 0.570 | -0.087 |
| RHSIM_Rhsim03G0152300 | 0.51 | 0.475 | 0.570 | -0.488 |
| RHSIM_Rhsim01G0232100 | 0.51 | 0.476 | 0.571 | 0.518 |
| novel.3264 | 0.51 | 0.476 | 0.571 | 0.563 |
| novel.471 | 0.51 | 0.476 | 0.571 | -0.400 |
| RHSIM_Rhsim08G0206300 | 0.51 | 0.476 | 0.571 | -1.078 |
| RHSIM_Rhsim08G0118700 | 0.51 | 0.476 | 0.571 | 0.094 |
| RHSIM_Rhsim11G0017600 | 0.51 | 0.476 | 0.571 | -0.362 |
| novel.3890 | 0.51 | 0.476 | 0.571 | -0.086 |
| RHSIM_Rhsim12G0115400 | 0.51 | 0.476 | 0.571 | 0.897 |
| RHSIM_Rhsim10G0142400 | 0.51 | 0.476 | 0.571 | -0.118 |
| RHSIM_Rhsim07G0086400 | 0.51 | 0.477 | 0.571 | 0.142 |
| novel.3077 | 0.51 | 0.477 | 0.571 | 0.717 |
| RHSIM_Rhsim13G0152300 | 0.51 | 0.477 | 0.571 | -1.549 |
| RHSIM_Rhsim08G0200200 | 0.51 | 0.477 | 0.571 | 0.408 |
| RHSIM_Rhsim01G0087400 | 0.51 | 0.477 | 0.572 | 1.936 |

| Gene | LRT statistic | P value | BH-adjusted P | log2FC (interaction) |
|---|---|---|---|---|
| novel.4121 | 0.50 | 0.477 | 0.572 | 0.911 |
| RHSIM_Rhsim10G0031600 | 0.50 | 0.477 | 0.572 | 0.908 |
| RHSIM_Rhsim09G0076200 | 0.50 | 0.478 | 0.572 | 0.102 |
| novel.486 | 0.50 | 0.478 | 0.572 | 0.645 |
| RHSIM_Rhsim06G0122900 | 0.50 | 0.478 | 0.573 | 1.316 |
| RHSIM_Rhsim05G0001400 | 0.50 | 0.478 | 0.573 | 0.505 |
| RHSIM_Rhsim11G0131300 | 0.50 | 0.478 | 0.573 | -1.557 |
| RHSIM_Rhsim01G0254600 | 0.50 | 0.478 | 0.573 | 0.485 |
| RHSIM_Rhsim08G0038700 | 0.50 | 0.478 | 0.573 | -1.429 |
| RHSIM_Rhsim10G0087100 | 0.50 | 0.478 | 0.573 | 1.518 |
| RHSIM_Rhsim01G0092100 | 0.50 | 0.478 | 0.573 | 1.593 |
| RHSIM_Rhsim06G0152100 | 0.50 | 0.478 | 0.573 | -0.464 |
| RHSIM_Rhsim13G0121600 | 0.50 | 0.478 | 0.573 | 1.384 |
| novel.2419 | 0.50 | 0.478 | 0.573 | 0.514 |
| RHSIM_Rhsim02G0148100 | 0.50 | 0.479 | 0.573 | 0.963 |
| novel.3439 | 0.50 | 0.479 | 0.573 | -0.594 |
| RHSIM_Rhsim08G0017300 | 0.50 | 0.479 | 0.574 | -0.118 |
| novel.2914 | 0.50 | 0.479 | 0.574 | -0.384 |
| RHSIM_RhsimPtG0002900 | 0.50 | 0.480 | 0.574 | 0.606 |
| RHSIM_Rhsim08G0167700 | 0.50 | 0.480 | 0.574 | 0.165 |
| RHSIM_Rhsim09G0062600 | 0.50 | 0.480 | 0.574 | -0.156 |
| novel.299 | 0.50 | 0.480 | 0.574 | -0.387 |
| RHSIM_Rhsim08G0009900 | 0.50 | 0.480 | 0.574 | 0.174 |
| RHSIM_Rhsim01G0274000 | 0.50 | 0.480 | 0.574 | 0.445 |
| RHSIM_Rhsim04G0002100 | 0.50 | 0.480 | 0.574 | -0.762 |
| RHSIM_Rhsim09G0048600 | 0.50 | 0.480 | 0.574 | -0.420 |
| RHSIM_Rhsim01G0112700 | 0.50 | 0.480 | 0.574 | -0.689 |
| RHSIM_Rhsim06G0181900 | 0.50 | 0.480 | 0.574 | -0.392 |
| RHSIM_Rhsim07G0113700 | 0.50 | 0.480 | 0.574 | -0.289 |
| novel.1714 | 0.50 | 0.480 | 0.574 | 0.786 |
| RHSIM_Rhsim13G0194500 | 0.50 | 0.480 | 0.574 | -0.616 |
| RHSIM_Rhsim03G0093000 | 0.50 | 0.480 | 0.575 | 0.267 |

| Gene | LRT statistic | P value | BH-adjusted P | log2FC (interaction) |
|---|---|---|---|---|
| RHSIM_Rhsim09G0143500 | 0.50 | 0.480 | 0.575 | -0.448 |
| RHSIM_Rhsim09G0122300 | 0.50 | 0.480 | 0.575 | -0.127 |
| RHSIM_Rhsim13G0033400 | 0.50 | 0.480 | 0.575 | -1.710 |
| RHSIM_Rhsim11G0000300 | 0.50 | 0.480 | 0.575 | 0.322 |
| novel.673 | 0.50 | 0.480 | 0.575 | -1.007 |
| RHSIM_Rhsim12G0201500 | 0.50 | 0.481 | 0.575 | 0.050 |
| RHSIM_Rhsim09G0085600 | 0.50 | 0.481 | 0.575 | -1.271 |
| RHSIM_Rhsim04G0160900 | 0.50 | 0.481 | 0.575 | 0.338 |
| RHSIM_Rhsim06G0243700 | 0.50 | 0.481 | 0.575 | -0.203 |
| novel.3789 | 0.50 | 0.481 | 0.575 | 0.418 |
| novel.3974 | 0.50 | 0.481 | 0.575 | -0.914 |
| RHSIM_Rhsim12G0100100 | 0.50 | 0.481 | 0.575 | 0.242 |
| RHSIM_RhsimUnG0155700 | 0.50 | 0.481 | 0.575 | 0.231 |
| RHSIM_Rhsim08G0121600 | 0.50 | 0.481 | 0.575 | -1.024 |
| RHSIM_RhsimUnG0043900 | 0.50 | 0.481 | 0.575 | -0.208 |
| RHSIM_Rhsim06G0023600 | 0.50 | 0.481 | 0.575 | -0.411 |
| RHSIM_Rhsim03G0181200 | 0.50 | 0.481 | 0.576 | -0.516 |
| RHSIM_Rhsim12G0069400 | 0.50 | 0.481 | 0.576 | -0.216 |
| RHSIM_Rhsim02G0249000 | 0.49 | 0.482 | 0.576 | 0.092 |
| RHSIM_Rhsim07G0208800 | 0.49 | 0.482 | 0.576 | 0.214 |
| RHSIM_Rhsim03G0025800 | 0.49 | 0.482 | 0.576 | -0.494 |
| novel.320 | 0.49 | 0.482 | 0.576 | 0.631 |
| RHSIM_Rhsim10G0076400 | 0.49 | 0.482 | 0.576 | -0.485 |
| RHSIM_Rhsim03G0116000 | 0.49 | 0.482 | 0.576 | -0.556 |
| RHSIM_Rhsim09G0050700 | 0.49 | 0.482 | 0.576 | -0.436 |
| RHSIM_Rhsim04G0182100 | 0.49 | 0.482 | 0.577 | 0.696 |
| RHSIM_Rhsim11G0149900 | 0.49 | 0.482 | 0.577 | 0.278 |
| RHSIM_Rhsim07G0241900 | 0.49 | 0.483 | 0.577 | -1.721 |
| RHSIM_Rhsim11G0003800 | 0.49 | 0.483 | 0.577 | 1.199 |
| RHSIM_Rhsim08G0048500 | 0.49 | 0.483 | 0.578 | 0.387 |
| novel.810 | 0.49 | 0.483 | 0.578 | -0.706 |
| novel.956 | 0.49 | 0.483 | 0.578 | 0.358 |

| Gene | LRT statistic | P value | BH-adjusted P | log2FC (interaction) |
|---|---|---|---|---|
| RHSIM_RhsimUnG0033800 | 0.49 | 0.483 | 0.578 | 0.479 |
| RHSIM_Rhsim01G0016000 | 0.49 | 0.484 | 0.578 | 0.067 |
| novel.1784 | 0.49 | 0.484 | 0.578 | -1.031 |
| RHSIM_Rhsim06G0021800 | 0.49 | 0.484 | 0.578 | 0.155 |
| RHSIM_Rhsim08G0168000 | 0.49 | 0.484 | 0.579 | 1.184 |
| RHSIM_Rhsim01G0111500 | 0.49 | 0.484 | 0.579 | -1.150 |
| novel.4476 | 0.49 | 0.484 | 0.579 | 1.657 |
| novel.3193 | 0.49 | 0.485 | 0.579 | -0.370 |
| RHSIM_Rhsim03G0213000 | 0.49 | 0.485 | 0.579 | -0.114 |
| RHSIM_Rhsim05G0199700 | 0.49 | 0.485 | 0.579 | 0.078 |
| novel.662 | 0.49 | 0.485 | 0.579 | 0.861 |
| RHSIM_Rhsim01G0257900 | 0.49 | 0.485 | 0.579 | -35.343 |
| RHSIM_Rhsim09G0097000 | 0.49 | 0.485 | 0.580 | 0.397 |
| RHSIM_Rhsim07G0242200 | 0.49 | 0.485 | 0.580 | -0.269 |
| RHSIM_Rhsim07G0191600 | 0.49 | 0.485 | 0.580 | -1.792 |
| RHSIM_Rhsim07G0210000 | 0.49 | 0.486 | 0.580 | 0.057 |
| RHSIM_Rhsim12G0211200 | 0.49 | 0.486 | 0.580 | 0.083 |
| RHSIM_Rhsim07G0081900 | 0.49 | 0.486 | 0.580 | 0.077 |
| novel.2040 | 0.49 | 0.486 | 0.580 | 1.609 |
| novel.3385 | 0.49 | 0.486 | 0.580 | -0.361 |
| RHSIM_Rhsim06G0025600 | 0.49 | 0.486 | 0.580 | 0.118 |
| RHSIM_Rhsim02G0057100 | 0.49 | 0.486 | 0.580 | 0.112 |
| RHSIM_Rhsim03G0216900 | 0.48 | 0.486 | 0.581 | -0.667 |
| novel.4277 | 0.48 | 0.486 | 0.581 | 0.965 |
| RHSIM_Rhsim05G0140000 | 0.48 | 0.486 | 0.581 | 0.341 |
| RHSIM_Rhsim09G0052600 | 0.48 | 0.486 | 0.581 | -35.278 |
| novel.989 | 0.48 | 0.486 | 0.581 | -0.388 |
| novel.263 | 0.48 | 0.487 | 0.581 | 1.408 |
| RHSIM_Rhsim07G0193900 | 0.48 | 0.487 | 0.581 | -0.380 |
| RHSIM_Rhsim01G0161300 | 0.48 | 0.487 | 0.581 | 1.689 |
| RHSIM_Rhsim12G0190100 | 0.48 | 0.487 | 0.581 | -0.155 |
| RHSIM_Rhsim08G0216000 | 0.48 | 0.487 | 0.581 | 0.744 |

| Gene | LRT statistic | P value | BH-adjusted P | log2FC (interaction) |
|---|---|---|---|---|
| RHSIM_Rhsim09G0014400 | 0.48 | 0.487 | 0.581 | 0.346 |
| RHSIM_Rhsim11G0119900 | 0.48 | 0.487 | 0.581 | -0.129 |
| RHSIM_Rhsim01G0024700 | 0.48 | 0.487 | 0.582 | 1.906 |
| RHSIM_Rhsim11G0132900 | 0.48 | 0.487 | 0.582 | -0.063 |
| RHSIM_Rhsim07G0125900 | 0.48 | 0.488 | 0.582 | 0.537 |
| novel.1559 | 0.48 | 0.488 | 0.582 | 1.786 |
| RHSIM_Rhsim09G0136000 | 0.48 | 0.488 | 0.582 | -35.146 |
| RHSIM_Rhsim07G0241000 | 0.48 | 0.488 | 0.582 | -0.345 |
| RHSIM_Rhsim01G0167900 | 0.48 | 0.488 | 0.582 | 0.368 |
| novel.108 | 0.48 | 0.488 | 0.582 | -0.352 |
| RHSIM_Rhsim01G0260700 | 0.48 | 0.488 | 0.583 | 0.615 |
| RHSIM_Rhsim06G0143800 | 0.48 | 0.489 | 0.583 | -0.264 |
| RHSIM_RhsimUnG0188600 | 0.48 | 0.489 | 0.583 | -0.158 |
| RHSIM_Rhsim06G0019100 | 0.48 | 0.489 | 0.583 | 1.585 |
| RHSIM_Rhsim01G0059600 | 0.48 | 0.489 | 0.583 | -0.108 |
| RHSIM_Rhsim06G0144900 | 0.48 | 0.489 | 0.583 | 0.277 |
| RHSIM_Rhsim10G0132300 | 0.48 | 0.489 | 0.583 | -0.080 |
| RHSIM_Rhsim06G0076700 | 0.48 | 0.489 | 0.583 | -0.069 |
| RHSIM_Rhsim09G0007600 | 0.48 | 0.489 | 0.583 | -0.133 |
| RHSIM_Rhsim09G0174200 | 0.48 | 0.489 | 0.583 | -1.101 |
| RHSIM_Rhsim06G0104900 | 0.48 | 0.490 | 0.584 | 1.970 |
| RHSIM_Rhsim07G0159600 | 0.48 | 0.490 | 0.584 | 0.784 |
| RHSIM_Rhsim03G0022500 | 0.48 | 0.490 | 0.584 | -1.057 |
| RHSIM_Rhsim01G0132300 | 0.48 | 0.490 | 0.584 | 0.075 |
| RHSIM_Rhsim09G0144200 | 0.48 | 0.490 | 0.584 | -0.170 |
| RHSIM_Rhsim06G0153300 | 0.48 | 0.490 | 0.585 | -0.452 |
| novel.2266 | 0.48 | 0.490 | 0.585 | 0.513 |
| RHSIM_Rhsim04G0170400 | 0.48 | 0.491 | 0.585 | -0.170 |
| RHSIM_RhsimUnG0113400 | 0.48 | 0.491 | 0.585 | 0.929 |
| RHSIM_Rhsim01G0240300 | 0.47 | 0.491 | 0.585 | 0.362 |
| RHSIM_RhsimUnG0119900 | 0.47 | 0.491 | 0.585 | 0.565 |
| RHSIM_Rhsim12G0068700 | 0.47 | 0.491 | 0.585 | 1.298 |

| Gene | LRT statistic | P value | BH-adjusted P | log2FC (interaction) |
|---|---|---|---|---|
| RHSIM_Rhsim12G0090800 | 0.47 | 0.491 | 0.586 | 0.797 |
| RHSIM_Rhsim01G0013800 | 0.47 | 0.491 | 0.586 | -0.606 |
| novel.3370 | 0.47 | 0.491 | 0.586 | 0.556 |
| RHSIM_Rhsim08G0110700 | 0.47 | 0.491 | 0.586 | -1.770 |
| RHSIM_Rhsim01G0107900 | 0.47 | 0.491 | 0.586 | 0.108 |
| RHSIM_RhsimUnG0030700 | 0.47 | 0.492 | 0.586 | -0.666 |
| RHSIM_RhsimUnG0107000 | 0.47 | 0.492 | 0.586 | -0.342 |
| RHSIM_Rhsim05G0104800 | 0.47 | 0.492 | 0.586 | -0.110 |
| novel.4572 | 0.47 | 0.492 | 0.586 | -0.357 |
| RHSIM_Rhsim09G0126600 | 0.47 | 0.492 | 0.586 | 0.668 |
| RHSIM_Rhsim08G0005400 | 0.47 | 0.492 | 0.586 | -0.056 |
| novel.1745 | 0.47 | 0.492 | 0.586 | 0.592 |
| novel.2731 | 0.47 | 0.492 | 0.587 | -0.668 |
| RHSIM_Rhsim08G0064700 | 0.47 | 0.493 | 0.587 | 0.689 |
| novel.3557 | 0.47 | 0.493 | 0.587 | 1.478 |
| RHSIM_Rhsim05G0002400 | 0.47 | 0.493 | 0.587 | 1.209 |
| RHSIM_Rhsim03G0256900 | 0.47 | 0.493 | 0.587 | -1.345 |
| novel.4128 | 0.47 | 0.493 | 0.587 | 0.881 |
| novel.186 | 0.47 | 0.493 | 0.587 | 0.124 |
| RHSIM_Rhsim07G0093200 | 0.47 | 0.493 | 0.587 | 0.542 |
| RHSIM_RhsimUnG0219400 | 0.47 | 0.493 | 0.587 | 0.094 |
| RHSIM_RhsimUnG0008700 | 0.47 | 0.493 | 0.587 | 0.595 |
| RHSIM_Rhsim13G0054300 | 0.47 | 0.493 | 0.587 | 0.162 |
| RHSIM_Rhsim02G0079100 | 0.47 | 0.493 | 0.587 | 0.278 |
| RHSIM_Rhsim09G0013000 | 0.47 | 0.493 | 0.587 | -0.594 |
| RHSIM_Rhsim13G0039700 | 0.47 | 0.493 | 0.587 | 0.125 |
| novel.1239 | 0.47 | 0.493 | 0.587 | 0.827 |
| RHSIM_Rhsim09G0006000 | 0.47 | 0.493 | 0.587 | -1.753 |
| RHSIM_Rhsim10G0155800 | 0.47 | 0.493 | 0.587 | -1.471 |
| RHSIM_Rhsim01G0136000 | 0.47 | 0.494 | 0.587 | 0.047 |
| RHSIM_Rhsim01G0199300 | 0.47 | 0.494 | 0.588 | -1.316 |
| RHSIM_Rhsim07G0074200 | 0.47 | 0.494 | 0.588 | -0.164 |

| Gene | LRT statistic | P value | BH-adjusted P | log2FC (interaction) |
|---|---|---|---|---|
| RHSIM_Rhsim04G0093000 | 0.47 | 0.494 | 0.588 | 1.204 |
| RHSIM_Rhsim02G0043200 | 0.47 | 0.494 | 0.588 | -0.875 |
| RHSIM_Rhsim09G0130000 | 0.47 | 0.494 | 0.588 | -0.140 |
| novel.64 | 0.47 | 0.494 | 0.588 | -0.238 |
| RHSIM_Rhsim02G0251200 | 0.47 | 0.494 | 0.588 | 35.418 |
| RHSIM_Rhsim02G0158000 | 0.47 | 0.495 | 0.588 | 1.243 |
| RHSIM_Rhsim07G0059500 | 0.47 | 0.495 | 0.588 | 0.225 |
| novel.97 | 0.47 | 0.495 | 0.588 | 0.212 |
| novel.1934 | 0.47 | 0.495 | 0.589 | -35.424 |
| RHSIM_Rhsim08G0155900 | 0.47 | 0.495 | 0.589 | 0.272 |
| RHSIM_Rhsim11G0141300 | 0.47 | 0.495 | 0.589 | 0.307 |
| novel.2359 | 0.47 | 0.495 | 0.589 | 0.380 |
| RHSIM_Rhsim07G0051700 | 0.47 | 0.495 | 0.589 | -34.958 |
| RHSIM_Rhsim06G0239300 | 0.47 | 0.495 | 0.589 | -0.196 |
| RHSIM_Rhsim04G0067100 | 0.46 | 0.495 | 0.589 | -0.369 |
| RHSIM_Rhsim01G0003600 | 0.46 | 0.495 | 0.589 | -0.186 |
| RHSIM_Rhsim11G0194500 | 0.46 | 0.495 | 0.589 | 0.105 |
| RHSIM_RhsimUnG0085500 | 0.46 | 0.496 | 0.589 | 1.144 |
| RHSIM_Rhsim12G0007600 | 0.46 | 0.496 | 0.590 | -35.245 |
| RHSIM_Rhsim05G0190100 | 0.46 | 0.496 | 0.590 | -0.615 |
| RHSIM_RhsimMtG0004100 | 0.46 | 0.496 | 0.590 | 0.561 |
| RHSIM_Rhsim06G0095200 | 0.46 | 0.496 | 0.590 | 1.260 |
| novel.1094 | 0.46 | 0.496 | 0.590 | 0.031 |
| RHSIM_Rhsim12G0049400 | 0.46 | 0.496 | 0.590 | -1.240 |
| RHSIM_Rhsim01G0226700 | 0.46 | 0.497 | 0.591 | -0.363 |
| RHSIM_Rhsim10G0196500 | 0.46 | 0.497 | 0.591 | -1.319 |
| novel.202 | 0.46 | 0.497 | 0.591 | 0.463 |
| novel.4144 | 0.46 | 0.497 | 0.591 | -0.665 |
| RHSIM_Rhsim11G0070100 | 0.46 | 0.497 | 0.591 | -0.132 |
| RHSIM_Rhsim02G0124900 | 0.46 | 0.497 | 0.591 | 0.458 |
| RHSIM_Rhsim13G0217500 | 0.46 | 0.497 | 0.591 | -0.150 |
| RHSIM_Rhsim06G0224100 | 0.46 | 0.497 | 0.591 | 0.064 |

| Gene | LRT statistic | P value | BH-adjusted P | log2FC (interaction) |
|---|---|---|---|---|
| RHSIM_Rhsim07G0193700 | 0.46 | 0.497 | 0.591 | 0.099 |
| RHSIM_Rhsim05G0126500 | 0.46 | 0.497 | 0.591 | 0.079 |
| RHSIM_Rhsim05G0028100 | 0.46 | 0.497 | 0.591 | 0.804 |
| RHSIM_Rhsim02G0237400 | 0.46 | 0.498 | 0.591 | -2.065 |
| novel.1097 | 0.46 | 0.498 | 0.591 | 0.077 |
| RHSIM_Rhsim13G0200500 | 0.46 | 0.498 | 0.591 | 0.100 |
| RHSIM_Rhsim05G0152000 | 0.46 | 0.498 | 0.591 | 0.101 |
| RHSIM_Rhsim09G0073500 | 0.46 | 0.498 | 0.591 | -0.067 |
| RHSIM_RhsimUnG0124400 | 0.46 | 0.498 | 0.591 | -0.400 |
| RHSIM_Rhsim08G0048800 | 0.46 | 0.498 | 0.591 | 0.106 |
| RHSIM_Rhsim13G0162500 | 0.46 | 0.498 | 0.592 | 35.191 |
| RHSIM_Rhsim01G0258600 | 0.46 | 0.498 | 0.592 | 0.334 |
| novel.1054 | 0.46 | 0.499 | 0.592 | -0.123 |
| RHSIM_Rhsim10G0146400 | 0.46 | 0.499 | 0.592 | 1.342 |
| novel.4360 | 0.46 | 0.499 | 0.592 | 0.661 |
| RHSIM_Rhsim13G0223500 | 0.46 | 0.499 | 0.593 | -0.724 |
| RHSIM_Rhsim13G0011900 | 0.46 | 0.499 | 0.593 | -0.268 |
| novel.460 | 0.46 | 0.499 | 0.593 | -0.051 |
| RHSIM_Rhsim06G0231400 | 0.46 | 0.499 | 0.593 | -35.177 |
| RHSIM_Rhsim06G0117600 | 0.46 | 0.499 | 0.593 | 1.827 |
| novel.4083 | 0.46 | 0.499 | 0.593 | -0.894 |
| RHSIM_Rhsim13G0200000 | 0.46 | 0.499 | 0.593 | 0.154 |
| RHSIM_Rhsim05G0130000 | 0.46 | 0.500 | 0.593 | 0.742 |
| RHSIM_Rhsim08G0077600 | 0.46 | 0.500 | 0.593 | 0.327 |
| RHSIM_Rhsim03G0191200 | 0.46 | 0.500 | 0.593 | 35.371 |
| novel.675 | 0.46 | 0.500 | 0.594 | 0.146 |
| RHSIM_Rhsim09G0210600 | 0.46 | 0.500 | 0.594 | -0.061 |
| novel.770 | 0.45 | 0.500 | 0.594 | 0.563 |
| RHSIM_Rhsim09G0109500 | 0.45 | 0.500 | 0.594 | 0.363 |
| RHSIM_RhsimUnG0066100 | 0.45 | 0.500 | 0.594 | -0.185 |
| RHSIM_Rhsim11G0127600 | 0.45 | 0.500 | 0.594 | -0.206 |
| RHSIM_Rhsim05G0010000 | 0.45 | 0.501 | 0.594 | -1.615 |

| Gene | LRT statistic | P value | BH-adjusted P | log2FC (interaction) |
|---|---|---|---|---|
| RHSIM_Rhsim04G0098400 | 0.45 | 0.501 | 0.594 | -0.114 |
| RHSIM_Rhsim02G0092300 | 0.45 | 0.501 | 0.594 | 1.314 |
| RHSIM_Rhsim09G0099700 | 0.45 | 0.501 | 0.594 | -0.273 |
| RHSIM_Rhsim13G0215100 | 0.45 | 0.501 | 0.594 | -1.020 |
| RHSIM_Rhsim10G0185000 | 0.45 | 0.501 | 0.594 | 0.301 |
| RHSIM_Rhsim06G0205700 | 0.45 | 0.501 | 0.594 | 0.235 |
| RHSIM_Rhsim10G0031700 | 0.45 | 0.501 | 0.594 | -0.838 |
| RHSIM_Rhsim05G0127100 | 0.45 | 0.501 | 0.594 | -0.177 |
| RHSIM_Rhsim01G0020600 | 0.45 | 0.501 | 0.594 | -0.213 |
| novel.588 | 0.45 | 0.501 | 0.595 | -35.024 |
| RHSIM_Rhsim08G0131700 | 0.45 | 0.501 | 0.595 | 0.097 |
| RHSIM_Rhsim05G0146700 | 0.45 | 0.501 | 0.595 | -0.437 |
| RHSIM_Rhsim08G0121100 | 0.45 | 0.501 | 0.595 | 0.120 |
| RHSIM_RhsimUnG0021200 | 0.45 | 0.501 | 0.595 | -0.472 |
| RHSIM_Rhsim01G0064300 | 0.45 | 0.501 | 0.595 | 0.094 |
| RHSIM_Rhsim03G0226800 | 0.45 | 0.501 | 0.595 | -0.177 |
| RHSIM_Rhsim10G0120300 | 0.45 | 0.501 | 0.595 | 0.128 |
| RHSIM_Rhsim03G0065900 | 0.45 | 0.502 | 0.595 | -1.469 |
| RHSIM_Rhsim11G0096500 | 0.45 | 0.502 | 0.595 | -0.872 |
| novel.2517 | 0.45 | 0.502 | 0.596 | 1.207 |
| RHSIM_Rhsim01G0118900 | 0.45 | 0.502 | 0.596 | 0.187 |
| RHSIM_Rhsim02G0068700 | 0.45 | 0.502 | 0.596 | 0.625 |
| RHSIM_Rhsim07G0071800 | 0.45 | 0.502 | 0.596 | 0.076 |
| RHSIM_Rhsim09G0095900 | 0.45 | 0.502 | 0.596 | 0.285 |
| RHSIM_Rhsim08G0166200 | 0.45 | 0.502 | 0.596 | 0.125 |
| novel.4076 | 0.45 | 0.502 | 0.596 | -1.338 |
| RHSIM_RhsimUnG0059100 | 0.45 | 0.503 | 0.596 | -0.204 |
| RHSIM_Rhsim01G0131600 | 0.45 | 0.503 | 0.596 | 0.253 |
| RHSIM_Rhsim04G0202300 | 0.45 | 0.503 | 0.596 | 0.113 |
| RHSIM_Rhsim07G0101800 | 0.45 | 0.504 | 0.597 | -0.204 |
| RHSIM_Rhsim10G0046700 | 0.45 | 0.504 | 0.597 | -0.118 |
| RHSIM_Rhsim09G0206200 | 0.45 | 0.504 | 0.597 | -0.245 |

| Gene | LRT statistic | P value | BH-adjusted P | log2FC (interaction) |
|---|---|---|---|---|
| RHSIM_Rhsim10G0041800 | 0.45 | 0.504 | 0.597 | 0.447 |
| novel.2519 | 0.45 | 0.504 | 0.597 | -0.144 |
| RHSIM_Rhsim06G0097600 | 0.45 | 0.504 | 0.598 | 1.089 |
| RHSIM_Rhsim07G0094500 | 0.45 | 0.504 | 0.598 | -1.704 |
| RHSIM_Rhsim02G0016800 | 0.45 | 0.504 | 0.598 | 0.063 |
| RHSIM_Rhsim10G0158400 | 0.45 | 0.504 | 0.598 | -0.238 |
| RHSIM_RhsimUnG0134300 | 0.45 | 0.504 | 0.598 | -0.348 |
| RHSIM_Rhsim09G0077300 | 0.45 | 0.505 | 0.598 | 1.821 |
| novel.892 | 0.45 | 0.505 | 0.598 | 0.698 |
| novel.674 | 0.45 | 0.505 | 0.598 | -35.241 |
| RHSIM_Rhsim13G0006100 | 0.44 | 0.505 | 0.598 | -0.088 |
| RHSIM_Rhsim05G0169600 | 0.44 | 0.505 | 0.598 | -0.951 |
| RHSIM_Rhsim02G0242400 | 0.44 | 0.505 | 0.599 | 0.580 |
| RHSIM_Rhsim02G0043600 | 0.44 | 0.505 | 0.599 | 0.679 |
| RHSIM_Rhsim13G0050300 | 0.44 | 0.505 | 0.599 | -1.528 |
| RHSIM_Rhsim07G0080500 | 0.44 | 0.505 | 0.599 | 0.091 |
| RHSIM_Rhsim02G0247500 | 0.44 | 0.506 | 0.599 | 0.865 |
| RHSIM_Rhsim01G0083300 | 0.44 | 0.506 | 0.599 | -0.080 |
| RHSIM_Rhsim02G0237800 | 0.44 | 0.506 | 0.599 | -0.113 |
| RHSIM_RhsimUnG0076700 | 0.44 | 0.506 | 0.599 | -1.651 |
| RHSIM_Rhsim11G0018200 | 0.44 | 0.506 | 0.599 | 0.891 |
| RHSIM_Rhsim11G0189800 | 0.44 | 0.506 | 0.599 | -35.159 |
| RHSIM_Rhsim05G0232700 | 0.44 | 0.506 | 0.599 | -1.839 |
| RHSIM_Rhsim01G0033600 | 0.44 | 0.506 | 0.599 | 0.067 |
| RHSIM_Rhsim04G0083200 | 0.44 | 0.506 | 0.600 | -0.095 |
| RHSIM_Rhsim11G0171300 | 0.44 | 0.506 | 0.600 | -0.268 |
| RHSIM_Rhsim08G0090600 | 0.44 | 0.506 | 0.600 | 1.127 |
| RHSIM_Rhsim01G0093600 | 0.44 | 0.506 | 0.600 | 0.607 |
| RHSIM_Rhsim03G0119300 | 0.44 | 0.506 | 0.600 | 0.096 |
| RHSIM_Rhsim01G0212200 | 0.44 | 0.507 | 0.600 | 1.510 |
| RHSIM_Rhsim07G0112000 | 0.44 | 0.507 | 0.600 | 0.558 |
| RHSIM_Rhsim06G0082000 | 0.44 | 0.507 | 0.600 | 0.078 |

| Gene | LRT statistic | P value | BH-adjusted P | log2FC (interaction) |
|---|---|---|---|---|
| RHSIM_Rhsim09G0032100 | 0.44 | 0.507 | 0.600 | 0.095 |
| RHSIM_Rhsim01G0153700 | 0.44 | 0.507 | 0.600 | -0.840 |
| RHSIM_RhsimUnG0086300 | 0.44 | 0.507 | 0.600 | -0.405 |
| RHSIM_Rhsim10G0113600 | 0.44 | 0.507 | 0.600 | 0.174 |
| RHSIM_Rhsim08G0240600 | 0.44 | 0.507 | 0.600 | 0.095 |
| RHSIM_Rhsim01G0291600 | 0.44 | 0.507 | 0.600 | 0.572 |
| RHSIM_Rhsim13G0160800 | 0.44 | 0.507 | 0.600 | -0.683 |
| RHSIM_RhsimUnG0252100 | 0.44 | 0.507 | 0.600 | -0.090 |
| RHSIM_Rhsim13G0055100 | 0.44 | 0.507 | 0.600 | -0.429 |
| novel.73 | 0.44 | 0.507 | 0.600 | 1.131 |
| RHSIM_Rhsim07G0093400 | 0.44 | 0.507 | 0.600 | 0.066 |
| novel.4189 | 0.44 | 0.507 | 0.600 | -1.543 |
| RHSIM_RhsimUnG0086700 | 0.44 | 0.507 | 0.600 | 0.490 |
| RHSIM_Rhsim06G0124800 | 0.44 | 0.507 | 0.600 | -0.234 |
| RHSIM_Rhsim02G0136100 | 0.44 | 0.507 | 0.600 | -1.374 |
| RHSIM_Rhsim13G0198900 | 0.44 | 0.508 | 0.601 | -0.195 |
| RHSIM_Rhsim09G0177900 | 0.44 | 0.508 | 0.601 | -0.068 |
| RHSIM_Rhsim02G0101200 | 0.44 | 0.508 | 0.601 | -0.940 |
| novel.2307 | 0.44 | 0.508 | 0.602 | -1.020 |
| RHSIM_Rhsim08G0069500 | 0.44 | 0.509 | 0.602 | -0.815 |
| novel.2381 | 0.44 | 0.509 | 0.602 | 0.227 |
| RHSIM_Rhsim08G0019000 | 0.44 | 0.509 | 0.602 | -0.107 |
| RHSIM_Rhsim13G0012900 | 0.44 | 0.509 | 0.602 | -0.998 |
| RHSIM_RhsimUnG0168600 | 0.44 | 0.509 | 0.602 | -0.691 |
| RHSIM_Rhsim02G0128000 | 0.44 | 0.509 | 0.602 | -0.158 |
| RHSIM_Rhsim09G0006300 | 0.43 | 0.510 | 0.603 | 0.290 |
| RHSIM_Rhsim03G0124000 | 0.43 | 0.510 | 0.603 | -0.458 |
| novel.4546 | 0.43 | 0.510 | 0.603 | 0.144 |
| RHSIM_Rhsim09G0168000 | 0.43 | 0.510 | 0.603 | 0.221 |
| novel.4177 | 0.43 | 0.510 | 0.603 | 1.318 |
| RHSIM_Rhsim05G0146200 | 0.43 | 0.510 | 0.603 | 0.068 |
| RHSIM_Rhsim12G0177100 | 0.43 | 0.510 | 0.603 | -0.577 |

| Gene | LRT statistic | P value | BH-adjusted P | log2FC (interaction) |
|---|---|---|---|---|
| RHSIM_Rhsim05G0061300 | 0.43 | 0.510 | 0.603 | 0.335 |
| RHSIM_Rhsim10G0032500 | 0.43 | 0.510 | 0.603 | 0.673 |
| RHSIM_Rhsim08G0180100 | 0.43 | 0.511 | 0.604 | -0.458 |
| novel.365 | 0.43 | 0.511 | 0.604 | 0.705 |
| RHSIM_Rhsim12G0090900 | 0.43 | 0.511 | 0.604 | -0.075 |
| RHSIM_Rhsim03G0034700 | 0.43 | 0.511 | 0.604 | -35.041 |
| novel.1091 | 0.43 | 0.511 | 0.604 | -1.018 |
| RHSIM_Rhsim13G0063900 | 0.43 | 0.511 | 0.604 | -0.113 |
| novel.524 | 0.43 | 0.511 | 0.604 | -0.242 |
| RHSIM_Rhsim03G0065600 | 0.43 | 0.511 | 0.604 | 0.236 |
| RHSIM_Rhsim04G0051200 | 0.43 | 0.511 | 0.604 | 1.237 |
| RHSIM_RhsimUnG0007700 | 0.43 | 0.511 | 0.604 | 1.396 |
| novel.2456 | 0.43 | 0.511 | 0.604 | -0.707 |
| RHSIM_Rhsim13G0068000 | 0.43 | 0.512 | 0.605 | -0.514 |
| RHSIM_Rhsim02G0061400 | 0.43 | 0.512 | 0.605 | -0.804 |
| RHSIM_Rhsim12G0145900 | 0.43 | 0.512 | 0.605 | 35.217 |
| RHSIM_Rhsim02G0097300 | 0.43 | 0.512 | 0.605 | -0.862 |
| novel.2990 | 0.43 | 0.512 | 0.605 | -0.048 |
| RHSIM_RhsimUnG0213600 | 0.43 | 0.512 | 0.605 | 1.750 |
| RHSIM_Rhsim01G0117200 | 0.43 | 0.513 | 0.606 | 0.476 |
| RHSIM_RhsimUnG0112100 | 0.43 | 0.513 | 0.606 | 1.582 |
| RHSIM_Rhsim06G0107900 | 0.43 | 0.513 | 0.606 | 1.241 |
| RHSIM_Rhsim02G0032300 | 0.43 | 0.513 | 0.606 | 0.088 |
| RHSIM_Rhsim12G0121100 | 0.43 | 0.513 | 0.606 | -0.089 |
| RHSIM_Rhsim04G0107500 | 0.43 | 0.513 | 0.606 | -0.445 |
| RHSIM_Rhsim06G0123700 | 0.43 | 0.513 | 0.606 | 1.800 |
| RHSIM_RhsimUnG0114600 | 0.43 | 0.513 | 0.606 | -1.360 |
| RHSIM_Rhsim04G0124100 | 0.43 | 0.513 | 0.606 | 0.149 |
| RHSIM_Rhsim04G0165700 | 0.43 | 0.513 | 0.606 | -0.559 |
| RHSIM_Rhsim04G0138900 | 0.43 | 0.514 | 0.607 | -1.855 |
| RHSIM_Rhsim03G0009100 | 0.43 | 0.514 | 0.607 | 0.054 |
| RHSIM_Rhsim12G0205200 | 0.43 | 0.514 | 0.607 | -1.001 |

| Gene | LRT statistic | P value | BH-adjusted P | log2FC (interaction) |
|---|---|---|---|---|
| RHSIM_Rhsim04G0035100 | 0.43 | 0.514 | 0.607 | 0.906 |
| RHSIM_Rhsim12G0166300 | 0.42 | 0.514 | 0.607 | 0.070 |
| RHSIM_Rhsim13G0091400 | 0.42 | 0.514 | 0.607 | 0.063 |
| RHSIM_Rhsim06G0174700 | 0.42 | 0.515 | 0.608 | -0.152 |
| RHSIM_Rhsim10G0146000 | 0.42 | 0.515 | 0.608 | -35.181 |
| novel.3244 | 0.42 | 0.515 | 0.608 | 0.420 |
| RHSIM_Rhsim12G0129300 | 0.42 | 0.515 | 0.609 | 0.105 |
| RHSIM_Rhsim10G0211100 | 0.42 | 0.516 | 0.609 | 0.126 |
| novel.3226 | 0.42 | 0.516 | 0.609 | 1.006 |
| RHSIM_RhsimUnG0082600 | 0.42 | 0.516 | 0.609 | 0.984 |
| RHSIM_Rhsim07G0062400 | 0.42 | 0.516 | 0.609 | 0.362 |
| RHSIM_Rhsim13G0106400 | 0.42 | 0.516 | 0.609 | 0.415 |
| RHSIM_RhsimUnG0125300 | 0.42 | 0.516 | 0.609 | -0.523 |
| RHSIM_Rhsim01G0018200 | 0.42 | 0.516 | 0.609 | 0.606 |
| RHSIM_Rhsim05G0100100 | 0.42 | 0.516 | 0.609 | 0.222 |
| RHSIM_Rhsim11G0066500 | 0.42 | 0.516 | 0.609 | 0.455 |
| RHSIM_Rhsim02G0081200 | 0.42 | 0.516 | 0.609 | -0.167 |
| RHSIM_Rhsim09G0136100 | 0.42 | 0.516 | 0.609 | -0.220 |
| RHSIM_Rhsim06G0103200 | 0.42 | 0.516 | 0.609 | 0.307 |
| RHSIM_Rhsim02G0027100 | 0.42 | 0.516 | 0.609 | 0.099 |
| RHSIM_Rhsim03G0125000 | 0.42 | 0.517 | 0.610 | 0.057 |
| novel.3402 | 0.42 | 0.517 | 0.610 | 0.128 |
| novel.4576 | 0.42 | 0.517 | 0.610 | 0.770 |
| RHSIM_Rhsim11G0078400 | 0.42 | 0.517 | 0.610 | 0.342 |
| novel.2376 | 0.42 | 0.517 | 0.610 | -0.959 |
| RHSIM_Rhsim03G0115300 | 0.42 | 0.517 | 0.610 | -0.233 |
| RHSIM_RhsimUnG0197700 | 0.42 | 0.517 | 0.610 | -0.099 |
| RHSIM_Rhsim08G0200800 | 0.42 | 0.518 | 0.611 | -0.219 |
| novel.1311 | 0.42 | 0.518 | 0.611 | -0.593 |
| RHSIM_Rhsim09G0185500 | 0.42 | 0.518 | 0.611 | -0.351 |
| RHSIM_Rhsim04G0119600 | 0.42 | 0.518 | 0.611 | 0.418 |
| RHSIM_Rhsim07G0253900 | 0.42 | 0.518 | 0.611 | 0.679 |

| Gene | LRT statistic | P value | BH-adjusted P | log2FC (interaction) |
|---|---|---|---|---|
| novel.572 | 0.42 | 0.518 | 0.611 | 0.502 |
| novel.2679 | 0.42 | 0.519 | 0.612 | 34.974 |
| novel.1532 | 0.42 | 0.519 | 0.612 | 0.241 |
| RHSIM_Rhsim03G0112000 | 0.42 | 0.519 | 0.612 | -0.046 |
| RHSIM_Rhsim01G0260000 | 0.42 | 0.519 | 0.612 | 1.884 |
| RHSIM_Rhsim08G0208900 | 0.42 | 0.519 | 0.612 | 1.041 |
| RHSIM_Rhsim07G0129600 | 0.42 | 0.519 | 0.612 | -1.211 |
| RHSIM_RhsimUnG0193200 | 0.42 | 0.519 | 0.612 | -1.059 |
| RHSIM_Rhsim09G0063600 | 0.41 | 0.520 | 0.612 | -1.269 |
| novel.4371 | 0.41 | 0.520 | 0.613 | 0.594 |
| novel.3411 | 0.41 | 0.520 | 0.613 | 0.145 |
| RHSIM_Rhsim08G0117000 | 0.41 | 0.520 | 0.613 | -0.163 |
| RHSIM_Rhsim08G0233100 | 0.41 | 0.520 | 0.613 | -0.069 |
| RHSIM_Rhsim04G0230700 | 0.41 | 0.520 | 0.613 | -0.124 |
| RHSIM_Rhsim11G0084600 | 0.41 | 0.520 | 0.613 | -0.652 |
| RHSIM_Rhsim11G0120100 | 0.41 | 0.520 | 0.613 | -1.153 |
| RHSIM_Rhsim07G0153200 | 0.41 | 0.520 | 0.613 | -0.166 |
| RHSIM_Rhsim02G0161200 | 0.41 | 0.521 | 0.614 | 0.121 |
| RHSIM_Rhsim03G0135300 | 0.41 | 0.521 | 0.614 | -0.214 |
| RHSIM_Rhsim13G0164000 | 0.41 | 0.521 | 0.614 | 0.115 |
| RHSIM_Rhsim05G0012500 | 0.41 | 0.521 | 0.614 | -0.976 |
| novel.3643 | 0.41 | 0.521 | 0.614 | 0.378 |
| novel.3657 | 0.41 | 0.521 | 0.614 | -0.333 |
| RHSIM_Rhsim06G0233600 | 0.41 | 0.522 | 0.614 | -0.495 |
| RHSIM_Rhsim05G0035300 | 0.41 | 0.522 | 0.614 | 0.079 |
| RHSIM_Rhsim10G0110400 | 0.41 | 0.522 | 0.615 | 0.493 |
| RHSIM_Rhsim10G0007000 | 0.41 | 0.522 | 0.615 | 0.110 |
| novel.2639 | 0.41 | 0.522 | 0.615 | -0.588 |
| RHSIM_Rhsim11G0038100 | 0.41 | 0.522 | 0.615 | -0.065 |
| RHSIM_Rhsim04G0190500 | 0.41 | 0.522 | 0.615 | -1.316 |
| RHSIM_Rhsim08G0100200 | 0.41 | 0.522 | 0.615 | -0.053 |
| RHSIM_Rhsim04G0049300 | 0.41 | 0.522 | 0.615 | -0.817 |

| Gene | LRT statistic | P value | BH-adjusted P | log2FC (interaction) |
|---|---|---|---|---|
| RHSIM_Rhsim10G0152500 | 0.41 | 0.522 | 0.615 | 35.193 |
| RHSIM_Rhsim12G0186400 | 0.41 | 0.522 | 0.615 | 0.912 |
| RHSIM_Rhsim07G0033700 | 0.41 | 0.522 | 0.615 | 0.294 |
| RHSIM_Rhsim10G0000300 | 0.41 | 0.522 | 0.615 | -0.142 |
| novel.3268 | 0.41 | 0.522 | 0.615 | -0.336 |
| novel.2469 | 0.41 | 0.523 | 0.615 | 0.805 |
| RHSIM_Rhsim06G0218900 | 0.41 | 0.523 | 0.615 | -0.070 |
| RHSIM_Rhsim03G0042200 | 0.41 | 0.523 | 0.616 | -34.853 |
| RHSIM_Rhsim09G0191000 | 0.41 | 0.523 | 0.616 | 1.567 |
| RHSIM_Rhsim07G0137200 | 0.41 | 0.523 | 0.616 | -0.063 |
| RHSIM_Rhsim08G0235900 | 0.41 | 0.523 | 0.616 | 0.396 |
| RHSIM_Rhsim13G0115000 | 0.41 | 0.523 | 0.616 | 0.782 |
| RHSIM_Rhsim10G0064400 | 0.41 | 0.523 | 0.616 | -1.200 |
| RHSIM_Rhsim08G0018300 | 0.41 | 0.524 | 0.616 | 0.493 |
| RHSIM_Rhsim04G0100200 | 0.41 | 0.524 | 0.616 | 0.649 |
| novel.1563 | 0.41 | 0.524 | 0.617 | 0.833 |
| RHSIM_Rhsim10G0024500 | 0.41 | 0.524 | 0.617 | -1.634 |
| novel.61 | 0.41 | 0.524 | 0.617 | 0.265 |
| RHSIM_Rhsim03G0124400 | 0.41 | 0.524 | 0.617 | -0.937 |
| RHSIM_RhsimUnG0113300 | 0.40 | 0.525 | 0.617 | 0.455 |
| RHSIM_Rhsim08G0182900 | 0.40 | 0.525 | 0.618 | 0.131 |
| novel.1783 | 0.40 | 0.525 | 0.618 | -1.005 |
| RHSIM_Rhsim06G0034200 | 0.40 | 0.525 | 0.618 | -0.224 |
| RHSIM_Rhsim03G0128400 | 0.40 | 0.525 | 0.618 | -0.417 |
| RHSIM_Rhsim10G0120600 | 0.40 | 0.526 | 0.619 | 0.399 |
| RHSIM_Rhsim06G0125000 | 0.40 | 0.526 | 0.619 | 0.569 |
| novel.1610 | 0.40 | 0.526 | 0.619 | -0.476 |
| novel.851 | 0.40 | 0.526 | 0.619 | 0.522 |
| RHSIM_Rhsim04G0051500 | 0.40 | 0.526 | 0.619 | 0.105 |
| RHSIM_Rhsim10G0213200 | 0.40 | 0.526 | 0.619 | 0.873 |
| RHSIM_Rhsim11G0158800 | 0.40 | 0.526 | 0.619 | 0.802 |
| RHSIM_Rhsim02G0248600 | 0.40 | 0.526 | 0.619 | -0.827 |

| Gene | LRT statistic | P value | BH-adjusted P | log2FC (interaction) |
|---|---|---|---|---|
| RHSIM_Rhsim04G0155600 | 0.40 | 0.526 | 0.619 | -0.586 |
| RHSIM_Rhsim01G0020200 | 0.40 | 0.527 | 0.620 | 0.698 |
| RHSIM_Rhsim03G0136200 | 0.40 | 0.527 | 0.620 | -0.929 |
| RHSIM_Rhsim09G0008100 | 0.40 | 0.527 | 0.620 | -0.777 |
| RHSIM_Rhsim11G0122600 | 0.40 | 0.527 | 0.620 | -0.113 |
| RHSIM_Rhsim07G0023400 | 0.40 | 0.527 | 0.620 | -1.061 |
| RHSIM_Rhsim04G0121100 | 0.40 | 0.527 | 0.620 | 0.473 |
| RHSIM_Rhsim04G0076100 | 0.40 | 0.528 | 0.620 | 0.090 |
| novel.1295 | 0.40 | 0.528 | 0.620 | 0.171 |
| RHSIM_RhsimUnG0090000 | 0.40 | 0.528 | 0.621 | -0.094 |
| novel.100 | 0.40 | 0.528 | 0.621 | 0.526 |
| RHSIM_Rhsim11G0179700 | 0.40 | 0.528 | 0.621 | -0.060 |
| RHSIM_Rhsim03G0167500 | 0.40 | 0.528 | 0.621 | 35.063 |
| RHSIM_RhsimUnG0180900 | 0.40 | 0.528 | 0.621 | -0.691 |
| RHSIM_Rhsim13G0156800 | 0.40 | 0.529 | 0.621 | -0.123 |
| RHSIM_Rhsim07G0077000 | 0.40 | 0.529 | 0.622 | -34.742 |
| RHSIM_Rhsim06G0135000 | 0.40 | 0.529 | 0.622 | 1.050 |
| RHSIM_Rhsim04G0121600 | 0.40 | 0.529 | 0.622 | -0.092 |
| novel.2024 | 0.40 | 0.529 | 0.622 | 0.379 |
| RHSIM_Rhsim09G0172900 | 0.40 | 0.529 | 0.622 | 0.613 |
| RHSIM_RhsimUnG0018900 | 0.40 | 0.529 | 0.622 | -0.120 |
| RHSIM_Rhsim01G0260500 | 0.40 | 0.529 | 0.622 | 0.066 |
| novel.3674 | 0.40 | 0.529 | 0.622 | -0.880 |
| novel.2140 | 0.40 | 0.529 | 0.622 | 0.666 |
| RHSIM_Rhsim02G0164500 | 0.40 | 0.529 | 0.622 | -0.217 |
| RHSIM_Rhsim01G0153800 | 0.40 | 0.529 | 0.622 | -0.901 |
| RHSIM_Rhsim07G0119800 | 0.40 | 0.529 | 0.622 | 0.306 |
| RHSIM_Rhsim09G0127200 | 0.39 | 0.530 | 0.622 | -0.055 |
| novel.423 | 0.39 | 0.530 | 0.622 | -0.770 |
| RHSIM_Rhsim04G0105600 | 0.39 | 0.530 | 0.622 | -1.358 |
| RHSIM_Rhsim13G0072300 | 0.39 | 0.530 | 0.622 | 0.178 |
| RHSIM_Rhsim09G0138100 | 0.39 | 0.530 | 0.623 | 1.574 |

| Gene | LRT statistic | P value | BH-adjusted P | log2FC (interaction) |
|---|---|---|---|---|
| RHSIM_Rhsim03G0213600 | 0.39 | 0.530 | 0.623 | 0.188 |
| RHSIM_Rhsim06G0142800 | 0.39 | 0.530 | 0.623 | -0.567 |
| novel.2883 | 0.39 | 0.530 | 0.623 | -0.311 |
| novel.2357 | 0.39 | 0.531 | 0.623 | 34.891 |
| RHSIM_Rhsim13G0029500 | 0.39 | 0.531 | 0.623 | -0.147 |
| RHSIM_Rhsim05G0097300 | 0.39 | 0.531 | 0.623 | 0.207 |
| novel.1790 | 0.39 | 0.531 | 0.623 | -0.354 |
| RHSIM_Rhsim04G0016100 | 0.39 | 0.531 | 0.624 | -0.084 |
| RHSIM_Rhsim08G0079300 | 0.39 | 0.532 | 0.624 | 0.084 |
| RHSIM_Rhsim06G0196900 | 0.39 | 0.532 | 0.624 | -0.187 |
| RHSIM_Rhsim06G0171300 | 0.39 | 0.532 | 0.624 | 0.431 |
| RHSIM_Rhsim06G0066000 | 0.39 | 0.532 | 0.624 | -0.578 |
| RHSIM_Rhsim01G0273000 | 0.39 | 0.532 | 0.624 | 0.579 |
| RHSIM_RhsimUnG0217200 | 0.39 | 0.532 | 0.624 | -0.245 |
| RHSIM_Rhsim06G0149500 | 0.39 | 0.532 | 0.624 | -0.127 |
| novel.4365 | 0.39 | 0.532 | 0.625 | -0.745 |
| RHSIM_Rhsim08G0157100 | 0.39 | 0.532 | 0.625 | 0.090 |
| RHSIM_Rhsim04G0182000 | 0.39 | 0.532 | 0.625 | -0.535 |
| RHSIM_Rhsim04G0115700 | 0.39 | 0.532 | 0.625 | -0.842 |
| novel.2568 | 0.39 | 0.532 | 0.625 | 0.151 |
| RHSIM_RhsimUnG0216400 | 0.39 | 0.532 | 0.625 | 0.413 |
| RHSIM_Rhsim04G0244700 | 0.39 | 0.532 | 0.625 | -0.404 |
| RHSIM_Rhsim10G0041400 | 0.39 | 0.533 | 0.625 | 0.699 |
| novel.1860 | 0.39 | 0.533 | 0.626 | 0.528 |
| RHSIM_Rhsim06G0056700 | 0.39 | 0.533 | 0.626 | 1.143 |
| novel.630 | 0.39 | 0.534 | 0.626 | -0.675 |
| RHSIM_RhsimUnG0072100 | 0.39 | 0.534 | 0.626 | 0.408 |
| RHSIM_Rhsim07G0132900 | 0.39 | 0.534 | 0.626 | 0.060 |
| RHSIM_Rhsim02G0227300 | 0.39 | 0.534 | 0.626 | -0.484 |
| RHSIM_RhsimUnG0193400 | 0.39 | 0.534 | 0.627 | -0.139 |
| RHSIM_Rhsim01G0034000 | 0.39 | 0.535 | 0.627 | 0.390 |
| RHSIM_Rhsim06G0015700 | 0.39 | 0.535 | 0.627 | 0.182 |

| Gene | LRT statistic | P value | BH-adjusted P | log2FC (interaction) |
|---|---|---|---|---|
| RHSIM_Rhsim02G0030700 | 0.39 | 0.535 | 0.627 | 0.862 |
| RHSIM_Rhsim02G0250200 | 0.38 | 0.535 | 0.628 | 1.019 |
| novel.615 | 0.38 | 0.535 | 0.628 | -0.382 |
| RHSIM_Rhsim05G0129700 | 0.38 | 0.535 | 0.628 | 0.475 |
| RHSIM_Rhsim05G0027800 | 0.38 | 0.535 | 0.628 | -34.897 |
| novel.4090 | 0.38 | 0.535 | 0.628 | 0.308 |
| RHSIM_Rhsim03G0042000 | 0.38 | 0.535 | 0.628 | -0.134 |
| novel.1817 | 0.38 | 0.536 | 0.628 | -0.350 |
| novel.2249 | 0.38 | 0.536 | 0.628 | 34.756 |
| RHSIM_Rhsim10G0103900 | 0.38 | 0.536 | 0.628 | 0.185 |
| novel.3094 | 0.38 | 0.536 | 0.628 | 0.831 |
| RHSIM_Rhsim03G0251600 | 0.38 | 0.536 | 0.628 | 0.120 |
| RHSIM_Rhsim08G0070100 | 0.38 | 0.536 | 0.628 | 0.133 |
| RHSIM_Rhsim05G0011400 | 0.38 | 0.536 | 0.628 | 0.288 |
| RHSIM_Rhsim10G0186900 | 0.38 | 0.536 | 0.628 | -0.414 |
| RHSIM_Rhsim03G0005400 | 0.38 | 0.536 | 0.628 | -0.092 |
| RHSIM_Rhsim04G0078500 | 0.38 | 0.536 | 0.629 | -0.966 |
| RHSIM_Rhsim08G0077200 | 0.38 | 0.536 | 0.629 | -0.050 |
| novel.4449 | 0.38 | 0.536 | 0.629 | 0.497 |
| RHSIM_Rhsim04G0208200 | 0.38 | 0.536 | 0.629 | -0.239 |
| RHSIM_Rhsim12G0082500 | 0.38 | 0.537 | 0.629 | -0.253 |
| RHSIM_Rhsim10G0083500 | 0.38 | 0.537 | 0.629 | 0.318 |
| RHSIM_Rhsim12G0186300 | 0.38 | 0.537 | 0.629 | -1.392 |
| RHSIM_Rhsim05G0205500 | 0.38 | 0.537 | 0.629 | -0.960 |
| novel.585 | 0.38 | 0.537 | 0.629 | -0.116 |
| RHSIM_Rhsim13G0014200 | 0.38 | 0.537 | 0.630 | -1.101 |
| RHSIM_Rhsim01G0128600 | 0.38 | 0.537 | 0.630 | -0.086 |
| novel.1941 | 0.38 | 0.537 | 0.630 | 0.320 |
| RHSIM_Rhsim13G0139400 | 0.38 | 0.538 | 0.630 | 1.023 |
| RHSIM_Rhsim04G0074600 | 0.38 | 0.538 | 0.630 | 0.979 |
| RHSIM_Rhsim04G0172500 | 0.38 | 0.538 | 0.631 | 1.346 |
| RHSIM_Rhsim02G0187800 | 0.38 | 0.538 | 0.631 | -35.857 |

| Gene | LRT statistic | P value | BH-adjusted P | log2FC (interaction) |
|---|---|---|---|---|
| RHSIM_Rhsim07G0018900 | 0.38 | 0.539 | 0.631 | 0.161 |
| RHSIM_Rhsim08G0129400 | 0.38 | 0.539 | 0.631 | 0.129 |
| novel.1968 | 0.38 | 0.539 | 0.631 | 0.658 |
| RHSIM_Rhsim03G0209300 | 0.38 | 0.539 | 0.631 | -1.531 |
| RHSIM_Rhsim11G0110600 | 0.38 | 0.539 | 0.631 | 1.763 |
| RHSIM_Rhsim12G0170800 | 0.38 | 0.539 | 0.631 | 0.509 |
| RHSIM_Rhsim06G0051600 | 0.38 | 0.539 | 0.631 | -0.090 |
| RHSIM_Rhsim02G0021500 | 0.38 | 0.539 | 0.632 | -0.156 |
| novel.1269 | 0.38 | 0.539 | 0.632 | -0.429 |
| RHSIM_Rhsim13G0224900 | 0.38 | 0.539 | 0.632 | -0.137 |
| RHSIM_Rhsim03G0100400 | 0.38 | 0.540 | 0.632 | 0.588 |
| RHSIM_RhsimUnG0095100 | 0.38 | 0.540 | 0.632 | -0.973 |
| RHSIM_Rhsim09G0053700 | 0.38 | 0.540 | 0.632 | 0.219 |
| novel.350 | 0.38 | 0.540 | 0.632 | -0.085 |
| RHSIM_Rhsim02G0157100 | 0.38 | 0.540 | 0.632 | -0.062 |
| RHSIM_Rhsim10G0052800 | 0.38 | 0.540 | 0.632 | -1.233 |
| RHSIM_Rhsim08G0177000 | 0.38 | 0.540 | 0.632 | -0.632 |
| RHSIM_Rhsim07G0016800 | 0.37 | 0.540 | 0.633 | -0.093 |
| RHSIM_Rhsim09G0145400 | 0.37 | 0.540 | 0.633 | 0.061 |
| RHSIM_Rhsim09G0200600 | 0.37 | 0.540 | 0.633 | 0.078 |
| RHSIM_Rhsim04G0126500 | 0.37 | 0.541 | 0.633 | -0.772 |
| RHSIM_Rhsim09G0048000 | 0.37 | 0.541 | 0.633 | 0.134 |
| novel.3510 | 0.37 | 0.541 | 0.633 | 0.239 |
| novel.613 | 0.37 | 0.541 | 0.633 | -0.400 |
| RHSIM_RhsimUnG0003400 | 0.37 | 0.541 | 0.633 | -0.477 |
| RHSIM_RhsimUnG0246400 | 0.37 | 0.541 | 0.633 | 0.196 |
| RHSIM_Rhsim08G0153200 | 0.37 | 0.541 | 0.633 | -0.080 |
| RHSIM_Rhsim06G0159600 | 0.37 | 0.541 | 0.633 | -1.379 |
| novel.2048 | 0.37 | 0.541 | 0.634 | -0.137 |
| RHSIM_Rhsim05G0204500 | 0.37 | 0.541 | 0.634 | -1.432 |
| novel.4056 | 0.37 | 0.542 | 0.634 | -0.379 |
| RHSIM_Rhsim01G0099100 | 0.37 | 0.542 | 0.634 | 1.111 |

| Gene | LRT statistic | P value | BH-adjusted P | log2FC (interaction) |
|---|---|---|---|---|
| novel.668 | 0.37 | 0.542 | 0.634 | 0.274 |
| RHSIM_Rhsim06G0089700 | 0.37 | 0.542 | 0.634 | 0.504 |
| RHSIM_Rhsim02G0214600 | 0.37 | 0.542 | 0.634 | 1.490 |
| novel.2795 | 0.37 | 0.542 | 0.634 | -0.172 |
| novel.2056 | 0.37 | 0.542 | 0.634 | -0.280 |
| RHSIM_Rhsim12G0004000 | 0.37 | 0.542 | 0.634 | 1.431 |
| novel.901 | 0.37 | 0.542 | 0.634 | 0.809 |
| RHSIM_Rhsim04G0229000 | 0.37 | 0.542 | 0.635 | -0.591 |
| RHSIM_Rhsim07G0079200 | 0.37 | 0.543 | 0.635 | -0.096 |
| RHSIM_Rhsim09G0045200 | 0.37 | 0.543 | 0.635 | 0.502 |
| RHSIM_Rhsim06G0010200 | 0.37 | 0.543 | 0.635 | 0.260 |
| RHSIM_Rhsim11G0155900 | 0.37 | 0.543 | 0.635 | -0.803 |
| RHSIM_Rhsim10G0047200 | 0.37 | 0.543 | 0.635 | 0.335 |
| novel.4042 | 0.37 | 0.544 | 0.636 | -0.437 |
| RHSIM_Rhsim07G0113100 | 0.37 | 0.544 | 0.636 | -34.753 |
| RHSIM_Rhsim01G0146400 | 0.37 | 0.544 | 0.636 | 0.799 |
| RHSIM_Rhsim06G0075400 | 0.37 | 0.544 | 0.636 | 0.197 |
| RHSIM_Rhsim08G0106600 | 0.37 | 0.544 | 0.636 | 1.741 |
| RHSIM_Rhsim03G0067900 | 0.37 | 0.544 | 0.636 | -0.228 |
| novel.1830 | 0.37 | 0.544 | 0.636 | -34.218 |
| RHSIM_Rhsim09G0144600 | 0.37 | 0.544 | 0.636 | -1.402 |
| RHSIM_Rhsim09G0152000 | 0.37 | 0.545 | 0.637 | -0.149 |
| RHSIM_RhsimUnG0233100 | 0.37 | 0.545 | 0.637 | 0.851 |
| RHSIM_Rhsim08G0095700 | 0.37 | 0.545 | 0.637 | 0.125 |
| RHSIM_Rhsim10G0004100 | 0.37 | 0.545 | 0.637 | -0.098 |
| RHSIM_Rhsim10G0111900 | 0.37 | 0.545 | 0.637 | 0.079 |
| RHSIM_Rhsim08G0012500 | 0.37 | 0.545 | 0.637 | -1.550 |
| novel.1250 | 0.37 | 0.545 | 0.637 | 0.458 |
| RHSIM_Rhsim06G0187900 | 0.37 | 0.545 | 0.637 | 0.990 |
| RHSIM_Rhsim12G0145300 | 0.37 | 0.545 | 0.637 | 0.639 |
| RHSIM_Rhsim03G0004500 | 0.37 | 0.545 | 0.637 | -0.523 |
| RHSIM_Rhsim01G0144700 | 0.37 | 0.545 | 0.637 | 0.089 |

| Gene | LRT statistic | P value | BH-adjusted P | log2FC (interaction) |
|---|---|---|---|---|
| RHSIM_RhsimUnG0202500 | 0.37 | 0.545 | 0.637 | -0.324 |
| novel.2845 | 0.37 | 0.546 | 0.638 | 0.221 |
| RHSIM_RhsimMtG0004300 | 0.36 | 0.546 | 0.638 | 0.922 |
| novel.55 | 0.36 | 0.546 | 0.638 | 0.174 |
| RHSIM_Rhsim05G0045200 | 0.36 | 0.546 | 0.638 | 0.300 |
| RHSIM_Rhsim02G0041000 | 0.36 | 0.546 | 0.638 | 0.447 |
| RHSIM_Rhsim12G0130200 | 0.36 | 0.546 | 0.638 | -34.453 |
| novel.627 | 0.36 | 0.546 | 0.638 | -0.676 |
| RHSIM_Rhsim11G0066300 | 0.36 | 0.546 | 0.638 | 1.364 |
| RHSIM_Rhsim11G0029000 | 0.36 | 0.546 | 0.638 | 0.084 |
| novel.3487 | 0.36 | 0.546 | 0.638 | -0.478 |
| RHSIM_Rhsim13G0189900 | 0.36 | 0.546 | 0.638 | -0.473 |
| RHSIM_Rhsim07G0254300 | 0.36 | 0.546 | 0.638 | 0.077 |
| novel.1851 | 0.36 | 0.547 | 0.638 | 0.045 |
| RHSIM_Rhsim01G0024600 | 0.36 | 0.547 | 0.638 | 0.162 |
| RHSIM_Rhsim05G0054900 | 0.36 | 0.547 | 0.638 | -0.418 |
| RHSIM_Rhsim03G0007200 | 0.36 | 0.547 | 0.638 | -0.162 |
| novel.2091 | 0.36 | 0.547 | 0.638 | -0.378 |
| RHSIM_Rhsim08G0247100 | 0.36 | 0.547 | 0.638 | 1.128 |
| RHSIM_Rhsim12G0069700 | 0.36 | 0.547 | 0.638 | -0.113 |
| RHSIM_Rhsim11G0050600 | 0.36 | 0.547 | 0.638 | -0.838 |
| RHSIM_Rhsim10G0048600 | 0.36 | 0.547 | 0.638 | 0.531 |
| novel.2624 | 0.36 | 0.547 | 0.639 | -34.814 |
| novel.4117 | 0.36 | 0.547 | 0.639 | -0.325 |
| RHSIM_Rhsim02G0163000 | 0.36 | 0.547 | 0.639 | 1.200 |
| RHSIM_RhsimUnG0231600 | 0.36 | 0.547 | 0.639 | 0.156 |
| RHSIM_Rhsim12G0148300 | 0.36 | 0.547 | 0.639 | -0.053 |
| RHSIM_Rhsim04G0135900 | 0.36 | 0.547 | 0.639 | -0.049 |
| RHSIM_Rhsim12G0033100 | 0.36 | 0.547 | 0.639 | -0.748 |
| RHSIM_Rhsim06G0093900 | 0.36 | 0.548 | 0.639 | 0.081 |
| RHSIM_Rhsim11G0041400 | 0.36 | 0.548 | 0.639 | -0.825 |
| RHSIM_Rhsim13G0168500 | 0.36 | 0.548 | 0.639 | -0.583 |

| Gene | LRT statistic | P value | BH-adjusted P | log2FC (interaction) |
|---|---|---|---|---|
| RHSIM_Rhsim06G0120500 | 0.36 | 0.548 | 0.639 | -0.367 |
| novel.3294 | 0.36 | 0.548 | 0.639 | 0.597 |
| RHSIM_Rhsim02G0230700 | 0.36 | 0.548 | 0.639 | 0.897 |
| novel.3234 | 0.36 | 0.548 | 0.639 | 0.081 |
| RHSIM_RhsimUnG0201600 | 0.36 | 0.548 | 0.640 | 0.596 |
| RHSIM_Rhsim08G0053800 | 0.36 | 0.548 | 0.640 | 0.161 |
| RHSIM_Rhsim03G0016300 | 0.36 | 0.548 | 0.640 | -0.254 |
| novel.2176 | 0.36 | 0.549 | 0.640 | -0.593 |
| RHSIM_Rhsim01G0139600 | 0.36 | 0.549 | 0.640 | 0.867 |
| RHSIM_Rhsim08G0039000 | 0.36 | 0.549 | 0.640 | 0.080 |
| novel.1160 | 0.36 | 0.549 | 0.640 | -34.812 |
| RHSIM_Rhsim06G0211800 | 0.36 | 0.549 | 0.640 | 0.226 |
| novel.1023 | 0.36 | 0.549 | 0.640 | 0.046 |
| RHSIM_Rhsim05G0177400 | 0.36 | 0.549 | 0.641 | 0.060 |
| RHSIM_Rhsim01G0121500 | 0.36 | 0.549 | 0.641 | -0.818 |
| RHSIM_RhsimUnG0171100 | 0.36 | 0.549 | 0.641 | 1.282 |
| RHSIM_Rhsim02G0015300 | 0.36 | 0.550 | 0.641 | -0.279 |
| novel.2705 | 0.36 | 0.550 | 0.641 | 0.665 |
| novel.2538 | 0.36 | 0.550 | 0.641 | 0.678 |
| RHSIM_Rhsim03G0106300 | 0.36 | 0.550 | 0.641 | -0.226 |
| RHSIM_Rhsim12G0165200 | 0.36 | 0.550 | 0.641 | 0.184 |
| novel.2947 | 0.36 | 0.550 | 0.641 | 0.284 |
| RHSIM_Rhsim12G0109200 | 0.36 | 0.550 | 0.641 | -0.710 |
| RHSIM_RhsimUnG0019400 | 0.36 | 0.550 | 0.641 | -34.759 |
| novel.2236 | 0.36 | 0.551 | 0.642 | -0.160 |
| RHSIM_Rhsim01G0241400 | 0.36 | 0.551 | 0.642 | -0.882 |
| novel.4410 | 0.36 | 0.551 | 0.642 | 34.857 |
| RHSIM_Rhsim04G0002200 | 0.36 | 0.551 | 0.642 | -0.192 |
| RHSIM_Rhsim08G0110600 | 0.36 | 0.551 | 0.642 | 0.460 |
| RHSIM_Rhsim03G0217900 | 0.36 | 0.551 | 0.642 | 0.107 |
| RHSIM_Rhsim09G0020800 | 0.36 | 0.551 | 0.642 | 1.681 |
| RHSIM_Rhsim13G0059900 | 0.36 | 0.551 | 0.642 | 0.042 |

| Gene | LRT statistic | P value | BH-adjusted P | log2FC (interaction) |
|---|---|---|---|---|
| RHSIM_Rhsim08G0042800 | 0.36 | 0.551 | 0.642 | -0.545 |
| RHSIM_Rhsim11G0032800 | 0.36 | 0.551 | 0.642 | -1.330 |
| RHSIM_Rhsim08G0135900 | 0.35 | 0.551 | 0.643 | 0.154 |
| novel.3178 | 0.35 | 0.551 | 0.643 | 0.174 |
| RHSIM_RhsimUnG0031700 | 0.35 | 0.552 | 0.643 | -0.464 |
| RHSIM_Rhsim01G0007000 | 0.35 | 0.552 | 0.643 | 0.046 |
| RHSIM_Rhsim02G0255200 | 0.35 | 0.552 | 0.643 | -0.443 |
| RHSIM_Rhsim05G0034200 | 0.35 | 0.552 | 0.643 | 0.396 |
| RHSIM_Rhsim08G0250200 | 0.35 | 0.552 | 0.643 | 0.443 |
| RHSIM_Rhsim05G0001200 | 0.35 | 0.552 | 0.643 | -0.079 |
| RHSIM_Rhsim08G0230200 | 0.35 | 0.552 | 0.643 | 0.096 |
| RHSIM_Rhsim03G0183200 | 0.35 | 0.552 | 0.643 | -0.451 |
| RHSIM_Rhsim03G0232300 | 0.35 | 0.552 | 0.643 | 0.384 |
| RHSIM_Rhsim13G0233100 | 0.35 | 0.552 | 0.643 | -0.516 |
| RHSIM_Rhsim10G0180700 | 0.35 | 0.552 | 0.644 | -0.234 |
| RHSIM_Rhsim03G0048600 | 0.35 | 0.553 | 0.644 | 0.151 |
| RHSIM_Rhsim03G0202400 | 0.35 | 0.553 | 0.644 | 1.512 |
| novel.370 | 0.35 | 0.553 | 0.644 | 0.777 |
| RHSIM_Rhsim02G0090800 | 0.35 | 0.553 | 0.644 | -0.292 |
| RHSIM_Rhsim04G0140700 | 0.35 | 0.553 | 0.644 | -1.230 |
| RHSIM_Rhsim10G0047900 | 0.35 | 0.553 | 0.644 | -0.766 |
| RHSIM_RhsimUnG0061600 | 0.35 | 0.553 | 0.644 | 0.200 |
| RHSIM_Rhsim05G0083200 | 0.35 | 0.553 | 0.644 | 0.051 |
| RHSIM_Rhsim11G0184000 | 0.35 | 0.553 | 0.644 | -0.041 |
| RHSIM_Rhsim06G0049400 | 0.35 | 0.553 | 0.644 | -1.496 |
| novel.732 | 0.35 | 0.554 | 0.644 | 0.129 |
| RHSIM_Rhsim08G0117800 | 0.35 | 0.554 | 0.645 | 0.093 |
| RHSIM_Rhsim03G0108500 | 0.35 | 0.554 | 0.645 | -0.424 |
| RHSIM_RhsimUnG0056600 | 0.35 | 0.554 | 0.645 | -0.379 |
| RHSIM_Rhsim04G0214600 | 0.35 | 0.554 | 0.645 | -1.240 |
| RHSIM_Rhsim02G0206200 | 0.35 | 0.554 | 0.645 | -0.075 |
| novel.1611 | 0.35 | 0.554 | 0.645 | 0.185 |

| Gene | LRT statistic | P value | BH-adjusted P | log2FC (interaction) |
|---|---|---|---|---|
| novel.1886 | 0.35 | 0.554 | 0.645 | -0.293 |
| novel.3227 | 0.35 | 0.555 | 0.646 | 0.559 |
| RHSIM_Rhsim07G0107600 | 0.35 | 0.555 | 0.646 | -0.387 |
| RHSIM_Rhsim12G0163200 | 0.35 | 0.555 | 0.646 | 0.079 |
| novel.944 | 0.35 | 0.555 | 0.646 | -1.413 |
| RHSIM_RhsimUnG0124500 | 0.35 | 0.555 | 0.646 | -0.129 |
| RHSIM_Rhsim10G0214000 | 0.35 | 0.555 | 0.646 | -34.733 |
| RHSIM_Rhsim07G0103500 | 0.35 | 0.555 | 0.646 | -35.044 |
| RHSIM_Rhsim07G0081000 | 0.35 | 0.555 | 0.646 | 0.081 |
| RHSIM_RhsimUnG0210300 | 0.35 | 0.555 | 0.646 | -0.914 |
| RHSIM_Rhsim02G0223000 | 0.35 | 0.556 | 0.646 | 0.140 |
| RHSIM_Rhsim05G0066500 | 0.35 | 0.556 | 0.647 | -0.688 |
| RHSIM_Rhsim08G0065700 | 0.35 | 0.556 | 0.647 | -0.423 |
| RHSIM_Rhsim06G0028800 | 0.35 | 0.556 | 0.647 | 1.049 |
| RHSIM_Rhsim12G0149900 | 0.35 | 0.556 | 0.647 | -0.586 |
| RHSIM_Rhsim04G0168500 | 0.35 | 0.556 | 0.647 | -0.192 |
| novel.2481 | 0.35 | 0.556 | 0.647 | 0.415 |
| RHSIM_Rhsim05G0230800 | 0.35 | 0.556 | 0.647 | 1.141 |
| RHSIM_Rhsim01G0255800 | 0.35 | 0.556 | 0.647 | 0.148 |
| novel.1946 | 0.35 | 0.556 | 0.647 | -0.085 |
| novel.1092 | 0.34 | 0.557 | 0.648 | -0.218 |
| RHSIM_Rhsim07G0009300 | 0.34 | 0.557 | 0.648 | 0.100 |
| RHSIM_Rhsim06G0163100 | 0.34 | 0.557 | 0.648 | 1.095 |
| RHSIM_RhsimUnG0254500 | 0.34 | 0.557 | 0.648 | 0.467 |
| RHSIM_RhsimUnG0179300 | 0.34 | 0.558 | 0.648 | -1.044 |
| novel.3870 | 0.34 | 0.558 | 0.648 | 0.069 |
| novel.3985 | 0.34 | 0.558 | 0.648 | 0.136 |
| RHSIM_Rhsim08G0214500 | 0.34 | 0.558 | 0.649 | -34.599 |
| RHSIM_Rhsim04G0201000 | 0.34 | 0.558 | 0.649 | 1.105 |
| RHSIM_Rhsim04G0001700 | 0.34 | 0.558 | 0.649 | 0.137 |
| RHSIM_Rhsim01G0222400 | 0.34 | 0.558 | 0.649 | 0.721 |
| RHSIM_Rhsim03G0259000 | 0.34 | 0.558 | 0.649 | -0.728 |

| Gene | LRT statistic | P value | BH-adjusted P | log2FC (interaction) |
|---|---|---|---|---|
| RHSIM_Rhsim02G0254200 | 0.34 | 0.558 | 0.649 | 0.900 |
| RHSIM_Rhsim08G0152300 | 0.34 | 0.558 | 0.649 | 0.204 |
| RHSIM_Rhsim07G0243200 | 0.34 | 0.558 | 0.649 | -0.178 |
| RHSIM_Rhsim10G0173300 | 0.34 | 0.559 | 0.650 | 0.188 |
| novel.3233 | 0.34 | 0.559 | 0.650 | -0.476 |
| RHSIM_Rhsim03G0058200 | 0.34 | 0.559 | 0.650 | 0.722 |
| RHSIM_Rhsim12G0103600 | 0.34 | 0.559 | 0.650 | 0.195 |
| RHSIM_Rhsim02G0069500 | 0.34 | 0.560 | 0.650 | -0.451 |
| RHSIM_Rhsim12G0072600 | 0.34 | 0.560 | 0.650 | 0.695 |
| RHSIM_Rhsim12G0212900 | 0.34 | 0.560 | 0.650 | -0.065 |
| RHSIM_Rhsim11G0138800 | 0.34 | 0.560 | 0.650 | 0.740 |
| novel.3403 | 0.34 | 0.560 | 0.651 | 0.374 |
| RHSIM_Rhsim03G0015600 | 0.34 | 0.560 | 0.651 | -0.903 |
| RHSIM_Rhsim08G0126100 | 0.34 | 0.560 | 0.651 | -0.361 |
| novel.3009 | 0.34 | 0.560 | 0.651 | 34.612 |
| RHSIM_Rhsim04G0141300 | 0.34 | 0.560 | 0.651 | 1.292 |
| RHSIM_Rhsim02G0033700 | 0.34 | 0.560 | 0.651 | -0.861 |
| novel.2584 | 0.34 | 0.560 | 0.651 | 0.379 |
| RHSIM_Rhsim08G0170700 | 0.34 | 0.560 | 0.651 | 0.795 |
| RHSIM_Rhsim07G0247300 | 0.34 | 0.560 | 0.651 | 0.183 |
| RHSIM_Rhsim01G0226800 | 0.34 | 0.560 | 0.651 | -0.429 |
| novel.3099 | 0.34 | 0.561 | 0.651 | -0.923 |
| RHSIM_Rhsim08G0203700 | 0.34 | 0.561 | 0.651 | -0.350 |
| RHSIM_Rhsim10G0111300 | 0.34 | 0.561 | 0.651 | -0.821 |
| RHSIM_RhsimUnG0168700 | 0.34 | 0.561 | 0.651 | -0.276 |
| RHSIM_Rhsim06G0048200 | 0.34 | 0.561 | 0.651 | -0.182 |
| RHSIM_Rhsim01G0176000 | 0.34 | 0.561 | 0.652 | 0.084 |
| RHSIM_Rhsim07G0212200 | 0.34 | 0.561 | 0.652 | 0.043 |
| novel.861 | 0.34 | 0.561 | 0.652 | 0.224 |
| RHSIM_Rhsim06G0037700 | 0.34 | 0.562 | 0.652 | 0.260 |
| RHSIM_Rhsim09G0110400 | 0.34 | 0.562 | 0.652 | -0.059 |
| RHSIM_Rhsim13G0217900 | 0.34 | 0.562 | 0.652 | 0.854 |

| Gene | LRT statistic | P value | BH-adjusted P | log2FC (interaction) |
|---|---|---|---|---|
| RHSIM_Rhsim06G0106600 | 0.34 | 0.562 | 0.652 | 0.477 |
| RHSIM_Rhsim06G0146600 | 0.34 | 0.562 | 0.653 | -34.882 |
| RHSIM_Rhsim08G0123000 | 0.34 | 0.562 | 0.653 | 1.033 |
| RHSIM_Rhsim01G0013200 | 0.34 | 0.562 | 0.653 | 1.570 |
| novel.2698 | 0.34 | 0.562 | 0.653 | -0.524 |
| RHSIM_RhsimUnG0157000 | 0.34 | 0.563 | 0.653 | -0.392 |
| novel.2857 | 0.33 | 0.563 | 0.654 | -0.962 |
| RHSIM_Rhsim13G0148500 | 0.33 | 0.563 | 0.654 | -0.459 |
| RHSIM_Rhsim13G0166300 | 0.33 | 0.563 | 0.654 | -0.234 |
| RHSIM_Rhsim07G0245300 | 0.33 | 0.564 | 0.654 | -0.117 |
| RHSIM_Rhsim06G0015600 | 0.33 | 0.564 | 0.654 | -0.183 |
| RHSIM_Rhsim06G0102600 | 0.33 | 0.564 | 0.654 | 0.606 |
| RHSIM_Rhsim03G0017300 | 0.33 | 0.564 | 0.654 | 0.272 |
| RHSIM_Rhsim08G0211800 | 0.33 | 0.564 | 0.654 | -0.202 |
| RHSIM_Rhsim13G0010800 | 0.33 | 0.564 | 0.654 | -1.654 |
| RHSIM_Rhsim06G0122500 | 0.33 | 0.564 | 0.654 | -0.318 |
| novel.4022 | 0.33 | 0.564 | 0.654 | 0.738 |
| RHSIM_RhsimUnG0059800 | 0.33 | 0.564 | 0.654 | -0.559 |
| RHSIM_Rhsim01G0010800 | 0.33 | 0.564 | 0.654 | 0.687 |
| RHSIM_Rhsim08G0220500 | 0.33 | 0.564 | 0.654 | -0.041 |
| novel.286 | 0.33 | 0.564 | 0.654 | 1.249 |
| RHSIM_Rhsim01G0017800 | 0.33 | 0.564 | 0.654 | 0.651 |
| RHSIM_Rhsim05G0003800 | 0.33 | 0.564 | 0.654 | 0.045 |
| novel.454 | 0.33 | 0.564 | 0.654 | -0.135 |
| RHSIM_Rhsim13G0050400 | 0.33 | 0.564 | 0.654 | -0.734 |
| RHSIM_Rhsim09G0007400 | 0.33 | 0.564 | 0.654 | 0.424 |
| RHSIM_Rhsim01G0264300 | 0.33 | 0.565 | 0.655 | 0.126 |
| RHSIM_Rhsim01G0132100 | 0.33 | 0.565 | 0.655 | 0.077 |
| RHSIM_Rhsim08G0125800 | 0.33 | 0.565 | 0.655 | 1.706 |
| RHSIM_Rhsim08G0019500 | 0.33 | 0.565 | 0.655 | -0.415 |
| novel.4225 | 0.33 | 0.565 | 0.655 | 0.715 |
| RHSIM_Rhsim01G0190500 | 0.33 | 0.565 | 0.655 | -0.066 |

| Gene | LRT statistic | P value | BH-adjusted P | log2FC (interaction) |
|---|---|---|---|---|
| RHSIM_Rhsim12G0088000 | 0.33 | 0.565 | 0.655 | 0.165 |
| RHSIM_Rhsim06G0229600 | 0.33 | 0.565 | 0.655 | 0.088 |
| RHSIM_Rhsim04G0092700 | 0.33 | 0.565 | 0.655 | -0.497 |
| RHSIM_Rhsim09G0167200 | 0.33 | 0.565 | 0.655 | 0.066 |
| RHSIM_Rhsim07G0065700 | 0.33 | 0.565 | 0.655 | 0.950 |
| RHSIM_Rhsim08G0024800 | 0.33 | 0.565 | 0.655 | 0.907 |
| RHSIM_Rhsim03G0027500 | 0.33 | 0.565 | 0.655 | 0.147 |
| RHSIM_Rhsim04G0174100 | 0.33 | 0.566 | 0.655 | 0.410 |
| RHSIM_Rhsim08G0167200 | 0.33 | 0.566 | 0.656 | 0.129 |
| RHSIM_Rhsim11G0187200 | 0.33 | 0.566 | 0.656 | 0.049 |
| RHSIM_Rhsim08G0018100 | 0.33 | 0.566 | 0.656 | -0.789 |
| novel.3358 | 0.33 | 0.566 | 0.656 | -0.552 |
| RHSIM_Rhsim12G0089000 | 0.33 | 0.566 | 0.656 | 0.977 |
| RHSIM_Rhsim02G0045400 | 0.33 | 0.566 | 0.656 | 1.650 |
| RHSIM_Rhsim04G0029700 | 0.33 | 0.566 | 0.656 | -0.178 |
| novel.3314 | 0.33 | 0.567 | 0.656 | 0.438 |
| novel.4209 | 0.33 | 0.567 | 0.656 | 0.371 |
| RHSIM_Rhsim03G0212200 | 0.33 | 0.567 | 0.656 | 0.060 |
| RHSIM_Rhsim03G0041400 | 0.33 | 0.567 | 0.657 | -34.542 |
| RHSIM_Rhsim04G0147700 | 0.33 | 0.567 | 0.657 | -0.064 |
| RHSIM_Rhsim13G0214500 | 0.33 | 0.567 | 0.657 | 0.193 |
| RHSIM_Rhsim10G0152900 | 0.33 | 0.567 | 0.657 | 0.972 |
| novel.394 | 0.33 | 0.567 | 0.657 | -0.680 |
| RHSIM_Rhsim04G0209300 | 0.33 | 0.567 | 0.657 | -0.957 |
| novel.1566 | 0.33 | 0.567 | 0.657 | 0.499 |
| RHSIM_Rhsim10G0006500 | 0.33 | 0.567 | 0.657 | -0.068 |
| RHSIM_Rhsim11G0078800 | 0.33 | 0.568 | 0.657 | -0.444 |
| RHSIM_Rhsim01G0130800 | 0.33 | 0.568 | 0.657 | -0.109 |
| RHSIM_Rhsim10G0201500 | 0.33 | 0.568 | 0.657 | -34.719 |
| RHSIM_Rhsim06G0137000 | 0.33 | 0.568 | 0.658 | -0.291 |
| RHSIM_Rhsim07G0039700 | 0.33 | 0.568 | 0.658 | 0.031 |
| RHSIM_Rhsim05G0229400 | 0.33 | 0.568 | 0.658 | 0.875 |

| Gene | LRT statistic | P value | BH-adjusted P | log2FC (interaction) |
|---|---|---|---|---|
| RHSIM_Rhsim03G0119200 | 0.33 | 0.568 | 0.658 | 0.079 |
| novel.2298 | 0.33 | 0.568 | 0.658 | -1.055 |
| RHSIM_Rhsim08G0095500 | 0.33 | 0.568 | 0.658 | 0.258 |
| RHSIM_Rhsim13G0202800 | 0.33 | 0.568 | 0.658 | -1.235 |
| novel.1872 | 0.33 | 0.568 | 0.658 | 0.360 |
| RHSIM_Rhsim07G0030300 | 0.33 | 0.569 | 0.658 | -0.124 |
| RHSIM_RhsimUnG0138200 | 0.33 | 0.569 | 0.658 | 0.300 |
| RHSIM_Rhsim04G0158700 | 0.33 | 0.569 | 0.658 | 1.023 |
| RHSIM_Rhsim05G0117100 | 0.32 | 0.569 | 0.658 | -0.096 |
| RHSIM_RhsimUnG0173300 | 0.32 | 0.569 | 0.658 | 1.357 |
| RHSIM_Rhsim05G0082300 | 0.32 | 0.569 | 0.658 | 0.248 |
| RHSIM_Rhsim06G0149300 | 0.32 | 0.569 | 0.658 | 0.038 |
| RHSIM_Rhsim06G0242600 | 0.32 | 0.569 | 0.659 | 0.072 |
| RHSIM_Rhsim02G0061200 | 0.32 | 0.569 | 0.659 | 0.405 |
| RHSIM_Rhsim12G0098500 | 0.32 | 0.569 | 0.659 | 0.772 |
| RHSIM_Rhsim05G0191100 | 0.32 | 0.569 | 0.659 | 0.121 |
| RHSIM_Rhsim05G0181200 | 0.32 | 0.569 | 0.659 | 0.136 |
| RHSIM_Rhsim05G0017800 | 0.32 | 0.569 | 0.659 | -0.149 |
| RHSIM_Rhsim04G0118100 | 0.32 | 0.569 | 0.659 | 0.342 |
| RHSIM_Rhsim13G0088100 | 0.32 | 0.570 | 0.659 | -0.678 |
| RHSIM_Rhsim06G0084600 | 0.32 | 0.570 | 0.659 | 0.040 |
| RHSIM_Rhsim08G0198300 | 0.32 | 0.570 | 0.659 | -0.213 |
| RHSIM_Rhsim02G0180700 | 0.32 | 0.570 | 0.659 | 0.068 |
| RHSIM_Rhsim01G0104300 | 0.32 | 0.570 | 0.659 | 0.375 |
| novel.53 | 0.32 | 0.570 | 0.659 | 0.574 |
| RHSIM_Rhsim02G0118200 | 0.32 | 0.570 | 0.659 | -0.264 |
| RHSIM_Rhsim02G0200100 | 0.32 | 0.570 | 0.659 | 0.057 |
| RHSIM_Rhsim12G0135200 | 0.32 | 0.570 | 0.659 | -1.163 |
| RHSIM_Rhsim11G0078000 | 0.32 | 0.570 | 0.659 | 0.714 |
| RHSIM_Rhsim07G0085000 | 0.32 | 0.570 | 0.659 | 0.293 |
| RHSIM_Rhsim07G0231600 | 0.32 | 0.570 | 0.660 | -0.911 |
| RHSIM_Rhsim10G0045300 | 0.32 | 0.570 | 0.660 | 0.337 |

| Gene | LRT statistic | P value | BH-adjusted P | log2FC (interaction) |
|---|---|---|---|---|
| RHSIM_Rhsim03G0061600 | 0.32 | 0.571 | 0.660 | -0.359 |
| RHSIM_Rhsim08G0213800 | 0.32 | 0.571 | 0.660 | 0.805 |
| RHSIM_Rhsim06G0044100 | 0.32 | 0.571 | 0.660 | 0.546 |
| novel.2841 | 0.32 | 0.571 | 0.660 | 0.237 |
| RHSIM_Rhsim13G0071800 | 0.32 | 0.571 | 0.660 | -34.493 |
| RHSIM_Rhsim12G0166400 | 0.32 | 0.571 | 0.660 | 0.115 |
| RHSIM_Rhsim02G0179500 | 0.32 | 0.571 | 0.661 | -0.093 |
| RHSIM_Rhsim03G0177400 | 0.32 | 0.571 | 0.661 | -34.756 |
| RHSIM_Rhsim04G0101600 | 0.32 | 0.572 | 0.661 | 0.329 |
| RHSIM_RhsimUnG0079500 | 0.32 | 0.572 | 0.661 | 0.682 |
| RHSIM_Rhsim08G0050400 | 0.32 | 0.572 | 0.661 | -0.773 |
| RHSIM_Rhsim01G0186700 | 0.32 | 0.572 | 0.661 | -0.087 |
| RHSIM_Rhsim02G0255000 | 0.32 | 0.572 | 0.662 | -0.242 |
| RHSIM_Rhsim04G0209200 | 0.32 | 0.572 | 0.662 | -1.472 |
| RHSIM_RhsimUnG0132600 | 0.32 | 0.573 | 0.662 | 0.429 |
| RHSIM_Rhsim01G0011000 | 0.32 | 0.573 | 0.662 | 1.146 |
| RHSIM_Rhsim06G0052700 | 0.32 | 0.573 | 0.662 | -0.036 |
| RHSIM_Rhsim07G0029100 | 0.32 | 0.573 | 0.663 | -0.516 |
| RHSIM_Rhsim13G0209200 | 0.32 | 0.573 | 0.663 | 0.078 |
| RHSIM_Rhsim01G0185600 | 0.32 | 0.574 | 0.663 | 0.355 |
| novel.913 | 0.32 | 0.574 | 0.663 | 0.543 |
| RHSIM_Rhsim07G0018600 | 0.32 | 0.574 | 0.663 | 0.209 |
| RHSIM_Rhsim02G0142200 | 0.32 | 0.574 | 0.663 | 1.621 |
| RHSIM_Rhsim02G0166200 | 0.32 | 0.574 | 0.664 | 0.728 |
| RHSIM_Rhsim09G0157900 | 0.32 | 0.574 | 0.664 | 0.052 |
| RHSIM_Rhsim03G0081800 | 0.32 | 0.574 | 0.664 | -0.053 |
| RHSIM_Rhsim05G0205800 | 0.32 | 0.574 | 0.664 | 1.315 |
| RHSIM_Rhsim12G0204700 | 0.32 | 0.575 | 0.664 | 0.037 |
| RHSIM_Rhsim02G0206000 | 0.32 | 0.575 | 0.664 | 0.124 |
| novel.2010 | 0.31 | 0.575 | 0.664 | 0.848 |
| RHSIM_Rhsim01G0239800 | 0.31 | 0.575 | 0.664 | -0.120 |
| RHSIM_Rhsim05G0125800 | 0.31 | 0.575 | 0.664 | 0.106 |

| Gene | LRT statistic | P value | BH-adjusted P | log2FC (interaction) |
|---|---|---|---|---|
| RHSIM_RhsimUnG0109200 | 0.31 | 0.575 | 0.664 | -0.117 |
| RHSIM_Rhsim10G0053000 | 0.31 | 0.575 | 0.664 | 0.219 |
| RHSIM_Rhsim04G0107200 | 0.31 | 0.575 | 0.664 | -0.617 |
| RHSIM_Rhsim04G0169300 | 0.31 | 0.575 | 0.664 | 0.092 |
| RHSIM_Rhsim05G0010200 | 0.31 | 0.575 | 0.664 | -34.646 |
| novel.3361 | 0.31 | 0.575 | 0.664 | -0.837 |
| RHSIM_Rhsim01G0164900 | 0.31 | 0.575 | 0.664 | 0.058 |
| novel.736 | 0.31 | 0.575 | 0.664 | -0.523 |
| RHSIM_Rhsim04G0103400 | 0.31 | 0.575 | 0.664 | -0.393 |
| RHSIM_RhsimUnG0220500 | 0.31 | 0.575 | 0.664 | 1.287 |
| RHSIM_Rhsim07G0099500 | 0.31 | 0.576 | 0.664 | -0.193 |
| RHSIM_Rhsim13G0230900 | 0.31 | 0.576 | 0.664 | -0.619 |
| RHSIM_Rhsim05G0179100 | 0.31 | 0.576 | 0.664 | 0.745 |
| RHSIM_Rhsim13G0126500 | 0.31 | 0.576 | 0.665 | -34.681 |
| RHSIM_Rhsim04G0069300 | 0.31 | 0.576 | 0.665 | 0.064 |
| RHSIM_Rhsim06G0136200 | 0.31 | 0.576 | 0.665 | -0.117 |
| RHSIM_Rhsim08G0045500 | 0.31 | 0.576 | 0.665 | 1.294 |
| RHSIM_Rhsim03G0055600 | 0.31 | 0.576 | 0.665 | -0.838 |
| RHSIM_Rhsim02G0227100 | 0.31 | 0.576 | 0.665 | 0.472 |
| RHSIM_Rhsim04G0204800 | 0.31 | 0.576 | 0.665 | -0.446 |
| RHSIM_Rhsim09G0014900 | 0.31 | 0.577 | 0.665 | 1.618 |
| novel.463 | 0.31 | 0.577 | 0.665 | 0.401 |
| RHSIM_Rhsim04G0061400 | 0.31 | 0.577 | 0.666 | -0.291 |
| novel.4231 | 0.31 | 0.577 | 0.666 | -0.524 |
| novel.2756 | 0.31 | 0.577 | 0.666 | 1.315 |
| RHSIM_Rhsim13G0207000 | 0.31 | 0.577 | 0.666 | -1.214 |
| RHSIM_Rhsim13G0173000 | 0.31 | 0.577 | 0.666 | -0.233 |
| RHSIM_Rhsim06G0221200 | 0.31 | 0.577 | 0.666 | -0.594 |
| RHSIM_Rhsim11G0094200 | 0.31 | 0.577 | 0.666 | 0.072 |
| RHSIM_Rhsim07G0092800 | 0.31 | 0.578 | 0.666 | 0.987 |
| novel.1397 | 0.31 | 0.578 | 0.667 | 0.312 |
| RHSIM_Rhsim12G0184100 | 0.31 | 0.578 | 0.667 | -0.353 |

| Gene | LRT statistic | P value | BH-adjusted P | log2FC (interaction) |
|---|---|---|---|---|
| RHSIM_Rhsim01G0138700 | 0.31 | 0.578 | 0.667 | 0.360 |
| RHSIM_Rhsim04G0118700 | 0.31 | 0.578 | 0.667 | 0.047 |
| RHSIM_Rhsim10G0205000 | 0.31 | 0.578 | 0.667 | -0.169 |
| RHSIM_RhsimUnG0166800 | 0.31 | 0.578 | 0.667 | 0.474 |
| RHSIM_Rhsim07G0116800 | 0.31 | 0.578 | 0.667 | -0.417 |
| novel.497 | 0.31 | 0.578 | 0.667 | -0.280 |
| RHSIM_RhsimUnG0204400 | 0.31 | 0.578 | 0.667 | -34.469 |
| RHSIM_Rhsim09G0036100 | 0.31 | 0.579 | 0.667 | -1.454 |
| RHSIM_Rhsim08G0247400 | 0.31 | 0.579 | 0.668 | 0.851 |
| RHSIM_Rhsim05G0016200 | 0.31 | 0.579 | 0.668 | -1.522 |
| RHSIM_Rhsim09G0134700 | 0.31 | 0.579 | 0.668 | 2.479 |
| RHSIM_Rhsim11G0128600 | 0.31 | 0.579 | 0.668 | -1.093 |
| novel.1326 | 0.31 | 0.580 | 0.668 | -0.281 |
| RHSIM_Rhsim12G0008500 | 0.31 | 0.580 | 0.668 | 0.152 |
| RHSIM_Rhsim02G0091200 | 0.31 | 0.580 | 0.668 | 0.486 |
| novel.17 | 0.31 | 0.580 | 0.668 | 0.531 |
| RHSIM_Rhsim03G0088000 | 0.31 | 0.580 | 0.668 | 0.266 |
| RHSIM_Rhsim03G0124500 | 0.31 | 0.580 | 0.668 | 0.156 |
| RHSIM_Rhsim03G0145300 | 0.31 | 0.580 | 0.668 | -34.567 |
| novel.1400 | 0.31 | 0.580 | 0.669 | -0.470 |
| RHSIM_Rhsim09G0003400 | 0.31 | 0.580 | 0.669 | -0.309 |
| RHSIM_Rhsim04G0081200 | 0.31 | 0.580 | 0.669 | 0.515 |
| RHSIM_Rhsim02G0038600 | 0.31 | 0.580 | 0.669 | -0.786 |
| RHSIM_Rhsim03G0035800 | 0.31 | 0.580 | 0.669 | 0.085 |
| RHSIM_Rhsim04G0148100 | 0.31 | 0.581 | 0.669 | 0.553 |
| RHSIM_Rhsim01G0138600 | 0.30 | 0.581 | 0.669 | 0.377 |
| RHSIM_Rhsim02G0176200 | 0.30 | 0.581 | 0.669 | 0.177 |
| RHSIM_Rhsim13G0025700 | 0.30 | 0.581 | 0.670 | -0.054 |
| RHSIM_Rhsim01G0103800 | 0.30 | 0.581 | 0.670 | 1.154 |
| RHSIM_Rhsim09G0003000 | 0.30 | 0.581 | 0.670 | -0.725 |
| RHSIM_Rhsim01G0216100 | 0.30 | 0.581 | 0.670 | -0.110 |
| RHSIM_Rhsim04G0165600 | 0.30 | 0.581 | 0.670 | -0.189 |

| Gene | LRT statistic | P value | BH-adjusted P | log2FC (interaction) |
|---|---|---|---|---|
| RHSIM_Rhsim10G0150400 | 0.30 | 0.581 | 0.670 | -0.666 |
| RHSIM_Rhsim03G0055000 | 0.30 | 0.582 | 0.670 | 1.290 |
| RHSIM_Rhsim08G0209500 | 0.30 | 0.582 | 0.670 | 0.236 |
| RHSIM_Rhsim08G0124500 | 0.30 | 0.582 | 0.670 | -0.084 |
| RHSIM_Rhsim02G0009400 | 0.30 | 0.582 | 0.670 | 0.525 |
| RHSIM_Rhsim13G0084300 | 0.30 | 0.582 | 0.670 | -0.061 |
| RHSIM_RhsimUnG0073800 | 0.30 | 0.583 | 0.671 | -0.220 |
| RHSIM_Rhsim04G0030400 | 0.30 | 0.583 | 0.671 | -0.251 |
| RHSIM_Rhsim01G0085500 | 0.30 | 0.583 | 0.671 | -0.357 |
| RHSIM_Rhsim01G0231900 | 0.30 | 0.583 | 0.671 | 0.977 |
| RHSIM_Rhsim13G0113300 | 0.30 | 0.583 | 0.671 | 0.063 |
| novel.2599 | 0.30 | 0.583 | 0.671 | -34.207 |
| RHSIM_Rhsim05G0183500 | 0.30 | 0.583 | 0.671 | -1.127 |
| RHSIM_Rhsim08G0202900 | 0.30 | 0.583 | 0.672 | 0.487 |
| RHSIM_RhsimUnG0084300 | 0.30 | 0.583 | 0.672 | -1.276 |
| RHSIM_Rhsim09G0121800 | 0.30 | 0.583 | 0.672 | 0.127 |
| novel.4447 | 0.30 | 0.584 | 0.672 | -0.492 |
| RHSIM_Rhsim09G0073200 | 0.30 | 0.584 | 0.672 | -0.058 |
| RHSIM_Rhsim08G0022300 | 0.30 | 0.584 | 0.672 | -0.034 |
| RHSIM_Rhsim08G0004900 | 0.30 | 0.584 | 0.672 | -0.068 |
| RHSIM_Rhsim08G0225600 | 0.30 | 0.584 | 0.672 | 0.070 |
| RHSIM_Rhsim10G0141800 | 0.30 | 0.584 | 0.672 | -0.081 |
| novel.2879 | 0.30 | 0.584 | 0.673 | 1.179 |
| novel.3726 | 0.30 | 0.584 | 0.673 | -0.396 |
| RHSIM_Rhsim03G0257900 | 0.30 | 0.584 | 0.673 | -0.103 |
| RHSIM_Rhsim02G0225700 | 0.30 | 0.585 | 0.673 | 0.638 |
| RHSIM_Rhsim11G0017400 | 0.30 | 0.585 | 0.673 | -0.144 |
| RHSIM_Rhsim05G0169200 | 0.30 | 0.585 | 0.673 | -0.677 |
| RHSIM_Rhsim11G0154700 | 0.30 | 0.585 | 0.673 | 0.867 |
| novel.1214 | 0.30 | 0.585 | 0.674 | -0.859 |
| RHSIM_Rhsim08G0038300 | 0.30 | 0.585 | 0.674 | -0.079 |
| RHSIM_Rhsim08G0244700 | 0.30 | 0.586 | 0.674 | -0.449 |

| Gene | LRT statistic | P value | BH-adjusted P | log2FC (interaction) |
|---|---|---|---|---|
| novel.1514 | 0.30 | 0.586 | 0.674 | -1.056 |
| RHSIM_Rhsim05G0222400 | 0.30 | 0.586 | 0.674 | -1.099 |
| RHSIM_Rhsim02G0253300 | 0.30 | 0.586 | 0.674 | -1.353 |
| RHSIM_Rhsim07G0233700 | 0.30 | 0.586 | 0.674 | 1.069 |
| RHSIM_Rhsim13G0039100 | 0.30 | 0.586 | 0.674 | -0.075 |
| RHSIM_Rhsim13G0060400 | 0.30 | 0.586 | 0.674 | 0.077 |
| RHSIM_Rhsim12G0159300 | 0.30 | 0.586 | 0.674 | -0.670 |
| RHSIM_Rhsim06G0157100 | 0.30 | 0.586 | 0.674 | -1.261 |
| RHSIM_Rhsim03G0249200 | 0.30 | 0.586 | 0.674 | -34.508 |
| novel.2844 | 0.30 | 0.586 | 0.674 | -0.627 |
| RHSIM_Rhsim01G0138800 | 0.30 | 0.586 | 0.674 | 0.069 |
| RHSIM_RhsimUnG0218100 | 0.30 | 0.586 | 0.674 | -34.264 |
| novel.1247 | 0.30 | 0.587 | 0.675 | 0.589 |
| novel.3991 | 0.30 | 0.587 | 0.675 | 0.418 |
| RHSIM_Rhsim13G0212700 | 0.29 | 0.587 | 0.675 | 0.270 |
| RHSIM_Rhsim02G0121200 | 0.29 | 0.587 | 0.675 | 0.745 |
| RHSIM_Rhsim12G0146100 | 0.29 | 0.587 | 0.675 | 0.197 |
| RHSIM_Rhsim08G0033700 | 0.29 | 0.587 | 0.675 | 0.560 |
| RHSIM_Rhsim05G0035500 | 0.29 | 0.587 | 0.675 | -0.137 |
| RHSIM_Rhsim01G0251600 | 0.29 | 0.587 | 0.675 | 0.739 |
| RHSIM_Rhsim02G0207000 | 0.29 | 0.588 | 0.676 | 1.020 |
| novel.3310 | 0.29 | 0.588 | 0.676 | 0.451 |
| novel.103 | 0.29 | 0.588 | 0.676 | -0.179 |
| RHSIM_Rhsim08G0118400 | 0.29 | 0.588 | 0.676 | -0.182 |
| RHSIM_Rhsim10G0177000 | 0.29 | 0.588 | 0.676 | 0.049 |
| RHSIM_Rhsim08G0028900 | 0.29 | 0.588 | 0.676 | -0.068 |
| RHSIM_Rhsim01G0085400 | 0.29 | 0.589 | 0.676 | -0.650 |
| RHSIM_Rhsim10G0201400 | 0.29 | 0.589 | 0.676 | 0.208 |
| RHSIM_Rhsim08G0109400 | 0.29 | 0.589 | 0.676 | -0.066 |
| novel.3219 | 0.29 | 0.589 | 0.676 | 0.184 |
| RHSIM_Rhsim04G0144700 | 0.29 | 0.589 | 0.676 | 1.236 |
| novel.2586 | 0.29 | 0.589 | 0.676 | 0.463 |

| Gene | LRT statistic | P value | BH-adjusted P | log2FC (interaction) |
|---|---|---|---|---|
| novel.3725 | 0.29 | 0.589 | 0.677 | -0.903 |
| RHSIM_RhsimUnG0098900 | 0.29 | 0.589 | 0.677 | -0.926 |
| RHSIM_Rhsim13G0132700 | 0.29 | 0.589 | 0.677 | -1.137 |
| RHSIM_Rhsim01G0221700 | 0.29 | 0.589 | 0.677 | 0.759 |
| novel.587 | 0.29 | 0.589 | 0.677 | -0.219 |
| RHSIM_Rhsim04G0005400 | 0.29 | 0.589 | 0.677 | -0.114 |
| RHSIM_Rhsim13G0094800 | 0.29 | 0.589 | 0.677 | -0.050 |
| RHSIM_Rhsim10G0043800 | 0.29 | 0.589 | 0.677 | -0.037 |
| novel.1124 | 0.29 | 0.589 | 0.677 | 0.178 |
| RHSIM_Rhsim07G0155500 | 0.29 | 0.589 | 0.677 | -0.495 |
| RHSIM_Rhsim12G0084600 | 0.29 | 0.590 | 0.677 | 1.590 |
| novel.317 | 0.29 | 0.590 | 0.677 | 0.256 |
| RHSIM_Rhsim13G0220300 | 0.29 | 0.590 | 0.677 | -0.608 |
| RHSIM_Rhsim04G0053600 | 0.29 | 0.590 | 0.677 | 0.546 |
| RHSIM_Rhsim05G0130700 | 0.29 | 0.590 | 0.677 | 0.599 |
| RHSIM_Rhsim10G0071900 | 0.29 | 0.590 | 0.677 | 1.043 |
| RHSIM_Rhsim13G0232500 | 0.29 | 0.590 | 0.678 | -0.442 |
| RHSIM_Rhsim05G0013000 | 0.29 | 0.590 | 0.678 | 0.132 |
| RHSIM_Rhsim02G0011200 | 0.29 | 0.590 | 0.678 | 1.519 |
| RHSIM_Rhsim08G0031300 | 0.29 | 0.591 | 0.678 | 0.055 |
| RHSIM_Rhsim12G0071200 | 0.29 | 0.591 | 0.678 | -0.777 |
| RHSIM_Rhsim12G0020100 | 0.29 | 0.591 | 0.678 | 0.486 |
| RHSIM_Rhsim01G0147800 | 0.29 | 0.591 | 0.679 | -0.234 |
| RHSIM_Rhsim04G0133900 | 0.29 | 0.591 | 0.679 | 0.095 |
| RHSIM_Rhsim03G0219200 | 0.29 | 0.591 | 0.679 | 0.630 |
| RHSIM_RhsimUnG0216600 | 0.29 | 0.591 | 0.679 | -0.197 |
| RHSIM_RhsimUnG0025700 | 0.29 | 0.591 | 0.679 | 0.067 |
| novel.963 | 0.29 | 0.592 | 0.679 | -0.692 |
| RHSIM_Rhsim04G0203600 | 0.29 | 0.592 | 0.679 | -0.052 |
| RHSIM_Rhsim12G0146400 | 0.29 | 0.592 | 0.679 | 0.947 |
| novel.3430 | 0.29 | 0.592 | 0.679 | -0.343 |
| RHSIM_Rhsim04G0007400 | 0.29 | 0.592 | 0.680 | -0.054 |

| Gene | LRT statistic | P value | BH-adjusted P | log2FC (interaction) |
|---|---|---|---|---|
| RHSIM_Rhsim11G0007300 | 0.29 | 0.592 | 0.680 | -0.416 |
| RHSIM_Rhsim03G0179500 | 0.29 | 0.592 | 0.680 | -0.033 |
| RHSIM_Rhsim13G0158700 | 0.29 | 0.593 | 0.680 | -34.502 |
| RHSIM_Rhsim11G0053900 | 0.29 | 0.593 | 0.680 | 0.123 |
| novel.4167 | 0.29 | 0.593 | 0.681 | 0.527 |
| RHSIM_Rhsim03G0184200 | 0.29 | 0.593 | 0.681 | 1.160 |
| RHSIM_RhsimUnG0134600 | 0.28 | 0.594 | 0.681 | 1.283 |
| RHSIM_RhsimUnG0158400 | 0.28 | 0.594 | 0.681 | -0.364 |
| RHSIM_Rhsim01G0091700 | 0.28 | 0.594 | 0.681 | 0.052 |
| novel.2427 | 0.28 | 0.594 | 0.681 | 0.413 |
| RHSIM_Rhsim06G0017500 | 0.28 | 0.594 | 0.681 | -34.274 |
| RHSIM_Rhsim11G0145000 | 0.28 | 0.594 | 0.681 | -0.203 |
| RHSIM_Rhsim01G0255900 | 0.28 | 0.594 | 0.681 | 0.047 |
| RHSIM_RhsimUnG0194000 | 0.28 | 0.594 | 0.681 | 0.050 |
| RHSIM_Rhsim08G0084700 | 0.28 | 0.594 | 0.681 | -0.526 |
| RHSIM_Rhsim12G0170900 | 0.28 | 0.594 | 0.681 | 34.235 |
| RHSIM_Rhsim07G0091200 | 0.28 | 0.594 | 0.682 | 0.306 |
| RHSIM_Rhsim07G0083700 | 0.28 | 0.594 | 0.682 | -0.086 |
| novel.3296 | 0.28 | 0.595 | 0.682 | 0.502 |
| RHSIM_Rhsim06G0009000 | 0.28 | 0.595 | 0.682 | -0.427 |
| novel.233 | 0.28 | 0.595 | 0.682 | -0.339 |
| RHSIM_Rhsim12G0031200 | 0.28 | 0.595 | 0.682 | 0.132 |
| RHSIM_Rhsim04G0137700 | 0.28 | 0.595 | 0.682 | 0.432 |
| RHSIM_Rhsim10G0125000 | 0.28 | 0.595 | 0.682 | 0.057 |
| RHSIM_Rhsim08G0127300 | 0.28 | 0.595 | 0.682 | 0.633 |
| RHSIM_Rhsim13G0142500 | 0.28 | 0.595 | 0.682 | -0.110 |
| RHSIM_Rhsim02G0163300 | 0.28 | 0.595 | 0.682 | -0.648 |
| RHSIM_Rhsim03G0159800 | 0.28 | 0.595 | 0.682 | -0.076 |
| RHSIM_Rhsim11G0145400 | 0.28 | 0.595 | 0.682 | 0.642 |
| RHSIM_Rhsim06G0217700 | 0.28 | 0.595 | 0.682 | 0.963 |
| RHSIM_Rhsim07G0041000 | 0.28 | 0.596 | 0.683 | -1.339 |
| RHSIM_Rhsim07G0156400 | 0.28 | 0.596 | 0.683 | 1.256 |

| Gene | LRT statistic | P value | BH-adjusted P | log2FC (interaction) |
|---|---|---|---|---|
| RHSIM_Rhsim04G0230200 | 0.28 | 0.596 | 0.683 | 0.067 |
| RHSIM_RhsimUnG0009500 | 0.28 | 0.596 | 0.683 | -0.089 |
| RHSIM_Rhsim01G0209300 | 0.28 | 0.597 | 0.684 | 0.051 |
| RHSIM_Rhsim12G0006900 | 0.28 | 0.597 | 0.684 | -0.486 |
| RHSIM_Rhsim05G0213600 | 0.28 | 0.597 | 0.684 | 0.575 |
| RHSIM_Rhsim03G0146600 | 0.28 | 0.597 | 0.684 | -0.055 |
| RHSIM_Rhsim05G0112000 | 0.28 | 0.597 | 0.684 | -0.135 |
| RHSIM_Rhsim05G0043100 | 0.28 | 0.597 | 0.684 | -34.508 |
| RHSIM_Rhsim10G0061700 | 0.28 | 0.597 | 0.684 | 0.518 |
| novel.597 | 0.28 | 0.597 | 0.684 | -0.172 |
| RHSIM_Rhsim04G0000600 | 0.28 | 0.598 | 0.685 | 0.343 |
| RHSIM_Rhsim05G0175100 | 0.28 | 0.598 | 0.685 | 0.055 |
| RHSIM_Rhsim07G0149300 | 0.28 | 0.598 | 0.685 | 1.559 |
| RHSIM_Rhsim04G0222700 | 0.28 | 0.598 | 0.685 | -0.073 |
| RHSIM_Rhsim08G0090400 | 0.28 | 0.598 | 0.685 | -0.208 |
| RHSIM_RhsimUnG0197400 | 0.28 | 0.598 | 0.685 | 0.837 |
| RHSIM_Rhsim01G0168600 | 0.28 | 0.598 | 0.685 | 1.262 |
| novel.2364 | 0.28 | 0.598 | 0.685 | -0.038 |
| novel.2065 | 0.28 | 0.598 | 0.685 | 0.248 |
| RHSIM_Rhsim11G0134800 | 0.28 | 0.599 | 0.685 | 0.034 |
| RHSIM_Rhsim08G0086500 | 0.28 | 0.599 | 0.685 | 0.099 |
| RHSIM_RhsimUnG0074200 | 0.28 | 0.599 | 0.686 | 0.537 |
| RHSIM_Rhsim11G0147100 | 0.28 | 0.599 | 0.686 | 0.302 |
| RHSIM_Rhsim01G0234000 | 0.28 | 0.599 | 0.686 | 0.047 |
| novel.3096 | 0.28 | 0.599 | 0.686 | -0.717 |
| novel.288 | 0.28 | 0.599 | 0.686 | -0.156 |
| RHSIM_Rhsim03G0229800 | 0.28 | 0.599 | 0.686 | -0.062 |
| novel.1809 | 0.28 | 0.599 | 0.686 | 0.642 |
| RHSIM_Rhsim12G0094400 | 0.28 | 0.599 | 0.686 | 0.454 |
| RHSIM_Rhsim10G0019100 | 0.28 | 0.600 | 0.686 | 0.389 |
| novel.3621 | 0.28 | 0.600 | 0.687 | -0.177 |
| RHSIM_Rhsim07G0213300 | 0.27 | 0.600 | 0.687 | 0.029 |

| Gene | LRT statistic | P value | BH-adjusted P | log2FC (interaction) |
|---|---|---|---|---|
| RHSIM_Rhsim02G0134100 | 0.27 | 0.600 | 0.687 | 0.134 |
| RHSIM_Rhsim09G0211600 | 0.27 | 0.600 | 0.687 | -0.426 |
| novel.1482 | 0.27 | 0.601 | 0.688 | 0.139 |
| novel.355 | 0.27 | 0.601 | 0.688 | -0.601 |
| RHSIM_Rhsim12G0034800 | 0.27 | 0.601 | 0.688 | 0.171 |
| RHSIM_Rhsim10G0152700 | 0.27 | 0.602 | 0.688 | 0.484 |
| RHSIM_Rhsim10G0193600 | 0.27 | 0.602 | 0.689 | 0.194 |
| RHSIM_Rhsim11G0166100 | 0.27 | 0.602 | 0.689 | 1.237 |
| RHSIM_Rhsim09G0102200 | 0.27 | 0.602 | 0.689 | -0.166 |
| RHSIM_Rhsim05G0150300 | 0.27 | 0.602 | 0.689 | 0.052 |
| RHSIM_Rhsim09G0058900 | 0.27 | 0.603 | 0.690 | 0.494 |
| novel.1690 | 0.27 | 0.603 | 0.690 | -0.336 |
| RHSIM_Rhsim04G0239500 | 0.27 | 0.603 | 0.690 | 1.106 |
| RHSIM_Rhsim11G0051200 | 0.27 | 0.603 | 0.690 | 0.250 |
| RHSIM_Rhsim03G0142300 | 0.27 | 0.603 | 0.690 | 0.054 |
| novel.1523 | 0.27 | 0.603 | 0.690 | -0.053 |
| novel.1641 | 0.27 | 0.604 | 0.690 | -0.343 |
| RHSIM_Rhsim03G0001000 | 0.27 | 0.604 | 0.690 | -0.126 |
| RHSIM_Rhsim03G0249300 | 0.27 | 0.604 | 0.691 | -1.295 |
| RHSIM_Rhsim01G0082600 | 0.27 | 0.604 | 0.691 | 0.949 |
| RHSIM_Rhsim10G0153400 | 0.27 | 0.604 | 0.691 | -0.133 |
| RHSIM_Rhsim11G0111900 | 0.27 | 0.604 | 0.691 | 1.174 |
| RHSIM_Rhsim01G0048800 | 0.27 | 0.604 | 0.691 | -0.569 |
| RHSIM_Rhsim09G0209800 | 0.27 | 0.604 | 0.691 | 0.721 |
| RHSIM_Rhsim12G0138700 | 0.27 | 0.605 | 0.691 | 0.384 |
| RHSIM_Rhsim13G0139600 | 0.27 | 0.605 | 0.692 | -0.752 |
| RHSIM_Rhsim07G0075400 | 0.27 | 0.605 | 0.692 | -0.043 |
| RHSIM_Rhsim08G0078800 | 0.27 | 0.605 | 0.692 | -0.075 |
| RHSIM_RhsimUnG0011300 | 0.27 | 0.605 | 0.692 | 0.849 |
| RHSIM_Rhsim06G0130400 | 0.27 | 0.605 | 0.692 | -0.068 |
| novel.294 | 0.27 | 0.605 | 0.692 | 0.371 |
| RHSIM_Rhsim13G0131800 | 0.27 | 0.605 | 0.692 | 0.113 |

| Gene | LRT statistic | P value | BH-adjusted P | log2FC (interaction) |
|---|---|---|---|---|
| RHSIM_RhsimUnG0202900 | 0.27 | 0.605 | 0.692 | 0.200 |
| RHSIM_Rhsim13G0084700 | 0.27 | 0.605 | 0.692 | -0.291 |
| RHSIM_Rhsim10G0128300 | 0.27 | 0.606 | 0.692 | 0.955 |
| RHSIM_Rhsim13G0103800 | 0.27 | 0.606 | 0.693 | -1.379 |
| RHSIM_Rhsim08G0169800 | 0.27 | 0.606 | 0.693 | 0.754 |
| RHSIM_Rhsim10G0196100 | 0.27 | 0.606 | 0.693 | -0.162 |
| RHSIM_Rhsim10G0109000 | 0.27 | 0.606 | 0.693 | -0.127 |
| RHSIM_Rhsim09G0182300 | 0.27 | 0.606 | 0.693 | 0.575 |
| novel.487 | 0.27 | 0.607 | 0.693 | -0.626 |
| novel.1201 | 0.27 | 0.607 | 0.693 | -0.193 |
| RHSIM_Rhsim03G0099400 | 0.26 | 0.607 | 0.694 | 0.191 |
| novel.1632 | 0.26 | 0.607 | 0.694 | -0.286 |
| RHSIM_Rhsim11G0112900 | 0.26 | 0.607 | 0.694 | 0.245 |
| novel.2193 | 0.26 | 0.608 | 0.694 | -0.075 |
| novel.1708 | 0.26 | 0.608 | 0.694 | 0.348 |
| RHSIM_Rhsim03G0265900 | 0.26 | 0.608 | 0.694 | -0.845 |
| RHSIM_Rhsim03G0136700 | 0.26 | 0.608 | 0.695 | 0.576 |
| novel.1562 | 0.26 | 0.608 | 0.695 | 0.654 |
| RHSIM_Rhsim01G0141600 | 0.26 | 0.608 | 0.695 | -0.053 |
| RHSIM_Rhsim03G0093500 | 0.26 | 0.608 | 0.695 | 0.074 |
| RHSIM_Rhsim04G0209800 | 0.26 | 0.608 | 0.695 | 0.346 |
| RHSIM_RhsimUnG0050400 | 0.26 | 0.609 | 0.695 | 0.198 |
| RHSIM_RhsimUnG0040600 | 0.26 | 0.609 | 0.695 | 0.108 |
| RHSIM_Rhsim05G0002700 | 0.26 | 0.609 | 0.696 | -1.220 |
| RHSIM_Rhsim01G0087300 | 0.26 | 0.609 | 0.696 | 0.807 |
| RHSIM_Rhsim05G0069400 | 0.26 | 0.609 | 0.696 | 0.104 |
| RHSIM_Rhsim03G0214100 | 0.26 | 0.609 | 0.696 | 1.045 |
| RHSIM_Rhsim04G0003300 | 0.26 | 0.609 | 0.696 | -0.262 |
| RHSIM_Rhsim07G0156600 | 0.26 | 0.609 | 0.696 | 0.379 |
| novel.111 | 0.26 | 0.609 | 0.696 | -0.350 |
| RHSIM_Rhsim06G0090100 | 0.26 | 0.610 | 0.696 | -34.319 |
| novel.4270 | 0.26 | 0.610 | 0.696 | 0.309 |

| Gene | LRT statistic | P value | BH-adjusted P | log2FC (interaction) |
|---|---|---|---|---|
| RHSIM_Rhsim06G0180100 | 0.26 | 0.610 | 0.696 | 0.595 |
| RHSIM_Rhsim09G0126700 | 0.26 | 0.610 | 0.696 | 0.086 |
| novel.2082 | 0.26 | 0.610 | 0.696 | -0.862 |
| RHSIM_Rhsim01G0107400 | 0.26 | 0.610 | 0.696 | 0.769 |
| RHSIM_Rhsim04G0099600 | 0.26 | 0.610 | 0.696 | -1.484 |
| novel.2250 | 0.26 | 0.610 | 0.696 | 0.235 |
| RHSIM_Rhsim01G0140600 | 0.26 | 0.610 | 0.696 | -0.369 |
| RHSIM_Rhsim11G0157300 | 0.26 | 0.610 | 0.696 | -0.302 |
| RHSIM_Rhsim01G0117900 | 0.26 | 0.610 | 0.696 | -0.540 |
| RHSIM_Rhsim06G0128100 | 0.26 | 0.610 | 0.696 | 0.360 |
| RHSIM_Rhsim09G0110800 | 0.26 | 0.610 | 0.697 | 0.383 |
| novel.1667 | 0.26 | 0.610 | 0.697 | 0.376 |
| RHSIM_Rhsim05G0150200 | 0.26 | 0.610 | 0.697 | -0.040 |
| RHSIM_Rhsim05G0081600 | 0.26 | 0.611 | 0.697 | 0.032 |
| RHSIM_Rhsim05G0112400 | 0.26 | 0.611 | 0.698 | 1.377 |
| RHSIM_Rhsim10G0160600 | 0.26 | 0.612 | 0.698 | -1.293 |
| RHSIM_Rhsim03G0098200 | 0.26 | 0.612 | 0.698 | -34.290 |
| novel.3638 | 0.26 | 0.612 | 0.698 | -0.451 |
| RHSIM_Rhsim01G0095000 | 0.26 | 0.612 | 0.698 | -0.369 |
| RHSIM_Rhsim04G0064400 | 0.26 | 0.612 | 0.698 | -0.720 |
| RHSIM_Rhsim07G0165900 | 0.26 | 0.612 | 0.698 | -0.346 |
| RHSIM_Rhsim02G0053300 | 0.26 | 0.612 | 0.698 | 0.212 |
| RHSIM_RhsimUnG0213900 | 0.26 | 0.612 | 0.698 | 0.178 |
| RHSIM_Rhsim13G0074000 | 0.26 | 0.612 | 0.698 | 0.127 |
| RHSIM_Rhsim02G0024100 | 0.26 | 0.612 | 0.699 | 0.130 |
| RHSIM_Rhsim05G0130200 | 0.26 | 0.613 | 0.699 | -1.287 |
| RHSIM_Rhsim08G0193800 | 0.26 | 0.613 | 0.699 | -33.923 |
| RHSIM_Rhsim11G0077700 | 0.26 | 0.613 | 0.699 | -0.880 |
| RHSIM_Rhsim11G0123100 | 0.26 | 0.613 | 0.699 | -0.678 |
| RHSIM_Rhsim08G0222000 | 0.26 | 0.613 | 0.699 | -0.159 |
| novel.3486 | 0.26 | 0.613 | 0.699 | -0.610 |
| RHSIM_Rhsim02G0049300 | 0.26 | 0.613 | 0.699 | 34.881 |

| Gene | LRT statistic | P value | BH-adjusted P | log2FC (interaction) |
|---|---|---|---|---|
| RHSIM_Rhsim03G0215200 | 0.26 | 0.614 | 0.700 | 0.020 |
| RHSIM_Rhsim03G0057400 | 0.25 | 0.614 | 0.700 | -0.071 |
| RHSIM_Rhsim11G0076100 | 0.25 | 0.614 | 0.700 | -34.194 |
| RHSIM_Rhsim05G0007700 | 0.25 | 0.614 | 0.700 | 0.127 |
| RHSIM_Rhsim01G0127200 | 0.25 | 0.614 | 0.701 | -0.954 |
| RHSIM_Rhsim10G0076600 | 0.25 | 0.615 | 0.701 | -1.326 |
| RHSIM_Rhsim10G0083400 | 0.25 | 0.615 | 0.701 | 1.036 |
| RHSIM_Rhsim08G0021400 | 0.25 | 0.615 | 0.701 | -1.122 |
| RHSIM_Rhsim07G0232500 | 0.25 | 0.615 | 0.701 | 1.073 |
| RHSIM_RhsimUnG0229200 | 0.25 | 0.615 | 0.701 | -0.051 |
| RHSIM_Rhsim12G0142900 | 0.25 | 0.615 | 0.702 | 0.061 |
| RHSIM_Rhsim07G0131200 | 0.25 | 0.615 | 0.702 | 0.068 |
| RHSIM_Rhsim08G0065300 | 0.25 | 0.616 | 0.702 | -0.119 |
| RHSIM_Rhsim04G0238500 | 0.25 | 0.616 | 0.702 | -0.089 |
| RHSIM_Rhsim07G0073800 | 0.25 | 0.616 | 0.702 | 0.538 |
| RHSIM_Rhsim11G0056800 | 0.25 | 0.616 | 0.702 | -0.489 |
| novel.1754 | 0.25 | 0.616 | 0.702 | 34.270 |
| RHSIM_Rhsim06G0132500 | 0.25 | 0.616 | 0.702 | 1.123 |
| novel.2549 | 0.25 | 0.616 | 0.702 | 0.318 |
| RHSIM_Rhsim09G0084400 | 0.25 | 0.617 | 0.703 | 0.087 |
| RHSIM_Rhsim09G0060600 | 0.25 | 0.617 | 0.703 | 0.488 |
| RHSIM_Rhsim04G0030800 | 0.25 | 0.617 | 0.703 | 0.049 |
| novel.2968 | 0.25 | 0.617 | 0.703 | -0.476 |
| novel.3993 | 0.25 | 0.617 | 0.703 | 0.224 |
| novel.2362 | 0.25 | 0.617 | 0.703 | -1.477 |
| novel.3526 | 0.25 | 0.617 | 0.703 | -0.194 |
| RHSIM_Rhsim08G0110200 | 0.25 | 0.617 | 0.703 | 0.378 |
| RHSIM_Rhsim02G0051400 | 0.25 | 0.617 | 0.703 | -0.595 |
| RHSIM_Rhsim09G0023500 | 0.25 | 0.617 | 0.703 | 0.460 |
| RHSIM_Rhsim02G0238200 | 0.25 | 0.617 | 0.703 | -0.491 |
| RHSIM_Rhsim08G0049500 | 0.25 | 0.618 | 0.703 | -0.145 |
| RHSIM_Rhsim02G0199900 | 0.25 | 0.618 | 0.703 | -0.067 |

| Gene | LRT statistic | P value | BH-adjusted P | log2FC (interaction) |
|---|---|---|---|---|
| RHSIM_Rhsim05G0084000 | 0.25 | 0.618 | 0.703 | -0.114 |
| RHSIM_Rhsim01G0263800 | 0.25 | 0.618 | 0.703 | 0.729 |
| RHSIM_Rhsim06G0078600 | 0.25 | 0.618 | 0.704 | 0.152 |
| RHSIM_Rhsim10G0027000 | 0.25 | 0.618 | 0.704 | -0.881 |
| RHSIM_Rhsim10G0151500 | 0.25 | 0.618 | 0.704 | -0.062 |
| novel.1283 | 0.25 | 0.618 | 0.704 | -0.298 |
| RHSIM_Rhsim03G0138100 | 0.25 | 0.618 | 0.704 | 0.498 |
| RHSIM_Rhsim06G0139600 | 0.25 | 0.618 | 0.704 | 0.225 |
| RHSIM_Rhsim03G0082700 | 0.25 | 0.618 | 0.704 | 0.567 |
| RHSIM_Rhsim03G0164700 | 0.25 | 0.619 | 0.704 | -34.227 |
| RHSIM_Rhsim06G0123100 | 0.25 | 0.619 | 0.705 | 0.277 |
| novel.3952 | 0.25 | 0.619 | 0.705 | -0.858 |
| RHSIM_Rhsim12G0204000 | 0.25 | 0.619 | 0.705 | -0.388 |
| RHSIM_Rhsim08G0093800 | 0.25 | 0.619 | 0.705 | 0.894 |
| RHSIM_Rhsim12G0181800 | 0.25 | 0.619 | 0.705 | -0.397 |
| RHSIM_Rhsim12G0199700 | 0.25 | 0.619 | 0.705 | -0.121 |
| RHSIM_Rhsim09G0173700 | 0.25 | 0.620 | 0.705 | 1.188 |
| novel.2455 | 0.25 | 0.620 | 0.705 | 0.313 |
| RHSIM_Rhsim01G0023600 | 0.25 | 0.620 | 0.705 | 0.199 |
| novel.3456 | 0.25 | 0.620 | 0.706 | 0.751 |
| RHSIM_Rhsim12G0141700 | 0.25 | 0.620 | 0.706 | -0.938 |
| RHSIM_Rhsim02G0031400 | 0.25 | 0.620 | 0.706 | -0.053 |
| RHSIM_Rhsim05G0101000 | 0.25 | 0.620 | 0.706 | 0.051 |
| RHSIM_Rhsim03G0099300 | 0.25 | 0.620 | 0.706 | -0.862 |
| RHSIM_Rhsim05G0208100 | 0.25 | 0.620 | 0.706 | -0.931 |
| RHSIM_Rhsim05G0076200 | 0.25 | 0.620 | 0.706 | -0.088 |
| RHSIM_Rhsim07G0029900 | 0.25 | 0.620 | 0.706 | 34.314 |
| RHSIM_Rhsim06G0139200 | 0.25 | 0.620 | 0.706 | -0.036 |
| RHSIM_Rhsim09G0170600 | 0.25 | 0.620 | 0.706 | -34.053 |
| RHSIM_Rhsim08G0135600 | 0.25 | 0.620 | 0.706 | -0.133 |
| novel.295 | 0.25 | 0.620 | 0.706 | 0.092 |
| RHSIM_RhsimUnG0135600 | 0.25 | 0.620 | 0.706 | -0.088 |

| Gene | LRT statistic | P value | BH-adjusted P | log2FC (interaction) |
|---|---|---|---|---|
| RHSIM_RhsimUnG0036600 | 0.25 | 0.620 | 0.706 | -0.583 |
| novel.1568 | 0.24 | 0.621 | 0.706 | 0.140 |
| RHSIM_Rhsim03G0191400 | 0.24 | 0.621 | 0.706 | -0.542 |
| novel.4484 | 0.24 | 0.621 | 0.706 | -0.489 |
| RHSIM_Rhsim03G0070100 | 0.24 | 0.621 | 0.706 | -0.108 |
| RHSIM_Rhsim06G0220600 | 0.24 | 0.621 | 0.706 | -0.610 |
| RHSIM_Rhsim06G0140300 | 0.24 | 0.621 | 0.706 | 1.222 |
| novel.676 | 0.24 | 0.621 | 0.707 | -34.211 |
| RHSIM_Rhsim06G0151100 | 0.24 | 0.622 | 0.707 | -0.922 |
| RHSIM_RhsimUnG0102700 | 0.24 | 0.622 | 0.707 | -0.052 |
| novel.2151 | 0.24 | 0.622 | 0.707 | -0.533 |
| RHSIM_RhsimUnG0220600 | 0.24 | 0.622 | 0.707 | -0.844 |
| RHSIM_Rhsim08G0027200 | 0.24 | 0.622 | 0.707 | -0.042 |
| novel.3297 | 0.24 | 0.622 | 0.707 | -0.466 |
| RHSIM_Rhsim04G0013200 | 0.24 | 0.622 | 0.707 | 0.641 |
| novel.2062 | 0.24 | 0.622 | 0.707 | 0.653 |
| novel.3438 | 0.24 | 0.623 | 0.708 | 0.529 |
| RHSIM_Rhsim07G0152800 | 0.24 | 0.623 | 0.708 | -0.147 |
| RHSIM_RhsimUnG0056000 | 0.24 | 0.623 | 0.708 | 1.200 |
| RHSIM_Rhsim10G0003700 | 0.24 | 0.623 | 0.708 | 0.948 |
| RHSIM_Rhsim06G0204400 | 0.24 | 0.623 | 0.708 | -0.064 |
| RHSIM_Rhsim05G0188000 | 0.24 | 0.623 | 0.708 | 0.356 |
| novel.3970 | 0.24 | 0.623 | 0.708 | 0.191 |
| novel.2090 | 0.24 | 0.623 | 0.708 | -0.276 |
| RHSIM_Rhsim11G0146300 | 0.24 | 0.623 | 0.708 | 0.040 |
| RHSIM_Rhsim07G0006000 | 0.24 | 0.623 | 0.708 | 0.030 |
| RHSIM_Rhsim06G0228700 | 0.24 | 0.623 | 0.708 | 0.089 |
| novel.540 | 0.24 | 0.623 | 0.708 | -0.272 |
| RHSIM_Rhsim11G0135200 | 0.24 | 0.624 | 0.708 | 0.055 |
| RHSIM_Rhsim01G0181400 | 0.24 | 0.624 | 0.708 | 0.734 |
| novel.3691 | 0.24 | 0.624 | 0.708 | -0.339 |
| RHSIM_Rhsim08G0224500 | 0.24 | 0.624 | 0.708 | 0.120 |

| Gene | LRT statistic | P value | BH-adjusted P | log2FC (interaction) |
|---|---|---|---|---|
| novel.3280 | 0.24 | 0.624 | 0.709 | 0.157 |
| RHSIM_RhsimMtG0003800 | 0.24 | 0.624 | 0.709 | -1.166 |
| novel.1853 | 0.24 | 0.624 | 0.709 | -0.550 |
| RHSIM_Rhsim06G0078400 | 0.24 | 0.624 | 0.709 | -0.326 |
| novel.2240 | 0.24 | 0.624 | 0.709 | 0.293 |
| RHSIM_Rhsim10G0082900 | 0.24 | 0.624 | 0.709 | 0.258 |
| RHSIM_Rhsim04G0155100 | 0.24 | 0.625 | 0.709 | 0.894 |
| RHSIM_Rhsim02G0012900 | 0.24 | 0.625 | 0.709 | -0.896 |
| RHSIM_Rhsim07G0104300 | 0.24 | 0.625 | 0.709 | 0.079 |
| RHSIM_Rhsim06G0062100 | 0.24 | 0.625 | 0.709 | 0.082 |
| novel.1911 | 0.24 | 0.625 | 0.709 | -0.170 |
| RHSIM_Rhsim09G0148200 | 0.24 | 0.625 | 0.710 | -0.095 |
| RHSIM_Rhsim04G0115300 | 0.24 | 0.626 | 0.710 | -0.092 |
| RHSIM_Rhsim02G0017000 | 0.24 | 0.626 | 0.710 | 0.063 |
| RHSIM_Rhsim05G0009100 | 0.24 | 0.626 | 0.710 | -0.697 |
| RHSIM_Rhsim04G0209700 | 0.24 | 0.626 | 0.710 | -0.628 |
| RHSIM_Rhsim02G0151500 | 0.24 | 0.626 | 0.711 | -0.142 |
| novel.1580 | 0.24 | 0.626 | 0.711 | -0.715 |
| RHSIM_Rhsim08G0172800 | 0.24 | 0.626 | 0.711 | -0.197 |
| RHSIM_Rhsim13G0052800 | 0.24 | 0.626 | 0.711 | -0.599 |
| novel.62 | 0.24 | 0.626 | 0.711 | -0.472 |
| RHSIM_Rhsim08G0191900 | 0.24 | 0.626 | 0.711 | -0.526 |
| RHSIM_Rhsim05G0054700 | 0.24 | 0.627 | 0.711 | -34.131 |
| novel.3028 | 0.24 | 0.627 | 0.711 | 0.385 |
| RHSIM_Rhsim02G0105900 | 0.24 | 0.627 | 0.711 | 0.074 |
| RHSIM_Rhsim09G0008500 | 0.24 | 0.627 | 0.712 | -0.385 |
| RHSIM_Rhsim06G0067300 | 0.24 | 0.627 | 0.712 | 0.706 |
| RHSIM_Rhsim10G0118100 | 0.24 | 0.627 | 0.712 | 0.519 |
| RHSIM_Rhsim08G0249600 | 0.24 | 0.627 | 0.712 | -0.081 |
| RHSIM_Rhsim08G0170800 | 0.24 | 0.627 | 0.712 | 1.211 |
| RHSIM_Rhsim09G0173000 | 0.24 | 0.627 | 0.712 | -0.392 |
| RHSIM_Rhsim13G0141000 | 0.24 | 0.627 | 0.712 | 0.862 |

| Gene | LRT statistic | P value | BH-adjusted P | log2FC (interaction) |
|---|---|---|---|---|
| RHSIM_Rhsim09G0139900 | 0.24 | 0.628 | 0.712 | 0.562 |
| RHSIM_Rhsim13G0103400 | 0.23 | 0.628 | 0.712 | 0.044 |
| RHSIM_Rhsim08G0012900 | 0.23 | 0.628 | 0.712 | -0.312 |
| novel.3751 | 0.23 | 0.628 | 0.713 | -0.649 |
| novel.2168 | 0.23 | 0.628 | 0.713 | -0.208 |
| RHSIM_Rhsim02G0194900 | 0.23 | 0.628 | 0.713 | 0.670 |
| RHSIM_Rhsim13G0087500 | 0.23 | 0.628 | 0.713 | 0.114 |
| RHSIM_Rhsim11G0149300 | 0.23 | 0.628 | 0.713 | 0.441 |
| RHSIM_Rhsim07G0254900 | 0.23 | 0.629 | 0.713 | 0.118 |
| RHSIM_Rhsim03G0235900 | 0.23 | 0.629 | 0.713 | -0.047 |
| novel.3781 | 0.23 | 0.629 | 0.713 | 0.291 |
| RHSIM_Rhsim05G0221100 | 0.23 | 0.629 | 0.713 | -0.166 |
| RHSIM_Rhsim01G0234800 | 0.23 | 0.629 | 0.713 | -1.279 |
| RHSIM_Rhsim12G0032400 | 0.23 | 0.629 | 0.713 | -0.760 |
| RHSIM_Rhsim09G0033000 | 0.23 | 0.629 | 0.713 | -0.116 |
| RHSIM_Rhsim07G0173100 | 0.23 | 0.629 | 0.713 | 0.173 |
| RHSIM_Rhsim04G0037200 | 0.23 | 0.629 | 0.713 | 1.206 |
| RHSIM_Rhsim08G0109100 | 0.23 | 0.629 | 0.713 | 0.046 |
| novel.1344 | 0.23 | 0.629 | 0.713 | -0.328 |
| novel.3977 | 0.23 | 0.629 | 0.713 | 0.963 |
| RHSIM_Rhsim08G0206800 | 0.23 | 0.629 | 0.713 | 0.792 |
| RHSIM_Rhsim13G0175600 | 0.23 | 0.629 | 0.713 | 0.226 |
| RHSIM_Rhsim12G0075400 | 0.23 | 0.630 | 0.714 | 34.090 |
| novel.1801 | 0.23 | 0.630 | 0.714 | 0.165 |
| RHSIM_Rhsim05G0022800 | 0.23 | 0.630 | 0.714 | 0.088 |
| RHSIM_Rhsim03G0052100 | 0.23 | 0.630 | 0.714 | -0.724 |
| novel.4548 | 0.23 | 0.630 | 0.714 | -0.455 |
| novel.1404 | 0.23 | 0.630 | 0.714 | 0.979 |
| RHSIM_Rhsim03G0158200 | 0.23 | 0.630 | 0.714 | 0.626 |
| RHSIM_Rhsim02G0210400 | 0.23 | 0.630 | 0.714 | -0.984 |
| RHSIM_Rhsim04G0015800 | 0.23 | 0.630 | 0.714 | -0.080 |
| RHSIM_Rhsim10G0191200 | 0.23 | 0.630 | 0.714 | -0.269 |

| Gene | LRT statistic | P value | BH-adjusted P | log2FC (interaction) |
|---|---|---|---|---|
| novel.3278 | 0.23 | 0.630 | 0.714 | 0.228 |
| novel.2162 | 0.23 | 0.631 | 0.714 | 0.199 |
| RHSIM_Rhsim11G0117600 | 0.23 | 0.631 | 0.714 | -0.317 |
| novel.4070 | 0.23 | 0.631 | 0.715 | -1.120 |
| RHSIM_Rhsim10G0028200 | 0.23 | 0.631 | 0.715 | -0.174 |
| RHSIM_Rhsim13G0153000 | 0.23 | 0.631 | 0.715 | 0.401 |
| RHSIM_Rhsim03G0005000 | 0.23 | 0.631 | 0.715 | 0.988 |
| RHSIM_Rhsim08G0227100 | 0.23 | 0.632 | 0.715 | -0.786 |
| RHSIM_Rhsim06G0056500 | 0.23 | 0.632 | 0.716 | 0.617 |
| RHSIM_Rhsim12G0207700 | 0.23 | 0.632 | 0.716 | -0.770 |
| RHSIM_Rhsim09G0123900 | 0.23 | 0.632 | 0.716 | -0.754 |
| novel.211 | 0.23 | 0.632 | 0.716 | -0.349 |
| novel.741 | 0.23 | 0.632 | 0.716 | 0.414 |
| RHSIM_Rhsim06G0014100 | 0.23 | 0.633 | 0.716 | -0.828 |
| RHSIM_Rhsim08G0072000 | 0.23 | 0.633 | 0.716 | 0.681 |
| RHSIM_Rhsim01G0150300 | 0.23 | 0.633 | 0.716 | 0.269 |
| RHSIM_Rhsim03G0042600 | 0.23 | 0.633 | 0.716 | 0.885 |
| RHSIM_Rhsim01G0235300 | 0.23 | 0.633 | 0.716 | 0.152 |
| RHSIM_Rhsim12G0202300 | 0.23 | 0.633 | 0.717 | 1.177 |
| RHSIM_Rhsim09G0053800 | 0.23 | 0.633 | 0.717 | -34.080 |
| novel.389 | 0.23 | 0.633 | 0.717 | 0.132 |
| RHSIM_Rhsim04G0102600 | 0.23 | 0.633 | 0.717 | -0.076 |
| RHSIM_Rhsim04G0063800 | 0.23 | 0.634 | 0.717 | 0.287 |
| RHSIM_RhsimUnG0081900 | 0.23 | 0.634 | 0.717 | -0.158 |
| RHSIM_RhsimUnG0233000 | 0.23 | 0.634 | 0.717 | -0.702 |
| RHSIM_Rhsim07G0058500 | 0.23 | 0.634 | 0.717 | 0.058 |
| RHSIM_Rhsim07G0155300 | 0.23 | 0.634 | 0.717 | 0.038 |
| RHSIM_RhsimUnG0247000 | 0.23 | 0.634 | 0.718 | -0.365 |
| RHSIM_Rhsim11G0043600 | 0.23 | 0.634 | 0.718 | -0.045 |
| RHSIM_Rhsim11G0016600 | 0.23 | 0.634 | 0.718 | 0.340 |
| RHSIM_Rhsim03G0077700 | 0.23 | 0.635 | 0.718 | 0.192 |
| RHSIM_Rhsim06G0139800 | 0.23 | 0.635 | 0.718 | -0.257 |

| Gene | LRT statistic | P value | BH-adjusted P | log2FC (interaction) |
|---|---|---|---|---|
| RHSIM_Rhsim13G0093900 | 0.23 | 0.635 | 0.718 | -0.049 |
| RHSIM_Rhsim09G0025800 | 0.23 | 0.635 | 0.718 | 0.244 |
| novel.643 | 0.23 | 0.635 | 0.719 | -0.198 |
| RHSIM_RhsimUnG0165800 | 0.23 | 0.635 | 0.719 | -0.754 |
| RHSIM_Rhsim13G0176800 | 0.22 | 0.635 | 0.719 | 0.145 |
| RHSIM_Rhsim12G0111200 | 0.22 | 0.636 | 0.719 | 0.424 |
| RHSIM_Rhsim12G0184400 | 0.22 | 0.636 | 0.719 | -0.417 |
| RHSIM_Rhsim02G0051600 | 0.22 | 0.636 | 0.719 | -0.409 |
| RHSIM_Rhsim01G0100800 | 0.22 | 0.636 | 0.719 | 0.083 |
| RHSIM_Rhsim01G0249700 | 0.22 | 0.636 | 0.720 | 1.034 |
| RHSIM_Rhsim13G0021000 | 0.22 | 0.636 | 0.720 | -0.046 |
| RHSIM_Rhsim12G0214900 | 0.22 | 0.636 | 0.720 | -0.516 |
| RHSIM_Rhsim07G0196200 | 0.22 | 0.636 | 0.720 | 0.279 |
| RHSIM_RhsimUnG0195600 | 0.22 | 0.637 | 0.720 | -0.844 |
| novel.2637 | 0.22 | 0.637 | 0.720 | 0.855 |
| RHSIM_Rhsim12G0192800 | 0.22 | 0.637 | 0.720 | -0.203 |
| RHSIM_Rhsim09G0043600 | 0.22 | 0.637 | 0.720 | -1.055 |
| RHSIM_Rhsim13G0013900 | 0.22 | 0.637 | 0.720 | 0.669 |
| novel.1081 | 0.22 | 0.637 | 0.721 | -0.117 |
| RHSIM_Rhsim12G0044100 | 0.22 | 0.638 | 0.721 | -0.441 |
| RHSIM_RhsimUnG0054000 | 0.22 | 0.638 | 0.721 | 0.468 |
| novel.3218 | 0.22 | 0.638 | 0.721 | -1.409 |
| RHSIM_RhsimUnG0122200 | 0.22 | 0.638 | 0.721 | -0.434 |
| RHSIM_RhsimUnG0206200 | 0.22 | 0.638 | 0.722 | 0.046 |
| RHSIM_Rhsim06G0123900 | 0.22 | 0.638 | 0.722 | 1.226 |
| RHSIM_Rhsim02G0062900 | 0.22 | 0.639 | 0.722 | 0.170 |
| novel.1733 | 0.22 | 0.639 | 0.722 | -1.052 |
| RHSIM_Rhsim06G0046900 | 0.22 | 0.639 | 0.722 | -0.043 |
| RHSIM_Rhsim08G0117200 | 0.22 | 0.639 | 0.723 | -0.071 |
| RHSIM_Rhsim08G0081500 | 0.22 | 0.640 | 0.723 | -0.025 |
| RHSIM_Rhsim06G0194300 | 0.22 | 0.640 | 0.723 | 0.308 |
| novel.2642 | 0.22 | 0.640 | 0.723 | -0.665 |

| Gene | LRT statistic | P value | BH-adjusted P | log2FC (interaction) |
|---|---|---|---|---|
| RHSIM_Rhsim04G0129600 | 0.22 | 0.640 | 0.723 | -0.145 |
| RHSIM_Rhsim12G0065200 | 0.22 | 0.640 | 0.723 | 0.110 |
| RHSIM_Rhsim04G0061300 | 0.22 | 0.640 | 0.723 | -0.239 |
| RHSIM_Rhsim03G0194900 | 0.22 | 0.640 | 0.723 | -0.597 |
| RHSIM_Rhsim13G0033200 | 0.22 | 0.640 | 0.723 | 0.306 |
| RHSIM_Rhsim07G0070800 | 0.22 | 0.640 | 0.723 | -0.229 |
| novel.116 | 0.22 | 0.640 | 0.723 | -0.572 |
| RHSIM_Rhsim02G0117600 | 0.22 | 0.640 | 0.724 | 0.216 |
| RHSIM_Rhsim13G0097700 | 0.22 | 0.641 | 0.724 | 0.099 |
| RHSIM_Rhsim12G0077300 | 0.22 | 0.641 | 0.724 | 0.100 |
| RHSIM_Rhsim13G0137600 | 0.22 | 0.641 | 0.724 | 0.057 |
| RHSIM_Rhsim13G0123800 | 0.22 | 0.641 | 0.724 | 0.197 |
| novel.1438 | 0.22 | 0.641 | 0.724 | -0.159 |
| RHSIM_Rhsim04G0182200 | 0.22 | 0.641 | 0.724 | -0.139 |
| RHSIM_Rhsim11G0128500 | 0.22 | 0.641 | 0.724 | 1.036 |
| novel.2444 | 0.22 | 0.641 | 0.724 | 33.878 |
| RHSIM_Rhsim04G0009600 | 0.22 | 0.641 | 0.724 | 0.555 |
| RHSIM_Rhsim13G0139300 | 0.22 | 0.641 | 0.724 | -0.883 |
| RHSIM_Rhsim09G0019500 | 0.22 | 0.642 | 0.725 | -1.113 |
| novel.738 | 0.22 | 0.642 | 0.725 | -0.486 |
| RHSIM_Rhsim01G0211000 | 0.22 | 0.642 | 0.725 | -0.157 |
| RHSIM_Rhsim04G0057300 | 0.22 | 0.642 | 0.725 | 0.301 |
| RHSIM_Rhsim09G0118300 | 0.22 | 0.642 | 0.725 | 0.164 |
| novel.4415 | 0.22 | 0.642 | 0.725 | 0.199 |
| RHSIM_Rhsim11G0053500 | 0.22 | 0.642 | 0.725 | 1.229 |
| RHSIM_Rhsim12G0067100 | 0.22 | 0.642 | 0.725 | -33.967 |
| RHSIM_Rhsim02G0114000 | 0.22 | 0.642 | 0.725 | 0.336 |
| RHSIM_Rhsim10G0055600 | 0.22 | 0.642 | 0.725 | 0.782 |
| RHSIM_Rhsim08G0103800 | 0.22 | 0.642 | 0.725 | 0.420 |
| RHSIM_Rhsim13G0005400 | 0.22 | 0.643 | 0.725 | -33.939 |
| RHSIM_Rhsim03G0019200 | 0.22 | 0.643 | 0.725 | 0.158 |
| RHSIM_Rhsim09G0057600 | 0.22 | 0.643 | 0.726 | 0.665 |

| Gene | LRT statistic | P value | BH-adjusted P | log2FC (interaction) |
|---|---|---|---|---|
| RHSIM_Rhsim12G0002300 | 0.21 | 0.643 | 0.726 | 0.101 |
| RHSIM_Rhsim07G0141200 | 0.21 | 0.643 | 0.726 | -0.131 |
| RHSIM_Rhsim07G0037700 | 0.21 | 0.643 | 0.726 | 0.122 |
| RHSIM_Rhsim02G0111200 | 0.21 | 0.643 | 0.726 | -0.250 |
| novel.4196 | 0.21 | 0.643 | 0.726 | -0.113 |
| RHSIM_Rhsim09G0004100 | 0.21 | 0.643 | 0.726 | -0.042 |
| novel.3673 | 0.21 | 0.643 | 0.726 | -0.250 |
| RHSIM_Rhsim12G0034900 | 0.21 | 0.644 | 0.726 | 0.100 |
| RHSIM_Rhsim09G0209700 | 0.21 | 0.644 | 0.726 | 0.645 |
| RHSIM_Rhsim06G0034500 | 0.21 | 0.644 | 0.726 | -0.170 |
| RHSIM_Rhsim04G0085100 | 0.21 | 0.644 | 0.726 | 0.665 |
| RHSIM_Rhsim09G0210000 | 0.21 | 0.644 | 0.727 | -0.049 |
| RHSIM_Rhsim05G0222800 | 0.21 | 0.644 | 0.727 | 0.061 |
| RHSIM_Rhsim10G0180900 | 0.21 | 0.644 | 0.727 | -0.906 |
| RHSIM_Rhsim12G0028300 | 0.21 | 0.644 | 0.727 | 0.041 |
| RHSIM_Rhsim07G0176000 | 0.21 | 0.644 | 0.727 | -0.785 |
| RHSIM_Rhsim02G0245800 | 0.21 | 0.645 | 0.727 | 0.235 |
| RHSIM_Rhsim11G0050800 | 0.21 | 0.645 | 0.728 | 0.239 |
| RHSIM_Rhsim07G0192400 | 0.21 | 0.645 | 0.728 | 0.345 |
| RHSIM_Rhsim13G0079000 | 0.21 | 0.645 | 0.728 | -34.468 |
| RHSIM_Rhsim09G0024100 | 0.21 | 0.645 | 0.728 | 1.041 |
| RHSIM_RhsimUnG0047500 | 0.21 | 0.645 | 0.728 | -0.652 |
| RHSIM_Rhsim04G0198600 | 0.21 | 0.645 | 0.728 | -1.087 |
| RHSIM_Rhsim02G0102300 | 0.21 | 0.645 | 0.728 | -0.486 |
| RHSIM_Rhsim12G0044500 | 0.21 | 0.645 | 0.728 | -0.251 |
| novel.4006 | 0.21 | 0.645 | 0.728 | 0.659 |
| RHSIM_Rhsim13G0234700 | 0.21 | 0.645 | 0.728 | 0.064 |
| RHSIM_Rhsim08G0188300 | 0.21 | 0.645 | 0.728 | 0.185 |
| RHSIM_Rhsim11G0185500 | 0.21 | 0.646 | 0.728 | -0.810 |
| RHSIM_Rhsim13G0000400 | 0.21 | 0.646 | 0.728 | 0.048 |
| novel.2007 | 0.21 | 0.646 | 0.728 | -0.970 |
| RHSIM_RhsimUnG0158100 | 0.21 | 0.646 | 0.728 | 0.066 |

| Gene | LRT statistic | P value | BH-adjusted P | log2FC (interaction) |
|---|---|---|---|---|
| RHSIM_Rhsim11G0025500 | 0.21 | 0.646 | 0.728 | -0.070 |
| RHSIM_Rhsim13G0220500 | 0.21 | 0.646 | 0.728 | -0.626 |
| RHSIM_Rhsim12G0094500 | 0.21 | 0.646 | 0.729 | -0.075 |
| RHSIM_Rhsim08G0153000 | 0.21 | 0.646 | 0.729 | -0.225 |
| RHSIM_Rhsim12G0071100 | 0.21 | 0.646 | 0.729 | -0.060 |
| RHSIM_Rhsim04G0141900 | 0.21 | 0.646 | 0.729 | 0.123 |
| RHSIM_Rhsim12G0007900 | 0.21 | 0.647 | 0.729 | -0.032 |
| RHSIM_Rhsim04G0243200 | 0.21 | 0.647 | 0.729 | 0.117 |
| novel.2238 | 0.21 | 0.647 | 0.729 | 0.512 |
| RHSIM_Rhsim07G0211200 | 0.21 | 0.647 | 0.729 | 1.387 |
| RHSIM_Rhsim09G0045100 | 0.21 | 0.647 | 0.729 | 0.103 |
| RHSIM_Rhsim07G0085200 | 0.21 | 0.647 | 0.729 | 1.058 |
| novel.416 | 0.21 | 0.647 | 0.729 | 0.166 |
| RHSIM_Rhsim11G0099500 | 0.21 | 0.647 | 0.729 | 0.175 |
| RHSIM_Rhsim01G0045700 | 0.21 | 0.647 | 0.729 | 1.321 |
| novel.2317 | 0.21 | 0.648 | 0.730 | 0.444 |
| novel.4087 | 0.21 | 0.648 | 0.730 | -0.811 |
| RHSIM_Rhsim12G0122800 | 0.21 | 0.648 | 0.730 | -0.767 |
| RHSIM_Rhsim11G0103000 | 0.21 | 0.648 | 0.730 | 0.163 |
| RHSIM_Rhsim09G0000600 | 0.21 | 0.648 | 0.730 | -0.155 |
| RHSIM_Rhsim13G0199900 | 0.21 | 0.648 | 0.730 | 0.242 |
| RHSIM_Rhsim10G0172100 | 0.21 | 0.648 | 0.730 | -0.252 |
| RHSIM_Rhsim07G0092200 | 0.21 | 0.648 | 0.730 | -0.393 |
| RHSIM_Rhsim01G0128800 | 0.21 | 0.648 | 0.730 | -1.508 |
| RHSIM_Rhsim12G0017500 | 0.21 | 0.648 | 0.730 | 0.039 |
| RHSIM_Rhsim06G0036700 | 0.21 | 0.648 | 0.730 | -0.498 |
| novel.2131 | 0.21 | 0.648 | 0.730 | -33.890 |
| novel.1439 | 0.21 | 0.648 | 0.730 | 0.433 |
| RHSIM_Rhsim02G0015900 | 0.21 | 0.648 | 0.730 | -0.143 |
| RHSIM_Rhsim03G0097500 | 0.21 | 0.648 | 0.730 | 0.034 |
| RHSIM_RhsimUnG0012100 | 0.21 | 0.649 | 0.730 | -0.757 |
| RHSIM_Rhsim01G0081400 | 0.21 | 0.649 | 0.730 | -0.401 |

| Gene | LRT statistic | P value | BH-adjusted P | log2FC (interaction) |
|---|---|---|---|---|
| RHSIM_Rhsim01G0147100 | 0.21 | 0.649 | 0.730 | 0.816 |
| RHSIM_Rhsim13G0025300 | 0.21 | 0.649 | 0.730 | -0.733 |
| RHSIM_Rhsim07G0066700 | 0.21 | 0.649 | 0.730 | 0.056 |
| RHSIM_Rhsim10G0191600 | 0.21 | 0.649 | 0.730 | 0.070 |
| RHSIM_Rhsim01G0043400 | 0.21 | 0.649 | 0.730 | 1.301 |
| RHSIM_Rhsim09G0083400 | 0.21 | 0.649 | 0.731 | 1.406 |
| novel.3930 | 0.21 | 0.649 | 0.731 | 0.283 |
| RHSIM_Rhsim04G0129400 | 0.21 | 0.649 | 0.731 | -0.469 |
| RHSIM_Rhsim07G0046100 | 0.21 | 0.649 | 0.731 | -0.457 |
| RHSIM_Rhsim03G0016400 | 0.21 | 0.649 | 0.731 | -0.198 |
| RHSIM_Rhsim03G0066000 | 0.21 | 0.649 | 0.731 | -0.304 |
| novel.2510 | 0.21 | 0.649 | 0.731 | -0.060 |
| RHSIM_Rhsim03G0148300 | 0.21 | 0.649 | 0.731 | 0.052 |
| RHSIM_Rhsim08G0145000 | 0.21 | 0.650 | 0.731 | 1.314 |
| RHSIM_Rhsim04G0051600 | 0.21 | 0.650 | 0.731 | -0.659 |
| RHSIM_Rhsim07G0066200 | 0.21 | 0.650 | 0.731 | -0.041 |
| novel.1852 | 0.21 | 0.650 | 0.731 | -0.547 |
| RHSIM_Rhsim12G0035300 | 0.21 | 0.650 | 0.732 | -0.810 |
| novel.4323 | 0.21 | 0.650 | 0.732 | 0.275 |
| RHSIM_Rhsim10G0187400 | 0.21 | 0.650 | 0.732 | -0.148 |
| RHSIM_Rhsim11G0195800 | 0.21 | 0.650 | 0.732 | 33.971 |
| RHSIM_Rhsim12G0178700 | 0.21 | 0.650 | 0.732 | 0.091 |
| RHSIM_Rhsim03G0084400 | 0.21 | 0.650 | 0.732 | 0.322 |
| RHSIM_Rhsim05G0092500 | 0.21 | 0.650 | 0.732 | 0.135 |
| RHSIM_Rhsim11G0050700 | 0.20 | 0.651 | 0.732 | 0.722 |
| RHSIM_Rhsim01G0002900 | 0.20 | 0.651 | 0.732 | 0.050 |
| novel.472 | 0.20 | 0.651 | 0.732 | -0.842 |
| novel.1252 | 0.20 | 0.651 | 0.732 | -0.599 |
| novel.2421 | 0.20 | 0.651 | 0.732 | -0.383 |
| RHSIM_Rhsim03G0214700 | 0.20 | 0.652 | 0.733 | 0.160 |
| RHSIM_Rhsim07G0060800 | 0.20 | 0.652 | 0.733 | -0.214 |
| RHSIM_Rhsim04G0039300 | 0.20 | 0.652 | 0.733 | 0.171 |

| Gene | LRT statistic | P value | BH-adjusted P | log2FC (interaction) |
|---|---|---|---|---|
| novel.4367 | 0.20 | 0.652 | 0.733 | -33.885 |
| RHSIM_Rhsim11G0119600 | 0.20 | 0.652 | 0.733 | 0.391 |
| RHSIM_Rhsim12G0061200 | 0.20 | 0.652 | 0.733 | 0.525 |
| RHSIM_Rhsim11G0064700 | 0.20 | 0.653 | 0.734 | -0.085 |
| RHSIM_Rhsim13G0041100 | 0.20 | 0.653 | 0.734 | 0.129 |
| RHSIM_Rhsim03G0021500 | 0.20 | 0.653 | 0.734 | -0.192 |
| RHSIM_Rhsim02G0079400 | 0.20 | 0.653 | 0.734 | -0.238 |
| RHSIM_Rhsim11G0044900 | 0.20 | 0.653 | 0.734 | 0.045 |
| novel.1985 | 0.20 | 0.653 | 0.734 | 0.037 |
| RHSIM_Rhsim10G0005800 | 0.20 | 0.654 | 0.735 | -0.355 |
| RHSIM_Rhsim12G0075900 | 0.20 | 0.654 | 0.735 | 0.597 |
| RHSIM_Rhsim11G0136100 | 0.20 | 0.654 | 0.735 | 0.067 |
| RHSIM_Rhsim06G0197800 | 0.20 | 0.654 | 0.735 | 0.271 |
| RHSIM_Rhsim04G0191400 | 0.20 | 0.654 | 0.735 | -0.534 |
| RHSIM_Rhsim05G0070300 | 0.20 | 0.654 | 0.735 | 0.044 |
| RHSIM_Rhsim01G0150500 | 0.20 | 0.654 | 0.735 | -0.239 |
| RHSIM_Rhsim01G0193800 | 0.20 | 0.654 | 0.735 | -0.714 |
| RHSIM_Rhsim03G0193100 | 0.20 | 0.654 | 0.735 | -1.126 |
| RHSIM_Rhsim07G0253600 | 0.20 | 0.655 | 0.736 | 0.338 |
| RHSIM_Rhsim12G0076000 | 0.20 | 0.655 | 0.736 | -0.388 |
| novel.768 | 0.20 | 0.655 | 0.736 | 0.562 |
| RHSIM_Rhsim05G0050200 | 0.20 | 0.655 | 0.736 | -0.701 |
| RHSIM_Rhsim05G0168600 | 0.20 | 0.655 | 0.736 | -0.338 |
| RHSIM_Rhsim03G0242200 | 0.20 | 0.655 | 0.736 | 0.329 |
| RHSIM_Rhsim08G0087400 | 0.20 | 0.655 | 0.736 | 0.731 |
| RHSIM_Rhsim01G0134400 | 0.20 | 0.655 | 0.736 | -0.057 |
| RHSIM_Rhsim03G0178100 | 0.20 | 0.656 | 0.736 | -0.203 |
| RHSIM_Rhsim08G0104900 | 0.20 | 0.656 | 0.736 | 0.107 |
| RHSIM_Rhsim05G0112900 | 0.20 | 0.656 | 0.737 | -0.303 |
| RHSIM_Rhsim11G0094900 | 0.20 | 0.656 | 0.737 | 33.707 |
| RHSIM_Rhsim06G0177100 | 0.20 | 0.656 | 0.737 | -0.081 |
| novel.425 | 0.20 | 0.656 | 0.737 | 0.633 |

| Gene | LRT statistic | P value | BH-adjusted P | log2FC (interaction) |
|---|---|---|---|---|
| RHSIM_Rhsim05G0022200 | 0.20 | 0.656 | 0.737 | 0.530 |
| RHSIM_Rhsim05G0097700 | 0.20 | 0.656 | 0.737 | 0.386 |
| novel.260 | 0.20 | 0.656 | 0.737 | -0.632 |
| RHSIM_Rhsim05G0104100 | 0.20 | 0.657 | 0.737 | -0.069 |
| RHSIM_Rhsim05G0217600 | 0.20 | 0.657 | 0.737 | -0.139 |
| RHSIM_RhsimUnG0120400 | 0.20 | 0.657 | 0.737 | -0.107 |
| novel.366 | 0.20 | 0.657 | 0.737 | -0.224 |
| RHSIM_Rhsim11G0178900 | 0.20 | 0.657 | 0.738 | 0.049 |
| RHSIM_Rhsim08G0088000 | 0.20 | 0.657 | 0.738 | -0.449 |
| novel.2939 | 0.20 | 0.657 | 0.738 | 0.156 |
| RHSIM_RhsimUnG0121800 | 0.20 | 0.657 | 0.738 | -0.853 |
| RHSIM_RhsimUnG0246600 | 0.20 | 0.658 | 0.738 | 0.490 |
| RHSIM_Rhsim08G0027500 | 0.20 | 0.658 | 0.738 | 0.057 |
| RHSIM_Rhsim04G0037300 | 0.20 | 0.658 | 0.738 | 0.814 |
| RHSIM_Rhsim01G0019200 | 0.20 | 0.658 | 0.738 | -0.506 |
| RHSIM_RhsimUnG0097100 | 0.20 | 0.658 | 0.739 | -0.056 |
| novel.3451 | 0.20 | 0.658 | 0.739 | -0.327 |
| RHSIM_Rhsim10G0023900 | 0.20 | 0.659 | 0.739 | -0.300 |
| novel.2462 | 0.20 | 0.659 | 0.739 | -33.550 |
| RHSIM_Rhsim03G0224900 | 0.20 | 0.659 | 0.739 | 0.807 |
| RHSIM_Rhsim12G0069200 | 0.19 | 0.659 | 0.740 | -0.339 |
| RHSIM_Rhsim02G0233000 | 0.19 | 0.659 | 0.740 | 0.294 |
| RHSIM_Rhsim04G0045700 | 0.19 | 0.659 | 0.740 | 1.078 |
| RHSIM_Rhsim13G0156400 | 0.19 | 0.660 | 0.740 | 0.302 |
| RHSIM_Rhsim09G0154700 | 0.19 | 0.660 | 0.740 | -0.615 |
| RHSIM_Rhsim03G0014700 | 0.19 | 0.660 | 0.740 | 1.173 |
| RHSIM_Rhsim10G0133500 | 0.19 | 0.660 | 0.740 | -1.297 |
| RHSIM_Rhsim02G0229600 | 0.19 | 0.660 | 0.740 | -0.318 |
| RHSIM_Rhsim07G0119000 | 0.19 | 0.660 | 0.740 | 0.047 |
| RHSIM_Rhsim13G0223700 | 0.19 | 0.660 | 0.740 | -0.093 |
| RHSIM_Rhsim09G0043200 | 0.19 | 0.660 | 0.740 | -0.268 |
| RHSIM_Rhsim02G0105100 | 0.19 | 0.660 | 0.741 | 0.060 |

| Gene | LRT statistic | P value | BH-adjusted P | log2FC (interaction) |
|---|---|---|---|---|
| RHSIM_Rhsim03G0015200 | 0.19 | 0.660 | 0.741 | -0.363 |
| RHSIM_Rhsim05G0144800 | 0.19 | 0.660 | 0.741 | 0.933 |
| RHSIM_Rhsim09G0125200 | 0.19 | 0.660 | 0.741 | -0.520 |
| RHSIM_Rhsim07G0080300 | 0.19 | 0.661 | 0.741 | 0.215 |
| RHSIM_Rhsim09G0166200 | 0.19 | 0.661 | 0.741 | 0.284 |
| RHSIM_Rhsim05G0002000 | 0.19 | 0.661 | 0.741 | -0.038 |
| RHSIM_Rhsim04G0079100 | 0.19 | 0.661 | 0.742 | -0.765 |
| RHSIM_Rhsim12G0085400 | 0.19 | 0.661 | 0.742 | -0.936 |
| RHSIM_Rhsim05G0180600 | 0.19 | 0.661 | 0.742 | 0.079 |
| RHSIM_Rhsim01G0142900 | 0.19 | 0.662 | 0.742 | -0.511 |
| RHSIM_Rhsim13G0179400 | 0.19 | 0.662 | 0.742 | 0.344 |
| RHSIM_Rhsim10G0038200 | 0.19 | 0.662 | 0.742 | 0.349 |
| RHSIM_Rhsim09G0019300 | 0.19 | 0.662 | 0.742 | -0.305 |
| RHSIM_Rhsim03G0117700 | 0.19 | 0.662 | 0.742 | 0.112 |
| novel.1211 | 0.19 | 0.662 | 0.742 | 0.331 |
| RHSIM_Rhsim11G0046100 | 0.19 | 0.662 | 0.742 | 0.040 |
| novel.3876 | 0.19 | 0.662 | 0.742 | -0.085 |
| RHSIM_Rhsim04G0120300 | 0.19 | 0.662 | 0.742 | 0.038 |
| RHSIM_Rhsim07G0132700 | 0.19 | 0.662 | 0.742 | 0.314 |
| RHSIM_RhsimUnG0069400 | 0.19 | 0.662 | 0.742 | -0.419 |
| RHSIM_Rhsim13G0042600 | 0.19 | 0.662 | 0.742 | -0.058 |
| RHSIM_Rhsim05G0010700 | 0.19 | 0.663 | 0.743 | 0.523 |
| RHSIM_Rhsim02G0230300 | 0.19 | 0.663 | 0.743 | -0.036 |
| RHSIM_Rhsim06G0119200 | 0.19 | 0.663 | 0.743 | 0.570 |
| RHSIM_Rhsim04G0004900 | 0.19 | 0.663 | 0.743 | -0.874 |
| novel.1003 | 0.19 | 0.663 | 0.743 | 0.095 |
| RHSIM_RhsimUnG0094300 | 0.19 | 0.663 | 0.743 | -0.054 |
| novel.1396 | 0.19 | 0.663 | 0.743 | -0.138 |
| RHSIM_Rhsim09G0140600 | 0.19 | 0.663 | 0.743 | 0.754 |
| RHSIM_Rhsim02G0114400 | 0.19 | 0.663 | 0.743 | 0.190 |
| RHSIM_Rhsim06G0039900 | 0.19 | 0.663 | 0.743 | -0.629 |
| RHSIM_Rhsim08G0164700 | 0.19 | 0.663 | 0.743 | 1.190 |

| Gene | LRT statistic | P value | BH-adjusted P | log2FC (interaction) |
|---|---|---|---|---|
| RHSIM_Rhsim04G0237700 | 0.19 | 0.663 | 0.743 | -0.696 |
| RHSIM_Rhsim13G0012500 | 0.19 | 0.664 | 0.743 | 0.097 |
| RHSIM_Rhsim13G0235000 | 0.19 | 0.664 | 0.743 | -0.329 |
| RHSIM_Rhsim11G0068000 | 0.19 | 0.664 | 0.744 | -1.257 |
| RHSIM_Rhsim07G0217100 | 0.19 | 0.664 | 0.744 | -0.614 |
| RHSIM_Rhsim03G0043700 | 0.19 | 0.664 | 0.744 | 0.302 |
| RHSIM_Rhsim08G0124800 | 0.19 | 0.664 | 0.744 | 0.068 |
| RHSIM_Rhsim08G0212200 | 0.19 | 0.664 | 0.744 | -0.040 |
| novel.3633 | 0.19 | 0.664 | 0.744 | 0.311 |
| RHSIM_Rhsim13G0086100 | 0.19 | 0.664 | 0.744 | 0.041 |
| novel.3263 | 0.19 | 0.664 | 0.744 | -0.165 |
| novel.2397 | 0.19 | 0.664 | 0.744 | -0.424 |
| RHSIM_Rhsim11G0090900 | 0.19 | 0.665 | 0.744 | -0.086 |
| novel.3926 | 0.19 | 0.665 | 0.744 | -0.485 |
| novel.4411 | 0.19 | 0.665 | 0.744 | 0.470 |
| RHSIM_Rhsim08G0002700 | 0.19 | 0.665 | 0.744 | -0.048 |
| novel.2895 | 0.19 | 0.665 | 0.744 | -0.463 |
| RHSIM_Rhsim06G0069700 | 0.19 | 0.665 | 0.744 | 0.525 |
| RHSIM_Rhsim05G0039300 | 0.19 | 0.665 | 0.744 | -0.194 |
| novel.3729 | 0.19 | 0.665 | 0.744 | 0.114 |
| RHSIM_Rhsim12G0089500 | 0.19 | 0.665 | 0.744 | -33.596 |
| RHSIM_Rhsim03G0088500 | 0.19 | 0.665 | 0.745 | -0.042 |
| RHSIM_Rhsim07G0232000 | 0.19 | 0.665 | 0.745 | 1.122 |
| RHSIM_Rhsim01G0229000 | 0.19 | 0.665 | 0.745 | 0.910 |
| RHSIM_Rhsim05G0174400 | 0.19 | 0.665 | 0.745 | -0.733 |
| novel.2862 | 0.19 | 0.665 | 0.745 | -0.205 |
| RHSIM_Rhsim06G0031800 | 0.19 | 0.665 | 0.745 | -0.084 |
| RHSIM_Rhsim11G0075600 | 0.19 | 0.665 | 0.745 | 0.563 |
| novel.4138 | 0.19 | 0.666 | 0.745 | 0.191 |
| RHSIM_Rhsim01G0137100 | 0.19 | 0.666 | 0.745 | 0.143 |
| RHSIM_Rhsim09G0002000 | 0.19 | 0.666 | 0.745 | -0.214 |
| novel.1340 | 0.19 | 0.666 | 0.745 | 0.592 |

| Gene | LRT statistic | P value | BH-adjusted P | log2FC (interaction) |
|---|---|---|---|---|
| RHSIM_Rhsim08G0094200 | 0.19 | 0.666 | 0.745 | -0.104 |
| RHSIM_Rhsim07G0086500 | 0.19 | 0.666 | 0.745 | 0.101 |
| RHSIM_Rhsim12G0196700 | 0.19 | 0.666 | 0.745 | 0.107 |
| novel.2437 | 0.19 | 0.666 | 0.745 | 0.234 |
| RHSIM_Rhsim11G0143200 | 0.19 | 0.667 | 0.746 | 0.448 |
| RHSIM_Rhsim03G0028000 | 0.19 | 0.667 | 0.746 | 0.256 |
| RHSIM_Rhsim09G0194300 | 0.19 | 0.667 | 0.746 | -0.455 |
| novel.2744 | 0.19 | 0.667 | 0.746 | 0.669 |
| RHSIM_Rhsim10G0059200 | 0.18 | 0.667 | 0.746 | 0.938 |
| RHSIM_Rhsim08G0018700 | 0.18 | 0.667 | 0.746 | 0.125 |
| novel.1085 | 0.18 | 0.667 | 0.746 | -0.218 |
| RHSIM_Rhsim13G0106900 | 0.18 | 0.667 | 0.746 | 0.060 |
| RHSIM_Rhsim06G0131600 | 0.18 | 0.667 | 0.746 | -0.063 |
| novel.2448 | 0.18 | 0.668 | 0.747 | 0.832 |
| RHSIM_Rhsim10G0150800 | 0.18 | 0.668 | 0.747 | -0.259 |
| RHSIM_Rhsim10G0027700 | 0.18 | 0.669 | 0.748 | 0.131 |
| RHSIM_Rhsim11G0031600 | 0.18 | 0.669 | 0.748 | 0.738 |
| RHSIM_Rhsim13G0104200 | 0.18 | 0.669 | 0.748 | -0.097 |
| novel.1346 | 0.18 | 0.669 | 0.748 | 0.633 |
| RHSIM_Rhsim04G0164900 | 0.18 | 0.669 | 0.748 | 0.101 |
| RHSIM_Rhsim08G0014300 | 0.18 | 0.669 | 0.748 | 1.317 |
| RHSIM_Rhsim13G0047800 | 0.18 | 0.669 | 0.748 | -0.060 |
| RHSIM_Rhsim01G0233300 | 0.18 | 0.669 | 0.748 | -0.100 |
| RHSIM_Rhsim11G0015300 | 0.18 | 0.669 | 0.748 | 0.354 |
| novel.161 | 0.18 | 0.669 | 0.748 | 0.893 |
| RHSIM_Rhsim05G0219100 | 0.18 | 0.669 | 0.748 | 0.900 |
| RHSIM_Rhsim08G0016100 | 0.18 | 0.669 | 0.748 | -0.585 |
| RHSIM_Rhsim07G0049400 | 0.18 | 0.670 | 0.748 | 0.547 |
| RHSIM_Rhsim09G0068400 | 0.18 | 0.670 | 0.749 | -0.054 |
| RHSIM_Rhsim09G0002600 | 0.18 | 0.670 | 0.749 | -1.330 |
| RHSIM_Rhsim01G0178100 | 0.18 | 0.670 | 0.749 | 0.510 |
| RHSIM_Rhsim01G0121100 | 0.18 | 0.670 | 0.749 | 0.044 |

| Gene | LRT statistic | P value | BH-adjusted P | log2FC (interaction) |
|---|---|---|---|---|
| RHSIM_Rhsim07G0059300 | 0.18 | 0.670 | 0.749 | -0.061 |
| RHSIM_Rhsim13G0227100 | 0.18 | 0.670 | 0.749 | -0.207 |
| RHSIM_Rhsim11G0090300 | 0.18 | 0.670 | 0.749 | -0.455 |
| RHSIM_Rhsim09G0039300 | 0.18 | 0.671 | 0.749 | 0.038 |
| RHSIM_Rhsim11G0102500 | 0.18 | 0.671 | 0.749 | 0.284 |
| RHSIM_Rhsim10G0004200 | 0.18 | 0.671 | 0.750 | -0.598 |
| RHSIM_Rhsim01G0070900 | 0.18 | 0.671 | 0.750 | -0.403 |
| RHSIM_Rhsim03G0028500 | 0.18 | 0.671 | 0.750 | -0.092 |
| RHSIM_Rhsim07G0178200 | 0.18 | 0.671 | 0.750 | -0.088 |
| RHSIM_Rhsim02G0018800 | 0.18 | 0.671 | 0.750 | 0.128 |
| RHSIM_Rhsim10G0143700 | 0.18 | 0.671 | 0.750 | -0.441 |
| RHSIM_Rhsim08G0238900 | 0.18 | 0.671 | 0.750 | -0.069 |
| RHSIM_Rhsim03G0078600 | 0.18 | 0.672 | 0.750 | 0.055 |
| RHSIM_Rhsim12G0189700 | 0.18 | 0.672 | 0.750 | -33.658 |
| RHSIM_RhsimUnG0157800 | 0.18 | 0.672 | 0.751 | -0.356 |
| RHSIM_Rhsim06G0220900 | 0.18 | 0.672 | 0.751 | -0.320 |
| RHSIM_Rhsim13G0009800 | 0.18 | 0.672 | 0.751 | 0.081 |
| novel.1066 | 0.18 | 0.672 | 0.751 | 0.096 |
| RHSIM_Rhsim13G0055700 | 0.18 | 0.672 | 0.751 | -0.040 |
| RHSIM_Rhsim09G0108300 | 0.18 | 0.673 | 0.751 | -0.622 |
| RHSIM_Rhsim13G0112000 | 0.18 | 0.673 | 0.751 | 0.038 |
| RHSIM_RhsimMtG0002300 | 0.18 | 0.673 | 0.751 | 0.216 |
| RHSIM_Rhsim06G0157300 | 0.18 | 0.673 | 0.751 | -0.836 |
| RHSIM_Rhsim02G0018700 | 0.18 | 0.673 | 0.751 | 0.814 |
| RHSIM_Rhsim08G0139200 | 0.18 | 0.673 | 0.752 | 0.489 |
| RHSIM_Rhsim08G0055300 | 0.18 | 0.673 | 0.752 | 0.151 |
| RHSIM_RhsimUnG0148500 | 0.18 | 0.673 | 0.752 | -0.206 |
| RHSIM_Rhsim11G0016800 | 0.18 | 0.673 | 0.752 | -1.123 |
| RHSIM_Rhsim06G0212000 | 0.18 | 0.674 | 0.752 | -0.082 |
| RHSIM_Rhsim11G0073000 | 0.18 | 0.674 | 0.752 | -0.241 |
| RHSIM_Rhsim01G0005800 | 0.18 | 0.674 | 0.753 | -0.113 |
| RHSIM_RhsimUnG0056800 | 0.18 | 0.675 | 0.753 | -0.139 |

| Gene | LRT statistic | P value | BH-adjusted P | log2FC (interaction) |
|---|---|---|---|---|
| novel.646 | 0.18 | 0.675 | 0.753 | 0.467 |
| RHSIM_Rhsim09G0210700 | 0.18 | 0.675 | 0.753 | 0.915 |
| novel.3341 | 0.18 | 0.675 | 0.753 | -0.584 |
| RHSIM_Rhsim09G0025700 | 0.18 | 0.675 | 0.753 | -0.076 |
| RHSIM_Rhsim01G0093300 | 0.18 | 0.675 | 0.753 | 1.022 |
| RHSIM_RhsimUnG0155600 | 0.18 | 0.675 | 0.753 | 0.584 |
| RHSIM_Rhsim01G0091300 | 0.18 | 0.675 | 0.753 | 0.472 |
| RHSIM_Rhsim12G0158400 | 0.18 | 0.675 | 0.754 | -0.109 |
| RHSIM_Rhsim09G0073400 | 0.18 | 0.676 | 0.754 | 0.061 |
| novel.640 | 0.18 | 0.676 | 0.754 | 0.392 |
| RHSIM_Rhsim02G0152400 | 0.18 | 0.676 | 0.754 | -0.243 |
| RHSIM_Rhsim12G0122900 | 0.18 | 0.676 | 0.754 | 0.243 |
| RHSIM_Rhsim04G0197700 | 0.18 | 0.676 | 0.754 | -0.111 |
| novel.3620 | 0.17 | 0.676 | 0.754 | 33.565 |
| RHSIM_Rhsim07G0214400 | 0.17 | 0.676 | 0.754 | 1.004 |
| RHSIM_Rhsim05G0022100 | 0.17 | 0.676 | 0.754 | -0.044 |
| RHSIM_Rhsim04G0116600 | 0.17 | 0.676 | 0.754 | -0.337 |
| RHSIM_Rhsim12G0014300 | 0.17 | 0.676 | 0.754 | 0.085 |
| RHSIM_RhsimUnG0038300 | 0.17 | 0.676 | 0.754 | 0.936 |
| novel.3825 | 0.17 | 0.677 | 0.755 | 0.565 |
| novel.495 | 0.17 | 0.677 | 0.755 | 0.143 |
| RHSIM_Rhsim02G0162500 | 0.17 | 0.677 | 0.755 | 0.700 |
| RHSIM_Rhsim03G0027800 | 0.17 | 0.677 | 0.755 | 0.192 |
| RHSIM_Rhsim06G0101000 | 0.17 | 0.677 | 0.755 | -0.195 |
| RHSIM_RhsimUnG0123500 | 0.17 | 0.677 | 0.755 | 0.526 |
| RHSIM_Rhsim01G0218300 | 0.17 | 0.678 | 0.755 | -0.527 |
| RHSIM_RhsimUnG0195800 | 0.17 | 0.678 | 0.756 | -0.256 |
| RHSIM_RhsimUnG0106900 | 0.17 | 0.678 | 0.756 | -0.127 |
| novel.3807 | 0.17 | 0.678 | 0.756 | 0.163 |
| RHSIM_Rhsim12G0189600 | 0.17 | 0.678 | 0.756 | -1.107 |
| RHSIM_RhsimUnG0243700 | 0.17 | 0.678 | 0.756 | -0.123 |
| RHSIM_Rhsim12G0180900 | 0.17 | 0.678 | 0.756 | 0.157 |

| Gene | LRT statistic | P value | BH-adjusted P | log2FC (interaction) |
|---|---|---|---|---|
| RHSIM_Rhsim05G0089500 | 0.17 | 0.679 | 0.757 | -0.293 |
| RHSIM_Rhsim03G0057100 | 0.17 | 0.679 | 0.757 | 0.496 |
| RHSIM_Rhsim10G0109400 | 0.17 | 0.679 | 0.757 | -0.725 |
| RHSIM_Rhsim06G0197500 | 0.17 | 0.679 | 0.757 | -0.045 |
| RHSIM_Rhsim05G0226000 | 0.17 | 0.679 | 0.757 | -0.092 |
| RHSIM_Rhsim12G0060500 | 0.17 | 0.679 | 0.757 | 0.419 |
| novel.733 | 0.17 | 0.680 | 0.757 | -0.407 |
| RHSIM_Rhsim09G0101500 | 0.17 | 0.680 | 0.757 | -0.181 |
| RHSIM_Rhsim03G0143100 | 0.17 | 0.680 | 0.757 | 0.098 |
| RHSIM_Rhsim11G0070600 | 0.17 | 0.680 | 0.758 | -0.057 |
| novel.1451 | 0.17 | 0.680 | 0.758 | 0.106 |
| RHSIM_Rhsim09G0116100 | 0.17 | 0.680 | 0.758 | -0.169 |
| novel.697 | 0.17 | 0.680 | 0.758 | -0.501 |
| RHSIM_Rhsim13G0092000 | 0.17 | 0.680 | 0.758 | 0.044 |
| novel.4021 | 0.17 | 0.680 | 0.758 | 0.100 |
| RHSIM_Rhsim01G0264200 | 0.17 | 0.680 | 0.758 | 0.040 |
| RHSIM_Rhsim11G0035800 | 0.17 | 0.681 | 0.758 | 0.050 |
| RHSIM_Rhsim04G0201100 | 0.17 | 0.681 | 0.758 | 0.417 |
| RHSIM_Rhsim01G0133800 | 0.17 | 0.681 | 0.759 | -0.253 |
| novel.2634 | 0.17 | 0.681 | 0.759 | 0.029 |
| novel.491 | 0.17 | 0.681 | 0.759 | 0.646 |
| RHSIM_Rhsim12G0174400 | 0.17 | 0.681 | 0.759 | 0.070 |
| RHSIM_RhsimUnG0157500 | 0.17 | 0.682 | 0.759 | 0.346 |
| RHSIM_Rhsim11G0145200 | 0.17 | 0.682 | 0.760 | -0.058 |
| novel.3299 | 0.17 | 0.682 | 0.760 | 0.397 |
| RHSIM_Rhsim03G0195700 | 0.17 | 0.682 | 0.760 | -0.139 |
| RHSIM_Rhsim07G0203400 | 0.17 | 0.682 | 0.760 | 33.443 |
| RHSIM_Rhsim11G0183500 | 0.17 | 0.683 | 0.760 | -33.604 |
| RHSIM_Rhsim04G0181600 | 0.17 | 0.683 | 0.761 | 0.065 |
| RHSIM_Rhsim08G0193600 | 0.17 | 0.683 | 0.761 | -0.491 |
| RHSIM_Rhsim11G0152000 | 0.17 | 0.684 | 0.761 | -1.341 |
| RHSIM_Rhsim01G0040900 | 0.17 | 0.684 | 0.761 | 1.078 |

| Gene | LRT statistic | P value | BH-adjusted P | log2FC (interaction) |
|---|---|---|---|---|
| RHSIM_Rhsim08G0022700 | 0.17 | 0.684 | 0.761 | 0.223 |
| RHSIM_Rhsim03G0035100 | 0.17 | 0.684 | 0.761 | -0.317 |
| RHSIM_Rhsim12G0180700 | 0.17 | 0.684 | 0.761 | -0.026 |
| RHSIM_RhsimUnG0160100 | 0.17 | 0.684 | 0.761 | 0.875 |
| RHSIM_Rhsim05G0087100 | 0.17 | 0.684 | 0.761 | 0.194 |
| RHSIM_Rhsim10G0061900 | 0.17 | 0.684 | 0.761 | -1.039 |
| RHSIM_Rhsim03G0025500 | 0.17 | 0.684 | 0.761 | 0.273 |
| RHSIM_Rhsim04G0149900 | 0.17 | 0.684 | 0.761 | 0.317 |
| RHSIM_Rhsim08G0150600 | 0.17 | 0.684 | 0.761 | -0.626 |
| RHSIM_RhsimUnG0216500 | 0.17 | 0.684 | 0.761 | -0.120 |
| RHSIM_Rhsim01G0051800 | 0.17 | 0.684 | 0.762 | 0.437 |
| RHSIM_Rhsim06G0222300 | 0.16 | 0.685 | 0.762 | 0.061 |
| novel.4026 | 0.16 | 0.685 | 0.762 | 1.105 |
| RHSIM_Rhsim02G0226500 | 0.16 | 0.685 | 0.762 | 0.256 |
| RHSIM_Rhsim02G0233800 | 0.16 | 0.685 | 0.762 | -0.453 |
| RHSIM_Rhsim12G0051600 | 0.16 | 0.685 | 0.762 | -1.246 |
| RHSIM_Rhsim06G0117200 | 0.16 | 0.685 | 0.762 | -0.637 |
| RHSIM_Rhsim07G0215700 | 0.16 | 0.685 | 0.762 | 0.997 |
| RHSIM_Rhsim04G0124800 | 0.16 | 0.685 | 0.763 | -0.223 |
| RHSIM_Rhsim01G0016900 | 0.16 | 0.686 | 0.763 | 0.296 |
| novel.878 | 0.16 | 0.686 | 0.763 | -0.152 |
| RHSIM_Rhsim11G0100400 | 0.16 | 0.686 | 0.763 | -0.726 |
| RHSIM_Rhsim02G0193500 | 0.16 | 0.686 | 0.763 | 0.386 |
| RHSIM_Rhsim04G0024800 | 0.16 | 0.686 | 0.763 | 0.046 |
| novel.1692 | 0.16 | 0.686 | 0.763 | 0.181 |
| RHSIM_RhsimUnG0159200 | 0.16 | 0.686 | 0.763 | 0.081 |
| novel.3857 | 0.16 | 0.686 | 0.763 | 0.308 |
| RHSIM_Rhsim03G0197700 | 0.16 | 0.687 | 0.764 | -0.053 |
| RHSIM_Rhsim06G0232800 | 0.16 | 0.687 | 0.764 | -0.073 |
| RHSIM_RhsimUnG0027700 | 0.16 | 0.687 | 0.764 | 0.190 |
| RHSIM_Rhsim06G0030000 | 0.16 | 0.687 | 0.764 | -0.916 |
| RHSIM_Rhsim01G0024100 | 0.16 | 0.687 | 0.764 | -0.417 |

| Gene | LRT statistic | P value | BH-adjusted P | log2FC (interaction) |
|---|---|---|---|---|
| RHSIM_Rhsim03G0002600 | 0.16 | 0.687 | 0.764 | 0.590 |
| novel.3878 | 0.16 | 0.687 | 0.764 | -0.336 |
| RHSIM_Rhsim12G0143400 | 0.16 | 0.687 | 0.764 | 0.069 |
| novel.2013 | 0.16 | 0.688 | 0.765 | 0.132 |
| RHSIM_Rhsim09G0038800 | 0.16 | 0.688 | 0.765 | -0.180 |
| RHSIM_Rhsim10G0020100 | 0.16 | 0.688 | 0.765 | 0.041 |
| RHSIM_Rhsim11G0120200 | 0.16 | 0.688 | 0.765 | 0.975 |
| RHSIM_Rhsim11G0186400 | 0.16 | 0.688 | 0.765 | 0.168 |
| novel.3818 | 0.16 | 0.688 | 0.765 | 0.409 |
| novel.4567 | 0.16 | 0.688 | 0.765 | -33.438 |
| RHSIM_Rhsim10G0067600 | 0.16 | 0.688 | 0.765 | 0.169 |
| RHSIM_Rhsim05G0158900 | 0.16 | 0.689 | 0.765 | -0.105 |
| RHSIM_Rhsim11G0147300 | 0.16 | 0.689 | 0.765 | -0.054 |
| RHSIM_Rhsim12G0101200 | 0.16 | 0.689 | 0.765 | -1.162 |
| RHSIM_Rhsim05G0090700 | 0.16 | 0.689 | 0.765 | 0.071 |
| RHSIM_Rhsim02G0102400 | 0.16 | 0.689 | 0.766 | -0.640 |
| RHSIM_Rhsim09G0117000 | 0.16 | 0.689 | 0.766 | -0.103 |
| RHSIM_Rhsim08G0125700 | 0.16 | 0.689 | 0.766 | -33.608 |
| RHSIM_Rhsim02G0027000 | 0.16 | 0.689 | 0.766 | 0.267 |
| RHSIM_Rhsim03G0078100 | 0.16 | 0.690 | 0.766 | -0.355 |
| RHSIM_Rhsim10G0027800 | 0.16 | 0.690 | 0.766 | -0.450 |
| RHSIM_Rhsim12G0187200 | 0.16 | 0.690 | 0.766 | -0.247 |
| novel.3846 | 0.16 | 0.690 | 0.766 | 0.173 |
| RHSIM_Rhsim02G0042700 | 0.16 | 0.690 | 0.767 | 0.028 |
| RHSIM_Rhsim06G0230900 | 0.16 | 0.690 | 0.767 | -0.307 |
| novel.4171 | 0.16 | 0.690 | 0.767 | -0.143 |
| RHSIM_RhsimUnG0117400 | 0.16 | 0.691 | 0.767 | 0.093 |
| RHSIM_Rhsim12G0105600 | 0.16 | 0.691 | 0.767 | 0.222 |
| novel.1221 | 0.16 | 0.691 | 0.767 | 0.762 |
| RHSIM_Rhsim12G0096000 | 0.16 | 0.691 | 0.767 | 0.237 |
| RHSIM_Rhsim11G0040300 | 0.16 | 0.691 | 0.767 | 0.062 |
| RHSIM_Rhsim13G0150300 | 0.16 | 0.691 | 0.767 | 0.061 |

| Gene | LRT statistic | P value | BH-adjusted P | log2FC (interaction) |
|---|---|---|---|---|
| RHSIM_Rhsim11G0025000 | 0.16 | 0.691 | 0.767 | 0.745 |
| novel.1789 | 0.16 | 0.691 | 0.767 | -0.515 |
| novel.2997 | 0.16 | 0.691 | 0.767 | -0.046 |
| RHSIM_RhsimMtG0002600 | 0.16 | 0.691 | 0.767 | 0.249 |
| RHSIM_Rhsim10G0035400 | 0.16 | 0.691 | 0.767 | 0.786 |
| RHSIM_Rhsim11G0185600 | 0.16 | 0.691 | 0.768 | 0.953 |
| novel.1044 | 0.16 | 0.692 | 0.768 | 0.207 |
| novel.694 | 0.16 | 0.692 | 0.768 | 0.263 |
| RHSIM_Rhsim13G0136700 | 0.16 | 0.692 | 0.768 | 0.052 |
| RHSIM_Rhsim02G0225900 | 0.16 | 0.692 | 0.768 | 0.125 |
| novel.121 | 0.16 | 0.692 | 0.768 | 0.336 |
| novel.124 | 0.16 | 0.692 | 0.768 | 0.590 |
| RHSIM_Rhsim09G0173300 | 0.16 | 0.692 | 0.768 | 0.122 |
| RHSIM_Rhsim13G0190200 | 0.16 | 0.692 | 0.768 | -0.271 |
| RHSIM_RhsimMtG0007400 | 0.16 | 0.692 | 0.768 | 0.267 |
| novel.1123 | 0.16 | 0.693 | 0.769 | 0.258 |
| RHSIM_Rhsim13G0047900 | 0.16 | 0.693 | 0.769 | 0.082 |
| RHSIM_Rhsim10G0215200 | 0.16 | 0.693 | 0.769 | 0.831 |
| RHSIM_Rhsim13G0052500 | 0.16 | 0.693 | 0.769 | 0.202 |
| RHSIM_Rhsim13G0129700 | 0.16 | 0.693 | 0.769 | 0.044 |
| RHSIM_Rhsim07G0225400 | 0.16 | 0.693 | 0.769 | -0.130 |
| RHSIM_Rhsim06G0001800 | 0.16 | 0.693 | 0.769 | -0.360 |
| RHSIM_Rhsim13G0173200 | 0.16 | 0.693 | 0.769 | -0.105 |
| RHSIM_Rhsim03G0242600 | 0.16 | 0.693 | 0.769 | 0.436 |
| RHSIM_RhsimUnG0137000 | 0.16 | 0.693 | 0.769 | -0.374 |
| RHSIM_Rhsim10G0056700 | 0.16 | 0.694 | 0.770 | 0.365 |
| RHSIM_Rhsim09G0182900 | 0.16 | 0.694 | 0.770 | -0.775 |
| RHSIM_Rhsim08G0202800 | 0.16 | 0.694 | 0.770 | -0.821 |
| RHSIM_Rhsim01G0259900 | 0.16 | 0.694 | 0.770 | 0.030 |
| RHSIM_Rhsim12G0109800 | 0.16 | 0.694 | 0.770 | 1.057 |
| RHSIM_Rhsim06G0216700 | 0.15 | 0.694 | 0.770 | -33.404 |
| RHSIM_Rhsim12G0025500 | 0.15 | 0.694 | 0.770 | -0.038 |

| Gene | LRT statistic | P value | BH-adjusted P | log2FC (interaction) |
|---|---|---|---|---|
| RHSIM_Rhsim02G0136200 | 0.15 | 0.694 | 0.770 | -0.527 |
| RHSIM_Rhsim03G0010100 | 0.15 | 0.694 | 0.770 | -0.726 |
| RHSIM_Rhsim13G0118900 | 0.15 | 0.694 | 0.770 | 0.496 |
| novel.606 | 0.15 | 0.694 | 0.770 | 0.117 |
| RHSIM_Rhsim01G0028500 | 0.15 | 0.694 | 0.770 | -0.120 |
| RHSIM_Rhsim10G0155000 | 0.15 | 0.694 | 0.770 | 0.113 |
| novel.784 | 0.15 | 0.694 | 0.770 | 0.238 |
| novel.452 | 0.15 | 0.695 | 0.770 | 0.399 |
| RHSIM_RhsimUnG0001300 | 0.15 | 0.695 | 0.770 | 0.943 |
| RHSIM_Rhsim01G0165800 | 0.15 | 0.695 | 0.771 | -0.898 |
| RHSIM_Rhsim10G0094100 | 0.15 | 0.695 | 0.771 | 33.406 |
| RHSIM_Rhsim08G0138700 | 0.15 | 0.695 | 0.771 | -0.588 |
| RHSIM_Rhsim11G0166700 | 0.15 | 0.695 | 0.771 | -0.396 |
| RHSIM_Rhsim04G0176100 | 0.15 | 0.695 | 0.771 | 0.044 |
| RHSIM_Rhsim10G0102500 | 0.15 | 0.695 | 0.771 | -0.062 |
| novel.3645 | 0.15 | 0.695 | 0.771 | -0.179 |
| RHSIM_Rhsim11G0184300 | 0.15 | 0.695 | 0.771 | -0.051 |
| novel.4161 | 0.15 | 0.695 | 0.771 | -0.290 |
| RHSIM_Rhsim08G0010000 | 0.15 | 0.696 | 0.771 | -0.488 |
| RHSIM_Rhsim07G0187400 | 0.15 | 0.696 | 0.771 | -0.314 |
| RHSIM_Rhsim07G0196400 | 0.15 | 0.696 | 0.772 | 0.144 |
| RHSIM_Rhsim01G0247700 | 0.15 | 0.696 | 0.772 | 0.078 |
| novel.3812 | 0.15 | 0.696 | 0.772 | -0.301 |
| RHSIM_Rhsim10G0183500 | 0.15 | 0.696 | 0.772 | -0.029 |
| novel.1213 | 0.15 | 0.696 | 0.772 | 0.481 |
| RHSIM_Rhsim08G0079100 | 0.15 | 0.696 | 0.772 | 0.184 |
| RHSIM_Rhsim08G0164900 | 0.15 | 0.697 | 0.772 | -0.380 |
| RHSIM_Rhsim01G0188000 | 0.15 | 0.697 | 0.772 | 0.953 |
| RHSIM_Rhsim07G0065800 | 0.15 | 0.697 | 0.772 | 0.320 |
| novel.4255 | 0.15 | 0.697 | 0.772 | -0.619 |
| novel.4174 | 0.15 | 0.697 | 0.772 | 0.195 |
| RHSIM_Rhsim09G0066200 | 0.15 | 0.697 | 0.772 | -0.036 |

| Gene | LRT statistic | P value | BH-adjusted P | log2FC (interaction) |
|---|---|---|---|---|
| novel.3698 | 0.15 | 0.697 | 0.772 | 0.207 |
| RHSIM_Rhsim12G0188100 | 0.15 | 0.697 | 0.772 | 0.460 |
| RHSIM_Rhsim02G0180200 | 0.15 | 0.697 | 0.772 | 0.615 |
| RHSIM_Rhsim13G0150100 | 0.15 | 0.697 | 0.772 | 0.295 |
| RHSIM_Rhsim06G0118400 | 0.15 | 0.697 | 0.772 | 0.172 |
| RHSIM_Rhsim07G0086000 | 0.15 | 0.697 | 0.772 | -0.532 |
| RHSIM_Rhsim01G0109000 | 0.15 | 0.697 | 0.772 | -1.226 |
| RHSIM_Rhsim03G0157100 | 0.15 | 0.698 | 0.773 | 0.302 |
| RHSIM_Rhsim06G0080900 | 0.15 | 0.698 | 0.773 | -0.151 |
| RHSIM_Rhsim09G0057500 | 0.15 | 0.698 | 0.773 | -0.142 |
| RHSIM_Rhsim06G0176200 | 0.15 | 0.698 | 0.773 | 0.082 |
| RHSIM_Rhsim13G0120300 | 0.15 | 0.698 | 0.773 | 0.047 |
| RHSIM_Rhsim09G0181700 | 0.15 | 0.699 | 0.774 | 0.401 |
| RHSIM_Rhsim11G0086200 | 0.15 | 0.699 | 0.774 | 0.596 |
| RHSIM_Rhsim03G0059900 | 0.15 | 0.699 | 0.774 | 0.339 |
| RHSIM_Rhsim10G0116100 | 0.15 | 0.699 | 0.774 | 0.342 |
| RHSIM_Rhsim13G0215400 | 0.15 | 0.699 | 0.774 | 0.100 |
| RHSIM_Rhsim01G0059000 | 0.15 | 0.699 | 0.774 | -0.957 |
| RHSIM_Rhsim06G0028900 | 0.15 | 0.699 | 0.774 | 0.180 |
| novel.1165 | 0.15 | 0.699 | 0.774 | -0.507 |
| RHSIM_RhsimUnG0165300 | 0.15 | 0.700 | 0.774 | -0.240 |
| RHSIM_Rhsim13G0167200 | 0.15 | 0.700 | 0.774 | 0.474 |
| novel.2905 | 0.15 | 0.700 | 0.774 | 0.467 |
| RHSIM_Rhsim01G0054000 | 0.15 | 0.700 | 0.775 | -0.369 |
| novel.2773 | 0.15 | 0.700 | 0.775 | 0.459 |
| RHSIM_Rhsim08G0135400 | 0.15 | 0.700 | 0.775 | 0.039 |
| RHSIM_Rhsim05G0231300 | 0.15 | 0.700 | 0.775 | -0.582 |
| RHSIM_Rhsim01G0253700 | 0.15 | 0.700 | 0.775 | 0.057 |
| RHSIM_Rhsim07G0240000 | 0.15 | 0.700 | 0.775 | -0.723 |
| novel.321 | 0.15 | 0.701 | 0.775 | 0.208 |
| novel.3701 | 0.15 | 0.701 | 0.776 | -0.177 |
| RHSIM_Rhsim04G0020400 | 0.15 | 0.701 | 0.776 | -0.183 |

| Gene | LRT statistic | P value | BH-adjusted P | log2FC (interaction) |
|---|---|---|---|---|
| novel.4157 | 0.15 | 0.701 | 0.776 | 0.235 |
| novel.2802 | 0.15 | 0.701 | 0.776 | -0.325 |
| RHSIM_Rhsim04G0200900 | 0.15 | 0.701 | 0.776 | -0.055 |
| RHSIM_Rhsim08G0008100 | 0.15 | 0.701 | 0.776 | 0.069 |
| RHSIM_Rhsim09G0213400 | 0.15 | 0.701 | 0.776 | -0.060 |
| RHSIM_Rhsim03G0169500 | 0.15 | 0.702 | 0.776 | -0.069 |
| RHSIM_Rhsim04G0010200 | 0.15 | 0.702 | 0.776 | 0.266 |
| RHSIM_Rhsim08G0004600 | 0.15 | 0.702 | 0.776 | -0.121 |
| RHSIM_Rhsim09G0031300 | 0.15 | 0.702 | 0.776 | -0.695 |
| RHSIM_Rhsim06G0229400 | 0.15 | 0.702 | 0.777 | -0.829 |
| RHSIM_Rhsim04G0062700 | 0.15 | 0.702 | 0.777 | 0.267 |
| RHSIM_RhsimUnG0204900 | 0.15 | 0.702 | 0.777 | 0.424 |
| RHSIM_Rhsim01G0096400 | 0.15 | 0.702 | 0.777 | -0.088 |
| novel.2647 | 0.15 | 0.703 | 0.777 | 0.374 |
| novel.1765 | 0.15 | 0.703 | 0.777 | -0.175 |
| RHSIM_RhsimUnG0109300 | 0.15 | 0.703 | 0.777 | 0.072 |
| RHSIM_Rhsim12G0205800 | 0.15 | 0.703 | 0.777 | 0.596 |
| novel.724 | 0.15 | 0.703 | 0.777 | 0.361 |
| RHSIM_Rhsim04G0126200 | 0.15 | 0.703 | 0.777 | 0.091 |
| novel.4280 | 0.15 | 0.703 | 0.777 | -0.097 |
| novel.1086 | 0.15 | 0.703 | 0.777 | -0.029 |
| novel.3848 | 0.15 | 0.703 | 0.777 | 0.267 |
| novel.3929 | 0.15 | 0.703 | 0.777 | -0.247 |
| RHSIM_Rhsim09G0085300 | 0.15 | 0.703 | 0.777 | 0.777 |
| RHSIM_Rhsim05G0034900 | 0.15 | 0.703 | 0.777 | 0.058 |
| RHSIM_Rhsim10G0172900 | 0.14 | 0.703 | 0.778 | -0.626 |
| RHSIM_Rhsim13G0089600 | 0.14 | 0.703 | 0.778 | -0.207 |
| RHSIM_Rhsim01G0098000 | 0.14 | 0.704 | 0.778 | -0.973 |
| RHSIM_Rhsim08G0004700 | 0.14 | 0.704 | 0.778 | 0.078 |
| RHSIM_Rhsim03G0206500 | 0.14 | 0.704 | 0.778 | 0.493 |
| RHSIM_Rhsim06G0217600 | 0.14 | 0.704 | 0.778 | 0.153 |
| RHSIM_Rhsim12G0035100 | 0.14 | 0.704 | 0.778 | -0.413 |

| Gene | LRT statistic | P value | BH-adjusted P | log2FC (interaction) |
|---|---|---|---|---|
| RHSIM_Rhsim12G0191600 | 0.14 | 0.704 | 0.778 | -0.054 |
| RHSIM_Rhsim08G0118200 | 0.14 | 0.704 | 0.778 | -0.236 |
| RHSIM_Rhsim13G0163300 | 0.14 | 0.704 | 0.778 | -0.899 |
| RHSIM_Rhsim05G0143200 | 0.14 | 0.704 | 0.778 | -0.419 |
| novel.3749 | 0.14 | 0.704 | 0.778 | 0.398 |
| novel.1163 | 0.14 | 0.704 | 0.778 | 0.236 |
| RHSIM_Rhsim12G0081700 | 0.14 | 0.704 | 0.778 | -0.340 |
| novel.4350 | 0.14 | 0.704 | 0.778 | -0.501 |
| RHSIM_Rhsim01G0128300 | 0.14 | 0.704 | 0.778 | -0.431 |
| RHSIM_Rhsim02G0178100 | 0.14 | 0.705 | 0.778 | 0.532 |
| RHSIM_Rhsim11G0103300 | 0.14 | 0.705 | 0.778 | -0.158 |
| RHSIM_Rhsim03G0184600 | 0.14 | 0.705 | 0.779 | -0.415 |
| RHSIM_Rhsim13G0027400 | 0.14 | 0.705 | 0.779 | -0.112 |
| RHSIM_Rhsim10G0214100 | 0.14 | 0.705 | 0.779 | 0.137 |
| RHSIM_Rhsim05G0201400 | 0.14 | 0.705 | 0.779 | -0.065 |
| novel.2748 | 0.14 | 0.706 | 0.779 | -0.374 |
| RHSIM_Rhsim11G0147700 | 0.14 | 0.706 | 0.779 | 0.398 |
| RHSIM_RhsimUnG0243200 | 0.14 | 0.706 | 0.779 | -0.513 |
| RHSIM_Rhsim03G0031100 | 0.14 | 0.706 | 0.780 | 0.056 |
| RHSIM_Rhsim04G0216400 | 0.14 | 0.706 | 0.780 | 0.085 |
| RHSIM_RhsimUnG0002200 | 0.14 | 0.706 | 0.780 | -0.094 |
| RHSIM_Rhsim04G0149600 | 0.14 | 0.706 | 0.780 | 0.406 |
| RHSIM_Rhsim02G0157900 | 0.14 | 0.706 | 0.780 | 0.265 |
| novel.4164 | 0.14 | 0.707 | 0.780 | 0.290 |
| RHSIM_Rhsim03G0231100 | 0.14 | 0.707 | 0.781 | -0.037 |
| RHSIM_RhsimUnG0220400 | 0.14 | 0.707 | 0.781 | -0.476 |
| RHSIM_Rhsim03G0156200 | 0.14 | 0.707 | 0.781 | 0.130 |
| RHSIM_Rhsim13G0171600 | 0.14 | 0.707 | 0.781 | -0.126 |
| RHSIM_Rhsim11G0191700 | 0.14 | 0.707 | 0.781 | 0.398 |
| RHSIM_Rhsim02G0037700 | 0.14 | 0.707 | 0.781 | 0.507 |
| RHSIM_Rhsim03G0105600 | 0.14 | 0.708 | 0.781 | -0.466 |
| RHSIM_Rhsim09G0096400 | 0.14 | 0.708 | 0.781 | 0.475 |

| Gene | LRT statistic | P value | BH-adjusted P | log2FC (interaction) |
|---|---|---|---|---|
| RHSIM_Rhsim09G0186000 | 0.14 | 0.708 | 0.781 | 0.208 |
| RHSIM_Rhsim07G0008800 | 0.14 | 0.708 | 0.781 | 0.951 |
| novel.3976 | 0.14 | 0.708 | 0.781 | -0.485 |
| RHSIM_Rhsim12G0149500 | 0.14 | 0.708 | 0.781 | -0.219 |
| RHSIM_Rhsim01G0134300 | 0.14 | 0.708 | 0.781 | 0.401 |
| RHSIM_Rhsim07G0083000 | 0.14 | 0.708 | 0.781 | -0.065 |
| RHSIM_RhsimUnG0241600 | 0.14 | 0.708 | 0.782 | -0.044 |
| RHSIM_Rhsim07G0258800 | 0.14 | 0.709 | 0.782 | 1.032 |
| RHSIM_Rhsim03G0174900 | 0.14 | 0.709 | 0.782 | -0.961 |
| RHSIM_RhsimUnG0165200 | 0.14 | 0.709 | 0.782 | 0.086 |
| novel.4346 | 0.14 | 0.709 | 0.782 | 0.224 |
| RHSIM_Rhsim10G0093200 | 0.14 | 0.709 | 0.782 | -0.276 |
| RHSIM_Rhsim05G0138500 | 0.14 | 0.709 | 0.782 | -0.131 |
| RHSIM_Rhsim01G0214700 | 0.14 | 0.709 | 0.782 | 0.933 |
| RHSIM_RhsimUnG0222900 | 0.14 | 0.709 | 0.783 | -0.222 |
| RHSIM_RhsimUnG0201500 | 0.14 | 0.709 | 0.783 | -0.886 |
| RHSIM_Rhsim12G0177900 | 0.14 | 0.710 | 0.783 | 0.029 |
| RHSIM_Rhsim12G0193200 | 0.14 | 0.710 | 0.783 | -32.581 |
| RHSIM_RhsimUnG0181200 | 0.14 | 0.710 | 0.783 | -0.374 |
| RHSIM_Rhsim13G0023200 | 0.14 | 0.710 | 0.783 | 0.117 |
| RHSIM_Rhsim02G0232700 | 0.14 | 0.710 | 0.783 | 0.038 |
| RHSIM_Rhsim01G0151200 | 0.14 | 0.710 | 0.783 | 0.188 |
| novel.95 | 0.14 | 0.710 | 0.783 | -0.075 |
| RHSIM_Rhsim04G0114200 | 0.14 | 0.711 | 0.784 | 0.061 |
| RHSIM_RhsimUnG0019700 | 0.14 | 0.711 | 0.784 | 0.535 |
| RHSIM_Rhsim13G0064100 | 0.14 | 0.711 | 0.784 | 0.045 |
| RHSIM_Rhsim04G0150400 | 0.14 | 0.711 | 0.784 | -0.082 |
| RHSIM_Rhsim05G0099100 | 0.14 | 0.711 | 0.784 | -0.983 |
| RHSIM_Rhsim12G0105100 | 0.14 | 0.711 | 0.784 | 0.063 |
| novel.1685 | 0.14 | 0.711 | 0.784 | -0.171 |
| RHSIM_Rhsim12G0162500 | 0.14 | 0.711 | 0.784 | -0.044 |
| novel.3311 | 0.14 | 0.711 | 0.784 | 0.174 |

| Gene | LRT statistic | P value | BH-adjusted P | log2FC (interaction) |
|---|---|---|---|---|
| RHSIM_Rhsim03G0020300 | 0.14 | 0.712 | 0.784 | -0.449 |
| RHSIM_Rhsim07G0066500 | 0.14 | 0.712 | 0.785 | 0.041 |
| RHSIM_RhsimUnG0046400 | 0.14 | 0.712 | 0.785 | -0.120 |
| RHSIM_RhsimUnG0129400 | 0.14 | 0.712 | 0.785 | 0.559 |
| RHSIM_Rhsim06G0184000 | 0.14 | 0.712 | 0.785 | 0.182 |
| RHSIM_Rhsim06G0226300 | 0.14 | 0.712 | 0.785 | 0.176 |
| RHSIM_Rhsim10G0177800 | 0.14 | 0.712 | 0.785 | -0.330 |
| RHSIM_Rhsim06G0031200 | 0.14 | 0.713 | 0.785 | -0.116 |
| RHSIM_Rhsim06G0240500 | 0.14 | 0.713 | 0.785 | 1.047 |
| novel.713 | 0.14 | 0.713 | 0.785 | -0.542 |
| RHSIM_Rhsim12G0057800 | 0.14 | 0.713 | 0.785 | -0.187 |
| RHSIM_Rhsim09G0028300 | 0.14 | 0.713 | 0.785 | -0.033 |
| RHSIM_Rhsim10G0015200 | 0.14 | 0.713 | 0.786 | -0.727 |
| RHSIM_Rhsim06G0231100 | 0.14 | 0.713 | 0.786 | -0.023 |
| RHSIM_Rhsim02G0031100 | 0.14 | 0.713 | 0.786 | 0.151 |
| RHSIM_Rhsim10G0155300 | 0.14 | 0.713 | 0.786 | -1.001 |
| novel.1771 | 0.14 | 0.713 | 0.786 | 0.229 |
| RHSIM_Rhsim11G0068100 | 0.14 | 0.713 | 0.786 | -0.387 |
| RHSIM_Rhsim12G0206700 | 0.13 | 0.713 | 0.786 | 0.043 |
| RHSIM_Rhsim01G0028900 | 0.13 | 0.714 | 0.786 | -0.732 |
| RHSIM_Rhsim10G0116900 | 0.13 | 0.714 | 0.786 | 0.483 |
| RHSIM_Rhsim12G0205100 | 0.13 | 0.714 | 0.786 | 0.820 |
| RHSIM_Rhsim13G0045700 | 0.13 | 0.714 | 0.786 | -0.525 |
| RHSIM_Rhsim07G0063500 | 0.13 | 0.714 | 0.786 | 0.675 |
| RHSIM_Rhsim08G0071200 | 0.13 | 0.714 | 0.786 | 0.221 |
| RHSIM_Rhsim04G0030900 | 0.13 | 0.714 | 0.786 | -0.867 |
| RHSIM_Rhsim11G0151300 | 0.13 | 0.714 | 0.786 | -0.311 |
| novel.1394 | 0.13 | 0.714 | 0.787 | 0.866 |
| RHSIM_Rhsim10G0097100 | 0.13 | 0.714 | 0.787 | -0.490 |
| RHSIM_Rhsim12G0182000 | 0.13 | 0.714 | 0.787 | -0.396 |
| RHSIM_Rhsim06G0230200 | 0.13 | 0.714 | 0.787 | 0.927 |
| RHSIM_Rhsim01G0081000 | 0.13 | 0.714 | 0.787 | 0.495 |

| Gene | LRT statistic | P value | BH-adjusted P | log2FC (interaction) |
|---|---|---|---|---|
| RHSIM_Rhsim12G0112000 | 0.13 | 0.715 | 0.787 | 0.220 |
| RHSIM_RhsimUnG0195500 | 0.13 | 0.715 | 0.787 | -0.697 |
| RHSIM_Rhsim11G0042700 | 0.13 | 0.715 | 0.787 | 0.560 |
| RHSIM_Rhsim07G0175500 | 0.13 | 0.715 | 0.787 | -0.061 |
| RHSIM_Rhsim03G0243100 | 0.13 | 0.715 | 0.787 | -0.520 |
| RHSIM_Rhsim04G0227600 | 0.13 | 0.715 | 0.787 | -0.911 |
| RHSIM_Rhsim10G0161500 | 0.13 | 0.715 | 0.788 | 1.009 |
| RHSIM_Rhsim07G0049500 | 0.13 | 0.716 | 0.788 | -0.188 |
| RHSIM_RhsimUnG0097200 | 0.13 | 0.716 | 0.788 | -0.206 |
| RHSIM_Rhsim03G0208500 | 0.13 | 0.716 | 0.789 | 0.037 |
| RHSIM_Rhsim06G0182400 | 0.13 | 0.717 | 0.789 | 0.157 |
| RHSIM_Rhsim07G0074800 | 0.13 | 0.717 | 0.789 | 0.545 |
| RHSIM_Rhsim04G0065300 | 0.13 | 0.717 | 0.789 | -0.239 |
| RHSIM_Rhsim04G0021800 | 0.13 | 0.717 | 0.789 | 0.027 |
| RHSIM_Rhsim13G0047000 | 0.13 | 0.717 | 0.789 | -0.113 |
| novel.4424 | 0.13 | 0.717 | 0.789 | 0.838 |
| RHSIM_Rhsim09G0069200 | 0.13 | 0.717 | 0.789 | 0.049 |
| RHSIM_Rhsim07G0025900 | 0.13 | 0.718 | 0.790 | 0.185 |
| RHSIM_Rhsim10G0014100 | 0.13 | 0.718 | 0.790 | -33.028 |
| RHSIM_Rhsim13G0045800 | 0.13 | 0.718 | 0.790 | -0.036 |
| RHSIM_Rhsim05G0046100 | 0.13 | 0.718 | 0.790 | 0.275 |
| RHSIM_Rhsim11G0088300 | 0.13 | 0.718 | 0.790 | -0.050 |
| RHSIM_Rhsim12G0062800 | 0.13 | 0.718 | 0.790 | 0.081 |
| RHSIM_Rhsim11G0178800 | 0.13 | 0.718 | 0.790 | -0.031 |
| RHSIM_Rhsim08G0044800 | 0.13 | 0.718 | 0.790 | -0.198 |
| RHSIM_Rhsim11G0027900 | 0.13 | 0.718 | 0.790 | 0.139 |
| novel.2613 | 0.13 | 0.718 | 0.790 | 0.114 |
| novel.2736 | 0.13 | 0.719 | 0.791 | -32.979 |
| RHSIM_Rhsim10G0137000 | 0.13 | 0.719 | 0.791 | -0.472 |
| novel.3642 | 0.13 | 0.719 | 0.791 | 0.156 |
| novel.1410 | 0.13 | 0.719 | 0.791 | -0.668 |
| novel.173 | 0.13 | 0.719 | 0.791 | 0.186 |

| Gene | LRT statistic | P value | BH-adjusted P | log2FC (interaction) |
|---|---|---|---|---|
| RHSIM_Rhsim03G0037300 | 0.13 | 0.719 | 0.791 | -0.544 |
| RHSIM_Rhsim03G0000300 | 0.13 | 0.720 | 0.791 | 0.248 |
| RHSIM_Rhsim12G0092700 | 0.13 | 0.720 | 0.792 | -0.449 |
| RHSIM_Rhsim12G0024000 | 0.13 | 0.720 | 0.792 | -0.036 |
| RHSIM_Rhsim02G0086700 | 0.13 | 0.720 | 0.792 | 0.052 |
| RHSIM_Rhsim12G0048000 | 0.13 | 0.720 | 0.792 | -0.755 |
| RHSIM_Rhsim03G0178800 | 0.13 | 0.720 | 0.792 | 0.529 |
| novel.322 | 0.13 | 0.721 | 0.792 | 0.227 |
| RHSIM_Rhsim03G0179700 | 0.13 | 0.721 | 0.792 | -0.065 |
| RHSIM_Rhsim10G0016100 | 0.13 | 0.721 | 0.792 | 0.115 |
| RHSIM_Rhsim01G0257700 | 0.13 | 0.721 | 0.792 | -0.031 |
| RHSIM_Rhsim02G0113300 | 0.13 | 0.721 | 0.792 | -0.353 |
| novel.3183 | 0.13 | 0.721 | 0.793 | 0.233 |
| novel.879 | 0.13 | 0.722 | 0.793 | -0.533 |
| RHSIM_Rhsim06G0225500 | 0.13 | 0.722 | 0.793 | -0.462 |
| novel.2775 | 0.13 | 0.722 | 0.793 | 0.289 |
| RHSIM_Rhsim12G0198600 | 0.13 | 0.722 | 0.793 | -0.110 |
| RHSIM_RhsimUnG0060700 | 0.13 | 0.722 | 0.793 | -0.290 |
| RHSIM_Rhsim08G0022200 | 0.13 | 0.722 | 0.793 | 0.408 |
| RHSIM_Rhsim02G0217500 | 0.13 | 0.722 | 0.793 | 0.044 |
| RHSIM_Rhsim07G0123900 | 0.13 | 0.722 | 0.793 | 0.239 |
| RHSIM_Rhsim11G0027700 | 0.13 | 0.722 | 0.793 | -0.128 |
| RHSIM_Rhsim11G0093100 | 0.13 | 0.723 | 0.794 | 0.038 |
| novel.684 | 0.13 | 0.723 | 0.794 | -0.072 |
| RHSIM_RhsimUnG0003600 | 0.13 | 0.723 | 0.794 | 33.062 |
| RHSIM_Rhsim07G0187200 | 0.13 | 0.723 | 0.794 | -0.502 |
| RHSIM_Rhsim01G0126300 | 0.13 | 0.723 | 0.794 | 0.236 |
| RHSIM_Rhsim07G0199600 | 0.13 | 0.723 | 0.794 | -0.565 |
| RHSIM_Rhsim05G0200200 | 0.13 | 0.723 | 0.794 | -0.625 |
| RHSIM_Rhsim07G0169800 | 0.13 | 0.723 | 0.794 | 0.547 |
| RHSIM_Rhsim07G0000400 | 0.13 | 0.723 | 0.794 | 0.498 |
| RHSIM_Rhsim07G0250000 | 0.13 | 0.723 | 0.794 | 0.371 |

| Gene | LRT statistic | P value | BH-adjusted P | log2FC (interaction) |
|---|---|---|---|---|
| RHSIM_Rhsim05G0100200 | 0.13 | 0.723 | 0.795 | -0.112 |
| RHSIM_Rhsim03G0147800 | 0.12 | 0.724 | 0.795 | 1.031 |
| RHSIM_Rhsim09G0055000 | 0.12 | 0.724 | 0.795 | 0.081 |
| RHSIM_Rhsim13G0138800 | 0.12 | 0.724 | 0.795 | -0.047 |
| RHSIM_Rhsim07G0051000 | 0.12 | 0.724 | 0.795 | 0.448 |
| RHSIM_Rhsim08G0102000 | 0.12 | 0.724 | 0.795 | 0.915 |
| RHSIM_Rhsim12G0101600 | 0.12 | 0.724 | 0.795 | 0.073 |
| novel.1848 | 0.12 | 0.724 | 0.795 | 0.184 |
| RHSIM_Rhsim07G0167100 | 0.12 | 0.724 | 0.795 | -33.072 |
| RHSIM_Rhsim10G0030300 | 0.12 | 0.724 | 0.795 | 0.196 |
| RHSIM_Rhsim05G0060500 | 0.12 | 0.724 | 0.795 | 0.106 |
| RHSIM_Rhsim02G0115300 | 0.12 | 0.725 | 0.795 | 0.160 |
| RHSIM_Rhsim09G0152900 | 0.12 | 0.725 | 0.795 | -0.103 |
| RHSIM_Rhsim03G0227600 | 0.12 | 0.725 | 0.795 | -0.021 |
| RHSIM_Rhsim07G0148700 | 0.12 | 0.725 | 0.796 | -0.621 |
| RHSIM_Rhsim04G0157900 | 0.12 | 0.725 | 0.796 | -0.061 |
| RHSIM_Rhsim09G0199400 | 0.12 | 0.725 | 0.796 | 0.069 |
| RHSIM_Rhsim09G0021700 | 0.12 | 0.725 | 0.796 | 0.083 |
| RHSIM_Rhsim08G0191300 | 0.12 | 0.725 | 0.796 | -0.808 |
| RHSIM_Rhsim10G0061000 | 0.12 | 0.725 | 0.796 | -33.120 |
| RHSIM_Rhsim11G0168600 | 0.12 | 0.726 | 0.796 | -0.052 |
| novel.3124 | 0.12 | 0.726 | 0.796 | 0.087 |
| RHSIM_Rhsim02G0017400 | 0.12 | 0.726 | 0.796 | 0.311 |
| novel.2313 | 0.12 | 0.726 | 0.797 | 0.460 |
| RHSIM_Rhsim02G0194600 | 0.12 | 0.726 | 0.797 | 0.051 |
| RHSIM_Rhsim01G0199000 | 0.12 | 0.726 | 0.797 | 0.178 |
| RHSIM_Rhsim03G0050800 | 0.12 | 0.726 | 0.797 | -0.226 |
| RHSIM_Rhsim12G0100400 | 0.12 | 0.726 | 0.797 | -0.265 |
| RHSIM_RhsimUnG0243000 | 0.12 | 0.726 | 0.797 | 0.113 |
| RHSIM_Rhsim10G0043300 | 0.12 | 0.726 | 0.797 | 0.064 |
| RHSIM_Rhsim08G0243200 | 0.12 | 0.726 | 0.797 | 0.114 |
| RHSIM_Rhsim03G0202300 | 0.12 | 0.727 | 0.797 | 0.291 |

| Gene | LRT statistic | P value | BH-adjusted P | log2FC (interaction) |
|---|---|---|---|---|
| RHSIM_RhsimUnG0202000 | 0.12 | 0.727 | 0.797 | -0.337 |
| RHSIM_Rhsim13G0158500 | 0.12 | 0.727 | 0.797 | -0.469 |
| RHSIM_Rhsim03G0163200 | 0.12 | 0.727 | 0.797 | -0.251 |
| RHSIM_Rhsim01G0073800 | 0.12 | 0.727 | 0.797 | -0.206 |
| novel.2936 | 0.12 | 0.727 | 0.797 | 0.501 |
| RHSIM_Rhsim08G0061700 | 0.12 | 0.727 | 0.798 | 0.223 |
| RHSIM_Rhsim01G0104600 | 0.12 | 0.727 | 0.798 | -0.884 |
| RHSIM_Rhsim07G0160700 | 0.12 | 0.728 | 0.798 | -0.669 |
| novel.2202 | 0.12 | 0.728 | 0.798 | 0.342 |
| RHSIM_Rhsim06G0014500 | 0.12 | 0.728 | 0.798 | 0.086 |
| RHSIM_Rhsim13G0038200 | 0.12 | 0.728 | 0.798 | -0.090 |
| RHSIM_Rhsim02G0176400 | 0.12 | 0.728 | 0.799 | 0.861 |
| RHSIM_Rhsim05G0122500 | 0.12 | 0.728 | 0.799 | 0.273 |
| RHSIM_Rhsim03G0121300 | 0.12 | 0.729 | 0.799 | 0.068 |
| RHSIM_Rhsim04G0204300 | 0.12 | 0.729 | 0.799 | 0.035 |
| RHSIM_Rhsim06G0050000 | 0.12 | 0.729 | 0.799 | -0.131 |
| novel.3832 | 0.12 | 0.729 | 0.799 | -0.136 |
| RHSIM_Rhsim03G0007100 | 0.12 | 0.729 | 0.799 | -0.040 |
| RHSIM_Rhsim02G0113200 | 0.12 | 0.730 | 0.800 | 0.903 |
| novel.1209 | 0.12 | 0.730 | 0.800 | -0.203 |
| RHSIM_RhsimUnG0077800 | 0.12 | 0.730 | 0.800 | -0.484 |
| RHSIM_Rhsim08G0039100 | 0.12 | 0.730 | 0.800 | 0.431 |
| RHSIM_Rhsim01G0112600 | 0.12 | 0.731 | 0.801 | 0.484 |
| novel.3230 | 0.12 | 0.731 | 0.801 | -0.075 |
| RHSIM_Rhsim01G0059700 | 0.12 | 0.731 | 0.801 | -0.069 |
| RHSIM_Rhsim02G0191600 | 0.12 | 0.731 | 0.801 | 0.228 |
| novel.3177 | 0.12 | 0.731 | 0.801 | -0.270 |
| RHSIM_Rhsim10G0106600 | 0.12 | 0.731 | 0.801 | -0.979 |
| RHSIM_RhsimUnG0233200 | 0.12 | 0.731 | 0.801 | 0.205 |
| novel.1336 | 0.12 | 0.731 | 0.801 | 0.243 |
| RHSIM_RhsimUnG0126400 | 0.12 | 0.731 | 0.801 | 0.563 |
| RHSIM_Rhsim03G0097700 | 0.12 | 0.731 | 0.801 | -0.428 |

| Gene | LRT statistic | P value | BH-adjusted P | log2FC (interaction) |
|---|---|---|---|---|
| RHSIM_Rhsim02G0152600 | 0.12 | 0.731 | 0.801 | -0.911 |
| RHSIM_Rhsim04G0090100 | 0.12 | 0.731 | 0.801 | -0.037 |
| RHSIM_Rhsim07G0099600 | 0.12 | 0.732 | 0.801 | -0.407 |
| novel.2878 | 0.12 | 0.732 | 0.801 | 0.572 |
| RHSIM_Rhsim05G0198200 | 0.12 | 0.732 | 0.802 | -0.665 |
| RHSIM_Rhsim07G0133600 | 0.12 | 0.732 | 0.802 | 0.208 |
| RHSIM_Rhsim03G0045600 | 0.12 | 0.732 | 0.802 | 0.113 |
| RHSIM_Rhsim02G0025000 | 0.12 | 0.732 | 0.802 | -0.265 |
| RHSIM_Rhsim04G0092900 | 0.12 | 0.732 | 0.802 | 0.077 |
| RHSIM_Rhsim11G0077300 | 0.12 | 0.733 | 0.802 | -0.054 |
| RHSIM_Rhsim07G0176400 | 0.12 | 0.733 | 0.802 | -0.139 |
| novel.938 | 0.12 | 0.733 | 0.802 | -0.697 |
| RHSIM_RhsimUnG0005800 | 0.12 | 0.733 | 0.802 | -0.152 |
| novel.1873 | 0.12 | 0.733 | 0.803 | -0.450 |
| RHSIM_Rhsim13G0085800 | 0.12 | 0.733 | 0.803 | -0.022 |
| RHSIM_RhsimUnG0113000 | 0.12 | 0.734 | 0.803 | 0.080 |
| novel.1307 | 0.12 | 0.734 | 0.803 | -0.060 |
| RHSIM_Rhsim13G0030300 | 0.12 | 0.734 | 0.804 | 0.883 |
| novel.137 | 0.12 | 0.734 | 0.804 | 0.288 |
| novel.3547 | 0.12 | 0.734 | 0.804 | 0.749 |
| novel.1063 | 0.11 | 0.735 | 0.804 | -0.024 |
| RHSIM_Rhsim06G0156700 | 0.11 | 0.735 | 0.805 | 0.662 |
| RHSIM_Rhsim07G0199900 | 0.11 | 0.735 | 0.805 | -0.312 |
| RHSIM_Rhsim07G0101500 | 0.11 | 0.735 | 0.805 | -0.106 |
| RHSIM_Rhsim05G0138600 | 0.11 | 0.735 | 0.805 | 0.022 |
| RHSIM_Rhsim03G0064000 | 0.11 | 0.735 | 0.805 | -0.033 |
| RHSIM_RhsimUnG0092300 | 0.11 | 0.735 | 0.805 | -0.032 |
| RHSIM_Rhsim04G0106100 | 0.11 | 0.735 | 0.805 | -0.061 |
| RHSIM_Rhsim01G0293400 | 0.11 | 0.736 | 0.805 | 0.607 |
| novel.3174 | 0.11 | 0.736 | 0.805 | 0.374 |
| RHSIM_Rhsim07G0134300 | 0.11 | 0.736 | 0.805 | -0.045 |
| novel.1819 | 0.11 | 0.736 | 0.805 | 0.055 |

| Gene | LRT statistic | P value | BH-adjusted P | log2FC (interaction) |
|---|---|---|---|---|
| novel.414 | 0.11 | 0.736 | 0.806 | 0.295 |
| RHSIM_Rhsim09G0042500 | 0.11 | 0.736 | 0.806 | -0.210 |
| novel.4058 | 0.11 | 0.736 | 0.806 | 0.047 |
| novel.63 | 0.11 | 0.737 | 0.806 | -0.327 |
| RHSIM_Rhsim04G0092200 | 0.11 | 0.737 | 0.807 | 0.094 |
| RHSIM_Rhsim09G0202800 | 0.11 | 0.737 | 0.807 | 0.684 |
| novel.4414 | 0.11 | 0.737 | 0.807 | -0.210 |
| RHSIM_Rhsim10G0076000 | 0.11 | 0.737 | 0.807 | 0.600 |
| RHSIM_RhsimUnG0163900 | 0.11 | 0.738 | 0.807 | 0.101 |
| RHSIM_Rhsim06G0120900 | 0.11 | 0.738 | 0.807 | 0.062 |
| RHSIM_Rhsim05G0202000 | 0.11 | 0.738 | 0.807 | 32.866 |
| novel.2079 | 0.11 | 0.738 | 0.807 | 0.299 |
| RHSIM_Rhsim10G0024300 | 0.11 | 0.738 | 0.807 | 0.726 |
| RHSIM_Rhsim08G0155300 | 0.11 | 0.738 | 0.807 | 0.691 |
| RHSIM_Rhsim07G0109200 | 0.11 | 0.738 | 0.807 | -0.325 |
| RHSIM_Rhsim03G0175200 | 0.11 | 0.738 | 0.807 | -0.106 |
| RHSIM_Rhsim02G0001600 | 0.11 | 0.738 | 0.807 | -0.046 |
| novel.556 | 0.11 | 0.738 | 0.807 | -0.485 |
| RHSIM_Rhsim10G0184700 | 0.11 | 0.738 | 0.807 | -0.235 |
| RHSIM_Rhsim08G0082700 | 0.11 | 0.739 | 0.808 | -0.168 |
| RHSIM_Rhsim12G0090100 | 0.11 | 0.739 | 0.808 | 0.042 |
| RHSIM_Rhsim03G0069900 | 0.11 | 0.739 | 0.808 | 0.341 |
| novel.1372 | 0.11 | 0.739 | 0.808 | -0.296 |
| RHSIM_Rhsim01G0260900 | 0.11 | 0.739 | 0.808 | 0.032 |
| novel.1821 | 0.11 | 0.739 | 0.808 | -0.290 |
| RHSIM_Rhsim01G0011300 | 0.11 | 0.740 | 0.809 | 0.261 |
| RHSIM_Rhsim09G0055200 | 0.11 | 0.740 | 0.809 | 0.255 |
| novel.2365 | 0.11 | 0.740 | 0.809 | -0.150 |
| RHSIM_Rhsim02G0020100 | 0.11 | 0.740 | 0.809 | 0.590 |
| RHSIM_Rhsim13G0187300 | 0.11 | 0.740 | 0.809 | -0.721 |
| RHSIM_Rhsim01G0060600 | 0.11 | 0.740 | 0.809 | 0.199 |
| novel.1621 | 0.11 | 0.740 | 0.809 | 0.107 |

| Gene | LRT statistic | P value | BH-adjusted P | log2FC (interaction) |
|---|---|---|---|---|
| RHSIM_Rhsim10G0206600 | 0.11 | 0.740 | 0.809 | -0.184 |
| novel.439 | 0.11 | 0.740 | 0.809 | -0.373 |
| RHSIM_Rhsim05G0030800 | 0.11 | 0.741 | 0.809 | 0.147 |
| RHSIM_Rhsim07G0227600 | 0.11 | 0.741 | 0.809 | -0.118 |
| RHSIM_Rhsim03G0168300 | 0.11 | 0.741 | 0.809 | -0.056 |
| RHSIM_Rhsim01G0218400 | 0.11 | 0.741 | 0.809 | -0.037 |
| RHSIM_Rhsim03G0127500 | 0.11 | 0.741 | 0.809 | 0.042 |
| RHSIM_Rhsim04G0146500 | 0.11 | 0.741 | 0.809 | 0.087 |
| RHSIM_Rhsim09G0063700 | 0.11 | 0.741 | 0.809 | 0.058 |
| RHSIM_Rhsim01G0106000 | 0.11 | 0.741 | 0.810 | 0.721 |
| novel.1429 | 0.11 | 0.741 | 0.810 | -0.176 |
| RHSIM_Rhsim02G0107500 | 0.11 | 0.741 | 0.810 | 0.639 |
| RHSIM_Rhsim03G0173600 | 0.11 | 0.742 | 0.810 | 0.082 |
| RHSIM_Rhsim04G0035300 | 0.11 | 0.742 | 0.810 | 0.881 |
| RHSIM_Rhsim07G0088000 | 0.11 | 0.742 | 0.810 | 0.210 |
| RHSIM_Rhsim12G0010200 | 0.11 | 0.742 | 0.810 | 0.068 |
| RHSIM_Rhsim01G0017400 | 0.11 | 0.742 | 0.810 | 0.048 |
| novel.1125 | 0.11 | 0.742 | 0.810 | -0.296 |
| novel.1330 | 0.11 | 0.742 | 0.810 | -0.096 |
| RHSIM_Rhsim13G0177200 | 0.11 | 0.742 | 0.811 | -0.178 |
| novel.3924 | 0.11 | 0.742 | 0.811 | -0.037 |
| RHSIM_Rhsim10G0095300 | 0.11 | 0.742 | 0.811 | 0.121 |
| RHSIM_Rhsim01G0265300 | 0.11 | 0.742 | 0.811 | -0.491 |
| RHSIM_Rhsim02G0218900 | 0.11 | 0.742 | 0.811 | -0.683 |
| RHSIM_Rhsim12G0041800 | 0.11 | 0.742 | 0.811 | -0.465 |
| RHSIM_Rhsim10G0177500 | 0.11 | 0.743 | 0.811 | 0.378 |
| RHSIM_Rhsim04G0197600 | 0.11 | 0.743 | 0.811 | 0.182 |
| novel.3695 | 0.11 | 0.743 | 0.811 | -0.192 |
| novel.4072 | 0.11 | 0.743 | 0.811 | -0.173 |
| RHSIM_Rhsim03G0144400 | 0.11 | 0.743 | 0.811 | 0.080 |
| RHSIM_Rhsim10G0066100 | 0.11 | 0.743 | 0.811 | -0.444 |
| RHSIM_Rhsim07G0102200 | 0.11 | 0.743 | 0.811 | 0.227 |

| Gene | LRT statistic | P value | BH-adjusted P | log2FC (interaction) |
|---|---|---|---|---|
| RHSIM_Rhsim06G0013700 | 0.11 | 0.743 | 0.811 | -0.439 |
| RHSIM_Rhsim05G0113700 | 0.11 | 0.743 | 0.811 | 0.440 |
| RHSIM_Rhsim13G0043600 | 0.11 | 0.743 | 0.811 | 0.668 |
| RHSIM_Rhsim04G0182500 | 0.11 | 0.743 | 0.811 | -0.149 |
| RHSIM_Rhsim09G0002800 | 0.11 | 0.744 | 0.811 | -0.760 |
| RHSIM_Rhsim09G0164600 | 0.11 | 0.744 | 0.812 | 0.046 |
| RHSIM_Rhsim06G0012300 | 0.11 | 0.744 | 0.812 | -0.062 |
| RHSIM_Rhsim12G0108200 | 0.11 | 0.744 | 0.812 | -32.769 |
| RHSIM_Rhsim13G0056500 | 0.11 | 0.744 | 0.812 | 0.037 |
| RHSIM_Rhsim06G0160400 | 0.11 | 0.744 | 0.812 | -0.216 |
| RHSIM_Rhsim04G0017300 | 0.11 | 0.744 | 0.812 | 0.681 |
| RHSIM_RhsimUnG0036500 | 0.11 | 0.744 | 0.812 | 0.923 |
| RHSIM_Rhsim12G0153000 | 0.11 | 0.744 | 0.812 | 0.288 |
| RHSIM_Rhsim09G0180900 | 0.11 | 0.744 | 0.812 | 0.634 |
| novel.794 | 0.11 | 0.745 | 0.812 | 0.106 |
| RHSIM_Rhsim06G0019900 | 0.11 | 0.745 | 0.812 | -0.029 |
| novel.2940 | 0.11 | 0.745 | 0.812 | -32.785 |
| RHSIM_Rhsim10G0067200 | 0.11 | 0.745 | 0.813 | 0.140 |
| novel.3327 | 0.11 | 0.745 | 0.813 | -0.340 |
| RHSIM_Rhsim10G0086000 | 0.11 | 0.745 | 0.813 | 0.140 |
| RHSIM_Rhsim01G0268000 | 0.11 | 0.745 | 0.813 | 0.035 |
| RHSIM_Rhsim04G0028700 | 0.11 | 0.745 | 0.813 | 0.494 |
| novel.1971 | 0.11 | 0.745 | 0.813 | 0.604 |
| RHSIM_Rhsim07G0156500 | 0.11 | 0.745 | 0.813 | -0.023 |
| novel.720 | 0.11 | 0.746 | 0.813 | 0.112 |
| RHSIM_Rhsim07G0054700 | 0.11 | 0.746 | 0.813 | 0.039 |
| RHSIM_Rhsim02G0144700 | 0.11 | 0.746 | 0.813 | -0.179 |
| novel.337 | 0.10 | 0.746 | 0.813 | -0.120 |
| novel.3892 | 0.10 | 0.746 | 0.813 | 0.423 |
| RHSIM_Rhsim04G0037400 | 0.10 | 0.746 | 0.814 | -0.108 |
| RHSIM_Rhsim04G0062200 | 0.10 | 0.746 | 0.814 | 0.443 |
| RHSIM_Rhsim02G0023700 | 0.10 | 0.746 | 0.814 | 0.641 |

| Gene | LRT statistic | P value | BH-adjusted P | log2FC (interaction) |
|---|---|---|---|---|
| novel.761 | 0.10 | 0.746 | 0.814 | 0.178 |
| novel.371 | 0.10 | 0.746 | 0.814 | 0.195 |
| RHSIM_Rhsim04G0060100 | 0.10 | 0.746 | 0.814 | -0.191 |
| novel.3480 | 0.10 | 0.747 | 0.814 | 0.181 |
| RHSIM_RhsimUnG0241700 | 0.10 | 0.747 | 0.814 | -0.053 |
| RHSIM_Rhsim06G0174400 | 0.10 | 0.747 | 0.814 | 0.030 |
| RHSIM_Rhsim11G0048800 | 0.10 | 0.747 | 0.814 | -0.036 |
| RHSIM_Rhsim13G0025900 | 0.10 | 0.747 | 0.814 | 0.028 |
| RHSIM_Rhsim02G0125400 | 0.10 | 0.747 | 0.814 | -0.064 |
| novel.3921 | 0.10 | 0.747 | 0.814 | -0.323 |
| novel.1134 | 0.10 | 0.747 | 0.815 | 0.259 |
| RHSIM_Rhsim05G0198300 | 0.10 | 0.747 | 0.815 | -0.397 |
| RHSIM_Rhsim08G0189400 | 0.10 | 0.748 | 0.815 | -0.083 |
| RHSIM_RhsimUnG0069800 | 0.10 | 0.748 | 0.815 | -0.397 |
| novel.3363 | 0.10 | 0.748 | 0.815 | -0.192 |
| RHSIM_Rhsim11G0037700 | 0.10 | 0.748 | 0.815 | -0.067 |
| RHSIM_Rhsim04G0079400 | 0.10 | 0.748 | 0.815 | 0.482 |
| novel.4060 | 0.10 | 0.748 | 0.815 | 0.449 |
| novel.2884 | 0.10 | 0.748 | 0.815 | 0.539 |
| RHSIM_Rhsim09G0189700 | 0.10 | 0.749 | 0.816 | -0.742 |
| RHSIM_Rhsim04G0192700 | 0.10 | 0.749 | 0.816 | 0.304 |
| RHSIM_Rhsim04G0001300 | 0.10 | 0.749 | 0.816 | -0.257 |
| RHSIM_Rhsim13G0133800 | 0.10 | 0.749 | 0.816 | 0.488 |
| novel.3340 | 0.10 | 0.749 | 0.816 | 0.103 |
| RHSIM_Rhsim08G0251200 | 0.10 | 0.749 | 0.816 | -0.906 |
| RHSIM_Rhsim08G0125600 | 0.10 | 0.749 | 0.816 | 0.029 |
| RHSIM_Rhsim12G0026500 | 0.10 | 0.749 | 0.816 | 0.101 |
| RHSIM_Rhsim12G0033200 | 0.10 | 0.749 | 0.816 | 0.549 |
| RHSIM_RhsimUnG0152900 | 0.10 | 0.749 | 0.816 | 1.220 |
| RHSIM_RhsimUnG0230900 | 0.10 | 0.749 | 0.816 | 0.723 |
| RHSIM_Rhsim12G0176700 | 0.10 | 0.749 | 0.816 | 0.826 |
| RHSIM_Rhsim08G0082800 | 0.10 | 0.750 | 0.816 | -0.040 |

| Gene | LRT statistic | P value | BH-adjusted P | log2FC (interaction) |
|---|---|---|---|---|
| RHSIM_Rhsim13G0232600 | 0.10 | 0.750 | 0.816 | -0.055 |
| RHSIM_Rhsim12G0069300 | 0.10 | 0.750 | 0.817 | -0.068 |
| RHSIM_Rhsim11G0174200 | 0.10 | 0.750 | 0.817 | -0.504 |
| RHSIM_Rhsim09G0150900 | 0.10 | 0.750 | 0.817 | 0.383 |
| novel.1074 | 0.10 | 0.750 | 0.817 | 0.133 |
| RHSIM_Rhsim01G0180000 | 0.10 | 0.751 | 0.817 | -0.669 |
| RHSIM_Rhsim10G0103000 | 0.10 | 0.751 | 0.817 | -0.452 |
| RHSIM_Rhsim04G0108600 | 0.10 | 0.751 | 0.817 | -0.155 |
| novel.3494 | 0.10 | 0.751 | 0.817 | -0.025 |
| novel.68 | 0.10 | 0.751 | 0.817 | -0.185 |
| RHSIM_Rhsim10G0122000 | 0.10 | 0.751 | 0.818 | 0.799 |
| RHSIM_Rhsim13G0218600 | 0.10 | 0.751 | 0.818 | 0.530 |
| novel.1515 | 0.10 | 0.751 | 0.818 | 0.052 |
| novel.2009 | 0.10 | 0.751 | 0.818 | 0.417 |
| RHSIM_Rhsim01G0286500 | 0.10 | 0.751 | 0.818 | 0.033 |
| RHSIM_Rhsim12G0040700 | 0.10 | 0.751 | 0.818 | -0.119 |
| RHSIM_Rhsim01G0180100 | 0.10 | 0.752 | 0.818 | 0.241 |
| novel.2031 | 0.10 | 0.752 | 0.818 | 0.139 |
| novel.647 | 0.10 | 0.752 | 0.818 | -0.363 |
| RHSIM_Rhsim10G0095700 | 0.10 | 0.752 | 0.818 | -0.736 |
| RHSIM_RhsimUnG0226900 | 0.10 | 0.752 | 0.819 | 0.030 |
| RHSIM_Rhsim10G0035500 | 0.10 | 0.753 | 0.819 | 0.882 |
| RHSIM_Rhsim04G0056500 | 0.10 | 0.753 | 0.819 | 0.423 |
| RHSIM_Rhsim12G0155700 | 0.10 | 0.753 | 0.819 | 0.026 |
| RHSIM_Rhsim12G0194400 | 0.10 | 0.753 | 0.819 | -0.207 |
| RHSIM_Rhsim12G0135300 | 0.10 | 0.753 | 0.819 | -0.149 |
| RHSIM_Rhsim04G0236400 | 0.10 | 0.753 | 0.819 | -0.428 |
| RHSIM_Rhsim11G0143500 | 0.10 | 0.753 | 0.819 | -0.160 |
| RHSIM_Rhsim01G0118600 | 0.10 | 0.753 | 0.819 | -0.040 |
| RHSIM_Rhsim10G0028300 | 0.10 | 0.753 | 0.819 | -0.301 |
| RHSIM_Rhsim05G0103700 | 0.10 | 0.753 | 0.819 | 0.237 |
| novel.397 | 0.10 | 0.753 | 0.819 | -0.049 |

| Gene | LRT statistic | P value | BH-adjusted P | log2FC (interaction) |
|---|---|---|---|---|
| RHSIM_Rhsim07G0079700 | 0.10 | 0.753 | 0.819 | 0.070 |
| novel.1037 | 0.10 | 0.753 | 0.819 | -32.711 |
| novel.562 | 0.10 | 0.754 | 0.820 | 0.257 |
| RHSIM_Rhsim06G0120400 | 0.10 | 0.754 | 0.820 | -0.611 |
| RHSIM_Rhsim04G0170100 | 0.10 | 0.754 | 0.820 | -0.533 |
| RHSIM_Rhsim06G0226200 | 0.10 | 0.754 | 0.820 | 0.133 |
| RHSIM_Rhsim01G0277000 | 0.10 | 0.754 | 0.820 | -0.029 |
| RHSIM_Rhsim02G0000500 | 0.10 | 0.754 | 0.820 | 0.095 |
| RHSIM_Rhsim03G0257800 | 0.10 | 0.754 | 0.820 | 0.792 |
| RHSIM_Rhsim09G0150700 | 0.10 | 0.754 | 0.820 | 0.469 |
| novel.3595 | 0.10 | 0.755 | 0.820 | -0.137 |
| RHSIM_Rhsim05G0015200 | 0.10 | 0.755 | 0.821 | -0.414 |
| RHSIM_Rhsim13G0167500 | 0.10 | 0.755 | 0.821 | -0.162 |
| RHSIM_Rhsim13G0018500 | 0.10 | 0.755 | 0.821 | -0.043 |
| RHSIM_Rhsim10G0094200 | 0.10 | 0.755 | 0.821 | -0.094 |
| novel.1421 | 0.10 | 0.756 | 0.821 | 0.252 |
| RHSIM_Rhsim01G0080400 | 0.10 | 0.756 | 0.821 | -0.700 |
| RHSIM_RhsimUnG0030300 | 0.10 | 0.756 | 0.821 | 0.437 |
| RHSIM_Rhsim06G0175600 | 0.10 | 0.756 | 0.821 | 0.324 |
| RHSIM_Rhsim06G0184900 | 0.10 | 0.756 | 0.822 | -0.239 |
| RHSIM_RhsimUnG0225900 | 0.10 | 0.756 | 0.822 | -0.243 |
| RHSIM_Rhsim11G0196900 | 0.10 | 0.756 | 0.822 | 0.039 |
| RHSIM_Rhsim09G0183200 | 0.10 | 0.756 | 0.822 | 0.072 |
| RHSIM_Rhsim06G0095000 | 0.10 | 0.756 | 0.822 | -0.585 |
| RHSIM_Rhsim01G0072900 | 0.10 | 0.756 | 0.822 | -0.030 |
| RHSIM_RhsimUnG0218200 | 0.10 | 0.757 | 0.822 | 0.421 |
| RHSIM_Rhsim04G0064500 | 0.10 | 0.757 | 0.823 | -0.097 |
| novel.3514 | 0.10 | 0.757 | 0.823 | 0.146 |
| RHSIM_Rhsim08G0091200 | 0.10 | 0.757 | 0.823 | 0.273 |
| RHSIM_Rhsim01G0224600 | 0.10 | 0.757 | 0.823 | -0.038 |
| novel.289 | 0.10 | 0.757 | 0.823 | 0.174 |
| RHSIM_Rhsim10G0192600 | 0.10 | 0.758 | 0.823 | 0.895 |

| Gene | LRT statistic | P value | BH-adjusted P | log2FC (interaction) |
|---|---|---|---|---|
| RHSIM_Rhsim05G0205400 | 0.10 | 0.758 | 0.823 | -0.133 |
| RHSIM_Rhsim12G0151600 | 0.09 | 0.759 | 0.824 | -0.057 |
| RHSIM_Rhsim03G0183000 | 0.09 | 0.759 | 0.824 | -0.464 |
| RHSIM_Rhsim12G0171600 | 0.09 | 0.759 | 0.824 | -0.053 |
| RHSIM_Rhsim13G0122300 | 0.09 | 0.759 | 0.824 | -0.315 |
| RHSIM_Rhsim12G0091200 | 0.09 | 0.759 | 0.824 | 0.250 |
| RHSIM_RhsimUnG0100200 | 0.09 | 0.759 | 0.824 | -0.342 |
| RHSIM_Rhsim10G0075200 | 0.09 | 0.759 | 0.825 | -0.249 |
| RHSIM_RhsimUnG0156300 | 0.09 | 0.760 | 0.825 | 0.321 |
| RHSIM_Rhsim02G0079500 | 0.09 | 0.760 | 0.825 | -0.125 |
| RHSIM_Rhsim06G0242200 | 0.09 | 0.760 | 0.825 | 0.253 |
| RHSIM_Rhsim10G0210000 | 0.09 | 0.760 | 0.825 | -0.797 |
| RHSIM_Rhsim10G0093800 | 0.09 | 0.760 | 0.826 | 0.264 |
| RHSIM_Rhsim12G0009000 | 0.09 | 0.760 | 0.826 | 0.348 |
| RHSIM_Rhsim13G0011100 | 0.09 | 0.761 | 0.826 | 0.025 |
| RHSIM_RhsimUnG0089300 | 0.09 | 0.761 | 0.826 | -0.077 |
| RHSIM_Rhsim01G0280300 | 0.09 | 0.761 | 0.826 | 0.030 |
| RHSIM_Rhsim13G0225900 | 0.09 | 0.761 | 0.826 | -0.120 |
| novel.4086 | 0.09 | 0.761 | 0.826 | -0.058 |
| novel.2663 | 0.09 | 0.761 | 0.826 | 0.037 |
| RHSIM_Rhsim05G0049400 | 0.09 | 0.761 | 0.827 | -0.130 |
| RHSIM_Rhsim10G0146100 | 0.09 | 0.762 | 0.827 | 0.881 |
| RHSIM_Rhsim11G0170700 | 0.09 | 0.762 | 0.827 | -0.109 |
| novel.1231 | 0.09 | 0.762 | 0.827 | -0.146 |
| RHSIM_Rhsim06G0066900 | 0.09 | 0.762 | 0.827 | -32.621 |
| RHSIM_Rhsim07G0096900 | 0.09 | 0.762 | 0.827 | 0.219 |
| RHSIM_Rhsim03G0215800 | 0.09 | 0.762 | 0.827 | -0.624 |
| RHSIM_Rhsim12G0062400 | 0.09 | 0.762 | 0.827 | 0.615 |
| RHSIM_Rhsim06G0084900 | 0.09 | 0.762 | 0.827 | 0.093 |
| RHSIM_Rhsim12G0146300 | 0.09 | 0.762 | 0.827 | -1.702 |
| RHSIM_RhsimUnG0128900 | 0.09 | 0.762 | 0.827 | 0.068 |
| RHSIM_Rhsim06G0157900 | 0.09 | 0.762 | 0.827 | -0.026 |

| Gene | LRT statistic | P value | BH-adjusted P | log2FC (interaction) |
|---|---|---|---|---|
| RHSIM_RhsimUnG0248400 | 0.09 | 0.762 | 0.827 | -0.070 |
| RHSIM_Rhsim04G0193200 | 0.09 | 0.762 | 0.827 | -0.046 |
| novel.3376 | 0.09 | 0.762 | 0.827 | -0.209 |
| novel.1227 | 0.09 | 0.763 | 0.827 | 32.563 |
| RHSIM_Rhsim03G0125300 | 0.09 | 0.763 | 0.827 | 0.313 |
| RHSIM_Rhsim01G0239200 | 0.09 | 0.763 | 0.827 | 0.514 |
| RHSIM_Rhsim06G0088600 | 0.09 | 0.763 | 0.827 | 0.476 |
| RHSIM_Rhsim10G0039500 | 0.09 | 0.763 | 0.827 | 0.262 |
| RHSIM_RhsimUnG0070700 | 0.09 | 0.763 | 0.827 | -0.106 |
| novel.3497 | 0.09 | 0.763 | 0.827 | 0.127 |
| RHSIM_Rhsim11G0133300 | 0.09 | 0.763 | 0.827 | -0.101 |
| RHSIM_Rhsim03G0018800 | 0.09 | 0.763 | 0.828 | 0.715 |
| novel.4151 | 0.09 | 0.763 | 0.828 | 0.554 |
| RHSIM_Rhsim10G0171600 | 0.09 | 0.763 | 0.828 | -0.299 |
| novel.2659 | 0.09 | 0.764 | 0.828 | 0.117 |
| novel.1587 | 0.09 | 0.764 | 0.828 | -0.200 |
| RHSIM_Rhsim07G0045900 | 0.09 | 0.764 | 0.829 | -0.072 |
| novel.581 | 0.09 | 0.764 | 0.829 | -0.446 |
| RHSIM_Rhsim01G0207400 | 0.09 | 0.764 | 0.829 | -0.569 |
| novel.4232 | 0.09 | 0.765 | 0.829 | -32.598 |
| RHSIM_Rhsim13G0134000 | 0.09 | 0.765 | 0.829 | 0.379 |
| RHSIM_Rhsim08G0001000 | 0.09 | 0.765 | 0.829 | 0.267 |
| novel.3104 | 0.09 | 0.765 | 0.829 | -0.771 |
| RHSIM_Rhsim05G0150700 | 0.09 | 0.765 | 0.829 | -0.039 |
| RHSIM_Rhsim07G0024500 | 0.09 | 0.765 | 0.829 | -0.081 |
| novel.3896 | 0.09 | 0.765 | 0.829 | -0.017 |
| RHSIM_Rhsim08G0191200 | 0.09 | 0.765 | 0.829 | -0.089 |
| RHSIM_Rhsim07G0147800 | 0.09 | 0.765 | 0.829 | 0.041 |
| RHSIM_RhsimUnG0179700 | 0.09 | 0.765 | 0.829 | 0.447 |
| RHSIM_Rhsim09G0190500 | 0.09 | 0.765 | 0.830 | -0.123 |
| RHSIM_Rhsim05G0057300 | 0.09 | 0.766 | 0.830 | -0.617 |
| RHSIM_Rhsim03G0193600 | 0.09 | 0.766 | 0.830 | 0.067 |

| Gene | LRT statistic | P value | BH-adjusted P | log2FC (interaction) |
|---|---|---|---|---|
| RHSIM_Rhsim04G0038500 | 0.09 | 0.766 | 0.830 | -0.445 |
| RHSIM_Rhsim12G0051900 | 0.09 | 0.766 | 0.830 | -0.106 |
| RHSIM_Rhsim06G0004500 | 0.09 | 0.766 | 0.830 | -0.068 |
| RHSIM_RhsimUnG0102500 | 0.09 | 0.766 | 0.830 | 0.030 |
| novel.919 | 0.09 | 0.766 | 0.830 | 0.273 |
| novel.703 | 0.09 | 0.766 | 0.830 | -0.212 |
| RHSIM_Rhsim07G0174800 | 0.09 | 0.767 | 0.830 | -0.516 |
| RHSIM_Rhsim04G0030500 | 0.09 | 0.767 | 0.831 | 0.508 |
| novel.811 | 0.09 | 0.767 | 0.831 | 0.296 |
| RHSIM_Rhsim13G0032200 | 0.09 | 0.767 | 0.831 | -0.071 |
| RHSIM_Rhsim03G0195400 | 0.09 | 0.767 | 0.831 | -0.146 |
| RHSIM_Rhsim12G0125000 | 0.09 | 0.767 | 0.831 | -0.469 |
| RHSIM_Rhsim01G0085300 | 0.09 | 0.767 | 0.831 | 0.276 |
| RHSIM_Rhsim03G0182100 | 0.09 | 0.767 | 0.831 | -0.568 |
| RHSIM_Rhsim07G0042100 | 0.09 | 0.768 | 0.831 | 0.237 |
| novel.52 | 0.09 | 0.768 | 0.831 | 0.109 |
| RHSIM_Rhsim09G0172800 | 0.09 | 0.768 | 0.831 | 0.259 |
| novel.3283 | 0.09 | 0.768 | 0.832 | -0.125 |
| RHSIM_Rhsim07G0093300 | 0.09 | 0.768 | 0.832 | -0.095 |
| RHSIM_Rhsim05G0080700 | 0.09 | 0.768 | 0.832 | -0.070 |
| RHSIM_Rhsim12G0112600 | 0.09 | 0.768 | 0.832 | -0.521 |
| novel.3995 | 0.09 | 0.768 | 0.832 | -0.118 |
| RHSIM_Rhsim01G0241500 | 0.09 | 0.768 | 0.832 | -0.256 |
| RHSIM_RhsimMtG0004200 | 0.09 | 0.769 | 0.832 | 0.188 |
| novel.508 | 0.09 | 0.769 | 0.832 | 0.195 |
| RHSIM_Rhsim10G0056600 | 0.09 | 0.769 | 0.832 | -0.573 |
| novel.3519 | 0.09 | 0.769 | 0.832 | -0.200 |
| novel.93 | 0.09 | 0.769 | 0.832 | -0.059 |
| RHSIM_Rhsim03G0104100 | 0.09 | 0.769 | 0.832 | -0.712 |
| RHSIM_RhsimUnG0124900 | 0.09 | 0.769 | 0.832 | -0.069 |
| RHSIM_Rhsim03G0086400 | 0.09 | 0.769 | 0.832 | 0.034 |
| RHSIM_Rhsim03G0077600 | 0.09 | 0.769 | 0.832 | -0.579 |

| Gene | LRT statistic | P value | BH-adjusted P | log2FC (interaction) |
|---|---|---|---|---|
| RHSIM_Rhsim11G0080400 | 0.09 | 0.769 | 0.833 | -0.576 |
| RHSIM_Rhsim05G0202100 | 0.09 | 0.769 | 0.833 | 0.419 |
| novel.4208 | 0.09 | 0.769 | 0.833 | 0.283 |
| RHSIM_Rhsim01G0247200 | 0.09 | 0.769 | 0.833 | -0.150 |
| RHSIM_Rhsim02G0048800 | 0.09 | 0.769 | 0.833 | -0.095 |
| RHSIM_Rhsim13G0220800 | 0.09 | 0.770 | 0.833 | -0.024 |
| RHSIM_Rhsim11G0088500 | 0.09 | 0.770 | 0.833 | -0.470 |
| RHSIM_Rhsim07G0143200 | 0.09 | 0.770 | 0.833 | 0.023 |
| RHSIM_Rhsim12G0073800 | 0.09 | 0.770 | 0.833 | -0.801 |
| RHSIM_Rhsim01G0290000 | 0.09 | 0.770 | 0.833 | -0.093 |
| RHSIM_Rhsim08G0120800 | 0.09 | 0.770 | 0.833 | 0.484 |
| RHSIM_Rhsim04G0236000 | 0.09 | 0.770 | 0.833 | -0.539 |
| RHSIM_Rhsim06G0219700 | 0.09 | 0.770 | 0.834 | -0.028 |
| novel.827 | 0.09 | 0.770 | 0.834 | 0.041 |
| RHSIM_Rhsim11G0034000 | 0.09 | 0.771 | 0.834 | 0.352 |
| RHSIM_Rhsim05G0066000 | 0.08 | 0.771 | 0.834 | 0.624 |
| RHSIM_Rhsim10G0209500 | 0.08 | 0.771 | 0.834 | 0.478 |
| RHSIM_Rhsim06G0235200 | 0.08 | 0.771 | 0.834 | 0.043 |
| RHSIM_Rhsim10G0067700 | 0.08 | 0.771 | 0.834 | -0.069 |
| RHSIM_Rhsim06G0063200 | 0.08 | 0.771 | 0.834 | 0.172 |
| RHSIM_Rhsim03G0179400 | 0.08 | 0.771 | 0.834 | -0.067 |
| RHSIM_Rhsim01G0095600 | 0.08 | 0.771 | 0.834 | 0.260 |
| novel.1383 | 0.08 | 0.772 | 0.835 | -0.521 |
| RHSIM_Rhsim10G0156500 | 0.08 | 0.772 | 0.835 | -0.151 |
| novel.2288 | 0.08 | 0.772 | 0.835 | 0.859 |
| RHSIM_Rhsim08G0059100 | 0.08 | 0.772 | 0.835 | 0.685 |
| RHSIM_Rhsim04G0179200 | 0.08 | 0.772 | 0.835 | -0.049 |
| novel.147 | 0.08 | 0.773 | 0.835 | -0.080 |
| RHSIM_RhsimUnG0054200 | 0.08 | 0.773 | 0.835 | -0.291 |
| RHSIM_Rhsim06G0234400 | 0.08 | 0.773 | 0.835 | -0.225 |
| novel.3406 | 0.08 | 0.773 | 0.836 | -0.478 |
| RHSIM_Rhsim11G0116400 | 0.08 | 0.773 | 0.836 | -0.589 |

| Gene | LRT statistic | P value | BH-adjusted P | log2FC (interaction) |
|---|---|---|---|---|
| RHSIM_Rhsim01G0223100 | 0.08 | 0.773 | 0.836 | -0.278 |
| RHSIM_Rhsim03G0016000 | 0.08 | 0.774 | 0.837 | -0.332 |
| RHSIM_Rhsim11G0068600 | 0.08 | 0.774 | 0.837 | -0.528 |
| RHSIM_Rhsim04G0100400 | 0.08 | 0.774 | 0.837 | 0.031 |
| novel.564 | 0.08 | 0.774 | 0.837 | -0.420 |
| RHSIM_Rhsim01G0085800 | 0.08 | 0.775 | 0.837 | -0.315 |
| RHSIM_Rhsim02G0058500 | 0.08 | 0.775 | 0.837 | 0.516 |
| RHSIM_Rhsim13G0069500 | 0.08 | 0.775 | 0.837 | 0.038 |
| RHSIM_Rhsim09G0042100 | 0.08 | 0.775 | 0.837 | 0.692 |
| RHSIM_Rhsim07G0215900 | 0.08 | 0.775 | 0.837 | 0.071 |
| RHSIM_Rhsim12G0036700 | 0.08 | 0.775 | 0.837 | 0.061 |
| RHSIM_Rhsim08G0114300 | 0.08 | 0.775 | 0.837 | 0.316 |
| RHSIM_Rhsim11G0155200 | 0.08 | 0.775 | 0.837 | 0.273 |
| RHSIM_Rhsim07G0105400 | 0.08 | 0.775 | 0.837 | -0.047 |
| RHSIM_Rhsim06G0097500 | 0.08 | 0.775 | 0.838 | 0.464 |
| RHSIM_Rhsim07G0012400 | 0.08 | 0.775 | 0.838 | 0.825 |
| novel.3623 | 0.08 | 0.775 | 0.838 | -0.049 |
| RHSIM_Rhsim04G0220300 | 0.08 | 0.775 | 0.838 | -0.065 |
| novel.3535 | 0.08 | 0.776 | 0.838 | -0.259 |
| RHSIM_Rhsim02G0118500 | 0.08 | 0.776 | 0.838 | 0.427 |
| RHSIM_Rhsim11G0088400 | 0.08 | 0.776 | 0.838 | 0.475 |
| RHSIM_Rhsim02G0128400 | 0.08 | 0.776 | 0.838 | 0.325 |
| novel.4495 | 0.08 | 0.776 | 0.838 | 0.245 |
| RHSIM_Rhsim12G0213500 | 0.08 | 0.776 | 0.838 | 0.433 |
| RHSIM_Rhsim02G0144800 | 0.08 | 0.776 | 0.838 | 0.019 |
| RHSIM_Rhsim03G0193500 | 0.08 | 0.776 | 0.838 | -0.628 |
| RHSIM_Rhsim09G0085800 | 0.08 | 0.776 | 0.838 | 0.117 |
| RHSIM_Rhsim11G0135400 | 0.08 | 0.776 | 0.838 | 0.878 |
| RHSIM_Rhsim09G0205300 | 0.08 | 0.776 | 0.838 | -0.077 |
| novel.1629 | 0.08 | 0.776 | 0.838 | 0.084 |
| RHSIM_Rhsim03G0059300 | 0.08 | 0.776 | 0.838 | -0.755 |
| novel.4503 | 0.08 | 0.776 | 0.838 | 0.224 |

| Gene | LRT statistic | P value | BH-adjusted P | log2FC (interaction) |
|---|---|---|---|---|
| RHSIM_Rhsim13G0166400 | 0.08 | 0.777 | 0.838 | 0.261 |
| RHSIM_Rhsim06G0161500 | 0.08 | 0.777 | 0.839 | -0.288 |
| RHSIM_Rhsim06G0144300 | 0.08 | 0.777 | 0.839 | 0.507 |
| novel.2378 | 0.08 | 0.777 | 0.839 | 0.177 |
| RHSIM_Rhsim08G0044900 | 0.08 | 0.777 | 0.839 | 0.072 |
| novel.3027 | 0.08 | 0.777 | 0.839 | 0.094 |
| RHSIM_Rhsim04G0137800 | 0.08 | 0.777 | 0.839 | -0.042 |
| RHSIM_Rhsim04G0156600 | 0.08 | 0.778 | 0.839 | -32.372 |
| RHSIM_Rhsim13G0086800 | 0.08 | 0.778 | 0.839 | -0.060 |
| RHSIM_Rhsim11G0119400 | 0.08 | 0.778 | 0.839 | -0.039 |
| RHSIM_Rhsim04G0032900 | 0.08 | 0.778 | 0.839 | 0.022 |
| RHSIM_Rhsim11G0139100 | 0.08 | 0.778 | 0.839 | -0.673 |
| RHSIM_Rhsim07G0136000 | 0.08 | 0.778 | 0.840 | 0.570 |
| novel.281 | 0.08 | 0.778 | 0.840 | -0.194 |
| RHSIM_Rhsim05G0167500 | 0.08 | 0.778 | 0.840 | 0.058 |
| RHSIM_Rhsim12G0042500 | 0.08 | 0.778 | 0.840 | 0.054 |
| RHSIM_Rhsim12G0214700 | 0.08 | 0.778 | 0.840 | -0.046 |
| RHSIM_Rhsim09G0097500 | 0.08 | 0.778 | 0.840 | -0.199 |
| RHSIM_Rhsim08G0036100 | 0.08 | 0.779 | 0.840 | 0.485 |
| RHSIM_Rhsim05G0145000 | 0.08 | 0.779 | 0.840 | 0.409 |
| RHSIM_Rhsim02G0025500 | 0.08 | 0.779 | 0.840 | 0.376 |
| novel.2181 | 0.08 | 0.779 | 0.840 | -0.230 |
| RHSIM_Rhsim08G0084800 | 0.08 | 0.779 | 0.841 | 0.036 |
| RHSIM_Rhsim09G0032400 | 0.08 | 0.779 | 0.841 | 0.454 |
| RHSIM_Rhsim07G0247900 | 0.08 | 0.779 | 0.841 | -0.093 |
| RHSIM_Rhsim10G0148400 | 0.08 | 0.780 | 0.841 | -0.087 |
| novel.1070 | 0.08 | 0.780 | 0.841 | 0.112 |
| RHSIM_Rhsim06G0064200 | 0.08 | 0.780 | 0.841 | -0.068 |
| novel.3521 | 0.08 | 0.780 | 0.841 | 0.095 |
| RHSIM_Rhsim06G0030700 | 0.08 | 0.780 | 0.842 | -0.019 |
| RHSIM_Rhsim10G0193300 | 0.08 | 0.780 | 0.842 | 0.153 |
| RHSIM_Rhsim02G0211700 | 0.08 | 0.780 | 0.842 | 0.148 |

| Gene | LRT statistic | P value | BH-adjusted P | log2FC (interaction) |
|---|---|---|---|---|
| novel.1917 | 0.08 | 0.780 | 0.842 | -0.145 |
| novel.529 | 0.08 | 0.780 | 0.842 | 0.279 |
| RHSIM_Rhsim13G0079900 | 0.08 | 0.781 | 0.842 | -0.097 |
| RHSIM_Rhsim06G0126400 | 0.08 | 0.781 | 0.842 | 0.233 |
| novel.622 | 0.08 | 0.781 | 0.842 | 0.125 |
| RHSIM_Rhsim13G0062600 | 0.08 | 0.781 | 0.842 | 0.072 |
| RHSIM_Rhsim02G0256100 | 0.08 | 0.781 | 0.842 | 0.108 |
| RHSIM_Rhsim02G0134200 | 0.08 | 0.781 | 0.842 | -0.195 |
| RHSIM_Rhsim01G0193200 | 0.08 | 0.781 | 0.842 | 0.242 |
| RHSIM_Rhsim02G0220800 | 0.08 | 0.781 | 0.842 | 0.081 |
| RHSIM_RhsimUnG0199400 | 0.08 | 0.782 | 0.842 | -0.620 |
| RHSIM_Rhsim04G0027700 | 0.08 | 0.782 | 0.842 | 0.536 |
| RHSIM_Rhsim09G0135100 | 0.08 | 0.782 | 0.842 | -0.243 |
| RHSIM_RhsimUnG0035800 | 0.08 | 0.782 | 0.843 | 0.061 |
| RHSIM_Rhsim01G0036300 | 0.08 | 0.782 | 0.843 | 0.234 |
| RHSIM_Rhsim10G0052100 | 0.08 | 0.782 | 0.843 | -0.680 |
| novel.2129 | 0.08 | 0.782 | 0.843 | 0.171 |
| novel.618 | 0.08 | 0.783 | 0.843 | 0.499 |
| RHSIM_Rhsim05G0052700 | 0.08 | 0.783 | 0.843 | 0.653 |
| RHSIM_Rhsim11G0166900 | 0.08 | 0.783 | 0.843 | 0.457 |
| RHSIM_Rhsim13G0206300 | 0.08 | 0.783 | 0.844 | -0.585 |
| RHSIM_Rhsim13G0137400 | 0.08 | 0.783 | 0.844 | 0.029 |
| RHSIM_Rhsim04G0106000 | 0.08 | 0.783 | 0.844 | -0.061 |
| RHSIM_Rhsim08G0027700 | 0.08 | 0.783 | 0.844 | -0.031 |
| novel.3787 | 0.08 | 0.783 | 0.844 | 0.024 |
| RHSIM_Rhsim02G0058400 | 0.08 | 0.783 | 0.844 | 0.238 |
| RHSIM_Rhsim11G0191100 | 0.08 | 0.783 | 0.844 | 0.848 |
| RHSIM_Rhsim10G0117300 | 0.08 | 0.783 | 0.844 | 0.148 |
| RHSIM_Rhsim02G0200900 | 0.08 | 0.783 | 0.844 | -0.032 |
| RHSIM_Rhsim03G0165900 | 0.08 | 0.783 | 0.844 | 0.087 |
| RHSIM_RhsimUnG0103600 | 0.08 | 0.783 | 0.844 | 0.024 |
| novel.4518 | 0.08 | 0.784 | 0.844 | -0.108 |

| Gene | LRT statistic | P value | BH-adjusted P | log2FC (interaction) |
|---|---|---|---|---|
| RHSIM_Rhsim13G0007300 | 0.08 | 0.784 | 0.844 | 0.198 |
| RHSIM_Rhsim05G0202600 | 0.08 | 0.784 | 0.844 | 0.033 |
| RHSIM_Rhsim05G0056000 | 0.08 | 0.784 | 0.845 | -0.737 |
| RHSIM_Rhsim08G0104100 | 0.08 | 0.784 | 0.845 | -0.243 |
| RHSIM_RhsimUnG0076800 | 0.08 | 0.784 | 0.845 | -0.496 |
| RHSIM_Rhsim02G0087000 | 0.07 | 0.784 | 0.845 | 0.047 |
| RHSIM_Rhsim02G0043100 | 0.07 | 0.784 | 0.845 | -0.171 |
| RHSIM_RhsimUnG0107800 | 0.07 | 0.784 | 0.845 | -0.647 |
| novel.1525 | 0.07 | 0.784 | 0.845 | -0.429 |
| RHSIM_Rhsim03G0060900 | 0.07 | 0.785 | 0.845 | -0.487 |
| RHSIM_Rhsim05G0140200 | 0.07 | 0.785 | 0.845 | 0.117 |
| RHSIM_Rhsim03G0112700 | 0.07 | 0.785 | 0.845 | -0.216 |
| RHSIM_Rhsim12G0102000 | 0.07 | 0.785 | 0.845 | -0.020 |
| RHSIM_Rhsim11G0186600 | 0.07 | 0.785 | 0.845 | 0.030 |
| novel.1662 | 0.07 | 0.785 | 0.845 | 0.134 |
| novel.3127 | 0.07 | 0.785 | 0.845 | 0.229 |
| RHSIM_RhsimUnG0198800 | 0.07 | 0.785 | 0.845 | 0.596 |
| RHSIM_RhsimUnG0153500 | 0.07 | 0.785 | 0.845 | 0.188 |
| RHSIM_Rhsim11G0059100 | 0.07 | 0.785 | 0.845 | -0.687 |
| RHSIM_Rhsim13G0109600 | 0.07 | 0.786 | 0.846 | 0.053 |
| RHSIM_Rhsim10G0100000 | 0.07 | 0.786 | 0.846 | 0.114 |
| RHSIM_Rhsim02G0173800 | 0.07 | 0.786 | 0.846 | 0.084 |
| RHSIM_Rhsim04G0141200 | 0.07 | 0.786 | 0.846 | -0.276 |
| RHSIM_Rhsim09G0105900 | 0.07 | 0.786 | 0.846 | -0.053 |
| RHSIM_Rhsim10G0115500 | 0.07 | 0.786 | 0.846 | -0.043 |
| RHSIM_Rhsim08G0030500 | 0.07 | 0.787 | 0.846 | 0.043 |
| novel.2796 | 0.07 | 0.787 | 0.846 | -0.023 |
| novel.2311 | 0.07 | 0.787 | 0.846 | 0.023 |
| RHSIM_Rhsim08G0148400 | 0.07 | 0.787 | 0.847 | -0.437 |
| RHSIM_Rhsim12G0009500 | 0.07 | 0.787 | 0.847 | 0.320 |
| RHSIM_Rhsim06G0194500 | 0.07 | 0.787 | 0.847 | 0.230 |
| RHSIM_Rhsim10G0032600 | 0.07 | 0.787 | 0.847 | -0.217 |

| Gene | LRT statistic | P value | BH-adjusted P | log2FC (interaction) |
|---|---|---|---|---|
| novel.1015 | 0.07 | 0.787 | 0.847 | 0.131 |
| RHSIM_RhsimUnG0194800 | 0.07 | 0.787 | 0.847 | -0.530 |
| RHSIM_Rhsim05G0204400 | 0.07 | 0.787 | 0.847 | -0.170 |
| RHSIM_Rhsim04G0223500 | 0.07 | 0.788 | 0.847 | 0.700 |
| RHSIM_RhsimUnG0253900 | 0.07 | 0.788 | 0.847 | -0.053 |
| RHSIM_Rhsim03G0132500 | 0.07 | 0.788 | 0.847 | -0.049 |
| RHSIM_Rhsim03G0082400 | 0.07 | 0.788 | 0.848 | 0.705 |
| novel.2870 | 0.07 | 0.789 | 0.848 | 0.098 |
| RHSIM_Rhsim04G0001500 | 0.07 | 0.789 | 0.848 | 0.040 |
| RHSIM_Rhsim07G0206900 | 0.07 | 0.789 | 0.848 | -0.166 |
| RHSIM_Rhsim07G0102600 | 0.07 | 0.789 | 0.848 | 0.337 |
| RHSIM_Rhsim12G0152200 | 0.07 | 0.789 | 0.849 | 0.043 |
| RHSIM_Rhsim07G0248600 | 0.07 | 0.789 | 0.849 | -0.056 |
| RHSIM_Rhsim11G0135300 | 0.07 | 0.789 | 0.849 | 0.207 |
| RHSIM_Rhsim02G0212900 | 0.07 | 0.789 | 0.849 | -0.074 |
| RHSIM_Rhsim04G0057000 | 0.07 | 0.789 | 0.849 | 0.045 |
| RHSIM_Rhsim03G0124100 | 0.07 | 0.790 | 0.849 | 0.779 |
| RHSIM_Rhsim04G0189600 | 0.07 | 0.790 | 0.849 | 0.231 |
| RHSIM_Rhsim07G0213200 | 0.07 | 0.790 | 0.849 | 0.132 |
| RHSIM_Rhsim12G0106400 | 0.07 | 0.790 | 0.849 | 0.275 |
| RHSIM_Rhsim08G0154400 | 0.07 | 0.790 | 0.849 | 0.175 |
| RHSIM_Rhsim03G0229300 | 0.07 | 0.790 | 0.849 | -0.067 |
| RHSIM_Rhsim02G0246500 | 0.07 | 0.790 | 0.849 | -0.031 |
| RHSIM_Rhsim02G0196600 | 0.07 | 0.790 | 0.849 | 0.528 |
| RHSIM_Rhsim13G0113200 | 0.07 | 0.790 | 0.849 | 0.250 |
| RHSIM_Rhsim12G0159700 | 0.07 | 0.790 | 0.849 | 0.798 |
| novel.1407 | 0.07 | 0.790 | 0.849 | -0.256 |
| novel.480 | 0.07 | 0.790 | 0.849 | -0.133 |
| novel.3205 | 0.07 | 0.791 | 0.850 | 0.583 |
| RHSIM_Rhsim10G0044600 | 0.07 | 0.791 | 0.850 | 0.173 |
| RHSIM_Rhsim11G0092400 | 0.07 | 0.791 | 0.850 | 0.034 |
| RHSIM_Rhsim11G0010600 | 0.07 | 0.791 | 0.850 | -0.232 |

| Gene | LRT statistic | P value | BH-adjusted P | log2FC (interaction) |
|---|---|---|---|---|
| RHSIM_Rhsim06G0141200 | 0.07 | 0.791 | 0.850 | 0.072 |
| RHSIM_Rhsim13G0128800 | 0.07 | 0.791 | 0.850 | -0.266 |
| RHSIM_Rhsim08G0087200 | 0.07 | 0.792 | 0.850 | 0.147 |
| RHSIM_Rhsim05G0118700 | 0.07 | 0.792 | 0.850 | -0.636 |
| RHSIM_Rhsim13G0050900 | 0.07 | 0.792 | 0.851 | 0.162 |
| RHSIM_RhsimUnG0179400 | 0.07 | 0.792 | 0.851 | 0.672 |
| RHSIM_Rhsim09G0197700 | 0.07 | 0.792 | 0.851 | -0.183 |
| RHSIM_Rhsim07G0219200 | 0.07 | 0.792 | 0.851 | -0.028 |
| RHSIM_Rhsim13G0219000 | 0.07 | 0.792 | 0.851 | -0.146 |
| RHSIM_Rhsim07G0021300 | 0.07 | 0.792 | 0.851 | -0.207 |
| novel.4351 | 0.07 | 0.792 | 0.851 | 0.340 |
| RHSIM_Rhsim03G0082100 | 0.07 | 0.792 | 0.851 | -0.532 |
| RHSIM_Rhsim02G0153400 | 0.07 | 0.792 | 0.851 | -0.035 |
| RHSIM_Rhsim11G0094800 | 0.07 | 0.792 | 0.851 | 0.039 |
| novel.1032 | 0.07 | 0.793 | 0.851 | 0.192 |
| novel.3891 | 0.07 | 0.793 | 0.851 | 0.290 |
| RHSIM_Rhsim11G0063700 | 0.07 | 0.793 | 0.851 | -0.222 |
| RHSIM_RhsimMtG0005500 | 0.07 | 0.793 | 0.851 | 0.185 |
| RHSIM_Rhsim13G0171800 | 0.07 | 0.793 | 0.851 | 0.170 |
| RHSIM_Rhsim03G0175800 | 0.07 | 0.793 | 0.851 | -0.447 |
| RHSIM_Rhsim04G0122500 | 0.07 | 0.793 | 0.851 | -0.632 |
| RHSIM_Rhsim12G0077000 | 0.07 | 0.793 | 0.851 | 0.033 |
| RHSIM_RhsimUnG0165000 | 0.07 | 0.793 | 0.851 | -0.311 |
| RHSIM_Rhsim05G0230000 | 0.07 | 0.793 | 0.851 | -0.466 |
| RHSIM_RhsimUnG0205500 | 0.07 | 0.794 | 0.852 | -0.086 |
| novel.3061 | 0.07 | 0.794 | 0.852 | -0.310 |
| novel.1027 | 0.07 | 0.794 | 0.852 | 0.077 |
| RHSIM_Rhsim12G0007500 | 0.07 | 0.794 | 0.852 | 0.243 |
| RHSIM_Rhsim05G0190600 | 0.07 | 0.794 | 0.852 | 0.446 |
| RHSIM_Rhsim04G0130300 | 0.07 | 0.794 | 0.852 | -0.031 |
| RHSIM_Rhsim11G0010200 | 0.07 | 0.794 | 0.852 | -0.255 |
| RHSIM_RhsimUnG0136500 | 0.07 | 0.794 | 0.852 | 0.681 |

| Gene | LRT statistic | P value | BH-adjusted P | log2FC (interaction) |
|---|---|---|---|---|
| RHSIM_Rhsim01G0275600 | 0.07 | 0.794 | 0.852 | 0.086 |
| novel.411 | 0.07 | 0.794 | 0.852 | -32.152 |
| RHSIM_Rhsim07G0165800 | 0.07 | 0.795 | 0.853 | -0.408 |
| RHSIM_Rhsim13G0127700 | 0.07 | 0.795 | 0.853 | 0.048 |
| RHSIM_Rhsim08G0008900 | 0.07 | 0.795 | 0.853 | 0.485 |
| RHSIM_Rhsim08G0226600 | 0.07 | 0.795 | 0.853 | -0.037 |
| RHSIM_Rhsim05G0233900 | 0.07 | 0.795 | 0.853 | 0.575 |
| RHSIM_RhsimUnG0128700 | 0.07 | 0.795 | 0.853 | -0.046 |
| RHSIM_Rhsim03G0246800 | 0.07 | 0.796 | 0.854 | 0.256 |
| novel.2321 | 0.07 | 0.796 | 0.854 | 0.115 |
| RHSIM_Rhsim01G0047200 | 0.07 | 0.796 | 0.854 | -0.411 |
| RHSIM_Rhsim04G0056800 | 0.07 | 0.796 | 0.854 | 0.559 |
| novel.2354 | 0.07 | 0.796 | 0.854 | -0.053 |
| RHSIM_Rhsim11G0014800 | 0.07 | 0.796 | 0.854 | -0.262 |
| novel.383 | 0.07 | 0.796 | 0.854 | 0.383 |
| RHSIM_Rhsim04G0037100 | 0.07 | 0.796 | 0.854 | -0.239 |
| RHSIM_Rhsim06G0162100 | 0.07 | 0.797 | 0.855 | -0.095 |
| RHSIM_Rhsim13G0122100 | 0.07 | 0.797 | 0.855 | 0.327 |
| RHSIM_Rhsim11G0032000 | 0.07 | 0.797 | 0.855 | -0.074 |
| RHSIM_Rhsim09G0192800 | 0.07 | 0.798 | 0.855 | -0.039 |
| RHSIM_Rhsim11G0199200 | 0.07 | 0.798 | 0.855 | -0.033 |
| RHSIM_RhsimUnG0099700 | 0.07 | 0.798 | 0.856 | 0.572 |
| RHSIM_Rhsim02G0158600 | 0.07 | 0.798 | 0.856 | -0.066 |
| RHSIM_Rhsim09G0179100 | 0.07 | 0.798 | 0.856 | 0.321 |
| RHSIM_Rhsim09G0038700 | 0.07 | 0.798 | 0.856 | 0.035 |
| RHSIM_Rhsim03G0049300 | 0.07 | 0.798 | 0.856 | 0.087 |
| novel.1861 | 0.07 | 0.798 | 0.856 | -0.154 |
| RHSIM_Rhsim03G0146500 | 0.07 | 0.798 | 0.856 | -0.101 |
| RHSIM_Rhsim06G0148400 | 0.07 | 0.799 | 0.856 | 0.103 |
| RHSIM_Rhsim01G0096700 | 0.07 | 0.799 | 0.856 | 0.517 |
| RHSIM_Rhsim07G0154000 | 0.06 | 0.799 | 0.856 | 0.107 |
| RHSIM_Rhsim03G0037200 | 0.06 | 0.799 | 0.856 | 0.244 |

| Gene | LRT statistic | P value | BH-adjusted P | log2FC (interaction) |
|---|---|---|---|---|
| RHSIM_Rhsim04G0108700 | 0.06 | 0.799 | 0.857 | 0.121 |
| RHSIM_Rhsim13G0127500 | 0.06 | 0.799 | 0.857 | 0.143 |
| RHSIM_Rhsim01G0247100 | 0.06 | 0.799 | 0.857 | -0.024 |
| RHSIM_Rhsim07G0238100 | 0.06 | 0.799 | 0.857 | 0.033 |
| RHSIM_Rhsim05G0073700 | 0.06 | 0.800 | 0.857 | 0.060 |
| RHSIM_Rhsim01G0174400 | 0.06 | 0.800 | 0.858 | -0.056 |
| novel.3184 | 0.06 | 0.800 | 0.858 | -0.040 |
| novel.138 | 0.06 | 0.800 | 0.858 | 0.078 |
| RHSIM_Rhsim08G0175200 | 0.06 | 0.801 | 0.858 | -0.201 |
| RHSIM_Rhsim08G0160000 | 0.06 | 0.801 | 0.858 | 0.209 |
| RHSIM_Rhsim11G0081000 | 0.06 | 0.801 | 0.858 | -0.563 |
| RHSIM_Rhsim12G0129700 | 0.06 | 0.801 | 0.859 | -0.064 |
| RHSIM_Rhsim04G0246000 | 0.06 | 0.802 | 0.859 | -0.167 |
| RHSIM_Rhsim08G0007700 | 0.06 | 0.802 | 0.859 | -0.396 |
| novel.4545 | 0.06 | 0.802 | 0.859 | 0.184 |
| RHSIM_RhsimUnG0032300 | 0.06 | 0.802 | 0.859 | -0.191 |
| RHSIM_Rhsim07G0091500 | 0.06 | 0.802 | 0.859 | -0.552 |
| RHSIM_Rhsim09G0030900 | 0.06 | 0.802 | 0.860 | 0.299 |
| novel.2374 | 0.06 | 0.802 | 0.860 | -0.717 |
| RHSIM_Rhsim02G0147600 | 0.06 | 0.803 | 0.860 | -0.718 |
| RHSIM_Rhsim12G0107200 | 0.06 | 0.803 | 0.860 | 0.121 |
| RHSIM_Rhsim11G0175800 | 0.06 | 0.803 | 0.860 | -0.181 |
| RHSIM_Rhsim10G0192100 | 0.06 | 0.803 | 0.860 | -0.032 |
| RHSIM_Rhsim01G0167000 | 0.06 | 0.803 | 0.860 | 0.069 |
| RHSIM_RhsimMtG0002500 | 0.06 | 0.803 | 0.860 | 0.433 |
| RHSIM_Rhsim08G0169000 | 0.06 | 0.804 | 0.861 | 0.200 |
| novel.433 | 0.06 | 0.804 | 0.861 | -0.026 |
| RHSIM_Rhsim04G0247000 | 0.06 | 0.804 | 0.861 | -0.030 |
| RHSIM_Rhsim08G0243000 | 0.06 | 0.804 | 0.861 | -0.015 |
| RHSIM_Rhsim05G0117600 | 0.06 | 0.804 | 0.861 | 0.166 |
| novel.4339 | 0.06 | 0.804 | 0.861 | -0.114 |
| RHSIM_Rhsim07G0059700 | 0.06 | 0.805 | 0.862 | 0.076 |

| Gene | LRT statistic | P value | BH-adjusted P | log2FC (interaction) |
|---|---|---|---|---|
| RHSIM_RhsimMtG0001200 | 0.06 | 0.805 | 0.862 | -0.336 |
| RHSIM_Rhsim12G0005900 | 0.06 | 0.805 | 0.862 | 0.094 |
| RHSIM_Rhsim13G0180400 | 0.06 | 0.805 | 0.862 | -31.819 |
| RHSIM_Rhsim07G0119500 | 0.06 | 0.806 | 0.862 | 0.051 |
| RHSIM_RhsimUnG0032900 | 0.06 | 0.806 | 0.862 | -0.076 |
| RHSIM_RhsimUnG0078600 | 0.06 | 0.806 | 0.862 | 0.566 |
| RHSIM_RhsimUnG0143200 | 0.06 | 0.806 | 0.862 | 0.139 |
| novel.1838 | 0.06 | 0.806 | 0.862 | 0.107 |
| RHSIM_Rhsim12G0106000 | 0.06 | 0.806 | 0.862 | -0.064 |
| RHSIM_Rhsim10G0131300 | 0.06 | 0.806 | 0.862 | 0.035 |
| RHSIM_Rhsim05G0033500 | 0.06 | 0.806 | 0.863 | 0.231 |
| RHSIM_RhsimUnG0089000 | 0.06 | 0.806 | 0.863 | 0.070 |
| RHSIM_Rhsim11G0183100 | 0.06 | 0.806 | 0.863 | -0.023 |
| RHSIM_Rhsim01G0191300 | 0.06 | 0.807 | 0.863 | -0.215 |
| RHSIM_RhsimUnG0185600 | 0.06 | 0.807 | 0.863 | 0.174 |
| RHSIM_Rhsim03G0158900 | 0.06 | 0.807 | 0.863 | 0.569 |
| RHSIM_Rhsim02G0082700 | 0.06 | 0.807 | 0.863 | 0.028 |
| RHSIM_Rhsim13G0025400 | 0.06 | 0.807 | 0.863 | 0.030 |
| RHSIM_Rhsim06G0162200 | 0.06 | 0.807 | 0.864 | -0.145 |
| RHSIM_Rhsim02G0064000 | 0.06 | 0.807 | 0.864 | 0.242 |
| RHSIM_Rhsim07G0066100 | 0.06 | 0.807 | 0.864 | 0.275 |
| RHSIM_Rhsim04G0010000 | 0.06 | 0.807 | 0.864 | -0.088 |
| novel.3780 | 0.06 | 0.807 | 0.864 | 0.165 |
| RHSIM_Rhsim05G0232500 | 0.06 | 0.808 | 0.864 | -0.326 |
| novel.4366 | 0.06 | 0.808 | 0.864 | -0.217 |
| RHSIM_RhsimUnG0252500 | 0.06 | 0.808 | 0.864 | 0.040 |
| novel.3349 | 0.06 | 0.808 | 0.864 | -0.086 |
| RHSIM_Rhsim11G0184600 | 0.06 | 0.808 | 0.864 | 0.023 |
| RHSIM_Rhsim13G0082300 | 0.06 | 0.809 | 0.865 | 0.212 |
| RHSIM_Rhsim08G0170900 | 0.06 | 0.809 | 0.865 | 0.250 |
| RHSIM_Rhsim04G0001200 | 0.06 | 0.809 | 0.865 | -0.074 |
| RHSIM_Rhsim06G0037100 | 0.06 | 0.809 | 0.866 | -0.594 |

| Gene | LRT statistic | P value | BH-adjusted P | log2FC (interaction) |
|---|---|---|---|---|
| novel.998 | 0.06 | 0.809 | 0.866 | -0.282 |
| RHSIM_Rhsim05G0052500 | 0.06 | 0.809 | 0.866 | -0.256 |
| RHSIM_RhsimUnG0253700 | 0.06 | 0.810 | 0.866 | -0.047 |
| RHSIM_RhsimUnG0083600 | 0.06 | 0.810 | 0.866 | -0.573 |
| RHSIM_Rhsim06G0035900 | 0.06 | 0.810 | 0.866 | -0.075 |
| RHSIM_Rhsim05G0197400 | 0.06 | 0.810 | 0.866 | 0.309 |
| RHSIM_Rhsim04G0155400 | 0.06 | 0.810 | 0.866 | 0.053 |
| RHSIM_Rhsim13G0083600 | 0.06 | 0.810 | 0.866 | -0.110 |
| RHSIM_RhsimUnG0106600 | 0.06 | 0.811 | 0.867 | -0.150 |
| novel.912 | 0.06 | 0.811 | 0.867 | 0.105 |
| novel.988 | 0.06 | 0.811 | 0.867 | 0.073 |
| novel.3646 | 0.06 | 0.811 | 0.867 | 0.329 |
| RHSIM_Rhsim04G0213700 | 0.06 | 0.811 | 0.867 | 0.250 |
| RHSIM_Rhsim07G0055100 | 0.06 | 0.811 | 0.867 | 0.178 |
| RHSIM_Rhsim11G0056200 | 0.06 | 0.812 | 0.867 | 0.282 |
| RHSIM_Rhsim05G0179300 | 0.06 | 0.812 | 0.867 | 0.417 |
| RHSIM_Rhsim05G0176400 | 0.06 | 0.812 | 0.868 | 0.531 |
| RHSIM_Rhsim06G0018500 | 0.06 | 0.812 | 0.868 | -0.443 |
| RHSIM_RhsimUnG0023800 | 0.06 | 0.812 | 0.868 | -0.375 |
| RHSIM_Rhsim03G0030000 | 0.06 | 0.813 | 0.868 | -0.063 |
| RHSIM_Rhsim05G0183400 | 0.06 | 0.813 | 0.869 | 0.422 |
| RHSIM_Rhsim03G0252500 | 0.06 | 0.813 | 0.869 | -0.306 |
| novel.3272 | 0.06 | 0.813 | 0.869 | 0.178 |
| RHSIM_Rhsim04G0216900 | 0.06 | 0.813 | 0.869 | 0.042 |
| novel.734 | 0.06 | 0.813 | 0.869 | -0.326 |
| RHSIM_Rhsim12G0129200 | 0.06 | 0.813 | 0.869 | 0.021 |
| novel.4324 | 0.06 | 0.813 | 0.869 | -0.082 |
| RHSIM_Rhsim02G0072900 | 0.06 | 0.814 | 0.869 | -0.729 |
| RHSIM_Rhsim07G0157400 | 0.06 | 0.814 | 0.869 | 0.064 |
| RHSIM_Rhsim10G0011300 | 0.06 | 0.814 | 0.869 | 0.027 |
| RHSIM_Rhsim07G0136300 | 0.06 | 0.814 | 0.870 | 0.668 |
| RHSIM_Rhsim03G0174600 | 0.06 | 0.814 | 0.870 | 0.304 |

| Gene | LRT statistic | P value | BH-adjusted P | log2FC (interaction) |
|---|---|---|---|---|
| RHSIM_Rhsim05G0145600 | 0.06 | 0.814 | 0.870 | 0.252 |
| RHSIM_Rhsim04G0036800 | 0.06 | 0.815 | 0.870 | -0.077 |
| RHSIM_Rhsim10G0051200 | 0.05 | 0.815 | 0.870 | -0.020 |
| RHSIM_Rhsim02G0111800 | 0.05 | 0.815 | 0.870 | -0.106 |
| RHSIM_Rhsim09G0110000 | 0.05 | 0.815 | 0.870 | -0.080 |
| RHSIM_Rhsim02G0040000 | 0.05 | 0.815 | 0.870 | 0.201 |
| RHSIM_Rhsim02G0035200 | 0.05 | 0.815 | 0.870 | 0.407 |
| RHSIM_Rhsim04G0071300 | 0.05 | 0.815 | 0.871 | 0.200 |
| novel.1203 | 0.05 | 0.815 | 0.871 | -0.182 |
| RHSIM_Rhsim09G0054300 | 0.05 | 0.815 | 0.871 | -0.087 |
| RHSIM_Rhsim01G0275700 | 0.05 | 0.816 | 0.871 | 0.126 |
| RHSIM_Rhsim09G0013700 | 0.05 | 0.816 | 0.871 | 0.515 |
| RHSIM_Rhsim09G0057000 | 0.05 | 0.816 | 0.871 | 0.223 |
| RHSIM_Rhsim08G0144700 | 0.05 | 0.816 | 0.871 | 0.333 |
| RHSIM_Rhsim08G0037600 | 0.05 | 0.816 | 0.871 | 0.022 |
| RHSIM_Rhsim02G0140800 | 0.05 | 0.816 | 0.871 | 0.343 |
| RHSIM_Rhsim10G0016500 | 0.05 | 0.816 | 0.871 | 0.531 |
| novel.3632 | 0.05 | 0.817 | 0.872 | -0.317 |
| RHSIM_Rhsim10G0187100 | 0.05 | 0.817 | 0.872 | 0.474 |
| RHSIM_Rhsim05G0084200 | 0.05 | 0.817 | 0.872 | -0.147 |
| RHSIM_Rhsim05G0017900 | 0.05 | 0.817 | 0.872 | -0.031 |
| RHSIM_Rhsim07G0147300 | 0.05 | 0.817 | 0.872 | -0.178 |
| RHSIM_Rhsim02G0190600 | 0.05 | 0.817 | 0.872 | -0.699 |
| RHSIM_Rhsim01G0217700 | 0.05 | 0.817 | 0.872 | -0.023 |
| novel.4306 | 0.05 | 0.817 | 0.872 | -0.227 |
| RHSIM_Rhsim10G0112200 | 0.05 | 0.817 | 0.872 | -0.430 |
| novel.396 | 0.05 | 0.817 | 0.872 | -0.082 |
| RHSIM_Rhsim13G0074900 | 0.05 | 0.818 | 0.872 | -0.074 |
| RHSIM_Rhsim06G0096300 | 0.05 | 0.818 | 0.873 | -0.030 |
| RHSIM_Rhsim04G0005800 | 0.05 | 0.818 | 0.873 | 0.037 |
| RHSIM_Rhsim08G0017500 | 0.05 | 0.818 | 0.873 | 0.635 |
| novel.2747 | 0.05 | 0.818 | 0.873 | -0.157 |

| Gene | LRT statistic | P value | BH-adjusted P | log2FC (interaction) |
|---|---|---|---|---|
| RHSIM_Rhsim03G0185500 | 0.05 | 0.818 | 0.873 | -0.159 |
| RHSIM_Rhsim12G0173100 | 0.05 | 0.818 | 0.873 | -0.029 |
| RHSIM_Rhsim11G0020000 | 0.05 | 0.818 | 0.873 | 0.091 |
| RHSIM_Rhsim07G0115200 | 0.05 | 0.818 | 0.873 | 0.022 |
| novel.3844 | 0.05 | 0.818 | 0.873 | -0.102 |
| RHSIM_Rhsim13G0076700 | 0.05 | 0.819 | 0.873 | -0.560 |
| RHSIM_Rhsim11G0005600 | 0.05 | 0.819 | 0.873 | -0.305 |
| RHSIM_Rhsim08G0128400 | 0.05 | 0.819 | 0.874 | 0.023 |
| RHSIM_Rhsim11G0105600 | 0.05 | 0.819 | 0.874 | 0.053 |
| RHSIM_Rhsim07G0105900 | 0.05 | 0.819 | 0.874 | 0.365 |
| RHSIM_Rhsim12G0128100 | 0.05 | 0.820 | 0.874 | -0.054 |
| RHSIM_Rhsim13G0093600 | 0.05 | 0.820 | 0.874 | 0.400 |
| RHSIM_Rhsim02G0166900 | 0.05 | 0.820 | 0.874 | -0.368 |
| RHSIM_RhsimUnG0214100 | 0.05 | 0.820 | 0.874 | -0.246 |
| RHSIM_Rhsim10G0143400 | 0.05 | 0.820 | 0.874 | 0.364 |
| novel.1205 | 0.05 | 0.820 | 0.875 | 0.200 |
| RHSIM_Rhsim01G0016400 | 0.05 | 0.820 | 0.875 | 0.112 |
| RHSIM_Rhsim02G0055100 | 0.05 | 0.821 | 0.875 | 0.082 |
| novel.783 | 0.05 | 0.821 | 0.875 | -0.236 |
| RHSIM_Rhsim09G0147400 | 0.05 | 0.821 | 0.875 | -0.508 |
| RHSIM_Rhsim01G0183200 | 0.05 | 0.821 | 0.875 | -0.574 |
| RHSIM_Rhsim03G0118300 | 0.05 | 0.821 | 0.875 | 0.206 |
| RHSIM_Rhsim03G0013500 | 0.05 | 0.821 | 0.876 | 0.116 |
| RHSIM_Rhsim12G0099100 | 0.05 | 0.822 | 0.876 | -0.047 |
| novel.1617 | 0.05 | 0.822 | 0.876 | -0.301 |
| RHSIM_Rhsim01G0083700 | 0.05 | 0.822 | 0.876 | -0.116 |
| RHSIM_Rhsim02G0226400 | 0.05 | 0.822 | 0.876 | 0.157 |
| RHSIM_Rhsim08G0084900 | 0.05 | 0.822 | 0.876 | -0.130 |
| RHSIM_Rhsim12G0118500 | 0.05 | 0.822 | 0.876 | 0.075 |
| novel.2546 | 0.05 | 0.823 | 0.877 | -0.545 |
| RHSIM_Rhsim11G0153600 | 0.05 | 0.823 | 0.877 | -0.042 |
| novel.4563 | 0.05 | 0.823 | 0.877 | 0.236 |

| Gene | LRT statistic | P value | BH-adjusted P | log2FC (interaction) |
|---|---|---|---|---|
| RHSIM_Rhsim01G0151300 | 0.05 | 0.823 | 0.877 | -0.103 |
| RHSIM_Rhsim13G0149100 | 0.05 | 0.823 | 0.877 | -0.033 |
| RHSIM_Rhsim13G0099300 | 0.05 | 0.823 | 0.877 | -0.440 |
| RHSIM_Rhsim08G0054000 | 0.05 | 0.823 | 0.877 | -0.497 |
| RHSIM_Rhsim01G0194200 | 0.05 | 0.823 | 0.877 | -0.188 |
| RHSIM_Rhsim05G0128400 | 0.05 | 0.823 | 0.877 | 0.062 |
| RHSIM_Rhsim09G0155600 | 0.05 | 0.823 | 0.877 | 0.185 |
| RHSIM_Rhsim10G0028400 | 0.05 | 0.823 | 0.877 | -0.063 |
| RHSIM_Rhsim09G0026000 | 0.05 | 0.824 | 0.877 | 0.087 |
| RHSIM_Rhsim07G0219900 | 0.05 | 0.824 | 0.878 | 0.033 |
| RHSIM_Rhsim05G0162400 | 0.05 | 0.824 | 0.878 | 0.199 |
| RHSIM_Rhsim06G0123500 | 0.05 | 0.824 | 0.878 | -0.268 |
| RHSIM_Rhsim10G0187500 | 0.05 | 0.824 | 0.878 | 0.364 |
| RHSIM_Rhsim08G0163900 | 0.05 | 0.824 | 0.878 | 0.183 |
| RHSIM_Rhsim05G0200900 | 0.05 | 0.824 | 0.878 | -0.196 |
| RHSIM_Rhsim01G0206300 | 0.05 | 0.824 | 0.878 | -0.184 |
| novel.2842 | 0.05 | 0.824 | 0.878 | 0.078 |
| RHSIM_Rhsim03G0091700 | 0.05 | 0.825 | 0.878 | -0.068 |
| RHSIM_Rhsim10G0056800 | 0.05 | 0.825 | 0.878 | 0.459 |
| RHSIM_RhsimUnG0092400 | 0.05 | 0.825 | 0.878 | -0.088 |
| RHSIM_Rhsim02G0096000 | 0.05 | 0.826 | 0.879 | -0.410 |
| novel.4454 | 0.05 | 0.826 | 0.880 | -0.352 |
| RHSIM_Rhsim07G0224700 | 0.05 | 0.826 | 0.880 | -0.095 |
| novel.1806 | 0.05 | 0.826 | 0.880 | 0.188 |
| RHSIM_Rhsim08G0182100 | 0.05 | 0.826 | 0.880 | -0.446 |
| novel.3365 | 0.05 | 0.826 | 0.880 | -0.069 |
| RHSIM_Rhsim09G0138500 | 0.05 | 0.827 | 0.880 | -0.014 |
| novel.1793 | 0.05 | 0.827 | 0.880 | -0.158 |
| RHSIM_Rhsim01G0206100 | 0.05 | 0.827 | 0.881 | 0.364 |
| RHSIM_Rhsim01G0020800 | 0.05 | 0.828 | 0.881 | -0.181 |
| RHSIM_Rhsim01G0045600 | 0.05 | 0.828 | 0.881 | 0.038 |
| RHSIM_Rhsim06G0064000 | 0.05 | 0.828 | 0.881 | 0.042 |

| Gene | LRT statistic | P value | BH-adjusted P | log2FC (interaction) |
|---|---|---|---|---|
| novel.325 | 0.05 | 0.828 | 0.881 | 0.333 |
| RHSIM_Rhsim05G0161800 | 0.05 | 0.828 | 0.881 | 0.369 |
| RHSIM_Rhsim12G0022700 | 0.05 | 0.828 | 0.881 | 0.279 |
| RHSIM_Rhsim08G0050500 | 0.05 | 0.828 | 0.881 | -0.269 |
| novel.2348 | 0.05 | 0.828 | 0.881 | -0.101 |
| RHSIM_Rhsim13G0026000 | 0.05 | 0.829 | 0.882 | -0.021 |
| novel.2484 | 0.05 | 0.829 | 0.882 | 0.184 |
| novel.2730 | 0.05 | 0.829 | 0.882 | 0.083 |
| RHSIM_Rhsim09G0140400 | 0.05 | 0.829 | 0.882 | -0.553 |
| RHSIM_Rhsim01G0061900 | 0.05 | 0.829 | 0.882 | -0.386 |
| novel.4513 | 0.05 | 0.829 | 0.882 | 0.166 |
| RHSIM_Rhsim08G0010800 | 0.05 | 0.829 | 0.882 | -0.274 |
| RHSIM_Rhsim12G0211100 | 0.05 | 0.829 | 0.882 | -0.140 |
| RHSIM_Rhsim06G0070900 | 0.05 | 0.829 | 0.882 | -0.034 |
| RHSIM_Rhsim04G0058500 | 0.05 | 0.829 | 0.882 | -0.048 |
| RHSIM_RhsimUnG0242200 | 0.05 | 0.830 | 0.882 | 0.020 |
| RHSIM_Rhsim13G0072800 | 0.05 | 0.830 | 0.883 | 0.272 |
| RHSIM_RhsimUnG0155900 | 0.05 | 0.830 | 0.883 | 0.032 |
| novel.2934 | 0.05 | 0.830 | 0.883 | -0.290 |
| RHSIM_Rhsim09G0080600 | 0.05 | 0.830 | 0.883 | 0.098 |
| RHSIM_Rhsim02G0209800 | 0.05 | 0.830 | 0.883 | 0.336 |
| RHSIM_Rhsim02G0255100 | 0.05 | 0.831 | 0.883 | 0.263 |
| RHSIM_Rhsim06G0212200 | 0.05 | 0.831 | 0.884 | 0.328 |
| RHSIM_Rhsim11G0094700 | 0.05 | 0.831 | 0.884 | 0.046 |
| RHSIM_Rhsim01G0058400 | 0.05 | 0.832 | 0.884 | -0.478 |
| RHSIM_Rhsim06G0199200 | 0.05 | 0.832 | 0.884 | 0.020 |
| RHSIM_Rhsim01G0225100 | 0.04 | 0.832 | 0.885 | -0.227 |
| RHSIM_Rhsim13G0226100 | 0.04 | 0.832 | 0.885 | -0.447 |
| RHSIM_Rhsim10G0040400 | 0.04 | 0.832 | 0.885 | 0.141 |
| novel.4007 | 0.04 | 0.832 | 0.885 | 0.290 |
| RHSIM_Rhsim05G0201200 | 0.04 | 0.832 | 0.885 | 0.506 |
| novel.3475 | 0.04 | 0.832 | 0.885 | -0.458 |

| Gene | LRT statistic | P value | BH-adjusted P | log2FC (interaction) |
|---|---|---|---|---|
| novel.1651 | 0.04 | 0.833 | 0.885 | 0.086 |
| RHSIM_Rhsim03G0257400 | 0.04 | 0.833 | 0.885 | 0.026 |
| RHSIM_Rhsim02G0016100 | 0.04 | 0.833 | 0.885 | 0.128 |
| RHSIM_Rhsim13G0229600 | 0.04 | 0.833 | 0.885 | -0.023 |
| RHSIM_Rhsim04G0225700 | 0.04 | 0.833 | 0.885 | -0.083 |
| RHSIM_Rhsim07G0229600 | 0.04 | 0.833 | 0.885 | 0.163 |
| RHSIM_Rhsim02G0125100 | 0.04 | 0.833 | 0.885 | 0.268 |
| RHSIM_Rhsim12G0038000 | 0.04 | 0.833 | 0.885 | -0.321 |
| RHSIM_Rhsim03G0039700 | 0.04 | 0.833 | 0.886 | -0.496 |
| RHSIM_Rhsim07G0243900 | 0.04 | 0.833 | 0.886 | -0.023 |
| RHSIM_Rhsim08G0163800 | 0.04 | 0.833 | 0.886 | 0.085 |
| RHSIM_Rhsim13G0128000 | 0.04 | 0.833 | 0.886 | 0.124 |
| RHSIM_Rhsim13G0064700 | 0.04 | 0.834 | 0.886 | 0.033 |
| RHSIM_Rhsim06G0144400 | 0.04 | 0.834 | 0.886 | 0.309 |
| RHSIM_Rhsim04G0202700 | 0.04 | 0.834 | 0.886 | -0.491 |
| RHSIM_Rhsim06G0132800 | 0.04 | 0.834 | 0.887 | -0.103 |
| RHSIM_Rhsim01G0237300 | 0.04 | 0.835 | 0.887 | 0.298 |
| RHSIM_Rhsim04G0151900 | 0.04 | 0.835 | 0.887 | -0.015 |
| novel.2932 | 0.04 | 0.835 | 0.887 | -31.457 |
| RHSIM_Rhsim05G0070100 | 0.04 | 0.835 | 0.887 | 0.012 |
| RHSIM_Rhsim02G0137700 | 0.04 | 0.835 | 0.887 | 0.029 |
| RHSIM_Rhsim12G0174300 | 0.04 | 0.835 | 0.887 | 0.072 |
| RHSIM_Rhsim13G0090500 | 0.04 | 0.835 | 0.887 | 0.079 |
| RHSIM_Rhsim13G0077800 | 0.04 | 0.835 | 0.887 | 0.079 |
| RHSIM_Rhsim06G0003000 | 0.04 | 0.835 | 0.887 | -0.029 |
| RHSIM_Rhsim09G0045300 | 0.04 | 0.835 | 0.887 | 0.291 |
| novel.1006 | 0.04 | 0.836 | 0.887 | 0.216 |
| RHSIM_Rhsim10G0178500 | 0.04 | 0.836 | 0.887 | -0.292 |
| RHSIM_Rhsim08G0120900 | 0.04 | 0.836 | 0.888 | 0.027 |
| RHSIM_Rhsim09G0183000 | 0.04 | 0.836 | 0.888 | 0.390 |
| RHSIM_Rhsim03G0117800 | 0.04 | 0.836 | 0.888 | -0.304 |
| RHSIM_Rhsim04G0147100 | 0.04 | 0.836 | 0.888 | -0.455 |

| Gene | LRT statistic | P value | BH-adjusted P | log2FC (interaction) |
|---|---|---|---|---|
| RHSIM_Rhsim07G0105700 | 0.04 | 0.837 | 0.888 | 0.116 |
| RHSIM_Rhsim03G0083800 | 0.04 | 0.837 | 0.888 | 0.018 |
| RHSIM_Rhsim12G0166500 | 0.04 | 0.837 | 0.888 | 0.062 |
| RHSIM_Rhsim08G0134600 | 0.04 | 0.837 | 0.888 | 0.400 |
| RHSIM_Rhsim06G0215600 | 0.04 | 0.837 | 0.888 | 0.499 |
| RHSIM_Rhsim07G0048900 | 0.04 | 0.837 | 0.888 | -0.330 |
| RHSIM_Rhsim01G0086900 | 0.04 | 0.837 | 0.889 | -0.067 |
| novel.1976 | 0.04 | 0.837 | 0.889 | 0.168 |
| RHSIM_Rhsim13G0067400 | 0.04 | 0.837 | 0.889 | -0.365 |
| novel.86 | 0.04 | 0.838 | 0.889 | -0.218 |
| RHSIM_Rhsim10G0140800 | 0.04 | 0.838 | 0.889 | 0.062 |
| RHSIM_Rhsim05G0040000 | 0.04 | 0.838 | 0.889 | 0.367 |
| RHSIM_Rhsim01G0233400 | 0.04 | 0.838 | 0.889 | -0.397 |
| RHSIM_Rhsim04G0028300 | 0.04 | 0.838 | 0.889 | -0.224 |
| RHSIM_Rhsim01G0204000 | 0.04 | 0.838 | 0.889 | -0.498 |
| RHSIM_Rhsim07G0254100 | 0.04 | 0.838 | 0.889 | 0.062 |
| RHSIM_Rhsim09G0030400 | 0.04 | 0.838 | 0.889 | -0.576 |
| RHSIM_Rhsim10G0042100 | 0.04 | 0.838 | 0.889 | -0.179 |
| RHSIM_Rhsim12G0161600 | 0.04 | 0.838 | 0.889 | 0.151 |
| novel.2774 | 0.04 | 0.838 | 0.889 | 0.296 |
| RHSIM_Rhsim10G0174800 | 0.04 | 0.838 | 0.890 | 0.168 |
| novel.641 | 0.04 | 0.839 | 0.890 | 0.083 |
| RHSIM_Rhsim02G0051300 | 0.04 | 0.839 | 0.890 | -0.181 |
| RHSIM_Rhsim10G0176900 | 0.04 | 0.839 | 0.890 | -0.012 |
| RHSIM_Rhsim13G0196500 | 0.04 | 0.840 | 0.891 | -0.038 |
| RHSIM_Rhsim02G0146100 | 0.04 | 0.840 | 0.891 | 0.233 |
| RHSIM_Rhsim08G0162000 | 0.04 | 0.840 | 0.891 | -0.030 |
| RHSIM_Rhsim06G0015200 | 0.04 | 0.840 | 0.891 | -31.384 |
| novel.962 | 0.04 | 0.840 | 0.891 | 0.063 |
| RHSIM_Rhsim13G0168300 | 0.04 | 0.840 | 0.891 | 0.089 |
| RHSIM_Rhsim13G0068800 | 0.04 | 0.840 | 0.891 | -0.104 |
| RHSIM_Rhsim03G0219800 | 0.04 | 0.840 | 0.891 | 0.170 |

| Gene | LRT statistic | P value | BH-adjusted P | log2FC (interaction) |
|---|---|---|---|---|
| novel.2608 | 0.04 | 0.840 | 0.891 | 0.445 |
| RHSIM_Rhsim04G0142800 | 0.04 | 0.840 | 0.891 | 0.524 |
| RHSIM_Rhsim10G0195600 | 0.04 | 0.840 | 0.891 | 0.039 |
| RHSIM_Rhsim01G0047300 | 0.04 | 0.841 | 0.891 | -0.100 |
| RHSIM_Rhsim13G0066300 | 0.04 | 0.841 | 0.892 | -0.084 |
| RHSIM_Rhsim11G0124900 | 0.04 | 0.841 | 0.892 | -0.036 |
| novel.1364 | 0.04 | 0.841 | 0.892 | 0.148 |
| RHSIM_Rhsim11G0095500 | 0.04 | 0.841 | 0.892 | 0.024 |
| RHSIM_Rhsim02G0245600 | 0.04 | 0.841 | 0.892 | 0.119 |
| RHSIM_Rhsim09G0202700 | 0.04 | 0.841 | 0.892 | 0.030 |
| RHSIM_Rhsim04G0095000 | 0.04 | 0.841 | 0.892 | -0.322 |
| RHSIM_Rhsim13G0144400 | 0.04 | 0.842 | 0.892 | -0.032 |
| novel.931 | 0.04 | 0.842 | 0.893 | 0.029 |
| RHSIM_Rhsim13G0086000 | 0.04 | 0.842 | 0.893 | 0.026 |
| RHSIM_RhsimUnG0118100 | 0.04 | 0.842 | 0.893 | 0.191 |
| RHSIM_Rhsim13G0016100 | 0.04 | 0.842 | 0.893 | -0.070 |
| RHSIM_Rhsim09G0130300 | 0.04 | 0.843 | 0.893 | -0.015 |
| novel.3011 | 0.04 | 0.843 | 0.893 | 0.204 |
| RHSIM_Rhsim03G0112600 | 0.04 | 0.843 | 0.893 | 0.325 |
| RHSIM_Rhsim07G0038800 | 0.04 | 0.843 | 0.894 | -0.205 |
| novel.2053 | 0.04 | 0.843 | 0.894 | -0.087 |
| RHSIM_Rhsim04G0174900 | 0.04 | 0.843 | 0.894 | -0.020 |
| RHSIM_Rhsim08G0018000 | 0.04 | 0.844 | 0.894 | 0.270 |
| RHSIM_Rhsim04G0168300 | 0.04 | 0.844 | 0.894 | 0.218 |
| RHSIM_Rhsim10G0212200 | 0.04 | 0.844 | 0.895 | 0.518 |
| RHSIM_Rhsim03G0050500 | 0.04 | 0.845 | 0.895 | 0.364 |
| RHSIM_Rhsim09G0093300 | 0.04 | 0.845 | 0.895 | -0.236 |
| RHSIM_Rhsim12G0110500 | 0.04 | 0.845 | 0.895 | -0.028 |
| novel.602 | 0.04 | 0.845 | 0.895 | -0.205 |
| RHSIM_Rhsim08G0071800 | 0.04 | 0.845 | 0.895 | 0.066 |
| RHSIM_Rhsim13G0091200 | 0.04 | 0.845 | 0.895 | -0.407 |
| novel.14 | 0.04 | 0.845 | 0.895 | 0.095 |

| Gene | LRT statistic | P value | BH-adjusted P | log2FC (interaction) |
|---|---|---|---|---|
| novel.1707 | 0.04 | 0.845 | 0.895 | 0.089 |
| RHSIM_Rhsim13G0059200 | 0.04 | 0.845 | 0.895 | 31.270 |
| RHSIM_Rhsim09G0145200 | 0.04 | 0.845 | 0.895 | -0.015 |
| RHSIM_Rhsim01G0203000 | 0.04 | 0.845 | 0.895 | 0.019 |
| RHSIM_Rhsim07G0004200 | 0.04 | 0.845 | 0.895 | 0.032 |
| novel.4130 | 0.04 | 0.846 | 0.895 | -0.172 |
| novel.2829 | 0.04 | 0.846 | 0.896 | -0.113 |
| novel.2159 | 0.04 | 0.846 | 0.896 | -0.113 |
| RHSIM_Rhsim05G0112700 | 0.04 | 0.846 | 0.896 | 0.431 |
| RHSIM_Rhsim09G0101300 | 0.04 | 0.846 | 0.896 | -0.029 |
| RHSIM_Rhsim09G0141200 | 0.04 | 0.846 | 0.896 | 0.226 |
| RHSIM_Rhsim02G0146400 | 0.04 | 0.847 | 0.896 | 0.017 |
| RHSIM_Rhsim10G0213800 | 0.04 | 0.847 | 0.896 | 0.122 |
| RHSIM_Rhsim09G0104700 | 0.04 | 0.847 | 0.896 | 0.104 |
| RHSIM_Rhsim06G0144000 | 0.04 | 0.847 | 0.896 | 0.077 |
| RHSIM_Rhsim05G0089000 | 0.04 | 0.847 | 0.896 | -0.138 |
| RHSIM_Rhsim01G0264000 | 0.04 | 0.847 | 0.897 | 0.012 |
| RHSIM_Rhsim05G0143400 | 0.04 | 0.847 | 0.897 | 0.327 |
| novel.4148 | 0.04 | 0.847 | 0.897 | -0.071 |
| RHSIM_Rhsim12G0002500 | 0.04 | 0.847 | 0.897 | -0.376 |
| RHSIM_Rhsim02G0072100 | 0.04 | 0.848 | 0.897 | 0.182 |
| RHSIM_Rhsim08G0100800 | 0.04 | 0.848 | 0.897 | -0.397 |
| novel.1491 | 0.04 | 0.848 | 0.897 | -0.080 |
| novel.1530 | 0.04 | 0.848 | 0.897 | -0.180 |
| RHSIM_Rhsim01G0027000 | 0.04 | 0.848 | 0.897 | -0.040 |
| novel.3911 | 0.04 | 0.848 | 0.897 | -0.219 |
| RHSIM_Rhsim04G0038000 | 0.04 | 0.848 | 0.897 | 0.360 |
| RHSIM_Rhsim01G0255400 | 0.04 | 0.848 | 0.897 | -0.180 |
| novel.187 | 0.04 | 0.848 | 0.897 | -0.176 |
| RHSIM_Rhsim04G0205100 | 0.04 | 0.848 | 0.898 | 0.155 |
| RHSIM_Rhsim04G0166100 | 0.04 | 0.848 | 0.898 | 0.194 |
| RHSIM_Rhsim08G0111700 | 0.04 | 0.849 | 0.898 | -0.026 |

| Gene | LRT statistic | P value | BH-adjusted P | log2FC (interaction) |
|---|---|---|---|---|
| novel.2161 | 0.04 | 0.849 | 0.898 | 0.056 |
| RHSIM_Rhsim06G0203900 | 0.04 | 0.849 | 0.898 | -0.028 |
| RHSIM_Rhsim08G0229500 | 0.04 | 0.849 | 0.898 | -0.025 |
| RHSIM_Rhsim07G0024600 | 0.04 | 0.849 | 0.898 | 0.019 |
| novel.3677 | 0.04 | 0.849 | 0.898 | -0.088 |
| RHSIM_Rhsim05G0000600 | 0.04 | 0.849 | 0.898 | -0.085 |
| novel.2636 | 0.04 | 0.849 | 0.898 | 0.277 |
| RHSIM_Rhsim04G0201800 | 0.04 | 0.850 | 0.899 | 0.037 |
| RHSIM_Rhsim05G0191900 | 0.04 | 0.850 | 0.899 | -0.270 |
| novel.796 | 0.04 | 0.850 | 0.899 | -0.240 |
| RHSIM_RhsimUnG0025400 | 0.04 | 0.850 | 0.899 | -0.236 |
| RHSIM_Rhsim06G0049000 | 0.04 | 0.850 | 0.899 | 0.141 |
| RHSIM_RhsimUnG0154800 | 0.04 | 0.850 | 0.899 | 0.103 |
| novel.42 | 0.04 | 0.850 | 0.899 | 0.142 |
| RHSIM_Rhsim09G0107200 | 0.04 | 0.850 | 0.899 | 0.025 |
| novel.149 | 0.04 | 0.850 | 0.899 | -0.089 |
| novel.2346 | 0.04 | 0.851 | 0.899 | 0.032 |
| novel.213 | 0.04 | 0.851 | 0.899 | 0.105 |
| RHSIM_Rhsim12G0054300 | 0.04 | 0.851 | 0.899 | 0.122 |
| novel.3304 | 0.04 | 0.851 | 0.899 | 0.136 |
| RHSIM_Rhsim12G0039100 | 0.04 | 0.851 | 0.899 | 0.020 |
| novel.1046 | 0.04 | 0.851 | 0.900 | -0.271 |
| novel.4200 | 0.04 | 0.851 | 0.900 | -0.081 |
| RHSIM_Rhsim11G0075100 | 0.04 | 0.851 | 0.900 | -0.131 |
| RHSIM_Rhsim05G0153900 | 0.04 | 0.851 | 0.900 | -0.499 |
| RHSIM_Rhsim03G0132900 | 0.04 | 0.851 | 0.900 | 0.030 |
| RHSIM_Rhsim03G0135400 | 0.04 | 0.851 | 0.900 | -0.373 |
| novel.3123 | 0.04 | 0.851 | 0.900 | -0.048 |
| novel.542 | 0.04 | 0.851 | 0.900 | 0.072 |
| novel.1034 | 0.03 | 0.852 | 0.900 | 0.083 |
| RHSIM_Rhsim10G0054800 | 0.03 | 0.852 | 0.900 | -0.205 |
| RHSIM_Rhsim07G0015200 | 0.03 | 0.852 | 0.901 | 0.110 |

| Gene | LRT statistic | P value | BH-adjusted P | log2FC (interaction) |
|---|---|---|---|---|
| RHSIM_Rhsim06G0007000 | 0.03 | 0.852 | 0.901 | -0.320 |
| novel.4228 | 0.03 | 0.853 | 0.901 | 0.113 |
| RHSIM_Rhsim02G0041800 | 0.03 | 0.853 | 0.901 | 0.202 |
| novel.1681 | 0.03 | 0.853 | 0.902 | 0.019 |
| novel.4517 | 0.03 | 0.853 | 0.902 | 0.383 |
| RHSIM_Rhsim04G0020200 | 0.03 | 0.853 | 0.902 | 0.176 |
| RHSIM_Rhsim10G0194100 | 0.03 | 0.854 | 0.902 | 0.174 |
| RHSIM_Rhsim12G0143600 | 0.03 | 0.854 | 0.902 | -0.053 |
| novel.4439 | 0.03 | 0.854 | 0.902 | -0.052 |
| RHSIM_Rhsim08G0166400 | 0.03 | 0.854 | 0.902 | 0.097 |
| RHSIM_Rhsim11G0161700 | 0.03 | 0.854 | 0.902 | 0.067 |
| RHSIM_Rhsim05G0215200 | 0.03 | 0.854 | 0.902 | -0.120 |
| RHSIM_Rhsim10G0194200 | 0.03 | 0.854 | 0.902 | -0.191 |
| RHSIM_Rhsim10G0114100 | 0.03 | 0.854 | 0.902 | 0.184 |
| RHSIM_Rhsim01G0268800 | 0.03 | 0.855 | 0.903 | -0.318 |
| RHSIM_Rhsim07G0067600 | 0.03 | 0.855 | 0.903 | 0.022 |
| RHSIM_Rhsim11G0154100 | 0.03 | 0.855 | 0.903 | -0.434 |
| RHSIM_Rhsim05G0117300 | 0.03 | 0.855 | 0.903 | 0.050 |
| RHSIM_Rhsim13G0035900 | 0.03 | 0.855 | 0.903 | -0.275 |
| RHSIM_Rhsim08G0206400 | 0.03 | 0.855 | 0.903 | -0.229 |
| RHSIM_RhsimUnG0028500 | 0.03 | 0.855 | 0.903 | -0.027 |
| novel.3837 | 0.03 | 0.855 | 0.903 | 0.074 |
| RHSIM_RhsimUnG0215000 | 0.03 | 0.855 | 0.903 | 0.055 |
| RHSIM_Rhsim08G0028100 | 0.03 | 0.855 | 0.903 | 0.421 |
| RHSIM_Rhsim01G0072800 | 0.03 | 0.856 | 0.903 | 0.241 |
| RHSIM_Rhsim08G0112300 | 0.03 | 0.856 | 0.903 | -0.037 |
| RHSIM_Rhsim13G0016700 | 0.03 | 0.856 | 0.903 | 0.024 |
| RHSIM_Rhsim10G0203800 | 0.03 | 0.856 | 0.904 | -0.079 |
| RHSIM_Rhsim10G0109100 | 0.03 | 0.856 | 0.904 | -0.073 |
| novel.4504 | 0.03 | 0.856 | 0.904 | -0.186 |
| RHSIM_Rhsim03G0187900 | 0.03 | 0.856 | 0.904 | 0.033 |
| novel.363 | 0.03 | 0.856 | 0.904 | -0.076 |

| Gene | LRT statistic | P value | BH-adjusted P | log2FC (interaction) |
|---|---|---|---|---|
| RHSIM_Rhsim05G0225300 | 0.03 | 0.856 | 0.904 | -0.375 |
| RHSIM_Rhsim06G0172900 | 0.03 | 0.856 | 0.904 | -0.027 |
| RHSIM_Rhsim13G0208600 | 0.03 | 0.857 | 0.904 | -0.414 |
| RHSIM_Rhsim08G0229700 | 0.03 | 0.857 | 0.904 | 0.021 |
| RHSIM_Rhsim09G0118200 | 0.03 | 0.857 | 0.904 | 0.315 |
| RHSIM_Rhsim06G0013000 | 0.03 | 0.857 | 0.904 | -0.097 |
| RHSIM_Rhsim11G0085500 | 0.03 | 0.857 | 0.904 | -0.016 |
| novel.2279 | 0.03 | 0.857 | 0.904 | -0.155 |
| RHSIM_Rhsim04G0193300 | 0.03 | 0.857 | 0.905 | 0.357 |
| RHSIM_Rhsim02G0188900 | 0.03 | 0.857 | 0.905 | -0.043 |
| RHSIM_Rhsim02G0088200 | 0.03 | 0.857 | 0.905 | 0.029 |
| RHSIM_Rhsim03G0204800 | 0.03 | 0.857 | 0.905 | -0.398 |
| RHSIM_Rhsim01G0214000 | 0.03 | 0.858 | 0.905 | -0.088 |
| novel.3873 | 0.03 | 0.858 | 0.905 | 0.146 |
| novel.1191 | 0.03 | 0.858 | 0.905 | 0.047 |
| RHSIM_Rhsim04G0080600 | 0.03 | 0.858 | 0.905 | 0.164 |
| novel.753 | 0.03 | 0.858 | 0.905 | -0.172 |
| novel.773 | 0.03 | 0.858 | 0.905 | -0.044 |
| RHSIM_Rhsim03G0173000 | 0.03 | 0.858 | 0.905 | -0.342 |
| RHSIM_Rhsim06G0032900 | 0.03 | 0.858 | 0.905 | -0.009 |
| novel.1777 | 0.03 | 0.858 | 0.905 | 0.280 |
| RHSIM_Rhsim12G0128300 | 0.03 | 0.859 | 0.906 | -0.087 |
| RHSIM_Rhsim04G0247400 | 0.03 | 0.859 | 0.906 | 0.147 |
| RHSIM_Rhsim01G0190700 | 0.03 | 0.859 | 0.906 | 0.020 |
| RHSIM_Rhsim12G0093300 | 0.03 | 0.859 | 0.906 | -0.130 |
| RHSIM_Rhsim03G0070800 | 0.03 | 0.859 | 0.906 | -0.316 |
| RHSIM_Rhsim03G0075100 | 0.03 | 0.859 | 0.906 | -0.039 |
| RHSIM_Rhsim07G0215600 | 0.03 | 0.859 | 0.906 | -0.021 |
| novel.176 | 0.03 | 0.859 | 0.906 | -0.200 |
| RHSIM_Rhsim10G0004400 | 0.03 | 0.859 | 0.906 | 0.183 |
| novel.2400 | 0.03 | 0.860 | 0.906 | -0.341 |
| RHSIM_Rhsim03G0252900 | 0.03 | 0.860 | 0.906 | 0.016 |

| Gene | LRT statistic | P value | BH-adjusted P | log2FC (interaction) |
|---|---|---|---|---|
| RHSIM_Rhsim03G0109700 | 0.03 | 0.860 | 0.906 | 0.018 |
| RHSIM_RhsimUnG0045000 | 0.03 | 0.860 | 0.906 | -0.039 |
| RHSIM_Rhsim06G0137100 | 0.03 | 0.860 | 0.906 | 0.011 |
| novel.3763 | 0.03 | 0.860 | 0.906 | 0.037 |
| novel.3671 | 0.03 | 0.860 | 0.906 | -0.396 |
| RHSIM_Rhsim02G0146500 | 0.03 | 0.860 | 0.906 | 0.032 |
| RHSIM_Rhsim11G0074700 | 0.03 | 0.860 | 0.906 | -30.988 |
| RHSIM_Rhsim03G0155300 | 0.03 | 0.860 | 0.907 | 0.050 |
| RHSIM_Rhsim08G0149500 | 0.03 | 0.860 | 0.907 | -0.019 |
| RHSIM_Rhsim04G0119000 | 0.03 | 0.860 | 0.907 | -0.341 |
| novel.1426 | 0.03 | 0.860 | 0.907 | 0.103 |
| RHSIM_RhsimUnG0005600 | 0.03 | 0.861 | 0.907 | -0.049 |
| RHSIM_Rhsim04G0012000 | 0.03 | 0.861 | 0.907 | 0.023 |
| RHSIM_Rhsim07G0144900 | 0.03 | 0.861 | 0.907 | -0.097 |
| RHSIM_Rhsim04G0096600 | 0.03 | 0.861 | 0.908 | -0.030 |
| RHSIM_Rhsim05G0183100 | 0.03 | 0.862 | 0.908 | -0.363 |
| RHSIM_Rhsim13G0131600 | 0.03 | 0.862 | 0.908 | 0.255 |
| RHSIM_Rhsim07G0185700 | 0.03 | 0.862 | 0.909 | 0.084 |
| RHSIM_Rhsim06G0162600 | 0.03 | 0.863 | 0.909 | 0.284 |
| RHSIM_Rhsim12G0165300 | 0.03 | 0.863 | 0.909 | 0.302 |
| RHSIM_Rhsim04G0090700 | 0.03 | 0.863 | 0.909 | -0.080 |
| RHSIM_Rhsim04G0113200 | 0.03 | 0.863 | 0.909 | 0.082 |
| RHSIM_Rhsim13G0012800 | 0.03 | 0.863 | 0.909 | 0.066 |
| RHSIM_Rhsim03G0224000 | 0.03 | 0.864 | 0.910 | -0.075 |
| novel.1504 | 0.03 | 0.864 | 0.910 | -0.096 |
| RHSIM_Rhsim09G0168200 | 0.03 | 0.864 | 0.910 | -0.257 |
| RHSIM_Rhsim11G0125900 | 0.03 | 0.864 | 0.910 | -0.020 |
| novel.2850 | 0.03 | 0.864 | 0.910 | 0.066 |
| RHSIM_Rhsim08G0236900 | 0.03 | 0.864 | 0.910 | 0.031 |
| novel.1281 | 0.03 | 0.864 | 0.910 | -0.069 |
| RHSIM_Rhsim06G0045600 | 0.03 | 0.864 | 0.910 | -0.445 |
| RHSIM_Rhsim01G0056100 | 0.03 | 0.865 | 0.910 | 0.142 |

| Gene | LRT statistic | P value | BH-adjusted P | log2FC (interaction) |
|---|---|---|---|---|
| RHSIM_Rhsim04G0065000 | 0.03 | 0.865 | 0.911 | 0.210 |
| RHSIM_RhsimUnG0245200 | 0.03 | 0.865 | 0.911 | -0.335 |
| novel.1715 | 0.03 | 0.865 | 0.911 | 0.037 |
| RHSIM_Rhsim01G0265600 | 0.03 | 0.865 | 0.911 | -0.144 |
| RHSIM_Rhsim07G0188700 | 0.03 | 0.865 | 0.911 | -0.499 |
| RHSIM_Rhsim11G0183700 | 0.03 | 0.865 | 0.911 | 0.037 |
| novel.1766 | 0.03 | 0.865 | 0.911 | 0.059 |
| RHSIM_Rhsim04G0067600 | 0.03 | 0.866 | 0.911 | -0.380 |
| RHSIM_RhsimUnG0175700 | 0.03 | 0.866 | 0.911 | 0.176 |
| RHSIM_Rhsim02G0071700 | 0.03 | 0.866 | 0.911 | -0.021 |
| novel.3146 | 0.03 | 0.866 | 0.911 | 0.013 |
| RHSIM_RhsimUnG0129600 | 0.03 | 0.866 | 0.911 | -0.378 |
| novel.3428 | 0.03 | 0.866 | 0.912 | -0.020 |
| RHSIM_Rhsim01G0236700 | 0.03 | 0.866 | 0.912 | -0.022 |
| RHSIM_Rhsim06G0196700 | 0.03 | 0.866 | 0.912 | -0.027 |
| RHSIM_Rhsim13G0121700 | 0.03 | 0.866 | 0.912 | -0.094 |
| novel.1980 | 0.03 | 0.867 | 0.912 | -0.155 |
| RHSIM_Rhsim03G0170600 | 0.03 | 0.867 | 0.912 | -0.143 |
| novel.1096 | 0.03 | 0.867 | 0.912 | 0.119 |
| RHSIM_Rhsim04G0209500 | 0.03 | 0.867 | 0.912 | -0.276 |
| RHSIM_Rhsim10G0001000 | 0.03 | 0.867 | 0.913 | 0.081 |
| novel.3039 | 0.03 | 0.868 | 0.913 | 0.017 |
| RHSIM_RhsimUnG0233600 | 0.03 | 0.868 | 0.913 | -0.056 |
| RHSIM_Rhsim02G0157000 | 0.03 | 0.868 | 0.913 | 0.016 |
| RHSIM_Rhsim13G0166100 | 0.03 | 0.868 | 0.913 | 0.251 |
| RHSIM_Rhsim09G0049600 | 0.03 | 0.868 | 0.913 | -0.089 |
| RHSIM_Rhsim04G0067200 | 0.03 | 0.868 | 0.914 | 0.036 |
| novel.2601 | 0.03 | 0.869 | 0.914 | 0.505 |
| novel.2454 | 0.03 | 0.869 | 0.914 | 0.209 |
| RHSIM_Rhsim12G0124400 | 0.03 | 0.869 | 0.914 | -0.052 |
| RHSIM_Rhsim04G0203400 | 0.03 | 0.869 | 0.914 | 0.137 |
| RHSIM_Rhsim04G0198400 | 0.03 | 0.869 | 0.914 | -0.032 |

| Gene | LRT statistic | P value | BH-adjusted P | log2FC (interaction) |
|---|---|---|---|---|
| RHSIM_Rhsim03G0178500 | 0.03 | 0.869 | 0.914 | 0.069 |
| RHSIM_Rhsim03G0072000 | 0.03 | 0.869 | 0.914 | -0.356 |
| RHSIM_Rhsim12G0098700 | 0.03 | 0.869 | 0.914 | -0.038 |
| RHSIM_Rhsim13G0147400 | 0.03 | 0.869 | 0.914 | 0.133 |
| RHSIM_Rhsim11G0091400 | 0.03 | 0.869 | 0.914 | -0.152 |
| RHSIM_Rhsim08G0014700 | 0.03 | 0.870 | 0.915 | 0.036 |
| RHSIM_Rhsim09G0103300 | 0.03 | 0.870 | 0.915 | -0.028 |
| RHSIM_Rhsim05G0196200 | 0.03 | 0.870 | 0.915 | 0.095 |
| RHSIM_Rhsim04G0033900 | 0.03 | 0.870 | 0.915 | -0.161 |
| RHSIM_Rhsim08G0108700 | 0.03 | 0.870 | 0.915 | -0.192 |
| novel.907 | 0.03 | 0.870 | 0.915 | 0.226 |
| RHSIM_Rhsim11G0053100 | 0.03 | 0.870 | 0.915 | -0.010 |
| novel.3614 | 0.03 | 0.870 | 0.915 | -0.112 |
| novel.2447 | 0.03 | 0.870 | 0.915 | -0.223 |
| RHSIM_Rhsim12G0091900 | 0.03 | 0.870 | 0.915 | 0.254 |
| RHSIM_Rhsim05G0025700 | 0.03 | 0.871 | 0.915 | -0.427 |
| RHSIM_Rhsim07G0213900 | 0.03 | 0.871 | 0.915 | -0.117 |
| RHSIM_Rhsim01G0192000 | 0.03 | 0.871 | 0.915 | -0.021 |
| RHSIM_Rhsim06G0212700 | 0.03 | 0.871 | 0.915 | -0.257 |
| RHSIM_Rhsim11G0120900 | 0.03 | 0.871 | 0.915 | 0.602 |
| novel.828 | 0.03 | 0.871 | 0.915 | -0.026 |
| RHSIM_Rhsim06G0066400 | 0.03 | 0.871 | 0.915 | -0.202 |
| RHSIM_Rhsim10G0136800 | 0.03 | 0.871 | 0.915 | -0.018 |
| RHSIM_RhsimUnG0023200 | 0.03 | 0.871 | 0.915 | -0.016 |
| RHSIM_Rhsim04G0065600 | 0.03 | 0.871 | 0.915 | -0.511 |
| RHSIM_Rhsim03G0065800 | 0.03 | 0.871 | 0.916 | 0.102 |
| RHSIM_Rhsim02G0030100 | 0.03 | 0.871 | 0.916 | -0.178 |
| RHSIM_Rhsim05G0136500 | 0.03 | 0.871 | 0.916 | 0.122 |
| RHSIM_Rhsim13G0136400 | 0.03 | 0.871 | 0.916 | 0.027 |
| RHSIM_Rhsim04G0207700 | 0.03 | 0.872 | 0.916 | 0.036 |
| RHSIM_Rhsim09G0190600 | 0.03 | 0.872 | 0.916 | -0.104 |
| RHSIM_Rhsim13G0162800 | 0.03 | 0.872 | 0.916 | -0.104 |

| Gene | LRT statistic | P value | BH-adjusted P | log2FC (interaction) |
|---|---|---|---|---|
| RHSIM_RhsimUnG0138800 | 0.03 | 0.872 | 0.916 | -0.042 |
| novel.745 | 0.03 | 0.872 | 0.916 | -0.323 |
| RHSIM_Rhsim06G0210200 | 0.03 | 0.872 | 0.916 | 0.016 |
| RHSIM_RhsimUnG0238900 | 0.03 | 0.873 | 0.917 | 0.291 |
| RHSIM_Rhsim03G0059800 | 0.03 | 0.873 | 0.917 | -0.428 |
| RHSIM_Rhsim09G0084600 | 0.03 | 0.873 | 0.917 | 0.042 |
| RHSIM_Rhsim04G0075200 | 0.03 | 0.873 | 0.917 | -0.054 |
| novel.3979 | 0.03 | 0.873 | 0.917 | 0.104 |
| RHSIM_Rhsim02G0136500 | 0.03 | 0.873 | 0.917 | -0.171 |
| RHSIM_Rhsim12G0063000 | 0.03 | 0.873 | 0.917 | -0.088 |
| RHSIM_Rhsim12G0168100 | 0.03 | 0.873 | 0.917 | -0.030 |
| novel.704 | 0.03 | 0.873 | 0.917 | 0.392 |
| RHSIM_Rhsim12G0209400 | 0.03 | 0.873 | 0.917 | -0.032 |
| novel.1314 | 0.03 | 0.873 | 0.917 | 0.092 |
| RHSIM_Rhsim07G0034900 | 0.03 | 0.874 | 0.917 | -0.084 |
| novel.1008 | 0.03 | 0.874 | 0.917 | -0.035 |
| RHSIM_Rhsim01G0261400 | 0.03 | 0.874 | 0.917 | 0.212 |
| RHSIM_RhsimUnG0159600 | 0.03 | 0.874 | 0.918 | -0.282 |
| RHSIM_RhsimUnG0218000 | 0.02 | 0.874 | 0.918 | -0.184 |
| RHSIM_Rhsim06G0228200 | 0.02 | 0.875 | 0.918 | 0.288 |
| RHSIM_Rhsim08G0029200 | 0.02 | 0.875 | 0.918 | -0.023 |
| RHSIM_Rhsim04G0076400 | 0.02 | 0.875 | 0.918 | 0.386 |
| RHSIM_Rhsim07G0028100 | 0.02 | 0.875 | 0.918 | -0.298 |
| RHSIM_Rhsim02G0167700 | 0.02 | 0.875 | 0.919 | -0.055 |
| RHSIM_Rhsim08G0217300 | 0.02 | 0.875 | 0.919 | 0.259 |
| RHSIM_Rhsim08G0051700 | 0.02 | 0.875 | 0.919 | 0.150 |
| RHSIM_Rhsim03G0182900 | 0.02 | 0.876 | 0.919 | -0.336 |
| RHSIM_Rhsim08G0064600 | 0.02 | 0.876 | 0.919 | -0.419 |
| RHSIM_Rhsim06G0031600 | 0.02 | 0.876 | 0.920 | -0.105 |
| RHSIM_Rhsim01G0078300 | 0.02 | 0.876 | 0.920 | -0.058 |
| RHSIM_Rhsim03G0039400 | 0.02 | 0.876 | 0.920 | -0.408 |
| RHSIM_Rhsim07G0076900 | 0.02 | 0.876 | 0.920 | 0.414 |

| Gene | LRT statistic | P value | BH-adjusted P | log2FC (interaction) |
|---|---|---|---|---|
| novel.1723 | 0.02 | 0.876 | 0.920 | 0.146 |
| RHSIM_Rhsim05G0124900 | 0.02 | 0.876 | 0.920 | -0.224 |
| RHSIM_Rhsim12G0084100 | 0.02 | 0.877 | 0.920 | 0.054 |
| RHSIM_Rhsim01G0270400 | 0.02 | 0.877 | 0.920 | -0.019 |
| RHSIM_Rhsim11G0088900 | 0.02 | 0.877 | 0.920 | 0.016 |
| RHSIM_Rhsim05G0125500 | 0.02 | 0.877 | 0.920 | -0.054 |
| RHSIM_Rhsim04G0075400 | 0.02 | 0.877 | 0.920 | -0.020 |
| novel.1717 | 0.02 | 0.877 | 0.920 | -0.106 |
| RHSIM_Rhsim06G0134500 | 0.02 | 0.877 | 0.920 | -0.070 |
| RHSIM_Rhsim06G0228000 | 0.02 | 0.877 | 0.920 | -0.339 |
| novel.3166 | 0.02 | 0.877 | 0.920 | 0.158 |
| RHSIM_Rhsim13G0195400 | 0.02 | 0.878 | 0.921 | 0.111 |
| RHSIM_Rhsim05G0212600 | 0.02 | 0.878 | 0.921 | 0.189 |
| RHSIM_Rhsim02G0231600 | 0.02 | 0.878 | 0.921 | -0.225 |
| novel.23 | 0.02 | 0.878 | 0.921 | -0.121 |
| novel.1436 | 0.02 | 0.879 | 0.921 | -0.152 |
| novel.568 | 0.02 | 0.879 | 0.921 | -0.098 |
| RHSIM_Rhsim09G0037200 | 0.02 | 0.879 | 0.921 | -0.164 |
| RHSIM_Rhsim06G0008700 | 0.02 | 0.879 | 0.921 | -0.317 |
| novel.3367 | 0.02 | 0.879 | 0.922 | -0.452 |
| RHSIM_Rhsim08G0027000 | 0.02 | 0.879 | 0.922 | -0.020 |
| RHSIM_Rhsim10G0151300 | 0.02 | 0.879 | 0.922 | 0.012 |
| RHSIM_RhsimUnG0079700 | 0.02 | 0.879 | 0.922 | 0.116 |
| RHSIM_RhsimPtG0004000 | 0.02 | 0.879 | 0.922 | 0.047 |
| RHSIM_Rhsim10G0025900 | 0.02 | 0.879 | 0.922 | 0.361 |
| RHSIM_Rhsim02G0046600 | 0.02 | 0.879 | 0.922 | 0.032 |
| RHSIM_Rhsim10G0026600 | 0.02 | 0.879 | 0.922 | -0.114 |
| RHSIM_Rhsim11G0071600 | 0.02 | 0.880 | 0.922 | 0.094 |
| RHSIM_Rhsim01G0153400 | 0.02 | 0.880 | 0.922 | 0.017 |
| RHSIM_Rhsim03G0208300 | 0.02 | 0.880 | 0.922 | 0.092 |
| RHSIM_Rhsim02G0253200 | 0.02 | 0.880 | 0.922 | -0.101 |
| RHSIM_Rhsim12G0068400 | 0.02 | 0.880 | 0.922 | -0.198 |

| Gene | LRT statistic | P value | BH-adjusted P | log2FC (interaction) |
|---|---|---|---|---|
| RHSIM_Rhsim05G0040800 | 0.02 | 0.880 | 0.923 | 0.018 |
| RHSIM_RhsimUnG0183600 | 0.02 | 0.880 | 0.923 | 0.042 |
| RHSIM_Rhsim06G0181500 | 0.02 | 0.880 | 0.923 | -0.054 |
| novel.2401 | 0.02 | 0.880 | 0.923 | -0.027 |
| RHSIM_Rhsim02G0146600 | 0.02 | 0.880 | 0.923 | -0.312 |
| RHSIM_Rhsim09G0144500 | 0.02 | 0.880 | 0.923 | -0.287 |
| novel.509 | 0.02 | 0.881 | 0.923 | -0.254 |
| RHSIM_Rhsim03G0003000 | 0.02 | 0.881 | 0.923 | -0.107 |
| RHSIM_Rhsim01G0094200 | 0.02 | 0.881 | 0.923 | 0.051 |
| RHSIM_Rhsim09G0056200 | 0.02 | 0.881 | 0.923 | 0.188 |
| RHSIM_Rhsim04G0142700 | 0.02 | 0.881 | 0.923 | -0.041 |
| RHSIM_Rhsim10G0206800 | 0.02 | 0.881 | 0.923 | -0.016 |
| RHSIM_Rhsim01G0076500 | 0.02 | 0.881 | 0.923 | -0.019 |
| RHSIM_Rhsim06G0074000 | 0.02 | 0.881 | 0.923 | 0.191 |
| novel.2866 | 0.02 | 0.881 | 0.923 | 0.213 |
| RHSIM_RhsimUnG0199900 | 0.02 | 0.881 | 0.923 | 0.171 |
| RHSIM_Rhsim03G0042900 | 0.02 | 0.881 | 0.923 | 0.371 |
| RHSIM_Rhsim12G0031000 | 0.02 | 0.881 | 0.923 | 0.340 |
| RHSIM_Rhsim05G0188500 | 0.02 | 0.882 | 0.923 | 0.146 |
| RHSIM_Rhsim07G0228300 | 0.02 | 0.882 | 0.923 | 0.023 |
| RHSIM_Rhsim12G0126900 | 0.02 | 0.882 | 0.923 | -0.061 |
| RHSIM_RhsimUnG0164000 | 0.02 | 0.882 | 0.923 | -0.352 |
| RHSIM_Rhsim04G0044200 | 0.02 | 0.882 | 0.924 | 0.091 |
| novel.2830 | 0.02 | 0.882 | 0.924 | 0.025 |
| RHSIM_Rhsim07G0221900 | 0.02 | 0.882 | 0.924 | 0.018 |
| RHSIM_Rhsim06G0012900 | 0.02 | 0.883 | 0.924 | 0.051 |
| RHSIM_Rhsim07G0245900 | 0.02 | 0.883 | 0.925 | 0.022 |
| novel.3383 | 0.02 | 0.883 | 0.925 | -0.028 |
| RHSIM_Rhsim12G0146900 | 0.02 | 0.883 | 0.925 | 0.026 |
| RHSIM_Rhsim04G0004800 | 0.02 | 0.884 | 0.925 | -0.033 |
| novel.2270 | 0.02 | 0.884 | 0.925 | -0.060 |
| RHSIM_Rhsim03G0153600 | 0.02 | 0.884 | 0.925 | -0.033 |

| Gene | LRT statistic | P value | BH-adjusted P | log2FC (interaction) |
|---|---|---|---|---|
| novel.2854 | 0.02 | 0.884 | 0.925 | -0.229 |
| novel.1208 | 0.02 | 0.884 | 0.925 | 0.050 |
| novel.4308 | 0.02 | 0.884 | 0.926 | -0.102 |
| RHSIM_RhsimUnG0135800 | 0.02 | 0.884 | 0.926 | -0.167 |
| RHSIM_Rhsim03G0054000 | 0.02 | 0.885 | 0.926 | 0.073 |
| novel.4271 | 0.02 | 0.885 | 0.926 | -0.108 |
| RHSIM_Rhsim07G0024300 | 0.02 | 0.885 | 0.926 | -0.081 |
| RHSIM_Rhsim01G0069500 | 0.02 | 0.885 | 0.926 | 0.177 |
| RHSIM_Rhsim12G0062500 | 0.02 | 0.885 | 0.926 | -0.069 |
| novel.2784 | 0.02 | 0.885 | 0.926 | -0.072 |
| RHSIM_Rhsim09G0024800 | 0.02 | 0.885 | 0.926 | -0.165 |
| RHSIM_Rhsim02G0164200 | 0.02 | 0.885 | 0.926 | 0.093 |
| RHSIM_Rhsim02G0161500 | 0.02 | 0.886 | 0.927 | 0.136 |
| RHSIM_Rhsim02G0004500 | 0.02 | 0.886 | 0.927 | 0.304 |
| RHSIM_Rhsim06G0186800 | 0.02 | 0.886 | 0.927 | 0.317 |
| novel.1242 | 0.02 | 0.886 | 0.927 | 30.406 |
| novel.4235 | 0.02 | 0.886 | 0.927 | -0.095 |
| RHSIM_Rhsim07G0248100 | 0.02 | 0.886 | 0.927 | -0.109 |
| RHSIM_Rhsim08G0186900 | 0.02 | 0.886 | 0.927 | -0.237 |
| RHSIM_Rhsim07G0004600 | 0.02 | 0.886 | 0.927 | -0.011 |
| RHSIM_Rhsim04G0189200 | 0.02 | 0.886 | 0.927 | 0.013 |
| RHSIM_Rhsim01G0267300 | 0.02 | 0.886 | 0.927 | -0.219 |
| RHSIM_Rhsim10G0195000 | 0.02 | 0.886 | 0.927 | -0.016 |
| RHSIM_Rhsim10G0153800 | 0.02 | 0.886 | 0.927 | 0.260 |
| RHSIM_Rhsim01G0173100 | 0.02 | 0.886 | 0.927 | 0.016 |
| RHSIM_Rhsim01G0043000 | 0.02 | 0.886 | 0.927 | 0.064 |
| novel.2039 | 0.02 | 0.887 | 0.927 | -0.316 |
| RHSIM_Rhsim09G0079900 | 0.02 | 0.887 | 0.927 | 0.363 |
| novel.4027 | 0.02 | 0.887 | 0.928 | -0.108 |
| RHSIM_Rhsim08G0181200 | 0.02 | 0.887 | 0.928 | 0.111 |
| RHSIM_Rhsim08G0009500 | 0.02 | 0.887 | 0.928 | 0.077 |
| novel.3958 | 0.02 | 0.887 | 0.928 | -0.128 |

| Gene | LRT statistic | P value | BH-adjusted P | log2FC (interaction) |
|---|---|---|---|---|
| RHSIM_Rhsim07G0198900 | 0.02 | 0.887 | 0.928 | -0.027 |
| RHSIM_Rhsim05G0085500 | 0.02 | 0.887 | 0.928 | -0.036 |
| RHSIM_Rhsim13G0047700 | 0.02 | 0.888 | 0.928 | -0.030 |
| novel.1740 | 0.02 | 0.888 | 0.928 | 0.008 |
| RHSIM_Rhsim11G0089500 | 0.02 | 0.888 | 0.928 | 0.017 |
| RHSIM_Rhsim08G0228200 | 0.02 | 0.888 | 0.928 | 0.023 |
| RHSIM_Rhsim13G0080500 | 0.02 | 0.888 | 0.928 | 0.057 |
| novel.523 | 0.02 | 0.888 | 0.928 | 0.048 |
| novel.809 | 0.02 | 0.888 | 0.929 | 0.033 |
| RHSIM_Rhsim04G0021900 | 0.02 | 0.888 | 0.929 | 0.012 |
| RHSIM_Rhsim08G0037300 | 0.02 | 0.888 | 0.929 | 0.037 |
| novel.1864 | 0.02 | 0.888 | 0.929 | -0.063 |
| novel.1353 | 0.02 | 0.888 | 0.929 | 0.154 |
| RHSIM_Rhsim09G0205100 | 0.02 | 0.888 | 0.929 | -0.017 |
| RHSIM_Rhsim03G0054200 | 0.02 | 0.889 | 0.929 | 0.405 |
| novel.3874 | 0.02 | 0.889 | 0.929 | 0.191 |
| RHSIM_Rhsim06G0105300 | 0.02 | 0.889 | 0.929 | -0.208 |
| RHSIM_Rhsim08G0081900 | 0.02 | 0.889 | 0.929 | -0.212 |
| RHSIM_Rhsim08G0211200 | 0.02 | 0.889 | 0.929 | -0.070 |
| novel.3982 | 0.02 | 0.889 | 0.929 | -0.166 |
| RHSIM_Rhsim03G0030700 | 0.02 | 0.889 | 0.929 | -0.174 |
| novel.2032 | 0.02 | 0.889 | 0.929 | -0.045 |
| novel.3126 | 0.02 | 0.890 | 0.929 | 0.205 |
| novel.2651 | 0.02 | 0.890 | 0.930 | 0.220 |
| RHSIM_Rhsim08G0034500 | 0.02 | 0.890 | 0.930 | -0.038 |
| RHSIM_Rhsim06G0216000 | 0.02 | 0.890 | 0.930 | -0.181 |
| novel.2873 | 0.02 | 0.890 | 0.930 | 0.035 |
| novel.873 | 0.02 | 0.890 | 0.930 | 0.074 |
| RHSIM_Rhsim13G0022000 | 0.02 | 0.890 | 0.930 | 0.020 |
| novel.4391 | 0.02 | 0.890 | 0.930 | 0.088 |
| novel.656 | 0.02 | 0.890 | 0.930 | -30.382 |
| RHSIM_Rhsim10G0159500 | 0.02 | 0.890 | 0.930 | -0.056 |

| Gene | LRT statistic | P value | BH-adjusted P | log2FC (interaction) |
|---|---|---|---|---|
| novel.2026 | 0.02 | 0.891 | 0.930 | -0.110 |
| RHSIM_Rhsim02G0179600 | 0.02 | 0.891 | 0.930 | -0.040 |
| novel.376 | 0.02 | 0.891 | 0.930 | -0.094 |
| RHSIM_Rhsim01G0057700 | 0.02 | 0.891 | 0.930 | 0.362 |
| novel.218 | 0.02 | 0.891 | 0.930 | 0.192 |
| RHSIM_RhsimUnG0122400 | 0.02 | 0.891 | 0.930 | 0.067 |
| RHSIM_Rhsim11G0141700 | 0.02 | 0.891 | 0.930 | 0.011 |
| RHSIM_Rhsim02G0184000 | 0.02 | 0.891 | 0.931 | -0.084 |
| novel.558 | 0.02 | 0.891 | 0.931 | 0.064 |
| RHSIM_Rhsim10G0062900 | 0.02 | 0.892 | 0.931 | 0.110 |
| RHSIM_Rhsim04G0034400 | 0.02 | 0.892 | 0.931 | -0.078 |
| RHSIM_Rhsim08G0163200 | 0.02 | 0.892 | 0.931 | -0.013 |
| RHSIM_RhsimUnG0185900 | 0.02 | 0.892 | 0.931 | -0.324 |
| RHSIM_Rhsim06G0135200 | 0.02 | 0.892 | 0.931 | -0.052 |
| RHSIM_Rhsim11G0008800 | 0.02 | 0.892 | 0.931 | -0.017 |
| RHSIM_Rhsim10G0070700 | 0.02 | 0.892 | 0.931 | 0.344 |
| novel.3507 | 0.02 | 0.893 | 0.932 | -0.010 |
| RHSIM_Rhsim11G0113300 | 0.02 | 0.893 | 0.932 | 0.015 |
| RHSIM_Rhsim12G0003700 | 0.02 | 0.893 | 0.932 | -0.097 |
| RHSIM_Rhsim11G0102600 | 0.02 | 0.893 | 0.932 | -0.081 |
| RHSIM_Rhsim05G0230300 | 0.02 | 0.893 | 0.932 | -0.448 |
| RHSIM_Rhsim08G0069900 | 0.02 | 0.893 | 0.932 | 0.022 |
| RHSIM_Rhsim03G0014200 | 0.02 | 0.893 | 0.932 | -0.146 |
| RHSIM_Rhsim03G0031300 | 0.02 | 0.894 | 0.933 | -0.015 |
| novel.4216 | 0.02 | 0.894 | 0.933 | -0.083 |
| RHSIM_Rhsim04G0067300 | 0.02 | 0.894 | 0.933 | 0.115 |
| RHSIM_Rhsim03G0245800 | 0.02 | 0.894 | 0.933 | 0.034 |
| novel.2488 | 0.02 | 0.894 | 0.933 | -0.266 |
| RHSIM_Rhsim08G0045100 | 0.02 | 0.894 | 0.933 | -0.046 |
| RHSIM_Rhsim03G0240900 | 0.02 | 0.894 | 0.933 | 0.046 |
| RHSIM_Rhsim04G0163500 | 0.02 | 0.894 | 0.933 | -0.072 |
| RHSIM_Rhsim08G0085000 | 0.02 | 0.894 | 0.933 | -0.044 |

| Gene | LRT statistic | P value | BH-adjusted P | log2FC (interaction) |
|---|---|---|---|---|
| RHSIM_Rhsim10G0162500 | 0.02 | 0.894 | 0.933 | -0.139 |
| RHSIM_RhsimUnG0077500 | 0.02 | 0.894 | 0.933 | 0.032 |
| RHSIM_Rhsim01G0200400 | 0.02 | 0.895 | 0.933 | 0.191 |
| RHSIM_Rhsim09G0182800 | 0.02 | 0.895 | 0.933 | -0.036 |
| RHSIM_Rhsim06G0110800 | 0.02 | 0.895 | 0.933 | -0.119 |
| RHSIM_Rhsim01G0250600 | 0.02 | 0.895 | 0.933 | 0.013 |
| RHSIM_Rhsim07G0009800 | 0.02 | 0.895 | 0.933 | -0.143 |
| RHSIM_Rhsim11G0085300 | 0.02 | 0.895 | 0.934 | -0.150 |
| RHSIM_Rhsim07G0062300 | 0.02 | 0.895 | 0.934 | 0.016 |
| RHSIM_Rhsim13G0191700 | 0.02 | 0.896 | 0.934 | 0.026 |
| RHSIM_Rhsim07G0146900 | 0.02 | 0.896 | 0.934 | 0.014 |
| RHSIM_Rhsim04G0022800 | 0.02 | 0.897 | 0.935 | 0.030 |
| RHSIM_Rhsim13G0079500 | 0.02 | 0.897 | 0.935 | 0.208 |
| novel.610 | 0.02 | 0.897 | 0.935 | 0.152 |
| RHSIM_Rhsim03G0003900 | 0.02 | 0.897 | 0.935 | -0.021 |
| RHSIM_RhsimUnG0079900 | 0.02 | 0.897 | 0.935 | -0.103 |
| RHSIM_Rhsim06G0067400 | 0.02 | 0.897 | 0.935 | -0.025 |
| novel.3716 | 0.02 | 0.897 | 0.935 | 0.084 |
| RHSIM_Rhsim03G0075600 | 0.02 | 0.897 | 0.935 | 0.015 |
| RHSIM_RhsimUnG0141800 | 0.02 | 0.897 | 0.935 | -0.191 |
| RHSIM_Rhsim12G0168500 | 0.02 | 0.897 | 0.935 | 0.018 |
| RHSIM_Rhsim10G0208900 | 0.02 | 0.897 | 0.935 | -0.252 |
| RHSIM_Rhsim03G0174500 | 0.02 | 0.897 | 0.935 | -0.414 |
| RHSIM_Rhsim13G0146000 | 0.02 | 0.897 | 0.935 | 0.256 |
| RHSIM_Rhsim06G0244800 | 0.02 | 0.897 | 0.935 | 0.011 |
| novel.2020 | 0.02 | 0.898 | 0.936 | 0.019 |
| RHSIM_RhsimUnG0066500 | 0.02 | 0.898 | 0.936 | -0.042 |
| RHSIM_Rhsim06G0062700 | 0.02 | 0.898 | 0.936 | -0.066 |
| RHSIM_Rhsim09G0145500 | 0.02 | 0.898 | 0.936 | -0.016 |
| RHSIM_Rhsim13G0051400 | 0.02 | 0.899 | 0.936 | 0.186 |
| novel.3813 | 0.02 | 0.899 | 0.937 | 0.242 |
| RHSIM_Rhsim11G0127700 | 0.02 | 0.899 | 0.937 | 0.006 |

| Gene | LRT statistic | P value | BH-adjusted P | log2FC (interaction) |
|---|---|---|---|---|
| novel.151 | 0.02 | 0.899 | 0.937 | -0.103 |
| RHSIM_Rhsim13G0142700 | 0.02 | 0.900 | 0.937 | -0.160 |
| RHSIM_Rhsim13G0218700 | 0.02 | 0.900 | 0.938 | -0.158 |
| novel.2164 | 0.02 | 0.900 | 0.938 | -0.100 |
| RHSIM_Rhsim13G0104500 | 0.02 | 0.900 | 0.938 | -0.016 |
| RHSIM_Rhsim12G0153800 | 0.02 | 0.900 | 0.938 | -0.264 |
| RHSIM_Rhsim06G0094200 | 0.02 | 0.901 | 0.938 | 0.115 |
| RHSIM_RhsimMtG0005300 | 0.02 | 0.901 | 0.938 | -0.318 |
| RHSIM_Rhsim10G0133300 | 0.02 | 0.901 | 0.938 | -0.027 |
| RHSIM_Rhsim10G0100900 | 0.02 | 0.901 | 0.938 | 0.283 |
| RHSIM_Rhsim08G0174800 | 0.02 | 0.901 | 0.939 | 0.036 |
| RHSIM_Rhsim03G0077100 | 0.02 | 0.901 | 0.939 | 0.016 |
| RHSIM_Rhsim03G0264000 | 0.02 | 0.901 | 0.939 | -0.172 |
| RHSIM_Rhsim04G0093800 | 0.02 | 0.901 | 0.939 | 0.017 |
| RHSIM_RhsimUnG0067800 | 0.02 | 0.902 | 0.939 | -0.067 |
| RHSIM_Rhsim08G0206200 | 0.02 | 0.902 | 0.939 | -0.014 |
| RHSIM_Rhsim06G0237400 | 0.02 | 0.902 | 0.939 | 0.212 |
| RHSIM_Rhsim07G0031700 | 0.02 | 0.902 | 0.939 | -0.015 |
| RHSIM_Rhsim06G0232000 | 0.02 | 0.902 | 0.940 | 0.254 |
| RHSIM_Rhsim13G0034800 | 0.02 | 0.902 | 0.940 | -0.025 |
| RHSIM_Rhsim10G0072500 | 0.01 | 0.903 | 0.940 | -0.202 |
| RHSIM_Rhsim03G0016800 | 0.01 | 0.903 | 0.940 | -0.115 |
| RHSIM_Rhsim12G0129100 | 0.01 | 0.903 | 0.940 | -0.017 |
| RHSIM_Rhsim11G0047800 | 0.01 | 0.903 | 0.940 | -0.144 |
| RHSIM_Rhsim07G0238800 | 0.01 | 0.903 | 0.940 | -0.020 |
| RHSIM_Rhsim03G0070900 | 0.01 | 0.903 | 0.940 | -0.262 |
| RHSIM_Rhsim03G0071800 | 0.01 | 0.903 | 0.940 | 0.307 |
| RHSIM_Rhsim07G0030000 | 0.01 | 0.903 | 0.940 | -0.058 |
| RHSIM_Rhsim10G0005700 | 0.01 | 0.904 | 0.941 | 0.016 |
| RHSIM_Rhsim04G0039200 | 0.01 | 0.904 | 0.941 | -0.078 |
| RHSIM_Rhsim07G0026500 | 0.01 | 0.904 | 0.941 | 0.009 |
| RHSIM_Rhsim11G0067200 | 0.01 | 0.904 | 0.941 | -0.052 |

| Gene | LRT statistic | P value | BH-adjusted P | log2FC (interaction) |
|---|---|---|---|---|
| RHSIM_Rhsim07G0125800 | 0.01 | 0.904 | 0.941 | -0.015 |
| RHSIM_Rhsim02G0220500 | 0.01 | 0.904 | 0.941 | 0.249 |
| RHSIM_Rhsim04G0099300 | 0.01 | 0.904 | 0.941 | -0.074 |
| novel.3129 | 0.01 | 0.904 | 0.941 | 0.059 |
| RHSIM_Rhsim07G0228800 | 0.01 | 0.904 | 0.941 | -0.219 |
| RHSIM_Rhsim11G0144800 | 0.01 | 0.904 | 0.941 | 0.321 |
| RHSIM_Rhsim12G0032000 | 0.01 | 0.904 | 0.941 | 0.192 |
| novel.3076 | 0.01 | 0.905 | 0.941 | -0.148 |
| novel.3159 | 0.01 | 0.905 | 0.941 | 0.059 |
| RHSIM_Rhsim11G0095000 | 0.01 | 0.905 | 0.942 | -0.025 |
| RHSIM_Rhsim04G0138600 | 0.01 | 0.905 | 0.942 | -0.018 |
| RHSIM_Rhsim08G0117700 | 0.01 | 0.905 | 0.942 | 0.035 |
| RHSIM_Rhsim06G0208300 | 0.01 | 0.905 | 0.942 | 0.009 |
| RHSIM_Rhsim11G0177700 | 0.01 | 0.906 | 0.942 | 0.067 |
| RHSIM_Rhsim03G0210100 | 0.01 | 0.906 | 0.942 | -0.206 |
| novel.2361 | 0.01 | 0.906 | 0.942 | 0.150 |
| RHSIM_Rhsim12G0022500 | 0.01 | 0.906 | 0.942 | -0.070 |
| RHSIM_Rhsim03G0112400 | 0.01 | 0.906 | 0.942 | -0.249 |
| RHSIM_Rhsim08G0205100 | 0.01 | 0.906 | 0.943 | -0.147 |
| RHSIM_Rhsim08G0020500 | 0.01 | 0.906 | 0.943 | -0.069 |
| RHSIM_Rhsim06G0171100 | 0.01 | 0.906 | 0.943 | -0.115 |
| RHSIM_Rhsim12G0076400 | 0.01 | 0.906 | 0.943 | -0.307 |
| RHSIM_Rhsim06G0040000 | 0.01 | 0.906 | 0.943 | -0.045 |
| RHSIM_Rhsim13G0155100 | 0.01 | 0.906 | 0.943 | 0.037 |
| RHSIM_Rhsim13G0000200 | 0.01 | 0.907 | 0.943 | -0.009 |
| RHSIM_RhsimUnG0015100 | 0.01 | 0.907 | 0.943 | -0.064 |
| novel.985 | 0.01 | 0.907 | 0.943 | -0.132 |
| RHSIM_Rhsim10G0181900 | 0.01 | 0.907 | 0.943 | 0.160 |
| novel.327 | 0.01 | 0.907 | 0.943 | 0.177 |
| RHSIM_Rhsim08G0076400 | 0.01 | 0.907 | 0.943 | -0.011 |
| RHSIM_Rhsim06G0074500 | 0.01 | 0.907 | 0.943 | 0.216 |
| novel.4519 | 0.01 | 0.907 | 0.943 | 0.144 |

| Gene | LRT statistic | P value | BH-adjusted P | log2FC (interaction) |
|---|---|---|---|---|
| RHSIM_Rhsim04G0024000 | 0.01 | 0.907 | 0.943 | 0.062 |
| RHSIM_Rhsim10G0051500 | 0.01 | 0.907 | 0.943 | 0.011 |
| RHSIM_Rhsim07G0035000 | 0.01 | 0.907 | 0.943 | 0.149 |
| RHSIM_Rhsim13G0073200 | 0.01 | 0.907 | 0.943 | -0.019 |
| RHSIM_Rhsim03G0042300 | 0.01 | 0.908 | 0.943 | 0.024 |
| RHSIM_Rhsim01G0116900 | 0.01 | 0.908 | 0.943 | 0.036 |
| novel.242 | 0.01 | 0.908 | 0.944 | -0.143 |
| novel.3703 | 0.01 | 0.908 | 0.944 | -0.139 |
| RHSIM_Rhsim11G0192400 | 0.01 | 0.908 | 0.944 | 0.009 |
| novel.3375 | 0.01 | 0.908 | 0.944 | 0.070 |
| novel.3756 | 0.01 | 0.908 | 0.944 | 0.164 |
| RHSIM_Rhsim12G0028500 | 0.01 | 0.908 | 0.944 | -0.011 |
| RHSIM_RhsimUnG0014400 | 0.01 | 0.909 | 0.944 | 0.085 |
| novel.579 | 0.01 | 0.909 | 0.944 | 0.095 |
| novel.2489 | 0.01 | 0.909 | 0.944 | 0.250 |
| RHSIM_Rhsim03G0216600 | 0.01 | 0.909 | 0.944 | 0.013 |
| novel.352 | 0.01 | 0.909 | 0.944 | 0.040 |
| RHSIM_Rhsim01G0224700 | 0.01 | 0.909 | 0.944 | 0.010 |
| RHSIM_Rhsim09G0047400 | 0.01 | 0.909 | 0.944 | -0.189 |
| RHSIM_Rhsim03G0042800 | 0.01 | 0.909 | 0.944 | -0.139 |
| RHSIM_Rhsim08G0189900 | 0.01 | 0.909 | 0.944 | -0.155 |
| RHSIM_Rhsim01G0271400 | 0.01 | 0.909 | 0.945 | 0.236 |
| novel.2043 | 0.01 | 0.909 | 0.945 | -0.180 |
| RHSIM_Rhsim02G0145600 | 0.01 | 0.909 | 0.945 | -0.267 |
| novel.3654 | 0.01 | 0.909 | 0.945 | 0.046 |
| RHSIM_Rhsim01G0126100 | 0.01 | 0.909 | 0.945 | -0.024 |
| novel.2907 | 0.01 | 0.910 | 0.945 | -0.008 |
| RHSIM_Rhsim09G0127900 | 0.01 | 0.910 | 0.945 | 0.101 |
| RHSIM_Rhsim02G0044000 | 0.01 | 0.910 | 0.945 | 0.011 |
| RHSIM_Rhsim09G0038900 | 0.01 | 0.910 | 0.945 | -0.037 |
| novel.4491 | 0.01 | 0.910 | 0.945 | 0.444 |
| RHSIM_Rhsim10G0117900 | 0.01 | 0.911 | 0.945 | -0.117 |

| Gene | LRT statistic | P value | BH-adjusted P | log2FC (interaction) |
|---|---|---|---|---|
| RHSIM_Rhsim06G0183800 | 0.01 | 0.911 | 0.945 | -0.208 |
| RHSIM_RhsimUnG0139400 | 0.01 | 0.911 | 0.946 | 0.021 |
| RHSIM_Rhsim07G0089700 | 0.01 | 0.911 | 0.946 | -0.238 |
| RHSIM_Rhsim09G0119300 | 0.01 | 0.911 | 0.946 | 0.027 |
| novel.3185 | 0.01 | 0.911 | 0.946 | -0.076 |
| RHSIM_Rhsim09G0066400 | 0.01 | 0.911 | 0.946 | 0.020 |
| novel.3966 | 0.01 | 0.911 | 0.946 | 0.198 |
| novel.4214 | 0.01 | 0.911 | 0.946 | -0.045 |
| RHSIM_RhsimUnG0134800 | 0.01 | 0.911 | 0.946 | -0.137 |
| RHSIM_Rhsim06G0170900 | 0.01 | 0.912 | 0.946 | -0.123 |
| RHSIM_RhsimUnG0246200 | 0.01 | 0.912 | 0.946 | -0.030 |
| RHSIM_Rhsim01G0046400 | 0.01 | 0.912 | 0.946 | 0.047 |
| RHSIM_Rhsim13G0187900 | 0.01 | 0.912 | 0.946 | 0.013 |
| RHSIM_RhsimUnG0138000 | 0.01 | 0.912 | 0.946 | -0.097 |
| RHSIM_Rhsim13G0042200 | 0.01 | 0.912 | 0.946 | 0.007 |
| novel.2122 | 0.01 | 0.912 | 0.946 | -0.134 |
| novel.1940 | 0.01 | 0.912 | 0.946 | -0.065 |
| RHSIM_Rhsim08G0053000 | 0.01 | 0.912 | 0.946 | -0.014 |
| RHSIM_Rhsim05G0016800 | 0.01 | 0.912 | 0.946 | 0.087 |
| novel.2609 | 0.01 | 0.912 | 0.946 | -28.318 |
| RHSIM_Rhsim11G0076600 | 0.01 | 0.912 | 0.946 | -0.072 |
| RHSIM_Rhsim01G0023800 | 0.01 | 0.912 | 0.947 | -0.010 |
| RHSIM_Rhsim04G0034700 | 0.01 | 0.912 | 0.947 | 0.210 |
| RHSIM_RhsimUnG0138400 | 0.01 | 0.913 | 0.947 | 0.317 |
| RHSIM_RhsimUnG0020000 | 0.01 | 0.913 | 0.947 | 0.214 |
| RHSIM_Rhsim10G0074000 | 0.01 | 0.913 | 0.947 | -0.165 |
| RHSIM_Rhsim01G0139800 | 0.01 | 0.913 | 0.947 | -0.295 |
| RHSIM_Rhsim08G0251500 | 0.01 | 0.913 | 0.947 | -0.019 |
| RHSIM_Rhsim06G0133500 | 0.01 | 0.913 | 0.947 | 0.070 |
| novel.1986 | 0.01 | 0.913 | 0.947 | 0.194 |
| RHSIM_Rhsim10G0211200 | 0.01 | 0.913 | 0.947 | -0.047 |
| RHSIM_Rhsim01G0013400 | 0.01 | 0.913 | 0.947 | 0.272 |

| Gene | LRT statistic | P value | BH-adjusted P | log2FC (interaction) |
|---|---|---|---|---|
| RHSIM_Rhsim12G0122300 | 0.01 | 0.914 | 0.947 | -0.088 |
| RHSIM_Rhsim08G0163000 | 0.01 | 0.914 | 0.948 | 0.025 |
| novel.3948 | 0.01 | 0.914 | 0.948 | 0.208 |
| RHSIM_Rhsim03G0020400 | 0.01 | 0.915 | 0.948 | 0.066 |
| RHSIM_Rhsim05G0091300 | 0.01 | 0.915 | 0.949 | 0.293 |
| RHSIM_Rhsim02G0205600 | 0.01 | 0.915 | 0.949 | -0.273 |
| RHSIM_Rhsim10G0147900 | 0.01 | 0.915 | 0.949 | 0.251 |
| RHSIM_Rhsim13G0211200 | 0.01 | 0.915 | 0.949 | 0.012 |
| RHSIM_Rhsim11G0174900 | 0.01 | 0.915 | 0.949 | -0.284 |
| RHSIM_RhsimUnG0013200 | 0.01 | 0.915 | 0.949 | -0.075 |
| RHSIM_Rhsim05G0043900 | 0.01 | 0.915 | 0.949 | -0.011 |
| novel.4417 | 0.01 | 0.915 | 0.949 | -0.044 |
| RHSIM_Rhsim02G0248100 | 0.01 | 0.915 | 0.949 | -0.290 |
| RHSIM_Rhsim02G0075100 | 0.01 | 0.916 | 0.949 | -0.234 |
| RHSIM_Rhsim10G0116200 | 0.01 | 0.916 | 0.949 | 0.110 |
| RHSIM_RhsimUnG0147300 | 0.01 | 0.916 | 0.949 | 0.129 |
| RHSIM_Rhsim13G0144500 | 0.01 | 0.916 | 0.949 | 0.064 |
| novel.2511 | 0.01 | 0.916 | 0.950 | 0.113 |
| RHSIM_Rhsim13G0015600 | 0.01 | 0.916 | 0.950 | -29.643 |
| RHSIM_RhsimUnG0126500 | 0.01 | 0.916 | 0.950 | -0.009 |
| RHSIM_Rhsim01G0195200 | 0.01 | 0.916 | 0.950 | -0.095 |
| novel.1967 | 0.01 | 0.917 | 0.950 | 0.086 |
| RHSIM_Rhsim10G0198100 | 0.01 | 0.917 | 0.950 | 0.085 |
| RHSIM_Rhsim01G0007800 | 0.01 | 0.917 | 0.950 | -0.016 |
| RHSIM_Rhsim05G0119000 | 0.01 | 0.917 | 0.950 | -0.116 |
| RHSIM_Rhsim02G0217400 | 0.01 | 0.917 | 0.950 | 0.009 |
| RHSIM_Rhsim06G0122100 | 0.01 | 0.917 | 0.950 | 0.022 |
| RHSIM_Rhsim06G0208000 | 0.01 | 0.917 | 0.950 | -0.014 |
| RHSIM_Rhsim09G0187700 | 0.01 | 0.917 | 0.950 | 0.228 |
| RHSIM_Rhsim04G0105400 | 0.01 | 0.917 | 0.950 | 0.110 |
| RHSIM_Rhsim03G0153100 | 0.01 | 0.917 | 0.950 | 0.040 |
| RHSIM_Rhsim04G0194700 | 0.01 | 0.917 | 0.950 | -0.008 |

| Gene | LRT statistic | P value | BH-adjusted P | log2FC (interaction) |
|---|---|---|---|---|
| RHSIM_Rhsim07G0112300 | 0.01 | 0.917 | 0.950 | 0.248 |
| RHSIM_Rhsim01G0130600 | 0.01 | 0.917 | 0.950 | 0.145 |
| RHSIM_Rhsim03G0089000 | 0.01 | 0.918 | 0.951 | 0.008 |
| novel.4446 | 0.01 | 0.918 | 0.951 | -0.101 |
| RHSIM_RhsimUnG0249300 | 0.01 | 0.918 | 0.951 | -0.008 |
| RHSIM_Rhsim08G0240200 | 0.01 | 0.918 | 0.951 | 0.012 |
| RHSIM_Rhsim05G0206200 | 0.01 | 0.918 | 0.951 | -0.026 |
| RHSIM_Rhsim07G0145800 | 0.01 | 0.918 | 0.951 | -0.033 |
| novel.3972 | 0.01 | 0.919 | 0.951 | -0.094 |
| RHSIM_Rhsim09G0196100 | 0.01 | 0.919 | 0.951 | -0.016 |
| RHSIM_RhsimUnG0070200 | 0.01 | 0.919 | 0.952 | 0.077 |
| RHSIM_Rhsim07G0150900 | 0.01 | 0.919 | 0.952 | -0.010 |
| novel.3723 | 0.01 | 0.919 | 0.952 | -0.113 |
| RHSIM_Rhsim01G0227800 | 0.01 | 0.919 | 0.952 | -0.017 |
| RHSIM_Rhsim06G0104400 | 0.01 | 0.919 | 0.952 | -0.024 |
| RHSIM_Rhsim11G0120700 | 0.01 | 0.919 | 0.952 | -0.147 |
| RHSIM_Rhsim04G0077800 | 0.01 | 0.919 | 0.952 | -0.143 |
| novel.3228 | 0.01 | 0.919 | 0.952 | 0.083 |
| RHSIM_Rhsim11G0058300 | 0.01 | 0.920 | 0.952 | 0.039 |
| novel.1423 | 0.01 | 0.920 | 0.952 | -0.092 |
| RHSIM_Rhsim04G0120100 | 0.01 | 0.920 | 0.952 | 0.151 |
| novel.760 | 0.01 | 0.920 | 0.952 | 0.106 |
| RHSIM_Rhsim03G0044800 | 0.01 | 0.920 | 0.952 | -0.292 |
| RHSIM_Rhsim01G0172400 | 0.01 | 0.920 | 0.953 | -0.138 |
| RHSIM_Rhsim04G0041900 | 0.01 | 0.920 | 0.953 | 0.028 |
| RHSIM_Rhsim05G0063600 | 0.01 | 0.921 | 0.953 | -29.302 |
| RHSIM_Rhsim01G0200000 | 0.01 | 0.921 | 0.953 | -0.012 |
| RHSIM_Rhsim13G0159800 | 0.01 | 0.921 | 0.953 | 0.053 |
| novel.844 | 0.01 | 0.921 | 0.953 | 0.183 |
| novel.1199 | 0.01 | 0.921 | 0.953 | 0.078 |
| RHSIM_Rhsim09G0117200 | 0.01 | 0.922 | 0.954 | 0.223 |
| RHSIM_Rhsim09G0052000 | 0.01 | 0.922 | 0.954 | 0.025 |

| Gene | LRT statistic | P value | BH-adjusted P | log2FC (interaction) |
|---|---|---|---|---|
| RHSIM_Rhsim01G0071700 | 0.01 | 0.922 | 0.954 | 0.162 |
| RHSIM_Rhsim06G0133000 | 0.01 | 0.922 | 0.954 | -0.315 |
| RHSIM_Rhsim06G0130800 | 0.01 | 0.922 | 0.954 | -0.024 |
| RHSIM_Rhsim02G0230800 | 0.01 | 0.922 | 0.954 | 0.070 |
| RHSIM_Rhsim11G0057500 | 0.01 | 0.922 | 0.954 | 0.277 |
| RHSIM_Rhsim11G0029700 | 0.01 | 0.922 | 0.954 | 0.077 |
| RHSIM_Rhsim01G0190100 | 0.01 | 0.922 | 0.954 | 0.015 |
| RHSIM_RhsimUnG0145300 | 0.01 | 0.922 | 0.954 | 0.538 |
| RHSIM_Rhsim07G0139300 | 0.01 | 0.922 | 0.954 | 0.244 |
| RHSIM_Rhsim04G0177900 | 0.01 | 0.923 | 0.954 | -0.205 |
| RHSIM_Rhsim03G0094900 | 0.01 | 0.923 | 0.954 | 0.278 |
| RHSIM_Rhsim10G0186000 | 0.01 | 0.923 | 0.955 | -0.010 |
| RHSIM_Rhsim02G0233600 | 0.01 | 0.923 | 0.955 | -0.035 |
| RHSIM_RhsimUnG0113900 | 0.01 | 0.923 | 0.955 | -0.014 |
| novel.3160 | 0.01 | 0.923 | 0.955 | 0.038 |
| RHSIM_Rhsim08G0037100 | 0.01 | 0.923 | 0.955 | -0.012 |
| RHSIM_Rhsim03G0265800 | 0.01 | 0.923 | 0.955 | -0.024 |
| RHSIM_Rhsim13G0019600 | 0.01 | 0.924 | 0.955 | 0.094 |
| RHSIM_Rhsim12G0072700 | 0.01 | 0.924 | 0.956 | 0.064 |
| RHSIM_Rhsim05G0147700 | 0.01 | 0.924 | 0.956 | -0.220 |
| RHSIM_Rhsim04G0240700 | 0.01 | 0.924 | 0.956 | -0.201 |
| RHSIM_Rhsim13G0150400 | 0.01 | 0.924 | 0.956 | 0.243 |
| novel.2069 | 0.01 | 0.924 | 0.956 | 0.114 |
| novel.372 | 0.01 | 0.924 | 0.956 | 0.102 |
| RHSIM_Rhsim12G0020300 | 0.01 | 0.924 | 0.956 | 0.049 |
| RHSIM_Rhsim04G0051900 | 0.01 | 0.925 | 0.956 | -0.047 |
| RHSIM_Rhsim04G0098500 | 0.01 | 0.925 | 0.957 | -0.030 |
| RHSIM_Rhsim05G0216600 | 0.01 | 0.925 | 0.957 | -0.006 |
| RHSIM_Rhsim08G0162300 | 0.01 | 0.926 | 0.957 | -0.065 |
| RHSIM_Rhsim06G0137700 | 0.01 | 0.926 | 0.957 | 0.276 |
| RHSIM_Rhsim04G0089100 | 0.01 | 0.926 | 0.957 | -0.028 |
| novel.3570 | 0.01 | 0.926 | 0.957 | -0.037 |

| Gene | LRT statistic | P value | BH-adjusted P | log2FC (interaction) |
|---|---|---|---|---|
| novel.3351 | 0.01 | 0.926 | 0.957 | 0.190 |
| RHSIM_Rhsim03G0114900 | 0.01 | 0.926 | 0.957 | 0.158 |
| RHSIM_Rhsim01G0103200 | 0.01 | 0.926 | 0.957 | 0.120 |
| novel.4147 | 0.01 | 0.926 | 0.957 | 0.078 |
| novel.3684 | 0.01 | 0.926 | 0.957 | 0.062 |
| RHSIM_Rhsim12G0071300 | 0.01 | 0.926 | 0.957 | -0.032 |
| RHSIM_Rhsim05G0030900 | 0.01 | 0.927 | 0.957 | -0.051 |
| RHSIM_Rhsim05G0105800 | 0.01 | 0.927 | 0.958 | -0.114 |
| RHSIM_Rhsim08G0130900 | 0.01 | 0.927 | 0.958 | 0.244 |
| RHSIM_Rhsim06G0225800 | 0.01 | 0.927 | 0.958 | 0.127 |
| RHSIM_Rhsim04G0122200 | 0.01 | 0.927 | 0.958 | -0.204 |
| novel.4373 | 0.01 | 0.927 | 0.958 | 0.219 |
| RHSIM_Rhsim13G0005500 | 0.01 | 0.927 | 0.958 | -0.089 |
| RHSIM_Rhsim09G0104100 | 0.01 | 0.927 | 0.958 | 0.011 |
| RHSIM_Rhsim10G0117700 | 0.01 | 0.927 | 0.958 | -0.041 |
| RHSIM_Rhsim13G0172500 | 0.01 | 0.927 | 0.958 | 0.048 |
| RHSIM_Rhsim06G0019200 | 0.01 | 0.927 | 0.958 | 0.157 |
| RHSIM_Rhsim06G0016300 | 0.01 | 0.928 | 0.958 | -0.059 |
| RHSIM_Rhsim02G0084400 | 0.01 | 0.928 | 0.958 | -0.010 |
| RHSIM_Rhsim03G0187100 | 0.01 | 0.928 | 0.958 | -0.056 |
| RHSIM_Rhsim13G0128700 | 0.01 | 0.928 | 0.958 | 0.213 |
| novel.3431 | 0.01 | 0.928 | 0.958 | -0.047 |
| RHSIM_Rhsim09G0023100 | 0.01 | 0.928 | 0.958 | -0.181 |
| RHSIM_Rhsim10G0026500 | 0.01 | 0.928 | 0.958 | -0.119 |
| RHSIM_Rhsim12G0007700 | 0.01 | 0.928 | 0.959 | -0.035 |
| novel.2064 | 0.01 | 0.929 | 0.959 | -0.026 |
| RHSIM_Rhsim10G0094000 | 0.01 | 0.929 | 0.959 | -0.088 |
| novel.1730 | 0.01 | 0.929 | 0.959 | -0.059 |
| RHSIM_Rhsim12G0168000 | 0.01 | 0.929 | 0.959 | -0.055 |
| RHSIM_Rhsim03G0006400 | 0.01 | 0.929 | 0.959 | -27.930 |
| RHSIM_Rhsim09G0005100 | 0.01 | 0.929 | 0.959 | -0.006 |
| RHSIM_Rhsim12G0007200 | 0.01 | 0.929 | 0.959 | -0.169 |

| Gene | LRT statistic | P value | BH-adjusted P | log2FC (interaction) |
|---|---|---|---|---|
| novel.2281 | 0.01 | 0.929 | 0.959 | -0.089 |
| RHSIM_Rhsim01G0127700 | 0.01 | 0.929 | 0.959 | 0.010 |
| RHSIM_Rhsim11G0084900 | 0.01 | 0.930 | 0.960 | -0.083 |
| novel.1520 | 0.01 | 0.930 | 0.960 | -0.081 |
| RHSIM_Rhsim13G0158900 | 0.01 | 0.930 | 0.960 | -0.049 |
| RHSIM_Rhsim05G0080500 | 0.01 | 0.930 | 0.960 | 0.140 |
| RHSIM_Rhsim03G0003700 | 0.01 | 0.930 | 0.960 | 0.075 |
| RHSIM_RhsimUnG0070600 | 0.01 | 0.930 | 0.960 | 0.027 |
| RHSIM_Rhsim13G0094600 | 0.01 | 0.930 | 0.960 | 0.018 |
| novel.3661 | 0.01 | 0.930 | 0.960 | -0.126 |
| novel.2337 | 0.01 | 0.931 | 0.960 | -0.091 |
| novel.3085 | 0.01 | 0.931 | 0.960 | 0.064 |
| RHSIM_Rhsim10G0064300 | 0.01 | 0.931 | 0.961 | 0.158 |
| RHSIM_Rhsim04G0186700 | 0.01 | 0.931 | 0.961 | -0.061 |
| RHSIM_Rhsim07G0183600 | 0.01 | 0.931 | 0.961 | 0.020 |
| RHSIM_Rhsim02G0196000 | 0.01 | 0.931 | 0.961 | -0.234 |
| novel.2811 | 0.01 | 0.932 | 0.961 | -0.034 |
| RHSIM_Rhsim06G0178900 | 0.01 | 0.932 | 0.962 | 0.066 |
| RHSIM_Rhsim07G0107900 | 0.01 | 0.932 | 0.962 | 0.229 |
| novel.4534 | 0.01 | 0.932 | 0.962 | -0.118 |
| RHSIM_Rhsim07G0139900 | 0.01 | 0.933 | 0.962 | -0.060 |
| RHSIM_Rhsim12G0165400 | 0.01 | 0.933 | 0.962 | 0.163 |
| RHSIM_Rhsim03G0217800 | 0.01 | 0.933 | 0.962 | 0.105 |
| RHSIM_Rhsim13G0105800 | 0.01 | 0.933 | 0.962 | 0.124 |
| RHSIM_Rhsim06G0107500 | 0.01 | 0.933 | 0.963 | 0.207 |
| RHSIM_Rhsim12G0212300 | 0.01 | 0.933 | 0.963 | -0.018 |
| RHSIM_Rhsim05G0154000 | 0.01 | 0.933 | 0.963 | 0.021 |
| RHSIM_Rhsim03G0158000 | 0.01 | 0.933 | 0.963 | 0.076 |
| novel.3114 | 0.01 | 0.934 | 0.963 | 0.122 |
| RHSIM_Rhsim07G0193300 | 0.01 | 0.934 | 0.963 | -0.091 |
| RHSIM_Rhsim02G0108300 | 0.01 | 0.934 | 0.963 | -0.018 |
| RHSIM_Rhsim06G0095600 | 0.01 | 0.934 | 0.963 | -0.012 |

| Gene | LRT statistic | P value | BH-adjusted P | log2FC (interaction) |
|---|---|---|---|---|
| RHSIM_Rhsim10G0009800 | 0.01 | 0.934 | 0.963 | 0.024 |
| RHSIM_Rhsim11G0030900 | 0.01 | 0.934 | 0.963 | 0.047 |
| RHSIM_Rhsim13G0074400 | 0.01 | 0.934 | 0.963 | 0.010 |
| novel.1365 | 0.01 | 0.934 | 0.963 | 0.074 |
| RHSIM_Rhsim06G0036000 | 0.01 | 0.934 | 0.963 | -0.172 |
| RHSIM_RhsimUnG0093400 | 0.01 | 0.934 | 0.963 | -0.012 |
| RHSIM_Rhsim05G0066200 | 0.01 | 0.935 | 0.964 | 0.111 |
| novel.145 | 0.01 | 0.935 | 0.964 | -0.034 |
| RHSIM_Rhsim05G0047400 | 0.01 | 0.935 | 0.964 | -0.115 |
| RHSIM_Rhsim08G0038900 | 0.01 | 0.935 | 0.964 | 0.006 |
| RHSIM_Rhsim09G0196000 | 0.01 | 0.935 | 0.964 | 0.021 |
| RHSIM_Rhsim04G0190900 | 0.01 | 0.935 | 0.964 | 0.014 |
| RHSIM_Rhsim05G0189900 | 0.01 | 0.935 | 0.964 | -0.029 |
| novel.4376 | 0.01 | 0.936 | 0.964 | 0.067 |
| RHSIM_Rhsim03G0135500 | 0.01 | 0.936 | 0.965 | -0.007 |
| RHSIM_Rhsim06G0114300 | 0.01 | 0.936 | 0.965 | 0.065 |
| RHSIM_RhsimUnG0103500 | 0.01 | 0.936 | 0.965 | -0.098 |
| RHSIM_Rhsim11G0032100 | 0.01 | 0.936 | 0.965 | -0.012 |
| RHSIM_Rhsim06G0112100 | 0.01 | 0.936 | 0.965 | -0.093 |
| RHSIM_Rhsim10G0158700 | 0.01 | 0.936 | 0.965 | 0.036 |
| RHSIM_Rhsim01G0180500 | 0.01 | 0.936 | 0.965 | 0.010 |
| novel.3586 | 0.01 | 0.936 | 0.965 | 0.020 |
| RHSIM_Rhsim01G0154700 | 0.01 | 0.936 | 0.965 | 0.019 |
| RHSIM_Rhsim03G0246700 | 0.01 | 0.936 | 0.965 | 0.098 |
| RHSIM_RhsimUnG0004000 | 0.01 | 0.937 | 0.965 | -0.022 |
| RHSIM_Rhsim02G0060800 | 0.01 | 0.937 | 0.965 | 0.007 |
| RHSIM_Rhsim06G0113700 | 0.01 | 0.937 | 0.965 | 0.016 |
| RHSIM_Rhsim08G0043900 | 0.01 | 0.937 | 0.966 | 0.007 |
| novel.687 | 0.01 | 0.937 | 0.966 | 0.025 |
| RHSIM_Rhsim11G0142400 | 0.01 | 0.937 | 0.966 | -0.017 |
| RHSIM_Rhsim08G0206700 | 0.01 | 0.937 | 0.966 | 0.084 |
| RHSIM_Rhsim08G0042400 | 0.01 | 0.938 | 0.966 | 0.022 |

| Gene | LRT statistic | P value | BH-adjusted P | log2FC (interaction) |
|---|---|---|---|---|
| RHSIM_Rhsim01G0238600 | 0.01 | 0.938 | 0.966 | 0.154 |
| novel.2130 | 0.01 | 0.938 | 0.966 | 0.013 |
| novel.3922 | 0.01 | 0.938 | 0.966 | -0.052 |
| novel.2894 | 0.01 | 0.938 | 0.966 | 0.018 |
| novel.1332 | 0.01 | 0.938 | 0.966 | -0.060 |
| RHSIM_Rhsim12G0069500 | 0.01 | 0.938 | 0.966 | -0.011 |
| RHSIM_Rhsim03G0112200 | 0.01 | 0.939 | 0.966 | 0.212 |
| RHSIM_Rhsim01G0292500 | 0.01 | 0.939 | 0.966 | -0.057 |
| RHSIM_Rhsim01G0223900 | 0.01 | 0.939 | 0.967 | 0.019 |
| RHSIM_Rhsim06G0076200 | 0.01 | 0.939 | 0.967 | 0.038 |
| RHSIM_Rhsim11G0008900 | 0.01 | 0.939 | 0.967 | 0.011 |
| RHSIM_Rhsim01G0282300 | 0.01 | 0.939 | 0.967 | -0.008 |
| RHSIM_Rhsim10G0097200 | 0.01 | 0.939 | 0.967 | 0.010 |
| RHSIM_Rhsim03G0152900 | 0.01 | 0.939 | 0.967 | -0.006 |
| RHSIM_RhsimUnG0180700 | 0.01 | 0.939 | 0.967 | -0.105 |
| RHSIM_Rhsim08G0178700 | 0.01 | 0.939 | 0.967 | 0.157 |
| RHSIM_RhsimUnG0180300 | 0.01 | 0.940 | 0.967 | 0.212 |
| RHSIM_Rhsim11G0032500 | 0.01 | 0.940 | 0.967 | -0.040 |
| RHSIM_Rhsim03G0051600 | 0.01 | 0.940 | 0.967 | 0.159 |
| RHSIM_Rhsim13G0174700 | 0.01 | 0.940 | 0.967 | 0.011 |
| novel.1170 | 0.01 | 0.940 | 0.968 | -0.051 |
| RHSIM_Rhsim12G0072300 | 0.01 | 0.941 | 0.968 | 0.023 |
| RHSIM_RhsimUnG0153200 | 0.01 | 0.941 | 0.968 | 0.135 |
| RHSIM_RhsimUnG0135300 | 0.01 | 0.941 | 0.968 | 0.215 |
| RHSIM_Rhsim08G0035900 | 0.01 | 0.941 | 0.968 | -0.025 |
| novel.2213 | 0.01 | 0.941 | 0.968 | -0.080 |
| RHSIM_Rhsim08G0156500 | 0.01 | 0.941 | 0.968 | -0.097 |
| RHSIM_Rhsim04G0102700 | 0.01 | 0.941 | 0.968 | 0.102 |
| RHSIM_Rhsim10G0206900 | 0.01 | 0.941 | 0.969 | 0.012 |
| RHSIM_Rhsim06G0072100 | 0.01 | 0.941 | 0.969 | 0.039 |
| RHSIM_Rhsim12G0104700 | 0.01 | 0.942 | 0.969 | 0.011 |
| novel.2025 | 0.01 | 0.942 | 0.969 | 0.012 |

| Gene | LRT statistic | P value | BH-adjusted P | log2FC (interaction) |
|---|---|---|---|---|
| RHSIM_Rhsim09G0048500 | 0.01 | 0.942 | 0.969 | 0.136 |
| RHSIM_Rhsim03G0096700 | 0.01 | 0.942 | 0.969 | -0.040 |
| RHSIM_RhsimUnG0210600 | 0.01 | 0.942 | 0.969 | 0.141 |
| RHSIM_Rhsim05G0167200 | 0.01 | 0.942 | 0.970 | 0.008 |
| RHSIM_Rhsim13G0156500 | 0.01 | 0.943 | 0.970 | -0.030 |
| RHSIM_Rhsim03G0259100 | 0.01 | 0.943 | 0.970 | -0.056 |
| novel.2673 | 0.01 | 0.943 | 0.970 | 0.082 |
| RHSIM_Rhsim08G0019900 | 0.01 | 0.943 | 0.970 | 0.036 |
| RHSIM_Rhsim11G0082400 | 0.01 | 0.943 | 0.970 | -0.168 |
| RHSIM_Rhsim13G0137800 | 0.01 | 0.944 | 0.970 | 0.008 |
| RHSIM_RhsimUnG0033100 | 0.01 | 0.944 | 0.970 | -0.080 |
| novel.2750 | 0.00 | 0.944 | 0.971 | -0.066 |
| RHSIM_Rhsim01G0263100 | 0.00 | 0.944 | 0.971 | 0.078 |
| RHSIM_Rhsim03G0250200 | 0.00 | 0.944 | 0.971 | 0.004 |
| RHSIM_Rhsim04G0042800 | 0.00 | 0.944 | 0.971 | 0.094 |
| RHSIM_Rhsim11G0154400 | 0.00 | 0.944 | 0.971 | 0.033 |
| novel.2453 | 0.00 | 0.944 | 0.971 | 0.066 |
| RHSIM_Rhsim03G0056900 | 0.00 | 0.944 | 0.971 | -0.015 |
| novel.4215 | 0.00 | 0.944 | 0.971 | 0.029 |
| RHSIM_Rhsim13G0114200 | 0.00 | 0.945 | 0.971 | -0.016 |
| novel.1339 | 0.00 | 0.945 | 0.971 | -0.123 |
| novel.1535 | 0.00 | 0.945 | 0.971 | -0.069 |
| RHSIM_Rhsim09G0205400 | 0.00 | 0.945 | 0.971 | 0.022 |
| novel.1026 | 0.00 | 0.945 | 0.972 | 0.012 |
| RHSIM_Rhsim07G0249300 | 0.00 | 0.945 | 0.972 | 0.019 |
| RHSIM_Rhsim08G0093700 | 0.00 | 0.945 | 0.972 | -0.015 |
| RHSIM_Rhsim02G0246000 | 0.00 | 0.946 | 0.972 | -0.088 |
| RHSIM_Rhsim02G0229800 | 0.00 | 0.946 | 0.972 | -0.099 |
| novel.3399 | 0.00 | 0.946 | 0.972 | 0.067 |
| RHSIM_Rhsim10G0053400 | 0.00 | 0.946 | 0.972 | -0.037 |
| novel.2745 | 0.00 | 0.946 | 0.972 | 0.116 |
| RHSIM_Rhsim09G0187200 | 0.00 | 0.946 | 0.972 | 0.011 |

| Gene | LRT statistic | P value | BH-adjusted P | log2FC (interaction) |
|---|---|---|---|---|
| RHSIM_Rhsim09G0009600 | 0.00 | 0.946 | 0.972 | 0.110 |
| RHSIM_Rhsim10G0138900 | 0.00 | 0.946 | 0.972 | -0.011 |
| RHSIM_Rhsim13G0039200 | 0.00 | 0.946 | 0.972 | 0.052 |
| RHSIM_Rhsim04G0058800 | 0.00 | 0.946 | 0.972 | 0.010 |
| RHSIM_RhsimUnG0046800 | 0.00 | 0.946 | 0.972 | -0.011 |
| RHSIM_Rhsim09G0057900 | 0.00 | 0.946 | 0.972 | 0.073 |
| RHSIM_Rhsim06G0121300 | 0.00 | 0.947 | 0.973 | -0.076 |
| novel.1287 | 0.00 | 0.947 | 0.973 | 0.050 |
| RHSIM_Rhsim09G0126000 | 0.00 | 0.947 | 0.973 | 0.015 |
| RHSIM_Rhsim08G0171100 | 0.00 | 0.947 | 0.973 | 0.012 |
| RHSIM_Rhsim05G0026200 | 0.00 | 0.948 | 0.974 | -0.099 |
| RHSIM_Rhsim04G0165200 | 0.00 | 0.948 | 0.974 | 0.015 |
| RHSIM_Rhsim13G0002500 | 0.00 | 0.948 | 0.974 | 0.006 |
| novel.2183 | 0.00 | 0.948 | 0.974 | -0.046 |
| RHSIM_Rhsim11G0020300 | 0.00 | 0.948 | 0.974 | 0.044 |
| novel.3915 | 0.00 | 0.948 | 0.974 | 0.006 |
| RHSIM_RhsimUnG0161600 | 0.00 | 0.948 | 0.974 | 0.200 |
| RHSIM_Rhsim07G0100000 | 0.00 | 0.948 | 0.974 | 0.049 |
| RHSIM_Rhsim01G0155700 | 0.00 | 0.948 | 0.974 | 0.029 |
| RHSIM_Rhsim10G0136700 | 0.00 | 0.949 | 0.974 | 0.013 |
| RHSIM_Rhsim12G0086000 | 0.00 | 0.949 | 0.974 | 0.071 |
| RHSIM_Rhsim09G0090000 | 0.00 | 0.949 | 0.974 | -0.061 |
| novel.2318 | 0.00 | 0.949 | 0.974 | 0.043 |
| novel.1306 | 0.00 | 0.949 | 0.974 | 0.064 |
| RHSIM_Rhsim08G0112400 | 0.00 | 0.949 | 0.975 | 0.014 |
| novel.3828 | 0.00 | 0.949 | 0.975 | -0.028 |
| novel.1045 | 0.00 | 0.949 | 0.975 | -0.060 |
| RHSIM_Rhsim05G0131100 | 0.00 | 0.949 | 0.975 | -0.133 |
| RHSIM_Rhsim07G0104800 | 0.00 | 0.949 | 0.975 | -0.011 |
| RHSIM_Rhsim09G0043800 | 0.00 | 0.950 | 0.975 | 0.012 |
| novel.254 | 0.00 | 0.950 | 0.975 | 0.016 |
| RHSIM_Rhsim04G0206400 | 0.00 | 0.950 | 0.975 | 0.006 |

| Gene | LRT statistic | P value | BH-adjusted P | log2FC (interaction) |
|---|---|---|---|---|
| RHSIM_RhsimUnG0064500 | 0.00 | 0.950 | 0.975 | 0.124 |
| RHSIM_RhsimUnG0153900 | 0.00 | 0.951 | 0.976 | -0.036 |
| RHSIM_Rhsim12G0024100 | 0.00 | 0.951 | 0.976 | -0.062 |
| RHSIM_Rhsim11G0105200 | 0.00 | 0.951 | 0.976 | -0.016 |
| RHSIM_Rhsim05G0111400 | 0.00 | 0.951 | 0.976 | 0.010 |
| novel.4348 | 0.00 | 0.951 | 0.976 | 0.030 |
| RHSIM_Rhsim06G0161400 | 0.00 | 0.951 | 0.976 | -0.089 |
| RHSIM_Rhsim13G0057400 | 0.00 | 0.951 | 0.976 | -0.006 |
| RHSIM_Rhsim06G0111500 | 0.00 | 0.952 | 0.977 | -0.028 |
| RHSIM_Rhsim11G0061700 | 0.00 | 0.952 | 0.977 | -0.032 |
| RHSIM_RhsimUnG0255800 | 0.00 | 0.952 | 0.977 | 0.099 |
| RHSIM_Rhsim08G0154900 | 0.00 | 0.952 | 0.977 | 0.010 |
| RHSIM_Rhsim10G0044400 | 0.00 | 0.952 | 0.977 | -0.037 |
| RHSIM_Rhsim11G0018500 | 0.00 | 0.952 | 0.977 | 0.077 |
| RHSIM_Rhsim07G0084100 | 0.00 | 0.953 | 0.977 | 0.011 |
| RHSIM_RhsimUnG0176900 | 0.00 | 0.953 | 0.978 | 0.064 |
| RHSIM_Rhsim08G0231800 | 0.00 | 0.953 | 0.978 | 0.071 |
| novel.993 | 0.00 | 0.954 | 0.978 | -0.031 |
| RHSIM_RhsimUnG0185400 | 0.00 | 0.954 | 0.978 | 0.028 |
| RHSIM_Rhsim03G0009800 | 0.00 | 0.954 | 0.978 | -0.010 |
| RHSIM_Rhsim05G0040200 | 0.00 | 0.954 | 0.978 | -0.029 |
| RHSIM_Rhsim05G0117000 | 0.00 | 0.954 | 0.978 | 0.160 |
| RHSIM_Rhsim12G0135100 | 0.00 | 0.954 | 0.979 | -0.127 |
| novel.1338 | 0.00 | 0.954 | 0.979 | -0.070 |
| RHSIM_Rhsim09G0053400 | 0.00 | 0.954 | 0.979 | 0.084 |
| RHSIM_Rhsim13G0066000 | 0.00 | 0.954 | 0.979 | 0.142 |
| novel.1844 | 0.00 | 0.955 | 0.979 | -0.071 |
| RHSIM_Rhsim08G0018600 | 0.00 | 0.955 | 0.979 | -0.006 |
| RHSIM_Rhsim09G0147300 | 0.00 | 0.955 | 0.979 | -0.007 |
| novel.2111 | 0.00 | 0.955 | 0.979 | 0.036 |
| novel.1938 | 0.00 | 0.955 | 0.979 | 0.063 |
| RHSIM_Rhsim11G0089300 | 0.00 | 0.955 | 0.979 | -0.118 |

| Gene | LRT statistic | P value | BH-adjusted P | log2FC (interaction) |
|---|---|---|---|---|
| RHSIM_Rhsim07G0116300 | 0.00 | 0.955 | 0.979 | 0.035 |
| RHSIM_Rhsim09G0136800 | 0.00 | 0.955 | 0.979 | -0.005 |
| RHSIM_Rhsim12G0108400 | 0.00 | 0.955 | 0.980 | 0.007 |
| RHSIM_Rhsim13G0155600 | 0.00 | 0.955 | 0.980 | -0.066 |
| novel.1246 | 0.00 | 0.956 | 0.980 | -0.065 |
| RHSIM_Rhsim13G0224700 | 0.00 | 0.956 | 0.980 | 0.024 |
| RHSIM_Rhsim04G0032100 | 0.00 | 0.956 | 0.980 | 0.006 |
| RHSIM_RhsimUnG0019900 | 0.00 | 0.956 | 0.980 | -0.029 |
| RHSIM_Rhsim11G0176700 | 0.00 | 0.956 | 0.980 | -0.068 |
| RHSIM_Rhsim13G0222500 | 0.00 | 0.956 | 0.980 | -0.090 |
| RHSIM_Rhsim05G0012700 | 0.00 | 0.957 | 0.980 | 0.033 |
| RHSIM_Rhsim08G0231400 | 0.00 | 0.957 | 0.981 | 0.004 |
| RHSIM_RhsimUnG0239000 | 0.00 | 0.957 | 0.981 | -0.012 |
| RHSIM_Rhsim06G0143100 | 0.00 | 0.957 | 0.981 | 0.082 |
| RHSIM_Rhsim02G0078900 | 0.00 | 0.957 | 0.981 | -0.043 |
| novel.2649 | 0.00 | 0.958 | 0.981 | 0.053 |
| RHSIM_Rhsim09G0092200 | 0.00 | 0.958 | 0.981 | 0.057 |
| RHSIM_Rhsim10G0073000 | 0.00 | 0.958 | 0.981 | 0.028 |
| RHSIM_Rhsim06G0019400 | 0.00 | 0.958 | 0.981 | -0.121 |
| novel.3329 | 0.00 | 0.958 | 0.981 | -0.054 |
| RHSIM_Rhsim08G0220700 | 0.00 | 0.958 | 0.981 | -0.131 |
| RHSIM_Rhsim11G0102900 | 0.00 | 0.958 | 0.982 | 0.064 |
| RHSIM_RhsimUnG0139300 | 0.00 | 0.958 | 0.982 | 0.010 |
| RHSIM_Rhsim08G0169300 | 0.00 | 0.958 | 0.982 | -0.010 |
| RHSIM_Rhsim05G0000900 | 0.00 | 0.958 | 0.982 | 0.005 |
| RHSIM_Rhsim11G0064600 | 0.00 | 0.958 | 0.982 | -0.010 |
| RHSIM_Rhsim13G0088600 | 0.00 | 0.959 | 0.982 | 0.011 |
| RHSIM_Rhsim02G0088300 | 0.00 | 0.959 | 0.983 | 0.011 |
| RHSIM_Rhsim04G0158900 | 0.00 | 0.959 | 0.983 | 0.069 |
| RHSIM_Rhsim04G0135500 | 0.00 | 0.960 | 0.983 | -0.154 |
| RHSIM_Rhsim13G0004300 | 0.00 | 0.960 | 0.983 | 0.008 |
| RHSIM_Rhsim07G0191100 | 0.00 | 0.960 | 0.983 | 0.101 |

| Gene | LRT statistic | P value | BH-adjusted P | log2FC (interaction) |
|---|---|---|---|---|
| RHSIM_Rhsim08G0037000 | 0.00 | 0.960 | 0.983 | -0.003 |
| RHSIM_Rhsim01G0237600 | 0.00 | 0.960 | 0.983 | -0.029 |
| RHSIM_Rhsim08G0189500 | 0.00 | 0.960 | 0.983 | -0.008 |
| novel.1804 | 0.00 | 0.961 | 0.984 | 0.035 |
| RHSIM_Rhsim04G0147500 | 0.00 | 0.961 | 0.984 | 0.012 |
| RHSIM_Rhsim08G0193200 | 0.00 | 0.961 | 0.984 | -0.010 |
| RHSIM_Rhsim08G0190600 | 0.00 | 0.961 | 0.984 | 0.007 |
| RHSIM_Rhsim01G0245500 | 0.00 | 0.961 | 0.984 | 0.006 |
| RHSIM_Rhsim03G0098800 | 0.00 | 0.961 | 0.984 | -0.033 |
| RHSIM_Rhsim04G0195500 | 0.00 | 0.961 | 0.984 | 0.109 |
| RHSIM_Rhsim09G0163100 | 0.00 | 0.962 | 0.984 | 0.031 |
| RHSIM_RhsimUnG0012700 | 0.00 | 0.962 | 0.985 | -0.095 |
| RHSIM_Rhsim01G0156200 | 0.00 | 0.962 | 0.985 | -0.025 |
| RHSIM_Rhsim12G0068000 | 0.00 | 0.962 | 0.985 | -0.003 |
| RHSIM_Rhsim07G0064300 | 0.00 | 0.962 | 0.985 | -0.112 |
| RHSIM_Rhsim13G0120400 | 0.00 | 0.962 | 0.985 | -0.144 |
| RHSIM_Rhsim02G0091300 | 0.00 | 0.962 | 0.985 | -0.042 |
| RHSIM_Rhsim08G0102700 | 0.00 | 0.962 | 0.985 | -0.037 |
| RHSIM_Rhsim02G0121400 | 0.00 | 0.962 | 0.985 | -0.085 |
| novel.3885 | 0.00 | 0.963 | 0.985 | 0.059 |
| RHSIM_Rhsim02G0227000 | 0.00 | 0.963 | 0.985 | -0.043 |
| RHSIM_Rhsim03G0032100 | 0.00 | 0.963 | 0.985 | 0.073 |
| RHSIM_Rhsim06G0134300 | 0.00 | 0.963 | 0.985 | -0.069 |
| RHSIM_Rhsim13G0008400 | 0.00 | 0.963 | 0.985 | -0.027 |
| RHSIM_Rhsim10G0053700 | 0.00 | 0.963 | 0.986 | -0.006 |
| novel.219 | 0.00 | 0.964 | 0.986 | 0.061 |
| RHSIM_Rhsim06G0002700 | 0.00 | 0.964 | 0.986 | -0.017 |
| RHSIM_Rhsim10G0023300 | 0.00 | 0.964 | 0.986 | 0.083 |
| RHSIM_Rhsim01G0074900 | 0.00 | 0.964 | 0.986 | -0.034 |
| RHSIM_Rhsim11G0171600 | 0.00 | 0.964 | 0.986 | 0.023 |
| RHSIM_Rhsim12G0192700 | 0.00 | 0.964 | 0.986 | -0.018 |
| RHSIM_RhsimUnG0191300 | 0.00 | 0.964 | 0.986 | 0.025 |

| Gene | LRT statistic | P value | BH-adjusted P | log2FC (interaction) |
|---|---|---|---|---|
| RHSIM_Rhsim01G0266000 | 0.00 | 0.964 | 0.986 | -0.008 |
| RHSIM_RhsimUnG0009400 | 0.00 | 0.964 | 0.986 | -0.110 |
| RHSIM_Rhsim01G0124700 | 0.00 | 0.964 | 0.987 | 0.006 |
| novel.2700 | 0.00 | 0.965 | 0.987 | -0.019 |
| novel.4220 | 0.00 | 0.965 | 0.987 | -0.062 |
| RHSIM_Rhsim02G0201700 | 0.00 | 0.965 | 0.987 | 0.014 |
| RHSIM_Rhsim12G0013700 | 0.00 | 0.965 | 0.987 | 0.111 |
| novel.4137 | 0.00 | 0.965 | 0.987 | -0.032 |
| novel.375 | 0.00 | 0.965 | 0.987 | -0.025 |
| novel.1565 | 0.00 | 0.965 | 0.987 | -0.029 |
| novel.3343 | 0.00 | 0.965 | 0.987 | -0.073 |
| RHSIM_Rhsim11G0108200 | 0.00 | 0.966 | 0.988 | 0.048 |
| RHSIM_Rhsim08G0017800 | 0.00 | 0.966 | 0.988 | 0.070 |
| RHSIM_Rhsim05G0039200 | 0.00 | 0.966 | 0.988 | 0.015 |
| RHSIM_Rhsim01G0100000 | 0.00 | 0.966 | 0.988 | 0.048 |
| RHSIM_Rhsim10G0197800 | 0.00 | 0.967 | 0.988 | 0.065 |
| novel.969 | 0.00 | 0.967 | 0.988 | 0.073 |
| RHSIM_Rhsim10G0029300 | 0.00 | 0.967 | 0.988 | 0.058 |
| novel.2080 | 0.00 | 0.967 | 0.988 | 0.011 |
| RHSIM_Rhsim06G0050600 | 0.00 | 0.967 | 0.989 | -0.076 |
| RHSIM_Rhsim09G0041400 | 0.00 | 0.967 | 0.989 | -0.059 |
| RHSIM_Rhsim05G0203400 | 0.00 | 0.967 | 0.989 | 0.083 |
| RHSIM_RhsimUnG0239900 | 0.00 | 0.968 | 0.989 | -0.014 |
| RHSIM_Rhsim06G0121400 | 0.00 | 0.968 | 0.989 | 0.018 |
| novel.920 | 0.00 | 0.968 | 0.989 | -0.035 |
| novel.3875 | 0.00 | 0.968 | 0.989 | -0.042 |
| RHSIM_Rhsim07G0184300 | 0.00 | 0.968 | 0.989 | 0.006 |
| RHSIM_Rhsim13G0152400 | 0.00 | 0.968 | 0.989 | 0.050 |
| RHSIM_Rhsim12G0185200 | 0.00 | 0.968 | 0.989 | -0.090 |
| RHSIM_Rhsim03G0192500 | 0.00 | 0.968 | 0.989 | -0.019 |
| RHSIM_Rhsim02G0028500 | 0.00 | 0.968 | 0.989 | 0.016 |
| RHSIM_Rhsim13G0120500 | 0.00 | 0.968 | 0.989 | -0.095 |

| Gene | LRT statistic | P value | BH-adjusted P | log2FC (interaction) |
|---|---|---|---|---|
| RHSIM_Rhsim08G0089800 | 0.00 | 0.969 | 0.990 | 0.050 |
| RHSIM_RhsimUnG0189100 | 0.00 | 0.969 | 0.990 | 0.011 |
| RHSIM_Rhsim02G0037800 | 0.00 | 0.969 | 0.990 | 0.049 |
| RHSIM_Rhsim03G0077900 | 0.00 | 0.969 | 0.990 | 0.009 |
| RHSIM_Rhsim03G0233700 | 0.00 | 0.969 | 0.990 | -0.022 |
| RHSIM_Rhsim04G0196200 | 0.00 | 0.969 | 0.990 | -0.046 |
| novel.669 | 0.00 | 0.969 | 0.990 | 0.011 |
| RHSIM_RhsimUnG0084200 | 0.00 | 0.970 | 0.991 | 0.092 |
| RHSIM_Rhsim07G0025400 | 0.00 | 0.970 | 0.991 | -0.045 |
| RHSIM_Rhsim03G0035200 | 0.00 | 0.970 | 0.991 | 0.040 |
| RHSIM_Rhsim07G0207700 | 0.00 | 0.970 | 0.991 | 0.016 |
| RHSIM_Rhsim02G0107400 | 0.00 | 0.970 | 0.991 | 0.046 |
| RHSIM_Rhsim12G0078600 | 0.00 | 0.970 | 0.991 | -0.016 |
| novel.1752 | 0.00 | 0.970 | 0.991 | -0.013 |
| RHSIM_RhsimUnG0165100 | 0.00 | 0.970 | 0.991 | 0.030 |
| RHSIM_Rhsim02G0193000 | 0.00 | 0.970 | 0.991 | -0.004 |
| RHSIM_Rhsim01G0060900 | 0.00 | 0.971 | 0.991 | 0.007 |
| novel.1029 | 0.00 | 0.971 | 0.991 | -0.035 |
| RHSIM_Rhsim09G0006100 | 0.00 | 0.971 | 0.991 | -0.053 |
| RHSIM_Rhsim10G0161600 | 0.00 | 0.971 | 0.991 | -0.021 |
| RHSIM_Rhsim06G0008100 | 0.00 | 0.971 | 0.992 | 0.032 |
| RHSIM_Rhsim03G0129800 | 0.00 | 0.972 | 0.992 | 0.002 |
| RHSIM_Rhsim06G0207800 | 0.00 | 0.972 | 0.992 | -0.005 |
| novel.2085 | 0.00 | 0.972 | 0.992 | -0.009 |
| novel.2432 | 0.00 | 0.972 | 0.992 | 0.045 |
| RHSIM_Rhsim12G0063900 | 0.00 | 0.972 | 0.992 | 0.055 |
| novel.175 | 0.00 | 0.972 | 0.992 | -0.051 |
| RHSIM_Rhsim10G0007600 | 0.00 | 0.972 | 0.992 | 0.023 |
| RHSIM_Rhsim08G0016600 | 0.00 | 0.972 | 0.993 | 0.040 |
| novel.1156 | 0.00 | 0.972 | 0.993 | 0.018 |
| RHSIM_Rhsim11G0144700 | 0.00 | 0.972 | 0.993 | 0.006 |
| RHSIM_Rhsim11G0047200 | 0.00 | 0.972 | 0.993 | -0.003 |

| Gene | LRT statistic | P value | BH-adjusted P | log2FC (interaction) |
|---|---|---|---|---|
| RHSIM_Rhsim13G0214200 | 0.00 | 0.973 | 0.993 | -0.009 |
| RHSIM_Rhsim03G0072800 | 0.00 | 0.973 | 0.993 | -0.032 |
| RHSIM_Rhsim13G0087800 | 0.00 | 0.973 | 0.993 | -0.085 |
| RHSIM_RhsimUnG0003700 | 0.00 | 0.973 | 0.993 | 0.030 |
| RHSIM_RhsimUnG0043700 | 0.00 | 0.973 | 0.993 | -0.013 |
| RHSIM_Rhsim03G0267200 | 0.00 | 0.973 | 0.993 | -0.003 |
| RHSIM_Rhsim12G0105500 | 0.00 | 0.973 | 0.993 | -0.005 |
| RHSIM_RhsimUnG0129700 | 0.00 | 0.973 | 0.993 | 0.071 |
| RHSIM_Rhsim11G0062400 | 0.00 | 0.973 | 0.993 | 0.007 |
| RHSIM_Rhsim10G0181200 | 0.00 | 0.973 | 0.993 | -0.030 |
| novel.1901 | 0.00 | 0.973 | 0.993 | 0.017 |
| RHSIM_Rhsim12G0103300 | 0.00 | 0.973 | 0.993 | 0.021 |
| RHSIM_Rhsim05G0094800 | 0.00 | 0.974 | 0.993 | -0.085 |
| RHSIM_Rhsim01G0217400 | 0.00 | 0.974 | 0.993 | 0.005 |
| RHSIM_Rhsim12G0067900 | 0.00 | 0.974 | 0.993 | 0.013 |
| novel.2543 | 0.00 | 0.974 | 0.993 | 0.037 |
| RHSIM_Rhsim06G0063400 | 0.00 | 0.974 | 0.993 | 0.007 |
| RHSIM_Rhsim04G0176600 | 0.00 | 0.974 | 0.993 | 0.094 |
| RHSIM_Rhsim04G0120500 | 0.00 | 0.974 | 0.993 | -0.005 |
| RHSIM_Rhsim02G0089500 | 0.00 | 0.974 | 0.993 | -0.007 |
| RHSIM_Rhsim04G0000300 | 0.00 | 0.974 | 0.993 | -0.081 |
| RHSIM_Rhsim07G0210300 | 0.00 | 0.974 | 0.993 | 0.009 |
| RHSIM_Rhsim02G0225500 | 0.00 | 0.974 | 0.993 | -0.053 |
| RHSIM_Rhsim08G0052900 | 0.00 | 0.974 | 0.993 | 0.010 |
| novel.4251 | 0.00 | 0.974 | 0.994 | 0.029 |
| RHSIM_Rhsim07G0143700 | 0.00 | 0.975 | 0.994 | 0.002 |
| RHSIM_Rhsim13G0081700 | 0.00 | 0.975 | 0.994 | 0.018 |
| RHSIM_Rhsim13G0199100 | 0.00 | 0.975 | 0.994 | 0.045 |
| RHSIM_RhsimUnG0090700 | 0.00 | 0.975 | 0.994 | -0.057 |
| RHSIM_Rhsim06G0215500 | 0.00 | 0.975 | 0.994 | -0.034 |
| novel.3021 | 0.00 | 0.975 | 0.994 | -0.014 |
| novel.4084 | 0.00 | 0.975 | 0.994 | 0.026 |

| Gene | LRT statistic | P value | BH-adjusted P | log2FC (interaction) |
|---|---|---|---|---|
| RHSIM_Rhsim09G0009800 | 0.00 | 0.975 | 0.994 | -0.048 |
| RHSIM_Rhsim09G0119600 | 0.00 | 0.975 | 0.994 | 0.003 |
| novel.1920 | 0.00 | 0.976 | 0.995 | 0.008 |
| RHSIM_Rhsim03G0206100 | 0.00 | 0.976 | 0.995 | -0.005 |
| novel.1589 | 0.00 | 0.976 | 0.995 | -0.034 |
| RHSIM_RhsimUnG0184900 | 0.00 | 0.976 | 0.995 | 0.053 |
| RHSIM_Rhsim13G0026600 | 0.00 | 0.976 | 0.995 | -0.006 |
| RHSIM_Rhsim01G0100200 | 0.00 | 0.976 | 0.995 | 0.061 |
| RHSIM_Rhsim09G0008700 | 0.00 | 0.976 | 0.995 | 0.004 |
| RHSIM_Rhsim06G0020300 | 0.00 | 0.976 | 0.995 | 0.039 |
| RHSIM_Rhsim03G0170000 | 0.00 | 0.976 | 0.995 | 0.023 |
| novel.3191 | 0.00 | 0.976 | 0.995 | -0.057 |
| RHSIM_Rhsim12G0052700 | 0.00 | 0.976 | 0.995 | -0.004 |
| RHSIM_Rhsim02G0080600 | 0.00 | 0.976 | 0.995 | 0.021 |
| RHSIM_Rhsim02G0014700 | 0.00 | 0.976 | 0.995 | 0.042 |
| RHSIM_Rhsim03G0096400 | 0.00 | 0.976 | 0.995 | 0.026 |
| RHSIM_Rhsim11G0143100 | 0.00 | 0.977 | 0.995 | -0.066 |
| RHSIM_Rhsim12G0073500 | 0.00 | 0.977 | 0.995 | 0.003 |
| RHSIM_Rhsim08G0219600 | 0.00 | 0.977 | 0.995 | 0.056 |
| RHSIM_Rhsim01G0146200 | 0.00 | 0.977 | 0.995 | 0.030 |
| RHSIM_Rhsim05G0175200 | 0.00 | 0.977 | 0.995 | -0.002 |
| RHSIM_Rhsim09G0034700 | 0.00 | 0.977 | 0.995 | -0.072 |
| RHSIM_Rhsim07G0148500 | 0.00 | 0.977 | 0.995 | -0.026 |
| RHSIM_Rhsim12G0177200 | 0.00 | 0.977 | 0.995 | 0.022 |
| RHSIM_Rhsim06G0182900 | 0.00 | 0.977 | 0.995 | 0.024 |
| RHSIM_Rhsim04G0016800 | 0.00 | 0.977 | 0.995 | 0.051 |
| novel.4535 | 0.00 | 0.977 | 0.996 | 0.031 |
| RHSIM_Rhsim02G0208600 | 0.00 | 0.978 | 0.996 | -0.010 |
| novel.992 | 0.00 | 0.978 | 0.996 | 0.038 |
| RHSIM_Rhsim02G0249800 | 0.00 | 0.978 | 0.996 | -0.011 |
| RHSIM_Rhsim01G0259500 | 0.00 | 0.978 | 0.996 | -0.002 |
| RHSIM_Rhsim05G0012800 | 0.00 | 0.978 | 0.996 | -0.036 |

| Gene | LRT statistic | P value | BH-adjusted P | log2FC (interaction) |
|---|---|---|---|---|
| RHSIM_Rhsim03G0111100 | 0.00 | 0.978 | 0.996 | 0.017 |
| RHSIM_Rhsim08G0017900 | 0.00 | 0.978 | 0.996 | 0.031 |
| RHSIM_Rhsim01G0171000 | 0.00 | 0.978 | 0.996 | 0.006 |
| novel.4322 | 0.00 | 0.978 | 0.996 | 0.062 |
| novel.1764 | 0.00 | 0.978 | 0.996 | -0.020 |
| RHSIM_Rhsim11G0051400 | 0.00 | 0.978 | 0.996 | 0.014 |
| RHSIM_Rhsim06G0086400 | 0.00 | 0.978 | 0.996 | -0.006 |
| RHSIM_Rhsim07G0085800 | 0.00 | 0.978 | 0.996 | 0.064 |
| RHSIM_Rhsim08G0130500 | 0.00 | 0.979 | 0.996 | 0.050 |
| RHSIM_Rhsim04G0023300 | 0.00 | 0.979 | 0.996 | 0.003 |
| novel.3819 | 0.00 | 0.979 | 0.996 | -0.021 |
| novel.4413 | 0.00 | 0.979 | 0.996 | 0.035 |
| RHSIM_Rhsim04G0193700 | 0.00 | 0.979 | 0.997 | 0.038 |
| RHSIM_Rhsim07G0210200 | 0.00 | 0.979 | 0.997 | -0.013 |
| RHSIM_Rhsim03G0091200 | 0.00 | 0.979 | 0.997 | 0.003 |
| RHSIM_Rhsim11G0062300 | 0.00 | 0.980 | 0.997 | -0.006 |
| novel.3776 | 0.00 | 0.980 | 0.997 | -0.007 |
| novel.2658 | 0.00 | 0.980 | 0.997 | 0.021 |
| RHSIM_Rhsim10G0128000 | 0.00 | 0.980 | 0.998 | 0.048 |
| novel.1747 | 0.00 | 0.980 | 0.998 | -0.015 |
| RHSIM_Rhsim07G0072600 | 0.00 | 0.981 | 0.998 | -0.042 |
| novel.2986 | 0.00 | 0.981 | 0.998 | 0.048 |
| RHSIM_Rhsim05G0005300 | 0.00 | 0.981 | 0.998 | 0.055 |
| RHSIM_Rhsim03G0045100 | 0.00 | 0.981 | 0.998 | 0.005 |
| novel.848 | 0.00 | 0.981 | 0.998 | -0.027 |
| RHSIM_Rhsim09G0029300 | 0.00 | 0.981 | 0.998 | -0.006 |
| RHSIM_Rhsim01G0247600 | 0.00 | 0.981 | 0.998 | -0.005 |
| novel.4375 | 0.00 | 0.981 | 0.998 | -0.042 |
| RHSIM_Rhsim01G0115600 | 0.00 | 0.982 | 0.999 | -0.015 |
| RHSIM_RhsimUnG0246900 | 0.00 | 0.982 | 0.999 | -0.031 |
| novel.3798 | 0.00 | 0.982 | 0.999 | -0.034 |
| RHSIM_Rhsim12G0043800 | 0.00 | 0.982 | 0.999 | 0.056 |

| Gene | LRT statistic | P value | BH-adjusted P | log2FC (interaction) |
|---|---|---|---|---|
| novel.2662 | 0.00 | 0.982 | 0.999 | 0.010 |
| RHSIM_Rhsim01G0208500 | 0.00 | 0.982 | 0.999 | -0.002 |
| novel.1706 | 0.00 | 0.982 | 0.999 | -0.011 |
| RHSIM_Rhsim13G0042100 | 0.00 | 0.983 | 0.999 | -0.004 |
| novel.2788 | 0.00 | 0.983 | 0.999 | 0.033 |
| RHSIM_Rhsim04G0134700 | 0.00 | 0.983 | 1.000 | 0.062 |
| novel.4102 | 0.00 | 0.983 | 1.000 | 0.002 |
| RHSIM_Rhsim04G0032400 | 0.00 | 0.983 | 1.000 | 0.014 |
| RHSIM_RhsimUnG0119400 | 0.00 | 0.983 | 1.000 | 0.021 |
| novel.1803 | 0.00 | 0.983 | 1.000 | -0.018 |
| RHSIM_Rhsim07G0153400 | 0.00 | 0.983 | 1.000 | 0.002 |
| RHSIM_Rhsim08G0183300 | 0.00 | 0.983 | 1.000 | -0.004 |
| novel.478 | 0.00 | 0.983 | 1.000 | -0.002 |
| RHSIM_Rhsim12G0147000 | 0.00 | 0.983 | 1.000 | -0.044 |
| RHSIM_Rhsim06G0161900 | 0.00 | 0.983 | 1.000 | -0.057 |
| RHSIM_Rhsim08G0146900 | 0.00 | 0.983 | 1.000 | -0.003 |
| RHSIM_Rhsim02G0242300 | 0.00 | 0.983 | 1.000 | 0.015 |
| RHSIM_Rhsim09G0025600 | 0.00 | 0.984 | 1.000 | -0.006 |
| RHSIM_Rhsim01G0190300 | 0.00 | 0.984 | 1.000 | -0.002 |
| RHSIM_Rhsim09G0051900 | 0.00 | 0.984 | 1.000 | -0.012 |
| novel.4406 | 0.00 | 0.984 | 1.000 | 0.022 |
| RHSIM_Rhsim09G0071700 | 0.00 | 0.984 | 1.000 | -0.012 |
| RHSIM_Rhsim02G0199100 | 0.00 | 0.984 | 1.000 | 0.034 |
| RHSIM_Rhsim01G0216500 | 0.00 | 0.984 | 1.000 | -0.012 |
| RHSIM_Rhsim02G0104300 | 0.00 | 0.984 | 1.000 | 0.031 |
| RHSIM_RhsimUnG0118300 | 0.00 | 0.985 | 1.000 | 0.046 |
| RHSIM_Rhsim11G0092300 | 0.00 | 0.985 | 1.000 | 0.003 |
| RHSIM_Rhsim03G0026100 | 0.00 | 0.985 | 1.000 | 0.026 |
| novel.2319 | 0.00 | 0.985 | 1.000 | -0.014 |
| RHSIM_Rhsim03G0250400 | 0.00 | 0.985 | 1.000 | 0.053 |
| novel.3020 | 0.00 | 0.985 | 1.000 | 0.017 |
| novel.1361 | 0.00 | 0.985 | 1.000 | -0.015 |

| Gene | LRT statistic | P value | BH-adjusted P | log2FC (interaction) |
|---|---|---|---|---|
| RHSIM_Rhsim05G0213800 | 0.00 | 0.985 | 1.000 | 0.006 |
| RHSIM_Rhsim11G0170800 | 0.00 | 0.985 | 1.000 | -0.002 |
| novel.957 | 0.00 | 0.986 | 1.000 | 0.007 |
| RHSIM_Rhsim07G0120500 | 0.00 | 0.986 | 1.000 | 0.002 |
| RHSIM_RhsimUnG0156500 | 0.00 | 0.986 | 1.000 | -0.037 |
| novel.3079 | 0.00 | 0.986 | 1.000 | 0.005 |
| RHSIM_RhsimUnG0205200 | 0.00 | 0.986 | 1.000 | -0.007 |
| novel.2611 | 0.00 | 0.986 | 1.000 | -0.001 |
| RHSIM_Rhsim12G0198300 | 0.00 | 0.986 | 1.000 | -0.034 |
| RHSIM_Rhsim12G0090200 | 0.00 | 0.986 | 1.000 | 0.043 |
| novel.2423 | 0.00 | 0.986 | 1.000 | 0.012 |
| RHSIM_Rhsim09G0141300 | 0.00 | 0.987 | 1.000 | 0.026 |
| novel.3359 | 0.00 | 0.987 | 1.000 | -0.007 |
| RHSIM_Rhsim12G0153300 | 0.00 | 0.987 | 1.000 | 0.044 |
| RHSIM_Rhsim03G0118100 | 0.00 | 0.988 | 1.000 | -0.008 |
| RHSIM_RhsimUnG0220800 | 0.00 | 0.988 | 1.000 | 0.003 |
| RHSIM_RhsimUnG0023400 | 0.00 | 0.988 | 1.000 | 0.005 |
| RHSIM_Rhsim03G0089200 | 0.00 | 0.988 | 1.000 | 0.028 |
| RHSIM_Rhsim06G0195700 | 0.00 | 0.988 | 1.000 | -0.010 |
| RHSIM_Rhsim07G0216500 | 0.00 | 0.988 | 1.000 | -0.012 |
| RHSIM_Rhsim13G0107200 | 0.00 | 0.988 | 1.000 | 0.012 |
| novel.595 | 0.00 | 0.988 | 1.000 | 0.006 |
| RHSIM_Rhsim06G0108900 | 0.00 | 0.988 | 1.000 | -0.015 |
| novel.3944 | 0.00 | 0.989 | 1.000 | -0.004 |
| RHSIM_Rhsim06G0118800 | 0.00 | 0.989 | 1.000 | 0.011 |
| RHSIM_Rhsim01G0158800 | 0.00 | 0.989 | 1.000 | 0.030 |
| RHSIM_Rhsim08G0095200 | 0.00 | 0.989 | 1.000 | 0.003 |
| RHSIM_Rhsim11G0116700 | 0.00 | 0.989 | 1.000 | 0.031 |
| RHSIM_Rhsim09G0081900 | 0.00 | 0.990 | 1.000 | 0.001 |
| novel.3496 | 0.00 | 0.990 | 1.000 | -0.010 |
| RHSIM_Rhsim05G0131400 | 0.00 | 0.990 | 1.000 | -0.024 |
| RHSIM_Rhsim01G0018100 | 0.00 | 0.990 | 1.000 | 0.023 |

| Gene | LRT statistic | P value | BH-adjusted P | log2FC (interaction) |
|---|---|---|---|---|
| RHSIM_RhsimUnG0230800 | 0.00 | 0.990 | 1.000 | -0.015 |
| RHSIM_Rhsim01G0058900 | 0.00 | 0.991 | 1.000 | 0.005 |
| novel.2406 | 0.00 | 0.991 | 1.000 | -0.010 |
| novel.492 | 0.00 | 0.991 | 1.000 | -0.004 |
| RHSIM_Rhsim12G0012800 | 0.00 | 0.991 | 1.000 | -0.002 |
| RHSIM_Rhsim06G0174300 | 0.00 | 0.991 | 1.000 | 0.002 |
| RHSIM_Rhsim04G0247500 | 0.00 | 0.991 | 1.000 | 0.002 |
| novel.1924 | 0.00 | 0.992 | 1.000 | -0.011 |
| RHSIM_Rhsim03G0217200 | 0.00 | 0.992 | 1.000 | 0.007 |
| RHSIM_Rhsim11G0178600 | 0.00 | 0.992 | 1.000 | -0.001 |
| novel.1716 | 0.00 | 0.992 | 1.000 | 0.003 |
| RHSIM_Rhsim02G0215700 | 0.00 | 0.992 | 1.000 | 0.023 |
| novel.1630 | 0.00 | 0.993 | 1.000 | -0.005 |
| RHSIM_Rhsim08G0249700 | 0.00 | 0.993 | 1.000 | -0.002 |
| RHSIM_Rhsim13G0177800 | 0.00 | 0.993 | 1.000 | -0.001 |
| RHSIM_RhsimUnG0212300 | 0.00 | 0.993 | 1.000 | -0.019 |
| RHSIM_Rhsim07G0214000 | 0.00 | 0.993 | 1.000 | 0.003 |
| RHSIM_Rhsim05G0047700 | 0.00 | 0.993 | 1.000 | 0.015 |
| RHSIM_RhsimUnG0106400 | 0.00 | 0.993 | 1.000 | 0.001 |
| RHSIM_Rhsim13G0128600 | 0.00 | 0.993 | 1.000 | 0.004 |
| RHSIM_Rhsim06G0102700 | 0.00 | 0.993 | 1.000 | 0.002 |
| RHSIM_Rhsim05G0039600 | 0.00 | 0.993 | 1.000 | 0.020 |
| RHSIM_Rhsim08G0158200 | 0.00 | 0.993 | 1.000 | 0.011 |
| RHSIM_Rhsim05G0063500 | 0.00 | 0.993 | 1.000 | -0.006 |
| RHSIM_Rhsim08G0028200 | 0.00 | 0.994 | 1.000 | 0.001 |
| RHSIM_Rhsim13G0125800 | 0.00 | 0.994 | 1.000 | -0.007 |
| RHSIM_Rhsim07G0162900 | 0.00 | 0.994 | 1.000 | 0.002 |
| RHSIM_Rhsim08G0173700 | 0.00 | 0.994 | 1.000 | -0.003 |
| novel.3347 | 0.00 | 0.994 | 1.000 | -0.012 |
| RHSIM_Rhsim01G0231300 | 0.00 | 0.995 | 1.000 | -0.019 |
| RHSIM_Rhsim12G0017200 | 0.00 | 0.995 | 1.000 | 0.001 |
| RHSIM_Rhsim09G0022500 | 0.00 | 0.995 | 1.000 | 0.013 |

| Gene | LRT statistic | P value | BH-adjusted P | log2FC (interaction) |
|---|---|---|---|---|
| RHSIM_Rhsim12G0180600 | 0.00 | 0.995 | 1.000 | 0.010 |
| RHSIM_Rhsim01G0192100 | 0.00 | 0.995 | 1.000 | 0.008 |
| RHSIM_Rhsim02G0075600 | 0.00 | 0.995 | 1.000 | 0.003 |
| RHSIM_Rhsim13G0035500 | 0.00 | 0.995 | 1.000 | -0.001 |
| RHSIM_Rhsim02G0207400 | 0.00 | 0.995 | 1.000 | -0.007 |
| RHSIM_Rhsim09G0027400 | 0.00 | 0.995 | 1.000 | -0.005 |
| RHSIM_Rhsim11G0156500 | 0.00 | 0.995 | 1.000 | 0.002 |
| RHSIM_Rhsim13G0086700 | 0.00 | 0.995 | 1.000 | 0.004 |
| RHSIM_Rhsim07G0241500 | 0.00 | 0.995 | 1.000 | -0.003 |
| RHSIM_Rhsim10G0070200 | 0.00 | 0.996 | 1.000 | -0.009 |
| RHSIM_Rhsim12G0150200 | 0.00 | 0.996 | 1.000 | -0.001 |
| RHSIM_Rhsim04G0233900 | 0.00 | 0.996 | 1.000 | 0.003 |
| RHSIM_RhsimUnG0010800 | 0.00 | 0.996 | 1.000 | 0.005 |
| novel.795 | 0.00 | 0.996 | 1.000 | -0.004 |
| novel.867 | 0.00 | 0.996 | 1.000 | 0.005 |
| novel.3153 | 0.00 | 0.997 | 1.000 | -0.002 |
| novel.3850 | 0.00 | 0.997 | 1.000 | 0.000 |
| RHSIM_RhsimUnG0182600 | 0.00 | 0.997 | 1.000 | -0.010 |
| RHSIM_Rhsim09G0133700 | 0.00 | 0.997 | 1.000 | 0.001 |
| novel.2759 | 0.00 | 0.998 | 1.000 | 0.001 |
| RHSIM_Rhsim12G0110000 | 0.00 | 0.998 | 1.000 | -0.001 |
| novel.2103 | 0.00 | 0.998 | 1.000 | -0.001 |
| RHSIM_Rhsim13G0012300 | 0.00 | 0.998 | 1.000 | -0.001 |
| RHSIM_Rhsim08G0015100 | 0.00 | 0.998 | 1.000 | -0.002 |
| RHSIM_Rhsim04G0212100 | 0.00 | 0.998 | 1.000 | 0.000 |
| RHSIM_Rhsim12G0102100 | 0.00 | 0.998 | 1.000 | -0.005 |
| RHSIM_Rhsim11G0058100 | 0.00 | 0.998 | 1.000 | 0.000 |
| RHSIM_RhsimUnG0209800 | 0.00 | 0.998 | 1.000 | -0.000 |
| RHSIM_Rhsim03G0268700 | 0.00 | 0.999 | 1.000 | -0.000 |
| RHSIM_Rhsim08G0123900 | 0.00 | 0.999 | 1.000 | 0.000 |
| RHSIM_Rhsim12G0093400 | 0.00 | 0.999 | 1.000 | -0.000 |
| RHSIM_Rhsim02G0073500 | 0.00 | 0.999 | 1.000 | -0.000 |

| Gene | LRT statistic | P value | BH-adjusted P | log2FC (interaction) |
|---|---|---|---|---|
| novel.3063 | 0.00 | 0.999 | 1.000 | 0.001 |
| RHSIM_Rhsim06G0189500 | 0.00 | 0.999 | 1.000 | -0.002 |
| RHSIM_Rhsim09G0031100 | 0.00 | 0.999 | 1.000 | -0.001 |
| RHSIM_Rhsim02G0068900 | 0.00 | 0.999 | 1.000 | 0.002 |
| novel.265 | 0.00 | 0.999 | 1.000 | -0.001 |
| RHSIM_Rhsim06G0055600 | 0.00 | 1.000 | 1.000 | 0.001 |
| novel.850 | 0.00 | 1.000 | 1.000 | -0.001 |
| RHSIM_Rhsim09G0051300 | 0.00 | 1.000 | 1.000 | -0.000 |
| RHSIM_Rhsim02G0095600 | 0.00 | 1.000 | 1.000 | 0.000 |
| RHSIM_Rhsim11G0043100 | 0.00 | 1.000 | 1.000 | 0.000 |
| RHSIM_Rhsim11G0109600 | 0.00 | 1.000 | 1.000 | -1.239 |
| novel.451 | 0.00 | 1.000 | 1.000 | 0.258 |
| RHSIM_Rhsim13G0027300 | 0.00 | 1.000 | 1.000 | -0.008 |
| RHSIM_Rhsim09G0024200 | 0.00 | 1.000 | 1.000 | -0.005 |
| RHSIM_Rhsim02G0131900 | 0.00 | 1.000 | 1.000 | -0.354 |
| RHSIM_Rhsim01G0032700 | 0.00 | 1.000 | 1.000 | -0.029 |
| novel.4541 | 0.00 | 1.000 | 1.000 | -0.079 |
| RHSIM_Rhsim03G0027300 | 0.00 | 1.000 | 1.000 | 0.068 |
| RHSIM_Rhsim09G0071400 | 0.00 | 1.000 | 1.000 | 0.084 |
| RHSIM_Rhsim01G0024400 | 0.00 | 1.000 | 1.000 | 0.001 |
| RHSIM_Rhsim03G0076500 | 0.00 | 1.000 | 1.000 | 0.166 |
| RHSIM_Rhsim05G0011900 | 0.00 | 1.000 | 1.000 | 0.006 |
| RHSIM_Rhsim06G0228400 | 0.00 | 1.000 | 1.000 | -0.094 |
| RHSIM_Rhsim06G0087000 | 0.00 | 1.000 | 1.000 | 0.106 |
| RHSIM_Rhsim05G0160000 | 0.00 | 1.000 | 1.000 | -0.011 |
| RHSIM_Rhsim01G0016600 | 0.00 | 1.000 | 1.000 | -0.720 |
| RHSIM_Rhsim01G0015400 | 0.00 | 1.000 | 1.000 | 23.295 |
| RHSIM_Rhsim13G0092500 | 0.00 | 1.000 | 1.000 | 1.475 |
| RHSIM_Rhsim08G0058600 | 0.00 | 1.000 | 1.000 | -0.333 |
| RHSIM_Rhsim06G0189400 | 0.00 | 1.000 | 1.000 | -2.074 |
| RHSIM_Rhsim08G0235600 | 0.00 | 1.000 | 1.000 | -1.907 |
| RHSIM_Rhsim06G0183100 | 0.00 | 1.000 | 1.000 | 3.301 |

| Gene | LRT statistic | P value | BH-adjusted P | log2FC (interaction) |
|---|---|---|---|---|
| RHSIM_Rhsim02G0189600 | 0.00 | 1.000 | 1.000 | -40.164 |
| novel.1292 | 0.00 | 1.000 | 1.000 | 43.300 |
| novel.1289 | 0.00 | 1.000 | 1.000 | 42.426 |
| RHSIM_RhsimUnG0044300 | 0.00 | 1.000 | 1.000 | 1.214 |
| novel.842 | 0.00 | 1.000 | 1.000 | 43.787 |
| RHSIM_Rhsim01G0015500 | 0.00 | 1.000 | 1.000 | 46.895 |
| RHSIM_Rhsim01G0015700 | 0.00 | 1.000 | 1.000 | 48.514 |
| RHSIM_Rhsim07G0219400 | 0.00 | 1.000 | 1.000 | 2.571 |
| novel.4181 | 0.00 | 1.000 | 1.000 | -2.757 |
| novel.4186 | 0.00 | 1.000 | 1.000 | -3.732 |
| RHSIM_Rhsim08G0223500 | 0.00 | 1.000 | 1.000 | 0.358 |
| RHSIM_Rhsim04G0116300 | 0.00 | 1.000 | 1.000 | 0.542 |
| novel.415 | 0.00 | 1.000 | 1.000 | 5.150 |
| RHSIM_Rhsim02G0189500 | 0.00 | 1.000 | 1.000 | -1.741 |
| RHSIM_Rhsim13G0142200 | 0.00 | 1.000 | 1.000 | 4.202 |
| RHSIM_Rhsim02G0053200 | 0.00 | 1.000 | 1.000 | 0.582 |
| RHSIM_Rhsim08G0150900 | 0.00 | 1.000 | 1.000 | 1.477 |
| RHSIM_Rhsim01G0048700 | 0.00 | 1.000 | 1.000 | -0.227 |
| RHSIM_Rhsim12G0192100 | 0.00 | 1.000 | 1.000 | 1.287 |
| RHSIM_Rhsim11G0093600 | 0.00 | 1.000 | 1.000 | 5.130 |
| RHSIM_Rhsim04G0223800 | 0.00 | 1.000 | 1.000 | 4.233 |
| novel.3238 | 0.00 | 1.000 | 1.000 | 1.107 |
| RHSIM_Rhsim07G0195000 | 0.00 | 1.000 | 1.000 | 1.245 |
| RHSIM_Rhsim12G0040400 | 0.00 | 1.000 | 1.000 | 2.383 |
| RHSIM_Rhsim12G0061000 | 0.00 | 1.000 | 1.000 | 2.931 |
| RHSIM_Rhsim12G0039500 | 0.00 | 1.000 | 1.000 | 1.670 |
| RHSIM_Rhsim09G0143100 | 0.00 | 1.000 | 1.000 | 0.967 |
| RHSIM_Rhsim03G0000800 | 0.00 | 1.000 | 1.000 | 3.422 |
| novel.1467 | 0.00 | 1.000 | 1.000 | -3.468 |
| novel.2547 | 0.00 | 1.000 | 1.000 | 4.331 |
| RHSIM_Rhsim09G0135000 | 0.00 | 1.000 | 1.000 | -1.157 |
| RHSIM_Rhsim12G0051200 | 0.00 | 1.000 | 1.000 | -0.289 |

| Gene | LRT statistic | P value | BH-adjusted P | log2FC (interaction) |
|---|---|---|---|---|
| RHSIM_Rhsim06G0038700 | 0.00 | 1.000 | 1.000 | 1.068 |
| RHSIM_Rhsim02G0131000 | 0.00 | 1.000 | 1.000 | 0.202 |
| novel.1312 | 0.00 | 1.000 | 1.000 | -2.053 |
| RHSIM_Rhsim01G0199700 | 0.00 | 1.000 | 1.000 | -0.629 |
| novel.1290 | 0.00 | 1.000 | 1.000 | 8.206 |
| RHSIM_Rhsim06G0039100 | 0.00 | 1.000 | 1.000 | 0.890 |
| RHSIM_Rhsim05G0201800 | 0.00 | 1.000 | 1.000 | -3.388 |
| RHSIM_Rhsim01G0008600 | 0.00 | 1.000 | 1.000 | -2.578 |
| RHSIM_Rhsim06G0058700 | 0.00 | 1.000 | 1.000 | 0.767 |
| novel.1794 | 0.00 | 1.000 | 1.000 | 2.546 |
| RHSIM_RhsimUnG0024700 | 0.00 | 1.000 | 1.000 | 9.454 |
| RHSIM_Rhsim07G0204800 | 0.00 | 1.000 | 1.000 | 40.845 |
| novel.1291 | 0.00 | 1.000 | 1.000 | 43.476 |
| novel.1253 | 0.00 | 1.000 | 1.000 | 7.194 |
| RHSIM_Rhsim03G0212000 | 0.00 | 1.000 | 1.000 | 41.138 |
| RHSIM_RhsimUnG0024900 | 0.00 | 1.000 | 1.000 | 43.605 |
| RHSIM_Rhsim06G0063100 | 0.00 | 1.000 | 1.000 | 2.131 |
| RHSIM_Rhsim01G0258000 | 0.00 | 1.000 | 1.000 | 0.846 |
| RHSIM_Rhsim06G0133600 | 0.00 | 1.000 | 1.000 | -40.158 |
| RHSIM_Rhsim06G0102000 | 0.00 | 1.000 | 1.000 | 3.070 |
| RHSIM_Rhsim11G0051600 | 0.00 | 1.000 | 1.000 | -41.182 |
| novel.3500 | 0.00 | 1.000 | 1.000 | 2.200 |
| RHSIM_Rhsim13G0118700 | 0.00 | 1.000 | 1.000 | 1.192 |
| RHSIM_Rhsim07G0042600 | 0.00 | 1.000 | 1.000 | -1.782 |
| RHSIM_Rhsim08G0013900 | 0.00 | 1.000 | 1.000 | -8.109 |
| RHSIM_Rhsim04G0070400 | 0.00 | 1.000 | 1.000 | 5.283 |
| RHSIM_Rhsim12G0215200 | 0.00 | 1.000 | 1.000 | 3.948 |
| RHSIM_Rhsim12G0001400 | 0.00 | 1.000 | 1.000 | 1.401 |
| RHSIM_Rhsim03G0088400 | 0.00 | 1.000 | 1.000 | 0.277 |
| RHSIM_Rhsim01G0208700 | 0.00 | 1.000 | 1.000 | 40.736 |
| RHSIM_Rhsim11G0107700 | 0.00 | 1.000 | 1.000 | -5.068 |
| RHSIM_Rhsim11G0107800 | 0.00 | 1.000 | 1.000 | -5.756 |

| Gene | LRT statistic | P value | BH-adjusted P | log2FC (interaction) |
|---|---|---|---|---|
| RHSIM_Rhsim10G0123900 | 0.00 | 1.000 | 1.000 | -0.715 |
| RHSIM_Rhsim07G0035500 | 0.00 | 1.000 | 1.000 | 4.889 |
| novel.347 | 0.00 | 1.000 | 1.000 | -4.082 |
| RHSIM_Rhsim07G0050300 | 0.00 | 1.000 | 1.000 | 0.534 |
| RHSIM_Rhsim02G0006400 | 0.00 | 1.000 | 1.000 | -0.526 |
| RHSIM_Rhsim08G0166100 | 0.00 | 1.000 | 1.000 | 2.269 |
| RHSIM_Rhsim04G0154600 | 0.00 | 1.000 | 1.000 | 2.417 |
| RHSIM_Rhsim04G0191300 | 0.00 | 1.000 | 1.000 | 0.056 |
| RHSIM_Rhsim01G0260200 | 0.00 | 1.000 | 1.000 | 1.340 |
| novel.2918 | 0.00 | 1.000 | 1.000 | 3.227 |
| RHSIM_Rhsim03G0164800 | 0.00 | 1.000 | 1.000 | 0.713 |
| RHSIM_Rhsim10G0058200 | 0.00 | 1.000 | 1.000 | -0.933 |
| RHSIM_Rhsim08G0001200 | 0.00 | 1.000 | 1.000 | 0.889 |
| RHSIM_Rhsim10G0196400 | 0.00 | 1.000 | 1.000 | 5.428 |
| RHSIM_Rhsim06G0139300 | 0.00 | 1.000 | 1.000 | 41.769 |
| RHSIM_Rhsim04G0075300 | 0.00 | 1.000 | 1.000 | 1.109 |
| RHSIM_RhsimUnG0134400 | 0.00 | 1.000 | 1.000 | 1.620 |
| RHSIM_Rhsim11G0193900 | 0.00 | 1.000 | 1.000 | 3.837 |
| RHSIM_Rhsim09G0031400 | 0.00 | 1.000 | 1.000 | -1.911 |
| RHSIM_Rhsim07G0161100 | 0.00 | 1.000 | 1.000 | 2.429 |
| RHSIM_Rhsim01G0148000 | 0.00 | 1.000 | 1.000 | 2.765 |
| RHSIM_Rhsim05G0065300 | 0.00 | 1.000 | 1.000 | -0.343 |
| RHSIM_Rhsim07G0244000 | 0.00 | 1.000 | 1.000 | 2.961 |
| novel.282 | 0.00 | 1.000 | 1.000 | 2.843 |
| RHSIM_Rhsim07G0146300 | 0.00 | 1.000 | 1.000 | 41.252 |
| RHSIM_Rhsim07G0146600 | 0.00 | 1.000 | 1.000 | 1.755 |
| RHSIM_Rhsim08G0175900 | 0.00 | 1.000 | 1.000 | 41.198 |
| RHSIM_Rhsim10G0054000 | 0.00 | 1.000 | 1.000 | 3.556 |
| RHSIM_Rhsim06G0219500 | 0.00 | 1.000 | 1.000 | 0.336 |
| RHSIM_Rhsim10G0062500 | 0.00 | 1.000 | 1.000 | 1.242 |
| RHSIM_Rhsim04G0152100 | 0.00 | 1.000 | 1.000 | -40.621 |
| RHSIM_Rhsim05G0182700 | 0.00 | 1.000 | 1.000 | -0.541 |

| Gene | LRT statistic | P value | BH-adjusted P | log2FC (interaction) |
|---|---|---|---|---|
| RHSIM_Rhsim10G0200600 | 0.00 | 1.000 | 1.000 | 3.390 |
| RHSIM_Rhsim09G0180400 | 0.00 | 1.000 | 1.000 | -2.930 |
| RHSIM_Rhsim11G0117100 | 0.00 | 1.000 | 1.000 | -0.528 |
| RHSIM_Rhsim12G0169700 | 0.00 | 1.000 | 1.000 | 3.605 |
| RHSIM_Rhsim01G0092800 | 0.00 | 1.000 | 1.000 | -2.964 |
| RHSIM_Rhsim11G0059200 | 0.00 | 1.000 | 1.000 | -1.349 |
| RHSIM_Rhsim05G0081100 | 0.00 | 1.000 | 1.000 | -40.907 |
| RHSIM_Rhsim13G0067000 | 0.00 | 1.000 | 1.000 | 3.868 |
| RHSIM_Rhsim05G0081700 | 0.00 | 1.000 | 1.000 | 2.658 |
| RHSIM_RhsimUnG0144100 | 0.00 | 1.000 | 1.000 | -5.057 |
| RHSIM_Rhsim13G0017000 | 0.00 | 1.000 | 1.000 | 3.936 |
| RHSIM_Rhsim12G0022100 | 0.00 | 1.000 | 1.000 | 3.722 |
| RHSIM_Rhsim04G0056400 | 0.00 | 1.000 | 1.000 | 1.118 |
| RHSIM_Rhsim07G0166200 | 0.00 | 1.000 | 1.000 | 0.861 |
| RHSIM_RhsimMtG0005800 | 0.00 | 1.000 | 1.000 | 2.467 |
| RHSIM_RhsimUnG0069900 | 0.00 | 1.000 | 1.000 | -2.924 |
| RHSIM_Rhsim13G0110500 | 0.00 | 1.000 | 1.000 | 4.821 |
| RHSIM_Rhsim06G0141400 | 0.00 | 1.000 | 1.000 | -0.656 |
| RHSIM_Rhsim04G0079700 | 0.00 | 1.000 | 1.000 | -1.959 |
| RHSIM_Rhsim08G0193000 | 0.00 | 1.000 | 1.000 | -40.314 |
| RHSIM_Rhsim01G0112100 | 0.00 | 1.000 | 1.000 | 41.029 |
| RHSIM_Rhsim03G0168000 | 0.00 | 1.000 | 1.000 | 40.695 |
| RHSIM_Rhsim07G0114400 | 0.00 | 1.000 | 1.000 | 3.367 |
| RHSIM_Rhsim07G0048400 | 0.00 | 1.000 | 1.000 | -1.762 |
| novel.3959 | 0.00 | 1.000 | 1.000 | 2.701 |
| RHSIM_Rhsim12G0137500 | 0.00 | 1.000 | 1.000 | 3.970 |
| RHSIM_Rhsim04G0137300 | 0.00 | 1.000 | 1.000 | 1.713 |
| RHSIM_Rhsim06G0216900 | 0.00 | 1.000 | 1.000 | 4.132 |
| RHSIM_Rhsim12G0175000 | 0.00 | 1.000 | 1.000 | 1.665 |
| RHSIM_Rhsim05G0121900 | 0.00 | 1.000 | 1.000 | 40.630 |
| RHSIM_Rhsim07G0048300 | 0.00 | 1.000 | 1.000 | -2.527 |
| RHSIM_Rhsim09G0044500 | 0.00 | 1.000 | 1.000 | -3.194 |

| Gene | LRT statistic | P value | BH-adjusted P | log2FC (interaction) |
|---|---|---|---|---|
| RHSIM_RhsimUnG0137600 | 0.00 | 1.000 | 1.000 | 1.896 |
| novel.1533 | 0.00 | 1.000 | 1.000 | 0.385 |
| RHSIM_Rhsim13G0213400 | 0.00 | 1.000 | 1.000 | 3.705 |
| RHSIM_RhsimUnG0128600 | 0.00 | 1.000 | 1.000 | 2.481 |
| RHSIM_Rhsim04G0194800 | 0.00 | 1.000 | 1.000 | 1.925 |
| RHSIM_Rhsim09G0167700 | 0.00 | 1.000 | 1.000 | 1.699 |
| RHSIM_Rhsim04G0009000 | 0.00 | 1.000 | 1.000 | 4.591 |
| novel.2344 | 0.00 | 1.000 | 1.000 | 7.171 |
| RHSIM_Rhsim05G0168800 | 0.00 | 1.000 | 1.000 | 4.038 |
| RHSIM_Rhsim10G0202700 | 0.00 | 1.000 | 1.000 | 40.850 |
| novel.2723 | 0.00 | 1.000 | 1.000 | -2.347 |
| RHSIM_Rhsim06G0235300 | 0.00 | 1.000 | 1.000 | 1.447 |
| RHSIM_Rhsim06G0152700 | 0.00 | 1.000 | 1.000 | -41.401 |
| RHSIM_Rhsim09G0210300 | 0.00 | 1.000 | 1.000 | 1.020 |
| RHSIM_Rhsim13G0076900 | 0.00 | 1.000 | 1.000 | -0.571 |
| RHSIM_Rhsim08G0023900 | 0.00 | 1.000 | 1.000 | 1.522 |
| RHSIM_Rhsim02G0097700 | 0.00 | 1.000 | 1.000 | 1.300 |
| RHSIM_Rhsim04G0086000 | 0.00 | 1.000 | 1.000 | 3.927 |
| RHSIM_Rhsim09G0016600 | 0.00 | 1.000 | 1.000 | 0.325 |
| RHSIM_Rhsim09G0014700 | 0.00 | 1.000 | 1.000 | -41.263 |
| RHSIM_RhsimUnG0145400 | 0.00 | 1.000 | 1.000 | 3.775 |
| RHSIM_Rhsim01G0109500 | 0.00 | 1.000 | 1.000 | -1.042 |
| RHSIM_Rhsim06G0142400 | 0.00 | 1.000 | 1.000 | 2.868 |
| RHSIM_Rhsim01G0041600 | 0.00 | 1.000 | 1.000 | 1.515 |
| RHSIM_Rhsim07G0057700 | 0.00 | 1.000 | 1.000 | -0.944 |
| RHSIM_Rhsim04G0141000 | 0.00 | 1.000 | 1.000 | -41.021 |
| RHSIM_Rhsim03G0089500 | 0.00 | 1.000 | 1.000 | 2.771 |
| RHSIM_Rhsim07G0044400 | 0.00 | 1.000 | 1.000 | 3.958 |
| novel.3758 | 0.00 | 1.000 | 1.000 | 1.272 |
| novel.3774 | 0.00 | 1.000 | 1.000 | -3.520 |
| novel.1408 | 0.00 | 1.000 | 1.000 | 4.390 |
| RHSIM_Rhsim11G0140300 | 0.00 | 1.000 | 1.000 | 1.353 |

| Gene | LRT statistic | P value | BH-adjusted P | log2FC (interaction) |
|---|---|---|---|---|
| RHSIM_Rhsim07G0027600 | 0.00 | 1.000 | 1.000 | 3.693 |
| RHSIM_Rhsim10G0104600 | 0.00 | 1.000 | 1.000 | -1.975 |
| RHSIM_Rhsim09G0203400 | 0.00 | 1.000 | 1.000 | 43.311 |
| novel.2377 | 0.00 | 1.000 | 1.000 | 6.385 |
| RHSIM_Rhsim10G0207000 | 0.00 | 1.000 | 1.000 | -1.208 |
| RHSIM_RhsimUnG0081400 | 0.00 | 1.000 | 1.000 | -0.253 |
| RHSIM_Rhsim09G0085700 | 0.00 | 1.000 | 1.000 | -41.248 |
| RHSIM_Rhsim02G0001000 | 0.00 | 1.000 | 1.000 | 5.238 |
| RHSIM_Rhsim04G0238600 | 0.00 | 1.000 | 1.000 | -1.725 |
| RHSIM_Rhsim06G0162800 | 0.00 | 1.000 | 1.000 | 3.658 |
| RHSIM_Rhsim06G0159200 | 0.00 | 1.000 | 1.000 | 41.687 |
| RHSIM_Rhsim03G0064100 | 0.00 | 1.000 | 1.000 | -40.276 |
| RHSIM_Rhsim01G0182100 | 0.00 | 1.000 | 1.000 | 0.493 |
| RHSIM_Rhsim09G0100100 | 0.00 | 1.000 | 1.000 | 2.401 |
| novel.654 | 0.00 | 1.000 | 1.000 | 43.236 |
| RHSIM_Rhsim02G0142500 | 0.00 | 1.000 | 1.000 | -0.349 |
| RHSIM_Rhsim04G0045400 | 0.00 | 1.000 | 1.000 | 0.211 |
| RHSIM_RhsimMtG0000900 | 0.00 | 1.000 | 1.000 | 1.704 |
| novel.2429 | 0.00 | 1.000 | 1.000 | 2.765 |
| RHSIM_Rhsim10G0168200 | 0.00 | 1.000 | 1.000 | 1.839 |
| novel.4290 | 0.00 | 1.000 | 1.000 | 0.583 |
| RHSIM_Rhsim13G0078900 | 0.00 | 1.000 | 1.000 | 2.255 |
| RHSIM_Rhsim01G0165600 | 0.00 | 1.000 | 1.000 | 3.435 |
| novel.4363 | 0.00 | 1.000 | 1.000 | 43.844 |
| novel.4347 | 0.00 | 1.000 | 1.000 | 5.049 |
| RHSIM_Rhsim05G0120300 | 0.00 | 1.000 | 1.000 | 1.455 |
| RHSIM_Rhsim08G0198200 | 0.00 | 1.000 | 1.000 | 2.039 |
| RHSIM_Rhsim11G0134100 | 0.00 | 1.000 | 1.000 | 3.164 |
| RHSIM_Rhsim10G0102000 | 0.00 | 1.000 | 1.000 | 1.504 |
| RHSIM_Rhsim13G0123600 | 0.00 | 1.000 | 1.000 | -41.489 |
| novel.3804 | 0.00 | 1.000 | 1.000 | 42.778 |
| RHSIM_Rhsim01G0203200 | 0.00 | 1.000 | 1.000 | 1.787 |

| Gene | LRT statistic | P value | BH-adjusted P | log2FC (interaction) |
|---|---|---|---|---|
| RHSIM_Rhsim10G0166500 | 0.00 | 1.000 | 1.000 | -2.787 |
| RHSIM_Rhsim01G0080600 | 0.00 | 1.000 | 1.000 | 0.892 |
| RHSIM_Rhsim04G0012400 | 0.00 | 1.000 | 1.000 | 0.847 |
| RHSIM_Rhsim02G0185500 | 0.00 | 1.000 | 1.000 | -2.657 |
| RHSIM_Rhsim05G0140700 | 0.00 | 1.000 | 1.000 | 1.667 |
| RHSIM_Rhsim04G0181300 | 0.00 | 1.000 | 1.000 | -43.937 |
| RHSIM_Rhsim07G0002500 | 0.00 | 1.000 | 1.000 | 1.864 |
| novel.655 | 0.00 | 1.000 | 1.000 | 41.926 |
| RHSIM_Rhsim07G0027100 | 0.00 | 1.000 | 1.000 | -0.869 |
| RHSIM_Rhsim01G0061300 | 0.00 | 1.000 | 1.000 | 1.759 |
| RHSIM_Rhsim13G0167800 | 0.00 | 1.000 | 1.000 | 2.903 |
| RHSIM_Rhsim10G0034100 | 0.00 | 1.000 | 1.000 | 0.602 |
| RHSIM_Rhsim08G0076800 | 0.00 | 1.000 | 1.000 | 1.660 |
| RHSIM_RhsimUnG0051600 | 0.00 | 1.000 | 1.000 | 41.045 |
| RHSIM_RhsimUnG0012900 | 0.00 | 1.000 | 1.000 | 0.100 |
| RHSIM_Rhsim09G0150600 | 0.00 | 1.000 | 1.000 | 3.842 |
| RHSIM_Rhsim12G0113500 | 0.00 | 1.000 | 1.000 | 0.320 |
| RHSIM_Rhsim03G0245300 | 0.00 | 1.000 | 1.000 | 3.479 |
| RHSIM_Rhsim04G0095700 | 0.00 | 1.000 | 1.000 | -2.641 |
| RHSIM_Rhsim13G0198400 | 0.00 | 1.000 | 1.000 | 2.151 |
| RHSIM_Rhsim01G0044700 | 0.00 | 1.000 | 1.000 | 40.520 |
| RHSIM_Rhsim05G0225400 | 0.00 | 1.000 | 1.000 | 3.621 |
| novel.4284 | 0.00 | 1.000 | 1.000 | 2.086 |
| RHSIM_Rhsim06G0203500 | 0.00 | 1.000 | 1.000 | 3.697 |
| RHSIM_Rhsim05G0231100 | 0.00 | 1.000 | 1.000 | 2.683 |
| RHSIM_Rhsim08G0040500 | 0.00 | 1.000 | 1.000 | 0.916 |
| novel.3093 | 0.00 | 1.000 | 1.000 | -3.187 |
| novel.1983 | 0.00 | 1.000 | 1.000 | 2.449 |
| RHSIM_Rhsim09G0012700 | 0.00 | 1.000 | 1.000 | -2.660 |
| RHSIM_Rhsim02G0161800 | 0.00 | 1.000 | 1.000 | 1.092 |
| novel.2675 | 0.00 | 1.000 | 1.000 | 5.941 |
| RHSIM_Rhsim06G0020900 | 0.00 | 1.000 | 1.000 | 41.640 |

| Gene | LRT statistic | P value | BH-adjusted P | log2FC (interaction) |
|---|---|---|---|---|
| RHSIM_Rhsim13G0042300 | 0.00 | 1.000 | 1.000 | -3.184 |
| novel.2703 | 0.00 | 1.000 | 1.000 | 5.417 |
| novel.2685 | 0.00 | 1.000 | 1.000 | -2.152 |
| RHSIM_Rhsim13G0041500 | 0.00 | 1.000 | 1.000 | 1.940 |
| RHSIM_Rhsim06G0020200 | 0.00 | 1.000 | 1.000 | 4.132 |
| RHSIM_Rhsim09G0103700 | 0.00 | 1.000 | 1.000 | 2.258 |
| RHSIM_Rhsim05G0196400 | 0.00 | 1.000 | 1.000 | 40.928 |
| RHSIM_Rhsim08G0066400 | 0.00 | 1.000 | 1.000 | -40.173 |
| novel.3323 | 0.00 | 1.000 | 1.000 | 1.400 |
| RHSIM_Rhsim11G0164600 | 0.00 | 1.000 | 1.000 | -43.693 |
| RHSIM_Rhsim10G0016600 | 0.00 | 1.000 | 1.000 | -0.217 |
| RHSIM_Rhsim03G0186500 | 0.00 | 1.000 | 1.000 | -0.848 |
| novel.3312 | 0.00 | 1.000 | 1.000 | 40.646 |
| RHSIM_Rhsim08G0069800 | 0.00 | 1.000 | 1.000 | -0.750 |
| RHSIM_Rhsim11G0080200 | 0.00 | 1.000 | 1.000 | 41.616 |
| RHSIM_Rhsim10G0015300 | 0.00 | 1.000 | 1.000 | 1.752 |
| novel.4249 | 0.00 | 1.000 | 1.000 | -1.535 |
| RHSIM_Rhsim02G0028800 | 0.00 | 1.000 | 1.000 | 6.162 |
| RHSIM_Rhsim02G0029000 | 0.00 | 1.000 | 1.000 | 41.815 |
| RHSIM_Rhsim06G0171400 | 0.00 | 1.000 | 1.000 | 1.981 |
| RHSIM_RhsimUnG0182700 | 0.00 | 1.000 | 1.000 | 2.594 |
| RHSIM_Rhsim03G0226200 | 0.00 | 1.000 | 1.000 | -0.726 |
| novel.3599 | 0.00 | 1.000 | 1.000 | 2.307 |
| novel.1842 | 0.00 | 1.000 | 1.000 | -3.482 |
| RHSIM_Rhsim08G0040100 | 0.00 | 1.000 | 1.000 | 4.418 |
| novel.1856 | 0.00 | 1.000 | 1.000 | -2.148 |
| RHSIM_Rhsim11G0077500 | 0.00 | 1.000 | 1.000 | 3.468 |
| RHSIM_Rhsim01G0239000 | 0.00 | 1.000 | 1.000 | -1.638 |
| RHSIM_RhsimUnG0175400 | 0.00 | 1.000 | 1.000 | 2.185 |
| novel.4269 | 0.00 | 1.000 | 1.000 | -40.965 |
| RHSIM_Rhsim07G0169000 | 0.00 | 1.000 | 1.000 | 42.063 |
| RHSIM_Rhsim07G0180100 | 0.00 | 1.000 | 1.000 | 2.143 |

| Gene | LRT statistic | P value | BH-adjusted P | log2FC (interaction) |
|---|---|---|---|---|
| RHSIM_Rhsim02G0195100 | 0.00 | 1.000 | 1.000 | 40.894 |
| RHSIM_Rhsim03G0211100 | 0.00 | 1.000 | 1.000 | 41.470 |
| RHSIM_Rhsim05G0165400 | 0.00 | 1.000 | 1.000 | 3.496 |
| novel.3734 | 0.00 | 1.000 | 1.000 | 2.453 |
| RHSIM_Rhsim05G0159000 | 0.00 | 1.000 | 1.000 | -1.435 |
| RHSIM_Rhsim11G0166200 | 0.00 | 1.000 | 1.000 | 0.065 |
| RHSIM_Rhsim07G0071600 | 0.00 | 1.000 | 1.000 | -2.625 |
| RHSIM_Rhsim05G0198500 | 0.00 | 1.000 | 1.000 | -3.321 |
| RHSIM_Rhsim13G0022900 | 0.00 | 1.000 | 1.000 | -4.395 |
| RHSIM_Rhsim09G0146000 | 0.00 | 1.000 | 1.000 | -2.819 |
| RHSIM_Rhsim08G0147900 | 0.00 | 1.000 | 1.000 | -2.945 |
| RHSIM_Rhsim02G0191100 | 0.00 | 1.000 | 1.000 | -0.231 |
| RHSIM_Rhsim08G0046800 | 0.00 | 1.000 | 1.000 | 3.411 |
| novel.1836 | 0.00 | 1.000 | 1.000 | -2.273 |
| RHSIM_Rhsim02G0223800 | 0.00 | 1.000 | 1.000 | 1.056 |
| RHSIM_Rhsim12G0131000 | 0.00 | 1.000 | 1.000 | -0.323 |
| novel.3285 | 0.00 | 1.000 | 1.000 | 0.793 |
| RHSIM_Rhsim02G0168500 | 0.00 | 1.000 | 1.000 | -1.585 |
| RHSIM_Rhsim02G0168600 | 0.00 | 1.000 | 1.000 | -0.319 |
| RHSIM_Rhsim11G0092000 | 0.00 | 1.000 | 1.000 | -2.141 |
| RHSIM_RhsimUnG0047200 | 0.00 | 1.000 | 1.000 | 0.763 |
| RHSIM_RhsimUnG0047300 | 0.00 | 1.000 | 1.000 | 0.381 |
| RHSIM_Rhsim03G0133500 | 0.00 | 1.000 | 1.000 | 0.359 |
| RHSIM_Rhsim07G0227000 | 0.00 | 1.000 | 1.000 | 1.723 |
| RHSIM_Rhsim07G0084200 | 0.00 | 1.000 | 1.000 | 6.369 |
| RHSIM_Rhsim07G0084400 | 0.00 | 1.000 | 1.000 | 0.880 |
| RHSIM_Rhsim08G0051300 | 0.00 | 1.000 | 1.000 | 40.709 |
| RHSIM_Rhsim05G0079500 | 0.00 | 1.000 | 1.000 | 41.177 |
| RHSIM_Rhsim05G0077700 | 0.00 | 1.000 | 1.000 | 0.010 |
| RHSIM_Rhsim05G0009700 | 0.00 | 1.000 | 1.000 | -2.068 |
| RHSIM_Rhsim06G0011600 | 0.00 | 1.000 | 1.000 | 1.113 |
| RHSIM_Rhsim01G0266100 | 0.00 | 1.000 | 1.000 | 0.299 |

| Gene | LRT statistic | P value | BH-adjusted P | log2FC (interaction) |
|---|---|---|---|---|
| RHSIM_Rhsim05G0008700 | 0.00 | 1.000 | 1.000 | -0.459 |
| novel.3899 | 0.00 | 1.000 | 1.000 | 5.175 |
| RHSIM_Rhsim01G0036600 | 0.00 | 1.000 | 1.000 | -42.081 |
| novel.464 | 0.00 | 1.000 | 1.000 | 2.745 |
| RHSIM_Rhsim09G0063300 | 0.00 | 1.000 | 1.000 | -2.340 |
| RHSIM_RhsimUnG0112700 | 0.00 | 1.000 | 1.000 | 42.449 |
| RHSIM_Rhsim08G0015200 | 0.00 | 1.000 | 1.000 | 0.862 |
| RHSIM_Rhsim02G0153300 | 0.00 | 1.000 | 1.000 | 41.278 |
| RHSIM_Rhsim03G0267900 | 0.00 | 1.000 | 1.000 | -1.967 |
| RHSIM_Rhsim02G0013500 | 0.00 | 1.000 | 1.000 | 2.628 |
| RHSIM_Rhsim09G0057200 | 0.00 | 1.000 | 1.000 | 0.313 |
| RHSIM_Rhsim01G0025400 | 0.00 | 1.000 | 1.000 | -0.890 |
| RHSIM_Rhsim04G0064900 | 0.00 | 1.000 | 1.000 | -2.967 |
| RHSIM_Rhsim02G0067400 | 0.00 | 1.000 | 1.000 | 2.564 |
| RHSIM_Rhsim12G0173200 | 0.00 | 1.000 | 1.000 | 1.554 |
| RHSIM_Rhsim10G0183100 | 0.00 | 1.000 | 1.000 | 1.857 |
| RHSIM_Rhsim06G0009400 | 0.00 | 1.000 | 1.000 | 3.736 |
| RHSIM_RhsimUnG0110300 | 0.00 | 1.000 | 1.000 | -2.309 |
| RHSIM_Rhsim13G0153700 | 0.00 | 1.000 | 1.000 | 48.263 |
| novel.2219 | 0.00 | 1.000 | 1.000 | -43.033 |
| RHSIM_Rhsim09G0183400 | 0.00 | 1.000 | 1.000 | -3.782 |
| RHSIM_Rhsim04G0222600 | 0.00 | 1.000 | 1.000 | 2.777 |
| RHSIM_Rhsim07G0076000 | 0.00 | 1.000 | 1.000 | -2.877 |
| RHSIM_Rhsim05G0107200 | 0.00 | 1.000 | 1.000 | -1.200 |
| RHSIM_Rhsim05G0200300 | 0.00 | 1.000 | 1.000 | 2.590 |
| RHSIM_Rhsim04G0116100 | 0.00 | 1.000 | 1.000 | -2.867 |
| RHSIM_Rhsim04G0117300 | 0.00 | 1.000 | 1.000 | -1.400 |
| RHSIM_Rhsim08G0216400 | 0.00 | 1.000 | 1.000 | 8.284 |
| RHSIM_RhsimUnG0207100 | 0.00 | 1.000 | 1.000 | 0.844 |
| RHSIM_Rhsim06G0189700 | 0.00 | 1.000 | 1.000 | -2.883 |
| RHSIM_Rhsim06G0069000 | 0.00 | 1.000 | 1.000 | -4.073 |
| RHSIM_Rhsim06G0037600 | 0.00 | 1.000 | 1.000 | 2.893 |

| Gene | LRT statistic | P value | BH-adjusted P | log2FC (interaction) |
|---|---|---|---|---|
| RHSIM_Rhsim09G0122500 | 0.00 | 1.000 | 1.000 | 1.707 |
| RHSIM_RhsimUnG0024400 | 0.00 | 1.000 | 1.000 | 7.449 |
| RHSIM_Rhsim09G0129200 | 0.00 | 1.000 | 1.000 | 4.509 |
| RHSIM_Rhsim05G0039100 | 0.00 | 1.000 | 1.000 | 1.612 |

Likelihood-ratio test (1 df) of the time × treatment interaction in a negative-binomial GLM (~ time + treatment + time:treatment) fitted with DESeq2 median-of-ratios size factors and full-design gene-wise dispersions. BH-adjusted *P*: Benjamini–Hochberg correction across all tested genes. $\log_2$FC (interaction) > 0: temporal trajectory amplified under alkali stress; < 0: buffered.
