# Supplementary material for: Integrated Physiological, Transcriptomic, and Proteomic Analyses Reveal Alkali Stress Response and Tolerance Mechanisms of Rhododendron obtusum ‘Fenzhuanglou’ Seedlings
Source: Plants (Basel). 2026 Oct 6;15(19):3053. doi: 10.3390/plants15193053 (PMC13666605; doi:10.3390/plants15193053)
Supplement: Supplementary file 1 [file plants-15-03053-s001.zip › Supplementary Table S4.pdf]

Supplementary Table S4. Benjamini–Hochberg FDR sensitivity analysis of DEP sets in the four pairwise comparisons.

Comparison	DEPs ($P \leq 0.05$): total	up	down	DEPs (BH q ≤ 0.05): total	up	down	DEPs (BH q ≤ 0.10): total	up	down
CK-40d vs CK-0d	852	400	452	476	228	248	712	332	380
Treat-0d vs CK-0d	267	180	87	48	36	12	119	81	38
Treat-40d vs CK-40d	1093	558	535	674	349	325	927	481	446
Treat-40d vs Treat-0d	1277	561	716	826	400	426	1174	525	649

DEPs were defined by FC ≥ 1.5 or ≤ 0.6667 together with the stated significance criterion. $P \leq 0.05$: uncorrected Student's t-test; q ≤ 0.05 / q ≤ 0.10: Benjamini–Hochberg-adjusted P values computed across all quantified proteins within each comparison (n = 7,406–7,439).
